# Early origin and global colonisation of foot-and-mouth disease virus

**DOI:** 10.1038/s41598-020-72246-6

**Published:** 2020-09-17

**Authors:** Pakorn Aiewsakun, Nakarin Pamornchainavakul, Chaidate Inchaisri

**Affiliations:** 1 Department of Microbiology, Faculty of Science, Mahidol University, 272, Rama VI Road, Ratchathewi, Bangkok, 10400 Thailand; 2 Pornchai Matangkasombut Center for Microbial Genomics (CENMIG), Department of Microbiology, Faculty of Science, Mahidol University, 272, Rama VI Road, Ratchathewi, Bangkok, 10400 Thailand; 3 Veterinary Epidemiology and Economics Group, Department of Veterinary Medicine, Faculty of Veterinary Science, Chulalongkorn University, Henri Dunant Road, Patumwan, Bangkok, 10330 Thailand

**Keywords:** Virology, Viral evolution, Evolution, Microbiology

## Abstract

In this study, we compiled 84-year worth (1934–2017) of genomic and epidemiological data of foot-and-mouth disease virus (FMDV), and performed comprehensive analyses to determine its early origin and transmission route. We found that recombination is a key feature of FMDV, and that the genomic regions coding for structural and non-structural proteins have markedly different evolutionary histories, and evolve at different rates. Despite all of these differences, analyses of both structural and non-structural protein coding regions consistently suggested that the most recent common ancestor of FMDV could be dated back to the Middle Age, ~ 200 to 300 years earlier than previously thought. The ancestors of the Euro-Asiatic and SAT strains could be dated back to the mid-seventeenth century, and to the mid-fifteenth to mid-sixteenth century, respectively. Our results implicated Mediterranean counties as an early geographical origin of FMDV before spreading to Europe and subsequently to Asia and South America.

## Introduction

Foot-and-mouth disease virus (FMDV) is a pathogen infecting a wide range of cloven-hoofed animals, many of which are domesticated and economically important animals, including pigs, cattle, sheep, and goats^1,2^. FMDV causes a disease called foot-and-mouth disease (FMD); although the disease itself is usually nonfatal in adult animals, it is highly contagious and can cause significant farming production losses, animal abortions, and high mortality rates in young infected animals^1,2^. Indeed, FMD outbreaks have caused severe economic losses worldwide^3,4^, making it a major threat to the global livestock industry. The global annual impact of FMDV has been estimated to be at least between 8–22.5 billion US dollars^4^. Despite this enormous global impact, where and when the disease originated, and how the disease spread globally still remind largely poorly characterised.

FMDV is a single-stranded, positive-sense RNA virus, belonging to the genus *Aphthovirus*, family *Picornaviridae*^5^. Its genome contains a single open reading frame (ORF), coding for a large polyprotein, which, after the translation, is processed into several mature proteins. The ORF can be divided into four regions based on the mature proteins’ functions, namely L, P1, P2 and P3. The P1 region codes for four structural proteins involved in capsid formation called VP1–VP4, while the L, P2, and P3 regions code for several non-structural proteins involved in virus genome replication and protein cleavage activities (see Ref.^6^ for review).

FMDV is currently classified into seven serotypes, namely A, O, Asia1, C, SAT1, SAT2, and SAT3. The former four are also known as Euro-Asiatic serotypes, circulating mainly in Europe, Asia, and South America, while the latter three are collectively known as South African Territories (SAT) serotypes, found mainly in Africa. This classification scheme was established based on immunological reactions^7^, but studies have shown that analyses of VP1 molecular sequences can reproduce this classification scheme well^8–10^. This is consistent with the fact that the VP1 protein is a major antigen which elicits protective humoral immunity^11,12^. Consequently, VP1 was quickly adopted and widely used by the scientific community for the purpose of FMDV identification and classification, outbreak assessment, and vaccination program design and evaluation (e.g. Refs.^13,14^).

Recent studies, however, showed that recombination is a common feature of FMDV, and different parts of its genome can have different evolutionary histories^15–19^. Most importantly, one of the major recombination regions could be mapped to the P1 region, containing the VP1 protein coding region. This means that analyses of VP1 alone cannot fully reveal how the virus evolves. Furthermore, most FMDV studies so far either focused on specific serotypes^20–23^, or those restricted to particular geographical regions^13,24,25^; thus, our understanding of FDMV evolution still remains largely fragmented and incomplete. Here, we set to investigate the global evolutionary history of FMDV across seven serotypes. Our results provided new insights into its origins and how it spread globally.

## Materials and methods

### Data collection

All publicly available whole genomes and complete coding sequences of FMDVs were retrieved from the National Center for Biotechnology Information (NCBI) database, using the search terms “(foot-and-mouth disease virus [Organism]) AND 6,000:9,000 [Sequence Length]”. Metadata associating with the sequences were compiled (Table S1), and examined to exclude experimental isolates. These included vaccine seed viruses, viruses from cell culture, and those with genomes modified for various research purposes. Duplicated sequences, sequences with significant portions of undetermined nucleotide sequences (≥ 200 ‘N’), and those without sampling dates were also removed from the dataset.

### Recombination detection

A manually-curated alignment of FMDVs’ ORFs was prepared using MAFFT^26^. Potential recombination events within the ORF were checked using RDP, GENECONV, Chimaera, MaxChi, BootScan, SiScan, and 3Seq, all implemented in Recombination Detection Program 4^27^. Only those that were detected by more than four programs were considered. The distribution of recombination breakpoints was manually examined, and the alignment was split according to the identified frequent breakpoints. The process was repeated until no frequent breakpoints were found. If small and sporadic recombination events were detected without any consistent breakpoint patterns, they were deleted to generate “recombination-free” alignments. Five recombination-free nucleotide alignments were constructed in total.

### Phylogeny reconstruction

Phylogenies were estimated from the five alignments under the maximum likelihood framework using RAxML V8^28^, all with the GTRCAT substitution model. The number of rate categories was set to 25. Bootstrap clade support values were calculated using Booster v.0.1.2^29^ from 1,000 bootstrap trees.

### Molecular dating

We regressed root-to-tip genetic divergence against sampling date to evaluate if the alignments had sufficient temporal signals using TempEst v.1.5.1^30^. The root placements were determined by minimising the heuristic residual mean squared errors. Given the estimated root placements, the root-to-tip regression analyses were repeated in R v.3.5.2 using the ‘*lm*’ function, and one-tail t-tests were used to assess the significance of the slopes (i.e. if the slopes were significantly positive). Examination revealed that there were several outliers (i.e. viruses with either too low or too high genetic distances given their sampling dates), and analyses were thus repeated again without them. For those with a significant temporal signal, i.e. the p-values of the estimated slopes were less than 0.05, the estimated models were used to calculate the dates of other divergent events by using the ‘*predict*’ function, also implemented in R v.3.5.2.

## Results and discussion

### Data

960 genomic sequences of FMDVs were retrieved from the NCBI database, 41 of which lacked serotype records. Out of the 919 sequences with serotype records, 91.19% of the sequences belonged to the Euro-Asiatic serotypes (O, A, Asia1, and C), and 8.81% belonged to the South African Territories strains (SAT1–3). All SAT strains had an African origin, except for one (AY593839.1; SAT1; collected in 1970), of which the origin was unclear. Serotype A, Asia1, C, and O FMDVs were from the rest of the world, with a few being from the African continent.

Upon manual inspection, we found a number of these sequences to be duplicates. Some belonged to vaccine or experimental strains, while some contained large strings of undetermined or missing nucleotides. Including these sequences could potentially bias our analyses, and thus they were removed from our dataset. In addition, we also excluded sequences without sampling dates as they were required for evolutionary dating analysis (see below). Incidentally, all of the sequences lacking the serotype records were also either obtained from experimental virus isolates, or did not have sampling dates, and thus were removed from the dataset. The curated dataset contained 769 sequences (Table 1). The proportion of the serotypes in the dataset did not change significantly (Chi-squared test: *χ*^2^ = 6.53, degree of freedom = 6, *p* value = 0.37), suggesting that the curation did not significantly change the data structure. Our data comprised epidemiological data and genomic sequences of FMDVs collected over a time span of 84 years (1934–2017).

**Table 1:** Summary of the FMDV sequences retrieved from the NCBI database.

Serotypes	O	A	Asia1	C	SAT1	SAT2	SAT3	Unclassified	Total
Before data curation	545 (56.77%)	200 (20.83%)	63 (6.56%)	30 (3.13%)	27 (2.81%)	30 (3.13%)	24 (2.50%)	41 (4.27%)	960
After data curation	476 (61.90%)	158 (20.55%)	48 (6.24%)	12 (1.56%)	27 (3.51%)	25 (3.25%)	23 (2.99%)	–	769

### Recombination detection

**Figure 1:**
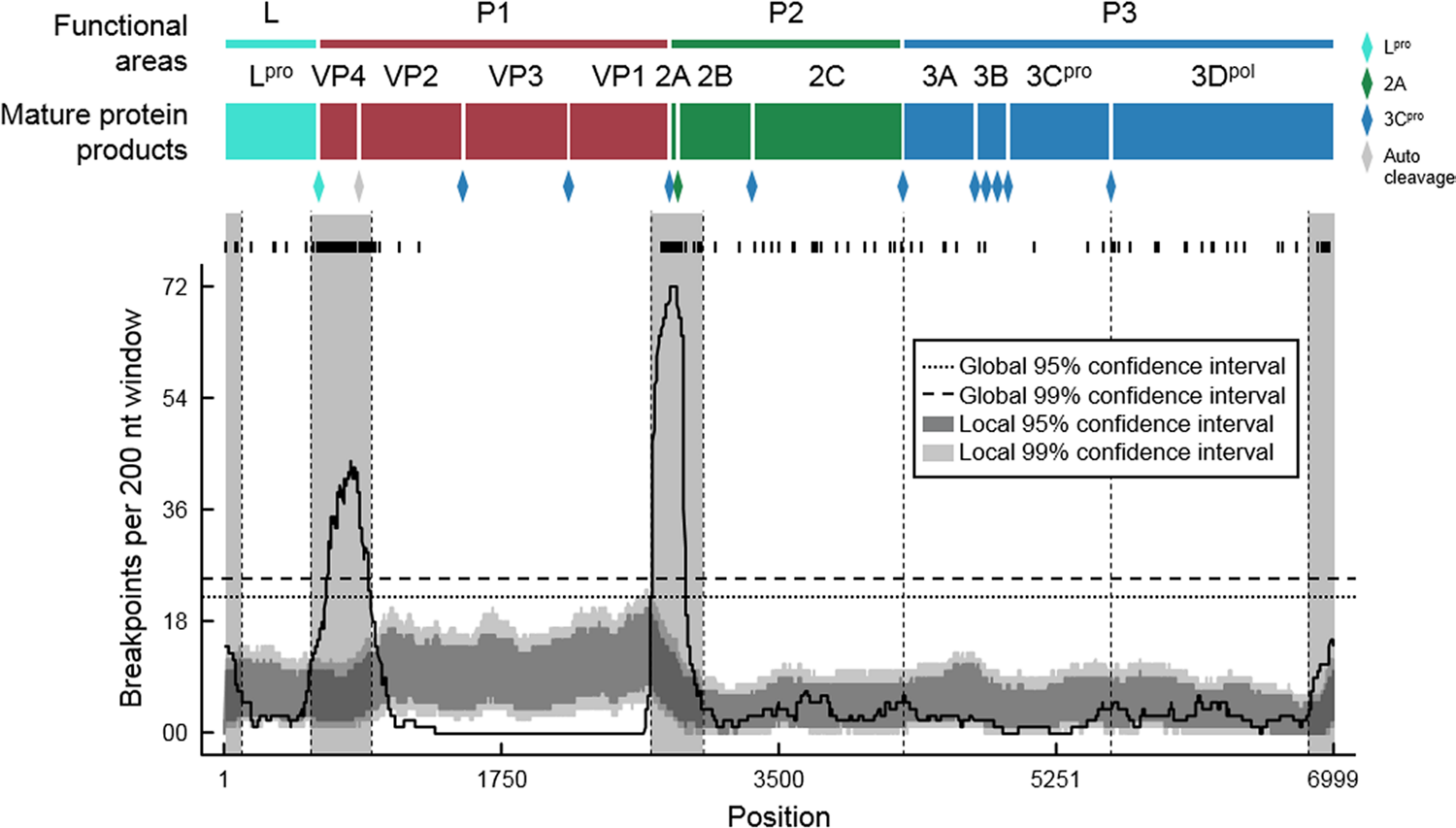
Recombination analysis. (Top) schematic diagrams of FMDV’s ORF. The polyprotein encoded by the ORF is processed into several protein products by proteases (Diamonds; see Keys for the names of proteases). Based on the functions of mature proteins, the ORF can be divided into four main functional areas (L: turquoise; P1: red; P2: green; and P3: blue). L region encodes a leader protease (L^pro^). The P1 region encodes four structural capsid proteins, VP1–4. The P2 and P3 regions encode several non-structure proteins, including 2A–C, and 3A–D. 3C and 3D are a viral protease (3C^pro^) and an RNA-dependent RNA polymerase (3D^pol^), respectively. (Bottom) number of inferred recombination breakpoints estimated per 200-nucleotide window. The tick marks above the plot show the locations of individual breakpoints inferred. Global and local 99% and 95% confidence intervals of the estimated recombination breakpoints are shown (see Keys). Regions with the inferred number of recombination breakpoints more than the 99% local confidence intervals are considered recombination hotspots. Four recombination hotspots were detected in total, indicated by vertical transparent strips. Vertical dotted lines are the locations at which the ORF alignment was split (Data S2–6).

An alignment of the sequences was made and checked for potential recombination. The alignment was manually curated, and was 6,999 nucleotide (nt) long after the curation (Data S1). Our analyses detected four major recombination hotspots (Fig. 1); two were located at the extreme ends of the ORF (nt 1–99, and nt 6,841–6,999), and the other two were mapped to the VP4 (nt 557–939), and 2A coding regions (nt 2,698–3,033).

These hotspots separated the ORF into three distinct regions, corresponding well to the established functional areas^6^. This suggested that recombination in FMDV is constrained by biological functions, and further supported the idea of mosaic structure of FMDV genome^17–19^. The genome of other viruses has also been observed to have a mosaic and modular nature, such as foamy virus^31^, and enteroviruses^32^, for example. Excluding the inferred recombination hotspots, the first region from the 5′ end contained the leader protease coding region (nt 100–556; L^pro^ alignment). The second region comprised almost the entire P1 area, covering the VP1–3 coding regions (nt 940–2,697; P1 alignment). The third region mapped to the P2 and P3 regions (nt 3,034–6,840; P2–3 alignment), excluding the 2A coding region. As previously noted, only a few recombination events were detected in the P1 region, which codes for capsid protein subunits^15–19^. A previous study of serotype O, A, and Asia‐1 FMDVs circulating in West Eurasia also showed that, while the VP1–3 phylogenies were the same, a phylogeny estimated from the VP4 coding region was different from the rest^33^. This finding was consistent and further supported that the VP4 region is a recombination hotspot, and that the VP1–3 regions share the same evolutionary history. This finding suggested that the interaction between protein subunits required to form a functional capsid protein shall, especially the VP1–3 proteins, is highly constrained, such that recombinants involving exchanging genomic materials in this region are strongly unfavoured by natural selection^19^. Potential recombination was then checked again within these three alignments. While we could not detect additional recombination events within the L^pro^, and P1 alignments, a number of recombination could still be found in the P2–3 alignment.

Inspection of the recombination breakpoints in the P2–3 alignment revealed that, although they were not hotspots, there were some aggregations of recombination breakpoints at the P2–P3 junction and the 3C-3D junction (Fig. 1). Again, this separated the ORFs into genomic regions coding for functionally related proteins, suggesting a role of epistatic interaction between them. We thus further split the P2–3 alignment into three alignments: (1) the P2 region (nt 3,034–4,275; P2 alignment), (2) the 3A–C region (nt 4,276–5,586; 3A–C alignment), and (3) the 3D region (nt 5,587–6,840; 3D alignment). Recombination detection analyses were performed again on these three alignments. A number of recombination events could still be detected, but there were only a few of them, and they were small and sporadic. The detected recombination regions were thus removed to create “recombination-free” alignments. Five recombination-free alignments were made in total, the L^pro^, P1, P2, 3A–C and 3D regions (Data S2–S6).

### Phylogenetic analyses

**Figure 2:**
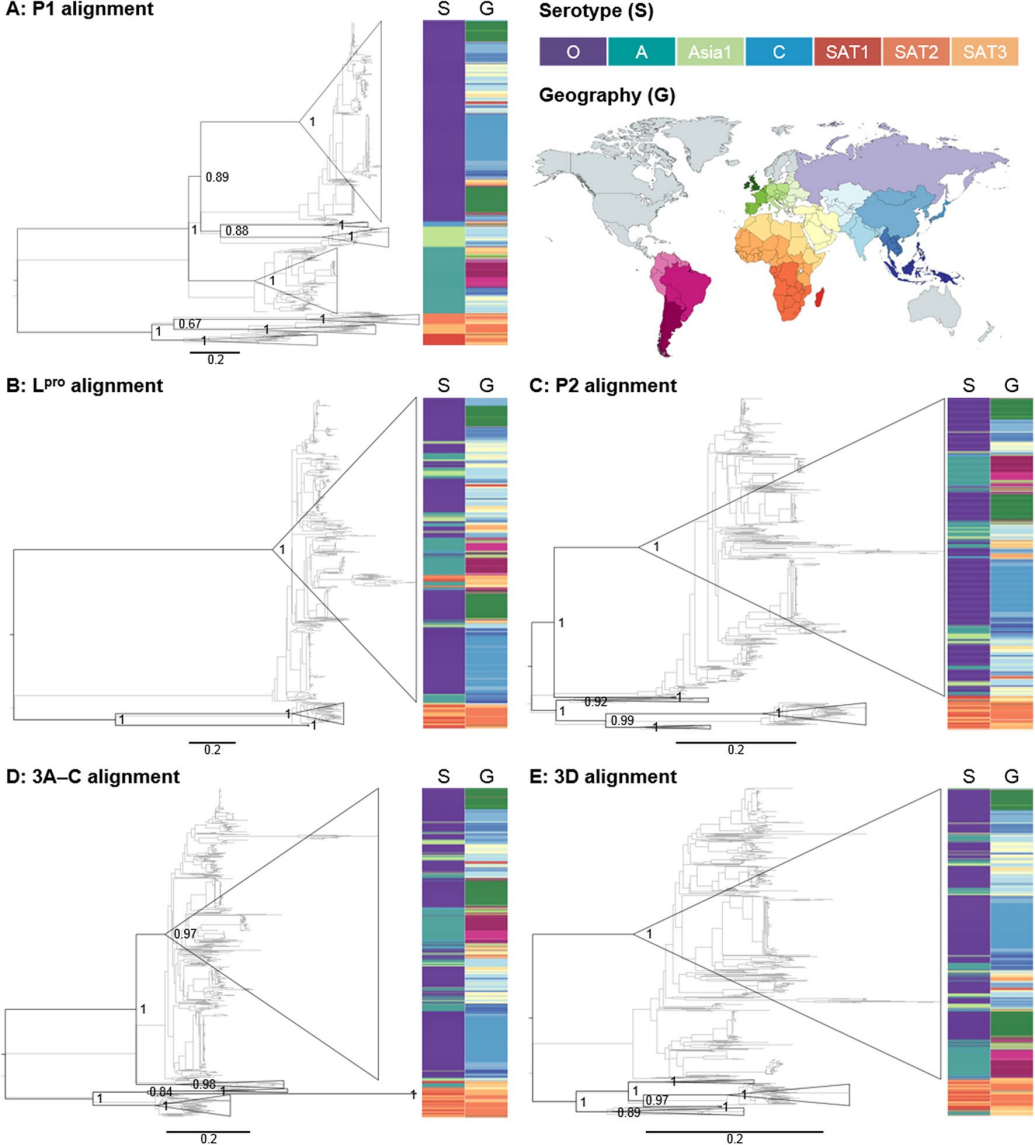
FMDV phylogenies. Five phylogenies were estimated from the five recombination-free alignments (**A**: P1; **B**: L^pro^; **C**: P2; **D**: 3A–C; and **E**: 3D) under the maximum likelihood framework. The numbers on nodes are bootstrap clade support values, shown for various distinct clades. Serotype (S) and geographical (G) information were mapped onto the trees (see Keys). Scale bars are in the units of nucleotide substitutions per site. The map (licence CC BY-SA 4.0) was created with mapchart.net.

Five maximum likelihood phylogenies were estimated from the curated alignments and were rooted by maximising the phylogenetic temporal signals. The results are shown in Fig. 2 (see Tree S1–5 for the trees in the Newick format).

The current classification of FMDVs divides the virus into seven serotypes^7^, and the P1 phylogeny (Fig. 2A) showed that FMDVs segregated into seven well-supported clades (100% bootstrap support), perfectly mirroring their conventional classification. This was expected however, given that the P1 region contains the VP1 coding region commonly used to define the serotype in the first place. A clear separation between the Euro-Asiatic serotypes and the SAT serotypes could also be seen, each forming a clade with 100% bootstrap support. Our analysis suggested that serotypes C and Asia1 were sisters (88% bootstrap support), forming a sister clade to serotype O (89% bootstrap support), while serotype A was the most basal serotype of all Euro-Asiatic serotypes. For the SAT serotypes, our analysis suggested that SAT1 was the most basal one, and SAT2 and SAT3 formed a clade, although not very well-supported (67% bootstrap support).

Four trees were estimated from non-structural protein coding regions (Fig. 2B–E). Again, we found that, although not absolute, Euro-Asiatic FMDVs tended to cluster together separately from the SAT stains (> 97% bootstrap support). However, in stark contrast to what we observed in the P1 tree, serotypes A, O, C and Asia1 appeared to intermix with one another in all of these trees, consistent with previous results^33^. Furthermore, a similar pattern was also found for the SAT1–3 serotypes. This finding strongly suggested that recombination among Euro-Asiatic serotypes and among SAT serotypes is very common. Recombination involving SAT and Euro-Asiatic serotypes could also be observed although rarer. This can be seen pictorially as clusters of green and orange colours in the S(erotype) columns in Fig. 2.

In terms of geographical separation, we found that, while FMDV sequences from the same country tended to cluster together, this phylogenetic pattern was not absolute. This finding was consistent with results previously reported, indicating that the spread of FMDV transcends country boundaries, perhaps as a consequence of international livestock trade. Nonetheless, we observed that African FMDVs tended to aggregate into a few large clades (Fig. 2; orange colour in the G(geography) columns). The same was observed for Asian FMDVs (Fig. 2; blue and yellow in G columns). South American FMDVs (Fig. 2; pink in G columns) also consistently appeared to be closely related to those from middle Europe in all trees (Fig. 2; light green in G columns). Together with our date estimates, these phylogenetic patterns gave new insight into the past transmission history of FMDVs.

### Molecular dating

**Figure 3:**
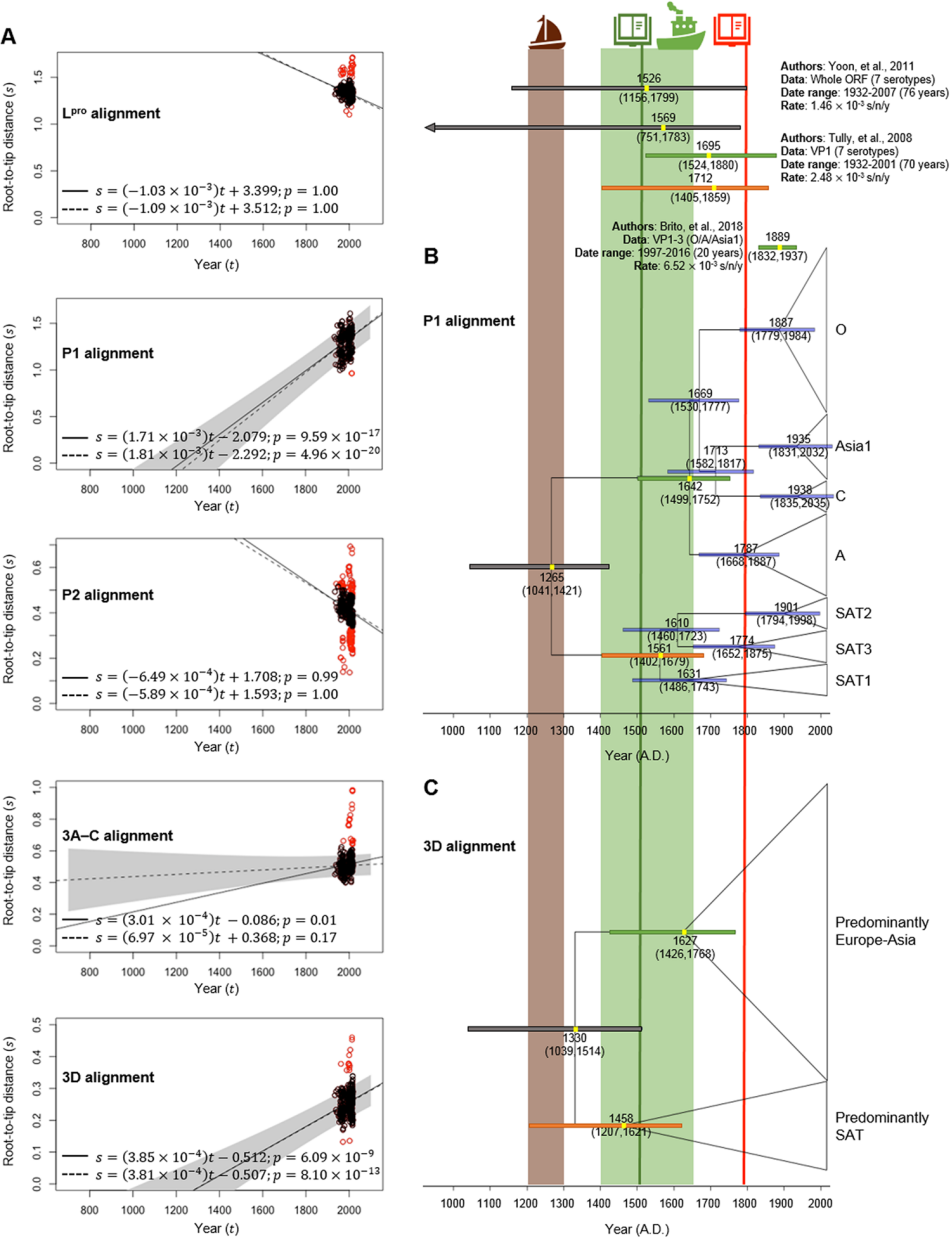
Molecular dating of FMDVs. Regression analyses of root-to-tip genetic distance (s) and sampling dates (t) were performed for the five genomic regions to evaluate their temporal signals (**A**). The solid lines are models estimated based on the entire dataset. The dotted lines are models estimated without outliers (red dots). P-values are reported next to the equations. Analyses suggested only the P1 and 3D regions had sufficient temporal signals for molecular dating (*p* values of the slopes < 0.05). Evolutionary timescales of FMDVs were computed by using the relationships between s and t estimated base on the P1 (**B**) and 3D (**C**) regions. Confidence intervals of the estimated dates are shown by the horizontal bars; grey: tMRCA of all FMDVs, green: tMRCA of Euro-Asiatic serotypes, orange: tMRCA of SAT serotypes, purple; others. Corresponding date estimates by other studies are shown for comparison (Top). The numbers on nodes are median date estimates and their associated confidence intervals. Coloured background and vertical line indicate historical events that might be relevant in the global transmission of FMDVs. The “sailboat” symbol: the rise of livestock transportation from the Mediterranean region to Northern Europe in the thirteenth century^35^; the green “book” symbol: early symptomatic record of FMD in Italy in 1514^34^; the orange “book” symbol: early symptomatic record of FMD in Africa in 1795^43^; the “ship” symbol: the age of discovery starting around early fifteenth century to mid-seventeenth century. Ship and book drawings were Microsoft PowerPoint ClipArts.

Temporal signal within each phylogeny was evaluated using root-to-tip regression analysis (Fig. 3A). The P1, 3A–C and 3D regions had statistically significant temporal signals while the L^pro^ and P2 region did not. Their substitution rates (i.e. the slopes of the regressions) were estimated to be 1.71 × 10^−3^, 3.01 × 10^−4^, and 3.85 × 10^−4^ substitutions/site/year (s/n/y), respectively. Manual inspection, however, showed that some virus isolates appeared to evolve much faster or slower than average (Fig. 3A, red dots), including FMDVs from Taiwan (1997–1999), China (2000–2002, 2005, 2013), and Nigeria (2015). We thus repeated the analyses again but without these “outliers”, and found that the 3A–C region no longer had a significant temporal signal, suggesting that the initially detected signal was an artefact, driven by a small group of viruses. The P1 and 3D regions, in contrast, retained the signals. The rate (i.e. the slope) estimates also changed only slightly for these two regions (P1: 1.81 × 10^−3^ s/n/y; 3D: 3.81 × 10^−4^ s/n/y; Fig. 3A), suggesting the signals were genuine and robust. The rate estimate of the P1 region, in particular, was also comparable to those previously reported (Fig. 3). We noted that the P1 region evolved about 1 order of magnitude faster than the 3D region. This marked difference in the rates could be explained by the fact that the P1 region codes for capsid proteins, which are known to be immunogenic and therefore are likely under a strong positive selection pressure, while the 3D region codes for a polymerase, which is generally known to be highly conserved.

The P1 and 3D regions were further analysed to compute the timings of the origins of various FMDV lineages, since they were the only two regions that had statistically significant and robust temporal signals for molecular dating. The root-to-tip regression analysis of the P1 region (Fig. 3B) suggested that the most recent common ancestor (MRCA) of FMDVs was ~ 750 (95% CI: 598–978) years old (the x-axis intercept), diversifying into the SAT and Euro-Asiatic lineages around 1,265 AD (95% CI: 1,041–1,421 AD). Thereafter, the ancestral SAT lineage diverged into the SAT1 lineage, and subsequently the SAT2 and SAT3 lineages in 1561 AD (95% CI: 1,402–1679 AD) and 1,610 AD (95% CI: 1,460–1723 AD), respectively. Their MRCAs started to diversify ~ 388 (1631 AD; 95% CI: 1,486–1743 AD), ~ 118 (1901 AD; 95% CI: 1794–1998 AD), and ~ 245 (1774 AD; 95% CI: 1652–1875 AD) years ago in their respective order. For the Euro-Asiatic serotypes, their MRCA was dated back to 1642 AD (95% CI: 1,499–1752 AD), with serotype A being the first lineage to branch out. Serotype O subsequently branched out into a separate lineage in 1669 AD (95% CI 1,530–1777 AD), and the last two serotypes, Asia1 and C, diverged from one another in 1713 AD (95% CI 1582–1817 AD). Furthermore, we estimated the time to MRCA (tMRCA) of serotypes A, O, Asia1 and C to be ~ 232 (1787 AD; 95% CI: 1668–1887 AD), ~ 132 (1887 AD; 95% CI: 1779–1984 AD), ~ 84 (1935 AD; 95% CI: 1831–2032 AD), and ~ 81 (1938 AD; 95% CI: 1835–2035 AD) years ago, respectively.

Analysis of the 3D region (Fig. 3C) suggested that the tMRCA of FMDV could be dated back to 1,330 AD (95% CI: 1,039–1514), comparable to the one yielded from the P1 analysis. Since the analysis could not recover a clear division among the seven serotypes of FMDV, we could not estimate the timescale for each one of them individually. Nevertheless, we could estimate the clade predominantly Euro-Asiatic to be ~ 392 years old, dated back to ~ 1627 AD (95% CI: 1,426–1768), and the clade dominated by SAT isolates to be ~ 560 years old, dated back to ~ 1,458 AD (95% CI: 1,207–1621). Remarkably, these results were also comparable to those obtained from the P1 analysis.

One of the earliest observations of FMD could be dated back to 1514 in Italy^34^. Coinciding with this record, Tully et al.^20^ and Yoon et al.^9^ dated the MRCA of FMDVs to early 1500 s based on analyses of the VP1 coding regions and virus whole genomes, respectively (Fig. 3). Based on this date estimate, which fell within the Age of Discovery, Tully et al.^20^ hypothesised that the origin of FMDVs was likely in Europe, and that the European exploration played a key role in the emergence of FMDV in Africa, Asia and the South America. In contrast, our analyses dated that the MRCA of FMDVs to the mid-thirteenth to mid-fourteenth century, being ~ 700–750 years old, which is ~ 200–300 years older than the previous age estimates^9,20^ (Fig. 3B, C). Our date estimates instead coincided with the increase in goods and livestock transportation from Mediterranean countries to Northern Europe in the Middle Age^35^, fitting very well with the early record of FMD in a Mediterranean country like Italy.

Regarding the Euro-Asiatic serotypes, our analyses consistently dated their MRCA to the mid-seventeenth century (Fig. 3). Our date estimates were comparable to that estimated by Tully et al.^20^ (~ 1695 AD), but were considerably older than that estimated by Brito et al.^15^ (~ 1889 AD). Our dates matched well with the end of the Age of Discovery, which, by the end of the era, the foundation for the European colonial empires and global trade between Europe and the rest of the world had been laid. This suggested that, following the introduction of FMD from Mediterranean countries, Europe then acted as a hub to spread the disease to Asia via exploration and trading. In addition, our analyses showed that South American serotype A FMDVs were closely related to European strains in all of the five trees, currently classified as belonging to the same topology, namely the Europe-South American topotype. Their basal diversification could be dated back to only ~ 1722–1858 AD. This phylogenetic pattern and date estimates were in line with the first record of FMD outbreak in the South America continent in 1,870, and could be explained by the historical record of cattle shipment from Europe to Argentina in the late 1860s by European immigrants. Again, this result implicated Europe as a major player in the spread of the disease^20,36,37^.

Regarding the SAT serotypes, they are very well confined to the African continent, and their MRCA could be dated back to the mid-1400 s and 1500 s, pre-dating and thus consistent with an early record of FMD in Africa in 1795 (Fig. 3). These date estimates were considerably earlier than that suggested by Tully et al.^20^, placing the MRCA of the SAT serotypes in early 1700s. Our older age estimates, however, agreed with the results from other studies, which, although did not estimate the tMRCA of SAT serotypes directly, calculated the ages of SAT1 and 2 to be at least ~ 400 to 540 years old^25,38^. These results further corroborated a well-received hypothesis of long-term persistent circulation of FMDVs in African wild animals, such as African buffalo and impala, which may have been acting as FMDV natural reservoirs, sporadically passing the disease to domesticated animals^20,25,39,40^.

As discussed above, Tully et al.^20^ suggested that the MRCA of FMDVs likely originated in Europe and later spread to the rest of world via exploration and trading routes. Our analyses, on the other hand, located the MRCA of FMDV to be the Mediterranean region, which lies between the Africa and Europe continents. As such, it is unclear whether the disease was first originally developed in the African or European continent. Indeed, it was originally generally believed that the disease might originate in the Africa due to the long-term FMDV infection of African wildlife and the greater genetic diversity of the SAT serotypes^41^. Analyses of additional FMDVs from wild animals or their virus relatives may help distinguish between these two completing hypotheses.

## Conclusion and final remarks

By integrating the genomic data with epidemiological information collected over 84 years, we performed comprehensive phylogenetic analyses to characterise how FMDV evolved and spread globally. Our analyses showed that recombination can readily occur in FMDV, especially among Euro-Asiatic serotypes and among SAT serotypes, but the process is likely largely constrained by epistatic interaction between regions coding for functionally related proteins, resulting in relatively clear mosaic genomic blocks of low recombination-rate regions as previously noted^17–19^. This phenomenon has in fact also been observed in other viruses^31,32^. Given how common it is, recombination likely plays an important role in survival and adaption of viruses in the face of new environments. In FMD endemic areas, especially those with high incidence rates of FMDV persistent infection, co-infection of FMDVs can indeed occur^42^, and intra-host recombination could play a significant role in increasing the genetic diversity of the virus, generating new virus variants^18,19^, and potentially altering the virus biology^33^. Although not directly shown in this study, it has been noted that recombination in the capsid protein coding region could occur, and this could potentially reduce the success of FMD vaccination programs^33^. Thus, antigenic diversities must be considered in the development of FMDV vaccine.

**Figure 4:**
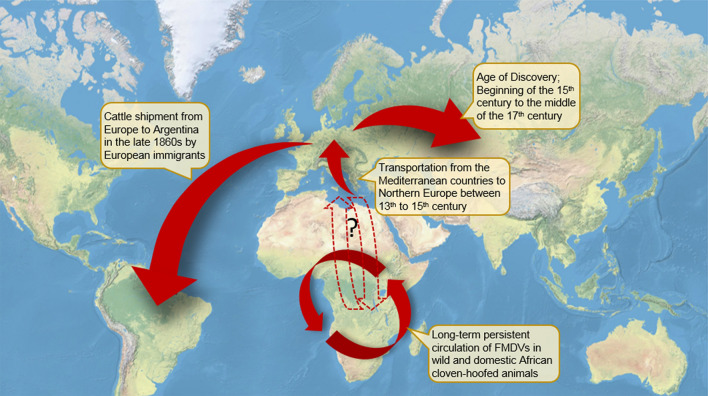
An updated hypothesis on the global colonisation of FMDV. The ancestor of Euro-Asiatic FMDV strains likely originated in the Mediterranean region, and spread to Europe, facilitated by the raise in goods and livestock transportation from the Mediterranean countries to Northern Europe between the thirteenth and fifteenth century. The foundation for the European global trade between Europe and the rest of the world was laid during the Age of Discovery (fifteenth–seventeenth century), and this likely helped the disease to disperse from Europe to Asia. The cattle shipment by European immigrants from Europe to Argentina in the late 1860s was likely responsible for the emergence of FMD in the South America continent. The SAT strains, on the other hand, are very well confined to the African continent. They likely have been circulating in wild and farm African animals for a long time, which play an important part in maintenance of the disease in the African continent. It is unclear, however, if the disease itself was first developed in the African continent then spread to Mediterranean countries (dotted upward arrow) or the other way around (dotted downward arrow). Further studies are required to distinguish between these two completing hypotheses. The world map was created by using *OpenStreetMap* V 0.3.4 R package (https://cran.r-project.org/package=OpenStreetMap).

Our evolutionary dating analyses suggested that FMDV are older than previously thought, dated back to the mid-thirteenth or mid-fourteenth century. By mapping our date estimates to historical events, we hypothesised that Euro-Asiatic strains had their early origin in the Mediterranean countries and subsequently spread to Europe and the rest of the world via European livestock trading and migration (Fig. 4). The SAT strains are also older than previously estimated. We calculated them to be ~ 450 to 550 years old, consistent with the hypothesis that FMDV has been circulating in African wild animals for a long time, playing an important role in maintaining FMD in the African continent^20,25,39,40^. The results from this study further our understanding and provided new insights into the early diversification, emergence, and global colonisation of FMDV.

## Supplementary information

Supplementary Information.

Supplementary Table S1.

Supplementary Data S1.

Supplementary Data S2.

Supplementary Data S3.

Supplementary Data S4.

Supplementary Data S5.

Supplementary Data S6.

Supplementary Tree S1.

Supplementary Tree S2.

Supplementary Tree S3.

Supplementary Tree S4.

Supplementary Tree S5.

## Data Availability

The data that supports the findings of this study are available in the supplementary material of this article.
