# Supplementary material for: Early origin and global colonisation of foot-and-mouth disease virus
Source: Sci Rep. 2020 Sep 17;10:15268. doi: 10.1038/s41598-020-72246-6 (PMC7498456; doi:10.1038/s41598-020-72246-6)
Supplement: Supplementary file 1 — Supplementary Information. [file 41598_2020_72246_MOESM1_ESM.doc]

# Supplementary Information

# Early origin and global colonisation of foot-and-mouth disease virus

# Authors

Pakorn Aiewsakun^1,2^*, Nakarin Pamornchainavakul^3^, Chaidate Inchaisri^3^*

# Author affiliations

1. Department of Microbiology, Faculty of Science, Mahidol University, 272, Rama VI Road, Ratchathewi, Bangkok, 10400, Thailand.

2. Center of Microbial Genomics (CENMIG), Faculty of Science, Mahidol University, 272, Rama VI Road, Ratchathewi, Bangkok, 10400, Thailand.

3. Veterinary Epidemiology and Economics Group, Faculty of Veterinary Science, Chulalongkorn University, Henri Dunant Road, Patumwan, Bangkok, 10330, Thailand

*** Corresponding author contacts**

Pakorn Aiewsakun: pakorn.aie@mahidol.ac.th

Chaidate Inchaisri: chaidate.i@chula.ac.th

# List of Supplementary Information files

**Table S1: FMDVs used in this study (.xlsx)**

Containing information associated with the sequences used in the study, including:

- Accession no.
- Serotype
- Isolate
- Host
- Country
- Country code
- Year of collection
- Study
- Length (nt)
- Topotype
- Lineage
- Exclusion criteria
- Taxon name in the trees

**Data S1: Full FMDV ORF alignment (.doc)**

A manually-curated nucleotide alignment of full-length FMDVs’ protein coding sequences (769 sequences, 6,999 nucleotides).

**Data S2: Lpro alignment (.doc)**

A manually-curated nucleotide alignment of the L^pro^ region (769 sequences, 456 nucleotides).

**Data S3: P1 alignment (.doc)**

A manually-curated nucleotide alignment of the P1 region, covering the VP1–3 coding sequences (769 sequences, 1,758 nucleotides).

**Data S4: P2 alignment (.doc)**

A manually-curated nucleotide alignment of the P2 region (769 sequences, 1,242 nucleotides).

**Data S5: 3A-C alignment (.doc)**

A manually-curated nucleotide alignment of the 3A-C protein coding region (769 sequences, 1,311 nucleotides).

**Data S6: 3D alignment (.doc)**

A manually-curated nucleotide alignment of the 3D protein coding region (769 sequences, 1,254 nucleotides).

**Tree S1: Lpro phylogeny (.doc)**

Maximum likelihood phylogeny estimated from the L^pro^ alignment (**Data S1**), under the GTRCAT substitution model with 25 rate categories, implemented in RAxML V.8. Bootstrap clade support values were computed using Booster v.0.1.2 with 1,000 bootstrap trees. The tree was rooted by maximising the phylogenetic temporal signal in TempEst v.1.5.1.

**Tree S2: P1 phylogeny (.doc)**

Maximum likelihood phylogeny estimated from the P1 alignment (**Data S2**)

**Tree S3: P2 phylogeny (.doc)**

Maximum likelihood phylogeny estimated from the P2 alignment (**Data S3**)

**Tree S4: 3A-C phylogeny (.doc)**

Maximum likelihood phylogeny estimated from the 3A-C alignment (**Data S4**)

**Tree S5: 3D phylogeny (.doc)**

Maximum likelihood phylogeny estimated from the 3D alignment (**Data S5**)
