# Supplementary material for: Early origin and global colonisation of foot-and-mouth disease virus
Source: Sci Rep. 2020 Sep 17;10:15268. doi: 10.1038/s41598-020-72246-6 (PMC7498456; doi:10.1038/s41598-020-72246-6)
Supplement: Supplementary file 3 — Supplementary Data S1. [file 41598_2020_72246_MOESM3_ESM.doc]

>AB079061.1_O_JPN_2000

atgagcacaactgactgtttcatcgctttgttgtacgctttcagagagattaaaacactgttcttatcacgagcacaaggaaagatggagttcacacttcacaacggtgagaagaaaacattctactccaggcccaacaaccacgacaactgctggctgaacaccgtcctccagttgtttaggtacgttgatgaacctttcttcgactgggtctaccactcacctgagaacctcacacttgatgctatcaaacaactggaagaaattactggtctcgagctccacgagggtggaccacccgctctcgttatttggaacattaaacacctgctcaacaccggaatcggcaccgcttcgcgacccagcgaagtgtgcatggtagacgggacggacatgtgtttggctgacttccacgctggcatcttcctgaaaggacaggaacacgctgtgttcgcctgcgttacctccaacgggtggtacgcgattgatgacgaggacttttacccctggacgccggacccgtccgacgttctggtgtttgtcccgtacgatcaagaaccgctcaacggagaatggaaagcaaaggttcagaaacgactcagaggcgccgggcaatccagcccggcgactgggtcacagaaccagtcgggcaacactggaagcattattaacaattactacatgcagcagtaccagaactccatggacacgcaacttggtgacaacgctattagcggaggctccaacgaggggtccacggacaccacctccacccacacaaccaacactcagaacaatgactggttttcaaagctggccagttccgcttttagcggtcttttcggcgctcttctcgccgacaagaaaaccgaggagaccactcttcttgaggaccgcatcctcactacccgcaacggacacacgacctcgacaacccagtcgagcgtcggagtcacttacgggtacgcaacagttgaggactttgtgagcggaccaaacacatctgggcttgagaccagggttgtgcaggcagagcggttcttcaaaacccacctgttcgactgggtcaccagtgacccgttcggacggtgccacctgctggaactcccaactgaccacaaaggtgtctacggcagcctgactgactcttatgcttacatgagaaacggttgggatgttgaggtcaccgcagtgggaaatcagttcaacggaggatgtctgttggtggccatggtgccagaactttgctctattgacaagagagagctgtaccagctcacgctctttccccaccagttcatcaacccccggacgaacatgacggcgcacatcactgtgccctttgttggcgtcaaccgctacgaccagtacaaggtacacaaaccttggaccctcgtggttatggttgtggccccgctgactgtcaacaccgaaggtgccccacagatcaaggtctatgccaacatcgcccctaccaacgtacacgttgcgggtgagttcccttctaaggaagggatcttccccgtggcatgtagcgacggttacggtggtctggtgaccactgacccaaagacggctgaccccgcctacgggaaagtgttcaatccacctcgcaacatgttgccggggcggttcaccaacttccttgatgtggctgaggcgtgccctacgttcctgcactttgagggcggcgtgccgtacgtgaccacaaagacggactcagacagggtgctcgcccagtttgacttgtctctggcagcaaagcacatgtcaaacaccttcctggcaggtctcgcccagtactacacacagtacagcggcaccatcaacctgcacttcatgttcacaggacccactgacgcgaaagcgcgttacatgattgcatacgccccccctggcatggagccgcccaaaacacctgaggcggccgcacactgcattcatgcggagtgggacacagggttgaattcaaaattcacattttcaatcccttacctttcggcggctgattacgcgtacaccgcgtctgacactgcggagaccacaaatgtacagggatgggtttgcctgtttcaaatcacacacgggaaggctgacggcgacgcactggtcgttctagctagcgccggtaaggactttgagctgcgtctgccagttgacgctcgcacgcagaccacctccacaggtgagtcggctgaccctgtgactgccactgttgagaactacggtggtgagacacaggtccagagacgccaacacacggatgtctcgttcatattagacagatttgtgaaagtaacaccaaaagaccaaattaatgtgttggacctgatgcaaacccctgcacacactttggtaggcgcgctcctccgtactgccacctactacttcgcagacctagaagtggcagtgaaacacgaggggaaccttacctgggtcccgaatggggcgcccgagacagcgttggacaacaccaccaatccaacggcttaccacaaggcaccgctcacccggcttgcactgccttacacggcaccacaccgtgtcttggctactgtttacaacgggaactgcaagtatggcgagagccccgtgaccaatgtgagaggtgacctgcaagtattggctcagaaggcggcaagaacgctgcctacctccttcaattacggcgccatcaaagccactcgggtgactgaactgctttaccgcatgaagagggccgaaacatactgcccccggcctcttttggctattcacccaagcgaagctagacacaaacaaaagattgtggcgcctgtgaaacagcttttgagctttgacctgctcaagttggcaggagacgtcgagtccaaccctgggcctttcttcttctctgacgtcaggtcaaatttttccaagttggttgaaaccatcaaccagatgcaggaggacatgtcaacaaaacacggacccgactttaaccggttggtgtctgcatttgaggaactggccactggagtgaaggctatcaggaccggtctcgatgaagccaaaccctggtacaagctcatcaagctcttgagccgcctgtcatgcatggccgctgtagcagcacggtcaaaggacccagtccttgtggccatcatgctggctgacaccggccttgagattctggacagcacctttgtcgtgaagaagatctccgactcgctctccagtctctttcacgtgccggcccccgtcttcagtttcggagccccgattttgttggccgggttggtaaaagtcgcctcgagtttcttccggtccacacccgaagaccttgagagagcggagaaacagctcaaagcacgtgacatcaatgacatattcgccattctcaagaacggcgagtggctggtcaagctgattcttgccatccgcgactggatcaaggcatggatcgcctcagaagaaaagtttgtcaccatgacagacctggtgcctggcatccttgaaaagcagcgggatctcaacgacccaagcaagtacaaggaggccaaggagtggctcgacaacgcgcgccaagcgtgtttgaagagcgggaacacccacatcgcaaacctttgcagagtggttgccccaacacccagcaggtcgaggcccgaacccgtggtcgtttgcctccgtggcaaatcgggccagggcaagagtttccttgcgaacgtgcttgcacaagcaatttcaacccacttcactggcagaaccgattcagtttggtactgcccacctgaccctgaccacttcgacggttacaaccagcagaccgttgtagtaatggatgatttgggccagaaccccgacgggaaggactttaagtacttcgcccaaatggtttcaactacgggggttatcccgcccatggcttcactcgaggacaaaggcaaacctttcaacagcaaggtcatcatcgccaccaccaacctgtactcgggcttcaccccgagaactatggtgtgccctgacgcactgaaccgaaggttccactttgacattgacgtgagcgccaaggacgggtacaaaattaacaacaaattggacatcatcaaagctcttgaagatacccacaccaacccagtggcaatgtttcaatacgactgtgcccttctcaacggcatggccgttgaaatgaagagaatgcgacaagatatgtttaagcctcaaccgcccctccagaacgtctaccagcttgttcaggaggtgattgaccgggtcgagctccacgagaaggtgtcgagccacccgattttcaagcagatctcaattccttcccaaaaggctgtactgtactttctcattgagaagggccagcacgaagcagcaattgaattctttgaggggatggtgcatgactccatcaaggaggagctccggcctctcatccaacagacctcatttgtgaagcgcgcttttaagcgcctgaaggaaaactttgagatagttgccctgtgtttgactcttttggcaaacatagtgatcatgatccgcgagactcgcaagagacagcagatggtggatgatgcagtgaacgagtacattgagagggcaaacatcaccacggatgacaagactcttgacgaggcggaaaagaaccctctggagaccagcggtgccaccactgttggtttcagagagaaaactctcccgggacacaaggcgggtgacgacgtgaactccgagcccgccaaacccgtggaagaacaaccacaagctgaaggaccctacaccggtccactcgagcgtcaaaaacccctgaaagtgagggccaagctcccacagcaggaggggccctacgctggcccgatggagagacagaaaccgctgaaagtgagagtgaaagccccggtcgttaaggaaggaccttacgaaggaccggtgaagaaacctgtcgctttgaaagtgaaagcaaagaacttgattgtcactgagagtggtgctcccccgactgacttgcaaaagatggtcatgggtaacaccaagcctgttgagctcatcctcgacgggaagacggtggccatctgctgcgccaccggagtgtttggtactgcctaccttgttcctcgtcatcttttcgcagagaagtatgacaagatcatgttggacggcagagccatgacagacagtgactacagagtgtttgagtttgagattaaagtgaaaggacaggacatgctctcagacgccgctctcatggtgcttcaccgtgggaatcgcgtgcgggacatcacgaagcacttccgtgatgtggcaagaatgaagaaaggcacccccgtcgtcggcgtgatcaacaacgctgatgttgggagactgatcttctctggtgaggcccttacctacaaggacattgtagtgtgcatggacggagacaccatgcccggtctcttcgcctacaaagctgccaccaaggcgggttactgtggaggagccgttcttgcaaaggacggagccgagactttcatcgtcggcactcactccgcaggcggcaatggagttggatactgctcatgcgtttccaggtccatgctgctcaaaatgaaggcacacatcgaccccgaaccacaccacgagggattgatagttgacaccagagatgttgaggagcgcgtacatgtcatgcgcaaaaccaagctcgcacccaccgtggcacacggtgtgtttaaccccgaatttgggcctgccgccttgtccaacaaggacccgcgcctgaatgagggggttgtccttgatgaagtcatcttctccaaacacaaaggaaacacaaagatgtctgaggaggacaaagcgctgttccgccgctgtgctgctgactacgcgtcgcgtctgcatagcgtgctgggtacggcaaatgccccactgagcgcttacgaggcaatcaagggcgtcgacggacttgacgccatggaaccagacaccgcgcctggtctcccctgggctctccaggggaaacgccgtggtgcgctcattgacttcgagaacggcactgtcggacccgaggttgaagctgccttgaagctcatggagaaaagagagtacaagtttgcatgccagaccttcctgaaggacgagattcgcccgatggagaaggtacgtgccggcaagactcgcattgtcgacgtcctgcccgttgaacacattctttacaccaggatgatgattggcagattttgtgctcaaatgcactcaaacaacggaccgcaaattggctcggcggttggttgtaatcctgatgttgattggcaaagatttggcacgcattttgctcagtatagaaacgtgtgggatgtggactattcggcctttgatgccaaccactgcagtgacgcaatgaacattatgtttgaggaggtgtttaacacggatttcggtttccacccaaacgctgagtggatcctgaaaactctcgtgaacactgaacacgcctatgagaacaaacgcatcactgttgaaggcgggatgccgtctggttgttccgcaacaagcatcatcaacacaattttgaacaacatctacgtgctctacgccttgcgtagacactatgagggagttgagctggactcttacaccatgatctcctacggagacgacatcgtggttgcaagtgattacgatctggactttgaggccctcaagcctcacttcaaatcccttggtcaaaccattactccagctgacaaaagcgacaaaggttttgttcttggtcactccattaccgatgtcactttcctcaaaagacacttccacatggactatggaactgggttttacaaacctgtgatggcttcgaagaccctcgaggctatcctctccttcgcacgccgtgggaccatacaggagaagttgacctccgtggcaggactcgccgtccactctggacctgacgagtaccggcgtctctttgagcctttccagggcctctttgagattccaagctacagatcactttacctgcgttgggtgaacgccgtgtgcggtgacgcataa

>AF026168.2_O_TAW_1997

atgaatacgaccgactgctttatcgctctgctatacgttctcagagagatcaaagcactgtttctgtcacgaacacaagggaagatggaattcacactttacaacggtgaaaagaaggtcttccactccagacccaacaaccacgacaactgttggctgaacgccatcctccaactgttcaggtacgttgacgagcccttcctcgaatgggtctacgactcacctgagaacctcactctcgaggcgatcaacaaactggaagaaatcacaggtcttgagctacacgagggcggaccgcccgcccttgtcgtctggaacatcaagcacttgctctacaccggaatcggcaccgcttcgcgacccagcgaggtgtgcatggtggacggtacagacatgtgcttggctgacttccacgccggtatatttctgaagggacaggaccacgccgtcttcgcctgcgtcacctccaacgggtggtacgcgattgacgacgaggacttttacccgtggacaccaaatccggccgacgttttggtgtttgttccgtacgatcaagaaccattcaacgcagaatggaaagcaaaggttcagaagcggctcaggggcgccgggcaatccagcccgacgaccggctcacaaaaccaatctggcaacactggcagcataattaacaattactacatgcagcagtaccagaactcaatggacccacaacttggcgacaacgccattagtggagggtccaacgagggcttcacggacactacctctacccacaccaacaacacccagaacaacgactggttttcgaaactggccaacaccgcttttatcggcctcttcggtgctcttcttgcagacaagaagacggaagaaaccaccctcctcgaagaccgcatcctcaccacccgcaacgggcacacgacctcgacaacccagtctagcgtcggggtgacttacgggtacgcaacggctgaagacttcgtgagtgggcctaacacctctggtcttgagaccagagttgttcaggccgaacggttcttcaaaacccacctgtttgactgggtcaccagtgacccgtttgggcggtgtcacttgttggagctaccgactgaccacaaaggcgtctacggtagcctgaccgactcgtacgcatacatgaggaatggttgggacgttgaagtcaccgcagtgggtaaccagttcaacggaggctgtttgctggtggcgatggtaccggagctctgttccatcagcaagagagagttgtaccagctcacgcttttcccccaccagttcatcaacccacggacgaatatgacggcacacatcaccgtgccctacctcggtgtcaacaggtacgaccagtacaaggtacacaaaccctggaccctcgtggtcatggttgtggcccccctgacggttaacaacgagggcgctccgcaaatcaaggtgtatgccaacatcgcccccaccaatgttcacgtcgcgggtgagctcccctctaaagagggaattttccccgtggcatgcagcgatggttacggtggcttggtgaccacggatccgaagacggcagaccccgtctacgggaaagtgttcaacccaccccgcaacctgttgccagggcggtttacaaacctccttgacgtggccgaggcgtgccccacattcctacacttcgacggtgacgttccgtacgtgaccacgaagacggattcggatagagtgctagcccggttcgatttgtccctcgcggcaaaacatatgtcgaacacttttctcgcgggtcttgcccagtactacacacagtacagcggcaccattaacctgcacttcatgttcacgggacccaccgacgcgaaggcacgctacatggttgcgtacgcccctcctggcatggaaccgccgaaaacgcctgaggcggctgcacattgcatccacgctgagtgggatacagggctgaattcgaagttcacgttttcaatcccatacctttcggcagctgactacccgtacaccgcgtccgacgtcgccgagaccacaaacgtacagggatgggtctgtttgttccagataacacacgggaaagccgacggtgacgccctggtcgtgctagctagtgctggcaaagactttgacttgcgtctgccggtcgacgccgaaccccaaaccacctctgcgggtgagtctgcggaccccgtgactgccaccgtcgagaactacggtggtgagacacaagtccagaggcgccaagacacggacattgcgttcatattggacaggttcgtgaaagtcaagccaaaggaacaagttaatgtgttggacctgatgcagatccctgcccacaccttggtaggggcgctcctgcgaacggccacctactacttctctgacctggagctggccgtcaagcacgagggcgatctcacctgggtcccaaacggcgcccctgagacagcactggacaacactaccaacccaacagcttaccacaaggaacccctcacacggctggcgctgccttacacggctccacaccgtgtcttagcgaccgtctacaacgggagcagtaagtacggtgacaccagcactaacaacgtgagaggtgaccttcaagtgttagctcagaaggcagaaagaactctgcctacctccttcaacttcggtgccatcaaggcaactcgtgttactgaactactctacagaatgaagagagccgagacatactgtcccaggccccttctcgccattcaaccgagtgacgctagacacaagcagaggattgtggcacccgcaaaacagcttctgaacttcgacctcctcaagttggcgggagacgttgagtccaaccctgggcccttcttcttctccgacgtcagggcgaacttcacgaagttggtggacactgtcaaccagatgcaggaggacatgtcaacgaaacacggacccgacttcaaccgactggtgtccgcgtttgagaattggcgc---tgggtcaaggccatcaggaccggtctcgacgaggccaagccctggtacaagctcatcaaacttctaagccgcctgtcgtgcatggccgctgtggcagcacggtccaaggacccagtccttgtggccatcatgcttggcgacaccggcctcgagattctggacagcaccttcgtggtaaagaagatctccgactcgctctccagtctcttccacgttgcggctcccgccttcagtttcgag---cccgatctgttggccgggttggtcaaagtcgcctcgagtttcttccagtccacacccgaagacctcgagagagcagaaaagcagctcaaagcacgtgacatcaacgacatatttgccgttcttaagaacggtgagtggctggtcaaactgatcctggccatccgcgactggattaaggcatggatcgcctcagaagagaagtttgtcaccatgacagacctggtgcctggcatccttgaaagtcaacgggatctcaatgaccccggcaaatacaaggaggccaaggaatggctggacaacgcgcgtcaagcgtgtttgaagagcgggaacgtgcacattgccaatctgtgtaaagtggtcgctccggcgcccagcaagtcgagacccgaaccagtggtcgtgtgccttcgcggcaaatccggcacaaggaaaagcatcctcgcgaacgtgctcgcgcaggcaatttccacacacttcactggtaggaccgactcggtctggtactgcccgcccgaccctgaccactttgacggttacaatcagcagaccgtcgtcgtgatggacgacttgggccaaaacccagacggcaaagacttcaagtactttgcccaaatggtctccaccacggggttcatcccgcctatggcctcgctcgaggataagggtaaacccttcaacagcaaggtcataatagctacaaccaacctgtactcgggattcaccccaaagaccatggtgtgccccgatgcgcttaaccggaggtttcactttgacatcgacgtgagcgccaaagacgggtacaagatcaacaacaaactggacatagtcaaagcacttgaagacacccacgctaacccggtggcgatgttccaatacgactgcgctcttctcaacggaatggccgttgaaatgaagagaatgcagcaagacatgttcaagcctcaaccacccttccagaacatctaccagctcgttcaggaggtgattgagcgggtggaactacacgaaaaggtgtcgagccacccgatatttaaacagatttcaatcccttcccagaagtccgtgttgtacttcctcattgagaagggtcagcacgaagcagcgatcgagttcttcgaggggatggtccacgattccatcaaagaggaactccgacccctcattcagcagacctcgttcgtaaaacgcgccttcaagcgcctgaaagagaactttgaagttgtagccctgtgtttgaccctcttggcaaacatagtgattatgctccgccaagcgcgcaagaggtaccaatcggtggatgacccactggac------------------------------ggcgacgtagctcttggcgacgcggaaaagaaccctctggagacgagtgccgctagcgctgtcggtttcagagagagatcccccaccgagcaagggacgcgcgaagacgcgaacgctgagcccgtcgtgttcggtagggaacaaccgcgagctgaaggaccctacgctgggccactcgagcgtcagaaacctcttaaagtgaaagccgagctgccacaacaggagggaccatacgccggcccaatggagagacagaaaccgctaaaggtgaaagcaaaagcccccgtcgtgaaggaaggaccttacgagggaccggtgaagaaacctgtcgctttaaaagtgaaagcaaagaacttgatagtcactgagagtggtgcgccaccgaccgacttgcaaaagatggtcatgggcaacactaagccagtcgagctcatcctcgacggcaagacggtagccatttgctgtgctaccggagtgttcggcactgcctacctcgtgcctcgtcatctcttcgcggaaaagtacgacaagatcatgttggacggcagagccttgacagacagtgactacagagtgtttgagtttgagattaaagtaaaaggacaggacatgctctcagacgccgctctcatggtgttgcaccgtgggaatcgcgtgcgtgacatcacgaaacactttcgtgacgtagcgagaatgaagaagggaacccccgtcgtcggtgtgatcaacaatgctgacgtcgggagactcatattctctggtgtagccctcacttacaaggacatcgtcgtgtgtatggatggagacaccatgcctgggctctttgcctacagggcatccaccaaggcaggctactgcggaggagccgtcctggcaaaggacggggccgaaacgttcatcgttggcacccactccgcaggtggaaacggcataggatactgttcgtgtgtttcccgatcaatgctcctgaagatgaaggcacacatcgaccctgaaccacaccacgaggggttgatcgtcgacaccagagatgtggaggagcgtgtccacgtgatgcgcaaaaccaagctcgcgcccaccgtggcgcacggtgtgttcaaccctgagttcgggcctgccgctctgtccaacaaggacccgcgcctgaacgaaggggttgtccttgacgatgtcattttctccaaacacaaaggagatacaaggatgtctgaagaggacaaagcgctgtttcggcgctgtgctgctgactacgcgtcgcgtctacacagtgtgttggggacagcaaacgccccactgagtgtgtatgaagccatcaaaggcgtcgacggacttgacgccatggagccggacacgcgccccggtctcccctgggctctccaagggaaacgccgcggcgtcctgatcgacttcgaaaacggcaccgtcgggcctgaggttgaggcagcactcaagctcatggaaagccgcgagtacaaattcgtctgccaaaccttcctgaaggacgaaattcggccgctagaaaaggtacgcgctggcaagacacgcattgtcgacgtgttgcctgttgaacacattctctacaccagaatgatgattggcagattctgtgctcagatgcattcaaacaacggaccgcaaattggatcagcggtcggttgtaaccctgacgttgattggcaaagatttggcacacatttcgcccagtacaagaacgtgtgggatgtggactactcagcctttgatgcaaaccactgcagcgatgcgatgaacatcatgttcgaggaagtgttccgcacggagttcggattccacccgaacgccgagtggattctgaagactctagtgaacacggagcacgcttacgagaacaagcgcattgttgttgaaggtggaatgccgtccggttgttccgcaacaagcatcatcaacacaattttgaacaacatctacgtgctttacgccctgcgtaggcactatgagggagtcgagctggacacttacaccatgatctcttatggagacgacatcgtggtggcaagtgactacgacctggactttgaggctctcaagccccacttcaagtcccttggtcagactatcactccggccgacaaaagcgacaaaggttttgttcttggtcactccataaccgacgtcactttcctcaaaagacacttccacatggactacggaactgggttttacaaacctgtgatggcctcgaagaccctcgaggccatcctctcctttgcacgccgtgggaccatacaggagaagttgatctccgtggcaggactcgccgtccactccggacctgacgaataccggcgcctctttgagcccttccaaggcctcttcgagattccaagctacagatcactttacctgcgatgggtgaacgccgtgtgcggtgacgcataa

>AF154271.1_O_TAW_1997

atgaatacgactgactgttttatcgctctgctatacgttctcagagagatcaaagcactgtttctgtcacgaacacaagggaagatggaattcacactttacaacggtgaaaagaaggtcttctactccagacccaacaaccacgacaactgttggctgaacgccatcctccaactgttcaggtacgttgacgagcccttcctcgaatgggtctacgactcacctgagaacctcactctcgaggcgatcaacaaactggaagaaatcacaggtcttgagctacacgagggcggaccgcccgcccttgtcgtctggagcatcaagcacttgctctacaccggaatcggcaccgcttcgcgacccagcgaggtgtgcatggtggacggtacagacatgtgcttggctgacttccacgccggtatatttctgaagggacaggaccacgccgtcttcgcctgcgtcacctctgacgggtggtacgcgattgacgacgaggacttttacccgtggacaccaaatccggccgacgttttggtttttgttccgtacgatcaagaaccattcaacgcagaatggaaagcaaaggttcagaagcggctcaggggcgccgggcaatccagcccgacgaccgggtcacaaaaccaatctggcaacactggcagcattattaacaattactacatgcagcagtaccagaactcaatggacacccaacttggcgacaacgccattagtggagggtccaacgagggctccacggacactacctctacccacaccaacaacacccagaacaacgactggttttcgaaactggccaacaccgcttttagcggcctcttcggtgctcttcttgcagacaagaagacggaagaaaccaccctcctcgaagaccgcatcctcaccacccgcaacgggcacacgacctcgacaacccagtctagcgtcggggtgacttacgggtacgcaacggctgaagacttcgtgagtgggcctaacacctctggtcttgagaccagagttgttcaggccgaacggttcttcaaaacccacctgtttgactgggtcaccagtgacccgtttgggcggtgtcacttgttggagctcccgactgaccacaaaggcgtctacggtagcctgaccgactcgtacgcatacatgaggaatggttgggacgttgaagtcaccgcagtgggtaaccagttcaacggaggctgtttgctggtggcgatggtaccggagctctgttccatcagcaagagagagttgtaccagctcacgcttttcccccaccagttcatcaacccacggacgaatatgacggcacacatcaccgtgccctacctcggtgtcaacaggtacgaccagtataaggtacacaaaccctggaccctggtggtcatggttgtggcccccctgacggttaacaacgagggcgctccgcaaatcaaggtgtatgccaacatcgcccccaccaatgttcacgtcgcgggtgagctcccttctaaagaggggattttccccgtggcatgcagcgatggttccggtggcttggtgaccacggatccgaagacggcagaccccgtctacgggaaagtgttcaacccaccccgcaacctgttgccagggcggtttacaaacctccttgacgtggccgaggcgtgccccacattcctacacttcgacggtgacgttccgtacgtgaccacgaagacggattcggacagggtgctagcccagttcgatttgtccctcgcggcaaaacatatgtcgaacacttttctcgcgggtcttgcccagtactacacacagtacagcggcaccattaacctgcacttcatgttcacgggacccaccgacgcgaaggcacgctacatggttgcgtacgcccctcctggcatggaaccgccgaaaacgcctgaggcggctgcacattgcatccacgctgagtgggatacagggctgaattcgaagttcacgttttcaatcccatacctttcggcagctgactacgcgtacaccgcgtccgacgtcgccgagaccacaaacgtacagggatgggtctgtttgttccagataacacacgggaaagccgacggtgacgccctggtcgtgctagctagtgctggcaaagactttgacttgcgtctgccggtcgacgcccgaacccaaaccacctctgcgggtgagtctgcggaccccgtgactgccaccgtcgagaactacggtggtgagacacaagtccagaggcgccagcacacggacattgcgttcatattggacaggttcgtgaaagtcaagccaaaggaacaagttaatgtgttggacctgatgcagatccctgcccacaccttggtaggggcgctcctgcgaacggccacctactacttctctgacctggagctggccgtcaagcacgagggcgatctcacctgggtcccaaacggcgcccctgagacagcactggacaacactaccaacccaacagcttaccacaaggaacccctcacacggctggcgctgccttacacggctccacaccgtgtcttagcgaccgtctacaacgggagcagtaagtacggtgacaccagcactaacaacgtgagaggtgaccttcaagtgttagctcagaaggcagaaagaactctgcctacctccttcaacttcggtgccatcaaggcaactcgtgttactgaactactctacagaatgaagagagccgagacatactgtcccaggccccttctcgccattcaaccgagtgacgctagacacaagcagaggattgtggcacccgcaaaacagcttctgaacttcgacctcctcaagttggcgggagacgttgagtccaaccctgggcccttcttcttctccgacgtcagggcgaacttcacgaagttggtggacactgtcaaccagatgcaggaggacatgtcaacgaaacacggacccgacttcaaccgactggtgtccgcgtttgaggaattggccgctggggtcaaggccatcaggaccggtctcgacgaggccaagccctggtacaagctcatcaaacttctaagccgcctgtcgtgcatggccgctgtggcagcacggtccaaggacccagtccttgtggccatcatgctggccgacaccggcctcgagattctggacagcaccttcgtggtaaagaagatctccgactcgctctccagtctcttccacgtgccggctcccgccttcagtttcagagccccgatcctgttggccgggttggtcaaagtcgcctcgagtttcttccagtccacacccgaagacctcgagagagcagaaaagcagctcaaagcacgtgacatcaacgacatatttgccgttcttaagaacggtgagtggctggtcaaactgatcctggccatccgcgactggattaaggcatggatcgcctcagaagagaagtttgtcaccatgacagacctggtgcctggcatccttgaaagacaacgggatctcaatgaccccggcaaatacaaggaggccaaggaatggctggacaacgcgcgtcaagcgtgtttgaagagcgggaacgtgcacattgccaatctgtgtaaagtggtcgctccggcgcccagcaagtcgagacccgaaccagtggtcgtgtgccttcgcggcaaatccggccaagggaaaagctttctcgcgaacgttctcgcgcaggcaatttccacacacttcactggtaggaccgactcggtctggtactgcccgcccgaccctgaccactttgacggttacaatcagcagaccgtcgtcgtgatggacgacttgggccaaaacccagacggcaaagacttcaagtactttgcccaaatggtctccaccacggggttcatcccgcctatggcctcgctcgaggataagggtaaacccttcaacagcaaggtcataatagctacaaccaacctgtactcgggattcaccccaaagaccatggtgtgccccgatgcgcttaaccggaggtttcactttgacatcgacgtgagcgccaaagacgggtacaagatcaacaacaaactggacatagtcaaagcacttgaagacacccacgctaacccggtggcgatgttccaatacgactgcgctcttctcaacggaatggccgttgaaatgaagagaatgcagcaagacatgttcaagcctcaaccacccctccagaacatctaccagctcgttcaggaggtgattgagcgggtggaactacacgaaaaggtgtcgagccacccgatatttaaacagatttcaatcccttcccagaagtccgtgttgtacttcctcattgagaagggtcagcacgaagcagcgatcgagttcttcgaggggatggtccacgattccatcaaagaggaactccgacccctcattcagcagacctcgttcgtaaaacgcgccttcaagcgcctgaaagagaactttgaagttgtagccctgtgtttgaccctcttggcaaacatagtgattatgctccgccaagcgcgcaagaggtaccaatcggtggatgacccactggac------------------------------ggcgacgtaactcttggcgacgcggaaaagaaccctctggagacgagtggcgctagcgctgtcggtttcagagagagatcccccaccgagcaagggacgcgcgaagacgcgaacgctgagcccgtcgtgttcggtagggaacaaccgcgagctgaaggaccctacgctgggccactcgagcgtcagaaacctcttaaagtgaaagccgagctgccacaacatgagggaccatacgccggcccaatggagagacagaaaccgctaaaggtgaaagcaaaagcccccgtcgtgaaggaaggaccttacgagggaccggtgaagaaacctgtcgctttgaaagtgaaagcaaagaacttgatagtcactgagagtggtgcgccaccgaccgacttgcaaaagatggtcatgggcaacactaagccagtcgagctcatcctcgacggcaagacggtagccatttgctgtgctaccggagtgttcggcactgcctacctcgtgcctcgtcatctcttcgcggaaaagtacgacaagatcatgttggacggcagagccttgacagacagtgactacagagtgtttgagtttgagattaaagtaaaaggacaggacatgctctcagacgccgctctcatggtgttgcaccgtgggaatcgcgtgcgtgacatcacgaaacactttcgtgacgtagcgagaatgaagaagggaacccccgtcgtcggtgtgatcaacaatgctgacgtcgggagactcatattctctggtgaagcccttacttacaaggacatcgtcgtgtgtatggatggagacaccatgcctgggctctttgcctacagggcatccaccaaggcaggctactgtggaggagccgtcctggcaaaggacggggccgaaacgttcatcgttggcacccactccgcaggtggaaacggcataggatactgttcgtgtgtttcccgatcaatgctcctgaagatgaaggcacacatcgaccctgaaccacaccacgaggggttgatcgtcgacaccagagatgtggaggagcgtgtccacgtgatgcgcaaaactaagctcgcgcccaccgtggcgcacggtgtgttcaaccctgagttcgggcctgccgctctgtccaacaaggacccgcgcctgaacgaaggggttgtccttgacgatgtcattttctccaaacacaaaggagatacaaggatgtctgaagaggacaaagcgctgtttcggcgctgtgctgctgactacgcgtcgcgtctacacagtgtgttggggacagcaaacgccccactgagtgtgtatgaagccatcaaaggcgtcgacggacttgacgccatggagccggacacggcgcccggtctcccctgggctctccaagggaaacgccgcggcgccctgatcgacttcgaaaacggcaccgtcgggcctgaggttgaggcagcactcaagctcatggaaagccgcgagtacaaattcgtctgccaaaccttcctgaaggacgaaattcggccgctagaaaaggtacgcgctggcaagacacgcattgtcgacgtgttgcctgttgaacacattctctacaccagaatgatgattggcagattctgtgctcagatgcattcaaacaacggaccgcaaattggatcagcggtcggttgtaaccctgacgttgattggcaaagatttggcacacatttcgcccagtacaagaacgtgtgggatgtggactactcagcctttgatgcaaaccactgcagcgatgcgatgaacatcatgttcgaggaagtgttccgcacggagttcggattccacccgaacgccgagtggattctgaagactctagtgaacacggagcacgcttacgggaacaagcgcattgttgttgaaggtggaatgccgtccggttgttccgcaacaagcatcatcaacacaattttgaacaacatctacgtgctttacgccctgcgtaggcactatgagggagtcgagctggacacttacaccatgatctcttatggagacgacatcgtggtggcaagtgactacgacctggactttgaggctctcaagccccacttcaagtcccttggtcagactatcactccggccgacaaaagcgacaaaggttttgttcttggtcactccataaccgacgtcactttcctcaaaagacacttccacatggactacggaactgggttttacaaacctgtgatggcctcgaagaccctcgaggccatcctctcctttgcacgccgtgggaccatacaggagaagttgatctccgtggcaggactcgccgtccactccggacctgatgaataccggcgcctctttgagcccttccaaggcctcttcgagattccaagctacagatcactttacctgcgatgggtgaacgccgtgtgcggtgacgcataa

>AF308157.1_O_TAW_1997

atgaatacgactgactgttttatcgctctgctatacgctctcagagagatcaaagcactgtttctgtcacgaacacaagggaagatggaattcacactttacaacggtgaaaagaaggtcttctactccagacccaacaaccacgacaactgttggctgaacgccatcctccaactgttcaggtacgttgacgagcccttcctcgaatgggtctacgactcacctgagaacctcactctcgaggcgatcaacaaactggaagaaatcacaggtcttgagctacacgagggcggaccgcccgcccttgtcgtctggaacatcaagcacttgctctacaccggaatcggcaccgcttcgcgacccagcgaggtgtgcatggtggacggtacagacatgtgcttggctgacttccacgccggtatatttctgaagggacaggaccacgccgtcttcgcctgcgtcacctctgacgggtggtacgcgattgacgacgaggacttttacccgtggacaccaaatccggccgacgttttggtttttgttccgtacgatcaagaaccattcaacgcagaatggaaagcaaaggttcagaagcggctcaggggcgccgggcaatccagcccgacgaccgggtcacaaaaccaatctggcaacactggcagcattattaacaattactacatgcagcagtaccagaactcaatggacacccaacttggcgacaacgccattagtggagggtccaacgagggctccacggacactacctctacccacaccaacaacacccagaacaacgactggttttcgaaactggccaacaccgcttttagcggcctcttcggtgctcttcttgcagacaagaagacggaagaaaccaccctcctcgaagaccgaatattgaccacccgtaacggccacacgacctcgacaacccagtctagcgtcggggtgacttacgggtacgcaacggctgaagacttcgtgagtgggcctaacacctctggtcttgagaccagagttgttcaggccgaacggttcttcaaaacccacctgtttgactgggtcaccagtgacccgtttgggcggtgtcacttgttggagctaccgactgaccacaaaggcgtctacggtagcctgaccgactcgtacgcatacatgaggaatggttgggacgttgaagtcaccgcagtgggtaaccaattcaacggaggctgtttgctggtggcgatggtaccggagctccgttccatcagcaagagagagttgtaccagcttacgcttttcccccaccagttcatcaacccacggacgaatatgacggcacacatcaccgtgccctacctcggtgtcaacaggtacgaccagtacaaggtacacaaaccctggaccctcgtggtcatggttgcggcccccttgaccgttaacaacgagggcgctccgcaaatcaaggtgtatgccaacatcgcccccaccaatgttcacgtcgcgggtgagctcccctctaaagaggggattttccccgtggcatgcagcgatggttacggtggcttggtgaccacggatccgaagacggcagaccccgtctacgggaaagtgttcaacccaccccgcaacctgttgccagggcggtttacaaacctccttgacgtggccgaggcgtgccccacattcctacacttcgacggtgacgttccgtacgtgaccacgaagacggattcggatagggtgctagcccagttcgatttgtccctcgcggcaaaacatatgtcgaacacttttctcgcgggtcttgcccagtactacacacagtacagcggcaccattaacctgcacttcatgttcacgggacccaccgacgcgaaggcacgctacatggttgcgtacgcccctcctggcatggaaccgccgaaaacgcctgaggcggctgcacattgcatccacgctgagtgggatacagggctgaattcgaagttcacgttttcaatcccatacctttcggcagctgactacgcgtacaccgcgtccgacgtcgccgagaccacaaacgtacagggatgggtctgtttgttccagataacacacgggaaagccgacggtgacgccctggtcgtgctagctagtgctggcaaagactttgacttgcgtctgccggtcgacgcccgaacccaaaccacctctgcgggtgagtctgcggaccccgtgactgccaccgtcgagaactacggtggtgagacacaagtccagaggcgccagcacacggacattgcgttcatactggacaggttcgtgaaagtcaagccaaaggaacaagttaatgtgttggacctgatgcagatccctgcccacaccttggtaggggcgctcctgcgaacggccacctactacttctctgacctggagctggccgtcaagcacgagggcgatctcacctgggtcccaaacggcgcccctgagacagcactggacaacactaccaacccaacagcttaccacaaggaacccctcacacggctggcgctgccttacacggctccacaccgtgtcttagcgaccgtctacaacgggagcagtaagtacggtgacaccagcactaacaacgtgagaggtgaccttcaagtgttagctcagaaggcagaaagaactctgcctacctccttcaacttcggtgccatcaaggcaactcgtgttactgaactactctacagaatgaagagagccgagacatactgtcccaggccccttctcgccattcaaccgagtgacgctagacacaagcagaggattgtggcacccgcaaaacagcttctgaacttcgacctcctcaagttggcgggagacgttgagtccaaccccgggcccttcttcttctccgacgtcagggcgaacttcacgaagttggtggacactgtcaaccagatgcaggaggacatgtcaacgaaacacggacccgacttcaaccgactggtgtccgcgtttgaggaattggccgctggggtcaaggccatcaggaccggtctcgacgaggccaagccctggtacaagctcatcaaacttctaagccgcctgtcgtgcatggccgctgtggcagcacggtccaaggacccagtccttgtggccatcatgctggccgacaccggcctcgagattctggacagcaccttcgtggtaaagaagatctccgactcgctctccagtctcttccacgtgccggctcccgccttcagtttcggagccccgatcctgttggccgggttggtcaaagtcgcctcgagtttcttccagtccacacccgaagacctcgagagagcagaaaagcagctcaaagcacgtgacatcaacgacatatttgccgttcttaagaacggtgagtggctggtcaaactgatcctggccatccgcgactggattaaggcatggatcgcctcagaagagaagtttgtcaccatgacagacctggtgcctggcatccttgaaagacaacgggatctcaatgaccccggcaaatacaaggaggccaaggaatggctggacaacgcgcgtcaagcgtgtttgaagagcgggaacgtgcacattgccaatctgtgtaaggtggtcgctccggcgcccagcaagtcgagacccgaaccagtggtcgtgtgccttcgcggcaaatccggccaagggaaaagctttctcgcgaacgttctcgcgcaggcaatttccacacacttcactggtaggaccgactcggtctggtactgcccgcccgaccctgaccactttgacggttacaatcagcagaccgtcgtcgtgatggacgacttgggccaaaacccagacggcaaagacttcaagtactttgcccaaatggtatccaccacggggttcatcccgcctatggcctcgctcgaggataagggtaaacccttcaacagcaaggtcataatagctacaaccaacctgtactcgggattcaccccaaagaccatggtgtgccccgatgcgcttaaccggaggtttcactttgacatcgacgtgagcgccaaagacgggtacaagatcaacaacaaactggacatagtcaaagcacttgaagacacccacgctaacccggtggcgatgttccaatacgactgcgctcttctcaacggaatggccgttgaaatgaagagaatgcagcaagacatgttcaagcctcaaccacccctccagaacatctaccagctcgttcaggaggtgattgagcgggtggaactacacgaaaaggtgtcgagccacctgatatttaaacagatttcaatcccttcccagaagtccgtgttgtacttcctcattgagaagggccagcacgaagcagcgatcgagttcttcgaggggatggtccacgattccatcaaagaggaactccgacccctcattcagcagacctcgttcgtaaaacgcgccttcaagcgcctgaaagagaactttgaagttgtagctctgtgtttgaccctcttggcaaacatagtgattatgctccgccaagcgcgcaagaggtaccaatcggtggatgacccactggac------------------------------ggcgacgtaactcttggcgacgcggaaaagaaccctctggagacgagtggcgctagcgctgtcggtttccgagagagatcccccaccgagcaagggacgcgcgaagacgcgaacgctgagcccgtcgtgttcggtagggaacaaccgcgagctgaaggaccctacgctgggccactcgagcgtcagaaacctcttaaagtgaaagccgagctgccacaacaggagggaccatacgccggcccaatggagagacagaaaccgctaaaggtgaaagcaaaagcccccgtcgtgaaggaaggaccttacgagggaccggtgaagaaacctgtcgctttgaaagtgaaagcaaagaacttgatagtcactgagagtggtgcgccaccgaccgacttgcaaaagatggtcatgggcaacactaagccagtcgagctcatcctcgacggcaagacggtagccatttgctgtgctaccggagtgttcggcactgcctacctcgtgcctcgtcatctcttcgcggaaaagtacgacaagatcatgttggacggcagagccttgacagacagtgactacagagtgtttgagtttgagattaaagtaaaaggacaggacatgctctcagacgccgctctcatggtgttgcaccgtgggaatcgcgtgcgtgacatcacgaaacactttcgtgacgtagcgagaatgaagaagggaacccccgtcgtcggtgtgatcaacaatgctgacgtcgggagactcatattctctggtgaagcccttacttacaaggacatcgtcgtgtgtatggatggagacaccatgcctgggctctttgcctacagggcatccaccaaggcaggctactgtggaggagccgtcctggcaaaggacggggccgaaacgttcatcgttggcacccactccgcaggtggaaacggcataggatactgttcgtgtgtttcccgatcaatgctcctgaagatgaaggcacacatcgaccctgaaccacaccacgaggggttgatcgtcgacaccagagatgtggaggagcgtgtccacgtgatgcgcaaaaccaagctcgcgcccaccgtggcgcacggtgtgttcaaccctgagttcgggcctgccgctctgtccaacaaggacccgcgcctgaacgaaggggttgtccttgacgatgtcattttctccaaacacaaaggagatacaaggatgtctgaagaggacaaagcgctgtttcggcgctgtgctgctgactacgcgtcgcgtctacacagtgtgttggggacagcaaacgccccactgagtgtgtatgaagccatcaaaggcgtcgacggacttgacgccatggagccggacacggcgcccggtctcccctgggctctccaagggaaacgccgcggcgccctgatcgacttcgaaaacggcaccgtcgggcctgaggttgaggcagcactcaagctcatggaaagccgcgagtacaaattcgtctgccaaaccttcctgaaggacgaaattcggccgctagaaaaggtacgcgctggcaagacacgcattgtcgacgtgttgcctgttgaacacattctctacaccagaatgatgattggcagattctgtgctcagatgcattcaaacaacggaccgcaaattggatcagcggtcggttgtaaccctgacgttgattggcaaagatttggcacacatttcgcccagtacaaaaacgtgtgggatgtggactactcagcctttgatgcaaaccactgcagcgatgcgatgaacatcatgttcgaggaagtgttccgcacggagttcggattccacccgaacgccgagtggattctgaagactctagtgaacacggagcacgcttacgagaacaagcgcattgttgttgaaggtggaatgccgtccggttgttccgcaacaagcatcatcaacacaattttgaacaacatctacgtgctttacgccctgcgtaggcactatgagggagtcgagctggacacttacaccatgatctcttatggagacgacatcgtggtggcaagtgactacgacctggactttgaggctctcaagccccacttcaagtcccttggtcagactatcactccggccgacaaaagcgacaaaggttttgttcttggtcactccataaccgacgtcactttcctcaaaagacacttccacatggactacggaactgggttttacaaacctgtgatggcctcgaagaccctcgaggccatcctctcctttgcacgccgtgggaccatacaggagaagttgatctccgtggcaggactcgccgtccactccggacctgatgaataccggcgcctctttgagcccttccaaggcctcttcgagattccaagctacagatcactttacctgcgatgggtgaacgccgtgtgcggtgacgcataa

>AF377945.1_O_SKR_2000

atgagcacaactgactgtttcaccgctttgttgtacgctttcagagagattaaaacgctgctcttatcacgagtacagggaaagatggagttcacacttcacaacggtgagaagaaaacattctactccaggcccaacaaccacgacaactgctggctgaacaccatcctccagttgtttaggtacgttgatgaacctttcttcgactgggtctactactcacctgagaacctcacactagatgctatcaaacaattggaagaaatcactggtctcgagctccacgagggtggaccacccgctctcgttatttggaacattaaacacctgctcaacaccggaatcggcaccgcttcgcgacccagcgaagtgtgcatgatagacgggacggacatgtgtttggctgacttccacgctggcatcttcctgaaaggacaggaacacgctgtgttcgcctgcgtcacctccaacgggtggtacgcgattgatgacgaggacttttacccctggacgccggacccgtccgacgttctggtgtttgtcccgtacgatcaagaaccgctcaacggagaatggaaagcaaaggtccagagaaaactgagaggcgccgggcaatccagcccggcgactgggtcacagaaccagtcaggcaacactggaagcattatcaacaattactacatgcagcagtaccaaaactccatggacacacagcttggtgacaacgctattagcggaggctccaacgagggatccacggacaccacctccacccacacaaccaacactcagaacaatgactggttttcaaagctggccagttccgcttttagcggtcttttcggcgctcttctcgccgacaagaaaaccgaggagaccactcttctcgaggaccgcatcctcactacccgcaacgggcacacgacctcgacaacccagtcgagcgttggagtcacctacgggtacgcaacagctgaggactttgtgagcggaccaaacacatctgggcttgagaccagggtggtgcaggcagagcggttcttcaaaacccattgttacgactgggtcaccagtgaccccttcggacggtgctacctgctggaactcccaactgaccacaaaggtgtctacggcagcctgactgactcttatgcttacatgagaaacggtggggatgttgaagtcactgcagttgggaaccagttcaacggagggtgtctgttggtggccatggtgccaaaactttgctctaatgacaagagagagctgtaccagctcacgctttttccccaccagttcatctacccccggacgaacatgacggcgcacatcactgtgccctttgttggcgtcaaccgctacgaccagtacaaggtacacaaaccttggaccctcgtggttatggttgtggccccgctgactatcaacaccgaaggtgccccacagatcaaggtctatgccaacatcgcccctaccaacgtgcacgttgcgggtgagttcccctctaaggaagggatcttccccgtggcatgtagcgacggttacggtggtatggtgaccactgacccaaagacggctgaccccgcctacgggaaagtgtttaatccaccccgtaacatgttgccggggtggttcaccaacttcctcgatgtggctgaggcgtgccctacgtttctgcactttgagggtgacgtgccgtacgtgaccacaaagacggactcagacagagtgctcgcccagtttgacctgtctctagcagcaaagcacatgtcaaacacctttctggcaggtctcgcccagtactacacacagtacagtggcacaatcaacctgcacttcatgttcacaggacccactgacgcgaacgcgcgttacatgattgcatacgccccccctggtatggagccgcccaaaacacctgaggcggccgctcactgcattcatgcggagtgggacacagggttgaattcaaaattcacattttcaatcccttacctttcggcggctgattacgcgtacaccgcgtctgacgttgcggagaccacaaatgtacagggatgggtttgcctgtttcaaattacacacgggaaggctgacggcgacgcactggtcgttctatccagcgccggcaaggactttgagctgcgtctgccagttgacgctcgcacgcaaaccacctccacaggtgagtccgctgaccccgtgactgccactgttgaaaactacggtggtgaaacacaggtccagagacgccaacacacggatgtctcgttcataatagacagatttgtgaaagtaacaccaaaagaccaaattaacgtgttggacctgatgcaaacccctgcacacactttggtaggcgcgctcctccgtactgccacttactacttcgcagatgtagaagtggcagtgaaacacgagggggaccttaccttggtcccgaatggggcgcccgagacaacgttggacaacaccaccaatccaacggcgtaccacaaggcaccgctcactcggtttgcactgccttacacggcaccacaccgtgtcttggctactgtttacaacgggaactgcaagtatggcgagagtcccgtgaccaatgtgagaggtgacctgcaagttttggcccagaaggcggcaagaacgctgcctacctccttcaattacggtgccatcaaagccactcgggtgactgaactgctttaccgcatgaagagggccgaaacatactgcccccggccacttttggccattcacccgagcgaagctagacacaaacaaaagattgtggcaccggtgaaacagcttttgagctttgacctgctcaagttggcaggggacgtcgagtccaaccctgggcctttcttcttctctgacgttaggtcaaatttttccaagttggttgaaaccatcaaccagatgcaggaggacatgtcaacaaaacacggacccgactttaaccggttggtgtctgcatttgaggaactggccaccggagtgaaggctatcaggaccggtctcgatgaggccaaaccctggtacaagctcatcaagctcttgagccgcctgtcatgtatggccgctgtagcagcacggtcaaaggacccagtccttgtggccatcatgctggctgacaccggccttgagattctggacagtacctttgtcgtgaagaagatctccgactcgctctccagtctctttcacgtaccggcccccgtcttcagtttcggagccccgattttgttggccgggttggtcaaggtcgcctcgagtttcttccggtccacacccgaagaccttgagagagcggagaaacagctcaaagcacgtgacatcaatgacatattcgccattctcaggaacggcgagtggctggtcaagctgattcttgccatccgcgactggattaaggcttggatcgcctcagaagaaaagtttgtcaccatgacagacctggtgcctggtatccttgaaaagcagcgggacctcaacgacccaagcaagtacaaggaggccaaggagtggctcgacaacacgcgccaagcgtgtttgaaaagcgggaacatccacatcgcaaacctttgcaaagtggttgccccagcacccagcaggtcgaggcccgaacccgtggtcgtttgcctccgtggcaaatcgggccagggcaagagtttccttgcgaacgtgcttgcacaagcaatatcaacccacttcactggtataaccgattcagtttggtactgcccacctgaccctgaccacttcgacggttacaaccagcagaccgttgtagtaatggatgacttgggccacaaccccgacgggaaggactccaagtacttcgcccaaatggtttcaactacggggtttatcccgcccatggcctcacttgaggacaaaggcaatcctttcaacagcaaggttatcatcgccaccaccaacctgtactcgggcttcaccccgagaaatatggtgtgccctgatgcactgaaccgaaggttccacgttgacattgacgtgagcgccaaggacgggtacaaaattagcaacaaattggacatcatcaaggctcttgaagatacccacaccaacccagtggcaatgtttcaatacgactgtgcccttctcaacggcatggccgttgaaatgaagagaatgcaacaagatatgttcaagcctcaaccgcccctccagaaagtttaccagcttgttcaggaggcgattgaccgggtcgagttccacgagaaggtgtcgagccacccgattttcaagcagatctcaattccttcccaaaaggctgtgctgtactttctcattgagaagggccagcacgaagcagcaattgaattctttgaggggatggtgcgtgactccgtcaaggaggagctccggcctctcatccagcagacctcatttgtgaggcgcgtttttaagcgcctgaaggaaaactttgagatagttgccctgtgtttgacccttatggcaaacatagtgatcatgatccgcgagactcgtaagagacagcagatggtggatgatgcagtgaacgagtacattgagaaggcaaacatcaccacggatgacaagactcttgatgaggcggaaaagaaccctctggagaccagcggtgccaccactgttggtttcagagagaaaactctcccgggacacaaagcgagtaatgacgtgaactccgagcccgccaaacccgtggaagaacaaccacaagctgaaggaccctacaccggtccactcgagcgtcagaaacctctgaaagtgagagccaagctcccacagcaggaggggccctacgctggtccgatggagagacagaaaccgctgaaagtgaaagtgaaaacctcggtcgtgaaggaaggaccttacgaaggaccggtgaagaaacctgtcgctttgaaagtgaaagctaagaatttgattgtcactgagagtggtgctcccccgactgacttgcaaaagatggtcatgggtaacaccaagcctgttgagctcatcctcgacgggaagacggtggccatttgctgcgccaccggagtgtttggtactgcctaccttgtccctcgtcatcttttcgcagagaagtatgacaagatcatgttggacggcagagccatgacagacagtgactacagagtgtttgagtttgagattaaagtgaaaggacaggacatgctctcagacgccgcgctcatggtgcttcaccgtgggaatcgcgtgcgggacatcacgaagcacttccgtgatgtggcaagaatgaagaaaggcacccccgtcgtcggcgtgatcaacaacgctgatgttgggagactaatcttctctggtgaggcccttacctacaaggacattgtagtgtgcatggacggagacaccatgcccggtctcttcgcctacaaagccgccaccaaggcgggttactgtggaggagccgttcttgccaaagacggagccgagactttcatcgtcggcactcactccgcaggcggcaacggagttggatactgctcatgcctttccaggtccatgcttcttaaaatgaaggcacacattgaccccgaaccacaccacgagggattgatagttgacaccagagatgttgaggagcgcgtacatgtcatgcgcaaaaccaagctcgcacccaccgtggcacacggtgtgtttaaccccgaatttgggcctgccgccttgtccaacaaggacccgcgcctgaatgagggggttgtcctcgatgaagccatcttctccaaacacaagggaaacacaaagatgtctgaggaggacaaagcgctgttccgccgctgtgctgctgactacgcgtcgcgactgcatagcgtgctgggtacggcaaacgccccactgagcatttacgaagcaatcaagggcgtcgacggacttgacgccatggaaccagacaccgcgcctggtttaccctgggctctccaggggaaacgccgtggtgccctcattgactttgagaacggcacaatcggacccgaagttgaagctgccttgaagctcatggagaaaagagagtacaagtttgtatgtcagaccttcctgaaggacgagattcgcccgatggagaaagtaagtgccggcaagactcgcattgtcgacgtcctgcctgttgaacacattctttacaccaggatgatgattggcagattttgtgctcaaatgcactccaacaacggaccgcaaattggctcggcggttgggtgtaatcctgatgttgattggcaaagatttggcacgcattttgctcagtacagaaacgtgtgggatgtagactattcggcctttgatgccaaccactgcagtgacgcaatgaacatcatgtttgaagaagtgttcaacacggatttcggtttccacccaaacgctgagtggatcctgaaaactctcgtgaacactgaacacgcctatgagaacaaaggcatcagtgttgaaggcgggatgccgtctggttgttccgcaacaagcatcttcaacacaatttcgaacaacatctacgtgctctacgccttgcgtaggcactatgagggagttgagctggactcttacaccatgatctcctacggagacgacatcgtggttgcaagtgattacgatctggactttgaggccttcaagcctcacttcaaatcccttggtcaaaccattactccagctgacaaaagcgacaaaggttttgttcttggtcactccattaccgatgtcactttcctcaaaagatctttccacatggactatggaactgggttttacaaacctgtgatggcttcgaagaccctcgaggctatcctctcctttgcacgccgtgggaccatacaggagaagttgatctccgtggcaggactcgccgtccactctggacctgacgagtaccggcgtctctttgagcccttccagggtctctttgagattccaagctacagatcactttacctgcgttgggtgaacgccgtgtgcggtgacgcataa

>AF506822.2_O_CHA_1999

atgagcacaactgactgtttcatcgctttgttgtacgctttcagagagattaaaacactgttcttatcacgagcacaaggaaagatggagttcacacttcacaacggtgagaagaaaacattctactccaggcccaacaaccacgataactgctggctgaacaccatcctccagttgtttaggtacgttgatgaacctttcttcgactgggtctactactcacctgagaacctcacacttgatgctatcaaacaattggaagaaattactggtctcgagctccacgagggtggaccacccgctctcgttatttggaacattaaacacctgctcaacaccggaatcggcaccgcttcgcgacccagcgaagtgtgcatggtagacgggacggacatgtgtttggctgacttccacgctggcatcttcctgaaaggacaggaacacgctgtgttcgcctgcgtcacctccaacgggtggtacgcgattgatgacgaggacttttacccctggacgccggacccgtccgacgttctggtgtttgtcccgtacgatcaagaaccgctcaacggagaatggaaagcaaaggttcagaaacgactcagaggcgccgggcaatccagcccggcgactgggtcacagaaccagtcaggcaacactggaagcattatcaacaattactacatgcagcagtaccagaactccatggacacgcaacttggtgataacgctattagcggaggctccaacgaggggtccacggacaccacctccacccacacaaccaacactcagaacaatgactggttttcaaagctggccagttccgcttttagcggtcttttcggcgctcttctcgccgacaagaaaaccgaggagaccactcttctcgaggaccgcatcctcactacccgcaacggacacacgacctcgacaacccagtcgagcgttggagtcacttacgggtacgcaacagctgaggactttgtgagcggaccaaacacatctgggcttgagaccagggttgtgcaggcagagcggttcttcaaaacccacttgttcgactgggtcaccggtgacccgttcggacggtgctacctgctggaactcccaactgaccacaaaggtgtctacggcagcctgactgactcttatgcttacatgagaaacggttgggatgttgaggtcactgcagtgggaaatcagttcaacggaggatgtctgttggtggccatggtgccagaactttgctctattgacaagagagagctgtaccagctcacgctctttccccaccagttcatcaacccccggacgaacatgacggcgcacatcactgtgccctttgttggtgtcaaccgctacgaccagtacaaggtacacaaaccttggaccctcgtggttatggttgtggccccgctgactgtcaacaccgaaggtgccccacagatcaaggtctatgccaacatcgcccctaccaacgtgcacgttgcgggtgagttcccttctaaggaagggatcttccccgtggcatgtagcgacggttacggtggtctggtgaccactgacccaaagacggctgaccccgcctacgggaaagtgttcaatccacctcgcaacatgttgccggggcggttcaccaacttccttgatgtggctgaggcgtgccctacgtttctgcactttgagggtgacgtgccgtacgtgaccacaaagacggactcagacagggtgctcgcccagtttgacttgtctctggcagcaaagcacatgtcaaacaccttcctggcaggtctcgcccagtactacacacagtacagcggcaccatcaacctgcacttcatgttcacaggacccactgacgcgaaagcgcgttacatgattgcatacgccccccctggcatggagccgcccaaaacacctgaggcggccgctcactgcattcatgcggagtgggacacagggttgaattcaaaattcacattttcaatcccttacctttcggcggctgattacgcgtacaccgcgtctgacgctgcggagaccacaaatgtacagggatgggtctgcctgtttcaaattacacacgggaaggctgacggcgacgcactggtcgttctagctagcgccggtaaggactttgagctgcgtctgccagttgacgctcgcacgcagaccacctccacaggtgagtcggctgaccccgtgactgccactgttgagaactacggtggtgagacacaggtccagagacgccaacacacggatgtctcgttcatattagacagatttgtgaaagtaacaccaaaagaccaaattaatgtgttggacctgatgcaaacccctgcacacactttggtaggcgcgctcctccgtactgccacctactacttcgcagatctagaagtggcagtgaaacacgaggggaaccttacctgggtcccgaatggggcgcccgagacagcgttggacaacaccaccaatccaacggcttaccacaaggcaccgctcacccggcttgcactgccttacacggcaccacaccgtgtcttggctactgtttacaacgggaactgcaagtatgacgagagccccgtgaccaatgtgagaggtgacctgcaagtgttggcccagaaggcggcaagaacgctgcctacctccttcaattacggtgccgtcaaagccactcgggtgactgaactgctttaccgcatgaagagggccgaaacatactgcccccggcctcttttggctattcacccgagcgaagctagacacaaacaaaagattgtggcgcctgtgaaacagcttttgaactttgacctgctcaagttggcaggagacgtcgagtccaaccctgggcctttcttcttctctgacgtcaggtcaaatttttccaagttggttgaaaccatcaaccagatgcaggaggacatgtcaacaaaacacggacccgactttaaccggttggtgtctgcatttgaggaactggccactggagtgaaggctatcaggaccggtctcgatgaggccaaaccctggtacaagctcatcaagctcttgagccgcctgtcatgcatggccgctgtagcagcacggtcaaaggacccagtccttgtggccatcatgctggctgacaccggccttgagattctggacagtacctttgtcgtgaagaagatctccgactcgctctccagtctctttcacgtgccggcccccgtcttcagtttcggagccccgattttgctggccgggttggtcaaagtcgcctcgagtttcttccggtccacacccgaagaccttgagagagcggagaaacagctcaaagcacgtgacatcaatgacatattcgccattctcaagaacggcgagtggctggtcaagctgattcttgccatccgcgactggatcaaggcatggatcgcctcagaagaaaagtttgtcaccatgacagacttggtgcctggcatccttgaaaagcagcgggatctcaacgacccaagcaagtacaaggaggccaaggagtggctcgacaacgcgcgccaagcgtgtttgaagagcgggaacatccacatcgcaaacctttgcaaagtggttgccccagcacccagcaggtcgaggcccgaacccgtggtcgtttgcctccgtggcaaatcgggccagggcaagagtttccttgcgaacgtgcttgcacaagcaatttcaacccacttcactggcagaaccgattcagtttggtactgcccacctgaccctgaccacttcgacggttacaaccagcagaccgttgtagtaatggatgatttgggccagaaccccgacgggaaggacttcaagtacttcgcccaaatggtttcaactacggggtttatcccgcccatggcttcactcgaggacaaaggcaaacctttcaacagcaaggtcatcatcgccaccaccaacctgtactcgggcttcaccccgagaactatggtgtgccctgatgcactgaaccgaaggttccactttgacattgacgtgagcgccaaggacgggtacaaaattaacaacaaattggacatcatcaaagctcttgaagatacccacaccaacccagtggcaatgtttcaatacgactgtgcccttctcaacggcatggccgttgaaatgaagagaatgcaacaagatatgttcaagcctcaaccgcccctccagaacgtctaccagcttgttcaggaggtgattgaccgggtcgagctccacgagaaggtgtcgagccacccgattttcaagcagatctcaattccttcccaaaaggctgtgctgtactttctcattgagaagggtcagcacgaagcagcaattgaattctttgaggggatggtgcatgactccatcaaggaggagctccggcctctcatccaacagacctcatttgtgaagcgcgcttttaagcgcctgaaggaaaactttgagatagttgccctgtgtttgactcttttggcaaacatagtgatcatgatccgcgagactcgcaagagacagcagatggtggatgatgcagtgaacgagtacattgagaaggcaaacatcaccacggatgacaagactcttgacgaggcggaaaagaaccctctggagaccagcggtgccgccactgttggtttcagagagaaaactctcccgggacacaaggcgagtgatgacgtgaactccgagcccgccaaacccgtggaagaacaaccacaagctgaaggaccctacaccggtccactcgagcgtcaaaaacctctgaaagtgagagccaagctcccacagcaggaggggccctacgctggtccgatggagagacagaaaccgctgaaagtgaaagtgaaagccccggtcgttaaggaaggaccttacgaaggaccggtgaagaaacctgtcgctttgaaagtgaaagcaaagaacttgattgtcactgagagtggtgctcccccgactgacttgcaaaagatggtcatgggtaacaccaagcctgttgagctcatcctcgacgggaagacggtggccatctgctgcgccaccggagtgtttggtactgcctaccttgttcctcgtcatcttttcgcagagaagtatgacaagatcatgttggacggcagagccatgacagacagtgactacagagtgtttgagtttgagattaaagtgaaaggacaggacatgctctcagacgccgcgctcatggtgcttcaccgtgggaatcgcgtgcgggacatcacgaagcacttccgtgatgtggcaagaatgaagaaaggcacccccgtcgtcggcgtgatcaacaacgctgatgttgggagactgatcttctctggtgaggcccttacctacaaggacattgtagtgtgcatggacggagacaccatgcccggtctcttcgcctacaaagccgccaccaaggcgggttactgtggaggagccgttcttgcaaaggacggagccgagactttcatcgtcggcactcactccgcaggcggcaacggagttggatactgctcatgcgtttccaggtctatgctgcttaaaatgaaggcacacatcgatcccgaaccacaccacgagggattgatagttgacaccagagatgttgaggagcgcgtacatgtcatgcgcaaaaccaagctcgcacccaccgtggcacacggtgtgtttaaccccgaatttgggcctgccgccttgtccaacaagggcccgcgcctgaatgagggggttgtcctcgatgaagccatcttctccaaacacaaaggaaacacaaagatgtctgaggaggacaaagcgctgttccgccgctgtgctgctgactacgcgtcgcgtctacatagcgtgctgggtacggcaaatgccccactgagcacttacgtggcaatcaagggcgtcgacggacttgacgccatggaaccagacaccgcgcctggtctcccctgggctctccaggggaaacgccgtggtgcgctcattgatttcgagaacggcactgtcggacccgaggttgaagctgccttgaagctcatggagaaaagagagtacaagtttgtatgccagaccttcctgaaggacgagattcgcccgatggagaaggtacgtgccggcaagactcgcattgtcgacgtcctgcctgttgaacacattctttacaccaggatgatgattggcagattttgtgctcaaatgcactcaaacaacggaccgcaaattggctcggcggttggttgtaatcctgatgttgattggcaaagatttggcacgcattttgctcagtacagaaacgtgtgggatgtggactattcggcctttgatgccaaccactgcagtgacgcaatgaacatcatgtttgaggaggtgttcaacacggatttcgggttccacccaaacgctgagtggatcctgaaaactctcgtgaacactgaacacgcctatgagaacaaacgcatcactgttgaaggcgggatgccgtctggttgttccgcaacaagcatcatcaacacaattttgaacaacatctacgtgctctacgccttgcgtagacactatgagggagttgagctggactcttacaccatgatctcctacggagacgacatcgtggttgcaagtgattacgatctggactttgaggccctcaagcctcacttcaaatcccttggtcaaaccattactccagctgacaaaagcgacaaaggttttgttcttggtcactccattaccgatgtcactttcctcaaaagacacttccacatggactatggaactgggttttacaaacctgtgatggcttcgaagaccctcgaggctatcctctcctttgcacgccgtgggaccatacaggagaagttgatctccgtggcaggactcgccgtccactctggacctgacgagtaccggcgtctctttgagcctttccagggcctctttgagattccaagttacagatcactttacctgcgttgggtgaacgccgtgtgcggtgacgcataa

>AH012984.2_O_SKR_2000

atgaacacaaccgactgtctcaccgctttgttgtacgccttcagagagatcaaaacactgttcttatcacgagcacaaggaaagatggagttcacacttcacaacggtgagaagaaaacattctactccaggcccaacaaccacgacaactgctggctgaacgccatcctccagttgtttaggtacgttgatgaacctttcttcgactgggtctactactcacctgagaacctcacgctcgatgctatcaaacaactggaaggaattactggtctcgagctccacgagggtggaccacccgctctcgttatttggaacattaaacacctgctcaacaccggaatcggcaccgcttcacgacccaacgaagtgtgcatggtagatgggacggacatgtgtttggctgacttccacgctggcatcttcctgaaaggacaggaacacgctgtgttcgcctgtgtcacctccaacgggtggtacgcgattgatgacgaggacttttacccctggacgccggacccgtctgacgttctggtgttcgtcccgtacgaccaacaaccgctcaacggagaatggaaagcgaaggttcagaaacgactcagaggcgccgggcaatccagcccggcgactgggtcgcagaaccagtcaggtaacactggaagcattatcaacaattactacatgcagcagtaccagaactccatggacacgcaacttggtgacaacgctattagtggaggctccaacgaggggtccacggacaccacctccacccacacaaccaacactcagaataatgactggttttcaaagctggccagttccgcttttagcggtcttttcggcgctctcctcgccgacaagaaaaccgaggagaccactcttctcgaggaccgcatcctcactacccgcaacggacacacgacctcgacaacccagtcgagcgttggagtcacttacgggtacgcaacagctgaggacttcgtgagcggaccaaacacatctgggcttgagaccagggttgtgcaggcagagcggttctttaaaacccacttgttcgactgggtcaccagtgacccgttcggacggtgctacctgctggaactcccaactgaccacaaaggtgtctacggcagcctgaccgactcttatgcttacatgagaaacggttgggacgttgaggtcactgcagtgggaaatcagttcaacggaggatgtttgttggtggtcatggtgccagaactttgctctattgacaagagagggctataccagctcacgctctttccccaccaattcatcaacccccagacgaacatgacggcgcacattactgtgccctttgttggcgtcaaccgctacgaccagtacaaagtacacaaaccttggaccctcgttgtcatggttgtggccccgctgactgtcaacaccgaaggtgccccacagatcaaggtctatgccaacatcgcccctactaacgtgcacgttgcgggtgagctcccttctaaggaagggatcttccccgtggcatgtagcgacggttacggtggtctggtgaccactgacccaaagacggctgaccccgcctacgggaaagtgttcaatccacctcgcaacatgttgccggggcggttcaccaacttccttgatgtggctgaggcgtgccctacgttcctgcactttgagggtgacgtgccgtacgtgaccacaaagacggactcagacagggtactcgcccagtttgacttgtctctggcagcaaagcacatgtcaaacaccttcctggcaggtctcgcccagtactacacacagtacagtggcaccatcaacctgcacttcatgttcacaggacccactgacgcgaaagcgcgttacatgattgcatacgccccccctggcatggagccgcccaaaacacccgaggcggccgctcactgcattcatgcggagtgggacacagggttgaattcaaaattcacattttcaatcccttacctttcggcggctgattatgcgtacaccgcgtctgacaccgcggagaccacaaatgtgcagggttgggtttgcctgtttcaaattacacacgggaaggctgacggcgacgcactggtcgttctagctagcgccggtaaggattttgagctgcgtctgccagttgacgctcgcacgcagaccacctccacaggtgagtcggctgaccccgtgactgccaccgttgagaactacggtggtgagacacaggtccagagacgccaacacacggatgtctcgttcatactagacagatttgtgaaagtaacaccaaaagaccaaattaatgtgttggacctgatgcaaatccctgcacacactttggtaggcgcgctcctccgtactgccacctactacttcgcagatctggaagtggcagtgaaacacgaggggaacctcacctgggtcccgaacggggcgcccgaggcagcgttggacaacaccaccaatccaacggcctatcacaaggcgccgctcacccggcttgcactgccttacacggcaccacaccgtgtcttggctactgtttacaacgggaactgcaagtatggcgagagccccgtgaccaatctgagaggtgacctgcaagtgttgacccagaaggcggcaagaacgctgcctacctccttcaattacggtgccatcaaagccactcgggtgactgaactgctttaccgcatgaagagggccgaaacatactgcccccggcctcttttggctattcacccgagcgaagctagacacaaacaaaagattgtggcgcctgtgaaacagctgttgaactttgacctgctcaagttggcaggagacgtcgagcccaaccctgggcccttcttcttctctgacgtcaggtcaaatttttccaagttggttgaaaccgtcaaccagatgcaggaggacatgtcaacaaaacacggacccgactttaaccggttggtgtctgcatttgaggaactggccactggagtgaaggccatcaggaccggtctcgacgaggccaaaccctggtacaagctcatcaagctcttgagccgcctgtcatgtatggccgctgtagcagcacggtcaaaggacccactccttgtggccatcatgctggctgacaccggccttgagatcctggacagtacctttgtcgtgaagaagatctccgactcgctctccagtctctttcacgtgccggcccccgtcttcagttccggagccccgattttgttggccgggttggtcaaagtcgcctcgagtttcttccggtccacacccgaagaccttgagagagcggagaaacagctcaaagcacgtgacatcaatgacatattcgccattcttaagaacggcgagtggccggtcaagctgattcttgccatccgcgactggatcaaggcatggatcgcctcagaagaaaagtttgtcaccatgacagacctggtgcctggcatccttgaaaagcagcgggatctcaacgacccaagcaagtacaaggaggccaaggagtggctcgacaacgcgcgccaagcgtgtctgaagagcgggaacatccacatcgcaaacctttgcaaagtggttgccccagcgcccagcaggtcgaggcccgaacccgtggtcgtttgcctccgtggcaagtcaggccagggcaagagtttccttgcgaacgtgcttgcacaagcaatttcaacccacttcactggcagaaccgattcagtttggtactgcccacctgaccctgaccacttcgacggttacaaccagcagaccgttgtagtaatggatgatttgggccacaaccccgacgggaaggacttcaagtacttcgcccaaatggtttcaactacggggtttatcccgcccatggcttcactagaggataaaggcaaacctttcaacagcaaggtcatcatcgccaccaccaacctgtactcgggcttcaccccgagaactatggtgtgccctgatgcactgaaccgaaggttccactttgacattgacgtgagcgccaaggacgggtacaaaattaacaacaaattggacatcatcaaagctcttgaagacacccaccccaacccagtggcaatgtttcaatacgactgtgcccttctcaacggcatggccgttgaaatgaagagaatgcaacaagatatgttcaagcctcaaccgcccctccagaacgtctaccagcttgttcaggaggtgattgaccgggtcgagctccacgagaaggtgtcgagtcacccgatcttcaagcagatctcaattccttcccaaaaggctgtgctgtactttctcattgagaaaggccagcacgaagcagcaattgaattctttgaggggatggtgtgtgactccatcaaggaggagctccggcctctcatccaacagacctcatttgtgaagcgcgcttttaagcgcctgaaggaaaactttgagatagttgccctgtgtttgactctaatggcaaacatagtgatcatgatccgcgagactcgcaagagacagcagatggtggatgatgcagtgaacgagtacactgagaaggtaaacatcaccacggatgacaagactcttgacgaggcggaaaagaaccctctggaaaccagcggtgccaccactgttggtttcagagagaaaactctcccggggcacaagacgggtgatgacgtgagctccgagcccaccaaacccgtggaaggacaaccacaagctgaaggaccctacaccggcccactcgagcgtcaaaaacctctgaaagtgagagccaagctcccgcagcaggaggggccttatgctggtcccatggagagacagaaaccactgaaagtgaaagcgaaagccccggtcgttaaggaaggaccttacgaaggaccggtgaagaaacctgtcgctttgaaagtgaaagcaaagaacttgattgtcactgagagtggtgctcccccgactgacttgcaaaagatggtcatgggtaacaccaagcctgttgagctcgtcctcgatgggaagacggtggccatctgctgcgccaccggagtgtttggtactgcctaccttgttcctcgtcatcttttcgcagagaagtatgacaagatcatgttggacggcagagccatgacagacagtgactacagagtgtttgagtttgagattaaagtgaaaggacaggacatgctctcagacgccgcgctcatggtgcttcaccgtgggaatcgagtgcgggacatcacgaagcacttccgtgatgtggcaagaatgaagaaaggcacccccgtcgtcggcgtgatcaacaacgctgatgttgggaggctgatcttctctggtgaggcccttacctacaaggacattgtagtgcgcatggacggagacaccatgcccggtctcttcgcctacaaagccgccaccaaggcgggctactgtggaggatccgttcttgcaaaggacggagccgatactttcatcgtcggcactcactccgcaggcggcaatggagttggatactgctcatgcgtttccaggtctatgctgcttaaaatgaaggcacacatcgaccccgaaccacaccacgagggattgatagttgacaccagagatgttgaggagcgcgtacatgtcatgcgcaaaaccaagctcgcacccaccgtggcacacggtgtgtttaaccccgaatttgggcctgccgccttgtccaacaagggcccgcgcctgaatgagggggttgtcctcgatgaagccatcttctccaaacacaaaggaaacacaaagatgtctgaggaggacaaagcgctgttccgccgctgtgctgctgattacgcgtcgcgtctgcacagtgtgctgggtacggcaaatgccccactgagcatttacgaggcaatcaagggcgtcgacggacttgacgccatggaaccagacaccgcgcctggtctcccttgggctctccaggggaaacgccggggtgcgctcattgacttcgaaaacggcactgtcggacccgaggttgaagctgccttgaagctcatggagaaaagagagtacaagtttgtgtgccagaccttcttgaaggacgagattcgcccgatggagaaggtacgtgccggtaagactcgcattgtcgacgtcctgcctgttgaacacattctttacaccaggatgatgattggcagattttgtgctcaaatgcactcaaacaacggaccgcaaattggctcggcggttggttgtaatcctgatgttgattggcaaagatttggcacgcactttgctcagtacagaaacgtgtgggatgtagactattcggccttcgacgccaaccactgcagtgacgcaatgaacatcatgcttgaggaggtgttcaacacggatttcggtttccacccaaacgctgagtggatcctgaaaaccctcgtgaacactgaacacgcctatgagaacaaacgcatcactgttgaaggcgggatgccgtctggttgttccgcgacaagcatcatcaacacaattttgaacaacatctacgtgctctacgccttgcgtagacactacgagggagtcgagctggactcttacaccatgatctcctacggagacgacatcgtggttgcaagtgatcacgatctggactttgaggccctcaagcctcacttcaaatcccttggtcaaaccatcactccagctgacaaaagcgacaaaggttttgttcttggtcactccattaccgatgtcactttcctcaaaagacacttccacatggactatggaactgggttttacaaacctgtgatggcttcgaagaccctcgaagctatcctctcctttgcacgtcgtgggaccatacaggagaagttgatctccgtggcaggacttgccgtccactctggacctgacgagtaccggcgtctctttgagcctttccagggtctctttgagattccaagctacagatcactttacctgcgttgggtgaaccccgtgtgcggtgacgcataa

>AH012985.2_O_SKR_2000

atgagcacaactgactgtttcatcgctttgttgtacgctttcagagagattaaaacactgttcttatcacgagcacaaggaaagatggagttcacacttcacaacggtgagaagaaaacattctactccaggcccaacaaccacgacaactgctggctgaacaccatcctccagttgtttaggtacgttgatgaacctttcttcgactgggtctactactcacctgagaacctcacgcttgatgctatcaaacaattggaagaaattactggtctcgaactccaggagggtggaccacccgctctcgttatttggaacattaaacacctgctcaacaccggaatcggcaccgcttcacgacccaacgaagtgtgcatggtagacgggacggacatgtgtttggctgacttccacgctggcatcttcctgaaaggacaggaacacgctgtgttcgcctgcgtcacctccaacgggtggtacgcgattgacgataaggacttttacccctggacgccggacccgtccgacgttctggtgtttgtcccgtacgatcaagaaccgctcaacggagaatggaaagcaagggttcagaaacgactcagaggcgccgggcaatccagcccggcgactgggtcacagaaccagtcaggcaacactggaagcatcatcaacaattactacatgcagcagtaccagaactccatggacacgcaacttggtgacgacgctattagcggaggctccaacgaggggtccacggacaccacttccactcacacaaccaacactcagaacaatgactggttctcaaagctggccagttccgcttttagcggtcttttcggcgctcttctcgccgataagaaaaccgaggagaccactcttctcgaggaccgcatcctcactacccgcaacgggcacacgacctcgacaacccagtcgagcgttggagtcacttacgggtacgcaacagccgaggactttgtgagcggaccaaacacatctgggctcgagaccagggttgtgcaggcagagcggttcttcaaaacccacttgttcgactgggtcaccagtgacccgttcggacggtgctacctgctggaactcccaactgaccacaaaggtgtctacggcagcctgactgactcttatgcttacatgagaaacggttgggttgttgaggtcactgcagtgggaaatcagttcaacggaggatgtccgttggtggccatggtgccagaacattgctctattgacaagagagagctgtaccagctcacgctctttccccaccagttcatcaacccccggacgaacatgacggcgcacatcactgtgccctttgttggtgtcaatcgctacgaccagtacaaggtacacaaaccttggaccctcgtggttatggttgtggccccgctgactgtcaacaccgaaggtgccccacagaccaaggtctacgccaacatcgcccctaccaacgtgtacgttgcgggtgagttcccttccaaggaagggatcttccccgtggcatgtagcgacggttacggtggtctggtgaccactgacccaaagacggctgaccccgcctacgggaaagtgttcaatccacctcgcaacatgttgccggggcggttcaccaacttccttgatgtggctgaggcgtgccctacgtttctgcactttgagggtgacgtgccgtacgtgaccacaaagacggactcagacagggtgctcgcccagtttgacttgtctctggcagcaaaacacatgtcaaacaccttcctggcgggtctcgcccagtactacacacagtacagtggcaccatcaacctgcacttcatgttcacaggacccactgacgcgaaagcgcgttacatgattgcatacgccccccctggcatggagccgcccaaaacacctgaggcggccgctcactgcattcatgcggagtgggacacagggttgaattcaaaattcacattttcaatcccttacctttcggcggctgattacgcgtacaccgcgtctgatgctgcggagaccacaaatgtacagggttgggtttgcctgtttcaaattacacacgggaaggctgacggcgacgcactggtcgttctagctagcgccggcaaggactttgagctgcgtctgccagttgacgctcgcacgcagaccacctccacaggtgagtcggctgaccccgtgactgccactgttgagaactacggtggtgagacacaggtccagagacgccaacacacggatgtctcgttcatattagacagatttgtgaaagtaacaccaaaagaccaaattaatgtgttggacctgatgcaaacccctgcacacactttggtaggcgcgctcctccgtactgccacctactacttcgcagatctagaagtggcagtgaaacacgaggggaaccttacctgggtcccgaacggggcgcccgagacagcgttggacaacaccaccaatccaacggcctaccacaaggcaccgctcacccggcttgcactgccttacacggcaccacaccgtgtcttggctactgtttacaacgggaactgcaagtatggcgagggccccgtgaccaatgtgagaggtgacctgcaagtattggcccagaaggcggcaagaacgctgcctacctccttcaactacggtgccatcaaagccactcgggtgactgaactgctttaccgcatgaagagggccgaaacatactgtccccggcctcttttggctattcacccgagcgaagctagacacaaacaaaagattgtggcgcctgtgaaacagcttctgaactttgacctgctcaagttggcaggagacgtcgagtccaaccctgggcctttcttcttctctgacgtcaggtcaaatttttccaagttggttgaaaccatcaaccagatgcaggaggacatgtcaacaaaacacggacccgactttaaccggttggtgtctgcatttgaggaactggccactggagtgaaggctatcaggaccggtctcgatgaggccaaaccctggtacaagctcatcaagctcttgagccgcctgtcatgcatggccgctgtagcagcacggtcaaaggacccagtccttgtggccatcatgctggctgacaccggccttgagattctggacagtacctttgtcgtgaagaagatctccgactcgctctccagtctctttcacgtgccggcccccgtcttcagtttcggagccccgattttgttggccgggttggtcaaagtcgcctcgactttcttccggtccacacccgaggaccttgagagagcggagaaacagctcaaagcacgtgacatcaatgacatattcgccattctcaagaacggcgagtggttggtcaagctgattcttgccatccgcgactggatcaaggcatggatcgcctcagaagaaaaatttgtcaccatgacagacctggtacctggcatccttgaaaagcagcgggatcttaacgacccaagcaagtacaaggaggccaaggagtggctcgacaacgcgcgccaagcgtgtttgaagagcgggaacatccacattgcaaacctttgtaaagtagttgccccagcacccagcaggtcgaggcctgaacccgtggtcgtttgcctccgtggcaaatcgggccagggcaagagtttccttgcgaacgtgcttgcacaagcaatttcaacccacttcactggcagaaccgattcagtttggtactgcccacctgaccctgaccacttcgacggttacaaccagcagaccgttgtagtaatggatgatttgggccagaaccccgacgggaaggacttcaaatacttcgcccaaatggtttcaactacggggtttatcccgcccatggcttcactcgaggacaaaggcaaacctttcaacagcaaggtcatcatcgccaccaccaacctgtactcgggcttcaccccgagaactatggtgtgccctgatgcactgaaccgaaggttccactttgacattgacgtgagcgccaaggacgggtacaaaattaacaacaaattggacatcaccaaagctcttgaagatacccacaccaacccagtggcaatgtttcaatacgactgtgcccttctcaacggcatggccgttgagatgaagagaatgcaacaagatatgttcaagcctcaaccgcccctccagaacgtctaccagcttgttcaggaggtgattgaccgggtcgagctccacgagaaggtgtcgagtcacccgatcttcaagcagatctcaataccttcccaaaaggctgtgctgtactttctcattgagaagggccagcacgatgcagcaattgaattctttgaggggatggtgcatgactccatcaaggaggagctccggcctctcatccaacagacctcatttgtgaagcgcgcttttaagcgcctgaaggaaaactttgaggtagttgccctgtgtttgacccttttggcaaacatagtgatcatgatccgcgagactcgcaagagacagcagatggtggatgacgcagtgaacgagtacattgagaaggcaaacatcaccacggatgacaagactcttgacgaggcggaaaagaaccctctggagaccagcggtgccaccactgttggttttagagagaaaactctcccgggacacaaggcgagtgacgacgtgaactccgagcccgccaaacccgcggaagaacaaccacaagctgaaggaccctacaccggtccactcgagcgtcaaaaacctctgaaagtgagagccaagctcccacagcaggaggggccctacgctggtccgatggagagacagaaaccgctgaaagtgaaagtgaaagccccggttgttaaggaaggaccttacgaaggaccggtgaagaaacctgtcgctttgaaagtgaaagcaaagaacttgattgtcactgagagtggtgctcccccgactgacttgcaaaagatggtcatgggtaacaccaagcctgttgagctcatcctcgacgggaagacggtggccatctgttgcgccaccggagtgtttggtactgcttaccttgtccctcgtcatcttttcgcagagaagtatgacaagatcatgttggacggcagagccatgacagacagtgactacagagtgtttgagtttgagattaaagtgaaaggacaggacatgctctcagacgccgcgctcatggtgcttcaccgtgggaatcgcgtgcgggacatcacgaagcacttccgtgatgtggcaagaatgaagaaaggcacccccgtcgtcggcgtggtcaacaacgctgatgttgggagactgatcttctctggtgaggcccttacctacaaggacattgtagtgtgcatggacggagacaccatgcccggtctcttcgcctacaaagccgccaccaaggcgggttactgtggaggagccgttcttgcaaaggacggagccgagactttcatcgtcggcactcactccgcaggcggcaatggggttggatactgctcatgcgtttccaggtctatgctgcttaaaatgaaggcacacatcgatcccgaaccacaccacgagggattgatagttgacaccagagatgttgaggagcgcgtacatgtcatgcgcaaaaccaagctcgcacccaccgtggcacacggtgtgtttaaccccgaatttgggcctgccgccttgtccaacaaggacccgcgcctgaatgagggggttgccctcgatgaagccatcttctccaaacacaagggaaacacaaagatgtctgaggaggacaaagcgctgttccgccgctgtgctgctgactacgcgtcgcgtctgcatagcgtgctgggtacggcaaacgccccactgagcatttacgaggcaatcaagggcgtcgacggacttgacgccatggaaccagacaccgcgcctggtttaccctgggctctccaggggaaacgccgtggtgcgctcattgactttgagaacggcacaatcggacccgaggttgaagctgccttgaagctcatggagaaaagagagtacaagtttgtatgtcagaccttcctgaaggacgagattcgcccgatggagaaggtacgtgccggcaagactcgcattgtcgacgtcctgcctgttgaacacactctttacaccaggatgatgattggcagattttgtgctcaaatgcactcaaacaacggaccgcaaattggctcggcggttgggtgtaatcctgatgttgattggcaaagatttggcacgcattttgctcagtacagaaacgtgtgggatgtagactattcggcctttgatgccaaccactgcagtgacgcaatgaacatcatgtttgaggaggtgttcaacacggatttcggtttccacccaaacgctgagtggatcctgaaaactctcgtgaacactgaacacgcctatgagaacaaacgcatcactgttgaaggcgggatgccgtctggttgttccgcaacaagcatcatcaacacaattttgaacaacatctacgtgctctacgccttgcgtaggcactatgagggagttgagctggactcttacaccatgatctcctacggagacgacatcgtggttgcaagtgattacgatctggactttgaggccctcaagcctcacttcaaatcccttggtcaaaccattactccagctgacaaaagcgacaaaggttttgttcttggtcactccattaccgatgtcactttcctcaaaagatctttccacatggactatggaactgggttttacaaacctgtgatggcttcgaagaccctcgaggctatcctctcctttgcacgccgtgggaccatacaggagaagttgatctccgtggcaggactcgccgtccactctggacctgacgagtaccggcgtctctttgagcccttccagggtctctttgagattccaagctacagatcgctttacctgcgttgggtgaacgccgtgtgcggtgacgcataa

>AJ539136.1_O_TAW_1999

atgagcacaactgactgtttcatcgctttgttgtacgcttttagagagattaaaacactgttcttatcacgagcacaaggaaagatggagttcacacttcacaacggtgagaggaaaacattctactccaggcccaacaaccacgacaactgctggctgaacaccatcctccagttgtttaggtacgttgatgaacctttcttcgactgggtctactactcacctgagaacctcacacttgatgctatcaaacaattggaagaaattactggtctcgagctccacgagggtggaccacccgctctcgttatttggaacattaaacacctgctcaacaccggaatcggcaccgcttcgcgacccagcgaagtgtgcatggtagacgggacggacatgtgtttggctgacttccacgctggcatcttcctgaaaggacaggaacacgctgtgttcgcctgcgtcacctccaacgggtggtacgcgattgatgacgaggacttttacccctggacgccggacccgtccgacgttctggtgtttgtcccgtacgatcaagaaccgctcaacggagaatggaaagcaaaggttcagaaacgactcagaggcgccgggcaatccagcccggcgactgggtcacagaaccagtcaggcaacactggaagcattatcaacaattactacatgcagcagtaccagaactccatggacacgcaacttggtgacaacgctattagcggaggctccaacgaggggtccacggacaccacctccacccacacaaccaacactcagaacaatgactggttttcaaagctggccagttccgcttttagcggtcttttcggcgctcttctcgccgacaagaaaaccgaggagaccactcttctcgaggaccgcatcctcactacccgcaacggacacacgacctcgacaacccagtcgagcgttggagtcacttacgggtacgcaacagctgaggactttgtgagcggaccaaacacatctgggcttgagaccagggttgtgcaggcagagcggttcttcaaaacccacttgttcgactgggtcaccagtgacccgttcggacggtactacctgctggaactcccaactgaccacaaaggtgtctacggcagcctgactgactcttatgcttacatgagaaacggttgggatgttgaggtcactgcagtgggaaatcagttcaacggaggatgtctcttggtggccatggtgccagaactttgctctattgacaagagagagctgtaccagctcacgctctttccccaccagttcatcaacccccggacgaacatgacggcgcacatcactgtgccctttgttggcgtcaaccgctacgaccagtacaaggtacacaaaccttggaccctcgtggttatggttgtggccccgctgactgtcaacaccgaaggtgccccacagatcaaggtctatgccaacatcgcccctaccaacgtgcacgttgcgggtgagttcccttctaaggaagggatcttccccgtggcatgtagcgacggttacggtggtctggtgaccactgacccaaagacggctgaccccgcctacgggaaagtgtttaatccacctcgcaacatgttgccggggcggttcaccaacttccttgatgtggctgaggcgtgtcctacgtttctgcactttgagggtgacgtgccgtacgtgaccacaaagacggactcagacagggtgctcgcccagtttgacttgtctctggcagcaaagcacatgtcaaacaccttcctggcaggtctcgcccagtactacacacagtacagcggcaccatcaacctgcacttcatgttcacaggacccactgacgcgaaagcgcgttacatgattgcatacgccccccctggcatggagccgcccaaaacacctgaggcggccgctcactgcattcatgcggagtgggacacagggttgaattcaaaattcacattttcaatcccttacctttcggcggctgattacgcgtacaccgcgtctgacgttgcggagaccacaaatgtacagggatgggtttgcctgtttcaaattacacacgggaaggctgacggcgacgcactggtcgttctagctagcgccggcaaggactttgagctgcgtctgccagttgacgctcgcacgcagaccacctccacaggtgagtcggctgaccccgtgactgccactgttgagaactacggtggtgagacacaggtccagagacgccaacacacggatgtctcgttcatattagacagatttgtgaaagtaacaccaaaagaccaaattaatgtgttggacctggtgcaaacccctgcacacactttggtaggcgcgctcctccgtactgccacctactacttcgcagatctagaagtggcagtgaaacacgaggggaaccttacctgggtcccgaatggggcgcccgagacagcgttggacaacaccaccaatccaacggcttaccacaaggcaccgctcacccggcttgcactgccttacacggcaccacaccgtgtcttggctactgtttacaacgggaactgcaagtatggcgagagccccgtgaccaacgtgagaggtgacctgcaagtattggcccagaaggcggcaagaacgctgcctacctccttcaattacggtgccatcaaagccactcgggtgactgaactgctttaccgcatgaagagggccgaaacatactgcccccggcctcttttggccattcacccgagcgaagctagacacaaacaaaagattgtggcgcctgtgaaacagcttttgaactttaacctgctcaagttggcaggagacgtcgagtccaaccctgggcctttcttcttctctgacgtcaggtcaaatttttccaagttggttgaaaccatcaaccagatgcaggaagacatgtcaacaaaacacggacccgactttaaccggttggtgtctgcatttgaggaactggccactggagtgaaggctatcaggaccggcctcgatgaggccaaaccctggtacaagctcatcaagctcttgagccgcctgtcatgcatggccgctgtagcagcacggtcaaaggacccagtccttgtggccatcatgctggctgacaccggccttgagattctggacagtacctttgtcgtgaagaagatctccgactcgctctccagtctctttcacgtgccggcccccgtcttcagtttcggagccccgattttgttggccgggttggtcaaagtcgcctcgagtttcttccggtccacacccgaagaccttgagagagcggagaaacagctcaaagcacgtgacatcaatgacatattcgccattctcaagaacggcgagtggctggtcaagctgattcttgccatccgcgactggatcaaggcatggatcgcctcagaagaaaagtttgtcaccatgacagacctggtgcctggcatccttgaaaagcagcgggatctcaacgacccaagcaagtacaaggaggccaaggagtggctcgacaacgcgcgccaagcgtgtttgaagagcgggaacatccacatcgcaaacctttgcaaagtggttgccccagcacccagcaggtcgaggcccgaacccgtggtcgtttgcctccgtggcaaatcgggccagggcaagagtttccttgcgaacgtgcttgcacaagcaatttcaacccacttcactggcagaaccgattcagtttggtactgcccacctgaccctgaccacttcgacggttacaaccagcagaccgttgtagtaatggatgatttgggccagaaccccgacgggaaggacttcaagtacttcgcccaaatggtttcaactacggggtttatcccgcccatggcttcactcgaggacaaaggcaaacctttcaacagcaaggtcatcatcgccaccaccaacctgtactcgggcttcaccccgagaactatggtgtgccctgatgcactgaaccgaaggttccactttgacattgacgtgagcgccaaggacgggtacaaaattaacaacaaactggacatcatcaaagctcttgaagacacccacaccaacccagtggcaatgtttcaatacgactgtgcccttctcaacggcatggccgttgaaatgaagagagtgcaacaagatgtgttcaagcctcaaccgcccctccagaacgtctaccagcttgttcaggaggtgattgaccgggtcgagctccacgagaaggtgtcgagccacccgattttcaagcagatctcaattccttcccaaaaggctgtgctgtactttctcattgagaagggccagcacgaagcagcaattgaattctttgaggggatggtgcatgactccatcaaggaggagctccggcctctcatccagcagacctcatttgtgaagcgcgcttttaagcgcctgaaggaaaactttgagatagttgccctgtgtttgactcttttggcaaacatagtgatcatgatccgcgagactcgcaagagacagcagatggtggatgatgcagtgaacgagtacattgagaaggcaagcatcaccacggatgacaagactcttgacgaggcggaaaagaaccctctggagaccagcggtgccaccactgttggtttcagagagaaaactctcccgggacacaaggcgagtgatgacgtgaactccgagcccgccaaacccgtggaagaacaaccacaagctgaaggaccctacaccggtccactcgagcgtcaaaaacctctgaaagtgagagccaagctcccacagcaggaggggccctacgctggtccgatggagagacagaaaccgctgaaagtgaaagtgaaagccccggtcgttaaggaaggaccttacgaaggaccggtgaagaaacctgtcgctttgaaagtgaaagcaaagaacttgatcgtcactgagagtggtgctcccccgactgacttgcaaaagatggtcatgggtaacaccaagcctgttgagctcatcctcgacgggaagacggtggccatctgctgcgccactggagtgtttggtactgcctaccttgttcctcgtcatcttttcgcagagaagtacgacaagatcatgttggacggtagagccatgacagacagtgactacagagtgtttgagtttgagattaaagtgaaaggacaggacatgctctcagacgccgcgctcatggtgcttcaccgtgggaatcgcgtgcgggacatcacgaagcacttccgtgatgtggcaagaatgaagaaaggcacccccgtcgtcggcgtgatcaacaacgctgatgttgggagactgatcttctctggtgaggcccttacctacaaggacattgtagtgtgcatggacggagacaccatgcccggtctcttcgcctacaaagccgccaccaaggcgggttactgtggaggagccgttcttgcaaaggacggagccgagactttcatcgtcggcactcactccgcaggcggcaatggagttggatactgctcatgcgtttccaggtctatgctgcttaaaatgaaggcacacatcgatcccgaaccacaccacgagggattgatagttgacaccagagatgttgaggagcgcgtacatgtcatgcgcaaaaccaagctcgcacccaccgtggcacacggtgtgtttaaccccgaatttgggcctgccgccttgtccaacaaggacccgcgcctgaatgagggggttgtcctcgatgaagccatcttctccaaacacaaaggaaacacaaagatgtctgaggaggacaaagcgctgttccgccgctgtgctgctgactacgcgtcgcgtctgcatagcgtgctgggtacggcaaatgccccactgagcacttacgaggcaatcaagggcgtcgacgggcttgacgccatggaaccggacaccgcgcctggtctcccctgggctctccaggggaaacgccgtggtgcgctcattgacttcgagaacggcactgttggacccgaggttgaagctgccttgaagctcatggagaaaagagagtacaagtttgtatgccagaccttcctgaaggacgagattcgcccgatggagaaggtacgtgccggcaagactcgcattgtcgacgtcctgcctgttgaacacattctttacaccaggatgatgattggcagattttgtgctcaaatgcacttaaacaacggaccgcaaattggctcggcggttggttgtaatcctgatgttgattggcaaagatttggcacgcattttgctcagtacagaaacgtgtgggatgtggactattcggcctttgatgccaaccactgcagtgacgcaatgaacatcatgtttgaggaggtgttcaacacggatttcggtttccacccaaacgctgagtggatcctgaaaactctcgtgaacactgaacacgcctatgagaacaaacgcatcactgttgaaggcgggatgccgtctggttgttccgcaacaagcatcatcaacacaattttgaacaacatctacgtgctctacgccttgcgtagacactatgagggagttgagctggactcttacaccatgatctcctacggggacgacatcgtggttgcaagtgattacgatctggactttgaggccctcaggcctcacttcaaatcccttggtcaaaccattactccagctgacaaaagcgacaaaggttttgttcttggtcactccattaccgatgtcactttcctcaaaagacacttccacatggactatggaactgggttttacaaacctgtgatggcttcgaagaccctcgaggctatcctctcctttgcacgccgtgggaccatacaggagaagttgatctccgtggcaggactcgccatccactctggacctgacgagtaccggcgtctctttgagcctttccagggcctctttgagattccaagctacagatcactttacctgcgttgggtgaacgccgtgtgcggtgacgcataa

>AJ539137.1_O_TAW_1999

atgagcacaactgactgtttcatcgctttgttgtacgcttttagagagattaaaacactgttcttatcacgagcacaaggaaagatggagttcacacttcacaacggtgagaggaaaacattctactccaggcccaacaaccacgacaactgctggctgaacaccatcctccagttgtttaggtacgttgatgaacctttcttcgactgggtctactactcacctgagaacctcacacttgatgctatcaaacaattggaagaaattactggtctcgagctccacgagggtggaccacccgctctcgttatttggaacattaaacacctgctcaacaccggaatcggcaccgcttcgcgacccagcgaagtgtgcatggtagacgggacggacatgtgtttggctgacttccacgctggcatcttcctgaaaggacaggaacacgctgtgttcgcctgcgtcacctccaacgggtggtacgcgattgatgacgaggacttttacccctggacgccggacccgtccgacgttctggtgtttgtcccgtacgatcaagaaccgctcaacggagaatggaaagcaaaggttcagaaacgactcagaggcgccgggcaatccagcccggcgactgggtcacagaaccagtcaggcaacactggaagcattatcaacaattactacatgcagcagtaccagaactccatggacacgcaacttggtgacaacgctattagcggaggctccaacgaggggtccacggacaccacctccacccacacaaccaacactcagaacaatgactggttttcaaagctggccagttccgcttttagcggtcttttcggcgctcttctcgccgacaagaaaaccgaggagaccactcttctcgaggaccgcatcctcactacccgcaacggacacacgacctcgacaacccagtcgagcgttggagtcacttacgggtacgcaacagctgaggactttgtgagcggaccaaacacatctgggcttgagaccagggttgtgcaggcagagcggttcttcaaaacccacttgttcgactgggtcaccagtgacccgttcggacggtgctacctgctggaactcccaactgaccacaaaggtgtctacggcagcctgactgactcttatgcttacatgagaaacggttgggatgttgaggtcactgcagtgggaaatcagttcaacggaggatgtctcttggtggccatggtgccagaactttgctctattgacaagagagagctgtaccagctcacgctctttccccaccagttcatcaacccccggacgaacatgacggcgcacatcactgtgccctttgttggcgtcaaccgctacgaccagtacaaggtacacaaaccttggaccctcgtggttatggttgtggccccgctgactgtcaacaccgaaggtgccccacagatcaaggtctatgccaacatcgcccctaccaacgtgcacgttgcgggtgagttcccttctaaggaagggatcttccccgtggcatgtagcgacggttacggtggtctggtgaccactgacccaaagacggctgaccccgcctacgggaaagtgtttaatccacctcgcaacatgttgccggggcggttcaccaacttccttgatgtggctgaggcgtgtcctacgtttctgcactttgagggtgacgtgccgtacgtgaccacaaagacggactcagacagggtgctcgcccagtttgacttgtctctggcagcaaagcacatgtcaaacaccttcctggcaggtctcgcccagtactacacacagtacagcggcaccatcaacctgcacttcatgttcacaggacccactgacgcgaaagcgcgttacatgattgcgtacgccccccctggcatggagccgcccaaaacacctgaggcggccgctcactgcattcatgcggagtgggacacagggttgaattcaaaattcacattttcaatcccttacctttcggcggctgattacgcgtacaccgcgtctgacgttgcggagaccacaaatgtacagggatgggtttgcctgtttcaaattacacacgggaaggctgacggcgacgcactggtcgttctagctagcgccggcaaggactttgagctgcgtctgccagttgacgctcgcacgcagaccacctccacaggtgagtcggctgaccccgtgactgccactgttgagaactacggtggtgagacacaggtccagagacgccaacacacggatgtctcgttcatattagacagatttgtgaaagtaacaccaaaagaccaaattaatgtgttggacctggtgcaaacccctgcacacactttggtaggcgcgctcctccgtactgccacctactacttcgcagatctagaagtggcagtgaaacacgaggggaaccttacctgggtcccgaatggggcgcccgagacagcgttggacaacaccaccaatccaacggcttaccacaaggcaccgctcacccggcttgcactgccttacacggcaccacaccgtgtcttggctactgtttacaacgggaactgcaagtatggcgagagccccgtgaccaacgtgagaggtgacctgcaagtattggcccagaaggcggcaagaacgctgcctacctccttcaattacggtgccatcaaagccactcgggtgactgaactgctttaccgcatgaagagggccgaaacatactgcccccggcctcttttggccattcacccgagcaaagctagacacaaacaaaagattgtggcgcctgtgaaacagcttttgaactttaacctgctcaagttggcaggagacgtcgagtccaaccctgggcctttcttcttctctgacgtcaggtcaaatttttccaagttggttgaaaccatcaaccagatgcaggaagacatgtcaacaaaacacggacccgactttaaccggttggtgtctgcatttgaggaactggccactggagtgaaggctatcaggaccggcctcgatgaggccaaaccctggtacaagctcatcaagctcttgagccgcctgtcatgcatggccgctgtagcagcacggtcaaaggacccagtccttgtggccatcatgctggctgacaccggccttgagattctggacagtacctttgtcgtgaagaagatctccgactcgctctccagtctctttcacgtgccggcccccgtcttcagtttcggagccccgattttgttggctgggttggtcaaagtcgcctcgagtttcttccggtccacacccgaagaccttgagagagcggagaaacagctcaaagcacgtgacatcaatgacatattcgccattctcaagaacggcgagtggctggtcaagctgattcttgccatccgcgactggatcaaggcatggatcgcctcagaagaaaagtttgtcaccatgacagacctggtgcctggcatccttgaaaagcagcgggatctcaacgacccaagcaagtacaaggaggccaaggagtggctcgacaacgcgcgccaagcgtgtttgaagagcgggaacatccacatcgcaaacctttgcaaagtggttgccccagcacccagcaggtcgaggcccgaacccgtggtcgtttgcctccgtggcaaatcgggccagggcaagagtttccttgcgaacgtgcttgcacaagcaatttcaacccacttcactggcagaaccgattcagtttggtactgcccacctgaccctgaccacttcgacggttacaaccagcagaccgttgtagtaatggatgatttgggccagaaccccgacgggaaggacttcaagtacttcgcccaaatggtttcaactacggggtttatcccgcccatggcttcactcgaggacaaaggcaaacctttcaacagcaaggtcatcatcgccaccaccaacctgtactcgggcttcaccccgagaactatggtgtgccctgatgcactgaaccgaaggttccactttgacattgacgtgagcgccaaggacgggtacaaaattaacaacaaactggacatcaacaaagctcttgaagacacccacaccaacccagtggcaatgtttcaatacgactgtgcccttctcaacggcatggccgttgaaatgaagagaatgcaacaagatatgttcaagcctcaaccgcccctccagaacgtctaccagcttgttcaggaggtgattgaccgggtcgagctccacgagaaggtgtcgagccacccgattttcaagcagatctcaattccttcccaaaaggctgtgctgtactttctcattgagaagggccagcacgaagcagcaattgaattctttgaggggatggtgcatgactccatcaaggaggagctccggcctctcatccagcagacctcatttgtgaagcgcgcttttaagcgcctgaaggaaaactttgagatagttgccctgtgtttgactcttttggcaaacatagtgatcatgatccgcgagactcgcaagagacagcagatggtggatgatgcagtgaacgagtacattgagaaggcaagcatcaccacggatgacaagactcttgacgaggcggaaaagaaccctctggagaccagcggtgccaccactgttggtttcagagagaaaactctcccgggacacaaggcgagtgatgacgtgaactccgagcccgccaaacccgtggaagaacaaccacaagctgaaggaccctacaccggtccactcgagcgtcaaaaacctctgaaagtgagagccaagctcccacagcaggaggggccctacgctggtccgatggagagacagaaaccgctgaaagtgaaagtgaaagccccggtcgttaaggaaggaccttacgaaggaccggtgaagaaacctgtcgctttgaaagtgaaagcaaagaacttgatcgtcactgagagtggtgctcccccgactgacttgcaaaagatggtcatgggtaacaccaagcctgttgagctcatcctcgacgggaagacggtggccatctgctgcgccactggagtgtttggtactgcctaccttgttcctcgtcatcttttcgcagagaagtacgacaagatcatgttggacggtagagccatgacagacagtgactacagagtgtttgagtttgagattaaagtgaaaggacaggacatgctctcagacgccgcgctcatggtgcttcaccgtgggaatcgcgtgcgggacatcacgaagcacttccgtgatgtggcaagaatgaagaaaggcacccccgtcgtcggcgtgatcaacaacgctgatgttgggagactgatcttctctggtgaggcccttacctacaaggacattgtagtgtgcatggacggagacaccatgcccggtctcttcgcctacaaagccgccaccaaggcgggttactgtggaggagccgttcttgcaaaggacggagccgagactttcatcgtcggcactcactccgcaggcggcaatggagttggatactgttcatgcgtttccaggtctatgctgcttaaaatgaaggcacacatcgatcccgaaccacaccacgagggattgatagttgacaccagagatgttgaggagcgcgtacatgtcatgcgcaaaaccaagctcgcacccaccgtggcacacggtgtgtttaaccccgaatttgggcctgccgccttgtccaacaaggacccgcgcctgaatgagggggttgtcctcgatgaagccatcttctccaaacacaaaggaaacacaaagatgtctgaggaggacaaagcgctgttccgccgctgtgctgctgactacgcgtcgcgtctgcatagcgtgctgggtacggcaaatgccccactgagcacttacgaggcaatcaagggcgtcgacgggcttgacgccatggaaccggacaccgcgcctggtctcccctgggctctccaggggaaacgccgtggtgcgctcattgacttcgagaacggcactgttggacccgaggttgaagctgccttgaagctcatggagaaaagagagtacaagtttgtatgccagaccttcctgaaggacgagattcgcccgatggagaaggtacgtgccggcaagactcgcattgtcgacgtcctgcctgttgaacacattctttacaccaggatgatgattggcagattttgtgctcaaatgcacttaaacaacggaccgcaaattggctcggcggttggttgtaatcctgatgttgattggcaaagatttggcacgcattttgctcagtacagaaacgtgtgggatgtggactattcggcctttgatgccaaccactgcagtgacgcaatgaacatcatgtttgaggaggtgttcaacacggatttcggtttccacccaaacgctgagtggatcctgaaaactctcgtgaacactgaacacgcctatgagaacaaacgcatcactgttgaaggcgggatgccgtctggttgttccgcaacaagcatcatcaacacaattttgaacaacatctacgtgctctacgccttgcgtagacactatgagggagttgagctggactcttacaccatgatctcctacggggacgacatcgtggttgcaagtgattacgatctggactttgaggccctcaggcctcacttcaaatcccttggtcaaaccattactccagctgacaaaagcgacaaaggttttgttcttggtcactccattaccgatgtcactttcctcaaaagacacttccacatggactatggaactgggttttacaaacctgtgatggcttcgaagaccctcgaggctatcctctcctttgcacgccgtgggaccatacaggagaagttgatctccgtggcaggactcgccatccactctggacctgacgagtaccggcgtctctttgagcctttccagggcctctttgagattccaagctacagatcactttacctgcgttgggtgaacgccgtgtgcggtgacgcataa

>AJ539138.1_O_CHA_1999

atgagcacaactgactgtttcatcgctttgttgtacgctttcagagagattaaaacactgttcttatcacgagcacaaggaaagatggagttcacacttcacaacggtgagaagaaaacattctactccaggcccaacaaccacgataactgctggctgaacaccatcctccagttgtttaggtacgttgatgaacctttcttcgactgggtctactactcacctgagaacctcacacttgatgctatcaaacaattggaagaaattactggtctcgagctccacgagggtggaccacccgctctcgttatttggaacattaaacacctgctcaacaccggaatcggcaccgcttcgcgacccagcgaagtgtgcatggtagacgggacggacatgtgtttggctgacttccacgctggcatcttcctgaaaggacaggaacacgctgtgttcgcctgcgtcacctccaacgggtggtacgcgattgatgacgaggacttttacccctggacgccggacccgtccgacgttctggtgtttgtcccgtacgatcaagaaccgctcaacggagaatggaaagcaaaggttcagaaacgactcagaggcgccgggcaatccagcccggcgactgggtcacagaaccagtcaggcaacactggaagcattatcaacaattactacatgcagcagtaccagaactccatggacacgcaacttggtgacaacgctattagcggaggctccaacgaggggtccacggacaccacctccacccacacaaccaacactcagaacaatgactggttttcaaagctggccagttccgcttttagcggtcttttcggcgctcttctcgccgacaagaaaaccgaggagaccactcttctcgaggaccgcatcctcactacccgcaacggacacacgacctcgacaacccagtcgagcgttggagtcacttacgggtacgcaacagctgaggactttgtgagcggaccaaacacatctgggcttgagaccagggttgtgcaggcagagcggttcttcaaaacccacttgttcgactgggtcaccagtgacccgttcggacggtnctacctgctggaactcccaactgaccacaaaggtgtctacggcagcctgactgactcttatgcttacatgagaaacggttgggatgttgaggtcactgcagtgggaaatcagttcaacggaggatgtctgttggtggccatggtgccagaactttgctctattgacaagagagagctgtaccagctcacgctctttccccaccagttcatcaacccccggacgaacatgacggcgcacatcactgtgccctttgttggtgtcaaccgctacgaccagtacaaggtacacaaaccttggaccctcgtggttatggttgtggccccgctgactgtcaacaccgaaggtgccccacagatcaaggtctatgccaacatcgcccctaccaacgtgcacgttgcgggtgagttcccttctaaggaagggatcttccccgtggcatgtagcgacggttacggtggtctggtgaccactgacccaaagacggctgaccccgcctacgggaaagtgttcaatccacctcgcaacatgttgccggggcggttcaccaacttccttgatgtggctgaggcgtgccctacgtttctgcactttgagggtgacgtgccgtacgtgaccacaaagacggactcagacagggtgctcgcccagtttgacttgtctctggcagcaaagcacatgtcaaacaccttcctggcaggtctcgcccagtactacacacagtacagcggcaccatcaacctgcacttcatgttcacaggacccactgacgcgaaagcgcgttacatgattgcatacgccccccctggcatggagccgcccaaaacacctgaggcggccgctcactgcattcatgcggagtgggacacagggttgaattcaaaattcacattttcaatcccttacctttcggcggctgattacgcgtacaccgcgtctgacgctgcggagaccacaaatgtacagggatgggtctgcctgtttcaaattacacacgggaaggctgacggcgacgcactggtcgttctagctagcgccggtaaggactttgagctgcgtctgccagttgacgctcgcacgcagaccacctccacaggtgagtcggctgaccccgtgactgccactgttgagaactacggtggtgagacacaggtccagagacgccaacacacggatgtctcgttcatattagacagatttgtgaaagtaacaccaaaagaccaaattaatgtgttggacctgatgcaaacccctgcacacactttggtaggcgcgctcctccgtactgccacctactacttcgcagatctagaagtggcagtgaaacacgaggggaaccttacctgggtcccgaatggggcgcccgagacagcgttggacaacaccaccaatccaacggcttaccacaaggcaccgctcacccggcttgcactgccttacacggcaccacaccgtgtcttggctactgtttacaacgggaactgcaagtatggcgagagccccgtgaccaatgtgagaggtgacctgcaagtgttggcccagaaggcggcaagaacgctgcctacctccttcaattacggtgccatcaaagccactcgggtgactgaactgctttaccgcatgaagagggccgaaacatactgcccccggcctcttttggctattcacccgagcgaagctagacacaaacaaaagattgtggcgcctgtgaaacagcttttgaactttgacctgctcaagttggcaggagacgtcgagtccaaccctgggcctttcttcttctctgacgtcaggtcaaatttttccaagttggttgaaaccatcaaccagatgcaggaggacatgtcaacaaaacacggacccgactttaaccggttggtgtctgcatttgaggaactggccactggagtgaaggctatcaggaccggtctcgatgaggccaaaccctggtacaagctcatcaagctcttgagccgcctgtcatgcatggccgctgtagcagcacggtcaaaggacccagtccttgtggccatcatgctggctgacaccggccttgagattctggacagtacctttgtcgtgaagaagatctccgactcgctctccagtctctttcacgtgccggcccccgtcttcagtttcggagccccgattttgctggccgggttggtcaaagtcgcctcgagtttcttccggtccacacccgaagaccttgagagagcggagaaacagctcaaagcacgtgacatcaatgacatattcgccattctcaagaacggcgagtggctggtcaagctgattcttgccatccgcgactggatcaaggcatggatcgcctcagaagaaaagtttgtcaccatgacagacttggtgcctggcatccttgaaaagcagcgggatctcaacgacccaagcaagtacaaggaggccaaggagtggctcgacaacgcgcgccaagcgtgtttgaagagcgggaacatccacatcgcaaacctttgcaaagtggttgccccagcacccagcaggtcgaggcccgaacccgtggtcgtttgcctccgtggcaaatcgggccagggcaagagtttccttgcgaacgtgcttgcacaagcaatttcaacccacttcactggcagaaccgattcagtttggtactgcccacctgaccctgaccacttcgacggttacaaccagcagaccgttgtagtaatggatgatttgggccagaaccccgacgggaaggacttcaagtacttcgcccaaatggtttcaactacggggtttatcccgcccatggcttcactcgaggacaaaggcaaacctttcaacagcaaggtcatcatcgccaccaccaacctgtactcgggcttcaccccgagaactatggtgtgccctgatgcactgaaccgaaggttccactttgacattgacgtgagcgccaaggacgggtacaaaattaacaacaaattggacatcatcaaagctcttgaagatacccacaccaacccagtggcaatgtttcaatacgactgtgcccttctcaacggcatggccgttgaaatgaagagaatgcaacaagatatgttcaagcctcaaccgcccctccagaacgtctaccagcttgttcaggaggtgattgaccgggtcgagctccacgagaaggtgtcgagccacccgattttcaagcagatctcaattccttcccaaaaggctgtgctgtactttctcattgagaagggtcagcacgaagcagcaattgaattctttgaggggatggtgcatgactccatcaaggaggagctccggcctctcatccaacagacctcatttgtgaagcgcgcttttaagcgcctgaaggaaaactttgagatagttgccctgtgtttgactcttttggcaaacatagtgatcatgatccgcgagactcgcaagagacagcagatggtggatgatgcagtgaacgagtacattgagaaggcaaacatcaccacggatgacaagactcttgacgaggcggaaaagaaccctctggagaccagcggtgccgccactgttggtttcagagagaaaactctcccgggacacaaggcgagtgatgacgtgaactccgagcccgccaaacccgtggaagaacaaccacaagctgaaggaccctacaccggtccactcgagcgtcaaaaacctctgaaagtgagagccaagctcccacagcaggaggggccctacgctggtccgatggagagacagaaaccgctgaaagtgaaagtgaaagccccggtcgttaaggaaggaccttacgaaggaccggtgaagaaacctgtcgctttgaaagtgaaagcaaagaacttgattgtcactgagagtggtgcycccccgactgacttgcaaaagatggtcatgggtaacaccaagcctgttgagctcatcctcgacgggaagacggtggccatctgctgcgccaccggagtgtttggtactgcctaccttgttcctcgtcatcttttcgcagagaagtatgacaagatcatgttggacggcagagccatgacagacagtgactacagagtgtttgagtttgagattaaagtgaaaggacaggacatgctctcagacgccgcgctcatggtgcttcaccgtgggaatcgcgtgcgggacatcacgaagcacttccgtgatgtggcaagaatgaagaaaggcacccccgtcgtcggcgtgatcaacaacgctgatgttgggagactgatcttctctggtgaggcccttacctacaaggacattgtagtgtgcatggacggagacaccatgcccggtctcttcgcctacaaagccgccaccaaggcgggttactgtggaggagccgttcttgcaaaggacggagccgagactttcatcgtcggcactcactccgcaggcggcaacggagttggatactgctcatgcgtttccaggtctatgctgcttaaaatgaaggcacacatcgatcccgaaccacaccacgagggattgatagttgacaccagagatgttgaggagcgcgtacatgtcatgcgcaaaaccaagctcgcacccaccgtggcacacggtgtgtttaaccccgaatttgggcctgccgccttgtccaacaaggacccgcgcctgaatgagggggttgtcctcgatgaagccatcttctccaaacacaaaggaaacacaaagatgtctgaggaggacaaagcgctgttccgccgctgtgctgctgactacgcgtcgcgtctgcatagcgtgctgggtacggcaaatgccccactgagcacttacgaggcaatcaagggcgtcgacggacttgacgccatggaaccagacaccgcgcctggtctcccctgggctctccaggggaaacgccgtggtgcgctcattgatttcgagaacggcactgtcggacccgaggttgaagctgccttgaagctcatggagaaaagagagtacaagtttgtatgccagaccttcctgaaggacgagattcgcccgatggagaaggtacgtgccggcaagactcgcattgtcgacgtcctgcctgttgaacacattctttacaccaggatgatgattggcagattttgtgctcaaatgcactcaaacaacggaccgcaaattggctcggcggttggttgtaatcctgatgttgattggcaaagatttggcacgcattttgctcagtacagaaacgtgtgggatgtggactattcggcctttgatgccaaccactgcagtgacgcaatgaacatcatgtttgaggaggtgttcaacacggatttcggtttccacccaaacgctgagtggatcctgaaaactctcgtgaacactgaacacgcctatgagaacaaacgcatcactgttgaaggcgggatgccgtctggttgttccgcaacaagcatcatcaacacaattttgaacaacatctacgtgctctacgccttgcgtagacactatgagggagttgagctggactcttacaccatgatctcctacggagacgacatcgtggttgcaagtgattacgatctggactttgaggccctcaagcctcacttcaaatcccttggtcaaaccattactccagctgacaaaagcgacaaaggttttgttcttggtcactccattaccgatgtcactttcctcaaaagacacttccacatggactatggaactgggttttacaaacctgtgatggcttcgaagaccctcgaggctatcctctcctttgcacgccgtgggaccatacaggagaagttgatctccgtggcaggactcgccgtccactctggacctgacgagtaccggcgtctctttgagcctttccagggcctctttgagattccaagttacagatcactttacctgcgttgggtgaacgccgtgtgcggtgacgcataa

>AJ539139.1_O_SKR_2000

atgagcacaactgactgtttcatcgctttgttgtacgctttcagagagattaaaacactgttcttatcacgagcacaaggaaagatggagttcacacttcacaacggtgagaagaaaacattctactccaggcccaacaaccacgacaactgctggctgaacaccatcctccagttgtttaggtacgttgatgaacctttcttcgactgggtctactactcacctgagaacctcacgcttgatgctatcaaacaattggaagaaattactggtctcgaactccacgagggtggaccacccgctctcgttatttggaacattaaacacctgctcaacaccggaatcggcaccgcttcgcgacccagcgaagtgtgcatggtagacgggacggacatgtgtttggctgacttccacgctggcatcttcctgaaaggacaggaacacgctgtgttcgcctgcgtcacctccaacgggtggtacgcgattgacgatgaggacttttacccctggacgccggacccgtccgacgttctggtgtttgtcccgtacgatcaagaaccgctcaacggagaatggaaagcaagggttcagaaacgactcagaggcgccgggcaatccagcccggcgactgggtcacagaaccagtcaggcaacactggaagcatcatcaacaattactacatgcagcagtaccagaactccatggacacgcaacttggtgacaacgctattagcggaggctccaacgaggggtccacggacaccacttccacccacacaaccaacactcagaacaatgactggttctcaaagctggccagttccgcttttagcggtcttttcggcgctcttctcgccgataagaaaaccgaggagaccactcttctcgaggaccgcatcctcactacccgcaacggacacacgacctcgacaacccagtcgagcgttggagtcacttacgggtacgcaacagccgaggactttgtgagcggaccaaacacatctgggctcgagaccagggttgtgcaggcagagcggttcttcaaaacccacttgttcgactgggtcaccagtgacccgttcggacggtgctacctgctggaactcccaactgaccacaaaggtgtctatggcagcctgactgactcttatgcttacatgagaaacggttgggatgttgaggtcactgcagtgggaaatcagttcaacggaggatgtctgttggtggccatggtgccagaactttgctctattgacaagagagagctgtaccagctcacgctctttccccaccagttcatcaacccccggacgaacatgacggcgcacatcactgtgccctttgttggcgtcaatcgctacgaccagtacaaggtacacaaaccttggaccctcgtggttatggttgtggccccgctgactgtcaacaccgaaggtgccccacagatcaaggtctacgccaacatcgcccctaccaacgtgcacgttgcgggtgagttcccttccaaggaagggatcttccccgtggcatgtagcgacggttacggtggtctggtgaccactgacccaaagacggctgaccccgcctacgggaaagtgttcaatccacctcgcaacatgttgccggggcggttcaccaacttccttgatgtggctgaggcgtgccctacgtttctgcactttgagggtgacgtgccgtacgtgaccacaaagacggactcagacagggtgctcgcccagtttgacttgtctctggcagcaaaacacatgtcaaacaccttcctggcgggtctcgcccagtactacacacagtacagcggcaccatcaacctgcacttcatgttcacaggacccactgacgcgaaagcgcgttacatgattgcatacgccccccctggcatggagccgcccaaaacacctgaggcggccgctcactgcattcatgcggagtgggacacagggttgaattcaaaattcacattttcaatcccttacctttcggcggctgattacgcgtacaccgcgtctgatgctgcggagaccacaaatgtacagggttgggtttgcctgtttcaaattacacacgggaaggctgacggcgacgcactggtcgttctagctagcgccggcaaggactttgagctgcgtctgccagttgacgctcgcacgcagaccacctccacaggtgagtcggctgaccccgtgactgccactgttgagaactacggtggtgagacacaggtccagagacgccaacacacggatgtctcgttcatattagacagatttgtgaaagtaacaccaaaagaccaaattaatgtgttggacctgatgcaaacccctgcacacactttggtaggcgcgctcctccgtactgccacctactacttcgcagatctagaagtggcagtgaaacacgaggggaaccttacctgggtcccgaacggggcgcccgagacagcgttggacaacaccaccaatccaacggcctaccacaaggcaccgctcacccggcttgcactgccttacacggcaccacaccgtgtcttggctactgtttacaacgggaactgcaagtatggcgagggccccgtgaccaatgtgagaggtgacctgcaagtattggcccagaaggcggcaagaacgctgcctacctccttcaactacggtgccatcaaagccactcgggtgactgaactgctttaccgcatgaagagggccgaaacatactgtccccggcctcttttggctattcacccgagcgaagctagacacaaacaaaagattgtggcgcctgtgaaacagcttctgaactttgacctgctcaagttggcaggagacgtcgagtccaaccctgggcctttcttcttctctgacgtcaggtcaaatttttccaagttggttgaaaccatcaaccanatgcaggaggacatgtcaacaaaacacggacccgactttaaccggttggtgtctgcatttgaggaactggccactggagtgaaggctatcaggaccggtctcgatgaggccaaaccctggtacaagctcatcaagctcttgagccgcctgtcatgcatggccgctgtagcagcacggtcaaaggacccagtccttgtggccatcatgctggctgacaccggccttgagattctggacagtacctttgtcgtgaagaagatctccgactcgctctccagtctctttcacgtgccggcccccgtcttcagtttcggagccccgattttgttggccgggttggtcaaagtcgcctcgactttcttccggtccacacccgaagaccttgagagagcggagaaacagctcaaagcacgtgacatcaatgacatattcgccattctcaagaacggcgagtggttggtcaagctgattcttgccatccgcgactggatcaaggcatggatcgcctcagaagaaaaatttgtcaccatgacagacctggtacctggcatccttgaaaagcagcgggatcttaacgacccaagcaagtacaaggaggccaaggagtggctcgacaacgcgcgccaagcgtgtttgaagagcgggaacatccacatcgcaaacctttgtaaagtagttgccccagcacccagcaggtcgaggcctgaacccgtggtcgtttgcctccgtggcaaatcgggccagggcaagagtttccttgcgaacgtgcttgcacaagcaatttcaacccacttcactggcagaaccgattcagtttggtactgcccacctgaccctgaccacttcgacggttacaaccagcagaccgttgtagtaatggatgatttgggccagaaccccgacgggaaggacttcaaatacttcgcccaaatggtttcaactacggggtttatcccgcccatggcttcactcgaggacaaaggcaaacctttcaacagcaaggtcatcatcgccaccaccaacctgtactcgggcttcaccccgagaactatggtgtgccctgatgcactgaaccgaaggttccactttgacattgacgtgagcgccaaggacgggtacaaaattaacaacaaattggacatcatcaaagctcttgaagatacccacaccaacccagtggcaatgtttcaatacgactgtgcccttctcaacggcatggccgttgagatgaagagaatgcaacaagatatgttcaagcctcaaccgcccctccagaacgtctaccagcttgttcaggaggtgattgaccgggtcgagctccacgagaaggtgtcgaaccacccgattttcaagcagatctcaataccttcccaaaaggctgtgctatactttctcattgagaagggccagcacgatgcagcaattgaattctttgaggggatggtgcatgactccatcaaggaggagctccggcctctcatccaacagacctcatttgtgaagcgcgcttttaagcgcctgaaggaaaactttgaggtagttgccctgtgtttgacccttttggcaaacatagtgatcatgatccgcgagactcgcaagagacagcagatggtggatgacgcagtgaacgagtacattgagaaggcaaacatcaccacggatgacaagactcttgacgaggcggaaaagaaccctctggagaccagcggtgccaccactgttggttttagagagaaaactctcccgggacacaaggcgagtgatgacgtgaactccgagcccgccaaacccgcggaagaacaaccacaagctgaaggaccctacaccggtccactcgagcgtcaaaaacctctgaaagtgagagccaagctcccacagcaggaggggccctacgctggtccgatggagagacagaaaccgctgaaagtgaaagtgaaagccccggttgttaaggaaggaccttacgaaggaccggtgaagaaacctgtcgctttgaaagtgaaagcaaagaacttgattgtcactgagagtggtgctcccccgactgacttgcaaaagatggtcatgggtaacaccaagcctgttgagctcatcctcgacgggaagacggtggccatctgttgcgccaccggagtgtttggtactgcttaccttgtccctcgtcatcttttcgcagagaagtatgacaagatcatgttggacggcagagccatgacagacagtgactacagagtgtttgagtttgagattaaagtgaaaggacaggacatgctctcagacgccgcgctcatggtgcttcaccgtgggaatcgcgtgcgggacatcacgaagcacttccgtgatgtggcaagaatgaagaaaggcacccccgtcgtcggcgtggtcaacaacgctgatgttgggagactgatcttctctggtgaggcccttacctacaaggacattgtagtgtgcatggacggagacaccatgcccggtctcttcgcctacaaagccgccaccaaggcgggttactgtgggggagccgttcttgcaaaggacggagccgagactttcatcgtcggcactcactccgcaggcggcaatggggttggatactgctcatgcgtttccaggtctatgctgcttaaaatgaaggcacacatcgatcccgaaccacaccacgagggattgatagttgacaccagagatgttgaggagcgcgtacatgtcatgcgcaaaaccaagctcgcacccaccgtggcacacggtgtgtttaaccccgaatttgggcctgccgccttgtccaacaaggacccgcgcctgaatgagggggttgtcctcgatgaagccatcttctccaaacacaagggaaacacaaagatgtctgaggaggacaaagcgctgttccgccgctgtgctgctgactacgcgtcgcgtctgcatagcgtgctgggtacggcaaacgccccactgagcatttacgaggcaatcaagggcgtcgacggacttgacgccatggaaccagacaccgcgcctggtttaccctgggctctccaggggaaacgccgtggtgcgctcattgactttgagaacggcacaatcggacccgaggttgaagctgccttgaagctcatggagaaaagagagtacaagtttgtatgtcagaccttcctgaaggacgagattcgcccgatggagaaggtacgtgccggcaagactcgcattgtcgacgtcctgcctgttgaacacattctttacaccaggatgatgattggcagattttgtgctcaaatgcactcaaacaacggaccgcaaattggctcggcggttgggtgtaatcctgatgttgattggcaaagatttggcacgcattttgctcagtacagaaacgtgtgggatgtagactattcggcctttgatgccaaccactgcagtgacgcaatgaacatcatgtttgaggaggtgttcaacacggatttcggtttccacccaaacgctgagtggatcctgaaaactctcgtgaacactgaacacgcctatgagaacaaacgcatcactgttgaaggcgggatgccgtctggttgttccgcaacaagcatcatcaacacaattttgaacaacatctacgtgctctacgccttgcgtaggcactatgagggagttgagctggactcttacaccatgatctcctacggagacgacatcgtggttgcaagtgattacgatctggactttgaggccctcaagcctcacttcaaatcccttggtcaaaccattactccagctgacaaaagcgacaaaggttttgttcttggtcactccattaccgatgtcactttcctcaaaagatctttccacatggactatggaactgggttttacaaacctgtgatggcttcgaagaccctcgaggctatcctctcctttgcacgccgtgggaccatacaggagaagttgatctccgtggcaggactcgccgtccactctggacctgacgagtaccggcgtctctttgagcccttccagggtctctttgagattccaagctacagatcactttacctgcgttgggtgaacgccgtgtgcggtgacgcataa

>AJ539140.1_O_SAR_2000

atgagcacaactgactgtttcatcgctttgttgtacgctttcagagagattaaaacactgttcttatcacgagcacaaggaaagatggagttcacacttcacaacggtgagaagaaaacattctactccaggcccaacaaccacgacaactgctggctgaacaccatcctccagttgtttaggtacgttgatgaacctttcttcgactgggtctactactcacctgagaacctcacacttgatgctatcaaacaattggaagagatcactggtctcgagctccacgagggtggaccacccgctctcgttatttggaacattaaacacttgctcaacaccggaatcggcaccgcttcgcgacccaacgaagtgtgcatggtagacgggacggatatgtgtttggctgacttccacgctggcatcttcctgaaaggacaggaacacgctgtgttcgcctgcgttacctccaacgggtggtacgcgattgatgacgaggacttttacccctggacgccggacccgtccgacgttctggtgtttgtcccgtacgatcaagaaccgctcaacggagaatggaaagcaaaggttcagaaacgactcagaggcgccgggcaatccagcccggcgactgggtcacagaaccagtcaggcaacactggaagcattatcaacaattactacatgcagcagtaccagaactccatggacacgcaacttggtgacaacgctattagcggaggctccaacgaggggtccacggacaccacctccacccacacaaccaacactcagaacaatgactggttttcaaagctggccagttccgcttttagcggtcttttcggcgctcttcttgctgacaagaaaaccgaggagaccactcttctcgaggaccgcatcctcactacccgcaacggacacacgacctcgacaacccagtcgagcgttggagtcacttacgggtacgcaacagctgaggactttgtgagcggaccaaacacatctgggcttgagaccagggttgtgcaggcagagcggttcttcaaaacccacttgttcgactgggtcaccagtgacccgtttggacggtnctatctgctggaactcccaactgaccacaaaggtgtctacggcagcctgaccgactcttatgcttacatgagaaacggttgggatgttgaggtcaccgcagtgggaaatcagttcaacggaggatgtctgttggtggccatggtgccagaactttgctctattgacaagagagagctgtaccagctcacgctctttccccaccagttcatcaacccccggacgaacatgacggcgcacatcactgtgccctttgttggcgtcaaccgctacgaccagtacaaggtacacaaaccttggaccctcgtggttatggttgtggccccgctgactgtcaacaccgaaggtgccccacagatcaaggtctatgccaacatcgcccctaccaacgtgcacgttgcgggtgagttcccttctaaggaagggatcttccccgtggcatgtagcgacggttacggtggtctggtgaccactgacccaaagacggctgaccccgcctacgggaaagtgttcaatccacctcgcaacatgttgccggggcggttcaccaacttccttgatgtggctgaggcgtgccctacgtttctgcactttgagggtggcgtgccgtacgtgaccacaaagacggactcagacagggtgctcgcccagtttgacttgtctctggcagcaaagcacatgtcaaacaccttcctggcaggtctcgcccagtactacacacagtacagcggcaccatcaacctgcacttcatgttcacaggacccactgacgcgaaagcgcgttacatgattgcatacgccccccctggtatggagccgcccaaaacacctgaggcggccgcccactgcattcatgcggagtgggacacagggttgaactcaaaattcacattttcaatcccttacctttcggcggctgattacgcgtacaccgcgtctgacgctgcggagaccacaaatgtacagggatgggtttgcctgtttcaaattacacacgggaaggctgacggcgacgcactggtcgttctagctagcgccggtaaggactttgagctgcgtctgccagttgacgctcgcacgcagaccacctccacaggtgagtcggctgaccccgtgactgccactgttgagaactacggtggtgagacacaggtccagagacgccaacacacggatgtctcgttcatattagacagatttgtgaaagtaacaccaaaagaccaaattaatgtgttggacctgatgcaaacccctgcacacactttggtaggcgcgctcctccgtactgccacctactacttcgcagatctagaagtggcagtgaaacacgaggggaaccttacctgggtcccgaatggggcgcccgagacagcgttggacaacaccaccaatccaacggcttaccacaaggcaccgctcacccggcttgcactgccttacacggcaccgcaccgtgtcttggctactgtttacaacgggaactgcaagtatggcgagagccccgtgaccaatgtgagaggtgacctgcaagtattggcccaaaaggcggcaagaacgctgcctacctccttcaattacggtgccatcaaagccactcgggtgactgaactgctttaccgcatgaagagggccgaaacatactgcccccggcctcttttggctattcacccaagcgaagctagacacaaacaaaagattgtggcgcctgtgaaacagcttttgaactttgacctgctcaagttggcaggagacgtcgagtccaaccctgggcctttcttcttctctgacgtcaggtcaaatttttccaagttggttgaaaccatcaaccagatgcaggaggacatgtcaacgaaacacggacccgactttaaccggttggtgtctgcatttgaggaactggccactggagtgaaggctatcaggaccggtctcgatgaagccaaaccctggtacaagctcatcaagctcttgagccgcctgtcatgcatggccgctgtagcagcacggtcaaaggacccagtccttgtggccatcatgctggctgacaccggccttgagattctggacagtacctttgtcgtgaagaagatctccgactcgctctccagtctctttcacgtgccggcccccgtcttcagtttcggagccccgattttgttggccgggttggtcaaagtcgcctcgagtttcttccggtccacacccgaagaccttgagagagcggagaaacagctcaaagcacgtgacatcaatgacatattcgccattctcaagaacggcgagtggctggtcaagctgattcttgccatccgcgactggatcaaggcatggatcgcctcagaagaaaagtttgtcaccatgacagacctggtgcctggcatccttgaaaagcagcgggatctcaacgacccaagcaagtacaaagaggccaaggagtggctcgacaacgcgcgccaagcgtgtttgaagagcgggaacatccacatcgcaaacctttgcaaagtggttgccccagcacccagcaggtcgaggcccgaacccgtggtcgtttgcctccgtggcaaatcgggccagggcaagagtttccttgcgaacgtgcttgcacaagcaatttcaacccacttcactggcagaaccgattcagtttggtactgcccacctgaccctgaccacttcgacggttacaaccagcagaccgttgtagtaatggatgatttgggccagaaccccgacgggaaggacttcaagtacttcgcccaaatggtttcaactacggggtttatcccgcccatggcttcactcgaggacaaaggcaaacctttcaacagcaaggtcatcatcgccaccaccaacctgtactcgggcttcaccccgagaactatggtgtgccctgacgcactgaaccgaaggttccactttgacattgacgtgagcgccaaggacgggtacaaaattaataacaaattggacatcatcaaagctcttgaagatacccacaccaacccagtggcaatgtttcaatacgactgtgcccttctcaacggcatggccgttgaaatgaagagaatgcaacaagatatgttcaagcctcaaccgcccctccagaacgtctaccagcttgttcaggaggtgattgaccgggtcgagctccacgagaaggtgtcgagccacccgattttcaagcagatctcaattccttcccaaaaggctgtgctgtactttctcattgagaagggccagcacgaagcagcaattgagttctttgaggggatggtgcatgactccatcaaggaggagctccggcctctcatccaacagacctcatttgtgaagcgcgcttttaagcgcctgaaggaaaactttgagatagttgccctgtgtttgactcttttggcaaacatagtgatcatgatccgcgagactcgcaagagacagcagatggtggatgatgcagtgaacgagtacattgagaaggcaaacatcaccacggatgacaagactcttgacgaggcggaaaagaaccctctggagaccagcggtgccaccactgttggtttcagagagaaaactctcccgggacacaaggcgggtgatgacgtgaactccgagcccaccaaacccgtggaagaacaaccacaagctgaaggaccctacaccggtccactcgagcgtcaaaaacccctgaaagtgaggaccaagctcccacagcaggaggggccctacgctggtccgatggagagacagaaaccgctgaaagtgaaagtgaaagccccggtcgttaaggaaggaccttacgaaggaccggtgaagaaacctgtcgctttgaaagtgaaagcaaagaacttgattgtcactgagagtggtgctcccccgactgacttgcaaaagatggtcatgggtaacaccaagcctgttgagctcatcctcgacgggaagacggtggccatctgctgcgccaccggagtgtttggtactgcctacctagttcctcgtcatcttttcgcagagaagtatgacaagatcatgttggacggcagagccatgacagacagtgactacagagtgtttgagtttgagactaaagtgaaaggacaggacatgctctcagacgccgctctcatggtgcttcaccgtgggaatcgcgtgcgggacatcacgaagcacttccgtgatgtggcaagaatgaagaaaggcacccccgtcgtcggcgtgatcaacaacgctgatgttgggagactgatcttctctggtgaggcccttacctacaaggacattgtagtgtgcatggacggagacaccatgcccggtctcttcgcctacaaagctgccaccaaggcgggttactgtggaggagccgttcttgcaaaggacggagccgagactttcatcgtcggcactcactccgcaggcggcaatggagttggatactgctcatgcgtttccaggtccatgctgcttaaaatgaaggcacacatcgatcccgaaccacaccacgagggattgatagttgacaccagagatgttgaggagcgcgtacatgtcatgcgcaaaaccaagctcgcacccaccgtggcacacggtgtgtttaaccccgaatttgggcctgccgccttgtccaacaaggacccgcgcctgaatgagggggttgtcctcgatgaagtcatcttctccaaacacaaaggaaacacaaagatgtctgaggaggacaaagcgctgttccgccgctgtgctgctgactacgcgtcgcgtctgcatagcgtgctgggtacggcaaatgccccactgagcacttacgaggcaatcaagggcgtcgacggacttgacgccatggaaccagacaccgcgcctggtctcccctgggctctccaggggaaacgccgtggtgcgctcatcgacttcgagaacggcacagtcggacccgaggttgaagctgccttgaagctcatggagaaaagagagtacaagtttacatgccagaccttcctgaaggacgagattcgcccgatggagaaggtacgtgccggcaagactcgcattgtcgacgtcctgcccgttgaacacattctttacactaggatgatgattggcagattttgtgctcaaatgcactcaaacaacggaccgcaaattggctcggcggttggttgtaatcctgatgttgattggcaaagatttggcacgcattttgctcagtatagaaacgtgtgggatgtggactattcggcctttgatgccaaccactgcagtgacgcaatgaacatcatgtttgaggaggtgtttaacacggacttcggtttccacccaaacgctgagtggatcctgaaaactctcgtgaacactgaacacgcctatgagaacaaacgcatcactgttgaaggcgggatgccgtctggttgttccgcaacaagcatcatcaacacaattttgaacaacatctacgtgctctacgccttgcgtagacactatgagggagttgagctggactcttacaccatgatctcctacggagacgacatcgtggttgcaagtgattacgatctggactttgaggccctcaagcctcacttcaaatcccttggtcaaaccattaccccagctgacaaaagcgacaaaggttttgttcttggtcactccattaccgatgtcactttcctcaaaagacacttccacatggactatggaactgggttttacaaacctgtgatggcttcgaagaccctcgaggctatcctctcctttgcacgccgtgggaccatacaggagaagttgacctccgtggcaggactcgccgtccactctggacctgacgagtaccggcgtctctttgagcctttccagggcctctttgagattccaagctacagatcactttacctgcgttgggtgaacgccgtgtgcggtgacgcataa

>AJ539141.1_O_UKG_2001

atgagcacaactgactgtttcatcgctttgttgtacgctttcagagagattaaaacactgttcttatcacgagcacaaggaaagatggagttcacacttcacaacggtgagaagaaaacattctactccaggcccaacaaacacgacaactgctggctgaacaccatcctccagttgtttaggtacgttgatgaaccttttttcgactgggtctactactcacctgagaacctcacacttgatgctatcaaacaattggaagaaattactggtctcgagctccacgagggtggaccacccgctctcgttatttggaacattaaacacttgctcaacaccggaatcggcaccgcctcgcgacccagcgaagtgtgcatggtagacgggacggatatgtgtttggctgacttccacgctggcatcttcctgaaaggacaggaacacgctgtgttcgcctgcgttacctccaacgggtggtacgcgattgatgacgaggacttttacccctggacgccggacccgtccgacgttctggtgtttgtcccgtacgatcaagaaccgctcaacggagaatggaaagcaaaggttcagaaacgactcagaggcgccgggcaatccagcccggcgactgggtcacagaaccagtcaggcaacactggaagcattatcaacaattactacatgcagcagtaccagaactccatggacacgcagcttggtgacaacgctattagcggaggctccaacgaggggtccacggacaccacctccactcacacaaccaacactcagaacaatgactggttttcaaagctggccagttccgcttttagcggtcttttcggcgctcttcttgctgacaagaaaaccgaggagaccactcttctcgaggaccgcatcctcactacccgcaacggacacacgacctcgacaacccagtcgagcgttggagtcacttacgggtacgcaacagctgaggactttgtgagcggaccaaacacatctgggcttgagaccagggttgtgcaggcagagcggttcttcaaaacccacttgttcgactgggtcaccagtgacccgtttggacggtgctatctgctggaactcccaactgaccacaaaggtgtctacggcagcctgaccgactcttatgcttacatgagaaacggttgggatgttgaggtcaccgcagtgggaaatcagttcaacggaggatgtctgttggtggccatggtgccagaactttgctctattgacaagagagagctgtaccagctcacgctctttccccaccagttcatcaacccccggacgaacatgacggcgcacatcactgtgccctttgttggcgtcaaccgctacgaccagtacaaggtacacaaaccttggaccctcgtggttatggttgtggccccgctgactgtcaacaccgaaggtgccccacagatcaaggtctatgccaacatcgcccctaccaacgtgcacgttgcgggtgagttcccttctaaggaagggatcttccccgtggcatgtagcgacggttacggtggtctggtgaccactgacccaaagacggctgaccccgcctacgggaaagtgttcaatccacctcgcaacatgttgccggggcggttcaccaacttccttgatgtggctgaggcgtgccctacgtttctgcactttgagggtggcgtgccgtacgtgaccacaaagacggactcagacagggtgctcgcccagttcgacttgtctctggcagcaaagcacatgtcaaacaccttcctggcaggtctcgcccagtactacacacagtacagcggcaccatcaacctgcacttcatgttcacaggacccactgacgcgaaagcgcgttacatgattgcatacgccccccctggtatggagccgcccaaaacacctgaggcggccgcccactgcattcatgcggagtgggacacagggttgaattcaaaattcacattttcaatcccttacctttcggcggctgattacgcgtacaccgcgtctgacgctgcggagaccacaaatgtacagggatgggtttgcctgtttcaaattacacacgggaaggctgacggcgacgcactggtcgttctagctagcgccggtaaggactttgagctgcgtctgccagttgacgctcgcacgcagaccacctccgcaggtgagtcggctgaccccgtgactgccactgttgagaactacggtggtgagacacaggtccagagacgccaacacacggatgtctcgttcatattagacagatttgtgaaagtaacaccaaaagaccaaattaatgtgttggacctgatgcaaacccctgcacacactttggtaggcgcgctcctccgtactgccacctactacttcgcagatctagaagtggcagtgaaacacgaggggaaccttacctgggtcccgaatggggcgcccgagacagcgttggacaacaccaccaatccaacggcttaccacaaggcaccgctcacccggcttgcactgccttacacggcaccgcaccgtgtcttggctactgtttacaacgggaactgcaagtatggcgagagccccgtgaccaatgtgagaggtgacctgcaagtattggcccaaaaggcggcaagaacgctgcctacctccttcaattacggtgccatcaaagccactcgggtgactgaactgctttaccgcatgaagagggccgaaacatactgcccccggcctcttttggctattcacccaagcgaagctagacacaaacaaaagattgttgcgcctgtgaaacagcttttgaactttgacctgctcaagttggcaggagacgtcgagtccaaccctgggcctttcttcttctctgacgtcaggtcaaatttttccaagttggttgaaaccatcaaccagatgcaggaggacatgtcaacgaaacacggacccgactttaaccggttggtgtctgcatttgaggaactggccactggagtgaaggctatcaggaccggtctcgatgaagccaaaccctggtacaagctcatcaagctcttgagccgcctgtcatgcatggccgctgtagcagcacggtcaaaggacccagtccttgtggccatcatgctggctgacaccggccttgagattctggacagtacctttgtcgtgaagaagatctccgactcgctctccagtctctttcacgtgccggcccccgtcttcagtttcggagccccgattttgttggccgggttggtcaaagtcgcctcgagtttcttccggtccacacccgaagaccttgagagagcggagaaacagctcaaagcacgtgacatcaatgacatattcgccattctcaagaacggcgagtggctggtcaagctgattcttgccatccgcgactggatcaaggcatggatcgcctcagaagaaaagtttgtcaccatgacagacctggtgcctggcatccttgaaaagcagcgggatctcaacgacccaagcaagtacaaagaggccaaggagtggctcgacaacgcgcgccaagcgtgtttgaagagcgggaacatccacatcgcaaacctttgcaaagtggttgccccagcacccagcaggtcgaggcccgaacccgtggtcgtttgcctccgtggcaagtcgggccagggcaagagtttccttgcgaacgtgcttgcacaagcaatttcaacccacttcactggcagaaccgactcagtttggtactgcccacctgaccctgaccacttcgacggttacaaccagcagaccgttgtagtaatggatgatttgggccagaaccccgacgggaaggacttcaagtacttcgcccaaatggtttcaactacggggtttatcccgcccatggcttcactcgaggacaaaggcaaacctttcaacagcaaggtcatcatcgccaccaccaacctgtactcgggcttcaccccgagaactatggtgtgccctgacgcactgaaccgaaggttccactttgacattgacgtgagcgccaaggacgggtacaaaattaacaacaaattggacatcatcaaagctcttgaagatacccacaccaacccagtggcaatgtttcaatacgactgtgcccttctcaacggcatggccgttgaaatgaagagaatgcaacaagatatgttcaagcctcaaccgcccctccagaacgtctaccagcttgttcaggaggtgattgaccgggtcgagctccacgagaaggtgtcgagccacccgattttcaagcagatctcaattccttcccaaaaggctgtgctgtactttctcattgagaagggccagcacgaagcagcaattgagttctttgaggggatggtgcatgactccatcaaggaggagctccggcctctcatccaacagacctcatttgtgaagcgcgcttttaagcgcctgaaggaaaactttgagatagttgccctgtgtttgactcttttggcaaacatagtgatcatgatccgcgagactcgcaagagacagcagatggtggatgatgcagtgaacgagtacattgagaaggcaaacatcaccacggatgacaagactcttgacgaggcggaaaagaaccctctggagaccagcggtgccaccactgttggcttcagagagaaaactctcccgggacacaaggcgggtgatgacgtgaactccgagcccgccaaacccgtggaagaacaaccacaagctgaaggaccctacaccggtccactcgagcgtcaaaaacccctgaaagtgagggccaagctcccacagcaggaggggccctacgctggtccgatggagagacagaaaccgctgaaagtgaaagtgaaagccccggtcgttaaggaaggaccttacgaaggaccggtgaagaaacctgtcgctttgaaagtgaaagcaaagaacttgattgtcactgagagtggtgctcccccgactgacttgcaaaagatggtcatgggtaacaccaagcctgttgagctcatcctcgacgggaagacggtggccatctgctgcgccaccggagtgtttggtactgcctacctagttcctcgtcatcttttcgcagagaagtatgacaagatcatgttggacggcagagccatgacagacagtgactacagagtgtttgagtttgagattaaagtgaaaggacaggacatgctctcagacgccgctctcatggtgcttcaccgtgggaatcgcgtgcgggacatcacgaagcacttccgtgatgtggcaagaatgaagaaaggcacccccgtcgtcggcgtgatcaacaacgctgatgttgggagactgatcttctctggtgaggcccttacctacaaggacattgtagtgtgcatggacggagacaccatgcccggtctcttcgcctacaaagctgccaccaaggcgggttactgtggaggagccgttcttgcaaaggacggagccgagactttcatcgtcggcactcactccgcaggcggcaatggagttggatactgctcatgcgtttccaggtccatgctgcttaaaatgaaggcacacatcgatcccgaaccacaccacgagggattgatagttgacaccagagatgttgaggagcgcgtacatgtcatgcgcaaaaccaagctcgcacccaccgtggcacacggtgtgtttaaccccgaatttgggcctgccgccttgtccaacaaggacccgcgcctgaatgagggggttgtcctcgatgaagtcatcttctccaaacacaaaggaaacacaaagatgtctgaggaggacaaagcgctgttccgccgctgtgctgctgactacgcgtcgcgtctgcatagcgtgctgggtacggcaaatgccccactgagcacttacgaggcaatcaagggcgtcgacggacttgacgccatggaaccagacaccgcgcctggtctcccctgggctctccaggggaaacgccgtggtgcgctcatcgacttcgagaacggcactgtcggacccgaggttgaagctgccttgaagctcatggagaaaagagagtacaagtttacatgccagaccttcctgaaggacgagattcgcccgatggagaaggtacgtgccggcaagactcgcattgtcgacgtcctgcccgttgaacacattctttacactaggatgatgattggcagattttgtgctcaaatgcactcaaacaacggaccgcaaattggctcggcggttggttgtaatcctgatgttgattggcaaagatttggcacgcattttgctcagtatagaaacgtgtgggatgtggactattcggcctttgatgccaaccactgcagtgacgcaatgaacatcatgtttgaggaggtgtttaacacggacttcggtttccacccaaacgctgagtggatcctgaaaactctcgtgaacactgaacacgcctatgagaacaaacgcatcactgttgaaggcgggatgccgtctggttgttccgcaacaagcatcatcaacacaattttgaacaacatctacgtgctctacgccttgcgtagacactatgagggggttgagctggactcttacaccatgatctcctacggagacgacatcgtggttgcaagtgattacgatctggactttgaggccctcaagcctcacttcaaatcccttggtcaaaccattaccccagctgacaaaagcgacaaaggttttgttcttggtcactccattaccgatgtcactttcctcaaaagacacttccacatggactatggaactgggttttacaaacctgtgatggcttcgaagaccctcgaggctatcctctcctttgcacgccgtgggaccatacaggagaagttgatctccgtggcaggactcgccgtccactctggacctgacgagtaccggcgtctctttgagcctttccagggcctctttgagattccaagctacagatcactttacctgcgttgggtgaacgccgtgtgcggtgacgcataa

>AJ633821.1_O_FRA_2001

atgagcacaactgactgtttcatcgctttgttgtacgctttcagagagattaaaacactgttcttatcacgagcacaaggaaagatggagttcacacttcacaacggtgagaagaaaacattctactccaggcccaacaaacacgacaactgctggctgaacaccatcctccagttgtttaggtacgttgatgaaccttttttcgactgggtctactactcacctgagaacctcacacttgatgctatcaaacaattggaagaaattactggtctcgagctccacgagggtggaccacccgctctcgttgtttggaacattaaacacttgctcaacaccggaatcggcaccgcctcgcgacccagcgaagtgtgcatggtagacgggacggatatgtgtttggctgacttccacgctggcatcttcctgaaaggacaggaacacgctgtgttcgcctgcgttacctccaacgggtggtacgcgattgatgacgaggacttttacccctggacgccggacccgtccgacgttctggtgtttgtcccgtacgatcaagaaccgctcaacggagaatggaaagcaaaggttcagaaacgactcagaggcgccgggcaatccagcccggcgactgggtcacagaaccagtcaggcaacactggaagcattatcaacaattactacatgcagcagtaccagaactccatggacacgcagcttggtgacaacgctattagcggaggctccaacgaggggtccacggacaccacctccactcacacaaccaacactcagaacaatgactggttttcaaagctggccagttccgcttttagcggtcttttcggcgctcttcttgctgacaagaaaaccgaggagaccactcttctcgaggaccgcatcctcactacccgcaacggacacacgacctcgacaacccagtcgagcgttggagtcacttacgggtacgcaacagctgaggactttgtgagcggaccaaacacatctgggcttgagaccagggttgtgcaggcagagcggttcttcaaaacccacttgttcgactgggtcaccagtgacccgtttggacggtgctatctgctggaactcccaactgaccacaaaggtgtctacggcagcctgaccgactcttatgcttacatgagaaacggttgggatgttgaggtcaccgcagtgggaaatcagttcaacggaggatgtctgttggtggccatggtgccagaactttgctctattgacaagagagagctgtaccagctcacgctctttccccaccagttcatcaacccccggacgaacatgacggcgcacatcactgtgccctttgttggcgtcaaccgctacgaccagtacaaggtacacaaaccttggaccctcgtggttatggttgtggccccgctgactgtcaacaccgaaggtgccccacagatcaaggtctatgccaacatcgcccctaccaacgtgcacgttgcgggtgagttcccttctaaggaagggatcttccccgtggcatgtagcgacggttacggtggtctggtgaccactgacccaaagacggctgaccccgcctacgggaaagtgttcaatccacctcgcaacatgttgccggggcggttcaccaacttccttgatgtggctgaggcgtgccctacgtttctgcactttgagggtggcgtgccgtacgtgaccacaaagacggactcagacagggtgctcgcccagtttgacttgtctctggcagcaaagcacatgtcaaacaccttcctggcaggtctcgcccagtactacacacagtacagcggcaccatcaacctgcacttcatgttcacaggacccactgacgcgaaagcgcgttacatgattgcatacgccccccctggtatggagccgcccaaaacacctgaggcggccgcccactgcattcatgcggagtgggacacagggttgaattcaaaattcacattttcaatcccttacctttcggcggctgattacgcgtacaccgcgtctgacgctgcggagaccacaaatgtacagggatgggtttgcctgtttcaaattacacacgggaaggctgacggcgacgcactggtcgttctagctagcgccggtaaggactttgagctgcgtctgccagttgacgctcgcacgcagaccacctccgcaggtgagtcggctgaccccgtgactgccactgttgagaactacggtggtgagacacaggtccagagacgccaacacacggatgtctcgttcatattagacagatttgtgaaagtaacaccaaaagaccaaattaatgtgttggacctgatgcaaacccctgcacacactttggtaggcgcgctcctccgtactgccacctactacttcgcagatctagaagtggcagtgaaacacgaggggaaccttacctgggtcccgaatggggcgcccgagacagcgttggacaacaccaccaatccaacggcttaccacaaggcaccgctcacccggcttgcactgccttacacggcaccgcaccgtgtcttggctactgtttacaacgggaactgcaagtatggcgagagccccgtgaccaatgtgagaggtgacctgcaagtattggcccaaaaggcggcaagaacgctgcctacctccttcaattacggtgccatcaaagccactcgggtgactgaactgctttaccgcatgaagagggccgaaacatactgcccccggcctcttttggctattcacccaagcgaagctagacacaaacaaaagattgttgcgcctgtgaaacagcttttgaactttgacctgctcaagttggcaggagacgtcgagtccaaccctgggcctttcttcttctctgacgtcaggtcaaatttttccaagttggttgaaaccatcaaccagatgcaggaggacatgtcaacgaaacacggacccgactttaaccggttggtgtctgcatttgaggaactggccactggagtgaaggctatcaggaccggtctcgatgaagccaaaccctggtacaagctcatcaagctcttgagccgcctgtcatgcatggccgctgtagcagcacggtcaaaggacccagtccttgtggccatcatgctggctgacaccggccttgagattctggacagtacctttgtcgtgaagaagatctccgactcgctctccagtctctttcacgtgccggcccccgtcttcagtttcggagccccgattttgttggccgggttggtcaaagtcgcctcgagtttcttccggtccacacccgaagaccttgagagagcggagaaacagctcaaagcacgtgacatcaatgacatattcgccattctcaagaacggcgagtggctggtcaagctgattcttgccatccgcgactggatcaaggcatggatcgcctcagaagaaaagtttgtcaccatgacagacctggtgcctggcatccttgaaaagcagcgggatctcaacgacccaagcaagtacaaagaggccaaggagtggctcgacaacgcgcgccaagcgtgtttgaagagcgggaacatccacatcgcaaacctttgcaaagtggttgccccagcacccagcaggtcgaggcccgaacccgtggtcgtttgcctccgtggcaaatcgggccagggcaagagtttccttgcgaacgtgcttgcacaagcaatttcaacccacttcactggcagaaccgactcagtttggtactgcccacctgaccctgaccacttcgacggttacaaccagcagaccgttgtagtaatggatgatttgggccagaaccccgacgggaaggacttcaagtacttcgcccaaatggtttcaactacggggtttatcccgcccatggcttcactcgaggacaaaggcaaacctttcaacagcaaggtcatcatcgccaccaccaacctgtactcgggcttcaccccgagaactatggtgtgccctgacgcactgaaccgaaggttccactttgacattgacgtgagcgccaaggacgggtacaaaattaacaacaaattggacatcatcaaagctcttgaagatacccacaccaacccagtggcaatgtttcaatacgactgtgcccttctcaacggcatggccgttgaaatgaagagaatgcaacaagatatgttcaagcctcaaccgcccctccagaacgtctaccagcttgttcaggaggtgattgaccgggtcgagctccacgagaaggtgtcgagccacccgattttcaagcagatctcaactccttcccaaaaggctgtgctgtactttctcattgagaagggccagcacgaagcagcaattgagttctttgaggggatggtgcatgactccatcaaggaggagctccggcctctcatccaacagacctcatttgtgaagcgcgcttttaagcgcctgaaggaaaactttgagatagttgccctgtgtttgactcttttggcaaacatagtgatcatgatccgcgagactcgcaagagacagcagatggtggatgatgcagtgaacgagtacattgagaaggcaaacatcaccacggatgacaagactcttgacgaggcggaaaagaaccctctggagaccagcggtgccaccactgttggtttcagagagaaaactctcccgggacacaaggcgggtgatgacgtgaactccgagcccgccaaacccgtggaagaacaaccacaagctgaaggaccctacaccggtccactcgagcgtcaaaaacccctgaaagtgagggccaagctcccacagcaggaggggccctacgctggtccgatggagagacagaaaccgctgaaagtgaaagtgaaagccccggtcgttaaggaaggaccttacgaaggaccggtgaagaaacctgtcgctttgaaagtgaaagcaaagaacttgattgtcactgagagtggtgctcccccgactgacttgcaaaagatggtcatgggtaacaccaagcctgttgagctcatcctcgacgggaagacggtggccatctgctgcgccaccggagtgtttggtactgcctacctagttcctcgtcatcttttcgcagagaagtatgacaagatcatgttggacggcagagccatgacagacagtgactacagagtgtttgagtttgagattaaagtgaaaggacaggacatgctctcagacgccgctctcatggtgcttcaccgtgggaatcgcgtgcgggacatcacgaagcacttccgtgatgtggcaagaatgaagaaaggcacccccgtcgtcggcgtgatcaacaacgctgatgttgggagactgatcttctctggtgaggcccttacctacaaggacattgtagtgtgcatggacggagacaccatgcccggtctcttcgcctacaaagctgccaccaaggcgggttactgtggaggagccgttcttgcaaaggacggagccgagactttcatcgtcggcactcactccgcaggcggcaatggagttggatactgctcatgcgtttccaggtccatgctgcttaaaatgaaggcacacatcgatcccgaaccacaccacgagggattgatagttgacaccagagatgttgaggagcgcgtacatgtcatgcgcaaaaccaagctcgcacccaccgtggcacacggtgtgtttaaccccgaatttgggcctgccgccttgtccaacaaggacccgcgcctgaatgagggggttgtcctcgatgaagtcatcttctccaaacacaaaggaaacacaaagatgtctgaggaggacaaagcgctgttccgccgctgtgctgctgactacgcgtcgcgtctgcatagcgtgctgggtacggcaaataccccactgagcacttacgaggcaatcaagggcgtcgacggacttgacgccatggaaccagacaccgcgcctggtctcccctgggctctccaggggaaacgccgtggtgcgctcatcgacttcgagaacggcactgtcggacccgaggttgaagctgccttgaagctcatggagaaaagagagtacaagtttacatgccagaccttcctgaaggacgagattcgcccgatggagaaggtacgtgccggcaagactcgcattgtcgacgtcctgcccgttgaacacattctttacactaggatgatgattggcagattttgtgctcaaatgcactcaaacaacggaccgcaaattggctcggcggttggttgtaatcctgatgttgattggcaaagatttggcacgcattttgctcagtatagaaacgtgtgggatgtggactattcggcctttgatgccaaccactgcagtgacgcaatgaacatcatgtttgaggaggtgtttaacacggacttcggtttccacccaaacgctgagtggatcctgaaaactctcgtgaacactgaacacgcctatgagaacaaacgcatcactgttgaaggcgggatgccgtctggttgttccgcaacaagcatcatcaacacaattttgaacaacatctacgtgctctacgccttgcgtagacactatgagggggttgagctggactcttacaccatgatctcctacggagacgacatcgtggttgcaagtgattacgatctggactttgaggccctcaagcctcacttcaaatcccttggtcaaaccattaccccagctgacaaaagcgacaaaggttttgttcttggtcactccattaccgatgtcactttcctcaaaagacacttccacatggactatggaactgggttttacaaacctgtgatggcttcgaagaccctcgaggctatcctctcctttgcacgccgtgggaccatacaggagaagttgatctccgtggcaggactcgccgtccactctggacctgacgagtaccggcgtctctttgagcctttccagggcctctttgagattccaagctacagatcactttacctgcgttgggtgaacgccgtgtgcggtgacgcataa

>AY317098.1_O_CHA_2002

atgaatacgactgactgttttatcgctctgctacacattctcagagagatcaaagcactgtttctgtcgcgaacacaagggaagatggaattcacactctacaacggggaaaagaaagtcttttactccagacccaacaaccacgacaattgttggttgaacgccatcctccaactgttcaggtacgttgacgaacccttcctcgaatgggtctacaactcacctgaggacctcactcttgaggcgattaacaacctggaagaagtcactggtcttgagctacgcgaaggcggaccacccgccctcgtcgtctggaacaccaagcacctgctctacaccggaatcggcaccgcctcgcggcccagcgaggtgtgcatgatcgacggcacagacatgtgcttggccgacttccacgccggtatatttctgaagggacaggaccacgccgtcttcgcctgcgtcacctctgacgggtggtacgcaatcgacgacgaagatttttatccgtggacaccagacccggctgacgtcttggtttttgttccgtacgatcaagaaccactcaacgcagaatggaaggcaaaagtccagaagcgactcaggggcgccgggcaatccagcccgaccaccggatcacaaaaccaatctggcaacaccggtagtatcattaacaattactacatgcagcagtaccagaactctatggacacccaacttggcgacaacgccattagtggagggtccaacgagggctccacggacactacatctactcacaccaacaacacccagaacaacgattggttttcgaaactggccaacaccgctttcagcggcctcttcggcgctcttcttgccgacaaaaagacggaggaaaccaccctcctcgaagaccgcatcctcacaacccgtaacggacacacgacctcgacaacccagtcaagtgtcggggtgacgtacggatatgcaacggctgaggactttgtaagcgggcccaacacttctggtcttgagaccagagttgttcaggccgaacggttcttcaagacccacctgttcgactggggcaccaacgactcgtttgggcggtgttacttgttggagctaccaactgaccacaaaggtgtctacggcagcctgaccgactcatacgcatacatgaggaacggttgggacgttgaggtcaccgcagtggggaaccagttcaacggaggttgtttggtagtggcgatggtaccggagctctgccccatcaccaagagagagctgtaccaactcacacttttccctcaccagttcatcaacccacggacgaacatgacggcacacatcaccgtgccctatctcggtgtcaacaggtacgaccagtacaaggtacacaaaccctggactctcgtggtcatggttgtggctcctttgacggtcaacaacgagggcgccccgcaaatcaaggtgtatgccaacatcgcccccaccaacgttcacgtcgcgggtgagctcccttccaaagaggggatcttccctgtggcatgtagcgacggttacggtggcttggtaaccacggacccgaagacggcagaccccgtctacgggaaagtgttcaacccaccccgcaacctgctgccagggcggttcacaaacctccttgatgtggctgaggcgtgtcctacgttcctgcacttcgatggtgacgttccctacgtgatcacgaagacggattcagacagggtgctggcccagttcgacttgtccctcgcggcaaagcacatgtcgaacacctttctcgcgggtcttgcccagtactacgcacagtacagcggcaccatcaacctgcacttcatgttcacggggcccaccgatgcaaaggcacgctacatggttgcgtatgcccctcctggcatggaaccacctaaaacgcctgaggcggctgcacactgcatccacgctgagtgggacactgggctgaactcgaaattcacgttttcgatcccatacctttcggcggcagactacgcatacaccgcgtccgacgttgccgagactacaaacgtgcagggatgggtctgtctgttccagataacacacgggaaagccgacggcgacgccctggtagtactagccagtgccggcaaggactttgacttgcgcctgccggttgacgcccgaacccaaaccacctctgcgggtgagtctgcggaccccgtgactaccaccgtcgaaaactacggcggcgagacacaagtccagaggcgccaacacacggacgttgcgttcatattggacaggttcgtgaaagtcaaaccacaggagcaagttaacgtgttggacctgatgcagatccctgcccacaccttggtaggggcactcctgcggacggccacctattacttctctgacctggaactagctgtcaagcacgagggcgatctcacctgggttccaaacggtgcccccgaggcagcactgaacaacaccaccaacccaacagcctaccacaaggaaccgctcacacggctggcgctgccttatacggctccgcaccgcgtcttagctaccgtctacaacgggagcagcaagtacggtgacaccagcactaacaacgtgagaggcgaccttcaggtgttggctcagaaggcagaaagagctctgcccacctccttcaactacggtgccatcaaggcaactcgtgtgactgaactactctacaggatgaaaagagccgagacgtactgtcccaggccccttctcgccattcaaccgagtactgccagacacaagcagaagattgtggcacccgcaaaacagcttctgaacttcgacctcctcaagttggcgggagatgttgagtccaaccctgggcccttcttcttctccgacgtcagggcgaacttcacgaaactggtggacactgtcaaccagatgcaggaagacatgtcaacgaaacacggacccgactttaaccgtctggtgtctgcgttcgaggaattggccgctggggtcaaggccatcaggactggtctcgacgaggccaagccctggtacaagctcatcaaactcctaagccgcctgtcgtgcatggccgctgttgcagcacggtccaaggacccagtccttgtggccatcatgttggccgacaccggtctcgagattctggacagcacctttgtggtaaagaagatctccgactcgctctccagtctcttccacgtgccggcccctgccttcagtttcggagccccgatcctgttggccggtttggtcaaagtcgcctcgagtttcttccagtcaacgcccgaagacctcgagagagcagaaaaacagctcaaagcacgtgacatcaacgacatatttgccgttctaaagaacggtgagtggctggtcaaactgatcctggccatccgcgactggattaaggcatggatcgcctcagaagaaaagttcgtcaccatgacagacctagtgcctggtatccttgaaaaacaacgggatctcaacgaccccggtaagtacaaggaggccaaggaatggctggacaacgcgcgccaagcgtgtctgaagagcgggaacgtccacattgccaacctgtgcaaagtggttgctccagcgcccagcaagtcgaggcccgaaccagtggtcgtgtgtcttcgcggcaaatccggccaagggaagagtttcctcgcgaacgttctcgcacaggcaatctccacccacttcactggcaggaccgactcagtctggtactgtccgcctgaccctgaccacttcgacggttacaaccagcagaccgtcgtcgtgatggacgacctgggccagaaccccgacggcaaagactttaagtacttcgcccagatggtctccactacggggttcatcccgccaatggcctcgctcgaggataaaggtaaacccttcaacagcaaggtcataatagccacaaccaacctgtactcgggattcaccccaagaaccatggtgtgccccgatgcgctcaaccggaggttccactttgacatcgacgtgagcgccaaagacgggtacaaggttaataacaaactggacatagtcaaagcactcgaagacacccacaccaacccggtggcgatgttccagtatgactgcgcccttctcaacggaatggccgttgaaatgaagagaatgcaacaagacatgttcaagcctcaaccgcccgtccagaacgtctaccaactcgttcaggaggtgattgagcgggtggagctgcacgaaaaggtatcgagccacccgatattcaaacagatttcaatcccttcccagaagtctgtgctgtacttcctcattgagaagggccagcacgaagcagcaatcgagttcttcgagggtatggtccacgactccatcaaggaggagctccggcccctcatccaacagacctcgtttgtaaaacgcgccttcaagcgcctgaaggagaactttgagatcgttgctctgtgtttaaccctcttggcaaacatagtaattatgctccgccaagcgcgcaagagacgccagtcggtggatgactcaccggac------------------------------agcgacataactcttggcggcgcggaaaagaaccctctggagacgagtggcgctagcgctgtcggtttcagagagaggccacccaccgagcaagagacgtgcgaagacgcgaacgctgagcccgtcgtgttcgggagggaacaaccgcgagctgaaggaccctacgctgggccacttgagcgtcagaaacctcttaaagtgaaagccaggctgccacaacaggagggaccctacgccggcccaatggagagacagaaaccgctaaaggtgaaagcaaaagtccccgtcgtgaaggaaggaccttacgaggggccggtgaagaaacctgtcgctttgaaagtgaaagcaaagaacttgatagtcactgagagtggtgcgccgccgaccgacttgcaaaagatggtcatgggcaacactaagccagtcgagcttatcctcgacggtaagacggtggccatctgttgtgccaccggagtgttcggcactgcctacctcgtgcctcgtcacctcttcgcggagaagtatgacaagatcatgttggacggtagagccttaacagacagcgactacagagtgttcgagtttgagattaaagtaaaaggacaggacatgctctcagacgccgctctcatggtgttgcaccgtgggaaccgcgtgcgcgatatcacgaagcacttccgtgatgtggcgagaatgaagaagggtacccccgtcgtcggtgtgatcaataatgccgacgtagggagactcatattctctggtgaagcccttacttataaggacattgtcgtgtgtatggacggagacaccatgcctgggctcttcgcctacagagcatccaccaaggcaggctactgtggaggagccgtcctagcaaaggacggtgccgagacattcatcgttggcactcactccgcaggtggtaacggtataggatactgttcatgcgtctcccgatcgatgctcatgaagatgaaggcacacatcgaccctgaaccacaccacgaggggttgatcgttgacaccagagatgtggaggaacgcgtccacgtgatgcgcaaaaccaagctcgcgcccaccgtagcacacggtgtgttcaatcctgagttcgggcctgctgctctgtccaacaaggacccgcgtctgaatgaaggggtcgtcctcgacgacgtcattttctcaaaacacaagggagacacgaggatgtctgaggaagacaaagcgctgttccggcgctgtgctgccgactacgcgtcgcgtctacacagcgtgctagggacggcaaacgccccactgagtgtatacgaagccatcaaaggcgtcgatggacttgacgccatggagccagacaccgcacccggtctccccggggctctccaaggaaaacgccgaggtgccctgatcgacttcgaaaacggtactgtcgggcccgaggttgaagcagcactcaagctcatggaaagccgtgagtataaattcgtctgccaaacctttctgaaagacgaaattcggccgctagagaaggtgcgcgccggtaagacacacattgtcgacgttttgcctgttgaacacattctctataccagaatgatgattggtagattctgtgctcagatgcactcaaacaacggaccgcaaattggctcagcggtcggttgcaaccctgatgttgattggcaaagatttggcacacatttcgcccagtacaagaacgtgtgggatgtggactactcagccttcgatgcaaaccactgcagcgatgcgatgaacatcatcttcgaagaggtgttccgcacggagtttgggttccacccgaacgccgagtggattctgaagactctggtgaacacggagcacgcttacgagaacaagcgcatcactgtggagggtggaatgccgtccggttgttccgcaacaagcatcatcaacacaattttgaacaacatctacgtgctctacgctctgcgtaggcactatgaaggagttgagctggacacctacacaatgatctcctatggagacgacatcgtggtggctagtgactacgacctggacttcgaggctctcaagccccacttcaagtccctcggtcagaccatcactccagccgacaaaagcgacaaaggttttgttcttggtcactccataaccgatgtcactttcctcaaaagacacttccacatggactacggaactgggttttacaaacctgtgatggcctcgaagaccctcgaggctatcctctcctttgcacgccgtgggaccatacaggagaagttgatctccgtggcaggactcgccgtccactccggacctgacgagtaccggcgtctctttgaacctttccaaggtctcttcgagattccaagctacagatcactttacctgcgatgggtgaacgccgtgtgcggtgacgcataa

>AY593751.1_A_NET_1942

atgaacacaactaattgttttatcgctttggtataccttatcagagagattaagacacttttccgttcaagaactacaggaaagatggaattcacactgcataacggtgaaaagaaaactttctactccaggcccaataaccacgacaactgctggttgaacaccatcctccagttgtttaggtacgtcgatgaacctttcttcgactgggtctataactcgcccgagaatcttacgcttgatgctatcaagcagttggaaaacttcaccgggcttgagttgcacgagggcggaccacctgcccttgtgatctggaacatcaaacacttgctccaaaccggtatcggtaccgcctcgcgacccagcgaggtgtgtatggtagacggcacggacatgtgtcttgctgatttccacgcaggcattttcatgaaaggacaggaacacgcagtgttcgcgtgtgtcacctcagacgggtggtacgcgattgacgacgaggacttttacccgtggacgcctgacccatcggacgtcttggtatttgtcccgtacgatcaagaaccactaaatggggactggaaaacactggttcagaggaagctcaagggtgctgggcagtccagcccagcaaccggctcgcagaaccagtctggcaacactggcagcataattaacaactactacatgcagcaataccagaactccatggacacacagcttggtgacaacgccatcagtggaggctccaacgagggctccacggacacaacttcaacacacacaaccaacacccaaaacaacgactggttttcaaaacttgccagttcggcttttaccggtctgttcggtgcacttctcgccgacaagaagacggaagagactacgcttctggaagaccgtatcctcactacccgcaacgggcacaccacttcgaccacccagtcgagtgtgggagtcacgtacgggtactccactgaggaagatcacgttgctgggcccaacacatcgggcttggagacgcgggtggtgcaggcagagagatttttcaagaagtttctgtttgactggacaacggacaaaccttttggacacttgacaaaactggagctccccaccgaccaccacggtgtcttcgggcacctggtggactcatacgcatatatgaggaacggctgggatgttgaggtatctgccgtcggcaaccagttcaacggcgggtgccttctggtggccatggtgccagagtggaaggaatttgacacacgtgaaaaataccagcttactcttttcccacaccagtttattagccccagaactaacatgactgcccacatcacggtaccgtatcttggtgtgaacaggtacgatcagtacaagaaacacaaaccctggacactggttgtcatggtattatcacccctcacggtcagcaacactgccgcaacacaaatcaaggtctacgccaacattgccccaacctacgttcacgtggctggagagctaccctcgaaagaggggattttcccagttgcatgcgcagacggttatggaggactggtgacaacagacccgaaaacagctgaccctgtttacggtaaggtgtataacccgcccaggaccaactaccccgggcgctttacaaacctattggacgtggccgaagcatgtcccaccttcctttgtttcgacgatgggaaaccgtacgtcgttacgcggacagacgacacccgtcttttggccaagtttgatgtctcccttgccgcaaaacacatgtccaacacatacctatcagggattgcacagtactacacacagtactctggtactatcaacctgcacttcatgttcacaggctccactgactcaaaagcccgctacatggtggcttacatcccgcctggggtggagccgccggacacacctgaagaagctgctcactgcattcatgctgagtgggacacaggactgaattccaaattcaccttttcaatcccttacgtgtctgccgcggattacgcgtacaccgcgtctgatacggcagagacaaccaatgtacagggatgggtctgtgtttaccaaattacacacgggaaggctgaaaatgacaccttggtagtgtcggctagcgccggcaaagactttgagttgcgcctcccaattgacccccggccacaaaccactgctactggggagtccgcagaccctgtcaccaccaccgtggagaactacggcggtgagacacaagtccagagacgccaccacacggacgtcggcttcatcatggaccgatttgtgaagataaacagcctgagccccacacatgtcattgacctcatgcacacccacaaacacgggatcgtgggtgcgttacttcgtgcagccacgtactacttctccgacttggagattgttgtgcggcacgatggtaatctgacctgggtgcccaacggtgcccccgaggcagccctgtcaaacaccagcaaccccactgcctacaacaaggcaccgttcacgagacttgctctcccttacactgcgccacaccgcgtgttggcaactgtgtacaacgggacaagcaagtactccgcgagcggttcg------agacgaggcgatctggggtccctcgcgacgcgagtcgcgacacaacttcctgcttcctttaactacggtgcaatcaaggcacaggccatccacgagcttctcgtgcgcatgaaacgggccgagctctactgtcccaggccacttctagcaatagaggcttcgcaagacaggtacaagcaaaagattattgcgcccgcaaaacagctcttgaactttgacctacttaagttggcgggtgacgttgagtccaaccctggacccttcttcttcgctgacgttaggtcaaacttttcgaagctggtagacaccatcaatcagatgcaggaggacatgtccacaaaacacggacctgactttaaccggttggtgtccgcttttgaggaattggccactggggttaaagctatcagaaccggtctcgatgaggccaaaccctggtacaagctcatcaagctcctaagccgcctgtcgtgcatggccgctgtggcagcacggtccaaggacccagtccttgtggccatcatgctggccgacaccggtctcgagattctggacagcactttcgtcgtgaagaaaatctccgactcgctctccagtctctttcacgtgccggcccccgccttcagtttcggagccccgattctgttggccgggttggtcaaggtcgcctcgagtttcttccggtccacacccgaagaccttgagagagcagagaaacagctcaaagcacgtgacattaacgacatcttcgccattctcaagaacggcgagtggctggtcaaactgatccttgccatccgcgactggattaaggcatggattgcctcagaagagaagttcgtcaccatgacggacttggtgcctggcatcctcgaaaagcagcgggaccttaacgacccgggcaagtacaaggaagccaaggagtggctcgacaacgcgcgccaggcgtgtttgaagagcgggaacgtccacattgccaacctgtgcaaagtggtcgccccagcacccagcaagtcgagacccgaacctgtggtcgtttgcctccgtggcaaatctggccagggtaagagtttccttgcgaacgtgctcgcacaagcaatttccacccactttactggcagaaccgactcggtttggtactgcccgcctgaccctgaccacttcgacggttacaaccaacagaccgtcgttgtgatggacgatttgggccagaaccctgatggcaaggacttcaagtactttgcccaaatggtttcgactacggggttcatcccgcccatggcatcacttgaggacaaaggtaaacctttcaacagtaaggtcatcattgcgaccaccaacttgtactcgggctttaccccgagaactatggtgtgccccgatgcactgaaccgaaggttccactttgacatcgacgtgagcgccaaggacgggtacaaaattaataacaaattggacatcatcaaagcacttgaagacacccacaccaacccagttgccatgttccagtacgattgtgcccttctcaacggtatggccgttgaaatgaagagattgcaacaagatatgtttaagcctcaaccacccctccaaaacgtgtaccaactcgttcaggaggtgattgaacgggtcgagctccatgagaaagtgtcgagccacccaattttcaagcagatctcgattccttcccaaaagtccgtgttgtacttcctcattgagaaaggccaacacgaggcagcaattgaattctttgagggcatggtacacgactccgtgaaggaggagctccggcctctcatccagcaaacctcatttgtgaaacgcgctttcaagcgcctgaaggagaactttgagattgttgccctatgtttgacccttctggctaacatagtgatcatgatccgcgaaacccgcaagaggcagaagatggtggatgatgcggtgaacgattacatcgagagggcaaacatcaccacggacgacaagactcttgacgaagcggagaagaaccctctggaaaccagcggtgccagtaccgttggattcagagagagaagcctcacaggccaaaaggcgcgcgatgacgtgaactccgagcccgcccagcctgctgaggatcaaccacaagctgaaggaccctacgccgggccactcgagcgtcagaaacctctgaaagtgagagccaagctcccacagcaggagggaccctacgctggcccgatggagagacagaaaccgctgagagtaaaagcaaaagccccggtcgttaaggaaggaccttacgagggaccggtgaagaagcctgtcgctttgaaagtgaaagctaggaacttgattgtcactgagagtggtgccccaccgaccgacttgcagaagctggtcatgggcaacacaaagcctgttgagcttatcctcgacgggaagacagtagccatctgctgtgctactggagtgtttggcactgcttacctcgtgcctcgtcatcttttcgcagagaagtatgacaagatcatgttggaaggcagagccatgacagacagtgattacagagtgtttgagtttgagattaaagtaaaaggacaggacatgctctcagacgcggcactcatggtgcttcaccgtgggaaccgcgtgagagacatcacgaaacactttcgtgatacagcaagaatgaagaaaggcacccccgtcgtcggtgttgtcaacaacgccgatgttgggagactgattttctctggtgaggcccttacctacaaggacattgtagtgtgcatggatggagacaccatgcccggcctctttgcctacaaagccgccaccaaggctggctactgtggaggagccgttcttgccaaggacggggctgacacatttatcgtcggcactcactctgcaggtggcaatggagttggatactgctcatgcgtttccaggtccatgcttcaaaagatgaaggctcacgtcgaccctgaaccacaccacgaggggttgattgttgataccagagatgtggaagagcgcgtccacgtgatgcgcaaaacaaagcttgcacccaccgttgcacacggcgtgttcaaccctgagtttgggcctgccgccttgtcaaacaaggacccgcgcctgaacgagggagttgttctcgatgaagtcattttctccaaacacaaaggagacgtaaagatgaccgaagaggacaaagcgctgttccgccgctgcgccgctgactacgcgtcacgcctgcacagcgtgctgggtacggcaaatgccccattgagcatctacgaggcaatcaagggcgttgatggactcgacgccatggagccggacactgcacctggcctcccctgggccctccagggaaaacgccgcggtgcgctcatcgacttcgagaacggcacggtcggacccgaagttgaggctgccttgaagctcatggagaaaagagaatacaagtttgcttgtcagaccttcctgaaggacgagattcgcccgatggagaaagtacgcgccggcaagactcgcatcgtcgatgttttgcctgttgaacacattctttacaccaggatgatgattggcaggttctgtgcacaaatgcactcgaacaacggaccacaaattggctctgcggtcggttgcaaccctgacgttgattggcaaagatttggcacacatttcgcccaatacagaaacgtgtgggatgtggattactcggcctttgatgcaaaccactgcagtgacgctatgaacatcatgtttgaggaggtgttccgcacagactttggcttccacccaaacgctgaatggatcctgaagactctcgtgaacacggaacacgcctatgagaacaagcgcatcactgttgaaggcgggatgccatctggttgttccgcaacaagcatcatcaacacaattttgaacaacatctacgtgctctacgccttgcgtagacactatgagggagttgagctggacacttacaccatgatctcctacggagacgacatcgtggtggcaagtgattacgatctggactttgaggctctcaagccccacttcaaatctcttggccaaaccatcactccagctgacaaaagcgacaaaggttttgttcttggtcactccattaccgatgtcactttcctcaaaagacacttccacatggattatggaactgggttttacaaacctgtgatggcctcaaagacccttgaggctatcctctcctttgcacgccgtgggaccatacaggagaagttgatctccgtggcaggactcgccgtccactctggaccagacgagtaccggcgtctctttgagcccttccagggcctctttgagatcccaagctacagatcactttacctgcgttgggtgaacgccgtgtgcggtgacgcataa

>AY593753.1_A_Brazil_1970

atgagcacaactgactgtcttatcgctttggtacacgttatcagagagatcagggcactttttctaccacgaaccacaggaaagatggaattcacactgtacaacggtgagagaaaagtgttttactccagacccaacagccacgacaactgttggttgaacaccatccttcagttgttcaggtacgttgatgaacctttcttcgactgggtctacaactcgcccgagaacctcacacttgaagccattgagcagttagaggaactcacagggctggagttacgcgagggcggaccacctgccctcgtagtctggaacatcaagcacctgctccaaactggcatcggtaccgcctcgcggcccagcgaggtgtgtatggtggacggtacggacatgtgtcttgctgacttccacgcaggcattttcatgaaaggacaggaacacgctgtgtttgcgtgtgtcacctccaatgggtggtacgcgattgacgacgaggacttctacccatggacaccggacccgtccgatgtcttggtgtttgtcccgtacgatcaagaaccactcaacggagaatggaaaactaaagttcagcagaagcttaagggagccgggcaatccagcccggtgaccggctcacagaaccaatctggcaacactggcagcataattaacaactattatatgcagcagtaccaaaactccatggacacacaacttggcgacaacgccatcagcgggggctccaacgagggctccacggacacaacttccacccacacaaccaacacacagaacaatgactggttttccaagcttgccagctctgccttcagtggtcttttcggcgcccttctcgccgataagaaaacggaagaaacaacacttctggaggaccgcatcctcaccacccgcaacgggcacaccacctcgaccacccagtcgagtgtgggtgtcacgtacgggtactccaccgcggaggaccacgttgctgggcccaacacatcgggtttggagacgcgggtgatacaggcagagagatacttcaagaagtttctgtttgagtggacaccggaaaagccttttggatacttggaaaaactggagcttcccactgtccaccacggcgttttcggacacctggtagactcgtatgcttatatgagaaatggctgggatgttgaggtgtctgctgttggcaaccagttcaacggcggatgtctcctggtggctatggtaccagagtggaaagaatttgacgcacgggagaaataccagcttacccttttcccacatcagttcatcagccccagaaccaacatgactgcccacatcacggtcccgtaccttggtgtgaacaggtatgatcagtacaaaaaccacaagccctggacactggttgtcatggttgtgtcgccacttacggttaacgccacgagtgcaacacaaatcaaggtctacgccaacattgctccaacttacgttcacgtggctggagaactcccctcgaaagaggggattttcccggtcgcatgtgcggacggttacggaggactggtgacgacagatccgaagacagctgaccccgcgtacggcaaggtgtacaacccgcccagaaccaactatcctgggcgcttcacaaacctattggacgtggccgaggcgtgtcccaccttcctctgcttcgacggcgggagaccgtacgtcgttacgcaggcgggtagcaaccgcctcctggccaaatttgatgtttcccttgccgcaaagcatatgtctaacacatacttgtcagggcttgcacagtacttcacacagtactctggcaccatcaacctgcacttcatgttcacaggctccactgactcaaaagcccgctacatggtggcctacgtaccgcctggggtggagccaccggaaacacccgagaaggccgcccactgcattcacgctgaatgggacacaggactgaactccaaattcacattctcaatcccgtacgtgtccgctgcagactatgcgtacactgcgtctgacacggcagaaacaaccaacgtacagggatgggtctgcatctaccaaattacacacgggaaggccgaagacgacactctggttgtgtcggtcagtgccggcaaggatttcgagctacgcctcccgattgacccccgttcacagaccacttctaccggggagtcagcagaccctgttaccaccaccgtagagaactacggcggtgagacacaagtccagaggcgtcaccacacggacgtcggtttcatcatggacagatttgtgaagataaacagtccaaaaccaacacatgtcattgacctcatgcagacccaccaacacggcttggtgggtgcgttgctgcgtgcagccacgtactacttctccgacctggagattgttgtgcagcacgacggcaacctgacctgggtgcccaacggtgcccctgaggcggccctactgaacaccagcaaccccaccgcctacaacaaggcaccgttcacgaggcttgctctcccctacactgcgccgcaccgcgtgctggcaactgtgtacaacgggacggacaagtaccccgtgagcgcttcg---ggaggacgaggtgattcggggcctcttgcggcgcgagccgcgaaacagctccctacttctttcaactacggtgcaatcaaggccactaccatccgcgagcttctcgtgcgtatgaaacgggccgagctctactgtcccaggccactcctggcgatagaggtttcccaaggcagatacaagcaaaagatcattgcacctgaaaaacagcttttgaactttgacctactcaagttggcgggcgacgttgagtccaaccctgggcccttcttcttctccgacgttaggtcaaacttttccaagctggtggacacaatcaaccagatgcaagaggacatgtccacaaaacacggacctgactttaaccggttggtgtccgcttttgaggagttggccactggagtgaaagccatcaggaccggtctcgatgaggccaaaccctggtacaagctcatcaagctcctgagccgcctgtcgtgcatggccgctgtagcagcacggtcaaaggacccagtccttgtggccatcatgctggctgacaccggtctcgagattctggacagcaccttcgtcgtgaagaagatttccgactctctctccagtctctttcacgtgccggcccccgccttcagtttcggagccccgattctgttagccgggctggtcaaggtcgcctcgagtttcttccggtccacacccgaagaccttgagagagcagagaaacagctcaaagcacgtgacatcaacgacattttcgccattctcaagaacggcgagtggctggtcaagctgatccttgccatccgcgactgggtcaaggcatggattgcctcagaagaaaagtttgtcaccatgacagacttggtgcctggcatccttgaaaaacaacgggatctcaacgacccaagcaagtacaaggaagccaaggagtggctcgacaacgcgcgccaaacgtgtttgaagaatgggaacattcacattgccaacctgtgcaaagtggtcgctccggcacccagcaagtcgcgacccgaacccgtggtcgtttgcctccgcggcaaatctggccagggcaagagtttccttgcaaacgtgctcgcacaagcaatctctacccacttcaccggcaggactgattcagtttggtactgcccgcctgaccctgaccacttcgacggttataaccaacagactgtcgtcgtgatggacgacctgggccagaaccccgacggcaaggacttcaagtacttcgcccagatggtttcaaccacggggttcatcccgcctatggcatcgcttgaggacaaaggcaaacctttcaacagtaaggtcatcattgcaaccaccaacttgtactcgggcttcaccccgaggaccatggtgtgtcctgacgccctgaaccggaggtttcactttgacatcgacgtgagcgccaaagacgggtacaaaattaacaacaaattggacatcatcaaagcacttgaagacacccacaccaatcccgtggcaatgtttcagtacgattgtgcccttctcaacggcatggctgtagaaatgaagagaatgcaacaagacgtgttcaaacctcagccacccctccagaacgtgtaccaacttgttcaggaggtgattgagcgggtggagctccacgagaaagtgtcgagccacccgattttcaagcagatctcaattccttcccaaaaatccgtgttgtacttcctcattgaaaaagggcagcacgaggcagcaattgaattctttgagggcatggtccacgactccatcaaggaggagctccggccccttatccagcgaacttcatttgtgaaacgcgctttcaagcgcttgaaggaaaactttgagattgttgccctatgtttgaccctcctggccaacatagtgatcatgatccgcgagactcgcaagagacagaagatggtggatgatgcggtgagtgagtacattgagaaagcaaacatcaccaccgacgacaagactcttgacgaggcggaaaagaaccctctggaaaccagcggtgccagcaccgtcggcttcagagagagaactcttccaggccagaaggcgcgtggtgaagagagctctgagcccgcccagcctattgaagagcaaccgcgagctgaaggaccctacgccgggccgcttgagcgtcagaaacctctgaaagtgagagccaagctcccacagcaagaggggccctacgctggcccgatggagagacagaaaccgcttaaggtgaaagcaaaagccccggtcattaaggaaggaccttacgagggaccggtgaagaagcctgtcgctttgaaagtgaaagctaagaacttgattgtcactgagagtggtgccccaccgactgacttgcaaaagatggtcatgggcaacactaagcctgttgagctcattctcgacgggaagacagtagccatctgctgtgctactggagtgtttggcaccgcctacctcgtgcctcgtcatcttttcgctgagaagtatgacaagattatggtggacggcagggccatgacagacagtgactacagagtgtttgagtttgagattaaagtaaaaggacaggacatgctctcagacgctgcgctcatggtgctccaccgtgggaaccgcgtgagagacatcacgaaacactttcgtgacacagcaagaatgaagaaaggcacccccgtcgttggcgtgatcaacaacgctgatgtcgggagactgattttctctggtgaagcccttacctacaaggacattgtagtgtgcatggatggtgacaccatgcctgggctctttgcctacaaagccgcaaccaaggctgggtattgcggaggagctgtccttgctaaggacggggccgacacgttcatcgtcggcactcactctgctggaggcaatggtgttggatactgctcttgcgtttccagatccatgctcctcaggatgaaggcccacattgaccctgaaccgcaccacgaggggttgattgttgacaccagagatgtggaagagcgcgtccacgtgatgcgcaaaaccaagcttgcacccaccgtcgcacacggtgtgtttaaccctgagttcgggcccgccgccttgtccaacaaggacccgcgcctgaacgatggtgttgtcctcgacgaaaccatcttctccaaacacaaaggagatacaaagatgtctgaagaggataaagcgctgttccgccgctgcgccgctgactacgcgtcacgcttgcacagtgtgttgggcacagcaaatgccccactgagcatcttcgaggcaatcaaaggtgttgatgggctcgacgcaatggagccagacactgcacccggcctcccttgggcactccaggggaagcgccgtggagcgcttatcgactttgagaacggcactgtcggacccgaagttgaggctgccttgaagctcatggagaaaagagagtacaagtttgtttgccaaaccttcctgaaggacgagattcgcccgatggagaaagtacgtgccggtaagactcgcattgtcgacgtcctgcctgttgaacacattctctacactaggatgatgattggcagattctgtgcacaaatgcactcaaacaacggaccccaaattggctcggcggtcggttgtaaccctgatgttgattggcaaagatttggcacacacttcgcccaatacagaaacgtgtgggatgtggactattcggcctttgatgctaaccattgcagcgatgccatgaacatcatgtttgaggaggtgttccgcacggagttcggcttccacccaaacgcggagtggattctgaagactctcgtgaacacggaacacgcctatgagaacaagcgcatcactgttgagggcgggatgccatctggctgttccgcaacaagcatcatcaacacaattctgaacaacatctacgtgctctacgctttgcgcagacactatgagggagttgagctggacacttacaccatgatctcctacggagacgacatcgtggtagcaagtgattacgatttggactttgaggctctcaagccccactttaaatcccttggtcaaaccatcactccagctgacaaaagcgacaaaggttttgttcttggtcactccattactgatgtcactttcctcaaaagacacttccacatggattacggaactgggttttacaaacctgtgatggcctcaaagacccttgaggctatcctctcctttgcacgccgtgggaccatacaggagaagttgatctccgtggcaggactcgctgtccactctggaccagacgagtaccggcgtctctttgagcccttccagggcctctttgagatcccaagctacagatcactttacctgcgttgggtgaacgccgtgtgcggtgacgcataa

>AY593754.1_A_SPA_1959

atgaatacaactgactgttttatcgctttggtgcacgctatcagagagatcagagcactttttctaccacgaaccacaggaaagatggaactcaccctgtacaacggcgagaaaaagactttttactcccgacccaacaaccacgacaactgttggttgaacaccatccttcagttgttcaggtatgtcgatgaacccttcttcgactgggtctacaattcgcccgagaacctcacgcttgaagccatcaaccaattggaggaactcacaggacttgagttgcacgagggcggaccacctgcccttgtgatctggaacatcaaacacttgctccacaccggcatcggcactgcctcacgacccagtgaggtgtgtatggtggacggcacggacatgtgtcttgctgacttccacgcaggcattttcctgaagggacaggaacacgcagtctttgcgtgtgtcacctccaacgggtggtacgcgattgacgacgaggaattttacccctggacgcctgacccgtcagacgtcctggtgtttgtcccgtacgatcaagaaccactcaacggggactggaaagcgatggttcagaggaagcttaagggtgccgggcaatccagcccggcgaccggctcccagaaccagtctggcaacactggcagcataattaacaactactatatgcaacagtaccagaactccatggacacacagcttggtgacaatgccattagtggaggctccaacgaaggctccacggacacaacttcaacacatacaaccaacacccaaaacaacgattggttttcaaaacttgccagttcagccttcaccggtctgttcggcgccttgctcgccgacaagaagacggaagagactacacttctggaagaccgcattctcaccacccgcaacgggcacactatctcgaccacccaatcgagcgtgggagtcacctacgggtactccactggagaagaccatgttgctgggcccaacacatcgggcctggagacgcgggtggtgcaggcagagagattttttaaaaagtttttgtttgactggacaacggacaaaccttttggacatttggaaaagctggaacttcccgccgaccaccacggcgttttcgggcacctggtggaatcgtatgcttacatgagaaatggttgggacgttgaggtgtctgctgttggcaaccagttcaacggcgggtgcctcctggtggctatggtaccggagtggaaagagtttgaacagcgcgagaaataccaactcaccctcttcccgcaccagttcatcagccccagaacaaacatgactgcccacatcacagtcccataccttggagtgaacaggtacgatcagtacaagaaacacaaaccttggacactggttgtcatggtagtgtcgcccctcacggttagcgacactgccgcgacacagattaaggtctacgccaatattgctccgacctacgttcacgtggctggggaactcccctcgaaagaggggattttcccagttgcatgctcggacggttacggaggactggtgacaacggacccgaaaacagctgaccccgcctacggtaaggtgtacaacccgcccaggaccaactaccctgggcggtttaccaacctgttggacgtggctgaagcgtgtcccactttcctctgtttcgacgacgggaaaccgtacgttgtcacgcggacagatgacacacgactattggccaagttcgacgtctcccttgctgcaaaacacatgtccaacacgtacctgtcaggggttgcacagtactacgcacagtactctggtaccatcaacttgcacttcatgttcacaggctcaactgactcaaaagcccgctacatggtggcctacatcccgcctggggtggaaccaccggacacacctgaaagggccgctcactgcatccacgctgaatgggacacaggactgaactccaaattcactttctcaatcccgtacgtgtccgccgcagattacgcgtataccgcgtctgacacggcagaaacaaccaacgtacagggctgggtttgcatataccagatcacacacgggaaggccgagaacgacacattggtggtgtcggccagcgccggcaaagactttgagttgcgcctcccgattgacccccgacagcaaactactgctgtcggggagtccgcagaccctgtcaccaccgccgtggagaactacggcggtgagacacaaacccggagacggcaccacacggatgtcggtttcatcatggacagatttgtgaagataaacagtttgagtcccacgcatgtcattgacctcatgcagacccaccagcacgggctggtgggtgcgctgctgcgtgcagccacgtactacttctctgacttggagattgttgtgcggcatgacggcaatttgacttgggtgcccaatggtgcccctgaagcagctttgtcaaacaccagcaaccccactgcctacaacaaggcaccgttcacgaggcttgctctcccttacactgcgccacaccgcgtgttggcaaccgtgtacaacgggacgaacaagtactccacaggcggtctg------agacgaggcgacacggggtcgctcgcggcgcgggccgcgaaacaacttcctgcctcttttaattacggtgcaattggggccgtcaccatccacgagcttctcgtgcgcatgaaacgggcagagctctactgccccaggccactactggcagtagaggctttacaagacaggcacaagcaaaagatcattgcacccgcaagacagttgctgaactttgacctacttaagttggctggagacgtggagtccaaccctgggcccttcttcttctctgacgttaggtcaaacttttctaagctggtggaaaccatcaaccagatgcaggaagacatgtcaacaaaacacgggcccgactttaaccggttggtgtccgcctttgaggaactggccgccggagtaaaagccatcaggaccggcctcgacgaggccaaaccctggtacaagcttatcaagctcctaagccgcctgtcgtgcatggccgctgtggcagcacggtccaaggacccggtccttgtggccatcatgctggccgacaccggtctcgagattctggacagcactttcgtcgtgaagaagatctccgactcgctctccagtctcttccacgtgccggcccccgtcttcagtttcggagccccgagtctgctagccgggttggtcaaggtcgcctcgagtttcttccggtccacgcccgaagaccttgagagagcagagaaacagctcaaagcacgtgacatcaacgacattttcgccattctcaagaacggcgagtggctggtcaaactgatccttgccatccgcgactggattaaggcgtggattgcctcagaagaaaagtttgtcactatgacagacttagtgcctggcatccttgaaaagcagcgggatctcaacgacccaagcaggtacaaggaggccaaggagtggctcgacaacgcgcgccaagcgtgtctgaagagcgggaacgtccacattgccaacctgtgcaaagtggtcgccccggcacccagcaagtcgagacccgaacccgtggtggtttgcctccgtggtaaatcaggccagggcaagagtttccttgcgaacgtgctcgcacaagcaatctctacccacttcaccgggcggactgactcagtctggtactgcccacctgaccctgaccacttcgacggttacaaccaacagactgttgttgtgatggacgatttgggccagaatcctgacggcaaggacttcaagtacttcgcccaaatggtctcgaccactgggttcatcccgcccatggcatcactcgaggacaaaggtaaacccttcaacagtaaggtcatcattgcaaccaccaacctgtactcgggcttcaccccgaggactatggtgtgccctgacgccctgaaccggaggtttcactttgacattgacgtgagcgccaaggatgagtacaaaattaacaacaaattggacattaccaaagcgcttgaagacacccacaccaacccagtagcaatgtttcagtacgactgcgcccttctcaacggcatggctgttgaaatgaagagactgcagcaagacatgttcaaacctcaaccacctctccagaacgtgtaccaactagttcaggaggtaattgaccgggtggcgctccacgagaaggtgtcaagccacccaatttttaaacagatctcaattccttcccaaaaatctgtgttgtacttcctcattgagaaagggcagcacgaggcagcaattgaattctttgagggcatggtgcatgactccgtcaaggaggagctccggccgctcatccaacaaacctcatttgtgaaacgcgcgtttaagcgcctgaaggaaaactttgagattgttgctctgtgcctaacacttttggccaacatcgtgatcatgatccgcgagacccgtaagagacagaagatggtggacgatgcggtgaacgagtacatcgagaaagcaaacatcaccaccgatgacaagactcttgacgaggcggaaaagaaccctctggagactagcggtgccagtaccgttggtttcagagagagaactcttccagatcagaaggcgcgtaatgacgtgaactccgagcccgcccaacctgctgaagaacaaccacaagctgaaggaccctacgccgggccacttgagcgtcagagacccctgaaggtgagaaccaagctcccacaacaggaaggaccctacgctggcccgatggagagacagaaaccgctgaaagtgaaagcaaaagccccggtcgtcaaggaaggaccttacgaaggaccggtgaagaaacctgtcgctttgaaagtgaaagctaagaatttgattgtcactgagagtggtgcccccccgaccgacctgcaaaagatggtcatgggcaacacaaagcccgttgagctcatcctcgacgggaagacagtagccatctgctgtgctactggagtgtttggtactgcctacctcgtgcctcgtcatcttttcgctgagaagtatgacaagatcatgttggacggcagagccatgacagacagtgactacagggtgtttgagttcgagatcagagtaaaaggacaggacatgctctcagacgccgcactcatggtgctccaccgtgggaaccgcgtgagagacatcacgaagcatttccgtgacacagcaagaatgaagaaaggcacccccattgtcggcgtgattaacaatgccgatgtcgggagactgattttctctggcgaagctcttacctacaaagacattgtagtgtgcatggacggagacaccatgcccgggctttttgcctacagagccgccactaaggctggctactgcgggggagccgttctcgctaaggacggggctgacacttttatcgttggcactcactctgcaggaggtaacggagttggatactgctcatgcgtttccaggtccatgcttctcaagatgaaggcacacattgaccctgagccgcaccacgaggggttgattgtagacaccagagatgtggaagagcgcgtccacgtgatgcgcaaaaccaagcttgcacccaccgttgcacacggtgtgttcaaccccgagtttgggccagctgccttgtccaacaaggacccgcgtctgaacgagggtgttgtccttgatgaagtcattttctccaaacacaagggagacacaaagatgtctgaggaggacaaagcgctgttccgccgatgtgctgctgactacgcgtcacgcctgcacagcgtgctgggtacggcaaatgccccattgagcatctacgaggcagtcaagggcgtcgacggactcgacgccatggagccagacaccgcacctggcctcccctgggctctccagggaaaacgccgtggtgcgctcatcgactttgagaacggcacggtcgggcccgaagtcgaggctgccttgaagctcatggagaaaagagagtacaagtttgtttgtcagaccttcctgaaggacgagattcgcccgatggagaaagtacgtgccggcaagactcgcattgtcgacgtcctgcccgttgaacacattctttacaccaggatgatgattggcagattttgtgcacaaatgcactcaaacaacggaccgcaaattggctcggcggtcggttgtaaccctgatgttgattggcaaagatttggcacacacttcgcccaatacagaaacgtgtgggatgtggactattcggcctttgatgctaaccactgcagtgacgccatgaacatcatgtttgaggaggtgttccgcacggacttcgggttccacccaaatgctgagtggatcttgaagactctcgtgcacacggaacatgcctatgagaacaaacgcatcactgttgaaggcgggatgccatctggttgttccgcaacgagcatcatcaacacaattttgaacaacatctacgtgctctacgccttgcgtagacactatgagggagttgagctggacacttacaccatgatctcctacggagacgacattgtggtggcaagtgattatgatctggactttgaggctctcaagcctcacttcaaatctcttggtcaaaccattactccagctgacaaaagcgacaaaggttttgttcttggtcactccatcaccgatgtcactttcctcaaaagacacttccacatggattatggaaccgggttttacaaacctgtgatggcctcaaagacccttgaggctatcctctcctttgcacgccgtgggaccatacaggagaagctgatctccgtggcaggactcgctgtccactctggaccagacgagtaccggcgtctctttgagccctttcagggcctctttgagattccaagctacagatcactttacctgcgttgggtgaacgccgtgtgtggcgacgcataa

>AY593755.1_A_TAI_1960

atggacacaactgactgctttgttgctttgctgcacgctctcagacagattaaggcactcttcctttcacagacacaagagaaaatggaattcacactctacaacggtgagaagaagaccttctactccaggcccaacaaccacgacaactgctggttgaacaccattttgcagctgtttaggtatgttgatgaaccgttctttgactgggtctacgactcgcccgagaatctcacgctcgaggcgatcagacagttggaggacatcactggtcttgacctgcacgacggcggaccacccgccctcgtcatttggaacatcaagcacctgctccacaccggcattggcactgcctcgcgacccagcgaggtgtgcatggtggacggtacggacatgtgcctggctgacttccacgctggcatcttcttgaagggacaggaacacgccgtgtttgcctgtgtcacttccaatgggtggtacgcgatcgacgacgaggacttctacccctggacaccggatccgtctgacgtcctagtttttgtcccgtacgatcaagaaccgctcaacggagaatggaaatcaaaggtccagaagcgactcaagggagccgggcaatccagcccggctaccggctcgcagaatcagtctggtaacacaggaagcatcattaacaactactacatgcagcagtaccagaactcaatggacacacaacttggtgacaatgccatcagtggagggtctaacgagggctccacggacacaacctccacacacacaaccaacactcagaacaacgactggttttcaaagcttgcaagttctgccttcaccggtcttttcggcgcgttgcttgccgacaagaagacggaggagaccactcttctggaagaccgaattctcaccacccgcaacggacacaccacctccacgacacagtcgagtgtgggagtcacgtacggatactccaccaaggaagatcacgtgtccgggcctaacacatctggcctggagacgcgggtggtgcaggcagagagattcttcaagaaacacttgtttgactggacaacagacaagccttttggacatttggaaaaactggaacttcccaccgaccacaaaggtgtctacgggcacctggttgactcatatgcatatatgagaaacggctgggacgtggaggtgtctgctgttggcaaccagttcaatggcgggtgtctcctggtggccatggtccctgagtggaaggaactcaccctgcgtgagaagtaccaactcacccttttcccgcaccagttcatcaaccccagaaccaacatgaccgcacacatcacggttccgtaccttggtgtgaaccggtacgaccagtacaagaagcacaaaccgtggaccctggtcgtgatggtggtgtcacctctcaccaccagcaccgttggtgcggaacaaatcaaggtctacgccaacatcgccccgacccacgttcacgtagccggcgaactcccttcaaaggagggaattgtaccggttgcttgttcagacggttacggtggtttggtgacaacagaccccaagacagctgaccctgtctacggcaaggtgtacaacccgcccaggaccaactaccccgggcgttacacaaacctgttggacgtagcagaggcctgcccaacctttctttgtttcgacgacgggaaaccgtatgttgtgacgaggacggacgggcagcgtctcctggctaagtttgacctttctcttgccgcaaaacacatgtccaacacctaccttgcaggtttagcacagtactacacacagtattctggcaccattaatctgcacttcaccttcactggttcaactgactcaaaagcccgctacatggtggcctacgttccgcctggcgtggaaccaccggacacacctgagaaggccgcacactgtatccatgctgagtgggacacaggactgaactccaaatttactttttcagtcccgtacatgtctgccgctgactacgcatacactgcgtcagatgaggcagagacaacaaatgtacagggatgggtctgcatttaccagattacacacgggaaagctgagggcgacactctggtcgtgtcggctagcgccggcaaagacttcgagttacgtctcccggttgatccccgcacacagaccaccaccactggggagtctgcagacccagtcaccaccactgttgagaactacggtggagagacacaagtccaaagacgacaacacacagatgtcggcttcataatggacagatttgtaaagataacaaacctgagtcccacacatgtcattgacctcatgcaaacccaccaacacggactggtgggtgccctgctgcgtgcggccacgtactacttctccgacctggagattgtcgtgaagcacgatggcaacctgacctgggtgcccaacggcgcgccagaagcagccttgggcaacacgagcaaccccaccgcctacaacaaggcgccatttacaagacttgccctcccttacaccgcgccacaccgcgtgctggcaacagtgtacaacgggacgaacaagtactctgcaagtggctcg---gccagacgaggtgacctggggtctctcgcggcgagagatgccgcgcaactccccgcctctttcaactttggtgcaattcgggccacaaccatccatgaacttcttgtgcgcatgaagcgtgccgagctttactgccccagaccactgttggcggtggaagcgtcgcaggacagacacaagcaaaggatcattgcaccagcaaagcaacttctgaatttcgacctgctcaagttggcaggagacgttgagtccaaccctgggcctttcttcttctccgacatcagggcgaacttttccaagctggtggacaccatcaaccagatgcaggaggacatgtcaacaaagcacggacccgactttaaccggttggtgtccgcatttgaggaattggccactggagtgaaggctatcaggacaggcctcgacgaggccaagccctggtacaagctcatcaaactcctgagccgcctgtcgtgcatggccgctgtggcagcacggtcaaaggatcccgtccttgtggccatcatgctagcagacaccggtctcgagattctggacagcacgtttgtcgtgaagaagatctccgactcgctctccagtctctttcacgtgccggcccccgccttcagtttcggagctccgattctgctggctgggttggtcaaagtcgcctcggggttcttccggtcaacgcccgaagaccttgagagagcagagaaacagctcaaagcacgtgacatcaacgacatcttcgccatcctcaagaatggcgagtggttggtcaagttgatccttgccatccgcgactggataaaggcatggatcgcctcagaagagaagtttgtcaccatgacagacctagtgcctggcatccttgaaaagcagcgggatctcaacgacccgagcaagtacaaggaggccaaggaatggctcgacaacacgcgccaggcgtgcttgaagagcgggaacacctgcattgccaatttatgcaaggtggtcgctccagcacccagcaagtcgagacccgaacctgtagtcgtttgcctccgtggcaagtccggccaaggtaagagtttccttgcgaacgtgctcgcgcaagcaatctccacccacttcaccggcagaactgattcagtttggtactgcccacccgaccctgaccacttcgacggttacaaccagcagaccgttgttgtgatggatgatttggggcagaaccccgacggcaaggactttaagtacttcgcccaaatggtctcaaccacggggttcatcccgcccatggcatcacttgaagacaaaggcaaacctttcaacagcaaagtcatcattgcaaccaccaatctctactcgggcttcaccccgaggaccatggtgtgccctgacgcgttgaaccggaggtttcactttgacatcgacgtgagcgccaaggacggttacacaattaacaacaaattggacatcatcaaagcacttgaagacacccacaccaacccagtggcaatgtttcaatatgactgtgcccttctcaacggcatggccgttgaaatgaaaagactccagcaagatgtgtttaagcctcaagcacccctccagaacgtgtaccagctcgtacaggaggtgattgaccgggttgaactccacgaaaaagtgttgagtcacccgatcttcaagcaaatctcgattccttcccaaaaatctgtgctttacttcctcattgagaaaggccaacacgaagcagcaattgaattctttgaggggatggtttgcgactctgtcaaggaggagctccggcccctcatccaacagacctcatttgtgaaacgcgctttcaagcgcctgaaggaaaactttgagattgttgccctgtgtttgacgcttctggcaaacatagtgatcatgatccgcgagactcgcaagagacaaaagatggtggatgatgcagtgaatgactacattgagagagcgaacatcaccacggatgacaagactcttgacgaggcggaaaagaaccctcaggggacgagcggtgccagcaccgttggtttcagagagagaaccctcccggggcaaaaggcgtgtgatgacgtgaacactgagcctgccaagcccgtgggggaacaaccacaagctgaaggaccctacgccggaccactcgagcgtcagaaacctcttaaagtgaaagccaagctgccacaacaagaggggccctacgctggtccgatggagagacaaaaaccactgaaagtgaaagcaaaagccccggtcgtgaaggaaggaccttacgagggaccggtgaagaaacctgtcgctttgaaagtgaaagcaaagaacttgattgtcactgagagtggtgccccaccgaccgacttgcaaaagatggtcatgggcaacacaaagcctgttgagctcattctcgacggcaagacagtagccatctgctgtgctactggagtgtttggcactgcttacctcgtgcctcgtcaccttttcgcggaaaagtatgacaaaatcatgttggacggcagagccatgacagacagtgactacagagtgtttgagtttgagattaaagtgaaaggacaggacatgctctcagacgccgccctcatggtgctgcaccgcgggaatcgcgtgcgtgacatcacgaaacacttccgtgatgtggcaaagatgaagaaaggaacccccgttgttggcgttatcaacaatgccgacgtcgggagactcatattctctggtgaggccctcacctacaaagacattgtagtgtgcatggatggagacaccatgcctggcctctttgcctacaaggccgccaccaaggcgggatactgtggaggagccgttctggcaaaggatggcgctgagactttcattgtcggcactcactctgcaggtggcaacggtgtgggatactgctcttgcgtttcccgatcgatgctccttaagatgaaggcacacgttgaccccgaaccacaccacgaggggttgattgtggataccagagatgtggaagagcgcgtccacgtgatgcgcaaaaccaagcttgcacccaccgtagcacacggtgtgttcaaccctgaattcgggcctgctgccttgtcaaacaaggacccgcgcctgaacgagggagttgtcctagatgaagttatcttctccaaacacaaaggagacacaaagatgactgatgaggacaaggcgctgttccgccgctgcgctgctgactacgcgtcacgcctccacagcgtgctggggacggcaaacgccccactgagcatttacgaggccatcaagggcgtcgacggactcgacgccatggagccggacaccgcgcccggcctcccctgggctctccaggggaaacgtcgtggtgcgctgattgactttgaaaacggcacagttggccctgagattgaggctgcccttgagctcatggagaagcgtgaatacaagtttgtttgtcagaccttcctgaaggacgagattcgcccgatggagaaagtgcgtgccggcaagactcgcatagtcgacgttttgcctgttgaacacattctttacaccaggatgatgataggcagattctgtgcacaaatgcactcaaacaacggaccgcaaattggctcagcggtcggttgcaatcctgacgttgactggcaaagatttggcacccattttgcccagtacagaaacgtgtgggatgtggactattcggcatttgatgctaaccactgcagcgatgcaatgaacatcatgtttgaggaggtctttcgcaccgagttcggtttccacccaaatgctgagtggatcctgaagactctggtgaacacggaacacgcctatgagaacaaacgcatcactgttgagggcgggatgccgtcaggctgttccgcaactagcatcatcaacacaatcttgaacaacatttacgtgctctacgccttgcgtagacactatgagggagttgagctggacacctacaccatgatctcctatggagacgatattgtggtggcaagtgattacgatttggacttcgaggccctcaagcctcattttaaatctcttggtcaaaccattaccccagctgacaaaagcgacaaaggttttgttcttggtcactccatcaccgatgtcactttcctcaaaagacacttccacatggactatggaactgggttttacaaacctgtgatggcttcgaagaccctcgaagctatcctctcctttgcacgccgtgggaccatacaggagaagttgatctccgtggcaggactcgctgtccactctgggcctgacgagtaccggcgcctcttcgaaccctttcagggtctctttgaggttccaagctacagatcactttacctgcgttgggtgaacgccgtgtgcggtgacgcgtaa

>AY593756.1_A_Brazil_1959

atgaatacaactgactgttttattgctttgatacacgctatcagagatatcataacggttttcgcctcacgaactacacaaaagatggagtttaaactgcacactggtgagaagaagactttttactccaggcccaataaccacgacaactgctggttgaacgccatactccagttgtttagatacgtcgatgaacctttcctcgactgggtctacaactcacccgagaaccttacgctggcagcaatcagacagctagaggacctcacagggcttgagttgcacgagggcggaccgcctgccctcgtaatttggaacatcaagcacttgctccaaaccggcatcggcaccgcctcgcgacccagcgaggtgtgcatggttgacggtacgaacatgtgtctggctgatttccacgcaggtatctttctaaaaggcaaagagcacgctgtgtttgcgtgtgtcacctccaacgggtggtacgcgattgatgacgaagacttttacccttggacaccggacccgtccgacgtcttggtgtttgtcccgtacgatcaagagccactcaatggagaatggaaagctaacgttcaacggaagctcaagggcgccggacaatccagcccgtcgaccggctcccagaaccaatctggcaacactggcagcataattaacaactactacatgcagcaatatcaaaattccatggacacacagctgggtgacaacgccatcagtggaggctccaacgagggctccacggacacaacatcaacacacacaaccaacactcaaaacaatgactggttctcaaagctcgccagttcagccttttctggcctgttcggtgcgttgctcgccgacaagaagacagaggagacaacacttcttgaggaccgcatcctcaccacccgcaatggacacaccacctcgacgacccaatcgagcgtgggtgtcacgtacgggtactccacagaggaggaccacgttgctgggcccaacacatcgggcctggagacacgggtggtgcaggcagagagattctacaaaaagtttctgtttgactggacaaaggaaaagccctttggacacctggtgaagctggagctcccggccgatcaccacggtgtctttggacacttggtggattcgtacgcctacatgagaaatggatgggatgttgaggtgtccgctgttggcaatcagttcaacggcgggtgcctcctggtggctatggtacctgagtggaaagaatttgacatacgggagaaataccaactcaccctcttcccgcaccagtttatcagccccagaaccaacatgactgcccacatcacggtcccctacctaggtgtgaacaggtatgaccagtacaaaaagcacaagccctggacactggttgtcatggtcgtgtcgccacttacggttaacgccactagtgcgacacagatcaaggtctacgccaacattgccccaacctacgttcacgtggccggtgaactcccctcgaaagaagggattttcccggtcgcatgcgcggacggttacgggggactggtaacgacagacccgaagacagctgaccctgtttacggcaaggtgtacaacccgcctaggaccaactaccctgggcgcttcaccaacctgttggacgtggccgaagcgtgtcccactttcctctgctttgacggcgggaaaccgtacgtctccacgcagataggtgagacccgacttttggccaagtttgacctttcccttgctgcaagacatatgtccaacacatacctgtcagggattgctcagtactacacacagtactctggcaccatcaacttgcacttcatgttcacaggtgccactgattcaaaggcccgatacatggtggcctacatcccgcctggggtggagccaccggacacacctgaggaggctgcccactgcattcacgctgaatgggacactgggctgaattccaaattcacattctcaatcccgtacgtatccgccgcagattacgcttacacagcgtctgacacggcagaaacaaccaacgtacagggatgggtctgcatctaccaaatcacacacgggaaggctgaaaacgacactctggtcgtatcggttagcgccggcaaagactttgagctacgcctcccgattgacccccgccagcagaccaccgctaccggggaatcagcagaccctgtcaccaccaccgtggagaactacggcggtgagacacaagtccagagacgtcaccacacggacattggtttcatcatggacagatttgtgaagatcaaagatttgagcccaacacatgtcattgacctcatgcagactcaccaacacggtctggtgggtgcgctgctgcgtgcagctacgtactacttttccgacctggaaattgtcgtacggcacgacggcaatctgacatgggtgcccaacggcgcccctgaatcagccctgttgaacaccagcaaccccactgcctacaacaaggaaccattcacgagactcgctctcccgtacactgcgccgcaccgcgtgctggcaacagtgtacaacgggacgagtgagtatgctgtgagtggtcca---gacagtcgtggcgacacggggcctatcgcggcgcgaaccgcgaaacagcttcctgcttcattcaactacggcgcaatcaaggccaaagtcatccacgaacttctcgtgcgcatgaaacgggccgagctctactgccccagaccgctgttagcagtagaagcttcgcaagaaaggtacaagcaaaagatcattgcacctgcaaagcagcttctgaattttgacctgctcaagttagcgggcgacgttgagtccaaccctgggcccttcttcttcgctgacgtcaggtcaaacttttcaaagttggtggaaacaatcaaccagatgcaggaggacatgtccacaaaacacgggcctgacttcaaccggttggtgtccgcttttgaggaattggccactggagtgagagctatcaggactggtcttgacgaggccaaaccctggtacaagctcattaagcttctgagccgcttgtcgtgcatggccgctgtagcagcacggtcaaaggaccccgtccttgtggccatcatgctggctgacaccggtctcgagattctggacagcaccttcgtcgtgaagaagatctccgactcgctctccagtctctttcacgtgccggcccccgtcttcagtttcggagccccgattctgttggccgggttggtcaaggtcgcctcaagtttcttccggtccacacccgaagaccttgagagagcagagaaacagctcaaagcacgtgacatcaacgacattttcgccattcttaagaacggcgagtggctggtcaaattgatccttgccatccgcgactggatcaaagcatggatcgccgcagaagagaagtttgtcaccatgacagacttagtgcctggcatccttgaaaagcagcgggatctcaacgacccgagcaagtacaaggaagccaaggagtggcttgacaacgcgcgtgaggtgtgtttgaagaacgggaacgtccacattgctaacctgtgcaaagtggtcgccccagcacccagcaagtcgagacccgagcccgtggtcgtctgcctccgtggcaagtccggccagggcaagagttttcttgcgaacgtgctcgcacaagcaatctctacccacttcaccggcagaaccgactctgtttggtactgcccacctgaccctgaccacttcgatggttacaaccaacagaccgtcgttgtgatggacgatttgggccagaaccctgacggcaaggacttcaagtacttcgcccaaatggtttcgaccacggggtttatcccgcccatggcatcactcgaggacaagggcaaacccttcaacagtaaggtcatcatcgcaaccaccaacctgtactcgggcttcaccccgaggaccatggtgtgccctgatgccctgaaccggaggtttcactttgacattgacgtgagcgccaaggacgggtacaaaattaacaacaaattggacatcatcaaagcacttgaagacacccacaccaacccagtggcaatgtttcagtacgactgtgcccttctcaacggcatggccgttgaaatgaagagaatgcaacaagacatgttcaaacctcaaccacctctccagaacgtataccaacttgttcaggaggtgattgaccgggtggagctccacgagaaagtgtcgagccacccgatttttaaacagatctcaattccttcccaaaaatccgtgttgtacttcctcattgagaaaggacagcacgaggcagcaattgaattctttgagggaatggtgcacgactccatcaaggaggagctccggcctctcatccaacgaacttcatttgtacaacgcgctttcaagcgcctgaaggaaaacttcgagattgttgccctatgtttgaccctcttggccaacatagtgatcatgatccgcgaaactcgcaagaggcagaagatggtggatgatgcggtgaatgagtacattgagaaagcaaacatcaccactgatgacaaaactcttgacgaggcggaaaagaaccctctggaaaccagcggtgccagcactgttggtttcagagagagaacacttccaggccagaaggtgcgcgatgacgtgaactctgagcccgcccaacctgttgaagaacaaccgcaagctgaaggaccctacgccggaccacttgagcgtcagatacctctgaaagtgagagccaagctcccgcaacaagagggaccttacgctggcccgatggagagacagaaaccgctgaaagtgaaagtaagagccccggtcgtcaaggaaggaccttacgagggaccggtgaagaagcctgtcgctttgaaagtgaaagccaagaatttgattgtcactgagagtggtgccccaccaaccgacttgcaaaagatggttatgggcaacactaagcctgttgagctcattctcgacgggaagacagtagccatctgctgtgctactggagtgtttggcactgcttacctcgtgcctcgtcatcttttcgcagagaagtatgacaagatcatgctggacggcagagccatgacagacagtgactacagagtgtttgagtttgagattaaagtaaaaggacaggacatgctctcagacgccgcgctcatggtgctccaccgtgggaaccgcgtgagagacatcacgaagcactttcgtgacacagcaagaatgaagaagggtacccccgtcgttggcgtaatcaacaacgctgatgtcgggagactgattttctctggtgaggccctcacctacaaggacattgtggtgtgcatggatggagacaccatgcccggtctctttgcctataaagccgcaaccaaggctggctattgcggaggagctgttcttgctaaggacggagctgacacgttcatcgtcggcactcactctgctggaggcaatggagttggttactgctcatgcgtttccaggtccatgcttctcaagatgaaggcacacattgaccctgagccacaccacgaggggttgatcattgacaccagagatgtggaagagcgcgtccatgtgatgcgcaaaaccaagcttgcacccaccgtcgcgcacggtgtgtttaaccctgagtttgggcccgccgccttgtctaacaaggacccgcgcctgaacgaaggtgttgtcctcgacgaagtcatcttctccaaacacaaaggggacacaaagatgtctgaggaggacaaagcgctgttccgccgctgcgccgccgactacgcgtcgcgcctgcacagcgtgctgggcacagcaaatgccccactgagcatttacgaggcaatcaagggcgttgacggactcgacgcaatggaaccggacaccgcgcccggtcttccctgggcgctccaggggaagcgccgcggtgcgctcatcgactttgagaacggcactgtcggacccgaagttgaggctgccttgaagctcatggagaaaagagaatacaaatttgtctgtcagaccttcctgaaggacgagattcgcccgatggagaaagtacgtgccggcaagactcgcattgtcgacgtcctgcccgttgaacacattctttacaccaggatgatgattggcagattttgtgcacaaatgcactcaaacaacggaccgcagattggctcggcggtcggttgcaaccctgatgttgattggcaaagatttggcacacacttcgcccaatacagaaacgtgtgggacgtggactattcggccttcgatgctaaccactgcagtgacgccatgaacatcatgtttgaggaggtgtttcgcacggactttgggttccacccgaatgctgagtggatcctgaaaactctcgtgaacacggaacacgcctatgagaacaaacgcatcactgttgagggcgggatgccatctggttgttctgcgacaagcatcatcaacacaatcctgaacaacatctacgtgctctacgccctgcgtagacactatgagggagttgagctggacacttacaccatgatctcctacggagacgacattgtggtggcaagtgattacgatttggactttgaggctctcaagccccactttaaatctcttggtcaaaccatcactccagctgacaaaagcgacaaaggttttgttcttggtcactccatcaccgatgtcactttcctcaaaagacacttccacatggattatggaactgggttttacaaacctgtgatggcctcaaagacccttgaggctatcctctcctttgcacgccgtgggaccatacaggagaagttgatctccgtggcaggactcgccgtccactctggacctgacgagtaccggcgtctctttgagcctttccagggcctctttgagattccaagctacagatcactttacctgcgttgggtgaacgccgtgtgcggtgacgcataa

>AY593757.1_A_Brazil_1967

atgagcacaactgactgtcttatcgctttggtacacgttatcagagagatcagggcactttttctaccacgaaccacaggaaagatggaattcacactgtacaacggtgagagaaaagtgttttactccagacccaacagccacgacaactgttggttgaacaccatccttcagctgttcaggtacgttgatgaacctttcttcgactgggtctacaactcgcccgagaacctcacacttgaagccattgagcagttagaggaactcacagggctggagttacgcgagggcggaccacctgccctcgtagtctggaacatcaagcacctgctccaaactggcatcggtaccgcctcgcggcccagcgaggtgtgtatggtggacggcacggacatgtgtcttgctgacttccacgcaggcattttcatgaaaggacaggaacacgctgtgttcgcgtgtgtcacttccaacgggtggtacgcgattgacgacgaggacttctacccatggacgccggacccgtccgatgtcttggtgtttgtcccgtacgatcaagaaccactcaacggagaatggaaaactaaagttcagcagaagcttaagggagccggacaatccagcccggcgaccggctcgcagaaccaatctggcaacactggcagcataattaacaactattacatgcagcagtaccaaaactccatggacacacaacttggtgacaacgccatcagcgggggctccaacgagggctccacggacacaacttccacccacacaactaacacccagaacaatgactggttttccaggcttgccaactctgccttcagtggccttttcggcgccctgctcgccgataagaaaacggaagagacaacacttctagaggatcgcatcctcaccacccgcaacgggcacaccacctcgaccacccagtcgagtgtgggtgtcacgtacgggtactccaccgcggaggaccacgttgccgggcccaacacatcgggtctggagacgcgggtgacacaggcagagagattcttcaagaagtttttgtttgaatggacaacggacaagccttttggacacttggaaaaactggagcttcccactgaccaccacggcgtcttcggacacctggtagactcgtatgcttatatgagaaatggctgggatgttgaggtgtccgccgttggcaaccagttcaacggtggatgtctcctggtggctatggtaccagagtggaaagaatttgacgcacgggagaaataccagcttacccttttcccacatcagttcatcagccccagaaccaacatgactgcccacatcacggtcccgtaccttggtgtgaacaggtatgatcagtacaaaaaccacaagccctggacattggttgtcatggttgtgtcgccactcacggttagcgccacgagtgcaacaaaaatcaaggtctacgccaacattgctccaacttacgttcacgtggctggggaactcccctcgaaagaggggattttcccggtcgcatgtgcggacggttacggaggactggtgacgacagacccgaagacagctgaccctgcatacggcaaggtgtacaacccgcccagaaccaactatcctgggcgcttcacaaacctattggatgtggccgaggcgtgtcccaccttcctctgcttcgacaacggaaaaccgtacgtcgttacgcgggcggacagcaaccgcctgctggccaaatttgatgtttcccttgccgcaaaacatatgtctaacacatacttgtcagggattgcacagtacttcacacagtactctggtaccatcaacctgcacttcatgttcacaggctccactgattcaaaagcccgctacatggtggcctacgtcccgcctggggtggagccaccggaaacacccgagatggccgcccactgcattcacgctgaatgggacacaggactgaactccaaattcacattctcaatcccgtacgtgtccgctgcagactatgcgtacactgcgtctgacacggcagaaacaaccaacgtacagggatgggtctgcatctaccaaattacacacgggaaggccgaagacgacactctggttgtgtcggtcagtgccggcaaggacttcgagctacgcctcccgattgacccccgttcacagaccactgctaccggggagtcagcagaccctgtcaccaccaccgtagagaactacggcggtgagacacaagtccagaggcgtcaccacacggacgtcggtttcatcatggacagatttgtgaagataaacaaactgaacccaacacatgttatcgacctcatgcagacccacgaacacggcttggtgggtgcgttgctgcgtgcagccacgtactacttctccgacctggagattgttgtgcggcacgaaggcaacctgacctgggtacccaacggtgcccctgaggcggccctactgaacaccagcaaccccaccgcctacaacaaggcaccgttcacgaggcttgctctcccctacactgcgccgcaccgcgtgctggcaactgtgtacaacgggacgaacaagtaccccgtgggcgcttcg---gggatacgaggtgatttggggcctcttgcggcgcaagccgcgaaacagctccctgcttctttcaactacggtgcaatcaaggccactaccatccacgagcttctcgtgcgtatgaaacgggccgagctctactgtcccaggccactcctggcgatagaggtttcccaagacagatacaagcaaaagatcattgcacctgcaaaacagcttttgaactttgacctactcaagttggcgggcgacgttgagtccaatcctgggcccttcttcttctccgacgttaggtcaaacttttccaagctggtagacacaatcaaccagatgcaagaggacatgtccacaaaacacggacctgactttaaccggttggtgtccgcttttgaggagttggccactggagtgaaagctatcaggaccggtctcgacgaggccaaaccctggtacaagctcatcaagctcctgagccgcctgtcgtgcatggccgctgtagcagcacggtcaaaggacccagtccttgtggccatcatgctggctgacaccggtctcgagattctggacagcactttcgtcgtgaagaagatttccgactctctctccagtctctttcacgtgccggcccccgtcttcagcttcggagccccgattctgttagccgggttggtcaaggtcgcctcgagtttcttccggtccacacccgaagaccttgagagagcagagaaacagctcaaagcacgtgacatcaatgacattttcgccattctcaagaacggcgagtggctggtcaagctgatccttgccatccgcgactggatcaaggcatggattgcctcagaagagaagtttgtcaccatgacagacttggtgcctggcatccttgaaaaacaacgggatctcaacgacccaagcaagtacaaggaagccaaggagtggctcgacaacgcgcgccaaacgtgtttgaagaatgggaacattcacattgccaacctgtgcaaagtggtcgctccggcacccagcaagtcgcgacccgagcccgtggtcgtttgcctccgcggcaaatccggccagggcaagagtttccttgcaaacgtgctcgcacaagcaatctctacccacttcaccggcaggactgactcagtttggtactgcccgcctgaccctgaccacttcgacggttacaaccaacagactgtcgttgtgatggacgatttgggccagaaccccgacggcaaggacttcaagtatttcgcccagatggtttcaaccacggggttcatcccgcctatggcatcgcttgaggacaaaggcaaacctttcaacagtaaggtcatcattgcaaccaccaacttgtactcgggcttcaccccgaggaccatggtgtgtcctgatgccctgaaccggaggtttcactttgacatcgacgtgagcgccaaagacgggtacaaaattaacaacaaattggacatcatcaaggcacttgaagacacccacaccaatcccgtggcaatgtttcagtacgattgtgcccttctcaacggcatggctgttgaaatgaagagaatgcaacaagacgtgttcaaacctcagccacccctccagaacgtataccaacttgttcaggaggtgattgagcgggtggagctccacgagaaagtgtcgagccacccgattttcaagcagatctcaattccttcccagaaatccgtgttgtacttcctcattgaaaaagggcagcacgaggcagcaattgaattctttgagggcatggtccacgactccatcaaggaggagctccggcccctcatccaacgaacttcatttgtgaaacgcgctttcaagcgcttgaaggaaaactttgagattgttgccctatgtttgaccctcctggccaacatagtgatcatgatccgcgagactcgcaagagacagaagatggtggatgatgcggtgaacgagtacattgagaaagcaaacatcaccaccgatgacaagactcttgacgaggcggaaaagaaccctctggaaaccagcggtgccagcaccgtcggcttcagagagagaactcttccaggccagaaggcgcgtggcgaagagaactctgagcccgcccagcctattgaagagcaaccacgagctgaaggaccctacgccgggccgcttgagcgtcagaaacctctgaaagtgagagccaagctcccacagcaagaggggccttacgctggcccgatggagagacagaaaccacttaaggtgaaagcaaaagccccggtcattaaggaaggaccttacgagggaccggtgaagaagcctgtcgctttgaaagtgaaagctaagaacctgattgtcactgagagtggtgccccaccgaccgacttgcaaaagatggtcatgggcaacactaagcctgttgagctcattctcgacgggaagacagtagccatctgctgtgctactggagtgtttggcaccgcctacctcgtgccacgtcatcttttcgctgagaagtatgacaagattatggtggatggcagggccatgacagacagtgactacagagtgtttgagtttgagattaaagtaaaaggacaggacatgctctcagacgctgcgctcatggtgctccaccgtgggaaccgcgtgagagacatcacgaaacactttcgtgacacagcaagaatgaagaaaggcacccccgtcgttggcgtgatcaacaacgctgatgtcgggagactgattttctctggtgaagcccttacctacaaggacattgtagtgtgcatggatggtgacaccatgcctgggctctttgcctacaaagcctcaaccaaggctgggtattgcggaggagctgtccttgctaaggacggggccgacacgttcatcgtcggcactcactctgctggaggcaatggtgttggatactgctcttgcgtttccagatccatgcttctcaggatgaaggcacacattgaccctgaaccacaccacgaggggttgattgttgacaccagagatgtggaagagcgcgtccacgtgatgcgcaaaaccaagcttgcacccaccgtcgcacacggtgtgtttaaccctgagttcgggcccgccgccttgtccaacaaggacccgcgcctgaacgatggtgttgtcctcgatgaaaccatcttctccaaacacaaaggagacacaaagatgtctgaggaggacaaagcgctgttccgccgctgtgctgctgactatgcgtcacgcttgcacagtgtgttgggcacagcaaatgccccactgagcatcttcgaggcaatcaaaggcgttgatggactcgacgcaatggagccagacactgcacccggcctcccctgggcactccaggggaagcgccgtggagcgcttatcgactttgagaacggcactgtcggacccgaagttgaggctgccttgaagctcatggagaaaagagagtacaagtttgtttgccaaaccttcctgaaggacgagattcgcccaatggagaaagtacgcgccggtaagactcgcattgtcgacgtcctacctgttgaacacattctttacactaggatgatgattggcagattctgtgcacaaatgcactcaaacaacggaccccaaattggctcggcggtcggttgtaaccctgatgttgattggcaaagatttggtacacacttcgcccaatacagaaacgtgtgggatgtggactactcggcctttgatgctaaccactgcagcgacgccatgaacatcatgtttgaggaggtgttccgcacggagttcggcttccacccaaacgcggagtggattctgaagactctcgtgaacacggaacacgcctatgagaacaagcgcatcactgttgagggcgggatgccatctggctgttccgcaacaagcatcatcaacacaattctgaacaacatctacgtgctctacgctttgcgcagacactatgagggagttgagctggacacttacaccatgatctcctacggagacgacatcgtggtagcaagtgattacgatttggactttgaggctctcaagccccactttaaatcccttggccaaaccatcactccagctgacaaaagcgacaaaggttttgttcttggtcactccattactgatgtcactttcctcaaaagacacttccacatggattacggaactgggttttacaaacctgtgatggcctcaaagacccttgaggctatcctctcctttgcacgccgtgggaccatacaggagaagttgatctccgtggcaggactcgctgtccactctggaccagacgagtaccggcgtctctttgagcccttccagggcctctttgagatcccaagctacagatcactttacctgcgttgggtgaacgccgtgtgcggtgacgcataa

>AY593758.1_A_VEN_1967

atgagcacaactgactgtcttatcgctttggtacacgttatcagagagatcagggcactttttctaccacgaaccacaggaaagatggaattcacactgtacaacggtgagagaaaagtgttttactccagacccaacagccacgacaactgttggttgaacaccatccttcagttgttcaggtacgttgatgaacctttcttcgactgggtctacaactcgcccgagaacctcacacttgaagccattgagcagttagaggaactcacagggctggagttacgcgagggcggaccacctgccctcgtagtctggaacatcaagcacctgctccaaactggcatcggtaccgcctcgcggcccagcgaggtgtgtatggtggacggtacggacatgtgtcttgctgacttccacgcaggcattttcatgaaaggacaggaacacgctgtgtttgcgtgtgtcacctccaatgggtggtacgcgattgacgacgaggacttttacccatggacaccggacccgtccgatgtcttggtgtttgtcccgtacgatcaagaaccactcaacggagaatggaaaactaaagttcagcagaagcttaagggagccgggcaatccagcccggtgaccggctcacagaaccaatctggcaacactggcagcataattaacaactattatatgcagcagtaccaaaactccatggacacacaacttggcgacaacgccatcagcgggggctccaacgagggctccacggacacaacttccacccacacaaccaacacacagaacaatgactggttttccaagcttgccagctctgccttcagtggtcttttcggcgcccttctcgccgataagaaaacggaagaaacaacacttctggaggaccgcatcctcaccacccgcaacgggcacaccacctcgaccacccagtcgagtgtgggtgtcacgtacgggtactccaccgcggaggaccacgttgctgggcccaacacatcgggtttggagacgcgggtgatacaggcagagagatacttcaagaagtttctgtttgagtggacaccggaaaagccttttggatacttggaaaaactggagcttcccactgtccaccacggcgttttcggacacctggtagactcgtatgcttatatgagaaatggctgggatgttgaggtgtctgctgttggcaaccagttcaacggcggatgtctcctggtggctatggtaccagagtggaaagaatttgacgcacgggagaaataccagcttacccttttcccacatcagttcatcagccccagaaccaacatgactgcccacatcacggtcccgtaccttggtgtgaacaggtatgatcagtacaaaaaccacaagccctggacactggttgtcatggttgtgtcgccacttacggttaacgccacgagtgcaacacaaatcaaggtctacgccaacattgctccaacttacgttcacgtggctggagaactcccctcgaaagaggggattttcccggtcgcatgcgcggacggttacggaggactggtgacgacagatccgaagacagctgaccccgcgtacggcaaggtgtacaacccgcccagaaccaactatcctgggcgcttcacaaacctattggacgtggccgaggcgtgtcccaccttcctctgcttcgacggcgggagaccgtacgtcgttacgcaggcgggtagcaaccgcctcctggccaaatttgatgtttcccttgccgcaaagcatatgtctaacacatacttgtcagggctcgcacagtacttcacacagtactctggcaccatcaacctgcacttcatgttcacaggctccactgactcaaaagcccgctacatggtggcctacgtaccgcctggggtggagccaccggaaacacccgagaaggccgcccactgcattcacgctgaatgggacacaggactgaactccaaattcacattctcaatcccgtacgtgtccgctgcagactatgcgtacactgcgtctgacacggcagaaacaaccaacgtacagggatgggtctgcatctaccaaattacacacgggaaggccgaagacgacactctggttgtgtcggtcagtgccggcaaggatttcgagctacgcctcccgattgacccccgttcacagaccacttctaccggggagtcagcagaccctgttaccaccaccgtagagaactacggcggtgagacacaagtccagaggcgtcaccacacggacgtcggtttcatcatggacagatttgtgaagataaacagtccaaaaccaacacatgtcattgacctcatgcagacccaccaacacggcttggtgggtgcgttgctgcgtgcagccacgtactacttctccgacctggagattgttgtgcagcacgacggcaacctgacctgggtgcccaacggtgcccctgaggcggccctactgaacaccagcaaccccaccgcctacaacaaggcaccgttcacgaggcttgctctcccctacactgcgccgcaccgcgtgctggcaactgtgtacaacgggacggacaagtaccccgtgagcgcttcg---ggaggacgaggtgatttggggcctcttgcggcgcgagccgcgaaacagctccctacttctttcaactacggtgcaatcaaggccactaccatccgcgagcttctcgtgcgtatgaaacgggccgagctctactgtcccaggccactcctggcgatagaggtttcccaaggcagatacaagcaaaagatcattgcacctgaaaaacagcttttgaactttgacctactcaagttggcgggcgacgttgagtccaaccctgggcccttcttcttctccgacgttaggtcaaacttttccaagctggtggacacaatcaaccagatgcaagaggacatgtccacaaaacacggacctgactttaaccggttggtgtccgcttttgaggagttggccactggagtgaaagccatcaggaccggtctcgatgaggccaaaccctggtacaagctcatcaagctcctgagccgcctgtcgtgcatggccgctgtagcagcacggtcaaaggacccagtccttgtggccatcatgctggctgacaccggtctcgagattctggacagcaccttcgtcgtgaagaagatttccgactctctctccagtctctttcacgtgccggcccccgccttcagtttcggagccccgattctgttagccgggctggtcaaggtcgcctcgagtttcttccggtccacacccgaagaccttgagagagcagagaaacagctcaaagcacgtgacatcaacgacattttcgccattctcaagaacggcgagtggctggtcaagctgatccttgccatccgcgactgggtcaaggcatggattgcctcagaagaaaagtttgtcaccatgacagacttggtgcctggcatccttgaaaaacaacgggatctcaacgacccaagcaagtacaaggaagccaaggagtggctcgacaacgcgcgccaaacgtgtttgaagaatgggaacattcacattgccaacctgtgcaaagtggtcgctccggcacccagcaagtcgcgacccgaacccgtggtcgtttgcctccgcggcaaatctggccagggcaagagtttccttgcaaacgtgctcgcacaagcaatctctacccacttcaccggcaggactgattcagtttggtactgcccgcctgaccctgaccacttcgacggttataaccaacagactgtcgtcgtgatggacgacctgggccagaaccccgacggcaaggacttcaagtacttcgcccagatggtttcaaccacggggttcatcccgcctatggcatcgcttgaggacaaaggcaaacctttcaacagtaaggtcatcattgcaaccaccaacttgtactcgggcttcaccccgaggaccatggtgtgtcctgacgccctgaaccggaggtttcactttgacatcgacgtgagcgccaaagacgggtacaaaattaacaacaaattggacatcatcaaagcacttgaagacacccacaccaatcccgtggcaatgtttcagtacgattgtgcccttctcaacggcatggctgtagaaatgaagagaatgcaacaagacgtgttcaaacctcagccacccctccagaacgtgtaccaacttgttcaggaggtgattgagcgggtggagctccacgagaaagtgtcgagccacccgattttcaagcagatctcaattccttcccaaaaatccgtgttgtacttcctcattgaaaaagggcagcacgaggcagcaattgaattctttgagggcatggtccacgactccatcaaggaggagctccggccccttatccaacgaacttcatttgtgaaacgcgctttcaagcgcttgaaggaaaactttgagattgttgccctatgtttgaccctcctggccaacatagtgatcatgatccgcgagactcgcaagagacagaagatggtggatgatgcggtgagtgagtacattgagaaagcaaacatcaccaccgacgacaagactcttgacgaggcggaaaagaaccctctggaaaccagcggtgccagcaccgtcggcttcagagagagaactcttccaggccagaaggcgcgtggtgaagagagctctgagcccgcccagcctattgaagagcaaccgcgagctgaaggaccctacgccgggccgcttgagcgtcagaaacctctgaaagtgagagccaagctcccacagcaagaggggccctacgctggcccgatggagagacagaaaccgcttaaggtgaaagcaaaagccccggtcattaaggaaggaccttacgagggaccggtgaagaagcctgtcgctttgaaagtgaaagctaagaacttgattgtcactgagagtggtgccccaccgactgacttgcaaaagatggtcatgggcaacactaagcctgttgagctcattctcgacgggaagacagtagccatctgctgtgctactggagtgtttggcaccgcctacctcgtgcctcgtcatcttttcgctgagaagtatgacaagattatggtggacggcagggccatgacagacagtgactacagagtgtttgagtttgagattaaagtaaaaggacaggacatgctctcagacgctgcgctcatggtgctccaccgtgggaaccgcgtgagagacatcacgaaacactttcgtgacacagcaagaatgaagaaaggcacccccgtcgttggcgtgatcaacaacgctgatgtcgggagactgattttctctggtgaagcccttacctacaaggacattgtagtgtgcatggatggtgacaccatgcctgggctctttgcctacaaagccgcaaccaaggctgggtattgcggaggagctgtccttgctaaggacggggccgacacgttcatcgtcggcactcactctgctggaggcaatggtgttggatactgctcttgcgtttccagatccatgctcctcaggatgaaggcccacattgaccctgaaccgcaccacgaggggttgattgttgacaccagagatgtggaagagcgcgtccacgtgatgcgcaaaaccaagcttgcacccaccgtcgcacacggtgtgtttaaccctgagttcgggcccgccgccttgtccaacaaggacccgcgcctgaacgatggtgttgtcctcgacgaaaccatcttctccaaacacaaaggagatacaaagatgtctgaagaggataaagcgctgttccgccgctgcgccgctgactacgcgtcacgcttgcacagtgtgttgggcacagcaaatgccccactgagcatcttcgaggcaatcaaaggtgttgatgggctcgacgcaatggagccagacactgcacccggcctcccttgggcactccaggggaagcgccgtggagcgcttatcgactttgagaacggcactgtcggacccgaagttgaggctgccttgaagctcatggagaaaagagagtacaagtttgtttgccaaaccttcctgaaggacgagattcgcccgatggagaaagtacgtgccggtaagactcgcattgtcgacgtcctgcctgttgaacacattctctacactaggatgatgattggcagattctgtgcacaaatgcactcaaacaacggaccccaaattggctcggcggtcggttgtaaccctgatgttgattggcaaagatttggcacacacttcgcccaatacagaaacgtgtgggatgtggactattcggcctttgatgctaaccattgcagcgatgccatgaacatcatgtttgaggaggtgttccgcacggagttcggcttccacccaaacgcggagtggattctgaagactctcgtgaacacggaacacgcctatgagaacaagcgcatcactgttgagggcgggatgccatctggctgttccgcaacaagcatcatcaacacaattctgaacaacatctacgtgctctacgctttgcgcagacactatgagggagttgagctggacacttacaccatgatctcctacggagacgacatcgtggtagcaagtgattacgatttggactttgaggctctcaagccccactttaaatcccttggtcaaaccatcactccagctgacaaaagcgacaaaggttttgttcttggtcactccattactgatgtcactttcctcaaaagacacttccacatggattacggaactgggttttacaaacctgtgatggcctcaaagacccttgaggctatcctctcctttgcacgccgtgggaccatacaggagaagttgatctccgtggcaggactcgctgtccactctggaccagacgagtaccggcgtctctttgagcccttccagggcctctttgagatcccaagctacagatcactttacctgcgttgggtgaacgccgtgtgcggtgacgcataa

>AY593759.1_A_GER_1971

atgaatacaactaactgctttatcgctttggtgtacgccatcagagagattaagacacttttcttttcacgagctacaggaaagatggaattcacactgcacaacggtgaaaagaaaactttttattccaggcccaatagccacgacaactgctggctgaacaccatcctccagttgtttaggtacgtcgatgaaccattcttcgactgggtctacaactcacccgagaaccttacgcatgatgctatcaagcagttggaagaactcaccgggcttgagttgcgcgagggcggaccgcccgcccttgtgatttggaacatcaaacacttgctccaaactggcatcggcaccgcctcgcgacccagcgaggtgtgtatggtggacggtacggacatgtgtcttgccgatttccacgcaggcattttcctgaaaggacaggaacacgcagtgtttgcgtgtgtcacctctgacgggtggtacgcgattgacgacgaggatttttacccgtggacgcctgacccatcggacgtcttggtatttgtcccgtacgatcaagaaccgctcaatggagactggaaaacactggttcagaggaagctcaagggtgctgggcaatccagcccagtaacaggctcgcagaaccagtcgggcaacaccggcagcataattaacaactactacatgcagcaataccagaactccatggacacacagcttggtgacaatgccatcagtggtggctccaacgagggctccacggacacaacctcaacacacacaaccaacacccaaaacaacgactggttttcaaaacttgccagttcagcttttaccggtctgttcggtgcacttctcgccgacaagaagacggaggagactacgcttctggaagaccgcatcctcaccactcgcaacggacacaccacctcgaccacccagtcgagtgtgggagtcacgtacgggtattccaccgaggaggatcacgttgctgggcccaacacatcgggcttggagacgcgggtggtgcaggcagagagattttttaaaaagtttctgtttgactggacaacagacaagccttttggacacttggcaaaactggagcttcccaccgaccaccgcggtgtcttcggacatctggtagactcatatgcgtacatgaggaatggctgggatgttgaggtgtctgccgttggcaaccagttcaacggcgggtgccttctggtggccatggtgccagagtggaaagattttgacgagcgtgaaagataccaactcacccttttcccacaccagttcatcagccccagaaccaacatgactgcccacatcacggtcccgtatcttggtgtgaacaggtacgatcagtacaagaaacacaagccttggacactggttgtcatggtggtatcacccctcacggtcagcaacactgccgcaacacaaatcaaggtctacgccaacattgccccaacctacgttcacgtggctggagagctcccctcgaaagaggggatcttcccagttgcgtgcgcggacggttatggagggctggtgacaacagacccgaaaacagctgaccctgtgtacggcaaggtgtacaacccgcccaggaccaactaccccgggcgttttacaaacctgttggacgtggccgaagcatgtcccacctttctctgtttcgacgatgggaaaccgtacgtcgttacgcggacagacgacacccgtcttttggccaagtttgacgtctccctagccgcaaaacacatgtccaacacatacctatcagggattgcacagtactacacacagtactctggtactatcaacctgcacttcatgttcacgggctccactgactcaaaagcccgctacatggtggcttacatcccacctggggtggagccaccggacacacctgaaaaagctgcccactgcattcatgctgaatgggacacaggactaaactccaaattcactttttcaatcccttacgtgtccgccgcggattacgcgtacaccgcgtctgatacggcggaaacaaccaacgtacagggatgggtctgtgtttaccaaattacacacgggaaggctgaaaacgacactttggtagtgtcggctagtgccggcaaagattttgagttgcgcctcccaattgacccccggccgcaaactaccactgctggggagtctgcagaccctgtcaccaccaccgtggagaactacggtggtgagacacaagtccagagacgtcaccacacggacgtcggtttcatcatggaccgatttgtgaggataaacaacctgaaccccacgcacgttattgacctcatgcagacccaccagcacgggctggtgggtgcgttgctgcgtgcagccacgtactacttctccgacttggagattgtagtgcggcacgatggtaatctgacctgggtacctaacggtgcccccgaagcagccctgttaaacaccagcaaccccactgcctacaacaaggcaccgttcacgagacttgctctcccttacactgcgccgcaccgcgtgttggcaactgtgtacaacgggacaagcaagtactccacgagcggttcg------ggacgaggcgatttggggaccctcgcggcgcgagccgcgacacagcttcctgcttctttcaattacggtgcaatcaaggcccagaccatccacgagcttctcgtgcgcatgaaacgagctgagctctactgtcctaggccgctgctggcaatagaggcttcgcaagacaggcacaagcaaaagattattgcacccgcaaagcagctgttgaacttcgacctactaaagttggcgggtgacgttgagcccaaccctgggcccttcttcttcgctgacgttaggtcgaacttttcaaagttggtagacaccatcaaccagatgcaggaagacatgtccacaaaacacggacccgactttaaccggttggtgtccgcatttgaggaactggccactggggttaaagccatcagaaccggtctcgatgaggccaaaccctggtacaagcttattaaactcctaagccgcctgtcgtgcatggccgctgtggcagcacggtccaaggacccagtccttgtggccatcatgctggccgacactggtctagagattctggacagcacctttgtcgtgaagaagatctccgactcactctccagtctctttcacgtgccggcccccgtcttcagtttcggagctccgatcctgttggccgggttggtcaaggtcgcctcgagtttcttccggtccacacccgaagaccttgagagggcagagaaacagctcaaagcacgtgacattaacgacattttcgccattctcaagaacggcgagtggctggtcaaactgatccttgccatccgcgactgggtcaaggcatggatcgcctcagaagagaagttcgtcaccatgacggacttggtgcctggcatcctcgaaaagcaacgggaccttaacgacccgagcaagtacaaggaagccaaggagtggctcgacaacgcgcgccaggcgtgcttgaagagcgggaacgtccacattgccaatttgtgcaaagtggtcgccccagcacccagcaagtcgagacccgagcctgtggtcgtttgcctccgtggcaagtctggccagggcaagagtttccttgcgaatgtactcgcacaagcaatttccacccactttactggcagaaccgactcggtctggtactgcccgcctgaccctgaccacttcgacggttacaaccagcagaccgtcgttgtgatggatgatttaggccagaaccctgatggtaaagactttaagtactttgcccaaatggtttcaaccacagggttcattccgcccatggcgtcgcttgaggacaaaggcaaacctttcaacagcaaggtcatcatcgcaactaccaacttgtactcgggcttcaccccgaggactatggtgtgccctgatgcactgaaccggcggtttcactttgacatcgacgtgagcgccaaggacgggtataaaatcaacaacaaattggacatcaacaaagcacttgaagacacccacaccaacccagtggcaatgtttcagtacgactgtgccctcctcaacggcatggccgttgaaatgaagagaatgcaacaagacatgttcaagccccaaccgcccctccagaacgtgtaccagctcgttcaggaggtgattgagcgggtcgagctccacgagaaagtgtcgagccacccaattttcaagcagatctcaattccttcccaaaaatctgtgttgtacttcctcattgagaaaggccaacacgaggcagcaattgaattctttgagggtatggtgcatgattccattaaggaagagctccggcccctcatccaacaaacctcatttgtgaaacgcgctttcaagcgcctgaaggaaaactttgagattgttgcactgtgcctgacccttctggccaacatagtgatcatggtccgcgaaactcgcaagaggcagaagatggtggatgatgctgtgaatgactacattgagagggcaaacatcaccacagatgacaaaacacttgacgaggcggagaagaaccctctggagaccagcggtgccagcaccgttggtttcagagagaaagctctggcaggacacaaagcgcgcgacgacgtgaaatctgagcccgcccagcctactgaagagcaaccacaagctgaaggaccctacgccgggccacttgagcgtcagaaacctctgaaagtgagagccaaactcccacaacaggaggggccctacgctggcccgatggagagacagaagccgctgaaagtaaaagcaagagccccggtcgtcagggaaggaccttacgagggaccggtgaagaagcctgtcgctttgaaagtgaaagccaagaacttgattgtcactgagagtggtgccccaccgaccgacttgcagaagttggtcatgggcaacacaaagcctgttgagctcatcctcgacgggaagacagtagccatttgctgtgctactggagtgttcggtactgcttaccttgtgcctcgtcatcttttcgcagagaagtatgacaagatcatgttggacggtagagccatgacagacagtgactacagagtgtttgagtttgagattaaagtaaaaggacaggacatgctctcagacgcggcactcatggtgcttcaccgtgggaaccgcgtgagagacatcacgaaacactttcgtgatacagcaagaatgaagaaaggtacccccgtcgtcggtgttattaacaacgccgatgtcgggagactgattttctctggtgaggcccttacctacaaggacattgtagtgtgtatggacggagacaccatgcccggcctcttcgcctacaaagccgccaccaaggctggctactgtggaggagccgtccttgccaaggacggggctgacacattcatcgtcggcacccactctgcaggtggcaacggagttggatactgttcatgtgtttccaggtccatgcttcaaaagatgaaggctcacgttgaccctgaaccgcaccacgaggggttgattgttgacaccagagatgtggaagagcgcgtccacgtgatgcgcaaaacaaaacttgcgcccaccgttgcacacggtgtgttcagccctgagtttgggcctgccgccttgtcaaacaaggacccgcgcctgaacgagggagtcgttctcgatgaagtcattttctccaaacacaagggagacacaaagatgaccgaggaggacaaagcgctgttccgacgctgtgccgctgactatgcgtcacacctgcacagtgtactgggtacggcaaatgccccactgagtatctatgaggcaatcaagggcgttgatggactcgacgccatggagccggacactgcacctggcctcccctgggccctccagggaaaacgccgcggtgcgcttattgacttcgagaacggcacggtcggacccgaagttgaggctgccttgaagctcatggagaaaagggaattcaagtttgtttgtcaaaccttcctgaaggacgagatccgcccgatggggaaagtacgcgccggcaagactcgcattgtcgatgttttgcctgttgaacatattctttataccaggatgatgattggtagattttgtgcgcaaatgcactcaaacaacggaccacacattggctctgcggtcggttgtaaccctgatgttgattggcaaagatttggcacacattttgcccaatacagaaacgtgtgggatgtggattattcggcctttgatgcgaaccactgcagtgacgctatgaacatcatgtttgaggaggtgttccgcacagactttggcttccacccaaatgcagagtggatcctgaaaaccctcgtgaacacggaacacgcctatgagaacaagcgcatcactgttgaaggcgggatgccatctggttgctccgcgacaagcatcatcaacacaattttgaacaacatttacgtgctctacgccttgcgtagacactatgagggagttgagctggacacctacaccatgatctcctatggagacgacatcgtggtggcaagtgactatgatctggactttgaggctctcaagccccacttcaaatctcttggccaaactatcactccagctgacaaaagcgacagaggttttgttcttggacattctattaccgatgtcaccttcctcaaaagacatttccacatggattatggaactgggttttacaaacctgtgatggcctcaaagacccttgaggctatcctctcctttgcacgccgtgggaccatacaggagaagttgatctctgtggcgggactcgccgtccactctggaccagacgagtaccggcgtctctttgaacccttccagggtctcttcgagattccaagctacagatcactttacctgcgttgggtgaacgccgtgtgcggtgacgcataa

>AY593760.1_A_USSR_1964

atgaacacaactgactgttttatcgctttggtgcacgctatcagagagatcagagcactttttctaccgcgaactacaggaaagatggaactcaccctgcacaacggcgagaaaaagactttttactccagacccaacaaccacgacaactgctggttgaacaccatccttcagttgttcaggtatgtcgatgaacccttcttcgactgggtctacaactcgcccgagaacctcacgcttgaagccatcaagcaattggaggaactcacggggcttgagttgcacgagggcggaccgcctgcccttgtgatctggaacatcaaacacttactccacaccggcatcggcaccgcctcacggcccagtgaggtgtgcatggtggacggtgcggacatgtgtcttgctgacttccacgcaggcattttcctgaagggacaggaacacgcagtttttgcgtgtgtcacctccaacgggtggtacgcgattgacgacgaggagttttacccctggacgcccgacccgtcggacgttctggtgtttgtcccgtacgatcaagagccactcaacggggactggaaggcgatggttcagaggaagcttaagggcgccggtcaatctagcccggcgaccggctcccagaaccagtccggtaacactggcagcataatcaacaactattacatgcagcagtaccagaactccatggacacacagcttggtgacaatgccattagtggaggctccaacgaaggctccacggacacaacttcaacacacacaaccaacacccaaaacaatgattggttttcaaaacttgccagctcagccttcaccggtctgttcggcgccctgctcgccgacaagaagacggaagagactacacttctggaagaccgcattcttaccacccgcaacgggcacaccatctcgaccacccaatcgagtgtgggagttacctacgggtactccactggggaagaccatgtcgctgggcccaacacatcgggcctggagacgcgggtggtccaggcagagagattttttaaaaagtttttgtttgactggacaacggacaaacctttcgggcatttggaaaagttggaacttcccaccggccaccacggcgttttcgggcacctggtggaatcatatgcttatatgagaaatggctgggacgttgaggtgtctgctgttggcaaccagttcaacggcgggtgcctcctggtggccatggtaccggagtggaaagagtttgagcagcgcgagaaataccaactcaccctcttcccgcaccagttcattagccccagaacaaacatgactgcccacatcacagtcccataccttggagtgaacaggtacgatcagtacaagaaacacaaaccttggacactggttgttatggtggtgtcgcccctcacggttagcgacactgccgcggcacagattaaggtctacgccaacattgctccaacctacgttcacgtggctggagaactcccctcgaaagaggggattttcccggttgcgtgttcggacggttatggaggactggtgacaacggacccgaaaacagctgaccccgcctacggcaaggtgtacaacccgcccaggactaactaccctgggcggtttaccaacttgttggacgtggctgaagcgtgtcccactctcctctgtttcgacgacgggaaaccgtatgttgtcacgcggacagatgacacacgactactagccaagtttgacgtctcccttgctgcaaaacacatgtccaacacgtacctgtcagggattgcacagtactacgcacagtactctggcaccatcaacctgcacttcatgttcacaggctcaactgactcaaaagcccgctacatggtggcctacatcccgcctggagtggaaccaccagacacacctgaaagggccgctcactgcatacacgctgaatgggacacaggactgaactccaaattcactttttcaatcccgtacgtgtccgccgcagattacgcgtacaccgcgtctgacacggcagaaacaaccaacgtacagggctgggtttgcatttaccagatcacacacgggaaggccgagaacgacacattggtggtatcagtcagcgccggtgcagactttgagttgcgcctcccgatcgacccccgacagcaaaccactgctgttggggagtccgcagaccctgtcaccaccaccgtggaggcctacggcggtgagacgcaagtccagagacggcaccacacggatgtcggttttatcatggacagatttgtgaagataaacagcttgagtcccacgcatgtcatcgacctcatgcagacccaccagcacgggttggtgggtgcgctgctgcgtgcagccacatactacttctctgacttggagattgtagtgcggcatgacggtaacttgacttgggtgcccaacggtgctcctgaagcagctttgttaaacaccagcaaccccactgcctacaacaaggcaccgttcacgaggctcgctctcccttacaccgcgccacaccgcgtgttggcaaccgtgtacaacgggacgaacaagtactccacgggcggtccg------ggacgaggcgacatggggtcgctcgcggcgcgggtcgcgaaacaacttcctgcctctttcaactacggtgcaattagggccgaaaacatccacgagcttctcgtgcgcatgaaacgggccgagctttactgccccaggccactactggcaatagaagcttcgcaggataggcacaagcaaaagatcattgcacccgcaaaacagctgctgaactttgacctgctcaagttggctggtgacgtggagtccaaccctgggcccttcttcttttctgatgttaggtcaaacttttctaagttggtggaaaccatcaaccagatgcaggaagacatgtcaacaaaacacgggcccgactttaaccggttggtgtccgcgtttgaggaattggccactggagtaaaggctatcaggaccggtcttgacgaggccaaaccctggtataaacttatcaagctcctgagccgtttgtcgtgcatggccgctgtagcagcacggtcaaaggatccagtccttgtggccattatgctggctgacaccggcctcgagattctggacagtacttttgtcgtgaaaaagatctccgactcactctccagtctctttcacgtgccggcccccgccttcagcttcggagccccgattctgctggccgggttggtcaaggtcgcctcgagtttcttccggtctacacccgaagaccttgagagagcagagaaacagctcaaagcgcgtgacattaatgacattttcgccattctcaagaacggcgagtggctggtcaagctgattcttgccatccgcgactggatcaaggcatgggtcgcctcagaagagaagtttgtcaccatgacagacttggtgcctggcattcttgaaaagcagcgggacctcaacgaccccagcaagtacaaggaggccaaggaatggctcgacaatgcgcgacaagcgtgtttgaagagcggaaacgtccacattgccaacctgtgcaaagtggtcgccccggcacccagcaagtcgagacccgagcccgtggtcgtttgcctccgcggcaagtccggccagggcaagagtttccttgcgaatgtgcttgcacaagcaatttccacccacttcactggcaaaaccgattcagtttggtattgcccacctgaccctgaccactttgacggttacaaccagcagactgtcgtagtgatggacgatttgggccagaaccctgacggcaaggacttcaagtactttgcccaaatggtctcaaccacggggttcatcccgcccatggcttcactcgaagacaaaggcaaaccttttaacagcaaggtcatcattgccaccaccaacctgtactcgggttttaccccgagaaccatggtgtgccctgacgcactgaaccgaaggtttcactttgacattgacgtgagtgccagggacgggtacaaaattaacaacaaattggacataaccaaagctcttgaggacacccacaccaacccagtggcaatgtttaagtacgattgtgcccttctcaacggcatggctgttgagatgaagagaatgcaacaagacatgttcaagcctcaaccacccctccagaacgtgtaccaacttgttcaggaggtgattgaacgggtcgagctccacgagaaagtgtcgagtcacccgattttcaagcagatctcaattccttcccaaaagtctgtgttgtacttcctcattgagaaaggccaacacgaagcagcaattgaattcttcgaggggatggtgcacgattccatcaaggaggaactccggcccctcatccaacagacctcatttgtgaaacgcgctttcaagcgcctgaaggaaaattttgagattgttgccctgtgtttgactcttttggcaaacatagtgatcatgattcgtgaaacccgcaagaggcagcagatggtggatgatgcagtgaatgagtacattgagagagcaaacatcaccacggatgataaaactcttgatgaagccgagaaaaaccctctggaaaccagcggtgtcagcactgttgggttcagagagagaaccctcccggggcag---gcgagtgatgacgtgaactccgagcccgtcaaacccgtggaggagcaaccacaagctgaaggaccatacgccggaccccttgagcgtcaaaaacctctgaaagtgagagccaggctaccacaacaggagggaccttacgctggtccgctggagagacagaaacctctgaaagtgaaagcaagagccccggtcgtcaaggaaggaccttatgaaggaccggtgaagaaacctgttgctttgaaagtgaaagctaagaatttgattgtcactgagagtggtgcccccccgaccgacttgcaaaagatggtcatgggcaacacaaagcctgttgagctcattctcgacgggaagacagtagccatttgctgtgctactggagtatttggcactgcttacctcgtacctcgtcatcttttcgcagagaaatatgacaagatcatgttggacggcagagctatgacagacagtgactacagagtgtttgagtttgagattaaagtgaagggacaggacatgctctcggacgccgctctcatggtacttcatcgtgggaatcgcgtgagagacatcacgaagcactttcgtgacacggcaagaatgaagaaaggcacccccgttgtcggcgtgattaacaatgccgatgtcgggagactgattttctccggtgaggccctcacctacaaggacattgtggtgtgcatggatggagacaccatgcccgggctttttgcctacagagccgccaccaaggctggctactgtggaggggccgttctcgccaaggacggagctgacacgttcatcgttggcactcactccgcaggtggcaatggagttggatactgctcgtgcgtttccagatccatgctcttgaaaatgaaggcacacattgaccctgagccacaccacgaggggttgattgtagacaccagagacgtggaagagcgcgtccacgtgatgcgcaaaaccaagcttgcacccaccgttgcacacggtgtgttcaaccctgagtttgggccagctgccttgtccaacaaggacccgcgtctgaacgagggtgttgttctcgatgaagtcatcttctccaaacacaagggagacacaaagatgtctgaggaggacaaagcgctgttccgccgctgtgctgctgattacgcgtcgcacctgcacagcgtactgggtacggcaaatgccccgttgagcatctacgaggcaattaaaggtgtcgacggactcgacgccatggagccagacaccgcacctggcctcccctgggctctccagggaaagcgccgtggtgcgctcatcgactttgagaacggtacggtcggacccgaagtcgaggctgcccttaagctcatggagaaaagagagtacaagtttgtttgtcagaccttcctgaaggacgagattcgtccgatggagaaagtacgtgccggcaagactcgcattgtcgacgtcctgcctgttgaacacattctttacaccaggatgatgattggcagattttgtgcacaaatgcactcaaacaacggaccgcaaattggttcggcggtcggttgtaaccctgatgttgattggcaaagatttggcacacacttcgcccaatacagaaacgtgtgggatgtggactattcggcctttgatgctaaccactgcagtgacgccatgaacatcatgtttgaggaggtgttccgcacggacttcgggttccacccaaatgccgagtggatcttgaagactctcgtgaatacggaacacgcctatgagaacaaacgcatcgctgttgaaggcgggatgccatctggttgttccgcaacgagcatcatcaacacaattttgaacaacatctatgtgctctacgccttgcgcagacactatgagggagttgagctggacacttacaccatgatctcctacggtgacgacatcgtggtggcgagtgattacgatctggactttgaggctctcaagcctcacttcaaatctcttggtcaaactattaccccagctgacaaaagcgacaaaggttttgttcttggtcactccattaccgatgtcactttcctcaaaagacacttccacatggactatggaactgggttttacaaacctgtgatggcttcgaagaccctcgaagctatcctctcctttgcacgccgtgggaccatacaggagaagttgatctccgtggcaggactcgcggtccactctggacctgacgagtaccggcgtctctttgagcctttccaaggcctctttgagattccaagctacagatcactttacctgcgttgggtgaacgccgtgtgcggtgacgcataa

>AY593761.1_A_KEN_1964

atggatacaacagactgttttatcgccttgatacgtgctatcagagagattaagacactgttttccctgagaacacctagagagatggagttcacactgcacaacggtgagaagaaaatcttttactctagacccaacaaccacgacaactgctggctgaacgcaattctccaactgttcaggtacgtcgatgagcctttcttcgactgggtctacgagtctcccgagaacctcacactacaggcaattgagcaattggaggaactcactggtcttgaactacacgagggtgggccgcccgctcttgttgtttggaacatcaaacacttgcttcacaccggcattggtactgcctcgcgacccagcgaggtgtgtatggtggatggtacagacatgtgcttggctgactttcatgctggaatcttcctaaaaggacaggaacacgctgtgtttgcttgcgtcacctccgaagggtggtacgcgattgacgacgaggatttctacccctggacaccggagccatccgacgtcttggtgtttgtcccgtacgatcaagagccacttaacggagactggaaagcaaaggttcagaggaaactcaagggggccgggcaatccagcccggcaaccggttcacagaaccaatctggtaacactggcagcattatcaacaactactacatgcagcagtaccagaactcaatggacacccagcttggtgacaatgccatcagcggtgggtccaacgagggttccacagacacaacctcaactcatacaaccagcactcaaaacaacgattggttttccaagcttgcaagttcagcattcacaggtctgttcggcgcactgctcgccgacaagaagactgaagagactactcttctggaggaccgcattctcaccacccgcaacggacacaccacctcgacgacacagtcgagcgtgggggtcacgtacgggtactccactggagaggaccacgttgccgggcccaacacatcgggcctggagacgcgggtggtgcaggcagaaaggtttttcaaaaagcacctctttgactggacaccggacaaagcttttggacacctggaaaaacttgaacttcccaccgaccacacaggtgtctacgggcacctggtgaactcgtatgcatatatgaggaacggttgggacgtggaggtgtctgccgttggaaaccagttcaacggcgggtgtctcctggtggccatggttccagagtggaaggaaccacaaccacgtgagaaatatcagctcactctctttccacaccagttcattagccccagaacaaacatgacagctcacatcacggtgccttaccttggtgtgaacaggtatgaccagtacaagaagcacaaaccctggacactggtagtgatggtagtgtcgccgctcacagtcagcacgacgtctgcggctcagatcaaggtctacgctaacatcgccccgacctacgtacatgtggctggggagctcccctcgaaacaggggatcgtcccggttgcgtgctcagacggttacggcggtttggtgacaacagacccgaagacagctgaccctgtctatggcaaggtgtacaacccgcccaggaccaactaccccgggcgttttacaaacttgttggatgtggccgaagcctgccctaccttcctccgtttcgacgacgggaaaccgtacgttgttacaaggacagatgaacggcggcttttggccaagttcgacgtttcccttgctgcaaaacacatgtccaacacctaccttgcagggcttgcacagtactacgcacagtactctggcaccatcaacttgcacttcatgttcactggctctgctgactcaaaagcccgctacatggtggcctatgttccacccggtgtggagcccccggacacacctgaggaggccgcccactgcatccacgcagaatgggacacaggactgaactccaaattcactttttctatcccgtacatatctgctgcagattacgcgtacaccgcgtctgacgtggcagagacaaccaacgtacagggatgggtctgcatctaccaaatcacacacgggaaggctgaaaacgacactctggtcgtgtcggtgagcgccggcaaagattttgagttacgcctcccgattgacccccgcgcacaaactactgctacgggggaatctgcagaccctgtcaccaccactgttgaaaactacggtggtgagacacaggtccagaggcggcaccacacggatgttggcttcatcatggacagatttgtaaaagtcaacagctctagtcccatgcatgtcatagacctcatgcagactcaccaacacgggctggtgggtgcgttgctgcgtgcagctacatactacttctctgacctggagattgtggtgaagcacgagggcaacctgacttgggtgcccaacggcgcccctgaggctgccctcctgaacacgagcaatcccacagcctaccacaaggaaccattcacgagacttgcacttccctacaccgcaccgcaccgtgtgctggcaacggtgtacaacgggacgagcaagtactccacaagtgtctca---agcaggcgcggtgagctggggcccctcgcggcgaggatcgccgcacagctccctgcatccttcaactacggtgcacttaaggccacgaacatccacgagcttctcgtgcgcatgaaacgggctgaactttactgccctaggccactactggcagtagaggcttcaccagacagacacaagcagaagatcattgcacccgcaaaacaacttctgaactttgacctcctcaagttggcaggagacgtcgagtccaaccctgggcccttcttcttctccgatgtcaggtcaaacttcaccaagctggtggaaaccatcaaccagatgcaggaagacatgtcaacaaaacacgggcccgactttagccggttggtgtccgcctttgaggaactggccactggagtaaaagccatcaggaacggtctcgacgaggctaagccctggtacaagctcatcaaactcctgagccgcttgtcgtgcatggccgctgtagcagcacggtccaaggacccagtccttgtggccatcatgctggctgacaccggtcttgagattctggacagcacctttgtcgtgaaaaagatctccgactcgctctccagtctctttcacgtgccggcccccgtcttcagtttcggagccccgatcctgttggccgggttggtcaaagtcgcctcgagtttcttccggtccacgcccgaagacctcgagagagcagagaaacagctcaaagcacgtgacatcaacgacattttcgccatactcaagaatggcgagtggctggtcaaactgatccttgccatccgcgactggattaaggcatggattgcctcagaagagaagtttgtcaccatgacagacttggtgcctggcatccttgagaaacagcatgacctcaacgacccgagcaagtacaaggaagccaaggaatggctcgacaacgcgcgtcaagcgtgtctgaagaacgggaacattcacattgctaacctgtgcagagtgattgccccagcacccagcaagtcgcgacctgagcccgtggtcgtttgtctccgtggcaaatccggccagggcaaaagtttccttgcaaacgtgctcgcacaggcaatttctactcacttcacaggcagaaccgactcggtttggtactgcccacctgaccctgaccacttcgacggttacaaccagcagaccgttgtcgtgatggatgatttgggccagaaccccgacggcaaggacttcaagtacttcgcccaaatggtttcgaccacggggttcatcccgcccatggcatcgctcgaggacaaaggcaaacccttcaacagcaaggtcatcatcgcaactaccaacctgtactcgggtttcaccccgaggactatggtgtgccctgatgcgttaaacaggaggtttcactttgacattgacgtgagcgccaaggacgggtacaaaattaacaacaaattggacatcatcaaagcacttgaggacacccacaccaacccggtggcaatgttccagtacgattgtgcccttctcaacggcatggccgttgagatgaagagaatgcaacaagatgtgtttaagcctcaaccgcccctcgagaacgtgtaccagctcgttcaagaggtgattgaacgggtcgagctccacgagaaagtgtcgagccacccgatcttcaaacagatctcaattccttcccaaaaatccgtgttgtactttctcattgagaagggccagcacgaggcagcaatcgagttctttgagggcatggttcacgactccatcaaagaagaactccgacccctcattcaacacacctcatttgcgaaacgcgctttcaagcgcctgaaggaaaattttgagatcgttgccctgtgtttggctctcttggccaacatagtgatcatgatccgcgagactcacaagaggcagaagatggtggatgacgcggtgaacgactacattgaaaaggcaaacatcaccacagatgacaaaacccttgatgaagctgcaaagaaccctctggaaatcagcggtgccagcaccgtcggtttcagagagaggactcccgcagggcggggtgcgtgtgacgacgtgaactccgagcccgcacagcccagcggagaccaaccgcaagctgaaggaccctacaccgggccactggagcgccagagacccctgaaggtgagagccaagctaccacaacaggagggaccctacgccggcccgatggagagacagaaaccactgaaggtgaaagtgaagacgcccgtcgtcaaggaaggtccttacgaggggccagtgaagaagcctgtcgctttgaaagtgaaagccaagaacctgattgtcactgagagtggtgccccaccgaccgacttgcaaaagatggtcatggggaacacaaagcctgttgagctcatcctcgacgggaagacagtagccatctgctgcgccactggagtgtttggcactgcctacctcgtgcctcgtcaccttttcgcagaaaagtacgacaagatcatgttggacggcagggccatgacagacagtgactacagagtgtttgagtttgagataaaagtaaaaggacaggacatgctctcggacgctgcgctcatggtgcttcaccgtgggaaccgcgtgcgagacatcacgaaacactttcgtgacacagcaagaatgaagaaaggcacccccgttgtcggtgttattaacaacgccgacgtcgggagactcattttctctggtgacgccctcacttacaaggacattgttgtgtgcatggacggggacaccatgcccggtctctttgcatacagagcagccaccaaggctggttactgcggaggagccgttcttgccaaggacggagccgacacatttatcgtgggcactcactccgcaggaggcaatggagttgggtattgctcctgcgtatccaggtccatgcttctcaagatgaaggcacacatcgaccctgaaccacaccacgaggggttgattgttgacactagagatgtggaagaacgcgtccacgtgatgcggaagacaaagcttgcacccaccgtcgcacacggtgtgttcaaccctgaattcgggcctgctgccttgtctaacaaggacccgcgcctgaacgaaggtgttgtcctcgatgaagtcatcttctccaagcacaagggagacacgaagatgtctgaggaggacaaagcgctgttccgtcgctgcgctgctgactacgcgtcacgcctgcacagcgtactgggcacggcaaatgccccattgagcatctacgaggcaatcaagggcgttgacggactcgacgccatggaaccagacactgcgcctggcctcccctgggccctccaggggaagcgccgtggcgcactcattgacttcgagaacggcacggtcggacccgaggtcgaggctgcactgaagctcatggagaacagagaatacaaatttgcttgtcaaaccttcctgaaggacgagattcgcccgatggagaaagtgcgtgccggcaagacccgcattgtcgacgttttgcctgttgaacatattctttacaccaggatgatgattggcagattttgtgcacaaatgcactcaaacaatggaccgcagattggttcagcggtcggatgcaaccctgatgttgattggcaaagatttggcacccacttcgcccagtacagaaacgtgtgggatgtggattattcggcctttgatgctaaccactgcagtgacgccatgaacatcatgttcgaggaggtcttccgcacggacttcggtttccacccaaacgctgagtggattctgaagactcttgtgaacacggagcacgcctacgagaacaagcgcatcactgttgagggcggaatgccgtctggttgttccgcgactagcatcatcaacacaattttgaacaacatctatgtgctctacgccctgcgtagacactatgagggagttgagctggacacttacaccatgatctcctacggagacgacatcgtggtagcaagtgactacgatttggacttcgaggccctcaagcctcactttaaatcccttggtcaaaccatcactccagctgacaaaagcgacaaaggttttgtacttggtcactccatcaccgatgtcactttcctcaaaagacacttccacatggattatggaactgggttttacaaacctgtgatggcctcgaagaccctcgaggctatcctctcctttgcacgccgtgggaccatacaggagaagttgatctccgtggcaggactcgccgtccactctggaccagacgagtaccggcgtctctttgagcccttccagggcctcttcgagataccaagctacagatcactttacctgcgttgggtgaacgccgtgtgcggtgacgcataa

>AY593764.1_A_IRQ_1970

atgaatacaactgactgttttatcgctttgctctacgctcttagagagatcaaagcgtttcttctttcacggacacaaggaaagatggaactcacactttacaacggtgaaaagaagactttctactccagacccaacaaccacgacaactgttggcttaacaccattctccagttgttcaggtacgtcgatgagcctttcttcgactgggtctatgactcgcctgagaacctcacctgtgaggcaattaggcagttggaagagataactggtcttgagctacacgagggtggaccacccgccctcgtcatctggaacatcaaacacttgctccacactgggatcggcactgcctcacgacctagtgaagtgtgcatggttgacggaacggacatgtgcttggctgacttccacgctggcattttcttgaaaggacaggaacatgctgtgtttgcctgcgtcacctccaacgggtggtacgcgatcgacgatgaggacttttacccctggacgccggacccgtccgatgtcttggtgtttgtcccgtatgaccaagaaccgctcaatggagagtggaaagcaaaagtccagaagcggctcaagggagccgggcaatccagtccggcgactgggtcgcagaaccagtcaggcaacactgggagtattattaacaactactacatgcaacagtaccagaactccatggacacccaattaggtgacaacgctataagcggaggctccaatgagggatccacggacacaacttccacccacacaaccaacactcagaacaacgactggttttcaaagcttgccagttctgctttcagcggtcttttcggcgcccttctcgccgataagaagaccgaggagaccactctcctcgaggaccgcattctcaccacccgcaacgggcacaccacctccacaacccaatcgagtgtgggagtcacgtacgggtactccacccaggaagatcatgtttccggacctaacacatctggtttggagacgcgggtggtgcaggcagaaagatttttcaagaagcacctgtttgattggacaccggacaaagcttttgggcacttagagaagttggaacttcccactgaccacaagggagtctacggacacttggtggactcatttgcatacatgagaaatggctgggacgtggaggtgtccgctgttggcaaccagtttaacggcgggtgtctcctggtggccatggtccctgaatggaaagagttcaccccgcgtgagaagtaccagctcactttgtttccacaccagttcatcagccccagaaccaacatgactgcccacatcgtagtcccgtaccttggtgtgaacaggtacgaccagtataagaagcacaaaccctggacgctggttgtgatggtggtctcaccgctcaccaccaacactgttagtgcaggacaaatcaaggtttatgccaacattgccccgactcacgttcacgtggccggcgagctcccctcgaaagaggggattgtaccggtcgcttgttcggacgggtacggtggcttggtgacaacagacccaaaaacagctgaccctgtttatggtatggtgtacaacccccccaggacaaactaccccgggcggttcacaaacctgttggatgtggctgaggcctgccccacctttctctgtttcgacgaagggaaaccgtacgttgtgacaagaacggacgagcagcgtcttctggccaagttcgacgtctctcttgctgcaaagcacatgtcaaacacctacctttcagggatagcacagtactacgcacagtactctggtaccatcaacctgcacttcatgtttaccggctccacggattcaaaagcccgctacatggtggcgtacgttccacccggtgtggagccgccggacacgcctgagaaagctgcacactgcatccatgctgagtgggacacagggttgaactccaagtttactttctctatcccgtacgtgtctgccgcagattacgcgtacactgcgtctgatgtggcagaaacaacaaacgtacagggatgggtctgcatataccaaattacacacgggaaagctgaacaagacactctggttgtgtcggttagcgccggcaaggactttgagttgcgcctcccgattgacccccgctcacaaaccactaccaccggggagtctgcagaccctgtcaccaccactgttgaaaactacggcggtgagacacaagtccaacgacgtcagcacaccgacgttactttcataatggacagatttgtaaagatacaaaacttgaaccccacacatgtcattgacctcatgcaaacccaccaacacgggttggtaggtgccctgttacgtgctgctacgtactacttctctgacctggagattgtggtacgccatgacggtaacctaacctgggtacccaatggagcacccgaggcagctctgtctaacacgggcaaccccaccgcctacctcaaggcaccatttacgaggctcgcgctcccctacaccgcgccacaccgcgtgttggcaacagtgtacaacgggacgagcaagtactccgcaggtggtacg---ggcagacggggcgacctagggcctctcgcggcgagggtcgccgctcagcttcctgcttctttcaactttggtgcaattcaagccacgaccatccacgagctcctcgtgcgcatgaagcgtgccgaactctactgccccagaccactgttggcagtggaggcgtctcaagacagacacaaacagaagatcattgcacctgcaaaacaacttttgaacttcgatttgctcaagttggcaggagacgttgagtccaaccccgggcccttcttcttctccgacgttaggtcaaatttttccaagctggtagagaccatcaaccagatgcaggaggacatgtcaacaaagcacggacccgactttaaccggttggtgtctgcgtttgaggaattggccactggagtgaaggctatcaggaccggtctcgatgaggccaaaccctggtacaagctcatcaagctcctgagccgcttgtcatgcatggccgctgtagcagcacggtcaaaggacccggtccttgtggctattatgctggctgacaccggccttgagattctggacagtacttttgtcgtgaagaagatttccgactcactctccagtctctttcacgtgccggcccccgtcttcagtttcggagccccgatcctgctggccgggttggtcaaagtcgcctcgagtttcttccggtctacgcccgaagacctcgagagagcagaaaaacagctcaaagcacgtgacatcaatgacatattcgccattctcaagaacggcgagtggttggtcaagctgattcttgctatccgcgactggattaaagcatggatcgcctcagaagaaaagtttgtcaccatgacagacttggtgcctggcattcttgaaaagcagcgggacctcaacgaccccagcaagtacaaggaggccaaggagtggctcgaaagcgcgcgtcaagcgtgcctgaagagtgggaatgtccacattgccaacctgtgcaaagtggtcaccccagcacctagcaagtcgagacctgaacccgtggtcgtttgcctccggggcaaatccggccagggaaagagtttccttgcaaacgtgctcgcacaggcaatttcaacgcattttactggcagaactgattcagtatggtactgtccacctgaccctgaccacttcgacggttacaaccaacagaccgttgttgtgatggatgatttgggccagaaccccgacggcaaggacttcaaatactttgcccaaatggtttcaaccacggggttcatcccgcccatggcctcgctcgaagacaaaggaaaacctttcaacagcaaagtcattatcaccactaccaacttatactcgggtttcaccccgagaaccatggtgtgccctgacgcgctgaaccggaggttccactttgacattgacgtgagcgccaaggacgggtacaaagttaacaacaaattggacataatcaaagctcttgaggacacccacaccaatccagtggcaatgtttcaatacgattgtgcccttcttaacggcatggccgttgagatgaagagaatgcaacaagacatgtttaagcctcaaccacccctccagaacgtgtaccaacttgttcaggaggtgattgaacgggtcgagctccacgagaaagtgtcgagccaccaaatttttaaacagatttcaattccttcccaaaagtctgtgttgtactttctcattgagaaaggccagcacgaagcagcaattgagttcttcgagggaatggtacacgactccatcaaggaggagctccgtcccctcatccaacaaacttcatttgtgaaacgcgcttttaagcgtctgaaggaaaactttgagattgttgccctgtgcttgactcttttggcaaacatagtgatcatgatccgcgagactcgcaagagacaacagatggtggatgatgcagtgaatgagtacattgagaaagcaaacatcaccacagacgacaaaactcttgacgaggcggaaaagaaccctttggagactagcggtgctagcactgttgggttcagagaaagaactctcccaggacacaaggcgagtggtgacgtgaactccgagcctgccagacctgtggaggaacaaccacaagctgagggaccctacgccgggccactcgagcgtcagaagcctctgaaagtgaaagccaagctgccacagcaggaaggaccttacgctggcccgatggagagacagaaaccactgaaagtgaaagtaaaagctccggtcgttaaggaaggaccttacgagggaccggtgaagaagcctgtcgctttgaaagtgaaagctaagaacttgattgtcactgagagtggagccccaccgaccgacttgcaaaagatggtcatgggcaacaccaagcctgttgagctcatcctcgacgggaagacggtggccatttgttgtgctaccggagtgtttggcactgcgtacctcgtgcctcgtcatctttttgcagaaaaatatgacaagatcatgctggacggcagagccatgacagacagtgactacagagtgtttgagtttgagattaaagtaaaaggacaggacatgctctcagacgctgcgctcatggtactccaccgtgggaatcgcgtgagagacatcacgaaacactttcgtgacacagcaagaatgaagaaaggcacccctgttgtcggagtgatcaacaatgccgacgtcgggagactgatcttctctggtgaggcccttacctacaaggacattgtagtgtgcatggatggagacaccatgcctggcctgtttgcctacaaagccgccaccaaggctggctactgtgggggagccgttcttgctaaggacggagctgacacattcatcgttggcactcactccgcaggcggcaatggagttggatactgctcatgcgtttccaggtccatgttgctgaaaatgaaggcgcacatcgaccccgaaccacaccacgaggggttgatcgttgacactagagatgttgaagagcgtgtgcatgtcatgcgcaaaaccaagcttgcacccaccgtggctcacggtgtgtttaatcctgaatttggtcccgccgccttgtccaacaaggacccgcggctgaatgaaggtgttgtcctcgatgaagtcattttctccaaacacaaaggagacacgaaaatgaccgaggaggacaaagcgctgttccgccgctgtgctgccgactacgcgtcgcgcttgcacaacgtgttgggtacggcaaatgccccactgagcatctatgaggcaataaaaggcgtcgacggccttgacgccatggaaccagacactgcgcctggccttccctgggccctccagggtaagcgccgcggcgcgttgattgacttcgagaacggcacggtcgggcccgaagtcgcggctgccttagagctcatggagaaaagacaatacaaatttgcttgtcagaccttcctgaaggacgaaattcgcccgatggaaaaagtacgtgccggcaagactcgcatcgtcgacgttttgcctgttgaacatattctttacaccaggatgatgattggcagattctgtgctcagatgcactcaaacaacggaccgcaaattggctcagcggttggctgcaatccagatgttgattggcagagatttggcacccattttgctcagtacaaaaacgtgtgggatgtggactattcggcctttgatgctaaccattgcagtgacgcaatgaacatcatgtttgaggaagtgttccgcacggagtttggtttccacccaaatgctgagtggatcctgaaaactctcgtgaacacggaacacgcctacgagaacaagcgcatcactgttgagggcgggatgccgtctggttgttccgcaacaagcatcatcaacacaattttgaacaacatctacgtgctctacgccttgcgtagacactatgagggagttgagctggacacctacaccatgatctcctacggagacgacatagtggtggcaagtgattacgatttggattttgaggctcttaagccgcactttaaatctcttggtcaaaccatcaccccagctgacaaaagcgacaaaggttttgttcttggtcactccattactgatgtcactttcctcaaaagacacttccgcatggactatggaactgggttttacaaacctgtgatggcttcgaagaccctcgaggccatcctctcctttgcacgccgtgggaccgtacaggagaagttgatctccgtggcagggctcgcagtccactctggacctgacgagtaccggcgtctctttgagccctttcagggcctctttgagattccgagctacagatcactttacctgcgttgggtgaacgccgtgtgcggtgacgcacaa

>AY593765.1_A_TUR_1965

atgaatacaattgactgttttatcgcgttgctctacgctcttagagagatcaaaacatttcttctttcacggacacaaggaaagatggaactcacactttacaacggtgaaaagaagaccttctactccagacccaacaaccacgacaactgttggcttaacaccattctccagttgtttaggtacgtcgatgagcctttcttcgactgggtctatgactcgcctgagaacctcacctgcgaggcaattaggcagctggaagagataactggtcttgagctacacgagggtggaccacccgccctcgtcatctggaacatcaaacacttgctccacactggaatcggcactgcctcacgacctagtgaagtgtgcatggttgacggaacggacatgtgcttggctgacttccacgctggcattttcttgaaaggacaggaacatgctgtgtttgcctgcgtcacctccaacgggtggtacgcgatcgacgatgaggacttttacccctggacgccggacccgtccgatgtcttggtgtttgtcccgtacgatcaagaaccgctcaatggagagtggaaagcaaaagtccagaagcggctcaagggagccgggcaatccagtccggcgactgggtcgcagaaccagtcaggcaacactgggagtattattaacaactactacatgcaacagtaccagaactccatggacacccaattaggtgacaacgctataagcggaggctccaatgagggatccacggacacaacttccacccacacaaccaacactcagaacaacgactggttttcaaagcttgccagttctgctttcagcggtcttttcggcgcccttctcgccgataagaagaccgaggagaccactctcctcgaggaccgcattctcaccacccgcaacgggcacaccacctccacaacccaatcgagtgtgggagtcacgtacgggtactccacccaggaagatcatgtttccggacctaacacatctggtttggagacgcgggtggtgcaggcagaaagatttttcaagaagcacctgtttgattggacaccggacaaagcttttgggcacttagagaagttggaacttcccactgaccacaagggagtctacggacacttggtggactcatttgcatacatgagaaatggctgggacgtggaggtgtccgctgttggcaaccagtttaacggcgggtgtctcctggtggccatggtccctgaatggaaagagctcaccccgcgtgagaagtaccagctcactttgtttccacaccagttcatcagccccagaaccaacatgactgcccacatcgtagtcccgtaccttggtgtgaacaggtacgaccagtataagaagcacaaaccctggacgctggttgtgatggtggtctcgccgctcaccaccaacactgttagtgcaggacaaatcaaggtttatgccaacattgccccgacccacgttcacgtggccggcgagctcccctcgaaagaggggatcgtgccggtcgcctgttcggacgggtatggtggcttggtgacaacagacccaaaaacagctgaccctgtttatggtatggtgtataacccccccaggacaaactaccccgggcggttcacaaacctgttggatgtggcagaggcctgccccacctttctctgcttcgacgacgggaaaccgtacgttgtgacaagaacggatgagcagcgtcttctggccaagttcgacgtctctcttgctgcaaagcacatgtcaaacacctacctttcagggatagcacagtactacgcacagtactctggtaccatcaacctgcacttcatgtttaccggctccactgactcaaaagcccgctacatggtggcgtacgttccgcccggtgtagagccgccggacacgcctgagaaagctgcacactgcatccatgctgagtgggacacagggttgaactctaaatttactttctctatcccgtacgtgtctgccgcagactacgcgtacactgcgtctgacgtggcagaaacaacaaacgtacagggatgggtctgcatataccaaattacacacgggaaagctgaacaagacactctggttgtgtcggttagcgccggcaaggactttgagttgcgcctcccgattgacccccgctcacaaaccactaccaccggggagtctgcagaccctgtcaccaccaccgttgaaaactacggcggtgagacacaagtccaacgacgtcagcacaccgacgttactttcataatggacagatttgtaaagatacaaaatttgaaccccatacatgtcattgacctcatgcaaacccaccaacacgggttggtaggtgccctgttacgtgctgctacgtactacttctctgacctggagattgtggtacgccatgacggtaacctaacctgggtacccaacggagcacccgaggcagctctgtctaacatgggcaaccccaccgcctacctcaaggcaccatttacgaggctcgcgctcccctacaccgcgccacaccgcgtgttggcaacagtgtacaacgggacgagcaagtactccgcaggtggtacg---ggcagacggggcgacctagggcctctcgcggcgagggtcgccgctcagcttcctgcttctttcaactttggtgcaattcaagccacgaccatccacgagctcctcgtgcgcatgaagcgtgccgaactctactgccctagaccactgttggcagtggaggcgtctcaagacagacacaaacagaagatcattgcacctgcaaaacaacttttgaactttgatttgctcaagttggcaggagatgttgagtccaaccccgggcccttcttcttctccgacgttaggtcaaatttttccaagctggtagagaccatcaaccagatgcaggaggacatgtcaacaaagcacggacccgactttaaccggttggtgtctgcgtttgaggaattggccactggagtgaaggctatcaggaccggtctcgatgaggccaaaccctggtacaagctcatcaagatcctgagccgcttgtcatgcatggccgccgtagcagcacggtcaaaggacccggtccttgtggctattatgctggctgacaccggccttgagattctggacagtacttttgtcgtgaagaagatctccgactcactctccagtctctttcacgtgccggcccccgtcttcagtttcggagccccgatcctgctggccgggttggtcaaagttgcctcgagtttcttccggtctacgcccgaagaccttgagagagcagaaaaacagctcaaagcacgtgacatcaatgacatattcgccattctcaagaacggcgagtggttggtcaagctgattcttgctatccgcgactggattaaagcatggatcgcctcagaagaaaagtttgtcaccatgacagacttggtgcctggcattcttgaaaagcagcgggacctcaacgaccccagcaagtacaaggaggccaaggagtggctcgaaagcgcgcgtcaagcgtgcctgaagagtgggaatgtccacattgccaacctgtgcaaagtggtcaccccagcacctagcaagtcgagacctgaacccgtggtcgtttgcctccggggcaaatccggccagggaaagagtttccttgcaaacgtgcttgcacaggcaatttcaacacattttactggcagaaccgattcagtttggtactgtccacctgaccctgaccacttcgacggttacaaccaacagaccgttgttgtgatggatgatttgggccagaaccccgacggcaaggacttcaaatactttgcccaaatggtttcaaccacggggtttatcccgcccatggcctcgctcgaagacaaaggaaaacccttcaacagcaaagtcattatcaccactaccaacttatactcgggtttcaccccgagaaccatggtgtgccctgacgcgctgaaccggaggttccactttgacattgacgtgagcgccaaggacgggtacaaagttaacaacaaattggacataatcaaagctcttgaggacacccacaccaatccagtggcaatgtttcaatacgattgtgcccttcttaacggcatggccgttgagatgaagagaatgcaacaagacatgtttaagcctcaaccacccctccagaacgtgtaccaacttgttcaggaggtgattgaacgggtcgagctccacgagaaagtgtcgagccaccaaatttttaaacagatttcaattccttcccaaaagtctgtgttgtactttctcattgagaaaggccagcacgaagcagcaattgagttcttcgagggaatggtacacgactccatcaaggaggagctccgtcccctcatccaacagacttcatttgtgaaacgcgcttttaagcgtctgaaggaaaactttgagattgttgccctatgtttgactctcttggcaaacatagtgatcatgatccgcgagactcgcaagagacaacagatggtggatgatgcagtgaatgagtacattgagaaagcaaacatcaccacagatgacaaaactcttgacgaggcggaaaagaaccctttggagactagcggtgccagcactgttgggttcagagaaagaactctcccaggacacaaggcgagtgatgacgtgaactccgagcccgccagacctgtggaggaacaaccacaagctgaaggaccctacgccgggccactcgagcgccagaagcctctgaaagtgaaagccaagctgccacagcaggaaggaccttacgctggcccgatggagagacagaaaccactgaaagtgaaagtaaaagctccggtcgttaaggaaggaccatacgagggaccggtgaagaagcctgtcgctttgaaagtgaaagctaagaacttgattgtcactgagagtggagccccaccgaccgacttgcaaaagatggtcatgggcaacaccaagcctgttgagctcatcctcgacgggaagacggtggccatctgttgtgctaccggagtgtttggcactgcgtacctcgtgcctcgtcatctttttgcagaaaaatatgacaagatcatgctggacggtagagccatgacagacagtgactacagagtgtttgagtttgagattaaagtaaaaggacaggacatgctctcagacgctgcgctcatggtactccaccgtgggaatcgcgtgagagacatcacgaaacactttcgtgacacagcaagaatgaagaaaggcacccctgttgtcggagtgatcaacaatgccgacgtcgggagactgattttctctggtgaggcccttacctacaaggacattgtagtatgcatggacggagacaccatgcctggcctgtttgcctacaaagccgccaccaaggctggctactgtgggggagccgttcttgttaaggacggagctgacacattcatcgttggcactcactccgcaggcggcaatggagttggatactgctcatgcgtttccaggtccatgttgctgaaaatgaaggcgcacatcgaccccgaaccacaccacgaggggttgatcgttgacactagagatgttgaagagcgtgtgcatgtcatgcgcaaaaccaagcttgcacccaccgtggctcacggtgtgtttaatcctgaatttggtcccgccgccttgtccaacaaggacccgcggctgaatgaaggtgttgtcctcgatgaagccattttctccaaacacaaaggagacacgaaaatgaccgaggaggacaaagcgctgttccgccgctgtgctgccgactacgcgtcgcgcttgcacaacgtgttgggtacggcaaatgccccactgagcatctacgaggcaataaaaggcgtcgacggccttgacgccatggaaccagacactgcgcctggccttccctgggccctccagggtaagcgccgcggcgcgttgattgacttcgagaacggcacggtcgggcccgaagtcgcggctgccttagagctcatggagaaaagacaatacaaatttgcttgccagaccttcctgaaagacgaaattcgcccgatggagaaagtacgtgccggcaagactcgcatcgtcgatgttttgcctgttgaacatattctttacaccaggatgatgattggcagattctgtgctcagatgcactcaaacaacggaccgcaaattggctcagcggttggttgtaatccagatgttgattggcagagatttggtacccattttgctcagtacaaaaacgtgtgggatgtggactattcggcctttgatgctaaccattgcagtgacgcaatgaacatcatgtttgaggaagtgttccgcacggaatttggtttccacccaaatgctgagtggatcctgaaaactctcgtgaacacggaacacgcctatgagaacaagcgcatcactgtcgagggcgggatgccgtctggttgttccgcaacaagcatcatcaacacaattttgaacaacatctacgtgctctacgccttgcgtagacactatgagggagttgagctggacacctacaccatgatctcctacggagatgacatagtggtggcaagtgattacgatttggattttgaggctcttaagccgcactttaaatctcttggtcaaaccatcaccccagctgacaaaagcgacaaaggttttgttcttggtcactccattactgatgtcactttcctcaaaagacacttccgcatggactatggaactgggttttacaaacctgtgatggcttcgaagaccctcgaggccatcctctcctttgcacgccgtgggaccatacaggagaagttgatctccgtggcaggactcgcagtccactctggacctgacgagtaccggcgtctctttgagccctttcagggcctctttgagattccgagctacagatcactttacctgcgttgggtgaacgccgtgtgcggtgacgcataa

>AY593766.1_A_KEN_1965

atgaacacaactgactgttttatcgctctgttacacgcccttagagagatcaaaacactctttctttcacggacacaaggaaagatggagctcacactctacaacggtgagaagaagacattttactcaagacccaacaaccacgataactgctggttgaacacaatcctccaactgttcaggtacgtcgaagaacctttcttcgactgggtttatgagtcccctgagaacctcacactgcaagcaattgaacaacttgaggacctaactggtcttgagctgcacgacggtggaccacccgccctcgtgatttggaacatcaaacacttgctctctactggtgtaggcactgcctcgcgacccagcgaggtgtgcatggtggacggcacagacatgtgtctggccgatttccacgctgggatcttcatgaaaggacaggaacacgctgtgttcgcttgtgtcacatccaacgggtggtgcgcgattgatgacgaggacttctacccctggacaccggacccctccgacgtcttggtgttcgtcccgtacgatcaagaaccgctcaatggtgaatggaaaacaaaggtccaaaagaacctcaagggagccgggcaatccagcccggcgaccggctcacaaaaccaatctggcaacactgggagcatcattaacaattactacatgcagcagtaccagaactccatggacacacagcttggagacaacgccatcagcggaggctccaatgaaggttctacagacacaacttctacccacacaaccaacacccagaacaacgactggttttcaaaacttgcaagctcggcgtttaccggcttgttcggggccctgcttgctgacaagaaaaccgaggagaccactcttttggaggaccgcatccttaccacccgcaacggccacaccacctcgacgacgcagtcgagtgtgggggtcacctacgggtactccactgcggaggaccacgttgccgggcctaacacatcgggcctggagacgcgggttgcgcaggctgaaaggttcttcaaaaagcacctgtttgactggacaacggacaaaccatttggacacattgaaaaactggagctccccaccgaccaccgcggtgtctacgggcacctagtggaatcatacgcatacatgaggaacggttgggatgtagaggtgtctgctgttggaaaccaattcaacggcggctgccttctggtggccatggtcccggagtggaaagagtttgacaaccgtgaaaagtaccaactcacccttttcccacaccaattcatcagccctaggacaaacatgacagcacacatcacggtaccgtaccttggtgtgaacaggtatgaccagtacaagaagcacaaaccctggaccctggtggtaatggtggtgtcaccactcacagtcagccagactgctgcaagccaaatcaaggtctacgccaacatcgccccaacccacgtgcacgtagccggggagctcccctcgaaagaggggattgtcccggttgcatgttccgacggttacggtggtttggtgacaacagacccgaagacagctgaccctgtttacggcaaggtgtacaacccgcccaggactaactaccctgggcgcttcacaaacttgttggacgtggctgaggcctgccccaccttcctctgtttcgacgacgggaaaccgtatgttgtcacgaggacagatgaacagcggctcttagctaagtttgacgtttcacttgctgcaaaacacatgtctaacacctatctctcagggattgcacagtactatgcccagtactctggtaccatcaacttgcatttcatgttcactggttcaactgactcaaaagcccgctacatggtggcttatgtcccgcccggtgtggaaccaccggacacgcctgagaaggctgcccactgcatccacgccgagtgggacacgggattgaattccaaattcactttctccatcccgtacgtttctgccgctgactacgcctacaccgcgtccgacgaggcagagacgaccaatgtgcaagggtgggtctgcatttaccagattacacacgggaaagctgagaacgacaccttggtcatctcggcaagcgcgggcaaagactttgaactgcgcctcccgattgacccccgcccgcagacaactgccaccggggagtccgcagaccctgtcaccaccaccgtcgaaaactacggtggtgagacacaagcccaaaggcggcaccacacggaggtcgccttcatcatggacagatttgtgaacatcaaagctcccagccccacgcacgtcattgacctcatgcaaacccaccagcacgggcttgtgggcgccttgttgcgcgctgccacgtactacttctccgacttggagatcgtggtacgacacgaaggcaatttgacttgggtgcccaacggtgcccctgagggcgctcttgcaaacacgggaaaccccaccgcctacaacaaggcaccattcacgagacttgcactcccttacaccgcgccgcaccgagtgctggcaacagtgtacaacgggacgaacaagtattccaggagtggtgcg---accaggcggggtgacatggcagccctcgcagcgagggttgccacccagcttcctgcatctttcaattacggagcacttcgggccaccaacatccatgaaatccttgtgcgcatgaagcgagctgaactctactgccccaggccactactggcggtggaagcagcccctgacaggcacaaacagaagatcattgcgcccgcaaaacagctcttgaattttgaccttctcaagttagccggagacgtagagtccaaccctgggcccttcttcttctccgacgtcaggtcaaacttcaccaaactggtggagaccatcaaccaaatgcaggaggacatgtcaacaaaacacggacccgactttaacaggttggtgtccgcatttgaggaattggccactggagtgaaagccatcaggaccggtctcgacgaagccaagccctggtacaaactcatcaagctcctgagccgtttgtcatgcatggccgctgtagcagcacggtcgaaggacccagtccttgtggccatcatgctggctgacaccggtcttgagattctggacagcaccttcgtcgtgaagaagatctccgactcgctctccagtctctttcacgtgccggcccccgtcttcagtttcggagccccgattctgctggccgggttggtcaaggtcgcctcgagtttcttccggtccacgcccgaagaccttgagagagcggaaaaacagctcaaagcacgtgacatcaatgacatattcgccattctcaagaacggcgagtggctggttaagctgattcttgccatccgcgactggatcaaggcatggatcgcctcagaagaaaagtttgtcaccatgacagacttagtgcctggcattcttgaaaagcagcgggacctcaacgaccccggcaagtacaaggaagctaaggagtggctcgacaatgcgcgccaagcgtgtttgaagagcgggaatgtccacattgccaacctgtgcaaagtggtcgccccagcacccagcaggtcgagacccgaacctgtggtcgtttgcctccgtggcaaatccggccagggaaagagtttccttgcgaacgtgctcgcacaagcaatttccacacacttcactggcagaacagactcagtctggtactgcccacctgaccctgaccactttgacggttacaaccaacaaaccgttgttgtgatggatgatctgggccaaaaccccgacggcaaggacttcaagtacttcgcccaaatggtgtcgaccacggggttcatcccacccatggcctcgctcgaggacaagggcaaacctttcaacagcaaagtcatcatcgccactaccaacctgtactcggggttcaccccgagaaccatggtgtgccccgatgcgctgaacagaaggtttcactttgacattgacgtgagtgccaaggatgggtacacaatcaacaacaaattggacataaccaaagctcttgaagacacccacaccaatccggtggcaatgtttcaatacgactgtgcccttctcaacggcatggctgttgagatgaagagaatgcaacaagacatgttcaaacctcaaccacccctccagaacgtttaccaacttgttcaggaggtaattgagcgggtcgagctccacgagaaggtgtcgagccacccgattttcaagcagatctcaattccttcccaaaaatctgtgttgtacttcctcattgagaaaggccaacacgaagcagcaattgaattctttgagggaatggtgcacgactccatcaaggaggagctccggcccctcatccaacaaacctcatttgtgaaacgcgctttcaagcgcttgaaggaaaactttgagatcgttgccctgtgtttgaccctcttggcaaacatagtgatcatgatccgcgagactcgcaagagacagaagatggtggatgatgcagtgaacgagtacattgagaaggcaaacatcaccacagatgacaagactcttgatgaggcggaaaagaacccgctggagaccagcggtgccagcaccgttggcttcagagagaggactctcccgggacacaaggcgagtgatgacgtgaactccgagcccgcccagcccctggagaagcaaccacaagctgaaggaccctacgccgggccactcgagcgtcagaaacctttgaaagtgagagccaagctaccacagcaggagggaccttacgctggcccgatggagagacaaaaaccactgaaggtgaaagcaaaagccccggtcgtcaaggaaggaccttacgagggaccggtgaagaagcctgtcgctttgaaagtgaaagccaagaatttgattgtcactgagagtggcgccccaccgactgacttgcaaaagatggtcatgggcaacaccaaacctgtcgaactcatcctcgacgggaagacggtagccatctgctgcgctaccggagtgtttggcactgcttacctcgtgcctcgtcacctcttcgcagagaagtatgacaagatcatgttggacggtagagccatgacagacagtgactacagagtgtttgagtttgagattaaagtaaaaggacaggacatgctctcagacgccgcgctcatggtgcttcaccgcgggaaccgtgtgagagacatcacgaaacactttcgtgacacagcaagaatgaagaaaggcacccccgtcgtcggagtgatcaacaacgccgatgtcgggagactgattttctctggtgaggccctcacctacaaggacattgtagtgtgcatggatggagacaccatgcctggcctctttgcctacagagccgccaccaaggctggctactgcgggggagccgttctcgctaaggacggagccgacacattcatcgttggcactcactccgcaggcggcaatggagtcggatactgctcatgtgtgtccaggtccatgctgctgaaaatgaaggcacacatcgaccctgagccgcaccacgaggggttgatcgttgacaccagagatgtggaagaacgcgtgcatgtcatgcgcaaaaccaagcttgcacccaccgtagcacacggtgtgttcaaccctgagttcggccccgctgccctgtccaacaaggacccgcgactgaacgaaggtgttgtcctcgatgaagtcatcttctccaaacacaaaggagacacaaagatgaccgaggaggacaaagcgctgttccgtcgctgtgctgctgactacgcgtcgcgcttgcacagtgtactgggtacggcaaatgccccactgagcatttacgaggcaatcaagggcgtcgacggcctcgacgccatggaaccagacaccgcgcctggtctcccctgggccctccaggggaaacgccgtggtgcgctgattgacttcgagaacggcacggttggacccgaggtcgcggctgccctgaagctcatggagaaaagagagtacaaatttgcttgtcagaccttcctgaaggacgagattcgcccgatggagaaagtacgtgccggcaagactcgcattgtcgacgttttgcctgttgaacatattctttacactaggatgatgattggtagattctgtgctcaaatgcactcaaacaacggaccgcaaattggctcagcggtcggttgtaacccagatgttgattggcaaagatttggcacacactttgcccagtacagaaatgtgtgggatgtggactattcggcctttgatgccaaccattgcagcgatgcgatgaacatcatgtttgaggaggtgtttcgcacggattttggtttccacccgaacgccgagtggatcctgaagactctcgtgaatacggagcacgcctatgaaaacaaacgcatcactgttgagggcgggatgccgtctggctgttccgcaacaagcatcatcaacacaattttgaacaacatctacgtgctctacgccctgcgtagacactatgagggagttgagctggacacttacaccatgatctcctacggagatgacatcgtggtggcaagtgattacgatctggactttgaggcccttaggccacacttcaaatctcttggtcaaaccattaccccagctgacaaaagcgacaaaggttttgttcttggccattccatcaccgacgtcactttcctcaaaagacacttccacatggattatggaactgggttttacaaacctgtgatggcctcgaagaccctcgaggctatcctctcctttgcacgccgtgggaccatacaggagaagttgatctccgtggcagggctcgccgtccattctggacctgacgagtaccggcgtctctttgagccttttcagggcctcttcgagattccaagctacagatcactttatctgcgttgggtgaacgccgtgtgcggcgacgcataa

>AY593767.1_A_ARG_1965

atgaacacaactgactgttttatcgctttggtgcacgcaatcagagagatcagagcacttttcctaccacgaaccacaggaaagatggaactcaccctgtacaacggcgagaaaaagactttctactccagacctaacaaccacgacaactgttggttgaacactgtccttcagttgttcaggtatgtcgatgagcccttcttcgactgggtctacaactcacctgagaacctcacgctcgaagccatcgagcaattggaggaactcacaggacttgagctgcacgaaggtgggccgcccgccctcgtgatctggaacatcaaacacttgctccacaccggcatcggcacagcctcacgacccagtgaggtgtgcatggtggacggtacggacatgtgtcttgccgacttccacgcaggcattttcctgaagggacaggaacacgcagtctttgcatgtgtcacctccaacgggtggtacgcgattgatgatgaggaattttacccctggacgcctgacccgtcagacgtcctggtgtttgtcccatacgaccaagagccactcaacggggactggaaagcgatggtccagaggaagcttaagggcgccgggcaatccagcccggcgaccggctctcagaaccagtctggcaacactggcagcataatcaataactactacatgcagcagtaccagaactccatggacacgcagcttggtgacaatgccatcagtggaggctccaacgaaggctccacggacacaacgtcaacacacacaaccaacacccaaaacaacgactggttttcgaaacttgccagctcagcctttaccggtctgttcggcgccttgcttgccgacaagaagacggaagagactacgcttctggaggaccgcattctcaccacccgcaacgggcacaccatctcgaccacccagtcgagtgtgggagtcacctacgggtactccactggagaagaccacgttgctgggcccaacacatcgggcctggagacgcgggtggtgcaggctgagagattttacaaaaagtttttgtttgattggacaacggataagccttttggacatttggaaaagttggaacttcccaccgaccaccacggtgttttcgggcacttggtggaatcgtatgcctacatgagaaacggttgggacgttgaggtgtctgctgttggcaaccagttcaacggcgggtgtctcctggtggctatggtaccggagtggaaggagtttgaacaacgtgagaagtaccagctcaccctctttccccaccagttcattagccccagaacaaacatgactgcccacattactgtcccataccttggagtgaacaggtacgaccagtacaagaaacacaaaccttggaccctggttgttatggtagtgtcgccccttacagttagcagcactgccgcggcacagattaaggtctacgccaacattgctccaacctacgttcacgtggccggggaactaccctcgaaggaggggattttcccggttgcatgttcggacggttacggaggactggtgacaacagacccgaaaacagctgaccctgcctacggcaaggtgtacaacccgcccaggaataactaccccgggcggttcaccaacttgttggacgtggctgaagcgtgtcccactttcctctgtttcgacgacgggaaaccgtacgtcgttacgcggacagatgacacacgactcttagccaagttcgacgtttcccttgccgcaaaacacatgtccaacacgtacctgtcagggatagcacagtactatacacagtactctggtaccatcaacttgcacttcatgtttacaggttcaacagattcaaaggcccgttacatggtggcctacatcccgcccggggtggaaccaccggacacacctgaaagggctgcccactgtatccacgctgaatgggacacaggactgaactccaaattcactttttcaatcccgtacgtgtccgccgcagattacgcgtacaccgcgtctgacacggcagaaacaaccaacgtacagggctgggtctgcatttaccagattacacacgggaaggccgagaacgacacactagtcgtgtcggccagcgccggcaaggactttgagttgcgcctcccgattgacccgcgacggcaaaccaccgctgttggggagtccgcagaccctgtcaccaccaccgtggagaactacggcggtgagacacagacccagaggcgacatcatacagatgtcagtttcatcatggacagatttgtgaaaataaacagcttgagtcccacacatgtcattgacctcatgcagacccaccaacacgggctggtgggcgcgctgctgcgtgcagccacgtactacttctccgacttggagattgttgtgcggcatgacggtaatttgacttgggtgcccaacggtgcgcctgaagcagctttgtcaaacaccagcaaccccactgcctacaacaaggcaccgttcacgaggctcgctctcccttacactgcgccacaccgcgtgttggcaacggtgtacaacgggacgaacaagtactccacgggcggtacg------ggaagaggcgacacgggttcgctcgcggcgcgggtcgcgaaacaacttcctgcttccttcaactacggtgcaatcagggctgacgccatcaacgagcttctcgtgcgcatgaaacgggcagagctctactgccctaggcccatattggcaatagaagcttcacaagacaggcacaaacaaaagatcattgcacccgcaaaacagttgttgaacttcgatctgcttaagttggctggagacgtggagtccaaccctgggcccttcttcttctctgacgtcaggtcgaacttttccaaactggtagaaaccatcaaccagatgcaggaagacatgtcaacaaaacacgggcccgactttaaccggttggtgtccgcctttgaggaactggccactggagtaaaagccatcaggaccggtcttgacgaggccaaaccctggtacaagctcatcaaactcctaagccgcctgtcgtgtatggccgctgtggcagcacggtccaaggacccagttcttgtggccatcatgctggctgacaccggcctcgagattctggacagcaccttcgtcgtgaagaagatctccgactcgctctccagtctcttccacgtgccggcccccgtcttcagtttcggagccccgatcttgctagccgggttggtcaaggtcgcctcgagtttcttccggtccacgcccgaagaccttgagagagcagagaaacagctcaaagcacgtgacatcaacgacatcttcgccattctcaagaacggcgagtggctggtcaaactgatccttgccatccgcgactggattaaggcttggatcgcctcagaagagaagtttgtcaccatgacagacttggtgcctggcatccttgaaaagcagcgggatctgaacgacccgagcaagtacaaggaagccaaggagtggctcgacaacgcgcgccaagcgtgtttgaagagcgggaacgtccacattgccaacctgtgtaaagtggtcgctccagcacccagcaagtcgaggcccgaacccgtggttgtttgcctccgcggcaaatctggccagggcaagagcttccttgcaaacgtgcttgcacaggcaatttccgcccacttcaccggcagaaccgactcagtgtggtactgcccacctgaccctgaccacttcgacggttacaaccagcaaaccgtcgttgtgatggatgatttgggccagaaccctgacggcaaggacttcaaatactttgcccaaatggtctcgaccacagggttcatcccgcccatggcatcactcgaggacaaaggtaaacctttcaacagcaaagtcatcatcgcgaccaccaacttgtactcgggcttcaccccgaggactatggtgtgtcccgacgcactgaaccggaggtttcactttgacatcgatgtgagtgctaaggatgggtacaaaattaacaacaaactggacattatcaaagcacttgaagacacccacaccaacccagtggcaatgtttcaatacgactgtgcccttctcaacggcatggccgttgaaatgaagagaatgcaacaagacatgttcaagcctcaaccacccctccagaatgtgtaccagcttgttcaggaggtgattgatcgggtcgagctccacgagaaagtgtcgagccacccgatcttcaagcagatctcaattccttcccaaaaatctgtgttgtactttctcattgagaagggccaacatgaggcagcaattgaattctttgagggcatggtccacgactccatcaaagaggaactccgacccctcatccaacaaacttcatttgtgaaacgcgctttcaagcgcctgaaggaaaattttgagattgttgctctgtgtttaacacttttggcgaacattgtgatcatgatccgtgagactcgcaagaggcagaaaatggtggatgatgcagtgaatgagtacattgagaaagcaaacatcaccacagatgacaagactcttgacgaggcggagaagagccctctagagaccagcggcgccagcaccgttggctttagagagagaactctcccaggtcaaaaggcacgcgatgacgtgaactccgagcctgcccaacctgttgaggagcaaccacaagctgaaggaccctacgccggaccactcgagcgtcagaaacctctgaaagtgagagccaagctcccacagcaggaggggccttacgctggtccgatggagagacagaaaccgctaaaagtgaaagcaaaagccccggtcgtgaaggaaggaccttacgagggaccggtgaagaagcctgtcgctttgaaagtaaaagctaagaacctgattgtcactgagagtggtgccccaccgaccgacttgcaaaagatggtcatgggcaacacaaagcctgttgagctcatcctcgacgggaagacagtagccatctgctgcgctactggagtgtttggcactgcttacctcgtgcctcgtcacctcttcgcagagaagtacgacaagatcatgttggacggcagagccatgacagacagtgactacagagtgtttgagtttgagatcaaagtaaaaggacaggacatgctctcagacgccgcgctcatggtgctccaccgtgggaaccgcgtgagggacatcacgaagcactttcgtgacacagcaagaatgaagaaaggcacccccgttgtcggtgtgattaacaacgccgatgtcgggagactgattttctctggtgaggcccttacttacaaggacattgtggtttgcatggacggagacaccatgcctggcctctttgcctacagagccgccaccaaggctggttactgcggaggagccgttcttgccaaagacggagctgacactttcatcgtcggcactcactctgcgggaggcaacggagttggatactgctcatgcgtttccaggtccatgcttcttaaaatgaaggcacacattgaccccgaaccacaccacgaggggttgattgtggacatcagagatgtggaagagcgcgttcacgtgatgcgcaaaaccaagcttgcacccaccgttgcacacggtgtgttcaaccccgagtttgggcccgctgccttgtccaacaaggacccgcgtctgaacgggggtgttgtcctcgatgaagtcatcttctccaaacacaagggagacacaaagatgtctgaggaggacaaagcgctgttccgccgctgcgctgctgactacgcgtcacgcctgcacagcgtgttgggcacagcaaatgccccactgagcatctacgaggcaatcaagggtgtcgacggactcgacgccatggaaccagacactgcgcccggcctcccctgggccctccagggtaaacgccgcggcgcgctcatcgacttcgagaacggcacggtcggacccgaagtcgaggctgccctgaagctcatggagaagagagaatacaaatttgtttgtcagaccttcctgaaggacgagatccgcccgttggagaaagtacgtgccggcaagactcgcattgtcgacgtcctgcccgttgagcatattctttacaccaggatgatgattggcagattttgtgcacagatgcactcaaacaacggaccgcaaattggctcagcggtcggttgcaaccctgatgttgattggcagagatttggcacacacttcgcccagtacagaaacgtgtgggatgtggactattcggcctttgatgctaatcactgcagtgatgccatgaacatcatgtttgaggaggtgtttcgcacggagttcggcttccacccgaatgccgagtggatcctgaagactcttgtgaacacggaacacgcctatgagaacaaacgcatcactgttgaaggcggaatgccgtctggttgttccgcgacaagcatcatcaacacaattttgaacaacatctacgtgctttacgctctgcgtagacactatgagggagttgagctggacacgtacaccatgatctcctacggagacgacatcgtggtggcaagtgattatgatttggacttcgaggctctcaagccccactttaaatcccttggtcaaaccatcactccagctgacaaaagcgacaaaggttttgttcttggtcactccattaccgatgtcactttcctcaaaagacacttccacatggactatggaactgggttttacaaacctgtgatggcctcaaagacccttgaggctatcctctcctttgcacgccgtgggaccatacaggagaagttgatctccgtggcaggactcgccgtccactctggaccagacgagtaccggcgtctctttgagcctttccaaggtctctttgagattccaagctacagatcactttacctgcgttgggtgaacgccgtgtgcggtgacgcataa

>AY593768.1_A_Brazil_1955

atgaacacaactgattgttttatcgctttggtacacgctatcagagagatcagagcatttttcctaccacgagccacaggaaggatggaattcacactgcacaacggtgagagaaaagtgttctattctagacccaacaaccacgacaactgttggttgaacaccatccttcagctgttcaggtacgtcggagaacccttcttcgactgggtctatgactcacccgagaacctcactcttgaagctatcgagcaactggaggagctcacagggttagagttgcacgagggcggaccacctgccctcgtgatctggaacatcaaacacctgcttcataccggcatcggcaccgcctcgcggcccagcgaggtgtgcatggtggacggcacgaacatgtgtcttgctgacttccacgcaggcattttcctgaaaggacaggaacacgctgtgtttgcgtgtgtcacctccaacgggtggtacgcgattgacgacgaggacttttacccatggacgccggacccgtccgacgttttggtgtttgttccgtacgatcaagagccacttaacggagaatggaaaaccaaggttcagcagaagctcaagggggccgggcaatccagtccggcgaccggctcgcagaaccaatctggcaacactggcagcataattaacaactactacatgcagcaataccagaactccatggacacacagttgggagacaatgccatcagtggaggctccaacgagggctccacggacacaacttcaacacacacaaccaacactcaaaacaatgactggttctcgaagctcgccagttcagcttttaccggtctgttcggtgcactgctcgccgacaagaagacagaggaaacgacacttcttgaggaccgcatcctcaccacccgcaacgggcacaccacctcgacgacccaatcgagtgtgggtgtcacacacgggtactccacagaggaggaccacgttgctgggcccaacacatcgggcctggagacgcgagtggtgcaggcagagagattctacaaaaagtacttgtttgactggacaacggacaaggcatttggacacctggaaaagctggagctcccgtccgaccaccacggtgtctttggacacttggtggactcgtacgcctatatgagaaatggctgggatgttgaggtgtccgctgttggcaaccagttcaacggcgggtgcctcctggtggccatggtacctgaatggaaggaatttgacacacgggagaaataccaactcacccttttcccgcaccagtttattagccccagaactaacatgactgcccacatcacggtcccctaccttggtgtgaacaggtatgatcagtacaagaagcataagccctggacattggttgtcatggtcgtgtcgccacttacggtcaacaacactagtgcggcacaaatcaaggtctacgccaacatagctccgacctatgttcacgtggccggtgaactcccctcgaaagaggggattttcccggttgcatgtgcggacggttacggaggattggtgacgacagacccgaagacagctgaccctgcttatggcaaggtgtacaacccgcctaggactaactaccctgggcgcttcaccaacctgttggacgtggccgaagcgtgtcccactttcctctgctttgacgacgggaaaccgtacgtcaccacgcggacggatgacacccgacttttggccaagtttgacctttcccttgccgcaaaacatatgtccaacacatacctgtcagggattgctcagtactacacacagtactctggcaccatcaatttgcatttcatgttcacaggttccactgattcaaaggcccgatacatggtggcctacatcccacctggggtggagccaccggacacacctgaaagggctgcccactgcattcacgctgaatgggacactggactaaactccaaattcactttctcaatcccgtacgtatccgccgcggattacgcgtacacagcgtctgacacggcagaaacaatcaacgtacagggatgggtctgcatctaccaaattacacacgggaaggctgaaaatgacaccttggtcgtgtcggttagcgccggcaaagactttgagttgcgcctcccgattgacccccgccagcagaccaccgctaccggggaatcagcagacccggtcaccaccaccgtggagaactacggcggtgagacacaaatccagagacgtcaccacacggacattggtttcatcatggacagatttgtgaagatccaaagcttgagcccaacacatgtcattgacctcatgcagactcaccaacacggtctggtgggtgccttgctgcgtgcagccacgtactacttttctgacctggaaattgttgtacggcacgaaggcaatctgacctgggtgcccaacggcgcccctgaatcagccctgttgaacaccagcaaccccactgcctacaacaaggcaccattcacgagactcgctctcccctacactgcgccgcaccgtgtgctggcaacagtgtacaacgggacgagtaagtatgctgtgggtggttca---ggcagaagaggcgacatggggtctctcgcggcgcgagtcgtgaaacagcttcctgcttcatttaactacggtgcaatcaaggccgacgccatccacgaacttctcgtgcgcatgaaacgggccgagctctactgccccagaccgctgttggcaatagaggcttcgcaagacaggcacaagcaaaagatcattgcaccagcaaagcagcttctgaattttgacctgctcaagttggccggagacgttgagtccaaccctgggcccttcttcttctccgacgttaggtcaaacttttccaagctggtagacacaatcaaccagatgcaggaagacatgtccacaaagcacggacctgactttaaccggttggtgtccgcttttgaggagttggccactggagtgaaagccatcaggaccggtcttgacgaggccaagccctggtacaagcttatcaagctcctgagccgcctgtcgtgcatggccgctgtggcagcacggtcaaaggacccagtccttgtggccatcatgctggctgacaccggtctcgagattctggacagcaccttcgtcgtgaagaagatctccgactcgctctccagtctcttccacgtgccggcccccgtcttcagtttcggagccccgattctgttagccgggttggtcaaggtcgcctcgagtttcttccggtccacgcccgaagaccttgagagagcagagaaacagctcaaagcacgtgacatcaacgacattttcgccattctcaagaacggcgagtggctggtcaaattgatccttgccatccgcgactggatcaaggcatggatagcctcagaagaaaagtttgtcaccacgacagacttggtacctggcatccttgaaaaacagcgggacctcaacgacccaagcaagtacaaggaagccaaggagtggctcgacaacgcgcgccaagcgtgtttgaagagcgggaacgtccacattgccaacctgtgcaaagtggtcgccccggcacccagcaggtcgagacccgagcccgtggtcgtttgcctccgtggcaagtccggtcagggcaagagtttccttgcaaacgtgctcgcacaagcaatctctacccatttcactggcaggaccgattcagtttggtactgcccgcctgaccctgaccacttcgacggttacaaccaacagactgtcgttgtgatggacgatttgggccagaaccccgacggcaaagacttcaagtacttcgcccaaatggtttcaacaacggggttcatcccgcccatggcatcgcttgaggataaaggcaaacccttcaacagtaaggtcatcatagcaaccaccaacctgtactcgggcttcaccccgaggactatggtgtgccctgatgccctgaaccggaggtttcactttgacatcgacgtgagcgccaaggacgggtacaaaattaacaacaaattggacatcatcaaagcacttgaagatactcacaccaacccagtggcaatgtttcagtacgactgtgcccttctcaacggcatggctgttgaaatgaagagaatgcaacaagatatgttcaagcctcaaccaccccttcagaacgtgtaccaactggttcaagaggtgattgagcgggtggagctccacgagaaggtgtcgagccacccgattttcaaacagatctcaattccttcccaaaaatccgtgttgtacttcctcattgagaaaggacagcacgaggcagcaattgaattctttgagggcatggtgcacgactccatcaaggaggagctccggccgctcatccaacaaacctcatttgtgaaacgcgcttttaagcgcctgaaggaaaactttgagattgttgccctatgtctgaccctcctggccaacatagtgatcatgatccgcgaaactcgcaagagacagaagatggtggacgatgcagtgagtgagtacattgagagagcaaacatcaccaccgacgacaagactcttgatgaggcggaaaagaaccctctggaaaccagcggtgccagcaccgtcggcttcagagagagacctctcccaggccaaaaggcgcgtaatgacgagaactccgagcccgcccagcctgctgaagagcaaccacaagctgaaggaccctacgccgggccgctagaacgacagaaacctctgaaagtgagagccaagctcccacaacaagagggaccttacgctggcccgatggagagacagaaaccactgaaagtgaaagcaaaagccccggtcgttaaggaaggaccttacgagggaccggtgaagaagcctgttgctttgaaagtgaaagctaagaacttgatcgtcactgagagtggtgccccaccgaccgacttgcaaaagttggtcatgggcaacaccaagcccgttgagctcatccttgacgggaagacggtagccatttgctgtgctactggagttttcggcactgcttacctcgtgcctcgtcatcttttcgcagaaaagtacgacaagatcatgttggacggcagagccatgacagatagtgactacagagtgtttgagtttgagattaaagtaaaaggacaggacatgctctcagacgctgcgctcatggtgctccaccgtgggaatcgcgtgagagacatcacgaaacactttcgtgacacagcaagaatgaagaaaggcacccccgtcgttggtgtgatcaacaacgccgatgtcgggagactgattttctctggtgaagcccttacctacaaggacattgtagtgtgcatggatggagacaccatgcctgggctctttgcctacaaagccgcaaccaaggctggttattgcggaggagccgtcctcgctaaggacggggctgacacgttcatcgttggcacccactccgctggaggcaatggcgttggatactgctcttgcgtttccaggtccatgcttctcaagatgaaggcacacgttgaccccgaaccacaccacgaggggttgattgttgacaccagagatgtggaagagcgcgttcacgtgatgcgcaaaaccaagcttgcacccaccgttgcgcacggtgtgttcaaccctgagttcgggcctgccgccttgtccaacaaggacccgcgcctgaacgacggtgttgtcctcgacgaagtcatcttctccaaacacaagggagacacaaagatgtctgaggaagacaaagcgctgttccgccgctgtgctgctgactacgcgtcacgcctgcacagcgtgttgggtacggcaaatgccccactgagcatctacgaggcaattaaaggcgttgatggactcgacgcaatggaaccagacaccgcacccggcctcccctgggcactccaggggaagcgccgtggcgcgctcatcgacttcgagaacggcactgttggacccgaagttgaggctgccttgaagctcatggagaaaagagaatacaagtttgcttgccaaaccttcctgaaggacgagattcgcccgatggagaaagtacgtgccggtaagactcgcattgtcgacgtcctacctgttgaacacatcctctacaccaggatgatgattggcagattttgtgcacaaatgcactcaaacaacggaccccaaattggctcggcggtcggttgtaaccctgatgttgattggcaaagatttggcacacacttcgcccaatacagaaacgtgtgggatgtggactattcggccttcgatgctaaccactgcagtgacgccatgaacatcatgtttgaggaagtgtttcgcacagaattcgggttccacccaaacgctgagtggatcctgaagactctcgtgaacacggaacacgcctatgagaacaaacgcatcactgttgaaggcgggatgccatctggttgttccgcaacaagcatcatcaacacaattttgaacaacatctacgtgctctacgctttgcgtagacactatgagggagttgagctggacacttacaccatgatctcttacggagacgatatcgtggtggcaagtgattacgatttggactttgaggctctcaagccccacttcaaatcccttggtcaaaccatcactccagctgacaaaagcgacaaaggttttgttcttggtcactccattactgatgtcactttcctcaaaagacacttccacatggattatggaactgggttttacaaacctgtgatggcctcaaagacccttgaggctatcctctcctttgcacgccgtgggaccatacaggagaagttgatctccgtggcaggactcgctgttcactctggaccagacgagtaccggcgtctcttcgagccctttcaaggcctcttcgagattccaagctacagatcactttacctgcgttgggtgaacgccgtgtgcggcgacgcataa

>AY593769.1_A_ARG_1959

atgaatacaactgattgtttcatcgctttggtgcacgccatcagagagatcataacacttctttttctacgaaccacaggaaagatggaattcacactgcacaacggtgagaagaaaactttttactctaggcccaacaaccacgacaactgttggctaaacgccattcttcagttgttcaggtacgtcgatgaacctttcttcgactgggtctacaactcgcccgaaaacctcacgcttgaagccatcaagcagttggaagaactcacagggcttgagttgcgcgagggcggaccacccgccctcgtggtctggaacatcaaacacttacttcacactggcattggtaccgcctcgcgacccagcgaggtgtgtatggtggatggcacggacatgtgtctcgctgacttccatgcaggcattttcctgaaaggatcggaacacgcagtgtttgcgtgtgtcacctccgatgggtggtacgcgatcgacgacgaggacttttacccctggactcctgacccatcagacgtcctggtattcgtcccgtacgatcaagaaccactcaacgggggttggagaacactggttcaaaggaggcttaagggcgccgggcaatccagcccggcgactggctcacaaaaccagtctggaaacactggtagcataatcaacaactactacatgcagcagtaccagaactccatggacacacagcttggtgacaatgccatcagtggaggctcaaacgagggctccacggacacaacctcaacacacacaaccaacacccaaaacaacgactggttctcaaaacttgccagttcagccttcaccggtctattcggcgcactgctcgccgataaaaagacagaagagaccacacttctggaagaccgcatcctcaccacccgcaatggacacaccacctcgaccacccagtcgagcgtgggggtcacctacgggtactccactggggaagaccacaccgcagggcccaacacatcgggcttggaaacgcgggtagtacaggctgaaaggttctttaagaaatttttgtttgactggacaacggacaaaccctttggacacttggaaaaactggaactccccaccgaccaccacggggtcttcggacacctggtggactcatatgcatacatgaggaacggttgggatgtcgaggtgtctgctgttggcaaccaattcaacggcgggtgcctcctggtggccatggtaccagaatggaaggaatttgacacgcgtgagaaataccaactcactctgtttccacaccagttcatcagccccagaacaaacatgaccgcccacatcacggtcccgtaccttggtgtgaacaggtatgaccagtacaaaaagcacaaaccctggacgctggttgtcatggtggtgtcgcccctcacggttagcaccactagtgcggcacagattaaggtctacgccaacattgccccaacctacgttcacgtggctggagagctcccttcgaaagaggggatttttcccgttgcgtgcgccgacggttacgggggactggtgacgacggacccgaagacagctgaccccgcctacggcaaggtgtacaatccgcccaggactaactaccccgggcgctttacaaacctgttggacgtggctgaggcgtgtcccacctttctttgtttcgacgacgggaaaccgtatgttgtcacgaagacagaacaagaccgacttctggccaagtttgacgtttcccttgccgcaaagcacatgtctaacacatacttgtcaggggttgcacagtactacgcacagtactctggtaccatcaacctgcactttatgttcacaggctctactgactcaaaggcccgctacatggtggcctacatcccgccaggggtggagccgccggacacacctgagaaagccgcacactgcatccacgctgaatgggacacagggttgaactccaagttcaccttttcaatcccgtacgtgtccgccgcggactacgcatacactgcgtccgacacggcagaaacaaccaacgtacagggatgggtttgcatttaccaaattacacacgggaaggctgagcaggacaccttggttgtgtcggttagcgccggcaaggactttgagctacgcctcccgattgacccccgtgcacaaaccactgccactggggaatctgcagaccctgtcaccaccaccgtggagaactacggcggtgagacacaagtccacagacgtcaccacacggacgtcagcttcatcatggacaggtttgtgaagatacagcctgtgaaccctatgcatgtcattgacctcatgcagacccaccaacacgggcttgtaggggcgttgctgcgtgcagccacgtactacttctctgacctggagattgtggtacgacacaacggcaacctgacctgggtacccaacggcgcccccgaggcagccctgtctaacaccagcaaccccactgcctacaacaaggcgccgttcaccagacttgccctcccctacactgcgccacaccgtgtgctggcaactgtgtacaacgggacgaacaagtacaccacaaacggtaca---ggtaggcgtgatgacacgggttctctcgcggcgagagtcgcgaaacatcttcctgcttcttttaattacggtgcaatcaaggccgacaccatccacgagcttctcgtgcgtatgaagcgggccgaactctactgccccagaccactgctggcaatagaggcctcacaagacaggcacaagcaaaagatcattgcacctgcaaagcagctgctgaactttgaccttctcaaactggcgggtgacgttgagtccaaccctgggcccttcttcttctccgacgttaggtcgaatttctccaaattggtggaaaccatcaaccaaatgcaggaagacatgtcaacaaagcacggacctgactttaaccggttagtgtccgcgtttgaggaattggccactggagtaaaagctatcagaaccggtctcgatgaggccaagccctggtacaagcttattaaactcctaagccgcctgtcgtgcatggccgctgtggcagcacggtccaaggacccagtccttgtggccatcatgctggccgacaccggtctcgagattctggacagcacctttgtcgtgaagaagatctccgactcgctctccagtctctttcacgtgccggcccccgtcttcagtttcggagctccgatcctgctggccgggttggtcaaagtcgcctcgagtttcttccggtccacacccgaagaccttgagagagcagagaaacagctcaaagcacgtgacatcaacgacatcttcgccattctcaagaacggcgagtggctggtcaaactgatcctcgctatccgcgactggattaaggcttggatcgcctcagaagagaagtttgtcaccatgacagacttggtgcctggcatccttgaaaagcagcgggatctcaacgacccgagcaagtacaaggaagccaaggaatggctcgacaacgcacgccaagcgtgcttgaagaacgggaacgtccacattgccaacctgtgcaaagtggtcgccccggcacccagcaagccgagacccgagcccgtggtcgtttgcctccgcggcaaatccggccagggcaagagtttccttgcgaacgtgctcgcgcaagcaatctccacccacttcaccggcagaaccgattcggtttggtactgcccgcctgaccccgaccacttcgacggttacaaccaacaggccgttgttgtgatggatgatttgggccagaaccctgacggcaaggacttcaagtacttcgcccaaatggtttcaaccacagggttcatcccgcccatggcatcgctcgaggacaaaggcaaacctttcaacagcaaggtcatcatcgccaccacaaacttgtactcgggtttcaccccgaggaccatggtgtgccctgatgcgctgaatcggaggtttcactttgacattgacgtgagcgccaaggacgggtacaaaattaatgagaaattggacatcaacaaagcacttgaagacacgcacactaacccagtggcgatgtttcagtacgattgtgcccttctcaacggtatggccgttgaaatgaagagaatgcaacagaatgtgttcaagcctctaccacccctccaaaacgtttaccagctcgttcaggaggtgattgaacgggtcgagctccacgagaaggtgtcgagccacccaatttttaaacaaatctcaattccttcccaaaaatccgtgttgtacttcctcattgagaaaggtcagcacgaagcagcaattgaattctttgagggaatggtgcatgactccatcaaggaggagctccggcccctcattcaacggacctcatttgtgaagcgcgctttcaagcgcctgaaggagaactttgagattgttgccctgtgtttgacccttttggccaacatagtgatcatgatccgcgagactcacaagagacagaaaatggtggatgatgcagtgaatgactacattgagaaagcaaacatcaccacagatgacaagactcttgacgaggcggaaaagaaccctctggagaccagcggagccagtaccgttggcttcagagagagaactctcacagggcacaagatgtgcgatgacgtgaactccgagcccatccaacct---gaagagcaaccacaagctgaaggaccctacgccgggccactcgagcgtcagaaacccctgaaagtgagagccaagctcccgcagcaggagggaccttacgctggcccgatggagagacagaaaccgctgaaggtaaaagtgaaagccccggtcgttaaggaaggaccttacgagggaccggtgaggaagcctgtcgctttgaaagtgaaagctaaaaacttgatagtcactgagagtggtgccccacctactgacttgcaaaagatggtcatgggcaacacaaagcctgttgagctcatcctcgacgggaagacagtagccatctgctgtgctactggagtgtttggtactgcctacctcgtgcctcgtcacctcttcgcagagaagtatgacaagatcatgctggatggtagagccatgacagacagtgactacagagtgtttgagtttgagattaaagtaaaaggacaggacatgctctcagacgccgcgctcatggtgctccaccgtgggaaccgcgtgagagacatcacgaaacactttcgtgatacagcaagaatgaagaaaggaacccccgtcgttggcgtgatcaacaacgccgatgttgggagactgattttctctggtgaggcccttacctacaaagacattgtggtgtgcatggatggagacaccatgcctggccttttcgcctacaaagccgccaccaaggctggctactgcggaggagccgttcttgccaaggacggggccgacactttcatcgttggcactcactccgcaggaggtaatggagttggatactgctcgtgcgtttccaggtccatgctcctcaagatgaaggcacacatcgaccccgaaccacaccacgaggggttgattgtggacaccagagatgtggaagagcgcgtccacgtgatgcgcaaaaccaagctcgcacccaccgttgcacacggtgtgttcaaccccgagttcgggcctgccgccttgtccaacaaggacccgcgcctgaacgagggtgttgttctcgatgaggtcatcttctccaaacacaagggagacacaaagatgtctgaagaggacaaagcgctgttccgccgctgcgccgctgactacgcgtcacgcctgcacagcgtgctgggcacagcaaatgccccactgagcatttatgaggcaattaagggtgtcgacggactcgacgccatggaaccagacactgcacccggcctcccctgggccctccaggggaaacgtcgtggtgcgctcatcgactttgagaacggcactgtcgggcccgaagtcgaggctgccctaaagctcatggagaaaagagaatacaagtttgcttgtcagaccttcctgaaggacgaaatccgcccgatggagaaagtacgtgccggcaagactcgcattgtcgatgttttgcctgttgaacacattctttacaccaggatgatgattggcagattctgtgcacaaatgcactcaaacaacggaccgcagattggctcagcggtcggctgtaaccctgatgttgattggcagagatttggcacacacttcgcccaatacagaaacgtgtgggatgtggactattcggccttcgatgctaaccactgcagtgacgcaatgaacatcatgttcgaggaggtgtttcgcacagactttggtttccacccaaatgctgagtggattctgaagactctcgtgaacacggagcacgcgtacgagaacaaacgcatcactgttgagggcgggatgccgtctggctgttccgcaacaagcatcatcaacacaattctgaacaacatctacgtgctctacgctctgcgtagacactatgagggagttgagctggacacttacaccatgatctcatacggagacgacatcgtggtggcaagtgattacgatttggacttcgaggctctcaagcctcactttaaatcccttggtcaaaccatcactccagctgacaaaagcgacaaaggttttgttcttggtcactccattaccgatgtcactttcctcaaaagacacttccacatggattatggaactgggttttacaaacctgtgatggcctcaaagacccttgaggctatcctctcctttgcacgccgtgggaccattcaggagaagttgatctcggtggcaggactcgccgtccactctggaccggacgagtaccgtcgtctctttgagccctttcagggcctctttgagattcctagctacagatcactttacctgcgttgggtgaacgccgtgtgcggcgacgcataa

>AY593770.1_A_ARG_1966

atgaatacaactgattgttttatcgctttggtacacgctatcagagagatcagaacatactttttgccacgaactacaggaaagatggaattcacactccacactggtgagaagaagactttttactccagacccaacaaccacgacaactgttggttgaacaccatactccagttgttcaggtatgttgacgaacctttctttgactgggtttacaactcgcccgagaacctcacactgacagcaatcaaacagttggaggaactcacaggacttgagttgcacgagggcggaccgcctgccctcgtaatctggaacatcaagcacttgctccacaccggcatcggcactgcctcgcgacccagcgaggtgtgcatggttgacggtacggacatgtgcctggctgacttccacgcaggcatcttcctaaaaggcaaagaacacgcagtgttcgcgtgtgtcacctccaacgggtggtacgcgattgatgacgaggacttttacccttggacaccggacccgtccgacgttttagtgtttgtcccgtacgatcaagaaccacttaacggggaatggaaaaacaacgttcaacggaagctcaagggcgccgggcagtccagcccggcgaccggctctcagaaccaatctggcaacactggcagcataattaacaactactacatgcagcagtaccaaaactccatggacacacagctaggcgacaacgccatcagtgggggctccaacgagggctccactgacacaacctcaacacacacaagcaacacacaaaacaatgactggttctcaaagcttgccagctcagctttcactggccttttcggtgctctgctcgccgacaagaagacagaagagacaacactccttgaggaccgcattctcaccactcgcaatggacacactacctcgacaacccaatcgagtgtgggtgtcacatacgggtactccacagaggaggaccacgttgctgggcccaacacatcgggcctagagacgagggtggtgcaggccgagagattctacaaaaagtttttgtttgattggacaaccgaaaaagcctttggacacattgtgaagctggaactcccggccgaccaccatggtgtcttcgggcacttggtggattcatacgcttacatgagaaatggctgggatgttgaggtatccgctgttggcaaccagttcaacggtgggtgcctcttggtggccatggtacctgagtggaaagaactcgacgcgcgggagaaataccaactaaccctcttcccgcatcagttcatcagtcccagaaccaacatgaccgcccacatcacggtcccctaccttggtgtgaacaggtacgaccagtacaaaaagcacaagccctggacactggttgtcatggtcgtgtcgccactcacggtcaacgccactagcgcgacacaaatcaaggtctacgccaacattgctccaacctacgttcacgtggccggggaacttccctcgaaagaggggattttccccgttgcgtgcgcggacggttacggaggactggtgacgacggacccgaagacagctgaccccgtttacggcaaggtgtacaacccgcccaggaccaactaccccgggcgcttcaccaacctgttagacgtggccgaagcgtgtcccaccttcctctgctttgacgacgggaaaccgtatgtcaccacgcggacggacgacactcgacttctggccaagtttgacctctcccttgccgcaaaacacatgtccaacacctacctggcaggcattgctcagtactacacccagtactctggcaccatcaacttgcacttcatgtttacaggttccacggactcaaaggcccgctacatggtggcctacatcccacctggggtggagccaccggacacacctgagagagctgcccactgcattcacgccgaatgggacactggactgaactccaaatttaccttctcaatcccgtacgtgtccgccgcggactacgcttacacagcgtctgacacggcagaaacaaccaatgtacagggatgggtctgtgtctaccaaattacacacgggaaggctgagaacgacactctggtcgtgacggttagcgccggtaaagactttgagttgcgcctcccgattgacccccgtcagcagaccaccgctacgggagaatcagcagaccccgtcactaccaccgtggaaaactacggcggtgagacacaagtccagaaacgtcaccacacagacatcggctttatcatggacagatttgtgaaggttagcgccttgagtccaatacacgtgattgacctcatgcagactcaccagcacggcctggtgggtgcgttgctgcgcgcggccacttactacttttcagacttggaaatagttgtgcgccacgacggcaatctgacctgggtgcccaacggcgcccccgaatcggccctgtcaaacaccagcaaccccactgtctacaggaaagaaccacttacgagactcgcactcccctacaccgcgccgcaccgcgtgctggcaactgtgtacaacgggacgagcaagtacaccacgagtgattca---agtaggcgtggtgacatgggggcccttgcggcacgggtcgcgaaacaacttcctgcttcattcaactacggtgcaattaaggccgacaccatccacgagcttctcgtgcgcatgaaacgggccgagctctactgccccagaccgctgttggctgtagaagcatcacaagacaggcacaagcaaaagatcattgcacctacaaagcaacttctgaactttgatctgcttaagttggctggggacgtggagtccaaccctggacccttcttcttctccgacgtcaggtcgaacttcaccaagctggtagagacaatcaaccagatgcaggaagacatgtcaacaaaacacggacccgactttagccggttggtgtccgcgtttgaggaattggccactggagtgaaggctatcaggaccggcctcgacgaggctaaaccctggtacaagctcattaagctcctgagccgcttgtcgtgcatggccgctgtagcagcacggtcaaaggacccggtccttgtggctatcatgctggctgacaccggtctcgagattctggacagcaccttcgtcgtgaagaagatctccgactcgctctccagtctcttccacgtgccggcccccgtcttcagtttcggagctccggttctgttagccgggttggttaaagtcgcctcgagtttcttccggtccacacccgaggagctcgagagagcagagaaacagctcaaagcacgtgacatcaacgacgttttcgccattctcaagaacggcgaatggctggtcaagttgatccttgccatccgcgactggatcaaggcatggatcgcctcagaagaaaagtttgtcaccatgacagacttggtgcctggaatccttgaaaagcagcgggacctcaacgacccgagcaagtacaaggaggccaaggagtggctcgacaacgcgcgccaggcgtgtctgaagagcgggaatatccacattgccaacctgtgcaaggtggtcgccccagcacccagcaagtcgagacctgaacccgtggtcgtttgcctccgtggcaaatctggccagggcaagagtttccttgcgaacgtgctcgcacaagcaatctccactcacttcaccggcagaaccgactccgtttggtactgcccacctgaccctgaccacttcgatggttacaaccaacagaccgtcgttgtgatggacgacctgggccagaaccctgatggcaaggacttcaagtactttgctcaaatggtttcaaccacggggttcatcccgcccatggcgtcgctcgaggacaaaggcaaacccttcaacagtagggtcattatcgcgaccaccaacttgtactcaggcttcaccccgaggaccatggtctgccctgatgccctgaaccggaggtttcactttgacatcgacgtgagcgccaaggacgggtacaaaaccaacaacagattggacatcaccaaagcacttgaagacacccacactaacccagtggcaatgtttcagtatgactgtgcccttctcaacggcatggctgttgaaatgaagagactgcaacaagacatgttcaagccccaaccacccctccagaacgtgtaccaacttgttcaggaggtgattgaacgggtggagctccacgagaaagtgtcgagccacccgattttcaagcagatctcaattccttcccaaaaatccgtgttgtacttcctcattgagaaaggtcagcacgaggcagcaattgaattctttgaggggatggtacacgactccatcaaggaggaactccgaccccttattcaacaaacttcatttgtgagacgcgctttcaagcgcctgaaggaaaactttgaggtcgttgccctatgtctgaccctcctggccaacatagtgatcatgatccgcgagactcgcaggagacagaaaatggtggatgatgcagtgaacgagtacattgagaaaacaaacatcaccacggacgacaagactcttgacgaggcggaaaagaaccctctggagaccagtggtgccagcaccgttggtttcagagagagaactcttccaggtcagaaggcgcgcgatgacgtgaactccgagcccgcccaacctgttgaagaacaaccacaagctgaaggaccctacgccgggccacttgagcgccagaaacctctgaaagtgagaaccaagctcccgcaacaagagggaccctacgctggcccgatggagagacagaaaccgctgaaagtaaaagcaaaggccccggtcgttaaggaaggaccttacgaaggaccggtgaagaaacctgtcgctttgaaagtgaaagctaggaacttgattgtcactgagagtggtgccccaccgaccgacttgcaaaagatggtcatgggcaacacgaagcccgttgagctcatcctcgacgggaagacggtagccatctgttgtgctactggagtgttcggcactgcctacctcgtgcctcgtcaccttttcgcagagaagtatgacaagatcatgttggacggcagagccttgacagacagtgactacagagtgtttgagtttgagattaaagtaaaaggacaggacatgctctcagacgccgcgctcatggtgctccaccgtgggaaccgcatgagagacatcacgaagcactttcgtgacacagcaagaatgaaaaagggtacccccgtcgttggcgtgatcaacaacgctgacgtcgggagactgattttctctggtgaggccctcacctacaaggacattgtagtgtgcatggacggagacaccatgcccggcctctttgcctacaaagccgccaccaaggccggctactgtggaggagccgttcttgccaaagacggagctgacacgttcatcgtcggcactcactccgctggaggcaatggagttggttactgctcatgcgtttccaggtccatgctactcaagatgaaggcacacatcgaccctgagccacaccacgaggggctgatcgttgacaccagagatgtggaagagcgcgtccacgtgatgcgcaaaaccaagcttgcacccaccgtcgcgcacggtgttttcaaccctgagtttgggcccgccgccttgtccaacaaggacccgcgcctgaatgaaggtgttgtcctcgatgaggtcatcttctccaagcacaagggagacacaaagatgtctgaggaggacaaagcgctgttccgccgctgtgctgctgactacgcgtcacgtttgcatagcgtgctgggtacagcaaacgccccattgagcatctacgaggcaatcaagggcgttgacggactcgacgccatggaaccagacaccgcgcctggccttccttgggccctccaggggaaacgccgtggtgcgctcatcgacttcgagaacggcacagtcggacccgaagttgatgctgccttgaagcttatggagaaaagagaatacaagtttgcttgccaaaccttcctgaaggacgagattcgcccgatggagaaggtgcgcgccggcaagactcgcattgtcgacgttttgcctgttgaacatattctttacaccaggatgatgattggtaggttctgtgcacaaatgcactcaaacaacggaccgcaaattggctcggcggtcggttgtaaccctgatgttgattggcaaagatttggcacacacttcgcccaatacagaaacgtgtgggatgtggactattcggcctttgatgccaaccactgcagtgacgcaatgaacatcatgtttgaggaggtgttccgcacggactttggtttccacccgaacgccgagtggattctgaagactctcgtgaacacggagcacgcctatgagaacaaacgcatcactgttgagggcgggatgccatccggttgttccgcgacaagcatcatcaacacaattttgaacaacatttacgtgctttacgccctgcgtaggcactatgaaggagttgagctggacacttacaccatgatctcttacggagacgacatcgtggtggcaagtgattacgatttggactttgaggctctcaagcctcacttcaaatcccttggtcagaccatcactccagctgacaaaagtgacaaaggtttcggtcttggtcactccatcaccgatgtcactttcctcaaaagacacttccacatggattacggaactgggttttacaaacctgtgatggcctcaaagacccttgaggctatcctctcctttgcacgccgtgggaccatacaggagaagttgatctccgtggcaggactcgccgtccactctgggccggacgagtaccggcgtctcttcgagcccttccagggcctctttgagattccaagctacagatcactttacctgcgttgggtgaacgccgtgtgcggcggcgcataa

>AY593771.1_A_COL_1967

atggacacaactgactgttttatcgctttggtacacgctatcagaaaactcagagcacttttcctaccgcgaatcacaggaaagatggaattcaccctgcacaacggcgagaaaaagactttctactcaagacccaacaaccacgacaactgctggttaaacaccattctgcagttgttcaggtatgtcgatgaacccttcttcgactgggtctacaactcgcctgagaacctcacacttgaagccatcaaacaactggaggaactcacaggacttgagctccgcgagggtgggccgcccgcccttgtgatttggaacatcaaacacctgctccacaccggcattggtaccgcctcacgacccagtgaggtgtgtatggtggacggtacagacatgtgtcttgctgacttccacgcaggcattttcctgaaaggtcaggagcacgcggtttttgcgtgtgtcacctccaacgggtggtacgcgattgatgacgaggaattctacccctggacacctgacccatcagatgtcttggtgtttgtcccgtacgatcaagaaccactcaacggagactggaaagcaatggttcagagaaagctcaaaggcgccgggcaatccagcccggcgaccgggtctcagaaccagtctggcaatactggcagcataattaacaactactacatgcagcagtaccagaactccatggacacacagcttggtgacaatgccatcagtggaggctccaatgaaggttccacagacacaacttcaacacacacaaccaacacccagaacaacgattggttttcaaagctcgccagctcagccttcaccggcttgtttggcgctctgcttgctgacaagaagacggaagagaccacacttttggaggaccgtatcctcaccacccgcaacgggcacaccacctcgaccactcaatcgagtgtgggagtcacctacgggtactccaccggtgaagaccacgtcgctgggcccaacacatcgggcctggagacgcgggtggtgcaggcagagaggtttttcaagaagtttttgtttgactggacaacggacaaaccttttggacacttggaaaagttggagctaccctccgaccaccatggtgttttcggacacctggtggactcatatgcttatatgaggaacggatgggacgttgaggtgtcagctgtcggcaaccagttcaacggcggttgtctcctggtggctatgataccggagtggaaagagtttgaacaacgcgagaagtaccaactcaccctcttcccacaccagtttattagccccagaacaaacatgactgcccacatcacagtcccataccttggagtgaacaggtacgatcagtacaaaaagcacaaaccttggacactggttgttatggtggtgtcgcccctcacggttagcaacactgccgcatcacaaattaaggtctacgccaacattgctccaacttacgttcacgtggccggggaactcccctcgaaagaggggattttcccagttgcatgttcggatggttacggagggttggtgacaacggacccaaagacagctgatcctgcctacggcaaggtgtacaatccgcccagaaccaactacccggggcggttcactaacctgttggacgtggccgaagcgtgccccactttcctctgtttcgacgacgggaaaccgtatgttgttacacggacagatgacacgcgactgctggccaagttcgacgtctcccttgctgcaaaacacatgtccaacacctacctgtcagggattgcacagtactacgcacagtactctggcaccatcaacttgcacttcatgttcacaggctcaactgactcaaaagcccgctacatggtggcctacatcccgccgggggtggaaccaccaaacacacccgaacgggctgcccactgcattcacgctgagtgggacacaggactaaattccaagttcactttttcaatcccgtacgtgtccgccgcagattacgcatacaccgcgtctgacacggcagaaacaaccaacgtacagggatgggtctgcatttaccagatcacacacgggaaggccgaaaacgacacactggtggtgtcggccagtgctggcaaagactttgagttgcgcctcccgatcgacccccggcagcaaaccactgctactggggagtccgcagaccctgttacaaccaccgtggaaaactacggcggtgagacacaagtccaaagacggcaccacacggatgtcgggttcattatggacagatttgtgaaaataagtaatttgagtcccacacatgtcattgacctcatgcagacccatcagcacgggttggtaggtgcgttgttgcgtgcagccacttactacttctctgacctggagattgttgtgcgccacgacggtaacctgacttgggtgcccaacggtgctcctgaggcagctctatcaaacaccagcaaccccactgcctacaacaaggcaccgttcacgagactcgctctcccgtacaccgcgccacaccgtgtgctggcaaccgtgtacaacgggacgaacaagtactccacgggtggtcag------aggccaggtgacatggggtcacttgcggcacgagtcgcaaagcaacttcctgcttctttcaactacggtgcaattagggcccagaccatccacgagcttctcgtgcgcatgaaaagggcggagctctactgccccagaccactgttggcaatagaggcttcgcaagacaggcacaagcagaagatcattgcacccgcaaagcagttgctgaattttgacctactcaagttagctggagacgtggagtccaaccctgggcccttcttcttctccgacgttaggtctaacttctccaaactggtggaaaccatcaaccagatgcaggaggacatgtcaacaaaacacggacccgactttaaccggttggtgtccgcctttgaggaattggccactggagtgaaggctatcagaaccggtctcgacgaggccaaaccctggtacaagctcatcaaactcctaagccgcctgtcgtgcatggccgctgtggcagcacggtccaaggacccagtccttgtggccatcatgctggccgacaccggccttgagattctggacagcacctttgtcgtgaagaagatctccgactcgctctccagtctctttcacgtgccggcccccgtcttcagtttcggagccccgattctgctagccgggctggtcaaggtcgcctcgactttcttccggtccacgcccgaagaccttgagagagcagagaagcaactcaaagcacgtgacatcaacgacattttcgccattctcaagaacggcgaatggctggtcaaactgatccttgccatccgcgactggatcaaggcttggattgcctcagaagaaaagtttgtcaccatgacagacttagtgcctggcatccttgaaaagcagcgggacctcaacgacccaggcaagtacaaggaagccaaggagtggcttgacaacgcgcgccaagcgtgtttgaagaacgggaacgtccacattgccaacctgtgcaaggtggtcaccccggcacccagcaagtcgagacccgaacccgtggtggtttgtctccgtggcaaatcaggccagggcaagagtttccttgcgaacgtgctcgcacaagcaatctctacccactttaccgggcggaccgattcagtttggtactgccctcctgaccctgaccacttcgatggttacaaccaacagaccgtcgtcgtgatggatgacttgggccagaaccctgacggcaaagactttaagtacttcgcccaaatggtttcgaccacggggttcatccctcccatggcatcactagaggacaaaggcaaacccttcaacagtaaggtcatcatcgcgaccaccaacctgtactcgggcttcaccccgaggactatggtgtgtcctgacgccctgaaccggaggtttcactttgacatcgacgtgagcgccaaggacgggtacaaaattaataacaaattggacatcatcaaagcacttgaagacacccacactaacccagtggcaatgttccagtacgactgtgcccttctcaatggcatggctgttgaaatgaagagaatgcaacaagatatgttcaagcctcaaccacccctccagaacgtataccaactggttcaggaggtgattgatcgggtggagctccacgagaaagtgtcgagccacccaattttcaagcagatttcaattccttcccaaaaatccgttttgtactttctcattgagaaagggcaacacgaggcagcaattgaattctttgagggcatggtgcatgactccattaaggaggagctccggccgctcatccaacgaacctcatttgtgaaacgcgccttcaagcgcctgaaggaaaactttgagattgttgccctttgcctaacacttttggccaacattgtgatcatgatccgcgagactcgcaagagacagaagatggtggatgatgcagtgaatgagtacattgagaaagcaaacatcaccaccgatgacaagacacttgacgaggcggaaaagaaccctctggagaccagcggtgccagcaccgttggtttcagagagagaagtcttccaggacagaaggcgcgcgatgacgtgaactctgagcccgcccaacctgttgaagaacaaccacaagctgaaggaccctacgccgggccactcgagcgtcagaggcccctgaaagtgagagccaagctcccgcaacaggagggaccctacgctggcccgatggagagacagaaaccgctgaaagtgaaagcaaaagccccggtcgttaaggaaggaccctacgaaggaccggtgaagaagcctgtcgccttgaaagtgaaagctaagaacttgattgtcactgagagtggtgcccctccgaccgacttgcaaaagatggtcatgggcaacacaaagcctgttgagctcatcctcgatgggaagacagtagccatctgctgcgctactggagtgtttggcactgcttacctcgtgccccgtcatcttttcgctgagaagtatgacaagatcatgttggacggcagagccatgacagacagtgactacagagtgtttgagtttgagattaaagtaaaaggacaggacatgctctcagacgccgcactcatggtgctccaccgtgggaaccgcgtgagagacatcacgaagcattttcgtgacacagcaagaatgaagaaaggcacccctgttgtcggcgtaatcaacaacgccgatgtcgggagactgattttctctggtgaagccctcacctacaaagacattgtggtgtgtatggacggagacaccatgcctgggctgtttgcctacagagctgccaccaaggctggctattgcggaggagccgtccttgctaaggacggagctgacaccttcatcgttggcactcactctgcaggaggcaatggagttggatactgctcatgcgtttccaggtccatgcttctcaaaatgaaggcacacattgaccctgagccacaccacgaggggttgattgtagacaccagagatgtggaagagcgcgtccacgtgatgcgcaaaaccaagcttgcacccaccgttgcacacggtgtgttcaaccctgagtttgggccagctgccttgtccaacaaggacccgcgtctgaacgagggtgtagtcctcgatgaagtcattttctccaaacacaaaggagacacaaagatgtctgaggaggacaaagcgctgttccgccgatgtgctgctgactacgcgtcacgcctgcacagcgtgctgggtacggcaaatgccccattgagcatttacgaggcaatcaagggcgtcgacggacttgacgccatggaaccagacaccgcgcctggtcttccctgggctctccagggaaaacgccgaggtgcactcattgacttcgagaacggcactgtcggacctgaagtcgcagaagccttgaagctcatggagaaaagagagtacaagtttgcttgccagaccttcctgaaggacgagattcgcccgatggagaaagtacgtgccggcaagactcgcattgtcgacgtcctgcccgttgaacacattctttacaccagaatgatgattggcagattctgcgcacaaatgcactcaaacaacggaccgcaaattggctcggcggtcggttgcaaccctgatgttgattggcaaagatttggcacacacttcgcccagtacagaaatgtgtgggatgtggactactcggcctttgatgctaaccattgtagtgatgccatgaacatcatgtttgaggaggtgttccgcacggaattcgggttccacccaaacgctgagtggatcttgaagactctcgtgaacacggagcacgcctatgagaacaaacgcatcactgttgaaggcgggatgccatccggctgttccgcaacgagcatcatcaacacaattttgaacaacatctacgtgctctacgccttgcgtagacactatgagggggttgagctggatacttacaccatgatctcctatggagacgacattgtggtggcaagtgattacgatttggactttgaggctctcaagcctcactttaaatcccttggtcaaaccatcactccagctgacaaaagcgacaaaggttttgttcttggtcactccatcaccgatgtcactttcctcaaaagacacttccacatggactatggaactgggttttacaaacctgtgatggcctcaaagacccttgaggctatcctctcctttgcacgccgtgggaccatacaggagaagttgatctccgtggcaggactcgctgtccactctggaccagacgagtaccggcgtctctttgagcccttccagggcctctttgagatcccaagctacagatcactttacctgcgttgggtgaacgccgtgtgcggtgacgcataa

>AY593772.1_A_TUR_1972

atgaatacaactgactgttttatcgctttgctctacgcccttagagaaattaaaacacttcttctttcacggacgcaaggaaagatggaactcacactttacaacggcgtaaagaagactttctactccagacccaacaaccacgacaactgctggcttaacaccattcttcagttgtttaggtacgtcgatgagcctttcttcgactgggtctatgactcgcctgagaacctcacctgtgaggcaattaagcagctggaagagataactggtcttgagctacacgagggtggaccacccgccctcgtcatttggaacatcaaacacttgctccacactggaatcggcactgcttcacgacctagtgaagtgtgcatggttgacggaacggacatgtgcttggctgacttccacgctggcatctttttgaaaggacaggaacacgctgtgtttgcctgcgtcacctccaacgggtggtacgcgatcgacgacgaggacttttacccctggacgccggacccgtccgatgttttagtgtttgtcccgtacgatcaggaaccgctcaatggggagtggaaagcaaaagtccagaagcggctcaagggagctgggcaatccagcccggcgactgggtcgcagaaccagtcgggcaacactgggagtatcattaacaactactacatgcaacagtaccagaactccatggacacccaactaggtgacaacgctataagcggaggctccaacgagggatccacggacacaacttccacccacacaaccaacactcagaataacgactggttttcaaagcttgccagttctgctttcagcggtcttttcggcgcccttctcgccgacaagaagaccgaggagaccactctcctcgaggaccgcatcctcaccacccgtaacgggcacaccacctccacaacccaatcgagtgtgggagtcacgtacgggtactctacccaggaagatcatgtttccggacccaacacatctggtttggagacgcgggtggtgcaggcagaaagatttttcaagaaacacctgtttgactggacaacagacaaagcttttgggcatctagagaaattggaactccccactgaacacaagggcgtctacggacacttggtggactcattcgcatacatgagaaatggctgggacgtggaggtgtccgctgttggcaaccagtttaacggcgggtgtctcctggtggccatggtccccgaatggaaagagttcacctcgcgtgagaagtaccagctcactttgtttccacaccagttcatcagccccagaaccaacatgactgcccacatcgtagtcccgtaccttggtgtgaacaggtatgaccagtacaagaagcacaaaccctggacgctggttgtgatggtggtttcaccgctcaccaccaacactgttagtgcaggacaaatcaaggtttatgtcaacattgccccgacccacgttcacgtggccggcgagctcccctcgaaggaggggatcgtgccggttgcttgttcggacgggtatggtggtttggtgacaacagacccaaaaacagctgaccctgtttatggtatggtgtacaacccccccagaacaaactaccccgggcggttcacgaacctgctggatgtggcggaggcctgccccacctttctctgtttcgacgacgggaaaccgtacgttgtgacaagagcggacgaacagcgtcttctggccaggttcgacgtttctcttgctgcaaagcacatgtcaaacacctacctttcagggatagcacagtactacgcacagtactctggtaccatcaacctccacttcatgtttactggctccactgactcaaaagcccgctacatggtggcgtacgttccgcccggtgtagagccgccggacacgcctgaggaagctgcacattgcatccatgctgagtgggacacggggttgaactccaaatttactttctctatcccgtacgtgtctgccgcggattacgcgtacaccgcgtctgacgtggcagaaacaacgaacgtacagggatgggtctgcatataccagattacacacgggaaagctgaacaagacactctggtcgtgtcggtcagtgccggcaaagactttgagttgcgcctcccgattgacccccgctcgcaaaccactaccaccggggagtctgcagaccctgtcaccaccaccgttgaaaactacggcggcgagacacaagtccaacgacgtcagcacaccgacgtcgccttcataatggacagatttgtgaagatacaaaacttgaaccccacacatgtcattgacctcatgcaaacccaccaacacgggttggtaggggccctgttacgtgctgctacgtactacttctctgacctggagattgtggtacgccatgatggcaacctaacctgggtacccaacggggcacctgaggcagctctgtctaacacgggcaaccccaccgcctacctcaaggcaccattcacgagactcgcacttccctacaccgcgccacaccgcgtgttggcaacagtgtacaacgggacgagcaagtactccacaggtggtacg---aacagacggggtgacctagggtctctcgcggcgagggtcgccgctcagctccctgcttctttcaactttggtgcaattcgagccacgaccatccacgagctcctcgtgcgcatgaagcgtgccgaactctactgccctagaccactgttggcagtggaggcgtctcaagataggcacaaacagaagatcattgcaccttcaaaacaacttttgaactttgacttgctcaagttggcaggagacgttgagtccaaccccgggcccttcttcttctccgatgttaggtcaaatttttccaagctggtagagaccatcaaccagatgcaggaggacatgtcaacaaagcacggacccgactttaaccggttggtgtctgcgtttgaggaactggccactggagtgaaggctatcaggaccggtctcgatgaggccaaaccctggtataaactcatcaagctcctgagccgcttgtcatgcatggccgctgtagcagcacggtcaaaggacccggtccttgtggctattatgctggctgacaccggccttgagattctggacagtacctttgtcgtgaagaagatctccgactcactctccagtctcttccacgtgccggcccccgtcttcagtttcggagctccgatcctgttggccggattggtcaaagtcgcctcgagtttcttccggtcaacgcccgaagaccttgagagagcagaaaaacagctcaaagcacgtgacatcaatgacatattcgccattctcaagaacggcgagtggttggtcaagctgattcttgctatccgcgactggattaaagcatggatcgcctcagaagaaaaatttgtcaccatgacagatttggtgcctggcatccttgaaaaacaacgggaccttaacgaccccagcaagtataaggaggccaaggagtggctcgagagcgcgcgccaagcgtgcctgaagagtgggaatgtccacattgccaacttgtgcaaagtgtccaccccagcacctagcaagtcgagacctgaacccgtggtcgtttgcctccggggcaaatccggccagggaaagagtttccttgcaaacgtgcttgcacaggcaatttcaacacactttactggcagaactgattcagtttggtactgtccgcctgaccctgaccacttcgacggttacaaccaacagaccgttgttgtgatggatgatttgggccagaaccccgacggcaaggacttcaaatacttcgcccaaatggtttcaaccacggggttcatcccgcccatggcctcgctcgaggacaaaggaaaacctttcaacagcaaagtcatcatcaccactaccaacttgtactcgggtttcaccccgagaactatggtgtgccctgacgcgctgaaccggaggttccactttgacatcgacgtgagtgccaaggacggatacaaagttaacaacaaattggacataaccaaagctcttgaggacacccacaccaatccagtggcaatgtttcagtacgattgtgcccttcttaacggcatggccgttgaaatgaagagaatgcaacaagacatgtttaaacctcaaccacccctccagaacgtgtaccaacttgttcaggaggtgattgaacgggtcgagctccacgagaaagtgtcgagccaccaaatttttaaacagatttcaattccttcccaaaagtctgtgttgtacttcctcattgagaaaggccaacacgaagcagcaattgagttctttgagggaatggtacacgactccatcaaggaggagctccgtcccctcatccagcggacttcatttgtgaaacgcgcttttaagcgtctgaaggaaaactttgagattgttgccttgtgtttgactcttttggcaaacatagtgatcatgatccgcgagactcgcaagagacaacagatggtggacgatgcagtgaatgagtatattgagaaagcaaacatcaccacagatgacaaaactcttgacgaggcggaaaagaaccctttggagactagcggtgccagcaccgttggattcagagagagaactctcccaagacacaaggcgagtgatgacgtgaactccgagcccgccggacctgtggaggaacaaccacaagctgaaggaccctacgccgggccactcgagcgtcagaaacctctgaaagtgaaagccaagctgccacagcaggaaggaccttacgctggcccgatggagagacagaaaccactgaaagtgaaagtgaaagccccggttgtcaaggaaggaccatacgagggaccggtgaagaagcctgtcgctttgaaagtgaaagctaagaatttgattgtcactgagagtggagccccaccgaccgacttgcaaaagatggtcatgggcaacaccaggcctgttgagctcattctcgacgggaagacggtggccatttgttgtgctaccggagtgtttggcactgcctatctcgtgcctcgtcatctttttgctgaaaaatatgacaagatcatgctggacggcagagccatgacagacagtgactacagagtgtttgagtttgagattaaagtaaaaggacaggacatgctctcagacgctgcgctcatggtactccaccgtgggaatcgcgtgagagacatcacgaaacactttcgtgacacagcaagaatgaagaaaggcacccccgttgtcggagtgatcaacaacgccgacgttgggagactgattttctctggtgaggcccttacctacaaggacattgtggtgtgcatggacggagacaccatgcctggcctgtttgcctacaaagccgccaccaaggctggctactgtgggggagccgttcttgctaaggacggagctgacacattcatcgttggcactcactccgcaggtggcaatggagttggatactgctcatgcgtttccaggtccatgttgctgaaaatgaaggcacacctcgatcccgaaccacaccacgaggggttgatcgttgacaccagagatgttgaggagcgcgtgcatgtcatgcgcaaaaccaagcttgcacccaccgtggcccacggtgtgttcaaccctgaatttggtcccgccgccttgtccagcaaggacccgcgactgaatgaaggtgttgtcctcgatgaggtcattttctccaaacacaagggagacacaaaaatgaccgaggaggacaaagcgctgttccgccgctgtgctgccgactacgcgtcacgcttgcacaacgtgttgggtacggcaaatgccccactgagcatctatgaggcgataaaaggcgttgacggcctcgacgccatggaaccagacaccgcgcctggccttccctgggccctccagggtaaacgccgcggtgcgttgattgactttgagaacggcacggtcgggcccgaagtcgcggctgccttagagctcatggagaaaagacaatacaaatttacttgtcagaccttcctgaaagacgaagttcgtccgatggagaaagtacgtgccggcaagactcgcatcgtcgatgttttgcctgttgaacacattctttacaccaggatgatgattggcagattttgtgcccagatgcactcaaacaacggaccgcaaattggctcggcggttggttgcaatccagatgttgattggcagagatttggtacccattttgctcagtacaaaaacgtgtgggatgtggactattcggcctttgatgctaaccattgtagtgacgcaatgaacatcatgtttgaggaagtgttccgcacggaatttggtttccacccaaatgctgagtggatcctgaaaactcttgtgaacacggaacacgcctacgagaacaagcgcatcactatcgagggcgggatgccgtctggttgttccgcaacaagcatcatcaacacaatcttgaacaacatctacgtgctctacgccttgcgtaggcactatgagggagttgagctggacacctacaccatgatctcctacggagatgacatagtggtggcaagtgatcacgatttggattttgaggctctcaagccgcactttaaatctcttggtcaaaccatcaccccagctgacaaaagcgacaaaggttttgttcttggtcactccatcaccgatgtcactttcctcaaaagacacttccacatggactatggaactgggttttacaaacctgtgatggcttcgaagaccctcgaggccatcctctcctttgcacgccgtgggaccatacaggagaagttgatctccgtggcaggactcgcagtccactccggacctgacgagtaccggcgtctctttgagccctttcagggcctctttgagattccgagctacagatcactttacctgcgttgggtgaacgccgtgtgcggtgacgcataa

>AY593773.1_A_PER_1969

atgaacacaactgattgttttatcgctttggtgcacgccatcagagagattaagacacttttcctaacacgaaccacagggaaaatggaattcacgctgcacaacggcgagaaaaagactttttactccagacccaacaaacacgataactgttggctgaacaccatcctccagttgtttagatacgtcgaagaacctttcttcgactgggtctacaactcacctgagaaccttacgctgtcagcaatcagacagctggaggaactcacagggcttgagttgcacgagggcggaccacctgctcttgtgatctggaacatcaaacacatgctccaaaccggtgtcggtactgcctcgcgacccagcgaggtatgtatggtcgatggtacggacatgtgtctggctgattttcacgcaggcatctttctaaaaggcaaggaacatgctgtctttgcgtgtgtcacctccaatgggtggtacgcgatcgatgatgaggacttttacccctggacaccggacccgtccgacgttctggtatttgttccgtacgatcaagagccgctcaacggggattggaaaaccaacgttcaacggaagctcaagggcgccgggcagtccagcccggcaactggctcccagaaccaatctggcaacactggtagcataattaataactactacatgcaacagtaccaaaattccatggacacacagcttggtgacaacgccatcagtggaggttccaacgaaggctccacagacacaacttcaacacacacaaccaacacccaaaacaatgactggttctcaaagcttgccagttcggctttcaccggcctgttcggggcgctgcttgccgacaaaaagacagaggagacaacactcctcgaggaccgcatcctcaccacccgcaatggacacaccacctcgacaacccagtccagtgtgggcgtcacatacgggtactccaccgtagaggaccacgttgccgggcccaacacatcgggcttggagacgcgggtggtgcaagcagagaggttctataaaaagtttttgtttgactggacaacggacaagccctttggacacttggtaaagctcgaccttccagccgaccaccacggtgttttcggacacttggtggactcatatgcttacatgagaaacggctgggacgttgaagtgtccgccgttggaaatcagttcaacggcgggtgcctcctggtggctatggtacccgagtggaaagaacttgacacacgggagaaataccaactcacccttttcccacaccagttcattagtcctagaaccaacatgactgcccacatcacggttccttaccttggtgtgaacaggtatgaccagtacaaaaagcacaaaccctggacactggttgtcatggtcgtgtctccacttacggttaacaccactggtgcgacacagatcaaggtctacgctaacattgctccaacctacgttcacgtggccggcgagctcccctcgaaggaggggattttccctgtcgcatgtgcggacggttacggaggactggtaacaacagacccgaagacagctgaccccgcttatggcaaggtgtacaacccgccccggaccaactaccctgggcgctttaccaacttgttggacgtggccgaagcgtgtcccactttcctctgttttgacgacgggaaaccgtatgtcaccacgcggacggatgagaccaggcttctggccaagtttgacgtttcccttgctgcaaagcacatgtccaacacatacctgtcagggattgcccagtactacgcacagtactctggtaccattaatctgcacttcatgtttactggttctactgattcaaaagcccggtacatggtggcttacatcccgcctggggtggagccgccggacacacccgagagggctgctcactgcatccacgccgagtgggatacaggactaaactccaaattcactttctcaatcccgtacgtgtccgctgcggattatgcctacacggcatctgacacggcggaaacaaccaacgtgcagggatgggtttgcatctaccaaattacacacgggaaggctgaaaatgacactctggttgtgtcggttagcgccggcaaagactttgagttgcgcctcccgattgaccctcgccagcagaccaccgctaccggggagtcggcagaccctgtcaccaccactgtagagaactacggtggtgagacacaagtccacagacgccaccacacggacattggcttcatcatggacagatttgtgaagataaaggatgtaagcccgacccatgtcattgacctcatgcaaactcaccaacacggcctggtgggtgcgctgctgcgtgcggccacctactacttctctgatttggaaattgtcgtgcgacacgacggcaatctgacttgggtgcctaacggtgcccctgaagcggccctgtcgaacaccagcaaccccaccgcctataacaaggcaccgttcacgagacttgctctcccctacactgcgccgcaccgtgtgctggcaaccgtgtacaacgggacgaataagtatactgtgagtggttca---ggcaggcgaggtgacatgggctctctcgcggcgcgggtcgcgaaacaacttcctgcctccttcaactacggtgcaattcaggccgtgaccatccacgagcttctcgtgcgcatgaaacgagctgagctctactgccctagaccactgttggcagtagaggctacacaagacaggcacaaacagaagatcattgcacctgcaaaacagctcctgaatttcgacctgctcaagttagctggggacgtggaatccaaccctggacccttcttcttctctgacgtcaggtcaaattttaccaagctggtggaaacaatcaaccagatgcaggaggacatgtcaacaaaacacgggcccgactttaaccggttggtgtccgcgtttgaggaattggccactggagtgaaggctatcagaactggtctcgacgaggccaaaccctggtacaagctcatcaagctcctaagccgcctgtcgtgtatggccgctgtggcagcacggtccaaggacccagtccttgtggccatcatgctggccgacaccggtctcgagattctggacagcaccttcgtcgtgaagaagatctccgactcgctctccagtctctttcacgtgccggcccccgtcttcagtttcggagcaccgatcctgttggccgggttggtcaaagtcgcctcgagtttcttccggtccacatccgaagaccttgagagagcagaaaaacagctcagagcacgtgacatcaacgacatcttcgccattctcaagaacggcgagtggctggtcaaattgatcctcgccatccgcgactggattaaggcttggatcgcctcagaagagaagtttgtcaccatgacagacttggtgcctggcatccttgaaaagcagcgggacctgaacgacccgagcaagtacaaggaagccaaggagtggctcgacaacgcgcgccaggcgtgtttgaagaacgggaatgtccacattgccaacctgtgcaaagtggtcgccccggcacccagcaagtcgagacccgagcctgtggtcgtttgcctccgtggcaaatctggccagggcaagagtttccttgcgaacgtgcttgcacaagcaatctccacccactttaccggcagaaccgactctgtttggtactgtccgcctgaccccgaccacttcgacggctacaaccaacagaccgtcgttgtgatggatgatttgggccagaatcccgatggcaaggatttcaagtacttcgcccaaatggtttccaccacagggttcattccgcccatggcgtcactcgaggataagggtaaacctttcaacagtaaggtcatcatcgcaaccaccaacctgtactcgggtttcaccccgaggactatggtgtgccctgacgccctgaaccggaggtttcactttgacattgacgtgactgccaaggacgggtacacaattaacaacaaattggacataattaaagcacttgaagacacccacaccaacccggtggcaatgttccaatatgattgtgcccttctcaacggcatggccgttgaaatgaagagattgcaacaagacatgttcaagcctcagccgcccctccagaacgtttaccaacttgttcaggaggtgattgaacgggttgagctccacgagaaagtgtcgagccacccgattttcaagcagatctcaattccttctcaaaaatctgtgctgtacttcctcattgagaaaggacaacacgaagcagcaattgaattctttgagggcatggtccacgactccatcaaggaggaactccgacccctcatccaacgaacttcatttgtgaaacgcgctttcaagcgcctgaaggaaaattttgagattgttgctctgtgcctaacacttttggcaaacattgtgatcatggtccgcgagactcgcaagaggcagaaaatggtggatgatgcagtgaatgagtacattgagaaagcgaacatcaccacagatgacaaaactcttgacgaggcggagaagaaccctctggaggccagcggcgctagcaccgttggctttagagagagaacccccccaggtcaaaaggcatgtgatgacgtgaaccccgagcctgcccaacctgttgaggatcaaccacaagctgaaggaccctacgccggaccactcgagcgtcagaaacctctgaaagtgagagccaagctcccacagcaggaggggccttacgctggtccgatggagagacagaaaccactgaaagtgaaagcaaaagccccggtcgtgaaggaaggaccctacgagggaccggtgaagaagcctgtcgctttgaaagtgaaagctaagaacttgattgtcactgagagtggtgccccaccgaccgacttgcaaaagatggtcatgggcaacacaaagcctgttgagctcatcctcgacgggaagacagtagccatctgctgcgctactggagtgttcggcactgcttacctcgtgcctcgtcacctcttcgcagagaagtatgacaagatcatgttggacggcagagccatgacagacagtgactacagagtgtttgagtttgagattaaagtaaaaggacaggacatgctctccgacgctgcgctcatggtgctccaccgtgggaatcgcgtgagggacatcacgaagcactttcgtgatacagcaagaatgaagaaaggcacccccgttgtcggtgtgatcaacaacgctgatgtcgggagactgattttctctggtgaggcccttacctacaaggacattgtagtttgcatggatggggacaccatgcctggcctctttgcctacaaagctgccactaaggctggctactgtggaggagccgttcttgctaaagatggagctaacactttcatcgttggcactcactctgcaggaggcaatggagttggatactgctcatgcgtttccaggtccatgcttcttaaaatgaaggcacacgtcgaccctgaaccacaccacgaggggttgattgtggacaccagagacgtggaggagcgcgtccacgtgatgcgcaaaaccaagcttgcacccaccgttgcacacggtgtgttcaaccccgagtttgggcccgctgccttgtccaacaaggactcgcgtctgaacgagggtgttgtcctcgatgaagtcatcttctccaaacacaagggagacacaaagatgactgaggaggataaagcgctgttccgccgctgcgctgctgactacgcgtcacgcctgcacagcgttctgggcacagcaaatgccccattgagcatctacgaggcaattaagggtgttgacggactcgacgctatggaaccagacactgcgcctggcctcccctgggccctccagggtaaacgccgcggtgcactcatcgactttgagaacggcacggtcggacccgaagtcgaggctgccctgaagctcatggagaagagagaatacaaatttgcttgtcagaccttcctgaaggacgagattcgcccgatggagaaagtacgtgccggcaagactcgcattgtcgacgtcctgcccgttgaacacattctttacaccaggatgatgattggcagattctgtgcacaaatgcactcaaacaacggaccgcaaattggctcagcggttggttgtaaccctgatgttgattggcagagatttggcacacacttcgcccaatacagaaacgtgtgggatgtggactattcggcctttgatgctaaccactgcagtgacgccatgaacatcatgtttgaggaagtgtttcgcacggagttcggtttccacccgaatgccgagtggattctgaagactcttgtgaacacggaacacgcctatgagaacaaacgcatcactgttgaaggcggaatgccgtctggctgttctgcaacaagcatcatcaacacaatcttgaacaacatctatgtgctctacgccctgcgtagacactatgagggagttgagctggacacatacaccatgatctcctacggagacgacatcgtggtggcaagtgattatgatttggacttcgaggctctcaagcctcactttaaatcccttggtcaaaccattactccagctgacaaaagcgacaaaggttttgttcttggtcactccattaccgacgtcactttcctcaaaagacatttccacatggactatggaactgggttttacaaacctgtgatggcctcaaagacccttgaggctatcctctcctttgcacgccgtgggaccatacaggagaagttgatctccgtggcaggactcgccgtccactctggaccagacgagtaccggcgtctctttgagcctttccaaggtctctttgagattccaagctacagatcactttacctgcgttgggtgaacgccgtgtgcggtgacgcataa

>AY593774.1_A_SPA_1969

atgaatacaactaactgttttatcgctttggtatacgctatcagagagattaagacacttttccttccacgagctacaggaaagatggaattcacactgcacaacggtgaaaagaaaactttctactccaggcccaacaaccacgacaactgctggttgaacaccatcctccagttgttcaggtacgtcgatgagcctttcttcgactgggtctacaactcgcccgagaatctcacgcttgatgctattaagcagttggaagaactcaccgggcttgagttacacgagggcggaccgcctgcccttgtgatctggaacatcaaacacttgctccaaaccggcattggtaccgcctcgcgacccagcgaggtgtgtatggttgacggcacggacatgtgtttggctgatttccatgcaggcatcttcctgaaaggaaaggaacacgccgtgtttgcgtgtgtcacctccaacgggtggtacgcgatcgatgatgaggacttttacccctggacgccggacccgtccgatgtcctggtgtttgtaccgtacgatcaagagccactcaacggaggatggaaagccaacgttcagcgaaagctcaagggagctgggcaatccagcccagcaaccggctcgcagaaccagtctggtaacaccggcagcataattaacaactactacatgcagcaataccagaattccatggacacacagcttggtgacaacgccatcagtggaggttccaacgagggctccacggacacaacttcaacacacacaaccaacacccaaaacaacgactggttttcaaaacttgccagttcagcttttaccggtctgttcggcgcactgctcgccgacaagaagacggaagagactacacttctggaagaccgcatcctcactacccgcaacgggcacaccacttcgactacccagtcgagtgtgggagtcacgtacgggtactccaccgaggaagaccacgttgctgggcccaacacatcgggcttggagacgcgggtggtgcaggcagagagatttttcaagaagtttctgtttgactggacaacggacaaaccttttggacacttgacaaaactggagcttcccaccgaccaccgcggtgtcttcggacacctggtggactcatatgcgtatatgaggaacggctgggatgttgaggtgtccgccgttggcaaccagtttaacggcgggtgccttctggtggccatggtgccagagtggaaagaatttgacgcacgtgaaaaataccaacttacccttttcccacaccagtttatcagccccagaactaacatgactgcccacatcacggttccgtatcttggtgtgaacaggtacgatcagtacaagaaacacaagccctggacactggttgtcatggtagtatcacccctcacggtcagcaacgctgccgcagcacaaatcaaggtctatgccaacattgccccaacctacgttcacgtggctggagagcttccctcgaaagaggggattttcccggttgcgtgcgcggacggttatggaggactagtgacaacagacccgaaaacagctgaccctgtttacggtaaggtgtacaacccgcccaggaccaactaccccgggcgctttacaaacctgttggacgtggccgaagcatgtcccactttcctctgtttcgacgatgggaaaccgtacgtcgttacgcggacagacaacacccgtcttttggccaagtttgacgtttcccttgccgcaaaacacatgtccaacacatacctatcagggattgcacagtactacacacagtactctggtactatcaacctgcacttcatgttcacaggctccactgactcaaaagcccgctacatggtggcttacatcccacctggggtggagccaccggacacacctgaaggggccgctcactgcattcatgctgaatgggacacaggactgaactccaaattcaccttttcaatcccttacgtgtccgccgcggattacgcgtacaccgcgtctgatacggcggagacaaccaatgtacagggatgggtttgtgtttaccaaattacacacgggaaggctgaaaatgacaccttggtagtgtcggctagcgccggcaaagactttgagttgcgcctcccaattgacccccggacacaaaccaccgctactggggagtccgcagaccctgtcaccaccaccgtggagaactacggtggtgagacgcaagtccagagacgtcaccacacggacgtcggcttcatcatggaccgatttgtgaagataaacagcctgaaccccacacacgtcattgacctcatgcagacccaccaacacgggctggtgggtgcgttgctgcgtgcagccacgtactacttctccgacttggagattgttgtgcggcatgatggtaatctgacctgggtgcctaacggtgcccccgaggcagccctgtcaaacaccagcaaccccactgcctacaacaaggcaccgttcacgagacttgctctcccttacactgcgccgcaccgcgtgttggcaactgtgtacaacgggacaagcaagtactccgcgagcggtttg------ggacgaggcgatctggggtccctcgcggcgcgagtcgcgacacagcttcctgcttctttcaactacggtgcaatcagggcccagaccatccacgagcttctcgtgcgcatgaaacgggccgagctctactgtcccaggccactgctggcaatagaggcttcgcaagacaggcacaagcaaaggatcattgcacccgcaaaacagctgttgaactttgacctacttcagttggcgggtgacgttgagtccaaccctggacccttcttctttgctgacgttaggtcaaacttttcaaagttggtagacaccatcaaccagatgcaggaggatatgtccacaaagcacggacccgactttaaccggttggtgtccgcatttgaggaattggccactggggttaaagctatcagaaccggtctcgatgaggccaaaccctggtacaagctcatcaaactcctaagccgcctgtcgtgcatggccgctgtggcagcacggtccaaggacccagtccttgtggccatcatgctggccgacaccggtcttgagattctggacagcacctttgtcgtgaagaagatttccgactcgctctccagtctctttcacgtgccggcccccgccttcagcttcggagccccgatcctgttggctgggttggtcaaggtcgcctcgagtttcttccggtccacacccgaagacctcgagagagcagagaaacagctcaaagcacgtgacatcaacgacattttcgccattctcaagaacggcgagtggctagtcaagttgatccttgccatccgcgactggatcaaggcatggattgcctcagaagagaagtttgtcaccatgacagacttggtacctggtatccttgaaaagcagcgggaccttaacgacccaagcaagtacacggaggccaaggagtggctcgacaacgcgcgccaggcgtgtttgaagagtgggaacgtccacattgccaacctgtgcaaagtggttgccccagcacccagcaagtcgagacccgaacccgtggtcgtttgcctccgtggcaaatccggccagggcaagagtttcctttcaaacgtgctcgctcaagcaatctctacccacttcaccggcaggaccgattcagtttggtactgtccgcctgaccctgaccacttcgacggttacaaccagcagaccgttgttgtgatggacgatttgggccagaaccctgatggcaaggacttcaagtactttgcccaaatggtttcaactacagggttcatcccgcccatggcgtcgcttgaggacaaaggcaaacctttcaacagcaaggtcatcattgcaaccaccaacctgtactcgggcttcaccccgaggactatggtgtgtcctgatgcactgaaccggaggtttcactttgacatcgacgtgagtgccaaggacgggtacaaaattaacaacaaattggacatcatcaaagcacttgaagacacccacaccaacccagtggcaatgtttcagtacgattgtgcccttctcaacggcatggctgttgaaatgaagagaatgcaacaagacgtgttcaagcctcaaccgcccctccagaacgtgtaccagctcgttcaggaggtgattgaacgggtcgagctccacgagaaagtgtcgagccacccaattttcaagcagatctcaattccttcccaaaaatctgtgttgtacttcctcattgagaaaggccaacatgaggcagcaattgaattctttgagggtatggtgcacgattccatcaaggaagagctccggcccctcatccaacaaacctcatttgtgaaacgcgctttcaagcgcctgaaggaaaattttgagattgttgctctgtgcctgacccttttggctaacatagtgatcatgatccgcgaaactcgcaagagacagaagatggtggatgatgccgtgaacaactacatcgagaaggcaaacatcaccacagatgacaaaacacttgacgaggcggaaaagaatcctctggagaccagtggtgccagtaccgttggtttcagagagaaaactctgtcaggccacaaagcgcgcgatgacgtgaactctgagcccgcccagcctgctgaagagcaaccacacgctgaaggaccctacgccgggccactcgagcgtcagaaacctctgaaagtgagagccaagctcccacagcaggagggaccctacgctggcccgatggagagacagaaaccgctgaaagtaaaagcaaaagccccggtcgttaaggaaggaccttacgagggaccggtgaagaagcctgtcgctttgaaagtgaaagctaagaacttgattgtcactgagagtggtgccccaccgaccgacttgcagaagatggtcatgggcaacacaaagcctgttgagcttatcctcgacgggaagacagtagccatctgctgcgctactggagtgtttggtactgcttacctcgtgcctcgtcatcttttcgcagagaagtatgacaagatcatgttggacggcagagccatgacagacagtgattacagagtgtttgagtttgagattaaagtaaaaggacaggacatgctctcagacgcggcactcatggtgcttcaccgtgggaaccgcgtgagggacatcacgaaacactttcgtgatacagcaagaatgaagaaaggcacccccgtcgtcggtgttatcaacaacgccgatgttgggagactgattttctctggtgaggcccttacctacaaggacattgtagtgtgcatggatggagacaccatgcccggcctctttgcctacaaagccgccaccaaggctggctactgtggaggagccgttcttgccaaggacggggctgacacattcatcgtcggcactcactctgcaggtggtaatggggttggatattgctcatgcgtttccaggtccatgcttcaaaagatgaaggctcacgtcgaccctgaaccacaccacgaggggttgattgttgataccagagatgtggaagagcgcgtccacgtgatgcgcaaaacaaagcttgcacccaccgttgcacacggtgtgttcaaccctgagtttgggcctgccgccttgtcaaacaaggacccgcgcctgaacgagggagttgttctcgatgaagtcattttctccaaacacaaaggagacgcaaagatgaccgaagaggacaaagcgctgttccgccgctgcgccgctgactacgcgtcacgcctgcacagcgtgctgggtacggcaaatgccccattgagcatctacgaggcaatcaagggcgttgacggactcgacgccatggagccggacactgcacctggcctcccctgggccctccaaggaaaacgccgcggtgcgctcatcgacttcgagaacggcacggtcggacccgaagttgaggctgccttgaagctcatggagaaaagagaatacaagtttgtttgtcagaccttcctgaaggacgagattcgcccgatggagaaagtacgcgccggcaagactcgcatcgtcgatgttttgcctgttgaacacattctttacaccaggatgatgattggcaggttctgtgcacaaatgcactcaaacaacggaccacaaattggctctgcggtcggttgcaaccctgacgttgattggcaaagatttggcacacatttcgctcaatacagaaacgtgtgggatgtggattactcggcctttgatgcaaaccactgcagtgacgccatgaacatcatgtttgaggaggtgttccgcacagactttggcttccacccaaatgctgagtggatcctgaagactctcgtgaacacggaacacgcctatgagaacaagcgcatcactgttgaaggcgggatgccatctggttgttccgcaacaagcatcatcaacacaattttgaacaacatctacgtgctctacgccttgcgtagacactatgagggagttgagctggacacttacaccatgatctcctacggagacgacatcgtggtggcaagtgattacgatctggactttgaggctctcaagccccacttcaaatctcttggccaaaccatcactccagctgacaaaagcgacaaaggttttgttcttggtcactccattaccgatgtcactttcctcaaaagacacttccacatggattatggaactgggttttacaaacctgtgatggcctcaaagacccttgaggctatcctctcctttgcacgccgtgggaccatacaggagaagttgatctccgtggcaggactcgccgtccactctggaccagacgagtaccggcgtctctttgagcccttccagggcctctttgagattccaagctacagatcactttacctgcgttgggtgaacgccgtgtgcggtgacgcataa

>AY593775.1_A_VEN_1970

atgaacacaactgattgttttatcgctttggtgcacgccatcagagagattaagacacttttcctaacacgaaccacagggaaaatggaattcacgctgcacaacggcgagaaaaagactttttactccagacccaacaaacacgataactgttggctgaacaccatcctccagttgtttagatacgtcgaagaacctttcttcgactgggtctacaactcacctgagaaccttacgctgtcagcaatcagacagctggaggaactcacagggcttgagttgcacgagggcggaccacctgctcttgtgatctggaacatcaaacacatgctccaaaccggtgtcggtactgcctcgcgacccagcgaggtatgtatggtcgatggtacggacatgtgtctggctgattttcacgcaggcatctttctaaaaggcaaggaacatgctgtctttgcgtgtgtcacctccaatgggtggtacgcgatcgatgatgaggacttttacccctggacaccggacccgtccgacgttctggtatttgttccgtacgatcaagagccgctcaacggggattggaaaaccaacgttcaacggaagctcaagggcgccgggcagtccagcccggcaactggctcccagaaccaatctggcaacactggtagcataattaataactactacatgcaacagtaccaaaattccatggacacacagcttggtgacaacgccatcagtggaggttccaacgaaggctccacagacacaacttcaacacacacaaccaacacccaaaacaatgactggttctcaaagcttgccagttcggctttcaccggcctgttcggggcgctgcttgccgacaaaaagacagaggagacaacactcctcgaggaccgcatcctcaccacccgcaatggacacaccacctcgacaacccagtccagtgtgggcgtcacatacgggtactccaccgtagaggaccacgttgccgggcccaacacatcgggcttggagacgcgggtggtgcaagcagagaggttctataaaaagtttttgtttgactggacaacggacaagccctttggacacttggtaaagctcgaccttccagccgaccaccacggtgttttcggacacttggtggactcatatgcttacatgagaaacggctgggacgttgaagtgtccgccgttggaaatcagttcaacggcgggtgcctcctggtggctatggtacccgagtggaaagaacttgacacacgggagaaataccaactcacccttttcccacaccagttcattagtcctagaaccaacatgactgcccacatcacggttccttaccttggtgtgaacaggtatgaccagtacaaaaagcacaaaccctggacactggttgtcatggtcgtgtctccacttacggttaacaccactggtgcgacacagatcaaggtctacgctaacattgctccaacctacgttcacgtggccggcgagctcccctcgaaggaggggattttccctgtcgcatgtgcggacggttacggaggactggtaacaacagacccgaagacagctgaccccgcttatggcaaggtgtacaacccgccccggaccaactaccctgggcgctttaccaacttgttggacgtggccgaagcgtgtcccactttcctctgttttgacgacgggaaaccgtatgtcaccacgcggacggatgagaccaggcttctggccaagtttgacgtttcccttgctgcaaagcacatgtccaacacatacctgtcagggattgcccagtactacgcacagtactctggtaccattaatctgcacttcatgtttactggttctactgattcaaaagcccggtacatggtggcttacatcccgcctggggtggagccgccggacacacccgagagggctgctcactgcatccacgccgagtgggatacaggactaaactccaaattcactttctcaatcccgtacgtgtccgctgcggattatgcctacacggcatctgacacggcggaaacaaccaacgtgcagggatgggtttgcatctaccaaattacacacgggaaggctgaaaatgacactctggttgtgtcggttagcgccggcaaagactttgagttgcgcctcccgattgaccctcgccagcagaccaccgctaccggggagtcggcagaccctgtcaccaccactgtagagaactacggtggtgagacacaagtccacagacgccaccacacggacattggcttcatcatggacagatttgtgaagataaaggatgtaagcccgacccatgtcattgacctcatgcaaactcaccaacacggcctggtgggtgcgctgctgcgtgcggccacctactacttctctgatttggaaattgtcgtgcgacacgacggcaatctgacttgggtgcctaacggtgcccctgaagcggccctgtcgaacaccagcaaccccaccgcctataacaaggcaccgttcacgagacttgctctcccctacactgcgccgcaccgtgtgctggcaaccgtgtacaacgggacgaataagtatactgtgagtggttca---ggcaggcgaggtgacatgggctctctcgcggcgcgggtcgcgaaacaacttcctgcctccttcaactacggtgcaattcaggccgtgaccatccacgagcttctcgtgcgcatgaaacgagctgagctctactgccctagaccactgttggcagtagaggctacacaagacaggcacaaacagaagatcattgcacctgcaaaacagctcctgaatttcgacctgctcaagttagctggggacgtggaatccaaccctggacccttcttcttctctgacgtcaggtcaaattttaccaagctggtggaaacaatcaaccagatgcaggaggacatgtcaacaaaacacgggcccgactttaaccggttggtgtccgcgtttgaggaattggccactggagtgaaggctatcagaactggtctcgacgaggccaaaccctggtacaagctcatcaagctcctaagccgcctgtcgtgtatggccgctgtggcagcacggtccaaggacccagtccttgtggccatcatgctggccgacaccggtctcgagattctggacagcaccttcgtcgtgaagaagatctccgactcgctctccagtctctttcacgtgccggcccccgtcttcagtttcggagcaccgatcctgttggccgggttggtcaaagtcgcctcgagtttcttccggtccacatccgaagaccttgagagagcagaaaaacagctcagagcacgtgacatcaacgacatcttcgccattctcaagaacggcgagtggctggtcaaattgatcctcgccatccgcgactggattaaggcttggatcgcctcagaagagaagtttgtcaccatgacagacttggtgcctggcatccttgaaaagcagcgggacctgaacgacccgagcaagtacaaggaagccaaggagtggctcgacaacgcgcgccaggcgtgtttgaagaacgggaatgtccacattgccaacctgtgcaaagtggtcgccccggcacccagcaagtcgagacccgagcctgtggtcgtttgcctccgtggcaaatctggccagggcaagagtttccttgcgaacgtgcttgcacaagcaatctccacccactttaccggcagaaccgactctgtttggtactgtccgcctgaccccgaccacttcgacggctacaaccaacagaccgtcgttgtgatggatgatttgggccagaatcccgatggcaaggatttcaagtacttcgcccaaatggtttccaccacagggttcattccgcccatggcgtcactcgaggataagggtaaacctttcaacagtaaggtcatcatcgcaaccaccaacctgtactcgggtttcaccccgaggactatggtgtgccctgacgccctgaaccggaggtttcactttgacattgacgtgactgccaaggacgggtacacaattaacaacaaattggacataattaaagcacttgaagacacccacaccaacccggtggcaatgttccaatatgattgtgcccttctcaacggcatggccgttgaaatgaagagattgcaacaagacatgttcaagcctcagccgcccctccagaacgtttaccaacttgttcaggaggtgattgaacgggttgagctccacgagaaagtgtcgagccacccgattttcaagcagatctcaattccttctcaaaaatctgtgctgtacttcctcattgagaaaggacaacacgaagcagcaattgaattctttgagggcatggtccacgactccatcaaggaggaactccgacccctcatccaacgaacttcatttgtgaaacgcgctttcaagcgcctgaaggaaaattttgagattgttgctctgtgcctaacacttttggcaaacattgtgatcatggtccgcgagactcgcaagaggcagaaaatggtggatgatgcagtgaatgagtacattgagaaagcgaacatcaccacagatgacaaaactcttgacgaggcggagaagaaccctctggaggccagcggcgctagcaccgttggctttagagagagaacccccccaggtcaaaaggcatgtgatgacgtgaaccccgagcctgcccaacctgttgaggatcaaccacaagctgaaggaccctacgccggaccgcttgagcgtcagaaacctctgaaagtgagagccaagctcccacagcaggaggggccttacgctggtccgatggagagacagaaaccactgaaagtgaaagcaaaagccccggtcgttaaggaagggccttacgagggaccggtgaagaagcctgtcgctttgaaagtgaaagctaagaacttgattgtcactgagagtggtgccccaccgaccgacttgcaaaagatggtcatgggcaacacaaagcctgttgagctcatcctcgacgggaagacagtagccatctgctgcgctactggagtgttcggcactgcttacctcgtgcctcgtcacctcttcgcagagaagtatgacaagatcatgttggacggcagagccatgacagacagtgactacagagtgtttgagtttgagattaaagtaaaaggacaggacatgctctccgacgctgcgctcatggtgctccaccgtgggaaccgcgtgagggacatcacgaagcactttcgtgatacagcaagaatgaagaaaggcacccccgttgtcggtgtgatcaacaacgctgatgtcgggagactgattttctctggtgaggcccttacctacaaggacattgtagtttgcatggacggggacaccatgcctggcctctttgcctacaaagctgccactaaggctggctactgcggaggggccgttcttgctaaagatggagctaacactttcatcgttggcactcactctgcaggaggcaatggagttggatactgctcatgcgtttccaggtccatgcttcttaaaatgaaggcacacgtcgaccctgaaccacaccacgaggggttgattgtggacaccagagacgtggaggagcgcgtccacgtgatgcgcaaaaccaagcttgcacccaccgttgcacacggtgtgttcaaccccgagtttgggcccgctgccttgtccaacaaggactcgcgtctgaacgagggtgttgtccttgatgaagtcatcttctccaaacacaagggagacacaaagatgactgaggaggataaagcgctgttccgccgctgcgctgctgactacgcgtcacgcctgcacagcgttctgggcacagcaaatgccccattgagcatctacgaggcaattaagggtgttgacggactcgacgctatggaaccagacactgcgcctggcctcccctgggccctccagggtaaacgccgcggtgcactcatcgactttgagaacggcacggtcggacccgaagtcgaggctgccctgaagctcatggagaagagagaatacaaatttgcttgtcagaccttcctgaaggacgagattcgcccgatggagaaagtacgtgccggcaagacgcgcattgtcgacgtcctgcccgttgaacacattctttacaccaggatgatgattggcagattctgtgcacaaatgcactcaaacaacggaccgcaaattggctcagcggttggttgtaaccctgatgttgattggcagaggtttggcacacacttcgcccaatacagaaacgtgtgggatgtggactattcggcctttgatgctaaccactgcagtgacgccatgaacatcatgtttgaggaagtgtttcgcacggagttcggtttccacccgaatgccgagtggattctgaagactcttgtgaacacggaacacgcctatgagaacaaacgcatcactgttgaaggcggaatgccgtctggctgttctgcaacaagcatcatcaacacaatcttgaacaacatctatgtgctctacgccctgcgtagacactatgagggagttgagctggacacatacaccatgatctcctacggagacgacatcgtggtggcaagtgattatgatttggacttcgaggctctcaagcctcactttaaatcccttggtcaaaccattactccagctgacaaaagcgacaaaggttttgttcttggtcactccattaccgacgtcactttccttaaaagacatttccacatggactatggaactgggttttacaaacctgtgatggcctcaaagacccttgaggctatcctctcctttgcacgccgtgggaccatacaggagaagttgatctccgtggcaggactcgccgtccactctggaccagacgagtaccggcgtctctttgagcctttccaaggtctctttgagattccaagctacagatcactttacctgcgttgggtgaacgccgtgtgcggtgacgcataa

>AY593776.1_A_GER_1968

atgaatacaactaactgttttatcgctttggtgtacgccatcagagagattaagacacttttcttttcaagagctacaggaaagatggaattcacactgcacaacggtgaaaagaaaactttttactccaggcccaacagccacgataactgctggttgaacaccatcctccagttgtttaggtacgtcgatgaacctttcttcgactgggtctacaactcgcccgagaaccttacgcttgatgccatcaagcagttggaagaacttactgggcttgagttgcacgagggcggaccgcctgccctcgtgatctggaacatcaaacacttgctccaaactggtatcggtaccgcctcgcgacccagcgaggtgtgtatggtagacggtacggacatgtgtcttgctgatttccacgctggcattttcttgaagggacaggaacacgcagtgtttgcgtgtgtcacttctgacgggtggtacgcgattgatgacgaggacttttacccgtggacgcctgacccatcggacgtcttggtatttgtcccgtacgatcaagaaccactcaatggggactggaaagcactggttcagagaaagctcaaaggtgctgggcattccagcccagcaaccggatcacagaaccagtctggcaacaccggtagcataattaacaactactacatgcagcaataccagaactccatggacacacagcttggtgacaacgccatcagtggtggctctaacgagggctccacggacacaacttctacacacacaaccagcacccaaaacaacgattggttttcgaaacttgccagctcagctttcaccggtctgtttggtgcacttctcgccgacaaaaagacggaagagaccacgcttctggaagatcgcatcctcaccacccgcaacgggcacaccacttcgaccacccagtcgagtgtgggagtcacgtacgggtactccaccgaggaagaccacgtcgctgggcccaacacatcgggcctggagactcgggtggtgcaggcagagagattttttaaaaagtttttgtttgactggacaacagataaaccttttggacacttggaaaaattggagctccccaccgaccaccgcggtgtcttcgggcacctggtggactcatatgcgtacatgaggaacggttgggacgttgaggtgtctgccgttggcaatcagtttaacggtgggtgccttctggtggccatgataccagagtggaaagattttgacaaacgtgaaaaataccaactcaccctttttccacaccagttcatcagccccagaaccaacatgactgcccacatcacggtcccgtaccttggtgtgaataggtatgatcagtacaagaagcacaagccttggacactagttgtcatggtggtatcacccctcacggtcagcgatactgctgcaccacaaatcaaggtctacgctaacattgccccaacctatgttcacgtggctggggagcttccctcgaaagaggggattttcccggttgcgtgcgcggacggttatggagggctggtgacaacagacccgaaaacagctgatcctgtgtacggcaaggtgtacaacccgcccaggaccaactaccctgggcgctttacaaacttgttggacgtggccgaagcatgtcccacttttctctgttttgacgatgggaaaccgtacgtcgttacgcggacagacaacacccgtcttttggccaagtttgacgtctcccttgccgcaaaacacatgtccaacacatacctatcagggattgcacagtactacacacaatactctggtactatcaacctgcacttcatgttcacaggctccactgactcaaaagcccgttacatggtggcctacatcccacccggggtggagccaccggacacacctgaaggggcagcccactgcattcatgctgaatgggacacaggactaaactccaaattcaccttttccatcccttacgtgtccgccgcggattacgcgtacaccgcgtctgacacggcggagacaaccaacgtgcagggatgggtctgtgtttaccaaattacacacggaaaggctgacggtgacaccttggtagtgtcggctagcgccggcaaagattttgagttacgcctcccaattgacccccggccacaaactaccgctgctggggagtctgcagaccctgtcaccaccaccgtggagaactacggtggtgagacacaagtccagaggcgtcaccacacggacgtcggcttcatcatggaccgatttgtgaagataaacagcctgaaccccacacacgtcattgacctcatgcagacccaccagcacgggctggtgggcgcgctgctgcgcgcagccacgtactacttctccgacttggagattgttgtgcggcatgatggcaatctgacctgggtgcctaacggcgcccctgaagcagccctgtcaaacaccagcaaccccactgcctacaacaaggcaccgttcacgagactcgctctcccttacactgcgccgcaccgcgtgttggcaactgtgtacaacgggacaagcaagtactccgtgagcggttcg------agacgaggcgacttggggaccctcgcggcgcgagtcgcgacacagcttcctacttctttcaactacggtgcaatcaaggcccagaccatccacgagcttctcgtgcgcatgaaacgggccgagctctactgtcccaggccgctgctggcaatagaggcttcacaagacaggcacaagcaaaagatcattgcacccgcaaaacagttgctgaactttgacctacttaagttggctggagacgtggagtccaaccctgggcccttcttcttctctgacgttaggtcaaacttttctaagctggtggaaaccatcaaccagatgcaggaagacatgtcaacaaaacacgggcccgactttaaccggttggtgtccgcctttgaggaactggccgctggagtaaaagccatcaggaccggcctcgacgaggccaaaccctggtacaagcttatcaaactcctaagccgcctgtcgtgcatggccgctgtggcagcacggtccaaggacccagtccttgtggccatcatgctggccgacaccggtctcgagattctggacagcactttcgtcgtgaagaagatctccgactcgctctccagtctcttccacgtgccggcccccgtcttcagtttcggagccccgattctgctagccgggctggtcaaggtcgcctcgagtttcttccggtccacacccgaagaccttgagagagcagagaaacagctcaaagcacgtgacatcaacgacattttcgccattctcaagaacggcgagtggctggtcaaactaatccttgccatccgcgactggatcaaggcttggattgcctcagaagaaaagtttgtcaccatgacagacttagtgcctggcatccttgaaaagcagcgggacctcaacgacccaagtaagtacaaggaggccaaggagtggctcgacaacgcgcgccaagcgtgtctgaagagcgggaacgtccacattgccaacctgtgcaaagtggtcgccccggcacccagcaagtcgagacccgaacccgtggtggtttgcctccgtggcaaatcaggccagggtaagagtttccttgcgaacgtgctcgcgcaagcaatctctacccacttcaccgggcggaccgattcagtctggtactgcccacctgaccctgatcacttcgacggttacaaccaacagactgtcgttgtgatggacgatttgggccagaaccctgacggcaaggacttcaagtacttcgcccaaatggtttcgaccacggggttcatcccgcccatggcatcactcgaggacaaaggtaaacccttcaacagtaaggtcatcattgcaaccaccaacctgtactcgggcttcaccccgaggactatggtgtgccctgacgccctgaaccggaggtttcactttgacattgacgtgagcgccaaggatgggtacaaaattaacaacaagttggacattatcaaagcacttgaagacacccacaccaacccagtggcaatgtttcagtacgactgtgcccttctcaacggcatggctgttgaaatgaagagaatgcagcaagacatgttcaaacctcaaccacccctccagaacgtgtaccaactagttcaggaggtgattgatcgggtggagctccacgagaaagtgtcgagccaccaaatttttaagcagatctcaattccttcccaaaaatctgtgttgtacttcctcattgagaaagggcagcacgaggcagcaattgaattctttgagggcatggtgcatgactccgtcaaggaggagctccggccgcttatccaacaaacctcatttgtgaaacgcgctttcaagcgcctgaaggaaaactttgagattgttgctctgtgcctaacacttttggccaacattgtgatcatgatccgcgaaactcgcaagagacagaagatggtggacgatgcggtgaacgagtacatcgagaaagcaaacatcaccaccgatgacaagacacttgacgaggcggaaaagaaccctctggagactagcggtgccagcaccgttggtttcagagagagaactcttccaggtcaaaaggcgcgtgatgacgtgaactccgagcccgcccaacctgctgaagaacaaccacaagctgaaggaccctacgccgggccacttgagcgtcagagacccctgaaggtgagagccaagctcccacaacaggaaggaccctacgctggcccgatggagagacagaaaccgctgaaagtgaaagcaaaagccccggtcgtcaaggaaggaccttatgaaggaccggtgaagagacctgtcgctttgaaagtgaaagctaagaatttgattgtcactgagagtggtgcccccccgaccgacttgcaaaagatggtcatgggcaacacaaagcctgttgagctcatcctcgacgggaagacagtagccatctgctgtgctactggagtgtttggcactgcctacctcgtacctcgtcatcttttcgctgagaagtatgacaagatcatgttggacggcagagccatgacagacagtgactacagagtgtttgagttcgagatcaaagtaaaaggacaggacatgctctcagacgccgcactcatggtgctccaccgtgggaaccgcgtgagagacatcacgaagcactttcgtgacacagcaaggatgaagaaaggcacccccgttgtcggcgtgatcaacaatgccgatgtcgggagactgattttctctggcgaagcccttacctacaaagacattgtagtgtgcatggacggagacaccatgcccgggctttttgcctacagagccgccactaaggctggctactgcgggggagccgttctcgctaaggacggggctgacactttcatcgttggcactcactctgcaggaggtaatggagttggatactgctcatgcgtttccaggtccatgcttctcaagatgaaggcacacattgaccctgagccgcaccacgaggggttgattgtagacaccagagatgtggaagagcgcgtccacgtgatgcgcaaaaccaagcttgcacccaccgttgcacacggtgtgttcaaccccgagtttgggccagctgccttgtccaacaaggacccgcgtctgaacgagggtgttgtcctcgatgaagtcattttctccaaacacaagggagacacaaagatgtctgaggaggacaaagcgctgttccgccgatgtgcagctgattacgcgtcgcgcctgcacagcgtgctgggtacggcaaatgccccattgagcatctacgaggcaatcaaaggcgtcgacggactcgacgccatggagccagacaccgcacctggcctcccctgggctctccagggaaaacgccgtggtgcgctcatcgactttgagaacggcacggtcgggcccgaagtcgaggatgccttgaagctcatggagaaaagagagtacaagtttgtttgtcagaccttcctgaaggacgagattcgcccgatggagaaagtacgtgccggcaagactcgcattgtcgacgtcctgcccgttgaacacattctttacaccaggatgatgattggcagattttgtgcacaaatgcactcaaacaacggaccgcaaattggctcggcggtcggttgcaaccctgatgttgattggcaaagatttggcacacatttcgcccagtacagaaacgtgtgggatgtggattattcggcctttgatgctaaccactgtagtgacgccatgaacatcatgtttgaggaggtgttccgcacagactttggcttccacccaaatgcagagtggatcctgaagactctcgtgaacacggaacacgcctatgagaacaagcgcatcactgttgaaggcgggatgccatctggttgttccgcaacaagcatcatcaacacaattttgaacaacatctacgtgctctacgccttgcgtagacactatgagggagttgagctggacacctacaccatgatctcctacggagacgacatcgtggtggcaagtgactacgatctggactttgaggctctcaagccccacttcaaatctcttggccaaactatcactccagctgacaaaagcgacaaaggttttgttcttggacactccattactgatgtcaccttcctcaaaagacacttccacatggattatgggactgggttttacaaacctgtgatggcctcaaagacccttgaggccatcctctcctttgcacgccgtgggaccatacaggagaagttgatctccgtggcgggactcgctgtccactctggaccagacgagtaccggcgtctctttgagcccttccagggcctcttcgagattccaagctacagatcactttacctgcgttgggtgaacgccgtgtgcggcgacgcataa

>AY593777.1_A_GER_1972

atgaatacaactaactgttttatcgctttggtatacgctatcagagagattaagacacttttccttccacgagctacaggaaagatggaattcacactgcacaacggtgaaaagaaaactttctactccaggcccaacaaccacgacaactgctggttgaacaccatcctccagttgttcaggtacgtcgatgagcctttcttcgactgggtctacaactcgcccgagaatctcacgcttgatgctattaagcagttggaagaactcaccgggcttgagttacacgagggcggaccgcctgcccttgtgatctggaacatcaaacacttgctccaaaccggcattggtaccgcctcgcgacccagcgaggtgtgtatggttgacggcacggacatgtgtttggctgatttccatgcaggcatcttcctgaaaggaaaggaacacgccgtgtttgcgtgtgtcacctccaacgggtggtacgcgatcgatgatgaggacttttacccctggacgccggacccgtccgatgtcctggtgtttgtaccgtacgatcaagagccactcaacggaggatggaaagccaacgttcagcgaaagctcaagggagctgggcaatccagcccagcaaccggctcgcagaaccagtctggtaacaccggcagcataattaacaactactacatgcagcaataccagaattccatggacacacagcttggtgacaacgccatcagtggaggttccaacgagggctccacggacacaacttcaacacacacaaccaacacccaaaacaacgactggttttcaaaacttgccagttcagcttttaccggtctgttcggcgcactgctcgccgacaagaagacggaagagactacacttctggaagaccgcatcctcactacccgcaacgggcacaccacttcgactacccagtcgagtgtgggagtcacgtacgggtactccaccgaggaagaccacgttgctgggcccaacacatcgggcttggagacgcgggtggtgcaggcagagagatttttcaagaagtttctgtttgactggacaacggacaaaccttttggacacttgacaaaactggagcttcccaccgaccaccgcggtgtcttcggacacctggtggactcatatgcgtatatgaggaacggctgggatgttgaggtgtccgccgttggcaaccagtttaacggcgggtgccttctggtggccatggtgccagagtggaaagaatttgacgcacgtgaaaaataccaacttacccttttcccacaccagtttatcagccccagaactaacatgactgcccacatcacggttccgtatcttggtgtgaacaggtacgatcagtacaagaaacacaagccctggacactggttgtcatggtagtatcacccctcacggtcagcaacgctgccgcagcacaaatcaaggtctatgccaacattgccccaacctacgttcacgtggctggagagcttccctcgaaagaggggattttcccggttgcgtgcgcggacggttatggaggactggtgacaacagacccgaaaacagctgaccctgtttacggtaaggtgtacaacccgcccaggaccaactaccccgggcgctttacaaacctgttggacgtggccgaagcatgtcccactttcctctgtttcgacgatgggaaaccgtacgtcgttacgcggacagacaacacccgtcttttggccaagtttgacgtttcccttgccgcaaaacacatgtccaacacatacctatcagggattgcacagtactacacacagtactctggtactatcaacctgcacttcatgttcacaggctccactgactcaaaagcccgctacatggtggcttacatcccacctggggtggagccaccggacacacctgaaggggccgctcactgcattcatgctgaatgggacacaggactgaactccaaattcaccttttcaatcccttacgtgtccgccgcggattacgcgtacaccgcgtctgatacggcggagacaaccaatgtacagggatgggtttgtgtttaccaaattacacacgggaaggctgaaaatgacaccttggtagtgtcggctagcgccggcaaagactttgagttgcgcctcccaattgacccccggacacaaaccaccgctactggggagtccgcagaccctgtcaccaccaccgtggagaactacggtggtgagacgcaagtccagagacgtcaccacacggacgtcggcttcatcatggaccgatttgtgaagataaacagcctgaaccccacacacgtcattgacctcatgcagacccaccaacacgggctggtgggtgcgttactgcgtgcagccacgtactacttctccgacttggagattgttgtgcggcatgatggtaatctgacctgggtgcctaacggtgcccccgaggcagccctgtcaaacaccagcaaccccactgcctacaacaaggcaccgttcacgagacttgctctcccttacactgcgccgcaccgcgtgttggcaactgtgtacaacgggacaagcaagtactccgcgagcggtttg------ggacgaggcgatctggggccccacgcggcgcgagtcgcgacacagcttcctgcttcttttaactacggtgcaatcagggcccagaccatccacgagcttctcgtgcgcatgaaacgggccgagctctactgtcccaggccactgctggcaatagaggcttcgcaagacaggcacaagcaaaagatcattgcacccgcaaaacagctgttgaactttgacctacttcagttggcgggtgacgttgagtccaaccctggacccttcttctttgctgacgttaggtcaaacttttcaaagttggtagacaccatcaaccagatgcaggaggatatgtccacaaagcacggacccgactttaaccggttggtgtccgcatttgaggaattggccactggggttaaagctatcagaaccggtctcgatgaggccaaaccctggtacaagctcatcaaactcctaagccgcctgtcgtgcatggccgctgtggcagcacggtccaaggacccagtccttgtggccatcatgctggccgacaccggtcttgagattctggacagcacctttgtcgtgaagaagatttccgactcgctctccagtctctttcacgtgccggcccccgccttcagcttcggagccccgatcctgttggctgggttggtcaaggtcgcctcgagtttcttccggtccacacccgaagacctcgagagagcagagaaacagctcaaagcacgtgacatcaacgacattttcgccattctcaagaacggcgagtggctagtcaagttgatccttgccatccgcgactggatcaaggcatggattgcctcagaagagaagtttgtcaccatgacagacttggtacctggtatccttgaaaagcagcgggaccttaacgacccaagcaagtacacggaggccaaggagtggctcgacaacgcgcgccaggcgtgtttgaagagtgggaacgtccacattgccaacctgtgcaaagtggttgccccagcacccagcaagtcgagacccgaacccgtggtcgtttgcctccgtggcaaatccggccagggcaagagtttccttgcaaacgtgctcgctcaagcaatctctacccacttcaccggcaggaccgattcagtttggtactgtccgcctgaccctgaccacttcgacggttataaccagcagaccgttgttgtgatggacgatttgggccagaaccctgatggcagggacttcaagtactttgcccaaatggtttcaactacagggttcatcccgcccatggcgtcgcttgaggacaaaggcaaacctttcaacagcaaggtcatcattgcaaccaccaacctgtactcgggcttcaccccgaggactatggtgtgtcctgatgcactgaaccggaggtttcactttgacatcgacgtgagtgccaaggacgggtacaaaattaacaacaaattggacatcatcaaagcacttgaagacacccacaccaacccagtggcaatgtttcagtacgattgtgcccttctcaacggcatggctgttgaaatgaagagaatgcaacaagacgtgttcaagcctcaaccgcccctccagaacgtgtaccagctcgttcaggaggtgattgaacgggtcgagctccacgagaaagtgtcgagccacccaattttcaagcagatctcaattccttcccaaaaatctgtgttgtacttcctcattgagaaaggccaacatgaggcagcaattgaattctttgagggtatggtgcacgattccatcaaggaagagctccggcccctcatccaacaaacctcatttgtgaaacgcgctttcaagcgcctgaaggaaaattttgagattgttgctctgtgcctgacccttttggctaacatagtgatcatgatccgcgaaactcgcaagagacagaagatggtggatgatgccgtgaacgactacatcgagaaggcaaacatcaccacagatgacaaaacacttgacgaggcggaaaagaatcctctggagaccagtggtgccagtaccgttggtttcagagagaaaactctgtcaggccacaaagcgcgcgatgacgtgaactctgagcccgcccagcctgctgaagagcaaccacacgctgaaggaccctacgccgggccactcgagcgtcagaaacctctgaaagtgagagccaagctcccacagcaggagggaccctacgctggcccgatggagagacagaaaccgctgaaagtaaaagcaaaagccccggtcgttaaggaaggaccttacgagggaccggtgaagaagcctgtcgctttgaaagtgaaagctaagaacttgattgtcactgagagtggtgccccaccgaccgacttgcagaagatggtcatgggcaacacaaagcctgttgagcttatcctcgacgggaagacagtagccatctgctgtgctactggagtgtttggtactgcttacctcgtgcctcgtcatcttttcgcagagaagtatgacaagatcatgttggacggcagagccatgacagacagtgattacagagtgtttgagtttgagattaaagtaaaaggacaggacatgctctcagacgcggcactcatggtgcttcaccgtgggaaccgcgtgagggacatcacgaaacactttcgtgatacagcaagaatgaagaaaggcacccccgtcgtcggtgttatcaacaacgccgatgttgggagactgattttctctggtgaggcccttacctacaaggacattgtagtgtgcatggatggagacaccatgcccggcctctttgcctacaaagccgccaccaaggctggctactgtggaggagccgttcttgccaaggacggggctgacacattcatcgtcggcactcactctgcaggtggtaatggggttggatattgctcatgcgtttccaggtccatgcttcaaaagatgaaggctcacgtcgaccctgaaccacaccacgaggggttgattgttgataccagagatgtggaagagcgcgtccacgtgatgcgcaaaacaaagcttgcacccaccgttgcacacggtgtgttcaaccctgagtttgggcctgccgccttgtcaaacaaggacccgcgcctgaacgagggagttgttctcgatgaagtcattttctccaaacacaaaggagacgcaaagatgaccgaagaggacaaagcgctgttccgccgctgcgccgctgactacgcgtcacgcctgcacagcgtgctgggtacggcaaatgccccattgagcatctacgaggcaatcaagggcgttgacggactcgacgccatggagccggacactgcacctggcctcccctgggccctccaaggaaaacgccgcggtgcgctcatcgacttcgagaacggcacggtcggacccgaagttgaggctgccttgaagctcatggagaaaagagaatacaagtttgtttgtcagaccttcctgaaggacgagattcgcccgatggagaaagtacgcgccggcaagactcgcatcgtcgatgttttgcctgttgaacacattctttacaccaggatgatgattggcaggttctgtgcacaaatgcactcaaacaacggaccacaaattggctctgcggtcggttgcaaccctgacgttgattggcaaagatttggcacacatttcgctcaatacagaaacgtgtgggatgtggattactcggcctttgatgcaaaccactgcagtgacgccatgaacatcatgtttgaggaggtgttccgcacagactttggcttccacccaaatgctgagtggatcctgaagactctcgtgaacacggaacacgcctatgagaacaagcgcatcactgttgaaggcgggatgccatctggttgttccgcaacaagcatcatcaacacaattttgaacaacatctacgtgctctacgccttgcgtagacactatgagggagttgagctggacacttacaccatgatctcctacggagacgacatcgtggtggcaagtgattacgatctggactttgaggctctcaagccccacttcaaatctcttggccaaaccatcactccagctgacaaaagcgacaaaggttttgttcttggtcactccattaccgatgtcactttcctcaaaagacacttccacatggattatggaactgggttttacaaacctgtgatggcctcaaagacccttgaggctatcctctcctttgcacgccgtgggaccatacaggagaagttgatctccgtggcaggactcgccgtccactctggaccagacgagtaccggcgtctctttgagcccttccagggcctctttgagattccaagctacagatcactttacctgcgttgggtgaacgccgtgtgcggtgacgcataa

>AY593778.1_A_SPA_1969

atgaatacaactgactgttttatcgctttggtgcacgctatcagagagatcagagcactttttctaccacgaaccacaggaaagatggaactcaccctgtacaacggcgagaaaaagactttttactcccgacccaacaaccacgacaactgttggttgaacaccatccttcagttgttcaggtatgtcgatgaacccttcttcgactgggtctacaattcgcccgagaacctcacgcttgaagccatcaaccaattggaggaactcacaggacttgagttgcacgagggcggaccacctgcccttgtgatctggaacatcaaacacttgctccacaccggcatcggcactgcctcacgacccagtgaggtgtgtatggtggacggcacggacatgtgtcttgctgacttccacgcaggcattttcctgaagggacaggaacacgcagtctttgcgtgtgtcacctccaacgggtggtacgcgattgacgacgaggaattttacccctggacgcctgacccgtcagacgtcctggtgtttgtcccgtacgatcaagaaccactcaacggggactggaaagcgatggttcagaggaagcttaagggtgccgggcaatccagcccggcgaccggctcccagaaccagtctggcaacactggcagcataattaacaactactatatgcaacagtaccagaactccatggacacacagcttggtgacaatgccattagtggaggctccaacgaaggctccacggacacaacttcaacacacacaaccaacacccaaaacaacgattggttttcaaaacttgccagttcagccttcaccggtctgttcggcgccttgctcgccgacaagaagacggaagagactacacttctggaagaccgcattctcaccacccgcaacgggcacactatctcgaccacccaatcgagcgtgggagtcacctacgggtactccactggagaagaccatgttgctgggcccaacacatcgggcctggagacgcgggtggtgcaggcagagagattttttaaaaagtttttgtttgactggacaacggacaaaccttttggacatttggaaaagctggaacttcccgccgaccaccacggcgttttcgggcacctggtggaatcgtatgcttacatgagaaatggttgggacgttgaggtgtctgctgttggcaaccagttcaacggcgggtgcctcctggtggctatggtaccggagtggaaagagtttgaacagcgcgagaaataccaactcaccctcttcccgcaccagttcatcagccccagaacaaacatgactgcccacatcacagtcccataccttggagtgaacaggtacgatcagtacaagaaacacaaaccttggacactggttgtcatggtagtgtcgcccctcacggttagcgacactgccgcgacacagattaaggtctacgccaatattgctccgacctacgttcacgtggctggggaactcccctcgaaagaggggattttcccagttgcatgctcggacggttacggaggactggtgacaacggacccgaaaacagctgaccccgcctacggtaaggtgtacaacccgcccaggaccaactaccctgggcggtttaccaacctgttggacgtggctgaagcgtgtcccactttcctctgtttcgacgacgggaaaccgtacgttgtcacgcggacagatgacacacgactattggccaagttcgacgtctcccttgctgcaaaacacatgtccaacacgtacctgtcaggggttgcacagtactacgcacagtactctggtaccatcaacttgcacttcatgttcacaggctcaactgactcaaaagcccgctacatggtggcctacatcccgcctggggtggaaccaccggacacacctgaaagggccgctcactgcatccacgctgaatgggacacaggactgaactccaaattcactttctcaatcccgtacgtgtccgccgcagattacgcgtataccgcgtctgacacggcagaaacaaccaacgtacagggctgggtttgcatataccagatcacacacgggaaggccgagaacgacacattggtggtgtcggccagcgccggcaaagactttgagttgcgcctcccgattgacccccgacagcaaactactgctgtcggggagtccgcagaccctgtcaccaccgccgtggagaactacggcggtgagacacaaacccggagacggcaccacacggatgtcggtttcatcatggacagatttgtgaagataaacagtttgagtcccacgcatgtcattgacctcatgcagacccaccagcacgggctggtgggtgcgctgctgcgtgcagccacgtactacttctctgacttggagattgttgtgcggcatgacggcaatttgacttgggtgcccaatggtgcccctgaagcagctttgtcaaacaccagcaaccccactgcctacaacaaggcaccgttcacgaggcttgctctcccttacactgcgccacaccgcgtgttggcaaccgtgtacaacgggacgaacaagtactccacaggcggtctg------agacgaggcgacacggggtcgcccgcggcgcgggccgcgaaacaacttcctgcctcttttaattacggtgcaattggggccgtcaccatccacgagcttctcgtgcgcatgaaacgggcagagctctactgccccaggccactactggcagtagaggctttacaagacaggcacaagcaaaagatcattgcacccgcaagacagttgctgaactttgacctacttaagttggctggagacgtggagtccaaccctgggcccttcttcttctctgacgttaggtcaaacttttctaagctggtggaaaccatcaaccagatgcaggaagacatgtcaacaaaacacgggcccgactttaaccggttggtgtccgcctttgaggaactggccgccggagtaaaagccatcaggaccggcctcgacgaggccaaaccctggtacaagcttatcaagctcctaagccgcctgtcgtgcatggccgctgtggcagcacggtccaaggacccggtccttgtggccatcatgctggccgacaccggtctcgagattctggacagcactttcgtcgtgaagaagatctccgactcgctctccagtctcttccacgtgccggcccccgtcttcagtttcggagccccggctctgctagccgggttggtcaaggtcgcctcgagtttcttccggtccacgcccgaagaccttgagagagcagagaaacagctcaaagcacgtgacatcaacgacattttcgccattctcaagaacggcgagtggctggtcaaactgatccttgccatccgcgactggattaaggcgtggattgcctcagaagaaaagtttgtcactatgacagacttagtgcctggcatccttgaaaagcagcgggatctcaacgacccaagcaggtacaaggaggccaaggagtggctcgacaacgcgcgccaagcgtgtctgaagagcgggaacgtccacattgccaacctgtgcaaagtggtcgccccggcacccagcaagtcgagacccgaacccgtggtggtttgcctccgtggtaaatcaggccagggcaagagtttccttgcgaacgtgctcgcacaagcaatctctacccacttcaccgggcggactgactcagtctggtactgcccacctgaccctgaccacttcgacggttacaaccaacagactgttgttgtgatggacgatttgggccagaatcctgacggcaaggacttcaagtacttcgcccaaatggtctcgaccactgggttcatcccgcccatggcatcactcgaggacaaaggtaaacccttcaacagtaaggtcatcattgcaaccaccaacctgtactcgggcttcaccccgaggactatggtgtgccctgacgccctgaaccggaggtttcactttgacattgacgtgagcgccaaggatgagtacaaaattaacaacaaattggacattaccaaagcgcttgaagacacccacaccaacccagtagcaatgtttcagtacgactgcgcccttctcaacggcatggctgttgaaatgaagagactgcagcaagacatgttcaaacctcaaccacctctccagaacgtgtaccaactagttcaggaggtaattgaccgggtggcgctccacgagaaggtgtcaagccacccaatttttaaacagatctcaattccttcccaaaaatctgtgttgtacttcctcattgagaaagggcagcacgaggcagcaattgaattctttgagggcatggtgcatgactccgtcaaggaggagctccggccgctcatccaacaaacctcatttgtgaaacgcgcgtttaagcgcctgaaggaaaactttgagattgttgctctgtgcctaacacttttggccaacatcgtgatcatgatccgcgagacccgtaagagacagaagatggtggacgatgcggtgaacgagtacatcgagaaagcaaacatcaccaccgatgacaagactcttgacgaggcggaaaagaaccctctggagactagcggtgccagtaccgttggtttcagagagagaactcttccagatcagaaggcgcgtaatgacgtgaactccgagcccgcccaacctgctgaagaacaaccacaagctgaaggaccctacgccgggccacttgagcgtcagagacccctgaaggtgagaaccaagctcccacaacaggaaggaccctacgctggcccgatggagagacagaaaccgctgaaagtgaaagcaaaagccccggtcgtcaaggaaggaccttacgaaggaccggtgaagaaacctgtcgctttgaaagtgaaagctaagaatttgattgtcactgagagtggtgcccccccgaccgacctgcaaaagatggtcatgggcaacacaaagcccgttgagctcatcctcgacgggaagacagtagccatctgctgtgctactggagtgtttggtactgcctacctcgtgcctcgtcatcttttcgctgagaagtatgacaagatcatgttggacggcagagccatgacagacagtgactacagggtgtttgagttcgagatcagagtaaaaggacaggacatgctctcagacgccgcactcatggtgctccaccgtgggaaccgcgtgagagacatcacgaagcatttccgtgacacagcaagaatgaagaaaggcacccccattgtcggcgtgattaacaatgccgatgtcgggaggctgattttctctggcgaagctcttacctacaaagacattgtagtgtgcatggacggagacaccatgcccgggctttttgcctacagagccgccactaaggctggctactgcgggggagccgttctcgctaaggacggggctgacacttttatcgttggcactcactctgcaggaggtaacggagttggatactgctcatgcgtttccaggtccatgcttctcaagatgaaggcacacattgaccctgagccgcaccacgaggggttgattgtagacaccagagatgtggaagagcgcgtccacgtgatgcgcaaaaccaagcttgcacccaccgttgcacacggtgtgttcaaccccgagtttgggccagctgccttgtccaacaaggacccgcgtctgaacgagggtgttgtccttgatgaagtcattttctccaaacacaagggagacacaaagatgtctgaggaggacaaagcgctgttccgccgatgtgctgctgactacgcgtcacgcctgcacagcgtgctgggtacggcaaatgccccattgagcatctacgaggcagtcaagggcgtcgacggactcgacgccatggagccagacaccgcacctggcctcccctgggctctccagggaaaacgccgtggtgcgctcatcgactttgagaacggcacggtcgggcccgaagtcgaggctgccttgaagctcatggagaaaagagagtacaagtttgtttgtcagaccttcctgaaggacgagattcgcccgatggagaaagtacgtgccggcaagactcgcattgtcgacgtcctgcccgttgaacacattctttacaccaggatgatgattggcagattttgtgcacaaatgcactcaaacaacggaccgcaaattggctcggcggtcggttgtaaccctgatgttgattggcaaagatttggcacacacttcgcccaatacagaaacgtgtgggatgtggactattcggcctttgatgctaaccactgcagtgacgccatgaacatcatgtttgaggaggtgttccgcacggacttcgggttccacccaaatgctgagtggatcttgaagactctcgtgcacacggaacatgcctatgagaacaaacgcatcactgttgaaggcgggatgccatctggttgttccgcaacgagcatcatcaacacaattttgaacaacatctacgtgctctacgccttgcgtagacactatgagggagttgagctggacacttacaccatgatctcctacggagacgacattgtggtggcaagtgattatgatctggactttgaggctctcaagcctcacttcaaatctcttggtcaaaccattactccagctgacaaaagcgacaaaggttttgttcttggtcactccatcaccgatgtcactttcctcaaaagacacttccacatggattatggaaccgggttttacaaacctgtgatggcctcaaagacccttgaggctatcctctcctttgcacgccgtgggaccatacaggagaagctgatctccgtggcaggactcgctgtccactctggaccagacgagtaccggcgtctctttgagccctttcagggcctctttgagattccaagctacagatcactttacctgcgttgggtgaacgccgtgtgtggcgacgcataa

>AY593779.1_A_GER_1972

atgaatacaactaactgttttatcgctttggtatacgctatcagagagattaagacacttttccttccacgagctacaggaaagatggaattcacactgcacaacggtgaaaagaaaactttctactccaggcccaacaaccacgacaactgctggttgaacaccatcctccagttgttcaggtacgtcgatgagcctttcttcgactgggtctacaactcgcccgagaatctcacgcttgatgctattaagcagttggaagaactcaccgggcttgagttacacgagggcggaccgcctgcccttgtgatctggaacatcaaacacttgctccaaaccggcattggtaccgcctcgcgacccagcgaggtgtgtatggttgacggcacggacatgtgtttggctgatttccatgcaggcatcttcctgaaaggaaaggaacacgccgtgtttgcgtgtgtcacctccaacgggtggtacgcgatcgatgatgaggacttttacccctggacgccggacccgtccgatgtcctggtgtttgtaccgtacgatcaagagccactcaacggaggatggaaagccaacgttcagcgaaagctcaagggagctgggcaatccagcccagcaaccggctcgcagaaccagtctggtaacaccggcagcataattaacaactactacatgcagcaataccagaattccatggacacacagcttggtgacaacgccatcagtggaggttccaacgagggctccacggacacaacttcaacacacacaaccaacacccaaaacaacgactggttttcaaaacttgccagttcagcttttaccggtctgttcggcgcactgctcgccgacaagaagacggaagagactacacttctggaagaccgcatcctcactacccgcaacgggcacaccacttcgactacccagtcgagtgtgggagtcacgtacgggtactccaccgaggaagaccacgttgctgggcccaacacatcgggcttggagacgcgggtggtgcaggcagagagatttttcaagaagtttctgtttgactggacaacggacaaaccttttggacacttgacaaaactggagcttcccaccgaccaccgcggtgtcttcggacacctggtggactcatatgcgtatatgaggaacggctgggatgttgaggtgtccgccgttggcaaccagtttaacggcgggtgccttctggtggccatggtgccagagtggaaagaatttgacgcacgtgaaaaataccaacttacccttttcccacaccagtttatcagccccagaactaacatgactgcccacatcacggttccgtatcttggtgtgaacaggtacgatcagtacaagaaacacaagccctggacactggttgtcatggtagtatcacccctcacggtcagcaacgctgccgcagcacaaatcaaggtctatgccaacattgccccaacctacgttcacgtggctggagagcttccctcgaaagaggggattttcccggttgcgtgcgcggacggttatggaggactggtgacaacagacccgaaaacagctgaccctgtttacggtaaggtgtacaacccgcccaggaccaactaccccgggcgctttacaaacctgttggacgtggccgaagcatgtcccactttcctctgtttcgacgatgggaaaccgtacgtcgttacgcggacagacaacacccgtcttttggccaagtttgacgtttcccttgccgcaaaacacatgtccaacacatacctatcagggattgcacagtactacacacagtactctggtactatcaacctgcacttcatgttcacaggctccactgactcaaaagcccgctacatggtggcttacatcccacctggggtggagccaccggacacacctgaaggggccgctcactgcattcatgctgaatgggacacaggactgaactccaaattcaccttttcaatcccttacgtgtccgccgcggattacgcgtacaccgcgtctgatacggcggagacaaccaatgtacagggatgggtttgtgtttaccaaattacacacgggaaggctgaaaatgacaccttggtagtgtcggctagcgccggcaaagactttgagttgcgcctcccaattgacccccggacacaaaccaccgctactggggagtccgcagaccctgtcaccaccaccgtggagaactacggtggtgagacgcaagtccagagacgtcaccacacggacgtcggcttcatcatggaccgatttgtgaagataaacagcctgaaccccacacacgtcattgacctcatgcagacccaccaacacgggctggtgggtgcgttactgcgtgcagccacgtactacttctccgacttggagattgttgtgcggcatgatggtaatctgacctgggtgcctaacggtgcccccgaggcagccctgtcaaacaccagcaaccccactgcctacaacaaggcaccgttcacgagacttgctctcccttacactgcgccgcaccgcgtgttggcaactgtgtacaacgggacaagcaagtactccgcgagcggtttg------ggacgaggcgatctggggccccacgcggcgcgagtcgcgacacagcttcctgcttcttttaactacggtgcaatcagggcccagaccatccacgagcttctcgtgcgcatgaaacgggccgagctctactgtcccaggccactgctggcaatagaggcttcgcaagacaggcacaagcaaaagatcattgcacccgcaaaacagctgttgaactttgacctacttcagttggcgggtgacgttgagtccaaccctggacccttcttctttgctgacgttaggtcaaacttttcaaagttggtagacaccatcaaccagatgcaggaggatatgtccacaaagcacggacccgactttaaccggttggtgtccgcatttgaggaattggccactggggttaaagctatcagaaccggtctcgatgaggccaaaccctggtacaagctcatcaaactcctaagccgcctgtcgtgcatggccgctgtggcagcacggtccaaggacccagtccttgtggccatcatgctggccgacaccggtcttgagattctggacagcacctttgtcgtgaagaagatttccgactcgctctccagtctctttcacgtgccggcccccgccttcagcttcggagccccgatcctgttggctgggttggtcaaggtcgcctcgagtttcttccggtccacacccgaagacctcgagagagcagagaaacagctcaaagcacgtgacatcaacgacattttcgccattctcaagaacggcgagtggctagtcaagttgatccttgccatccgcgactggatcaaggcatggattgcctcagaagagaagtttgtcaccatgacagacttggtacctggtatccttgaaaagcagcgggaccttaacgacccaagcaagtacacggaggccaaggagtggctcgacaacgcgcgccaggcgtgtttgaagagtgggaacgtccacattgccaacctgtgcaaagtggttgccccagcacccagcaagtcgagacccgaacccgtggtcgtttgcctccgtggcaaatccggccagggcaagagtttccttgcaaacgtgctcgctcaagcaatctctacccacttcaccggcaggaccgattcagtttggtactgtccgcctgaccctgaccacttcgacggttacaaccagcagaccgttgttgtgatggacgatttgggccagaaccctgatggcaaggacttcaagtactttgcccaaatggtttcaactacagggttcatcccgcccatggcgtcgcttgaggacaaaggcaaacctttcaacagcaaggtcatcattgcaaccaccaacctgtactcgggcttcaccccgaggactatggtgtgtcctgatgcactgaaccggaggtttcactttgacatcgacgtgagtgccaaggacgggtacaaaattaacaacaaattggacatcatcaaagcacttgaagacacccacaccaacccagtggcaatgtttcagtacgattgtgcccttctcaacggcatggctgttgaaatgaagagaatgcaacaagacgtgttcaagcctcaaccgcccctccagaacgtgtaccagctcgttcaggaggtgattgaacgggtcgagctccacgagaaagtgtcgagccacccaattttcaagcagatctcaattccttcccaaaaatctgtgttgtacttcctcattgagaaaggccaacatgaggcagcaattgaattctttgagggtatggtgcacgattccatcaaggaagagctccggcccctcatccaacaaacctcatttgtgaaacgcgctttcaagcgcctgaaggaaaattttgagattgttgctctgtgcctgacccttttggctaacatagtgatcatgatccgcgaaactcgcaagagacagaagatggtggatgatgccgtgaacgactacatcgagaaggcaaacatcaccacagatgacaaaacacttgacgaggcggaaaagaatcctctggagaccagtggtgccagtaccgttggtttcagagagaaaactctgtcaggccacaaagcgcgcgatgacgtgaactctgagcccgcccagcctgctgaagagcaaccacacgctgaaggaccctacgccgggccactcgagcgtcagaaacctctgaaagtgagagccaagctcccacagcaggagggaccctacgctggcccgatggagagacagaaaccgctgaaagtaaaagcaaaagccccggtcgttaaggaaggaccttacgagggaccggtgaagaagcctgtcgctttgaaagtgaaagctaagaacttgattgtcactgagagtggtgccccaccgaccgacttgcagaagatggtcatgggcaacacaaagcctgttgagcttatcctcgacgggaagacagtagccatctgctgtgctactggagtgtttggtactgcttacctcgtgcctcgtcatcttttcgcagagaagtatgacaagatcatgttggacggcagagccatgacagacagtgattacagagtgtttgagtttgagattaaagtaaaaggacaggacatgctctcagacgcggcactcatggtgcttcaccgtgggaaccgcgtgagggacatcacgaaacactttcgtgatacagcaagaatgaagaaaggcacccccgtcgtcggtgttatcaacaacgccgatgttgggagactgattttctctggtgaggcccttacctacaaggacattgtagtgtgcatggatggagacaccatgcccggcctctttgcctacaaagccgccaccaaggctggctactgtggaggagccgttcttgccaaggacggggctgacacattcatcgtcggcactcactctgcaggtggtaatggggttggatattgctcatgcgtttccaggtccatgcttcaaaagatgaaggctcacgtcgaccctgaaccacaccacgaggggttgattgttgataccagagatgtggaagagcgcgtccacgtgatgcgcaaaacaaagcttgcacccaccgttgcacacggtgtgttcaaccctgagtttgggcctgccgccttgtcaaacaaggacccgcgcctgaacgagggagttgttctcgatgaagtcattttctccaaacacaaaggagacgcaaagatgaccgaagaggacaaagcgctgttccgccgctgcgccgctgactacgcgtcacgcctgcacagcgtgctgggtacggcaaatgccccattgagcatctacgaggcaatcaagggcgttgacggactcgacgccatggagccggacactgcacctggcctcccctgggccctccaaggaaaacgccgcggtgcgctcatcgacttcgagaacggcacggtcggacccgaagttgaggctgccttgaagctcatggagaaaagagaatacaagtttgtttgtcagaccttcctgaaggacgagattcgcccgatggagaaagtacgcgccggcaagactcgcatcgtcgatgttttgcctgttgaacacattctttacaccaggatgatgattggcaggttctgtgcacaaatgcactcaaacaacggaccacaaattggctctgcggtcggttgcaaccctgacgttgattggcaaagatttggcacacatttcgctcaatacagaaacgtgtgggatgtggattactcggcctttgatgcaaaccactgcagtgacgccatgaacatcatgtttgaggaggtgttccgcacagactttggcttccacccaaatgctgagtggatcctgaagactctcgtgaacacggaacacgcctatgagaacaagcgcatcactgttgaaggcgggatgccatctggttgttccgcaacaagcatcatcaacacaattttgaacaacatctacgtgctctacgccttgcgtagacactatgagggagttgagctggacacttacaccatgatctcctacggagacgacatcgtggtggcaagtgattacgatctggactttgaggctctcaagccccacttcaaatctcttggccaaaccatcactccagctgacaaaagcgacaaaggttttgttcttggtcactccattaccgatgtcactttcctcaaaagacacttccacatggattatggaactgggttttacaaacctgtgatggcctcaaagacccttgaggctatcctctcctttgcacgccgtgggaccatacaggagaagttgatctccgtggcaggactcgccgtccactctggaccagacgagtaccggcgtctctttgagcccttccagggcctctttgagattccaagctacagatcactttacctgcgttgggtgaacgccgtgtgcggtgacgcataa

>AY593780.1_A_FRA_1960

atgaatgcaactgactgttttatcgctttggtacacgctatcagagagatcagagcactttttctaccacgaaccacaggaaagatggaactcaccctgtacaacggcgagaaaaagactttttactctagacccaacaaccacgacaactgctggttgaacaccatccttcagctgttcaggtatgtcgatgaacccttcttcgactgggtctacaactcgcccgagaacctcacgcttgaagccatcaaccaattggaggaactcacaggacttgagttgcacgagggcggaccgcctgcccttgtgatctggaacatcaagcacttgctccacaccggcatcggcactgcctcacgacccagtgaggtgtgtatggtggacggcacggacatgtgtcttgctgacttccacgcaggcattttcctgaagggacaggaacacgcagtctttgcgtgtgtcacctccaacgggtggtacgcgattgacgacgaggaattttacccctggacgcctgacccgtcagacgtcctggtgtttgtcccgtacgatcaagaaccactcaacggggactggaaagcgatggttcagaggaagcttaagggtgccgggcaatccagcccggcgaccggctcccagaaccagtctggcaacactggcagcataattaacaactactacatgcagcagtatcagaactccatggacacacagcttggtgacaatgccatcagtggaggctccaacgaaggctccacggacacaacttcaacacacacaaccaacacccaaaacaacgattggttttcaaaacttgccagttcagccttcaccggtctgttcggcgccctgctcgccgacaagaagacggaagagactacacttctggaagaccgcattctcaccacccgcaacgggcacaccatctcgaccacccaatcgagcgtgggagtcacctacgggtactccactggagaagaccatgttgctgggcccaacacatcgggcctggagacgcgggtggtgcaggcagagagattttttaaaaagtttttgtttgactggacaacggacaaaccttttggacatttggaaaagctggaacttcccgccgaccaccacggcgttttcgggcacctggtggaatcatatgcttacatgagaaatggttgggacgttgaggtgtctgctgttggcaaccagttcaacggcgggtgcctcctggtggctatggtaccggagtggaaagagtttgaacagcgcgagaaataccaactcaccctcttcccgcaccagttcatcagccccagaacaaacatgactgctcacatcacagtcccataccttggagtgaacaggtacgatcagtacaagaaacacaaaccttggacactggttgttatggtagtgtcgcccctcacggttagcgacactgccgcggcacagattaaggtctacgccaatattgctccgacctacgttcacgtggctggggaactcccctcgaaagaggggattttcccagttgcatgttcggacggttacggaggactggtgacaacggacccgaaaacagctgaccccgcctacggcaaggtgtacaacccgcccaggaccaactaccctgggcggtttaccaacttgttggatgtggctgaagcgtgtcccactttcctctgtttcgacgacgggaaaccgtacgttgtcacgcggacagatgacacacgactattggccaagttcgacgtctcccttgctgcaaaacacatgtccaacacgtacctgtcagggattgcacagtactacgcacagtactctggtaccatcaacttgcacttcatgttcacaggctcaactgactcaaaagcccgctacatggtggcctacatcccgcccggggtggaaccaccggacacacctgaaagggccgctcactgcatccacgctgaatgggacacaggactgaactccaaattcactttctcaatcccgtacgtgtccgccgcagattacgcgtacaccgcgtctgacacggcagaaacaaccaacgtacagggctgggtctgcatctaccagatcacacacgggaaggccgagaacgacacattggtggtgtcggccagcgccggcaaagactttgagttgcgcctcccgatcgacccccgacagcaaactactgctgttggggagtccgcagaccctgtcaccaccaccgtggagaactacggcggtgagacacaaacccagagacggcaccacacggatgtcggtttcatcatggacagatttgtgaagataaacagtttgagtcctacgcatgtcattgacctcatgcagacccaccagcacgggctggtgggtgcgctgttgcgtgcagccacgtactacttctctgacttggagattgttgtgcggcatgacggcaatttgacttgggtgcccaacggtgcccctgaagcagctttgtcaaacaccagcaaccccactgcctacaacaaggcaccgttcacgaggctcgctctcccttacactgcgccacaccgcgtgttggcaaccgtgtacaacgggacgaacaagtactccacggacggtccg------agacgaggcgacatggggtcgctcgcggcgcgggccgcgaaacaacttcctgcctcttttaactacggtgcaatcagggccgtcaccatccacgagcttctcgtgcgcatgaaacgggcagagctctactgccccaggccactattggcaatagaggcttcacaagacaggcacaagcaaaagatcattgcacctgcaagacagttgctgaactttgacctacttaagttggctggagacgtggagtccaaccctgggcccttcttcttctctgacgttaggtcaaacttttctaagctggtggaaaccatcaaccagatgcaggaagacatgtcaacaaaacacgggcccgactttaaccggttggtgtccgcctttgaggaactggccgctggagtgaaagccatcaggaccggcctcgacgaggccaaaccctggtacaagcttatcaaactcctaagccgcctgtcgtgcatggccgctgtggcagcacggtccaaggacccggtccttgtggccatcatgctggccgacaccggtctcgagattctggacagcactttcgtcgtgaagaagatctccgactcgctctccagtctctttcacgtgccggcccccgtcttcagtttcggagccccgattctgctagccgggctggtcaaggtcgcctcgagtttcttccggtccacgcccgaagaccttgagagagcagagaaacagctcaaagcacgtgacatcaacgacattttcgccattctcaagaacggcgagtggctggtcaaactgatccttgccatccgcgactggattaaggcttggattgcctcagaagaaaagtttgtcaccatgacagacttagtgcctggcatccttgaaaagcagcgggacctcaacgacccaagcaagtacaaggaggccaaggagtggctcgacaacgcgcgccaagcgtgtctgaagagcgggaacgtccacattgccaacctgtgcaaagtggtcgccccggcacccagcaagtcgagacccgaacccgtggtggtttgcctccgtggcaaatcaggccagggcaagagtttccttgcgaacgtgctcgcacaagcaatctctacccacttcaccgggcggactgattcagtctggtactgcccacctgaccctgaccacttcgacggttacaaccaacagactgtcgttgtgatggacgatttgggccagaaccctgacggcaaggacttcaagtacttcgcccaaatggtctcgaccacggggttcatcccgcccatggcatcacttgaggacaaaggtaaacccttcaacagtaaggtcatcattgcaaccaccaacctgtactcgggcttcaccccgaggactatggtgtgccctgacgccctgaaccggaggtttcactttgacattgacgtgagcgccaaggatgggtacaaaattaacaacaaattggacattatcaaagcacttgaagacacccacaccaacccagtggcaatgtttcagtacgactgcgcccttctcaacggcatggctgttgaaatgaagagacttcagcaagacatgttcaaacctcaaccacccctccagaacgtgtaccaactagttcaggaggtgattgatcgggtggagctccacgagaaagtgtcgagccacccaatttttaagcagatctcaattccttcccaaaaatctgtgttgtacttcctcattgagaaagggcagcacgaggcagcaattgaattctttgagggcatggtgcatgactccgtcaaggaggagctccggccgctcatccaacaaacctcatttgtgaaacgcgctttcaagcgcctgaaggaaaactttgagattgttgctctgtgcctaacacttttggccaacattgtgatcatgatccgcgagacccgcaagagacagaagatggtggacgatgcggtgaacgagtacatcgagaaagcaaacatcaccaccgatgacaagacacttgacgaggcggaaaagaaccctctggagactagcggtgccagcaccgttggtttcagagagagaactcttccaggtcagaaggcgcgtaatgacgtgaattccgagcccgcccaacctgctgaagaacaaccacaagctgaaggaccctacgccgggccacttgagcgtcagagacccctgaaggtgagagccaagctcccacaacaggaaggaccctacgctggcccgatggagagacagaaaccgctgaaagtgaaagcaaaagccccggtcgtcaaggaaggaccttacgaaggaccggtgaagaaacctgtcgctttgaaagtgaaagctaagaatttgattgtcactgagagtggtgcccccccgaccgacttgcaaaagatggtcatgggcaacacaaagcctgttgagctcattctcgacgggaagacagtagccatctgctgtgctactggagtgtttggcactgcctacctcgtgcctcgtcatcttttcgctgagaagtatgacaagatcatgttggacggcagagccatgacagacagtgactacagagtgtttgagttcgagatcaaagtaaaaggacaggacatgctctcagacgccgcactcatggtgctccaccgtgggaaccgcgtgagagacatcacgaagcatttccgtgacacagcaagaatgaagaaaggcacccccattgtcggcgtgatcaacaatgccgatgtcgggagactgattttctctggcgaagctcttacctacaaagacattgtagtgtgcatggacggagataccatgcccgggctttttgcctacagagccgccactaaggctggctactgcgggggagccgttctcgctaaggacggggctgacactttcatcgttggcactcactctgcaggaggtaatggagttggatactgctcatgcgtttccaggtccatgcttctcaagatgaaggcacacattgaccctgagccgcaccacgaggggttgattgtagacaccagagatgtggaagagcgcgtccacgtaatgcgcaagaccaagcttgcacccaccgttgcacacggtgtgtttaaccccgagtttgggccagctgccttgtccaacaaggacccgcgtctgaacgagggtgttgtccttgatgaagtcattttctccaaacacaagggagacacaaagatgtctgaggaggacaaagcgctgttccgtcgatgtgctgctgactacgcgtcacgcctgcacagcgtgctgggtacggcaaacgccccattgagcatctacgaggcaatcaaaggcgtcgacggactcgacgccatggagccagacaccgcacctggcctcccctgggctctccagggaaaacgccgtggtgcgctcatcgactttgagaacggcacggtcgggcccgaagtcgaggctgccttgaagctcatggagagaagagagtacaagtttgtttgtcagaccttcctgaaggacgagattcgcccgatggagaaaatacgtgccggcaagactcgcattgtcgacgtcctgcccgttgaacacattctttacaccaggatgatgattggcagattttgtgcacaaatgcactcaaacaacggaccgcaaattggctcggcggtaggttgtaaccctgatgttgattggcaaagatttggcacacacttcgcccaatacagaaatgtgtgggatgtggactattcggcctttgatgctaaccactgcagtgacgccatgaacatcatgtttgaggaggtgttccacacggacttcgggttccacccaaatgctgagtggatcttgaagactctcgtgaacacggaacatgcctatgagaacaaacgcatcactgttgaaggcgggatgccatctggttgttccgcaacgagcatcatcaacacaattttgaacaacatctacgtgctctacgccttgcgtagacactatgagggagttgagctggacacttacaccatgatctcctacggagacgacattgtggtggcaagtgattacgatctggactttgaggctctcaagccccacttcaaatctcttggtcaaaccattactccagctgacaaaagcgacaaaggttttgttcttggtcactccatcaccgatgtcactttcctcaaaagacacttccacatggattatggaactgggttttacaaacctgtgatggcctcaaagacccttgaggctatcctctcctttgcacgccgtgggaccatgcaggagaagctgatttccgtggcaggactcgctgtccactctggaccagacgagtaccggcgtctctttgagccctttcagggcctctttgagattccaagctacagatcactttacctgcgttgggtgaacgccgtgtgcggcgacgcataa

>AY593781.1_A_GER_1951

atgcatacaactgactgttttatcgctttggtgcacgctatcagagagatcagagcactttttctaccacgaaccacaggaaagatggaactcaccctgcacaacggcgagaaaaagactttttactctagacccaacaaccacgacaactgctggttgaacaccatccttcagttgttcaggtatgtcgatgaacccttcttcgactgggtctacaactcgcccgagaacctcacgcttgaagccatcaaccaattggaggaactcacaggacttgagttgcacgagggcggaccgcctgcccttgtgatctggaacatcaaacacttgctccacaccggcatcggcaccgcctcacgacccagtgaggtgtgtatggtggacggcacggacatgtgtcttgctgacttccacgcaggcattttcctgaagggacaggaacacgcagtctttgcgtgtgtcacctccaacgggtggtacgcgattgacgacgaggaattttacccctggacgcctgacccgtcagacgtcctggtgtttgtcccgtacgatcaagaaccactcaacggggactggaaagcgatggttcagaggaagcttaagggtgccgggcaatccagcccggcgaccggctcccagaaccagtctggcaatactggcagcataattaacaactactacatgcagcagtaccagaactccatggacacacagcttggtgacaatgccattagtggaggctccaacgaaggctccacggacacaacttcaacacacacaaccaacacccaaaacaacgattggttttcaaaacttgccagttcagccttcaccggtctgttcggcgccctgctcgccgacaagaagacggaagagactacacttctggaagaccgcattctcaccacccgcaacgggcacaccatctcgaccacccaatcgagtgtgggagtcacctacgggtactccactggagaagaccatgtcgctgggcccaacacatcgggcctggagacgcgggtggtgcaggcagagagatttttcaaaaagtttttgtttgactggacaacggacaaaccttttggacatttggaaaagctggaacttcccgccgaccaccacggcgttttcgggcacctggtggaatcatatgcttacatgagaaatggttgggacgttgaggtgtctgctgttggcaaccagttcaacggcgggtgcctcctggtggctatggtaccggagtggaaagagtttgaacagcgcgagaaataccaactcaccctcttcccgcaccagttcatcagccccagaacaaacatgactgctcacatcacagtcccataccttggagtgaacaggtacgatcagtacaagaaacacaaaccttggacactggttgttatggtagtgtcgcccctcacggttagcgacactgccgcggcacagattaaggtctacgccaacattgctccaacctacgttcacgtggctggggaactcccctcgaaagaggggattttcccagtcgcatgttcggacggttacggaggattggtgacaacggacccgaaaacagctgaccccgcctacggcaaggtgtacaacccgcccaggaccaactaccctgggcggtttaccaacttgttggacgtggctgaagcgtgtcccactttcctctgtttcgacgacgggaaaccgtacgttgtcacgcggacagatgacacacgactattggccaagttcgacgtctcccttgctgcaaaacacatgtccaacacgtacctgtcagggattgcacagtactacgcacagtactctggtaccatcaacttgcacttcatgttcacaggctcaactgactcaaaagcccgctacatggtggcctacatcccgcctggggtggaaccaccggacacacctgaaagggccgctcactgcatccacgctgaatgggacacaggactgaactccaaattcactttttcaatcccgtacgtgtccgccgcagattacgcgtataccgcgtctgacacggcagaaacaaccaacgtacagggctgggtctgcatctaccagatcacacacgggaaggccgagaacgacacattggtggtgtcggccagcgccggcaaagactttgagttgcgcctcccgatcgacccccgacagcaaaccactgctgttggggagtccgcagaccctgtcaccaccaccgtggagaactacggcggtgagacacaaacccagagacggcaccacacggatgtcggtttcatcatggacagatttgtgaagataaacagtttgagtcccacgcatgtcattgacctcatgcagacccaccagcacgggctggtaggtgcgctgttgcgtgcagccacgtactacttctctgacttggagattgttgtgcggcatgacggcaatttgacttgggtgcccaatggtgcccctgaagcagctttgtcaaacaccagcaaccccactgcctacaacaaggcaccgttcacgaggctcgctctcccttacactgcgccacaccgcgtgttggcaaccgtgtacaacgggacgaacaagtactccacgggcggtccg------agacgaggcgacacggggtcgcccgcggcgcgggccgcgaaacaacttcctgcctcttttaactacggtgcaatcagggccgtcaccatccacgagcttctcgtgcgcatgaaacgggcagagctctactgccccaggccactattggcaatagaggcttcacaagacaggcacaagcaaaagatcattgcacccgcaagacagttgctgaactttgacctacttaagttggctggagacgtggagtccaaccctgggcccttcttcttctctgacgttaggtcaaacttttctaagctggtggaaaccatcaaccagatgcaggaagacatgtcaacaaaacacgggcccgactttaaccggttggtgtccgcctttgaggaactggccgctggagtaaaagccatcaggaccggcctcgacgaggccaaaccctggtacaagcttatcaaactcctaagccgcctgtcgtgcatggccgctgtggcagcacggtccaaggacccagtccttgtggccatcatgctggccgacaccggtctcgagattctggacagcactttcgtcgtgaagaagatctccgactcgctctccagtctcttccacgtgccggcccccgtcttcagtttcggagccccgattctgctagccgggctggtcaaggtcgcctcgagtttcttccggtccacgcccgaagaccttgagagagcagagaaacagctcaaagcacgtgacatcaacgacattttcgccattctcaagaacggcgagtggctggtcaaactgatccttgccatccgcgactggattaaggcttggattgcctcagaagaaaagtttgtcaccatgacagacttagtgcctggcatccttgaaaagcagcatgacctcaacgacccaagcaagtacaaggaggccaaggagtggctcgacaacgcgcgccaagcgtgtctgaagagcgggaacgtccacattgccaacctgtgcaaagtggtcgccccggcacccagcaagccgagacccgaacccgtggtggtttgcctccgtggcaaatcaggccagggcaagagtttccttgcgaacgtgctcgcacaagcaatctctacccacttcaccgggcggaccgattcagtctggtactgcccacctgaccctgaccacttcgacggttacaaccaacagactgtcgttgtgatggacgatttgggccagaaccctgacggcaaggacttcaagtacttcgcccaaatggtttcgaccacggggttcatcccgcccatggcatcactcgaggacaaaggtaaacccttcaacagtaaggtcatcattgcaaccaccaacctgtactcgggcttcaccccgaggactatggtgtgccctgacgccctgaaccggaggtttcactttgacattgacgtgagcgccaaggatgggtacaaaattaacaacaaattggacattaccaaagcacttgaagacacccacaccaacccagtggcaatgtttcagtacgactgtgcccttctcaacggcatggctgttgaaatgaagagaatgcagcaagacatgttcaaacctcaaccacccctccagaacgtgtaccaactagttcaggaggtgattgatcgggtggagctccacgagaaagtgtcgagccacccaatttttaagcagatctcaattccttcccaaaaatctgtgttgtacttcctcattaagaaagggcagcacgaggcagcaattgaattctttgagggcatggtgcatgactccgtcaaggaggagctccggccgctcatccaacaaacctcatttgtgaaacgcgctttcaagcgcctgaaggaaaactttgagattgttgctctgtgcctaacacttttggccaacattgtgatcatgatccgcgaaacccgcaagagacagaagatggtggacgatgcggtgaacgagtacatcgagaaagcaaacatcaccaccgatgacaagacacttgacgaggcggaaaagaaccctctggagactagcggtgccagcaccgttggtttcagagagagaactcttccaggtcagaaggcgcgtgatgacgtgaactccgagcccgcccaacctgctgaagaacaaccacaagctgaaggaccctacgccgggccacttgagcgtcagagacccctgaaggtgagagccaagctcccacaacaggaaggaccctacgctggcccgatggagagacagaaaccgctgaaagtgaaagcaaaagccccggtcgtcaaggaaggaccttacgaaggaccggtgaagaaacctgtcgctttgaaagtgagagctaagaatttgattgtcactgagagtggtgcccccccgaccgacttgcaaaagatggtcatgggcaacacaaagcctgttgagctcatcctcgacgggaagacagtagccatctgctgtgctactggagtgtttggcactgcctacctcgtgcctcgtcatcttttcgctgagaagtatgacaagatcatgttggacggcagagccatgacagacagtgactacagagtgtttgagttcgagatcaaagtaaaaggacaggacatgctctcagacgccgcactcatggtgctccaccgtgggaaccgcgtgagagacatcacgaagcactttcgtgacacagcaagaatgaagaaaggcacccccgttgtcggcgtgatcaacaatgccgatgtcgggagactgattttctctggcgaagcccttacctacaaagacattgtagtgtgcatggacggagacaccatgcccgggctttttgcctacagagccgccactaaggcaggctactgcgggggagccgttctcgctaaggacggggctgacactttcatcgttggcactcactctgcaggaggtaatggagttggatactgctcatgcgtttccaggtccatgcttctcaagatgaaggcacacattgaccctgagccgcaccacgaggggttgattgtagacaccagagatgcggaagagcgcgtccacgtgatgcgcaaaaccaagcttgcacccaccgttgcacacggtgtgttcaaccccgagtttgggccagctgccttgtccaacaaggacccgcgtctgaacgagggtgttgtcctcgatgaagtcattttctccaaacacaagggagacacaaagatgtctgaggaggacaaagcgctgttccgccgatgtgctgctgactacgcgtcacgcctgcacagcgtgctgggtacggcaaatgccccattgagcatctacgaggcaatcaaaggcgtcgacggactcgacgccatggagccagacaccgcacctggcctcccctgggctctccagggaaaacgccgtggtgcgctcatcgactttgagaacggcacggtcgggcccgaagtcgaggctgccttgaagctcatggagaaaagagagtacaagtttgtttgtcagaccttcctgaaggacgagattcgcccgatggagaaagtacgtgccggcaagactcgcattgtcgacgtcctgcccgttgaacacattctttacaccaggatgatgattggcagattttgtgcacaaatgcactcaaacaacggaccgcaaattggctcggcggtcggttgtaaccctgatgttgattggcaaagatttggcacacacttcgcccaatacagaaacgtgtgggatgtggactattcggcctttgatgctaaccactgcagtgacgccatgaacatcatgtttgaggaggtgttccgcacggacttcgggttccacccaaatgctgagtggatcttgaagactctcgtgaacacggaacatgcctatgagaacaaacgccacactgttgaaggcgggatgccatctggttgttccgcaacgagcatcatcaacacaattttgaacaacatctacgtgctctacgccttgcgtagacactatgagggagttgagctggacacttacaccatgatctcctacggagacgacattgtggtggcaagtgattacgatctggactttgaggctctcaagcctcacttcaaatctcttggtcaaaccattactccagctgacaaaagcgacaaaggttttgttcttggtcactccatcaccgatgtcactttcctcaaaagacacttccacatggattatggaactgggttttacaaacctgtgatggcctcaaagacccttgaggctatcctctcctttgcacgccgtgggaccatacaggagaagctgatctccgtggcaggactcgctgtccactctggaccagacgagtaccggcgtctctttgagccctttcagggcctctttgagattccaagctacagatcactttacctgcgttgggtgaacgccgtgtgcggcgacgcataa

>AY593782.1_A_ARG_2000

atgaacacaactgactgccttatcgctttggtgtttgcaatcagagagatcaaggggcttttcacaaaacgaactagaggagaaatggaactcacgctgcacaacggcgacagaaagactttctactcaagacccaacaaacacgacaactgttggctgaacgccatccttcagttgttcaggtatgtcgacgaaccattcttcgactgggtctacagctcgcccgaaaacctcacgctcaaggccattgagcagctggagagacttactgggcttgagctacacgagggtggaccgcccgctctcgtcatttggaacatcaagcacttgctccaaaccggcatcggcactgcctcacgacccagtgaggtgtgcatggtggacgggacggatatgtgtcttgcagactttcacgcaggcatcttcctgaaaggcgaagaacatgctgtcttcgcatgtgtcacctctgatgggtggtacgcgattgacgatgaggacttttacccctggacaccggacccgtccgacgtcttggtgtttgttccttacgatcaagagccactcaacggagaatggaaagccaaggtacagcggaagctaaagggagctgggcaatccagcccagctactggctcccagaaccaatctggtaacactggcagtataatcaacaactattacatgcagcagtaccagaactccatggacacacaacttggtgacaacgccatcagtggaggctctaacgagggttccacggacacaacttcaacccatacaaccaacactcaaaacaacgactggttctcaaaacttgccagctcggccttctccggcttgttcggggcgttgcttgccgacaagaagacggaggagacgacgctacttgaggaccgcattctcaccacccgcaacgggcacaccacctcgactacccagtccagcgtaggcgtcacgtacgggtactccacggcggaagatcacgtcgccgggcccaacacatcgggcttggagacccgggtggtacaagcagagagattttacaaaaagtttttgtttgactggacaaaggacaaggcttttggacatgtggaaaagttggaactgcccgccgaccaccacggtgttttcggacacttggtggactcatatgcctacatgaggaacggttgggatgttgaggtgtctgctgttggcaaccagttcaacggcggctgtctcttggtggctatggtacctgagtggaaagaatttgacacacgggagaaataccaacttacacttttcccacatcaattcattaaccccagaaccaacatgactgcccacatcacggtcccttacctcggtgtgaacaggtacgaccagtacaagaagcacaaaccctggacattggttgttatggttgtatccccactcacagtcagctccaatggtgcagcacagattaaggtctatgctaacatcgcgccaacctacgtccacgtggccggtgagctcccgtcgaaagaggggatctttcctgttgcgtgcgcggacggttatggcgggctggtgacgacagacccgaaaacagctgaccccgcctacggcaaggtgtacaacccgccccggaccaactaccccgggcgtttcaccaatttgttggacgtggccgaggcgtgtcctaccttcctttgttttgacgacgggaaaccgtacgtcgttacgaggacagacggcacgcgcctcttggccaagttcgacctttcccttgctgcaaagcacatgtccaacacttacttgtcaggaattgcccagtactacgcacagtactcaggtaccatcaatttgcacttcatgttcacaggttcaactgattcaaaggcccggtacatggtggcctacatcccgcctggggtggagccaccggacacacctgagcgtgcggcccactgcatccacgccgaatgggacacaggactgaactccaagttcactttttcaatcccgtacgtgtctgctgcggattacgcctacacggcgtctgacgaggcagaagcaacaaacgtacagggatgggtttgcatttaccaaatcacacacgggaaggccgaagacgacactcttgttgtgtcagtcagtgctggcaaggacttcgagctgcgcctcccgattgacccccgccagcaaaccaccgccactggggaatcagcagaccctgtcaccaccacagtggagaactacggcggtgaaacacaagtccagagacgccaccacacagacgttggcttcatcatggacagatttgtgaaagtgacaaccacagctcccacccacgttattgacctcatgcaaacacaccaacacggcctagtgggtgcgctgctgcgggcggcaacctactacttctccgatctggagattgtcgtgcgacatgaaggcaacctgacgtgggtacccaatggtgctcctgaatcggccctgtccaacacaagcaaccccaccgcctacaagaaggcaccgttcacgagacttgctctaccttacaccgcgccgcaccgagtgctggcaactgtgtacaacggagtaagcaagtacaccgcgaatggttca---aacaggcggggtgacatggccgctctcgcggcacgagtcgcgaaggcacttcctgcttctttcaactacggtgcaatcaaggccactaacatacacgagcttctcgtgcgcatgaaacgggccgagctctactgcccccgaccactgctggcaatagaggcatcacaggacagacacaaacaaaagatcattgcacccgcaaaacagtctctgaacttcgacctgctcaagctggcgggagacgttgagtccaaccccgggcccttcttcttctccgacgtcaggtcgaactttaccaagttagtggagactctcaaccaaatgcaggaggacatgtccacaaagcacggacccgactttaaccggttggtgtctgcgtttgaggaattggccactggagtcaaggctatcaggactggtctcgacgaggccaaaccttggtacaaactgatcaaactcctgagccgcttgtcgtgcatggccgctgtagcagcacggtcaaaggatccagtccttgtggccatcatgctggctgacaccggtctcgagattctggacagcacatttgtcgtgaagaaaatctccgactcgctctccagtctctttcacgtgccggcccccgtcttcagtttcggagccccgatcctgctggccgggttggtcaaggtcgcctcgagtttcttccggtcaacacccgaagaccttgagagagcagagaaacagctcaaagcacgtgacattaacgacattttcgccattctcaagaacggcgagtggctggtcaaattgatccttgccatccgcgactggatcaaggcatggatcgcctcagaagagaagtttgtcaccgtgacagacttggtgcctggcatccttgaaaagcaaagggacctcaacgacccaagcaagtacaaggaggccaaggagtggctcgacaacgcgcgccaggcgtgtttgaagagcggaaatgtccacattgccaacctgtgcaaagtggtcgccccagcacccagcaggtcgagacccgagcctgtggtcgtttgcctccgcggcaaatctggccagggcaagagtttccttgccaacgtgcttgcacaagcaatctccacccactttactggcagaaccgattcggtttggtactgcccccctgaccccgatcactttgacggttacaaccaacagaccgttgtcgtgatggatgatttgggccagaaccctgacggcaaggacttcaagtacttcgcccaaatggtttcgaccacagggttcatcccgcctatggcgtcactcgaggacaaaggcaaacccttcaacagtaaggtcatcattgcaaccaccaacctgtacgcgggctttaccccgaggactatggtctgccctgatgccctgaaccggaggtttcactttgacatcgacgtgagtgctaaagacgggtacaaaattaacaacaaattggacattataaaagcacttgaagacactcacaccaacccagtggcaatgtttcaatacgactgtgcccttctcaacggcatggccgttgaaatgaagagaatgcaacaaaacgtgtttgagccccaaccacccctccagaacctctaccagcttgttcaggaggtgattgaacgggtagagctccacgagaaagtgtcgagccacccaattttcaagcaaatctcaattccttcccaaaagtctgtgttgtacttcctcattgagaaaggacaacatgaggcagcaattgaattctttgaggggatggtacacgactccatcaaggaggagctccgacctctcatccaacaaacctcatttgtgaaacgcgccttcaagcgcttgaaggaaaattttgagatggttgctttgtgcctgactcttctggccaacatagtgatcatgatccgcgagactcgcaagagacaaaagatggtggatgacgcagtgggtgagtacattgagaagacaaacatcaccaccgacgacaagactcttgacgaggcggaaaagaaccctctggagactagtggtgccagcaccgttggtttcagagagagaactctcccagggcagaaagcgagtgatgacgtgaacactgaacccgcccagcctgccggagaggaaccaaaagctgaaggaccctacagcgggccacttgagcgtcagaaacctctgaaagtgagagccaaattcccacagcaagaggggccctacgctggtccgttggagagacagaaaccactgaaagtgaaagcaaaagccccggtcgttaaggaaggaccttacgaagggccggtgaagaaacctgtcgctttgaaagtgaaagccaagaacttgatagttactgagagtggtgcccccccgaccgacttgcaaaagatggtcatgggcaacacaaagcctgttgagctcatccttgacgggaagacagtggccatctgctgtgctactggagtgtttggtactgcttacctcgtgcctcgtcatcttttcgcagagaagtatgacaagatcatgttgggcggtagagccatgacagacagtgactacagagtgtttgagtttgagattaaagtaaaaggacaggacatgctctcagacgccgcactcatggtgcttcaccgtgggaaccgcgtgagagacatcacgaaacactttcgtgatactgcaagaatgaagaaaggaacccccgttgttggcgtgatcaacaacgccgacgtcgggagactgattttctctggtgaggcccttacctacaaggacattgtagtgtgcatggacggagacaccatgcccgggctttttgcctacagagctgcaaccaaggcaggttactgcggaggagccgttctggccaaggacggcgctgacacgttcatcgtcggcactcactccgctggaggcaatggagttggatactgctcgtgcgtttccaggtccatgcttctcaagatgaaggcacacatcgaccctgaaccacaccacgagggtttgatagttgacaccagagatgtggaagagcgcgtccacgtgatgcgcaaaaccaagcttgcacccaccgttgcacacggtgtgttcaaccctgaattcgggcccgctgccttgtccaacaaggacccgcgcctgaacgagggtgttgtcctcgatgaagtcatcttttccaaacacaagggagacacaaggatgtctgaggaggataaagcgctgttccgccgctgcgcggctgactacgcgtcacgcctgcacagtgtgctgggtacagcaaatgccccattgagtatttacgaggcgatcaagggcgtcgacgggctcgacgccatggaaccagacaccgcacccggtctcccttgggccctccaggggaagcgccgcggcgcactcatcgactttgagaacggcacggtcggacctgaagtcgaggctgccttgaaacttatggagaaaagagaatacaagtttgcttgccagaccttcctgaaggacgaaattcgcccgatggagaaagtgcgcgccggcaagactcgcatcgtcgatgtcttgcctgttgaacatattctttacaccaggatgatgattggcagattttgtgcacaaatgcacatgaacaacggtccgcagattggctcggcggtcggttgtaaccctgatgttgattggcaaagattcggcacacacttcgcccaatacaaaaacgtgtgggatgtggactattcggcctttgatgctaaccattgcagtgacgccatgaacatcatgtttgaggaggtgttccgcacggatttcgggttccacccaaacgctgagtggattctgaagacccttgtgaacacggaacacgcttatgaaaacaaacgcatcactgttgagggcgggatgccgtctggctgttccgcgacaagcatcatcaacacaattatgaacaacatctacgtgctctacgccctgcgcagacactatgagggagttgagctggacacctacactatgatctcctacggagacgacatagttgtggcaagtgattatgatttggactttgaggctctcaagccccactttaaatctcttggtcaaaccatcaccccagctgacaagagcgacaaaggttttgttgttggtcactccattactgatgtcactttcctcaaaagacacttccacatggattatggaactgggttttacaaacctgtgatggcctcaaagacccttgaggctatcctctcctttgcacgccgtgggaccatacaagagaagttgatctccgtggcaggactcgccgtccactctggacctgacgagtaccggcgtctctttgagcctttccaaggcctctttgagattccaagctacagatcactttacttgcgttgggtgaacgccgtgtgtggcgacgcataa

>AY593784.1_A_ARG_2001

atgaacacaactgactgttttatcgctttggtgcacgccatcagagagattaagacgtttttcttcacacggcacacaggaagaatggaattcacactgtacaacggtgagaagaagacattttactccagacccaacaaccacgacaactgttggttgaacgccatcctccagttgttcaggtacgtcgacgaacctttcttcgactgggtctacaactcgcctgagaacctcacgctctcggccatcgaacagctggaggaaattaccgggcttgagttgcacgagggcggaccacccgcgctcgtggtttggaacatcaaacacatgctccacactggcatcggcaccgcctcgcgacccagcgaggtgtgcatggtcgacggtacggacatgtgtttggctgatttccatgctggcattttcctgaaaggtcaggagcacgctgtgtttgcatgtgtcacctctgacgggtggtacgcgatcgacgacgaggacttctacccttggacaccagacccgtctgacgtcctggtgtttgtcccgtacgaccaagaaccgctcaacggagaatggaaggctaaggttcagcgcaagctcaagggagctgggcaatccagcccagctactggctcgcagaaccaatctggtaacacaggtagcataatcaacaactactacatgcaacagtaccaaaactccatggacacacagcttggtgacaatgccatcagtggaggctctaacgagggctccacggacacaacttcaactcacacaaccaacacccaaaacaatgactggttttcaagactcgccggttcggccttctccggtttgtttggggccttgcttgccgacaagaagacggaggagacgacactccttgaggaccgcattctcaccactcgcaatgggcacaccacctccacgacccagtccagcgtaggcgttacatacgggtactccacaacagaggaccacgttgctggacccaacacatcaggtttggagacacgagtggtacaggcagagagattctacaaaaagtttttgtttgattggacaacggacaagccttttggacacctgcacaaactggagttgcccaccgaccaccacggtgttttcggacacttggtggactcatacgcctacatgaggaacggttgggacgttgaggtgtctgctgttggcaaccagttcaacggcggatgcctcctagtggccatggtacccgaatggaaagagtttgaaacgcgggagaagtaccagctcacgcttttcccgcaccagttcattagccccagaaccaacatgaccgcccacatcacggttccttaccttggtgtgaatagatatgatcagtacaaaaaacacaaaccctggacactggttgtcatggtcgtgtccccgctcacggtcaacgccacgagcgcggcacagatcaaggtctatgccaacatcgctccgacctacgttcatgtggccggcgagctcccctcgaaagaggggatcttccctgtcgcgtgcgcggacggttacggaggactggtgacaacggacccgaaaacagctgaccccgcctacggcaaggtgtacaatccgccccggactaactaccccgggcgtttcactaacttgttggacgtggctgaggcatgtcccacctttctgtgttttgacgacgggaaaccgtacgttaccacacagacaggtgagtctcgtcttctggccaagttcgacctttcccttgccgcgaagcacatgtctaacacatacttggcaggaattgcccagtactacacacagtactcaggcaccatcaatttgcatttcatgttcacaggttcaactgattcaaaagcccgctacatggtggcttacatcccgcctggggtggaaccaccggacacacctgagagggcagcccactgcatccatgctgagtgggacacagggctgaattccaaattcacattctcaatcccgtacgtgtctgccgcggattacgcctacacggcgtctgatgaggcagagacaacaaacgtacagggatgggtctgcgtttaccagatcacacacgggaaggctgacaacgacactctggtcgtgtcggttagcgccggcaaggacttcgagttgcgcctccccattgacccccgaccgcagaccaccgctactggggaatcagcagaccctgtcaccaccactgtagagaactacggcggtgagacacaagttcagagacgccaccacaccgacgttggcttcatcatggacagatttgtgaaaataaacagcccaaaatccacccatgttattgacctcatgcaaacccaccaacacggtctagtgggtgcgctgctgcgtgcggcgacctactacttctcagatctggaaattgttgtgcggcatgacggcaacctaacttgggtgcccaatggtgctcccgtgtcagccttgtccaacaccagcaaccccaccgcctacaacaaggcaccgttcacgagacttgccctcccctacaccgcgccacaccgcgtgttggcgactgtgtacaacgggacgagcaagtacactgtgagtgggtca---agcagacgaggcgacttgggttccctcgcggcacgagtcgtgaaggcacttcctgcttctttcaactacggtgcaatcaaggccgacaacgtgcacgagcttctcgtgcgcatgaaacgggccgaactctactgccctagaccactgttggcactagaggtctcacaagacaggcgcaaacagaagatcattgcacccgaaaaacagcttttgaatttcgacctgctcaagttggcgggagacgttgagtccaaccctgggcccttcttcttctccgacgtcaggtcaaatttctccaagctggtggaaaccatcaaccagatgcaggaggacatgtcaacaaaacacggacccgactttaaccggttggtgtctgcgtttgaggaactggccgctggagttaaggctatcaggaccggtctcgacgaggccaaaccctggtacaagctgattaagctcctgagccgcttgtcgtgcatggccgctgtagcagcacggtcaaaggacccagtccttgtggctatcatgctggctgacaccggtcttgagattctggacagcacgtttgtcgtgaagaagatctccgactcgctctccagtctctttcacgtgccggcccccgtcttcagtttcggagccccgattctgttggccgggctggtcaaggtcgcctcgagtttcttccggtccacacccgaggatctcgagagagcagaaaaacagctcaaagcacgtgacatcaacgacattttcgccattctcaagaacggcgagtggctggtcaagctgattcttgccatccgcgactggattaaggcatggatcgcctcagaagaaaagtttgtcaccacgacagacttggtgcctggcatccttgagaagcaacgggacctcaacgacccggccaagtacaaggaagccaaggagtggctcgacaacgcgcgccaggcgtgtttgaagagcgggaacgtccacatcgccaacctgtgcaaggtggtcgccccagcacccagcaagtcgagacccgaacccgtggtcgtctgcctccgtggcaagtctggccagggcaagagtttccttgctaacgtgcttgcacaagcaatttccactcacttcaccggcagaaccgactcggtttggtactgcccccctgaccctgaccacttcgacggttacaaccaacagaccgttgtagtgatggatgatttgggccagaaccctgacggcaaggacttcaagtactttgcccaaatggtgtcaaccacagggttcatcccgcccatggcgtcactcgaggacaaaggcaaacccttcaacagcaaggtcatcatcgcgaccaccaacttgtactcgggcttcaccccgaggaccatggtctgccctgacgccctgaatcgaaggtttcacttcgacattgacgtgacagccaaagacgggtacaaagttaacaacaaattggacatcattaaagcactcgaagacacccacaccaatcctgtggcaatgtttcagtatgactgtgcccttctcaacggcatggctgttgaaatgaagagaatgcaacaagacctcttcaaacctcaaccacccctccagaacgtctaccagctcgtccaggaggtgattgaccgggtagagctccacgaaaaagtgtcgagccacccgatcttcaagcagatctcaattccttcccaaaaatctgtgttgtactttctcattgagaaagggcagcacgaagcagcaattgatttctttgagggcatggtgcatgactccatcaaggaggaactccggcccctcatccaacaaacctcatttgtgaaacgcgctttcaaacgcttgaaggagaactttgagattgttgccctatgtttgaccctgctggccaacatagtgatcatgatccgcgagactcgcaagagacagaagatggtggacgatgcagtgaacgagtacatcgagaaggcaaacatcaccaccgacgacaagactcttgacgaggcggaaaagaaccctctggagactagcggtgccagtaccgttggtttcagagagaaaactctcccaggtcacaacgcgcgtgatgacgtgaactccgagcccgcccaacgtgacacagagcaaccacaagctgaaggaccctacgccgggccactcgagcgtcagaaacctctgaaagtgagagccaagctaccacagcaggagggaccttacgctggcccgatggagagacagaaaccactgaaagtgaaagcaaaagccccggtcgttaaggaagggccgtacgaaggaccggtcaagaaacctgtcgctttgaaagtgaaagctaagaacttgattgtcactgagagtggtgccccaccgaccgacctgcagaagatggtcatgggcaacacaaagcctgttgagcttatcctcgacgggaagacagtagccatctgttgcgctactggagtgttcggtactgcctacctcgtgcctcgtcatcttttcgctgagaagtacgacaagatcatgctggacggcagagccctgacagacagtgactacagagtgtttgagtttgagattaaagtaaaaggacaggacatgctctcagacgctgcgctcatggtgcttcaccgtgggaaccgcgtgagagacatcacgaaacactttcgtgacacagctagactgaagaaaggcacccccgttgttggcgttattaacaatgctgacgtcgggagactgatcttctctggtgaggcccttacctacaaggacattgtagtgtgcatggacggagataccatgccagggctttttgcctacaaagccgcaactaaggctggctattgcggaggggccgttctcgcaaaggacggagccgacactttcatcgttggcacccactctgctggaggcaatggagttggttactgctcatgcgtttccaggtccatgctccaaaagatgaaggcacacgtcgacccggagccacaccacgaagggttgattgttgacaccagagatgtggaagaacgcgtccacgtgatgcgcaaaaccaagcttgcacccaccgtggctcacggtgtgttcaaccctgagttcgggcccgctgccttgtccagcaaggatccgcggctgaacgagggtgttgtcctcgatgaagtcattttctccaaacacaaaggagacacaaaaatgtctgaggaggacaaagcgctgttccgccattgtgccgccgactacgcgtcgcgcttacacaatgtgttgggcacggcaaacgccccattgagcgtttacgaagcaatcaaaggcatcgacggtctcgatgcgatggaaccagacaccgcgcccggcctgccatgggcactccagggaaaacgccgcggtgcgctcatcgacttcgagaacggcactgtcgggcccgaggttgaagctgctttgaagctcatggagaacagagaatacaaatttgcttgccagaccttcctgaaggacgagattcgcccgatggagaaagtacgtgccggcaagactcgcatcgtcgatgtcctgcctgttgaacacattctttacaccaggatgatgattggcagattttgtgcacaaatgcactcaaacaacggaccgcagattggctcagcggtcggttgcaaccctgatgttgattggcaaagatttggcacccatttcgcccagtacagaaacgtgtgggacgtggattattcggccttcgatgctaaccactgcagtgatgccatgaacatcatgtttgaggaggtgttccgcacggaatttggattccaccccaacgctgagtggattctaaagactctcgtgaacacagaacacgcctatgagaacaaacgcattgtggttgaaggcgggatgccctccggctgttccgcgacaagcatcatcaacacgattttgaacaacatctacgtgctctacgccttgcgtagacactatgagggagttgagctggacacctacaccatgatctcctacggagacgacatcgtggtggcaagtgattatgatttggactttgaggctctcaggccccactttaaatctcttggtcaaaccattactccagctgacaaaagcgacaaaggttttgttcttggtcactccatcaccgatgtcactttcctcaaaagacacttccacatggactacggaactgggttttacaaacctgtgatggcctcaaagacccttgaagccatcctctccttcgcacgccgtgggaccatacaggagaagttgatctctgtggcaggactcgccgtccactctggacctgacgagtaccggcgtctctttgagccttttcaaggcctctttgagattccaagctacagatcactttacctgcgttgggtgaacgccgtgtgcggtgacgcataa

>AY593785.1_A_ARG_2001

atgaacacaactgactgttttatcgctttggtgcacgccatcagagagattaagacgtttttcttcacacggcacacaggaagaatggaattcacactgtacaacggtgagaagaagacattttactccagacccaacaaccacgacaactgttggttgaacgccatcctccagttgttcaggtacgtcgacgaacctttcttcgactgggtctacaactcgcctgagaacctcacgctctcggccatcgaacagctggaggaaattaccgggcttgagttgcacgagggcggaccacccgcgctcgtggtttggaacatcaaacacatgctccacactggcatcggcaccgcctcgcgacccagcgaggtgtgcatggtcgacggtacggacatgtgtttggctgatttccatgctggcattttcctgaaaggtcaggagcacgctgtgtttgcatgtgtcacctctgacgggtggtacgcgatcgacgacgaggacttctacccttggacaccagacccgtctgacgtcctggtgtttgtcccgtacgaccaagaaccgctcaacggagaatggaaggctaaggttcagcgcaagctcaagggagctgggcaatccagcccagctactggctcgcagaaccaatctggtaacacaggtagcataatcaacaactactacatgcaacagtaccaaaactccatggacacacagcttggtgacaatgccatcagtggaggctctaacgagggctccacggacacaacttcaactcacacaaccaacacccaaaacaatgactggttttcaagactcgccggttcggccttctccggtttgtttggggccttgcttgccgacaagaagacggaggagacgacactccttgaggaccgcattctcaccactcgcaatgggcacaccacctccacgacccagtccagcgtaggcgttacatacgggtactccacaacagaggaccacgttgctggacccaacacatcaggtttggagacacgagtggtacaggcagagagattctacaaaaagtttttgtttgattggacaacggacaagccttttggacacctgcacaaactggagttgcccaccgaccaccacggtgttttcggacacttggtggactcatacgcctacatgaggaacggttgggacgttgaggtgtctgctgttggcaaccagttcaacggcggatgcctcctagtggccatggtacccgaatggaaagagtttgaaacgcgggagaagtaccagctcacgcttttcccgcaccagttcattagccccagaaccaacatgaccgcccacatcacggttccttaccttggtgtgaatagatatgatcagtacaaaaaacacaaaccctggacactggttgtcatggtcgtgtccccgctcacggtcaacgccacgagcgcggcacagatcaaggtctatgccaacatcgctccgacctacgttcatgtggccggcgagctcccctcgaaagaggggatcttccctgtcgcgtgcgcggacggttacggaggactggtgacaacggacccgaaaacagctgaccccgcctacggcaaggtgtacaatccgccccggactaactaccccgggcgtttcactaacttgttggacgtggctgaggcatgtcccacctttctgtgttttgacgacgggaaaccgtacgttaccacacagacaggtgagtctcgtcttctggccaagttcgacctttcccttgccgcgaagcacatgtctaacacatacttggcaggaattgcccagtactacacacagtactcaggcaccatcaatttgcatttcatgttcacaggttcaactgattcaaaagcccgctacatggtggcttacatcccgcctggggtggaaccaccggacacacctgagagggcagcccactgcatccatgctgagtgggacacagggctgaattccaaattcacattctcaatcccgtacgtgtctgccgcggattacgcctacacggcgtctgataaggcagagacaacaaacgtacagggatgggtctgcgtttaccagatcacacacgggaaggctgacaacgacactctggtcgtgtcggttagcgccggcaaggacttcgagttgcgcctccccattgacccccgaccgcagaccaccgctactggggaatcagcagaccctgtcaccaccactgtagagaactacggcggtgagacacaagttcagagacgccaccacaccgacgttggcttcatcatggacagatttgtgaaaataaacagcccaaaatccacccatgttattgacctcatgcaaacccaccaacacggtctagtgggtgcgctgctgcgtgcggcgacctactacttctcagatctggaaattgttgtgcggcatgacggcaacctaacttgggtgcccaatggtgctcccgtgtcagccttgtccaacaccagcaaccccaccgcctacaacaaggcaccgttcacgagacttgccctcccctacaccgcgccacaccgcgtgttggcgactgtgtacaacgggacgagcaagtacactgtgagtgggtca---agcagacgaggcgacttgggttccctcgcggcacgagtcgtgaaggcacttcctgcttctttcaactacggtgcaatcaaggccgacaacgtgcacgagcttctcgtgcgcatgaaacgggccgaactctactgccctagaccactgttggcactagaggtctcacaagacaggcgcaaacagaagatcattgcacccgaaaaacagcttttgaatttcgacctgctcaagttggcgggagacgttgagtccaaccctgggcccttcttcttctccgacgtcaggtcaaatttctccaagctggtggaaaccatcaaccagatgcaggaggacatgtcaacaaaacacggacccgactttaaccggttggtgtctgcgtttgaggaactggccgctggagttaaggctatcaggaccggtctcgacgaggccaaaccctggtacaagctgattaagctcctgagccgcttgtcgtgcatggccgctgtagcagcacggtcaaaggacccagtccttgtggctatcatgctggctgacaccggtcttgagattctggacagcacgtttgtcgtgaagaagatctccgactcgctctccagtctctttcacgtgccggcccccgtcttcagtttcggagccccgattctgttggccgggctggtcaaggtcgcctcgagtttcttccggtccacacccgaggatctcgagagagcagaaaaacagctcaaagcacgtgacatcaacgacattttcgccattctcaagaacggcgagtggctggtcaagctgattcttgccatccgcgactggattaaggcatggatcgcctcagaagaaaagtttgtcaccacgacagacttggtgcctggcatccttgagaagcaacgggacctcaacgacccggccaagtacaaggaagccaaggagtggctcgacaacgcgcgccaggcgtgtttgaagagcgggaacgtccacatcgccaacctgtgcaaggtggtcgccccagcacccagcaagtcgagacccgaacccgtggtcgtctgcctccgtggcaagtctggccagggcaagagtttccttgctaacgtgcttgcacaagcaatttccactcacttcaccggcagaaccgactcggtttggtactgcccccctgaccctgaccacttcgacggttacaaccaacagaccgttgtagtgatggatgatttgggccagaaccctgacggcaaggacttcaagtactttgcccaaatggtgtcaaccacagggttcatcccgcccatggcgtcactcgaggacaaaggcaaacccttcaacagcaaggtcatcatcgcgaccaccaacttgtactcgggcttcaccccgaggaccatggtctgccctgacgccctgaatcgaaggtttcacttcgacattgacgtgacagccaaagacgggtacaaagttaacaacaaattggacatcattaaagcactcgaagacacccacaccaatcctgtggcaatgtttcagtatgactgtgcccttctcaacggcatggctgttgaaatgaagagaatgcaacaagacctcttcaaacctcaaccacccctccagaacgtctaccagctcgtccaggaggtgattgaccgggtagagctccacgaaaaagtgtcgagccacccgatcttcaagcagatctcaattccttcccaaaaatctgtgttgtactttctcattgagaaagggcagcacgaagcagcaattgatttctttgagggcatggtgcatgactccatcaaggaggaactccggcccctcatccaacaaacctcatttgtgaaacgcgctttcaaacgcttgaaggagaactttgagattgttgccctatgtttgaccctgctggccaacatagtgatcatgatccgcgagactcgcaagagacagaagatggtggacgatgcagtgaacgagtacatcgagaaggcaaacatcaccaccgacgacaagactcttgacgaggcggaaaagaaccctctggagactagcggtgccagtaccgttggtttcagagagaaaactctcccaggtcacaacgcgcgtgatgacgtgaactccgagcccgcccaacgtgacacagagcaaccacaagctgaaggaccctacgccgggccactcgagcgtcagaaacctctgaaagtgagagccaagctaccacagcaggagggaccttacgctggcccgatggagagacagaaaccactgaaagtgaaagcaaaagccccggtcgttaaggaagggccgtacgaaggaccggtcaagaaacctgtcgctttgaaagtgaaagctaagaacttgattgtcactgagagtggtgccccaccgaccgacctgcagaagatggtcatgggcaacacaaagcctgttgagcttatcctcgacgggaagacagtagccatctgttgcgctactggagtgttcggtactgcctacctcgtgcctcgtcatcttttcgctgagaagtacgacaagatcatgctggacggcagagccctgacagacagtgactacagagtgtttgagtttgagattaaagtaaaaggacaggacatgctctcagacgctgcgctcatggtgcttcaccgtgggaaccgcgtgagagacatcacgaaacactttcgtgacacagctagactgaagaaaggcacccccgttgttggcgttattaacaatgctgacgtcgggagactgatcttctctggtgaggcccttacctacaaggacattgtagtgtgcatggacggagataccatgccagggctttttgcctacaaagccgcaactaaggctggctattgcggaggggccgttctcgcaaaggacggagccgacactttcatcgttggcacccactctgctggaggcaatggagttggttactgctcatgcgtttccaggtccatgctccaaaagatgaaggcacacgtcgacccggagccacaccacgaagggttgattgttgacaccagagatgtggaagaacgcgtccacgtgatgcgcaaaaccaagcttgcacccaccgtggctcacggtgtgttcaaccctgagttcgggcccgctgccttgtccagcaaggatccgcggctgaacgagggtgttgtcctcgatgaagtcattttctccaaacacaaaggagacacaaaaatgtctgaggaggacaaagcgctgttccgccattgtgccgccgactacgcgtcgcgcttacacaatgtgttgggcacggcaaacgccccattgagcgtttacgaagcaatcaaaggcatcgacggtctcgatgcgatggaaccagacaccgcgcccggcctgccatgggcactccagggaaaacgccgcggtgcgctcatcgacttcgagaacggcactgtcgggcccgaggttgaagctgctttgaagctcatggagaacagagaatacaaatttgcttgccagaccttcctgaaggacgagattcgcccgatggagaaagtacgtgccggcaagactcgcatcgtcgatgtcctgcctgttgaacacattctttacaccaggatgatgattggcagattttgtgcacaaatgcactcaaacaacggaccgcagattggctcagcggtcggttgcaaccctgatgttgattggcaaagatttggcacccatttcgcccagtacagaaacgtgtgggacgtggattattcggccttcgatgctaaccactgcagtgatgccatgaacatcatgtttgaggaggtgttccgcacggaatttggattccaccccaacgctgagtggattctaaagactctcgtgaacacagaacacgcctatgagaacaaacgcattgtggttgaaggcgggatgccctccggctgttccgcgacaagcatcatcaacacgattttgaacaacatctacgtgctctacgccttgcgtagacactatgagggagttgagctggacacctacaccatgatctcctacggagacgacatcgtggtggcaagtgattatgatttggactttgaggctctcaggccccactttaaatctcttggtcaaaccattactccagctgacaaaagcgacaaaggttttgttcttggtcactccatcaccgatgtcactttcctcaaaagacacttccacatggactacggaactgggttttacaaacctgtgatggcctcaaagacccttgaagccatcctctccttcgcacgccgtgggaccatacaggagaagttgatctctgtggcaggactcgccgtccactctggacctgacgagtaccggcgtctctttgagccttttcaaggcctctttgagattccaagctacagatcactttacctgcgttgggtgaacgccgtgtgcggtgacgcataa

>AY593786.1_A_ARG_2001

atgaacacaactgactgttttatcgctttggtgcacgccatcagagagattaagacgtttttcttcacacggcacacaggaagaatggaattcacactgtacaacggtgagaagaagacattttactccagacccaacaaccacgacaactgttggttgaacgccatccttcagttgttcaggtacgtcgacgaacctttcttcgactgggtctacaactcgcctgagaacctcacgctctcggccatcgagcagctggaggaaattaccgggcttgagttgcacgagggcggaccacccgcgctcgtggtttggaacatcaaacacatgctccacactggcatcggcaccgcctcgcgacccagcgaggtgtgcatggtcgacggtacggacatgtgtttggctgatttccatgctggcattttcctgaaaggtcgggagcacgctgtgtttgcatgtgtcacctctgacgggtggtacgcgatcgacgacgaggacttctacccttggacaccagacccgtctgacgtcctggtgtttgtcccgtacgaccaagaaccgctcaacggagaatggaaggctaaggttcagcgcaagcttaagggagctgggcaatccagcccagctactggctcgcagaaccaatctggtaacacaggtagcataatcaacaactactacatgcaacagtaccaaaactccatggacacacagcttggtgacaatgccatcagtggaggctctaacgagggctccacggacacaacttcaactcacacagccaacacccaaaacaatgactggttttcaagactcgccagttcggccttctccggtttgtttggggccttgcttgccgacaagaagacggaggagacgacactccttgaggaccgcattctcaccactcgtaatgggcacaccacctccacgacccagtccagcgtaggcgttacatacgggtactccacaacagaggaccacgttgctggacccaacacatcaggtttggagacacgagtggtacaggcagagagattctacaaaaagtttttgtttgattggacaacggacaagccttttggacacctgcacaaactggagttgcccaccgaccaccacggtgttttcggacacttggtggactcatacgcctacatgaggaacggttgggacgttgaggtgtctgctgttggtaaccagttcaacggcggatgcctcctagtggccatggtacccgaatggaaagagtttgaaacgcgggaaaagtaccagctcacgcttttcccgcaccagttcattagccccagaaccaacatgactgcccacatcacggttccttaccttggtgtgaatagatatgatcagtacagaaaacacaaaccctggacactggttgtcatggtcgtgtccccgctcacggtcaacgccacgagcgcggcacagatcaaggtctatgccaacatcgctccgacctacgttcatgtggccggcgagctcccctcgaaagaggggatcttccctgtcgcgtgcgcggacggttacggaggactggtgacaacggacccgaaaacagctgaccccgcctacggcaaggtgtacaatccgccccggactaactaccccgggcgtttcaccaacttgttggacgtggctgaggcatgtcccacctttctgtgttttgacgacgggaaaccgtacgttaccacacagacaggtgagtctcgtcttctggccaagttcgacctttcccttgctgcgaagcacatgtctaacacatatttggcaggaattgcccagtactacacacagtactcgggcaccatcaatttgcatttcatgttcacaggttcaactgattcaaaagcccgctacatggtggcttacatcccgcctggggtggaaccaccggacacacctgagagggcagcccactgcatccatgctgagtgggacacagggctgaattccaaattcacattctcaatcccgtacgtgtctgccgcggattacgcctacacggcgtctgatgaggcagagacaacaaacgtacagggatgggtctgcgtttaccagatcacacacgggaaggctgacaacgacactctggtcgtgtcggttagcgccggcaaggacttcgagttgcgcctccccattgacccccgaccgcagaccaccgctactggggaatcagcagaccctgtcaccaccactgtagagaactacggcggtgagacacaagttcagagacgccaccacaccgacgttggcttcatcatggacagatttgtgaaaataaacagcccaaaatccacccatgtcattgacctcatgcaaacccaccaacacggtctagtgggtgcgctgctgcgtgcggcgacctactacttctcagatctggaaatcgttgtgcggcatgacggtaacctaacttgggtgcccaatggtgctcccgtgtcagccttgtccaacaccagcaaccccaccgcctacaacaaggcaccgttcacgagacttgccctcccctacaccgcgccacaccgcgtgttggcgactgtgtacaacgggacgagcaagtatactgtgagtgggtca---agcagacgaggcgacttgggttccctcgcggcacgagtcgcgaaggcacttcctgcttctttcaactacggtgcaatcaaggccgacaacgtgcacgagcttctcgtgcgcatgaaacgggccgaactctactgccctagaccactgttggcactagaggtctcacaagacagacacaaacagaagatcattgcacccgaaaaacagcttttgaatttcgacctgctcaagttggcgggagacgttgagtccaaccctgggcccttcttcttctccgacgtcaggtcaaatttctccaagctggtggaaaccatcaaccagatgcaggaggacatgtcaacaaaacacggacccgactttaaccggttggtgtctgcgtttgaggaactggccgctggagttaaggctatcaggaccggtctcgacgaggccaaaccctggtacaagctgatcaagctcctgagccgcttgtcgtgcatggccgctgtagcagcacggtcaaaggacccagtccttgtggctatcatgctggctgacaccggtcttgagattctggacagcacgtttgtcgtgaagaagatctccgactcgctctccagtctctttcacgtgccggcccccgtcttcagtttcggagccccgattctgttggccgggctggtcaaggtcgcctcgagtttcttccggtccacacccgaggatctcgagagagcagaaaaacagctcaaagcacgtgacatcaacgacattttcgccattctcaagaacggcgagtggctggtcaagctgattcttgccatccgcgactggatcaaggcatggatcgcctcagaagaaaagtttgtcaccatgacagacttggtgcctggcatccttgagaagcaacgggacctcaacgacccggccaagtacaaggaagccaaggagtggctcgacaacgcgcgccaggcgtgtttgaagagcgggaacgtccacatcgccaacctgtgcaaggtggtcgccccagcacccagcaagtcgagacccgaacccgtggtcgtctgcctccgtggtaagtctggccagggcaagagtttccttgctaacgtgcttgcacaagcaatttccactcacttcaccggcagaaccgactcggtttggtactgcccccctgaccctgaccacttcgacggttacaaccaacagaccgttgtagtgatggatgatttgggccagaaccctgacggcaaggacttcaagtactttgcccaaatggtgtcaaccacagggttcatcccgcccatggcgtcactcgaggacaaaggcaaacccttcaacagcaaggtcatcatcgcgaccaccaacttgtactcgggcttcaccccgaggaccatggtctgccctgacgccctgaaccgaaggtttcacttcgacattgacgtgacagccaaagacgggtacaaagttaacaacaaattggacatcattaaagcactcgaagacacccacaccaatcctgtggcaatgtttcagtatgactgtgcccttctcaacggcatggctgttgaaatgaagagaatgcaacaagacctcttcaaacctcaaccacccctccagaacgtctaccagctcgtccaggaggtgattgaccgggtagagctccacgaaaaagtgtcgagccacccgattttcaagcagatctcaattccttcccaaaaatctgtgttgtactttctcattgagaaagggcagcacgaagcagcaattgatttctttgagggcatggtgcatgactccatcaaggaggaactccggcccctcatccaacaaacctcatttgtgaaacgcgcttttaaacgcttgaaggagaactttgagattgttgccctatgtttgaccctgctggccaacatagtgatcatgatccgcgagactcgcaagagacagaagatggtggatgatgcagtgaacgagtacatcgagaaggcaaacatcaccaccgacgacaagactcttgacgaggcggaaaagaaccctctggagactagcggtgccagtaccgttggtttcagagagaaaactctcccaggtcacaacgcgcgtgatgacgtgaaatccgagcccgcccaacgtgacacagagcaaccacaagctgagggaccctacgccgggccactcgagcgtcagaaacctctgaaagtgagagccaagctaccacagcaggagggaccttacgctggcccgatggagagacagaaaccactgaaagtgaaagcaaaagccccggtcgtcaaggaaggaccgtacgaaggaccggtcaagaaacctgtcgctttgaaagtgaaagctaagaacttgattgtcactgagagtggtgccccaccgaccgacctgcagaagatggtcatgggcaacacaaagcctgttgagcttatcctcgacgggaagacagtagccatctgctgcgctactggagtgttcggcactgcctacctcgtgcctcgtcatcttttcgctgagaagtacgacaagatcatgctggacggcagagccctgacagacagtgactacagagtgtttgagtttgagattaaagtaaaaggacaggacatgctctcagacgctgcgctcatggtgcttcaccgtgggaaccgcgtgagagacatcacgaaacactttcgtgacacagctagaatgaagaaaggcacccccgttgttggcgttatcaacaatgctgacgtcgggagactgatcttctctggtgaggcccttacttacaaggacattgtagtgtgcatggacggagacaccatgccagggctttttgcctacaaagccgcaactaaggctggctattgcggaggggccgttctcgcaaaggacggagccgacactttcatcgttggcacccactctgctggaggcaatggagttggttactgctcatgcgtttccaggtccatgctccaaaagatgaaggcacacgtcgacccggagccacaccacgaagggttgattgttgacaccagagatgtggaagaacgcgtccacgtgatgcgcaaaaccaagcttgcacccaccgtggctcacggtgtgttcaaccctgagttcgggcccgctgccttgtccagcaaggatccgcggctgaacgaaggtgttgtcctcgatgaagtcattttctccaaacacaaaggagacacgaaaatgtctgaggaggacaaagcgctgttccgccgttgtgctgccgactacgcgtcgcgcttacacaatgtgctgggtacggcaaacgccccattgagcgtttatgaagcaatcaaaggcatcgacggtctcgatgcgatggaaccagacaccgcgcccggcctgccatgggcactccagggaaaacgccgcggtgcgctcatcgacttcgagaacggcactgtcgggcccgaggttgaagctgctttgaagctcatggagaacagagaatacaaatttgcttgccagaccttcctgaaggacgagattcgcccgatggagaaagtacgtgccggcaagactcgcatcgtcgatgtcctgcctgttgaacacattctttacaccaggatgatgataggcagattttgtgcacaaatgcactcaaacaacggaccgcagattggctcagcggtcggttgcaaccccgatgttgattggcaaagatttggcacccatttcgcccaatacagaaacgtgtgggacgtggattattcggccttcgatgctaaccactgcagtgatgccatgaacatcatgtttgaggaggtgttccgcacggaatttggattccaccccaacgctgagtggatactaaagactctcgtgaacacagaacacgcctatgagaacaaacgcattgtggttgaaggcgggatgccctccggctgttccgcgacaagcatcatcaacacgattttgaacaacatctacgtgctctacgccttgcgtagacactatgagggagttgagctggatacctacaccatgatctcctacggagacgacatcgtggtggcaagtgattacgatttggactttgaggctctcaggccccactttaaatctcttggtcaaaccattactccagctgacaaaagcgacaaaggttttgttcttggtcactccatcaccgatgtcactttcctcaaaagacacttccacatggactatggaactgggttttacaaacctgtgatggcctcaaagacccttgaagccatcctctcctttgcacgccgtgggaccatacaggagaagttgatctctgtggcaggactcgccgtccattctggacctgacgagtaccggcgtctctttgagccttttcaaggcctctttgagataccaagctacagatcactttacctgcgttgggtgaacgccgtgtgcggtgacgcataa

>AY593787.1_A_Brazil_1977

atggacgcaactgattgatttattgctttggtacacgctatcagagagatcaaaacacttctcttttcaaggcacacaggaaaaatggaattcacactccacaacggtgaaaagaagactttctactccagacccaacaaccacgacaactgttggctgaacaccatccttcagctgtttaggtacgtcgatgaacccttcttcgactgggtctacaactcgcccgagaacctgacgctgtctgccatcaggcagctggaagaactcacgggacttgagttgcacgagggcggaccacctgccctcgtgatctggaacatcaaacacttgctccataccggcattggcactgcctcgcgacccagcgaggtgtgcatggttgacggtacggacatgtgtctagctgatttccacgcaggcattttcctgaaaggcaaagaacacgctgtgttcgcgtgtgtcacctccaacgggtggtacgcgatcgacgacgaggacttctacccttggacaccggacccgtccgacgttctggtgtttgtcccgtacgatcaggaaccactcaatggagaatggaaagctaacgttcaacggaagcttaagggtgcagggcaatccagcccggcaaccggttcccagaatcagtctggcaacactggcagcataattaacaactactacatgcagcagtaccagaattccatggacacacagctgggtgacaatgccatcagtggaggatctaacgaaggctccactgacacaacctcgacacacactaccaacacacaaaacaacgactggttttcaaaacttgccagttcagctttcactggtctgttcggcgctctgctcgccgacaagaagacggaggaaacaacgcttcttgaggaccggatcctgaccacccgcaatgggcacaccacctcgacgacccagtcgagtgttggtgtcacgtacgggtattccacaggagaagaccacgttgcagggcccaacacatcgggcctggagacacgggtggtacaggcagagagattttacaaaaaatttttgtttgactggacaacggacaaggcttttggacacctggagaagctcggacttccaaccgaccaccacggtgttttcggacacttggtggactcatacgcctacatgagaaatggttgggatatcgaggtgtctgccgttggcaaccagttcaacggcgggtgtctcctggtggccatggtgcccgaatggaaggattttgacgcgcgggagaaataccaactcactcttttcccgcaccagttcattagccccagaaccaacatgactgcccacatcacggtcccctaccttggtgtgaacaggtatgaccagtacaaaaagcacaagccttggaccttggttgtcatggtcgtgtctccgctaacggttaacaccgctggcgcgtcacagatcaaggtctacgccaacattgctccgacctacgttcacgtggctggtgagctcccctcgaaagaggggattttcccggttgcgtgcgcggacggttatggaggactggtgacaacagacccaaagacagctgaccctgtttacggcaaggtgtacaacccgcccaggaccaactaccctgggcggttcactaacttgttggacgtggccgaagcgtgtcccaccttcctctgctttgacgacgggaaaccgtacgtcaccacgcggacggaccaaactcgacttctggccaagtttgacctttcccttgccgcaaaacacatgtccaacacatacctggcaggacttgcccagtactacacacagtactcgggcaccatcaatttgcacttcatgttcacaggctccactgattcaaaggcccgctacatggtggcctacatcccacctggggtgcagccacctgaaacacctgagatggctgcccactgcatacacgccgagtgggacactggactgaactccaaattcactttttcaatcccgtacgtgtctgccgcagactacgcctacacagcgtctgacacggcagaaacaaccaatgtgcagggctgggtctgcatttaccagattacacacgggaaggctgaaaatgacgccttggtcgtgtcggtcagtgccggcagagactttgagttgcgcctcccgattgacccccgcacgcagactaccgccaccggggagtcagcagaccctgtcaccaccaccgtggagaactacggcggtgagacacaagttcagagacgccaccacactgacatcggcttcatcatggacaggtttgtgaagattaaggacgtgcaaccgacgcacgtcattgacctcatgcagactcaccaacacggcctggtgggtgcaatgctgcgtgcagctacgtactacttttctgacttggaaattgttgtacggcacgacggcaatctgacttgggtgcccaacggcgcccctgagtcagccctagacaacactggcaatcccaccgcctacaacaaggcaccattcacgagacttgctctcccttacacggcaccacaccgtgtgctggcaacagtgtacaacgggacaagcaaatacaccgtgggtggttca---ggcaggcgtggtgacatggggtccctcgcggcacgagtcgcgaaacagcttcctgcttcattcaactacggtgcaattaaggccaccgacatccacgagcttctcgtgcgcatgaaacgggccgaactctactgccccaggccactcctggcggtggaagcgtcgcaagaccggcacaaacagaagattattgcacctgcaaaacagcttttgaactttgacctgctcaagttggctggggacgtggagtccaatcctggacccttcttcttctccgacgttaggtcgaacttcacgaagctggtggagacaatcaaccagatgcaggaggacatgtccacaaaacacgggcccgactttaaccggttggtgtccgcatttgaggaactggccactggagttaaagccatcaggaccggtctcgatgaggcaaagccctggtacaagctcatcaagctcctgagccgcctgtcgtgcatggccgctgtagcagcacggtcaaaggatccagtccttgtggccatcatgctggctgacaccggtctcgagattctggacagcacttttgtcgtgaagaagatctccgactcgctctccagtctctttcacgtgccggcccccgtcttcagtttcggagccccgattctgttggccgggttggtcaaggtcgcctcgagtttcttccggtctacacccgaagaccttgagagagcagagaaacagctcaaagcacgtgacattaacgacattttcgccattctcaagaacggcgagtggctggtcaaactgattcttgccatccgcgactggatcaaggcatggatcgcctcagaagagaagtttgtcaccatgacagacttggtgcctggcatccttgaaaagcagagggacctcaacgacccaagcaagtacaaggaggccaaggagtggctcgacaatgcgcgccaagcgtgtttgaagagcgggaacgtccacatcgccaacctgtgcaaagtggtcgccccggcacccagcaagtcgagacccgagcctgtggtcgtttgtctccgcggcaaatccggccagggtaagagtttccttgcaaacgtgctagcacaagcaatttccacccacttcactggcagaaccgactctgtttggtactgtccacctgaccctgaccacttcgacggttacaaccaacagaccgttgttgtgatggatgatttgggccagaatcccgacggcaaggacttcaagtactttgcccagatggtttcaaccacggggttcatcccgcccatggcatcgctcgaggacaagggcaaacccttcaacagtaaggtcatcatcgcgaccaccaacttgtactcgggtttcaccccgaggactatggtgtgccctgatgccctgaaccggaggtttcactttgacatcgacgtgagtgccaaggacgggtacaaaattaacaacaaattggacatcatcaaagcacttgaagacacccacaccaacccagtggcaatgtttcagtacgattgtgcccttctcaacggcatggctgttgaaatgaagagaatgcaacaagacatgttcaagccccaaccacccctccagaacgtctaccaacttgttcaggaggtgattgaacgggtggagctccacgagaaagtgtcgagccacccaattttcaagcagatctcaattccttcccaaaaatccgtgctgtacttcctcattgagaaagggcaacacgaggctgcaattgaattctttgagggaatggtgcatgactccatcaaggaggaactccggccccttatacaacaaacttcatttgtgaaacgcgcttttaagcgcctgaaggaaaattttgagattgttgccctatgtttgacccttctggccaacatagtgatcatgatccgcgagacacgcaagagacagcagatggtggacgaagcagtcaatgaatacattgagaaagcaaacatcaccaccgacgacaagactcttgatgaggcggaaaagaaccctctagagaccagcggtgccagcacggttggtttcagagagagaactcttccaggtcaaaaggcgcgcgatgacgtgaactccgagcccgccaaacctgctgaggagcaaccacaagctgaaggaccctacgccgggccactcgagcgtcagaaacctctgaaagtgagagctaagctcccacagcaggagggaccctacgctggcccgatggagagacagaaaccgcttaaagtgaaagcaaaagcccccgtcgtcaaggaaggaccttacgaggggccggtgaagaagcctgtcgctttgaaagtgaaagccaagaatttgattgtcactgagagtggtgccccaccgactgatcttcaaaagatggtcatgggtaacaccaagcccgttgagctcatccttgacgggaagacagtagccatctgctgtgctactggagtgtttggcactgcttacctcgtgcctcgtcacctttttgcagaaaagtacgacaagatcatgttggacggcagagccatgacagacagtgactacagagtgtttgagtttgagattaaagtaaaaggacaggacatgctctcagacgctgcgctcatggtgcttcaccgcgggaaccgcgtgagagacatcacgaaacactttcgtgatacagcaagaatgaagaaaggcacccccgtcgttggtgtgatcaacaacgctgatgtcgggagactgattttctctggtgaggctcttacctacaaggatattgtagtgtgcatggatggagacaccatgcctggcctctttgcctacaaagctgcaaccaaagcagggtactgcggaggagccgttctggccaaggacggggctgacacgttcatcgtcggcactcactccgctggaggcaatggagttggatactgctcgtgcgtttccaggtccatgcttctcaaaatgaaggcacacattgaccccgaaccacaccacgaggggttgattgttgacaccagagatgtggaagagcgcgtccacgtgatgcgcaagaccaagcttgcacccaccgtcgcacacggtgtgttcaaccctgaatttgggcccgccgccttgtctaacaaggacccgcgcctgaatgaaggtgttgtcctcgatgaagtcatcttctccaaacacaaaggagacacaaagatgtctgacgaggacaaagcgctgtttcgccgctgcgctgccgactacgcgtcgcgcctgcacagcgtgctgggtacagcaaatgccccattgagcatttacgaggcaatcaaaggcgtcgacggactcgacgccatggagccagacactgcacctggccttccctgggcactccaggggaaacgccgcggtgcactcatcgacttcgagaacggcactgtcggacccgaagttgaggctgccctgaagctcatggagaaaagagagtacaagtttgcttgtcagaccttcctgaaggacgagattcgcccgatggagaaagtacgtgccggtaagactcgcattgtcgacgtcctgcctgttgaacacattctttacaccaggatgatgattggcagattttgtgcccaaatgcactcaaacaacggaccgcagattggctcagcggtcggatgcaaccctgatgttgattggcaaagatttggtacacacttcgcccaatacagaaacgtgtgggacgtggactattcggcctttgatgctaaccactgcagtgacgctatgaacatcatgtttgaggaggtgttccgcacggagttcgggttccaccccaacgccgagtggatcttgaagactctcgtgaacacggagcacgcctatgagaacaaacgcatcactgttgagggcgggatgccgtctggttgttccgcgacaagcatcatcaacacaattctgaacaacatctacgtgctctacgccctgcgtaggcactatgagggagttgagctggacacttacaccatgatctcctacggagacgacatcgtggtggcgagtgactatgatttggactttgaggccctcaagccccactttaaatctcttggccaaactatcactccagctgacaaaagcgacaaaggttttgttcttggtcactccattaccgatgtcactttcctcaaaagacacttccacatggattatggaactgggttttacaaacctgtgatggcctcaaagacccttgaggctatcctctcctttgcacgccgtgggaccatacaggagaagttgatctcggtggcaggactcgccgtccactctggaccagacgagtaccggcgtctctttgagcccttccaaggcctctttgagattccgagctacagatcactttacctgcgttgggtgaacgccgtgtgcggtgacgcataa

>AY593788.1_A_Brazil_1979

atggacacaactgattgttttattgctttggtgcacgtcatcagagagatcaaagcacttttcttttcaaggcgcacaggaaaaatggaatttacactccacaacggtgaaaagaagactttctactccagacccaacaaccacgacaactgttggctgaacaccatccttcagctgtttaggtacgtcgacgaaccgttcctcgactgggtctacaactcgcccgagaacctgacgctgtctgccgtcaggcagctggaagaaatcacgggacttgagttgcacgagggtggaccacctgccctcgtgatctggaacatcaaacacttgcttcataccggcattggcactgcctcgcgacccagcgaggtgtgcatggttgacggtacggacatgtgtctggctgatttccacgcaggcattttcctgaaaggcaaagaacacgctgtgttcgcatgtgtcacctccaacgggtggtacgcgatcgatgacgaggacttctacccttggacaccggacccgtccgacgttctggtgtttgtcccgtacgatcaggagccactcaatggagaatggaaagctaacgttcaacggaagcttaagggtgcagggcaatccagtccggcaaccggttcccagaatcagtctggcaacactggcagcataattaacaactactacatgcagcagtaccagaattccatggacacacagctgggtgacaatgccatcagtggaggatctaacgaaggctccactgacacaacctcaacacacactaccaacacacaaaacaacgactggttctcaaaacttgccagttcagctttcactggtctgttcggcgctctgctcgccgacaagaagacggaggaaacaacgctccttgaggaccggatcctgaccacccgcaatgggcacaccacctcgacgactcagtcgagtgttggtgtcacgtacgggtattccacaggagaagaccacgttgcagggcccaacacatcgggcctggagacacgggtggtacaggcagagagattttataaaaaatttctgtttgactggacaacggacagggcttttggacacctggagaaactcgaacttccaacagaccaccacggtgttttcggacacttggtggactcatacgcctacatgagaaatggttgggatgttgaggtgtctgccgttggcaaccagttcaacggcgggtgtctcctggtggccatggtgcccgaatggaaggattttgacgcgcgggagaaataccaactcactcttttccctcaccagttcattagccccagaaccaacatgactgcccacatcacggtcccctaccttggcgtgaacaggtatgaccagtacaaaaagcacaagccttggaccttggttgtcatggtcgtgtctccgctaacggttaacaccgctggcgcgtcacagatcaaggtttacgccaacattgctccgacctacgtacacgtggctggtgagctcccctcgaaagaggggattttcccggttgcgtgcgcggacggttacggaggactagtgacaacagacccaaagacagctgaccctgtttacggcaaggtgtacaacccgcccaggaccaactaccctgggcggttcactaacttgttggacgtggccgaagcgtgtcccaccttcctctgctttgacgacgggaaaccgtacgtcaccacgcggacggaccaaactcgacttctggccaagtttgacctttcccttgccgcaaaacacatgtccaatacatacctggcaggacttgcccaatactacacacagtactcgggcaccatcaatttacacttcatgttcacaggctccactgattcaaaggcccgctacatggtggcctacatcccacctggggtgcagccacctgaaacacctgagatggctgcccactgcatacacgccgagtgggacactggactgaactccaaattcactttttcaatcccgtacgtgtctgccgcagactacgcctacacagcgtctgacacggcagaaacaaccaatgtgcagggctgggtctgcatttaccagattacacacgggaaggctgaaaatgacgccttggtcgtatcggtcagtgccggcagagactttgagttgcgcctcccgattgacccccgcacgcagactaccgccaccggggagtcagcagaccctgtcaccaccaccgtggagaactacggcggtgagacacaggttcagagacgctaccacactgacatcggcttcatcatggacaggtttgtgaagattaaggacgtgcaaccgacgcatgtcattgaccttatgcagactcaccaatacggcctggtgggtgcaatgctgcgtgcagctacgtactacttttctgacttggaaattgttgtacggcacgacggcaacctgacttgggtgcccaacggcgcccctgagtcagccctagacaacactggcaatcccaccgcctacaacaaggcaccattcacgagacttgctctcccttacacggcaccacaccgtgtgctggcaacagtgtacaacgggacaagcaaatacaccgtgggtggttca---ggcaggcgtggtgacatggggtccctcgcggcacgagtcgcgaaacaacttcctgcttcattcaactatggtgcaattaaggccaccgccatccacgagctcctcgtgcgcatgaaacgggccgaactctactgtcccaggccactcctggcggtggaagcgtcacaagaccggcacaaacagaagattattgcacctgcaaaacagcttttgaactttgacctactcaagttggctggggacgtggagtccaatcctggacccttcttcttctccgacgttaggtcgaacttcacgaagctggtggagacaatcaaccagatacaggaggacatgtccacaaaacacgggcccgactttaaccggttggtgtccgcatttgaggaactggccactggagttaaagccatcaggaccggtctcgatgaggcaaagccctggtacaagctcatcaagctcctgagccgcctgtcgtgcatggccgctgtagcagcacggtcaaaggatccagtccttgtggccatcatgctggctgacaccggtctcgagattctggacagcacttttgtcgtgaagaagatctccgactcgctctccagtctctttcacgtgccggcccccgtcttcagtttcggagccccgattctgttggccgggttggtcaaggtcgcctcgagtttcttccggtccacacccgaagaccttgagagagcagagaaacagctcaaagcacgtgacattaacgacattttcgccattctcaagaacggcgagtggctggtcaaactgattcttgccatccgcgactggatcaaggcatggatcgcctcagaagagaagtttgtcaccatgacagacttggtgcctggcatccttgaaaagcagagggacctcaacgacccaagcaagtacaaggaggccaaggagtggctcgacaatgcgcgccaagcgtgtttgaagagcgggaacgtccacatcgccaacctgtgcaaagtggtcgccccggcacccagcaagtcgagacccgagcctgtggtcgtttgtctccgcggcaaatccggccagggtaagagtttccttgcaaacgtgctggcacaagcaatttctacccacttcactggcagaaccgactctgtttggtactgtccacctgaccctgaccacttcgacggttacaaccaacagaccgttgttgtgatggatgatttgggacagaatcccgatggcaaggacttcaagtactttgcccagatggtttcaaccacggggttcatcccgcccatggcatcgctcgaggacaagggcaaacccttcaacagtaaggtcatcatcgcgaccaccaacttgtactcgggtttcaccccgaggaccatggtgtgccccgatgccctgaaccggaggtttcactttgacatcgacgtgagtgccaaggacgggtacaaaattaacaacaaactggacatcatcagagcacttgaagacacccataccaacccagtggcaatgttccaatacgattgtgcccttctcaacggtatggctgttgaaatgaagagaatgcaacaagacctgttcaagccccaaccacccctccagaacgtctaccaacttgttcaggaggtgattgaacgggtggagctccacgagaaagtgtcgagccacccaatttttaaacagatctcaattccttcccaaaaatccgtgctgtacttcctcattgagaaagggcagcacgaggctgctattgaattctttgagggaatggtgcatgactccatcaaggaggaactccggccccttatacaacaaacttcatttgtgaaacgcgcttttaagcgcctgaaggaaaattttgagattgttgccctatgtttgacccttctggccaacatagtgatcatgatccgcgagacacgcaagagacagcagatggtggacgaagcagtcaatgaatacattgagaaagcaaacatcaccaccgacgacaagactcttgatgaagcggaaaagaaccctctagagaccagtggtgccagcacggttggtttcagagagagaactcttccaggtcaaaaggcgcgcgacgacgtgaactctgagcccgccaaacctgttgaggggcaaccacaagctgaaggaccctacgccgggccactcgagcgtcagaaacctctgaaagtgagagctaagctcccacagcaggaggggccctacgctggcccgatggagagacagaaaccgcttaaagtgaaaacaaaagcccccgtcgtcaaggaaggaccttatgaggggccggtgaagaagcctgtcgctttgaaagtgaaggccaagaatctgattgtcactgagagtggtgctccaccgactgatcttcaaaagatggtcatgggtaacactaagcccgttgagctcatccttgacgggaagacagtagctatctgctgtgctactggagtgtttggcactgcttacctcgtgcctcgtcacctttttgcagaaaagtatgacaagatcatgttggacggcagagccatgacagacagtgactacagagtgtttgagtttgagattaaagtaaaaggacaggacatgctctcagacgctgcgctcatggtgcttcaccgcgggaaccgcgtgagagacatcacgaaacattttcgtgatacagcaagaatgaagaaaggcacccccgtcgttggtgtgatcaacaacgctgatgtcgggagactgatcttctctggtgaggctcttacctacaaggatattgtagtgtgcatggatggagacaccatgcctggcctctttgcctacaaagctgcaaccaaagcagggtactgcggaggagccgttctggccaaggacggggctgacacgttcatcgtcggcactcactccgctggaggcaatggagttggatactgctcgtgcgtttccaggtccatgcttctcaaaatgaaggcacacattgaccccgaaccacaccacgaggggttgattgttgacaccagagatgtggaagagcgcgtccacgtgatgcgcaagaccaagcttgcacccaccgtcgcacacggtgtgttcaaccctgaatttgggcccgccgccttgtctaacaaggacccgcgcctgaatgaaggtgttgtcctcgatgaagtcatcttctccaaacacaaaggggacacaaagatgtctgaggaggacaaagcgctgttccgccgctgcgctgccgattacgcatcgcgcctacacagcgtgctgggcacagcaaacactccattgagcatttacgaggcaatcaaaggtgtcgacggcctcgacgccatggagccagacactgcacctggccttccctgggcactccaggggaaacgccgcggtgcactcatcgacttcgagaacggcactgtcggacccgaagttgaggctgccctgaagctcatggaaaaaagagaatacaagtttgcttgtcagaccttcctgaaggacgagattcgcccgatggagaaagtacgtgccggcaagactcgcattgtcgacgtcctgcctgttgaacacattctttacaccaggatgatgattggcagattttgtgcccaaatgcactcaaacaacggaccgcagattggctcagcggtcggatgcaaccctgatgttgactggcaaagatttggtacacacttcgcccaatacagaaacgtgtgggacgtggactactcggcctttgatgccaaccactgcagtgacgctatgaacatcatgtttgaggaggtgttccgcacggagttcgggttccaccccaacgccgagtggatcttgaagactctcgtgaacacggagcacgcctatgagaacaaacgcatcactgttgagggcgggatgccgtctggttgttccgcgacaagcatcatcaacacaattttgaacaacatctacgtgctctacgctctgcgtaggcactatgagggagtcgagctggacacttacaccatgatctcctacggagacgacatcgtggtggcgagtgactatgatttggactttgaggccctcaagccccacttcaaatctcttggccaaaccatcactccagctgacaaaagcgacaaaggttttgttcttggtcactccattaccgatgtcactttcctcaaaagacacttccacatggattatggaactgggttttacaaacctgtgatggcctcaaagacccttgaggctatcctctcctttgcacgccgtgggaccatacaggagaagttgatctcggtggcaggactcgccgtccactctggaccagacgagtaccggcgtctctttgagcccttccaaggcctctttgagattccgagctacagatcactttacctgcgttgggtgaacgccgtgtgcggcgacgcataa

>AY593789.1_A_ARG_1961

atgaatacaactgattgtttcatcgctttggtgcacgccatcagagagatcataacacttctttttctacgaaccacaggaaagatggaattcacactgcacaacggtgagaagaaaactttttactctaggcccaacaaccacgacaactgttggctaaacgccattcttcagttgttcaggtacgtcgatgaacctttcttcgactgggtctacaactcgcccgaaaacctcacgcttgaagccatcaagcagttggaagaactcacagggcttgagttgcgcgagggcggaccacccgccctcgtggtctggaacatcaaacacttacttcacactggcattggtaccgcctcgcgacccagcgaggtgtgtatggtggatggcacggacatgtgtctcgctgacttccatgcaggcattttcctgaaaggatcggaacacgcagtgtttgcgtgtgtcacctccgatgggtggtacgcgatcgacgacgaggacttttacccctggactcctgacccatcagacgtcctggtattcgtcccgtacgatcaagaaccactcaacgggggttggagaacactggttcaaaggaggcttaagggcgccgggcaatccagcccggcgactggctcacaaaaccagtctggaaacactggtagcataatcaacaactactacatgcagcagtaccagaactccatggacacacagcttggtgacaatgccatcagtggaggctcaaacgagggctccacggacacaacctcaacacacacaaccaacacccaaaacaacgactggttctcaaaacttgccagttcagccttcaccggtctattcggcgcactgctcgccgataaaaagacagaagagaccacacttctggaagaccgcatcctcaccacccgcaatggacacaccacctcgaccacccagtcgagcgtgggggtcacctacgggtactccactggggaagaccacaccgcagggcccaacacatcgggcttggaaacgcgggtagtacaggctgaaaggttctttaagaaatttttgtttgactggacaacggacaaaccctttggacacttggaaaaactggaactccccaccgaccaccacggggtcttcggacacctggtggactcatatgcatacatgaggaacggttgggatgtcgaggtgtctgctgttggcaaccaattcaacggcgggtgcctcctggtggccatggtaccagaatggaaggaatttgacacgcgtgagaaataccaactcactctgtttccacaccagttcatcagccccagaacaaacatgaccgcccacatcacggtcccgtaccttggtgtgaacaggtatgaccagtacaaaaagcacaaaccctggacgctggttgtcatggtggtgtcgcccctcacggttagcaccactagtgcggcacagattaaggtctacgccaacattgccccaacctacgttcacgtggctggagagctcccttcgaaagaggggatttttcccgttgcgtgcgccgacggttacgggggactggtgacgacggacccgaagacagctgaccccgcctacggcaaggtgtacaatccgcccaggactaactaccccgggcgctttacaaacctgttggacgtggctgaggcgtgtcccacctttctttgtttcgacgacgggaaaccgtatgttgtcacgaagacagaacaagaccgacttctggccaagtttgacgtttcccttgccgcaaagcacatgtctaacacatacttgtcaggggttgcacagtactacgcacagtactctggtaccatcaacctgcactttatgttcacaggctctactgactcaaaggcccgctacatggtggcctacatcccgccaggggtggagccgccggacacacctgagaaagccgcacactgcatccacgctgaatgggacacagggttgaactccaagttcaccttttcaatcccgtacgtgtccgccgcggactacgcatacactgcgtccgacacggcagaaacaaccaacgtacagggatgggtttgcatttaccaaattacacacgggaaggctgagcaggacaccttggttgtgtcggttagcgccggcaaggactttgagctacgcctcccgattgacccccgtgcacaaaccactgccactggggaatctgcagaccctgtcaccaccaccgtggagaactacggcggtgagacacaagtccacagacgtcaccacacggacgtcagcttcatcatggacaggtttgtgaagatacagcctgtgaaccctatgcatgtcattgacctcatgcagacccaccaacacgggcttgtaggggcgttgctgcgtgcagccacgtactacttctctgacctggagattgtggtacgacacaacggcaacctgacctgggtacccaacggcgcccccgaggcagccctgtctaacaccagcaaccccactgcctacaacaaggcgccgttcaccagacttgccctcccctacactgcgccacaccgtgtgctggcaactgtgtacaacgggacgaacaagtacaccacaaacggtaca---ggtaggcgtgatgacatgggttctctcgcggcgagagtcgcgaaacatcttcctgcttcttttaattacggtgcaatcaaggccgacaccatccacgagcttctcgtgcgtatgaagcgggccgaactctactgccccagaccactgctggcaatagaggcctcacaagacaggcacaagcaaaagatcattgcacctgcaaagcagctgctgaactttgaccttctcaaactggcgggtgacgttgagtccaaccctgggcccttcttcttctccgacgttaggtcgaatttctccaaattggtggaaaccatcaaccaaatgcaggaagacatgtcaacaaagcacggacctgactttaaccggttagtgtccgcgtttgaggaattggccactggagtaaaagctatcagaaccggtctcgatgaggccaagccctggtacaagcttattaaactcctaagccgcctgtcgtgcatggccgctgtggcagcacggtccaaggacccagtccttgtggccatcatgctggccgacaccggtctcgagattctggacagcacctttgtcgtgaagaagatctccgactcgctctccagtctctttcacgtgccggcccccgtcttcagtttcggagctccgatcctgctggccgggttggtcaaagtcgcctcgagtttcttccggtccacacccgaagaccttgagagagcagagaaacagctcaaagcacgtgacatcaacgacatcttcgccattctcaagaacggcgagtggctggtcaaactgatcctcgctatccgcgactggattaaggcttggatcgcctcagaagagaagtttgtcaccatgacagacttggtgcctggcatccttgaaaagcagcgggatctcaacgacccgagcaagtacaaggaagccaaggaatggctcgacaacgcacgccaagcgtgcttgaagagcgggaacgtccacattgccaacctgtgcaaagtggtcgccccggcacccagcaagccgagacccgagcccgtggtcgtttgcctccgcggcaaatccggccagggcaagagtttccttgcgaacgtgctcgcgcaagcaatctccacccacttcaccggcagaaccgattcggtttggtactgcccgcctgaccccgaccacttcgacggttacaaccaacaggccgttgttgtgatggatgatttgggccagaaccctgacggcaaggacttcaagtacttcgcccaaatggtttcaaccacagggttcatcccgcccatggcatcgctcgaggacaaaggcaaacctttcaacagcaaggtcatcatcgccaccacaaacttgtactcgggtttcaccccgaggaccatggtgtgccctgatgcgctgaatcggaggtttcactttgacattgacgtgagcgccaaggacgggtacaaaattaatgagaaattggacatcatcaaagcacttgaagacacgcacactaacccagtggcgatgtttcagtacgattgtgcccttctcaacggtatggccgttgaaatgaagagaatgcaacagaatgtgttcaagcctctaccacccctccaaaacgtttaccagctcgttcaggaggtgattgaacgggtcgagctccacgagaaggtgtcgagccacccaatttttaaacaaatctcaattccttcccaaaaatccgtgttgtacttcctcattgagaaaggtcagcacgaagcagcaattgaattctttgagggaatggtgcatgactccatcaaggaggagctccggcccctcattcaacagacctcatttgtgaagcgcgctttcaagcgcctgaaggagaactttgagattgttgccctgtgtttgacccttttggccaacatagtgatcatgatccgcgagactcacaagagacagaaaatggtggatgatgcagtgaatgactacattgagaaagcaaacatcaccacagatgacaagactcttgacgaggcggaaaagaaccctctggagaccagcggagccagtaccgttggcttcagagagagaactctcacagggcacaagatgtgcgatgacgtgaactccgagcccatccaacct---gaagagcaaccacaagctgaaggaccctacgccgggccactcgagcgtcagaaacccctgaaagtgagagccaagctcccgcagcaggagggaccttacgctggcccgatggagagacagaaaccgctgaaggtaaaagtgaaagccccggtcgttaaggaaggaccttacgagggaccggtgaggaagcctgtcgctttgaaagtgaaagctaaaaacttgatagtcactgagagtggtgccccacctactgacttgcaaaagatggtcatgggcaacacaaagcctgttgagctcatcctcgacgggaagacagtagccatctgctgtgctactggagtgtttggtactgcctacctcgtgcctcgtcacctcttcgcagagaagtatgacaagatcatgctggatggtagagccatgacagacagtgactacagagtgtttgagtttgagattaaagtaaaaggacaggacatgctctcagacgccgcgctcatggtgctccaccgtgggaaccgcgtgagagacatcacgaaacactttcgtgatacagcaagaatgaagaaaggaacccccgtcgttggcgtgatcaacaacgccgatgttgggagactgattttctctggtgaggcccttacctacaaagacattgtggtgtgcatggatggagacaccatgcctggccttttcgcctacaaagccgccaccaaggctggctactgcggaggagccgttcttgccaaggacggggccgacactttcatcgttggcactcactccgcaggaggtaatggagttggatactgctcgtgcgtttccaggtccatgctcctcaagatgaaggcacacatcgaccccgaaccacaccacgaggggttgattgtggacaccagagatgtggaagagcgcgtccacgtgatgcgcaaaaccaagctcgcacccaccgttgcacacggtgtgttcaaccccgagtttgggcctgccgccttgtccaacaaggacccgcgcctgaacgagggtgttgttctcgatgaggtcatcttctccaaacacaagggagacacaaagatgtctgaagaggacaaagcgctgttccgccgctgcgccgctgactacgcgtcacgcctgcacagcgtgctgggcacagcaaatgccccactgagcatttatgaggcaattaagggtgtcgacggactcgacgccatggaaccagacactgcacccggcctcccctgggccctccaggggaaacgtcgtggtgcgctcatcgactttgagaacggcactgtcgggcccgaagtcgaggctgccctaaagctcatggagaaaagagaatacaagtttgcttgtcagaccttcctgaaggacgaaatccgcccgatggagaaagtacgtgccggcaagactcgcattgtcgatgttttgcctgttgaacacattctttacaccaggatgatgattggcagattctgtgcacaaatgcactcaaacaacggaccgcagattggctcagcggtcggctgtaaccctgatgttgattggcagagatttggcacacacttcgcccaatacagaaacgtgtgggatgtggactattcggccttcgatgctaaccactgcagtgacgcaatgaacatcatgttcgaggaggtgtttcgcacagactttggtttccacccaaatgctgagtggattctgaagactctcgtgaacacggagcacgcgtacgagaacaaacgcatcactgttgagggcgggatgccgtctggctgttccgcaacaagcatcatcaacacaattctgaacaacatctacgtgctctacgctctgcgtagacactatgagggagttgagctggacacttacaccatgatctcatacggagacgacatcgtggtggcaagtgattacgatttggacttcgaggctctcaagcctcactttaaatcccttggtcaaaccatcactccagctgacaaaagcgacaaaggttttgttcttggtcactccattaccgatgtcactttcctcaaaagacacttccacatggattatggaactgggttttacaaacctgtgatggcctcaaagacccttgaggctatcctctcctttgcacgccgtgggaccattcaggagaagttgatctcggtggcaggactcgccgtccactctggaccggacgagtaccgtcgtctctttgagccctttcagggcctctttgagattcctagctacagatcactttacctgcgttgggtgaacgccgtgtgcggcgacgcataa

>AY593790.1_A_ARG_2001

atgaacacaactgactgttttatcgctttggtgcacgccatcagagagattaagacgtttttcttcacacggcacacaggaagaatggaattcacactgtacaacggtgagaagaagacattttactccagacccaacaaccacgacaactgttggttgaacgccatccttcagttgttcaggtacgtcgacgaacctttcttcgactgggtctacaactcgcctgagaacctcacgctctcggccatcgagcagctggaggaaattaccgggcttgagttgcacgagggcggaccacccgcgctcgtggtttggaacatcaaacacatgctccacactggcatcggcaccgcctcgcgacccagcgaggtgtgcatggtcgacggtacggacatgtgtttggctgatttccatgctggcattttcctgaaaggtcgggagcacgctgtgtttgcatgtgtcacctctgacgggtggtacgcgatcgacgacgaggacttctacccttggacaccagacccgtctgacgtcctggtgtttgtcccgtacgaccaagaaccgctcaacggagaatggaaggctaaggttcagcgcaagcttaagggagctgggcaatccagcccagctactggctcgcagaaccaatctggtaacacaggtagcataatcaacaactactacatgcaacagtaccaaaactccatggacacacagcttggtgacaatgccatcagtggaggctctaacgagggctccacggacacaacttcaactcacacaaccaacacccaaaacaatgactggttttcaagactcgccagttcggccttctccggtttgtttggggccttgcttgccgacaagaagacggaggagacgacactccttgaggaccgcattctcaccactcgtaatgggcacaccacctccacgacccagtccagcgtaggcgttacatacgggtactccacaacagaggaccacgttgctggacccaacacatcaggtttggagacacgagtggtacaggcagagagattctacaaaaagtttttgtttgattggacaacggacaagccttttggacacctgcacaaactggagttgcccaccgaccaccacggtgttttcggacacttggtggactcatacgcctacatgaggaacggttgggacgttgaggtgtctgctgttggtaaccagttcaacggcggatgcctcctagtggccatggtacccgaatggaaagagtttgaaacgcgggagaagtaccagctcacgcttttcccgcaccagttcattagccccagaaccaacatgactgcccacatcacggttccttaccttggtgtgaatagatatgatcagtacagaaaacacaaaccctggacactggttgtcatggtcgtgtccccgctcacggtcaacgccacgagcgcggcacagatcaaggtctatgccaacatcgctccgacctacgttcatgtggccggcgagctcccctcgaaagaggggatcttccctgtcgcgtgcgcggacggttacggaggactggtgacaacggacccgaaaacagctgaccccgcctacggcaaggtgtacaatccgccccggactaactaccccgggcgtttcaccaacttgttggacgtggctgaggcatgtcccacctttctgtgttttgacgacgggaaaccgtacgttaccacacagacaggtgagtctcgtcttctggccaagttcgacctttcccttgccgcgaagcacatgtctaacacatatttggcaggaattgcccagtactacacacagtactcgggcaccatcaatttgcatttcatgttcacaggttcaactgattcaaaagcccgctacatggtggcttacatcccgcctggggtggaaccaccggacacacctgagagggcagcccactgcatccatgctgagtgggacacagggctgaattccaaattcacattctcaatcccgtacgtgtctgccgcggattacgcctacacggcgtctgatgaggcagagacaacaaacgtacagggatgggtctgcgtttaccagatcacacacgggaaggctgacaacgacactctggtcgtgtcggttagcgccggcaaggacttcgagttgcgcctccccattgacccccgaccgcagaccaccgctactggggaatcagcagaccctgtcaccaccactgtagagaactacggcggtgagacacaagttcagagacgccaccacaccgacgttggcttcatcatggacagatttgtgaaaataaacagcccaaaatccacccatatcattgacctcatgcaaacccaccaacacgggctagtgggtgcgctgctgcgtgcggcgacctactacttctcagatctggaaattgttgtgcggcatgacggtaacctaacttgggtgcccaatggtgctcccgtgtcagccttgtccaacaccagcaaccccaccgcctacaacaaggcaccgttcacgagacttgccctcccctacaccgcgccacaccgcgtgttggcgactgtgtacaacgggacgagcaagtatactgtgagtgggtca---agcagacgaggcgacttgggttccctcgcggcacgagtcgcgaaggcacttcctgcttctttcaactacggtgcaatcaaggccgacaacgtgcacgagcttctcgtgcgcatgaaacgggccgaactctactgccctagaccactgctggcactagaggtctcacaagacagacacaaacagaagatcattgcacccgaaaaacagcttttgaatttcgacctgctcaagttggcgggagacgttgagtccaaccctgggcctttcttcttctccgacgtcaggtcaaatttctccaagctggtggaaaccatcaaccagatgcaggaggacatgtcaacaaaacacggacccgactttaaccggttggtgtctgcgtttgaggaactggccgctggagttaaggctatcaggaccggtctcgacgaggccaaaccctggtacaagctgatcaagctcctgagccgcttgtcgtgcatggccgctgtagcagcacggtcaaaggacccagtccttgtggctatcatgctggctgacaccggtcttgagattctggacagcacgtttgtcgtgaagaagatctccgactcgctctccagtctctttcacgtgccggcccccgtcttcagtttcggagccccgattctgttggccgggctggtcaaggtcgcctcgagtttcttccggtccacacccgaggatctcgagagagcagaaaaacagctcaaagcacgtgacatcaacgacattttcgccattctcaagaacggcgagtggctggtcaagctgattcttgccatccgcgactggatcaaggcatggatcgcctcagaagaaaagtttgtcaccatgacagacttggtgcctggcatccttgagaagcaacgggacctcaacgacccggccaagtacaaggaagccaaggagtggctcgacaacgcgcgccaggcgtgtttgaagagcgggaacgtccatatcgccaacctgtgcaaggtggtcgccccagcacccagcaagtcgagacccgaacccgtggtcgtctgcctccgtggcaagtctggccagggcaagagtttccttgctaacgtgcttgcacaagcaatttccactcacttcaccggcagaaccgactcggtttggtactgcccccctgaccctgaccacttcgacggttacaaccaacagaccgttgtagtgatggatgatttgggccagaaccctgacggcaaggacttcaagtactttgcccaaatggtgtcaaccacagggttcatcccgcccatggcatcactcgaggacaaaggcaaacccttcaacagcaaggtcatcatcgcgaccaccaacttgtactcgggcttcaccccgaggaccatggtctgccctgacgccctgaaccgaaggtttcacttcgacattgacgtgacagccaaagacgggtacaaagttaacaacaaattggacatcattaaagcactcgaagacacccacaccaatcctgtggcaatgtttcagtatgactgtgcccttctcaacggcatggctgttgaaatgaagagaatgcaacaagacctcttcaaacctcaaccacccctccagaacgtctaccagctcgtccaggaggtgattgaccgggtagagctccacgaaaaagtgtcgagccacccgattttcaagcagatctcaattccttcccaaaaatctgtgttgtactttctcattgagaaagggcagcacgaagcagcaattgatttctttgagggcatggtgcatgactccatcaaggaggaactccggcccctcatccaacaaacctcatttgtgaaacgcgcttttaaacgcttgaaggagaactttgagattgttgccctatgtttgaccctgctggccaacatagtgatcatgatccgcgagactcgcaagagacagaagatggtggacgatgcagtgaacgagtacatcgagaaggcaaacatcaccaccgacgacaagactcttgacgaggcggaaaagaaccctctggagactagcggtgccagtaccgttggtttcagagagaaaactctcccaggtcacaacgcgcgtgatgacgtgaactccgagcccgcccaacgtgacacagagcaaccacaagctgagggaccctacgccgggccactcgagcgtcagaaacctctgaaagtgagagccaagctaccacagcaggagggaccttacgctggcccgatggagagacagaaaccactgaaagtgaaagcaaaagccccggtcgtcaaggaaggaccgtacgaaggaccggtcaagaaacctgccgctttgaaagtgaaagccaagaacttgattgtcactgagagtggtgccccaccgaccgacctgcagaagatggtcatgggcaacacaaagcctgttgagcttatcctcgacgggaagacagtagccatctgctgcgctactggagtgttcggcactgcctacctcgtgcctcgtcatcttttcgctgagaagtacgacaagatcatgctggacggcagagccctgacagacagtgactacagagtgtttgagtttgagattaaagtaaaaggacaggacatgctctcagacgctgcgctcatggtgcttcaccgtgggaaccgcgtgagagacatcacgaaacactttcgtgacacagctagaatgaagaaaggcacccccgttgttggcgttatcaacaatgctgacgtcgggagactgatcttctctggtgaggcccttacttacaaggacattgtagtgtgcatggacggagacaccatgccagggctttttgcctacaaagccgcaactaaggctggctattgcggaggggccgttctcgcaaaggacggagccgacactttcatcgttggcacccactctgctggaggcaatggagttggttactgctcatgcgtttccaggtccatgctccaaaagatgaaggcacacgtcgacccggagccacaccacgaagggttgattgttgacaccagagatgtggaagaacgcgtccacgtgatgcgcaaaaccaagcttgcacccaccgtggctcacggtgtgttcaaccctgagttcgggcccgctgccttgtccagcaaggatccgcggctgaacgaaggtgttgtcctcgatgaagtcattttctccaaacacaaaggagacacgaaaatgtctgaggaggacaaagcgctgttccgccgttgtgctgccgactacgcgtcgcgcttacacaatgtgctgggtacggcaaacgccccattgagcgtttacgaagcaatcaaaggcatcgacggtctcgatgcgatggaaccagacaccgcgcccggcctgccatgggcactccagggaaaacgccgcggtgcgctcatcgacttcgagaacggcactgtcgggcccgaggttgaagctgctttgaagctcatggagaacagagaatacaaatttgcttgccagaccttcctgaaggacgagattcgcccgatggagaaagtacgtgccggcaagactcgcatcgtcgatgtcctgcctgttgaacacattctttacaccaggatgatgataggcagattttgtgcacaaatgcactcaaacaacggaccgcagattggctcagcggtcggttgcaaccctgatgttgattggcaaagatttggcacccatttcgcccaatacagaaacgtgtgggacgtggattattcggccttcgatgctaaccactgcagtgatgccatgaacatcatgtttgaggaggtgttccgcacggaatttggattccaccccaacgctgagtggattctaaagactctcgtgaacacagaacacgcctatgagaacaaacgcattgtggttgaaggcgggatgccctccggctgttccgcgacaagcatcatcaacacgattttgaacaacatctacgtgctctacgccttgcgtagacactatgagggagttgagctggatacctacaccatgatctcctacggagacgacatcgtggtggcaagtgattatgatttggactttgaggctctcaggccccactttaaatctcttggtcaaaccattactccagctgacaaaagcgacaaaggttttgttcttggtcactccatcaccgatgtcactttcctcaaaagacacttccacatggactatggaactgggttttacaaacctgtgatggcctcaaagacccttgaagccatcctctcctttgcacgccgtgggaccatacaggagaagttgatctctgtggcaggactcgccgtccactctggacctgacgagtaccggcgtctctttgaaccttttcaaggcctctttgagataccaagctacagatcactttacctgcgttgggtgaacgccgtgtgcggtgacgcataa

>AY593791.1_A_IRN_1998

atggatacaactgactgttttaccgctctgttccacgctctcagggagattaaaacactgtttctttcacgaacacaaggaaagatggaattcacactccacaatggagagaaaaagacattttactctaggcccaacagccacgataactgctggttgaacaccatccttcagttgtttaggtacgtcgatgaacctttcttcgactgggtctatgattcgcctgaaaacctcacacttgaggccataaggcaactggaagaactcactggtcttgaactgcacgagggtggaccgcccgccctcgtcatctggaacatcaaacaccttctccacaccggaatcggtaccgcctcgcgacccagcgaggtgtgcatggtagacggaacggatatgtgtctggctgactttcacgctggtattttcctgaaaggacaagagcatgctgtgtttgcctgcgtcacatctaacgggtggtacgcgatcgacgacgaggacttttacccctggacaccggacccgtccgacgtcctggtattcgtcccgtacgatcaggaaccactcaacggagagtggaaggcaaaggttcaaaagcggctcaagggagccggacaatccagtccggcaaccgggtcgcaaaaccaatcaggcaacactggaagcatcatcaacaactactacatgcaacaataccagaattccatggacacacaacttggagacaacgccatcagcggaggctccaacgagggatccacagacaccacctccacccacacaaccaacacccaaaacaatgactggttttcaaaactggccagctctgcctttagcgggctctttggtgctcttcttgctgacaagaagacagaggaaaccaccctcttggaagaccgcatcctcactacccgcaacggacataccacctcaacaacccagtcgagtgtgggagtcacctacgggtattccactggagaagaccacgtttccgggcccaacacgtctggcttggaaacgcgggtgacacaggcagagagatttttcaagaaacacttgtttaattggacaactgacaaaccttttgggtacttggaaaagctggagcttcccactgaccacaagggtgtttacggacacctagtggattcttttgcatacatgagaaacggctgggacgtggaggtgtccgccgttggcaatcagttcaacggtggatgcctcctagtggccatggtgcctgaatggaaagagttcactccacgtgagaagtaccagctcaccttgttcccgcaccagttcatcagccccagaaccaacatgactgctcacatcacggtcccgtaccttggtgtgaatagatatgaccagtacaagaaacacaagccctggacgctggtcgtgatggtggtttcgccgcttaccaacagcagcattggtgccacagaaatcaaggtctacgccaatatcgccccaacccacgttcacgtagccggtgaactcccgtcgaaagaggggatcgtaccggttgcttgttcggatgggtacggcggtttggtgacaacggacccgaaaacagctgaccctgtctacggtaaggtgtacaacccgcctaggacaaactatcctgggcgcttcacaaacttgttggacgtggccgaggcttgcccaaccttcctctgtttcgacgacgggaaaccgtacgttgtgacaagagaggacgggcagcgtctactggccaagttcgacgtctctcttgctgcaaagcacatgtcaaacacctacctatcagggatagcgcagtactatgcacagtactctggcaccatcaacctccacttcatgttcactggttctactgactcaaaagcccgctacatggtggcgtacgtcccgcccggtgtggaaccgccggatacgcctgagagagctgcacactgcatccacgctgagtgggacacagggctgaactccaaattcactttttctatcccgtacgtgtccgccgcggattacgcgtacaccgcgtccgatgtggccgaaacaacaaacgtacaggggtgggtctgcatctaccagatcacacacgggaaggctcaaaacgacactctggttgtgtcggttagcgccggcaaggactttgagttgcgtctcccggttgacccccgcacacagaccacatctgccggggagtctgcagacccagtcaccaccactgttgaaaactacggcggtgagacacaagtccagcggcgtcaccacactgatgtcggcttcataatggacagatttgtgaagattaacaacaccagccccacacacgtcattgacctcatgcaaacccaccaacacgggttggtgggcgctctcctgcgtgctgccacgtactacttctcagacctggagattgtggtgtgccacgaaggcaatctaacgtgggtgcccaatggagcaccagaggcagccctgagcaacgcgggcaaccccaccgcatacaacaaagcaccattcacgaggctagcactcccctacactgcaccgcaccgcgtgctggcgacggtgtacaacgggacgagcaagtactcgacaactggtggg---cacacacggggtgacttgggagctcttgcggcgagggccgccgcacaactccctgcctctttcaactttggcgcaatccgggccactgacatcagtgagcttcttgtgcgcatgaagcgtgctgagctctactgccccaggccactactggcagtggaggcagcgcaagataggcacaaacagaagatcattacgcctgtgaaacagctcctgaactttgacctgctcaagttggcgggagacgttgagtccaaccctgggcccttcttcttctccgacgtcaggacaaacttctccaaactggtagagaccatcaaccagatgcaggaggacatgtcaacaaaacacggacccgactttaaccggttagtttccgcgtttgaggaattggccacaggagtgaaggccatcaggaccggcctcgacgaggccaaaccctggtacaaactcattaagctcctgagccgcttatcatgcatggccgctgtagcagcacggtcaaaggacccggtccttgtggccatcatgctggctgacaccggtcttgagattctggacagcacgtttgtcgtgaaaaagatctctgactcgctctccagtctctttcacgtgccggcccctgtcttcagtttcggagccccgattctgttggccggactggtcaaagtcgcctcgagcttcttccggtctacacccgaagatcttgagagagcagagaaacagctcaaagcacgtgatattaatgacattttcgccattctcaagaacggcgagtggctggtcaaactgattcttgccatccgcgactggatcaaggcatggatcgcctcagaagaaaagtttgtcaccatgacagacttggtgcctggtatccttgagaagcaacgggatctcaacgaccccagcaaatacaaggaggccaaggagtggctcgacaacgcgcgccaagcgtgtttgaagagcgggaacgtccacattgccaacctgtgcaaagtggtcgccccagcacccagcaagtcgagaccagagcccgtggtcgtctgcctccgtggcagatccggccaggggaagagtttccttgcgaacgtgcttgcacaagcaatctccacccacttcactggcagaactgactctgtttggtactgcccgcctgaccctgaccacttcgacggttacaatcaacaaaccgttgttgtgatggatgatttgggccagaaccctgacggcaaggacttcaagtacttcgcccagatggtctctaccacggggttcatcccgcccatggcgtcgctcgaggacaaaggcaaacctttcaacagcaaggtcatcatcgccaccaccaacctgtactcgggtttcaccccgagaaccatggtgtgccctgacgcgctgaaccgaaggtttcactttgacattgacgtgagtgccaaggacgggtacaaaattaacaacaaattggacataatcaaagctcttgaagacacccacaccaacccagtggcaatgttccaatacgactgtgcccttctcaacggcatggccgttgaaatgaagagaatgcaacaagacatgttcaagccacagccacccctccagaacgtataccagctcgttcaggaggtgattgagagggtcgcgctccacgagaaagtgtcgagccacccgatcttcaagcagatctcaattccttcccaaaaatctgtgttgtacttcctcattgagaaaggccaacacgaagcagcaattgaattctttgaggggatggtgcatgactccatcaaggaggaactccggcccctcatccaacagacctcatttgtaaaacgcgctttcaaacgcctgaaggagaactttgagatcgttgccctatgtttgactcttctagcaaacatagtgatcatgatccgcgagactcgcaagagacagcagatggtggatgatgcagtgaacgagtacatcgagaaggcaaacatcaccacagacgacaagactcttgacgaggcggaaaagaaccctctggaaactagcggtgccagcaccgtcggtttcagagagagagccctcccgggacacaaggcgagtggtgacgtgaactcggagcccgccaaacccgcggaggaacaaccacaagctgaaggaccctacgccgggccacttgagcgtcagaagcctctgaaagtgagagccaagctaccacagcaagaggggccttacgccggcccgatggagagacagaaaccactgaaagtgaaagcaaaagccccggtcgttaaggaaggaccttacgagggaccggtgaagaagcctgtcgctttgaaagtgaaagcgaagaacttgattgtcactgagagtggtgccccaccgaccgacttgcaaaagatggttatgggtaacactaagcctgttgagctcattctcgacgggaagacagtagccatctgctgtgctactggagtgtttggtactgcctacctcgtacctcgtcaccttttcgcggagaagtacgacaagatcatgctggacggcagagccatgacagatagtgactacagagtgtttgagtttgagattaaagtaaaagggcaggacatgctctcagacgccgcgctcatggtgctccaccgtgggaatcgcgtgcgtgacattacgaaacactttcgtgatgtagcaaaaatgaagaaaggcacccccgtcgtcggtgtgattaacaacgctgatgtcgggagactgattttctctggtgaggcccttacctacaaagacattgtagtgtgcatggatggcgacaccatgcctggcctctttgcctacagagccgccaccaaggctggttactgcggaggggctgttcttgccaaagacggtgctgaaacattcatcgttggcacacactccgcaggtggcaatggagttggatactgctcatgcgtttccaggtccatgctcttgaagatgaaggcacacatcgaccctgaaccacaccatgagggtttgattgttgacaccagagatgtggaggagcgcgtgcatgtcatgcgcaaaaccaagcttgcacccaccgtggcacacggtgtgttcaaccccgaatttgggcctgctgccttgtccaacaaggacccgcgcttgagtgagggagttgtcctcgatgaagtcatcttctctaaacacaagggagacaccaagatgtctgaggaggacaaagcgctgttccgccgctgtgctgctgactacgcgtcacgcttacacagtgtgctgggcacggcaaatgccccactgagcatctacgaggcaatcaaaggcgttgacggcctcgacgccatggaaccagacaccgcgcctggcctcccctgggctctccaggggaaacgccgtggcgcgctcatcgacttcgagaatggcacagtcggacccgaggttgaagctgccttgaagctcatggagaaaaaggaatacaagtttgcatgccagaccttcttgaaagacgagatccgtccgatggagaaagtacgtgccggcaagacacgcattgtcgacgtcttgccagttgaacacattctttacaccaggatgatgattggcagattctgtgctcagatgcactcaaacaacggaccgcagattggctcggcagtcggttgtaaccctgatgttgactggcaaagatttggcacacacttcgctcagtacagaaacgtgtgggatgtggactattcggcctttgatgctaatcactgcagcgatgcgatgaacatcatgttcgaggaagtgttctccacggaatttggtttccacccaaacgctgagtggatcctgaaaacactcgtgaacacggagcacgcctatgaaaacaaacgcatcactgtcgagggcgggatgccgtctggttgttccgcgacaagcatcatcaacacaattctgaacaacatctacgttctctacgctttgcgtagacactatgagggagttgagctggacacttacaccatgatctcctacggagacgacatcgtggttgcaagcgattacgatctggactttgaggccctcaagcctcacttcaaatctcttggtcaaaccatcactccagctgacaaaagcgacaaaggttttgttcttggtcactctattactgatgtcactttcctcaaaagacacttccacatggattatggaactgggttttacaaacctgtgatggcctcgaagaccctcgaggctatcctctcctttgcacgccgtgggaccatacaggagaagttgatctccgtggcaggactcgccgtccactctggacctgacgagtaccggcgtctcttcgagcccttccagggtctctttgagattccaagctacagatcactttacctgcgttgggtgaacgccgtgtgcggcgacgcataa

>AY593792.1_A_ITL_1962

atgaatacaattgactgttttatcgctttggtacacgctatcagagagatcagagcactttttctaccacgaaccacaggaaaaatggaactcaccctgcacaacggcgagaaaaagactttttactctagacccaacaaccacgacaactgctggttgaacaccatccttcagttgttcaggtatatcgatgaacctttcttcgactgggtctacaactcgcccgagaacctcacgcttgaagccatcaagcaattggaggaactcacagggcttgagttgcacgagggcggaccgcctgcccttgtgatctggaacatcaaacacttgctccacaccggcatcggcaccgcctcacgacccagtgaggtgtgtatggtggacggcacggacatgtgtcttgctgacttccacgcaggcattttcctgaagggacaggaacacgcagtctttgcgtgtgtcacctccaacgggtggtacgcgattgacgacgaggaattttacccctggacgcctgacccgtcagacgtcttggtgtttgtcccgtacgatcaagaaccactcaacggggactggaaagcgatggttcagaggaagcttaagggtgccgggcaatccagcccggcgaccggctcccagaaccagtctggcaatactggcagcataattaacaactactacatgcagcagtaccagaactccatggacacacagcttggtgacaatgccattagtggaggctccaacgaaggctccacggacacaacttctacacacacaaccaacacccaaaacaacgattggttttcaaaacttgccagttcagccttcaccggtctgttcggcgccctgctcgccgacaagaagacggaagagactacacttctggaagaccgcattctcaccacccgcaacgggcacaccatctcgaccacccaatcgagtgtgggagtcacctacgggtactccactggagaagaccatgtcgctgggcccaacacatcgggcctggagacgcgggttgtgcaggcagagagatttttcaaaaagtttttgtttgactggacaaaggacaaaccttttggacatttggaaaagctggaacttcccgccgaccaccacggcgttttcgggcacctggtggaatcatatgcttacatgagaaatggttgggacgttgaggtgtctgctgttggcaaccagttcaacggcgggtgcctcctggtagctatggtaccggagtggaaagagtttgaacagcgcgagaaataccaactcaccctcttcccacaccagttcatcagccccagaacaaacatgactgctcacatcacggtcccataccttggagtgaacaggtacgatcagtacaagaaacacaaaccttggacactggttgttatggtagtgtcgcccctcacggttagcgacactgccgcggcacagattaaggtctacgccaacattgctccaacctacgttcacgtggctggggaactcccctcgaaagaggggattttcccagttgcatgttcggacggttacggaggactggtgacaacggacccgaaaacagctgaccccgcctacggcaaggtgtacaacccgcccaggaccaactaccctgggcggtttaccaacttgttggacgtggctgaagcgtgtcccactttcctctgtttcgacggcgggaaaccgtacgttgtcacgcggacagatgacacacgactattggccaagttcgacgtctcccttgctgcaaaacacatgtccaacacgtacctgtcagggattgcacagtactacgcacagtactctggcaccatcaacttgcacttcatgttcacaggctcaactgactcaaaagcccgctacatggtggcctacatcccgcctggggtggaaccaccggacacacctgaaagggccgctcactgcatccacgcagaatgggacacaggactgaactccaaattcactttttcaatcccgtacgtgtccgccgcagattacgcttataccgcgtctgacacggcagaaacaaccaacgtacagggctgggtctgcatctaccagatcacacacgggaaggccgagaacgacacattggtggtgtcggccagcgccggcagagactttgagttgcgcctcccgatcgacccccgacaacaaaccactgctgttggggagtccgcagaccctgtcaccaccaccgtggagaactacggtggtgagacacaaacccagagacggcaccacacggatgtcggtttcatcatggacagatttgtgaagataaacagtttgagtcccacacatgtcattgacctcatgcagacccaccaacacgggctggtaggtgcgctgttgcgtgcagccacgtactacttctctgacttggagattgttgtgcggcacgaaggcaatttgacttgggtgcccaatggtgcccctgaagcagctttgtcaaacaccagcaaccccaccgcctacaacaaggcaccgttcacgaggctcgctctcccttacactgcgccacaccgcgtgttggcaaccgtgtacaacgggacgaacaagtactccacgggcggtccg------ggacgaggcgacatggggtcgctcgcggcgcgggtcgcgaaacaacttcctgcctctttcaactacggtgcaatcagggccgacaccatccacgagcttctcgtgcgcatgaaacggacagagctctactgccccaggccactattggcaatagaggcttcacaagacaggcacaagcaaaagatcattgcacccgcaaaacagttgctgaactttgacctacttaagttggctggagacgtggagtccaaccctgggcccttcttcttctctgacgttaggtcaaacttttctaagctggtggaaaccatcaaccagatgcaggaagacatgtcaacaaaacacgggcccgacttcaaccggttggtgtccgcctttgaggaactggccgctggagtaaaagccatcaggaccggcctcgacgaggccaaaccctggtacaagcttatcaaactcctaagccgcctgtcgtgcatggccgctgtggcagcacggtccaaggacccagtccttgtggccatcatgctggccgacaccggtctcgagattctggacagcactttcgtcgtgaagaagatctccgactcgctctccagtctcttccacgtgccggcccccgctttcagtttcggagccccgattctgctggccgggttggtcaaggtcgcctcgagtttcttccggtccacgcccgaagaccttgagagggcagagaaacagctcaaagcacgtgacatcaacgacattttcgccattctcaagaacggcgagtggctggtcaaactgatccttgccatccgcgactggattaaggcttggattgcctcagaagaaaagtttgtcaccatggcagacttagtgcctggcatccttgaaaagcagcgggacctcaacgacccaagtaagtacaaggaggccaaggagtggctcgacaacgcgcgccaagcgtgtctgaagagcgggaacgtccacattgccaacctgtgcaaggtggtcgccccggcccccagcaagtcgagacccgaacccgtggtggtttgcctccgtggcaaatcaggccagggtaagagtttccttgcgaacgtgctcgcacaagcaatctctacccacttcaccgggcggaccgattcagtctggtactgcccacctgaccctgatcacttcgacggttacaaccaacagactgtcgttgtgatggacgatttgggccagaaccctgacggcaaggacttcaagtacttcgcccaaatggtttcgaccacggggttcatcccgcccatggcatcactcgaggacaaaggtaaacccttcaacagtaaggtcatcattgcaaccaccaacctgtactcgggcttcaccccgaggactatggtgtgccctgacgccctgaaccggaggtttcactttgacattgacgtgagcgccaaggatgggtacaaaattaacaacaaattggacattatcaaagcacttgaagacacccacaccaacccagtggcaatgtttcagtacgactgtgcccttctcaacggcatggctgttgaaatgaagagaatgcagcaagacatgttcaaacctcaaccacccctccagaacgtgtaccaactagttcaggaggtgattgatcgggtggagctccacgagaaagtgtcgagccacccaatttttaagcagatctcaattccttcccaaaaatctgtgttgtacttcctcattgagaaagggcagcacgaggcagcaattgaattctttgagggcatggtgcatgactccgtcaaggaggagctccggccgctcatccaacagacctcatttgtgaaacgcgctttcaagcgcctgaaggaaaactttgagattgttgctctgtgcctaacacttttggccaacattgtgatcatgatccgcgaaactcgcaagagacagaagatggtggacgatgcggtgaacgagtacatcgagaaagcaaacatcaccaccgatgacaagacacttgacgaggcggaaaagaaccctctggagactagcggtgccagcaccgttggtttcagagagagaactcttccaggccagaaggcgcgtgatgacgtgaactccgagcccgcccaacctgctgaagaacaaccacaagctgaaggaccctacgccgggccacttgagcgtcagagacccctgaaggtgagagccaagctcccacaacaggaaggaccctacgctggcccgatggagagacagaaaccgctgaaagtgaaagcaaaagccccggtcgtcaaggaaggaccttacgaaggaccggtgaagaaacctgtcgctttgaaagtgaaagctaagaatttgattgtcactgagagtggtgcccccccgaccgacttgcaaaagatggtcatgggcaacacaaagcctgttgagctcatcctcgacgggaagacagtagccatctgctgtgctactggagtgtttggcactgcctacctcgtgcctcgtcatcttttcgctgagaagtatgacaagatcatgttggacggcagagccatgacagacagtgactacagagtgtttgagttcgagatcaaagtaaaaggacaggacatgctctcagacgccgcactcatggtgctccaccgtgggaaccgtgtgagagacatcacgaagcactttcgtgacacagcaagaatgaagaaaggcacccccgttgtcggcgtgatcaacaatgccgatgtcgggagactgattttctctggcgaagcccttacctacaaagacattgtagtgtgcatggacggagacaccatgcccgggctttttgcctacagagccgccactaaggctggctactgcgggggagccgttctcgctaaggacggggctgacactttcatcgttggcactcactctgcaggaggtaatggagttggatactgctcatgcgtttccaggtccatgcttctcaagatgaaggcacacattgaccctgagccgcaccacgaggggttgattgtagacaccagagatgtggaagagcgcgtccacgtgatgcgcaaaaccaagcttgcacccaccgttgcacacggtgtgttcaaccccgagtttgggccagctgccttgtccaacaaggacccgcgtctgaacgagggtgttgtcctcgatgaagtcattttctccaaacacaagggagacacaaagatgtctgaggaggacaaagcgctgttccgccgatgtgctgctgattacgcgtcacgcctgcacagcgtgctgggtacggcaaatgccccactgagcatctacgaggcaatcaaaggcgtcgacggactcgacgccatggagccagacaccgcacctggcctcccctgggctctccagggaaaacgccgtggtgcgctcatcgactttgagaacggcacggtcgggcccgaagtcgaggctgccttgaagctcatggagaaaagagagtacaagtttgtttgtcagaccttcctgaaggacgagattcgcccgatggagaaagtacgtgccggcaagactcgcattgtcgacgtcctgcccgttgaacacattctttacaccaggatgatgattggcagattttgtgcacaaatgcactcaaacaatggaccgcaaattggctcggcggtcggttgtaaccctgatgttgattggcaaagatttggcacacacttcgcccaatacagaaacgtgtgggatgtggactattcggcctttgatgctaaccactgcagtgacgccatgaacatcatgtttgaggaggtgttccgcacggacttcgggttccacccaaatgctgagtggatcttgaagactctcgtgaacacggaacatgcctatgagaacaaacgcatcactgttgaaggcgggatgccatctggttgttccgcaacgagcatcatcaacacaattttgaacaacatctacgtgctctacgccttgcgtagacactatgagggagttgagctggacacttacaccatgatctcctacggagacgacattgtggtggcaagtgattacgatctggactttgaggctctcaagcctcacttcagatctcttggtcaaaccattactccagctgacaaaagcgacaaaggttttgttcttggtcactccatcaccgatgtcactttcctcaaaagacactttcacatggattatggaactgggttttacaaacctgtgatggcctcaaagacccttgaggctatcctctcctttgcacgccgtgggaccgtacaggagaagttgatctccgtggcaggactcgctgtccactctggaccagacgagtaccggcgtctctttgagccctttcagggcctctttgagattccaagctacagatcactttacctgcgttgggtgaacgccgtgtgcggcgacgcataa

>AY593793.1_A_PHI_1975

atgaacacaactgactgttttatcgctttggtgcacgccatcagagagatcaaaacacttttcttcccaaagactacaagaaagatggaactcacactgtacaacggtgagagaaaggttttctactctaggcccaacaaccacgacaactgttggctaaacactatcctccagttgtttaggtatgtcgatgaacctttcttcgactgggtctacaactcgcccaagaacctcacgctcagagcgatagaacagctagaggaagccactgggcttgagttgcacgagggtggaccgcctgccctcgtgatttggaacattaaacacttgctccacactggtattggcaccgcctcgcgacctagtgaggtgtgcatggtggacggcacaaacatgtgtttggccgacttccacgcaggcatattccttaagggacaagaacacgctgtgtttgcgtgtgtcacttccaacgggtggtacgcgattgatgacgaagacttctacccgtggacgccggacccgtccgacgttctggttttcgtcccgtacgatcaagaaccactcaatgggagttggaaagccaatgttcagtggaaactcaaaggagccggacaatccagcccggcaacaggttcgcagaaccaatctggcaacactggcagcataatcaacaactactacatgcagcagtaccaaaactccatggacacacaacttggtgataacgccattagcggaggctctaacgaaggctccacggacacaacttcaacacacacaaccaacacccaaaacaacgattggttttcaaagcttgccagttcggctttcactggcctgttcggggcactgcttgccgacaaaaagacagaggagacaacactccttgaggaccgcattctcaccacccgtaacggacacaccacctcgacaacacagtccagcgtgggcgttacatacgggtattccactgcggaggaccacgttgccgggcccaacacatcgggcctggagactcgggtagtgcaggcagaaaggttcttcaagaagtttttgtttgattggacaacggacaagccctttggacatttggaaaagctggagcttccaaccgatcacagcggtgttttcggacacttggtggactcatatgcttatatgagaaacggttgggacgttgaggtgtccgctgttggcaatcagttcaatggcggttgcctcctggtggctatggtgcccgagtggaaaaaactcgacacacgggagaaataccagcttacccttttcccacaccagtttatcagtcctagaaccaacatgactgcccacatcacggtcccttaccttggtgtgaacagatatgaccagtacaaaaagcacaaaccctggacactggttgtcatggtcgtgtctccacttacggttaacaccactagtgcgacacagatcaaggtctacgccaacattgccccgacctacgttcacgtggccggtgaactcccctcgaaggaggggattttccctgttgcatgtgcggatggttacgggggattggtgacaacagacccaaaaaccgctgatcctgcttacggcaaggtgtacaacccgccccggaccaactaccctgggcgctttaccaacttgttggacgtggctgaagcgtgtcccactttcctctgcttcgacgacgggaaaccgtatgtcaccacgcgggcggatgagacccggcttttggccaagtttgatgtttcccttgccgcaaagcacatgtctaacacgtacctgtcaggaattgcccagtactacgcacagtactctggtaccatcaacttgcacttcatgttcactggttctactgattcaaaagcccggtacatggtggcctacatcccgcctggggtggagccaccggacacacctgagaaggctgcccactgcatccacgctgaatgggacacaggactaaattctaaatttactttctcaatcccgtacgtgtctgccgcagactacgcctatacagcgtccgacacggcagagacaaccaacgtacagggatgggtctgcatttatcagatcacacacgggaaggctgaaaacgacactctggtcgtgtcggtcagcgccggcaaagactttgagttgcgcctcccgatcgacccccgccagcagaccactgctaccggggagtcggcagacccagtcaccaccactgtggagaactacggcggtgagacacaagtccacagacgccaccacacggacattggctttattatggacagatttgtgaagataaaggaagtgcggtcaactcatgccattgatctcatgcaaactcaccaacacggcctagtgggtgcgctgctacgcgcggccacctactacttctctgatttggaaatcgtcgtgcgacacgacggcaacctgacttgggtgcccaacggtgctcctgaatcggccctgcagaacaccagcaaccccactgcctaccacaaggcaccattcacgagacttgctctcccctacactgcgccgcaccgtgtgttggcaactgtgtacaacgggacgaacaagtatactgtgagtggttca---ggcaggcgaggtgacatggggtccctcgcggcgcgagtcgcgaaacaactccctgcctcttttaactacggtgcaatcaaggccgacaacatccacgagcttctcgtgcgcatgaaacgggctgagctttactgccccagaccactgttggcaatagaggcttcgcaagataggcacaagcagaagatcattgcacccgaaaaacagcttttgaattttgacctgctcaagttagctggggacgtggaatccaaccctgggcccttctttttctctgacgttaggtcaaattttaccaagctagtggaaacaatcaaccagatgcaggaggacatgtcaacaaaacacgggcccgactttaaccggttggtgtccgcttttgaggaattggccactggagtgaaagctatcaggactggtcttgacgaggccaaaccctggtacaaacttatcaagctcctaagccgcctgtcgtgtatggccgctgtggcggcacggtccaaggacccagtccttgtggccatcatgctggccgacaccggccttgagattctggacagcacatttgtcgtgaagaagatttccgactcgctctccagtctctttcacgtgccggcccccgtctttagcttcggatcaccgatcctgctagccgggttggtcaaagtcgcctcgagtttcttccggtccacgcccgaagaccttgagagagcagagaaacagctcaaagcacgtgacatcaacgacattttcgccattcttaagaacggcgagtggctggtcaaactcatccttgccatccgcgactggatcaaggcatggatcgcctcagaagagaagtttgtcactatgacagacctggtgcctggcattcttgaaaagcagcgggacctgaacgacccgagtaagtacaaggaagccaaggaatggctcgacaacgcgcgccaggcgtgtttgaagagcgggaacacccacattgccaacctgtgcaaagtggttgccccagcacccagcaagtcgagacccgaacctgtggttgtttgcctccgtggtaaatccggccagggtaagagtttccttgcgaacgtgctcgcacaagcaatctccacccactttaccggcagaaccgactctgtttggtactgcccacctgaccccgaccacttcgacggttacaaccaacaaactgtcgttgtgatggatgatctgggccagaaccctgacggcaaggactttaagtactttgcccaaatggtttcgaccacagggttcatcccgcctatggcatcacttgaagacaagggcaaacctttcaacagcaaggtcatcatcgcgaccaccaacttgtacgcgggcttcaccccgaggactatggtgtgtcccgacgcgctgaaccgaaggtttcacttcgacatcgatgtgagtgccaaggacgggtacaaaattaacaacaaattggacatcaccaaagcacttgaagacacccacaccaacccagtggcaatgttccagtacgattgtgcccttctcaacggtatggccgttgaaatgaagagaatgcaacaagatgtgttcaagcctcaaccgcccctccagaacatttaccagctcgttcaggaggtgattgaacgggtcgagctacacgagaaagtgtcgagccacccgatcttcaagcagatttcaattccttcccaaaagtccgtgttgtacttcctcattgagaaagggcaacacgaagcagcaattgaattctttgaggggatggtacacgactccatcaaggaggagctccgacctctcctccaacacacctcatttgtgaaacgcgcttttaagcgcctgaaggaaaatttcgagattgttgccctgtgtctgaccctcttggccagtatagtggttatgatccgcgcgactcacaagagacagaagatggtggatgatgcagtgaatgagtacattgagaaagcaaacatcaccactgacgacaagactcttgatgaggcggaaaagaaccctctggaaactagcggtgccagcaccgttggcttcagagagagaactctcccaggccagaaagcgcgcgatgacgtgaactctgagcccgcccaacctgttggagaacaaccacaagctgaaggaccctacgccgggccacttgaacgtcagaaacctctgaaagtgagagccaagctcccgcaacaagagggaccttacgctggcccgatggagagacagaaaccgctgaaagtgaaagcaaaagccccggtcgttaaggaaggaccctacgagggaccggtgaagaagcctgtcgctttgaaagtgaaagctaagaatttgattgtcactgagagtggtgccccaccgaccgacttacagaagatggtgatgggtaacaccaagcctgtcgagctcatccttgacgggaagacagtagccatctgctgtgctactggggtgtttggcactgcttaccttgttccccgtcatctttttgcggaaaagtatgacaagatcatgttggacggcagagccatgacagacagtgactacagagtgtttgagtttgagattaaagtaaaaggacaggacatgctctcggacgccgcgctcatggtgcttcaccgtgggaaccgcgtgagagacatcacgaaacactttcgtgatgtagcaagaatgaagaaaggaacccctgtcgttggcgtgatcaacaacgctgatgtcgggagactaattttctctggtgaggccctcacctacaaggacattgtagtgtgcatggatggagacaccatgcctggcctctttgcctacagagctgcaaccaaggcaggttattgtggaggagccgttcttgccaaggacggagccgagacgttcatcgtcggcacgcactccgcaggaggcaatggagttggatactgctcgtgcgtttccaggtccatgcttctgaagatgaaggcacacatcgaccctgaaccacaccacgaggggttgattgttgacaccagagatgtggaggagcgtgttcacgtgatgcgcaaaaccaagcttgcacccaccgtcgcgcacggtgtttttaaccccgaattcgggcctgctgccttgtccaacaaggacccacgcctgaatgaaggtgttgtcctcgatgaagtcatcttctccaaacacaagggagacacaaagatgtctgaggaggacaaagcgctgttccgccgctgtgccgctgactacgcgtcacgtctacactgtgtgctgggtacagcaaatgccccactgagcatttacgaggcaatcaagggcgtcgacggactcgacgcaatggaaccggacactgcacctggcctcccttgggcactccaggggaaacgccgcggtgccctcattgactttgagaacggcactgtcggacccgaagttgaagctgccttgaagctcatggagaaaagagagtacaaatttgcttgtcagaccttcctgaaggacgagatccgcccgatggagaaagtacgtgccggcaagactcgcattgtcgatgtcctgcccgttgaacacattctttacaccaggatgatgattggcagattctgtgcacaaatgcactcaaacaacggaccgcaaattggctcggcggtcggttgtaaccctgatgttgattggcagagatttggcacacacttcgcccaatacagaaacgtgtgggacgtggactattcggcctttgatgctaaccactgcagtgacgccatgaacatcatgtttgaggaggtgttccgcacggagttcggcttccacccaaacgctgagtggatcttgaagactctcgtgaacacggagcacgcctatgagaacaaacgcatcactgtcgagggcggaatgccgtccggttgctccgccacaagcatcatcaacacaattttgaacaacatctacgtgctctacgccctgcgtagacactatgagggagttgagctggacacctacaccatgatctcctatggagacgacatcgtggtggcaagtgattacgatttggactttgaggctctcaagccccactttaaatctcttggtcaaaccatcactccagctgacaaaagcgacaaaggttttgttcttggtcactccattaccgatgtcactttcctcaaaagacacttccacatggactatggaactgggttttacaaacctgtgatggcctcaaagacccttgaggctatcctctcctttgcacgccgtgggaccatacaggagaagttgatctccgtggcgggactcgccgtccactctggacctgacgagtaccggcgtctctttgagccctttcagggcctcttcgagattccaagctacagatcactttacctgcgctgggtgaacgccgtgtgcggtgacgcatga

>AY593794.1_A_COL_1985

atgaacacaactgactgttttatcgctttggtgcacgctatcagagagatcagaacatttttcctagcgcgacgcacaggaaagatggagttcactttgcacaacggcgagaagaaaactttctattctagacccaacaaccacgacaactgttggttgaacaccatcctccagttgttcaggtacgtcgacgaacctttcttcgactgggtctacaactcgcccgagaacctcacgcttgaagctatcaagcagttggaggaactcacagggcttgagttgcacgagggcggaccgcctgccctcgtaatttggaacatcaagcacttgctccacaccggcatcggcaccgcttcgcgacctagcgaggtgtgcatggtggacggcacggacatgtgtcttgctgacttccacgcaggcattttcctgaaaggacaggaacacgcagtgtttgcgtgtgtcacctccaacggatggtacgcgatcgacgacgaggacttttacccctggacccctgatccgtcagacgtcctggtgtttgttccgtacgatcaagaaccactcaatggatcctggaaggcgctggtccagcggaagctcaagggcgccgggcaatccagcccggcgactggctcgcagaaccagtctggtaacacaggcagtataatcaacaactactacatgcagcagtaccaaaattctatggacacacaacttggtgacaatgccatcagtggaggatccaacgaaggatccaccgacacgacttcaacacacacaaccaacacccaaaataatgattggttttcaaagcttgccagttcagctttcaccggcctgttcggcgcactgctcgccgacaagaagacagaagaaacaacacttcttgaggaccgcatcctcaccacccgtaacgggcacaccacctcaacaacccagtcgagcgtgggtgtcacgtatggatactccacagaagaggaccacgttgctgggcccaacacatcgggcctggaaacacgggtggtgcaggcagaaagattctacaaaaagtacttgtttgattggacacctgacaaaccattcggggaactggttaagctggagcttccgtccgaacataacggcgtttttggacacttggtggactcatacgcctacatgagaaacggatgggacattgaggtgtccgctgttggcaaccagttcaacggtgggtgcctcctggtggccatggtacctgagtggaaggaatttgacacacgggagaagtaccaactcacccttttcccgcaccaatttatcagccctagaaccaacatgactgcccacatcacggtcccctatataggggtgaacaggtatgaccaatacaagaagcacaggccctggacactggttgtgatggttgtgtcaccacttacggtcaacaacaccggtgcgcagcaaatcaaggtttacgccaacatagccccgacctacgtccacgtggcaggtgagctcccctcgaaagaggggattttcccagtcgcgtgtgcggacggctacggaggactggtgaccacggacccgaagacagctgatcctgcttatggcaaggtgtacaatccgcctaggaccaactaccctgggcgcttcaccaacctgttggacgtggccgaagcgtgtccaaccttcctctgttttgacggcgggaaaccgtacgtcaccacactgacgggagaaacacgatgtctggccaaattcgacctttcccttgccgcaaagcacatgtccaacacatacctgtctggcattgctcagtactacgcacagtactctggcaccattaacttgcacttcatgttcacaggctccactgactcaaaggctcggtacatggtggcctacatcccgcccggggtggagccaccggacacacctgaaaaggctgcccactgcatccacgctgagtgggacactggacttaactccaaattcaccttctcgatcccgtacgtgtccgccgcggactatgcctacacagcatctgacacggcagaaacaacaaacgtacagggatgggtctgcatttaccaaattacacacgggaaggctgaaaatgacactctggtcgtgtcggttagcgccggcaaagactttgagctgcgcctcccgattgaccctcgccagcagaccacggctactggggaatcagcagacccggtcaccaccactgtggagaactatggtggtgagacgcaaatccagagacgtcatcacacggatgttgggttcatcatggacaggtttgtaaaaatcacacaacacagcccgacacacgtcattgacctcatgcagactcaccaacacggtctggtgggcgccttgctgcgtgctgccacgtactacttttccgacctggaaattgttgtacggcacgacggcaatctgacctgggtgcccaacggtgcccctgtctcggccttgtcgaacaccagcaaccccactgcctacaataaggcaccgttcacaagactcgctctcccatacactgcgccacaccgcgtgttggcaacggtgtacaatggggtgagcaagtacgctgtgggcgattca---ggcagacgtggtgatctaggagctctcgcggcgcgagtcgcgaaacaacttcctgcctcatttaactacggtgcaatcaaggctgataccatccacgagcttctcgtgcgcatgaaacgagccgagctctactgccccaggccactactggcggtagaggcctcacaagacaggtacaaacaacagatcattgcaccggcaaaacagcttttgaactttgacctgctcaagttggctggggacgtcgagtccaaccctggacccttcttcttctccgatgtcaggtcgaatttttccaagctggtggagacaatcaaccaaatgcaggaagacatgtcaacaaaacacggtcctgactttaaccggttggtgtccgcgtttgaagagttggccactggagtgaaggccatcagaaccgggctcgatgaggctaaaccctggtacaagctgatcaaactcctgagccgcctgtcgtgcatggccgctgtggcagcacggtccaaggacccagtccttgtggccatcatgctggccgacaccggtctcgagattctggacagcacctttgtcgtgaagaaaatctccgactcgctctccagtctctttcacgtgccggcccccgtcttcagtttcggtgccccgattctgttggccgggttggtcaaggtcgcctcgagcttcttccggtccacacccgaagatcttgagagagcagagaaacagctcaaagcacgtgacataaacgacatcttcgccattctcaagaacggcgagtggctggtcaaactgattcttgccatccgcgactggattaaggcatggatcgcctcagaagagaagttcgtcaccatgacggacttggtgcctggcattcttgaaaagcagcgggacctcaacgacccgagcaagtacaaggaagccaaggagtggctcgacaacgcgcgccaagcgtgtttgaagagcgggaacgtccacattgcgaacctctgcaaagtggtcgccccagcacccagcaagtcgagacccgaacctgtggtcgtttgcctccgtggcaagtctggccagggcaagagtttccttgcgaacgtgctcgcacaagcaatctccactcactttaccggcaggactgattcggtttggtactgcccccctgaccccgaccacttcgacggctacaaccagcagaccgttgttgtgatggatgatttgggccagaaccctgacggcaaggacttcaagtacttcgcccagatggtctcgaccacagggttcatcccgcccatggcatcacttgaggacaaagggaaacctttcaacagcaaggtcatcattgcaaccaccaacttgtactcgggtttcaccccgaggactatggtgtgtcctgacgctttgaaccggaggtttcactttgacatcgatgtgtgtgccaaggacgggtacaaaaccaacaacaaattggacatcatcaaagcacttgaagacacccacaccaacccagtggcaatgtttcagtacgattgtgcccttctcaacggcatggccgtagaaatgaagagactgcaacaagacatgttcaaaccacaaccacccctccagaacgtgtaccaacttgttcaagaggtgattgaacgggtcgaactccacgagaaagtgtcgagtcacccgatcttcaaacagatctcaattccttcccaaaaatccgtgttgtacttcctcattgagaaaggtcaacatgaggcagcaattgaattctttgagggtatggtgcatgactccatcaaggaggagctccgaccgctcatccaacagacctcatttgtgaaacgcgcttttaagcgcttgaaggaaaactttgagatcgtggccttgtgtctgacactcctggccaacattgtcatcatgatccgcgaaactcgcaagagacagaagatggtggacgatgccgtgaacgattacatcgagagggcaaacatcaccacagatgacaagacacttgacgaggcggaaaagaaccctctggagaccaacggtgccagcgctgttggcttcagagagaaaaccctcccaggccacgcggcacgcaatgacgtgaactctgagcctgcccagcctgctgaggagcaaccacaagctgaaggaccctacgccgggccactcgagcgtcagaaacccctgaaggtgagagccaagctcccacaacaggagggaccctacgctggcccgatggagagacagaaaccgcttaaagtgaaagcaagagccccggtcgttaaggaaggaccctacgagggaccggtgaagagacctgtcgctttgaaagtgaaagaaaaaagtttgattgtcactgagagtggtgccccgccgaccgacttacaaaagatggtcatgggcaacacaaagcccgttgagctcatccttgacgggaagacggtagccatctgctgtgctactggagtgtttggtactgcttacctcgtgcctcgtcatcttttcgcagaaaagtatgacaagatcatgttggacggcagagccatgacagacagtgactacagagtgtttgagtttgagattaaagtaaaaggacaggacatgctctcagacgcggcactcatggtgctccaccgtgggaaccgcgtgagagacatcacgaagcactttcgtgatacagcaagactgaagaaaggcacccccgtcgtcggcgttatcaacaatgctgatgtcgggagactgattttctctggtgaggcccttacctacaaggacattgtagtgtgcatggatggagacaccatgcccgggctttttgcctacaaagctgccaccaaggctggctactgtggaggagctgttctcgcaaaggacggagctgacacgttcatcgtcggcactcactctgcaggtggcaatggggttggatactgctcatgcgtttccaggtccatgctacagaggatgaaagcacacgtcgaccctgaaccacaccacgaggggttgatcgttgacaccagagatgtggaagagcgcgtccatgttatgcgcaaaacaaagcttgcacccaccgtcgcgcacggtgtgttcaaccctgagtttgggcccgctgccttgtcaaacaaagacccgcgcctgaatgagggagttgtcctcgacgaagtcatcttttccaagcacaagggagacacaaagatgaccgaggaagacaaagcgctgttccgccgctgcgccgctgactacgcgtcacgtctgcatagcgttttgggcacagcaaatgccccactgagcatctatgaggcaatcaaaggcgttgacggactcgacgccatggagcctgacacagcgccaggcctcccttgggccctccaggggaagcgccgcggtgcgctcatcgactttgagaacggcacggtcggacccgaggttgaagctgccttgaagctcatggagaaaagagaatacaagtttgcttgtcagaccttcctgaaagacgaaattcgtccgatggagaaggtgcgcgctggcaaaactcgcattgtcgacgtcctgcctgtcgagcacattctctacaccaggatgatgattggcaggttctgcgcgcaaatgcactccaacaacggaccgcaaattggctctgcggtcggttgtaaccctgatgttgattggcaaagattcggcacacatttcgcccaatacaggaacgtgtgggatgtggactattcggcctttgatgcaaaccactgcagtgacgccatgaacatcatgtttgaggaggtgtttcgcacggactttggtttccacccaaacgctgagtggattctgaagactctcgtgaacacggaacacgcctatgaaaacaagcgcatcactgttgagggcgggatgccatctggttgttccgcaacaagcattatcaacacaattctgaacaacatctacgtgctctacgccctacgtagacactatgaaggagttgagctggacacttacaccatgatctcctacggagacgacatcgtggtggcaagtgattatgatctggactttgaggcccttaagcctcacttcaaatctcttggccagaccatcactccagctgacaaaagcgacaaaggttttgttcttggtcactccattaccgatgtcactttcctaaaaagacacttccacatggattatggaactgggttttataaacctgtgatggcctcaaagacccttgaggccatcctctcctttgcacgccgtgggaccatacaggagaagttgatctccgtggcaggactcgccgtccactctggaccagacgagtaccggcgtctctttgagcccttccaaggcctcttcgagattccaagctacagatcactttacctgcgttgggtgaacgccgtgtgcggtgacgcataa

>AY593795.1_Asia1_PAK_1954
[truncated: 5,009,270 more chars]
