# Supplementary material for: Early origin and global colonisation of foot-and-mouth disease virus
Source: Sci Rep. 2020 Sep 17;10:15268. doi: 10.1038/s41598-020-72246-6 (PMC7498456; doi:10.1038/s41598-020-72246-6)
Supplement: Supplementary file 4 — Supplementary Data S2. [file 41598_2020_72246_MOESM4_ESM.doc]

>AB079061.1_O_JPN_2000

cacaacggtgagaagaaaacattctactccaggcccaacaaccacgacaactgctggctgaacaccgtcctccagttgtttaggtacgttgatgaacctttcttcgactgggtctaccactcacctgagaacctcacacttgatgctatcaaacaactggaagaaattactggtctcgagctccacgagggtggaccacccgctctcgttatttggaacattaaacacctgctcaacaccggaatcggcaccgcttcgcgacccagcgaagtgtgcatggtagacgggacggacatgtgtttggctgacttccacgctggcatcttcctgaaaggacaggaacacgctgtgttcgcctgcgttacctccaacgggtggtacgcgattgatgacgaggacttttacccctggacgccggacccgtccgacgttctggtgtttgtcccgtacgatcaa

>AF026168.2_O_TAW_1997

tacaacggtgaaaagaaggtcttccactccagacccaacaaccacgacaactgttggctgaacgccatcctccaactgttcaggtacgttgacgagcccttcctcgaatgggtctacgactcacctgagaacctcactctcgaggcgatcaacaaactggaagaaatcacaggtcttgagctacacgagggcggaccgcccgcccttgtcgtctggaacatcaagcacttgctctacaccggaatcggcaccgcttcgcgacccagcgaggtgtgcatggtggacggtacagacatgtgcttggctgacttccacgccggtatatttctgaagggacaggaccacgccgtcttcgcctgcgtcacctccaacgggtggtacgcgattgacgacgaggacttttacccgtggacaccaaatccggccgacgttttggtgtttgttccgtacgatcaa

>AF154271.1_O_TAW_1997

tacaacggtgaaaagaaggtcttctactccagacccaacaaccacgacaactgttggctgaacgccatcctccaactgttcaggtacgttgacgagcccttcctcgaatgggtctacgactcacctgagaacctcactctcgaggcgatcaacaaactggaagaaatcacaggtcttgagctacacgagggcggaccgcccgcccttgtcgtctggagcatcaagcacttgctctacaccggaatcggcaccgcttcgcgacccagcgaggtgtgcatggtggacggtacagacatgtgcttggctgacttccacgccggtatatttctgaagggacaggaccacgccgtcttcgcctgcgtcacctctgacgggtggtacgcgattgacgacgaggacttttacccgtggacaccaaatccggccgacgttttggtttttgttccgtacgatcaa

>AF308157.1_O_TAW_1997

tacaacggtgaaaagaaggtcttctactccagacccaacaaccacgacaactgttggctgaacgccatcctccaactgttcaggtacgttgacgagcccttcctcgaatgggtctacgactcacctgagaacctcactctcgaggcgatcaacaaactggaagaaatcacaggtcttgagctacacgagggcggaccgcccgcccttgtcgtctggaacatcaagcacttgctctacaccggaatcggcaccgcttcgcgacccagcgaggtgtgcatggtggacggtacagacatgtgcttggctgacttccacgccggtatatttctgaagggacaggaccacgccgtcttcgcctgcgtcacctctgacgggtggtacgcgattgacgacgaggacttttacccgtggacaccaaatccggccgacgttttggtttttgttccgtacgatcaa

>AF377945.1_O_SKR_2000

cacaacggtgagaagaaaacattctactccaggcccaacaaccacgacaactgctggctgaacaccatcctccagttgtttaggtacgttgatgaacctttcttcgactgggtctactactcacctgagaacctcacactagatgctatcaaacaattggaagaaatcactggtctcgagctccacgagggtggaccacccgctctcgttatttggaacattaaacacctgctcaacaccggaatcggcaccgcttcgcgacccagcgaagtgtgcatgatagacgggacggacatgtgtttggctgacttccacgctggcatcttcctgaaaggacaggaacacgctgtgttcgcctgcgtcacctccaacgggtggtacgcgattgatgacgaggacttttacccctggacgccggacccgtccgacgttctggtgtttgtcccgtacgatcaa

>AF506822.2_O_CHA_1999

cacaacggtgagaagaaaacattctactccaggcccaacaaccacgataactgctggctgaacaccatcctccagttgtttaggtacgttgatgaacctttcttcgactgggtctactactcacctgagaacctcacacttgatgctatcaaacaattggaagaaattactggtctcgagctccacgagggtggaccacccgctctcgttatttggaacattaaacacctgctcaacaccggaatcggcaccgcttcgcgacccagcgaagtgtgcatggtagacgggacggacatgtgtttggctgacttccacgctggcatcttcctgaaaggacaggaacacgctgtgttcgcctgcgtcacctccaacgggtggtacgcgattgatgacgaggacttttacccctggacgccggacccgtccgacgttctggtgtttgtcccgtacgatcaa

>AH012984.2_O_SKR_2000

cacaacggtgagaagaaaacattctactccaggcccaacaaccacgacaactgctggctgaacgccatcctccagttgtttaggtacgttgatgaacctttcttcgactgggtctactactcacctgagaacctcacgctcgatgctatcaaacaactggaaggaattactggtctcgagctccacgagggtggaccacccgctctcgttatttggaacattaaacacctgctcaacaccggaatcggcaccgcttcacgacccaacgaagtgtgcatggtagatgggacggacatgtgtttggctgacttccacgctggcatcttcctgaaaggacaggaacacgctgtgttcgcctgtgtcacctccaacgggtggtacgcgattgatgacgaggacttttacccctggacgccggacccgtctgacgttctggtgttcgtcccgtacgaccaa

>AH012985.2_O_SKR_2000

cacaacggtgagaagaaaacattctactccaggcccaacaaccacgacaactgctggctgaacaccatcctccagttgtttaggtacgttgatgaacctttcttcgactgggtctactactcacctgagaacctcacgcttgatgctatcaaacaattggaagaaattactggtctcgaactccaggagggtggaccacccgctctcgttatttggaacattaaacacctgctcaacaccggaatcggcaccgcttcacgacccaacgaagtgtgcatggtagacgggacggacatgtgtttggctgacttccacgctggcatcttcctgaaaggacaggaacacgctgtgttcgcctgcgtcacctccaacgggtggtacgcgattgacgataaggacttttacccctggacgccggacccgtccgacgttctggtgtttgtcccgtacgatcaa

>AJ539136.1_O_TAW_1999

cacaacggtgagaggaaaacattctactccaggcccaacaaccacgacaactgctggctgaacaccatcctccagttgtttaggtacgttgatgaacctttcttcgactgggtctactactcacctgagaacctcacacttgatgctatcaaacaattggaagaaattactggtctcgagctccacgagggtggaccacccgctctcgttatttggaacattaaacacctgctcaacaccggaatcggcaccgcttcgcgacccagcgaagtgtgcatggtagacgggacggacatgtgtttggctgacttccacgctggcatcttcctgaaaggacaggaacacgctgtgttcgcctgcgtcacctccaacgggtggtacgcgattgatgacgaggacttttacccctggacgccggacccgtccgacgttctggtgtttgtcccgtacgatcaa

>AJ539137.1_O_TAW_1999

cacaacggtgagaggaaaacattctactccaggcccaacaaccacgacaactgctggctgaacaccatcctccagttgtttaggtacgttgatgaacctttcttcgactgggtctactactcacctgagaacctcacacttgatgctatcaaacaattggaagaaattactggtctcgagctccacgagggtggaccacccgctctcgttatttggaacattaaacacctgctcaacaccggaatcggcaccgcttcgcgacccagcgaagtgtgcatggtagacgggacggacatgtgtttggctgacttccacgctggcatcttcctgaaaggacaggaacacgctgtgttcgcctgcgtcacctccaacgggtggtacgcgattgatgacgaggacttttacccctggacgccggacccgtccgacgttctggtgtttgtcccgtacgatcaa

>AJ539138.1_O_CHA_1999

cacaacggtgagaagaaaacattctactccaggcccaacaaccacgataactgctggctgaacaccatcctccagttgtttaggtacgttgatgaacctttcttcgactgggtctactactcacctgagaacctcacacttgatgctatcaaacaattggaagaaattactggtctcgagctccacgagggtggaccacccgctctcgttatttggaacattaaacacctgctcaacaccggaatcggcaccgcttcgcgacccagcgaagtgtgcatggtagacgggacggacatgtgtttggctgacttccacgctggcatcttcctgaaaggacaggaacacgctgtgttcgcctgcgtcacctccaacgggtggtacgcgattgatgacgaggacttttacccctggacgccggacccgtccgacgttctggtgtttgtcccgtacgatcaa

>AJ539139.1_O_SKR_2000

cacaacggtgagaagaaaacattctactccaggcccaacaaccacgacaactgctggctgaacaccatcctccagttgtttaggtacgttgatgaacctttcttcgactgggtctactactcacctgagaacctcacgcttgatgctatcaaacaattggaagaaattactggtctcgaactccacgagggtggaccacccgctctcgttatttggaacattaaacacctgctcaacaccggaatcggcaccgcttcgcgacccagcgaagtgtgcatggtagacgggacggacatgtgtttggctgacttccacgctggcatcttcctgaaaggacaggaacacgctgtgttcgcctgcgtcacctccaacgggtggtacgcgattgacgatgaggacttttacccctggacgccggacccgtccgacgttctggtgtttgtcccgtacgatcaa

>AJ539140.1_O_SAR_2000

cacaacggtgagaagaaaacattctactccaggcccaacaaccacgacaactgctggctgaacaccatcctccagttgtttaggtacgttgatgaacctttcttcgactgggtctactactcacctgagaacctcacacttgatgctatcaaacaattggaagagatcactggtctcgagctccacgagggtggaccacccgctctcgttatttggaacattaaacacttgctcaacaccggaatcggcaccgcttcgcgacccaacgaagtgtgcatggtagacgggacggatatgtgtttggctgacttccacgctggcatcttcctgaaaggacaggaacacgctgtgttcgcctgcgttacctccaacgggtggtacgcgattgatgacgaggacttttacccctggacgccggacccgtccgacgttctggtgtttgtcccgtacgatcaa

>AJ539141.1_O_UKG_2001

cacaacggtgagaagaaaacattctactccaggcccaacaaacacgacaactgctggctgaacaccatcctccagttgtttaggtacgttgatgaaccttttttcgactgggtctactactcacctgagaacctcacacttgatgctatcaaacaattggaagaaattactggtctcgagctccacgagggtggaccacccgctctcgttatttggaacattaaacacttgctcaacaccggaatcggcaccgcctcgcgacccagcgaagtgtgcatggtagacgggacggatatgtgtttggctgacttccacgctggcatcttcctgaaaggacaggaacacgctgtgttcgcctgcgttacctccaacgggtggtacgcgattgatgacgaggacttttacccctggacgccggacccgtccgacgttctggtgtttgtcccgtacgatcaa

>AJ633821.1_O_FRA_2001

cacaacggtgagaagaaaacattctactccaggcccaacaaacacgacaactgctggctgaacaccatcctccagttgtttaggtacgttgatgaaccttttttcgactgggtctactactcacctgagaacctcacacttgatgctatcaaacaattggaagaaattactggtctcgagctccacgagggtggaccacccgctctcgttgtttggaacattaaacacttgctcaacaccggaatcggcaccgcctcgcgacccagcgaagtgtgcatggtagacgggacggatatgtgtttggctgacttccacgctggcatcttcctgaaaggacaggaacacgctgtgttcgcctgcgttacctccaacgggtggtacgcgattgatgacgaggacttttacccctggacgccggacccgtccgacgttctggtgtttgtcccgtacgatcaa

>AY317098.1_O_CHA_2002

tacaacggggaaaagaaagtcttttactccagacccaacaaccacgacaattgttggttgaacgccatcctccaactgttcaggtacgttgacgaacccttcctcgaatgggtctacaactcacctgaggacctcactcttgaggcgattaacaacctggaagaagtcactggtcttgagctacgcgaaggcggaccacccgccctcgtcgtctggaacaccaagcacctgctctacaccggaatcggcaccgcctcgcggcccagcgaggtgtgcatgatcgacggcacagacatgtgcttggccgacttccacgccggtatatttctgaagggacaggaccacgccgtcttcgcctgcgtcacctctgacgggtggtacgcaatcgacgacgaagatttttatccgtggacaccagacccggctgacgtcttggtttttgttccgtacgatcaa

>AY593751.1_A_NET_1942

cataacggtgaaaagaaaactttctactccaggcccaataaccacgacaactgctggttgaacaccatcctccagttgtttaggtacgtcgatgaacctttcttcgactgggtctataactcgcccgagaatcttacgcttgatgctatcaagcagttggaaaacttcaccgggcttgagttgcacgagggcggaccacctgcccttgtgatctggaacatcaaacacttgctccaaaccggtatcggtaccgcctcgcgacccagcgaggtgtgtatggtagacggcacggacatgtgtcttgctgatttccacgcaggcattttcatgaaaggacaggaacacgcagtgttcgcgtgtgtcacctcagacgggtggtacgcgattgacgacgaggacttttacccgtggacgcctgacccatcggacgtcttggtatttgtcccgtacgatcaa

>AY593753.1_A_Brazil_1970

tacaacggtgagagaaaagtgttttactccagacccaacagccacgacaactgttggttgaacaccatccttcagttgttcaggtacgttgatgaacctttcttcgactgggtctacaactcgcccgagaacctcacacttgaagccattgagcagttagaggaactcacagggctggagttacgcgagggcggaccacctgccctcgtagtctggaacatcaagcacctgctccaaactggcatcggtaccgcctcgcggcccagcgaggtgtgtatggtggacggtacggacatgtgtcttgctgacttccacgcaggcattttcatgaaaggacaggaacacgctgtgtttgcgtgtgtcacctccaatgggtggtacgcgattgacgacgaggacttctacccatggacaccggacccgtccgatgtcttggtgtttgtcccgtacgatcaa

>AY593754.1_A_SPA_1959

tacaacggcgagaaaaagactttttactcccgacccaacaaccacgacaactgttggttgaacaccatccttcagttgttcaggtatgtcgatgaacccttcttcgactgggtctacaattcgcccgagaacctcacgcttgaagccatcaaccaattggaggaactcacaggacttgagttgcacgagggcggaccacctgcccttgtgatctggaacatcaaacacttgctccacaccggcatcggcactgcctcacgacccagtgaggtgtgtatggtggacggcacggacatgtgtcttgctgacttccacgcaggcattttcctgaagggacaggaacacgcagtctttgcgtgtgtcacctccaacgggtggtacgcgattgacgacgaggaattttacccctggacgcctgacccgtcagacgtcctggtgtttgtcccgtacgatcaa

>AY593755.1_A_TAI_1960

tacaacggtgagaagaagaccttctactccaggcccaacaaccacgacaactgctggttgaacaccattttgcagctgtttaggtatgttgatgaaccgttctttgactgggtctacgactcgcccgagaatctcacgctcgaggcgatcagacagttggaggacatcactggtcttgacctgcacgacggcggaccacccgccctcgtcatttggaacatcaagcacctgctccacaccggcattggcactgcctcgcgacccagcgaggtgtgcatggtggacggtacggacatgtgcctggctgacttccacgctggcatcttcttgaagggacaggaacacgccgtgtttgcctgtgtcacttccaatgggtggtacgcgatcgacgacgaggacttctacccctggacaccggatccgtctgacgtcctagtttttgtcccgtacgatcaa

>AY593756.1_A_Brazil_1959

cacactggtgagaagaagactttttactccaggcccaataaccacgacaactgctggttgaacgccatactccagttgtttagatacgtcgatgaacctttcctcgactgggtctacaactcacccgagaaccttacgctggcagcaatcagacagctagaggacctcacagggcttgagttgcacgagggcggaccgcctgccctcgtaatttggaacatcaagcacttgctccaaaccggcatcggcaccgcctcgcgacccagcgaggtgtgcatggttgacggtacgaacatgtgtctggctgatttccacgcaggtatctttctaaaaggcaaagagcacgctgtgtttgcgtgtgtcacctccaacgggtggtacgcgattgatgacgaagacttttacccttggacaccggacccgtccgacgtcttggtgtttgtcccgtacgatcaa

>AY593757.1_A_Brazil_1967

tacaacggtgagagaaaagtgttttactccagacccaacagccacgacaactgttggttgaacaccatccttcagctgttcaggtacgttgatgaacctttcttcgactgggtctacaactcgcccgagaacctcacacttgaagccattgagcagttagaggaactcacagggctggagttacgcgagggcggaccacctgccctcgtagtctggaacatcaagcacctgctccaaactggcatcggtaccgcctcgcggcccagcgaggtgtgtatggtggacggcacggacatgtgtcttgctgacttccacgcaggcattttcatgaaaggacaggaacacgctgtgttcgcgtgtgtcacttccaacgggtggtacgcgattgacgacgaggacttctacccatggacgccggacccgtccgatgtcttggtgtttgtcccgtacgatcaa

>AY593758.1_A_VEN_1967

tacaacggtgagagaaaagtgttttactccagacccaacagccacgacaactgttggttgaacaccatccttcagttgttcaggtacgttgatgaacctttcttcgactgggtctacaactcgcccgagaacctcacacttgaagccattgagcagttagaggaactcacagggctggagttacgcgagggcggaccacctgccctcgtagtctggaacatcaagcacctgctccaaactggcatcggtaccgcctcgcggcccagcgaggtgtgtatggtggacggtacggacatgtgtcttgctgacttccacgcaggcattttcatgaaaggacaggaacacgctgtgtttgcgtgtgtcacctccaatgggtggtacgcgattgacgacgaggacttttacccatggacaccggacccgtccgatgtcttggtgtttgtcccgtacgatcaa

>AY593759.1_A_GER_1971

cacaacggtgaaaagaaaactttttattccaggcccaatagccacgacaactgctggctgaacaccatcctccagttgtttaggtacgtcgatgaaccattcttcgactgggtctacaactcacccgagaaccttacgcatgatgctatcaagcagttggaagaactcaccgggcttgagttgcgcgagggcggaccgcccgcccttgtgatttggaacatcaaacacttgctccaaactggcatcggcaccgcctcgcgacccagcgaggtgtgtatggtggacggtacggacatgtgtcttgccgatttccacgcaggcattttcctgaaaggacaggaacacgcagtgtttgcgtgtgtcacctctgacgggtggtacgcgattgacgacgaggatttttacccgtggacgcctgacccatcggacgtcttggtatttgtcccgtacgatcaa

>AY593760.1_A_USSR_1964

cacaacggcgagaaaaagactttttactccagacccaacaaccacgacaactgctggttgaacaccatccttcagttgttcaggtatgtcgatgaacccttcttcgactgggtctacaactcgcccgagaacctcacgcttgaagccatcaagcaattggaggaactcacggggcttgagttgcacgagggcggaccgcctgcccttgtgatctggaacatcaaacacttactccacaccggcatcggcaccgcctcacggcccagtgaggtgtgcatggtggacggtgcggacatgtgtcttgctgacttccacgcaggcattttcctgaagggacaggaacacgcagtttttgcgtgtgtcacctccaacgggtggtacgcgattgacgacgaggagttttacccctggacgcccgacccgtcggacgttctggtgtttgtcccgtacgatcaa

>AY593761.1_A_KEN_1964

cacaacggtgagaagaaaatcttttactctagacccaacaaccacgacaactgctggctgaacgcaattctccaactgttcaggtacgtcgatgagcctttcttcgactgggtctacgagtctcccgagaacctcacactacaggcaattgagcaattggaggaactcactggtcttgaactacacgagggtgggccgcccgctcttgttgtttggaacatcaaacacttgcttcacaccggcattggtactgcctcgcgacccagcgaggtgtgtatggtggatggtacagacatgtgcttggctgactttcatgctggaatcttcctaaaaggacaggaacacgctgtgtttgcttgcgtcacctccgaagggtggtacgcgattgacgacgaggatttctacccctggacaccggagccatccgacgtcttggtgtttgtcccgtacgatcaa

>AY593764.1_A_IRQ_1970

tacaacggtgaaaagaagactttctactccagacccaacaaccacgacaactgttggcttaacaccattctccagttgttcaggtacgtcgatgagcctttcttcgactgggtctatgactcgcctgagaacctcacctgtgaggcaattaggcagttggaagagataactggtcttgagctacacgagggtggaccacccgccctcgtcatctggaacatcaaacacttgctccacactgggatcggcactgcctcacgacctagtgaagtgtgcatggttgacggaacggacatgtgcttggctgacttccacgctggcattttcttgaaaggacaggaacatgctgtgtttgcctgcgtcacctccaacgggtggtacgcgatcgacgatgaggacttttacccctggacgccggacccgtccgatgtcttggtgtttgtcccgtatgaccaa

>AY593765.1_A_TUR_1965

tacaacggtgaaaagaagaccttctactccagacccaacaaccacgacaactgttggcttaacaccattctccagttgtttaggtacgtcgatgagcctttcttcgactgggtctatgactcgcctgagaacctcacctgcgaggcaattaggcagctggaagagataactggtcttgagctacacgagggtggaccacccgccctcgtcatctggaacatcaaacacttgctccacactggaatcggcactgcctcacgacctagtgaagtgtgcatggttgacggaacggacatgtgcttggctgacttccacgctggcattttcttgaaaggacaggaacatgctgtgtttgcctgcgtcacctccaacgggtggtacgcgatcgacgatgaggacttttacccctggacgccggacccgtccgatgtcttggtgtttgtcccgtacgatcaa

>AY593766.1_A_KEN_1965

tacaacggtgagaagaagacattttactcaagacccaacaaccacgataactgctggttgaacacaatcctccaactgttcaggtacgtcgaagaacctttcttcgactgggtttatgagtcccctgagaacctcacactgcaagcaattgaacaacttgaggacctaactggtcttgagctgcacgacggtggaccacccgccctcgtgatttggaacatcaaacacttgctctctactggtgtaggcactgcctcgcgacccagcgaggtgtgcatggtggacggcacagacatgtgtctggccgatttccacgctgggatcttcatgaaaggacaggaacacgctgtgttcgcttgtgtcacatccaacgggtggtgcgcgattgatgacgaggacttctacccctggacaccggacccctccgacgtcttggtgttcgtcccgtacgatcaa

>AY593767.1_A_ARG_1965

tacaacggcgagaaaaagactttctactccagacctaacaaccacgacaactgttggttgaacactgtccttcagttgttcaggtatgtcgatgagcccttcttcgactgggtctacaactcacctgagaacctcacgctcgaagccatcgagcaattggaggaactcacaggacttgagctgcacgaaggtgggccgcccgccctcgtgatctggaacatcaaacacttgctccacaccggcatcggcacagcctcacgacccagtgaggtgtgcatggtggacggtacggacatgtgtcttgccgacttccacgcaggcattttcctgaagggacaggaacacgcagtctttgcatgtgtcacctccaacgggtggtacgcgattgatgatgaggaattttacccctggacgcctgacccgtcagacgtcctggtgtttgtcccatacgaccaa

>AY593768.1_A_Brazil_1955

cacaacggtgagagaaaagtgttctattctagacccaacaaccacgacaactgttggttgaacaccatccttcagctgttcaggtacgtcggagaacccttcttcgactgggtctatgactcacccgagaacctcactcttgaagctatcgagcaactggaggagctcacagggttagagttgcacgagggcggaccacctgccctcgtgatctggaacatcaaacacctgcttcataccggcatcggcaccgcctcgcggcccagcgaggtgtgcatggtggacggcacgaacatgtgtcttgctgacttccacgcaggcattttcctgaaaggacaggaacacgctgtgtttgcgtgtgtcacctccaacgggtggtacgcgattgacgacgaggacttttacccatggacgccggacccgtccgacgttttggtgtttgttccgtacgatcaa

>AY593769.1_A_ARG_1959

cacaacggtgagaagaaaactttttactctaggcccaacaaccacgacaactgttggctaaacgccattcttcagttgttcaggtacgtcgatgaacctttcttcgactgggtctacaactcgcccgaaaacctcacgcttgaagccatcaagcagttggaagaactcacagggcttgagttgcgcgagggcggaccacccgccctcgtggtctggaacatcaaacacttacttcacactggcattggtaccgcctcgcgacccagcgaggtgtgtatggtggatggcacggacatgtgtctcgctgacttccatgcaggcattttcctgaaaggatcggaacacgcagtgtttgcgtgtgtcacctccgatgggtggtacgcgatcgacgacgaggacttttacccctggactcctgacccatcagacgtcctggtattcgtcccgtacgatcaa

>AY593770.1_A_ARG_1966

cacactggtgagaagaagactttttactccagacccaacaaccacgacaactgttggttgaacaccatactccagttgttcaggtatgttgacgaacctttctttgactgggtttacaactcgcccgagaacctcacactgacagcaatcaaacagttggaggaactcacaggacttgagttgcacgagggcggaccgcctgccctcgtaatctggaacatcaagcacttgctccacaccggcatcggcactgcctcgcgacccagcgaggtgtgcatggttgacggtacggacatgtgcctggctgacttccacgcaggcatcttcctaaaaggcaaagaacacgcagtgttcgcgtgtgtcacctccaacgggtggtacgcgattgatgacgaggacttttacccttggacaccggacccgtccgacgttttagtgtttgtcccgtacgatcaa

>AY593771.1_A_COL_1967

cacaacggcgagaaaaagactttctactcaagacccaacaaccacgacaactgctggttaaacaccattctgcagttgttcaggtatgtcgatgaacccttcttcgactgggtctacaactcgcctgagaacctcacacttgaagccatcaaacaactggaggaactcacaggacttgagctccgcgagggtgggccgcccgcccttgtgatttggaacatcaaacacctgctccacaccggcattggtaccgcctcacgacccagtgaggtgtgtatggtggacggtacagacatgtgtcttgctgacttccacgcaggcattttcctgaaaggtcaggagcacgcggtttttgcgtgtgtcacctccaacgggtggtacgcgattgatgacgaggaattctacccctggacacctgacccatcagatgtcttggtgtttgtcccgtacgatcaa

>AY593772.1_A_TUR_1972

tacaacggcgtaaagaagactttctactccagacccaacaaccacgacaactgctggcttaacaccattcttcagttgtttaggtacgtcgatgagcctttcttcgactgggtctatgactcgcctgagaacctcacctgtgaggcaattaagcagctggaagagataactggtcttgagctacacgagggtggaccacccgccctcgtcatttggaacatcaaacacttgctccacactggaatcggcactgcttcacgacctagtgaagtgtgcatggttgacggaacggacatgtgcttggctgacttccacgctggcatctttttgaaaggacaggaacacgctgtgtttgcctgcgtcacctccaacgggtggtacgcgatcgacgacgaggacttttacccctggacgccggacccgtccgatgttttagtgtttgtcccgtacgatcag

>AY593773.1_A_PER_1969

cacaacggcgagaaaaagactttttactccagacccaacaaacacgataactgttggctgaacaccatcctccagttgtttagatacgtcgaagaacctttcttcgactgggtctacaactcacctgagaaccttacgctgtcagcaatcagacagctggaggaactcacagggcttgagttgcacgagggcggaccacctgctcttgtgatctggaacatcaaacacatgctccaaaccggtgtcggtactgcctcgcgacccagcgaggtatgtatggtcgatggtacggacatgtgtctggctgattttcacgcaggcatctttctaaaaggcaaggaacatgctgtctttgcgtgtgtcacctccaatgggtggtacgcgatcgatgatgaggacttttacccctggacaccggacccgtccgacgttctggtatttgttccgtacgatcaa

>AY593774.1_A_SPA_1969

cacaacggtgaaaagaaaactttctactccaggcccaacaaccacgacaactgctggttgaacaccatcctccagttgttcaggtacgtcgatgagcctttcttcgactgggtctacaactcgcccgagaatctcacgcttgatgctattaagcagttggaagaactcaccgggcttgagttacacgagggcggaccgcctgcccttgtgatctggaacatcaaacacttgctccaaaccggcattggtaccgcctcgcgacccagcgaggtgtgtatggttgacggcacggacatgtgtttggctgatttccatgcaggcatcttcctgaaaggaaaggaacacgccgtgtttgcgtgtgtcacctccaacgggtggtacgcgatcgatgatgaggacttttacccctggacgccggacccgtccgatgtcctggtgtttgtaccgtacgatcaa

>AY593775.1_A_VEN_1970

cacaacggcgagaaaaagactttttactccagacccaacaaacacgataactgttggctgaacaccatcctccagttgtttagatacgtcgaagaacctttcttcgactgggtctacaactcacctgagaaccttacgctgtcagcaatcagacagctggaggaactcacagggcttgagttgcacgagggcggaccacctgctcttgtgatctggaacatcaaacacatgctccaaaccggtgtcggtactgcctcgcgacccagcgaggtatgtatggtcgatggtacggacatgtgtctggctgattttcacgcaggcatctttctaaaaggcaaggaacatgctgtctttgcgtgtgtcacctccaatgggtggtacgcgatcgatgatgaggacttttacccctggacaccggacccgtccgacgttctggtatttgttccgtacgatcaa

>AY593776.1_A_GER_1968

cacaacggtgaaaagaaaactttttactccaggcccaacagccacgataactgctggttgaacaccatcctccagttgtttaggtacgtcgatgaacctttcttcgactgggtctacaactcgcccgagaaccttacgcttgatgccatcaagcagttggaagaacttactgggcttgagttgcacgagggcggaccgcctgccctcgtgatctggaacatcaaacacttgctccaaactggtatcggtaccgcctcgcgacccagcgaggtgtgtatggtagacggtacggacatgtgtcttgctgatttccacgctggcattttcttgaagggacaggaacacgcagtgtttgcgtgtgtcacttctgacgggtggtacgcgattgatgacgaggacttttacccgtggacgcctgacccatcggacgtcttggtatttgtcccgtacgatcaa

>AY593777.1_A_GER_1972

cacaacggtgaaaagaaaactttctactccaggcccaacaaccacgacaactgctggttgaacaccatcctccagttgttcaggtacgtcgatgagcctttcttcgactgggtctacaactcgcccgagaatctcacgcttgatgctattaagcagttggaagaactcaccgggcttgagttacacgagggcggaccgcctgcccttgtgatctggaacatcaaacacttgctccaaaccggcattggtaccgcctcgcgacccagcgaggtgtgtatggttgacggcacggacatgtgtttggctgatttccatgcaggcatcttcctgaaaggaaaggaacacgccgtgtttgcgtgtgtcacctccaacgggtggtacgcgatcgatgatgaggacttttacccctggacgccggacccgtccgatgtcctggtgtttgtaccgtacgatcaa

>AY593778.1_A_SPA_1969

tacaacggcgagaaaaagactttttactcccgacccaacaaccacgacaactgttggttgaacaccatccttcagttgttcaggtatgtcgatgaacccttcttcgactgggtctacaattcgcccgagaacctcacgcttgaagccatcaaccaattggaggaactcacaggacttgagttgcacgagggcggaccacctgcccttgtgatctggaacatcaaacacttgctccacaccggcatcggcactgcctcacgacccagtgaggtgtgtatggtggacggcacggacatgtgtcttgctgacttccacgcaggcattttcctgaagggacaggaacacgcagtctttgcgtgtgtcacctccaacgggtggtacgcgattgacgacgaggaattttacccctggacgcctgacccgtcagacgtcctggtgtttgtcccgtacgatcaa

>AY593779.1_A_GER_1972

cacaacggtgaaaagaaaactttctactccaggcccaacaaccacgacaactgctggttgaacaccatcctccagttgttcaggtacgtcgatgagcctttcttcgactgggtctacaactcgcccgagaatctcacgcttgatgctattaagcagttggaagaactcaccgggcttgagttacacgagggcggaccgcctgcccttgtgatctggaacatcaaacacttgctccaaaccggcattggtaccgcctcgcgacccagcgaggtgtgtatggttgacggcacggacatgtgtttggctgatttccatgcaggcatcttcctgaaaggaaaggaacacgccgtgtttgcgtgtgtcacctccaacgggtggtacgcgatcgatgatgaggacttttacccctggacgccggacccgtccgatgtcctggtgtttgtaccgtacgatcaa

>AY593780.1_A_FRA_1960

tacaacggcgagaaaaagactttttactctagacccaacaaccacgacaactgctggttgaacaccatccttcagctgttcaggtatgtcgatgaacccttcttcgactgggtctacaactcgcccgagaacctcacgcttgaagccatcaaccaattggaggaactcacaggacttgagttgcacgagggcggaccgcctgcccttgtgatctggaacatcaagcacttgctccacaccggcatcggcactgcctcacgacccagtgaggtgtgtatggtggacggcacggacatgtgtcttgctgacttccacgcaggcattttcctgaagggacaggaacacgcagtctttgcgtgtgtcacctccaacgggtggtacgcgattgacgacgaggaattttacccctggacgcctgacccgtcagacgtcctggtgtttgtcccgtacgatcaa

>AY593781.1_A_GER_1951

cacaacggcgagaaaaagactttttactctagacccaacaaccacgacaactgctggttgaacaccatccttcagttgttcaggtatgtcgatgaacccttcttcgactgggtctacaactcgcccgagaacctcacgcttgaagccatcaaccaattggaggaactcacaggacttgagttgcacgagggcggaccgcctgcccttgtgatctggaacatcaaacacttgctccacaccggcatcggcaccgcctcacgacccagtgaggtgtgtatggtggacggcacggacatgtgtcttgctgacttccacgcaggcattttcctgaagggacaggaacacgcagtctttgcgtgtgtcacctccaacgggtggtacgcgattgacgacgaggaattttacccctggacgcctgacccgtcagacgtcctggtgtttgtcccgtacgatcaa

>AY593782.1_A_ARG_2000

cacaacggcgacagaaagactttctactcaagacccaacaaacacgacaactgttggctgaacgccatccttcagttgttcaggtatgtcgacgaaccattcttcgactgggtctacagctcgcccgaaaacctcacgctcaaggccattgagcagctggagagacttactgggcttgagctacacgagggtggaccgcccgctctcgtcatttggaacatcaagcacttgctccaaaccggcatcggcactgcctcacgacccagtgaggtgtgcatggtggacgggacggatatgtgtcttgcagactttcacgcaggcatcttcctgaaaggcgaagaacatgctgtcttcgcatgtgtcacctctgatgggtggtacgcgattgacgatgaggacttttacccctggacaccggacccgtccgacgtcttggtgtttgttccttacgatcaa

>AY593784.1_A_ARG_2001

tacaacggtgagaagaagacattttactccagacccaacaaccacgacaactgttggttgaacgccatcctccagttgttcaggtacgtcgacgaacctttcttcgactgggtctacaactcgcctgagaacctcacgctctcggccatcgaacagctggaggaaattaccgggcttgagttgcacgagggcggaccacccgcgctcgtggtttggaacatcaaacacatgctccacactggcatcggcaccgcctcgcgacccagcgaggtgtgcatggtcgacggtacggacatgtgtttggctgatttccatgctggcattttcctgaaaggtcaggagcacgctgtgtttgcatgtgtcacctctgacgggtggtacgcgatcgacgacgaggacttctacccttggacaccagacccgtctgacgtcctggtgtttgtcccgtacgaccaa

>AY593785.1_A_ARG_2001

tacaacggtgagaagaagacattttactccagacccaacaaccacgacaactgttggttgaacgccatcctccagttgttcaggtacgtcgacgaacctttcttcgactgggtctacaactcgcctgagaacctcacgctctcggccatcgaacagctggaggaaattaccgggcttgagttgcacgagggcggaccacccgcgctcgtggtttggaacatcaaacacatgctccacactggcatcggcaccgcctcgcgacccagcgaggtgtgcatggtcgacggtacggacatgtgtttggctgatttccatgctggcattttcctgaaaggtcaggagcacgctgtgtttgcatgtgtcacctctgacgggtggtacgcgatcgacgacgaggacttctacccttggacaccagacccgtctgacgtcctggtgtttgtcccgtacgaccaa

>AY593786.1_A_ARG_2001

tacaacggtgagaagaagacattttactccagacccaacaaccacgacaactgttggttgaacgccatccttcagttgttcaggtacgtcgacgaacctttcttcgactgggtctacaactcgcctgagaacctcacgctctcggccatcgagcagctggaggaaattaccgggcttgagttgcacgagggcggaccacccgcgctcgtggtttggaacatcaaacacatgctccacactggcatcggcaccgcctcgcgacccagcgaggtgtgcatggtcgacggtacggacatgtgtttggctgatttccatgctggcattttcctgaaaggtcgggagcacgctgtgtttgcatgtgtcacctctgacgggtggtacgcgatcgacgacgaggacttctacccttggacaccagacccgtctgacgtcctggtgtttgtcccgtacgaccaa

>AY593787.1_A_Brazil_1977

cacaacggtgaaaagaagactttctactccagacccaacaaccacgacaactgttggctgaacaccatccttcagctgtttaggtacgtcgatgaacccttcttcgactgggtctacaactcgcccgagaacctgacgctgtctgccatcaggcagctggaagaactcacgggacttgagttgcacgagggcggaccacctgccctcgtgatctggaacatcaaacacttgctccataccggcattggcactgcctcgcgacccagcgaggtgtgcatggttgacggtacggacatgtgtctagctgatttccacgcaggcattttcctgaaaggcaaagaacacgctgtgttcgcgtgtgtcacctccaacgggtggtacgcgatcgacgacgaggacttctacccttggacaccggacccgtccgacgttctggtgtttgtcccgtacgatcag

>AY593788.1_A_Brazil_1979

cacaacggtgaaaagaagactttctactccagacccaacaaccacgacaactgttggctgaacaccatccttcagctgtttaggtacgtcgacgaaccgttcctcgactgggtctacaactcgcccgagaacctgacgctgtctgccgtcaggcagctggaagaaatcacgggacttgagttgcacgagggtggaccacctgccctcgtgatctggaacatcaaacacttgcttcataccggcattggcactgcctcgcgacccagcgaggtgtgcatggttgacggtacggacatgtgtctggctgatttccacgcaggcattttcctgaaaggcaaagaacacgctgtgttcgcatgtgtcacctccaacgggtggtacgcgatcgatgacgaggacttctacccttggacaccggacccgtccgacgttctggtgtttgtcccgtacgatcag

>AY593789.1_A_ARG_1961

cacaacggtgagaagaaaactttttactctaggcccaacaaccacgacaactgttggctaaacgccattcttcagttgttcaggtacgtcgatgaacctttcttcgactgggtctacaactcgcccgaaaacctcacgcttgaagccatcaagcagttggaagaactcacagggcttgagttgcgcgagggcggaccacccgccctcgtggtctggaacatcaaacacttacttcacactggcattggtaccgcctcgcgacccagcgaggtgtgtatggtggatggcacggacatgtgtctcgctgacttccatgcaggcattttcctgaaaggatcggaacacgcagtgtttgcgtgtgtcacctccgatgggtggtacgcgatcgacgacgaggacttttacccctggactcctgacccatcagacgtcctggtattcgtcccgtacgatcaa

>AY593790.1_A_ARG_2001

tacaacggtgagaagaagacattttactccagacccaacaaccacgacaactgttggttgaacgccatccttcagttgttcaggtacgtcgacgaacctttcttcgactgggtctacaactcgcctgagaacctcacgctctcggccatcgagcagctggaggaaattaccgggcttgagttgcacgagggcggaccacccgcgctcgtggtttggaacatcaaacacatgctccacactggcatcggcaccgcctcgcgacccagcgaggtgtgcatggtcgacggtacggacatgtgtttggctgatttccatgctggcattttcctgaaaggtcgggagcacgctgtgtttgcatgtgtcacctctgacgggtggtacgcgatcgacgacgaggacttctacccttggacaccagacccgtctgacgtcctggtgtttgtcccgtacgaccaa

>AY593791.1_A_IRN_1998

cacaatggagagaaaaagacattttactctaggcccaacagccacgataactgctggttgaacaccatccttcagttgtttaggtacgtcgatgaacctttcttcgactgggtctatgattcgcctgaaaacctcacacttgaggccataaggcaactggaagaactcactggtcttgaactgcacgagggtggaccgcccgccctcgtcatctggaacatcaaacaccttctccacaccggaatcggtaccgcctcgcgacccagcgaggtgtgcatggtagacggaacggatatgtgtctggctgactttcacgctggtattttcctgaaaggacaagagcatgctgtgtttgcctgcgtcacatctaacgggtggtacgcgatcgacgacgaggacttttacccctggacaccggacccgtccgacgtcctggtattcgtcccgtacgatcag

>AY593792.1_A_ITL_1962

cacaacggcgagaaaaagactttttactctagacccaacaaccacgacaactgctggttgaacaccatccttcagttgttcaggtatatcgatgaacctttcttcgactgggtctacaactcgcccgagaacctcacgcttgaagccatcaagcaattggaggaactcacagggcttgagttgcacgagggcggaccgcctgcccttgtgatctggaacatcaaacacttgctccacaccggcatcggcaccgcctcacgacccagtgaggtgtgtatggtggacggcacggacatgtgtcttgctgacttccacgcaggcattttcctgaagggacaggaacacgcagtctttgcgtgtgtcacctccaacgggtggtacgcgattgacgacgaggaattttacccctggacgcctgacccgtcagacgtcttggtgtttgtcccgtacgatcaa

>AY593793.1_A_PHI_1975

tacaacggtgagagaaaggttttctactctaggcccaacaaccacgacaactgttggctaaacactatcctccagttgtttaggtatgtcgatgaacctttcttcgactgggtctacaactcgcccaagaacctcacgctcagagcgatagaacagctagaggaagccactgggcttgagttgcacgagggtggaccgcctgccctcgtgatttggaacattaaacacttgctccacactggtattggcaccgcctcgcgacctagtgaggtgtgcatggtggacggcacaaacatgtgtttggccgacttccacgcaggcatattccttaagggacaagaacacgctgtgtttgcgtgtgtcacttccaacgggtggtacgcgattgatgacgaagacttctacccgtggacgccggacccgtccgacgttctggttttcgtcccgtacgatcaa

>AY593794.1_A_COL_1985

cacaacggcgagaagaaaactttctattctagacccaacaaccacgacaactgttggttgaacaccatcctccagttgttcaggtacgtcgacgaacctttcttcgactgggtctacaactcgcccgagaacctcacgcttgaagctatcaagcagttggaggaactcacagggcttgagttgcacgagggcggaccgcctgccctcgtaatttggaacatcaagcacttgctccacaccggcatcggcaccgcttcgcgacctagcgaggtgtgcatggtggacggcacggacatgtgtcttgctgacttccacgcaggcattttcctgaaaggacaggaacacgcagtgtttgcgtgtgtcacctccaacggatggtacgcgatcgacgacgaggacttttacccctggacccctgatccgtcagacgtcctggtgtttgttccgtacgatcaa

>AY593795.1_Asia1_PAK_1954

cataacggtgagaagaagactttttactctagacccaacaaccacgacaactgctggttgaacaccatccttcagctgttcaggtacgtcgatgagcctttctttgactgggtttatgattcacctgaaaacctcacgtttgaggcgatcaggcaactggaggaggttactggtcttgaactgcacgagggcggaccacctgccctcgttatttggaacatcaaacacttgctccacactggcattggcaccgcctcgcgacccagcgaggtgtgtatggtggatggcacggacatgtgtttggctgacttccatgctggcattttcttgaaaggacaggaacacgccgtgtttgcctgcgtcacctccaacgggtggtacgcgatcgacgacgaagacttttacccttggacgccggatccgtccgatgtcctggtgtttgtcccgtatgatcaa

>AY593796.1_Asia1_ISR_1963

tacaacggtgagaggaagactttctactccaggcccaacaaccacgacaactgctggttgaacaccatccttcagctgttcagatatgtcgatgaacctttcttcgactgggtctatgaatcgcccgagaatcttactcttgaggcgatcaggcaactggaagaggttactggtcttgagctgcacgagggtgggccacccgctctcgtcatttggaacattaagcacttgctccacaccggaatcggtactgcctcacgacccagtgaggtgtgcatggtggacggtacggacatgtgcctggctgacttccacgctggcattttcctgaaaggacaagaacatgctgtgtttgcctgtgtcacctccaaagggtggcacgcgatcgacgacgaggacttttacccttggacgccggacccatccgatgtcctggtatttgttccgtacgaccag

>AY593797.1_Asia1_ISR_1963

tacaacggtgagaagaagactttctactccaggcccaacaaccacgacaactgctggttgaacaccatcctccagttgtttaggtacgtcgatgaacctttcttcgactgggtctatgaatcgcctgagaatctcactcttgaggcgatcaggcaactggaagaggttactggtcttgagctgcacgagggtggaccacccgctctcgttatttggaacattaaacacttgctccacaccggaatcggtactgcctcgcgacccagcgaggtgtgtatggtggacggtacggacatgtgcctggctgactttcacgctggtattttcctgaaaggacaagaacacgctgtgtttgcctgtgtcacctccaatgggtggtacgcgatcgacgacgaagacttttacccttggacgccggacccgtccgatgtcctggtatttgtgccgtacgatcaa

>AY593799.1_Asia1_LEB_1983

tacaacggtgagaagaagaccttttactccaggcccaacaaccacgacaactgttggttgaacaccatcctccagctgtttaggtacgttgatgagcctttctttgactgggtttatgactcgcccgagaacctcacccttgctgcaattgaacagttggaagaggttaccggtcttgagctgcacgaaggcgggccacccgctctcgtcatttggaacatcaaacacttgctccacactggaatcggtactgcttcgcgacccagcgaggtgtgtatggtggacggcacggacatgtgtttggctgacttccacgccggcattttcctgaaaggtcaggaacatgccgtgtttgcctgtgtcacctccaacgggtggtatgcgattgatgatgaggacttttacccttggacgccggacccgtccgacgttctggtgtttgttccgtacgatcag

>AY593800.1_Asia1_LEB_1983

tacaacggtgagaagaagaccttttactccaggcccaacaaccacgacaactgctggttgaacaccatcctccagctgtttaggtacgttgatgagcctttctttgactgggtttatgactcgcccgagaacctcacccttgctgcaattgaacagttggaagaggttaccggtcttgagctgcacgaaggcgggccacccgctctcgtcatttggaacatcaaacacttgctccacactggaatcggtactgcttcgcgacccagcgaggtgtgtatggtggacggcacggacatgtgtttggctgacttccacgccggcattttcctgaaaggtcaggaacatgccgtgtttgcctgtgtcacctccaacgggtggtatgcgattgatgatgaggacttttacccttggacgccggacccgtccgacgttctggtgtttgttccgtacgatcag

>AY593802.1_A_URU_2001

tacaacggtgagaagaagacattttactccagacccaacaaccacgacaactgttggttgaacgccatcctccagttgttcaggtacgtcgacgaacctttcttcgactgggtctacaactcgcctgagaacctcacgctctcggccatcgagcagctggaggaaatcaccgggcttgagttgcacgagggcggaccacccgcgctcgtggtttggaacatcaaacacatgctccacactggcatcggcaccgcctcgcgacccagcgaggtgtgcatggtcgacggtacggacatgtgtttggctgatttccatgctggcattttcctgaaaggtcaggagcacgctgtgtttgcatgtgtcacctctgacgggtggtacgcgatcgacgacgaggacttctacccttggacaccagacccgtctgacgtcctggtgtttgtcccgtacgaccaa

>AY593803.1_A_Brazil_1979

cacaacggtgaaaagaaggttttctactccagacccaacaaacacgacaactgttggctgaacaccatcctgcagttgtttaggtatgtcgacgaacctttcttcgactgggtctacaactcacccgagaacctgacgctgtctgccatcaggcagctggaagagctcacgggacttgagttgcacgagggcggaccacctgccctcgtgatctggaacatcaaacacttgctccacaccggcatcggcaccgcctcgcgacccagtgaggtgtgcatggttgacggtacggacatgtgtctggctgatttccacgcaggcattttcctgaaaggcaaagaacacgctgtgttcgcgtgtgtcacctccaacgggtggtacgcggtcgacgacgaggacttctacccttggacaccggacccgtccgacgttctggtgtttgtcccgtacgatcag

>AY593804.1_C_SWZ_1965

cacgacggcgagaaaaaggtcttttactccagacccaacaaccacgacaactgctggttgaacaccatccttcagttgttcaggtacgtcgatgaacctttcttcgactgggtctacaattcgcccgagaacctcacgcttgaagccatcaagcaactggaggaactcacagggttggagttgcgcgagggtggaccgcccgcccttgtgatttggaacatcaagcacctgctccacactggcatcggtaccgcttcgcgacccagcgaggtgtgtatggtggacggtacggacatgtgtcttgctgactttcatgcaggcattttcatgaaaggacaggaacacgctgtctttgcgtgtgtcacctccaacgggtggtacgcgattgatgacgaggacttctacccatggacgccagacccgtctgatgtcctggtatttgttccatacgatcaa

>AY593805.1_C_GER_1960

cacgacggcgagaaaaaggtcttttactccagacccaacaaccacgacaactgctggttgaacaccatccttcagttgttcaggtacgtcgatgaacctttcttcgactgggtctacaattcgcccgagaacctcacgcttgaagccatcaagcaactggaggaactcacagggttggagttgcgcgagggtggaccgcccgcccttgtgatttggaacatcaagcacctgctccacactggcatcggtaccgcttcgcgacccagcgaggtgtgtatggtggacggtacggacatgtgtcttgctgactttcatgcaggcattttcatgaaaggacaggaacacgctgtctttgcgtgtgtcacctccaacgggtggtacgcgattgatgacgaggacttctacccatggacgccagacccgtctgatgtcctggtatttgttccatacgatcaa

>AY593806.1_C_Brazil_1971

cacaacggtgagaagaaaatcttttattccagacccaacaaccacgacaactgttggttgaacaccatcctccagttgttcaggtatgtcgatgaacccttcttcgactgggtctacaactcgcccgagaacctcacgctgtcggccattgaacaactagagaaactcactgggctcgagttgcgcgagggcggaccacccgctctcgtgatttggaacatcaaacacctgctccacaccggcatcggtactgcctcgcgacccagtgaggtgtgtatggttgacggtacggacatgtgtctggctgatttccatgctggcattttcctgaaaggccaggaacacgctgtgtttgcgtgtgtcacctctgacgggtggtacgcgattgacgacgaagacttttacccctggacgccagatccgtctgatgtcctggtatttgtcccgtacgatcaa

>AY593807.1_C_Brazil_1955

tacaccggcgagaagaagactttttactccaggcccaacaaccacgacaactgttggttgaacgccatcctccagttgtttaggtatgtcgatgaaccattcttcgactgggtctacaactcgcccgagaacctgacgcttgaagccatcaagcagctggaagaactcacagggcttgagttgcacgagggtgggccccccgcccttgtgatctggaacatcaaacacttactcaataccggcatcggtacagcctcgcgacccagcgaggtgtgcatggttgacggtacggatatgtgcttggctgatttccacgcaggaattttcctgaaaggacaggaacacgctgtgttcgcgtgtgtcacctccaatgggtggtacgcgattgacgacgaggacttttacccatggacaccggacccgtccgacgtcttggtgtttgtcccgtacgatcaa

>AY593808.1_C_ARG_1966

cacaacggcgagagaaaggtcttttactctagacccaataaccatgacaactgctggttgaacaccatccttcagttgttcaggtatgttgatgaacctttcttcgactgggtctacaactcgcccgagaacctcacacttgaagccattaaacagctagaggaactcacggggttagagctgcacgagggtggaccgcccgcccttgtgatttggaacatcaagcacctgcttcacactggcatcggtaccgcttcgcgacccagcgaggtgtgtatggtggacggtacggacatgtgtcttgctgactttcatgcaggcattttcatgaaaggacaggaacacgctgtgtttgcgtgtgtcacctccaacgggtggtacgcgatcgatgacgaggacttttacccatggacaccagacccgtctgatgttctggtatttgttccgtacgatcaa

>AY593809.1_C_ARG_1969

cacaccggcgagaagaagactttttactccaggcccaacaaccacgacaactgttggttgaacaccatcctccagttgtttaggtatgtcgatgaaccattcttcgactgggtctacaactcgcccgagaacctgacgctcgaagccattaagcagctggaagaactcacagggcttgagttgcacgagggtgggccccccgcccttgtgatctggaacatcaaacacttactcaataccggcatcggtacagcctcgcgacccagcgaggtgtgcatggtcgacggtacggatatgtgcttggctgatttccacgcaggaattttcctgaaaggacaggaacacgccgtgttcgcgtgtgtcacctccaatgggtggtacgcgattgacgacgaggacttttacccatggacaccggacccgtccgacgtcttggtgtttgtcccgtacgatcaa

>AY593810.1_C_UKG_1970

cacaacggcgagaagaagactttttactccaggcccaacaatcacgacaactgttggttgaacaccatcctccagttgtttaggtacgtcgacgaacctttcttcgactgggtctacaactcgcctgagaacctcacactagcagccatcaaacagctggaggaactcacagggcttgagttgcacgagggcggaccacctgctctcgtgatctggaacatcaaacacttgctccaaaccggcattggtaccgcctcgcgacccagcgaggtgtgtatggttgacggcacggacatgtgtctggctgatttccatgcaggcattttcctgaagggacaggaacacgccgtgtttgcgtgtgtcacctctaacgggtggtacgcgatcgacgacgaggacttttacccctggacaccggacccgtccgatgtcctggtgtttgttccgtacgaccaa

>AY593812.1_O_PHI_1958

cacaacggtgagaagaaaaccttttactccagacccaacaaccacgacaactgttggcttaacaccattctccagttgtttaggtatgttgatgagcctttctttgactgggtttacaactcgcctgagaacctcactcttgatgcaattaggcagttggaagagataactggtcttgagctgcacgagggtggaccacccgctctcgtcatttggaacatcaaacatttgctccacaccggaatcggcactgcctcgcgtcctagtgaggtgtgtatggtggacggaacggacatgtgtttggccgatttccacgcaggcatctttctgaaaggacaggaacatgccgtgtttgcctgtgtcacctccaaagggtggtacgcgattgacgacgaggacttctacccttggacaccggacccgtccgacgtcctggtttttgttccgtacgatcaa

>AY593813.1_O_ISA_1962

cacaacggtgagaagaagacattttattcaagacccaacaaccacgacaactgctggttgaacacaatcctccagctgttcaggtacgtcgatgagccgttcttcgactgggtctacaactcgccagagaacctcacgctcgccgccatcgcgcagcttgaggagctcaccgggcttgagttgggtgagggcggtccgcccgccctcgtcatctggaacatcaagcacttgctgcacaccggaatcggcactgcttcgcgacctagcgaggtgtgcatggtagacggaacagacatgtgcttggcagactttcacgctggcattttcctaaagggacaggaacacgctgtgtttgcatgtgtcacacacaatgggtggtacgcgattgacgacgaggacttttacccttggacgccagacccgtccgacgttctggtctttgtcccgtacgatcaa

>AY593814.1_O_ARG_1965

tacaacggtgagaagaagaccttttactccaggcccaacaaccacgacaactgctggttgaacgccatcctccagttgttcaggtacgttgaagaaccattcttcgactgggtctatagttcgcctgagaacctcacgcttgaagccatcaagcagttggaggatctcacagggcttgaactacacgagggtggaccacctgctctcgtgatctggaacatcaagcacttgctccacaccggcattggcaccgcctcgcgacccagcgaggtgtgcatggtggatggtacggacatgtgcttggctgatttccatgcaggcattttccttaaggggcaagaacacgctgtgttcgcgtgtgtcacctccaacgggtggtacgcgattgatgatgaggacttttacccctggacgccggacccgtccgacgttctggtgtttgtcccgtacgatcaa

>AY593815.1_O_UKG_1967

tacaacggtgagaagaagaccttttactccaggcccaacaaccacgacaactgctggttgaacgccatcctccagttgttcaggtacgttgaagaaccattcttcgactgggtctacagttcgcctgagaacctcacgcttgaagccatcaagcagttggaggacctcacagggcttgaactgcacgagggtggaccacctgctctcgtgatctggaacatcaagcacttgctccacaccggcattggcaccgcctcgcgacccagcgaggtgtgcatggtggatggtacggacatgtgcttggctgatttccatgcaggcattttccttaaggggcaagaacacgctgtgttcgcgtgtgtcacctccaacgggtggtacgcgattgatgatgaggacttctacccctggacgccggacccatccgacgttctggtgtttgtcccgtacgatcaa

>AY593816.1_O_UKG_1967

tacaacggtgagaagaagaccttttactccaggcccaacaaccacgacaactgctggttgaacgccatcctccagttgttcaggtacgttgaagaaccattcttcgactgggtctacagttcgcctgagaacctcacgcttgaagccatcaagcagttggaggacctcacagggcttgaactgcacgagggtggaccacctgctctcgtgatctggaacatcaagcacttgctccacaccggcattggtaccgcctcgcgacccagcgaggtgtgcatggtggatggtacggacatgtgcttggctgatttccatgcaggcattttccttaaggggcaagaacacgctgtgttcgcgtgtgtcacctccaacgggtggtacgcgattgatgatgaggacttctacccctggacgccggacccatccgacgttctggtgtttgtcccgtacgatcaa

>AY593817.1_O_Belgium_1973

tacaacggtgagaagaagaccttttactccaggcccaacaaccacgacaactgctggttgaacgccatcctccagttgttcaggtacgtcgaagaaccattcttcgactgggtctacagttcgcctgagaacctcacgcttgaagccatcaagcagttggaggatctcacagggcttgaactgcacgagggtggaccacctgctctcgtgatctggaacatcaagcacttgctccacaccggcatcggcaccgcctcgcgacccagcgaggtgtgcatggtggatggtacggacatgtgcttggctgatttccatgcaggcattttccttaaggggcaagaacacgctgtgtttgcgtgtgtcacctccaacgggtggtacgcgattgatgatgaggacttctacccctggacgccggacccgtccgacgttctggtgtttgtcccgtacgatcaa

>AY593818.1_O_ARG_1958

tacaacggtgagaagaagaccttttactccaggcccaacaaccacgacaactgctggttgaacgccatcctccagttgttcaggtacgttgaagaaccattcttcgactgggtctacagttcgcctgagaacctcacgcttgaagccatcaagcagttggaggatctcacagggcttgaactgcacgagggtggaccacctgctctcgtgatctggaacatcaagcacttgctccacaccggcattggcaccgcctcgcgacccagcgaggtgtgcatggtggatggtacggacatgtgcttggctgatttccatgcaggcattttccttaaggggcaagaacacgctgtgttcgcgtgtgtcacctccaacgggtggtacgcgattgatgatgaggacttctacccctggacgccggacccgtccgacgttctggtgtttgtcccgtacgatcaa

>AY593819.1_O_ARG_1994

tacaacggtgagaagaagaccttttactccaggcccaacaaccacgacaactgctggttgaacgccatcctccagttgttcaggtacgttgaagaaccattcttcgactgggtctacagttcgcctgagaacctcacgcttgaagccatcaagcagttggaggatctcacagggcttgaactgcacgagggtggaccacctgctctcgtgatctggaacatcaagcacttgctccacaccggcattggcaccgcctcgcgacccagcgaggtgtgcatggtggatggtacggacatgtgcttggctgatttccatgcaggcattttccttaaggggcaagaacacgctgtgttcgcgtgtgtcacctccaacgggtggtacgcgattgatgatgaggacttctacccctggacgccggacccgtccgacgttctggtgtttgtcccgtacgatcaa

>AY593820.1_O_ARG_1964

tacaacggtgagaagaagaccttttactccaggcccaacaaccacgacaactgctggttgaacgccatcctccagttgttcaggtacgttgaagaaccattcttcgactgggtctacagttcgcctgagaacctcacgcttgaagccatcaagcagttggaggatctcacagggcttgaactgcacgagggtggaccacctgctctcgtgatctggaacatcaagcacttgctccacaccggcattggcaccgcctcgcgacccagcgaggtgtgcatggtggatggtacggacatgtgcttggctgatttccatgcaggcattttccttaaggggcaagaacacgctgtgttcgcgtgtgtcacctccaacgggtggtacgcgattgatgatgaggacttctacccctggacgccggacccgtccgacgttctggtgtttgtcccgtacgatcaa

>AY593821.1_O_ARG_1967

cacaacggcgagaagaaaaccttctactcaaggcccaacaaccacgacaactgttggctgaacaccatcctccagttgtttaggtatgtcgatgaacctttcttcgactgggtctacagctcacctgagaacctcacgcttgaagcaatcaaacagctggaggaacttacagggcttgagctgcacgagggtggaccacccgcccttgtgatctggaacatcaaacacttgctccaaactggcatcggtaccgcctcgcgacccagcgaggtttgtatggttgatggtacggacatgtgtttggctgacttccacgcaggcatcttcctgaaaggacaagaacacgctgtgttcgcgtgtgtcacctccaacgggtggtatgcgatcgacgacgaggacttttacccgtggacgccggacccgtccggcgttctggtgtttgttccctacgatcag

>AY593823.1_O_TUR_1969

tacaacggtgagaagaaaaccttctactccagacccaacaaccacgacaactgctggcttaacaccattctccagttgttcaggtatgttgatgagcctttctttgactgggtctacgactcgcctgaaaacctcactcttgaggcaatcaaacagttggaagagacaaccggtcttgagctgcacgagggtggaccacccgctctcgtcatctggaacatcaaacacttgcttcacaccggaatcggcactgcctcacgccctagcgaggtgtgtatggtggacggaacggacatgtgtttagctgattttcatgctggcattttcctgaaaggacaggaacatgctgtgttcgcctgtgtcacctccaacgggtggtacgcgattgatgacgaggacttttacccttggacaccggacccgtccgacgttctggtgtttgtcccgtacgatcaa

>AY593824.1_O_SKR_2000

cacaacggtgagaagaaaacattctactccaggcccaacaaccacgacaactgctggctgaacaccatcctccagttgtttaggtacgttgatgaacctttcttcgactgggtctactactcacctgagaacctcacgcttgatgctatcaaacaattggaagaaattactggtctcgaactccacgagggtggaccacccgctctcgttatttggaacattaaacacctgctcaacaccggaatcggcaccgcttcgcgacccagcgaagtgtgcatggtagacgggacggacatgtgtttggctgacttccacgctggcatcttcctgaaaggacaggaacacgctgtgttcgcctgcgtcacctccaacgggtggtacgcgattgacgatgaggacttttacccctggacgccggacccgtccgacgttctggtgtttgtcccgtacgatcaa

>AY593825.1_O_ARG_1939

cacaacggcgaaaagaagacttttttctccaggcccaacaaccacgacaactgttggttgaacaccatccttcagttgttcaggtacgtcgatgaacccttcttcgactgggtctacagctcgcctgagaacctcacgcttgatgccatcaagcagctggaggaactcacagggcttgagttgcgcgagggcggaccacctgcccttgtgatctggaatatcaaacacttgctccaaaccggcatcggtactgcctcgcgacccagcgaggtgtgtatggtggacggcacggacatgtgtctggctgatttccacgcgggcattttcttgaaaggacaggagcacgctgtgtttgcttgtgtcaccaccaacgggtggtacgcgattgatgacgaggacttctacccgtggacgccggacccgtccgacgttctggtgtttgttccgtacgatcaa

>AY593826.1_O_ITL_1947

tacaacggtgaaaagaaaactttctactccaggcccaacacccacgacaactgttggttgaacaccatcctccagttgtttaggtacgtcgatgagcctttcttcgactgggtctacaactcgcccgagaatctcacgcttgatgctatcaagcagttggaagaattcaccgggctcgagttacacgagggcggcccgcctgcccttgtgatctggaacatcaaacacttactccaaaccggcatcggcaccgcctcgcgacctagtgaggtgtgcatggtggacggtacggacatgtgtcttgctgacttccacgcaggcattttcctgaaaggacaggaacacgcagtgtttgcgtgtgtcacctctgacgggtggtacgcgattgacgacgaggacttttacccgtggacgcccgacccatcggacgtcttggtatttgtcccgtacgaccaa

>AY593827.1_O_VEN_1971

tacaacggtgaaaagaaaactttctactccaggcccaataaccacgacaactgttggttgaacgccatcctccagttgtttaggtacgtcgatgagcctttcttcgactgggtctacaactcgcccgagaatctcacgcttgatgctatcaagcagttggaagaactcaccgggcttgagttacacgagggcggcccgcctgcccttgtgatctggaacatcaaacacttactccaaaccggcatcggcaccgcctcgcgacccagcgaggtgtgcatggtggacggtacggacatgtgtcttgctgatttccacgcaggcattttcctgaaaggacaggaacacgcagtgtttgcgtgtgtcacctctgacgggtggtacgcgattgacgacgaggacttttacccgtggacgcctgacccatcagacgtcttggtatttgtcccgtacgaccaa

>AY593828.1_O_IND_1962

tacaacggtgagaagaaaacattttactccagacccaacaaccacgacaactgttggctcaacaccattcttcagttgtttaggtatgttgatgaacctttctttgactgggtttacgactcgcctgagaacctcactctcgaggcaattaggcagttggaagaaataactggtcttgagctgcacgagggtggaccacccgctctcgtcatttggaacatcaaacacttgctccacaccggaatcggtactgcctcacgtcccagtgaggtgtgtatggtggacggaacggacatgtgtttggccgacttccatgctggcatctttctgaaaggacaggaacatgctgtgttcgcctgcgtcacctccaaagggtggtacgcgatcgacgacgaggacttttacccttggacaccggacccgtccgacgtcctggtttttgttccgtacgatcag

>AY593830.1_O_POL_1959

tataacggtgagaagaagaccttttactccaggcccaacaaccacgacaactgctggttgaacgccatcctccagttgttcaggtacgttgaagaaccgttcttcgactgggtctacagttcgcctgagaacctcacgcttgaagccatcaagcagttggaggatctcacagggcttgaactgcacgagggtggaccacctgctctcgtgatctggaacatcaagcacttgctccacaccggcattggcaccgcctcgcgacccagcgaggtgtgcatggtggatggtacggacatgtgcttggccgatttccatgcaggcattttccttaaggggcacgaacacgctgtgttcgcgtgtgtcacctccaacgggtggtacgcgattgatgatgaggacttctacccctggacgccggacccgtccgacgttctggtgtttgtcccgtacgatcag

>AY593831.1_O_UKG_2002

cacaacggtgagaagaaaacattctactccaggcccaacaaacacgacaactgctggctgaacaccatcctccagttgtttaggtacgttgatgaaccttttttcgactgggtctactactcacctgagaacctcacacttgatgctatcaaacaattggaagaaattactggtctcgagctccacgagggtggaccacccgctctcgttatttggaacattaaacacttgctcaacaccggaatcggcaccgcctcgcgacccagcgaagtgtgcatggtagacgggacggatatgtgtttggctgacttccacgctggcatcttcctgaaaggacaggaacacgctgtgttcgcctgcgttacctccaacgggtggtacgcgattgatgacgaggacttttacccctggacgccggacccgtccgacgttctggtgtttgtcccgtacgatcaa

>AY593833.1_O_TAW_1999

tacaacggtgaaaagaaggtcttctactccagacccaacaaccacgacaactgttggctgaacgccatcctccaactgttcaggtacgttgacgagcccttcctcgaatgggtctacgactcacctgagaacctcactctcgaggcgatcaacaaactggaagaaatcacaggtcttgagctacacgagggcggaccgcccgcccttgtcgtctggaacatcaagcacttgctctacaccggaatcggcaccgcttcgcggcccagcgaggtgtgcatggtggacggtacagacatgtgcttggctgacttccacgccggtatatttctgaagggacaggaccacgccgtcttcgcctgcgtcacctctgacgggtggtacgcgattgacgacgaggacttttacccgtggacaccaaatccggccgacgttttggtttttgttccgtacgaccaa

>AY593834.1_O_IRN_1966

cacaacggtgaaaagaagaccttttattccaggcccaacaaccatgacaactgctggttgaacaccatccttcagctgttcaggtatgttgatgaacccttcttcgactgggtttatgactcgcctgagaacctcactcttgaggcaattaggcagttggaagaggtcactggtcttgagttgcacgagggtggaccgcccgcccttgtcatctggaacattaaacacctgctccacaccggagtcggtaccgcctcacgccccagcgaagtgtgtatggtggatggtacggacatgtgtttggctgattttcacgctggcatcttcctgaaaggacaagaacacgctgtgtttgcctgtgtcacctccaacgggtggtacgcgatcgacgacgaggacttttacccctggacgccggacccgtccgatgtcttggtgtttgtcccgtacgatcaa

>AY593835.1_O_TAW_1997

tacaacggtgaaaagaaggtcttctactccagacccaacaaccacgacaactgttggctgaacgccatcctccaactgttcaggtacgttgacgagcccttcctcgaatgggtctacgactcacctgagaacctcactctcgaggcgatcaacaaactggaagaaatcacaggtcttgagctacacgagggcggaccgcccgcccttgtcgtctggaacatcaagcacttgctctacaccggaatcggcaccgcttcgcgacccagcgaggtgtgcatggtggacggtacagacatgtgcttggctgacttccacgccggtatatttctgaagggacaggaccacgccgtcttcgcctgcgtcacctctgacgggtggtacgcgattgacgacgaggacttttacccgtggacaccaaatccggccgacgttttggtttttgttccgtacgatcaa

>AY593836.1_O_UKG_2001

cacaacggtgagaagaaaacattctactccaggcccaacaaacacgacaactgctggttgaacaccatcctccagttgtttaggtacgttgatgaaccttttttcgactgggtctactactcacctgagaacctcacacttgatgctatcaaacaattggaagaaattactggtctcgagctccacgagggtggaccacccgctctcgttatttggaacattaaacacttgctcaacaccggaatcggcaccgcctcgcgacccagcgaagtgtgcatggtagacgggacggatatgtgtttggctgacttccacgctggcatcttcctgaaaggacaggaacacgctgtgttcgcctgcgttacctccaacgggtggtacgcgattgatgacgaggacttttacccctggacgccggacccgtccgacgttctggtgtttgtcccgtacgatcaa

>AY593837.1_O_URU_1963

tacaacggtgagaagaagaccttttactccaggcccaacaaccacgacaactgctggttgaacgccatcctccagttgttcaggtacgttgaagaaccattcttcgactgggtctacagttcgcctgagaacctcacgcttgaagccatcaagcagttggaggatctcacagggcttgaactgcacgagggtggaccacctgctctcgtgatctggaacatcaagcacttgctccacaccggcattggcaccgcctcgcgacccagcgaggtgtgcatggtggatggtacggacatgtgcttggctgatttccatgcaggcattttccttaaggggcaagaacacgctgtgttcgcgtgtgtcacctccaacgggtggtacgcgattgatgatgaggacttctacccctggacgccggacccgtccgacgttctggtgtttgtcccgtacgatcaa

>AY593838.1_SAT1_BOT_1970

tacaacggagagaagaagaccttctacagcaggcccaatacgcacgggaattgctggctcaactcgttgttgcagctctttcgatacgtcgatgagcctctcttcgagtctgagtacctgtcacctgaaaataagacattggatatgatcaaacaactttctgattacactaaactagacttgtcagacggtgggccacccgccctcgtcctgtggctgatcaaggactgtcttcaaactggcgttggcaccagcactcgccctagcgagatctgtgtgatcaacggggttgtcatgaccctggctgatttccacgccggcattttcatcaagggtaccgaacacgctgtgttcgccctcaacacatctgagggctggtacgccattgatgatgaggtgttctacccttggacacctgaccctgagaacgtgctcgtgtatgtgccctacgaccaa

>AY593839.1_SAT1_UKG_1970

tacaacggagagaagaagaccttctacagcaggcccaacacacacggcaactgctggctcaactctttactgcagctctttcgatacgtcgatgagccgctctttgagtctgagtacctctccccggagaacaagacattggacatgatcaaacaactgactgattataccaaacttgacctttcagacggtgggccaccggcactcgtgctttggctcatcaaagactgtcttcagaccggcgttggcaccagcactcgccccagcgagatctgtgtgatcaacggagtagccatgactctggctgacttccacgccggtattttcatcaaaggcgaagaacacgccgtgttcgccctcaacacatctgagggctggtacgccattgatgatgaagtgttttacccatggacacccgaccccgaaaacgtactcgcgtacgtgccctacgaccag

>AY593840.1_SAT1_NMB_1949

tacaacggagagaagaagaccttctacagcagacccaacacacacgggaactgttggctcaactcgttgctgcagctctttcgatacgtcgatgagcccctcttcgagtctgagtacttgtcacctgaaaacaagacattggacatgatcaaacaactttctgattacaccaaattggacttgtcggacggtgggcctcccgctctcgtcctttggctgatcaaagactgtcttcaaactggtgttggcaccagcactcgccccagcgagatctgtgtgatcaacggggtcgtcatgaccctggctgacttccacgccggtatcttcatcaaaggcaccgaacacgccgtgttcgccctcaacacatccgagggctggtacgccattgatgacgagggtttttacccatggacacccgaccctgcgaacgtactcgcgtacgtcccctacgaccag

>AY593841.1_SAT1_ZIM_1958

tacaacggtgagaagaagaccttctacagcagacccaacacacacgggaactgttggctcaactcgttgctacagctctttcgatacgtcgatgagccgctcttcgagtctgagtacctgtcacctgaaaacaagacattggacatgatcaaacaactctctgattacaccaaattggacttgtcggacggtgggcccccagccctcgtcctttggctgatcaaggattgtcttcagaccggcgttggcaccagcactcgccccagcgagatctgtgtgatcaacggggttgtcatgaccctggctgacttccacgctggcattttcatcaagggtaccgagcacgctgtgttcgctctcaacacatctgagggctggtatgccattgatgatgaggtgttctacccctggacacccgaccctgagaacgtacttgcgtacgtcccctacgaccag

>AY593842.1_SAT1_SAR_1961

tacaacggagagaagaagatcttctacagcaggcccaacaaacatgggaactgttggctcaactcgctgttgcagctctttcgatacgtcgatgagccactctttgagtcagagtatttgtcacctgaaaacaagacattggatatgatcaaacaactctctgattacaccaaattggatttgtcggacggtgggccacccgctctcgtcctctggctgatcaaggattgtcttcaaactggcgttggcactagcactcgccccagcgagatctgtgtcatcaacggggttgtcatgaccctggctgattttcacgccggcatcttcatcaaaggtactgagcacgctgtgttcgccctcaacacatccgagggctggtacgccattgatgatgagatattctacccatggacacctgaccctgagaacgtgctcgcgtacgttccctacgaccag

>AY593843.1_SAT1_NMB_1940

tacaacggagagaagaagaccttctacagcagacccaacaaacacgggaactgttggctcaactcattactgcagctctttcgatacgtcgacgagccgctctttgagtctgagtatttgtcacctgaaaacaagacattggacatgatcaaacaactatctgattacaccaaattggacttgtcggacggagggccccccgctctcgtcctttggctgatcaaagactgtcttcaaaccggtgttggcaccagtactcgccccagcgagatctgtgtcatcaacggggttgtcatgaccctggctgatttccacgccggtatcttcatcaaaggcactgaacacgctgtgttcgccctcaacacatccgagggatggtacgccattgatgatgaggtgttctacccgtggacacctgaccctgaaaacgtactcgcgtacgtgccctacgaccag

>AY593844.1_SAT1_ISR_1962

tacaacggtgagaagaaaacattctactccagacccaaccgccatgataactgctggttgaacaccatcctacagttgttcaggtatgtcgatgaaccattcttcgactgggtttacaactcgcccgagaacctcacactccaagcaattgaacaactggaggaactcactgggcttgagttgcgcgagggtggacctcccgctctcgtgatttggagcatcaaacacttgctctacaccggaatcggcactgcctcgcgacccagtgaggtgtgcatggtagacggtactgacatgtgtcttgctgatttccatgcaggaatcttcctgaaaggtactgaacacgccgtgtttgcctgtttgacctccaacggctggtatgccatcgacgatgaggacttttacccgtggacaccagacccatctgatgtcctgtgttttgtcccatacgacatg

>AY593845.1_SAT1_BOT_1968

tacaacggagagaagaagaccttctacagcagacccaacaaacacgggaactgctggctcaactcactgctgcagctctttcgatacgtcgatgagccgctctttgagtctgagtacttgtcacctgaaaacaagacattggacatgatcaaacaactatctgattacaccaaattggacttgtcggacggagggccccccgctctcgtcctttggctgatcaaagactgtcttcaaaccggcgttggcactagcactcgccccagcgagatctgtgttatcaacggggttgtcatgaccctggctgactttcacgccggcatttttatcaaaggcactgaacacgctgtgttcgccctcaacacatccgagggctggtacgccattgatgatgaggtgttctatccatggacacccgaccctgagaacgtgctcgcgtacgtcccctacgaccag

>AY593846.1_SAT1_ZIM_1966

tacaacggagagaagaagaccttttacagcaggcccaacacccacgggaactgttggctcaactcactcctgcagctctttcgatacgtcgacgagccgctgtttgaggctgagtatctttcacctgaaaacaagacactggacatgatcaagcaattatctgattacaccaaacttgacctttcagatggtgggccaccggcacttgtgctctggctcatcaaagactgtcttcaaaccggcgttggcaccagcactcgtcccagcgagatctgtgtgatcaacggggtcaccatgactctggctgatttccacgctggtattttcatcaagggcacagaacacgctgtgttcgctctcaacacatctgagggctggtacgccattgatgacgaggtgttctatccgtggacacccgaccctgagaacgtgctcgcgtacgtcccctacgaccag

>AY593847.1_SAT2_ZIM_1948

tacaacggtgaaaagaagaccttctacagcaggcccaacacacacggcaactgctggctcaactctctactgcagctctttcgatacgtcgatgagccgctgtttgagtctgagtacctctcaccagaaaacaagacattggacatgatcaaacaactttcagattacaccaagcttgacctttctaatggtgggccacctgcactcgtgttgtggctcatcaaagactgtcttcagaccggtgttggcaccagcactcgccccagcgagatctgtgtgatcaacggagtcaccatgactctggctgatttccacgccggcattttcatcaaaggcaccgaacacgcagtgtttgccctcaacacatccgagggatggtacgccattgatgatgaggtgttctacccgtggacacccgaccccgagaacgtgctcgcgtacgtcccctacgaccag

>AY593848.1_SAT2_u_1967

tacaacggagagaagaagaccttctacagcaggcctaacacacacggcaactgctggctcaactcattgttgcagctctttcgatacgtcgacgagccgctctttgagagtgagtacctttcaccagagaacaagacattggacatgatcaaacaactatctgactacaccaaacttgacctctcagacggcggaccaccagcacttgtgctttggctcatcaaagaccgtcttcagaccggcgtcggcaccagtactcgccccagcgagatctgtgtgatcaacggagtcaccatgactctggctgactttcacgccggcatcttcatcaaaggcactgaacacgctgtgttcgccctcaacacttctgagggctggtacgccattgatgacgaggtgtactacccgtggacacctgaccctgagaacgtactcgcatacgtgccctacgaccag

>AY593849.1_SAT2_KEN_1960

tacaacggtgagaagaaaactttctactccagacccaatacgcacgacaactgttggctgaataccattttacagttgtttcgttacgtcgatgaacctttcttcgactgggtctacaactcgcccgagaacctcacgctgcgagcgattgaacagctcgaggaactcactgggcttgagttgcgcgagggaggaccacccgccctcgtgatttggaacatcaagcacttgctctacactggcatcggcacggcctcgcggcccagcgaggtatgcatggttgacggtactgacatgtgtcttgctgatttccacgcaggtatcttcttgaaaggtactgagcatgccgtgttcgccctgttgacttccgacggatggtacgccatagacgatgaggacttttacccatggaccccggacccgtccgatgtcctgtgtttcgtcccatacgacatg

>AY593850.1_SAT3_SAR_1959

tacaacggtgagaagaagaccttctacagcaggcccaacacacgcgggaactgttggctcaactcactcttacagctctttcgatacgtcgatgagccgctgtttgagtctgaatatctgtcacccgaagacaagacattggacatgatcaaacaactttctgattacaccaaacttgacctttcggacggtgggccacccgcactcgtgctgtggctcatcaaggactgtctgcaaaccggcgttggcaccagcactcgccccagcgagatctgtgtgatcaacggggtcaccatgactctggccgatttccacgccggaattttcatcaagggtactgaacacgccgtgttcgctctcaacacatccgagggctggtacgccatagatgatgaggtgttctacccgtggacacccgaccctgacagcgtgcttgcgtacgtgccctacgaccag

>AY593851.1_SAT3_BOT_1961

tacaacggagagaagaagactttctacagcaggcccaacacacacgggaactgctggctcaactctctgctgcagctctttcgatacgtcgatgagcctctctttgagtctgagtacctgtcacctgagaataagacattggacatgatcaaacagctttctgactacacgaaactggacttgacggacggtgggcctcccgccctcgttctttggctgattaaggactgtcttcagaccggtgttggcaccagcactcgccccagcgagatctgtgtgatcaacggggttgtcatgaccctggctgacttccacgccggtatcttcatcaaaggcactgaacacgctgtgttcgccctcaacacgtctgagggttggtacgctattgatgatgaggtgttttacccctggacacctgaccctgagaacgtgctcgcgtacgtaccatacgaccag

>AY593852.1_SAT3_KEN_1960

tacaacggagagaagaagactttctacagcaggcccaacacacacgggaactgctggctcaactctctgctgcagctctttcgatacgtcgatgagcctctctttgagtctgagtacctgtcacctgagaataagacattggacatgatcaaacagctttctgactacacgaaactggacttgacggacggtgggcctcccgccctcgttctttggctgattaaggactgtcttcagaccggtgttggcaccagcactcgccccagcgagatctgtgtgatcaacggggttgtcatgaccctggctgacttccacgccggtatcttcatcaaaggcactgaacacgctgtgttcgccctcaacacgtctgagggttggtacgctattgatgatgaggtgttttacccctggacacctgaccctgagaacgtgctcgcgtacgtaccatacgaccag

>AY593853.1_SAT3_BOT_1965

tacaacggagagaagaagaccttctacagcagacccaacacacacgggaactgttggctcaactcgttgttgcagctctttcgatacgtcgatgagcccctcttcgagtctgagtacctatcacctgagaacaagacattggatatgatcaaacaactctctgattacactaaactggacttgtcggacggtgggcctccagccctcgtcctttggctgattaaagactgtcttcaaaccggcgttggcaccagcactcgccccagcgagatctgcgtgatcaacggggttgtcatgaccctggctgacttccacgctggcatttttatcaagggtaccgagcacgctgtgttcgctctcaatacatctgagggctggtacgccattgatgatgaggtgttctacccctggacacccgacccagagaacgtacttgcgtacgtcccctacgaccag

>AY686687.1_O_CHA_2001

tacaacggtgagaagaaggtcttctactccagacccaacaaccacgacaactgttggctgaacgccatcctccaactgttcaggtatgttgacgaacctttcctcgagtgggtctacgactcacctgaggacctcactctcgaggcgatcagcaagctggaagaaaacaccggtcttgagctacacgagggcggaccacccgcccttgttgtctggaacatcaaacacttgctctacaccggaatcggcaccgcttcgcggcccagcgaggtgtgtatggtggacggcacagacatgtgtttggctgacttccacgccggtatatttctaaagggacaggatcacgccgtgttcgcctgcgtcacctccgacgggtggtacgcggttgacgacgaggatttttacccgtggacaccagatccggccgatgttttggtttttgttccgtacgaccag

>AY687333.1_Asia1_IND_2001

tacaacggcgagaagaagactttttactctagacccaacaaccatgacaactgttggctgaacaccatcctccagttgttcaggtacgtcgacgaacctttcttcgactgggtctatgactcacctgaaaacctcactcttgatgcaatcaaacaactggaagaactcactggtcttgagctgcacgagggtggaccgcccgccctagtcatttggaacatcaagcacttgctccacaccggaatcggtaccgcctcgcgacccagcgaggtgtgtatggtggacgaaacagacatgtgcttggctgacttccatgctggcatcttcttgaaaggacaagagcatgctgtgtttgcctgtgtcacctccaacgggtggtacgcgatcgacgacgaagacttttacccttggacgccagatccgtccgacgttctggtatttgtcccgtacgatcaa

>DQ404158.1_O_UKG_2001

cacaacggtgagaagaaaacattctactccaggcccaacaaacacgacaactgctggctgaacaccatcctccagttgtttaggtacgttgatgaaccttttttcgactgggtctactactcacctgagaacctcacacttgatgctatcaaacaactggaagaaattactggtctcgagctccacgagggtggaccacccgctctcgttatttggaacattaaacacttgctcaacaccggaatcggcaccgcctcgcgacccagcgaagtgtgcatggtagacgggacggatatgtgtttggctgacttccacgctggcatcttcctgaaaggacaggaacacgctgtgttcgcctgcgttacctccaacgggtggtacgcgattgatgacgaggacttttacccctggacaccggacccgtccgacgttctggtctttgtcccgtacgatcaa

>DQ404159.1_O_UKG_2001

cacaacggtgagaagaaaacattctactccaggcccaacaaacacgacaactgctggctgaacaccatcctccagttgtttaggtacgttgatgaaccttttttcgactgggtctactactcacctgagaacctcacacttgatgctatcaaacaactggaagaaattactggtctcgagctccacgagggtggaccacccgctctcgttatttggaacattaaacacttgctcaacaccggaatcggcaccgcctcgcgacccagcgaagtgtgcatggtagacgggacggatatgtgtttggctgacttccacgctggcatcttcctgaaaggacaggaacacgctgtgttcgcctgcgttacctccaacgggtggtacgcgattgatgacgaggacttttacccctggacaccggacccgtccgacgttctggtctttgtcccgtacgatcaa

>DQ404160.1_O_UKG_2001

cacaacggtgagaagaaaacattctactccaggcccaacaaacacgacaactgctggctgaacaccatcctccagttgtttaggtaygttgatgaaccttttttcgactgggtctactactcacctgagaacctcacacttgatgctatcaaacaactggaagaaattactggtctcgagctccacgagggtggaccacccgctctcgttatttggaacattaaacacttgctcaacaccggaatcggcaccgcctcgcgacccagcgaagtgtgcatggtagacgggacggatatgtgtttggctgacttccacgctggcatcttcctgaaaggacaggaacacgctgtgttcgcctgcgttacctccaacgggtggtacgcgattgatgacgaggacttttacccctggacaccggacccgtccgacgttctggtctttgtcccgtacgatcaa

>DQ404161.1_O_UKG_2001

cacaacggtgagaagaaaacattctactccaggcccaacaaacacgacaactgctggctgaacaccatcctccagttgtttaggtacgttgatgaaccttttttcgactgggtctactactcacctgagaacctcacacttgatgctatcaaacaactggaagaaattactggtctcgagctccacgagggtggaccacccgctctcgttatttggaacattaaacacttgctcaacaccggaatcggcaccgcctcgcgacccagcgaagtgtgcatggtagacgggacggatatgtgtttggctgacttccacgctggcatcttcctgaaaggacaggaacacgctgtgttcgcctgcgttacctccaacgggtggtacgcgattgatgacgaggacttttacccctggacgccggacccggccgacgttctggtgtttgtcccgtacgatcaa

>DQ404162.1_O_UKG_2001

cacaacggtgagaagaagacattctactccaggcccaacaaacacgacaactgctggctgaacaccatcctccagttgtttaggtacgttgatgaaccttttttcgactgggtctactactcacctgagaacctcacacttgatgctatcaaacaattggaagaaattactggtctcgagctccacgagggtggaccacccgctctcgttatttggaacattaaacacttgctcaacaccggaatcggcaccgcctcgcgacccagcgaagtgtgcatggtagacgggacggacatgtgtttggctgacttccacgctggcatcttcctgaaaggacaggaacacgctgtgttcgcctgcgttacctccaacgggtggtacgcgattgatgacgaggacttttacccctggacgccggacccgtccgacgttctggtgtttgtcccgtacgatcaa

>DQ404163.1_O_UKG_2001

cacaacggtgagaggaaaacattctactccaggcccaacaaacacgacaactgctggctgaacaccatcctccagttgtttaggtacgttgatgaaccttttttcgactgggtctactactcacctgagaacctcacacttgatgctatcaaacaattggaagaaattactggtctcgagctccacgagggtggaccacccgctctcgttatatggaacattaaacacttgctcaacaccggaatcggcaccgcctcgcgacccagcgaagtgtgcatggtagacgggacggatatgtgtttggctgacttccacgctggcatcttcctgaaaggacaggaacacgctgtgttcgcctgcgttacctccaacgggtggtacgcgattgatgacgaggacttttacccctggacgccggacccgtccgacgtcctggtgtttgtcccgtacgatcaa

>DQ404164.1_O_UKG_2001

cacaacggtgagaagaaaacattctactccaggcccaacaaacacgacaactgctggctgaacaccatcctccagttgttcaggtacgttgatgaaccttttttcgactgggtctactactcacctgagaacctcacacttgatgctatcaaacaattggaagaaattactggtctcgagctccacgagggtggaccacccgctctcgttatttggaacattaaacacttgctcaacaccggaatcggcaccgcctcgcgacccagcgaagtgtgcatggtagacgggacggatatgtgtttggctgacttccacgctggcatcttcatgaaaggacaggaacacgctgtgttcgcctgcgttacctccaacgggtggtacgcgattgatgacgaggacttttacccctggacgccggacccgtccgacgttctggtgtttgtcccgtacgatcaa

>DQ404165.1_O_UKG_2001

cacaacggtgagaagaaaacattctactccaggcccaacaaacacgacaactgctggctgaacaccatcctccagttgtttaggtacgttgatgaaccttttttcgactgggtctaccactcacctgagaacctcacacttgatgctatcaaacaattggaagaaattactggtctcgagctccacgagggtggaccacccgctctcgttatttggaacattaaacacttgctcaacaccggaatcggcaccgcctcgcgacccagcgaagtgtgcatggtagacgggacggacatgtgtttggctgacttccacgctggcatcttcctgaaaggacaggaacacgctgtgttcgcctgcgttacctccaacgggtggtacgcgattgatgacgaggacttttacccctggacgccggacccgtccgacgttctggtgtttgtcccgtacgatcaa

>DQ404166.1_O_UKG_2001

cacaacggtgagaagaaaacattctactccaggcccaacaaacacgacaactgctggctgaacaccatcctccagttgtttaggtacgttgatgaaccttttttcgactgggtctaccactcacctgagaacctcacacttgatgctatcaaacaattggaagaaattactggtctcgagctccacgagggtggaccacccgctctcgttatttggaacattaaacacttgctcaacaccggaatcggcaccgcctcgcgacccagcgaagtgtgcatggtagacgggacggacatgtgtttggctgacttccacgctggcatcttcctgaaaggacaggaacacgctgtgttcgcctgcgttacctccaacgggtggtacgcgattgatgacgaggacttttacccctggacgccggacccgtccgacgttctggtgtttgtcccgtacgatcaa

>DQ404167.1_O_UKG_2001

cacaacggtgagaagaaaacattctactccaggcccaacaaacacgacaactgctggctgaacaccatcctccagttgtttaggtacgttgatgaaccttttttcgactgggtctaccactcacctgagaacctcacacttgatgctatcaaacaattggaagaaattactggtctcgagctccacgagggtggaccacccgctctcgttatttggaacattaaacacttgctcaacaccggaatcggcaccgcctcgcgacccagcgaagtgtgcatggtagacgggacggacatgtgtttggctgacttccacgctggcatcttcctgaaaggacaggaacacgctgtgttcgcctgcgttacctccaacgggtggtacgcgattgatgacgaggacttttacccctggacgccggacccgtccgacgttctggtgtttgtcccgtacgatcaa

>DQ404168.1_O_UKG_2001

cacaacggtgagaagaaaacattctactccaggcccaacaaacacgacaactgctggctgaacaccatcctccagttgtttaggtacgttgatgaaccttttttcgactgggtctactactcacctgagaacctcacacttgatgctatcaaacaattggaagaaattactggtctcgagctccacgagggtggaccacccgctctcgttatttggaacattaaacacttgctcaacaccggaatcggcaccgcctcgcgacccagcgaagtgtgcatggtagacgggacggatatgtgtttggctgacttccacgctggcatcttcctgaaaggacaggaacacgctgtgttcgcctgcgttacctccaacgggtggtacgcgattgatgacgaggacttttacccctggacgccggacccgtccgacgttctggtgtttgtcccgtacgatcaa

>DQ404169.1_O_UKG_2001

cacaacggtgagaagaaaacattctactccaggcccaacaaacacgacaactgctggctgaacaccatcctccagttgtttaggtacgttgatgaaccttttttcgactgggtctactactcacctgagaacctcacacttgatgctatcaaacaattggaagaaattactggtctcgagctccacgagggtggaccacccgctctcgttatttggaacattaaacacttgctcaacaccggaatcggcaccgcctcgcgacccagcgaagtgtgcatggtagacgggacggacatgtgtttggctgacttccacgctggcatcttcctgaaaggacaggaacacgctgtgttcgcctgcgttacctccaacgggtggtacgcgattgatgacgaggacttttacccctggacgccggacccgtccgacgttctggtgtttgtcccgtacgatcaa

>DQ404170.1_O_UKG_2001

cacaacggtgagaagaaaacattctactccaggcccaacaaacacgacaactgctggctgaacaccatcctccagttgtttaggtacgttgatgaaccttttttcgactgggtctaccactcacctgagaacctcacacttgatgctatcaaacaattggaagaaattactggtctcgagctccacgagggtggaccacccgctctcgttatttggaacattaaacacttgctcaacaccggaatcggcaccgcctcgcgacccagcgaagtgtgcatggtagacgggacggacatgtgtttggctgacttccacgctggcatcttcctgaaaggacaggaacacgctgtgttcgcctgcgttacctccaacgggtggtacgcgattgatgacgaggacttttacccctggacgccggacccgtccgacgttctggtgtttgtcccgtacgatcaa

>DQ404171.1_O_UKG_2001

cacaacggtgagaagaaaacattctactccaggcccaacaaacacgacaactgctggctgaacaccatcctccagttgtttaggtacgttgatgaaccttttttcgactgggtctactactcacctgagaacctcacacttgatgctatcaaacaattggaagaaattactggtctcgagctccacgagggtggaccacccgctctcgttatttggaacattaaacacttgctcaacaccggaatcggcaccgcctcgcgacccagcgaagtgtgcatggtagacgggacggatatgtgtttggctgacttccacgctggcatcttcctgaaaggacaggaacacgctgtgttcgcctgcgttacctccaacgggtggtacgcgattgatgacgaggacttttacccctggacgccggacccgtccgacgttctggtgtttgtcccgtacgatcaa

>DQ404172.1_O_UKG_2001

cacaacggtgagaagaaaacattctactccaggcccaacaaacacgacaactgctggctgaacaccatcctccagttgtttaggtacgttgatgaaccttttttcgactgggtctactactcacctgagaacctcacacttgatgctatcaaacaattggaagaaattactggtctcgagctccacgagggtggaccacccgctctcgttatttggaacattaaacacttgctcaacaccggaatcggcaccgcctcgcgacccagcgaagtgtgcatggtagacgggacggatatgtgtttggctgacttccacgctggcatcttcctgaaaggacaggaacacgctgtgttcgcctgcgttacctccaacgggtggtacgcgattgatgacgaggacttttacccctggacgccggacccgtccgacgttctggtgtttgtcccgtacgatcaa

>DQ404173.1_O_UKG_2001

cacaacggtgagaagaaaacattctactccaggcccaacaaacacgacaactgctggctgaacaccatcctccagttgtttaggtacgttgatgaaccttttttcgactgggtctactactcacctgagaacctcacacttgatgctatcaaacaattggaagaaattactggtctcgagctccacgagggtggaccacccgctctcgttatttggaacattaaacacttgctcaacaccggaatcggcaccgcctcgcgacccagcgaagtgtgcatggtagacgggacggatatgtgtttggctgacttccacgctggcatcttcctgaaaggacaggaacacgctgtgttcgcctgcgttacctccaacgggtggtacgcgattgatgacgaggacttttacccctggacgccggacccgtccgacgttctggtgtttgtcccgtacgatcaa

>DQ404174.1_O_UKG_2001

cacaacggtgagaagaaaacattctactccaggcccaacaaacacgacaactgctggctgaacaccatcctccagttgtttaggtacgttgatgaaccttttttcgactgggtctactactcacctgagaacctcacacttgatgctatcaaacaattggaagaaattactggtctcgagctccacgagggtggaccacccgctctcgttatttggaacattaaacacttgctcaacaccggaatcggcaccgcctcgcgacccagcgaagtgtgcatggtagacgggacggatatgtgtttggctgacttccacgctggcatcttcctgaaaggacaggaacacgctgtgttcgcctgcgttacctccaacgggtggtacgcgattgatgacgaggacttttacccctggacgccggacccgtccgacgttctggtgtttgtcccgtacgatcaa

>DQ404175.1_O_UKG_2001

cacaacggtgagaagaaaacattctactccaggcccaacaaacacgacaactgctggctgaacaccatcctccagttgtttaggtacgttgatgaaccttttttcgactgggtctactactcacctgagaacctcacacttgatgctatcaaacaattggaagaaattactggtctcgagctccacgagggtggaccacccgctctcgttatttggaacattaaacacttgctcaacaccggaatcggcaccgcctcgcgacccagcgaagtgtgcatggtagacgggacggatatgtgtttggctgacttccacgctggcatcttcctgaaaggacaggaacacgctgtgttcgcctgcgttacctccaacgggtggtacgcgattgatgacgaggacttttacccctggacgccggacccgtccgacgttctggtgtttgtcccgtacgatcaa

>DQ404176.1_O_UKG_2001

cacaacggtgagaagaaaacattctactccaggcccaacaaacacgacaactgctggctgaacaccatcctccagttgtttaggtacgttgatgaaccttttttcgactgggtctactactcacctgagaacctcacacttgatgctatcaaacaattggaagaaattactggtctcgagctccacgagggtggaccacccgctctcgttatttggaacattaaacacttgctcaacaccggaatcggcaccgcctcgcgacccagcgaagtgtgcatggtagacgggacggatatgtgtttggctgacttccacgctggcatcttcctgaaaggacaggaacacgctgtgttcgcctgcgttacctccaacgggtggtacgcgattgatgacgaggacttttacccctggacgccggacccgtccgacgttctggtgtttgtcccgtacgatcaa

>DQ404177.1_O_UKG_2001

cacaacggtgagaagaaaacattctactccaggcccaacaaacacgacaactgctggctgaacaccatcctccagttgtttaggtacgttgatgaaccttttttcgactgggtctactactcacctgagaacctcacacttgatgctatcaaacaattggaagaaattactggtctcgagctccacgagggtggaccacccgctctcgttatttggaacattaaacacttgctcaacaccggaatcggcaccgcctcgcgacccagcgaagtgtgcatggtagacgggacggatatgtgtttggctgacttccacgctggcatcttcctgaaaggacaggaacacgctgtgttcgcctgcgttacctccaacgggtggtacgcgattgatgacgaggacttttacccctggacgccggacccgtccgacgttctggtgtttgtcccgtacgatcaa

>DQ404178.1_O_UKG_2001

cacaacggtgagaagaaaacattctactccaggcccaacaaacacgacaactgctggctgaacaccatcctccagttgtttaggtacgttgatgaaccttttttcgactgggtctactactcacctgagaacctcacacttgatgctatcaaacaattggaagaaattactggtctcgagctccacgagggtggaccacccgctctcgttatttggaacattaaacacttgctcaacaccggaatcggcaccgcctcgcgacccagcgaagtgtgcatggtagacgggacggatatgtgtttggctgacttccacgctggcatcttcctgaaaggacaggaacacgctgtgttcgcctgcgttacctccaacgggtggtacgcgattgatgacgaggacttttacccctggacgccggacccgtccgacgttctggtgtttgtcccgtacgatcaa

>DQ404179.1_O_UKG_2001

cacaacggtgagaagaaaacattctactccaggcccaacaaacacgacaactgctggctgaacaccatcctccagttgtttaggtacgttgatgaaccttttttcgactgggtctactactcacctgagaacctcacacttgatgctatcaaacaattggaagaaattactggtctcgagctccacgagggtggaccacccgctctcgttatttggaacattaaacacttgctcaacaccggaatcggcaccgcctcgcgacccagcgaagtgtgcatggtagacgggacggatatgtgtttggctgacttccacgctggcatcttcctgaaaggacaggaacacgctgtgttcgcctgcgttacctccaacgggtggtacgcgattgatgacgaggacttttacccctggacgccggacccgtccgacgttctggtgtttgtcccgtacgatcaa

>DQ404180.1_O_UKG_2001

cacaacggtgagaagaaaacattctactccaggcccaacaaacacgacaactgctggctgaacaccatcctccagttgtttaggtacgttgatgaaccttttttcgactgggtctactactcacctgagaacctcacacttgatgctatcaaacaattggaagaaattactggtctcgagctccacgagggtggaccacccgctctcgttatttggaacattaaacacttgctcaacaccggaatcggcaccgcctcgcgacccagcgaagtgtgcatggtagacgggacggatatgtgtttggctgacttccacgctggcatcttcctgaaaggacaggaacacgctgtgttcgcctgcgttacctccaacgggtggtacgcgattgatgacgaggacttttacccctggacgccggacccgtccgacgttctggtgtttgtcccgtacgatcaa

>DQ989303.1_Asia1_IND_1993

tacaacggtgagaagaagaccttttactccaggcccaacaactacgacaactgttggttgaacactatccttcagttgtttagatacgtcgatgatcctttcttcgactgggtctatgaatcacccgagaaccttacccttgaggcgatcaatcaattggaagaggttactggtcttgaactgcacgagggtgggccacctgctctcgtcatctggaacatcaagcacttgctccacaccggaatcggtaccgcctcgcgaccaagcgaagtgtgtatgggggacggcacggacatgtgcctggcagaccaccatgcgggcattttcctgaaaggacaggaacatgctgtgtttgcttgggacacctccaacgggtggtacgctattgacgacgaggattttaccctctggacgccggacccgtccgatgttctggtatatgtgccgtacgatcaa

>DQ989304.1_Asia1_IND_2000

tacaacggtgagaagaagaccttctactcgaggcccaacaaccacgacaactgttggttgaacaccatcctccagttgtttaggtacgtcgatgaacctttcttcgactgggtctatgaatcacctgagaacctcactcttgaggcgatcaaacaattggaagaggttactggtcttgaactcacggagggtgggccacccgctctcgtcatctggaacatcaagcacttgcttcacaccggaatcggtaccgcctcgcgaccaagcgaagtgtgtatggtggacggcacggacatgtgcctggctgacttccacgctggcattttcctgaaaggacaggaacatgctgtgtttgcctttgtcacctccaacgggtggtacgcgattgacgacgaggacttttacccctggacgccggacccgtccgatgtcctggtatatgttccgtacgatcaa

>DQ989305.1_Asia1_IND_1990

cacaacggtgagaagaaaaccttctactctagacccaacaaccacgacaactgttggttgaacaccatactccagttgtttaggtacgtcgatgagcccttcttcgactgggtttatgactcacctgagaaccttacccttgatgccatcaaacaattggaagagatcactggtcttgagttgacggagggtggaccacccgctcttgttatctggaacatcaaacacttgctccacaccggaatcggcaccgcctcacgccccagtgaggtgtgcatggttgacggaacggacatgtgtctggctgactttcacgccggcattttcttgaaaggacaggaacacgctgtgttcgcctgcatcacctccaaagggtggtacgcgatcgatgacgaggacttctacccctggacaccggacccgtccgacgtcctggtatttgttccgtacgatcaa

>DQ989306.1_Asia1_IND_1986

tacaacggtgagaagaagaccttctactctaggcccaacaaccacgacaactgttggttgaacaccatcccccagttgtttaggtacgtcgatgaaccattcttcgactgggtctatgagtcacctgcgaacctcactcttgaggcgatcaaacaattggaaaaggttactggtcttgaactcaccgagggtggcccacccgctctcgtaatctggaacatcaggcacttgcttcacaccggaatcggtaccgcctcgcgcccaagcgaagtgtgtatggtcggcggcacggacatgtgcctggctgacttccacgctggcattttcctgaaaggacaggaacacgctgtgtttgcctgtgtcacctccaacgggtggtacgcgattgacgacgaggacttttacccctggacgccggacccgtccgatgtcctggtatatgttccgtacgatcaa

>DQ989307.1_Asia1_IND_1992

cacaacggtgagaagaaaaccttctactctagacccaacaaccacgacaactgttggttgaacaccatactccagttgtttaggtacgtcgatgagcccttcttcgactgggtttatgactcacctgagaaccttacccttgatgccatcaaacaattggaagagatcactggtcttgagttgcacgagggtggaccacccgctcttgttatttggaacatcaaacacttgctccacaccggaatcggcaccgcctcacggcccagtgaggtgtgcatggttgacggaacggacatgtgtctggctgactttcacgccggcattttcttgaaaggacaggaacacgctgtgttcgcctgcatcacctccaaagggtggtacgcgatcgatgacgaggacttttacccttggacaccggacccgtccgacgtcctggtatttgttccgtacgatcaa

>DQ989308.1_Asia1_IND_1994

tacaacggtgagaaaaagaccttctactctaggcccaacaaccacgacaactgttggttgaacaccatcctccagttgttcaggtatgtcgatgaacctttcttcgactgggtctacgagtcgcctgagaacctcactcttgaggcgattaggcagctggaggaagttactggtcttgaactacacgagggtggaccgcccgcccttgtcatttggaacattaaacacttgctccacaccggaatcggcactgcttcgcgacccagcgaggtgtgtatggttgacggaacggacatgtgtttggctgacttccacgctggcattttcttgaaaggacaagagcatgctgtgtttgcttgtgtcacctccaacgggtggtacgcgatcgacgacgaggacttctacccttggacgccggacccgtccgacgttctggtgtttgtcccttatgatcaa

>DQ989309.1_Asia1_IND_1996

tacaacggtgagaaaaagaccttctactctaggcccaacaaccacgacaactgttggttgaacaccatcctccagttgttcaggtatgttgatgaacctttcttcgactgggtctacgagtcgcctgagaacctcactcttgaggcgattaggcagctggaggaagttactggtcttgaactacacgagggtggaccgcccgcccttgtcatttggaacattaaacacttgctccacaccggaatcggcactgcttcgcgacccagcgaggtgtgtatggttgacggaacggacatgtgtttggctgacttccacgctggcattttcttgaaaggacaagagcatgctgtgtttgcttgtgtcacctccaacgggtggtacgcgatcgacgacgaggacttctacccttggacgccggacccgtccgacgttctggtgtttgtcccttatgatcaa

>DQ989310.1_Asia1_IND_1999

tacaacggtgagaaaaagactttctactctaggcccaacagccacgacaactgttggctgaacaccatcctacagttgttcaggtatgtcgatgaaccaatcttcgactgggtttacgaatcacctgtgaacctcacccttgaggcaatcaggcaattggaggaactcactggccttgaactgcacgagggtggcccacctgccctcgtcatctggaacatcaagcacctgctccacaccggaatcggtaccgcctcgcgacccagcgaggtgtgcatggttgacggtacggacatgtgtttggctgactttcacgctggcattttcctgaaaggacaagaacatgctgtgtttgcttgcgtcacctccaacgggtggtacgcgatcgacgacgaggacttttacccttggacgccggacccgtccgacgttctggtgtttgtcccttacgatcaa

>DQ989311.1_Asia1_IND_2002

cacaacggagagaagaaaactttttactctagacccaacaaccatgacaactgttggttgaacaccatcctccagctgtttaggtacgtcgatgagcctttcttcgactgggtttacgagtcacctgagaacctcaccgttgaggcaatcaggcaactggaggaactcactggccttgaactgcacgagggtggaccacctgccctcgtcatctggaacatcaagcacctgctccacaccggaatcggcaccgcctcgcgacccagcgaggtgtgcatggttgatggcacggacatgtgtttggctgactttcacgctggcatcttcctgaaaggacaagaacatgctgtgtttgcttgtgtcacctccaacgggtggtacgcgatcgacgatgaggacttctacccttggacaccggatccgtccgacgttctggtgtttgtcccttacgatcaa

>DQ989312.1_Asia1_IND_1990

cacaacggtgagaagaaaactttctactctagacccaacaaccatgacaactgttggttgaacaccatcctccagttgttcaggtacgtcgatgagcccttcctcgactgggtctatgactcacctgaaaacctcactcttgatgcaatcaaacaattggaagaaattactggtcttgagctgcacgagggtgggccacccgctctcgtcatttggaacatcaagcacttgctccacaccggaatcggtactgcctcgcgacccagcgaggtgtgtatggtggacggcacggacatgtgcttggctgacttccatgctggcatcttcctgaagggacaggaacatgctgtgtttgcctgcgtcacctccaacgggtggtacgcgattgacgacgaggatttctacccctggacgccgtacccgtccgatgtcctggtgtttgtaccgtacgatcat

>DQ989313.1_Asia1_IND_1986

cacagcggtgagaagaaaactttttactctagacccaacaaccacgacaactgttggttgaacaccatccttcagttgtttaggtacgtcgacgagcccttcttcgactgggtctacaactcacctgaaaaccttactcttgatgccatcaaacaactggaagagatcactggtcttgaactgcacgagggtggaccacccgctctcgtcatctggaacatcaaacacttgcttcaaaccggaatcggcaccgcctcgcgtcctagtgaggtatgcatggttgacgggacggacatgtgtttggctgattttcatgctggcattttcttgaagggacaggaacacgctgtgttcgcctgcatcacctccaatgggtggtacgcgattgacgatgaggacttctacccctggacaccggacccgtccgacgtcctggtgtttgtcccgtacgatcaa

>DQ989314.1_Asia1_IND_2001

tacaacggtgagaaaaagaccttctattctaggcccaacaaccacgacaattgttggttgaacaccatcctccagttgttcaggtatgtcgatgaacctttcttcgactgggtctatgagtcgcccgagaacctcactcttgaggcgattgggcaactcgaggaactcactggtcttgagctgcacgaaggcggaccacccgctctcgtcatttggaacatcaaacacttgctccacaccggaatcggcactgcttcgcgacccagcgaggtatgcatggttgacggtacggacatgtgcttggcggacttccacgctggcattttcttgaaaggacaagagcacgctgtgtttgcttgcgtcacctccaacgggtggtacgcgatcgatgacgaagacttttacccctggacgcctgacccgtcagacgttttggtgtttgtcccgtacgatcaa

>DQ989315.1_Asia1_IND_1993

tacaacggagagaaaaaggtcttctactctagacccaacaaccacgacaactgttggctgaacaccatcctccagttgttcaggtatgtcgatgagcctttctttgactgggtctatgagtcgcctgagaacctcactcttgaggcgattaggcagcttgaggaagttactggtcttgagttgcacgagggcgggccacccgccctcgtcatttggaatatcaagcacttgctccacaccggaatcggcactgcttcgcgacctagcgaggtatgcatggttgacggcacggacatgtgtttggctgacttccacgctggcattttcttgaaaggacaagaacatgctgtgtttgcttgtgtcacctctaacggatggtacgcgatcgacgacgaggacttctacccttggacgccggacccgtccgacgttctggtgtttgtcccgtacgaccaa

>DQ989317.1_Asia1_IND_2000

tacaacggtgagaaaaagaccttctattctaggcccaacaaccacgacaattgttggttgaacaccatcctccagttgttcaggtatgtcgatgaacctttcttcgactgggtctatgagtcgcccgagaacctcactcttgaggcgattgggcaactcgagaaactcactggtcttgagctgcacgaaggcggaccacccgctctcgtcatttggaacatcaaacacttactccacaccggaatcggcactgcttcgcgacccagcgaggtatgcatggttgacggtacggacatgtgcttggcggacttccacgctggcattttcctgaaaggacaagagcacgctgtgtttgcttgcgtcacctccaacgggtggtacgcgatcgatgacgaagacttttacccctggacgcctgacccgtcagacgctttggtgtttgtcccgtacgatcaa

>DQ989318.1_Asia1_IND_2002

tacaacggtgagaaaaagaccttctattctaggcccaacaaccacgacaattgttggttgaacaccatcctccagttgttcaggtatgtcgatgaacctttcttcgactgggtctatgagtcgcccgagaacctcactcttgaggcgattgggcaactcgaggaactcactggtcttgagctgcacgaaggcggaccacccgctctcgtcatttggaacatcaaacacttgctccacaccggaattggcactgcttcgcgacccagcgaggtatgcatggttgacggtacggacatgtgcttggcggacttccacgctggcattttcctgaagggacaagagcacgctgtgtttgcttgcgccacctccaacgggtggaacgcgatcgatgatgaagacttttacccctggacgcctgacccgtcagacgtgttggtgttggtcccgtacgatcaa

>DQ989319.1_Asia1_IND_2001

tacaacggtgagaaaaagaccttctattctaggcccaacaaccacgacaattgttggttgaacaccatcctccagttgttcaggtatgtcgatgaacctttcttcgactgggtctatgagtcgcccgagaacctcactctcgaggcgattgggcaactcgaggaactcactggtcttgagctgcacgaaggcggaccacccgctctcgtcatttggaacatcaagcacttgcttcacaccggaatcggcactgcttcgcgacccagcgaggtatgcatggttgacggtacggacatgtgcttggcggacttccacgctggcattttcctgaaaggacaagagcacgctgtgtttgcttgcgtcacctccaacgggtggtacgcgatcgatgacgaagatttttacccctggacgcccgacccgtcagacgctttggtgtttgtcccgtacgatcaa

>DQ989320.1_Asia1_IND_2002

tacaacggtgagaaaaagaccttctattctaggcccaacaaccacgacaattgttggttgaacaccatcctccagttgttcaggtatgtcgatgaacctttcttcgactgggtctatgagtcgcccgaaaacctcactcttgaggcgattgggcaactcgaggaactcactggtcttgagctgcacgaaggcggaccacccgctctcgtcatttggaacatcaaacacttgctccacactggaatcggcactgcttcgcgacccagcgaggtatgcatggttgacggtacggacatgtgcttggcggacttccacgctggcattttcctgaaaggacaagagcacgctgtgtttgcttgcgtcacctccaacggctggtacgcgatcgatgacgaagacttttacccctggacgcctgacccgtcagacgttttggtgtttgtcccgtacgatcaa

>DQ989321.1_Asia1_IND_2001

tacaacggtgagaaaaagaccttctattctaggcccaacaaccacgacaattgttggttgaacaccatcctccagttgttcaggtatgtcgatgaacctttcttcgactgggtctatgagtcgcccgagaacctcactcttgaggcgattgggcaactcgaggaactcactggtcttgagctgcacgaaggcggaccacccgctctcgtcatttggaacatcaaacacttgctccacaccggaatcggcactgcttcgcgacccagcgaggtatgcatggttgacggtacggacatgtgcttggcggacttccacgctggcattttcttgaaaggacaagagcacgctgtgtttgcttgtgtcacctccaacgggtggtacgcgatcgatgacgaagacttttacccctggacgcctgacccgtcagacgttttggtgtttgtcccgtacgatcaa

>DQ989322.1_Asia1_IND_2002

tacaacggtgagaaaaagaccttctattctaggcccaacaaccacgacaattgttggttgaacaccatcctccagttgttcaggtatgtcgatgaacctttcttcgactgggtctatgagtcgcccgagaacctcactcttgaggcgattgggcaactcgaggaactcactggtcttgagctgcacgaaggcggaccacccgctctcgtcatttggaacatcaaacacttgctccacaccggaatcggcactgcttcgcgacccagcgaggtatgcatggttgacggtacggacatgtgcttggcggacttccacgctggcattttcctgaaaggacaagagcacgctgtgtttgcttgcgtcacctccaacgggtggtacgcggtcgatgacgaagacttttacccctggacgcctgacccgtcagacgttttggtgtttgtcccgtacgatcaa

>DQ989323.1_Asia1_IND_2002

tacaacggtgagaaaaagaccttctattctaggcccaacaaccacgacaattgttggttgaacaccatcctccagttgttcaggtatgtcgatgaacctttcttcgactgggtctatgagtcgcccgagaacctcactcttgaggcgattgggcaactcgaggaactcactggtcttgagctgcacgaaggcggaccacccgctctcgtcatttggaacatcaaacacttgctccacaccggaatcggcactgcttcgcgacccagcgaggtatgcgtggttgacggtacggacatgtgcttggcggacttccacgctggcattttcctgaaaggacaagagcacgctgtgtttgcttgcgtcacctccaacgggtggtacgcgatcgatgacgaagacttttacccctggacgcctgacccgtcagacgttttggtgtttgtcccgtacgatcaa

>EF117837.1_A_PAK_2006

cacaacggtgagaagaagacctttttctctaggcccaacaaccatgacaactgttggttgaacaccatcctccagttgttcaggtacgttgatgagcctttcttcgactgggtctacgactctcctgagaatctcaccctcaaggcaatacaacaattggaggagattactggcctagagctgcacgagggtggaccgcccgctctcgtcatctggaacatcaaacacttgctcaacaccgggatcggcactgcttcacggcccagtgaggtgtgcatggtcgacgggacagacatgtgtttggctgatttccatgctggcattttcttgaaaggacaggaacacgctgtgtttgcctgtgtcacctccaacgggtggtacgcgatcgacgacgaggacttctacccgtggacgccggatccgtctgacgtcctggtgtttgttccgtacgatcaa

>EF149009.1_Asia1_CHA_2005

cacaacggtgagaagaagaccttttactccagacccaacaaccatgacaactgctggctgaacactatcctccagttgttcaggtatgtcgatgagcctttcttcgactgggtctacgactcgcctgaaaacctcactctcgaggcgattaagcagttggaggaagttactggtcttgagctgcatgagggtggaccacccgcccttgtcatctggaacatcaagcatttgctccacaccggagtcggtaccgcttcgcgccctagcgaagtgtgtatggtagacggcacggacatgtgtttggctgattcccatgctggcattttcttgaaaggacaagaacatgctgtgtttgcctgtgtcacctccaacgggtggtacgcgatcgatgatgaggacttttacccctggacaccggatccgtccgatgttctggtgtttgttccgtacgaccaa

>EF149010.1_Asia1_CHA_2005

cacaacggtgagaagaagactttctactccaggcccaacaaccacgacaactgctggttgaacaccatcctccagttgtttaggtacgtcgatgaacctttcttcgactgggtctacgactcgcccgagaatctcacacttgatgccatcaaacaactggaagaaattactggtcttgagctgcacgagggtggaccacccgctctcgttatttggaacatcaaacacctgctccacaccggaatcggcaccgcttcgcgacccagcgaagtgtgcatggtagacgggacggacatgtgtttggctgacttccatgctggcattttcctgaaaggacaggaacacgctgtgtttgcctgcgtcacctccaatgggtggtacgcgattgacgacgaagacttttacccctggacgccggacccgtccgacgttctggtgtttgtcccgtacgatcaa

>EF494486.1_A_TUR_2005

cacaacggtgagaagaagacctttttctctaggcccaacaaccatgacaactgttggttgaacaccatcctccagttattcaggtacgttgatgagcctttcttcgactgggtctacgactctcctgagaatctcaccctcaaggcaatacaacaattggaggagattactggcctagagctgcacgagggtgggccgcccgctctcgtcatctggaacatcaaacacttgctcaacaccgggatcggcactgcttcacggcccagtgaggtgtgcatggtcgacgggacagacatgtgtttggccgacttccatgctggcattttcttgaaaggacaggaacacgctgtgtttgcctgtgtcacctccaacgggtggtatgcgatcgacgacgaggacttctacccgtggacgccggatccgtctgacgtcctggtgtttgttccgtacgatcaa

>EF494487.1_A_PAK_2006

cacaacggtgagaagaagacctttttctctaggcccaacaaccatgacaactgttggttgaacaccatcctccagttgttcaggtacgttgatgagcctttcttcgactgggtctacgactctcctgagaatctcaccctcaaggcaatacaacaattggaggagattactggcctagagctgcacgagggtggaccgcccgctctcgtcatctggaacatcaaacacttgctcaacaccgggatcggcactgcttcacggcccagtgaggtgtgcatggtcgacgggacagacatgtgtttggctgatttccatgctggcattttcttgaaaggacaggaacacgctgtgtttgcctgtgtcacctccaacgggtggtacgcgatcgacgacgaggacttctacccgtggacgccggatccgtctgacgtcctggtgtttgttccgtacgatcaa

>EF494488.1_A_PAK_2006

cacaacggtgagaagaagacctttttctctaggcccaacaaccatgacaactgttggttgaacaccatcctccagttgttcaggtatgttgatgagcctttcttcgactgggtctacgactctcctgagaatctcaccctcaaggcaatacaacaattggaagagattactggcctagagttgcacgagggtgggccgcccgctctcgtcatctggaatatcaaacacttgctcaacaccgggatcggcactgcttcacggcccagtgaggtgtgcatggtcgacgggacagacatgtgtttggccgatttccatgctggcattttcttgaaaggacaagaacacgctgtgtttgcctgtgtcacctccaacgggtggtacgcgatcgacgacgaggacttctacccgtggacgccggatccgtctgacgtcctggtgtttgttccgtacgatcaa

>EF552688.1_O_UKG_2001

cacaacggtgagaagaaaacattctactccaggcccaacaaacacgacaactgctggctgaacaccatcctccagttgtttaggtacgttgatgaaccttttttcgactgggtctactactcacctgagaacctcacacttgatgctatcaaacaattggaagaaattactggtctcgagctccacgagggtggaccacccgctctcgttatttggaacattaaacacttgctcaacaccggaatcggcaccgcctcgcgacccagcgaagtgtgcatggtagacgggacggatatgtgtttggctgacttccacgctggcatcttcctgaaaggacaggaacacgctgtgttcgcctgcgttacctccaacgggtggtacgcgattgatgacgaggacttttacccctggacgccggacccgtccgacgttctggtgtttgtcccgtacgatcaa

>EF552689.1_O_UKG_2001

cacaacggtgagaagaaaacattctactccaggcccaacaaacacgacaactgctggctgaacaccatcctccagttgtttaggtacgttgatgaaccttttttcgactgggtctactactcacctgagaacctcacacttgatgctatcaaacaattggaagaaattactggtctcgagctccacgagggtggaccacccgctctcgttatttggaacattaaacacttgctcaacaccggaatcggcaccgcctcgcgacccagcgaagtgtgcatggtagacgggacggacatgtgtttggctgacttccacgctggcatcttcctgaaaggacaggaacacgctgtgttcgcctgcgttacctccaacgggtggtacgcgattgatgacgaggacttttacccctggacgccggacccgtccgacgttctggtgtttgtcccgtacgatcaa

>EF552690.1_O_UKG_2001

cacaacggtgagaagaaaacattctactccaggcccaacaaacacgacaactgctggctgaacaccatcctccagttgtttaggtacgttgatgaaccttttttcgactgggtctactactcacctgagaacctcacacttgatgctatcaaacaattggaagaaattactggtctcgagctccacgagggtggaccacccgctctcgttatttggaacattaaacacttgctcaacaccggaatcggcaccgcctcgcgacccagcgaagtgtgcatggtagacgggacggacatgtgtttggctgacttccacgctggcatcttcctgaaaggacaggaacacgctgtgttcgcctgcgttacctccaacgggtggtacgcgattgatgacgaggacttttacccctggacgccggacccgtccgacgttctggtgtttgtcccgtacgatcaa

>EF552691.1_O_UKG_2001

cacaacggtgagaagaaaacattctactccaggcccaacaaacacgacaactgctggctgaacaccatcctccagttgtttaggtacgttgatgaaccttttttcgactgggtctactactcacctgagaacctcacacttgatgctatcaaacaattggaagaaattactggtctcgagctccacgagggtggaccacccgctctcgttatttggaacattaaacacttgctcaacaccggaatcggcaccgcctcgcgacccagcgaagtgtgcatggtagacgggacggacatgtgtttggctgacttccacgctggcatcttcctgaaaggacaggaacacgctgtgttcgcctgcgttacctccaacgggtggtacgcgattgatgacgaggacttttacccctggacgccggacccgtccgacgttctggtgtttgtcccatacgatcaa

>EF552692.1_O_UKG_2001

cacaacggtgagaagaaaacattctactccaggcccaacaaacacgacaactgctggctgaacaccatcctccagttgtttaggtacgttgatgaaccttttttcgactgggtctaccactcacctgagaacctcacacttgatgctatcaaacaattggaagaaattactggtctcgagctccacgagggtggaccacccgctctcgttatttggaacattaaacacttgctcaacaccggaatcggcaccgcctcgcgacccagcgaagtgtgcatggtagacgggacggacatgtgtttggctgacttccacgctggcatcttcctgaaaggacaggaacacgctgtgttcgcctgcgttacctccaacgggtggtacgcgattgatgacgaggacttttacccctggacgccggacccgtccgacgttctggtgtttgtcccgtacgatcaa

>EF552693.1_O_UKG_2001

cacaacggtgagaagaaaacattctactccaggcccaacaaacacgacaactgctggctgaacaccatcctccagttgtttaggtacgttgatgaaccttttttcgactgggtctaccactcacctgagaacctcacacttgatgctatcaaacaattggaagaaattactggtctcgagctccacgagggtggaccacccgctctcgttatttggaacattaaacacttgctcaacaccggaatcggcaccgcctcgcgacccagcgaagtgtgcatggtagacgggacggacatgtgtttggctgacttccacgctggcatcttcctgaaaggacaggaacacgctgtgttcgcctgcgttacctccaacgggtggtacgcgattgatgacgaggacttttacccctggacgccggacccgtccgacgttctggtgtttgtcccgtacgatcaa

>EF552695.1_O_UKG_2001

cacaacggtgagaagaaaacattctactccaggcccaacaaacacgacaactgctggctgaacaccatcctccagttgtttaggtacgttgatgaaccttttttcgactgggtctactactcacctgagaacctcacacttgatgctatcaaacaattggaagaaattactggtctcgagctccacgagggtggaccacccgctctcgttatttggaacattaaacacttgctcaacaccggaatcggcaccgcctcgcgacccagcgaagtgtgcatggtagacgggacggacatgtgtttggctgacttccacgctggcatcttcctgaaaggacaggaacacgctgtgttcgcctgcgttacctccaacgggtggtacgcgattgatgacgaggacttttacccctggacgccggacccgtccgacgttctggtgtttgtcccgtacgatcaa

>EF552696.1_O_UKG_2001

cacaacggtgagaagaaaacattctactccaggcccaacaaacacgacaactgctggctgaacaccatcctccagttgtttaggtacgttgatgaaccttttttcgactgggtctactactcacctgagaacctcacacttgatgctatcaaacaattggaagaaattactggtctcgagctccacgagggtggaccacccgctctcgttatttggaacattaaacacttgctcaacaccggaatcggcaccgcctcgcgacccagcgaagtgtgcatggtagacgggacggatatgtgtttggctgacttccacgctggcatcttcctgaaaggacaggaacacgctgtgttcgcctgcgttacctccaacgggtggtacgcgattgatgacgaggacttttacccctggacgccggacccgtccgacgttctggtgtttgtcccgtacgatcaa

>EF552697.1_O_UKG_2001

cacaacggtgagaagaaaacattctactccaggcccaacaaacacgacaactgctggctgaacaccatcctccagttgtttaggtacgttgatgaaccttttttcgactgggtctactactcacctgagaacctcacacttgatgctatcaaacaattggaagaaattactggtctcgagctccacgagggtggaccacccgctctcgttatttggaacattaaacacttgctcaacaccggaatcggcaccgcctcgcgacccagcgaagtgtgcatggtagacgggacggacatgtgtttggctgacttccacgctggcatcttcctgaaaggacaggaacacgctgtgttcgcctgcgttacctccaacgggtggtacgcgattgatgacgaggacttttacccctggacgccggacccgtccgacgttctggtgtttgtcccgtacgatcaa

>EF611987.1_O_UGA_2006

tacaacggtgagaaaaagacattttactctaggcctaacaaccacgacaactgctggcttaacgccatcctgcagctgtttaggtatgttgatgaacctttctttgactgggtctatgactcacctgagaaccttactgttgaagcaatcaggcagctggagggcctaactggtcttgagctgcacgagggcggaccacccgctctcgtcatttggaacatcaagcacttgctccacaccggcatcggcacggcttcacgacctagtgaggtgtgcatggtagacgggacagacatgtgcttggctgacttccatgctggcattttcctgaaaggacaggaacacgctgtgtttgcatgtgtcacctccgacgggtggtttgcgattgatgacgaggacttctacccctggacgccggacccgtccgacgttttggtttttgtcccgtacgatcaa

>EF614457.1_O_SKR_2002

cacaacggtgagaagaaaacattctactccaggcccaacaaccacgacaactgctggctgaacgccatcctccagttgtttaggtacgttgatgaacctttcttcgactgggtctactactcacctgagaacctcacgctcgatgctatcaaacaattggaaggaattactggtctcgagctccacgagggtggaccacccgctctcgttatttggaacattaaacacctgctcaacaccggaatcggcaccgcttcgcgacccagcgaagtgtgcatggtagatgggacggacatgtgtttggctgacttccacgctggcatcttcctgaaaggacaggaacacgctgtgttcgcctgtgtcacctccaacgggtggtacgcgattgatgacgaggacttttacccctggacgccggacccgtctgacgttctggtgttcgtcccgtacgaccaa

>EU214601.1_O_UKG_2001

cacaacggtgagaagaaaacattctactccaggcccaacaaacacgacaactgctggctgaacaccatcctccagttgtttaggtacgttgatgaaccttttttcgactgggtctactactcacctgagaacctcacacttgatgctatcaaacaattggaagaaattactggtctcgagctccacgagggtggaccacccgctctcgttatttggaacattaaacacttgctcaacactggaatcggcaccgcctcgcgacccagcgaagtgtgcatggtagacgggacggacatgtgtttggctgacttccacgctggcatcttcctgaaaggacaggaacacgctgtgttcgcctgcgttacctccaacgggtggtacgcgattgatgacgaggacttttacccctggacgccggacccgtccgacgttctggtgtttgtcccgtacgatcaa

>EU400597.1_O_CHA_2001

tacaacggggaaaagaaagtcttttactccagacccaacaaccacgacaattgttggttgaacgccatcctccaactgttcaggtacgttgacgaacccttcctcgaatgggtctacaactcacctgaggacctcactcttgaggcgattaacaacctggaagaagtcactggtcttgagctacacgaaggcggaccacccgccctcgtcgtctggaacaccaagcacctgctctacaccggaatcggcaccgcctcgcggcccagcgaggtgtgcatgatcgacggcacagacatgtgcttggctgacttccacgccggtatatttctgaagggacaggaccacgccgtcttcgcctgcgtcacctctgacgggtggtacgcaatcgacgacgaagatttttatccgtggacaccagacccggctgacgtcttggtttttgttccatacgatcaa

>EU448368.1_O_UKG_1967

tacaacggtgagaagaagaccttttactccaggcccaacaaccacgacaactgctggttgaacgccatcctccagttgttcaggtacgttgaagaaccattcttcgactgggtctacagttcgcctgagaacctcacgcttgaagccatcaagcagttggaggacctcacagggcttgaactgcacgagggtggaccacctgctctcgtgatctggaacatcaagcacttgctccacaccggcattggcaccgcctcgcgacccagcgaggtgtgcatggtggatggtacggacatgtgcttggctgatttccatgcaggcattttccttaaggggcaagaacacgctgtgttcgcgtgtgtcacctccaacgggtggtacgcgattgatgatgaggacttctacccctggacgccggacccatccgacgttctggtgtttgtcccgtacgatcaa

>EU448369.1_O_UKG_1967

tacaacggtgagaagaagaccttttactccaggcccaacaaccacgacaactgctggttgaacgccatcctccagttgttcaggtacgttgaagaaccattcttcgactgggtctacagttcgcctgagaacctcacgcttgaagccatcaagcagttggaggacctcacagggcttgaactgcacgagggtggaccacctgctctcgtgatctggaacatcaagcacttgctccacaccggcattggcaccgcctcgcgacccagcgaggtgtgcatggtggatggtacggacatgtgcttggctgatttccatgcaggcattttccttaaggggcaagaacacgctgtgttcgcgtgtgtcacctccaacgggtggtacgcgattgatgatgaggacttctacccctggacgccggacccatccgacgttctggtgtttgtcccgtacgatcaa

>EU448370.1_O_UKG_1967

tacaacggtgagaagaagaccttttactccaggcccaacaaccacgacaactgctggttgaacgccatcctccagttgtycaggtacgttgaagaaccattcttcgactgggtctacagttcgcctgagaacctcacgcttgaagccatcaagcagttggaggacctcacagggcttgaactgcacgagggtggaccacctgctctcgtgatctggaacatcaagcacttgctccacaccggcattggcaccgcctcgcgacccagcgaggtgtgcatggtggatggtacggacatgtgcttggctgatttccatgcaggcattttccttaaggggcaagaacacgctgtgttcgcgtgtgtcacctccaacgggtggtacgcgattgatgatgaggacttctacccctggacgccggacccatccgacgttctggtgtttgtcccgtacgatcaa

>EU448371.1_O_UKG_2007

tacaacggtgagaagaagaccttttactccaggcccaacaaccacgacaactgctggttgaacgccatcctccagttgttcaggtacgttgaagaaccattcttcgactgggtctacagttcgcctgagaacctcacgcttgaagccatcaagcagttggaggacctcacagggcttgaactgcacgagggtggaccacctgctctcgtgatctggaacatcaagcacttgctccacaccggcattggcaccgcctcgcgacccagcgaggtgtgcatggtggatggtacggacatgtgcttggctgatttccatgcaggcattttccttaaggggcaagaacacgctgtgttcgcgtgtgtcacctccaacgggtggtacgcgattgatgatgaggacttctacccctggacgccggacccatccgacgttctggtgtttgtcccgtacgatcaa

>EU448372.1_O_UKG_2007

tacaacggtgagaagaagaccttttactccaggcccaacaaccacgacaactgctggttgaacgccatcctccagttgttcaggtacgttgaagaaccattcttcgactgggtctacagttcgcctgagaacctcacgcttgaagccatcaagcagttggaggacctcacagggcttgaactgcacgagggtggaccacctgctctcgtgatctggaacatcaagcacttgctccacaccggcattggcaccgcctcgcgacccagcgaggtgtgcatggtggatggtacggacatgtgcttggctgatttccatgcaggcattttccttaaggggcaagaacacgctgtgttcgcgtgtgtcacctccaacgggtggtacgcgattgatgatgaggacttctacccctggacgccggacccatccgacgttctggtgtttgtcccgtacgatcaa

>EU448373.1_O_UKG_2007

tacaacggtgagaagaagaccttttactccaggcccaacaaccacgacaactgctggttgaacgccatcctccagttgttcaggtacgttgaagaaccattcttcgactgggtctacagttcgcctgagaacctcacgcttgaagccatcaagcagttggaggacctcacagggcttgaactgcacgagggtggaccacctgctctcgtgatctggaacatcaagcacttgctccacaccggcattggcaccgcctcgcgacccagcgaggtgtgcatggtggatggtacggacatgtgcttggctgatttccatgcaggcattttccttaaggggcaagaacacgctgtgttcgcgtgtgtcacctccaacgggtggtacgcgattgatgatgaggacttctacccctggacgccggacccatccgacgttctggtgtttgtcccgtacgatcaa

>EU448374.1_O_UKG_2007

tacaacggtgagaagaagaccttttactccaggcccaacaaccacgacaactgctggttgaacgccatcctccagttgttcaggtacgttgaagaaccattcttcgactgggtctacagttcgcctgagaacctcacgcttgaagccatcaagcagttggaggacctcacagggcttgaactgcacgagggtggaccacctgctctcgtgatctggaacatcaagcacttgctccacaccggcattggcaccgcctcgcgacccagcgaggtgtgcatggtggatggtacggacatgtgcttggctgatttccatgcaggcattttccttaaggggcaagaacacgctgtgttcgcgtgtgtcacctccaacgggtggtacgcgattgatgatgaggacttctacccctggacgccggacccatccgacgttctggtgtttgtcccgtacgatcaa

>EU448375.1_O_UKG_2007

tacaacggtgagaagaagaccttttactccaggcccaacaaccacgacaactgctggttgaacgccatcctccagttgttcaggtacgttgaagaaccattcttcgactgggtctacagttcgcctgagaacctcacgcttgaagccatcaagcagttggaggacctcacagggcttgaactgcacgagggtggaccacctgctctcgtgatctggaacatcaagcacttgctccacaccggcattggcaccgcctcgcgacccagcgaggtgtgcatggtggatggtacggacatgtgcttggctgatttccatgcaggcattttccttaaggggcaagaacacgctgtgttcgcgtgtgtcacctccaacgggtggtacgcgattgatgatgaggacttctacccctggacgccggacccatccgacgttctggtgtttgtcccgtacgatcaa

>EU448376.1_O_UKG_2007

tacaacggtgagaagaagaccttttactccaggcccaacaaccacgacaactgctggttgaacgccatcctccagttgttcaggtacgttgaagaaccattcttcgactgggtctacagttcgcctgagaacctcacgcttgaagccatcaagcagttggaggacctcacagggcttgaactgcacgagggtggaccacctgctctcgtgatctggaacatcaagcacttgctccacaccggcattggcaccgcctcgcgacccagcgaggtgtgcatggtggatggtacggacatgtgcttggctgatttccatgcaggcattttccttaaggggcaagaacacgctgtgttcgcgtgtgtcacctccaacgggtggtacgcgattgatgatgaggacttctacccctggacgccggacccatccgacgttctggtgtttgtcccgtacgatcaa

>EU448377.1_O_UKG_2007

tacaacggtgagaagaagaccttttactccaggcccaacaaccacgacaactgctggttgaacgccatcctccagttgttcaggtacgttgaagaaccattcttcgactgggtctacagttcgcctgagaacctcacgcttgaagccatcaagcagttggaggacctcacagggcttgaactgcacgagggtggaccacctgctctcgtgatctggaacatcaagcacttgctccacaccggcattggcaccgcctcgcgacccagcgaggtgtgcatggtggatggtacggacatgtgcttggctgatttccatgcaggcattttccttaaggggcaagaacacgctgtgttcgcgtgtgtcacctccaacgggtggtacgcgattgatgatgaggacttctacccctggacgccggacccatccgacgttctggtgtttgtcccgtacgatcaa

>EU448378.1_O_UKG_2007

tacaacggtgagaagaagaccttttactccaggcccaacaaccacgacaactgctggttgaacgccatcctccagttgttcaggtacgttgaagaaccattcttcgactgggtctacagttcgcctgagaacctcacgcttgaagccatcaagcagttggaggacctcacagggcttgaactgcacgagggtggaccacctgctctcgtgatctggaacatcaagcacttgctccacaccggcattggcaccgcctcgcgacccagcgaggtgtgcatggtggatggtacggacatgtgcttggctgatttccatgcaggcattttccttaaggggcaagaacacgctgtgttcgcgtgtgtcacctccaacgggtggtacgcgattgatgatgaggacttctacccctggacgccggacccatccgacgttctggtgtttgtcccgtacgatcaa

>FJ175661.1_O_ISR_2007

tacaacggtgagaaaaagactttttactctaggcccaacagacacgacaactgttggttgaacaccatccttcagttgttcaggtacgtcgatgaaccattcttcgactgggtttatgattcacctgaaaaccttactcttgaagcaatcagacaactggaggaactcactggtcttgaactgcacgagggtggaccacctgctctcgtcatctggaacatcaaacatctgctccacaccggaatcggcaccgcctcgcgacccagtgaggtgtgtatggtcgacggtacggacatgtgtttggctgactttcatgctggcattttcctgaaaggacaagagcatgctgtgttcgcctgtgtcacatccgacgggtggtacgcgattgacgacgaagacttctacccctggacgccggacccgtccgacgttctggtttttgtcccgtacgatcaa

>FJ175662.1_O_ISR_2007

tacaacggtgagaaaaagactttctactctaggcccaacagacacgacaactgttggttgaacaccatccttcagttgttcaggtacgtcgatgaaccatttttcgactgggtttatgattcacctgaaaaccttactcttgaagcaatcagacaactggaggaactcactggtcttgaactgcacgagggtggaccacctgctctcgtcatctggaacatcaaacatctgctccacaccggaatcggcaccgcctcgcgacccagtgaggtgtgtatggtcgacggtacggacatgtgtttggctgactttcatgctggcattttcctgaaaggacaagagcatgctgtgttcgcctgtgtcacatccgacgggtggtacgcgattgacgacgaagacttctacccctggacgccggacccgtccgacgttctggtttttgtcccgtacgatcaa

>FJ175663.1_O_ISR_2007

tacaacggtgagaaaaagactttctactctaggcccaacagacacgacaactgttggttgaacaccatccttcagttgttcaggtacgtcgatgaaccattcttcgactgggtttatgattcacctgaaaaccttactcttgaagcaatcagacagctggaggaactcactggtcttgaactgcacgagggtggaccacctgctctcgtcatctggaacatcaaacatctgctccacaccggaatcggcaccgcctcgcgacccagtgaggtgtgtatggtcgacggtacggacatgtgtttggctgactttcatgctggcattttcctgaaaggacaagagcatgctgtgttcgcctgcgtcacatccgacgggtggtacgcgattgacgacgaagacttctacccctggacgccggacccgtccgacgttctggtttttgtcccgtacgatcaa

>FJ175664.1_O_ISR_2007

tacaacggtgagaaaaagactttctactctaggcccaacagacacgacaactgttggttgaacaccatccttcagttgttcaggtacgtcgatgaaccattcttcgactgggtttatgattcacctgaaaaccttactcttgaagcaatcagacaactggaggaactcactggtcttgaactgcacgagggtggaccacctgctctcgtcatctggaacatcaaacatctgctccacaccggaatcggcaccgcctcgcgacccagtgaggtgtgtatggtcgacggtacggacatgtgtttggctgactttcatgctggcattttcctgaaaggacaagagcatgctgtgttcgcctgcgtcacatccgacgggtggtacgcgattgacgacgaagacttctacccctggacgccggacccgtccgacgttctggtttttgtcccgtacgatcaa

>FJ175665.1_O_ISR_2007

tacaacggtgagaaaaagactttctactccaggcccaacagacacgacaactgttggttgaacaccatccttcagttgttcaggtatgtcgatgaaccattcttcgactgggtttatgattcacctgaaaaccttactcttgaagcaatcagacaactggaggaactcacaggtcttgaactgcgcgagggtggaccacctgctctcgtcatctggaacatcaaacatctgctccacaccgggatcggcaccgcctcgcgacccagtgaggtgtgtatggtcgacggtacggacatgtgtttggctgactttcatgctggcattttcctgaaaggacaggagcatgctgtgttcgcctgtgtcacatccgacgggtggtacgcgattgacgacgaagacttctacccctggacgccggacccgtccgacgttctggtttttgtcccgtacgatcaa

>FJ175666.1_O_ISR_2007

tacaacggtgagaaaaagactttctactctaggcccaacagacacgacaactgttggttgaacaccatccttcagttgttcaggtacgtcgatgaaccattcttcgactgggtttatgattcacctgaaaaccttactcttgaagcaatcagacaactggaggaactcactggtcttgaactgcacgagggtggaccacctgctctcgtcatctggaacatcaaacatctgctccacaccggaatcggcaccgcctcgcggcccagtgaggtgtgtatggtcgacggtacggacatgtgtttggctgactttcatgctggcattttcctgaaaggacaagagcatgctgtgttcgcctgcgtcacatccgacgggtggtacgcgattgacgacgaagacttctacccctggacgccggacccgtccgacgttctggtttttgtcccgtacgatcaa

>FJ461344.1_O_UGA_2002

tacaacggtgagaaaaagacattttactctaggcccaacaaccacgacaactgttggcttaacgccatcctacagctgtttaggtatgttgatgagcctttctttgactgggtctaygactcacctgagaacctcactgctgaagcaatcaggcagttggagggtctaactggccttgagctgcacgagggcggaccacccgctcttgtcatttggaacatcaagcacttgctccacaccggtgtcggcacggcttcacgacccagtgaggtgtgcatggtagacgggacagacatgtgcttggctgacttccacgctggcattttcctgaaaggacaggaacacgctgtgtttgcatgcgtcacgtccgacgggtggttcgcgattgacgacgaggacttttacccctggacgccggacccgtccgacgttctggtttttgtcccgtacgatcaa

>FJ461345.1_O_UGA_2002

tacaacggtgagaaaaagacattttactctaggcccaacaaccacgacaactgctggctcaacgccatcctgcagctgtttaggtatgttgatgagcccttctttgactgggtctacgactcacctgagaacctcactgctgaagcaatcaagcagttggagggcctaactggtcttgagctgcacgagggcggaccacccgctcttgtcatttggaacatcaagcacctgctccacaccggtatcggcacggcctcacgacccagtgaggtgtgcatggtagacgggacagacatgtgcttggctgacttccacgctggcattttcctaaaaggacaggaacacgctgtgtttgcatgtgtcacatctgacgggtggttcgcgattgacgacgaggacttttacccctggacgccggacccgtccgacgttctcgtttttgtcccgtacgatcaa

>FJ461346.1_SAT2_UGA_2002

cacaacggtgagaagaagactttctactccagacccaacaagcacgacaactgttggcttaacaccatcctacagttgttccgttacgtcgatgaaccattcttcgactgggtctacaactcgcccgagaacctcackctgcaagcaattgaacarctggaggaactcaccgggctcgagttacacgagggaggcccgcctgccctcgtgatttggaacatyaagcacttgctctacaccggaatcggtaccgcctcgcgacccagcgaggtgtgtatggttgacggtactgacatgtgtcttgctgatttccacgcaggaattttcctgaaaggtactgaacacgcagtgttcgcctgtttgacctccgacgggtggtatgccattgacgacgaggacttttatccgtggaccccggacccgtccgatgtcctgtgttttgtcccgtatgatatg

>FJ542365.1_O_UKG_2001

cacaacggtgagaagaaaacattctactccaggcccaacaaacacgacaactgctggctgaacaccatcctccagttgtttaggtacgttgatgaaccttttttcgactgggtctactactcacctgagaacctcacacttgatgctatcaaacaattggaagaaattactggtctcgagctccacgagggtggaccacccgctctcgttatttggaacattaaacacttgctcaacaccggaatcggcaccgcctcgcgacccagcgaagtgtgcatggtagacgggacggatatgtgtttggctgacttccacgctggcatcttcctgaaaggacaggaacacgctgtgttcgcctgcgttacctccaacgggtggtacgcgattgatgacgaggacttttacccctggacgccggacccgtccgacgttctggtgtttgtcccgtacgatcaa

>FJ542368.1_O_UKG_2001

cacaacggtgagaagaaaacattctactccaggcccaacaaacacgacaactgctggctgaacaccatcctccagttgtttaggtacgttgatgaaccttttttcgactgggtctactactcacctgagaacctcacacttgatgctatcaaacaattggaagaaattactggtctcgagctccacgagggtggaccacccgctctcgttatttggaacattaaacacttgctcaacaccggaatcggcaccgcctcgcgacccagcgaagtgtgcatggtagacgggacggatatgtgtttggctgacttccacgctggcatcttcctgaaaggacaggaacacgctgtgttcgcctgcgttacctccaacgggtggtacgcgattgatgacgaggacttttacccctggacgccggacccgtccgacgttctggtgtttgtcccgtacgatcaa

>FJ542369.1_O_UKG_2001

cacaacggtgagaagaaaacattctactccaggcccaacaaacacgacaactgctggctgaacaccatcctccagttgtttaggtacgttgatgaaccttttttcgactgggtctactactcacctgagaacctcacacttgatgctatcaaacaattggaagaaattactggtctcgagctccacgagggtggaccacccgctctcgttatttggaacattaaacacttgctcaacaccggaatcggcaccgcctcgcgacccagcgaagtgtgcatggtagacgggacggatatgtgtttggctgacttccacgctggcatcttcctgaaaggacaggaacacgctgtgttcgcctgcgttacctccaacgggtggtacgcgattgatgacgaggacttttacccctggacgccggacccgtccgacgttctggtgtttgtcccgtacgatcaa

>FJ542370.1_O_UKG_2001

cacaacggtgagaagaaaacattctactccaggcccaacaaacacgacaactgctggctgaacaccatcctccagttgtttaggtacgttgatgaaccttttttcgactgggtctactactcacctgagaacctcacacttgatgctatcaaacaattggaagaaattactggtctcgagctccacgagggtggaccacccgctctcgttatttggaacattaaacacttgctcaacaccggaatcggcaccgcctcgcgacccagcgaagtgtgcatggtagacgggacggatatgtgtttggctgacttccacgctggcatcttcctgaaaggacaggaacacgctgtgttcgcctgcgttacctccaacgggtggtacgcgattgatgacgaggacttttacccctggacgccggacccgtccgacgttctggtgtttgtcccgtacgatcaa

>FJ542371.1_O_UKG_2001

cacaacggtgagaagaaaacattctactccaggcccaacaaacacgacaactgctggctgaacaccatcctccagttgtttaggtacgttgatgaaccttttttcgactgggtctactactcacctgagaacctcacacttgatgctatcaaacaattggaagaaattactggtctcgagctccacgagggtggaccacccgctctcgttatttggaacattaaacacttgctcaacaccggaatcggcaccgcctcgcgacccagcgaagtgtgcatggtagacgggacggatatgtgtttggctgacttccacgctggcatcttcctgaaaggacaggaacacgctgtgttcgcctgcgttacctccaacgggtggtacgcgattgatgacgaggacttttacccctggacgccggacccgtccgacgttctggtgtttgtcccgtacgatcaa

>FJ542372.1_O_UKG_2001

cacaacggtgagaagaaaacattctactccaggcccaacaaacacgacaactgctggctgaacaccatcctccagttgtttaggtacgttgatgaaccttttttcgactgggtctactactcacctgagaacctcacacttgatgctatcaaacaattggaagaaattactggtctcgagctccacgagggtggaccacccgctctcgttatttggaacattaaacacttgctcaacaccggaatcggcaccgcctcgcgacccagcgaagtgtgcatggtagacgggacggatatgtgtttggctgacttccacgctggcatcttcctgaaaggacaggaacacgctgtgttcgcctgcgttacctccaacgggtggtacgcgattgatgacgaggacttttacccctggacgccggacccgtccgacgttctggtgtttgtcccgtacgatcaa

>FJ623456.1_A_KAZ_1999

tacaacggtgaaaagaagactttctactccagacccaacaaccacgacaactgttggcttaacaccattctccagttgttcaggtacgtcgatgagcctttcttcgactgggtctatgactcgccagagaacctcacctgtgaggcaattaggcagttggaagagataactggtcttgagctacacgagggtggaccacccgccctcgtcatctggaacatcaaacacttgctccacactggaatcggcactgcctcacgacctagtgaagtgtgcatggttgacggaacggacatgtgcttggctgactttcacgctggcattttcttgaaaggacaggaacatgctgtgtttgcctgcgtcacctccaacgggtggtacgcgatcgacgatgaggacttttacccctggacgccggacccgtccgatgtcttggtgtttgtcccgtatgatcaa

>FJ824812.1_C_SPA_2009

cacgacggcgagaaaaaggtcttttactccagacccaacaactacgacaactgctggttgaacaccatccttcagttgttcaggtacgtcgatgaacctttcttcgactgggtctacaattcgcccgagaacctcacgcttgaagccatcaagcaactggaggaactcacagggttggagttgcgcgagggtggaccgcccgcccttgtgatttggaacatcaaacacctgctccacactggcatcggtaccgcttcgcgacccagcgaggtgtgtatggtggacggcacggacatgtgtcttgctgactttcatgcaggcattttcatgaaaggacgggaacacgctgtctttgcgtgtgtcacctccaacgggtggtacgcgattgatgacgaggacttctacccatggacgccagacccgtctgatgtcctggtatttgttccatacgaccaa

>FJ906802.1_Asia1_CHA_2006

cacaacggtgagaagaagaccttttactccagacccaacaaccatgacaactgctggctgaacactatcctccagttgttcaggtatgtcgatgagcctttcttcgactgggtctacgactcgcctgaaaacctcactctcgaggcgattaggcagttggaggaagttactggtcttgagctgcacgagggtggaccacccgcccttgtcatctggaacatcaagcatttgctccacaccggagtcggtaccgcttcgcgccctagcgaagtgtgtatggtagacggcacggacatgtgtttggctgatttccatgctggcattttcctgaaaggacaagaacatgctgtgtttgcctgtgtcacctccaacgggtggtacgcgatcgatgacgaggacttttacccctggacaccggacccgtccgacgtcttggtgtttgttccgtacgaccaa

>GQ406247.1_A_VIT_2009

tacaacggtgagaagaaaactttttactctaggcccaacaaccacgacaactgttggttgaacaccatcctccaactgttcaggtacgtcgacgaaccattcttcgactgggtctatgaatcacctgagaacctcactcttgaggcgattgagcaattggaacaggtcactggtcttgagctgcacgagggtggcccacccgctctcgtaatttggaacattaagcacttgctccacaccggaatcggtaccgcctcgcgacctagtgaggtgtgtatggtagacgggacggacatgtgtctggctgatttccatgctggcattttcctgaaagggcaggaacacgcagtgtttgcctgtgttacctccaacgggtggtacgcgattgacgacgaggacttttacccctggacaccagacccgtctgatgtcctggtgtttgtcccgtatgatcaa

>GQ406248.1_A_VIT_2009

tacaacggtgagaagaaaactttttactctaggcccaacaaccacgacaactgttggttgaacaccatcctccaactgttcaggtacgtcgacgaaccattcttcgactgggtctatgaatcacctgagaacctcactcttgaggcgattgagcaattggaacaggtcactggtcttgagctgcacgagggtggcccacccgctctcgtaatttggaacattaagcacttgctccacaccggaatcggtaccgcctcgcgacctagtgaggtgtgtatggtagacgggacggacatgtgtctggctgatttccatgctggcattttcctgaaagggcaggaacacgcagtgtttgcctgtgttacctccaacgggtggtacgcgattgacgacgaggacttttacccctggacaccagacccgtctgatgtcctggtgtttgtcccgtatgatcaa

>GQ406249.1_A_VIT_2009

tacaacggtgagaagaaaactttttactctaggcccaacaaccacgacaactgttggttgaacaccatcctccaactgttcaggtacgtcgacgaaccattcttcgactgggtctatgaatcacctgagaacctcactcttgaggcgattaagcaattggaacaggtcactggtcttgagctgcacgagggtggcccacccgccctcgtaatttggaacatcaagcacttgctccacaccggaatcggtaccgcctcgcgacctagtgaggtgtgtatggtagacgggacggacatgtgtctggctgatttccatgctggcattttcctgaaagggcaggaacacgcagtgtttgcctgtgttacctccaacgggtggtacgcgattgacgacgaggacttttacccctggacaccagacccgtctgatgtcctggtgtttgtcccgtatgatcaa

>GQ406250.1_A_VIT_2009

tacaacggtgagaagaaaattttttactctaggcccaacaaccacgacaactgttggttgaacaccatcctccaactgttcaggtacgtcgacgaaccattcttcgactgggtctatgaatcacctgagaacctcactcttgaggcgattaagcaattggaacaggtcactggtcttgagctgcacgagggtggcccacccgccctcgtaatttggaacatcaagcacttgctccacaccggaatcggtaccgcctcgcgacctagtgaggtgtgtatggtagacgggacggacatgtgtctggctgatttccatgctggcattttcctgaaagggcaggaacacgcagtgtttgcctgtgttacctccaacgggtggtacgcgattgacgacgaggacttttacccctggacaccagacccgtctgatgtcctggtgtttgtcccgtatgatcaa

>GQ406251.1_A_VIT_2009

tacaacggtgagaagaaaactttttactctaggcccaacaaccacgacaactgttggttgaacaccatcctccaactgttcaggtacgtcgacgaaccattcttcgactgggtctatgaatcacctgagaacctcacgcttgaggcgattgagcaattggaacaggtcactggtcttgagctgcacgagggtggcccacccgccctcgtaatttggaacatcaagcacttgctccacaccggaatcggtaccgcctcgcgacctagtgaggtgtgtatggtagacgggacggacatgtgtctggctgatttccatgctggcattttcctgaaagggcaggaacacgcagtgtttgcctgtgttacctccaacgggtggtacgcgattgacgacgaggacttttacccctggacaccagacccgtctgacgtcctggtgtttgtcccgtatgatcaa

>GQ406252.1_A_VIT_2009

tacaacggtgagaagaaaacttttttctctaggcccaacaaccacgacaactgttggttgaacaccatcctccaactgttcaggtacgtcgacgaaccatttttcgactgggtctatgaatcacctgagaacctcactcttgaggcgattaagcaattggaacaggtcactggtcttgagctgcacgagggtggcccacccgccctcgtaatttggaacatcaagcacttgctccacaccggaatcggtaccgcctcgcgacctagtgaggtgtgtatggtagacgggacggacatgtgtctggctgacttccatgctggcattttcctgaaagggcaggaacacgcagtgtttgcctgtgttacctccaacgggtggtacgcgattgacgacgaggacttttacccctggacaccagacccgtctgatgtcctggtgtttgtcccgtatgatcaa

>GQ452295.1_Asia1_VIT_2007

cacaacggtgagaagaagaccttttactccagacccaacaaccatgacaactgctggctgaacactatcctccagttgttcaggtatgtcgatgagcctttcttcgactgggtctacgactcgcctgaaaacctcactctcgaggcgattaggcagttggaggaagttactggtcttgagctgcacgagggtggaccacccgcccttgtcatctggaacatcaagcatttgctccacaccggagtcggtaccgcttcgcgccctagcgaagtgtgtatggtagacggcacggacatgtgtttggctgatttccatgctggcattttcctgaaaggacaagaacacgctgtgtttgcctgtgtcacctccaacgggtggtacgcgatcgacgacgaggacttttacccctggacaccggacccgtccgacgtcttggtgtttgttccgtacgaccaa

>GU125645.1_Asia1_VIT_2007

cacaacggtgagaagaagaccttttactccagacccaacaaccatgacaactgctggctgaacactatcctccagttgttcaggtatgtcgatgagcctttcttcgactgggtctacgactcgcctgaaaacctcactctcgaggcgattaggcagttggaggaagttactggtcttgagctgcacgagggtggaccacccgcccttgtcatctggaacatcaagcatttgctccacaccggagtcggtaccgcttcgcgccctagcgaagtgtgtatggtagacggcacggacatgtgtttggctgatttccatgctggcattttcctgaaaggacaagaacacgctgtgtttgcctgtgtcacctccaacgggtggtacgcgatcgacgacgaggacttttacccctggacaccggacccgtccgacgtcttggtgtttgttccgtacgaccaa

>GU125646.1_Asia1_VIT_2005

tacaacggtgagaagaaaatcttctactccagacccaacaaccatgacaactgctggctgaacaccatcctccagttgttcaggtacgttgatgaacctttcttcgactgggtctatgaatcacctgaaaaccttactcttgaggcaattcagcaactggaagaggttactggtcttgagctgcacgagggcggtccacccgctctcgtcatttggaacattaagcacctgctccacaccggaatcggtaccgcctcgcgacctagcgaggtgtgtatggtagacggtaccgacatgtgtctggctgacttccatgctggcatctttctgaaaggacaagaacacgctgtgtttgcctgtgtcacctccaacgggtggtacgcgattgatgacgaggaattttacccctggacgccggacccgtccgacgtcctggtgtttgtcccgtatgatcaa

>GU125647.1_O_VIT_2006

cacaacggtgagaagaagaccttttactctaggcccaacaacaacgacaactgttggctgaatgccatcctccagctgtttaggtatgtcgatgaacctttcttcgactgggtctacgaatcacctgagaacctcactcttgaggcgatcaaacaactggaagaaatcaccggccttgaactgcacgagggcggtccacccgctctagttgtctggaacatcaagcacttgctccaaaccggaatcggtaccgcctcgcgacccagcgaggtgtgtatggtggacggtacggacatgtgtttggctgactttcacgctggcatcttcctgaaaggacaggaacacgccgtgtttgcctgtgtcacctccaatgggtggtacgcgattgatgacgaggacttttacccctggacgccggacccgtccgacgtgctggtgtttgtcccgtacgatcaa

>GU125648.1_O_VIT_2006

cacaacggtgagaagaagaccttttactctaggcccaacaacaacgacaactgttggctgaatgccatcctccagctgtttaggtatgtcgatgaacctttcttcgactgggtctacgaatcacctgagaacctcactcttgaggcgatcaaacaactggaagaaatcaccggccttgaactgcacgagggcggtccacccgctctagttgtctggaacatcaagcacttgctccaaaccggaatcggtaccgcctcgcgacccagcgaggtgtgtatggtggacggtacggacatgtgtttggctgactttcacgctggcatcttcctgaaaggacaggaacacgccgtgtttgcctgtgtcacctccaatgggtggtacgcgattgatgacgaggacttttacccctggacgccggacccgtccgacgtgctggtgtttgtcccgtacgatcaa

>GU125649.1_O_VIT_2006

cacaacggtgagaagaagaccttttactctaggcccaacaacaacgacaactgttggctgaatgccatcctccagctgtttaggtatgtcgatgaacctttcttcgactgggtctacgaatcacctgagaacctcactcttgaggcgatcaaacaactggaagaaatcaccggccttgaactgcacgagggcggtccacccgctctagttgtctggaacatcaagcacttgctccaaaccggaatcggtaccgcctcgcgacccagcgaggtgtgtatggtggacggtacggacatgtgtttggctgactttcacgctggcatcttcctgaaaggacaggaacacgccgtgtttgcctgtgtcacctccaatgggtggtacgcgattgatgacgaggacttttacccctggacgccggacccgtccgacgtgctggtgtttgtcccgtacgatcaa

>GU125650.1_O_VIT_2006

cacaacggtgagaagaagaccttttactctaggcccaacaacaacgacaactgttggttgaacgctatcctacagctgttcaggtatgtcgatgaaccgttctttgactgggtctatgaatcacctgagaacctcactcttgaggcgatcaagcaactggaaggaatcactggtcttgagctgcacgagggcggcccacccgctctcgttatctggaacatcaaacacttgctccacaccggaatcggtaccgcctcgcgacccagcgaggtgtgtatggtggacggtacggacatgtgcctggctgactttcacgctggcatcttcctgaaaggacaggaacacgctgtgtttgcctgcgtcacctccaacgggtggtacgcgattgacgacgaggacttttacccctggacgccggacccgtctgacgtgctggtgtttgtcccgtacgaccaa

>GU384682.1_O_PAK_2008

tacaacggtgagaaaaagactttctactctaggcccaacagacacgacaactgttggttgaacaccatccttcagttgttcaggtatgttgatgaaccattcttcgactgggtctatgattcacctgaaaatctcactcttgaagcaatcaggcaactggaagaactcactggtcttgaactgcacgagggtggaccacctgctctcgtcatctggaacatcaaacacctgcttcataccggaatcggtaccgcctcgcgacctagtgaggtgtgtatggttgacggcacggacatgtgcttggctgactttcacgctggcattttcctgaaaggacaagagcatgctgtgttcgcctgtgtcacatccgatgggtggtacgcgattgatgacgaggacttttacccttggacgccggacccgtccgacgttctggtgtttgtcccgtacgatcaa

>GU384683.1_O_PAK_2008

tacaacggtgagaaaaagactttctactctaggcccaacagacacgacaactgttggttgaacaccatccttcagttgttcaggtatgttgatgaaccattcttcgactgggtctatgattcacctgaaaatctcactcttgaagcaatcaggcaactggaagaactcactggtcttgaactgcacgagggtggaccacctgctctcgtcatctggaacatcaaacacctgcttcataccggaatcggtaccgcctcgcgacctagtgaggtgtgtatggttgacggcacggacatgtgcttggctgactttcacgctggcattttcctgaaaggacaagagcatgctgtgttcgcctgtgtcacatccgatgggtggtacgcgattgatgacgaggacttttacccttggacgccggacccgtccgacgttctggtgtttgtcccgtacgatcaa

>GU582115.1_O_VIT_2009

tacaacggtgagaggaagaccttttactctaggcccaacaacaatgacaactgttggctgaacgccatcctgcagttgtttaggtatgtcgatgaacctttcttcgactgggtctacgaatcgcctgagaaccgcactctcgaggcaattgaacaattagagggaattactggtcttgaactgcacgagggcggtccacccgctctcgtggtttggaacatcaaacacttgctccacaccgggattggcaccgcctcgcgacccagcgaggtgtgcatggttgacggtacggacatgtgcctggctgacttccacgctggcatcttcctgaaaggacaggaacacgctgtgtttgcctgcgtcacctccaacgggtggtacgcgattgatgacgaggacttttacccctggacgccggatccgtccgacgtgctggtgttcgtcccgtacgatcaa

>GU582116.1_O_VIT_2009

tacaacggtgagaggaagaccttttactctaggcccaacaacaatgacaactgttggctgaacgccatcctgcagttgtttaagtatgtcgatgaacctttcttcgactgggtctacgaatcgcctgagaaccgcactctcgaggcaattgaacaattagagggaattactggtcttgaactgcacgagggcggtccacccgctctcgtggtttggaacatcaaacacttgctccacaccgggattggcaccgcctcgcgacccagcgaggtgtgcatggttgacggtacggacatgtgcctggctgacttccacgctggcatcttcctgaaaggacaggaacacgctgtgtttgcctgcgtcacctccaacgggtggtacgcgattgatgacgaggacttttacccctggacgccggatccgtccgacgtgctggggttcgtcccgtacgatcaa

>GU931682.1_Asia1_CHA_2005

cacaacggtgagaagaagaccttttactccagacccaacaaccatgacaactgctggctgaacactatcctccagttgttcaggtatgtcgatgagcctttcttcgactgggtctacgactcgcctgaaaacctcactctcgaggcgattaggcagttggaggaagttactggtcttgagctgcatgagggtggaccacccgcccttgtcatctggaacatcaagcatttgctccacaccggagtcggtaccgcttcgcgccctagcgaagtgtgtatggtagacggcacggacatgtgtttggctgatttccatgctggcattttcctgaaaggacaagaacatgctgtgtttgcctgtgtcacctccaacgggtggtacgcgatcgatgacgaggacttttacccctggacaccggacccgtccgacgtcctggtgtttgttccgtacgaccaa

>HM008917.1_O_CHA_2005

cacaacggtgagaagaaaacattctactccagacccaacaaccacgacaactgctggttgaacgccatcctccagctgtttaggtacgttgatgaacctttcttcgactgggtctactgttcacacgagaacctcacactcaatgctataaaacaattggaagaaattactggtctcgagctccacgagggtggaccacccgctctcgttatttggaacatcaaacacctgctcaacaccggaataggcaccgcttcgcgacccagcgaagtgtgcatggtagacgggacggacatgtgcttggctgacttccatgctggcatcttcctgcaaggacaggaacacgctgtgttcgcctgcgtcacctccaacgggtggtacgcaatcgatgacgaggacttttacccctggacgccggacccgtccgacgttctggtgttcgtcccgtacgaccaa

>HM055510.1_O_VIT_2009

tacaacggtgagaagaagatcttctactccaggcccaacaaccacgacaactgttggctgaacgccatccttcagctgttcaggtacgtcgatgaacctttcttcgactgggtatatgaatcacctgaaaacctcacccttgaggcgatcagacaactggagaacattactggtcttgagctgcacgagggtggtccgcccgccctcgtcatttggaacatcaaacacttgctccacaccgggatcggcaccgcctcgcgacccagcgaggtgtgcatggtggacggtacggacatgtgcctggctgacttccacgctggcatcttcctgaaaggacaggaacacgccgtgtttgcctgcgtcacctccaacgggtggtacgcgatcgacgacgaagaattctacccctggacgccagatccgtctgacgtgctggtctttgtcccgtacgaccaa

>HM067704.1_SAT2_UGA_2007

cacaacggtgagaagaaaactttttactcaagacccaacaaccatgacaactgttggttaaacaccatcttgcagttgttcaggtacgtcgacgagccattcttcgactgggtttacaactcgcccgagaacctcaccctccaagcaattgagcagcttgaggaactcaccgggcttgagttgcacgaaggtggaccccccgccctcgtgatttggaacatcaaacacttgctctacaccggagtcggcaccgcctcgcgacccagcgaggtgtgcatggtggacggtactgagatgtgtcttgctgatttccatgcaggaatattcctgaaaggtgctgaacacgccgtgttcgcctgcctgacctccgacgggtggtacgccattgacgacgagggcttctacccttggaccccggatccatccgacgtcctttgttttgtcccgtacgatatg

>HM067705.1_SAT2_UGA_2007

tacaacggtgagaagaagactttctactctaggcccaataaacacgacaactgttggttgaacaccatcctgcagttgttcaggtacgtcgatgaaccattcttcgactgggtctacaactcgcccgagaacctcactcttcgggcaattgaacaactggaggaactcaccgggcttgagttgcacgagggcggaccacccgccctcgtgatttggaacattaaacacctgctctacactggaattggcactgcctcgcgacccagcgaggtgtgcatggtggacggcacagaaatgtgtctcgccgacttccacgcagggattttcctcaagggcactgaacacgccgtgttcgcctgtctgacctccgacgggtggtacgccatcgacgacgaggacttctacccatggacaccggatccgtccgacgtcctgtgttttgtcccgtacgatatg

>HM067706.1_SAT1_UGA_2007

tacaacggtgaaaagaaaactttctactcaagacccaacaaacacgacaactgttggttgaacaccatcctacagttgtttaggtacgtcgacgaaccgttcttcgactgggtctacaactcgcccgaaaacctcactttacaagcaattgaacagttagaggaactcacaaggctcgagttacacgagggcggcccacctgctcttgtgatttggaacatcaaacacttgctctacaccggaataggcactgcctcgcgacccagcgaggtgtgcatggttgacggtaccgacatgtgtcttgctgatttccacgcgggaatcttcctgaaaggtactgagcacgccgtgttcgcctgcttgacctctgacgggtggtacgccattgatgacgaggacttctacccttggaccccggaaccgtccgacgtcctctgttttgtcccgtacgatatg

>HM191257.1_O_UGA_2006

tacaacggtgagaaaaagacattttactctaggcctaacaaccacgacaactgctggcttaacgccatcctgcagctgtttaggtatgttgatgaacctttctttgactgggtctatgactcacctgagaaccttactgttgaagcaatcaggcggctggagggcctaactggtcttgagctgcacgagggcggaccacccgctctcgtcatttggaacatcaagcacttgctccacaccggcattggcacggcttcacgacctagtgaggtgtgcatggtagacgggacagacatgtgcttggctgacttccatgctggcattttcctgaaaggacaggaacacgctgtgtttgcatgtgtcacctccgacgggtggtttgcgattgatgacgaggacttctacccctggacgccggacccgtccgacgttttggtttttgtcccgtacgatcaa

>HM229661.1_O_HKN_2010

tacaacggtgagaagaagatcttctactccaggcccaacaaccacgacaactgttggctgaacgccatccttcagctgttcaggtacgtcgatgaacctttcttcgactgggtatatgaatcacctgaaaacctcacccttgaggcgatcagacaactggagaacattactggtcttgagctgcacgagggtggtccgcccgccctcgtcatttggaacatcaaacacttgctccacaccgggatcggcaccgcctcgcgacccagcgaggtgtgcatggtggacggtacggacatgtgcctggctgacttccacgctggcatcttcctgaaaggacaggaacacgccgtgtttgcctgcgtcacctccaacgggtggtacgcgatcgacgacgaagaattctacccctggacgccagatccgtccgacgtgctggtctttgtcccgtacgatcaa

>HM854021.1_A_IND_2000

tacaacggtgagaaaaagactttctattctagacccaacaaccacgacaactgttggctgaacaccatccttcagttattcagatatgtcgatgaaccattcttcgactgggtttatgattcacctgagaacctcactcttgaggcgatcaaacaattggaggaactcactggtcttgagctgcacgagggcggaccacctgctctcgtcatttggaacatcaagcatctgctccacaccggaatcggcaccgcctcgcgacccagcgaggtgtgtatggttgatggtacggacatgtgcttggctgacttccatgctggcatcttcctgaaagggcaagagcatgctgtgttcgcctgtgtcacctccgatgggtggtacgcgattgatgacgaggacttttacccttggacaccggacccgtccgatgttctggtttttgtcccgtacgatcaa

>HM854022.1_A_IND_1977

tacaacggtgagaagaagactttctactccaggcccaacaaccacgacaactgttggctgaacaccatccttcagttgtttaggtatgtcgatgaacctttcttcgactgggtctatgactcgcctgaaaacctcacgcttgaggccataaggcaactggaagaagttactggtcttgaactacacgagggtggaccgcccgctctcgtcatctggaacatcaaacaccttctccacaccggaatcggcactgcctcgcgccccagcgaggtgtgtatggtggacggaacggacatgtgtttggctgacttccacgctggcattttcctgaaaggacaagagcatgctgtgttcgcctgtgtcacctccaacgggtggtacgcgatcgacgacgaggacttttacccctggacaccggacccgtccgacgtcctggtatttgttccgtacgatcaa

>HM854023.1_A_IND_1999

tacaacggtgagaaaaagactttctattccagacccaacaaccacgacaactgttggttgaacaccatcctgcagttgttcaggtacgtcgatgaacctttcttcgactgggtctatggatcgcctgagaacctcactctcgaggcaatcaggcagttggaagaggttactggccttgagctgcacgagggtggaccaccggctctcgtcatttggaacatcaaacacttgctcaacaccggaatcgggacttcttcgcgacccagcgaggtgtgcatggttgacggtacggacatgtgtttggctgacttccacgctggcatcttcctgaaaggacaggagcacgctgtgtttgcctgtatcacctccaacggttggtacgcgatcgacgacgaggacttctacccttggacgccggatccgtccgacgttctggtgtttgttccgtacgatcaa

>HQ009509.1_O_CHA_1999

cacaacggtgagaagaaaacattctactccaggcccaacaaccacgacaactgctggttgaacgccatccttcagctgtttaggtacgttgatgaacctttcctcgactgggtctactcttcacacgagaacctcacactcagtgctatcaaacaattggaagaaattactggccttgagctccacgagggcggaccacccgctctcgttatttggaacatcaaacacctgctcaacaccggaataggcaccgcttcgcgacccagcgaagtgtgcatggtaggcgggacggacatgtgcttggctgacttccacgctggcatcttcctgcagggacacgaacacgctgtgttcgcctgcgtcacctctaacgggtggtacgcgattgatgacgaggatttttacccctggacgccggatccgtccgacgttctggtgttcgtcccgtacgaccaa

>HQ113232.1_O_PAK_2009

cacaacggtgagaaaaagactttttactctaggcccaacagacacgacaactgttggttgaacaccatccttcagttgttcaggtatgttgatgaaccattcttcgactgggtctatgattcacctgaaaacctcactcttgaagcaatcaggcaactggaagaactcactggtcttgaactgcacgagggcggaccacctgcccttgtcatctggaacatcaaacacctgcttcataccggaatcggtaccgcctcacgacccagtgaggtgtgtatggttgatggcacggacatgtgtttggctgactttcacgctggcattttcctgaaaggacaagagcatgctgtgtttgcctgtgtcacatccaatgggtggtacgcgattgatgacgaggacttttacccttggacgccggacccgtccgacgttctggtatttgtcccgtacgatcaa

>HQ113233.1_Asia1_AFG_2009

cacaacggtgagaagaaaacattctactccaggcccaacaaccacgacaactgctggttgaacaccatcctccagttgtttaggtacgtcgatgaacctttcttcgactgggtctacaactcacccgagaatctcacacttgatgccatcaaacaactggaagaaattactggtcttgaactgcacgagggtggaccacccgctctcgttatttggaacatcaaacacctgctcagcaccggaatcggcaccgcttcgcgacctagcgaagtatgcatggtagacgggacggacatgtgtttggctgacttccatgctggcattttcctgaaagggcaggaacacgctgtgttcgcctgcgtcacctccaacgggtggtacgcgattgacgacgaggacttttacccctggacgccggacccgtccgacgttctggtgtttgtcccgtacgatcaa

>HQ268509.2_A_VIT_2004

cacaacggtgaaaagaagactttctactctaggcccaacaactacgacaactgttggttgaacaccatcctccaattgttcaggtacgtcgacgaaccattcttcgactgggtctatgaatcacctgagaacctcacccttgaagcgatcaggcaattggaagagatcactggtcttgagctgcacgagggtggtccacccgccctcgtgatttggaacatcaagcacttgctccacaccggaatcggcaccgcttcgcgacccagtgaggtgtgtatggtagacggtacggacatgtgcttggctgatttccatgctggcatcttcctgaaaggacaggaacacgcagtgtttgcctgtgtcacctccaacgggtggtacgcgattgacgacgaggacttttacccctggacaccagatccgtctgatgtcctggtgtttgtcccgtatgatcaa

>HQ268524.1_O_BHU_2004

tacaacggtgagaaaaagactttctactctaggcccaacagacacgacaactgttggttgaacaccatccttcagttgttcaggtatgtcgatgaaccattcctcgactgggtctatgattcacctgaaaacctcacttttgaagcaatcaggcaattggaggaactcactggtcttgaactgcacgagggtggaccacctgctctcgtcatctggaacatcaaacatctgctccacaccggaatcggcaccgcctcgcgacccagtgaggtgtgtatggttgacggtacggacatgtgtttggctgactttcacgctggcattttcctgaaaggacaagagcatgctgtgttcgcctgtgtcacatccgatgggtggtatgcgattgacgacgaggacttttacccttggacgccggacccgtccgacgttctggtttttgtcccgtacgatcaa

>HQ412603.1_O_CHA_2000

tacaacggcgaaaagaaggtgttttactccagacccaacaaccacgacaactgctggttgaacgccatcctccagttgttcaggtacgttgacgagcccttcctcgaatgggtctacgactcacccgagaacctcactctcgaggcgatcaacaaactggaagaaatcactggtcttgagctgcacgagggtggaccacccgcccttgttgtttggaacatcaaacacttgctctgtaccggaatcggcaccgcttcgcggcccagcgaagtgtgtatggtggacggcacagacatgtgtttggctgacttccacgccggtatatttctgaaggggcaagaccacgccgtattcgcctgccttacctctcatggttggtatgcgatcgacgacgaggacttctacccgtggacaccagatccggctgacgtcttggtttttgttccgtacgaccag

>HQ631363.1_Asia1_CHA_2006

cacaacggtgagaagaagaccttttactccagacccaacaaccatgacaactgctggctgaacactatcctccagttgttcaggtatgtcgatgagcctttcttcgactgggtctacgactcgcctgaaaacctcactctcgaggcgattaggcagttggaggaagttactggtcttgagctgcacgagggtggaccacccgcccttgtcatctggaacatcaagcatttgctccacaccggagtcggtaccgcttcgcgccctagcgaagtgtgtatggtagacggcacggacatgtgtttggctgatttccatgctggcattttcctgaaaggacaagaacatgctgtgtttgcctgtgtcacctccaacgggtggtacgcgatcgatgacgaggacttttacccctggacaccggacccgtccgacgtcttggtgtttgttccgtacgaccaa

>HQ632768.1_O_MAY_2000

cacaacggtgagaagaaaacattctactccaggcccaacaaccacgacaactgctggctgaacaccatcctccagttgtttaggtacgttgatgaacctttcttcgactgggtctactactcacctgagaacctcacacttgatgctatcaaacaattggaagaaattactggtctcgagctccacgagggtggaccacccgctctcgttatttggaacattaaacacctgctcaacaccggaatcggcactgcttcgcgacccagcgaagtgtgcatggtagacgggacggacatgtgtttggctgacttccacgctggcatcttcctgaaaggacaggaacacgctgtgttcgcctgcgtcacctccaacgggtggtacgcgattgatgacgaggacttttacccctggacgccggacccgtccgacgttctggtgtttgtcccgtacgatcaa

>HQ632769.1_O_MAY_2001

cacaacggtgaaaagaaaaccttttactctaggcccaacacccacgataactgttggttgaataccatcctacagctgttcaggtacgtcgatgaacctttcttcgactgggtctatgaatcgcccgagaacctcactcttgaggcgattaaacaactggaagagattactggtcttgaactgcgcgagggtggtccgcccgccctcgtcatttggaacatcaaacacttgctccacactggcatcggtaccgcctcgcgacccagcgaggtgtgtatggtggacggtacggacatgtgtctggctgacttccatgctggtattttcctgaaaggacatgaacacgccgtgtttgcctgtgtcacctccgacgggtggtacgcgattgatgacgaggacttctacccctggacgccagaaccctctgacgtcttggtgtttgtcccgtacgatcaa

>HQ632770.1_O_MAY_2004

tacaacggtgagaaaaagactttctactctaggcccaacagacacgacaactgttggttgaacaccatccttcagttgttcaggtatgtcgacgaaccattcttcgactgggtctatgattcacctgaaaacctcactcttgaagcaatcaggcaattggaggaactcactggtcttgaactgcacgagggtggaccacctgctctcgtcatctggaacatcaaacacctgctccacaccggaatcggcaccgcctcgcgacccagtgaggtgtgtatggttgacggtacggacatgtgtttggctgactttcacgctggcattttcctgaaaggacaagagcatgctgtgttcgcctgtgtcacatccgatggttggtacgcgattgatgacgaggacttttacccttggacgccggacccgtccgacgttctggtttttgtcccgtacgatcaa

>HQ632771.1_O_MAY_2005

cacaacggtgaaaagaaggtcttctactccagacccaacaaccacgacaactgttggctgaacgccatcctccaattgttcaggtacgtcgacgaacccttccttgagtgggtctacgactcgcctgagaacctcaccctcgaagcaattaacaagctggaagacatcactggtctagagctgcacgagggtgggccacctgccctcgtcatctggcacatcaaacacttgctctacaccggaatcggtaccgcctcacggcccagcgaggtgtgtatggtggacggcacggacatgtgtctggctgacttccatgccggcatatttctgaagggacaggaccacgccgtgttcgcttgtgtcacctctgacgggtggtacgcgatcgacgacgaggatttctatccgtggacaccagacccggttgacgtcctggttttcgtcccgtacgatcaa

>HQ632772.1_O_MAY_2007

tacaacggtgagaagaagaccttttactctaggcccaacaacaatgacaactgttggctgaatgccatcctgcagttgtttaggtatgtcgatgaacctttcttcgactgggtctatgaatcgcctgagaaccgcactcttgaggcgattaaacaattagagggaattactggtcttgaactgcacgagggcggtccacccgctctcgttgtttggaacatcaaacacttgctccacaccggaattggtaccgcctcgcgacccagcgaggtgtgtatggttgacggtacggacatgtgcctggctgacttccacgctggcatcttcctgaaaggacaggaacacgctgtgtttgcctgcgtcacctccaacgggtggtacgcgattgatgacgaggacttttacccctggacgccggatccgtccgacgtgctggtgttcgtcccgtacgatcaa

>HQ632773.1_A_MAY_2007

cacaacggtgaaaagaaaactttctactctaggcccaacaaccacgacaactgttggttgaacaccattctccaactgttcaggtacgtcgacgaaccattcttcgactgggtctatgaatcacctgagaacctcacccttgaagcgatcaggcaattggaacagatcactggtcttgagctgcacgagggtggcccacccgccctcgtaatttggaacatcaagcacttgctccacaccggaatcggtaccgcctcgcgacctagtgaggtgtgtatggtagacgggacggacatgtgtctggctgatttccatgctggtattttcctgaaagggcaggaacatgcagtatttgcctgtgttacctccaacgggtggtacgcaattgacgacgaggacttttacccctggacaccagacccgtctgatgtcctggtgtttgtcccgtatgatcaa

>HQ632774.1_Asia1_MAY_1999

tacaacggtgaaaagaagatctttttctctagacccaacaaccacgacaactgttggttgaacaccatccttcagctgttcaggtacgtcgatgagcctttctttgactgggtttatgaatcacctgagaacctcactcttgaagcaattaaacagctggaagagattactggtcttgagctgcgtgagggtggtccacccgccctcgtcatttggaacatcaagcacttgctccacaccggaatcggtaccgcctcacgacccagtgaggtgtgtatggtagatggtacggacatgtgtctggctgacttccacgctggcattttcctgaaaggacaggaacatgctgtgtttgcctgtgtcacctccgatggatggtacgcgattgacgacgaagacttctacccctggacgccggatccgtccgacgtcctggtgtttgtcccgtacgatcag

>HQ832576.1_A_IND_1990

cacaacggtgagaagaagactttctactccagacccaacaaccatgacaactgttggttgaacgccattctacagttgttcaggtatgtcgatgagcctttctttgactgggtctatgaatcacctgaaaaccttactctggatgcaatcaaacaactggaagagattactggccttgagctgcacgagggtggaccacccgcccttgtcgtctggaacatcaaacacctgctccacaccggaatcggcaccgcctcgcgccccagtgaggtgtgcatggttgacggaacgaacatgtgtctggctgactttcacgctggcatcttcctgaaagggcaagagcacgccgtgttcgcctgcgtcacctccaacgggtggtacgcgatcgatgacgaggacttctacccctggacaccggacccgtccgacgtcctggtatttgttccgtacgatcaa

>HQ832577.1_A_IND_1999

tacaacggtgagaaaaagactttctattccagacccaacaaccacgacaactgttggttgaacaccatcctgcagttgttcaggtacgtcgatgaacctttcttcgactgggtctatgaatcgcctgagaacctcactctcgaggcaatcaggcagttggaagaggttactggccttgagctgcacgagggtggaccacccgctctcgtcatttggaacatcaaacacttgctcaacaccggaatcggcactgcctcgcgacccagcgaggtgtgcatggttgacggcacggacatgtgtttggctgacttccacgctggcatcttcctgaaaggacaggagcacgctgtgtttgcctgtaacacctccaacgggtggtacgggatcgacgacgaggacttctacccttggacggcggatccgtccgacgttctggtgtttgttccgtacgatcaa

>HQ832578.1_A_IND_2003

tacaacggtgaaaagaaaaccttctattctagacccaacaaccacgacaactgttggttgaacaccatcctccagttgtttaggtacgttgatgaacccttcttcgactgggtctatgagtcgcctgaaaacctcaccctcgaggcgatcaggcagttggaagaagttactggtcttgaactgcacgagggtggaccacccgcccttgtcatttggatcatcaagcacctgctccacaccggaatcggcactgtctcgcgacccagcgaggtgtgcatggttgacggtacggacatgtgtttggccgacttccacgctggcatcttcatgagagtgcaggaacacgctgtgtttgcctgtgtcacctccaatggttggtacgcgattgacgacgaggacttttacccttggacgccggatccgtccgacgttttggtgtttgttccgtacgatcaa

>HQ832579.1_A_IND_2003

tacaacggtgaaaagaaaaccttctattctagacccaacaaccacgacaactgttggttgaacaccatcctccagttgtttaggtacgttgatgaacccttcttcgactgggtctatgagtcgcctgaaaacctcaccctcgaggcgatcaggcaattggaagaagttactggacttgaactgcacgagggtggaccacccgcccttgtcatttggaacatcaagcacctgctccacaccggaatcggcactgcctcgcgacccagcgaggtgtgcatggttgacggtacggacatgtgtttggccgacttccacgctggcatcttcatgaaagggcaggaacacgctgtgtttgcctgtgtcacctccaatggttggtacgcgattgacgacgaggacttttacccttggacgccggatccgtccgacgttttggtgtttgttccgtacgatcaa

>HQ832580.1_A_IND_2003

tacaacggtgaaaagaaaaccttctattctagacccaacaaccacgacaactgttggttgaacaccatcctccagttgtttaggtacgttgatgaacccttcttcgactgggtctatgagtcgcctgaaaacctcaccctcgaggcgatcaggcagttggaagaagttactggacttgaactgcacgagggtggaccacccgcccttgtcatttggaacatcaagcacctgctccacaccggaatcggcactgcctcgcgacccagcgaggtgtgcatggttgacggtacggacatgtgtttggccgacttccacgctggcatcttcatgaaagggcaggaacacgccgtgtttgcctgtgtcacctccaatggttggtacgcgattgacgacgaggacttttacccttggacgccggatccgtccgacgttttggtgtttgttccgtacgatcaa

>HQ832581.1_A_IND_2004

tacaacggtgaaaagaaaaccttctattccagacccaacaaccacgacaactgttggttgaacaccatcctccagttgtttaggtacgttgatgaacccttcttcgactgggtctatgagtcacctgaaaacctcaccctcgaggcgatcaggcagttggaagaagttactggacttgaactgcacgagggtggaccacccgcccttgtcatttggaacatcaagcacctgctccacaccggaatcggcactgcctcgcgacccagcgaggtgtgcatggttgacggtacggacatgtgcttggccgacttccacgctggcatcttcatgaaagggcaggaacacgccgtatttgcctgtgtcacctccaatggctggtacgcgattgacgacgaggacttttacccttggacgccggatccgtctgacgttttggtgtttgttccgtacgatcaa

>HQ832582.1_A_IND_2004

tacaacggtgaaaagaaaaccttctattctagacccaacaaccacgacaactgttggttgaacaccatcctccagttatttaggtacgttgatgaacctttcttcgactgggtttatgagtcgcctgagaacctcaccctcgaggcgatcaggcagttagaagaagttactggactcgaactgcacgagggtggaccacccgcccttgtcatttggaacatcaagcacctgctccacaccggaattggcactgcctcgcgacccagcgaggtgtgcatggttgacggtacggacatgtgtttggccgacttccacgctggcatctttatgaaagggcaggaacacgctgtgtttgcctgtgtcacctccaatggttggtacgcgattgacgacgaggacttttacccttggacgccggatccgtccgacgttttggtgtttgttccgtacgatcaa

>HQ832583.1_A_IND_2005

tacaacggtgaaaagaaaaccttctattctagacccaacaaccacgacaactgttggttgaacaccatcctccagttgtttaggtacgttgatgaacccttcttcgactgggtctatgagtcgcctgaaaacctcaccctcgaggcgatcaggcagttggaagaagttactggacttgaactgcacgagggtggaccacccgcccttgtcatttggaacatcaagcacctgctccacaccggaatcggcacttcctcgcgacccagcgaggtgtgcatggttgacggtacggacatgtgtttggccgacttccacgctggcatcttcatgaaagggcaggaacacgctgtgtttgcctgtgtcacctccaacggttggtacgcgattgacgacgaggacttttacccttggacgccggatccgtccgacgttttggtgtttgttccgtacgatcaa

>HQ832584.1_A_IND_2005

cacaacggtgagaaaaagactttctattctagacccaacaaacacgacaactgttggctgaacaccatccttcagctattcaggtatgtcgatgaaccattcttcaactgggtttatgattcacctgagaacctcactcttgaggcgatcaaacaattggaagaactaactggtcttgagttgcacgagggcgggccacccgctctcgtcatttggaacatcaagcatctgctccacaccggaatcggcaccgcctcacgacccagtgaggtgtgcatggtcgacggtacggacatgtgcttggctgactttcacgctggcatcttcctgaaaggacaagaacatgctgtgttcgcctgtgtcacctctgacgggtggtacgcgattgacgacgaggacttttacccttggacaccggacccgtccgatgttctggtttttgtcccgtacgatcaa

>HQ832585.1_A_IND_2005

tacaacggtgagaagaagactttttactccagacccaacaaccatgacaactgttggttaaacaccatccttcagttgttcaggtacgttgatgaaccattctttgactgggtttatgattcacctgagaacctcacccttgaggcaatcaagcaattggaagaacttactggtcttgaactgcatgagggcggaccacctgctctcgtcatttggaatattaagcatctgctccacaccggtatcggcaccgcctcgcgacccagtgaagtgtgcatggtagatggtacggacatgtgtttggctgactttcacgctggcattttcctgaaaggacaagagcatgctgtgttcgcctgtgtcacatctgacgggtggtacgcgattgacgacgaggaattctacccctggacaccggacccgtccgatgttctggtttttgtcccgtacgatcaa

>HQ832586.1_A_IND_2006

cacaacggtgagaagaagactttttactctagacccaacaaccatgacaactgttggttgaacaccatccttcagttgtttaggtacgtcgacgaacctttcttcgactgggtctatgagtcacctgagaacctcacgttagaggcgatcaagcaattggaagaactcactggtcttgagctgcacgagggcggaccacccgctctcgtcatttggaacattaagcacctgctccacaccgggatcggtactgcctcgcgacccagcgaggtgtgcatggttgatggtacggacatgtgcttggctgactttcatgctggcatcttcctgaaagggcaagaacacgctgtgttcgcctgtgtcacctccgacgggtggtacgcgattgacgacgaggacttttacccttggacaccggatccgtccgatgttctggtttttgtcccgtacgatcaa

>HQ832587.1_A_IND_2005

tacaacggtgagaacaagatcttctactctagacccaacaaccacgacaactgttggctgaacaccatcctccagctgttcaggtacgtcgatgagcctttcttcgactgggtctatgaatcacctgagaacctcactcttgaggcgatcaaacaactggaagaagtcactggtcttgaactgcacgagggtggaccgcccgcccttgtcatctggaacattaaacacttgctccacaccggaatcggtactgcttcgcgacccagcgaggtgtgtatggttgacggaacggacatgtgtttggctgactttcatgctggcattttcctgaaaggacaagagcacgctgtgttcgcctgtgtcacctccaacgggtggtacgcgattgatgacgaggacttttacccttggacgccggatccgtccgatgttctggtttttgtcccgtatgatcaa

>HQ832588.1_A_IND_2005

cacaatggtgagaagaaaacattctactccaggcccaacaaccacgacaactgctggctgaacaccatccttcagttgttcaggtacgttgatgaacctttcttcgactgggtctacaactcacctgagaacctcacacttgatgctatcaaacaattggaggaaatcactggccttgagctgcacgagggcggaccacccgctctcgttatttggaacattaaacacctgctcaacaccggaatcggcaccgcttcgcgacccagcgaagtgtgcatggtagacgggacggacatgtgtttggctgacttccacgctggcatcttcctgaaaggacaggaacacgccgtgtttgcctgcgtcacctctaacgggtggtacgcgattgacgacgaggacttttacccctggacgccggatccatccgacgttctggtgttcgtcccgtacgatcaa

>HQ832589.1_A_IND_2006

tacaacggtgagaaaaagactttctactctagacccaacaaccacgacaactgttggctgaacaccatccttcagttgtttaggtacgttgatgaaccattcttcgactgggtttatgattcacctgaaaacctcacttctgaggcgatcaaacaactggaggaactcactggtcttgagctgcacgagggcggaccacctgctcttgtaaattggaacatcaagcacctgctccacaccggaattggcaccgcctcgcgacccagcgaggtgtgcatggttgatggtacggacatgtgcttggctgactttcatgctggcatcttcctgaaagggcaagagcacgctgtgttcgcctgtatcacctccaatgggtggtacgcgattgatgacgaggacttttacccttggacaccggacccgtccgatgttctggtttttgtcccgtacgatcaa

>HQ832590.1_A_IND_2007

cacaacggtgagaaaaagaccttctactctaggcccaacaaccacgacaactgttggttgaacaccatcctccagttgtttaggtacgtcgacgaacccttcttcgactgggtctatgagtcgcctgaaaacctcactcttgaggcaattaggcaactagaagaaatcactggtcttgagctgcacgagggtggcccgcccgctctcgtcatttggaacatcaagcacttgctccacaccggaatcggcaccgcttcgcgacccagcgaggtgtgcatggttgatggcacggacatgtgtttggccgacttccacgctggcatcttcctgaaagggcaagaacacgccgtgttcgcctgtgtcacctccaacgggtggtacgcgatcgatgacgaggacttttacccctggacgccggatccgtctgacgttctggtgtttgtcccgtacgatcaa

>HQ832591.1_A_IND_2008

cacaacggtgagaaaaagaccttctactctaggcccaacaaccacgacaactgttggttgaataccatcctccagttgtttaggtacgtcggcgaacccttcttcgactgggtctatgagtcgcctgaaaacctcactcttgaggcgattaggcaactagaagaaatcactggtcttgaactgcacgagggtggcccgcccgctctcgtcatttggaacatcaagcacttgctccacaccggaatcggcactgcttcgcgacccagcgaggtgtgcatggttgatggcacggacatgtgtttggccgacttccacgctggcatcttcctgaaagggcaagaacatgccgtgttcgcctgtgtcacctccaacgggtggtacgcgatcgacgacgaggacttttacccctggacaccggatccgtctgatgttctggtgtttgtcccgtacgatcaa

>HQ832592.1_A_IND_2009

cacaacggtgagaaaaagaccttctactctaggcccaacaaccacgacaactgttggttgaacaccatcctccagttgtttaggtacgtcgacgaacccttcttcgactgggtctatgagtcgcctgaaaacctcactcttgaggcaattaggcaactagaagaaatcactggtcttgagctgcacgagggtggcccgcccgctctcgtcatttggaacatcaagcacttgctccacaccggaatcggcactgcttcgcgacccagcgaggtgtgcatggttgatggcacggacatgtgtttggccgacttccacgctggcatcttcctgaaagggcaagaacacgccgtgttcgcctgtgtcacctccaacgggtggtacgcgatcgatgacgaggacttttacccctggacgccggatccgtctgacgttctggtgtttgtcccgtacgatcaa

>JF749841.1_A_TUR_2006

cacaacggtgagaagaagacctttttctctaggcccaacaaccacgacaactgttggttgaacaccatcctccagttgttcaggtacgttgatgagcctttcttcgactgggtctacgactctcctgagaacctcaccctcaaggcaatacagcaattggaggagattactggcctagagctgcacgagggtgggccgcccgctctcgtcatctggaacatcaaacacttgctcaacaccgggattggcactgcttcacggcccagtgaggtgtgcatggtcgacgggacagacatgtgtttggccgatttccatgctggcattttcttgaaaggacaggaacacgctgtgtttgcctgtgtcacctctaacgggtggtacgcgatcgacgacgaggacttctacccgtggacgccggacccgtctgacgtcctggtgtttgttccgtacgatcaa

>JF749843.1_A_EGY_2006

tacaacggtgacaagaagaccttctactccaggcccaacaaccacgacaactgttggttgaatgctgtcctgcaactttttaggtatgttgatgaacctttctttgactgggtttatgagtcccccgaaaacctcacccttcaagcaattgaacaactggaggaactcaccggtcttgaactccacgagggtggaccacccgctctcgtcatttggaacattaaacacttgctccacactggcatcggcaccgcctcgcgccccagcgaggtgtgcatggtggatggcacggacatgtgtttggctgactttcatgctggaatctttctgaaagggcaggaacacgctgtgtttgcttgtgtcacctctgaagggtggtacgcgattgacgacgaggacttttacccttggacaccggacccttccgacgtcctagtcttcgtcccctacgatcaa

>JF749848.1_A_TUR_2003

tacaacggagagaagaagacattttactccagacccaacaaccacgacaactgctggttgaacgccatcctccagttgttcaggtacgtcgatgaacctttcttcgactgggtctataattcgcctgaaaacctcacacttgaggccataaggcagctggaagagcttactggtcttgaactgcacgagggtggaccgcccgctctcgttgtctggaacatcaaacaccttctccacaccggaatcggtaccgcctcgcgacccagcgaggtgtgtacggtagacggaacggacatgtgtttggctgactttcacgctggtattttcttgaaaggacaagagcatgctgtgtttgcctgcgtcacatctaacgggtggtacgcgatcgacgacgaggacttttacccctggacgccggacccgtccgacgtcctggtatttgtcccgtacgatcag

>JF749849.1_Asia1_PAK_2002

cacaacggtgagaagaaaacattctactccaggcccaacaaccacgacaactgttggttgaacaccatcctccagttgtttaggtacgtcgatgaacctttcttcgactgggtctacaactcacccgagaacctcacacttgatgccatcaaacaactggaagaaatcactggtcttgcgctgcacgagggtggaccacccgctctcgttatttggaacatcaaacacctgctcagcaccggaatcggcaccgcttcgcgacccagcgaagtgtgcatggtagacgggacggacatgtgtttggctgacttccatgctggcattttcctgaaaggacaggaacacgctgtgtttgcttgcgtcacctccaacgggtggtacgcaattgacgacgaagacttttacccctggacgccggacccgtccgacgttctggtgtttgtcccgtacgatcaa

>JF749851.1_O_IRN_2001

cacaacggtgagaagaaaacattctactccaggcccaacaaccacgacaactgttggctgaacaccatcctccagttgtttaggtacgtcgatgaaccgttcttcgactgggtctacaactcacctgaaaacctcacacttgatgccatcaaacaactggaagaaaccactggtcttgagctgcacgagggtggaccacccgctctcgttatttggaacatcaaacacctgctcagcaccggaatcggcaccgcttcgcgacccagcgaagtgtgcatggtagacgggacggacatgtgtttggctgacttccatgctggcattttcctgaaaggacaggaacacgctgtgttcgcctgcgtcacctccaacgggtggtacgcgattgacgacgaggacttttacccctggacgccggacccgtccgacgttctagtgtttgtcccgtacgatcaa

>JF749852.1_O_MAY_2004

tacaacggtgagaaaaagactttctactctaggcccaacagacacgacaactgttggttgaacaccatccttcagttgttcaggtatgtcgatgaaccattcttcgactgggtctatgattcacctgaaaacctcactcttgaagcaatcaggcaattggaggaactcactggtcttgaactgcacgagggtggaccacctgctctcgtcatctggaacatcaaacacctgctccacaccggaatcggcaccgcctcgcgacccagtgaggtgtgtatggttgacggtacggacatgtgtttggctgactttcacgctggcattttcctgaaaggacaagagcatgctgtgttcgcctgtgtcacatccgatggttggtacgcgattgatgacgaggacttttacccttggacgccggacccgtccgacgttctggtttttgtcccgtacgatcaa

>JF749860.1_SAT1_KEN_2002

tacaacggtgagaagaagacattctacagcaggcccaacacacacggcaactgctggctcaactcactgctgcagctcttccgctacgtggatgagccactctttgagtctgagtacttgtcaccggagaacaagactttggacatgattagacagctgtctgattacaccaaacttgatctctcggacggtgggccaccggcactcgtgctctggctcatcaaggactgccttcaaaccggcgttggtacgagcacccgtcccagcgaggtttgtgtcatcaacggggtcaccatgactctggctgatttccacgccggtattttcctcaaaggcactgaacacgctgtcttcgccctcaacacctccgagggctggtacgcaattgatgatgaggtgttctacccatggacgcccgaccctgcagacgtactcgcgtacgtaccctacgaccaa

>JF749861.1_SAT2_KEN_2002

tataacggtgagaagaagaccttctacagcaggcctaacacatacggcaactgctggctcaactcgctgctgcagctctttcgatacgtcgatgaaccgcttttcgagtctgagtacctgtcacctgagaacaagacactggacatgatcaaacagctttctgactacaccggacttgacctctcagacggtgggccacccgcacttgtgctttggctcatcaaagattgtctcaacactggcgttggcaccagcactcgccccagcgagatctgcgtgatcaacggggttgtaatgacactggctgacttccacgctggcatcttcatcaaaggtaccgaacacgcagtgtttgccctcaacacatcagagggctggtacgccattgatgatgaggttttctacccatggacacctgaccccgcggacgtgcttgcgtacgtaccgtacgaccaa

>JF749862.1_SAT2_UGA_2002

tacaacggtgagaagaagactttctattctaggcccaacaaacacgacaactgctggttgaacaccatcctgcaattgttcaggtacgtcgacgaaccattcttcgactgggtctacaactcgcccgagaacctcactctccaagcaattgaacaactggaggaactcactgggcttgagttgcacgaaggcggaccccccgccctcgtgatttggaacatcaaacacctgctctacactgggattggtactgcctcgcgacccagcgaggtgtgcatggtggacggcacagagatgtgtctcgccgacttccacgcagggattttcctcaagggtactgaacacgcagtgttcgcctgtctgacctccgacggatggtacgccatagacgacgaggacttctacccatggacaccggatccgtccgacgtcctgtgttttgtcccgtacgacatg

>JF749864.1_SAT2_ZIM_2003

tacaacggagagaagaagaccttctacagcagacccaacaaacacgggaactgctggctcaattcgctgttgcagctctttcgatacgtcgatgagccgcttttcgaatctgagtatctgtcacctgaaaacaagacattggacatgatcaaacagctatccgattacaccaaactggatttgtcggacggagggccccccgctctcgtcctttggctgatcaaagactgtcttcagactggcgtcggcaccagcactcgccccagcgagatctgtgtcatcaatggggttgtcatgaccctggctgatttccacgccggtattttcatcaaaggcactgaacacgctgtgtttgccctcaacacatctgagggctggtacgccattgatgatgaggtgttctacccgtggacacctgaccctgaaaacgtacttgcgtacgttccctacgaccag

>JN006719.1_Asia1_PAK_2008

cacaacggtgagaagaagactttctactccaggcccaacaaccacgacaactgctggctgaacaccatcctccagttgttcaggtacgtcgatgaacctttcttcgactgggtctacaactcacctgagaacctcacacttgatgccatcaaacaattggaagaaatcactggccttgagctgcacgagggtggaccacccgctctcgtcatttggaacattaaacacttgctcaacaccggaatcggcaccgcttcgcgacccagcgaagtgtgcatggtagacgggacggacatgtgtttggctgacttccacgctggcattttcctgaaaggacaggaacacgctgtgttcgcctgcgtcacctccaacgggtggtacgcgatcgacgacgaggacttttacccctggacgccggacccgtccgacgttctggtgtttgtcccgtacgatcaa

>JN006720.1_Asia1_PAK_2009

cataacggtgagaaaaagactttctactctaggcccaatagacatgacaactgttggttgaacaccatccttcagttgtttaggtacgtcgatgaaccattcttcgactgggtctatgattcacctgaaaaccttactcttgaagcaatcaggcaactggaggaactcactggtcttgaactgcacgagggtggaccacctgctctcgtcatttggaacatcaaacacctgctccacaccggaatcggcaccgcctcgcgacccagtgaggtgtgtatggttgacggtacggacatgtgtttggctgactttcatgctggtattttcctgaaaggacaagagcatgctgtgttcgcctgtgtcacatccgatggatggtacgcgattgacgacgaggacttctatccttggacgccggacccgtctgacgttctggtttttgtcccgtacgatcaa

>JN006722.1_A_PAK_2008

cataacggtgagaaaaagactttctactctaggcccaacagacacgacaactgttggttgaacaccatccttcagttgtttaggtatgtcgatgaaccattcttcgactgggtctatgattcacctgaaaaccttactcttgaagcaatcaggcaactggaggaactcactggtcttgaactgcacgagggtggaccacctgctctcgtcatttggaacatcaaacacctgctccacaccggaatcggcaccgcctcgcgacccagtgaggtgtgtatggttgacggtacggacatgtgtttggctgactttcatgctggcattttcctgaaaggacaagagcatgctgtgttcgcctgtgtcacatccgatggatggtacgcgattgacgacgaggacttctatccttggacgccggacccgtctgacgttctggtttttgtcccgtacgatcaa

>JN099688.1_A_IRQ_2009

cacaacggtgagaagaagactttcttttctaggcccaacaaacacgacaactgttggttgaacaccatcctccagttgttcaggtacgttgatgagcctttctttgactgggtctacaactctcctgagaacctcaccctcaaggcaatacaacaattggaagagattactggtctggagttacacgagggtgggccgcccgctctcgtcatctggaacatcaaacacttgcttaacaccgggatcggcaccgcttcacgtcccagtgaggtgtgcatggtcgacgggacagacatgtgtttagctgatttccatgctggtattttcttgaaaggacaagagcatgctgtgtttgcctgtgtcacctccaacgggtggtacgcgatcgacgacgaggacttctatccgtggacgccagatccgtctgacgtcctggtgtttgttccgtacgatcaa

>JN099694.1_A_IRQ_2009

cacaacggtgagaagaagactttcttttctaggcccaacaaccacgacaactgttggttgaacaccatccttcagttgttcaggtacgttgatgagcctttcttcgactgggtctacaactctcctgagaacctcaccctcaaggcaatacaacaattggaagagattactggtctggagttacacgagggtgggccgcccgctctcgtcatctggaacatcaaacacttgcttaacaccgggatcggcaccgcttcacgtcccagtgaggtgtgcatggtcgacgggacagacatgtgtttagctgatttccatgctggtattttcttgaaaggacaagagcatgctgtgtttgcctgtgtcacctccaacgggtggtacgcgatcgacgacgaggacttctatccgtggacgccagatccgtctgacgtcctggtgtttgttccgtacgatcaa

>JN099695.1_A_IRQ_2009

cacaacggtgagaagaagactttcttttctaggcccaacaaccacgacaactgttggttgaacaccatccttcagttgttcaggtacgttgatgagcctttcttcgactgggtctacaactctcctgagaacctcaccctcaaggcaatacaacaattggaagagattactggtctggagttacacgagggtgggccgcccgctctcgtcatctggaacatcaaacacttgcttaacaccgggatcggcaccgcttcacgtcccagtgaggtgtgcatggtcgacgggacagacatgtgtttagctgatttccatgctggtattttcttgaaaggacaagagcatgctgtgtttgcctgtgtcacctccaacgggtggtacgcgatcgacgacgaggacttctatccgtggacgccagatccgtctgacgtcctggtgtttgttccgtacgatcaa

>JN099697.1_A_IRQ_2009

cacaacggtgagaagaagactttcttttctaggcccaacaaccacgacaactgttggttgaacaccatcctccagttgttcaggtacgttgatgagcctttcttcgactgggtctacaactctcctgagaacctcaccctcaaggcaatacaacaattggaagagattactggtctagagttacacgagggtgggccgcccgctctcgtcatctggaacatcaaacacttgcttaacaccgggatcggcaccgcttcacgtcccagtgaggtgtgcatggtcgacgggacagacatgtgtttggctgatttccatgctggtattttcttgaaaggacaagagcatgctgtgtttgcctgtgtcacctccaacgggtggtacgcgatcgacgacgaggacttctatccgtggacgccagatccgtctgacgtcctggtgtttgttccgtacgatcaa

>JN099698.1_A_IRQ_2009

cacaacggtgagaagaagactttcttttctaggcccaacaaccacgacaactgttggttgaacaccatcctccagttgttcaggtacgttgatgagcctttctttgactgggtctacaactctcctgagaacctcaccctcaaggcaatacaacaattggaagagattactggtctagagttacacgagggtgggccgcccgctctcgtcatctggaacatcaaacacttgcttaacaccgggatcggcaccgcttcacgtcccagtgaggtgtgcatggtcgacgggacagacatgtgtttggctgatttccatgccggtattttcttgaaaggacaagagcatgctgtgtttgcctgtgtcacctccaacgggtggtacgcgatcgacgacgaggacttctatccgtggacgccagatccgtctgacgtcctggtgtttgttccgtacgatcaa

>JN099699.1_A_IRQ_2009

cacaacggtgagaagaagactttcttttctaggcccaacaaccacgacaactgttggttgaacaccatcctccagttgttcaggtacgttgatgagcctttctttgactgggtctacaactctcctgagaacctcaccctcaaggcaatacaacaattggaagagattactggtctagagttacacgagggtgggccgcccgctctcgtcatctggaacatcaaacacttgcttaacaccgggatcggcaccgcttcacgtcccagtgaggtgtgcatggtcgacgggacagacatgtgtttggctgatttccatgctggtattttcttgaaaggacaagagcatgctgtgtttgcctgtgtcacctccaacgggtggtacgcgatcgacgacgaggacttctatccgtggacgccagatccgtctgacgtcctggtgtttgttccgtacgatcaa

>JN998085.1_O_CHA_2010

tacaacggtgagaagaagatcttctactccaggcccaacaaccacgacaactgttggctgaacgccatccttcagctgttcaggtacgtcgatgaacctttcttcgactgggtatatgaatcacctgaaaacctcacccttgaggcgatcagacaactggagaacattactggttttgagctgcacgagggtggcccgcccgccctcgtcatttggaacatcaaacacttgctccacaccgggatcggcaccgcctcgcgacccagcgaggtgtgcatggtggacggcacggacatgtgcctggctgacttccacgctggcatcttcctgaaaggacaggaacacgccgtgtttgcctgcgtcacctccaacgggtggtacgcgatcgacgacgaagaattctacccctggacgccagatccgtccgacgtgctggtctttgtcccgtacgatcaa

>JN998086.1_O_CHA_2010

tacaacggtgagaagaagatcttctactccaggcccaacaaccatgacaactgttggctgaacgccatccttcagctgttcaggtacgtcgatgaacctttcttcgactgggtatatgaatcacctgaaaacctcacccttgaggcgatcagacaactggagaacattactggtcttgagctgcacgagggtggtccgcccgccctcgtcatttggaacatcaaacacttgctccacaccgggatcggcaccgcctcgcgacccagcgaggtgtgcatggtggacggtacggacatgtgcctggctgacctccacgctggcatcttcctgaaaggacaggaacacgccgtgtttgcctgcgtcacctccaacgggtggtacgcgatcgacgacgaagaattctacccctggacgccagatccgtccgacgtgctggtctttgtcccgtacgaccaa

>JQ900581.1_O_CHA_2010

tacaacggtgagaagaagatcttgtactccaggcccaacaaccacgacaactgttggctgaacgccatccttcagctgttcaggtacgtcgatgaacctttcttcgactgggtatatgaatcacctgaaaacctcacccttgaggcgatcagacaactggagaacattactggtcttgagctgcacgagggtggtccgcccgccctcgtcatttggaacatcaaacacttgctccacaccgggatcggcaccgcctcgcgacccagcgaggtgtgcatggtggacggtacggacatgtgcctggctgacttccacgctggcatcttcctgaaaggacaggaacacgccgtgtttgcctgcgtcacctccaacgggtggtacgcgatcgacgacgaagaattctacccctggacgccagatccgtccgacgtgctggtctttgtcccgtacgatcaa

>JQ973889.1_O_CHA_2010

tacaacggtgagaagaagatcttttactccaggcccaacaaccacgacaactgttggctgaacgccatccttcagctgttcaggtacgtcgatgaacctttcttcgactgggtatatgaatcacctgaaaacctcacccttgaggcgatcggacaactggagaacattactggtcttgagctgcacgagggtggtccgcccgccctcgtcatttggaacatcaaacacttgctccacaccgggatcggcaccgcctcgcgacccagcgaggtgtgcatggtggacggtacggacatgtgcctggctgacttccacgctggcatcttcctgaaaggacgggaacacgccgtgtttgcctgcgtcacctccaacgggtggtacgcgatcgacgacgaagaattctacccctggacgccagatccgtccgacgtgctggtctttgtcccgtacgatcaa

>JX014255.1_SAT2_EGY_2012

tacaacggtgagaaaaagaccttctactcaaggcccaaccgtcacgacaactgctggttgaacaccatactgcagctgttcaggtacgtcgatgaaccattcttcgactgggtctacaattcacctgagaacctcacgcttcaagcaattgagcaacttgaggagctcacaggtctcaacctacgcgagggtggccctcctgccctcgtgatttggaacatcaagcacctgttgtacaccggaatcggcactgcctcacgacccagtgaggtgtgcatggttgacggtactgacatgtgtcttgctgatttccacgcaggaattttcctcaaaggtgctgaacacgccgtgttcgcctgcttgacctccaacggatggtatgctattgatgacgaggacttctacccatggacaccggacccgtccgacgtcctgtgttttgtcccgtatgatgta

>JX014256.1_SAT2_PAT_2012

tacaacggtgagaaaaagaccttctactcaaggcccaaccgtcacgacaactgctggttgaacaccatactgcagctgttcaggtacgtcgatgaaccattcttcgactgggtctacaattcacctgagaacctcacgcttcaagcaattgagcaacttgaggagctcacaggtctcaacctacgcgagggtggccctcctgccctcgtgatttggaacatcaagcacctgttgtacaccggaattggcactgcctcacgacccagtgaggtgtgcatggttgacggtactgacatgtgtcttgctgatttccacgcaggaattttcctcaaaggtgctgaacacgccgtgttcgcctgcttgacctccaacggatggtatgctattgatgacgaggacttctacccatggacaccggacccgtccgacgtcctgtgttttgtcccgtatgatgta

>JX040485.1_O_BUL_2010

cacaatggtgagaagaagactttctactccaggcccaacaaccacgacaactgctggctgaacaccatcctccagttgttcaggtatgtcgatgaacctttcttcgactgggtctacaactcacctgagaacctcacactcgaagccatcaaacaattggaagaaattactggccttgagctgcacgagggtggaccacccgctctcgtcatctggaacatcaaacacttgctcaacaccggaatcggcaccgcctcgcgacccagcgaggtgtgcatggtagacgggacggacatgtgtttggctgacttccacgctggcattttcctgaaaggacaggaacacgctgtgttcgcctgcgtcaccaccaacgggtggtacgcgatcgacgatgaggatttttacccctggacgccggacccgtccgacgttctggtgtttgtcccgtacgatcaa

>JX040486.1_O_BUL_2011

cacaatggtgagaagaagactttctactccaggcccaacaaccacgacaactgctggctgaacaccatcctccagttgttcaggtatgtcgatgaacctttcttcgactgggtctacaactcacctgagaacctcacactcgaagccatcaaacaattggaagaaattactggccttgagctgcacgagggtggaccacccgctctcgtcatctggaacatcaaacacttgctcaacaccggaatcggcaccgcctcgcgacccagcgaggtgtgcatggtagacgggacggacatgtgtttggctgacttccacgctggcattttcctgaaaggacaggaacacgctgtgttcgcctgcgtcaccaccaacgggtggtacgcgatcgacgacgaggatttttacccctggacgccggacccgtccgacgttctggtgtttgtcccgtacgatcaa

>JX040487.1_O_BUL_2011

cacaatggtgagaagaaaactttctactccaggcccaacaaccacgacaactgctggctgaacgccatcctccagttgttcaggtatgtcgatgaacctttcttcgactgggtctacaactcacctgagaacctcacactcgaagccatcaaacaattggaagaaattactggccttgagctgcacgagggtggaccacccgctctcgtcatctggaacatcaaacacttgctcaacaccggaatcggcaccgcctcgcgacccagcgaggtgtgcatggtagacgggacggacatgtgtttggctgacttccacgctggcattttcctgaaaggacaggaacacgctgtgttcgcctgcgtcaccaccaacgggtggtacgcgatcgacgacgaggatttttacccctggacgccggacccgtccgacgttctggtgtttgtcccgtacgatcaa

>JX040488.1_O_BUL_2011

cacaatggtgagaagaaaactttctactccaggcccaacaaccacgacaactgctggctgaacaccatcctccagttgttcaggtatgtcgatgaacctttcttcgactgggtctacaactcacctgagaacctcacactcgaagccatcaaacgattggaagaaattactggccttgagctgcacgagggtggaccacccgctctcgtcatctggaacatcaaacacttgctcaacaccggaatcggcaccgcctcgcgacccagcgaggtgtgcatggtagacgggacggacatgtgtttggctgacttccacgctggcattttcctgaaaggacaggaacacgctgtgttcgcctgcgtcaccaccaacgggtggtacgcgatcgacgacgaggatttttacccctggacgccggacccgtccgacgttctggtgtttgtcccgtacgatcaa

>JX040489.1_O_BUL_2011

cacaatggtgagaagaaaactttctactccaggcccaacaaccacgacaactgctggctgaacaccatcctccagttgttcaggtatgtcgatgaacctttcttcgactgggtctacaactcacctgagaacctcacactcgaagccatcaaacaattggaagaaattactggccttgagctgcacgagggtggaccacccgctctcgtcatctggaacatcaaacacttgctcaacaccggaatcggcaccgcctcgcgacccagcgaggtgtgcatggtagacgggacggacatgtgtttggctgacttccacgctggcattttcctgaaaggacaggaacacgctgtgttcgcctgcgtcaccaccaacgggtggtacgcgatcgacgacgaggatttttacccctggacgccggacccgtccgacgttctggtgtttgtcccgtacgatcaa

>JX040490.1_O_BUL_2011

cacaatggtgagaagaaaactttctactccaggcccaacaaccacgacaactgctggctgaacgccatcctccagttgttcaggtatgtcgatgaacctttcttcgactgggtctacaactcacctgagaacctcacactcgaagccatcaaacaattggaagaaattactggccttgagctgcacgagggtggaccacccgctctcgtcatctggaacatcaaacacttgctcaacaccggaatcggcaccgcctcgcgacccagcgaggtgtgcatggtagacgggacggacatgtgtttggctgacttccacgctggcattttcctgaaaggacaggaacacgctgtgttcgcctgcgtcaccaccaacgggtggtacgcgatcgacgacgaggatttttacccctggacgccggacccgtccgacgttctggtgtttgtcccgtacgatcaa

>JX040491.1_O_TUR_2010

cacaacggtgagaagaagactttctactccaggcccaacaaccacgacaactgctggctgaacaccatcctccagttattcaggtatgtcgatgaacctttcttcgactgggtctacaactcacctgagaacctcacactcgaagccatcaaacaattggaagaaattactggccttgagctgcacgagggtggaccacccgctctcgtcatctggaacatcaaacacttgctcaacaccggaatcggcaccgcctcgcgacccagcgaggtgtgcatggtagacgggacggacatgtgtttggctgacttccacgctggcattttcctgaaaggacaggaacacgctgtgttcgcctgcgtcaccaccaacgggtggtacgcgatcgacgacgaggaattttacccctggacgccggacccgtccgacgttctggtgtttgtcccgtacgatcaa

>JX040492.1_O_TUR_2010

cacaacggtgagaagaagactttctactccaggcccaacaaccacgacaactgctggctgaacaccatcctccagttgttcaggtatgtcgatgaacctttcttcgactgggtctacaactcacctgaggacctcacactcgaagccatcaaacaattggaagaaattactggccttgagctgcacgagggtggaccacccgctctcgtcatctggaacatcaaacacttgctcaacaccggaatcggcaccgcctcgcgacccagcgaggtgtgcatggtagacgggacggacatgtgtttggctgacttccacgctggcattttcctgaaaggacaggaacacgctgtgttcgcctgcgtcaccaccaacgggtggtacgcgatcgacgacgaggatttttacccctggacgccggacccgtccgacgttctggtgtttgtcccgtacgatcaa

>JX040493.1_O_TUR_2010

cacaacggtgagaagaagactttctactccaggcccaacaaccacgacaactgctggctgaacaccatcctccagttgttcaggtatgtcgatgaacccttcttcgactgggtctacaactcacctgagaacctcacactcgaagccatcaaacaattggaagaaattactggccttgagctgcacgagggtggaccacccgctctcgtcatctggaacatcaaacacttgctcaacaccggaatcggcaccgcctcgcgacccagcgaggtgtgcatggtagacgggacggacatgtgtttggctgacttccacgctggcattttcctgaaaggacaggaacacgccgtgttcgcctgcgtcaccaccaacgggtggtacgcgatcgacgacgaggatttttacccctggacgccggacccgtccgacgttctggtgtttgtcccgtacgatcaa

>JX040494.1_O_TUR_2010

cacaacggtgagaagaagactttctactccaggcccaacaaccacgacaactgctggctgaacaccatcctccagttgttcaggtatgtcgatgaacctttcttcgactgggtctacaactcacctgagaacctcacactcgaagccatcaaacaattggaagaaattactggccttgagctgcacgagggtggaccacccgctctcgtcatctggaacatcaaacacttgctcaacaccggaatcggcaccgcctcgcgacccagcgaggtgtgcatggtagacgggacggacatgtgtttggctgacttccacgctggcattttcctgaaaggacaggaacacgctgtgttcgcctgcgtcaccaccaacgggtggtacgcgatcgacgacgaggatttttacccctggacgccggacccgtccgacgttctggtgtttgtcccgtacgatcaa

>JX040495.1_O_TUR_2010

cacaacggtgagaagaagactttctactccaggcccaacaaccacgacaactgctggctgaacaccatcctccagttgttcaggtatgtcgatgaacctttcttcgactgggtctacaactcacctgagaacctcacactcgaagccatcaaacaattggaagaaattactggccttgagctgcacgagggtggaccacccgctctcgtcatctggaacatcaaacacttgctcaacaccggaattggcaccgcctcgcgacccagcgaggtgtgcatggtagacgggacggacatgtgtttggctgacttccacgctggcattttcctgaaaggacaggaacacgctgtgttcgcctgcgtcaccaccaacgggtggtacgcgatcgacgacgaggatttttacccctggacgccggacccgtccgacgttctggtgtttgtcccgtacgatcaa

>JX040496.1_O_TUR_2010

cacaacggtgagaagaagactttctactccaggcccaacaaccacgacaactgctggctgaacaccatcctccagttgttcaggtatgtcgatgaacctttcttcgactgggtctacaactcacctgagaacctcacactcgaagccatcaaacaattggaagaaattactggccttgagctgcacgagggtggaccacccgctctcgtcatctggaacatcaaacacttgctcaacaccggaatcggcaccgcctcgcgacccagcgaggtgtgcatggtagacgggacggacatgtgtttggctgacttccacgctggcattttcctgaaaggacaggaacacgctgtgttcgcctgcgtcaccaccaacgggtggtacgcgatcgacgacgaggatttttacccctggacgccggacccgtccgacgttctggtgtttgtcccgtacgatcaa

>JX040497.1_O_TUR_2010

cacaacggtgagaagaagactttctactccaggcccaacaaccacgacaactgctggctgaacaccatcctccagttattcaggtatgtcgatgaacctttcttcgactgggtctacaactcacctgagaacctcacactcgaagccatcaaacaattggaagaaattactggccttgagctgcacgagggtggaccacccgctctcgtcatctggaacatcaaacacttgctcaacaccggaatcggcaccgcctcgcgacccagcgaggtgtgcatggtagacgggacggacatgtgtttggctgacttccacgctggcattttcctgaaaggacaggaacacgctgtgttcgcctgcgtcaccaccaacgggtggtacgcgatcgacgacgaggatttttacccctggacgccggacccgtccgacgttctggtgtttgtcccgtacgatcaa

>JX040498.1_O_TUR_2010

cacaacggtgagaagaagactttctactccaggcccaacaaccacgacaactgctggctgaacaccatcctccagttgttcaggtatgtcgatgaacctttcttcgactgggtctacaactcacctgagaacctcacactcgaagccatcaaacaattggaagaaattactggccttgagctgcacgagggtggaccacccgctctcgtcatctggaacatcaaacacttgctcaacaccggaatcggcaccgcctcgcgacccagcgaggtgtgcatggtagacgggacggacatgtgtttggctgacttccacgctggcattttcctgaaaggacaggaacacgctgtgttcgcctgcgtcaccaccaacgggtggtacgcgatcgacgacgaggatttttacccctggacgccggacccgtccgacgttctggtgtttgtcccgtacgatcaa

>JX040499.1_O_TUR_2011

cacaacggtgagaagaagactttctactccaggcccaacaaccacgacaactgctggctgaacaccatcctccagttgttcaggtatgtcgatgaacctttcttcgactgggtctacaactcacctgagaacctcacactcgaagccatcaaacaattggaagaaattactggccttgagctgcacgagggtggaccacccgctctcgtcatctggaacatcaaacacttgctcaacaccggaatcggcaccgcctcgcgacccagcgaggtgtgcatggtagacgggacggacatgtgtttggctgacttccacgctggcattttcctgaaaggacacgaacacgctgtgttcgcctgcgtcaccaccaacgggtggtacgcgatcgacgacgaggatttttacccctggacgccggacccgtccgacgttctggtgtttgtcccgtacgatcaa

>JX040500.1_O_TUR_2011

cacaacggtgagaagaagactttctactccaggcccaacaaccacgacaactgctggctgaacaccatcctccagttgttcaggtatgtcgatgaacctttcttcgactgggtctacaactcacctgagaacctcacactcgaagccatcaaacaactggaagaaattactggccttgagctgcacgagggtggaccacccgctctcgtcatctggaacatcaaacacttgctcaacaccggaatcggcaccgcctcgcgacccagcgaggtgtgcatggtagacgggacggacatgtgtttggctgacttccacgctggcattttcctgaaaggacaggaacacgctgtgttcgcctgcgtcaccaccaacgggtggtacgcgatcgacgacgaggatttttacccctggacgccggacccgtccgacgttctggtgtttgtcccgtacgatcaa

>JX040501.1_O_ISR_2011

cacaacggtgagaagaagactttctactccaggcccaacaaccacgacaactgttggttgaacaccatcctccagttgttcaggtacgtcgatgaacctttcttcgactgggtctacaactcacctgagaacctcacactcgaagccatcaaccaattggaagagattactggccttgagttgcacgagggtggaccacccgctctcgtcatctggaacatcaaacacttgctcaacaccggaatcggcaccgcctcgcgacccagcgaggtgtgtatggtagacgggacggacatgtgtttggctgacttccacgctggcattttcctgaaaggacaggaacacgctgtgttcgcctgtgtcaccaccaacgggtggtacgcgatcgacgacgaggatttttacccctggacgccggacccgtccgacgttttggtgtttgtcccgtacgatcaa

>JX066664.1_O_BUL_2011

cacaatggtgagaagaagactttctactccaggcccaacaaccacgacaactgctggctgaacaccatcctccagttgttcaggtatgtcgatgaacctttcttcgactgggtctacaactcacctgagaacctcacactcgaagccatcaaacaattggaagaaattactggccttgagctgcacgagggtggaccacccgctctcgtcatctggaacatcaaacacttgctcaacaccggaatcggcaccgcctcgcgacccagcgaggtgtgcatggtagacgggacggacatgtgtttggctgacttccacgctggcattttcctgaaaggacaggaacacgctgtgttcgcctgcgtcaccaccaacgggtggtacgcgatcgacgacgaggatttttacccctggacgccggacccgtccgacgttctggtgtttgtcccgtacgatcaa

>JX066665.1_O_BUL_2011

cacaatggtgagaagaagactttctactccaggcccaacaaccacgacaactgctggctgaacaccatcctccagttgttcaggtatgtcgatgaacctttcttcgactgggtctacaactcacctgagaacctcacactcgaagccatcaaacaattggaagaaattactggccttgagctgcacgagggtggaccacccgctctcgtcatctggaacatcaaacacttgctcaacaccggaatcggcaccgcctcgcgacccagcgaggtgtgcatggtagacgggacggacatgtgtttggctgacttccacgctggcattttcctgaaaggacaggaacacgctgtgttcgcctgcgtcaccaccaacgggtggtacgcgatcgacgacgaggatttttacccctggacgccggacccgtccgacgttctggtgtttgtcccgtacgatcaa

>JX570638.1_O_UKG_2007

tacaacggtgagaagaagaccttttactccaggcccaacaaccacgacaactgctggttgaacgccatcctccagttgttcaggtacgttgaagaaccattcttcgactgggtctacagttcgcctgagaacctcacgcttgaagccatcaagcagttggaggacctcacagggcttgaactgcacgagggtggaccacctgctctcgtgatctggaacatcaagcacttgctccacaccggcattggcaccgcctcgcgacccagcgaggtgtgcatggtggatggtacggacatgtgcttggctgatttccatgcaggcattttccttaaggggcaagaacacgctgtgttcgcgtgtgtcacctccaacgggtggtacgcgattgatgatgaggacttctacccctggacgccggacccatccgacgttctggtgtttgtcccgtacgatcaa

>JX570639.1_O_UKG_2007

tacaacggtgagaagaagaccttttactccaggcccaacaaccacgacaactgctggttgaacgccatcctccagttgttcaggtacgttgaagaaccattcttcgactgggtctacagttcgcctgagaacctcacgcttgaagccatcaagcagttggaggacctcacagggcttgaactgcacgagggtggaccacctgctctcgtgatctggaacatcaagcacttgctccacaccggcattggcaccgcctcgcgacccagcgaggtgtgcatggtggatggtacggacatgtgcttggctgatttccatgcaggcattttccttaaggggcaagaacacgctgtgttcgcgtgtgtcacctccaacgggtggtacgcgattgatgatgaggacttctacccctggacgccggacccatccgacgttctggtgtttgtcccgtacgatcaa

>JX570640.1_O_UKG_2007

tacaacggtgagaagaagaccttttactccaggcccaacaaccacgacaactgctggttgaacgccatcctccagttgttcaggtacgttgaagaaccattcttcgactgggtctacagttcgcctgagaacctcacgcttgaagccatcaagcagttggaggacctcacagggcttgaactgcacgagggtggaccacctgctctcgtgatctggaacatcaagcacttgctccacaccggcattggcaccgcctcgcgacccagcgaggtgtgcatggtggatggtacggacatgtgcttggctgatttccatgcaggcattttccttaaggggcaagaacacgctgtgttcgcgtgtgtcacctccaacgggtggtacgcgattgatgatgaggacttctacccctggacgccggacccatccgacgttctggtgtttgtcccgtacgatcaa

>JX570641.1_O_UKG_2007

tacaacggtgagaagaagaccttttactccaggcccaacaaccacgacaactgctggttgaacgccatcctccagttgttcaggtacgttgaagaaccattcttcgactgggtctacagttcgcctgagaacctcacgcttgaagccatcaagcagttggaggacctcacagggcttgaactgcacgagggtggaccacctgctctcgtgatctggaacatcaagcacttgctccacaccggcattggcaccgcctcgcgacccagcgaggtgtgcatggtggatggtacggacatgtgcttggctgatttccatgcaggcattttccttaaggggcaagaacacgctgtgttcgcgtgtgtcacctccaacgggtggtacgcgattgatgatgaggacttctacccctggacgccggacccatccgacgttctggtgtttgtcccgtacgatcaa

>JX570642.1_O_UKG_2007

tacaacggtgagaagaagaccttttactccaggcccaacaaccacgacaactgctggttgaacgccatcctccagttgttcaggtacgttgaagaaccattcttcgactgggtctacagttcgcctgagaacctcacgcttgaagccatcaagcagttggaggacctcacagggcttgaactgcacgagggtggaccacctgctctcgtgatctggaacatcaagcacttgctccacaccggcattggcaccgcctcgcgacccagcgaggtgtgcatggtggatggtacggacatgtgcttggctgatttccatgcaggcattttccttaaggggcaagaacacgctgtgttcgcgtgtgtcacctccaacgggtggtacgcgattgatgatgaggacttctacccctggacgccggacccatccgacgttctggtgtttgtcccgtacgatcaa

>JX570643.1_O_UKG_2007

tacaacggtgagaagaagaccttttactccaggcccaacaaccacgacaactgctggttgaacgccatcctccagttgttcaggtacgttgaagaaccattcttcgactgggtctacagttcgcctgagaacctcacgcttgaagccatcaagcagttggaggacctcacagggcttgaactgcacgagggtggaccacctgctctcgtgatctggaacatcaagcacttgctccacaccggcattggcaccgcctcgcgacccagcgaggtgtgcatggtggatggtacggacatgtgcttggctgatttccatgcaggcattttccttaaggggcaagaacacgctgtgttcgcgtgtgtcacctccaacgggtggtacgcgattgatgatgaggacttctacccctggacgccggacccatccgacgttctggtgtttgtcccgtacgatcaa

>JX570644.1_O_UKG_2007

tacaacggtgagaagaagaccttttactccaggcccaacaaccacgacaactgctggttgaacgccatcctccagttgttcaggtacgttgaagaaccattcttcgactgggtctacagttcgcctgagaacctcacgcttgaagccatcaagcagttggaggacctcacagggcttgaactgcacgagggtggaccacctgctctcgtgatctggaacatcaagcacttgctccacaccggcattggcaccgcctcgcgacccagcgaggtgtgcatggtggatggtacggacatgtgcttggctgatttccatgcaggcattttccttaaggggcaagaacacgctgtgttcgcgtgtgtcacctccaacgggtggtacgcgattgatgatgaggacttctacccctggacgccggacccatccgacgttctggtgtttgtcccgtacgatcaa

>JX570645.1_O_UKG_2007

tacaacggtgagaagaagaccttttactccaggcccaacaaccacgacaactgctggttgaacgccatcctccagttgttcaggtacgttgaagaaccattcttcgactgggtctacagttcgcctgagaacctcacgcttgaagccatcaagcagttggaggacctcacagggcttgaactgcacgagggtggaccacctgctctcgtgatctggaacatcaagcacttgctccacaccggcattggcaccgcctcgcgacccagcgaggtgtgcatggtggatggtacggacatgtgcttggctgatttccatgcaggcattttccttaaggggcaagaacacgctgtgttcgcgtgtgtcacctccaacgggtggtacgcgattgatgatgaggacttctacccctggacgccggacccatccgacgttctggtgtttgtcccgtacgatcaa

>JX570646.1_O_UKG_2007

tacaacggtgagaagaagaccttttactccaggcccaacaaccacgacaactgctggttgaacgccatcctccagttgttcaggtacgttgaagaaccattcttcgactgggtctacagttcgcctgagaacctcacgcttgaagccatcaagcagttggaggacctcacagggcttgaactgcacgagggtggaccacctgctctcgtgatctggaacatcaagcacttgctccacaccggcattggcaccgcctcgcgacccagcgaggtgtgcatggtggatggtacggacatgtgcttggctgatttccatgcaggcattttccttaaggggcaagaacacgctgtgttcgcgtgtgtcacctccaacgggtggtacgcgattgatgatgaggacttctacccctggacgccggacccatccgacgttctggtgtttgtcccgtacgatcaa

>JX570647.1_O_UKG_2007

tacaacggtgagaagaagaccttttactccaggcccaacaaccacgacaactgctggttgaacgccatcctccagttgttcaggtacgttgaagaaccattcttcgactgggtctacagttcgcctgagaacctcacgcttgaagccatcaagcagttggaggacctcacagggcttgaactgcacgagggtggaccacctgctctcgtgatctggaacatcaagcacttgctccacaccggcattggcaccgcctcgcgacccagcgaggtgtgcatggtggatggtacggacatgtgcttggctgatttccatgcaggcattttccttaaggggcaagaacacgctgtgttcgcgtgtgtcacctccaacgggtggtacgcgattgatgatgaggacttctacccctggacgccggacccatccgacgttctggtgtttgtcccgtacgatcaa

>JX570648.1_O_UKG_2007

tacaacggtgagaagaagaccttttactccaggcccaacaaccacgacaactgctggttgaacgccatcctccagttgttcaggtacgttgaagaaccattcttcgactgggtctacagttcgcctgagaacctcacgcttgaagccatcaagcagttggaggacctcacagggcttgaactgcacgagggtggaccacctgctctcgtgatctggaacatcaagcacttgctccacaccggcattggcaccgcctcgcgacccagcgaggtgtgcatggtggatggtacggacatgtgcttggctgatttccatgcaggcattttccttaaggggcaagaacacgctgtgttcgcgtgtgtcacctccaacgggtggtacgcgattgatgatgaggacttctacccctggacgccggacccatccgacgttctggtgtttgtcccgtacgatcaa

>JX570649.1_O_UKG_2007

tacaacggtgagaagaagaccttttactccaggcccaacaaccacgacaactgctggttgaacgccatcctccagttgttcaggtacgttgaagaaccattcttcgactgggtctacagttcgcctgagaacctcacgcttgaagccatcaagcagttggaggacctcacagggcttgaactgcacgagggtggaccacctgctctcgtgatctggaacatcaagcacttgctccacaccggcattggcaccgcctcgcgacccagcgaggtgtgcatggtggatggtacggacatgtgcttggctgatttccatgcaggcattttccttaaggggcaagaacacgctgtgttcgcgtgtgtcacctccaacgggtggtacgcgattgatgatgaggacttctacccctggacgccggacccatccgacgttctggtgtttgtcccgtacgatcaa

>JX570650.1_O_UKG_2007

tacaacggtgagaagaagaccttttactccaggcccaacaaccacgacaactgctggttgaacgccatcctccagttgttcaggtacgttgaagaaccattcttcgactgggtctacagttcgcctgagaacctcacgcttgaagccatcaagcagttggaggacctcacagggcttgaactgcacgagggtggaccacctgctctcgtgatctggaacatcaagcacttgctccacaccggcattggcaccgcctcgcgacccagcgaggtgtgcatggtggatggtacggacatgtgcttggctgatttccatgcaggcattttccttaaggggcaagaacacgctgtgttcgcgtgtgtcacctccaacgggtggtacgcgattgatgatgaggacttctacccctggacgccggacccatccgacgttctggtgtttgtcccgtacgatcaa

>JX570651.1_O_UKG_2007

tacaacggtgagaagaagaccttttactccaggcccaacaaccacgacaactgctggttgaacgccatcctccagttgttcaggtacgttgaagaaccattcttcgactgggtctacagttcgcctgagaacctcacgcttgaagccatcaagcagttggaggacctcacagggcttgaactgcacgagggtggaccacctgctctcgtgatctggaacatcaagcacttgctccacaccggcattggcaccgcctcgcgacccagcgaggtgtgcatggtggatggtacggacatgtgcttggctgatttccatgcaggcattttccttaaggggcaagaacacgctgtgttcgcgtgtgtcacctccaacgggtggtacgcgattgatgatgaggacttctacccctggacgccggacccatccgacgttctggtgtttgtcccgtacgatcaa

>JX570652.1_O_UKG_2007

tacaacggtgagaagaagaccttttactccaggcccaacaaccacgacaactgctggttgaacgccatcctccagttgttcaggtacgttgaagaaccattcttcgactgggtctacagttcgcctgagaacctcacgcttgaagccatcaagcagttggaggacctcacagggcttgaactgcacgagggtggaccacctgctctcgtgatctggaacatcaagcacttgctccacaccggcattggcaccgcctcgcgacccagcgaggtgtgcatggtggatggtacggacatgtgcttggctgatttccatgcaggcattttccttaaggggcaagaacacgctgtgttcgcgtgtgtcacctccaacgggtggtacgcgattgatgatgaggacttctacccctggacgccggacccatccgacgttctggtgtttgtcccgtacgatcaa

>JX570653.1_O_UKG_2007

tacaacggtgagaagaagaccttttactccaggcccaacaaccacgacaactgctggttgaacgccatcctccagttgttcaggtacgttgaagaaccattcttcgactgggtctacagttcgcctgagaacctcacgcttgaagccatcaagcagttggaggacctcacagggcttgaactgcacgagggtggaccacctgctctcgtgatctggaacatcaagcacttgctccacaccggcattggcaccgcctcgcgacccagcgaggtgtgcatggtggatggtacggacatgtgcttggctgatttccatgcaggcattttccttaaggggcaagaacacgctgtgttcgcgtgtgtcacctccaacgggtggtacgcgattgatgatgaggacttctacccctggacgccggacccatccgacgttctggtgtttgtcccgtacgatcaa

>JX570654.1_O_UKG_2007

tacaacggtgagaagaagaccttttactccaggcccaacaaccacgacaactgctggttgaacgccatcctccagttgttcaggtacgttgaagaaccattcttcgactgggtctacagttcgcctgagaacctcacgcttgaagccatcaagcagttggaggacctcacagggcttgaactgcacgagggtggaccacctgctctcgtgatctggaacatcaagcacttgctccacaccggcattggcaccgcctcgcgacccagcgaggtgtgcatggtggatggtacggacatgtgcttggctgatttccatgcaggcattttccttaaggggcaagaacacgctgtgttcgcgtgtgtcacctccaacgggtggtacgcgattgatgatgaggacttctacccctggacgccggacccatccgacgttctggtgtttgtcccgtacgatcaa

>JX570655.1_O_UKG_2007

tacaacggtgagaagaagaccttttactccaggcccaacaaccacgacaactgctggttgaacgccatcctccagttgttcaggtacgttgaagaaccattcttcgactgggtctacagttcgcctgagaacctcacgcttgaagccatcaagcagttggaggacctcacagggcttgaactgcacgagggtggaccacctgctctcgtgatctggaacatcaagcacttgctccacaccggcattggcaccgcctcgcgacccagcgaggtgtgcatggtggatggtacggacatgtgcttggctgatttccatgcaggcattttccttaaggggcaagaacacgctgtgttcgcgtgtgtcacctccaacgggtggtacgcgattgatgatgaggacttctacccctggacgccggacccatccgacgttctggtgtttgtcccgtacgatcaa

>JX869177.1_O_UKG_1967

tacaacggtgagaagaagaccttttactccaggcccaacaaccacgacaactgctggttgaacgccatcctccagttgttcaggtacgttgaagaaccattcttcgactgggtctacagttcgcctgagaacctcacgcttgaagccatcaagcagctggaggatctcacagggcttgaattgcacgagggtggaccacctgctctcgtgatctggaacatcaagcacttgctccacaccggcattggcaccgcctcgcgacccagcgaggtgtgcatggtggatggtacggacatgtgcttggctgatttccatgcaggcattttccttaaggggcaagaacacgctgtgttcgcgtgtgtcacctccaacgggtggtacgcgattgatgatgaggacttctacccctggacgccggacccgtccgacgttctggtgtttgtcccgtacgatcaa

>JX869178.1_O_UKG_1967

tacaacggtgagaagaaggccttttactccaggcccaacaaccacgacaactgctggttgaacgccatcctccagttattcaggtacgttgaagaaccattcttcgactgggtctacagttcacctgagaacctcacgcttgaagctatcaagcagttggaagatctcacagggcttgaattgcacgagggtggaccacctgctctcgtgatctggaacatcaagcacttgctccacaccggcattggcaccgcctcgcgacccagcgaggtgtgcatggtggatggtacggacatgtgcttggctgattttcatgcaggcattttccttaaggggcaagaacacgctgtgttcgcgtgtgtcacctccaacgggtggtacgcgattgatgatgaggacttctacccctggacgccggacccgtccgacgttctggtatttgtcccgtacgatcaa

>JX869179.1_O_UKG_1967

tacaacggtgagaagaagaccttttactccaggcccaacaaccacgacaactgctggttgaacgccatcctccagttgttcaggtacgttgaagaaccattcttcgactgggtctacagttcgcctgagaacctcacgcttgaagccatcaagcagttggaggacctcacagggcttgaactgcacgagggtggaccacctgctctcgtgatctggaacatcaagcacttgctccacaccggcattggcaccgcctcgcgacccagcgaggtgtgcatggtggatggtacggacatgtgcttggctgatttccatgcaggcattttccttaaggggcaagaacacgctgtgttcgcgtgtgtcacctccaacgggtggtacgcgattgatgatgaggacttctacccctggacgccggacccatccgacgttctggtgtttgtcccgtacgatcaa

>JX869180.1_O_UKG_1967

tacaacggtgagaagaagaccttttactccaggcccaacaaccacgacaactgctggttgaacgccatcctccagttgttcaggtacgttgaagaaccattcttcgactgggtctacagttcgcctgagaacctcacgcttgaagccatcaagcagttggaggacctcacagggcttgaactgcacgagggtggaccacctgctctcgtgatctggaacatcaagcacttgctccacaccggcattggcaccgcctcgcgacccagcgaggtgtgcatggtggatggtacggacatgtgcttggctgatttccatgcaggcattttccttaaggggcaagaacacgctgtgttcgcgtgtgtcacctccaacgggtggtacgcgattgatgatgaggacttctacccctggacgccggacccatccgacgttctggtgtttgtcccgtacgatcaa

>JX869181.1_O_UKG_1967

tacaacggtgagaagaagaccttttactccaggcccaacaaccacgacaactgctggttgaacgccatcctccagttgttcaggtacgttgaagaaccattcttcgactgggtctacagttcgcctgagaacctcacgcttgaagccatcaagcagttggaggacctcacagggcttgaactgcacgagggtggaccacctgctctcgtgatctggaacatcaagcacttgctccacaccggcattggcaccgcctcgcgacccagcgaggtgtgcatggtggatggtacggacatgtgcttggctgatttccatgcaggcattttccttaaggggcaagaacacgctgtgttcgcgtgtgtcacctccaacgggtggtacgcgattgatgatgaggacttctacccctggacgccggacccatccgacgttctggtatttgtcccgtacgatcaa

>JX869182.1_O_UKG_1968

tacaacggtgagaagaagaccttttactccaggcccaacaaccacgacaactgctggttgaacgccatcctccagttgttcaggtacgttgaagaaccattcttcgactgggtctacagttcgcctgagaacctcacgcttgaagccatcaagcagttggaggacctcacagggcttgaactgcacgagggtggaccacctgctctcgtgatctggaacatcaaacacttgctccacaccggcattggcaccgcctcgcgacccagcgaggtgtgcatggtggatggtacggacatgtgcttggctgatttccatgcaggcattttccttaaggggcaagaacacgctgtgttcgcgtgtgtcacctccaacgggtggtacgcgattgatgatgaggacttctacccctgggcgccggacccatccgacgttctggtgtttgtcccgtacgatcaa

>JX869183.1_O_UKG_1968

tacaacggtgagaagaagaccttttactccaggcccaacaaccacgacaactgctggttgaacgccatcctccagttgttcaggtacgttgaagaaccattcttcgactgggtctacagttcgcctgagaacctcacgcttgaagccatcaagcagttggaggacctcacagggcttgaactgcacgagggtggaccacctgctctcgtgatctggaacatcaagcacttgctccacaccggcattggcaccgcctcgcgacccagcgaggtgtgcatggtggatggtacggacatgtgcttggctgatttccatgcaggcattttccttaaggggcaagaacacgctgtgttcgcgtgtgtcacctccaacgggtggtacgcgattgatgatgaggacttctacccctggacgccggacccatccgacgttctggtgtttgtcccgtacgatcaa

>JX869184.1_O_UKG_1968

tacaacggtgagaagaagaccttttactccaggcccaacaaccacgacaactgctggttgaacgccatcctccagttgttcaggtacgttgaagaaccattcttcgactgggtctacagttcgcctgagaacctcacgcttgaagccatcaagcagttggaggacctcacagggcttgaactgcacgagggtggaccacctgctctcgtgatctggaacatcaagcacttgctccacaccggcattggcaccgcctcgcgacccagcgaggtgtgcatggtggatggtacggacatgtgcttggctgatttccatgcaggcattttccttaaggggcaagaacacgctgtgttcgcgtgtgtcacctccaacgggtggtacgcgattgatgatgaggacttctacccctggacgccggacccatccgacgttctggtgtttgtcccgtacgatcaa

>JX869185.1_O_UKG_1968

tacaacggtgagaagaagaccttttactccaggcccaacaaccacgacaactgctggttgaacgccatcctccagttgttcaggtacgttgaagaaccattcttcgactgggtctacagttcgcctgagaacctcacgcttgaagccatcaagcagttggaggacctcacagggcttgaactgcacgagggtgggccacctgctctcgtgatctggaacatcaagcacttgctccacaccggcattggcaccgcctcgcgacccagcgaggtgtgcatggtggatggtacggacatgtgcttggctgatttccatgcaggcattttccttaaggggcaagaacacgctgtgttcgcgtgtgtcacctccaacgggtggtacgcgattgatgatgaggacttctacccctggacgccggacccatccgacgttctggtgtttgtcccgtacgatcaa

>JX869186.1_O_UKG_1968

tacaacggtgagaagaagaccttttactccaggcccaacaaccacgacaactgctggttgaacgccatcctccagttgttcaggtacgttgaagaaccattcttcgactgggtctacagttcgcctgagaacctcacgcttgaagccatcaagcagttggaggacctcacagggcttgaactgcacgagggtggaccacctgctctcgtgatctggaacatcaagcacttgctccacaccggcattggcaccgcctcgcgacccagcgaggtgtgcatggtggatggtacggacatgtgcttggctgatttccatgcaggcattttccttaaggggcaagaacacgctgtgttcgcgtgtgtcacctccaacgggtggtacgcgattgatgatgaggacttctacccctggacgccggacccatccgacgttctggtgtttgtcccgtacgatcaa

>JX869187.1_O_UKG_1968

tacaacggtgagaagaagaccttttactccaggcccaacaaccacgacaactgctggttgaacgccatcctccagttgttcaggtacgttgaagaaccattcttcgactgggtctacagttcgcctgagaacctcacgcttgaagccatcaagcagttggaggacctcacagggcttgaactgcacgagggtggaccacctgctctcgtgatctggaacatcaagcacttgctccacaccggcattggcaccgcctcgcgacccagcgaggtgtgcatggtggatggtacggacatgtgcttggctgatttccatgcaggcattttccttaaggggcaagaacacgctgtgttcgcgtgtgtcacctccaacgggtggtacgcgattgatgatgaggacttctacccctggacgccggacccatccgacgttctggtgtttgtcccgtacgatcaa

>JX869188.1_O_UKG_1968

tacaacggtgagaagaagaccttttactccaggcccaacaaccacgacaactgctggttgaacgccatcctccagttgttcaggtacgttgaagaaccattcttcgactgggtctacagttcgcctgagaacctcacgcttgaagccatcaagcagttggaggacctcacagggcttgaactgcacgagggtggaccacctgctctcgtgatctggaacatcaagcacttgctccacaccggcattggcaccgcctcgcgacccagcgaggtgtgcatggtggatggtacggacatgtgcttggctgatttccatgcaggcattttccttaaggggcaagaacacgctgtgttcgcgtgtgtcacctccaacgggtggtacgcgattgatgatgaggacttctacccctggacgccggacccatccgacgttctggtgtttgtcccgtacgatcaa

>KC412634.1_Asia1_CHA_2006

cacaacggtgagaagaagaccttttactccagacccaacaaccatgataactgctggctgaacactatcctccagttgttcaggtatgtcgacgagcctttcttcgactgggtctacgactcgcctgaaaacctcactctcgaggcgattaggcagttggaggaagttactggtcttgagctgcatgagggtggaccacccgcccttgtcatctggaacatcaagcatttgctccacaccggagtcggtaccgcttcgcgccctagcgaagtgtgtatggtagacggcacggacatgtgtttggctgatttccatgctggcattttcctgaaaggacaagaacatgctgtgtttgcctgtgtcacctccaacgggtggtacgcgatcgatgacgaggacttttacccctggacaccggacccgtccgacgtcttggtgtttgttccgtacgaccaa

>KC440881.1_A_EGY_2011

tacaacggcgagaagaagactttctattcaaggcccaaccgtcacgacaactgctggttgaacaccattctgcagctgttcagatatgtcgacgaaccattcttcgactgggtctatgactcacctgagaacctcacgctccaagcaattgagcaactcgaggggttcacaggtcttgacctacgcgagggcggaccacccgctctcgtgatttggaacatcaggcacttgttgtacactggaattggcactgcttcacggcccagtgaggtgtgtatggttgatggcactgacatgtgtctggctgatttccacgcaggaatcttccttaaaggtactgaacacgccgtgtttgcctgtttgacctccgatggttggtacgccatcgacgacgaggacttttacccatggactccggatccgtctgatgtcttgtgttttgtcccatacgacatg

>KC440882.1_A_EGY_2012

cacaacggtgagaagaagactttcttttctcggcccaacaaccacgacaactgttggttgaacaccatcctccagttgttcaggtatgttgatgagcctttctttgactgggtctacaactctcctgaaaacctcaccctcaaggcaatacaacaattggaagagattactggtctagagttgcacgagggtgggccgcccgctctcgtcatctggaacatcaaacacttgcttaacaccgggatcggcaccgcttcacgtcccagtgaggtgtgcatggtcgacgggacagacatgtgtttggcagatttccatgctggtattttcttgaaagggcaagagcatgctgtgtttgcctgtgtcacctccaacgggtggtacgcgatcgacgacgaggacttctatccgtggacgccagatccgtctgacgtcctggtgtttgttccgtacgatcaa

>KC440883.1_O_EGY_2011

tacaacggggaaagaaaaactttctactccaggcccaatagacacgacaactgttggttgaacaccatccttcagttgttcaggtacgttgatgaaccattctttgactgggtttatgattcacctgaaaaccttactctagaagcaatcagacaactggaggaacttactggtcttgaactacacgagggtggaccacctgctctcgtcatctggaacatcaaacatctgcttcacaccggaatcggcaccgcctcgcgacccagtgaggtgtgtatggttgacggtacggacatgtgtttggctgatttccacgctggcattttcctaaaaggacaggagcatgctgtgttcgcctgtgtcacatccgacgggtggtacgcgattgacgacgaggacttctacccctggacaccggacccgtccgacgttctggtttttgtcccgtacgaccaa

>KC440884.1_SAT2_EGY_2012

tacaacggtgagaaaaagaccttctactcaaggcccaaccgtcacgacaactgctggttgaacaccatactgcagctgttcaggtacgtcgatgaaccattcttcgactgggtctacaattcacctgagaacctcacgcttcaagcaattgagcaacttgaggagctcacaggtctcaacctacgcgagggtggccctcctgccctcgtgatttggaacatcaagcacctgttgtacaccggaatcggcactgcctcacgacccagtgaggtgtgcatggttgacggtactgacatgtgtcttgctgatttccacgcaggaattttcctcaaaggtgctgaacacgccgtgttcgcctgcttgacctccaacggatggtatgctattgatgacgaggacttctacccatggacaccggacccgtccgacgtcctgtgttttgtcccgtatgatgta

>KC462884.1_Asia1_CHA_2006

cacaacggtgagaagaagaccttttactccagacccaacaaccatgataactgctggctgaacactatcctccagttgttcaggtatgtcgacgagcctttcttcgactgggtctacgactcgcctgaaaacctcactctcgaggcgattaggcagttggaggaagttactggtcttgagctgcatgagggtggaccacccgcccttgtcatctggaacatcaagcatttgctccacaccggagtcggtaccgcttcgcgccctagcgaagtgtgtatggtagacggcacggacatgtgtttggctgatttccatgctggcattttcctgaaaggacaagaacatgctgtgtttgcctgtgtcacctccaacgggtggtacgcgatcgatgacgaggacttttacccctggacaccggacccgtccgacgtcttggtgtttgttccgtacgaccaa

>KC503937.1_O_SKR_2010

tacaacggtgagaagaagatcttctactccaggcccaacaaccacgacaactgttggctgaacaccatccttcagctgttcaggtacgtcgatgaacctttcttcgactgggtatatgaatcacctgagaacctcacccttgaggcgatcagacaactggagaacattactggtcttgagctgcacgagggtggtccgcccgccctcgtcatttggaacatcaaacacttgctccacaccgggatcggcaccgcctcgcgacccagcgaggtgtgcatggtggacggtacggacatgtgcctggctgacttccacgctggcatcttcctgaaaggacaggaacacgccgtgtttgcctgcgtcacctccaacgggtggtacgcgatcgacgacgaagaattttacccctggacgccagatccgtccgacgtgctggtctttgtcccgtacgatcaa

>KC588943.1_A_SKR_2010

tacaacggtgagaagaaaactttttactctaggcccaacaaccacgacaactgttggttgaacaccatcctccaactgtttaggtacgtcgacgaaccattcttcgactgggtctatgaatcacctgagaacctcacttttgaggcgattaaacaattggaacaggtcactggtcttgagctgcgcgagggtggcccacccgccctcgtaatttggaacattaagcacttgctccacaccggaatcggtaccgcctcgcgacctagtgaggtgtgtatggtagacgggacggacatgtgtctggctgatttccatgctggcattttcctgaaagggcaggaacacgcagtgtttgcctgtgttacctccaacgggtggtacgcaattgacgacgaggacttttacccctggacaccagacccgtctgatgtcctggtgtttgtcccgtatgatcaa

>KF112879.1_O_TAI_2009

tacaacggtgagaagaagatcttctattccaggcccaacaaccacgacaactgttggctgaacgccatccttcagctgttcaggtacgtcgatgaacctttcttcgactgggtatatcaatcacctgaaaacctcacccttgaggcgatcaggcaactggagaacattactggtcttgagctgcacgagggtggtccgcccgccctcgtcatttggaacatcaaacacttgctccacaccgggatcggcaccgcctcgcgacccagcgaggtgtgcatggtggacggtgcggacatgtgcctggctgacttccacgctggcatcttcctgaaaggacgggaacacgccgtgtttgcctgcgtcacctccaacgggtggtacgcgatcgacgacgaagaattctacccctggacgccggatccgtccgacgtgttggtctttgtcccgtacgatcaa

>KF112880.1_O_MYA_2009

tacaacggtgagaagaagatcttctactccaggcccaacaaccacgacaactgttggctgaacgccatccttcagctgtttaggtacgtcgatgaacctttcttcgactgggtatatgaatcacctgaaaacctcacccttgaggcgatcaggcaactggagaacattactggtcttgagctgcacgagggtggtccgcccgccctcgtcatttggaacatcaaacacttgctccacaccgggatcggcaccgcctcgcgacccagcgaggtgtgcatggtggacggtacggacatgtgcctggctgacttccacgctggcatcttcctgaaaggacaggaacacgccgtgtttgcctgcgtcacctccaacgggtggtacgcgatcgacgacgaagaattttacccctggacgccagatccgtccgacgtgctggtctttgtcccgtacgatcaa

>KF112881.1_O_MOG_2010

tacaacggtgagaggaagaccttttactctaggcccaacaacaatgacaactgttggctgaacgccatcctgcagttgttcaggtatgtcgatgaacctttcttcgactgggtctacgaatcgcctgagaaccgcactctcgaggcaattgaacaattagagggaatcactggtcttgaactgcacgagggcggtccacccgctctcgtggtttggaacatcaaacacttgctccacaccgggattggcaccgcctcgcgacccagcgaggtgtgcatggttgacggtacggacatgtgcctggctgacttccacgctggcatcttcctgaaaggacaggaacacgctgtgtttgcctgcgtcacctctaacgggtggtacgcgattgatgacgaggacttttacccctggacgccggatccgtccgacgtgctggtgttcgtcccgtacgatcaa

>KF112882.1_O_MOG_2010

tacaacggtgagaggaagaccttttactctaggcccaacaacaatgacaactgttggctgaacgccatcctgcagttgttcaggtatgtcgatgaacctttcttcgactgggtctacgaatcgcctgagaaccgcactctcgaggcaattgaacaattagagggaatcactggtcttgaactgcacgagggcggtccacccgctctcgtggtttggaacatcaaacacttgctccacaccgggattggcaccgcctcgcgacccagcgaggtgtgcatggttgacggtacggacatgtgcctggctgacttccacgctggcatcttcctgaaaggacaggaacacgctgtgtttgcctgcgtcacctctaacgggtggtacgcgattgatgacgaggacttttacccctggacgccggatccgtccgacgtgctggtgttcgtcccgtacgatcaa

>KF112883.1_O_RUS_2010

tacaacggtgagaagaagatcttctactccaggcccaacaaccacgacaactgttggctgaacaccatccttcagctgttcaggtacgtcgatgaacctttcttcgactgggtatatgaatcacctgaaaacctcacccttgaggcgatcagacaactggagaacattactggtcttgagctgcacgagggtggtccgcccgccctcgtcatttggaacatcaaacacttgctccacaccgggatcggcaccgcctcgcgacccagcgaggtgtgcatggtggacggtacggacatgtgtctggctgacttccacgctggcatcttcctgaaaggacaggaacacgccgtgtttgcctgcgtcacctccaacgggtggtacgcgatcgacgacgaagaattttacccctggacgccagatccgtccgacgtgctggtctttgtcccgtacgatcaa

>KF112884.1_O_RUS_2010

tacaacggtgagaggaagaccttttactctaggcccaacaacaatgacaactgttggctgaacgccatcctgcagttgttcaggtatgtcgatgaacctttcttcgactgggtctacgaatcgcctgagaaccgcactctcgaggcaattgaacaattagagggaatcactggtcttgaactgcacgagggcggtccacccgctctcgtggtttggaacatcaaacacttgctccacaccgggattggcaccgcctcgcgacccagcgaggtgtgcatggttgacggtacggacatgtgcctggctgacttccacgctggcatcttcctgaaaggacaggaacacgctgtgtttgcctgcgtcacctctaacgggtggtacgcgattgatgacgaggacttttacccctggacgccggatccgtccgacgtgctggtgttcgtcccgtacgatcaa

>KF112885.1_O_JPN_2010

tacaacggtgagaagaagatcttctactccaggcccaacaaccacgacaactgttggctgaacgccatccttcagctgttcaggtacgtcgatgaacctttcttcgactgggtatatgaatcacctgaaaacctcacccttgaggcgatcagacaactggagaacattactggtcttgagctgcacgagggtggtccgcccgccctcgtcatttggaacatcaaacacttgctccacaccgggatcggcaccgcctcgcgacccagcgaggtgtgcatggtggacggtacggacatgtgcctggctgacttccacgctggcatcttcctgaaaggacaggaacacgccgtgtttgcctgcgtcacctccaacgggtggtacgcgatcgacgacgaagaattctacccctggacgccagatccgtccgacgtgctggtctttgtcccgtacgatcaa

>KF112886.1_O_SKR_2010

tacaacggtgagaagaagatcttttactccaggcccaacaaccacgacaactgttggctgaacgtcatccttcagctgttcaggtacgtcgatgaacctttcttcgactgggtgtatgaatcacctgaaaacctcacccttgaggcgatcagacaactggagaacattactggtcttgagctgcacgagggtggtccgcccgccctcgtcgtttggaacatcaaacacttgctccacaccgggatcggcaccgcctcgcgacccagtgaggtgtgcatggtggacggtacggacatgtgcctggctgacttccacgctggcatcttcatgaaaggacgggaacacgccgtgtttgcctgcgtcacctccaacgggtggtacgcgatcgacgacgaagaattctacccctggacgccagatccgtccgacgtgctggtctttgtcccgtacgaccaa

>KF112887.1_O_SKR_2010

tacaacggtgagaagaagatcttctactccaggcccaacaaccacgacaactgttggctgaacaccatccttcagctgttcaggtacgtcgatgaacctttcttcgactgggtatatgaatcacctgagaacctcacccttgaggcgatcagacaactggagaacattactggtcttgagctgcacgagggtggtccgcccgccctcgtcatttggaacatcaaacacttgctccacaccgggatcggcaccgcctcgcgacccagcgaggtgtgcatggtggacggtacggacatgtgcctggctgacttccacgctggcatcttcctgaaaggacaggaacacgccgtgtttgcctgcgtcacctccaacgggtggtacgcgatcgacgacgaagaattttacccctggacgccagatccgtccgacgtgctggtctttgtcccgtacgatcaa

>KF112888.1_O_DRK_2011

tacaacggtgagaagaagatcttctactccaggcccaacaaccacgacaactgttggctgaacaccatccttcagctgttcaggtacgtcgatgaacctttcttcgactgggtgtatgaatcacctgaaaatctcacccttgaggcgatcagacaactggagaacattactggtcttgagctgcacgaaggtggtccgcccgccctcgtcatttggaacatcaaacacttgctccacaccgggatcggcaccgcctcgcgacccagcgaggtgtgcatggtggacggtacggacatgtgcctggctgacttccacgctggcatcttcctgaaaggacaggaacatgccgtgtttgcctgcgtcacctccaacgggtggtacgcgatcgacgacgaagaattttacccctggacgccagatccgtccgacgtgctggtctttgtcccgtacgatcaa

>KF112889.1_O_HKN_2010

tacaacggtgagaagaagatcttctactccaggcccaacaaccacgacaactgttggctgaacgccatccttcagctgttcaggtacgtcgatgaacctttcttcgactgggtatatgaatcacctgaaaacctcacccttgaggcgatcagacaactggagaacattactggtcttgagctgcacgagggtggtccgcccgccctcgtcatttggaacatcaaacacttgctccacaccgggatcggcaccgcctcgcgacccagcgaggtgtgcatggtggacggtacggacatgtgcctggctgacttccacgctggcatcttcctgaaaggacaggaacacgccgtgtttgcctgcgtcacctccaacgggtggtacgcgatcgacgacgaagaattctacccctggacgccagatccgtccgacgtgctggtctttgtcccgtacgatcaa

>KF501486.1_O_SKR_2010

tacaacggtgagaagaagatcttctactccaggcccaacaaccacgacaactgttggctgaacaccatccttcagctgttcaggtacgtcgatgaacctttcttcgactgggtatatgaatcacctgagaacctcacccttgaggcgatcagacaactggagaacattactggtcttgagctgcacgagggtggtccgcccgccctcgtcatttggaacatcaaacacttgctccacaccgggatcggcaccgcctcgcgacccagcgaggtgtgcatggtggacggtacggacatgtgcctggctgacttccacgctggcatcttcctgaaaggacaggaacacgccgtgtttgcctgcgtcacctccaacgggtggtacgcgatcgacgacgaagaattttacccctggacgccagatccgtccgacgtgctggtctttgtcccgtacgatcaa

>KF501487.1_O_SKR_2010

tacaacggtgagaagaagatcttctactccaggcccaacaaccacgacaactgttggctgaacaccatccttcagctgttcaggtacgtcgatgaacctttcttcgactgggtatatgaatcacctgagaacctcacccttgaggcgatcagacaactggagaacattactggtcttgagctgcacgagggtggtccgcccgccctcgtcatttggaacatcaaacacttgctccacaccgggatcggcaccgcctcgcgacccagcgaggtgtgcatggtggacggtacggacatgtgcctggctgacttccacgctggcatcttcctgaaaggacaggaacacgccgtgttcgcctgcgtcacctccaacgggtggtacgcgatcgacgacgaagaattttacccctggacgccagatccgtccgacgtgttggtctttgtcccgtacgaccaa

>KF501488.1_O_SKR_2010

tacaacggtgagaagaagatcttctactccaggcccaacaaccacgacaactgttggctgaacaccatccttcagctgttcaggtacgtcgatgaacctttcttcgactgggtatatgagtcacctgagaacctcacccttgaggcgatcagacaactggagaacattactggtcttgagctgcacgagggtggtccgcccgccctcgtcatttggaacatcaaacacttgctccacaccgggatcggcaccgcctcgcgacccagcgaggtgtgcatggtggacggtacggacatgtgcctggctgacttccacgctggcatcttcctgaaaggacaggaacacgccgtgttcgcctgcgtcacctccaacgggtggtacgcgatcgacgacgaagaattttacccctggacgccagatccgtccgacgtgttggtctttgtcccgtacgaccaa

>KF694731.1_O_SKR_2002

cacaacggtgagaagaaaacattctactccaggcccaacaaccacgacaactgctggctgaacgccatcctccagttgtttaggtacgttgatgaacctttcttcgactgggtctactactcacctgagaacctcacgctcgatgctatcaaacaattggaaggaattactggtctcgagctccacgagggtggaccacccgctctcgttatttggaacattaaacacctgctcaacaccggaatcggcaccgcttcgcgacccagcgaagtgtgcatggtagatgggacggacatgtgtttggctgacttccacgctggcatcttcctgaaaggacaggaacacgctgtgttcgcctgtgtcacctccaacgggtggtacgcgattgatgacgaggacttttacccctggacgccggacccgtctgacgttctggtgttcgtcccgtacgaccaa

>KF694732.1_O_SKR_2002

cacaacggtgagaagaaaacattctactccaggcccaacaaccacgacaactgctggctgaacgccatcctccagttgttcaggtacgttgatgaacctttcttcgactgggtctactactcacctgagaacctcacgctcgatgctatcaaacaactggaagaaattactggtctcgcgctccacgagggtggaccacccgctctcgttatttggaacattaaacacctgctcaacaccggaatcggcaccgcttcgcgacccagcgaagtgtgcatggtagacgggacggacatgtgtttggctgacttccacgctggcatcttcctgaaaggacaggaacacgctgtgttcgcctgtgtcacctccaacgggtggtacgcgattgatgacgaggacttttacccctggacgccggacccgtctgacgttctggtgttcgtcccgtacgaccaa

>KF694734.1_O_SKR_2002

cacaacggtgagaagaaaacattctactccaggcccaacaaccacgacaactgctggctgaacgccatcctccagttgtttaggtacgttgatgaacctttcttcgactgggtctactactcacctgagaacctcacgctcgatgctatcaaacaattggaaggaattactggtctcgagctccacgagggtggaccacccgctctcgttatttggaacattaaacacctgctcaacaccggaatcggcaccgcttcgcgacccagcgaagtgtgcatggtagatgggacggacatgtgtttggctgacttccacgctggcatcttcctgaaaggacaggaacacgctgtgttcgcctgtgtcacctccaacgggtggtacgcgattgatgacgaggacttttacccctggacgccggacccgtctgacgttctggtgttcgtcccgtacgaccaa

>KF694735.1_O_SKR_2002

cacaacggtgagaagaaaacattctactccaggcccaacaaccacgacaactgctggctgaacgccatcctccagttgtttaggtacgttgatgaacctttcttcgactgggtctactactcacctgagaacctcacgctcgatgctatcaaacaattggaaggaattactggtctcgagctccacgagggtggaccacccgctctcgttatttggaacattaaacacctgctcaacaccggaatcggcaccgcttcgcgacccagcgaagtgtgcatggtagatgggacggacatgtgtttggctgacttccacgctggcatcttcctgaaaggacaggaacacgctgtgttcgcctgtgtcacctccaacgggtggtacgcgattgatgacgaggacttttacccctggacgccggacccgtctgacgttctggtgttcgtcccgtacgaccaa

>KF694736.1_O_SKR_2002

cacaacggtgagaagaaaacattctactccaggcccaacaaccacgacaactgctggctgaacgccatcctccagttgtttaggtacgttgatgaacctttcttcgactgggtctactactcacctgagaacctcacgctcgatgctatcaaacaattggaaggaattactggtctcgagctccacgagggtggaccacccgctctcgttatttggaacattaaacacctgctcaacaccggaatcggcaccgcttcgcgacccagcgaagtgtgcatggtagatgggacggacatgtgtttggctgacttccacgctggcatcttcctgaaaggacaggaacacgctgtgttcgcctgtgtcacctccaacgggtggtacgcgattgatgacgaggacttttacccctggacgccggacccgtctgacgttctggtgttcgtcccgtacgaccaa

>KF694737.1_O_SKR_2002

cacaacggtgagaagaaaacattctactccaggcccaacaaccacgacaactgctggctgaacgccatcctccagttgtttaggtacgttgatgaacctttcttcgactgggtctactactcacctgagaacctcacgctcgatgctatcaaacaattggaaggaattactggtctcgagctccacgagggtggaccacccgctctcgttatttggaacattaaacacctgctcaacaccggaatcggcaccgcttcgcgacccagcgaagtgtgcatggtagatgggacggacatgtgtttggctgacttccacgctggcatcttcctgaaaggacaggaacacgctgtgttcgcctgtgtcacctccaacgggtggtacgcgattgatgacgaggacttttacccctggacgccggacccgtctgacgttctggtgttcgtcccgtacgaccaa

>KF694738.1_O_SKR_2002

cacaacggtgagaagaaaacattctactccaggcccaacaaccacgacaactgctggctgaacgccatcctccagttgttcaggtacgttgatgaacctttcttcgactgggtctactactcacctgagaacctcacgctcgatgctatcaaacaactggaagaaattactggtctcgcgctccacgagggtggaccacccgctctcgttatttggaacattaaacacctgctcaacaccggaatcggcaccgcttcgcgacccagcgaagtgtgcatggtagacgggacggacatgtgtttggctgacttccacgctggcatcttcctgaaaggacaggaacacgctgtgttcgcctgtgtcacctccaacgggtggtacgcgattgatgacgaggacttttacccctggacgccggacccgtctgacgttctggtgttcgtcccgtacgaccaa

>KF694739.1_O_SKR_2002

cacaacggtgagaagaaaacattctactccaggcccaacaaccacgacaactgctggctgaacgccatcctccagttgtttaggtacgttgatgaacctttcttcgactgggtctactactcacctgagaacctcacgctcgatgctatcaaacaattggaaggaattactggtctcgagctccacgagggtggaccacccgctctcgttatttggaacattaaacacctgctcaacaccggaatcggcaccgcttcgcgacccagcgaagtgtgcatggtagatgggacggacatgtgtttggctgacttccacgctggcatcttcctgaaaggacaggaacacgctgtgttcgcctgtgtcacctccaacgggtggtacgcgattgatgacgaggacttttacccctggacgccggacccgtctgacgttctggtgttcgtcccgtacgaccaa

>KF694740.1_O_SKR_2002

cacaacggtgagaagaaaacattctactccaggcccaacaaccacgacaactgctggctgaacgccatcctccagttgtttaggtacgttgatgaacctttcttcgactgggtctactactcacctgagaacctcacgctcgatgctatcaaacaattggaaggaattactggtctcgagctccacgagggtggaccacccgctctcgttatttggaacattaaacacctgctcaacaccggaatcggcaccgcttcgcgacccagcgaagtgtgcatggtagatgggacggacatgtgtttggctgacttccacgctggcatcttcctgaaaggacaggaacacgctgtgttcgcctgtgtcacctccaacgggtggtacgcgattgatgacgaggacttttacccctggacgccggacccgtctgacgttctggtgttcgtcccgtacgaccaa

>KF694741.1_O_SKR_2002

cacaacggtgagaagaaaacattctactccaggcccaacaaccacgacaactgctggctgaacgccatcctccagttgtttaggtacgttgatgaacctttcttcgactgggtctactactcacctgagaacctcacgctcgatgctatcaaacaattggaaggaattactggtctcgagctccacgagggtggaccacccgctctcgttatttggaacattaaacacctgctcaacaccggaatcggcaccgcttcgcgacccagcgaagtgtgcatggtagatgggacggacatgtgtttggctgacttccacgctggcatcttcctgaaaggacaggaacacgctgtgttcgcctgtgtcacctccaacgggtggtacgcgattgatgacgaggacttttacccctggacgccggacccgtctgacgttctggtgttcgtcccgtacgaccaa

>KF694742.1_O_SKR_2002

cacaacggtgagaagaaaacattctactccaggcccaacaaccacgacaactgctggctgaacgccatcctccagttgtttaggtacgttgatgaacctttcttcgactgggtctactactcacctgagaacctcacgctcgatgctatcaagcaattggaaggaattactggtctcgagctccacgagggtggaccacccgctctcgttatttggaacattaaacacctgctcaacaccggaatcggcaccgcttcgcgacccagcgaagtgtgcatggtagatgggacggacatgtgtttggctgacttccacgctggcatcttcctgaaaggacaggaacacgctgtgttcgcctgtgtcacctccaacgggtggtacgcgattgatgacgaggacttttacccctggacgccggacccgtctgacgttctggtgttcgtcccgtacgaccaa

>KF694743.1_O_SKR_2002

cacaacggtgagaagaaaacattctactccaggcccaacaaccacgacaactgctggctgaacgccatcctccagttgtttaggtacgttgatgaacctttcttcgactgggtctactactcacctgagaacctcacgctcgatgctatcaaacaattggaaggaattactggtctcgagctccacgagggtggaccacccgctctcgttatttggaacattaaacacctgctcaacaccggaatcggcaccgcttcgcgacccagcgaagtgtgcatggtagatgggacggacatgtgtttggctgacttccacgctggcatcttcctgaaaggacaggaacacgctgtgttcgcctgtgtcacctccaacgggtggtacgcgattgatgacgaggacttttacccctggacgccggacccgtctgacgttctggtgttcgtcccgtacgaccaa

>KF694744.1_O_SKR_2002

cacaacggtgagaagaaaacattctactccaggcccaacaaccacgacaactgctggctgaacgccatcctccagttgtttaggtacgttgatgaacctttcttcgactgggtctactactcacctgagaacctcacgctcgatgctatcaaacaattggaaggaattactggtctcgagctccacgagggtggaccacccgctctcgttatttggaacattaaacacctgctcaacaccggaatcggcaccgcttcgcgacccagcgaagtgtgcatggtagatgggacggacatgtgtttggctgacttccacgctggcatcttcctgaaaggacaggaacacgctgtgttcgcctgtgtcacctccaacgggtggtacgcgattgatgacgaggacttttacccctggacgccggacccgtctgacgttctggtgttcgtcccgtacgaccaa

>KF694745.1_O_SKR_2002

cacaacggtgagaagaaaacattctactccaggcccaacaaccacgacaactgctggctgaacgccatcctccagttgtttaggtacgttgatgaacctttcttcgactgggtctactactcacctgagaacctcacgctcgatgctatcaaacaattggaaggaattactggtctcgagctccacgagggtggaccacccgctctcgttatttggaacattaaacacctgctcaacaccggaatcggcaccgcttcgcgacccagcgaagtgtgcatggtagatgggacggacatgtgtttggctgacttccacgctggcatcttcctgaaaggacaggaacacgctgtgttcgcctgtgtcacctccaacgggtggtacgcgattgatgacgaggacttttacccctggacgccggacccgtctgacgttctggtgttcgtcccgtacgaccaa

>KF985189.1_O_BAN_2013

cacaacggtgagaaaaagaccttctattctaggcccaacagccacgacaattgttggttgaacaccatccttcagttgtttaggtacgtcgacgaacctttcttcgactgggtctatgagtcgcctgaaaacctcacccttgaggcgattaggcaactagaagaagttactggtcttgagctgcacgagggtggaccgcccgctctcgtcatttggaacatcaagcacttgctccacaccggagtcggcactgcttcgcgacccagcgaggtgtgcatggttgatggcacggacatgtgtttggccgacttccacgctggcatcttcctgaaagggcaagaacacgctgtgttcgcctgcgtcacctccaacgggtggtacgcgatcgacgacgaggacttctacccctggacgccggacccgtccgacgttctggtgtttgtcccgtacgatcaa

>KJ206908.1_O_BHU_2013

tacaacggtgagaaaaagaccttctactctaggcccaacaaccacgacaactgttggttgaacgccatccttcagttgtttaggtacgtcgacgagcctttcttcgactgggtctatgaatcgcccgaaaaccacactcttgaggcgattagacaactggaggagatcactggtcttgagctgcacgagggtggaccgcccgctctcgtcatttggaacactaaacacttgctccacaccggaatcggcactgcttcgcgacccagcgaggtgtgcatggttgatggcacggacatgtgtttggccgacttccacgctggcatcttcctgaaagggcaagaacacgctgtgttcgcctgcgtcacctccaacgggtggtacgcgatcgacgacgaggacttctacccctggacgccggacccgtccgacgttttggtgtttgtcccgtacgatcaa

>KJ206909.1_O_LIB_2013

cacaacggtgagaaaaagaccttctactctaggcccaacaaccacgacaactgttggttaaacaccatccttcagttgtttaggtacgtcgacgaacctttcttcgactgggtctatgaatcgcctgaaaacctcactcttgaggcgattaggcaactggaagagatcactggccttgagctgcacgagggtggaccgcccgctctcgtcatctggaacatcaaacacttgctccacaccggaatcggcactgcttcgcgacccagtgaggtgtgcatggttgatggtacagacatgtgtttggccgacttccacgctggcatcttcctgaaagggcaagaacacgctgtgttcgcctgcgtcacctccaacgggtggtacgcgatcgacgacgaggacttctacccctggacgccggacccgtccgacgttctggtgtttgtcccgtacgatcaa

>KJ206910.1_O_SAU_2013

cacaacggtgagaaaaagaccttctactctaggcccaataaccacgacaactgttggttgaacaccatccttcagttgttcaggtacgtcgacgaacctttcttcgactgggtctatgaatcgcctgaaaacctcactcttgaggcgattaggcaattggaagagatcactggccttgagctgcacgagggtggaccgcccgctctcgtcatctggaacatcaaacacttgctccacaccggaatcggcactgcttcgcgacccagcgaggtgtgcatggttgatggcacagacatgtgtttggccgacttccacgctggtatcttcctgaaagggcaagaacacgctgtgttcgcctgcgtcacctccaacgggtggtacgcgatcgacgacgaggacttctacccctggacgccggacccgtccgacgttctggtgtttgtcccgtacgatcaa

>KJ560276.1_O_UKG_2007

tacaacggtgagaagaagaccttttactccaggcccaacaaccacgacaactgctggttgaacgccatcctccagttgttcaggtacgttgaagaaccattcttcgactgggtctacagttcgcctgagaacctcacgcttgaagccatcaagcagttggaggacctcacagggcttgaactgcacgagggtggaccacctgctctcgtgatctggaacatcaagcacttgctccacaccggcattggcaccgcctcgcgacccagcgaggtgtgcatggtggatggtacggacatgtgcttggctgatttccatgcaggcattttccttaaggggcaagaacacgctgtgttcgcgtgtgtcacctccaacgggtggtacgcgattgatgatgaggacttctacccctggacgccggacccatccgacgttctggtgtttgtcccgtacgatcaa

>KJ560277.1_O_UKG_2007

tacaacggtgagaagaagaccttttactccaggcccaacaaccacgacaactgctggttgaacgccatcctccagttgttcaggtacgttgaagaaccattcttcgactgggtctacagttcgcctgagaacctcacgcttgaagccatcaagcagttggaggacctcacagggcttgaactgcacgagggtggaccacctgctctcgtgatctggaacatcaagcacttgctccacaccggcattggcaccgcctcgcgacccagcgaggtgtgcatggtggatggtacggacatgtgcttggctgatttccatgcaggcattttccttaaggggcaagaacacgctgtgttcgcgtgtgtcacctccaacgggtggtacgcgattgatgatgaggacttctacccctggacgccggacccatccgacgttctggtgtttgtcccgtacgatcaa

>KJ560281.1_O_UKG_2007

tacaacggtgagaagaagaccttttactccaggcccaacaaccacgacaactgctggttgaacgccatcctccagttgttcaggtacgttgaagaaccattcttcgactgggtctacagttcgcctgagaacctcacgcttgaagccatcaagcagttggaggacctcacagggcttgaactgcacgagggtggaccacctgctctcgtgatctggaacatcaagcacttgctccacaccggcattggcaccgcctcgcgacccagcgaggtgtgcatggtggatggtacggacatgtgcttggctgatttccatgcaggcattttccttaaggggcaagaacacgctgtgttcgcgtgtgtcacctccaacgggtggtacgcgattgatgatgaggacttctacccctggacgccggacccatccgacgttctggtgtttgtcccgtacgatcaa

>KJ560283.1_O_UKG_2007

tacaacggtgagaagaagaccttttactccaggcccaacaaccacgacaactgctggttgaacgccatcctccagttgttcaggtacgttgaagaaccattcttcgactgggtctacagttcgcctgagaacctcacgcttgaagccatcaagcagttggaggacctcacagggcttgaactgcacgagggtggaccacctgctctcgtgatctggaacatcaagcacttgctccacaccggcattggcaccgcctcgcgacccagcgaggtgtgcatggtggatggtacggacatgtgcttggctgatttccatgcaggcattttccttaaggggcaagaacacgctgtgttcgcgtgtgtcacctccaacgggtggtacgcgattgatgatgaggacttctacccctggacgccggacccatccgacgttctggtgtttgtcccgtacgatcaa

>KJ560285.1_O_UKG_2007

tacaacggtgagaagaagaccttttactccaggcccaacaaccacgacaactgctggttgaacgccatcctccagttgttcaggtacgttgaagaaccattcttcgactgggtctacagttcgcctgagaacctcacgcttgaagccatcaagcagttggaggacctcacagggcttgaactgcacgagggtggaccacctgctctcgtgatctggaacatcaagcacttgctccacaccggcattggcaccgcctcgcgacccagcgaggtgtgcatggtggatggtacggacatgtgcttggctgatttccatgcaggcattttccttaaggggcaagaacacgctgtgttcgcgtgtgtcacctccaacgggtggtacgcgattgatgatgaggacttctacccctggacgccggacccatccgacgttctggtgtttgtcccgtacgatcaa

>KJ560287.1_O_UKG_2007

tacaacggtgagaagaagamcttttactccaggcccaacaaccacgacaactgctggttgaacgccatcctccagttgttcaggtacgttgaagaaccattcttcgactgggtctacagttcgcctgagaacctcacgcttgaagccatcaagcagttggaggacctcacagggcttgaactgcacgagggtggaccacctgctctcgtgatctggaacatcaagcacttgctccacaccggcattggcaccgcctcgcgacccagcgaggtgtgcatggtggatggtacggacatgtgcttggctgatttccatgcaggcattttccttaaggggcaagaacacgctgtgttcgcgtgtgtcacctccaacgggtggtacgcgattgatgatgaggacttctacccctggacgccggacccatccgacgttctggtgtttgtcccgtacgatcaa

>KJ560294.1_O_UKG_2007

tacaacggtgagaagaagaccttttactccaggcccaacaaccacgacaactgctggttgaacgccatcctccagttgttcaggtacgttgaagaaccattcttcgactgggtctacagttcgcctgagaacctcacgcttgaagccatcaagcagttggaggacctcacagggcttgaactgcacgagggtggaccacctgctctcgtgatctggaacatcaagcacttgctccacaccggcattggcaccgcctcgcgacccagcgaggtgtgcatggtggatggtacggacatgtgcttggctgatttccatgcaggcattttccttaaggggcaagaacacgctgtgttcgcgtgtgtcacctccaacgggtggtacgcgattgatgatgaggacttctacccctggacgccggacccatccgacgttctggtgtttgtcccgtacgatcaa

>KJ560296.1_O_UKG_2007

tacaacggtgagaagaagaccttttactccaggcccaacaaccacgacaactgctggttgaacgccatcctccagttgttcaggtacgttgaagaaccattcttcgactgggtctacagttcgcctgagaacctcacgcttgaagccatcaagcagttggaggacctcacagggcttgaactgcacgagggtggaccacctgctctcgtgatctggaacatcaagcacttgctccacaccggcattggcaccgcctcgcgacccagcgaggtgtgcatggtggatggtacggacatgtgcttggctgatttccatgcaggcattttccttaaggggcaagaacacgctgtgttcgcgtgtgtcacctccaacgggtggtacgcgattgatgatgaggacttctacccctggacgccggacccatccgacgttctggtgtttgtcccgtacgatcaa

>KJ560297.1_O_UKG_2007

tacaacggtgagaagaagaccttttactccaggcccaacaamcacgacaactgctggttgaacgccatcctccagttgttcaggtacgttgaagaaccattcttcgactgggtctacagttcgcctgagaacctcacgcttgaagccatcaagcagttggaggacctcacagggcttgaactgcacgagggtggaccacctgctctcgtgatctggaacatcaagcacttgctccacaccggcattggcaccgcctcgcgacccagcgaggtgtgcatggtggatggtacggacatgtgcttggctgatttccatgcaggcattttccttaaggggcaagaacatgctgtgttcgcgtgtgtcacctccaacgggtggtacgcgattgatgatgaggacttctacccctggacgccggacccatccgacgttctggtgtttgtcccgtacgatcaa

>KJ560298.1_O_UKG_2007

tacaacggtgagaagaagaccttttactccaggcccaacaaccacgacaactgctggttgaacgccatcctccagttgttcaggtacgttgaagaaccattcttcgactgggtctacagttcgcctgagaacctcacgcttgaagccatcaagcagttggaggacctcacagggcttgaactgcacgagggtggaccacctgctctcgtgatctggaacatcaagcacttgctccacaccggcattggcaccgcctcgcgacccagcgaggtgtgcatggtggatggtacggacatgtgcttggctgatttccatgcaggcattttccttaaggggcaagaacacgctgtgttcgcgtgtgtcacctccaacgggtggtacgcgattgatgatgaggacttctacccctggacgccggacccatccgacgttctggtgtttgtcccgtacgatcaa

>KJ560299.1_O_UKG_2007

tacaacggtgagaagaagaccttttactccaggcccaacaaccacgacaactgctggttgaacgccatcctccagttgttcaggtacgttgaagaaccattcttcgactgggtctacagttcgcctgagaacctcacgcttgaagccatcaagcagttggaggacctcacagggcttgaactgcacgagggtggaccacctgctctcgtgatctggaacatcaagcacttgctccacaccggcattggcaccgcctcgcgacccagcgaggtgtgcatggtggatggtacggacatgtgcttggctgatttccatgcaggcattttccttaaggggcaagaacacgctgtgttcgcgtgtgtcacctccaacgggtggtacgcgattgatgatgaggacttctacccctggacgccggacccatccgacgttctggtgtttgtcccgtacgatcaa

>KJ560300.1_O_UKG_2007

tacaacggtgagaagaagaccttttactccaggcccaacaaccacgacaactgctggttgaacgccatcctccagttgttcaggtacgttgaagaaccattcttcgactgggtctacagttcgcctgagaacctcacgcttgaagccatcaagcagttggaggacctcacagggcttgaactgcacgagggtggaccacctgctctcgtgatctggaacatcaagcacttgctccacaccggcattggcaccgcctcgcgacccagcgaggtgtgcatggtggatggtacggacatgtgcttggctgatttccatgcaggcattttccttaaggggcaagaacacgctgtgttcgcgtgtgtcacctccaacgggtggtacgcgattgatgatgaggacttctacccctggacgccggacccatccgacgttctggtgtttgtcccgtacgatcaa

>KJ560302.1_O_UKG_2007

tacaacggtgagaagaagaccttttactccaggcccaacaaccacgacaactgctggttgaacgccatcctccagttgttcaggtacgttgaagaaccattcttcgactgggtctacagttcgcctgagaacctcacgcttgaagccatcaagcagttggaggacctcacagggcttgaactgcacgagggtggaccacctgctctcgtgatctggaacatcaagcacttgctccacaccggcattggcaccgcctcgcgacccagcgaggtgtgcatggtggatggtacggacatgtgcttggctgatttccatgcaggcattttccttaaggggcaagaacacgctgtgttcgcgtgtgtcacctccaacgggtggtacgcgattgatgatgaggacttctacccctggacgccggacccatccgacgttctggtgtttgtcccgtacgatcaa

>KJ560303.1_O_UKG_2007

tacaacggtgagaagaagaccttttactccaggcccaacaaccacgacaactgctggttgaacgccatcctccagttgttcaggtacgttgaagaaccattcttcgactgggtctacagttcgcctgagaacctcacgcttgaagccatcaagcagttggaggacctcacagggcttgaactgcacgagggtggaccacctgctctcgtgatctggaacatcaagcacttgctccacaccggcattggcaccgcctcgcgacccagcgaggtgtgcatggtggatggtacggacatgtgcttggctgatttccatgcaggcattttccttaaggggcaagaacacgctgtgttcgcgtgtgtcacctccaacgggtggtacgcgattgatgatgaggacttctacccctggacgccggacccatccgacgttctggtgtttgtcccgtacgatcaa

>KJ560304.1_O_UKG_2007

tacaacggtgagaagaagaccttttactccaggcccaacaaccacgacaactgctggttgaacgccatcctccagttgttcaggtacgttgaagaaccattcttcgactgggtctacagttcgcctgagaacctcacgcttgaagccatcaagcagttggaggacctcacagggcttgaactgcacgagggtggaccacctgctctcgtgatctggaacatcaagcacttgctccacaccggcattggcaccgcctcgcgacccagcgaggtgtgcatggtggatggtacggacatgtgcttggctgatttccatgcaggcattttccttaaggggcaagaacacgctgtgttcgcgtgtgtcacctccaacgggtggtacgcgattgatgatgaggacttctacccctggacgccggacccatccgacgttctggtgtttgtcccgtacgatcaa

>KJ560307.1_O_UKG_2007

tacaacggtgagaagaagaccttttactccaggcccaacaaccacgacaactgctggttgaacgccatcctccagttgttcaggtacgttgaagaaccattcttcgactgggtctacagttcgcctgagaacctcacgcttgaagccatcaagcagttggaggacctcacagggcttgaactgcacgagggtggaccacctgctctcgtgatctggaacatcaagcacttgctccacaccggcattggcaccgcctcgcgacccagcgaggtgtgcatggtggatggtacggacatgtgcttggctgatttccatgcaggcattttccttaaggggcaagaacacgctgtgttcgcgtgtgtcacctccaacgggtggtacgcgattgatgatgaggacttctacccctggacgccggacccatccgacgttctggtgtttgtcccgtacgatcaa

>KJ560308.1_O_UKG_2007

tacaacggtgagaagaagaccttttactccaggcccaacaaccacgacaactgctggttgaacgccatcctccagttgttcaggtacgttgaagaaccattcttcgactgggtctacagttcgcctgagaacctcacgcttgaagccatcaagcagttggaggacctcacagggcttgaactgcacgagggtggaccacctgctctcgtgatctggaacatcaagcacttgctccacaccggcattggcaccgcctcgcgacccagcgaggtgtgcatggtggatggtacggacatgtgcttggctgatttccatgcaggcattttccttaaggggcaagaacacgctgtgttcgcgtgtgtcacctccaacgggtggtacgcgattgatgatgaggacttctacccctggacgccggacccatccgacgttctggtgtttgtcccgtacgatcaa

>KJ608371.1_A_VIT_2013

cacaacggtgaaaagaagaccttctactctaggcccaacaaccacgacaactgctggctgaacaccattctccaactgttcaggtacgtcgatgaaccattctttgattgggtctatgaatcacctgaaaaccttactcttgaagcaattaggcaattggaagagatcactggtcttgagctgcacgagggtggcccgcccgctctcgtaatttggaacatcaagcacttgctccataccggaatcggtactgcttcgcgacccagcgaggtgtgtatggtagacggtacggacatgtgtttggctgatttccatgctggcatcttcctgaaaggacaggaacatgcagtgtttgcctgtgtcacctccaacgggtggtacgcgattgatgacgaggacttttacccctggacaccagatccgtctgacgtcctggtgtttgtcccgtacgatcaa

>KJ754939.1_A_BAN_2013

cacaacggtgagaaaaagaccttttactctaggcccaacaaccacgacaactgttggttgaacaccatcctccaattgtttaggtatgtcgacgaacctttcttcgactgggtctatgagtcacctgagaacctcactctcgaggcgattaggcaactagaagaaatcactggtcttgagctgcacgagggtggtccgcccgctctcgtcatttggaacattaagcacttgctccacaccggaatcggcactgcttcgcgacccagcgaggtgtgcatggttgatggcacggacatgtgcttggcagacttccacgctggcatcttcctgaaagggcaagagcacgctgtgttcgcctgtgtcacctccaacgggtggtatgcgatcgatgacgaggacttttacccctggacgccggatccgtccgacgttctggtgtttgtcccgtacgatcaa

>KJ820999.1_SAT3_UGA_2013

tacaacggtgagaagaaaactttctactcaagacccaacaagcacgacaactgctggttgaacaccatcctacagttgttcaggtacgtcgatgaaccattcttcgactgggtctacaactcgcccgagaaccttactctccgtgccattgaacagctcgaggaactcactgggcttgagttgcatgagggtggaccccccgctctcgtgatttggaacatcaaacacttactctataccggaatcggtaccgcttcgcgacccagcgaagtgtgtatggtggacggtactgagatgtgtcttgctgatttccacgcagggatcttcctgaaaggtactgaacacgccgtgttcgcctgcctgacctccgacggttggtacgccattgacgacgaggacttttacccttggactccggacccatccgacgtcctttgttttgtcccctatgacatg

>KJ825801.1_O_IND_2013

tacaacggtgagaaaaagaccttctactctaggcccaacaaccacgacaactgttggttgaacaccatccttcagttgtttaggtatgtcgacgaaccttttttcgactgggtctatgaatcgcctgaaaacctcactcttgaggcgattaggcaactggaagagatcactggtcttgagctgcacgagggtggaccgcccgctctcgtcatctggaacatcaaacacttgctccacaccggaatcggcactgcttcgcggcccagcgaggtgtgcatggttgatggcacggacatgtgtctggccgacttccacgctggcatcttcctgaaagggcaagaacacgctgtgttcgcctgcgtcacctctaacgggtggtacgcgattgacgacgaggacttctacccctggacgccggacccgtccgacgttctggtgtttgtcccgtacgatcaa

>KJ825802.1_O_IND_2013

cacaacggtgagaaaaagaccttctactctaggcccaacaaccacgacaactgttggttgaacaccatccttcagttgtttaggtacgtcgacgaacctttcttcgactgggtctatgaatcgcctgaaaacctcactcttgaggcgattaggcaactggaagagatcactggtcttgagctgcacgagggtggaccgcccgctctcgtcatctggaacatcaaacacttgctccacaccggaatcggcactgcttcgcgacccagcgaggtgtgcatggttgatggcacagacatgtgtttggccgacttccacgctggcatcttcctgaaagggcaagaacacgctgtgttcgcctgcgtcacctccaacgggtggtacgcgatcgacgacgaggacttctacccctggacgccggacccgtccgacgttctggtgtttgtcccgtacgatcaa

>KJ825803.1_O_IND_2013

tacaacggtgagaaaaagaccttctactctaggcccaacaaccacgacaactgttggttgaacaccatccttcagttgtttaggtatgtcgacgaaccttttttcgactgggtctatgaatcgcctgaaaacctcactcttgaggcgattaggcaactggaagagatcactggtcttgagctgcacgagggtggaccacccgctctcgtcatctggaacattaaacacttgctccacaccgggatcggcactgcttcgcgacccagcgaggtgtgcatggttgatggcacggacatgtgtttggccgacttccacgctggcatcttcctgaaagggcaagaacacgctgtgttcgcctgcgtcacctccaacgggtggtacgcgattgacgacgaggacttctacccttggacgccggacccgtccgacgttctggtgtttgtcccgtacgatcaa

>KJ825804.1_O_IND_2013

tacaacggtgagaaaaagaccttctactctaggcccaacaaccacgacaactgttggttgaacaccatccttcagttgtttaggtatgtcgacgaaccttttttcgactgggtctatgaatcgcctgaaaacctcactcttgaggcgattaggcaactggaagagatcactggtcttgagctgcacgagggtggaccgcccgctctcgtcatctggaacattaaacacttgctccacaccggaatcggcactgcttcgcgacccagcgaggtgtgcatggttgacggcacggacatgtgtttggctgacttccacgctggcatcttcctgaaagggcaagaacacgctgtgttcgcctgcgtcacctccaacgggtggtatgcgattgacgacgaggacttctacccctggacgccggacccgtccgacgttctggtgtttgtcccgtacgatcaa

>KJ825805.1_O_IND_2013

tacaacggtgagaaaaagaccttctactctaggcccaacaaccacgacaactgttggttgaacaccatccttcagttgtttaggtatgtcgacgaaccttttttcgactgggtctatgaatcgcctgaaaacctcactcttgaggcgattaggcaactggaagagatcactggtctcgagctgcacgagggtggaccgcccgctctcgttatctggaacattaaacacttgctccacaccggaatcggcactgcttcgcgacccagcgaggtgtgcatggttgatggcacggacatgtgtttggctgacttccacgctggcatcttcctgaaagggcaagaacacgctgtgttcgcctgcgtcacctccaacgggtggtatgcgattgacgacgaggacttctacccctggacgccggacccgtccgacgttctggtgtttgtcccgtacgatcaa

>KJ825806.1_O_IND_2013

tacaacggtgagaaaaagaccttctactctaggcccaacaaccacgacaactgttggttgaacaccatccttcagttgtttaggtatgtcgacgaaccttttttcgactgggtctatgaatcgcctgaaaacctcactcttgaggcgattaggcaactggaagagatcactggtctcgagctgcacgagggtggaccgcccgctctcgtcatctggaacattaaacacttgctccacaccggaatcggcactgcttcgcgacccagcgaggtgtgcatggttgatggcacggacatgtgtttggctgacttccacgctggcatcttcctgaaagggcaagaacacgctgtgttcgcctgcgtcacctccaacgggtggtatgcgattgacgacgaggacttctacccctggacgccggacccgtccgacgttctggtgtttgtcccgtacgaccaa

>KJ825807.1_O_IND_2014

tacaacggtgagaaaaagaccttttactctaggcccaacaaccacgacaactgttggttgaacaccatccttcagttgtttaggtatgtcgacgaaccttttttcgactgggtctatgaatcgcctgaaaacctcactcttgaggcgattaggcaactggaagagatcactggtcttgagctgcacgagggtggaccgcccgctctcgtcatctggaacatcaaacacttgctccacaccggaatcggcactgcttcgcgacccagcgaggtgtgcatggttgatggcacggacatgtgtttggctgacttccacgctggcatcttcctgaaagggcaagaacacgctgtgttcgcctgcgtcacctccaacgggtggtatgcgattgacgacgaggacttctacccctggacgccggacccgtccgacgttctggtgtttgtcccgtacgatcaa

>KJ825808.1_O_IND_2013

tacaacggtgagaaaaagaccttctactctaggcccaacaaccacgacaactgttggttgaacaccatccttcagttgtttaggtatgtcgacgaaccttttttcgactgggtctatgaatcgcctgaaaacctcactcttgaggcgattaggcaactggaagagatcactggtcttgagctgcacgagggtggaccgcccgctctcgtcatctggaacatcaaacacttgctccacaccggaatcggcactgcttcgcggcccagcgaggtgtgcatggttgatggcacggacatgtgtctggccgacttccacgctggcatcttcctgaaagggcaagaacacgctgtgttcgcctgcgtcacctctaacgggtggtacgcgattgacgacgaggacttctacccctggacgccggacccgtccgacgttctggtgtttgtcccgtacgatcaa

>KJ825809.1_O_IND_2013

tacaacggtgagaaaaagaccttctactctaggcccaacaaccacgacaactgttggttgaacaccatccttcagttgtttaggtatgtcgacgaaccttttttcgactgggtctatgaatcgcctgaaaacctcactcttgaggcgattaggcaactggaagagatcactggtcttgagctgcacgagggtggaccgcccgctctcgtcatctggaacattaaacacttgctccacaccggaatcggcactgcttcgcgacccagcgaggtgtgcatggttgatggcacggacatgtgtttggctgacttccacgctggcatcttcctgaaagggcaagaacacgctgtgttcgcctgcgtcacctccaacgggtggtatgcgattgacgacgaggacttctacccctggacgccggacccgtccgacgttctggtgtttgtcccgtacgatcaa

>KJ933864.1_A_MAY_1997

cacaacggtgaaaagaagaccttctattccaggcccaacaaccacgacaactgttggctgaacaccatcctccagttgttcaggtacgtcgacgaacctttcttcgactgggtctatgaatcacctgagaacctcactcttgaggcgatcagacaactggaagagatcactggtcttgaactgcacgagggtggtccacccgccctcgtaatttggaacatcaagcacttgcttcacaccggaatcggtaccgcctcgcgacccagcgaggtgtgtatggtagacggcacggacatgtgcttggctgatttccatgctggcatcttcctgaaaggacaggaacacgcagtgtttgcctgtgtcacctccaacgggtggtacgcgattgacgacgaggacttttacccctggacaccagatccgtctgatgtcctggtgtttgtcccgtacgatcaa

>KM257061.1_O_UKG_2001

cacaacggtgagaagaaaacattctactccaggcccaacaaacacgacaactgctggctgaacaccatcctccagttgtttaggtacgttgatgaaccttttttcgactgggtctactactcacctgagaacctcacacttgatgctatcaaacaattggaagaaattactggtctcgagctccacgagggtggaccacccgctctcgttatttggaacattaaacacttgctcaacaccggaatcggcaccgcctcgcgacccagcgaagtgtgcatggtagacgggacggatatgtgtttggctgacttccacgctggcatcttcctgaaaggacaggaacacgctgtgttcgcctgcgttacctccaacgggtggtacgcgattgatgacgaggacttttacccctggacgccggacccgtccgacgttctggtgtttgtcccgtacgatcaa

>KM257062.1_O_UKG_2001

cacaacggtgagaagaaaacattctactccaggcccaacaaacacgacaactgctggctgaacaccatcctccagttgtttaggtacgttgatgaaccttttttcgactgggtctactactcacctgagaacctcacacttgatgctatcaaacaattggaagaaattactggtctcgagctccacgagggtggaccacccgctctcgttatttggaacattaaacacttgctcaacaccggaatcggcaccgcctcgcgacccagcgaagtgtgcatggtagacgggacggatatgtgtttggctgacttccacgctggcatcttcctgaaaggacaggaacacgctgtgttcgcctgcgttacctccaacgggtggtacgcgattgatgacgaggacttttacccctggacgccggacccgtccgacgttctggtgtttgtcccgtacgatcaa

>KM257063.1_O_UKG_2001

cacaacggtgagaagaaaacattctactccaggcccaacaaacacgacaactgctggctgaacaccatcctccagttgtttaggtacgttgatgaaccttttttcgactgggtctactactcacctgagaacctcacacttgatgctatcaaacaattggaagaaattactggtctcgagctccacgagggtggaccacccgctctcgttatttggaacattaaacacttgctcaacaccggaatcggcaccgcctcgcgacccagcgaagtgtgcatggtagacgggacggatatgtgtttggctgacttccacgctggcatcttcctgaaaggacaggaacacgctgtgttcgcctgcgttacctccaacgggtggtacgcgattgatgacgaggacttttacccctggacgccggacccgtccgacgttctggtgtttgtcccgtacgatcaa

>KM257064.1_O_UKG_2001

cacaacggtgagaagaaaacattctactccaggcccaacaaacacgacaactgctggctgaacaccatcctccagttgtttaggtacgttgatgaaccttttttcgactgggtctaccactcacctgagaacctcacacttgatgctatcaaacaattggaagaaattactggtctcgagctccacgagggtggaccacccgctctcgttatttggaacattaaacacttgctcaacaccggaatcggcaccgcctcgcgacccagcgaagtgtgcatggtagacgggacggacatgtgtttggctgacttccacgctggcatcttcctgaaaggacaggaacacgctgtgttcgcctgcgttacctccaacgggtggtacgcgattgatgacgaggacttttacccctggacgccggacccgtccgacgttctggtgtttgtcccgtacgatcaa

>KM257065.1_O_UKG_2001

cacaacggtgagaagaaaacattctactccaggcccaacaaacacgacaactgctggctgaacaccatcctccagttgtttaggtacgttgatgaaccttttttcgactgggtctactactcacctgagaacctcacacttgatgctatcaaacaactggaagaaattactggtctcgagctccacgagggtggaccacccgctctcgttatttggaacattaaacacttgctcaacaccggaatcggcaccgcctcgcgacccagcgaagtgtgcatggtagacgggacggatatgtgtttggctgacttccacgctggcatcttcctgaaaggacaggaacacgctgtgttcgcctgcgttacctccaacgggtggtacgcgattgatgacgaggacttttacccctggacaccggacccgtccgacgttctggtctttgtcccgtacgatcaa

>KM268895.1_O_TUR_2013

cacaacggtgagaaaaagactttctattctaggcccaacagacacgacaactgttggttgaacaccatccttcagttgttcagatacgtcgacgaaccgttcttcgactgggtctatgattcacctgaaaacctcactcttgaagcaatcacacaactggaagaactcactggtcttgaactgcacgagggtggaccacctgctctcgtcatctggaacatcaaacacctgctccacaccggaatcggtaccgcctcgcgacccagtgaggtgtgtatggttgacggcacggacatgtgtctagctgactttcatgctggtattttcctgaaaggacaagagcatgcagtgttcgcctgtgtcacatccgacgggtggtacgcgattgacgacgaggacttctacccttggacgccggacccgtccgacgttctggtttttgtcccgtacgatcaa

>KM268896.1_A_TUR_2013

cataacggtgagagaaagaccttctactctaggcccaacaaacacgacaactgttggttgaacaccatccttcagttgttcaggtatgttgatgaaccattctttgactgggtctatgattcacctgaaaacctcactctcgaagcaatcagacaactggaagaactcactggttttgaactgcacgagggtgggccacctgctctcgtcatttggaatatcaaacacctgcttcacaccggaatcggtaccgcctcgcgacccagtgaggtgtgtatggttgacggcacggacatgtgtttggctgactttcacgctggcattttcttgaaagggcaagagcatgctgtgtttgcctgtgtcacatccgatgggtggtacgcgattgacgacgaggacttttacccttggacgccggacccgtccgacgttctggtgtttgtcccgtacgatcaa

>KM268897.1_C_KEN_2004

tataacggtgagaagaaaacattctactccagacccaacaaccacgacaactgttggcttaacgccatccttcaattgttcaggtacgttgatgagcctttcttcgattgggtttacaactcgcctgagaaccttactgttgaggcaatcagacagctggaggatgtgactggtcttgagctacacgagggtgggccgcccgctctcgtcgtctggaacattaaacacctgctccacaccggaatcggcaccgcctcacgccccagtgaggtgtgcatggtagacggtacggacatgtgtttggctgatttccacgctggcatcttcttgaaaggccaagaacacgcagtgtttgcgtgtgtcacctcccaagggtggtacgcgattgatgacgaggacttttacccctggacacccgacccaacggatgtcttggtgtttgtcccgtatgatcag

>KM268898.1_Asia1_TUR_2013

cacaacggtgagaagaagactttctactccaggcccaacaaccacgacaactgctggttgaacaccatcctccagttgttcaggtacgtcgatgaacctttcttcgactgggtctacaactcacctgagaacctcacacttgatgccatcaaacaattagaagaagtcactggcctagaactacacgagggtggaccacccgctctcgtcatttggaacatcaaacacttactcagcaccggaatcggtaccgcttcgcggcctagcgaagtgtgtatggtagacggaactgacatgtgtttggctgacttccacgctggcattttcctgaaaggacaggaacacgctgtgttcgcctgcgtcacctccaacggatggtacgcgatcgacgacgaggacttttacccgtggacgccggacccgtccgacgttttggtgtttgttccgtacgatcaa

>KM268899.1_SAT1_TAN_2012

tacaacggtgagaagaagaccttctacagcaggccaaacacacacggcaactgctggctcaactcgttactgcagctctttcgatacgtcgatgaaccacttttcgagtctgagtatctgtcacctgagaacaagacactggatatgatcaaacaactttctgactacaccggacttgacctctcagatggtgggccacccgcacttgtgctttggctcatcaaggattgtctcagtactggcgttggcaccagcactcggcccagtgagatctgtgtgatcaacggcgttgtgatgacattggctgacttccacgctggcatcttcatcaagggcaccgaacacgcagtgttcgccctcaacacatcagagggctggtacgccattgatgatgaggtgttctacccatggacacccgaccccgcggacgtactcgcgtacgtgccgtacgaccaa

>KM268900.1_SAT2_TAN_2012

tacaacggtgagaagaagacattctacagcaggcccaacacgcacggcaactgctggctcaactcgttattgcagctctttcgctatgtcgatgaaccgctcttcgagtctgagtatttgtcacctgagaacaagacactggacatgatcaaacaactttctgactacactggacttgacctctcagacggtgggccacctgcacttgtgctttggctcatcaaggactgtctccacaccggcgttggcaccagcactcgtccaagcgagatctgcgtgatcaacggagtcgtaatgacactggctgatttccacgctggcattttcctcaagggtaccgaacacgcagtgtttgctctcaacacatcagagggctggtacgccattgacgatgaagtcttctacccatggacacccgaccctgcggacgtactcgcgtacgtaccgtatgaccaa

>KM268901.1_SAT3_ZIM_1991

tacaacggcgagaagaagaccttctacagcaggcccaacacccacgggaactgttggctcaactcgcttttgcagctctttcgatacgtcgatgagccgctctttgagtctgaatatctgtcaccagagaacaagacattggacatgatcaaacaactttctgattacaccaaacttgatctttctgatggtgggccacccgcacttgtgctctggctcatcaaggactgcttgcagactggcgttggcaccagcactcgccccagcgagatctgtgtgatcaacggggtcgtcatgaccctcgctgacttccacgccggtattttcatcaaaggtactgaacacgctgtgttcgccctcaacacatccgagggttggtacgccattgatgatgaggtgttctatccatggacacccgaccctgcggacgtactcgcgtacgtgccatacgaccag

>KP940473.1_O_EGY_2014

tacaacggtgagaagaaaaccttttactccaggcccaacaaccacgacaactgttggttgaacgccatcctccagttgttcaggtacgttgaagaaccgttcttcgactgggtctacagttcgcctgagaacctcacgcttgaagccatcaagcagttggaggatctcacagggcttgagctgcacgagggtggaccacctgctctcgtcatttggaacattaaacacttactccacaccggaatcggtaccgcctcgcgccctagcgaggtgtgtatggtggacggaacagacatgtgtttagctgactttcatgccggcatctttctgaaaggacaggaacatgctgtgtttgcctgtgttacctccaacgggtggtacgcgattgatgacgaggacttttacccttggacgccggacccgtccgacgttctggtatttgttccgtacgatcaa

>KP940474.1_A_EGY_2014

tacaacggcgagaagaagactttctattcaaggcccaaccgtcacgacaactgctggttgaacaccattctgcagctgttcagatatgtcgacgaaccattcttcgactgggtctatgactcacctgagaacctcacgctccaagcaattgagcaactcgaggggttcacaggtcttgacctacgcgagggcggaccacccgctctcgtgatttggaacatcaggcacttgttgtacactggaattggcactgcttcacggcccagtgaggtgtgtatggttgatggcactgacatgtgtctggctgatttccacgcaggaatcttccttaaaggtactgaacacgccgtgtttgcctgtttgacctccgatggttggtacgccatcgacgacgaggacttttacccatggactccggatccgtctgatgtcttgtgttttgtcccatacgacatg

>KR108948.1_SAT1_SAR_2009

tacaacggtgagaagaagactttctacagcaggcccaacacacacggcaactgctggctcaactcgctgctgcagctctttcgatacgtcgacgagccgctcttcgagagtgagtacctttcaccagagaacaagacwttggacatgatcaaacaactgwctgactacaccaaacttgacctctcagacggtgggccaccagcactcgtgctttggctcatcaaggactgtcttcaaaccggtgttggcaccagcactcgccccagcgagatctgtgtcatcaacggagtcaccatgactctggctgacttccacgccggtattttcatcaagggtactgaacacgctgtgttcgccctcaacacrtctgagggctggtaygcyattgatgatgaggtgttctayccgtggacaccsgaccctgaaaacgtactcgcgtacgtgccctacgaccag

>KR108949.1_SAT2_SAR_2009

tacaacggagaaaagaagaccttctacagcaggccaaacaaacacggcaactgctggctcaattcattgctgcagctctttcgatacgtcgatgagccgctctttgaatctgagtacctctcaccagagaacaagacattggacatgatcaaacaactgtctgactacaccaaacttgacctctcagacggtgggccaccggcactcgtgctttggctcatcaaggactgtcttcaaaccggcgttggtaccagcactcgccccagcgagatctgtgtcatcaacggagttaccatgactctggctgacttccacgccggtattttcatcaaaggcaccgaacacgctgtgttcgccctcaacacatctgagggctggtacgccattgatgacgaggtgttttacccatggacacccgaccctgagaacgtgctcgcgtacgtgccatacgaccag

>KR108950.1_SAT3_SAR_2009

tacaacggtgagaagaagaccttttacagcaggcccaacacacacgggaactgttggctcaactcgcttttgcagctctttcgatacgtcgatgagccgctatttgagtctgagtatttatcacctgaggacaagacattggacatgatcaaacagctctctgactacaccaaacttgacctctcagatggtgggccacctgcacttgtgctctggctcatcaaggactgtcttcaaactggcgtcggcaccagtactcgccccagcgagatctgtgtgatcaacggggttgtcatgaccctggctgacttccacgccggtattttcatcaagggtactgaacacgctgtgtttgctctcaacacatctgagggctggtacgccattgatgatgaggtgttttatccatggacacccgaccccgagaacgtactcgcgtacgtaccctacgaccag

>KR401152.1_O_MYA_2009

cacaacggtgagaagaagatcttctactccaggcccaacaaccacgacaactgttggctgaacaccatccttcagctgttcaggtacgtcgatgaacctttcttcgactgggtatacgaatcacctgaaaacctcacccttgaggcgatcaggcaactggagaacattactggtcttgagctgcacgagggtggtccgcccgccctcgtcatttggaacatcaaacacttgctccacaccgggatcggcaccgcctcgcgacccagcgaggtgtgcatggtggacggtacggacatgtgcctggctgacttccacgctggcatcttcctgaaaggacaggaacacgccgtgtttgcctgcgtcacctccaacgggtggtacgcgatcgacgacgaagaattctacccctggacgccagatccgtccgacgtgctggtctttgtcccgtacgatcaa

>KR401153.1_O_MYA_2009

tacaacggtgagaagaagatcttctactccaggcccaacaaccacgacaactgttggctgaatgccatccttcagctgttcaggtacgtcgatgaacctttcttcgactgggtatatgaatcacctgaaaacctcacccttgaggcgatcaggcaactggagaacattactggtcttgagctgcacgagggtggtccgcccgccctcgtcatttggaacatcaaacacttgctccacaccgggatcggcaccgcctcgcgacccagcgaggtgtgcatggtggacggtacggacatgtgcctggctgacttccacgctggcatcttcctgaaaggacaggaacacgccgtgtttgcctgcgtcacctccaacgggtggtacgcgattgacgacgaagaattctacccctggacgccagatccgtccgacgtgctggtcttcgtcccgtacgatcaa

>KR401154.1_O_MYA_1998

cacaacggtgagaagaagaccttttactccaggcccaacaaccacgacaactgttggctgaatgccatccttcagttgttcaggtacgtcgatgaacctttcttcgactggatctatgaatcacctgaaaacctcactcttgaggcgatcagacaactggagaacattactggtcttgaactgcacgagggtggtccgcccgctctcgtcatttggaacatcaaacacttgctccacaccggaatcggcaccgcctcgcgacccagcgaggtgtgcatggtggacggtacggacatgtgcctggctgacttccacgctggcatcttcctgaaaggacaggaacacgccgtgtttgcctgcgtcacctccaacgggtggtacgcgattgacgacgaggaattctacccctggacgccggatccgtccgacgtgctggtctttgtcccgtacgatcaa

>KR401155.1_O_MYA_2007

tacaacggtgagaagaagatcttctactccaggcccaacaaccacgacaactgttggctgaatgccatccttcagctgttcaggtacgtcgatgaacctttcttcgactgggtatatgaatcacctgaaaacctcacccttgaggcgatcaggcaactggagaacattactggtcttgagctgcacgagggtggtccgcccgccctcgtcatttggaacatcaaacacttgctccacaccgggatcggcaccgcctcgcgacccagcgaggtgtgcatggtggacggtacggacatgtgcctggctgacttccacgctggcatcttcctgaaaggacaggaacacgccgtgtttgcctgcgtcacctccaacgggtggtacgcgattgacgacgaagaattctacccctggacgccagatccgtccgacgtgctggtcttcgtcccgtacgatcaa

>KR401156.1_O_MYA_2009

tacaacggtgagaagaagatcttctactccaggcccaacaaccacgacaactgttggctgaacgccatccttcagctgtttaggtacgtcgatgaacctttcttcgactgggtatatgaatcacctgaaaacctcacccttgaggcgatcaggcaactggagaacattactggtcttgagctgcacgagggtggtccgcccgctctcgtcatttggaacatcaaacacttgctccacaccgggatcggcaccgcctcgcgacccagcgaggtgtgcatggtggacggtacggacatgtgcctggctgacttccacgctggcatcttcctgaaaggacaggaacacgccgtgtttgcctgcgtcacctccaacgggtggtacgcgattgacgacgaggaattctacccctggacgccggatccgtccgacgtgctggtctttgtcccgtacgatcaa

>KR401157.1_O_MYA_2009

tacaacggtgagaagaagatcttctactccaggcccaacaaccacgacaactgttggctgaacgccatccttcagctgtttaggtacgtcgatgaacctttcttcgactgggtatatgaatcacctgaaaacctcacccttgaggcgatcaggcaactggagaacattactggtcttgagctccacgagggtggtccgcccgctctcgtcatttggaacatcaaacacttgctccacaccgggatcggcaccgcctcgcgacccagcgaggtgtgcatggtggacggtacggacatgtgcctggctgacttccacgctggcatcttcctgaaaggacaggaacacgccgtgtttgcctgcgtcacctccaacgggtggtacgcgattgacgacgaggaattctacccctggacgccggatccgtccgacgtgctggtctttgtcccgtacgatcaa

>KR401158.1_O_SKR_2010

tacaacggtgagaagaagatcttttactccaggcccaacaaccacgacaactgttggctgaacgtcatccttcagctgttcaggtacgtcgatgaacctttcttcgactgggtgtatgaatcacctgaaaacctcacccttgaggcgatcagacaactggagaacattactggtcttgagctgcacgagggtggtccgcccgccctcgtcatttggaacatcaaacacttgctccacaccgggatcggcaccgcctcgcgacccagcgaggtgtgcatggtggacggtacggacatgtgcctggctgacttccacgctggcatcttcctgaaaggacaggaacacgccgtgtttgcctgcgtcacctccaacgggtggtacgcgatcgacgacgaagaattctacccctggacgccagatccgtccgacgtgctggtctttgtcccgtacgaccaa

>KR401159.1_O_SKR_2010

tacaacggtgagaagaagatcttctactccaggcccaacaaccacgacaactgttggctgaacaccatccttcagctgttcaggtacgtcgatgaacctttcttcgactgggtatatgaatcacctgagaacctcacccttgaggcgatcagacaactggagaacattactggtcttgagctgcacgagggtggtccgcccgccctcgtcatttggaacatcaaacacttgctccacaccgggatcggcaccgcctcgcgacccagcgaggtgtgcatggtggacggtacggacatgtgcctggctgacttccacgctggcatcttcctgaaaggacaggaacacgccgtgtttgcctgcgtcacctccaacgggtggtacgcgatcgacgacgaagaattttacccctggacgccagatccgtccgacgtgctggtctttgtcccgtacgatcaa

>KR401160.1_O_SKR_2011

tacaacggtgagaagaagatcttctactccaggcccaacaaccacgacaactgttggctgaacaccatccttcagctgttcaggtacgtcgatgaacctttcttcgactgggtatatgaatcacctgaaaacctcacccttgaggcgatcagacaactggagaacattactggtcttgagctgcacgagggtggtccgcccgccctcgtcatttggaacatcaaacacttgctccacaccgggatcggcaccgcctcgcgacccagcgaggtgtgcatggtggacggtacggacatgtgcctggctgacttccacgctggcatcttcctgaaaggacaggaacacgccgtgttcgcctgcgtcacctccaacgggtggtacgcgatcgacgacgaagaattttacccctggacgccagatccgtccgacgtgttggtctttgtcccgtacgaccaa

>KT003716.1_O_PAK_2005

cacaacggtgagaagaagactttttactctagacccaacaaccatgacaactgttggctgaataccattcttcagctgtttaggtatgtcgatgagcctttcttcgactgggtctatgactcacctgaaaacctcactcttgatgctatcaaacaactggaagaaattactggtcttgaactgcacgagggcggaccacccgcgctcgtcatctggaacattaaacacttgctccacaccgggatcggcactgcctcacgccccagtgaggtgtgcatgattgacgggacggacatgtgtttggctgacttccacgccggcatctttttgaagggacaggaacatgccgtgtttgcctgcatcacctctaacgggtggtacgcgatcgacgacgaggacttttacccctggacaccggacccgtccgacgttctggtatttgttccgtacgatcaa

>KT968663.1_A_CHA_2013

cacaacggtaaaaagaagaccttttactctaggcccaacagccatgacaactgctggctgaacaccatcctccaattgttcaggtacgttgatgaaccattctttgattgggtctatgaatcacctgaaaaccttactcttgaagcaattaggcaattggaagagatcactggtctcgagcttcacgagggtggcccgcccgctctcgtaatttggaacatcaagcacttgctccataccggaatcggtaccgcttcgcgacctagcgaggtgtgtatggtagacggtacggacatgtgtttggctgacttccatgctggcatcttcctgaaagggcaggaacacgcagtgttcgcctgtgtcacctccaacggatggtacgcgattgacgacgaggacttttacccctggacaccggatccgtctgacgtcctggtgtttgttccgtacgatcaa

>KU127247.1_A_SAU_2015

cacaacggtgagaaaaagaccttctactctaggcccaacaaccacgacaactgttggttgaacaccatcctccagttgtttaggtatgttgatgaacctttcttcgactgggtctatgagtcacctgaaaacctcactcttgaggcgattaggcaactggaagaaattactggtcttgaactgcacgagggtggtccgcccgccctcgtcatttggaacatcaagcacttgctccacaccggaatcggcactgcttcgcgacccagcgaggtgtgcatggttgacggcacggacatgtgtttggcagacttccacgctggcattttcctgaaaggacaagagcacgctgtgttcgcctgtgtcacctccaacgggtggtatgcgattgatgacgaggacttttacccctggacgccggacccgtccgacgttctggtgtttgtcccgtacgatcaa

>KU204893.1_O_CHA_2013

tacaacggggaaaagaaactcttttactccagacccaacaactacgacaactgttggttgaacgccgtccttcaactgttcagatacgtcgacgaacccttcctcgagtgggtctacaactcgcccgaggacctcactcttgaggcgatcgacaacctggaagaggtcactggactcgaactgcacgaaggcggaccgcccgccctcgttgtctggaacatcaagcacttgctctacaccggtatcggcacagcttcgcggcccagcgaggtgtgtatgatcgatggtacggacatgtgcctggctgatttccacgccggtatattcctgaagggacaggaccacgccgttttcgcctgcgtcacctctaacgggtggtacgcgatcgacgacgaagacttttacccgtggacaccggacccgaccgacgttttggtttttgtcccatacgaccaa

>KU204894.1_O_CHA_2013

tacaacggggaaaagaaactcttttactccagacccaacaaccacgacaactgttggctgaacgccgtccttcaactgttcaggtacgtcgacgaacccttcctcgagtgggtctacaactcgcccgaggacctcactcttgaagcgatcgacaacctggaagaggtcactggtctcgaactacgcgaaggcggaccgcccgccctcgttgtctggaacatcaagcacctgctctacaccggaatcggcaccgcttcgcggcccagcgaggtgtgcatgatcgacggcacggacatgtgtctggctgattttcacgccggtatattcctgaagggacaggatcacgccgtcttcgcctgcgtcacctctaacgggtggtacgcgatcgacgacgaagacttttatccgtggacaccggacccggctgacgttttggtttttgttccatacgaccaa

>KU291242.1_O_MOR_2015

cacaacggtgagaaaaagaccttctactctaggcccaacaaccacgacaactgttggttaaacaccatccttcagttgtttaggtacgtcgacgaacctttcttcgactgggtctatgactcgcctgaaaacctcactcttgaggcgattaggcaactggaagagatcactggccttgagttgcacgagggtggaccgcccgctctcgtcatctggaacatcaaacacttgctccacaccggaatcggcactgcttcgcgacccagcgaggtgtgcatggttgatggcacagacatgtgtttggccgacttccacgctggcatcttcctgaaagggcaagaacacgctgtgttcgcctgcgtcacctccaacgggtggtacgcgatcgacgacgaggacttctacccctggacgccggacccgtccgacgttctggtgtttgtcccgtacgatcaa

>KU360085.1_Asia1_CHA_2015

cacaacggtgagaagaagaccttttactccagacccaacaaccatgataactgctggctgaacactatcctccagttgttcaggtatgtcgacgagcctttcttcgactgggtctacgactcgcctgaaaacctcactctcgaggcgattaggcagttggaggaagttactggtcttgagctgcatgagggtggaccacccgcccttgtcatctggaacatcaagcatttgctccacaccggagtcggtaccgcttcgcgccctagcgaagtgtgtatggtagacggcacggacatgtgtttggctgatttccatgctggcattttcctgaaaggacaagaacatgctgtgtttgcctgtgtcacctccaacgggtggtacgcgatcgatgacgaggacttttacccctggacaccggacccgtccgacgtcttggtgttcgttccgtacgaccaa

>KU726614.1_O_GRE_1994

cacagcggtgagaagaaaactttctactctaggcccaataaccacgacaactgttggttgaataccatcctccagttgttcaggtacgtcgatgagcccttcttcgactgggtctatgactcacctgaaaacctcactcttgatgcaattaaacaactggaagaaattactggtcttgagctgcacgagggtgggccacccgctctcgtcatttggaacattaagcacttgctccacaccggaatcggtaccgcctcgcgacccagtgaggtgtgtatggtggacggtacggacatgtgcttggctgacttccatgctggcatcttcctgaaaggacaggaacatgctgtgtttgcctgtgtcacctccaacgggtggtacgcgattgacgacgaggacttctacccctggacaccggacccgtccgatgtcttggtgtttgtcccgtacgatcaa

>KU821590.1_SAT1_NMB_2010

tacaacggagagaagaagaccttctacagcaggcccaacaaacacgggaactgttggctcaactcgctgttgcagctctttcgatacgtcgatgagccgctattcgagtctgagtacttgtcacctgaaaacaagacattggacatgattaaacaactatctgattacaccaaactggacttgtcggacggagggccacccgctctcgtcctttggctgatcaaagactgccttcagaccggcgttggcaccagcactcgccccagcgagatctgtgtcatcaacggggttgtcatgaccctggctgatttccatgccggcattttcatcaagggtactgaacacgccgtgttcgccctcaacacatctgagggctggtacgccattgatgatgaggtgttctacccatggacacccgaccctgaaaacgtgctcgcatacgttccctacgaccag

>KU821591.1_O_ZAM_2010

tacaacggtgagaaaaagacattctactctaggcctaacaaccacgacaactgctggctcaacgccatcctgcagctgtttaggtatgttgatgaacctttctttgattgggtctatgattcacctgagaacctcactactgaagcaatcaagcagctggagggcctaactggtctcgagctgcacgagggcggaccacccgctctcgtcatttggaacatcaagcacttgctccacaccggcatcggcacggcttcacgacccagtgaggtgtgcatggtagacgggacagacatgtgcttggctgacttccatgctggcattttcctgaagggacaggaacacgctgtctttgcatgtgtcacctccaatgggtggtttgcgattgatgacgaggacttctacccctggacgccggatccgtccgacgttttggtttttgtcccttacgatcaa

>KU821592.1_SAT2_ZAM_2009

tacaacggagagaagaaaaccttctacagcagacccaacaaacacgggaactgttggctcaattcattgttgcagctctttcgatacgtcgacgagccgctctttgagtctgagtatttgtcacctgaaaacaagacattggacatgatcaaacaactatctgattacaccaaactggacttgtcggacggagggcctcccgctctcgtcctttggctgatcaaagattgtcttcaaaccggtgttggcaccagcactcgccccagcgagatctgtgtgatcaacggggttaccatgaccctggcagacttccacgccggtattttcctcaaaggtactgaacacgctgtgttcgccctcaacacatctgagggctggtacgcaattgatgatgaggtgttctacccatggacgcccgaccctgaaaacgtactcgcgtacgttccctacgaccag

>KX002176.1_A_ARG_2001

tacaacggtgagaaaaagacattttactccagacccaacaaccacgacaactgttggttgaacgccatccttcagttgttcaggtacgtcgacgagcctttcttcgactgggtctacaactcgcctgagaacctcacgctctcggccatcgagcagctggaggaaattaccgggcttgagttgcacgagggcggaccacccgcgctcgtggtttggaacatcaaacacatgctccacactggcatcggcaccgcctcgcgacccagcgaggtgtgcatggtcgacggtacggacatgtgtttggctgatttccatgctggcattttcctgaaaggtcgggagcacgctgtgtttgcatgtgtcacctctgacgggtggtacgcgatcgacgacgaggacttctacccttggacaccagacccgtctgacgtcctggtgtttgtcccgtacgaccaa

>KX002177.1_A_ARG_2001

tacaacggtgagaagaagacattttactccagacccaacaaccacgacaactgttggttgaacgccatcctccagttgttcaggtacgtcgacgaacctttcttcgactgggtctacaactcgcctgagaacctcacgctctcggccatcgagcagctggaggaaattaccgggcttgagttgcacgagggcggaccacccgcgctcgtggtttggaacatcaaacacatgctccacactggcatcggcaccgcctcgcgacccagcgaggtgtgcatggtcgacggtacggacatgtgtttggctgatttccatgctggcattttcctgaaaggtcaggagcacgctgtgtttgcatgtgtcacctctgacgggtggtacgcgatcgacgacgaggacttctacccttggacaccagacccgtctgacgtcctggtgtttgtcccgtacgaccaa

>KX002178.1_A_ARG_2001

tacaacggtgagaagaagacattttactccagacccaacaaccacgacaactgttggttgaacgccatccttcagttgttcaggtacgtcgacgaacccttcttcgactgggtctacaactcgcctgagaacctcacgctctcggccatcgagcagctggaggaaattaccgggcttgagttgcacgagggcggaccacccgcgctcgtggtttggaacatcaaacacacgctccacactggcatcggcaccgcctcgcgacccagcgaggtgtgcatggtcgacggtacggacatgtgtttggctgatttccatgctggcattttcctgaaaggtcgggagcacgctgtgtttgcatgtgtcacctctgacgggtggtacgcgatcgacgacgaggacttctacccttggacaccagacccgtctgacgtcctggtgtttgtcccgtacgaccaa

>KX002179.1_A_ARG_2001

tacaacggtgagaagaagacattttactccagacccaacaaccacgacaactgttggttgaacgccatcctccagttgttcaggtacgtcgacgaacctttcttcgactgggtctacaactcgcctgagaacctcacgctctcggccatcgagcagctggaggaaattaccgggcttgagttgcacgagggcggaccacccgcgctcgtggtttggaacatcaaacacatgctccacactggcatcggcaccgcctcgcgacccagcgaggtgtgcatggtcgacggtacggacatgtgtttggctgatttccatgctggcattttcctgaaaggtcaggagcacgctgtgtttgcatgtgtcacctctgacgggtggtacgcgatcgacgacgaggacttctacccttggacaccagacccgtctgacgtcctggtgtttgtcccgtacgaccaa

>KX002180.1_A_ARG_2001

tacaacggtgagaaaaagacattttactccagacccaacaaccacgacaactgttggttgaacgccatccttcagttgttcaggtacgtcgacgaacctttcttcgactgggtctacaactcgcctgagaacctcacgctctcggccatcgagcagctggaggaaattaccgggcttgagttgcacgagggcggaccacccgcgctcgtggtttggaacatcaaacacatgctccacactggcatcggcaccgcctcgcgacccagcgaggtgtgcatggtcgacggtacggacatgtgtttggctgatttccatgctggcattttcctgaaaggtcgggagcacgctgtgtttgcatgtgtcacctctgacgggtggtacgcgatcgacgacgaggacttctacccttggacaccagacccgtctgacgtcctggtgtttgtcccgtacgaccaa

>KX002181.1_A_ARG_2001

tacaacggtgagaagaagacattttactccagacccaacaaccacgacaactgttggttgaacgccatcctccagttgttcaggtacgtcgacgaacctttcttcgactgggtctacaactcgcctgagaacctcacgctctcggccatcgagcagctggaggaaattaccgggcttgagttacacgagggcggaccacccgcgctcgtggtttggaacatcaaacacatgctccacactggcatcggcaccgcctcgcgacccagcgaggtgtgcatggtcgacggtacggacatgtgtttggctgatttccatgctggcatcttcctgaaaggtcaggagcacgctgtgtttgcatgtgtcacctctgaagggtggtacgcgatcgacgacgaggacttctacccttggacaccagacccgtctgacgtcctggtgtttgtcccgtacgaccaa

>KX002182.1_A_ARG_2001

tacaacggtgagaaaaagacattttactccagacccaacaaccacgacaactgttggttgaacgccatccttcagttgttcaggtacgtcgacgaacctttcttcgactgggtctacaactcgcctgagaacctcacgctctcggccatcgagcagctggaggaaattaccgggcttgagttgcacgagggcggaccacccgcgctcgtggtttggaacatcaaacacatgctccacactggcatcggcaccgcctcgcgacccagcgaggtgtgcatggtcgacggtacggacatgtgtttggctgatttccatgctggcattttcctgaaaggtcgggagcacgctgtgtttgcatgtgtcacctctgacgggtggtacgcgatcgacgacgaggacttctacccttggacaccagacccgtctgacgtcctggtgtttgtcccgtacgaccaa

>KX002183.1_A_ARG_2001

tacaacggtgagaagaagacattttactccagacccaacaaccacgacaactgttggttgaacgccatccttcagttgttcaggtacgtcgacgaacctttcttcgactgggtctacaactcgcctgagaacctcacgctctcggccattgagcagctggaggaaattaccgggcttgagttgcacgagggcggaccgcccgcgctcgtggtttggaacatcaaacacatgctccacactggcatcggcaccgcctcgcgacccagcgaggtgtgcatggtcgacggtacggacatgtgtttggctgatttccatgctggcattttcctgaaaggtcgggagcacgctgtgtttgcatgtgtcacctctgacgggtggtacgcgatcgacgatgaggacttctacccttggacaccagacccgtctgacgtcctagtgtttgtcccgtacgaccaa

>KX002184.1_A_ARG_2001

tacaacggtgagaaaaagacattttactccagacccaacaaccacgacaactgttggttgaacgccatccttcagttgttcaggtacgtcgacgaacctttcttcgactgggtctacaactcgcctgagaacctcacgctctcggctatcgagcagctggaggaaattaccgggcttgagttgcacgagggcggaccacccgcgctcgtggtttggaacatcaaacacatgctccacactggcatcggcaccgcctcgcgacccagcgaggtgtgcatggtcgacggtacggacatgtgtttggctgatttccatgctggcattttcctgaaaggtcgggagcacgctgtgtttgcatgtgtcacctctgacgggtggtacgcgatcgacgacgaggacttctacccttggacaccagacccgtctgacgtcctggtgtttgtcccgtacgaccaa

>KX002185.1_A_ARG_2001

tacaacggtgagaagaagacattttactccagacccaacaaccacgacaactgttggttgaacgccatcctccagttgttcaggtacgtcgacgaacctttcttcgactgggtctacaactcgcctgagaacctcacgctctcggccatcgagcagctggaggaaattaccgggcttgagttgcacgagggcggaccacccgcgctcgtggtttggaacatcaaacacatgctccacactggcatcggcaccgcctcgcgacccagcgaggtgtgcatggtcgacggtacggacatgtgtttggctgatttccatgctggcattttcctgaaaggtcaggagcacgctgtgtttgcatgtgtcacctctgacgggtggtacgcgatcgacgacgaggacttctacccttggacaccagacccgtctgacgtcctggtgtttgtcccgtacgaccaa

>KX002186.1_A_ARG_2001

tacaacggtgagaagaagacattttactccagacccaacaaccacgacaactgttggttgaacgccatcctccagttgttcaggtacgtcgacgaacctttcttcgactgggtctacaactcgcctgagaacctcacgctctcggccatcgagcagctggaggaaattaccgggtttgagttgcacgagggcggaccacccgcgctcgtggtttggaacatcaaacacatgctccacactggcatcggcaccgcctcgcgacccagcgaggtgtgcatggtcgacggtacggacatgtgtttggctgatttccatgctggcattttcctgaaaggtcaggagcacgctgtgtttgcatgtgtcacctctgacgggtggtacgcgatcgacgacgaggacttctacccttggacaccagacccgtctgacgtcctggtgtttgtcccgtacgaccaa

>KX002187.1_A_ARG_2001

tacaacggtgagaagaagacattttactccagacccaacaaccacgacaactgttggttgaacgccatcctacagttgttcaggtacgtcgacgaacctttcttcgactgggtctacaactcgcctgagaacctcacgctctcggccatcgagcaactggaggaaattaccgggcttgagttgcacgagggcggaccacccgcgctcgtggtttggaacatcaaacacatgctccacactggcatcggcaccgcctcgcgacccagcgaggtgtgcatggtcgacggtacggacatgtgtttggctgatttccatgctggcattttcctgaaaggtcgggaccacgctgtgtttgcatgtgtcacctctgacgggtggtacgcgatcgacgacgaggacttctacccttggacaccagacccgtctgacgtcctggtgtttgtcccgtacgaccaa

>KX002188.1_A_ARG_2001

tacaacggtgagaagaagacattttactccagacccaacaaccacgacaactgttggttgaacgccatcctccagttgttcaggtacgtcgacgaacctttcttcgactgggtctacaactcgcctgagaacctcacgctctcggccattgagcagctggaggaaattaccgggcttgagttgcacgagggcggaccacccgcgctcgtggtttggaacatcaaacacatgctccacactggcatcggcaccgcctcgcgacccagcgaggtgtgcatggtcgacggtacggacatgtgtttggctgacttccatgctggcattttcctgaaaggtcaggagcacgctgtgtttgcatgtgtcacctctgacgggtggtacgcgatcgacgacgaggacttctacccttggacaccagacccgtctgacgtcctggtgtttgtcccgtacgaccaa

>KX002189.1_A_ARG_2001

tacaacggtgagaagaagacattttactccagacccaacaaccacgacaactgttggttgaacgccatccttcagttgttcaggtacgtcgacgaacctttcttcgactgggtctacaactcgcctgagaacctcacgctctcggccatcgagcagctggaggaaattaccgggcttgagttgcacgagggcggaccacccgcgctcgtggtttggaacatcaaacacatgctccacactggcatcggcaccgcctcgcgacccagcgaggtgtgcatggtcgacggtacggacatgtgcttggctgatttccatgctggcattttcctgaaaggtcgggagcacgctgtgtttgcatgtgtcacctctgacgggtggtacgcgatcgacgacgaggacttctacccttggacaccagacccgtctgacgtcctggtgtttgtcccgtacgaccaa

>KX002190.1_A_ARG_2001

tacaacggtgagaagaagacattttactccagacccaacaaccacgacaactgttggttgaacgccatcctccagttgttcaggtacgtcgacgaacctttcttcgactgggtctacaactcgcctgagaacctcacgctctcggccatcgagcagctggaggaaattaccgggcttgagttgcacgagggcggaccacccgcgctcgtggtttggaacatcaaacacatgctccacactggcatcggcaccgcctcgcgacccagcgaggtgtgcatggtcgacggtacggacatgtgtttggctgacttccatgctggcattttcctgaaaggtcaggagcacgctgtgtttgcgtgtgtcacctctgacgggtggtacgcgatcgacgacgaggacttctacccttggacaccagacccgtctgacgtcctggtgtttgtcccgtacgaccaa

>KX002191.1_A_ARG_2001

tacaacggtgagaagaagacattttactccagacccaacaaccacgacaactgttggttgaacgccatcctccagttgttcaggtacgtcgacgaacctttcttcgattgggtctacaactcgcctgagaacctcacgctctcggccatcgagcagctggagggaattaccgggcttgagttgcacgaaggcggaccacccgcgctcgtggtttggaacatcaaacacatgctccacactggcatcggcaccgcctcgcgacccagcgaggtgtgcatggtcgacggtacggacatgtgtttggctgatttccatgctggcatcttcctgaaaggtcaggagcacgctgtgtttgcgtgtgtcacctctgacgggtggtacgcgatcgacgacgaggacttctacccctggacaccagacccgtctgacgtcctggtgtttgtcccgtacgatcaa

>KX002192.1_A_ARG_2001

tacaacggtgagaagaagacattttactccagacccaacaaccacgacaactgttggttgaacgccatccttcagttgttcaggtacgtcgacgaacctttcttcgactgggtctacaactcgcctgagaacctcacgctctcggccatcgagcagctggaggaaattaccgggcttgagttgcacgagggcggaccacccgcgctcgtggtttggaacatcaaacacatgctccacactggcatcggcaccgcctcgcgacccagcgaagtgtgcatggtcgacggtacggacatgtgcttggctgatttccatgctggcattttcctgaaaggacgggagcacgctgtgtttgcatgtgtcacctctgacgggtggtacgcgatcgacgacgaggacttctacccttggacaccagacccgtctgacgtcctggtgtttgtcccgtacgaccaa

>KX002193.1_A_ARG_2001

tacaacggtgagaagaagacattttactccagacccaacaaccacgacaactgttggttgaacgccatcctccagttgttcaggtacgtcgacgagcctttcttcgactgggtctacaactcgcctgagaacctcacgctctcggccatcgagcagctggagggaattaccgggcttgagttgcacgagggcggaccacccgcgctcgtggtttggaacatcaaacacatgctccacactggcatcggcaccgcctcgcgacccagcgaggtgtgcatggtcgacggtacggacatgtgtttggctgatttccatgctggcattttcctgaaaggtcaggagcacgctgtgtttgcgtgtgtcacctctgacgggtggtacgccatcgacgacgaggacttctacccctggacaccagacccgtctgacgtcctggtgtttgtcccgtacgatcaa

>KX002194.1_A_ARG_2001

tacaacggtgagaagaagacattttactccagacccaacaaccacgacaactgttggttgaacgccatcctccagttgttcaggtacgtcgacgaacctttcttcgactgggtctacaactcgcctgagaacctcacgctctcggccatcgagcagctggaggaaattaccgggcttgagttgcacgagggcggaccacccgcgctcgtggtttggaacatcaaacacatgctccacactggcatcggcaccgcctcgcgacccagcgaggtgtgcatggtcgacggtacggacatgtgtttggctgatttccatgctggcattttcctgaaaggtcaggagcacgctgtgtttgcatgtgtcacctctgacgggtggtacgcgatcgacgacgaggacttctacccttggacaccagacccgtctgatgtcctggtgtttgtcccgtacgaccaa

>KX002195.1_A_ARG_2001

tacaacggtgagaagaagacattttactccagacccaacaaccacgacaactgttggttgaacgccatcctccagttgttcaggtacgtcgacgaacctttcttcgactgggtctacaactcgcctgagaacctcacgctctcggccatcgagcagctggaggaaattaccgggcttgagttgcacgagggcggaccacccgcgctcgtggtttggaacatcaaacacatgctccacactggcatcggcaccgcctcgcgacccagcgaggtgtgcatggtcgacggtacggacatgtgtttggctgatttccatgctggcattttcctgaaaggtcaggagcacgctgtgtttgcatgtgtcacctctgacgggtggtacgcgatcgacgacgaggacttctacccttggacaccagacccgtctgacgtcctggtgtttgtcccgtacgaccaa

>KX002196.1_A_ARG_2001

tacaacggtgagaagaagacattttactccagacccaacaaccacgacaactgttggttgaacgccatcctccagttgttcaggtacgtcgacgaacctttcttcgactgggtctacaactcgcctgagaacctcacgctctcggccatcgagcagctggagggaattaccgggcttgagttgcacgagggcggaccacccgcgctcgtggtttggaacatcaaacacatgctccacactggcatcggcaccgcctcgcgacccagcgaggtgtgtatggtcgacggtacggacatgtgtttggctgatttccatgctggcattttcctgaaaggtcaggagcacgctgtgtttgcatgtgtcacctctgacgggtggtacgcgatcgacgacgaggacttctacccctggacaccagacccgtctgacgtcctggtgtttgtcccgtacgatcaa

>KX002197.1_A_ARG_2001

tacaacggtgagaagaagacattttactccagacccaacaaccacgacaactgttggttgaacgccatcctccagttgttcaggtacgtcgacgaacctttcttcgactgggtctacaactcgcctgagaacctcacgctctcggccatcgagcagctggaggaaattaccgggcttgagttgcacgagggcggaccacccgcgcttgtggtttggaacatcaaacacatgctccacactggcatcggcaccgcctcgcgacccagcgaggtgtgcatggtcgacggtacggacatgtgtttggctgatttccatgctggcattttcctgaaaggtcaggagcacgctgtgtttgcatgtgtcacctctgacgggtggtacgcgatcgacgacgaggacttctacccttggacaccagacccgtctgacgtcctggtgtttgtcccgtacgaccaa

>KX002198.1_A_ARG_2001

tacagcggtgagaaaaagacattttactccagacccaacaaccacgacaactgttggttgaacgccatccttcagttgttcaggtacgtcgacgaacctttcttcgactgggtctacaactcgcctgagaacctcacgctctcggccatcgagcagctggaggaaattaccgggcttgagttgcacgagggcggaccacccgcgctcgtggtttggaacatcaaacacatgctccacactggcatcggcaccgcctcgcgacccagcgaggtgtgcatggtcgacggtacggacatgtgtttggctgatttccatgctggcattttcctgaaaggtcgggagcacgctgtgtttgcatgtgtcacctctgacgggtggtacgcgatcgacgacgaggacttctacccttggacaccagacccgtctgacgtcctggtgtttgtcccgtacgaccaa

>KX002199.1_A_ARG_2001

tacaacggtgagaagaagacattttactccagacccaacaaccacgacaactgttggctgaacgccatcctccagttgttcaggtacgtcgacgaacctttcttcgactgggtctacaactcgcctgagaacctcacgctctcggccatcgagcagctggaggaaattaccgggcttgagttgcacgagggcggaccacccgcgctcgtggtttggaacatcaaacacatgctccacactggcatcggcaccgccacgcgacccagcgaggtgtgcatggtcgacggtacggacatgtgtttggctgatttccatgctggcattttcctgaaaggtcaggagcacgctgtgtttgcatgtgtcacctctgacgggtggtacgcgatcgacgacgaggacttctacccttggacaccagacccgtctgatgtcctggtgtttgtcccgtacgaccaa

>KX002200.1_A_ARG_2001

tacaacggtgagaagaagacattttactccagacccaacaaccacgacaactgttggttgaacgccatcctccagttgttcaggtacgttgacgaacctttcttcgactgggtctacaactcgcctgagaacctcacgctctcggccatcgagcagctggaggaaattaccgggcttgagttgcacgagggcggaccacccgcgctcgtggtttggaacatcaaacacatgctccacactggcatcggcaccgcctcgcgacccagcgaggtgtgcatggtcgacggtacggacatgtgtttggctgatttccatgctggcattttcctgaaaggtcaggagcacgctgtgtttgcatgtgtcacctctgacgggtggtacgcgatcgacgacgaggacttctacccttggacaccagacccgtctgacgtcctggtgtttgtcccgtacgaccaa

>KX002201.1_A_ARG_2001

tacaacggtgagaagaagacattttactccagacccaacaaccacgacaactgttggttgaacgccatcctccagttgttcaggtacgtcgacgaacctttcttcgactgggtctacaactcgcctgagaacctcacgctctcggccatcgagcagctggaggaaattaccgggcttgagttgcacgagggcggaccacccgcgctcgtggtctggaacatcaaacacatgctccacactggcatcggcaccgcctcgcgacccagcgaggtgtgcatggtcgacggtacggacatgtgtttggctgatttccatgctggcattttcctgaaaggtcaggagcacgctgtgtttgcatgtgtcacctctgacgggtggtacgcgatcgacgacgaggacttctacccttggacaccagacccgtctgacgtcctggtgtttgtcccgtacgaccaa

>KX002202.1_A_ARG_2001

tacaacggtgagaagaagacattttactccagacccaacaaccacgacaactgttggttgaacgccatcctccagttgttcaggtacgtcgacgaacctttcttcgactgggtctacaactcgcctgagaacctcacgctctcggccatcgagcagctggaggaaattaccgggcttgagttgcacgagggcgggccacccgcgctcgtggtttggaacatcaaacacgtgctccacactggcgtcggcaccgcctcgcgacccagcgaggtgtgcatggtcgacggtacggacatgtgtttggctgatttccatgctggcattttcctgaaaggtcaggagcacgctgtgtttgcatgtgtcacctctgacgggtggtacgcgatcgacgacgaggacttctacccttggacaccagacccgtctgacgtcttggtgtttgtcccgtacgaccaa

>KX002203.1_A_ARG_2001

tacaacggtgagaagaagacattttactccagacccaacaaccacgacaactgttggttgaacgccatcctccagttgttcaggtacgtcgacgaacctttcttcgactgggtctacaactcgcctgagaacctcacgctctcggccatcgaacagctggaggaaattaccgggcttgagttgcacgagggcggaccacccgcgctcgtggtttggaacatcaaacacatgctccacactggcatcggcaccgcctcgcgacccagcgaggtgtgcatggtcgacggtacggacatgtgtttggctgatttccatgctggcattttcctgaaaggtcaggagcacgctgtgtttgcatgtgtcacctctgacgggtggtacgcgatcgacgacgaggacttctacccttggacaccagacccgtctgacgtcctggtgtttgtcccgtacgaccaa

>KX002204.1_A_ARG_2001

tacaacggtgagaagaagacattttactccagacccaacaaccacgacaactgttggttgaacgccatccttcagttgttcaggtacgtcgacgaacctttcttcgactgggtctacaactcgcctgagaacctcacgctctcggccatcgagcagctggaggaaattaccgggcttgagttgcacgagggcggaccacccgcgctcgtggtttggaacatcaaacacatgctccacactggcatcggcaccgcctcgcgacccagcgaggtgtgcatggtcgacggtacggacatgtgtttggctgatttccatgctggcattttcctgaaaggtcgggagcacgctgtgtttgcatgtgtcacctctgacgggtggtacgcgatcgacgacgaggacttctacccttggacaccagacccgtctgacgtcctggtgtttgtcccgtacgaccaa

>KX002205.1_A_ARG_2001

tacaacggtgagaagaagacattttactccagacccaacaaccacgacaactgttggttgaacgccatccttcagttgttcaggtacgtcgacgaacctttcttcgactgggtctacaactcgcctgagaacctcacgctctcggccatcgagcagctggaggaaattaccgggcttgagttgcacgagggcggaccacccgcgctcgtggtttggaacatcaaacacatgctccacactggcatcggcaccgcctcgcgacccagcgaggtgtgcatggtcgacggtacggacatgtgtttggctgatttccatgctggcattttcctgaaaggtcgggagcacgctgtgtttgcatgtgtcacctctgacgggtggtacgcgatcgacgacgaggacttctacccttggacaccagacccgtctgacgtcctggtgtttgtcccgtacgaccaa

>KX162590.1_O_SKR_2014

tacaacggcgagaagaatatcttctactccgggccaaaccaccacgaccactgttggctgaacgccatcctccaggtgctcaggtatatcgatgagcctttcttcgactgggtgtatgaatcatctgaaaacctcacccttgaggcgatcagacaactggagaacattactggttttgagctgcacgagggtggcccgcccgccctcgttatttggaacatcaaacacttgcttcacactgggatcggcaccgcctcgcgacccagcgaggtgtgcatggtggacggtacagacatgtgcctggcagacttccacgctggcatcttcctgaaaggacaggaacacgccgtgtttgcctgcgtcacctctaacgggtggtacgcgatcgatgacgaagagttctacccctggacgccggatccgtccgacgtgttggtctttgtcccctacgatcaa

>KX375417.1_SAT3_ZIM_1981

tacaacggagagaagaagaccttctacagcaggcccaacactcacgggaactgttggctcaactcactcctgcagctctttcgatacgtcgatgagccgctttttgagtctgagtacctctcacctgaaaacaagacattggacatgatcaaacaactgtctgattacaccaaacttgacctttcagacggtgggccaccggcactcgtgctctggctcatcaaggactgccttcagaccggcgttggcaccagtactcgcccaagcgagatctgtgtcatcaacggggtcgtcatgaccctggctgatttccacgccggcattttcatcaagggcacggaacacgccgtgttcgccctcaacacatccgagggctggtatgccattgatgatgaggtgttttatccatggacacctgaccctgagaacgtgctcgcgtacgttccctacgaccag

>KX534089.1_O_SKR_2016

tacaacggcgagaagaagatcttctactccaggcccaacaatcacgacaactgttggctgaacgccatcctccagctgttcaggtatgtcgatgagcctttcttcgactgggtatatgaatcatctgaaaacctcacccttgaggcgatcagacaactggagagcattactggttttgagctgcacgagggtggcccgcccgccctcgttatttggaacatcaaacacttgcttcacactgggatcggcaccgcctcgcgacccagcgaggtgtgcatggtggacggtacggacatgtgcctggcagacttccacgctggcatcttcctgaaaggacaggaacacgccgtgtttgcctgcgtcacctctaacgggtggtacgcgatcgacgacgaagagttttacccctggacgccggatccgtccgacgtgttggtctttgtcccctacgatcaa

>KX712091.1_O_BAN_2015

cacaacggtgagaaaaagactttctattctaggcccaacaaccacgacaattgttggctgaacaccatcctccaattgtttaggtacgtcgatgaacctttcttcgactgggtctatgaatcgcctgaaaacctcactcttgaggcgattaggcaattggaagaactcactggtcttgagctgcacgagggtgggccacccgctctcgtcatttggaacatcaagcatttgctccacaccggaatcggcactgcttcgcgacccagcgaggtgtgcatggttgatggcacggacatgtgtttggctgacttccacgctggcatcttcctgaaagggcaagagcacgctgtgttcgcctgcgtcacctccaacgggtggtacgcgatcgacgacgaggacttctacccctggacgccggatccgtccgacgttctggtgtttgtcccgtacgatcaa

>KY072818.1_O_CHA_1959

tacaacggtgagaagaaaactttctactcccggcctaacaacaatgacaactgttggctgaacaccattttgcagttgtttaggtacgtcgacgagccgttctttgattgggtctatgactcacctgagaacctcaccctcgacgcgatcagacagctggaagagattactggacttgaactacatgagggcggtccacctgctcttgtcatctggaacatcaagcatttgctcaacaccggcatcggtactgcttcacgacccagtgaagtttgtatggtggacggcacggacatgtgtttggctgattttcacgctggcattttccagaaaggaacagaacacgctgtgttcgcttgtgccacctccaacgggtggtacgcgattgatgacgaggacttttacccctggacgccggatccgtctgacgttttggtgtttgtcccgtacgatcaa

>KY086465.1_O_SKR_2016

tacaacggcgagaagaagatcttctactccaggcccaacaatcacgacaactgttggctgaacgccatcctccagctgttcaggtatgtcgatgagcctttcttcgactgggtatatgaatcatctgaaaacctcacccttgaggcgatcagacaactggagagcattactggttttgagctgcacgagggtggcccgcccgccctcgttatttggaacatcaaacacttgcttcacacagggatcggcaccgcctcgcgacccagcgaggtgtgcatggtggacggtacggacatgtgcctggcagacttccacgctggcatcttcctgaaaggacaggaacacgccgtgtttgcctgcgtcacctctaacgggtggtacgcgatcgacgacgaagagttttacccctggacgccggatccgtccgacgtgttggtctttgtcccctacgatcaa

>KY086466.1_O_SKR_2016

tacaacggcgagaaaaagatcttctactccaggcccaacaaccacgacaactgttggctgaacgccatcctccagctgttcaggtatgtcgatgagcctttcttcgactgggtgtatgaatcatctgaaaacctcacccttgaggcgatcagacaactggagaacattactggttttgagttgcacgagggtggcccgcccgccctcgttatttggaacatcaaacacttgcttcacactgggatcggcaccgcctcgcgacccagcgaggtgtgcatggtggacggtacggacatgtgcctggcagacttccacgctggcatcttcctgaaaggacaggaacacgccgtgtttgcctgcgtcacctctaacgggtggtacgcgatcgatgacgaagagttctacccctggacgccggatccgtccgacgtgttggtctttgtcccctacgatcaa

>KY234501.1_O_CHA_2011

cacaacggtgaaaagaagacattctactccaggcccaacaaccacgacaactgctggctgaacaccatcctccagttgtttaggtacgttgatgaacccttcttcgactgggtttacaactcgcctgagaacctcacacttgacgctattgagcaattggaagaaattacaggccttgaactccacgagggtggtccgcccgctctcgttatctggaacattaaacacctgctcaacaccggaatcggcaccgcttcgcgacccagcgaagtgtgcatggtagacgggacggacatgtgtttggctgacttccacgctggcattttcctgaaagggcaggagcacgctgtgttcgcctgtgttacctccaacgggtggtacgcgattgatgacgaggacttttacccctggacgccggacccgtccgacgttctggtgtttgttccgtacgatcaa

>KY234502.1_O_CHA_2015

cacaacggtgaaaagaagacattctactccaggcccaacaaccacgacaactgctggctgaacaccatcctccagttgtttaggtacgttgacgaacccttctttgactgggtttacaactcgcccgagaacctcacacttgatgctattgagcaattggaagaaattacaggtcttgaactccgcgagggcggtccacccgccctcgtcatctggaacattaaacacctgctcaataccggaatcggcaccgcttcgcgccccagcgaagtgtgcatggtagacgggacggacatgtgtttggctgacttccacgctggcattttcctgaaaggacaggaacacgctgtgttcgcctgtgtcacctccaacgggtggtacgcgattgatgacgaggacttttacccctggacaccggacccgtccgacgtcctggtgtttgttccgtacgatcag

>KY322670.1_O_LAO_2013

tacaacggtgagaggaagaccttttattctaggcccaacaacaacgacaactgttggctgaatgccatcctgcagttgttcaggtacgtcgatgaacctttcttcgactgggtctatgaatcgcctgagaaccgcactcttgaggcaattgaacaattagagggaattactggtcttgaactgcacgagggcggtccacctgctctcgtgatttggaacatcaaacacttgctccataccgggatcggcaccgcctcgcgacctagcgaggtgtgcatggttgacggtacggacatgtgcctggctgacttccacgctggcatcttcctgaaaggacaggagcacgctgtgtttgcctgtgtcacctccaacgggtggtacgcgattgatgacgaggacttttacccctggacgccggatccgtccgacgtgctggtgtttgtcccgtacgatcaa

>KY322671.1_O_MAY_2014

tacaacggtgagaggaagaccttttattcaaggcccaacaataacgacaactgttggctgaatgccatcctgcagttgttcaggtacgtcgatgaacctttcttcgactgggtctatgaatcgcctgagaaccgcactcttgaggcaatcgaacaattagagggaattactggtcttgagctgcacgagggcggtccacctgctctcgtgatttggaacatcaaacacttgctccacaccgggatcggcaccgcctcgcgacccagcgaggtgtgcatggttgacggtacggacatgtgcctggctgacttccacgctggcatcttcctgaaaggacaggagcacgcagtgtttgcctgtgtcacctccaatgggtggtacgcgattgatgacgaggacttttacccctggacgccggatccgtccgacgtgctggtgtttgtcccgtacgatcaa

>KY322672.1_O_MAY_2014

cacaacggtgagaagaaaatcttttactccagacccaacacccacgacaactgttggttgaacgccatccttcagttgtttaggtacgtcgatgaacctttcttcgactgggtatacgaatcacctgaaaacctcactcttgaggcgatcagacaactggagaacattactggtcttgaactgcacgagggtggcccgcccgccctcgtcatttggaacatcaagcatttgctccacaccggaatcggcaccgcctcgcgacccagcgaggtgtgcatggtagacggtacggacatgtgcttggctgactttcacgctggcattttcctgaaaggacaggaacacgctgtgtttgcctgcgtcacctccgaagggtggtacgcgatcgacgacgaagaattctacccctggacgccggatccgtccgacgtgctggtctttgtcccgtacgatcag

>KY322673.1_O_MAY_2014

tacaatggtgagaggaagaccttttactctaggcccaacaacaacgacaattgttggttgaacgccatcctgcagttgtttaggtacgtcgacgaacctttcttcgactgggtttatgaatcgcctgagaactgcactcttgaggcgattgaacaactagagggaattactggccttgaactgcacgagggcggtccacccgctctcgttgtttggaacatcaaacatttgctccatactgggattggcactgcctcgcgacctagcgaggtgtgcatggttgacggcacggacatgtgcctggctgacttccacgctggtatctttctgaaaggacaggaacacgctgtgttcgcctgcgtcacctccaatgggtggtacgcgattgatgacgaggacttttacccctggacgccggatccgtccgacgtgctggtgttcgtcccgtacgatcaa

>KY322674.1_O_SKR_2014

tacaacggtgagaagaagatcttctaccccaagcccaacaaccacgacaactgttggctgaacgccatccttcagctgttcaggtacgtcgatgaacctttcttcgactgggtgtatgaatcacctgaaaacctcacccttgaggcgatcaggcaactggagaacattactggttttgagctgcacgagggtggtccgcccgccctcgttatttggaacatcaaacacttgctccacactgggatcggcaccgcctcgcgacccagcgaggtgtgcgtggtggacggtacggacatgtgcctggcagacttccacgctggcattttcctgaaaggacaggagcacgccgtgtttgcctgcgttacctccaacgggtggtacgcgatcgacgacgaagagttctacccctggacgccggatccgtccgacgtgttggtctttgtcccgtacgatcaa

>KY322675.1_A_LAO_2014

cacaacggtgagaagaagactttctactctaggcccaacaaccacgacaactgctggttgaacaccatcctccagttgttcaggtacgtcgacgagccattcttcgactgggtctatgaatcacctgagaacctcactcttgaagctattagacaactggaggatatcactggtcttgaactgcacgagggtggaccacctgccctcgtgatytggaacatcaagcacttgctccacaccggaatcggtaccgcctcgcgacccagcgaggtgtgtatggtggacggtacggacatgtgtttggctgactttcatgccggcatyttcctgaaaggacaggaacacgcagtgtttgcctgtgttacctccaacgggtggtacgcgattgacgacgaggacttctacccctggacgccagatccgtctgacgtcctggtgtttgtcccgtacgatcaa

>KY322676.1_A_MAY_2013

tacaacggtgtaaagaagaccttctactctaggcccaacagccacgacaactgttggctgaataccatcctccaattgttcaggtacgttgatgaaccattctttgattgggtctatgaatcacctgaaaacctcactcttgaagcaattaggcaattggaagagatcactggtcttgagctgcacgagggtggcccacccgctctcgtaatttggaacatcaagcacttgctccacaccggaatcggtaccgcttcgcgacccagcgaggtgtgtatggtagacggtacagacatgtgtttggctgacttccacgctggcatcttcctgaaaggacaggaacacgcagtgttcgcctgtgtcacctccaacgggtggtacgcgattgacgacgaggacttttacccctggacgccagacccgtctgacgtcctggtgtttgtcccgtacgatcaa

>KY322677.1_A_MAY_2013

cacaacggtgagaagaagactttctactctaggcccaacaaccacgacaactgctggttgaacaccatcctccagttgttcaggtacgtcgacgagccattcttcgactgggtctatgaatcacctgagaacctcactcttgaagctattagacaactggaggatatcactggtcttgaactgcacgagggtggaccacctgccctcgtgatttggaacatcaagcacttgctccacaccggaatcggtaccgcctcgcgacccagcgaggtgtgtatggtggacggtacggacatgtgtttggctgactttcatgccggcatcttcctgaaaggacaggaacacgcagtgtttgcctgtgttacctccaacgggtggtacgcgattgacgacgaggacttctacccctggacgccagatccgtctgacgtcctggtgtttgtcccgtacgatcaa

>KY322678.1_A_MAY_2013

cacaacggtgaaaagaagaccttttactctaggcccaacagccacgacaactgctggctgaacaccatcctccaactgttcaggtacgtcgatgagccattcttcgactgggtctacgaatcacctgagaacctcactcttgaagcaattagacaattggaagagatcactggtctggagctgcacgagggtggcccacccgctctcgtaatttggaacatcaagcacttgctccataccggaatcggtaccgcttcgcgacccagcgaggtgtgtatggtggacggtacggacatgtgtttggctgatttccatgctggcatctttctaaaaggacaggaacacgcagtgttcgcctgtgtcacctccaacgggtggtacgcgattgatgacgaggacttttacccctggacaccagatccgtctgacgtcctggtgtttgtcccgtacgaccaa

>KY322679.1_A_TAI_2014

cacaacggtgagaagaagactttctactctaggcccaacaaccacgacaactgctggttaaacaccatcctccagttgttcaggtacgtcgacgagccattcttcgactgggtctatgaatcacctgagaacctcactcttgaagctattagacaactggaggatatcactggtctcgaactacacgagggtggaccacctgccctcgtgatttggaacattaagcacttgctccacaccggaatcggtaccgcctcgcgacccagcgaggtgtgtatggtagacggtacggacatgtgtttggctgactttcatgccggcatcttcctgaaaggacaggaacacgcagtgtttgcctgtgttacctccaacgggtggtacgcgattgacgacgaggacttctacccctggacgccagatccgtctgacgtcctggtgtttgtcccgtacgatcaa

>KY322680.1_A_VIT_2013

cacaacggtgagaagaagactttctactctaggcccaacaaccacgacaactgctggttgaacaccatcctccagttgttcaggtacgtcgacgagccattcttcgactgggtctatgaatcacctgagaacctcactcttgaagctattggacaactggaggatatcactggtctcgaactgcacgagggtggaccacctgccctcgtgatttggaacatcaagcacttgctccacaccggaatcggtaccgcctcgcgacccagcgaggtgtgtatggtagacggtacggacatgtgtttggctgactttcatgccggcatcttcctgaaaggacaggaacacgcagtgtttgcctgtgttacctccaacgggtggtacgcgattgacgacgaggacttctacccctggacgccagatccgtctgacgtcctggtgtttgtcccgtacgatcaa

>KY446901.1_Asia1_PAK_2006

cacaacggtgagaagaagaccttttactccagacccaacaaccatgacaactgctggctgaacactatcctccagttgttcaggtatgtcgatgagcctttcttcgactgggtctacgactcgcctgaaaacctcactctcgaggcgattaggcagttggaggaagttactggtcttgagctgcacgagggtggaccacccgcccttgtcatctggaacatcaagcatttgctccacaccggagtcggtaccgcttcgcgccctagcgaagtgtgtatggtagacggcacggacatgtgtttggctgatttccatgctggcattttcctgaaaggacaagaacatgctgtgtttgcctgtgtcacctccaacgggtggtacgcgatcgatgacgaggacttttacccctggacaccggacccgtccgacgtcttggtgtttgttccgtacgaccaa

>KY446902.1_A_PAK_2005

cacaacggtgagaagaagactttctactctaggcccaacaaccacgacaactgttggttgaacaccatccttcagttgttcaggtacgtcgatgaacctttcttcgactgggtctatgactcacctgaaaacctcactcttgatgcaattaaacagctggaagaaatcactggccttgagttgcacgagggtggaccacccgctcgcgtcatctggaacatcaaacacttgctctgcaccggagtcggcactgcctcacgccccagtgaggtatgcatggttgatggaacggacatgtgcttggctgacttccacgctggcatctttttgaaagggcaggaacacgctgtgttcgcctgcgtcacctccaatgggtggtacgcgattgacgacgaggacttttacccatggacgccggatccgtccgatgtcctggtatttgttccctacgatcaa

>KY446903.1_O_PAK_2005

cacaacggtgagaagaagactttttactctagacccaacaaccatgacaactgttggctgaataccattcttcagctgtttaggtatgtcgatgagcctttcttcgactgggtctatgactcacctgaaaacctcactcttgatgctatcaaacaactggaagaaattactggtcttgaactgcacgagggcggaccacccgcgctcgtcatctggaacattaaacacttgctccacaccgggatcggcactgcctcacgccccagtgaggtgtgcatgattgacgggacggacatgtgtttggctgacttccacgccggcatctttttgaagggacaggaacatgccgtgtttgcctgcatcacctctaacgggtggtacgcgatcgacgacgaggacttttacccctggacaccggacccgtccgacgttctggtatttgttccgtacgatcaa

>KY657269.1_O_VIT_2015

tacaacggtgagaaaaagaccttctactctaggcccaacaaccacgacaactgttggttgaacaccatccttcagttgtttaggtatgtcgacgaacctttcttcgactgggtctatgaatcgcctgaaaacctcactcttgaggcgattaagcaactggaagagatcactggtctggagttacacgagggtggaccgcccgctctcgtcatctggaacattaaacacttgctccacaccggaatcggcactgcttcgcgacccagcgaggtttgcatggttgatggcacggacatgtgtttggctgacttccacgctggcatcttcctgaaagggcaagaacacgctgtgttcgcctgcgtcacctccaacgggtggtacgcgattgacgacgaggacttctacccctggacgccggacccgtccgacgttctggtatttgtcccgtacgatcag

>KY825718.1_Asia1_ISR_1989

tacaacggtgagaagaagaccttttactccaggcccaacaaccacgacaactgttggttgaacaccatcctccagctgtttaggtacgttgatgagcctttctttgactgggtttatgactcgcccgagaacctcacccttgctgcaattgaacagttggaagaggttaccggtcttgagctgcacgaaggcgggccacccgctctcgtcatttggaacatcaaacacttgctccacactggaatcggtactgcttcgcgacccagcgaggtgtgtatggtggacggcacggacatgtgtttggctgacttccacgccggcattttcctgaaaggtcaggaacatgccgtgtttgcctgtgtcacctccaacgggtggtatgcgattgatgatgaggacttttacccttggacgccggacccgtccgacgttctggtgtttgttccgtacgatcag

>LC036265.1_O_JPN_2010

tacaacggtgagaagaagatcttctactccaggcccaacaaccacgacaactgttggctgaacgccatccttcagctgttcaggtacgtcgatgaacctttcttcgactgggtatatgaatcacctgaaaacctcacccttgaggcgatcagacaactggagaacattactggtcttgagctgcacgagggtggtccgcccgccctcgtcatttggaacatcaaacacttgctccacaccgggatcggcaccgcctcgcgacccagcgaggtgtgcatggtggacggtacggacatgtgcctggctgacttccacgctggcatcttcctgaaaggacaggaacacgccgtgtttgcctgcgtcacctccaacgggtggtacgcgatcgacgacgaagaattctacccctggacgccagatccgtccgacgtgctggtctttgtcccgtacgatcaa

>LC149617.1_O_JPN_2010

tacaacggtgagaagaagatcttctactccaggcccaacaaccacgacaactgttggctgaacgccatccttcagctgttcaggtacgtcgatgaacctttcttcgactgggtatatgaatcacctgaaaacctcacccttgaggcgatcagacaactggagaacattactggtcttgagctgcacgagggtggtccgcccgccctcgtcatttggaacatcaaacacttgctccacaccgggatcggcaccgcctcgcgacccagcgaggtgtgcatggtggacggtacggacatgtgcctggctgacttccacgctggcatcttcctgaaaggacaggaacacgccgtgtttgcctgcgtcacctccaacgggtggtacgcgatcgacgacgaagaattctacccctggacgccagatccgtccgacgtgctggtctttgtcccgtacgatcaa

>LC149618.1_O_JPN_2010

tacaacggtgagaagaagatcttctactccaggcccaacaaccacgacaactgttggctgaacgccatccttcagctgttcaggtacgtcgatgaacctttcttcgactgggtatatgaatcacctgaaaacctcacccttgaggcgatcagacaactggagaacattactggtcttgagctgcacgagggtggtccgcccgccctcgtcatttggaacatcaaacacttgctccacaccgggatcggcaccgcctcgcgacccagcgaggtgtgcatggtggacggtacggacatgtgcctggctgacttccacgctggcatcttcctgaaaggacaggaacacgccgtgtttgcctgcgtcacctccaacgggtggtacgcgatcgacgacgaagaattctacccctggacgccagatccgtccgacgtgctggtctttgtcccgtacgatcaa

>LC149619.1_O_JPN_2010

tacaacggtgagaagaagatcttctactccaggcccaacaaccacgacaactgttggctgaacgccatccttcagctgttcaggtacgtcgatgaacctttcttcgactgggtatatgaatcacctgaaaacctcacccttgaggcgatcagacaactggagaacattactggtcttgagctgcacgagggtggtccgcccgccctcgtcatttggaacatcaaacacttgctccacaccgggatcggcaccgcctcgcgacccagcgaggtgtgcatggtggacggtacggacatgtgcctggctgacttccacgctggcatcttcctgaaaggacaggaacacgccgtgtttgcctgcgtcacctccaacgggtggtacgcgatcgacgacgaagaattctacccctggacgccagatccgtccgacgtgctggtctttgtcccgtacgatcaa

>LC149620.1_O_JPN_2010

tacaacggtgagaagaagatcttctactccaggcccaacaaccacgacaactgttggctgaacgccatccttcagctgttcaggtacgtcgatgaacctttcttcgactgggtatatgaatcacctgaaaacctcacccttgaggcgatcagacaactggagaacattactggtcttgagctgcacgagggtggtccgcccgccctcgtcatttggaacatcaaacacttgctccacaccgggatcggcaccgcctcgcgacccagcgaggtgtgcatggtggacggtacggacatgtgcctggctgacttccacgctggcatcttcctgaaaggacaggaacacgccgtgtttgcctgcgtcacctccaacgggtggtacgcgatcgacgacgaagaattctacccctggacgccagatccgtccgacgtgctggtctttgtcccgtacgatcaa

>LC149621.1_O_JPN_2010

tacaacggtgagaagaagatcttctactccaggcccaacaaccacgacaactgttggctgaacgccatccttcagctgttcaggtacgtcgatgaacctttcttcgactgggtatatgaatcacctgaaaacctcacccttgaggcgatcagacaactggagaacattactggtcttgagctgcacgagggtggtccgcccgccctcgtcatttggaacatcaaacacttgctccacaccgggatcggcaccgcctcgcgacccagcgaggtgtgcatggtggacggtacggacatgtgcctggctgacttccacgctggcatcttcctgaaaggacaggaacacgccgtgtttgcctgcgtcacctccaacgggtggtacgcgatcgacgacgaagaattctacccctggacgccagatccgtccgacgtgctggtctttgtcccgtacgatcaa

>LC149622.1_O_JPN_2010

tacaacggtgagaagaagatcttctactccaggcccaacaaccacgacaactgttggctgaacgccatccttcagctgttcaggtacgtcgatgaacctttcttcgactgggtatatgaatcacctgaaaacctcacccttgaggcgatcagacaactggagaacattactggtcttgagctgcacgagggtggtccgcccgccctcgtcatttggaacatcaaacacttgctccacaccgggatcggcaccgcctcgcgacccagcgaggtgtgcatggtggacggtacggacatgtgcctggctgacttccacgctggcatcttcctgaaaggacaggaacacgccgtgtttgcctgcgtcacctccaacgggtggtacgcgatcgacgacgaagaattctacccctggacgccagatccgtccgacgtgctggtctttgtcccgtacgatcaa

>LC149623.1_O_JPN_2010

tacaacggtgagaagaagatcttctactccaggcccaacaaccacgacaactgttggctgaacgccatccttcagctgttcaggtacgtcgatgaacctttcttcgactgggtatatgaatcacctgaaaacctcacccttgaggcgatcagacaactggagaacattactggtcttgagctgcacgagggtggtccgcccgccctcgtcatttggaacatcaaacacttgctccacaccgggatcggcaccgcctcgcgacccagcgaggtgtgcatggtggacggtacggacatgtgcctggctgacttccacgctggcatcttcctgaaaggacaggaacacgccgtgtttgcctgcgtcacctccaacgggtggtacgcgatcgacgacgaagaattctacccctggacgccagatccgtccgacgtgctggtctttgtcccgtacgatcaa

>LC149624.1_O_JPN_2010

tacaacggtgagaagaagatcttctactccaggcccaacaaccacgacaactgttggctgaacgccatccttcagctgttcaggtacgtcgatgaacctttcttcgactgggtatatgaatcacctgaaaacctcacccttgaggcgatcagacaactggagaacattactggtcttgagctgcacgagggtggtccgcccgccctcgtcatttggaacatcaaacacttgctccacaccgggatcggcaccgcctcgcgacccagcgaggtgtgcatggtggacggtacggacatgtgcctggctgacttccacgctggcatcttcctgaaaggacaggaacacgccgtgtttgcctgcgtcacctccaacgggtggtacgcgatcgacgacgaagaattctacccctggacgccagatccgtccgacgtgctggtctttgtcccgtacgatcaa

>LC149625.1_O_JPN_2010

tacaacggtgagaagaagatcttctactccaggcccaacaaccacgacaactgttggctgaacgccatccttcagctgttcaggtacgtcgatgaacctttcttcgactgggtatatgaatcacctgaaaacctcacccttgaggcgatcagacaactggagaacattactggtcttgagctgcacgagggtggtccgcccgccctcgtcatttggaacatcaaacacttgctccacaccgggatcggcaccgcctcgcgacccagcgaggtgtgcatggtggacggtacggacatgtgcctggctgacttccacgctggcatcttcctgaaaggacaggaacacgccgtgtttgcctgcgtcacctccaacgggtggtacgcgatcgacgacgaagaattctacccctggacgccagatccgtccgacgtgctggtctttgtcccgtacgatcaa

>LC149626.1_O_JPN_2010

tacaacggtgagaagaagatcttctactccaggcccaacaaccacgacaactgttggctgaacgccatccttcagctgttcaggtacgtcgatgaacctttcttcgactgggtatatgaatcacctgaaaacctcacccttgaggcgatcagacaactggagaacattactggtcttgagctgcacgagggtggtccgcccgccctcgtcatttggaacatcaaacacttgctccacaccgggatcggcaccgcctcgcgacccagcgaggtgtgcatggtggacggtacggacatgtgcctggctgacttccacgctggcatcttcctgaaaggacaggaacacgccgtgtttgcctgcgtcacctccaacgggtggtacgcgatcgacgacgaagaattctacccctggacgccagatccgtccgacgtgctggtctttgtcccgtacgatcaa

>LC149627.1_O_JPN_2010

tacaacggtgagaagaagatcttctactccaggcccaacaaccacgacaactgttggctgaacgccatccttcagctgttcaggtacgtcgatgaacctttcttcgactgggtatatgaatcacctgaaaacctcacccttgaggcgatcagacaactggagaacattactggtcttgagctgcacgagggtggtccgcccgccctcgtcatttggaacatcaaacacttgctccacaccgggatcggcaccgcctcgcgacccagcgaggtgtgcatggtggacggtacggacatgtgcctggctgacttccacgctggcatcttcctgaaaggacaggaacacgccgtgtttgcctgcgtcacctccaacgggtggtacgcgatcgacgacgaagaattctacccctggacgccagatccgtccgacgtgctggtctttgtcccgtacgatcaa

>LC149628.1_O_JPN_2010

tacaacggtgagaagaagatcttctactccaggcccaacaaccacgacaactgttggctgaacgccatccttcagctgttcaggtacgtcgacgaacctttcttcgactgggtatatgaatcacctgaaaacctcacccttgaggcgatcagacaactggagaacattactggtcttgagctgcacgagggtggtccgcccgccctcgtcatttggaacatcaaacacttgctccacaccgggatcggcaccgcctcgcgacccagcgaggtgtgcatggtggacggtacggacatgtgcctggctgacttccacgctggcatcttcctgaaaggacaggaacacgccgtgtttgcctgcgtcacctccaacgggtggtacgcgatcgacgacgaagaattctacccctggacgccagatccgtccgacgtgctggtctttgtcccgtacgatcaa

>LC149629.1_O_JPN_2010

tacaacggtgagaagaagatcttctactccaggcccaacaaccacgacaactgttggctgaacgccatccttcagctgttcaggtacgtcgatgaacctttcttcgactgggtatatgaatcacctgaaaacctcacccttgaggcgatcagacaactggagaacattactggtcttgagctgcacgagggtggtccgcccgccctcgtcatttggaacatcaaacacttgctccacaccgggatcggcaccgcctcgcgacccagcgaggtgtgcatggtggacggtacggacatgtgcctggctgacttccacgctggcatcttcctgaaaggacaggaacacgccgtgtttgcctgcgtcacctccaacgggtggtacgcgatcgacgacgaagaattctacccctggacgccagatccgtccgacgtgctggtctttgtcccgtacgatcaa

>LC149630.1_O_JPN_2010

tacaacggtgagaagaagatcttctactccaggcccaacaaccacgacaactgttggctgaacgccatccttcagctgttcaggtacgtcgatgaacctttcttcgactgggtatatgaatcacctgaaaacctcacccttgaggcgatcagacaactggagaacattactggtcttgagctgcacgagggtggtccgcccgccctcgtcatttggaacatcaaacacttgctccacaccgggatcggcaccgcctcgcgacccagcgaggtgtgcatggtggacggtacggacatgtgcctggctgacttccacgctggcatcttcctgaaaggacaggaacacgccgtgtttgcctgcgtcacctccaacgggtggtacgcgatcgacgacgaagaattctacccctggacgccagatccgtccgacgtgctggtctttgtcccgtacgatcaa

>LC149631.1_O_JPN_2010

tacaacggtgagaagaagatcttctactccaggcccaacaaccacgacaactgttggctgaacgccatccttcagctgttcaggtacgtcgatgaacctttcttcgactgggtatatgaatcacctgaaaacctcacccttgaggcgatcagacaactggagaacattactggtcttgagctgcacgagggtggtccgcccgccctcgtcatttggaacatcaaacacttgctccacaccgggatcggcaccgcctcgcgacccagcgaggtgtgcatggtggacggtacggacatgtgcctggctgacttccacgctggcatcttcctgaaaggacaggaacacgccgtgtttgcctgcgtcacctccaacgggtggtacgcgatcgacgacgaagaattctacccctggacgccagatccgtccgacgtgctggtctttgtcccgtacgatcaa

>LC149632.1_O_JPN_2010

tacaacggtgagaagaagatcttctactccaggcccaacaaccacgacaactgttggctgaacgccatccttcagctgttcaggtacgtcgatgaacctttcttcgactgggtatatgaatcacctgaaaacctcacccttgaggcgatcagacaactggagaacattactggtcttgagctgcacgagggtggtccgcccgccctcgtcatttggaacatcaaacacttgctccacaccgggatcggcaccgcctcgcgacccagcgaggtgtgcatggtggacggtacggacatgtgcctggctgacttccacgctggcatcttcctgaaaggacaggaacacgccgtgtttgcctgcgtcacctccaacgggtggtacgcgatcgacgacgaagaattctacccctggacgccagatccgtccgacgtgctggtctttgtcccgtacgatcaa

>LC149633.1_O_JPN_2010

tacaacggtgagaagaagatcttctactccaggcccaacaaccacgacaactgttggctgaacgccatccttcagctgttcaggtacgtcgatgaacctttcttcgactgggtatatgaatcacctgaaaacctcacccttgaggcgatcagacaactggagaacattactggtcttgagctgcacgagggtggtccgcccgccctcgtcatttggaacatcaaacacttgctccacaccgggatcggcaccgcctcgcgacccagcgaggtgtgcatggtggacggtacggacatgtgcctggctgacttccacgctggcatcttcctgaaaggacaggaacacgccgtgtttgcctgcgtcacctccaacgggtggtacgcgatcgacgacgaaaaattctacccctggacgccagatccgtccgacgtgctggtctttgtcccgtacgatcaa

>LC149634.1_O_JPN_2010

tacaacggtgagaagaagatcttctactccaggcccaacaaccacgacaactgttggctgaacgccatccttcagctgttcaggtacgtcgatgaacctttcttcgactgggtatatgaatcacctgaaaacctcacccttgaggcgatcagacaactggagaacattactggtcttgagctgcacgagggtggtccgcccgccctcgtcatttggaacatcaaacacttgctccacaccgggatcggcaccgcctcgcgacccagcgaggtgtgcatggtggacggtacggacatgtgcctggctgacttccacgctggcatcttcctgaaaggacaggaacacgccgtgtttgcctgcgtcacctccaacgggtggtacgcgatcgacgacgaagaattctacccctggacgccagatccgtccgacgtgctggtctttgtcccgtacgatcaa

>LC149635.1_O_JPN_2010

tacaacggtgagaagaagatcttctactccaggcccaacaaccacgacaactgttggctgaacgccatccttcagctgttcaggtacgtcgatgaacctttcttcgactgggtatatgaatcacctgaaaacctcacccttgaggcgatcagacaactggagaacattaccggtcttgagctgcacgagggtggtccgcccgccctcgtcatttggaacatcaaacacttgctccacaccgggatcggcaccgcctcgcgacccagcgaggtgtgcatggtggacggtacggacatgtgcctggctgacttccacgctggcatcttcctgaaaggacaggaacacgccgtgtttgcctgcgtcacctccaacgggtggtacgcgatcgacgacgaagaattctacccctggacgccagatccgtccgacgtgctggtctttgtcccgtacgatcaa

>LC149636.1_O_JPN_2010

tacaacggtgagaagaagatcttctactccaggcccaacaaccacgacaactgttggctgaacgccatccttcagctgttcaggtacgtcgatgaacctttcttcgactgggtatatgaatcacctgaaaacctcacccttgaggcgatcagacaactggagaacattactggtcttgagctgcacgagggtggtccgcccgccctcgtcatttggaacatcaaacacttgctccacaccgggatcggcaccgcctcgcgacccagcgaggtgtgcatggtggacggtacggacatgtgcctggctgacttccacgctggcatcttcctgaaaggacaggaacacgccgtgtttgcctgcgtcacctccaacgggtggtacgcgatcgacgacgaagaattctacccctggacgccagatccgtccgacgtgctggtctttgtcccgtacgatcaa

>LC149637.1_O_JPN_2010

tacaacggtgagaagaagatcttctactccaggcccaacaaccacgacaactgttggctgaacgccatccttcagctgttcaggtacgtcgacgaacctttcttcgactgggtatatgaatcacctgaaaacctcacccttgaggcgatcagacaactggagaacattactggtcttgagctgcacgagggtggtccgcccgccctcgtcatttggaacatcaaacacttgctccacaccgggatcggcaccgcctcgcgacccagcgaggtgtgcatggtggacggtacggacatgtgcctggctgacttccacgctggcatcttcctgaaaggacaggaacacgccgtgtttgcctgcgtcacctccaacgggtggtacgcgatcgacgacgaagaattctacccctggacgccagatccgtccgacgtgctggtctttgtcccgtacgatcaa

>LC149638.1_O_JPN_2010

tacaacggtgagaagaagatcttctactccaggcccaacaaccacgacaactgttggctgaacgccatccttcagctgttcaggtacgtcgatgaacctttcttcgactgggtatatgaatcacctgaaaacctcacccttgaggcgatcagacaactggagaacattactggtcttgagctgcacgagggtggtccgcccgccctcgtcatttggaacatcaaacacttgctccacaccgggatcggcaccgcctcgcgacccagcgaggtgtgcatggtggacggtacggacatgtgcctggctgacttccacgctggcatcttcctgaaaggacaggaacacgccgtgtttgcctgcgtcacctccaacgggtggtacgcgatcgacgacgaagaattctacccctggacgccagatccgtccgacgtgctggtctttgtcccgtacgatcaa

>LC149639.1_O_JPN_2010

tacaacggtgagaagaagatcttctactccaggcccaacaaccacgacaactgttggctgaacgccatccttcagctattcaggtacgtcgatgaacctttcttcgactgggtatatgaatcacctgaaaacctcacccttgaggcgatcagacaactggagaacattactggtcttgagctgcacgagggtggtccgcccgccctcgtcatttggaacatcaaacacttgctccacaccgggatcggcaccgcctcgcgacccagcgaggtgtgcatggtggacggtacggacatgtgcctggctgacttccacgctggcatcttcctgaaaggacaggaacacgccgtgtttgcctgcgtcacctccaacgggtggtacgcgatcgacgacgaagaatyctacccctggacgccagatccgtccgacgtgctggtctttgtcccgtacgatcaa

>LC149640.1_O_JPN_2010

tacaacggtgagaagaagatcttctactccaggcccaacaaccacgacaactgttggctgaacgccatccttcagctgttcaggtacgtcgatgaacctttcttcgactgggtatatgaatcacctgaaaacctcacccttgaggcgatcagacaactggagaacattactggtcttgagctgcacgagggtggtccgcccgccctcgtcatttggaacatcaaacacttgctccacaccgggatcggcaccgcctcgcgacccagcgaggtgtgcatggtggacggtacggacatgtgcctggctgacttccacgctggcatcttcctgaaaggacaggaacacgccgtgtttgcctgcgtcacctccaacgggtggtacgcgatcgacgacgaagaattctacccctggacgccagatccgtccgacgtgctggtctttgtcccgtacgatcaa

>LC149641.1_O_JPN_2010

tacaacggtgagaagaagatcttctactccaggcccaacaaccacgacaactgttggctgaacgccatccttcagctgttcaggtacgtcgatgaacctttcttcgactgggtatatgaatcacctgaaaacctcacccttgaggcgatcagacaactggagaacattactggtcttgagctgcacgagggtggtccgcccgccctcgtcatttggaacatcaaacacttgctccacaccgggatcggcaccgcctcgcgacccagcgaggtgtgcatggtggacggtacggacatgtgcctggctgacttccacgctggcatcttcctgaaaggacaggaacacgccgtgtttgcctgcgtcacctccaacgggtggtacgcgatcgacgacgaagaattctacccctggacgccagatccgtccgacgtgctggtctttgtcccgtacgatcaa

>LC149642.1_O_JPN_2010

tacaacggtgagaagaagatcttctactccaggcccaacaaccacgacaactgttggctgaacgccatccttcagctgttcaggtacgtcgatgaacctttcttcgactgggtatatgaatcacctgaaaacctcacccttgaggcgatcagacaactggagaacattactggtcttgagctgcacgagggtggtccgcccgccctcgtcatttggaacatcaaacacttgctccacaccgggatcggcaccgcctcgcgacccagcgaggtgtgcatggtggacggtacggacatgtgcctggctgacttccacgctggcatcttcctgaaaggacaggaacacgccgtgtttgcctgcgtcacctccaacgggtggtacgcgatcgacgacgaagaattctacccctggacgccagatccgtccgacgtgctggtctttgtcccgtacgatcaa

>LC149643.1_O_JPN_2010

tacaacggtgagaagaagatcttctactccaggcccaacaaccacgacaactgttggctgaacgccatccttcagctgttcaggtacgtcgatgaacctttcttcgactgggtatatgaatcacctgaaaacctcacccttgaggcgatcagacaactggagaacattactggtcttgagctgcacgagggtggtccgcccgccctcgtcatttggaacatcaaacacttgctccacaccgggatcggcaccgcctcgcgacccagcgaggtgtgcatggtggacggtacggacatgtgcctggctgacttccacgctggcatcttcctgaaaggacaggaacacgccgtgtttgcctgcgtcacctccaacgggtggtacgcgatcgacgacgaagaattctacccctggacgccagatccgtccgacgtgctggtctttgtcccgtacgatcaa

>LC149644.1_O_JPN_2010

tacaacggtgagaagaagatcttctactccaggcccaacaaccacgacaactgttggctgaacgccatccttcagctgttcaggtacgtcgatgaacctttcttcgactgggtatatgaatcacctgaaaacctcacccttgaggcgatcagacaactggagaacattactggtcttgagctgcacgagggtggtccgcccgccctcgtcatttggaacatcaaacacttgctccacaccgggatcggcaccgcctcgcgacccagcgaggtgtgcatggtggacggtacggacatgtgcctggctgacttccacgctggcatcttcctgaaaggacaggaacacgccgtgtttgcctgcgtcacctccaacgggtggtacgcgatcgacgacgaagaattctacccctggacgccagatccgtccgacgtgctggtctttgtcccgtacgatcaa

>LC149645.1_O_JPN_2010

tacaacggtgagaagaagatcttctactccaggcccaacaaccacgacaactgttggctgaacgccatccttcagctgttcaggtacgtcgatgaacctttcttcgactgggtatatgaatcacctgaaaacctcacccttgaggcgatcagacaactggagaacattactggtcttgagctgcacgagggtggtccgcccgccctcgtcatttggaacatcaaacacttgctccacaccgggatcggcaccgcctcgcgacccagcgaggtgtgcatggtggacggtacggacatgtgcctggctgacttccacgctggcatcttcctgaaaggacaggaacacgccgtgtttgcctgcgtcacctccaacgggtggtacgcgatcgacgacgaagaattctacccctggacgccagatccgtccgacgtgctggtctttgtcccgtacgatcaa

>LC149646.1_O_JPN_2010

tacaacggtgagaagaagatcttctactccaggcccaacaaccacgacaactgttggctgaacgccatccttcagctgttcaggtacgtcgatgaacctttcttcgactgggtatatgaatcacctgaaaacctcacccttgaggcgatcagacaactggagaacattactggtcttgagctgcacgagggtggtccgcccgccctcgtcatttggaacatcaaacacttgctccacaccgggatcggcaccgcctcgcgacccagcgaggtgtgcatggtggacggtacggacatgtgcctggctgacttccacgctggcatcttcctgaaaggacaggaacacgccgtgtttgcctgcgtcacctccaacgggtggtacgcgatcgacgacgaagaattctacccctggacgccagatccgtccgacgtgctggtctttgtcccgtacgatcaa

>LC149647.1_O_JPN_2010

tacaacggtgagaagaagatcttctactccaggcccaacaaccacgacaactgttggctgaacgccatccttcagctgttcaggtacgtcgatgaacctttcttcgactgggtatatgaatcacctgaaaacctcacccttgaggcgatcagacaactggagaacattactggtcttgagctgcacgagggtggtccgcccgccctcgtcatttggaacatcaaacacttgctccacaccgggatcggcaccgcctcgcgacccagcgaggtgtgcatggtggacggtacggacatgtgcctggctgacttccacgctggcatcttcctgaaaggacaggaacacgccgtgtttgcctgcgtcacctccaacgggtggtacgcgatcgacgacgaaaaattctacccctggacgccagatccgtccgacgtgctggtctttgtcccgtacgatcaa

>LC149648.1_O_JPN_2010

tacaacggtgagaagaagatcttctactccaggcccaacaaccacgacaactgttggctgaacgccatccttcagctgttcaggtacgtcgatgaacctttcttcgactgggtatatgaatcacctgaaaacctcacccttgaggcgatcagacaactggagaacattactggtcttgagctgcacgagggtggtccgcccgccctcgtcatttggaacatcaaacacttgctccacaccgggatcggcaccgcctcgcgacccagcgaggtgtgcatggtggacggtacggacatgtgcctggctgacttccacgctggcatcttcctgaaaggacaggaacacgccgtgtttgcctgcgtcacctccaacgggtggtacgcgatcgacgacgaagaattctacccctggacgccagatccgtccgacgtgctggtctttgtcccgtacgatcaa

>LC149649.1_O_JPN_2010

tacaacggtgagaagaagatcttctactccaggcccaacaaccacgacaactgttggctgaacgccatccttcagctgttcaggtacgtcgatgaacctttcttcgactgggtatatgaatcacctgaaaacctcacccttgaggcgatcagacaactggagaacattactggtcttgagctgcacgagggtggtccgcccgccctcgtcatttggaacatcaaacacttgctccacaccgggatcggcaccgcctcgcgacccagcgaggtgtgcatggtggacggtacggacatgtgcctggctgacttccacgctggcatcttcctgaaaggacaggaacacgccgtgtttgcctgcgtcacctccaacgggtggtacgcgatcgacgacgaagaattctacccctggacgccagatccgtccgacgtgctggtctttgtcccgtacgatcaa

>LC149650.1_O_JPN_2010

tacaacggtgagaagaagatcttctactccaggcccaacaaccacgacaactgttggctgaacgccatccttcagctgttcaggtacgtcgatgaacctttcttcgactgggtatatgaatcacctgaaaacctcacccttgaggcgatcagacaactggagaacattactggtcttgagctgcacgagggtggtccgcccgccctcgtcatttggaacatcaaacacttgctccacaccgggatcggcaccgcctcgcgacccagcgaggtgtgcatggtggacggtacggacatgtgcctggctgacttccacgctggcatcttcctgaaaggacaggaacacgccgtgtttgcctgcgtcacctccaacgggtggtacgcgatcgacgacgaagaattctacccctggacgccagatccgtccgacgtgctggtctttgtcccgtacgatcaa

>LC149651.1_O_JPN_2010

tacaacggtgagaagaagatcttctactccaggcccaacaaccacgacaactgttggctgaacgccatccttcagctgttcaggtacgtcgatgaacctttcttcgactgggtatatgaatcacctgaaaacctcacccttgaggcgatcagacaactggagaacattactggtcttgagctgcacgagggtggtccgcccgccctcgtcatttggaacatcaaacacttgctccacaccgggatcggcaccgcctcgcgacccagcgaggtgtgcatggtggacggtacggacatgtgcctggctgacttccacgctggcatcttcctgaaaggacaggaacacgccgtgtttgcctgcgtcacctccaacgggtggtacgcgatcgacgacgaagaattctacccctggacgccagatccgtccgacgtgctggtctttgtcccgtacgatcaa

>LC149652.1_O_JPN_2010

tacaacggtgagaagaagatcttctactccaggcccaacaaccacgacaactgttggctgaacgccatccttcagctgttcaggtacgtcgatgaacctttcttcgactgggtatatgaatcacctgaaaacctcacccttgaggcgatcagacaactggagaacattactggtcttgagctgcacgagggtggtccgcccgccctcgtcatttggaacatcaaacacttgctccacaccgggatcggcaccgcctcgcgacccagcgaggtgtgcatggtggacggtacggacatgtgcctggctgacttccacgctggcatcttcctgaaaggacaggaacacgccgtgtttgcctgcgtcacctccaacgggtggtacgcgatcgacgacgaagaattctacccctggacgccagatccgtccgacgtgctggtctttgtcccgtacgatcaa

>LC149653.1_O_JPN_2010

tacaacggtgagaagaagatcttctactccaggcccaacaaccacgacaactgttggctgaacgccatccttcagctgttcaggtacgtcgatgaacctttcttcgactgggtatatgaatcacctgaaaacctcacccttgaggcgatcagacaactggagaacattactggtcttgagctgcacgagggtggtccgcccgccctcgtcatttggaacatcaaacacttgctccacaccgggatcggcaccgcctcgcgacccagcgaggtgtgcatggtggacggtacggacatgtgcctggctgacttccacgctggcatcttcctgaaaggacaggaacacgccgtgtttgcctgcgtcacctccaacgggtggtacgcgatcgacgacgaagaattctacccctggacgccagatccgtccgacgtgctggtctttgtcccgtacgatcaa

>LC149654.1_O_JPN_2010

tacaacggtgagaagaagatcttctactccaggcccaacaaccacgacaactgttggctgaacgccatccttcagctgttcaggtacgtcgatgaacctttcttcgactgggtatatgaatcacctgaaaacctcacccttgaggcgatcagacaactggagaacattactggtcttgagctgcacgagggtggtccgcccgccctcgtcatttggaacatcaaacacttgctccacaccgggatcggcaccgcctcgcgacccagcgaggtgtgcatggtggacggtacggacatgtgcctggctgacttccacgctggcatcttcctgaaaggacaggaacacgccgtgtttgcctgcgtcacctccaacgggtggtacgcgatcgacgacgaagaattctacccctggacgccagatccgtccgacgtgctggtctttgtcccgtacgatcaa

>LC149655.1_O_JPN_2010

tacaacggtgagaagaagatcttctactccaggcccaacaaccacgacaactgttggctgaacgccatccttcagctgttcaggtacgtcgatgaacctttcttcgactgggtatatgaatcacctgaaaacctcacccttgaggcgatcagacaactggagaacattactggtcttgagctgcacgagggtggtccgcccgccctcgtcatttggaacatcaaacacttgctccacaccgggatcggcaccgcctcgcgacccagcgaggtgtgcatggtggacggtacggacatgtgcctggctgacttccacgctggcatcttcctgaaaggacaggaacacgccgtgtttgcctgcgtcacctccaacgggtggtacgcgatcgacgacgaaaaattctacccctggacgccagatccgtccgacgtgctagtctttgtcccgtacgatcaa

>LC149656.1_O_JPN_2010

tacaacggtgagaagaagatcttctactccaggcccaacaaccaggacaactgttggctgaacgccatccttcagctgttcaggtacgtcgatgaacctttcttcgactgggtatatgaatcacctgaaaacctcacccttgaggcgatcagacaactggagaacattactggtcttgagctgcacgagggtggtccgcccgccctcgtcatttggtacatcaaacacttgctccacaccgggatcggcaccgcctcgcgacccagcgaggtgtgcatggtggacggtacggacatgtgcctggctgacttccacgctggcatcttcctgaaaggacaggaacacgccgtgtttgcctgcgtcacctccaacgggtggtacgcgatcgacgacgaagaattctacccctggacgccagatccgtccgacgtgctggtctttgtcccgtacgatcaa

>LC149657.1_O_JPN_2010

tacaacggtgagaagaagatcttctactccaggcccaacaaccacgacaactgttggctgaacgccatccttcagctgttcaggtacgtcgatgaacctttcttcgactgggcatatgaatcacctgaaaacctcacccttgaggcgatcagacractggagaacattactggtcttgagctgcacgagggtggtccgcccgccctcgtcatttggaacatcaaacacttgctccacaccgggatcggcaccgcctcgcgacccagcgaggtgtgcatggtggacggtacggacatgtgcctggctgacttccacgctggcatcttcctgaaaggacaggaacacgccgtgtttgcctgcgtcacctccaacgggtggtacgcgatcgacgacgaagaattctacccctggacgccagatccgtccgacgtgctggtctttgtcccgtacgatcaa

>LC149658.1_O_JPN_2010

tacaacggtgagaagaagatcttctactccaggcccaacaaccacgacaactgttggctgaacgccatccttcagctgttcaggtacgtcgatgaacctttcttcgactgggtatatgaatcacctgaaaacctcacccttgaggcgatcagacaactggagaacattactggtcttgagctgcacgagggtggtccgcccgccctcgtcatttggaacatcaaacacttgctccacaccgggatcggcaccgcctcgcgacccagcgaggtgtgcatggtggacggtacggacatgtgcctggctgacttccacgctggcatcttcctgaaaggacaggaacacgccgtgtttgcctgcgtcacctccaacgggtggtacgcgatcgacgacgaagaattctacccctggacgccagatccgtccgacgtgctggtctttgtcccgtacgatcaa

>LC149659.1_O_JPN_2010

tacaacggtgagaagaagatcttctactccaggcccaacaaccacgacaactgttggctgaacgccatccttcagctgttcaggtacgtcgatgaacctttcttcgactgggtatatgaatcacctgaaaacctcacccttgaggcgatcagacaactggagaacattactggtcttgagctgcacgagggtggtccgcccgccctcgtcatttggtacatcaaacacttgctccacaccgggatcggcaccgcctcgcgacccagcgaggtgtgcatggtggacggtacggacatgtgcctggctgacttccacgctggcatcttcctgaaaggacaggaacacgccgtgtttgcctgcgtcacctccaacgggtggtacgcgatcgacgacgaagaattctacccctggacgccagatccgtccgacgtgctggtctttgtcccgtacgatcaa

>LC149660.1_O_JPN_2010

tacaacggtgagaagaagatcttctactccaggcccaacaaccacgacaactgttggctgaacgccatccttcagctgttcaggtacgtcgatgaacctttcttcgactgggtatatgaatcacctgaaaacctcacccttgaggcgatcagacaactggagaacattactggtcttgagctgcacgagggtggtccgcccgccctcgtcatttggaacatcaaacacttgctccacaccgggatcggcaccgcctcgcgacccagcgaggtgtgcatggtggacggtacggacatgtgcctggctgacttccacgctggcatcttcctgaaaggacaggaacacgccgtgtttgcctgcgtcacctccaacgggtggtacgcgatcgacgacgaagaattctacccctggacgccagatccgtccgacgtgctggtctttgtcccgtacgatcaa

>LC149661.1_O_JPN_2010

tacaacggtgagaagaagatcttctactccaggcccaacaaccacgacaactgttggctgaacgccatccttcagctattcaggtacgtcgatgaacctttcttcgactnggtatatgaatcacctgaaaacctcacccttgaggcgatcagacaactggagaacattactggtcttgagctgcacgagggtggtccgcccgccctcgtcatttggaacatcaaacacttgctccacaccgggatcggcaccgcctcgcgacccagcgaggtgtgcatggtggacggtacggacatgtgcctggctgacttccacgctggcatcttcctgaaaggacaggaacacgccgtgtttgcctgcgtcacctccaacgggtggtacgcgatcgacgacgaagaattctacccctggacgccagatccgtccgacgtgctggtctttgtcccgtacgatcaa

>LC149662.1_O_JPN_2010

tacaacggtgagaagaagatcttctactccaggcccaacaaccacgacaactgttggctgaacgccatccttcagctgttcaggtacgtcgatgaacctttcttcgactgggtatatgaatcacctgaaaacctcacccttgaggcgatcagacaactggagaacattactggtcttgagctgcacgagggtggtccgcccgccctcgtcatttggaacatcaaacacttgctccacaccgggatcggcaccgcctcgcgacccagcgaggtgtgcatggtggacggtacggacatgtgcctggctgacttccacgctggcatcttcctgaaaggacaggaacacgccgtgtttgcctgcgtcacctccaacgggtggtacgcgatcgacgacgaagaattctacccctggacgccagatccgtccgacgtgctggtctttgtcccgtacgatcaa

>LC149663.1_O_JPN_2010

tacaacggtgagaagaagatcttctactccaggcccaacaaccacgacaactgttggctgaacgccatccttcagctgttcaggtacgtcgatgaacctttcttcgactgggtatatgaatcacctgaaaacctcacccttgaggcgatcagacaactggagaacattactggtcttgagctgcacgagggtggtccgcccgccctcgtcatttggaacatcaaacacttgctccacaccgggatcggcaccgcctcgcgacccagcgaggtgtgcatggtggacggtacggacatgtgcctggctgacttccacgctggcatcttcctgaaaggacaggaacacgctgtgtttgcctgcgtcacctccaacgggtggtacgcgatcgacgacgaagaattctacccctggacgccagatccgtccgacgtgctggtctttgtcccgtacgatcaa

>LC149664.1_O_JPN_2010

tacaacggtgagaagaagatcttctactccaggcccaacaaccacgacaactgttggctgaacgccatccttcagctgttcaggtacgtcgatgaacctttcttcgactgggtatatgaatcacctgaaaacctcacccttgaggcgatcagacaactggagaacattactggtcttgagctgcacgagggtggtccgcccgccctcgtcatttggaacatcaaacacytgctccacaccgggatcggcaccgcctcgcgacccagcgaggtgtgcatggtggacggtacggacatgtgcctggctgacttccacgctggcatcttcctgaaaggacaggaacacgccgtgtttgcctgcgtcacctccaacgggtggtacgcgatcgacgacgaagaattctacccctggacgccagatccgtccgacgtgctggtctttgtcccgtacgatcaa

>LC149665.1_O_JPN_2010

tacaacggtgagaagaagatcttctactccaggcccaacaaccacgacaactgttggctgaacgccatccttcagctgttcaggtacgtcgatgaacctttcttcgactgggtatatgaatcacctgaaaacctcacccttgaggcgatcagacaactggagaacattactggtcttgagctgcacgagggtggtccgcccgccctcgtcatttggaacatcaaacacttgctccacaccgggatcggcaccgcctcgcgacccagcgaggtgtgcatggtggacggtacggacatgtgcctggctgacttccacgctggcatcttcctgaaaggacaggaacacgccgtgtttgcctgcgtcacctccaacgggtggtacgcgatcgacgacgaagaattctacccctggacgccagatccgtccgacgtgctggtctttgtcccgtacgatcaa

>LC149666.1_O_JPN_2010

tacaacggtgagaagaagatcttctactccaggcccaacaaccacgacaactgttggctgaacgccatccttcagctgttcaggtacgtcgatgaacctttcttcgactgggtatatgaatcacctgaaaacctcacccttgaggcgatcagacaactggagaacattactggtcttgagctgcacgagggtggtccgcccgccctcgtcatttggaacatcaaacacttgctccacaccgggatcggcaccgcctcgcgacccagcgaggtgtgcatggtggacggtacggacatgtgcctggctgacttccacgctggcatcttcctgaaaggacaggaacacgccgtgtttgcctgcgtcacctccaacgggtggtacgcgatcgacgacgaagaattctacccctggacgccagatccgtccgacgtgctggtctttgtcccgtacgatcaa

>LC149667.1_O_JPN_2010

tacaacggtgagaagaagatcttctactccaggcccaacaaccacgacaactgttggctgaacgccatccttcagctgttcaggtacgtcgatgaacctttcttcgactgggtatatgaatcacctgaaaacctcacccttgaggcgatcagacaactggagaacattactggtcttgagctgcacgagggtggtccgcccgccctcgtcatttggaacatcaaacacttgctccacaccgggatcggcaccgcctcgcgacccagcgaggtgtgcatggtggacggtacggacatgtgcctggctgacttccacgctggcatcttcctgaaaggacaggaacacgccgtgtttgcctgcgtcacctccaacgggtggtacgcgatcgacgacgaagaattctacccctggacgccagatccgtccgacgtgctggtctttgtcccgtacgatcaa

>LC149668.1_O_JPN_2010

tacaacggtgagaagaagatcttctactccaggcccaacaaccacgacaactgttggctgaacgccatccttcagctgttcaggtacgtcgatgaacctttcttcgactgggtatatgaatcacctgaaaacctcacccttgaggcgatcagacaactggagaacattactggtcttgagctgcacgagggtggtccgcccgccctcgtcatttggaacatcaaacacttgctccacaccgggatcggcaccgcctcgcgacccagcgaggtgtgcatggtggacggtacggacatgtgcctggctgacttccacgctggcatcttcctgaaaggacaggaacacgccgtgtttgcctgcgtcacctccaacgggtggtacgcgatcgacgacgaagaattctacccctggacgccagatccgtccgacgtgctggtctttgtcccgtacgatcaa

>LC149669.1_O_JPN_2010

tacaacggtgagaagaagatcttctactccaggcccaacaaccacgacaactgttggctgaacgccatccttcagctgttcaggtacgtcgatgaacctttcttcgactgggtatatgaatcacctgaaaacctcacccttgaggcgatcagacaactggagaacattactggtcttgagctgcacgagggtggtccgcccgccctcgtcatttggaacatcaaacacttgctccacaccgggatcggcaccgcctcgcgacccagcgaggtgtgcatggtggacggtacggacatgtgcctggctgacttccacgctggcatcttcctgaaaggacaggaacacgccgtgtttgcctgcgtcacctccaacgggtggtacgcgatcgacgacgaagaattctacccctggacgccagatccgtccgacgtgctggtctttgtcccgtacgatcaa

>LC149670.1_O_JPN_2010

tacaacggtgagaagaagatcttctactccaggcccaacaaccacgacaactgttggctgaacgccatccttcagctgttcaggtacgtcgatgaacctttcttcgactgggtatatgaatcacctgaaaacctcacccttgaggcgatcagacaactggagaacattactggtcttgagctgcacgagggtggtccgcccgccctcgtcatttggaacatcaaacacttgctccacaccgggatcggcaccgcctcgcgacccagcgaggtgtgcatggtggacggtacggacatgtgcctggctgacttccacgctggcatcttcctgaaaggacaggaacacgccgtgtttgcctgcgtcacctccaacgggtggtacgcgatcgacgacgaagaattctacccctggacgccagatccgtccgacgtgctggtctttgtcccgtacgatcaa

>LC149671.1_O_JPN_2010

tacaacggtgagaagaagatcttctactccaggcccaacaaccacgacaactgttggctgaacgccatccttcagctgttcaggtacgtcgatgaacctttcttcgactgggtatatgaatcacctgaaaacctcacccttgaggcgatcagacaactggagaacattactggtcttgagctgcacgagggtggtccgcccgccctcgtcatttggaacatcaaacacttgctccacaccgggatcggcaccgcctcgcgacccagcgaggtgtgcatggtggacggtacggacatgtgcctggctgacttccacgctggcatcttcctgaaaggacaggaacacgccgtgtttgcctgcgtcacctccaacgggtggtacgcgatcgacgacgaagaattctacccctggacgccagatccgtccgacgtgctggtctttgtcccgtacgatcaa

>LC149672.1_O_JPN_2010

tacaacggtgagaagaagatcttctactccaggcccaacaaccacgacaactgttggctgaacgccatccttcagctgttcaggtacgtcgatgaacctttcttcgactgggtatatgaatcacctgaaaacctcacccttgaggcgatcagacaactggagaacattactggtcttgagctgcacgagggtggtccgcccgccctcgtcatttggaacatcaaacacttgctccacaccgggatcggcaccgcctcgcgacccagcgaggtgtgcatggtggacggtacggacatgtgcctggctgacttccacgctggcatcttcctgaaaggacaggaacacgccgtgtttgcctgcgtcacctccaacgggtggtacgcgatcgacgacgaagaattctacccctggacgccagatccgtccgacgtgctggtctttgtcccgtacgatcaa

>LC149673.1_O_JPN_2010

tacaacggtgagaagaagatcttctactccaggcccaacaaccacgacaactgttggctgaacgccatccttcagctgttcaggtacgtcgatgaacctttcttcgactgggtatatgaatcacctgaaaacctcacccttgaggcgatcagacaactggagaacattactggtcttgagctgcacgagggtggtccgcccgccctcgtcatttggaacatcaaacacttgctccacaccgggatcggcaccgcctcgcgacccagcgaggtgtgcatggtggacggtacggacatgtgcctggctgacttccacgctggcatcttcctgaaaggacaggaacacgccgtgtttgcctgcgtcacctccaacgggtggtacgcgatcgacgacgaagaattctacccctggacgccagatccgtccgacgtgctggtctttgtcccgtacgatcaa

>LC149674.1_O_JPN_2010

tacaacggtgagaagaagatcttctactccaggcccaacaaccacgacaactgttggctgaacgccatccttcagctgttcaggtacgtcgatgaacctttcttcgactgggtatatgaatcacctgaaaacctcacccttgaggcgatcagacaactggagaacattactggtcttgagctgcacgagggtggtccgcccgccctcgtcatttggaacatcaaacacttgctccacaccgggatcggcaccgcctcgcgacccagcgaggtgtgcatggtggacggtacggacatgtgcctggctgacttccacgctggcatcttcctgaaaggacaggaacacgccgtgtttgcctgcgtcacctccaacgggtggtacgcgatcgacgacgaagaattctacccctggacgccagatccgtccgacgtgctggtctttgtcccgtacgatcaa

>LC149675.1_O_JPN_2010

tacaacggtgagaagaagatcttctactccaggcccaacaaccacgacaactgttggctgaacgccatccttcagctgttcaggtacgtcgatgaacctttcttcgactgggtatatgaatcacctgaaaacctcacccttgaggcgatcagacaactggagaacattactggtcttgagctgcacgagggtggtccgcccgccctcgtcatttggaacatcaaacacttgctccacaccgggatcggcaccgcctcgcgacccagcgaggtgtgtatggtggacggtacggacatgtgcctggctgacttccacgctggcatcttcctgaaaggacaggaacacgccgtgtttgcctgcgtcacctccaacgggtggtacgcgatcgacgacgaagaattctacccctggacgccagatccgtccgacgtgctggtctttgtcccgtacgatcaa

>LC149676.1_O_JPN_2010

tacaacggtgagaagaagatcttctactccaggcccaacaaccacgacaactgttggctgaacgccatccttcagctgttcaggtacgtcgatgaacctttcttcgactgggtatatgaatcacctgaaaacctcacccttgaggcgatcagacaactggagaacattactggtcttgagctgcacgagggtggtccgcccgccctcgtcatttggaacatcaaacacttgctccacaccgggatcggcaccgcctcgcgacccagcgaggtgtgcatggtggacggtacggacatgtgcctggctgacttccacgctggcatcttcctgaaaggacaggaacacgccgtgtttgcctgcgtcacctccaacgggtggtacgcgatcgacgacgaagaattctacccctggacgccagatccgtccgacgtgctggtctttgtcccgtacgatcaa

>LC149677.1_O_JPN_2010

tacaacggtgagaagaagatcttctactccaggcccaacaaccacgacaactgttggctgaacgccatccttcagctgttcaggtacgtcgatgaacctttcttcgactgggtatatgaatcacctgaaaacctcacccttgaggcgatcagacaactggagaacattactggtcttgagctgcacgagggtggtccgcccgccctcgtcatttggaacatcaaacacttgctccacaccgggatcggcaccgcctcgcgacccagcgaggtgtgcatggtggacggtacggacatgtgcctggctgacttccacgctggcatcttcctgaaaggacaggaacacgccgtgtttgcctgcgtcacctccaacgggtggtacgcgatcgacgacgaagaattctacccctggacgccagatccgtccgacgtgctggtctttgtcccgtacgatcaa

>LC149678.1_O_JPN_2010

tacaacggtgagaagaagatcttctactccaggcccaacaaccacgacaactgttggctgaacgccatccttcagctgttcaggtacgtcgatgaacctttcttcgactgggtatatgaatcacctgaaaacctcacccttgaggcgatcagacaactggagaacattactggtcttgagctgcacgagggtggtccgcccgccctcgtcatttggaacatcaaacacttgctccacaccgggatcggcaccgcctcgcgacccagcgaggtgtgcatggtggacggtacggatatgtgcctggctgacttccacgctggcatcttcctgaaaggacaggaacacgccgtgtttgcctgcgtcacctccaacgggtggtacgcgatcgacgacgaagaattctacccctggacgccagatccgtccgacgtgctggtctttgtcccgtacgatcaa

>LC149679.1_O_JPN_2010

tacaacggtgagaagaagatcttctactccaggcccaacaaccacgacaactgttggctgaacgccatccttcagctgttcaggtacgtcgatgaacctttcttcgactgggtatatgaatcacctgaaaacctcacccttgaggcgatcagacaactggagaacattactggtcttgagctgaacgagggtggtccgcccgccctcgtcatttggaacatcaaacacttgctccacaccgggatcggcaccgcctcgcgacccagcgaggtgtgcatggtggacggtacggacatgtgcctggctgacttccacgctggcatcttcctgaaaggacaggaacacgccgtgtttgcctgcgtcacctccaacgggtggtacgcgatcgacgacgaagaattctacccctggacgccagatccgtccgacgtgctggtctttgtcccgtacgatcaa

>LC149680.1_O_JPN_2010

tacaacggtgagaagaagatcttctactccaggcccaacaaccacgacaactgttggctgaacgccatccttcagctgttcaggtacgtcgatgaacctttcttcgactgggtatatgaatcacctgaaaacctcacccttgaggcgatcagacaactggagaacattactggtcttgagctgcacgagggtggtccgcccgccctcgtcatttggaacatcaaacacttgctccacaccgggatcggcaccgcctcgcgacccagcgaggtgtgcatggtggacggtacggacatgtgcctggctgacttccacgctggcatcttcctgaaaggacaggaacacgccgtgtttgcctgcgtcacctccaacgggtggtacgcgatcgacgacgaagaattctacccctggacgccagatccgtccgacgtgctggtctttgtcccgtacgatcaa

>LC149681.1_O_JPN_2010

tacaacggtgagaagaagatcttctactccaggcccaacaaccacgacaactgttggctgaacgccatccttcagctgttcaggtacgtcgatgaacctttcttcgactgggtatatgaatcacctgaaaacctcacccttgaggcgatcagacaactggagaacattactggtcttgagctgcacgagggtggtccgcccgccctcgtcatttggaacatcaaacacttgctccacaccgggatcggcaccgcctcgcgacccagcgaggtgtgcatggtggacggtacggacatgtgcctggctgacttccacgctggcatcttcctgaaaggacaggaacacgccgtgtttgcctgcgtcacctccaacgggtggtacgcgatcgacgacgaagaattctacccctggacgccagatccgtccgacgtgctggtctttgtcccgtacgatcaa

>LC149682.1_O_JPN_2010

tacaacggtgagaagaagatcttctactccaggcccaacaaccacgacaactgttggctgaacgccatccttcagctgttcaggtacgtcgatgaacctttcttcgactgggtatatgaaycacctgaaaacctcacccttgaggcgatcagacaactggagaacattactggtcttgagctgcacgagggtggtccgcccgccctcgtcatttggaacatcaaacacttgctccacaccgggatcggcaccgcctcgcgacccagcgaggtgtgcatggtggacggtacggacatgtgcctggctgacttccacgctggcatcttcctgaaaggacaggaacacgccgtgtttgcctgcgtcacctccaacgggtggtacgcgatcgacgacgaagaattctacccctggacgccagatccgtccgacgtgctggtctttgtcccgtacgatcaa

>LC149683.1_O_JPN_2010

tacaacggtgagaagaagatcttctactccaggcccaacaaccacgacaactgttggctgaacgccatccttcagctgttcaggtacgtcgatgaacctttcttcgactgggtatatgaatcacctgaaaacctcacccttgaggcgatcagacaactggagaacattactggtcttgagctgcacgagggtggtccgcccgccctcgtcatttggaacatcaaacacttgctccacaccgggatcggcaccgcctcgcgacccagcgaggtgtgcatggtggacggtacggacatgtgcctggctgacttccacgctggcatcttcctgaaaggacaggaacacgccgtgtttgcctgcgtcacctccaacgggtggtacgcgatcgacgacgaagaattctacccctggacgccagatccgtccgacgtgctggtctttgtcccgtacgatcaa

>LC149684.1_O_JPN_2010

tacaacggtgagaagaagatcttctactccaggcccaacaaccacgacaactgttggctgaacgccatccttcagctgttcaggtacgtcgatgaacctttcttcgactgggtatatgaatcacctgaaaacctcacccttgaggcgatcagacaactggagaacattactggtcttgagctgcacgagggtggtccgcccgccctcgtcatttggaacatcaaacacttgctccacaccgggatcggcaccgcctcgcgacccagcgaggtgtgcatggtggacggtacggacatgtgcctggctgacttccacgctggcatcttcctgaaaggacaggaacacgccgtgtttgcctgcgtcacctccaacgggtggtacgcgatcgacgacgaagaattctacccctggacgccagatccgtccgacgtgctggtctttgtcccgtacgatcaa

>LC149685.1_O_JPN_2010

tacaacggtgagaagaagatcttctactccaggcccaacaaccacgacaactgttggctgaacgccatccttcagctattcaggtacgtcgatgaacctttcttcgactgggtatatgaatcacctgaaaacctcacccttgaggcgatcagacaactggagaacattactggtcttgagctgcacgagggtggtccgcccgccctcgtcatttggaacatcaaacacttgctccacaccgggatcggcaccgcctcgcgacccagcgaggtgtgcatggtggacggtacggacatgtgcctggctgacttccacgctggcatcttcctgaaaggacaggaacacgccgtgtttgcctgcgtcacctccaacgggtggtacgcgatcgacgacgaagaattctacccctggacgccagatccgtccgacgtgctggtctttgtcccgtacgatcaa

>LC149686.1_O_JPN_2010

tacaacggtgagaagaagatcttctactccaggcccaacaaccacgacaactgttggctgaacgccatccttcagctgttcaggtacgtcgatgaacctttcttcgactgggtatatgaatcacctgaaaacctcacccttgaggcgatcagacaactggagaacattactggtcttgagctgcacgagggtggtccgcccgccctcgtcatttggaacatcaaacacttgctccacaccgggatcggcaccgcctcgcgacccagcgaggtgtgcatggtggacggtacggacatgtgcctggctgacttccacgctggcatcttcctgaaaggacaggaacacgctgtgttcgcctgcgtcacctccaacgggtggtacgcgatcgacgacgaagaattctacccctggacgccagatccgtccgacgtgctggtctttgtcccgtacgatcaa

>LC149687.1_O_JPN_2010

tacaacggtgagaagaagatcttctactccaggcccaacaaccacgacaactgttggctgaacgccatccttcagctgttcaggtacgtcgatgaacctttcttcgactgggtatatgaatcacctgaaaacctcacccttgaggcgatcagacaactggagaacattactggtcttgagctgcacgagggtggtccgcccgccctcgtcatttggaacatcaaacacttgctccacaccgggatcggcaccgcctcgcgacccagcgaggtgtgcatggtggacggtacggacatgtgcctggctgacttccacgctggcatcttcctgaaaggacaggaacacgccgtgtttgcctgcgtcacctccaacgggtggtacgcgatcgacgacgaagaattctacccctggacgccagatccgtccgacgtgctggtctttgtcccgtacgatcaa

>LC149688.1_O_JPN_2010

tacaacggtgagaagaagatcttctactccaggcccaacaaccacgacaactgttggctgaacgccatccttcagctgttcaggtacatcgatgaacctttcttcgactgggtatatgaatcacctgaaaacctcacccttgaggcgatcagacaactggagaacattactggtcttgagctgcacgagggtggtccgcccgccctcgtcatttggaacatcaaacacttgctccacaccgggatcggcaccgcctcgcgacccagcgaggtgtgcatggtggacggtacggacatgtgcctggctgacttccacgctggcatcttcctgaaaggacaggaacacgccgtgtttgcctgcgtcacctccaacgggtggtacgcgatcgacgacgaagaattctacccctggacgccagatccgtccgacgtgctggtctttgtcccgtacgatcaa

>LC149689.1_O_JPN_2010

tacaacggtgagaagaagatcttctactccaggcccaacaaccacgacaactgttggctgaacgccatccttcagctgttcaggtacgtcgatgaacctttcttcgactgggtatatgaatcacctgaaaacctcacccttgaggcgatcagacaactggagaacattactggtcttgagctgcacgagggtggtccgcccgccctcgtcatttggaacatcaaacacttgctccacaccgggatcggcaccgcctcgcgacccagcgaggtgtgcatggtggacggtacggacatgtgcctggctgacttccacgctggcatcttcctgaaaggacaggaacacgctgtgtttgcctgcgtcacctccaacgggtggtacgcgatcgacgacgaagaattctacccctggacgccagatccgtccgacgtgctggtctttgtcccgtacgatcaa

>LC149690.1_O_JPN_2010

tacaacggtgagaagaagatcttctactccaggcccaacaaccacgacaactgttggctgaacgccatccttcagctgttcaggtacgtcgatgaacctttcttcgactgggtatatgaatcacctgaaaacctcacccttgaggcgatcagacaactggagaacattactggtcttgagctgcacgagggtggtccgcccgccctcgtcatttggaacatcaaacacttgctccacaccgggatcggcaccgcctcgcgacccagcgaggtgtgcatggtggacggtacggacatgtgcctggctgacttccacgctggcatcttcctgaaaggacaggaacacgccgtgtttgcctgcgtcacctccaacgggtggtacgcgatcgacgacgaagaattctacccctggacgccagatccgtccgacgtgctggtctttgtcccgtacgatcaa

>LC149691.1_O_JPN_2010

tacaacggtgagaagaagatcttctactccaggcccaacaaccacgacaactgttggctgaacgccatccttcagctgttcaggtacgtcgatgaacctttcttcgactgggtatatgaatcacctgaaaacctcacccttgaggcgatcagacaactggagaacattactggtcttgagctgcacgagggtggtccgcccgccctcgtcatttggaacatcaaacacttgctccacactgggatcggcaccgcctcgcgacccagcgaggtgtgcatggtggacggtacggacatgtgcctggctgacttccacgctggcatcttcctgaaaggacaggaacacgccgtgtttgcctgcgtcacctccaacgggtggtacgcgatcgacgacgaagaattctacccctggacgccagatccgtccgacgtgctggtctttgtcccgtacgatcaa

>LC149692.1_O_JPN_2010

tacaacggtgaaaagaagatcttctactccaggcccaacaaccacgacaactgttggctgaacgccatccttcagctgttcaggtacgtcgatgaacctttcttcgactgggtatatgaatcacctgaaaacctcacccttgaggcgatcagacaactggagaacattactggtcttgagctgcacgagggtggtccgcccgccctcgtcatttggaacatcaaacacttgctccacaccgggatcggcaccgcctcgcgacccagcgaggtgtgcatggtggacggtacggacatgtgcctggctgacttccacgctggcatcttcctgaaaggacaggaacacgccgtgtttgcctgcgtcacctccaacgggtggtacgcgatcgacgacgaagaattctacccctggacgccagatccgtccgacgtgctggtctttgtcccgtacgatcaa

>LC149693.1_O_JPN_2010

tacaacggtgagaagaagatcttctactccaggcccaacaaccacgacaactgttggctgaacgccatccttcagctgttcaggtacgtcgatgaacctttcttcgactgggtatatgaatcacctgaaaacctcacccttgaggcgatcagacaactggagaacattactggtcttgagctgcacgagggtggtccgcccgccctcgtcatttggaacatcaaacacttgctccacaccgggatcggcaccgcctcgcgacccagcgaggtgtgcatggtggacggtacggacatgtgcctggctgacttccacgctggcatcttcctgaaaggacaggaacacgccgtgtttgcctgcgtcacctccaacgggtggtacgcgatcgacgacgaagaattctacccctggacgccagatccgtccgacgtgctggtctttgtcccgtacgatcaa

>LC149694.1_O_JPN_2010

tacaacggtgagaagaagatcttctactccaggcccaacaaccacgacaactgttggctgaacgccatccttcagctgttcaggtacgtcgatgaacctttcttcgactgggtatatgaatcacctgaaaacctcacccttgaggcgatcagacaactggagaacattactggtcttgagctgcacgagggtggtccgcccgccctcgtcatttggaacatcaaacacttgctccacaccgggatcggcaccgcctcgcgacccagcgaggtgtgcatggtggacggtacggacatgtgcctggctgacttccacgctggcatcttcctgaaaggacaggaacacgccgtgtttgcctgcgtcacctccaacgggtggtacgcgatcgacgacgaagaattctacccctggacgccagatccgtccgacgtgctggtctttgtcccgtacgatcaa

>LC149695.1_O_JPN_2010

tacaacggtgagaagaagatcttctactccaggcccaacaaccacgacaactgttggctgaacgccatccttcagctgttcaggtacgtcgatgaacctttcttcgactgggtatatgaatcacctgaaaacctcacccttgaggcgatcagacaactggagaacattactggtcttgagctgcacgagggtggtccgcccgccctcgtcatttggaacatcaaacacttgctccacaccgggatcggcaccgcctcgcgacccagcgaggtgtgcatggtggacggtacggacatgtgcctggctgacttccacgctggcatcttcctgaaaggacaggaacacgccgtgtttgcctgcgtcacctccaacgggtggtacgcgatcgacgacgaagaattctacccctggacgccagatccgtccgacgtgctggtctttgtcccgtacgatcaa

>LC149696.1_O_JPN_2010

tacaacggtgagaagaagatcttctactccaggcccaacaaccacgacaactgttggctgaacgccatccttcagctgttcaggtacgtcgatgaacctttcttcgactgggtatatgaatcacctgaaaacctcacccttgaggcgatcagacaactggagaacattactggtcttgagctgcacgagggtggtccgcccgccctcgtcatttggaacatcaaacacttgctccacaccgggatcggcaccgcctcgcgacccagcgaggtgtgcatggtggacggtacggacatgtgcctggctgacttccacgctggcatcttcctgaaaggacaggaacacgccgtgtttgcctgcgtcacctccaacgggtggtacgcgatcgacgacgaagaattctacccctggacgccagatccgtccgacgtgctggtctttgtcccgtacgatcaa

>LC149697.1_O_JPN_2010

tacaacggtgagaagaagatcttctactccaggcccaacaaccacgacaactgttggctgaacgccatccttcagctgttcaggtacgtcgatgaacctttcttcgactgggtatatgaatcacctgaaaacctcacccttgaggcgatcagacaactggagaacattactggtcttgagctgcacgagggtggtccgcccgccctcgtcatttggaacatcaaacacttgctccacaccgggatcggcaccgcctcgcgacccagcgaggtgtgcatggtggacggtacggacatgtgcctggctgacttccacgctggcatcttcctgaaaggacaggaacacgccgtgtttgcctgcgtcacctccaacgggtggtacgcgatcgacgacgaagaattctacccctggacgccagatccgtccgacgtgctggtctttgtcccgtacgatcaa

>LC149698.1_O_JPN_2010

tacaacggtgagaagaagatcttctactccaggcccaacaaccacgacaactgttggctgaacgccatccttcagctgttcaggtacgtcgatgaacctttcttcgactgggtatatgaatcacctgaaaacctcacccttgaggcgatcagacaactggagaacattactggtcttgagctgcacgagggtggtccgcccgccctcgtcatttggaacatcaaacacttgctccacaccgggatcggcaccgcctcgcgacccagcgaggtgtgcatggtggacggtacggacatgtgcctggctgacttccacgctggcatcttcctgaaaggacaggaacacgccgtgtttgcctgcgtcacctccaacgggtggtacgcgatcgacgacgaagaattctacccctggacgccagatccgtccgacgtgctggtctttgtcccgtacgatcaa

>LC149699.1_O_JPN_2010

tacaacggtgagaagaagatcttctactccaggcccaacaaccacgacaactgttggctgaacgccatccttcagctgttcaggtacgtcgatgaacctttcttcgactgggtatatgaatcacctgaaaacctcacccttgaggcgatcagacaactggagaacattactggtcttgagctgcacgagggtggtccgcccgccctcgtcatttggaacatcaaacacttgctccacaccgggatcggcaccgcctcgcgacccagcgaggtgtgcatggtggacggtacggacatgtgcctggctgacttccacgctggcatcttcctgaaaggacaggaacacgccgtgtttgcctgcgtcacctccaacgggtggtacgcgatcgacgacgaagaattctacccctggacgccagatccgtccgacgtgctggtctttgtcccgtacgatcaa

>LC149700.1_O_JPN_2010

tacaacggtgagaagaagatcttctactccaggcccaacaaccacgacaactgttggctgaacgccatccttcagctgttcaggtacgtcgatgaacctttcttcgactgggtatatgaatcacctgaaaacctcacccttgaggcgatcagacaactggagaacattactggtcttgagctgcacgagggtggtccgcccgccctcgtcatttggaacatcaaacacttgctccacaccgggatcggcaccgcctcgcgacccagcgaggtgtgcatggtggacggtacggacatgtgcctggctgacttccacgctggcatcttcctgaaaggacaggaacacgccgtgtttgcctgcgtcacctccaacgggtggtacgcgatcgacgacgaagaattctacccctggacgccagatccgtccgacgtgctggtctttgtcccgtacgatcaa

>LC149701.1_O_JPN_2010

tacaacggtgagaagaagatcttctactccaggcccaacaaccacgacaactgttggctgaacgccatccttcagctgttcaggtacgtcgatgaacctttcttcgactgggtatatgaatcacctgaaaacctcacccttgaggcgatcagacaactggagaacattactggtcttgagctgcacgagggtggtccgcccgccctcgtcatttggaacatcaaacacttgctccacaccgggatcggcaccgcctcgcgacccagcgaggtgtgcatggtggacggtacggacatgtgcctggctgacttccacgctggcatcttcctgaaaggacaggaacacgccgtgtttgcctgcgtcacctccaacgggtggtacgcgatcgacgacgaagaattctacccctggacgccagatccgtccgacgtgctggtctttgtcccgtacgatcaa

>LC149702.1_O_JPN_2010

tacaacggtgagaagaagatcttctactccaggcccaacaaccacgacaactgttggctgaacgccatccttcagctgttcaggtacgtcgatgaacctttcttcgactgggtatatgaatcacctgaaaacctcacccttgaggcgatcagacaactggagaacattactggtcttgagctgcacgagggtggtccgcccgccctcgtcatttggaacatcaaacacttgctccacaccgggatcggcaccgcctcgcgacccagcgaggtgtgcatggtggacggtacggacatgtgcctggctgacttccacgctggcatcttcctgaaaggacaggaacacgccgtgtttgcctgcgtcacctccaacgggtggtacgcgatcgacgacgaagaattctacccctggacgccagatccgtccgacgtgytggtctttgtcccgtacgatcaa

>LC149703.1_O_JPN_2010

tacaacggtgagaagaagatcttctactccaggcccaacaaccacgacaactgttggctgaacgccatccttcagctgttcaggtacgtcgatgaacctttcttcgactgggtatatgaatcacctgaaaacctcacccttgaggcgatcagacaactggagaacgttactggtcttgagctgcacgagggtggtccgcccgccctcgtcatttggaacatcaaacacttgctccacaccgggatcggcaccgcctcgcgacccagcgaggtgtgcatggtggacggtacggacatgtgcctggctgacttccacgctggcatcttcctgaaaggacaggaacacgccgtgtttgcctgcgtcacctccaacgggtggtacgcgatcgacgacgaagaattctacccctggacgccagatccgtccgacgtgctggtctttgtcccgtacgatcaa

>LC149704.1_O_JPN_2010

tacaacggtgagaagaagatcttctactccaggcccaacaaccacgacaactgttggctgaacgccatccttcagctgttcaggtacgtcgatgaacctttcttcgactgggtatatgaatcacctgaaaacctcacccttgaggcgatcagacaactggaggacattactggtcttgagctgcacgagggtggtccgcccgccctcgtcatttggaacatcaaacacttgctccacaccgggatcggcaccgcctcgcgacccagcgaggtgtgcatggtggacggtacggacatgtgcctggctgacttccacgctggcatcttcctgaaaggacaggaacacgccgtgtttgcctgcgtcacctccaacgggtggtacgcgatcgacgacgaagaattctacccctggacgccagatccgtccgacgtgctggtctttgtcccgtacgatcaa

>LC149705.1_O_JPN_2010

tacaacggtgagaagaagatcttctactccaggcccaacaaccacgacaactgttggctgaacgccatccttcagctgttcaggtacgtcgacgaacctttcttcgactgggtatatgaatcacctgaaaacctcacccttgaggcgatcagacaactggagaacattactggtcttgagctgcacgagggtggtccgcccgccctcgtcatttggaacatcaaacacttgctccacaccgggatcggcaccgcctcgcgacccagcgaggtgtgcatggtggacggtacggacatgtgcctggctgacttccacgctggcatcttcctgaaaggacaggaacacgccgtgtttgcctgcgtcacctccaacgggtggtacgcgatcgacgacgaagaattctacccctggacgccagatccgtccgacgtgctggtctttgtcccgtacgatcaa

>LC149706.1_O_JPN_2010

tacaacggtgagaagaagatcttctactccaggcccaacaaccacgacaactgttggctgaacgccatccttcagctgttcaggtacgtcgatgaacctttcttcgactgggtatatgaatcacctgaaaacctcacccttgaggcgatcagacaactggagaacattactggtcttgagctgcacgagggtggtccgcccgccctcgtcatttggaacatcaaacacttgctccacaccgggatcggcaccgcctcgcgacccagcgaggtgtgcatggtggacggtacggacatgtgcctggctgacttccacgctggcatcttcctgaaaggacaggaacacgccgtgtttgcctgcgtcacctccaacgggtggtacgcgatcgacgacgaagaattctacccctggacgccagatccgtccgacgtgctggtctttgtcccgtacgatcaa

>LC149707.1_O_JPN_2010

tacaacggtgagaagaagatcttctactccaggcccaacaaccacgacaactgttggctgaacgccatccttcagctgttcaggtacgtcgatgaacctttcttcgactgggtatatgaatcacctgaaaacctcacccttgaggcgatcagacaactggagaacattactggtcttgagctgcacgagggtggtccgcccgccctcgtcatttggaacatcaaacacttgctccacaccgggatcggcaccgcctcgcgacccagcgaggtgtgcatggtggacggtacggacatgtgcctggctgacttccacgctggcatcttcctgaaaggacaggaacacgccgtgtttgcctgcgtcacctccaacgggtggtacgcgatcgacgacgaagaattctacccctggacgccagatccgtccgacgtgctggtctttgtcccgtacgaccaa

>LC149708.1_O_JPN_2010

tacaacggtgagaagaagatcttctactccaggcccaacaaccacgacaactgttggctgaacgccatccttcagctgttcaggtacgtcgatgaacctttcttcgactgggtatatgaatcacctgaaaacctcacccttgaggcgatcagacaactggagaacattactggtcttgagctgcacgagggtggtccgcccgccctcgtcatttggaacatcaaacacttgctccacaccgggatcggcaccgcctcgcgacccagcgaggtgtgcatggtggacggtacggacatgtgcctggctgacttccacgctggcatcttcctgaaaggacaggaacacgccgtgtttgcctgcgtcacctccaacgggtggtacgcgatcgacgacgaagaattctacccctggacgccagatccgtccgacgtgctggtctttgtcccgtacgatcaa

>LC149709.1_O_JPN_2010

tacaacggtgagaagaagattttctactccaggcccaacaaccacgacaactgttggctgaacgccatccttcagctgttcaggtacgtcgatgaacctttcttcgactgggtatatgaatcacctgaaaacctcacccttgaggcgatcagacaactggagaacattactggtcttgagctgcacgagggtggtccgcccgccctcgtcatttggaacatcaaacacttgctccacaccgggatcggcaccgcctcgcgacccagcgaggtgtgcatggtggacggtacggacatgtgcctggctgacttccacgctggcatcttcctgaaaggacaggaacacgccgtgtttgcctgcgtcacctccaacgggtggtacgcgatcgacgacgaagaattctacccctggacgccagatccgtccgacgtgctggtctttgtcccgtacgatcaa

>LC149710.1_O_JPN_2010

tacaacggtgagaagaagatcttctactccaggcccaacaaccacgacaactgttggctgaacgccatccttcagctgttcaggtacgtcgatgaacctttcttcgactgggtatatgaatcacctgaaaacctcacccttgaggcgatcagacaactggagaacattactggtcttgagctgcacgagggtggtccacccgccctcgtcatttggaacatcaaacacttgctccacaccgggatcggcaccgcctcgcgacccagcgaggtgtgcatggtggacggtacggacatgtgcctggctgacttccacgctggcatcttcctgaaaggacaggaacacgccgtgtttgcctgcgtcacctccaacgggtggtacgcgatcgacgacgaagaattctacccctggacgccagatccgtccgacgtgctggtctttgtcccgtacgatcaa

>LC149711.1_O_JPN_2010

tacaacggtgagaagaagatcttctactccaggcccaacaaccacgacaactgttggctgaacgccatccttcagctgttcaggtacgtcgatgaacctttcttcgactgggtatatgaatcacctgaaaacctcacccttgaggcgatcagacaactggagaacattactggtcttgagctgcacgagggtggtccgcccgccctcgtcatttggaacatcaaacacttgctccacaccgggatcggcaccgcctcgcgacccagcgaggtgtgcatggtggacggtacggacatgtgcctggctgacttccacgctggcatcttcctgaaaggacaggaacacgccgtgtttgcctgcgtcacctccaacgggtggtacgcgatcgacgacgaagaattctacccctggacgccagatccgtccgacgtgctggtctttgtcccgtacgatcaa

>LC149712.1_O_JPN_2010

tacaacggtgagaagaagatcttctactccaggcccaacaaccacgacaactgttggctgaacgccatccttcagctgttcaggtacgtcgatgaacctttcttcgactgggtatatgaatcacctgaaaacctcacccttgaggcgatcagacaactggagaacattactggtcttgagctgcacgagggtggtccgcccgccctcgtcatttggaacatcaaacacttgctccacaccgggatcggcaccgcctcgcgacccagcgaggtgtgcatggtggacggtacggacatgtgcctggctgacttccacgctggcatcttcctgaaaggacaggaacacgccgtgtttgcctgcgtcacctccaacgggtggtacgcgatcgacgacgaagaattctacccctggacgccagatccgtccgacgtgctggtctttgtcccgtacgatcaa

>LC149713.1_O_JPN_2010

tacaacggtgagaagaagatcttctactccaggcccaacaaccacgacaactgttggctgaacgccatccttcagctgttcaggtacgtcgatgaacctttcttcgactgggtatatgaatcacctgaaaacctcacccttgaggcgatcagacaactggagaacattactggtcttgagctgcacgagggtggtccgcccgccctcgtcatttggaacatcaaacacttgctccacaccgggatcggcaccgcctcgcgacccagcgaggtgtgcatggtggacggtacggacatgtgcctggctgacttccacgctggcatcttcctgaaaggacaggaacacgccgtgtttgcctgcgtcacctccaacgggtggtacgcgatcgacgacgaagaattctacccctggacgccagatccgtccgacgtgctggtctttgtcccgtacgatcaa

>LC149714.1_O_JPN_2010

tacaacggtgagaagaagatcttctactccaggcccaacaaccacgacaactgttggctgaacgccatccttcagctgttcaggtacgtcgatgaacctttcttcgactgggtatatgaatcacctgaaaacctcacccttgaggcgatcagacaactggagaacattactggtcttgagctgcacgagggtggtccgcccgccctcgtcatttggaacatcaaacacttgctccacaccgggatcggcaccgcctcgcgacccagcgaggtgtgcatggtggacggtacggacatgtgcctggctgacttccacgctggcatcttcctgaaaggacaggaacacgccgtgtttgcctgcgtcacctccaacgggtggtacgcgatcgacgacgaagaattctacccctggacgccagatccgtccgacgtgctggtctttgtcccgtacgatcaa

>LC149715.1_O_JPN_2010

tacaacggtgagaagaagatcttctactccaggcccaacaaccacgacaactgttggctgaacgccatccttcagctattcaggtacgtcgatgaacctttcttcgactgggtatatgaatcacctgaaaacctcacccttgaggcgatcagacaactggagaacattactggtcttgagctgcacgagggtggtccgcccgccctcgtcatttggaacatcaaacacttgctccacaccgggatcggcaccgcctcgcgacccagcgaggtgtgcatggtggacggtacggacatgtgcctggctgacttccacgctggcatcttcctgaaaggacaggaacacgccgtgtttgcctgcgtcacctccaacgggtggtacgcgatcgacgacgaagaattctacccctggacgccagatccgtccgacgtgctggtctttgtcccgtacgatcaa

>LC149716.1_O_JPN_2010

tacaacggtgagaagaagatcttctactccaggcccaacaaccacgacaactgttggctgaacgccatccttcagctgttcaggtacgtcgatgaacctttcttcgactgggtatatgaatcacctgaaaacctcacccttgaggcgatcagacaactggagaacattactggtcttgagctgcacgagggtggtccgcccgccctcgtcatttggaacatcaaacacttgctccacaccgggatcggcaccgcctcgcgacccagcgaggtgtgcatggtggacggtacggacatgtgcctggctgacttccacgctggcatcttcctgaaaggacaggaacacgccgtgtttgcctgcgtcacctccaacgggtggtacgcgatcgacgacgaagaattctacccctggacgccagatccgtccgacgtgctggtctttgtcccgtacgatcaa

>LC149717.1_O_JPN_2010

tacaacggtgagaagaagatcttctactccaggcccaacaaccacgacaactgttggctgaacgccatccttcagctgttcaggtacgtcgatgaacctttcttcgactgggtatatgaatcacctgaaaacctcacccttgaggcgatcagacaactggagaacattactggtcttgagctgcacgagggtggtccgcccgccctcgtcatttggaacatcaaacacttgctccacaccgggatcggcaccgcctcgcgacccagcgaggtgtgcatggtggacggtacggacatgtgcctggctgacttccacgctggcatcttcctgaaaggacaggaacacgccgtgtttgcctgcgtcacctccaacgggtggtacgcgatcgacgacgaagaattctacccctggacgccagatccgtccgacgtgttggtctttgtcccgtacgatcaa

>LC149718.1_O_JPN_2010

tacaacggtgagaagaagatcttctactccaggcccaacaaccacgacaactgttggctgaacgccatccttcagctgttcaggtacgtcgatgaacctttcttcgactgggtatatgaatcacctgaaaacctcacccttgaggcgatcagacaactggagaacattactggtcttgagctgcacgagggtggtccgcccgccctcgtcatttggaacatcaaacacttgctccacaccgggatcggcaccgcctcgcgacccagcgaggtgtgcatggtggacggtacggacatgtgcctggctgacttccacgctggcatcttcctgaaaggacaggaacacgccgtgtttgcctgcgtcacctccaacgggtggtacgcgatcgacgacgaagaattctacccctggacgccagatccgtccgacgtgctggtctttgtcccgtacgatcaa

>LC149719.1_O_JPN_2010

tacaacggtgagaagaagatcttctactccaggcccaacaaccacgacaactgttggctgaacgccatccttcagctgttcaggtacgtcgatgaacctttcttcgactgggtatatgaatcacctgaaaacctcacccttgaggcgatcagacaactggagaacattactggtcttgagctgcacgagggtggtccgcccgccctcgtcatttggaacatcaaacacttgctccacaccgggatcggcaccgcctcgcgacccagcgaggtgtgcatggtggacggtacggacatgtgcctggctgacttccacgctggcatcttcctgaaaggacaggaacacgccgtgtttgcctgcgtcacctccaacgggtggtacgcgatcgacgacgaagaattctacccctggacgccagatccgtccgacgtgctggtctttgtcccgtacgatcaa

>LC149720.1_O_JPN_2010

tacaacggtgagaagaagatcttctactccaggcccaacaaccacgacaactgttggctgaacgccatccttcagctgttcaggtacgtcgatgaacctttcttcgactgggtatatgaatcacctgaaaacctcacccttgaggcgatcagacaactggagaacattactggtcttgagctgcacgagggtggtccgcccgccctcgtcatttggaacatcaaacacttgctccacaccgggatcggcaccgcctcgcgacccagcgaggtgtgcatggtggacggtacggacatgtgcctggctgacttccacgctggcatcttcctgaaaggacaggaacacgccgtgtttgcctgcgtcacctccaacgggtggtacgcgatcgacgacgaagaattctacccctggacgccagatccgtccgacgtgctggtctttgtcccgtacgatcaa

>LC320038.1_O_MOG_2015

cacaacggtgagaaaaagactttctattctaggcccaacaaccacgacaattgttggttgaacaccatcctccaattgtttaggtacgtcgatgaacctttcttcgactgggtctatgaatcacctgaaaacctcactcttgaggcgattgggcaattggaagaactcactggtcttaagctgcacgagggtgggccacccgctctcgtcatttggaacatcaaacatttgctccacaccggaattggcactgcctcgcgacccagcgaggtgtgcatggtcgatggcacggacatgtgtttggctgacttccacgctggcatcttcctgaaagggcaagagcacgctgtgttcgcctgcgtcacctccaacgggtggtacgcgatcgacgacgaggacttctacccctggacgcctgatccgtccgacgttctggtgtttgtcccgtacgatcaa

>LC438822.1_O_MYA_2016

tacaacggtgagaaaaagactttctattctagacccaacaaccacgacaattgttggctgaacaccatcctccaattgtttaggtacgtcgatgaacctttcttcgactgggtctatgaatcacctgaaaacctcactcttgaggcgattggacaattggaagaactcactggtcttaagctgcacgagggtgggccacccgctctcgtcatttggaacatcaagcatttgctccacaccggaattggcactgcctcgcgacccagcgaggtgtgcatggttgatggcacggacatgtgtttggctgacttccacgctggcatcttcctgaaagggcaagagcacgctgtgttcgcctgcgtcacctccaacgggtggtacgcgatcgacgatgaggacttctacccctggacgcctgatccgtccgacgttctggtgtttgtcccgtacgatcaa

>LC438823.1_O_MYA_2016

cacaacggtgagaaaaagactttctattctaggcccaacaaccacgacaactgttggctgaacaccatcctccaattgtttaggtacgtcgatgaacctttcttcgactgggtctatgaatcacctgaaaacctcactcttgaggcgattgggcaattggaagaactcactggtcttaagctgcacgagggtgggccacccgctctcgtcatttggaacatcaagcatttgctccacaccggaattggcactgcctcgcgacccagcgaggtatgcatggttgatggcacggacatgtgtytggctgacttccacgctggcatcttcctgaaagggcaagagcacgctgtgttcgcctgcgtcacctccaacgggtggtacgcgatcgacgacgaggacttctacccctggacgcctgatccgtccgacgttctggtgtttgtcccgtacgatcaa

>MF143572.1_O_VIT_2012

cacaacggtgaaaagaagacattctactccaggcccaacaaccacgacaactgctggctgaacaccatcctccagttgtttaggtacgttgatgaaccctttttcgactgggtttacaactcgcctgagaacctcacacttgatgctattgagcaattggaagaaattacaggccttgaactccacgagggtggtccgcccgccctcgtcatctggaacatcaaacacctgctaaacaccggaatcggcaccgcttcgcgacccagcgaagtgtgcatggtagacgggacggacatgtgtttggctgacttccacgctggcattttcctgaaagggcaggaacacgctgtgttcgcctgtgttacctccaacgggtggtacgcgattgatgacgaggacttttacccctggacgccggacccgtccgacgttctggtgtttgttccgtacgatcaa

>MF143573.1_O_VIT_2012

cacaacggtgaaaagaagacattctactccaggcccaacaaccacgacaactgctggttgaacaccatcctccagttgtttaggtacgttgatgaacccttcttcggctgggtttacaactcgcctgagaacctcacacttgatgctattgagcaattggaagaaattacaggccttgaactccacgagggtggtccgcccgctctcgtcatctggaacatcaaacacctgctaaacaccggaatcggcaccgcttcgcgacccagcgaagtgtgcatggtagacgggacggacatgtgtttggctgacttccacgctggcattttcctgaaagggcaggaacacgctgtgttcgcctgtcttacctccaacgggtggtacgcgattgatgacgaggacttttacccctggacgccggacccgtccgacgttctggtgtttgttccgtacgatcaa

>MF143574.1_O_VIT_2012

cacaacggtgaaaagaagacattctactccaggcccaacaaccacgacaactgctggttgaacaccatcctccagttgtttaggtacgttgatgaacccttcttcgactgggtttacaactcgcctgagaacctcacacttgatgccattgagcaattggaagaaattacaggccttgaactccacgagggtggtccgcccgctctcgttatctggaacattaaacacctgctaaacaccggaatcggcaccgcttcgcgacccagcgaagtgtgcatggttgacgggacgaacatgtgtttggctgacttccacgctggcattttcctgaaagggcaggaacacgctgtgttcgcctgtgttacctccaacgggtggtacgcgattgatgacgaggacttttacccctggacgccggacccgtccgacgttctggtgtttgttccgtacgatcaa

>MF143575.1_O_VIT_2012

cacaacggtgaaaagaagacattctactccaggcccaacaaccacgacaactgctggctgaacaccatcctccagttgtttaggtacgttgatgaacccttcttcgactgggtttacaactcgcctgagaacctcacacttgacgccattgagcaactggaagaaattacaggccttgaactccacgagggtggtccgcccgctctcgttatctggaacattaaacacctgctcaacaccggaatcggcaccgcttcgcgacccagcgaagtgtgcatggtagacgggacgaacatgtgtttggctgacttccacgctggcattttcctgaaaggacaggaacatgctgtgttcgcctgtgttacctccaacgggtggtacgcgattgatgacgaggacttttacccctggacgccggacccgtccgacgttctggtgtttgttccgtacgatcaa

>MF143576.1_O_VIT_2013

cacaacggtgaaaagaagacattctactccaggcccaacaaccacgacaactgctggctgaacaccatcctccagttgtttaggtacgttgatgaacccttcttcgactgggtttacaactcgcctgagaacctcacacttgacgccattgagcaactggaagaaattacaggccttgaactccacgagggtggtccgcccgctctcgttatctggaacattaaacacctgctcaacaccggaatcggcaccgcttcgcgacccagcgaagtgtgcatggttgacgggacgaacatgtgtttggctgacttccacgctggcattttcctgaaaggacaggaacacgctgtgttcgcctgtgttacctccaacgggtggtacgcgattgatgacgaggacttttacccctggacgccggacccgtccgacgttctggtgtttgttccgtacgatcaa

>MF143577.1_O_VIT_2013

cacaacggtgaaaagaagacattctactccaggcccaacaaccacgacaactgctggctgaacaccatcctccagttgtttaggtacgttgatgaacccttcttcgactgggtttacaactcgcctgagaacctcacacttgacgccattgagcaactggaagaaattacaggccttgaactccacgagggtggtccgcccgctctcgttatctggaacattaaacacctgctcaacaccggaatcggcaccgcttcgcgacccagcgaagtgtgcatggttgacgggacgaacatgtgtttggctgacttccacgctggcattttcctgaaaggacaggaacacgctgtgttcgcctgtgtcacctccaacgggtggtacgcgattgatgacgaggacttttacccctggacgccggacccgtccgacgttctggtgtttgttccgtacgatcaa

>MF143578.1_O_VIT_2013

cacaacggtgaaaagaagacattctactccaggcccaacaaccacgacaactgctggctgaacaccatcctccagttgtttaggtacgttgatgaacccttcttcgactgggtttacaactcgcctgagaacctcacacttgacgccattgagcaactggaagaaattacaggccttgaactccacgagggtggtccgcccgctctcgttatctggaacattaaacacctgctcaacaccggaatcggcaccgcttcgcgacccagcgaagtgtgcatggttgacgggacgaacatgtgtttggctgacttccacgctggcattttcctgaaaggacaggaacacgctgtgttcgcctgtgtcacctccaacgggtggtacgcgattgatgacgaggacttttacccctggacgccggacccgtccgacgttctggtgtttgttccgtacgatcaa

>MF372125.1_Asia1_IND_2016

tacaacggtgagaaaaagactttctactctaggcccaacagccacgacaactgttggctgaacaccatcctacagttgttcaggtatgtcgatgaaccaatcttcgactgggtttacgaatcacctgtgaacctcacccttgaggcaatcaggcaattggaggaactcactggccttgaactgcacgagggtggcccacctgccctcgtcatctggaacatcaagcacctgctccacaccggaatcggtaccgcctcgcgacccagcgaggtgtgcatggttgacggtacggacatgtgtttggctgactttcacgctggcattttcctgaaaggacaagaacatgctgtgtttgcttgcgtcacctccaacgggtggtacgcgatcgacgacgaggacttttacccttggacgccggacccgtccgacgttctggtgtttgtcccttacgatcaa

>MF372126.1_Asia1_IND_1994

tacaacggtgagaaaaagaccttctactctaggcccaacaaccacgacaactgttggttgaacaccatcctccagttgttcaggtatgttgatgaacctttcttcgactgggtctacgagtcgcctgagaacctcactcttgaggcgattaggcagctggaggaagttactggtcttgaactacacgagggtggaccgcccgcccttgtcatttggaacattaaacacttgctccacaccggaatcggcactgcttcgcgacccagcgaggtgtgtatggttgacggaacggacatgtgtttggctgacttccacgctggcattttcttgaaaggacaagagcatgctgtgtttgcttgtgtcacctccaacgggtggtacgcgatcgacgacgaggacttctacccttggacgccggacccgtccgacgttctggtgtttgtcccttatgatcaa

>MF461724.1_O_CHA_2017

cacaacggtgagaaaaagactttctattctaggcccaacaaccacgacaattgttggttgaacaccatcctccaattgtttaggtacgtcgatgaacctttcttcgactgggtctatgaatcacctgaaaacctcactcttgaggcgattgggcaattggaagaactcactggtcttaagctgcacgagggtgggccacccgctctcgtcatttggaacatcaagcatttgctccacaccggaattggcactgcctcgcgacccagcgaggtgtgcatggtcgatggcacggacatgtgtttggctgacttccacgctggcatcttcctgaaagggcaagagcacgctgtgttcgcctgcgtcacctccaacgggtggtacgcgatcgacgacgaggacttctacccctggacgcctgatccgtccgacgttctggtgtttgtcccgtacgatcaa

>MF678823.1_SAT1_NIG_2015

ttcaacggtaagaagaagaccttctacagcagaccaaacacgcacgacaactgctggttgaacgcaatcttgcaactcttccgatacgttgacgagccgctgtttgaaagtgagtaccttgcacccgagaacctcacgggtcgcatgattgagcaactcatggaacacactggcatggacttacgcgagggcggaccacctgctctcgtcatttgggccatcaaggacatactgatgaccggtgtcggcacatcaactcgccccagcgagatttgtgtcgtcaatggtgttcagatgtgcttggccgacttccacgccggtatcttcctcaaaggtactgaacatgctgtcttcgcactgctcacaagtgagggctggtacgcgatagacgacgagcacttctatccctggactcccaacccagacgatgtgcttgcctacgtcccgtacacgccc

>MF678824.1_SAT1_NIG_2015

ttcaacggtaagaagaagaccttctacagcagaccaaacacgcacgacaactgctggttgaacgcaatcttgcaactcttccgatacgttgacgagccgctgtttgaaagtgagtaccttgcacccgagaacctcacgggtcgcatgattgagcaactcatggaacacactggcatggacttacgcgagggcggaccacctgctctcgtcatttgggccatcaaggacatactgatgaccggtgtcggcacatcaactcgccccagcgagatttgtgtcgtcaatggtgttcagatgtgcttggccgacttccacgccggtatcttcctcaaaggtgctgaacatgctgtcttcgcactgctcacaagtgagggctggtacgcgatagacgacgagcacttctatccctggactcccaacccagacgatgtgcttgcctacgtcccgtacacgccc

>MF678825.1_SAT1_NIG_2015

ttcaacggtgagaagaagaccttctacagcagaccaaacacgcacgacaactgctggttgaacgcaatcttgcaactcttccgatacgttgacgagccgctgtttgaaagtgagtaccttgcacccgagaacctcacgggtcgcatgattgagcaactcatggaacacactggcatggacttacgcgagggcggaccacctgctctcgtcatttgggccatcaaggacatactgatgaccggtgtcggcacatcaacccgccccagcgagatttgtgtcgttaatggtgttcagatgtgcttggccgacttccacgccggtatcttcctcaaaggtactgaacatgctgtcttcgcactgctcacaagtgagggctggtacgcgatagacgacgagcacttctatccctggactcccaacccagacgatgtgcttgcctacgtcccgtacacgccc

>MF678826.1_SAT1_NIG_2015

ttcaacggtgagaagaagaccttctacagcagaccaaacacgcacgacaactgctggttgaacgcaatcttgcaactcttccgatacgttgacgagccgctgtttgaaagtgagtaccttgcacccgagaacctcacgggtcgcatgattgagcaactcatggaacacactggcatggacttacgcgagggcggaccacctgctctcgtcatttgggccatcaaggacatactgatgaccggtgtcggcacatcaacccgccccagcgagatttgtgtcgttaatggtgttcagatgtgcttggccgacttccacgccggtatcttcctcaaaggtactgaacatgctgtcttcgcactgctcacaagtgagggctggtacgcgatagacgacgagcacttctatccctggactcccaacccagacgatgtgcttgcctacgtcccgtacacgccc

>MF782478.1_Asia1_BAN_2013

tacaacggtgagaaaaagatcttttactctaggcccaacaaccatgataactgctggttgaacaccatcctgcagttgttcaggtatgtcgacgagccattcttcgactgggtttacgagtcgcccgagaatctcaccctcgaggcaatcatgcaattggaggaacttactggccttgaactacacgagggagggccacctgccctcgtcatctggaacatcaagcacttgctccacactgggatcggcaccgcctcgcgacccagcgaggtgtgcatggtcgatggtacggacatgtgtttggcagactttcacgctggcattttcctgaaaggacaagaacacgctgtgtttgcttgcatcacctccaacgggtggtacgcgattgatgatgaagacttttacccctggacgcctgacccgtctgacgttctggtatttgtcccttacgatcaa

>MF947123.1_O_VIT_2011

cacaacggtgaaaagaagacattctactccaggcccaacaaccacgacaactgctggctgaacaccatcctccagttgtttaggtacgttgatgaacccttcttcgactgggtttacaactcgcctgagaacctcacacttgacgctattgagcaattggaagaaattacaggccttgaactccacgagggtggtccgcccgctctcgttatctggaacattaaacacctgctcaacaccggaatcggcaccgcttcgcgacccagcgaagtgtgcatggtagacgggacggacatgtgtttggctgacttccacgctggcattttcctgaaagggcaggagcacgctgtgttcgcctgtgttacctccaacgggtggtacgcgattgatgacgaggacttttacccctggacgccggacccgtccgacgttctggtgtttgttccgtacgatcaa

>MF947124.1_O_VIT_2012

cacaacggtgaaaagaagacattctactccaggcccaacaaccacgacaactgctggttgaacaccatcctccagttgtttaggtacgttgatgaacccttcttcgactgggtttacaactcgcctgagaacctcacacttgacgctattgagcagttggaagaatttacaggccttgaactccacgagggtggtccgcccgctctcgttatctggaacattaaacacctgctcaacaccggaatcggcaccgcttcgcgacccagcgaagtgtgcatggtagacgggacggacatgtgtttggctgacttccacgctggcattttcctgaaagggcaggagcacgctgtgttcgcctgtgttacctccaacgggtggtacgcgattgatgacgaggacttttacccctggacgccagacccgtccgacgttctggtgtttgttccgtatgatcaa

>MF947126.1_O_VIT_2011

cacaacggtgaaaagaagacattctactccaggcccaacaaccacgacaactgctggctgaacaccatcctccagttgtttaggtacgttgatgaacccttcttcgactgggtttacaactcgcctgagaacctcacacttgacgctattgagcaattggaagaaattacaggccttgaactccacgagggtggtccgcccgctctcgttatctggaacattaaacacctgctcaacaccggaatcggcaccgcttcgcgacccagcgaagtgtgtatggtagacgggacggacatgtgtttggctgactttcacgctggcattttcctgaaagggcaggagcacgctgtgttcgcctgtgttacctccaacgggtggtacgcgattgatgacgaggacttttacccctggacgccggacccgtccgacgttctggtgtttgttccgtacgatcaa

>MF947127.1_O_VIT_2012

cacaacggtgaaaagaagacattctactccaggcccaacaaccacgacaactgctggctgaacacgatcctccagttgtttaggtacgttgatgaacccttcttcgactgggtttacaactcgcctgagaacctcacacttgacgctattgagcaattggaagaaattacaggccttgaactccacgagggtggtccgcccgctctcgttatctggaacattaaacacctgctcaacaccggaatcggcaccgcttcgcgacccagcgaagtgtgcatggtagacgggacggacatgtgtttggctgacttccacgctggcattttcctgaaaggacaggagcacgctgtgttcgcctgtgttacctccaatgggtggtatgcgattgatgacgaagacttttacccctggacgccggacccgtctgacgttctggtgtttgttccgtatgatcaa

>MF947128.1_O_VIT_2010

cacaacggtgaaaagaagacattctactccaggcccaacaaccacgacaactgctggctgaacaccatcctccagttgtttaggtacgttgatgaacccttcttcgactgggtttacaactcgcctgagaacctcacacttgacgctattgagcaattggaagaaattacaggccttgaactccacgagggtggtccgcccgctctcgttatctggaacattaaacacctgctcaacaccggaatcggcaccgcttcgcgacccagcgaagtgtgcatggtagacgggacggacatgtgtttggctgacttccacgctggcattttcctgaaagggcaggagcacgctgtgttcgcctgtgttacctccaacgggtggtacgcgattgatgacgaggacttttacccctggacgccggacccgtccgacgttctggtgtttgttccgtacgatcaa

>MF947129.1_O_VIT_2014

cacaacggtgaaaagaagacattctactccaggctcaacaaccacgacaactgctggttgaacgccatcctccagttgtttaggtacgttgatgaacccttcttcgactgggtttacaactcgcctgagaacctcacacttgacgctattgagcagttggaagaaattacaggtcttgaactccacgagggtggtccgcccgctctcgtcatctggaacattaaacacctgctcaacaccggaatcggcaccgcttcgcgacccagcgaagtgtgcttggtagacgggacggacatgtgtttggctgatttccacgctggcattttcctgaaagggcaggaacacgctgtgttcgcctgtgttacctccaacgggtggtacgcgattgatgacgaggacttttacccctggacgccagacccgtccgacgttctagtgtttgttccgtatgatcaa

>MF947130.1_O_VIT_2014

tacaacggtgagaggaagaccttttattcaaggcccaacaataacgacaactgttggctgaatgccatcctgcagttgttcaggtacgtcgatgaacctttcttcgactgggtctatgaatcgcctgagaaccgcactcttgaggcaatcgaacaactagagggaattactggtcttgagctacacgagggcggtccacctgctctcgtgatttggaacatcaaacacttgctccacaccgggatcggcaccgcctcgcgacccagcgaggtgtgcatggttgacggtacggacatgtgcctggctgacttccacgctggcatcttcctgaaaggacaggagcacgctgtgtttgcctgtgtcacctccaatgggtggtacgcgattgatgacgaggacttttacccctggacgccggatccgtccgacgtgctggtgtttgtcccgtacgatcaa

>MF947131.1_O_VIT_2013

cacaacggtgaaaagaagacattctactccaggcccaacaaccacgacaactgctggctgaacaccatcctccagttgtttaggtacgttgatgaacccttctttgactgggtttacaactcgcctgagaacctcacacttgatgctattgagcaattggaagaaattacaggccttgaactccacgagggtggtccgcccgccctcgtcatctggaacattaaacacctgctcaacaccggaatcggcaccgcttcgcgacccagcgaagtgtgcatggtagacgggacggacatgtgtttggctgacttccacgctggcattttcctgaaaggacaggaacacgctgtgttcgcctgtgttacctccaacgggtggtacgcgattgatgacgaggacttttacccctggacgccggacccgtccgacgttctggtgtttgttccgtacgatcaa

>MF947132.1_O_VIT_2015

cacaacggtgaaaagaagatattctactccaggcccaacaaccacgacaactgctggctgaacaccatcctccagttgttcagatacgttgatgagcccttctttgactgggtttacaactcgcctgagaacctcacacttgatgctattgagcaattggaagaaattacaggtcttgaactccgcgagggtggcccacccgccctcgtcatctggaacattaaacacctgctcaacaccggaatcggcaccgcttcgcgccccagcgaagtgtgcatggtagacggaacggacatgtgtttggctgacttccacgctggcattttcctgaaaggacaggaacacgctgtattcgcctgtgtcacctccaacgggtggtacgcgattgatgatgaggacttttacccctggacaccggacccgtccgacgttctggtgtttgttccatacgatcag

>MF947137.1_O_VIT_2012

cacaacggtgaaaagaagacattctactccaggcccaacaaccacgacaactgctggctgaacacgatcctccagttgtttaggtacgttgatgaacccttcttcgactgggtttacaactcgcctgagaacctcacacttgacgctattgagcaattggaagaaattacaggccttgaactccacgagggtggtccgcccgctctcgttatctggaacattaaacacctgctcaacaccggaatcggcaccgcttcgcgacccagcgaagtgtgcatggtagacgggacggacatgtgtttggctgacttccacgctggcattttcctgaaaggacaggagcacgctgtgttcgcctgtgttacctccaatgggtggtatgcgattgatgacgaagacttttacccctggacgccggacccgtctgacgttctggtgtttgttccgtatgatcaa

>MF947141.1_O_VIT_2012

cacaacggtgaaaagaagacattctactccagacccaacaaccacgacaactgctggctgaacaccatcctccagttgtttaggtacgttgatgaacccttcttcgactgggtttacaactcgcctgagaacctcacacttgacgctattgagcaattggaagaaattacaggccttgaactccacgagggtggtccgcccgctctcgttatctggaacatcaaacacctgctcaacaccggaatcggcaccgcttcgcggcccagcgaagtgtgcatggtagacgggacggacatgtgtttggctgacttccacgctggcattttcctgaaagggcaggagcacgctgtgttcgcctgtgttacctccaacgggtggtacgcgattgacgacgaggacttttacccctggacgccggacccgtccgacgttctggtgtttgttccgtacgatcaa

>MF947142.1_O_VIT_2013

cacaacggtgaaaagaagacattctactccaggcccaacaaccacgacaactgctggctgaacaccatcctccagttgtttaggtacgttgatgaacccttctttgactgggtttacaactcacctgagaacctcacacttgatgctattgagcaattggaagaaattacaggccttgaactccacgagggtggtccgcccgccctcgtcatctggaacattaaacacctgctcaacaccggaatcggcaccgcttcgcgacccagcgaagtgtgcatggtagacgggacggacatgtgtttggctgacttccacgctggcattttcctgaaaggacaggaacacgctgtgttcgcctgtgttacctccaacgggtggtacgcgattgatgacgaggacttttacccctggacgccggacccgtccgacgttctggtgtttgttccgtacgatcaa

>MF947143.1_O_VIT_2013

cacaacggtgaaaagaagacattctactccaggcccaacaaccacgacaactgctggctgaacaccatcctccagttgtttaggtacgttgatgaacccttctttgactgggtttacaactcacctgagaacctcacacttgatgctattgagcaattggaagaaattacaggccttgaactccacgagggtggtccgcccgccctcgtcatctggaacattaaacacctgctcaacaccggaatcggcaccgcttcgcgacccagcgaagtgtgcatggtagacgggacggacatgtgtttggctgacttccacgctggcattttcctgaaaggacaggaacacgctgtgttcgcctgtgttacctccaacgggtggtacgcgattgatgacgaggacttttacccctggacgccggacccgtccgacgttctggtgtttgttccgtacgatcaa

>MG372730.1_O_SKR_2000

cacaacggtgagaagaaaacattctactccaggcccaacaaccacgacaactgctggctgaacaccatcctccagttgtttaggtacgttgatgaacctttcttcgactgggtctactactcacctgagaacctcacgcttgatgctatcaaacaattggaagaaattactggtctcgaactccacgagggtggaccacccgctctcgttatttggaacattaaacacctgctcaacaccggaatcggcaccgcttcgcgacccagcgaagtgtgcatggtagacgggacggacatgtgtttggctgacttccacgctggcatcttcctgaaaggacaggaacacgctgtgttcgcctgcgtcacctccaacgggtggtacgcgattgacgatgaggacttttacccctggacgccggacccgtccgacgttctggtgtttgtcccgtacgatcaa

>MG725872.1_A_NIG_2013

cataacggtgagaagaagactttttactcaaggcccaaccgccacgataactgttggttgaataccattctacagttgttcaggtatgtcgacgaaccattcttcgactgggtctacaactcacccgagaacctcacgcttcaagcgattgaacaacttgaggcgattacaggccttgaactgcacaacggcggtcctcccgcccttgtgatctggaacatcagacacttgttgtacactgggatcggcaccgcctcgcgacccagcgaggtgtgcatggttgatggtaccgacatgtgtcttgctgactttcatgcaggaatctttcttaaaggtactgaacacgccgtgttcgcctgtttgacctccaacgggtggtacgccattgacgacgaggacttctacccatggactccggacccgtccgacgtcctgtgttttgtcccgtacgacatg

>MG725873.1_A_NIG_2015

cacaacggtgagaaaaagaccttttactcaagacccaaccgccacgacaactgctggttgaacaccatcctacagttgttcaggtacgtcgacgaaccattctttgactgggtctacaactcacctgaaaacctcacgcttcaagcgattgagcaacttgaggcgatcacaggccttgaactgcacgatggtggccctcccgccctcgtgatctggaacatcaggcacttgttgtacactgggatcggcactgcctcgcgacccagcgaggtgtgtatggttgatggtaccgacatgtgtcttgctgatttccacgcaggaatcttcctcaaaggtactgaacacgccgtgttcgcctgcttgacctccaacgggtggtacgccattgacgacgaggacttttacccatggactccggacccgtctgatgtcctgtgttttgtcccgtacgacatg

>MG725874.1_A_NIG_2015

cacaacggtgagaagaagactttctactcaagacccaaccgccacgataactgttggttgaacaccatcctacagttgttcaggtatgtcgacgaaccattcttcgactgggtctacaactcacccgagaacctcacactccaagccattgaacagctcgaggcagttacaggcctcgaactgcacaacggtggtccccccgcccttgtgatctggaacatcaggcacttgttgtacactgggatcggcactgcttcgcgacccagcgaggtgtgcatggttgatggtaccgacatgtgtcttgctgacttccatgcaggaatcttcctcaaaggtactgaacacgccgtgttcgcctgtttgacctccaacgggtggtacgccattgacgacgaagacttttacccatggaccccggatccgtccgacgtcctgtgttttgtcccgtacgacatg

>MG725875.1_A_NIG_2015

cacaacggtgagaaaaagaccttttactcaagacccaaccgccacgacaactgctggttgaacaccatcctacagttgttcaggtacgtcgacgaaccattctttgactgggtctacaactcacctgaaaacctcacgcttcaagcgattgagcaacttgaggcgatcacaggccttgaactgcacgatggtggccctcccgccctcgtgatctggaacatcaggcacttgttgtacactgggatcggcactgcctcgcgacccagcgaggtgtgtatggttgatgggaccgacatgtgtcttgctgatttccacgcaggaattttcctcaaaggtactgaacacgctgtgttcgcctgcttgacctccaacgggtggtacgccattgacgacgaagacttttacccatggactccggacccgtctgatgtcctgtgttttgtcccgtacgacatg

>MG725876.1_A_NIG_2015

cacaacggtgagaaaaagaccttttactcaagacccaaccgccacgacaactgctggttgaacaccatcctacagttgttcaggtacgtcgacgaaccattctttgactgggtctacaactcacctgaaaacctcacgcttcaagcgattgagcaacttgaggcgatcacaggccttgaactgcacgatggtggccctcccgccctcgtgatctggaacatcaggcacttgttgtacactgggatcggcactgcctcgcgacccagcgaggtgtgtatggttgatgggaccgacatgtgtcttgctgatttccacgcaggaattttcctcaaaggtactgaacacgctgtgttcgcctgcttgacctccaacgggtggtacgccattgacgacgaggacttttacccatggactccggacccgtctgatgtcctgtgttttgtcccgtacgacatg

>MG913340.1_A_ALG_2017

cacaacggtgagaaaaagaccttttactcaagacccaaccgccacgacaactgctggttgaacaccatcctgcagctgttcaggtacgtcgacgaaccattctttgactgggtctacaactcacctgaaaaccttacgcttcaagcgattgagcaacttgaggcgatcacaggcctcgaactgcacgatggtggccctcccgccctcgtgatctggaacatcaggcacttgttgtacactgggatcggcactgcctcgcgacccagcgaggtgtgcatggttgatggtactgacatgtgtcttgctgacttccacgcaggaattttcctcaagggtactgaacacgccgtgttcgcctgcttgacctccaacggttggtacgccattgacgacgaggacttttacccatggactccggacccgtctgatgtcctgtgttttgtcccgtacgacatg

>MG923579.1_A_ALG_2017

cacaacggtgagaaaaagaccttttactcaagacccaaccgccacgacaactgctggttgaacaccatcctgcagctgttcaggtacgtcgacgaaccattctttgactgggtctacaactcacctgaaaaccttacgcttcaagcgattgagcaacttgaggcgatcacaggcctcgaactgcacgatggtggccctcccgccctcgtgatctggaacatcaggcacttgttgtacactgggatcggcactgcctcgcgacccagcgaggtgtgcatggttgatggtactgacatgtgtcttgctgacttccacgcaggaattttcctcaagggtactgaacacgccgtgttcgcctgcttgacctccaacggttggtacgccattgacgacgaggacttttacccatggactccggacccgtctgatgtcctgtgttttgtcccgtacgacatg

>MG923580.1_A_ALG_2017

cacaacggtgagaaaaagaccttttactcaagacccaaccgccacgacaactgctggttgaacaccatcctgcagctgttcaggtacgtcgacgaaccattctttgactgggtctacaactcacctgaaaaccttacgcttcaagcgattgagcaacttgaggcgatcacaggcctcgaactgcacgatggtggccctcctgccctcgtggtctggaacatcaggcacttgttgtacactgggatcggcactgcctcgcgacccagcgaggtgtgcatggttgatggtactgacatgtgtcttgctgacttccacgcaggaattttcctcaagggtactgaacacgccgtgttcgcctgcttgacctccaacggttggtacgccattgacgacgaggacttttacccatggactccggacccgtctgatgtcctgtgttttgtcccgtacgacatg

>MG983683.1_O_ALG_2014

cacaacggtgagaaaaagaccttctactctaggcccaacaaccacgacaactgttggttaaacaccatccttcagttgtttaggtacgtcgacgaacctttcttcgactgggtctatgaatcgcctgaaaacctcactcttgaggcgattaggcaactggaagagatcactggccttgagctgcacgagggtggaccgcccgctctcgtcatctggaacatcaaacacttgctccacaccggaatcggcactgcttcgcgacccagcgaggtgtgcatggttgatggcacagacatgtgtttggccgacttccacgctggcatcttcctgaaagggcaagaacacgctgtgttcgcctgcgtcacctccaacgggtggtacgcgatcgacgacgaggacgtctacccctggacgccggacccgtccgacgttctggtgtttgtcccgtacgatcaa

>MG983684.1_O_BAN_2009

tacaacggtgagaaaaagaccttctattctaggcccaacaaccacgacaactgttggttgaacaccatcctccagttgtttaggtacgtcgacgaacctttcttcgactgggtctatgaatcgcctgaaaacctcacttttgaggcgattaggcaactagaagaaattactggtcttgagctgcacgagggtggaccgcccgctctcgtcatctggaacatcaaacatttgctccacaccggaattggcactgcttcgcgacccagcgaggtgtgcatggttgatggcacggacatgtgtttggccgacttccacgctggcatcttcctgaaaggacaagaacacgctgtgttcgcctgcgtcacctccaacggatggtacgcgatcgacgacgaggatttctacccctggacgccggacccgtccgacgttctggtgtttgtcccgtacgatcaa

>MG983685.1_O_BAR_2015

tacaacggtgagaaaaagaccttctactctaggcccaacaaccacgacaactgttggttgaacaccatccttcagttgtttaggtatgtcgacgaaccttttttcgactgggtctatgaatcgcctgaaaaccgcactcttgaggcgattaggcaactggaagaaatcactggtcttgagctgcacgagggtggaccgcccgctctcgtcatctggaacattaaacacttgctccacaccggaatcggcactgcatcgcgacccagcgaggtgtgcatggttgatggcacggacatgtgtttggctgacttccacgctggcatcttcctgaaagggcaagaacatgctgtgttcgcctgcgtcacctctaacgggtggtatgcgattgacgacgaggacttctacccctggacgccggacccgtccgacgttctggtgtttgtcccgtacgatcaa

>MG983686.1_O_BAR_2015

tacaacggtgagaaaaagaccttctactctaggcccaacaaccacgacaactgttggttgaacactatccttcagttgtttaggtatgtcgacgaacctttcttcgactgggtctatgaatcgcctgaaaacctcactcttgaggcgatcaggcaactggaagagatcactggtcttgagctgcacgagggtggaccgcccgctctcgtcatctggaacattaaacacttgctccacaccggaatcggcaccgcttcgcgacctagcgaggtgtgcatggttgatggcacagacatgtgtttggctgacttccacgctggcatcttcctgaaagggcaagaacacgctgtgttcgcctgtgtcacctccgacgggtggtatgcgattgacgacgaggacttctacccctggacgccggacccgtccgacgttctggtgtttgttccgtacgatcaa

>MG983687.1_O_BHU_2009

tacaacggtgagaaaaagaccttttattctaggcccaacaaccacgacaactgttggttgaacaccatcctccagttgtttaggtacgtcgacgaacctttcttcgactgggtctatgaatcgcctgaaaacctcactcttgaggcgattaggcaactagaagaaattactggtcttgagctgcacgagggtggaccgcccgctctcgtcatctggaacatcaaacatttgctccacaccggaatcggcactgcttcgcgacccagcgaggtgtgcatggttgatggcacggacatgtgtttggccgacttccacgctggcatcttcctgaaagggcaagaacacgctgtgttcgcctgcgtcacctccaacggatggtacgcgatcgacgacgaggatttctacccctggacgccggacccgtccgacgttctggtgtttgtcccgtacgatcaa

>MG983688.1_O_BHU_2009

tacaacggtgagaaaaagaccttctattctaggcccaacaaccacgacaactgttggttgaacaccatcctccagttgtttaggtacgtcgacgaacctttcttcgactgggtctatgaatcgcctgaaaacctcactcttgaggcgattagacaactagaagaaattactggtcttgagctgcacgagggtggaccgcccgctctcgtcatctggaacatcaaacatttgctccacaccggaatcggcactgcttcgcgacccagcgaggtgtgcatggttgatggcacggacatgtgtttggccgacttccacgctggcatcttcctgaaagggcaagaacacgctgtgttcgcctgcgtcacctccaacggatggtacgcgatcgacgacgaggatttctacccctggacgccggacccgtccgacgttctggtgtttgtcccgtacgatcaa

>MG983689.1_O_BHU_2012

cacaacggtgagaaaaagactttctattctaggcccaacaaccacgacaactgctggttgaacaccatcctccagttgtttaggtacgtcgatgaacctttcttcgactgggtctatgaatcgcctgaaaacctcactcttgaggcgattaggcaattagaagaaatcactggtcttgagctgcacgaaggtggaccgcccgctctcgtcatttggaacatcaagcatttgctccacaccggaatcggcactgcttcgcgacccagcgaggtgtgcatggttgatggcacggacatgtgtttggctgacttccacgctggcatcttcctaaaagggcaagagcacgctgtgttcgcctgcgtcacctccaacgggtggtacgcgatcgacgacgaggacttctacccctggacgccggatccgtccgacgtcctggtgtttgttccgtacgatcaa

>MG983690.1_O_BHU_2016

tacaacggtgagaaaaagaccttctactctaggcccaacaaccacgacaactgttggttgaacactatccttcagttgtttaggtatgtcgacgaacctttcttcgactgggtctatgaatcgcctgaaaacctcactcttgaggcgattaggcaactggaagagattactggtcttgagctgcacgagggtggaccgcccgctctcgtcatctggaacattaaacacttgctccacaccggaatcggcaccgcttcgcgacctagcgaggtgtgcatggttgatggcacggacatgtgtttggctgacttccacgcaggcatcttcctgaaagggcaggaacacgctgtgttcgcctgtgtcacctccgacgggtggtatgcgattgacgacgaggacttctacccctggacgccggacccgtccgacgttctggtgtttgttccgtacgatcaa

>MG983691.1_O_BHU_2016

cacaacggtgagaaaaagactttctattctaggcccaacaaccacgacaattgctggctgaacaccatcctccagttgtttaggtacgtcgatgaacctttcttcgactgggtctatgagtcgcctgaaaacctcactcttgaggcgattaggcaattggaagaactcactggtcttgagctgcacgagggtgggccacccgctctcgtcatttggaacatcaagcatttgctccacaccggaatcggcactgcttcgcgacccagcgaggtgtgcatggttgatggcacggacatgtgtttggctgacttccacgctggcatcttcctgaaagggcaagagcacgctgtgttcgcctgcgtcacctccaacgggtggtacgcgatcgacgacgaggacttctacccctggacgccggatccgtccgacgttctggtgtttgtcccgtacgatcaa

>MG983692.1_O_IRN_2009

tacaacggtgagaaaaagaccttctattctaggcccaacaaccacgacaactgttggttgaacaccatcctccagttgtttaggtacgtcgacgaacctttcttcgactgggtctatgaatcgcctgaaaacctcactcttgaggcgattaggcaactagaaggaattactggtcttgagctgcatgagggtggaccgcccgctctcgtcatctggaacatcaaacatttgctccacaccggaatcggcactgcttcgcgccccagcgaggtgtgcatggttgatggcacggacatgtgtttggccgacttccacgctggcatcttcctgaaagggcaagaacacgctgtgttcgcctgcgtcacctccaacggatggtacgcgatcgacgacgaggatttctacccctggacgccggacccgtccgacgttctggtgtttgtcccgtacgatcaa

>MG983693.1_O_LAO_2015

tacaacggtgagaaaaagaccttctactctaggcccaacaaccacgacaactgttggttgaacaccatccttcagttgtttaggtatgtcgacgaacctttcttcgactgggtctatgaatcgcctgaaaacctcactcttgaggcgattaagcaactggaagagatcactggtctggagttgcacgagggtggaccgcccgctctcgtcatctggaacattaaacacttgctccacaccggaatcggcactgcttcgcgacccagcgaggtttgcatggttgatggcacggacatgtgtttggctgacttccacgctggcatcttcctgaaagggcaagaacacgctgtgttcgcctgcgtcacctccaacgggtggtacgcgattgacgacgaggacttctacccctggacgccggacccgtccgacgttctggtgtttgtcccgtacgatcaa

>MG983694.1_O_LIB_2013

cacaacggtgagaaaaagaccttctactctaggcccaacaaccacgacaactgttggttaaacaccatccttcagttgtttaggtacgtcgacgaacctttcttcgactgggtctatgaatcgcctgaaaacctcactcttgaggcgattaggcaactggaagagatcactggccttgagctgcacgagggtggaccgcccgctctcgtcatctggaacatcaaacacttgctccacaccggaatcggcactgcttcgcgacccagtgaggtgtgcatggttgatggtacagacatgtgtttggccgacttccacgctggcatcttcctgaaagggcaagaacacgctgtgttcgcctgcgtcacctccaacgggtggtacgcgatcgacgacgaggacttctacccctggacgccggacccgtccgacgttctggtgtttgtcccgtacgatcaa

>MG983695.1_O_LIB_2013

cacaacggtgagaaaaagaccttctactctaggcccaacaaccacgacaactgttggttaaacaccatccttcagttgtttaggtacgtcgacgaacctttcttcgactgggtctatgaatcgcctgaaaacctcactcttgaggcaattaggcaactggaagagatcactggccttgagctgcacgagggtggaccgcccgctctcgtcatctggaacatcaaacacttgctccacaccggaatcggcactgcttcgcgacccagcgaggtgtgcatggttgatggtacagacatgtgtttggccgacttccacgctggcatcttcctgaaagggcaagaacacgctgtgttcgcctgcgtcacctccaacgggtggtacgcgatcgacgacgaggacttctacccctggacgccggacccgtccgacgttctggtgtttgtcccgtacgatcaa

>MG983696.1_O_LIB_2013

cacaacggtgagaaaaagaccttctactctaggcccaacaaccacgacaactgttggttaaataccatccttcagttgtttaggtacgtcgacgaacctttcttcgactgggtctatgaatcgcctgaaaacctcactcttgaggcgattaggcaactggaagagatcactggccttgagctgcacgagggtggaccgcccgctctcgtcatctggaacatcaaacacttgctccacaccggaatcggcactgcttcgcgacccagcgaggtgtgcatggttgatggtacagacatgtgtttggccgacttccacgctggcatcttcctgaaagggcaagaacacgctgtgttcgcctgcgtcacctccaacgggtggtacgcgatcgacgacgaggacttctacccctggacgccggacccgtccgacgttctggtgtttgtcccgtacgatcaa

>MG983697.1_O_LIB_2013

cacaatggtgagaaaaagaccttctactctaggcccaacaaccacgacaactgttggttaaacaccatccttcagttgtttaggtacgtcgacgaacctttcttcgactgggtctatgaatcgcctgaaaacctcactcttgaggcgattaggcaactggaagagatcactggccttgagctgcacgagggtggaccgcccgctctcgtcatctggaacatcaaacacttgctccacaccggaatcggcactgcttcgcgacccagcgaggtgtgcatggttgatggtacggacatgtgtttggccgacttccacgctggcatcttcctgaaagggcaagaacacgctgtgttcgcctgcgtcacctccaacgggtggtacgcgatcgacgacgaggacttctacccctggacgccggacccgtccgacgttctggtgtttgtcccgtacgaccaa

>MG983698.1_O_MUR_2016

cacaacggtgagaaaaagactttctattctaggcccaacaaccacgacaattgctggctgaacaccatcctccagttgtttaggtatgtcgatgaacctttcttcgactgggtctatgaatcgcctgaaaacctcactcttgaggcgattaggcaattggaagaactcactggtcttgagctgcacgagggtgggccacccgctctcgttatttggaacatcaagcatttgctccacaccggaatcggcactgcttcgcgacccagcgaggtgtgcatggttgacggcacggacatgtgtttggctgacttccacgctggcatcttcctgaaagggcaagagcacgctgtgttcgcctgcgtcacctccaacgggtggtacgcgattgacgacgaggacttctacccctggacgccggatccgtccgacgttctggtgtttgtcccgtacgatcaa

>MG983699.1_O_MUR_2016

cacaacggtgagaaaaagactttctattctaggcccaacaaccacgacaattgctggctgaacaccatcctccagttgtttaggtatgtcgatgaacctttcttcgactgggtctatgaatcgcctgaaaacctcactcttgaggcgattaggcaattggaagaactcactggtcttgagctgcacgagggtgggccacccgctctcgttatttggaacatcaagcatttgctccacaccggaatcggcactgcttcgcgacccagcgaggtgtgcatggttgacggcacggacatgtgtttggctgacttccacgctggcatcttcctgaaagggcaagagcacgctgtgttcgcctgcgtcacctccaacgggtggtacgcgattgacgacgaggacttctacccctggacgccggatccgtccgacgttctggtgtttgtcccgtacgatcaa

>MG983700.1_O_MUR_2016

cacaacggtgagaaaaagactttctattctaggcccaacaaccacgacaattgctggctgaacaccatcctccagttgtttaggtatgtcgatgaacctttcttcgactgggtctatgaatcgcctgaaaacctcactcttgaggcgattaggcaattggaagaactcactggtcttgagctgcacgagggtgggccacccgctctcgttatttggaacatcaagcatttgctccacaccggaatcggcactgcttcgcgacccagcgaggtgtgcatggttgacggcacggacatgtgtttggctgacttccacgctggcatcttcctgaaagggcaagagcacgctgtgttcgcctgcgtcacctccaacgggtggtacgcgattgacgacgaggacttctacccctggacgccggatccgtccgacgttctggtgtttgtcccgtacgatcaa

>MG983701.1_O_MUR_2016

cacaacggtgagaaaaagactttctattctaggcccaacaaccacgacaattgctggctgaacaccatcctccagttgtttaggtatgtcgatgaacctttcttcgactgggtctatgaatcgcctgaaaacctcactcttgaggcgattaggcaattggaagaactcactggtcttgagctgcacgagggtgggccacccgctctcgttatttggaacatcaagcatttgctccacaccggaatcggcactgcttcgcgacccagcgaggtgtgcatggttgacggcacggacatgtgtttggctgacttccacgctggcatcttcctgaaagggcaagagcacgctgtgttcgcctgcgtcacctccaacgggtggtacgcgattgacgacgaggacttctacccctggacgccggatccgtccgacgttctggtgtttgtcccgtacgatcaa

>MG983702.1_O_MUR_2016

cacaacggtgagaaaaagactttctattctaggcccaacaaccacgacaattgttggctgaacaccatcctccagttgtttaggtatgtcgatgaacctttcttcgactgggtctatgaatcgcctgaaaacctcactcttgaggcgattaggcaattggaagaactcactggtcttgagctgcacgagggtgggccacccgctctcgttatttggaacatcaagcatttgctccacaccggaatcggcactgcttcgcgacccagcgaggtgtgcatggttgacggcacggacatgtgtttggctgacttccacgctggcatcttcctgaaagggcaagagcacgctgtgttcgcctgcgtcacctccaacgggtggtacgcgattgacgacgaggacttctacccctggacgccggatccgtccgacgttctggtgtttgtcccgtacgatcaa

>MG983703.1_O_MYA_2016

cacaacggtgagaaaaagactttctattctaggcccaacaaccacgacaactgttggctgaacaccatcctccagttgtttaggtacgtcgatgagcctttcttcgactgggtctatgaatcgcctgaaaacctcactcttgaggcgattaggcaattggaagaactcactggtcttgagctgcacgagggtgggccacccgctctcgtcatttggaacatcaagcatttgctccacaccggaatcggtactgcttcgcgacccagcgaggtgtgcatggttgatggcacggacatgtgtttggctgacttccacgctggcatcttcctgaaagggcaagagcacgctgtgttcgcctgcgtcacctccaacgggtggtacgcgatcgacgacgaggacttctacccctggacgccggatccgtccgacgttctggtgtttgtcccgtacgatcaa

>MG983704.1_O_NEP_2008

tacaacggtgagaaaaagaccttctattctaggcccaacaaccacgacaactgttggttgaacaccatcctccagttgtttaggtacgtcgacgaacctttcttcgactgggtctatgaatcgcctgaaaacctcacttttgaggcgattaggcaactagaagaaattactggtcttgagctgcacgagggtggaccgcccgctctcgtcatttggaacatcaagcatttgctccacaccggaatcggcactgcttcgcgacccagcgaggtgtgcatggttgatggcacggacatgtgtttggccgacttccacgctggcatcttcctgaaagggcaagaacacgctgtgttcgcctgcgtcacctccaacgggtggtacgcgatcgacgacgaggacttctacccctggacgccggacccgtccgacgttctggtgtttgtcccgtacgatcaa

>MG983705.1_O_NEP_2010

tacaacggtgagaaaaagaccttctattctaggcccaacaaccacgacaactgttggttgaacactatcctccagttgtttaggtacgtcgacgaacctttcttcgactgggtctatgaatcgcctgaaaacctcactcttgaggcgattagacaactagaagaaattactggtcttgagctgcacgagggtggaccgcccgctctcgtcatctggaacatcaagcatttgctccacaccggaatcggcactgcttcgcgacccagcgaggtgtgcatggttgatggcacggacatgtgtttggccgacttccacgctggcatcttcctgaaagggcaagaacatgctgtgttcgcctgcgtcacctccaacggatggtacgcgatcgacgacgaggatttctacccctggacgccggacccgtccgacgttctggtgtttgtcccgtacgatcaa

>MG983706.1_O_NEP_2010

tacaacggtgagaaaaagaccttctattctaggcccaacaaccacgacaactgttggttgaacaccatcctccagttgttcaggtacgtcgacgaacctttcttcgactgggtctatgaatcgcctgaaaacctcactcttgaggcgattaggcaactagaagaaattactggtcttgagctgcatgagggtggaccgcccgctctcgtcatctggaacatcaaacatttgctccacaccggaatcggcactgcttcgcgacccagcgaggtgtgcatggttgatggcacggacatgtgtttggccgacttccacgccggcatcttcctgaaaggacaagaacacgctgtgttcgcctgcgtcacctccaacggatggtacgcgatcgacgacgaggatttctacccctggacgccggacccgtccgacgttcttgtgtttgtcccgtacgatcaa

>MG983707.1_O_NEP_2012

cacaacggtgagagaaagactttctattctaggcccaacaaccacgacaactgctggttgaacaccatcctccagttgtttaggtacgtcgatgaacctttcttcgactgggtctatgaatcgcctgaaaacctcactcttgaggcgattaggcaattagaagaaatcactggtcttgagctgcacgagggtggaccgcccgctctcgtcatttggaacatcaaacatttgctccacaccggaatcggcactgcttcgcgacccagcgaggtgtgcatggttgatggcacggacatgtgtttggccgacttccacgctggcatcttcctaaaagggcaagagcacgctgtgttcgcctgcgtcacctccaacgggtggtacgcgatcgacgacgaagacttctacccctggacgccggatccgtccgacgttctggtgtttgttccgtacgatcaa

>MG983708.1_O_NEP_2012

tacaacggtgagaaaaagaccttctactctaggcccaacaaccacgacaactgttggttgaacaccatccttcagttgtttaggtacgtcgacgaacctttcttcgactgggtttatgaatcgcctgaaaacctcactcttgaggcgattaggcaactggaagagaccactggtcttgagctgcacgagggtggaccgcccgctctcgtcatctggaacattaaacacttgctccacaccggaatcggcactgcttcgcgacctagcgaggtgtgcatggttgatggcacggacatgtgtttggccgacttccacgctggcatcttcctgaaagggcaagaacacgctgtgttcgcctgcgtcacctccaacgggtggtacgcgatcgacgacgaggacttctacccctggacgccggacccgtccgacgttctggtgtttgtcccgtacgatcaa

>MG983709.1_O_NEP_2012

tacaacggtgagaaaaagaccttctactctaggcccaacaaccacgacaactgttggttgaacaccatccttcagttgtttaggtacgtcgacgaacctttcttcgactgggtttatgaatcgcctgaaaacctcactcttgaggcgattaggcaactggaagagatcactggtcttgagctgcacgagggtggaccgcccgctctcgtcatctggaacattaaacacttgctccacaccggaatcggcactgcttcgcgacctagcgaggtgtgcatggttgatggcacggacatgtgtttggccgacttccacgctggcatcttcctgaaagggcaagaacacgctgtgttcgcctgcgtcacctccaacgggtggtacgcgatcgacgacgaggacttctacccctggacgccggacccgtccgacgttctggtgtttgtcccgtacgatcaa

>MG983710.1_O_NEP_2012

cacaacggtgagaaaaagactttctattctaggcccaacagacacgacaactgctggttgaacaccatcctccagttgtttaggtacgtcgatgaacctttcttcgactgggtttatgagtcgcctgaaaacctcactcttgaggcgattaggcaattagaagagatcactggtcttgagctgcacgagggtggaccgcccgctctcgtcatttggaacatcaagcatttgctccacaccggaatcggcactgcttcgcgacccagcgaggtgtgcatggttgatggcacggacatgtgtttggctgacttccacgctggcatcttcctaaaagggcaagagcacgctgtgttcgcctgcgtcacctccaacgggtggtacgcgatcgacgacgaggacttctacccctggacgccggatccgtccgacgttctggtgtttgttccgtacgatcaa

>MG983711.1_O_NEP_2013

tacaacggtgagaaaaagaccttctactctaggcccaacacccacgacaactgttggttgaacaccatccttcagttgtttaggtatgtcgacgaaccttttttcgactgggtctatgaatcgcctgaaaacctcactcttgaggcgattaggcaactggaaaagatcactggtcttgagctgcacgagggtggaccgcccgctctcgtcatctggaacattaaacacttgctccacaccggaatcggcactgcttcgcgacccagcgaggtgtgcatggttgatggcacggacatgtgtttggccgatttccacgctggcatcttcctgaaagggcaggaacacgctgtgttcgcctgcgtcacctccaacgggtggtatgcgattgacgacgaggacttctacccctggacgccggacccgtccgacgttctggtgtttgtcccgtacgatcaa

>MG983712.1_O_NEP_2013

tacaacggtgagaaaaagaccttctactctaggcccaacaaccacgacaactgttggttgaacaccatccttcagttgtttaggtacgtcgacgaacctttcttcgactgggtttatgaatcgcctgaaaacctcactcttgaggcgattaggcaactggaagagatcactggtcttgagctgcacgagggcggaccgcccgctctcgtcatctggaacattaaacacttgctccacaccggaatcggcactgcttcgcgacccagcgaggtgtgcatggttgatggcacggacatgtgtttggccgacttccacgctggcatcttcctgaaagggcaagaacacgctgtgttcgcctgcgtcacctccaacgggtggtacgcgattgacgacgaggacttctacccctggacgccggacccgtccgacgttctggtgtttgtcccgtacgatcaa

>MG983713.1_O_NEP_2013

tacaacggtgagaaaaagaccttctactctaggcccaacaaccacgacaactgttggttgaacaccatccttcagttgtttaggtacgtcgacgaacctttcttcgactgggtttatgaatcgcctgaaaacctcactcttgaggcgattaggcaactggaagagatcactggtcttgagctgcacgagggcggaccgcccgctctcgtcatctggaacattaaacacttgctccacaccggaatcggcactgcttcgcgacccagcgaggtgtgcatggttgatggcacggacatgtgtttggccgacttccacgctggcatcttcctgaaagggcaagaacacgctgtgttcgcctgcgtcacctccaacgggtggtacgcgattgacgacgaggacttctacccctggacgccggacccgtccgacgttctggtgtttgtcccgtacgatcaa

>MG983714.1_O_NEP_2013

tacaacggtgagaaaaagaccttctactctaggcccaacaaccacgacaactgttggttgaacaccatccttcagttgtttaggtatgtcgacgaacctttctttgactgggtctatgaatcgcctgaaaacctcactctcgaggcgattaggcaactggaagagatcactggtcttgagctgcacgagggtggaccgcccgctctcgtcatctggaacattaaacacttgctccacaccggaatcggcactgcttcgcgacccagcgaggtgtgcatggttgatggcacggacatgtgtttggctgacttccacgctggcatcttcctgaaagggcaagaacacgctgtgttcgcctgcgtcacctccaacgggtggtatgcgattgacgacgaggacttctacccctggacgccggacccgtccgacgttctggtgtttgtcccgtacgaccaa

>MG983715.1_O_NEP_2014

tacaacggtgagaaaaagaccttctactctaggcccaacaaccacgacaactgttggttgaacaccatccttcagttgtttaggtatgtcgacgaaccttttttcgactgggtctatgaatcgcctgaaaacctcactcttgaggcgattaggcgactggaagagatcactggtcttgagctgcacgagggtggaccgcccgctctcgtcatctggaacattaaacacttgctccacaccggaatcggcactgcttcgcgacccagcgaggtgtgcatggttgatggcacggacatgtgtttggctgacttccacgctggcatcttcctgaaagggcaagaacacgctgtgttcgcctgcgtcacctccaacgggtggtatgcgattgacgacgaggacttctacccctggacgccggacccgtccgacgttctggtgtttgtcccgtacgatcaa

>MG983716.1_O_NEP_2014

tacaacggtgagaaaaagaccttctactctaggcccaacaaccacgacaactgttggttgaacaccatccttcagttgtttaggtatgtcgacgaaccttttttcgactgggtctatgaatcgcctgaaaacctcactcttgaggcgattaggcaactggaagagatcactggtcttgagctgcacgagggtggaccgcccgctctcgtcatctggaacattaaacacttgctccacaccggaatcggcactgcttcgcgacccagcgaggtgtgcatggttgatggcacggacatgtgtttggctgacttccacgctggcatcttcctgaaaggacaagaacacgctgtgttcgcctgcgtcacctccaacgggtggtatgcgattgacgacgaggacttctacccctggacgccggacccgtccgacgttctggtgtttgtcccgtacgatcaa

>MG983717.1_O_NEP_2015

tacaacggtgagaaaaagaccttctactctaggcccaacaatcacgacaactgttggttgaacaccatccttcagttgtttaggtacgtcgacgaaccttttttcgactgggtctatgaatcgcctgaaaaccacactcttgaggcgattaggcaactggaagagatcactggtcttgagctgcacgagggtggaccgcccgctctcgtcatttggaacattaaacacttgctccacaccggaatcggcactgcctcgcgacccagcgaggtgtgcatggttgatggcacggacatgtgtttggccgacttccacgctggcatcttcctgaaagggcaagaacacgctgtgttcgcctgcgtcacctccaacgggtggtacgcgatcgacgacgaggacttctacccctggacgccagacccgtccgacgttctggtgtttgtcccgtacgatcaa

>MG983718.1_O_NEP_2015

tacaacggtgagaaaaagactttctattctaggcccaacaaccacgacaattgctggctgaacaccatcctccagttgtttaggtacgtcgatgaacctttcttcgactgggtctatgaatcgcctgaaaacctcactcttgaggcgattaggcaattggaagaactcactggtcttgagctgcacgagggtgggccacccgctctcgtcatttggaacatcaagcatttgctccacaccggaatcggcactgcttcgcgacccagcgaggtgtgcatggttgatggcacggacatgtgtttggctgacttccacgctggcatcttcctgaaagggcaagagcacgctgtgttcgcctgcgtcacctccaacgggtggtacgcgatcgacgacgaggacttctacccctggacgccagatccgtccgacgttctggtgtttgtcccgtacgatcaa

>MG983719.1_O_NEP_2015

cacaacggtgagaaaaagactttctattctaggcccaacaaccacgacaattgctggctgaacaccatcctccagttgtttaggtacgtcgatgaacctttcttcgactgggtttatgaatcgcctgaaaacctcactcttgaggcgattaggcaattggaagaactcactggtcttgagctgcacgagggtgggccacccgctctcgtcatttggaacatcaaacatttgctccacaccggaatcggcactgcttcgcgacccagcgaggtgtgcatggttgatggcacggacatgtgtttggctgacttccacgccggcatcttcctgaaagggcaagagcacgctgtgttcgcctgcgtcacctccaacgggtggtacgcgatcgacgacgaggacttctacccctggacaccggatccgtccaacgttctggtgtttgtcccgtacgatcaa

>MG983720.1_O_RUS_2016

cacaacggtgagaaaaagactttctattctaggcccaacaaccacgacaattgttggttgaacaccatcctccaattgtttaggtacgtcgatgaacctttcttcgactgggtctatgaatcacctgaaaacctcactcttgaggcgattgggcaattggaagaactcactggtcttaagctgcacgagggtgggccacccgctctcgtcatttggaacatcaagcatttgctccacaccggaattggcactgcctcgcgacccagcgaggtgtgcatggtcgatggcacggacatgtgtttggctgacttccacgctggcatcttcctgaaagggcaagagcacgctgtgttcgcctgcgtcacctccaacgggtggtacgcgatcgacgacgaggacttctacccctggacgcctgatccgtccgacgttctggtgtttgtcccgtacgatcaa

>MG983721.1_O_SAU_2013

cacaacggtgagaaaaagaccttctactctaggcccaataaccacgacaactgttggttgaacaccatccttcagttgttcaggtacgtcgacgaacctttcttcgactgggtctatgaatcgcctgaaaacctcactcttgaggcgattaggcaattggaagagatcactggccttgagctgcacgagggtggaccgcccgctctcgtcatctggaacatcaaacacttgctccacaccggaatcggcactgcttcgcgacccagcgaggtgtgcatggttgatggcacagacatgtgtttggccgacttccacgctggtatcttcctgaaagggcaagaacacgctgtgttcgcctgcgtcacctccaacgggtggtacgcgatcgacgacgaggacttctacccctggacgccggacccgtccgacgttctggtgtttgtcccgtacgatcaa

>MG983722.1_O_SAU_2013

cacaacggtgagaaaaagaccttctactctaggcccaataaccacgacaactgttggttgaacaccatccttcagttgttcaggtacgtcgacgaacctttcttcgactgggtctatgaatcgcctgaaaacctcactcttgaggcgattaggcaattggaagagatcactggccttgagctgcacgagggtggaccgcccgctctcgtcatctggaacatcaaacacttgctccacaccggaatcggcactgcttcgcgacccagcgaggtgtgcatggttgatggcacagacatgtgtttggccgacttccacgctggtatcttcctgaaagggcaagaacacgctgtgttcgcctgcgtcacctccaacgggtggtacgcgatcgacgacgaggacttctacccctggacgccggacccgtccgacgttctggtgtttgtcccgtacgatcaa

>MG983723.1_O_SAU_2013

cacaacggtgagaaaaagaccttttactctaggcccaacaaccacgacaactgttggctgaacaccatccttcagttgtttaggtacgtcgacgaacctttcttcgactgggtctatgaatcgcctgaaaacctcacttttgaggcgattaggcaactggaagagatcactggccttgagctgcacgagggtggaccgcccgctcttgtcatctggaacatcaaacacttgctccacaccggaatcggcactgcttcgcgacccagcgaggtgtgcatggttgatggcacagacatgtgtttggccgacttccacgctggtatcttcctgaaagggcaagaacacgctgtgttcgcctgcgtcacctccaacgggtggtacgcgatcgacgacgaggacttctacccctggacgccggacccgtccgacgttctggtgtttgtcccgtacgatcaa

>MG983724.1_O_SAU_2013

cacaacggtgagaaaaagaccttttactctaggcccaacaaccacgacaactgttggctgaacaccatccttcagttgtttaggtacgtcgacgaacctttcttcgactgggtctatgaatcgcctgaaaacctcacttttgaggcgattaggcaactggaagagatcactggccttgagctgcacgagggtggaccgcccgctcttgtcatctggaacatcaaacacttgctccacaccggaatcggcactgcttcgcgacccagcgaggtgtgcatggttgatggcacagacatgtgtttggccgacttccacgctggtatcttcctgaaagggcaagaacacgctgtgttcgcctgcgtcacctccaacgggtggtacgcgatcgacgacgaggacttctacccctggacgccggacccgtccgacgttctggtgtttgtcccgtacgatcaa

>MG983725.1_O_SAU_2014

cacaacggtgagaaaaagaccttctactctaggcccaacaaccacgacaactgttggttgaacaccatccttcagttgtttaggtacgtcgacgaacctttcttcgactgggtctatgaatcacctgaaaacctcactcttgaggcgattaggcaactggaagagatcactggccttgagctgcacgagggtggaccgcccgctctcgtcatctggaacatcaaacacttgctccacaccggaatcggcactgcttcgcgacctagcgaggtgtgcatggttgatggcactgacatgtgtttggccgacttccacgctggtatcttcctgaaagggcaagaacacgctgtgttcgcctgcgtcacctccaacgggtggtacgcgatcgacgacgaggacttctacccctggacgccggacccgtccgacgttctggtgtttgtcccgtacgatcaa

>MG983726.1_O_SAU_2014

cacaacggtgagaaaaagaccttctactctaggcccaacaaccacgacaactgttggttgaacaccatccttcagttgtttaggtacgtcgacgaacctttcttcgactgggtctatgaatcgcctgaaaacctcactcttgaggcgattaggcaactggaagagatcactggccttgagctgcacgagggtggaccgcccgctcttgtcatctggaacatcaaacacttgctccacaccggaatcggcactgcttcgcgacccagcgaggtgtgcatggttgatggcacagacatgtgtttggccgacttccacgctggtatcttcctgaaagggcaagaacacgctgtgttcgcctgcgtcacctccaacgggtggtacgcgatcgacgacgaggacttctacccctggacgccggacccgtccgacgttctggtgtttgtcccgtacgatcaa

>MG983727.1_O_SAU_2015

cacaacggtgagaaaaagactttctattctaggcccaacaaccacgacaattgctggctgaacaccatcctccagttgtttaggtacgtcgatgaacctttcttcgactgggtctatgaatcgcctgaaaacctcacccttgaggcgattaggcaattggaagaactcactggtcttgagctgcacgagggtgggccacccgctctcgtcatttggaacatcaagcacttgctccacaccggaattggcactgcttcgcgacccagcgaggtgtgcatggttgatggcacggacatgtgtttggctgacttccacgctggcatcttcctgaaggggcaagagcacgctgtgttcgcctgcgtcacctccaacgggtggtacgcgatcgacgacgaggacttctacccctggacgccggatccgtccgacgttctggtgtttgtcccgtacgatcaa

>MG983728.1_O_SAU_2016

cacaacggtgagaaaaagactttctattctaggcccaacaaccacgacaattgctggctgaacaccatcctccagttgtttaggtacgtcgatgaacctttcttcgactgggtctatgaatcgcctgaaaacctcactcttgaggcgattaggcaattggaagaactcactggtcttgagctgcacgagggtgggccacccgctctcgtcatttggaacatcaagcacttgctccacaccggaattggcactgcttcgcgacccagcgaggtgtgcatggttgatggcacggacatgtgtttggctgacttccacgctggcatcttcctgaaagggcaagagcacgctgtgttcgcctgcgtcacctccaacgggtggtacgcgatcgacgacgaggacttctacccctggacgccggatccgtccgacgttctggtgtttgtcccgtacgatcaa

>MG983729.1_O_SAU_2016

cacaacggtgagaaaaagactttctattctaggcccaataaccacgacaattgctggctgaacaccatcctccagttgtttaggtacgtcgatgaacctttcttcgactgggtctatgaatcgcctgaaaacctcactcttgaggcgattaggcaattggaagaactcactggtcttgagctgcacgagggtgggccacccgctctcgtcatttggaacatcaagcatttgctccacaccggaatcggcactgcttcacgacccagcgaggtgtgcatggttgatggcacggacatgtgtttggctgacttccacgctggcatcttcctgaaagggcaagagcacgctgtgttcgcctgcgtcacctccaacgggtggtacgcgatcgacgacgaggacttctacccctggacgccggatccgtccgacgttctggtgtttgtcccgtacgatcaa

>MG983730.1_O_SKR_2017

cacaacggtgagaaaaagactttctattctaggcctaacaaccacgacaattgttggctgaacaccatcctccaattgtttaggtacgtcgatgaacctttcttcgactgggtctatgaatcacctgaaaacctcactcttgaggcgattgggcaactggaaggactcactggtcttaagctgcacgagggtgggccacccgctctcgtcatttggaacatcaagcatttgctccacaccggaattggcactgcctcgcgacccagcgaggtgtgcatggtcgatggcacggacatgtgtttggctgacttccacgctggcatcttcctgaaagggcaagagcacgctgtgttcgcctgcgtcacctccaacgggtggtacgcgatcgacgacgaggacttctacccctggacgcctgatccgtccgacgttctggtgtttgtcccgtacgatcaa

>MG983731.1_O_SRL_2013

tacaacggtgagaaaaagaccttctactctaggcccaacaaccacgacaactgttggttgaacaccatccttcagttgtttaggtatgtcgacgaaccttttttcgaatgggtctatggatcgcctgaaaacctcactcttgaggcgattaggcaactggaagagatcactggtcttgagctgcacgagggtggaccgcccgctctcgtcatctggaacattaaacacttgctccacaccggaatcggcaccgcttcgcgacccagcgaggtgtgcatggttgatggcacggacatgtgtttggctgacttccacgctggcatcttcctgaaagggcaagaacacgctgtgttcgcctgcgtcacctccaacgggtggtatgcgattgacgacgaggacttctacccctggacgccggacccgtccgacgttctggtgtttgtcccgtacgatcaa

>MG983732.1_O_SRL_2014

tacaacggtgagaaaaagaccttctactctaggcccaacaaccacgacaactgttggttgaacaccatccttcagttgtttaggtatgtcgacgaaccttttttcgactgggtctatgaatcgcctgaaaacctcactcttgaggcgattaggcaactggaagagatcactggtcttgagctgcacgagggtggaccgcccgctctcgtcatctggaacattaaacacttgctccacaccggaatcggcactgcttcgcgacccagcgaggtgtgcatggttgatggcacggacatgtgtttggctgacttccacgctggcatcttcctgaaagggcaagaacacgctgtgttcgcctgcgtcacctccaacgggtggtatgcgattgacgacgaggacttctacccctggacgccggacccgtccgacgttctggtgtttgtcccgtacgatcaa

>MG983733.1_O_SRL_2014

tacaacggtgagaaaaagaccttctactctaggcccaacaaccacgacaactgttggttgaacaccatccttcagttgtttaggtatgtcgacgaaccttttttcgactgggtctatgaatcgcctgaaaacctcactcttgaggcgattaggcaactggaagaaatcactggtcttgagctgcacgcgggtggaccgcccgctctcgtcatctggaacattaaacacttgctccacaccggaatcggcactgcttcgcgacccagcgaggtgtgcatggttgatggcacggacatgtgtttggctgacttccacgctggcatcttcctgaaagggcaagaacacgctgtgttcgcctgcgtcacctccaacgggtggtatgcgattgacgacgaggacttctacccctggacgccggacccgtccgacgttctggtgtttgtcccgtacgatcaa

>MG983734.1_O_SRL_2014

tacaacggtgagaaaaagaccttctactctaggcccaacaaccacgacaactgttggttgaacaccatccttcagttgtttaggtatgtcgacgaaacttttttcgactgggtctatgaatcgcctgaaaacctcactcttgaggcgattaggcaactggaagagatcactggtcttgagctgcacgagggtggaccgcccgctctcgtcatctggaacattaaacacttgctccacaccggaatcggcactgcttcgcgacccagcgaggtgtgcatggttgatggcacggacatgtgtttggctgacttccacgctggcatcttcctgaaagggcaagaacacgctgtgttcgcctgcgtcacctccaacgggtggtatgcgattgacgacgaggacttctacccctggacaccggacccgtccgacgttctggtgtttgtcccgtacgatcaa

>MG983735.1_O_TUN_2014

cacaacggtgagaaaaagaccttctactctaggcccaacaaccacgacaactgttggttaaacaccatccttcagttgtttaggtacgtcgacgagcctttcttcgactgggtctatgaatcgcctgaaaacctcactcttgaggcgattaggcaactggaagagatcactggccttgagctgcacgagggtggaccgcccgctctcgtcatctggaacatcaaacacttgctccacaccggaatcggcactgcttcgcgacccagcgaggtgtgcatggttgatggcacagacatgtgtttggccgacttccacgctggcatcttcctgaaagggcaagaacacgctgtgttcgcctgcgtcacctccaacgggtggtacgcgatcgacgacgaggacttctacccctggacgccggacccgtccgacgttctggtgtttgtcccgtacgatcaa

>MG983736.1_O_UAE_2014

tacaacggtgagaaaaagaccttctactctaggcccaacaaccacgacaactgttggttgaacaccatccttcagttgtttaggtatgtcgacgaaccttttttcgactgggtctatgaatcgcctgaaaacctcactcttgaggcgattaggcaactggaagagatcactggtcttgagctgcacgagggtggaccgcccgctctcgtcatctggaacattaaacacttgctccacaccggaatcggcactgcttcgcgacccagcgaggtgtgcatggttgatggcacggacatgtgtttggctgacttccacgctggcatcttcctgaaaggacaagaacacgctgtgttcgcctgcgtcacctccaacgggtggtatgcgattgacgacgaggacttctacccctggacgccggacccgtccgacgttctggtgtttgtcccgtacgatcaa

>MG983738.1_O_UAE_2016

cacaacggtgagaaaaagactttctattctaggcccaacaaccacgacaattgctggctgaacaccatcctccagttgtttaggtacgtcgatgaacctttcttcgactgggtctatgagtcgcctgaaaacctcactcttgaggcgattaggcaattggaagaactcactggtcttgagctgcacgagggtgggccacccgctctcgtcatttggaacatcaagcatttgctccacaccggaatcggcactgcttcgcgacccagcgaggtgtgtatggttgatggcacggacatgtgtttggctgacttccacgctggcatcttcctgaaagggcaagagcacgctgtgttcgcctgcgtcacctccaacgggtggtacgcgatcgacgacgaggacttctacccctggacgccggatccgtccgacgttctggtgtttgtcccgtacgatcaa

>MG983739.1_O_UAE_2016

cacaacggtgagaaaaagactttctattctaggcccaacaaccacgacaattgctggctgaacaccatcctccagttgtttaggtacgtcgatgaacctttcttcgactgggtctatgagtcgcctgaaaacctcactcttgaggcgattaggcaattggaagaactcactggtcttgagctgcacgagggtgggccacccgctctcgtcatttggaacatcaagcatttgctccacaccggaatcggcactgcttcgcgacccagcgaggtgtgtatggttgatggcacggacatgtgtttggctgacttccacgctggcatcttcctgaaagggcaagagcacgctgtgttcgcctgcgtcacctccaacgggtggtacgcgatcgacgacgaggacttctacccctggacgccggatccgtccgacgttctggtgtttgtcccgtacgatcaa

>MG983740.1_O_VIT_2015

tacaacggtgagaaaaagaccttctactctaggcccaacaaccacgacaactgttggttgaacaccatccttcagttgtttaggtatgtcgacgaacctttcttcgactgggtctatgaatcgcctgaaaacctcactcttgaggcgatcaagcaactggaagagatcactggtctggagttgcacgagggtggaccgcccgctctcgtcatctggaacatcaaacacttgctccacaccggaatcggcactgcttcgcgacccagcgaggtttgcatggttgatggcacggacatgtgtttggctgacttccacgctggcatcttcctgaaagggcaagaacacgctgtgttcgcctgcgtcacctccaacgggtggtacgcgattgacgacgaggacttctacccctggacgccggacccgtccgacgttctggtgtttgtcccgtacgatcaa

>MG983741.1_O_VIT_2016

cacaacggtgagaaaaagactttctattctaggcccaacaaccacgacaattgttggctgaacaccatcctccaattgtttaggtacgtcgatgaacctttcttcgactgggtctatgaatcacctgaaaacctcactcttgaggcgattgggcaattggaagaactcactggtcttaagctgcacgagggtgggccacccgctctcgtcatttggaacatcaagcatttgctccacaccggaattggcactgcctcgcggcccagcgaggtgtgcatggttgatggcacggacatgtgtttggctgacttccacgctggcatcttcctgaaagggcaagagcacgctgtgttcgcttgcgtcacctccaacgggtggtacgcgatcgacgacgaggacttctacccctggacgcctgatccgtccgacgttctggtgtttgtcccgtacgatcaa

>MH053305.1_A_EGY_1972

cacaacggtgagaagaaaacattctactctagacccaacaatcacgacaactgctggttgaacgcaattctccaattgttcaggtacgtcgatgaaccgttcttcgactgggtctacgagtctcccgagaacctcacactacaggcaattaggcaattggaggagctcactggccttgaactccacgagggtggacctcccgctctcgtagtttggaacatcaagcatttgctccataccggcattggcaccgcctcgcgacccagcgaggtgtgtatggtggatggtacggacatgtgtttggccgactttcatgctggaatcttcctaaaaggacaggaacatgctgtgtttgcttgtgtcacctccaaagggtggtacgcgatcgacgatgaggatttttacccctggacaccggatccgtccgacgtcctagtgtttgtcccgtacgatcaa

>MH053306.1_A_TCH_1973

tacaacggtgaaaagaagactttcttttctagacccaacaaccatgacaactgttggttgaacgcaattctccaactgttcaggtacgtcgacgaaccctttttcgactgggtctatgagtctcctgagaacctcacattgcaggcaatcagacaactggaggaacttactggtcttgaactgcacgagggcggcccgcctgccctcgttgtttggaacatcaaacacctgctccacaccggcattggtaccgcctcgcgacccagcgaggtgtgtatggtggacggtacggacatgtgcttggctgactttcacgctggaattttcctgaaaggacaggaacacgctgtgtttgcttgcgtcacctccgatgggtggtacgcgatcgacgacgaggacttttacccctggacaccagacccttctgacgtcctggtgtttgtcccgtacgatcaa

>MH053307.1_A_ZAM_1990

tacaacggtgagaaaaagacattctactccaggcccaacaaccacgacaactgttggctcaatgccatcctgcagttgttcaggtacgttgatgaacctttctttgactgggtctacgactcgcctgagaacctcactgctgaggcgattaggcagttggaggacctgactggtcttgaactacacgagggcggaccacctgctcttgtcatctggaacatcaaacacttgctccacaccggtatcggcacggcctcacgccccagtgaggtgtgcatggtagacgggacggacatgtgcctggctgacttccacgctggcatcttcctgaagggacaggaacacgccgtgtttgcatgtgtcacatccaacgggtggtatgcgatcgatgacgaggacttttacccttggacgccagatccgtccgatgttctggtttttgtcccgtacgatcaa

>MH053308.1_C_ETH_1971

tacaacggtgagaagaaaacgttctactccagacccaacaaccatgacaactgttggcttaacaccatcctccagttgttcagatacgttgatgagcctttctttgattgggtttacaactcgcctgagaaccttactgttgaggcaattagacagttggaggatgtgactggtctcgagctacacgagggtggaccgcccgctctcgtcatctggaacatcaaacacctgctccaaaccgggatcggcactgcctcacgacccagtgaggtgtgtatggtagacggtacagacatgtgtttggctgacttccacgctggcattttcttgaaaggccaagaacacgcagtgtttgcgtgtgtcacctcacaagggtggtacgcgattgacgacgaggacttttatccctggacgcccgacccatcggacgtcctggtgtttgtcccgtacgatcag

>MH053309.1_C_KEN_1967

tataacggtgagaagaaaacattctactccagacccaacaaccacgacaactgttggcttaacgccatccttcaattgttcaggtacgttgatgagcctttcttcgattgggtttacaactcgcctgagaaccttactgttgaggcaatcagacagctggaggatgtgactggtcttgagctacacgagggtgggccgcccgctctcgtcgtctggaacattaaacacctgctccacaccggaatcggcaccgcctcacgccccagtgaggtgtgcatggtagacggtacggacatgtgtttggctgatttccacgctggcatcttcttgaaaggccaagaacacgcagtgtttgcgtgtgtcacctcccaagggtggtacgcgattgatgacgaggacttttacccctggacacccgacccaacggatgtcttggtgtttgtcccgtatgatcag

>MH053310.1_C_UGA_1970

cacaatggtgagaagaagattttcttctctagacccaacaaccacgacaactgttggttgaacaccatcctccaactttttaggtatgtcgatgaacctttcttcgactgggtctacaactcacccgagaacctcacgctcaaggccatcgagcagctggaggaagttacagggcttgagctacacgatggtgggccacccgccctcgtgatctggaacatcaaacacttgcttcaaaccggcatcggaaccgcctcgcgacccagcgaggtgtgcatggtggacggtacggacatgtgtttggccgacttccacgctggtatctttctgaaaggccaagaacacgcagtgtttgcgtgtgtcacctcccaagggtggtacgcgattgatgacgaggacttttacccctggacgcccgatccgtcggacgttttggtgtttgtcccgtatgatcag

>MH053311.1_O_ETH_2004

tacaacggtgagaagaagacattttactccaggcccaacaaccacgacaactgttggcttaacaccattctccagttgtttaggtacgttgatgaaccattctttgattgggtctatgactcacctgagaacctcactgccgaagcaatcaggcagttggaagatgtgactggtcttgaactgcaagagggtggaccacctgcccttgttgtttggaacataaaacacctgctccacaccggaatcggcacggcttcgcgacccagcgaggtgtgtgttgttgacgggacggacatgtgtttggctgacttccatgctggcatctttctgaaagggcaagagcacgctgtgttcgcctgcgtcacctccaatgggtggtacgcgattgatgacgaggacttctacccgtggacaccggacccgtccgatgttctggtgtttgttccgtacgatcaa

>MH053312.1_O_ETH_2005

tacaacggtgtaaagaagaccttttactccaggcccaacaaccacgacaattgctggctgaacgccattttgcagttgttcaggtacgtcgatgaacctttcttcgactgggtctatgagtctcctgagaacctcactttgcaagcaatcaaccaattggaggaactcaccagccttgaactccacgagggaggcccacccgccctcgtcatttggaacattaaacacctgctccacaccggcatcggcactgcctcgcgacccagcgaggtgtgcatggtggatggtacggacatgtgcttggctgatttccacgctggaatcttcttaaaaggaccagaacacgctgtgttcgcctgcgtcacctccggagggtggtacgcgatcgacgatgaggacttctacccctggacaccggatccttccgatgttctggtgtttgtcccgtacgatcaa

>MH053313.1_O_ETH_2006

tacaacggtgagaagaagacattctactcaaggcccaacaaccacgataactgttggcttaacaccattctacagttgtttaggtacgttggtgaacctctttttgattgggtttacgactcacctgaaaacctcactgctgaagcaatcaagcagctggaagatgtaactggtcttgacctgcaagagggcggaccacctgctctcgttgtttggaacataaaacacctgctccacaccggagtcggcacagcctcgcgccctagcgagatatgtgtcgtagacgggacagacatgtgtttggctgacttccacgctggcattttcctgaaaggacaagaacacgctgtgtttgcctgcgtcacctccaacgggtggtacgcgattgacgacgaggacttttacccctggacaccggacccgtccgatgttctggtatttgtcccgtacgatcag

>MH053314.1_O_ETH_2007

tacaacggtgagaagaaaatgttctactctaggcccaacaaccacgacaactgctggcttaacaccattcttcagttgtttaggtatgttgatgaaccattctttgattgggtctacgactcacctgagaacctcactgctgaagcgattaggcagctggaagacgtgactggtcttggactgcaagagggtggaccacctgctctcgttgtctggaacataaaacacctactccacaccggaatcggcacggcttcgcgacccagcgaggtatgtgttgttgacggaacggacatgtgtttggctgacttccatgctggcatcttcctgaagggacaagaacacgctgtgttcgcctgtgtcacctccaacgggtggtacgcgattgatgacgaggacttctacccatggacaccggacccgtctgatgttctggtgtttgttccgtacgatcaa

>MH053315.1_O_SUD_1976

tacaacggtgagaagaaaacattttactccaggccaaacaaccatgacaactgttggcttaacaccattctacagttgtttaggtacgtggatgagcctttctttgactgggtctacaattcacctgaaaacctcactgttgaggcaatcgaccagttggaggaagtgactggtctcgagcttcgtgagggcggaccgcccgctctcgtcatttggaacatcaagcatctgctccacaccggaatcggcacggcttcacgccccagtgaggtgtgcatggtagacgggacagacatgtgtttggctgatttccacgctggcattttcatgaaaggacacgagcacgccgtgtttgcctgtgtcacctccaacgggtggtacgcgattgacgacgaggacttttacccctggacgccggacccgtccgacgtcctggtatttgttccgtacgatcaa

>MH053316.1_O_UGA_1996

tacaacggtgagaaaaagacattttactccaggcccaacaaccatgacaactgttggctcaacgccatcctccagttgtttaggtacgttgatgagcctttctttgactgggtctacgactcacctgagaacctcaccactgaggcgatcaggcagttggaggaactaactggtcttgaactgcacgagggcggaccacctgctcttgtaatttggaacatcaaacacttgctccacaccggcatcggcactgcctcacgacccagtgaggtgtgcatggtagacgggacggacatgtgcttggctgacttccacgctggcatcttcctgaaaggacaggaacacgccgtgtttgcctgcatcacctccaacgggtggtacgcgatcgatgacgaggacttttacccctggacaccggacccgtccgacgtcctggtgtttgtcccgtacgatcaa

>MH053317.1_O_UGA_1998

tacaacggtgaaaagaagatcttctactccagacccaacaaccacgacaactgttggttaaacgccatcctgcagctgttcaggtacgtcgatgaacctttcttcgactgggtctacgagtctcctgagaacctcaccttgcaagcaattaaccaactggaggaactcaccagcctggaactccacgagggaggtccgcctgcccttgtcatatggaacatcaagcacctgctccacaccggcatcggcaccgcctcacgacccagtgaggtgtgcatggtggatggtacagacatgtgtctggccgacttccacgctggaatcttcctgaaaggacacgaacacgctgtgttcgcctgtgtcacctccgaagggtggtacgcgattgatgacgaggacttctacccctggacaccggacccttccgatgttctggtatttgttccgtatgatcaa

>MH053318.1_O_UGA_2002

tacaacggtgagaaaaagacattctactctaggcccaacaaccacgacaactgctggctcaacgccatcctgcagctgtttaggtatgttgacgagcctttctttgactgggtctacgactcacctgagaacctcactgctgaagcaatcaggcagttggagggcctaactggtcttgagctgcacgagggcggaccacccgctcttgtcatttggaacatcaagcacttgctccacaccggtatcggcacggcttcacgacccagtgaggtgtgcatggtagacgggacagacatgtgcttggctgacttccacgctggcattttcctgaaaggacaggaacacgctgtgtttgcatgcgtcacgtccgacgggtggttcgcgattgacgacgaggacttttacccctggacgccggacccggccgacgttctggtttttgtcccgtacgatcaa

>MH053319.1_SAT1_BOT_1974

tacaacggagagaagaagaccttctacagcaggcccaacaaacatgggaactgttggctcaactcgctgttgcagctcttccgatacgtcgacgagccgctcttcgagtcagagtatttgtcacctgaaaacaagacattggacatgatcaaacaattatctgattataccaaattggacttgtcggacggagggccccccgccctcgtcctttggctgatcaaagactgtcttcaaaccggcgttggcaccagcactcgccccagcgagatctgtgtcatcaacggggtcgccatgaccctggcagatttccacgccggcattttcatcaaaggcaccgaacacgcagtgttcgccctcaacacaaccgagggctggtacgccattgatgacgaggtgttctacccatggacacctgaccctgagaacgtactcgcttacgtcccctacgaccag

>MH053320.1_SAT1_KEN_1983

tacaacggagagaagaagacattctacagcaggcccaacacacacggcaactgctggctcaattcattgttgcagctcttccactacgtagatgagccgctctttgagtccgagtatttgtcaccagagaacaagacgctggacatgattagacagctttctgactacaccaaacttgacctctcggatggtgggccaccagcactcgtgctttggcttatcaaggattgtctcaacaccggcgttggcacaagcactcgccccagcgagatttgtgttatcaacggggtcaccatgaccctggctgacttccatgccggtatcttcattaaaggtactgaacacgctgtcttcgccctcaacacttccgagggttggtatgcaattgatgatgaggtgttctacccgtggacacccgaccctgagtgcgtgctcgcgtacgtgccctacgaccaa

>MH053321.1_SAT1_MOZ_1981

tacaacggagagaagaagaccttttacagcagacccaacacacacgggaactgttggctcaattcactcctgcagctctttcgatacgtcgatgagccgctgtttgaatctgagtatttgtcacccgaaaacaagacattggacatgatcaaacaactttctgattacaccaaacttgacctttcggacggtgggccgcccgcactcgtgctgtggctcatcaaggactgtctgcagaccggcgttggcacaagcactcgccccagcgagatctgtgtgatcaacggggtcgcaatgaccctggccgacttccacgccggaattttcatcaaaggtaccgaacacgctgtgttcgctctcaacacatctgagggctggtacgccatagatgatgaagtgttctacccatggacacctgaccctgacaacgtactcgcgtacgtgccttacgaccag

>MH053322.1_SAT1_NMB_1989

tacaacggtgagaagaagaccttctacagcagacccaacaaacacgggaactgctggctcaactcgctactgcagctctttcgatacgtcgatgagccgctcttcgagtctgagtacctatcacctgaaaacaagacattggacatgatcaaacaactttctgattacaccaaattggacttgtcagacggcggaccgcccgccctcgtcctttggctgatcaaagactgtcttcagaccggtgttggcaccagcactcgccccagcgagatctgtgtcatcaacggggtcgtcatgaccttggctgatttccacgccggtatcttcatcaaaggtactgaacacgctgtgtttgccctcaacacatccgagggctggtacgccattgatgatgaggtgttctacccttggacacctgaccctgagaatgtgctcgcgtacgttccctacgaccag

>MH053323.1_SAT1_TCH_1972

tacaacggtgagaagaaaactttctactctagacccaaccgtcacgacaactgttggttgaacaccatcctgcagttgtttcgttacgtcgatgaaccattcttcgactgggtctacaactcgcccgagaacctcacgctacaagcaattgaacagctagaggaactcactgggctcgagttgcacgagggtggaccccccgctctcgtcatttggaacatcaagcacctgctctacaccggaatcggtaccgcctcgcgacccagtgaggtgtgtatggttgacggtacagagatgtgtcttgctgattttcatgcaggaatcttcctgaaaggtactgagcacgctgtgtttgcctgcctgacctccgaagggtggtatgccattgacgacgaggcattctacccatggacaccggatccgtccgatgtcctgtgttttgtcccgtacgacatg

>MH053324.1_SAT1_UGA_1971

tataacggtgagaagaagactttctattccagacccaacaagcacgacaactgttggttaaacaccatcctacagttgttccgctatgtcgacgagccattcttcgactgggtctacaactcgcctgagaacctcacgctccaagcaattaagcaactggaggaactcaccgggctcgagttacacgagggcggtccacctgcccttgtgatttggaacatcaagcacctgctgtacaccggtatcggtaccgcctcgcgacccagcgaggtgtgcatggttgacggaacagagatgtgcttggctgatttccatgcaggaatcttcctgaaaggtactgaacacgcagtgttcgcctgtttgacctccgacggatggtacgccatcgacgacgaggacttctacccatggacaccggacccgtccgacgtcttgtgttttgtcccgtacgacatg

>MH053325.1_SAT1_UGA_1978

tacaacggtgaaaagaaaattttctactccagacctaaccgccacgacaactgctggctgaacaccatcctccagttgttcaggtacgtcgacgaaccattctttgactgggtctacaactcgcctgagaacctcacgctccaagcaattgaacagctcgagggactcactgggcttgaattacacgagggtggcccgcctgctctcgttatctggaacatcagacacttgctctacaccggaattggtactgcctcgcgacctagcgaggtgtgcatggttgacgggacagagatgtgtttagctgacttccatgctgggattttcctgaaaggtactgaacatgccgtgtttgcctgtttgacctcagaagggtggtatgccattgacgacgaggacttctacccatggacgccggatcccaccgatgtcctgtgttttgtcccgtacgacatg

>MH053326.1_SAT1_UGA_1970

cacaacggagagaaaaagaccttctattccagacccaaccgccacgacaactgttggttgaacaccatcctacaattgtttaggtatgttgacgaacctttcttcgactgggtctacaactcacctgagaacctcacgctccaagcaattcaacagcttgaggagttcactggaatgcacctacgcgaaggcggaccacctgccctcgtgatctggaacatcaaacacttgctctacaccggaattggtacagcctcacgacccagtgaggtttgtatggttgacggtacggacatgtgtcttgctgattttcacgcaggaattttcctcaaaggtactgaacacgccgtgtttgcttgtttgacctccgaaggatggtacgccattgacgacgaggacttctacccatggacgccagacccgtctgacgtgttatgttttgtcccgtacgacatg

>MH053327.1_SAT1_UGA_1970

tacaacggtgagaagaaaactttctactcaagacccaacaamcaygacaactgttggttgaacaccatcctacagctgttcaggtacgtcgatgaaccattcttcgactgggtctacaactcgcccgagaacctcacyctccaagcaatcgagcrgttagaggaactcactgggcttgaactacacgaaggcggcccccccgctctcgtgatttggaacatcaaacacttgctctacaccggaatcggcaccgcctcgcgacccagcgaggtgtgcatggtagacggtaccgacatgtgtcttgctgatttccacgcaggaatcttyctgaaaggtacygagcacgctgtgttcgcctgcctgacctctgacggatggtacgccattgacgacgaggacttttacccctggaccccggacccrtccgatgtcctgtgttttgtcccgtatgacatg

>MH053328.1_SAT2_BOT_1968

tacaacggagagaagaagaccttctacagtagacccaacaaacacgggaactgttggctcaactcactgttgcagctctttcgatacgtcgacgagccgctctttgagtctgaatatttgtcacctgaaaacaagacattggacatgatcaaacaactatctgattacaccaaattggacttgtcggacggagggccccccgctctcgtcctctggctgatcaaagattgtcttcaaaccggtgttggcaccagcactcgccccagcgagatctgtgtcatcaacggggttgtaatgaccctggctgatttccacgccggtatcttcatcaaaggcactgaacacgccgtgtttgctctcaatacatccgagggctggtatgctattgatgatgaggtgttctacccgtggacacccgaccctgggaacgtgctcgcgtacgtcccctacgaccag

>MH053329.1_SAT2_BOT_1969

tacaacggagagaagaagaccttctacagcagacccaacaaacacgggaactgttggctcaactcgctgttgcagctctttcgatacgtcgacgagccgctctttgagtctgagtacttgtcacctgaaaacaagacattggacatgatyaaacaattatctgattacaccaaattggacttgtcggacggagggccycctgctctcgtcctttggytgattaaagaytgtcttcaaaccggcgttggcaccagcactcgccccagcgagatctgtgtcatcaacggggttgtaatgaccctggctgactttcacgccggtatcttcatcaaaggcactgaacacgccgtgttcgccctcaacacatctgagggctggtacgccattgatgatgaggtgttctayccatggacacctgaccctgagaacgtactcgcrtacgtcccctacgaccag

>MH053330.1_SAT2_BOT_1969

tacaacggagaaaagaagaccttctacagcagacctaacacacacgggaactgctggctcaactcgttgcttcagctctttcgatacgtcgatgagccgctcttcgagtctgagtacttgtcacctgagaacaagacattggacatgatcaaacaactctctgactacaccaaactggacttgtcagacggtgggccccccgccctcgtcctatggctcatcaaggattgtcttcaaaccggcgttggcaccagcactcgccccagcgagatctgtgtgatcaacggggttgccatgaccctggctgacttccacgccggtattttcatcaaaggcaccgaacacgccgtgttcgctctcaacacatccgagggctggtacgccattgatgatgaggtgttctacccatggacacccgaccctgagaacgtgctcgcgtacgttccctacgaccag

>MH053331.1_SAT2_BOT_1972

tacaacggagagaagaagaccttctacagcagacccaacacacacgggaattgctggctcaactcactgttgcagctctttcgatacgtcgacgagccgctctttgagtctgagtacttgtcacctgaaaacaagacattggacatgatcaaacagctatctgattacaccaaattggacttgtcggacggagggccccccgctctcgtcctctggctgattaaagattgtcttcaaaccggtgttggtaccagcactcgccccagcgagatctgtgtcatcaacggggtcaccatgaccctggctgatttccacgccggcatcttcatcaaaggcaccgaacacgccgtgttcgctctcaacacatctgagggctggtacgccattgatgatgaggtgttctacccatggacacccgaccctgagaacgtgctcgcgtacgttccctacgaccag

>MH053332.1_SAT2_BOT_1974

tacaacggagaaaagaagaccttctacagcagacccaacaaacacgggaactgttggctcaactcgctgctgcagctctttcggtacgtcgatgagccgctctttgagtctgagtatttgtcacctgaaaacaagacattggacatgatcaaacagctttctgattacaccaaattggatttgtcggatggtgggccacctgccctcgtcctttggctgatcaaagattgtcttcaaaccggcgttggcaccagcactcgccccagcgagatctgtgtgatcaacggggttgtcatgaccctggccgatttccacgctggtattttcatcaagggtaccgaacacgctgtgttcgccctcaacacatctgagggctggtacgccattgatgacgaggtgttctacccgtggacacccgaccctaaggacgtgctcgcgtacgtcccctacgaccag

>MH053333.1_SAT2_ETH_1989

tacaacggtgagaagaagatcttctacagcaggcctaacacacacggcaactgctggctcaactcgctgctccagctctttcactatgttgatgagccgctcttcgagtctgagtacatgtcacctgagaacaagacattggacatgataaaacagctctctgactacaccaaacttgacctctcagacggtggaccaccagcacttgtgctgtggctcatcaaggactgtctccaaaccggcgtcggcacaagcactcgccccagcgagatttgtgtaatcaacggggttgtgatgaccctggctgacttccatgccggcatcttcatcaaaggtactgaacatgctgtctttgcccttaatacatctgagggctggtatgcaattgatgatgagatgttctatccttggacacctgaccctaacaacgtgctcgcgtacgtgccttacgaccaa

>MH053334.1_SAT2_ZAM_1964

tacaacggagagaagaagaccttctacagcagacctaacactcacgggaactgctggctcaactcattactacagctttttcgatacgtcgatgagccgttgtttgagtctgagtacctctcacctgaaaacaggacattggacatgatcaaacaactttcagattacaccaagcttgacctttcagacggcggtccacccgcactcgtactgtggctcatcaaggactgcctccagaccggcgttggcaccagcactcgccccagcgagatctgcgtgatcaacggagtcgtcatgactctggcggactttcacgccggcattttcatcaaaggcaccgaacatgcggtatttgccctcaacacatctgagggatggtacgctattgatgatgaggtgttctacccatggacacccgaccctgagaacgtgctcgcgtacgtgccctacgaccag

>MH053335.1_SAT2_ZIM_1965

tacaacggtgagaagaagactttttacagcagacccaatacacacgggaactgttggctcaactcactcctgcagctctttcgatacgtcgatgagccgctgtttgagtctgagtatttgtcacctgaaaacaagacattggacatgatcaaacaactatctgattacaccaagcttgacctttcagacggtgggcccccagcgctagtgctctggctcatcaaagactgtcttcagaccggcgtaggtaccagcactcgccccagcgagatctgtgtgatcaacggagtcgtcatgactcttgctgatttccacgccggcattttcatcaaaggtactgaacacgcagtgttcgccctcaacacagctgagggctggtacgccattgatgatgaggtgttttacccatggacacccgatcccaacaacgtgctcgcgtacgtgccctacgaccaa

>MH053336.1_SAT2_UGA_1970

cacaacggtgagaagaaaactttctactcaagacccaacaarcacgacaactgttggttgaacaccatcctgcagttgttcagataygtcgacgagccattcttcgactgggtctacaactcgcctgagaacctcactctccaagcaatygagcagcttgaggaactcaccgggcttgagttgcgcgaaggtggtccccccgccctcgtgatttggaacatcaaacacttgctttacaccggaatcggtactgcttcgcgacccagcgaagtgtgcatggtagacggaactgcaatgtgtctttctgatttccacgcaggaattttcctcaagggtactgarcacgccgtgttcgcctgtttgacctccgacggatggtacgccattgacgacgaggaattytacccatggacaccggatccgtccgatgttctgtgttttgtcccgtacgatatg

>MH053337.1_SAT2_UGA_1970

tacaacggtgagaagaaaaccttctactcaagacccaacaagcacgacaactgytggttgaacaccatcttgcagytgttcaggtaygtcgatgaaccattcttcgactgggtctayaactcgcctgagaacctcactctccaagcaattgagcagctagaggaactcactgggcttgaactacacgaaggtggtccccccgctctcgtgatttggaacatcaaacacctgctctacaccggaatcggcaccgcctcgcgacccagcgaggtgtgcatggtrgacggtactgagatgtgtcttgctgatttccaygcaggaatyttcctgaaaggtactgagcacgccgtgttcgcctgcctgacctctgatggatggtacgccattgacgacgaggacttctacccctggactccggacccgtccgacgtcctttgttttgtcccgtaygatatg

>MH053338.1_SAT3_BOT_1966

tacaacggagagaagaagaccttctacagcagacccaacacacacgggaactgttggctcaactcgttgttacagctctttcgatacgtcgatgagcccctcttcgagtctgagtacctatcacctgagaacaagacattggacatgatcaaacaactctctgattacactaaactggacttgtcggacggcgggcctccagccctcgtcctttggctgattaaagactgtcttcaaaccggcgttggcaccagcactcgccccagcgagatttgcgtgatcaacggggttgttatgaccctggctgacttccacgctggcatttttatcaagggtaccgagcacgctgtgttcgctctcaatacatctgagggctggtacgccattgatgatgaggtgttctacccctggacacccgacccagagaacgtacttgtgtacgtcccctacgaccag

>MH053339.1_SAT3_BOT_1970

tacaacggtgagaagaagaccttctacagcagacccaacacgcacgggaactgctggctcaactcgctgctgcagctctttcgatacgtcgatgagccgctcttcgagtctgagtacttatcacctgaaaacaagacattggatatgatcaaacaactctctgattacaccaaattggacttgtcggacggcgggcccccagccctcgtcctttggctgattaaggactgtcttcaaaccggygttggtaccagcactcgccccagcgagatctgtgtgatcaacggggttgtaatgaccctggcagacttccaygccggtatcttcatcaagggtaccgaacacgctgtgttcgccctcaacacatctgagggttggtacgccattgatgatgaggtgttctacccatggacacccgaccctgagaacgtgctcgcgtacgtcccctacgaccag

>MH053340.1_SAT3_MAL_1976

cacaacggtgagaagaaaaccttctacagcagacccaacacccacgggaactgctggctcaactctctgttgcagctctttcgatacgtcgatgagccgttctttgagtctgagtacttgtcaccagagaacaagacattggacatgatcaaacaactctctgactataccaaacttgacctctctgatggtgggccaccggcacttgtgctgtggcttatcaaggactgtttgcagaccggtgtcggcaccagtactcgcccaagcgagatctgtgtcatcaacggagtcaccatgactctggctgacttccacgccggaatcttcatcaagggtaccgaacacgccgtgttcgccctcaacacatctgagggctggtacgccattgatgatgaggtgttctacccttggacgcctgaccctgagaacgtactcgcgtacgtaccctacgaccag

>MH053341.1_SAT3_UGA_1970

tacaacggtgaaaagaagaccttctactcaagacccaacaaacacgacaactgttggttgaacaccatcctacagctgttcaggtacgtcgatgaaccattctttgactgggtctacaactcgcccgagaacctcacactcaaagcaatcgagcaacttgaggagcttactgggcttgagttgcatgagggcggacctcccgccctcgtgatctggaacatcaarcacctactctacaccggaatcggcaccgcttcgcgacccagtgaagtgtgyatggtagacggtactgagatgtgtcttgctgatttccatgcaggaattttcctgaaaggtactgaacacgccgtgttcgcctgtctgacctctgaaggctggtacgccattgacgacgaggacttctacccctggactccggacccgtccgatgtcctgtgttttgtcccgtacgacatg

>MH053342.1_SAT3_ZAM_1996

tacaacggagagaagaagaccttctacagcagacccaacacacacgggaactgttggctcaactcgcttttgcagctctttcgatacgtcgacgagccgctgttcgagtctgagtacttgtcacctgaaaacaagacattggacatgatcaaacaactgtctgaytacactaaacttgacctytcagacggtgggccacccgcactygtgctttggctcatyaaggartgtcttcagaccggtgttggcaccagtactcgccccagcgagatctgtgtgatcaacggagttgtcatgactctggctgatttccacgccggcatcttcatcaagggtacygaacacgcggtgttcgctctcaacacatccgagggctggtacgccattgatgatgaggtgttctacccatggacacctgacccggagaacgtactcgcgtacgtgccctacgaccag

>MH053343.1_SAT3_ZIM_1934

tacaacggtgagaagaagaccttctacagcaggcccaacaaacacgggaattgctggctcaactcgcttctgcagctctttcgatacgtcgatgagccgctcttcgagtctgagtatctgtcaccagagaacaagacattggacatgatcaaacaactttctgattacaccaaacttgacctctcagacggtgggccacccgcacttgtgctctggctcatcaaagactgtcttcagactggcgttggcaccagcactcgccccagcgagatctgtgtgatcaatggggttgtcatgaccctggctgatttccacgccggtattttcatcaaaggtactgaacacgcggtgttcgctctcaacacatccgagggctggtacgctattgatgacgaggtgttctacccatggacacccgaccccgaaaacgtacttgcgtacgtcccctacgaccag

>MH053344.1_SAT3_ZIM_1974

tacaacggtgaaaagaagactttttacagcaggcccaacacacacggcaactgctggctcaactcgttgctgcagctctttcgatacgtcgacgagccactcttcgaatctgagtatttgtcaccagagaacaagacgctggatatgatcaaacaactgtctgattacaccaagcttgacctctcggacggtgggccaccggcacttgtgttgtggctaatcaaagactgtcttcaaaccggtgttggcaccagcactcgccccagcgagatctgtgtgatcaacggagtgaccatgactctggctgacttccacgccggcattttcatcaagggcactgaacacgctgtgttcgctctcaacacatctgagggctggtacgccattgatgatgaggtgttctacccctggacgcctgaccctgacaacgtgctcgcgtacgttccctacgaccag

>MH053345.1_SAT3_ZIM_1975

tacaacggtgagaagaagaccttctacagcagacccaacaaacacgggaactgctggctcaactcacttctgcagctctttcgatacgtcgatgagccgcttttcgagtctgagtatctgtcaccagaaaacaaaacattggatatgatcaaacaactttctgattacacccaacttgatctttctgatggtgggccacctgcacttgtgctttggctcatcaaggactgtttgcagactggcgtcggcacaagtactcgccccagcgagatctgtgtgatcaacggggttgtcatgacccttgctgactttcacgccggtatcttcatcaaaggcaccgaacacgctgtgttcgctctcaacacatctgagggctggtacgctattgatgatgaggtgttctacccgtggacaccggaccctgagaacgtactcgcgtacgtgccttacgaccag

>MH053346.1_SAT3_ZIM_1976

tacaacggtgagaagaagaccttctacagcagacccaacacccacgggaactgttggctcaactcacttctgcagctctttcgatacgtcgacgagccgctttttgagtctgaatatctctcacctgaaaacaagacactggacatgatcagacaactgtctgattacaccaaacttgacctttcagacggtgggccaccagcacttgtgctttggctcatcaaggactgccttcagaccggcgtcggcaccagcactcgcccaagcgagatctgtgtcatcaacggggttgtcatgaccctggctgacttccacgctggtatcttcatcaagggcacggaacacgccgtgtttgccctcaacacatccgagggctggtacgccattgatgatgaggtgttttacccatggacacccgaccctggaaacgtactcgcgtacgtcccctacgaccag

>MH053347.1_SAT3_ZIM_1976

tacaacggtgagaagaagaccttctacagcagacccaacaaacacgggaactgctggctcaactcacttctgcagctctttcgatacgtcgatgagccgcttttcgagtctgagtatctgtcaccagaaaacaaaacattggacatgatcaaacaactttctgattacacccaacttgatctttctgatggtgggccacctgcacttgtgctttggctcatcaaggactgtttgcagactggcgtcggcacaagtactcgccccagcgagatctgtgtgatcaacggggttgtcatgacccttgctgactttcacgccggtatcttcatcaaaggcaccgaacacgctgtgttcgctctcaacacatctgagggctggtacgctattgatgatgaggtgttctacccgtggacaccggaccctgagaacgtactcgcgtacgtgccttacgaccag

>MH053348.1_SAT3_ZIM_1977

tacaacggtgagaagaagaccttctacagcagacccaacacctacgggaactgttggctcaactcacttctgcagctctttcgatacgtcgacgagccgctttttgagtctgaatatctctcacctgaaaacaagacactggacatgatcagacaactgtctgattacaccaaacttgacctttcagacggtgggccaccagcacttgtgctttggctcatcaaggactgccttcagaccggcgtcggcaccagcactcgcccaagcgagatctgtgtcatcaacggggttgtcatgaccctggctgacttccacgctggtatcttcatcaagggcacggaacacgccgtgtttgccctcaacacatccgagggctggtacgccattgatgatgaggtgttttacccatggacacccgaccctggaaacgtactcgcgtacgtcccctacgaccag

>MH053349.1_SAT3_ZIM_1983

tacaacggtgagaagaagaccttctacagcagacccaacaaacacgggaactgttggctcaactcattgctgcagctctttcgatacgtcgatgagccactttttgagtctgagtatttgtcaccagaaaacaaaacattggatatgatcaaacaactctctgattacaccaaacttgacctttctgatggtgggccacctgcacttgtgctttggctcatcaaggactgtytgcagaccggtgttggcacaagcactcgccccagcgagatttgtgtaatcaacggggttgtcatgacccttgctgatttccacgccggtatcttcatcaaaggcaccgaacacgctgtgttcgccctcaacacatccgagggctggtacgccattgatgatgaggtgttctacccrtggacaccggaccctgagaacgtactcgcgtacgtgccttacgaccag

>MH053350.1_SAT3_ZIM_1983

tacaacggtgagaagaagaccttctacagcaggcccaacaaacacgggaactgttggctcaactcattgctgcagctctttcgatacgtcgatgagccactttttgagtctgagtatttgtcaccagaaaacaaaacattggatatgatcaaacaactctctgattacaccaaacttgacctttctgatggtgggccacctgcacttgtgctttggctcatcaaggactgtctgcagaccggtgttggcacaagcactcgccccagcgagatttgtgtaatcaacggggttgtcatgacccttgctgatttccacgccggtatcttcatcaaaggcaccgaacacgctgtgttcgccctcaacacatccgagggctggtacgccattgatgatgaggtgttctacccatggacaccggaccctgagaacgtactcgcgtacgtgccttacgaccag

>MH053351.1_SAT3_ZIM_1984

tacaacggagaaaagaaaaccttctacagcaggcccaacacccacgggaactgttggctcaactcattgctgcagctctttcgatacgtcgatgagccgctctttgagtctgagtacttgtcacctgaaaacaagacattggacatgatcaaacaactatctgattacaccaaattggacttgtcggacggagggccccccgctctcgtcctttggctgatcaaggactgtcttcaaaccggtgttggcaccagcactcgccccagcgagatctgtgtcatcaacggggttgtcatgaccctggctgactttcacgccggtatcttcatcaagggtactgaacacgctgtgttcgccctcaacacatctgagggctggtacgccattgatgatgaggtgttctacccatggacacctgaccctgaaaacgtgctcgcgtacgttccttacgaccgg

>MH053352.1_SAT3_ZIM_1990

tacaacggtgaaaagaagaccttctacagcaggcccaacacacacgggaactgttggctcaactcactgctgcagctcttccgatacgtcgatgagccgctctttgagtctgagtatttgtcacctgaaaacaagacattggacatgatcaaacaactctctgattacaccaagcttgatctttcagatggtgggccaccagcacttgtgctttggctcatcaaagactgcctccaaaccggcgttggtaccagcactcgccctagcgagatctgtgtgatcaacggggtcgccatgaccctggctgatttccacgccggcattttcatcaagggcactgagcacgccgtgttcgcactcaacacgtctgagggctggtacgccattgatgatgaggtgttctacccatggacacccgaccctgagaacgtactcgcgtacgtaccttacgaccag

>MH559780.1_A_Brazil_2016

cacaacggtgagagaaaagtgttctattctagacccaacaaccacgacaactgttggttgaacaccatccttcagctgttcaggtacgtcggagaacccttcttcgactgggtctatgactcacccgagaacctcactcttgaagctatcgagcaactggaggagctcacagggttagagttgcacgagggcggaccacctgccctcgtgatctggaacatcaaacacctgcttcataccggcatcggcaccgcctcgcggcccagcgaggtgtgcatggtggacggcacgaacatgtgtcttgctgacttccacgcaggcattttcctgaaaggacaggaacacgctgtgtttgcgtgtgtcacctccaacgggtggtacgcgattgacgacgaggacttttacccatggacgccggacccgtccgacgttttggtgtttgttccgtacgatcaa

>MH559781.1_A_Brazil_2016

cacaacggtgagagaaaagtgttctattctagacccaacaaccacgacaactgttggttgaacaccatccttcagctgttcaggtacgtcggagaacccttcttcgactgggtctatgactcacccgagaaccttactcttgaagctatcgagcaactggaggagctcacagggttagagttgcacgagggcggaccacctgccctcgtgatctggaacatcaaacacctgcttcataccggcatcggcaccgcctcgcggcccagcgaggtgtgcatggtggacggcacgaacatgtgtcttgctgacttccacgcaggcattttcctgaaaggacaggaacacgctgtgtttgcgtgtgtcacctccaacgggtggtacgcgattgacgacgaggacttttacccatggacgccggacccgtccgacgttttggtgtttgttccgtacgatcaa

>MH559783.1_A_Brazil_2016

cacaacggtgagagaaaagtgttctattctagacccaacaaccacgacaactgttggttgaacaccatccttcagctgttcaggtacgtcggagaacccttcttcgactgggtctatgactcacccgagaacctcactcttgaagctatcgagcaactggaggagctcacagggttagagttgcacgagggcggaccacctgccctcgtgatctggaacatcaaacacctgcttcataccggcatcggcaccgcctcgcggcccagcgaggtgtgcatggtggacggcacgaacatgtgtcttgctgacttccacgcaggcattttcctgaaaggacaggaacacgctgtgtttgcgtgtgtcacctccaacgggtggtacgcgattgacgacgaggacttttacccatggacgccggacccgtccgacgttttggtgtttgttccgtacgatcaa

>MH559785.1_A_Brazil_2016

cacaacggtgagagaaaagtgttctattctagacccaacaaccacgacaactgttggttgaacaccatccttcagctgttcaggtacgtcggagaacccttcttcgactgggtctatgactcacccgagaacctcactcttgaagctatcgagcaactggaggagctcacagggttagagttgcacgagggcggaccacctgccctcgtgatctggaacatcaaacacctgcttcataccggcatcggcaccgcctcgcggcccagcgaggtgtgcatggtggacggcacgaacatgtgtcttgctgacttccacgcaggcattttcctgaaaggacaggaacacgctgtgtttgcgtgtgtcacctccaacgggtggtacgcgattgacgacgaggacttttacccatggacgccggacccgtccgacgttttggtgtttgttccgtacgatcaa

>MH559786.1_A_Brazil_2016

cacaacggtgagagaaaagtgttctattctagacccaacaaccacgacaactgttggttgaacaccatccttcagctgttcaggtacgtcggagaacccttcttcgactgggtctatgactcacccgagaacctcactcttgaagctatcgagcaactggaggagctcacagggttagagttgcacgagggcggaccacctgccctcgtgatctggaacatcaaacacctgcttcataccggcatcggcaccgcctcgcggcccagcgaggtgtgcatggtggacggcacgaacatgtgtcttgctgacttccacgcaggcattttcctgaaaggacaggaacacgctgtgtttgcgtgtgtcacctccaacgggtggtacgcgattgacgacgaggacttttacccatggacgccggacccgtccgacgttttggtgtttgttccgtacgatcaa

>MH559788.1_A_Brazil_2016

cacaacggtgagagaaaagtgttctattctagacccaacaaccacgacaactgttggttgaacaccatccttcagctgttcaggtacgtcggagaacccttcttcgactgggtctatgactcacccgagaacctcactcttgaagctatcgagcaactggaggagctcacagggttagagttgcacgagggcggaccacctgccctcgtgatctggaacatcaaacacctgcttcataccggcatcggcaccgcctcgcggcccagcgaggtgtgcatggtggacggcacgaacatgtgtcttgctgacttccacgcaggcattttcctgaaaggacaggaacacgctgtgtttgcgtgtgtcacctccaacgggtggtacgcgattgacgacgaggacttttacccatggacgccggacccgtccgacgttttggtgtttgttccgtacgatcaa

>MH559791.1_A_Brazil_2016

cacaacggtgagagaaaagtgttctattctagacccaacaaccacgacaactgttggttgaacaccatccttcagctgttcaggtacgtcggagaacccttcttcgactgggtctatgactcacccgagaacctcactcttgaagctatcgagcaactggaggagctcacagggttagagttgcacgagggcggaccacctgccctcgtgatctggaacatcaaacacctgcttcataccggcatcggcaccgcctcgcggcccagcgaggtgtgcatggtggacggcacgaacatgtgtcttgctgacttccacgcaggcattttcctgaaaggacaggaacacgctgtgtttgcgtgtgtcacctccaacgggtggtacgcgattgacgacgaggacttttacccatggacgccggacccgtccgacgttttggtgtttgttccgtacgatcaa

>MH559793.1_A_Brazil_2016

cacaacggtgagagaaaagtgttctattctagacccaacaaccacgacaactgttggttgaacaccatccttcagctgttcaggtacgtcggagaacccttcttcgactgggtctatgactcacccgagaacctcactcttgaagctatcgagcaactggaggagctcacagggttagagttgcacgagggcggaccacctgccctcgtgatctggaacatcaaacacctgcttcataccggcatcggcaccgcctcgcggcccagcgaggtgtgcatggtggacggcacgaacatgtgtcttgctgacttccacgcaggcattttcctgaaaggacaggaacacgctgtgtttgcgtgtgtcacctccaacgggtggtacgcgattgacgacgaggacttttacccatggacgccggacccgtccgacgttttggtgtttgttccgtacgatcaa

>MH559796.1_A_Brazil_2016

cacaacggtgagagaaaagtgttctattctagacccaacaaccacgacaactgttggttgaacaccatccttcagctgttcaggtacgtcggagaacccttcttcgactgggtctatgactcacccgagaacctcactcttgaagctatcgagcaactggaggagctcacagggttagagttgcacgagggcggaccacctgccctcgtgatctggaacatcaaacacctgcttcataccggcatcggcaccgcctcgcggcccagcgaggtgtgcatggtggacggcacgaacatgtgtcttgctgacttccacgcaggcattttcctgaaaggacaggaacacgctgtgtttgcgtgtgtcacctccaacgggtggtacgcgattgacgacgaggacttttacccatggacgccggacccgtccgacgttttggtgtttgttccgtacgatcaa

>MH559798.1_A_Brazil_2016

cacaacggtgagagaaaagtgttctattctagacccaacaaccacgacaactgttggttgaacaccatccttcagctgttcaggtacgtcggagaacccttcttcgactgggtctatgactcacccgagaacctcactcttgaagctatcgagcaactggaggagctcacagggttagagttgcacgagggcggaccacctgccctcgtgatctggaacatcaaacacctgcttcacaccggcatcggcaccgcctcgcggcccagcgaggtgtgcatggtggacggcacgaacatgtgtcttgctgacttccacgcaggcattttcctgaaaggacaggaacacgctgtgtttgcgtgtgtcacctccaacgggtggtacgcgattgacgacgaggacttttacccatggacgccggacccgtccgacgttttggtgtttgttccgtacgatcaa

>MH559799.1_A_Brazil_2016

cacaacggtgagagaaaagtgttctattctagacccaacaaccacgacaactgttggttgaacaccatccttcagctgttcaggtacgtcggagaacccttcttcgactgggtctatgactcacccgagaacctcactcttgaagctatcgagcaactggaggagctcacagggttagagttgcacgagggcggaccacctgccctcgtgatctggaacatcaaacacctgcttcataccggcatcggcaccgcctcgcggcccagcgaggtgtgcatggtggacggcacgaacatgtgtcttgctgacttccacgcaggcattttcctgaaaggacaggaacacgctgtgtttgcgtgtgtcacctccaacgggtggtacgcgattgacgacgaggacttttacccatggacgccggacccgtccgacgttttggtgtttgttccgtacgatcaa

>MH559800.1_A_Brazil_2016

cacaacggtgagagaaaagtgttctattctagacccaacaaccacgacaactgttggttgaacaccatccttcagctgttcaggtacgtcggagaacccttcttcgactgggtctatgactcacccgagaacctcactcttgaagctatcgagcaactggaggagctcacagggttagagttgcacgagggcggaccacctgccctcgtgatctggaacatcaaacacctgcttcataccggcatcggcaccgcctcgcggcccagcgaggtgtgcatggtggacggcacgaacatgtgtcttgctgacttccacgcaggcattttcctgaaaggacaggaacacgctgtgtttgcgtgtgtcacctccaacgggtggtacgcgattgacgacgaggacttttacccatggacgccggacccgtccgacgttttggtgtttgttccgtacgatcaa

>MH559801.1_A_Brazil_2016

cacaacggtgagagaaaagtgttctattctagacccaacaaccacgacaactgttggttgaacaccatccttcagctgttcaggtacgtcggagaacccttcttcgactgggtctatgactcacccgagaaccttactcttgaagctatcgagcaactggaggagctcacagggttagagttgcacgagggcggaccacctgccctcgtgatctggaacatcaaacacctgcttcataccggcatcggcaccgcctcgcggcccagcgaggtgtgcatggtggacggcacgaacatgtgtcttgctgacttccacgcaggcattttcctgaaaggacaggaacacgctgtgtttgcgtgtgtcacctccaacgggtggtacgcgattgacgacgaggacttttacccatggacgccggacccgtccgacgttttggtgtttgttccgtacgatcaa

>MH559804.1_A_Brazil_2016

cacaacggtgagagaaaagtgttctattctagacccaacaaccacgacaactgttggttgaacaccatccttcagctgttcaggtacgtcggagaacccttcttcgactgggtctatgactcacccgagaacctcactcttgaagctatcgagcaactggaggagctcacagggttagagttgcacgagggcggaccacctgccctcgtgatctggaacatcaaacacctgcttcataccggcatcggcaccgcctcgcggcccagcgaggtgtgcatggtggacggcacgaacatgtgtcttgctgacttccacgcaggcattttcctgaaaggacaggaacacgctgtgtttgcgtgtgtcacctccaacgggtggtacgcgattgacgacgaggacttttacccatggacgccggacccgtccgacgttttggtgtttgttccgtacgatcaa

>MH559805.1_A_Brazil_2016

cacaacggtgagagaaaagtgttctattctagacccaacaaccacgacaactgttggttgaacaccatccttcagctgttcaggtacgtcggagaacccttcttcgactgggtctatgactcacccgagaacctcactcttgaagctatcgagcaactggaggagctcacagggttagagttgcacgagggcggaccacctgccctcgtgatctggaacatcaaacacctgcttcataccggcatcggcaccgcctcgcggcccagcgaggtgtgcatggtggacggcacgaacatgtgtcttgctgacttccacgcaggcattttcctgaaaggacaggaacacgctgtgtttgcgtgtgtcacctccaacgggtggtacgcgattgacgacgaggacttttacccatggacgccggacccgtccgacgttttggtgtttgttccgtacgatcaa

>MH784403.1_O_PAK_2016

tacaacggtgagaagaagactttctactccagacccaacaaccacgacaactgttggctgaacaccattcttcagttgttcaggtacgtcgatgagcctttcttcgactgggtttacaactcacctgagaacctcacacttgaagccatcagacaactggaggaagttactggccttgagctgcacgagggtgggccacccgccctcgtcatttggaacatcaaacacttgctcaacaccggaatcggcaccgcctcgcgacccagcgaggtgtgcatggtagacgggacggacatgtgtttggctgacttccacgctggcattttcctgaagggacaggaacacgctgtgttcgcctgcatcaccgccaacgggtggtacgcgatcgacgacgaggacttttacccctggacgccggacccgtccgacgttctggtgtttgtcccgtacgatcaa

>MH784404.1_O_PAK_2017

tacaacggtgagaagaagactttctactccagacccaacaaccacgacaactgttggctgaacaccattcttcagttgttcaggtacgtcgatgagcctttcttcgactgggtttacaactcacctgagaacctcacacttgaagccatcagacaactggaggaagttactggccttgagctgcacgagggtgggccacccgccctcgtcatttggaacatcaaacacctgctcaacaccggaatcggcaccgcctcgcgacccagcgaggtgtgcatggtagacgggacggacatgtgtttggctgacttccacgctggcattttcctgaagggacaggaacacgctgtgttcgcctgcatcaccgccaacgggtggtacgcgatcgacgacgaggacttttacccctggacgccggacccgtccgacgttctggtgtttgtcccgtacgatcaa

>MH784405.1_O_PAK_2017

tacaacggtgagaagaagactttctactccaggcccaacaaccacgacaactgttggctgaacaccattcttcagttgttcaagtacgtcgatgagcctttcttcgactgggtttacaactcacctgagaacctcacacttgaagccatcagacaactggaggaaattactggccttgagctgcacgagggtggaccacccgctctcgtcatttggaacatcaaacacctgctcaacaccggaatcggcaccgcctcgcgacccagcgaggtgtgcatggtagacgggacggacatgtgtttggctgacttccacgctggcattttcctgaagggacaggaacacgctgtgttcgcctgcgtcaccgccaacgggtggtacgcgatcgacgacgaggacttttacccctggacgccggacccgtccgacgttctggtgtttgtcccgtacgatcaa

>MH845413.2_O_VIT_2014

tacaacggcgagaagaagatcttctactccaggcccaacaaccacgacaactgttggctgaacgccatccttcagctgttcaggtacgtcgatgaacctttcttcgactgggtgtatgaatcacctgaaaacctcacccttgaggcgatcagacaactggagaacattactggttttgagctgcacgagggtgggccgcccgccctcgttatttggaacatcaaacacttactccacactgggatcggcactgcctcgcgacccagcgaggtgtgcatggtggacggtacagacatgtgcctggcagactttcacgctggcatcttcctgaaaggacaggaacacgccgtgtttgcctgcgtcacctccaacgggtggtacgcgatcgatgacgaagagttctacccctggacgccggatccgtccgacgtgttggtctttgtcccgtacgatcaa

>MH891503.1_O_VIT_2017

cacaacggtgagaaaaagactttctattctagacccaacaaacacgacaactgttggctgaacaccatcctccaattgtttaggtacgtcgatgaacctttcttcgactgggtctatgaatcacctgaaaacctcactcttgaggcgattgggcaattggaagaactcactggtcttaagctgcacgagggtgggccacccgctctcgtcatttggaacatcaaacatttgctccacaccggaattggcactgcctcgcgacccagcgaggtgtgcatggttgatggcacggacatgtgtttggctgacttccacgctggcatcttcctgaaagggcaagagcacgctgtgttcgcctgcgtcacctccaacgggtggtacgcgatcgacgacgaggacttctacccctggacgcctgatccgtccgacgtcctggtgtttgtcccgtacgatcag

>NC_039210.1_O_UKG_1965

tacaacggtgaaaagaagaccttttactccaggcccaacaaccacgacaactgttggttgaacaccatccttcagttgttcaggtacgtcgatgagcctttcttcgactgggtctacaactcaccccagaacctcacgcttgaagccatcaaacagctggaagaactcacagggcttgagttacacgaaggcggaccgcctgccgctgttgtttggaacatcaaacacttgcttcaaaccggtattggtaccgcctcgcgacctagcgaggtgtgtgtggtggacggtacgaacatgtgtctggctgatttccatgcaggcatcttcctgaaaggacaggaacatgccgtgtttgcttgtgtcacctccaacgggtggtacgcgattgacgatgaggacttctacccatggacgccggacccgtccgacgttctggtgttcgttccatatgatcaa
