# Supplementary material for: Early origin and global colonisation of foot-and-mouth disease virus
Source: Sci Rep. 2020 Sep 17;10:15268. doi: 10.1038/s41598-020-72246-6 (PMC7498456; doi:10.1038/s41598-020-72246-6)
Supplement: Supplementary file 5 — Supplementary Data S3. [file 41598_2020_72246_MOESM5_ESM.doc]

>AB079061.1_O_JPN_2000

tcgagcgtcggagtcacttacgggtacgcaacagttgaggactttgtgagcggaccaaacacatctgggcttgagaccagggttgtgcaggcagagcggttcttcaaaacccacctgttcgactgggtcaccagtgacccgttcggacggtgccacctgctggaactcccaactgaccacaaaggtgtctacggcagcctgactgactcttatgcttacatgagaaacggttgggatgttgaggtcaccgcagtgggaaatcagttcaacggaggatgtctgttggtggccatggtgccagaactttgctctattgacaagagagagctgtaccagctcacgctctttccccaccagttcatcaacccccggacgaacatgacggcgcacatcactgtgccctttgttggcgtcaaccgctacgaccagtacaaggtacacaaaccttggaccctcgtggttatggttgtggccccgctgactgtcaacaccgaaggtgccccacagatcaaggtctatgccaacatcgcccctaccaacgtacacgttgcgggtgagttcccttctaaggaagggatcttccccgtggcatgtagcgacggttacggtggtctggtgaccactgacccaaagacggctgaccccgcctacgggaaagtgttcaatccacctcgcaacatgttgccggggcggttcaccaacttccttgatgtggctgaggcgtgccctacgttcctgcactttgagggcggcgtgccgtacgtgaccacaaagacggactcagacagggtgctcgcccagtttgacttgtctctggcagcaaagcacatgtcaaacaccttcctggcaggtctcgcccagtactacacacagtacagcggcaccatcaacctgcacttcatgttcacaggacccactgacgcgaaagcgcgttacatgattgcatacgccccccctggcatggagccgcccaaaacacctgaggcggccgcacactgcattcatgcggagtgggacacagggttgaattcaaaattcacattttcaatcccttacctttcggcggctgattacgcgtacaccgcgtctgacactgcggagaccacaaatgtacagggatgggtttgcctgtttcaaatcacacacgggaaggctgacggcgacgcactggtcgttctagctagcgccggtaaggactttgagctgcgtctgccagttgacgctcgcacgcagaccacctccacaggtgagtcggctgaccctgtgactgccactgttgagaactacggtggtgagacacaggtccagagacgccaacacacggatgtctcgttcatattagacagatttgtgaaagtaacaccaaaagaccaaattaatgtgttggacctgatgcaaacccctgcacacactttggtaggcgcgctcctccgtactgccacctactacttcgcagacctagaagtggcagtgaaacacgaggggaaccttacctgggtcccgaatggggcgcccgagacagcgttggacaacaccaccaatccaacggcttaccacaaggcaccgctcacccggcttgcactgccttacacggcaccacaccgtgtcttggctactgtttacaacgggaactgcaagtatggcgagagccccgtgaccaatgtgagaggtgacctgcaagtattggctcagaaggcggcaagaacgctgcctacctccttcaattacggcgccatcaaagccactcgggtgactgaa

>AF026168.2_O_TAW_1997

tctagcgtcggggtgacttacgggtacgcaacggctgaagacttcgtgagtgggcctaacacctctggtcttgagaccagagttgttcaggccgaacggttcttcaaaacccacctgtttgactgggtcaccagtgacccgtttgggcggtgtcacttgttggagctaccgactgaccacaaaggcgtctacggtagcctgaccgactcgtacgcatacatgaggaatggttgggacgttgaagtcaccgcagtgggtaaccagttcaacggaggctgtttgctggtggcgatggtaccggagctctgttccatcagcaagagagagttgtaccagctcacgcttttcccccaccagttcatcaacccacggacgaatatgacggcacacatcaccgtgccctacctcggtgtcaacaggtacgaccagtacaaggtacacaaaccctggaccctcgtggtcatggttgtggcccccctgacggttaacaacgagggcgctccgcaaatcaaggtgtatgccaacatcgcccccaccaatgttcacgtcgcgggtgagctcccctctaaagagggaattttccccgtggcatgcagcgatggttacggtggcttggtgaccacggatccgaagacggcagaccccgtctacgggaaagtgttcaacccaccccgcaacctgttgccagggcggtttacaaacctccttgacgtggccgaggcgtgccccacattcctacacttcgacggtgacgttccgtacgtgaccacgaagacggattcggatagagtgctagcccggttcgatttgtccctcgcggcaaaacatatgtcgaacacttttctcgcgggtcttgcccagtactacacacagtacagcggcaccattaacctgcacttcatgttcacgggacccaccgacgcgaaggcacgctacatggttgcgtacgcccctcctggcatggaaccgccgaaaacgcctgaggcggctgcacattgcatccacgctgagtgggatacagggctgaattcgaagttcacgttttcaatcccatacctttcggcagctgactacccgtacaccgcgtccgacgtcgccgagaccacaaacgtacagggatgggtctgtttgttccagataacacacgggaaagccgacggtgacgccctggtcgtgctagctagtgctggcaaagactttgacttgcgtctgccggtcgacgccgaaccccaaaccacctctgcgggtgagtctgcggaccccgtgactgccaccgtcgagaactacggtggtgagacacaagtccagaggcgccaagacacggacattgcgttcatattggacaggttcgtgaaagtcaagccaaaggaacaagttaatgtgttggacctgatgcagatccctgcccacaccttggtaggggcgctcctgcgaacggccacctactacttctctgacctggagctggccgtcaagcacgagggcgatctcacctgggtcccaaacggcgcccctgagacagcactggacaacactaccaacccaacagcttaccacaaggaacccctcacacggctggcgctgccttacacggctccacaccgtgtcttagcgaccgtctacaacgggagcagtaagtacggtgacaccagcactaacaacgtgagaggtgaccttcaagtgttagctcagaaggcagaaagaactctgcctacctccttcaacttcggtgccatcaaggcaactcgtgttactgaa

>AF154271.1_O_TAW_1997

tctagcgtcggggtgacttacgggtacgcaacggctgaagacttcgtgagtgggcctaacacctctggtcttgagaccagagttgttcaggccgaacggttcttcaaaacccacctgtttgactgggtcaccagtgacccgtttgggcggtgtcacttgttggagctcccgactgaccacaaaggcgtctacggtagcctgaccgactcgtacgcatacatgaggaatggttgggacgttgaagtcaccgcagtgggtaaccagttcaacggaggctgtttgctggtggcgatggtaccggagctctgttccatcagcaagagagagttgtaccagctcacgcttttcccccaccagttcatcaacccacggacgaatatgacggcacacatcaccgtgccctacctcggtgtcaacaggtacgaccagtataaggtacacaaaccctggaccctggtggtcatggttgtggcccccctgacggttaacaacgagggcgctccgcaaatcaaggtgtatgccaacatcgcccccaccaatgttcacgtcgcgggtgagctcccttctaaagaggggattttccccgtggcatgcagcgatggttccggtggcttggtgaccacggatccgaagacggcagaccccgtctacgggaaagtgttcaacccaccccgcaacctgttgccagggcggtttacaaacctccttgacgtggccgaggcgtgccccacattcctacacttcgacggtgacgttccgtacgtgaccacgaagacggattcggacagggtgctagcccagttcgatttgtccctcgcggcaaaacatatgtcgaacacttttctcgcgggtcttgcccagtactacacacagtacagcggcaccattaacctgcacttcatgttcacgggacccaccgacgcgaaggcacgctacatggttgcgtacgcccctcctggcatggaaccgccgaaaacgcctgaggcggctgcacattgcatccacgctgagtgggatacagggctgaattcgaagttcacgttttcaatcccatacctttcggcagctgactacgcgtacaccgcgtccgacgtcgccgagaccacaaacgtacagggatgggtctgtttgttccagataacacacgggaaagccgacggtgacgccctggtcgtgctagctagtgctggcaaagactttgacttgcgtctgccggtcgacgcccgaacccaaaccacctctgcgggtgagtctgcggaccccgtgactgccaccgtcgagaactacggtggtgagacacaagtccagaggcgccagcacacggacattgcgttcatattggacaggttcgtgaaagtcaagccaaaggaacaagttaatgtgttggacctgatgcagatccctgcccacaccttggtaggggcgctcctgcgaacggccacctactacttctctgacctggagctggccgtcaagcacgagggcgatctcacctgggtcccaaacggcgcccctgagacagcactggacaacactaccaacccaacagcttaccacaaggaacccctcacacggctggcgctgccttacacggctccacaccgtgtcttagcgaccgtctacaacgggagcagtaagtacggtgacaccagcactaacaacgtgagaggtgaccttcaagtgttagctcagaaggcagaaagaactctgcctacctccttcaacttcggtgccatcaaggcaactcgtgttactgaa

>AF308157.1_O_TAW_1997

tctagcgtcggggtgacttacgggtacgcaacggctgaagacttcgtgagtgggcctaacacctctggtcttgagaccagagttgttcaggccgaacggttcttcaaaacccacctgtttgactgggtcaccagtgacccgtttgggcggtgtcacttgttggagctaccgactgaccacaaaggcgtctacggtagcctgaccgactcgtacgcatacatgaggaatggttgggacgttgaagtcaccgcagtgggtaaccaattcaacggaggctgtttgctggtggcgatggtaccggagctccgttccatcagcaagagagagttgtaccagcttacgcttttcccccaccagttcatcaacccacggacgaatatgacggcacacatcaccgtgccctacctcggtgtcaacaggtacgaccagtacaaggtacacaaaccctggaccctcgtggtcatggttgcggcccccttgaccgttaacaacgagggcgctccgcaaatcaaggtgtatgccaacatcgcccccaccaatgttcacgtcgcgggtgagctcccctctaaagaggggattttccccgtggcatgcagcgatggttacggtggcttggtgaccacggatccgaagacggcagaccccgtctacgggaaagtgttcaacccaccccgcaacctgttgccagggcggtttacaaacctccttgacgtggccgaggcgtgccccacattcctacacttcgacggtgacgttccgtacgtgaccacgaagacggattcggatagggtgctagcccagttcgatttgtccctcgcggcaaaacatatgtcgaacacttttctcgcgggtcttgcccagtactacacacagtacagcggcaccattaacctgcacttcatgttcacgggacccaccgacgcgaaggcacgctacatggttgcgtacgcccctcctggcatggaaccgccgaaaacgcctgaggcggctgcacattgcatccacgctgagtgggatacagggctgaattcgaagttcacgttttcaatcccatacctttcggcagctgactacgcgtacaccgcgtccgacgtcgccgagaccacaaacgtacagggatgggtctgtttgttccagataacacacgggaaagccgacggtgacgccctggtcgtgctagctagtgctggcaaagactttgacttgcgtctgccggtcgacgcccgaacccaaaccacctctgcgggtgagtctgcggaccccgtgactgccaccgtcgagaactacggtggtgagacacaagtccagaggcgccagcacacggacattgcgttcatactggacaggttcgtgaaagtcaagccaaaggaacaagttaatgtgttggacctgatgcagatccctgcccacaccttggtaggggcgctcctgcgaacggccacctactacttctctgacctggagctggccgtcaagcacgagggcgatctcacctgggtcccaaacggcgcccctgagacagcactggacaacactaccaacccaacagcttaccacaaggaacccctcacacggctggcgctgccttacacggctccacaccgtgtcttagcgaccgtctacaacgggagcagtaagtacggtgacaccagcactaacaacgtgagaggtgaccttcaagtgttagctcagaaggcagaaagaactctgcctacctccttcaacttcggtgccatcaaggcaactcgtgttactgaa

>AF377945.1_O_SKR_2000

tcgagcgttggagtcacctacgggtacgcaacagctgaggactttgtgagcggaccaaacacatctgggcttgagaccagggtggtgcaggcagagcggttcttcaaaacccattgttacgactgggtcaccagtgaccccttcggacggtgctacctgctggaactcccaactgaccacaaaggtgtctacggcagcctgactgactcttatgcttacatgagaaacggtggggatgttgaagtcactgcagttgggaaccagttcaacggagggtgtctgttggtggccatggtgccaaaactttgctctaatgacaagagagagctgtaccagctcacgctttttccccaccagttcatctacccccggacgaacatgacggcgcacatcactgtgccctttgttggcgtcaaccgctacgaccagtacaaggtacacaaaccttggaccctcgtggttatggttgtggccccgctgactatcaacaccgaaggtgccccacagatcaaggtctatgccaacatcgcccctaccaacgtgcacgttgcgggtgagttcccctctaaggaagggatcttccccgtggcatgtagcgacggttacggtggtatggtgaccactgacccaaagacggctgaccccgcctacgggaaagtgtttaatccaccccgtaacatgttgccggggtggttcaccaacttcctcgatgtggctgaggcgtgccctacgtttctgcactttgagggtgacgtgccgtacgtgaccacaaagacggactcagacagagtgctcgcccagtttgacctgtctctagcagcaaagcacatgtcaaacacctttctggcaggtctcgcccagtactacacacagtacagtggcacaatcaacctgcacttcatgttcacaggacccactgacgcgaacgcgcgttacatgattgcatacgccccccctggtatggagccgcccaaaacacctgaggcggccgctcactgcattcatgcggagtgggacacagggttgaattcaaaattcacattttcaatcccttacctttcggcggctgattacgcgtacaccgcgtctgacgttgcggagaccacaaatgtacagggatgggtttgcctgtttcaaattacacacgggaaggctgacggcgacgcactggtcgttctatccagcgccggcaaggactttgagctgcgtctgccagttgacgctcgcacgcaaaccacctccacaggtgagtccgctgaccccgtgactgccactgttgaaaactacggtggtgaaacacaggtccagagacgccaacacacggatgtctcgttcataatagacagatttgtgaaagtaacaccaaaagaccaaattaacgtgttggacctgatgcaaacccctgcacacactttggtaggcgcgctcctccgtactgccacttactacttcgcagatgtagaagtggcagtgaaacacgagggggaccttaccttggtcccgaatggggcgcccgagacaacgttggacaacaccaccaatccaacggcgtaccacaaggcaccgctcactcggtttgcactgccttacacggcaccacaccgtgtcttggctactgtttacaacgggaactgcaagtatggcgagagtcccgtgaccaatgtgagaggtgacctgcaagttttggcccagaaggcggcaagaacgctgcctacctccttcaattacggtgccatcaaagccactcgggtgactgaa

>AF506822.2_O_CHA_1999

tcgagcgttggagtcacttacgggtacgcaacagctgaggactttgtgagcggaccaaacacatctgggcttgagaccagggttgtgcaggcagagcggttcttcaaaacccacttgttcgactgggtcaccggtgacccgttcggacggtgctacctgctggaactcccaactgaccacaaaggtgtctacggcagcctgactgactcttatgcttacatgagaaacggttgggatgttgaggtcactgcagtgggaaatcagttcaacggaggatgtctgttggtggccatggtgccagaactttgctctattgacaagagagagctgtaccagctcacgctctttccccaccagttcatcaacccccggacgaacatgacggcgcacatcactgtgccctttgttggtgtcaaccgctacgaccagtacaaggtacacaaaccttggaccctcgtggttatggttgtggccccgctgactgtcaacaccgaaggtgccccacagatcaaggtctatgccaacatcgcccctaccaacgtgcacgttgcgggtgagttcccttctaaggaagggatcttccccgtggcatgtagcgacggttacggtggtctggtgaccactgacccaaagacggctgaccccgcctacgggaaagtgttcaatccacctcgcaacatgttgccggggcggttcaccaacttccttgatgtggctgaggcgtgccctacgtttctgcactttgagggtgacgtgccgtacgtgaccacaaagacggactcagacagggtgctcgcccagtttgacttgtctctggcagcaaagcacatgtcaaacaccttcctggcaggtctcgcccagtactacacacagtacagcggcaccatcaacctgcacttcatgttcacaggacccactgacgcgaaagcgcgttacatgattgcatacgccccccctggcatggagccgcccaaaacacctgaggcggccgctcactgcattcatgcggagtgggacacagggttgaattcaaaattcacattttcaatcccttacctttcggcggctgattacgcgtacaccgcgtctgacgctgcggagaccacaaatgtacagggatgggtctgcctgtttcaaattacacacgggaaggctgacggcgacgcactggtcgttctagctagcgccggtaaggactttgagctgcgtctgccagttgacgctcgcacgcagaccacctccacaggtgagtcggctgaccccgtgactgccactgttgagaactacggtggtgagacacaggtccagagacgccaacacacggatgtctcgttcatattagacagatttgtgaaagtaacaccaaaagaccaaattaatgtgttggacctgatgcaaacccctgcacacactttggtaggcgcgctcctccgtactgccacctactacttcgcagatctagaagtggcagtgaaacacgaggggaaccttacctgggtcccgaatggggcgcccgagacagcgttggacaacaccaccaatccaacggcttaccacaaggcaccgctcacccggcttgcactgccttacacggcaccacaccgtgtcttggctactgtttacaacgggaactgcaagtatgacgagagccccgtgaccaatgtgagaggtgacctgcaagtgttggcccagaaggcggcaagaacgctgcctacctccttcaattacggtgccgtcaaagccactcgggtgactgaa

>AH012984.2_O_SKR_2000

tcgagcgttggagtcacttacgggtacgcaacagctgaggacttcgtgagcggaccaaacacatctgggcttgagaccagggttgtgcaggcagagcggttctttaaaacccacttgttcgactgggtcaccagtgacccgttcggacggtgctacctgctggaactcccaactgaccacaaaggtgtctacggcagcctgaccgactcttatgcttacatgagaaacggttgggacgttgaggtcactgcagtgggaaatcagttcaacggaggatgtttgttggtggtcatggtgccagaactttgctctattgacaagagagggctataccagctcacgctctttccccaccaattcatcaacccccagacgaacatgacggcgcacattactgtgccctttgttggcgtcaaccgctacgaccagtacaaagtacacaaaccttggaccctcgttgtcatggttgtggccccgctgactgtcaacaccgaaggtgccccacagatcaaggtctatgccaacatcgcccctactaacgtgcacgttgcgggtgagctcccttctaaggaagggatcttccccgtggcatgtagcgacggttacggtggtctggtgaccactgacccaaagacggctgaccccgcctacgggaaagtgttcaatccacctcgcaacatgttgccggggcggttcaccaacttccttgatgtggctgaggcgtgccctacgttcctgcactttgagggtgacgtgccgtacgtgaccacaaagacggactcagacagggtactcgcccagtttgacttgtctctggcagcaaagcacatgtcaaacaccttcctggcaggtctcgcccagtactacacacagtacagtggcaccatcaacctgcacttcatgttcacaggacccactgacgcgaaagcgcgttacatgattgcatacgccccccctggcatggagccgcccaaaacacccgaggcggccgctcactgcattcatgcggagtgggacacagggttgaattcaaaattcacattttcaatcccttacctttcggcggctgattatgcgtacaccgcgtctgacaccgcggagaccacaaatgtgcagggttgggtttgcctgtttcaaattacacacgggaaggctgacggcgacgcactggtcgttctagctagcgccggtaaggattttgagctgcgtctgccagttgacgctcgcacgcagaccacctccacaggtgagtcggctgaccccgtgactgccaccgttgagaactacggtggtgagacacaggtccagagacgccaacacacggatgtctcgttcatactagacagatttgtgaaagtaacaccaaaagaccaaattaatgtgttggacctgatgcaaatccctgcacacactttggtaggcgcgctcctccgtactgccacctactacttcgcagatctggaagtggcagtgaaacacgaggggaacctcacctgggtcccgaacggggcgcccgaggcagcgttggacaacaccaccaatccaacggcctatcacaaggcgccgctcacccggcttgcactgccttacacggcaccacaccgtgtcttggctactgtttacaacgggaactgcaagtatggcgagagccccgtgaccaatctgagaggtgacctgcaagtgttgacccagaaggcggcaagaacgctgcctacctccttcaattacggtgccatcaaagccactcgggtgactgaa

>AH012985.2_O_SKR_2000

tcgagcgttggagtcacttacgggtacgcaacagccgaggactttgtgagcggaccaaacacatctgggctcgagaccagggttgtgcaggcagagcggttcttcaaaacccacttgttcgactgggtcaccagtgacccgttcggacggtgctacctgctggaactcccaactgaccacaaaggtgtctacggcagcctgactgactcttatgcttacatgagaaacggttgggttgttgaggtcactgcagtgggaaatcagttcaacggaggatgtccgttggtggccatggtgccagaacattgctctattgacaagagagagctgtaccagctcacgctctttccccaccagttcatcaacccccggacgaacatgacggcgcacatcactgtgccctttgttggtgtcaatcgctacgaccagtacaaggtacacaaaccttggaccctcgtggttatggttgtggccccgctgactgtcaacaccgaaggtgccccacagaccaaggtctacgccaacatcgcccctaccaacgtgtacgttgcgggtgagttcccttccaaggaagggatcttccccgtggcatgtagcgacggttacggtggtctggtgaccactgacccaaagacggctgaccccgcctacgggaaagtgttcaatccacctcgcaacatgttgccggggcggttcaccaacttccttgatgtggctgaggcgtgccctacgtttctgcactttgagggtgacgtgccgtacgtgaccacaaagacggactcagacagggtgctcgcccagtttgacttgtctctggcagcaaaacacatgtcaaacaccttcctggcgggtctcgcccagtactacacacagtacagtggcaccatcaacctgcacttcatgttcacaggacccactgacgcgaaagcgcgttacatgattgcatacgccccccctggcatggagccgcccaaaacacctgaggcggccgctcactgcattcatgcggagtgggacacagggttgaattcaaaattcacattttcaatcccttacctttcggcggctgattacgcgtacaccgcgtctgatgctgcggagaccacaaatgtacagggttgggtttgcctgtttcaaattacacacgggaaggctgacggcgacgcactggtcgttctagctagcgccggcaaggactttgagctgcgtctgccagttgacgctcgcacgcagaccacctccacaggtgagtcggctgaccccgtgactgccactgttgagaactacggtggtgagacacaggtccagagacgccaacacacggatgtctcgttcatattagacagatttgtgaaagtaacaccaaaagaccaaattaatgtgttggacctgatgcaaacccctgcacacactttggtaggcgcgctcctccgtactgccacctactacttcgcagatctagaagtggcagtgaaacacgaggggaaccttacctgggtcccgaacggggcgcccgagacagcgttggacaacaccaccaatccaacggcctaccacaaggcaccgctcacccggcttgcactgccttacacggcaccacaccgtgtcttggctactgtttacaacgggaactgcaagtatggcgagggccccgtgaccaatgtgagaggtgacctgcaagtattggcccagaaggcggcaagaacgctgcctacctccttcaactacggtgccatcaaagccactcgggtgactgaa

>AJ539136.1_O_TAW_1999

tcgagcgttggagtcacttacgggtacgcaacagctgaggactttgtgagcggaccaaacacatctgggcttgagaccagggttgtgcaggcagagcggttcttcaaaacccacttgttcgactgggtcaccagtgacccgttcggacggtactacctgctggaactcccaactgaccacaaaggtgtctacggcagcctgactgactcttatgcttacatgagaaacggttgggatgttgaggtcactgcagtgggaaatcagttcaacggaggatgtctcttggtggccatggtgccagaactttgctctattgacaagagagagctgtaccagctcacgctctttccccaccagttcatcaacccccggacgaacatgacggcgcacatcactgtgccctttgttggcgtcaaccgctacgaccagtacaaggtacacaaaccttggaccctcgtggttatggttgtggccccgctgactgtcaacaccgaaggtgccccacagatcaaggtctatgccaacatcgcccctaccaacgtgcacgttgcgggtgagttcccttctaaggaagggatcttccccgtggcatgtagcgacggttacggtggtctggtgaccactgacccaaagacggctgaccccgcctacgggaaagtgtttaatccacctcgcaacatgttgccggggcggttcaccaacttccttgatgtggctgaggcgtgtcctacgtttctgcactttgagggtgacgtgccgtacgtgaccacaaagacggactcagacagggtgctcgcccagtttgacttgtctctggcagcaaagcacatgtcaaacaccttcctggcaggtctcgcccagtactacacacagtacagcggcaccatcaacctgcacttcatgttcacaggacccactgacgcgaaagcgcgttacatgattgcatacgccccccctggcatggagccgcccaaaacacctgaggcggccgctcactgcattcatgcggagtgggacacagggttgaattcaaaattcacattttcaatcccttacctttcggcggctgattacgcgtacaccgcgtctgacgttgcggagaccacaaatgtacagggatgggtttgcctgtttcaaattacacacgggaaggctgacggcgacgcactggtcgttctagctagcgccggcaaggactttgagctgcgtctgccagttgacgctcgcacgcagaccacctccacaggtgagtcggctgaccccgtgactgccactgttgagaactacggtggtgagacacaggtccagagacgccaacacacggatgtctcgttcatattagacagatttgtgaaagtaacaccaaaagaccaaattaatgtgttggacctggtgcaaacccctgcacacactttggtaggcgcgctcctccgtactgccacctactacttcgcagatctagaagtggcagtgaaacacgaggggaaccttacctgggtcccgaatggggcgcccgagacagcgttggacaacaccaccaatccaacggcttaccacaaggcaccgctcacccggcttgcactgccttacacggcaccacaccgtgtcttggctactgtttacaacgggaactgcaagtatggcgagagccccgtgaccaacgtgagaggtgacctgcaagtattggcccagaaggcggcaagaacgctgcctacctccttcaattacggtgccatcaaagccactcgggtgactgaa

>AJ539137.1_O_TAW_1999

tcgagcgttggagtcacttacgggtacgcaacagctgaggactttgtgagcggaccaaacacatctgggcttgagaccagggttgtgcaggcagagcggttcttcaaaacccacttgttcgactgggtcaccagtgacccgttcggacggtgctacctgctggaactcccaactgaccacaaaggtgtctacggcagcctgactgactcttatgcttacatgagaaacggttgggatgttgaggtcactgcagtgggaaatcagttcaacggaggatgtctcttggtggccatggtgccagaactttgctctattgacaagagagagctgtaccagctcacgctctttccccaccagttcatcaacccccggacgaacatgacggcgcacatcactgtgccctttgttggcgtcaaccgctacgaccagtacaaggtacacaaaccttggaccctcgtggttatggttgtggccccgctgactgtcaacaccgaaggtgccccacagatcaaggtctatgccaacatcgcccctaccaacgtgcacgttgcgggtgagttcccttctaaggaagggatcttccccgtggcatgtagcgacggttacggtggtctggtgaccactgacccaaagacggctgaccccgcctacgggaaagtgtttaatccacctcgcaacatgttgccggggcggttcaccaacttccttgatgtggctgaggcgtgtcctacgtttctgcactttgagggtgacgtgccgtacgtgaccacaaagacggactcagacagggtgctcgcccagtttgacttgtctctggcagcaaagcacatgtcaaacaccttcctggcaggtctcgcccagtactacacacagtacagcggcaccatcaacctgcacttcatgttcacaggacccactgacgcgaaagcgcgttacatgattgcgtacgccccccctggcatggagccgcccaaaacacctgaggcggccgctcactgcattcatgcggagtgggacacagggttgaattcaaaattcacattttcaatcccttacctttcggcggctgattacgcgtacaccgcgtctgacgttgcggagaccacaaatgtacagggatgggtttgcctgtttcaaattacacacgggaaggctgacggcgacgcactggtcgttctagctagcgccggcaaggactttgagctgcgtctgccagttgacgctcgcacgcagaccacctccacaggtgagtcggctgaccccgtgactgccactgttgagaactacggtggtgagacacaggtccagagacgccaacacacggatgtctcgttcatattagacagatttgtgaaagtaacaccaaaagaccaaattaatgtgttggacctggtgcaaacccctgcacacactttggtaggcgcgctcctccgtactgccacctactacttcgcagatctagaagtggcagtgaaacacgaggggaaccttacctgggtcccgaatggggcgcccgagacagcgttggacaacaccaccaatccaacggcttaccacaaggcaccgctcacccggcttgcactgccttacacggcaccacaccgtgtcttggctactgtttacaacgggaactgcaagtatggcgagagccccgtgaccaacgtgagaggtgacctgcaagtattggcccagaaggcggcaagaacgctgcctacctccttcaattacggtgccatcaaagccactcgggtgactgaa

>AJ539138.1_O_CHA_1999

tcgagcgttggagtcacttacgggtacgcaacagctgaggactttgtgagcggaccaaacacatctgggcttgagaccagggttgtgcaggcagagcggttcttcaaaacccacttgttcgactgggtcaccagtgacccgttcggacggtnctacctgctggaactcccaactgaccacaaaggtgtctacggcagcctgactgactcttatgcttacatgagaaacggttgggatgttgaggtcactgcagtgggaaatcagttcaacggaggatgtctgttggtggccatggtgccagaactttgctctattgacaagagagagctgtaccagctcacgctctttccccaccagttcatcaacccccggacgaacatgacggcgcacatcactgtgccctttgttggtgtcaaccgctacgaccagtacaaggtacacaaaccttggaccctcgtggttatggttgtggccccgctgactgtcaacaccgaaggtgccccacagatcaaggtctatgccaacatcgcccctaccaacgtgcacgttgcgggtgagttcccttctaaggaagggatcttccccgtggcatgtagcgacggttacggtggtctggtgaccactgacccaaagacggctgaccccgcctacgggaaagtgttcaatccacctcgcaacatgttgccggggcggttcaccaacttccttgatgtggctgaggcgtgccctacgtttctgcactttgagggtgacgtgccgtacgtgaccacaaagacggactcagacagggtgctcgcccagtttgacttgtctctggcagcaaagcacatgtcaaacaccttcctggcaggtctcgcccagtactacacacagtacagcggcaccatcaacctgcacttcatgttcacaggacccactgacgcgaaagcgcgttacatgattgcatacgccccccctggcatggagccgcccaaaacacctgaggcggccgctcactgcattcatgcggagtgggacacagggttgaattcaaaattcacattttcaatcccttacctttcggcggctgattacgcgtacaccgcgtctgacgctgcggagaccacaaatgtacagggatgggtctgcctgtttcaaattacacacgggaaggctgacggcgacgcactggtcgttctagctagcgccggtaaggactttgagctgcgtctgccagttgacgctcgcacgcagaccacctccacaggtgagtcggctgaccccgtgactgccactgttgagaactacggtggtgagacacaggtccagagacgccaacacacggatgtctcgttcatattagacagatttgtgaaagtaacaccaaaagaccaaattaatgtgttggacctgatgcaaacccctgcacacactttggtaggcgcgctcctccgtactgccacctactacttcgcagatctagaagtggcagtgaaacacgaggggaaccttacctgggtcccgaatggggcgcccgagacagcgttggacaacaccaccaatccaacggcttaccacaaggcaccgctcacccggcttgcactgccttacacggcaccacaccgtgtcttggctactgtttacaacgggaactgcaagtatggcgagagccccgtgaccaatgtgagaggtgacctgcaagtgttggcccagaaggcggcaagaacgctgcctacctccttcaattacggtgccatcaaagccactcgggtgactgaa

>AJ539139.1_O_SKR_2000

tcgagcgttggagtcacttacgggtacgcaacagccgaggactttgtgagcggaccaaacacatctgggctcgagaccagggttgtgcaggcagagcggttcttcaaaacccacttgttcgactgggtcaccagtgacccgttcggacggtgctacctgctggaactcccaactgaccacaaaggtgtctatggcagcctgactgactcttatgcttacatgagaaacggttgggatgttgaggtcactgcagtgggaaatcagttcaacggaggatgtctgttggtggccatggtgccagaactttgctctattgacaagagagagctgtaccagctcacgctctttccccaccagttcatcaacccccggacgaacatgacggcgcacatcactgtgccctttgttggcgtcaatcgctacgaccagtacaaggtacacaaaccttggaccctcgtggttatggttgtggccccgctgactgtcaacaccgaaggtgccccacagatcaaggtctacgccaacatcgcccctaccaacgtgcacgttgcgggtgagttcccttccaaggaagggatcttccccgtggcatgtagcgacggttacggtggtctggtgaccactgacccaaagacggctgaccccgcctacgggaaagtgttcaatccacctcgcaacatgttgccggggcggttcaccaacttccttgatgtggctgaggcgtgccctacgtttctgcactttgagggtgacgtgccgtacgtgaccacaaagacggactcagacagggtgctcgcccagtttgacttgtctctggcagcaaaacacatgtcaaacaccttcctggcgggtctcgcccagtactacacacagtacagcggcaccatcaacctgcacttcatgttcacaggacccactgacgcgaaagcgcgttacatgattgcatacgccccccctggcatggagccgcccaaaacacctgaggcggccgctcactgcattcatgcggagtgggacacagggttgaattcaaaattcacattttcaatcccttacctttcggcggctgattacgcgtacaccgcgtctgatgctgcggagaccacaaatgtacagggttgggtttgcctgtttcaaattacacacgggaaggctgacggcgacgcactggtcgttctagctagcgccggcaaggactttgagctgcgtctgccagttgacgctcgcacgcagaccacctccacaggtgagtcggctgaccccgtgactgccactgttgagaactacggtggtgagacacaggtccagagacgccaacacacggatgtctcgttcatattagacagatttgtgaaagtaacaccaaaagaccaaattaatgtgttggacctgatgcaaacccctgcacacactttggtaggcgcgctcctccgtactgccacctactacttcgcagatctagaagtggcagtgaaacacgaggggaaccttacctgggtcccgaacggggcgcccgagacagcgttggacaacaccaccaatccaacggcctaccacaaggcaccgctcacccggcttgcactgccttacacggcaccacaccgtgtcttggctactgtttacaacgggaactgcaagtatggcgagggccccgtgaccaatgtgagaggtgacctgcaagtattggcccagaaggcggcaagaacgctgcctacctccttcaactacggtgccatcaaagccactcgggtgactgaa

>AJ539140.1_O_SAR_2000

tcgagcgttggagtcacttacgggtacgcaacagctgaggactttgtgagcggaccaaacacatctgggcttgagaccagggttgtgcaggcagagcggttcttcaaaacccacttgttcgactgggtcaccagtgacccgtttggacggtnctatctgctggaactcccaactgaccacaaaggtgtctacggcagcctgaccgactcttatgcttacatgagaaacggttgggatgttgaggtcaccgcagtgggaaatcagttcaacggaggatgtctgttggtggccatggtgccagaactttgctctattgacaagagagagctgtaccagctcacgctctttccccaccagttcatcaacccccggacgaacatgacggcgcacatcactgtgccctttgttggcgtcaaccgctacgaccagtacaaggtacacaaaccttggaccctcgtggttatggttgtggccccgctgactgtcaacaccgaaggtgccccacagatcaaggtctatgccaacatcgcccctaccaacgtgcacgttgcgggtgagttcccttctaaggaagggatcttccccgtggcatgtagcgacggttacggtggtctggtgaccactgacccaaagacggctgaccccgcctacgggaaagtgttcaatccacctcgcaacatgttgccggggcggttcaccaacttccttgatgtggctgaggcgtgccctacgtttctgcactttgagggtggcgtgccgtacgtgaccacaaagacggactcagacagggtgctcgcccagtttgacttgtctctggcagcaaagcacatgtcaaacaccttcctggcaggtctcgcccagtactacacacagtacagcggcaccatcaacctgcacttcatgttcacaggacccactgacgcgaaagcgcgttacatgattgcatacgccccccctggtatggagccgcccaaaacacctgaggcggccgcccactgcattcatgcggagtgggacacagggttgaactcaaaattcacattttcaatcccttacctttcggcggctgattacgcgtacaccgcgtctgacgctgcggagaccacaaatgtacagggatgggtttgcctgtttcaaattacacacgggaaggctgacggcgacgcactggtcgttctagctagcgccggtaaggactttgagctgcgtctgccagttgacgctcgcacgcagaccacctccacaggtgagtcggctgaccccgtgactgccactgttgagaactacggtggtgagacacaggtccagagacgccaacacacggatgtctcgttcatattagacagatttgtgaaagtaacaccaaaagaccaaattaatgtgttggacctgatgcaaacccctgcacacactttggtaggcgcgctcctccgtactgccacctactacttcgcagatctagaagtggcagtgaaacacgaggggaaccttacctgggtcccgaatggggcgcccgagacagcgttggacaacaccaccaatccaacggcttaccacaaggcaccgctcacccggcttgcactgccttacacggcaccgcaccgtgtcttggctactgtttacaacgggaactgcaagtatggcgagagccccgtgaccaatgtgagaggtgacctgcaagtattggcccaaaaggcggcaagaacgctgcctacctccttcaattacggtgccatcaaagccactcgggtgactgaa

>AJ539141.1_O_UKG_2001

tcgagcgttggagtcacttacgggtacgcaacagctgaggactttgtgagcggaccaaacacatctgggcttgagaccagggttgtgcaggcagagcggttcttcaaaacccacttgttcgactgggtcaccagtgacccgtttggacggtgctatctgctggaactcccaactgaccacaaaggtgtctacggcagcctgaccgactcttatgcttacatgagaaacggttgggatgttgaggtcaccgcagtgggaaatcagttcaacggaggatgtctgttggtggccatggtgccagaactttgctctattgacaagagagagctgtaccagctcacgctctttccccaccagttcatcaacccccggacgaacatgacggcgcacatcactgtgccctttgttggcgtcaaccgctacgaccagtacaaggtacacaaaccttggaccctcgtggttatggttgtggccccgctgactgtcaacaccgaaggtgccccacagatcaaggtctatgccaacatcgcccctaccaacgtgcacgttgcgggtgagttcccttctaaggaagggatcttccccgtggcatgtagcgacggttacggtggtctggtgaccactgacccaaagacggctgaccccgcctacgggaaagtgttcaatccacctcgcaacatgttgccggggcggttcaccaacttccttgatgtggctgaggcgtgccctacgtttctgcactttgagggtggcgtgccgtacgtgaccacaaagacggactcagacagggtgctcgcccagttcgacttgtctctggcagcaaagcacatgtcaaacaccttcctggcaggtctcgcccagtactacacacagtacagcggcaccatcaacctgcacttcatgttcacaggacccactgacgcgaaagcgcgttacatgattgcatacgccccccctggtatggagccgcccaaaacacctgaggcggccgcccactgcattcatgcggagtgggacacagggttgaattcaaaattcacattttcaatcccttacctttcggcggctgattacgcgtacaccgcgtctgacgctgcggagaccacaaatgtacagggatgggtttgcctgtttcaaattacacacgggaaggctgacggcgacgcactggtcgttctagctagcgccggtaaggactttgagctgcgtctgccagttgacgctcgcacgcagaccacctccgcaggtgagtcggctgaccccgtgactgccactgttgagaactacggtggtgagacacaggtccagagacgccaacacacggatgtctcgttcatattagacagatttgtgaaagtaacaccaaaagaccaaattaatgtgttggacctgatgcaaacccctgcacacactttggtaggcgcgctcctccgtactgccacctactacttcgcagatctagaagtggcagtgaaacacgaggggaaccttacctgggtcccgaatggggcgcccgagacagcgttggacaacaccaccaatccaacggcttaccacaaggcaccgctcacccggcttgcactgccttacacggcaccgcaccgtgtcttggctactgtttacaacgggaactgcaagtatggcgagagccccgtgaccaatgtgagaggtgacctgcaagtattggcccaaaaggcggcaagaacgctgcctacctccttcaattacggtgccatcaaagccactcgggtgactgaa

>AJ633821.1_O_FRA_2001

tcgagcgttggagtcacttacgggtacgcaacagctgaggactttgtgagcggaccaaacacatctgggcttgagaccagggttgtgcaggcagagcggttcttcaaaacccacttgttcgactgggtcaccagtgacccgtttggacggtgctatctgctggaactcccaactgaccacaaaggtgtctacggcagcctgaccgactcttatgcttacatgagaaacggttgggatgttgaggtcaccgcagtgggaaatcagttcaacggaggatgtctgttggtggccatggtgccagaactttgctctattgacaagagagagctgtaccagctcacgctctttccccaccagttcatcaacccccggacgaacatgacggcgcacatcactgtgccctttgttggcgtcaaccgctacgaccagtacaaggtacacaaaccttggaccctcgtggttatggttgtggccccgctgactgtcaacaccgaaggtgccccacagatcaaggtctatgccaacatcgcccctaccaacgtgcacgttgcgggtgagttcccttctaaggaagggatcttccccgtggcatgtagcgacggttacggtggtctggtgaccactgacccaaagacggctgaccccgcctacgggaaagtgttcaatccacctcgcaacatgttgccggggcggttcaccaacttccttgatgtggctgaggcgtgccctacgtttctgcactttgagggtggcgtgccgtacgtgaccacaaagacggactcagacagggtgctcgcccagtttgacttgtctctggcagcaaagcacatgtcaaacaccttcctggcaggtctcgcccagtactacacacagtacagcggcaccatcaacctgcacttcatgttcacaggacccactgacgcgaaagcgcgttacatgattgcatacgccccccctggtatggagccgcccaaaacacctgaggcggccgcccactgcattcatgcggagtgggacacagggttgaattcaaaattcacattttcaatcccttacctttcggcggctgattacgcgtacaccgcgtctgacgctgcggagaccacaaatgtacagggatgggtttgcctgtttcaaattacacacgggaaggctgacggcgacgcactggtcgttctagctagcgccggtaaggactttgagctgcgtctgccagttgacgctcgcacgcagaccacctccgcaggtgagtcggctgaccccgtgactgccactgttgagaactacggtggtgagacacaggtccagagacgccaacacacggatgtctcgttcatattagacagatttgtgaaagtaacaccaaaagaccaaattaatgtgttggacctgatgcaaacccctgcacacactttggtaggcgcgctcctccgtactgccacctactacttcgcagatctagaagtggcagtgaaacacgaggggaaccttacctgggtcccgaatggggcgcccgagacagcgttggacaacaccaccaatccaacggcttaccacaaggcaccgctcacccggcttgcactgccttacacggcaccgcaccgtgtcttggctactgtttacaacgggaactgcaagtatggcgagagccccgtgaccaatgtgagaggtgacctgcaagtattggcccaaaaggcggcaagaacgctgcctacctccttcaattacggtgccatcaaagccactcgggtgactgaa

>AY317098.1_O_CHA_2002

tcaagtgtcggggtgacgtacggatatgcaacggctgaggactttgtaagcgggcccaacacttctggtcttgagaccagagttgttcaggccgaacggttcttcaagacccacctgttcgactggggcaccaacgactcgtttgggcggtgttacttgttggagctaccaactgaccacaaaggtgtctacggcagcctgaccgactcatacgcatacatgaggaacggttgggacgttgaggtcaccgcagtggggaaccagttcaacggaggttgtttggtagtggcgatggtaccggagctctgccccatcaccaagagagagctgtaccaactcacacttttccctcaccagttcatcaacccacggacgaacatgacggcacacatcaccgtgccctatctcggtgtcaacaggtacgaccagtacaaggtacacaaaccctggactctcgtggtcatggttgtggctcctttgacggtcaacaacgagggcgccccgcaaatcaaggtgtatgccaacatcgcccccaccaacgttcacgtcgcgggtgagctcccttccaaagaggggatcttccctgtggcatgtagcgacggttacggtggcttggtaaccacggacccgaagacggcagaccccgtctacgggaaagtgttcaacccaccccgcaacctgctgccagggcggttcacaaacctccttgatgtggctgaggcgtgtcctacgttcctgcacttcgatggtgacgttccctacgtgatcacgaagacggattcagacagggtgctggcccagttcgacttgtccctcgcggcaaagcacatgtcgaacacctttctcgcgggtcttgcccagtactacgcacagtacagcggcaccatcaacctgcacttcatgttcacggggcccaccgatgcaaaggcacgctacatggttgcgtatgcccctcctggcatggaaccacctaaaacgcctgaggcggctgcacactgcatccacgctgagtgggacactgggctgaactcgaaattcacgttttcgatcccatacctttcggcggcagactacgcatacaccgcgtccgacgttgccgagactacaaacgtgcagggatgggtctgtctgttccagataacacacgggaaagccgacggcgacgccctggtagtactagccagtgccggcaaggactttgacttgcgcctgccggttgacgcccgaacccaaaccacctctgcgggtgagtctgcggaccccgtgactaccaccgtcgaaaactacggcggcgagacacaagtccagaggcgccaacacacggacgttgcgttcatattggacaggttcgtgaaagtcaaaccacaggagcaagttaacgtgttggacctgatgcagatccctgcccacaccttggtaggggcactcctgcggacggccacctattacttctctgacctggaactagctgtcaagcacgagggcgatctcacctgggttccaaacggtgcccccgaggcagcactgaacaacaccaccaacccaacagcctaccacaaggaaccgctcacacggctggcgctgccttatacggctccgcaccgcgtcttagctaccgtctacaacgggagcagcaagtacggtgacaccagcactaacaacgtgagaggcgaccttcaggtgttggctcagaaggcagaaagagctctgcccacctccttcaactacggtgccatcaaggcaactcgtgtgactgaa

>AY593751.1_A_NET_1942

tcgagtgtgggagtcacgtacgggtactccactgaggaagatcacgttgctgggcccaacacatcgggcttggagacgcgggtggtgcaggcagagagatttttcaagaagtttctgtttgactggacaacggacaaaccttttggacacttgacaaaactggagctccccaccgaccaccacggtgtcttcgggcacctggtggactcatacgcatatatgaggaacggctgggatgttgaggtatctgccgtcggcaaccagttcaacggcgggtgccttctggtggccatggtgccagagtggaaggaatttgacacacgtgaaaaataccagcttactcttttcccacaccagtttattagccccagaactaacatgactgcccacatcacggtaccgtatcttggtgtgaacaggtacgatcagtacaagaaacacaaaccctggacactggttgtcatggtattatcacccctcacggtcagcaacactgccgcaacacaaatcaaggtctacgccaacattgccccaacctacgttcacgtggctggagagctaccctcgaaagaggggattttcccagttgcatgcgcagacggttatggaggactggtgacaacagacccgaaaacagctgaccctgtttacggtaaggtgtataacccgcccaggaccaactaccccgggcgctttacaaacctattggacgtggccgaagcatgtcccaccttcctttgtttcgacgatgggaaaccgtacgtcgttacgcggacagacgacacccgtcttttggccaagtttgatgtctcccttgccgcaaaacacatgtccaacacatacctatcagggattgcacagtactacacacagtactctggtactatcaacctgcacttcatgttcacaggctccactgactcaaaagcccgctacatggtggcttacatcccgcctggggtggagccgccggacacacctgaagaagctgctcactgcattcatgctgagtgggacacaggactgaattccaaattcaccttttcaatcccttacgtgtctgccgcggattacgcgtacaccgcgtctgatacggcagagacaaccaatgtacagggatgggtctgtgtttaccaaattacacacgggaaggctgaaaatgacaccttggtagtgtcggctagcgccggcaaagactttgagttgcgcctcccaattgacccccggccacaaaccactgctactggggagtccgcagaccctgtcaccaccaccgtggagaactacggcggtgagacacaagtccagagacgccaccacacggacgtcggcttcatcatggaccgatttgtgaagataaacagcctgagccccacacatgtcattgacctcatgcacacccacaaacacgggatcgtgggtgcgttacttcgtgcagccacgtactacttctccgacttggagattgttgtgcggcacgatggtaatctgacctgggtgcccaacggtgcccccgaggcagccctgtcaaacaccagcaaccccactgcctacaacaaggcaccgttcacgagacttgctctcccttacactgcgccacaccgcgtgttggcaactgtgtacaacgggacaagcaagtactccgcgagcggttcg------agacgaggcgatctggggtccctcgcgacgcgagtcgcgacacaacttcctgcttcctttaactacggtgcaatcaaggcacaggccatccacgag

>AY593753.1_A_Brazil_1970

tcgagtgtgggtgtcacgtacgggtactccaccgcggaggaccacgttgctgggcccaacacatcgggtttggagacgcgggtgatacaggcagagagatacttcaagaagtttctgtttgagtggacaccggaaaagccttttggatacttggaaaaactggagcttcccactgtccaccacggcgttttcggacacctggtagactcgtatgcttatatgagaaatggctgggatgttgaggtgtctgctgttggcaaccagttcaacggcggatgtctcctggtggctatggtaccagagtggaaagaatttgacgcacgggagaaataccagcttacccttttcccacatcagttcatcagccccagaaccaacatgactgcccacatcacggtcccgtaccttggtgtgaacaggtatgatcagtacaaaaaccacaagccctggacactggttgtcatggttgtgtcgccacttacggttaacgccacgagtgcaacacaaatcaaggtctacgccaacattgctccaacttacgttcacgtggctggagaactcccctcgaaagaggggattttcccggtcgcatgtgcggacggttacggaggactggtgacgacagatccgaagacagctgaccccgcgtacggcaaggtgtacaacccgcccagaaccaactatcctgggcgcttcacaaacctattggacgtggccgaggcgtgtcccaccttcctctgcttcgacggcgggagaccgtacgtcgttacgcaggcgggtagcaaccgcctcctggccaaatttgatgtttcccttgccgcaaagcatatgtctaacacatacttgtcagggcttgcacagtacttcacacagtactctggcaccatcaacctgcacttcatgttcacaggctccactgactcaaaagcccgctacatggtggcctacgtaccgcctggggtggagccaccggaaacacccgagaaggccgcccactgcattcacgctgaatgggacacaggactgaactccaaattcacattctcaatcccgtacgtgtccgctgcagactatgcgtacactgcgtctgacacggcagaaacaaccaacgtacagggatgggtctgcatctaccaaattacacacgggaaggccgaagacgacactctggttgtgtcggtcagtgccggcaaggatttcgagctacgcctcccgattgacccccgttcacagaccacttctaccggggagtcagcagaccctgttaccaccaccgtagagaactacggcggtgagacacaagtccagaggcgtcaccacacggacgtcggtttcatcatggacagatttgtgaagataaacagtccaaaaccaacacatgtcattgacctcatgcagacccaccaacacggcttggtgggtgcgttgctgcgtgcagccacgtactacttctccgacctggagattgttgtgcagcacgacggcaacctgacctgggtgcccaacggtgcccctgaggcggccctactgaacaccagcaaccccaccgcctacaacaaggcaccgttcacgaggcttgctctcccctacactgcgccgcaccgcgtgctggcaactgtgtacaacgggacggacaagtaccccgtgagcgcttcg---ggaggacgaggtgattcggggcctcttgcggcgcgagccgcgaaacagctccctacttctttcaactacggtgcaatcaaggccactaccatccgcgag

>AY593754.1_A_SPA_1959

tcgagcgtgggagtcacctacgggtactccactggagaagaccatgttgctgggcccaacacatcgggcctggagacgcgggtggtgcaggcagagagattttttaaaaagtttttgtttgactggacaacggacaaaccttttggacatttggaaaagctggaacttcccgccgaccaccacggcgttttcgggcacctggtggaatcgtatgcttacatgagaaatggttgggacgttgaggtgtctgctgttggcaaccagttcaacggcgggtgcctcctggtggctatggtaccggagtggaaagagtttgaacagcgcgagaaataccaactcaccctcttcccgcaccagttcatcagccccagaacaaacatgactgcccacatcacagtcccataccttggagtgaacaggtacgatcagtacaagaaacacaaaccttggacactggttgtcatggtagtgtcgcccctcacggttagcgacactgccgcgacacagattaaggtctacgccaatattgctccgacctacgttcacgtggctggggaactcccctcgaaagaggggattttcccagttgcatgctcggacggttacggaggactggtgacaacggacccgaaaacagctgaccccgcctacggtaaggtgtacaacccgcccaggaccaactaccctgggcggtttaccaacctgttggacgtggctgaagcgtgtcccactttcctctgtttcgacgacgggaaaccgtacgttgtcacgcggacagatgacacacgactattggccaagttcgacgtctcccttgctgcaaaacacatgtccaacacgtacctgtcaggggttgcacagtactacgcacagtactctggtaccatcaacttgcacttcatgttcacaggctcaactgactcaaaagcccgctacatggtggcctacatcccgcctggggtggaaccaccggacacacctgaaagggccgctcactgcatccacgctgaatgggacacaggactgaactccaaattcactttctcaatcccgtacgtgtccgccgcagattacgcgtataccgcgtctgacacggcagaaacaaccaacgtacagggctgggtttgcatataccagatcacacacgggaaggccgagaacgacacattggtggtgtcggccagcgccggcaaagactttgagttgcgcctcccgattgacccccgacagcaaactactgctgtcggggagtccgcagaccctgtcaccaccgccgtggagaactacggcggtgagacacaaacccggagacggcaccacacggatgtcggtttcatcatggacagatttgtgaagataaacagtttgagtcccacgcatgtcattgacctcatgcagacccaccagcacgggctggtgggtgcgctgctgcgtgcagccacgtactacttctctgacttggagattgttgtgcggcatgacggcaatttgacttgggtgcccaatggtgcccctgaagcagctttgtcaaacaccagcaaccccactgcctacaacaaggcaccgttcacgaggcttgctctcccttacactgcgccacaccgcgtgttggcaaccgtgtacaacgggacgaacaagtactccacaggcggtctg------agacgaggcgacacggggtcgctcgcggcgcgggccgcgaaacaacttcctgcctcttttaattacggtgcaattggggccgtcaccatccacgag

>AY593755.1_A_TAI_1960

tcgagtgtgggagtcacgtacggatactccaccaaggaagatcacgtgtccgggcctaacacatctggcctggagacgcgggtggtgcaggcagagagattcttcaagaaacacttgtttgactggacaacagacaagccttttggacatttggaaaaactggaacttcccaccgaccacaaaggtgtctacgggcacctggttgactcatatgcatatatgagaaacggctgggacgtggaggtgtctgctgttggcaaccagttcaatggcgggtgtctcctggtggccatggtccctgagtggaaggaactcaccctgcgtgagaagtaccaactcacccttttcccgcaccagttcatcaaccccagaaccaacatgaccgcacacatcacggttccgtaccttggtgtgaaccggtacgaccagtacaagaagcacaaaccgtggaccctggtcgtgatggtggtgtcacctctcaccaccagcaccgttggtgcggaacaaatcaaggtctacgccaacatcgccccgacccacgttcacgtagccggcgaactcccttcaaaggagggaattgtaccggttgcttgttcagacggttacggtggtttggtgacaacagaccccaagacagctgaccctgtctacggcaaggtgtacaacccgcccaggaccaactaccccgggcgttacacaaacctgttggacgtagcagaggcctgcccaacctttctttgtttcgacgacgggaaaccgtatgttgtgacgaggacggacgggcagcgtctcctggctaagtttgacctttctcttgccgcaaaacacatgtccaacacctaccttgcaggtttagcacagtactacacacagtattctggcaccattaatctgcacttcaccttcactggttcaactgactcaaaagcccgctacatggtggcctacgttccgcctggcgtggaaccaccggacacacctgagaaggccgcacactgtatccatgctgagtgggacacaggactgaactccaaatttactttttcagtcccgtacatgtctgccgctgactacgcatacactgcgtcagatgaggcagagacaacaaatgtacagggatgggtctgcatttaccagattacacacgggaaagctgagggcgacactctggtcgtgtcggctagcgccggcaaagacttcgagttacgtctcccggttgatccccgcacacagaccaccaccactggggagtctgcagacccagtcaccaccactgttgagaactacggtggagagacacaagtccaaagacgacaacacacagatgtcggcttcataatggacagatttgtaaagataacaaacctgagtcccacacatgtcattgacctcatgcaaacccaccaacacggactggtgggtgccctgctgcgtgcggccacgtactacttctccgacctggagattgtcgtgaagcacgatggcaacctgacctgggtgcccaacggcgcgccagaagcagccttgggcaacacgagcaaccccaccgcctacaacaaggcgccatttacaagacttgccctcccttacaccgcgccacaccgcgtgctggcaacagtgtacaacgggacgaacaagtactctgcaagtggctcg---gccagacgaggtgacctggggtctctcgcggcgagagatgccgcgcaactccccgcctctttcaactttggtgcaattcgggccacaaccatccatgaa

>AY593756.1_A_Brazil_1959

tcgagcgtgggtgtcacgtacgggtactccacagaggaggaccacgttgctgggcccaacacatcgggcctggagacacgggtggtgcaggcagagagattctacaaaaagtttctgtttgactggacaaaggaaaagccctttggacacctggtgaagctggagctcccggccgatcaccacggtgtctttggacacttggtggattcgtacgcctacatgagaaatggatgggatgttgaggtgtccgctgttggcaatcagttcaacggcgggtgcctcctggtggctatggtacctgagtggaaagaatttgacatacgggagaaataccaactcaccctcttcccgcaccagtttatcagccccagaaccaacatgactgcccacatcacggtcccctacctaggtgtgaacaggtatgaccagtacaaaaagcacaagccctggacactggttgtcatggtcgtgtcgccacttacggttaacgccactagtgcgacacagatcaaggtctacgccaacattgccccaacctacgttcacgtggccggtgaactcccctcgaaagaagggattttcccggtcgcatgcgcggacggttacgggggactggtaacgacagacccgaagacagctgaccctgtttacggcaaggtgtacaacccgcctaggaccaactaccctgggcgcttcaccaacctgttggacgtggccgaagcgtgtcccactttcctctgctttgacggcgggaaaccgtacgtctccacgcagataggtgagacccgacttttggccaagtttgacctttcccttgctgcaagacatatgtccaacacatacctgtcagggattgctcagtactacacacagtactctggcaccatcaacttgcacttcatgttcacaggtgccactgattcaaaggcccgatacatggtggcctacatcccgcctggggtggagccaccggacacacctgaggaggctgcccactgcattcacgctgaatgggacactgggctgaattccaaattcacattctcaatcccgtacgtatccgccgcagattacgcttacacagcgtctgacacggcagaaacaaccaacgtacagggatgggtctgcatctaccaaatcacacacgggaaggctgaaaacgacactctggtcgtatcggttagcgccggcaaagactttgagctacgcctcccgattgacccccgccagcagaccaccgctaccggggaatcagcagaccctgtcaccaccaccgtggagaactacggcggtgagacacaagtccagagacgtcaccacacggacattggtttcatcatggacagatttgtgaagatcaaagatttgagcccaacacatgtcattgacctcatgcagactcaccaacacggtctggtgggtgcgctgctgcgtgcagctacgtactacttttccgacctggaaattgtcgtacggcacgacggcaatctgacatgggtgcccaacggcgcccctgaatcagccctgttgaacaccagcaaccccactgcctacaacaaggaaccattcacgagactcgctctcccgtacactgcgccgcaccgcgtgctggcaacagtgtacaacgggacgagtgagtatgctgtgagtggtcca---gacagtcgtggcgacacggggcctatcgcggcgcgaaccgcgaaacagcttcctgcttcattcaactacggcgcaatcaaggccaaagtcatccacgaa

>AY593757.1_A_Brazil_1967

tcgagtgtgggtgtcacgtacgggtactccaccgcggaggaccacgttgccgggcccaacacatcgggtctggagacgcgggtgacacaggcagagagattcttcaagaagtttttgtttgaatggacaacggacaagccttttggacacttggaaaaactggagcttcccactgaccaccacggcgtcttcggacacctggtagactcgtatgcttatatgagaaatggctgggatgttgaggtgtccgccgttggcaaccagttcaacggtggatgtctcctggtggctatggtaccagagtggaaagaatttgacgcacgggagaaataccagcttacccttttcccacatcagttcatcagccccagaaccaacatgactgcccacatcacggtcccgtaccttggtgtgaacaggtatgatcagtacaaaaaccacaagccctggacattggttgtcatggttgtgtcgccactcacggttagcgccacgagtgcaacaaaaatcaaggtctacgccaacattgctccaacttacgttcacgtggctggggaactcccctcgaaagaggggattttcccggtcgcatgtgcggacggttacggaggactggtgacgacagacccgaagacagctgaccctgcatacggcaaggtgtacaacccgcccagaaccaactatcctgggcgcttcacaaacctattggatgtggccgaggcgtgtcccaccttcctctgcttcgacaacggaaaaccgtacgtcgttacgcgggcggacagcaaccgcctgctggccaaatttgatgtttcccttgccgcaaaacatatgtctaacacatacttgtcagggattgcacagtacttcacacagtactctggtaccatcaacctgcacttcatgttcacaggctccactgattcaaaagcccgctacatggtggcctacgtcccgcctggggtggagccaccggaaacacccgagatggccgcccactgcattcacgctgaatgggacacaggactgaactccaaattcacattctcaatcccgtacgtgtccgctgcagactatgcgtacactgcgtctgacacggcagaaacaaccaacgtacagggatgggtctgcatctaccaaattacacacgggaaggccgaagacgacactctggttgtgtcggtcagtgccggcaaggacttcgagctacgcctcccgattgacccccgttcacagaccactgctaccggggagtcagcagaccctgtcaccaccaccgtagagaactacggcggtgagacacaagtccagaggcgtcaccacacggacgtcggtttcatcatggacagatttgtgaagataaacaaactgaacccaacacatgttatcgacctcatgcagacccacgaacacggcttggtgggtgcgttgctgcgtgcagccacgtactacttctccgacctggagattgttgtgcggcacgaaggcaacctgacctgggtacccaacggtgcccctgaggcggccctactgaacaccagcaaccccaccgcctacaacaaggcaccgttcacgaggcttgctctcccctacactgcgccgcaccgcgtgctggcaactgtgtacaacgggacgaacaagtaccccgtgggcgcttcg---gggatacgaggtgatttggggcctcttgcggcgcaagccgcgaaacagctccctgcttctttcaactacggtgcaatcaaggccactaccatccacgag

>AY593758.1_A_VEN_1967

tcgagtgtgggtgtcacgtacgggtactccaccgcggaggaccacgttgctgggcccaacacatcgggtttggagacgcgggtgatacaggcagagagatacttcaagaagtttctgtttgagtggacaccggaaaagccttttggatacttggaaaaactggagcttcccactgtccaccacggcgttttcggacacctggtagactcgtatgcttatatgagaaatggctgggatgttgaggtgtctgctgttggcaaccagttcaacggcggatgtctcctggtggctatggtaccagagtggaaagaatttgacgcacgggagaaataccagcttacccttttcccacatcagttcatcagccccagaaccaacatgactgcccacatcacggtcccgtaccttggtgtgaacaggtatgatcagtacaaaaaccacaagccctggacactggttgtcatggttgtgtcgccacttacggttaacgccacgagtgcaacacaaatcaaggtctacgccaacattgctccaacttacgttcacgtggctggagaactcccctcgaaagaggggattttcccggtcgcatgcgcggacggttacggaggactggtgacgacagatccgaagacagctgaccccgcgtacggcaaggtgtacaacccgcccagaaccaactatcctgggcgcttcacaaacctattggacgtggccgaggcgtgtcccaccttcctctgcttcgacggcgggagaccgtacgtcgttacgcaggcgggtagcaaccgcctcctggccaaatttgatgtttcccttgccgcaaagcatatgtctaacacatacttgtcagggctcgcacagtacttcacacagtactctggcaccatcaacctgcacttcatgttcacaggctccactgactcaaaagcccgctacatggtggcctacgtaccgcctggggtggagccaccggaaacacccgagaaggccgcccactgcattcacgctgaatgggacacaggactgaactccaaattcacattctcaatcccgtacgtgtccgctgcagactatgcgtacactgcgtctgacacggcagaaacaaccaacgtacagggatgggtctgcatctaccaaattacacacgggaaggccgaagacgacactctggttgtgtcggtcagtgccggcaaggatttcgagctacgcctcccgattgacccccgttcacagaccacttctaccggggagtcagcagaccctgttaccaccaccgtagagaactacggcggtgagacacaagtccagaggcgtcaccacacggacgtcggtttcatcatggacagatttgtgaagataaacagtccaaaaccaacacatgtcattgacctcatgcagacccaccaacacggcttggtgggtgcgttgctgcgtgcagccacgtactacttctccgacctggagattgttgtgcagcacgacggcaacctgacctgggtgcccaacggtgcccctgaggcggccctactgaacaccagcaaccccaccgcctacaacaaggcaccgttcacgaggcttgctctcccctacactgcgccgcaccgcgtgctggcaactgtgtacaacgggacggacaagtaccccgtgagcgcttcg---ggaggacgaggtgatttggggcctcttgcggcgcgagccgcgaaacagctccctacttctttcaactacggtgcaatcaaggccactaccatccgcgag

>AY593759.1_A_GER_1971

tcgagtgtgggagtcacgtacgggtattccaccgaggaggatcacgttgctgggcccaacacatcgggcttggagacgcgggtggtgcaggcagagagattttttaaaaagtttctgtttgactggacaacagacaagccttttggacacttggcaaaactggagcttcccaccgaccaccgcggtgtcttcggacatctggtagactcatatgcgtacatgaggaatggctgggatgttgaggtgtctgccgttggcaaccagttcaacggcgggtgccttctggtggccatggtgccagagtggaaagattttgacgagcgtgaaagataccaactcacccttttcccacaccagttcatcagccccagaaccaacatgactgcccacatcacggtcccgtatcttggtgtgaacaggtacgatcagtacaagaaacacaagccttggacactggttgtcatggtggtatcacccctcacggtcagcaacactgccgcaacacaaatcaaggtctacgccaacattgccccaacctacgttcacgtggctggagagctcccctcgaaagaggggatcttcccagttgcgtgcgcggacggttatggagggctggtgacaacagacccgaaaacagctgaccctgtgtacggcaaggtgtacaacccgcccaggaccaactaccccgggcgttttacaaacctgttggacgtggccgaagcatgtcccacctttctctgtttcgacgatgggaaaccgtacgtcgttacgcggacagacgacacccgtcttttggccaagtttgacgtctccctagccgcaaaacacatgtccaacacatacctatcagggattgcacagtactacacacagtactctggtactatcaacctgcacttcatgttcacgggctccactgactcaaaagcccgctacatggtggcttacatcccacctggggtggagccaccggacacacctgaaaaagctgcccactgcattcatgctgaatgggacacaggactaaactccaaattcactttttcaatcccttacgtgtccgccgcggattacgcgtacaccgcgtctgatacggcggaaacaaccaacgtacagggatgggtctgtgtttaccaaattacacacgggaaggctgaaaacgacactttggtagtgtcggctagtgccggcaaagattttgagttgcgcctcccaattgacccccggccgcaaactaccactgctggggagtctgcagaccctgtcaccaccaccgtggagaactacggtggtgagacacaagtccagagacgtcaccacacggacgtcggtttcatcatggaccgatttgtgaggataaacaacctgaaccccacgcacgttattgacctcatgcagacccaccagcacgggctggtgggtgcgttgctgcgtgcagccacgtactacttctccgacttggagattgtagtgcggcacgatggtaatctgacctgggtacctaacggtgcccccgaagcagccctgttaaacaccagcaaccccactgcctacaacaaggcaccgttcacgagacttgctctcccttacactgcgccgcaccgcgtgttggcaactgtgtacaacgggacaagcaagtactccacgagcggttcg------ggacgaggcgatttggggaccctcgcggcgcgagccgcgacacagcttcctgcttctttcaattacggtgcaatcaaggcccagaccatccacgag

>AY593760.1_A_USSR_1964

tcgagtgtgggagttacctacgggtactccactggggaagaccatgtcgctgggcccaacacatcgggcctggagacgcgggtggtccaggcagagagattttttaaaaagtttttgtttgactggacaacggacaaacctttcgggcatttggaaaagttggaacttcccaccggccaccacggcgttttcgggcacctggtggaatcatatgcttatatgagaaatggctgggacgttgaggtgtctgctgttggcaaccagttcaacggcgggtgcctcctggtggccatggtaccggagtggaaagagtttgagcagcgcgagaaataccaactcaccctcttcccgcaccagttcattagccccagaacaaacatgactgcccacatcacagtcccataccttggagtgaacaggtacgatcagtacaagaaacacaaaccttggacactggttgttatggtggtgtcgcccctcacggttagcgacactgccgcggcacagattaaggtctacgccaacattgctccaacctacgttcacgtggctggagaactcccctcgaaagaggggattttcccggttgcgtgttcggacggttatggaggactggtgacaacggacccgaaaacagctgaccccgcctacggcaaggtgtacaacccgcccaggactaactaccctgggcggtttaccaacttgttggacgtggctgaagcgtgtcccactctcctctgtttcgacgacgggaaaccgtatgttgtcacgcggacagatgacacacgactactagccaagtttgacgtctcccttgctgcaaaacacatgtccaacacgtacctgtcagggattgcacagtactacgcacagtactctggcaccatcaacctgcacttcatgttcacaggctcaactgactcaaaagcccgctacatggtggcctacatcccgcctggagtggaaccaccagacacacctgaaagggccgctcactgcatacacgctgaatgggacacaggactgaactccaaattcactttttcaatcccgtacgtgtccgccgcagattacgcgtacaccgcgtctgacacggcagaaacaaccaacgtacagggctgggtttgcatttaccagatcacacacgggaaggccgagaacgacacattggtggtatcagtcagcgccggtgcagactttgagttgcgcctcccgatcgacccccgacagcaaaccactgctgttggggagtccgcagaccctgtcaccaccaccgtggaggcctacggcggtgagacgcaagtccagagacggcaccacacggatgtcggttttatcatggacagatttgtgaagataaacagcttgagtcccacgcatgtcatcgacctcatgcagacccaccagcacgggttggtgggtgcgctgctgcgtgcagccacatactacttctctgacttggagattgtagtgcggcatgacggtaacttgacttgggtgcccaacggtgctcctgaagcagctttgttaaacaccagcaaccccactgcctacaacaaggcaccgttcacgaggctcgctctcccttacaccgcgccacaccgcgtgttggcaaccgtgtacaacgggacgaacaagtactccacgggcggtccg------ggacgaggcgacatggggtcgctcgcggcgcgggtcgcgaaacaacttcctgcctctttcaactacggtgcaattagggccgaaaacatccacgag

>AY593761.1_A_KEN_1964

tcgagcgtgggggtcacgtacgggtactccactggagaggaccacgttgccgggcccaacacatcgggcctggagacgcgggtggtgcaggcagaaaggtttttcaaaaagcacctctttgactggacaccggacaaagcttttggacacctggaaaaacttgaacttcccaccgaccacacaggtgtctacgggcacctggtgaactcgtatgcatatatgaggaacggttgggacgtggaggtgtctgccgttggaaaccagttcaacggcgggtgtctcctggtggccatggttccagagtggaaggaaccacaaccacgtgagaaatatcagctcactctctttccacaccagttcattagccccagaacaaacatgacagctcacatcacggtgccttaccttggtgtgaacaggtatgaccagtacaagaagcacaaaccctggacactggtagtgatggtagtgtcgccgctcacagtcagcacgacgtctgcggctcagatcaaggtctacgctaacatcgccccgacctacgtacatgtggctggggagctcccctcgaaacaggggatcgtcccggttgcgtgctcagacggttacggcggtttggtgacaacagacccgaagacagctgaccctgtctatggcaaggtgtacaacccgcccaggaccaactaccccgggcgttttacaaacttgttggatgtggccgaagcctgccctaccttcctccgtttcgacgacgggaaaccgtacgttgttacaaggacagatgaacggcggcttttggccaagttcgacgtttcccttgctgcaaaacacatgtccaacacctaccttgcagggcttgcacagtactacgcacagtactctggcaccatcaacttgcacttcatgttcactggctctgctgactcaaaagcccgctacatggtggcctatgttccacccggtgtggagcccccggacacacctgaggaggccgcccactgcatccacgcagaatgggacacaggactgaactccaaattcactttttctatcccgtacatatctgctgcagattacgcgtacaccgcgtctgacgtggcagagacaaccaacgtacagggatgggtctgcatctaccaaatcacacacgggaaggctgaaaacgacactctggtcgtgtcggtgagcgccggcaaagattttgagttacgcctcccgattgacccccgcgcacaaactactgctacgggggaatctgcagaccctgtcaccaccactgttgaaaactacggtggtgagacacaggtccagaggcggcaccacacggatgttggcttcatcatggacagatttgtaaaagtcaacagctctagtcccatgcatgtcatagacctcatgcagactcaccaacacgggctggtgggtgcgttgctgcgtgcagctacatactacttctctgacctggagattgtggtgaagcacgagggcaacctgacttgggtgcccaacggcgcccctgaggctgccctcctgaacacgagcaatcccacagcctaccacaaggaaccattcacgagacttgcacttccctacaccgcaccgcaccgtgtgctggcaacggtgtacaacgggacgagcaagtactccacaagtgtctca---agcaggcgcggtgagctggggcccctcgcggcgaggatcgccgcacagctccctgcatccttcaactacggtgcacttaaggccacgaacatccacgag

>AY593764.1_A_IRQ_1970

tcgagtgtgggagtcacgtacgggtactccacccaggaagatcatgtttccggacctaacacatctggtttggagacgcgggtggtgcaggcagaaagatttttcaagaagcacctgtttgattggacaccggacaaagcttttgggcacttagagaagttggaacttcccactgaccacaagggagtctacggacacttggtggactcatttgcatacatgagaaatggctgggacgtggaggtgtccgctgttggcaaccagtttaacggcgggtgtctcctggtggccatggtccctgaatggaaagagttcaccccgcgtgagaagtaccagctcactttgtttccacaccagttcatcagccccagaaccaacatgactgcccacatcgtagtcccgtaccttggtgtgaacaggtacgaccagtataagaagcacaaaccctggacgctggttgtgatggtggtctcaccgctcaccaccaacactgttagtgcaggacaaatcaaggtttatgccaacattgccccgactcacgttcacgtggccggcgagctcccctcgaaagaggggattgtaccggtcgcttgttcggacgggtacggtggcttggtgacaacagacccaaaaacagctgaccctgtttatggtatggtgtacaacccccccaggacaaactaccccgggcggttcacaaacctgttggatgtggctgaggcctgccccacctttctctgtttcgacgaagggaaaccgtacgttgtgacaagaacggacgagcagcgtcttctggccaagttcgacgtctctcttgctgcaaagcacatgtcaaacacctacctttcagggatagcacagtactacgcacagtactctggtaccatcaacctgcacttcatgtttaccggctccacggattcaaaagcccgctacatggtggcgtacgttccacccggtgtggagccgccggacacgcctgagaaagctgcacactgcatccatgctgagtgggacacagggttgaactccaagtttactttctctatcccgtacgtgtctgccgcagattacgcgtacactgcgtctgatgtggcagaaacaacaaacgtacagggatgggtctgcatataccaaattacacacgggaaagctgaacaagacactctggttgtgtcggttagcgccggcaaggactttgagttgcgcctcccgattgacccccgctcacaaaccactaccaccggggagtctgcagaccctgtcaccaccactgttgaaaactacggcggtgagacacaagtccaacgacgtcagcacaccgacgttactttcataatggacagatttgtaaagatacaaaacttgaaccccacacatgtcattgacctcatgcaaacccaccaacacgggttggtaggtgccctgttacgtgctgctacgtactacttctctgacctggagattgtggtacgccatgacggtaacctaacctgggtacccaatggagcacccgaggcagctctgtctaacacgggcaaccccaccgcctacctcaaggcaccatttacgaggctcgcgctcccctacaccgcgccacaccgcgtgttggcaacagtgtacaacgggacgagcaagtactccgcaggtggtacg---ggcagacggggcgacctagggcctctcgcggcgagggtcgccgctcagcttcctgcttctttcaactttggtgcaattcaagccacgaccatccacgag

>AY593765.1_A_TUR_1965

tcgagtgtgggagtcacgtacgggtactccacccaggaagatcatgtttccggacctaacacatctggtttggagacgcgggtggtgcaggcagaaagatttttcaagaagcacctgtttgattggacaccggacaaagcttttgggcacttagagaagttggaacttcccactgaccacaagggagtctacggacacttggtggactcatttgcatacatgagaaatggctgggacgtggaggtgtccgctgttggcaaccagtttaacggcgggtgtctcctggtggccatggtccctgaatggaaagagctcaccccgcgtgagaagtaccagctcactttgtttccacaccagttcatcagccccagaaccaacatgactgcccacatcgtagtcccgtaccttggtgtgaacaggtacgaccagtataagaagcacaaaccctggacgctggttgtgatggtggtctcgccgctcaccaccaacactgttagtgcaggacaaatcaaggtttatgccaacattgccccgacccacgttcacgtggccggcgagctcccctcgaaagaggggatcgtgccggtcgcctgttcggacgggtatggtggcttggtgacaacagacccaaaaacagctgaccctgtttatggtatggtgtataacccccccaggacaaactaccccgggcggttcacaaacctgttggatgtggcagaggcctgccccacctttctctgcttcgacgacgggaaaccgtacgttgtgacaagaacggatgagcagcgtcttctggccaagttcgacgtctctcttgctgcaaagcacatgtcaaacacctacctttcagggatagcacagtactacgcacagtactctggtaccatcaacctgcacttcatgtttaccggctccactgactcaaaagcccgctacatggtggcgtacgttccgcccggtgtagagccgccggacacgcctgagaaagctgcacactgcatccatgctgagtgggacacagggttgaactctaaatttactttctctatcccgtacgtgtctgccgcagactacgcgtacactgcgtctgacgtggcagaaacaacaaacgtacagggatgggtctgcatataccaaattacacacgggaaagctgaacaagacactctggttgtgtcggttagcgccggcaaggactttgagttgcgcctcccgattgacccccgctcacaaaccactaccaccggggagtctgcagaccctgtcaccaccaccgttgaaaactacggcggtgagacacaagtccaacgacgtcagcacaccgacgttactttcataatggacagatttgtaaagatacaaaatttgaaccccatacatgtcattgacctcatgcaaacccaccaacacgggttggtaggtgccctgttacgtgctgctacgtactacttctctgacctggagattgtggtacgccatgacggtaacctaacctgggtacccaacggagcacccgaggcagctctgtctaacatgggcaaccccaccgcctacctcaaggcaccatttacgaggctcgcgctcccctacaccgcgccacaccgcgtgttggcaacagtgtacaacgggacgagcaagtactccgcaggtggtacg---ggcagacggggcgacctagggcctctcgcggcgagggtcgccgctcagcttcctgcttctttcaactttggtgcaattcaagccacgaccatccacgag

>AY593766.1_A_KEN_1965

tcgagtgtgggggtcacctacgggtactccactgcggaggaccacgttgccgggcctaacacatcgggcctggagacgcgggttgcgcaggctgaaaggttcttcaaaaagcacctgtttgactggacaacggacaaaccatttggacacattgaaaaactggagctccccaccgaccaccgcggtgtctacgggcacctagtggaatcatacgcatacatgaggaacggttgggatgtagaggtgtctgctgttggaaaccaattcaacggcggctgccttctggtggccatggtcccggagtggaaagagtttgacaaccgtgaaaagtaccaactcacccttttcccacaccaattcatcagccctaggacaaacatgacagcacacatcacggtaccgtaccttggtgtgaacaggtatgaccagtacaagaagcacaaaccctggaccctggtggtaatggtggtgtcaccactcacagtcagccagactgctgcaagccaaatcaaggtctacgccaacatcgccccaacccacgtgcacgtagccggggagctcccctcgaaagaggggattgtcccggttgcatgttccgacggttacggtggtttggtgacaacagacccgaagacagctgaccctgtttacggcaaggtgtacaacccgcccaggactaactaccctgggcgcttcacaaacttgttggacgtggctgaggcctgccccaccttcctctgtttcgacgacgggaaaccgtatgttgtcacgaggacagatgaacagcggctcttagctaagtttgacgtttcacttgctgcaaaacacatgtctaacacctatctctcagggattgcacagtactatgcccagtactctggtaccatcaacttgcatttcatgttcactggttcaactgactcaaaagcccgctacatggtggcttatgtcccgcccggtgtggaaccaccggacacgcctgagaaggctgcccactgcatccacgccgagtgggacacgggattgaattccaaattcactttctccatcccgtacgtttctgccgctgactacgcctacaccgcgtccgacgaggcagagacgaccaatgtgcaagggtgggtctgcatttaccagattacacacgggaaagctgagaacgacaccttggtcatctcggcaagcgcgggcaaagactttgaactgcgcctcccgattgacccccgcccgcagacaactgccaccggggagtccgcagaccctgtcaccaccaccgtcgaaaactacggtggtgagacacaagcccaaaggcggcaccacacggaggtcgccttcatcatggacagatttgtgaacatcaaagctcccagccccacgcacgtcattgacctcatgcaaacccaccagcacgggcttgtgggcgccttgttgcgcgctgccacgtactacttctccgacttggagatcgtggtacgacacgaaggcaatttgacttgggtgcccaacggtgcccctgagggcgctcttgcaaacacgggaaaccccaccgcctacaacaaggcaccattcacgagacttgcactcccttacaccgcgccgcaccgagtgctggcaacagtgtacaacgggacgaacaagtattccaggagtggtgcg---accaggcggggtgacatggcagccctcgcagcgagggttgccacccagcttcctgcatctttcaattacggagcacttcgggccaccaacatccatgaa

>AY593767.1_A_ARG_1965

tcgagtgtgggagtcacctacgggtactccactggagaagaccacgttgctgggcccaacacatcgggcctggagacgcgggtggtgcaggctgagagattttacaaaaagtttttgtttgattggacaacggataagccttttggacatttggaaaagttggaacttcccaccgaccaccacggtgttttcgggcacttggtggaatcgtatgcctacatgagaaacggttgggacgttgaggtgtctgctgttggcaaccagttcaacggcgggtgtctcctggtggctatggtaccggagtggaaggagtttgaacaacgtgagaagtaccagctcaccctctttccccaccagttcattagccccagaacaaacatgactgcccacattactgtcccataccttggagtgaacaggtacgaccagtacaagaaacacaaaccttggaccctggttgttatggtagtgtcgccccttacagttagcagcactgccgcggcacagattaaggtctacgccaacattgctccaacctacgttcacgtggccggggaactaccctcgaaggaggggattttcccggttgcatgttcggacggttacggaggactggtgacaacagacccgaaaacagctgaccctgcctacggcaaggtgtacaacccgcccaggaataactaccccgggcggttcaccaacttgttggacgtggctgaagcgtgtcccactttcctctgtttcgacgacgggaaaccgtacgtcgttacgcggacagatgacacacgactcttagccaagttcgacgtttcccttgccgcaaaacacatgtccaacacgtacctgtcagggatagcacagtactatacacagtactctggtaccatcaacttgcacttcatgtttacaggttcaacagattcaaaggcccgttacatggtggcctacatcccgcccggggtggaaccaccggacacacctgaaagggctgcccactgtatccacgctgaatgggacacaggactgaactccaaattcactttttcaatcccgtacgtgtccgccgcagattacgcgtacaccgcgtctgacacggcagaaacaaccaacgtacagggctgggtctgcatttaccagattacacacgggaaggccgagaacgacacactagtcgtgtcggccagcgccggcaaggactttgagttgcgcctcccgattgacccgcgacggcaaaccaccgctgttggggagtccgcagaccctgtcaccaccaccgtggagaactacggcggtgagacacagacccagaggcgacatcatacagatgtcagtttcatcatggacagatttgtgaaaataaacagcttgagtcccacacatgtcattgacctcatgcagacccaccaacacgggctggtgggcgcgctgctgcgtgcagccacgtactacttctccgacttggagattgttgtgcggcatgacggtaatttgacttgggtgcccaacggtgcgcctgaagcagctttgtcaaacaccagcaaccccactgcctacaacaaggcaccgttcacgaggctcgctctcccttacactgcgccacaccgcgtgttggcaacggtgtacaacgggacgaacaagtactccacgggcggtacg------ggaagaggcgacacgggttcgctcgcggcgcgggtcgcgaaacaacttcctgcttccttcaactacggtgcaatcagggctgacgccatcaacgag

>AY593768.1_A_Brazil_1955

tcgagtgtgggtgtcacacacgggtactccacagaggaggaccacgttgctgggcccaacacatcgggcctggagacgcgagtggtgcaggcagagagattctacaaaaagtacttgtttgactggacaacggacaaggcatttggacacctggaaaagctggagctcccgtccgaccaccacggtgtctttggacacttggtggactcgtacgcctatatgagaaatggctgggatgttgaggtgtccgctgttggcaaccagttcaacggcgggtgcctcctggtggccatggtacctgaatggaaggaatttgacacacgggagaaataccaactcacccttttcccgcaccagtttattagccccagaactaacatgactgcccacatcacggtcccctaccttggtgtgaacaggtatgatcagtacaagaagcataagccctggacattggttgtcatggtcgtgtcgccacttacggtcaacaacactagtgcggcacaaatcaaggtctacgccaacatagctccgacctatgttcacgtggccggtgaactcccctcgaaagaggggattttcccggttgcatgtgcggacggttacggaggattggtgacgacagacccgaagacagctgaccctgcttatggcaaggtgtacaacccgcctaggactaactaccctgggcgcttcaccaacctgttggacgtggccgaagcgtgtcccactttcctctgctttgacgacgggaaaccgtacgtcaccacgcggacggatgacacccgacttttggccaagtttgacctttcccttgccgcaaaacatatgtccaacacatacctgtcagggattgctcagtactacacacagtactctggcaccatcaatttgcatttcatgttcacaggttccactgattcaaaggcccgatacatggtggcctacatcccacctggggtggagccaccggacacacctgaaagggctgcccactgcattcacgctgaatgggacactggactaaactccaaattcactttctcaatcccgtacgtatccgccgcggattacgcgtacacagcgtctgacacggcagaaacaatcaacgtacagggatgggtctgcatctaccaaattacacacgggaaggctgaaaatgacaccttggtcgtgtcggttagcgccggcaaagactttgagttgcgcctcccgattgacccccgccagcagaccaccgctaccggggaatcagcagacccggtcaccaccaccgtggagaactacggcggtgagacacaaatccagagacgtcaccacacggacattggtttcatcatggacagatttgtgaagatccaaagcttgagcccaacacatgtcattgacctcatgcagactcaccaacacggtctggtgggtgccttgctgcgtgcagccacgtactacttttctgacctggaaattgttgtacggcacgaaggcaatctgacctgggtgcccaacggcgcccctgaatcagccctgttgaacaccagcaaccccactgcctacaacaaggcaccattcacgagactcgctctcccctacactgcgccgcaccgtgtgctggcaacagtgtacaacgggacgagtaagtatgctgtgggtggttca---ggcagaagaggcgacatggggtctctcgcggcgcgagtcgtgaaacagcttcctgcttcatttaactacggtgcaatcaaggccgacgccatccacgaa

>AY593769.1_A_ARG_1959

tcgagcgtgggggtcacctacgggtactccactggggaagaccacaccgcagggcccaacacatcgggcttggaaacgcgggtagtacaggctgaaaggttctttaagaaatttttgtttgactggacaacggacaaaccctttggacacttggaaaaactggaactccccaccgaccaccacggggtcttcggacacctggtggactcatatgcatacatgaggaacggttgggatgtcgaggtgtctgctgttggcaaccaattcaacggcgggtgcctcctggtggccatggtaccagaatggaaggaatttgacacgcgtgagaaataccaactcactctgtttccacaccagttcatcagccccagaacaaacatgaccgcccacatcacggtcccgtaccttggtgtgaacaggtatgaccagtacaaaaagcacaaaccctggacgctggttgtcatggtggtgtcgcccctcacggttagcaccactagtgcggcacagattaaggtctacgccaacattgccccaacctacgttcacgtggctggagagctcccttcgaaagaggggatttttcccgttgcgtgcgccgacggttacgggggactggtgacgacggacccgaagacagctgaccccgcctacggcaaggtgtacaatccgcccaggactaactaccccgggcgctttacaaacctgttggacgtggctgaggcgtgtcccacctttctttgtttcgacgacgggaaaccgtatgttgtcacgaagacagaacaagaccgacttctggccaagtttgacgtttcccttgccgcaaagcacatgtctaacacatacttgtcaggggttgcacagtactacgcacagtactctggtaccatcaacctgcactttatgttcacaggctctactgactcaaaggcccgctacatggtggcctacatcccgccaggggtggagccgccggacacacctgagaaagccgcacactgcatccacgctgaatgggacacagggttgaactccaagttcaccttttcaatcccgtacgtgtccgccgcggactacgcatacactgcgtccgacacggcagaaacaaccaacgtacagggatgggtttgcatttaccaaattacacacgggaaggctgagcaggacaccttggttgtgtcggttagcgccggcaaggactttgagctacgcctcccgattgacccccgtgcacaaaccactgccactggggaatctgcagaccctgtcaccaccaccgtggagaactacggcggtgagacacaagtccacagacgtcaccacacggacgtcagcttcatcatggacaggtttgtgaagatacagcctgtgaaccctatgcatgtcattgacctcatgcagacccaccaacacgggcttgtaggggcgttgctgcgtgcagccacgtactacttctctgacctggagattgtggtacgacacaacggcaacctgacctgggtacccaacggcgcccccgaggcagccctgtctaacaccagcaaccccactgcctacaacaaggcgccgttcaccagacttgccctcccctacactgcgccacaccgtgtgctggcaactgtgtacaacgggacgaacaagtacaccacaaacggtaca---ggtaggcgtgatgacacgggttctctcgcggcgagagtcgcgaaacatcttcctgcttcttttaattacggtgcaatcaaggccgacaccatccacgag

>AY593770.1_A_ARG_1966

tcgagtgtgggtgtcacatacgggtactccacagaggaggaccacgttgctgggcccaacacatcgggcctagagacgagggtggtgcaggccgagagattctacaaaaagtttttgtttgattggacaaccgaaaaagcctttggacacattgtgaagctggaactcccggccgaccaccatggtgtcttcgggcacttggtggattcatacgcttacatgagaaatggctgggatgttgaggtatccgctgttggcaaccagttcaacggtgggtgcctcttggtggccatggtacctgagtggaaagaactcgacgcgcgggagaaataccaactaaccctcttcccgcatcagttcatcagtcccagaaccaacatgaccgcccacatcacggtcccctaccttggtgtgaacaggtacgaccagtacaaaaagcacaagccctggacactggttgtcatggtcgtgtcgccactcacggtcaacgccactagcgcgacacaaatcaaggtctacgccaacattgctccaacctacgttcacgtggccggggaacttccctcgaaagaggggattttccccgttgcgtgcgcggacggttacggaggactggtgacgacggacccgaagacagctgaccccgtttacggcaaggtgtacaacccgcccaggaccaactaccccgggcgcttcaccaacctgttagacgtggccgaagcgtgtcccaccttcctctgctttgacgacgggaaaccgtatgtcaccacgcggacggacgacactcgacttctggccaagtttgacctctcccttgccgcaaaacacatgtccaacacctacctggcaggcattgctcagtactacacccagtactctggcaccatcaacttgcacttcatgtttacaggttccacggactcaaaggcccgctacatggtggcctacatcccacctggggtggagccaccggacacacctgagagagctgcccactgcattcacgccgaatgggacactggactgaactccaaatttaccttctcaatcccgtacgtgtccgccgcggactacgcttacacagcgtctgacacggcagaaacaaccaatgtacagggatgggtctgtgtctaccaaattacacacgggaaggctgagaacgacactctggtcgtgacggttagcgccggtaaagactttgagttgcgcctcccgattgacccccgtcagcagaccaccgctacgggagaatcagcagaccccgtcactaccaccgtggaaaactacggcggtgagacacaagtccagaaacgtcaccacacagacatcggctttatcatggacagatttgtgaaggttagcgccttgagtccaatacacgtgattgacctcatgcagactcaccagcacggcctggtgggtgcgttgctgcgcgcggccacttactacttttcagacttggaaatagttgtgcgccacgacggcaatctgacctgggtgcccaacggcgcccccgaatcggccctgtcaaacaccagcaaccccactgtctacaggaaagaaccacttacgagactcgcactcccctacaccgcgccgcaccgcgtgctggcaactgtgtacaacgggacgagcaagtacaccacgagtgattca---agtaggcgtggtgacatgggggcccttgcggcacgggtcgcgaaacaacttcctgcttcattcaactacggtgcaattaaggccgacaccatccacgag

>AY593771.1_A_COL_1967

tcgagtgtgggagtcacctacgggtactccaccggtgaagaccacgtcgctgggcccaacacatcgggcctggagacgcgggtggtgcaggcagagaggtttttcaagaagtttttgtttgactggacaacggacaaaccttttggacacttggaaaagttggagctaccctccgaccaccatggtgttttcggacacctggtggactcatatgcttatatgaggaacggatgggacgttgaggtgtcagctgtcggcaaccagttcaacggcggttgtctcctggtggctatgataccggagtggaaagagtttgaacaacgcgagaagtaccaactcaccctcttcccacaccagtttattagccccagaacaaacatgactgcccacatcacagtcccataccttggagtgaacaggtacgatcagtacaaaaagcacaaaccttggacactggttgttatggtggtgtcgcccctcacggttagcaacactgccgcatcacaaattaaggtctacgccaacattgctccaacttacgttcacgtggccggggaactcccctcgaaagaggggattttcccagttgcatgttcggatggttacggagggttggtgacaacggacccaaagacagctgatcctgcctacggcaaggtgtacaatccgcccagaaccaactacccggggcggttcactaacctgttggacgtggccgaagcgtgccccactttcctctgtttcgacgacgggaaaccgtatgttgttacacggacagatgacacgcgactgctggccaagttcgacgtctcccttgctgcaaaacacatgtccaacacctacctgtcagggattgcacagtactacgcacagtactctggcaccatcaacttgcacttcatgttcacaggctcaactgactcaaaagcccgctacatggtggcctacatcccgccgggggtggaaccaccaaacacacccgaacgggctgcccactgcattcacgctgagtgggacacaggactaaattccaagttcactttttcaatcccgtacgtgtccgccgcagattacgcatacaccgcgtctgacacggcagaaacaaccaacgtacagggatgggtctgcatttaccagatcacacacgggaaggccgaaaacgacacactggtggtgtcggccagtgctggcaaagactttgagttgcgcctcccgatcgacccccggcagcaaaccactgctactggggagtccgcagaccctgttacaaccaccgtggaaaactacggcggtgagacacaagtccaaagacggcaccacacggatgtcgggttcattatggacagatttgtgaaaataagtaatttgagtcccacacatgtcattgacctcatgcagacccatcagcacgggttggtaggtgcgttgttgcgtgcagccacttactacttctctgacctggagattgttgtgcgccacgacggtaacctgacttgggtgcccaacggtgctcctgaggcagctctatcaaacaccagcaaccccactgcctacaacaaggcaccgttcacgagactcgctctcccgtacaccgcgccacaccgtgtgctggcaaccgtgtacaacgggacgaacaagtactccacgggtggtcag------aggccaggtgacatggggtcacttgcggcacgagtcgcaaagcaacttcctgcttctttcaactacggtgcaattagggcccagaccatccacgag

>AY593772.1_A_TUR_1972

tcgagtgtgggagtcacgtacgggtactctacccaggaagatcatgtttccggacccaacacatctggtttggagacgcgggtggtgcaggcagaaagatttttcaagaaacacctgtttgactggacaacagacaaagcttttgggcatctagagaaattggaactccccactgaacacaagggcgtctacggacacttggtggactcattcgcatacatgagaaatggctgggacgtggaggtgtccgctgttggcaaccagtttaacggcgggtgtctcctggtggccatggtccccgaatggaaagagttcacctcgcgtgagaagtaccagctcactttgtttccacaccagttcatcagccccagaaccaacatgactgcccacatcgtagtcccgtaccttggtgtgaacaggtatgaccagtacaagaagcacaaaccctggacgctggttgtgatggtggtttcaccgctcaccaccaacactgttagtgcaggacaaatcaaggtttatgtcaacattgccccgacccacgttcacgtggccggcgagctcccctcgaaggaggggatcgtgccggttgcttgttcggacgggtatggtggtttggtgacaacagacccaaaaacagctgaccctgtttatggtatggtgtacaacccccccagaacaaactaccccgggcggttcacgaacctgctggatgtggcggaggcctgccccacctttctctgtttcgacgacgggaaaccgtacgttgtgacaagagcggacgaacagcgtcttctggccaggttcgacgtttctcttgctgcaaagcacatgtcaaacacctacctttcagggatagcacagtactacgcacagtactctggtaccatcaacctccacttcatgtttactggctccactgactcaaaagcccgctacatggtggcgtacgttccgcccggtgtagagccgccggacacgcctgaggaagctgcacattgcatccatgctgagtgggacacggggttgaactccaaatttactttctctatcccgtacgtgtctgccgcggattacgcgtacaccgcgtctgacgtggcagaaacaacgaacgtacagggatgggtctgcatataccagattacacacgggaaagctgaacaagacactctggtcgtgtcggtcagtgccggcaaagactttgagttgcgcctcccgattgacccccgctcgcaaaccactaccaccggggagtctgcagaccctgtcaccaccaccgttgaaaactacggcggcgagacacaagtccaacgacgtcagcacaccgacgtcgccttcataatggacagatttgtgaagatacaaaacttgaaccccacacatgtcattgacctcatgcaaacccaccaacacgggttggtaggggccctgttacgtgctgctacgtactacttctctgacctggagattgtggtacgccatgatggcaacctaacctgggtacccaacggggcacctgaggcagctctgtctaacacgggcaaccccaccgcctacctcaaggcaccattcacgagactcgcacttccctacaccgcgccacaccgcgtgttggcaacagtgtacaacgggacgagcaagtactccacaggtggtacg---aacagacggggtgacctagggtctctcgcggcgagggtcgccgctcagctccctgcttctttcaactttggtgcaattcgagccacgaccatccacgag

>AY593773.1_A_PER_1969

tccagtgtgggcgtcacatacgggtactccaccgtagaggaccacgttgccgggcccaacacatcgggcttggagacgcgggtggtgcaagcagagaggttctataaaaagtttttgtttgactggacaacggacaagccctttggacacttggtaaagctcgaccttccagccgaccaccacggtgttttcggacacttggtggactcatatgcttacatgagaaacggctgggacgttgaagtgtccgccgttggaaatcagttcaacggcgggtgcctcctggtggctatggtacccgagtggaaagaacttgacacacgggagaaataccaactcacccttttcccacaccagttcattagtcctagaaccaacatgactgcccacatcacggttccttaccttggtgtgaacaggtatgaccagtacaaaaagcacaaaccctggacactggttgtcatggtcgtgtctccacttacggttaacaccactggtgcgacacagatcaaggtctacgctaacattgctccaacctacgttcacgtggccggcgagctcccctcgaaggaggggattttccctgtcgcatgtgcggacggttacggaggactggtaacaacagacccgaagacagctgaccccgcttatggcaaggtgtacaacccgccccggaccaactaccctgggcgctttaccaacttgttggacgtggccgaagcgtgtcccactttcctctgttttgacgacgggaaaccgtatgtcaccacgcggacggatgagaccaggcttctggccaagtttgacgtttcccttgctgcaaagcacatgtccaacacatacctgtcagggattgcccagtactacgcacagtactctggtaccattaatctgcacttcatgtttactggttctactgattcaaaagcccggtacatggtggcttacatcccgcctggggtggagccgccggacacacccgagagggctgctcactgcatccacgccgagtgggatacaggactaaactccaaattcactttctcaatcccgtacgtgtccgctgcggattatgcctacacggcatctgacacggcggaaacaaccaacgtgcagggatgggtttgcatctaccaaattacacacgggaaggctgaaaatgacactctggttgtgtcggttagcgccggcaaagactttgagttgcgcctcccgattgaccctcgccagcagaccaccgctaccggggagtcggcagaccctgtcaccaccactgtagagaactacggtggtgagacacaagtccacagacgccaccacacggacattggcttcatcatggacagatttgtgaagataaaggatgtaagcccgacccatgtcattgacctcatgcaaactcaccaacacggcctggtgggtgcgctgctgcgtgcggccacctactacttctctgatttggaaattgtcgtgcgacacgacggcaatctgacttgggtgcctaacggtgcccctgaagcggccctgtcgaacaccagcaaccccaccgcctataacaaggcaccgttcacgagacttgctctcccctacactgcgccgcaccgtgtgctggcaaccgtgtacaacgggacgaataagtatactgtgagtggttca---ggcaggcgaggtgacatgggctctctcgcggcgcgggtcgcgaaacaacttcctgcctccttcaactacggtgcaattcaggccgtgaccatccacgag

>AY593774.1_A_SPA_1969

tcgagtgtgggagtcacgtacgggtactccaccgaggaagaccacgttgctgggcccaacacatcgggcttggagacgcgggtggtgcaggcagagagatttttcaagaagtttctgtttgactggacaacggacaaaccttttggacacttgacaaaactggagcttcccaccgaccaccgcggtgtcttcggacacctggtggactcatatgcgtatatgaggaacggctgggatgttgaggtgtccgccgttggcaaccagtttaacggcgggtgccttctggtggccatggtgccagagtggaaagaatttgacgcacgtgaaaaataccaacttacccttttcccacaccagtttatcagccccagaactaacatgactgcccacatcacggttccgtatcttggtgtgaacaggtacgatcagtacaagaaacacaagccctggacactggttgtcatggtagtatcacccctcacggtcagcaacgctgccgcagcacaaatcaaggtctatgccaacattgccccaacctacgttcacgtggctggagagcttccctcgaaagaggggattttcccggttgcgtgcgcggacggttatggaggactagtgacaacagacccgaaaacagctgaccctgtttacggtaaggtgtacaacccgcccaggaccaactaccccgggcgctttacaaacctgttggacgtggccgaagcatgtcccactttcctctgtttcgacgatgggaaaccgtacgtcgttacgcggacagacaacacccgtcttttggccaagtttgacgtttcccttgccgcaaaacacatgtccaacacatacctatcagggattgcacagtactacacacagtactctggtactatcaacctgcacttcatgttcacaggctccactgactcaaaagcccgctacatggtggcttacatcccacctggggtggagccaccggacacacctgaaggggccgctcactgcattcatgctgaatgggacacaggactgaactccaaattcaccttttcaatcccttacgtgtccgccgcggattacgcgtacaccgcgtctgatacggcggagacaaccaatgtacagggatgggtttgtgtttaccaaattacacacgggaaggctgaaaatgacaccttggtagtgtcggctagcgccggcaaagactttgagttgcgcctcccaattgacccccggacacaaaccaccgctactggggagtccgcagaccctgtcaccaccaccgtggagaactacggtggtgagacgcaagtccagagacgtcaccacacggacgtcggcttcatcatggaccgatttgtgaagataaacagcctgaaccccacacacgtcattgacctcatgcagacccaccaacacgggctggtgggtgcgttgctgcgtgcagccacgtactacttctccgacttggagattgttgtgcggcatgatggtaatctgacctgggtgcctaacggtgcccccgaggcagccctgtcaaacaccagcaaccccactgcctacaacaaggcaccgttcacgagacttgctctcccttacactgcgccgcaccgcgtgttggcaactgtgtacaacgggacaagcaagtactccgcgagcggtttg------ggacgaggcgatctggggtccctcgcggcgcgagtcgcgacacagcttcctgcttctttcaactacggtgcaatcagggcccagaccatccacgag

>AY593775.1_A_VEN_1970

tccagtgtgggcgtcacatacgggtactccaccgtagaggaccacgttgccgggcccaacacatcgggcttggagacgcgggtggtgcaagcagagaggttctataaaaagtttttgtttgactggacaacggacaagccctttggacacttggtaaagctcgaccttccagccgaccaccacggtgttttcggacacttggtggactcatatgcttacatgagaaacggctgggacgttgaagtgtccgccgttggaaatcagttcaacggcgggtgcctcctggtggctatggtacccgagtggaaagaacttgacacacgggagaaataccaactcacccttttcccacaccagttcattagtcctagaaccaacatgactgcccacatcacggttccttaccttggtgtgaacaggtatgaccagtacaaaaagcacaaaccctggacactggttgtcatggtcgtgtctccacttacggttaacaccactggtgcgacacagatcaaggtctacgctaacattgctccaacctacgttcacgtggccggcgagctcccctcgaaggaggggattttccctgtcgcatgtgcggacggttacggaggactggtaacaacagacccgaagacagctgaccccgcttatggcaaggtgtacaacccgccccggaccaactaccctgggcgctttaccaacttgttggacgtggccgaagcgtgtcccactttcctctgttttgacgacgggaaaccgtatgtcaccacgcggacggatgagaccaggcttctggccaagtttgacgtttcccttgctgcaaagcacatgtccaacacatacctgtcagggattgcccagtactacgcacagtactctggtaccattaatctgcacttcatgtttactggttctactgattcaaaagcccggtacatggtggcttacatcccgcctggggtggagccgccggacacacccgagagggctgctcactgcatccacgccgagtgggatacaggactaaactccaaattcactttctcaatcccgtacgtgtccgctgcggattatgcctacacggcatctgacacggcggaaacaaccaacgtgcagggatgggtttgcatctaccaaattacacacgggaaggctgaaaatgacactctggttgtgtcggttagcgccggcaaagactttgagttgcgcctcccgattgaccctcgccagcagaccaccgctaccggggagtcggcagaccctgtcaccaccactgtagagaactacggtggtgagacacaagtccacagacgccaccacacggacattggcttcatcatggacagatttgtgaagataaaggatgtaagcccgacccatgtcattgacctcatgcaaactcaccaacacggcctggtgggtgcgctgctgcgtgcggccacctactacttctctgatttggaaattgtcgtgcgacacgacggcaatctgacttgggtgcctaacggtgcccctgaagcggccctgtcgaacaccagcaaccccaccgcctataacaaggcaccgttcacgagacttgctctcccctacactgcgccgcaccgtgtgctggcaaccgtgtacaacgggacgaataagtatactgtgagtggttca---ggcaggcgaggtgacatgggctctctcgcggcgcgggtcgcgaaacaacttcctgcctccttcaactacggtgcaattcaggccgtgaccatccacgag

>AY593776.1_A_GER_1968

tcgagtgtgggagtcacgtacgggtactccaccgaggaagaccacgtcgctgggcccaacacatcgggcctggagactcgggtggtgcaggcagagagattttttaaaaagtttttgtttgactggacaacagataaaccttttggacacttggaaaaattggagctccccaccgaccaccgcggtgtcttcgggcacctggtggactcatatgcgtacatgaggaacggttgggacgttgaggtgtctgccgttggcaatcagtttaacggtgggtgccttctggtggccatgataccagagtggaaagattttgacaaacgtgaaaaataccaactcaccctttttccacaccagttcatcagccccagaaccaacatgactgcccacatcacggtcccgtaccttggtgtgaataggtatgatcagtacaagaagcacaagccttggacactagttgtcatggtggtatcacccctcacggtcagcgatactgctgcaccacaaatcaaggtctacgctaacattgccccaacctatgttcacgtggctggggagcttccctcgaaagaggggattttcccggttgcgtgcgcggacggttatggagggctggtgacaacagacccgaaaacagctgatcctgtgtacggcaaggtgtacaacccgcccaggaccaactaccctgggcgctttacaaacttgttggacgtggccgaagcatgtcccacttttctctgttttgacgatgggaaaccgtacgtcgttacgcggacagacaacacccgtcttttggccaagtttgacgtctcccttgccgcaaaacacatgtccaacacatacctatcagggattgcacagtactacacacaatactctggtactatcaacctgcacttcatgttcacaggctccactgactcaaaagcccgttacatggtggcctacatcccacccggggtggagccaccggacacacctgaaggggcagcccactgcattcatgctgaatgggacacaggactaaactccaaattcaccttttccatcccttacgtgtccgccgcggattacgcgtacaccgcgtctgacacggcggagacaaccaacgtgcagggatgggtctgtgtttaccaaattacacacggaaaggctgacggtgacaccttggtagtgtcggctagcgccggcaaagattttgagttacgcctcccaattgacccccggccacaaactaccgctgctggggagtctgcagaccctgtcaccaccaccgtggagaactacggtggtgagacacaagtccagaggcgtcaccacacggacgtcggcttcatcatggaccgatttgtgaagataaacagcctgaaccccacacacgtcattgacctcatgcagacccaccagcacgggctggtgggcgcgctgctgcgcgcagccacgtactacttctccgacttggagattgttgtgcggcatgatggcaatctgacctgggtgcctaacggcgcccctgaagcagccctgtcaaacaccagcaaccccactgcctacaacaaggcaccgttcacgagactcgctctcccttacactgcgccgcaccgcgtgttggcaactgtgtacaacgggacaagcaagtactccgtgagcggttcg------agacgaggcgacttggggaccctcgcggcgcgagtcgcgacacagcttcctacttctttcaactacggtgcaatcaaggcccagaccatccacgag

>AY593777.1_A_GER_1972

tcgagtgtgggagtcacgtacgggtactccaccgaggaagaccacgttgctgggcccaacacatcgggcttggagacgcgggtggtgcaggcagagagatttttcaagaagtttctgtttgactggacaacggacaaaccttttggacacttgacaaaactggagcttcccaccgaccaccgcggtgtcttcggacacctggtggactcatatgcgtatatgaggaacggctgggatgttgaggtgtccgccgttggcaaccagtttaacggcgggtgccttctggtggccatggtgccagagtggaaagaatttgacgcacgtgaaaaataccaacttacccttttcccacaccagtttatcagccccagaactaacatgactgcccacatcacggttccgtatcttggtgtgaacaggtacgatcagtacaagaaacacaagccctggacactggttgtcatggtagtatcacccctcacggtcagcaacgctgccgcagcacaaatcaaggtctatgccaacattgccccaacctacgttcacgtggctggagagcttccctcgaaagaggggattttcccggttgcgtgcgcggacggttatggaggactggtgacaacagacccgaaaacagctgaccctgtttacggtaaggtgtacaacccgcccaggaccaactaccccgggcgctttacaaacctgttggacgtggccgaagcatgtcccactttcctctgtttcgacgatgggaaaccgtacgtcgttacgcggacagacaacacccgtcttttggccaagtttgacgtttcccttgccgcaaaacacatgtccaacacatacctatcagggattgcacagtactacacacagtactctggtactatcaacctgcacttcatgttcacaggctccactgactcaaaagcccgctacatggtggcttacatcccacctggggtggagccaccggacacacctgaaggggccgctcactgcattcatgctgaatgggacacaggactgaactccaaattcaccttttcaatcccttacgtgtccgccgcggattacgcgtacaccgcgtctgatacggcggagacaaccaatgtacagggatgggtttgtgtttaccaaattacacacgggaaggctgaaaatgacaccttggtagtgtcggctagcgccggcaaagactttgagttgcgcctcccaattgacccccggacacaaaccaccgctactggggagtccgcagaccctgtcaccaccaccgtggagaactacggtggtgagacgcaagtccagagacgtcaccacacggacgtcggcttcatcatggaccgatttgtgaagataaacagcctgaaccccacacacgtcattgacctcatgcagacccaccaacacgggctggtgggtgcgttactgcgtgcagccacgtactacttctccgacttggagattgttgtgcggcatgatggtaatctgacctgggtgcctaacggtgcccccgaggcagccctgtcaaacaccagcaaccccactgcctacaacaaggcaccgttcacgagacttgctctcccttacactgcgccgcaccgcgtgttggcaactgtgtacaacgggacaagcaagtactccgcgagcggtttg------ggacgaggcgatctggggccccacgcggcgcgagtcgcgacacagcttcctgcttcttttaactacggtgcaatcagggcccagaccatccacgag

>AY593778.1_A_SPA_1969

tcgagcgtgggagtcacctacgggtactccactggagaagaccatgttgctgggcccaacacatcgggcctggagacgcgggtggtgcaggcagagagattttttaaaaagtttttgtttgactggacaacggacaaaccttttggacatttggaaaagctggaacttcccgccgaccaccacggcgttttcgggcacctggtggaatcgtatgcttacatgagaaatggttgggacgttgaggtgtctgctgttggcaaccagttcaacggcgggtgcctcctggtggctatggtaccggagtggaaagagtttgaacagcgcgagaaataccaactcaccctcttcccgcaccagttcatcagccccagaacaaacatgactgcccacatcacagtcccataccttggagtgaacaggtacgatcagtacaagaaacacaaaccttggacactggttgtcatggtagtgtcgcccctcacggttagcgacactgccgcgacacagattaaggtctacgccaatattgctccgacctacgttcacgtggctggggaactcccctcgaaagaggggattttcccagttgcatgctcggacggttacggaggactggtgacaacggacccgaaaacagctgaccccgcctacggtaaggtgtacaacccgcccaggaccaactaccctgggcggtttaccaacctgttggacgtggctgaagcgtgtcccactttcctctgtttcgacgacgggaaaccgtacgttgtcacgcggacagatgacacacgactattggccaagttcgacgtctcccttgctgcaaaacacatgtccaacacgtacctgtcaggggttgcacagtactacgcacagtactctggtaccatcaacttgcacttcatgttcacaggctcaactgactcaaaagcccgctacatggtggcctacatcccgcctggggtggaaccaccggacacacctgaaagggccgctcactgcatccacgctgaatgggacacaggactgaactccaaattcactttctcaatcccgtacgtgtccgccgcagattacgcgtataccgcgtctgacacggcagaaacaaccaacgtacagggctgggtttgcatataccagatcacacacgggaaggccgagaacgacacattggtggtgtcggccagcgccggcaaagactttgagttgcgcctcccgattgacccccgacagcaaactactgctgtcggggagtccgcagaccctgtcaccaccgccgtggagaactacggcggtgagacacaaacccggagacggcaccacacggatgtcggtttcatcatggacagatttgtgaagataaacagtttgagtcccacgcatgtcattgacctcatgcagacccaccagcacgggctggtgggtgcgctgctgcgtgcagccacgtactacttctctgacttggagattgttgtgcggcatgacggcaatttgacttgggtgcccaatggtgcccctgaagcagctttgtcaaacaccagcaaccccactgcctacaacaaggcaccgttcacgaggcttgctctcccttacactgcgccacaccgcgtgttggcaaccgtgtacaacgggacgaacaagtactccacaggcggtctg------agacgaggcgacacggggtcgcccgcggcgcgggccgcgaaacaacttcctgcctcttttaattacggtgcaattggggccgtcaccatccacgag

>AY593779.1_A_GER_1972

tcgagtgtgggagtcacgtacgggtactccaccgaggaagaccacgttgctgggcccaacacatcgggcttggagacgcgggtggtgcaggcagagagatttttcaagaagtttctgtttgactggacaacggacaaaccttttggacacttgacaaaactggagcttcccaccgaccaccgcggtgtcttcggacacctggtggactcatatgcgtatatgaggaacggctgggatgttgaggtgtccgccgttggcaaccagtttaacggcgggtgccttctggtggccatggtgccagagtggaaagaatttgacgcacgtgaaaaataccaacttacccttttcccacaccagtttatcagccccagaactaacatgactgcccacatcacggttccgtatcttggtgtgaacaggtacgatcagtacaagaaacacaagccctggacactggttgtcatggtagtatcacccctcacggtcagcaacgctgccgcagcacaaatcaaggtctatgccaacattgccccaacctacgttcacgtggctggagagcttccctcgaaagaggggattttcccggttgcgtgcgcggacggttatggaggactggtgacaacagacccgaaaacagctgaccctgtttacggtaaggtgtacaacccgcccaggaccaactaccccgggcgctttacaaacctgttggacgtggccgaagcatgtcccactttcctctgtttcgacgatgggaaaccgtacgtcgttacgcggacagacaacacccgtcttttggccaagtttgacgtttcccttgccgcaaaacacatgtccaacacatacctatcagggattgcacagtactacacacagtactctggtactatcaacctgcacttcatgttcacaggctccactgactcaaaagcccgctacatggtggcttacatcccacctggggtggagccaccggacacacctgaaggggccgctcactgcattcatgctgaatgggacacaggactgaactccaaattcaccttttcaatcccttacgtgtccgccgcggattacgcgtacaccgcgtctgatacggcggagacaaccaatgtacagggatgggtttgtgtttaccaaattacacacgggaaggctgaaaatgacaccttggtagtgtcggctagcgccggcaaagactttgagttgcgcctcccaattgacccccggacacaaaccaccgctactggggagtccgcagaccctgtcaccaccaccgtggagaactacggtggtgagacgcaagtccagagacgtcaccacacggacgtcggcttcatcatggaccgatttgtgaagataaacagcctgaaccccacacacgtcattgacctcatgcagacccaccaacacgggctggtgggtgcgttactgcgtgcagccacgtactacttctccgacttggagattgttgtgcggcatgatggtaatctgacctgggtgcctaacggtgcccccgaggcagccctgtcaaacaccagcaaccccactgcctacaacaaggcaccgttcacgagacttgctctcccttacactgcgccgcaccgcgtgttggcaactgtgtacaacgggacaagcaagtactccgcgagcggtttg------ggacgaggcgatctggggccccacgcggcgcgagtcgcgacacagcttcctgcttcttttaactacggtgcaatcagggcccagaccatccacgag

>AY593780.1_A_FRA_1960

tcgagcgtgggagtcacctacgggtactccactggagaagaccatgttgctgggcccaacacatcgggcctggagacgcgggtggtgcaggcagagagattttttaaaaagtttttgtttgactggacaacggacaaaccttttggacatttggaaaagctggaacttcccgccgaccaccacggcgttttcgggcacctggtggaatcatatgcttacatgagaaatggttgggacgttgaggtgtctgctgttggcaaccagttcaacggcgggtgcctcctggtggctatggtaccggagtggaaagagtttgaacagcgcgagaaataccaactcaccctcttcccgcaccagttcatcagccccagaacaaacatgactgctcacatcacagtcccataccttggagtgaacaggtacgatcagtacaagaaacacaaaccttggacactggttgttatggtagtgtcgcccctcacggttagcgacactgccgcggcacagattaaggtctacgccaatattgctccgacctacgttcacgtggctggggaactcccctcgaaagaggggattttcccagttgcatgttcggacggttacggaggactggtgacaacggacccgaaaacagctgaccccgcctacggcaaggtgtacaacccgcccaggaccaactaccctgggcggtttaccaacttgttggatgtggctgaagcgtgtcccactttcctctgtttcgacgacgggaaaccgtacgttgtcacgcggacagatgacacacgactattggccaagttcgacgtctcccttgctgcaaaacacatgtccaacacgtacctgtcagggattgcacagtactacgcacagtactctggtaccatcaacttgcacttcatgttcacaggctcaactgactcaaaagcccgctacatggtggcctacatcccgcccggggtggaaccaccggacacacctgaaagggccgctcactgcatccacgctgaatgggacacaggactgaactccaaattcactttctcaatcccgtacgtgtccgccgcagattacgcgtacaccgcgtctgacacggcagaaacaaccaacgtacagggctgggtctgcatctaccagatcacacacgggaaggccgagaacgacacattggtggtgtcggccagcgccggcaaagactttgagttgcgcctcccgatcgacccccgacagcaaactactgctgttggggagtccgcagaccctgtcaccaccaccgtggagaactacggcggtgagacacaaacccagagacggcaccacacggatgtcggtttcatcatggacagatttgtgaagataaacagtttgagtcctacgcatgtcattgacctcatgcagacccaccagcacgggctggtgggtgcgctgttgcgtgcagccacgtactacttctctgacttggagattgttgtgcggcatgacggcaatttgacttgggtgcccaacggtgcccctgaagcagctttgtcaaacaccagcaaccccactgcctacaacaaggcaccgttcacgaggctcgctctcccttacactgcgccacaccgcgtgttggcaaccgtgtacaacgggacgaacaagtactccacggacggtccg------agacgaggcgacatggggtcgctcgcggcgcgggccgcgaaacaacttcctgcctcttttaactacggtgcaatcagggccgtcaccatccacgag

>AY593781.1_A_GER_1951

tcgagtgtgggagtcacctacgggtactccactggagaagaccatgtcgctgggcccaacacatcgggcctggagacgcgggtggtgcaggcagagagatttttcaaaaagtttttgtttgactggacaacggacaaaccttttggacatttggaaaagctggaacttcccgccgaccaccacggcgttttcgggcacctggtggaatcatatgcttacatgagaaatggttgggacgttgaggtgtctgctgttggcaaccagttcaacggcgggtgcctcctggtggctatggtaccggagtggaaagagtttgaacagcgcgagaaataccaactcaccctcttcccgcaccagttcatcagccccagaacaaacatgactgctcacatcacagtcccataccttggagtgaacaggtacgatcagtacaagaaacacaaaccttggacactggttgttatggtagtgtcgcccctcacggttagcgacactgccgcggcacagattaaggtctacgccaacattgctccaacctacgttcacgtggctggggaactcccctcgaaagaggggattttcccagtcgcatgttcggacggttacggaggattggtgacaacggacccgaaaacagctgaccccgcctacggcaaggtgtacaacccgcccaggaccaactaccctgggcggtttaccaacttgttggacgtggctgaagcgtgtcccactttcctctgtttcgacgacgggaaaccgtacgttgtcacgcggacagatgacacacgactattggccaagttcgacgtctcccttgctgcaaaacacatgtccaacacgtacctgtcagggattgcacagtactacgcacagtactctggtaccatcaacttgcacttcatgttcacaggctcaactgactcaaaagcccgctacatggtggcctacatcccgcctggggtggaaccaccggacacacctgaaagggccgctcactgcatccacgctgaatgggacacaggactgaactccaaattcactttttcaatcccgtacgtgtccgccgcagattacgcgtataccgcgtctgacacggcagaaacaaccaacgtacagggctgggtctgcatctaccagatcacacacgggaaggccgagaacgacacattggtggtgtcggccagcgccggcaaagactttgagttgcgcctcccgatcgacccccgacagcaaaccactgctgttggggagtccgcagaccctgtcaccaccaccgtggagaactacggcggtgagacacaaacccagagacggcaccacacggatgtcggtttcatcatggacagatttgtgaagataaacagtttgagtcccacgcatgtcattgacctcatgcagacccaccagcacgggctggtaggtgcgctgttgcgtgcagccacgtactacttctctgacttggagattgttgtgcggcatgacggcaatttgacttgggtgcccaatggtgcccctgaagcagctttgtcaaacaccagcaaccccactgcctacaacaaggcaccgttcacgaggctcgctctcccttacactgcgccacaccgcgtgttggcaaccgtgtacaacgggacgaacaagtactccacgggcggtccg------agacgaggcgacacggggtcgcccgcggcgcgggccgcgaaacaacttcctgcctcttttaactacggtgcaatcagggccgtcaccatccacgag

>AY593782.1_A_ARG_2000

tccagcgtaggcgtcacgtacgggtactccacggcggaagatcacgtcgccgggcccaacacatcgggcttggagacccgggtggtacaagcagagagattttacaaaaagtttttgtttgactggacaaaggacaaggcttttggacatgtggaaaagttggaactgcccgccgaccaccacggtgttttcggacacttggtggactcatatgcctacatgaggaacggttgggatgttgaggtgtctgctgttggcaaccagttcaacggcggctgtctcttggtggctatggtacctgagtggaaagaatttgacacacgggagaaataccaacttacacttttcccacatcaattcattaaccccagaaccaacatgactgcccacatcacggtcccttacctcggtgtgaacaggtacgaccagtacaagaagcacaaaccctggacattggttgttatggttgtatccccactcacagtcagctccaatggtgcagcacagattaaggtctatgctaacatcgcgccaacctacgtccacgtggccggtgagctcccgtcgaaagaggggatctttcctgttgcgtgcgcggacggttatggcgggctggtgacgacagacccgaaaacagctgaccccgcctacggcaaggtgtacaacccgccccggaccaactaccccgggcgtttcaccaatttgttggacgtggccgaggcgtgtcctaccttcctttgttttgacgacgggaaaccgtacgtcgttacgaggacagacggcacgcgcctcttggccaagttcgacctttcccttgctgcaaagcacatgtccaacacttacttgtcaggaattgcccagtactacgcacagtactcaggtaccatcaatttgcacttcatgttcacaggttcaactgattcaaaggcccggtacatggtggcctacatcccgcctggggtggagccaccggacacacctgagcgtgcggcccactgcatccacgccgaatgggacacaggactgaactccaagttcactttttcaatcccgtacgtgtctgctgcggattacgcctacacggcgtctgacgaggcagaagcaacaaacgtacagggatgggtttgcatttaccaaatcacacacgggaaggccgaagacgacactcttgttgtgtcagtcagtgctggcaaggacttcgagctgcgcctcccgattgacccccgccagcaaaccaccgccactggggaatcagcagaccctgtcaccaccacagtggagaactacggcggtgaaacacaagtccagagacgccaccacacagacgttggcttcatcatggacagatttgtgaaagtgacaaccacagctcccacccacgttattgacctcatgcaaacacaccaacacggcctagtgggtgcgctgctgcgggcggcaacctactacttctccgatctggagattgtcgtgcgacatgaaggcaacctgacgtgggtacccaatggtgctcctgaatcggccctgtccaacacaagcaaccccaccgcctacaagaaggcaccgttcacgagacttgctctaccttacaccgcgccgcaccgagtgctggcaactgtgtacaacggagtaagcaagtacaccgcgaatggttca---aacaggcggggtgacatggccgctctcgcggcacgagtcgcgaaggcacttcctgcttctttcaactacggtgcaatcaaggccactaacatacacgag

>AY593784.1_A_ARG_2001

tccagcgtaggcgttacatacgggtactccacaacagaggaccacgttgctggacccaacacatcaggtttggagacacgagtggtacaggcagagagattctacaaaaagtttttgtttgattggacaacggacaagccttttggacacctgcacaaactggagttgcccaccgaccaccacggtgttttcggacacttggtggactcatacgcctacatgaggaacggttgggacgttgaggtgtctgctgttggcaaccagttcaacggcggatgcctcctagtggccatggtacccgaatggaaagagtttgaaacgcgggagaagtaccagctcacgcttttcccgcaccagttcattagccccagaaccaacatgaccgcccacatcacggttccttaccttggtgtgaatagatatgatcagtacaaaaaacacaaaccctggacactggttgtcatggtcgtgtccccgctcacggtcaacgccacgagcgcggcacagatcaaggtctatgccaacatcgctccgacctacgttcatgtggccggcgagctcccctcgaaagaggggatcttccctgtcgcgtgcgcggacggttacggaggactggtgacaacggacccgaaaacagctgaccccgcctacggcaaggtgtacaatccgccccggactaactaccccgggcgtttcactaacttgttggacgtggctgaggcatgtcccacctttctgtgttttgacgacgggaaaccgtacgttaccacacagacaggtgagtctcgtcttctggccaagttcgacctttcccttgccgcgaagcacatgtctaacacatacttggcaggaattgcccagtactacacacagtactcaggcaccatcaatttgcatttcatgttcacaggttcaactgattcaaaagcccgctacatggtggcttacatcccgcctggggtggaaccaccggacacacctgagagggcagcccactgcatccatgctgagtgggacacagggctgaattccaaattcacattctcaatcccgtacgtgtctgccgcggattacgcctacacggcgtctgatgaggcagagacaacaaacgtacagggatgggtctgcgtttaccagatcacacacgggaaggctgacaacgacactctggtcgtgtcggttagcgccggcaaggacttcgagttgcgcctccccattgacccccgaccgcagaccaccgctactggggaatcagcagaccctgtcaccaccactgtagagaactacggcggtgagacacaagttcagagacgccaccacaccgacgttggcttcatcatggacagatttgtgaaaataaacagcccaaaatccacccatgttattgacctcatgcaaacccaccaacacggtctagtgggtgcgctgctgcgtgcggcgacctactacttctcagatctggaaattgttgtgcggcatgacggcaacctaacttgggtgcccaatggtgctcccgtgtcagccttgtccaacaccagcaaccccaccgcctacaacaaggcaccgttcacgagacttgccctcccctacaccgcgccacaccgcgtgttggcgactgtgtacaacgggacgagcaagtacactgtgagtgggtca---agcagacgaggcgacttgggttccctcgcggcacgagtcgtgaaggcacttcctgcttctttcaactacggtgcaatcaaggccgacaacgtgcacgag

>AY593785.1_A_ARG_2001

tccagcgtaggcgttacatacgggtactccacaacagaggaccacgttgctggacccaacacatcaggtttggagacacgagtggtacaggcagagagattctacaaaaagtttttgtttgattggacaacggacaagccttttggacacctgcacaaactggagttgcccaccgaccaccacggtgttttcggacacttggtggactcatacgcctacatgaggaacggttgggacgttgaggtgtctgctgttggcaaccagttcaacggcggatgcctcctagtggccatggtacccgaatggaaagagtttgaaacgcgggagaagtaccagctcacgcttttcccgcaccagttcattagccccagaaccaacatgaccgcccacatcacggttccttaccttggtgtgaatagatatgatcagtacaaaaaacacaaaccctggacactggttgtcatggtcgtgtccccgctcacggtcaacgccacgagcgcggcacagatcaaggtctatgccaacatcgctccgacctacgttcatgtggccggcgagctcccctcgaaagaggggatcttccctgtcgcgtgcgcggacggttacggaggactggtgacaacggacccgaaaacagctgaccccgcctacggcaaggtgtacaatccgccccggactaactaccccgggcgtttcactaacttgttggacgtggctgaggcatgtcccacctttctgtgttttgacgacgggaaaccgtacgttaccacacagacaggtgagtctcgtcttctggccaagttcgacctttcccttgccgcgaagcacatgtctaacacatacttggcaggaattgcccagtactacacacagtactcaggcaccatcaatttgcatttcatgttcacaggttcaactgattcaaaagcccgctacatggtggcttacatcccgcctggggtggaaccaccggacacacctgagagggcagcccactgcatccatgctgagtgggacacagggctgaattccaaattcacattctcaatcccgtacgtgtctgccgcggattacgcctacacggcgtctgataaggcagagacaacaaacgtacagggatgggtctgcgtttaccagatcacacacgggaaggctgacaacgacactctggtcgtgtcggttagcgccggcaaggacttcgagttgcgcctccccattgacccccgaccgcagaccaccgctactggggaatcagcagaccctgtcaccaccactgtagagaactacggcggtgagacacaagttcagagacgccaccacaccgacgttggcttcatcatggacagatttgtgaaaataaacagcccaaaatccacccatgttattgacctcatgcaaacccaccaacacggtctagtgggtgcgctgctgcgtgcggcgacctactacttctcagatctggaaattgttgtgcggcatgacggcaacctaacttgggtgcccaatggtgctcccgtgtcagccttgtccaacaccagcaaccccaccgcctacaacaaggcaccgttcacgagacttgccctcccctacaccgcgccacaccgcgtgttggcgactgtgtacaacgggacgagcaagtacactgtgagtgggtca---agcagacgaggcgacttgggttccctcgcggcacgagtcgtgaaggcacttcctgcttctttcaactacggtgcaatcaaggccgacaacgtgcacgag

>AY593786.1_A_ARG_2001

tccagcgtaggcgttacatacgggtactccacaacagaggaccacgttgctggacccaacacatcaggtttggagacacgagtggtacaggcagagagattctacaaaaagtttttgtttgattggacaacggacaagccttttggacacctgcacaaactggagttgcccaccgaccaccacggtgttttcggacacttggtggactcatacgcctacatgaggaacggttgggacgttgaggtgtctgctgttggtaaccagttcaacggcggatgcctcctagtggccatggtacccgaatggaaagagtttgaaacgcgggaaaagtaccagctcacgcttttcccgcaccagttcattagccccagaaccaacatgactgcccacatcacggttccttaccttggtgtgaatagatatgatcagtacagaaaacacaaaccctggacactggttgtcatggtcgtgtccccgctcacggtcaacgccacgagcgcggcacagatcaaggtctatgccaacatcgctccgacctacgttcatgtggccggcgagctcccctcgaaagaggggatcttccctgtcgcgtgcgcggacggttacggaggactggtgacaacggacccgaaaacagctgaccccgcctacggcaaggtgtacaatccgccccggactaactaccccgggcgtttcaccaacttgttggacgtggctgaggcatgtcccacctttctgtgttttgacgacgggaaaccgtacgttaccacacagacaggtgagtctcgtcttctggccaagttcgacctttcccttgctgcgaagcacatgtctaacacatatttggcaggaattgcccagtactacacacagtactcgggcaccatcaatttgcatttcatgttcacaggttcaactgattcaaaagcccgctacatggtggcttacatcccgcctggggtggaaccaccggacacacctgagagggcagcccactgcatccatgctgagtgggacacagggctgaattccaaattcacattctcaatcccgtacgtgtctgccgcggattacgcctacacggcgtctgatgaggcagagacaacaaacgtacagggatgggtctgcgtttaccagatcacacacgggaaggctgacaacgacactctggtcgtgtcggttagcgccggcaaggacttcgagttgcgcctccccattgacccccgaccgcagaccaccgctactggggaatcagcagaccctgtcaccaccactgtagagaactacggcggtgagacacaagttcagagacgccaccacaccgacgttggcttcatcatggacagatttgtgaaaataaacagcccaaaatccacccatgtcattgacctcatgcaaacccaccaacacggtctagtgggtgcgctgctgcgtgcggcgacctactacttctcagatctggaaatcgttgtgcggcatgacggtaacctaacttgggtgcccaatggtgctcccgtgtcagccttgtccaacaccagcaaccccaccgcctacaacaaggcaccgttcacgagacttgccctcccctacaccgcgccacaccgcgtgttggcgactgtgtacaacgggacgagcaagtatactgtgagtgggtca---agcagacgaggcgacttgggttccctcgcggcacgagtcgcgaaggcacttcctgcttctttcaactacggtgcaatcaaggccgacaacgtgcacgag

>AY593787.1_A_Brazil_1977

tcgagtgttggtgtcacgtacgggtattccacaggagaagaccacgttgcagggcccaacacatcgggcctggagacacgggtggtacaggcagagagattttacaaaaaatttttgtttgactggacaacggacaaggcttttggacacctggagaagctcggacttccaaccgaccaccacggtgttttcggacacttggtggactcatacgcctacatgagaaatggttgggatatcgaggtgtctgccgttggcaaccagttcaacggcgggtgtctcctggtggccatggtgcccgaatggaaggattttgacgcgcgggagaaataccaactcactcttttcccgcaccagttcattagccccagaaccaacatgactgcccacatcacggtcccctaccttggtgtgaacaggtatgaccagtacaaaaagcacaagccttggaccttggttgtcatggtcgtgtctccgctaacggttaacaccgctggcgcgtcacagatcaaggtctacgccaacattgctccgacctacgttcacgtggctggtgagctcccctcgaaagaggggattttcccggttgcgtgcgcggacggttatggaggactggtgacaacagacccaaagacagctgaccctgtttacggcaaggtgtacaacccgcccaggaccaactaccctgggcggttcactaacttgttggacgtggccgaagcgtgtcccaccttcctctgctttgacgacgggaaaccgtacgtcaccacgcggacggaccaaactcgacttctggccaagtttgacctttcccttgccgcaaaacacatgtccaacacatacctggcaggacttgcccagtactacacacagtactcgggcaccatcaatttgcacttcatgttcacaggctccactgattcaaaggcccgctacatggtggcctacatcccacctggggtgcagccacctgaaacacctgagatggctgcccactgcatacacgccgagtgggacactggactgaactccaaattcactttttcaatcccgtacgtgtctgccgcagactacgcctacacagcgtctgacacggcagaaacaaccaatgtgcagggctgggtctgcatttaccagattacacacgggaaggctgaaaatgacgccttggtcgtgtcggtcagtgccggcagagactttgagttgcgcctcccgattgacccccgcacgcagactaccgccaccggggagtcagcagaccctgtcaccaccaccgtggagaactacggcggtgagacacaagttcagagacgccaccacactgacatcggcttcatcatggacaggtttgtgaagattaaggacgtgcaaccgacgcacgtcattgacctcatgcagactcaccaacacggcctggtgggtgcaatgctgcgtgcagctacgtactacttttctgacttggaaattgttgtacggcacgacggcaatctgacttgggtgcccaacggcgcccctgagtcagccctagacaacactggcaatcccaccgcctacaacaaggcaccattcacgagacttgctctcccttacacggcaccacaccgtgtgctggcaacagtgtacaacgggacaagcaaatacaccgtgggtggttca---ggcaggcgtggtgacatggggtccctcgcggcacgagtcgcgaaacagcttcctgcttcattcaactacggtgcaattaaggccaccgacatccacgag

>AY593788.1_A_Brazil_1979

tcgagtgttggtgtcacgtacgggtattccacaggagaagaccacgttgcagggcccaacacatcgggcctggagacacgggtggtacaggcagagagattttataaaaaatttctgtttgactggacaacggacagggcttttggacacctggagaaactcgaacttccaacagaccaccacggtgttttcggacacttggtggactcatacgcctacatgagaaatggttgggatgttgaggtgtctgccgttggcaaccagttcaacggcgggtgtctcctggtggccatggtgcccgaatggaaggattttgacgcgcgggagaaataccaactcactcttttccctcaccagttcattagccccagaaccaacatgactgcccacatcacggtcccctaccttggcgtgaacaggtatgaccagtacaaaaagcacaagccttggaccttggttgtcatggtcgtgtctccgctaacggttaacaccgctggcgcgtcacagatcaaggtttacgccaacattgctccgacctacgtacacgtggctggtgagctcccctcgaaagaggggattttcccggttgcgtgcgcggacggttacggaggactagtgacaacagacccaaagacagctgaccctgtttacggcaaggtgtacaacccgcccaggaccaactaccctgggcggttcactaacttgttggacgtggccgaagcgtgtcccaccttcctctgctttgacgacgggaaaccgtacgtcaccacgcggacggaccaaactcgacttctggccaagtttgacctttcccttgccgcaaaacacatgtccaatacatacctggcaggacttgcccaatactacacacagtactcgggcaccatcaatttacacttcatgttcacaggctccactgattcaaaggcccgctacatggtggcctacatcccacctggggtgcagccacctgaaacacctgagatggctgcccactgcatacacgccgagtgggacactggactgaactccaaattcactttttcaatcccgtacgtgtctgccgcagactacgcctacacagcgtctgacacggcagaaacaaccaatgtgcagggctgggtctgcatttaccagattacacacgggaaggctgaaaatgacgccttggtcgtatcggtcagtgccggcagagactttgagttgcgcctcccgattgacccccgcacgcagactaccgccaccggggagtcagcagaccctgtcaccaccaccgtggagaactacggcggtgagacacaggttcagagacgctaccacactgacatcggcttcatcatggacaggtttgtgaagattaaggacgtgcaaccgacgcatgtcattgaccttatgcagactcaccaatacggcctggtgggtgcaatgctgcgtgcagctacgtactacttttctgacttggaaattgttgtacggcacgacggcaacctgacttgggtgcccaacggcgcccctgagtcagccctagacaacactggcaatcccaccgcctacaacaaggcaccattcacgagacttgctctcccttacacggcaccacaccgtgtgctggcaacagtgtacaacgggacaagcaaatacaccgtgggtggttca---ggcaggcgtggtgacatggggtccctcgcggcacgagtcgcgaaacaacttcctgcttcattcaactatggtgcaattaaggccaccgccatccacgag

>AY593789.1_A_ARG_1961

tcgagcgtgggggtcacctacgggtactccactggggaagaccacaccgcagggcccaacacatcgggcttggaaacgcgggtagtacaggctgaaaggttctttaagaaatttttgtttgactggacaacggacaaaccctttggacacttggaaaaactggaactccccaccgaccaccacggggtcttcggacacctggtggactcatatgcatacatgaggaacggttgggatgtcgaggtgtctgctgttggcaaccaattcaacggcgggtgcctcctggtggccatggtaccagaatggaaggaatttgacacgcgtgagaaataccaactcactctgtttccacaccagttcatcagccccagaacaaacatgaccgcccacatcacggtcccgtaccttggtgtgaacaggtatgaccagtacaaaaagcacaaaccctggacgctggttgtcatggtggtgtcgcccctcacggttagcaccactagtgcggcacagattaaggtctacgccaacattgccccaacctacgttcacgtggctggagagctcccttcgaaagaggggatttttcccgttgcgtgcgccgacggttacgggggactggtgacgacggacccgaagacagctgaccccgcctacggcaaggtgtacaatccgcccaggactaactaccccgggcgctttacaaacctgttggacgtggctgaggcgtgtcccacctttctttgtttcgacgacgggaaaccgtatgttgtcacgaagacagaacaagaccgacttctggccaagtttgacgtttcccttgccgcaaagcacatgtctaacacatacttgtcaggggttgcacagtactacgcacagtactctggtaccatcaacctgcactttatgttcacaggctctactgactcaaaggcccgctacatggtggcctacatcccgccaggggtggagccgccggacacacctgagaaagccgcacactgcatccacgctgaatgggacacagggttgaactccaagttcaccttttcaatcccgtacgtgtccgccgcggactacgcatacactgcgtccgacacggcagaaacaaccaacgtacagggatgggtttgcatttaccaaattacacacgggaaggctgagcaggacaccttggttgtgtcggttagcgccggcaaggactttgagctacgcctcccgattgacccccgtgcacaaaccactgccactggggaatctgcagaccctgtcaccaccaccgtggagaactacggcggtgagacacaagtccacagacgtcaccacacggacgtcagcttcatcatggacaggtttgtgaagatacagcctgtgaaccctatgcatgtcattgacctcatgcagacccaccaacacgggcttgtaggggcgttgctgcgtgcagccacgtactacttctctgacctggagattgtggtacgacacaacggcaacctgacctgggtacccaacggcgcccccgaggcagccctgtctaacaccagcaaccccactgcctacaacaaggcgccgttcaccagacttgccctcccctacactgcgccacaccgtgtgctggcaactgtgtacaacgggacgaacaagtacaccacaaacggtaca---ggtaggcgtgatgacatgggttctctcgcggcgagagtcgcgaaacatcttcctgcttcttttaattacggtgcaatcaaggccgacaccatccacgag

>AY593790.1_A_ARG_2001

tccagcgtaggcgttacatacgggtactccacaacagaggaccacgttgctggacccaacacatcaggtttggagacacgagtggtacaggcagagagattctacaaaaagtttttgtttgattggacaacggacaagccttttggacacctgcacaaactggagttgcccaccgaccaccacggtgttttcggacacttggtggactcatacgcctacatgaggaacggttgggacgttgaggtgtctgctgttggtaaccagttcaacggcggatgcctcctagtggccatggtacccgaatggaaagagtttgaaacgcgggagaagtaccagctcacgcttttcccgcaccagttcattagccccagaaccaacatgactgcccacatcacggttccttaccttggtgtgaatagatatgatcagtacagaaaacacaaaccctggacactggttgtcatggtcgtgtccccgctcacggtcaacgccacgagcgcggcacagatcaaggtctatgccaacatcgctccgacctacgttcatgtggccggcgagctcccctcgaaagaggggatcttccctgtcgcgtgcgcggacggttacggaggactggtgacaacggacccgaaaacagctgaccccgcctacggcaaggtgtacaatccgccccggactaactaccccgggcgtttcaccaacttgttggacgtggctgaggcatgtcccacctttctgtgttttgacgacgggaaaccgtacgttaccacacagacaggtgagtctcgtcttctggccaagttcgacctttcccttgccgcgaagcacatgtctaacacatatttggcaggaattgcccagtactacacacagtactcgggcaccatcaatttgcatttcatgttcacaggttcaactgattcaaaagcccgctacatggtggcttacatcccgcctggggtggaaccaccggacacacctgagagggcagcccactgcatccatgctgagtgggacacagggctgaattccaaattcacattctcaatcccgtacgtgtctgccgcggattacgcctacacggcgtctgatgaggcagagacaacaaacgtacagggatgggtctgcgtttaccagatcacacacgggaaggctgacaacgacactctggtcgtgtcggttagcgccggcaaggacttcgagttgcgcctccccattgacccccgaccgcagaccaccgctactggggaatcagcagaccctgtcaccaccactgtagagaactacggcggtgagacacaagttcagagacgccaccacaccgacgttggcttcatcatggacagatttgtgaaaataaacagcccaaaatccacccatatcattgacctcatgcaaacccaccaacacgggctagtgggtgcgctgctgcgtgcggcgacctactacttctcagatctggaaattgttgtgcggcatgacggtaacctaacttgggtgcccaatggtgctcccgtgtcagccttgtccaacaccagcaaccccaccgcctacaacaaggcaccgttcacgagacttgccctcccctacaccgcgccacaccgcgtgttggcgactgtgtacaacgggacgagcaagtatactgtgagtgggtca---agcagacgaggcgacttgggttccctcgcggcacgagtcgcgaaggcacttcctgcttctttcaactacggtgcaatcaaggccgacaacgtgcacgag

>AY593791.1_A_IRN_1998

tcgagtgtgggagtcacctacgggtattccactggagaagaccacgtttccgggcccaacacgtctggcttggaaacgcgggtgacacaggcagagagatttttcaagaaacacttgtttaattggacaactgacaaaccttttgggtacttggaaaagctggagcttcccactgaccacaagggtgtttacggacacctagtggattcttttgcatacatgagaaacggctgggacgtggaggtgtccgccgttggcaatcagttcaacggtggatgcctcctagtggccatggtgcctgaatggaaagagttcactccacgtgagaagtaccagctcaccttgttcccgcaccagttcatcagccccagaaccaacatgactgctcacatcacggtcccgtaccttggtgtgaatagatatgaccagtacaagaaacacaagccctggacgctggtcgtgatggtggtttcgccgcttaccaacagcagcattggtgccacagaaatcaaggtctacgccaatatcgccccaacccacgttcacgtagccggtgaactcccgtcgaaagaggggatcgtaccggttgcttgttcggatgggtacggcggtttggtgacaacggacccgaaaacagctgaccctgtctacggtaaggtgtacaacccgcctaggacaaactatcctgggcgcttcacaaacttgttggacgtggccgaggcttgcccaaccttcctctgtttcgacgacgggaaaccgtacgttgtgacaagagaggacgggcagcgtctactggccaagttcgacgtctctcttgctgcaaagcacatgtcaaacacctacctatcagggatagcgcagtactatgcacagtactctggcaccatcaacctccacttcatgttcactggttctactgactcaaaagcccgctacatggtggcgtacgtcccgcccggtgtggaaccgccggatacgcctgagagagctgcacactgcatccacgctgagtgggacacagggctgaactccaaattcactttttctatcccgtacgtgtccgccgcggattacgcgtacaccgcgtccgatgtggccgaaacaacaaacgtacaggggtgggtctgcatctaccagatcacacacgggaaggctcaaaacgacactctggttgtgtcggttagcgccggcaaggactttgagttgcgtctcccggttgacccccgcacacagaccacatctgccggggagtctgcagacccagtcaccaccactgttgaaaactacggcggtgagacacaagtccagcggcgtcaccacactgatgtcggcttcataatggacagatttgtgaagattaacaacaccagccccacacacgtcattgacctcatgcaaacccaccaacacgggttggtgggcgctctcctgcgtgctgccacgtactacttctcagacctggagattgtggtgtgccacgaaggcaatctaacgtgggtgcccaatggagcaccagaggcagccctgagcaacgcgggcaaccccaccgcatacaacaaagcaccattcacgaggctagcactcccctacactgcaccgcaccgcgtgctggcgacggtgtacaacgggacgagcaagtactcgacaactggtggg---cacacacggggtgacttgggagctcttgcggcgagggccgccgcacaactccctgcctctttcaactttggcgcaatccgggccactgacatcagtgag

>AY593792.1_A_ITL_1962

tcgagtgtgggagtcacctacgggtactccactggagaagaccatgtcgctgggcccaacacatcgggcctggagacgcgggttgtgcaggcagagagatttttcaaaaagtttttgtttgactggacaaaggacaaaccttttggacatttggaaaagctggaacttcccgccgaccaccacggcgttttcgggcacctggtggaatcatatgcttacatgagaaatggttgggacgttgaggtgtctgctgttggcaaccagttcaacggcgggtgcctcctggtagctatggtaccggagtggaaagagtttgaacagcgcgagaaataccaactcaccctcttcccacaccagttcatcagccccagaacaaacatgactgctcacatcacggtcccataccttggagtgaacaggtacgatcagtacaagaaacacaaaccttggacactggttgttatggtagtgtcgcccctcacggttagcgacactgccgcggcacagattaaggtctacgccaacattgctccaacctacgttcacgtggctggggaactcccctcgaaagaggggattttcccagttgcatgttcggacggttacggaggactggtgacaacggacccgaaaacagctgaccccgcctacggcaaggtgtacaacccgcccaggaccaactaccctgggcggtttaccaacttgttggacgtggctgaagcgtgtcccactttcctctgtttcgacggcgggaaaccgtacgttgtcacgcggacagatgacacacgactattggccaagttcgacgtctcccttgctgcaaaacacatgtccaacacgtacctgtcagggattgcacagtactacgcacagtactctggcaccatcaacttgcacttcatgttcacaggctcaactgactcaaaagcccgctacatggtggcctacatcccgcctggggtggaaccaccggacacacctgaaagggccgctcactgcatccacgcagaatgggacacaggactgaactccaaattcactttttcaatcccgtacgtgtccgccgcagattacgcttataccgcgtctgacacggcagaaacaaccaacgtacagggctgggtctgcatctaccagatcacacacgggaaggccgagaacgacacattggtggtgtcggccagcgccggcagagactttgagttgcgcctcccgatcgacccccgacaacaaaccactgctgttggggagtccgcagaccctgtcaccaccaccgtggagaactacggtggtgagacacaaacccagagacggcaccacacggatgtcggtttcatcatggacagatttgtgaagataaacagtttgagtcccacacatgtcattgacctcatgcagacccaccaacacgggctggtaggtgcgctgttgcgtgcagccacgtactacttctctgacttggagattgttgtgcggcacgaaggcaatttgacttgggtgcccaatggtgcccctgaagcagctttgtcaaacaccagcaaccccaccgcctacaacaaggcaccgttcacgaggctcgctctcccttacactgcgccacaccgcgtgttggcaaccgtgtacaacgggacgaacaagtactccacgggcggtccg------ggacgaggcgacatggggtcgctcgcggcgcgggtcgcgaaacaacttcctgcctctttcaactacggtgcaatcagggccgacaccatccacgag

>AY593793.1_A_PHI_1975

tccagcgtgggcgttacatacgggtattccactgcggaggaccacgttgccgggcccaacacatcgggcctggagactcgggtagtgcaggcagaaaggttcttcaagaagtttttgtttgattggacaacggacaagccctttggacatttggaaaagctggagcttccaaccgatcacagcggtgttttcggacacttggtggactcatatgcttatatgagaaacggttgggacgttgaggtgtccgctgttggcaatcagttcaatggcggttgcctcctggtggctatggtgcccgagtggaaaaaactcgacacacgggagaaataccagcttacccttttcccacaccagtttatcagtcctagaaccaacatgactgcccacatcacggtcccttaccttggtgtgaacagatatgaccagtacaaaaagcacaaaccctggacactggttgtcatggtcgtgtctccacttacggttaacaccactagtgcgacacagatcaaggtctacgccaacattgccccgacctacgttcacgtggccggtgaactcccctcgaaggaggggattttccctgttgcatgtgcggatggttacgggggattggtgacaacagacccaaaaaccgctgatcctgcttacggcaaggtgtacaacccgccccggaccaactaccctgggcgctttaccaacttgttggacgtggctgaagcgtgtcccactttcctctgcttcgacgacgggaaaccgtatgtcaccacgcgggcggatgagacccggcttttggccaagtttgatgtttcccttgccgcaaagcacatgtctaacacgtacctgtcaggaattgcccagtactacgcacagtactctggtaccatcaacttgcacttcatgttcactggttctactgattcaaaagcccggtacatggtggcctacatcccgcctggggtggagccaccggacacacctgagaaggctgcccactgcatccacgctgaatgggacacaggactaaattctaaatttactttctcaatcccgtacgtgtctgccgcagactacgcctatacagcgtccgacacggcagagacaaccaacgtacagggatgggtctgcatttatcagatcacacacgggaaggctgaaaacgacactctggtcgtgtcggtcagcgccggcaaagactttgagttgcgcctcccgatcgacccccgccagcagaccactgctaccggggagtcggcagacccagtcaccaccactgtggagaactacggcggtgagacacaagtccacagacgccaccacacggacattggctttattatggacagatttgtgaagataaaggaagtgcggtcaactcatgccattgatctcatgcaaactcaccaacacggcctagtgggtgcgctgctacgcgcggccacctactacttctctgatttggaaatcgtcgtgcgacacgacggcaacctgacttgggtgcccaacggtgctcctgaatcggccctgcagaacaccagcaaccccactgcctaccacaaggcaccattcacgagacttgctctcccctacactgcgccgcaccgtgtgttggcaactgtgtacaacgggacgaacaagtatactgtgagtggttca---ggcaggcgaggtgacatggggtccctcgcggcgcgagtcgcgaaacaactccctgcctcttttaactacggtgcaatcaaggccgacaacatccacgag

>AY593794.1_A_COL_1985

tcgagcgtgggtgtcacgtatggatactccacagaagaggaccacgttgctgggcccaacacatcgggcctggaaacacgggtggtgcaggcagaaagattctacaaaaagtacttgtttgattggacacctgacaaaccattcggggaactggttaagctggagcttccgtccgaacataacggcgtttttggacacttggtggactcatacgcctacatgagaaacggatgggacattgaggtgtccgctgttggcaaccagttcaacggtgggtgcctcctggtggccatggtacctgagtggaaggaatttgacacacgggagaagtaccaactcacccttttcccgcaccaatttatcagccctagaaccaacatgactgcccacatcacggtcccctatataggggtgaacaggtatgaccaatacaagaagcacaggccctggacactggttgtgatggttgtgtcaccacttacggtcaacaacaccggtgcgcagcaaatcaaggtttacgccaacatagccccgacctacgtccacgtggcaggtgagctcccctcgaaagaggggattttcccagtcgcgtgtgcggacggctacggaggactggtgaccacggacccgaagacagctgatcctgcttatggcaaggtgtacaatccgcctaggaccaactaccctgggcgcttcaccaacctgttggacgtggccgaagcgtgtccaaccttcctctgttttgacggcgggaaaccgtacgtcaccacactgacgggagaaacacgatgtctggccaaattcgacctttcccttgccgcaaagcacatgtccaacacatacctgtctggcattgctcagtactacgcacagtactctggcaccattaacttgcacttcatgttcacaggctccactgactcaaaggctcggtacatggtggcctacatcccgcccggggtggagccaccggacacacctgaaaaggctgcccactgcatccacgctgagtgggacactggacttaactccaaattcaccttctcgatcccgtacgtgtccgccgcggactatgcctacacagcatctgacacggcagaaacaacaaacgtacagggatgggtctgcatttaccaaattacacacgggaaggctgaaaatgacactctggtcgtgtcggttagcgccggcaaagactttgagctgcgcctcccgattgaccctcgccagcagaccacggctactggggaatcagcagacccggtcaccaccactgtggagaactatggtggtgagacgcaaatccagagacgtcatcacacggatgttgggttcatcatggacaggtttgtaaaaatcacacaacacagcccgacacacgtcattgacctcatgcagactcaccaacacggtctggtgggcgccttgctgcgtgctgccacgtactacttttccgacctggaaattgttgtacggcacgacggcaatctgacctgggtgcccaacggtgcccctgtctcggccttgtcgaacaccagcaaccccactgcctacaataaggcaccgttcacaagactcgctctcccatacactgcgccacaccgcgtgttggcaacggtgtacaatggggtgagcaagtacgctgtgggcgattca---ggcagacgtggtgatctaggagctctcgcggcgcgagtcgcgaaacaacttcctgcctcatttaactacggtgcaatcaaggctgataccatccacgag

>AY593795.1_Asia1_PAK_1954

tcgagtgttggcgtgacatacggttacgccgtgactgaggacgcggtatcaggacccaacacttcaggcttggaaacccgcgtgacacaagctgagcggttcttcaagaaacacttgtttgactggacaccgaatctggcatttggacactgtcactacctggaactccccactgaacacaaaggtgtgtacggcagtcttatggactcctatgcatacatgaggaacggatgggacatcgaggtgaccgctgttggcaaccagtttaacggcggatgtctccttgtcgcacttgtaccagaattgaaggaacttgacacaaggcaaaagtaccagttgacccttttcccccaccagtttataaacccacgcaccaacatgacggctcacatcaacgtgccgtttgtgggtgtcaacagatacgaccagtacgcgctccacaaaccgtggacgcttgttgtgatggtagtggcaccacttaccgtcaagactggtggttctgaacaaattaaggtttacatgaatgcagcaccgacctacgtgcacgtggcaggagaactgccctcgaaagaggggatagtccccgtcgcgtgtgcggacggttatggcaacatggtgaccacagacccgaagacggctgaccccgtttacggaaaagtgttcaacccccccaggacgaaccttcctggacgcttcacgaacttccttgatgtagcagaggcatgtccaaccttcctccgcttcggagaa---gttccatttgtgaagacggtgaactccggtgaccgcttgctcgccaagtttgacgtttcgctcgctgcagggcacatgtccaacacctacttggccggcttagcgcagtactacacacagtatagcggcaccatgaacatccactttatgttcactggacccacagatgccaaggcccgctacatggtggcctacgtacctcccggtatgacgccgcccacggatcctgagcgggctgcccactgcattcactctgaatgggacactggtctcaactccaagtttactttttctataccctacctttctgctgctgactatgcttacactgcttctgacgtggctgaggccacgagtgtgcagggatgggtttgcatctaccagatcacgcacggcaaagccgagggcgacgcgttagttgtctccgccagtgccggcaaggattttgagtttcgcttaccagttgacgcacgccagcagaccaccaccactggcgagtcggcggatccagtcaccaccacagtggagaactacggtggcgagacccaaacggcgcggcggctacacaccgacgttgcctttgttctcgacaggtttgtgaaattcacg---cccaagaacacccagactcttgacctcatgcagatcccgtcacacacactggttggagcgctcctccggtctgcaacgtactacttttcagacctggaaattgcgcttgttcacacaggtccggtcacgtgggtgcccaatggtgcgcccaagactgcgctggacaatcagaccaacccaactgcttaccacaagcaacccatcacgcgtttggcacttccttacaccgcgccccaccgtgtgctggcaacagtgtacaacgggaagacgacgtacggggaagagcctacg------atgcgtggtgatcgcgccgtgcttgcgagcaaggtgaacaagcaactgcccacctcctttaactacggtgcagtgaaggctgagaacatcacggag

>AY593796.1_Asia1_ISR_1963

tcgagcgttggcgtgacgtacggttacgctgtggccgaggacgcggtatctgggcctaacacttcaggcttggagacccgtgtggtgcaggctgaacggtttttcaagaaacatctgtttgattggacacagaatttgtcatttggacactgtcactacctggaactcccctctgaacacaaaggcgtgtacggtggcctcatggacttgtacgcgtacatgaggaacgggtgggacattgaggtgaccgctgttggaaatcagttcaacggtggttgcctcctcgtcgcactcgtcccggagctgaaaagccttgacacgcggcagaagtaccagttgacccttttcccacaccagttcattaacccacgcaccaacatgacggctcacattaacgtgccgttcgtgggtgtcaacaggtacgaccagtacgcgctccacaaaccgtggacgcttgttgtgatggtggtggctccacttaccgtcaaaactggtggttccgagcaaatcaaggtttacatgaatgcagcaccgacctacgtgcacgtggcaggggagctgccctcgaaagagggaatagttcctgtggcgtgtgcggacggttacggcaacatggtgaccacagacccgaagacggctgaccccgtatacgggaaagtgttcaacccccccagaacaaacctccctgggcgtttcacaaacttccttgatgtagcggaggcatgtccaaccttccttcgcttcggagaa---gtaccatttgtgaagacggtgaactctggtgaccgcttgcttgccaagttcgacgtgtcgctcgctgcggggcacatgtccaacacctacttggcaggcttggcgcagtactacacgcagtacagcggtaccatgaacattcacttcatgttcaccgggcctacggatgccaaagcccgctacatggtggcttacatacctcctggtatgacgccacccacggaccctgagcgggccgcacaccgcatacactctgagtgggacactggtcttaattccaaatttaccttttctatcccttacctctctgctgctgaccatgcttacactgcttctgacgtggccgagaccacaagtgtgcaaggatgggtgtgcatttaccaaatcacacacggtaaggctgaaggtgatgcactggtcgtgtccgtcagtgccggcaaggactttgagttccggctgccagtggatgctcgccgacagaccaccactgctggcgaatctgcggacccagtcactaccacggttgagaactacggaggagagactcagacagcccgacggttccacactgatgttgcctttgttctcgacaggtttgtgaaactcacccagcccaagagcacccagactcttgatctcatgcagatcccaccgcacacactggttggggcgttgctccggtccgcgacatactacttctcagacctggaggttgcgcttgtccacacaggctcggtcacatgggtgcccaacggcgcgcccaaggacgccttggacaatcacaccaacccgactgcctaccagaagcaacccatcacccgcttggcgctcccttacactgctccccaccgtgtgctggcaacagtgtacaacgggaagacaacgtacggagagcagtccacg------cgacgtggtgaacttgctacccttgcacagagggtgagcaaccggctgcccacctccttcaactacggtgctgtgaaggctgacaccatcacggag

>AY593797.1_Asia1_ISR_1963

tcgagcgttggcgtgacgtacggttacgctgtgaccgaggacgcggtatccgggcctaacacttcaggcttggagacccgtgtgatacaggccgaacggtttttcaagaaacatctgtttgactggacacaggatctgtcatttggacactgtcactacctggaactcccctctgaacacaaaggcgtatacggcggcctcatggactcgtacgcgtacatgaggaacgggtgggacattgaggtgaccgctgttggaaaccagttcaatggtggttgcctcctcgtcgcactcgtcccggagctgaaaagccttgacacgcggcagaagtaccagttgaccctcttcccacaccagttcattaacccacgcaccaacatgacggcccacatcaacgtgccgttcgtgggtgttaacaggtacgaccagtacgcgctccacaaaccgtggacgcttgttgtgatggtggtggctccacttaccgtcaaaactggtggttctgaacagattaaggtttacatgaacgcagcgccgacctacgtgcacgtggcaggggagctgccctcgaaggagggaatagttcctgtagcgtgtgtggacggttacggcaacatggtaaccacagacccgaagacggctgaccccgtatacgggaaagtgttcaacccccccagaacaaatcttcctgggcgcttcacgaacttccttgatgtagcggaggcatgtccaaccttcctccgcttcggagaa---gtaccatttgtgaagacggtgaactctggtgaccgcttgcttgccaagttcgacatgtcgctcgctgcggggcacatgtccaacacctacttggcaggcttggcgcagtactacacacagtacagcggtaccatgaacattcactttatgttcaccgggcctacggatgccaaagcccgctacatggtggcttacataccccctggcatgacgccacccacggaccctgagcgggccgcacactgcatacattctgagtgggacactggtcttaattccaaatttaccttttccatcccttacctctctgctgctgactatgcttacactgcctctgacgtggccgagaccacgagtgtgcagggatgggtgtgcatttaccagatcacgcacggtaaagctgaaggtgacgcactggtcgtatccgtcagtgccggcaaagactttgagttccgactgccagtggatgctcgccggcagaccaccactgctggcgaatctgcagacccagtcactaccacagttgagaactacggaggagagactcaggcggcccgacggcttcacactgatgttgcctttgttctcgacagatttgtgaaacttacccagcccaagaacacccagactcttgatctcatgcagatcccctcgcacacactggttggggcgttactccggtccgcgacgtactacttctcggacctggaggttgcgcttgtccacacaggctcggttacgtgggtgcccaacggcgcgcccaaggatgccttggacaaccacaccaacccgactgcctaccagaagcaacccatcacccgcttggcgcttccttacaccgctccccaccgtgtgctggcaacagtgtacaacgggaagacaacgtatggggaacagtccacg------cgacatggtgatcttgccacccttgcacaaggggtgagcaaccggctgcccacctccttcaactacggtgctgtgaaggctgacaccatcacggag

>AY593799.1_Asia1_LEB_1983

tcgagcgttggcgtaacatacggttacgctgtggccgaggacgcggtgtctggacccaatacctcgggtctagagactcgtgttcaacaggcagaacggtttttcaagaaacacctgtttgactggacaccgaacttggcatttggacactgttactacctggaacttcccactgaacacaaaggcgtgtacggcagtctcatgggctcgtacgcctacatgagaaatggatgggacatagaggtgactgctgttggaaaccaattcaacggtggttgtctccttgtcgcgctcgtgccagagctgaaggaactcgacacgcgacagaagtaccagctgaccctctttccccaccagttcatcaacccacgcaccaacatgacggcccacatcaacgtgccgtacgtgggtatcaacaggtacgaccagtacgccctccacaagccgtggacgcttgttgtgatggtggtagccccactcaccgtcaaaactggtggttctgaacagatcaaggtttacatgaatgcagcgccaacctacgtgcatgtggcgggagagctgccctcgaaagagggaatagttcccgtcgcgtgtgcggacggttacggcaacatggtgaccacggacccgaagacggccgatccagtttacgggaaagtgttcaacccccccaggacaaacctccctgggcgcttcacgaacttccttgatgttgcggaggcatgtccaactttcctccgctttggagaa---gtaccatttgtgaagacggtgaactctggtgaccgcttgctggccaagttcgacgtgtccctcgctgcagggcacatgtccaacacctacttggctggcctggcgcagtactacacacagtacagcggcaccatgaacgtccacttcatgttcaccgggcccacggatgctaaagcccgatacatggtggcttatgtcccccctggcatgacaccgcccacggaccctgagcacgccgcacactgcattcactctgagtgggatactggtcttaactctaagtttaccttttccataccttacctctctgctgctgactatgcctacactgcttctgacgtggcggagaccacgagtgtgcagggatgggtgtgtatctatcagatcacccacggcaaggctgagggagacgcactggtcgtttctgtcagcgccggcaaagactttgagtttcgcttgcctgttgacgcacgccagcaaaccaccaccactggcgaatcagcagatccagtcacaaccacggttgagaactatggaggagagactcagacagccagacggcttcacactgacgtcgccttcattcttgacaggtttgtgaaactcactgctcccaagaacatccaaaccctcgatctcatgcagatcccctcacacacgctggttggagcactacttcgttctgcgacgtactacttctcagacctggaggtcgcgcttgtccacacaggcccggtcacctgggtgcccaacggcgcgcccaaggatgctctaaacaaccagaccaacccaactgcctatcagaagcaacccatcacccgcctggcactcccctacaccgccccccatcgtgtgctggcaacagtgtacaacgggaagacggcgtacggggaaacgacctca------aggcgcggcgacatggcggccctcgcacaaaggttgagcgctcggctgcccacctccttcaactacggcgccgtgaaggccgacaccatcactgag

>AY593800.1_Asia1_LEB_1983

tcgagcgttggcgtaacatacggttacgctgtggccgaggacgcggtgtctggacccaatacctcgggtctagagactcgtgttcaacaggcagaacggtttttcaagaaacacctgtttgactggacaccgaacttggcatttggacactgttactacctggaacttcccactgaacacaaaggcgtgtacggcagtctcatgggctcgtacgcctacatgagaaatggatgggacatagaggtgactgctgttggaaaccaattcaacggtggttgtctccttgtcgcgctcgtgccagagctgaaggaactcgacacgcgacagaagtaccagctgaccctctttccccaccagttcatcaacccacgcaccaacatgacggcccacatcaacgtgccgtacgtgggtatcaacaggtacgaccagtacgccctccacaagccgtggacgcttgttgtgatggtggtagccccactcaccgtcaaaactggtggttctgaacagatcaaggtttacatgaatgcagcgccaacctacgtgcatgtggcgggagagctgccctcgaaagagggaatagttcccgtcgcgtgtgcggacggttacggcaacatggtgaccacggacccgaagacggccgatccagtttacgggaaagtgttcaacccccccaggacaaacctccctgggcgcttcacgaacttccttgatgttgcggaggcatgtccaactttcctccgctttggagaa---gtaccatttgtgaagacggtgaactctggtgaccgcttgctggccaagttcgacgtgtccctcgctgcagggcacatgtccaacacctacttggctggcctggcgcagtactacacacagtacagcggcaccatgaacgtccacttcatgttcaccgggcccacggatgctaaagcccgatacatggtggcttatgtcccccctggcatgacaccgcccacggaccctgagcacgccgcacactgcattcactctgagtgggatactggtcttaactctaagtttaccttttccataccttacctctctgctgctgactatgcctacactgcttctgacgtggcggagaccacgagtgtgcagggatgggtgtgtatctatcagatcacccacggcaaggctgagggagacgcactggtcgtttctgtcagcgccggcaaagactttgagtttcgcttgcctgttgacgcacgccagcaaaccaccaccactggcgaatcagcagatccagtcacaaccacggttgagaactatggaggagagactcagacagccagacggcttcacactgacgtcgccttcattcttgacaggtttgtgaaactcactgctcccaagaacatccaaaccctcgatctcatgcagatcccctcacacacgctggttggagcactacttcgttctgcgacgtactacttctcagacctggaggtcgcgcttgtccacacaggcccggtcacctgggtgcccaacggcgcgcccaaggatgctctaaacaaccagaccaacccaactgcctatcagaagcaacccatcacccgcctggcactcccctacaccgccccccatcgtgtgctggcaacagtgtacaacgggaagacggcgtacggggaaacgacctca------aggcgcggcgacatggcggccctcgcacaaaggttgagcgctcggctgcccacctccttcaactacggcgccgtgaaggccgacaccatcactgag

>AY593802.1_A_URU_2001

tccagcgtaggcgttacatacgggtactccacaacagaggaccacgttgctggacccaacacatcaggtttggagacacgagtggtacaggcagagagattctacaaaaagtttttgtttgattggacaacggacaagccttttggacacctgcacaaactggagttgcccaccgaccaccacggtgttttcggacacttggtggactcatacgcctacatgaggaacggttgggacgttgaggtgtctgctgttggcaaccagttcaacggcggatgcctcatagtggccatggtacccgaatggaaagagtttgaaacgcgggagaagtaccagctcacgcttttcccgcaccagttcattagccccagaaccaacatgactgcccacatcacggttccttaccttggtgtgaatagatatgatcagtacaaaaaacacaaaccctggacactggttgtcatggtcgtgtccccgctcacggtcaacgccacgagcgcggcacagatcaaggtctatgccaacatcgctccgacctacgttcatgtggccggcgagctcccctcgaaagaggggatcttccctgtcgcgtgcgcggacggttacggaggactggtgacaacggacccgaaaacagctgaccccgcctacggcaaggtgtacaatccgccccggactaactaccccgggcgtttcactaacttgttggacgtggctgaggcatgtcccacctttctgtgttttgacggcgggaaaccgtacgttaccacacagacaggtgagtctcgtcttctggccaagttcgacctttcccttgccgcgaagcacatgtctaacacatacttggcaggaattgcccagtactacacacagtactcaggcaccatcaatttgcatttcatgttcacaggttcaactgattcaaaagcccgctacatggtggcttacatcccgcctggggtggaaccaccggacacacctgagagggcagcccactgcatccatgctgagtgggacacagggctgaattccaaattcacattctcaatcccgtacgtgtctgccgcggattacgcctacacggcgtctgatgaggcagagacaacaaacgtacagggatgggtctgcgtttaccagatcacacacgggaaggctgacaacgacactctggtcgtgtcggttagcgccggcaaggacttcgagttgcgcctccccattgacccccgaccgcagaccaccgctactggggaatcagcagaccctgtcaccaccactgtagagaactacggcggtgagacacaagttcagagacgccaccacaccgacgttggcttcatcatggacagatttgtgaaaataaacagcccaaaatccacccatgttattgacctcatgcaaacccaccaacacggtctagtgggtgcgctgctgcgtgcggcgacctactacttctcagatctggaaattgttgtgcggcatgacggcaacctaacttgggtgcccaatggtgctcccgtgtcagccttgtccaacaccagcaaccccaccgcctacaacaaggcaccgttcacgagacttgccctcccctacaccgcgccgcaccgcgtgttggcgactgtgtacaacgggacgagcaagtacactgtgagtgggtca---agcagacgaggcgacttgggttccctcgcggcacgagtcgtgaaggcacttcctgcttctttcaactacggtgcaatcaaggccgacaacgtgcacgag

>AY593803.1_A_Brazil_1979

tcgagtgttggcgttacgtacgggtattccacaggggaagaccacgttgcagggcctaacacatcgggcctagaaacgcgggtggtacaggcagagagattttacaaaaagtttttgtttgactggacaacggacaggccttttggacacctggagaagctcgaacttccaaccgaccaccacggtgttttcgggcacttggtggactcatacgcctacatgagaaatggttgggatgtcgaggtgtctgccgttggaaaccagttcaacggcgggtgtctcctggtggccatggtgcccgaatggaaggattttgacacgcgggagaaataccaacttactcttttcccgcatcagttcattagccccagaaccaacatgactgcccacatcacggtcccctaccttggtgtgaacaggtatgaccagtacaaaaagcacaagccttggactttggttgtcatggtcgtgtcgccgctaacggtcaacactgctggcgcgtcgcagatcaaggtctacgccaacattgctccgacctacgttcacgtggccggtgagctcccctcgaaggaggggattttcccggttgcgtgcgcggacggttatggaggtctggtgacgacagacccaaagacagctgaccctgtttacggcaaggtgtacaacccgcccaggaccaactaccctgggcgcttcaccaacctgttggacgtggccgaagcgtgtcccaccttcctctgctttgacgacggaaaaccgtatgtcaccacgcggacggaccaaacccgacttctggccaagtttgacctttcccttgctgcaaaacacatgtccaacacatacctggcagggcttgcccagtactacacacagtactctggcaccatcaacttgcatttcatgttcacaggctctactgattcaaaggcccgctacatggtggcttacatcccacctggggtggagccacctgaaacacctgagatggccgcccactgcattcacgccgagtgggacactggactgaactccaaatttactttctcaatcccgtacgtgtctgccgcggactacgcctacaccgcgtctgacgtggcagaaacaaccaacgtgcagggatgggtctgcatttaccagatcacacacgggaaggctgaaaacgacgccttggtcgtgtcggtcagcgccggcaaagactttgagttgcgcctcccgattgacccccgcttgcagaccaccgccaccggggagtcagcagaccctgtcaccaccaccgtggagaactacggtggtgagacacaagttcagagacgccatcacactgacattggcttcatcatggataggtttgtgaagattaaggacgtgacaccaacacacgtcattgacctcatgcagactcaccaacatggcctggtgggtgcaatgctgcgtgcagctacgtactacttttctgacttggaaattgttgtacagcacgacggcaacctgacttgggtgcccaacggcgcccccgtgtcagccctagagaatactagcaatcccactgcctacaacaaggcaccattcacgagacttgctctcccttacacggcaccacaccgtgtgctggcaacagtgtacaacgggacaagcaaatacaccgtgagtggttca---ggcaggcgtggtgacatggggtctctcgcggcacgtgtcgcgaaacagcttcctgcttcatttaactatggtgcaatcaaggccaccgccatccacgag

>AY593804.1_C_SWZ_1965

tcgagcgtcggagtcacattcgggtatgcaactgctgaagatagcacgtctggacccaatacatctggtctagagacgcgcgttcatcaggcagagaggtttttcaaaatggcactttttgattgggttccttcacaaaattttggacacatgcacaaggttgttctgccccatgaaccaaaaggtgtttacgggggtctcgtcaagtcatacgcgtacatgcgcaatggctgggacgtcgaggtgactgctgttggaaaccagttcaacggcggctgcctcctggtggcgctcgtccccgagatgggcgacatcagtgacagggaaaagtaccaactaaccctttacccccaccagttcatcaacccacgcaccaacatgacggcacacatcactgtgccctacgtgggtgtcaacaggtatgaccagtacaaacagcacaggccctggaccctcgtggtcatggttgtcgcgccactcaccacaaacacagcaggtgcccaacagatcaaagtgtatgccaacatagccccaaccaacgtgcacgtggcaggtgagctcccctccaaggaggggatcttccccgttgcgtgttctgccggttacggcaacatggtgacaactgacccgaaaacggctgaccctgcctacgggaaggtttacaacccccctcggactgctctgccggggcggttcacaaactacctggatgttgccgaggcttgtcccaccttcctgatgttcgagaac---gtaccttacgtctcaacacgaactgacgggcaaaggctactggccaagttcgacgtgtcgctggcagcgaaacacatgtcaaacacctacttggccggcttggcccagtactacacacagtacactgggacaatcaacctacacttcatgttcactgggccgaccgacgcgaaagctcggtacatggtggcgtacgtgccccctggcatggacgcaccagacaacccagaagaggctgcccactgcatacacgcagaatgggacactggtctgaactctaagttcacattttcaatcccgtacatctcggccgctgactacgcgtacaccgcgtcccacgaggctgaaacaacatgtgtacaggggtgggtctgtgtgtaccaaatcactcacggcaaggcagacgccgacgcgctcgtcgtctccgcatcagcggggaaagactttgagctccggctacctgtggacgctagacaacaaactacggccactggtgaatctgctgaccccgtcaccactaccgttgagaactacggaggagagacccaagtccaacgtcgccaccacaccgacgttgccttcgttcttgaccggtttgtgaaggtcacagtgtcgggtaaccaacacacactcgacgtgatgcaggcacacaaagacaatatcgtgggcgcgcttcttcgcgcagccacgtactacttttctgatttggaaatagcagtgacccacactgggaagctcacatgggtgcccaacggtgcaccagtttctgcacttgacaacacaaccaatcccactgcgtaccacaagggcccgttgactcgactggctctcccatacaccgcgccacaccgtgtgttggctacggcgtacactggcactacgacctacaccgccagtaca------------cgcggggattcggctcacctaacggcgacgcatgctcggcatttgccgacatcgttcaactttggtgcagttaaagcagaaacaatcactgag

>AY593805.1_C_GER_1960

tcgagcgtcggagtcacattcgggtatgcaactgctgaagatagcacgtctggacccaatacatctggtctagagacgcgcgttcatcaggcagagaggtttttcaaaatggcactttttgattgggttccttcacaaaattttggacacatgcacaaggttgttctgccccatgaaccaaaaggtgtttacgggggtctcgtcaagtcatacgcgtacatgcgcaatggctgggacgtcgaggtgactgctgttggaaaccagttcaacggcggctgcctcctggtggcgctcgtccccgagatgggcgacatcagtgacagggaaaagtaccaactaaccctttacccccaccagttcatcaacccacgcaccaacatgacggcacacatcactgtgccctacgtgggtgtcaacaggtatgaccagtacaaacagcacaggccctggaccctcgtggtcatggttgtcgcgccactcaccacaaacacagcaggtgcccaacagatcaaagtgtatgccaacatagccccaaccaacgtgcacgtggcaggtgagctcccctccaaggaggggatcttccccgttgcgtgttctgacggttacggcaacatggtgacaactgacccgaaaacggctgaccctgcctacgggaaggtttacaacccccctcggactgctctgccggggcggttcacaaactacctggatgttgccgaggcttgtcccaccttcctgatgttcgagaac---gtaccttacgtctcaacacgaactgacgggcaaaggctactggccaagttcgacgtgtcgctggcagcgaaacacatgtcaaacacctacttggccggcttggcccagtactacacacagtacactgggacaatcaacctacacttcatgttcactgggccgaccgacgcgaaagctcggtacatggtggcgtacgtgccccctggcatggacgcaccagacaacccagaagaggctgcccactgcatacacgcagaatgggacactggtctgaactctaagttcacattttcaatcccgtacatctcggccgctgactacgcgtacaccgcgtcccacgaggctgaaacaacatgtgtacaggggtgggtctgtgtgtaccaaatcactcacggcaaggcagacgccgacgcgctcgtcgtctccgcatcagcggggaaagactttgagctccggctacctgtggacgctagacaacaaactacggccactggtgaatctgctgaccccgtcaccactaccgttgagaactacggaggagagactcaagtccaacgtcgccaccacaccgacgttgccttcgttcttgaccggtttgtgaaggtcacagtgtcgggtaaccaacacacactcgacgtgatgcaggcacacaaagacaatatcgtgggcgcgcttcttcgcgcagccacgtactacttttctgatttggaaatagcagtgacccacactgggaagctcacatgggtgcccaacggtgcaccagtttctgcacttgacaacacaaccaatcccactgcgtaccacaagggcccgttgactcgactggctctcccatacaccgcgccacaccgtgtgttggctacggcgtacactggcactacgacctacaccgccagtaca------------cgcggggattcggctcacctaacggcgacgcgtgctcggcatttgccgacatcgttcaactttggtgcagttaaagcagaaacaatcactgag

>AY593806.1_C_Brazil_1971

tcgagcgttggggtcacatacgggtacgcaacaactgaggatagcacgtcagggcccaacacatccggccttgagacacgtgttcaccaggcagaacggtttttcaagatgacactctttgaatgggttccctcccagagttttggacacatgcacaaggtcgttctgccctcagaaccgaaaggtgtctatgggggtctcgtcaagtcatacgcgtacatgcgcaatggctgggacgttgaggtgactgctgttggaaaccagttcaacggcggttgtctcctggtggcgctcgttcctgaaatgggtgacatcagtgacagagagaagtaccaactgactctctacccccaccaattcatcaacccacgcactaacatgacggcacacatcaccgtgccttacgtgggtgtcaacagatacgaccaatacaaccaacacaagccctggactcttgtcgtcatggtcgttgctccacttactgtgaacacatcaggtgcccagcagatcaaggtgtatgccaacatagccccaaccaacgttcacgttgctggtgaacttccctccaaggaggggatcttccccgttgcgtgtgccgacggctatggcaacatggtgacaactgacccgaagacagctgaccctgcctacgggaaagtctacaatccacccaggaccgccctgccgggccggttcacaaactacctggatgttgctgaggcttgccccactctcctgacgttcgagaac---gtgccttacgtttcaacacggactgatggacaaaggctgttggccaagttcgacgtgtcattggcagcgaaacacatgtcaaacacttacttggctggcttggcccagtactacacacagtacgctgggacaatcaacctgcacttcatgttcactgggccaaccgacgcgaaagctcggtacatggtggcatacgtgccccctggcatggaagcaccagacaacccagaggaggctgcccactgcatacacgcagagtgggacactggtttgaactctaagttcacattttcaatcccgtacatctcggccgctgactacgcatacaccgcgtccagcgaggctgaaacaacaagcgtacagggatgggtttgtgtgtaccagatcactcacggcaaggcagacgctgacgcgctcgtcgtctccgcttcggcggggaaagactttgagctccggctacctgtggacgctagacagcaaactacgaccactggcgaatctgccgaccccgtcaccactaccgttgagaactacggaggagaaacacaaactcaacgtcgccaccacactgacgttgccttcgttcttgaccggtttgtgaaggtccaggtgtcgggcaaccaacacacactggacgttatgcaggtacacaaggacagtattgtgggtgcactcctacgcgcagccacatactacttctctgacttggaaatagcagtgactcacactgggaagctcacatgggtgcccaacggcgccccagtttctgcacttgacaacacaaccaaccccactgcctaccacaaggggccgctgactcggctggctctcccatacaccgcaccacaccgcgtgctggccacggcgtacaccggtacaacggcctacactaccggtgta---------cgcaggggagacctagcccacttggcggcggcgcacgctcggcacctgccgacgtcgttcaactttggtgcagttaaagcagagacaatcacagag

>AY593807.1_C_Brazil_1955

tcgagtgtcggagttacatacgggtatgcaacagctgaggacagctcgtcagggcctaacacgtctggccttgagacgcgtgtccatcaggcagagcggtttttcaaaatgacgctttttgattgggttccctcgcaaaattttggacacatgcacaaggttgttctgcccacagacccgaaaggtgtctatgggggtctcgtcaagtcatatgcgtacatgcgcaatggctgggacgttgaggtgactgccgtcggaaaccagttcaacggcggttgtctcttggtggcgctcgtccccgagatgggcgacatcagtgacagggaaaagtaccaactgaccctttacccccaccagttcatcaacccacgtactaacatgacggcacacatcaccgtgccctacgtgggtgtcaacagatacgaccagtacaagcaacacaagccctggaccctcgtggttatggtcgttgctccactcactgtgaacacatcaggcgcccagcagattaaagtgtatgccaacatagccccaaccaacgtccacgttgcaggtgagctcccctccaaggaggggatctttcccgttgcgtgtgccgacggttatggcaacatggtgacaactgacccgaaaacggctgatcctgcctacgggaaagtgtacaaccccccccggactgctttgccggggcggttcacaaactacctggatgttgctgaggcttgtcccactttcctggtgttcgagaac---gtgccctacgtctcaacacggactgatggacaaaggttactggccaagttcgacgtgtcgctggcagcgagacacatgtcaaacacctacttggctggtctggcccagtactacacacagtacgctgggacaatcaacctacactttatgttcactgggccgaccgacgcgaaagctcggtacatggtagcatacgtgccccccggcatggaagcaccagaaaacccagaagaggctgcccactgcatacacgcagagtgggacactggtctgaactccaagttcacattttcaatcccgtacatttcggccgctgactacgcgtacaccgcgtccaacgaggctgaaacaacatgtgtacagggatgggtttgtgtgtaccaaatcacccacggcaaggcagatgccgacgcgctcgtcatctccgcatctgcggggaaagactttgagctccggctgcctgtggacgctaggcaacaaactacgaccactggtgaatctgccgaccctgttaccactaccgttgagaactacggaggagagacgcaagtccaacgtcgtcaccacactgacgttgccttcgttcttgaccggtttgtgaaggtccctgtgtacagacaacaacacacactggacgtgatgcaggtacacaaggacagtattgtgggagcgcttctccgcgcagccacgtactacttctctgatctggaaatagcggtgacccacaccgggaagctcacctgggtgcccaacggtgcaccggtttctgcacttgacaacacaaccaaccccactgcataccacaagggaccgctgactcgactggctctcccatacaccgcgccacaccgcgtgttggccacgacgtacactggtacaacaacctacactaccagtgca---------cgtagaggagattcggcccatttggcggcagcacacgctcggcatttgccgacgtcgttcaactttggtgcagttaaagcagaaacagtcactgag

>AY593808.1_C_ARG_1966

tcgagcgtcggagtcacatacgggtatgcaactgctgaagacagctcgtctggacccaacacatccggtctggagacgcgtgttcatcaggcagaaaggtttttcaaaatgacactttttgattgggatccctcgcaaaaatttggatacatgcacaaggttgttctgcccactgaaccaaaaggtatttacgggggtctcgtcaagtcatacgcgtacatgcgcaatggctgggacgtcgaggtgactgctgttggaaatcagttcaacggcggttgcctcctggtggcgcttgtccccgagatgggcgacatcagtgacagggaaaagtaccaactaaccctttacccccaccagtttattaacccacgcaccaacatgacggcacacatcactgtgccctacgtgggtgtcaacaggtacgaccagtacaatcaacacaagccctggacccttgtggtcatggttcttgcaccactcactgtgaacacagcaggtgcccagcagatcaaagtgtacgccaacatagccccaaccaacgtgcacgtggcaggtgagctcccctccaaggaggggatctttcctgttgcgtgctctgacggttacggcaacatggtgacaactgacccgaaaacggctgaccctgcctacgggaaggtttacaaccccccccggaccgctttgccggggcggttcacaaactacttggatgttgccgaggcttgtcccaccttcctgatgttcgagaac---gtgccttacgtctcaacacgagctgacggacaaaggctactggccaagttcgacgtgtcgctggcagcgaaacacatgtcaaacacctacttggccggcttggcccagtactacacacagtacgctgggacaatcaacttgcacttcatgttcactgggccgaccgacgcgaaagctcggtacatggtagcgtacgtgccccccggcatggatgcaccagacaacccagaagaggctgcccactgcatacacgcagagtgggacactggtctgaactctaagttcacattttcaatcccgtacatttcggccgctgattacgcatacaccgcgtctaatgaggctgaaacaacgtgtgtacagggatgggtctgtgtgtaccaaatcacccgcggtaaggaaggcgccgatgcgctcgtcgtctccgcatcagcggggaaagacttcgagctccggctacctgtggacgctagacgacaaactacggccactggtgaatctgctgaccctgttaccactaccgtcgagaactacggaggagagacgcaagtccaacgtcgccaccacactgacgttgccttcgttcttgaccggtttgtgaaggtcaaagcgtcgggtaaccaacacacacttgacgtgatgcaggtacacaaagacagcatcgtgggcgcgcttcttcgcgcggccacgtactacttttctgatttggaaatagcagtgacccacactggcaagctcacatgggtgcccaacggtgcaccagtttctgcgcttgacaacacaagcaaccccactgcgtaccacaaggggccgttgactcgactggctctcccgtacaccgcgccacaccgtgtgttggccacgacgtacactggcactactacctacaccaccagtgca---------cgcggaggggatctggctcacctggcggcaacgcatgctcagcgcttgccgacatcgttcaactttggtgcagttaaagcagaaacaattactgag

>AY593809.1_C_ARG_1969

tcgagtgtcggagttacatacgggtatgcaacagctgaggacagctcgtcagggcctaacacgtctggtcttgagacgcgtgtccatcaggcagagcggtttttcaaaatgacgctttttgattgggttccctcgcaagagtttggacacatgcacaaggttgttctgcccacagacccgaaaggtgtctatgggggtctcgtcaagtcatacgcgtacatgcgcaatggctgggacgttgaggtgactgccgtcggaaatcagttcaacggcggttgtctcttggtggcgctcgtccccgagatgggcgacatcaatgacagggaaaagtaccaactgaccctttacccccaccagttcatcaacccacgtactaacatgacggcacacatcaccgtgccctacgtgggtgtcaacagatacgaccagtacaagcaacacaagccctggaccctcgtggttatggttgttgccccactcaccgtgaacacatcaggcgcccagcagattaaggtgtatgccaatatagccccaaccaacgtccacgttgcaggtgagctcccctccaaggaggggatctttcccgttgcgtgtgccgacggttatggcaacatggtgacaactgacccgaaaacggctgatcctgcctacgggaaagtgtacaaccccccccggactgctttgccggggcggttcacaaactacctggatgttgctgaggcttgtcccactttcctggtgttcgggaac---gtgccctacgtctcaacacggactgatggacaaaggttactggccaagttcgacgtgtcgctggcagcgagacacatgtcaaacacctacttggctggtctggcccagtattacacgcagtacgctgggacaatcaacctacactttatgttcactgggccgaccgacgcgaaagcccggtacatggtagcatacgtgccccccggcatggaggcaccagaaaacccagaagaggctgcacactgcatacacgcagagtgggacactggtctgaactccaagttcacattttcgatcccgtacatttcggccgctgattacgcgtacaccgcgtccaacgaggctgaaacaacatgtgtacagggatgggtttgtgtgtaccaaatcacccacggcaaggcagatgccgacgcgctcgtcatctgcgcatctgcggggaaagactttgagctccggctgcctgtggacgctaggcgacaaactacgaccactggtgaatctgccgaccctgttaccactaccgttgagaactacggaggagagacgcaagtccaacgtcgtcaccacactgacgttgccttcgttcttgatcggtttgtgaaggttcctgtgtgcggacaacaacacacactggacgtgatgcaggtacacaaagacagtattgtgggagcgcttctccgcgcagccacgtactacttctctgatctggaaatagcggtgacccacaccgggaagctcacctgggtgcccaacggtgcaccggtttctgcacttgacaacacaaccaaccccactgcataccacaagggaccgctgactcgactggccctcccgtacaccgcgccacaccgcgtgttggccacgacgtacactggtacaacaacctacaccaccagtgca---------cgtacaggagatttggcccagttggcggcagcacacgctcggcacttgccgacgtcgttcaactttggtgcagttaaagcagaaacagtcactgag

>AY593810.1_C_UKG_1970

tctagtgtgggagtcacctacgggtacgcaactggtgaagacagctcgtcgggacctaacacatcaggtctcgagacgcgtgttcaacaggctgagcggttctttaagatttcactttttgagtgggtcccatcacaaaactttggacacatgcacaaggttgttctgcccacagacccgaaaggtgtctacgggggtctcgtcaagtcatacgcgtacatgcgcaatggctgggacgtcgaggtgaccgcggtcggaaaccagttcaacggcggttgtctcctggtggcgctcgtcccggagatgggcgacatcagcgagagggagaaataccaactcaccctctacccccatcagttcatcaacccacgtactaacatgacggcacacatcaccgtgccctacgtgggtgtcaacaggtatgaccaatacaaacaacacagaccttggaccctcgtggtcatggttgttgcaccactcactgtgaacgcatcaggtgctcagcagatcaaggtgtatgccaacatagccccaaccaatgtacacgttgcaggtgagctcccctccaaggaggggatcttcccggttgcgtgctctgacggttacggcaacatggtgacaactgacccgaagacgtctgaccccgcctacgggaaagtttacaaccctcccagaactgctctgccggggcggtttacaaactacctggatgttgccgaggcctgccccaccctcctgacgtttgagaac---gtaccttacgtctcaacacggactgatggacagaggttgctagccaagttcgacgtctctctggcagcgagacacatgtctaacacctacttggccggtttggcccagtactacacacagtacgctgggacaatcaacctgcacttcatgttcactgggccgacagacgcgaaagcgcggtacatggtggcgtacgtgccccccggcatggaa-caccagaaaacccagaagaggctgcccactgcatacacgctgagtgggacactggtttgaactccaagttcacattctcaatcccgtacatctcggccgctgactacgcgtacaccgcgtctaacgaggccgagacaacatgtgtgcagggatgggtttgcgtgtaccaagtcacccacggcaaggcagatgccgatgcacttgttgtctccgcttcggcggggaaggactttgagcttcggctaccggtggacgctagacagcagactacgactgctggcgaatctgctgaccctgttaccactaccgttgagaactacggaggcgagacgcaagtccaacgtcgccatcacactgacgttgcctttgttcttgatcggttcgtgaaggtccaagtgtcggacaaccaacacacactggacatgatgcaggtgcacaaggacagtattgtgggtgcgcttctccgcgcggccacttactacttctccgacttggaaatcgctgtgacccacactgggaagctcacgtgggtgcccaacggcgcaccggtctctgcgcttgaaaacacaagcaatcccactgcgtaccacaaggggccgctgactcggctggctctcccgtacaccgcgccacaccgcgtgttggccacggcgtacactggtactacaacttacaccagtac-------------cgtggaggggatctgactcacctggcagcggcgcacgctcggcgcctgccgacgtcgttcaactttggtgcagtcaaagcagaaaccattactgag

>AY593812.1_O_PHI_1958

tcgagtgttggagtcacgtacgggtacgcaacagctgaggattttgtgagtggaccaaacacctctggtcttgagaccagagttgtccaggcggaacgattttttaaaacccacctgttcgattgggtcactagtgactcatttggacggtgccaccttttggagcttccaactgaccacaaaggtgtctatggtagtctgaccgactcgtatgcttatatgagaaacggttgggatgttgaagtcactgcagtgggaaaccagtttaatggaggatgtctgctggtggccatggtgcctgagctttgttccatcgaagggagagagctgtatcagcttacgctctttccccaccagttcatcaacccacggacgaacatgacagctcacatcactgtgccctttgttggcgtcaatcgctacgaccagtacaaggtacacaagccctggaccctcgtggtcatggttgtagctcctttgaccgtcaataatgaaggtgccccacagatcaaagtgtacgccaacatcgcccccaccaacgtacacgttgcgggtgagttcccttccaaagagggaattttccccgtggcctgtagcgacggttacggcggtttggtgaccacagacccaaagacggctgaccccgcctacgggaaggtgttcaaccccccccgcaacatgttgccggggcgtttcaccaactttcttgatgtggctgaggcgtgccctacgtttctgcacttcgacggtggcgtgccatacgtgaccacgaagacggattcagacagggtgctcgctcagtttgacttgtctttggcagcaaagcacatgtcgaacaccttccttgcgggccttgcccagtactacacacagtacagcggcaccatcaacctgcacttcatgttcacaggtcccactgacgcaaaggcgcgttacatggttgcatatgccccacctggcatggaaccgcccaaaacacccgaggcggctgcccactgcattcatgctgaatgggacacaggtttgaactcaaaattcacattttcaatcccttacctctcggcggccgactacgcgtacaccgcgtctgacgctgccgagaccacaaatgtacagggatgggtttgcttgttccaaattacacacgggaaagctgacggcgatgcactggttgtgctggctagcgccggcaaggactttgagcttcgtctaccagtggacgcccgaacacagaccacctccacgggtgagtcggctgaccccgtgaccgccaccgttgagaactacggcggcgagacacaggtccagaggcgccagcacacggacgtctcatttatattagacagatttgtgaaagtcacacctaaagaccaaattaatgtactggacctgatgcaaacccctgctcacaccctagtgggtgggctccttcgtgctgccacttactacttcgctgatttagaagtggcagtgaaacacgaggggaacctcacttgggtcccaaatggggcgcctgagacagccttggacaacaccaccaacccaacggcataccacaaggcaccacttacccggcttgcactgccctacacggcaccacaccgtgttttggctaccgtttacaatgggaattgcaagtacgcgcacggtcccgtgaccagagtaagaggtgacctacaagtgttggcacagaaagcggcgagagcgctgcccacctcctttaactacggtgccattaaagccacccgggtgactgaa

>AY593813.1_O_ISA_1962

tcgagtgttggagtcacctacgggtacgcaacagctgaggattttgtcagcgggccaaacacttctggtctggagaccagagttgtacaggcagaacgcttcttcaaaactcacttgtttgactgggtcaccagcgacccatttggacgctgtcacttgctggaactcccaacagaccacaagggtgtctacggcggactgaccgaatcatatgcttatatgagaaacggttgggacgttgaagtcaccgcggtcgggaaccagttcaatggagggtgtttgttggtggccatggtgccagaactctgctccttgcagaagagggaactttaccagcttactcttttcccgcaccagttcatcaacccgcgcacgaatatgactgcgcacatcactgtgcccttcgttggtgtcaatcgctacgaccagtacaaggtacacaaaccgtggacccttgtggtaatggtcgtggcccccctgactgtgaacagtgaaggtgccccccagatcaaggtgtatgccaacattgcccccaccaacgtgcatgtcgcaggtgagttcccttccaaagagggaatatttccagtggcttgtagcgacggttacggcggtttggtgactactgacccgaagacggctgaccccgtttacgggaaagttttcaacccaccccgtacgctgttgccggggcgttttaccaactttcttgatgtggccgaggcgtgccctacctttctgcacttcgagggcgacgtgccatacgtgaccacgaaaacggactcggacagggtgcttgctcagtttgacttgtctttggcagcaaagcatatgtcgaccacctttcttgcgggcttggcacagtactacgctcagtacagtggcacgataaacctgcatttcatgttcacagggcccactgacgcgaaggcgcgttacatggttgcttatgccccccctggcatggagccgcctaaaacacctgaggccgctgcccattgcatccatgctgagtgggacacagggttgaactccaaatttacattttcaatcccctacctttcggctgctgactacgcctacaccgcgtctgacacggccgagaccacaaatgtgcaaggatgggtctgcctttttcaaataactcacggcaaagccgacggtgatgctttggtcgtactggctagcgccggtaaggactttgagctgcgtctgccggtggatgcccgcacgcaaaccacctccacaggcgagtcagctgatcccgtgaccgctaccgttgagaactacggtggtgagacacaggtccagaggcgccaacacactgacgtttcgtttatattggatagatttgttaaggttacgccacatgaccaaattaatgttttggaccttatgcagattccagcccacaccctagtaggggcgctcctgcgcacagctacttactacttctctgacttggagctggcggtggcacacgagggaaacctcacttgggtccccaacggggcgccagaggcggccctgaacaacaccaccaatccaacagcctaccacaaggcacccctcactcgacttgccttgccctacacggcgccgcaccgcgtgttggcgactgcgtacaacggggcctgcaagtacggcacagacaccgcacccaatgtgagaggggaccttcaagtgctggctcagaaggcagcgcggccgctgcccacctccttcaattacggtgccataaaggccacacgggtgactgaa

>AY593814.1_O_ARG_1965

tcaagcgttggagtcacatacgggtacgcaacagctgaggattttgtgagcggaccgaacacttctggtctcgagaccagagttgtgcaggcagaacggtttttcaaaacccacctcttcgactgggtcaccagtgactcattcggacgttgccacctcctggaactcccgaccgaccacaaaggtgtctacggcagcctgaccgactcgtatgcatatatgagaaacggctgggatgtcgaggtcaccgcggttggcaaccagttcaacggagggtgcctgctggtcgcaatggtgccagagctttgttctatccaaaagagggaactgtaccagctcacacttttccctcaccagttcatcaacccacgcacgaacatgactgcgcacatcacagtgccctttgttggcgtcaaccgctacgaccagtacaaggttcacaagccttggacccttgtggttatggttgtagcccctctgaccgtcaacactgaaggtgcccctcagatcaaggtgtatgccaacattgccccaaccaatgtgcacgtcgcgggtgagtttccttccaaggagggaatattccccgtggcctgtagcgacggctatggtggcctggtgaccacggacccgaagacggctgaccccgtttatgggaaagtgttcaaccccccccgcaaccagttgccggggcgttttaccaacctccttgatgtggctgaggcatgcccgacgtttctgcacttcgagggtgacgtaccgtacgtgaccacgaaaacagactcggacagggtgcttgctcagtttgacatgtctttggcagcaaaacacatgtcaaacaccttcctcgcaggtcttgcgcagtactacacacagtacagtggcaccatcaacctgcacttcatgttcacaggacccactgacgcgaaggcgcgttacatgattgcctatgccccaccgggcatggagccgcccaagacacctgaggcggccgcgcactgcatccatgctgaatgggacactgggttgaactcaaagttcactttttccatcccctacctctcggccgccgattacgcgtacaccgcgtctgacgtggccgagaccacaaatgtgcagggatgggtctgcttgtttcaaattacacatggcaaggccgacggcgacgctctggtcgtactggctagtgctggtaaagactttgagctaaggctgccggtggacgcccgtgcggaaaccacttctgcgggcgagtcggcggatcctgtcaccaccactgttgaaaactacggtggcgaaacacagatccagaggcgccaacacacggacgtctcgttcatcatggacagatttgtgaaagtgacaccgcaaaaccaaattaacattttggacctcatgcagattccatcacacactttggtgggagcgctcctacgcgcgtccacttactacttctctgacttggagatagcagtaaaacacgagggagacctcacttgggttccaaatggagcgcctgaaaaggcgttggacaacaccaccaacccaactgcttaccacaaggcaccactcacccggcttgccctgccctacaccgcgccccaccgcgtgttggcaaccgtgtacaacggtgagtgcaggtacagcagaaatgctgtgcccaactcgagaggtgaccttcaggtgttggctcaaaaggtggcacggacgctgcctacctccttcaactacggtgccatcaaagcgacccgggtcaccgag

>AY593815.1_O_UKG_1967

tcaagcgttggagtcacatacgggtacgcaacagctgaggattttgtgagcggaccgaacacttctggtctcgagaccagagttgtgcaggcagaacggtttttcaaaacccacctcttcgactgggtcaccagtgactcattcggacgttgccacctcctggaactcccgaccgaccacaaaggtgtctacggcagcctgaccgactcgtatgcatatatgaggaacggctgggatgtcgaggtcaccgcggttggcaaccagttcaacggagggtgcctgctggtcgcaatggtaccagagctttgttctatccaaaagagggaactgtaccagctcacacttttccctcaccagttcatcaacccacgcacgaacatgactgcgcacattacagtgccctttgttggcgtcaaccgctacgaccagtacaaggttcacaagccttggacccttgtggttatggttgtagcccctctgaccgtcaacactgaaggtgcccctcagatcaaggtgtatgccaacattgccccaactaacgtgcacgtcgcgggtgagtttccttccaaagagggaatattccccgtggcctgtagcgacggctatggtggcctggtgaccacggacccgaagacggctgaccccgtttatgggaaagtgttcaaccccccccgcaaccagttgccggggcgttttaccaacctccttgatgtggctgaggcatgcccgacgtttctgcacttcgagggtgacgtaccgtacgtgaccacgaaaacagactcggacagggtgcttgctcagtttgatatgtctttggcagcaaaacacatgtcaaacaccttcctcgcaggtcttgcgcagtactacacacagtacagtggcaccatcaacctgcacttcatgttcacaggacccactgacgcgaaggcgcgttacatgattgcctacgccccaccgggcatggagccgcccaagacacctgaggcggccgcgcactgcattcatgctgaatgggacactgggttgaactcaaagtttactttttccatcccctacctctcggccgccgattacgcgtacaccgcgtctgacgtggccgagaccacaaatgtgcagggatgggtctgcttgtttcaaattacacatggcaaggccgacggcgacgctctggtcgtactggctagtgctggtaaagactttgagctaaggctgccggtggacgcccgtgcggaaaccacttctgcgggcgagtcagcggatcctgtcaccaccactgttgaaaactacggtggcgaaacacagatccagaggcgccaacacacggacgtctcgttcattatggacagatttgtgaaagtgacaccgcaaaaccaaattaacattttggacctcatgcaggttccatcacacactttggtgggagcgctcctacgcgcgtccacttactacttctctgacttggagatagcagtaaaacacgagggagacctcacctgggttccaaatggagcgcctgaaaaggcgttggacaacaccaccaacccaactgcttaccacaaggcaccactcacccggcttgccctgccctacactgcgccccaccgcgtgttggcaaccgtgtacaacggtgagtgcaggtacagcagaaatgctgtgcccaacttgagaggtgaccttcaagtgttggctcaaaaggtggcacggacgctgcctacctccttcaactacggtgccatcaaagcgacccgggtcaccgag

>AY593816.1_O_UKG_1967

tcaagcgttggagtcacatacgggtacgcaacagctgaggattttgtgagcggaccgaacacttctggtctcgagaccagagttgtgcaggcagaacggtttttcaaaacccacctcttcgactgggtcaccagtgactcattcggacgttaccacctcctggaactcccgaccgaccacaaaggtgtctacggcagcctgaccgactcgtatgcatatatgaggaacggctgggatgtcgaggtcaccgcggttggcaaccagttcaacggagggtgcctgctggtcgcaatggtaccagagctttgttctatccaaaagagggaactgtaccagctcacacttttccctcaccagttcatcaacccacgcacgaacatgactgcgcacattacagtgccctttgttggcgtcaaccgctacgaccagtacaaggttcacaagccttggacccttgtggttatggttgtagcccctctgaccgtcaacactgaaggtgcccctcagatcaaggtgtatgccaacattgccccaactaacgtgcacgtcgcgggtgagtttccttccaaagagggaatattccccgtggcctgtagcgacggctatggtggcctggtgaccacggacccgaagacggctgaccccgtttatgggaaagtgttcaaccccccccgcaaccagttgccggggcgttttaccaacctccttgatgtggctgaggcatgcccgacgtttctgcacttcgagggtgacgtaccgtacgtgaccacgaaaacagactcggacagggtgcttgctcagtttgatatgtctttggcagcaaaacacatgtcaaacaccttcctcgcaggtcttgcgcagtactacacacagtacagtggcaccatcaacctgcacttcatgttcacaggacccactgacgcgaaggcgcgttacatgattgcctacgccccaccgggcatggagccgcccaagacacctgaggcggccgcgcactgcattcatgctgaatgggacactgggttgaactcaaagtttactttttccatcccctacctctcggccgccgattacgcgtacaccgcgtctgacgtggccgagaccacaaatgtgcagggatgggtctgcttgtttcaaattacacatggcaaggccgacggcgacgctctggtcgtactggctagtgctggcaaagactttgagctaaggctgccggtggacgcccgtgcggaaaccacttctgcgggcgagtcagcggatcctgtcaccaccactgttgaaaactacggtggcgaaacacagatccagaggcgccaacacacggacgtctcgttcattatggacagatttgtgaaagtgacaccgcaaaaccaaattaacattttggacctcatgcaggttccatcacacactttggtgggagcgctcctacgcgcgtccacttactacttctctgacttggagatagcagtaaaacacgagggagacctcacctgggttccaaatggagcgcctgaaaaggcgttggacaacaccaccaacccaactgcttaccacaaggcaccactcacccggcttgccctgccctacactgcgccccaccgcgtgttggcaaccgtgtacaacggtgagtgcaggtacagcagaaatgctgtgcccaacttgagaggtgaccttcaagtgttggctcaaaaggtggcacggacgctgcctacctccttcaactacggtgccatcaaagcgacccgggtcaccgag

>AY593817.1_O_Belgium_1973

tcaagcgttggagtcacatacgggtacgcaacagctgaggattttgtgagcggaccgaacacttccggtctcgaaaccagagttgtgcaggcagaacggtttttcaaaacccacctcttcgactgggtcaccagtgactcattcggacgttgccacctcctggaactcccgaccgaccacaaaggtgtctacggcagcctgaccgactcgtatgcatatatgagaaacggctgggatgtcgaggtcaccgcggttggcaaccagttcaacggagggtgcctgctggtcgcaatggtaccagagctttgttctatccaaaagagggaaatgtaccagctcacacttttccctcaccagttcatcaacccacgcacgaacatgactgcgcacatcacagtgccctttgttggcgtcaaccgctacgaccagtacaaggttcacaagccttggacccttgtggttatggttgtagcccctctgaccgtcaacactgaaggtgcccctcagatcaaggtgtatgccaacattgccccaaccaacgtgcacgtcgcgggtgagtttccttccaaggagggaatattccccgtggcctgtagcgacggctatggtggcctggtgaccacggacccgaagacggctgaccccgtttatgggaaagtgttcaaccccccccgcaaccagttgccggggcgttttaccaacctccttgatgtggctgaggcatgcccgacgtttctgcacttcgagggtgacgtaccgtacgtgaccacgaaaacggactcggacagggtgcttgctcagtttgacatgtctttggcagcaaaacacatgtcaaacaccttcctcgcaggtcttgcgcagtactacacacagtacagtggcaccatcaacctgcacttcatgttcacaggacccaccgacgcgaaggcgcgttacatgattgcctacgccccaccgggcatggagccgcccaagacacctgaggcggccgcgcactgcattcatgctgaatgggacactgggttgaactcgaagtttactttttccatcccctacctctcggccgccgattacgcgtacaccgcgtctgacgcggccgagaccacaaatgtgcagggatgggtctgcttgtttcaaattacacatggcaaggccgacggcgacgctctggtcgtactggctagtgctggtaaagactttgagctaaggctgccggtggacgcccgtgtggaaaccacttctgcgggcgagtcagcggatcctgtcaccaccaccgttgaaaactacggtggcgaaacacagatccagaggcgccaacacacggacgtctcgttcatcatggacagatttgtgaaagtgacaccgcaaaaccaaattaacattttggacctcatgcagattccatcacacactttggtgggagcgctcctacgcgcgtccacttactacttctctgacttggagatagcagtaaaacacgagggagacctcacctgggttccaaatggagcgcctgaaaaggcgttggacaacaccaccaacccaactgcttaccacaaggcaccactcacccggcttgccctgccctacaccgcgcctcaccgcgtgttggcaaccgtgtacaacggtgggtgcaggtacagcggaaatgctgtgcccaacttgagaggtgaccttcaggtgttggctcaaaaggtggcacggacgctgcctacctccttcaactacggtgccatcaaagcgacccgggtcaccgag

>AY593818.1_O_ARG_1958

tcaagcgttggagtcacatacgggtacgcaacagctgaggattttgtgagcggaccgaacacttctggtctcgagaccagagttgtgcaggcagaacggtttttcaaaacccacctcttcgactgggtcaccagtgactcattcggacgttgccacctcctggaactcccgaccgaccacaaaggtgtctacggcagcctgaccgactcgtatgcatatatgagaaacggctgggatgtcgaggtcaccgcggttggcaaccagttcaacggagggtgcctgctggtcgcaatggtaccagagctttgttctatccaaaagagggaactgtaccagctcacacttttccctcaccagttcatcaacccacgcacgaacatgactgcgcacatcacagtgccctttgttggcgtcaaccgctacgaccagtacaaggttcacaagccttggacccttgtggttatggttgtagcccctctgaccgtcaacactgaaggtgcccctcagatcaaggtgtatgccaacattgccccaaccaacgtgcacgtcgcgggtgagtttccttccaaggagggaatattccccgtggcctgtagcgacggctatggtggcctggtgaccacggacccgaagacggctgaccccgtttatgggaaagtgttcaaccccccccgcaaccagttgccggggcgttttaccaacctccttgatgtggctgaggcatgcccgacgtttctgcgcttcgagggtggcgtaccgtacgtgaccacgaaaacagactcggacagggtgcttgctcagtttgatatgtctttggcagcaaaacacatgtcaaacaccttcctcgcaggtcttgcgcagtactacacacagtacagtggcaccatcaacccgcacttcatgttcacaggacccactgacgcgaaggcgcgttacatgattgcctacgccccaccgggcatggagccgcccaagacacctgaggcggccgcgcactgcattcatgctgaatgggacactgggttgaactcaaagtttactttttccatcccctacctctcggccgccgattacgcgtataccgcgtctgacgtggccgagaccacaaatgtgcagggatgggtctgcttgtttcaaattacacatggcaaggccgacggcgacgctctggtcgtactggctagtgctggtaaagactttgagctaaggctgccggtggacgcccgtgcggaaaccacttctgcgggcgagtcagcggatcctgtcaccaccactgttgaaaactacggtggcgaaacacagatccagaggcgccaacacacggacgtctcgttcatcatggacagatttgtgaaagtgacaccgcaaaaccaaattaacattttggacctcatgcagattccatcacacactttggtgggagcgctcctacgtgcgtccacttactacttctctgacttggagatagcagtaaaacacgagggagacctcacctgggttccaaatggagcgcctgaaaaggcgttggacaacaccaccaacccaactgcttaccacaaggcaccactcacccggcttgccctgccctacaccgcgccccaccgcgtgttggcaaccgtgtacaacggtgagtgcaggtacagcagaaatgctgtgcccaacgtgagaggtgaccttcaggtgttggctcaaaaggtggtacggacgctgcctacctccttcaactacggtgccatcaaagcgacccgggtcaccgag

>AY593819.1_O_ARG_1994

tcaagcgttggagtcacatacgggtacgcaacagctgaggattttgtgagcggaccgaacacttctggtctcgagaccagagttgtgcaggcagaacggtttttcaaaacccacctcttcgactgggtcaccagtgactcattcggacgttgccacctcctggaactcccgaccgaccacaaaggtgtctacggcagcctgaccgactcgtatgcatatatgagaaacggctgggatgtcgaggtcaccgcggttggcaaccagttcaacggagggtgcctgctggtcgcaatggtaccagagctttgttctatccaaaagagggaactgtaccagctcacacttttccctcaccagttcatcaacccacgcacgaacatgactgcgcacatcacagtgccctttgttggcgtcaaccgctacgaccagtacaaggttcacaagccttggacccttgtggttatggttgtagcccctctgaccgtcaacactgaaggtgcccctcagatcaaggtgtatgccaacattgccccaaccaacgtgcacgtcgcgggtgagtttccttccaaggagggaatattccccgtggcctgtagcgacggctatggtggcctggtgaccacggacccgaagacggctgaccccgtttatgggaaagtgttcaaccccccccgcaaccagttgccggggcgttttaccaacctccttgatgtggctgaggcatgcccgacgtttctgcacttcgagggtgacgtaccgtacgtgaccacgaaaacagactcggacagggtgcttgctcagtttgatatgtctttggcagcaaaacacatgtcaaacaccttcctcgcaggtcttgcgcagtactacacacagtacagtggcaccatcaacctgcacttcatgttcacaggacccactgacgcgaaggcgcgttacatgattgcctacgccccaccgggcatggagccgcccaagacacctgaggcggccgcgcactgcattcatgctgaatgggacactgggttgaactcaaagtttactttttccatcccctacctctcggccgccgattacgcgtacaccgcgtctgacatggccgagaccacaaatgtgcagggatgggtctgcttgtttcaaattacacatggcaaggccgacggcgacgctctggtcgtactggctagtgctggtaaagactttgagctaaggctgccggtggacgcccgtgcggaaaccacttctgcgggcgagtcagcggatcctgtcaccgccactgttgaaaactacggtggcgaaacacagatccagaggcgccaacacacggacgtctcgttcatcatggacagatttgtgaaagtgacaccgcaaaaccaaattaacattttggacctcatgcagattccatcacacactttggtgggagcgctcctacgcgcgtccacttactacttctctgacttggagatagcagtaaaacacgagggagacctcacctgggttccaaatggagcgcctgaaaaggcgttggacaacaccaccaacccaactgcttaccacaaggcaccactcacccggcttgccctgccctacaccgcgccccaccgcgtgttggcaaccgtgtacaacggtgagtgcaggtacagcagaaatgctgtgcccaacgtgagaggtgaccttcaggtgttggctcaaaaggtggcacggacgctgcctacctccttcaactacggtgccatcaaagcgacccgggtcaccgag

>AY593820.1_O_ARG_1964

tcaagcgttggagtcacatacgggtacgcaacagctgaggatttcgtgagcggaccgaacacttctggtctcgagaccagagttgtgcaggcagaacggtttttcaaaacccacctcttcgactgggtcaccagtgactcattcggacgttgccacctcctggaactcccgaccgaccacaaaggtgtctacggcagcctgaccgactcgtatgcatatatgagaaacggctgggatgtcgaggtcaccgcggttggcaaccagttcaacggagggtgcctgctggtcgcaatggtaccagagctttgttctatccaaaagagggaactgtaccagctcacacttttccctcaccagttcatcaacccacgcacgaacatgactgcgcacatcacagtgccctttgttggcgtcaaccgctacgaccagtacaaggttcacaagccttggacccttgtggttatggttgtagcccctctgaccgtcaacactgaaggtgcccctcagatcaaggtgtatgccaacattgccccaaccaacgtgcacgtcgcgggtgagtttccttccaaggagggaatattccccgtggcctgtagcgacggctatggtggcctggtgaccacggacccgaagacggctgaccccgtttatgggaaagtgttcaaccccccccgcaaccagttgccggggcgttttaccaacctccttgatgtggctgaggcatgcccgacgtttctgcacttcgagggtgacgtaccgtacgtgaccacgaaaacagactcggacagggtgcttgctcagtttgatatgtctttggcagcaaaacacatgtcaaacaccttcctcgcaggtcttgcgcagtactacacacagtacagtggcaccatcaacctgcacttcatgttcacaggacccactgacgcgaaggcgcgttacatgattgcctacgccccaccgggcatggagccgcccaagacacctgaggcggccgcgcactgcattcatgctgaatgggacactgggttgaactcaaagtttactttttccatcccctacctctcggccgccgattacgcgtacaccgcgtctgacgtggccgagaccacaaatgtgcagggatgggtctgcttgtttcaaattacacatggcaaggccgacggcgacgctctggtcgtactggctagtgctggtaaagactttgagctaaggctgccggtggacgcccgtgcggaaaccacttctgcgggcgagtcagcggatcctgtcaccaccactgttgaaaactacggtggcgaaacacagatccagaggcgccaacacacggacgtctcgttcatcatggacagatttgtgaaagtgacaccgcaaaaccaaattaacattttggacctcatgcagattccatcacacactttggtgggagcgctcctacgcgcgtccacttactacttctctgacttggagatagcagtaaaacacgagggagacctcacctgggttccaaatggagcgcctgaaaaggcgttggacaacaccaccaacccaactgcttaccacaaggcaccactcacccggcttgccctgccctacaccgcgccccaccgcgtgttggcaaccgtgtacaacggtgagtgcaggtacagcagaaatgctgtgcccaacgggagaggtgaccttcaggtgttggatcaaaaggtggcacggacgctgcctacctccttcaactacggtgccatcaaagcgacccgggtcaccgag

>AY593821.1_O_ARG_1967

tcgagtgtcggagtcacttacgggtacgcaacagctgaggactttgtgaacgggccgaacacctctggtctcgagaccagagttgtccaggcagaacgtttcttcaaaacccacctgttcgactgggtcactagtgactcgttcggacgctgccaccttctggagcttccaactgaccacaaaggtgtctacggcagcctgaccgaatcttatgcgtacatgagaaacggctgggacgtcgaggttaccgcagttggtaaccagttcaacggaggctgcctgttggtggcgatggtgccagagctttgctccctccagaagagggaattgtaccaactcacactcttcccacaccagttcatcaacccacgcacgaacatgactgcacacctgactgtaccctttgtcggcgtcaaccgctacgaccaatacaaagtacacaaaccctggaccctcgtggttatggttgtagctcctctgacagtcaacactgagggtgcgccacaaatcaaggtgtacgccaacattgccccaaccaacgtgcatgtcgcgggcgagtttccctccaaggagggaatctttcctgtggcttgcgccgacggttatggcggcctggtgaccacggaccctaagaccgctgacccggtctacgggaaagtgttcaaccctccccgcagcttgttgcctgggcgtttcaccaacctccttgatgtggctgaggcctgcccgacattcctgcacttcgagggtgacgtgccatacgttaccacgaaaactgactcggacagggtgcttactcggttcgacatgtctctggcagcgaaacacatgtcaaacacctttcttgcaggtctcgcgcagtactacacccagtacagcggcaccatcaacctgcatttcatgttcacagggcccactgacgccaaagcgcgttacatgattgcttatgccccgccgggcatggaaccgcccaaaacaccggaggcggctgcccactgcattcatgctgagtgggacactgggttgaattcaaaattcactttctctatcccctacctgtcggccgctgactacgcgtacaccgcgtctgacacggccgaaaccacaaatgtacaggggtggatctgcttgtttcaaatcacgcacggcaaggccgacggtgacgctctggtcgtgctggctagcgctggcaaggactttgagttgagactgccggtggacgcccgcgcacaaacaacctccacgggtgaatcagcagaccctgtgacctccactgttgaaaactacggtggtgaaacacaggtccagaggcgccaccacacggacgtctcattcatcatggacagatttgtgaaagtcacaccaaaagaccaaattaatgttttggacctgatgcagattccagcacacaccctggtgggagcgctcctccgcgcttccacgtactacttttcagacttggagatagcagtgaaacatgagggagacctcacctgggtcccgaacggggcgcctgaaaaggcgttggacaacatcaccaacccgacggcctaccacaaggcgccgctcacccggcttgccttgccttacacggcgccacaccgcgtgctggcaaccgtgtacaacggtaggtgtaggtacggcgacggtgctgtacctaatgtgagaggagatctccaagcgctggctcaaaaagcagcgcgaccactgcccacctccttcaattacggggccatcaaagcaacccgagtcactgag

>AY593823.1_O_TUR_1969

tcgagcgttggagtcacgtacgggtatgcaacagctgaggatttcgtgagcgggccaaacacctctggtctcgagaccagggttgcccaggcagagcggttctttaaaacccacctgttcgactgggtcaccagtgacccgttcggacggtgccacctgctggaacttccaactgaccacaaaggtgtctacggcagcctgaccgactcgtatgcttatatgaggaacggctgggatgttgaagtcactgcagtgggaaaccagttcaatggaggatgcctgttggtggccatggtgccagaactttgctccatacagaagagggagctgtaccagctcacgctctttcctcaccagttcatcaaccctcggacgaacatgacagcacacatcactgtgccctttgttggcgtcaaccgttatgaccagtacaaggtacacaaaccttggaccctcgtggttatggttgtagcccccctgaccgtcaacagtgaaggtgccccgcaaatcaaggtgtatgccaacatcgcacctaccaacgtacacgtcgcgggtgagttcccttccaaagaggggatcttccctgtggcttgcagcgatggttatggcggtctggtgaccactgacccgaaaacggctgaccccgcttacgggaaagtgtttaaccccccccgcaacatgttgccggggcggttcaccaattttcttgacgtggctgaggcgtgccccacgtttctccacttcgagggtgacgtgccatacgtgaccacgaagacggattcagacagggtgctcgctcagttcgacttgtctttggcagcaaagcacatgtcgaacaccttccttgcaggtctcgcccagtactacacacagtacagcggcaccatcaacctgcacttcatgttcacagggcctactgacgcgaaggcgcgttacatgattgcgtatgctcctcctggcatggaaccacctaaaacgccagaggcggctgcccactgcattcatgctgaatgggacacagggttgaactcaaaattcacattttcaatcccttacctttcggcggctgattacgcttacacagcgtctgacactgctgagaccacaaatgtacagggatgggtttgcctgtttcaaataacacacgggaaagctgacggcgacgcactggtcgttttggctagcgccggaaaggactttgagctgcgcctgccggtggatgctcgcacacagactacctccgcgggcgagtcagctgaccccgtgaccgccaccgttgagaattacggtggcgagacacaggtccagaggcgccaacacacggacgtctcatttatattagacagatttgtgaaagtgacaccaaaagaccaaattaatgtattggacctgatgcaaacccctgctcacactttggtgggagcactccttcgtactgccacttactatttcgctgacttagaggtggcagtgaagcacgagggaaacctcacctgggtcccgaacggggcgcctgaagcggcgttggacaacaccaccaacccaacagcttaccacaaggcaccactcacccgacttgcactgccttacacggcgccacaccgcgtgttggctactgtttacaacgggaacagcaagtatggtgacggcacggtggccaatgtgagaggtgacctgcaagtgttggcccagaaggcggcgagagcgctgcctacctccttcaactacggtgccattaaagctactcgggtgactgaa

>AY593824.1_O_SKR_2000

tcgagcgttggagtcacttacgggtacgcaacagccgaggactttgtgagcggaccaaacacatctgggctcgagaccagggttgtgcaggcagagcggttcttcaaaacccacttgttcgactgggtcaccagtgacccgttcggacggtgctacctgctggaactcccaactgaccacaaaggtgtctatggcagcctgactgactcttatgcttacatgagaaacggttgggatgttgaggtcactgcagtgggaaatcagttcaacggaggatgtctgttggtggccatggtgccagaactttgctctattgacaagagagagctgtaccagctcacgctctttccccaccagttcatcaacccccggacgaacatgacggcgcacatcactgtgccctttgttggcgtcaatcgctacgaccagtacaaggtacacaaaccttggaccctcgtggttatggttgtggccccgctgactgtcaacaccgaaggtgccccacagatcaaggtctacgccaacatcgcccctaccaacgtgcacgttgcgggtgagttcccttccaaggaagggatcttccccgtggcatgtagcgacggttacggtggtctggtgaccactgacccaaagacggctgaccccgcctacgggaaagtgttcaatccacctcgcaacatgttgccggggcggttcaccaacttccttgatgtggctgaggcgtgccctacgtttctgcactttgagggtgacgtgccgtacgtgaccacaaagacggactcagacagggtgctcgcccagtttgacttgtctctggcagcaaaacacatgtcaaacaccttcctggcgggtctcgcccagtactacacacagtacagcggcaccatcaacctgcacttcatgttcacaggacccactgacgcgaaagcgcgttacatgattgcatacgccccccctggcatggagccgcccaaaacacctgaggcggccgctcactgcattcatgcggagtgggacacagggttgaattcaaaattcacattttcaatcccttacctttcggcggctgattacgcgtacaccgcgtctgatgctgcggagaccacaaatgtacagggttgggtttgcctgtttcaaattacacacgggaaggctgacggcgacgcactggtcgttctagctagcgccggcaaggactttgagctgcgtctgccagttgacgctcgcacgcagaccacctccacaggtgagtcggctgaccccgtgactgccactgttgagaactacggtggtgagacacaggtccagagacgccaacacacggacgtctcgttcatattagacagatttgtgaaagtaacaccaaaagaccaaattaatgtgttggacctgatgcaaacccctgcacacactttggtaggcgcgctcctccgtactgccacctactacttcgcagatctagaagtggcagtgaaacacgaggggaaccttacctgggtcccgaacggggcgcccgagacagcgttggacaacaccaccaatccaacggcctaccacaaggcaccgctcacccggcttgcactgccttacacggcaccacaccgtgtcttggctactgtttacaacgggaactgcaagtatggcgagggccccgtgaccaatgtgagaggtgacctgcaagtattggcccagaaggcggcaagaacgctgcctacctccttcaactacggtgccatcaaagccactcgggtgactgaa

>AY593825.1_O_ARG_1939

tcgagtgtcggggtcacgtacgggtacgcaacagctgaggactttgtgagtgggccaaacacttctggtctcgagaccagagttgtccaggcagaacgtttcttcaaaacccacctctttgattgggtcaccagcgatccgttcggacgttgtcacttgttggagcttccaactgaccacaaaggtgtctacggcagcctgaccgactcgtatgcatacatgagaaatggttgggacgttgaagtcaccgcggttggcaaccagttcaacggaggatgcttgctggtggcgatggtgccagagctttgttccatccagaagagggagctgtaccagctcacgctcttcccccatcagttcatcaacccacgcacaaacatgactgcgcacatcactgtgccctttgttggcgttaaccgttacgaccagtataaggtgcacaaaccctggaccctcgtggttatggtcgtagctcctctgactgtcaacaccgaaggtgccccacaaatcaaggtgtatgccaacattgccccaaccaacgtgcacgtcgcgggcgagcttccttccaaggagggaatcttccccgtagcttgtagtgacggctacggtggcctggtgaccacggacccgaagacggctgaccccgtttacgggaaagtgtttaacccccctcgcaacctcttgcctgggcgtttcaccaatctccttgatgtggctgaggcgtgcccgacgtttctgcacttcgagggtgacgtgccgtacgtgaccacgaaaacagattcagacagggtgcttgcccagtttgacatgtccttggcagcaaagcacatgtcgaacacctttcttgcgggcctcgcgcagtactacgcacagtacagtggcaccatcaacctgcacttcatgtttacagggcccactgacgcgaaagcgcgttacatgattgcttacgccccaccgggtatggaaccgcctcgaacacctgaggcggctgcacactgcattcatgctgagtgggacactgggttgaactcaaaatttacattctccatcccctacctttcggccgctgactacgcatacaccgcgtcggatgtggctgaaaccacaaatgtacaaggatgggtctgcttgtttcaaatcacgcatggcaaagccgacggcgacgctctggttgtgctggctagcgctggcaaggactttgagctgaggctgccggtggacgcccgcagacaaaccacctccgcgggtgaatcagcagaccccgtgactgccactgttgagaattacggtggcgagacacaggtccagaggcgccaacacacagacgtctcgttcatcatggacagatttgtgaaagtggcaccacaagatcaaatcaatgtcttggacctcatgcagattccagcctacacactggtgggggcgctcctgcgcgcgtccacgtactacttctctgacttagagatagcagtaaagcatgagggaaacctcacctgggttccgaacggggcgcctgaaacagcgctggagaatgccaccaacccaacagcttaccacaaggcgccactcacccggcttgccctgccctacacggcaccgcaccgtgtactggcaaccgcgtacaacggtgagtgcaagtacagtaaaaacgctgtacccagtgtgagaggtgacctccaggtgttggctcagaaggcggcgcgaacgctgcccacttcctttaactacggtgccatcagagcaactcgggtcaccgag

>AY593826.1_O_ITL_1947

tcgagtgtcggggtcacgtatgggtacgcaacggttgaggattttgtgagcggaccaaacacttctggtctcgagaccagagttgtccaggcagaacgcttcttcaaaacccaccttttcgattgggtcaccagtgacccattcggacgctgccacctcctggaactcccgactgatcacaaaggtgtctacggcagcttgaccgactcgtatgcatacatgagaaacggttgggacgtcgaagttaccgcggttggcaaccagttcaacggaggatgcttactggtggcaatggtaccagagctttgttccatacagaagagggagctgtaccagctcacacttttccctcaccagttcatcaacccgcgcacaaacatgactgcacacatcaccgtgccctttgttggcgtcaaccgctacgaccagtacaaggttcacaaaccgtggaccctagtggtcatggttgtggcgcctttgactgtcaacaatgaaggtgccccacaaatcaaggtgtacgccaacattgccccaaccaacgtacacgttgcgggcgagttcccttccaaagagggaattttccccgtggcctgtagcgacggttacggtggcctggtgaccacggacccaaagacggctgaccccgtttacgggaaagtgttcaaccccccccgcaacttgttgcctgggcgtttcaccaattttcttgatgtggctgaggcttgtccgacgtttctgcacttcgagggtgacgtaccgtacgtgaccacgaaaacagattcagacaggctactcgctcagtttgacatgtccttggcagcaaagcacatgtcgaacacctttcttgcaggtctcgcacagtactacacacagtacagcggcaccatcaacctgcacttcatgtttacagggcccactgacgcgaaagcgcgttacatgattgcttatgccccaccaggcatggagccaccccgaacacctgaggcggctgcacactgcattcatgctgagtgggacactgggttgaattcaaagttcacatttcccatcccttatctctcagctgctgattacgcgtacactgcgtctgacgtggctgaaaccacaaacgtgcaaggatgggtctgcttgtttcaaatcacacatggcaaggccgacggtgacgcactggtcgtgctggctagcgctggcaaagactttgagctgagactgccggtggacgcccgcagacaaaccacttccgcgggtgaatcagcagatcccgtgactgccaccgttgagagctacggtggagagacacaggtccagaggcgccaacacacggacgtctcgttcatcatggacagattcgtgaaagtcacaccaacagaccaaaccaatgttttgaacctcatgcagatcccatcccacacactggtgggagcactcctgcgtacatccacatactacttctctgacttagagatagcagtaaagcacgagggagatctcacctgggtcccgaatggggctcctgaagctgcgctggagaacaccaccaacccaacagcttaccacaaagctccactcacccggctcgccctgccttacacggcaccacaccgtgtgctggcaactgtgtacaacggtgagtccaggtacagcaaaaacgctgtgcccaatttgagaggtgacctccaggcgctggcccaaaaggcggcgagaacgctgcccacctctttcaactacggtgccattagagcaactcgggtcactgag

>AY593827.1_O_VEN_1971

tcgagtgtcggggtcacgtacgggtacgcaacagctgaggactttgtgagtggaccaaacacttctggtctcgagaccagagttgtccaggcagaacgctttttcaaaacccaccttttcgattgggtcaccagcgactcattcggacggtgccacctcctggaactcccgactgaccacaaaggtgtctacggcagcttgaccgactcgtatgcatatatgagaaacggatgggatgtcgaagtcaccgcggttggcaaccagttcaacggaggatgcctactggtggcaatggtgccagagctttgttctatgcagaagagggaactctaccaactcacacttttccctcaccagttcatcaacccacgcacgaacatgactgcacacatcactgtgccctttgttggcatcaatcgctacgaccaatacaaggttcacaaaccttggaccctagtggtcatggttgtggcgcctttgactgtcaataatgaaggtgccccacaaatcaaggtgtacgccaacattgccccaaccaacgtacacgttgcgggtgagctcccttccaaagagggaatcttccccgtggcctgtagcgacggttacggtggcctggtgaccacggacccgaagacggctgaccccgtttacgggaaagtgttcaacccccctcgcaacttgttgcctgggcgtttcaccaatctccttgacgtggctgaggcttgcccgacgtttctgcacttcgagggtgacgtgccgtacgtgaccacgaaaacagattcagacagggtactcgctcagtttgacatgtccttggcagcaaagcacatgtcgaacacctttcttgcaggtctcgcgcagtactacacacagtacagcggcaccattaacctgcatttcatgtttacagggcccactgacgcgaaagcgcgttacatgattgcttatgccccaccgggcatggagccacccaaaacacctgaggcggctgcacactgcattcatgccgagtgggacaccgggttgaattcaaagttcacatttcccattccttacctctcagctgctgactacgcgtacaccgcgtctgatgcggctgaaaccacaaatgtgcaaggatgggtctgcttgtttcaaatcacacatggcaaggccgacggtgacgcgctggtcgtgctggctagcgctggcaaagactttgagctgagactgccggtggacgcccgcagacaaaccacctccgcgggtgaatcagcagatcccgtggctgccaccgttgagaactacggtggggagacacaggtccagaggcgccaacacacggacgtctcgttcatcatggacagatttgtgaaagttacaccaaaagaccaaaccaatgttttggacctcatgcagattccatcctacacactggtgggagcactcctgcgtgcatccacgtactacttctctgacttagagatagcagtaaagcacgagggagatctcacctgggtcccaaatggggctcctgaatctgcgctggagaacaccaccaacccaacagcttaccacaaagctccactcacccgactcgccctgccttacacggcaccacaccgtgtgttggcaacagtgtacaacggtgagtgtaggtacagcagaaacactgtgcccaatgtgagaggtgacctccaggtgctgacccaaaaggcggcgcgaacgctgcccacctctttcaactacggtgccatcagggcaactcgggtcactgag

>AY593828.1_O_IND_1962

tcgagcgttggagtcacatacgggtacgcaacagctgaggattttgtgagcggaccaaacacctctggtctcgagactagggttgtccaggcagaacggtttttcaaaacccacctgttcgactgggtcactagtgatccgtttggacggtgccacctgctggaacttccaactgaccacaaaggtgtctacggcagcttgaccgactcgtatgcttatatgagaaacggttgggatgtcgaagtcactgcagtggggaatcagttcaacggaggttgcctgttggtggccatggtgcctgagctttgttccattcagaagagagagctgtaccagcttacgctctttccccaccagttcatcaaccctcggacgaacatgacagctcacattactgtgccctttgttggcgtcaaccgctacgaccagtacaaggtacacaagccttggaccctcgtggtcatggttgtagcccctttgactgtcaacactgaaggtgccccacagatcaaggtgtacgccaacatcgctcccaccaacgtacacgttgcgggtgagttcccttccaaagagggaattttccccgtggcttgtagcgacggttacggcggtttggtgaccacagacccgaagacggctgaccccgcctacgggaaagtgtttaaccccccccgcaacatgttgccggggcggttcaccaacctccttgatgtggctgaggcgtgccccacgtttctgcgcttcgaaggtggcgtgccatacgtgaccacgaagacggattcagacagggtgctcgctcagtttgacttgtctttggcagcaaagcacatgtcgaacaccttccttgcgggccttgcccagtactacacacagtacagcggcaccatcaacctgcacttcatgttcacaggtcccactgacgcgaaggcgcgttacatgattgcatacgccccgccaggcatggagccgcctaaaacacccgaggcggctgcccactgcattcatgctgagtgggacacaggtctgaactcaaaattcacattttcaatcccttacctctcggcggctgactacgcgtacaccgcgtctgacactgctgagaccacaaatgtacagggatgggtttgcttgtttcaaataacacatgggaaagctgaaggtgacgcgctggttgtgctggctagtgccggcaaggactttgagctgcgcctaccagtggatgctcgcacacagaccacctccacgggcgagtcggctgaccccgtgaccgccaccgttgagaactacggcggtgagacacaggtccagaggcgtcagcacacggacgtctcattcatactggacagatttgtgaaagtcacaccaaaagaccaaattaatgtactggacctgatgcaaacccctgctcacactctggtgggagcgctccttcgtactgccacttactatttcgctgatttggaagtggcagtaaaacacgaggggaacctcacttgggtcccgaatggggcgcctgagacagccttggacaacaccaccaatccaacggcataccacaaggcaccacttacccggcttgcactgccttacacggcaccacaccgtgttttggctaccgtttacaacgggaattgcaagtacgctgatggcccggtggccaatgtaagaggcgacctgcaagtgttggcccagaaggcggcgagagcgctgcctacctcctttaactacggtgccatcaaagccacccgggtgactgaa

>AY593830.1_O_POL_1959

tcaagcgttggagtcacatacgggtacgcaacagctgaggattttgtgagcggaccgaacacttctggtctcgagaccagagttgtgcaggcagaacggtttttcaaaacccacctcttcgactgggtcaccagtgactcattcggacgttgccacctcctggaactcccgaccgaccacaaaggtgtctacggcagcctgaccgactcgtatgcatacatgagaaacggctgggatgtcgaggtcaccgcggtcggcaaccagttcaacggagggtgcctgctggtcgcaatggtaccagagctttgttctatccaaaagagggagctgtaccagctcacacttttccctcaccagttcatcaacccacgcacgaacatgactgcgcacatcacagtgccctttgttggcgtcaaccgctacgaccagtacaaggttcacaagccttggacccttgtggttatggttgtagcccctctgaccgtcaacactgaaggtgcccctcagatcaaggtgtatgccaacattgccccaaccaacgtgcacgtcgcgggtgagtttccttccaaggagggaatattccccgtggcctgtagcgacggctatggtggcctggtgaccacggacccgaagacggctgaccccgtttatgggaaagtgttcaaccccccccgcaaccagttgccggggcgttttaccaacctccttgatgtggctgaggcatgcccgacgtttctgcacttcgagggtgacgtaccgtacgtgaccacgaaaacagactcggacagggtgcttgctcagtttgatatgtctttggcagcaaaacacatgtcaaacaccttcctcgcaggtcttgcgcagtactacacacagtacagtggcaccatcaacctgcacttcatgttcacaggacccactgacgcgaaggcgcgttacatgatcgcctacgccccaccgggcatggagccgcccaagacacctgaggcggccgcgcactgcattcatgctgaatgggacactgggttgaactcaaagtttactttttccatcccctacctctcggccgccgattacgcgtacaccgcgtctgacgtggccgagaccacaaatgtgcagggatgggtctgcttgtttcaaattacacatggcaaggccgacggcgacgctctggtcgtactggctagtgctggtaaagactttgagctaaggctgccggtggacgcccgtgcggaaaccacttctgcgggcgagtcagcggaacctgtcaccgccactgttgaaaactacggtggcgaaacacagatccagaggcgccaacacacggacgtctcgttcatcatggacagatttgtgaaagtgacaccgcaaaaccaaattaacattttggacctcatgcagattccatcacacactttggtgggagcgctcctacgcgcgtccacttactacttctctgacttggagatagcagtaaaacacgagggagacctcacctgggttccaaatggagcgcctgaaaaggcgttggacaacaccaccaacccaactgcttaccacaaggcaccactcacccggcttgccctgccctacaccgcgccccaccgcgtgttggcaaccgtgtacaacggtgagtgcaggtacagcagaaatgctgtgcccaacaggagaggtgaccttcagttgttggctcaaaaggtggcacggacgctgcctacctccttcaactacggtgccatcaaagcgacccgggtcaccgag

>AY593831.1_O_UKG_2002

tcgagcgttggagtcacttacgggtacgcaacagctgaggactttgtgagcggaccaaacacatctgggcttgagaccagggttgtgcaggcagagcggttcttcaaaacccacttgttcgactgggtcaccagtgacccgtttggacggtgctatctgctggaactcccaactgaccacaaaggtgtctacggcagcctgaccgactcttatgcttacatgagaaacggttgggatgttgaggtcaccgcagtgggaaatcagttcaacggaggatgtctgttggtggccatggtgccagaactttgctctattgacaagagagagctgtaccagctcacgctctttccccaccagttcatcaacccccggacgaacatgacggcgcacatcactgtgccctttgttggcgtcaaccgctacgaccagtacaaggtacacaaaccttggaccctcgtggttatggttgtggccccgctgactgtcaacaccgaaggtgccccacagatcaaggtctatgccaacatcgcccctaccaacgtgcacgttgcgggtgagttcccttctaaggaagggatcttccccgtggcatgtagcgacggttacggtggtctggtgaccactgacccaaagacggctgaccccgcctacgggaaagtgttcaatccacctcgcaacatgttgccggggcggttcaccaacttccttgatgtggctgaggcgtgccctacgtttctgcactttgagggtggcgtgccgtacgtgaccacaaagacggactcagacagggtgctcgcccagtttgacttgtctctggcagcaaagcacatgtcaaacaccttcctggcaggtctcgcccagtactacacacagtacagcggcaccatcaacctgcacttcatgttcacaggacccactgacgcgaaagcgcgttacatgattgcatacgccccccctggtatggagccgcccaaaacacctgaggcggccgcccactgcattcatgcggagtgggacacagggttgaattcaaaattcacattttcaatcccttacctttcggcggctgattacgcgtacaccgcgtctgacgctgcggagaccacaaatgtacagggatgggtttgcctgtttcaaattacacacgggaaggctgacggcgacgcactggtcgttctagctagcgccggtaaggactttgagctgcgtctgccagttgacgctcgcacgcagaccacctccgcaggtgagtcggctgaccccgtgactgccactgttgagaactacggtggtgagacacaggtccagagacgccaacacacggatgtctcgttcatattagacagatttgtgaaagtaacaccaaaagaccaaattaatgtgttggacctgatgcaaacccctgcacacactttggtaggcgcgctcctccgtactgccacctactacttcgcagatctagaagtggcagtgaaacacgaggggaaccttacctgggtcccgaatggggcgcccgagacggcgttggacaacaccaccaatccaacggcttaccacaaggcaccgctcacccggcttgcactgccttacacggcaccgcaccgtgtcttggctactgtttacaacgggaactgcaagtatggcgagagccccgtgaccaatgtgagaggtgacctgcaagtattggcccaaaaggcggcaagaacgctgcccacctccttcaattacggtgccatcaaagccactcgggtgactgaa

>AY593833.1_O_TAW_1999

tctagcgtcggggtgacttacgggtacgcaacggctgaagacttcgtgagtgggcctaacacctctggtcttgagaccagagttgttcaggccgaacggttcttcaaaacccacctgtttgactgggtcaccagtgacccgtttgggcggtgtcacttgttggagctaccgactgaccacaaaggcgtctacggtagcctgaccgactcgtacgcatacatgaggaatggttgggacgttgaagtcaccgcagtgggtaaccagttcaacggaggctgtttgctggtggcgatggtaccggagctccgttccatcagcaagagagagttgtaccagctcacgcttttcccccaccagttcatcaacccacggacgaatatgacggcacacatcaccgtgccctacctcggtgtcaacaggtacgaccagtacaaggtacacaaaccctggaccctcgtggtcatggttgtggcccccctgacggttaacaacgagggcgctccgcaaatcaaggtgtatgccaacatcgcccccaccaatgttcacgtcgcgggtgagctcccctctaaagaggggattttccccgtggcatgcagcgatggttacggtggcttggtgaccacggatccgaagacggcagaccccgtctacgggaaagtgttcaacccaccccgcaacctgttgccagggcggtttacaaacctccttgacgtggccgaggcgtgccccacattcctacacttcgacggtgacgttccgtacgtgaccacgaagacggattcggataggatgctagcccagttcgatttgtccctcgcggcaaaacatatgtcgaacacttttctcgcgggtcttgcccagtactacacacagtacagcggcaccattaacctgcacttcatgttcacgggacccaccgacgcgaaggcacgctacatggttgcgtacgcccctcctggcatggaaccgccgaaaacgcctgaggcggctgcacattgcatccacgctgagtgggatacagggctgaattcgaagttcacgttttcaatcccatacctttcggcagctgactacgcgtacaccgcgtccgacatcgccgagaccacaaacgtacagggatgggtctgtttgttccagataacacacgggaaagccgacggtgacgccctggtcgtgctagctagtgctggcaaagactttgacttgcgtctgccggtcgacgcccgaacccaaaccacctctgcgggtgagtctgcggaccccgtgaccgccaccgtcgagaactacggtggtgagacacaagtccagaggcgccagcacacggacattgcgttcatattggacaggttcgtgaaagtcaagccaaaggaacaagttaatgtgttggacctgatgcagatccctgcccacaccttggtaggggcgctcctgcgaacggccacctactacttctctgacctggagctggccgtcaagcacgagggcgatctcacctgggtcccaaacggcgcccctgagacagcactggacaacactactaacccaacagcttaccacaaggaacccctcacacggctggcgctgccttacacggctccacaccgtgtcttagcgaccgtctacaacgggagcagtaagtacggtgacaccagcactaacaacgtgagaggtgaccttcaagtgttagctcagaaggcagaaagaactctgcctacctccttcaacttcggtgccatcaaggcaactcgtgttactgaa

>AY593834.1_O_IRN_1966

tcgagtgttggagttacgtacgggtacgcaacaaccgaggattttgtaagcggaccaaacacctctggtctcgagactagagtggttcaggcggaacgcttctttaaaacccacctgttcgactgggtcaccagcgaccccttcggacggtgtcacctactggaactcccaactgaccacaaaggtgtctacggcagcctgactgactcatatgcatacatgagaaatggttgggacgttgaagtcactgctgtgggaaatcagtttaatggaggatgcctgttggtggccatggtgccagagctttgttccatccaaaagcgagagttgtaccagctcacgctctttccccaccagttcatcaacccacgaacgaacatgacggcacacatcactgtgccctttgttggcgtcaaccgttatgaccagtacaaggtacacaagccttggaccctcgtggtcatggtcgtagcccccctgactgtcaacactgaaggtgccccacagatcaaggtgtacgccaacatcgcccccaccaacgtgcacgttgcgggtgaattcccctccaaggaggggattttccctgtggcttgcagtgacggttacggcggtttggtgaccacggacccgaagacggctgaccccgcctatgggaaagtgtttaaccccccccgaaacatgttaccggggcggttcaccaacttccttgatgtggctgaggcgtgccctacgtttctgcacttcgagggtgacgtgccatacgtgactacgaagacagattcggacagggtgcttgctcagtttgacttgtctttggcagcgaagcacatgtcgaacaccttccttgctggtcttgcccagtactacacacagtacagcggcaccattaacctacacttcatgttcacaggtcccactgacgcgaaagcgcgctacatgattgcatatgccccacccggcatggagccgccacgcacacctgaagcggctgcccactgcattcatgctgaatgggacacagggttgaattcaaaatttacattctcaattccctacctctcggcggctgactacgcgtacaccgcgtctgacacagctgagaccacaaatgtgcagggatgggtctgcttgttccaaataacacacgggaaagctgacggcgacgccctggttgtgctggccagcgccggcaaggactttgagctgcgcctaccagtggacgcccgcacacagaccacctccccgggtgagtcggctgaccccgtgaccgccaccgttgagaactacggcggcgaaacacaggtccagaggcgccaacacacggacgtctcattcatattggacaggtttgtgaaagtaacaccacaagaccaaattaatgtattggacctgatgcagacccctgctcacaccttggtgggtgcgctccttcgcaccgccacttactatttcgctgatctagaagtggcagtgaagcacgagggaaacctcacgtgggtcccgaacggggctcctgagaccgcgctggacaacaccaccaacccaacagcataccacaaagcacctcttacccgccttgccctgccatacacagcgccacaccgcgtgttggcaaccgtttacaacgggaactgcaagtacggcactggcccggtggccaatgtgaggggtgacctccaagtgttggctcagaaggcggcgagaccgctgcctacctcctttaactacggtgccatcaaggctgtccgggtgactgaa

>AY593835.1_O_TAW_1997

tctagcgtcggggtgacttacgggtacgcaacggctgaagacttcgtgagtgggcctaacacctctggtcttgagaccagagttgttcaggccgaacggttcttcaaaacccacctgtttgactgggtcaccagtgacccgtttgggcggtgtcacttgttggagctaccgactgaccacaaaggcgtctacggtagcctgaccgactcgtacgcatacatgaggaatggttgggacgttgaagtcaccgcagtgggtaaccaattcaacggaggctgtttgctggtggcgatggtaccggagctctgttccatcagcaagagagagttgtaccagcttacgcttttcccccaccagttcatcaacccacggacgaatatgacggcacacatcaccgtgccctacctcggtgtcaacaggtacgaccagtacaaggtacacaaaccctggaccctcgtggtcatggttgtggcccccttgacggttaacaacgagggcgctccgcaaatcaaggtgtatgccaacatcgcccccaccaatgttcacgtcgcgggtgagctcccctctaaagaggggattttccccgtggcatgcagcgatggttacggtggcttggtgaccacggatccgaagacggcagaccccgtctacgggaaagtgttcaacccaccccgcaacctgttgccagggcggtttacaaacctccttgacgtggccgaggcgtgccccacattcctacacttcgacggtgacgttccgtacgtgaccacgaagacggattcggatagggtgctagcccagttcgatttgtccctcgcggcaaaacatatgtcgaacacttttctcgcgggtcttgcccagtactacacacagtacagcggcaccattaacctgcacttcatgttcacgggacccaccgacgcgaaggcacgctacatggttgcgtacgcccctcctggcatggaaccgccgaaaacgcctgaggcggctgcacattgcatccacgctgagtgggatacagggctgaattcgaagttcacgttttcaatcccatacctttcggcagctgactacgcgtacaccgcgtccgacgtcgccgagaccacaaacgtacagggatgggtctgtttgttccagataacacacgggaaagccgacggtgacgccctggtcgtgctagctagtgctggcaaagactttgacttgcgtctgccggtcgacgcccgaacccaaaccacctctgcgggtgagtctgcggaccccgtgactgccaccgtcgagaactacggtggtgagacacaagtccagaggcgccagcacacggacattgcgttcatattggacaggttcgtgaaagtcaagccaaaggaacaagttaatgtgttggacctgatgcagatccctgcccacaccttggtaggggcgctcctgcgaacggccacctactacttctctgacctggagctggccgtcaagcacgagggcgatctcacctgggtcccaaacggcgcccctgagacagcactggacaacactaccaacccaacagcttaccacaaggaacccctcacacggctggcgctgccttacacggctccacaccgtgtcttagcgaccgtctacaacgggagcagtaagtacggtgacaccagcactaacaacgtgagaggtgaccttcaagtgttagctcagaaggcagaaagaactctgcctacctccttcaacttcggtgccatcaaggcaactcgtgttactgaa

>AY593836.1_O_UKG_2001

tcgagcgttggagtcacttacgggtacgcaacagctgaggactttgtgagcggaccaaacacatctgggcttgagaccagggttgtgcaggcagagcggttcttcaaaacccacttgttcgactgggtcaccagtgacccgtttggacggtgctatctgctggaactcccaactgaccacaaaggtgtctacggcagcctgaccgactcttatgcttacatgagaaacggttgggatgttgaggtcaccgcagtgggaaatcagttcaacggaggatgtctgttggtggccatggtgccagaactttgctctattgacaagagagagctgtaccagctcacgctctttccccaccagttcatcaacccccggacgaacatgacggcgcacatcactgtgccctttgttggcgtcaaccgctacgaccagtacaaggtacacaaaccttggaccctcgtggttatggttgtggccccgctgactgtcaacaccgaaggtgccccacagatcaaggtctatgccaacatcgcccctaccaacgtgcacgttgcgggtgagttcccttctaaggaagggatcttccccgtggcatgtagcgacggttacggtggtctggtgaccactgacccaaagacggctgaccccgcctacgggaaagtgttcaatccacctcgcaacatgttgccggggcggttcaccaacttccttgatgtggctgaggcgtgccctacgtttctgcactttgagggtggcgtgccgtacgtgaccacaaagacggactcagacagggtgctcgcccagtttgacttgtctctggcagcaaagcacatgtcaaacaccttcctggcaggtctcgcccagtactacacacagtacagcggcaccatcaacctgcacttcatgttcacaggacccactgacgcgaaagcgcgttacatgattgcatacgccccccctggtatggagccgcccaaaacacctgaggcggccgcccactgcattcatgcggagtgggacacagggttgaattcaaaattcacattttcaatcccttacctttcggcggctgattacgcgtacaccgcgtctgacgctgcggagaccacaaatgtacagggatgggtttgcctgtttcaaattacacacgggaaggctgacggcgacgcactggtcgttctagctagcgccggtaaggactttgagctgcgtctgccagttgacgctcgcacgcagaccacctccgcaggtgagtcggctgaccccgtgactgccactgttgagaactacggtggtgagacacaggtccagagacgccaacacacggatgtctcgttcatattagacagatttgtgaaagtaacaccaaaagaccaaattaatgtgttggacctgatgcaaacccctgcacacactttggtaggcgcgctcctccgtactgccacctactacttcgcagatctagaagtggcagtgaaacacgaggggaaccttacctgggtcccgaatggggcgcccgagacggcgttggacaacaccaccaatccaacggcttaccacaaggcaccgctcacccggcttgcactgccttacacggcaccgcaccgtgtcttggctactgtttacaacgggaactgcaagtatggcgagagccccgtgaccaatgtgagaggtgacctgcaagtattggcccaaaaggcggcaagaacgctgcccacctccttcaattacggtgccatcaaagccactcgggtgactgaa

>AY593837.1_O_URU_1963

tcaagcgttggagtcacatacgggtacgcaacagctgaggattttgtgagcggaccgaacacttctggtctcgagaccagagttgtgcaggcagaacggtttttcaaaacccacctcttcgactgggtcaccagtgactcattcggacgttgccacctcctggaactcccgaccgaccacaaaggtgtctacggcagcctgaccgactcgtatgcatatatgagaaacggctgggatgtcgaggtcaccgcggttggcaaccagttcaacggagggtgcctgctggtcgcaatggtaccagagctttgttctatccaaaagagggaactgtaccagctcacacttttccctcaccagttcatcaacccacgcacgaacatgactgcgcacatcacagtgccctttgttggcgtcaaccgctacgaccagtacaaggttcacaagccttggacccttgtggttatggttgtagcccctctgaccgtcaacactgaaggtgcccctcagatcaaggtgtatgccaacattgccccaaccaacgtgcacgtcgcgggtgagtttccttccaaggagggaatattccccgtggcctgtagcgacggctatggtggcctggtgaccacggacccgaagacggctgaccccgtttatgggaaagtgttcaaccccccccgcaaccagttgccggggcgttttaccaacctccttgatgtggctgaggcatgcccgacgtttctgcacttcgagggtgacgtaccgtacgtgaccacgaaaacagactcggacagggtgcttgctcagtttgatatgtctttggcagcaaaacacatgtcaaacaccttcctcgcaggtcttgcgcagtactacacacagtacagtggcaccatcaacctgcacttcatgttcacaggacccactgacgcgaaggcgcgttacatgattgcctacgccccaccgggcatggagccgcccaagacacctgaggcggccgcgcactgcattcatgctgaatgggacactgggttgaactcaaagtttaccttttccatcccctacctctcggccgccgattacgcgtacaccgcgtctgacgtggccgagaccacaaatgtgcagggatgggtctgcttgtttcaaattacacatggcaaggccgacggcgacgctctggtcgtactggctagtgctggtaaagactttgagctaaggctgccggtggacgcccgtgcggaaaccacttctgcgggcgagtcagcggatcctgtcaccgccactgttgaaaactacggtggcgaaacacagatccagaggcgccaacacacggacgtctcgttcatcatggacagatttgtgaaagtgacaccgcaaaaccaaattaacattttggacctcatgcagattccatcacacactttggtgggagcgctcctacgcgcgtccacttactacttctctgacttggagatagcagtaaaacacgagggagacctcacctgggttccaaatggagcgcctgaaaaggcgttggacaacaccaccaacccaactgcttaccacaaggcaccactcacccggcttgccctgccctacaccgcgccccaccgcgtgttggcaaccgtgtacaacggtgagtgcaggtacagcagaaatgctgtgcccaacgcgagaggtgaccttcaggtgttggctcaaaaggtggcacggacgctgcctacctccttcaactacggtgccatcaaagcgacccgggtcaccgag

>AY593838.1_SAT1_BOT_1970

agctcggtcggcgtcacctatgggtacgcgtcagccgacaaattcttgcctggcccaaacaccaacgggctggaaacaagagtggaacaagcagagaggtttttcaaacacaagctttttgattggacactcgaacagcaatttggcacaacgcacattttggaactccccacagatcataagggcatctatgggcagctggttgactcacacgcgtacatccgaaacggatgggacgtgcaagtgtcggccactgccacacagtttaacggaggctgcctgttggtggccatggtgcctgagctgtgtaaactggctgacagagagaaataccaactcactctcttcccacaccaattcttgaacccacgcaccaacaccaccgcacacattcaggtaccatacctgggtgttgacagacacgaccagggaactcgtcacaaggcgtggaccctcgtcgtgatggtggtggcaccatacactaacgacacaattggctcactaaaagctgaggtctacgttaacattgcaccaaccaatgtgtacgtggccggtgagaaacccgccaaacagggaattttccctgtggccgttgctgacggctacggtggttttcagaacaccgaccccaagacctcggaccccatatacgggcacgtgtacaacccggcacgcacgctctaccctggcaagtttaccaacctgctggacgtggcagaagcgtgcccgacactgctcgacttcaac---ggggttccgtatgtcaagaccacaggtaattctgcaaaagtgctcacacgttttgatttggcctttggacacaaaaacatgaaaaacacctacatgtctggtctggcccagtactttgcacagtacagtggcaccctcaatcttcactttatgtacactggccctaccaataccaaggctaagtacatggtagcatacatcccacctggtacaagccttcctgaaacaccagagatggcctcacactgctatcacgctgagtgggatactgggttgaactcaacatttaccttcaccgtgccgtacatttcagcagcggattacgcctacacctactctgatgagcctgaacaggcttcagtacaaggttgggttggtgtgtatcagatcactgacacacacgagacggacgcggcagtcatcgttactgtcagtgcaggtccggactttgagttcagaatgcccatcagcccatcgcgccaaacaacctccgcaggtgaaggcgcagacccagtcaccacagacgtctctgagcacggaggcgtctccagaaccgcacgccgggcacacaccgacgtcgctttccttctcgaccggttcactctggttgggaccatggaaaacaaaatggttctggatttgttgaccacgaaggagaaatcactggttggcgcactcctgcgtgcggccacgtattacttctctgatttggaggtggcagtcgggaacaacaaatgggtaggctgggctcccaatggtagcccagtgctgattgaggtgggc---gacaatccagtcgtcttctcacacaatggtacaacccgttttgcactcccgtacactgcaccccaccgggtgctcgccacagtttacaacggtgactgcaagtacaagcccactggcactggaaacatccgcggggacctcgccgctgtggctgggaggatcgccagccacatcccgacgacatttaatttcgggatgatttacacagaggcagaggtggac

>AY593839.1_SAT1_UKG_1970

agctcagtgggcattacctacgggtatgccgactcggacaaattcctgcccggtccaaacaccaacgggttggaaacacgtgtggaacaagccgaaaggttctttaagcacaagctatttgattggacaactgaccaagaatttggaacaacccacgttttggagctgcccacagaccacaaaggcatctacggccaactcgttgactctcactcttacatccgtaacgggtgggacgtcgaggtctccgcgaccgcgactcaattcaacgggggctgcctcttggtggcaatggtgcccgagttgtgcaaacttgaggacagagagaaataccaacttactctctttcctcaccagtttctgaacccaaggaccaacaccacagcacacattcaggtgccttacctgggtgtggaccgccacgatcaggggacgcgccacaaagcgtggaccttggtcgtaatggtggtggcaccttataccaacgacacaattggttcaaacaaagccgaggtgtacgtgaacatcgctcccacgaacgtttacgtcgctggtgagaagcccgcaaaacagggcattctccccgtagccgtctctcacggctacggcggtttccaaaacacagaccccaaaacttcggaccccgtgtacgggcacgtgtacaacccagcacgcactggtctgccaggaaggttcacgaacctcctggacgtggctgaagcgtgtcccacactgcttgacttcaac---gggcgtccgtacgtgaccacccaggccaactctgcgaaagtactgactcgttttgatttggcttttggacacaagaatttgaaaaatactttcatgtccggtctcgcccagtactacacacagtacagtggcacactcaatctgcacttcatgtacacaggcccaacaaacaacaaggcaaagtacatggtggcctacatcccaccagggacacacctcccggaaactccggagatggcgtcccactgctaccacgctgaatgggacacaggcctaaactcaaccttcactttcaccgtgccgtacgtgtcgaccgctgatttcgcgtacacctactctgacgagcccgaacaggctacggtgcagggttgggtgggcgtgtaccaggtaactgacacgcacgagaaggatggagcggttgtggtgtccgtcagtgctggacccgacttcgagttcagaatgcccatcagcccctcacgccagaccacgtctgttggcgagggtgcagagcccgtcacgactgacgcctcccagcatggtggcgacaggcgcgcggcgcgcaggtaccacactgatgtgagcttcttgctcgatcggttcacactggtcggcacacagaacaacaggctgacactagacctgctccagactaaggagaaagcgctggtcggcgcaatcctgcgtgcggccacgtactatttctctgatttggaagttgcggtgggcaccaacaagtgggtcggctggactcccaacggagcgccggagcttagtgaagtgggc---gacaacccagtcatcttctccaccaatgacaccacccgctttgcgctgccctacactgcaccacacagatgcttggcaacatcctacaacggtgactgcaagtacaagcccgctggcacagagaacatccgcggggacctcgcgacccttgcgcagaggattgcaagacacatcccaaccactttcaattatggcaggatttacacagaagccgaggtagac

>AY593840.1_SAT1_NMB_1949

agttcggtaggtgttacctacgggtacgcactggccgacaagttcctccccggcccgaacaccaacgggttggagacgcgggtggaacaggcggagaggttctttaaacacaagctttgcgattggacacctgaccaagaattcggtacaactcacatcctggaactccccacagaccacaagggcatctatgggcagttggttgattcgcacgcatacatccgcaacggatgggatgtccaggtttcggccactgccacccagttcaacggaggctgcctgttggtggccatggtgcctgaactctgcaaattggacaacagggaaaagtaccagctcacactttttccccaccagttcttgaacccacgcaccaacaccactgcacacatccaggtaccgtacctgggtgttgacagacacgaccagggaacacgccacaaagcgtggaccctcgttgtgatggtactggcaccatacaccaacgacaccattggatcaacaaaagctgaggtttacgtgaacattgcaccaaccaacgtttacgtggccggtgagaaacccgccaaagaagggattctccccgtagccgtctctgacggttacggtggcttccaaaacactgaccccaaaacctcggaccccatatacgggcacgtgtacaacccggcacgaacgctctaccctggcagattcaccaacctgctagacgtggcagaagcgtgcccaacactgctcgactttaat---ggggtaccatatgtccaaacccagaacaattctgcaaaggttctcgcacgcttcgatttggcttttgggcacaaaaacatgaagaacacatacatgtctggtctggcccagtactttgcacaatacagtggtactctcaatcttcacttcatgtacactggccccaccaacaacaaggccaagtacatggtggcatacatcccacctggcacacacctccccgaaacaccggagcaggcgtcacactgttaccatgccgagtgggacactgggttgaactcgacattcaccttcaccgtgccgtacatttcggcagcggactacgcctatacctacgctgatgaacctgaacaggcttcagtgcaaggttgggttggcgtgtaccagataactgacacgcacgagaaggacggggccgttgtcatcactgtcagcgcaggtcccgacttcgagttcagaatgcccatcagcccatcgcgccagacaacctctgcaggtgaaggcgcagatccagtcaccacggacgtttccgaacacggaggcgtttccagaaccacccgtcgggcccacaccgacgtggcattccttcttgaccggttcaccaaggttgggaccaaggatcgcaaaatggttttggacctgttgaccaccaacgagaaatcactggtcggcgcactcctgcgtgcggccacgtactatttctccgacctggaagtggcagtcgggaccaacaaatgggtgggatgggctcccaatggtagcccggtgtttagtgaagtgggc---gacaatccagttgtcttctcacgcaacgacaccacccgtttcgcactcccttacacggcgccccacagggtgctcgccacaacctacaacggtgactgcaagtacaagcccactggcactgagaacatccgcggcgacctcgcaaccttggccgcgcgaattgccagccacatcccaacaacattcaactacgggatgatatacacagaggcagaggtggac

>AY593841.1_SAT1_ZIM_1958

agctcggtgggcgttacttacgggtacgccctggctgacaggtttctccctggcccgaacactaacgggctggagacacgagtggaacaagcagaaaggtttttcaaacaaaagctctttgattggacaactggacaggagtttggtacaacacacgtgctggaactccccacggaccacaagggtatctatggacaactggttgactcacacgcgtacatccgcaacggatgggacgtccaggtctcagccactgccacccagttcaacggaggctgtctactggtagccatggtacccgagctctgcaaactggacaacagggagaagtaccaactcacacttttcccgcatcagttcttgaacccacgtaccaacaccacagcacacatccaagtaccctacctgggtgtcgacagacacgaccaagggacccgccacaaggcatggactcttgttgtcatggtgttggcaccatacaccaatgacaccattggatcaacaaaagcagaggtttacgtgaacattgcaccaaccaacgtttacgtagccggtgagaaacccgcaaaacaggggattctccccgtggctgtctctgacggctacggcggcttccaaaacactgatcccaaaacctcggaccccatatacgggcacgtgtacaatccggcacgcacgctctaccccggtaggttcaccaacctgctggatgtggcagaagcctgccccacactgctcgatttcaac---ggggttccatacgtccaaacccagaacaactctgcaaaagttctcgcatgttttgatttggcctttggacacaagaacatgaaaaacacatacatgtctggtctggcccagtactttgcacagtacagtggcactctcaaccttcatttcatgtacacaggccccaccaacaacaaggccaagtacatggtggcatacatcccacctggcacacacctccctgaaacaccggagatggcgtcacactgctatcatgccgagtgggacactggattgaactcaacattcaccttcaccgtgccgtacatctcggcagcggactacgcctacacctatgctgacgagcccgaacaggcctcagtacaaggttgggttggtgtgtatcagatcactgacacacacgagaaggacggggctgtcattgttaccgtcagtgcaggccccgactttgagttcagaatgcccatcagtccatcgcgccaaacaacctccgcaggtgaaggcgcggacccagtcaccactgacacgtccgaacacggaggtgcccccagaaccgcccgtcgggttcacactgatgtggcgttccttctcgaccggttcactctggttgggaccgagggcaacaaattagtgttggacctgttgaccacgaaggagaaatcactggtcggcgcactcctgcgtgcggccacgtactacttctccgacatggaagtggcggtcgggactaacaaatgggtgggctgggctcccaacggcagcccggtgctgagtgaagtgggc---gacaatccagtcgtcttctcacacaacaacaccacccgctttgcaatcccctacacagcgccccaccgggtgctcgccacagtctacaatggtgactgtaagtacaaacccactaacgctgagaacattcgcggagacctcgccaccctggctgagcggattgctagccacatcccaacaacattcaattacgggataatttacacagaggcagaagtggac

>AY593842.1_SAT1_SAR_1961

agctcggtgggcgtcacctacgggtatgctctggccgataagttcctccctggcccaaacaccaacgggctggagacgagagtggaacaagcagagaggttcttcaaacacaagctttttgattggacacttgaacagaaatttggcacaacctacgttctggaacttcccacagatcataagggtatctacggacaactggttgactcacatgcgtacatccgcaacggatgggacgtccaggtctcagccactgccacccagttcaacggaggctgcctgttggtagccatggtgcctgagctttgcaaattggccgacagggagaagtaccaacttactcttttcccacaccagttcctgaacccacgcaccaacaccacagcacacatccaggtaccgtacttgggtgttgacagacacgaccaggggactcgccacaaggcgtggactctcgtcgtgatggtgctggcaccatacaccaacgacaccattggatcaacaaaagctgaggtctacgtgaacattgcaccaaccaacgtttacgtggccggtgagaaacccgccaagcaagggattctccccgtggccgtttccgacggttacggtggcttccaaaacactgaccccaagacatcggaccccatttacgggcacgtgtacaacccggcacgcacgctctaccccggcaggttcaccaacctgttggacgtagcagaagcctgccccacactgcttgatttcaac---ggggtcccatatgtccaaacccagaacaactctgcaaaagttctcacacgtttcgatttggcttttgggcataaaaccatgaagaatacatacatgtctggtctggcccagtacttcgcacagtacagtggcactctcaatctgcatttcatgtacactggccccaccaacaacaaggccaagtacatggtggcgtacatcccacctggcacacacctccccgacacaccggagatggcgtcacactgctaccacgctgagtgggatactggactgaactcaacattcaccttcaccgtgccgtacatttcggcagcggactacgcctacacctacgctgacgagcccgaacaggcttcagtgcaaggttgggttggtgtgtatcagatcactgacacacatgaaaaggacggagccgtcatcgtcaccgtgagtgcaggccccgactttgagttcaggatgcccatcagtccatcgcgccagacaacctctgcaggtgaaggcgcggacccagtcaccacagacacgtccgaacacggaggtgcctccagagtcgcccgtcgggcccacaccgacgtggcgttccttcttgaccggttcactctggtcgggaccaagaacaaccaattggttctggacctcttggacaccaaagagaaatcactggtcggcgcactcctgcgcgcggccacgtactacttctctgacttggaagtggcagttgggaccaatacatgggtgggctggacgcccaatggtagtccagtgatgagcgaagtgggc---gacaacccagtcgtcttctcgcacaacggcaccactcgttttgcactcccttacactgcaccccaccgggtgcttgccacagtctacaatggtgactgcaagtacaaacccactggcaccgaaaacatccgcggtgacctcgcaactcttgctgcgcggattgctagccacatcccgacgacatttaactacgggatgatttacacacaggcagaggtggac

>AY593843.1_SAT1_NMB_1940

agctcggtgggcgtcacctacgggtatgccctgatcgacaagtttctccccggtccaaacaccaacgggctggagacaagagtggaacaagcagagaggttcttcaaacacaaactttttgattggacacttgaccaacagtttggcacaacctacgtgctggaattgcccacagaccacaagggtatctacggacagctggttgattcacacgcgtacatccgcaacggatgggacgtccaggtctcagccaccgccacccagttcaacggaggctgcctgttggtagccatggtacccgagctctgcaaattggacaccagggagaagtaccaactcactcttttcccgcaccagttcttgaacccacgcactaacaccacagcacacatccaggtaccctacctgggtgttgacagacacgatcaggggactcgccacaaggcgtggaccctcgtcgtgatggtgctggcaccatacaccaacgacaccattggatcaacaaaagccgaggtctacgtgaacattgcaccaaccaacgtttacgtggccggtgagagacccgtcaaacaagggattctccccgtggccgtttccgacggctacggtggcttccaaaacaccgatcccaagacctcggaccccatttacgggcacgtgtacaacccggcacgcacgctctaccccggcaggttcaccaacttgctggacgtggcagaagcttgtcccacgctgcttgatttcaat---ggggtaccgtatgtccagacccagagcaactctgcaaaagtactcgcatgttttgatttggcattcggacacaaaaacatgaaaaacacatacatgtctggtctggcccagtacttcgcacagtacagtggcaccctcaatcttcacttcatgtacactggccccaccaacaacaaggctaagtacatggtggcatacatcccacctggcacacacctccccagcacaccggagatggcgtcacactgctaccatgctgagtgggatactggactgaactcgacattcaccttcaccgtgccgtacatctcggcagcggactacgcctacacctacgctgacgagcctgaacaggcttcagtgcaaggttgggttggtgtgtaccagatcactgacacacatgagaaggacggggctgtcatcgtcaccgtgagtgcgggccctgactttgagttcaggatgcccatcagtccatgccgccagacaacctccgcaggtgaaggcgcggacccagtcaccacagatgcatccgcacacggaggtgataccagaaccgcccgtcgggcccacaccgacgtggcgttccttcttgaccggttcactctggttgggaccaaagacaacaaattggttctggacctcctgagcaccaaggagaaatcgctggtcggcgcacttctgcgtgcggccacgtactacttctccgacctggaagtggcggttgggaccaacgcttgggtaggttggactcctaacggtagtccagagctgacggaagtgggc---gacaacccagtcgtcttctcacacggaggcactactcgcttcgcgctcccttacaccgcaccccaccgggtgcttgccacagtctacaatggtgattgcaagtacaaacccactggcaccgaaaacatccgcggtgacctcgcaaccctggctgcgcggattgctagccacatcccaacaacattcaactatggaatgatttacacacaggcagaagtggac

>AY593844.1_SAT1_ISR_1962

agttctgtgggggtgacctacggttatgcagcatcagacaagtttctcccagggccaaataccaacgggcttgagacacgtgtggaacaggccgaacgctacttcaagcagaagctgttcgactgggacaccacccaaaaatttggtgtgacacacgtgttggaacttcccaccgaccacaagggtgtctacggacaacttgtggattcatacacgtacatgagaaacggttgggacgtccaggtttcagcaactgccacacagttcaacggaggatgcttgttggttgcaatggtgcctgagttgtgtaaactggacgagagagagaagtaccaactcacgctctttccacaccagtacatcaaccctcggaccaacaccactgcacacattcaggtcccgtacctgggtgtcgaccgacatgatcaagggaagagacacaaggcttggacccttgtggtgatggtcgtggctccatatacgaacgacaccatcggctcctccaaagctgaagtctatgtgaacatagctccaacaaatgtctatgtcgctggcgaacgaccagcgaaggagggcattgtcccagtagccgtggctgacggttacggaggtttccaaaacactgaccccaaaacttcagatccaatctacggtcacgtgtacaacgccccacgcacaggctatcccggtaggtacaccaacctgttggacgtagccgaggcgtgccccacgtttctcgacttcaac---ggagtcccatacgtgacaacccagaacaactctgccaaagtaatggcatgttttgatttggcatttggacacaaaaacctcaagaacaccttcctttcagggttggcccagtactacacacagtacagtggcaccttgaatctgcacttcatgtactctggcccaacaaacaacaaggccaaatacatggttgcatacatcccacctggcacacacctgcccaacacgcccgaacaggcttctcactgctaccacgcagagtgggatacaggactcaactccacctttaccttcacagtgccgtacgtgtctggggcggacttcgcctacacacacgctgatgaaccagagcaggcttcagtgcagggatgggtcggagtgtatcagatcactgacacacacgaaaaggacggtgctctcatcgtcactgtcagtgcaggccctgacctggagttccgcttgcccatcagcccctcgcgccagacaaccagcgcaggcgaaggggcagacgtggtgactgttgacgccactgtacacggtggcaaccagagacggactcgccgcgtccacacggatgtagcgttccttctagacagattcacactagtgggaacacaggacaacaggatggtacttgacatgctcaagactaaggagaaagcactggtaggcgcaatcctgcgctctgccacgtactacttcgctgatcttgaggtagcggtgggtacgaacaagtgggttggttggctgccaaatggtgcgcccgtcccaaaggaggttggc---gacaatccggtcgtcttctctcacaatggaacaacccgcttcgccttgcccttcaccgctccacaccgtgttctggcaacagtgtacaacggtggttgcaagtataaaccaaccaacgagaccaacatcaggggtgacctcgcaacactggctgaacgcatccgcgaacacattccaacaactttcaactatggaatcgttctgaccgaggcacaggtggac

>AY593845.1_SAT1_BOT_1968

agctcggtgggcgttacctacgggtacgccttgactgacaagttcctccccggcccaaacaccaacgggctggagacaagagtggaacaggcagagaggttcttcaagcacaaactttttgattggacgcttgaccaacagttcggcacaacctacgtgctggagctccctacagatcacaagggtatctacgggcagctggttgattcacacgcgtacatccgcaacggatgggacgtccaagtctcagccaccgccacccagttcaacggaggctgcctgctggtggccatggtacccgagctgtgcaaattggacgacagggagaaataccagctcacccttttcccacaccagttcttgaacccacgcaccaacaccacagcacacatccaggtaccctacttgggtgttgacaggcacgaccaggggactcgccacaaggcgtggaccctcgttgtgatggtgctggcaccatacaccaacgacaccattggatcaacaaaagccgaagtctacgtaaacattgcaccaaccaacgtttacgtggccggtgagaaacccgtcaaacaagggattctccctgtggccgtttccgacggctacggtggcttccaaaatactgaccctaagacctcggaccccgtttacggacacgtgtacaatccggcacgcacgctctaccccggcaggttcaccaacttgctggacgtagcagaagcttgccccacgctgctcgatttcaac---ggggtaccgtatgtccaaacccagagcaactctgcaaaagttctcgcatgttttgatttggcattcggacacaaaaacatgaaaaacacatacatgtctggtctggcccagtactttgcacagtacagtggcactctcaaccttcatttcatgtacactggccccaccaacaacaaggccaagtacatggtggcatacatcccacccggcacacacctccctgaaacaccggagatggcgtcacactgctaccacgccgagtgggacactggactgaactcgactttcaccttcaccgtgccgtacatttcggcggcggattacgcctacacctacgctgacgagcccgaacaggcctcagtacaaggttgggtcggtgtgtaccagatcactgacacacatgagaaagacggggccgtcatcgtcaccgtgagcgccggtcccgactttgagttcaggatgcccatcagtccatcgcgccagacaacctctgcaggcgaaggcgcggacccagtcaccacagacgcatccgcacacggaggtgacaccagaaccacccgtcgggcccacaccgacgtgacgttccttcttgaccggttcactctggttgggaccaacgacaacaaactggttttggacctcctgagcaccaaagagaaatcactggtcggcgcactcctgcgcgcggccacgtattacttctctgacctggaggtggcggttgggaccaacgcatgggtgggttggactcctaacggtagtccagtgctgacggaagtgggc---gacaacccagtcgtcttctcacgtagaggcaccactcgtttcgcgctcccttacaccgcaccccaccgggtgctcgccacagtctacaacggtgactgtaagtacaaacccactggcaccgaaaacatccgcggcgacctcgcaaccctggcagcgcggattgctagccacatcccaacaacattcaactacgggatgatttacacacaggcagaagtggac

>AY593846.1_SAT1_ZIM_1966

agctcagtgggcgtcacccgcgggtacgccgactcagacaaattcctacccggcccaaacaccaacgggttggaaacacgtgtggaacaagccgaaagatttttcaaacacaaattatttgattggacaaccgaccaacaatttggggtggcccacatcttggagctgcccacagatcacaaaggcatctacggccaacttgtggactcccactcttacatccgtaacgggtgggatgtcgaggtctccgcgaccgcgacgcagtttaacggaggctgcctgctagtggcaatggtgcccgagttgtgcaaacttgaggacagagagaaataccaactcactctcttcccacaccagttcctaaatccaaggaccaacaccacggcacacatccaggtaccctacttgggtgtggatcgccacgaccagggaatgcgccacaaggcgtggactttggttgtgatggtggtggcaccttacaccaatgacacaattggttcaaataaagccgaggtgtacgtgaacatcgctcccacgaacgtttacgtcgccggcgagaaacccgcaaaacagggcattctccccgtagctgtctcccacggttacggtggcttccaaaacacagaccccaaaacctcggatcccgtgtacgggcacgtgtacaatccagcacgcactggcctgccaggaaggttctcgaacctcctggacgtggctgaagcatgccccacactgctcgatttcagc---ggacgtccgtacgtgatcacccaggccaactccgcaaaagtgctcacactctttgatttggcttttggacacaaaaatctgaaaaacaccttcatgtctggtctcgcccagtactacacacagtacagtggcaccctcaacttgcacttcatgtacacaggcccaacaaacaacaaggcaaagtacatggtggcctacatcccgccagggacacacctcccggaaacaccggagatggcgtcccattgctaccatgccgagtgggacacaggcctaaattcaactttcaccttcaccgtaccgtacgtgtcggccgccgatttcgcgtacacctactctgacgagcctgaacaggcttcagtgcagggttgggtgggcgtgtaccaggtaactgacacacacgagaaggatggagcagtggtggtatctgtcagcgctggacccgacttcgagttcagaatgcccatcagcccctcacgccagactacgtctgctggtgagggtgcagagcccgtcacgactgacgcttcccaacacggtggcgacagacgtacggcacgcaggtaccataccgacgtgagttttctgctcgaccggttcacactggttggtacacagaactgcaggttgacactggatttgctccaaactaaggagaaagcactagtcggcgcaatcctgcgcgcggccacgtactacttctctgatttggaagtcgcagtcggcaccaacgagtgggttggatggactcctaacggagcgccggaattgagtgaagtgggc---gacaaccccgtcgtcttctccaccaataacacaacccgctttgcgctgccctacactgcgccacacagatgtctggcaacatcctacaacggtgactgcaagtacaagcccgctggcacagagaacatccgcggtgacctcgcaactcttgcggggaggattgcaagccacatccctaccacctttaactatggcaggatatacacacaagccgatgttgac

>AY593847.1_SAT2_ZIM_1948

agctcggtcggcattacctacgggtatgccgatgccgactccttcagacctggtccgaacacatccggactggaaacgagggttgaacaggcggaacggtttttcaaggaaaaattgtttgattggacttctgacaaaccatttggcacacttcacgtgttggaacttcccaaagaccataaggggatctacggcagcttgaccgacgcgtatgcttacatgcgcaacggttgggatgtccaggtcactgccactagcacccaattcaacggtggctccctgttggtggccatggtgcctgaactctgctctctcaaagaacgagaagagttccagctcaccctctacccacaccagttcatcaacccaaggactaacacaactgcccacatccaggtgccctatctgggtgtcaacagacacgaccaaggcaagcgccaccaagcgtggtccctggttgtgatggtcctgacgcctctcaccacagagcagatgaactccgggactgttgaggtgtacgccaacatcgctccaacgaacgtgttcgttgcgggtgaaatgccagccaagcagggcatcatacctgttgcctgtgcagacggctatggcgggttccaaaacacagacccgaagaccgcagaccccatctacggttatgtctacaacccgtcgcggaatgactgtcacggcaggtactccaacctgttggacgtcgctgaggcgtgccccacattgctgaactttgac---gggaaaccctatgtcgtcaccaagaacaacggtgacaaagtcatggcctgttttgatgtagcattcacacacaaggcacacaagaacacctttcttgcgggcctcgctgattactacactcagtatcaaggatcattgaactaccacttcatgtacacagggccaacccatcacaaggctaagttcatggtggcgtacatcccacctggtgttgaactgccgaaaacacccgaggacgcagcacactgctatcactcggagtgggacaccggattgaactcacagttcacgttcgctgttccatacgtctcggccagtgacttctcatacacccacacggacacgccggcgatggctaccaccaatgggtggatagcggtctaccaagtgaccgatacccactcagcggaggcggccgttgtggtgtccgtgagtgctggacccgacctggaattccggttcccgatcgaccctgttcggcaaaccaccagcgtaggcgagggcgcggacgttgtcacgaccgacccttccacacacggtggcagtgtaattgagaagaaacgaatgcacacagatgttgcgttcgtgcttgacagattcacacacgtgcacacttcaaaaaccacgtttaacgtggatctgatggacaccaaggaaaagactctagtaggtgccctgctgagggcttccacctactacttctgtgacctggaaattgcggttggcgaacacagccgtgtcttctggcagcctaacggcgcgccccggacaacacaactggga---gacaacccaatggttttctcacacaatggagtgacacggttcgccatcccttacaccgcaccacaccggttgcttgcaaccaggtacaacggcaagtgtaagtacacacaggaggcgaaa---gccatccgtggtgatcgggcagtgctggcggccaagtacgccggcgcgctcccttccaccttcaactttggccacgtcaccgccgacaaggcagtggat

>AY593848.1_SAT2_u_1967

agctcagttggtatcacctacgggtatgccgacgccgactccttcaggcccggacccaacacgtctggacttgaaacaagggttcagcaggctgagcgcttcttcaaggagaaactttttgactggacatcggaaaaaccatttgggactttgcatgtgctggaactccccaaggaccacaaaggcatctacggcagcctgaccgacgcgtacacatacatgcgcaacggatgggatgtccaagtgaccgccactagcactcaattcaacggcggttcgcttcttgtggccatggtaccagaactctgttcactacgggacagggaagaattccagctctccctgtacccgcaccagttcatcaaccctaggaccaacaccacggcacacatccaggtgccttacctgggtgtcaaccgacacgaccaaggcaaacgccaccaagcgtggtccttggttgtgatggtcctcactcctctcacaacggagcagatgaactctgggactgttgaggtctacgccaacatcgccccgacgaacgtgttcgttgcaggcgaaaaaccggccaaacaaggcatcataccagttgcctgttctgacggttacggcggattccagaacacagatcccaaaaccgcagacccgatctacggctatgtctacaacccgtctcggaacgactgtcacggcaggtactccaacctgttggacgtcgctgaggcgtgcccaaccttcttgaactttgac---gggaaaccgtatgttgtcaccaagaacaacggtgacaaggtgatgacctactttgacgtggcattcacacacaaggtccacaagaacacctttttggccggactcgctgactactacacacagtaccaggggtccttgaattaccacttcatgtacacaggtcccactcaccacaaagcaaagttcatggtggcgtacatcccgccaggcattgaactgcccaagacccccgaggacgcagcacactgctaccactcggaatgggacacaggcctgaactcccagttcacgtttgccgtcccgtacgtctcagccagtgacttctcctacacccacactgacacacccgcaatggccacaaccaacgggtgggtggcagtctaccaagtaactgacacccattcggcagaagcggcagttgtcgtttctgtgagcgccgggcccgatttggaattcaggttcccgatcgacccagtgcgccagactaccagtgccggagagggtgctgaagttgtgaccactgacccctcaacccacggagggcaagtcaaggagaagagacggatgcacacagacgttgcattcgtgctcgacaggttcacgcacgtccacacaaacaagacgactttcaacgtggatctcatggacaccaaggaaaagaccttagtaggtgccctcctgagggcatccacctactacttctgtgacctggaaattgccgtgggcacgcacaagcgcgtgtactggcaacccaacggcgcacccagaaccacccagctcggg---gacaaccccatggttttctcgaacaatggtgtcactcggttcgccgtgccatacactgcgccacaccgtctgttgtcaactgtttacaacggtgagtgcgagtacggaacaaccgttacc---gccattcgcggtgaccgcgctgtgttagcggccaaatacgcaaacacgctcccttccacgttcaactttggacatgttaccgcagaccaaccagttgac

>AY593849.1_SAT2_KEN_1960

agttctgttgggatcacctacgggtatgccgacagtgactctttcaggcctggccccaatacctcgggcctcgagactcgtgtggaacaggctgaacgcttcttcaaggagaagttgttcgactggactagtgacaaacccttcggaacgctgtacgtgttggagttgcccaaagatcacaagggtatttacggtaaactcaccgactcctacacgtacatgcgcaacgggtgggacgtccaggtcagcgccaccagcactcaattcaatggaggatgtttgttggtggcaatggtcccggaactgtgcagtttgaaagcccgagaggagtaccagctcacactctacccacaccagttcatcaatcctaggaccaacactactgctcacctccaggtgccatacttgggcgtgaacagacatgaccagggtaaacgacaccaaacctggtccttggtagtgatggtcctgacgcctctgactactgagcagatgaatagcggaaccgtcgaggtatacgctaatattgcaccaaccaacgtgcatgtggcaggcgagctccctgggaaacagggaatcgtacctgttgcgtgtgctgacggttatggtggtttccaaaacactgaccccaagtccgcagatccaatctacggacatgtgtacaacccctcaaggaacgactgccacggtaggttttctaacctgctggacgtcgcagaggcgtgtcccacactcctcgacttcgat---gggaagccatacgtggtgaccaaaaacagcggggacaaagtcatggcagcctttgacgtggcttttacccacaaggtacacaaaaacacgtacctggctgggcttgcccagtactacacacagtactccggcagtcttaactaccacttcatgtacacgggcccaacgcaccacaaagcaaagttcatggtcgcgtacgtaccaccgggcattgagctacccaaaacacctgaggatgcagcacactgttaccattctgaatgggacacaggtctgaactcgagctacacgttcgcggtaccatacctgtcgtccggtgacttctcctacacgcacaccgacaccccagccatggccacaaccaacggctgggtggtggtgttgcaagtgactgacacccactctgcggaggcggcggtagtcgtctcggtaagtgcgggtccagacctggagttccggtttcccattgacccggtgagacaaacaacatccgccggtgagggagctgaagtggtcaccactgacccaaccactcacggtgggaaggtcacgacaccccgccgggtccataccgatgtggcgtttctccttgaccgcagcacacatgtgcacacgaacaagaccacctttgcggtggacctgatggacaccaaggagaaggcgctagtgggagcaatcctgcgctcagccacatactacttttgtgacctggaaatcacagtgggcgaacacaagcgcgtgttttggcagcctaacggtgcgccacgcacaacccagttgggc---gacaacccaatggtgttctcccacaacaaagtgacccgctttgcaatcccgttcacagcgcctcacaggttgttgtccacggtctacaacggggagtgcgagtacacaaaaacagtcacc---gcaattcgcggtgatagagaggtgttggcgcggaaatactcttcctctctgccatccaccttcaacttcgggtttgtgaccgccgacgagccagtcgac

>AY593850.1_SAT3_SAR_1959

agttctgttggcgtgacatacggttacgcctcggcggaccgtttcttgcctggacccaacaccagtgggctcgagacacgcgtcgaacaagcggagagattcttcaaggagaaactcttcacttggaccgctagtcaagagtacgcacatgtgcatctgttggaactgccggtggaccacaagggcatctacggtgccatcctggacggccacgcatacgtgcgcaatggctgggacgtgcaggtttcggcaaccagcacgcagttcaacggcggcactcttcttgtcgccatggtccccgagctacaagagttggagaagcgtgacgtttcacaactcacgctctttcctcaccagttcatcaacccgcgcaccaacaccaccgcccacattgtggtgccatacgtgggtgtcaacagacacgaccaggcaaagatgcacaaggcgtggacactcgtggtggcggtgctcgcaccgctaaccact---aacatgggacaggacaacgtcgaggtgtatgcaaacatcgcacccaccaacgtgcacgttgctggagagaggccaatgaaacaaggcatcattcccgtggcctgcaacgatggctacggtggcttccagaacaccgatcccaagaccgcagatccaatctacggtctagtgtccaacccaccgcgcacagcgtttcccggtaggttcactaacctgctagacgtcgctgaagcgtgtccaacattcctggactttgac---ggcacaccttacgtcaaaaccaggcacaacagtggcaagatactagcacacattgacttggcttttgggcacaagtgcttcaagagcacttacttggcaggactcgcacagtactacgcccagtacagtggatctttgaatctacacttcatgtacaccggtcccacgcaatcgaaagctcgctttatggttgcatacatcccaccagggaccgtagtgcccaatacccctgagggtgcagcacactgctatcactcagagtgggacactggactgaactccaaattcacgttcacagtcccgtacgtgtcagcagcagactttgcctatacatattgtgatgaacccgaacaggcttcagcacaaggatgggtgacactgtaccaaatcacagacacgcacgaccctgactcggcggtgcttgtctcggtcagcgctggcgctgatcttgaatttcggctcccgattaaccctgcgacccaaacaactagtgcaggtgagggtggtgacgttgtgacaaccgatgtcacgacacatggcggagttgtggacacaccgcgacgccaacacaccaacgtggagttcctgctggacaggttcacacacattggtacgatcaccgcctcaaagacaatcgacctcatggacacgaaggaacacacattggtgggcgcaatcctgcgctcggctacgtactacttctgtgacctggaggttgctttgggctcggaaaaatggactgcgtgggttcccaacggttgcccacacaccgaacgcgtggag---gacaacccagttgttcacgcgaagaacggtgtagcccgttttgccctgccatacacagcaccacacagggttcttgctacagtgtacaatggtaactgcaaatactccaaaaccgttgta---gcacgccgtggcgacatggcagtgttggcacaacgcgttgagagatgcaggcccacaacattcaacttcgggagactgttgtgtgaa---acaggtgag

>AY593851.1_SAT3_BOT_1961

agttctgttggagtgacttacggttatgtctcggccgaccgtttcctccctgggcctaacacgagcgggctcgagtcacgcgttgaacaggcagagagatttttcaaggagagattgttcacctggaccgcgtcacaggagtatgctcatgtccacctgttggagctgcccaccgaccacaaaggcatctacggcgtcatggttgacagccacgcgtatgtgcgcaacggttgggacgtgcaggtcaccgcgaccagcacgcagttcaatggaggaacactcctagtggcaatggtgcctgagctccactcgatggacacacgtgatgtgtcacagcttacgctcttcccacaccaatttattaacccccgcactaacaccacggctcacattgttgtgccgtacgtgggggttaacagacacgaccaggtgcaaatgcacaaagcttggactttggtcgtggcggtgatggcaccactcaccacc---agcatgggtcaggacaacgttgaggtttacgctaacatcgcgcccaccaacgtttacgtggctggggaacggccctccaagcaagggatcatccccgttgcgtgcaacgacgggtatggaggattccagaacactgacccaaagaccgcagaccccatctacggtcttgtgtccaacccaccgcgcacggctttcccgggcaggttcactaatctgctggatgtggccgaagcgtgtcccactttcctggacttcgac---ggggtgccttacgtcaagaccacgcacaacagtggtaaaattctcacccacattgatttggcttttggacacaaatctttcaagaacacgtaccttgctgggctggctcaatactacgcccagtacagtggctccatcaacctgcacttcatgtacaccgggccaacgcagtcaaaagctcgcttcatggttgcgtacattccaccgggcactacagtcccaaacacacctgagcaggcggcacactgctaccactcggaatgggacacaggtctgaactccaagttcacctttacggtgccgtacatgtcagcagcagattttgcatacacctactgtgatgagcctgaacaagcatcagcacaaggttgggtgactctgtaccaaattacagacacacatgaccccaattcggccgtacttgtctcagtcagtgctggcgctgactttgagctcaggttgccaataaaccccaccgcccagacaactagtgcgggtgagggagccgatgttgtcaccactgacgtcaccacacacggtggtgaggtgagtgtgccacgacgtcagcacaccaacgtggagtttctgctggacagattcacacatattggcacaatcaatggacacagaacaatttgcctcctggacacgaaggaacacacgctggtgggagcaatcctgcgttctgccacgtattacttttgtgacctggaagttgctttgggtaacgccaagtacgcggcctgggtacccaacgggtgcccgcacaccgacagagtggaa---gacaatccagtcgttcactcaaaaggttcggttgtccgctttgcgctgccctacaccgcgccacacggtgttttggcaactgtctacaacggcaactgcaagtacagcacaaccgtggct---ccgcgccggggcgatctgggcgcgttgagccgacgagttgagaaatgcatccccacaacgttcaacttcgggagactgttgtgtgaa---tcaggtgat

>AY593852.1_SAT3_KEN_1960

agttctgttggagtgacttacggttatgtctcggccgaccgtttcctccctgggcctaacacgagcgggctcgagtcacgcgttgaacaggcagagagatttttcaaggagagattgttcacctggaccgcgtcacaggagtatgctcatgtccacctgttggagctgcccaccgaccacaaaggcatctacggcgccatggttgacagccacgcgtatgtgcgcaacggttgggacgtgcaggtcaccgcgaccagcacgcagttcaatggaggaacactcctagtggcaatggtgcctgagctccactcgatagacacacgtgatgtgtcacagcttacgctcttcccacaccaatttattaacccccgcactaacaccacggctcacattgttgtgccgtacgtgggggttaacagacacgaccaggtgcaaatgcacaaagcttggactttggtcgtggcggtgatggcaccactcaccacc---agcatgggtcaggacaacgttgaggtttacgctaacatcgcgcccaccaacgtttacgtggctggggaacggccctccaagcaagggatcatccccgttgcgtgcaacgacgggtatggaggattccagaacactgacccaaagaccgcagaccccatctacggtcttgtgtccaacccaccgcgcacggctttcccgggcaggttcactaatctgctggatgtggccgaagcgtgtcccactttcctggacttcgac---ggggtgccttacgtcaagaccacgcacaacagtggtaaaattctcacccacattgatttggcttttggacacaaatctttcaagaacacgtaccttgctgggctggctcaatactacgcccagtacagtggctccatcaacctgcacttcatgtacaccgggccaacgcagtcaaaagctcgcttcatggttgcgtacattccaccgggcactacagtcccaaacacacctgagcaggcggcacactgctaccactcggaatgggacacaggtctgaactccaagttcacctttacggtgccgtacatgtcagcagcagattttgcatacacctactgtgatgagcctgaacaagcatcagcacaaggttgggtgactctgtaccaaattacagacacacatgaccccaattcggccgtacttgtctcagtcagtgctggcgctgactttgagctcaggttgccaataaaccccaccgcccagacaactagtgcgggtgagggagccgatgttgtcaccactgacgtcaccacacacggtggtgaggtgagtgtgccacgacgtcagcacaccaacgtggagtttctgctggacagattcacacatattggcacaatcaatggacacagaacaatttgcctcctggacacgaaggaacacacgctggtgggagcaatcctgcgttctgccacgtattacttttgtgacctggaagttgctttgggtaacgccaagtacgcggcctgggtacccaacgggtgcccgcacaccgacagagtggaa---gacaatccagtcgttcactcaaaaggttcggttgtccgctttgcgctgccctacaccgcgccacacggtgttttggcaactgtctacaacggcaactgcaagtacagcacaaccgtggct---ccgcgccggggcgacctgggcgcgttgagccgacgagttgagaaatgcatccccacaacgttcaacttcgggagactgttgtgtgaa---tcaggtgat

>AY593853.1_SAT3_BOT_1965

agttctgtcggagtgacctacggttacgcttcggctgaccgtttcctccccgggcccaacacgagcgggcttgagtcacgcgtcgaacaggcagagagattcttcaaggagaagctgttcacctggaccgcgtcacaagagttcgcccacgtccacctgttggagttgcccaccgaccacaaaggtatctacggtgccatggtcgagagccacgcatacgtgcgcaatggctgggacgtgcaggtctccgcgaccagcacacagttcaatggaggaacactcctggtggcaatggtgcccgagctccactcgctcgacaagcgcgatgtgtcgcagctcacacttttcccacaccagtacatcaacccccgcaccaacaccacagctcacattgttgtgccgtacgtgggggttaacagacacgaccaagtgcagatgcacaaggcctggacactggtcgtggcagtgatggcaccgctcaccact---aacatgggtcaagacaacgttgaggtctacgctaacatcgcgcccaccaacgtttacgtggccggagaacggccctcaaaacaaggaatcatccccgtcgcgtgcaacgacgggtacggagggttccagaacactgacccaaagaccgcagatcccatctacggtctcgtgtccaacgcaccacgcacagcctttcccggcaggttcaccaacttgttggacgtggctgaggcgtgtcccactttcctggactttgac---ggaacaccctacgtcaagaccaggcacaatagtggcaaaattctcgcccacattgatctggcttttgggcacaaatcatttaagaacacataccttgctgggcttgcacaatactacgcccagtatagtggctccatcaatctgcatttcatgtacaccgggccaacgcaatcaaaagctcgctttatggttgcgtatatcccgccaggcacctcagtcccagatacacctgaaaaagcagcacactgctaccactcagaatgggatactggtttgaactccaagttcactttcactgtgccatacatgtcagcagcagactttgcatacacctactgtgatgagcctgaacaagcatcagcacagggatgggtcactctgtaccaaatcacagacacacacgaccctgattcagctgttctcgtctcggtcagtgctggtgctgatttcgagctcaggttgccgataaaccccgccacccagacaactagcgcgggtgagggagccgacgtcgtcaccactgacgtcaccacccatggtggtgaggtgagtgttccaagacgtcagcacaccaacgtggagtttctgctggacagattcacacacgtcggcaaagtcaacgagagcagaaccatttccctgatggacacgaaggaacacacgctggtgggagcaatcctgcgttccgccacgtattacttttgtgacctggaagttgccttgggcaccgcgccgtgggcggcctgggtgcccaacgggtgcccacacaccggtagagtggag---gacaacccagttgttcactcaaaaggatcggtcgtccgcttcgcgttgccctacacagcgccacatggcgttttggcaactgtttacaatggcaactgtaagtacagtgaaaccgtgact---tcacgccgaggcgatctggccgtgttggcgcaacgcgttgagaaatgtttgcccacgacattcaacttcggaagattgttgtgtgaa---gagggtgat

>AY686687.1_O_CHA_2001

tctagcgtcggggtgacgtacgggtacgcaacggctgaagacttcgtgagtgggcctaacacctccggtcttgagaccagagtcgttcaggccgaacggttcttcaaaacccacctgttcgactgggtcaccagtgacccgtttggacggtgtcacttgttggagctaccgaccgaccacaaaggtgtctacggtagcctgaccgactcttacgcgtacatgaggaatggttgggacgttgaagtcaccgcagtggggaaccagttcaacggaggctgtttgctggtagcgatggtaccggagctctgttctatcaacaagagagagttgtaccaactcacgcttttcccccaccagttcatcaacccacggacgaacatgacggcacacatcaccgtgccctaccccggtgttaacaggtacgaccagtacaaggtacacaaaccctggaccctcgtggtcatggttgtggctcccctgacggttaacaacgagggcgctccgcaaatcaaggtgtatgccaacatcgcccccaccaacgttcacgtcgcaggcgagctcccttccaaagaggggatcttccccgtggcatgcagcgatggttacggtggcttggtgaccacggatccgaagacggcagaccccgtctacgggaaagtgttcaacccaccccgtaacctgttgccagggcggttcacgaacctcctcgacgtggccgaggcgtgccctacgttcctacacttcgacggcgacgttccgtacgtgaccacaaagacggactcagacagggtgttggcccagttcgacttgtccctcgcggcgaaacacatgtcgaacacctttctcgcgggtcttgcccagtactacacacagtacagtggcaccattaacctacacttcatgttcacggggcccaccgacgcgaaggcacgctacatggttgcgtatgcccctcctggcatggaaccgccgaaaacgcctgaggcggctgcacactgtatccacgctgagtgggacacagggttgaattcgaagttcacgttttcaatcccatacctttcggcagccgactacgcgtacaccgcgtccgatgtcgccgagaccactaacgttcagggatgggtctgtctgttccagataacacacgggaaagcagacggtgatgctctggtcgtgctggctagtgccggcaaagactttgacttgcgcctgccggttgacgcccggacccaaaccacctctgcgggtgagtccgcggaccccgtgactgccaccgttgagaactacggtggcgagacacaagcccagagacgccaacacacggacatttcgttcatactagacaggttcgtgaaagtcaagccaaaggaacaagtcaacgtgttggacctgatgcagatccctgcccacaccttggtgggggcgctcctgcgaacggccacctactacttctctgacctggaactggctgtcaagcacgagggcgatctcacctgggtcccaaacggtgcccctgagacagcactggacaacactaccaacccaacagcttaccacaaggaaccgctcacacggctggcactgccttacacggctccacaccgtgtcttggcgaccgtctacaacgggagcagtaagtacggtgacaccagcactaacaacgtgagaggtgaccttcaagtgttggctcagaagacggaaagaactctgcctacttccttcaacttcggtgccattaaggctactcgtgtgactgaa

>AY687333.1_Asia1_IND_2001

tcgagcgttggcgtgacatacggttacgctgtggctgaggacgcggtgtcagggcctaacacctcaggcctggagacccgcgttcaacaagcggaacggttcttcaaaaagcacttgtttgactggacaacaaatttggcatttggacactgtcactacttggaactacccactgaacacaagggcgtgtacggcagtctcatggactcgtacgcctacatgcgaaatgggtgggacatagaggtgactgctgttggaaaccagtttaacggcggctgcctccttgtcgcacttgtgccagagctgaagagccttgacacgcggcagaagtaccaactgacccttttcccccatcagttcatcaacccacgcaccaacatgacggctcacattaacgtgccttttgtgggtgttaacaggtatgaccagtacgtgctccacaaaccatggacgctcgttgtgatggtggtggccccactcaccgtcaagactggtggctctgaacagatcaaggtctacatgaatgcagcgccgacctacgtgcatgtggcaggggagctgccctcgaaagagggaatagttcccgtcgcgtgcgcggacggttatggcaacatggtgaccacagacccgaaaactgctgacccagtgtacgggaaagtgttcaatcctccccggacgaatcttcccgggcgcttcacgaacttccttgatgtcgcggaggcgtgtccaaccttcctccgcttcggagaa---gtaccatttgtgaagacagtgaactctggtgaccgtctgctagccaagtttgacgtttcgctcgctgcgggacacatggctaacacctacttggccggtttggcacagtactacacacagtacagtggcaccatgaatgttcacttcatgtttaccgggcccacagatgctaaggctcggtacatggtggcctacattccccctggcatgacaccgcccacggaccctgagcgcgccgcgcactgcatccactctgagtgggatactggtcttaactccaagttcaccttttccataccttacctctctgctgctgactacgcatacaccgcttctgacacagcggagactacaagtgtgcaaggatgggtgtgcatctaccagatcacccacggcaaagctgaaggagacgcactggtcgtttctgtcagcgccggcaaagactttgagtttcgcctgcccgttgacgcgcgccggcaaaccaccgctaccggcgagtcagcggacccggtaacaaccacggtcgagaactacggaggagagactcaggtagccagacggcttcacactgatgttgcctccgttctggacaggtttgtgaaactcactgcacccaaaaacactcaggttcttgacctcatgcagatcccctcacacacgctggttggagcactgcttcggtctgcgacgtactacttctcagacctggaggttgcgcttgtccacacaggcccagtcacctgggtgcccaacggctcacccaaggatgctctagacaaccagaccaatccaactgcctaccagaagcagcccatcacccgcctggcgctcccctacaccgctccccaccgtgtgctggcgacagtatacaacgggaagacgacgtacggggaaacaactcca------cgccgtggcgacatggcggcccttgcacaaagactgagcgggcagctacccacttctttcaactacggcgctgtaaaggctgataccatcactgag

>DQ404158.1_O_UKG_2001

tcgagcgttggagtcacttacgggtacgcaacagctgaggactttgtgagcggaccaaacacatctgggcttgagaccagggttgtgcaggcagagcggttcttcaaaacccacttgttcgactgggtcaccagtgacccgtttggacggtgctatctgctggaactcccaactgaccacaaaggtgtctacggcagcctgaccgactcttatgcttacatgagaaacggctgggatgttgaggtcaccgcagtgggaaatcagttcaacggaggatgtctgttggtggccatggtgccagaactttgctctattgacaagagagagctgtaccagctcacgctctttccccaccagttcatcaacccccggacgaacatgacggcgcacatcactgtgccctttgttggcgtcaaccgctacgaccagtacaaggtacacaaaccttggaccctcgtggttatggttgtggccccgctgactgtcaacaccgaaggtgccccacagatcaaggtctatgccaacatcgcccctaccaacgtgcacgttgcgggtgagttcccttctaaggaagggatcttccccgtggcatgtagcgacggttacggtggtctggtgaccactgacccaaagacggctgaccccgcctacgggaaagtgtttaatccacctcgcaacatgttgccggggcggttcaccaacttccttgatgtggctgaggcgtgccccacgtttctgcactttgagggtggcgtgccgtacgtgaccacaaagacggactcagacagggtgctcgcccagtttgacttgtctctggcagcaaagcacatgtcaaacaccttcctggcaggtctcgcccagtactacacacagtacagcggcaccatcaacctgcacttcatgttcacaggacccactgacgcgaaagcgcgttacatgattgcatacgccccccccggcatggagccgcccaaaacacctgaggcggccgcccactgcattcatgcggagtgggacacagggttgaattcaaaattcacatttccaatcccttacctctcggcggctgattacgcgtacaccgcgtctgacgctgcggagaccacaaatgtacagggatgggtttgcctgtttcaaattacacacgggaaggctgacggcgacgcactggtcgttctagctagcgccggtaaggactttgagctgcgtctgccagttgacgctcgcacgcagaccacctccgcaggtgagtcggctgaccccgtgactgccactgttgagaactacggtggtgagacacaggtccagagacgccaacacacggatgtctcgttcatactagacagatttgtgaaagcaacaccaaaagatcaaattaatgtgttggacctgatgcaaacccccgcacacactttggtaggcgcgctcctccgtactgccacctactacttcgcagatctagaagtggcagtgaaacacgaggggaaccttacttgggtcccgaatggggcgcccgagacagcgttggacaacaccaccaatccaacggcttaccacaaggcaccgctcacccggcttgcactgccttacacggcaccgcaccgtgtcttggctactgtttacaacgggaactgcaagtatggcgagagccccgtgaccaatgtgagaggtgacctgcaagtattggcccaaaaggcggcaagaacgctgcctacctccttcaattacggtgccatcaaagccactcgggtgactgaa

>DQ404159.1_O_UKG_2001

tcgagcgttggagtcacttacgggtacgcaacagctgaggactttgtgagcggaccaaacacatctgggcttgagaccagggttgtgcaggcagagcggttcttcaaaacccacttgttcgactgggtcaccagtgacccgtttggacggtgctatctgctggaactcccaactgaccacaaaggtgtctacggcagcctgaccgactcttatgcttacatgagaaacggctgggatgttgaggtcaccgcagtgggaaatcagttcaacggaggatgtctgttggtggccatggtgccagaactttgctctattgacaagagagagctgtaccagctcacgctctttccccaccagttcatcaacccccggacgaacatgacggcgcacatcactgtgccctttgttggcgttaaccgctacgaccagtacaaggtacacaaaccttggaccctcgtggttatggttgtggccccgctgactgtcaacaccgaaggtgccccacagatcaaggtctatgccaacatcgcccctaccaacgtgcacgttgcgggtgagttcccttctaaggaagggatcttccccgtggcatgtagcgacggttacggtggtctggtgaccactgacccaaagacggctgaccccgcctacgggaaagtgttcaatccacctcgcaacatgttgccggggcggttcaccaacttccttgatgtggctgaggcgtgccccacgtttctgcactttgagggtggcgtgccgtacgtgaccacaaagacggactcagacagggtgctcgcccagtttgacttgtctctggcagcaaagcacatgtcaaacaccttcctggcaggtctcgcccagtattacacacagtacagcggcaccatcaacctgcatttcatgttcacaggacccactgacgcgaaagcgcgttacatgattgcatacgccccccccggcatggagccgcccaaaacacctgaggcggccgcccactgcattcatgcggagtgggacacagggttgaattcaaaattcacatttccaatcccttacctctcggcggctgattacgcgtacaccgcgtctgacgctgcggagaccacaaatgtacagggatgggtttgcctgtttcaaattacacacgggaaggctgacggcgacgcactggtcgttctagctagcgccggtaaggactttgagctgcgtctgccagttgacgctcgcacgcagaccacctccgcaggtgagtcggctgaccccgtgactgccactgttgagaactacggtggtgagacacaggtccagagacgccaacacacggatgtctcgttcatattagacagatttgtgaaagcaacaccaaaagatcaaattaatgtgttggacctgatgcaaacccctgcacacactttggtaggcgcgctcctccgtactgccacctactacttcgcagatctagaagtggcagtgaaacacgaggggaaccttacttgggtcccgaatggggcgcccgagacagcgttggacaacaccaccaatccaacggcttaccacaaggcaccgctcacccggcttgcactgccttacacggcaccgcaccgtgtcttggctactgtttacaacgggaactgcaagtatggcgagagccccgtgaccaatgtgagaggtgacctgcaagtattggcccaaaaggcggcaagaacgctgcctacctccttcaattacggtgccatcaaagccactcgggtgactgaa

>DQ404160.1_O_UKG_2001

tcgagcgttggagtcacttacgggtacgcaacagctgaggactttgtgagcggaccaaacacatctgggcttgagaccagggttgtgcaggcagagcggttcttcaaaacccacttgttcgactgggtcaccagtgacccgtttggacggtgctatctgctggaactcccaactgaccacaaaggtgtctacggcagcctgaccgactcttatgcttacatgagaaacggctgggatgttgaggtcaccgcagtgggaaatcagttcaacggaggatgtctgttggtggccatggtgccagaactttgctctattgacaagagagagctgtaccagctcacgctctttccccaccagttcatcaacccccggacgaacatgacggcgcacatcactgtgccctttgttggcgtcaaccgctacgaccagtacaaggtacacaaaccttggaccctcgtggttatggttgtggccccgctgactgtcaacaccgaaggtgccccacagatcaaggtctatgccaacatcgcccctaccaacgtgcacgttgcgggtgagttcccttctaaggaagggatcttccccgtggcatgtagcgacggttacggtggtctggtgaccactgacccaaagacggctgaccccgcctacgggaaagtgttcaatccacctcgcaacatgttgccggggcggttcaccaacttccttgatgtggctgaggcgtgccccacgtttctgcacttcgagggtggcgtgccgtacgtgaccacaaagacggactcagacagggtgctcgcccagtttgacttgtctctggcagcaaagcacatgtcaaacaccttcctggcaggtctcgcccagtactacacacagtacagcggcaccatcaacctgcacttcatgttcacaggacccactgacgcgaaagcgcgttacatgattgcatacgccccccccggcatggagccgcccaaaacacctgaggcggccgcccactgcattcatgcggagtgggacacagggttgaattcaaaattcacatttccaatcccttacctctcggcggctgattacgcgtacaccgcgtctgacgctgcggagaccacaaatgtacagggatgggtttgcctgtttcaaattacacacgggaaggctgacggcgacgcactggtcgttctagctagcgccggtaaggactttgagctgcgtctgccagttgacgctcgcacgcagaccacctccgcaggtgagtcggctgaccccgtgactgccactgttgagaactacggtggtgagacacaggtccagagacgccaacacacggatgtctcgttcatattagacagatttgtgaaagcaacaccaaaagatcaaattaatgtgttggacctgatgcaaacccctgcacacactttggtaggcgcgctcctccgtactgccacctactacttcgcagatctagaagtggcagtgaaacacgaggggaaccttacttgggtcccgaatggggcgcccgagacagcgttggacaacaccaccaatccaacggcttaccacaaggcaccgctcacccggcttgcactgccttacacggcaccgcaccgtgtcttggctactgtttacaacgggaactgcaagtatggcgagagccccgtgaccaatgtgagaggtgacctgcaagtattggcccaaaaggcagcaagaacgctgcctacctccttcaattacggtgccatcaaagccactcgggtgactgaa

>DQ404161.1_O_UKG_2001

tcgagcgttggagtcacttacgggtacgcaacagctgaggactttgtgagcggaccaaacacatctgggcttgagaccagggttgtgcaggcagagcggttcttcaaaacccacttgttcgactgggtcaccagtgacccgtttggacggtgctatctgctggaactcccaactgaccacaaaggtgtctacggcagcctgaccgactcttatgcttacatgagaaacggctgggatgttgaggtcaccgcagtgggaaatcagttcaacggaggatgtctgttggtggccatggtgccagaactttgctctattgacaagagagagctgtaccagctcacgctctttccccaccagttcatcaacccccggacgaacatgacggcgcacatcactgtgccctttgttggcgtcaaccgctacgaccagtacaaggtacacaaaccttggaccctcgtggttatggttgtggccccgctgactgtcaacaccgaaggtgccccacagatcaaggtctatgccaacatcgcccctaccaacgtgcacgtagcgggtgagttcccttctaaggaagggatcttccccgtggcatgtagcgacggttacggtggtctggtgaccactgacccaaagacggctgaccccgcctacgggagagtgttcaatccacctcgcaacatgttgccggggcggttcaccaacttccttgatgtggctgaggcgtgccccacgtttctgcactttgagggtggcgtgccgtacgtgaccacaaagacggactcagacagggtgctcgcccagtttgacttgtctctggcagcaaagcacatgtcaaacaccttcctggcaggtctcgcccagtactacacacagtacagcggcaccatcaacctgcacttcatgttcacaggacccactgacgcgaaagcgcgttacatgattgcatacgccccccctggcatggagccgcccaaaacacctgaggcggccgcccactgcattcatgcggagtgggacacagggttgaattcaaaattcacatttccaatcccttacctttcggcggctgattacgcgtacaccgcgtctgacgctgcggagaccacaaatgtacagggatgggtttgcctgtttcaaattacacacgggaaggctgacggcgacgcactggtcgttctagctagcgccggtaaggactttgagctgcgtctgccagttgacgctcgcacgcagaccacctccgcaggtgagtcggctgaccccgtgactgccactgttgagaactacggtggtgagacacaggtccagagacgccaacacacggatgtctcgttcatattagacagatttgtgaaagtaacaccaaaagaccaaattaatgtgttggacctgatgcaaacccctgcacacactttggtaggcgcgctcctccgtactgccacctactacttcgcagatctagaagtggcagtgaaacacgaggggaaccttacctgggtccccaatggggcgcccgagacagcgttggacaacaccaccaatccaacggcttaccacaaggcaccgctcacccggcttgcactgccttacacggcaccgcaccgtgtcttggctactgtttacaacgggaactgcaagtatggcgagagccccgtgaccaatgtgagaggtgacctgcaagtattggcccaaaaggcggcaagaacgctgcctacctccttcaattacggtgccatcaaagccactcgggtgactgaa

>DQ404162.1_O_UKG_2001

tcgagcgttggagtcacttacgggtacgcaacagctgaggactttgtgagcggaccaaacacatctgggcttgagaccagggttgtgcaggcagagcggttcttcaaaacccacttgttcgactgggtcaccagtgacccgtttggacggtgctatctgctggaactcccaactgaccacaaaggtgtctacggcagcctgaccgactcttatgcttacatgagaaacggctgggatgttgaggtcaccgcagtgggaaatcagttcaacggaggatgtctgttggtggccatggtgccagaactttgctctattgacaagagagagctgtaccagctcacgctctttccccaccagttcatcaacccccggacgaacatgacggcgcacatcactgtgccctttgttggcgtcaaccgctacgaccagtacaaggtacacaaaccttggaccctcgtggttatggttgtggccccgctgactgtcaacaccgaaggtgccccacagatcaaggtctatgccaacatcgcccctaccaacgtgcacgttgcgggtgagttcccttctaaggaagggatcttccccgtggcatgtagcgacggttacggtggtctggtgaccactgacccaaagacggctgaccccgcctacgggaaagtgtccaatccacctcgcaacatgttgccggggcggttcaccaacttccttgatgtggctgaggcgtgccctacgtttctgcactttgagggtggcgtgccgtacgtgaccacaaagacggacacagacagggtgctcgcccagtttgacttgtctctggcagcaaagcacatgtcaaacaccttcctggcaggtctcgcccagtactacacacagtacagcggcaccatcaacctgcacttcatgttcacaggacccactgacgcgaaagcgcgttacatgattgcatacgccccccctggcatggagccgcccaaaacacctgaggcggccgcacactgcattcatgcggagtgggacacagggttgaattcaaaattcacatttccaatcccttacctttcggcggctgattacgcgtacaccgcgtctgacgctgcggagaccacaaatgtacagggatgggtttgcctgtttcaaattacacacgggaaggctgacggcgacgcactggtcgttctagctagcgccggtaaggactttgagctgcgtctgccagttgacgctcgcacgcagaccacctccgcaggtgagtcggctgaccccgtgactgccactgttgagaactacggtggtgagacacaggtccagagacgccaacacacggatgtctcgttcatattagacagatttgtgaaagtaacaccaaaagaccaaattaacgtgttggacctgatgcaaacccctgcacacactttggtaggcgcgctcctccgtactgccacctactacttcgcagatctagaagtggcagtgaaacacgaggggaaccttacctgggtcccgaatggggcgcccgagacagcgttggacaacaccaccaatccaacggcttaccacaaggcaccgctcacccggcttgcactgccttacacggcaccgcaccgtgtcttggctactgtttacaacgggaactgcaagtatggcgagagccccgtgaccaacgtgagaggtgacctgcaagtattggcccaaaaggcggcaagaacgctgcctacctccttcaattacggtgccatcaaagccactcgggtgactgaa

>DQ404163.1_O_UKG_2001

tcgagcgttggagtcacttacgggtacgcaacagctgaggactttgtgagcggaccaaacacatctgggcttgagaccagggttgtgcaggcagagcggttcttcaaaacccacttgttcgactgggtcaccagcgacccgtttggacggtgctatctgctggaactcccaactgaccacaaaggtgtctacggcagcctgaccgactcttatgcttacatgagaaacggttgggatgttgaggtcaccgcagtgggaaatcagttcaacggaggatgtctgttggtggccatggtgccagaactttgctctattgacaagagagagctgtaccagctcacgctcttcccccaccagttcatcaacccccggacgaacatgacggcgcacatcactgtgccctttgttggcgtcaaccgctacgaccagtacaaggtacacaaaccttggaccctcgtggttatggttgtggccccgctgactgtcaacaccgaaggtgccccacagatcaaggtctatgccaacatcgcccctaccaacgtgtacgttgcgggtgagttcccttctaaggaagggatcttccccgtggcatgtagcgacggttacggtggtctggtgaccactgacccaaagacggctgaccccgcctacgggaaagtgttcaatccacctcgcaacatgttgccggggcggttcaccaacttccttgatgtggctgaggcgtgccctacgtttctgcactttgagggtggcgtgccgtacgtgaccacaaggacggactcagacagggtgctcgcccagtttgacttgtctctggcagcaaagcacatgtcaaacaccttcctggcaggtctcgcccagtactacacacagtacaccggcaccatcaacctgcacttcatgttcacaggacccactgacgcgaaagcgcgttacatgattgcatacgccccccctggtatggagccgcccaaaacacctgaggcggccgcccactgcattcatgcggagtgggacacagggttgaattcaaaattcacattttcaatcccttacctttcggcggctgattacgcgtacaccgcgtctgacgctgcggagaccacaaatgtacagggatgggtttgcctgtttcaaattacacacgggaaggctgacggcgacgcactggtcgttctagctagcgccggtaaggactttgagttgcgtctgccagttgacgctcgcacacagaccacctccgcaggtgagtcggctgaccccgtgactgccactgttgagaactacggtggtgagacacaggtccagagacgccaacacacggatgtctcgttcatattagacagatttgtgaaagtaacaccaaaagaccaaactaacgtgttggacctgatgcagacccctgcacacactttggtaggcgcgctcctccgtactgccacctactacttcgcagatctagaagtggcagtgaaacacgaggggaaccttacctgggtcccgaatggggcgcccgagacagcgttggacaacaccaccaatccaacggcttaccacaaggcaccgctcacccggcttgcactgccttacacggcaccgcaccgtgtcttggctactgtttacaacgggaactgcaagtatggcgagagccccgtgaccaatgtgagaggtgacctgcaagtattggcccaaaaggcggcaagaacgctgcctacctccttcaattacggtgccatcaaagccactcgggtgactgaa

>DQ404164.1_O_UKG_2001

tcgagcgttggagtcacttacgggtacgcaacagctgaggactttgtgagcggaccaaacacatctgggcttgagaccagggttgtgcaggcagagcggttcttcaaaacccacttgttcgactgggtcaccagtgacccgtttggacggtgctatctgctggaactcccaactgaccacaaaggtgtctacggcagcctgaccgactcttatgcttacatgagaaacggttgggatgttgaggtcaccgcagtgggaaatcagttcaacggaggatgtctgttggtggccatggtgccagaactttgctctattgacaagagagagctgtaccagctcacgctctttccccaccagttcatcaacccccggacgaacatgacggcgcacatcactgtgccctttgttggcgtcaaccgctacgaccagtacaaggtacacaaaccttggaccctcgtggttatggttgtggccccgctgactgtcaacaccgaaggtgccccacagatcaaggtctatgccaacatcgcccctaccaacgtgcacgttgcgggtgagttcccttctaaggaagggatcttccccgtggcatgtagcgacggttacggtggtctggtgaccactgacccaaagacggctgaccccgcctacgggaaagtgttcaatccacctcgcaacatgttgccggggcggttcaccaacttccttgacgtggctgaggcgtgccctacgtttctgcactttgagggtggcgtgccgtacgtgaccacaaagacggactcagacagggtgctcgcccagtttgacttgtctctggcagcaaagcacatgtcaaacaccttcctggcaggtctcgcccagtactacacacagtacagcggcaccatcaacctgcacttcatgttcacaggacccactgacgcgaaagcgcgttacatgattgcatacgccccccctggtatggagccgcccaaaacacctgaggcggccgcccactgcattcatgcggagtgggacacagggttgaattcgaaattcacattttcaatcccttacctttcggcggctgattacgcgtacaccgcgtctgacgctgcggagaccacaaatgtacagggatgggtttgcctgtttcaaattacacacgggaaggctgacggcgacgcactggtcgttctagctagcgccggtaaggactttgagctgcgtctgccagttgacgctcgcacgcagaccacctccgcaggtgagtcggctgaccccgtgactgccactgttgagaactacggtggtgagacacaggtccagagacgccaacacacggatgtctcgttcatattagacagatttgtgaaaataacaccaaaagaccaaattaatgtgttggacctgatgcaaacccctgcacacactttggtaggcgcgctcctccgtactgccacctactacttcgcagatctagaagtggcagtgaaacacgaggggaaccttacctgggtcccgaatggggcgcccgagacagcgttggacaacaccaccaatccaacggcttaccacaaggcaccgctcacccggcttgcactgccttacacggcaccgcaccgtgtcttggctactgtttacaacgggaactgcaagtatggcgagagccccgtgaccaacgtgagaggtgacctgcaagtattggcccaaaaggcggcaagaacgctgcctacctccttcaattacggtgccatcaaagccactcgggtgactgaa

>DQ404165.1_O_UKG_2001

tcgagcgttggagtcacttacgggtacgcaacagctgaggactttgtgagcggaccaaacacatctgggcttgagaccagggttgtgcaggcagagcggttcttcaaaacccacttgttcgactgggtcaccagtgacccgtttggacggtgctatctgctggaactcccaactgaccacaaaggtgtctacggcagcctgaccgactcttatgcttacatgagaaacggctgggatgttgaggtcaccgcagtgggaaatcagttcaacggaggatgtctgttggtggccatggtgccagaactttgctctattgacaagagagagctgttccagctcacgctctttccccaccagttcatcaacccccggacgaacatgacggcgcacatcactgtgccctttgttggtgtcaaccgctacgaccagtacaaggtacacaaaccttggaccctcgtggttatggttgtggccccgctgactgtcaacaccgaaggtgccccacagatcaaggtctatgccaacatcgcccctaccaacgtgcacgttgcgggtgagttcccttctaaggaagggatcttccccgtggcatgtagcgacggttacggtggtctggtgaccactgacccaaagacggctgaccccgcctacgggaaagtgttcaatccacctcgcaacaagttgccggggcggttcaccaacttccttgatgtggctgaggcgtgccctacgtttctgcactttgagggtggcgtgccgtacgtgaccacaaagacggactcagacagggtgctcgcccagtttgacttgtctctggcagcaaagcacatgtcaaacaccttcctggcaggtctcgcccagtactacacacagtacagcggcaccatcaacctgcacttcatgttcacaggacccactgacgcgaaagcgcgttacatggttgcatacgccccccctggcatggagccgcccaaaacacctgaggcggccgcccactgcattcatgcggagtgggacacagggttgaattcaaaattcacattttcaatcccttacctttcggcggctgattacgcgtacaccgcgtctgacgccgcggagaccacaaatgtacagggatgggtttgcctgtttcaaattacacacgggaaggctgacggcgacgcactggtcgttctagctagcgccggtaaggactttgagctgcgtctgccagttgacgctcgcacgcagaccacctccgcaggtgagtcggctgaccccgtgactgccactgttgagaactacggtggtgagacacaggtccagagacgccaacacacggatgtctcgttcatattagacagatttgtgaaagtaacaccaaaagaccaaattaatgtgttggacctgatgcaaacccctgcacacactttggtaggcgcgctcctccgtactgccacctactacttcgcagatctagaagtggcagtgaaacacgaggggaaccttacctgggtcccgaatggggcgcccgagacagcgttggacaacaccaccaatccaacggcttaccacaaggcaccgctcacccggcttgcactgccttacacggcaccgcaccgtgtcttggctactgtttacaacgggaactgcaagtatggcgagagccccgtgaccaatgtgagaggtgacctgcaagtattggcccaaaaggcggcaagaacgctgcctacctccttcaattacggtgccatcaaagccactcgggtgactgaa

>DQ404166.1_O_UKG_2001

tcgagcgttggagtcacttacgggtacgcaacagctgaggactttgtgagcggaccaaacacatctgggcttgagaccagggttgtgcaggcagagcggttcttcaaaacccacttgttcgactgggtcaccagtgacccgtttggacggtgctatctgctggaactcccaactgaccacaaaggtgtctacggcagcctgaccgactcttatgcttacatgagaaacggctgggatgttgaggtcaccgcagtgggaaatcagttcaacggaggatgtctgttggtggccatggtgccagaactttgctctattgacaagagagagctgttccagctcacgctctttccccaccagttcatcaacccccggacgaacatgacggcgcacatcactgtgccctttgttggtgtcaaccgctacgaccagtacaaggtacacaaaccttggaccctcgtggttatggttgtggccccgctgactgtcaacaccgaaggtgccccacagatcaaggtctatgccaacatcgcccctaccaacgtgcacgttgcgggtgagttcccttctaaggaagggatcttccccgtggcatgtagcgacggttacggtggtctggtgaccactgacccaaagacggctgaccccgcctacgggaaagtgttcaatccacctcgcaacaagttgccggggcggttcaccaacttccttgatgtggctgaggcgtgccctacgtttctgcactttgagggtggcgtgccgtacgtgaccacaaagacggactcagacagggtgctcgcccagtttgacttgtctctggcagcaaagcacatgtcaaacaccttcctggcaggtctcgcccagtactacacacagtacagcggcaccatcaacctgcacttcatgttcacaggacccactgacgcgaaagcgcgttacatggttgcatacgccccccctggcatggagccgcccaaaacacctgaggcggccgcccactgcattcatgcggagtgggacacagggttgaattcaaaattcacattttcaatcccttacctttcggcggctgattacgcgtacaccgcgtctgacgccgcggagaccacaaatgtacagggatgggtttgcctgtttcaaattacacacgggaaggctgacggcgacgcactggtcgttctagctagcgccggtaaggactttgagctgcgtctgccagttgacgctcgcacgcagaccacctccgcaggtgagtcggctgaccccgtgactgccactgttgagaactacggtggtgagacacaggtccagagacgccaacacacggatgtctcgttcatattagacagatttgtgaaagtaacaccaaaagaccaaattaatgtgttggacctgatgcaaacccctgcacacactttggtaggcgcgctcctccgtactgccacctactacttcgcagatctagaagtggcagtgaaacacgaggggaaccttacctgggtcccgaatggggcgcccgagacagcgttggacaacaccaccaatccaacggcttaccacaaggcaccgctcacccggcttgcactgccttacacggcaccgcaccgtgtcttggctactgtttacaacgggaactgcaagtatggcgagagccccgtgaccaatgtgagaggtgacctgcaagtattggcccaaaaggcggcaagaacgctgcctacctccttcaattacggtgccatcaaagccactcgggtgactgaa

>DQ404167.1_O_UKG_2001

tcgagcgttggagtcacttacgggtacgcaacagctgaggactttgtgagcggaccaaacacatctgggcttgagaccagggttgtgcaggcagagcggttcttcaaaacccacttgttcgactgggtcaccagtgacccgtttggacggtgctatctgctggaactcccaactgaccacaaaggtgtctacggcagcctgaccgactcttatgcttacatgagaaacggctgggatgttgaggtcaccgcagtgggaaatcagttcaacggaggatgtctgttggtggccatggtgccagaactttgctctattgacaagagagagctgttccagctcacgctctttccccaccagttcatcaacccccggacgaacatgacggcgcacatcactgtgccctttgttggtgtcaaccgctacgaccagtacaaggtacacaaaccttggaccctcgtggttatggttgtggccccgctgactgtcaacaccgaaggtgccccacagatcaaggtctatgccaacatcgcccctaccaacgtgcacgttgcgggtgagttcccttctaaggaagggatcttccccgtggcatgtagcgacggttacggtggtctggtgaccactgacccaaagacggctgaccccgcctacgggaaagtgttcaatccacctcgcaacaagttgccggggcggttcaccaacttccttgatgtggctgaggcgtgccctacgtttctgcactttgagggtggcgtgccgtacgtgaccacaaagacggactcagacagggtgctcgcccagtttgacttgtctctggcagcaaagcacatgtcaaacaccttcctggcaggtctcgcccagtactacacacagtacagcggcaccatcaacctgcacttcatgttcacaggacccactgacgcgaaagcgcgttacatggttgcatacgccccccctggcatggagccgcccaaaacacctgaggcggccgcccactgcattcatgcggagtgggacacagggttgaattcaaaattcacattttcaatcccttacctttcggcggctgattacgcgtacaccgcgtctgacgccgcggagaccacaaatgtacagggatgggtttgcctgtttcaaattacacacgggaaggctgacggcgacgcactggtcgttctagctagcgccggtaaggactttgagctgcgtctgccagttgacgctcgcacgcagaccacctccgcaggtgagtcggctgaccccgtgactgccactgttgagaactacggtggtgagacacaggtccagagacgccaacacacggatgtctcgttcatattagacagatttgtgaaagtaacaccaaaagaccaaattaatgtgttggacctgatgcaaacccctgcacacactttggtaggcgcgctcctccgtactgccacctactacttcgcagatctagaagtggcagtgaaacacgaggggaaccttacctgggtcccgaatggggcgcccgagacagcgttggacaacaccaccaatccaacggcttaccacaaggcaccgctcacccggcttgcactgccttacacggcaccgcaccgtgtcttggctactgtttacaacgggaactgcaagtatggcgagagccccgtgaccaatgtgagaggtgacctgcaagtattggcccaaaaggcggcaagaacgctgcctacctccttcaattacggtgccatcaaagccactcgggtgactgaa

>DQ404168.1_O_UKG_2001

tcgagcgttggagtcacttacgggtacgcaacagctgaggactttgtgagcggaccaaacacatctgggcttgagaccagggttgtgcaggcagagcggttcttcaaaacccacttgttcgactgggtcaccagtgacccgtttggacggtgctatctgctggaactcccaactgaccacaaaggtgtctacggcagcctgaccgactcttatgcttacatgagaaacggctgggatgttgaggtcaccgcagtgggaaatcagttcaacggaggatgtctgttggtggccatggtgccagaactttgctctattgacaagagagagctgtaccagctcacgctctttccccaccagttcatcaacccccggacgaacatgacggcgcacatcactgtgccctttgttggcgtcaaccgctacgaccagtacaaggtacacaaaccttggaccctcgtggttatggttgtggccccgctgactgtcaacaccgaaggtgccccacagatcaaggtctatgccaacatcgcccctaccaacgtgcacgttgcgggtgagttcccttctaaggaagggatcttccccgtggcatgtagcgacggttacggtggtctggtgaccactgacccaaagacggctgaccccgcctacgggaaagtgttcaatccacctcgcaacatgttgccggggcggttcaccaacttccttgatgtggctgaggcgtgccctacgtttctgcactttgagggtggcgtgccgtacgtgaccacaaagacggactcagacagggtgctcgcccagtttgacttgtctctggcagcaaagcacatgtcaaacaccttcctggcaggtctcgcccagtactacacacagtacagcggcaccatcaacctgcacttcatgttcacaggacccactgacgcgaaagcgcgttacatgattgcatacgccccccctggcatggagccgcccaaaacacctgaggcggccgcccactgcattcatgcggagtgggacacagggttgaattcaaaattcacatttccaatcccttacctttcggcggctgattacgcgtacaccgcgtctgacgctgcggagaccacaaatgtacagggatgggtttgcctgtttcaaattacacacgggaaggctgacggcgacgcactggtcgttctagctagcgccggtaaggactttgagctgcgtctgccagttgacgctcgcacgcagaccacctccgcaggtgagtcggctgaccccgtgactgccactgttgagaactacggtggtgagacacaggtccagagacgccaacacacggatgtctcgttcatattagacagatttgtgaaagtaacaccaaaagaccaaattaacgtgttggacctgatgcaaacccctgcacacactttggtaggcgcgctcctccgtactgccacctactacttcgcagatctagaagtggcagtgaaacacgaggggaaccttacctgggtcccgaatggggcgcccgagacagcgttggacaacaccaccaatccaacggcttaccacaaggcaccgctcacccggcttgcactgccttacacggcaccgcaccgtgtcttggctactgtttacaacgggaactgcaagtatggcgagagccccgtgaccaatgtgagaggtgacctgcaagtattggcccaaaaggcggcaagaacgctgcctacctccttcaattacggtgccatcaaagccactcgggtgactgaa

>DQ404169.1_O_UKG_2001

tcgagcgttggagtcacttacgggtacgcaacagctgaggactttgtgagcggaccaaacacatctgggcttgagaccagggttgtgcaggcagagcggttcttcaaaacccacttgttcgactgggtcaccagtgacccgtttggacggtgctatctgctggaactcccaactgaccacaaaggtgtctacggcagcctgaccgactcttatgcttacatgagaaacggctgggatgttgaggtcaccgcagtgggaaatcagttcaacggaggatgtctgttggtggccatggtgccagaactttgctctattgacaagagagagctgtaccagctcacgctctttccccaccagttcatcaacccccggacgaacatgacggcgcacatcactgtgccctttgttggcgtcaaccgctacgaccagtacaaggtacacaaaccttggaccctcgtggttatggttgtggccccgctgactgtcaacaccgaaggtgccccacagatcaaggtctatgccaacatcgcccctaccaacgtgcacgttgcgggtgagttcccttctaaggaagggatcttccccgtggcatgtagcgacggttacggtggtctggtgaccactgacccaaagacggctgaccccgcctacgggaaagtgttcaatccacctcgcaacatgttgccggggcggttcaccaacttccttgatgtggctgaggcgtgccctacgtttctgcactttgagggtggcgtgccgtacgtgaccacaaagacggactcagacagggtgctcgcccagtttgacttgtctctggcagcaaagcacatgtcaaacaccttcctggcaggtctcgcccagtactacacacagtacagcggcaccatcaacctgcacttcatgttcacaggacccactgacgcgaaagcgcgttacatgattgcatacgccccccctggcatggagccgcccaaaacacctgaggcggccgcccactgcattcatgcggagtgggacacagggttgaattcaaaattcacattttcaatcccttacctttcggcggctgattacgcgtacaccgcgtctgacgccgcggagaccacaaatgtacagggatgggtttgcctgtttcaaattacacacgggaaggctgacggcgacgcactggtcgttctagctagcgccggtaaggactttgagctgcgtctgccagttgacgctcgcacgcagaccacctccgcaggtgagtcggctgaccccgtgactgccactgttgagaactacggtggtgagacacaggtccagagacgccaacacacggatgtctcgttcatattagacagatttgtgaaagtaacaccaaaagaccaaattaatgtgttggacctgatgcaaacccctgcacacactttggtaggcgcgctcctccgtactgccacctactacttcgcagatctagaagtggcagtgaaacacgaggggaaccttacctgggtcccgaatggggcgcccgagacagcgttggacaacaccaccaatccaacggcttaccacaaggcaccgctcacccggcttgcactgccttacacggcaccgcaccgtgtcttggctactgtttacaacgggaactgcaagtatggcgagagccccgtgaccaatgtgagaggtgacctgcaagtattggcccaaaaggcggcaagaacgctgcctacctccttcaattacggtgccatcaaagccactcgggtgactgaa

>DQ404170.1_O_UKG_2001

tcgagcgttggagtcacttacgggtacgcaacagctgaggactttgtgagcggaccaaacacatctgggcttgagaccagggttgtgcaggcagagcggttcttcaaaacccacttgttcgactgggtcaccagtgacccgtttggacggtgctatctgctggaactcccaactgaccacaaaggtgtctacggcagcctgaccgactcttatgcttacatgagaaacggctgggatgttgaggtcaccgcagtgggaaatcagttcaacggaggatgtctgttggtggccatggtgccagaactttgctctattgacaagagagagctgtaccagctcacgctctttccccaccagttcatcaacccccggacgaacatgacggcgcacatcactgtgccctttgttggcgtcaaccgctacgaccagtacaaggtacacaaaccttggaccctcgtggttatggttgtggccccgctgactgtcaacaccgaaggtgccccacagatcaaggtctatgccaacatcgcccctaccaacgtgcacgttgcgggtgagttcccttctaaggaagggatcttccccgtggcatgtagcgacggttacggtggtctggtgaccactgacccaaagacggctgaccccgcctacgggaaagtgttcaatccacctcgcaacaagttgccggggcggttcaccaacttccttgatgtggctgaggcgtgccctacgtttctgcactttgagggtggcgtgccgtacgtgaccacaaagacggactcagacagggtgctcgcccagtttgacttgtctctggcagcaaagcacatgtcaaacaccttcctggcaggtctcgcccagtactacacacagtacagcggcaccatcaacctgcacttcatgttcacaggacccactgacgcgaaagcgcgttacatgattgcatacgccccccctggcatggagccgcccaaaacacctgaggcggccgcccactgcattcatgcggagtgggacacagggttgaattcaaaattcacattttcaatcccttacctttcggcggctgattacgcgtacaccgcgtctgacgccgcggagaccacaaatgtacagggatgggtttgcctgtttcaaattacacacgggaaggctgacggcgacgcactggtcgttctagctagcgccggtaaggactttgagctgcgtctgccagttgacgctcgcacgcagaccacctccgcaggtgagtcggctgaccccgtgactgccactgttgagaactacggtggtgagacacaggtccagagacgccaacacacggatgtctcgttcatattagacagatttgtgaaagtaacaccaaaagaccaaattaatgtgttggacctgatgcaaacccctgcacacactttggtaggcgcgctcctccgtactgccacctactacttcgcagatctagaagtggcagtgaaacacgaggggaaccttacctgggtcccgaatggggcgcccgagacagcgttggacaacaccaccaatccaacggcttaccacaaggcaccgctcacccggcttgcactgccttacacggcaccgcaccgtgtcttggctactgtttacaacgggaactgcaagtatggcgagagccccgtgaccaatgtgagaggtgacctgcaagtattggcccaaaaggcggcaagaacgctgcctacctccttcaattacggtgccatcaaagccactcgggtgactgaa

>DQ404171.1_O_UKG_2001

tcgagcgttggagtcacttacgggtacgcaacagctgaggactttgtgagcggaccaaacacatctgggcttgagaccagggttgtgcaggcagagcggttcttcaaaacccacttgttcgactgggtcaccagtgacccgtttggacggtgctatctgctggaactcccaactgaccacaaaggtgtctacggcagcctgaccgactcttatgcttacatgagaaacggttgggatgttgaggtcaccgcagtgggaaatcagttcaacggaggatgtctgttggtggccatggtgccagaactttgctctattgacaagagagagctgtaccagctcacgctctttccccaccagttcatcaacccccggacgaacatgacggcgcacatcactgtgccctttgttggcgtcaaccgctacgaccagtacaaggtacacaaaccttggaccctcgtggttatggttgtggccccgctgactgtcaacaccgaaggtgccccacagatcaaggtctatgccaacatcgcccctaccaacgtgcacgttgcgggtgagttcccttctaaggaagggatcttccccgtggcatgtagcgacggttacggtggtctggtgaccactgacccaaagacggctgaccccgcctacgggaaagtgttcaatccacctcgcaacatgttgccggggcggttcaccaacttccttgatgtggctgaggcgtgccctacgtttctgcactttgagggtggcgtgccgtacgtgaccacaaagacggactcagacagggtgctcgcccagtttgacttgtctctggcagcaaagcacatgtcaaacaccttcctggcaggtctcgcccagtactacacacagtacagcggcaccatcaacctgcacttcatgttcacaggacccactgacgcgaaagcgcgttacatgattgcatacgccccccctggtatggagccgcccaaaacacctgaggcggccgcccactgcattcatgcggagtgggacacagggttgaattcaaaattcacattttcaatcccttacctttcggcggctgattacgcgtacaccgcgtctgacgctgcggagaccacaaatgtacagggatgggtttgcctgtttcaaattacacacgggaaggctgacggcgacgcactggtcgttctagctagcgccggtaaggactttgagctgcgtctgccagttgacgctcgcacgcagaccacctccgcaggtgagtcggctgaccccgtgactgccactgttgagaactacggtggtgagacacaggtccagagacgccaacacacggatgtctcgttcatattagacagatttgtgaaagtaacaccaaaagaccaaattaatgtgttggacctgatgcaaacccctgcacacactttggtaggcgcgctcctccgtactgccacttactacttcgcagatctagaagtggcagtgaaacacgaggggaaccttacctgggtcccgaatggggcgcccgagacagcgttggacaacaccaccaatccaacggcttaccacaaggcaccgctcacccggcttgcactgccttacacggcaccgcaccgtgtcttggctactgtttacaacgggaactgcaagtatggcgagagccccgtgaccaatgtgagaggtgacctgcaagtattggcccaaaaggcggcaagaacgctgcctacctccttcaattacggtgccatcaaagccactcgggtgactgaa

>DQ404172.1_O_UKG_2001

tcgagcgttggagtcacttacgggtacgcaacagctgaggactttgtgagcggaccaaacacatctgggcttgagaccagggttgtgcaggcagagcggttcttcaaaacccacttgttcgactgggtcaccagtgacccgtttggacggtgctatctgctggaactcccaactgaccacaaaggtgtctacggcagcctgaccgactcttatgcttacatgagaaacggttgggatgttgaggtcaccgcagtgggaaatcagttcaacggaggatgtctgttggtggccatggtgccagaactttgctctattgacaagagagagctgtaccagctcacgctctttccccaccagttcatcaacccccggacgaacatgacggcgcacatcactgtgccctttgttggcgtcaaccgctacgaccagtacaaggtacacaaaccttggaccctcgtggttatggttgtggccccgctgactgtcaacaccgaaggtgccccacagatcaaggtctatgccaacatcgcccctaccaacgtgcacgttgcgggtgagttcccttctaaggaagggatcttccccgtggcatgtagcgacggttacggtggtctggtgaccactgacccaaagacggctgaccccgcctacgggaaagtgttcaatccacctcgcaacatgttgccggggcggttcaccaacttccttgatgtggctgaggcgtgccctacgtttctgcactttgagggtggcgtgccgtacgtgaccacaaagacggactcagacagggtgctcgcccagtttgacttgtctctggcagcaaagcacatgtcaaacaccttcctggcaggtctcgcccagtactacacacagtacagcggcaccatcaacctgcacttcatgttcacaggacccactgacgcgaaagcgcgttacatgattgcatacgccccccctggtatggagccgcccaaaacacctgaggcggccgcccactgcattcatgcggagtgggacacagggttgaattcaaaattcacattttcaatcccttacctttcggcggctgattacgcgtacaccgcgtctgacgctgcggagaccacaaatgtacagggatgggtttgcctgtttcaaattacacacgggaaggctgacggcgacgcactggtcgttctagctagcgccggtaaggactttgagctgcgtctgccagttgacgctcgcacgcagaccacctccgcaggtgagtcggctgaccccgtgactgccactgttgagaactacggtggtgagacacaggtccagagacgccaacacacggatgtctcgttcatattagacagatttgtgaaagtaacaccaaaagaccaaattaatgtgttggacctgatgcagacccctgcacacactttggtaggcgcgctcctccgtactgccacctactacttcgcagatctagaagtggcagtgaaacacgaggggaaccttacctgggtcccgaatggggcgcccgagacagcgttggacaacaccaccaatccaacggcttaccacaaggcaccgctcacccggcttgcactgccttacacggcaccgcaccgtgtcttggctactgtttacaacgggaactgcaagtatggcgagagccccgtgaccaatgtgagaggtgacctgcaagtattggcccaaaaggcggcaagaacgctgcctacctccttcaattacggtgccatcaaagccactcgggtgactgaa

>DQ404173.1_O_UKG_2001

tcgagcgttggagtcacttacgggtacgcaacagctgaggactttgtgagcggaccaaacacatctgggcttgagaccagggttgtgcaggcagagcggttcttcaaaacccacttgttcgactgggtcaccagtgacccgtttggacggtgctatctgctggaactcccaactgaccacaaaggtgtctacggcagcctgaccgactcttatgcttacatgagaaacggttgggatgttgaggtcaccgcagtgggaaatcagttcaacggaggatgtctgttggtggccatggtgccagaactttgctctattgacaagagagagctgtaccagctcacgctctttccccaccagttcatcaacccccggacgaacatgacggcgcacatcactgtgccctttgttggcgtcaaccgctacgaccagtacaaggtacacaaaccttggaccctcgtggttatggttgtggccccgctgactgtcaacaccgaaggtgccccacagatcaaggtctatgccaacatcgcccctaccaacgtgcacgttgcgggtgagttcccttctaaggaagggatcttccccgtggcatgtagcgacggttacggtggtctggtgaccactgacccaaagacggctgaccccgcctacgggaaagtgttcaatccacctcgcaacatgttgccggggcggttcaccaacttccttgatgtggctgaggcgtgccctacgtttctgcactttgagggtggcgtgccgtacgtgaccacaaagacggactcagacagggtgctcgcccagtttgacttgtctctggcagcaaagcacatgtcaaacaccttcctggcaggtctcgcccagtactacacacagtacagcggcaccatcaacctgcacttcatgttcacaggacccactgacgcgaaagcgcgttacatgattgcatacgccccccctggtatggagccgcccaaaacacctgaggcggccgcccactgcattcatgcggagtgggacacagggttgaattcaaaattcacattttcaatcccttacctttcggcggctgattacgcgtacaccgcgtctgacgctgcggagaccacaaatgtacagggatgggtttgcctgtttcaaattacacacgggaaggctgacggcgacgcactggtcgttctagctagcgccggtaaggactttgagctgcgtctgccagttgacgctcgcacgcagaccacctccgcaggtgagtcggctgaccccgtgactgccactgttgagaactacggtggtgagacacaggtccagagacgccaacacacggatgtctcgttcatattagacagatttgtgaaagtaacaccaaaagaccaaattaatgtgttggacctgatgcagacccctgcacacactttggtaggcgcgctcctccgtactgccacctactacttcgcagatctagaagtggcagtgaaacacgaggggaaccttacctgggtcccgaatggggcgcccgagacagcgttggacaacaccaccaatccaacggcttaccacaaggcaccgctcacccggcttgcactgccttacacggcaccgcaccgtgtcttggctactgtttacaacgggaactgcaagtatggcgagagccccgtgaccaatgtgagaggtgacctgcaagtattggcccaaaaggcggcaagaacgctgcctacctccttcaattacggtgccatcaaagccactcgggtgactgaa

>DQ404174.1_O_UKG_2001

tcgagcgttggagtcacttacgggtacgcaacagctgaggactttgtgagcggaccaaacacatctgggcttgagaccagggttgtgcaggcagagcggttcttcaaaacccacttgttcgactgggtcaccagtgacccgtttggacggtgctatctgctggaactcccaactgaccacaaaggtgtctacggcagcctgaccgactcttatgcttacatgagaaacggttgggatgttgaggtcaccgcagtgggaaatcagttcaacggaggatgtctgttggtggccatggtgccagaactttgctctattgacaagagagagctgtaccagctcacgctctttccccaccagttcatcaacccccggacgaacatgacggcgcacatcactgtgccctttgttggcgtcaaccgctacgaccagtacaaggtacacaaaccttggaccctcgtggttatggttgtggccccgctgactgtcaacaccgaaggtgccccacagatcaaggtctatgccaacatcgcccctaccaacgtgcacgttgcgggtgagttcccttctaaggaagggatcttccccgtggcatgtagcgacggttatggtggtctggtgaccactgacccaaagacggctgaccccgcctacgggaaagtgttcaatccacctcgcaacatgttgccggggcggttcaccaacttccttgatgtggctgaggcgtgccctacgtttctgcactttgagggtggcgtgccgtacgtgaccacaaagacggactcagacagggtgctcgcccagtttgacttgtctctggcagcaaagcacatgtcaaacaccttcctggcaggtctcgcccagtactacacacagtacagcggcaccatcaacctgcacttcatgttcacaggacccactgacgcgaaagcgcgttacatgattgcatacgccccccctggtatggagccgcccaaaacacctgaggcggccgcccactgcattcatgcggagtgggacacagggttgaattcaaaattcacattttcaatcccttacctttcggcggctgattacgcgtacaccgcgtctgacgctgcggagaccacaaatgtacagggatgggtttgcctgtttcaaattacacacgggaaggctgacggcgacgcactggtcgttctagctagcgccggtaaggactttgagctgcgtctgccagttgacgctcgcacgcagaccacctccgcaggtgagtcggctgaccccgtgactgccactgttgagaactacggtggtgagacacaggtccagagacgccaacacacggatgtctcgttcatattagacagatttgtgaaagtaacaccaaaagaccaaattaatgtgttggacctgatgcaaacccctgcacacactttggtaggcgcgctcctccgtactgccacctactacttcgcagatctagaagtggcagtgaaacacgaggggaaccttacctgggtcccgaatggggcgcccgagacagcgttggacaacaccaccaatccaacggcttaccacaaggcaccgctcacccggcttgcactgccttacacggcaccgcaccgtgtcttggctactgtttacaacgggaactgcaagtatggcgagagccccgtgaccaatgtgagaggtgacctgcaagtattggcccaaaaggcggcaagaacgctgcctacctccttcaattacggtgccatcaaagccactcgggtgactgaa

>DQ404175.1_O_UKG_2001

tcgagcgttggagtcacttacgggtacgcaacagctgaggactttgtgagcggaccaaacacatctgggcttgagaccagggttgtgcaggcagagcggttcttcaaaacccacttgttcgactgggtcaccagtgacccgtttggacggtgctatctgctggaactcccaactgaccacaaaggtgtctacggcagcctgaccgactcttatgcttacatgagaaacggttgggatgttgaggtcaccgcagtgggaaatcagttcaacggaggatgtctgttggtggccatggtgccagaactttgctctattgacaagagagagctgtaccagctcacgctctttccccaccagttcatcaacccccggacgaacatgacggcgcatatcactgtgccctttgttggcgtcaaccgctacgaccagtacaaggtacacaaaccttggaccctcgtggttatggttgtggccccgctgactgtcaacaccgaaggtgccccacagatcaaggtctatgccaacatcgcccctaccaacgtgcacgttgcgggtgagttcccttctaaggaagggatcttccccgtggcatgtagcgacggttacggtggtctggtgaccactgacccaaagacggctgaccccgcctacgggaaagtgttcaatccacctcgcaacatgttgccggggcggttcaccaacttccttgatgtggctgaggcgtgccctacgtttctgcactttgagggtggcgtgccgtacgtgaccacaaagacggactcagacagggtgctcgcccagtttgacttgtctctggcagcaaagcacatgtcaaacaccttcctggcaggtctcgcccagtactacacacagtacagcggcaccatcaacctgcacttcatgttcacaggacccactgacgcgaaagcgcgttacatgattgcatacgccccccctggtatggagccgcccaaaacacctgaggcggccgcccactgcattcatgcggagtgggacacagggttgaattcaaaattcacattttcaatcccttacctttcggcggctgattacgcgtacaccgcgtctgacgctgcggagaccacaaatgtacagggatgggtttgcctgtttcaaattacacacgggaaggctgacggcgacgcactggtcgttctagctagcgccggtaaggactttgagctgcgtctgccagttgacgctcgcacgcagaccacctccgcaggtgagtcggctgaccccgtgactgccactgttgagaactacggtggtgagacacaggtccagagacgccaacacacggatgtctcgttcatattagacagatttgtgaaagtaacaccaaaagaccaaattaatgtgttggacctgatgcaaacccctgcacacactttggtaggcgcgctcctccgtactgccacctactacttcgcagatctagaagtggcagtgaaacacgaggggaaccttacctgggtcccgaatggggcgcccgagacagcgttggacaacaccaccaatccaacggcttaccacaaggcaccgctcacccggcttgcactgccttacacggcaccgcaccgtgtcttggctactgtttacaacgggaactgcaagtatggcgagagccccgtgaccaatgtgagaggtgacctgcaagtattggcccaaaaggcggcaagaacgctgcctacctccttcaattacggtgccatcaaagccactcgggtgactgaa

>DQ404176.1_O_UKG_2001

tcgagcgttggagtcacttacgggtacgcaacagctgaggactttgtgagcggaccaaacacatctgggcttgagaccagggttgtgcaggcagagcggttcttcaaaacccacttgttcgactgggtcaccagtgacccgtttggacggtgctatctgctggaactcccaactgaccacaaaggtgtctacggcagcctgaccgactcttatgcttacatgagaaacggttgggatgttgaggtcaccgcagtgggaaatcagttcaacggaggatgtctgttggtggccatggtgccagaactttgctctattgacaagagagagctgtaccagctcacgctctttccccaccagttcatcaacccccggacgaacatgacggcgcacatcactgtgccctttgttggcgtcaaccgctacgaccagtacaaggtacacaaaccttggaccctcgtggttatggttgtggccccgctgactgtcaacaccgaaggtgccccacagatcaaggtctatgccaacatcgcccctaccaacgtgcacgttgcgggtgagttcccttctaaggaagggatcttccccgtggcatgtagcgacggttacggtggtctggtgaccactgacccaaagacggctgaccccgcctacgggaaagtgttcaatccacctcgcaacatgttgccggggcggttcaccaacttccttgatgtggctgaggcgtgccctacgtttctgcactttgagggtggcgtgccgtacgtgaccacaaagacggactcagacagggtgctcgcccagtttgacttgtctctggcagcaaagcacatgtcaaacaccttcctggcaggtctcgcccagtactacacacagtacagcggcaccatcaacctgcacttcatgttcacaggacccactgacgcgaaagcgcgttacatgattgcatacgccccccctggtatggagccgcccaaaacacctgaggcggccgcccactgcattcatgcggagtgggacacagggttgaattcaaaattcacattttcaatcccttacctttcggcggctgattacgcgtacaccgcgtctgacgctgcggagaccacaaatgtacagggatgggtttgcctgtttcaaattacacacgggaaggctgacggcgacgcactggtcgttctagctagcgccggtaaggactttgagctgcgtctgccagttgacgctcgcacgcagaccacctccgcaggtgagtcggctgaccccgtgactgccactgttgagaactacggtggtgagacacaggtccagagacgccaacacacggatgtctcgttcatattagacagatttgtgaaagtaacaccaaaagaccaaattaatgtgttggacctgatgcaaacccctgcacacactttggtaggcgcgctcctccgtactgccacctactacttcgcagatctagaagtggcagtgaaacacgaggggaaccttacctgggtcccgaatggggcgcccgagacagcgttggacaacaccaccaatccaacggcttaccacaaggcaccgctcacccggcttgcactgccttacacggcaccgcaccgtgtcttggctactgtttacaacgggaactgcaagtatggcgagagccccgtgaccaatgtgagaggtgacctgcaagtattggcccaaaaggcggcaagaacgctgcctacctccttcaattacggtgccatcaaagccactcgggtgactgaa

>DQ404177.1_O_UKG_2001

tcgagcgttggagtcacttacgggtacgcaacagctgaggactttgtgagcggaccaaacacatctgggcttgagaccagggttgtgcaggcagagcggttcttcaaaacccacttgttcgactgggtcaccagtgacccgtttggacggtgctatctgctggaactcccaactgaccacaaaggtgtctacggcagcctgaccgactcttatgcttacatgagaaacggttgggatgttgaggtcaccgcagtgggaaatcagttcaacggaggatgtctgttggtggccatggtgccagaactttgctctattgacaagagagagctgtaccagctcacgctctttccccaccagttcatcaacccccggacgaacatgacggcgcacatcactgtgccctttgttggcgtcaaccgctacgaccagtacaaggtacacaaaccttggaccctcgtggttatggttgtggccccgctgactgtcaacaccgaaggtgccccacagatcaaggtctatgccaacatcgcccctaccaacgtgcacgttgcgggtgagttcccttctaaggaagggatcttccccgtggcatgtagcgacggttacggtggtctggtgaccactgacccaaagacggctgaccccgcctacgggaaagtgttcaatccacctcgcaacatgttgccggggcggttcaccaacttccttgatgtggctgaggcgtgccctacgtttctgcactttgagggtggcgtgccgtacgtgaccacaaagacggactcagacagggtgctcgcccagtttgacttgtctctggcagcaaagcacatgtcaaacaccttcctggcaggtctcgcccagtactacacacagtacagcggcaccatcaacctgcacttcatgttcacaggacccactgacgcgaaagcgcgttacatgattgcatacgccccccctggtatggagccgcccaaaacacctgaggcggccgcccactgcattcatgcggagtgggacacagggttgaattcaaaattcacattttcaatcccttacctttcggcggctgattacgcgtacaccgcgtctgacgctgcggagaccacaaatgtacagggatgggtttgcctgtttcaaattacacacgggaaggctgacggcgacgcactggtcgttctagctagcgccggtaaggactttgagctgcgtctgccagttgacgctcgcacgcagaccacctccgcaggtgagtcggctgaccccgtgactgccactgttgagaactacggtggtgagacacaggtccagagacgccaacacacggatgtctcgttcatattagacagatttgtgaaagtaacaccaaaagaccaaattaatgtgttggacctgatgcaaacccctgcacacactttggtaggcgcgctcctccgtactgccacctactacttcgcagatctagaagtggcagtgaaacacgaggggaaccttacctgggtcccgaatggggcgcccgagacagcgttggacaacaccaccaatccaacggcttaccacaaggcaccgctcacccggcttgcactgccttacacggcaccgcaccgtgtcttggctactgtttacaacgggaactgcaagtatggcgagagccccgtgaccaatgtgagaggtgacctgcaagtattggcccaaaaggcggcaagaacgctgcctacctccttcaattacggtgccatcaaagccactcgggtgactgaa

>DQ404178.1_O_UKG_2001

tcgagcgttggagtcacttacgggtacgcaacagctgaggactttgtgagcggaccaaacacatctgggcttgagaccagggttgtgcaggcagagcggttcttcaaaacccacttgttcgactgggtcaccagtgacccgtttggacggtgctatctgctggaactcccaactgaccacaaaggtgtctacggcagcctgaccgactcttatgcttacatgagaaacggttgggatgttgaggtcaccgcagtgggaaatcagttcaacggaggatgtctgttggtggccatggtgccagaactttgctctattgacaagagagagctgtaccagctcacgctctttccccaccagttcatcaacccccggacgaacatgacggcgcacatcactgtgccctttgttggcgtcaaccgctacgaccagtacaaggtacacaaaccttggaccctcgtggttatggttgtggccccgctgactgtcaacaccgaaggtgccccacagatcaaggtctatgccaacatcgcccctaccaacgtgcacgttgcgggtgagttcccttctaaggaagggatcttccccgtggcatgtagcgacggttacggtggtctggtgaccactgacccaaagacggctgaccccgcctacgggaaagtgttcaatccacctcgcaacatgttgccggggcggttcaccaacttccttgatgtggctgaggcgtgccctacgtttctgcactttgagggtggcgtgccgtacgtgaccacaaagacggactcagacagggtgctcgcccagtttgacttgtctctggcagcaaagcacatgtcaaacaccttcctggcaggtctcgcccagtactacacacagtacagcggcaccatcaacctgcacttcatgttcacaggacccactgacgcgaaagcgcgttacatgattgcatacgccccccctggtatggagccgcccaaaacacctgaggcggccgcccactgcattcatgcggagtgggacacagggttgaattcaaaattcacattttcaatcccttacctttcggcggctgattacgcgtacaccgcgtctgacgctgcggagaccacaaatgtacagggatgggtttgcctgtttcaaattacacacgggaaggctgacggcgacgcactggtcgttctagctagcgccggtaaggactttgagctgcgtctgccagttgacgctcgcacgcagaccacctccgcaggtgagtcggctgaccccgtgactgccactgttgagaactacggtggtgagacacaggtccagagacgccaacacacggatgtctcgttcatattagacagatttgtgaaagtaacaccaaaagaccaaattaatgtgttggacctgatgcaaacccctgcacacactttggtaggcgcgctcctccgtactgccacctactacttcgcagatctagaagtggcagtgaaacacgaggggaaccttacctgggtcccgaatggggcgcccgagacagcgttggacaacaccaccaatccaacggcttaccacaaggcaccgctcacccggcttgcactgccttacacggcaccgcaccgtgtcttggctactgtttacaacgggaactgcaagtatggcgagagccccgtgaccaatgtgagaggtgacctgcaagtattggcccaaaaggcggcaagaacgctgcctacctccttcaattacggtgccatcaaagccactcgggtgactgaa

>DQ404179.1_O_UKG_2001

tcgagcgttggagtcacttacgggtacgcaacagctgaggactttgtgagcggaccaaacacatctgggcttgagaccagggttgtgcaggcagagcggttcttcaaaacccacttgttcgactgggtcaccagtgacccgtttggacggtgctatctgctggaactcccaactgaccacaaaggtgtctacggcagcctgaccgactcttatgcttacatgagaaacggttgggatgttgaggtcaccgcagtgggaaatcagttcaacggaggatgtctgttggtggccatggtgccagaactttgctctattgacaagagagagctgtaccagctcacgctctttccccaccagttcatcaacccccggacgaacatgacggcgcacatcactgtgccctttgttggcgtcaaccgctacgaccagtacaaggtacacaaaccttggaccctcgtggttatggttgtggccccgctgactgtcaacaccgaaggtgccccacagatcaaggtctatgccaacatcgcccctaccaacgtgcacgttgcgggtgagttcccttctaaggaagggatcttccccgtggcatgtagcgacggttacggtggtctggtgaccactgacccaaagacggctgaccccgcctacgggaaagtgttcaatccacctcgcaacatgttgccggggcggttcaccaacttccttgatgtggctgaggcgtgccctacgtttctgcactttgagggtggcgtgccgtacgtgaccacaaagacggactcagacagggtgctcgcccagtttgacttgtctctggcagcaaagcacatgtcaaacaccttcctggcaggtctcgcccagtactacacacagtacagcggcaccatcaacctgcacttcatgttcacaggacccactgacgcgaaagcgcgttacatgattgcatacgccccccctggtatggagccgcccaaaacacctgaggcggccgcccactgcattcatgcggagtgggacacagggttgaattcaaaattcacattttcaatcccttacctttcggcggctgattacgcgtacaccgcgtctgacgctgcggagaccacaaatgtacagggatgggtttgcctgtttcaaattacacacgggaaggctgacggcgacgcactggtcgttctagctagcgccggtaaggactttgagctgcgtctgccagttgacgctcgcacgcagaccacctccgcaggtgagtcggctgaccccgtgactgccactgttgagaactacggtggtgagacacaggtccagagacgccaacacacggatgtctcgttcatattagacagatttgtgaaagtaacaccaaaagaccaaattaatgtgttggacctgatgcaaacccctgcacacactttggtaggcgcgctcctccgtactgccacctactacttcgcagatctagaagtggcagtgaaacacgaggggaaccttacctgggtcccgaatggggcgcccgagacagcgttggacaacaccaccaatccaacggcttaccacaaggcaccgctcacccggcttgcactgccttacacggcaccgcaccgtgtcttggctactgtttacaacgggaactgcaagtatggcgagagccccgtgaccaatgtgagaggtgacctgcaagtattggcccaaaaggcggcaagaacgctgcctacctccttcaattacggtgccatcaaagccactcgggtgactgaa

>DQ404180.1_O_UKG_2001

tcgagcgttggagtcacttacgggtacgcaacagctgaggactttgtgagcggaccaaacacatctgggcttgagaccagggttgtgcaggcagagcggttcttcaaaacccacttgttcgactgggtcaccagtgacccgtttggacggtgctatctgctggaactcccaactgaccacaaaggtgtctacggcagcctgaccgactcttatgcttacatgagaaacggttgggatgttgaggtcaccgcagtgggaaatcagttcaacggaggatgtctgttggtggccatggtgccagaactttgctctattgacaagagagagctgtaccagctcacgctctttccccaccagttcatcaacccccggacgaacatgacggcgcacatcactgtgccctttgttggcgtcaaccgctacgaccagtacaaggtacacaaaccttggaccctcgtggttatggttgtggccccgctgactgtcaacaccgaaggtgccccacagatcaaggtctatgccaacatcgcccctaccaacgtgcacgttgcgggtgagttcccttctaaggaagggatcttccccgtggcatgtagcgacggttacggtggtctggtgaccactgacccaaagacggctgaccccgcctacgggaaagtgttcaatccacctcgcaacatgttgccggggcggttcaccaacttccttgatgtggctgaggcgtgccctacgtttctgcactttgagggtggcgtgccgtacgtgaccacaaagacggactcagacagggtgctcgcccagttygacttgtctctggcagcaaagcacatgtcaaacaccttcctggcaggtctcgcccagtactacacacagtacagcggcaccatcaacctgcacttcatgttcacaggacccactgacgcgaaagcgcgttacatgattgcatacgccccccctggtatggagccgcccaaaacacctgaggcggccgcccactgcattcatgcggagtgggacacagggttgaattcaaaattcacattttcaatcccttacctttcggcggctgattacgcgtacaccgcgtctgacgctgcggagaccacaaatgtacagggatgggtttgcctgtttcaaattacacacgggaaggctgacggcgacgcactggtcgttctagctagcgccggtaaggactttgagctgcgtctgccagttgacgctcgcacgcagaccacctccgcaggtgagtcggctgaccccgtgactgccactgttgagaactacggtggtgagacacaggtccagagacgccaacacacggatgtctcgttcatattagacagatttgtgaaagtaacaccaaaagaccaaattaatgtgttggacctgatgcaaacccctgcacacactttggtaggcgcgctcctccgtactgccacctactacttcgcagatctagaagtggcagtgaaacacgaggggaaccttacctgggtcccgaatggggcgcccgagacagcgttggacaacaccaccaatccaacggcttaccacaaggcaccgctcacccggcttgcactgccttacacggcaccgcaccgtgtcttggctactgtttacaacgggaactgcaagtatggcgagagccccgtgaccaatgtgagaggtgacctgcaagtattggcccaaaaggcggcaagaacgctgcctacctccttcaattacggtgccatcaaagccactcgggtgactgaa

>DQ989303.1_Asia1_IND_1993

tcaagcgtcggcgtgacttacggttacgctgtggccgaagacgctgtttctgggcccaacacctcaggcttggagacccgcgtgacacaggctgaacggtttttcaagaaacacctgtttgattggacaccaaatctatcgtttggacactgtcactacctgggactcccctccgaacacaaaggcgtgttcggcagcctcatggactcctacgcctacatgaggaacgggtgggacattgaggtgaccgctgttggaaaccagttcaatggtggttgcctcctcgtcgcactcgtcccggagctgaaagaacttgacacgcggcagaagtaccagttgaccctcttcccacaccagttcatcaacccacgcaccaacatgacggctcacatcaacgtgccgttcgtgggtgtcaacaggtacgaccaatacaagctccacaagccgtggacgcttgttgtgatggtggtggctccacttaccgtcaaaaccggtggttccgaacagatcaaggtttacatgaatgcagcaccaacccacgtgcatgtggcaggggaactgccctcgaaagaggggatagtacccgttgcgtgtgcggccggttatggcaacatggtgaccacagacccgaagacggctgaccccgtttacgggaaagtgttcaacccccccagaacaaatctccctgggcgcttcacaaacttccttgatgtagcggaggcatgcccaaccttcctccgcttcggagaa---gtaccatttgtgaagacggggaactctggtgaccgcttgcttgccaagtttgacgtgtcgctcgctgcggggcacatgtccaacacctacttggcaggcttggcgcagtactacacacagtacagcggcaccatgaacatccacttcatgttcaccgggcccacggatgccaaagctcgctacatggtggcttacgtacctcctggtatggagccacccacagaacccgagcgggccgcgcactgtatacattctgagtgggacactggtcttaattccaagttcaccttttccattccttacctctctgctgctgactacgcttacactgcttctgacgtggccgagaccacgagtgtgcagggatgggtgtgcatttatcagattacgcacggcaaagctgaaggcgacgcgctggtcgtgtctgtcagtgccggcaaggactttgagtttcgactgccagtggatgctcgccgagagactaccaccgctggcgagtccgcagacccagtcaccaccacagttgagaactacggaggagagactcagtcggcccgacggctacacactgacgttgcttttgttctcgacaggtttgtgaaactcacc---cccaagaacacccagattcttgatctcatgcagatcccctcacacacgctggttggagcgttactccggtccgcgacgtactacttctcggacctggaggttgcgcttgttcacacaggctcagtcacatgggtgcccaatggcgcgcccaaggacgccttggacaaccacaccaacccgactgcctaccagaagaaacccatcacccgcctggcgctcccctacaccgctccccaccgtgtgctggcaacagtgtacaacgggaagacaacgtacgggacacaacccacg------cggcgtggtgaccttgctgttcttgcacagcgggtaagcaacaggctgcccacctccttcaactacggtgctgtgaaggctgacaccatcacggag

>DQ989304.1_Asia1_IND_2000

tcaagcgtcggcgtgacttacggttacgctgtggccgaagacgctgtttctgggcccaacacctcaggcttggagacccgcgtgacacaggctgaacggtttttcaagaaacacctgtttgattggacaccaaatctatcgtttggacactgtcactacctggaactcccctccgaacacaaaggcgtgttcggcagcctcatggactcctacgcctacatgaggaacgggtgggacattgaggtgaccgctgttggaaaccagttcaatggtggttgcctcctcgtcgcactcgtcccggagctgaaagaacttgacacgcggcagaagtaccagttgaccctcttcccacaccagttcatcaacccacgcaccaacatgacggctcacatcaacgtgccgttcgtgggtgtcaacaggtacgaccaatacaagctccacaagccgtggacgcttgttgtgatggtggtggctccacttaccgtcaaaaccggtggttccgaacagatcaaggtttacatgaatgcagcaccaacccacgtgcatgtggcaggggaactgccctcgaaagaggggatagtacccgttgcgtgtgcggccggttatggcaacatggtgaccacagacccgaagacggctgaccccgtttacgggaaagtgttcaacccccccagaacaaatctccctgggcgcttcacaaacttccttgatgtagcggaggcatgtccaaccttcctccgcttcggagaa---gtaccatttgtgaagacggggaactctggtgaccgcttgcttgccaagtttgacgtgtcgctcgctgcggggcacatgtccaacacctacttggcaggcttggcgcagtactacacacagtacagcggcaccatgaacatccacttcatgttcaccgggcccacggatgccaaagctcgctacatggtggcttacgtacctcctggtatggagccacccacagaacccgagcgggccgcgcactgtatacattctgagtgggacactggtcttaattccaagttcaccttttccattccttacctctctgctgctgactacgcttacactgcttctgacgtggccgagaccacgagtgtgcagggatgggtgtgcatttatcagattacgcacggcaaagctgaaggcgacgcgctggtcgtgtctgtcagtgccggcaaggactttgagtttcgactgccagtggatgctcgccgagagactaccaccgctggcgagtccgcagacccagtcaccaccacagttgagaactacggaggagagactcagtcggcccgacggctacacactgacgttgcttttgttctcgacaggtttgtgaaactcacc---cctaagaacacccagattcttgatctcatgcagatcccctcacacacgctggttggagcgttactccggtccgcgacgtactacttctcggacctggaggttgcgcttgttcacacaggctcagtcacatgggtgcccaatggcgcgcccaaggacgccttggacaaccacaccaacccgactgcctaccagaagaaacccatcacccgcctggcgctcccctacaccgctccccaccgtgtgctggcaacagtgtacaacgggaagacaacgtacgggacacaacccacg------cggcgtggtgaccttgctgttcttgcacagcgggtaagcaacaggctgcccacctccttcaactacggtgctgtgaaggctgacaccatcacggag

>DQ989305.1_Asia1_IND_1990

tcgagcgtcggcgtaacgtacggttacgctgtggctgaggacgcggtgtcaggacctaacacttcaggtcttgagacccgtgttcaacaggcggaacggttctttaaaaagcacttgtttgactggacaccgaatttggcatttggacactgtcactacctggaactccccactgaacacaaaggcgtgtacggcagtctcatggactcgtacgctacaatgaggaatggatgggatatagaggtgactgctgttggaaaccaattcaacggcggttgtctccttgtcgcacttgtgccagagctgaagagccttgacacgcggcagaaataccaactgactctctttccccaccagttcatcaacccacgcaccaacatgacggcccacatcaacgtgccgttcgtgggtgtcaacagatacgaccagtacgcgcttcacaaaccgtggacgctcgttgtgatggtgttggccccactcaccgtcaagactggtggttctgaacagattaaggtttacatgaatgcagcaccgacctacgtgcacgtggcgggagagctgccctcgaaagagggaatagttcccgttgcgtgtgcggacggttatggcaacatggtgaccacggacccgaagactgccgacccagtttacgggaaagtgtacaacccccccaggacaaacctccctgggcgcttcacaaacttccttgatgttgcggaggcatgtccaaccttcctccgcttcggagaa---gtgccatttgtgaagacggtgaactctggtgaccgcctgctggccaagttcgatgtctcgcttgccgcggggcacatgtccaacacctacttggctggtttggcgcagtactacacacagtacagcggcaccataaacgttcacttcatgttcaccgggcccacagacgccaaagcccgctacatggtggcctacatccctcccggcatgacaccgcccacagaccctgagcgcgctgcgcactgcattcactctgagtgggacactggtcttaactccaagttcaccttttctataccttacctttctgctgctgactacgcctacactgcttctgacacggcggagaccacaagtgtgcagggatgggtgtgcatctaccagatcacccacggcaaggctgaaggagatgcactggtcgtttctgtcagcgccggcaaagattttgagttccgcttgcccgttgacgcgtgccggcaaaccaccacaaccggcgagtcagcggacccagtgacaaccacggtcgagaactacggaggagaaactcagacggccagacggcttcacactgacgttgccttcgttcttgacaggtttgtgaaactcactacacccaagagcacccagacccttgatctcatgcagatcccctcacacacgttggttggagcactgcttcggtctgcgacgtactacttctcagacctagaggttgcgcttgtccacacaggcccggttacctgggtgcccaacggctcgcccaaggatgccctagacaaccaaactaacccaactgcctatcagaagcagcccatcacccgcttggcactcccctacactgccccccaccgtgtgctggcaacagtgtacaacgggaagacgacgtacggggaaacaacctca------cggcgtggtgatatggcggcccttgcacaaaggctgagtgggcggctgcccacctccttcaactacggcgctgtgaaggctgaaaccatcactgag

>DQ989306.1_Asia1_IND_1986

tcaagcgtcggcgtgacttacggttacgctgtggccgaagacgctgtttctgggcccaacacctcaggcttggagacccgcgtgacacaggctgaacggtttttcaagaaacacctgtttgattggacaccaaatctatcgtttggacactgtcactacctggaactcccctccgaacacaaaggcgtgttcggcagcctcatggactcctacgctacaatgaggaacgggtgggacattgaggtgaccgctgttggaaaccagttcaatggtggttgcctcctcgtcgcactcgtcccggagctgaaagaacttgacacgcggcagaagtaccagttgaccctcttcccacaccagttcatcaacccacgcaccaacatgacggctcacatcaacgtgccgttcgtgggtgtaaacaggtacgaccattacaagctccacaagccgtggacgcttgttgtgatggtggtggctccacttaccgtcaaaaccggtggctccgaacagatcaaggtttacatgaatgcagcaccaacccacgtgcatgtggcaggggaactgccctcgaaagaggggatagtacccgttgcgtgtgcggacggttatggcaacatggtgaccacagacccgaagacggctgaccccgtttacggcaaagtgttcaacccccccagaacgaatcttcctgggcgcttcacaaacttccttgatgtagcggaggcatgtccaaccttcctccgcttcggagaa---gtaccatttgtgaagacggtgaactctggtgaccgcttgcttgccaagtttgacgtgtcgctcgctgcggggcacatgtccaacatctacttggcaggcttggcgcagtactacacacagtacagcggcaccatgaacatccatttcatgttcaccgggcccacggatgccaaagctcgctacatggtggcttacgtacctcctggtatggagccacccacagaacccgagcgggccgcgcactgtatacattctgagtgggacactggtcttaattccaagttcaccttttccattccttacctctctgctgctgactacgcttacactgcctctgaggtggccgagactacgagtgtgcagggatgggtgtgtatttatcagattacgcaggttaaagctgaaggcgacgcgctggtcgtgtctgtcagtgccggcaaggactttgagtttcgactgccagtggatgctcgccgagagactaccaccgctggcgagtccgcagacccagtcaccaccacagttgagaactacggaggagagactcagtcggcccgacggctacacactgacgttgcttttgttctcgacaggtttgtgaaactcacc---cccaagaacacccagattcttgatctcatgcagatcccctcacacacgctggttggagcgttactccggtccgcgacgtactacttctcggacctggaggttgcgcttgttcacacaggctcagtcacatgggtgcccaatggcgcgcccaaaggacgcctggacaaccacaccaacccgactgcctaccagaagaaacccatcacccgcctggcgctcccctacaccggtccccaccgtgtgctggcaacagtgtacaacgggaagacaacgtacgggacacaacccacg------cggcgtggtgacctagctgctctggcacagcgggtaagcaacaggttgcccacctccttcaactacggagctgtgaaagctgacaccatcacggag

>DQ989307.1_Asia1_IND_1992

tcgagcgtcggcgtaacgtacggttacgctgtggctgaggacgcggtgtcaggacctaacacttcaggtcttgagacccgtgttcaacaggcggaacggttctttaaaaagcacttgtttgactggacaccgaatttggcatttggacactgtcactacctggaactccccactgaacacaaaggcgtgtacggcagtctcatggactcgtacgcctacatgaggaatggatgggatatagaggtgactgctgttggaaaccaattcaacggcggttgtctccttgtcgcacttgtgccagagctgaagagccttgacacgcggcagaaataccaactgactctctttccccaccagttcatcaacccacgcaccaacatgacggcccacatcaacgtgccgttcgtgggtgtcaacagatacgaccagtacgcgcttcacaaaccgtggacgctcgttgtgatggtgttggccccactcaccgtcaaaactggtggttctgaacagattaaggtttacatgaatgcagcaccgacctacgtgcacgtggcgggagagctgccctcgaaagagggaatagttcccgttgcgtgtgcggacggttatggcaacatggtgaccacggacccgaagactgccgacccagtttacgggaaagtgtacaacccccccagaacaaacctccctgggcgcttcacaaacttccttgatgttgcggaggcatgtccaaccttcctccgcttcggagaa---gtgccatttgtgaagacggtgaactctggtgaccgcctgctggccaagttcgatgtctcgcttgccgcggggcacatgtccaacacctacttggctggtttggcgcagtactacacacagtacagcggcaccataaacattcacttcatgttcaccgggcccacagacgccaaagcccgctacatggtggcctacatccctcccggcatgagaccgcccacagaccctgagcgcgctgcgcactgcattcactctgagtgggacactggtcttaactccaagttcaccttttctatacctcacctttctgctgctgactacgcctacactgcttctgacacggcggagaccacaagtgtgcagggttgggtgtgcatctaccagatcactcacggcaaggctgaaggagacgcactggtcgtttctgtcagcgccggcaaagattttgagttccgcttgcccgttgacgcgcgccggcaaaccaccacaaccggcgagtcagcggacccagtgacaaccacggtcgagaactacggaggagaaactcagacggccagacggcttcacactgacgttgccttcgttcttgacaggtttgtgaaactcactacacccaagagcacccagacccttgatctcatgcagatcccctcacacacgttggttggagcactgcttcggtctgcgacgtactacttctcagacctagaggttgcgcttgtccacacaggcccggttacctgggtgcccaacggctcgcccaaggatgccctagacaaccaaactaacccaactgcctatcagaagcagcccatcacccgcttggcactcccctacactgccccccaccgtgtgctggcaacagtgtacaacgggaagacgacgtacggggaaacaacctca------cggcgtggtgatatggcggcccttgcacaaaggctgagtaggcggctgcccacctccttcaactacggcgctgtgaaggctgaaaccatcactgag

>DQ989308.1_Asia1_IND_1994

tcgagcgttggcgtgacatacggttacgctgtggctgaggacgcggtgtcaggacctaacacctcaggcctggagacccgtgttcaacaagcggaacggttctttaaaaagcacttgtttgactggacaccgaatttggcatttggacattgtcactacctggaactccccactgaacacaaaggcgtgtacggcagtcttatggattcgtacgcctacatgagaaatgggtgggacatagaggtgactgctgttggaaaccagttcaacggcggttgtctccttgttgcacttgtgccagagctgaagagccttgacacgcggcagaagtaccaactgaccctcttccctcaccagttcatcaacccacgcaccaacatgacggcccacatcaacgtgccttctgtggttgttaacaggtatgaccagtacgcgctccacaaaccgtggacgctcgttgtgatggtggtggccccactcactgtcaagactggaggctctgaacagatcaaggtctacatgaatgcagcaccgacctacgtgcacgtggcaggggagctgccctcgaaagagggaatagttcccgttccgtgtgcggacggttatggcaacatggtgaccacggacccgaaaactgctgacccagtgtacgggaaagtgttcaaccctcccaggacgaatcttcccgggcgcttcacgaacttccttgatgtcgcggaggcgtgtccaaccttcctccgcttcggagaa---gtaccatttgtgaagacagtgaactctggtgaccgtctgctagccaagtttgatgtctcgctcgctgcggggcacatgtccaacacctacttggctggcctggcacagtactacacacagtatagtggaaccatgaacgtccacttcatgttcaccgggcccacagacgccaaagctcggtacatggtggcctacattcctcccggcatgacaccgcccacagaccctgagcgcgccgcgcactgcatccactctgagtgggacactggtcttaactccaagttcaccttttccataccctacctctctgctgctgactacgcatacaccgcttctgacacggcggagaccacaagtgtgcaaggatgggtgtgcatctaccagatcacccatggcaaagctgaaggtgacgcactggtcgtttctgtcagcgccggcaaagactttgagtttcgcctgcccgtggacgcgcgccggcaaactaccacaaccggcgagtcagcggacccagtaacaaccacggttgagaactacggaggagaaactcagacagccagacggcttcacactgacgttgccttcgttcttgacaggtttgtgaaactcactgcacccaagaacacccagacccttgatctcatgcagattccctcacacacgctggttggagcactgcttcggtctgcgacgtactacttctcagacctggaggttgcgcttgtacacacaggcccagcaacctgggtgcccaacggctcacccaaagatgctctagacaaccagaccaatccaactgcctaccagaagcagcccatcacgcgcttggcgctcccctacaccgcgccccaccgcgtgctggcgacagtgtacaatgggaagacgacgtacggggaaacaacttca------cggcgcggtgatatggcggctcttgcaacaaaggtcagtgggcagctgcccacctctttcaactacggcgctgtaaaggctgaaaccatcactgag

>DQ989309.1_Asia1_IND_1996

tcgagcgttggcgtgacatacggttacgctgtggctgaggacgcggtgtcaggacctaacacctcaggcctggagacccgtgttcaacaagcggaacggttctttaaaaagcacttgtttgactggacaccgaatttggcatttggacattgtcactacctgggactccccactgaacacaaaggcgtgtacggcagtctcatggattcgtacgcctacatgagaaacgggtgggacatagaggtgactgctgttggaaaccagttcaacggcggttgtctccttgttgcacttgtgccagagctaaagagccttgacacccggcagaagtaccaactgaccctcttcccccaccagttcatcaacccacgcaccaacatgacggcccacattaacgtgccttttgtgggtgttaacaggtatgaccagtacgcgctccacaaaccgtggacgctcgttgtgatggtgttggccccactcactgtcaagactggcggctctgaacagatcaaggtctacatgaatgcagcaccgacctacgtgcacgtggcaggggagctgccctcgaaagagggaatagtccccgttgcgtgtgcggacggttatggcaacatggtgaccacggacccgaaaactgctgacccagtgtacgggaaagtgttcaaccctccccggacaaatcttcccgggcgcttcacgaacttccttgatgtcgcggaggcgtgtccaaccttcctccgcttcggagaa---gtaccatttgtgaagacagtgaactctggtgaccgtctgctagccaagtttgatgtctcgctcgctgcggggcacatgtccaacacctacttggctggtctggcacagtactacacacagtatagtggcaccatgaacgtccacttcatgttcaccgggcccacagacgccaaagctcggtacatggtggcctacattcctcccggcatgacaccgcccacagaccctgagcgcgccgcggactgcatccactctgagtgggacactggtcttaactccaagttcaccttttccataccctacctctctgctgctgactacgcatacaccgcttctgacacggcggagaccacaagtgtgcaaggatgggtgtacatctaccagatcacccacggcaaagctgaaggagacgcactggtcgtttctgtcagcgccggcaaagactttgagtttcgcctgcccgtcgacgcgcgccggcaaactaccactaccggcgagtcagcggacccggtaacaaccacggttgagaactacggaggagaaactcagacagccagacggcttcacactgacgttgccttcgttctcgacaggtttgtgaaactcactgcgcccaagaacactcagacccttgacctcatgcaaatcccctcacacacgctggttggagcactgcttcggtctgcgacgtactacttctcagacctggaggttgcgcttgtccacacaggcccagccacctgggtgcccaacggctcaccaaaagatgctctggacaaccagaccaatccaactgcctaccagaaacagcccatcacccgcttggcgctcccctacaccgccccccatcgtgtgctggcgacagtgtacaacgggaagacgacgtacggggaaacaactcca------cggcgcggtgatatggcggctcttgcacaaagactgagtgggcagctgcccacctctttcaactacggcgctgtaaaggctgacaccatcactgag

>DQ989310.1_Asia1_IND_1999

tcgagcgttggcgtgacatatggttacgctgtggctgaggacgcggtgtcaggacctaacacctcaggcctggagacccgtgttcaacaagcggaacggttctttaagaagcacttgtttgactggacaccgaacttggcatttgggcattgtcactacctggaactccccactgaacacaaaggcgtgtacggcagtctcatggcttcgtacgcctacatgagaaacgggtgggacatagaggtgactgctgttggaaaccaattcaacggcggctgtctccttgttgcacttgtgccagaattgaaggagcttgacacgcggcagaagtaccaactgactctctacccccaccagttcatcaacccacgcaccaacatgacggcccacatcagtgtaccttttgtgggtgttaacaggtatgatcagtacgcgctccacaaaccgtggacgcttgttgtgatggtggtggccccactcactgtcaagactggtggctctgaacagattaaggtttacatgaatgcagcaccgacctacgtgcacgtggcaggggagctgccctcgaaagagggaatagttcccgttgcgtgtgcggacggatatggcaacatggtgaccaccgacccgaaaactgctgacccagtgtacgggaaagtgttcaaccctcccaggacaaatctccccgggcgcttcacgaacttccttgatgttgcggaggcgtgtcccaccttcctccgcttcggagaa---gtaccatttgtgaagacagtgaactctggtgaccgtttgctggccaagtttgatgtctcgcttgctgcggggcacatgtccaacacctacttggctggtctggcacagtactacacacagtacagtggcaccatgaatggtcacttcatgttcaccgggcccacagatgccaaagctcggtacatggtagcctacatccctcccggcatgacaccggccacggaccccgagcacgccgcgcactgcatccactctgagtgggacactggccttaactccaagttcaccttttccataccctacctctctgctgctgactacgcatacaccgcttctgacacggcggagaccacaagtgtgcaaggatgggtgtgcatctatcagatcacccacggcaaagctgaaggagacgcactggtcgtttctgtcagcgccggcaaagactttgagtttcgcctgcccgttgacgcgcgccgacaaaccaccactaccggcgagtcagcggacccggtaacaaccacggttgagaactacggaggagaaactcaggcagccagacggcttcacactgatgtcgctttcgttcttgacaggttggtgaaactcactgcacccaagaacattcagactcttgacctcatgcaaatccctgcacacacgctggttggagcactgcttcggtctgcaacgtactacttctcagacctggaggttgcgcttgtccacacgggcccagccacctgggtgcccaatggctcacccaaagatgctctggacaaccagaccaacccaactgcctaccagaagcaacccatcacccgcctggcgctcccctacaccgccccccaccgtgtactggcgacagtgtacaacgggaggacgacgtacggggaaacaacttca------cggcgcggtgatatggcagcccttgcacagaggctgagtgggcaactgcccacctccttcaactacggcgctgtaaaggctgacaccatcactgag

>DQ989311.1_Asia1_IND_2002

tcgagcgttggcgtgacatatggttacgccgtggctgaggacgcggtgtcaggacccaatacctcaggcctggagacccgtgttcaacaagcggaacggttctttaaaaagcacttgtttgattggacaccggatttggcatttggtcattgtcactacctggaactccccactgaacacaaaggcgtgtacggcagtctcatggcctcgtacgcctacatgagaaacgggtgggatatagaggtgactgctgttggaaatcagttcaacggcggctgtctccttgttgcacttgtaccagaattgaagagccttgacacgcggcagaaataccaactgactctcttcccccaccagtttatcaacccacgcaccaacatgacggcccacatcagtgtgccttttgtgggtgttaacaggtatgatcagtatgcgctccacaaaccgtggacgcttgttgtgatggtggtggccccacttactgtcaagactggtggctctgaacagattaaggtttacatgaacgcggcaccaacctacgtgcacgtggcaggggagctgccctcgaaagaggggatagttcccgttgcgtgtgcggacggttatggcaacatggtgaccaccgacccgaaaactgctgacccagtgtacgggaaagtgttcaatcctccccggacaaacctccccgggcgcttcacgaacttccttgatgttgcggaggcgtgtcccaccttcctccgcttcggagaa---gtgccatttgtgaggacggtgaactctggtgaccgtttgctggccaagtttgatgtctcgcttgctgcggggcacatgtccaacacctacttggctggtctggcacagtactacacacagtacagtggcaccatgaacgttcacttcatgttcaccgggcccacagatgccaaagcccggtacatggtagcctacatccctcctggcatgacaccgcccacggaccctgagcacgccgcgcactgcatccactctgagtgggacactggccttaactccaagttcaccttttccataccctacctctccgctgctgactatgcatacaccgcttctgacacagcggagaccacaagtgtgcaaggatgggtgtgcatctaccagatcacccacggcaaagctgaaggagacgcactggtcgtttctgttagcgccggcaaagactttgagtttcgcctgcccgttgacgcgcgccggcaaaccaccactaccggcgagtcagccgacccggtaacaaccacggttgagaactacggaggagaaactcagacagccagacggcttcacactgacgttgccttcgttcttgaaaggtttgtgaaactcactgcacccaagaacattcagactcttgacctcatgccaatccctgcacacacgctggttggagcactgcttcggtctgcgacgtactacttctcagacctggaggttgcgcttgtccacacgggcccagccacctgggtgcccaatggctcacccaaaaccgctctcgacaaccagaccaacccaactgcctaccagaagcaacccattacccgcctggcgctcccctacaccgccccccaccgtgtactggcgacagtgtacaacgggaggacgacgtacggggaaacaacttca------cggcgcggtgacatggcaaccctcgcacagaggctgagtgggcaactgcccacctcattcaactacggcgctgtaaaggctgacactatcactgag

>DQ989312.1_Asia1_IND_1990

tcgagcgttggcgtgacgtatggttacgctgtggccgaggacgcggtgtcaggacctaacacatcaggtcttgagacccgtgttcaacaagcagaacggttctttaaaaagcacttgtttgactggacaccgaatctggcatttggacattgttgctacctggaactcccaactgagcacaaaggcgtgtatggcagtctcatggactcgtacgcctacatgaggaatggatgggacatagaggtgactgctgttggaaaccaattcaacggcggttgtcttcttgttgcacttgtgccggaactgaagagccttgacacgcggcagaaataccagctgactctctttccccaccagttcattaacccacgcaccaacatgacggcccacatcaacgtgccgttcgtgggtgtcaacagatacgaccagtacgcgcttcacaaaccgtggacgctcgtagtgatggtggtggccccactcactgtcaaaactggtggttctgaacagattaaggtttacatgaatgcagcaccgacctacgtgcacgtggcgggagagctgccctcgaaagagggaatagtccccgttgcgtgcgtggacggttatggcaacatggtgaccacggacccgaagactgctgacccagtttacgggaaagtctacaacccccccagaacaaacctcccagggcgttttacaaacttccttgatgttgcggaggcatgcccaaccttcctccgcttcggagaa---gtgccatttgtgaagacggtgaactctggtgaccgcctactggccaagttcgatgtctcgcttgccgcggggcacatgtccaacacctacttggctggtttggcgcagtactacacacagtacagcggcaccataaatattcacttcatgttcaccgggcccacagatgccaaagcccgctgtatggtggcctacatccctcccggcatgacaccgcccacagaccctgagcgcgctgcgcattgcatccactctgagtgggacactggtctcaactccaagttcaccttttctataccgtacctttctgctgctgactacgcctacactgcttctgacgcggcggaggccacaagcgtgcaggggtgggtgtgcatctaccaaatcactcacggcaaggctgaaggcgacgccctggtcgtttctgtcagcgccggcaaggattttgagttccgactgcccgttgacgcgcgacggcaaaccaccacaaccggtgagtcagcggatccagtgacaaccacagtcgagaactacggaggagaaactcaggcggtcagacggcttcacactgatgttgcctttgttcttgacaggtttgtgaaactcactacacctaagagcacccagacccttgatctcatgcaggtgccttcacacacgttggtaggagcactgcttcggtctgcaacgtactacttctcagacctagaggttgcgcttgtccacacaggcccggttacctgggtgcccaacggctcgcccaagaatgccctggacaaccaaactaacccaactgcttaccagaagcagcccatcacccgcctagcgctcccctacaccgccccccaccgtgtgctggcaacagtgtacaacgggaagacgacgtacggggaaacaacctca------cggcgcggtgacatggcggcccttgcacaaagggtgatggggcaactgcccacctctttcaattacggcgctgtaaaggctgaaaccatcactgag

>DQ989313.1_Asia1_IND_1986

tcgagtgtcggcgtgacctacggttacgctgtgaccgaggatgcggtgtcagggcccaacacttcgggcctcgagacccgcgttcagcaggctgagcggttcttcaagaaacacctgtttgattggacaccgaacctggcatttggacactgtcactatctggaacttcccactgaacacaagggcgtgtacggcggtctcatggcctcgtacgcgtacatgagaaacgggtgggacgtcgaggtgaccgctgttggaaatcagttcaacggtggttgtctccttgtcgcgctcgtaccagagttgaagagtctcgacacacggcaaaagtaccagttgactctcttcccccaccagttcatcaacccacgcaccaacatgacggcgcacattagcgtgccgtttgtgggtgtcaacaggtatgatcagtacgcgttacacaaaccgtggacgctcgttgtgatggtagtggccccacttaccgtcaagactggtggttctgagcaaattaaggtttacatgaatgcagcaccaacctatgtgcatgtggcgggagagttgccctcgaaagagggcatcgtacccgttgcgtgtgcggacggctatggcaacatggtgaccacagacccgaagacggctgaccccgtttacgggaaggtgttcaacccccccaggacaaatctccctgggcgcttcacaaacttccttgatgtcgcggaggcttgcccaaccttcctccgcttcggagaa---gtgccctttgtgaagacggtgaactctggtgaccgcttgcttgccaagtttgacgtgtcgctcgctgcggggcacatgtccaacacttacttggctgggctagcacagtactacacacagtacagtggcaccatgaatatccacttcatgttcaccggtcccacggacggcaaagcgcgctacatggtggcttacgtcccccctggcatgacaccgcccactgaccctgagcacgctgcgcactgcattcactctgaatgggacactggtcttaattccaaattcactttttccataccctacctttctgctgctgactacgcttacactgcttctgatgtggcggagaccacgagtgtacagggatgggtgtgcatctaccagatcacacacggcaaagctgagggagacgcactggtcgtttctgttagcgccggtaaggactttgagtttcgcctaccagtggacgcgcgccagcaaaccaccactgctgccgaatcggcagatccagtcacaaccacggttgagaactacggaggagagactcagacggccagacggcttcacactgatgttgcctttgttcttgacaggtttgtgaaactcactagccccaagaacacccagacccttgatctcatgcagatccccttacacacgctggttggggcgctgcttcggtctgcgacgtactacttttcagacctggaggttgcgcttgtccacacaggcccggtcacgtgggtgcccaacggcgcgcccaagactgccttgtacaaccaaaccaacccaaccgcataccagaagcagcccatcacacgcctggcgctcccttacaccgctccccaccgtgtgctggcgacagtgtacaacgggaagacggcatacgggacggagacccca------gggcgcggtgatctagcggccattgcacaaagggtgaacaacagcttacccacctccttcaactacggtgctgtcagggctgacaccatcactgag

>DQ989314.1_Asia1_IND_2001

tcgagcgtcggcgtgacgtatggttatgctgtggctgaggatgcggtgtcagggcctaacacctcaggcctggagacccgcgttcaccaagcggaacggttctttaagaagcacttgtttgactggacaccgaatttggcatttggacactgtcactacctggaacttcctactgaacacaaaggggtgtacggcagtctcatggactcgtacgcctacatgaggaacggctgggacattgaggtgaccgctgttggaaaccaattcaacggcggttgtctcctcgtcgcacttgtgccagagctgaagagccttgacacgcggcagaaataccagctgacactctttccccaccaattcatcaacccacgcaccaacatgacggctcacatcaacgtgcctttcgtgggtgtcaacaggtacgaccagtacgcgcttcacaagccgtggacgctcgttgtaatggtagtggccccactcaccgtcaagactggtggttctgaacaaatcaaggtttacatgaacgctgcaccgacctacgtgcacgtggcaggcgaactgccctcgaaagagggaatagtacccgttgcatgcgcggacggttatggcaacatggtgaccactgacccaaagactgctgacccagtgtacggaaaagtgttcaacccccccaggacgaacctccctgggcgcttcacaaacttccttgatgtcgcggaggcttgcccaactttcctccgcttcggagag---gtgccatttgtgaagacagtgaattctggtgaccgtttgctggccaagtttgatgtctcgcttgctgcggggcacatgtctaacacctacttggccggcctggcgcagtactacacacagtacagtggtaccatgaatgttcacttcatgttcaccgggcctacggacgccaaggctcgttacatggtggcatacatcccccccggcatgacaccgccaactgaccccgagcgcgccgcccactgcattcactctgagtgggacactggtcttaactccaagttcaccttttccataccctacctctctgctgctgactatgcgtacactgcttctgacacggcggagaccacaagtgtgcaaggttgggtgtgcatctaccagatcacccacggcaaggctgaaggagacgccctggttgtttctgtcagtgccggcaaagactttgagttccgcttgcctgtcgacgcacgccggcaaaccaccacaaccggcgagtcggcggacccggtaacaaccacggttgagaactacggaggagaaactcagacggccagacggctccacactgacgttgccttcgttctcgacaggtttgtgaaactcactgcacccaagaacgctcagaccctcgacctcatgcagatcccctcacacacgctggtcggggcactgcttcggtctgcgacgtactacttctcagacctggaggttgcgcttgttcacacaggcccgatcacctgggtgcccaacggctcacccaaggacgctctggacaatcaaaccaacccaactgcctaccagaaacagcccatcacccgcctagcactcccctacaccgccccccatcgtgtgctggcaacagtgtacaacgggaagacgacgtacggggaaacgccctca------cggcgcggtgacatggcagcccttacacagagactgagcgagcggctgcccacctccttcaactacggcgctgtaaaggctgaaaccatcaatgag

>DQ989315.1_Asia1_IND_1993

tcgagcgttggcgtgacgtacggttacgctgtggctgaggatgcggtgtcaggacctaacacctcaggtctggagacccgtgttcaccaagcagaacggttcttcaaaaagcacttgtttgactggacaccgaatttggcatttggacactgtcactacctggaactccctactgaacacaaaggcgtgtacggtagtctcatggactcgtacgcctacatgaggaacgggtgggacatagaggtgactgctgttggaaaccaattcaacggcggttgtctccttgttgcacttgtgccagagctgaagaaccttgacacacggcagaaataccagctgaccctctttccccaccagttcatcaacccacgtaccaacatgacggctcacatcaacgtgccttttgtgggtgttaacaggtacgaccagtacgcgcttcacaaaccgtggacgctcgttgtgatggtgttggccccacttaccgttaagactggtggttctgaacagatcagggtttacatgaatgctgcaccaacctacgtgcacgtggcaggggagctgccctcgaaagaggggatagttcccgttgcgtgtgcggacggttacggcaacatggtgaccacggacccgaagactgctgacccagtgtacgggaaagtgttcaacccccccaggacgaaccttcccgggcgcttcacaaacttccttgatgtcgcggaggcgtgtccaaccttcctccgcttcggagaa---gtgccatttgtgaagacagtgaactctggcgaccgtctgctggccaagttcgatgtctcgctcgccgcggggcatatgtccaacacctacttggccggactggcacagtactacacacagtacagtggcaccataaatgttcacttcatgttcaccgggcccacggacgccaaggctcgttacatggtggcctacattccccccggcatgacaccgcccactgaccctgagcgcgccgcccattgcattcactctgagtgggatactggtcttaactccaagttcaccttttccataccctacctctctgctgctgactacgcgtacactgcttctgacacggcggagaccacaagtgtgcaaggatgggtgtgcatctatcagatcacccacggcaaggctgaaggagacgccctggtcgtgtctgtcagcgccggcaaagactttgagttccgcctgccagtggatgctcgccgacaaaccaccacaaccggcgagtcggcggacccggtcacaaccacggtcgagaagtacggaggagaaactcagacggccagacggcttcacacggatgttgccttcggtcttgacaggtttgtgaaactcacttcacccaagagcacccagaccctagaccttattcagatccccccacacacgttggtcggggcactacttcggtcttcgacgtactacttctcagacctggaggtcgcgcttgtccacacaggcccggtcacctgggtgcccaacggctcacccaaggatgctctagacaaccagaccaacccaactgcctaccagaaacagcccatcacccgcctggcactcccctacaccgccccccaccgtgtgctggcgacagtgtacaacgggaagacgacgtacggggaaacaactcca------cgccgtggtgatatggcggcccttgcgcagaggctgagcgggcggctgcccacctccttcaactacggcgctgtaaaggctgaaagcattactgag

>DQ989317.1_Asia1_IND_2000

tcgagcgtcggcgtgacgtatggttatgctgtggctgaggatgcggtgtcagggcctaacacctcaggcctggagacccgcgttcaccaagcggaacggttcttcaagaagcacttgtttgactggacaccgaatttggcatttggacactgtcactacctggaacttcctactgaacacaaaggggtgtacggcagtctcatggactcgtacgcctacatgaggaacggctgggacattgaggtgaccgctgttggaaaccaattcaacggcggttgtctcctcgtcgcacttgtgccagagctgaagagccttgacacgcggcagaaataccagctgacactctttccccaccaattcatcaacccacgcaccaacatgacggctcacatcaacgtgcctttcgtgggtgtcaacaggtacgaccagtacgtgcttcacaaaccgtggacgctcgttgtgatggtagtggccccactcaccgtcaagactggtggttctgaacaaatcaaggtttacatgaatgctgcaccgacctacgtgcacgtggcaggcgaactgccctcgaaagagggaatagtacccgttgcatgcgcggacggctatggcaacatggtgaccactgacccaaagactgctgacccagtgtacggaaaagtgttcaacccccccaggacgaacctccctgggcgcttcacaaacttccttgatgtcgcggaagcttgcccaactttcctccgcttcggagaa---gtgccatttgtgaagacagtgaattctggtgaccgtttgctggccaagtttgatgtctcgctcgctgcggggcacatgtctaacacctacttggccggcctggcgcagtactacacacagtacagtggtaccatgaatgttcacttcatgttcaccgggcctacggacgccaaggctcgttacatggtggcatacatcccccccggcatgacaccgccaactgacccggagcgcgccgcccattgcattcactctgagtgggacactggtcttaactccaagttcaccttttccataccctacctctctgctgctgactatgcgtacactgcttctgacacggcggagaccacaagtgtgcaaggttgggtgtgcatctaccagatcacccacggcaaggctgaaggagacgccctggttgtttctgtcagtgccggcaaagattttgagttccgcttgcctgtcgacgcacgccggcaaaccaccacaaccggcgagtcggcggacccggtaacaaccacggttgagaactacggaggagaaactcagacgccagacggctcccacactgacgttgccttcgttctcgacaggtttgtgaaactcactgcacccaagaacactcagaccctcgacctcatgcagatcccctcacacacgctggtcggggcactgcttcggtctgcgacgtactacttctcagacctggaggttgcgcttgttcacacaggcccggtcacctgggtgcccaacggctcacccaaggacgctctggacaatcagaccaacccaactgcctaccagaaacagcccatcacccgcctagcactcccctacaccgccccccaccgtgtgctggcaacagtgtacaacgggaagacgacgtacggggaaacgccctca------cggcgcggtgacatggcagcccttgcacagagactgagcgagcggctgcccacctccttcaactacggcgctgtaaaggctgaaaccatcactgag

>DQ989318.1_Asia1_IND_2002

tcgagcgtcggcgtgacgtatggttatgctgtggctgaggatgcggtgtcagggcctaacacctcaggcctggagacacgcgttcaccaagcggaacggttcttcaagaagcacttgtttgactggacaccgaatttggcatttggacactgtcactacctggaacttcctactgaacacaaaggggtgtacggcagtctcatgggctcgtacgcctacatgagggacggctgggacattgaggtgaccgctgttgggaaccaattcaacggcggttgtcttctcgtcgcacttgtgccagagctgaagagccttgacacgcggcagaaataccagctgacactctttccccaccaattcatcaacccacgcaccaacatgacggctcacatcaacgtgcctttcgtggttgtcaacaggtacgaccagtacgcgcttcacaagccgtggacgctcgttgtgatggtagtggccccactcaccgtcaagactggtggttctgaacaaatcaaggtttacatgaatgctgcaccgacctacgtgcacgtggcaggcgaactaccctcgaaagagggaatagtacccgttgcatgcgcggacggttatggcaacatggtgaccactgacccaaagactgctgacccagtgtacggaaaagtgttcaacccccccaggacgaacctccctgggcgcttcacaaacttccttgatgtcgcggaggcttgcccaactttcctccgcttcggagag---gtgccatttgtgaagacagtgaattctggtgaccgtttgctggccaagtttgatgtctcgcttgctgcggggcacatgtctaacacctacttggccggcctggcgcagtactacacacagtacagtggtaccatgaatgttcacttcatgttcactgggcctacggatgccaaggctcgttacatggtggcatacatcccccccggcatgacaccgccaactgaccccgagcgcgccgcccactgcattcactctgagtgggacactggtcttaactccaagttcaccttttccataccctacctctctgctgctgactatgcgtacactgcttctgacacggcggagaccacaagtgtgcaaggttgggtgtgcatctaccagatcacccacggcaaggctgaaggagacgccctggttgtttctgtcagtgccggcaaagattttgagttccgcttgcctgtcgacgcacgccggcaaaccactacaaccggcgagtcggcggacccggtaacaaccacggttgagaactacggaggagaaactcagacggccagacggctccacactgacgttgccttcgttttcgacaggtttgtgaaactcactgcacccaagagcgctcagaccctcgacctcatgcagatcccctcacacacgctggtcggagcactgcttcggtctgcgacgtactacttctcagacctggaggttgcgcttgttcacacaggcccggtcacctgggtgcccaacggctcacccaaggacgctctggacaatcagaccaacccaactgcctaccagaaacagcccatcacccgcctagcactcccctacaccgccccccatcgtgtgctggcaacagtgtacaacgggaagacgacgtatggggaaacgccctca------cggcgcggtgacatggcagcccttacacagagactgagcgagcggctgcccacctccttcaactacggcgctgtaaaggctgaaaccatcactgag

>DQ989319.1_Asia1_IND_2001

tcgagcgtcggcgtgacgtatggttatgctgtggctgaggatgcggtgtcagggcctaacacctcaggcctggagacccgcgttcaccaagcggaacggttctttaaaaagcacttgtttgactggacaccgaatttggcatttggacactgtcactacctggaacttcctactgaacacaaaggggtgtacggcagtctcatggactcgtacgcctacatgaggaacggctgggacattgaggtgaccgctgttggaaaccaattcaacggcggttgtctcctcgtcgcacttgtgccagagctgaagagccttgacacacggcagaaataccagctgacactctttccccaccaattcatcaacccacgcaccaacatgacggctcacatcaacgtgcctttcgtgggtgtcaacaggtacgaccagtacgcgcttcacaagccgtggacgctcgttgtgatggtagtggccccactcaccgtcaagactggtggttctgaacaaatcaaggtttacatgaatgctgcaccgacctacgtgcacgtggcaggcgaactgccctcgaaagagggaatagtgcccgttgcatgcgcggacggttatggcaacatggtgaccactgacccaaagactgctgacccagtgtacggaaaagtgttcaacccccccaggacgaacctccctgggcgcttcacaaacttccttgatgtcgcggaggcttgcccaactttcctccgcttcggagag---gtgccatttgtgaagacggtgaattctggtgaccgtttgctggccaagtttgatgtctcgcttgctgcggggcacatgtctaacacctacttggccggcctggcgcagtactacacacagtacagtggtaccatgaatgttcacttcatgttcactgggcctacggacgccaaggctcgttacatggtggcatacatcccccccggcatgacaccgccaactgaccccgagcgcgccgcccactgcattcactctgagtgggacactggtcttaactccaagttcaccttttccataccctacctctctgctgctgactatgcgtacactgcttctgacacggcggagaccacaagtgtgcaaggttgggtgtgcatctaccagatcacgcacggacatgctgaaggagacgccctggttgtttctgtcagtgccggcaaagattttgagttccgcttgcctgtcgacgcacgccggcaaaccactacaaccggcgagtcggccgacccggtgaccaccacggttgagaactacggaggtgaaactcaaacggccagacggctccacactgacgttcgcttcgttctcgacaggtttgtgaaactcactgcacccaagaacgctcagaccctcgacctcatgcagatcccctcacacacgctggtcggagcactgcttcggtctgcgacgtactacttctcagacctggaggttgcacttgttcacacaggcccggtcacctgggtgcccaacggctcacctaaggacgctctggacaatcagaccaacccaactgcctaccagaaacagcccatcacccgcctagcactcccctacaccgccccccatcgtgtgctggcaacggtgtacaacgggaagacgacgtacggggaaacgccctca------cggcgcggtgacatggcggcccttgcacagagactgagcgagcggctgcccacctctttcaactacggcgctgtgaaggctgaaaccatcactgag

>DQ989320.1_Asia1_IND_2002

tcgagcgtcggcgtgacgtatggttatgctgtggctgaggatgcggtgtcagggcctaacacctcaggcctggagacccgcgttcaccaggcggaacggttcttcaagaagcacctgtttgactggacaccgaatttggcatttggacactgtcactacctggaacttccttctgaacacaaaggggtgtacggcagtctcatggactcgtacgctacaatgaggaacggctgggacattgaggtgaccgctgttggaaaccaattcaacggcggttgtctcctcgtcgcacttgtgccagagctgaaagagcttgacacgcggcagaaataccagctgacactctttccccaccaattcatcaacccacgcaccaacatgacggctcacatcaacgtgcctttcgtggttgtcaacaggtacgaccagtacgcgcttcacaagccgtggacgctcgttgtgatggtagtggccccactcaccgtcaagactggtggttctgaacaaatcaaggtttacatgaatgctgcaccgacccacgtgcacgtggcaggcgaactgccctcgaaagagggaatagtacccgttgcatgcgcggacggttatggcaacatggtgaccactgacccaaagactgctgacccagtgtacggaaaagtgttcaacccccccaggacgaacctccctgggcgcttcacaaacttccttgatgtcgcggaggcttgcccaactttcctccgcttcggagag---gtgccatttgtgaagacagtgaattctggtgaccgtttgctggccaagtttgatgtctcgcttgctgcggggcacatgtctaacacctacttggccggcctggcgcagtactatacacagtacagtggtaccatgaatgtccacttcatgttcactgggcctacggacgccaaggctcgttacatggtggcatacatccctcccggcatgacaccgccaactgaccccgagcgcgccgcccactgcattcactctgagtgggacactggtcttaactccaagttcaccttttccataccctacctctctgctgctgactatgcgtacactgcttctaacacggcggagaccacaagtgtgcaaggttgggtgtgcatctaccagatcacccacggcaaggctgaaggagacgccctggttgtttctgtcagtgccggcaaagattttgagttccgcttgcctgtcgacgcacgccggcaaaccactacaaccggcgagtcggcggacccggtcaccactacggttgcgaactacggcggagaaactcaaacggccagacggctccacactgaagttgcctttggtctcgacaggtttgtgaaactcactgcacctaagaaagctcagaccctcgaccttatgcagatcccttcacaaacgctggtcggggcgctgcttcggtctgcgacgtactacttctcagacctggaggttgcgcttgttcacacaggcctggtcacctgggtgcccaacggctcacccaaggacgctctggacaatcagaccaacccaactgcctaccagaaacagcccataacccgcttggcactcccctacaccgccccccaccgtgtgctggcaacagtgtacaacgggaagacgacgtacggggaaacgccctca------cggcgcggtgacatggcagcccttacacagagactgagcgagcggctgcccacctccttcaactacggcgctgtaaaggctgaaaccatcactgag

>DQ989321.1_Asia1_IND_2001

tcgagcgtcggcgtgacgtatggttatgctgtggctgaggatgcggtgtcagggcctaacacctcaggcctggagacccgcgttcaccaagcggaacggttcttcaagaagcacttgtttgactggacaccgaatttggcatttggacactgtcactacctggaacttcctactgaacacaaaggggtgtacggcagtctcatggactcgtacgcctacatgaggaacggctgggacattgaggtgaccgctgttggaaaccaattcaacggcggttgtctcctcgtcgcacttgtgccagagctgaagagccttgacacgcggcagaaataccagctgacactctttccccaccaattcatcaacccacgcaccaacatgacggctcacatcaacgtgcctttcgtgggtgtcaacaggtacgaccagtacgcgcttcacaagccgtggacgctcgttgtgatggtagtggccccactcaccgtcaagactggtggttctgaacaaatcaaggtttacatgaatgctgcaccgacctacgtgcacgtggcaggcgaactgccctcgaaagagggaatagtacccgttgcatgcgcggacggctatggcaacatggtgaccactgacccaaagactgctgacccagtgtacggaaaagtgttcaacccccccaggacgaacctccctgggcgcttcacaaacttccttgatgtcgcggaggcttgcccaactttcctccgcttcggagag---gtgccatttgtgaagacagtgaattctggtgaccgtttgctggccaagtttgatgtctcgcttgctgcggggcacatgtctaacacctacttggccggcctggcgcagtactacacacagtacagtggtaccatgaatgttcacttcatgttcactgggcctacggacgccaaggctcgttacatggtggcatacatcccccccggcatgacaccgccaactgacccggagcgcgccgcccactgcattcactctgagtgggacactggtcttaactccaagttcaccttttccataccctacctctctgctgctgactatgcgtacactgcttctgacacggcggagaccacaagtgtgcaaggttgggtgtgcatctaccagataacgacggtcaaggccgaaggagacgccctggttgtatctgtcagtgccggcaaagattttgagttccgcttgcctgtcgacgcacgtcggcaaaccaccacaaccggcgagtcggcggacccggtaacaaccacggttgagaactacggaggagaaacgcagacggccagacgtcgccacacagacgtcgccttcgttcttgacaggtttgtgaaactcactgcacccaagaacgctcagaccctcgacctcatgcagatcccctcacacacgctggtcggggcactgcttcggtctgcgacgtactacttctcagacctggaggttgcgcttgttcacacaggcccggtcacctgggtgcccaacggctcacccaaggacgctctggacaatcagaccaacccaactgcctaccagaaacagcccatcacccgcctagcactcccctacaccgccccccatcgtgtgctggcaacagtgtacaacgggaagacgacgtacggggaaacgccctca------cggcgcggtgacatggcggcccttacacagagactgagcgagcggctgcccacctccttcaactacggcgctgtaaaggctgacaccatcactgag

>DQ989322.1_Asia1_IND_2002

tcgagcgtcggcgtgacgtacggttatgctgtggctgaggatgcggtgtccgggcctaatacctcaggcctggagacccgcgttcaccaagcggaacggttcttcaagaagcacttgtttgactggacaccgaatttggcatttggacactgtcactacctggaacttcctactgaacacaaaggggtgtacggcagtctcatggactcgtacgccaacatgaggaacggctgggacatagaggtgaccgctgtgggaaaccaattcaacggcggttgtctcctcgtcgcacttgtgccagagctgaagagccttgacacgcggcagaaataccagctgacactcttcccccaccaattcatcaacccacgcaccaacatgacggctcacatcaacgtgccttccgtgggtgccaacaggtacgaccagtacgcgcttcacaagccgtggacgctcgttgtgatggtagtggccccactcaccgtcaagactggtggttctgagcaaatcaaggtttacatgaatgctgcaccgacctacgtgcacgtggcaggcgaactgccctcgaaagagggaatagtacccgttgcatgcgcggacggttatggcaacagggtgaccactgacccaaagacggctgacccagtgtacggaaaagtgttcaacccccccaggacgaacctccctgggcgcttcacaaatcttcctgatgtcgcggaggcttgcccaactttcctccgcttcggagag---gtgccatttgtgaagacagtgaattctggtgaccggttactggccaagtttgatgtgtcgcttgctgcggggcacatgtctaacacctactttgccggcctggcgcagtactacacacaatacagtggtaccatgaatattcacttcatgttcactgggcctacggacgccaaggctcgttacatggtggcatacatgccccccggcatgacaccgcaactggaccccgagcgggccgcccactgcattcactctgagtgggacactggtcttaactccaatttcacctttccatacccttacctctctgctgctgactatgcttacactgcttctgacacggccgagaccacaagtgtgcaaggttgggtgtgcatctaccaaatcacccacgtcaaagctgaaggagacgccctggttgtttctgtcagtgccggcaaagattttgagtttcgtttgcctgtggacgcacgccgccaaactactactaccggcgagtcggcggacccggtaaccaccacggttgagaactacggaggagaaactcagacagcaagacggctccacactgacgttgccttcgttctcgacaggtttgtgaaactcactgcacccaagaacgctcagaccctcgacctcatgcagatcccctcacacacgctggtcggggcactgcttcggtctgcgacgtactacttctcagacctggaggttgcgcttgttcacacaggcccggtcacctgggtgcccaacggctcacccaaggacgctctggacaatcagaccaacccaactgcctaccagaaacagcccatcacccgcctagcactcccctacaccgccccccatcgtgtgctggcaacagtgtacaacgggaagacaacgtacggggaaacgccctca------cggcgcggtgacatggcagcccttacacagagactgagcgagcggctgcccacctccttcaactacggcgctgtaaaggctgaaaccatcactgag

>DQ989323.1_Asia1_IND_2002

tcgagcgtcggcgtgacgtatggttatgctgtggctgaggatgcggtgtcagggcctaacacctcaggcctggagacccgcgttcaccaagcggaacggttcttcaagaagcacttgtttgactggacaccgaatttggcatttggacactgtcactacctggagcttcctactgaacacaaaggggtgtacggcagtctcatggactcgtacgctacaatgaggaacggctgggacattgaggtgaccgccgttggaaaccaattcaacggcggttgtctcctcgtcgcacttgtgccagagctgaaggagcttgacacgcggcagaaataccagctgacactcttcccccaccaattcatcaacccacgcaccaacatgacggctcacatcaacgtgccgttcgtgggtgtcaacaggtacgaccagtacgcgctccacaagccgtggacgctcgttgtgatggtagtggctccactcaccgtcaaaactggtggttctgaacaaatcaaggtttacatgaatgctgcgccgacccacgtgcacgtggcaggcgaactgccctcgaaagaggggatagtacccgttgcatgcgcggacggttatggcaacatggtgaccactgacccaaagactgctgacccagtgtacggaaaagtgtacaacccccccaggacgaacctccctgggcgcttcacaaacttccttgatgtcgcggaggcttgcccaactttcctccgcttcggagag---gtgccatttgtgaagacagtgaattctggtgaccgtttgctggccaagtttgatgtctcgcttgctgcggggcacatgtctaacacctacttggccggcctggcgcagtactacacacagtacagtggtaccatgaatgttcacttcatgttcactgggcctacggacgccaaggctcgttacatggtggcatacatcccccccggcatgacaccgccaactgaccccgagcgcgccgcccactgcattcactctgagtgggacactggtcttaactccaagttcaccttttccataccctacctctctgctgctgactatgcgtacactgcttctaacacggcggagaccacaagtgtgcaaggttgggtgtgcatctaccagatcacccacggcaaggctgaaggagacgccctggttgtttctgtcagtgccggcaaagattttgagttccgcttgcctgtcgacgcacgccggcaaaccaccacaaccggcgagtcggcggacccggtaacaaccacggttgagaactacggaggagaaactcagacggccagacggctccacactgacgttgccttcgttctcgacaggtttgtgaaactcactgcacccaagaacgctcagaccctcgacctcatgcagatcccctcacacacgctggtcggggcactgcttcggtctgcgacgtactacttctcagacctggaggttgcgcttgttcacacaggcccggtcacctgggtgcccaacggctcacccaaggacgccctggacaatcagaccaacccaactgcctaccagaaacagcccatcacccgcctagcactcccctacaccgccccccatcgcgtgctggcaacagtgtacaacgggaagacgacgtacggggaaacgccctca------cggcgcggtgacatggcagcccttacacagagactaagcgagcggctgcccacctccttcaactacggcgctgtaaaggctgaaaccatcactgag

>EF117837.1_A_PAK_2006

tcgagtgtgggagtcacctacgggtactccactggggaagaccatgtctccggacctaacacatctggcctggagacgcgagtggtacaggcagagagattcttcaagaagtatttgtttgattggacaactgataaagcttttggacacctggaaaaactggaactccccaccgaacacaagggtgtctacgggcacttggtggactctttcgcatacatgagaaatggctgggacgtggaggtgaccgccgttggcaaccagttcaacggtgggtgtctcctggtggccatggtacctgagtggaaagagtttacccctcgtgagaaataccagctcaccttgtttccacaccaatttatcaaccccagaaccaacatgacagcccacatcacggtcccgtaccttggtgtcaataggtatgaccagtacaaacagcacaaaccctggacactggtcgtgatggtggtttcgccgctgaccaccagcagcattggagcctcacagatcaaggtgtatgccaacattgccccaaccttcgttcacgtggccggcgagctcccgtcgaaagaagggatcgtgccggttgcttgttcagacgggtacggtggcctggtgacaacagacccgaaaacagctgaccctgtttatggtatggtgtacaacccgcccagaaccaactaccctgggcgctttacaaacttgttggacgtggccgaggcttgcccgaccttcctctgttttgacgacgggaaaccgtacgttgtgacaaggacggacgaccaacgcctcctggccaagttcgacgtttctcttgctgcaaagcacatgtcaaacacctacctctcagggatagcacagtactacacgcagtactctggcactatcaacctgcacttcatgttcactggctctactgaatcaaaggcccggtacatggtggcgtacattccacctggcatggacccaccagacacacctgagaaggctgcacattgcatccacgccgagtgggacaccgggctgaactccaaatttactttttctatcccgtacgtgtctgctgcagactacgcatacaccgcgtctgacgtggcagaaacaacaaacgtacaggggtgggtctgcatataccaaatcactcacgggaaggctgagcaggacactctggtcgtgtcggtcagcgccggcaaggactttgaactgcgcctcccaattgacccccgcacgcaaaccaccactgccggggagtcagcagaccctgtcaccaccaccgttgagaactacggtggtgagacacaggctcggcgacgtcagcacactgacgtcggcttcatcatggacaggtttgtgaaaatcagccccgggagccccacgcacgtcattgacctcatgcaaacacaccaacacgcgttggtgggtgcccttttgcgtgcagccacgtactacttctccgatctggagattgtggtgcgtcatgatggcaacttgacgtgggtgcccaatggagcacctgtagaagccttggccaacacaagcaaccccaccgcctaccacaagcagccatttacgagacttgcgctcccttacaccgcgccgcaccgagtgttggcaacagtgtataacggagtaagcaagtactctacaactggtggt---ggtagaaggggtgacctgggacctcttgcggcgcgggtcgccacacagctccccagctctttcaactttggtgcaattcgggccacgaccatccacgag

>EF149009.1_Asia1_CHA_2005

tcgagtgttggcgtaacatatggttacgctgtggctgaggacgcggtatctgggcctaacacctcaggcctggagacccgcgtgacacaggctgaacggttcttcaagaaacacctgtttgactggacgccggatttgtcatttggacactgtcactacttggaactcccctctgaacacaagggcgtgtttggcagcctcatgagctcttatgcttacatgaggaacgggtgggacattgaggtgaccgctgttggaaatcagttcaatggtggttgtctcctcgtcgcactcgtgccggagctgaaagagctcgacacgcggcagaagtatcagttaaccctcttcccacaccagttcattaacccgcgcactaacatgacggctcacattaacgtgccgtacgtgggtgtcaacaggtacgaccagtacgagctccacaaaccgtggacgcttgtggtgatggtggtggccccgcttaccgtcaaaactggtggttctgaacagatcaaggtctacatgaatgcagcgccgacctacgtgcacgtggcaggagaactgccctcgaaagaggggatagttcctgtggcgtgtgtggacggttacggcaacatggtaaccacggacccgaagacggctgaccccgtctacgggaaagtgtctaacccccccagaacaagcttccctgggcgcttcacaaacttccttgatgtagcggaggcgtgtccaaccttcctccgcttcggagaa---gtaccatttgtgaagacggtgaactctggtgaccgcttgcttgccaagtttgacgtgtccctcgctgcggggcacatgtccaacacctacttggcaggtttggcgcagtactacacacagtacagcggcactatgaatatccacttcatgttcaccggacccacggatgccaaagcccgctacatggtggcttacatacctcctggtatgacgccgccaacggacccggagcgggctgcacactgcattcattctgagtgggacactggactcaattctaaatttaccttttctatcccttacctttctgctgcagactatgcttacactgcttctgacgtggctgagaccacgagtgtgcagggatgggtgtgtatttaccagatcacccacggaaaagctgaaggtgacgcgctggtcgtgtccgtcagcgctggcaaggactttgagtttcgactgccggtggatgcccgccaacagactaccaccactggcgagtccgcggacccagtcaccaccacggttgagaactacggaggagagacccagacggcccgacggcttcacactgatgtcgccttcgttctcgacaggttcgtgaaactcacccagcccaagagcacccaaacccttgatctcatgcagatcccctcacacacactggtcggggcgcttctccggtctgcgacgtactacttctcagacctggaggttgcgctcgtccacacaggaccggtcacgtgggtgcccaatggtgcgcccaagaccgccttgaacaaccacaccaacccgactgcttaccagaagcagcctatcacccgcttggcactcccctacaccgctccccaccgtgtgctgtcaacagtgtacaacgggaagacaacgtacggagaagaatcctcg------cggcgtggtgatctcgccgcccttgcacgcagagtgaacaaccggctgcccacttccttcaactacggcgctgtgaaggccgacaccatcacggag

>EF149010.1_Asia1_CHA_2005

tcgagcgttggcgtgacatacggttacgctgtggctgaggacgcggtatcaggacctaacacctcaggtctggagacccgtgttcaacaggcggaacggttcttcaaaaaacacttgtttgactggacaccgaatttggcatttggacactgtcactacctggaactccccactgaacacaaaggtgtgtacggcagtctcatggactcgtacgcctacatgagaaacgggtgggacatagaggtgactgctgttggaaaccagttcaacggcggttgtctccttgtcgcacttgtgccagagctgaagagccttgacacgcggcagaagtaccagctgacccttttcccccatcagttcatcaacccacgcaccaacatgacggcccacatcaacgtgccctttgtgggtgttaacaggtatgaccagtacatgctccacaaaccgtggacgcttgttgtgatggtggtggccccactcaccgtcaagactggtggttctgaacagatcaaggtctacatgaatgcagcaccgacctacgtgcacgtggcaggggagctcccctcgaaagaggggatagttcccgttgcgtgtgcggacggttacggtaacatggtgaccacggacccgaagactgccgacccagtgtacgggaaagtgttcaaccccccccggacgaatctccccgggcgcttcacaaacttccttgatgtcgcggaggcgtgtccaaccttcctccgcttcggagaa---gtaccatttgtgaagacggtgaactctggtgaccgtttgctagccaagtttgatgtgtcgctcgctgcgggccacatgtccaacacctacttggctggtctggcgcagtactacacacagtacagtggtaccatgaatgttcacttcatgttcaccgggcccacggatgccaaggcccggtacatggtggcctacattcctcccggcatgacaccgcccacggaccctgagcgcgccgcccactgcatccactctgagtgggacactggtctaaactccaaattcaccttttccataccctacctctctgctgctgactacgcatacaccgcttctgacacggcggagaccacaagtgtgcaaggatgggtgtgcatctaccagatcacccacggcaaggctgaaggagacgcactggtcgtttctgtcagcgccggcaaagactttgagtttcgcctgcccgttgacgcgcgccggcaaaccaccactaccggcgagtcagcagacccggtaacaaccacggtcgagaactacggaggagaaactcagacagccaggcggcttcacaccgacgttgcctttgttcttgacaggtttgtgaaactcactgcacccaagaacatccagacccttgaccttatgcaaattccctcacacacgctggttggagcactgctgcggtctgcgacgtactacttctcggacctagaggttgcgattgtccacacaggcccaatcacctgggtgcccaacggctcgcccaaggatgccctagacaaccagaccaacccaactgcttaccagaagcaacctgtcacccgcctggcgctcccctacaccgccccccaccgtgtgctggcgacagtgtacaacgggaagacgacgtacggggaaacaaccgag------cggcgtggcgatatggcggcccttgcacaaagattgagtgggcggttgcccacctcatttaactacggcgctgtaaaggctgaaaccatcactgag

>EF494486.1_A_TUR_2005

tcgagtgtgggagtcacctacgggtactccactggggaagaccacgtctctggacctaacacatctggcctggagacgcgagtggtacaggcagagagattcttcaagaaacacttgtttgattggacaaccgataaagcttttggacacctggaaaaactggaactccccactgaacacaagggtgtctacgggcacttggtggactctttcgcatacatgagaaatggctgggacgtggaggtgaccgccgttggcaaccagttcaacggtgggtgtctcctggtggccatggtacctgagtggaaagagtttacccttcgtgagaaataccagctcaccctgtttccacaccaatttatcaaccccagaaccaacatgacagcccacatcacggtcccgtaccttggtgtcaataggtatgaccagtacaaacagcacaaaccctggacactggtcgtgatggtggtttcgccactgaccaccagcagcattggagcttcacagatcaaggtctacgccaacattgccccaaccttcgttcacgtggccggcgagctcccatcgaaggaagggatcgtgccggttgcttgttcagacgggtacggtggcctggtgacaacagacccgaaaacagctgaccctgtttatggtatggtgtacaacccgcccagaaccaactaccctgggcgctttacaaacttgttggacgtggccgaggcttgcccgaccttcctctgttttgacgacgggaaaccgtacgttgtgacaaggacggacgaccaacgcctcctggccaagtttgacgtttctcttgctgcaaagcacatgtcaaacacctacctctcagggatagcacagtactacacacagtactctggcactatcaatctgcacttcatgttcactggctctactgaatcaaaggcccggtacatggtggcgtacattccacctggcatggacccaccggacacacctgagaaggctgcacattgcatccacgccgagtgggacaccgggctgaactccaaatttactttttctatcccgtacgtgtctgctgcagactacgcatacactgcgtctgacgtggcagaaacaacaaacgtacaggggtgggtctgcatataccaaatcacccacgggaaggctgagcaggacactctggtcgtgtcggtcagcgccggcaaggactttgaactgcgcctcccaattgacccccgcacccaaaccaccactgccggggagtcagcagaccctgtcaccaccaccgttgagaactacggtggtgagacacaggctcagcgacgtcagcacactgacgtcggcttcatcatggacaggtttgtgaaaatcagccccgtgagccccacgcacgtcattgacctcatgcaaacacaccaacacgcgttggtgggtgcccttttgcgtgcagccacgtactacttctccgatctggagatcgtggtgcgtcatgatggcaacttgacgtgggtgcccaatggagcacctgtagaagccttggccaacacaagcaaccccaccgcctaccacaagcagccatttacgagacttgcgctcccttacaccgcgccgcaccgagtgttggcaacagtgtataacggagtaagcaagtactctacaactggtaat---ggtagaaggggtgacctggggcctcttgcggcgcgggtcgccgcacagctccccagctctttcaactttggtgcaattcgggccacgaccatccacgag

>EF494487.1_A_PAK_2006

tcgagtgtgggagtcacctacgggtactccactggggaagaccatgtctccggacctaacacatctggcctggagacgcgagtggtacaggcagagagattcttcaagaagtatttgtttgattggacaactgataaagcttttggacacctggaaaaactggaactccccaccgaacacaagggtgtctacgggcacttggtggactctttcgcatacatgagaaatggctgggacgtggaggtgaccgccgttggcaaccagttcaacggtgggtgtctcctggtggccatggtacctgagtggaaagagtttacccctcgtgagaaataccagctcaccttgtttccacaccaatttatcaaccccagaaccaacatgacagcccacatcacggtcccgtaccttggtgtcaataggtatgaccagtacaaacagcacaaaccctggacactggtcgtgatggtggtttcgccgctgaccaccagcagcattggagcctcacagatcaaggtttacgccaacattgccccaaccttcgttcacgtggccggcgagctcccgtcgaaagaagggatcgtgccggttgcttgttcagacgggtacggtggcctggtgacaacagacccgaaaacagctgaccctgtttatggtatggtgtacaacccgcccagaaccaactaccctgggcgctttacaaacttgttggacgtggccgaggcttgcccgaccttcctctgttttgacgacgggaaaccgtacgttgtgacaaggacggacgaccaacgcctcctggccaagttcgacgtttctcttgctgcaaagcacatgtcaaacacctacctctcagggatagcacagtactacacgcagtactctggcactatcaatctgcatttcatgttcactggctctactgaatcaaaggcccggtacatggtggcgtacattccacctggcatggacccaccagacacacctgagaaggctgcacattgcatccacgccgagtgggacaccgggctgaactccaaatttactttttctatcccgtacgtgtctgctgcagactacgcatacaccgcgtctgacgtggcagaaacaacaaacgtacaggggtgggtctgcatataccaaatcactcacgggaaggctgagcaggacactctggtcgtgtcggtcagcgccggcaaggactttgaactgcgcctcccaattgacccccgcacgcaaaccaccactgccggggagtcagcagaccctgtcaccaccaccgttgagaactacggtggtgagacacaggctcggcgacgtcagcacactgacgtcggcttcatcatggacaggtttgtgaaaatcagccccgggagccccacgcacgtcattgacctcatgcaaacacaccaacacgcgttggtgggtgcccttttgcgtgcagccacgtactacttctccgatctggagattgtggtgcgtcatgatggcaacttgacgtgggtgcccaatggagcacctgtagaagccttggccaacacaagcaaccccaccgcctaccacaagcagccatttacgagacttgcgctcccttacaccgcgccgcaccgagtgttggcaacagtgtataacggagtaagcaagtactctacaactggtggt---ggtagaaggggtgacctgggacctcttgcggcgcgggtcgccacacagctccccagctctttcaactttggtgcaattcgggccacgaccatccacgag

>EF494488.1_A_PAK_2006

tcgagtgtgggagtcacctacgggtactccaccagggaagaccatgtctctggacccaacacatctggcctggagacgcgagtggtacaggcagagagattcttcaagaaacacttgtttgattggacaactgataaagcttttggacacctggaaaaactggaactccccactgaacacaagggtgtctacgggcacttggtggactctttcgcatacatgagaaatggctgggacgtggaggtgaccgccgttggcaaccagttcaacggtggatgtctcctggtggccatggtacctgagtggaaagagtttacccttcgtgagaaataccagctcaccctgtttccacatcaatttatcaaccccagaaccaacatgacagcccacatcacggtcccgtaccttggtgtcaataggtatgaccagtacaaacagcataaaccctggacactggtcgtgatggtggtttcgccactgaccaccagcagcattggagcctcacagatcaaggtctacgccaacatcgccccgaccttcgttcacgtggccggcgagctcccatcgaaagaggggatcgtgccggtcgcctgttcagacgggtacggtggcctggtgacaacagacccgaaaacagctgaccctatttatggtatggtgtacaacccgcccagaaccaactaccctgggcgcttcacaaacttgttggacgtggccgaggcttgcccgaccttcctctgttttgacgacgggaaaccgtacgttgtgacaaggacggacgcccaacgcctcctggccaagtttgacgtttctcttgctgcaaagcacatgtcaaacacctacctctcagggatagcacagtactacacgcagtactctggcactatcaacctgcatttcatgttcactggctctactgaatcaaaggcccggtacatggtggcgtacattccacctggcatggacccaccggatacacctgaggaagctgcacattgcatccacgccgagtgggacaccgggctgaactccaaatttactttttctatcccgtacgtgtccgctgcagactacgcatacactgcgtctgacgtggcagaaacaacaaacgtacaggggtgggtctgcatataccaaattactcacgggaaggctgaacaggacactctggtcgtgtcggtcagcgccggcaaggactttgaactgcgcctcccaattgacccccgcacgcaaaccaccactgccggggagtcagcagaccctgtcaccaccaccgttgagaactacggtggtgagacacaggctcagcgacgtcatcacactgacgtcggcttcatcatggacaggtttgtgaaaatcagccccgtgagccccacgcacgtcattgacctcatgcaaacacaccagcacgcgttggtgggtgcccttttgcgtgcagccacgtactacttctccgatctggagattgtggtgcgtcacgatggcaacttgacgtgggtgcccaatggagcacctgtagaagccttggccaacacaagcaaccccaccgcctaccacaagcagccatttacgagacttgcgctcccttacaccgcgccgcaccgagtgttggcaacagtgtacaacggagtaagcaagtactctacaactggtggt---ggtagaaggggtgacctggggtctcttgcggcgcgggtcgccgcacagctacccagctctttcaactttggtgcaattcgggccacgaccatccacgag

>EF552688.1_O_UKG_2001

tcgagcgttggagtcacttacgggtacgcaacagctgaggactttgtgagcggaccaaacacatctgggcttgagaccagggttgtgcaggcagagcggttctttaaaacccacttgttcgactgggtcaccagtgacccgtttggacggtgctatctgctggaactcccaactgaccacaaaggtgtctacggcagcctgaccgactcttatgcttacatgagaaacggttgggatgttgaggtcaccgcagtgggaaatcagttcaacggaggatgtctgttggtggccatggtgccagaactttgctctattgacaagagagagctgtaccagctcacgctctttccccaccagttcatcaacccccggacgaacatgacggcgcacatcactgtgccctttgttggcgtcaaccgctacgaccagtacaaggtacacaaaccttggaccctcgtggttatggttgtggccccgctgactgtcaacaccgaaggtgccccacagatcaaggtctatgccaacatcgcccctaccaacgtgcacgtcgcgggtgagttcccttctaaggaagggatcttccccgtggcatgtagcgacggttacggtggtctggtgaccactgacccaaagacggctgaccccgcctacgggaaagtgttcaatccacctcgcaacatgttgccggggcggttcaccaacttccttgatgtggctgaggcgtgccctacgtttctgcactttgagggtggcgtgccgtacgtgaccacaaagacggactcagacagggtgctcgcccagtttgacttgtctctggcagcaaagcacatgtcaaacaccttcctggcaggtctcgcccagtactacacacagtacagcggcaccatcaacctgcacttcatgttcacaggacccactgacgcgaaagcgcgttacatgattgcatacgccccccctggtatggagccgcccaaaacacctgaggcggccgcccactgcattcatgcggagtgggacacagggttgaattcaaaattcacattttcaatcccttacctttcggcggctgattacgcgtacaccgcgtctgacgctgcggagaccacaaatgtacagggatgggtttgcctgtttcaaattacacacgggaaggctgacggcgacgcactggtcgttctagctagcgccggtaaggactttgagctgcgtctgccagttgacgctcgcacgcagaccacctccgcaggtgagtcggctgaccccgtgactgccactgttgagaactatggtggtgagacacaggtccagagacgccaacacacggatgtctcgttcatattagacagatttgtgaaagtaacaccaaaagaccaaattaatgtgttggacctgatgcaaacccctgcacacactttggtaggcgcgctcctccgtactgccacctactacttcgcagatctagaagtggcagtgaaacacgaggggaaccttacctgggtcccgaatggggcgcccgagacagcgttggacaacaccaccaatccaacggcttaccacaaggcaccgctcacccggcttgcactgccttacacggcaccgcaccgtgtcttggctactgtttacaacgggaactgcaagtatggcgagatccccgtgaccaatgtgagaggtgacctgcaagtattggcccaaaaggcggcaagaacgctgcctacctccttcaattacggtgccatcaaagccactcgggtgactgaa

>EF552689.1_O_UKG_2001

tcgagcgttggagtcacttacgggtacgcaacagctgaggactttgtgagcggaccaaacacatctgggcttgagaccagggttgtgcaggcagagcggttcttcaaaacccacttgttcgactgggtcaccagtgacccgtttggacggtgctatctgctggaactcccaactgaccacaaaggtgtctacggcagcctgaccgactcttatgcttacatgagaaacggctgggatgttgaggtcaccgcagtgggaaatcagttcaacggaggatgtctgttggtggccatggtgccagaactttgctctattgacaagagagagctgtaccagctcacgctctttccccaccagttcatcaacccccggacgaacatgacggcgcacatcactgtgccctttgttggcgtcaaccgctacgaccagtacaaggtacacaaaccttggaccctcgtggttatggttgtggccccgctgactgtcaacaccgaaggtgccccacagatcaaggtctatgccaacatcgcccctaccaacgtgcacgttgcgggtgagttcccttctaaggaagggatcttccccgtggcatgtagcgacggttacggtggtctggtgaccactgacccaaagacggctgaccccgcctacgggaaagtgttcaatccacctcgcaacatgttgccggggcggttcaccaacttccttgatgtggctgaggcgtgccctacgtttctgcactttgagggtggcgtgccgtacgtgaccacaaagacggactcagacagggtgctcgcccagtttgacttgtctctggcagcaaagcacatgtcaaacaccttcctggcaggtctcgcccagtactacacacagtacagcggcaccatcaacctgcacttcatgttcacaggacccactgacgcgaaagcgcgttacatgattgcatacgccccccctggcatggagccgcccaaaacacctgaggcggccgcccactgcattcatgcggagtgggacacagggttgaattcaaaattcacattttcaatcccctacctttcggcggctgattacgcgtacaccgcgtctgacgccgcggagaccacaaatgtacagggatgggtttgcctgtttcaaattacacacgggaaggccgacggcgacgcactggtcgttctagctagcgccggtaaggactttgagctgcgtctgccagttgacgctcgcacgcagaccacctccgcaggtgagtcggctgaccccgtgactgccactgttgagaactacggtggtgagacacaggtccagagacgccaacacacggatgtctcgttcatattagacagatttgtgaaagtaacaccaaaagaccaaattaatgtgttggacctgatgcaaacccctgcacacactttggtaggcgcgctcctccgtactgccacctactacttcgcagatctagaagtggcagtgaaacacgaggggaaccttacctgggtcccgaatggggcgcccgagacagcgttggacaacaccaccaatccaacggcttaccacaaggcaccgctcacccggcttgcactgccttacacggcaccgcaccgtgtcttggctactgtttacaacgggaactgcaagtatggcgagagccccgtgaccaatgtgagaggtgacctgcaagtattggcccaaaaggcggcaagaacgctgcctacctccttcaattacggtgccatcaaagccactcgggtgactgaa

>EF552690.1_O_UKG_2001

tcgagcgttggagtcacttacgggtacgcaacagctgaggactttgtgagcggaccaaacacatctgggcttgagaccagggttgtgcaggcagagcggttcttcaaaacccacttgttcgactgggtcaccagtgacccgtttggacggtgctatctgctggaactcccaactgaccacaaaggtgtctacggcagcctgaccgactcttatgcttacatgagaaacggctgggatgttgaggtcaccgcagtgggaaatcagttcaacggaggatgtctgttggtggccatggtgccagaactttgctctattgacaagagagagctgtaccagctcacgctctttccccaccagttcatcaacccccggacgaacatgacggcgcacatcactgtgccctttgttggcgtcaaccgctacgaccagtacaaggtacacaaaccttggaccctcgtggttatggttgtggccccgctgactgtcaacaccgaaggtgccccacagatcaaggtctatgccaacatcgcccctaccaacgtgcacgttgcgggtgagttcccttctaaggaagggatcttccccgtggcatgtagcgacggttacggtggtctggtgaccactgacccaaagacggctgaccccgcctacgggaaagtgttcaatccacctcgcaacatgttgccggggcggttcaccaacttccttgatgtggctgaggcgtgccctacgtttctgcactttgagggtggcgtgccgtacgtgaccacaaagacggactcagacagggtgctcgcccagtttgacttgtctctggcagcaaagcacatgtcaaacaccttcctggcaggtctcgcccagtactacacacagtacagcggcaccatcaacctgcacttcatgttcacaggacccactgacgcgaaagcgcgttacatgattgcatacgccccccctggcatggagccgcccaaaacacctgaggcggccgcccactgcattcatgcggagtgggacacagggttgaattcaaaattcacattttcaatcccttacctttcggcggctgattacgcgtacaccgcgtctgacgccgcggagaccacaaatgtacagggatgggtttgcctgtttcaaattacacacgggaaggctgacggcgacgcactggtcgttctagctagcgccggtaaggactttgagctgcgtctgccagttgacgctcgcacgcagaccacctccgcaggtgagtcggctgaccccgtgactgccactgttgagaactacggtggtgagacacaggtccagagacgccaacacacggatgtctcgttcatattagacagatttgtgaaagtaacaccaaaagaccaaattaatgtgttggacctgatgcaaacccctgcacacactttggtaggcgcgctcctccgtactgccacctactacttcgcagatctagaagtggcagtgaaacacgaggggaaccttacctgggtcccgaatggggcgcccgagacagcgttggacaacaccaccaatccaacggcttaccacaaggcaccgctcacccggcttgcactgccttacacggcaccgcaccgtgtcttggctactgtttacaacgggaactgcaagtatggcgagagccccgtgaccaatgtgagaggtgacctgcaagtattggcccaaaaggcggcaagaacgctgcctacctccttcaattacggtgccatcaaagccactcgggtgactgaa

>EF552691.1_O_UKG_2001

tcgagcgttggagtcacttacgggtacgcaacagctgaggactttgtgagcggaccaaacacatctgggcttgagaccagggttgtgcaggcagagcggttcttcaaaacccacttgttcgactgggtcaccagtgacccgtttggacggtgctatctgctggaactcccaactgaccacaaaggtgtctacggcagcctgaccgactcttatgcttacatgagaaacggctgggatgttgaggtcaccgcagtgggaaatcagttcaacggaggatgtctgttggtggccatggtgccagaactttgctctattgacaagagagagctgtaccagctcacgctctttccccaccagttcatcaacccccggacgaacatgacggcgcacatcactgtaccctttgttggcgtcaaccgctacgaccagtacaaggtacacaaaccttggaccctcgtggttatggttgtggccccgctgactgtcaacaccgaaggtgccccacagatcaaggtctatgccaacatcgcccctaccaacgtgcacgttgcgggtgagttcccttctaaggaagggatcttccccgtggcatgtagcgacggttacggtggtctggtgaccactgacccaaagacggctgaccccgcctacgggaaagtgttcaatccacctcgcaacatgttgccggggcggttcaccaacttccttgatgtggctgaggcgtgccctacgtttctgcactttgagggtggcgtgccgtacgtgaccacaaagacggactcagacagggtgctcgcccagtttgacttgtctctggcagcaaagcacatgtcaaacaccttcctggcaggtctcgcccagtactacacacagtacagcggcaccatcaacctgcacttcatgttcacaggacccactgacgcgaaagcgcgttacatgattgcatacgccccccctggcatggagccgcccaaaacacctgaggcggccgcccactgcattcatgcggagtgggacacagggttgaattcaaaattcacattttcaatcccttacctttcggcggctgattacgcgtacaccgcgtctgacgccgcggagaccacaaatgtacagggatgggtttgcctgtttcaaattacacacgggaaggctgacggcgacgcactggtcgttctagctagcgccggtaaggactttgagctgcgtctgccagttgacgctcgcacgcagaccacctccgcaggtgagtcggctgaccccgtgactgccactgttgagaactacggtggtgagacacaggtccagagacgccaacacacggatgtctcgttcatattagacagatttgtgaaagtaacaccaaaagaccaaattaatgtgttggacctgatgcaaacccctgcacacactttggtaggcgcgctcctccgtactgccacctactacttcgcagatctagaagtggcagtgaaacacgaggggaaccttacctgggtcccgaatggggcgcccgagacagcgttggacaacaccaccaatccaacggcttaccacaaggcaccgctcacccggcttgcactgccttacacggcaccgcaccgtgtcttggctactgtttacaacgggaactgcaagtatggcgagagccccgtgaccaatatgagaggtgacctgcaagtattggcccaaaaggcggcaagaacgctgcctacctccttcaattacggtgccatcaaagccactcgggtgactgaa

>EF552692.1_O_UKG_2001

tcgagcgttggagtcacttacgggtacgcaacagctgaggactttgtgagcggaccaaacacatctgggcttgagaccagggttgtgcaggcagagcggttcttcaaaacccacttgttcgactgggtcaccagtgacccgtttggacggtgttatctgctggaactcccaactgaccacaaaggtgtctacggcagcctgaccgactcttatgcttacatgagaaacggctgggatgttgaggtcaccgcagtgggaaatcagttcaacggaggatgtctgttggtggccatggtgccagaactttgctctattgacaagagagagctgtaccagctcacgctctttccccaccagttcatcaacccccggacgaacatgacggcgcacatcactgtgccctttgttggcgtcaaccgctacgaccagtacaaggtacacaaaccttggaccctcgtggttatggttgtggccccgctgactgtcaacaccgaaggtgccccacagatcaaggtctatgccaacatcgcccctaccaacgtgcacgttgcgggtgagttcccttctaaggaagggatcttccccgtggcatgtagcgacggctacggtggtctggtgaccactgacccaaagacggctgaccccgcctacgggaaagtgttcaatccacctcgcaacaagttgccggggcggttcaccaacttccttgatgtggctgaggcgtgccctacgtttctgcactttgagggtggcgtgccgtacgtgaccacaaagacggactcagacagggtgctcgcccagtttgacttgtctctggcagcaaagcacatgtcaaacaccttcctggcaggtctcgcccagtactacacacagtacagcggcaccatcaacctgcacttcatgttcacaggacccactgacgcgaaagcgcgttacatgattgcatacgccccccctggcatggagccgcccaaaacacctgaggcggccgcccactgcattcatgcggagtgggacacagggttgaattcaaaattcacattttcaatcccttacctttcggcggctgattacgcgtacaccgcgtctgacgccgcggagaccacaaatgtacagggatgggtttgcctgtttcaaattacacacgggaaggctgacggcgacgcactggtcgttctagctagcgccggtaaggactttgagctgcgtctgccagttgacgctcgcacgcagaccacctccgcaggtgagtcggctgaccccgtgactgccactgttgagaactacggtggtgagacacaggtccagagacgccaacacacggatgtctcgttcatattagacagatttgtgaaagtaacaccaaaagaccaaattaatgtgttggacctgatgcaaacccctgcacacactttggtaggcgcgctcctccgtactgccacctactacttcgcagatctagaagtggcagtgaaacacgaggggaaccttacctgggtcccgaatggggcgcccgagacagcgttggacaacaccaccaatccaacggcttaccacaaggcaccgctcacccggcttgcactgccttacacggcaccgcaccgtgtcttggctactgtttacaacgggaactgcaagtatggcgagagccccgtgaccaatgtgagaggtgacctgcaagtattggctcaaaaggcggcaagaacgctgcctacctccttcaattacggtgccatcaaagccactcgggtgactgaa

>EF552693.1_O_UKG_2001

tcgagcgttggagtcacttacgggtacgcaacagctgaggactttgtgagcggaccaaacacatctgggcttgagaccagggttgtgcaggcagagcggttcttcaaaacccacttgttcgactgggtcaccagtgacccgtttggacggtgctatctgctggaactcccaactgaccacaaaggtgtctacggcagcctgaccgactcttatgcttacatgagaaacggctgggatgttgaggtcaccgcagtgggaaatcagttcaacggaggatgtctgttggtggccatggtgccagaactttgctctattgacaagagagagctgtaccagctcacgctctttccccaccagttcatcaacccccggacgaacatgacggcgcacatcactgtgccctttgttggcgtcaaccgctacgaccagtacaaggtacacaaaccttggaccctcgtggttatggttgtggccccgctgactgtcaacaccgaaggtgccccacagatcaaggtctacgccaacatcgcccctaccaacgtgcacgttgcgggtgagttcccttctaaggaagggatcttccccgtggcatgtagcgacggttacggtggtctggtgaccactgacccaaagacggctgaccccgcctacgggaaagtgttcaatccacctcgcaacaagttgccggggcggttcaccaacttccttgatgtggctgaggcgtgccctacgtttctgcactttgagggtggcgtgccgtacgtgaccacaaagacggactcagacagggtgctcgcccagtttgacttgtctctggcagcaaagcacatgtcaaacaccttcctggcaggtctcgcccagtactacacacagtacagcggcaccatcaacctgcacttcatgttcacaggacccactgacgcgaaagcgcgttacatgattgcatacgccccccctggcatggagccgcccaaaacacctgaggcggccgcccactgcattcatgcggagtgggacacagggttgaattcaaaattcacattttcaatcccttacctttcggcggctgattacgcgtacaccgcgtctgacgccgcggagaccacaaatgtacagggatgggtttgcctgtttcaaattacacacgggaaggctgacggcgacgcactggtcgttctagctagcgccggtaaggactttgagctgcgtctgccagttgacgctcgcacgcagaccacctccgcaggtgagtcggctgaccccgtgactgccactgttgagaactacggtggtgagacacaggtccagagacgccaacacacggatgtctcgttcatattagacagatttgtgaaagtaacaccaaaagaccaaattaatgtgttggacctgatgcaaacccctgcacacactttggtaggcgcgctcctccgtactgccacctactacttcgcagatctagaagtggcagtgaaacacgaggggaaccttacctgggtcccgaatggggcgcccgagacagcgttggacaacaccaccaatccaacggcttaccacaaggcaccgctcacccggcttgcactgccttacacggcaccgcaccgtgtcttggctactgtttacaacgggaactgcaagtatggcgagagccccgtgaccaatgtgagaggtgacctgcaagtattggcccaaaaggcggcaagaacgctgcctacctccttcaattacggtgccatcaaagccactcgggtgactgaa

>EF552695.1_O_UKG_2001

tcgagcgttggagtcacttacgggtacgcaacagctgaggactttgtgagcggaccaaacacatctgggcttgagaccagggttgtgcaggcagagcggttcttcaaaacccacttgttcgactgggtcaccagtgacccgtttggacggtgctatctgctggaactcccaactgaccacaaaggtgtctacggcagcctgaccgactcttatgcttacatgagaaacggctgggatgttgaggtcaccgcagtgggaaatcagttcaacggaggatgtctgttggtggccatggtgccagaactttgctctattgacaagagagagctgtaccagctcacgctctttccccaccagttcatcaacccccggacgaacatgacggcgcacatcactgtaccctttgttggcgtcaaccgctacgaccagtacaaggtacacaaaccttggaccctcgtggttatggttgtggccccgctgactgtcaacaccgaaggtgccccacagatcaaggtctatgccaacatcgcccctaccaacgtgcacgttgcgggtgagttcccttctaaggaagggatcttccccgtggcatgtagcgacggttacggtggtctggtgaccactgacccaaagacggctgaccccgcctacgggaaagtgttcaatccacctcgcaacatgttgccggggcggttcaccaacttccttgatgtggctgaggcgtgccctacgtttctgcactttgagggtggcgtgccgtacgtgaccacaaagacggactcagacagggtgctcgcccagtttgacttgtctctggcagcaaagcacatgtcaaacaccttcctggcaggtctcgcccagtactacacacagtacagcggcaccatcaacctgcacttcatgttcacaggacccactgacgcgaaagcgcgttacatgattgcatacgccccccctggcatggagccgcccaaaacacctgaggcggccgcccactgcattcatgcggagtgggacacagggttgaattcaaaattcacattttcaatcccttacctttcggcggctgattacgcgtacaccgcgtctgacgccgcggagaccacaaatgtacagggatgggtttgcctgtttcaaattacacacgggaaggctgacggcgacgcactggtcgttctagctagcgccggtaaggactttgagctgcgtctgccagttgacgctcgcacgcagaccacctccgcaggtgagtcggctgaccccgtgactgccactgttgagaactacggtggtgagacacaggtccagagacgccaacacacggatgtctcgttcatattagacagatttgtgaaagtaacaccaaaagaccaaattaatgtgttggacctgatgcaaacccctgcacacactttggtaggcgcgctcctccgtactgccacctactacttcgcagatctagaagtggcagtgaaacacgaggggaaccttacctgggtcccgaatggggcgcccgagacagcgttggacaacaccaccaatccaacggcttaccacaaggcaccgctcacccggcttgcactgccttacacggcaccgcaccgtgtcttggctactgtttacaacgggaactgcaagtatggcgagagccccgtgaccaatatgagaggtgacctgcaagtattggcccaaaaggcggcaagaacgctgcctacctccttcaattacggtgccatcaaagccactcgggtgactgaa

>EF552696.1_O_UKG_2001

tcgagcgttggagtcacttacgggtacgcaacagctgaggactttgtgagcggaccaaacacatctgggcttgagaccagggttgtgcaggcagagcggttctttaaaacccacttgttcgactgggtcaccagtgacccgtttggacggtgctatctgctggaactcccaactgaccacaaaggtgtctacggcagcctgaccgactcttatgcttacatgagaaacggttgggatgttgaggtcaccgcagtgggaaatcagttcaacggaggatgtctgttggtggccatggtgccagaactttgctctattgacaagagagagctgtaccagctcacgctctttccccaccagttcatcaacccccggacgaacatgacggcgcacatcactgtgccctttgttggcgtcaaccgctacgaccagtacaaggtacacaaaccttggaccctcgtggttatggttgtggccccgctgactgtcaacaccgaaggtgccccacagatcaaggtctatgccaacatcgcccctaccaacgtgcacgtcgcgggtgagttcccttctaaggaagggatcttccccgtggcatgtagcgacggttacggtggtctggtgaccactgacccaaagacggctgaccccgcctacgggaaagtgttcaatccacctcgcaacatgttgccggggcggttcaccaacttccttgatgtggctgaggcgtgccctacgtttctgcactttgagggtggcgtgccgtacgtgaccacaaagacggactcagacagggtgctcgcccagtttgacttgtctctggcagcaaagcacatgtcaaacaccttcctggcaggtctcgcccagtactacacacagtacagcggcaccatcaacctgcacttcatgttcacaggacccactgacgcgaaagcgcgttacatgattgcatacgccccccctggtatggagccgcccaaaacacctgaggcggccgcccactgcattcatgcggagtgggacacagggttgaattcaaaattcacattttcaatcccttacctttcggcggctgattacgcgtacaccgcgtctgacgctgcggagaccacaaatgtacagggatgggtttgcctgtttcaaattacacacgggaaggctgacggcgacgcactggtcgttctagctagcgccggtaaggactttgagctgcgtctgccagttgacgctcgcacgcagaccacctccgcaggtgagtcggctgaccccgtgactgccactgttgagaactatggtggtgagacacaggtccagagacgccaacacacggatgtctcattcatattagacagatttgtgaaagtaacaccaaaagaccaaattaatgtgttggacctgatgcaaacccctgcacacactttggtaggcgcgctcctccgtactgccacctactacttcgcagatctagaagtggcagtgaaacacgaggggaaccttacctgggtcccgaatggggcgcccgagacagcgttggacaacaccaccaatccaacggcttaccacaaggcaccgctcacccggcttgcactgccttacacggcaccgcaccgtgtcttggctactgtttacaacgggaactgcaagtatggcgagatccccgtgaccaatgtgagaggtgacctgcaagtattggcccaaaaggcggcaagaacgctgcctacctccttcaattacggtgccatcaaagccactcgggtgactgaa

>EF552697.1_O_UKG_2001

tcgagcgttggagtcacttacgggtacgcaacagctgaggactttgtgagcggaccaaacacatctgggcttgagaccagggttgtgcaggcagagcggttcttcaaaacccacttgttcgactgggtcaccagtgacccgtttggacggtgctatctgctggaactcccaactgaccacaaaggtgtctacggcagcctgaccgactcttatgcttacatgagaaacggctgggatgttgaggtcaccgcagtgggaaatcagttcaacggaggatgtctgttggtggccatggtgccagaactttgctctattgacaagagagagctgtaccagctcacgctctttccccaccagttcatcaacccccggacgaacatgacggcgcacatcactgtgccctttgttggcgtcaaccgctacgaccagtacaaggtacacaaaccttggaccctcgtggttatggttgtggccccgctgactgtcaacaccgaaggtgccccacagatcaaggtctatgccaacatcgcccctaccaacgtgcacgttgcgggtgagttcccttctaaggaagggatcttccccgtggcatgtagcgacggttacggtggtctggtgaccactgacccaaagacggctgaccccgcctacgggaaagtgttcaatccacctcgcaacatgttgccggggcggttcaccaacttccttgatgtggctgaggcgtgccctacgtttctgcactttgagggtggcgtgccgtacgtgaccacaaagacggactcagacagggtgctcgcccagtttgacttgtctctggcagcaaagcacatgtcaaacaccttcctggcaggtctcgcccagtactacacacagtacagcggcaccatcaacctgcacttcatgttcacaggacccactgacgcgaaagcgcgttacatgattgcatacgccccccctggcatggagccgcccaaaacacctgaggcggccgcccactgcattcatgcggagtgggacacagggttgaattcaaaattcacattttcaatcccttacctttcggcggctgattacgcgtacaccgcgtctgacgccgcggagaccacaagtgtacagggatgggtttgcctgtttcaaattacacacgggaaggctgacggcgacgcactggtcgttttagctagcgccggtaaggactttgagctgcgtctgccagttgacgctcgcacgcagaccacctccgcaggtgagtcggctgaccccgtgactgccactgttgagaactacggtggtgagacacaggtccagagacgccaacacacggatgtctcgttcatattagacagatttgtgaaagtaacaccaaaagaccaaattaatgtgttggacctgatgcaaacccctgcacacactttggtaggcgcgctcctccgtactgccacctactacttcgcagatctagaagtggcagtgaaacacgaggggaaccttacctgggtcccgaatggggcgcccgagacagcgttggacaacaccaccaatccaacggcttaccacaaggcaccgctcacccggcttgcactgccttacacggcaccgcaccgtgtcttggctactgtttacaacgggaactgcaagtatggcgagagccccgtgaccaatgtgagaggtgacctgcaagtattggcccaaaaggcggcaagaacgctgcctacctccttcaattacggtgccatcaaagccactcgggtgactgaa

>EF611987.1_O_UGA_2006

tcgagtgttggggtcacgtacgggtatgcaacagctgaggactttgtgagtggaccgaacacctctggtctcgagaccagagttgcccaagcagagcggttcttcaagacccatttgttcgactgggtcaccagtgacycattcgggcgatgccacttgctggagctcccaactgaccacaaaggtatctacggcggcctgatcgactcatatgcttacatgagaaacggttgggacgtcgaggtgaccgctgtggggaaccagttcaacggaggctgtttgctagtggcaatggtaccagagctttgttccatccagaagagggaactgtaccaactcacactcttcccccatcagttcattaacccccggacgaacatgacggcacacatcacagtgccctttgttggtgtcaaccgctacgaccagtacaaggtacacaaaccttggacccttgtggtcatggttgtggcacctttgactgtcaacaccgagggtgccccacagatcaaggtgtatgctaacatcgccccaaccaacgtgcacgttgcgggtgagttcccttccaaagagggaattttccccgtggcttgcagcgacggttacggcggtttggtgaccacggacccgaaaacggctgaccccgcttacgggaaagtgttcaaccccccgcgcaacatgttgccggggcgtttcaccaatctccttgatgtggctgaggcgtgtcccacgtttctgcacttcgagggtgacgttccttacgtgaccacgaagacagactctgacagggtgctcactcagtttgatttgtctttggccgcaaagcacatgtcaaacaccttccttgcaggtctcgcccaatactacactcagtatagcggcaccattaacctgcacttcatgttcacaggtcccactgatgcgaaagcacgctacatgattgcatacgccccccctgggatggaaccgcccaagacacctgaaacggcggctcactgcattcatgctgagtgggacacggggctgaactcaaaatttacattctccattccctacctctcggcggctgactacgcgtacaccgcgtcaagcactgccgaaaccaccaacgtgcagggatgggtctgccttttccaaataacacacgggaaagccgacggggatgctttggtcgtgctggccagtgccggcaaggacttcgacctgcgtctcccagtggacgctcgcacacaaaccacctccccgggtgagtcggccgaccctgtgactgccacagtggagaactacggtggtgtaactcaggcccagaggcgccaacacacggacgtctcgttcattttggatagatttgtgaaggttacaccccaagaccaaatcaatgttttggacctgatgcaaatccctgcccacacactggtgggcgcgctcttgcgcacatccacttactactttgctgacttggaactggcagtgaaacacgagggtaacctcacttgggtcccgaacggagcacccgaagccgcactggacaacaccaccaacccaacagtgtaccacaaggcacctctcactcgccttgcactgccttacaccgcaccacaccgcgtgttggcaaccgtgtacaacgggaactgcaagtacagtggttcctcagccactaatgtgaggggtgacctccaagtgctggcccagagggctgcgagaacactgcccacctccttcaactacggtgccatcaaggccactcgggtgacagaa

>EF614457.1_O_SKR_2002

tcgagcgttggagtcacttacgggtacgcaacagctgaggacttcgtgagcggaccaaacacatctgggcttgagaccagggttgtgcaggcagagcggttctttaaaacccacttgttcgactgggtcaccagtgacccgttcggacggtgctacctgctggaactcccaactgaccacaaaggtgtctacggcagcctgaccgactcttatgcttacatgagaaacggttgggacgttgaggtcactgcggtgggaaatcagttcaacggaggatgcttgttggtggccatggtgccagaactttgctctattgacaagagagggctgtaccagctcacgctctttccccaccagttcatcaacccccggacgaacatgacggcgcacattactgtgccctttgttggcgtcaaccgctacgaccagtacaaagtacacaaaccttggaccctcgttgtcatggttgtggccccgctgactgtcaacaccgaaggtgccccacagatcaaggtctatgccaacatcgcccctactaacgtgcacgttgcgggtgagctcccttctaaggaagggatcttccccgtggcatgtagcgacggttacggtggtctggtgaccactgacccaaagacggctgaccccgcctacgggaaagtgttcaatccacctcgcaacatgttgccggggcggttcaccaacttccttgatgtggctgaggcgtgccctacgttcctgcactttgagggtgacgtgccgtacgtgaccacaaagacggactcagacagggtactcgcccagtttgacttgtctctggcagcaaagcacatgtcaaacaccttcctggcaggtctcgcccagtactacacacagtacagcggcaccatcaacctgcacttcatgtttacaggacccactgacgcgaaagcgcgttacatgattgcatacgccccccctggcatggagccgcccaaaacacccgaggcggccgctcactgcattcacgcggagtgggacacagggttgaattcaaaattcacattttcaatcccttacctttcggcggctgattatgcgtacaccgcgtctgacgccgcggagaccacaaatgtgcagggttgggtttgcctgtttcaaattacacacgggaaggctgacggcgacgcactggtcgttctagctagcgccggtaaggactttgagctgcgtctgccagttgacgctcgcacgcagaccacctccacaggtgagtcggctgaccccgtgactgccaccgttgagaactacggtggtgagacacaggtccagagacgccaacacacggatgtctcgttcatactagacagatttgtgaaagtaacaccaaaagaccaaattaatgtgttggacctgatacaaatccctgcacacactttggtaggcgcgctcctccgtactgccacctactacttcgcagatctggaagtggcagtgaaacacaagggggacctcacctgggtcccgaacggggcgcccgaggcagcgttggacaacaccaccaatccaacggcctatcacaaggcgccgctcacccggcttgcactgccttacacggcaccacaccgtgtcttggctactgtttacaacgggaactgcaagtatggcgagagccccgtgaccaatccgagaggtgacctgcaagtgttgacccagaaggcggcaagaacgctgcctacctccttcaattacggtgccatcaaagccactcgggtgattgaa

>EU214601.1_O_UKG_2001

tcgagcgttggagtcacttacgggtacgcaacagctgaggactttgtgagcggaccaaacacatctgggcttgagaccagggttgtgcaggcagagcggttcttcaaaacccacttgttcgactgggtcaccagtgacccgtttggacggtgctatctgctggaactcccaactgaccacaaaggtgtctacggcagcctgaccgactcttatgcttacatgagaaacggctgggatgttgaggtcaccgcagtgggaaatcagttcaacggaggatgtctgttggtggccatggtgccagaactttgctctattgacaagagagagctgtaccagctcacgctctttccccaccagttcatcaacccccggacgaacatgacggcgcacatcactgtgccctttgttggcgtcaaccgctacgaccagtacaaggtacacaaaccttggaccctcgtggttatggttgtggccccgctgactgtcaacaccgaaggtgccccacagatcaaggtctatgccaacatcgcccctaccaacgtgcacgttgcgggtgagttcccttctaaggaagggatcttccccgtggcatgtagcgacggttacggtggtctggtgaccactgacccaaagacggctgaccccgcctacgggaaagtgttcaatccacctcgcaacatgttgccggggcggttcaccaacttccttgatgtggctgaggcgtgccctacgtttctgcactttgagggtggcgtgccgtacgtgaccacaaagacggactcagacagggtgctcgcccagtttgacttgtctctggcagcaaagcacatgtcaaacaccttcctggcaggtctcgcccagtactacacacagtacagcggcaccatcaacctgcacttcatgttcacaggacccactgacgcgaaagcgcgttacatgattgcatacgccccccctggcatggagccgcccaaaacacctgaggcggccgcccactgcattcatgcggagtgggacacagggttgaattcaaaattcacattttcaatcccttacctttcggcggctgattacgcgtacaccgcgtctgacgccgcggagaccacaaatgtacagggatgggtttgcctgtttcaaattacacacgggaaggctgacggcgacgcactggtcgttctagctagcgccggtaaggactttgagctgcgtctgccagttgacgctcgcacgcagaccacctccgcaggtgagtcggctgaccccgtgactgccactgttgagaactacggtggtgagacacaggtccagagacgccaacacacggatgtctcgttcatattagacagatttgtgaaagtaacaccaaaagaccaaattaatgtgttggacctgatgcaaacccctgcacacactttggtaggcgcgctcctccgtactgccacctactacttcgcagatctagaagtggcagtgaaacacgaggggaaccttacctgggtcccgaatggggcgcccgagacagcgttggacaacaccaccaatccaacggcttaccacaaggcaccgctcacccggcttgcactgccttacacggcaccgcaccgtgtcttggctactgtttacaacgggaactgcaagtatggcgagagccccgtgaccaatgtgagaggtgacctgcaagtattggcccaaaaggcggcaagaacgctgcctacctccttcaattacggtgccatcaaagccactcgggtgactgaa

>EU400597.1_O_CHA_2001

tcaagtgtcggggtgacgtacgggtatgcaacggctgaggactttgtaagcgggcccaacacttctggtcttgagaccagagttgttcaggccgaacggttcttcaagacccacctgttcgactggggcaccaacgactcgtttgggcggtgtcacttgttggagctaccaactgaccacaaaggtgtctacggcagcctgaccgactcatacgcatacatgaggaacggttgggacgttgaggtcaccgcagtggggaaccagttcaacggaggttgcttgttagtggcgatggtgccggagctctgttccatcaccaagagagagctgtaccaactcacacttttccctcaccagttcatcaacccacggacgaacatgacggcacacatcaccgtgccctatctcggtgtcaacaggtacgaccagtacaaggtacacaaaccctggactctcgtggtcatggttgtagctcctttgacggtcaacaacgagggcgccccgcaaatcaaggtgtatgccaacatcgcccccaccaacgttcacgtcgcgggcgagctcccttccaaagaggggatcttccctgtggcatgcagcgacggctacggtggcttggtaaccacggacccgaagacggcagaccccgtctacgggaaagtgttcaacccaccccgcaacctgctgccagggcggttcacaaacctccttgatgtggccgaggcgtgtcctacgttcctgcacttcgatggtgacgttccctacgtgatcacgaagacggattcagacagagtgctggcccagttcgacttgtccctcgcggcaaagcacatgtcgaacacctttctcgcgggtcttgcccagtactacgcacagtacagcggtaccatcaacctgcacttcatgttcacggggccaaccgatgcaaaggcacgctacatggttgcgtatgcccctcctggcatggaaccacctaaaacgcctgaggcggctgcacactgcatccacgctgagtgggacactgggctgaactcgaaattcacgttttcgatcccatacctctcggcggcagactacgcatacaccgcgtccgacgttgccgagactacaaacgtgcagggatgggtctgtctgttccagataacacacgggaaagccgacggcgacgccctggtagtactagccagtgccggcaaggactttgatttgcgcctgccggttgacgcccgaacccagaccacctctgcgggcgagtctgcggaccccgtgactaccaccgtcgaaaactacggcggcgagacacaagtccagaggcgccaacacacggacgttgcgttcatattggacaggttcgtgaaagtcaaaccacaggagcaagttaacgtgttggacctgatgcagatccctgcccacaccttggtaggggcactcctgcggacggccacatattacttctctgacctggaactagctgtcaagcacgagggcgatctcacctgggttccaaatggtgcccccgagacagcactggacaacactaccaacccaacagtctaccacaaggaaccgctcacacggctggtgctgccttatacggctccgcaccgcgtcttagctaccgtctacaacgggagcagcaagtacggtgacaccagcactaacaacgtgagaggcgacctgcaggtgttggctcagaaggcagaaagagctctgcccacctccttcaactacggtgccatcaaggcagctcgtgtgactgaa

>EU448368.1_O_UKG_1967

tcaagcgttggagtcacatacgggtacgcaacagctgaggattttgtgagcggaccgaacacttctggtctcgagaccagagttgtgcaggcagaacggtttttcaaaacccacctcttcgactgggtcaccagtgactcattcggacgttaccacctcctggaactcccgaccgaccacaaaggtgtctacggcagcctgaccgactcgtatgcatatatgaggaacggctgggatgtcgaggtcaccgcggttggcaaccagttcaacggagggtgcctgctggtcgcaatggtaccagagctttgttctatccaaaagagggaactgtaccagctcacacttttccctcaccagttcatcaacccacgcacgaacatgactgcgcacattacagtgccctttgttggcgtcaaccgctacgaccagtacaaggttcacaagccttggacccttgtggttatggttgtagcccctctgaccgtcaacactgaaggtgcccctcagatcaaggtgtatgccaacattgccccaactaacgtgcacgtcgcgggtgagtttccttccaaagagggaatattccccgtggcctgtagcgacggctatggtggcctggtgaccacggacccgaagacggctgaccccgtttatgggaaagtgttcaaccccccccgcaaccagttgccggggcgttttaccaacctccttgatgtggctgaggcatgcccgacgtttctgcacttcgagggtgacgtaccgtacgtgaccacgaaaacagactcggacagggtgcttgctcagtttgatatgtctttggcagcaaaacacatgtcaaacaccttcctcgcaggtcttgcgcagtactacacacagtacagtggcaccatcaacctgcacttcatgttcacaggacccactgacgcgaaggcgcgttacatgattgcctacgccccaccgggcatggagccgcccaagacacctgaggcggccgcgcactgcattcatgctgaatgggacactgggttgaactcaaagtttactttttccatcccctacctctcggccgccgattacgcgtacaccgcgtctgacgtggccgagaccacaaatgtgcagggatgggtctgcttgtttcaaattacacatggcaaggccgacggcgacgctctggtcgtactggctagtgctggtaaagactttgagctaaggctgccggtggacgcccgtgcggaaaccacttctgcgggcgagtcagcggatcctgtcaccaccactgttgaaaactacggtggcgaaacacagatccagaggcgccaacacacggacgtctcgttcattatggacagatttgtgaaagtgacaccgcaaaaccaaattaacattttggacctcatgcaggttccatcacacactttggtgggagcgctcctacgcgcgtccacttactacttctctgacttggagatagcagtaaaacacgagggagacctcacctgggttccaaatggagcgcctgaaaaggcgttggacaacaccaccaacccaactgcttaccacaaggcaccactcacccggcttgccctgccctacactgcgccccaccgcgtgttggcaaccgtgtacaacggtgagtgcaggtacagcagaaatgctgtgcccaacttgagaggtgaccttcaagtgttggctcaaaaggtggcacggacgctgcctacctccttcaactacggtgccatcaaagcgacccgggtcaccgag

>EU448369.1_O_UKG_1967

tcaagcgttggagtcacatacgggtacgcaacagctgaggattttgtgagcggaccgaacacttctggtctcgagaccagagttgtgcaggcagaacggtttttcaaaacccacctcttcgactgggtcaccagtgactcattcggacgttgccacctcctggaactcccgaccgaccacaaaggtgtctacggcagcctgaccgactcgtatgcatatatgaggaacggctgggatgtcgaggtcaccgcggttggcaaccagttcaacggagggtgcctgctggtcgcaatggtaccagagctttgttctatccaaaagagggaactgtaccagctcacacttttccctcaccagttcatcaacccacgcacgaacatgactgcgcacattacagtgccctttgttggcgtcaaccgctacgaccagtacaaggttcacaagccttggacccttgtggttatggttgtagcccctctgaccgtcaacactgaaggtgcccctcagatcaaggtgtatgccaacattgccccaactaacgtgcacgtcgcgggtgagtttccttccaaagagggaatattccccgtggcctgtagcgacggctatggtggcctggtgaccacggacccgaagacggctgaccccgtttatgggaaagtgttcaaccccccccgcaaccagttgccggggcgttttaccaacctccttgatgtggctgaggcatgcccgacgtttctgcgcttcgagggtggcgtaccgtacgtgaccacgaaaacagactcggacagggtgcttgctcagtttgatatgtctttggcagcaaaacacatgtcaaacaccttcctcgcaggtcttgcgcagtactacacacagtacagtggcaccatcaacctgcacttcatgttcacaggacccactgacgcgaaggcgcgttacatgattgcctacgccccaccgggcatggagccgcccaagacacctgaggcggccgcgcactgcattcatgctgaatgggacactgggttgaactcaaagtttactttttccatcccctacctctcggccgccgattacgcgtacaccgcgtctgacgtggccgagaccacaaatgtgcagggatgggtctgcttgtttcaaattacacatggcaaggccgacggcgacgctctggtcgtactggctagtgctggtaaagactttgagctaaggctgccggtggacgcccgtgcggaaaccacttctgcgggcgagtcagcggatcctgtcaccaccactgttgaaaactacggtggcgaaacacagatccagaggcgccaacacacggacgtctcgttcattatggacagatttgtgaaagtgacaccgcaaaaccaaattaacattttggacctcatgcaggttccatcacacactttggtgggagcgctcctacgcgcgtccacttactacttctctgacttggagatagcagtaaaacacgagggagacctcacctgggttccaaatggagcgcctgaaaaggcgttggacaacaccaccaacccaactgcttaccacaaggcaccactcacccggcttgccctgccctacactgcgccccaccgcgtgttggcaaccgtgtacaacggtgagtgcaggtacagcagaaatgctgtgcccaacttgagaggtgaccttcaagtgttggctcaaaaggtggcacggacgctgcctacctccttcaactacggtgccatcaaagcgacccgggtcaccgag

>EU448370.1_O_UKG_1967

tcaagcgttggagtcacatacgggtacgcaacagctgaggattttgtgagcggaccgaacacttctggtctcgagaccagagttgtgcaggcagaacggtttttcaaaacccacctcttcgactgggtcaccagtgactcattcggacgttgccacctcctggaactcccgaccgaccacaaaggtgtctacggcagcctgaccgactcgtatgcatatatgaggaacggctgggatgtcgaggtcaccgcggttggcaaccagttcaacggagggtgcctgctggtcgcaatggtaccagagctttgttctatccaaaagagggaactgtaccagctcacacttttccctcaccagttcatcaacccacgcacgaacatgactgcgcacattacagtgccctttgttggcgtcaaccgctacgaccagtacaaggttcacaagccttggacccttgtggttatggttgtagcccctctgaccgtcaacactgaaggtgcccctcagatcaaggtgtatgccaacattgccccaactaacgtgcacgtcgcgggtgagtttccttccaaagagggaatattccccgtggcctgtagcgacggctatggtggcctggtgaccacggacccgaagacggctgaccccgtttatgggaaagtgttcaaccccccccgcaaccagttgccggggcgttttaccaacctccttgatgtggctgaggcatgcccgacgtttctgcgcttcgagggtggcgtaccgtacgtgaccacgaaaacagactcggacagggtgcttgctcagtttgatatgtctttggcagcaaaacacatgtcaaacaccttcctcgcaggtcttgcgcagtactacacacagtacagtggcaccatcaacctgcacttcatgttcacaggacccactgacgcgaaggcgcgttacatgattgcctacgccccaccgggcatggagccgcccaagacacctgaggcggccgcgcactgcattcatgctgaatgggacactgggttgaactcaaagtttactttttccatcccctacctctcggccgccgattacgcgtacaccgcgtctgacgtggccgagaccacaaatgtgcagggatgggtctgcttgtttcaaattacacatggcaaggccgacggcgacgctctggtcgtactggctagtgctggtaaagactttgagctaaggctgccggtggacgcccgtgcggaaaccacttctgcgggcgagtcagcggatcctgtcaccaccactgttgaaaactacggtggcgaaacacagatccagaggcgccaacacacggacgtctcgttcattatggacagatttgtgaaagtgacaccgcaaaaccaaattaacattttggacctcatgcaggttccatcacacactttggtgggagcgctcctacgcgcgtccacttactacttctctgacttggagatagcagtaaaacacgagggagacctcacctgggttccaaatggagcgcctgaaaaggcgttggacaacaccaccaacccaactgcttaccacaaggcaccactcacccggcttgccctgccctacactgcgccccaccgcgtgttggcaaccgtgtacaacggtgagtgcaggtacagcagaaatgctgtgcccaacttgagaggtgaccttcaagtgttggctcaaaaggtggcacggacgctgcctacctccttcaactacggtgccatcaaagcgacccgggtcaccgag

>EU448371.1_O_UKG_2007

tcaagcgttggagtcacatacgggtacgcaacagctgaggattttgtgagcggaccgaacacttctggtctcgagaccagagttgtgcaggcagaacggtttttcaaaacccacctcttcgactgggtcaccagtgactcattcggacgttgccacctcctggaactcccgaccgaccacaaaggtgtctacggcagcctgaccgactcgtatgcatatatgaggaacggctgggatgtcgaggtcaccgcggttggcaaccagttcaacggagggtgcctgctggtcgcaatggtaccagagctttgttctatccaaaagagggaactgtaccagctcacactcttccctcaccagttcatcaacccacgcacgaacatgactgcgcacattacagtgccctttgttggcgtcaaccgctacgaccagtacaaggttcacaagccttggacccttgtggttatggttgtagcccctctgaccgtcaacactgaaggtgcccctcagatcaaggtgtatgccaacattgccccaactaacgtgcacgtcgcgggtgagtttccttccaaagagggaatattccccgtggcctgtagcgacggctatggtggcctggtgaccacggacccgaagacggctgaccccgtttatgggaaagtgttcaaccccccccgcaaccagttgccggggcgttttaccaacctccttgatgtggctgaggcatgcccgacgtttctgcacttcgagggtggcgtaccgtacgtgaccacgaaaacagactcggacagggtgcttgctcagtttgatatgtctttggcagcaaaacacatgtcaaacaccttcctcgcaggtcttgcgcagtactacacacagtacagtggcaccatcaacctgcacttcatgttcacaggacccactgacgcgaaggcgcgttacatgattgcctacgccccaccgggcatggagccgcccaagacacctgaggcggccgcgcactgcattcatgctgaatgggacactgggttgaactcaaagtttactttttccatcccctacctctcggccgccgattacgcgtacaccgcgtctgacgtggccgagaccacaaatgtgcagggatgggtctgcttgtttcaaattacacatggcaaggccgacggcgacgctctggtcgtactggctagtgctggtaaagactttgagctaaggctgccggtggacgcccgtgcggaaaccacttctgcgggcgagtcagcggatcctgtcaccaccactgttgaaaactacggtggcgaaacacagatccagaggcgccaacacacggacgtctcgttcattatggacagatttgtgaaagtgacaccgcaaaaccaaattaacattttggacctcatgcaggtcccatcacacactttggtgggagcgctcctacgcgcgtccacttactacttctctgacttggagatagcagtaaaacacgagggagacctcacctgggttccaaatggagcgcctgaaaaggcgttggacaacaccaccaacccaactgcttaccacaaggcaccactcacccggcttgccctgccctacactgcgccccaccgcgtgttggcaaccgtgtacaacggtgagtgcaggtacagcagaaatgctgtgcccaacttgagaggtgaccttcaagtgttggctcaaaaggtggcacggacgctgcctacctccttcaactacggtgccatcaaagcgacccgggtcaccgag

>EU448372.1_O_UKG_2007

tcaagcgttggagtcacatacgggtacgcaacagctgaggattttgtgagcggaccgaacacttctggtctcgagaccagagttgtgcaggcagaacggtttttcaaaacccacctcttcgactgggtcaccagtgactcattcggacgttgccacctcctggaactcccgaccgaccacaaaggtgtctacggcagcctgaccgactcgtatgcatatatgaggaacggctgggatgtcgaggtcaccgcggttggcaaccagttcaacggagggtgcctgctggtcgcaatggtaccagagctttgttctatccaaaagagggaactgtaccagctcacactcttccctcaccagttcatcaacccacgcacgaacatgactgcgcacattacagtgccctttgttggcgtcaaccgctacgaccagtacaaggttcacaagccttggacccttgtggttatggttgtagcccctctgaccgtcaacactgaaggtgcccctcagatcaaggtgtatgccaacattgccccaactaacgtgcacgtcgcgggtgagtttccttccaaagagggaatattccccgtggcctgtagcgacggctatggtggcctggtgaccacggacccgaagacggctgaccccgtttatgggaaagtgttcaaccccccccgcaaccagttgccggggcgttttaccaacctccttgatgtggctgaggcatgcccgacgtttctgcacttcgagggtggcgtaccgtacgtgaccacgaaaacagactcggacagggtgcttgctcagtttgatatgtctttggcagcaaaacacatgtcaaacaccttcctcgcaggtcttgcgcagtactacacacagtacagtggcaccatcaacctgcacttcatgttcacaggacccactgacgcgaaggcgcgttacatgattgcctacgccccaccgggcatggagccgcccaagacacctgaggcggccgcgcactgcattcatgctgaatgggacactgggttgaactcaaagtttactttttccatcccctacctctcggccgccgattacgcgtacaccgcgtctgacgtggccgagaccacaaatgtgcagggatgggtctgcttgtttcaaattacacatggcaaggccgacggcgacgctctggtcgtactggctagtgctggtaaagactttgagctaaggctgccggtggacgcccgtgcggaaaccacttctgcgggcgagtcagcggatcctgtcaccaccactgttgaaaactacggtggcgaaacacagatccagaggcgccaacacacggacgtctcgttcattatggacagatttgtgaaagtgacaccgcaaaaccaaattaacattttggacctcatgcaggtcccatcacacactttggtgggagcgctcctacgcgcgtccacttactacttctctgacttggagatagcagtaaaacacgagggagacctcacctgggttccaaatggagcgcctgaaaaggcgttggacaacaccaccaacccaactgcttaccacaaggcaccactcacccggcttgccctgccctacactgcgccccaccgcgtgttggcaaccgtgtacaacggtgagtgcaggtacagcagaaatgctgtgcccaacttgagaggtgaccttcaagtgttggctcaaaaggtggcacggacgctgcctacctccttcaactacggtgccatcaaagcgacccgggtcaccgag

>EU448373.1_O_UKG_2007

tcaagcgttggagtcacatacgggtacgcaacagctgaggattttgtgagcggaccgaacacttctggtctcgagaccagagttgtgcaggcagaacggtttttcaaaacccacctcttcgactgggtcaccagtgactcattcggacgttgccacctcctggaactcccgaccgaccacaaaggtgtctacggcagcctgaccgactcgtatgcatatatgaggaacggctgggatgtcgaggtcaccgcggttggcaaccagttcaacggagggtgcctgctggtcgcaatggtaccagagctttgttctatccaaaagagggaactgtaccagctcacactcttccctcaccagttcatcaacccacgcacgaacatgactgcgcacattacagtgccctttgttggcgtcaaccgctacgaccagtacaaggttcacaagccttggacccttgtggttatggttgtagcccctctgaccgtcaacactgaaggtgcccctcagatcaaggtgtatgccaacgttgccccaactaacgtgcacgtcgcgggtgagtttccttccaaagagggaatattccccgtggcctgtagcgacggctatggtggcctggtgaccacggacccgaagacggctgaccccgtttatgggaaagtgttcaaccccccccgcaaccagttgccggggcgttttaccaacctccttgatgtggctgaggcatgcccgacgtttctgcacttcgagggtggcgtaccgtacgtgaccacgaaaacagactcggacagggtgcttgctcagtttgatatgtctttggcagcaaaacacatgtcaaacaccttcctcgcaggtcttgcgcagtactacacacagtacagtggcaccatcaacctgcacttcatgttcacaggacccactgacgcgaaggcgcgttacatgattgcctacgccccaccgggcatggagccgcccaagacacctgaggcggccgcgcactgcattcatgctgaatgggacactgggttgaactcaaagtttactttttccatcccctacctctcggccgccgattacgcgtacaccgcgtctgacgtggccgagaccacaaatgtgcagggatgggtctgcttgtttcaaattacacatggcaaggccgacggcgacgctctggtcgtactggctagtgctggtaaagactttgagctaaggctgccggtggacgcccgtgcggaaaccacttctgcgggcgagtcagcggatcctgtcaccaccactgttgaaaactacggtggcgaaacacagatccagaggcgccaacacacggacgtctcgttcattatggacagatttgtgaaagtgacaccgcaaaaccaaattaacattttggacctcatgcaggtcccatcacacactttggtgggagcgctcctacgcgcgtccacttactacttctctgacttggagatagcagtaaaacacgagggagacctcacctgggttccaaatggagcgcctgaaaaggcgttggacaacaccaccaacccaactgcttaccacaaggcaccactcacccggcttgccctgccctacactgcgccccaccgcgtgttggcaaccgtgtacaacggtgagtgcaggtacagcagaaatgctgtgcccaacttgagaggtgaccttcaagtgttggctcaaaaggtggcacggacgctgcctacctccttcaactacggtgccatcaaagcgacccgggtcaccgag

>EU448374.1_O_UKG_2007

tcaagcgttggagtcacatacgggtacgcaacagctgaggattttgtgagcggaccgaacacttctggtctcgagaccagagttgtgcaggcagaacggtttttcaaaacccacctcttcgactgggtcaccagtgactcattcggacgttgccacctcctggaactcccgaccgaccacaaaggtgtctacggcagcctgaccgactcgtatgcatatatgaggaacggctgggatgtcgaggtcaccgcggttggcaaccagttcaacggagggtgcctgctggtcgcaatggtaccagagctttgttctatccaaaagagggaactgtaccagctcacactcttccctcaccagttcatcaacccacgcacgaacatgactgcgcacattacagtgccctttgttggcgtcaaccgctacgaccagtacaaggttcacaagccttggacccttgtggttatggttgtagcccctctgaccgtcaacactgaaggtgcccctcagatcaaggtgtatgccaacattgccccaactaacgtgcacgtcgcgggtgagtttccttccaaagagggaatattccccgtggcctgtagcgacggctatggtggcctggtgaccacggacccgaagacggctgaccccgtttatgggaaagtgttcaaccccccccgcaaccagttgccggggcgttttaccaacctccttgatgtggctgaggcatgcccgacgtttctgcacttcgagggtggcgtaccgtacgtgaccacgaaaacagactcggacagggtgcttgctcagtttgatatgtctttggcagcaaaacacatgtcaaacaccttcctcgcaggtcttgcgcagtactacacacagtacagtggcaccatcaacctgcacttcatgttcacaggacccactgacgcgaaggcgcgttacatgattgcctacgccccaccgggcatggagccgcccaagacacctgaggcggccgcgcactgcattcatgctgagtgggacactgggttgaactcaaagtttactttttccatcccctacctctcggccgccgattacgcgtacaccgcgtctgacgtggccgagaccacaaatgtgcagggatgggtctgcttgtttcaaattacacatggcaaggccgacggcgacgctctggtcgtactggctagtgctggtaaagactttgagctaaggctgccggtggacgcccgtgcggaaaccacttctgcgggcgagtcagcggatcctgtcaccaccactgttgaaaactacggtggcgaaacacagatccagaggcgccaacacacggacgtctcgttcattatggacagatttgtgaaagtgacaccgcaaaaccaaattaacattttggacctcatgcaggtcccatcacacactttggtgggagcgctcctacgcgcgtccacttactacttctctgacttggagatagcagtaaaacacgagggagacctcacctgggttccaaatggagcgcctgaaaaggcgttggacaacaccaccaacccaactgcttaccacaaggcaccactcacccggcttgccctgccctacactgcgccccaccgcgtgttggcaaccgtgtacaacggtgagtgcaggtacagcagaaatgctgtgcccaacttgagaggtgaccttcaagtgttggctcaaaaggtggcacggacgctgcctacctccttcaactacggtgccatcaaagcgacccgggtcaccgag

>EU448375.1_O_UKG_2007

tcaagcgttggagtcacatacgggtacgcaacagctgaggattttgtgagcggaccgaacacttctggtctcgagaccagagttgtgcaggcagaacggtttttcaaaacccacctcttcgactgggtcaccagtgactcattcggacgttgccacctcctggaactcccgaccgaccacaaaggtgtctacggcagcctgaccgactcgtatgcatatatgaggaacggctgggatgtcgaggtcaccgcggttggcaaccagttcaacggagggtgcctgctggtcgcaatggtaccagagctttgttctatccaaaagagggaactgtaccagctcacactcttccctcaccagttcatcaacccacgcacgaacatgactgcgcacattacagtgccctttgttggcgttaaccgctacgaccagtacaaggttcacaagccttggacccttgtggttatggttgtagcccctctgaccgtcaacactgaaggtgcccctcagatcaaggtgtatgccaacattgccccaactaacgtgcacgtcgcgggtgagtttccttccaaagagggaatattccccgtggcctgtagcgacggctatggtggcctggtgaccacggacccgaagacggctgaccccgtttatgggaaagtgttcaaccccccccgcaaccagttgccggggcgttttaccaacctccttgatgtggctgaggcatgcccgacgtttctgcacttcgagggtggcgtaccgtacgtgaccacgaaaacagactcggacagggtgcttgctcagtttgatatgtctttggcagcaaaacacatgtcaaacaccttcctcgcaggtcttgcgcagtactacacacagtacagtggcaccatcaacctgcacttcatgttcacaggacccactgacgcgaaggcgcgttacatgattgcctacgccccaccgggcatggagccgcccaagacacctgaggcggccgcgcactgcattcatgctgaatgggacactgggttgaactcaaagtttactttttccatcccctacctctcggccgccgattacgcgtacaccgcgtctgacgtggccgagaccacaaatgtgcagggatgggtctgcttgtttcaaattacacatggcaaggccgacggcgacgctctggtcgtactggctagtgctggtaaagactttgagctaaggctgccggtggacgcccgtgcggaaaccacttctgcgggcgagtcagcggatcctgtcaccaccactgttgaaaactacggtggcgaaacacagatccagaggcgccaacacacggacgtctcgttcattatggacagatttgtgaaagtgacaccgcaaaaccaaattaacattttggacctcatgcaggtcccatcacacactttggtgggagcgctcctacgcgcgtccacttactacttctctgacttggagatagcagtaaaacacgagggagacctcacctgggttccaaatggagcgcctgaaaaggcgttggacaacaccaccaacccaactgcttaccacaaggcaccactcacccggcttgccctgccctacactgcgccccaccgcgtgttggcaaccgtgtacaacggtgagtgcaggtacagcagaaatgctgtgcccaacttgagaggtgaccttcaagtgttggctcaaaaggtggcacggacgctgcctacctccttcaactacggtgccatcaaagcgacccgggtcaccgag

>EU448376.1_O_UKG_2007

tcaagcgttggagtcacatacgggtacgcaacagctgaggattttgtgagcggaccgaacacttctggtctcgagaccagagttgtgcaggcagaacggtttttcaaaacccacctcttcgactgggtcaccagtgactcattcggacgttgccacctcctggaactcccgaccgaccacaaaggtgtctacggcagcctgaccgactcgtatgcatatatgaggaacggctgggatgtcgaggtcaccgcggttggcaaccagttcaacggagggtgcctgctggtcgcaatggtaccagagctttgttctatccaaaagagggaactgtaccagctcacactcttccctcaccagttcatcaacccacgcacgaacatgactgcgcacattacagtgccctttgttggcgttaaccgctacgaccagtacaaggttcacaagccttggacccttgtggttatggttgtagcccctctgaccgtcaacactgaaggtgcccctcagatcaaggtgtatgccaacattgccccaactaacgtgcacgtcgcgggtgagtttccttccaaagagggaatattccccgtggcctgtagcgacggctatggtggcctggtgaccacggacccgaagacggctgaccccgtttatgggaaagtgttcaaccccccccgcaaccagttgccggggcgttttaccaacctccttgatgtggctgaggcatgcccgacgtttctgcacttcgagggtggcgtaccgtacgtgaccacgaaaacagactcggacagggtgcttgctcagtttgatatgtctttggcagcaaaacacatgtcaaacaccttcctcgcaggtcttgcgcagtactacacacagtacagtggcaccatcaacctgcacttcatgttcacaggacccactgacgcgaaggcgcgttacatgattgcctacgccccaccgggcatggagccgcccaagacacctgaggcggccgcgcactgcattcatgctgaatgggacactgggttgaactcaaagtttactttttccatcccctacctctcggccgccgattacgcgtacaccgcgtctgacgtggccgagaccacaaatgtgcagggatgggtttgcttgtttcaaattacacatggcaaggccgacggcgacgctctggtcgtactggctagtgctggtaaagactttgagctaaggctgccggtggacgcccgtgcggaaaccacttctgcgggcgagtcagcggatcctgtcaccaccactgttgaaaactacggtggcgaaacacagatccagaggcgccaacacacggacgtctcgttcattatggacagatttgtgaaagtgacaccgcaaaaccaaattaacattttggacctcatgcaggtcccatcacacactttggtgggagcgctcctacgcgcgtccacttactacttctctgacttggagatagcagtaaaacacgagggagacctcacctgggttccaaatggagcgcctgaaaaggcgttggacaacaccaccaacccaactgcttaccacaaggcaccactcacccggcttgccctgccctacactgcgccccaccgcgtgttggcaaccgtgtacaacggtgagtgcaggtacagcagaaatgctgtgcccaacttgagaggtgaccttcaagtgttggctcaaaaggtggcacggacgctgcctacctccttcaactacggtgccatcaaagcgacccgggtcaccgag

>EU448377.1_O_UKG_2007

tcaagcgttggagtcacatacgggtacgcaacagctgaggattttgtgagcggaccgaacacttctggtctcgagaccagagttgtgcaggcagaacggtttttcaaaacccacctcttcgactgggtcaccagtgactcattcggacgttgccacctcctggaactcccgaccgaccacaaaggtgtctacggcagcctgaccgactcgtatgcatatatgaggaacggctgggatgtcgaggtcaccgcggttggcaaccagttcaacggagggtgcctgctggtcgcaatggtaccagagctttgttctatccaaaagagggaactgtaccagctcacactcttccctcaccagttcatcaacccacgcacgaacatgactgcgcacattacagtgccctttgttggcgttaaccgctacgaccagtacaaggttcacaagccttggacccttgtggttatggttgtagcccctctgaccgtcaacactgaaggtgcccctcagatcaaggtgtatgccaacattgccccaactaacgtgcacgtcgcgggtgagtttccttccaaagagggaatattccccgtggcctgtagcgacggctatggtggcctggtgaccacggacccgaagacggctgaccccgtttatgggaaagtgttcaaccccccccgcaaccagttgccggggcgttttaccaacctccttgatgtggctgaggcatgcccgacgtttctgcacttcgagggtggcgtaccgtacgtgaccacgaaaacagactcggacagggtgcttgctcagtttgatatgtctttggcagcaaaacacatgtcaaacaccttcctcgcaggtcttgcgcagtactacacacagtacagtggcaccatcaacctgcacttcatgttcacaggacccactgacgcgaaggcgcgttacatgattgcctacgccccaccgggcatggagccgcccaagacacctgaggcggccgcgcactgcattcatgctgaatgggacactgggttgaactcaaagtttactttttccatcccctacctctcggccgccgattacgcgtacaccgcgtctgacgtggccgagaccacaaatgtgcagggatgggtctgcttgtttcaaattacacatggcaaggccgacggcgacgctctggtcgtactggctagtgctggtaaagactttgagctaaggctgccggtggacgcccgtgcggaaaccacttctgcgggcgagtcagcggatcctgtcaccaccactgttgaaaactacggtggcgaaacacagatccagaggcgccaacacacggacgtctcgttcattatggacagatttgtgaaagtgacaccgcaaaaccaaattaacattttggacctcatgcaggtcccatcacacactttggtgggagcgctcctacgcgcgtccacttactacttctctgacttggagatagcagtaaaacacgagggagacctcacctgggttccaaatggagcgcctgaaaaggcgttggacaacaccaccaacccaactgcttaccacaaggcaccactcacccggcttgccctgccctacactgcgccccaccgcgtgttggcaaccgtgtacaacggtgagtgcaggtacagcagaaatgctgtgcccaacttgagaggtgaccttcaagtgttggctcaaaaggtggcacggacgctgcctacctccttcaactacggtgccatcaaagcgacccgggtcaccgag

>EU448378.1_O_UKG_2007

tcaagcgttggagtcacatacgggtacgcaacagctgaggattttgtgagcggaccgaacacttctggtctcgagaccagagttgtgcaggcagaacggtttttcaaaacccacctcttcgactgggtcaccagtgactcattcggacgttgccacctcctggaactcccgaccgaccacaaaggtgtctacggcagcctgaccgactcgtatgcatatatgaggaacggctgggatgtcgaggtcaccgcggttggcaaccagttcaacggagggtgcctgctggtcgcaatggtaccagagctttgttctatccaaaagagggaactgtaccagctcacactcttccctcaccagttcatcaacccacgcacgaacatgactgcgcacattacagtgccctttgttggcgttaaccgctacgaccagtacaaggttcacaagccttggacccttgtggttatggttgtagcccctctgaccgtcaacactgaaggtgcccctcagatcaaggtgtatgccaacattgccccaactaacgtgcacgtcgcgggtgagtttccttccaaagagggaatattccccgtggcctgtagcgacggctatggtggcctggtgaccacggacccgaagacggctgaccccgtttatgggaaagtgttcaaccccccccgcaaccagttgccggggcgttttaccaacctccttgatgtggctgaggcatgcccgacgtttctgcacttcgagggtgacgtaccgtacgtgaccacgaaaacagactcggacagggtgcttgctcagtttgatatgtctttggcagcaaaacacatgtcaaacaccttcctcgcaggtcttgcgcagtactacacacagtacagtggcaccatcaacctgcacttcatgttcacaggacccactgacgcgaaggcgcgttacatgattgcctacgccccaccgggcatggagccgcccaagacacctgaggcggccgcgcactgcattcatgctgaatgggacactgggttgaactcaaagtttactttttccatcccctacctctcggccgccgattacgcgtacaccgcgtctgacgtggccgagaccacaaatgtgcagggatgggtctgcttgtttcaaattacacatggcaaggccgacggcgacgctctggtcgtactggctagtgctggtaaagactttgagctaaggctgccggtggacgcccgtgcggaaaccacttctgcgggcgagtcagcggatcctgtcaccaccactgttgaaaactacggtggcgaaacacagatccagaggcgccaacacacggacgtctcgttcattatggacagatttgtgaaagtgacaccgcaaaaccaaattaacattttggacctcatgcaggtcccatcacacactttggtgggagcgctcctacgcgcgtccacttactacttctctgacttggagatagcagtaaaacacgagggagacctcacctgggttccaaatggagcgcctgaaaaggcgttggacaacaccaccaacccaactgcttaccacaaggcaccactcacccggcttgccctgccctacactgcgccccaccgcgtgttggcaaccgtgtacaacggtgagtgcaggtacagcagaaatgctgtgcccaacttgagaggtgaccttcaagtgttggctcaaaaggtggcacggacgctgcctacctccttcaactacggtgccatcaaagcgacccgggtcaccgag

>FJ175661.1_O_ISR_2007

tcgagcgttggggtcacttacgggtacgcaacaaccgaggactttgtgagcgggccaaacacatccggtcttgagactagggttgtgcaagcagagcggttcttcaaaacccacttgttcgactgggtcaccagtgactcgttcggacgatgccacctgctggaacttccaactgaccacaaaggtgtctacggtagcctgactgattcttatgcttacatgagaaacggttgggatgtcgaggtcactgcagtgggaaaccagttcaacggaggatgcctgttggtagccatggtgccagaactctgctctatcagcaaaagagagctgtaccagctcacgctctttccccaccaattcatcaacccccggacgaacatgacggcacacatcaccgtgccctttgtcggcgtcaatcgctacgaccagtacaaggtacacaaaccttggaccctcgtggtcatggtcgtggccccgctgactgtcaatactgaaggtgctccacagatcaaggtttatgccaacatcgcccccaccaacgtgcacgtcgcgggtgagttcccttccaaggaagggatcttccccgtggcatgtagtgacggttacggcggtcttgtgaccactgacccaaagacggctgaccccgcctacgggaaagttttcaacccccctcgcaacatgttgccagggcggttcaccaacttccttgatgtggctgaggcgtgccctacgtttctgcactttgagggtgacgtgccatacgtgaccacaaagacggattcggacagggttcttgcccagtttgacttgtctttggcagcgaagcacatgtcaaacacctttctggcaggtctcgcccagtactacacgcagtacagcggcaccatcaacctgcacttcatgttcacaggacccactgacgcgaaagcgcgttacatgattgcatatgccccccccggcatggagccgcccaaaacacctgaggcagccgcccattgcattcatgcggagtgggatactgggttgaattcaaaattcacattttcaatcccttacctttcggcggctgactacgcgtacaccgcgtctgacactgctgagaccacaaatgtacagggatgggtttgcctgtttcaaatcacacacgggaaggccgatggtgacgcacttgtcgttctggctagcgccggtaaggacttcgagctgcggttgccagttgacgctcgcacgcagaccacctccacaggtgagtcagctgaccccgtgactgccactgttgagaactacggtggcgagacacaggtccagagacgccagcacacggatgtctcgttcatattggacagatttgtgaaagtgacaccaaaagaccaaattaatgtgttggacttgatgcaaacccccgcccacactttggtaggcgcgctcctccgcaccgccacctactacttcgcagatctagaggtggcagtgaaacatgaggggaaccttacctgggtcccgaatggggcgcccgagacagcgttggacaacaccactaatccaacggcttaccacaaagcaccgctcacccgacttgcactgccttacacggcaccacaccgtgtcttggctaccgtatacaacgggaactgcaagtatggcgagagccacacaaccaacgtgagaggtgacctgcaagtgttggcccagaaggcggcgataacgctgcctacctccttcaactacggtgccatcaaagctacccgggtgactgaa

>FJ175662.1_O_ISR_2007

tcgagcgttggggtcacttacgggtacgcaacaaccgaggactttgtgagcgggccaaacacatccggtcttgagactagggttgtgcaagcagagcggttcttcaaaacccacttgttcgactgggtcaccagtgactcgttcggacgatgccacctgctggaacttccaactgaccacaaaggtgtctacggtagcctgactgattcttatgcttacatgagaaacggttgggatgtcgaggtcactgcagtgggaaaccagttcaacggaggatgcctgttggtagccatggtgccagaactctgctctatcagcaaaagagagctgtaccagctcacgctctttccccaccaattcatcaacccccggacgaacatgacggcacacatcaccgtgccctttgtcggcgtcaatcgctacgaccagtacaaggtacacaaaccttggaccctcgtggtcatggtcgtggccccgctgactgtcaatactgaaggtgctccacagatcaaggtttatgccaacatcgcccccaccaacgtgcacgtcgcgggtgagttcccttccaaggaagggatcttccccgtggcatgtagtgacggttacggcggtcttgtgaccactgacccaaagacggctgaccccgcctacgggaaagttttcaacccccctcgcaacatgttgccagggcggttcaccaacttccttgatgtggctgaggcgtgccctacgtttctgcactttgagggtgacgtgccatacgtgaccacaaagacggattcggacagggttcttgcccagtttgacttgtctttggcagcgaagcacatgtcaaacacctttctggcaggtctcgcccagtactacacacagtacagcggcaccatcaacctgcatttcatgttcacaggacccactgacgcgaaagcgcgttacatgattgcatatgccccccccggcatggagccgcccaaaacacctgaggcagccgcccattgcattcatgcggagtgggacactgggttgaattcaaaattcacattttcaatcccttacctttcggcggctgactacgcgtacaccgcgtctgacactgctgagaccacaaatgtacagggatgggtttgcctgtttcaaatcacacacgggaaggccgatggtgacgcacttgtcgttctggctagcgccggtaaggacttcgagctgcggttgccagttgacgctcgcacgcagaccacctccacaggtgagtcagctgaccccgtgactgccactgttgagaactacggtggcgagacacaggtccagagacgccagcacacggatgtctcgttcatattggacagatttgtgaaagtgacaccaaaagaccaaattaatgtgttggacttgatgcaaacccccgcccacactttggtaggcgcgctcctccgcgccgccacctactacttcgcagatctagaggtggcagtgaaacatgaggggaaccttacctgggtcccgaatggggcgcccgagacagcgttggataacaccactaatccaacggcttaccacaaagcaccgctcacccgacttgcactgccttacacggcaccacaccgtgtcttggctaccgtatacaacgggaactgcaagtatggcgagagccacacaaccaacgtgagaggtgacctgcaagtgttggcccagaaggcggcgataacgctgcctacctccttcaactacggtgccatcaaagctacccgggtgactgaa

>FJ175663.1_O_ISR_2007

tcgagcgttggggtcacttacgggtacgcaacaaccgaggactttgtgagcgggccaaacacatccggtcttgagactagggttgtgcaagcagagcggttctttaaaacccacttgttcgattgggtcaccagtgactcgttcggacgatgccacctgctggaacttccaactgaccacaaaggtgtctacggtagcctgactgattcttatgcttacatgagaaacggttgggatgtcgaggtcactgcagtgggaaaccagttcaacggaggatgcttgttggtagccatggtgccagaactctgctctatcagcaaaagagagctgtaccagatcacgctctttccccaccaattcatcaacccccggacgaacatgacggcgcacatcaccgtgccctttgtcggcgtcaatcgctacgaccagtacaaggtacacaaaccttggaccctcgtggtcatggtcgtggccccgctgactgtcaatactgagggtgctccacagatcaaggtgtatgccaacatcgcccctaccaacgtgcacgtcgcgggtgagttcccttccaaggaagggatcttccccgtggcatgtagtgacggctacggcggtcttgtgaccactgacccaaagacggctgaccccgcctacgggaaagttttcaacccccctcgcaacatgttgccagggcggttcaccaacttccttgatgtggctgaggcgtgccctacgtttctgcactttgagggtgacgtgccatacgtgaccacaaagacggattcggacagggttcttgctcaatttgacttgtctttggcagcgaagcacatgtcaaacacctttctggcaggtctcgcccagtactacacacagtacagcggcaccatcaacctgcacttcatgttcacaggacccactgacgcgaaagcacgttacatgattgcatatgccccccccggcatggagccgcccaaaacacctgaggcagccgctcattgcattcatgcggagtgggatactgggttgaattcaaaattcacattttcaatcccttacctttcggcggctgactacgcgtacaccgcgtctgacactgccgagaccacaaatgtacagggatgggtttgcctgtttcaaatcacacacgggaaggccgatggtgacgcacttgtcgttctggctagcgccggtaaggacttcgagctgcggttgccagttgacgctcgcacgcagaccacctccacaggcgagtcagctgaccccgtgactgccactgttgagaactacggtggcgagacacaggtccagagacgccagcatacggatgtctcgttcatattggacagatttgtgaaagtgacaccaaaagaccaaattaatgtgttggacttgatgcaaacccccgcccacactttggtaggcgcgctcctccgcaccgccacctactacttcgcagatctagaggtggcagtgaaacatgaggggaaccttacctgggtcccgaacggggcgcccgagacagcgttggataacaccactaatccaacggcttaccacaaagcaccgctcacccgacttgcactgccttacacggcaccacaccgtgtcttggctaccgtatacaacgggaactgcaagtatggcgagagccacacaaccaacgtgagaggtgacctgcaggtgttggcccagaaggcggcgataacgctgcctacctcctttaactacggtgccatcaaagctacccgggtgactgaa

>FJ175664.1_O_ISR_2007

tcgagcgttggggtcacttacgggtacgcaacaaccgaggactttgtgagcgggccaaacacatccggtcttgagactagggttgtgcaagcagagcggttctttaaaacccacttgttcgactgggtcaccagtgactcgttcggacgatgccacctgctggaacttccaactgaccacaaaggtgtctacggtagcctgactgattcttatgcttacatgagaaacggttgggatgtcgaggtcactgcagtgggaaaccagttcaacggaggatgcttgttggtagccatggtgccagaactctgctctatcagcaaaagagagctgtaccagctcacgctctttccccaccaattcatcaacccccggacgaacatgacggcacacatcaccgtgccctttgtcggcgtcaatcgctacgaccagtacaaggtacacaaaccttggaccctcgtggtcatggtcgtggccccgctgactgtcaatactgagggtgctccacagatcaaggtgtatgccaacatcgcccctaccaacgtgcacgtcgcgggtgagttcccttccaaggaagggatcttccccgtggcatgtagtgacggctacggcggtcttgtgaccactgacccaaagacggctgaccccgcctacgggaaagttttcaacccccctcgcaacatgttgccagggcggttcaccaacttccttgatgtggctgaggcgtgccctacgtttctgcactttgagggtgacgtgccatacgtgaccacaaagacggattcggacagggttcttgctcagtttgacttgtctttggcagcgaagcacatgtcaaacacctttctggcaggtctcgcccagtactacacacagtacagcggcaccatcaacctgcacttcatgttcacaggacccactgacgcgaaagcacgttacatgattgcatatgccccccccggcatggagccgcccaaaacacctgaggcagccgctcattgcattcatgcggagtgggatactgggttgaattcaaaattcacattttcaatcccttacctttcggcggctgactacgcgtacaccgcgtctgacactgccgagaccacaaatgtacagggatgggtttgcctgtttcaaatcacacacgggaaggccgatggtgacgcacttgtcgttctggctagcgccggtaaggacttcgagctgcggttgccagttgacgctcgcacgcagaccacctccacaggcgagtcagctgaccccgtgactgccactgttgagaactacggtggcgagacacaggtccagagacgccagcacacggatgtctcgttcatattggacagatttgtgaaagtgacaccaaaagaccaaattaatgtgttggacttgatgcaaacccccgcccacactttggtaggcgcgctcctccgcaccgccacctactacttcgcagatctagaggtggcagtgaaacatgaggggaaccttacctgggtcccgaatggggcgcccgagacagcgttggataacaccactaatccaacggcttaccacaaagcaccgctcacccgacttgcactgccttacacggcaccacaccgtgtcttggctaccgtatacaacgggaactgcaagtatggcgagagccacacaaccaacgtgagaggtgacctgcaggtgttggcccagaaggcggcgataacgctgcctacctcctttaactacggtgccatcaaagctacccgggtgactgaa

>FJ175665.1_O_ISR_2007

tcgagcgttggggtcacttacgggtacgcaacaaccgaggactttgtgagcgggccaaacacatccggtcttgagactagggttgtgcaagcagagcggttctttaaaacccacttgttcgactgggtcaccagtgactcgttcggacgatgccacctgctggaacttccaactgaccacaaaggtgtctacggtagcctgactgattcttatgcttacatgagaaacggttgggatgtcgaggtcactgcagtgggaaaccagttcaacggagggtgcctgttggtagccatggtgccagaactctgctctatcagcaaaagagagctgtaccagctcacgctctttccccaccaattcatcaacccccggacgaacatgacggcacacatcaccgtgccctttgtcggcgtcaatcgctacgaccagtacaaggtacacaaaccttggaccctcgtggtcatggtcgtggccccgctgactgtcaatactgaaggtgctccacagatcaaggtttatgccaacatcgcccctaccaacgtgcacgtcgcgggtgagttcccttccaaggaagggatcttccccgtggcatgtagtgacggttacggcggtcttgtgaccactgacccaaagacggctgaccccgcctacgggaaagttttcaacccccctcgcaacatgttgccagggcggttcaccaacttccttgatgtggctgaggcgtgccctacgtttctgcactttgagggtgacgtgccatacgtgaccacaaagacggattcggacagggttcttgctcagtttgacttgtctttggcagcgaagcacatgtcgaacacctttctggcaggtctcgcccagtactacacacagtacagcggcaccatcaacctgcacttcatgttcacaggacccactgacgcgaaagcgcgttacatgattgcatatgccccccccggcatggagccacccaaaacacctgaggcagccgctcattgcattcatgcggagtgggatactgggttgaattcaaaattcacattttcaatcccttacctttcggcggctgactacgcgtacaccgcgtccgacactgctgagaccacaaatgtacagggatgggtttgcctgtttcaaatcacacacgggaaggccgatggtgacgcacttgtcgttctggctagcgccggtaaggacttcgagctgcggttgccagttgacgctcgcacgcagaccacctccacaggtgagtcagctgaccccgtgactgccactgttgagaactacggtggcgagacacaggtccagagacgccagcacacggatgtctcgttcatattggacagatttgtgaaagtgacaccaaaagaccaaattaatgtgttggacttgatgcaaacccccgcccacactttggtaggcgcgctcctccgcaccgccacctactacttcgcagatctagaggtggcggtgaaacatgaggggaaccttacctgggtcccgaatggggcgcccgagacagcgttggataacaccactaatccaacggcttaccacaaagcgccgctcacccgacttgcactgccttacacggcaccacaccgtgtcttggctaccgtatacaacgggaactgcaagtatggcgagagtcacacaactaacgtgagaggtgacctgcaagtgttggcccagaaggcggcgataacgctgcctacctccttcaactacggtgccatcaaagctacccgggtgactgaa

>FJ175666.1_O_ISR_2007

tcgagcgttggggtcacttacgggtacgcaacaaccgaggactttgtgagcgggccaaacacatccggtcttgagactagggttgtgcaagcagagcggttctttaaaacccacttgttcgactgggtcaccagtgactcgttcggacgatgccacctgctggaacttccaactgaccacaaaggtgtctacggtagcctgactgattcttatgcttacatgagaaacggttgggatgtcgaggtcactgcagtgggaaaccagttcaacggaggatgcttgttggtagccatggtgccagaactctgctctatcagcaaaagagagctgtaccagctcacgctctttccccaccaattcatcaacccccggacgaacatgacggcacacatcaccgtgccctttgtcggcgtcaatcgctacgaccagtacaaggtacacaaaccttggaccctcgtggtcatggtcgtggccccgctgactgtcaatactgagggtgctccacagatcaaggtgtatgccaacatcgcccctaccaacgtgcacgtcgcgggtgagttcccttccaaggaagggatcttccccgtggcatgtagtgacggctacggcggtcttgtgaccactgacccaaagacggctgaccccgcctacgggaaagttttcaacccccctcgcaacatgttgccagggcggttcaccaacttccttgatgtggctgaggcgtgccctacgtttctgcactttgagggtgacgtgccatacgtgaccacaaagacggattcggacagggttcttgctcagtttgacttgtctttggcagcgaagcacatgtcaaacacctttctggcaggtctcgcccagtactacacacagtacagcggcaccatcaacctgcacttcatgttcacaggacccactgacgcgaaagcacgttacatgattgcatatgccccccccggcatggagccgcccaaaacacctgaggcagccgctcattgcattcatgcggagtgggatactgggttgaattcaaaattcacattttcaatcccttacctttcggcggctgactacgcgtacaccgcgtctgacactgccgagaccacaaatgtacagggatgggtttgcctgtttcaaatcacacacgggaaggccgatggtgacgcacttgtcgttctggctagcgccggtaaggacttcgagctgcggttgccagttgacgctcgcacgcagaccacctccacaggcgagtcagctgaccccgtgactgccactgttgagaactacggtggcgagacacaggtccagagacgccagcacacggatgtctcgttcatattggacagatttgtgaaagtgacaccaaaagaccaaattaatgtgttggacttgatgcaaacccccgcccacactttggtaggcgcgctcctccgcaccgccacctactacttcgcagatctagaggtggcagtgaaacatgaggggaaccttacctgggtcccgaatggggcgcccgagacagcgttggataacaccactaatccaacggcttaccacaaagcaccgctcacccgacttgcactgccttacacggcaccacaccgtgtcttggctaccgtatacaacgggaactgcaagtatggcgagagccacacaaccaacgtgagaggtgacctgcaggtgttggcccagaaggcggcgataacgctgcctacctcctttaactacggtgccatcaaagctacccgggtgactgaa

>FJ461344.1_O_UGA_2002

tcgagtgttggggttacgtacgggtatgcaacggctgaggactttgtgagtgggccaaacacctctggtcttgagactagagttgcccaggcagagcggttctttaagactcacctgttcgactgggtcaccagtgacccattcgggcgatgccacttgctggagctcccaactgaccacaaaggtgtctacggcggcctgatcgactcgtatgcttatatgagaaacggttgggacgtcgaggtgaccgctgtggggaaccagttcaacggaggttgcttgttagtggcgatggtaccagagctttgttccatccagaagagggaactgtaccaactcacactcttccctcatcagttcatcaatccccggacgaacatgacggcacacatcactgtgccctttgttggcgtcaaccgctacgaccagtacaaagtacacaaaccctggacccttgtggtcatggtcgtggcccccttgactgtcaacaacgagggtgctccacagatcaaggtgtatgctaacatcgccccaaccaacgtacacgtcgcgggtgagttcccttccaaagagggaattttccccgtggcttgcagcgacggttacggcggtttggtgaccacggacccgaaaacggctgaccccgcttacgggaaagtgtttaaccccccgcgcaacatgttgccggggcgtttcaccaatctccttgatgtggctgaggcgtgccctacgtttctgcacttcgaaggtgacgttccctacgtgaccacgaagacagagtctgacagggtgcttgctcagtttgatttgtctttggccgcaaagcacatgtcaaacaccttccttgcaggtcttgcccagtactacacccagtacagcggcaccatcaacctgcacttcatgttcacaggtcccactgacgcgaaagcgcgttacatgattgcatatgccccccccggcatggagccgcccaagacacctgaaacggcggctcactgcattcatgctgagtgggacacggggttgaactcaaaattcacattctccattccctacctctcggcggctgactacgcgtacaccgcgtcaagcactgccgaaaccaccaacgtgcagggatgggtctgccttttccaaataacacacgggaaagccgacggggatgccttggtcgtgctggccagtgccggcaaggacttcgacctgcgtctcccagtggacgctcgcacacaaaccacctccctgggtgagtcggctgaccctgtgactgctactgtggagaactacggtggcgcaactcaggcccagaaacgccaacacacggatgtctcgttcattctggacagatttgtgaaggtcacaccccaagaccaaattaatgttctggacctgatgcagatccctgcccacacactggtgggcgcgctcttgcgcgcatccacttactactttgctgatttggaagtggcagtgaaacacgagggcaacctcacttgggtcccgaacggagcacccgaagctgcactggacaacaccaccaacccaacagcatactacaaggcacctctcactcgccttgcactgccttacacggcaccacaccgtgtgctggcaaccgtatacaacgggaactgcaagtacagtggctcctcagttactaacgtgaggggtgaccttcaagtgttggcccagaaggctgcgagagcgctgcccacctccttcaactacggtgccgtcaaggctactcgggtgacagag

>FJ461345.1_O_UGA_2002

tcgagtgttggggttacgtacgggtatgcaacagctgaggactttgtgagcgggccaaacacctctggtcttgagactagagttgcccaagcagagcggttctttaagacccacctgttcgactgggtcaccagtgactcattcgggcgatgccacttgctggagcttccaactgaccacaaaggtgtctacggcggcctgatcgactcgtatgcttatatgagaaacggttgggacgtcgaggtgaccgctgtggggaaccagttcaacggaggttgtttgttagtggcaatggtaccagagctttgttccatccagaagagggaactgtaccaacttacactcttccctcatcagttcatcaacccccggacgaacatgacggcacacatcaccgtgccctttgttggcgtcaaccgctacgaccagtacaaggtacacaaaccctggacccttgtggtcatggttgtggccccgttgactgtcaacaccgaaggtgctccacagatcaaggtgtatgctaacatcgccccaaccaacgtgcacgtcgcgggtgagttcccttccaaagagggaattttccccgtggcttgcagcgacggttacggcggtttggtgaccacggacccgaaaacggctgaccccgtttacgggaaagtgtttaaccccccgcgcaacatgttgccggggcgtttcaccaatctccttgatgtggctgaggcatgccctacgtttctgcacttcgaaggtgacgttccctacgtgaccacgaagacagactctgacagggtgctcacccagtttgatttgtctttggccgcaaagcacatgtcaaacaccttcctcgcaggtctcgcccagtactacacccagtacagcggcaccatcaacctgcacttcatgttcacaggtcccactgacgcgaaagcgcgttacatgattgcatatgcccctcctggtatggagccgcccaaaacacctgaaacagcggctcactgcattcatgctgagtgggacacggggttgaactcaaagttcacattctccattccctacctctcggcggctgactacgcgtacaccgcgtcagacactgccgaaaccaccaacgtgcagggatgggtctgccttttccaaataacacacgggaaagccgacggggatgctttggtcgtgctggccagtgccggcaaggacttcgacctgcgtctcccagtggacgctcgcacacaaaccacctccccgggtgagtcggctgaccctgtgactgctactgtggagaactacggtggcgcaactcaggtccaaagacgccaacacacggacgtctcgttcattttggacagatttgtgaaggttacaccccaagaccagatcaatgttctggacctgatgcagatccctgcccacacactggtgggcgcgctcttgcgcgcatccacttactactttgctgacttggaagtggcagtgaaacatgagggcgacctcacttgggtcccgaatggagcacccgaagctgcactgaacaacaccaccaacccaacagcataccacaaggcacctctcactcgccttgcactgccttacacggcaccacaccgcgtgttggcaaccgtgtacaacgggaactgcaagtacagtgactcctcagtgactaacgtgaggggtgaccttcaggtattggctcagaaggctgcgagagcgctgcctacctccttcaactacggtgccgtcaaggccactcgggtgacagaa

>FJ461346.1_SAT2_UGA_2002

agctctgttggcgtgaccctgggttaygccgacgctgacgctttccgaccgggrcccaacacttccggtttggaaacacgcgtccaacaggctgaacgcttcttcaaggagaagctgtttgactggaccagcgacaaaccgttcggcacgctgtacgtcctggagttgcccaaggatcacaagggcatttacgggaaacttaccgactcctacacgtacatgygcaacggatgggatgtycaggtcagygccaccagcacacagtttaacggcgggtcgctactcgtagcaatggtacccgagttgtgcagcctgaaaagcakagaagagttccaacttactctctacccacatcagttcatcaacccgcgcacaaacacaacagcgcacatccaggtcccttacctgggcgtgaacagrcacgatcagggtaaacgccaccaggcgtggtcgctggtcgtgatggtgctcactcccctcaccacggagcagatgaatagcgggaccgtcgaggtctacgccaacattgcaccaacaaacgttgttgtagcgggtgagttgccgggaaaacaaggcattgtcccggtggctgccgccgatgggtacggtgggttccaaaacaccgatccaaagacggccgaccccatctacgggtatgtgtacaacccgtccagaaatgattgccacgggcgattttccaacctcatggatgtcgcggaagcgtgcccaacgctcctgaattttgatggc---aaaccgtacgttgtgaccaggaacagcggcgacaaagtcatgacggcttttgacgtcgccttcacacacaaggtacacaagaacaccttcctggcgggtttggccgattactacacacagtactcgggcagcctgaactaccacttcatgtacacaggcccaactcatcacaaggcaaaattcatggtagcatacgtaccaccgggcgtaagccttcccaagacaccggaagatgccgcccactgctaccacgcagagtgggacactggattgaactcctcgttctcctttgctgtaccctacatctctgcatcagactttacttacactcacacagacacaccagccatggccactaccaacggttgggtggttgtgctgcaggtcactgacacacactctgctgaagctgccgtagtggtgtctgtcagcgccgggccagatttggagtttcgattccctatcgatccggtgcgacagaccacctcagcgggagaaggagcggatgttgtcaccacagacccgtcgacccacggcggggctgtgcgtgaagggagccgcaaacacactgaagtggctttcctgcttgatcgcagcacacacgttcacacaaacaagacatccttcgctgtggacctcatggacaccaaggaaggggcgctcgttggagcaatcttgcgggcttctacctactacttctgtgaccttgagatcgccgtgggtgaccacaagagagtcttctggcaacccaacggagcaccgcggaccacacaacttggc---gataaccctatggtcttcgccacaaacggggtcacgcggttcgctgttccgttcacggcgccacacagactattgtccactgtttacaacggtgagtgtgagtacaagacaacagtctct---gccatacgtggtgaccgcscagtgctcgcgaataagtacgctggcgccttgccgtcaaccttcaacttcgggttcgtgaccgtcgacaaaccagtagac

>FJ542365.1_O_UKG_2001

tcgagcgttggagtcacttacgggtacgcaacagctgaggactttgtgagcggaccaaacacatctgggcttgagaccagggttgtgcaggcagagcggttcttcaaaacccacttgttcgactgggtcaccagtgacccgtttggacggtgctatctgctggaactcccaactgaccacaaaggtgtctacggcagcctgaccgactcttatgcttacatgagaaacggttgggatgttgaggtcaccgcagtgggaaatcagttcaacggaggatgtctgttggtggccatggtgccagaactttgctctattgacaagagagagctgtaccagctcacgctctttccccaccagttcatcaacccccggacgaacatgacggcgcacatcactgtgccctttgttggcgtcaaccgctacgaccagtacaaggtacacaaaccttggaccctcgtggttatggttgtggccccgctgactgtcaacaccgaaggtgccccacagatcaaggtctatgccaacatcgcccctaccaacgtgcacgttgcgggtgagttcccttctaaggaagggatcttccccgtggcatgtagcgacggttacggtggtctggtgaccactgacccaaagacggctgaccccgcctacgggaaagtgttcaatccacctcgcaacatgttgccggggcggttcaccaacttccttgatgtggctgaggcgtgccctacgtttctgcactttgagggtggcgtgccgtacgtgaccacaaagacggactcagacagggtgctcgcccagtttgacttgtctctggcagcaaagcacatgtcaaacaccttcctggcaggtctcgcccagtactacacacagtacagcggcaccatcaacctgcacttcatgttcacaggacccactgacgcgaaagcgcgttacatgattgcatacgccccccctggtatggagccgcccaaaacacctgaggcggccgcccactgcattcatgcggagtgggacacagggttgaattcaaaattcacattttcaatcccttacctttcggcggctgattacgcgtacaccgcgtctgacgctgcggagaccacaaatgtacagggatgggtttgcctgtttcaaattacacacgggaaggctgacggcgacgcactggtcgttctagctagcgccggtaaggactttgagctgcgtctgccagttgacgctcgcacgcagaccacctccgcaggtgagtcggctgaccccgtgactgccactgttgagaactacggtggtgagacacaggtccagagacgccaacacacggatgtctcgttcatattagacagatttgtgaaagtaacaccaaaagaccaaattaatgtgttggacctgatgcaaacccctgcacacactttggtaggcgcgctcctccgtactgccacctactacttcgcagatctagaagtggcagtgaaacacgaggggaaccttacctgggtcccgaatggggcgcccgagacagcgttggacaacaccaccaatccaacggcttaccacaaggcaccgctcacccggcttgcactgccttacacggcaccgcaccgtgtcttggctactgtttacaacgggaactgcaagtatggcgagagccccgtgaccaatgtgagaggtgacctgcaagtattggcccaaaaggcggcaagaacgctgcctacctccttcaattacggtgccatcaaagccactcgggtgactgaa

>FJ542368.1_O_UKG_2001

tcgagcgttggagtcacttacgggtacgcaacagctgaggactttgtgagcggaccaaacacatctgggcttgagaccagggttgtgcaggcagagcggttcttcaaaacccacttgttcgactgggtcaccagtgacccgtttggacggtgctatctgctggaactcccaactgaccacaaaggtgtctacggcagcctgaccgactcttatgcttacatgagaaacggttgggatgttgaggtcaccgcagtgggaaatcagttcaacggaggatgtctgttggtggccatggtgccagaactttgctctattgacaagagagagctgtaccagctcacgctctttccccaccagttcatcaacccccggacgaacatgacggcgcacatcactgtgccctttgttggcgtcaaccgctacgaccagtacaaggtacacaaaccttggaccctcgtggttatggttgtggccccgctgactgtcaacaccgaaggtgccccacagatcaaggtctatgccaacatcgcccctaccaacgtgcacgttgcgggtgagttcccttctaaggaagggatcttccccgtggcatgtagcgacggttacggtggtctggtgaccactgacccaaagacggctgaccccgcctacgggaaagtgttcaatccacctcgcaacatgttgccggggcggttcaccaacttccttgatgtggctgaggcgtgccctacgtttctgcactttgagggtggcgtgccgtacgtgaccacaaagacggactcagacagggtgctcgcccagtttgacttgtctctggcagcaaagcacatgtcaaacaccttcctggcaggtctcgcccagtactacacacagtacagcggcaccatcaacctgcacttcatgttcacaggacccactgacgcgaaagcgcgttacatgattgcatacgccccccctggtatggagccgcccaaaacacctgaggcggccgcccactgcattcatgcggagtgggacacagggttgaattcaaaattcacattttcaatcccttacctttcggcggctgattacgcgtacaccgcgtctgacgctgcggagaccacaaatgtacagggatgggtttgcctgtttcaaattacacacgggaaggctgacggcgacgcactggtcgttctagctagcgccggtaaggactttgagctgcgtctgccagttgacgctcgcacgcagaccacctccgcaggtgagtcggctgaccccgtgactgccactgttgagaactacggtggtgagacacaggtccagagacgccaacacacggatgtctcgttcatattagacagatttgtgaaagtaacaccaaaagaccaaattaatgtgttggacctgatgcaaacccctgcacacactttggtaggcgcgctcctccgtactgccacctactacttcgcagatctagaagtggcagtgaaacacgaggggaaccttacctgggtcccgaatggggcgcctgagacagcgttggacaacaccaccaatccaacggcttaccacaaggcaccgctcacccggcttgcactgccttacacggcaccgcaccgtgtcttggctactgtttacaacgggaactgcaagtatggcgagagccccgtgaccaatgtgagaggtgacctgcaagtattggcccaaaaggcggcaagaacgctgcctacctccttcaattacggtgccatcaaagccactcgggtgactgaa

>FJ542369.1_O_UKG_2001

tcgagcgttggagtcacttacgggtacgcaacagctgaggactttgtgagcggaccaaacacatctgggcttgagaccagggttgtgcaggcagagcggttcttcaaaacccacttgttcgactgggtcaccagtgacccgtttggacggtgctatctgctggaactcccaactgaccacaaaggtgtctacggcagcctgaccgactcttatgcttacatgagaaacggttgggatgttgaggtcaccgcagtgggaaatcagttcaacggaggatgtctgttggtggccatggtgccagaactttgctctattgacaagagagagctgtaccagctcacgctctttccccaccagttcatcaacccccggacgaacatgacggcgcacatcactgtgccctttgttggcgtcaaccgctacgaccagtacaaggtacacaaaccttggaccctcgtggttatggttgtggccccgctgactgtcaacaccgaaggtgccccacagatcaaggtctatgccaacatcgcccctaccaacgtgcacgttgcgggtgagttcccttctaaggaagggatcttccccgtggcatgtagcgacggttacggtggtctggtgaccactgacccaaagacggctgaccccgcctacgggaaagtgttcaatccacctcgcaacatgttgccggggcggttcaccaacttccttgatgtggctgaggcgtgccctacgtttctgcactttgagggtggcgtgccgtacgtgaccacaaagacggactcagacagggtgctcgcccagtttgacttgtctctggcagcaaagcacatgtcaaacaccttcctggcaggtctcgcccagtactacacacagtacagcggcaccatcaacctgcacttcatgttcacaggacccactgacgcgaaagcgcgttacatgattgcatacgccccccctggtatggagccgcccaaaacacctgaggcggccgcccactgcattcatgcggagtgggacacagggttgaattcaaaattcacattttcaatcccttacctttcggcggctgattacgcgtacaccgcgtctgacgctgcggagaccacaaatgtacagggatgggtttgcctgtttcaaattacacacgggaaggctgacggcgacgcactggtcgttctagctagcgccggtaaggactttgagctgcgtctgccagttgacgctcgcacgcagaccacctccgcaggtgagtcggctgaccccgtgactgccactgttgagaactacggtggtgagacacaggtccagagacgccaacacacggatgtctcgttcatattagacagatttgtgaaagtaacaccaaaagaccaaattaatgtgttggacctgatgcaaacccctgcacacactttggtaggcgcgctcctccgtactgccacctactacttcgcagatctagaagtggcagtgaaacacgaggggaaccttacctgggtcccgaatggggcgcccgagacagcgttggacaacaccaccaatccaacggcttaccacaaggcaccgctcacccggcttgcactgccttacacggcaccgcaccgtgtcttggctactgtttacaacgggaactgcaagtatggcgagagccccgtgaccaatgtgagaggtgacctgcaagtattggcccaaaaggcggcaagaacgctgcctacctccttcaattacggtgccatcaaagccactcgggtgactgaa

>FJ542370.1_O_UKG_2001

tcgagcgttggagtcacttacgggtacgcaacagctgaggactttgtgagcggaccaaacacatctgggcttgagaccagggttgtgcaggcagagcggttcttcaaaacccacttgttcgactgggtcaccagtgacccgtttggacggtgctatctgctggaactcccaactgaccacaaaggtgtctacggcagcctgaccgactcttatgcttacatgagaaacggttgggatgttgaggtcaccgcagtgggaaatcagttcaacggaggatgtctgttggtggccatggtgccagaactttgctctattgacaagagagagctgtaccagctcacgctctttccccaccagttcatcaacccccggacgaacatgacggcgcacatcactgtgccctttgttggcgtcaaccgctacgaccagtacaaggtacacaaaccttggaccctcgtggttatggttgtggccccgctgactgtcaacaccgaaggtgccccacagatcaaggtctatgccaacatcgcccctaccaacgtgcacgttgcgggtgagttcccttctaaggaagggatcttccccgtggcatgtagcgacggttacggtggtctggtgaccactgacccaaagacggctgaccccgcctacgggaaagtgttcaatccacctcgcaacatgttgccggggcggttcaccaacttccttgatgtggctgaggcgtgccctacgtttctgcactttgagggtggcgtgccgtacgtgaccacaaagacggactcagacagggtgctcgcccagtttgacttgtctctggcagcaaagcacatgtcaaacaccttcctggcaggtctcgcccagtactacacacagtacagcggcaccatcaacctgcacttcatgttcacaggacccactgacgcgaaagcgcgttacatgattgcatacgccccccctggtatggagccgcccaaaacacctgaggcggccgcccactgcattcatgcggagtgggacacagggttgaattcaaaattcacattttcaatcccttacctttcggcggctgattacgcgtacaccgcgtctgacgctgcggagaccacaaatgtacagggatgggtttgcctgtttcaaattacacacgggaaggctgacggcgacgcactggtcgttctagctagcgccggtaaggactttgagctgcgtctgccagttgacgctcgcacgcagaccacctccgcaggtgagtcggctgaccccgtgactgccactgttgagaactacggtggtgagacacaggtccagagacgccaacacacggatgtctcgttcatattagacagatttgtgaaagtaacaccaaaagaccaaattaatgtgttggacctgatgcaaacccctgcacacactttggtaggcgcgctcctccgtactgccacctactacttcgcagatctagaagtggcagtgaaacacgaggggaaccttacctgggtcccgaatggggcgcccgagacagcgttggacaacaccaccaatccaacggcttaccacaaggcaccgctcacccggcttgcactgccttacacggcaccgcaccgtgtcttggctactgtttacaacgggaactgcaagtatggcgagagccccgtgaccaatgtgagaggtgacctgcaagtattggcccaaaaggcggcaagaacgctgcctacctccttcaattacggtgccatcaaagccactcgggtgactgaa

>FJ542371.1_O_UKG_2001

tcgagcgttggagtcacttacgggtacgcaacagctgaggactttgtgagcggaccaaacacatctgggcttgagaccagggttgtgcaggcagagcggttcttcaaaacccacttgttcgactgggtcaccagtgacccgtttggacggtgctatctgctggaactcccaactgaccacaaaggtgtctacggcagcctgaccgactcttatgcttacatgagaaacggttgggatgttgaggtcaccgcagtgggaaatcagttcaacggaggatgtctgttggtggccatggtgccagaactttgctctattgacaagagagagctgtaccagctcacgctctttccccaccagttcatcaacccccggacgaacatgacggcgcacatcactgtgccctttgttggcgtcaaccgctacgaccagtacaaggtacacaaaccttggaccctcgtggttatggttgtggccccgctgactgtcaacaccgaaggtgccccacagatcaaggtctatgccaacatcgcccctaccaacgtgcacgttgcgggtgagttcccttctaaggaagggatcttccccgtggcatgtagcgacggttacggtggtctggtgaccactgacccaaagacggctgaccccgcctacgggaaagtgttcaatccacctcgcaacatgttgccggggcggttcaccaacttccttgatgtggctgaggcgtgccctacgtttctgcactttgagggtggcgtgccgtacgtgaccacaaagacggactcagacagggtgctcgcccagtttgacttgtctctggcagcaaagcacatgtcaaacaccttcctggcaggtctcgcccagtactacacacagtacagcggcaccatcaacctgcacttcatgttcacaggacccactgacgcgaaagcgcgttacatgattgcatacgccccccctggtatggagccgcccgaaacacctgaggcggccgcccactgcattcatgcggagtgggacacagggttgaattcaaaattcacattttcaatcccttacctttcggcggctgattacgcgtataccgcgtctgacgctgcggagaccacaaatgtacagggatgggtttgcctgtttcaaattacacacgggaaggctgacggcgacgcactggtcgttctagctagcgccggtaaggactttgagctgcgtctgccagttgacgctcgcacgcagaccacctccgyaggtgagtcggctgaccccgtgactgccactgttgagaactacggtggtgagacacaggtccagagacgccaacacacggatgtctcgttcatattagacagatttgtgaaagtaacaccaaaagaccaaattaatgtgttggacctgatgcaaacccctgcacacactttggtaggcgcgctcctccgtactgccacctactacttcgcagatctagaagtggcagtgaaacacgaggggaaccttacctgggtcccgaatggggcgcccgagacagcgttggacaacaccaccaatccaacggcttaccacaaggcaccgctcacccggcttgcactgccttacacggcaccgcaccgtgtcttggctactgtttacaacgggaactgcaagtatggcgagagccccgtgaccaatgtgagaggtgacctgcaagtattggcccaaaaggcggcaagaacgctgcctacctccttcaattacggtgccatcaaagccactcgggtgactgaa

>FJ542372.1_O_UKG_2001

tcgagcgttggagtcacttacgggtacgcaacagctgaggactttgtgagcggaccaaacacatctgggcttgagaccagggttgtgcaggcagagcggttcttcaaaacccacttgttcgactgggtcaccagtgacccgtttggacggtgctatctgctggaactcccaactgaccacaaaggtgtctacggcagcctgaccgactcttatgcttacatgagaaacggttgggatgttgaggtcaccgcagtgggaaatcagttcaacggaggatgtctgttggtggccatggtgccagaactttgctctattgacaagagagagctgtaccagctcacgctctttccccaccagttcatcaacccccggacgaacatgacggcgcacatcactgtgccctttgttggcgtcaaccgctacgaccagtacaaggtacacaaaccttggaccctcgtggttatggttgtggccccgctgactgtcaacaccgaaggtgccccacagatcaaggtctatgccaacatcgcccctaccaacgtgcacgttgcgggtgagttcccttctaaggaagggatcttccccgtggcatgtagcgacggttacggtggtctggtgaccactgacccaaagacggctgaccccgcctacgggaaagtgttcaatccacctcgcaacatgttgccggggcggttcaccaacttccttgatgtggctgaggcgtgccctacgtttctgcactttgagggtggcgtgccgtacgtgaccacaaagacggactcagacagggtgctcgcccagtttgacttgtctctggcagcaaagcacatgtcaaacaccttcctggcaggtctcgcccagtactacacacagtacagcggcaccatcaacctgcacttcatgttcacaggacccactgacgcgaaagcgcgttacatgattgcatacgccccccctggtatggagccgcccaaaacacctgaggcggccgcccactgcattcatgcggagtgggacacagggttgaattcaaaattcacattttcaatcccttacctttcggcggctgattacgcgtacaccgcgtctgacgctgcggagaccacaaatgtacagggatgggtttgcctgtttcaaattacacacgggaaggctgacggcgacgcactggtcgttctagctagcgccggtaaggactttgagctgcgtctgccagttgacgctcgcacgcagaccacctccgcaggtgagtcggctgaccccgtgactgccactgttgagaactacggtggtgagacacaggtccagagacgccaacacacggatgtctcgttcatattagacagatttgtgaaagtaacaccaaaagaccaaattaatgtgttggacctgatgcaaacccctgcacacactttggtaggcgcgctcctccgtactgccacctactacttcgcagatctagaagtggcagtgaaacacgaggggaaccttacctgggtcccgaatggggcgcccgagacagcgttggacaacaccaccaatccaacggcttaccacaaggcaccgctcacccggcttgcactgccttacacggcaccgcaccgtgtcttggctactgtttacaacgggaactgcaagtatggcgagagccccgtgaccaatgtgagaggtgacctgcaagtattggcccaaaaggcggcaagaacgctgcctacctccttcaattacggtgccatcaaagccactcgggtgactgaa

>FJ623456.1_A_KAZ_1999

tcgagtgtgggagtcacgtacgggtactccacccaggaagatcatgtttccggacctaacacatctggtttggagacgcgggtggtgcaggcagaaagatttttcaagaagtacctgtttgattggacaccggacaaagcttttgggcacttagagaaattggaacttcccactgaccacaagggagtctacggacacttggtgggctcatttgcatacatgagaaatggctgggacgtggaggtgtccgctgttggcaaccagtttaacggcgggtgtctcctggtggccatggtccctgaatggaaagagctcaccccgcgtgagaagtaccagctcactttgtttccacaccagttcatcagccccagaaccaacatgactgcccacatcgtagtcccgtaccttggtgtgaacaggtacgaccagtataagaagcacaaaccctggacgctggttgtgatggtggtctcaccgctcaccaccaacactgttagtgcaggacaaatcaaggtttatgccaacattgccccgacccacgttcacgtggccggcgagctcccctcgaaagaggggatcgtgccggtcgcttgttcggacgggtatggtggcttggtgacaacagacccaaaaacagctgaccctgtttatggtatggtgtacaacccccccaggacaaactaccccgggcggttcacaaacctgttggatgtggcagaggcctgccccacctttctctgtttcgacgacgggaaaccgtacgttgtgacaagaacggacgagcagcgtcttctggccaagttcgacctctctcttgctgcaaagcacatgtcaaacacctacctttcagggatagcacagtactacgcacagtactctggtaccatcaacctgcacttcatgtttaccggctccactgattcaaaagcccgctacatggtggcgtacgttccgcccggtgtgaagccgccggacacgcctgagaaagctgcacactgcatccatgctgagtgggacacagggttgaactccaaatttactttctctatcccgtacgtgtctgccgcagattacgcgtacactgcgtctgacgtggcagaaacaacaaacgtacagggatgggtctgcatataccaaattacacacgggaaagctgaacaagacactctggttgtgtcggttagcgccggtaaggactttgaattgcgcctcccgattgacccccgttcacaaaccacttccaccggggagtctgcagaccctgtcaccaccaccgttgaaaactacggcggtgagacacaagtccaacgacgtcagcacaccgatgttactttcataatggacagatttgtaaagatacaaaatttgaaacccatacatgtcattgacctcatgcaaacccaccaacacgggttggtaggtgccctgttacgtgctgctacgtactacttctctgacctggagattgtggtacgccatgacggtaacctaacctgggtacccaatggagcacccgaggcagctctgtctaacatgggcaaccccaccgcctaccccaaggcaccatttacgaggctcgcgctcccctacaccgcgccacaccgcgtattggcaacagtgtacaacgggacgggcaagtactccgcaggtggtatg---ggcagacggggcgacctagagcctctcgcggcgagggtcgccgctcagcttcctacttctttcaactttggtgcaattcaagccacgaccatccacgag

>FJ824812.1_C_SPA_2009

tcgagcgtcggagtcacattcgggtatgcaaccgctgaagatagcacgtctggacccaatacatctggtctagagacgcgcgttcatcaggcagagaggtttttcaaaatggcactttttgattgggttccttcacaaaattttggacacatgcacaaggttgttctgccccacgaaccaaaaggtgtttacgggggtctcgtcaagtcatacgcgtacatgcgcaatggctgggacgtcgaggtgaccgctgttggaaaccagttcaacggcggttgcctcctggtggcgctcgtccccgagatgggcgacatcagtgacagggaaaagtaccaactaactctttacccccaccagttcatcaacccacgcaccaacatgacggcacacatcactgtgccctatgtgggtgtcaacaggtatgaccagtacaaacagcacaggccctggaccctcgtggtcatggttgtcgcgccactcaccacaaacacagcaggtgcccaacagatcaaagtgtatgccaacatagccccaaccaacgtgcacgtggcgggtgagctcccctccaaggaggggatcttccccgttgcgtgttctgacggttacggcaacatggtgacaactgacccgaaaacggctgaccctgtctacgggaaggtttacaacccccctcggactgctctgccggggcggttcacaaactacctggatgttgccgaggcttgtcccaccttcctgatgttcgagaac---gtaccttacgtctcaacacgaactgacgggcaaaggctactggccaagttcgacgtgtcgctggcagcgaaacacatgtcaaacacctacttggccggcttggcccagtactacacacagtacaccgggacaatcaacctacacttcatgttcactgggccgaccgacgcgaaagctcggtacatggtggcgtacgtgccccctggcatggacgcaccagacaacccagaagaggctgcccactgcatacacgcagaatgggacactggtctgaactccaagttcacgttttcaatcccgtacatctcggccgctgactacgcgtacaccgcgtccgacaaggctgaaacaacatgtgtacaggggtgggtctgtgtgtaccaaatcactcacggcaaggcagacgccgacgcgctcgtcgtctccgcatcagcggggaaagactttgagctccggctacctgtggacgctagaaaacaaactacgaccactggtgaatctgctgaccccgtcaccactaccgttgagaactacggaggagagactcaggtccaacgtcgccaccacaccgacgttgccttcgttcttgaccggtttgtggaggtcacagtgtcgggtaaccaacacacactcgacgtgatgcaggcacacaaagacaacatcgtgggcgcgcttcttcgcgcagccacgtactacttttctgatctggaaatagcagtgacccacactgggaagctcacatgggtgcccaacggtgcaccagtttctgcacttaacaacacaaccaatcccactgcctaccacaagggcccggtgactcgactggctctcccatacaccgcgccacaccgtgtgttggctacggcgtacactggcactacgacctacaccgccagtgca------------cgcggggatttggctcacctaacgacgacgcatgctcggcatttgccgacatcgttcaactttggtgcagttaaagcagaaacaatcactgag

>FJ906802.1_Asia1_CHA_2006

tcgagtgttggcgtaacatatggttacgccgtggctgaggacgcggtatctgggcctaacacctcaggcctggagacccgcgtgacacaggctgaacggttcttcaagaaacacctgtttgactggacgccgggtttgtcatttggacactgtcactacttggaactcccctctgaacacaagggcgtgtttggcagcctcatgagctcttatgcttacatgaggaacgggtgggatattgaggtgaccgctgttggaaatcagttcaatggtggttgtctcctcgtcgcactcgtgccggagctgaaagagctcgacacgcggcagaagtatcagttaaccctcttcccacaccagttcattaacccgcgcactaacatgacggctcacattaacgtgccgtacgtgggtgtcaacaggtacgaccagtacgagctccacaaaccgtggacgcttgtggcgatggtggtggccccgcttaccgtcaaaactggtggttctgaacagatcaaggtctacatgaatgcagcgccgacctacgtgcacgtggcaggagaactgccctcgaaagaggggatagttcctgtggcgtgtgtggacggttacggcaacatggtaaccacggacccgaagacggctgaccccgtctacgggaaagtgtctaacccccccagaacaagcttccctgggcgcttcacaaacttccttgatgtagcggaggcgtgtccaaccttcctccgcttcggagaa---gtaccatttgtgaagacggtgaactctggtgaccgcttgcttgccaagtttgacgtgtccctcgctgcggggcacatgtccaacacctacttggcaggtttggcacagtactacacacagtacagcggcactatgaatatccacttcatgttcaccggacccacggatgccaaagcccgctacatggtggcttacatacctcctggtatgacgccgccaacggacccggagcgggctgcacactgcattcattctgagtgggacactggactcaattctaaatttaccttttctatcccttacctttctgctgcagactatgcttacactgcttctgacgtggctgagaccacgagtgtgcagggatgggtgtgtatttaccagatcacccacggtaaagctgaaggtgacgcgctggtcgtgtccgtcagcgctggcaaggactttgagtttcgactgccggtggatgcccgccaacagacaaccaccactggcgagtccgcggacccagtcaccaccacggttgagaactacggaggagagacccagacggcccgacggcttcacactgatgtcgccttcgttctcgacaggttcgtgaaactcacccagcccaagagcacccaaacccttgatctcatgcagatcccctcacacacgctggtcggggcgcttctccggtctgcgacgtactacttctcagacctggaggttgcgctcgtccacacaggaccggtcacgtgggtgcccaatggtgcgcccaagaccgccttgaacaaccacaccaacccgactgcctaccagaagcagcctatcacccgcttggcactcccctacaccgctccccaccgtgtgttgtcaacagtgtacaacgggaagacaacgtacggagaagaatcctcg------cggcgtggtgatcttgccgcccttgcacgcagagtggacaaccggctgcccacttccttcaactacggcgctgtgaaggccgacaccatcacggag

>GQ406247.1_A_VIT_2009

tcgagtgtgggggtcacctacgggtattcaactggtgaggaccacgtttctggacctaacacatcaggtttggagacgcgggtggtacaagctgaaaggttcttcaagaagcacttgtttgattggacaacggacaaaccctttggtcacattgaaaagctggaacttcccactgatcacaaaggtgtctacggacagctggtggactcctttgcatacatgagaaatggctgggacgtggaggtgtctgctgttggcaaccagttcaacggcgggtgccttctcgtggccatggtacctgagtttaaggagttcaccacacgtgaaaagtaccagctcaccctgttcccccaccagttcattagccccagaaccaacatgaccgcgcacatcacggtcccgtaccttggtgtgaacaggtatgaccagtacaacaaacacaaaccctggacgttggtggtgatggtggtttcgccacttaccactagctccattggtgcatcacagattaaggtctacaccaacatcgccccgacccacgttcacgtggctggcgagctcccgtcgaaagaggggatcgtgccggtcgcctgctcggacgggtacggtggcctggtgacaacagaccctaaaacagctgaccctgcttacggtatggtgtacaacccacctaggaccaactaccccgggcggtttacaaacttgttggacgtggcagaggcgtgtcccaccttcctctgtttcgacgacgggaaaccgtatgttgtgacaagagcggacgagcagcgcctcttggccaagtttgacctttcccttgctgcgaagcacatgtcaaacacctacctttcagggatagcacagtactacgcacagtactctggcaccattaatttgcacttcatgtttactggttccactgactcaaaggcccgttgcatggtggcttacgtcccgcccggcgtgacaccaccggacacgcctgagagagctgcgcactgcatccacgcagaatgggacacggggctaaactccaaattcactttttcaatcccgtacgtatctgctgcagattacgcgtacacagcgtccgatgtggcagacacaacaaacgtacagggatgggtttgcatctaccaaatcacccatgggaaggccgaacaagacactctggttgtgtcggtcagcgccggcaaagactttgagctgcgcctccccattgacccccgtgcgcaaaccaccgccaccggggaatcagcagaccccgtcacaaccaccgtcgagaactacggtggtgagacacaagtgcagcgacgccaccacaccgacgtcagcttcataatggacaggtttgtgcaaatcaagcctgtgagccccacacatgtcattgacctcatgcaaacacaccaacacgggctggtgggcgctatgttgcgcgcggccacctactacttttctgatcttgagattgtggtgaaccacacgggtcgcctaacgtgggtacccaatggagcacctgaggcagcactggacaacacgagcaaccccactgcttaccacaaagcaccgttcacaaggcttgcactcccttacaccgcgccacaccgcgtgttggcaactgtgtacaacgggactagcaagtactctgcgcccgcaaca------cggcgaggtgacttggggtctctcgcggcgaggctcgccgcacagcttcctgcctccttcaactacggcgcgattcgagccacggagatccaagaa

>GQ406248.1_A_VIT_2009

tcgagtgtgggggtcacctacgggtattcaactggtgaggaccacgtttctggacctaacacatcaggtttggagacgcgggtggtacaagctgaaaggttcttcaagaagcacttgtttgattggacaacggacaaaccctttggtcacattgaaaagctggaacttcccactgatcacaaaggtgtctacggacagctggtggactcctttgcatacatgagaaatggctgggacgtggaggtgtctgctgttggcaaccagttcaacggcgggtgccttctcgtggccatggtacctgagtttaaggagttcaccacacgtgaaaagtaccagctcaccctgttcccccaccagttcattagccccagaaccaacatgaccgcgcacatcacggtcccgtaccttggtgtgaacaggtatgaccagtacaacaaacacaaaccctggacgttggtggtgatggtggtttcgccacttaccactagctccattggtgcatcacagattaaggtctacaccaacatcgccccgacccacgttcacgtggctggcgagctcccgtcgaaagaggggatcgtgccggtcgcctgctcggacgggtacggtggcctggtgacaacagaccctaaaacagctgaccctgcttacggtatggtgtacaacccacctaggaccaactaccccgggcggtttacaaacttgttggacgtggcagaggcgtgtcccaccttcctctgtttcgacgacgggaaaccgtatgttgtgacaagagcggacgagcagcgcctcttggccaagtttgacctttcccttgctgcgaagcacatgtcaaacacctacctttcagggatagcacagtactacgcacagtactctggcaccattaatttgcacttcatgtttactggttccactgactcaaaggcccgttacatggtggcttacgtcccgcccggcgtgacaccaccggacacgcctgagagagctgcgcactgcatccacgcagaatgggacacggggctaaactccaaattcactttttcaatcccgtacgtatctgctgcagattacgcgtacacagcgtccgatgtggcagacacaacaaacgtacagggatgggtttgcatctaccaaatcacccatgggaaggccgaacaagacactctggttgtgtcggtcagcgccggcaaagactttgagctgcgcctccccattgacccccgtgcgcaaaccaccgccaccggggaatcagcagaccccgtcacaaccaccgtcgagaactacggtggtgagacacaagtgcagcgacgccaccacaccgacgtcagcttcataatggacaggtttgtgcaaatcaagcctgtgagccccacacatgtcattgacctcatgcaaacacaccaacacgggctggtgggcgctatgttgcgcgcggccacctactacttttctgatcttgagattgtggtgaaccacacgggtcgcctaacgtgggtacccaatggagcacctgaggcagcactggacaacacgagcaaccccactgcttaccacaaagcaccgttcacaaggcttgcactcccttacaccgcgccacaccgcgtgttggcaactgtgtacaacgggactagcaagtactctgcgcctgcaaca------cggcgaggtgacttggggtctctcgcggcgaggctcgccgcacagcttcctgcctccttcaactacggcgcgattcgagccacggagatccaagaa

>GQ406249.1_A_VIT_2009

tcgagtgtgggggtcacctacgggtattcaactggtgaggaccacgtttctggacctaacacatcaggtttggagacgcgagtggtacaagctgaaaggttcttcaagaagcacttgtttgattggacaacggacaaaccctttggtcacattgaaaagctggaacttcccactgatcacaaaggtgtctacggacagctggtggactcctttgcatacatgagaaatggctgggacgtggaggtgtctgctgttggcaaccagttcaacggcgggtgccttctcgtggccatggtacctgagtttaaggagttcaccacacgtgaaaagtaccagctcaccctgttcccccaccagttcattagccccagaaccaacatgaccgcgcacatcacggtcccgtaccttggtgtgaacaggtatgaccagtacaacaaacacaaaccctggacgttggtggtgatggtggtttcgccacttaccactagctccattggtgcatcacagattaaggtctacaccaacatcgccccgacccacgttcacgtggctggcgagctcccgtcgaaagaggggatcgtgccggtcgcctgctcggacgggtacggtggcctggtgacaacagaccctaaaacagctgaccctgcttacggtatggtgtacaacccacctaggaccaactaccccgggcggtttacaaacttgttggacgtggcagaggcgtgtcccaccttcctctgtttcgacgacgggaaaccgtacgttgtgacaagaacggacgagcagcgcctcttggccaagtttgacctttcccttgctgcaaagcacatgtcaaacacctacctttcagggatagcacagtactacgcacagtactctggcaccatcaatttgcacttcatgtttactggttccactgactcaaaggcccgttacatggtggcttacgtcccgcccggcgtgacaccaccggacacgcctgagagagctgcgcactgcatccacgcagaatgggacacggggctaaactccaaattcactttttcaatcccgtacgtatctgctgcagattacgcgtacacagcgtccgatgtggcggacacaacaaacgtacagggatgggtttgcatctaccaaatcacccatgggaaggccgaacaagacactctggttgtgtcggtcagcgccggcaaagactttgagctgcgcctccccattgacccccgtgcgcaaaccaccgccaccggggaatcagcagaccccgtcacaaccaccgtcgagaactacggtggtgagacacaagtgcagcgacgccaccacaccgacgtcagcttcataatggacaggtttgtgcaaatcaagcctgtgagccccacacatgtcattgacctcatgcaaacacaccaacacgggctggtgggcgctatgttgcgcgcggccacctactacttttctgatcttgagattgtggtgaaccacacgggtcgcctaacgtgggtacccaatggagcacctgaggcagcactggacaacacgagcaaccccactgcttaccacaaagcaccgttcacaaggcttgcactcccttacaccgcgccacaccgcgtgttggcaactgtgtacaacgggactagcaagtactctgcgcctgcaaca------cggcgaggtgacttggggtctctcgcggcgaggctcgccgcacagcttcctgcctccttcaactacggcgcgattcgagccacggagatccaagaa

>GQ406250.1_A_VIT_2009

tcgagtgtgggggtcacctacgggtattcaactggtgaggaccacgtttctggacctaacacatcaggtttggagacgcgggtggtgcaagctgaaaggttcttcaagaagcacttgtttgattggacaacggacaaaccctttggtcacattgaaaagctggaacttcccactgatcacaaaggtgtctacggacagctggtggactcctttgcatacatgagaaatggctgggacgtggaggtgtctgctgttggcaaccagttcaacggcgggtgccttctcgtggccatggtacctgagtttaaggagttcaccacacgtgaaaagtaccagctcaccctgttcccccaccagttcattagccccagaaccaacatgaccgcgcacatcacggtcccgtaccttggtgtgaacaggtatgaccagtacaacaaacacaaaccctggacgttggtggtgatggtggtttcgccacttaccactagctccattggtgcatcacagattaaggtctacaccaacatcgccccgacccacgttcacgtggctggcgagctcccgtcgaaagaggggatcgtgccggtcgcctgctcggacgggtacggtggcctggtgacaacagaccctaaaacagctgaccctgcttacggtatggtgtacaacccacctaggaccaactaccccgggcggtttacaaacttgttggacgtggcagaggcgtgtcccaccttcctctgtttcgacgacgggaaaccgtacgttgtgacaagagcggacgagcagcgcctcttggccaagtttgacctttcccttgctgcaaagcacatgtcaaacacctacctttcagggatagcacagtactacgcacagtactctggcaccatcaatttgcacttcatgtttactggttccactgactcaaaggcccgttacatggtggcttacgtcccgcccggcgtgacaccaccggacacgcctgagagagctgcgcactgcatccacgcagaatgggacacggggctaaactccaaattcactttttcaatcccgtacgtatctgctgcagattacgcgtacacagcgtccgatgtggcagacacgacaaacgtacagggatgggtttgcatctaccaaatcacccatgggaaggccgaacaagacactctggttgtgtcggtcagcgccggcaaagactttgagctgcgcctccccattgacccccgtgcgcaaaccaccgccaccggggaatcagcagaccccgtcacaaccaccgtcgagaactacggtggtgagacacaagtgcagcgacgccaccacaccgacgtcagcttcataatggacaggtttgtgcaaatcaagcctgtgagccccacacatgtcattgatctcatgcaaacacaccaacacgggctggtgggcgctatgttgcgcgcggccacctactacttttctgatcttgagattgtggtgaaccacacgggtcgcctaacgtgggtacccaatggagcacctgaggcagcactggacaacacgagcaaccccactgcttaccacaaagcaccgttcacaaggcttgcactcccttacaccgcgccacaccgcgtgttggcaactgtgtacaacgggactagcaagtactctgcgcctgcaaca------cggcgaggtgacttggggtctctcgcggcgaggctcgccgcacagcttcctgcctccttcaactacggcgcgattcgagccacggagatccaagaa

>GQ406251.1_A_VIT_2009

tcgagtgtgggggtcacctacgggtattcaactggtgaggaccacgcttctggacctaacacatcaggtttggagacgcgggtggtacaagctgaaaggttcttcaagaagcacttgtttgactggacaacggacaaaccctttggtcacattgaaaagctggaacttcccactgatcacaagggtgtctacggacagctggtggactcctttgcatacatgagaaatggctgggacgtggaggtgtctgctgttggcaaccagttcaacggcgggtgccttctcgtggccatggtacctgagtttaaggagttcaccacacgtgaaaagtaccagctcaccctgttcccccaccagttcattagccccagaaccaacatgaccgcgcacatcacggtcccgtaccttggtgtgaacaggtatgaccagtacaacaaacacaaaccctggacgttggtggtgatggtggtctcgccacttaccactagctccattggtgcatcacagattaaggtctacaccaacatcgccccgacccacgttcacgtggctggcgagctcccgtcgaaagaggggatcgtgccggtcgcctgctcggacgggtacggtggcttggtgacaacagaccctaaaacagctgaccctgcttacggtatggtgtacaacccacctaggaccaactaccccgggcggtttacaaacttgttggacgtggcagaggcgtgtcccaccttcctctgtttcgacgacgggaaaccgtacgttgtgacaagaacggacgagcagcgcctcttggccaagtttgacctttcccttgctgcaaagcacatgtcaaacacctacctttcagggatagcacagtactacgcacagtactctggcaccatcaatttgcacttcatgtttactggttccactgactcaaaggcccgttacatggtggcctacgtcccgcccggcgtgacaccaccggacacgcctgagagagctgcacactgcatccatgcagaatgggacacggggctaaactccaaattcactttttcaatcccgtacgtatctgctgctgattacgcgtacacagcgtccgatgtggcagacacaacaaacgtacagggatgggtttgcatctaccaaatcacccatgggaaggccgaacaagacactctggttgtgtcggtcagcgccggcaaagactttgagctgcgcctccccattgacccccgtgcgcaaaccaccgccaccggggaatcagcagaccccgtcacaaccgccgtcgagaactacggtggtgagacacaagtacagcgacgccaccacaccgacgtcagcttcataatggacaggtttgcgcaaatcaagcctgtgagccccacacatgtcattgacctcatgcaaacacaccaatacgggctggtgggcgctatgttgcgcgcggccacttactacttttctgatcttgagattgtggtgaaccacacgggtcgcctaacgtgggtacccaatggagcacctgaggcagcactggacaacacgagcaaccccactgcttaccacaaagcaccgttcacaaggcttgcactcccttacaccgcgccacaccgcgtgttggcaactgtgtacaacgggactagcaagtactctgcgcctgcaaca------cggcgaggtgacttggggtctctcgcggcgaggctcgccgcacagcttcctgcctccttcaactacggcgcgattcgagccacggagatccaagaa

>GQ406252.1_A_VIT_2009

tcgagtgtgggggtcacctacgggtattcaactggtgaggaccacgtttctggacctaacacatcaggtttggagacgcgggtggtacaagctgaaaggttcttcaagaagcacttgtttgattggacaacggacaaaccctttggtcacattgaaaagctggaacttcccactgatcacaagggtgtctacggacagctggtggactcctttgcatacatgagaaatggctgggacgtggaggtgtctgctgttggcaaccagttcaacggcgggtgccttctcgtggccatggtacctgagtttaaggagttcaccacacgtgaaaagtaccagctcaccctgttcccccaccagttcattagccccagaaccaacatgaccgcgcacatcacggtcccgtaccttggtgtgaacaggtatgaccagtacaacaaacacaaaccctggacgttggtggtgatggtggtttcgccacttaccactagctccattggtgcatcccagattaaggtctacaccaacatcgccccgacccacgttcacgtggctggcgagctcccgtcgaaagaggggatcgtgccggtcgcttgctcggacgggtacggtggcctggtgacaacagaccctaaaacagctgaccctgcttacggtatggtgtacaacccacctaggaccaactaccccgggcggtttacaaacttgttggacgtggcagaggcgtgtcccaccttcctctgtttcgacgacgggaaaccgtacgttgtgacaagaacggacgagcagcgcctcttggccaagtttgacctttcccttgctgcaaagcacatgtcaaacacctacctttcagggatagcacagtactacgcacagtactctggcaccatcaatttgcacttcatgtttactggttccactgactcaaaggcccgttacatggtggcttacgtcccgcccggcgtgacaccaccggacacgcctgagagagctgcgcactgcatccacgcagaatgggacacggggctaaactccaaattcactttttcaatcccgtacgtatctgctgcagattacgcgtacacagcgtccgatgtggcagacacaacaaacgtacagggatgggtttgcatctaccaaatcacccatgggaaggccgaacaagacactctggttgtgtcggtcagcgccggcaaagactttgagctgcgcctccccattgacccccgtgcgcaaaccaccgccaccggggaatcagcagaccccgtcacaaccaccgtcgagaactacggtggtgagacacaagtgcagcgacgccaccacaccgacgtcagcttcgtaatggacaggtttgtgcaaatcaagcctgtgagccccacacatgtcattgacctcatgcaaacacaccaacacgggttggtgggcgctatgttgcgcgcggccacctactacttttctgatcttgagattgtggtgaaccacacgggtcgcctaacgtgggtacccaatggagcacctgaggcagcactggacaacacgagcaaccccactgcttaccacaaagcaccgttcacaaggcttgcactcccttacaccgcgccacaccgcgtgttggcaactgtgtacaacgggactagcaagtactccgcgcctgcaaca------cggcgaggtgacttggggtctctcgcggcgaggctcgccgcacagcttcctgcctccttcaactacggcgcgattcgagccacggagatccaagaa

>GQ452295.1_Asia1_VIT_2007

tcgagtgttggcgtaacatatggttacgctgtggctgaggacgcggtatctgggcctaacacctcaggcctggagacccgcgtgacacaggctgaacggttcttcaagaaacacctgtttgactggacgccgggtttgccatttggacactgtcactacttggaactcccctctgaacacaagggcgtgtttggcagcctcatgagctcttatgcttacatgaggaacgggtgggatattgaggtgaccgctgttggaaaccagttcaatggtggttgtctcctcgtcgcactcgtgccggagctgaaagagctcgacacgcggcagaagtatcagttaaccctcttcccacaccagttcattaacccgcgtactaacatgacggctcacattaacgtgccgtacgtgggtgtcaacaggtacgaccagtatgagctccacaaaccgtggacgcttgtggtgatggtggtggccccgcttaccgtcaaaactggtggctccgaacagatcaaggtctacatgaatgcagcgccgacctacgtgcacgtggcaggagaactgccctcgaaagaggggatagttcctgtggcgtgtgtggacggttacggcaacatggtaaccacggacccgaagacggctgaccccgtctacgggaaagtgtctaacccccccagaacaagcttccctgggcgcttcacaaacttccttgatgtagcggaggcgtgtccaaccttcctccgcttcggagaa---gtaccatttgtgaagacggtgaactctggtgaccgcttgcttgccaagtttgacgtgtccctcgctgcggggcacatgtccaacacctacttggcagggttggcacagtactacacacagtacagcggcactatgaatatccacttcatgttcaccggacccacggatgccaaagcccgctacatggtggcttacatacctcctggtatgacgccgccaacggacccggagcgggctgcacactgcattcattctgagtgggacactggactcaattctaaatttaccttttctatcccttacctttctgctgcagactatgcttacactgcttctgacgtggctgagaccacgagtgtgcagggatgggtgtgtatttaccagatcacccacggtaaagctgaaggtgacgcgctggtcgtgtccgtcagcgctggcaaggactttgagtttcgactgccggtggacgcccgccaacagactaccaccactggcgagtccgcggacccagtcaccaccacggttgagaactacggaggagagacccagacggcccgacggcttcacactgatgtcgcattcgttctcgacaggttcgtgaaactcacccagcccaagagcacccaaacccttgatctcatgcagatcccctcacacacactggtcggggcgcttctccggtctgcgacgtactacttctcagacctggaggttgcgctcgtccacacaggaccggtcacgtgggtgcccaatggtgcgcccaagaccgccttgaacaaccacaccaacccgactgcctaccagaagcagcctatcacccgcttggcactcccctacaccgctccccaccgtgtgctgtcaacagtgtacaacgggaagacaacgtacggagaagaatcctcg------cggcgtggtgatcttgccgcccttgcacgcagagtgaacaaccggctgcccacttccttcaactacggcgctgtgaaggccgacaccatcacggag

>GU125645.1_Asia1_VIT_2007

tcgagtgttggcgtaacatatggttacgctgtggctgaggacgcggtatctgggcctaacacctcaggcctggagacccgcgtgacacaggctgaacggttcttcaagaaacacctgtttgactggacgccgggtttgccatttggacactgtcactacctggaactcccctctgaacacaagggcgtgtttggcagcctcatgagctcttatgcttacatgaggaacgggtgggatattgaggtgaccgctgttggaaaccagttcaatggtggttgtctcctcgtcgcactcgtgccggagctgaaagagctcgacacgcggcagaagtatcagttaaccctcttcccacaccagttcattaacccgcgtactaacatgacggctcacattaacgtgccgtacgtgggtgtcaacaggtacgaccagtatgagctccacaaaccgtggacgcttgtggtgatggtggtggccccgcttaccgtcaaaactggtggctccgaacagatcaaggtctacatgaatgcagcgccgacctacgtgcacgtggcaggagaactgccctcgaaagaggggatagttcctgtggcgtgtgtggacggttacggcaacatggtaaccacggacccgaagacggctgaccccgtctacgggaaagtgtctaacccccccagaacaagcttccctgggcgcttcacaaacttccttgatgtagcggaggcgtgtccaaccttcctccgcttcggagaa---gtaccatttgtgaagacggtgaactctggtgaccgcttgcttgccaagtttgacgtgtccctcgctgcggggcacatgtccaacacctatttggcagggttggcacagtactacacacagtacagcggcactatgaatattcacttcatgttcaccggacccacggatgccaaagcccgctacatggtggcttacatacctcctggtatgacgccgccaacggacccggagcgggctgcacactgcattcattctgagtgggacactggactcaattctaaatttaccttttctatcccttacctttctgctgcagactatgcttacactgcttctgacgtggctgagaccacgagtgtgcagggatgggtgtgtatttaccagatcacccacggtaaagctgaaggtgacgcgctggtcgtgtccgtcagcgctggcaaggactttgagtttcgactgccggtggacgcccgccaacagactaccaccactggcgagtccgcggacccagtcaccaccacggttgagaactacggaggagagacccagacggcccgacggcttcacactgatgtcgcattcgttctcgacaggttcgtgaaactcacccagcccaagagcacccaaacccttgatctcatgcagatcccctcacacacactggtcggggcgcttctccggtctgcgacgtactacttctcagacctggaggttgcgctcgtccacacaggaccggtcacgtgggtgcccaatggtgcgcccaagaccgccttgaacaaccacaccaacccgactgcctaccagaagcagcctatcacccgcttggcactcccctacaccgctccccaccgtgtgctgtcaacagtgtacaacgggaagacaacgtacggagaagaatcctcg------cggcgtggtgatcttgccgcccttgcacgcagagtgaacaaccggctgcccacttccttcaactacggcgctgtgaaggccgacaccatcacggag

>GU125646.1_Asia1_VIT_2005

tcgagcgtcggcgttacctacggttacgccatggccgaagacgcggtgtcagggcccaacacctcgggcctagagactcgtgtgttacaagctgagcggttcttcaagaaacacctgtttgattggacaccgaatttggaatttgggcactgtcattacctggaactcccctctgaacacaaaggcgtctatggaagcctccagaactcttatgcttacatgaggaacgggtgggacattgaggttaccgctgttggaaaccaattcaacggtggttgtcttcttgtcgcgcttgtgccagaattgaaaacccttggaacacggcaaaagtaccaactgactcttttcccccaccagtttgtaaacccacgcaccaacatgacggctcacatcaacgtgccgtacgtgggtgttaacaggtatgatcaatacgcgctccacaaaccatggacgctcgtagtgatggtggtggcgccactcactgtcaaaactggtggatctgaacaaatcaaggtttacatgaatgcagcaccgacctacgtgcacgtggcgggagagctaccctcgaaggagggaatagtccccgttgcgtgtgcggatggatatggcaacatggtgaccacggacccgaagacggctgaccccgtttatgggaaagtgttcaaccccccccggacgaacctgcccgggcggttcacgaacttccttgatgttgcggaggcctgtccaaccttcctccgcttcggagaa---gttccgtttgtgaagacggtgaacaccggtgaccgtttgcttgccaagtttgacgtgtccctctctgcagggcacatggctaacacctacttggctggcttggcgcagtactacacacagtacagcggcaccatgaacgttcacttcatgtttaccggccccacagatgccaaagcccgctatatggtggcttacgtgcccccgggcatggaaccacccactgaccctgagcgggctgcacactgcattcattctgagtgggacaccggactcaactccaaattcactttctccataccatacctttctgctgctgactacgggtacactgcttccaacgtggccgagaccacaagtgtgcagggctgggtgtgcatctaccagatcacacatggcaaagctgagggtgacgccttggtcgtttccgtcagcgccggcaaggactttgagtttcgactgccagtcgatgctcgccagcaaaccacaaccacaggcgagtcagcagaccccgtcaccactacggtggaaaactacgggggagtgacgcaggcggcccgacgtctccacaccgacgttgccttcgttctcgacaggtttgtgaaacttacccagcccaagagcactcaggttcttgacctcatgcagataccctcacacacactggttggagccctgcttcggtctgcgacgtactacttctcagacctggagattgcgcttgtccacacaggcccggtcacgtgggtgcccaacggcgcacctaaggttgccctggacaaccagaccaatcccactgcctaccaaaagcagcccattacccgtctggcgctcccttacaccgccccccaccgtgtgctggcaacagtgtacaacgggaagacggcatacggggagacgaccacg------aggcgtggtgatcttgctgccctagcacaaagggtgagcaggcagttgccaacctccttcaactacggcgcagtgaaggccgaaagcatcacagag

>GU125647.1_O_VIT_2006

tcgagtgttggtatcacgtacgggtacgcgacagctgaggactttgtgagcgggccaaacacctccggtcttgagaccagggttgtccaggcagaacggtttttcaaaacccacttgttcgactgggtcaccagtgactcgttcggacggtgccaccttttggagctcccgactgaccacaaaggtgtctacggtggcctgaccgactcatatgcctacatgaggaacggttgggatgtcgaagtcaccgctgtggggaaccagttcaacgggggctgcctgttggtggccatggtgcctgagctttgttccatccaaaagagagagctgtaccagctcacgctctttccccaccagttcatcaacccacggacgaacatgacagcccacatcaaggtgcccttcgttggcgtcaaccgctacgaccagtacaaggtgcataagccttggacccttgtggttatggttgtagctcccctgactgtcaacaccgaaggcgctccgcagatcaaggtgtatgccaacatcgcaccaaccaacgtgcacgtcgcaggtgaattcccttccaaagaggggattttccctgtggcttgtagcgatggttacggcggtttggtgaccactgatccgaagacggctgaccccgtctacggcaaagtgttcaaccccccccgcagcatgttgccggggcggttcaccaacctcctggatgtggctgaggcgtgtcccacgtttctgcacttcgaaggtgacgtaccatacgtgactacgaagacggattcggacagggtgctcgcgcaatttgatttgtctttggcagcaaagcacatgtcaaacacctttcttgcgggtctcgcccagtactacgcacagtacagtggcacaatcaacctgcacttcatgttcacgggtcccactgacgcgaaagcgcgttacatgattgcatatgctccaccgggcatggagccgcccaaaacacctgaggccgctgcccattgcatccatgcagagtgggatacgggtttgaactcaaagttcactttttccatcccctacctctcggcggctgactacgcgtataccgcgtctgacactgccgagaccacaaatgtccaggggtgggtctgtttgttccagataacacacggcaaagctgatggtgatgctcttgttgtgctggccagcgctggcaaagactttgagctgcgcctgcctgtggacgcccgccagcagaccacttcgacaggcgagtcagccgatcccgtgactaccaccgttgagaactacggtggtgagacacaggtccagaggcgtcaacacacagacgtctcatttatattggatagatttgtgaaagttacaccaaaagaccaaattaatgtgttggacctgatgcagaccccctcccacaccctggtgggggcgctccttcgtactgccacttactacttcgctgatttagaagtggcggtgaaacacgaggggaacctcacttgggtaccaaatggagcacctgaaacagctttggaaaacaccaccaatccaactgcctaccacaaagcaccactcacccggcttgcgctgccttacacggcaccacaccgtgttttggctaccgtttacaacgggaactgcagatacgccgagggtcccttgaccaacgtgagaggtgatctccaggtgctggctcagaaggcggcgaggccgctgcctacctctttcaactacggtgccatcaaagccactcgggtgacagaa

>GU125648.1_O_VIT_2006

tcgagtgttggtatcacgtacgggtacgcgacagctgaggactttgtgagcgggccaaacacctccggtcttgagaccagggttgtccaggcagaacggtttttcaaaacccacttgttcgactgggtcaccagtgactcgttcggacggtgccaccttttggagctcccgactgaccacaaaggtgtctacggtggcctgaccgactcatatgcctacatgaggaacggttgggatgtcgaagtcaccgctgtggggaaccagttcaacgggggctgcctgttggtggccatggtgcctgagctttgttccatccaaaagagagagctgtaccagctcacgctctttccccaccagttcatcaacccacggacgaacatgacagcccacatcaaggtgcccttcgttggcgtcaaccgctacgaccagtacaaggtgcataagccttggacccttgtggttatggttgtagctcccctgactgtcaacaccgaaggcgctccgcagatcaaggtgtatgccaacatcgcaccaaccaacgtgcacgtcgcaggtgaattcccttccaaagaggggattttccctgtggcttgtagcgatggttacggcggtttggtgaccactgatccgaagacggctgaccccgtctacggcaaagtgttcaaccccccccgcagcatgttgccggggcggttcaccaacctcctggatgtggctgaggcgtgtcccacgtttctgcacttcgaaggtgacgtaccatacgtgactacgaagacggattcggacagggtgctcgcgcaatttgatttgtctttggcagcaaagcacatgtcaaacacctttcttgcgggtctcgcccagtactacgcacagtacagtggcacaatcaacctgcacttcatgttcacgggtcccactgacgcgaaagcgcgttacatgattgcatatgctccaccgggcatggagccgcccaaaacacctgaggccgctgcccattgcatccatgcagagtgggatacgggtttgaactcaaagttcactttttccatcccctacctctcggcggctgactacgcgtataccgcgtctgacactgccgagaccacaaatgtccaggggtgggtctgtttgttccagataacacacggcaaagctgatggtgatgctcttgttgtgctggccagcgctggcaaagactttgagctgcgcctgcctgtggacgcccgccagcagaccacttcgacaggcgagtcagccgatcccgtgactaccaccgttgagaactacggtggtgagacacaggtccagaggcgtcaacacacagacgtctcatttatattggatagatttgtgaaagttacaccaaaagaccaaattaatgtgttggacctgatgcagaccccctcccacaccctggtgggggcgctccttcgtactgccacttactacttcgctgatttagaagtggcggtgaaacacgaggggaacctcacttgggtaccaaatggagcacctgaaacagctttggaaaacaccaccaatccaactgcctaccacaaagcaccactcacccggcttgcgctgccttacacggcaccacaccgtgttttggctaccgtttacaacgggaactgcagatacgccgagggtcccttgaccaacgtgagaggtgatctccaggtgctggctcagaaggcggcgaggccgctgcctacctctttcaactacggtgccatcaaagccactcgggtgacagaa

>GU125649.1_O_VIT_2006

tcgagtgttggtatcacgtacgggtacgcgacagctgaggactttgtgagcgggccaaacacctccggtcttgagaccagggttgtccaggcagaacggtttttcaaaacccacttgttcgactgggtcaccagtgactcgttcggacggtgccaccttttggagctcccgactgaccacaaaggtgtctacggtggcctgaccgactcatatgcctacatgaggaacggttgggatgtcgaagtcaccgctgtggggaaccagttcaacgggggctgcctgttggtggccatggtgcctgagctttgttccatccaaaagagagagctgtaccagctcacgctctttccccaccagttcatcaacccacggacgaacatgacagcccacatcaaggtgcccttcgttggcgtcaaccgctacgaccagtacaaggtgcataagccttggacccttgtggttatggttgtagctcccctgactgtcaacaccgaaggcgctccgcagatcaaggtgtatgccaacatcgcaccaaccaacgtgcacgtcgcaggtgaattcccttccaaagaggggattttccctgtggcttgtagcgatggttacggcggtttggtgaccactgatccgaagacggctgaccccgtctacggcaaagtgttcaaccccccccgcagcatgttgccggggcggttcaccaacctcctggatgtggctgaggcgtgtcccacgtttctgcacttcgaaggtgacgtaccatacgtgactacgaagacggattcggacagggtgctcgcgcaatttgatttgtctttggcagcaaagcacatgtcaaacacctttcttgcgggtctcgcccagtactacgcacagtacagtggcacaatcaacctgcacttcatgttcacgggtcccactgacgcgaaagcgcgttacatgattgcatatgctccaccgggcatggaaccgcccaaaacacctgaggccgctgcccattgcatccatgcagagtgggatacgggtttgaactcaaagttcactttttccatcccctacctctcggcggctgactacgcgtataccgcgtctgacactgccgagaccacaaatgtccaggggtgggtctgtttgttccagataacacacggcaaagctgatggtgatgctcttgttgtgctggccagcgctggcaaagactttgagctgcgcctgcctgtggacgcccgccagcagaccacttcgacaggcgagtcagccgatcccgtgactaccaccgttgagaactacggtggtgagacacaggtccagaggcgtcaacacacagacgtctcatttatattggatagatttgtgaaagttacaccaaaagaccaaattaatgtgttggacctgatgcagaccccctcccacaccctggtgggggcgctccttcgtactgccacttactacttcgctgatttagaagtggcggtgaaacacgaggggaacctcacttgggtaccaaatggagcacctgaaacagctttggaaaacaccaccaatccaactgcctaccacaaagcaccactcacccggcttgcgctgccttacacggcaccacaccgtgttttggctaccgtttacaacgggaactgcagatacgccgagggtcccttgaccaacgtgagaggtgatctccaggtgctggctcagaaggcggcgaggccgctgcctacctctttcaactacggtgccatcaaagccactcgggtgacagaa

>GU125650.1_O_VIT_2006

---------------------------------------------------------------------------------------------------------------------------------------------------cggtgccaccttttggagctcccgactgaccacaaaggtgtctacggtggcctgaccgactcatatgcctacatgaggaacggttgggatgtcgaagtcaccgctgtggggaaccagttcaacgggggctgcctgttggtggccatggtgcctgagctttgttccatccaaaagagagagctgtaccagctcacgctctttccccaccagttcatcaacccacggacgaacatgacagcccacatcaaggtgcccttcgttggcgtcaaccgctacgaccagtacaaggtgcataagccttggacccttgtggttatggttgtagctcccctgactgtcaacaccgaaggcgctccgcagatcaaggtgtatgccaacatcgcaccaaccaacgtgcacgtcgcaggtgaattcccttccaaagaggggattttccctgtggcttgtagcgatggttacggcggtttggtgaccactgatccgaagacggctgaccccgtctacggcaaagtgttcaaccccccccgcagcatgttgccggggcggttcaccaacctcctggatgtggctgaggcgtgtcccacgtttctgcacttcgaaggtgacgtaccatacgtgactacgaagacggattcggacagggtgctcgcgcaatttgatttgtctttggcagcaaagcacatgtcaaacacctttcttgcgggtctcgcccagtactacgcacagtacagtggcacaatcaacctgcacttcatgttcacgggtcccactgacgcgaaagcgcgttacatgattgcatatgctccaccgggcatggagccgcccaaaacacctgaggccgctgcccattgcatccatgcagagtgggatacgggtttgaactcaaagttcactttttccatcccctacctctcggcggctgactacgcgtataccgcgtctgacactgccgagaccacaaatgtccaggggtgggtctgtttgttccagataacacacggcaaagctgatggtgatgctcttgttgtgctggccagcgctggcaaagactttgagctgcgcctgcctgtggacgcccgccagcagaccacttcgacaggcgagtcagccgatcccgtgactaccaccgttgagaactacggtggtgagacacaggtccagagacgccaccacacagacgtctcatttatattggacagatttgtgaaagttacaccacaagaccaaattaatgtgttggacctgatgcagacccccccccacaccctggtaggggctctccttcgtactgccacttactactttgctgatctagaagtggcagtgaaacacgaggggaacctcacctgggtgccaaatggagcacctgaggcggccttggaaaacaccaccaacccaacggcgtaccacaaagcgccactcacccggcttgcactgccttacacggccccacaccgtgttttggctaccgtttacaacgggaactgcaaatacgccgagggtccactgaccaacgtgagaggtgacctccaggtgctggctcagaaggcggcgaggccgctgcctacctctttcaactatggtgccattaaagccactcgggtgacagaa

>GU384682.1_O_PAK_2008

tcgagcgttggagtcacttacgggtacgcaacagctgaggactttgtgagcgggccaaacacatccggtcttgagaccagggttgtgcaagcagagcggttcttcaaaacccacttgttcgactgggtcactagcgacccgttcggacggtgccacctgctggaacttccaactgaccacaaaggtgtctacggcagcctgaccgattcttatgcttacatgagaaacggttgggatgttgaggtcactgcagtgggaaaccagttcaacggaggatgcctgttggtagccatggtgccagaactttgctctattgacaaaagagagctgtaccagctcacgctctttccccaccaattcatcaacccccggacgaacatgacggcgcacatcaccgtgccctttgttggcgtcaatcgctacgaccagtacaaggtacacaagccttggaccctcgtggtcatggtcgtggccccgctgactgtcaacactgaaggtgctccacagatcaaggtttatgccaacatcgcccctaccaacgtgcacgtcgcgggtgagttcccttccaaggaagggatcttccccgtggcatgtagcgacggttatggcggtcttgtgaccactgacccaaagacggctgaccccgcctacgggaaagttttcaatccccctcgcaacatgttgccagggcggttcaccaacttccttgacgtggctgaggcgtgccctacgtttctgcactttgagggtgacgtgccatacgtgaccacaaagacggattcggacagggttcttgctcagtttgacttgtctttggcagcgaagcacatgtcgaacacctttctggcaggtctcgcccagtactacacacagtacagcggcaccatcaacctgcacttcatgttcacagggcccactgacgcgaaagcgcgttacatgattgcatacgccccccctggcatggaaccgcccagaacacctgaggcggccgctcactgcattcatgcggagtgggacactgggttgaattcaaaattcacattttcaatcccttacctttcggcggctgactacgcgtacaccgcgtctgacactgctgagaccacaaatgtacagggatgggtttgcctgtttcagatcacacacgggaaggctgacggtgacgcacttgtcgttctggctagcgccggtaaggacttcgagctgcggttgccagttgacgctcgcacgcagaccacctccacaggtgagtcagctgaccccgtgactgccactgttgagaactacggtggcgagacgcaggtccagagacgccagcacacggacgtctcgttcatactggacagatttgtgaaagtgacaccaaaagaccaaattaatgtgttggacctgatgcagacccccgcccacactttggtaggtgcgcttctccgcaccgccacctactacttcgcagacctagaggtggcagtgaaacacgaggggaaccttacctgggtcccgaatggggcgcccgagacagcgttggataacaccaccaatccaacggcttaccacaaggcacctctcacccggcttgcgctgccttacacggcaccacaccgtgtcttggctactgtttacaacgggaactgcaagtatggcgagagctccacaaccaacgtgagaggtgacctgcaagtgttggcccagaaagcggcgagagcgctgcctacctcctttaactacggtgccattaaggccactcgggtgactgaa

>GU384683.1_O_PAK_2008

tcgagcgttggagtcacttacgggtacgcaacagctgaggactttgtgagcgggccaaacacatccggtcttgagaccagggttgtgcaagcagagcggttcttcaaaacccacttgttcgactgggtcactagcgacccgttcggacggtgccacctgctggaacttccaactgaccacaaaggtgtctacggcagcctgaccgattcttatgcttacatgagaaacggttgggatgttgaggtcactgcagtgggaaaccagttcaacggaggatgcctgttggtagccatggtgccagaactttgctctattgacaaaagagagctgtaccagctcacgctctttccccaccaattcatcaacccccggacgaacatgacggcgcacatcaccgtgccctttgttggcgtcaatcgctacgaccagtacaaggtacacaagccttggaccctcgtggtcatggtcgtggccccgctgactgtcaacactgaaggtgctccacagatcaaggtttatgccaacatcgcccctaccaacgtgcacgtcgcgggtgagttcccttccaaggaagggatcttccccgtggcatgtagcgacggttatggcggtcttgtgaccactgacccaaagacggctgaccccgcctacgggaaagttttcaatccccctcgcaacatgttgccagggcggttcaccaacttccttgacgtggctgaggcgtgccctacgtttctgcactttgagggtgacgtgccatacgtgaccacaaagacggattcggacagggttcttgctcagtttgacttgtctttggcagcgaagcacatgtcgaacacctttctggcaggtctcgcccagtactacacacagtacagcggcaccatcaacctgcacttcatgttcacagggcccactgacgcgaaagcgcgttacatgattgcatacgccccccctggcatggaaccgcccagaacacctgaggcggccgctcactgcattcatgcggagtgggacactgggttgaattcaaaattcacattttcaatcccttacctttcggcggctgactacgcgtacaccgcgtctgacactgctgagaccacaaatgtacagggatgggtttgcctgtttcagatcacacacgggaaggctgacggtgacgcacttgtcgttctggctagcgccggtaaggacttcgagctgcggttgccagttgacgctcgcacgcagaccacctccacaggtgagtcagctgaccccgtgactgccactgttgagaactacggtggcgagacgcaggtccagagacgccagcacacggacgtctcgttcatactggacagatttgtgaaagtgacaccaaaagaccaaattaatgtgttggacctgatgcagacccccgcccacactttggtaggtgcgcttctccgcaccgccacctactacttcgcagacctagaggtggcagtgaaacacgaggggaaccttacctgggtcccgaatggggcgcccgagacagcgttggataacaccaccaatccaacggcttaccacaaggcacctctcacccggcttgcgctgccttacacggcaccacaccgtgtcttggctactgtttacaacgggaactgcaagtatggcgagagctccacaaccaacgtgagaggtgacctgcaagtgttggcccagaaagcggcgagagcgctgcctacctcctttaactacggtgccattaaggccactcgggtgactgaa

>GU582115.1_O_VIT_2009

tcgagcgttggcgttacgtacgggtatgcaacagctgaggactttgtgagcgggccaaacacctctggtctcgagaccagggttgttcaggcagaacggtttttcaaaacccacttgttcgactgggtcaccagcgacccgttcggacggtgccacctcctggaactcccgactgaccacaaaggtgtctacggcggcctgaccgactcatatgcctacatgaggaatggctgggacgtcgaagttaccgctgtggggaaccagttcaacgggggctgcctattggtggccatggtgcctgagctttgttccatccaaaagagagagctgtaccaactcacgctcttcccccatcagttcatcaaccctcggacgaacatgacagcccacatcaaggtgccctttgttggcgtcaaccgctatgaccagtacaaggtacacaaaccttggacccttgtggttatggttgtagcccccctgactgtcaacaccgaaggcgctccacagatcaaggtgtacgccaacatcgcacctaccgacgtacacgttgcaggcgagttcccttccaaagaggggattttccctgtggcatgtagcgacggttacggcggtttggtgaccactgacccaaagacggctgaccccgtctacggcaaggtgtttaaccccccccgtaacatgttgccggggcggttcaccaacttcttggatgtggccgaggcgtgccccacgtttctgcacttcgaaggtgacgtgccatacgtgaccacgaagacggactcagacaggattctcgcgcaatttgacttgtctctggcagcaaaacacatgtcaaacacctttcttgcaggtcttgcccagtactacacgcagtacagtggtacgatcaacctgcacttcatgttcacaggtcccactgacgcgaaagcgcgttacatgattgcatacgccccaccgggtatggagccgcctcgcacgcctgaggccgctgcccattgcattcatgctgagtgggacacgggtttgaattcaaagttcaccttttccatcccatatctctcagcagctgactacgcatacaccgcgtctgacactgccgagaccacaaatgttcagggatgggtctgcttgttccagataacacacgggaaagctgacggcgacgctcttgtcgtgctggccagcgctggtaaagactttgagctgcgcttgcctgtggacgcccgccaacagaccacttcgacgggcgagtcagccgaccccgtgaccgctaccgttgagaactacggtggcgagacacaggtccagaggcgtcaccacacagacgtctcatttatattggacagatttgtgaaagtcacaccacaagaccaaattaatgttttggacctgatgcagacccccccccacactctggtgggagcgctccttcgtactgccacttactactttgctgatctagaggtggcagtgaaacacgagggggatctcacctgggtaccaaatggagcacctgaggcagccttgggtaataccaccaacccaacggcataccacaaagcgccactcactcggcttgcactgccttacacggcaccacaccgtgttctggctaccgtttacaacgggaactgcaaatacgctgggggtccactgaccaacgtgagaggcgatctccaggtgctggctcagaaggcggcgaggccgctgcctacctccttcaactatggtgccatcaaagccacccgggtgacagaa

>GU582116.1_O_VIT_2009

tcgagtgttggcgttacgtacgggtatgcaacagctgaggactttgtgagcgggccaaacacctctggtctcgagaccagggttgttcaggcagaacggtttttcaaaacccacttgttcgactgggtcaccagcgacccgttcggacggtgccacctcctggaactcccgactgaccacaaaggtgtctacggcggcctgaccgactcatatgcctacatgaggaacggctgggacgtcgaagttaccgctgtggggaaccagttcaacgggggctgcctattggtggccatggtgcctgagctttgttccatccaaaagagagagctgtaccaacttacgctcttcccccatcagttcatcaaccctcggacgaacatgacagcccacatcaaggtgccctttgttggcgtcaaccgctatgaccagtacaaggtacacaaaccttggacccttgtggttatggttgtagcccccctgactgtcaacaccgaaggcgctccacagatcaaggtgtacgccaacatcgcacctaccgacgtacacgttgcaggcgagttcccttccaaagaggggattttccctgtggcatgtagcgacggttacggcggtttggtgaccactgacccaaagacggctgaccccgtctacggcaaggtgtttaaccccccccgcaacatgctgccggggcggttcaccaacttcttggatgtggccgaggcgtgccccacgtttctgcacttcgaaggcgacgtgccatacgtgaccacgaagacggactcagacaggattctcgcgcaatttgacttgtctctggcagcaaaacacatgtcaaacacctttcttgcaggtcttgcccagtactacacgcagtacagtgggacgatcaacctgcacttcatgttcacaggtcccactgacgcgaaagcgcgttacatgattgcatacgccccaccgggtatggagccgcctcgcacgcctgaggccgctgcccattgcattcatgctgagtgggacacgggtttgaattcaaagttcaccttttccatcccatatctctcagcagctgactacgcatacaccgcgtctgacactgccgagaccacaaatgttcagggatgggtctgcttgttccagataacacacgggaaagctgacggcgacgctcttgtcgtgctggccagcgctggtaaagactttgagctgcgcttgcctgtggacgcccgccaacagaccacttcgacgggcgagtcagccgaccccgtgaccgctaccgttgagaactacggtggcgagacacaggtccagaggcgtcaccacacagacgtctcatttatactggacagatttgtgaaagtcacaccacaagaccaaattaatgttttggacctgatgcagacccccccccacactctggtgggagcgctccttcgtactgccacttactactttgctgatctagaggtggcagtgaaacacgagggggatctcacctgggtaccaaatggagcacctgaggcagccttgggtaataccaccaacccaacggcataccacaaagcgccactcactcggcttgcattgccttacacggcaccacaccgtgttctggctaccgtttacaacgggaactgcaaatacgctgggggtccactgaccaacgtgagaggcgatctccaggtgctggctcagaaggcggcgaggccgctgcctacctccttcaactatggtgccatcaaagccacccgggtgacagaa

>GU931682.1_Asia1_CHA_2005

tcgagtgttggcgtaacatatggttacgctgtggctgaggacgcggtatctgggcctaacacctcaggcctggagacccgcgtgacacaggctgaacggttcttcaagaaacacctgtttgactggacgccggatttgtcatttggacactgtcactacttggaactcccctctgaacacaagggcgtgtttggcagcctcatgagctcttatgcttacatgaggaacgggtgggacgttgaggtgaccgctgttggaaatcagttcaatggtggttgtctcctcgtcgcactcgtgccggagctgaaagagctcgacacgcggcagaagtatcagttaaccctcttcccacaccagttcattaacccgcgcactaacatgacggctcacattaacgtgccgtacgtgggtgtcaacaggtacgaccagtacgagctccacaaaccgtggacgcttgtggtgatggtggtggccccgcttaccgtcaaaactggtggttctgaacagatcaaggtctacatgaatgcagcgccgacctacgtgcacgtggcaggagaactgccctcgaaagaggggatagttcctgtggcgtgtgtggacggttacggcaacatggtaaccacggacccgaagacggctgaccccgtctacgggaaagtgtctaacccccccagaacaagcttccctgggcgcttcacaaacttccttgatgtagcggaggcgtgtccaaccttcctccgcttcggagaa---gtaccatttgtgaagacggtgaactctggtgaccgcttgcttgccaagtttgacgtgtccctcgctgcggggcacatgtccaacacctacttggcaggtttggcacagtactacacacagtacagcggcactatgaatatccacttcatgttcaccggacccacggatgccaaagcccgctacatggtggcttacatacctcctggtatgacaccgccaacggacccggagcgggctgcacactgcattcattctgagtgggacactggactcaattctaaatttaccttttctatcccttacctttctgctgcagactatgcttacactgcttctgacgtggctgagaccacgagtgtgcagggatgggtgtgtatttaccagatcacccacggtaaagctgaaggtgacgcgctggtcgtgtccgtcagcgctggcaaggactttgagtttcgactaccggtggatgcccgccaacagactaccaccactggcgagtccgcggacccagtcaccaccacggttgagaactacggaggagagacccagacggcccgacggcttcacactgatgtcgccttcgttctcgacaggttcgtgaaactcacccagcccaagagcacccaaacccttgatctcatgcagatcccctcacacacactggtcggggcgcttctccggtctgcgacgtactacttctcagacctggaggttgcgctcgtccacacaggaccggtcacgtgggtgcccaatggtgcgcctaagaccgccttgaacaaccacaccaacccgactgcctaccagaagcagcctatcacccgcttggcactcccctacaccgctccccaccgtgtgctgtcaacagtgtacaacgggaagacaacgtacggagaagaatcctcg------cggcgtggtgatcttgccgccctcgcacgcagagtgagcaaccggctgcccacttccttcaactacggcgctgtgaaggccgacaccatcacggag

>HM008917.1_O_CHA_2005

tcaagcgttggagtcacttacgggtacgcgacagctgaggactttgtgagcggaccgaacacgtctgggcttgagaccagggttgtgcaggcagagcggttcttcaaaacccacttgttcgactgggtcaccagtgacccgttcggacggtgctacctgctggaactcccaactgaccacaaaggtgtctacggtagcctaactgactcttatgcttacatgagaaacggttgggatgtagaggttactgcagtggggaatcagttcaacggaggatgtctgttggtggctatggtaccagaactttgctctattgacaagagagggctttaccaactcacgctcttcccccaccagttcatcaacccccggacgaacatgacggcgcacatcactgtgccttttgttggcgtcaaccgctacgaccagtacaaggtacacagaccttggactctcgtggtcatggttgtggccccgctgactgtcaacactgaaggtgccccacagatcaaggtttacgccaacatcgcccctactaacgtgcacgtcgcgggtgagctcccttctaaggaagggatcttccccgtggcatgtagcgacggttacggtggcctggtgaccactgacccaaagacggctgaccccgcctacgggaaagtgttcaatccacctcgcaacatgttgccggggcggttcaccaacttccttgatgtggctgaggcgtgtcctacgtttctgcattttgagggtgacgtaccgtacgtgaccacaaagacggactcagacagggtgctcgcccagtttgacttgtctctggcagcaaaacacatgtcaaacaccttcctggcaggtctcgcccagtattacacacagtacagcggcaccatcaacctgcacttcatgttcactggacccactgacgcgaaagcgcgttacatgattgcatacgccccccctggcatggagccgcccaaaacacccgaggcggccgctcactgcattcatgcggagtgggacacagggttgaactcaaaattcacattttcaatcccttacctttcggcggctgactacgcgtacaccgcgtctgactccgcggagaccacaaacgtgcagggatgggtttgcctgtttcaaatcacacacgggaaggctgacggcgacgcgctggtcgttctagctagtgccggtaaggactttgaactgcgtttgccagttgatgctcgcacgcagaccacctctacaggtgagtcggctgaccccgtaactgccaccgttgagaactacggtggtgagacacaggtccagagacgccagcacacggatgtctcgttcatactagacagatttgtgaaagtaacaccaaaagaccaaatcaatgtgttggacctgatgcaaacccctgcacacactttggtaggcgcgctcctccgtactgccacttactactttgcagatctagaagtggcagtgaaacacgaggggaaccttacctgggtcccgaatggggcgcccgaggcagcattggacaacaccaccaatccaacggcctaccacaaggcgccgctcacccggcttgcactgccttacacggcaccacaccgtgtcttggctactgtttacaacgggaactgtaagtacggcaagagccccgtggccaacgcgagaggtgacctgcaagtgttgaccccgaaggcggcaagaacgctgcctacctccttcaattacggcgccatcaaagccactcgggtgactgaa

>HM055510.1_O_VIT_2009

tcgagtgttggcataacgcacgggtacgcaacagctgaggattttgtgagcgggccaaacacctctggtcttgagaccagagttgtccaggcggaacggttctttaaaacccacctgttcgactgggtcaccagtgatccgttcggacggtgccacttgttggagctcccgactgaccacaaaggtgtctacggcagcctgaccgactcatacgcctacatgagaaacggttgggatgttgaagtcaccgctgtggggaatcagttcaacggaggctgcctactggtggccatggtgcctgaactttgttccatcgagcggagagagctgttccagcttacgctcttcccccaccagttcatcaacccccggacgaacatgacagcccacatcaaggtgccctttgttggcgtcaaccgttacgatcagtacaaggtacacaagccgtggacccttgtggttatggtcgtagccccactaactgtcaacaccgaaggcgctccgcagatcaaggtgtatgccaacatcgcacccaccaacgtgcacgtcgcgggtgagttcccttccaaagaggggattttccctgtggcctgtagcgacggttatggcggcttggtgacaactgacccaaagacggctgaccccgtttacggcaaagtgttcaaccccccccgcaacatgttgccggggcggttcaccaacctcctggacgtggctgaggcttgccccacgtttctgcacttcgatggtgacgtaccgtatgtgaccactaagacggattcggacagggtgctcgcacaatttgacttgtctttggcagcaaaacacatgtcaaacaccttccttgcaggtcttgcccagtactacacgcagtacagcggcaccatcaacctgcacttcatgttcacaggtcccactgacgcgaaagcgcgttacatgattgcgtatgcccctccgggcatggagccgcccaaaacacctgaggctgctgctcactgcattcacgcagagtgggacacgggtctgaactcaaagtttaccttttccatcccctacctctcggcggctgattacgcgtacaccgcgtctgacgctgctgagaccacaaatgttcagggatgggtctgcttattccaaataacacacgggaaagctgagggtgacgctcttgccgtgctggccagtgctggcaaagactttgagctgcgcctgcctgtggacgctcggcaacagaccacttcgacaggcgagtcggctgaccccgtgactgccaccgttgagaattacggcggcgagacacaggtccagaggcgccaccacacagacgtctcattcatattggacagatttgtgaaagtcacaccaaaagactcaataaatgtattggacctgatgcagacccccccccacaccctagtaggggcgctcctccgcactgccacttactatttcgctgatctagaggtggcagtgaaacacgagggggaccttacctgggtgccaaatggagcacctgaagcagccttggacaacaccaccaacccaacggcgtaccataaggcgccgcttacccggcttgcattgccctacacggcaccacaccgtgttttggccaccgtttacaacgggaactgcaaatacgccgggggctcactgaccaacgtgagaggcgatctccaagtgctggctcagaaggcggcgaggccgctgcctacttctttcaactacggtgccatcaaagccactcgggtgacagaa

>HM067704.1_SAT2_UGA_2007

agctctgttggcattacctacgggtatgctgacagtgactccttcaggtccgggccaaacacctctggactagagacgcgggttgaacaagctgagcgtttcttcaaggagaaattgtttgactggacaagcgacaaaccgttcgggacgctgtacgtgttggagctgcccaaagatcacaagggcatctacggcaaacttgctgactcctacacgtacatgcgcaatggatgggatgtccaagtcagtgctacaagcacccagttcaacggaggttcattgctagtggcgatggtccccgaactctgcagcctcagggatagagaagagtttcaactcacgctctacccccaccagttcattaaccctcgaaccaacaccaccgcacacatccaagtgccttacctgggtgtgaaccggcatgaccagggcaaacgacaccagtcatggtcgttggtggtaatggtcttgacacctctaacaacagagcagatgaacagcgggactgtggaagtttacgccaacattgccccgaccaacgtccacgtggcgggcgagctccccaccaaacaagggatcgtgcccgttgcctgcgccgatggttatggcggctttcagaacactgacccaaagagtgcggacccaatctacggccatgtgtacaatccatcacggaacgactgtcacggtaggttctccaacttacttgacgttgcagaagcgtgtcccacgctcctcaactttgacggc---aaaccgtacgtggtgaccaagaacaacggtgataagatcatgactgcatttgatgtggctttcactcacaaagttcacaaaaacacgtacctggccgggctggcggactactacacacagtactccggcagcctgaactaccactttatgttcacgggccccacccaccacaaggcaaagttcatggtggcgtacgttcctcccggtgtagacttacccaaaacacctgaggacgccgctcactgctaccactcagaatgggacacaggtctgaactcgagctttacgttcgcagtaccatacctgtcgtcagctgacttttcttacacacacactgacacgcctgccatggccaccaccaacggctgggtgctggtgctccaggtcactgacacccactctgccgaggctgctgttgtagtctcagtgagtgctggaccagatttggaattccggttccccattgacccagtgcgccagacgacctctgctggtgagggcgctgatgtggtaaccactgaccccaccacccacggtggatcggtgtgtgcgccgcgacgcgttcacacagacgtcgctttcctccttgaccgcagcacgcacgtccacaccaacaaaactgcgtttgcggttgacctcatggacaccaaggaaaaggcgcttgtaggagcaatccttaggtcagcaacttactatttctgtgatttggagatcgccgtgggtaaccacacgcgcgtgttttggcaacccaacggtgccccgcgaacgacacaacttggg---gacaacccaatggtgttctcgcacaacaacgtcacccgttttgccattccattcacagccccacacagactgctgtctactgtctacaacggagagtgcaattacaacaccaaagtcacc---gccattcgtggtgacaggcaggttctggctcagaagtacgcgtccgcgttgccatccacgttcaacttcggatttgtgaccgccgaccaacccgtcgac

>HM067705.1_SAT2_UGA_2007

agttctgttggcatcacttacgggtacgccgacgacgactccttcagacccggacctaacacctctgggttggacacacgtgtcaaacaggccgagcgtttcttcaaggagaaactgttcgactggaccagcgacaaaccgttcggaacactgtatgtgttggagttgcccaaagatcacaagggtatctatggtaaactcaccgactcttacacgtacatgcgtaacgggtgggatgttcaagtcagtgctacaagcactcaattcaatggaggttcactgttggttgcgatggttcccgaactatgcaacctcagacagagggaagagttccaacttacactctacccacaccaattcatcaacccaaggactaacaccacagcacacatccaggtaccctacctgggcgtgaaccgttatgaccagggtaaacggcaccaatcctggtcgctagtggtgatggtattaacgcccctaacaacagagcagatgaacagcgggaccgtggaggtctacgccaacattgcaccaaccaacgtccacgtggcgggcgagctccctggcaaacagggtattgtgcccgtcgcctgtgctgatggctatggtgggttccaaaacactgacccaaagaccgcagatccaatctacgggcatgtgtacaacccatcgaggaatgactgccacggcaggttctcaaacctgctcgatgtggccgaagcgtgccccacgcttctcaacttcgatggt---aaaccatacgtggtgaccaagaacaacggtgacaaggtcatgactgcatttgacgtggcctttacacacaaggtacacaggaacacgtacctggctggacttgcagactactacacacagtattctggcagtctcaactaccacttcatgttcacgggtcctacccaccacaaagccaaattcatggtagcatatgtccctccgggcatagacctacccaaaacacctgaggatgcagcacactgttaccattctgaatgggacacaggtctgaactcgagcttcacgttcgcggtgccatacctgtcgtccgctgattactcctacacacatactgacaccccggccatggctaccaccaatggctgggtggtggtcctccaagtcaccgacacccactctgctgaggctgcggttgtagtctcagtcagtgcggggccggacttggagttccgctttcccattgacccaatccgccagaccacttctgccggagagggtgctgacgtggtcaccaccgatcctaccacccacggtggcacggtcagtgcgccacgccgcgtccacacggacgtcgccttcctcctcgatcgtagcactcacgtgcacaccaacaaaaccagttttgcggtcgacctcatggacaccaaggagaaggcgcttgtgggagccattctgagatcagcaacctactacttttgtgacctggaaatcgcagtgggtgaacacggccgggtgttctggcaacccaacggtgcgccacgaactacacagcttgga---gacaacccgatggtgttttcacacaacggtgtcacccgctttgccattccgttcacagccccacaccggttgttgtccacagtctacaacggggtatgtgaatacaacaacaaagtcgcc---gccatccgtggcgacaggcaggttttagctcaaaagtacgcgtccgcgttaccatccacgttcaacttcgggtttgtgaccgccgacaggccagtcgac

>HM067706.1_SAT1_UGA_2007
[truncated: 972,526 more chars]
