# Supplementary material for: Early origin and global colonisation of foot-and-mouth disease virus
Source: Sci Rep. 2020 Sep 17;10:15268. doi: 10.1038/s41598-020-72246-6 (PMC7498456; doi:10.1038/s41598-020-72246-6)
Supplement: Supplementary file 6 — Supplementary Data S4. [file 41598_2020_72246_MOESM6_ESM.doc]

>AB079061.1_O_JPN_2000

CCCTGGTACAAGCTCATCAAGCTCTTGAGCCGCCTGTCATGCATGGCCGCTGTAGCAGCACGGTCAAAGG

ACCCAGTCCTTGTGGCCATCATGCTGGCTGACACCGGCCTTGAGATTCTGGACAGCACCTTTGTCGTGAA

GAAGATCTCCGACTCGCTCTCCAGTCTCTTTCACGTGCCGGCCCCCGTCTTCAGTTTCGGAGCCCCGATT

TTGTTGGCCGGGTTGGTAAAAGTCGCCTCGAGTTTCTTCCGGTCCACACCCGAAGACCTTGAGAGAGCGG

AGAAACAGCTCAAAGCACGTGACATCAATGACATATTCGCCATTCTCAAGAACGGCGAGTGGCTGGTCAA

GCTGATTCTTGCCATCCGCGACTGGATCAAGGCATGGATCGCCTCAGAAGAAAAGTTTGTCACCATGACA

GACCTGGTGCCTGGCATCCTTGAAAAGCAGCGGGATCTCAACGACCCAAGCAAGTACAAGGAGGCCAAGG

AGTGGCTCGACAACGCGCGCCAAGCGTGTTTGAAGAGCGGGAACACCCACATCGCAAACCTTTGCAGAGT

GGTTGCCCCAACACCCAGCAGGTCGAGGCCCGAACCCGTGGTCGTTTGCCTCCGTGGCAAATCGGGCCAG

GGCAAGAGTTTCCTTGCGAACGTGCTTGCACAAGCAATTTCAACCCACTTCACTGGCAGAACCGATTCAG

TTTGGTACTGCCCACCTGACCCTGACCACTTCGACGGTTACAACCAGCAGACCGTTGTAGTAATGGATGA

TTTGGGCCAGAACCCCGACGGGAAGGACTTTAAGTACTTCGCCCAAATGGTTTCAACTACGGGGGTTATC

CCGCCCATGGCTTCACTCGAGGACAAAGGCAAACCTTTCAACAGCAAGGTCATCATCGCCACCACCAACC

TGTACTCGGGCTTCACCCCGAGAACTATGGTGTGCCCTGACGCACTGAACCGAAGGTTCCACTTTGACAT

TGACGTGAGCGCCAAGGACGGGTACAAAATTAACAACAAATTGGACATCATCAAAGCTCTTGAAGATACC

CACACCAACCCAGTGGCAATGTTTCAATACGACTGTGCCCTTCTCAACGGCATGGCCGTTGAAATGAAGA

GAATGCGACAAGATATGTTTAAGCCTCAACCGCCCCTCCAGAACGTCTACCAGCTTGTTCAGGAGGTGAT

TGACCGGGTCGAGCTCCACGAGAAGGTGTCGAGCCACCCGATTTTCAAGCAG

>AF026168.2_O_TAW_1997

CCCTGGTACAAGCTCATCAAACTTCTAAGCCGCCTGTCGTGCATGGCCGCTGTGGCAGCACGGTCCAAGG

ACCCAGTCCTTGTGGCCATCATGCTTGGCGACACCGGCCTCGAGATTCTGGACAGCACCTTCGTGGTAAA

GAAGATCTCCGACTCGCTCTCCAGTCTCTTCCACGTTGCGGCTCCCGCCTTCAGTTTCGAG---CCCGAT

CTGTTGGCCGGGTTGGTCAAAGTCGCCTCGAGTTTCTTCCAGTCCACACCCGAAGACCTCGAGAGAGCAG

AAAAGCAGCTCAAAGCACGTGACATCAACGACATATTTGCCGTTCTTAAGAACGGTGAGTGGCTGGTCAA

ACTGATCCTGGCCATCCGCGACTGGATTAAGGCATGGATCGCCTCAGAAGAGAAGTTTGTCACCATGACA

GACCTGGTGCCTGGCATCCTTGAAAGTCAACGGGATCTCAATGACCCCGGCAAATACAAGGAGGCCAAGG

AATGGCTGGACAACGCGCGTCAAGCGTGTTTGAAGAGCGGGAACGTGCACATTGCCAATCTGTGTAAAGT

GGTCGCTCCGGCGCCCAGCAAGTCGAGACCCGAACCAGTGGTCGTGTGCCTTCGCGGCAAATCCGGCACA

AGGAAAAGCATCCTCGCGAACGTGCTCGCGCAGGCAATTTCCACACACTTCACTGGTAGGACCGACTCGG

TCTGGTACTGCCCGCCCGACCCTGACCACTTTGACGGTTACAATCAGCAGACCGTCGTCGTGATGGACGA

CTTGGGCCAAAACCCAGACGGCAAAGACTTCAAGTACTTTGCCCAAATGGTCTCCACCACGGGGTTCATC

CCGCCTATGGCCTCGCTCGAGGATAAGGGTAAACCCTTCAACAGCAAGGTCATAATAGCTACAACCAACC

TGTACTCGGGATTCACCCCAAAGACCATGGTGTGCCCCGATGCGCTTAACCGGAGGTTTCACTTTGACAT

CGACGTGAGCGCCAAAGACGGGTACAAGATCAACAACAAACTGGACATAGTCAAAGCACTTGAAGACACC

CACGCTAACCCGGTGGCGATGTTCCAATACGACTGCGCTCTTCTCAACGGAATGGCCGTTGAAATGAAGA

GAATGCAGCAAGACATGTTCAAGCCTCAACCACCCTTCCAGAACATCTACCAGCTCGTTCAGGAGGTGAT

TGAGCGGGTGGAACTACACGAAAAGGTGTCGAGCCACCCGATATTTAAACAG

>AF154271.1_O_TAW_1997

CCCTGGTACAAGCTCATCAAACTTCTAAGCCGCCTGTCGTGCATGGCCGCTGTGGCAGCACGGTCCAAGG

ACCCAGTCCTTGTGGCCATCATGCTGGCCGACACCGGCCTCGAGATTCTGGACAGCACCTTCGTGGTAAA

GAAGATCTCCGACTCGCTCTCCAGTCTCTTCCACGTGCCGGCTCCCGCCTTCAGTTTCAGAGCCCCGATC

CTGTTGGCCGGGTTGGTCAAAGTCGCCTCGAGTTTCTTCCAGTCCACACCCGAAGACCTCGAGAGAGCAG

AAAAGCAGCTCAAAGCACGTGACATCAACGACATATTTGCCGTTCTTAAGAACGGTGAGTGGCTGGTCAA

ACTGATCCTGGCCATCCGCGACTGGATTAAGGCATGGATCGCCTCAGAAGAGAAGTTTGTCACCATGACA

GACCTGGTGCCTGGCATCCTTGAAAGACAACGGGATCTCAATGACCCCGGCAAATACAAGGAGGCCAAGG

AATGGCTGGACAACGCGCGTCAAGCGTGTTTGAAGAGCGGGAACGTGCACATTGCCAATCTGTGTAAAGT

GGTCGCTCCGGCGCCCAGCAAGTCGAGACCCGAACCAGTGGTCGTGTGCCTTCGCGGCAAATCCGGCCAA

GGGAAAAGCTTTCTCGCGAACGTTCTCGCGCAGGCAATTTCCACACACTTCACTGGTAGGACCGACTCGG

TCTGGTACTGCCCGCCCGACCCTGACCACTTTGACGGTTACAATCAGCAGACCGTCGTCGTGATGGACGA

CTTGGGCCAAAACCCAGACGGCAAAGACTTCAAGTACTTTGCCCAAATGGTCTCCACCACGGGGTTCATC

CCGCCTATGGCCTCGCTCGAGGATAAGGGTAAACCCTTCAACAGCAAGGTCATAATAGCTACAACCAACC

TGTACTCGGGATTCACCCCAAAGACCATGGTGTGCCCCGATGCGCTTAACCGGAGGTTTCACTTTGACAT

CGACGTGAGCGCCAAAGACGGGTACAAGATCAACAACAAACTGGACATAGTCAAAGCACTTGAAGACACC

CACGCTAACCCGGTGGCGATGTTCCAATACGACTGCGCTCTTCTCAACGGAATGGCCGTTGAAATGAAGA

GAATGCAGCAAGACATGTTCAAGCCTCAACCACCCCTCCAGAACATCTACCAGCTCGTTCAGGAGGTGAT

TGAGCGGGTGGAACTACACGAAAAGGTGTCGAGCCACCCGATATTTAAACAG

>AF308157.1_O_TAW_1997

CCCTGGTACAAGCTCATCAAACTTCTAAGCCGCCTGTCGTGCATGGCCGCTGTGGCAGCACGGTCCAAGG

ACCCAGTCCTTGTGGCCATCATGCTGGCCGACACCGGCCTCGAGATTCTGGACAGCACCTTCGTGGTAAA

GAAGATCTCCGACTCGCTCTCCAGTCTCTTCCACGTGCCGGCTCCCGCCTTCAGTTTCGGAGCCCCGATC

CTGTTGGCCGGGTTGGTCAAAGTCGCCTCGAGTTTCTTCCAGTCCACACCCGAAGACCTCGAGAGAGCAG

AAAAGCAGCTCAAAGCACGTGACATCAACGACATATTTGCCGTTCTTAAGAACGGTGAGTGGCTGGTCAA

ACTGATCCTGGCCATCCGCGACTGGATTAAGGCATGGATCGCCTCAGAAGAGAAGTTTGTCACCATGACA

GACCTGGTGCCTGGCATCCTTGAAAGACAACGGGATCTCAATGACCCCGGCAAATACAAGGAGGCCAAGG

AATGGCTGGACAACGCGCGTCAAGCGTGTTTGAAGAGCGGGAACGTGCACATTGCCAATCTGTGTAAGGT

GGTCGCTCCGGCGCCCAGCAAGTCGAGACCCGAACCAGTGGTCGTGTGCCTTCGCGGCAAATCCGGCCAA

GGGAAAAGCTTTCTCGCGAACGTTCTCGCGCAGGCAATTTCCACACACTTCACTGGTAGGACCGACTCGG

TCTGGTACTGCCCGCCCGACCCTGACCACTTTGACGGTTACAATCAGCAGACCGTCGTCGTGATGGACGA

CTTGGGCCAAAACCCAGACGGCAAAGACTTCAAGTACTTTGCCCAAATGGTATCCACCACGGGGTTCATC

CCGCCTATGGCCTCGCTCGAGGATAAGGGTAAACCCTTCAACAGCAAGGTCATAATAGCTACAACCAACC

TGTACTCGGGATTCACCCCAAAGACCATGGTGTGCCCCGATGCGCTTAACCGGAGGTTTCACTTTGACAT

CGACGTGAGCGCCAAAGACGGGTACAAGATCAACAACAAACTGGACATAGTCAAAGCACTTGAAGACACC

CACGCTAACCCGGTGGCGATGTTCCAATACGACTGCGCTCTTCTCAACGGAATGGCCGTTGAAATGAAGA

GAATGCAGCAAGACATGTTCAAGCCTCAACCACCCCTCCAGAACATCTACCAGCTCGTTCAGGAGGTGAT

TGAGCGGGTGGAACTACACGAAAAGGTGTCGAGCCACCTGATATTTAAACAG

>AF377945.1_O_SKR_2000

CCCTGGTACAAGCTCATCAAGCTCTTGAGCCGCCTGTCATGTATGGCCGCTGTAGCAGCACGGTCAAAGG

ACCCAGTCCTTGTGGCCATCATGCTGGCTGACACCGGCCTTGAGATTCTGGACAGTACCTTTGTCGTGAA

GAAGATCTCCGACTCGCTCTCCAGTCTCTTTCACGTACCGGCCCCCGTCTTCAGTTTCGGAGCCCCGATT

TTGTTGGCCGGGTTGGTCAAGGTCGCCTCGAGTTTCTTCCGGTCCACACCCGAAGACCTTGAGAGAGCGG

AGAAACAGCTCAAAGCACGTGACATCAATGACATATTCGCCATTCTCAGGAACGGCGAGTGGCTGGTCAA

GCTGATTCTTGCCATCCGCGACTGGATTAAGGCTTGGATCGCCTCAGAAGAAAAGTTTGTCACCATGACA

GACCTGGTGCCTGGTATCCTTGAAAAGCAGCGGGACCTCAACGACCCAAGCAAGTACAAGGAGGCCAAGG

AGTGGCTCGACAACACGCGCCAAGCGTGTTTGAAAAGCGGGAACATCCACATCGCAAACCTTTGCAAAGT

GGTTGCCCCAGCACCCAGCAGGTCGAGGCCCGAACCCGTGGTCGTTTGCCTCCGTGGCAAATCGGGCCAG

GGCAAGAGTTTCCTTGCGAACGTGCTTGCACAAGCAATATCAACCCACTTCACTGGTATAACCGATTCAG

TTTGGTACTGCCCACCTGACCCTGACCACTTCGACGGTTACAACCAGCAGACCGTTGTAGTAATGGATGA

CTTGGGCCACAACCCCGACGGGAAGGACTCCAAGTACTTCGCCCAAATGGTTTCAACTACGGGGTTTATC

CCGCCCATGGCCTCACTTGAGGACAAAGGCAATCCTTTCAACAGCAAGGTTATCATCGCCACCACCAACC

TGTACTCGGGCTTCACCCCGAGAAATATGGTGTGCCCTGATGCACTGAACCGAAGGTTCCACGTTGACAT

TGACGTGAGCGCCAAGGACGGGTACAAAATTAGCAACAAATTGGACATCATCAAGGCTCTTGAAGATACC

CACACCAACCCAGTGGCAATGTTTCAATACGACTGTGCCCTTCTCAACGGCATGGCCGTTGAAATGAAGA

GAATGCAACAAGATATGTTCAAGCCTCAACCGCCCCTCCAGAAAGTTTACCAGCTTGTTCAGGAGGCGAT

TGACCGGGTCGAGTTCCACGAGAAGGTGTCGAGCCACCCGATTTTCAAGCAG

>AF506822.2_O_CHA_1999

CCCTGGTACAAGCTCATCAAGCTCTTGAGCCGCCTGTCATGCATGGCCGCTGTAGCAGCACGGTCAAAGG

ACCCAGTCCTTGTGGCCATCATGCTGGCTGACACCGGCCTTGAGATTCTGGACAGTACCTTTGTCGTGAA

GAAGATCTCCGACTCGCTCTCCAGTCTCTTTCACGTGCCGGCCCCCGTCTTCAGTTTCGGAGCCCCGATT

TTGCTGGCCGGGTTGGTCAAAGTCGCCTCGAGTTTCTTCCGGTCCACACCCGAAGACCTTGAGAGAGCGG

AGAAACAGCTCAAAGCACGTGACATCAATGACATATTCGCCATTCTCAAGAACGGCGAGTGGCTGGTCAA

GCTGATTCTTGCCATCCGCGACTGGATCAAGGCATGGATCGCCTCAGAAGAAAAGTTTGTCACCATGACA

GACTTGGTGCCTGGCATCCTTGAAAAGCAGCGGGATCTCAACGACCCAAGCAAGTACAAGGAGGCCAAGG

AGTGGCTCGACAACGCGCGCCAAGCGTGTTTGAAGAGCGGGAACATCCACATCGCAAACCTTTGCAAAGT

GGTTGCCCCAGCACCCAGCAGGTCGAGGCCCGAACCCGTGGTCGTTTGCCTCCGTGGCAAATCGGGCCAG

GGCAAGAGTTTCCTTGCGAACGTGCTTGCACAAGCAATTTCAACCCACTTCACTGGCAGAACCGATTCAG

TTTGGTACTGCCCACCTGACCCTGACCACTTCGACGGTTACAACCAGCAGACCGTTGTAGTAATGGATGA

TTTGGGCCAGAACCCCGACGGGAAGGACTTCAAGTACTTCGCCCAAATGGTTTCAACTACGGGGTTTATC

CCGCCCATGGCTTCACTCGAGGACAAAGGCAAACCTTTCAACAGCAAGGTCATCATCGCCACCACCAACC

TGTACTCGGGCTTCACCCCGAGAACTATGGTGTGCCCTGATGCACTGAACCGAAGGTTCCACTTTGACAT

TGACGTGAGCGCCAAGGACGGGTACAAAATTAACAACAAATTGGACATCATCAAAGCTCTTGAAGATACC

CACACCAACCCAGTGGCAATGTTTCAATACGACTGTGCCCTTCTCAACGGCATGGCCGTTGAAATGAAGA

GAATGCAACAAGATATGTTCAAGCCTCAACCGCCCCTCCAGAACGTCTACCAGCTTGTTCAGGAGGTGAT

TGACCGGGTCGAGCTCCACGAGAAGGTGTCGAGCCACCCGATTTTCAAGCAG

>AH012984.2_O_SKR_2000

CCCTGGTACAAGCTCATCAAGCTCTTGAGCCGCCTGTCATGTATGGCCGCTGTAGCAGCACGGTCAAAGG

ACCCACTCCTTGTGGCCATCATGCTGGCTGACACCGGCCTTGAGATCCTGGACAGTACCTTTGTCGTGAA

GAAGATCTCCGACTCGCTCTCCAGTCTCTTTCACGTGCCGGCCCCCGTCTTCAGTTCCGGAGCCCCGATT

TTGTTGGCCGGGTTGGTCAAAGTCGCCTCGAGTTTCTTCCGGTCCACACCCGAAGACCTTGAGAGAGCGG

AGAAACAGCTCAAAGCACGTGACATCAATGACATATTCGCCATTCTTAAGAACGGCGAGTGGCCGGTCAA

GCTGATTCTTGCCATCCGCGACTGGATCAAGGCATGGATCGCCTCAGAAGAAAAGTTTGTCACCATGACA

GACCTGGTGCCTGGCATCCTTGAAAAGCAGCGGGATCTCAACGACCCAAGCAAGTACAAGGAGGCCAAGG

AGTGGCTCGACAACGCGCGCCAAGCGTGTCTGAAGAGCGGGAACATCCACATCGCAAACCTTTGCAAAGT

GGTTGCCCCAGCGCCCAGCAGGTCGAGGCCCGAACCCGTGGTCGTTTGCCTCCGTGGCAAGTCAGGCCAG

GGCAAGAGTTTCCTTGCGAACGTGCTTGCACAAGCAATTTCAACCCACTTCACTGGCAGAACCGATTCAG

TTTGGTACTGCCCACCTGACCCTGACCACTTCGACGGTTACAACCAGCAGACCGTTGTAGTAATGGATGA

TTTGGGCCACAACCCCGACGGGAAGGACTTCAAGTACTTCGCCCAAATGGTTTCAACTACGGGGTTTATC

CCGCCCATGGCTTCACTAGAGGATAAAGGCAAACCTTTCAACAGCAAGGTCATCATCGCCACCACCAACC

TGTACTCGGGCTTCACCCCGAGAACTATGGTGTGCCCTGATGCACTGAACCGAAGGTTCCACTTTGACAT

TGACGTGAGCGCCAAGGACGGGTACAAAATTAACAACAAATTGGACATCATCAAAGCTCTTGAAGACACC

CACCCCAACCCAGTGGCAATGTTTCAATACGACTGTGCCCTTCTCAACGGCATGGCCGTTGAAATGAAGA

GAATGCAACAAGATATGTTCAAGCCTCAACCGCCCCTCCAGAACGTCTACCAGCTTGTTCAGGAGGTGAT

TGACCGGGTCGAGCTCCACGAGAAGGTGTCGAGTCACCCGATCTTCAAGCAG

>AH012985.2_O_SKR_2000

CCCTGGTACAAGCTCATCAAGCTCTTGAGCCGCCTGTCATGCATGGCCGCTGTAGCAGCACGGTCAAAGG

ACCCAGTCCTTGTGGCCATCATGCTGGCTGACACCGGCCTTGAGATTCTGGACAGTACCTTTGTCGTGAA

GAAGATCTCCGACTCGCTCTCCAGTCTCTTTCACGTGCCGGCCCCCGTCTTCAGTTTCGGAGCCCCGATT

TTGTTGGCCGGGTTGGTCAAAGTCGCCTCGACTTTCTTCCGGTCCACACCCGAGGACCTTGAGAGAGCGG

AGAAACAGCTCAAAGCACGTGACATCAATGACATATTCGCCATTCTCAAGAACGGCGAGTGGTTGGTCAA

GCTGATTCTTGCCATCCGCGACTGGATCAAGGCATGGATCGCCTCAGAAGAAAAATTTGTCACCATGACA

GACCTGGTACCTGGCATCCTTGAAAAGCAGCGGGATCTTAACGACCCAAGCAAGTACAAGGAGGCCAAGG

AGTGGCTCGACAACGCGCGCCAAGCGTGTTTGAAGAGCGGGAACATCCACATTGCAAACCTTTGTAAAGT

AGTTGCCCCAGCACCCAGCAGGTCGAGGCCTGAACCCGTGGTCGTTTGCCTCCGTGGCAAATCGGGCCAG

GGCAAGAGTTTCCTTGCGAACGTGCTTGCACAAGCAATTTCAACCCACTTCACTGGCAGAACCGATTCAG

TTTGGTACTGCCCACCTGACCCTGACCACTTCGACGGTTACAACCAGCAGACCGTTGTAGTAATGGATGA

TTTGGGCCAGAACCCCGACGGGAAGGACTTCAAATACTTCGCCCAAATGGTTTCAACTACGGGGTTTATC

CCGCCCATGGCTTCACTCGAGGACAAAGGCAAACCTTTCAACAGCAAGGTCATCATCGCCACCACCAACC

TGTACTCGGGCTTCACCCCGAGAACTATGGTGTGCCCTGATGCACTGAACCGAAGGTTCCACTTTGACAT

TGACGTGAGCGCCAAGGACGGGTACAAAATTAACAACAAATTGGACATCACCAAAGCTCTTGAAGATACC

CACACCAACCCAGTGGCAATGTTTCAATACGACTGTGCCCTTCTCAACGGCATGGCCGTTGAGATGAAGA

GAATGCAACAAGATATGTTCAAGCCTCAACCGCCCCTCCAGAACGTCTACCAGCTTGTTCAGGAGGTGAT

TGACCGGGTCGAGCTCCACGAGAAGGTGTCGAGTCACCCGATCTTCAAGCAG

>AJ539136.1_O_TAW_1999

CCCTGGTACAAGCTCATCAAGCTCTTGAGCCGCCTGTCATGCATGGCCGCTGTAGCAGCACGGTCAAAGG

ACCCAGTCCTTGTGGCCATCATGCTGGCTGACACCGGCCTTGAGATTCTGGACAGTACCTTTGTCGTGAA

GAAGATCTCCGACTCGCTCTCCAGTCTCTTTCACGTGCCGGCCCCCGTCTTCAGTTTCGGAGCCCCGATT

TTGTTGGCCGGGTTGGTCAAAGTCGCCTCGAGTTTCTTCCGGTCCACACCCGAAGACCTTGAGAGAGCGG

AGAAACAGCTCAAAGCACGTGACATCAATGACATATTCGCCATTCTCAAGAACGGCGAGTGGCTGGTCAA

GCTGATTCTTGCCATCCGCGACTGGATCAAGGCATGGATCGCCTCAGAAGAAAAGTTTGTCACCATGACA

GACCTGGTGCCTGGCATCCTTGAAAAGCAGCGGGATCTCAACGACCCAAGCAAGTACAAGGAGGCCAAGG

AGTGGCTCGACAACGCGCGCCAAGCGTGTTTGAAGAGCGGGAACATCCACATCGCAAACCTTTGCAAAGT

GGTTGCCCCAGCACCCAGCAGGTCGAGGCCCGAACCCGTGGTCGTTTGCCTCCGTGGCAAATCGGGCCAG

GGCAAGAGTTTCCTTGCGAACGTGCTTGCACAAGCAATTTCAACCCACTTCACTGGCAGAACCGATTCAG

TTTGGTACTGCCCACCTGACCCTGACCACTTCGACGGTTACAACCAGCAGACCGTTGTAGTAATGGATGA

TTTGGGCCAGAACCCCGACGGGAAGGACTTCAAGTACTTCGCCCAAATGGTTTCAACTACGGGGTTTATC

CCGCCCATGGCTTCACTCGAGGACAAAGGCAAACCTTTCAACAGCAAGGTCATCATCGCCACCACCAACC

TGTACTCGGGCTTCACCCCGAGAACTATGGTGTGCCCTGATGCACTGAACCGAAGGTTCCACTTTGACAT

TGACGTGAGCGCCAAGGACGGGTACAAAATTAACAACAAACTGGACATCATCAAAGCTCTTGAAGACACC

CACACCAACCCAGTGGCAATGTTTCAATACGACTGTGCCCTTCTCAACGGCATGGCCGTTGAAATGAAGA

GAGTGCAACAAGATGTGTTCAAGCCTCAACCGCCCCTCCAGAACGTCTACCAGCTTGTTCAGGAGGTGAT

TGACCGGGTCGAGCTCCACGAGAAGGTGTCGAGCCACCCGATTTTCAAGCAG

>AJ539137.1_O_TAW_1999

CCCTGGTACAAGCTCATCAAGCTCTTGAGCCGCCTGTCATGCATGGCCGCTGTAGCAGCACGGTCAAAGG

ACCCAGTCCTTGTGGCCATCATGCTGGCTGACACCGGCCTTGAGATTCTGGACAGTACCTTTGTCGTGAA

GAAGATCTCCGACTCGCTCTCCAGTCTCTTTCACGTGCCGGCCCCCGTCTTCAGTTTCGGAGCCCCGATT

TTGTTGGCTGGGTTGGTCAAAGTCGCCTCGAGTTTCTTCCGGTCCACACCCGAAGACCTTGAGAGAGCGG

AGAAACAGCTCAAAGCACGTGACATCAATGACATATTCGCCATTCTCAAGAACGGCGAGTGGCTGGTCAA

GCTGATTCTTGCCATCCGCGACTGGATCAAGGCATGGATCGCCTCAGAAGAAAAGTTTGTCACCATGACA

GACCTGGTGCCTGGCATCCTTGAAAAGCAGCGGGATCTCAACGACCCAAGCAAGTACAAGGAGGCCAAGG

AGTGGCTCGACAACGCGCGCCAAGCGTGTTTGAAGAGCGGGAACATCCACATCGCAAACCTTTGCAAAGT

GGTTGCCCCAGCACCCAGCAGGTCGAGGCCCGAACCCGTGGTCGTTTGCCTCCGTGGCAAATCGGGCCAG

GGCAAGAGTTTCCTTGCGAACGTGCTTGCACAAGCAATTTCAACCCACTTCACTGGCAGAACCGATTCAG

TTTGGTACTGCCCACCTGACCCTGACCACTTCGACGGTTACAACCAGCAGACCGTTGTAGTAATGGATGA

TTTGGGCCAGAACCCCGACGGGAAGGACTTCAAGTACTTCGCCCAAATGGTTTCAACTACGGGGTTTATC

CCGCCCATGGCTTCACTCGAGGACAAAGGCAAACCTTTCAACAGCAAGGTCATCATCGCCACCACCAACC

TGTACTCGGGCTTCACCCCGAGAACTATGGTGTGCCCTGATGCACTGAACCGAAGGTTCCACTTTGACAT

TGACGTGAGCGCCAAGGACGGGTACAAAATTAACAACAAACTGGACATCAACAAAGCTCTTGAAGACACC

CACACCAACCCAGTGGCAATGTTTCAATACGACTGTGCCCTTCTCAACGGCATGGCCGTTGAAATGAAGA

GAATGCAACAAGATATGTTCAAGCCTCAACCGCCCCTCCAGAACGTCTACCAGCTTGTTCAGGAGGTGAT

TGACCGGGTCGAGCTCCACGAGAAGGTGTCGAGCCACCCGATTTTCAAGCAG

>AJ539138.1_O_CHA_1999

CCCTGGTACAAGCTCATCAAGCTCTTGAGCCGCCTGTCATGCATGGCCGCTGTAGCAGCACGGTCAAAGG

ACCCAGTCCTTGTGGCCATCATGCTGGCTGACACCGGCCTTGAGATTCTGGACAGTACCTTTGTCGTGAA

GAAGATCTCCGACTCGCTCTCCAGTCTCTTTCACGTGCCGGCCCCCGTCTTCAGTTTCGGAGCCCCGATT

TTGCTGGCCGGGTTGGTCAAAGTCGCCTCGAGTTTCTTCCGGTCCACACCCGAAGACCTTGAGAGAGCGG

AGAAACAGCTCAAAGCACGTGACATCAATGACATATTCGCCATTCTCAAGAACGGCGAGTGGCTGGTCAA

GCTGATTCTTGCCATCCGCGACTGGATCAAGGCATGGATCGCCTCAGAAGAAAAGTTTGTCACCATGACA

GACTTGGTGCCTGGCATCCTTGAAAAGCAGCGGGATCTCAACGACCCAAGCAAGTACAAGGAGGCCAAGG

AGTGGCTCGACAACGCGCGCCAAGCGTGTTTGAAGAGCGGGAACATCCACATCGCAAACCTTTGCAAAGT

GGTTGCCCCAGCACCCAGCAGGTCGAGGCCCGAACCCGTGGTCGTTTGCCTCCGTGGCAAATCGGGCCAG

GGCAAGAGTTTCCTTGCGAACGTGCTTGCACAAGCAATTTCAACCCACTTCACTGGCAGAACCGATTCAG

TTTGGTACTGCCCACCTGACCCTGACCACTTCGACGGTTACAACCAGCAGACCGTTGTAGTAATGGATGA

TTTGGGCCAGAACCCCGACGGGAAGGACTTCAAGTACTTCGCCCAAATGGTTTCAACTACGGGGTTTATC

CCGCCCATGGCTTCACTCGAGGACAAAGGCAAACCTTTCAACAGCAAGGTCATCATCGCCACCACCAACC

TGTACTCGGGCTTCACCCCGAGAACTATGGTGTGCCCTGATGCACTGAACCGAAGGTTCCACTTTGACAT

TGACGTGAGCGCCAAGGACGGGTACAAAATTAACAACAAATTGGACATCATCAAAGCTCTTGAAGATACC

CACACCAACCCAGTGGCAATGTTTCAATACGACTGTGCCCTTCTCAACGGCATGGCCGTTGAAATGAAGA

GAATGCAACAAGATATGTTCAAGCCTCAACCGCCCCTCCAGAACGTCTACCAGCTTGTTCAGGAGGTGAT

TGACCGGGTCGAGCTCCACGAGAAGGTGTCGAGCCACCCGATTTTCAAGCAG

>AJ539139.1_O_SKR_2000

CCCTGGTACAAGCTCATCAAGCTCTTGAGCCGCCTGTCATGCATGGCCGCTGTAGCAGCACGGTCAAAGG

ACCCAGTCCTTGTGGCCATCATGCTGGCTGACACCGGCCTTGAGATTCTGGACAGTACCTTTGTCGTGAA

GAAGATCTCCGACTCGCTCTCCAGTCTCTTTCACGTGCCGGCCCCCGTCTTCAGTTTCGGAGCCCCGATT

TTGTTGGCCGGGTTGGTCAAAGTCGCCTCGACTTTCTTCCGGTCCACACCCGAAGACCTTGAGAGAGCGG

AGAAACAGCTCAAAGCACGTGACATCAATGACATATTCGCCATTCTCAAGAACGGCGAGTGGTTGGTCAA

GCTGATTCTTGCCATCCGCGACTGGATCAAGGCATGGATCGCCTCAGAAGAAAAATTTGTCACCATGACA

GACCTGGTACCTGGCATCCTTGAAAAGCAGCGGGATCTTAACGACCCAAGCAAGTACAAGGAGGCCAAGG

AGTGGCTCGACAACGCGCGCCAAGCGTGTTTGAAGAGCGGGAACATCCACATCGCAAACCTTTGTAAAGT

AGTTGCCCCAGCACCCAGCAGGTCGAGGCCTGAACCCGTGGTCGTTTGCCTCCGTGGCAAATCGGGCCAG

GGCAAGAGTTTCCTTGCGAACGTGCTTGCACAAGCAATTTCAACCCACTTCACTGGCAGAACCGATTCAG

TTTGGTACTGCCCACCTGACCCTGACCACTTCGACGGTTACAACCAGCAGACCGTTGTAGTAATGGATGA

TTTGGGCCAGAACCCCGACGGGAAGGACTTCAAATACTTCGCCCAAATGGTTTCAACTACGGGGTTTATC

CCGCCCATGGCTTCACTCGAGGACAAAGGCAAACCTTTCAACAGCAAGGTCATCATCGCCACCACCAACC

TGTACTCGGGCTTCACCCCGAGAACTATGGTGTGCCCTGATGCACTGAACCGAAGGTTCCACTTTGACAT

TGACGTGAGCGCCAAGGACGGGTACAAAATTAACAACAAATTGGACATCATCAAAGCTCTTGAAGATACC

CACACCAACCCAGTGGCAATGTTTCAATACGACTGTGCCCTTCTCAACGGCATGGCCGTTGAGATGAAGA

GAATGCAACAAGATATGTTCAAGCCTCAACCGCCCCTCCAGAACGTCTACCAGCTTGTTCAGGAGGTGAT

TGACCGGGTCGAGCTCCACGAGAAGGTGTCGAACCACCCGATTTTCAAGCAG

>AJ539140.1_O_SAR_2000

CCCTGGTACAAGCTCATCAAGCTCTTGAGCCGCCTGTCATGCATGGCCGCTGTAGCAGCACGGTCAAAGG

ACCCAGTCCTTGTGGCCATCATGCTGGCTGACACCGGCCTTGAGATTCTGGACAGTACCTTTGTCGTGAA

GAAGATCTCCGACTCGCTCTCCAGTCTCTTTCACGTGCCGGCCCCCGTCTTCAGTTTCGGAGCCCCGATT

TTGTTGGCCGGGTTGGTCAAAGTCGCCTCGAGTTTCTTCCGGTCCACACCCGAAGACCTTGAGAGAGCGG

AGAAACAGCTCAAAGCACGTGACATCAATGACATATTCGCCATTCTCAAGAACGGCGAGTGGCTGGTCAA

GCTGATTCTTGCCATCCGCGACTGGATCAAGGCATGGATCGCCTCAGAAGAAAAGTTTGTCACCATGACA

GACCTGGTGCCTGGCATCCTTGAAAAGCAGCGGGATCTCAACGACCCAAGCAAGTACAAAGAGGCCAAGG

AGTGGCTCGACAACGCGCGCCAAGCGTGTTTGAAGAGCGGGAACATCCACATCGCAAACCTTTGCAAAGT

GGTTGCCCCAGCACCCAGCAGGTCGAGGCCCGAACCCGTGGTCGTTTGCCTCCGTGGCAAATCGGGCCAG

GGCAAGAGTTTCCTTGCGAACGTGCTTGCACAAGCAATTTCAACCCACTTCACTGGCAGAACCGATTCAG

TTTGGTACTGCCCACCTGACCCTGACCACTTCGACGGTTACAACCAGCAGACCGTTGTAGTAATGGATGA

TTTGGGCCAGAACCCCGACGGGAAGGACTTCAAGTACTTCGCCCAAATGGTTTCAACTACGGGGTTTATC

CCGCCCATGGCTTCACTCGAGGACAAAGGCAAACCTTTCAACAGCAAGGTCATCATCGCCACCACCAACC

TGTACTCGGGCTTCACCCCGAGAACTATGGTGTGCCCTGACGCACTGAACCGAAGGTTCCACTTTGACAT

TGACGTGAGCGCCAAGGACGGGTACAAAATTAATAACAAATTGGACATCATCAAAGCTCTTGAAGATACC

CACACCAACCCAGTGGCAATGTTTCAATACGACTGTGCCCTTCTCAACGGCATGGCCGTTGAAATGAAGA

GAATGCAACAAGATATGTTCAAGCCTCAACCGCCCCTCCAGAACGTCTACCAGCTTGTTCAGGAGGTGAT

TGACCGGGTCGAGCTCCACGAGAAGGTGTCGAGCCACCCGATTTTCAAGCAG

>AJ539141.1_O_UKG_2001

CCCTGGTACAAGCTCATCAAGCTCTTGAGCCGCCTGTCATGCATGGCCGCTGTAGCAGCACGGTCAAAGG

ACCCAGTCCTTGTGGCCATCATGCTGGCTGACACCGGCCTTGAGATTCTGGACAGTACCTTTGTCGTGAA

GAAGATCTCCGACTCGCTCTCCAGTCTCTTTCACGTGCCGGCCCCCGTCTTCAGTTTCGGAGCCCCGATT

TTGTTGGCCGGGTTGGTCAAAGTCGCCTCGAGTTTCTTCCGGTCCACACCCGAAGACCTTGAGAGAGCGG

AGAAACAGCTCAAAGCACGTGACATCAATGACATATTCGCCATTCTCAAGAACGGCGAGTGGCTGGTCAA

GCTGATTCTTGCCATCCGCGACTGGATCAAGGCATGGATCGCCTCAGAAGAAAAGTTTGTCACCATGACA

GACCTGGTGCCTGGCATCCTTGAAAAGCAGCGGGATCTCAACGACCCAAGCAAGTACAAAGAGGCCAAGG

AGTGGCTCGACAACGCGCGCCAAGCGTGTTTGAAGAGCGGGAACATCCACATCGCAAACCTTTGCAAAGT

GGTTGCCCCAGCACCCAGCAGGTCGAGGCCCGAACCCGTGGTCGTTTGCCTCCGTGGCAAGTCGGGCCAG

GGCAAGAGTTTCCTTGCGAACGTGCTTGCACAAGCAATTTCAACCCACTTCACTGGCAGAACCGACTCAG

TTTGGTACTGCCCACCTGACCCTGACCACTTCGACGGTTACAACCAGCAGACCGTTGTAGTAATGGATGA

TTTGGGCCAGAACCCCGACGGGAAGGACTTCAAGTACTTCGCCCAAATGGTTTCAACTACGGGGTTTATC

CCGCCCATGGCTTCACTCGAGGACAAAGGCAAACCTTTCAACAGCAAGGTCATCATCGCCACCACCAACC

TGTACTCGGGCTTCACCCCGAGAACTATGGTGTGCCCTGACGCACTGAACCGAAGGTTCCACTTTGACAT

TGACGTGAGCGCCAAGGACGGGTACAAAATTAACAACAAATTGGACATCATCAAAGCTCTTGAAGATACC

CACACCAACCCAGTGGCAATGTTTCAATACGACTGTGCCCTTCTCAACGGCATGGCCGTTGAAATGAAGA

GAATGCAACAAGATATGTTCAAGCCTCAACCGCCCCTCCAGAACGTCTACCAGCTTGTTCAGGAGGTGAT

TGACCGGGTCGAGCTCCACGAGAAGGTGTCGAGCCACCCGATTTTCAAGCAG

>AJ633821.1_O_FRA_2001

CCCTGGTACAAGCTCATCAAGCTCTTGAGCCGCCTGTCATGCATGGCCGCTGTAGCAGCACGGTCAAAGG

ACCCAGTCCTTGTGGCCATCATGCTGGCTGACACCGGCCTTGAGATTCTGGACAGTACCTTTGTCGTGAA

GAAGATCTCCGACTCGCTCTCCAGTCTCTTTCACGTGCCGGCCCCCGTCTTCAGTTTCGGAGCCCCGATT

TTGTTGGCCGGGTTGGTCAAAGTCGCCTCGAGTTTCTTCCGGTCCACACCCGAAGACCTTGAGAGAGCGG

AGAAACAGCTCAAAGCACGTGACATCAATGACATATTCGCCATTCTCAAGAACGGCGAGTGGCTGGTCAA

GCTGATTCTTGCCATCCGCGACTGGATCAAGGCATGGATCGCCTCAGAAGAAAAGTTTGTCACCATGACA

GACCTGGTGCCTGGCATCCTTGAAAAGCAGCGGGATCTCAACGACCCAAGCAAGTACAAAGAGGCCAAGG

AGTGGCTCGACAACGCGCGCCAAGCGTGTTTGAAGAGCGGGAACATCCACATCGCAAACCTTTGCAAAGT

GGTTGCCCCAGCACCCAGCAGGTCGAGGCCCGAACCCGTGGTCGTTTGCCTCCGTGGCAAATCGGGCCAG

GGCAAGAGTTTCCTTGCGAACGTGCTTGCACAAGCAATTTCAACCCACTTCACTGGCAGAACCGACTCAG

TTTGGTACTGCCCACCTGACCCTGACCACTTCGACGGTTACAACCAGCAGACCGTTGTAGTAATGGATGA

TTTGGGCCAGAACCCCGACGGGAAGGACTTCAAGTACTTCGCCCAAATGGTTTCAACTACGGGGTTTATC

CCGCCCATGGCTTCACTCGAGGACAAAGGCAAACCTTTCAACAGCAAGGTCATCATCGCCACCACCAACC

TGTACTCGGGCTTCACCCCGAGAACTATGGTGTGCCCTGACGCACTGAACCGAAGGTTCCACTTTGACAT

TGACGTGAGCGCCAAGGACGGGTACAAAATTAACAACAAATTGGACATCATCAAAGCTCTTGAAGATACC

CACACCAACCCAGTGGCAATGTTTCAATACGACTGTGCCCTTCTCAACGGCATGGCCGTTGAAATGAAGA

GAATGCAACAAGATATGTTCAAGCCTCAACCGCCCCTCCAGAACGTCTACCAGCTTGTTCAGGAGGTGAT

TGACCGGGTCGAGCTCCACGAGAAGGTGTCGAGCCACCCGATTTTCAAGCAG

>AY317098.1_O_CHA_2002

CCCTGGTACAAGCTCATCAAACTCCTAAGCCGCCTGTCGTGCATGGCCGCTGTTGCAGCACGGTCCAAGG

ACCCAGTCCTTGTGGCCATCATGTTGGCCGACACCGGTCTCGAGATTCTGGACAGCACCTTTGTGGTAAA

GAAGATCTCCGACTCGCTCTCCAGTCTCTTCCACGTGCCGGCCCCTGCCTTCAGTTTCGGAGCCCCGATC

CTGTTGGCCGGTTTGGTCAAAGTCGCCTCGAGTTTCTTCCAGTCAACGCCCGAAGACCTCGAGAGAGCAG

AAAAACAGCTCAAAGCACGTGACATCAACGACATATTTGCCGTTCTAAAGAACGGTGAGTGGCTGGTCAA

ACTGATCCTGGCCATCCGCGACTGGATTAAGGCATGGATCGCCTCAGAAGAAAAGTTCGTCACCATGACA

GACCTAGTGCCTGGTATCCTTGAAAAACAACGGGATCTCAACGACCCCGGTAAGTACAAGGAGGCCAAGG

AATGGCTGGACAACGCGCGCCAAGCGTGTCTGAAGAGCGGGAACGTCCACATTGCCAACCTGTGCAAAGT

GGTTGCTCCAGCGCCCAGCAAGTCGAGGCCCGAACCAGTGGTCGTGTGTCTTCGCGGCAAATCCGGCCAA

GGGAAGAGTTTCCTCGCGAACGTTCTCGCACAGGCAATCTCCACCCACTTCACTGGCAGGACCGACTCAG

TCTGGTACTGTCCGCCTGACCCTGACCACTTCGACGGTTACAACCAGCAGACCGTCGTCGTGATGGACGA

CCTGGGCCAGAACCCCGACGGCAAAGACTTTAAGTACTTCGCCCAGATGGTCTCCACTACGGGGTTCATC

CCGCCAATGGCCTCGCTCGAGGATAAAGGTAAACCCTTCAACAGCAAGGTCATAATAGCCACAACCAACC

TGTACTCGGGATTCACCCCAAGAACCATGGTGTGCCCCGATGCGCTCAACCGGAGGTTCCACTTTGACAT

CGACGTGAGCGCCAAAGACGGGTACAAGGTTAATAACAAACTGGACATAGTCAAAGCACTCGAAGACACC

CACACCAACCCGGTGGCGATGTTCCAGTATGACTGCGCCCTTCTCAACGGAATGGCCGTTGAAATGAAGA

GAATGCAACAAGACATGTTCAAGCCTCAACCGCCCGTCCAGAACGTCTACCAACTCGTTCAGGAGGTGAT

TGAGCGGGTGGAGCTGCACGAAAAGGTATCGAGCCACCCGATATTCAAACAG

>AY593751.1_A_NET_1942

CCCTGGTACAAGCTCATCAAGCTCCTAAGCCGCCTGTCGTGCATGGCCGCTGTGGCAGCACGGTCCAAGG

ACCCAGTCCTTGTGGCCATCATGCTGGCCGACACCGGTCTCGAGATTCTGGACAGCACTTTCGTCGTGAA

GAAAATCTCCGACTCGCTCTCCAGTCTCTTTCACGTGCCGGCCCCCGCCTTCAGTTTCGGAGCCCCGATT

CTGTTGGCCGGGTTGGTCAAGGTCGCCTCGAGTTTCTTCCGGTCCACACCCGAAGACCTTGAGAGAGCAG

AGAAACAGCTCAAAGCACGTGACATTAACGACATCTTCGCCATTCTCAAGAACGGCGAGTGGCTGGTCAA

ACTGATCCTTGCCATCCGCGACTGGATTAAGGCATGGATTGCCTCAGAAGAGAAGTTCGTCACCATGACG

GACTTGGTGCCTGGCATCCTCGAAAAGCAGCGGGACCTTAACGACCCGGGCAAGTACAAGGAAGCCAAGG

AGTGGCTCGACAACGCGCGCCAGGCGTGTTTGAAGAGCGGGAACGTCCACATTGCCAACCTGTGCAAAGT

GGTCGCCCCAGCACCCAGCAAGTCGAGACCCGAACCTGTGGTCGTTTGCCTCCGTGGCAAATCTGGCCAG

GGTAAGAGTTTCCTTGCGAACGTGCTCGCACAAGCAATTTCCACCCACTTTACTGGCAGAACCGACTCGG

TTTGGTACTGCCCGCCTGACCCTGACCACTTCGACGGTTACAACCAACAGACCGTCGTTGTGATGGACGA

TTTGGGCCAGAACCCTGATGGCAAGGACTTCAAGTACTTTGCCCAAATGGTTTCGACTACGGGGTTCATC

CCGCCCATGGCATCACTTGAGGACAAAGGTAAACCTTTCAACAGTAAGGTCATCATTGCGACCACCAACT

TGTACTCGGGCTTTACCCCGAGAACTATGGTGTGCCCCGATGCACTGAACCGAAGGTTCCACTTTGACAT

CGACGTGAGCGCCAAGGACGGGTACAAAATTAATAACAAATTGGACATCATCAAAGCACTTGAAGACACC

CACACCAACCCAGTTGCCATGTTCCAGTACGATTGTGCCCTTCTCAACGGTATGGCCGTTGAAATGAAGA

GATTGCAACAAGATATGTTTAAGCCTCAACCACCCCTCCAAAACGTGTACCAACTCGTTCAGGAGGTGAT

TGAACGGGTCGAGCTCCATGAGAAAGTGTCGAGCCACCCAATTTTCAAGCAG

>AY593753.1_A_Brazil_1970

CCCTGGTACAAGCTCATCAAGCTCCTGAGCCGCCTGTCGTGCATGGCCGCTGTAGCAGCACGGTCAAAGG

ACCCAGTCCTTGTGGCCATCATGCTGGCTGACACCGGTCTCGAGATTCTGGACAGCACCTTCGTCGTGAA

GAAGATTTCCGACTCTCTCTCCAGTCTCTTTCACGTGCCGGCCCCCGCCTTCAGTTTCGGAGCCCCGATT

CTGTTAGCCGGGCTGGTCAAGGTCGCCTCGAGTTTCTTCCGGTCCACACCCGAAGACCTTGAGAGAGCAG

AGAAACAGCTCAAAGCACGTGACATCAACGACATTTTCGCCATTCTCAAGAACGGCGAGTGGCTGGTCAA

GCTGATCCTTGCCATCCGCGACTGGGTCAAGGCATGGATTGCCTCAGAAGAAAAGTTTGTCACCATGACA

GACTTGGTGCCTGGCATCCTTGAAAAACAACGGGATCTCAACGACCCAAGCAAGTACAAGGAAGCCAAGG

AGTGGCTCGACAACGCGCGCCAAACGTGTTTGAAGAATGGGAACATTCACATTGCCAACCTGTGCAAAGT

GGTCGCTCCGGCACCCAGCAAGTCGCGACCCGAACCCGTGGTCGTTTGCCTCCGCGGCAAATCTGGCCAG

GGCAAGAGTTTCCTTGCAAACGTGCTCGCACAAGCAATCTCTACCCACTTCACCGGCAGGACTGATTCAG

TTTGGTACTGCCCGCCTGACCCTGACCACTTCGACGGTTATAACCAACAGACTGTCGTCGTGATGGACGA

CCTGGGCCAGAACCCCGACGGCAAGGACTTCAAGTACTTCGCCCAGATGGTTTCAACCACGGGGTTCATC

CCGCCTATGGCATCGCTTGAGGACAAAGGCAAACCTTTCAACAGTAAGGTCATCATTGCAACCACCAACT

TGTACTCGGGCTTCACCCCGAGGACCATGGTGTGTCCTGACGCCCTGAACCGGAGGTTTCACTTTGACAT

CGACGTGAGCGCCAAAGACGGGTACAAAATTAACAACAAATTGGACATCATCAAAGCACTTGAAGACACC

CACACCAATCCCGTGGCAATGTTTCAGTACGATTGTGCCCTTCTCAACGGCATGGCTGTAGAAATGAAGA

GAATGCAACAAGACGTGTTCAAACCTCAGCCACCCCTCCAGAACGTGTACCAACTTGTTCAGGAGGTGAT

TGAGCGGGTGGAGCTCCACGAGAAAGTGTCGAGCCACCCGATTTTCAAGCAG

>AY593754.1_A_SPA_1959

CCCTGGTACAAGCTTATCAAGCTCCTAAGCCGCCTGTCGTGCATGGCCGCTGTGGCAGCACGGTCCAAGG

ACCCGGTCCTTGTGGCCATCATGCTGGCCGACACCGGTCTCGAGATTCTGGACAGCACTTTCGTCGTGAA

GAAGATCTCCGACTCGCTCTCCAGTCTCTTCCACGTGCCGGCCCCCGTCTTCAGTTTCGGAGCCCCGAGT

CTGCTAGCCGGGTTGGTCAAGGTCGCCTCGAGTTTCTTCCGGTCCACGCCCGAAGACCTTGAGAGAGCAG

AGAAACAGCTCAAAGCACGTGACATCAACGACATTTTCGCCATTCTCAAGAACGGCGAGTGGCTGGTCAA

ACTGATCCTTGCCATCCGCGACTGGATTAAGGCGTGGATTGCCTCAGAAGAAAAGTTTGTCACTATGACA

GACTTAGTGCCTGGCATCCTTGAAAAGCAGCGGGATCTCAACGACCCAAGCAGGTACAAGGAGGCCAAGG

AGTGGCTCGACAACGCGCGCCAAGCGTGTCTGAAGAGCGGGAACGTCCACATTGCCAACCTGTGCAAAGT

GGTCGCCCCGGCACCCAGCAAGTCGAGACCCGAACCCGTGGTGGTTTGCCTCCGTGGTAAATCAGGCCAG

GGCAAGAGTTTCCTTGCGAACGTGCTCGCACAAGCAATCTCTACCCACTTCACCGGGCGGACTGACTCAG

TCTGGTACTGCCCACCTGACCCTGACCACTTCGACGGTTACAACCAACAGACTGTTGTTGTGATGGACGA

TTTGGGCCAGAATCCTGACGGCAAGGACTTCAAGTACTTCGCCCAAATGGTCTCGACCACTGGGTTCATC

CCGCCCATGGCATCACTCGAGGACAAAGGTAAACCCTTCAACAGTAAGGTCATCATTGCAACCACCAACC

TGTACTCGGGCTTCACCCCGAGGACTATGGTGTGCCCTGACGCCCTGAACCGGAGGTTTCACTTTGACAT

TGACGTGAGCGCCAAGGATGAGTACAAAATTAACAACAAATTGGACATTACCAAAGCGCTTGAAGACACC

CACACCAACCCAGTAGCAATGTTTCAGTACGACTGCGCCCTTCTCAACGGCATGGCTGTTGAAATGAAGA

GACTGCAGCAAGACATGTTCAAACCTCAACCACCTCTCCAGAACGTGTACCAACTAGTTCAGGAGGTAAT

TGACCGGGTGGCGCTCCACGAGAAGGTGTCAAGCCACCCAATTTTTAAACAG

>AY593755.1_A_TAI_1960

CCCTGGTACAAGCTCATCAAACTCCTGAGCCGCCTGTCGTGCATGGCCGCTGTGGCAGCACGGTCAAAGG

ATCCCGTCCTTGTGGCCATCATGCTAGCAGACACCGGTCTCGAGATTCTGGACAGCACGTTTGTCGTGAA

GAAGATCTCCGACTCGCTCTCCAGTCTCTTTCACGTGCCGGCCCCCGCCTTCAGTTTCGGAGCTCCGATT

CTGCTGGCTGGGTTGGTCAAAGTCGCCTCGGGGTTCTTCCGGTCAACGCCCGAAGACCTTGAGAGAGCAG

AGAAACAGCTCAAAGCACGTGACATCAACGACATCTTCGCCATCCTCAAGAATGGCGAGTGGTTGGTCAA

GTTGATCCTTGCCATCCGCGACTGGATAAAGGCATGGATCGCCTCAGAAGAGAAGTTTGTCACCATGACA

GACCTAGTGCCTGGCATCCTTGAAAAGCAGCGGGATCTCAACGACCCGAGCAAGTACAAGGAGGCCAAGG

AATGGCTCGACAACACGCGCCAGGCGTGCTTGAAGAGCGGGAACACCTGCATTGCCAATTTATGCAAGGT

GGTCGCTCCAGCACCCAGCAAGTCGAGACCCGAACCTGTAGTCGTTTGCCTCCGTGGCAAGTCCGGCCAA

GGTAAGAGTTTCCTTGCGAACGTGCTCGCGCAAGCAATCTCCACCCACTTCACCGGCAGAACTGATTCAG

TTTGGTACTGCCCACCCGACCCTGACCACTTCGACGGTTACAACCAGCAGACCGTTGTTGTGATGGATGA

TTTGGGGCAGAACCCCGACGGCAAGGACTTTAAGTACTTCGCCCAAATGGTCTCAACCACGGGGTTCATC

CCGCCCATGGCATCACTTGAAGACAAAGGCAAACCTTTCAACAGCAAAGTCATCATTGCAACCACCAATC

TCTACTCGGGCTTCACCCCGAGGACCATGGTGTGCCCTGACGCGTTGAACCGGAGGTTTCACTTTGACAT

CGACGTGAGCGCCAAGGACGGTTACACAATTAACAACAAATTGGACATCATCAAAGCACTTGAAGACACC

CACACCAACCCAGTGGCAATGTTTCAATATGACTGTGCCCTTCTCAACGGCATGGCCGTTGAAATGAAAA

GACTCCAGCAAGATGTGTTTAAGCCTCAAGCACCCCTCCAGAACGTGTACCAGCTCGTACAGGAGGTGAT

TGACCGGGTTGAACTCCACGAAAAAGTGTTGAGTCACCCGATCTTCAAGCAA

>AY593756.1_A_Brazil_1959

CCCTGGTACAAGCTCATTAAGCTTCTGAGCCGCTTGTCGTGCATGGCCGCTGTAGCAGCACGGTCAAAGG

ACCCCGTCCTTGTGGCCATCATGCTGGCTGACACCGGTCTCGAGATTCTGGACAGCACCTTCGTCGTGAA

GAAGATCTCCGACTCGCTCTCCAGTCTCTTTCACGTGCCGGCCCCCGTCTTCAGTTTCGGAGCCCCGATT

CTGTTGGCCGGGTTGGTCAAGGTCGCCTCAAGTTTCTTCCGGTCCACACCCGAAGACCTTGAGAGAGCAG

AGAAACAGCTCAAAGCACGTGACATCAACGACATTTTCGCCATTCTTAAGAACGGCGAGTGGCTGGTCAA

ATTGATCCTTGCCATCCGCGACTGGATCAAAGCATGGATCGCCGCAGAAGAGAAGTTTGTCACCATGACA

GACTTAGTGCCTGGCATCCTTGAAAAGCAGCGGGATCTCAACGACCCGAGCAAGTACAAGGAAGCCAAGG

AGTGGCTTGACAACGCGCGTGAGGTGTGTTTGAAGAACGGGAACGTCCACATTGCTAACCTGTGCAAAGT

GGTCGCCCCAGCACCCAGCAAGTCGAGACCCGAGCCCGTGGTCGTCTGCCTCCGTGGCAAGTCCGGCCAG

GGCAAGAGTTTTCTTGCGAACGTGCTCGCACAAGCAATCTCTACCCACTTCACCGGCAGAACCGACTCTG

TTTGGTACTGCCCACCTGACCCTGACCACTTCGATGGTTACAACCAACAGACCGTCGTTGTGATGGACGA

TTTGGGCCAGAACCCTGACGGCAAGGACTTCAAGTACTTCGCCCAAATGGTTTCGACCACGGGGTTTATC

CCGCCCATGGCATCACTCGAGGACAAGGGCAAACCCTTCAACAGTAAGGTCATCATCGCAACCACCAACC

TGTACTCGGGCTTCACCCCGAGGACCATGGTGTGCCCTGATGCCCTGAACCGGAGGTTTCACTTTGACAT

TGACGTGAGCGCCAAGGACGGGTACAAAATTAACAACAAATTGGACATCATCAAAGCACTTGAAGACACC

CACACCAACCCAGTGGCAATGTTTCAGTACGACTGTGCCCTTCTCAACGGCATGGCCGTTGAAATGAAGA

GAATGCAACAAGACATGTTCAAACCTCAACCACCTCTCCAGAACGTATACCAACTTGTTCAGGAGGTGAT

TGACCGGGTGGAGCTCCACGAGAAAGTGTCGAGCCACCCGATTTTTAAACAG

>AY593757.1_A_Brazil_1967

CCCTGGTACAAGCTCATCAAGCTCCTGAGCCGCCTGTCGTGCATGGCCGCTGTAGCAGCACGGTCAAAGG

ACCCAGTCCTTGTGGCCATCATGCTGGCTGACACCGGTCTCGAGATTCTGGACAGCACTTTCGTCGTGAA

GAAGATTTCCGACTCTCTCTCCAGTCTCTTTCACGTGCCGGCCCCCGTCTTCAGCTTCGGAGCCCCGATT

CTGTTAGCCGGGTTGGTCAAGGTCGCCTCGAGTTTCTTCCGGTCCACACCCGAAGACCTTGAGAGAGCAG

AGAAACAGCTCAAAGCACGTGACATCAATGACATTTTCGCCATTCTCAAGAACGGCGAGTGGCTGGTCAA

GCTGATCCTTGCCATCCGCGACTGGATCAAGGCATGGATTGCCTCAGAAGAGAAGTTTGTCACCATGACA

GACTTGGTGCCTGGCATCCTTGAAAAACAACGGGATCTCAACGACCCAAGCAAGTACAAGGAAGCCAAGG

AGTGGCTCGACAACGCGCGCCAAACGTGTTTGAAGAATGGGAACATTCACATTGCCAACCTGTGCAAAGT

GGTCGCTCCGGCACCCAGCAAGTCGCGACCCGAGCCCGTGGTCGTTTGCCTCCGCGGCAAATCCGGCCAG

GGCAAGAGTTTCCTTGCAAACGTGCTCGCACAAGCAATCTCTACCCACTTCACCGGCAGGACTGACTCAG

TTTGGTACTGCCCGCCTGACCCTGACCACTTCGACGGTTACAACCAACAGACTGTCGTTGTGATGGACGA

TTTGGGCCAGAACCCCGACGGCAAGGACTTCAAGTATTTCGCCCAGATGGTTTCAACCACGGGGTTCATC

CCGCCTATGGCATCGCTTGAGGACAAAGGCAAACCTTTCAACAGTAAGGTCATCATTGCAACCACCAACT

TGTACTCGGGCTTCACCCCGAGGACCATGGTGTGTCCTGATGCCCTGAACCGGAGGTTTCACTTTGACAT

CGACGTGAGCGCCAAAGACGGGTACAAAATTAACAACAAATTGGACATCATCAAGGCACTTGAAGACACC

CACACCAATCCCGTGGCAATGTTTCAGTACGATTGTGCCCTTCTCAACGGCATGGCTGTTGAAATGAAGA

GAATGCAACAAGACGTGTTCAAACCTCAGCCACCCCTCCAGAACGTATACCAACTTGTTCAGGAGGTGAT

TGAGCGGGTGGAGCTCCACGAGAAAGTGTCGAGCCACCCGATTTTCAAGCAG

>AY593758.1_A_VEN_1967

CCCTGGTACAAGCTCATCAAGCTCCTGAGCCGCCTGTCGTGCATGGCCGCTGTAGCAGCACGGTCAAAGG

ACCCAGTCCTTGTGGCCATCATGCTGGCTGACACCGGTCTCGAGATTCTGGACAGCACCTTCGTCGTGAA

GAAGATTTCCGACTCTCTCTCCAGTCTCTTTCACGTGCCGGCCCCCGCCTTCAGTTTCGGAGCCCCGATT

CTGTTAGCCGGGCTGGTCAAGGTCGCCTCGAGTTTCTTCCGGTCCACACCCGAAGACCTTGAGAGAGCAG

AGAAACAGCTCAAAGCACGTGACATCAACGACATTTTCGCCATTCTCAAGAACGGCGAGTGGCTGGTCAA

GCTGATCCTTGCCATCCGCGACTGGGTCAAGGCATGGATTGCCTCAGAAGAAAAGTTTGTCACCATGACA

GACTTGGTGCCTGGCATCCTTGAAAAACAACGGGATCTCAACGACCCAAGCAAGTACAAGGAAGCCAAGG

AGTGGCTCGACAACGCGCGCCAAACGTGTTTGAAGAATGGGAACATTCACATTGCCAACCTGTGCAAAGT

GGTCGCTCCGGCACCCAGCAAGTCGCGACCCGAACCCGTGGTCGTTTGCCTCCGCGGCAAATCTGGCCAG

GGCAAGAGTTTCCTTGCAAACGTGCTCGCACAAGCAATCTCTACCCACTTCACCGGCAGGACTGATTCAG

TTTGGTACTGCCCGCCTGACCCTGACCACTTCGACGGTTATAACCAACAGACTGTCGTCGTGATGGACGA

CCTGGGCCAGAACCCCGACGGCAAGGACTTCAAGTACTTCGCCCAGATGGTTTCAACCACGGGGTTCATC

CCGCCTATGGCATCGCTTGAGGACAAAGGCAAACCTTTCAACAGTAAGGTCATCATTGCAACCACCAACT

TGTACTCGGGCTTCACCCCGAGGACCATGGTGTGTCCTGACGCCCTGAACCGGAGGTTTCACTTTGACAT

CGACGTGAGCGCCAAAGACGGGTACAAAATTAACAACAAATTGGACATCATCAAAGCACTTGAAGACACC

CACACCAATCCCGTGGCAATGTTTCAGTACGATTGTGCCCTTCTCAACGGCATGGCTGTAGAAATGAAGA

GAATGCAACAAGACGTGTTCAAACCTCAGCCACCCCTCCAGAACGTGTACCAACTTGTTCAGGAGGTGAT

TGAGCGGGTGGAGCTCCACGAGAAAGTGTCGAGCCACCCGATTTTCAAGCAG

>AY593759.1_A_GER_1971

CCCTGGTACAAGCTTATTAAACTCCTAAGCCGCCTGTCGTGCATGGCCGCTGTGGCAGCACGGTCCAAGG

ACCCAGTCCTTGTGGCCATCATGCTGGCCGACACTGGTCTAGAGATTCTGGACAGCACCTTTGTCGTGAA

GAAGATCTCCGACTCACTCTCCAGTCTCTTTCACGTGCCGGCCCCCGTCTTCAGTTTCGGAGCTCCGATC

CTGTTGGCCGGGTTGGTCAAGGTCGCCTCGAGTTTCTTCCGGTCCACACCCGAAGACCTTGAGAGGGCAG

AGAAACAGCTCAAAGCACGTGACATTAACGACATTTTCGCCATTCTCAAGAACGGCGAGTGGCTGGTCAA

ACTGATCCTTGCCATCCGCGACTGGGTCAAGGCATGGATCGCCTCAGAAGAGAAGTTCGTCACCATGACG

GACTTGGTGCCTGGCATCCTCGAAAAGCAACGGGACCTTAACGACCCGAGCAAGTACAAGGAAGCCAAGG

AGTGGCTCGACAACGCGCGCCAGGCGTGCTTGAAGAGCGGGAACGTCCACATTGCCAATTTGTGCAAAGT

GGTCGCCCCAGCACCCAGCAAGTCGAGACCCGAGCCTGTGGTCGTTTGCCTCCGTGGCAAGTCTGGCCAG

GGCAAGAGTTTCCTTGCGAATGTACTCGCACAAGCAATTTCCACCCACTTTACTGGCAGAACCGACTCGG

TCTGGTACTGCCCGCCTGACCCTGACCACTTCGACGGTTACAACCAGCAGACCGTCGTTGTGATGGATGA

TTTAGGCCAGAACCCTGATGGTAAAGACTTTAAGTACTTTGCCCAAATGGTTTCAACCACAGGGTTCATT

CCGCCCATGGCGTCGCTTGAGGACAAAGGCAAACCTTTCAACAGCAAGGTCATCATCGCAACTACCAACT

TGTACTCGGGCTTCACCCCGAGGACTATGGTGTGCCCTGATGCACTGAACCGGCGGTTTCACTTTGACAT

CGACGTGAGCGCCAAGGACGGGTATAAAATCAACAACAAATTGGACATCAACAAAGCACTTGAAGACACC

CACACCAACCCAGTGGCAATGTTTCAGTACGACTGTGCCCTCCTCAACGGCATGGCCGTTGAAATGAAGA

GAATGCAACAAGACATGTTCAAGCCCCAACCGCCCCTCCAGAACGTGTACCAGCTCGTTCAGGAGGTGAT

TGAGCGGGTCGAGCTCCACGAGAAAGTGTCGAGCCACCCAATTTTCAAGCAG

>AY593760.1_A_USSR_1964

CCCTGGTATAAACTTATCAAGCTCCTGAGCCGTTTGTCGTGCATGGCCGCTGTAGCAGCACGGTCAAAGG

ATCCAGTCCTTGTGGCCATTATGCTGGCTGACACCGGCCTCGAGATTCTGGACAGTACTTTTGTCGTGAA

AAAGATCTCCGACTCACTCTCCAGTCTCTTTCACGTGCCGGCCCCCGCCTTCAGCTTCGGAGCCCCGATT

CTGCTGGCCGGGTTGGTCAAGGTCGCCTCGAGTTTCTTCCGGTCTACACCCGAAGACCTTGAGAGAGCAG

AGAAACAGCTCAAAGCGCGTGACATTAATGACATTTTCGCCATTCTCAAGAACGGCGAGTGGCTGGTCAA

GCTGATTCTTGCCATCCGCGACTGGATCAAGGCATGGGTCGCCTCAGAAGAGAAGTTTGTCACCATGACA

GACTTGGTGCCTGGCATTCTTGAAAAGCAGCGGGACCTCAACGACCCCAGCAAGTACAAGGAGGCCAAGG

AATGGCTCGACAATGCGCGACAAGCGTGTTTGAAGAGCGGAAACGTCCACATTGCCAACCTGTGCAAAGT

GGTCGCCCCGGCACCCAGCAAGTCGAGACCCGAGCCCGTGGTCGTTTGCCTCCGCGGCAAGTCCGGCCAG

GGCAAGAGTTTCCTTGCGAATGTGCTTGCACAAGCAATTTCCACCCACTTCACTGGCAAAACCGATTCAG

TTTGGTATTGCCCACCTGACCCTGACCACTTTGACGGTTACAACCAGCAGACTGTCGTAGTGATGGACGA

TTTGGGCCAGAACCCTGACGGCAAGGACTTCAAGTACTTTGCCCAAATGGTCTCAACCACGGGGTTCATC

CCGCCCATGGCTTCACTCGAAGACAAAGGCAAACCTTTTAACAGCAAGGTCATCATTGCCACCACCAACC

TGTACTCGGGTTTTACCCCGAGAACCATGGTGTGCCCTGACGCACTGAACCGAAGGTTTCACTTTGACAT

TGACGTGAGTGCCAGGGACGGGTACAAAATTAACAACAAATTGGACATAACCAAAGCTCTTGAGGACACC

CACACCAACCCAGTGGCAATGTTTAAGTACGATTGTGCCCTTCTCAACGGCATGGCTGTTGAGATGAAGA

GAATGCAACAAGACATGTTCAAGCCTCAACCACCCCTCCAGAACGTGTACCAACTTGTTCAGGAGGTGAT

TGAACGGGTCGAGCTCCACGAGAAAGTGTCGAGTCACCCGATTTTCAAGCAG

>AY593761.1_A_KEN_1964

CCCTGGTACAAGCTCATCAAACTCCTGAGCCGCTTGTCGTGCATGGCCGCTGTAGCAGCACGGTCCAAGG

ACCCAGTCCTTGTGGCCATCATGCTGGCTGACACCGGTCTTGAGATTCTGGACAGCACCTTTGTCGTGAA

AAAGATCTCCGACTCGCTCTCCAGTCTCTTTCACGTGCCGGCCCCCGTCTTCAGTTTCGGAGCCCCGATC

CTGTTGGCCGGGTTGGTCAAAGTCGCCTCGAGTTTCTTCCGGTCCACGCCCGAAGACCTCGAGAGAGCAG

AGAAACAGCTCAAAGCACGTGACATCAACGACATTTTCGCCATACTCAAGAATGGCGAGTGGCTGGTCAA

ACTGATCCTTGCCATCCGCGACTGGATTAAGGCATGGATTGCCTCAGAAGAGAAGTTTGTCACCATGACA

GACTTGGTGCCTGGCATCCTTGAGAAACAGCATGACCTCAACGACCCGAGCAAGTACAAGGAAGCCAAGG

AATGGCTCGACAACGCGCGTCAAGCGTGTCTGAAGAACGGGAACATTCACATTGCTAACCTGTGCAGAGT

GATTGCCCCAGCACCCAGCAAGTCGCGACCTGAGCCCGTGGTCGTTTGTCTCCGTGGCAAATCCGGCCAG

GGCAAAAGTTTCCTTGCAAACGTGCTCGCACAGGCAATTTCTACTCACTTCACAGGCAGAACCGACTCGG

TTTGGTACTGCCCACCTGACCCTGACCACTTCGACGGTTACAACCAGCAGACCGTTGTCGTGATGGATGA

TTTGGGCCAGAACCCCGACGGCAAGGACTTCAAGTACTTCGCCCAAATGGTTTCGACCACGGGGTTCATC

CCGCCCATGGCATCGCTCGAGGACAAAGGCAAACCCTTCAACAGCAAGGTCATCATCGCAACTACCAACC

TGTACTCGGGTTTCACCCCGAGGACTATGGTGTGCCCTGATGCGTTAAACAGGAGGTTTCACTTTGACAT

TGACGTGAGCGCCAAGGACGGGTACAAAATTAACAACAAATTGGACATCATCAAAGCACTTGAGGACACC

CACACCAACCCGGTGGCAATGTTCCAGTACGATTGTGCCCTTCTCAACGGCATGGCCGTTGAGATGAAGA

GAATGCAACAAGATGTGTTTAAGCCTCAACCGCCCCTCGAGAACGTGTACCAGCTCGTTCAAGAGGTGAT

TGAACGGGTCGAGCTCCACGAGAAAGTGTCGAGCCACCCGATCTTCAAACAG

>AY593764.1_A_IRQ_1970

CCCTGGTACAAGCTCATCAAGCTCCTGAGCCGCTTGTCATGCATGGCCGCTGTAGCAGCACGGTCAAAGG

ACCCGGTCCTTGTGGCTATTATGCTGGCTGACACCGGCCTTGAGATTCTGGACAGTACTTTTGTCGTGAA

GAAGATTTCCGACTCACTCTCCAGTCTCTTTCACGTGCCGGCCCCCGTCTTCAGTTTCGGAGCCCCGATC

CTGCTGGCCGGGTTGGTCAAAGTCGCCTCGAGTTTCTTCCGGTCTACGCCCGAAGACCTCGAGAGAGCAG

AAAAACAGCTCAAAGCACGTGACATCAATGACATATTCGCCATTCTCAAGAACGGCGAGTGGTTGGTCAA

GCTGATTCTTGCTATCCGCGACTGGATTAAAGCATGGATCGCCTCAGAAGAAAAGTTTGTCACCATGACA

GACTTGGTGCCTGGCATTCTTGAAAAGCAGCGGGACCTCAACGACCCCAGCAAGTACAAGGAGGCCAAGG

AGTGGCTCGAAAGCGCGCGTCAAGCGTGCCTGAAGAGTGGGAATGTCCACATTGCCAACCTGTGCAAAGT

GGTCACCCCAGCACCTAGCAAGTCGAGACCTGAACCCGTGGTCGTTTGCCTCCGGGGCAAATCCGGCCAG

GGAAAGAGTTTCCTTGCAAACGTGCTCGCACAGGCAATTTCAACGCATTTTACTGGCAGAACTGATTCAG

TATGGTACTGTCCACCTGACCCTGACCACTTCGACGGTTACAACCAACAGACCGTTGTTGTGATGGATGA

TTTGGGCCAGAACCCCGACGGCAAGGACTTCAAATACTTTGCCCAAATGGTTTCAACCACGGGGTTCATC

CCGCCCATGGCCTCGCTCGAAGACAAAGGAAAACCTTTCAACAGCAAAGTCATTATCACCACTACCAACT

TATACTCGGGTTTCACCCCGAGAACCATGGTGTGCCCTGACGCGCTGAACCGGAGGTTCCACTTTGACAT

TGACGTGAGCGCCAAGGACGGGTACAAAGTTAACAACAAATTGGACATAATCAAAGCTCTTGAGGACACC

CACACCAATCCAGTGGCAATGTTTCAATACGATTGTGCCCTTCTTAACGGCATGGCCGTTGAGATGAAGA

GAATGCAACAAGACATGTTTAAGCCTCAACCACCCCTCCAGAACGTGTACCAACTTGTTCAGGAGGTGAT

TGAACGGGTCGAGCTCCACGAGAAAGTGTCGAGCCACCAAATTTTTAAACAG

>AY593765.1_A_TUR_1965

CCCTGGTACAAGCTCATCAAGATCCTGAGCCGCTTGTCATGCATGGCCGCCGTAGCAGCACGGTCAAAGG

ACCCGGTCCTTGTGGCTATTATGCTGGCTGACACCGGCCTTGAGATTCTGGACAGTACTTTTGTCGTGAA

GAAGATCTCCGACTCACTCTCCAGTCTCTTTCACGTGCCGGCCCCCGTCTTCAGTTTCGGAGCCCCGATC

CTGCTGGCCGGGTTGGTCAAAGTTGCCTCGAGTTTCTTCCGGTCTACGCCCGAAGACCTTGAGAGAGCAG

AAAAACAGCTCAAAGCACGTGACATCAATGACATATTCGCCATTCTCAAGAACGGCGAGTGGTTGGTCAA

GCTGATTCTTGCTATCCGCGACTGGATTAAAGCATGGATCGCCTCAGAAGAAAAGTTTGTCACCATGACA

GACTTGGTGCCTGGCATTCTTGAAAAGCAGCGGGACCTCAACGACCCCAGCAAGTACAAGGAGGCCAAGG

AGTGGCTCGAAAGCGCGCGTCAAGCGTGCCTGAAGAGTGGGAATGTCCACATTGCCAACCTGTGCAAAGT

GGTCACCCCAGCACCTAGCAAGTCGAGACCTGAACCCGTGGTCGTTTGCCTCCGGGGCAAATCCGGCCAG

GGAAAGAGTTTCCTTGCAAACGTGCTTGCACAGGCAATTTCAACACATTTTACTGGCAGAACCGATTCAG

TTTGGTACTGTCCACCTGACCCTGACCACTTCGACGGTTACAACCAACAGACCGTTGTTGTGATGGATGA

TTTGGGCCAGAACCCCGACGGCAAGGACTTCAAATACTTTGCCCAAATGGTTTCAACCACGGGGTTTATC

CCGCCCATGGCCTCGCTCGAAGACAAAGGAAAACCCTTCAACAGCAAAGTCATTATCACCACTACCAACT

TATACTCGGGTTTCACCCCGAGAACCATGGTGTGCCCTGACGCGCTGAACCGGAGGTTCCACTTTGACAT

TGACGTGAGCGCCAAGGACGGGTACAAAGTTAACAACAAATTGGACATAATCAAAGCTCTTGAGGACACC

CACACCAATCCAGTGGCAATGTTTCAATACGATTGTGCCCTTCTTAACGGCATGGCCGTTGAGATGAAGA

GAATGCAACAAGACATGTTTAAGCCTCAACCACCCCTCCAGAACGTGTACCAACTTGTTCAGGAGGTGAT

TGAACGGGTCGAGCTCCACGAGAAAGTGTCGAGCCACCAAATTTTTAAACAG

>AY593766.1_A_KEN_1965

CCCTGGTACAAACTCATCAAGCTCCTGAGCCGTTTGTCATGCATGGCCGCTGTAGCAGCACGGTCGAAGG

ACCCAGTCCTTGTGGCCATCATGCTGGCTGACACCGGTCTTGAGATTCTGGACAGCACCTTCGTCGTGAA

GAAGATCTCCGACTCGCTCTCCAGTCTCTTTCACGTGCCGGCCCCCGTCTTCAGTTTCGGAGCCCCGATT

CTGCTGGCCGGGTTGGTCAAGGTCGCCTCGAGTTTCTTCCGGTCCACGCCCGAAGACCTTGAGAGAGCGG

AAAAACAGCTCAAAGCACGTGACATCAATGACATATTCGCCATTCTCAAGAACGGCGAGTGGCTGGTTAA

GCTGATTCTTGCCATCCGCGACTGGATCAAGGCATGGATCGCCTCAGAAGAAAAGTTTGTCACCATGACA

GACTTAGTGCCTGGCATTCTTGAAAAGCAGCGGGACCTCAACGACCCCGGCAAGTACAAGGAAGCTAAGG

AGTGGCTCGACAATGCGCGCCAAGCGTGTTTGAAGAGCGGGAATGTCCACATTGCCAACCTGTGCAAAGT

GGTCGCCCCAGCACCCAGCAGGTCGAGACCCGAACCTGTGGTCGTTTGCCTCCGTGGCAAATCCGGCCAG

GGAAAGAGTTTCCTTGCGAACGTGCTCGCACAAGCAATTTCCACACACTTCACTGGCAGAACAGACTCAG

TCTGGTACTGCCCACCTGACCCTGACCACTTTGACGGTTACAACCAACAAACCGTTGTTGTGATGGATGA

TCTGGGCCAAAACCCCGACGGCAAGGACTTCAAGTACTTCGCCCAAATGGTGTCGACCACGGGGTTCATC

CCACCCATGGCCTCGCTCGAGGACAAGGGCAAACCTTTCAACAGCAAAGTCATCATCGCCACTACCAACC

TGTACTCGGGGTTCACCCCGAGAACCATGGTGTGCCCCGATGCGCTGAACAGAAGGTTTCACTTTGACAT

TGACGTGAGTGCCAAGGATGGGTACACAATCAACAACAAATTGGACATAACCAAAGCTCTTGAAGACACC

CACACCAATCCGGTGGCAATGTTTCAATACGACTGTGCCCTTCTCAACGGCATGGCTGTTGAGATGAAGA

GAATGCAACAAGACATGTTCAAACCTCAACCACCCCTCCAGAACGTTTACCAACTTGTTCAGGAGGTAAT

TGAGCGGGTCGAGCTCCACGAGAAGGTGTCGAGCCACCCGATTTTCAAGCAG

>AY593767.1_A_ARG_1965

CCCTGGTACAAGCTCATCAAACTCCTAAGCCGCCTGTCGTGTATGGCCGCTGTGGCAGCACGGTCCAAGG

ACCCAGTTCTTGTGGCCATCATGCTGGCTGACACCGGCCTCGAGATTCTGGACAGCACCTTCGTCGTGAA

GAAGATCTCCGACTCGCTCTCCAGTCTCTTCCACGTGCCGGCCCCCGTCTTCAGTTTCGGAGCCCCGATC

TTGCTAGCCGGGTTGGTCAAGGTCGCCTCGAGTTTCTTCCGGTCCACGCCCGAAGACCTTGAGAGAGCAG

AGAAACAGCTCAAAGCACGTGACATCAACGACATCTTCGCCATTCTCAAGAACGGCGAGTGGCTGGTCAA

ACTGATCCTTGCCATCCGCGACTGGATTAAGGCTTGGATCGCCTCAGAAGAGAAGTTTGTCACCATGACA

GACTTGGTGCCTGGCATCCTTGAAAAGCAGCGGGATCTGAACGACCCGAGCAAGTACAAGGAAGCCAAGG

AGTGGCTCGACAACGCGCGCCAAGCGTGTTTGAAGAGCGGGAACGTCCACATTGCCAACCTGTGTAAAGT

GGTCGCTCCAGCACCCAGCAAGTCGAGGCCCGAACCCGTGGTTGTTTGCCTCCGCGGCAAATCTGGCCAG

GGCAAGAGCTTCCTTGCAAACGTGCTTGCACAGGCAATTTCCGCCCACTTCACCGGCAGAACCGACTCAG

TGTGGTACTGCCCACCTGACCCTGACCACTTCGACGGTTACAACCAGCAAACCGTCGTTGTGATGGATGA

TTTGGGCCAGAACCCTGACGGCAAGGACTTCAAATACTTTGCCCAAATGGTCTCGACCACAGGGTTCATC

CCGCCCATGGCATCACTCGAGGACAAAGGTAAACCTTTCAACAGCAAAGTCATCATCGCGACCACCAACT

TGTACTCGGGCTTCACCCCGAGGACTATGGTGTGTCCCGACGCACTGAACCGGAGGTTTCACTTTGACAT

CGATGTGAGTGCTAAGGATGGGTACAAAATTAACAACAAACTGGACATTATCAAAGCACTTGAAGACACC

CACACCAACCCAGTGGCAATGTTTCAATACGACTGTGCCCTTCTCAACGGCATGGCCGTTGAAATGAAGA

GAATGCAACAAGACATGTTCAAGCCTCAACCACCCCTCCAGAATGTGTACCAGCTTGTTCAGGAGGTGAT

TGATCGGGTCGAGCTCCACGAGAAAGTGTCGAGCCACCCGATCTTCAAGCAG

>AY593768.1_A_Brazil_1955

CCCTGGTACAAGCTTATCAAGCTCCTGAGCCGCCTGTCGTGCATGGCCGCTGTGGCAGCACGGTCAAAGG

ACCCAGTCCTTGTGGCCATCATGCTGGCTGACACCGGTCTCGAGATTCTGGACAGCACCTTCGTCGTGAA

GAAGATCTCCGACTCGCTCTCCAGTCTCTTCCACGTGCCGGCCCCCGTCTTCAGTTTCGGAGCCCCGATT

CTGTTAGCCGGGTTGGTCAAGGTCGCCTCGAGTTTCTTCCGGTCCACGCCCGAAGACCTTGAGAGAGCAG

AGAAACAGCTCAAAGCACGTGACATCAACGACATTTTCGCCATTCTCAAGAACGGCGAGTGGCTGGTCAA

ATTGATCCTTGCCATCCGCGACTGGATCAAGGCATGGATAGCCTCAGAAGAAAAGTTTGTCACCACGACA

GACTTGGTACCTGGCATCCTTGAAAAACAGCGGGACCTCAACGACCCAAGCAAGTACAAGGAAGCCAAGG

AGTGGCTCGACAACGCGCGCCAAGCGTGTTTGAAGAGCGGGAACGTCCACATTGCCAACCTGTGCAAAGT

GGTCGCCCCGGCACCCAGCAGGTCGAGACCCGAGCCCGTGGTCGTTTGCCTCCGTGGCAAGTCCGGTCAG

GGCAAGAGTTTCCTTGCAAACGTGCTCGCACAAGCAATCTCTACCCATTTCACTGGCAGGACCGATTCAG

TTTGGTACTGCCCGCCTGACCCTGACCACTTCGACGGTTACAACCAACAGACTGTCGTTGTGATGGACGA

TTTGGGCCAGAACCCCGACGGCAAAGACTTCAAGTACTTCGCCCAAATGGTTTCAACAACGGGGTTCATC

CCGCCCATGGCATCGCTTGAGGATAAAGGCAAACCCTTCAACAGTAAGGTCATCATAGCAACCACCAACC

TGTACTCGGGCTTCACCCCGAGGACTATGGTGTGCCCTGATGCCCTGAACCGGAGGTTTCACTTTGACAT

CGACGTGAGCGCCAAGGACGGGTACAAAATTAACAACAAATTGGACATCATCAAAGCACTTGAAGATACT

CACACCAACCCAGTGGCAATGTTTCAGTACGACTGTGCCCTTCTCAACGGCATGGCTGTTGAAATGAAGA

GAATGCAACAAGATATGTTCAAGCCTCAACCACCCCTTCAGAACGTGTACCAACTGGTTCAAGAGGTGAT

TGAGCGGGTGGAGCTCCACGAGAAGGTGTCGAGCCACCCGATTTTCAAACAG

>AY593769.1_A_ARG_1959

CCCTGGTACAAGCTTATTAAACTCCTAAGCCGCCTGTCGTGCATGGCCGCTGTGGCAGCACGGTCCAAGG

ACCCAGTCCTTGTGGCCATCATGCTGGCCGACACCGGTCTCGAGATTCTGGACAGCACCTTTGTCGTGAA

GAAGATCTCCGACTCGCTCTCCAGTCTCTTTCACGTGCCGGCCCCCGTCTTCAGTTTCGGAGCTCCGATC

CTGCTGGCCGGGTTGGTCAAAGTCGCCTCGAGTTTCTTCCGGTCCACACCCGAAGACCTTGAGAGAGCAG

AGAAACAGCTCAAAGCACGTGACATCAACGACATCTTCGCCATTCTCAAGAACGGCGAGTGGCTGGTCAA

ACTGATCCTCGCTATCCGCGACTGGATTAAGGCTTGGATCGCCTCAGAAGAGAAGTTTGTCACCATGACA

GACTTGGTGCCTGGCATCCTTGAAAAGCAGCGGGATCTCAACGACCCGAGCAAGTACAAGGAAGCCAAGG

AATGGCTCGACAACGCACGCCAAGCGTGCTTGAAGAACGGGAACGTCCACATTGCCAACCTGTGCAAAGT

GGTCGCCCCGGCACCCAGCAAGCCGAGACCCGAGCCCGTGGTCGTTTGCCTCCGCGGCAAATCCGGCCAG

GGCAAGAGTTTCCTTGCGAACGTGCTCGCGCAAGCAATCTCCACCCACTTCACCGGCAGAACCGATTCGG

TTTGGTACTGCCCGCCTGACCCCGACCACTTCGACGGTTACAACCAACAGGCCGTTGTTGTGATGGATGA

TTTGGGCCAGAACCCTGACGGCAAGGACTTCAAGTACTTCGCCCAAATGGTTTCAACCACAGGGTTCATC

CCGCCCATGGCATCGCTCGAGGACAAAGGCAAACCTTTCAACAGCAAGGTCATCATCGCCACCACAAACT

TGTACTCGGGTTTCACCCCGAGGACCATGGTGTGCCCTGATGCGCTGAATCGGAGGTTTCACTTTGACAT

TGACGTGAGCGCCAAGGACGGGTACAAAATTAATGAGAAATTGGACATCAACAAAGCACTTGAAGACACG

CACACTAACCCAGTGGCGATGTTTCAGTACGATTGTGCCCTTCTCAACGGTATGGCCGTTGAAATGAAGA

GAATGCAACAGAATGTGTTCAAGCCTCTACCACCCCTCCAAAACGTTTACCAGCTCGTTCAGGAGGTGAT

TGAACGGGTCGAGCTCCACGAGAAGGTGTCGAGCCACCCAATTTTTAAACAA

>AY593770.1_A_ARG_1966

CCCTGGTACAAGCTCATTAAGCTCCTGAGCCGCTTGTCGTGCATGGCCGCTGTAGCAGCACGGTCAAAGG

ACCCGGTCCTTGTGGCTATCATGCTGGCTGACACCGGTCTCGAGATTCTGGACAGCACCTTCGTCGTGAA

GAAGATCTCCGACTCGCTCTCCAGTCTCTTCCACGTGCCGGCCCCCGTCTTCAGTTTCGGAGCTCCGGTT

CTGTTAGCCGGGTTGGTTAAAGTCGCCTCGAGTTTCTTCCGGTCCACACCCGAGGAGCTCGAGAGAGCAG

AGAAACAGCTCAAAGCACGTGACATCAACGACGTTTTCGCCATTCTCAAGAACGGCGAATGGCTGGTCAA

GTTGATCCTTGCCATCCGCGACTGGATCAAGGCATGGATCGCCTCAGAAGAAAAGTTTGTCACCATGACA

GACTTGGTGCCTGGAATCCTTGAAAAGCAGCGGGACCTCAACGACCCGAGCAAGTACAAGGAGGCCAAGG

AGTGGCTCGACAACGCGCGCCAGGCGTGTCTGAAGAGCGGGAATATCCACATTGCCAACCTGTGCAAGGT

GGTCGCCCCAGCACCCAGCAAGTCGAGACCTGAACCCGTGGTCGTTTGCCTCCGTGGCAAATCTGGCCAG

GGCAAGAGTTTCCTTGCGAACGTGCTCGCACAAGCAATCTCCACTCACTTCACCGGCAGAACCGACTCCG

TTTGGTACTGCCCACCTGACCCTGACCACTTCGATGGTTACAACCAACAGACCGTCGTTGTGATGGACGA

CCTGGGCCAGAACCCTGATGGCAAGGACTTCAAGTACTTTGCTCAAATGGTTTCAACCACGGGGTTCATC

CCGCCCATGGCGTCGCTCGAGGACAAAGGCAAACCCTTCAACAGTAGGGTCATTATCGCGACCACCAACT

TGTACTCAGGCTTCACCCCGAGGACCATGGTCTGCCCTGATGCCCTGAACCGGAGGTTTCACTTTGACAT

CGACGTGAGCGCCAAGGACGGGTACAAAACCAACAACAGATTGGACATCACCAAAGCACTTGAAGACACC

CACACTAACCCAGTGGCAATGTTTCAGTATGACTGTGCCCTTCTCAACGGCATGGCTGTTGAAATGAAGA

GACTGCAACAAGACATGTTCAAGCCCCAACCACCCCTCCAGAACGTGTACCAACTTGTTCAGGAGGTGAT

TGAACGGGTGGAGCTCCACGAGAAAGTGTCGAGCCACCCGATTTTCAAGCAG

>AY593771.1_A_COL_1967

CCCTGGTACAAGCTCATCAAACTCCTAAGCCGCCTGTCGTGCATGGCCGCTGTGGCAGCACGGTCCAAGG

ACCCAGTCCTTGTGGCCATCATGCTGGCCGACACCGGCCTTGAGATTCTGGACAGCACCTTTGTCGTGAA

GAAGATCTCCGACTCGCTCTCCAGTCTCTTTCACGTGCCGGCCCCCGTCTTCAGTTTCGGAGCCCCGATT

CTGCTAGCCGGGCTGGTCAAGGTCGCCTCGACTTTCTTCCGGTCCACGCCCGAAGACCTTGAGAGAGCAG

AGAAGCAACTCAAAGCACGTGACATCAACGACATTTTCGCCATTCTCAAGAACGGCGAATGGCTGGTCAA

ACTGATCCTTGCCATCCGCGACTGGATCAAGGCTTGGATTGCCTCAGAAGAAAAGTTTGTCACCATGACA

GACTTAGTGCCTGGCATCCTTGAAAAGCAGCGGGACCTCAACGACCCAGGCAAGTACAAGGAAGCCAAGG

AGTGGCTTGACAACGCGCGCCAAGCGTGTTTGAAGAACGGGAACGTCCACATTGCCAACCTGTGCAAGGT

GGTCACCCCGGCACCCAGCAAGTCGAGACCCGAACCCGTGGTGGTTTGTCTCCGTGGCAAATCAGGCCAG

GGCAAGAGTTTCCTTGCGAACGTGCTCGCACAAGCAATCTCTACCCACTTTACCGGGCGGACCGATTCAG

TTTGGTACTGCCCTCCTGACCCTGACCACTTCGATGGTTACAACCAACAGACCGTCGTCGTGATGGATGA

CTTGGGCCAGAACCCTGACGGCAAAGACTTTAAGTACTTCGCCCAAATGGTTTCGACCACGGGGTTCATC

CCTCCCATGGCATCACTAGAGGACAAAGGCAAACCCTTCAACAGTAAGGTCATCATCGCGACCACCAACC

TGTACTCGGGCTTCACCCCGAGGACTATGGTGTGTCCTGACGCCCTGAACCGGAGGTTTCACTTTGACAT

CGACGTGAGCGCCAAGGACGGGTACAAAATTAATAACAAATTGGACATCATCAAAGCACTTGAAGACACC

CACACTAACCCAGTGGCAATGTTCCAGTACGACTGTGCCCTTCTCAATGGCATGGCTGTTGAAATGAAGA

GAATGCAACAAGATATGTTCAAGCCTCAACCACCCCTCCAGAACGTATACCAACTGGTTCAGGAGGTGAT

TGATCGGGTGGAGCTCCACGAGAAAGTGTCGAGCCACCCAATTTTCAAGCAG

>AY593772.1_A_TUR_1972

CCCTGGTATAAACTCATCAAGCTCCTGAGCCGCTTGTCATGCATGGCCGCTGTAGCAGCACGGTCAAAGG

ACCCGGTCCTTGTGGCTATTATGCTGGCTGACACCGGCCTTGAGATTCTGGACAGTACCTTTGTCGTGAA

GAAGATCTCCGACTCACTCTCCAGTCTCTTCCACGTGCCGGCCCCCGTCTTCAGTTTCGGAGCTCCGATC

CTGTTGGCCGGATTGGTCAAAGTCGCCTCGAGTTTCTTCCGGTCAACGCCCGAAGACCTTGAGAGAGCAG

AAAAACAGCTCAAAGCACGTGACATCAATGACATATTCGCCATTCTCAAGAACGGCGAGTGGTTGGTCAA

GCTGATTCTTGCTATCCGCGACTGGATTAAAGCATGGATCGCCTCAGAAGAAAAATTTGTCACCATGACA

GATTTGGTGCCTGGCATCCTTGAAAAACAACGGGACCTTAACGACCCCAGCAAGTATAAGGAGGCCAAGG

AGTGGCTCGAGAGCGCGCGCCAAGCGTGCCTGAAGAGTGGGAATGTCCACATTGCCAACTTGTGCAAAGT

GTCCACCCCAGCACCTAGCAAGTCGAGACCTGAACCCGTGGTCGTTTGCCTCCGGGGCAAATCCGGCCAG

GGAAAGAGTTTCCTTGCAAACGTGCTTGCACAGGCAATTTCAACACACTTTACTGGCAGAACTGATTCAG

TTTGGTACTGTCCGCCTGACCCTGACCACTTCGACGGTTACAACCAACAGACCGTTGTTGTGATGGATGA

TTTGGGCCAGAACCCCGACGGCAAGGACTTCAAATACTTCGCCCAAATGGTTTCAACCACGGGGTTCATC

CCGCCCATGGCCTCGCTCGAGGACAAAGGAAAACCTTTCAACAGCAAAGTCATCATCACCACTACCAACT

TGTACTCGGGTTTCACCCCGAGAACTATGGTGTGCCCTGACGCGCTGAACCGGAGGTTCCACTTTGACAT

CGACGTGAGTGCCAAGGACGGATACAAAGTTAACAACAAATTGGACATAACCAAAGCTCTTGAGGACACC

CACACCAATCCAGTGGCAATGTTTCAGTACGATTGTGCCCTTCTTAACGGCATGGCCGTTGAAATGAAGA

GAATGCAACAAGACATGTTTAAACCTCAACCACCCCTCCAGAACGTGTACCAACTTGTTCAGGAGGTGAT

TGAACGGGTCGAGCTCCACGAGAAAGTGTCGAGCCACCAAATTTTTAAACAG

>AY593773.1_A_PER_1969

CCCTGGTACAAGCTCATCAAGCTCCTAAGCCGCCTGTCGTGTATGGCCGCTGTGGCAGCACGGTCCAAGG

ACCCAGTCCTTGTGGCCATCATGCTGGCCGACACCGGTCTCGAGATTCTGGACAGCACCTTCGTCGTGAA

GAAGATCTCCGACTCGCTCTCCAGTCTCTTTCACGTGCCGGCCCCCGTCTTCAGTTTCGGAGCACCGATC

CTGTTGGCCGGGTTGGTCAAAGTCGCCTCGAGTTTCTTCCGGTCCACATCCGAAGACCTTGAGAGAGCAG

AAAAACAGCTCAGAGCACGTGACATCAACGACATCTTCGCCATTCTCAAGAACGGCGAGTGGCTGGTCAA

ATTGATCCTCGCCATCCGCGACTGGATTAAGGCTTGGATCGCCTCAGAAGAGAAGTTTGTCACCATGACA

GACTTGGTGCCTGGCATCCTTGAAAAGCAGCGGGACCTGAACGACCCGAGCAAGTACAAGGAAGCCAAGG

AGTGGCTCGACAACGCGCGCCAGGCGTGTTTGAAGAACGGGAATGTCCACATTGCCAACCTGTGCAAAGT

GGTCGCCCCGGCACCCAGCAAGTCGAGACCCGAGCCTGTGGTCGTTTGCCTCCGTGGCAAATCTGGCCAG

GGCAAGAGTTTCCTTGCGAACGTGCTTGCACAAGCAATCTCCACCCACTTTACCGGCAGAACCGACTCTG

TTTGGTACTGTCCGCCTGACCCCGACCACTTCGACGGCTACAACCAACAGACCGTCGTTGTGATGGATGA

TTTGGGCCAGAATCCCGATGGCAAGGATTTCAAGTACTTCGCCCAAATGGTTTCCACCACAGGGTTCATT

CCGCCCATGGCGTCACTCGAGGATAAGGGTAAACCTTTCAACAGTAAGGTCATCATCGCAACCACCAACC

TGTACTCGGGTTTCACCCCGAGGACTATGGTGTGCCCTGACGCCCTGAACCGGAGGTTTCACTTTGACAT

TGACGTGACTGCCAAGGACGGGTACACAATTAACAACAAATTGGACATAATTAAAGCACTTGAAGACACC

CACACCAACCCGGTGGCAATGTTCCAATATGATTGTGCCCTTCTCAACGGCATGGCCGTTGAAATGAAGA

GATTGCAACAAGACATGTTCAAGCCTCAGCCGCCCCTCCAGAACGTTTACCAACTTGTTCAGGAGGTGAT

TGAACGGGTTGAGCTCCACGAGAAAGTGTCGAGCCACCCGATTTTCAAGCAG

>AY593774.1_A_SPA_1969

CCCTGGTACAAGCTCATCAAACTCCTAAGCCGCCTGTCGTGCATGGCCGCTGTGGCAGCACGGTCCAAGG

ACCCAGTCCTTGTGGCCATCATGCTGGCCGACACCGGTCTTGAGATTCTGGACAGCACCTTTGTCGTGAA

GAAGATTTCCGACTCGCTCTCCAGTCTCTTTCACGTGCCGGCCCCCGCCTTCAGCTTCGGAGCCCCGATC

CTGTTGGCTGGGTTGGTCAAGGTCGCCTCGAGTTTCTTCCGGTCCACACCCGAAGACCTCGAGAGAGCAG

AGAAACAGCTCAAAGCACGTGACATCAACGACATTTTCGCCATTCTCAAGAACGGCGAGTGGCTAGTCAA

GTTGATCCTTGCCATCCGCGACTGGATCAAGGCATGGATTGCCTCAGAAGAGAAGTTTGTCACCATGACA

GACTTGGTACCTGGTATCCTTGAAAAGCAGCGGGACCTTAACGACCCAAGCAAGTACACGGAGGCCAAGG

AGTGGCTCGACAACGCGCGCCAGGCGTGTTTGAAGAGTGGGAACGTCCACATTGCCAACCTGTGCAAAGT

GGTTGCCCCAGCACCCAGCAAGTCGAGACCCGAACCCGTGGTCGTTTGCCTCCGTGGCAAATCCGGCCAG

GGCAAGAGTTTCCTTTCAAACGTGCTCGCTCAAGCAATCTCTACCCACTTCACCGGCAGGACCGATTCAG

TTTGGTACTGTCCGCCTGACCCTGACCACTTCGACGGTTACAACCAGCAGACCGTTGTTGTGATGGACGA

TTTGGGCCAGAACCCTGATGGCAAGGACTTCAAGTACTTTGCCCAAATGGTTTCAACTACAGGGTTCATC

CCGCCCATGGCGTCGCTTGAGGACAAAGGCAAACCTTTCAACAGCAAGGTCATCATTGCAACCACCAACC

TGTACTCGGGCTTCACCCCGAGGACTATGGTGTGTCCTGATGCACTGAACCGGAGGTTTCACTTTGACAT

CGACGTGAGTGCCAAGGACGGGTACAAAATTAACAACAAATTGGACATCATCAAAGCACTTGAAGACACC

CACACCAACCCAGTGGCAATGTTTCAGTACGATTGTGCCCTTCTCAACGGCATGGCTGTTGAAATGAAGA

GAATGCAACAAGACGTGTTCAAGCCTCAACCGCCCCTCCAGAACGTGTACCAGCTCGTTCAGGAGGTGAT

TGAACGGGTCGAGCTCCACGAGAAAGTGTCGAGCCACCCAATTTTCAAGCAG

>AY593775.1_A_VEN_1970

CCCTGGTACAAGCTCATCAAGCTCCTAAGCCGCCTGTCGTGTATGGCCGCTGTGGCAGCACGGTCCAAGG

ACCCAGTCCTTGTGGCCATCATGCTGGCCGACACCGGTCTCGAGATTCTGGACAGCACCTTCGTCGTGAA

GAAGATCTCCGACTCGCTCTCCAGTCTCTTTCACGTGCCGGCCCCCGTCTTCAGTTTCGGAGCACCGATC

CTGTTGGCCGGGTTGGTCAAAGTCGCCTCGAGTTTCTTCCGGTCCACATCCGAAGACCTTGAGAGAGCAG

AAAAACAGCTCAGAGCACGTGACATCAACGACATCTTCGCCATTCTCAAGAACGGCGAGTGGCTGGTCAA

ATTGATCCTCGCCATCCGCGACTGGATTAAGGCTTGGATCGCCTCAGAAGAGAAGTTTGTCACCATGACA

GACTTGGTGCCTGGCATCCTTGAAAAGCAGCGGGACCTGAACGACCCGAGCAAGTACAAGGAAGCCAAGG

AGTGGCTCGACAACGCGCGCCAGGCGTGTTTGAAGAACGGGAATGTCCACATTGCCAACCTGTGCAAAGT

GGTCGCCCCGGCACCCAGCAAGTCGAGACCCGAGCCTGTGGTCGTTTGCCTCCGTGGCAAATCTGGCCAG

GGCAAGAGTTTCCTTGCGAACGTGCTTGCACAAGCAATCTCCACCCACTTTACCGGCAGAACCGACTCTG

TTTGGTACTGTCCGCCTGACCCCGACCACTTCGACGGCTACAACCAACAGACCGTCGTTGTGATGGATGA

TTTGGGCCAGAATCCCGATGGCAAGGATTTCAAGTACTTCGCCCAAATGGTTTCCACCACAGGGTTCATT

CCGCCCATGGCGTCACTCGAGGATAAGGGTAAACCTTTCAACAGTAAGGTCATCATCGCAACCACCAACC

TGTACTCGGGTTTCACCCCGAGGACTATGGTGTGCCCTGACGCCCTGAACCGGAGGTTTCACTTTGACAT

TGACGTGACTGCCAAGGACGGGTACACAATTAACAACAAATTGGACATAATTAAAGCACTTGAAGACACC

CACACCAACCCGGTGGCAATGTTCCAATATGATTGTGCCCTTCTCAACGGCATGGCCGTTGAAATGAAGA

GATTGCAACAAGACATGTTCAAGCCTCAGCCGCCCCTCCAGAACGTTTACCAACTTGTTCAGGAGGTGAT

TGAACGGGTTGAGCTCCACGAGAAAGTGTCGAGCCACCCGATTTTCAAGCAG

>AY593776.1_A_GER_1968

CCCTGGTACAAGCTTATCAAACTCCTAAGCCGCCTGTCGTGCATGGCCGCTGTGGCAGCACGGTCCAAGG

ACCCAGTCCTTGTGGCCATCATGCTGGCCGACACCGGTCTCGAGATTCTGGACAGCACTTTCGTCGTGAA

GAAGATCTCCGACTCGCTCTCCAGTCTCTTCCACGTGCCGGCCCCCGTCTTCAGTTTCGGAGCCCCGATT

CTGCTAGCCGGGCTGGTCAAGGTCGCCTCGAGTTTCTTCCGGTCCACACCCGAAGACCTTGAGAGAGCAG

AGAAACAGCTCAAAGCACGTGACATCAACGACATTTTCGCCATTCTCAAGAACGGCGAGTGGCTGGTCAA

ACTAATCCTTGCCATCCGCGACTGGATCAAGGCTTGGATTGCCTCAGAAGAAAAGTTTGTCACCATGACA

GACTTAGTGCCTGGCATCCTTGAAAAGCAGCGGGACCTCAACGACCCAAGTAAGTACAAGGAGGCCAAGG

AGTGGCTCGACAACGCGCGCCAAGCGTGTCTGAAGAGCGGGAACGTCCACATTGCCAACCTGTGCAAAGT

GGTCGCCCCGGCACCCAGCAAGTCGAGACCCGAACCCGTGGTGGTTTGCCTCCGTGGCAAATCAGGCCAG

GGTAAGAGTTTCCTTGCGAACGTGCTCGCGCAAGCAATCTCTACCCACTTCACCGGGCGGACCGATTCAG

TCTGGTACTGCCCACCTGACCCTGATCACTTCGACGGTTACAACCAACAGACTGTCGTTGTGATGGACGA

TTTGGGCCAGAACCCTGACGGCAAGGACTTCAAGTACTTCGCCCAAATGGTTTCGACCACGGGGTTCATC

CCGCCCATGGCATCACTCGAGGACAAAGGTAAACCCTTCAACAGTAAGGTCATCATTGCAACCACCAACC

TGTACTCGGGCTTCACCCCGAGGACTATGGTGTGCCCTGACGCCCTGAACCGGAGGTTTCACTTTGACAT

TGACGTGAGCGCCAAGGATGGGTACAAAATTAACAACAAGTTGGACATTATCAAAGCACTTGAAGACACC

CACACCAACCCAGTGGCAATGTTTCAGTACGACTGTGCCCTTCTCAACGGCATGGCTGTTGAAATGAAGA

GAATGCAGCAAGACATGTTCAAACCTCAACCACCCCTCCAGAACGTGTACCAACTAGTTCAGGAGGTGAT

TGATCGGGTGGAGCTCCACGAGAAAGTGTCGAGCCACCAAATTTTTAAGCAG

>AY593777.1_A_GER_1972

CCCTGGTACAAGCTCATCAAACTCCTAAGCCGCCTGTCGTGCATGGCCGCTGTGGCAGCACGGTCCAAGG

ACCCAGTCCTTGTGGCCATCATGCTGGCCGACACCGGTCTTGAGATTCTGGACAGCACCTTTGTCGTGAA

GAAGATTTCCGACTCGCTCTCCAGTCTCTTTCACGTGCCGGCCCCCGCCTTCAGCTTCGGAGCCCCGATC

CTGTTGGCTGGGTTGGTCAAGGTCGCCTCGAGTTTCTTCCGGTCCACACCCGAAGACCTCGAGAGAGCAG

AGAAACAGCTCAAAGCACGTGACATCAACGACATTTTCGCCATTCTCAAGAACGGCGAGTGGCTAGTCAA

GTTGATCCTTGCCATCCGCGACTGGATCAAGGCATGGATTGCCTCAGAAGAGAAGTTTGTCACCATGACA

GACTTGGTACCTGGTATCCTTGAAAAGCAGCGGGACCTTAACGACCCAAGCAAGTACACGGAGGCCAAGG

AGTGGCTCGACAACGCGCGCCAGGCGTGTTTGAAGAGTGGGAACGTCCACATTGCCAACCTGTGCAAAGT

GGTTGCCCCAGCACCCAGCAAGTCGAGACCCGAACCCGTGGTCGTTTGCCTCCGTGGCAAATCCGGCCAG

GGCAAGAGTTTCCTTGCAAACGTGCTCGCTCAAGCAATCTCTACCCACTTCACCGGCAGGACCGATTCAG

TTTGGTACTGTCCGCCTGACCCTGACCACTTCGACGGTTATAACCAGCAGACCGTTGTTGTGATGGACGA

TTTGGGCCAGAACCCTGATGGCAGGGACTTCAAGTACTTTGCCCAAATGGTTTCAACTACAGGGTTCATC

CCGCCCATGGCGTCGCTTGAGGACAAAGGCAAACCTTTCAACAGCAAGGTCATCATTGCAACCACCAACC

TGTACTCGGGCTTCACCCCGAGGACTATGGTGTGTCCTGATGCACTGAACCGGAGGTTTCACTTTGACAT

CGACGTGAGTGCCAAGGACGGGTACAAAATTAACAACAAATTGGACATCATCAAAGCACTTGAAGACACC

CACACCAACCCAGTGGCAATGTTTCAGTACGATTGTGCCCTTCTCAACGGCATGGCTGTTGAAATGAAGA

GAATGCAACAAGACGTGTTCAAGCCTCAACCGCCCCTCCAGAACGTGTACCAGCTCGTTCAGGAGGTGAT

TGAACGGGTCGAGCTCCACGAGAAAGTGTCGAGCCACCCAATTTTCAAGCAG

>AY593778.1_A_SPA_1969

CCCTGGTACAAGCTTATCAAGCTCCTAAGCCGCCTGTCGTGCATGGCCGCTGTGGCAGCACGGTCCAAGG

ACCCGGTCCTTGTGGCCATCATGCTGGCCGACACCGGTCTCGAGATTCTGGACAGCACTTTCGTCGTGAA

GAAGATCTCCGACTCGCTCTCCAGTCTCTTCCACGTGCCGGCCCCCGTCTTCAGTTTCGGAGCCCCGGCT

CTGCTAGCCGGGTTGGTCAAGGTCGCCTCGAGTTTCTTCCGGTCCACGCCCGAAGACCTTGAGAGAGCAG

AGAAACAGCTCAAAGCACGTGACATCAACGACATTTTCGCCATTCTCAAGAACGGCGAGTGGCTGGTCAA

ACTGATCCTTGCCATCCGCGACTGGATTAAGGCGTGGATTGCCTCAGAAGAAAAGTTTGTCACTATGACA

GACTTAGTGCCTGGCATCCTTGAAAAGCAGCGGGATCTCAACGACCCAAGCAGGTACAAGGAGGCCAAGG

AGTGGCTCGACAACGCGCGCCAAGCGTGTCTGAAGAGCGGGAACGTCCACATTGCCAACCTGTGCAAAGT

GGTCGCCCCGGCACCCAGCAAGTCGAGACCCGAACCCGTGGTGGTTTGCCTCCGTGGTAAATCAGGCCAG

GGCAAGAGTTTCCTTGCGAACGTGCTCGCACAAGCAATCTCTACCCACTTCACCGGGCGGACTGACTCAG

TCTGGTACTGCCCACCTGACCCTGACCACTTCGACGGTTACAACCAACAGACTGTTGTTGTGATGGACGA

TTTGGGCCAGAATCCTGACGGCAAGGACTTCAAGTACTTCGCCCAAATGGTCTCGACCACTGGGTTCATC

CCGCCCATGGCATCACTCGAGGACAAAGGTAAACCCTTCAACAGTAAGGTCATCATTGCAACCACCAACC

TGTACTCGGGCTTCACCCCGAGGACTATGGTGTGCCCTGACGCCCTGAACCGGAGGTTTCACTTTGACAT

TGACGTGAGCGCCAAGGATGAGTACAAAATTAACAACAAATTGGACATTACCAAAGCGCTTGAAGACACC

CACACCAACCCAGTAGCAATGTTTCAGTACGACTGCGCCCTTCTCAACGGCATGGCTGTTGAAATGAAGA

GACTGCAGCAAGACATGTTCAAACCTCAACCACCTCTCCAGAACGTGTACCAACTAGTTCAGGAGGTAAT

TGACCGGGTGGCGCTCCACGAGAAGGTGTCAAGCCACCCAATTTTTAAACAG

>AY593779.1_A_GER_1972

CCCTGGTACAAGCTCATCAAACTCCTAAGCCGCCTGTCGTGCATGGCCGCTGTGGCAGCACGGTCCAAGG

ACCCAGTCCTTGTGGCCATCATGCTGGCCGACACCGGTCTTGAGATTCTGGACAGCACCTTTGTCGTGAA

GAAGATTTCCGACTCGCTCTCCAGTCTCTTTCACGTGCCGGCCCCCGCCTTCAGCTTCGGAGCCCCGATC

CTGTTGGCTGGGTTGGTCAAGGTCGCCTCGAGTTTCTTCCGGTCCACACCCGAAGACCTCGAGAGAGCAG

AGAAACAGCTCAAAGCACGTGACATCAACGACATTTTCGCCATTCTCAAGAACGGCGAGTGGCTAGTCAA

GTTGATCCTTGCCATCCGCGACTGGATCAAGGCATGGATTGCCTCAGAAGAGAAGTTTGTCACCATGACA

GACTTGGTACCTGGTATCCTTGAAAAGCAGCGGGACCTTAACGACCCAAGCAAGTACACGGAGGCCAAGG

AGTGGCTCGACAACGCGCGCCAGGCGTGTTTGAAGAGTGGGAACGTCCACATTGCCAACCTGTGCAAAGT

GGTTGCCCCAGCACCCAGCAAGTCGAGACCCGAACCCGTGGTCGTTTGCCTCCGTGGCAAATCCGGCCAG

GGCAAGAGTTTCCTTGCAAACGTGCTCGCTCAAGCAATCTCTACCCACTTCACCGGCAGGACCGATTCAG

TTTGGTACTGTCCGCCTGACCCTGACCACTTCGACGGTTACAACCAGCAGACCGTTGTTGTGATGGACGA

TTTGGGCCAGAACCCTGATGGCAAGGACTTCAAGTACTTTGCCCAAATGGTTTCAACTACAGGGTTCATC

CCGCCCATGGCGTCGCTTGAGGACAAAGGCAAACCTTTCAACAGCAAGGTCATCATTGCAACCACCAACC

TGTACTCGGGCTTCACCCCGAGGACTATGGTGTGTCCTGATGCACTGAACCGGAGGTTTCACTTTGACAT

CGACGTGAGTGCCAAGGACGGGTACAAAATTAACAACAAATTGGACATCATCAAAGCACTTGAAGACACC

CACACCAACCCAGTGGCAATGTTTCAGTACGATTGTGCCCTTCTCAACGGCATGGCTGTTGAAATGAAGA

GAATGCAACAAGACGTGTTCAAGCCTCAACCGCCCCTCCAGAACGTGTACCAGCTCGTTCAGGAGGTGAT

TGAACGGGTCGAGCTCCACGAGAAAGTGTCGAGCCACCCAATTTTCAAGCAG

>AY593780.1_A_FRA_1960

CCCTGGTACAAGCTTATCAAACTCCTAAGCCGCCTGTCGTGCATGGCCGCTGTGGCAGCACGGTCCAAGG

ACCCGGTCCTTGTGGCCATCATGCTGGCCGACACCGGTCTCGAGATTCTGGACAGCACTTTCGTCGTGAA

GAAGATCTCCGACTCGCTCTCCAGTCTCTTTCACGTGCCGGCCCCCGTCTTCAGTTTCGGAGCCCCGATT

CTGCTAGCCGGGCTGGTCAAGGTCGCCTCGAGTTTCTTCCGGTCCACGCCCGAAGACCTTGAGAGAGCAG

AGAAACAGCTCAAAGCACGTGACATCAACGACATTTTCGCCATTCTCAAGAACGGCGAGTGGCTGGTCAA

ACTGATCCTTGCCATCCGCGACTGGATTAAGGCTTGGATTGCCTCAGAAGAAAAGTTTGTCACCATGACA

GACTTAGTGCCTGGCATCCTTGAAAAGCAGCGGGACCTCAACGACCCAAGCAAGTACAAGGAGGCCAAGG

AGTGGCTCGACAACGCGCGCCAAGCGTGTCTGAAGAGCGGGAACGTCCACATTGCCAACCTGTGCAAAGT

GGTCGCCCCGGCACCCAGCAAGTCGAGACCCGAACCCGTGGTGGTTTGCCTCCGTGGCAAATCAGGCCAG

GGCAAGAGTTTCCTTGCGAACGTGCTCGCACAAGCAATCTCTACCCACTTCACCGGGCGGACTGATTCAG

TCTGGTACTGCCCACCTGACCCTGACCACTTCGACGGTTACAACCAACAGACTGTCGTTGTGATGGACGA

TTTGGGCCAGAACCCTGACGGCAAGGACTTCAAGTACTTCGCCCAAATGGTCTCGACCACGGGGTTCATC

CCGCCCATGGCATCACTTGAGGACAAAGGTAAACCCTTCAACAGTAAGGTCATCATTGCAACCACCAACC

TGTACTCGGGCTTCACCCCGAGGACTATGGTGTGCCCTGACGCCCTGAACCGGAGGTTTCACTTTGACAT

TGACGTGAGCGCCAAGGATGGGTACAAAATTAACAACAAATTGGACATTATCAAAGCACTTGAAGACACC

CACACCAACCCAGTGGCAATGTTTCAGTACGACTGCGCCCTTCTCAACGGCATGGCTGTTGAAATGAAGA

GACTTCAGCAAGACATGTTCAAACCTCAACCACCCCTCCAGAACGTGTACCAACTAGTTCAGGAGGTGAT

TGATCGGGTGGAGCTCCACGAGAAAGTGTCGAGCCACCCAATTTTTAAGCAG

>AY593781.1_A_GER_1951

CCCTGGTACAAGCTTATCAAACTCCTAAGCCGCCTGTCGTGCATGGCCGCTGTGGCAGCACGGTCCAAGG

ACCCAGTCCTTGTGGCCATCATGCTGGCCGACACCGGTCTCGAGATTCTGGACAGCACTTTCGTCGTGAA

GAAGATCTCCGACTCGCTCTCCAGTCTCTTCCACGTGCCGGCCCCCGTCTTCAGTTTCGGAGCCCCGATT

CTGCTAGCCGGGCTGGTCAAGGTCGCCTCGAGTTTCTTCCGGTCCACGCCCGAAGACCTTGAGAGAGCAG

AGAAACAGCTCAAAGCACGTGACATCAACGACATTTTCGCCATTCTCAAGAACGGCGAGTGGCTGGTCAA

ACTGATCCTTGCCATCCGCGACTGGATTAAGGCTTGGATTGCCTCAGAAGAAAAGTTTGTCACCATGACA

GACTTAGTGCCTGGCATCCTTGAAAAGCAGCATGACCTCAACGACCCAAGCAAGTACAAGGAGGCCAAGG

AGTGGCTCGACAACGCGCGCCAAGCGTGTCTGAAGAGCGGGAACGTCCACATTGCCAACCTGTGCAAAGT

GGTCGCCCCGGCACCCAGCAAGCCGAGACCCGAACCCGTGGTGGTTTGCCTCCGTGGCAAATCAGGCCAG

GGCAAGAGTTTCCTTGCGAACGTGCTCGCACAAGCAATCTCTACCCACTTCACCGGGCGGACCGATTCAG

TCTGGTACTGCCCACCTGACCCTGACCACTTCGACGGTTACAACCAACAGACTGTCGTTGTGATGGACGA

TTTGGGCCAGAACCCTGACGGCAAGGACTTCAAGTACTTCGCCCAAATGGTTTCGACCACGGGGTTCATC

CCGCCCATGGCATCACTCGAGGACAAAGGTAAACCCTTCAACAGTAAGGTCATCATTGCAACCACCAACC

TGTACTCGGGCTTCACCCCGAGGACTATGGTGTGCCCTGACGCCCTGAACCGGAGGTTTCACTTTGACAT

TGACGTGAGCGCCAAGGATGGGTACAAAATTAACAACAAATTGGACATTACCAAAGCACTTGAAGACACC

CACACCAACCCAGTGGCAATGTTTCAGTACGACTGTGCCCTTCTCAACGGCATGGCTGTTGAAATGAAGA

GAATGCAGCAAGACATGTTCAAACCTCAACCACCCCTCCAGAACGTGTACCAACTAGTTCAGGAGGTGAT

TGATCGGGTGGAGCTCCACGAGAAAGTGTCGAGCCACCCAATTTTTAAGCAG

>AY593782.1_A_ARG_2000

CCTTGGTACAAACTGATCAAACTCCTGAGCCGCTTGTCGTGCATGGCCGCTGTAGCAGCACGGTCAAAGG

ATCCAGTCCTTGTGGCCATCATGCTGGCTGACACCGGTCTCGAGATTCTGGACAGCACATTTGTCGTGAA

GAAAATCTCCGACTCGCTCTCCAGTCTCTTTCACGTGCCGGCCCCCGTCTTCAGTTTCGGAGCCCCGATC

CTGCTGGCCGGGTTGGTCAAGGTCGCCTCGAGTTTCTTCCGGTCAACACCCGAAGACCTTGAGAGAGCAG

AGAAACAGCTCAAAGCACGTGACATTAACGACATTTTCGCCATTCTCAAGAACGGCGAGTGGCTGGTCAA

ATTGATCCTTGCCATCCGCGACTGGATCAAGGCATGGATCGCCTCAGAAGAGAAGTTTGTCACCGTGACA

GACTTGGTGCCTGGCATCCTTGAAAAGCAAAGGGACCTCAACGACCCAAGCAAGTACAAGGAGGCCAAGG

AGTGGCTCGACAACGCGCGCCAGGCGTGTTTGAAGAGCGGAAATGTCCACATTGCCAACCTGTGCAAAGT

GGTCGCCCCAGCACCCAGCAGGTCGAGACCCGAGCCTGTGGTCGTTTGCCTCCGCGGCAAATCTGGCCAG

GGCAAGAGTTTCCTTGCCAACGTGCTTGCACAAGCAATCTCCACCCACTTTACTGGCAGAACCGATTCGG

TTTGGTACTGCCCCCCTGACCCCGATCACTTTGACGGTTACAACCAACAGACCGTTGTCGTGATGGATGA

TTTGGGCCAGAACCCTGACGGCAAGGACTTCAAGTACTTCGCCCAAATGGTTTCGACCACAGGGTTCATC

CCGCCTATGGCGTCACTCGAGGACAAAGGCAAACCCTTCAACAGTAAGGTCATCATTGCAACCACCAACC

TGTACGCGGGCTTTACCCCGAGGACTATGGTCTGCCCTGATGCCCTGAACCGGAGGTTTCACTTTGACAT

CGACGTGAGTGCTAAAGACGGGTACAAAATTAACAACAAATTGGACATTATAAAAGCACTTGAAGACACT

CACACCAACCCAGTGGCAATGTTTCAATACGACTGTGCCCTTCTCAACGGCATGGCCGTTGAAATGAAGA

GAATGCAACAAAACGTGTTTGAGCCCCAACCACCCCTCCAGAACCTCTACCAGCTTGTTCAGGAGGTGAT

TGAACGGGTAGAGCTCCACGAGAAAGTGTCGAGCCACCCAATTTTCAAGCAA

>AY593784.1_A_ARG_2001

CCCTGGTACAAGCTGATTAAGCTCCTGAGCCGCTTGTCGTGCATGGCCGCTGTAGCAGCACGGTCAAAGG

ACCCAGTCCTTGTGGCTATCATGCTGGCTGACACCGGTCTTGAGATTCTGGACAGCACGTTTGTCGTGAA

GAAGATCTCCGACTCGCTCTCCAGTCTCTTTCACGTGCCGGCCCCCGTCTTCAGTTTCGGAGCCCCGATT

CTGTTGGCCGGGCTGGTCAAGGTCGCCTCGAGTTTCTTCCGGTCCACACCCGAGGATCTCGAGAGAGCAG

AAAAACAGCTCAAAGCACGTGACATCAACGACATTTTCGCCATTCTCAAGAACGGCGAGTGGCTGGTCAA

GCTGATTCTTGCCATCCGCGACTGGATTAAGGCATGGATCGCCTCAGAAGAAAAGTTTGTCACCACGACA

GACTTGGTGCCTGGCATCCTTGAGAAGCAACGGGACCTCAACGACCCGGCCAAGTACAAGGAAGCCAAGG

AGTGGCTCGACAACGCGCGCCAGGCGTGTTTGAAGAGCGGGAACGTCCACATCGCCAACCTGTGCAAGGT

GGTCGCCCCAGCACCCAGCAAGTCGAGACCCGAACCCGTGGTCGTCTGCCTCCGTGGCAAGTCTGGCCAG

GGCAAGAGTTTCCTTGCTAACGTGCTTGCACAAGCAATTTCCACTCACTTCACCGGCAGAACCGACTCGG

TTTGGTACTGCCCCCCTGACCCTGACCACTTCGACGGTTACAACCAACAGACCGTTGTAGTGATGGATGA

TTTGGGCCAGAACCCTGACGGCAAGGACTTCAAGTACTTTGCCCAAATGGTGTCAACCACAGGGTTCATC

CCGCCCATGGCGTCACTCGAGGACAAAGGCAAACCCTTCAACAGCAAGGTCATCATCGCGACCACCAACT

TGTACTCGGGCTTCACCCCGAGGACCATGGTCTGCCCTGACGCCCTGAATCGAAGGTTTCACTTCGACAT

TGACGTGACAGCCAAAGACGGGTACAAAGTTAACAACAAATTGGACATCATTAAAGCACTCGAAGACACC

CACACCAATCCTGTGGCAATGTTTCAGTATGACTGTGCCCTTCTCAACGGCATGGCTGTTGAAATGAAGA

GAATGCAACAAGACCTCTTCAAACCTCAACCACCCCTCCAGAACGTCTACCAGCTCGTCCAGGAGGTGAT

TGACCGGGTAGAGCTCCACGAAAAAGTGTCGAGCCACCCGATCTTCAAGCAG

>AY593785.1_A_ARG_2001

CCCTGGTACAAGCTGATTAAGCTCCTGAGCCGCTTGTCGTGCATGGCCGCTGTAGCAGCACGGTCAAAGG

ACCCAGTCCTTGTGGCTATCATGCTGGCTGACACCGGTCTTGAGATTCTGGACAGCACGTTTGTCGTGAA

GAAGATCTCCGACTCGCTCTCCAGTCTCTTTCACGTGCCGGCCCCCGTCTTCAGTTTCGGAGCCCCGATT

CTGTTGGCCGGGCTGGTCAAGGTCGCCTCGAGTTTCTTCCGGTCCACACCCGAGGATCTCGAGAGAGCAG

AAAAACAGCTCAAAGCACGTGACATCAACGACATTTTCGCCATTCTCAAGAACGGCGAGTGGCTGGTCAA

GCTGATTCTTGCCATCCGCGACTGGATTAAGGCATGGATCGCCTCAGAAGAAAAGTTTGTCACCACGACA

GACTTGGTGCCTGGCATCCTTGAGAAGCAACGGGACCTCAACGACCCGGCCAAGTACAAGGAAGCCAAGG

AGTGGCTCGACAACGCGCGCCAGGCGTGTTTGAAGAGCGGGAACGTCCACATCGCCAACCTGTGCAAGGT

GGTCGCCCCAGCACCCAGCAAGTCGAGACCCGAACCCGTGGTCGTCTGCCTCCGTGGCAAGTCTGGCCAG

GGCAAGAGTTTCCTTGCTAACGTGCTTGCACAAGCAATTTCCACTCACTTCACCGGCAGAACCGACTCGG

TTTGGTACTGCCCCCCTGACCCTGACCACTTCGACGGTTACAACCAACAGACCGTTGTAGTGATGGATGA

TTTGGGCCAGAACCCTGACGGCAAGGACTTCAAGTACTTTGCCCAAATGGTGTCAACCACAGGGTTCATC

CCGCCCATGGCGTCACTCGAGGACAAAGGCAAACCCTTCAACAGCAAGGTCATCATCGCGACCACCAACT

TGTACTCGGGCTTCACCCCGAGGACCATGGTCTGCCCTGACGCCCTGAATCGAAGGTTTCACTTCGACAT

TGACGTGACAGCCAAAGACGGGTACAAAGTTAACAACAAATTGGACATCATTAAAGCACTCGAAGACACC

CACACCAATCCTGTGGCAATGTTTCAGTATGACTGTGCCCTTCTCAACGGCATGGCTGTTGAAATGAAGA

GAATGCAACAAGACCTCTTCAAACCTCAACCACCCCTCCAGAACGTCTACCAGCTCGTCCAGGAGGTGAT

TGACCGGGTAGAGCTCCACGAAAAAGTGTCGAGCCACCCGATCTTCAAGCAG

>AY593786.1_A_ARG_2001

CCCTGGTACAAGCTGATCAAGCTCCTGAGCCGCTTGTCGTGCATGGCCGCTGTAGCAGCACGGTCAAAGG

ACCCAGTCCTTGTGGCTATCATGCTGGCTGACACCGGTCTTGAGATTCTGGACAGCACGTTTGTCGTGAA

GAAGATCTCCGACTCGCTCTCCAGTCTCTTTCACGTGCCGGCCCCCGTCTTCAGTTTCGGAGCCCCGATT

CTGTTGGCCGGGCTGGTCAAGGTCGCCTCGAGTTTCTTCCGGTCCACACCCGAGGATCTCGAGAGAGCAG

AAAAACAGCTCAAAGCACGTGACATCAACGACATTTTCGCCATTCTCAAGAACGGCGAGTGGCTGGTCAA

GCTGATTCTTGCCATCCGCGACTGGATCAAGGCATGGATCGCCTCAGAAGAAAAGTTTGTCACCATGACA

GACTTGGTGCCTGGCATCCTTGAGAAGCAACGGGACCTCAACGACCCGGCCAAGTACAAGGAAGCCAAGG

AGTGGCTCGACAACGCGCGCCAGGCGTGTTTGAAGAGCGGGAACGTCCACATCGCCAACCTGTGCAAGGT

GGTCGCCCCAGCACCCAGCAAGTCGAGACCCGAACCCGTGGTCGTCTGCCTCCGTGGTAAGTCTGGCCAG

GGCAAGAGTTTCCTTGCTAACGTGCTTGCACAAGCAATTTCCACTCACTTCACCGGCAGAACCGACTCGG

TTTGGTACTGCCCCCCTGACCCTGACCACTTCGACGGTTACAACCAACAGACCGTTGTAGTGATGGATGA

TTTGGGCCAGAACCCTGACGGCAAGGACTTCAAGTACTTTGCCCAAATGGTGTCAACCACAGGGTTCATC

CCGCCCATGGCGTCACTCGAGGACAAAGGCAAACCCTTCAACAGCAAGGTCATCATCGCGACCACCAACT

TGTACTCGGGCTTCACCCCGAGGACCATGGTCTGCCCTGACGCCCTGAACCGAAGGTTTCACTTCGACAT

TGACGTGACAGCCAAAGACGGGTACAAAGTTAACAACAAATTGGACATCATTAAAGCACTCGAAGACACC

CACACCAATCCTGTGGCAATGTTTCAGTATGACTGTGCCCTTCTCAACGGCATGGCTGTTGAAATGAAGA

GAATGCAACAAGACCTCTTCAAACCTCAACCACCCCTCCAGAACGTCTACCAGCTCGTCCAGGAGGTGAT

TGACCGGGTAGAGCTCCACGAAAAAGTGTCGAGCCACCCGATTTTCAAGCAG

>AY593787.1_A_Brazil_1977

CCCTGGTACAAGCTCATCAAGCTCCTGAGCCGCCTGTCGTGCATGGCCGCTGTAGCAGCACGGTCAAAGG

ATCCAGTCCTTGTGGCCATCATGCTGGCTGACACCGGTCTCGAGATTCTGGACAGCACTTTTGTCGTGAA

GAAGATCTCCGACTCGCTCTCCAGTCTCTTTCACGTGCCGGCCCCCGTCTTCAGTTTCGGAGCCCCGATT

CTGTTGGCCGGGTTGGTCAAGGTCGCCTCGAGTTTCTTCCGGTCTACACCCGAAGACCTTGAGAGAGCAG

AGAAACAGCTCAAAGCACGTGACATTAACGACATTTTCGCCATTCTCAAGAACGGCGAGTGGCTGGTCAA

ACTGATTCTTGCCATCCGCGACTGGATCAAGGCATGGATCGCCTCAGAAGAGAAGTTTGTCACCATGACA

GACTTGGTGCCTGGCATCCTTGAAAAGCAGAGGGACCTCAACGACCCAAGCAAGTACAAGGAGGCCAAGG

AGTGGCTCGACAATGCGCGCCAAGCGTGTTTGAAGAGCGGGAACGTCCACATCGCCAACCTGTGCAAAGT

GGTCGCCCCGGCACCCAGCAAGTCGAGACCCGAGCCTGTGGTCGTTTGTCTCCGCGGCAAATCCGGCCAG

GGTAAGAGTTTCCTTGCAAACGTGCTAGCACAAGCAATTTCCACCCACTTCACTGGCAGAACCGACTCTG

TTTGGTACTGTCCACCTGACCCTGACCACTTCGACGGTTACAACCAACAGACCGTTGTTGTGATGGATGA

TTTGGGCCAGAATCCCGACGGCAAGGACTTCAAGTACTTTGCCCAGATGGTTTCAACCACGGGGTTCATC

CCGCCCATGGCATCGCTCGAGGACAAGGGCAAACCCTTCAACAGTAAGGTCATCATCGCGACCACCAACT

TGTACTCGGGTTTCACCCCGAGGACTATGGTGTGCCCTGATGCCCTGAACCGGAGGTTTCACTTTGACAT

CGACGTGAGTGCCAAGGACGGGTACAAAATTAACAACAAATTGGACATCATCAAAGCACTTGAAGACACC

CACACCAACCCAGTGGCAATGTTTCAGTACGATTGTGCCCTTCTCAACGGCATGGCTGTTGAAATGAAGA

GAATGCAACAAGACATGTTCAAGCCCCAACCACCCCTCCAGAACGTCTACCAACTTGTTCAGGAGGTGAT

TGAACGGGTGGAGCTCCACGAGAAAGTGTCGAGCCACCCAATTTTCAAGCAG

>AY593788.1_A_Brazil_1979

CCCTGGTACAAGCTCATCAAGCTCCTGAGCCGCCTGTCGTGCATGGCCGCTGTAGCAGCACGGTCAAAGG

ATCCAGTCCTTGTGGCCATCATGCTGGCTGACACCGGTCTCGAGATTCTGGACAGCACTTTTGTCGTGAA

GAAGATCTCCGACTCGCTCTCCAGTCTCTTTCACGTGCCGGCCCCCGTCTTCAGTTTCGGAGCCCCGATT

CTGTTGGCCGGGTTGGTCAAGGTCGCCTCGAGTTTCTTCCGGTCCACACCCGAAGACCTTGAGAGAGCAG

AGAAACAGCTCAAAGCACGTGACATTAACGACATTTTCGCCATTCTCAAGAACGGCGAGTGGCTGGTCAA

ACTGATTCTTGCCATCCGCGACTGGATCAAGGCATGGATCGCCTCAGAAGAGAAGTTTGTCACCATGACA

GACTTGGTGCCTGGCATCCTTGAAAAGCAGAGGGACCTCAACGACCCAAGCAAGTACAAGGAGGCCAAGG

AGTGGCTCGACAATGCGCGCCAAGCGTGTTTGAAGAGCGGGAACGTCCACATCGCCAACCTGTGCAAAGT

GGTCGCCCCGGCACCCAGCAAGTCGAGACCCGAGCCTGTGGTCGTTTGTCTCCGCGGCAAATCCGGCCAG

GGTAAGAGTTTCCTTGCAAACGTGCTGGCACAAGCAATTTCTACCCACTTCACTGGCAGAACCGACTCTG

TTTGGTACTGTCCACCTGACCCTGACCACTTCGACGGTTACAACCAACAGACCGTTGTTGTGATGGATGA

TTTGGGACAGAATCCCGATGGCAAGGACTTCAAGTACTTTGCCCAGATGGTTTCAACCACGGGGTTCATC

CCGCCCATGGCATCGCTCGAGGACAAGGGCAAACCCTTCAACAGTAAGGTCATCATCGCGACCACCAACT

TGTACTCGGGTTTCACCCCGAGGACCATGGTGTGCCCCGATGCCCTGAACCGGAGGTTTCACTTTGACAT

CGACGTGAGTGCCAAGGACGGGTACAAAATTAACAACAAACTGGACATCATCAGAGCACTTGAAGACACC

CATACCAACCCAGTGGCAATGTTCCAATACGATTGTGCCCTTCTCAACGGTATGGCTGTTGAAATGAAGA

GAATGCAACAAGACCTGTTCAAGCCCCAACCACCCCTCCAGAACGTCTACCAACTTGTTCAGGAGGTGAT

TGAACGGGTGGAGCTCCACGAGAAAGTGTCGAGCCACCCAATTTTTAAACAG

>AY593789.1_A_ARG_1961

CCCTGGTACAAGCTTATTAAACTCCTAAGCCGCCTGTCGTGCATGGCCGCTGTGGCAGCACGGTCCAAGG

ACCCAGTCCTTGTGGCCATCATGCTGGCCGACACCGGTCTCGAGATTCTGGACAGCACCTTTGTCGTGAA

GAAGATCTCCGACTCGCTCTCCAGTCTCTTTCACGTGCCGGCCCCCGTCTTCAGTTTCGGAGCTCCGATC

CTGCTGGCCGGGTTGGTCAAAGTCGCCTCGAGTTTCTTCCGGTCCACACCCGAAGACCTTGAGAGAGCAG

AGAAACAGCTCAAAGCACGTGACATCAACGACATCTTCGCCATTCTCAAGAACGGCGAGTGGCTGGTCAA

ACTGATCCTCGCTATCCGCGACTGGATTAAGGCTTGGATCGCCTCAGAAGAGAAGTTTGTCACCATGACA

GACTTGGTGCCTGGCATCCTTGAAAAGCAGCGGGATCTCAACGACCCGAGCAAGTACAAGGAAGCCAAGG

AATGGCTCGACAACGCACGCCAAGCGTGCTTGAAGAGCGGGAACGTCCACATTGCCAACCTGTGCAAAGT

GGTCGCCCCGGCACCCAGCAAGCCGAGACCCGAGCCCGTGGTCGTTTGCCTCCGCGGCAAATCCGGCCAG

GGCAAGAGTTTCCTTGCGAACGTGCTCGCGCAAGCAATCTCCACCCACTTCACCGGCAGAACCGATTCGG

TTTGGTACTGCCCGCCTGACCCCGACCACTTCGACGGTTACAACCAACAGGCCGTTGTTGTGATGGATGA

TTTGGGCCAGAACCCTGACGGCAAGGACTTCAAGTACTTCGCCCAAATGGTTTCAACCACAGGGTTCATC

CCGCCCATGGCATCGCTCGAGGACAAAGGCAAACCTTTCAACAGCAAGGTCATCATCGCCACCACAAACT

TGTACTCGGGTTTCACCCCGAGGACCATGGTGTGCCCTGATGCGCTGAATCGGAGGTTTCACTTTGACAT

TGACGTGAGCGCCAAGGACGGGTACAAAATTAATGAGAAATTGGACATCATCAAAGCACTTGAAGACACG

CACACTAACCCAGTGGCGATGTTTCAGTACGATTGTGCCCTTCTCAACGGTATGGCCGTTGAAATGAAGA

GAATGCAACAGAATGTGTTCAAGCCTCTACCACCCCTCCAAAACGTTTACCAGCTCGTTCAGGAGGTGAT

TGAACGGGTCGAGCTCCACGAGAAGGTGTCGAGCCACCCAATTTTTAAACAA

>AY593790.1_A_ARG_2001

CCCTGGTACAAGCTGATCAAGCTCCTGAGCCGCTTGTCGTGCATGGCCGCTGTAGCAGCACGGTCAAAGG

ACCCAGTCCTTGTGGCTATCATGCTGGCTGACACCGGTCTTGAGATTCTGGACAGCACGTTTGTCGTGAA

GAAGATCTCCGACTCGCTCTCCAGTCTCTTTCACGTGCCGGCCCCCGTCTTCAGTTTCGGAGCCCCGATT

CTGTTGGCCGGGCTGGTCAAGGTCGCCTCGAGTTTCTTCCGGTCCACACCCGAGGATCTCGAGAGAGCAG

AAAAACAGCTCAAAGCACGTGACATCAACGACATTTTCGCCATTCTCAAGAACGGCGAGTGGCTGGTCAA

GCTGATTCTTGCCATCCGCGACTGGATCAAGGCATGGATCGCCTCAGAAGAAAAGTTTGTCACCATGACA

GACTTGGTGCCTGGCATCCTTGAGAAGCAACGGGACCTCAACGACCCGGCCAAGTACAAGGAAGCCAAGG

AGTGGCTCGACAACGCGCGCCAGGCGTGTTTGAAGAGCGGGAACGTCCATATCGCCAACCTGTGCAAGGT

GGTCGCCCCAGCACCCAGCAAGTCGAGACCCGAACCCGTGGTCGTCTGCCTCCGTGGCAAGTCTGGCCAG

GGCAAGAGTTTCCTTGCTAACGTGCTTGCACAAGCAATTTCCACTCACTTCACCGGCAGAACCGACTCGG

TTTGGTACTGCCCCCCTGACCCTGACCACTTCGACGGTTACAACCAACAGACCGTTGTAGTGATGGATGA

TTTGGGCCAGAACCCTGACGGCAAGGACTTCAAGTACTTTGCCCAAATGGTGTCAACCACAGGGTTCATC

CCGCCCATGGCATCACTCGAGGACAAAGGCAAACCCTTCAACAGCAAGGTCATCATCGCGACCACCAACT

TGTACTCGGGCTTCACCCCGAGGACCATGGTCTGCCCTGACGCCCTGAACCGAAGGTTTCACTTCGACAT

TGACGTGACAGCCAAAGACGGGTACAAAGTTAACAACAAATTGGACATCATTAAAGCACTCGAAGACACC

CACACCAATCCTGTGGCAATGTTTCAGTATGACTGTGCCCTTCTCAACGGCATGGCTGTTGAAATGAAGA

GAATGCAACAAGACCTCTTCAAACCTCAACCACCCCTCCAGAACGTCTACCAGCTCGTCCAGGAGGTGAT

TGACCGGGTAGAGCTCCACGAAAAAGTGTCGAGCCACCCGATTTTCAAGCAG

>AY593791.1_A_IRN_1998

CCCTGGTACAAACTCATTAAGCTCCTGAGCCGCTTATCATGCATGGCCGCTGTAGCAGCACGGTCAAAGG

ACCCGGTCCTTGTGGCCATCATGCTGGCTGACACCGGTCTTGAGATTCTGGACAGCACGTTTGTCGTGAA

AAAGATCTCTGACTCGCTCTCCAGTCTCTTTCACGTGCCGGCCCCTGTCTTCAGTTTCGGAGCCCCGATT

CTGTTGGCCGGACTGGTCAAAGTCGCCTCGAGCTTCTTCCGGTCTACACCCGAAGATCTTGAGAGAGCAG

AGAAACAGCTCAAAGCACGTGATATTAATGACATTTTCGCCATTCTCAAGAACGGCGAGTGGCTGGTCAA

ACTGATTCTTGCCATCCGCGACTGGATCAAGGCATGGATCGCCTCAGAAGAAAAGTTTGTCACCATGACA

GACTTGGTGCCTGGTATCCTTGAGAAGCAACGGGATCTCAACGACCCCAGCAAATACAAGGAGGCCAAGG

AGTGGCTCGACAACGCGCGCCAAGCGTGTTTGAAGAGCGGGAACGTCCACATTGCCAACCTGTGCAAAGT

GGTCGCCCCAGCACCCAGCAAGTCGAGACCAGAGCCCGTGGTCGTCTGCCTCCGTGGCAGATCCGGCCAG

GGGAAGAGTTTCCTTGCGAACGTGCTTGCACAAGCAATCTCCACCCACTTCACTGGCAGAACTGACTCTG

TTTGGTACTGCCCGCCTGACCCTGACCACTTCGACGGTTACAATCAACAAACCGTTGTTGTGATGGATGA

TTTGGGCCAGAACCCTGACGGCAAGGACTTCAAGTACTTCGCCCAGATGGTCTCTACCACGGGGTTCATC

CCGCCCATGGCGTCGCTCGAGGACAAAGGCAAACCTTTCAACAGCAAGGTCATCATCGCCACCACCAACC

TGTACTCGGGTTTCACCCCGAGAACCATGGTGTGCCCTGACGCGCTGAACCGAAGGTTTCACTTTGACAT

TGACGTGAGTGCCAAGGACGGGTACAAAATTAACAACAAATTGGACATAATCAAAGCTCTTGAAGACACC

CACACCAACCCAGTGGCAATGTTCCAATACGACTGTGCCCTTCTCAACGGCATGGCCGTTGAAATGAAGA

GAATGCAACAAGACATGTTCAAGCCACAGCCACCCCTCCAGAACGTATACCAGCTCGTTCAGGAGGTGAT

TGAGAGGGTCGCGCTCCACGAGAAAGTGTCGAGCCACCCGATCTTCAAGCAG

>AY593792.1_A_ITL_1962

CCCTGGTACAAGCTTATCAAACTCCTAAGCCGCCTGTCGTGCATGGCCGCTGTGGCAGCACGGTCCAAGG

ACCCAGTCCTTGTGGCCATCATGCTGGCCGACACCGGTCTCGAGATTCTGGACAGCACTTTCGTCGTGAA

GAAGATCTCCGACTCGCTCTCCAGTCTCTTCCACGTGCCGGCCCCCGCTTTCAGTTTCGGAGCCCCGATT

CTGCTGGCCGGGTTGGTCAAGGTCGCCTCGAGTTTCTTCCGGTCCACGCCCGAAGACCTTGAGAGGGCAG

AGAAACAGCTCAAAGCACGTGACATCAACGACATTTTCGCCATTCTCAAGAACGGCGAGTGGCTGGTCAA

ACTGATCCTTGCCATCCGCGACTGGATTAAGGCTTGGATTGCCTCAGAAGAAAAGTTTGTCACCATGGCA

GACTTAGTGCCTGGCATCCTTGAAAAGCAGCGGGACCTCAACGACCCAAGTAAGTACAAGGAGGCCAAGG

AGTGGCTCGACAACGCGCGCCAAGCGTGTCTGAAGAGCGGGAACGTCCACATTGCCAACCTGTGCAAGGT

GGTCGCCCCGGCCCCCAGCAAGTCGAGACCCGAACCCGTGGTGGTTTGCCTCCGTGGCAAATCAGGCCAG

GGTAAGAGTTTCCTTGCGAACGTGCTCGCACAAGCAATCTCTACCCACTTCACCGGGCGGACCGATTCAG

TCTGGTACTGCCCACCTGACCCTGATCACTTCGACGGTTACAACCAACAGACTGTCGTTGTGATGGACGA

TTTGGGCCAGAACCCTGACGGCAAGGACTTCAAGTACTTCGCCCAAATGGTTTCGACCACGGGGTTCATC

CCGCCCATGGCATCACTCGAGGACAAAGGTAAACCCTTCAACAGTAAGGTCATCATTGCAACCACCAACC

TGTACTCGGGCTTCACCCCGAGGACTATGGTGTGCCCTGACGCCCTGAACCGGAGGTTTCACTTTGACAT

TGACGTGAGCGCCAAGGATGGGTACAAAATTAACAACAAATTGGACATTATCAAAGCACTTGAAGACACC

CACACCAACCCAGTGGCAATGTTTCAGTACGACTGTGCCCTTCTCAACGGCATGGCTGTTGAAATGAAGA

GAATGCAGCAAGACATGTTCAAACCTCAACCACCCCTCCAGAACGTGTACCAACTAGTTCAGGAGGTGAT

TGATCGGGTGGAGCTCCACGAGAAAGTGTCGAGCCACCCAATTTTTAAGCAG

>AY593793.1_A_PHI_1975

CCCTGGTACAAACTTATCAAGCTCCTAAGCCGCCTGTCGTGTATGGCCGCTGTGGCGGCACGGTCCAAGG

ACCCAGTCCTTGTGGCCATCATGCTGGCCGACACCGGCCTTGAGATTCTGGACAGCACATTTGTCGTGAA

GAAGATTTCCGACTCGCTCTCCAGTCTCTTTCACGTGCCGGCCCCCGTCTTTAGCTTCGGATCACCGATC

CTGCTAGCCGGGTTGGTCAAAGTCGCCTCGAGTTTCTTCCGGTCCACGCCCGAAGACCTTGAGAGAGCAG

AGAAACAGCTCAAAGCACGTGACATCAACGACATTTTCGCCATTCTTAAGAACGGCGAGTGGCTGGTCAA

ACTCATCCTTGCCATCCGCGACTGGATCAAGGCATGGATCGCCTCAGAAGAGAAGTTTGTCACTATGACA

GACCTGGTGCCTGGCATTCTTGAAAAGCAGCGGGACCTGAACGACCCGAGTAAGTACAAGGAAGCCAAGG

AATGGCTCGACAACGCGCGCCAGGCGTGTTTGAAGAGCGGGAACACCCACATTGCCAACCTGTGCAAAGT

GGTTGCCCCAGCACCCAGCAAGTCGAGACCCGAACCTGTGGTTGTTTGCCTCCGTGGTAAATCCGGCCAG

GGTAAGAGTTTCCTTGCGAACGTGCTCGCACAAGCAATCTCCACCCACTTTACCGGCAGAACCGACTCTG

TTTGGTACTGCCCACCTGACCCCGACCACTTCGACGGTTACAACCAACAAACTGTCGTTGTGATGGATGA

TCTGGGCCAGAACCCTGACGGCAAGGACTTTAAGTACTTTGCCCAAATGGTTTCGACCACAGGGTTCATC

CCGCCTATGGCATCACTTGAAGACAAGGGCAAACCTTTCAACAGCAAGGTCATCATCGCGACCACCAACT

TGTACGCGGGCTTCACCCCGAGGACTATGGTGTGTCCCGACGCGCTGAACCGAAGGTTTCACTTCGACAT

CGATGTGAGTGCCAAGGACGGGTACAAAATTAACAACAAATTGGACATCACCAAAGCACTTGAAGACACC

CACACCAACCCAGTGGCAATGTTCCAGTACGATTGTGCCCTTCTCAACGGTATGGCCGTTGAAATGAAGA

GAATGCAACAAGATGTGTTCAAGCCTCAACCGCCCCTCCAGAACATTTACCAGCTCGTTCAGGAGGTGAT

TGAACGGGTCGAGCTACACGAGAAAGTGTCGAGCCACCCGATCTTCAAGCAG

>AY593794.1_A_COL_1985

CCCTGGTACAAGCTGATCAAACTCCTGAGCCGCCTGTCGTGCATGGCCGCTGTGGCAGCACGGTCCAAGG

ACCCAGTCCTTGTGGCCATCATGCTGGCCGACACCGGTCTCGAGATTCTGGACAGCACCTTTGTCGTGAA

GAAAATCTCCGACTCGCTCTCCAGTCTCTTTCACGTGCCGGCCCCCGTCTTCAGTTTCGGTGCCCCGATT

CTGTTGGCCGGGTTGGTCAAGGTCGCCTCGAGCTTCTTCCGGTCCACACCCGAAGATCTTGAGAGAGCAG

AGAAACAGCTCAAAGCACGTGACATAAACGACATCTTCGCCATTCTCAAGAACGGCGAGTGGCTGGTCAA

ACTGATTCTTGCCATCCGCGACTGGATTAAGGCATGGATCGCCTCAGAAGAGAAGTTCGTCACCATGACG

GACTTGGTGCCTGGCATTCTTGAAAAGCAGCGGGACCTCAACGACCCGAGCAAGTACAAGGAAGCCAAGG

AGTGGCTCGACAACGCGCGCCAAGCGTGTTTGAAGAGCGGGAACGTCCACATTGCGAACCTCTGCAAAGT

GGTCGCCCCAGCACCCAGCAAGTCGAGACCCGAACCTGTGGTCGTTTGCCTCCGTGGCAAGTCTGGCCAG

GGCAAGAGTTTCCTTGCGAACGTGCTCGCACAAGCAATCTCCACTCACTTTACCGGCAGGACTGATTCGG

TTTGGTACTGCCCCCCTGACCCCGACCACTTCGACGGCTACAACCAGCAGACCGTTGTTGTGATGGATGA

TTTGGGCCAGAACCCTGACGGCAAGGACTTCAAGTACTTCGCCCAGATGGTCTCGACCACAGGGTTCATC

CCGCCCATGGCATCACTTGAGGACAAAGGGAAACCTTTCAACAGCAAGGTCATCATTGCAACCACCAACT

TGTACTCGGGTTTCACCCCGAGGACTATGGTGTGTCCTGACGCTTTGAACCGGAGGTTTCACTTTGACAT

CGATGTGTGTGCCAAGGACGGGTACAAAACCAACAACAAATTGGACATCATCAAAGCACTTGAAGACACC

CACACCAACCCAGTGGCAATGTTTCAGTACGATTGTGCCCTTCTCAACGGCATGGCCGTAGAAATGAAGA

GACTGCAACAAGACATGTTCAAACCACAACCACCCCTCCAGAACGTGTACCAACTTGTTCAAGAGGTGAT

TGAACGGGTCGAACTCCACGAGAAAGTGTCGAGTCACCCGATCTTCAAACAG

>AY593795.1_Asia1_PAK_1954

CCCTGGTACAAGCTTATCAAACTCCTGAGCCGCCTGTCGTGTATGGCCGCTGTAGCAGCACGGTCCAAGG

ACCCAGTCCTTGTGGCTATAATGCTGGCTGACACCGGTCTTGAGATTCTGGACAGCACGTTTGTCGTGAA

GAAAATCTCCGACTCGCTCTCCAGTCTCTTTCACGTGCCGGCCCCCGTCTTCAGTTTCGGAGCACCGATT

CTGTTGGCCGGGTTGGTCAAGGTCGCCTCGAGTTTCTTCCGGTCGACGCCCGAAGACCTCGAGAGAGCAG

AGAAACAGCTCAAAGCACGTGACATTAACGACATTTTCGCCATTCTCAAGAACGGCGAGTGGCTGGTCAA

ACTGATCCTTGCCATCCGCGACTGGATCAAGGCATGGATCGCCTCAGAAGAGAAGTTTGTCACTATGACA

GACTTGGTGCCTGGCATCCTTGAAAAGCAGCGGGACCTTAACGACCCGAGCAAGTACAAGGAAGCCAAGG

AATGGCTCGACAATGCGCGGCAAGCGTGTTTGAAGAGTGGGAACGTCCACATTGCCAACTTGTGCAAAGT

GGTCGCCCCAGCACCTAGCAAGTCGAGACCCGAACCCGTGGTCGTTTGCCTCCGTGGCAAATCCGGCCAG

GGCAAGAGTTTCCTCGCGAACGTGCTCGCACAGGCAATCTCCACCCATTTCACCGGCAGGACTGATTCAG

TTTGGTACTGCCCGCCTGACCCTGACCACTTCGACGGTTATAACCAACAAACCGTTGTTGTGATGGATGA

TTTGGGCCAGAACCCTGACGGCAAGGACTTTAAGTACTTCGCCCAGATGGTCTCGACCACAGGGTTCATC

CCGCCCATGGCGTCACTCGAGGACAAAGGTAAACCTTTCAACAGCAAGGTTATCATTGCAACCACCAACC

TGTACTCGGGGTTCACTCCGAGGACCATGATGTGCCCTGATGCACTGAACCGCAGGTTTCACTTTGACAT

TGACGTGAGCGCCAAGGATGGGTACACAATTAACAACAGATTGGACATAATCAAAGCTCTTGAAGACACC

CACACCAATCCAGTGGCAATGTTTCAATATGACTGTGCCCTTCTCAACGGCATGGCCGTTGAAATGAAGA

GAATGCAACAAGACTTGTTCAAGCCTCAACCACCCCTCCAGAACGTGTACCAGCTTGTTCAGGAGGTGAT

TGAACGGGTCGAGCTCCACGAGAAAGTGTCGAGCCACCCAATTTTTAAACAG

>AY593796.1_Asia1_ISR_1963

CCCTGGTATAAGCTCATCAAACTCCTAAGCCGCCTGTCATGCATGGCCGCTGTAGCAGCACGGTCCAAGG

ACCCAGTCCTTGTGGCCATCATGCTGGCTGACACCGGTCTCGAGATTCTGGACAGCACGTTTGTTGTGAA

GAAGATCTCCGACTCGCTCTCCAGTCTCTTTCACGTGCCGGCCCCCGTCTTCAGTTTCGGAGCCCCGATT

CTGTTGGCTGGGTTGGTCAAGGTCGCCTCGAGTTTCTTCCGGTCAACACCCGAAGAGCTTGAGAGAGCAG

AGAAACAGCTCAAAGCACGTGACATCAATGACATTTTCGCCATTCTCAAGAACGGCGAGTGGCTGGTCAA

GCTGATCCTTGCCATCCGCGACTGGATTAAGGCATGGATCGCCTCAGAAGAGAAGTTTGTCACCATGACA

GACTTGGTGCCTGGCATACTTGAAAAGCAGCGGGATCTCAACGACCCCAGCAAGTACAAGGAAGCCAAGG

AGTGGCTCGACAACACGCGCCAAGCGTGTCTGAAGAGCGGGAACGTCCATATTGCCAACCTGTGCAGAGT

GGTCGCCCCGGCACCTAGCAAGTCGAGACCCGAACCCGTGGTCGTTTGCCTCCGTGGCAAGTCCGGCCAG

GGAAAGAGTTTCCTTGCGAACGTGCTCGCACAAGCAATTTCTACCCACTTTACTGGCAAAACCGACTCAG

TTTGGTACTGCCCGCCTGATCCTGACCACTTCGACGGTTACAACCAACAGACCGTTGTTGTGATGGATGA

TCTGGGCCAGAACCCCGACGGCAAGGACTTCAAGTACTTCGCCCAGATGGTTTCAACCACGGGGTTCATC

CCGCCCATGGCTTCACTCGAGGACAAAGGCAAGCCCTTCAACAGCAAAGTCATCATTGCCACCACTAACC

TGTACTCGGGCTTCACCCCGAGAACCATGGTGTGCCCTGACGCGCTGAACCGAAGGTTCCACTTTGACAT

TGACGTGAGTGCCAAGGACGGGTACAAAATCAACAACAAATTGGACATAACCAAAGCTCTTGAAGACACC

CACACTAACCCGGTGGCAATGTTTCAATACGACTGTGCCCTCCTCAATGGCATGGCCGTTGAGATGAAGA

GAATGCAACAAGACATGTTCAAGCCTCAACCACCTCTCCAGAACGTGTACCAACTTGTTCAGGAGGTGAT

TGATCGGGTTGAGCTCCACGAGAAGGTGTCGAGCCAACCGATCTTCAAGCAA

>AY593797.1_Asia1_ISR_1963

CCCTGGTACAAGCTCATCAAACTCCTAAGCCGCCTGTCATGCATGGCCGCTGTAGCAGCACGGTCAAAGG

ACCCAGTCCTTGTGGCCATCATGCTGGCTGACACCGGCCTTGAGATTCTGGACAGCACGTTTGTCGTGAA

GAAGATCTCCGACTCGCTCTCCAGTTTCTTTCACGTGCCGGCCCCCGTCTTCAGTTTCGGAGCCCCGACT

CTGTTGGCTGGGTTGGTCAAGGTCGCCTCGAATTTCTTCCGGTCAACACCCGAAGACCTTGAGAGAGCAG

AGAAACAGCTCAAAGCACGTGACATCAATGACATTTTCGCCATTCTCAAGAACGGCGAGTGGCTGGTCAA

GCTGATCCTTGCCATCCGCGACTGGATTAAGGCATGGATCGCCTCAGAAGAGAAGTTTGTCACCATGACA

GACTTGGTGCCTGGCATACTTGAAAAGCAGCGGGATCTCAACGACCCCAGCAAGTACAAGGAGGCCAAGG

AGTGGCTCGACAACGCACGCCAAGCGTGCTTGAAGAGCGGGAACGTCCACATTGCCAACCTGTGCAAAGT

GGCCACCCCGGCACCTAGCGAGTCGAGACCCGAACCCGTGGTCGTTTGCCTCCGTGGCAAGTCCGGTCAG

GGAAAGAGTTTCCTTGCGAACGTGCTCGCACAAGCAATTTCTACCCACTTCACAGGCAAAACCGATTCAG

TTTGGTACTGTCCGCCTGACCCTGACCACTTCGACGGTTACAACCAACAGACCGTTGTTGTGATGGATGA

TTTGGGTCAGAACCCCGACGGCAAGGACTTCAAGTACTTCGCCCAGATGGTTTCAACTACGGGGTTCATC

CCGCCTATGGCTTCACTCGAAGACAAAGGCAAGCCTTTCAACAGCAAAGTCATCATCGCCACCACCAACC

TGTACTCGGGTTTCACCCCGAGAACCATGGTGTGTCCTGACGCGCTGAACCGAAGGTTCCACTTTGACAT

TGACGTGAGTGCCAAGGACGGGTACAAAACTAACAACAAATTGGACATAATCAAAGCTCTTGAAGACACC

CACACCAACCCGGTGGCAATGTTTCAATATGACTGTGCCCTTCTCAATGGAATGGCCGTTGAGATGAAGA

GAATGCAACAAGATGTGTTCAAGCCCCAACCACCTCTCCAGAACGTGTACCAACTTGTTCAGGAGGTGAT

TGATCGGGTCGAGCTCCACGAGAAAGTGTCGAGCCACCCGATTTTCAAGCAA

>AY593799.1_Asia1_LEB_1983

CCCTGGTACAAGCTCATCAAACTCCTAAGCCGCCTGTCGTGCATGGCCGCTGTAGCAGCACGGTCCAAGG

ACCCAGTCCTTGTGGCCATCATGCTGGCTGACACCGGTCTTGAGATTCTGGACAGCACGTTCGTCGTGAA

GAAGATCTCCGACTCGCTCTCCAGTCTCTTTCACGTGCCGGCCCCCGTCTTCAGTTTCGGAGCTCCGATT

CTGTTGGCTGGGTTGGTCAAAGTCGCCTCGAGTTTCTTCCGGTCCACACCCGAAGACCTTGAGAGAGCAG

AGAAACAGCTCAAAGCACGTGACATCAACGACATATTCGCCATTCTTAAAAACGGCGAGTGGCTGGTCAA

GCTGATTCTTGCCATCCGCGACTGGATAAAGGCATGGATCGCCTCAGAAGAAAAGTTTGTTACCATGACA

GACTTGGTGCCTGGCATCCTTGAAAAGCAGCGGGACCTCAACGACCCGAGCAAGTACAAAGAAGCCAAGG

AGTGGCTCGACAACGCGCGCCAAGCGTGTCTGAAGAGCGGGAACGTCCACATTGCCAACCTGTGCAAAGT

GGTTGCCCCGGCACCCAGCAAGTCGAGACCCGAACCTGTGGTCGTTTGCCTACGTGGTAAATCCGGCCAG

GGAAAGAGTTTCCTTGCGAACGTGCTCGCACAAGCAATCTCCACCCACTTCACTGGCAGAACTGATTCAG

TTTGGTACTGCCCGCCTGACCCTGACCACTTCGACGGTTACAACCAGCAAACCGTTGTTGTGATGGACGA

TTTGGGCCAGAACCCCGATGGCAAGGACTTTAAATACTTTGCCCAGATGGTTTCAACCACGGGGTTCATC

CCGCCCATGGCCTCGCTAGAGGACAAAGGCAAACCTTTCAACAGCAAGGTCATCATTGCCACCACCAATC

TGTACTCGGGATTCACCCCGAGAACTATGGTGTGCCCTGATGCACTGAACCGGAGGTTTCACTTTGACAT

CGACGTGAGCGCCAAGGACGAGTACAAAATTAACAACAAATTGGACATAATCAAAGCCCTTGAAGACACC

CACACTAACCCTGTGGCAATGTTTCAGTACGACTGTGCCCTTCTCAACGGCATGGCCGTTGAAATGAAGA

GAATGCAACAAGACGTATTCAAGCCTTTGCCACCCCTCCAGAACGTGTACCAACTCGTACAGGAGGTGAT

TGACCGGGTCGAGCTTCACGAGAAAGTGTCGAGCCGGCCGATCTTTAAGCAG

>AY593800.1_Asia1_LEB_1983

CCCTGGTACAAGCTCATCAAACTCCTAAGCCGCCTGTCGTGCATGGCCGCTGTAGCAGCACGGTCCAAGG

ACCCAGTCCTTGTGGCCATCATGCTGGCTGACACCGGTCTTGAGATTCTGGACAGCACGTTCGTCGTGAA

GAAGATCTCCGACTCGCTCTCCAGTCTCTTTCACGTGCCGGCCCCCGTCTTCAGTTTCGGAGCTCCGATT

CTGTTGGCTGGGTTGGTCAAAGTCGCCTCGAGTTTCTTCCGGTCCACACCCGAAGACCTTGAGAGAGCAG

AGAAACAGCTCAAAGCACGTGACATCAACGACATATTCGCCATTCTTAAAAACGGCGAGTGGCTGGTCAA

GCTGATTCTTGCCATCCGCGACTGGATAAAGGCATGGATCGCCTCAGAAGAAAAGTTTGTTACCATGACA

GACTTGGTGCCTGGCATCCTTGAAAAGCAGCGGGACCTCAACGACCCGAGCAAGTACAAAGAAGCCAAGG

AGTGGCTCGACAACGCGCGCCAAGCGTGTCTGAAGAGCGGGAACGTCCACATTGCCAACCTGTGCAAAGT

GGTTGCCCCGGCACCCAGCAAGTCGAGACCCGAACCTGTGGTCGTTTGCCTACGTGGTAAATCCGGCCAG

GGAAAGAGTTTCCTTGCGAACGTGCTCGCACAAGCAATCTCCACCCACTTCACTGGCAGAACTGATTCAG

TTTGGTACTGCCCGCCTGACCCTGACCACTTCGACGGTTACAACCAGCAAACCGTTGTTGTGATGGACGA

TTTGGGCCAGAACCCCGATGGCAAGGACTTTAAATACTTTGCCCAGATGGTTTCAACCACGGGGTTCATC

CCGCCCATGGCCTCGCTAGAGGACAAAGGCAAACCTTTCAACAGCAAGGTCATCATTGCCACCACCAATC

TGTACTCGGGATTCACCCCGAGAACTATGGTGTGCCCTGATGCACTGAACCGGAGGTTTCACTTTGACAT

CGACGTGAGCGCCAAGGACGAGTACAAAATTAACAACAAATTGGACATAATCAAAGCCCTTGAAGACACC

CACACTAACCCTGTGGCAATGTTTCAGTACGACTGTGCCCTTCTCAACGGCATGGCCGTTGAAATGAAGA

GAATGCAACAAGACGTATTCAAGCCTTTGCCACCCCTCCAGAACGTGTACCAACTCGTACAGGAGGTGAT

TGACCGGGTCGAGCTTCACGAGAAAGTGTCGAGCCGGCCGATCTTTAAGCAG

>AY593802.1_A_URU_2001

CCCTGGTACAAGCTGATTAAGCTCCTGAGCCGCTTGTCGTGCATGGCCGCTGTAGCAGCACGGTCAAAGG

ACCCAGTCCTTGTGGCTATCATGCTGGCTGACACCGGTCTTGAGATTCTGGACAGCACGTTTGTCGTGAA

GAAGATCTCCGACTCGCTCTCCAGTCTCTTTCACGTGCCGGCCCCCGTCTTCAGTTTCGGAGCCCCGATT

CTGTTGGCCGGGCTGGTCAAGGTCGCCTCGAGTTTTTTCCGGTCCACACCCGAGGATCTCGAGAGAGCGG

AAAAACAGCTCAAAGCACGTGACATCAACGACATTTTCGCCATTCTCAAGAACGGCGAGTGGCTGGTCAA

GCTGATTCTTGCCATCCGCGACTGGATTAAGGCATGGATCGCCTCAGAAGAAAAGTTTGTCACCACGACA

GACTTGGTGCCTGGCATCCTTGAGAAGCAACGGGACCTCAACGACCCGGCCAAGTACAAGGAAGCCAAGG

AGTGGCTCGACAACGCGCGCCAGGCGTGTTTGAAGAGCGGGAACGTCCACATCGCCAACCTGTGCAAGGT

GGTCGCCCCAGCACCCAGCAAGTCGAGACCCGAACCCGTGGTCGTCTGCCTCCGTGGCAAGTCTGGCCAG

GGCAAGAGTTTCCTTGCTAACGTGCTTGCACAAGCAATTTCCACTCACTTCACCGGCAGAACCGACTCGG

TTTGGTACTGCCCCCCTGACCCTGACCACTTCGACGGTTACAACCAACAGACCGTTGTAGTGATGGATGA

TTTGGGCCAGAACCCTGACGGCAAGGACTTCAAGTACTTTGCCCAAATGGTGTCAACCACAGGGTTCATC

CCGCCCATGGCGTCACTCGAGGACAAAGGCAGACCCTTCAACAGCAAGGTCATCATCGCGACCACCAACT

TGTACTCGGGCTTCACCCCGAGGACCATGGTCTGCCCTGACGCCCTGAATCGAAGGTTTCACTTCGACAT

TGACGTGACAGCCAAAGACGGGTACAAAGTTAACAACAAATTGGACATCATTAAAGCACTCGAAGACACC

CACACCAATCCTGTGGCAATGTTTCAGTATGACTGTGCCCTTCTCAACGGCATGGCTGTTGAAATGAAGA

GAATGCAACAAGACCTCTTCAAACCTCAACCACCCCTCCAGAACGTCTACCAGCTCGTCCAGGAGGTGAT

TGACCGGGTAGAGCTCCACGAAAAAGTGTCGAGCCACCCGATCTTCAAGCAG

>AY593803.1_A_Brazil_1979

CCCTGGTACAAGCTCATCAAGCTCCTGAGCCGCCTGTCGTGCATGGCCGCTGTAGCAGCACGGTCAAAGG

ACCCAGTCCTTGTGGCCATCATGCTGGCTGACACCGGTCTCGAGATTCTGGACAGCACTTTTGTTGTGAA

GAAGATCTCCGACTCGCTCTCCAGTCTCTTTCACGTGCCGGCCCCCGTCTTCAGTTTCGGAGCCCCGATT

CTGTTGGCCGGGTTGGTCAAGGTCGCCTCGAGTTTCTTCCGGTCCACACCCGAAGATCTTGAGAGAGCAG

AGAAACAGCTCAAAGCACGTGACATCAACGACATTTTCGCCATTCTCAAGAACGGCGAGTGGCTGGTCAA

ACTGATTCTTGCCATCCGCGACTGGATCAAGGCATGGATCGCCTCAGAAGAGAAGTTTGTCACCATGACA

GACTTGGTGCCTGGCATCCTTGAAAAGCAGAGGGACCTTGACGACCCAAGCAAGTACAAGGAGGCCAAGG

AGTGGCTCGACAACGCGCGCCAAGCGTGTCTGAAGAGCGGGAACGTCCACATTGCCAACCTGTGTAAAGT

GGTCACCCCGGCACCAAGCAAGTCGAGGCCCGAACCTGTGGTCGTTTGTCTCCGCGGCAAATCCGGCCAG

GGCAAGAGTTTCCTTGCGAACGTGCTAGCACAAGCAATTTCCACCCACTTCACCGGCAGAACCGACTCCG

TCTGGTACTGCCCGCCTGACCCTGACCACTTCGACGGTTACAACCAACAGACCGTTGTTGTGATGGATGA

TTTGGGCCAGAACCCTGATGGCAAAGACTTCAAGTACTTCGCCCAGATGGTTTCAACCACAGGGTTCATC

CCGCCCATGGCATCGCTCGAGGACAAGGGCAAACCCTTCAACAGTAAGGTCATCATCGCGACCACCAACT

TGTACTCGGGTTTCACCCCGAGGACCATGGTGTGCCCTGATGCCCTGAACCGGAGGTTTCACTTTGACAT

CGATGTGAGCGCTAAGGACGGGTACAAAGTTAATAACAAATTGGACATCATCAAAGCACTTGAAGACACC

CACACCAATCCGGTGGCAATGTTTCAGTACGACTGTGCCCTTCTCAACGGCATGGCTGTTGAAATGAAGA

GAATGCAACAAGATATGTTCAAGCCTCAACCACCCCTCCAGAACGTGTATCAGCTCGTCCAGGAGGTGAT

TGAACGGGTCGAGCTCCACGAGAAAGTGTCGAGCCACCCGATTTTCAAGCAA

>AY593804.1_C_SWZ_1965

CCCTGGTACAAGCTCATCAAACTCCTGAGCCGCTTGTCGTGCATGGCCGCTGTAGCAGCACGGTCAAAGG

ACCCAGTCCTTGTGGCCATCATGCTGGCTGACACCGGTCTTGAGATTCTGGACAGCACGTTTGTCGTGAA

GAAGATCTCCGACTCGCTCTCCAGTCTCTTTCACGTGCCGGCCCCCGCCTTCAGCTTCGGAGCCCCGATT

CTGTTGGCCGGGTTGGTCAAGGTCGCCTCGAGTTTCTTCCGGTCCACACCCGAAGACCTTGAGAGAGCAG

AGAAACAGCTCAAAGCACGTGACATCAACGACATCTTCGCCATTCTCAAGAACGGCGAGTGGCTGGTCAA

GCTGATCCTTGCCATCCGCGACTGGATTAAGGCATGGATTGCCTCAGAAGAGAAGTTTGTCACCATGACA

GACCTGGTGCCTGGCATCCTTGAAAAACAGCGGGACCTCAACGACCCAAGCAAGTACAAGGATGCCAAGG

AATGGCTCGACAACACGCGCCAAGCGTGTTTGAAGAGCGGGAACGTCCATATTGCCAACCTTTGCAAAGT

GGTCGCCCCAGCACCCAGCAAGTCGAGACCCGAGCCCGTGGTCGTTTGCCTCCGCGGCAAGTCTGGCCAG

GGCAAGAGTTTCCTTGCGAACGTGCTTGCACAAGCAATCTCCACCCACCTCACCGGCAGAACCGACTCTG

TTTGGTACTGCCCGCCTGACCCTGACCACTTCGACGGTTACAACCAGCAGACCGTTGTTGTGATGGACGA

TTTGGGCCAGAACCCTGACGGCAAGGACTTCAAGTACTTCGCCCAAATGGTTTCAACCACGGGGTTCATC

CCGCCCATGGCATCACTTGAGGACAAAGGCAAACCCTTCAGCAGTAAGGTCATCATTGCAACCACCAACC

TGTACTCGGGCTTCACCCCGAAGACCATGGTGTGCCCTGATGCTCTGAACCGGAGGTTTCACTTTGACAT

CGACGTGAGCGCCAAGGACGGGTACAAAATCAACAACAAATTGGACATCATCAAAGCACTTGAAGACACC

CACACCAACCCAGTGGCAATGTTTCAGTACGACTGTGCCCTTCTCAACGGCATGGCTGTTGAAATGAAGA

GATTGCAACAGGATATGTTCAAGCCTCAACCACCCCTCCAGAACGTGTACCAACTTGTTCAGGAGGTGAT

TGAACGGGTCGAGCTTCACGAGAAAGTGTCAAGCCACCCAATTTTCAAGCAG

>AY593805.1_C_GER_1960

CCCTGGTACAAGCTTATTAAGCTCCTGAGCCGCTTGTCGTGCATGGCCGCTGTAGCAGCACGGTCAAAGG

ACCCAGTCCTTGTGGCCATCATGCTGGCTGACACCGGTCTCGAGATTCTGGACAGCACGTTTGTCGTGAA

GAAGATCTCCGACTCGCTCTCCAGTCTCTTTCACGTGCCGGCCCCCGCCTTCAGCTTCGGAGCCCCGATT

CTGTTGGCCGGGTTGGTCAAGGTCGCCTCGAGTTTCTTCCGGTCCACACCCGAAGACCTTGAGAGAGCAG

AGAAACAGCTCAAAGCACGTGACATCAACGACATCTTCGCCATTCTCAAGAACGGCGAGTGGCTGGTCAA

GCTGATCCTTGCCATCCGCGACTGGATTAAGGCATGGATTGCCTCAGAAGAGAAGTTTGTCACCATGACA

GACCTGGTGCCTGGCATCCTTGAAAAACAGCGGGACCTCAACGACCCAAGCAAGTACAAGGATGCCAAGG

AATGGCTCGACAACACGCGCCAAGCGTGTTTGAAGAGCGGGAACGTCCATATTGCCAACCTTTGCAAAGT

GGTCGCCCCAGCACCCAGCAAGTCGAGACCCGAGCCCGTGGTCGTTTGCCTCCGCGGCAAGTCTGGCCAG

GGCAAGAGTTTCCTTGCGAACGTGCTTGCACAAGCAATCTCCACCCACCTCACCGGCAGAACCGACTCTG

TTTGGTACTGCCCGCCTGACCCTGACCACTTCGACGGTTACAACCAGCAGACCGTTGTTGTGATGGACGA

TTTGGGCCAGAACCCTGACGGCAAGGACTTCAAGTACTTCGCCCAAATGGTTTCAACCACGGGGTTCATC

CCGCCCATGGCATCACTTGAGGACAAAGGCAAACCCTTCAGCAGTAAGGTCATCATTGCAACCACCAACC

TGTACTCGGGCTTCACCCCGAAGACCATGGTGTGCCCTGATGCTCTGAACCGGAGGTTTCACTTTGACAT

CGACGTGAGCGCCAAGGACGGGTACAAAATCAACAACAAATTGGACATCATCAAAGCACTTGAAGACACC

CACACCAACCCAGTGGCAATGTTTCAGTACGACTGTGCCCTTCTCAACGGCATGGCTGTTGAAATGAAGA

GATTGCAACAGGATATGTTCAAGCCTCAACCACCCCTCCAGAACGTGTACCAACTTGTTCAGGAGGTGAT

TGAACGGGTCGAGCTTCACGAGAAAGTGTCAAGCCACCCAATTTTCAAGCAG

>AY593806.1_C_Brazil_1971

CCCTGGTACAAGCTCATTAAGCTCCTAAGCCGCCTGTCATGCATGGCCGCTGTAGCAGCACGGTCAAAGG

ACCCAGTCCTTGTGGCCATCATGCTAGCTGACACCGGTCTTGAGATTCTGGACAGCACGTTTGTCGTGAA

GAAGATCTCCGACTCGCTCTCCAGTCTCTTTCACGTGCCGGCCCCCGTCTTCAGTTTCGGAGCCCCGATC

CTGTTAGCCGGGTTGGTCAAGGTCGCCTCGAGCTTCTTCCGGTCAACACCCGAAGACCTCGAGAGAGCAG

AGAAACAGCTCAAAGCACGTGACATCAATGACATTTTCGCCATTCTCAAGAACGGCGAGTGGCTGGTCAA

GCTGATCCTTGCCATCCGCGACTGGATTAAGGCTTGGATCGCCTCAGAAGAGAAGTTTGTCACCATGACA

GACTTGGTGCCTGGCATTCTTGAAAAGCAGAGGGACCTTAACGACCCGAGCAAGTACAAGGAAGCCAAGG

AGTGGCTCGACAACGCGCGCCAAGCGTGTTTGAAGAACGGGAACACTCACATTGCCAACCTGTGTAAAGT

GGTCGCCCCAGCACCTAGCAAGTCGAGGCCCGAGCCTGTGGTCGTTTGCCTTCGCGGCAAATCTGGCCAG

GGCAAGAGCTTCCTTGCGAACGTGCTCGCGCAGGCGATTTCCACCCACTTCACTGGCAGAACTGATTCGG

TTTGGTACTGCCCACCAGACCCTGACCACTTTGACGGTTACAACCAACAGACCGTTGTCGTAATGGATGA

TTTAGGCCAGAACCCTGACGGTAAGGACTTCAAGTACTTCGCCCAGATGGTCTCAACCACAGGGTTCATC

CCGCCCATGGCATCACTTGAAGACAAAGGCAAACCCTTCAACAGTAAGGTCATCATTGCAACCACCAATC

TGTACTCGGGTTTCACCCCAAGGACTATGGTGTGCCCTGATGCCCTGAACCGGAGGTTCCACTTTGATAT

CGACGTGAGTGCTAAGGACGGGTACAAAATTAACAACAAATTGGACATTATCAAAGCACTTGAGGACACC

CACACCAACCCAGTGGCAATGTTTCAGTACGATTGTGCCCTTCTCAATGGCATGGCCGTTGAAATGAAGA

GAATGCAACAAGATATGTTCAAGCCTCAACCACCCCTCCAGAACGTGTACCAGCTCGTTCAGGAGGTGAT

TGAACGGGTAGAGCTCCACGAGAAAGTGTCGAGCCACCCCATTTTCAAACAG

>AY593807.1_C_Brazil_1955

CCCTGGTACAAGCTTATCAAGCTCCTGAGCCGCTTGTCGTGCATGGCCGCTGTAGCAGCACGTTCAAAGG

ACCCAGTCCTTGTGGCCATCATGCTGGCTGACACCGGCCTTGAGATTCTGGACAGCACCTTCGTCGTGAA

GAAAATCTCCGACTCGCTCTCCAGTCTCTTTCACGTGCCGGCCCCCGTCTTCAGTTTCGGAGCCCCGATT

CTGTTGGCCGGGTTGGTCAAGGTCGCCTCGAGTTTCTTCCGGTCCACACCCGAGGAGCTTGAGAGAGCAG

AGAAACAGCTCAAAGCACGTGACATCAACGACATTTTCGCCATTCTCAAGAACGGCGAGTGGCTGGTTAA

GTTGATCCTTGCCATCCGCGACTGGATAAAAGCATGGATCGCCTCAGAAGAAAAGTTTGTCACCATGACA

GACTTGGTGCCTGGCATCCTTGAAAAGCAGCGGGACCTTAACGACCCAAGCAAGTACAAGGAAGCCAAGG

AGTGGCTCGACAACGCGCGCCAGGCGTGTTTGAAGAGCGGGAACGTCCACATTGCCAACCTGTGCAAAGT

GGTCGCCCCAGCACCCAGCAAGTCGAGACCCGAGCCTGTGGTCGTTTGCCTCCGTGGTAAATCCGGCCAG

GGTAAGAGTTTCCTTGCGAACGTGCTCGCACAAGCAATCTCTACCCACTTTACCGGCAGAACTGACTCTG

TTTGGTACTGCCCACCTGACCCCGACCACTTCGACGGTTACAACCAACAGACTGTCGTTGTGATGGATGA

TTTGGGCCAGAACCCTGACGGCAAGGACTTCAAGTACTTCGCCCAAATGGTTTCGACCACGGGGTTCATC

CCGCCCATGGCGTCACTTGAGGACAAAGGTAAACCCTTCAATAGTAAGGTCATCATTGCAACTACCAACC

TGTACTCGGGCTTCACCCCGAGGACCATGGTGTGCCCTGATGCCCTGAATCGGAGGTTTCACTTTGACAT

CGACGTGAGCGCTAAGGACGGGTACAAAATTAACAACAAATTGGACATTATCAAAGCACTTGAAGACACC

CACACCAACCCAGTGGCAATGTTTCAGTACGATTGTGCCCTTCTCAACGGCATGGCCGTTGAAATGAAGA

GAATGCAACAAGACGTGTTCAAGCCTCAGCCACCCCTCCAGAACGTCTACCAACTTGTTCAGGAGGTGAT

TGAGCGGGTGGAGCTCCACGAGAAAGTGTCGAGCCACCCGATTTTTAAGCAG

>AY593808.1_C_ARG_1966

CCCTGGTACAAGCTTATCAAGCTCCTGAGCCGCTTGTCGTGCATGGCCGCTGTAGCAGCACGGTCAAAGG

ACCCAGTCCTTGTGGCCATTATGCTGGCTGACACCGGTCTCGAGATTCTGGACAGCACGTTTGTCGTGAA

GAAGATCTCCGACTCGCTCTCCAGTCTCTTTCACGTGCCGGCCCCCGCCTTCAGCTTCGGAGCCCCGATT

CTGTTGGCCGGGTTGGTCAAGGTCGCCTCGAGTTTCTTCCGGTCTACGCCCGAAGACCTTGAGAGAGCAG

AGAAACAGCTCAGAGCACGTGACATCAACGACATCTTTGCCATTCTCAAGAACGGCGAGTGGCTGGTCAA

GCTGATCCTTGCCATCCGCGACTGGATTAAGGCATGGATTGCCTCAGAAGAGAAGTTTGTCACCATGACA

GACCTGGTGCCTGGCATTCTTGAAAAGCAGCGGGACCTCAACGACCCGAGCAAGTATCAGGAAGCCAAGG

AATGGCTCGACAACGCGCGCCAAGCGTGTTTGAAGAGTGGCAACGTCCACATTGCCAACTTGTGCAAAGT

GGTCGCCCCAGCACCCAGCAAGTCGAGACCCGAGCCCGTGGTCGTTTGCCTCCGCGGCAAATCTGGCCAG

GGCAAGAGTTTCCTTGCGAACGTGCTCGCACAAGCAATCTCCACCCACTTCACCGGTAGAACCGACTCTG

TTTGGTACTGCCCGCCTGACCCTGACCACTTCGACGGTTACAACCAACAGACCGTTGTTGTGATGGACGA

TTTGGGCCAGAACCCTGACGGCAAGGACTTTAAGTACTTCGCCCAAATGGTTTCAACCACGGGGTTCATC

CCGCCCATGGCATCACTTGAGGACAAAGGCAAACCCTTCAACAGCAAGGTCATTATTGCAACCACCAACC

TGTACTCGGGCTTTACCCCGAGGACCATGGTGTGCCCTGATGCCCTGAACCGGAGGTTTCACTTTGACAT

CGACGTGAGTGCCAAGGATGGGTACAAAATTAACAACAAATTGGACATTATCAAAGCACTTGAAGACACC

CATACCAACCCAGTGGCAATGTTTCAGTACGATTGTGCCCTTCTCAACGGCATGGCTGTTGAAATGAAGA

GAATGCAACAGGATATGTTCAAGCCTCAACCACCCCTCCAGAACGTGTACCAACTTGTTCAGGAGGTGAT

TGAACGGGTCGAGCTTCACGAGAAAGTGTCAAGCCACCCAATTTTCAAGCAG

>AY593809.1_C_ARG_1969

CCCTGGTACAAGCTTATTAAGCTCCTGAGCCGCTTGTCGTGCATGGCCGCTGTAGCAGCACGTTCAAAGG

ACCCGGTCCTTGTGGCCATCATGCTGGCTGACACCGGCCTTGAGATTCTGGACAGCACCTTCGTCGTGAA

GAAAATCTCCGACTCGCTCTCCAGTCTCTTTCACGTGCCGGCCCCCGTCTTCAGTTTCGGAGCCCCGATT

CTGTTGGCCGGGTTGGTCAAGGTTGCCTCGAGTTTCTTCCGTTCCACACCCGAGGAGCTTGAGAGAGCAG

AGAAACAGCTCAAAGCACGTGACATCAACGACATTTTCGCCATTCTCAAGAACGGCGAGTGGCTGGTTAA

GTTGATCCTTGCCATCCGCGACTGGATAAAAGCATGGATTGCCTCAGAAGAAAAGTTTGTCACCATGACA

GACTTGGTGCCTGGCATCCTTGAAAAACAGCGGGACCTCAACGACCCAAGTAAGTACAAGGAAGCCAAGG

AGTGGCTCGACAACGCGCGTCAGGCGTGTTTGAAGAGCGGGAACGTCCACATTGCCAACCTGTGCAAAGT

GGTCGCCCCAGCACCCAGCAAGTCGAGACCCGAGCCTGTGGTCGTTTGCCTCCGTGGTAAATCCGGCCAG

GGTAAGAGTTTCCTTGCGAACGTGCTCGCACAAGCAATCTCTACCCACTTTACCGGCAGAACTGACTCTG

TTTGGTACTGCCCACCTGACCCCGACCACTTCGACGGTTACAACCAACAGACTGTCGTTGTGATGGATGA

TTTGGGCCAGAACCCTGACGGCAAGGACTTCAAGTACTTCGCCCAAATGGTTTCGACCACGGGGTTCATC

CCGCCCATGGCGTCACTTGAGGACAAAGGTAAACCCTTCAATAGTAAGGTCATCATTGCAACTACCAACC

TGTACTCGGGCTTTACCCCGAGGACCATGGTGTGCCCTGATGCCCTGAATCGGAGGTTTCACTTTGACAT

CGACGTGAGCGCAAAGGACGGGTACAAAATTAACAACAAATTGGACATTATCAAAGCACTTGAAGACACC

CACACCAACCCAGTGGCAATGTTTCAGTACGATTGTGCCCTTCTCAACGGCATGGCCGTTGAAATGAAGA

GAATGCAACAAGACGTGTTCAAGCCTCAGCCACCCCTCCAGAACGTCTACCAACTTGTTCAGGAGGTGAT

TGAGCGGGTGGAGCTCCACGAGAAAGTGTCGAGCCACCCGATTTTTAAGCAG

>AY593810.1_C_UKG_1970

CCCTGGTACAAGCTTATCAAACTCCTAAGCCGCCTGTCGTGCATGGCCGCTGTGGCAGCACGGTCCAAGG

ACCCAGTCCTTGTGGCCATCATGCTGGCCGACACCGGCCTCGAGATTCTGGACAGCACCTTTGTCGTGAA

GAAGATTTCCGACTCGCTCTCCAGTCTCTTTCACGTGCCGGCCCCCGTCTTCAGTTTCGGAGCCCCGATC

CTGTTGGCTGGGTTGGTCAAGGTCGCCTCGAGTTTCTTCCGGTCCACACCCGAAGACCTCGAGAGAGCAG

AAAAACAGCTCAAAGCACGTGACATTAACGACATTTTCGCCATTCTCAAGAATGGCGAGTGGCTGGTCAA

GTTGATCCTTGCCATCCGCGACTGGATCAAGGCATGGATCGCCTCAGAAGAGAAGTTTGTCACCATGACA

GACTTGGTACCTGGTATCCTTGAAAAGCAGCGGGACCTCAGCGACCCAAGCAAGTACAAGGAGGCTAAGG

AGTGGCTCGACAACGCGCGCCAGGCGTGTTTGAAGAGCGGGAACGTCCACATTGCCAACTTGTGCAAAGT

GGTCGCCCCAGCACCTAGCAAGTCGAGACCCGAACCTGTGGTCGTTTGCCTCCGTGGCAAATCTGGCCAG

GGGAAGAGTTTCCTTGCAAACGTGCTCGCTCAAGCAATCTCCACCCACTTCACCGGCAGAACCGATTCAG

TTTGGTACTGCCCGCCTGACCCTGACCACTTCGACGGTTACAACCAGCAGACCGTTGTCGTGATGGATGA

TTTGGGCCAGAACCCTGATGGCAAAGACTTCAAGTACTTTGCCCAAATGGTTTCAACTACGGGGTTCATC

CCGCCCATGGCATCACTTGAGGACAAAGGTAAACCTTTCAACAGCAAGGTCATCATTGCGACCACCAACC

TGTACTCGGGCTTTACCCCGAGAACTATGGTGTGCCCTGATGCACTGAACCGGAGGTTCCACTTTGACAT

CGATGTGAGCGCCAAGGACGGGTACAAAATTAACAACAAATTGGACATCATCAAAGCGCTTGAAGACACC

CACACCAACCCAGTGGCAATGTTTCAGTACGATTGTGCCCTTCTCAACGGCATGGCCGTTGAAATGAAGA

GAATGCAACAAGATGTGTTCAAGCCTCAACCGCCCCTCCAGAACGTATACCAACTCGTTCAGGAGGTGGT

TGAACGGGTCGAGCTCCACGAGAAAGTGTCGAGCCACCCAATTTTCAAGCAG

>AY593812.1_O_PHI_1958

CCCTGGTACAAGCTCATCAAGCTCCTGAGCCGCTTGTCATGTATGGCCGCTGTAGCAGCACGGTCAAAGG

ACCCAGTCCTCGTGGCCATTATGCTGGCTGACACCGGTCTCGAGATTCTGGACAGCACCTTTGTCGTGAA

GAAGATTTCCGACTCGCTCTCCAGTCTCTTTCACGTGCCGGCCCCCGTCTTCAGTTTCGGAGCTCCGATT

CTGTTGGCCGGGTTGGTCAAAGTCGCCTCAAGTTTCTTCCGGTCCACACCCGAAGACCTTGAGAGAGCAG

AGAAACAGCTCAAAGCACGTGACATTAACGACATATTCGCCATTCTTAAGAACGGCGAGTGGCTGGTCAA

ACTGATTCTTGCCATCCGCGACTGGATCAAGGCATGGATCACCTCAGAGGAAAAGTTTGTTACCATGACA

GACTTGGTGCCTGGAATTCTTGAAAAGCAGCGGGACCTCAACGACCCCAGCAAGTACAAGGAGGCCAAGG

AGTGGCTCGATAACGCGCGCCAGGCGTGTCTGAAGAGCGGGAACACTTGCATCGCCAACCTGTGCAAAGT

GGTCACTCCAGCGCCCAGCAGGTCGAGACCCGAACCCGTGGTCGTTTGCCTCCGTGGCAAATCTGGCCAG

GGAAAGAGTTTCCTTGCGAACGTGCTCGCACAAGCAATTTCCACCCATTTCACTGGCAGAACCGATTCAG

TTTGGTACTGTCCACCTGACCCTGACCACTTCGACGGTTACAACCAACAGACCGTTGTTGTGATGGATGA

TTTGGGCCAGAACCCTGACGGCAAGGACTTTAAGTACTTCGCCCAGATGGTTTCAACCACGGGGTTCATC

CCGCCCATGGCTTCACTCGAAGACAAAGGCAAACCTTTTAACAGCAAAGTCATCATTGCAACCACCAACC

TGTACTCGGGCTTTACCCCGAGGACCATGGTGTGCCCTGACGCACTGAACCGAAGGTTTCACTTTGACAT

TGACGTGAGTGCCAGGGACGGGTACAAAATTAACAACAAATTGGACATAATTAAAGCTCTTGAAGATACC

CACACTAACCCAGTGGCAATGTTTCAATACGACTGTGCCCTTCTCAACGGCATGGCTGTTGAAATGAAGA

GAATGCAACAAGATGTTTTCAAGCCTCAACCACCCCTCCTGAACGTGTACCAACTTGTTCAGGAGGTGAT

TGAAAGGGTCGAGCTCCACGAGAAAGTGTCGAGCCACCCAATCTTCAAGCAG

>AY593813.1_O_ISA_1962

CCATGGTACAAGCTTATCAAACTCCTAAGCCGCCTGTCGTGCATGGCCGCTGTAGCAGCACGGTCAAAGG

ACCCTGTCCTTGTGGCCATCATGCTGGCTGACACCGGCCTCGAGATTCTAGACAGCACGTTCGTCGTGAA

GAAGATCTCCGACTCGCTCTCCAGTCTCTTTCACGTGCCGGCCCCGGTTTTCAGTTTCGGAGCTCCGATT

CTGTTAGCCGGACTGGTCAAAGTCGCCTCGAGTTTCTTCCGGTCCACACCCGAAGACCTTGAGAGAGCAG

AAAAACAGCTCAAAGCACGTGACATTAACGACATTTTCGCCATTCTCAAGAACGGCGAATGGCTGGTCAA

GCTGATTCTTGCCATCCGCGACTGGATTAAGGCATGGATCGCCTCAGAAGAGAAGTTTGTCACCATGACA

GACTTGGTGCCTGGCATCCTTGAGAAACAACGGGACCTCAACGATCCTGCTAAGTACAAGGAGGCCAAGG

AGTGGCTCGACAACACGCGCCAGGCGTGTTTGAAGAGCGGAAACACTCACATTGCTAACCTCTGCAAAGT

GGTTGCCCCAGCACCCAGCAAGTCGAGACCCGAGCCCGTGGTCGTGTGCCTCCGTGGCAAATCCGGCCAG

GGCAAGAGTTTCCTTGCGAACGTGCTTGCACAAGCAATCTCCACCCACTTCACTGGCAGAACTGACTCAG

TTTGGTACTGCCCGCCAGACCCTGACCACTTCGACGGTTACAGCCAACAGACCGTAGTAGTGATGGATGA

TTTGGGCCAGAACCCTGACGGCAAGGACTTTAAGTACTTTGCCCAAATGGTTTCGACCACGGGGTTTATC

CCACCGATGGCCTCACTTGAGGACAAAGGGAAACCTTTTAACAGCAAGGTCATCATTGCTACAACCAACC

TGTACTCAGGCTTCACCCCGAGAACTATGGTTTGCCCTGATGCACTGAACCGTAGGTTTCACTTTGACAT

CGATGTGAGCGCTAAGGATGGGTACAAAACTAACAACAAATTGGACATCATCAAAGCTCTTGAAGACACA

CACACCAACCCAGTGGCAATGTTTCAATACGACTGCGCCCTTCTCAACGGCATGGCGGTCGAGATGAAAA

GGATGCAACAAGATGTGTTTAAGCCTCTGCCACCCCTCCAGAACGTGTACCAACTTGTCCAGGAGGTGAT

TGAGCGGGTTGAGCTCCACGAGAAAGTGTCGAGCCACCCGATCTTCAAACAG

>AY593814.1_O_ARG_1965

CCCTGGTACAAGCTCATCAAGCTCCTAAGCCGCCTGTCGTGCATGGCCGCTGTGGCAGCACGGTCCAAGG

ACCCAGTCCTTGTGGCCATCATGCTGGCCGACACCGGTCTCGAGATTCTGGACAGCACCTTCGTCGTGAA

GAAGATCTCCGACTCGCTCTCCAGTCTCTTTCACGTGCCGGCCCCCGTCTTCAGTTTCGGAGCACCGGTC

CTGTTGGCCGGGTTGGTCAAAGTCGCCTCGAGTTTCTTCCGGTCCACACCCGAAGACCTTGAGAGAGCAG

AGAAACAGCTCAAAGCACGTGACATCAACGACATCTTCGCCATTCTCAAGAACGGCGAGTGGCTGGTCAA

ACTGATCCTTGCCATCCGCGACTGGATTAAGGCTTGGATCGCCTCAGAAGAGAAGTTTGTCACCATGACA

GACTTGGTGCCTGGCATCCTTGAAAAGCAGCGGGATCTGAACGACCCGAGCAAGTACAAGGAGGCCAAGG

AGTGGCTCGACAACGCGCGCCAAGCGTGTTTGAAGAACGGGAACGTCCACATTGCCAACTTGTGTAAAGT

GGTCGCTCCAGCACCCAGCAAGTCGAGGCCCGAACCCGTGGTTGTTTGCCTCCGCGGCAAATCTGGCCAG

GGCAAGAGCTTCCTTGCAAACGTGCTTGCACAGGCAATTTCCGCCCACTTCACCGGCAGAACCGACTCAG

TGTGGTACTGCCCACCTGACCCTGACCACTTCGACGGTTACAACCAGCAAACCGTCGTTGTGATGGATGA

TTTGGGCCAGAACCCTGACGGCAAGGACTTCAAATACTTTGCCCAAATGGTCTCGACCACAGGGTTCATC

CCGCCCATGGCATCACTCGAGGACAAAGGTAAACCTTTCAACAGCAAAGTCATCATCGCGACCACCAACT

TGTACTCGGGCTTCACCCCGAGGACTATGGTGTGTCCCGACGCACTGAACCGGAGGTTTCACTTTGACAT

CGATGTGAGTGCTAAGGATGGGTACAAAATTAACAACAAACTGGACATTATCAAAGCACTTGAAGACACC

CACACCAACCCAGTGGCAATGTTTCAATACGACTGTGCCCTTCTCAACGGCATGGCCGTTGAAATGAAGA

GAATGCAACAAGACATGTTCAAGCCCCAACCACCCCTCCAGAATGTGTACCAGCTTGTTCAGGAGGTGAT

TGATCGGGTCGAGCTCCACGAGAAAGTGTCGAGCCACCCGATCTTCAAGCAG

>AY593815.1_O_UKG_1967

CCCTGGTACAAGCTTATCAAGCTCCTAAGCCGCCTGTCGTGCATGGCCGCTGTGGCAGCACGGTCCAAGG

ACCCAGTCCTTGTGGCCATCATGCTGGCCGACACCGGTCTCGAGATTCTGGACAGCACCTTCGTCGTGAA

GAAGATCTCCGACTCGCTCTCCAGTCTCTTTCACGTGCCGGCCCCCGTCTTCAGTTTCGGAGCACCGGTC

CTGTTGGCCGGGTTGGTCAAAGTCGCCTCGAGTTTCTTCCGGTCTACACCCGAAGACCTTGAGAGAGCAG

AGAAACAGCTCAAAGCACGTGACATCAACGACATCTTCGCCATTCTCAAGAACGGCGAGTGGCTGGTCAA

ACTGATCCTTGCCATCCGCGACTGGATTAAGGCTTGGATCGCCTCAGAAGAGAAGTTTGTCACCATGACA

GACTTGGTGCCTGGCATCCTTGAAAAGCAGCGGGATCTGAACGACCCGAGCAAGTACAAGGAAGCCAAGG

AGTGGCTCGACAACGCGCGCCAAGCGTGTTTGAAGAGCGGGAACGTCCACATTGCCAACCTGTGTAAAGT

GGTCGCTCCAGCACCCAGCAAGTCGAGGCCCGAACCCGTGGTTGTTTGTCTCCGCGGCAAATCTGGCCAG

GGCAAGAGCTTCCTTGCAAACGTGCTTGCACAGGCAATTTCCGCCCACTTCACCGGCAGAACCGACTCAG

TGTGGTACTGCCCACCTGACCCTGACCACTTCGACGGTTACAACCAGCAAACCGTCGTTGTGATGGATGA

TTTGGGCCAGAACCCTGACGGCAAGGACTTCAAATACTTTGCCCAAATGGTCTCGACCACAGGGTTCATC

CCGCCCATGGCATCACTCGAGGACAAAGGTAAACCTTTCAACAGCAAAGTCATCATCGCGACCACCAACT

TGTACTCGGGCTTCACCCCGAGGACTATGGTGTGTCCCGACGCACTGAACCGGAGGTTTCACTTTGACAT

CGATGTGAGTGCTAAGGATGGGTACAAAATTAACAACAAATTGGACATTATCAAAGCACTTGAAGACACC

CACACCAACCCAGTGGCAATGTTTCAATACGACTGTGCCCTTCTCAACGGCATGGCCGTTGAAATGAAGA

GAATGCAACAAGACATGTTCAAGCCTCAACCACCCCTCCAGAATGTGTACCAGCTTGTTCAGGAGGTGAT

TGATCGGGTCGAGCTCCACGAGAAAGTGTCGAGCCACCCGATCTTCAAGCAG

>AY593816.1_O_UKG_1967

CCCTGGTACAAGCTTATCAAGCTCCTAAGCCGCCTGTCGTGCATGGCCGCTGTGGCAGCACGGTCCAAGG

ACCCAGTCCTTGTGGCCATCATGCTGGCCGACACCGGTCTCGAGATTCTGGACAGCACCTTCGTCGTGAA

GAAGATCTCCGACTCGCTCTCCAGTCTCTTTCACGTGCCGGCCCCCGTCTTCAGTTTCGGAGCACCGGTC

CTGTTGGCCGGGTTGGTCAAAGTCGCCTCGAGTTTCTTCCGGTCTACACCCGAAGACCTTGAGAGAGCAG

AGAAACAGCTCAAAGCACGTGACATCAACGACATCTTCGCCATTCTCAAGAACGGCGAGTGGCTGGTCAA

ACTGATCCTTGCCATCCGCGACTGGATTAAGGCTTGGATCGCCTCAGAAGAGAAGTTTGTCACCATGACA

GACTTGGTGCCTGGCATCCTTGAAAAGCAGCGGGATCTGAACGACCCGAGCAAGTACAAGGAAGCCAAGG

AGTGGCTCGACAACGCGCGCCAAGCGTGTTTGAAGAGCGGGAACGTCCACATTGCCAACCTGTGTAAAGT

GGTCGCTCCAGCACCCAGCAAGTCGAGGCCCGAACCCGTGGTTGTTTGTCTCCGCGGCAAATCTGGCCAG

GGCAAGAGCTTCCTTGCAAACGTGCTTGCACAGGCAATTTCCGCCCACTTCACCGGCAGAACCGACTCAG

TGTGGTACTGCCCACCTGACCCTGACCACTTCGACGGTTACAACCAGCAAACCGTCGTTGTGATGGATGA

TTTGGGCCAGAACCCTGACGGCAAGGACTTCAAATACTTTGCCCAAATGGTCTCGACCACAGGGTTCATC

CCGCCCATGGCATCACTCGAGGACAAAGGTAAACCTTTCAACAGCAAAGTCATCATCGCGACCACCAACT

TGTACTCGGGCTTCACCCCGAGGACTATGGTGTGTCCCGACGCACTGAACCGGAGGTTTCACTTTGACAT

CGATGTGAGTGCTAAGGATGGGTACAAAATTAACAACAAATTGGACATTATCAAAGCACTTGAAGACACC

CACACCAACCCAGTGGCAATGTTTCAATACGACTGTGCCCTTCTCAACGGCATGGCCGTTGAAATGAAGA

GAATGCAACAAGACATGTTCAAGCCTCAACCACCCCTCCAGAATGTGTACCAGCTTGTTCAGGAGGTGAT

TGATCGGGTCGAGCTCCACGAGAAAGTGTCGAGCCACCCGATCTTCAAGCAG

>AY593817.1_O_Belgium_1973

CCCTGGTACAAGCTTATCAAGCTCCTAAGCCGCCTGTCGTGCATGGCCGCTGTGGCAGCACGGTCCAAGG

ACCCAGTCCTTGTGGCCATCATGCTGGCCGACACCGGTCTCGAGATTCTGGACAGCACCTTCGTCGTGAA

GAAGATCTCCGACTCGCTCTCCAGTCTCTTTCACGTGCCGGCCCCCGTCTTCAGTTTCGGAGCACCGGTC

CTGTTGGCCGGGTTGGTCAAAGTTGCCTCGAGTTTCTTCCGGTCCACACCCGAAGACCTTGAGAGAGCAG

AGAAACAACTCAAAGCACGTGACATCAACGACATCTTCGCCATTCTCAAGAACGGCGAGTGGCTGGTCAA

ACTGATCCTTGCCATCCGCGACTGGATTAAGGCTTGGATCGCCTCAGAAGAGAAGTTTGTCACCATGACA

GACTTGGTGCCTGGCATCCTTGAAAAGCAGCGGGACCTGAACGACCCGAGCAAGTACAAAGAAGCCAAGG

AGTGGCTCGACAACGCGCGCCAAGCGTGTTTGAAGAGCGGGAACGTCCACATTGCCAACCTGTGTAAAGT

GGTCGCTCCAGCACCCAGCAAGTCGAGGCCCGAACCCGTGGTTGTTTGCCTCCGCGGCAAATCTGGCCAG

GGCAAGAGCTTCCTTGCAAACGTGCTTGCACAGGCAATTTCCGCCCACTTCACCGGCAGAACCGACTCAG

TGTGGTACTGCCCACCTGACCCTGACCACTTCGACGGTTACAACCAGCAAACCGTTGTTGTGATGGATGA

TTTGGGCCAGAACCCTGACGGCAAGGACTTCAAATACTTTGCCCAAATGGTCTCGACCACAGGGTTCATC

CCGCCCATGGCATCACTCGAGGACAAAGGTAAACCTTTCAACAGCAAAGTCATCATCGCGACCACCAACT

TGTACTCGGGCTTCACCCCGAGGACTATGGTGTGTCCCGACGCACTGAACCGGAGGTTTCACTTTGACAT

CGATGTGAGTGCTAAGGATGGGTACAAAATTAACAACAAATTGGACATTATCAAAGCACTTGAAGACACC

CACACCAACCCAGTGGCAATGTTCCAATACGACTGTGCCCTTCTCAACGGCATGGCCGTTGAAATGAAGA

GAATGCAACAAGACATGTTCAAGCCTCAACCACCCCTCCAGAATGTGTACCAGCTTGTTCAGGAGGTGAT

TGATCGGGTCGAGCTCCACGAGAAAGTGTCGAGCCACCCGATCTTCAAGCAG

>AY593818.1_O_ARG_1958

CCCTGGTACAAGCTTATCAAGCTCCTAAGCCGCCTGTCGTGCATGGCCGCTGTGGCAGCACGGTCCAAGG

ACCCAGTCCTTGTGGCCATCATGCTGGCCGACACCGGTCTCGAGATTCTGGACAGCACCTTCGTCGTGAA

GAAGATCTCCGACTCGCTCTCCAGTCTCTTTCACGTGCCGGCCCCCGTCTTCAGTTTCGGAGCACCGATC

CTGTTGGCCGGGTTGGTCAAAGTCGCCTCGAGTTTCTTCCGGTCCACACCCGAAGACCTTGAGAGAGCAG

AGAAACAGCTCAAAGCACGTGACATCAACGACATCTTCGCCATTCTCAAGAACGGCGAGTGGCTGGTCAA

GCTGATCCTTGCCATCCGCGACTGGATTAAGGCTTGGATCGCCTCAGAAGAGAAGTTTGTCACCATGACA

GACTTGGTGCCTGGCATCCTTGAAAAGCAGCGGGATCTGAACGACCCGAGCAAGTACAAGGAAGCCAAGG

AGTGGCTCGACAACGCGCGCCAAGCGTGTTTGAAGAGCGGGAACGTCCACATTGCCAACCTGTGTAAAGT

GGTCGCTCCAGCACCCAGCAAGTCGAGGCCCGAACCCGTGGTTGTTTGCCTCCGCGGCAAATCTGGCCAG

GGCAAGAGCTTCCTTGCAAACGTGCTTGCACAGGCAATTTCCGCCCACTTCACCGGCAGAACCGACTCAG

TGTGGTACTGCCCACCTGACCCTGACCACTTCGACGGTTACAACCAGCAAACCGTCGTTGTGATGGATGA

TTTGGGCCAGAACCCTGACGGCAAGGACTTCAAATACTTTGCCCAAATGGTCTCGACCACAGGGTTCATC

CCGCCCATGGCATCACTCGAGGACAAAGGTAAACCTTTCAACAGCAAAGTCATCATCGCGACCACCAACT

TGTACTCGGGCTTCACCCCGAGGACTATGGTGTGTCCCGACGCACTGAACCGGAGGTTTCACTTTGACAT

CGATGTGAGTGCTAAGGATGGGTACAAAATTAACAACAAATTGGACATTATCAAAGCACTTGAAGACACC

CACACCAACCCAGTGGCAATGTTTCAATACGACTGTGCCCTTCTCAACGGCATGGCCGTTGAAATGAAGA

GAATGCAACAAGACATGTTCAAGCCTCAACCACCCCTCCAGAATGTGTACCAGCTTGTTCAGGAGGTGAT

TGATCGGGTCGAGCTCCACGAGAAAGTGTCGAGCCACCCGATCTTCAAGCAG

>AY593819.1_O_ARG_1994

CCCTGGTACAAGCTTATCAAGCTCCTAAGCCGCCTGTCGTGCATGGCCGCTGTGGCAGCACGGTCCAAGG

ACCCAGTCCTTGTGGCCATCATGCTGGCCGACACCGGTCTCGAGATTCTGGACAGCACCTTCGTCGTGAA

GAAGATCTCCGACTCGCTCTCCAGTCTCTTTCACGTGCCGGCCCCCGTCTTCAGTTTCGGAGCACCGATC

CTGTTGGCCGGGTTGGTCAAAGTCGCCTCGAGTTTCTTCCGGTCCACACCCGAAGACCTTGAGAGAGCAG

AGAAACAGCTCAAAGCACGTGACATCAACGACATCTTCGCCATTCTCAAGAACGGCGAGTGGCTGGTCAA

GCTGATCCTTGCCATCCGCGACTGGATTAAGGCTTGGATCGCCTCAGAAGAGAAGTTTGTCACCATGACA

GACTTGGTGCCTGGCATCCTTGAAAAGCAGCGGGATCTGAACGACCCGAGCAAGTACAAGGAAGCCAAGG

AGTGGCTCGACAACGCGCGCCAAGCGTGTTTGAAGAGCGGGAACGTCCACATTGCCAACCTGTGTAAAGT

GGTCGCTCCAGCACCCAGCAAGTCGAGGCCCGAACCCGTGGTTGTTTGCCTCCGCGGCAAATCTGGCCAG

GGCAAGAGCTTCCTTGCAAACGTGCTTGCACAGGCAATTTCCGCCCACTTCACCGGCAGAACCGACTCAG

TGTGGTACTGCCCACCTGACCCTGACCACTTCGACGGTTACAACCAGCAAACCGTCGTTGTGATGGATGA

TTTGGGCCAGAACCCTGACGGCAAGGACTTCAAATACTTTGCCCAAATGGTCTCGACCACAGGGTTCATC

CCGCCCATGGCATCACTCGAGGACAAAGGTAAACCTTTCAACAGCAAAGTCATCATCGCGACCACCAACT

TGTACTCGGGCTTCACCCCGAGGACTATGGTGTGTCCCGACGCACTGAACCGGAGGTTTCACTTTGACAT

CGATGTGAGTGCTAAGGATGGGTACAAAATTAACAACAAATTGGACATTATCAAAGCACTTGAAGACACC

CACACCAACCCAGTGGCAATGTTTCAATACGACTGTGCCCTTCTCAACGGCATGGCCGTTGAAATGAAGA

GAATGCAACAAGACATGTTCAAGCCTCAACCACCCCTCCAGAATGTGTACCAGCTTGTTCAGGAGGTGAT

TGATCGGGTCGAGCTCCACGAGAAAGTGTCGAGCCACCCGATCTTCAAGCAG

>AY593820.1_O_ARG_1964

CCCTGGTACAAGCTTATCAAGCTCCTAAGCCGCCTGTCGTGCATGGCCGCTGTGGCAGCACGGTCCAAGG

ACCCAGTCCTTGTGGCCATCATGCTGGCCGACACCGGTCTCGAGATTCTGGACAGCACCTTCGTCGTGAA

GAAGATCTCCGACTCGCTCTCCAGTCTCTTTCACGTGCCGGCCCCCGTCTTCAGTTTCGGAGCACCGATC

CTGTTGGCCGGGTTGGTCAAAGTCGCCTCGAGTTTCTTCCGGTCCACACCCGAAGACCTTGAGAGAGCAG

AGAAACAGCTCAAAGCACGTGACATCAACGACATCTTCGCCATTCTCAAGAACGGCGAGTGGCTGGTCAA

GCTGATCCTTGCCATCCGCGACTGGATTAAGGCTTGGATCGCCTCAGAAGAGAAGTTTGTCACCATGACA

GACTTGGTGCCTGGCATCCTTGAAAAGCAGCGGGATCTGAACGACCCGAGCAAGTACAAGGAAGCCAAGG

AGTGGCTCGACAACGCGCGCCAAGCGTGTTTGAAGAGCGGGAACGTCCACATTGCCAACCTGTGTAAAGT

GGTCGCTCCAGCACCCAGCAAGTCGAGGCCCGAACCCGTGGTTGTTTGCCTCCGCGGCAAATCTGGCCAG

GGCAAGAGCTTCCTTGCAAACGTGCTTGCACAGGCAATTTCCGCCCACTTCACCGGCAGAACCGACTCAG

TGTGGTACTGCCCACCTGACCCTGACCACTTCGACGGTTACAACCAGCAAACCGTCGTTGTGATGGATGA

TTTGGGCCAGAACCCTGACGGCAAGGACTTCAAATACTTTGCCCAAATGGTCTCGACCACAGGGTTCATC

CCGCCCATGGCATCACTCGAGGACAAAGGTAAACCTTTCAACAGCAAAGTCATCATCGCGACCACCAACT

TGTACTCGGGCTTCACCCCGAGGACTATGGTGTGTCCCGACGCACTGAACCGGAGGTTTCACTTTGACAT

CGATGTGAGTGCTAAGGATGGGTACAAAATTAACAACAAATTGGACATTATCAAAGCACTTGAAGACACC

CACACCAACCCAGTGGCAATGTTTCAATACGACTGTGCCCTTCTCAACGGCATGGCCGTTGAAATGAAGA

GAATGCAACAAGACATGTTCAAGCCTCAACCACCCCTCCAGAATGTGTACCAGCTTGTTCAGGAGGTGAT

TGATCGGGTCGAGCTCCACGAGAAAGTGTCGAGCCACCCGATCTTCAAGCAG

>AY593821.1_O_ARG_1967

CCCTGGTACAAGCTCATCAAACTCCTAAGCCGCCTGTCGTGCATGGCCGCTGTGGCAGCACGGTCCAAGG

ACCCAGTCCTTGTGGCCATCATGCTGGCCGACACCGGTCTTGAGATTCTGGACAGCACCTTCGTCGTGAA

GAAGATCTCCGACTCGCTCTCCAGTCTCTTTCACGTGCCGGCCCCCGTCTTCAGTTTCGGAGCCCCGATT

CTGTTGGCCGGGTTGGTCAAGGTCGCCTCGAGTTTCTTCCGGTCCACACCCGAGGAGCTTGAGAGAGCAG

AGAAACAGCTCAAAGCACGTGACATCAACGACATTTTCGCCATTCTCAAGAACGGCGAGTGGCTGGTCAA

GCTGATCCTTGCCATCCGCGACTGGATCAAAGCATGGATCGCCTCAGAGGAAAAGTTTGTCACCATGACA

GACTTGGTGCCTGGCATCCTTGAAAAGCAGCGGGACCTTAACGACCCGAGCAAGTACAAGGAGGCCAAGG

AGTGGCTCGACAACGCGCGCCAGGCGTGTCTGAAGAGCGGGAATGTTCACATTGCCAACCTCTGCAAAGT

GGTCGCACCAGCACCCAGCAAGTCGAGACCCGAACCTGTGGTCGTTTGCCTCCGTGGCAAATCCGGCCAG

GGCAAGAGTTTCCTTGCGAACGTGCTTGCGCAAGCAATCTCCACCCACTTCACCGGCAGGACCGATTCTG

TTTGGTATTGTCCACCTGACCCCGACCACTTCGACGGCTACAACCAGCAGACCGTTGTTGTGATGGATGA

TTTGGGCCAGAACCCCGACGGCAAGGACTTTAAGTACTTCGCCCAGATGGTCTCGACCACGGGGTTCATC

CCACCCATGGCCTCGCTCGAAGACAAGGGTAAACCTTTCAACAGTAAGGTCATCATAGCAACCACCAACC

TGTACTCGGGTTTCACCCCGAGGACCATGGTGTGCCCTGATGCCCTGAACCGAAGGTTTCACTTTGACAT

TGACGTGAGCGCCAAGGACGGGTACAAAATTAACAACAAATTGGACATCACCAAAGCTCTTGAAGACACC

CACACCAACCCGGTGGCCATGTTTCAATACGATTGTGCCCTTCTCAACGGCATGGCCGTTGAAATGAAGA

GAATGCAGCAAGATATGTTCAAACCACAACCACCCCTCCAGAATGTCTACCAACTTGTTCAGGAGGTGAT

TGAGCGGGTAGAGCTCCACGAGAAAGTGTCGAGCCACCCGATTTTCAAGCAG

>AY593823.1_O_TUR_1969

CCCTGGTACAAACTCATCAAGCTCCTGAGCCGCTTGTCATGCATGGCCGCTGTAGCAGCACGGTCAAAGG

ACCCAGTCCTTGTGGCCATCATGCTGGCTGACACCGGTCTTGAGATTCTGGACAGCACCTTTGTCGTGAA

GAAGATCTCCGACTCGCTCTCCAGTCTCTTTCACGTGCCGGCCCCCGTCTTCAGTTTCGGAGCCCCGATT

CTGTTGGCCGGGTTGGTCAAAGTCGCCTCGAGTTTCTTCCGGTCCACACCCGAAGACCTTGAGAGAGCAG

AAAAACAGCTCAAAGCACGTGACATTAACGACATATTCGCCATTCTCAAGAACGGCGAGTGGCTGGTCAA

GCTGATCCTTGCCATCCGCGACTGGATCAAAGCGTGGATCGCCTCAGAAGAAAAGTTTGTCACCATGACG

GACTTGGTGCCTGGTATCCTTGAAAAGCAGCGGGATCTCAACGACCCGAGTAAGTACAAGGAAGCCAAGG

AGTGGCTCGACAACGCGCGCCAGGCGTGTTTGAAGAGCGGGAACGTTCACATTGCCAATTTGTGCAAAGT

GGTCGCCCCGGCACCCAGCAAGTCGAGACCCGAACCCGTGGTCGTTTGCCTCCGCGGCAAATCCGGCCAG

GGGAAGAGTTTCCTTGCGAACGTGCTCGCGCAAGCAATCTCCACCCACTTCACCGGCAGAACTGATTCGG

TTTGGTACTGCCCGCCTGACCCTGACCACTTCGACGGTTACAACCAGCAGACCGTTGTCGTGATGGACGA

TTTGGGCCAGAACCCCGATGGCAAGGACTTCAAGTACTTCGCCCAGATGGTTTCGACCACGGGGTTCATC

CCGCCCATGGCCTCGCTTGAGGACAAAGGCAAGCCTTTCAACAGCAAAGTCATCATTGCTACCACCAACC

TGTACTCGGGTTTCACCCCGAGAACAATGGTGTGTCCTGACGCGCTGAACCGGAGGTTCCACTTTGACAT

CGACGTGAGTGCCAAGGACGGGTACAAAGTTAACAACAAATTGGACATAATCAAAGCTCTTGAAGACACC

CACACCAACCCAGTGGCGATGTTCCAATACGACTGTGCCCTTCTAAACGGTATGGCAGTTGAAATGAAGA

GAATGCAACAGGATATGTTCAAGCCTCAACCACCCCTCCAGAACGTGTACCAACTCGTTCACGAGGTGAT

TGAACGGGTCGAGCTCCACGAGAAGGTGTCGAGCCACCCGATTTTCAAACAG

>AY593824.1_O_SKR_2000

CCCTGGTACAAGCTCATCAAGCTCTTGAGCCGCCTGTCATGCATGGCCGCTGTAGCAGCACGGTCAAAGG

ACCCAGTCCTTGTGGCCATCATGCTGGCTGACACCGGCCTTGAGATTCTGGACAGTACCTTTGTCGTGAA

GAAGATCTCCGACTCGCTCTCCAGTCTCTTTCACGTGCCGGCCCCCGTCTTCAGTTTCGGAGCCCCGATT

TTGTTGGCCGGGTTGGTCAAAGTCGCCTCGACTTTCTTCCGGTCCACACCCGAAGACCTTGAGAGAGCGG

AGAAACAGCTCAAAGCACGTGACATCAATGACATATTCGCCATTCTCAAGAACGGCGAGTGGTTGGTCAA

GCTGATTCTTGCCATCCGCGACTGGATCAAGGCATGGATCGCCTCAGAAGAAAAATTTGTCACCATGACA

GACCTGGTACCTGGCATCCTTGAAAAGCAGCGGGATCTTAACGACCCAAGCAAGTACAAGGAGGCCAAGG

AGTGGCTCGACAACGCGCGCCAAGCGTGTTTGAAGAGCGGGAACATCCACATCGCAAACCTTTGTAAAGT

AGTTGCCCCAGCACCCAGCAGGTCGAGGCCTGAACCCGTGGTCGTTTGCCTCCGTGGCAAATCGGGCCAG

GGCAAGAGTTTCCTTGCGAACGTGCTTGCACAAGCAATTTCAACCCACTTCACTGGCAGAACCGATTCAG

TTTGGTACTGCCCACCTGACCCTGACCACTTCGACGGTTACAACCAGCAGACCGTTGTAGTAATGGATGA

TTTGGGCCAGAACCCCGACGGGAAGGACTTCAAATACTTCGCCCAAATGGTTTCAACCACGGGGTTTATC

CCGCCCATGGCTTCACTCGAGGACAAAGGCAAACCTTTCAACAGCAAGGTCATCATCGCCACCACCAACC

TGTACTCGGGCTTCACCCCGAGAACTATGGTGTGCCCTGATGCACTGAACCGAAGGTTCCACTTTGACAT

TGACGTGAGCGCCAAGGACGGGTACAAAATTAACAACAAATTGGACATCATCAAAGCTCTTGAAGATACC

CACACCAACCCAGTGGCAATGTTTCAATACGACTGTGCCCTTCTCAACGGCATGGCCGTTGAGATGAAGA

GAATGCAACAAGATATGTTCAAGCCTCAACCGCCCCTCCAGAACGTCTACCAGCTTGTTCAGGAGGTGAT

TGACCGGGTCGAGCTCCACGAGAAGGTGTCGAACCACCCGATTTTCAAGCAG

>AY593825.1_O_ARG_1939

CCTTGGTACAAGCTTATTAAGCTCCTAAGCCGCCTGTCGTGCATGGCCGCTGTAGCAGCACGGTCCAAGG

ACCCAGTCCTTGTGGCCATCATGCTGGCTGACACCGGTCTCGAGATTCTGGACAGCACCTTTGTCGTGAG

GAAAATCTCCGACTCGCTCTCCAGTCTCTTTCACGTGCCGGCCCCCGTCTTCAGTTTCGGAGCTCCGATC

CTGTTGGCCGGATTGGTCAAGGTCGCCTCGAGTTTCTTCCGGTCCACGCCCGAAGATCTTGAGAGAGCTG

AGAAGCAGCTCAAAGCACGTGACATTAACGATGTTTTCGCCATTCTCAAGAATGGCGAGTGGTTGGTCAA

GTTGATCCTTGCCATCCGCGACTGGATCAAGGCATGGATCGCCTCAGAAGAGAAGTTTGTCACCATGACG

GACTTGGTACCTGGAATCCTTGAAAAGCAGCGAGACCTTAACGACCCAAGCAAGTACAAGGAAGCCAAGG

AATGGCTCGACAACGCGCGCCAAGCGTGTTTGAAGAGTGGGAACGTCCACATTGCCAACCTGTGCAAAGT

GGTCGCCCCAGCACCCAGCAAGTCGAGACCCGAACCCGTGGTCGTTTGCCTCCGTGGTAAATCCGGCCAG

GGCAAGAGTTTCCTTGCAAACGTACTCGCGCAAGCAATCTCCACTCACTTCACTGGCAGAACCGATTCAG

TTTGGTACTGCCCGCCTGACCCTGACCACTTCGACGGCTACAACCAGCAGGCCGTTGTCGTGATGGATGA

TTTGGGCCAGAACCCCGATGGCAAGGACTTTAAGTACTTCGCCCAAATGGTTTCAACCACGGGGTTTATC

CCGCCCATGGCATCACTTGAGGACAAAGGCAAACCGTTCAACAGCAAGGTCATTATCGCAACCACCAACC

TGTACTCGGGCTTCACCCCGAGGACTATGGTCTGCCCCGATGCGTTGAACAGGAGGTTCCACTTTGACAT

CGACGTGAGCGCCAAGGACGAGTACAAGATCAACAACAAATTGGATATCATTAAAGCTCTTGAAGACACC

CACACCAACCCGGTGGCAATGTTTCAGTACGACTGTGCCCTCCTCAACGGCATGGCCGTTGAAATGAAGA

GAATGCAACAGGATGTGTTCAAGCCTCAACCACCCCTCCAGAACGTGTACCAGCTCGTTCAGGAGGTGAT

TGAACGGGTTGAGCTCCACGAAAAAGTGTCGAGCCACCCAATCTTCAAGCAG

>AY593826.1_O_ITL_1947

CCCTGGTACAAGCTTATCAAGCTCCTAAGCCGCCTGTCGTGCATGGCCGCTGTGGCAGCACGGTCCAAGG

ACCCAGTTCTTGTGGCCATCATGCTGGCCGACACCGGTCTCGAGATTCTGGACAGCACTTTCGTCGTGAA

GAAAATCTCCGACTCGCTCTCCAGTCTCTTTCACGTGCCGGCCCCCGCCTTCAGTTTCGGAGCTCCGATT

CTGTTGGCCGGGTTGGTCAAGGTCGCCTCGAGTTTCTTCCGGTCCACACCCGAAGACCTTGAGAGAGCAG

AGAAACAGCTCAAAGCACGTGACATCAACGACATTTTCGCCATTCTCAAGAACGGTGAGTGGCTGGTCAA

ACTGATCCTTGCCATCCGCGACTGGATTAAGGCATGGATTGCCTCAGAAGAGAAGTTCGTCACCATGACG

GACTTGGTGCCTGGCATCCTCGAAAAGCAGCGGGACCTTAACGACCCGAGCAAGTACAAGGAAGCCAAGG

AGTGGCTCGACAACGCGCGCCAGGCGTGTTTGAAGAGCGGGAACGTCCACATTGCCAACTTGTGCAAAGT

GGTCGCCCCAGCACCCAGCAAGTCGAGACCCGAACCTGTGGTCGTTTGCCTCCGTGGCAAGTCTGGCCAG

GGAAAGAGTTTCCTTGCGAACGTGCTCGCACAAGCAATTTCCACCCACTTCACTGGCAGAACCGACTCGG

TTTGGTACTGCCCGCCTGACCCTGACCACTTCGACGGTTACAACCAGCAGACCGTCGTTGTGATGGACGA

TTTGGGCCAGAACCCTGATGGCAAGGACTTTAAGTACTTTGCCCAAATGGTTTCAACCACAGGGTTCATC

CCGCCCATGGCATCTCTCGAGGACAAAGGCAAGCCTTTCAACAGCAAGGTCATCATTGCAACCACCAACC

TGTACTCGGGCTTCACCCCGAGGACAATGGTGTGCCCTGACGCACTGAACCGGAGGTTTCACTTTGACAT

CGACGTGAGCGCCAAGGACGGGTACAAAATTAACAACAAACTGGACATTACCAAAGCACTTGAAGACACC

CACACCAACCCAGTGGCAATGTTCAAGTACGATTGTGCCCTTCTCAACGGCATGGCTGTTGAAATGAAGA

GAATGCAACAAGATGTGTTCAAGCCTCAACCGCCCCTCCAAAACGTGTACCAGCTCGTTCAGGAGGTGAT

TGAACGGGTCGAGCTCCACGAGAAAGTGTCGAGCCACCAAATTTTCAAGCAG

>AY593827.1_O_VEN_1971

CCCTGGTACAAGCTTATCAAGCTCCTAAGCCGCCTGTCGTGCATGGCCGCTGTGGCAGCACGGTCCAAGG

ACCCAGTCCTTGTGGCCATCATGCTGGCCGACACCGGTCTCGAGATTCTGGACAGCACTTTCATTGTGAA

GAAAATCTCCGACTCGCTCTCCAGTCTCTTTCACGTGCCGGCCCCCGTCTTCAGTTTCGGAGCTCCGATT

CTGTTGGCCGGGTTGGTCAAGGTCGCCTCGAGTTTCTTCCGGTCCACACCCGAAGACCTTGAGAGAGCAG

AGAAACAGCTCAAAGCACGTGACATCAACGACATTTTCGCCATTCTCAAGAACGGTGAGTGGCTGGTCAA

ACTGATCCTTGCCATCCGCGACTGGATTAAGGCATGGATTGCCTCAGAAGAGAAGTTCGTCACCATGACG

GACTTGGTGCCTGGCATCCTCGAAAAGCAGCGGGACCTTAACGACCCGAGCAAGTACAAGGAAGCCAAGG

AGTGGCTCGACAACGCGCGCCAGGCGTGTTTGAAGAGCGGGAACGTCCACATTGCCAACCTGTGCAAAGT

GGTCGCCCCAGCACCCAGCAAGTCGAGACCCGAACCTGTGGTCGTTTGCCTCCGTGGCAAGTCTGGCCAG

GGTAAGAGTTTCCTTGCGAACGTGCTCGCACAAGCAATTTCCACCCACTTCACTGGCAGAACCGACTCGG

TTTGGTACTGCCCGCCTGACCCTGACCACTTCGACGGTTACAACCAGCAGACCGTCGTTGTGATGGACGA

TTTGGGCCAGAACCCTGATGGCAAGGACTTTAAGTACTTTGCCCAAATGGTTTCAACTACAGGGTTCATC

CCGCCCATGGCGTCTCTTGAGGACAAAGGCAAACCTTTCAACAGCAAGGTCATCATTGCAACTACCAACC

TGTACTCGGGCTTCACCCCGAGGACTATGGTGTGCCCTGACGCACTGAACCGGAGGTTTCACTTTGACAT

CGACGTGAGCGCCAAGGACGGGTACAAAATTAACAACAAATTGGACATTACCAAAGCACTTGAAGACACC

CACACCAACCCGGTGGCAATGTTTAAGTACGATTGTGCCCTTCTCAACGGCATGGCTGTTGAAATGAAGA

GAATGCAACAAGATGTGTTCAAGCCTCAACCGCCCCTCCAGAACGTGTACCAGCTCGTTCAGGAGGTGAT

TGAACGGGTCGAGCTCCACGAGAAAGTGTCGGGCCACCAAATTTTCAAGCAG

>AY593828.1_O_IND_1962

CCCTGGTACAAGCTCATCAAGCTCCTGAGCCGCTTGTCATGCATGGCCGCTGTAGCAGCACGGTCACAGG

ACCCAGTCCTTGTGGCCATCATGCTGGCTGACACCGGTCTCGAGATTCTGGACAGCACTTTTGTCGTGAA

GAAGATCTCCGACTCGCTCTCCAGTCTCTTTCACGTGCCGGCCCCCGTCTTCAGCTTCGGAGCCCCAATT

CTGTTGGCCGGGTTGGTCAAAGTCGCCTCGAGTTTCTTCCGGTCCACACCCGAAGACCTTGAGAGAGCAG

AGAAACAGCTCAAAGCACGTGACATCAATGACATATTCGCCATTCTCAAGAACGGCGAGTGGCTGGTCAA

GCTGATTCTTGCCATCCGCGACTGGATCAAGGCATGGATCGCCTCAGAAGAAAAGTTTGTCACCATGACA

GACTTGGTGCCTGGCATTCTTGAAAAGCAGCGGGACCTCAACGACCCCAGCAAGTACAAGGAGGCCAAGG

AGTGGCTCGACAACGCGCGCCAAGCGTGCTTGAAGAACGGGAACGTCCACATCGCCAACCTGTGCAAAGT

GGTCGCCCCGGCACCTAGCAAGTCGAGACCCGAACCCGTGGTCGTTTGCCTCCGTGGCAAATCCGGCCAG

GGAAAGAGTTTCCTTGCGAACGTGCTCGCACAAGCAATTTCTACCCACTTCACTGGCAGAACCGATTCAG

TTTGGTACTGTCCGCCTGACCCTGACCACTTCGACGGTTACAACCAACAGACCGTTGTTGTGATGGATGA

TTTGGGCCAGAACCCCGACGGCAAGGACTTCAAGTACTTCGCCCAGATGGTTTCAACCACGGGGTTCATC

CCGCCCATGGCTTCACTTGAGGACAAAGGCAAGCCTTTCAACAGCAAAGTCATCATCGCCACCACCAACT

TGTACTCGGGTTTCACCCCGAGGACCATGGTGTGCCCTGACGCACTGAACCGAAGGTTCCACTTTGACAT

CGATGTGAGTGCCAAGGACGGGTACAAAATTAACAACAAATTGGACATAACCAAAGCCCTTGAAGACACC

CACACCAACCCGGTGGCAATGTTTCAATACGACTGTGCCCTTCTCAACGGCATGGCTGTTGAGATGAAGA

GAATGCAACAAGACATGTTCAAGCCCCAACCACCTCTACAGAATGTGTACCAACTCGTTCAGGAGGTGAT

TGATCGGGTCGAGCTCCACGAGAAAGTGTCGAGCCACCCGATTTTCAAGCAA

>AY593830.1_O_POL_1959

CCCTGGTACAAGCTTATCAAGCTCCTAAGCCGCCTGTCGTGCATGGCCGCTGTGGCAGCACGGTCCAAGG

ACCCAGTCCTTGTGGCCATCATGCTGGCCGACACCGGTCTCGAGATTCTGGACAGCACCTTCGTCGTGAA

GAAGATCTCCGACTCGCTCTCCAGTCTCTTTCACGTGCCGGCCCCCGTCTTCAGTTTCGGAGCACCGGTC

CTGTTGGCCGGGTTGGTCAAAGTCGCCTCGAGTTTCTTCCGGTCCACACCCGAAGACCTTGAGAGAGCAG

AGAAACAGCTCAAAGCACGTGACATCAACGACATCTTCGCCATTCTCAAGAACGGCGAGTGGCTGGTCAA

GCTGATCCTTGCCATCCGCGACTGGATTAAGGCTTGGATCGCCTCAGAAGAGAAGTTTGTCACCATGACA

GACTTGGTGCCTGGCATCCTTGAAAAGCAGCGGGATCTGAACGACCCGAGCAAGTACAAGGAAGCCAAGG

AGTGGCTCGACAACGCGCGCCAAGCGTGTTTGAAGAGCGGGAACGTCCACATTGCCAACCTGTGTAAAGT

GGTCGCTCCAGCACCCAGCAAGTCGAGGCCCGAACCCGTGGTTGTTTGCCTCCGCGGCAAATCTGGCCAG

GGCAAGAGCTTCCTTGCAAACGTGCTTGCACAGGCAATTTCCACCCACTTCACCGGCAGAACCGACTCAG

TGTGGTACTGCCCACCTGACCCTGACCACTTCGACGGTTACAACCAGCAAACCGTCGTTGTGATGGATGA

TTTGGGCCAGAACCCTGACGGCAAGGACTTTAAATACTTTGCCCAAATGGTCTCGACCACAGGGTTCATC

CCGCCCATGGCATCACTCGAGGACAAAGGTAAACCTTTCAACAGCAAAGTCATCATCGCGACCACCAACT

TGTACTCGGGCTTCACCCCGAGGACTATGGTGTGTCCCGACGCACTGAACCGGAGGTTTCACTTTGACAT

CGATGTGAGTGCTAAGGATGGGTACAAAATTAACAACAAATTGGACATTAACAAAGCACTTGAAGACACC

CACACCAACCCAGTGGCAATGTTTCAATACGACTGTGCCCTTCTCAACGGCATGGCCGTTGAAATGAAGA

GAATGCAACAAGACATGTTCAAGCCTCAACCACCCCTCCAGAATGTGTACCAGCTTGTTCAGGAGGTGAT

TGATCGGGTCGAGCTCCACGAGAAAGTGTCGAGCCACCCGATCTTCAAGCAG

>AY593831.1_O_UKG_2002

CCCTGGTACAAGCTCATCAAGCTCTTGAGCCGCCTGTCATGCATGGCCGCTGTAGCAGCACGGTCAAAGG

ACCCAGTCCTTGTGGCCATCATGCTGGCTGACACCGGCCTTGAGATTCTGGACAGTACCTTTGTCGTGAA

GAAGATCTCCGACTCGCTCTCCAGTCTCTTTCACGTGCCGGCCCCCGTCTTCAGTTTCGGAGCCCCGATT

TTGTTGGCCGGGTTGGTCAAAGTCGCCTCGAGTTTCTTCCGGTCCACACCCGAAGACCTTGAGAGAGCGG

AGAAACAGCTCAAAGCACGTGACATCAATGACATATTCGCCATTCTCAAGAACGGCGAGTGGCTGGTCAA

GCTGATTCTTGCCATCCGCGACTGGATCAAGGCATGGATCGCCTCAGAAGAAAAGTTTGTCACCATGACA

GACCTGGTGCCTGGCATCCTTGAAAAGCAGCGGGATCTCAACGACCCAAGCAAGTACAAAGAGGCCAAGG

AGTGGCTCGACAACGCGCGCCAAGCGTGTTTGAAGAGCGGGAACATCCACATCGCAAACCTTTGCAAAGT

GGTTGCCCCAGCACCCAGCAGGTCGAGGCCCGAACCCGTGGTCGTTTGCCTCCGTGGCAAATCGGGCCAG

GGCAAGAGTTTCCTTGCGAACGTGCTTGCACAAGCAATTTCAACCCACTTCACTGGCAGAACCGACTCAG

TTTGGTACTGCCCACCTGACCCTGACCACTTCGACGGTTACAACCAGCAGACCGTTGTAGTAATGGATGA

TTTGGGCCAGAACCCCGACGGGAAGGACTTCAAGTACTTCGCCCAAATGGTTTCAACTACGGGGTTTATC

CCGCCCATGGCTTCACTCGAGGACAAAGGCAAACCTTTCAACAGCAAGGTCATCATCGCCACCACCAACC

TGTACTCGGGCTTCACCCCGAGAACTATGGTGTGCCCTGACGCACTGAACCGAAGGTTCCACTTTGACAT

TGACGTGAGCGCCAAGGACGGGTACAAAATTAACAACAAATTGGACATCATCAAAGCTCTTGAAGATACC

CACACCAACCCAGTGGCAATGTTTCAATACGACTGTGCCCTTCTCAACGGCATGGCCGTTGAAATGAAGA

GAATGCAACAAGATATGTTCAAGCCTCAACCGCCCCTCCAGAACGTCTACCAGCTTGTTCAGGAGGTGAT

TGACCGGGTCGAGCTCCACGAGAAGGTGTCGAGCCACCCGATTTTCAAGCAG

>AY593833.1_O_TAW_1999

CCCTGGTACAAGCTCATCAAACTTCTAAGCCGCCTGTCGTGCATGGCCGCTGTGGCAGCACGGTCCAAGG

ACCCAGTCCTTGTGGCCATCATGCTGGCCGACACCGGCCTCGAGATTCTGGACAGCACCTTCGTGGTAAA

GAAGATCTCCGACTCGCTCTCCAGTCTCTTCCACGTGCCGGCTCCCGCCTTCAGTTTCGGAGCCCCGATC

CTGTTGGCCGGGTTGGTCAAAGTCGCCTCGAGTTTCTTCCAGTCCACACCCGAAGACCTCGAGAGAGCAG

AAAAGCAGCTCAAAGCACGTGACATCAACGACATATTTGCCGTTCTTAAGAACGGTGAGTGGCTGGTCAA

ACTGATCCTGGCCATCCGCGACTGGATTAAGGCATGGATCGCCTCAGAAGAGAAGTTTGTCACCATGACA

GACCTGGTGCCTGGCATCCTTGAAAGACAACGGGATCTCAATGACCCCGGCAAATACAAGGAGGCCAAGG

AATGGCTGGACAACGCGCGTCAAGCGTGTTTGAAAAGCGGGAACGTGCACATTGCCAATCTGTGTAAAGT

GGTCGCTCCGGCGCCCAGCAAGTCGAGACCCGAACCAGTGGTCGTGTGCCTTCGCGGCAAATCCGGCCAA

GGGAAAAGCTTTCTCGCGAACGTTCTCGCGCAGGCAATTTCCACACACTTCACTGGTAGGACCGACTCGG

TCTGGTACTGCCCGCCCGACCCTGACCACTTTGACGGTTACAATCAGCAGACCGTCGTCGTGATGGACGA

CCTGGGCCAAAACCCAGACGGCAAAGACTTCAAGTACTTTGCCCAAATGGTATCCACCACGGGGTTCATC

CCGCCTATGGCCTCGCTCGAGGATAAGGGTAAACCCTTCAACAGCAAGGTCATAATAGCTACAACCAACC

TGTACTCGGGATTCACCCCAAAGACCATGGTGTGCCCCGATGCGCTTAACCGGAGGTTTCACTTTGACAT

CGACGTGAGCGCCAAAGACGGGTACAAGATCAACAACAAACTGGACATAGTCAAAGCACTTGAAGACACC

CACGCTAACCCGGTGGCGATGTTCCAATACGACTGCGCTCTTCTCAACGGAATGGCCGTTGAAATGAAGA

GAATGCAGCAAGACATGTTCAAGCCTCAACCACCCCTCCAGAACATCTACCAGCTCGTTCAGGAGGTGAT

TGAGCGGGTGGAACTACACGAAAAGGTGTCGAGCCACCCGATATTTAAACAG

>AY593834.1_O_IRN_1966

CCCTGGTACAAGCTCATTAAACTCCTGAGCCGCCTGTCGTGCATGGCCGCTGTAGCAGCACGGTCCAAGG

ACCCAGTTCTTGTGGCCATCATGCTGGCTGACACCGGCCTCGAGATTCTGGACAGCACGTTTGTCGTGAA

AAAGATCTCCGACTCGCTCTCCAGTCTCTTTCACGTGCCGGCCCCCGTCTTCAGTTTCGGAGCCCCGATT

CTGCTGGCCGGGTTGGTCAAAGTCGCCTCGAGTTTCTTCCGGTCGACACCCGAAGACCTCGAGAGAGCAG

AGAAACAGCTCAAAGCACGTGACATCAACGACATTTTCGCCATTCTCAAGAACGGCGAGTGGCTGGTCAA

ACTGATTCTTGCCATCCGCGACTGGATCAAGGCATGGATCGCCTCAGAAGAGAAGTTTGTCACCATGACA

GACTTGGTGCCTGGCATCCTTGAAAAGCAACGGGATCTCAACGACCCAAGCAAGTACAAGGAGGCCAAGG

AGTGGCTCGACAACGCGCGCCAGGCGTGTTTGAAGAGCGGGAACGTCCACATTGCCAACCTGTGCAAGGT

GGTCGCCCCGGCACCCAGCAAGTCGAGACCCGAACCTGTGGTCGTTTGTCTCCGTGGCAAGTCCGGCCAG

GGCAAGAGTTTCCTTGCGAACGTGCTCGCCCAAGCAATTTCCACACACTTCACCGGCAGAACTGATTCAG

TTTGGTACTGCCCACCTGACCCTGACCACTTCGACGGTTACAACCAACAGACCGTCGTTGTGATGGACGA

TTTGGGCCAGAACCCCGACGGCAAGGACTTTAAGTACTTTGCCCAAATGGTTTCGACCACGGGGTTCATC

CCGCCCATGGCGTCACTCGAGGACAAAGGTAAGCCTTTCAACAGCAAGGTCATCATTGCAACTACCAACT

TGTACTCGGGCTTTACCCCGAGGACCATGGTATGCCCTGATGCACTGAACCGGAGGTTTCACTTTGACAT

CGACGTGAGTGCTAAGGACGGGTACAAAATTAATAACAAATTGGACATTATCAAAGCACTTGAAGACACC

CACACTAACCCAGTGGCAATGTTTCAGTACGACTGTGCCCTTCTCAACGGCATGGCCGTTGAAATGAAGA

GAATGCAACAGGACATGTTCAAGCCTCAACCGCCCCTCCAGAACGTGTACCAACTCGTACAGGAGGTGAT

TGAACGGGTCGAGCTCCACGAGAAGGCGGCGAGCCACCCGATTTTCAAACAA

>AY593835.1_O_TAW_1997

CCCTGGTACAAGCTCATCAAACTTCTAAGCCGCCTGTCGTGCATGGCCGCTGTGGCAGCACGGTCCAAGG

ACCCAGTCCTTGTGGCCATCATGCTGGCCGACACCGGCCTCGAGATTCTGGACAGCACCTTCGTGGTAAA

GAAGATCTCCGACTCGCTCTCCAGTCTCTTCCACGTGCCGGCTCCCGCCTTCAGTTTCGGAGCCCCGATC

CTGTTGGCCGGGTTGGTCAAAGTCGCCTCGAGTTTCTTCCAGTCCACACCCGAAGACCTCGAGAGAGCAG

AAAAGCAGCTCAAAGCACGTGACATCAACGACATATTTGCCGTTCTTAAGAACGGTGAGTGGCTGGTCAA

ACTGATCCTGGCCATCCGCGACTGGATTAAGGCATGGATCGCCTCAGAAGAGAAGTTTGTCACCATGACA

GACCTGGTGCCTGGCATCCTTGAAAGACAACGGGATCTCAATGACCCCGGCAAATACAAGGAGGCCAAGG

AATGGCTGGACAACGCGCGTCAAGCGTGTTTGAAGAGCGGGAACGTGCACATTGCCAATCTGTGTAAGGT

GGTCGCTCCGGCGCCCAGCAAGTCGAGACCCGAACCAGTGGTCGTGTGCCTTCGCGGCAAATCCGGCCAA

GGGAAAAGCTTTCTCGCGAACGTTCTCGCGCAGGCAATTTCCACACACTTCACTGGTAGGACCGACTCGG

TCTGGTACTGCCCGCCCGACCCTGACCACTTTGACGGTTACAATCAGCAGACCGTCGTCGTGATGGACGA

CTTGGGCCAAAACCCAGACGGCAAAGACTTCAAGTACTTTGCCCAAATGGTATCCACCACGGGGTTCATC

CCGCCTATGGCCTCGCTCGAGGATAAGGGTAAACCCTTCAACAGCAAGGTCATAATAGCTACAACCAACC

TGTACTCGGGATTCACCCCAAAGACCATGGTGTGCCCCGATGCGCTTAACCGGAGGTTTCACTTTGACAT

CGACGTGAGCGCCAAAGACGGGTACAAGATCAACAACAAACTGGACATAGTCAAAGCACTTGAAGACACC

CACGCTAACCCGGTGGCGATGTTCCAATACGACTGCGCTCTTCTCAACGGAATGGCCGTTGAAATGAAGA

GAATGCAGCAAGACATGTTCAAGCCTCAACCACCCCTCCAGAACATCTACCAGCTCGTTCAGGAGGTGAT

TGAGCGGGTGGAACTACACGAAAAGGTGTCGAGCCACCTGATATTTAAACAG

>AY593836.1_O_UKG_2001

CCCTGGTACAAGCTCATCAAGCTCTTGAGCCGCCTGTCATGCATGGCCGCTGTAGCAGCACGGTCAAAGG

ACCCAGTCCTTGTGGCCATCATGCTGGCTGACACCGGCCTTGAGATTCTGGACAGTACCTTTGTCGTGAA

GAAGATCTCCGACTCGCTCTCCAGTCTCTTTCACGTGCCGGCCCCCGTCTTCAGTTTCGGAGCCCCGATT

TTGTTGGCCGGGTTGGTCAAAGTCGCCTCGAGTTTCTTCCGGTCCACACCCGAAGACCTTGAGAGAGCGG

AGAAACAGCTCAAAGCACGTGACATCAATGACATATTCGCCATTCTCAAGAACGGCGAGTGGCTGGTCAA

GCTGATTCTTGCCATCCGCGACTGGATCAAGGCATGGATCGCCTCAGAAGAAAAGTTTGTCACCATGACA

GACCTGGTGCCTGGCATCCTTGAAAAGCAGCGGGATCTCAACGACCCAAGCAAGTACAAAGAGGCCAAGG

AGTGGCTCGACAACGCGCGCCAAGCGTGTTTGAAGAGCGGGAACATCCACATCGCAAACCTTTGCAAAGT

GGTTGCCCCAGCACCCAGCAGGTCGAGGCCCGAACCCGTGGTCGTTTGCCTCCGTGGCAAATCGGGCCAG

GGCAAGAGTTTCCTTGCGAACGTGCTTGCACAAGCAATTTCAACCCACTTCACTGGCAGAACCGACTCAG

TTTGGTACTGCCCACCTGACCCTGACCACTTCGACGGTTACAACCAGCAGACCGTTGTAGTAATGGATGA

TTTGGGCCAGAACCCCGACGGGAAGGACTTCAAGTACTTCGCCCAAATGGTTTCAACTACGGGGTTTATC

CCGCCCATGGCTTCACTCGAGGACAAAGGCAAACCTTTCAACAGCAAGGTCATCATCGCCACCACCAACC

TGTACTCGGGCTTCACCCCGAGAACTATGGTGTGCCCTGACGCACTGAACCGAAGGTTCCACTTTGACAT

TGACGTGAGCGCCAAGGACGGGTACAAAATTAACAACAAATTGGACATCATCAAAGCTCTTGAAGATACC

CACACCAACCCAGTGGCAATGTTTCAATACGACTGTGCCCTTCTCAACGGCATGGCCGTTGAAATGAAGA

GAATGCAACAAGATATGTTCAAGCCTCAACCGCCCCTCCAGAACGTCTACCAGCTTGTTCAGGAGGTGAT

TGACCGGGTCGAGCTCCACGAGAAGGTGTCGAGCCACCCGATTTTCAAGCAG

>AY593837.1_O_URU_1963

CCCTGGTACAAGCTTATCAAGCTCCTAAGCCGCCTGTCGTGCATGGCCGCTGTGGCAGCACGGTCCAAGG

ACCCAGTCCTTGTGGCCATCATGCTGGCCGACACCGGTCTCGAGATTCTGGACAGCACCTTCGTCGTGAA

GAAGATCTCCGACTCGCTCTCCAGTCTCTTTCACGTGCCGGCCCCCGTCTTCAGTTTCGGAGCACCGATC

CTGTTGGCCGGGTTGGTCAAAGTCGCCTCGAGTTTCTTCCGGTCCACACCCGAAGACCTTGAGAGAGCAG

AGAAACAGCTCAAAGCACGTGACATCAACGACATCTTCGCCATTCTCAAGAACGGCGAGTGGCTGGTCAA

GCTGATCCTTGCCATCCGCGACTGGATTAAGGCTTGGATCGCCTCAGAAGAGAAGTTTGTCACCATGACA

GACTTGGTGCCTGGCATCCTTGAAAAGCAGCGGGATCTGAACGACCCGAGCAAGTACAAGGAAGCCAAGG

AGTGGCTCGACAACGCGCGCCAAGCGTGTTTGAAGAGCGGGAACGTCCACATTGCCAACCTGTGTAAAGT

GGTCGCTCCAGCACCCAGCAAGTCGAGGCCCGAACCCGTGGTTGTTTGCCTCCGCGGCAAATCTGGCCAG

GGCAAGAGCTTCCTTGCAAACGTGCTTGCACAGGCAATTTCCGCCCACTTCACCGGCAGAACCGACTCAG

TGTGGTACTGCCCACCTGACCCTGACCACTTCGACGGTTACAACCAGCAAACCGTCGTTGTGATGGATGA

TTTGGGCCAGAACCCTGACGGCAAGGACTTCAAATACTTTGCCCAAATGGTCTCGACCACAGGGTTCATC

CCGCCCATGGCATCACTCGAGGACAAAGGTAAACCTTTCAACAGCAAAGTCATCATCGCGACCACCAACT

TGTACTCGGGCTTCACCCCGAGGACTATGGTGTGTCCCGACGCACTGAACCGGAGGTTTCACTTTGACAT

CGATGTGAGTGCTAAGGATGGGTACAAAATTAACAACAAATTGGACATTATCAAAGCACTTGAAGACACC

CACACCAACCCAGTGGCAATGTTTCAATACGACTGTGCCCTTCTCAACGGCATGGCCGTTGAAATGAAGA

GAATGCAACAAGACATGTTCAAGCCTCAACCACCCCTCCAGAATGTGTACCAGCTTGTTCAGGAGGTGAT

TGATCGGGTCGAGCTCCACGAGAAAGTGTCGAGCCACCCGATCTTCAAGCAG

>AY593838.1_SAT1_BOT_1970

CCCTGGTACAAAGTCATTAAACTCCTCAGCCGCCTGTCGTGCATGGCCGCTGTCGCAGCTCGCTCCAAGG

ACCCTGTCCTTGTGGCGATCATGCTAGCTGACACCGGCCTCGAGATTCTGGACAGCACCTTTGTCGTGAA

GAAAATCTCCGACGCGCTCTCCAGCGTTTTCCACGTCCCGGCCCCTGTCTTCAGTTTCGGAGCCCCGGTC

CTGCTGGCAGGTTTGGTCAAGGTCGCCTCCACGTTTTTCCGGTCCACGCCCGAAGACTTGGAGAGAGCAG

AGAAACAACTCAAGGCACGTGACATCAACGACATCTTCGCCATCCTCAAGAACGGCGAATGGCTGGTCAA

ACTTATCCTGGCTATCCGCGACTGGATCAAAGCCTGGATTTCCTCGGAGGAGAAGTACATCTCCATGACG

GATCTTGTGCCCCGCATTCTCGAATGCCAGCACAACCTGAACGACCCGTCAAAATACCAGGAAAGCAAAG

AGTGGCTGGAAAACGCTCGCGAGGCTTGCCTCAAGAACGGAAACCACCACATTGCTAACCTGTGTAAAGT

GAATGCACCAGCACCCAGCAAGTCGAGACCCGAACCCGTGGTCGTTTGCCTCCGTGGCAAATCCGGCCAG

GGCAAGAGCTTCCTTGCTAATGTGCTCGCACAAGCAATCTCAACCCACTTCACTGGCAGAACCGACTCTG

TCTGGTACTGCCCACCTGACCCCGACCACTTCGACGGTTACAACCAACAAACTGTCGTTGTCATGGACGA

CTTGGGTCAGAACCCTGACGGCAAAGACTTTAAGTACTTTGCCCAGATGGTTTCCACCACAGGGTTCATC

CCTCCAATGGCTTCGCTTGAAGACAAGGGAAAACCGTTCAACAGCAAGGTCATCATTGCGACTAGCAACC

TCTACTCTGGGTTCACACCCAGAACGATGGTTTGCCCGGACGCTTTGAACCGACGGTTTCACTTCGACAT

CGACGTGAGTGCCAAGGGTGGGTACAAAGTTAACAACAGATTGGACATCATCAAAGCATTGGAGGACACA

CACACAAATGCGCCCGCCATGTTCAACTATGACTGTGCCCTTCTCAATGGCTCCGCCGTTGAAATGAAGA

GACTGCAACAGGACGTGTTCAAGCCTCTGCCACCGCTCAACAGCCTGTACCAACTGGTTGACGAGGTGAT

AGAGAGGGTGAAGCTCCACGAGAAGGTGTCGAGCCACCCGATTTTCAAGCAG

>AY593839.1_SAT1_UKG_1970

CCCTGGTACAAAGTCATCAAACTCCTCAGCCGCCTGTCGTGCATGGCCGCTGTCGCAGCTCGCTCCAAGG

ATCCCGTCCTCGTTGCGATCATGCTAGCTGACACCGGTCTCGAGATTCTGGACAGCACCTTCGTCGTGAA

GAAAATCTCCGACGCGCTCTCCAGCGTTTTCCACGTCCCGGCCCCTGTCTTCAGTTTCGGAGCACCGATT

CTGTTGGCAGGCTTGGTCAAGGTCGCCTCCACGTTCTTCCGGTCCACACCCGAAGACTTGGAGAGAGCAG

AAAAACAGCTCAAGGCACGTGACATCAACGACATCTTCGCCATCCTCAAGAACGGCGAATGGCTGGTCAA

ACTCATCCTGGCTATCCGCGACTGGATCAAAGCCTGGATTTCCTCAGAGGAGAAATACATCTCCATGACG

GATCTTGTGCCACGCATTCTCGAATGCCAGCGCAATCTCAACGACCCGTCAAAATACCAGGAAAGCAAGG

AGTGGCTCGAAAACGCTCGCGAGGCTTGCCTCAAGAACGGCAACGTCCACATTGCTAACCTGTGTAAAGT

CAACGCACCAGCACCCAGCAAGTCGAGACCCGAACCCGTGGTCGTTTGCCTCCGCGGCAAGTCCGGCCAG

GGCAAGAGTTTCCTTGCCAACGTGCTTGCTCAAGCAATCTCTACCCACTTCACTGGACGCGTGGATTCAG

TCTGGTACTGTCCACCTGACCCCGACCACTTCGACGGCTACAACCAACAGGCCGTTGTTGTGATGGACGA

TTTGGGCCAAAACCCTGACGGCAAGGACTTCAAGTACTTTGCCCAAATGGTCTCGACCACAGGGTTCATC

CCGCCCATGGCTTCGCTCGAAGACAAGGGGAAACCGTTCAACAGCAAGGTGATCATTGCGACGAGCAACC

TTTATTCTGGGTTCACACCTAGGACAATGGTCTGCCCCGACGCTTTGAACCGACGGTTCCACTTTGACAT

TGACGTGAGTGCCAAGGACGGGTACAAAGTGAACAACAGATTGGACATCATCAAAGCACTGGAGGACACA

CACACAAATGCGCCCGCCATGTTCAACTACGATTGTGCCCTTCTCAATGGTTCTGCCGTTGAAATGAAGA

GACTGCAACAAGATATGTTCAAGCCTCTACCACCTCTCAACAGCCTGTACCAACTGGTTGATGAAGTGAT

AGAGAGGGTGAAACTCCACGAGAAAGTGTCGAGCCACCCGATCTTCAAACAG

>AY593840.1_SAT1_NMB_1949

CCCTGGTACAAAGTCATCAAACTCCTCAGCCGTCTGTCGTGCATGGCCGCTGTCGCAGCTCGCTCCAAGG

ACCCCGTCCTTGTGGCGATCATGCTAGCTGACACCGGTCTCGAGATCCTGGACAGCACTTTCGTCGTGAA

GAAAATCTCCGACGCGCTCTCCAGTGTTTTCCACGTCCCGGCCCCTGTCTTCAGTTTCGGAGCCCCGATC

CTGATGGCAGGTCTGGTCAAGGTCGCCTCCACGTTCTTCCGGTCCACACCCGAAGACTTGGAGAGAGCAG

AAAAACAGCTCAAGGCACGTGACGTCAACGACATCTTCGCCATCCTCAAGAACGGCGAATGGCTGGTCAA

ACTCATCCTGGCTATCCGCGACTGGATCAAAGCCTGGATTTCCTCAGAGGAGAAGTACATCTCCATGACG

GACCTTGTGCCGCGCATTCTCGAGTGCCAGCGCAACCTGAACGATCCGTCAAAATACCAGGAGAGCAAAG

AGTGGTTGGAAAACGCTCGCGAGGCCTGCCTCAAGAACGGAAACCACCACATTGCTAACCTGTGTAAAGT

GACTGCACCAGCACCCAGCAAGTCGAGACCCGAGCCCGTGGTCGTTTGCCTCCGTGGCAAATCCGGCCAG

GGCAAGAGTTTCCTTGCTAACGTGCTCGCACAAGCAATCTCAACCCACTTCACTGGCAGAACCGACTCCG

TTTGGTACTGCCCACCTGACCCCGACCACTTCGATGGCTACAACCAACAAGCTGTCGTTGTCATGGATGA

TTTGGGCCAGAACCCTGACGGCAAGGACTTCAAGTACTTCGCCCAGATGGTCTCCACCACAGGGTTCATC

CCTCCAATGGCCTCTCTCGAAGACAAGGGGAAACCGTTCAACAGCAAGGTCATCATTGCGACGAGCAACC

TTTATTCTGGGTTCACACCTAGGACGATGGTCTGCCCCGACGCTCTGAACCGACGGTTCCACTTCGACAT

CGACGTGAGTGCCAAGGATGGGTACAAAGTTAACAACAGATTGGATATCATCAAAGCACTGGAGGACACA

CACACAAACGCGCCCGCCATGTTCAACTATGACTGTGCCCTTCTCAACGGCTCGGCCGTTGAAATGAAGA

GACTGCAACAAGATGTTTTCAAGCCTCTGCCACCTCTCAACAGCCTGTACCAACTGGTTGATGAAGTGAT

AGAGAGAGTGAAGCTCCACGAGAAAGTGTCGAGCCACCCGATTTTCAAGCAG

>AY593841.1_SAT1_ZIM_1958

CCCTGGTACAAGGTCATCAAACTCCTCAGCCGCCTGTCGTGCATGGCCGCTGTCGCAGCTCGCTCCAAGG

ACCCTGTCCTCGTGGCGATTATGCTAGCTGACACCGGTCTCGAGATTCTGGACAGCACCTTCGTCGTGAA

GAAAATCTCCGACGCGCTCTCCAGCGTTTTCCACGTCCCGGCCCCTGTCTTCAGTTTCGGAGCCCCGATT

CTGCTGGCAGGTTTGGTCAAGGTCGCCTCCACGTTCTTCCGGTCCACACCCGAAGACTTGGAGAGAGCAG

AAAAACAGCTCAAGGCACGTGACATCAACGACATTTTCGCCATCCTCAAGAACGGCGAATGGTTGGTCAA

ACTCATCCTGGCTATCCGCGACTGGATCAAAGCTTGGATTTCCTCAGAGGAGAAGTACATCTCCATGACG

GATCTGGTGCCGCGCATTCTCGAGTGCCAGCGCAATCTGAATGACCCGTCAAAATACCAGGAAAGCAAGG

AGTGGCTGGAGAACGCCCGCGAGGCCTGCCTCAAGAACGGAAACCACCACATTGCCAACCTGTGTAAAGT

GAATGCACCAGCACCCAGCAAGTCGAGACCCGAACCCGTGGTCGTTTGCCTCCGCGGCAAGTCCGGCCAG

GGCAAGAGTTTCCTTGCCAACGTGCTCGCACAAGCAATCTCATCCCACTTCACGGGTAGAACCGACTCTG

TTTGGTACTGCCCGCCTGATCCTGACCACTTCGATGGCTACAACCAACAAGCTGTCGTTGTCATGGATGA

TTTGGGCCAGAACCCTGACGGCAAGGACTTCAAGTACTTCGCCCAGATGGTCTCCACCACAGGGTTCATC

CCTCCCATGGCCTCACTCGAAGACAAGGGAAAACCGTTCAACAGCAAGGTCATCATTGCGACAAGCAACC

TTTATTCTGGGTTCACACCTAGGACGATGGTCTGCCCCGACGCTCTGAACCGACGGTTTCACTTTGACAT

TGACGTGAGTGCCAAGGAAGGGTACAAAGTCAACAACAGATTGGACATCATCAAAGCACTGGAGGACACG

CACACAAACGCGCCCGCCATGTTCAACTACGACTGTGCCCTTCTCAATGGCTCCGCCGTTGAAATGAAGA

GACTGCAACAAGATGTGTTCAAGCCTCTGCCACCTCTCAACAGCCTGTACCAACTGGTTGATGAAGTGAT

AGAGAGGGTGAAGCTCCACGAGAAAGTGTCGAGTCACCCGATTTTCAAACAG

>AY593842.1_SAT1_SAR_1961

CCCTGGTACAAAGTCATCAAACTCCTCAGCCGCCTGTCGTGCATGGCCGCTGTCGCAGCTCGCTCCAAGG

ATCCCGTCCTTGTGGCGATCATGCTAGCTGACACCGGTCTCGAGATTCTGGACAGCACTTTCGTCGTGAA

GAAAATCTCCGACGCGCTCTCCAGCGTTTTCCACGTCCCGGCCCCTGTTTTCAGTTTCGGAGCCCCGATC

CTGCTGGCAGGTTTGGTCAAGGTCGCCTCCACGTTCTTCCGGTCCACACCCGAAGACTTGGAGAGAGCAG

AAAAACAGCTCAAGGCACGTGACATTAACGACATCTTCGCCATCCTCAAGAACGGCGAATGGCTGGTCAA

ACTCATCCTGGCTATCCGCGACTGGATCAAAGCCTGGATTTCCTCAGAGGAGAAGTACATCTCCATGACG

GATCTTGTGCCACGCATTCTCGAGTGCCAGCGCAACCTGAATGATCCGTCAAAATACCAGGAGAGCAAAG

AGTGGCTGGAGAACGCTCGCGAGGCCTGCCTCAAGAATGGAAACCATCACATTGCCAACCTGTGTAAAGT

GAACGCACCAGCGCCCAGCAAGTCGAGACCCGAGCCCGTGGTCGTTTGCCTCCGTGGTAAATCCGGCCAG

GGCAAGAGCTTCCTTGCCAACGTGCTTGCACAAGCAATCTCAACCCACTTCACTGGCAGAACCGACTCTG

TCTGGTACTGCCCACCTGACCCCGACCACTTCGATGGCTACAACCAACAAGCTGTCGTTGTCATGGATGA

TTTGGGCCAGAACCCTGACGGCAAGGACTTCAAGTACTTCGCCCAGATGGTCTCCACCACGGGGTTCATC

CCTCCTATGGCCTCACTTGAAGACAAGGGAAAACCGTTCAACAGCAAGGTCATTATTGCGACGAGCAACC

TTTATTCTGGGTTCACGCCCAGGACGATGGTCTGCCCCGACGCTCTGAACCGACGGTTCCACTTTGACAT

CGACGTGAGTGCCAAGGACGGATACAAAGTTAACAACAGACTGGACATCATCAAAGCATTGGAGGACACG

CACACAAACGCGCCCGCCATGTTCAACTATGACTGTGCCCTTCTCAATGGCTCCGCCGTTGAAATGAAGA

GACTGCAACAAGATGTGTTCAAGCCTCTGCCACCTCTCAACAGCCTGTACCAACTGGTTGATGAAGTGAT

AGAGAGGGTGAAGCTCCACGAGAAAGTGTCAAGCCACCCGATTTTCAAGCAG

>AY593843.1_SAT1_NMB_1940

CCCTGGTACAAAGTCATCAAACTCCTCAGCCGCCTGTCGTGCATGGCCGCTGTCGCAGCTCGCTCCAAGG

ATCCCGTCCTTGTGGCGATTATGCTAGCTGACACCGGTCTCGAGATTCTGGACAGCACTTTCGTCGTGAA

GAAAATCTCCGACGCGCTCTCCAGCGTTTTCCACGTCCCGGCCCCTGTCTTCAGTTTCGGAGCTCCGATC

CTGTTGGCAGGCTTGGTCAAGGTCGCCTCCACGTTCTTCCGGTCCACACCCGAAGACCTGGAGAGAGCAG

AGAAACAGCTCAAGGCACGTGACATCAACGACATCTTCGCCATCCTCAAGAACGGCGAATGGCTGGTCAA

ACTCATCCTGGCTATCCGCGACTGGATCAAAGCCTGGATTTCCTCAGAGGAGAAGTACATCTCCATGACG

GATCTTGTGCCGCGTATTCTCGAGTGCCAGCGCAACCTGAACGACCCGTCAAAATACCAGGAAAGCAAGG

AGTGGCTGGAGAACGCTCGCGAGGCTTGCCTCAAGAACGGGAACCACCACATCGCCAACCTGTGTAAAGT

GAATGCACCAGCACCCAGCAAGTCGAGACCCGAACCCGTGGTCGTTTGCCTCCGCGGCAAGTCCGGCCAG

GGCAAGAGTTTCCTTGCCAACGTGCTCGCACAAGCAATCTCAACCAACTTCACTGGCAGAACTGACTCTG

TTTGGTACTGCCCACCTGACCCCGATCACTTCGATGGCTACAACCAGCAAGCTGTCGTTGTCATGGATGA

TTTGGGCCAGAACCCTGACGGCAAAGACTTCAAGTACTTCGCCCAGATGGTCTCCACCACAGGGTTCATC

CCTCCTATGGCCTCACTCGAAGATAAGGGAAAACCGTTTAACAGCAAGGTCATCATTGCGACGAGCAACC

TTTATTCTGGGTTCACACCTAGGACAATGGTCTGCCCCGACGCTTTGAACCGACGGTTTCACTTTGACAT

CGATGTGAGTGCTAGGGACGGGTACAAAGTTAACAACAGATTGGACATCATCAAAGCACTGGAGGACACG

CACACAAACGCGCCCGCTATGTTCAACTACGACTGTGCCCTTCTCAATGGCTCCGCCGTTGAAATGAAGA

GACTGCAACAAGATGTGTTCAAGCCTCTACCACCTCTCAACAGCCTGTACCAACTGGTTGATGAAGTGAT

AGAGAGGGTGAAGCTTCACGAGAAAGTGTCGAGTCACCCGATTTTCAAGCAG

>AY593844.1_SAT1_ISR_1962

CCTTGGTACAAAGTCATCAAACTCTTCAGCCGCCTGTCGTGTATGGCCGCTGTAGCAGCACGGTCCAAGG

ACCCAGTCCTTGTGGCTATCATGCTAGCTGACACCGGTCTTGAGATTCTGGACAGTACATTTGTGGTCAA

GAAAATTGCTGACTCCCTCTCCAGTGTCTTTCACGTGCCGGCCCCTGTCTTCAGTTTTGGAGCCCCGATA

CTGCTGGCAGGTTTGGTCAAGGTCGCCTCGAGTTTCTTCCGGTCAACACCCGAAGAGCTTGAGAGAGCAG

AGAAACAGCTCAAAGCACGTGACATCAACGACATTTTCGCCATCCTTAAGAACGGCGAATGGCTGGTCAA

ACTCATCCTGGCAATCCGCGACTGGATTAAAGCCTGGATCTCCTCAGAAGAGAAGTTTGTCACCATGACA

GACCTAGTGCCTGGTATCTTGGAGAAACAGCACGATCTCAACGATCCCTCCAAGTACCATGAAGCAAAGG

AATGGCTCGAGAACGCCCGCCAGGCGTGTCTCAAGAATGGCAACATCCATATTGCCAATCTCTGCAAGGT

CACAGCACCGGCACCAAGCAAGTCGAGACCCGAACCAGTGGTCGTTTGCCTCCGTGGCAAATCCGGCCAA

GGCAAGAGTTTCCTCGCGAACGTGCTTGCACAAGCCATTTCCACACATTTCACTGGCAGAACCGACTCTG

TTTGGTACTGTCCACCTGACCCTGATCACTTTGATGGCTACAACCAACAGGCCGTCGTGGTCATGGATGA

CCTCGGACAGAATCCTGATGGCAAGGACTTCAAATACTTTGCCCAGATGGTCTCCACAACCGGGTTCATC

CCGCCCATGGCTTCTCTCGAGGACAAAGGGAAACCTTTCAACAGCAAGGTCATCATCGCGACCTCAAACT

TGTACTCTGGGTTCACCCCGCGTACAATGGTCTGCCCTGATGCACTGAACCGTAGGTTCCACTTTGACAT

CGATGTAAGCGCCAAGGACGGGTACAAAGTCAACAACAAATTGGACATCATCAAAGCACTTGAGGACACC

CACACAAACCCAGTGGCAATGTTCCAATACGATTGTGCCCTTCTGAACGGCATGGCTGTCGAGATGAAGA

GACTGCAACAAGATTTGTTCAAGCCTCAACCGCCGATTCTTAACGTGTACCAGCTCGTTGATGAGGTGAT

TGAGAGAGTCAACCTGCACGAGAAGGTTGCCTCCCAACCAATTTTCAAACAG

>AY593845.1_SAT1_BOT_1968

CCATGGTACAAAGTCATCAAACTCCTCAGCCGCCTGTCGTGCATGGCCGCTGTCGCAGCTCGCTCCAAGG

ATCCCGTCCTCGTGGCGATCATGCTAGCTGACACCGGTCTCGAGATCCTGGACAGCACTTTCGTCGTGAA

GAAAATCTCCGACGCGCTCTCCAGTGTTTTCCACGTCCCGGCCCCTGTCTTCAGTTTCGGAGCCCCGATC

CTGTTGGCAGGTTTGGTCAAGGTCGCCTCCACGTTCTTCCGGTCCACACCCGAAGACTTGGAGAGAGCAG

AAAAGCAGCTCAAGGCACGTGACATCAACGACATCTTCGCCATCCTCAAGAATGGTGAATGGCTGGTCAA

ACTCATCCTGGCTATCCGCGACTGGATTAAAGCTTGGATTTCCTCAGAGGAGAAGTACATCTCCATGACG

GACCTTGTGCCGCACATTCTCGAGTGCCAGCGCAACCTGAACGATCCGTCAAAATACCAGGAGAGCAAGG

AGTGGTTGGAGAACGCTCGTGAGGCCTGCCTCAAAGTCGGGAACCACCACATCGCTAATCTGTGTAAAGT

GGCAGCACCAGCACCCAGCAAGTCGAGACCCGAACCCGTGGTCGTTTGCCTCCGCGGCAAGTCCGGCCAG

GGTAAGAGTTTCCTTGCTAACGTGCTTGCACAAGCAATCTCAACCCACTTCACTGGCAGAACCGACTCAG

TTTGGTACTGTCCACCTGACCCCGACCACTTCGATGGCTACAACCAACAGGCCGTCGTTGTCATGGATGA

TTTGGGCCAGAACCCTGACGGCAAGGACTTCAAGTACTTCGCCCAAATGGTTTCCACCACAGGGTTCATC

CCTCCCATGGCCTCACTCGAAGACAAGGGCAAACCGTTCAACAGCAAGGTCATCATTGCGACGAGCAACC

TTTATTCTGGGTTCACACCCAGGACGATGGTCTGCCCTGACGCTCTGAACCGACGGTTCCACTTTGACAT

TGACGTGAGTGCCAAGGACGGGTACAAAGTTAACAACAGATTGGACATCATCAAAGCACTGGAGGACACG

CACACAAACGCGCCCGCCATGTTCAACTATGACTGTGCCCTTCTCAACGGTTCCGCCGTTGAAATGAAGA

GACTGCAACAAGATGTGTTCAAGCCTCTGCCACCTCTCAACAGCCTGTACCAACTGGTTGATGAAGTGAT

AGAGAGGGTGAAGCTCCACGAGAAGGTGTCGAGACACCCGATTTTCAAGCAG

>AY593846.1_SAT1_ZIM_1966

CCCTGGTACAAGGTCATCAAACTCCTCAGCCGCCTGTCGTGCATGGCCGCTGTCGCAGCTCGCTCCAAGG

ATCCCGTCCTTGTGGCGATCATGCTAGCTGACACCGGTCTCGAGATTCTGGACAGCACTTTCGTCGTGAA

GAAAATCTCCGACGCGCTCTCCAGCGTTTTCCACGTCCCGGCCCCTGTCTTCAGTTTCGGAGCCCCGATC

CTGCTGGCAGGTTTGGTCAAGGTCGCCTCCACGTTCTTCCGGTCCACACCCGAAGACCTGGAGAGAGCAG

AGAAACAGCTCAAGGCACGTGACATCAACGACATCTTCGCCATCCTCAAGAACGGCGAATGGCTGGTCAA

ACTCATCCTGGCTATCCGCGACTGGATCAAGGCTTGGATTTCCTCAGAAGAGAAATACATCTCCATGACG

GACCTTGTGCCGCGCATTCTCGAATGCCAGCGCAACCTCAACGATCCGTCCAAATACCAGGAAAGCAAAG

AGTGGCTCGAAAACGCTCGCGAGGCTTGCCTCAAGAACGGGAACGTCCACATTGCCAACCTGTGTAAAGT

GAATGCACCGGCACCCAGCAAGTCGAGACCCGAACCCGTGGTCGTTTGCCTCCGCGGCAAATCCGGCCAG

GGTAAGAGTTTCCTTGCCAACGTGCTCGCTCAAGCAATCTCAACCCACTTCACTGGACGTGTGGATTCAG

TCTGGTACTGTCCACCTGACCCCGACCACTTCGACGGTTACAACCAACAGGCCGTTGTTGTGATGGATGA

TTTGGGCCAGAACCCTGACGGCAAGGACTTCAAGTACTTCGCTCAGATGGTCTCGACCACAGGGTTCATC

CCGCCCATGGCCTCACTCGAAGATAAGGGGAAACCGTTCAACAGCAAAGTGATCATTGCGACGAGCAACC

TTTATTCTGGGTTCACGCCCAGGACAATGGTCTGCCCCGACGCTCTGAACCGACGGTTCCACTTTGACAT

CGATGTGAGTGCCAAGGACGGGTACAAAGTGAATAACAGATTGGACATCATCAAAGCACTGGAGGACACG

CACACAAATGCACCCGCCATGTTCAACTATGACTGTGCCCTTCTCAACGGCTCTGCCGTTGAAATGAAGA

GACTACAACAAGACATGTTCAAGCCTCTGCCACCTCTCAACAGCCTGTACCAACTGGTTGATGAAGTGAT

AGAGAGGGTGAAGCTCCACGAAAAGGTGTCGAGCCACCCGATTTTCAAACAG

>AY593847.1_SAT2_ZIM_1948

CCCTGGTACAAAGTCATCAAACTCCTCAGCCGCCTGTCGTGCATGGCCGCTGTCGCAGCTCGTTCCAAGG

ACCCTGTCCTTGTCGCGATCATGCTAGCTGACACCGGTCTCGAGATTCTGGACAGCACCTTCGTCGTGAA

GAAAATCTCCGACGCGCTCTCCAGCGTTTTCCACGTCCCGGCCCCAGTCTTCAGTTTCGGAGCCCCGATT

CTGTTGGCTGGTTTGGTCAAGGTCGCCTCCACGTTCTTCCGGTCCACACCCGAGGACTTGGAGAGAGCAG

AAAAGCAGCTCAAGGCACGTGACATCAACGACATCTTTGCCATCCTCAAGAACGGCGAATGGCTGGTCAA

ACTCATCCTGGCTATCCGCGACTGGATCAAGGCCTGGATTTCCTCAGAGGAGAAGTACATCTCCATGACG

GATCTTGTGCCCCGCATTCTCGAGTGCCAGCACAACCTGAACGATCCGTCCAAGTACCAGGAAAGCAAAG

AGTGGCTTGACAACGCTCGCGAGGTTTGCCTCAAGAACGGAAACACCCACATTGCTAACCTGTGTAAAGT

GAATGCACCAGCACCCAACAAGTCGAGACCCGAACCCGTGGTCGTTTGCCTCCGCGGCAAATCCGGCCAG

GGCAAGAGCTTCCTTGCCAACGTGCTTGCACAAGCAATCTCAACCCACTTCACCGGACGTGTGGACTCAG

TCTGGTACTGTCCACCTGACCCCGATCACTTCGATGGCTACAACCAACAGGTCGTTGTTGTGATGGATGA

TCTGGGCCAGAACCCGGACGGCAAAGACTTCAAGTACTTCGCCCAAATGGTGTCAACCACCGGGTTTATC

CCCCCCATGGCCTCACTCGAAGACAAGGGAAAACCGTTCAACAGCAAGGTAATCATTGCGACGAGCAACC

TTTATTCTGGGTTCACGCCCAGAACAATGGTCTGCCCCGACGCTTTGAACCGACGGTTTCACTTTGACAT

TGACGTGAGTGCCAGGGACGGGTACAAAGTTAACAACAGATTGGACATAATCAAAGCACTGGAGGACACG

CACACAAACGCGCCCGCCATGTTTAACTATGACTGTGCCCTTCTCAACGGTTCCGCCGTTGAAATGAAGA

GACTGCAACAGGATGTGTTCAAGCCTCTGCCACCTCTCAACAGCCTGTACCAACTGGTTGATGAAGTGAT

AGAGAGGGTGAAGCTCCACGAGAAGGTGTCGAGCCACCCGATTTTCAAACAG

>AY593848.1_SAT2_u_1967

CCCTGGTATAAAGTCATCAAACTCCTCAGCCGCCTGTCGTGTATGGCCGCTGTCGCAGCTCGCTCCAAGG

ACCCTGTCCTCGTTGCGATCATGCTAGCTGACACCGGTCTCGAGATTCTGGACAGCACCTTCGTCGTGAA

GAAAATCTCCGACGCGCTCTCCAGCGTTTTCCACGTCCCGGCCCCTGTCTTCAGTTTCGGAGCCCCGATT

CTGCTGGCAGGCTTGGTCAAAGTCGCCTCCACGTTCTTCCGGTCCACGCCCGAAGACTTGGAGAGAGCAG

AGAAACAGCTCAAGGCACGTGACATCAACGACATTTTCGCCATCCTCAAGAACGGCGAATGGCTGGTCAA

ACTCATCCTGGCTATCCGCGACTGGATTAAAGCTTGGATTTCCTCAGAGGAGAAGTACATCTCCATGACG

GACCTTGTGCCGCGCATTCTCGAGTGCCAGCGCAATCTGAACGATCCGTCCAAGTACCAGGAGAGCAAAG

AGTGGCTCGAAAACGCTCGCGAGGCTTGCCTCAAGAACGGAAACGTCCACATTGCTAACCTGTGTAAAGT

CAATGCACCAGCACCAAGCAAGTCGAGACCCGAGCCTGTGGTCGTTTGCCTCCGCGGTAAATCCGGCCAG

GGTAAGAGTTTCCTTGCGAACGTGCTTGCACAAGCAATCTCAACCCACTTCACTGGACGCGTGGATTCAG

TCTGGTACTGTCCACCTGACCCTGACCACTTCGACGGCTACAACCAACAAGCTGTTGTTGTGATGGATGA

TTTGGGCCAGAACCCTGACGGCAAGGACTTTAAGTACTTCGCCCAGATGGTCTCGACCACGGGGTTCATC

CCACCCATGGCTTCACTTGAAGACAAGGGGAAACCGTTCAACAGTAAGGTCATCATTGCGACGAGCAACC

TTTATTCTGGGTTCACACCTAGGACAATGGTCTGCCCCGACGCTTTGAACCGACGGTTCCACTTTGACAT

CGACGTGAGTGCCAGGGACGGGTACAAAGTTAACAACAGATTGGACATCATCAAAGCTCTGGAGGACACG

CACACAAACGCGCCCGCCATGTTCAACTATGACTGTGCCCTTCTCAATGGCTCCGCCGTTGAAATGAAGA

GACTGCAACAAGATGTGTTCAAGCCTCTACCACCTCTCAACAGCCTGTACCAACTGGTTGATGAAGTGAT

AGAAAGGGTGAAGCTCCACGAGAAAGTGTCGAGTCACCCGATTTTCAAGCAG

>AY593849.1_SAT2_KEN_1960

CCCTGGTACAAAATCATCAAACTCCTGAGCCGCCTGTCGTGCATGGCCGCTGTAGCAGCACGGTCAAAGG

ACCCTGTCCTTGTGGCCATCATGCTAGCTGACACCGGTCTCGAAATCCTGGACAGCACTTTTGTCGTGAA

GAAAATCGCCGACGCGCTCTCCAGTGTTTTCCACGTGCCAGCCCCTGTCTTCAGTTTCGGGGCTCCGATC

CTGTTGGCAGGTTTGGTCAAGGTCGCCTCGAGTTTCTTCCGGTCGACACCCGAAGAGCTTGAGAGAGCAG

AGAAACAGCTCAAGGCACGTGACATCAACGACATTTTTGCCATTCTCAAGAATGGCGAGTGGCTTGTCAA

ACTCATCCTCGCCATCCGCGACTGGATTAAGGCGTGGATCTCCTCAGAAGAGAAGTTCGTCACCATGACG

GACCTTGTGCCTGGTATCTTGGAGAAACAGCGTGATCTCAATGATCCGTCCAAGTATCATGAAGCAAAGG

AGTGGCTCGAGAATGCCCGCCAGGCATGCCTCAAGAACGGCAATGTCCACATCGCCAACCTTTGCAAAGT

CACAGCACCAGCACCAAGCAAGTCAAGACCAGAACCAGTGGTCGTTTGCCTCCGTGGTAAATCTGGCCAA

GGTAAAAGTTTCCTTGCGAACGTGCTTGCACAGGCAATCTCCACCCACTTCACGGGCAGAACCGACTCTG

TGTGGTACTGTCCACCAGACCCTGACCACTTCGACGGCTATAACCAACAAGCTGTCGTGGTGATGGATGA

TCTGGGACAGAATCCCGATGGCAAGGACTTCAAGTACTTCGCCCAAATGGTATCAACCACGGGATTCATC

CCGCCCATGGCGTCCCTCGAGGACAAGGGGAAACCTTTCAACAGCAAGGTCATCATTGCAACCTCCAATC

TGTATTCTGGGTTCACCCCACGTACGATGGTTTGCCCTGATGCACTGAACCGTAGGTTTCACTTTGACAT

CGACGTGAGCGCCAAGGACGGATACAGGGTTAATAACAAACTGGACATCACCAAAGCACTTGAGGACACC

CACACAAACCCAGTGGCGATGTTCCAGTATGATTGTGCCCTTCTCAACGGCATGGCCGTTGAAATGAAGA

GACTGCAACAAGATATGTTCAAGCCTCAACCACCAATTCTCAACGTCTACCAACTTGTGGATGAGGTGAT

TGAGAGAGTCAACCTCCACGAGAAGGTTGCCTCGCAGCCAATCTTCAAACAG

>AY593850.1_SAT3_SAR_1959

CCATGGTACAAAGTCATCAAACTCCTCAGCCGCCTGTCGTGCATGGCCGCTGTCGCAGCTCGCTCCAAGG

ACCCTGTCCTTGTGGCGATCATGCTAGCTGACACCGGTCTCGAGATTCTGGACAGCACCTTCGTCGTGAA

GAAAATCTCCGACGCGCTCTCCAGCGTTTTCCACGTCCCGGCCCCTGTCTTCAGTTTCGGAGCCCCGATT

CTGTTGGCAGGTTTGGTCAAGGTCGCCTCCACGTTCTTCCGGTCCACACCCGAAGACTTGGAGAGAGCAG

AGAAACAGCTCAAGGCACGTGACATCAACGACATTTTCGCCATCCTCAAGAACGGCGAATGGCTGGTCAA

ACTTATCCTGGCTATCCGCGACTGGATCAAAGCCTGGATTTCCTCAGAGGAGAAATACATCTCCATGACG

GACCTTGTGCCGCGCATTCTTGAATGCCAGCGCAACCTCAACGATCCGTCAAAGTACCAGGAGAGCAAAG

AGTGGCTCGAAAACGCTCGCGAGGCTTGTCTCAAGAACGGAAACGTCCACATCGCTAACCTGTGTAAAGT

GAACGCACCAGCACCAAGCAAGTCGAGACCCGAACCCGTGGTCGTTTGCCTCCGGGGTAAATCTGGCCAG

GGCAAGAGTTTCCTTGCCAACGTGCTTGCACAAGCAATCTCAACCCACTTCACTGGACGTGTGGACTCAG

TCTGGTACTGTCCACCTGACCCTGACCACTTCGACGGCTACAACCAACAGGCCGTTGTTGTGATGGATGA

TTTGGGCCAGAACCCTGACGGCAAGGACTTCAAGTACTTCGCCCAGATGGTCTCCACCACAGGGTTCATC

CCACCCATGGCTTCGCTCGAAGATAAGGGAAAACCGTTCAACAGCAAGGTCATCATTGCGACGAGCAACC

TTTATTCTGGGTTCACACCTAGGACGATGGTCTGCCCCGACGCTCTGAACCGACGGTTCCACTTTGACAT

CGATGTGAGTGCCAGGGATGGGTACAGAGTTAACAACAGATTGGACATCATCAAAGCACTGGAGGACACG

CACACAAACGCGCCCGCCATGTTCAACTATGACTGCGCCCTTCTCAACGGTTCCGCCGTTGAAATGAAGA

GACTGCAACAAGATGTGTTCAAGCCTCTGCCACCTCTCAACAGCCTGTACCAACTGGTTGATGAAGTGAT

AGAGAGGGTGAAGCTCCACGAGAAAGTGTCGAGCCACCCGATTTTCAAGCAG

>AY593851.1_SAT3_BOT_1961

CCCTGGTACAAAATCATCAAACTCCTCAGCCGCCTGTCGTGCATGGCCGCTGTCGCAGCTCGCTCCAAGG

ACCCTGTCCTCGTGGCGATCATGCTAGCTGACACCGGTCTTGAGATCCTGGACAGCACCTTCGTCGTGAA

GAAAATCTCCGACGCGCTCTCCAGTGTTTTCCACGTCCCGGCCCCTGTCTTCAGTTTCGGAGCCCCGGTC

CTGTTGGCAGGTTTGGTCAAAGTCGCCTCCACGTTCTTCCGGTCCACACCCGAAGACTTGGAGAGAGCAG

AAAAACAGCTCAAGGCGCGTGACATCAACGACGTTTTCGCCATCCTCAAGAACGGCGAATGGCTGGTCAA

ACTCATCCTGGCTATCCGCGACTGGATCAAAGCCTGGATTTCCTCAGAGGAGAAGTACATCTCCATGACG

GATCTTGTGCCGCGCATTCTCGAGTGCCAGCACAACCTGAACGATCCGTCCAAGTACCAGGAAAGCAAAG

AGTGGTTGGAAAACGCCCGCGAGGCTTGCCTCAAGAACGGAAACCACCACATCGCTAACCTGTGTAAAGT

GAATGCACCAGCACCCAGCAAGTCGAGACCCGAACCCGTGGTCGTTTGCCTCCGCGGCAAGTCCGGCCAG

GGCAAGAGTTTCCTTGCCAACGTGCTCGCACAAGCAATCTCAACCCACTTCACTGGCAGAACCGACTCTG

TTTGGTACTGCCCACCTGACCCCGACCACTTCGATGGCTACAACCAACAAGCTGTCGTTGTTATGGACGA

TTTGGGCCAGAACCCTGACGGCAAGGACTTCAAGTACTTCGCCCAAATGGTTTCCACCACAGGGTTCATC

CCTCCCATGGCCTCACTTGAAGATAAGGGAAAACCGTTCAACAGCAAGGTCATCATTGCGACGAGCAACC

TTTATTCTGGGTTCACGCCTAGGACGATGGTCTGCCCCGACGCTCTGAACCGACGGTTCCACTTCGACAT

CGACGTGAGTGCCAGGGATGGGTACAAAGTTAACAACAGATTGGACATCATCAAAGCACTGGAGGACACG

CACACAAACGCGCCTGCCATGTTCAACTATGACTGTGCCCTTCTCAATGGCTCCGCCGTTGAAATGAAGA

GACTGCAACAAGATGTGTTCAAGCCTCTGCCACCTCTCAACAGCCTGTACCAACTGGTTGATGAAGTGAT

AGAGAGGGTGAAGCTCCACGAGAAAGTGTCGAGCCACCCGATTTTCAAGCAG

>AY593852.1_SAT3_KEN_1960

CCCTGGTACAAAATCATCAAACTCCTCAGCCGCCTGTCGTGCATGGCCGCTGTCGCAGCTCGCTCCAAGG

ACCCTGTCCTCGTGGCGATCATGCTAGCTGACACCGGTCTTGAGATCCTGGACAGCACCTTCGTCGTGAA

GAAAATCTCCGACGCGCTCTCCAGTGTTTTCCACGTCCCGGCCCCTGTCTTCAGTTTCGGAGCCCCGGTC

CTGTTGGCAGGTTTGGTCAAAGTCGCCTCCACGTTCTTCCGGTCCACACCCGAAGACTTGGAGAGAGCAG

AAAAACAGCTCAAGGCGCGTGACATCAACGACGTTTTCGCCATCCTCAAGAACGGCGAATGGCTGGTCAA

ACTCATCCTGGCTATCCGCGACTGGATCAAAGCCTGGATTTCCTCAGAGGAGAAGTACATCTCCATGACG

GATCTTGTGCCGCGCATTCTCGAGTGCCAGCACAACCTGAACGATCCGTCCAAGTACCAGGAAAGCAAAG

AGTGGTTGGAAAACGCCCGCGAGGCTTGCCTCAAGAACGGAAACCACCACATCGCTAACCTGTGTAAAGT

GAATGCACCAGCACCCAGCAAGTCGAGACCCGAACCCGTGGTCGTTTGCCTCCGCGGCAAGTCCGGCCAG

GGCAAGAGTTTCCTTGCCAACGTGCTCGCACAAGCAATCTCAACCCACTTCACTGGCAGAACCGACTCTG

TTTGGTACTGCCCACCTGACCCCGACCACTTCGATGGCTACAACCAACAAGCTGTCGTTGTTATGGACGA

TTTGGGCCAGAACCCTGACGGCAAGGACTTCAAGTACTTCGCCCAAATGGTTTCCACCACAGGGTTCATC

CCTCCCATGGCCTCACTTGAAGATAAGGGAAAACCGTTCAACAGCAAGGTCATCATTGCGACGAGCAACC

TTTATTCTGGGTTCACGCCTAGGACGATGGTCTGCCCCGACGCTCTGAACCGACGGTTCCACTTCGACAT

CGACGTGAGTGCCAGGGATGGGTACAAAGTTAACAACAGATTGGACATCATCAAAGCACTGGAGGACACG

CACACAAACGCGCCTGCCATGTTCAACTATGACTGTGCCCTTCTCAATGGCTCCGCCGTTGAAATGAAGA

GACTGCAACAAGATGTGTTCAAGCCTCTGCCACCTCTCAACAGCCTGTACCAACTGGTTGATGAAGTGAT

AGAGAGGGTGAAGCTCCACGAGAAAGTGTCGAGCCACCCGATTTTCAAGCAG

>AY593853.1_SAT3_BOT_1965

CCCTGGTACAAAGTCATCAAACTCCTCAGCCGCCTGTCGTGCATGGCCGCTGTCGCAGCTCGCTCCAAGG

ACCCCGTCCTCGTGGCGATCATGCTAGCTGACACCGGTCTCGAGATTCTGGACAGCACCTTCGTCGTGAA

GAAAATCTCCGACGCGCTCTCCAGCGTTTTCCACGTCCCGGCCCCTGTCTTCAGTTTCGGAGCCCCGATC

CTGTTGGCAGGTTTGGTCAAGGTCGCCTCCACGTTCTTCCGGTCCACACCCGAAGACTTGGAGAGAGCAG

AGAAACAGCTCAAGGCACGTGACATTAACGACATCTTCGCCATCCTTAAGAACGGCGAATGGCTGGTCAA

ACTCATCCTGGCTATCCGCGACTGGATCAAAGCCTGGATTTCCTCAGAAGAGAAGTACATCTCCATGACG

GACCTTGTGCCGCGCATTCTCGAGTGCCAGCACAACCTGAACGATCCGTCAAAATACCAGGAGAGCAAAG

AGTGGTTGGAGAACGCCCGCGAGGCCTGCCTCAAGAACGGAAACCACCACATCGCTAACCTGTGTAAAGT

GAATGCACCAGCACCTAGTAAGTCGAGACCCGAGCCCGTGGTCGTTTGCCTCCGCGGCAAATCCGGCCAG

GGCAAGAGTTTCCTTGCCAACGTGCTAGCACAAGCAATCTCAACCCACTTCACTGGCAAAACCGACTCTG

TTTGGTACTGTCCACCTGACCCCGATCACTTCGACGGCTACAACCAACAAGCTGTCGTTGTCATGGACGA

TTTGGGCCAAAACCCTGACGGTAAGGACTTTAAGTACTTCGCCCAAATGGTTTCCACCACGGGGTTCATC

CCTCCCATGGCCTCACTCGAAGACAAGGGAAAACCGTTCAACAGCAAGGTCATCATTGCGACGAGCAACC

TTTATTCTGGGTTCACACCTAGGACGATGGTCTGCCCCGACGCTCTGAACCGACGGTTCCACTTTGACAT

CGACGTGAGTGCCAAGGACGGGTACAAAGTTAACAACAGATTGGACATCATCAAAGCACTGGAGGACACG

CACACAAACGCGCCCGCCATGTTCAACTATGATTGTGCCCTTCTCAATGGCTCCGCCGTTGAAATGAAGA

GACTGCAACAGGATGTGTTCAAGCCTCTGCCACCTCTCAACAGCCTGTACCAACTGGTTGATGAAGTGAT

AGAGAGGGTGAAGCTCCATGAGAAAGTGTCGAGCCACCCGATTTTCAAGCAA

>AY686687.1_O_CHA_2001

CCCTGGTACAAGCTCATCAAACTCCTAAGCCGCCTGTCGTGCATGGCCGCTGTGGCAGCACGGTCCAAGG

ACCCAGTCCTTGTGGCCATCATGCTGGCTGACACCGGTCTCGAAATTCTGGACAGCACTTTTGTGGTAAA

GAAGATCTCCGATTCGCTCTCCAGTCTCTTCCACGTGCCGGCTCCCGCCCTCAGTTTCGGAGCCCCGATC

CTGTTGGCCGGGTTGGTCAAAGTCGCCTCGAGTTTCTTCCAGTCCACACCCGAAGACCTCGAGAGAGCAG

AAAAGCAGCTCAAAGCACGCGATATCAACGACGTATTTGCCGTTCTTAAGAACGGCGAGTGGCTGGTCAA

ACTGATCCTGGCCATCCGCGACTGGATCAAGGCATGGATCGCCTCAGAAGAAAAGTTTGTCACTATGACA

GACCTGGTGCCTGGCATCCTTGAAAAGCAACGGGATCTCAATGACCCCGGCAAATACAAGGAGGCCAAGG

AGTGGCTGGACAACGCGCGTCAAGCGTGTCTGAAGAGCGGGAACGTGCACATTGCCAACCTGTGCAAAGT

AGTCGCTCCGGCGCCCAGCAAGTCGAGACCCGAACCAGTGGTCGTTTGTCTTCGCGGCAAATCTGGTCAG

GGGAAAAGCTTCCTCGCGAACGTTCTCGCGCAGGCAATTTCTACACACTTCACTGGTAGGACCGACTCGG

TCTGGTACTGCCCACCCGACCCTGACCACTTCGACGGGTACAACCAGCAGACCGTCGTCGTGATGGACGA

CTTGGGCCAGAACCCAGACGGCAAAGACTTCAAGTACTTCGCCCAAATGGTCTCCACTACGGGATTCATC

CCACCTATGGCCTCGCTCGAGGATAAGGGCAAACCCTTCAACAGCAAGGTCATAATAGCCACAACTAACC

TGTACTCAGGATTTACCCCAAGAACCATGGTGTGTCCCGATGCGCTTAACAGGCGGTTCCACTTCGACCT

CGACGTGAGTGCCAAGGACGGGTACAGGGTCAACAACAAACTGGACATAGTCAAAGCTCTTGAAGACACC

CACACCGACCCTGTGGCGATGTTCCAGTACGACTGCGCCCTTCTCAACGGAATGGCTGTTGAAATGAAGA

GAATGCAACAAGACATGTTCAAACCTCAACCGCCTCTCCAGAACGTCTACCAGCTTGTTCAAGAGGTGAT

TGAGCGGGTGGAGCTGCACGAAAAGGTGTCGAGCCACCCAATATTCAAGCAG

>AY687333.1_Asia1_IND_2001

CCCTGGTACAAGCTCATCAAGCTCCTAAGCCGCCTGTCATGTATGGCCGCTGTAGCAGCACGGTCAAAGG

ACCCAGTCCTTGTGGCCATCATGCTGGCTGACACCGGCCTTGAGATTCTGGACAGCACCTTCGTCGTGAA

GAAGATCTCCGACTCGCTCTCCAGTCTCTTTCACGTGCCGGCCCCCGCCTTCAGTTTCGGAGCCCCGATT

CTGCTGGCCGGGTTGGTCAAAGTCGCCTCGAGTTTCTTCCGGTCCACACCCGAAGACCTTGAGAGAGCAG

AGAAACAGCTCAAAGCACGTGACATCAATGACATATTCGCCATTCTCAAGAACGGCGAGTGGCTGGTCAA

ACTGATTCTTGCCATCCGCGACTGGATTAAGGCATGGATCGCCTCAGAAGAAAAGTTTGTCACCATGACG

GACTTGGTGCCTGGCATCCTCGAAAAGCAGCGGGACCTTAATGACCCAAGCAAGTACAAGGAGGCCAAGG

AGTGGCTTGAAAACGCGCGACAAGCGTGTCTGAAGAGTGGGAACGTCCACATTGCTAACCTCTGCAAAGT

GGTCGCCCGGCACCCCAGCAAGTCGAGACCTGAACCTGTGGTCGTTTGCCTCCGTGGCAAATCCGGCCAG

GGAAAGAGTTTCCTTGCGAACGTGCTCGCACAGGCAATATCCACCCACTTCACTGGCAGAACAGACTCAG

TTTGGTACTGCCCACCTGATCCTGACCACTTCGACGGTTACAACCAACAGACCGTTGTTGTGATGGATGA

TTTGGGCCAGAATCCTAACGCGAAGGACTTTAAGTACTTTGCCCAAATGGTGTCAACCACAGGGTTCATC

CCGCCCATGGCGTCACTCGAGGACAAAGGTAAACCTTTCAACAGCAAGGTCATCATTGCAACCACCAACC

TGTACTCGGGCTTCACCCCGAGGACTATGGTGTGCCCTGATGCACTGAACCGTAGGTTTCACTTTGACAT

TGACGTGAGTGCCAAGGACGGGTACAAAATCAACAACAAATTGGACATTATCAAAGCACTTGAGGACACC

CACACCAACCCAGTGGCAATGTTTCAATATGACTGTGCCCTTCTCAACGGCATGGCCGTTGAGATGAAGA

GAATGCAACAGGATATGTTCAAGCCTCAACCGCCCCTCCAGAACGTATACCAACTTGTTCAGGAGGTGAT

TGAACGGGTCGAGCTCCACGAGAAAGTGTCGAGCCACCCAATTTTTAAACAG

>DQ404158.1_O_UKG_2001

CCCTGGTACAAGCTCATCAAGCTCTTGAGCCGCCTGTCATGCATGGCCGCTGTAGCAGCACGGTCAAAGG

ACCCAGTCCTTGTGGCCATCATGCTGGCTGACACCGGCCTTGAGATTCTGGATAGTACCTTTGTCGTGAA

GAAGATCTCCGACTCGCTCTCCAGTCTCTTTCACGTGCCGGCCCCCGTCTTCAGTTTCGGAGCCCCGATT

TTGTTGGCCGGGTTGGTCAAAGTTGCCTCGAGTTTCTTCCGGTCCACGCCCGAAGACCTTGAGAGAGCGG

AGAAACAGCTCAAAGCACGTGACATCAATGACATATTCGCCATTCTCAAGAACGGCGAGTGGCTGGTCAA

GCTGATTCTTGCCATCCGCGACTGGATCAAGGCATGGATTGCCTCAGAAGAAAAGTTTGTCACCATGACA

GACCTGGTGCCTGGCATCCTTGAAAAGCAGCGGGATCTCAACGACCCAAGCAAGTACAAAGAGGCCAAGG

AGTGGCTCGACAACGCGCGCCAAGCGTGTTTGAAGAGCGGGAACATCCACATCGCAAACCTTTGCAAAGT

GGTTGCCCCAGCACCCAGCAGGTCGAGGCCCGAACCCGTGGTCGTTTGCCTCCGTGGCAAATCGGGCCAG

GGCAAGAGTTTCCTTGCGAACGTGCTTGCACAAGCAATTTCAACCCACTTCACTGGCAGAACCGACTCAG

TTTGGTACTGCCCACCTGACCCTGACCACTTCGACGGTTACAACCAGCAGACCGTTGTAGTAATGGATGA

TTTGGGCCAGAACCCCGACGGGAAGGACTTCAAGTACTTCGCCCAAATGGTTTCAACCACGGGGTTTATC

CCGCCCATGGCTTCACTCGAGGACAAAGGCAAACCCTTCAACAGCAAGGTCATCATCGCCACCACCAACC

TGTACTCGGGCTTCACCCCGAGAACTATGGTGTGCCCTGACGCACTGAACCGAAGGTTTCACTTTGACAT

TGACGTGAGCGCCAAGGACGGGTACAAAATTAACAACAAATTGGACATCATCAAAGCTCTTGAAGATACC

CACACCAACCCAGTGGCAATGTTTCAATACGACTGTGCCCTTCTCAACGGCATGGCCGTTGAAATGAAGA

GAATGCAACAGGATATGTTCAAGCCTCAACCGTCCCTCCAGAACGTCTACCAGCTTGTTCAGGAGGTGAT

TGACCGGGTCGAGCTCCACGAGAAGGTGTCGAGCCACCCGATTTTCAAGCAG

>DQ404159.1_O_UKG_2001

CCCTGGTACAAGCTCATCAAGCTCTTGAGCCGCCTGTCATGCATGGCCGCTGTAGCAGCACGGTCAAAGG

ACCCAGTCCTTGTGGCCATCATGCTGGCTGACACCGGCCTTGAGATTCTGGACAGTACCTTTGTCGTGAA

GAAGATCTCCGACTCGCTCTCCAGTCTCTTTCACGTGCCGGCCCCCGTCTTCAGTTTCGGAGCCCCGATT

TTGTTGGCCGGGTTGGTCAAAGTTGCCTCGAGTTTCTTCCGGTCCACGCCCGAAGACCTTGAGAGAGCGG

AGAAACAGCTCAAAGCACGTGACATCAATGACATATTCGCCATTCTCAAGAACGGCGAGTGGCTGGTCAA

GCTGATTCTTGCCATCCGCGACTGGATCAAGGCATGGATCGCCTCAGAAGAAAAGTTTGTCACCATGACA

GACCTGGTGCCTGGCATCCTTGAAAAGCAGCGGGATCTCAACGACCCAAGCAAGTACAAAGAGGCCAAGG

AGTGGCTCGACAACGCGCGCCAAGCGTGTTTGAAGAGCGGGAACATCCACATCGCAAACCTTTGCAAAGT

GGTTGCCCCAGCACCCAGCAGGTCGAGGCCCGAACCCGTGGTCGTTTGCCTCCGTGGCAAATCGGGCCAG

GGCAAGAGTTTCCTTGCGAACGTGCTTGCACAAGCAATTTCAACCCACTTCACTGGCAGAACCGACTCAG

TTTGGTACTGCCCACCTGACCCTGACCACTTCGACGGTTACAACCAGCAGACCGTTGTAGTAATGGATGA

TTTGGGCCAGAACCCCGACGGGAAGGACTTCAAGTACTTCGCCCAAATGGTTTCAACCACGGGGTTTATC

CCGCCCATGGCTTCACTCGAGGACAAAGGCAAACCCTTCAACAGCAAGGTCATCATCGCCACCACCAACC

TGTACTCGGGCTTCACCCCGAGAACTATGGTGTGCCCTGACGCACTGAACCGAAGGTTTCACTTTGACAT

TGACGTGAGCGCCAAGGACGGGTACAAAATTAACAACAAATTGGACATCATCAAAGCTCTTGAAGATACC

CATACCAACCCAGTGGCAATGTTTCAATACGACTGTGCCCTTCTCAACGGCATGGCCGTTGAAATGAAGA

GAATGCAACAGGATATGTTCAAGCCTCAACCGTCCCTCCAGAACGTCTACCAGCTTGTTCAGGAGGTGAT

TGACCGGGTCGAGCTCCACGAGAAGGTGTCGAGCCACCCGATTTTCAAGCAG

>DQ404160.1_O_UKG_2001

CCCTGGTACAAGCTCATCAAGCTCTTGAGCCGCCTGTCATGCATGGCCGCTGTAGCAGCACGGTCAAAGG

ACCCAGTCCTTGTGGCCATCATGCTGGCTGACACCGGCCTTGAGATTCTGGACAGTACCTTTGTCGTGAA

GAAGATCTCCGACTCGCTCTCCAGTCTCTTTCACGTGCCGGCCCCCGTCTTCAGTTTCGGAGCCCCGATT

TTGTTGGCCGGGTTGGTCAAAGTTGCCTCGAGTTTCTTCCGGTCCACGCCCGAAGACCTTGAGAGAGCGG

AGAAACAGCTCAAAGCACGTGACATCAATGACATATTCGCCATTCTCAAGAACGGCGAGTGGCTGGTCAA

GCTGATTCTTGCCATCCGCGACTGGATCAAGGCATGGATCGCCTCAGAAGAAAAGTTTGTCACCATGACA

GACCTGGTGCCTGGCATCCTTGAAAAGCAGCGGGATCTCAACGATCCAAGCAAGTACAAAGAGGCCAAGG

AGTGGCTCGACAACGCGCGCCAAGCGTGTTTGAAGAGCGGGAACATCCACATCGCAAACCTTTGCAAAGT

GGTTGCCCCAGCGCCCAGCAGGTCGAGGCCCGAACCCGTGGTCGTTTGCCTCCGTGGCAAATCGGGCCAG

GGCAAGAGTTTCCTTGCGAACGTGCTTGCACAAGCAATTTCAACCCACTTCACTGGCAGAACCGACTCAG

TTTGGTACTGCCCACCTGACCCTGACCACTTCGACGGTTACAACCAGCAGACCGTTGTAGTAATGGATGA

TTTGGGCCAGAACCCCGACGGGAAGGACTTCAAGTACTTCGCCCAAATGGTTTCAACCACGGGGTTTATC

CCGCCCATGGCTTCACTCGAGGACAAAGGCAAACCCTTCAACAGCAAGGTCATCATCGCCACCACCAACC

TGTACTCGGGCTTCACCCCGAGAACTATGGTGTGCCCTGACGCACTGAACCGAAGGTTTCACTTTGACAT

TGACGTGAGCGCCAAGGACGGGTACAAAATTAACAACAAATTGGACATCATCAAAGCTCTTGAAGATACC

CACACCAACCCAGTGGCAATGTTTCAATACGACTGTGCCCTTCTCAACGGCATGGCCGTTGAAATGAAGA

GAATGCAACAGGATATGTTCAAGCCTCAACCGTCCCTCCAGAACGTCTACCAGCTTGTTCAGGAGGTGAT

TGACCGGGTCGAGCTCCACGAGAAGGTGTCGAGCCACCCGATTTTCAAGCAG

>DQ404161.1_O_UKG_2001

CCCTGGTACAAGCTCATCAAGCTCTTGAGCCGCCTGTCATGCATGGCCGCTGTAGCAGCACGGTCAAAGG

ACCCAGTCCTTGTGGCCATCATGCTGGCTGACACCGGCCTTGAGATTCTGGACAGTACCTTTGTCGTGAA

GAAGATCTCCGACTCGCTCTCCAGTCTCTTTCACGTGCCGGCCCCCGTCTTCAGTTTCGGAGCCCCGATT

TTGTTGGCCGGGTTGGTCAAAGTTGCCTCGAGTTTCTTCCGGTCCACGCCCGAAGACCTTGAGAGAGCGG

AGAAACAGCTCAAAGCACGTGACATCAATGACATATTCGCCATTCTCAAGAACGGCGAGTGGCTGGTCAA

GCTGATTCTTGCCATCCGCGACTGGATCAAGGCATGGATCGCCTCAGAAGAAAAGTTTGTCACCATGACA

GACCTGGTGCCTGGCATCCTTGAAAAGCAGCGGGATCTCAACGACCCAAGCAAGTACAAAGAGGCCAAGG

AGTGGCTCGACAACGCGCGCCAAGCGTGTTTGAAGAGCGGGAACATCCACATCGCAAACCTTTGCAAAGT

GGTTGCCCCAGCACCCAGCAGGTCGAGGCCCGAACCCGTGGTCGTTTGCCTCCGTGGCAAATCGGGCCAG

GGCAAGAGTTTCCTTGCGAACGTGCTTGCACAAGCAATTTCAACCCACTTCACTGGCAGAACCGACTCAG

TTTGGTACTGCCCACCTGACCCTGACCACTTCGACGGTTACAACCAACAGACCGTTGTAGTAATGGATGA

TTTAGGCCAGAACCCCGACGGGAAGGACTTCAAGTACTTCGCCCAAATGGTTTCAACCACGGGGTTTATC

CCGCCCATGGCTTCACTCGAGGACAAAGGCAAACCTTTCAACAGCAAGGTCATCATCGCCACCACCAACC

TGTACTCGGGCTTCACCCCGAGAACTATGGTGTGCCCTGACGCACTGAACCGAAGGTTTCACTTTGACAT

TGACGTGAGCGCCAAGGACGGGTACAAAATTAACAACAAATTGGACATCATCAAAGCTCTTGAAGATACC

CACACCAACCCAGTGGCAATGTTTCAATACGACTGTGCCCTTCTCAACGGCATGGCCGTTGAAATGAAGA

GAATGCAACAGGATATGTTCAAGCCTCAACCGTCCCTCCAGAACGTCTACCAGCTTGTTCAGGAGGTGAT

TGACCGGGTCGAGCTCCACGAGAAGGTGTCGAGCCACCCGATTTTCAAGCAG

>DQ404162.1_O_UKG_2001

CCCTGGTACAAGCTCATCAAGCTCTTGAGCCGCCTGTCATGCATGGCCGCTGTAGCAGCACGGTCAAAGG

ACCCAGTCCTTGTGGCCATCATGCTGGCTGACACCGGCCTTGAGATTCTGGACAGTACCTTTGTCGTGAA

GAAGATCTCCGACTCGCTCTCCAGTCTCTTTCACGTGCCGGCCCCCGTCTTCAGTTTCGGAGCCCCGATT

TTGTTGGCCGGGTTGGTCAAAGTCGCCTCGAGTTTCTTCCGGTCCACGCCCGAAGACCTTGAGAGAGCGG

AGAAACAGCTCAAAGCACGTGACATCAATGACATATTCGCCATTCTCAAGAACGGCGAGTGGCTGGTCAA

GCTGATTCTTGCCATCCGCGACTGGATCAAGGCATGGATCGCCTCAGAAGAAAAGTTTGTCACCACGACA

GACCTGGTGCCTGGCATCCTTGAAAAGCAGCGGGATCTCAACGACCCAAGCAAGTACAAAGAGGCCAAGG

AGTGGCTCGACAACGCGCGCCAAGCGTGTTTGAAGAGCGGGAACATCCACATCGCAAACCTTTGCAAAGT

GGTTGCCCCAGCACCCAGCAGGTCGAGGCCCGAACCCGTGGTCGTTTGCCTCCGTGGCAAATCGGGCCAG

GGCAAGAGTTTCCTTGCGAACGTGCTTGCACAAGCAATTTCAACCCACTTCACTGGCAGAACCGACTCAG

TTTGGTACTGCCCACCTGACCCTGACCACTTCGACGGTTACAACCAGCAGACCGTTGTAGTAATGGATGA

TTTGGGCCAGAACCCCGACGGGAAGGACTTCAAGTACTTCGCCCAAATGGTTTCAACCACGGGGTTTATC

CCGCCCATGGCTTCACTCGAGGACAAAGGCAAACCTTTCAACAGCAAGGTCATCATCGCCACCACCAACC

TGTACTCGGGCTTCACCCCGAGAACTATGGTGTGCCCTGACGCACTGAACCGAAGGTTCCACTTTGACAT

CGACGTGAGCGCCAAGGACGGGTACAAAATTAACAACAAATTGGACATCATCAAAGCTCTTGAAGATACC

CACACCAACCCAGTGGCAATGTTTCAATACGACTGTGCCCTTCTCAACGGCATGGCCGTTGAAATGAAGA

GAATGCAACAAGATATGTTCAAGCCTCAACCGTCCCTCCAGAACGTCTACCAGCTTGTTCAGGAGGTGAT

TGACCGGGTCGAGCTCCACGAGAAGGTGTCGAGCCACCCGATTTTCAAGCAG

>DQ404163.1_O_UKG_2001

CCCTGGTACAAGCTCATCAAGCTCTTGAGCCGCCTGTCATGCATGGCCGCTGTAGCAGCACGGTCAAAGG

ACCCAGTCCTTGTGGCCATCATGCTGGCTGACACCGGCCTTGAGATTCTGGACAGTACCTTTGTCGTGAA

GAAGATCTCCGACTCGCTCTCCAGTCTCTTTCACGTGCCGGCCCCCGTCTTCAGTTTCGGAGCCCCGATT

TTGTTGGCCGGGTTGGTCAAAGTCGCCTCGAGTTTCTTCCGGTCCACGCCCGAAGACCTTGAGAGAGCGG

AGAAACAGCTCAAAGCACGTGACATCAATGACATATTCGCCATTCTCAAGAACGGCGAGTGGCTGGTCAA

GCTGATTCTTGCCATCCGCGACTGGATCAAGGCATGGATCGCCTCAGAAGAAAAGTTTGTCACCATGACA

GACCTGGTGCCTGGCATCCTTGAAAAGCAGCGGGATCTCAACGACCCAAGCAAGTACAAAGAGGCCAAGG

AGTGGCTCGACAACGCGCGCCAAGCGTGTTTGAAGAGCGGGAACATCCACATCGCAAACCTTTGCAAAGT

GGTTGCCCCAGCACCCAGCAGGTCGAGGCCCGAACCCGTAGTCGTTTGCCTCCGTGGCAAATCGGGCCAG

GGCAAGAGTTTCCTTGCGAACGTGCTTGCACAAGCAATTTCAACCCACTTCACCGGCAGAACCGACTCAG

TTTGGTACTGCCCACCTGACCCTGACCACTTCGACGGTTACAACCAGCAGACCGTTGTAGTAATGGATGA

TTTGGGCCAGAACCCCGACGGGAAGGACTTCAAGTACTTCGCCCAAATGGTTTCAACTACGGGGTTTATC

CCGCCCATGGCTTCACTCGAGGACAAAGGCAAACCTTTCAACAGCAAGGTCATCATCGCCACCACCAACC

TGTACTCGGGCTTCACCCCGAGAACTATGGTGTGCCCTGACGCACTGAACCGAAGGTTCCACTTTGACAT

TGACGTGAGCGCCAAGGACGGGTACAAAATTAACAACAAATTGGACATCATCAAAGCTCTTGAAGATACC

CACACCAACCCAGTGGCAATGTTTCAATACGACTGTGCCCTTCTCAACGGCATGGCCGTTGAAATGAAGA

GAATGCAACAAGATATGTTCAAGCCTCAACCGTCCCTCCAGAACGTCTACCAGCTTGTCCAGGAGGTGAT

TGACCGGGTCGAGCTCCACGAGAAGGTGTCGAGCCACCCGATTTTCAAGCAG

>DQ404164.1_O_UKG_2001

CCCTGGTACAAGCTCATCAAGCTCTTGAGCCGCCTGTCATGCATGGCCGCTGTAGCAGCACGGTCAAAGG

ACCCAGTCCTTGTGGCCATCATGCTGGCTGACACCGGCCTTGAGATTCTGGACAGTACCTTTGTCGTGAA

GAAGATCTCCGACTCGCTCTCCAGTCTCTTTCACGTGCCGGCCCCCGTCTTCAGTTTCGGAGCCCCGATT

TTGTTGGCCGGGTTGGTCAAAGTCGCCTCGAGTTTCTTCCGGTCCACGCCCGAAGACCTTGAGAGAGCGG

AGAAACAGCTCAAAGCACGTGACATCAATGACATATTCGCCATTCTCAAGAACGGCGAGTGGCTGGTCAA

GCTGATTCTTGCCATCCGCGACTGGATCAAGGCATGGATCGCCTCAGAAGAAAAGTTTGTCACCATGACA

GACCTGGTGCCTGGCATCCTTGAAAAGCAGCGGGATCTCAACGACCCAAGCAAGTACAAAGAGGCCAAGG

AGTGGCTCGACAACGCGCGCCAAGCGTGTTTGAAGAGCGGGAACATCCACATCGCAAACCTTTGTAAAGT

GGTTGCCCCAGCACCCAGCAGGTCGAGGCCCGAACCCGTGGTCGTTTGCCTCCGTGGCAAATCGGGCCAG

GGCAAGAGTTTCCTTGCGAACGTGCTTGCACAAGCAATTTCAACCCACTTCACTGGCAGAACTGACTCAG

TTTGGTACTGCCCACCTGACCCTGACCACTTCGACGGTTACAACCAGCAGACCGTTGTAGTAATGGATGA

TTTGGGCCAGAACCCCGACGGGAAGGACTTCAAGTACTTCGCCCAAATGGTTTCAACTACGGGGTTTATC

CCGCCCATGGCTTCACTCGAGGACAAAGGCAAACCTTTCAACAGCAAGGTCATCATCGCCACCACCAACC

TGTACTCGGGCTTCACCCCGAGAACTATGGTGTGCCCTGACGCACTGAACCGAAGGTTCCACTTTGACAT

TGACGTGAGCGCCAAGGACGGGTACAAAATTAACAACAAATTGGACATCATCAAAGCTCTTGAAGATACC

CACACCAACCCAGTGGCAATGTTTCAATACGACTGTGCCCTTCTCAACGGCATGGCCGTTGAAATGAAGA

GAATGCAACAAGATATGTTCAAGCCTCAACCGTCCCTCCAGAACGTCTACCAGCTTGTTCAGGAGGTGAT

TGACCGGGTCGAGCTCCACGAGAAGGTGTCGAGCCACCCGATTTTCAAGCAG

>DQ404165.1_O_UKG_2001

CCCTGGTACAAGCTCATCAAGCTCTTAAGCCGCCTGTCATGCATGGCCGCTGTAGCAGCACGGTCAAAGG

ACCCAGTCCTTGTGGCCATCATGCTGGCTGATACCGGCCTTGAGATTCTGGACAGTACCTTTGTCGTGAA

GAAGATCTCCGACTCGCTCTCCAGTCTCTTTCACGTGCCGGCCCCCGTCTTCAGTTTCGGAGCCCCGATT

TTGTTGGCCGGGTTGGTCAAAGTCGCCTCGAGTTTCTTCCGGTCCACGCCCGAAGACCTTGAGAGAGCGG

AGAAACAGCTCAAAGCACGTGACATCAATGACATATTCGCCATTCTCAAGAACGGCGAGTGGCTGGTCAA

GCTGATTCTTGCCATCCGCGACTGGATCAAGGCATGGATCGCCTCAGAAGAAAAGTTTGTCACCATGACA

GACCTGGTGCCTGGCATCCTTGAAAAGCAGCGGGATCTCAACGACCCAAGCAAGTACAAAGAGGCCAAGG

AGTGGCTCGACAACGCGCGCCAAGCGTGTTTGAAGAGCGGGAACATCCACATCGCAAACCTTTGCAAAGT

GGTTGCCCCAGCACCCAGCAGGTCGAGGCCCGAACCCGTGGTCGTTTGCCTCCGTGGCAAATCGGGCCAG

GGCAAGAGTTTCCTTGCGAACGTGCTTGCACAAGCAATTTCAACCCACTTCACTGGCAGAACCGACTCAG

TTTGGTACTGCCCACCTGACCCTGACCACTTCGACGGTTACAACCAGCAGACCGTTGTAGTAATGGATGA

TTTGGGCCAGAACCCCGACGGGAAGGACTTCAAGTACTTCGCCCAAATGGTTTCAACTACGGGGTTTATC

CCGCCCATGGCTTCACTCGAGGACAAAGGCAAACCTTTCAACAGCAAGGTCATCATCGCCACCACCAACC

TGTACTCGGGCTTCACCCCGAGAACTATGGTGTGCCCTGACGCACTGAACCGAAGGTTCCACTTTGACAT

TGACGTGAGCGCCAAGGACGGGTACAAAATTAACAACAAATTGGACATCATCAAAGCTCTTGAAGATACC

CACACCAACCCAGTGGCAATGTTTCAATACGACTGTGCCCTTCTCAACGGCATGGCCGTTGAAATGAAGA

GAATGCAACAAGATATGTTCAAGCCTCAACCGTCCCTCCAGAACGTCTACCAGCTTGTTCAGGAGGTGAT

TGACCGGGTCGAGCTCCACGAGAAGGTGTCGAGCCACCCGATTTTCAAGCAG

>DQ404166.1_O_UKG_2001

CCCTGGTACAAGCTCATCAAGCTCTTAAGCCGCCTGTCATGCATGGCCGCTGTAGCAGCACGGTCAAAGG

ACCCAGTCCTTGTGGCCATCATGCTGGCTGATACCGGCCTTGAGATTCTGGACAGTACCTTTGTCGTGAA

GAAGATCTCCGACTCGCTCTCCAGTCTCTTTCACGTGCCGGCCCCCGTCTTCAGTTTCGGAGCCCCGATT

TTGTTGGCCGGGTTGGTCAAAGTCGCCTCGAGTTTCTTCCGGTCCACGCCCGAAGACCTTGAGAGAGCGG

AGAAACAGCTCAAAGCACGTGACATCAATGACATATTCGCCATTCTCAAGAACGGCGAGTGGCTGGTCAA

GCTGATTCTTGCCATCCGCGACTGGATCAAGGCATGGATCGCCTCAGAAGAAAAGTTTGTCACCATGACA

GACCTGGTGCCTGGCATCCTTGAAAAGCAGCGGGATCTCAACGACCCAAGCAAGTACAAAGAGGCCAAGG

AGTGGCTCGACAACGCGCGCCAAGCGTGTTTGAAGAGCGGGAACATCCACATCGCAAACCTTTGCAAAGT

GGTTGCCCCAGCACCCAGCAGGTCGAGGCCCGAACCCGTGGTCGTTTGCCTCCGTGGCAAATCGGGCCAG

GGCAAGAGTTTCCTTGCGAACGTGCTTGCACAAGCAATTTCAACCCACTTCACTGGCAGAACCGACTCAG

TTTGGTACTGCCCACCTGACCCTGACCACTTCGACGGTTACAACCAGCAGACCGTTGTAGTAATGGATGA

TTTGGGCCAGAACCCCGACGGGAAGGACTTCAAGTACTTCGCCCAAATGGTTTCAACTACGGGGTTTATC

CCGCCCATGGCTTCACTCGAGGACAAAGGCAAACCTTTCAACAGCAAGGTCATCATCGCCACCACCAACC

TGTACTCGGGCTTCACCCCGAGAACTATGGTGTGCCCTGACGCACTGAACCGAAGGTTCCACTTTGACAT

TGACGTGAGCGCCAAGGACGGGTACAAAATTAACAACAAATTGGACATCATCAAAGCTCTTGAAGATACC

CACACCAACCCAGTGGCAATGTTTCAATACGACTGTGCCCTTCTCAACGGCATGGCCGTTGAAATGAAGA

GAATGCAACAAGATATGTTCAAGCCTCAACCGTCCCTCCAGAACGTCTACCAGCTTGTTCAGGAGGTGAT

TGACCGGGTCGAGCTCCACGAGAAGGTGTCGAGCCACCCGATTTTCAAGCAG

>DQ404167.1_O_UKG_2001

CCCTGGTACAAGCTCATCAAGCTCTTAAGCCGCCTGTCATGCATGGCCGCTGTAGCAGCACGGTCAAAGG

ACCCAGTCCTTGTGGCCATCATGCTGGCTGATACCGGCCTTGAGATTCTGGACAGTACCTTTGTCGTGAA

GAAGATCTCCGACTCGCTCTCCAGTCTCTTTCACGTGCCGGCCCCCGTCTTCAGTTTCGGAGCCCCGATT

TTGTTGGCCGGGTTGGTCAAAGTCGCCTCGAGTTTCTTCCGGTCCACGCCCGAAGACCTTGAGAGAGCGG

AGAAACAGCTCAAAGCACGTGACATCAATGACATATTCGCCATTCTCAAGAACGGCGAGTGGCTGGTCAA

GCTGATTCTTGCCATCCGCGACTGGATCAAGGCATGGATCGCCTCAGAAGAAAAGTTTGTCACCATGACA

GACCTGGTGCCTGGCATCCTTGAAAAGCAGCGGGATCTCAACGACCCAAGCAAGTACAAAGAGGCCAAGG

AGTGGCTCGACAACGCGCGCCAAGCGTGTTTGAAGAGCGGGAACATCCACATCGCAAACCTTTGCAAAGT

GGTTGCCCCAGCACCCAGCAGGTCGAGGCCCGAACCCGTGGTCGTTTGCCTCCGTGGCAAATCGGGCCAG

GGCAAGAGTTTCCTTGCGAACGTGCTTGCACAAGCAATTTCAACCCACTTCACTGGCAGAACCGACTCAG

TTTGGTACTGCCCACCTGACCCTGACCACTTCGACGGTTACAACCAGCAGACCGTTGTAGTAATGGATGA

TTTGGGCCAGAACCCCGACGGGAAGGACTTCAAGTACTTCGCCCAAATGGTTTCAACTACGGGGTTTATC

CCGCCCATGGCTTCACTCGAGGACAAAGGCAAACCTTTCAACAGCAAGGTCATCATCGCCACCACCAACC

TGTACTCGGGCTTCACCCCGAGAACTATGGTGTGCCCTGACGCACTGAACCGAAGGTTCCACTTTGACAT

TGACGTGAGCGCCAAGGACGGGTACAAAATTAACAACAAATTGGACATCATCAAAGCTCTTGAAGATACC

CACACCAACCCAGTGGCAATGTTTCAATACGACTGTGCCCTTCTCAACGGCATGGCCGTTGAAATGAAGA

GAATGCAACAAGATATGTTCAAGCCTCAACCGTCCCTCCAGAACGTCTACCAGCTTGTTCAGGAGGTGAT

TGACCGGGTCGAGCTCCACGAGAAGGTGTCGAGCCACCCGATTTTCAAGCAG

>DQ404168.1_O_UKG_2001

CCCTGGTACAAGCTCATCAAGCTCTTGAGCCGCCTGTCATGCATGGCCGCTGTAGCAGCACGGTCAAAGG

ACCCAGTCCTTGTGGCCATCATGCTGGCTGACACCGGCCTTGAGATTCTGGACAGTACCTTTGTCGTGAA

GAAGATCTCCGACTCGCTCTCCAGTCTCTTTCACGTGCCGGCCCCCGTCTTCAGTTTCGGAGCCCCGATT

TTGTTGGCCGGGTTGGTCAAAGTCGCCTCGAGTTTCTTCCGGTCCACGCCCGAAGACCTTGAGAGAGCGG

AGAAACAGCTCAAAGCACGTGACATCAATGACATATTCGCCATTCTCAAGAACGGCGAGTGGCTGGTCAA

GCTGATTCTTGCCATCCGCGACTGGATCAAGGCATGGATCGCCTCAGAAGAAAAGTTTGTCACCATGACA

GACCTGGTGCCTGGCATCCTTGAAAAGCAGCGGGATCTCAACGACCCAAGCAAGTACAAAGAGGCCAAGG

AGTGGCTCGACAACGCGCGCCAAGCGTGTTTGAAGAGCGGGAACATCCACATCGCAAACCTTTGCAAAGT

GGTTGCCCCAGCACCCAGCAGGTCGAGGCCCGAACCCGTGGTCGTTTGCCTCCGTGGCAAATCGGGCCAG

GGCAAGAGTTTCCTTGCGAACGTGCTTGCACAAGCAATTTCAACCCACTTCACTGGCAGAACCGACTCAG

TTTGGTACTGCCCACCTGACCCTGACCACTTCGACGGTTACAACCAGCAGACCGTTGTAGTAATGGACGA

TTTGGGCCAGAACCCCGACGGGAAGGACTTCAAGTACTTCGCCCAAATGGTTTCAACCACGGGGTTTATC

CCGCCCATGGCTTCACTCGAGGACAAAGGCAAACCTTTCAACAGCAAGGTCATCATCGCCACCACCAACC

TGTACTCGGGCTTCACCCCGAGAACTATGGTGTGCCCTGACGCACTGAACCGAAGGTTCCACTTTGACAT

TGACGTGAGCGCCAAGGACGGGTACAAAATTAACAACAAATTGGACATCATCAAAGCTCTTGAAGATACC

CACACCAACCCAGTGGCAATGTTTCAATACGACTGTGCCCTTCTCAACGGCATGGCCGTTGAAATGAAGA

GAATGCAACAAGATATGTTCAAGCCTCAACCGTCCCTCCAGAACGTCTACCAGCTTGTTCAGGAGGTGAT

TGACCGGGTCGAGCTCCACGAGAAGGTGTCGAGCCACCCGATTTTCAAGCAG

>DQ404169.1_O_UKG_2001

CCCTGGTACAAGCTCATCAAGCTCTTGAGCCGCCTGTCATGCATGGCCGCTGTAGCAGCACGGTCAAAGG

ACCCAGTCCTTGTGGCCATCATGCTGGCTGACACCGGCCTTGAGATTCTGGACAGTACCTTTGTCGTGAA

GAAGATCTCCGACTCGCTCTCCAGTCTCTTTCACGTGCCGGCCCCCGTCTTCAGTTTCGGAGCCCCGATT

TTGTTGGCCGGGTTGGTCAAAGTCGCCTCGAGCTTCTTCCGGTCCACGCCCGAAGACCTTGAGAGAGCGG

AGAAACAGCTCAAAGCACGTGACATCAATGACATATTCGCCATTCTCAAGAACGGCGAGTGGCTGGTCAA

GCTGATTCTTGCCATCCGCGACTGGATCAAGGCATGGATCGCCTCAGAAGAAAAGTTTGTCACCATGACA

GACCTGGTGCCTGGCATCCTTGAAAAGCAGCGGGATCTCAACGACCCAAGCAAGTACAAAGAGGCCAAGG

AGTGGCTCGACAACGCGCGCCAAGCGTGTTTGAAGAGCGGGAACATCCACATCGCAAACCTTTGCAAAGT

GGTTGCCCCAGCACCCAGCAGGTCGAGGCCCGAACCCGTGGTCGTTTGCCTCCGTGGCAAATCGGGCCAG

GGCAAGAGTTTCCTTGCGAACGTGCTTGCACAAGCAATTTCAACCCACTTCACTGGCAGAACCGACTCAG

TTTGGTACTGCCCACCTGACCCTGACCACTTCGACGGTTACAACCAGCAGACCGTTGTAGTAATGGATGA

TTTGGGCCAGAACCCCGACGGGAAGGACTTCAAGTACTTCGCCCAAATGGTTTCAACTACGGGGTTTATC

CCGCCCATGGCTTCACTCGAGGACAAAGGCAAACCTTTCAACAGCAAGGTCATCATCGCCACCACCAACC

TGTACTCGGGCTTCACCCCGAGAACTATGGTGTGCCCTGACGCACTGAACCGAAGGTTCCACTTTGACAT

TGACGTGAGCGCCAAGGACGGGTACAAAATTAACAACAAATTGGACATCATCAAAGCTCTTGAAGATACC

CACACCAACCCAGTGGCAATGTTTCAATACGACTGTGCCCTTCTCAACGGCATGGCCGTTGAAATGAAGA

GAATGCAACAAGATATGTTCAAGCCTCAACCGTCCCTCCAGAACGTCTACCAGCTTGTTCAGGAGGTGAT

TGACCGGGTCGAGCTCCACGAGAAGGTGTCGAGCCACCCGATTTTCAAGCAG

>DQ404170.1_O_UKG_2001

CCCTGGTACAAGCTCATCAAGCTCTTGAGCCGCCTGTCATGCATGGCCGCTGTAGCAGCACGGTCAAAGG

ACCCAGTCCTTGTGGCCATCATGCTGGCTGATACCGGCCTTGAGATTCTGGACAGTACCTTTGTCGTGAA

GAAGATCTCCGACTCGCTCTCCAGTCTCTTTCACGTGCCGGCCCCCGTCTTCAGTTTCGGAGCCCCGATT

TTGTTGGCCGGGTTGGTCAAAGTCGCCTCGAGTTTCTTCCGGTCCACGCCCGAAGACCTTGAGAGAGCGG

AGAAACAGCTCAAAGCACGTGACATCAATGACATATTCGCCATTCTCAAGAACGGCGAGTGGCTGGTCAA

GCTGATTCTTGCCATCCGCGACTGGATCAAGGCATGGATCGCCTCAGAAGAAAAGTTTGTCACCATGACA

GACCTGGTGCCTGGCATCCTTGAAAAGCAGCGGGATCTCAACGACCCAAGCAAGTACAAAGAGGCCAAGG

AGTGGCTCGACAACGCGCGCCAAGCGTGTTTGAAGAGCGGGAACATCCACATCGCAAACCTTTGCAAAGT

GGTTGCCCCAGCACCCAGCAGGTCGAGGCCCGAACCCGTGGTCGTTTGCCTCCGTGGCAAATCGGGCCAG

GGCAAGAGTTTCCTTGCGAACGTGCTTGCACAAGCAATTTCAACCCACTTCACTGGCAGAACCGACTCAG

TTTGGTACTGCCCACCTGACCCTGACCACTTCGACGGTTACAACCAGCAGACCGTTGTAGTAATGGATGA

TTTGGGCCAGAACCCCGACGGGAAGGACTTCAAGTACTTCGCCCAAATGGTTTCAACTACGGGGTTTATC

CCGCCCATGGCTTCACTCGAGGACAAAGGCAAACCTTTCAACAGCAAGGTCATCATCGCCACCACCAACC

TGTACTCGGGCTTCACCCCGAGAACTATGGTGTGCCCTGACGCACTGAACCGAAGGTTCCACTTTGACAT

TGACGTGAGCGCCAAGGACGGGTACAAAATTAACAACAAATTGGACATCATCAAAGCTCTTGAAGATACC

CACACCAACCCAGTGGCAATGTTTCAATACGACTGTGCCCTTCTCAACGGCATGGCCGTTGAAATGAAGA

GAATGCAACAAGATATGTTCAAGCCTCAACCGTCCCTCCAGAACGTCTACCAGCTTGTTCAGGAGGTGAT

TGACCGGGTCGAGCTCCACGAGAAGGTGTCGAGCCACCCGATTTTCAAGCAG

>DQ404171.1_O_UKG_2001

CCCTGGTACAAGCTCATCAAGCTCTTGAGCCGCCTGTCATGCATGGCCGCTGTAGCAGCACGGTCAAAGG

ACCCAGTCCTTGTGGCCATCATGCTGGCTGACACCGGCCTTGAGATTCTGGACAGTACCTTTGTCGTGAA

GAAGATCTCCGACTCGCTCTCCAGTCTCTTTCACGTGCCGGCCCCCGTCTTCAGTTTCGGAGCCCCGATT

TTGTTGGCCGGGTTGGTCAAAGTCGCCTCGAGTTTCTTCCGGTCCACGCCCGAAGACCTTGAGAGAGCGG

AGAAACAGCTCAAAGCACGTGACATCAATGACATATTCGCCATTCTCAAGAACGGCGAGTGGCTGGTCAA

GCTGATTCTTGCCATCCGCGACTGGATCAAGGCATGGATCGCCTCAGAAGAAAAGTTTGTCACCATGACA

GACCTGGTGCCTGGCATCCTTGAAAAGCAGCGGGATCTCAACGACCCAAGCAAGTACAAAGAGGCCAAGG

AGTGGCTCGACAACGCGCGCCAAGCGTGTTTGAAGAGCGGGAACATCCACATCGCAAACCTTTGCAAAGT

GGTTGCCCCAGCACCCAGCAGGTCGAGGCCCGAACCCGTGGTCGTTTGCCTCCGTGGCAAATCGGGCCAG

GGCAAGAGTTTCCTTGCGAACGTGCTTGCACAAGCAATTTCAACCCACTTCACTGGCAGAACCGACTCAG

TTTGGTACTGCCCACCTGACCCTGACCACTTCGACGGTTACAACCAGCAGACCGTTGTAGTAATGGATGA

TTTGGGCCAGAACCCCGACGGGAAGGACTTCAAGTACTTCGCCCAAATGGTTTCAACTACGGGGTTTATC

CCGCCCATGGCTTCACTCGAGGACAAAGGCAAACCTTTCAACAGCAAGGTCATCATCGCCACCACCAACC

TGTACTCGGGCTTCACCCCGAGAACTATGGTGTGCCCTGACGCACTGAACCGAAGGTTCCACTTTGACAT

TGACGTGAGCGCCAAGGACGGGTACAAAATTAACAACAAATTGGACATCATCAAAGCTCTTGAAGATACC

CACACCAACCCAGTGGCAATGTTTCAATACGACTGTGCCCTTCTCAACGGCATGGCCGTTGAAATGAAGA

GAATGCAACAAGATATGTTCAAGCCTCAACCGTCCCTCCAGAACGTCTACCAGCTTGTTCAGGAGGTGAT

TGACCGGGTCGAGCTCCACGAGAAGGTGTCGAGCCACCCGATTTTCAAGCAG

>DQ404172.1_O_UKG_2001

CCCTGGTACAAGCTCATCAAGCTCTTGAGCCGCCTGTCATGCATGGCCGCTGTAGCAGCACGGTCAAAGG

ACCCAGTCCTTGTGGCCATCATGCTGGCTGACACCGGCCTTGAGATTCTGGACAGTACCTTTGTCGTGAA

GAAGATCTCCGACTCGCTCTCCAGTCTCTTTCACGTGCCGGCCCCCGTCTTCAGTTTCGGAGCCCCGATT

TTGTTGGCCGGGTTGGTCAAAGTCGCCTCGAGTTTCTTCCGGTCCACGCCCGAAGACCTTGAGAGAGCGG

AGAAACAGCTCAAAGCACGTGACATCAATGACATATTCGCCATTCTCAAGAACGGCGAGTGGCTGGTCAA

GCTGATTCTTGCCATCCGCGACTGGATCAAGGCATGGATCGCCTCAGAAGAAAAGTTTGTCACCATGACA

GACCTGGTACCTGGCATCCTTGAAAAGCAGCGGGATCTCAACGACCCAAGCAAGTACAAAGAGGCCAAGG

AGTGGCTCGACAACGCGCGCCAAGCGTGTTTGAAGAGCGGGAACATCCACATCGCAAACCTTTGCAAAGT

GGTTGCCCCAGCACCCAGCAGGTCGAGGCCCGAACCCGTGGTCGTTTGCCTCCGTGGCAAATCGGGCCAG

GGCAAGAGTTTCCTTGCGAACGTGCTTGCACAAGCAATTTCAACCCACTTCACCGGCAGAACCGACTCAG

TTTGGTACTGCCCACCTGACCCTGACCACTTCGACGGTTACAACCAGCAGACCGTTGTAGTAATGGATGA

TTTGGGCCAGAACCCCGACGGGAAGGACTTCAAGTACTTCGCCCAAATGGTTTCAACTACGGGGTTTATC

CCGCCCATGGCTTCACTTGAGGACAAAGGCAAACCTTTCAACAGCAAGGTCATCATCGCCACCACCAACC

TGTACTCGGGCTTCACCCCGAGAACTATGGTGTGCCCTGACGCACTGAACCGAAGGTTCCACTTTGACAT

TGACGTGAGCGCCAAGGACGGGTACAAAATTAACAACAAATTGGACATCATCAAAGCTCTTGAAGATACC

CACACCAACCCAGTGGCAATGTTTCAATACGACTGTGCCCTTCTCAACGGCATGGCCGTTGAAATGAAGA

GAATGCAACAAGATATGTTCAAGCCTCAACCGTCCCTCCAGAACGTCTACCAGCTTGTCCAGGAGGTGAT

TGACCGGGTCGAGCTCCACGAGAAGGTGTCGAGCCACCCGATTTTCAAGCAG

>DQ404173.1_O_UKG_2001

CCCTGGTACAAGCTCATCAAGCTCTTGAGCCGCCTGTCATGCATGGCCGCTGTAGCAGCACGGTCAAAGG

ACCCAGTCCTTGTGGCCATCATGCTGGCTGACACCGGCCTTGAGATTCTGGACAGTACCTTTGTCGTGAA

GAAGATCTCCGACTCGCTCTCCAGTCTCTTTCACGTGCCGGCCCCCGTCTTCAGTTTCGGAGCCCCGATT

TTGTTGGCCGGGTTGGTCAAAGTCGCCTCGAGTTTCTTCCGGTCCACGCCCGAAGACCTTGAGAGAGCGG

AGAAACAGCTCAAAGCACGTGACATCAATGACATATTCGCCATTCTCAAGAACGGCGAGTGGCTGGTCAA

GCTGATTCTTGCCATCCGCGACTGGATCAAGGCATGGATCGCCTCAGAAGAAAAGTTTGTCACCATGACA

GACCTGGTGCCTGGCATCCTTGAAAAGCAGCGGGATCTCAACGACCCAAGCAAGTACAAAGAGGCCAAGG

AGTGGCTCGACAACGCGCGCCAAGCGTGTTTGAAGAGCGGGAACATCCACATCGCAAACCTTTGCAAAGT

GGTTGCCCCAGCACCCAGCAGGTCGAGGCCCGAACCCGTGGTCGTTTGCCTCCGTGGCAAATCGGGCCAG

GGCAAGAGTTTCCTTGCGAACGTGCTTGCACAAGCAATTTCAACCCACTTCACCGGCAGAACCGACTCAG

TTTGGTACTGCCCACCTGACCCTGACCACTTCGACGGTTACAACCAGCAGACCGTTGTAGTAATGGATGA

TTTGGGCCAGAACCCCGACGGGAAGGACTTCAAGTACTTCGCCCAAATGGTTTCAACTACGGGGTTTATC

CCGCCCATGGCTTCACTCGAGGACAAAGGCAAACCTTTCAACAGCAAGGTCATCATCGCCACCACCAACC

TGTACTCGGGCTTCACCCCGAGAACTATGGTGTGCCCTGACGCACTGAACCGAAGGTTCCACTTTGACAT

TGACGTGAGCGCCAAGGACGGGTACAAAATTAACAACAAATTGGACATCATCAAAGCTCTTGAAGATACC

CACACCAACCCAGTGGCAATGTTTCAATACGACTGTGCCCTTCTCAACGGCATGGCCGTTGAAATGAAGA

GAATGCAACAAGATATGTTCAAGCCTCAACCGTCCCTCCAGAACGTCTACCAGCTTGTTCAGGAGGTGAT

TGACCGGGTCGAGCTCCACGAGAAGGTGTCGAGCCACCCGATTTTCAAGCAG

>DQ404174.1_O_UKG_2001

CCCTGGTACAAGCTCATCAAGCTCTTGAGCCGCCTGTCATGCATGGCCGCTGTAGCAGCACGGTCAAAGG

ACCCAGTCCTTGTGGCCATCATGCTGGCTGACACCGGCCTTGAGATTCTGGACAGTACCTTTGTCGTGAA

GAAGATCTCCGACTCGCTCTCCAGTCTCTTTCACGTGCCGGCCCCCGTCTTCAGTTTCGGAGCCCCGATT

TTGTTGGCCGGGTTGGTCAAAGTCGCCTCGAGTTTCTTCCGGTCCACGCCCGAAGACCTTGAGAGAGCGG

AGAAACAGCTCAAAGCACGTGACATCAATGACATATTCGCCATTCTCAAGAACGGCGAGTGGCTGGTCAA

GCTGATTCTTGCCATCCGCGACTGGATCAAGGCATGGATCGCCTCAGAAGAAAAGTTTGTCACCATGACA

GACCTGGTGCCTGGCATCCTTGAAAAGCAGCGGGATCTCAACGACCCAAGCAAGTACAAAGAGGCCAAGG

AGTGGCTCGACAACGCGCGCCAAGCGTGTTTGAAGAGCGGGAACATCCACATCGCAAACCTTTGCAAAGT

GGTTGCCCCAGCACCCAGCAGGTCGAGGCCCGAACCCGTGGTCGTTTGCCTCCGTGGCAAATCGGGCCAG

GGCAAGAGTTTCCTTGCGAACGTGCTTGCACAAGCAATTTCAACCCACTTCACTGGCAGAACCGACTCAG

TTTGGTACTGCCCACCTGACCCTGACCACTTCGACGGTTACAACCAGCAGACCGTTGTAGTAATGGATGA

TTTGGGCCAGAACCCCGACGGGAAGGACTTCAAGTACTTCGCCCAAATGGTTTCAACTACGGGGTTTATC

CCGCCCATGGCTTCACTCGAGGACAAAGGCAAACCTTTCAACAGCAAGGTCATCATCGCCACCACCAACC

TGTACTCGGGCTTCACCCCGAGAACTATGGTGTGCCCTGACGCACTGAACCGAAGGTTCCACTTTGACAT

TGACGTGAGCGCCAAGGACGGGTACAAAATTAACAACAAATTGGACATCATCAAAGCTCTTGAAGATACC

CACACCAACCCAGTGGCAATGTTTCAATACGACTGTGCCCTTCTCAACGGCATGGCCGTTGAAATGAAGA

GAATGCAACAAGATATGTTCAAGCCTCAACCGTCCCTCCAGAACGTCTACCAGCTTGTTCAGGAGGTGAT

TGACCGGGTCGAGCTCCACGAGAAGGTGTCGAGCCACCCGATTTTCAAGCAG

>DQ404175.1_O_UKG_2001

CCCTGGTACAAGCTCATCAAGCTCTTGAGCCGCCTGTCATGCATGGCCGCTGTAGCAGCACGGTCAAAGG

ACCCAGTCCTTGTGGCCATCATGCTGGCTGACACCGGCCTTGAGATTCTGGACAGTACCTTTGTCGTGAA

GAAGATCTCCGACTCGCTCTCCAGTCTCTTTCACGTGCCGGCCCCCGTCTTCAGTTTCGGAGCCCCGATT

TTGTTGGCCGGGTTGGTCAAAGTCGCCTCGAGTTTCTTCCGGTCCACGCCCGAAGACCTTGAGAGAGCGG

AGAAACAGCTCAAAGCACGTGACATCAATGACATATTCGCCATTCTCAAGAACGGCGAGTGGCTGGTCAA

GCTGATTCTTGCCATCCGCGACTGGATCAAGGCATGGATCGCCTCAGAAGAAAAGTTTGTCACCATGACA

GACCTGGTGCCTGGCATCCTTGAAAAGCAGCGGGATCTCAACGACCCAAGCAAGTACAAAGAGGCCAAGG

AGTGGCTCGACAACGCGCGCCAAGCGTGTTTGAAGAGCGGGAACATCCACATCGCAAACCTTTGCAAAGT

GGTTGCCCCAGCACCCAGCAGGTCGAGGCCCGAACCCGTGGTCGTTTGCCTCCGTGGCAAATCGGGCCAG

GGCAAGAGTTTCCTTGCGAACGTGCTTGCACAAGCAATTTCAACCCACTTCACTGGCAGAACCGACTCAG

TTTGGTACTGCCCACCTGACCCTGACCACTTCGACGGTTACAACCAGCAGACCGTTGTAGTAATGGATGA

TTTGGGCCAGAACCCCGACGGGAAGGACTTCAAGTACTTCGCCCAAATGGTTTCAACTACGGGGTTTATC

CCGCCCATGGCTTCACTCGAGGACAAAGGCAAACCTTTCAACAGCAAGGTCATCATCGCCACCACCAACC

TGTACTCGGGCTTCACCCCGAGAACTATGGTGTGCCCTGACGCACTGAACCGAAGGTTCCACTTTGACAT

TGACGTGAGCGCCAAGGACGGGTACAAAATTAACAACAAATTGGACATCATCAAAGCTCTTGAAGATACC

CACACCAACCCAGTGGCAATGTTTCAATACGACTGTGCCCTTCTCAACGGCATGGCCGTTGAAATGAAGA

GAATGCAACAAGATATGTTCAAGCCTCAACCGTCCCTCCAGAACGTCTACCAGCTTGTTCAGGAGGTGAT

TGACCGGGTCGAGCTCCACGAGAAGGTGTCGAGCCACCCGATTTTCAAGCAG

>DQ404176.1_O_UKG_2001

CCCTGGTACAAGCTCATCAAGCTCTTGAGCCGCCTGTCATGCATGGCCGCTGTAGCAGCACGGTCAAAGG

ACCCAGTCCTTGTGGCCATCATGCTGGCTGACACCGGCCTTGAGATTCTGGACAGTACCTTTGTCGTGAA

GAAGATCTCCGACTCGCTCTCCAGTCTCTTTCACGTGCCGGCCCCCGTCTTCAGTTTCGGAGCCCCGATT

TTGTTGGCCGGGTTGGTCAAAGTCGCCTCGAGTTTCTTCCGGTCCACACCCGAAGACCTTGAGAGAGCGG

AGAAACAGCTCAAAGCACGTGACATCAATGACATATTCGCCATTCTCAAGAACGGCGAGTGGCTGGTCAA

GCTGATTCTTGCCATCCGCGACTGGATCAAGGCATGGATCGCCTCAGAAGAAAAGTTTGTCACCATGACA

GACCTGGTGCCTGGCATCCTTGAAAAGCAGCGGGATCTCAACGACCCAAGCAAGTACAAAGAGGCCAAGG

AGTGGCTCGACAACGCGCGCCAAGCGTGTTTGAAGAGCGGGAACATCCACATCGCAAACCTTTGCAAAGT

GGTTGCCCCAGCACCCAGCAGGTCGAGGCCCGAACCCGTGGTCGTTTGCCTCCGTGGCAAATCGGGCCAG

GGCAAGAGTTTCCTTGCGAACGTGCTTGCACAAGCAATTTCAACCCACTTCACTGGCAGAACCGACTCAG

TTTGGTACTGCCCACCTGACCCTGACCACTTCGACGGTTACAACCAGCAGACCGTTGTAGTAATGGATGA

TTTGGGCCAGAACCCCGACGGGAAGGACTTCAAGTACTTCGCCCAAATGGTTTCAACTACGGGGTTTATC

CCGCCCATGGCTTCACTCGAGGACAAAGGCAAACCTTTCAACAGCAAGGTCATCATCGCCACCACCAACC

TGTACTCGGGCTTCACCCCGAGAACTATGGTGTGCCCTGACGCACTGAACCGAAGGTTCCACTTTGACAT

TGACGTGAGCGCCAAGGACGGGTACAAAATTAACAACAAATTGGACATCATCAAAGCTCTTGAAGATACC

CACACCAACCCAGTGGCAATGTTTCAATACGACTGTGCCCTTCTCAACGGCATGGCCGTTGAAATGAAGA

GAATGCAACAAGATATGTTCAAGCCTCAACCGCCCCTCCAGAACGTCTACCAGCTTGTTCAGGAGGTGAT

TGACCGGGTCGAGCTCCACGAGAAGGTGTCGAGCCACCCGATTTTCAAGCAG

>DQ404177.1_O_UKG_2001

CCCTGGTACAAGCTCATCAAGCTCTTGAGCCGCCTGTCATGCATGGCCGCTGTAGCAGCACGGTCAAAGG

ACCCAGTCCTTGTGGCCATCATGCTGGCTGACACCGGCCTTGAGATTCTGGACAGTACCTTTGTCGTGAA

GAAGATCTCCGACTCGCTCTCCAGTCTCTTTCACGTGCCGGCCCCCGTCTTCAGTTTCGGAGCCCCGATT

TTGTTGGCCGGGTTGGTCAAAGTCGCCTCGAGTTTCTTCCGGTCCACACCCGAAGACCTTGAGAGAGCGG

AGAAACAGCTCAAAGCACGTGACATCAATGACATATTCGCCATTCTCAAGAACGGCGAGTGGCTGGTCAA

GCTGATTCTTGCCATCCGCGACTGGATCAAGGCATGGATCGCCTCAGAAGAAAAGTTTGTCACCATGACA

GACCTGGTGCCTGGCATCCTTGAAAAGCAGCGGGATCTCAACGACCCAAGCAAGTACAAAGAGGCCAAGG

AGTGGCTCGACAACGCGCGCCAAGCGTGTTTGAAGAGCGGGAACATCCACATCGCAAACCTTTGCAAAGT

GGTTGCCCCAGCACCCAGCAGGTCGAGGCCCGAACCCGTGGTCGTTTGCCTCCGTGGCAAATCGGGCCAG

GGCAAGAGTTTCCTTGCGAACGTGCTTGCACAAGCAATTTCAACCCACTTCACTGGCAGAACCGACTCAG

TTTGGTACTGCCCACCTGACCCTGACCACTTCGACGGTTACAACCAGCAGACCGTTGTAGTAATGGATGA

TTTGGGCCAGAACCCCGACGGGAAGGACTTCAAGTACTTCGCCCAAATGGTTTCAACTACGGGGTTTATC

CCGCCCATGGCTTCACTCGAGGACAAAGGCAAACCTTTCAACAGCAAGGTCATCATCGCCACCACCAACC

TGTACTCGGGCTTCACCCCGAGAACTATGGTGTGCCCTGACGCACTGAACCGAAGGTTCCACTTTGACAT

TGACGTGAGCGCCAAGGACGGGTACAAAATTAACAACAAATTGGACATCATCAAAGCTCTTGAAGATACC

CACACCAACCCAGTGGCAATGTTTCAATACGACTGTGCCCTTCTCAACGGCATGGCCGTTGAAATGAAGA

GAATGCAACAAGATATGTTCAAGCCTCAACCGCCCCTCCAGAACGTCTACCAGCTTGTTCAGGAGGTGAT

TGACCGGGTCGAGCTCCACGAGAAGGTGTCGAGCCACCCGATTTTCAAGCAG

>DQ404178.1_O_UKG_2001

CCCTGGTACAAGCTCATCAAGCTCTTGAGCCGCCTGTCATGCATGGCCGCTGTAGCAGCACGGTCAAAGG

ACCCAGTCCTTGTGGCCATCATGCTGGCTGACACCGGCCTTGAGATTCTGGACAGTACCTTTGTCGTGAA

GAAGATCTCCGACTCGCTCTCCAGTCTCTTTCACGTGCCGGCCCCCGTCTTCAGTTTCGGAGCCCCGATT

TTGTTGGCCGGGTTGGTCAAAGTCGCCTCGAGTTTCTTCCGGTCCACACCCGAAGACCTTGAGAGAGCGG

AGAAACAGCTCAAAGCACGTGACATCAATGACATATTCGCCATTCTCAAGAACGGCGAGTGGCTGGTCAA

GCTGATTCTTGCCATCCGCGACTGGATCAAGGCATGGATCGCCTCAGAAGAAAAGTTTGTCACCATGACA

GACCTGGTGCCTGGCATCCTTGAAAAGCAGCGGGATCTCAACGACCCAAGCAAGTACAAAGAGGCCAAGG

AGTGGCTCGACAACGCGCGCCAAGCGTGTTTGAAGAGCGGGAACATCCACATCGCAAACCTTTGCAAAGT

GGTTGCCCCAGCACCCAGCAGGTCGAGGCCCGAACCCGTGGTCGTTTGCCTCCGTGGCAAATCGGGCCAG

GGCAAGAGTTTCCTTGCGAACGTGCTTGCACAAGCAATTTCAACCCACTTCACTGGCAGAACCGACTCAG

TTTGGTACTGCCCACCTGACCCTGACCACTTCGACGGTTACAACCAGCAGACCGTTGTAGTAATGGATGA

TTTGGGCCAGAACCCCGACGGGAAGGACTTCAAGTACTTCGCCCAAATGGTTTCAACTACGGGGTTTATC

CCGCCCATGGCTTCACTCGAGGACAAAGGCAAACCTTTCAACAGCAAGGTCATCATCGCCACCACCAACC

TGTACTCGGGCTTCACCCCGAGAACTATGGTGTGCCCTGACGCACTGAACCGAAGGTTCCACTTTGACAT

TGACGTGAGCGCCAAGGACGGGTACAAAATTAACAACAAATTGGACATCATCAAAGCTCTTGAAGATACC

CACACCAACCCAGTGGCAATGTTTCAATACGACTGTGCCCTTCTCAACGGCATGGCCGTTGAAATGAAGA

GAATGCAACAAGATATGTTCAAGCCTCAACCGCCCCTCCAGAACGTCTACCAGCTTGTTCAGGAGGTGAT

TGACCGGGTCGAGCTCCACGAGAAGGTGTCGAGCCACCCGATTTTCAAGCAG

>DQ404179.1_O_UKG_2001

CCCTGGTACAAGCTCATCAAGCTCTTGAGCCGCCTGTCATGCATGGCCGCTGTAGCAGCACGGTCAAAGG

ACCCAGTCCTTGTGGCCATCATGCTGGCTGACACCGGCCTTGAGATTCTGGACAGTACCTTTGTCGTGAA

GAAGATCTCCGACTCGCTCTCCAGTCTCTTTCACGTGCCGGCCCCCGTCTTCAGTTTCGGAGCCCCGATT

TTGTTGGCCGGGTTGGTCAAAGTCGCCTCGAGTTTCTTCCGGTCCACACCCGAAGACCTTGAGAGAGCGG

AGAAACAGCTCAAAGCACGTGACATCAATGACATATTCGCCATTCTCAAGAACGGCGAGTGGCTGGTCAA

GCTGATTCTTGCCATCCGCGACTGGATCAAGGCATGGATCGCCTCAGAAGAAAAGTTTGTCACCATGACA

GACCTGGTGCCTGGCATCCTTGAAAAGCAGCGGGATCTCAACGACCCAAGCAAGTACAAAGAGGCCAAGG

AGTGGCTCGACAACGCGCGCCAAGCGTGTTTGAAGAGCGGGAACATCCACATCGCAAACCTTTGCAAAGT

GGTTGCCCCAGCACCCAGCAGGTCGAGGCCCGAACCCGTGGTCGTTTGCCTCCGTGGCAAATCGGGCCAG

GGCAAGAGTTTCCTTGCGAACGTGCTTGCACAAGCAATTTCAACCCACTTCACTGGCAGAACCGACTCAG

TTTGGTACTGCCCACCTGACCCTGACCACTTCGACGGTTACAACCAGCAGACCGTTGTAGTAATGGATGA

TTTGGGCCAGAACCCCGACGGGAAGGACTTCAAGTACTTCGCCCAAATGGTTTCAACTACGGGGTTTATC

CCGCCCATGGCTTCACTCGAGGACAAAGGCAAACCTTTCAACAGCAAGGTCATCATCGCCACCACCAACC

TGTACTCGGGCTTCACCCCGAGAACTATGGTGTGCCCTGACGCACTGAACCGAAGGTTCCACTTTGACAT

TGACGTGAGCGCCAAGGACGGGTACAAAATTAACAACAAATTGGACATCATCAAAGCTCTTGAAGATACC

CACACCAACCCAGTGGCAATGTTTCAATACGACTGTGCCCTTCTCAACGGCATGGCCGTTGAAATGAAGA

GAATGCAACAAGATATGTTCAAGCCTCAACCGCCCCTCCAGAACGTCTACCAGCTTGTTCAGGAGGTGAT

TGACCGGGTCGAGCTCCACGAGAAGGTGTCGAGCCACCCGATTTTCAAGCAG

>DQ404180.1_O_UKG_2001

CCCTGGTACAAGCTCATCAAGCTCTTGAGCCGCCTGTCATGCATGGCCGCTGTAGCAGCACGGTCAAAGG

ACCCAGTCCTTGTGGCCATCATGCTGGCTGACACCGGCCTTGAGATTCTGGACAGTACCTTTGTCGTGAA

GAAGATCTCCGACTCGCTCTCCAGTCTCTTTCACGTGCCGGCCCCCGTCTTCAGTTTCGGAGCCCCGATT

TTGTTGGCCGGGTTGGTCAAAGTCGCCTCGAGTTTCTTCCGGTCCACACCCGAAGACCTTGAGAGAGCGG

AGAAACAGCTCAAAGCACGTGACATCAATGACATATTCGCCATTCTCAAGAACGGCGAGTGGCTGGTCAA

GCTGATTCTTGCCATCCGCGACTGGATCAAGGCATGGATCGCCTCAGAAGAAAAGTTTGTCACCATGACA

GACCTGGTGCCTGGCATCCTTGAAAAGCAGCGGGATCTCAACGACCCAAGCAAGTACAAAGAGGCCAAGG

AGTGGCTCGACAACGCGCGCCAAGCGTGTTTGAAGAGCGGGAACATCCACATCGCAAACCTTTGCAAAGT

GGTTGCCCCAGCACCCAGCAGGTCGAGGCCCGAACCCGTGGTCGTTTGCCTCCGTGGCAA-TCGGGCCAG

GGCAAGAGTTTCCTTGCGAACGTGCTTGCACAAGCAATTTCAACCCACTTCACTGGCAGAACCGACTCAG

TTTGGTACTGCCCACCTGACCCTGACCACTTCGACGGTTACAACCAGCAGACCGTTGTAGTAATGGATGA

TTTGGGCCAGAACCCCGACGGGAAGGACTTCAAGTACTTCGCCCAAATGGTTTCAACTACGGGGTTTATC

CCGCCCATGGCTTCACTCGAGGACAAAGGCAAACCTTTCAACAGCAAGGTCATCATCGCCACCACCAACC

TGTACTCGGGCTTCACCCCGAGAACTATGGTGTGCCCTGACGCACTGAACCGAAGGTTCCACTTTGACAT

TGACGTGAGCGCCAAGGACGGGTACAAAATTAACAACAAATTGGACATCATCAAAGCTCTTGAAGATACC

CACACCAACCCAGTGGCAATGTTTCAATACGACTGTGCCCTTCTCAACGGCATGGCCGTTGAAATGAAGA

GAATGCAACAAGATATGTTCAAGCCTCAACCGCCCCTCCAGAACGTCTACCAGCTTGTTCAGGAGGTGAT

TGACCGGGTCGAGCTCCACGAGAAGGTGTCGAGCCACCCGATTTTCAAGCAG

>DQ989303.1_Asia1_IND_1993

CCCTGGTACAAGCTCATCAAACTCCTAAGCCGCCTGTCATGTATGGCCGCTGTAGCAGCACGGTCCAAGG

ACCCAGTCCTTGTGGCCATCATGCTGGCTGATACCGGCCTTGAGATTCTGGACAGCACGTTTGTCGTGAA

GAAGATCTCCGACTCGCTCTCCAGTCTCTTTCACGTGCCGGCCCCCGTCTTCAGTTTCGGAGCCCCGATT

CTGTTGGCTGGGTTGGTCAAGGTCGCCTCGAGTTTCTTCCGGTCAACACCCGAAGACCTCGAGAGAGCAG

AGAAACAGCTCAAAGCACGTGACATCAACGACATTTTCGCCATTCTCAAGAACGGCGAGTGGCTGGTCAA

GCTGATCCTTGCCATCCGCGACTGGATTAAGGCGTGGATCGCCTCAGAAGAGAAGTTTGTCACCATGACA

GACTTAGTGCCTGGCATACTTGAAAAGCAGCGGGACCTCAACGACCCCAGCAAGTACAAGGAGGCCAAGG

AGTGGCTCGACAACGCGCGCCAAGCGTGTCTGAAGAGCGGGAACATCCACATTGCCAACTTGTGCAAAGT

GGTCGCCCCGGCACCAAGCAAGTCGGGACCCGAACCCGTGGTCGTTTGTCTCCGTGGCAAATCCGGCCAG

GGTAAGAGTTTTCTTGCGAACGTGCTCGCACAAGCGATTTCTACCCACTTCACTGGCAGAACCGATTCAG

TTTGGTACTGCCCGCCTGACCCTGACCACTTCGACGGTTACAACCAACAGACCGTTGTTGTGATGGATGA

TCTGGGTCAGAACCCCGACGGCAAGGACTTCAAGTACTTCGCCCAGATGGTTTCAACCACGGGGTTCATC

CCGCCCATGGCTTCACTCGAAGATAAAGGCAAGCCTTTCAACAGCAAAGTCATCATTGCCACCACCAACC

TGTATTCGGGTTTCACCCCGAGGACCATGGTGTGCCCTGACGCGCTGAACCGGAGGTTCCACTTTGACAT

TGATGTGAGTGCCAAGGACGGGTACAAAACTAACAACAAATTGGACATAATCAAAGCTCTTGAAGACACC

CACACCAACCCGGTGGCGATGTTCCAATATGATTGTGCCCTTCTCAATGGCATGGCCGTTGAGATGAAGA

GAATGCAACAAGATATGTTTAAGCCCCAACCACCTCTACAGAATGTCTACCAACTTGTTCAGGAGGTGAT

TGACCGGGTCGAGCTCCACGAGAAAGTGTCGAACCACCCGATCTTCAAACAA

>DQ989304.1_Asia1_IND_2000

CCCTGGTACAAGCTCATCAAACTCCTAAGCCGCCTGTCATGTATGGCCGCTGTAGCAGCACGGTCCAAGG

ACCCAGTCCTTGTGGCCATCATGCTGGCTGATACCGGCCTTGAGATTCTGGACAGCACGTTTGTCGTGAA

GAAGATCTCCGACTCGCTCTCCAGTCTCTTTCACGTGCCGGCCCCCGTCTTCAGTTTCGGAGCCCCGATT

CTGTTGGCTGGGTTGGTCAAGGTCGCCTCGAGTTTCTTCCGGTCAACACCCGAAGACCTCGAGAGAGCAG

AGAAACAGCTCAAAGCACGTGACATCAATGACATTTTCGCCATTCTCAAGAACGGCGAGTGGCTGGTCAA

GCTGATCCTTGCCATCCGCGACTGGATTAAGGCGTGGATCGCCTCAGAAGAGAAGTTTGTCACCATGACA

GACTTAGTGCCTGGCATACTTGAAAAGCAGCGGGACCTCAACGACCCCAGCAAGTACAAGGAGGCCAAGG

AGTGGCTCGACAACGCGCGCCAAGCGTGTCTGAAGAGCGGGAACATCCACATTGCCAACTTGTGCAAAGT

GGTCGCCCCGGCACCAAGCAAGTCGAGACCCGAACCCGTGGTCGTTTGTCTCCGTGGCAAATCCGGCCAG

GGTAAGAGTTTCCTTGCGAACGTGCTTGCACAAGCGATTTCTACCCACTTCACTGGCAGAACCGATTCAG

TTTGGTACTGCCCGCCTGACCCTGACCACTTCGACGGTTACAACCAACAGACCGTTGTTGTGATGGATGA

TCTGGGTCAGAACCCCGACGGCAAGGACTTCAAGTACTTCGCCCAGATGGTTTCAACCACGGGGTTCATC

CCGCCCATGGCTTCACTCGAAGATAAAGGCAAGCCTTTCAACAGCAAAGTCATCATTGCCACCACCAACC

TGTATTCGGGTTTCACCCCGAGGACCATGGTGTGCCCTGACGCGCTGAACCGGAGGTTCCACTTTGACAT

TGATGTGAGTGCCAAGGACGGGTACAAAACTAACAACAAATTGGACATAATCAAAGCTCTTGAAGACACC

CACACCAACCCGGTGGCGATGTTCCAATATGATTGTGCCCTTCTCAATGGCATGGCCGTTGAGATGAAGA

GAATGCAACAAGATATGTTTAAGCCCCAACCACCTCTACAGAATGTCTACCAACTTGTTCAGGAGGTGAT

TGACCGGGTCGAGCTCCACGAGAAAGTGTCGAACCACCCGATCTTCAAACAA

>DQ989305.1_Asia1_IND_1990

CCCTGGTACAAGCTCATCAAGCTCCTGAGCCGCCTGTCGTGCATGGCCGCTGTAGCAGCACGGTCCAAGG

ACCCGGTCCTTGTGGCTATCATGCTGGCTGACACCGGCCTTGAGATTCTGGACAGCACGTTTGTCGTGAA

GAAGATCTCCGACTCGCTCTCCAGTCTCTTTCACGTGCCGGCCCCCGTCTTCAGTTTCGGAGCCCCGATT

CTGTTGGCCGGGTTGGTCAAAGTCGCCTCGAGCTTCTTCCGGTCCACGCCCGAAGACCTCGAGAGAGCAG

AGAAACAGCTCAAAGCACGTGACATCAACGACATATTCGCCATTCTCAAGAACGGCGAGTGGCTGGTCAA

GCTGATCCTTGCCATCCGCGACTGGATCAAGGCATGGATCGCCTCAGAAGAAAAGTTTGTCACCATGACA

GACTTGGTGCCTGGCATCCTTGAAAAGCAACGGGACCTTAACGACCCGAGCAAGTACAAGGAGGCCAAGG

AGTGGCTCGACAACGCGCGCCAAGCGTGTTTGAAGAGTGGGAACGTCCACATTGCCAATCTGTGCAAAGT

GGTCGCCCCGGCACCTAGCAAGTCGAGACCCGAACCTGTGGTCGTTTGCCTCCGTGGCAAATCCGGCCAG

GGGAAGAGTTTCCTTGCGAACGTGCTCGCACAGGCAATCTCCACCCACTTCACCGGCAGAACTGATTCAG

TTTGGTACTGCCCACCTGACCCTGACCACTTCGACGGTTACAACCAGCAGACCGTTGTTGTGATGGACGA

TTTGGGCCAGAACCCTGATGGCAAGGACTTCAAGTACTTCGCCCAGATGGTGTCAACTACAGGGTTCATC

CCGCCCATGGCCTCACTTGAGGACAAAGGCAAACCTTTCAACAGCAAGGTCATCATCGCCACCACCAACC

TGTACTCGGGTTTCACCCCGAGAACCATGGTGTGCCCTGACGCGCTGAACCGGAGGTTCCACTTTGACAT

CGATGTGAGTGCCAAGGACGGGTACAAAATTAACAACAAATTGGACATAATCAAGGCTCTTGAAGATACC

CACACCAACCCCGTGGCAATGTTTCAATACGACTGTGCCCTTCTCAACGGCATGGCCGTTGAAATGAAGA

GAATGCAACAAGACATGTTCAAGCCTCAACCGCCCCTCCAGAACGTTTACCAGCTCGTTCAGGAGGTGAT

TGAACGGGTCGAGCTCCACGAGAAAGTGTCGAGCCACCCGATCTTCAAGCAG

>DQ989306.1_Asia1_IND_1986

CCCTGGTACAAGCTCATCAAACTCCTAAGCCGCCTGTCATGTATGGCCGCTGTAGCAGCACGGTCCAAGG

ACCCAGTCCTTGTGGCCATCATGCTGGCTGATACCGGCCTTGAGATTCTGGACAGCACGTTTGTCGTGAA

GAAGATCTCCGACTCGCTCTCCAGTCTCTTTCACGTGCCGGCCCCCGTCTTCAGTTTCGGAGCCCCGATT

CTGTTGGCTGGGTTGGTCAAGGTCGCCTCGAGTTTCTTCCGGTCAACACCCGAAGACCTCGAGAGAGCAG

AGAAACAGCTCAAAGCACGTGACATCAATGACATTTTCGCCATTCTCAAGAACGGCGAGTGGCTGGTCAA

GCTGATCCTTGCCATCCGCGACTGGATTAAGGCGTGGATCGCCTCAGAAGAGAAGTTTGTCACCATGACA

GACTTAGTGCCTGGCATACTTGAAAAGCAGCGGGACCTCAACGACCCCAGCAAGTACAAGGAGGCCAAGG

AGTGGCTCGACAACGCGCGCCAAGCGTGTCTGAAGAGCGGGAACGTCCACATTGCCAACTTGTGCAAAGT

GGTCGCCCCGGCACCAAGCAAGTCGAGACCCGAACCCGTGGTCGTTTGTCTCCGTGGCAAATCCGGCCAG

GGTAAGAGTTTCCTTGCGAACGTGCTCGCACAAGCAATTTCTACCCACTTCACTGGCAGAACCGATTCAG

TTTGGTACTGCCCGCCTGACCCTGACCACTTCGACGGTTACAACCAACAGACCGTTGTTGTGATGGATGA

TCTGGGTCAGAACCCCGACGGCAAGGACTTCAAGTACTTCGCCCAGATGGTTTCAACCACGGGGTTCATC

CCGCCCATGGCTTCACTCGAAGATAAAGGCAAGCCTTTCAACAGCAAAGTCATCATTGCCACCACCAACC

TGTATTCGGGTTTCACCCCGAGGACCATGGTGTGCCCTGACGCGCTGAACCGGAGGTTCCACTTTGACAT

TGATGTGAGTGCCAAGGACGGGTACAAAACTAACAACAAATTGGACATAACCAAAGCTCTTGAAGACACC

CACACCAACCCGGTGGCGATGTTCCAATATGATTGTGCCCTTCTCAATGGCATGGCCGTTGAGATGAAGA

GAATGCAACAAGATATGTTTAAGCCCCAACCACCTCTACAGAATGTGTACCAACTTGTTCAGGAGGTGAT

TGACCGGGTCGAGCTCCACGAGAAAGTGTCGAACCACCCGATCTTCAAACAA

>DQ989307.1_Asia1_IND_1992

CCCTGGTACAAGCTCATCAAACTCCTGAGCCGCCTGTCATGCATGGCCGCTGTAGCAGCACGGTCAAAGG

ACCCAGTCCTTGTGGCTATCATGCTAGCTGACACCGGTCTCGAGATTCTGGACAGCACCTTCGTCGTGAA

GAAGATCTCCGACTCGCTCTCCAGTCTCTTTCACGTGCCGGCCCCCGTCTTCAGCTTCGGAGCCCCGATT

CTGTTGGCCGGGTTGGTCAAAGTCGCCTCGAGTTTCTTCCGGTCCACACCCGAAGACCTTGAGAGAGCAG

AAAAACAGCTCAAAGCACGTGACATCAATGACATATTCGCCATTCTCAAGAACGGCGAGTGGCTGGTCAA

GCTGATCCTTGCCATCCGCGACTGGATCAAGGCATGGATCGCCTCCGAAGAAAAGTTTGTCACCATGACA

GACTTGGTTCCTGGCATCCTCGAAAAGCAGCGGGACCTCAACGACCCGAGCAAGTACAAGGAAGCCAAGG

AGTGGCTCGACAACGCGCGCCAGGCGTGCTTGAAGAGCGGGAACGTCCACATTGCCAACCTGTGTAAAGT

GGTTGCCCCAGCACCCAGCAAGTCGAGACCCGAACCCGTGGTCGTTTGCCTCCGTGGTAAATCCGGCCAG

GGAAAGAGTTTCCTCGCGAACGTGCTTGCACAGGCAATTTCAACCCACTTCACCGGCAGAACTGATTCAG

TTTGGTACTGCCCACCTGACCCTGACCACTTCGACGGTTACAACCAGCAGACCGTTGTTGTGATGGACGA

TTTGGGCCAGAACCCTGATGGCAAGGACTTCAAGTACTTCGCCCAGATGGTGTCAACTACAGGGTTCATC

CCGCCCATGGCCTCACTTGAGGACAAAGGCAAACCTTTCAACAGCAAGGTCATCATCGCCACCACCAACC

TGTACTCGGGTTTCACCCCGAGAACCATGGTGTGCCCTGACGCGCTGAACCGGAGGTTCCACTTTGACAT

CGATGTGAGTGCCAAGGACGGGTACAAAATTAACAACAAATTGGACATAATCAAGGCTCTTGAAGATACC

CACACCAACCCCGTGGCAATGTTTCAATACGACTGTGCCCTTCTCAACGGCATGGCCGTTGAAATGAAGA

GAATGCAACAAGACATGTTCAAGCCTCAACCGCCCCTCCAGAACGTTTACCAGCTCGTTCAGGAGGTGAT

TGAACGGGTCGAGCTCCACGAGAAAGTGTCGAGCCACCCGATCTTCAAGCAG

>DQ989308.1_Asia1_IND_1994

CCCTGGTACAAGCTCATCAAACTCCTAAGCCGCCTGTCGTGCATGGCCGCTGTAGCAGCACGGTCTAAGG

ACCCAGTTCTTGTGGCCATCATGCTGGCTGACACCGGCCTTGAGATTCTGGACAGCACGTTCGTTGTGAA

GAAGATCTCCGACTCGCTCTCCAGTCTCTTCCACGTGCCGGCCCCCGTCTTCAGTTTCGGAGCCCCGATT

CTGTTGGCCGGGTTGGTCAAAGTCGCCTCGAGCTTCTTCCGGTCCACACCCGAAGACCTCGAGAGAGCAG

AGAAACAGCTCAAAGCACGTGACATCAACGACATATTCGCCATTCTCAAGAACGGCGAGTGGCTGGTCAA

ACTGATTCTTGCCATCCGCGACTGGGTTAAGGCATGGATCGCCTCAGAAGAAAAGTTTGTCACCATGACA

GACTTGGTGCCTGGCATTCTTGAAAAGCAACGGGACCTTAACGACCCGAGCAAGTACAAGGAGGCCAAGG

AATGGCTCGACAACGCGCGTCAAGCGTGTCTGAAGAGCGGGAACGTCCACATTGCCAACCTGTGTAAAGT

GGTCGCCCCGGCACCAAGCAAGTCGAGACCCGAACCCGTGGTCGTTTGTCTCCGTGGCAAATCCGGCCAG

GGTAAGAGTTTCCTTGCGAACGTGCTCGCACAAGCGATTTCTACCCACTTCACTGGCAGAACCGATTCAG

TTTGGTACTGCCCGCCTGACCCTGACCACTTCGACGGTTACAACCAACAGACCGTTGTTGTGATGGATGA

TCTGGGTCAGAACCCCGACGGCAAGGACTTCAAGTACTTCGCCCAGATGGTTTCAACCACGGGGTTCATC

CCGCCCATGGCTTCACTCGAGGATAAAGGCAAGCCTTTCAACAGCAAAGTCATCATTGCCACCACCAACC

TGTATTCGGGTTTCACCCCGAGGACCATGGTGTGCCCTGACGCGCTGAACCGGAGGTTCCACTTTGACAT

TGATGTGAGTGCCAAGGACGGGTACAAAACTAACAACAAATTGGACATAATCAAAGCTCTTGAAGACACC

CACACCAACCCGGTGGCGATGTTCCAATATGATTGTGCCCTTCTCAATGGCATGGCCGTTGAGATGAAGA

GAATGCAACAAGATATGTTTAAGCCCCAACCACCTCTACAGAATGTCTACCAACTTGTTCAGGAGGTGAT

TGACCGGGTCGAGCTCCACGAGAAAGTGTCGAACCACCCGATCTTCAAACAG

>DQ989309.1_Asia1_IND_1996

CCCTGGTACAAGCTCATCAAACTCCTAAGTCGCCTGTCGTGCATGGCCGCTGTAGCAGCACGGTCTAAGG

ACCCAGTCCTTGTGGCCATCATGTTGGCTGACACCGGCCTTGAGATTCTGGACAGCACGTTCGTCGTGAA

AAAGATCTCCGACACGCTCTCCGGTCTCTTCCACGTGCCGGCCCCCGTCTTCAGCTTCGGAGCCCCGATC

CTGTTGGCCGGGTTGGTCAAAGTCGCCTCGAGTTTCTTCCGGTCCACACCCGAAGACCTCGAGAGAGCAG

AGAGACAGCTCAAAGCACGTGACATCAACGACGTATTCGCCATTCTCAAGAACGGCGAGTGGCTGGTCAA

ACTGATTCTTGCCATCCGCGACTGGATTAAGGCATGGATCGCCTCAGAAGAAAAGTTTGTCACCATGACA

GACTTGGTGCCTGGCATCCTTGAAAAGCAACGGGACCTCAACGACCCGAGCAAGTACAAGGAGGCCAAGG

AATGGCTCGACAACGCGCGTCAAGCGTGTCTGAAGAGCGGGAACGTCCATATTGCCAACCTGTGCAAAGT

GGTCGCCCCGGCACCGAGCAAGTCGAGACCCGAACCTGTGGTCGTTTGCCTCCGTGGCAAATCCGGCCAG

GGGAAGAGTTTCCTGGCGAACGTGCTTGCACAAGCAATCTCCACCCACTTCACTGGCAGAACCGATTCTG

TTTGGTACTGCCCGCCTGACCCCGACCACTTCGACGGTTACAACCAACAAACTGTTGTTGTGATGGATGA

TTTGGGCCAGAACCCTGATGGCAAGGATTTCAAATACTTCGCTCAAATGGTTTCAACCACAGGGTTCATC

CCGCCCATGGCTTCACTAGAGGACAAAGGCAAACCTTTCAACAGCAAGGTCATCATTGCCACCACCAACC

TGTACTCGGGCTTCACCCCGAGAACAATGGTGTGCCCTGATGCACTGAACCGAAGGTTCCACTTTGACAT

CGACGTGAGCGCCAAGGATGGGTACAAAATTAACAACAAATTGGACATAATCAAAGCTCTTGAGGACACC

CACACCAACCCCGTGGCAATGTTCCGGTACGACTGTGCCCTTCTCAATGGCATGGCCGTTGAAATGAAGA

GAATGCAACAAGACATGTTCAAGCCTCAACCACCCCTCCAGAACGTGTACCAACTAGTTCAGGAGGTGAT

TGAACGGGTCGAGCTCCACGAGAAAGTGTCGAGCCACCCGATCTTCAAGCAG

>DQ989310.1_Asia1_IND_1999

CCTTGGTACAAGCTCATCAAACTCCTGAGCCGCTTGTCATGCATGGCCGCTGTAGCAGCACGGTCAAAGG

ACCCAGTCCTTGTGGCTATCATGCTAGCTGACACCGGTCTCGAGATTCTGGACAGCACCTTTGTCGTGAA

GAAAATCTCCGACTCACTCTCCAGTCTCTTTCACGTGCCGGCCCCCGTCTTCAGTTTCGGAGCTCCGATT

CTGTTGGCCGGGTTGGTCAAGGTCGCCTCGAGTTTCTTCCGGTCTACGGCCGAAGACCTCGAGAGAGCAG

AAAAACAGCTCAAAGCGCGTGACATCAATGACATATTCGCCATTCTAAAGAACGGCGAGTGGCTGGTCAA

GCTGATTCTTGCTATCCGCGACTGGATTAAAGCATGGATCGCCTCAGAAGAGAAGTTTGTCACCATGACA

GACTTGGTGCCCGGCATCCTTGAAAAGCAACGAGACCTCAACGACCCGAGTAAGTACAAGGAGGCCAAGG

ATTGGCTCGACAACGCGCGCCAAGCGTGTCTGAAGAGCGGGAACGTCCACATTGCCAACTTGTGCAAAGT

GGTCGCCCCGGCACCGAGTAAGTCGAGACCCGAACCCGTGGTCGTTTGCCTCCGGGGCAAATCCGGCCAG

GGTAAGAGTTTCCTTGCGAACGTACTCGCACAAGCAATTTCCACCCACTTTACTGGCAGAACTGATTCAG

TCTGGTACTGCCCACCTGACCCTGACCACTTCGACGGTTACAACCAACAGACCGTTGTTGTGATGGATGA

TTTGGGACAGAACCCTGATGGCAAGGACTTCAAGTACTTCGCCCAAATGGTATCAACCACAGGGTTCATC

CCGCCCATGGCGTCACTCGAAGACAAAGGCAAACCTTTCAACAGCAAGGTCATCATTGCCACCACCAACC

TGTACTCTGGCTTCACCCCGAGAACAATGGTGTGCCCTGACGCACTGAACCGAAGGTTCCACTTTGACAT

TGACGTGAGTGCCAAGGACGGGTACAAAAACAACAACAAATTGGACATAATCAAAGCTCTTGAGGACACC

CACACCAACCCCGTGGCAATGTTCCAGTACGACTGTGCCCTTCTCAATGGCATGGCCGTTGAAATGAAGA

GAATGCAACAAGACATGTTCAAGCCCCAACCACCCCTCCAGAACGTGTACCAACTGGTTCAGGAAGTGAT

TGAACGGGTCGAGCTCCACGAGAAAGTATCGAGCCACCCAATCTTCAAGCAG

>DQ989311.1_Asia1_IND_2002

CCCTGGTACAAGCTCATTAAACTCCTGAGTCGTCTGTCATGTATGGCCGCTGTAGCAGCACGGTCCAAGG

ACCCAGTCCTTGTGGCCATTATGCTGGCTGACACCGGTCTCGAGATTCTGGACAGCACTTTTGTCGTGAA

GAAGATCTCCGACTCGCTCTCCAGTCTCTTTCACGTGCCGGCCCCCGTCTTCAGTTTCGGAGCCCCGATT

CTGTTGGCCGGGTTGGTCAAAGTCGCCTCGAGTTTCTTCCGGTCAACGCCCGAGGACCTTGAGAGAGCAG

AAAAACAGCTCAAAGCACGTGACATAAATGACATTTTCGCCATTCTTAAGAACGGCGAGTGGCTGGTCAA

GCTGATTCTTGCCATCCGCGACTGGATCAAGGCATGGATCGCCTCAGAAGAAAAGTTTGTCACCATGACA

GACTTGGTGCCTGGCATCCTTGAAAAGCAGCGGGACCTCAACGACCCGAGCAAGTACAAGGAGGCCAAGG

AGTGGCTCGACAACGCGCGCCAAGCGTGTTTGAAGAGTGGGAACGTCCACATTGCCAACCTGTGCAAAGT

GGCTCGCCCAGCACCTAGCAAGTCGAGACCCGAACCTGTGGTCGTTTGCCTCCGTGGCAAATCCGGCCAG

GGGAAGAGTTTCCTCGCGAACGTGCTCGCACAAGCAATCTCCACCCACTTCACTGGCAGAACTGATTCAG

TTTGGTACTGCCCGCCTGACCCTGACCACTTCGACGGCTACAACCAGCAGACCGTTGTTGTGATGGATGA

TTTGGGCCAGAACCCTGATGGTAAGGACTTCAAGTACTTTGCCCAAATGGTTTCGACCACGGGGTTCATC

CCGCCCATGGCTTCACTAGAGGACAAAGGTAAACCTTTCAACAGCAAGGTCATCATTGCCACTACAAACC

TGTACTCGGGGTTCACCCCGAGAACAATGGTGTGCCCTGATGCACTGAACCGAAGGTTTCACTTTGACAT

CGACGTGAGCGCCAAGGACGGGTACAAAATTAACAACAAATTGGACATCATCAAAGCTCTTGAGGACACC

CACACCAACCCAGTGGCAATGTTTCAATACGACTGTGCCCTTCTCAACGGCATGGCCGTTGAAATGAAGA

GAATGCAACAAGATATGTTCAAGCCCCAGCCACCCCTCCAGAACGTGTACCAGCTTGTTCAGGAGGTGAT

TGAACGGGTCGAGCTCCACGAGAAGGTGTCGAGCCACCCCATTTTCAAGCAG

>DQ989312.1_Asia1_IND_1990

CCCTGGTACAAGCTTATAAAGCTCCTGAGCCGCCTGTCGTGCATGGCCGCTGTAGCAGCACGGTCGAAGG

ACCCAGTCCTTGTGGCCATCATGTTAGCTGACACCGGCCTTGAGATTCTAGACAGCACCTTTGTCGTGAA

GAAGATCTCCGACTCGCTCTCTAGTCTCTTTCACGTGCCGGCCCCCGTCTTCAGTTTCGGAGCCCCGATT

CTGCTGGCCGGGTTGGTCAAAGTCGCCTCGAGTTTCTTCCGGTCCACACCCGAAGACCTTGAGAGAGCAG

AAAAACAGCTCAAAGCACGTGACATTAACGACATTTTCGCCATTCTCAAGAACGGCGAGTGGCTGGTCAA

ACTGATTCTTGCCATCCGCGACTGGATAAAGGCATGGATCGCCTCAGAAGAGAAGTTTGTCACCATGACA

GACTTGGTGCCTGGCATCCTTGAGAAGCAGCGGGACCTTAACGACCCCAGCAAGTACAAGGAGGCTAAGG

AGTGGCTCGAAAACGCGCGCCAGGCGTGCTTGAAGAGCGGGAACGTCCACATTGCTAACCTGTGCAAGGT

GGTTGCCCCGGCACCCAGTAAGTCGAGACCCGAACCCGTGGTCGTTTGCCTCCGGGGCAAATCCGGCCAG

GGTAAGAGTTTCCTTGCGAACGTGCTCGCACAAGCAATCTCTACCCACTTCACCGGCAGAACTGATTCAG

TTTGGTACTGCCCACCTGACCCTGACCACTTCGACGGTTACAACCAACAGACCGTTGTTGTGATGGATGA

TTTGGGCCAAAACCCCGACGGCAAGGACTTCAAGTACTTCGCCCAGATGGTGTCTACCACGGGGTTTATC

CCGCCCATGGCCTCGCTCGAAGACAAAGGCAAGCCCTTCAACAGCAAAGTCATCATCGCTACCACCAACC

TGTACTCGGGCTTCACCCCGAGAACCATGGTGTGTCCTGATGCGCTGAACCGGAGGTTTCACTTTGACAT

TGATGTGAGTGCCAAGGATGGGTACAAAATTAACAACAAATTGGACATAACCAAAGCTCTTGAAGACACC

CACACCAACCCTGTGGCAATGTTTCAGTACGACTGTGCCCTTCTCAACGGCATGGCCGTTGAAATGAAGA

GAATGCAACAAGATGTGTTCAAGCCCCAACCGCCTCTGGAAAACGTGTACCAACTCGTCCAGGAGGTGAT

TGAACGGGTCGAGCTCCACGAGAAAGTGTCGAGCCACCCGATTTTCAAGCAG

>DQ989313.1_Asia1_IND_1986

CCCTGGTACAAGCTCATCAAGCTCCTGAGCCGCTTGTCATGCATGGCCGCTGTAGCAGCACGGTCAAAGG

ACCCAGTCCTTGTGGCTATCATGCTGGCTGACACCGGCCTTGAGATTCTGGACAGCACGTTTGTCGTGAA

GAAGATCTCCGACTCGCTCTCCAGTCTCTTTCACGTGCCGGCCCCCGTCTTCAGTTTCGGAGCTCCGATT

CTGTTGGCCGGGTTGGTCAAAGTCGCCTCGAGTTTCTTCCGGTCCACGGCCGAAGACCTTGAGAGAGCAG

AGAAACAGCTCAAAGCACGTGATATCAATGACATATTCGCCATTCTCAAGAACGGCGAGTGGCTGGTCAA

ACTGATTCTTGCCATCCGCGACTGGATCAAGGCATGGATCGCCTCAGAAGAAAAGTTTGTCACCATGACA

GACTTGGTGCCTGGCATCCTTGAAAAACAGCGGGACCTTAACGACCCAAGTAAGTACAAGGAAGCCAAGG

AGTGGCTCGACAACGCGCGCCAAGCGTGTCTGAAGAGCGGGAATGTCCACATTGCCAACCTGTGTAAAGT

GGTCGCCCCGGCACCCAGCAAGTCGAGACCCGAACCCGTGGTCGTTTGCCTCCGTGGCAAATCCGGCCAG

GGAAAGAGTTTCCTTGCGAACGTGCTTGCACAAGCAATTTCCACCCACTTCACTGGCAGAACCGACTCGG

TCTGGTACTGCCCGCCTGACCCTGACCACTTCGACGGTTATAACCAACAGGCTGTTGTTGTGATGGATGA

CTTGGGCCAGAACCCTGATGGCAAGGACTTCAAGTACTTTGCCCAGATGGTCTCCACCACAGGGTTCATC

CCGCCCATGGCTTCACTCGAGGACAAAGGCAAGCCCTTCAACAGCAAGGTCATCATTGCTACCACCAACC

TGTACTCGGGTTTCACCCCGAGAACCATGGTGTGCCCTGATGCACTGAACCGGAGGTTTCACTTTGACAT

CGACGTGAGCGCCAAGGACGGGTACAAAATTAACAACAAATTGGACATAATCAAAGCTCTTGAAGACACC

CACACCAACCCTGTGGCAATGTTCCAGTATGACTGTGCCCTTCTCAACGGCATGGCTGTTGAAATGAAGA

GAATGCAACAAGACATGTTCAAGCCTCAACCACCCCTCCAGAACGTATACCAACTTGTTCAGGAGGTGAT

TGAACGGGTCGAGCTCCACGAGAAGGTGTCGAGCCACCCGATTTTCAAGCAA

>DQ989314.1_Asia1_IND_2001

CCCTGGTACAAGCTCATTAAACTCCTGAGCCGCCTGTCATGCATGGCCGCTGTAGCAGCACGGTCAAAGG

ACCCAGTCCTTGTGGAAAAAATGCTAGCTGACACCGGTCTCGAGATTCTGGACAGCACCTTTGTCGTGAA

GAAGATCTCCGACTCGCTCTCCAGTCTCTTTCACGTGCCGGCCCCCGTCTTCAGTTTCGGAGCCCCGATC

CTGTTGGCTGGGTTGGTCAAAGTCGCCTCGAGTTTCTTCCGGTCCACACCCGAAGACCTTGAGAGAGCAG

AGAAACAGCTCAAAGCACGTGACATCAATGACATCTTCGCCATTCTCAAGAACGGCGAGTGGCTGGTAAA

GCTGATCCTTGCTATCCGGGACTGGATCAAAGCATGGATTGCCTCAGAAGAGAAGTTTGTCACCATGACA

GACTTGGTGCCTGGCATCCTTGAAAAGCAGCGGGACCTCAACGACCCGAGCAAGTACAAGGAGGCCAAGG

AGTGGCTCGACAACGCGCGCCAAGCGTGCTTGAAGAGTGGGAACGTCCACATTGCCAACCTCTGCAAAGT

GGTCGCCCCGGCACCCAGCAAGTCGAGACCCGAACCTGTGGTCGTTTGCCTCCGTGGTAAGTCTGGCCAA

GGGAAGAGTTTCCTTGCGAACGTGCTCGCACAAGCAATTTCCACCCACTTCACTGGCAGAACCGACTCAG

TTTGGTACTGCCCGCCTGACCCCGACCACTTCGACGGTTACAACCAGCAGACCGTCGTTGTGATGGACGA

TTTAGGCCAGAACCCTGACGGCAAGGACTTCAAGTACTTTGCCCAGATGGTTTCCACCACAGGGTTCATC

CCGCCCATGGCCTCACTCGAAGACAAAGGTAAACCTTTCAACAGCAAGGTCATCATTGCAACAACCAACC

TGTACTCGGGGTTTACTCCGAGAACCATGGTGTGCCCTGATGCACTGAACCGTAGGTTTCACTTTGACAT

TGACGTGAGTGCCAAAGACGGGTACAAAATTAACAACAAATTGGACATCATCAAAGTTCTTGAAGACAAC

CACACCAATCCAGTGGCAATGTTTCAGTACGATTGTGCCCTTCTCAACGGCATGGCCGTTGAAATGAAGA

GAATGCAACAAGATATGTTCAAGCCTCAACCACCCCTTCAGAACGGGTACCAACTAGTCCAGGAGGTGAT

TGAACGGGTTGAGCTCCACGAGAAAGTGTCGAGCCACCCGATCTTTAAGCAA

>DQ989315.1_Asia1_IND_1993

CCCTGGTACAAGCTCATCAAGCTCCTGAGCCGCTTGTCATGCATGGCCGCTGTAGCAGCACGGTCAAAGG

ACCCAGTCCTTGTGGCTATCATGCTAGCTGACACCGGTCTCGAGATTCTGGACAGCACCTTTGTCGTGAA

GAAAATCTCCGACTCGCTCTCCAGTCTCTTTCACGTGCCGGCCCCCGTCTTCAGTTTCGGAGCCCCGATT

CTGTTGGCCGGGTTGGTCAAGGTCGCCTCGAGTTTCTTCCGGTCCACGGCCGAAGACCTCGAGAGAGCAG

AAAAGCAGCTCAAAACACGTGACATCAATGACATATTCGCCATTCTCAAGAACGGCGAGTGGCTGGTCAA

GCTGATTCTTGCCATCCGCGACTGGATTAAGGCATGGATCGCCTCAGAAGAAAAGTTTGTCACCATGACA

GACTTGGTGCCTGGCATCCTTGAAAAGCAGCGGGATCTCAACGACCCAAGCAAGTACAAGGAGGCCAAGG

AGTGGCTCGACAACGCGCGCCAAGCGTGTTTGAAGAGCGGGAACGTCCACATTGCCAACCTCTGCAAAGT

GGTCGCCCCGGCACCCAGCAAGTCGAGACCCGAACCCGTGGTCGTTTGCCTCCGTGGCAAGTCCGGCCAG

GGCAAGAGTTTCCTTGCAAACGTGCTTGCACAAGCAATCTCCACCCACTTCACTGGCAGAACTGATTCAG

TTTGGTACTGCCCACCTGACCCTGACCACTTCGACGGCTACAACCAACAGACCGTTGTAGTGATGGATGA

TTTGGGCCAGAACCCTGATGGCAAAGACTTCAAGTACTTCGCCCAGATGGTGTCAACCACGGGGTTCATC

CCGCCCATGGCTTCACTCGAGGACAAAGGTAAGCCTTTCAACAGCAAGGTCATCATTGCCACCACCAACT

TGTACTCGGGCTTCACCCCGAGGACCATGGTGTGCCCTGATGCACTGAACCGTAGGTTTCACTTTGACAT

CGATGTGAGCGCCAAGGATGGGTACAAAATTAACAACAAATTGGACATTATCAAAGCACTTGAAGACACC

CACACCAATCCGGTGGCAATGTTTCAATATGACTGTGCCCTTCTCAACGGCATGGCCGTTGAAATGAAGA

GAATGCAACAAGATGTGTTTGCGCCTCAGCCACCCCTCCAGAACGTGTACCAACTTGTTCAGGAGGTGAT

TGAACGGGTCGAGCTCCACGAGAAGGTGTCGAGTCACCCGATCTTCAAACAG

>DQ989317.1_Asia1_IND_2000

CCCTGGTACAAGCTCATCAAACTCCTGAGCCGCCTGTCATGCATGGCCGCTGTAGCAGCACGGTCAAAGG

ACCCAGTCCTTGTGGCCATCATGCTAGCTGACACCGGTCTCGAGATTCTGGACAGCACCTTTGTCGTGAA

GAAGATCTCCGACTCGCTCTCCAGTCTCTTTCACGTGCCGGCCCCCGTCTTCAGTTTCGGAGCCCCGATC

CTGTTGGCTGGGTTGGTCAAAGTCGCCTCGAGTTTCTTCCGGTCCACACCCGAAGACCTTGAGAGAGCAG

AGAAACAGCTCAGAGCACGTGACATCAATGACATCTTCGCCATTCTCAAGAACGGCGAGTGGCTGGTCAA

GCTGATCCTTGCTATCCGCGACTGGATCAAAGCATGGATTGCCTCAGAAGAGAAGTTTGTCACCATGACA

GACTTGGTGCCTGGCATCCTTGAAAAGCAGCGGGACCTCAACGACCCAAGCAAGTACAAGGAGGCCAAGG

AGTGGCTCGACAACGCGCGCCAAGCGTGCTTGAAGAGTGGGAACGTCCACATTGCCAACCTCTGCAAAGT

GGTCGCCCCGGCACCCAGCAAGTCGAGACCCGAACCTGTGGTCGTTTGCCTCCGTGGTAAATCCGGCCAG

GGTAAGAGTTTCCTTGCGAACGTGCTCGCACAAGCAATCTCCACCCACTTCACTGGCAGAACCGACTCAG

TTTGGTACTGCCCGCCTGACCCCGACCACTTCGACGGTTACAACCAGCAGACCGTCGTTGTGATGGACGA

TTTAGGCCAGAACCCTGACGGCAAGGACTTCAAGTACTTTGCCCAGATGGTTTCCACCACAGGGTTCATC

CCGCCCATGGCCTCACTCGAAGACAAAGGTAAACCTTTCAACAGCAAGGTCATCATTGCAACAACCAACC

TGTACTCGGGGTTTACTCCGAGGACCATGGTGTGCCCTGATGCACTGAACCGTAGGTTTCACTTTGACAT

TGACGTGAGTGCCAAAGACGGGTACAAAGTTAACAACAAATTGGACATCATCAAAGCTCTTGAAGACACC

CACACCAATCCAGTGGCAATGTTTCAGTACGATTGTGCCCTTCTCAACGGCATGGCCGTTGAAATGAAGA

GAATGCAACAAGATCTGTTCAAGCCTCAACCACCCCTCCAGAACGTGTACCAACTAGTCCAGGAGGTGAT

TGAACGGGTTGAGCTCCACGAGAAAGTGTCGAGCCACCCGATCTTCAAGCAA

>DQ989318.1_Asia1_IND_2002

CCCTGGTACAAGCTCATTAAACTCCTGAGCCGCCTGTCATGCATGGCCGCTGTAGCAGCACGGTCAAAGG

ACCCAGTCCTTGTGGCCATCATGCTAGCTGACACCGGTCTCGAGATTCTGGACAGCACCTTTGTCGTGAA

GAAGATCTCCGACTCGCTCTCCAGTCTCTTTCACGTGCCGGCCCCCGTCTTCAGTTTCGGAGCCCCGATC

CTGTTGGCTGGGTTGGTCAAAGTCGCCTCGAGTTTCTTCCGGACCACACCCGAAGACCTTGAGAGAGCAG

AGAAACAGCTCAAAGCACGTGACATCAATGACATCTTCGCCATTCTCAAGAACGGCGAGTGGCTGGTCAA

GCTGATCCTTGCTATCCGCGACTGGATCAAAGCATGGATTGCCTCAGAAGAGAAGTTTGTTACCATGACA

GACTTGGTGCCTGGCATCCTTGAAAAGCAGCGGGACCTTAACGACCCAAGCAAGTACAAGGAGGCCAAGG

AGTGGCTCGACAACGCGCGCCAAGCGTGCTTGAAGAGTGGGAACGTCCACATTGCCAACCTCTGCAAAGT

GGTCGCCCCGGCACCCAGCAAGTCGAGACCCGAACCTGTGGTCGTTTGCCTCCGTGGTAAGTCTGGCCAG

GGAAAGAGTTTCCTTGCGAACGTGCTCGCACAAGCAATTTCCACCCACTTCACTGGCAGAACCGACTCAG

TTTGGTACTGCCCGCCTGACCCCGACCACTTCGACGGTTACAACCAGCAGACCGTCGTTGTGATGGACGA

TTTAGGCCAGAACCCTGACGGCAAGGACTTCAAGTACTTTGCCCAGATGGTTTCCACCACAGGGTTCATC

CCGCCCATGGCCTCACTCGAAGACAAAGGTAAACCTTTCAACAGCAAGGTCATCATTGCAACAACCAACC

TGTACTCGGGGTTTACTCCGAGAACCATGGTGTGCCCTGATGCACTGAACCGTAGGTTTCACTTTGACAT

TGACGTGAGTGCCAAAGACGGGTACAAAATTAACAACAAATTGGACATCATCAAAGCTCTTGAAGACACC

CACACCAATCCAGTGGCAATGTTTCAGTACGATTGTGCCCTTCTCAACGGCATGGCCGTTGAAATGAAGA

GAATGCAACAAGATATGTTCAAGCCTCAACCACCCCTCCAGAACGTGTACCAACTAGTTCAGGAGGTGAT

TGAACGGGTTGAGCTCCACGAGAAAGTGTCGAGCCACCCGATCTTTAAGCAA

>DQ989319.1_Asia1_IND_2001

CCCTGGTACAAGCTCATCAAACTCCTGAGCCGCCTGTCATGCATGGCCGCTGTAGCAGCACGGTCAAAGG

ACCCAGTCCTTGTGGCCATAATGCTAGCTGACACCGGTCTCGAGATTCTGGACAGCACCTTTGTCGTGAA

GAAGATCTCCGACTCGCTCTCCAGTCTCTTTCACGTGCCGGCCCCCGTCTTCAGTTTCGGAGCCCCGATC

CTCCTGGCTGGGTTGGTCAAAGTCGCCTCGAGTTTCTTCCGGTCCACACCCGAAGACCTTGAGAGAGCGG

AGAAACAGCTCAAAGCACGTGACATCAATGACATCTTCGCCATTCTCAAGAACGGCGAGTGGCTGGTCAA

GCTTATCCTTGCTATCCGCGACTGGCTCAAAGCATGGATTGCCTCAGAGGAGAAGTTTGTCACCGTGACA

GACTTGGTGCCTGGTATCCTCGAAAAGCAGCGGGACCTTAACGACCCAAGCAAATACAAGGAAGCCAAGG

AGTGGCTCGACAACGCGCGTCAAGCGTGCTTGAAGAGTGGGAACGCCCACATTGCCAACCTTTGCAAAGT

GGTCGCCCCGGCACCCAGCAAGTCGAGACCCGAACCTGTGGTCGTTTGCCTCCGCGGCAAATCCGGCCAG

GGTAAGAGTTTCCTTGCGAATGTGCTCGCACAAGCAATCTCCACCCACTTCACTGGCAGAACCGATTCAG

TTTGGTACTGCCCGCCTGACCCCGACCACTTCGACGGTTACAACCAGCAGACCGTTGTTGTGATGGACGA

TTTGGGCCAGAACCCTGATGGCAAGGACTTCAAGTACTTTGCCCAGATGGTTTCCACCACAGGGTTCATC

CCGCCCATGGCTTCACTCGAGGACAAAGGTAAGCCTTTCAACAGCAAGGTCATCATAGCCACCACCAACC

TGTACTCGGGGTTCACCCCGAGAACTATGGTGTGCCCTGATGCACTGAACCGCAGGTTTCACTTTGACAT

TGATGTGAGCGCCAAGGACGGGTACAAAAATAACAACAAATTGGACATAATCAAAGCTCTTGAAGACACC

CACACCAATCCTGTGGCAATGTTTCAATATGACTGTGCCCTTCTCAACGGCATGGCCGTTGAAATGAAGA

GAATGCAACAAGACATGTTTAAGCCTCAGCCACCCCTCCAGAACGTGTACCAGCTTGTTCAGGAGGTGAT

TGAACGGGTCGAGCTCCACGAGAAAGTGTCGAGCCACCCGATCTTCAAGCAA

>DQ989320.1_Asia1_IND_2002

CCCTGGTACAAGCTCATTAAACTCCTGAGCCGCCTGTCATGCATGGCCGCTGTAGCAGCACGGTCAAAGG

ACCCAGTCCTTGTGGCAAACATGCTAGCTGACACCGGTCTCGAGATTCTGGACAGCACCTTTGTCGTGAA

GAAGATCTCCGACTCGCTCTCCAGTCTATTTCACGTGCCGGCCCCCGTCTTCAGTTTCGGAGCCCCGATC

CTGTTGGCTGGGTTGGTCAAAGTCGCCTCGAGTTTCTTCCGGTCCACACCCGAAGACCTTGAGAGAGCAG

AGAAACAGCTCAAAGCACGTGACATCAATGACATCTTCGCCATTCTCAAGAACGGCGAGTGGCTGGTCAA

GCTGATCCTTGCTATCCGCGACTGGATCAAAGCATGGATTGCCTCAGAAGAGAAGTTTGTCACCATGACA

GACTTGGTGCCTGGCATCCTTGAAAAGCAGCGGGACCTCAACGACCCAAGCAAATACAAGGAGGCCAAGG

AGTGGCTCGACAACGCGCGCCAAGCGTGCTTGAAGAGCGGGAACGTCCACATTGCCAACCTCTGCAAAGT

GGTCGCCCCGGCACCCAGCAAGTCGAGACCCGAACCTGTGGTCGTTTGCCTCCGTGGTAAGTCTGGCCAG

GGAAAGAGTTTCCTTGCGAACGTGCTCGCACAAGCAATTTCCACCCACTTCACTGGCAGAACCGACTCAG

TTTGGTACTGCCCGCCTGACCCCGACCACTTCGACGGTTACAACCAGCAGACCGTCGTTGTGATGGACGA

TTTAGGCCAGAACCCTGACGGCAAGGACTTCAAGTACTTCGCCCAGATGGTTTCCACCACAGGGTTCATC

CCGCCCATGGCCTCACTCGAAGACAAAGGTAAACCTTTCAACAGCAAGGTCATCATTGCAACAACCAACC

TGTATTCGGGGTTTACTCCGAGAACCATGGTGTGCCCTGATGCACTGAACCGTAGGTTTCACTTTGACAT

TGACGTGAGTGCCAAAGACGGGTACAAAATTAACAACAAATTGGATATCATCAAAGCTCTTGAAGACACC

CACACCAATCCAGTGGCAATGTTTCAGTACGATTGTGCCCTTCTCAACGGCATGGCCGTTGAAATGAAGA

GAATGCAACAAGATATATTCAAGCCTCAATCACCCCTCCAGAACGTGTACCAACTAGTCCAGGAGGTGAT

TGAACGGGTTGAGCTCCACGAGAAAGTGTCGAGCCACCCGATCTTTAAGCAA

>DQ989321.1_Asia1_IND_2001

CCCTGGTACAAGCTCATTAAACTCCTGAGCCGCCTGTCATGCATGGCCGCTGTGGCAGCACGGTCAAAGG

ACCCAGTCCTTGTGGCCATCATGCTAGCTGACACCGGTCTCGAGATTCTGGACAGCACCTTTGTCGTAAA

GAAGATCTCCGACTCGCTCTCCAGTCTCTTTCACGTGCCGGCCCCCGTCTTCAGTTTCGGAGCCCCGATC

CTGTTGGCTGGTTTGGTCAAAGTCGCCTCGAGTTTCTTCCGGTCCACACCCGAAGACCTTGAGAGAGCAG

AGAAACAGCTCAAAGCACGTGACATCAACGACATCTTCGCCATTCTCAAGAACGGCGAGTGGCTGGTCAA

GCTGATCCTTGCTATCCGCGACTGGATCAAAGCATGGATTGCCTCAGAAGAGAAGTTTGTCACCATGACA

GACTTGGTGCCTGGCATCCTTGAAAAGCAGCGGGACCTCAACGACCCAAGCAAGTACAAGGAGGCCAAGG

AGTGGCTCGACAACGCGCGCCAAGCGTGCTTGAAGAGTGGGAACGTCCACATTGCCAACCTCTGCAAAGT

GGTCGCCCCGGCACCCAGCAAGTCGAGACCCGAACCTGTGGTCGTTTGCCTCCGTGGTAAGTCTGGCCAG

GGAAAGAGTTTCCTTGCGAACGTGCTCGCACAAGCAATTTCCACCCACTTCACTGGCAGAACCGACTCAG

TTTGGTACTGCCCGCCTGACCCCGACCACTTCGACGGTTACAACCAGCAGACCGTCGTTGTGATGGACGA

TTTAGGCCAGAACCCTGACGGCAAGGACTTCAAGTACTTTGCCCATATGGTTTCCACCACAGGGTTCATC

CCGCCCATGGCCTCACTCGAAGACAAAGGTAAACCTTTCAACAGCAAGGTCATCATTGCAACAACCAACC

TGTACTCGGGGTTTACTCCGAGAACCATGGTGTGCCCTGATGCACTGAACCGTAGGTTTCACTTTGACAT

TGACGTGAGTGCCAAAGACGGGTACAAAATTAACAACAAATTGGACATCATCAAAGCTCTTGAAGACACC

CACACCAATCCAGTGGCAATGTTTCAGTACGATTGTGCCCTTCTCAACGGCATGGCCGTTGAAATGAAGA

GAATGCAACAAGATATGTTCAAGCCTCAACCACCCCTCCAGAACGTGTACCAACTAGTCCAGGAGGTGAT

TGAACGGGTTGAGCTCCACGAGAAAGTGTCGAGCCACCCGATCTTTAAGCAA

>DQ989322.1_Asia1_IND_2002

CCCTGGTACAAGCTCGTTAAACTCCTGAGCCGCCTGTCATGCATGGCCGCTGTAGCAGCACGGTCAAAGG

ACCCAGTCCTTGTGGCAAAAATGCTAGCTGACACCGGTCTCGAGATTCTGGACAGCACCTTCGTTGTGAA

GAAGATCTCCGACTCGCTCTCCAGTCTCTTTCACGTGCCGGCCCCCGTCTTCAGTTTCGGAGCCCCGATC

CTGTTGGCTGGGTTGGTCAAAGTCGCCTCGAGTTTCTTCCGGTCCACACCCGAAGACCTTGAGAGAGCAG

AGAAACAGCTCAAAGCACGTGACATCAATGACATCTTCGCCATTCTCAAGAACGGCGAGTGGCTGGTCAA

GCTGATCCTTGCTATCCGCGACTGGATCAAAGCATGGATTGCCTCAGAAGAGAAGTTTGTCACCATGACA

GACTTGGTGCCTGGCATCCTTGAAAGGCAGCGGGACCTCAACGACCCAAGCAAGTACAAGGAGGCCAAGG

AGTGGCTCGACAACGCGCGCCAAGCGTGCTTGAAGAGTGGGAACGTCCACATTGCCAACCTCTGCAAAGT

GGTCGCCCCGGCACCCAGCAAGTCGAGACCCGAACCTGTGGTCGTTTGCCTCCGTGGTAAGTCTGGCCAG

GGAAAGAGTTTCCTTGCGAACGTGCTCGCACAAGCAATTTCCACCCACTTCACTGGCAGAACCGATTCAG

TTTGGTACTGCCCGCCTGACCCCGACCACTTCGACGGTTACAACCAGCAGACCGTTGTTGTGATGGACGA

TTTGGGCCAGAACCCTGATGGCAAGGACTTCAAGTACTTTGCCCAGATGGTTTCCACCACAGGGTTCATC

CCGCCCATGGCTTCACTCGAGGACAAAGGTAAGCCTTTCAACAGCAAGGTCATCATAGCCACCACCAACC

TGTACTCGGGGTTCACCCCGAGAACTATGGTGTGCCCTGATGCACTGAACCGCAGGTTTCACTTTGACAT

TGATGTGAGCGCCAAGGACGGGTACAAAATTAACAACAAATTGGACATAATCAAAGCTCTTGAAGACACC

CACACCAATCCTGTGGCAATGTTTCAATATGACTGTGCCCTTCTCAACGGCATGGCCGTTGAAATGAAGA

GAATGCAACAAGACATGTTTAAGCCTCAGCCACCCCTCCAGAACGTGTACCAGCTTGTTCAGGAGGTGAT

TGAACGGGTTGAGCTCCACGAGAAAGTGTCGAGCCACCCGATCTTTAAGCAA

>DQ989323.1_Asia1_IND_2002

CCCTGGTACAAGCTCATTAAACTCCTGAGCCGCCTGTCGTGCATGGCCGCTGTAGCAGCACGGTCAAAGG

ACCCAGTCCTCGTGGCCATCATGTTAGCTGACACCGGTCTCGAGATTCTGGACAGCACCTTTGTCGTGAA

GAAGATCTCCGACTCGCTCTCCAGTCTCTTTCACGTGCCGGCCCCCGTCTTCAGTTTCGGAGCCCCGATC

CTGTTGGCTGGGTTGGTCAAAGTCGCCTCGAGTTTCTTCCGGTCCACACCTGAAGACCTTGAGAGAGCAG

AGAAACAGCTCAAAGCACGTGACATCAATGACATCTTCGCCATTCTCAAGAACGGCGAGTGGCTGGTCAA

GCTGATCCTTGCTATCCGCGACTGGATCAAAGCATGGATTGCCTCAGAAGAGAAGTTTGTCACCATGACA

GACTTGGTGCCTGGCATCCTTGAAAAGCAGCGGGACCTCAACGACCCAAGCAAGTACAAGGAGGCCAAGG

AGTGGCTCGACAACGCGCGCCAAGCGTGCTTGAAGAGTGGGAACGTCCACATTGCCAACCTTTGCAAAGT

GACCGCCCCGGCACCCAGCAAGTCGAGACCCGAACCTGTGGTCGTTTGCCTCCGTGGTAAGTCTGGCCAG

GGAAAGAGTTTCCTTGCGAACGTGCTCGCACAAGCAATTTCCACCCACTTCACTGGCAGAACCGACTCAG

TTTGGTACTGCCCGCCTGACCCCGACCACTTCGACGGTTACAACCAGCAGACTGTCGTTGTGATGGACGA

TTTAGGCCAGAACCCTGACGGCAAGGACTTCAAGTACTTTGCCCAGATGGTTTCCACCACAGGGTTCATC

CCGCCCATGGCCTCACTCGAAGACAAAGGTAAACCTTTCAACAGCAAGGTCATCATTGCAACAACCAACC

TGTACTCGGGGTTTACTCCGAGAACCATGGTGTGCCCTGATGCACTGAACCGTAGGTTTCACTTTGACAT

TGACGTGAGTGCCAAAGACGGGTACAAAATTAACAACAAATTGGACATCATCAAAGCTCTTGAAGACACC

CACACCAATCCAGTGGCAATGTTTCAGTACGATTGTGCCCTTCTTAACGGCACGGCCGTTGAAATGAAGA

GAATGCAACAAGATATGTTTAAGCCTCAACCACCCCTCCAGAACGTGTACCAACTAGTCCAGGAGGTGAT

TGAACGGGTTGAGCTCCACGAGAAAGTGTCGAGCCACCCGATCTTTAAGCAA

>EF117837.1_A_PAK_2006

CCCTGGTACAAGCTCATCAAGCTCCTGAGCCGCCTGTCATGCATGGCCGCTGTAGCAGCACGGTCAAAGG

ACCCAGTTCTTGTGGCCATCATGCTAGCTGACACCGGTCTCGAGATTCTGGACAGCACCTTTGTCGTGAA

GAAGATCTCCGACTCGCTCTCCAGTCTCTTTCACGTGCCGGCCCCCGTCTTCAGTTTCGGAGCTCCGATT

CTGCTGGCCGGGTTGGTCAAAGTCGCCTCGAATTTCTTCCGGTCCACACCCGAAGACCTTGAGAGAGCAG

AGAAACAGCTCAGAGCACGTGACATCAATGACATTTTCGCCATTCTCAAGAACGGCGAGTGGCTGGTCAA

ACTGATCCTTGCCATCCGCGACTGGATAAAGGCTTGGATCGCCTCAGAAGAGAAGTTCGTCACCATGACA

GACTTGGTGCCCGGCATCCTTGAAAAGCAACGGGATCTCAACGACCCGAGCAAGTACGAGGAGGCCAAGG

AGTGGCTCGACAACGCGCGCCAGGCGTGTCTGAAGAGCGGGAACGTCCACATTGCCAACTTGTGCAAAGT

GGTCGCCCCGGCACCCAGCAAGTCGAGACCCGAACCTGTGGTCGTTTGCCTCCGCGGTAAATCCGGCCAG

GGGAAGAGTTTCCTTGCGAACGTGCTTGCACAAGCAATCTCCACCCACTTCACTGGCAGAACCGATTCAG

TTTGGTACTGCCCACCTGACCCTGACCACTTCGACGGTTACAACCAGCAGACCGTTGTTGTGATGGACGA

TTTGGGCCAGAATCCCGACGGCAAGGACTTCAAGTACTTCGCCCAGATGGTTTCGACCACGGGGTTCATC

CCGCCCATGGCGTCACTTGAGGACAAAGGCAAACCCTTCAACAGCAAGGTCATCATCGCCACCACCAACC

TGTACTCGGGATTTACCCCGAGAACTATGGTGTGCCCTGATGCCCTGAACCGGAGGTTTCACTTTGACAT

CGACGTGAGCGCCAAGGACGGGTACAAAATTAACAACAAATTGGACATAATCAAAGCTCTTGAAGACACC

CACACCAACCCTGTGGCAATGTTTCAATATGACTGTGCCCTTCTCAACGGCATGGCCGTTGAAATGAAGA

GAATGCAACAAGATGTGTTCAAGCCTCAACCGCCCCTCCAGAACGTATACCAACTCGTTCAGGAGGTGAT

CGAACGGGTCGAGCTCCACGAGAAAGTGTCGAGCCACCCGATCTTCAAGCAG

>EF149009.1_Asia1_CHA_2005

CCCTGGTACAAACTCATCAAACTCCTGAGCCGCTTGTCATGCATGGCCGCTGTAGCAGCACGGTCAAAGG

ACCCAGTCCTTGTGGCCATCATGCTGGCTGACACCGGCCTTGAGATTCTGGACAGCACATTCGTCGTGAA

GAAGATCTCCGACTCACTCTCCAGTCTCTTTCACGTGCCGGCCCCCGTCTTCAGTTTCGGAGCCCCGGTT

CTGTTGGCCGGGTTGGTCAAAGTCGCCTCGAGTTTCTTCCGGTCCACGCCCGAAGAACCTGAGAGAGCAG

AGAAGCAGCTCAAAGCACGTGACATCAATGACATCTTCGCCATTCTCAAGAACGGCGAGTGGTTGGTCAA

ATTGATTCTTGCCATCCGCGACTGGATTAAGGCATGGATTGCCCCCGAAGAAAAGTTTGTCACTATGACA

GACTTGGTGCCTGGCATCCTTGAAAAGCAGCCGGACCTCAACGGCCCAAGCAAGTATAAAGAAGCCAAGG

AGTGGCTCGACAATGCACGGCAAGCGTGCCTGAAGAGTGGGAACGTCCACATTGCCAACTTGTGCAAAGT

GGTTGCCCCGGCACCTAGCAAGTCGAGACCTGAACCCGTGGTCGTTTGCCTCCGTGGCAAATCCGGCCAG

GGAAAGAGTTTCCTTGCGGACGTGCTCGCACAGGCAATTTCCACACACTACACTGGCAGAATTGATTCAG

TTTGGTACTGCCCGCCTGACCTTGACCACTTCGACGGTTACAACCAACAGACCGTTGTTGTGATGGATGA

TTTGGGCCAGAACCCCGACGGCAAGGACTTTAAGTACTTCGCCCAGATGGTTTCAACCACGGGGTTCATC

CCGCCCATGGCCTCGCTCGAAGACAAAGGCAAACCTTTCAACAGCAAGGTCATCATCGCCACCACCAACC

TGTACTCGGGCTTCACCCCGAGGACCATGGTGTGCCCTGATGCACTGAACCGGAGGTTTCACTTTGACAT

TGACGTGAGTGCCAAGGACGGGTACAAAATTAACAACAAGTTGGACATTATCAAAGCACTTGAAGACACC

CACACCAACCCGGTGGCAATGTTTCAGTACGATTGTGCCCTTCTCAACGGCATGGCCGTTGAAATGAAGA

GAATGCAACAAGACATGTTCAAGCCTCAACCGCCCCTCCAGAACGTGTACCAACTTGTTCAGGAGGTGAT

TGATCGGGTCGAGCTCCACGAGAAAGTGTCGAGCCACCCGATTTTCAAGCAG

>EF149010.1_Asia1_CHA_2005

CCCTGGTACAAGCTCATCAAGCTCCTGAGTCGCCTGTCATGCATGGCCGCTGTAGCAGCACGGTCAAAGG

ACCCAGTCCTTGTGGCCATCATGCTGGCTGACACCGGCCTTGAGATACTGGACAGCACTTTTGTCGTGAA

GAAGATCTCCGACTCGCTCTCCAGTCTCTTTCACGTGCCGGCCCCCGTCTTCAGTTTCGGAGCTCCGATT

CTGTTGGCCGGGTTGGTCAAAGTCGCCTCGAGTTTCTTCCGGTCCACACCCGAAGAACTTGAGAGAGCGG

AGAAACAGCTCAGAGCACGTGACATCAATGACATATTCGCCATTCTTAAGAACGGCGAGTGGCTGGTCAA

ACTGATTCTGGCCATCCGCGACTGGATCCAGGCTTGGATCGCCTCAGAAGAAAAGTTGGTCACCATGACA

GACCTGGTGCCTGGCATCCTTGAAAAGCAGCAGGACCTCAACGGCCCAAGCAAGTATAAAGAAGCCAAGG

AGTGGCTCGACAATGCACGGCAAGCGTGCCTGAAGAGTGGGAACATTCACATTGCCAACTTGTGCAAAGT

GGTTGCCCCAGCACCCAGTAGGTCGAGGCCCGAACCTGTGTTCGTTTGTTTCCGTGGCAAATCGGGCCAG

GGTAAGAGCTTTCTTGCGAACGTGCTAGCACAAGCAATTTCAACCCATTTCACTGGCAGAACTGATTCAG

TTTGGTACTGCCCACCTGACCCTGACCACTTCGACGGTTACAACCAGCAGACCGTTGTAGTGATGGATGA

TTTGGGCCAGAACCCCGACGGGAAGGACTTCAAGTACTTCGCCCAAATGGTTTCAACCACGGGGTTCATC

CCGCCCATGGCTTCACTCGAAGACAAAGGCAAACCTTTCAACAGCAAGGTCATCATCGCCACCACCAACC

TGTACTCGGGCTTCACCCCGAGAACTATGGTGTGCCCTGATGCACTGAACCGAAGGTTCCACTTTGACAT

TGACGTGAGCGCCAAGGACGGGTACAAAATTAACAACAAACTGGACATTATCAAAGCTCTTGAAGACACC

CACACCAACCCTGTGGCAATGTTTCAATACGACTGTGCCCTTCTCAACGGCACAGCCGTTGAAATGAAGA

GAATGCAACAAGATCTGTTCAAGCCTCAACCGCCCCTCCAGAACGTCTACCAGCTTGTTCAGGAGGTGAT

TGACCGGGTCGAGCTCCATGAGAAGGTGTCGAGCCACCCGATTTTTAAACAG

>EF494486.1_A_TUR_2005

CCCTGGTACAAGCTCATTAAGCTCCTGAGCCGCCTGTCATGCATGGCCGCTGTAGCAGCACGGTCGAAGG

ACCCAGTCCTTGTGGCCATCATGCTAGCTGACACCGGTCTCGAGATTCTGGACAGCACCTTTGTCGTGAA

GAAGATCTCCGACTCGCTCTCCAGTCTCTTTCACGTGCCGGCCCCCGTCTTCAGTTTCGGAGCTCCGATT

CTGCTGGCCGGGTTGGTCAAAGTCGCCTCGAGTTTCTTCCGGTCCACACCCGAAGACCTTGAGAGAGCAG

AGAAACAGCTCAAAGCACGTGACATCAATGACATTTTCGCCATTCTCAAGAACGGCGAGTGGCTGGTCAA

ACTGATCCTCGCCATCCGCGACTGGATAAAGGCTTGGATCGCCTCAGAAGAGAAGTTTGTCACCATGACA

GACTTGGTGCCCGGCATCCTTGAAAAGCAACGGGATCTCAACGACCCGAGCAAGTACGAGGAGGCCAAGG

AGTGGCTCGACAACGCGCGCCAGGCGTGTCTGAAGAGCGGGAACGTCCACATTGCCAACTTGTGCAAAGT

GGTCGCCCCGGCACCCAGCAAGTCGAGACCCGAACCTGTGGTCGTTTGCCTCCGTGGTAAATCCGGCCAG

GGGAAGAGTTTCCTTGCGAACGTGCTTGCACAAGCAATCTCCACCCACTTCACTGGCAGAACCGATTCAG

TTTGGTACTGCCCACCTGACCCTGACCACTTCGACGGTTACAACCAGCAGACCGTTGTTGTGATGGACGA

CTTGGGCCAGAATCCCGACGGCAAGGACTTCAAGTACTTCGCCCAGATGGTTTCGACTACGGGGTTCATC

CCGCCCATGGCGTCACTTGAGGACAAAGGCAAACCCTTCAACAGCAAGGTCATCATCGCCACCACCAACC

TGTACTCGGGATTTACCCCGAGAACTATGGTGTGCCCTGATGCCCTGAACCGGAGGTTTCACTTTGACAT

CGACGTGAGCGCCAAGGACGGGTACAAAATTAACAACAAATTGGACATAATCAAAGCTCTTGAAGACACC

CACACCAACCCTGTGGCAATGTTTCAATATGACTGTGCCCTTCTCAACGGCATGGCCGTTGAAATGAAGA

GAATGCAACAAGACGTGTTCAAGCCTCAACCACCCCTCCAGAACGTATACCAACTCGTTCAGGAGGTGAT

CGAACGGGTCGAGCTCCACGAGAAAGTGTCGAGCCACCCGATCTTCAAGCAG

>EF494487.1_A_PAK_2006

CCCTGGTACAAGCTCATCAAGCTCCTGAGCCGCCTGTCATGCATGGCCGCTGTAGCAGCACGGTCAAAGG

ACCCAGTTCTTGTGGCCATCATGCTAGCTGACACCGGTCTCGAGATTCTGGACAGCACCTTTGTCGTGAA

GAAGATCTCCGACTCGCTCTCCAGTCTCTTTCACGTGCCGGCCCCCGTCTTCAGTTTCGGAGCTCCGATT

CTGCTGGCCGGGTTGGTCAAAGTCGCCTCGAATTTCTTCCGGTCCACACCCGAAGACCTTGAGAGAGCAG

AGAAACAGCTCAGAGCACGTGACATCAATGACATTTTCGCCATTCTCAAGAACGGCGAGTGGCTGGTCAA

ACTGATCCTTGCCATCCGCGACTGGATAAAGGCTTGGATCGCCTCAGAAGAGAAGTTCGTCACCATGACA

GACTTGGTGCCCGGCATCCTTGAAAAGCAACGGGATCTCAACGACCCGAGCAAGTACGAGGAGGCCAAGG

AGTGGCTCGACAACGCGCGCCAGGCGTGTCTGAAGAGCGGGAACGTCCACATTGCCAACTTGTGCAAAGT

GGTCGCCCCGGCACCCAGCAAGTCGAGACCCGAACCTGTGGTCGTTTGCCTCCGCGGTAAATCCGGCCAG

GGGAAGAGTTTCCTTGCGAACGTGCTTGCACAAGCAATCTCCACCCACTTCACTGGCAGAACCGATTCAG

TTTGGTACTGCCCACCTGACCCTGACCACTTCGACGGTTACAACCAGCAGACCGTTGTTGTGATGGACGA

TTTGGGCCAGAATCCCGACGGCAAGGACTTCAAGTACTTCGCCCAGATGGTTTCGACCACGGGGTTCATC

CCGCCCATGGCGTCACTTGAGGACAAAGGCAAACCCTTCAACAGCAAGGTCATCATCGCCACCACCAACC

TGTACTCGGGATTTACCCCGAGAACTATGGTGTGCCCTGATGCCCTGAACCGGAGGTTTCACTTTGACAT

CGACGTGAGCGCCAAGGACGGGTACAAAATTAACAACAAATTGGACATAATCAAAGCTCTTGAAGACACC

CACACCAACCCTGTGGCAATGTTTCAATATGACTGTGCCCTTCTCAACGGCATGGCCGTTGAAATGAAGA

GAATGCAACAAGATGTGTTCAAGCCTCAACCGCCCCTCCAGAACGTATACCAACTCGTTCAGGAGGTGAT

CGAACGGGTCGAGCTCCACGAGAAAGTGTCGAGCCACCCGATCTTCAAGCAG

>EF494488.1_A_PAK_2006

CCCTGGTACAAGCTCATCAAGCTCCTGAGCCGCCTGTCATGCATGGCCGCTGTAGCAGCACGGTCAAAGG

ACCCAGTCCTTGTGGCCATCATGCTAGCTGACACCGGTCTCGAGATTCTGGACAGCACCTTTGTCGTGAA

GAAGATCTCCGACTCGCTCTCCAGTCTCTTTCACGTGCCGGCCCCCGTCTTCAGTTTCGGAGCCCCGATT

CTGTTGGCCGGGTTGGTCAAAGTCGCCTCGAGTTTCTTCCGGTCCACACCCGAAGACCTTGAGAGAGCAG

AGAAACAGCTCAAAGCACGTGACATCAATGACATTTTCGCCATTCTCAAGAACGGCGAGTGGCTGGTCAA

ACTGATCCTTGCCATCCGCGACTGGATAAAGGCTTGGATCGCCTCAGAAGAGAAGTTTGTCACCATGACA

GACTTGGTGCCCGGCATCCTTGAAAAACAACGGGATCTCAACGACCCGAGCAAGTACAAGGAGGCCAAGG

AGTGGCTCGACAACGCGCGCCAGGCGTGTCTGAAGAGCGGGAACGTCCACATTGCCAACTTGTGCAGAGT

GGTCGCCCCGGCACCCAGCAAGTCGAGACCCGAACCTGTGGTCGTTTGCCTCCGTGGTAAATCCGGCCAG

GGGAAGAGTTTCATTGCGAACGTGCTTGCACAAGCAATCTCCACCCACTTCACTGGCAGAACCGATTCAG

TTTGGTACTGCCCACCTGACCCTGACCACTTCGACGGTTACAACCAACAGACCGTTGTTGTGATGGACGA

TTTGGGCCAGAATCCCGACGGCAAGGACTTCAAGTACTTCGCCCAGATGGTTTCGACCACGGGGTTCATC

CCGCCCATGGCGTCACTTGAGGACAAAGGCAAACCCTTCAACAGCAAGGTCATCATCGCCACCACCAACC

TGTACTCGGGATTCACCCCGAGAACTATGGTGTGCCCTGATGCCCTGAACCGGAGGTTTCACTTTGACAT

CGACGTGAGCGCCAAGGACGGGTACAAAATTAACAACAAATTGGACATAATCAAAGCTCTTGAAGACACC

CACACCAACCCTGTGGCAATGTTTCAATATGACTGTGCCCTTCTCAACGGCATGGCCGTTGAAATGAAGA

GAATGCAACAAGATGTGTTCAAGCCTCAGCCACCCCTCCAGAACGTATACCAACTCGTTCAGGAGGTGAT

TGAACGGGTTGAGCTCCACGAGAAAGTGTCGAGCCACCCGATCTTCAAGCAG

>EF552688.1_O_UKG_2001

CCCTGGTACAAGCTCATCAAGCTCTTGAGCCGCCTGTCATGCATGGCCGCTGTAGCAGCACGGTCAAAGG

ACCCAGTCCTTGTGGCCATCATGCTGGCTGACACCGGCCTTGAGATTCTGGACAGTACCTTTGTCGTGAA

GAAGATCTCCGACTCGCTCTCCAGTCTCTTTCACGTGCCGGCCCCCGTCTTCAGTTTCGGAGCCCCGATT

TTGTTGGCCGGGTTGGTCAAAGTCGCCTCGAGTTTCTTCCGGTCCACACCCGAAGACCTTGAGAGAGCGG

AGAAACAGCTCAAAGCACGTGACATCAATGACATATTCGCCATTCTCAAGAACGGCGAGTGGCTGGTCAA

GCTGATTCTTGCCATCCGCGACTGGATCAAGGCATGGATCGCCTCAGAAGAAAAGTTTGTCACCATGACA

GACCTGGTGCCTGGCATCCTTGAAAAGCAGCGGGATCTCAACGACCCAAGCAAGTACAAAGAGGCCAAGG

AGTGGCTCGACAACGCGCGCCAAGCGTGTTTGAAGAGCGGGAACATCCACATCGCAAACCTTTGCAAAGT

GGTTGCCCCAGCACCCAGCAGGTCGAGGCCCGAACCCGTGGTCGTTTGCCTCCGTGGCAAATCGGGCCAG

GGCAAGAGTTTCCTTGCGAACGTGCTTGCACAAGCAATTTCAACCCACTTCACTGGCAGAACCGACTCAG

TTTGGTACTGCCCACCTGACCCTGACCACTTCGACGGTTACAACCAGCAGACCGTTGTAGTAATGGATGA

TTTGGGCCAGAACCCCGACGGGAAGGACTTCAAGTACTTCGCCCAAATGGTTTCAACTACGGGGTTTATC

CCGCCCATGGCTTCACTCGAGGACAAAGGCAAACCTTTCAACAGCAAGGTCATCATCGCCACCACCAACC

TGTACTCGGGCTTCACCCCGAGAACTATGGTGTGCCCTGACGCACTGAACCGAAGGTTCCACTTTGACAT

TGACGTGAGCGCCAAGGACGGGTACAAAATTAACAACAAATTGGACATCATCAAAGCTCTTGAAGATACC

CACACCAACCCAGTGGCAATGTTTCAATACGACTGTGCCCTTCTCAACGGCATGGCCGTTGAAATGAAGA

GAATGCAACAAGATATGTTCAAGCCTCAACCGCCCCTCCAGAACGTCTACCAGCTTGTTCAGGAGGTGAT

TGACCGGGTCGAGCTCCACGAGAAGGTGTCGAGCCACCCGATTTTCAAGCAG

>EF552689.1_O_UKG_2001

CCCTGGTACAAGCTCATCAAGCTCTTGAGCCGCCTGTCATGCATGGCCGCTGTAGCAGCACGGTCAAAGG

ACCCAGTCCTTGTGGCCATCATGCTGGCTGACACCGGCCTTGAGATTCTGGACAGTACCTTTGTCGTGAA

GAAGATCTCCGACTCGCTCTCCAGTCTCTTTCACGTGCCGGCCCCCGTCTTCAGTTTCGGAGCCCCGATT

TTGTTGGCCGGGTTGGTCAAAGTCGCCTCGAGTTTCTTCCGGTCCACGCCCGAAGACCTTGAGAGAGCGG

AGAAACAGCTCAAAGCACGTGACATCAATGACATATTCGCCATTCTCAAGAACGGCGAGTGGCTGGTCAA

GCTGATTCTTGCCATCCGCGACTGGATCAAGGCATGGATCGCCTCAGAAGAAAAGTTTGTCACCATGACA

GACCTGGTGCCTGGCATCCTTGAAAAGCAGCGGGATCTCAACGACCCAAGCAAGTACAAAGAGGCCAAGG

AGTGGCTCGACAACGCGCGCCAAGCGTGTTTGAAGAGCGGGAACATCCACATCGCAAACCTTTGCAAAGT

GGTTGCCCCAGCACCCAGCAGGTCGAGGCCCGAACCCGTGGTCGTTTGCCTCCGTGGCAAATCGGGCCAG

GGCAAGAGTTTCCTTGCGAACGTGCTTGCACAAGCAATTTCAACCCACTTCACTGGCAGAACCGACTCAG

TTTGGTACTGCCCACCTGACCCTGACCACTTCGACGGTTACAACCAGCAGACCGTTGTAGTAATGGATGA

TTTGGGCCAGAACCCCGACGGGAAGGACTTCAAGTACTTCGCCCAAATGGTTTCAACTACGGGGTTTATC

CCGCCCATGGCTTCACTCGAGGACAAAGGCAAACCTTTCAACAGCAAGGTCATCATCGCCACCACCAACC

TGTACTCGGGCTTCACCCCGAGAACTATGGTGTGCCCTGACGCACTGAACCGAAGGTTCCACTTTGACAT

TGACGTGAGCGCCAAGGACGGGTACAAAATTAACAACAAATTGGACATCATCAAAGCTCTTGAAGATACC

CACACCAACCCAGTGGCAATGTTTCAATACGACTGTGCCCTTCTCAACGGCATGGCCGTTGAAATGAAGA

GAATGCAACAAGATATGTTCAAGCCTCAACCGTCCCTCCAGAACGTCTACCAGCTTGTTCAGGAGGTGAT

TGACCGGGTCGAGCTCCACGAGAAGGTGTCGAGCCACCCGATTTTCAAGCAG

>EF552690.1_O_UKG_2001

CCCTGGTACAAGCTCATCAAGCTCTTGAGCCGCCTGTCATGCATGGCCGCTGTAGCAGCACGGTCAAAGG

ACCCAGTCCTTGTGGCCATCATGCTGGCTGACACCGGCCTTGAGATTCTGGACAGTACCTTTGTCGTGAA

GAAGATCTCCGACTCGCTCTCCAGTCTCTTTCACGTGCCGGCCCCCGTCTTCAGTTTCGGAGCCCCGATT

TTGTTGGCCGGGTTGGTCAAAGTCGCCTCGAGCTTCTTCCGGTCCACGCCCGAAGACCTTGAGAGAGCGG

AGAAACAGCTCAAAGCACGTGACATCAATGACATATTCGCCATTCTCAAGAACGGCGAGTGGCTGGTCAA

GCTGATTCTTGCCATCCGCGACTGGATCAAGGCATGGATCGCCTCAGAAGAAAAGTTTGTCACCATGACA

GACCTGGTGCCTGGCATCCTTGAAAAGCAGCGGGATCTCAACGACCCAAGCAAGTACAAAGAGGCCAAGG

AGTGGCTCGACAACGCGCGCCAAGCGTGTTTGAAGAGCGGGAACATCCACATCGCAAACCTTTGCAAAGT

GGTTGCCCCAGCACCCAGCAGGTCGAGGCCCGAACCCGTGGTCGTTTGCCTCCGTGGCAAATCGGGCCAG

GGCAAGAGTTTCCTTGCGAACGTGCTTGCACAAGCAATTTCAACCCACTTCACTGGCAGAACCGACTCAG

TTTGGTACTGCCCACCTGACCCTGACCACTTCGACGGTTACAACCAGCAGACCGTTGTAGTAATGGATGA

TTTGGGCCAGAACCCCGACGGGAAGGACTTCAAGTACTTCGCCCAAATGGTTTCAACTACGGGGTTTATC

CCGCCCATGGCTTCACTCGAGGACAAAGGCAAACCTTTCAACAGCAAGGTCATCATCGCCACCACCAACC

TGTACTCGGGCTTCACCCCGAGAACTATGGTGTGCCCTGACGCACTGAACCGAAGGTTCCACTTTGACAT

TGACGTGAGCGCCAAGGACGGGTACAAAATTAACAACAAATTGGACATCATCAAAGCTCTTGAAGATACC

CACACCAACCCAGTGGCAATGTTTCAATACGACTGTGCCCTTCTCAACGGCATGGCCGTTGAAATGAAGA

GAATGCAACAAGATATGTTCAAGCCTCAACCGTCCCTCCAGAACGTCTACCAGCTTGTTCAGGAGGTGAT

TGACCGGGTCGAGCTCCACGAGAAGGTGTCGAGCCACCCGATTTTCAAGCAG

>EF552691.1_O_UKG_2001

CCCTGGTACAAGCTCATCAAGCTCTTGAGCCGCCTGTCATGCATGGCCGCTGTAGCAGCACGGTCAAAGG

ACCCAGTCCTTGTGGCCATCATGCTGGCTGACACCGGCCTTGAGATTCTGGACAGTACCTTTGTCGTGAA

GAAGATCTCCGACTCGCTCTCCAGTCTCTTTCACGTGCCGGCCCCCGTCTTCAGTTTCGGAGCCCCGATT

TTGTTGGCCGGGTTGGTCAAAGTCGCCTCGAGTTTCTTCCGGTCCACGCCCGAAGACCTTGAGAGAGCGG

AGAAACAGCTCAAAGCACGTGACATCAATGACATATTCGCCATTCTCAAGAACGGCGAGTGGCTGGTCAA

GCTGATTCTTGCCATCCGCGACTGGATCAAGGCATGGATCGCCTCAGAAGAAAAGTTTGTCACCATGACA

GACCTGGTGCCTGGCATCCTTGAAAAGCAGCGGGATCTCAACGACCCAAGCAAGTACAAAGAGGCCAAGG

AGTGGCTCGACAACGCGCGCCAAGCGTGTTTGAAGAGCGGGAACATCCACATCGCGAACCTTTGCAAAGT

GGTTGCCCCAGCACCCAGCAGGTCGAGGCCCGAACCCGTGGTCGTTTGCCTCCGTGGCAAATCGGGCCAG

GGCAAGAGTTTCCTTGCGAACGTGCTTGCACAAGCAATTTCAACCCACTTCACTGGCAGAACCGACTCAG

TTTGGTACTGCCCACCTGACCCTGACCACTTCGACGGTTACAACCAGCAGACCGTTGTAGTAATGGATGA

TTTGGGCCAGAACCCCGACGGGAAGGACTTCAAGTACTTCGCCCAAATGGTTTCAACTACGGGGTTTATC

CCGCCCATGGCTTCACTCGAGGACAAAGGCAAACCTTTCAACAGCAAGGTCATCATCGCCACCACCAACC

TGTACTCGGGCTTCACCCCGAGAACTATGGTGTGCCCTGACGCACTGAACCGAAGGTTCCACTTTGACAT

TGACGTGAGCGCCAAGGACGGGTACAAAATTAACAACAAATTGGACATCATCAAAGCTCTTGAAGATACC

CACACCAACCCAGTGGCAATGTTTCAATACGACTGTGCCCTTCTCAACGGCATGGCCGTTGAAATGAAGA

GAATGCAACAAGATATGTTCAAGCCTCAACCGTCCCTCCAGAACGTCTACCAGCTTGTTCAGGAGGTGAT

TGACCGGGTCGAGCTCCACGAGAAGGTGTCGAGCCACCCGATTTTCAAGCAG

>EF552692.1_O_UKG_2001

CCCTGGTACAAGCTCATCAAGCTCTTGAGCCGCCTGTCATGCATGGCCGCTGTAGCAGCACGGTCAAAGG

ACCCAGTCCTTGTGGCCATCATGCTGGCTGACACCGGCCTTGAGATTCTGGACAGTACCTTTGTCGTGAA

GAAGATCTCCGACTCGCTCTCCAGTCTCTTTCACGTGCCGGCCCCCGTCTTCAGTTTCGGAGCCCCGATT

TTGTTGGCCGGGTTGGTCAAAGTCGCCTCGAGTTTCTTCCGGTCCACGCCCGAAGACCTTGAGAGAGCGG

AGAAACAGCTCAAAGCACGTGACATCAATGACATATTCGCCATTCTCAAGAACGGCGAGTGGCTGGTCAA

GCTGATTCTTGCCATCCGCGACTGGATCAAGGCATGGATCGCCTCAGAAGAAAAGTTTGTCACCATGACA

GACCTGGTGCCTGGCATCCTTGAAAAGCAGCGGGATCTCAACGACCCAAGCAAGTACAAAGAGGCCAAGG

AGTGGCTCGACAACGCGCGCCAAGCGTGTTTGAAGAGCGGGAACATCCACATCGCAAACCTTTGCAAAGT

GGTTGCCCCAGCACCCAGCAGGTCGAGGCCCGAACCCGTGGTCGTTTGCCTCCGTGGCAAATCGGGCCAG

GGCAAGAGTTTCCTTGCGAACGTGCTTGCACAAGCAATTTCAACCCACTTCACTGGCAGAACCGACTCAG

TTTGGTACTGCCCACCTGACCCTGACCACTTCGACGGTTACAACCAGCAGACCGTTGTAGTAATGGATGA

TTTGGGCCAGAACCCCGACGGGAAGGACTTCAAGTACTTCGCCCAAATGGTTTCAACTACGGGGTTTATC

CCGCCCATGGCTTCACTCGAGGACAAAGGCAAACCTTTCAACAGCAAGGTCATCATCGCCACCACCAACC

TGTACTCGGGCTTCACCCCGAGAACTATGGTGTGCCCTGACGCACTGAACCGAAGGTTCCACTTTGACAT

TGACGTGAGCGCCAAGGACGGGTACAAAATTAACAACAAATTGGACATCATCAAAGCTCTTGAAGATACC

CACACCAACCCAGTGGCAATGTTTCAATACGACTGTGCCCTTCTCAACGGCATGGCCGTTGAAATGAAGA

GAATGCAACAAGATATGTTCAAGCCTCAACCGTCCCTCCAGAACGTCTACCAGCTTGTTCAGGAGGTGAT

TGACCGGGTCGAGCTCCACGAGAAGGTGTCGAGCCACCCGATTTTCAAGCAG

>EF552693.1_O_UKG_2001

CCCTGGTACAAGCTCATCAAGCTCTTGAGCCGCCTGTCATGCATGGCCGCTGTAGCAGCACGGTCAAAGG

ACCCAGTCCTTGTGGCCATCATGCTGGCTGACACCGGCCTTGAGATTCTGGACAGTACCTTTGTCGTGAA

GAAGATCTCCGACTCGCTCTCCAGTCTCTTTCACGTGCCGGCCCCCGTCTTCAGTTTCGGAGCCCCGATT

TTGTTGGCCGGGTTGGTCAAAGTCGCCTCGAGTTTCTTCCGGTCCACGCCCGAAGACCTTGAGAGAGCGG

AGAAACAGCTCAAAGCACGTGACATCAATGACATATTCGCCATTCTCAAGAACGGCGAGTGGCTGGTCAA

GCTGATTCTTGCCATCCGCGACTGGATCAAGGCATGGATCGCCTCAGAAGAAAAGTTTGTCACCATGACA

GACCTGGTGCCTGGCATCCTTGAAAAGCAGCGGGATCTCAACGACCCAAGCAAGTACAAAGAGGCCAAGG

AGTGGCTCGACAACGCGCGCCAAGCGTGTTTGAAGAGCGGGAACATCCACATCGCAAACCTTTGCAAAGT

GGTTGCCCCAGCACCCAGCAGGTCGAGGCCCGAACCCGTGGTCGTTTGCCTCCGTGGCAAATCGGGCCAG

GGCAAGAGTTTCCTTGCGAACGTGCTTGCACAAGCAATTTCAACCCACTTCACTGGCAGAACCGACTCAG

TTTGGTACTGCCCACCTGACCCTGACCACTTCGACGGTTACAACCAGCAGACCGTTGTAGTAATGGATGA

TTTGGGCCAGAACCCCGACGGGAAGGACTTCAAGTACTTCGCCCAAATGGTTTCAACTACGGGGTTTATC

CCGCCCATGGCTTCACTCGAGGACAAAGGCAAACCTTTCAACAGCAAGGTCATCATCGCCACCACCAACC

TGTACTCGGGCTTCACCCCGAGAACTATGGTGTGCCCTGACGCACTGAACCGAAGGTTCCACTTTGACAT

TGACGTGAGCGCCAAGGACGGGTACAAAATTAACAACAAATTGGACATCATCAAAGCTCTTGAAGATACC

CACACCAACCCAGTGGCAATGTTTCAATACGACTGTGCCCTTCTCAACGGCATGGCCGTTGAAATGAAGA

GAATGCAACAAGATATGTTCAAGCCTCAACCGTCCCTCCAGAACGTCTACCAGCTTGTTCAGGAGGTGAT

TGACCGGGTCGAGCTCCACGAGAAGGTGTCGAGCCACCCGATTTTCAAGCAG

>EF552695.1_O_UKG_2001

CCCTGGTACAAGCTCATCAAGCTCTTGAGCCGCCTGTCATGCATGGCCGCTGTAGCAGCACGGTCAAAGG

ACCCAGTCCTTGTGGCCATCATGCTGGCTGACACCGGCCTTGAGATTCTGGACAGTACCTTTGTCGTGAA

GAAGATCTCCGACTCGCTCTCCAGTCTCTTTCACGTGCCGGCCCCCGTCTTCAGTTTCGGAGCCCCGATT

TTGTTGGCCGGGTTGGTCAAAGTCGCCTCGAGTTTCTTCCGGTCCACGCCCGAAGACCTTGAGAGAGCGG

AGAAACAGCTCAAAGCACGTGACATCAATGACATATTCGCCATTCTCAAGAACGGCGAGTGGCTGGTCAA

GCTGATTCTTGCCATCCGCGACTGGATCAAGGCATGGATCGCCTCAGAAGAAAAGTTTGTCACCATGACA

GACCTGGTGCCTGGCATCCTTGAAAAGCAGCGGGATCTCAACGACCCAAGCAAGTACAAAGAGGCCAAGG

AGTGGCTCGACAACGCGCGCCAAGCGTGTTTGAAGAGCGGGAACATCCACATCGCGAACCTTTGCAAAGT

GGTTGCCCCAGCACCCAGCAGGTCGAGGCCCGAACCCGTGGTCGTTTGCCTCCGTGGCAAATCGGGCCAG

GGCAAGAGTTTCCTTGCGAACGTGCTTGCACAAGCAATTTCAACCCACTTCACTGGCAGAACCGACTCAG

TTTGGTACTGCCCACCTGACCCTGACCACTTCGACGGTTACAACCAGCAGACCGTTGTAGTAATGGATGA

TTTGGGCCAGAACCCCGACGGGAAGGACTTCAAGTACTTCGCCCAAATGGTTTCAACTACGGGGTTTATC

CCGCCCATGGCTTCACTCGAGGACAAAGGCAAACCTTTCAACAGCAAGGTCATCATCGCCACCACCAACC

TGTACTCGGGCTTCACCCCGAGAACTATGGTGTGCCCTGACGCACTGAACCGAAGGTTCCACTTTGACAT

TGACGTGAGCGCCAAGGACGGGTACAAAATTAACAACAAATTGGACATCATCAAAGCTCTTGAAGATACC

CACACCAACCCAGTGGCAATGTTTCAATACGACTGTGCCCTTCTCAACGGCATGGCCGTTGAAATGAAGA

GAATGCAACAAGATATGTTCAAGCCTCAACCGTCCCTCCAGAACGTCTACCAGCTTGTTCAGGAGGTGAT

TGACCGGGTCGAGCTCCACGAGAAGGTGTCGAGCCACCCGATTTTCAAGCAG

>EF552696.1_O_UKG_2001

CCCTGGTACAAGCTCATCAAGCTCTTGAGCCGCCTGTCATGCATGGCCGCTGTAGCAGCACGGTCAAAGG

ACCCAGTCCTTGTGGCCATCATGCTGGCTGACACCGGCCTTGAGATTCTGGACAGTACCTTTGTCGTGAA

GAAGATCTCCGACTCGCTCTCCAGTCTCTTTCACGTGCCGGCCCCCGTCTTCAGTTTCGGAGCCCCGATT

TTGTTGGCCGGGTTGGTCAAAGTCGCCTCGAGTTTCTTCCGGTCCACACCCGAAGACCTTGAGAGAGCGG

AGAAACAGCTCAAAGCACGTGACATCAATGACATATTCGCCATTCTCAAGAACGGCGAGTGGCTGGTCAA

GCTGATTCTTGCCATCCGCGACTGGATCAAGGCATGGATCGCCTCAGAAGAAAAGTTTGTCACCATGACA

GACCTGGTGCCTGGCATCCTTGAAAAGCAGCGGGATCTCAACGACCCAAGCAAGTACAAAGAGGCCAAGG

AGTGGCTCGACAACGCGCGCCAAGCGTGTTTGAAGAGCGGGAACATCCACATCGCAAACCTTTGCAAAGT

GGTTGCCCCAGCACCCAGCAGGTCGAGGCCCGAACCCGTGGTCGTTTGCCTCCGTGGCAAATCGGGCCAG

GGCAAGAGTTTCCTTGCGAACGTGCTTGCACAAGCAATTTCAACCCACTTCACTGGCAGAACCGACTCAG

TTTGGTACTGCCCACCTGACCCTGACCACTTCGACGGTTACAACCAGCAGACCGTTGTAGTAATGGATGA

TTTGGGCCAGAACCCCGACGGGAAGGACTTCAAGTACTTCGCCCAAATGGTTTCAACTACGGGGTTTATC

CCGCCCATGGCTTCACTCGAGGACAAAGGCAAACCTTTCAACAGCAAGGTCATCATCGCCACCACCAATC

TGTACTCGGGCTTCACCCCGAGAACTATGGTGTGCCCTGACGCACTGAACCGAAGGTTCCACTTTGACAT

TGACGTGAGCGCCAAGGACGGGTACAAAATTAACAACAAATTGGACATCATCAAAGCTCTTGAAGATACC

CACACCAACCCAGTGGCAATGTTTCAATACGACTGTGCCCTTCTCAACGGCATGGCCGTTGAAATGAAGA

GAATGCAACAAGATATGTTCAAGCCTCAACCGCCCCTCCAGAACGTCTACCAGCTTGTTCAGGAGGTGAT

TGACCGGGTCGAGCTCCACGAGAAGGTGTCGAGCCACCCGATTTTCAAGCAG

>EF552697.1_O_UKG_2001

CCCTGGTACAAGCTCATCAAGCTCTTGAGCCGCCTGTCATGCATGGCCGCTGTAGCAGCACGGTCAAAGG

ACCCAGTCCTTGTGGCCATCATGCTGGCTGACACCGGCCTTGAGATTCTGGACAGTACCTTTGTCGTGAA

GAAGATCTCCGACTCGCTCTCCAGTCTCTTTCACGTGCCGGCCCCCGTCTTCAGTTTCGGAGCCCCGATT

TTGTTGGCCGGGTTGGTCAAAGTCGCCTCGAGTTTCTTCCGGTCCACGCCCGAAGACCTTGAGAGAGCGG

AGAAACAGCTCAAAGCACGTGACATCAATGACATATTCGCCATTCTCAAGAACGGCGAGTGGCTGGTCAA

GCTGATTCTTGCCATCCGCGACTGGATCAAGGCATGGATCGCCTCAGAAGAAAAGTTTGTCACCATGACA

GACCTGGTGCCTGGCATCCTTGAAAAGCAGCGGGATCTCAACGACCCAAGCAAGTACAAAGAGGCCAAGG

AGTGGCTCGACAACGCGCGCCAAGCGTGTTTGAAGAGCGGGAACATCCACATCGCAAACCTTTGCAAAGT

GGTTGCCCCAGCACCCAGCAGGTCGAGGCCCGAACCCGTGGTCGTTTGCCTCCGTGGCAAATCGGGCCAG

GGCAAGAGTTTCCTTGCGAACGTGCTTGCACAAGCAATTTCAACCCACTTCACTGGCAGAACCGACTCAG

TTTGGTACTGCCCACCTGACCCTGACCACTTCGACGGTTACAACCAGCAGACCGTTGTAGTAATGGATGA

TTTGGGCCAGAACCCCGACGGGAAGGACTTCAAGTACTTCGCCCAAATGGTTTCAACTACGGGGTTTATC

CCGCCCATGGCTTCACTCGAGGACAAAGGCAAACCTTTCAACAGCAAGGTCATCATCGCCACCACCAACC

TGTACTCGGGCTTCACCCCGAGAACTATGGTGTGCCCTGACGCACTGAACCGAAGGTTCCACTTTGACAT

TGACGTGAGCGCCAAGGACGGGTACAAAATTAACAACAAATTGGACATCATCAAAGCTCTTGAAGATACC

CACACCAACCCAGTGGCAATGTTTCAATACGACTGTGCCCTTCTCAACGGCATGGCCGTTGAAATGAAGA

GAATGCAACAAGATATGTTCAAGCCTCAACCGTCCCTCCAGAACGTCTACCAGCTTGTTCAGGAGGTGAT

TGACCGGGTCGAGCTCCACGAGAAGGTGTCGAGCCACCCGATTTTCAAGCAG

>EF611987.1_O_UGA_2006

CCCTGGTACAAGCT-ATCAAACTCCTGAGCCGCTTGTCATGTATGGCCGCTGTAGCAGCACGGTCCAAGG

ACCCAGTCCTTGTGGCCATCATGCTGGCTGACACCGGCCT-GAGATTCTGGACAGCACGTTCGTCGTGAA

AAAGATCTCCGACTCGCTCTCCAGTCTCTTTCACGTGCCGGCCCCCGCTTTCAGTTTCGGAGCCCCGTTG

CTGTTGGCCGGGTTGGTCAAGGTCGCCTCGAGTTTCTTCCGGTCTACACCCGAAGAACTCGAGAGAGC-G

AGAAGCAGCTCAAAGCACGTGACATCAATGACATATTCGCCATTCTCAAGAACGGCGAGTGGCTGGTCAA

ATTGATCCTTGCCATCCGCGACTGGATTAAGGCATGGATCGCCTCAGAAGAAAAGTTTGTCACCATGACA

GACTTAGTGCCTGGCATCCTCGAAAAGCAGCGGGACCTCAACGACCCCAGCAAGTACAAGGAGGCCAAGG

AGTGGCTCGACAACGCGCGCCAAGCGTGTCTGAAGAGCGGGAACGTCCACATTGCTAACCTTTGCAAAGT

GGTCGCCCCGGCACCTAGCAGGTCGAGACCCGAGCCCGTGGTCGTTTGCCTCCGTGGCAAGTCCGGCCAG

GGAAAGAGTTTCCTTGCGAACGTGCTCGCACAAGCAATCTCTACCCACTTCACAGGTAGAACAGACTCAG

TCTGGTACTGCCCACCTGACCCTGACCACTTCGATGGTTACAACCAACAGACCGTCGTTGTGATGGATGA

TTTGGGCCAGAACCCCGACGGCAAGGACTTCAAGTACTTTGCCCAGATGGTGTCAACCACGGGGTTCATC

CCGCCAATGGCCTCTCTCGAGGACAAAGGAAAACCTTTTAACAGCAAGGTTATCATTGCTACCACCAACC

TTTACTCGGGTTTCACCCCGAGAACCATGGTGTGCCCTGATGCATTGAACCGGAGGTTTCACTTTGACAT

TGACGTGAGCGCCAAAGACGGGTACAAAATTAACAACAAATTGGACATAATCAAAGCTCTTGAAGATACA

CACACCAACCCTGTGGCAATGTTTCAATACGACTGTGCCCTTCTCAACGGCATGGCCGTTGAAATGAAGA

GAATGCAACAAGACATGTTCAAGCCTCAACCGCCCCTCCAGAACGTGTACCAACTTGTTCAGGAGGTGAT

TGAACGGGTGGAGCTCCACGAGAAAGTGTCGAACCACCCAATCTTCAAACAA

>EF614457.1_O_SKR_2002

CCCTGGTACAAGCTCATCAAGCTCTTGAGCCGCCTGTCATGTATGGCCGCTGTAGCAGCACGGTCAAAGG

ACCCACTCCTTGTGGCCATCATGCTGGCTGACACCGGCCTTGAGATCCTGGACAGTACCTTTGTCGTGAA

GAAGATCTCCGACTCGCTCTCCAGTCTCTTTCACGTGCCGGCCCCCGTCTTCAGTTTCGGAGCCCCGATT

TTGTTGGCCGGGTTGGTCAAAGTCGCCTCGAGTTTCTTCCGGTCCACACCCGAAGACCTTGAGAGAGCGG

AGAAACAGCTCAAAGCACGTGACATCAATGACATATTCGCCATTCTTAAGAACGGCGAGTGGCTGGTCAA

GCTGATTCTTGCCATCCGCGACTGGATCAAGGCATGGATCGCCTCAGAAGAAAAGTTTGTCACCATGACA

GACCTGGTGCCTGGCATCCTTGAAAAGCAGCGGGATCTCAACGACCCAAGCAAGTACAAGGAGGCCAAGG

AGTGGCTCGACAACGCGCGCCAAGCGTGTCTGAAGAGCGGGAACATCCACATCGCAAACCTTTGCAAAGT

GGTTGCCCCAGCGCCCAGCAGGTCGAGGCCCGAACCCGTGGTCGTTTGCCTCCGTGGCAAGTCAGGCCAG

GGCAAGAGTTTCCTTGCGAACGTGCTTGCACAAGCAATTTCAACCCACTTCACTGGCAGCACCGATTCAG

TTTGGTACTGCCCACCTGACCCTGACCACTTCGACGGTTACAACCAGCAGACCGTTGTAGTAATGGATGA

TTTGGGCCAGAACCCCGACGGGAAGGACTTCAAGTACTTTGCCCAAATGGTTTCAACTACGGGGTTTATC

CCGCCCATGGCTTCACTCGAGGACAAAGGCAAGCCTTTTAACAGCAAGGTCATCATCGCCACCACCAACC

TGTACTCGGGCTTCACCCCGAGAACTATGGTGTGCCCTGATGCACTGAACCGAAGGTTCCACTTTGACAT

TGACGTGAGCGCCAAGGACGGGTACAAAATTAACAACAAATTGGACATCATCAAAGCTCTTGAAGACACC

CACACCAACCCAGTGGCAATGTTTCAATACGACTGTGCCCTTCTCAACGGCATGGCCGTTGAAATGAAGA

GAATGCAACAAAACATGTTCAAGCCCCAACCGCCCCTCCAGAACGTCTACCAGCTTGTTCAGGAGGTGAT

TGACCAGGTCGAGCTCCACGAGAAGGTGTCGGACCACCCGATTTTCAAGCAG

>EU214601.1_O_UKG_2001

CCCTGGTACAAGCTCATCAAGCTCTTGAGCCGCCTGTCATGCATGGCCGCTGTAGCAGCACGGTCAAAGG

ACCCAGTCCTTGTGGCCATCATGCTGGCTGACACCGGTCTTGAGATTCTGGACAGTACCTTTGTCGTGAA

GAAGATCTCCGACTCGCTCTCCAGTCTCTTTCACGTGCCGGCCCCCGTCTTCAGTTTCGGAGCCCCGATT

TTGTTGGCCGGGTTGGTCAAAGTCGCCTCGAGTTTCTTCCGGTCCACGCCCGAAGACCTTGAGAGAGCGG

AGAAACAGCTCAAAGCACGTGACATCAATGACATATTCGCCATTCTCAAGAACGGCGAGTGGCTGGTCAA

GCTGATTCTTGCCATCCGCGACTGGATCAAGGCATGGATCGCCTCAGAAGAAAAGTTTGTCACCATGACA

GACCTGGTGCCTGGCATCCTTGAAAAGCAGCGGGATCTCAACGACCCAAGCAAGTACAAAGAGGCCAAGG

AGTGGCTCGACAACGCGCGCCAAGCGTGTTTGAAGAGCGGGAACATCCACATCGCAAACCTTTGCAAAGT

GGTTGCCCCAGCACCCAGCAGGTCGAGGCCCGAACCCGTGGTCGTTTGCCTCCGTGGCAAATCGGGCCAG

GGCAAGAGTTTCCTTGCGAACGTGCTTGCACAAGCAATTTCAACCCACTTCACTGGCAGAACCGACTCAG

TTTGGTACTGCCCACCTGACCCTGACCACTTCGACGGTTACAACCAGCAGACCGTTGTAGTAATGGATGA

TTTGGGCCAGAACCCCGACGGGAAGGACTTCAAGTACTTCGCCCAAATGGTTTCAACTACGGGGTTTATC

CCGCCCATGGCTTCACTCGAGGACAAAGGCAAACCTTTCAACAGCAAGGTCATCATCGCCACCACCAACC

TGTACTCGGGCTTCACCCCGAGAACTATGGTGTGCCCTGACGCACTGAACCGAAGGTTCCACTTTGACAT

TGACGTGAGCGCCAAGGACGGGTACAAAATTAACAACAAATTGGACATCATCAAAGCTCTTGAAGATACC

CACACCAACCCAGTGGCAATGTTTCAATACGACTGTGCCCTTCTCAACGGCATGGCCGTTGAAATGAAGA

GAATGCAACAAGATATGTTCAAGCCTCAACCGTCCCTCCAGAACGTCTACCAGCTTGTTCAGGAGGTGAT

TGACCGGGTCGAGCTCCACGAGAAGGTGTCGAGCCACCCGATTTTCAAGCAG

>EU400597.1_O_CHA_2001

CCCTGGTACAAGCTCATCAAACTCCTAAGCCGCCTGTCGTGCATGGCCGCTGTTGCAGCACGGTCCAAGG

ACCCAGTCCTTGTGGCCATCATGCTGGCCGACACCGGTCTTGAGATTCTGGACAGCACCTTTGTGGTAAA

GAAGATCTCCGATTCGCTCTCCAGTCTCTTCCACGTGCCGGCCCCTGCCTTCAGTTTCGGAGCCCCGATC

CTGTTGGCCGGTTTGGTCAAAGTCGCCTCGAGTTTCTTCCAGTCAACGCCCGAGGACCTCGAGAGAGCAG

AAAAACAGCTCAAAGCACGTGACATCAACGACATATTTGCCGTTCTAAAGAACGGTGAGTGGCTGGTCAA

ACTGATCCTGGCCATCCGCGACTGGATTAAGGCATGGATCGCCTCAGAAGAAAAGTTCGTCACCATGACA

GACCTAGTGCCTGGTATCCTTGAAAAACAACGGGATCTCAACGACCCCGGTAAGTACAAGGAGGCCAAGG

AATGGCTGGACAACGCGCGCCAAGCGTGTCTGAAGAGCGGGAACGTGCACATTGCCAACCTGTGCAAAGT

GGTTGCTCCAGCGCCCAGCAAGTCGAGGCCCGAACCAGCGGTCGTGTGTCTTCGCGGCAAATCCGGCCAA

GGGAAGAGTTTCCTCGCAAACGTTCTCGCACAGGCAATCTCCACCCACTTCACTGGCAGGACCGACTCAG

TCTGGTACTGTCCGCCCGACCCTGACCACTTCGACGGTTACAACCAGCAGACCGTCGTCGTGATGGACGA

CTTGGGCCAGAACCCCGACGGCAAAGACTTTAAGTACTTCGCCCAGATGGTCTCCACTACGGGGTTCATC

CCGCCAATGGCCTCGCTCGAGGATAAAGGTAAGCCCTTCAACAGCAAGGTCATAATAGCCACAACCAACC

TGTACTCGGGATTCACCCCAAGAACCATGGTGTGCCCCGATGCGCTCAACCGGAGGTTCCACTTTGACAT

CGACGTGAGCGCCAAAGACGGGTACAAGGTTAACAACAAACTGGACATAGTCAAGGCACTCGAAGACACC

CACACCAACCCGGTGGCAATGTTCCAGTATGATTGCGCCCTTCTCAACGGAATGGCCGTTGAAATGAAGA

GAATGCAACAAGACATGTTCAAGCCTCAACCGCCCATCCAGAACGTCTACCAACTCGTTCAGGAGGTGAT

TGAGCGGGTGGAGCTGCACGAAAAGGTATCGAGCCACCCGATATTCAAACAG

>EU448368.1_O_UKG_1967

CCCTGGTACAAGCTTATCAAGCTCCTAAGCCGCCTGTCGTGCATGGCCGCTGTGGCAGCACGGTCCAAGG

ACCCAGTCCTTGTGGCCATCATGCTGGCCGACACCGGTCTCGAGATTCTGGACAGCACCTTCGTCGTGAA

GAAGATCTCCGACTCGCTCTCCAGTCTCTTTCACGTGCCGGCCCCCGTCTTCAGTTTCGGAGCACCGGTC

CTGTTGGCCGGGTTGGTCAAAGTCGCCTCGAGTTTCTTCCGGTCTACACCCGAAGACCTTGAGAGAGCAG

AGAAACAGCTCAAAGCACGTGACATCAACGACATCTTCGCCATTCTCAAGAACGGCGAGTGGCTGGTCAA

ACTGATCCTTGCCATCCGCGACTGGATTAAGGCTTGGATCGCCTCAGAAGAGAAGTTTGTCACCATGACA

GACTTGGTGCCTGGCATCCTTGAAAAGCAGCGGGATCTGAACGACCCGAGCAAGTACAAGGAAGCCAAGG

AGTGGCTCGACAACGCGCGCCAAGCGTGTTTGAAGAGCGGGAACGTCCACATTGCCAACCTGTGTAAAGT

GGTCGCTCCAGCACCCAGCAAGTCGAGGCCCGAACCCGTGGTTGTTTGTCTCCGCGGCAAATCTGGCCAG

GGCAAGAGCTTCCTTGCAAACGTGCTTGCACAGGCAATTTCCGCCCACTTCACCGGCAGAACCGACTCAG

TGTGGTACTGCCCACCTGACCCTGACCACTTCGACGGTTACAACCAGCAAACCGTCGTTGTGATGGATGA

TTTGGGCCAGAACCCTGACGGCAAGGACTTCAAATACTTTGCCCAAATGGTCTCGACCACAGGGTTCATC

CCGCCCATGGCATCACTCGAGGACAAAGGTAAACCTTTCAACAGCAAAGTCATCATCGCGACCACCAACT

TGTACTCGGGCTTCACCCCGAGGACTATGGTGTGTCCCGACGCACTGAACCGGAGGTTTCACTTTGACAT

CGATGTGAGTGCTAAGGATGGGTACAAAATTAACAACAAATTGGACATTATCAAAGCACTTGAAGACACC

CACACCAACCCAGTGGCAATGTTTCAATACGACTGTGCCCTTCTCAACGGCATGGCCGTTGAAATGAAGA

GAATGCAACAAGACATGTTCAAGCCTCAACCACCCCTCCAGAATGTGTACCAGCTTGTTCAGGAGGTGAT

TGATCGGGTCGAGCTCCACGAGAAAGTGTCGAGCCACCCGATCTTCAAGCAG

>EU448369.1_O_UKG_1967

CCCTGGTACAAGCTTATCAAGCTCCTAAGCCGCCTGTCGTGCATGGCCGCTGTGGCAGCACGGTCCAAGG

ACCCAGTCCTTGTGGCCATCATGCTGGCCGACACCGGTCTCGAGATTCTGGACAGCACCTTCGTCGTGAA

GAAGATCTCCGACTCGCTCTCCAGTCTCTTTCACGTGCCGGCCCCCGTCTTCAGTTTCGGAGCACCGGTC

CTGTTGGCCGGGTTGGTCAAAGTCGCCTCGAGTTTCTTCCGGTCTACACCCGAAGACCTTGAGAGAGCAG

AGAAACAGCTCAAAGCACGTGACATCAACGACATCTTCGCCATTCTCAAGAACGGCGAGTGGCTGGTCAA

ACTGATCCTTGCCATCCGCGACTGGATTAAGGCTTGGATCGCCTCAGAAGAGAAGTTTGTCACCATGACA

GACTTGGTGCCTGGCATCCTTGAAAAGCAGCGGGATCTGAACGACCCGAGCAAGTACAAGGAAGCCAAGG

AGTGGCTCGACAACGCGCGCCAAGCGTGTTTGAAGAGCGGGAACGTCCACATTGCCAACCTGTGTAAAGT

GGTCGCTCCAGCACCCAGCAAGTCGAGGCCCGAACCCGTGGTTGTTTGTCTCCGCGGCAAATCTGGCCAG

GGCAAGAGCTTCCTTGCAAACGTGCTTGCACAGGCAATTTCCGCCCACTTCACCGGCAGAACCGACTCAG

TGTGGTACTGCCCACCTGACCCTGACCACTTCGACGGTTACAACCAGCAAACCGTCGTTGTGATGGATGA

TTTGGGCCAGAACCCTGACGGCAAGGACTTCAAATACTTTGCCCAAATGGTCTCGACCACAGGGTTCATC

CCGCCCATGGCATCACTCGAGGACAAAGGTAAACCTTTCAACAGCAAAGTCATCATCGCGACCACCAACT

TGTACTCGGGCTTCACCCCGAGGACTATGGTGTGTCCCGACGCACTGAACCGGAGGTTTCACTTTGACAT

CGATGTGAGTGCTAAGGATGGGTACAAAATTAACAACAAATTGGACATTATCAAAGCACTTGAAGACACC

CACACCAACCCAGTGGCAATGTTTCAATACGACTGTGCCCTTCTCAACGGCATGGCCGTTGAAATGAAGA

GAATGCAACAAGACATGTTCAAGCCTCAACCACCCCTCCAGAATGTGTACCAGCTTGTTCAGGAGGTGAT

TGATCGGGTCGAGCTCCACGAGAAAGTGTCGAGCCACCCGATCTTCAAGCAG

>EU448370.1_O_UKG_1967

CCCTGGTACAAGCTTATCAAGCTCCTAAGCCGCCTGTCGTGCATGGCCGCTGTGGCAGCACGGTCCAAGG

ACCCAGTCCTTGTGGCCATCATGCTGGCCGACACCGGTCTCGAGATTCTGGACAGCACCTTCGTCGTGAA

GAAGATCTCCGACTCGCTCTCCAGTCTCTTTCACGTGCCGGCCCCCGTCTTCAGTTTCGGAGCACCGGTC

CTGTTGGCCGGGTTGGTCAAAGTCGCCTCGAGTTTCTTCCGGTCTACACCCGAAGACCTTGAGAGAGCAG

AGAAACAGCTCAAAGCACGTGACATCAACGACATCTTCGCCATTCTCAAGAACGGCGAGTGGCTGGTCAA

ACTGATCCTTGCCATCCGCGACTGGATTAAGGCTTGGATCGCCTCAGAAGAGAAGTTTGTCACCATGACA

GACTTGGTGCCTGGCATCCTTGAAAAGCAGCGGGATCTGAACGACCCGAGCAAGTACAAGGAAGCCAAGG

AGTGGCTCGACAACGCGCGCCAAGCGTGTTTGAAGAGCGGGAACGTCCACATTGCCAACCTGTGTAAAGT

GGTCGCTCCAGCACCCAGCAAGTCGAGGCCCGAACCCGTGGTTGTTTGTCTCCGCGGCAAATCTGGCCAG

GGCAAGAGCTTCCTTGCAAACGTGCTTGCACAGGCAATTTCCGCCCACTTCACCGGCAGAACCGACTCAG

TGTGGTACTGCCCACCTGACCCTGACCACTTCGACGGTTACAACCAGCAAACCGTCGTTGTGATGGATGA

TTTGGGCCAGAACCCTGACGGCAAGGACTTCAAATACTTTGCCCAAATGGTCTCGACCACAGGGTTCATC

CCGCCCATGGCATCACTCGAGGACAAAGGTAAACCTTTCAACAGCAAAGTCATCATCGCGACCACCAACT

TGTACTCGGGCTTCACCCCGAGGACTATGGTGTGTCCCGACGCACTGAACCGGAGGTTTCACTTTGACAT

CGATGTGAGTGCTAAGGATGGGTACAAAATTAACAACAAATTGGACATTATCAAAGCACTTGAAGACACC

CACACCAACCCAGTGGCAATGTTTCAATACGACTGTGCCCTTCTCAACGGCATGGCCGTTGAAATGAAGA

GAATGCAACAAGACATGTTCAAGCCTCAACCACCCCTCCAGAATGTGTACCAGCTTGTTCAGGAGGTGAT

TGATCGGGTCGAGCTCCACGAGAAAGTGTCGAGCCACCCGATCTTCAAGCAG

>EU448371.1_O_UKG_2007

CCCTGGTACAAGCTTATCAAGCTCCTAAGCCGCCTGTCGTGCATGGCCGCTGTGGCAGCACGGTCCAAGG

ACCCAGTCCTTGTGGCCATCATGCTGGCCGACACCGGTCTCGAGATTCTGGACAGCACCTTCGTCGTGAA

GAAGATCTCCGACTCGCTCTCCAGTCTCTTTCACGTGCCGGCCCCCGTCTTCAGTTTCGGAGCACCGGTC

CTGTTGGCCGGGTTGGTCAAAGTCGCCTCGAGTTTCTTCCGGTCTACACCCGAAGACCTTGAGAGAGCAG

AGAAACAGCTCAAAGCACGTGACATCAACGACATCTTCGCCATTCTCAAGAACGGCGAGTGGCTGGTCAA

ACTGATCCTTGCCATCCGCGACTGGATTAAGGCTTGGATCGCCTCAGAAGAGAAGTTTGTCACCATGACA

GACTTGGTGCCTGGCATCCTTGAAAAGCAGCGGGATCTGAACGACCCGAGCAAGTACAAGGAAGCCAAGG

AGTGGCTCGACAACGCGCGCCAAGCGTGTTTGAAGAGCGGGAACGTCCACATTGCCAACCTGTGTAAAGT

GGTCGCTCCAGCACCCAGCAAGTCGAGGCCCGAACCCGTGGTTGTTTGTCTCCGCGGCAAATCTGGCCAG

GGCAAGAGCTTCCTTGCAAACGTGCTTGCACAGGCAATTTCCGCCCACTTCACCGGCAGAACCGACTCAG

TGTGGTACTGCCCACCTGACCCTGACCACTTCGACGGTTACAACCAGCAAACCGTCGTTGTGATGGATGA

TTTGGGCCAGAACCCTGACGGCAAGGACTTCAAATACTTTGCCCAAATGGTCTCGACCACAGGGTTCATC

CCGCCCATGGCATCACTCGAGGACAAAGGTAAACCTTTCAACAGCAAAGTCATCATCGCGACCACCAACT

TGTACTCGGGCTTCACCCCGAGGACTATGGTGTGTCCCGACGCACTGAACCGGAGGTTTCACTTTGACAT

CGATGTGAGTGCTAAGGATGGGTACAAAATTAACAACAAATTGGACATTATCAAAGCACTTGAAGACACC

CACACCAACCCAGTGGCAATGTTTCAATACGACTGTGCCCTTCTCAACGGCATGGCCGTTGAAATGAAGA

GAATGCAACAAGACATGTTCAAGCCTCAACCACCCCTCCAGAATGTGTACCAGCTTGTTCAGGAGGTGAT

TGATCGGGTCGAGCTCCATGAGAAAGTGTCGAGCCACCCGATCTTCAAGCAG

>EU448372.1_O_UKG_2007

CCCTGGTACAAGCTTATCAAGCTCCTAAGCCGCCTGTCGTGCATGGCCGCTGTGGCAGCACGGTCCAAGG

ACCCAGTCCTTGTGGCCATCATGCTGGCCGACACCGGTCTCGAGATTCTGGACAGCACCTTCGTCGTGAA

GAAGATCTCCGACTCGCTCTCCAGTCTCTTTCACGTGCCGGCCCCCGTCTTCAGTTTCGGAGCACCGGTC

CTGTTGGCCGGGTTGGTCAAAGTCGCCTCGAGTTTCTTCCGGTCTACACCCGAAGACCTTGAGAGAGCAG

AGAAACAGCTCAAAGCACGTGACATCAACGACATCTTCGCCATTCTCAAGAACGGCGAGTGGCTGGTCAA

ACTGATCCTTGCCATCCGCGACTGGATTAAGGCTTGGATCGCCTCAGAAGAGAAGTTTGTCACCATGACA

GACTTGGTGCCTGGCATCCTTGAAAAGCAGCGGGATCTGAACGACCCGAGCAAGTACAAGGAAGCCAAGG

AGTGGCTCGACAACGCGCGCCAAGCGTGTTTGAAGAGCGGGAACGTCCACATTGCCAACCTGTGTAAAGT

GGTCGCTCCAGCACCCAGCAAGTCGAGGCCCGAACCCGTGGTTGTTTGTCTCCGCGGCAAATCTGGCCAG

GGCAAGAGCTTCCTTGCAAACGTGCTTGCACAGGCAATTTCCGCCCACTTCACCGGCAGAACCGACTCAG

TGTGGTACTGCCCACCTGACCCTGACCACTTCGACGGTTACAACCAGCAAACCGTCGTTGTGATGGATGA

TTTGGGCCAGAACCCTGACGGCAAGGACTTCAAATACTTTGCCCAAATGGTCTCGACCACAGGGTTCATC

CCGCCCATGGCATCACTCGAGGACAAAGGTAAACCTTTCAACAGCAAAGTCATCATCGCGACCACCAACT

TGTACTCGGGCTTCACCCCGAGGACTATGGTGTGTCCCGACGCACTGAACCGGAGGTTTCACTTTGACAT

CGATGTGAGTGCTAAGGATGGGTACAAAATTAACAACAAATTGGACATTATCAAAGCACTTGAAGACACC

CACACCAACCCAGTGGCAATGTTTCAATACGACTGTGCCCTTCTCAACGGCATGGCCGTTGAAATGAAGA

GAATGCAACAAGACATGTTCAAGCCTCAACCACCCCTCCAGAATGTGTACCAGCTTGTTCAGGAGGTGAT

TGATCGGGTCGAGCTCCATGAGAAAGTGTCGAGCCACCCGATCTTCAAGCAG

>EU448373.1_O_UKG_2007

CCCTGGTACAAGCTTATCAAGCTCCTAAGCCGCCTGTCGTGCATGGCCGCTGTGGCAGCACGGTCCAAGG

ACCCAGTCCTTGTGGCCATCATGCTGGCCGACACCGGTCTCGAGATTCTGGACAGCACCTTCGTCGTGAA

GAAGATCTCCGACTCGCTCTCCAGTCTCTTTCACGTGCCGGCCCCCGTCTTCAGTTTCGGAGCACCGGTC

CTGTTGGCCGGGTTGGTCAAAGTCGCCTCGAGTTTCTTCCGGTCTACACCCGAAGACCTTGAGAGAGCAG

AGAAACAGCTCAAAGCACGTGACATCAACGACATCTTCGCCATTCTCAAGAACGGCGAGTGGCTGGTCAA

ACTGATCCTTGCCATCCGCGACTGGATTAAGGCTTGGATCGCCTCAGAAGAGAAGTTTGTCACCATGACA

GACTTGGTGCCTGGCATCCTTGAAAAGCAGCGGGATCTGAACGACCCGAGCAAGTACAAGGAAGCCAAGG

AGTGGCTCGACAACGCGCGCCAAGCGTGTTTGAAGAGCGGGAACGTCCACATTGCCAACCTGTGTAAAGT

GGTCGCTCCAGCACCCAGCAAGTCGAGGCCCGAACCCGTGGTTGTTTGTCTCCGCGGCAAATCTGGCCAG

GGCAAGAGCTTCCTTGCAAACGTGCTTGCACAGGCAATTTCCGCCCACTTCACCGGCAGAACCGACTCAG

TGTGGTACTGCCCACCTGACCCTGACCACTTCGACGGTTACAACCAGCAAACCGTCGTTGTGATGGATGA

TTTGGGCCAGAACCCTGACGGCAAGGACTTCAAATACTTTGCCCAAATGGTCTCGACCACAGGGTTCATC

CCGCCCATGGCATCACTCGAGGACAAAGGTAAACCTTTCAACAGCAAAGTCATCATCGCGACCACCAACT

TGTACTCGGGCTTCACCCCGAGGACTATGGTGTGTCCCGACGCACTGAACCGGAGGTTTCACTTTGACAT

CGATGTGAGTGCTAAGGATGGGTACAAAATTAACAACAAATTGGACATTATCAAAGCACTTGAAGACACC

CACACCAACCCAGTGGCAATGTTTCAATACGACTGTGCCCTTCTCAACGGCATGGCCGTTGAAATGAAGA

GAATGCAACAAGACATGTTCAAGCCTCAACCACCCCTCCAGAATGTGTACCAGCTTGTTCAGGAGGTGAT

TGATCGGGTCGAGCTCCATGAGAAAGTGTCGAGCCACCCGATCTTCAAGCAG

>EU448374.1_O_UKG_2007

CCCTGGTACAAGCTTATCAAGCTCCTAAGCCGCCTGTCGTGCATGGCCGCTGTGGCAGCACGGTCCAAGG

ACCCAGTCCTTGTGGCCATCATGCTGGCCGACACCGGTCTCGAGATTCTGGACAGCACCTTCGTCGTGAA

GAAGATCTCCGACTCGCTCTCCAGTCTCTTTCACGTGCCGGCCCCCGTCTTCAGTTTCGGAGCACCGGTC

CTGTTGGCCGGGTTGGTCAAAGTCGCCTCGAGTTTCTTCCGGTCTACACCCGAAGACCTTGAGAGAGCAG

AGAAACAGCTCAAAGCACGTGACATCAACGACATCTTCGCCATTCTCAAGAACGGCGAGTGGCTGGTCAA

ACTGATCCTTGCCATCCGCGACTGGATTAAGGCTTGGATCGCCTCAGAAGAGAAGTTTGTCACCATGACA

GACTTGGTGCCTGGCATCCTTGAAAAGCAGCGGGATCTGAACGACCCGAGCAAGTACAAGGAAGCCAAGG

AGTGGCTCGACAACGCGCGCCAAGCGTGTTTGAAGAGCGGGAACGTCCACATTGCCAACCTGTGTAAAGT

GGTCGCTCCAGCACCCAGCAAGTCGAGGCCCGAACCCGTGGTTGTTTGTCTCCGCGGCAAATCTGGCCAG

GGCAAGAGCTTCCTTGCAAACGTGCTTGCACAGGCAATTTCCGCCCACTTCACCGGCAGAACCGACTCAG

TGTGGTACTGCCCACCTGACCCTGACCACTTCGACGGTTACAACCAGCAAACCGTCGTTGTGATGGATGA

TTTGGGCCAGAACCCTGACGGCAAGGACTTCAAATACTTTGCCCAAATGGTCTCGACCACAGGGTTCATC

CCGCCCATGGCATCACTCGAGGACAAAGGTAAACCTTTCAACAGCAAAGTCATCATCGCGACCACCAACT

TGTACTCGGGCTTCACCCCGAGGACTATGGTGTGTCCCGACGCACTGAACCGGAGGTTTCACTTTGACAT

CGATGTGAGTGCTAAGGATGGGTACAAAATTAACAACAAATTGGACATTATCAAAGCACTTGAAGACACC

CACACCAACCCAGTGGCAATGTTTCAATACGACTGTGCCCTTCTCAACGGCATGGCCGTTGAAATGAAGA

GAATGCAACAAGACATGTTCAAGCCTCAACCACCCCTCCAGAATGTGTACCAGCTTGTTCAGGAGGTGAT

TGATCGGGTCGAGCTCCATGAGAAAGTGTCGAGCCACCCGATCTTCAAGCAG

>EU448375.1_O_UKG_2007

CCCTGGTACAAGCTTATCAAGCTCCTAAGCCGCCTGTCGTGCATGGCCGCTGTGGCAGCACGGTCCAAGG

ACCCAGTCCTTGTGGCCATCATGCTGGCCGACACCGGTCTCGAGATTCTGGACAGCACCTTCGTCGTGAA

GAAGATCTCCGACTCGCTCTCCAGTCTCTTTCACGTGCCGGCCCCCGTCTTCAGTTTCGGAGCACCGGTC

CTGTTGGCCGGGTTGGTCAAAGTCGCCTCGAGTTTCTTCCGGTCTACACCCGAAGACCTTGAGAGAGCAG

AGAAACAGCTCAAAGCACGTGACATCAACGACATCTTCGCCATTCTCAAGAACGGCGAGTGGCTGGTCAA

ACTGATCCTTGCCATCCGCGACTGGATTAAGGCTTGGATCGCCTCAGAAGAGAAGTTTGTCACCATGACA

GACTTGGTGCCTGGCATCCTTGAAAAGCAGCGGGATCTGAACGACCCGAGCAAGTACAAGGAAGCCAAGG

AGTGGCTCGACAACGCGCGCCAGGCGTGTTTGAAGAGCGGGAACGTCCACATTGCCAACCTGTGTAAAGT

GGTCGCTCCAGCACCCAGCAAGTCGAGGCCCGAACCCGTGGTTGTTTGTCTCCGCGGCAAATCTGGCCAG

GGCAAGAGCTTCCTTGCAAACGTGCTTGCACAGGCAATTTCCGCCCACTTCACCGGCAGAACCGACTCAG

TGTGGTACTGCCCACCTGACCCTGACCACTTCGACGGTTACAACCAGCAAACCGTCGTTGTGATGGATGA

TTTGGGCCAGAACCCTGACGGCAAGGACTTCAAATACTTTGCCCAAATGGTCTCGACCACAGGGTTCATC

CCGCCCATGGCATCACTCGAGGACAAAGGTAAACCTTTCAACAGCAAAGTCATCATCGCGACCACCAACT

TGTACTCGGGCTTCACCCCGAGGACTATGGTGTGTCCCGACGCACTGAACCGGAGGTTTCACTTTGACAT

CGATGTGAGTGCTAAGGATGGGTACAAAATTAACAACAAATTGGACATTATCAAAGCACTTGAAGACACC

CACACCAACCCAGTGGCAATGTTTCAATACGACTGTGCCCTTCTCAACGGCATGGCCGTTGAAATGAAGA

GAATGCAACAAGACATGTTCAAGCCTCAACCACCCCTCCAGAATGTGTACCAGCTTGTTCAGGAGGTGAT

TGATCGGGTCGAGCTCCATGAGAAAGTGTCGAGCCACCCGATCTTCAAGCAG

>EU448376.1_O_UKG_2007

CCCTGGTACAAGCTTATCAAGCTCCTAAGCCGCCTGTCGTGCATGGCCGCTGTGGCAGCACGGTCCAAGG

ACCCAGTCCTTGTGGCCATCATGCTGGCCGACACCGGTCTCGAGATTCTGGACAGCACCTTCGTCGTGAA

GAAGATCTCCGACTCGCTCTCCAGTCTCTTTCACGTGCCGGCCCCCGTCTTCAGTTTCGGAGCACCGGTC

CTGTTGGCCGGGTTGGTCAAAGTCGCCTCGAGTTTCTTCCGGTCTACACCCGAAGACCTTGAGAGAGCAG

AGAAACAGCTCAAAGCACGTGACATCAACGACATCTTCGCCATTCTCAAGAACGGCGAGTGGCTGGTCAA

ACTGATCCTTGCCATCCGCGACTGGATTAAGGCTTGGATCGCCTCAGAAGAGAAGTTTGTCACCATGACA

GACTTGGTGCCTGGCATCCTTGAAAAGCAGCGGGATCTGAACGACCCGAGCAAGTACAAGGAAGCCAAGG

AGTGGCTCGACAACGCGCGCCAAGCGTGTTTGAAGAGCGGGAACGTCCACATTGCCAACCTGTGTAAAGT

GGTCGCTCCAGCACCCAGCAAGTCGAGGCCCGAACCCGTGGTTGTTTGTCTCCGCGGCAAATCTGGCCAG

GGCAAGAGCTTCCTTGCAAACGTGCTTGCACAGGCAATTTCCGCCCACTTCACCGGCAGAACCGACTCAG

TGTGGTACTGCCCACCTGACCCTGACCACTTCGACGGTTACAACCAGCAAACCGTCGTTGTGATGGATGA

TTTGGGCCAGAACCCTGACGGCAAGGACTTCAAATACTTTGCCCAAATGGTCTCGACCACAGGGTTCATC

CCGCCCATGGCATCACTCGAGGACAAAGGTAAACCTTTCAACAGCAAAGTCATCATCGCGACCACCAACT

TGTACTCGGGCTTCACCCCGAGGACTATGGTGTGTCCCGACGCACTGAACCGGAGGTTTCACTTTGACAT

CGATGTGAGTGCTAAGGATGGGTACAAAATTAACAACAAATTGGACATTATCAAAGCACTTGAAGACACC

CACACCAACCCAGTGGCAATGTTTCAATACGACTGTGCCCTTCTCAACGGCATGGCCGTTGAAATGAAGA

GAATGCAACAAGACATGTTCAAGCCTCAACCACCCCTCCAGAATGTGTACCAGCTTGTTCAGGAGGTGAT

TGATCGGGTCGAGCTCCATGAGAAAGTGTCGAGCCACCCGATCTTCAAGCAG

>EU448377.1_O_UKG_2007

CCCTGGTACAAGCTTATCAAGCTCCTAAGCCGCCTGTCGTGCATGGCCGCTGTGGCAGCACGGTCCAAGG

ACCCAGTCCTTGTGGCCATCATGCTGGCCGACACCGGTCTCGAGATTCTGGACAGCACCTTCGTCGTGAA

GAAGATCTCCGACTCGCTCTCCAGTCTCTTTCACGTGCCGGCCCCCGTCTTCAGTTTCGGAGCACCGGTC

CTGTTGGCCGGGTTGGTCAAAGTCGCCTCGAGTTTCTTCCGGTCTACACCCGAAGACCTTGAGAGAGCAG

AGAAACAGCTCAAAGCACGTGACATCAACGACATCTTCGCCATTCTCAAGAACGGCGAGTGGCTGGTCAA

ACTGATCCTTGCCATCCGCGACTGGATTAAGGCTTGGATCGCCTCAGAAGAGAAGTTTGTCACCATGACA

GACTTGGTGCCTGGCATCCTTGAAAAGCAGCGGGATCTGAACGACCCGAGCAAGTACAAGGAAGCCAAGG

AGTGGCTCGACAACGCGCGCCAAGCGTGTTTGAAGAGCGGGAACGTCCACATTGCCAACCTGTGTAAAGT

GGTCGCTCCAGCACCCAGCAAGTCGAGGCCCGAACCCGTGGTTGTTTGTCTCCGCGGCAAATCTGGCCAG

GGCAAGAGCTTCCTTGCAAACGTGCTTGCACAGGCAATTTCCGCCCACTTCACCGGCAGAACCGACTCAG

TGTGGTACTGCCCACCTGACCCTGACCACTTCGACGGTTACAACCAGCAAACCGTCGTTGTGATGGATGA

TTTGGGCCAGAACCCTGACGGCAAGGACTTCAAATACTTTGCCCAAATGGTCTCGACCACAGGGTTCATC

CCGCCCATGGCATCACTCGAGGACAAAGGTAAACCTTTCAACAGCAAAGTCATCATCGCGACCACCAACT

TGTACTCGGGCTTCACCCCGAGGACTATGGTGTGTCCCGACGCACTGAACCGGAGGTTTCACTTTGACAT

CGATGTGAGTGCTAAGGATGGGTACAAAATTAACAACAAATTGGACATTATCAAAGCACTTGAAGACACC

CACACCAACCCAGTGGCAATGTTTCAATACGACTGTGCCCTTCTCAACGGCATGGCCGTTGAAATGAAGA

GAATGCAACAAGACATGTTCAAGCCTCAACCACCCCTCCAGAATGTGTACCAGCTTGTTCAGGAGGTGAT

TGATCGGGTCGAGCTCCATGAGAAAGTGTCGAGCCACCCGATCTTCAAGCAG

>EU448378.1_O_UKG_2007

CCCTGGTACAAGCTTATCAAGCTCCTAAGCCGCCTGTCGTGCATGGCCGCTGTGGCAGCACGGTCCAAGG

ACCCAGTCCTTGTGGCCATCATGCTGGCCGACACCGGTCTCGAGATTCTGGACAGCACCTTCGTCGTGAA

GAAGATCTCCGACTCGCTCTCCAGTCTCTTTCACGTGCCGGCCCCCGTCTTCAGTTTCGGAGCACCGGTC

CTGTTGGCCGGGTTGGTCAAAGTCGCCTCGAGTTTCTTCCGGTCTACACCCGAAGACCTTGAGAGAGCAG

AGAAACAGCTCAAAGCACGTGACATCAACGACATCTTCGCCATTCTCAAGAACGGCGAGTGGCTGGTCAA

ACTGATCCTTGCCATCCGCGACTGGATTAAGGCTTGGATCGCCTCAGAAGAGAAGTTTGTCACCATGACA

GACTTGGTGCCTGGCATCCTTGAAAAGCAGCGGGATCTGAACGACCCGAGCAAGTACAAGGAAGCCAAGG

AGTGGCTCGACAACGCGCGCCAAGCGTGTTTGAAGAGCGGGAACGTCCACATTGCCAACCTGTGTAAAGT

GGTCGCTCCAGCACCCAGCAAGTCGAGGCCCGAACCCGTGGTTGTTTGTCTCCGCGGCAAATCTGGCCAG

GGCAAGAGCTTCCTTGCAAACGTGCTTGCACAGGCAATTTCCGCCCACTTCACCGGCAGAACCGACTCAG

TGTGGTACTGCCCACCTGACCCTGACCACTTCGACGGTTACAACCAGCAAACCGTCGTTGTGATGGATGA

TTTGGGCCAGAACCCTGACGGCAAGGACTTTAAATACTTTGCCCAAATGGTCTCGACCACAGGGTTCATC

CCGCCCATGGCATCACTCGAGGACAAAGGTAAACCTTTCAACAGCAAAGTCATCATCGCGACCACCAACT

TGTACTCGGGCTTCACCCCGAGGACTATGGTGTGTCCCGACGCACTGAACCGGAGGTTTCACTTTGACAT

CGATGTGAGTGCTAAGGATGGGTACAAAATTAACAACAAATTGGA-ATTATCAAAGCACTTGAAGACACC

CACACCAACCCAGTGGCAATGTTTCAATACGACTGTGCCCTTCTCAACGGCATGGCCGTTGAAATGAAGA

GAATGCAACAAGACATGTTCAAGCCTCAACCACCCCTCCAGAATGTGTACCAGCTTGTTCAGGAGGTGAT

TGATCGGGTCGAGCTCCATGAGAAAGTGTCGAGCCACCCGATCTTCAAGCAG

>FJ175661.1_O_ISR_2007

CCCTGGTACAAGCTCATCAAGCTCCTGAGCCGCCTGTCATGCATGGCCGCTGTAGCAGCACGGTCAAAGG

ACCCAATCCTTGTGGCCATCATGCTAGCTGACACCGGTCTCGAGATTCTGGACAGCACCTTTGTCGTGAA

GAAAATCTCCGACTCGCTCTCTAGTCTCTTCCACGTGCCGGCCCCCGTCTTCAGTTTCGGAGCCCCGATT

TTGTTGGCCGGGTTGGTCAAGGTTGCCTCGAGTTTCTTCCGGTCCACGCCCGAAGACCTTGAGAGAGCAG

AAAAACAGCTCAAAGCACGTGACATCAATGACATATTCGCCATTCTTAAGAACGGCGAGTGGCTGGTCAA

GCTGATCCTTGCCATCCGCGACTGGATTAAGGCATGGATTGCCTCAGAAGAAAAGTTTGTCACCATGACG

GACCTAGTGCCTGGCATCCTCGAAAAGCAGCGGGACCTCAACGACCCAAGCAAGTACAAGGAGGCCAAGG

AGTGGCTCGACAACGCGCGCCAAGCGTGTCTGAAGAGCGGGAATGTCCACATTGCCAACCTTTGCAAAGT

GGTCGCCCCGGCACCCAGCAAGTCGAGACCCGAACCTGTGGTCGTTTGCCTCCGCGGCAAATCCGGCCAG

GGTAAGAGTTTCCTCGCGAACGTGCTCGCACAAGCAATCTCCACCCACTTCACTGGTAGAACTGATTCAG

TTTGGTACTGCCCGCCTGACCCTGACCACTTCGACGGCTACAACCAGCAGGCCGTTGTCGTGATGGACGA

TTTGGGCCAGAACCCTGATGGCAAGGACTTCAAGTACTTTGCCCAGATGGTGTCAACCACGGGGTTCATC

CCGCCAATGGCGTCACTCGAAGACAAAGGTAAACCTTTCAACAGCAAGGTCATCATTGCAACCACCAACT

TGTACTCGGGGTTCACTCCGAGGACCATGGTGTGCCCTGATGCACTGAACCGCAGGTTTCACTTTGACAT

TGACGTGAGTGCCAAAGACGGGTACAAAATTAACAACAAATTGGACATCATCAAAGCTCTTGAAGACACC

CACACCAACCCAGTGGCAATGTTTCAGTACGATTGTGCCCTTCTCAACGGCATGGCCGTTGAAATGAAGA

GAATGCAACAAGATATGTTCAAGCCTCAACCGCCCCTCCAGAACGTGTACCAGCTAGTTCAGGAGGTGAT

TGAACGGGTCGAACTCCACGAGAAAGTGTCGAGCCACCCGATTTTCAAGCAA

>FJ175662.1_O_ISR_2007

CCCTGGTACAAGCTCATCAAGCTCCTGAGCCGCCTGTCATGCATGGCCGCTGTAGCAGCACGGTCAAAGG

ACCCAGTCCTTGTGGCCATCATGCTAGCTGACACCGGTCTCGAGATTCTGGACAGCACCTTTGTCGTGAA

GAAAATCTCCGACTCGCTCTCTAGTCTCTTCCACGTGCCGGCCCCCGTCTTCAGTTTCGGAGCCCCGATT

TTGTTGGCCGGGTTGGTCAAGGTTGCCTCGAGTTTCTTCCGGTCCACGCCCGAAGACCTTGAGAGAGCAG

AAAAACAGCTCAAAGCACGTGACATCAATGACATATTCGCCATTCTTAAGAACGGCGAGTGGCTGGTCAA

GCTGATCCTTGCCATCCGCGACTGGATTAAGGCATGGATTGCCTCAGAAGAAAAGTTTGTCACCATGACG

GACCTAGTGCCTGGCATCCTCGAAAAGCAGCGGGACCTCAACGACCCAAGCAAGTACAAGGAGGCCAAGG

AGTGGCTCGACAACGCGCGCCAAGCGTGTCTGAAGAGCGGGAATGTCCACATTGCCAACCTTTGCAAAGT

GGTCGCCCCGGCACCCAGCAAGTCGAGACCCGAACCTGTGGTCGTTTGCCTCCGCGGCAAATCCGGCCAG

GGTAAGAGTTTCCTCGCGAACGTGCTCGCACAAGCAATCTCCACCCACTTCACTGGTAGAACTGATTCAG

TTTGGTACTGCCCGCCTGACCCTGACCACTTCGACGGCTACAACCAGCAGGCCGTTGTCGTGATGGACGA

TTTGGGCCAGAACCCTGATGGCAAGGACTTCAAGTACTTTGCCCAGATGGTGTCAACCACGGGGTTCATC

CCGCCAATGGCGTCACTCGAAGACAAAGGTAAACCTTTCAACAGCAAGGTCATCATTGCAACCACCAACT

TGTACTCGGGGTTCACTCCGAGGACCATGGTGTGCCCTGATGCACTGAACCGCAGGTTTCACTTTGACAT

TGACGTGAGTGCCAAAGACGGGTACAAAATTAACAACAAATTGGACATCGTCAAAGCTCTTGAAGACACC

CACACCAACCCAGTGGCAATGTTTCAGTACGATTGTGCCCTTCTCAACGGCATGGCCGTTGAAATGAAGA

GAATGCAACAAGATATGTTCAAGCCTCAACCGCCCCTCCAGAACGTGTACCAGCTAGTTCAGGAGGTGAT

TGAACGGGTCGAACTCCACGAGAAAGTGTCGAGCCACCCGATTTTCAAGCAA

>FJ175663.1_O_ISR_2007

CCCTGGTACAAGCTCATCAAGCTCCTGAGCCGCCTGTCATGCATGGCCGCTGTAGCAGCACGGTCAAAGG

ACCCAGTCCTTGTGGCCATCATGCTAGCTGACACCGGTCTCGAGATTCTGGACAGCACCTTTGTCGTGAA

GAAAATCTCCGACTCGCTCTCTAGTCTCTTCCACGTGCCGGCCCCCGTCTTCAGTTTCGGAGCCCCGATT

TTGTTGGCCGGGTTGGTCAAGGTTGCCTCGAGTTTCTTCCGGTCCACGCCCGAAGACCTTGAGAGAGCAG

AAAAACAGCTCAAAGCACGTGACATCAATGACATATTCGCCATTCTCAAGAACGGCGAGTGGCTGGTCAA

GCTGATCCTTGCCATCCGCGACTGGATTAAGGCATGGATTGCCTCAGAAGAAAAGTTTGTCACCATGACG

GACTTGGTGCCTGGCATCCTTGAAAAGCAGCGGGACCTCAACGACCCAAGCAAGTACAAGGAGGCCAAGG

AGTGGCTCGACAACGCGCGCCAAGCGTGTCTGAAGAGCGGGAATGTCCACATTGCCAACCTTTGCAAAGT

GGTCGCCCCGGCACCCAGCAAGTCGAGACCCGAACCTGTGGTCGTTTGCCTCCGCGGCAAATCCGGCCAG

GGTAAGAGTTTCCTCGCGAACGTGCTCGCACAAGCAATCTCCACCCACTTCACTGGCAGAACTGATTCAG

TTTGGTACTGTCCGCCTGACCCTGACCACTTCGACGGCTACAACCAGCAGGCCGTTGTCGTGATGGACGA

TTTGGGCCAGAACCCTGATGGCAAGGACTTCAAGTACTTTGCCCAGATGGTGTCAACCACAGGGTTCATC

CCGCCAATGGCGTCACTCGAAGACAAAGGTAAACCTTTCAACAGCAAGGTCATCATTGCAACCACCAACT

TGTACTCGGGGTTCACCCCGAGGACCATGGTGTGCCCTGATGCACTGAACCGCAGGTTTCACTTTGACAT

TGACGTGAGTGCCAAAGACGGGTACAAAATTAACAACAAATTGGACATCATCAAAGCTCTTGAAGACACC

CACACCAACCCAGTGGCAATGTTTCAGTATGATTGTGCCCTTCTCAACGGCATGGCCGTTGAAATGAAGA

GAATGCAACAAGATATGTTCAAGCCTCAACCGCCCCTCCAGAACGTGTACCAGCTAGTTCAGGAGGTGAT

TGAACGGGTCGAGCTCCACGAGAAAGTGTCGAGCCACCCGATTTTCAAGCAA

>FJ175664.1_O_ISR_2007

CCCTGGTACAAGCTCATCAAGCTCCTGAGCCGCCTGTCATGCATGGCCGCTGTAGCAGCACGGTCAAAGG

ACCCAGTCCTTGTGGCCATCATGCTAGCTGACACCGGTCTCGAGATTCTGGACAGCACCTTTGTCGTGAA

GAAAATCTCCGACTCGCTCTCTAGTCTCTTCCACGTGCCGGCCCCCGTCTTCAGTTTCGGAGCCCCGATT

TTGTTGGCCGGGTTGGTCAAGGTTGCCTCGAGTTTCTTCCGGTCCACGCCCGAAGACCTTGAGAGAGCAG

AAAAACAGCTCAAAGCACGTGACATCAATGACATATTCGCCATTCTCAAGAACGGCGAGTGGCTGGTCAA

GCTGATCCTTGCCATCCGCGACTGGATTAAGGCATGGATTGCCTCAGAAGAAAAGTTTGTCACCATGACG

GACTTGGTGCCTGGCATCCTTGAAAAGCAGCGGGACCTCAACGACCCAAGCAAGTACAAGGAGGCCAAGG

AGTGGCTCGACAACGCGCGCCAAGCGTGTCTGAAGAGCGGGAATGTCCACATTGCCAACCTTTGCAAAGT

GGTCGCCCCGGCACCCAGCAAGTCGAGACCCGAACCTGTGGTCGTTTGCCTCCGCGGCAAATCCGGCCAG

GGTAAGAGTTTCCTCGCGAACGTGCTCGCACAAGCAATCTCCACCCACTTCACTGGCAGAACTGATTCAG

TTTGGTACTGTCCGCCTGACCCTGACCACTTCGACGGCTACAACCAGCAGGCCGTTGTCGTGATGGACGA

TTTGGGCCAGAACCCTGATGGCAAGGACTTCAAGTACTTTGCCCAGATGGTGTCAACCACAGGGTTCATC

CCGCCAATGGCGTCACTCGAAGACAAAGGTAAACCTTTCAACAGCAAGGTCATCATTGCAACCACCAACT

TGTACTCGGGGTTCACCCCGAGGACCATGGTGTGCCCTGATGCACTGAACCGCAGGTTTCACTTTGACAT

TGACGTGAGTGCCAAAGACGGGTACAAAATTAACAACAAATTGGACATCATCAAAGCTCTTGAAGACACC

CACACCAACCCAGTGGCAATGTTTCAGTATGATTGTGCCCTTCTCAACGGCATGGCCGTTGAAATGAAGA

GAATGCAACAAGATATGTTCAAGCCTCAACCGCCCCTCCAGAACGTGTACCAGCTAGTTCAGGAGGTGAT

TGAACGGGTCGAGCTCCACGAGAAAGTGTCGAGCCACCCGATTTTCAAGCAA

>FJ175665.1_O_ISR_2007

CCCTGGTACAAGCTCATCAAGCTCCTGAGCCGCCTGTCATGCATGGCCGCTGTAGCAGCACGGTCAAAGG

ACCCAGTCCTTGTGGCCATCATGCTGGCTGACACCGGTCTCGAGATTCTGGACAGCACCTTTGTCGTGAA

GAAAATCTCCGACTCGCTCTCTAGTCTCTTCCACGTGCCGGCCCCCGTCTTCAGTTTCGGAGCCCCGATT

TTGTTGGCCGGGTTGGTCAAGGTTGCCTCGAGTTTCTTCCGGTCCACGCCCGAAGACCTTGAGAGAGCAG

AAAAACAGCTCAAAGCACGTGACATCAATGACATATTCGCCATTCTCAAGAACGGCGAGTGGCTGGTCAA

GCTGATCCTTGCCATCCGCGACTGGATTAAGGCATGGATTGCCTCAGAAGAAAAGTTTGTCACCATGACG

GACTTAGTACCTGGCATCCTTGAAAAGCAGCGGGACCTCGACGACCCAAGCAAGTACAAGGAGGCCAAGG

AGTGGCTCGACAACGCGCGCCAAGCGTGTCTGAAGAGCGGGAATGTCCACATTGCCAACCTTTGCAAAGT

GGTCGCCCCGGCACCCAGCAAGTCGAGACCCGAACCTGTGGTCGTTTGCCTCCGCGGCAAATCCGGCCAG

GGTAAGAGTTTCCTCGCGAACGTGCTCGCACAAGCAATCTCCACCCACTTCACTGGCAGAACTGATTCAG

TTTGGTACTGTCCGCCTGACCCTGACCACTTCGACGGCTACAACCAGCAGGCCGTTGTCGTGATGGACGA

TTTGGGCCAGAACCCTGATGGCAAGGACTTCAAGTACTTTGCCCAGATGGTGTCAACCACAGGGTTCATC

CCGCCAATGGCGTCACTCGAAGACAAAGGTAAACCTTTCAACAGCAAGGTCATCATTGCAACCACCAACT

TGTACTCGGGGTTCACTCCGAGGACCATGGTGTGCCCTGATGCACTGAACCGCAGGTTTCACTTTGACAT

TGACGTGAGTGCCAAAGACGGGTACAAAATTAACAACAAACTGGACATCATCAAAGCTCTTGAAGACACC

CACACCAACCCAGTGGCAATGTTTCAGTATGATTGTGCCCTTCTCAACGGCATGGCCGTTGAAATGAAGA

GAATGCAACAAGATATGTTCAAGCCTCAACCGCCCCTCCAGAACGTGTACCAGCTAGTTCAGGAGGTGAT

TGAACGGGTCGAGCTCCACGAGAAAGTGTCGAGCCACCCGATTTTCAAGCAA

>FJ175666.1_O_ISR_2007

CCCTGGTACAAGCTCATCAAGCTCCTGAGCCGCCTGTCATGCATGGCCGCTGTAGCAGCACGGTCAAAGG

ACCCAGTCCTTGTGGCCATCATGCTAGCTGACACCGGTCTCGAGATTCTGGACAGCACCTTTGTCGTGAA

GAAAATCTCCGACTCGCTCTCTAGTCTCTTCCACGTGCCGGCCCCCGTCTTCAGTTTCGGAGCCCCGATT

TTGTTGGCCGGGTTGGTCAAGGTTGCCTCGAGTCTCTTCCGGTCCACGCCCGAAGACCTTGAGAGAGCAG

AAAAACAGCTCAAAGCACGTGACATCAATGACATATTCGCCATTCTCAAGAACGGCGAGTGGCTGGTCAA

GCTGATCCTTGCCATCCGCGACTGGATTAAGGCATGGATTGCCTCAGAAGAAAAGTTTGTCACCATGACG

GACTTGGTGCCTGGCATCCTTGAAAAGCAGCGGGACCTCAACGACCCAAGCAAGTACAAGGAGGCCAAGG

AGTGGCTCGACAACGCGCGCCAAGCGTGTCTGAAGAGCGGGAATGTCCACATTGCCAACCTTTGCAAAGT

GGTCGCCCCGGCACCCAGCAAGTCGAGACCCGAACCTGTGGTCGTTTGCCTCCGCGGCAAATCCGGCCAG

GGTAAGAGTTTCCTCGCGAACGTGCTCGCACAAGCAATCTCCACCCACTTCACTGGCAGAACTGATTCAG

TTTGGTACTGTCCGCCTGACCCTGACCACTTCGACGGCTACAACCAGCAGGCCGTTGTCGTGATGGACGA

TTTGGGCCAGAACCCTGATGGCAAGGACTTCAAGTACTTTGCCCAGATGGTGTCAACCACAGGGTTCATC

CCGCCAATGGCGTCACTCGAAGACAAAGGTAAACCTTTCAACAGCAAGGTCATCATTGCAACCACCAACT

TGTACTCGGGGTTCACCCCGAGGACCATGGTGTGCCCTGATGCACTGAACCGCAGGTTTCACTTTGACAT

TGACGTGAGTGCCAAAGACGGGTACAAAATTAACAACAAATTGGACATCATCAAAGCTCTTGAAGACACC

CACACCAACCCAGTGGCAATGTTTCAGTATGATTGTGCCCTTCTCAACGGCATGGCCGTTGAAATGAAGA

GAATGCAACAAGATATGTTCAAGCCTCAACCGCCCCTCCAGAACGTGTACCAGCTAGTTCAGGAGGTGAT

TGAACGGGTCGAGCTCCACGAAAAAGTGTCGAGCCACCCGATTTTCAAGCAA

>FJ461344.1_O_UGA_2002

CCCTGGTACAAGCTCATCAAGCTCCTGAGCCGCTTGTCATGCATGGCCGCTGTAGCAGCACGGTCCAAGG

ACCCAGTCCTTGTGGCCATCATGCTGGCTGACACCGGCCTTGAGATTCTGGACAGCACGTTTGTCGTGAA

AAAGATCTCCGACTCGCTCTCCAGTCTCTTCCACGTGCCGGCCCCCGTCTTCAGTTTCGGAGCCCCGCTA

CTGTTGGCCGGGTTGGTCAAGGTCGCCTCGAGTTTCTTCCGGTCCACACCCGAAGACCTTGAGAGAGCTG

AAAAGCAGCTCAAAGCACGTGACATCAATGACATATTCGCCATTCTCAAGAACGGCGAGTGGCTGGTCAA

ATTGATCCTTGCCATCCGCGACTGGATTAAGGCATGGATCGCCTCAGAAGAAAAGTTTGTCACCATGACA

GACTTGGTGCCTGGCATTCTTGAAAAGCAGCGGGACCTCAACGACCCCAGCAAGTACAAGGAGGCCAAGG

AATGGCTTGACAACGCGCGCCAAGCGTGTTTGAAGAGCGGGAACGTCCACATTGCTAACCTTTGCAAAGT

GGTCGCCCCGGCACCCAGCAGGTCGAGACCCGAGCCCGTGGTCGTTTGCCTCCGTGGCAAGTCCGGCCAG

GGAAAGAGTTTCCTTGCGAACGTGCTCGCACAAGCAATCTCTACCCACTTCACAGGTAGAACAGACTCAG

TCTGGTACTGCCCACCTGACCCTGACCACTTCGACGGTTACAACCAACAGACCGTTGTTGTGATGGATGA

TTTGGGCCAGAACCCCGACGGCAAGGACTTCAAGTACTTTGCCCAGATGGTGTCAACCACGGGGTTTATC

CCGCCAATGGCCTCTCTCGAGGACAAGGGGAAACCTTTCAACAGCAAGGTTATCATTGCTACCACCAATC

TTTACTCGGGTTTCACCCCGAGAACTATGGTGTGTCCTGATGCATTGAACCGAAGGTTTCACTTTGACAT

TGACGTGAGCGCCAAAGACGGGTACAAAATCAACAACAAATTGGACATAATCAAGGCTCTTGAGGACACT

CACACCAACCCTGTGGCAATGTTTCAATACGACTGTGCCCTTCTCAACGGCATGGCCGTTGAAATGAAGA

GAATGCAACAAGACATATTTAAGCCTCAACTGCCCCTCCAGAACGTGTACCAACTTGTTCAGGAGGTGAT

TGAACGGGTGGAGCTCCACGAGAAAGTGTCGAGCCACCCAATTTTCAAACAA

>FJ461345.1_O_UGA_2002

CCCTGGTACAAACTCATCAAGCTCCTGAGCCGCTTGTCATGCATGGCCGCTGTAGCAGCACGGTCCAAGG

ACCCAGTCCTTGTGGCCATCATGCTGGCTGACACCGGCCTTGAGATTCTGGACAGCACGTTTGTCGTGAA

AAAGATCTCCGACTCGCTCTCCAGTCTCTTCCACGTGCCGGCCCCCGTCTTCAGTTTCGGAGCCCCGCTA

CTGTTGGCCGGATTGGTCAAGGTCGCCTCGAGTTTCTTCCGGTCCACACCCGAAGACCTTGAGAGAGCTG

AAAAGCAGCTCAAAGCACGTGACATCAATGACATATTCGCCATTCTCAAGAACGGCGAGTGGCTGGTCAA

ATTGATCCTTGCCATCCGCGACTGGATCAAGGCATGGATCGCCTCAGAAGAAAAGTTTGTCACCATGACA

GACTTGGTGCCTGGCATCCTTGAAAAGCAGCGGGACCTCAACGACCCCAGTAAGTACAAGGAGGCCAAGG

AATGGCTTGACAACGCGCGCCAAGCGTGTTTGAAGAGCGGGAACGTCCACATTGCTAACCTGTGCAAAGT

GGTCGCCCCGGCACCTAGCAGGTCGAGACCCGAGCCCGTGGTCGTTTGCCTCCGTGGCAAGTCTGGCCAG

GGAAAGAGTTTCCTTGCGAACGTGCTCGCACAAGCAATCTCCACCCACTTCACAGGCAGAACAGATTCAG

TCTGGTACTGCCCACCTGACCCTGACCACTTCGATGGTTACAACCAGCAGACCGTCGTTGTGATGGATGA

TTTGGGCCAGAACCCCGACGGCAAGGACTTCAAGTACTTCGCCCAGATGGTGTCAACCACGGGGTTCATC

CCGCCAATGGCCTCTCTCGAGGACAAAGGGAAACCTTTCAACAGCAAGGTTATCATTGCCACCACCAATC

TCTACTCGGGTTTCACCCCGAGAACTATGGTGTGCCCTGACGCATTGAACCGGAGGTTTCACTTTGACAT

TGACGTGAGCGCCAAAGACGGGTACAAAATTAATAACAAATTGGACATAATCAAAGCTCTTGAGGACACT

CACACCAACCCTGTGGCAATGTTTCAATACGACTGTGCCCTTCTCAACGGCATGGCCGTTGAAATGAAGA

GAATGCAGCAAGACATGTTCAAGCCTCAACCGCCCCTCCAGAACGTGTACCAACTTGTTCAGGAGGTGAT

TGACCGGGTGGAGCTCCACGAGAAAGTGTCGAGCCACCCAATTTTCAAACAA

>FJ461346.1_SAT2_UGA_2002

CCCTGGTACAAAGTCATCAAACTCCTCAGCCGCCTGTCGTGCATGGC-G-TGTAGCAGCACGGTCCAAGG

ATCCCGTCCTTGTGGCTATCATG-TAGCTGACACCGGTCTCGAAAT-CTGGACAGCACTTTTGTCGTGAA

GAAAATCGCCGACTCGCTCTCCAGTGTTTTCCACGTGCCGGCCCCCGTCTTCAGTTTCGG-GCCCCGATT

CTGTTGGCAGGGTTGGTCAA-GTCGCCTCGAGTTTCTTCCGGTCGACACCCGAAGAGCTTGAGAGAGCTG

AGAAACAGCTCAAGGCACGTGACATCAACGACATCTTCGCCATTCTCAAGAACGGCGAGTGGCTGGTCAA

ACTCATCCTGGCTATCCGCGACTGGATCAAAGCTTGGATCTCCTCAGAAGAGAAGTTCGTCACCATGACG

GACTTGGTGCCTGGTATCTTGGAGAAACAGCG-GATCTCAACGATCCCTCTAAGTACCAAGAAGCGAAGG

AATGGCTCGAGAACGCTCGCCAGGCGTGTCTCAAGAATGGAAACATCCACATTGCCAACCTTTGCAAAGT

GACAGCACCAGCACCGAGCAAGTCGAGACCCGAACCCGTGGT-GTTTGCCTCCGTGG-AAGTCCGGCCAG

GGCAAGAGTTTCCTTGCAAACGTGCTCGC-CAAGCTATCTCGACCCACTTCACCGGCAGAACCGACTCTG

TGTGGTACTGCCCACCTGACCCCGACCACTTCGATGGCTACAACCAACAAGCTGTCGTTGTCATGGATGA

TTTGGGCCAGAACCCTGACGGCAAGGACTTCAAGTACTT-GCCCAGATGGTTTCCACCACAGGGTTCATC

CCGCCCATGGCCTCACTCGAGGACAAAGGGAAACCTTTCAACAGCAAGGTCATCATTGCAACTTCCAAC-

TGTACTCCGGGTTCACCCCGCGTACTATGGTCTGCCCTGATGCACTGAACCGCAGGTTTCACTTTGACAT

TGA-GTGAGTGCCAAGGATGGGTACAAAGTTAACAACAAATTGGACATCATCAAAGCTCTTGAGGACACC

CACACCAACCCAGTGGCAATGTTCCAGTACGACTGTGCCCTTCTGAACGGCATGGCTGTTGAGATGAAGA

GACTGCAACAAGATGTGTTCAAGCCTCAACCACCAATCCTCAACGTGTACCAGCTTGTTGACGAGGTGAT

TGAGAGAGTCAACCTTCACGAGAAGGTTGCCTCGCAACCAATTTT-AAACAG

>FJ542365.1_O_UKG_2001

CCCTGGTACAAGCTCATCAAGCTCTTGAGCCGCCTGTCATGCATGGCCGCTGTAGCAGCACGGTCAAAGG

ACCCAGTCCTTGTGGCCATCATGCTGGCTGACACCGGCCTTGAGATTCTGGACAGTACCTTTGTCGTGAA

GAAGATCTCCGACTCGCTCTCCAGTCTCTTTCACGTGCCGGCCCCCGTCTTCAGTTTCGGAGCCCCGATT

TTGTTGGCCGGGTTGGTCAAAGTCGCCTCGAGTTTCTTCCGGTCCACGCCCGAAGACCTTGAGAGAGCGG

AGAAACAGCTCAAAGCACGTGACATCAATGACATATTCGCCATTCTCAAGAACGGCGAGTGGCTGGTCAA

GCTGATTCTTGCCATCCGCGACTGGATCAAGGCATGGATCGCCTCAGAAGAAAAGTTTGTCACCATGACA

GACCTGGTGCCTGGCATCCTTGAAAAGCAGCGGGATCTCAACGACCCAAGCAAGTACAAAGAGGCCAAGG

AGTGGCTCGACAACGCGCGCCAAGCGTGTTTAAAGAGCGGGAACATCCACATCGCAAACCTTTGCAAAGT

GGTTGCCCCAGCACCCAGCAGGTCGAGGCCCGAACCCGTGGTCGTTTGCCTCCGTGGCAAATCGGGCCAG

GGCAAGAGTTTCCTTGCGAACGTGCTTGCACAAGCAATTTCAACCCACTTCACTGGCAGAACCGACTCAG

TTTGGTACTGCCCACCTGACCCTGACCACTTCGACGGTTACAACCAGCAGACCGTTGTAGTAATGGATGA

TTTGGGCCAGAACCCCGACGGGAAGGACTTCAAGTACTTCGCCCAAATGGTTTCAACTACGGGGTTTATC

CCGCCCATGGCTTCACTCGAGGACAAAGGCAAACCTTTCAACAGCAAGGTCATCATCGCCACCACCAACC

TGTACTCGGGCTTCACCCCGAGAACTATGGTGTGCCCTGACGCACTGAACCGAAGGTTCCACTTTGACAT

TGACGTGAGCGCCAAGGACGGGTACAAAATTAACAACAAATTGGACATCATCAAAGCTCTTGAAGATACC

CACACCAACCCAGTGGCAATGTTTCAATACGACTGTGCCCTTCTCAACGGCATGGCCGTTGAAATGAAGA

GAATGCAACAAGATATGTTCAAGCCTCAACCGTCCCTCCAGAACGTCTACCAGCTTGTTCAGGAGGTGAT

TGACCGGGTCGAGCTCCACGAGAAGGTGTCGAGCCACCCGATTTTCAAGCAG

>FJ542368.1_O_UKG_2001

CCCTGGTACAAGCTCATCAAGCTCTTGAGCCGCCTGTCATGCATGGCCGCTGTAGCAGCACGGTCAAAGG

ACCCAGTCCTTGTGGCCATCATGCTGGCTGACACCGGCCTTGAGATTCTGGACAGTACCTTTGTCGTGAA

GAAGATCTCCGACTCGCTCTCCAGTCTCTTTCACGTGCCGGCCCCCGTCTTCAGTTTCGGAGCCCCGATT

TTGTTGGCCGGGTTGGTCAAAGTCGCCTCGAGTTTCTTCCGGTCCACGCCCGAAGACCTTGAGAGAGCGG

AGAAACAGCTCAAAGCACGTGACATCAATGACATATTCGCCATTCTCAAGAACGGCGAGTGGCTGGTCAA

GCTGATTCTTGCCATCCGCGACTGGATCAAGGCATGGATCGCCTCAGAAGAAAAGTTTGTCACCATGACA

GACCTGGTGCCTGGCATCCTTGAAAAGCAGCGGGATCTCAACGACCCAAGCAAGTACAAAGAGGCCAAGG

AGTGGCTCGACAACGCGCGCCAAGCGTGTTTGAAGAGCGGGAACATCCACATCGCAAACCTTTGCAAAGT

GGTTGCCCCAGCACCCAGCAGGTCGAGGCCCGAACCCGTGGTCGTTTGCCTCCGTGGCAAATCGGGCCAG

GGCAAGAGTTTCCTTGCGAACGTGCTTGCACAAGCAATTTCAACCCACTTCACTGGCAGAACCGACTCAG

TTTGGTACTGCCCACCTGACCCTGACCACTTCGACGGTTACAACCAGCAGACCGTTGTAGTAATGGATGA

TTTGGGCCAGAACCCCGACGGGAAGGACTTCAAGTACTTTGCCCAAATGGTTTCAACTACGGGGTTTATC

CCGCCCATGGCTTCACTCGAGGACAAAGGCAAACCTTTCAACAGCAAGGTCATCATCGCCACCACCAACC

TGTACTCGGGCTTCACCCCGAGAACTATGGTGTGCCCTGACGCACTGAACCGAAGGTTCCACTTTGACAT

TGACGTGAGCGCCAAGGACGGGTACAAAATTAACAACAAATTGGACATCATCAAAGCTCTTGAAGATACC

CACACCAACCCAGTGGCAATGTTTCAATACGACTGTGCCCTTCTCAACGGCATGGCCGTTGAAATGAAGA

GAATGCAACAAGATATGTTCAAGCCTCAACCGTCCCTCCAGAACGTCTACCAGCTTGTTCAGGAGGTGAT

TGACCGGGTCGAGCTCCACGAGAAGGTGTCGAGCCACCCGATTTTCAAGCAG

>FJ542369.1_O_UKG_2001

CCCTGGTACAAGCTCATCAAGCTCTTGAGCCGCCTGTCATGCATGGCCGCTGTAGCAGCACGGTCAAAGG

ACCCAGTCCTTGTGGCCATCATGCTGGCTGACACCGGCCTTGAGATTCTGGACAGTACCTTTGTCGTGAA

GAAGATCTCCGACTCGCTCTCCAGTCTCTTTCACGTGCCGGCCCCCGTCTTCAGTTTCGGAGCCCCGATT

TTGTTGGCCGGGTTGGTCAAAGTCGCCTCGAGTTTCTTCCGGTCCACGCCCGAAGACCTTGAGAGAGCGG

AGAAACAGCTCAAAGCACGTGACATCAATGACATATTCGCCATTCTCAAGAACGGCGAGTGGCTGGTCAA

GCTGATTCTTGCCATCCGCGACTGGATCAAGGCATGGATCGCCTCAGAAGAAAAGTTTGTCACCATGACA

GACCTGGTGCCTGGCATCCTTGAAAAGCAGCGGGATCTCAACGACCCAAGCAAGTACAAAGAGGCCAAGG

AGTGGCTCGACAACGCGCGCCAAGCGTGTTTAAAGAGCGGGAACATCCACATCGCAAACCTTTGCAAAGT

GGTTGCCCCAGCACCCAGCAGGTCGAGGCCCGAACCCGTGGTCGTTTGCCTCCGTGGCAAATCGGGCCAG

GGCAAGAGTTTCCTTGCGAACGTGCTTGCACAAGCAATTTCAACCCACTTCACTGGCAGAACCGACTCAG

TTTGGTACTGCCCACCTGACCCTGACCACTTCGACGGTTACAACCAGCAGACCGTTGTAGTAATGGATGA

TTTGGGCCAGAACCCCGACGGGAAGGACTTCAAGTACTTCGCCCAAATGGTTTCAACTACGGGGTTTATC

CCGCCCATGGCTTCACTCGAGGACAAAGGCAAACCTTTCAACAGCAAGGTCATCATCGCCACCACCAACC

TGTACTCGGGCTTCACCCCGAGAACTATGGTGTGCCCTGACGCACTGAACCGAAGGTTCCACTTTGACAT

TGACGTGAGCGCCAAGGACGGGTACAAAATTAACAACAAATTGGACATCATCAAAGCTCTTGAAGATACC

CACACCAACCCAGTGGCAATGTTTCAATACGACTGTGCCCTTCTCAACGGCATGGCCGTTGAAATGAAGA

GAATGCAACAAGATATGTTCAAGCCTCAACCGTCCCTCCAGAACGTCTACCAGCTTGTTCAGGAGGTGAT

TGACCGGGTCGAGCTCCACGAGAAGGTGTCGAGCCACCCGATTTTCAAGCAG

>FJ542370.1_O_UKG_2001

CCCTGGTACAAGCTCATCAAGCTCTTGAGCCGCCTGTCATGCATGGCCGCTGTAGCAGCACGGTCAAAGG

ACCCAGTCCTTGTGGCCATCATGCTGGCTGACACCGGCCTTGAGATTCTGGACAGTACCTTTGTCGTGAA

GAAGATCTCCGACTCGCTCTCCAGTCTCTTTCACGTGCCGGCCCCCGTCTTCAGTTTCGGAGCCCCGATT

TTGTTGGCCGGGTTGGTCAAAGTCGCCTCGAGTTTCTTCCGGTCCACACCCGAAGACCTTGAGAGAGCGG

AGAAACAGCTCAAAGCACGTGACATCAATGACATATTCGCCATTCTCAAGAACGGCGAGTGGCTGGTCAA

GCTGATTCTTGCCATCCGCGACTGGATCAAGGCATGGATCGCCTCAGAAGAAAAGTTTGTCACCATGACA

GACCTGGTGCCTGGCATCCTTGAAAAGCAGCGGGATCTCAACGACCCAAGCAAGTACAAAGAGGCCAAGG

AGTGGCTCGACAACGCGCGCCAAGCGTGTTTGAAGAGCGGGAACATCCACATCGCAAACCTTTGCAAAGT

GGTTGCCCCAGCACCCAGCAGGTCGAGGCCCGAACCCGTGGTCGTTTGCCTCCGTGGCAAATCGGGCCAG

GGCAAGAGTTTCCTTGCGAACGTGCTTGCACAAGCAATTTCAACCCACTTCACTGGCAGAACCGACTCAG

TTTGGTACTGCCCACCTGACCCTGACCACTTCGACGGTTACAACCAGCAGACCGTTGTAGTAATGGATGA

TTTGGGCCAGAACCCCGACGGGAAGGACTTCAAGTACTTCGCCCAAATGGTTTCAACTACGGGGTTTATC

CCGCCCATGGCTTCACTCGAGGACAAAGGCAAACCTTTCAACAGCAAGGTCATCATCGCCACCACCAACC

TGTACTCGGGCTTCACCCCGAGAACTATGGTGTGCCCTGACGCACTGAACCGAAGGTTCCACTTTGACAT

TGACGTGAGCGCCAAGGACGGGTACAAAATTAACAACAAATTGGACATCATCAAAGCTCTTGAAGATACC

CACACCAACCCAGTGGCAATGTTTCAATACGACTGTGCCCTTCTCAACGGCATGGCCGTTGAAATGAAGA

GAATGCAACAAGATATGTTCAAGCCTCAACCGCCCCTCCAGAACGTCTACCAGCTTGTTCAGGAGGTGAT

TGACCGGGTCGAGCTCCACGAGAAGGTGTCGAGCCACCCGATTTTCAAGCAG

>FJ542371.1_O_UKG_2001

CCCTGGTACAAGCTCATCAAGCTCTTGAGCCGCCTGTCATGCATGGCCGCTGTAGCAGCACGGTCAAAGG

ACCCAGTCCTTGTGGCCATCATGCTGGCTGACACCGGCCTTGAGATTCTGGACAGTACCTTTGTCGTGAA

GAAGATCTCCGACTCGCTCTCCAGTCTCTTTCACGTGCCGGCCCCCGTCTTCAGTTTCGGAGCCCCGATT

TTGTTGGCCGGGTTGGTCAAAGTCGCCTCGAGTTTCTTCCGGTCCACGCCCGAAGACCTTGAGAGAGCGG

AGAAACAGCTCAAAGCACGTGACATCAATGACATATTCGCCATTCTCAAGAACGGCGAGTGGCTGGTCAA

GCTGATTCTTGCCATCCGCGACTGGATCAAGGCATGGATCGCCTCAGAAGAAAAGTTTGTCACCATGACA

GACCTGGTGCCTGGCATCCTTGAAAAGCAGCGGGATCTCAACGACCCAAGCAAGTACAAAGAGGCCAAGG

AGTGGCTCGACAACGCGCGCCAAGCGTGTTTAAAGAGCGGGAACATCCACATCGCAAACCTTTGCAAAGT

GGTTGCCCCAGCACCCAGCAGGTCGAGGCCCGAACCCGTGGTCGTTTGCCTCCGTGGCAAATCGGGCCAG

GGCAAGAGTTTCCTTGCGAACGTGCTTGCACAAGCAATTTCAACCCACTTCACTGGCAGAACCGACTCAG

TTTGGTACTGCCCACCTGACCCTGACCACTTCGACGGTTACAACCAGCAGACCGTTGTAGTAATGGATGA

TTTGGGCCAGAACCCCGACGGGAAGGACTTCAAGTACTTCGCCCAAATGGTTTCAACTACGGGGTTTATC

CCGCCCATGGCTTCACTCGAGGACAAAGGCAAACCTTTCAACAGCAAGGTCATCATCGCCACCACCAACC

TGTACTCGGGCTTCACCCCGAGAACTATGGTGTGCCCTGACGCACTGAACCGAAGGTTCCACTTTGACAT

TGACGTGAGCGCCAAGGACGGGTACAAAATTAACAACAAATTGGACATCATCAAAGCTCTTGAAGATACC

CACACCAACCCAGTGGCAATGTTTCAATACGACTGTGCCCTTCTCAACGGCATGGCCGTTGAAATGAAGA

GAATGCAACAAGATATGTTCAAGCCTCAACCGTCCCTCCAGAACGTCTACCAGCTTGTTCAGGAGGTGAT

TGACCGGGTCGAGCTCCACGAGAAGGTGTCGAGCCACCCGATTTTCAAGCAG

>FJ542372.1_O_UKG_2001

CCCTGGTACAAGCTCATCAAGCTCTTGAGCCGCCTGTCATGCATGGCCGCTGTAGCAGCACGGTCAAAGG

ACCCAGTCCTTGTGGCCATCATGCTGGCTGACACCGGCCTTGAGATTCTGGACAGTACCTTTGTCGTGAA

GAAGATCTCCGACTCGCTCTCCAGTCTCTTTCACGTGCCGGCCCCCGTCTTCAGTTTCGGAGCCCCGATT

TTGTTGGCCGGGTTGGTCAAAGTCGCCTCGAGTTTCTTCCGGTCCACGCCCGAAGACCTTGAGAGAGCGG

AGAAACAGCTCAAAGCACGTGACATCAATGACATATTCGCCATTCTCAAGAACGGCGAGTGGCTGGTCAA

GCTGATTCTTGCCATCCGCGACTGGATCAAGGCATGGATCGCCTCAGAAGAAAAGTTTGTCACCATGACA

GACCTGGTGCCTGGCATCCTTGAAAAGCAGCGGGATCTCAACGACCCAAGCAAGTACAAAGAGGCCAAGG

AGTGGCTCGACAACGCGCGCCAAGCGTGTTTAAAGAGCGGGAACATCCACATCGCAAACCTTTGCAAAGT

GGTTGCCCCAGCACCCAGCAGGTCGAGGCCCGAACCCGTGGTCGTTTGCCTCCGTGGCAAATCGGGCCAG

GGCAAGAGTTTCCTTGCGAACGTGCTTGCACAAGCAATTTCAACCCACTTCACTGGCAGAACCGACTCAG

TTTGGTACTGCCCACCTGACCCTGACCACTTCGACGGTTACAACCAGCAGACCGTTGTAGTAATGGATGA

TTTGGGCCAGAACCCCGACGGGAAGGACTTCAAGTACTTCGCCCAAATGGTTTCAACTACGGGGTTTATC

CCGCCCATGGCTTCACTCGAGGACAAAGGCAAACCTTTCAACAGCAAGGTCATCATCGCCACCACCAACC

TGTACTCGGGCTTCACCCCGAGAACTATGGTGTGCCCTGACGCACTGAACCGAAGGTTCCACTTTGACAT

TGACGTGAGCGCCAAGGACGGGTACAAAATTAACAACAAATTGGACATCATCAAAGCTCTTGAAGATACC

CACACCAACCCAGTGGCAATGTTTCAATACGACTGTGCCCTTCTCAACGGCATGGCCGTTGAAATGAAGA

GAATGCAACAAGATATGTTCAAGCCTCAACCGTCCCTCCAGAACGTCTACCAGCTTGTTCAGGAGGTGAT

TGACCGGGTCGAGCTCCACGAGAAGGTGTCGAGCCACCCGATTTTCAAGCAG

>FJ623456.1_A_KAZ_1999

CCCTGGTACAAGCTCATCAAGCTCCTGAGCCGTTTGTCATGCATGGCCGCTGTAGCAGCACGGTCAAAGG

ACCCGGTCCTTGTGGCTATTATGCTGGCTGACACCGGCCTTGAGATTCTGGACAGTACTTTTGTCGTGAA

GAAGATCTCCGACTCACTCTCCAGTCTCTTTCACGTGCCGGCCCCCGTCTTCAGTTTCGGAGCCCCGATC

CTGCTGGCCGGGTTGGTCAAAGTCGCCTCGAGTTTCTTCCGGTCTACGCCCGAAGACCTTGAGAGAGCAG

AAAAACAGCTCAAAGCACGTGACATCAATGACATATTCGCCATTCTCAAGAACGGCGAGTGGTTGGTCAA

GCTGATTCTTGCTATCCGCGACTGGATTAAAGCATGGATCGCCTCAGAAGAAAAGTTTGTCACCATGACA

GACTTGGTGCCTGGCATTCTTGAAAAGCAGCGGGACCTCAACGACCCCAGCAAGTACAAGGAGGCCAAGG

AGTGGCTCGAAAGCGCGCGTCAAGCGTGCCTGAAGAATGGGAATGTCCACATTGCCAACCTGTGCAAAGT

GGTCACCCCAGCACCTAGCAAGTCGAGACCTGAACCTGTGGTCGTTTGCCTCCGGGGCAAATCCGGCCAG

GGAAAGAGTTTCCTTGCAAACGTGCTCGCACAGGCAATTTCAACACATTTTACTGGCAGAATTGATTCAG

TTTGGTACTGTCCACCTGACCCTGACCACTTCGACGGTTACAACCAACAGACCGTTGTTGTGATGGATGA

TTTGGGCCAGAACCCTGACGGCAAGGACTTCAAATACTTTGCCCAAATGGTTTCAACCACGGGGTTCATC

CCGCCCATGGCCTCGCTCGAAGACAAAGGAAAACCTTTCAACAGCAAAGTCATTATCACCACTACCAACT

TGTACTCGGGTTTCACCCCGAGAACCATGGTGTGTCCTGACGCGCTGAACCGGAGGTTCCACTTTGACAT

TGACGTGAGCGCCAAGGACGGGTACAAAGTTAACAACAAATTGGACATAACCAAAGCTCTTGAGGACACC

CACACAAATCCAGTGGCAATGTTTAAATACGATTGTGCCCTTCTTAACGGCATGGCCGTTGAGATGAAGA

GAATGCAGCAAGACATGTTTAAGCCTCAACCACCCCTCCAGAACGTGTACCAACTTGTTCAGGAGGTGAT

TGAACGGGTCGAGCTCCACGAGAAAGTGTCGAGCCACCAAATTTTTAAACAG

>FJ824812.1_C_SPA_2009

CCCTGGTACAAGCTTATTAAGCTCCTGAGCCGCTTGTCGTGCATGGCCGCTGTAGCAGCGCGGTCAAAGG

ACCCAGTCCTTGTGGCCATCATGCTGGCTGACACCGGTCTCGAGATTCTGGACAGCACGTTTGTCGTGAA

GAAGATCTCCGACTCGCTCTCCAGTCTCTTTCACGTGCCGGCCCCCGCCTTCAGCTTCGGAGCCCCGATT

CTGTTGGCCGGGTTGGTCAAGGTCGCCTCGAGTTTCTTCCGGTCCACACCCGAAGACCTTGAGAGAGCAG

AGAAACAACTCAAAGCACGTGACATCAACGACATCTTCGCCATTCTCAAGAACGGCGAGTGGCTGGTCAA

GCTGATCCTTGCCATCCGCGACTGGATTAAGGCATGGATCGCCTCAGAAGAGAAGTTTGTCACCATGACA

GACCTGGTGCCTGGTATCCTTGAAAAACAGCGGGACCTCAACGACCCAAGCAAGTACAAGGATGCCAAGG

AATGGCTCGACAACACGCGCCAAGTGTGTTTGAAGAACGGGAACGTCCATATTGCCAACCTTTGCAAAGT

GGTCGCCCCAGCACCCAGCAAGTCGAGACCCGAGCCCGTGGTCGTTTGCCTCCGCGGCAAGTCTGGCCAG

GGCAAGAGTTTCCTTGCGAACGTGCTTGCACAAGCAATCTCCACCCACCTCACCGGCAGGACCGATTCTG

TTTGGTACTGCCCGCCTGACCCTGACCACTTCGACGGTTACAACCAGCAGACCGTTGTTGTGATGGACGA

TTTGGGCCAGAACCCTGACGGCAAGGACTTCAAGTACTTCGCCCAAATGGTTTCAACCACGGGGTTCATC

CCGCCCATGGCATCACTTGAGGACAAAGGCAAACCCTTCAGCAGTAAGGTCATCATTGCAACCACCAACC

TGTACTCGGGCTTCACCCCGAAGACCATGGTGTGTCCTGATGCTCTGAACCGGAGGTTTCACTTTGACAT

CGACGTGAGCGCCAAGGACGGGTACAAAATCAACAACAAATTGGACATCATCAAAGCACTTGAAGACACC

CACGCCAATCCAGTGGCAATGTTTCACTACGACTGTGCCCTTCTCAACGGCATGGCTGTTGAAATGAAGA

GATTGCAACAGGATGTGTTCAAGCCTCAACCACCCCTCCAGAACGTGTACCAACTTGTTCAGGAGGTGAT

TGAGCGGGTCGAGCTTCACGAGAAAGTGTCAAGCCACCCAATTTTTAAGCAA

>FJ906802.1_Asia1_CHA_2006

CCCTGGTACAAACTCATCAAACTCCTGAGCCGCTTGTCATGCATGGCCGCTGTAGCAGCACGGTCAAAGG

ACCCAGTCCTTGTGGCCATCATGCTGGCTGACACCGGCCTTGAGATTCTGGACAGCACATTCGTCGTGAA

GAAGATCTCCGACTCACTCTCCAGTCTCTTTCACGTGCCGGCCCCCGTCTTCAGTTTCGGAGCCCCGGTT

CTGTTAGCCGGGTTGGTCAAAGTCGCCTCGAGTTTCTTCCGGTCCACGCCCGAAGACCTTGAGAGAGCAG

AGAAACAGCTCAAAGCACGTGACATCAATGACATCTTCGCCATTCTCAAGAACGGCGAGTGGCTGGTCAA

ATTGATTCTTGCCATCCGCGACTGGATTAAGGCATGGATCGCCTCAGAAGAAAAGTTTGTCACCATGACA

GACTTGGTGCCTGGCATCCTCGAAAAGCAGCGGGACCTCAACGGCCCAAGCAAGTATGAGGAAGCCAAGG

AGTGGCTCGACAACGCGCGTCAAGCGTGTCTGAAGAGCGGGAACGTCCACATTGCCAACCTGTGCAAAGT

GACCGCCCCGGCACCCAGCAAGTCGAGACCTGAACCCGTGGTCGTTTGCCTCCGAGGCAAATCCGGCCAG

GGAAAGAGTTTCCTTGCGAACGTGCTCGCACAAGCAATCTCCACACACTTCACTGGCAGAACTGATTCAG

TTTGGTACTGCCCGCCCGACCCTGACCACTTCGACGGTTACAACCAACAGACCGTTGTTGTGATGGATGA

TTTGGGCCAGAACCCCGACGGCAAGGACTTTAAGTACTTCGCCCAGATGGTTTCAACCACGGGGTTCATC

CCGCCCATGGCCTCGCTCGAAGACAAAGGCAAACCCTTCAACAGCAAGGTCATCATCGCCACCACCAACC

TGTACTCGGGCTTCACCCCGAGGACCATGGTGTGCCCTGATGCACTGAACCGGAGGTTTCACTTTGACAT

TGACGTGAGTGCCAAGGACGGGTACAAAATTAACAACAAGTTGGACATTATCAAAGCACTTGAAGACACC

CACACCAACCCGGTGGCAATGTTTCAGTACGACTGTGCCCTTCTCAACGGCATGGCCGTTGAAATGAAGA

GAATGCAACAAGACATGTTCAAGCCTCAACCGCCCCTCCAGAACGTGTACCAACTTGTTCAGGAGGTGAT

TGATCGGGTCAAGCTCCACGAGAAAGTGTCGAGCCACCCGATTTTCAAGCAG

>GQ406247.1_A_VIT_2009

CCCTGGTACAAGCTCATCAAACTCCTAAGCCGCTTGTCATGCATGGCCGCTGTAGCAGCACGGTCCAAGG

ACCCAGTCCTTGTGGCCATCATGCTGGCTGACACCGGCCTTGAGATTCTGGACAGCACGTTTGTCGTGAA

GAAGATCTCCGACTCGCTCTCCAGTCTCTTTCACGTGCCGGCCCCCGTCTTTAGTTTCGGAGCTCCGATC

CTATTGGCTGGGTTGGTCAAGGTCGCCTCGAGTTTCTTCCGGTCCACGCCCGAAGACCTTGAGAGAGCAG

AAAAACAGCTCAAAGCACGTGACATCAATGACATATTCGCCATTCTCAAGAACGGCGAGTGGCTGGTCAA

GCTGATCCTAGCTATCCGCGACTGGATTAAAGCATGGATCGCCTCAGAAGAAAAGTTTGTCACCATGACA

GACTTGGTGCCTGGTATCCTTGAAAAGCAGCGGGATCTCAACGACCCGAGCAAGTACAAGGCAGCCAAGG

AGTGGCTCGACAACGCGCGTCAAGCGTGCCTGAAGAGTGGGAACGTCCACATTGCCAACCTTTGCAAAGT

GGTCGCCCCAGCGCCAAGCAAGTCGAGACCCGAACCCGTGGTCGTTTGCCTCCGTGGCAAATCCGGCCAG

GGCAAGAGTTTCCTTGCGAACGTGCTCGCACAAGCAATTTCAACCCACTTCACTGGCAGAACCGATTCAG

TTTGGTACTGCCCACCTGACCCCGACCACTTCGACGGTTACAACCAACAAACCGTTGTTGTGATGGACGA

TTTGGGCCAGAACCCCGACGGCAAGGACTTTAAGTACTTCGCCCAGATGGTTTCAACCACGGGGTTCATC

CCGCCCATGGCCTCACTTGAGGACAAGGGGAAACCCTTCAACAGCAAAGTCATTATTACCACCACCAACC

TCTACTCGGGCTTCACCCCGAGAACCATGGTGTGTCCTGACGCGCTGAACCGGAGGTTCCACTTTGACAT

TGATGTGAGTGCCAAGGACGGGTACAAAATTAACAACAAATTGGACATAATCAAAGCACTTGAAGACACC

CACACCAACCCGGTGGCAATGTTCCAGTACGACTGCGCCCTCCTCAACGGCATGGCAGTCGAGATGAAGA

GATTGCAACAGGATGTGTTCACTCCCCAACCACCCATACAGAATGTTTATCAACTTGTTCAAGAGGTAAT

TGATCGGGTGGAGCTCCACGAGAAAGTGTCGAGCCACCCGATTTTCAAACAG

>GQ406248.1_A_VIT_2009

CCCTGGTACAAGCTCATCAAACTCCTAAGCCGCTTGTCATGCATGGCCGCTGTAGCAGCACGGTCCAAGG

ACCCAGTCCTTGTGGCCATCATGCTGGCTGACACCGGCCTTGAGATTCTGGACAGCACGTTTGTCGTGAA

GAAGATCTCCGACTCGCTCTCCAGTCTCTTTCACGTGCCGGCCCCCGTCTTTAGTTTCGGAGCTCCGATC

CTATTGGCTGGGTTGGTCAAGGTCGCCTCGAGTTTCTTCCGGTCCACGCCCGAAGACCTTGAGAGAGCAG

AAAAACAGCTCAAAGCACGTGACATCAATGACATATTCGCCATTCTCAAGAACGGCGAGTGGCTGGTCAA

GCTGATCCTAGCTATCCGCGACTGGATTAAAGCATGGATCGCCTCAGAAGAAAAGTTTGTCACCATGACA

GACTTGGTGCCTGGTATCCTTGAAAAGCAGCGGGATCTCAACGACCCGAGCAAGTACAAGGCAGCCAAGG

AGTGGCTCGACAACGCGCGTCAAGCGTGCCTGAAGAGTGGGAACGTCCACATTGCCAACCTTTGCAAAGT

GGTCGCCCCAGCGCCAAGCAAGTCGAGACCCGAACCCGTGGTCGTTTGCCTCCGTGGCAAATCCGGCCAG

GGCAAGAGTTTCCTTGCGAACGTGCTCGCACAAGCAATTTCAACCCACTTCACTGGCAGAACCGATTCAG

TTTGGTACTGCCCACCTGACCCCGACCACTTCGACGGTTACAACCAACAAACCGTTGTTGTGATGGACGA

TTTGGGCCAGAACCCCGACGGCAAGGACTTTAAGTACTTCGCCCAGATGGTTTCAACCACGGGGTTCATC

CCGCCCATGGCCTCACTTGAGGACAAGGGGAAACCCTTCAACAGCAAAGTCATTATTACCACCACCAACC

TCTACTCGGGCTTCACCCCGAGAACCATGGTGTGTCCTGACGCGCTGAACCGGAGGTTCCACTTTGACAT

TGATGTGAGTGCCAAGGACGGGTACAAAATTAACAACAAATTGGACATAATCAAAGCACTTGAAGACACC

CACACCAACCCGGTGGCAATGTTCCAGTACGACTGCGCCCTCCTCAACGGCATGGCAGTCGAGATGAAGA

GATTGCAACAGGATGTGTTCACTCCCCAACCACCCATACAGAATGTTTATCAACTTGTTCAAGAGGTAAT

TGATCGGGTGGAGCTCCACGAGAAAGTGTCGAGCCACCCGATTTTCAAACAG

>GQ406249.1_A_VIT_2009

CCCTGGTACAAGCTCATCAAACTCCTAAGCCGCTTGTCATGCATGGCCGCTGTAGCAGCACGGTCCAAGG

ACCCAGTCCTTGTGGCCATCATGCTGGCTGACACCGGCCTTGAGATTCTGGACAGCACGTTTGTCGTGAA

GAAGATCTCCGACTCGCTCTCCAGTCTCTTTCACGTGCCGGCCCCCGTCTTTAGTTTCGGAGCTCCGGTC

CTATTGGCTGGGTTGGTCAAGGTCGCCTCGAGTTTCTTCCGGTCCACGCCCGAAGACCTTGAGAGAGCAG

AAAAACAGCTCAAAGCACGTGACATCAATGACATATTCGCCATTCTCAAGAACGGCGAGTGGCTGGTCAA

GCTGATCCTAGCTATCCGCGACTGGATTAAAGCATGGATCGCCTCAGAAGAAAAGTTTGTCACCATGACA

GACTTGGTGCCTGGTATCCTTGAAAAGCAGCGGGATCTCAACGACCCGAGCAAGTACAAGGCAGCCAAGG

AGTGGCTCGACAACGCGCGTCAAGCGTGCTTGAAGAGCGGGAACGTCCACATTGCCAACCTTTGCAAAGT

GGTCGCCCCAGCGCCAAGCAAGTCGAGACCCGAACCCGTGGTCGTTTGCCTCCGTGGCAAATCCGGCCAG

GGCAAGAGTTTCCTTGCGAACGTGCTCGCACAAGCAATTTCAACCCACTTCACTGGCAGAACCGATTCAG

TTTGGTACTGCCCACCTGACCCCGACCACTTCGACGGTTACAACCAACAAACCGTTGTTGTGATGGACGA

TTTGGGCCAGAACCCCGACGGCAAGGACTTTAAGTACTTCGCCCAGATGGTTTCAACCACGGGGTTCATC

CCGCCCATGGCCTCACTTGAGGACAAGGGGAAACCCTTCAACAGCAAAGTCATCATTACCACCACCAACC

TCTACTCGGGCTTTACCCCGAGAACCATGGTGTGTCCTGACGCGCTGAACCGGAGGTTCCACTTTGACAT

TGATGTGAGTGCCAAGGACGGGTACAAAATTAACAACAAATTGGACATAATCAAAGCACTTGAAGACACC

CACACCAACCCGGTGGCAATGTTCCAGTACGACTGCGCCCTCCTCAACGGCATGGCAGTCGAGATGAAGA

GACTGCAACAGGATGTGTTCACTCCCCAACCACCCATACAGAATGTTTATCAACTTGTTCAAGAGGTGAT

TGATCGGGTGGAGCTCCACGAGAAAGTGTCGAGCCACCCGATTTTCAAACAG

>GQ406250.1_A_VIT_2009

CCCTGGTACAAGCTCATCAAACTCCTAAGCCGCTTGTCATGCATGGCCGCTGTAGCAGCACGGTCCAAGG

ACCCAGTCCTTGTGGCCATCATGCTGGCTGACACCGGCCTTGAGATTCTGGACAGCACGTTTGTCGTGAA

GAAGATCTCCGACTCGCTCTCCAGTCTCTTTCACGTGCCGGCCCCCGTCTTTAGTTTCGGAGCTCCGGTC

CTATTGGCTGGGTTGGTCAAGGTCGCCTCGAGTTTCTTCCGGTCCACGCCCGAAGACCTTGAGAGAGCAG

AAAAACAGCTCAAAGCACGTGACATCAATGACATATTCGCCATTCTCAAGAACGGCGAGTGGCTGGTCAA

GCTGATCCTAGCTATCCGCGACTGGATTAAAGCATGGATCGCCTCAGAAGAAAAGTTTGTCACCATGACA

GACTTGGTGCCTGGTATCCTTGAAAAGCAGCGGGATCTCAACGACCCGAGCAAGTACAAGGCAGCCAAGG

AGTGGCTCGACAACGCGCGCCAAGCGTGCTTGAAGAGCGGGAACGTCCACATTGCCAACCTTTGCAAAGT

GGTCGCCCCAGCGCCAAGCAAGTCGAGACCCGAACCCGTGGTCGTTTGCCTCCGTGGCAAATCCGGCCAG

GGCAAGAGTTTCCTTGCGAACGTGCTCGCACAAGCAATTTCAACCCACTTCACTGGCAGAACCGATTCAG

TTTGGTACTGCCCACCTGACCCCGACCACTTCGACGGTTACAACCAACAAACCGTTGTTGTGATGGACGA

TTTGGGCCAGAACCCCGACGGCAAGGACTTTAAGTACTTCGCCCAGATGGTTTCAACCACGGGGTTCATC

CCGCCCATGGCCTCACTTGAGGACAAGGGGAAACCCTTCAACAGCAAAGTCATCATTACCACCACCAACC

TCTACTCGGGCTTCACCCCGAGAACCATGGTGTGTCCTGACGCGCTGAACCGGAGGTTCCACTTTGACAT

TGATGTGAGTGCCAAGGACGGGTACAAAATTAACAACAAATTGGACATAATCAAAGCACTTGAAGACACC

CACACCAACCCGGTGGCAATGTTCCAGTACGACTGCGCCCTCCTCAACGGCACGGCAGTCGAGATGAAGA

GATTACAACAGGATGTGTTCACTCCCCAACCACCCATACAGAATGTTTATCAACTTGTTCAAGAGGTGAT

TGATCGGGTGGAGCTCCACGAGAAAGTGTCGAGCCACCCGATTTTCAAACAG

>GQ406251.1_A_VIT_2009

CCCTGGTACAAGCTCATCAAACTCCTAAGCCGCTTGTCATGCATGGCCGCTGTAGCAGCACGGTCCAAGG

ACCCAGTCCTTGTGGCCATCATGCTGGCTGACACCGGCCTTGAGATTCTGGACAGCACGTTTGTCGTGAA

GAAGATCTCCGACTCGCTCTCCAGTCTCTTTCACGTGCCGGCCCCCGTCTTTAGTTTCGGAGCTCCGATC

CTATTGGCTGGGTTGGTCAAGGTCGCCTCGAGTTTCTTCCGGTCCACGCCCGAAGACCTTGAGAGAGCAG

AAAAACAGCTCAAAGCACGTGACATCAATGACATATTCGCCATTCTCAAGAACGGCGAGTGGCTGGTCAA

GCTGATCCTAGCTATCCGCGACTGGATTAAAGCATGGATCGCCTCAGAAGAAAAGTTTGTCACCATGACA

GACTTGGTGCCTGGTATCCTTGAAAAGCAGCGGGATCTCAACGACCCGAGCAAGTACAAGGCAGCCAAGG

AGTGGCTCGACAACGCGCGTCAAGCGTGCTTGAAGAGCGGGAACGTCCACATTGCCAACCTTTGCAAAGT

GGTCGCCCCAGCGCCAAGCAAGTCGAGACCCGAACCCGTGGTCGTTTGCCTCCGTGGCAAATCCGGCCAG

GGCAAGAGTTTCCTTGCGAACGTGCTCGCACAAGCAATTTCAACCCACTTCACTGGCAGAACCGATTCAG

TTTGGTACTGCCCACCTGACCCCGACCACTTCGACGGTTACAACCAACAAACCGTTGTTGTGATGGACGA

TTTGGGCCAGAACCCCGACGGCAAGGACTTTAAGTACTTCGCCCAGATGGTTTCAACCACGGGGTTCATC

CCGCCCATGGCCTCACTTGAGGACAAGGGGAAACCCTTCAACAGCAAAGTCATCATTACCACCACCAACC

TCTACTCGGGCTTCACCCCGAGAACCATGGTGTGTCCTGACGCGCTGAACCGGAGGTTCCACTTTGACAT

TGATGTGAGTGCCAAGGACGGGTACAAAATTAACAACAAATTGGACATAATCAAAGCACTTGAAGACACC

CACACCAACCCGGTGGCAATGTTCCAGTACGACTGCGCCCTCCTCAACGGCATGGCAGTCGAGATGAAGA

GATTGCAACAGGATGTGTTCACTCCCCAACCACCCATACAGAATGTTTATCAACTTGTTCAAGAGGTGAT

TGATCGGGTGGAGCTCCACGAGAAAGTGTCGAGCCACCCGATTTTCAAACAG

>GQ406252.1_A_VIT_2009

CCCTGGTACAAGCTCATCAAACTCCTAAGCCGCTTGTCATGCATGGCCGCTGTAGCAGCACGGTCCAAGG

ACCCGGTCCTTGTGGCCATCATGCTGGCTGACACCGGCCTTGAGATTCTGGACAGCACGTTTGTCGTGAA

GAAGATCTCCGACTCGCTCTCCAGTCTCTTTCACGTGCCGGCCCCCGTCTTTAGTTTCGGAGCTCCGGTC

CTATTGGCTGGGTTGGTCAAGGTCGCCTCGAGTTTCTTCCGGTCCACGCCCGAAGACCTTGAGAGAGCAG

AAAAACAGCTCAAAGCACGTGACATCAATGACATATTCGCCATTCTCAAGAACGGCGAGTGGCTGGTCAA

GCTGATCCTAGCTATCCGCGACTGGATTAAAGCATGGATCGCCTCAGAAGAAAAGTTTGTCACCATGACA

GACTTGGTGCCTGGTATCCTTGAAAAGCAGCGGGATCTCAACGACCCGAGCAAGTACAAGGCAGCCAAGG

AGTGGCTCGACAACGCGCGTCAAGCGTGCTTGAAGAGCGGGAACGTCCACATTGCCAACCTTTGTAAAGT

GGTCGCCCCAGCGCCAAGCAAGTCGAGACCCGAACCCGTGGTCGTTTGCCTCCGTGGCAAATCCGGCCAG

GGCAAGAGTTTCCTTGCGAACGTGCTCGCACAAGCAATTTCAACCCACTTCACTGGCAGAACCGATTCAG

TTTGGTACTGCCCACCTGACCCCGACCACTTCGACGGTTACAACCAACAAACCGTTGTTGTGATGGACGA

TTTGGGCCAGAACCCCGACGGCAAGGACTTTAAGTACTTCGCCCAGATGGTTTCAACCACGGGGTTCATC

CCGCCCATGGCCTCACTTGAGGACAAGGGGAAACCCTTCAACAGCAAAGTCATCATTACCACCACCAACC

TCTACTCGGGCTTCACCCCGAGAACCATGGTGTGTCCTGACGCGCTGAACCGGAGGTTCCACTTTGACAT

TGATGTGAGTGCCAAGGACGGGTACAAAATTAACAACAAATTGGACATAATCAAAGCACTTGAAGACACC

CACACCAACCCGGTGGCAATGTTCCAGTACGACTGCGCCCTCCTCAACGGCATGGCAGTTGAGATGAAGA

GATTGCAACAGGATGTGTTCACTCCCCAACCACCCATACAGAATGTTTATCAACTTGTTCAAGAGGTGAT

TGATCGGGTGGAGCTCCACGAGAAAGTGTCGAGCCACCCGATTTTCAAACAG

>GQ452295.1_Asia1_VIT_2007

CCCTGGTACAAACTCATCAAACTCCTGAGCCGTTTGTCATGCATGGCCGCTGTAGCAGCACGGTCAAAGG

ACCCAGTCCTTGTGGCCATCATGCTGGCTGACACCGGCCTTGAGATTCTGGACAGCACATTCGTCGTGAA

GAAGATCTCCGACTCACTCTCCAGTCTCTTTCACGTGCCGGCCCCCGTCTTCAGTTTCGGAGCCCCGGTT

CTGTTGGCCGGGTTGGTCAAAGTCGCCTCGAGTTTCTTCCGGTCCACGCCCGAAGACCTTGAGAGAGCAG

AGAAACAGCTCAAAGCACGTGACATCAATGACATCTTCGCCATTCTCAAGAACGGCGAGTGGCTGGTCAA

ATTGATTCTTGCCATCCGCGACTGGATTAAGGCATGGATCGCCTCAGAAGAAAAGTTTGTCACCATGACA

GACTTGGTGCCTGGCATCCTAGAAAAGCAGCGGGACCTCAACGACCCGAGCAAGTACGAGGAAGCCAAGG

AGTGGCTCGACAACGCGCGTCAAGCGTGTCTGAAGAGCGGGAACGTCCACATTGCTAACCTGTGCAAAGT

GACCGCCCCGGCACCCAGCAAGTCGAGACCCGAACCCGTGGTCGTTTGCCTCCGAGGCAAATCCGGCCAG

GGAAAGAGTTTCCTTGCGAACGTGCTCGCACAAGCAATCTCCACACACTTCACTGGCAGAACTGATTCAG

TTTGGTACTGCCCGCCTGACCCTGACCACTTCGACGGTTACAACCAACAGACCGTTGTTGTGATGGATGA

TTTGGGCCAGAACCCCGACGGCAAGGACTTTAAGTACTTCGCCCAGATGGTTTCAACCACGGGGTTCATC

CCGCCCATGGCCTCGCTCGAAGACAAAGGCAAACCCTTCAACAGCAAGGTCATCATCGCCACCACCAACC

TGTACTCGGGCTTCACCCCGAGGACCATGGTGTGCCCTGATGCACTGAACCGGAGGTTTCACTTTGACAT

TGACGTGAGTGCCAAGGACGAGTACAAAATTAACAACAAGTTGGACATTATCAAAGCACTTGAAGACACC

CACACCAACCCGGTGGCAATGTTTCAGTACGACTGTGCCCTTCTCAACGGCATGGCCGTTGAAATGAAGA

GAATGCAACAAGACATGTTCAAGCCTCAACCGCCCCTCCAGAACGTGTACCAACTTGTTCAGGAGGTGAT

TGATCGGGTCGAGCTCCACGAGAAAGTGTCGAGCCACCCGATTTTCAAGCAG

>GU125645.1_Asia1_VIT_2007

CCCTGGTACAAACTCATCAAACTCCTGAGCCGTTTGTCATGCATGGCCGCTGTAGCAGCACGGTCAAAGG

ACCCAGTCCTTGTGGCCATCATGCTGGCTGACACCGGCCTTGAGATTCTGGACAGCACATTCGTCGTGAA

GAAGATCTCCGACTCACTCTCCAGTCTCTTTCACGTGCCGGCCCCCGTCTTCAGTTTCGGAGCCCCGGTT

CTGTTGGCCGGGTTGGTCAAAGTCGCCTCGAGTTTCTTCCGGTCCACGCCCGAAGACCTTGAGAGAGCAG

AGAAACAGCTCAAAGCACGTGACATCAATGACATCTTCGCCATTCTCAAGAACGGCGAGTGGCTGGTCAA

ATTGATTCTTGCCATCCGCGACTGGATTAAGGCATGGATCGCCTCAGAAGAAAAGTTTGTCACCATGACA

GACTTGGTGCCTGGCATCCTAGAAAAGCAGCGGGACCTCAACGACCCGAGCAAGTACGAGGAAGCCAAGG

AGTGGCTCGACAACGCGCGTCAAGCGTGTCTGAAGAGCGGGAACGTCCACATTGCTAACCTGTGCAAAGT

GACCGCCCCGGCACCCAGCAAGTCGAGACCCGAACCCGTGGTCGTTTGCCTCCGAGGCAAATCCGGCCAG

GGAAAGAGTTTCCTTGCGAACGTGCTCGCACAAGCAATCTCCACACACTTCACTGGCAGAACTGATTCAG

TTTGGTACTGCCCGCCTGACCCTGACCACTTCGACGGTTACAACCAACAGACCGTTGTTGTGATGGATGA

TTTGGGCCAGAACCCCGACGGCAAGGACTTTAAGTACTTCGCCCAGATGGTTTCAACCACGGGGTTCATC

CCGCCCATGGCCTCGCTCGAAGACAAAGGCAAACCCTTCAACAGCAAGGTCATCATCGCCACCACCAACC

TGTACTCGGGCTTCACCCCGAGGACCATGGTGTGCCCTGATGCACTGAACCGGAGGTTTCACTTTGACAT

TGACGTGAGTGCCAAGGACGAGTACAAAATTAACAACAAGTTGGACATTATCAAAGCACTTGAAGACACC

CACACCAACCCGGTGGCAATGTTTCAGTACGACTGTGCCCTTCTCAACGGCATGGCCGTTGAAATGAAGA

GAATGCAACAAGACATGTTCAAGCCTCAACCGCCCCTCCAGAACGTGTACCAACTTGTTCAGGAGGTGAT

TGATCGGGTCGAGCTCCACGAGAAAGTGTCGAGCCACCCGATTTTCAAGCAG

>GU125646.1_Asia1_VIT_2005

CCCTGGTACAAGCTTATCAAACTCCTAAGCCGCTTGTCATGCATGGCCGCTGTAGCAGCACGGTCAAAGG

ACCCAGTCCTTGTGGCCATCATGCTGGCTGACACCGGCCTTGAGATTCTGGACAGCACATTCGTCGTGAA

GAAGATCTCCGACTCACTCTCCAGTCTCTTTCACGTGCCGGCCCCCGTCTTCAGTTTCGGAGCCCCGGTT

CTGTTGGCCGGGTTGGTCAAAGTCGCCTCGAGTTTCTTCCGGTCCACGCCCGAAGACCTTGAGAGAGCAG

AGAAACAGCTCAAAGCACGTGACATCAATGACATCTTCGCCATTCTCAAGAACGGCGAGTGGCTGGTCAA

GCTGATCCTGGCTATCCGCGACTGGATTAAAGCATGGATCGCCTCGGAAGAGAAGTTTGTCACCATGACA

GACCTGGTGCCTGGTATCCTTGAAAAGCAACGGGACCTCAACGACCCAAGCAAGTACAAGGAAGCCAAGG

AATGGCTCGACAACGCGCGCCAAGCGTGTCTGAAGAGCGGAAACGTCCACATTGCCAACCTGTGCAAAGT

GGTCGCTCCGGCACCAAGCAAGTCGAGACCCGAGCCTGTGGTCGTTTGCCTCCGCGGCAAATCTGGCCAG

GGCAAGAGTTTCCTTGCGAACGTGCTCGCACAAGCAATCTCTTCCCACTTCACTGGCAGAACCGATTCAG

TTTGGTACTGCCCACCAGACCCTGACCACTTCGACGGTTACAACCAACAGACCGTTGTTGTGATGGATGA

TTTGGGCCAGAACCCCGACGGCAAGGACTTCAAGTACTTTGCCCAAATGGTCTCAACCACCGGGTTCATC

CCGCCCATGGCCTCACTTGAAGACAAGGGCAAACCTTTCAACAGCAAAGTCATCATCGCCACCACCAACC

TGTACTCGGGCTTCACCCCGAGGACCATGGTGTGCCCTGATGCACTGAACCGGAGGTTTCACTTTGACAT

TGACGTGAGTGCCAAGGACGAGTACAAAATTAACAACAAGTTGGACATTATCAAAGCTCTTGAGGACACG

CACACCAACCCTGTCGCGATGTTCCAATACGACTGTGCCCTTCTCAATGGCATGGCCGTTGAAATGAAGA

GAATGCAACAAGACATGTTCAAGCCTCAACCGCCCCTCCAGAACGTGTACCAGCTTGTTCAAGAGGTGAT

TGACCGGGTCGAGCTCCACGAGAAAGTGTCGAGCCACCCGATTTTCAAGCAG

>GU125647.1_O_VIT_2006

CCCTGGTACAAGCTCATCAAACTCCTAAGCCGCCTGTCATGTATGGCCGCTGTGGCAGCACGGTCCAAGG

ACCCAGTCCTTGTGGCCATCATGCTGGCCGACACCGGTCTTGAGATTCTGGACAGCACGTTTGTCGTGAA

GAAGATCTCCGACTCGCTCTCCAGTCTCTTTCACGTGCCGGCCCCCGTCTTCAGTTTCGGAGCCCCGATT

CTGCTGGCCGGGTTGGTCAAAGTCGCCTCGAGTTTCTTCCGGTCAACACCCGAAGACCTCGAGAGAGCAG

AAAAGCAGCTCAAAGCACGTGACATTAATGACATTTTCGCCATTCTCAAGAACGGCGAGTGGCTGGTCAA

GTTGATCCTAGCTATCCGCGACTGGATTAAAGCATGGATCGCCTCAGAAGAGAAGTTTGTCACCATGACA

GACTTAGTGCCTGGCATTCTTGAAAAGCAGCGGGACCTCAACGACCCGAGCAAGTACAAGGAAGCCAAGG

AATGGCTCGACAACGCGCGCCAAGCGTGTTTGAAGAGCGGGAACGTCCACATTGCCAACCTTTGCAAAGT

GGTCGCCCCGGCACCGAGCAAGTCGAGACCCGAGCCCGTGGTCGTTTGCCTCCGAGGCAAATCCGGTCAG

GGCAAGAGTTTCCTTGCGAACGTGCTTGCACAAGCAATCTCCACCCACTTCACTGGCAGAACCGATTCAG

TTTGGTACTGCCCGCCAGACCCTGACCACTTCGACGGTTACAACCAACAGACCGTTGTAGTGATGGATGA

TTTGGGCCAGAACCCTGATGGCAAGGACTTTAAGTACTTTGCCCAAATGGTTTCCACCACGGGGTTCATC

CCGCCCATGGCCTCGCTCGAAGACAAGGGAAAACCCTTCAACAGCAAAGTCATCATTGCCACCACCAACC

TGTACTCGGGTTTCACCCCGAGGACCATGGTGTGCCCAGACGCGCTGAACCGAAGGTTTCACTTTGACAT

CGACGTGAGTGCCAAGGATGGGTACAAAATTAACAACAAACTGGACATAATCAAAGCTCTTGAGGACACC

CACACCAACCCAGTGGCGATGTTCCAGTACGACTGCGCCCTTCTCAACGGCATGGCAGTCGAGATGAAGA

GAATGCAACAAGACATGTTCAAGCCCCAACCGCCTCTGCAGAACGTGTACCAGCTCGTTCAGGAGGTGAT

TGATCGGGTCGAGCTCCACGAGAAAGTGTCGAGCCACCCGATTTTCAAACAA

>GU125648.1_O_VIT_2006

CCCTGGTACAAGCTCATCAAACTCCTAAGCCGCCTGTCATGTATGGCCGCTGTGGCAGCACGGTCCAAGG

ACCCAGTCCTTGTGGCCATCATGCTGGCCGACACCGGTCTTGAGATTCTGGACAGCACGTTTGTCGTGAA

GAAGATCTCCGACTCGCTCTCCAGTCTCTTTCACGTGCCGGCCCCCGTCTTCAGTTTCGGAGCCCCGATT

CTGCTGGCCGGGTTGGTCAAAGTCGCCTCGAGTTTCTTCCGGTCAACACCCGAAGACCTCGAGAGAGCAG

AAAAGCAGCTCAAAGCACGTGACATTAATGACATTTTCGCCATTCTCAAGAACGGCGAGTGGCTGGTCAA

GTTGATCCTAGCTATCCGCGACTGGATTAAAGCATGGATCGCCTCAGAAGAGAAGTTTGTCACCATGACA

GACTTAGTGCCTGGCATTCTTGAAAAGCAGCGGGACCTCAACGACCCGAGCAAGTACAAGGAAGCCAAGG

AATGGCTCGACAACGCGCGCCAAGCGTGTTTGAAGAGCGGGAACGTCCACATTGCCAACCTTTGCAAAGT

GGTCGCCCCGGCACCGAGCAAGTCGAGACCCGAGCCCGTGGTCGTTTGCCTCCGAGGCAAATCCGGTCAG

GGCAAGAGTTTCCTTGCGAACGTGCTTGCACAAGCAATCTCCACCCACTTCACTGGCAGAACCGATTCAG

TTTGGTACTGCCCGCCAGACCCTGACCACTTCGACGGTTACAACCAACAGACCGTTGTAGTGATGGATGA

TTTGGGCCAGAACCCTGATGGCAAGGACTTTAAGTACTTTGCCCAAATGGTTTCCACCACGGGGTTCATC

CCGCCCATGGCCTCGCTCGAAGACAAGGGAAAACCCTTCAACAGCAAAGTCATCATTGCCACCACCAACC

TGTACTCGGGTTTCACCCCGAGGACCATGGTGTGCCCAGACGCGCTGAACCGAAGGTTTCACTTTGACAT

CGACGTGAGTGCCAAGGATGGGTACAAAATTAACAACAAACTGGACATAATCAAAGCTCTTGAGGACACC

CACACCAACCCAGTGGCGATGTTCCAGTACGACTGCGCCCTTCTCAACGGCATGGCAGTCGAGATGAAGA

GAATGCAACAAGACATGTTCAAGCCCCAACCGCCTCTGCAGAACGTGTACCAGCTCGTTCAGGAGGTGAT

TGATCGGGTCGAGCTCCACGAGAAAGTGTCGAGCCACCCGATTTTCAAACAA

>GU125649.1_O_VIT_2006

CCCTGGTACAAGCTCATCAAACTCCTAAGCCGCCTGTCATGTATGGCCGCTGTGGCAGCACGGTCCAAGG

ACCCAGTCCTTGTGGCCATCATGCTGGCCGACACCGGTCTTGAGATTCTGGACAGCACGTTTGTCGTGAA

GAAGATCTCCGACTCGCTCTCCAGTCTCTTTCACGTGCCGGCCCCCGTCTTCAGTTTCGGAGCCCCGATT

CTGCTGGCCGGGTTGGTCAAAGTCGCCTCGAGTTTCTTCCGGTCAACACCCGAAGACCTCGAGAGAGCAG

AAAAGCAGCTCAAAGCACGTGACATTAATGACATTTTCGCCATTCTCAAGAACGGCGAGTGGCTGGTCAA

GTTGATCCTAGCTATCCGCGACTGGATTAAAGCATGGATCGCCTCAGAAGAGAAGTTTGTCACCATGACA

GACTTAGTGCCTGGCATTCTTGAAAAGCAGCGGGACCTCAACGACCCGAGCAAGTACAAGGAAGCCAAGG

AATGGCTCGACAACGCGCGCCAAGCGTGTTTGAAGAGCGGGAACGTCCACATTGCCAACCTTTGCAAAGT

GGTCGCCCCGGCACCGAGCAAGTCGAGACCCGAGCCCGTGGTCGTTTGCCTCCGAGGCAAATCCGGTCAG

GGCAAGAGTTTCCTTGCGAACGTGCTTGCACAAGCAATCTCCACCCACTTCACTGGCAGAACCGATTCAG

TTTGGTACTGCCCGCCAGACCCTGACCACTTCGACGGTTACAACCAACAGACCGTTGTAGTGATGGATGA

TTTGGGCCAGAACCCTGATGGCAAGGACTTTAAGTACTTTGCCCAAATGGTTTCCACCACGGGGTTCATC

CCGCCCATGGCCTCGCTCGAAGACAAGGGAAAACCCTTCAACAGCAAAGTCATCATTGCCACCACCAACC

TGTACTCGGGTTTCACCCCGAGGACCATGGTGTGCCCAGACGCGCTGAACCGAAGGTTTCACTTTGACAT

CGACGTGAGTGCCAAGGATGGGTACAAAATTAACAACAAACTGGACATAATCAAAGCTCTTGAGGACACC

CACACCAACCCAGTGGCGATGTTCCAGTACGACTGCGCCCTTCTCAACGGCATGGCAGTCGAGATGAAGA

GAATGCAACAAGACATGTTCAAGCCCCAACCGCCTCTGCAGAACGTGTACCAGCTCGTTCAGGAGGTGAT

TGATCGGGTCGAGCTCCACGAGAAAGTGTCGAGCCACCCGATTTTCAAACAA

>GU125650.1_O_VIT_2006

CCCTGGTACAAGCTCATCAAACTCCTAAGCCGCCTGTCATGTATGGCCGCTGTAGCAGCACGGTCCAAGG

ACCCAGTCCTTGTGGCTATCATGCTGGCTGACACCGGCCTTGAGATTCTGGACAGTACGTTTGTCGTGAA

GAAGATCTCCGACTCACTCTCCAGTCTCTTTCACGTGCCGGCCCCCGTCTTCAGTTTCGGAGCCCCGATT

CTGCTGGCCGGGTTGGTCAAAGTCGCCTCGAGTTTCTTCCGGTCAACACCCGAAGACCTTGAGAGAGCGG

AAAAACAGCTCAAAGCACGTGACATCAATGACATTTTCGCCATTCTCAAGAACGGCGAGTGGCTGGTCAA

GCTGATCCTAGCTATCCGCGACTGGATTAAAGCATGGATCGCCTCAGAAGAGAAGTTTGTCACCATGACA

GACTTGGTGCCTGGCATCCTTGAAAAGCAGCGGGACCTCAACGACCCGAGCAAGTACAAGGAAGCCAAGG

AGTGGCTCGACAGCGCGCGCCAAGCGTGCTTGAAGAGTGGGAACGTTCACATCGCCAACCTTTGCAAAGT

GACCGCCCCGGCACCGAGCAGGTCGAGACCCGAGCCCGTGGTCGTTTGCCTCCGAGGCAAGTCCGGCCAG

GGCAAGAGTTTCCTTGCGAACGTGCTTGCACAAGCAATCTCCACCCACTTCACTGGCAGAACCGATTCAG

TTTGGTACTGCCCACCAGACCCTGACCACTTCGACGGTTACAACCAACAAACCGTTGTGGTGATGGACGA

TTTGGGCCAAAACCCCGATGGCAAAGACTTCAAGTACTTCGCCCAAATGGTTTCAACCACGGGGTTCATC

CCACCCATGGCCTCGCTCGAAGACAAGGGAAAACCTTTCAACAGCAAAGTCATCATTGCCACCACCAACC

TGTACTCGGGCTTCACCCCGAGGACTATGGTGTGCCCAGACGCGCTGAACCGAAGGTTTCACTTTGACAT

TGACGTGAGTGCCAAGGATGGGTACAAAATTAACAACAAATTGGACATTATCAAAGCTCTTGAGGACACC

CACACCAACCCAGTGGCGATGTTCCAATACGACTGCGCCCTTCTCAACGGCATGGCAGTCGAAATGAAGA

GAATGCAACAAGACACGTTCAAGCCCCAACCACCCCTGCAGAACGTGTACCAACTCGTTCAAGAGGTGAT

TGACCGGGTCGAGCTCCACGAAAAGGTGTCGAGCCACCCGATTTTCAAGCAG

>GU384682.1_O_PAK_2008

CCCTGGTACAAGCTCATCAAGCTCCTGAGTCGCCTGTCATGCATGGCCGCTGTAGCAGCACGGTCAAAGG

ACCCAGTCCTTGTGGCCATCATGCTAGCTGACACCGGTCTTGAGATTCTGGACAGCACCTTTGTCGTGAA

GAAAATCTCCGACTCGCTCTCCAGTCTCTTCCACGTGCCGGCCCCCGCCTTCAGTTTCGGAGCCCCGATT

CTGTTGGCCGGGTTGGTCAAGGTCGCCTCGAGTTTCTTCCGGTCCACACCCGAAGACCTTGAGAGAGCAG

AAAAACAGCTCAAGGCACGTGACGTCAATGACATATTCGCCATTCTCAAGAACGGCGAGTGGCTGGTCAA

GCTGATCCTTGCCATCCGCGACTGGATTAAGGCATGGATTGCCTCAGAAGAAAAGTTTGTCACCATGACG

GACTTGGTGCCTGGCATCCTTGAAAAGCAGCGGGACCTCAACGACCCAAGCAAGTACAAGGAGGCCAAGG

AGTGGCTCGACAACGCGCGCCAAGCGTGTCTGAAGAGCGGGAATGTCCACATTGCCAACCTTTGCAAGGT

GGTCGCCCCGGCACCCAGCAAGTCGAGACCCGAACCTGTGGTCATTTGCCTCCGTGGCAAATCCGGCCAG

GGTAAGAGTTTCCTTGCGAACGTGCTTGCACAAGCAATCTCCACCCACTTCACTGGCAGAACTGATTCAG

TTTGGTACTGCCCGCCTGACCCTGACCACTTCGACGGTTACAACCAACAGACCGTTGTCGTGATGGACGA

TTTGGGCCAGAACCCTGATGGCAAGGACTTCAAGTACTTTGCCCAGATGGTGTCAACCACAGGGTTCATC

CCGCCAATGGCGTCACTCGAAGACAAAGGTAAACCTTTCAACAGCAAGGTCATCATTGCAACCACCAACC

TGTACTCGGGGTTCACTCCGAGGACCATGGTGTGCCCTGATGCACTGAACCGTAGGTTTCACTTTGACAT

TGACGTGAGTGCCAAAGACGGGTACAAAGTTAACAACAAACTGGACATCATCAAAGCTCTTGAAGACACC

CACACCAAACCAGTGGCAATGTTTCAGTACGATTGTGCCCTTCTCAACGGCATGGCCGTTGAAATGAAGA

GAATGCAACAAGATGTGTTCAAGCCTCAACCACCCCTCCAGAACGTGTACCAGCTAGTTCAGGAGGTGAT

TGAACGGGTCGATCTCCACGAGAAAGTGTCGAGCCACCCGATTTTTAAACAA

>GU384683.1_O_PAK_2008

CCCTGGTACAAGCTCATCAAGCTCCTGAGTCGCCTGTCATGCATGGCCGCTGTAGCAGCACGGTCAAAGG

ACCCAGTCCTTGTGGCCATCATGCTAGCTGACACCGGTCTTGAGATTCTGGACAGCACCTTTGTCGTGAA

GAAAATCTCCGACTCGCTCTCCAGTCTCTTCCACGTGCCGGCCCCCGCCTTCAGTTTCGGAGCCCCGATT

CTGTTGGCCGGGTTGGTCAAGGTCGCCTCGAGTTTCTTCCGGTCCACACCCGAAGACCTTGAGAGAGCAG

AAAAACAGCTCAAGGCACGTGACGTCAATGACATATTCGCCATTCTCAAGAACGGCGAGTGGCTGGTCAA

GCTGATCCTTGCCATCCGCGACTGGATTAAGGCATGGATTGCCTCAGAAGAAAAGTTTGTCACCATGACG

GACTTGGTGCCTGGCATCCTTGAAAAGCAGCGGGACCTCAACGACCCAAGCAAGTACAAGGAGGCCAAGG

AGTGGCTCGACAACGCGCGCCAAGCGTGTCTGAAGAGCGGGAATGTCCACATTGCCAACCTTTGCAAGGT

GGTCGCCCCGGCACCCAGCAAGTCGAGACCCGAACCTGTGGTCATTTGCCTCCGTGGCAAATCCGGCCAG

GGTAAGAGTTTCCTTGCGAACGTGCTTGCACAAGCAATCTCCACCCACTTCACTGGCAGAACTGATTCAG

TTTGGTACTGCCCGCCTGACCCTGACCACTTCGACGGTTACAACCAACAGACCGTTGTCGTGATGGACGA

TTTGGGCCAGAACCCTGATGGCAAGGACTTCAAGTACTTTGCCCAGATGGTGTCAACCACAGGGTTCATC

CCGCCAATGGCGTCACTCGAAGACAAAGGTAAACCTTTCAACAGCAAGGTCATCATTGCAACCACCAACC

TGTACTCGGGGTTCACTCCGAGGACCATGGTGTGCCCTGATGCACTGAACCGTAGGTTTCACTTTGACAT

TGACGTGAGTGCCAAAGACGGGTACAAAGTTAACAACAAACTGGACATCATCAAAGCTCTTGAAGACACC

CACACCAAACCAGTGGCAATGTTTCAGTACGATTGTGCCCTTCTCAACGGCATGGCCGTTGAAATGAAGA

GAATGCAACAAGATGTGTTCAAGCCTCAACCACCCCTCCAGAACGTGTACCAGCTAGTTCAGGAGGTGAT

TGAACGGGTCGATCTCCACGAGAAAGTGTCGAGCCACCCGATTTTTAAACAA

>GU582115.1_O_VIT_2009

CCCTGGTACAAACTCATCAAACTTCTGAGCCGTTTGTCATGTATGGCCGCTGTAGCAGCACGGTCCAAGG

ACCCAGTCCTTGTGGCCATCATGCTGGCTGACACCGGCCTTGAGATTCTGGACAGTACGTTCGTCGTGAA

AAAGATCTCCGACTCACTCTCCAGTCTCTTTCACGTGCCGGCCCCCGTTTTCAGTTTCGGAGCCCCGATT

CTGTTGGCCGGGTTGGTCAAGGTCGCCTCAGGTTTCTTCCGGTCAACACCCGAAGATCTTGAGAGAGCAG

AAAAACAGCTCAAAGCACGTGACATCAATGACATTTTCGCCATTCTCAAGAACGGCGAGTGGCTGGTCAA

GCTGATCCTAGCTATCCGCGACTGGATTAAAGCATGGATTGCCTCAGAGGAGAAGTTTGTCACCATGACA

GACTTGGTGCCTGGCATTCTTGAAAAGCAGCGGGACCTTAACGACCCGAGCAAGTACAAGGAAGCCAAGG

AGTGGCTCGACAACGCGCGCCAAGCGTGCTTGAAGAGCGGTAACGTCCACATTGCCAACCTCTGCAAAGT

GGTTGCCCCGGCACCGAGCAAGTCGAGACCCGAGCCCGTGGTCGTTTGCCTCCGAGGCAAATCCGGCCAG

GGCAAGAGTTTCCTTGCGAACGTGCTTGCGCAAGCAATTTCCACCCACTTCACTGGCAGAACCGATTCAG

TTTGGTACTGCCCACCAGACCCTGACCACTTTGACGGTTACAACCAGCAAACTGTTGTAGTGATGGATGA

TTTGGGCCAGAACCCTGATGGCAAGGACTTTAAGTACTTTGCCCAAATGGTGTCAACAACAGGGTTCATC

CCGCCCATGGCCTCGCTCGAAGACAAAGGGAAACCCTTCAACAGCAAAGTCATTATCGCCACCACCAACT

TGTACTCGGGTTTCACCCCGAGGACCATGGTGTGTCCAGACGCGCTGAACCGAAGGTTCCACTTTGACAT

TGATGTGAGTGCCAAGGATGGGTACAAAATTAACAACAAATTGGACATAATCAAAGCTCTTGAGGACACC

CACACCAACCCAGTGGCGATGTTCCAATACGACTGCGCCCTTCTCAACGGCATGGCAGTCGAAATGAAGA

GAATGCAACAAGACATGTTCAAGCCCCAACCGCCTCTTCAGAATGTGTATCAACTCGTTCAAGAGGTGAT

TGACCGGGTCGAGCTCCACGAGAAAGTGTCGAGCCACCCGATTTTTAAGCAA

>GU582116.1_O_VIT_2009

CCCTGGTACAAACTCATCAAACTTCTGAGCCGCTTGTCATGTATGGCCGCTGTAGCAGCACGGTCCAAGG

ACCCAGTCCTTGTGGCCATCATGCTGGCCGACACCGGCCTTGAGATTCTGGACAGTACGTTTGTCGTGAA

AAAGATCTCCGACTCACTCTCCAGTCTCTTTCACGTGCCGGCCCCCGTTTTCAGTTTCGGAGCCCCGATT

CTGTTGGCCGGGTTGGTCAAGGTCGCCTCAGGTTTCTTCCGGTCAACACCCGAAGATCTTGAGAGAGCAG

AAAAACAGCTCAAAGCACGTGACATCAATGACATTTTCGCCATTCTCAAGAACGGCGAGTGGCTGGTCAA

GCTGATCCTAGCTATCCGCGACTGGATTAAAGCATGGATTGCCTCAGAGGAGAAGTTTGTCACCATGACA

GACTTGGTGCCTGGCATTCTTGAAAAGCAGCGGGACCTTAACGACCCGAGCAAGTACAAGGAAGCCAAGG

AGTGGCTCGACAACGCGCGCCAAGCGTGCCTGAAGAGCGGTAACGTCCACATTGCCAACCTCTGCAAAGT

GGTTGCCCCGGCACCGAGCAAGTCGAGACCCGAGCCCGTGGTCGTTTGCCTCCGAGGCAAATCCGGCCAG

GGCAAGAGTTTCCTTGCGAACGTGCTTGCGCAAGCAATTTCCACCCACTTCACTGGCAGAACCGATTCAG

TTTGGTACTGCCCACCAGACCCTGACCACTTTGACGGTTACAACCAGCAAACTGTTGTAGTGATGGATGA

TTTGGGCCAGAACCCTGATGGCAAGGACTTTAAGTACTTTGCCCAAATGGTGTCAACAACAGGGTTCATC

CCACCCATGGCCTCGCTCGAAGACAAAGGGAAACCCTTCAACAGCAAAGTTATTATCGCCACCACCAACT

TGTACTCGGGTTTCACCCCGAGGACCATGGTGTGTCCAGACGCGCTGAACCGAAGGTTCCACTTTGACAT

TGATGTGAGTGCCAAGGATGGGTACAAAATTAACAACAAATTGGACATAATCAAAGCTCTTGAGGACACC

CACACCAACCCAGTGGCGATGTTCCAATACGACTGCGCCCTTCTCAACGGCATGGCAGTCGAAATGAAGA

GAATGCAACAAGACATGTTCAAGCCCCAACCGCCTCTTCAGAATGTGTATCAACTCGTTCAAGAGGTGAT

TGACCGGGTCGAGCTCCACGAGAAAGTGTCGAGCCACCCGATTTTTAAGCAA

>GU931682.1_Asia1_CHA_2005

CCCTGGTACAAACTCATCAAACTCCTGAGCCGCTTGTCATGCATGGCCGCTGTAGCAGCACGGTCAAAGG

ACCCAGTCCTTGTGGCCATCATGCTGGCTGACACCGGCCTTGAGATTCTGGACAGCACATTCGTCGTGAA

GAAGATCTCCGACTCACTCTCCAGTCTCTTTCACGTGCCGGCCCCCGTCTTCAGTTTCGGAGCCCCGGTT

CTGTTGGCCGGGTTGGTCAAAGTCGCCTCGAGTTTCTTCCGGTCCACGCCCGAAGACCTTGAGAGAGCAG

AGAAACAGCTCAAAGCACGTGACATCAATGACATCTTCGCCATTCTCAAGAACGGCGAGTGGTTGGTCAA

ATTGATTCTTGCCATCCGCGACTGGATTAAGGCATGGATCGCCTCAGAAGAAAAGTTTGTCACCATGACA

GACTTGGTGCCTGGCATCCTCGAAAAGCAGCGGGACCTCAACGACCCGAGCAAGTACGAGGAAGCCAAGG

AGTGGCTCGACAACGCGCGTCAAGCGTGTCTGAAGAGCGGGAACGTCCACATTGCCAACCTGTGCAAGGT

GGTCGCCCCGGCACCCAGCAAGTCGAGACCCGAACCCGTGGTCGTTTGCCTCCGAGGCAAATCCGGCCAG

GGAAAGAGTTTCCTTGCGAACGTGCTCGCACAAGCAATCTCCACACACTTCACTGGCAGAACTGATTCAG

TTTGGTACTGCCCGCCTGACCCTGACCACTTCGACGGTTACAACCAACAGACCGTTGTTGTGATGGATGA

TTTGGGCCAGAACCCCGACGGCAAGGACTTTAAGTACTTCGCCCAGATGGTTTCAACCACGGGGTTCATC

CCGCCCATGGCCTCGCTCGAAGACAAAGGCAAACCTTTCAACAGCAAGGTCATCATCGCCACCACCAACC

TGTACTCGGGCTTCACCCCGAGGACCATGGTGTGCCCTGATGCACTGAACCGGAGGTTTCACTTTGACAT

TGACGTGAGTGCCAAGGACGGGTACAAAATTAACAACAAGTTGGACATTATCAAAGCACTTGAAGACACC

CACACCAACCCGGTGGCAATGTTTCAGTACGATTGTGCCCTTCTCAACGGCATGGCCGTTGAAATGAAGA

GAATGCAACAAGACATGTTCAAGCCTCAACCGCCCCTCCAGAACGTGTACCAACTTGTTCAGGAGGTGAT

TGATCGGGTCGAGCTCCACGAGAAAGTGTCGAGCCACCCGATTTTCAAGCAG

>HM008917.1_O_CHA_2005

CCCTGGTACAAGCTCATCAAGCTACTGAGCCGCCTGTCATGCATGGCCGCTGTAGCAGCACGGTCAAAGG

ACCCAGTCCTTGTGGCCATCATGCTGGCTGACACCGGTCTCGAGATCCTGGACAGTACCTTTGTCGTGAA

GAAGATCTCCGACTCGCTCTCCAGTCTCTTTCACGTGCCGGCCCCCGTCTTCAGTTTCGGAGCCCCGATT

TTGTTGGCCGGGTTGGTCAAGGTCGCCTCGAGTTTCTTCCGGTCCACGCCCGAAGACCTTGAGAGAGCGG

AGAAACAGCTCAAAGCACGTGACATCAATGACATATTCGCCATTCTCAAGAACGGCGAGTGGCTGGTCAA

GCTGATCCTTGCCATCCGCGACTGGATCAAGGCATGGATCGCCTCAGAGGAAAAGTTTGTCACCATGACA

GACCTGGTGCCCGGTATCCTTGAAAAGCAGCGGGATCTCAACGACCCAAGCAAGTACGAGGAGGCCAAGG

AGTGGCTCGACAACGCGCGCCAAGCGTGTTTGAAGAGCGGGAACATCCACATCGCAAACCTTTGCAAAGT

GGCTGCCCCAGCACCCAGCAGGTCGAGGCCCGAACCCGTGGTCGTTTGCCTTCGTGGCAAATCAGGCCAG

GGCAAGAGTTTCCTTGCGAACGTGCTTGCACAAGCAATTTCAACCCACTTCACTGGCAGAACCGATTCAG

TTTGGTACTGCCCACCTGACCCTGACCACTTCGACGGTTACAACCAGCAGACCGTTGTAGTAATGGATGA

CCTGGGCCAGAACCCCGACGGGAAGGACTTTAAGTACTTCGCCCAAATGGTTTCAACTACGGGGTTTATC

CCGCCCATGGCTTCACTTGAGGACAAAGGCAAACCTTTCAACAGCAAGGTCATCATCGCCACCACCAACC

TGTACTCGGGCTTCACCCCGAGAACTATGGTGTGCCCTGATGCGCTGAACCGGAGGTTCCACTTTGACAT

CGACGTGAGCGCTAAGGACGGATACAAAATTAACAACAAATTGGACATCATCAAAGCTCTTGAAGACACC

CACACCAACCCAGTGGCAATGTTTCAATACGACTGTGCCCTTCTCAACGGCATGGCCGTTGAAATGAAGA

GAATGCAACAAGACATGTTCAAGCCTCAACCGCCCCTCCAGAACGTCTACCAGCTTGTTCAGGAGGTGAT

TGACCGGGTCGAGCTCCACGAAAAGGTGTCGAACCACCCGATCTTCAAGCAG

>HM055510.1_O_VIT_2009

CCCTGGTACAAGCTCATCAAGCTCCTGAGCCGCTTGTCATGCATGGCCGCTGTAGCAGCACGGTCCAAGG

ACCCAGTCCTTGTGGCCATCATGCTGGCTGACACCGGTCTTGAGATTCTGGACAGCACATTTGTCGTGAA

GAAAATCTCCGACTCCCTCTCCAGTCTCTTTCACGTGCCGGCCCCCGTCTTCAGTTTCGGAGCTCCGATT

CTGCTAGCCGGGTTGGTCAAGGTCGCCTCGAGCTTCTTCCGGTCCACACCCGAGGATCTCGAGAGAGCAG

AGAAACAGCTCAAAGCACGTGACATCAATGACATCTTCGCCATTCTTAAGAACGGCGAGTGGCTGGTCAA

GTTGATCCTAGCCATCCGCGACTGGATTAAAGCATGGATCGCCTCAGAAGAGAAGTTTGTCACCATGACA

GACTTGGTGCCTGGCATCCTTGAAAAGCAGCGGGACCTTAACGACCCGGCCAAGTACAAGGAAGCCAAGG

AATGGCTCGACAACGCGCGCCAAACGTGTTTGAAGAGCGGGAACGTCCACATTGCCAACCTGTGCAAAGT

GGTCGCCCCAGCGCCGAGCAAGTCGAGACCTGAACCCGTGGTCGTTTGCCTCCGCGGCAAATCCGGCCAG

GGTAAGAGTTTCCTTGCGAACGTGCTGGCACAAGCCATCTCTACCCACTTTACCGGCAGGACTGATTCAG

TTTGGTACTGTCCGCCAGACCCTGACCACTTCGACGGTTACAACCAGCAGACCGTTGTTGTGATGGATGA

TTTGGGCCAGAATCCCGACGGCAAGGACTTCAAGTACTTCGCCCAGATGGTCTCGACCACGGGGTTCATC

CCGCCCATGGCTTCACTTGAGGACAAAGGCAAGCCTTTCAACAGCAAAGTCATCATTGCCACCACCAACT

TGTACTCGGGCTTCACCCCGAGAACCATGGTGTGCCCCGATGCGCTGAACCGAAGGTTTCACTTTGACAT

TGACGTGAGTGCCAAGGACGGGTACAAAATTAACAACAAATTGGACATAATCAAAGCTCTCGAGGACACC

CACACCAACCCTGTGGCAATGTTCCAATACGACTGTGCCCTTCTCAACGGCATGGCCGTTGAAATGAAGA

GAATGCAACAAGACATGTTCAAACCCCAACCGCCTTTGCAGAACATATACCAACTTGTGCAAGAGGTGAT

TGACCGGGTCGAGCTCCACGAGAAAGTGTCGAGCCACCCGATTTTCAAGCAG

>HM067704.1_SAT2_UGA_2007

CCTTGGTACAAGGTCATCAAGCTCCTCAGCCGCTTGTCATGCATGGCCGCTGTAGCAGCACGCTCAAAGG

ATCCAGTCCTCGTAGCAATAATGCTGGCTGACACCGGTCTTGAAATACTGGACAGCACATTTGTAGTCAA

GAAAATCTCTGACTCCCTCTCCAGTGTTTTCCACGTGCCGGCCCCCGTCTTCAGTTTCGGAGCCCCGATT

CTGTTGGCGGGATTGGTCAAGGTCGCCTCCACGTTCTTCCGGTCAACACCCGAAGACTTGGAGAGAGCTG

AGAAGCAGCTCAAAGCACGTGACATCAATGACATTTTCGCCATCCTCAAGAATGGCGAGTGGCTAGTCAA

ACTCATTCTGGCAATCCGCGACTGGATTAAAGCCTGGATCTCCTCAGAAGAAAAGTACATCAACATGACA

GACCTGGTCCCGCGCATTCTCGAGTGCCAGCGGAACTTGAACGACCCGTCCAAGTACCAGGAATCAAAGC

AGTGGCTTGAGCACGCGCGCGAGACGTGCCTCAAGAACGGCAACATTCACATCGCCAACCTCTGCAAAGT

CAACGCGCCAGCACCAAGCAAATCGAGACCCGAACCAGTGGTCGTTTGCCTCCGCGGCAAATCTGGCCAG

GGTAAGAGTTTCCTTGCAAACGTGCTTGCACAGGCAATCTCAACCCACTTCACTGGGCGTACTGACTCGG

TCTGGTACTGCCCACCTGACCCTGACCACTTTGACGGCTACAACCAACAGGCTGTCGTGGTCATGGACGA

CCTGGGACAGAATCCCGACGGCAAGGATTTCAAGTACTTTGCCCAAATGGTGTCAACCACGGGGTTCATC

CCGCCCATGGCCTCACTCGAGGACAAGGGGAAGCCTTTCAACAGCAAGGTCATCATTGCGACCTCCAATC

TGTACTCCGGATTCACTCCGCGTACAATGGTCTGTCCTGATGCACTGAACCGTAGGTTTCACTTTGACAT

TGACGTGAGTGCCAAAGACGGGTACAAAATCAACAACAAATTGGACATCATCAAAGCACTCGAGGACACC

CACACCAACCCTGTGGCAATGTTCCAGTATGATTGTGCCCTTCTGAACGGCATGGCTGTCGAGATGAAGA

GACTGCAACAAGATATGTTCAAGCCTCAACCACCGATTCAGAGTCTGTACCAATTGGTTGATGAGGTGAT

TGAGAGAGTCAACCTTCACGAGAAAGTTGCCTCGCACCCAATTTTCAAGCAA

>HM067705.1_SAT2_UGA_2007

CCCTGGTACAAAGTCATCAAACTCCTCAGCCGACTGTCATGCATGGCCGCTGTAGCAGCACGCTCAAAGG

ACCCAGTCCTCGTAGCGATAATGCTGGCTGACACCGGTCTCGAAATACTGGACAGCACATTTGTGGTCAA

GAAGATCTCTGACTCCCTCTCCAGTGTTTTCCACGTGCCGGCCCCTGTCTTCAGCTTCGGAGCCCCGATT

CTGTTGGCAGGTTTGGTCAAAGTCGCCTCCACGTTCTTCCGCTCAACACCCGAAGACCTGGAGAGAGCCG

AAAAGCAGCTCAAAGCACGTGACATTAATGACATTTTCGCCATTCTCAAGAACGGCGAGTGGCTGGTTAA

ACTCATCCTGGCTATCCGCGACTGGATAAAAGCCTGGATCTCCTCAGAAGAGAAGTACATAAACATGACT

GACCTGGTCCCGCGCATCCTCGAATGCCAGCGGAACTTGAACGACCCGTCCAAATACCAGGAGTCAAAAC

AGTGGCTTGAGAACGCTCGAGAGACGTGTCTCAAGAATGGCAACATCCATATTGCCAACCTCTGTAAAGT

CAGCGCACCAGCACCGAGCAAGTCGAGACCCGAGCCGGTGGTCGTCTGCCTCCGTGGCAAGTCTGGCCAA

GGCAAGAGTTTCCTTGCTAACGTGCTTGCACAAGCAGTTTCAACCCACTTCACTGGACGTACTGACTCAG

TCTGGTACTGTCCGCCTGACCCTGATCATTTCGATGGCTACAACCAACAAGCTGTCGTGGTCATGGACGA

CTTGGGCCAGAACCCTGACGGCAAGGACTTCAAGTACTTTGCTCAAATGGTGTCAACTACGGGGTTTATC

CCGCCCATGGCTTCACTTGAAGACAAGGGGAAACCATTCAACAGCAAGGTCATCATTGCTACCTCCAACT

TGTACTCCGGGTTCACCCCGCGTACAATGGTATGCCCTGATGCACTGAACCGTAGGTTTCACTTTGACAT

CGACGTGAGTGCTAAGGACGGGTACAAAATTAACAACAAATTGGACATAATCAAAGCACTTGAAGACACA

CACACCAACCCTGTGGCAATGTTTCAGTACGATTGCGCCCTTCTGAACGGCATGGCTGTCGAGATGAAGA

GACTGCAACAGGACATGTTCAAGCCTCAACCACCACTACAGAGCCTGTACCAATTGGTTGATGAGGTGAT

TGAGAGAGTCAACCTACACGAAAAGGTCGCCTCGCACCCAATCTTCAAACAA

>HM067706.1_SAT1_UGA_2007

CCCTGGTACAAAGTCATCAAGCTCCTAAGCCGCCTGTCATGCATGGCCGCTGTAGCAGCACGCTCAAAGG

ATCCAGTCCTCGTAGCAATAATGCTGGCTGACACCGGTCTCGAAATACTGGACAGCACATTTGTAGTCAA

GAAGATCTCTGACTCCCTCTCCAGTGTTTTCCACGTGCCGGCCCCTGTCTTCAGTTTCGGAGCCCCGATC

CTGTTGGCAGGTTTGGTCAAGGTCGCCTCCACGTTCTTCCGCTCAACACCCGAAGACTTGGAGAGGGCTG

AAAAACAGCTCAAAGCACGTGACATCAATGACATTTTCGCCATTCTCAAAAACGGCGAATGGCTGGTCAA

ACTCATCCTGGCAATTCGCGACTGGATTAAAGCCTGGATCTCTTCAGAAGAGAAGTACATAAATATGACT

GACCTGGTCCCGCGCATTCTCGAATGTCAGCGGAACTTGAACGACCCGTCCAAGTACCAGGAGTCAAAGC

AGTGGCTTGAGAACGCACGCGAGACGTGTCTCAAGAATGGCAACATCCACATCGCCAACCTCTGCAAAGT

CAACGCACCGGCACCAAGCAAGTCGAGACCCGAACCAGTGGTCGTCTGCCTCCGCGGCAAATCTGGCCAG

GGCAAGAGTTTCCTTGCAAACGTGCTTGCACAAGCAATCTCAACCCATTTCACTGGGCGTACCGACTCGG

TTTGGTATTGTCCACCTGATCCTGACCACTTTGACGGCTACAACCAACAGACTGTCGTGGTCATGGACGA

TCTGGGACAGAATCCCGATGGCAAGGACTTCAAGTACTTCGCCCAAATGGTGTCAACCACGGGATTCATC

CCGCCCATGGCTTCACTCGAGGACAAGGGAAAACCTTTCAACAGCAAGGTCATCATCGCGACCTCCAACT

TGTACTCCGGATTCACTCCGCGCACAATGGTCTGCCCTGATGCATTGAACCGTAGGTTCCACTTTGACAT

CGACGTGAGTGCCAAGGACGGATACAGAGTTAATAACAAATTGGACATCATCAAAGCACTTGAAGACACC

CACACTAACCCTGTGGCGATGTTCCAGTATGATTGTGCCCTTCTGAACGGCATGGCTGTCGAGATGAAGA

GACTGCAACAAGATATGTTCAAGCCTCAACCACCGCTTCAGAGCCTGTACCAACTGGTCGATGAGGTGAT

TGAGAGGGTCAACCTTCACGAGAAGGTTGCCTCGCACCCAATTTTCAAACAG

>HM191257.1_O_UGA_2006

CCCTGGTACAAGCTCATCAAACTCCTGAGCCGCTTGTCATGTATGGCCGCTGTAGCAGCACGGTCCAAGG

ACCCAGTCCTTGTGGCCATCATGCTGGCTGACACCGGCCTCGAGATTCTGGACAGCACGTTCGTCGTGAA

AAAGATCTCCGACTCGCTCTCCAGTCTCTTTCACGTGCCGGCCCCCGTCTTCAGTTTCGGAGCCCCGTTG

CTGTTGGCCGGGTTGGTCAAGGTCGCCTCGAGTTTCTTCCGGTCTACACCCGAAGAACTCGAGAGAGCTG

AGAAGCAGCTCAAAGCACGTGACATCAATGACATATTCGCCATTCTCAAGAACGGCGAGTGGCTGGTCAA

ATTGATCCTTGCCATCCGCGACTGGATCAAGGCATGGATCGCCTCAGAAGAAAAGTTTGTCACCATGACA

GACTTAGTGCCTGGCATCCTCGAAAAGCAGCGGGACCTCAACGACCCCAGCAAGTACAAGGAGGCCAAGG

AGTGGCTCGACAACGCGCGCCAAGCGTGTCTGAAGAGCGGGAACGTCCACATTGCTAACCTTTGCAAAGT

GGTCGCCCCGGCACCTAGCAGGTCGAGACCCGAGCCCGTGGTCGTTTGCCTCCGTGGCAAGTCCGGCCAG

GGAAAGAGTTTCCTTGCGAACGTGCTCGCACAAGCAATCTCTACCCACTTCACAGGTAGAACAGACTCAG

TCTGGTACTGCCCACCTGACCCTGACCACTTCGATGGTTACAACCAACAGACCGTCGTTGTGATGGATGA

TTTGGGCCAGAACCCCGACGGCAAGGACTTCAAGTACTTTGCCCAGATGGTGTCAACCACGGGGTTCATC

CCGCCAATGGCCTCTCTCGAGGACAAAGGAAAACCTTTTAACAGCAAGGTTATCATTGCTACCACCAACC

TTTACTCGGGTTTCACCCCGAGAACCATGGTGTGCCCTGACGCATTGAACCGGAGGTTTCACTTTGACAT

TGACGTGAGCGCCAAAGACGGGTACAAAATTAACAACAAATTGGACATAATCAAAGCTCTTGAAGATACA

CACACCAACCCTGTGGCAATGTTTCAATACGACTGTGCCCTTCTCAACGGCATGGCCGTTGAAATGAAGA

GAATGCAACAAGACATGTTCAAGCCTCAACCGCCCCTCCAGAACGTGTACCAACTTGTTCAGGAGGTGAT

TGAACGGGTGGAGCTCCACGAGAAAGTGTCGAACCACCCAATCTTCAAACAA

>HM229661.1_O_HKN_2010

CCCTGGTACAAGCTCATCAAGCTCCTGAGCCGCTTGTCATGCATGGCCGCTGTAGCAGCACGGTCCAAGG

ACCCAGTCCTTGTGGCCATCATGCTGGCTGACACCGGTCTTGAGATTCTGGACAGCACATTTGTCGTGAA

GAAAATCTCCGACTCCCTCTCCAGTCTCTTTCACGTGCCGGCCCCCGTCTTCAGTTTCGGAGCTCCGATT

CTGCTAGCCGGGTTGGTCAAGGTCGCCTCGAGCTTCTTCCGGTCCACACCCGAGGATCTCGAGAGAGCAG

AGAAACAGCTCAAAGCACGTGACATCAATGACATCTTCGCCATTCTCAAGAACGGCGAGTGGCTGGTCAA

GTTGATCCTAGCCATCCGCGACTGGATTAAAGCATGGATCGCCTCAGAAGAGAAGTTTGTCACCATGACA

GACTTGGTGCCTGGCATCCTTGAAAAGCAGCGGGACCTCAACGACCCGGCCAAGTACAAGGAGGCCAAGG

AATGGCTCGACAACGCGCGCCAAACGTGTTTGAAGAGCGGGAACGTCCACATTGCCAACCTGTGCAAAGT

GGTCGCCCCAGCGCCGAGCAAGTCGAGACCTGAACCCGTGGTCGTTTGCCTCCGCGGCAAATCCGGCCAG

GGTAAGAGTTTCCTTGCGAACGTTCTGGCACAAGCCATCTCTACCCACTTTACCGGCAGGACTGACTCAG

TTTGGTACTGTCCGCCAGACCCTGACCACTTCGACGGTTACAACCAGCAGACCGTTGTTGTGATGGATGA

TTTGGGCCAGAATCCCGACGGCAAGGACTTCAAGTACTTCGCCCAGATGGTCTCGACCACGGGGTTCATC

CCGCCCATGGCTTCACTTGAGGACAAAGGCAAGCCTTTCAACAGCAAAGTCATCATTGCCACCACCAACC

TGTACTCGGGCTTCACCCCGAGAACCATGGTGTGCCCCGATGCGCTGAACCGAAGGTTTCACTTTGACAT

TGACGTGAGTGCCAAGGACGGGTACAAAATTAACAACAAATTGGACATAATCAAAGCTCTCGAGGACACC

CACACCAACCCTGTGGCAATGTTCCAATACGACTGTGCCCTTCTCAACGGCATGGCCGTTGAAATGAAGA

GAATGCAACAAGACATGTTTAAACCCCAACCGCCTTTGCAGAACATATACCAACTTGTGCAAGAGGTGAT

TGACCGGGTCGAGCTCCACGAGAAAGTGTCGAGCCACCCGATTTTCAAGCAG

>HM854021.1_A_IND_2000

CCCTGGTACAAACTTATCAAACTCCTGAGCCGTCTGTCGTGCATGGCCGCTGTAGCAGCACGGTCAAAGG

ACCCAGTCCTTGTGGCCATCATGCTAGCTGACACCGGTCTCGAGATTCTGGACAGCACCTTTGTCGTGAA

GAAAATCTCCGACTCGCTCTCCAGTCTCTTTCACGTGCCGGCCCCCGTCTTCAGTTTTGGAGCTCCAATC

CTGTTGGCCGGGTTGGTCAAGGTCGCCTCGAGTTTCTTCCGGTCCACACCCGAAGACCTTGAGAGAGCAG

AAAAACAGCTCAAAGCACGTGACATCAACGACATATTCGCCATTCTCAAGAACGGCGAGTGGCTGGTCAA

GCTGATTCTTGCCATCCGCGACTGGATTAAGGCATGGATCGCCTCAGAAGAAAAGTTTGTCACCATGACA

GACTTGGTGCCTGGCATCCTTGAAAAGCAGCGGGACCTCAACGACCCGAGCAAGTACAAGGAGGCCAAGG

AGTGGCTCGACAACGCGCGCCAAGCGTGCTTGAAGAGCGGGAACGTCCACATTGCCAACCTTTGCAAAGT

GGTCGCCCCGGCACCCAGCAAGTCGAGACCCGAACCTGTGGTCGTTTGCCTCCGTGGCAAGTCCGGTCAG

GGTAAGAGTTTCCTTGCGAACGTGCTCGCGCAAGCAATCTCCACCCACTTCACTGGCAGAACCGATTCAG

TTTGGTACTGCCCGCCTGACCCCGACCACTTCGACGGTTACAACCAGCAGACCGTTGTTGTGATGGATGA

TTTGGGCCAAAACCCTGATGGCAAGGACTTTAAGTACTTTGCCCAAATGGTTTCAACCACGGGGTTCATC

CCGCCCATGGCTTCACTCGAGGACAAAGGCAAGCCTTTCAACAGCAAGGTCATCATTGCCACCACCAACT

TGTACTCGGGCTTCACCCCGAGGACCATGGTGTGCCCTGATGCACTGAACCGCAGGTTTCACTTTGACAT

CGATGTGAGCGCTAAGGATGGGTACAAAATTAACAACAAATTGGACATAATCAAAGCACTTGAGGACACC

CACACCAATCCAGTGGCAATGTTTCAATACGACTGTGCCCTTCTCAACGGCATGGCCGTTGAAATGAAGA

GAATGCAACACGACATGTTTAAGCCTCAGCCACCCCTCCAGAACGTGTACCAACTTGTTCAGGAGGTGAT

TGACCGGGTCGAGCTCCACGAGAAAGTGTCGAGCCACCCAATCTTCAAGCAG

>HM854022.1_A_IND_1977

CCCTGGTACAAGCTCATCAAGCTCCTAAGCCGTCTGTCGTGTATGGCCGCTGTAGCAGCACGGTCCAAGG

ACCCAGTCCTTGTGGCCATCATGCTGGCTGACACCGGCCTTGAGATTCTGGACAGCACGTTTGTCGTGAA

GAAGATCTCCGACTCGCTCTCAAGTCTCTTTCACGTGCCGGCCCCCGTCTTCAGTTTCGGAGCCCCGATT

CTGCTGGCCGGGTTGGTCAAAGTCGCCTCGAGTTTCTTCCGGTCCACACCCGAAGAGCTTGAGAGAGCAG

AAAAGCAGCTCAAAGCACGTGACATCAATGACATATTCGCCATTCTCAAGAACGGCGAGTGGCTGGTCAA

ACTGATCCTTGCCATCCGTGACTGGATCAAGGCATGGATCGCCTCAGAAGAAAAGTTTGTCACCATGACA

GACTTGGTGCCTGGCATCCTTGAAAAACAACACGACCTCAACGACCCGAGCAAGTACAAGGAAGCCAAGG

AGTGGCTCGACAACGCGCGCCAAGCGTGTCTGAAGAACGGGAACATCCACATTGCCAACCTGTGCAAAGT

GGTTGCCCCGGCGCCCAGTAAGTCGAGGCCCGAACCTGTGGTCGTTTGCCTCCGCGGTAAATCCGGCCAG

GGAAAGAGCTTCCTTGCAAACGTGCTCGCACAAGCGATCTCCACACACTTTACTGGCAGAACCGACTCGG

TTTGGTACTGCCCACCTGACCCTGACCACTTCGACGGTTACAACCAACAGACCGTTGTTGTGATGGATGA

TTTGGGCCAGAACCCCGATGGCAAGGACTTCAAGTACTTTGCCCAGATGGTTTCAACTACGGGGTTCATC

CCGCCCATGGCTTCACTAGAGGACAAAGGCAAACCTTTCAACAGCAAGGTCATCATTGCCACCACCAACC

TGTACTCGGGCTTCACCCCGAGAACTATGGTTTGCCCTGATGCACTGAACCGTAGGTTCCACTTTGACAT

CGACGTGAGCGCCAAGGACGGGTACAAAACTAACAACAAATTGGACATAATCAAAGCTCTCGAAGACACC

CACACCAACCCTGTGGCAATGTTCCAATACGACTGTGCCCTTCTCAACGGCATGGCCGTTGAGATGAAGA

GAATGCAACAAGACATTTTCGAGCCCCAACCGCCTCTACAGAATGTGTACCAACTCGTTCAGGAGGTGAT

TGATCGGGTCGAGCTCCACGAGAAAGTGTCGAGCCACCCGATTTTCAAGCAG

>HM854023.1_A_IND_1999

CCCTGGTACAAGCTCATCAAACTCCTGAGCCGCCTGTCATGTATGGCCGCTGTAGCAGCACGGTCAAAGG

ACCCAGTCCTTGTGGCTATCATGCTGGCTGATACCGGTCTCGAGATTCTGGACAGCACCTTTGTCGTGAA

GAAGATCTCCGACTCGCTCTCCAGTCTCTTTCACGTGCCGGCCCCCGTCTTCAGTTTCGGAGCCCCGATT

CTGTTGGCCGGGTTGGTCAAAGTCGCCTCGAGTTTCTTCCAGTCAACACCCGAAGAACTTGAGAGAGCAG

AAAAACAGCTCAAAGCACGTGACATCAATGACGTGTTCGCCATTCTCAAGAACGGCGAGTGGCTGGTCAA

GCTGATTCTTGCCATCCGCGACTGGATCAAGGCATGGATCGCCTCAGAAGAGAAGTTTGTCACCATGACA

GACTTGGTGCCTGGCATCCTTGAAAAGCAGCGGGACCTCAACGACCCGAGCAAGTACAAGGAAGCCAAGG

AGTGGCTCGACAACGCGCGCCAGGCGTGTTTGAAGAGCGGGAACGTCCACATTGCCAACCTCTGCAAAGT

GGTCGCCCCGGCACCCAGCAAGTCGAGACCCGAGCCCGTTGTCGTTTGCCTCCGTGGCAAATCCGGCCAG

GGTAAGAGTTTCCTTGCTAACGTGCTTGCACAAGCAATTTCAACCCACTTTACTGGCAGAACTGATTCAG

TTTGGTACTGCCCACCTGACCCCGACCACTTCGACGGTTACAACCAACAGACCGTTGTAGTGATGGATGA

TTTGGGCCAGAACCCCGATGGCAAGGACTTCAAGTACTTCGCCCAGATGGTGTCAACCACGGGGTTCATC

CCGCCCATGGCTTCACTCGAGGACAAAGGCAAGCCTTTCAACAGCAAGGTCATCATTGCCACCACCAACT

TGTACTCGGGCTTCACCCCGAGGACCATGGTGTGCCCTGATGCACTGAACCGCAGGTTTCACTTTGACAT

CGATGTGAGCGCCAAGGATGGGTACAAAATTAACAACAAATTGGACATAATCAAAGCACTTGAAGACACC

CACACCAATCCAGTGGCAATGTTTCAATACGACTGTGCCCTTCTCAACGGCATGGCCGTTGAAATGAAGA

GAATGCAACACGACATGTTTAAGCCTCAGCCACCCCTCCAGAACGTGTACCAACTTGTTCAGGAGGTGAT

TGACCGGGTCGAGCTCCACGAGAAAGTGTCGAGCCACCCAATCTTCAAGCAG

>HQ009509.1_O_CHA_1999

CCCTGGTACAAGCTCATCAAGCTCTTAAGCCGCTTGTCATGCATGGCCGCTGTAGCGGCACGGTCAAAGG

ACCCGGTTCTTGTGGCCATCATGCTGGCTGACACCGGTCTTGAGATCCTGGACAGTACCTTTGTCGTGAA

GAAGATCTCCGACTCGCTCTCCAGTCTCTTTCACGTACCAGCCCCCGTATTCAGTTTCGGAGCCCCGATT

TTGTTGGCCGGGTTGGTCAAAGTCGCCTCGAGCTTCTTCCGGTCCACGCCCGAAGACCTTGAGAGAGCGG

AGAAACAGCTCAAAGCACGTGACATCAATGACATATTCGCCATTCTCAAGAACGGCGAGTGGCTGGTTAA

GCTGATCCTCGCCATCCGCGACTGGATCAAGGCATGGATCGCCTCAGAGGAAAAGTTTGTCACCATGACA

GACCTGGTGCCTGGTATCCTTGAAAAGCAGCGGGATCTCAACGACCCAAGCAAGTACGAGGAAGCCAAGG

AGTGGCTCGACAACGCGCGCCAAGCGTGTTTGAAGAGCGGGAACATCCACATCGCAAACCTTTGCAAAGT

GGCTGCCCCAGCACCCAGCAGGTCGAGGCCCGAACCCGTGGTGGTTTGCCTTCGTGGTAAATCAGGCCAG

GGTAAGAGTTTCCTTGCGAACGTGCTTGCACAAGCAATTTCAACCCACTTCACTGGCAGAACCGATTCAG

TCTGGTACTGCCCACCTGACCCTGACCACTTCGACGGTTACAACCAGCAGACCGTTGTAGTAATGGACGA

CCTGGGCCAGAACCCCGACGGGAAGGACTTTAAGTACTTCGCCCAAATGGTTTCAACTACGGGGTTTATC

CCACCCATGGCTTCACTCGAGGACAAAGGTAAACCTTTTAACAGCAAGGTCATCATCGCCACCACCAACC

TGTACTCGGGTTTCACCCCGAGAACTATGGTGTGCCCTGATGCACTGAACCGGAGGTTCCACTTTGACAT

CGACGTGAGCGCTAAGGATGGGTACAAAACTAACAACAAACTGGACATCATCAAAGCTCTTGAAGACACT

CACACCAACCCAGTAGCAATGTTTCAATACGACTGTGCCCTTCTTAACGGCATGGCCGTTGAAATGAAGA

GAATGCAACAAGACATGTTCAAGCCTCAACCGCCCCTCCAGAACGTCTACCAACTTGTTCAGGAGGTGAT

TGATCGGGTTGAACTCCACGAGAAGGTGTCGAATCACCCGATCTTCAAACAG

>HQ113232.1_O_PAK_2009

CCCTGGTACAAGCTCATCAAACTCCTGAGCCGCCTGTCATGTATGGCCGCTGTAGCAGCACGGTCAAAGG

ACCCAGTCCTTGTGGCCATCATGCTAGCTGACACCGGTCTCGAGATTCTGGACAGCACCTTTGTCGTGAA

GAAGATCTCCGACTCACTCTCCAGTCTCTTTCACGTGCCGGCCCCCGTCTTCAGTTTCGGAGCCCCGATT

CTGTTGGCCGGGTTGGTCAAAGTCGCCTCGAGTTTCTTCCGGTCTACACCCGAAGACCTTGAGAGAGCGG

AGAAACAGCTCAAAGCACGTGACATCAATGACATTTTTGCCATTCTCAAGAACGGCGAGTGGCTGGTCAA

ACTGATCCTTGCCATCCGCGACTGGATAAAGGCTTGGATCGCCTCAGAAGAGAAGTTTGTCACCATGACA

GACTTGGTGCCTGGCATCCTTGAGAAGCAGCGGGACCTTAACGACCCAAGCAAGTACAAGGAGGCCAAGG

AGTGGCTCGACAACGCGCGCCAAGCGTGTCTGAAGAGCGGGAATGTCCACATTGCCAACCTTTGCAAGGT

GGTCGCCCCGGCACCCAGCAAGTCGAGACCCGAACCCGTGGTCGTTTGCCTCCGTGGCAAATCCGGCCAG

GGTAAGAGTTTCCTTGCGAACGTGCTCGCACAAGCAATCTCCACCCACTTCACTGGCAGAACTGATTCAG

TTTGGTACTGTCCGCCTGACCCTGACCACTTCGACGGTTACAACCAACAGACCGTTGTCGTGATGGATGA

TTTGGGCCAAAACCCTGATGGCAAGGACTTCAAGTACTTTGCCCAGATGGTGTCAACCACAGGGTTCATC

CCGCCAATGGCGTCACTCGAAGACAAAGGTAAACCTTTCAACAGCAAGGTCATCATTGCAACCACCAACC

TGTACTCGGGGTTTACTCCGAGGACCATGGTGTGCCCTGACGCACTGAACCGTAGGTTTCACTTTGACAT

TGACGTGAGTGCCAAAGACGGGTACAAAATTAACAACAAATTGGACATCATCAAAGCTCTTGAAGACACC

CACACCAACCCAGTGGCAATGTTTCAGTACGATTGTGCCCTTCTCAACGGCATGGCCGTTGAAATGAAGA

GAATGCAACAAGATGTGTTCAAGCCTCAGCCACCCCTCCAGAACGTGTACCAACTCGTTCAGGAGGTGAT

TGAACGGGTCGAGCTCCACGAGAAAGTGTCGAGCCACCCGATCTTCAAGCAG

>HQ113233.1_Asia1_AFG_2009

CCCTGGTACAAGCTCATCAAGCTCCTGAGCCGCCTGTCATGTATGGCCGCTGTGGCAGCACGGTCAAAGG

ACCCAATCCTTGTGGCCATCATGCTGGCTGACACCGGCCTTGAGATTCTGGACAGCACCTTTGTCGTGAA

GAAGATCTCCGACTCGCTCTCCAGTCTCTTTCACGTGCCGGCCCCCGTTTTCAGTTTCGGAGCTCCGATC

CTGTTGGCCGGGTTGGTCAAAGTCGCCTCGAGTTTCTTCCGGTCCACACCCGAAGACCTTGAGAGAGCGG

AGAAACAGCTCAAAGCACGTGACATCAATGACATATTCGCTATTCTCAAGAATGGCGAGTGGCTGGTCAA

ACTGATTCTTGCCATCCGCGACTGGATCAAGGCATGGATCGCCTCAGAAGAAAAGTTTGTCACCATGACA

GACCTGGTGCCTGGCATCCTTGAAAAGCAGCGGGACCTCAACGACCCAAGCAAGTACAAGGAGGCCAAGG

AATGGCTCGACAACGCGCGCCAAGCGTGTCTGAAGAGCGGGAATGTCCACATTGCCAACCTTTGCAAAGT

GGTCGCCCCGGCACCCAGCAAGTCGAGACCCGAGCCCGTGGTCGTTTGCCTCCGCGGCAAATCTGGCCAG

GGCAAGAGTTTCCTTGCGAACGTGCTCGCACAAGCAATCTCCTCCCACTTTACTGGCAGAACTGATTCAG

TTTGGTACTGCCCGCCTGACCCTGACCACTTCGACGGTTACAACCAACAGACCGTTGTCGTGATGGATGA

TTTGGGCCAGAACCCTGATGGCAAGGACTTTAAGTACTTTGCCCAGATGGTGTCAACCACAGGGTTCATC

CCGCCAATGGCGTCACTCGAAGACAAAGGTAAACCTTTCAACAGCAAGGTCATCATTACAACCACCAACT

TGTACTCGGGGTTCACTCCGAGGACCATGGTGTGCCCTGATGCACTGAACCGCAGGTTTCACTTTGACAT

TGACGTGAGTGCCAAAGACGGGTATAAAATTAACAACAAATTGGACATCATCAAAGCTCTTGAAGACACC

CACACCAACCCAGTGGCAATGTTTCAGTATGATTGTGCCCTTCTCAACGGCATGGCCGTTGAAATGAAGA

GAATGCAACAAGATATGTTCAAGCCTCAACCGCCCCTCCAGAACGTGTACCAACTAGTTCAGGAGGTGAT

TGAACGGGTCGAGCTCCACGAGAAAGTGTCGAGCCACCCGATTTTCAAGCAA

>HQ268509.2_A_VIT_2004

CCCTGGTACAAGCTTATCAAGCTCCTAAGCCGCTTGTCATGCATGGCCGCTGTAGCAGCACGGTCCAAGG

ACCCAGTCCTTGTGGCCATCATGCTGGCTGACACCGGCCTTGAGATTCTGGACAGCACGTTTGTCGTGAA

GAAGATCTCCGACTCGCTCTCCAGTCTCTTTCACGTGCCGGCCCCCGTCTTTAGTTTCGGAGCCCCGATC

CTATTGGCTGGGTTGGTCAAGGTCGCCTCGAGTTTCTTCCGGTCCACGCCCGAAGACCTTGAGAGAGCAG

AAAAACAGCTCAAAGCACGTGACATTAATGACATATTCGCCATTCTCAAGAACGGCGAGTGGCTGGTCAA

GCTGATCCTAGCTATCCGCGACTGGATTAAAGCATGGATCGCCTCAGAAGAAAAGTTTGTCACCATGACA

GACTTGGTGCCTGGCATCCTTGAAAAGCAGCGGGATCTCAACGACCCGAGCAAATACAAGGCAGCTAAGG

AGTGGCTTGACAACGCGCGTCAAGCGTGCTTGAAGAGCGGGAACGTCCACATTGCCAACCTTTGCAAAGT

GGTCGCCCCAGCACCAAGCAAGTCGAGACCCGAACCCGTGGTTATTTGCCTCCGTGGCAAATCCGGCCAG

GGCAAGAGTTTCCTTGCGAACGTGCTCGCACAAGCAATTTCAACCCACTTCACTGGCAGAACCGATTCAG

TTTGGTACTGCCCGCCTGATCCCGACCACTTCGACGGTTACAACCAACAAACCGTTGTTGTGATGGACGA

TTTGGGCCAAAATCCCGACGGCAAGGACTTCAAGTACTTCGCCCAGATGGTCTCAACCACGGGGTTCATC

CCGCCCATGGCCTCGCTTGAGGACAAGGGGAAACCTTTCAACAGCAAAGTCATCATTGCCACCACCAACC

TCTACTCGGGCTTCACCCCGAGAACCATGGTGTGCCCTGACGCGCTGAACCGAAGGTTCCACTTTGATAT

CGACGTGAGTGCCAAGGACGGGTACAGAATTGGCAACAAATTGGACATAATCAAAGCACTTGAAGACACC

CACACCAACCCGGTGGCAATGTTCCAGTACGACTGCGCCCTCCTCAACGGCATGGCAGTCGAGATGAAGA

GATTGCAACAGGATGTGTTCAGTCCCCAACCACCCCTACAGAATGTTTACCAACTTGTTCAAGAGGTGAT

TGACCGGGTGGAGCTCCACGAGAAAGTGTCGAGCCACCCGATTTTCAAGCAG

>HQ268524.1_O_BHU_2004

CCCTGGTACAAGCTCATCAAGCTCCTGAGCCGCCTGTCATGCATGGCCGCTGTAGCAGCACGGTCAAAGG

ACCCGGTCCTTGTGGCCATCATGCTAGCTGACACCGGTCTCGAGATTCTAGACAGCACCTTTGTCGTGAA

GAAAATCTCCGACTCGCTCTCTAGTCTCTTTCACGTGCCGGCCCCCGTCTTCAGTTTCGGAGCCCCGATT

CTGTTGGCCGGGCTGGTCAAGGCCGCCTCGAGTTTCTTCCGGTCCACGCCCGAAGACCTCGAGAGAGCAG

AAAAACAGCTCAAAGCACGTGACATCAATGACATATTCGCCATTCTCAAGAACGGCGAGTGGCTGGTCAA

GCTGATCCTTGCCATCCGCGACTGGATTAAGGCATGGATCGCCTCAGAAGAAAAGTTTGTCACCATGACG

GACTTGGTGCCTGGCATCCTTGAAAAACAGCGGGACCTCAACGACCCAAGCAAGTACAAGGAGGCCAAGG

AGTGGCTCGACAACGCGCGCCAAGCGTGTCTGAAGAGCGGGAATGTCCACATTGCCAACCTCTGCAAAGT

GGTCGCCCCGGCGCCCAGCAAGTCGAGACCCGAACCTGTAGTCGTTTGCCTCCGCGGCAAATCCGGCCAG

GGTAAAAGTTTCCTTGCGAACGTGCTCGCACAAGCAATCTCCACCCACTTCACTGGCAGAACTGATTCAG

TTTGGTACTGCCCGCCTGACCCTGACCACTTTGACGGCTACAACCAACAGGCCGTTGTCGTGATGGATGA

TTTGGGCCAGAACCCTGATGGCAAGGACTTCAAGTACTTTGCCCAGATGGTGTCAACCACAGGGTTCATC

CCGCCAATGGCGTCACTCGAAGACAAAGGCAAACCTTTCAACAGCAAGGTCATCATTGCAACCACCAACT

TGTACTCGGGGTTCACCCCGAGGACCATGGTGTGCCCTGATGCACTGAACCGTAGGTTTCACTTTGACAT

TGACGTGAGTGCCAAAGACGGGTACAAAATTAACAACAAATTGGACATCATCAAAGCTCTCGAAGACACC

CACACCAACCCAGTGGCAATGTTTCAGTACGATTGTGCCCTTCTCAACGGCATGGCCGTTGAAATGAAGA

GAATGCAACAAGATGTGTTCAAGCCTCAACCACCCCTCCAGAACGTGTACCAGCTAGTTCAGGAGGTGAT

TGAACGGGTCGAGCTCCACGAGAAAGTGTCGAGCCACCCGATTTTCAAGCAA

>HQ412603.1_O_CHA_2000

CCCTGGTACAAGCTCATCAAACTCCTAAGCCGCCTGTCGTGCATGGCCGCTGTGGCAGCACGGTCCAAGG

ACCCAGTCCTCGTGGCCATCATGTTGGCCGACACTGGCCTCGAGATTCTGGACAGCACCTTTGTGGTAAA

GAAGATATCCGACTCGCTCTCCAGTCTCTTCCACGTGCCGGCTCCCGCCTTCAGTTTCGGAGCCCCGATC

CTGTTGGCCGGGTTGGTCAAAGTCGCCTCGAGTTTCTTCCAGTCCACGCCCGAAGATCTCGAGAGAGCAG

AAAAACAGCTCAAAGCACGTGACATCAACGACATATTTGCCGTCCTTAAGAACGGTGAGTGGCTGGTCAA

ACTGATCCTGGCCATCCGCGACTGGATTAAGGCATGGATCGCCTCAGAAGAAAAGTTTGTCACCATGACA

GACCTGGTGCCTGGCATCCTTGAAAGACAACGGGACCTCAATGACCCCGGCAAGTACAAGGAGGCCAAGG

AATGGCTGGACAACGCGCGTCAAGCGTGTTTGAAGAGCGGGAACGTACACATTGCCAACCTGTGCAAAGT

GGTCGCTCCGGCGCCCAGCAAGTCGAGACCCGAACCAGTGGTCGTGTGTCTTCGCGGCAAGTCCGGCCAA

GGGAAAAGCTTCCTTGCGAACGTTCTCGCACAGGCGATCTCCACACACTTCACTGGTAGGACCGACTCGG

TCTGGTACTGCCCGCCCGACCCTGATCACTTCGACGGTTACAACCAGCAGACCGTCGTCGTAATGGACGA

CTTGGGCCAGAACCCAGATGGCAAAGATTTCAAGTACTTCGCCCAAATGGTCTCCACCACGGGTTTCATT

CCGCCCATGGCCTCACTCGAGGACAAGGGTAAACCTTTTAACAGCAAGGTCATAATAGCCACAACTAACT

TGTACTCAGGATTCACCCCAAGAACTATGGTGTGTCCCGATGCGCTTAATAGGAGGTTCCACTTTGACCT

CGACGTGAGCGCTAAGGACGGATACAAGACCAACAACAAACTGGACATAGTCAAAGCTCTTGAAGACACC

CACACCAACCCGGTGGCGATGTTCCAGTACGACTGCGCCCTTCTCAACGGAATGGCTGTTGAAATGAAGA

GAATGCAACAAGACATGTTCAAGCCTCAACCACCTCTCCAGAACGTCTACCAGCTTGTTCAAGAGGTGAT

TGAGCGGGTGGAACTGCACGAAAAGGTGTCGAGCCACCCAATATTCAAGCAG

>HQ631363.1_Asia1_CHA_2006

CCCTGGTACAAACTCATCAAACTCCTGAGCCGCTTGTCATGCATGGCCGCTGTAGCAGCACGGTCAAAGG

ACCCAGTCCTTGTGGCCATCATGCTGGCTGACACCGGCCTTGAGATTCTGGACAGCACATTCGTCGTGAA

GAAGATCTCCGACTCACTCTCCAGTCTCTTTCACGTGCCGGCCCCCGTCTTCAGTTTCGGAGCCCCGGTT

CTGTTGGCCGGGTTGGTCAAAGTCGCCTCGAGTTTCTTCCGGTCCACGCCCGAAGACCTTGAGAGAGCAG

AGAAACAGCTCAAAGCACGTGACATCAATGACATCTTCGCCATTCTCAAGAACGGCGAGTGGCTGGTCAA

ATTGATTCTTGCCATCCGCGACTGGATTAAGGCATGGATCGCCTCAGAAGAAAAGTTTGTCACCATGACA

GACTTGGTGCCTGGCATCCTCGAAAAGCAGCGGGACCTCAACGACCCGAGCAAGTACGAGGAAGCCAAGG

AGTGGCTCGACAACGCGCGTCAAGCGTGTCTGAAGAGCGGGAACGTCCACATTGCCAACCTGTGCAAAGT

GACCGCCCCGGCACCCAGCAAGTCGAGACCCGAACCCGTGGTCGTTTGCCTCCGAGGCAAATCCGGCCAG

GGAAAGAGTTTCCTTGCGAACGTGCTCGCACAAGCAATCTCCACACACTTCACTGGCAGAACTGATTCAG

TTTGGTACTGCCCGCCTGACCCTGACCACTTCGACGGTTACAACCAACAGACCGTTGTTGTGATGGATGA

TTTGGGCCAGAACCCCGACGGCAAGGACTTTAAGTACTTCGCCCAGATGGTTTCAACCACGGGGTTCATC

CCGCCCATGGCCTCGCTCGAAGACAAAGGCAAACCCTTCAACAGCAAGGTCATCATCGCCACCACCAACC

TGTACTCGGGCTTCACCCCGAGGACCATGGTGTGCCCTGATGCACTGAACCGGAGGTTTCACTTTGACAT

TGACGTGAGTGCCAAGGACGGGTACAAAATTAACAACAAGTTGGACATTATCAAAGCACTTGAAGACACC

CACACCAACCCGGTGGCAATGTTTCAGTACGACTGTGCCCTTCTCAACGGCATGGCCGTTGAAATGAAGA

GAATGCAACAAGACATGTTCAAGCCTCAACCGCCCCTCCAGAACGTGTACCAACTTGTTCAGGAGGTGAT

TGATCGGGTCGAGCTCCACGAGAAAGTGTCGAGCCACCCGATTTTCAAGCAG

>HQ632768.1_O_MAY_2000

CCCTGGTACAAGCTCATCAAGCTCTTGAGCCGCCTGTCATGTATGGCCGCTGTAGCAGTACGGTCAAAGG

ACCCAGTCCTTGTGGCCATCATGCTGGCTGACACCGGCCTTGAGATTCTGGACAGTACCTTTGTCGTGAA

GAAGATCTCCGACTCGCTCTCCAGTCTCTTTCACGTGCCGGCCCCCGTCTTCAGTTTCGGAGCCCCGATT

TTGTTGGCCGGGTTGGTCAAAGTCGCCTCGAGTTTCTTCCGGTCCACACCCGAAGACCTTGAGAGAGCGG

AGAAACAGCTCAAAGCACGTGACATCAATGACATATTCGCCATTCTCAAGAACGGCGAGTGGCTGGTCAA

GCTGATTCTTGCCATCCGCGACTGGATCAAGGCATGGATCGCCTCAGAAGAAAAATTTGTCACCATGACA

GACCTGGTGCCTGGCATCCTTGAAAAGCAGCGGGATCTCAACGACCCAAGCAAATACAAGGAGGCCAAGG

AGTGGCTCGACAACGCTCGCCAAGCGTGTTTGAAGAGCGGGAACATCCACATCGCAAACCTTTGCAAAGT

GGTTGCCCCAGCACCCAGCAGGTCGAGGCCCGAACCCGTGGTCGTTTGCCTCCGTGGCAAATCGGGCCAG

GGCAAGAGTTTCCTTGCGAACGTGCTTGCACAAGCAATTTCAACCCACTTCACTGGCAGAACCGATTCAG

TTTGGTACTGCCCACCTGACCCTGATCACTTCGACGGTTACAACCAGCAGACCGTTGTAGTAATGGATGA

TTTGGGCCAGAACCCCGACGGGAAGGACTTCAAGTACTTCGCCCAAATGGTTTCAACTACGGGGTTTATC

CCGCCCATGGCTTCACTCGAGGACAAAGGCAAACCTTTCAACAGCAAGGTCATCATCGCCACCACCAACC

TGTACTCGGGCTTCACCCCGAGAACTATGGTGTGCCCCGATGCACTGAACCGAAGGTTCCACTTTGACAT

TGACGTGAGCGCCAAGGACGGGTACAAAATTAATAACAAATTGGACATCATCAAAGCTCTTGAAGACACC

CACACCAACCCAGTGGCAATGTTTCAATACGACTGTGCCCTTCTCAACGGCATGGCCATTGAAATGAAGA

GAATGCAACAAGACATGTTCAAGCCTCAACCGCCCCTCCAGAACGTCTACCAGCTTGTTCAGGAGGTGAT

TGACCGGGTCGAGCTCCACGAGAAGGTGTCGAGCCACCCGATTTTCAAGCAG

>HQ632769.1_O_MAY_2001

CCCTGGTACAAGCTCATCAAACTCCTAAGCCGCCTGTCATGTATGGCCGCTGTAGCAGCACGCTCCAAGG

ACCCAGTCCTTGTGGCTATCATGCTGGCTGACACCGGCCTTGAGATTCTGGACAGTACATTTGTTGTGAA

GAAGATCTCCGACTCACTCTCCAGTCTCTTTCACGTGCCGGCCCCCGCCTTCAGCTTCGGAGCCCCGATT

CTGTTGGCCGGGTTGGTCAAGGTCGCCTCGAGTTTCTTCCGGTCCACACCCGAAGACCTTGAGAGAGCAG

AGAAACAGCTCAAAGCACGTGACATCAATGACATATTCGCCATTCTCAAGAACGGTGAGTGGCTGGTCAA

GTTGATCCTTGCTATCCGCGACTGGATCAAAGCATGGATCGCCTCAGAAGAAAAGTTTGTCACCATGACA

GATTTGGTGCCTGGCATTCTTGAAAAGCAGCGGGACCTCAACGACCCGAGCAAGTACAAGGAAGCCAAGG

AGTGGCTCGACAACGCGCGTCAAGCGTGTCTGAAGAGCGGAAACGTCCACATTGCTAACCTCTGCAAAGT

GGTCGCCCCAGCACCGAGCAAGTCGAGACCCGAACCCGTGGTCGTTTGTCTCCGCGGCAAATCCGGCCAG

GGTAAGAGTTTCCTTGCGAACGTGCTCGCGCAGGCAATCTCCACACACTTCACTGGCAGAACCGATTCAG

TTTGGTACTGCCCACCAGACCCTGACCACTTTGACGGTTACAACCAACAAACCGTCGTAGTGATGGATGA

TTTGGGCCAGAACCCTGACGGCAAGGACTTCAAGTACTTCGCCCAAATGGTCTCAACCACGGGGTTCATC

CCGCCCATGGCCTCACTCGAAGACAAGGGAAAACCTTTCAACAGCAAAGTTATCATTGCCACCACCAACC

TGTACTCGGGTTTCACCCCGAGGACTATGGTGTGCCCAGACGCGCTGAATAGAAGGTTTCACTTTGACAT

TGACGTGAGTGCCAAGGACGGGTACAAAATTAACAACAAATTGGACATAATCAAAGCTCTTGAGGACACT

CACACCAACCCGGTGGCGATGTTCCAATACGACTGCGCCCTCCTCAACGGCATGGCAGTCGAAATGAAGA

GAATGCAACAGGACATGTTCAAGCCCCAACCGCCTTTGCAGAATGTTTACCAACTTGTTCAAGAGGTGAT

TGATCGGGTCGAGCTCCACGAGAAAGTGTCGAGCCACCCGATCTTTAAGCAA

>HQ632770.1_O_MAY_2004

CCCTGGTACAAGCTCATCAAGCTCCTGAGCCGCCTGTCATGCATGGCCGCTGTAGCAGCACGGTCAAAGG

ACCCAGTCCTTGTGGCCATCATGCTAGCTGACACCGGTCTCGAGATTCTAGACAGCACCTTTGTCGTGAA

GAAAATCTCCGACTCGCTCTCTAGTCTCTTCCACGTGCCGGCCCCCGTCTTCAGTTTCGGAGTCCCGATT

CTGTTGGCCGGGTTGGTCAAGGTCGCCTCGAGTTTCTTCCAGTCCACGCCCGAAGACCTCGAGAGAGCAG

AAAAACAGCTCAAAGCACGTGACATCAATGACGTATTCGCCATTCTCAAGAACGGCGAGTGGCTGGTCAA

GCTGATCCTTGCCATCCGCGACTGGATTAAGGCATGGATCGCCTCAGAAGAAAAGTTTGTCACCATGACG

GACTTGGTGCCTGGCATCCTTGAAAAACAGCGGGACCTCAACGACCCAAGCAAGTACAAGGAGGCCAAGG

AGTGGCTCGACAACGCACGCCAAGCGTGTCTGAAGAGCGGGAACGTCCACATTGCCAACCTCTGCAAAGT

GGTCGCCCCGGCGCCCAGCAAGTCGAGGCCCGAACCTGTGGTCGTTTGCCTCCGCGGCAAATCCGGCCAG

GGTAAGAGTTTCCTTGCGAACGTGCTCGCACAAGCAATCTCCACCCACTTCACTGGCAGAACTGATTCAG

TTTGGTACTGCCCGCCTGACCCTGACCACTTCGACGGCTACAACCAACAGGCCGTTGTCGTGATGGACGA

CTTGGGCCAGAACCCTGATGGCAAGGACTTCAAGTACTTTGCCCAGATGGTGTCAACCACAGGGTTCATC

CCGCCAATGGCGTCACTCGAAGACAAAGGTAAACCTTTCAACAGCAAGGTCATCATTGCAACCACCAACT

TGTACTCGGGGTTCACTCCGAGGACCATGGTGTGCCCTGATGCACTGAACCGTAGGTTTCACTTTGACAT

TGACGTGAGTGCCAAAGACGGGTACAAAATTAACAACAAATTGGACATCATCAAAGCTCTTGAAGACACC

CACACCAACCCAGTGGCAATGTTCCAGTACGATTGTGCCCTTCTCAACGGCATGGCCGTTGAAATGAAGA

GAATGCAACAAGATGTGTTCAAGCCTCAACCACCCCTCCAGAACGTGTACCAGCTAGTTCAGGAGGTGAT

TGAACGGGTCGAGCTCCACGAGAAAGTGTCGAGCCACCCGATTTTTAAGCAA

>HQ632771.1_O_MAY_2005

CCCTGGTACAAACTCATCAAACTCCTAAGCCGCCTGTCGTGCATGGCCGCTGTGGCAGCACGGTCCAAGG

ACCCAGTCCTCGTGGCAATCATGCTGGCCGACACCGGTCTCGAAATCCTGGACAGCACCTTTGTGGTAAA

GAAAATCTCCGACTCGCTCTCCAGTCTTTTCCACGTGCCGGCGCCTGCCTTCAGTTTCGGAGCCCCGATC

CTGTTGGCTGGGTTAGTCAAGGTCGCCTCGAGTTTCTTCCAGTCCACACCCGAAGACCTCGAGAGAGCGG

AAAAACAGCTCAAGGCACGTGACATCAACGACGTGTTCGCCGTCCTCAAGAACGGCGAGTGGTTGGTCAA

ACTGATCCTGGCCATTCGCGACTGGATCAAAGCATGGATCGCCTCAGAAGAAAAGTTTGTCACCATGACA

GACCTGGTGCCAGGCATCCTTGAAAAACAACGGGACCTCAACGACCCCGGCAAATACAAGGAGGCCAAAG

AATGGTTGGACAACGCGCGTCAAGCGTGTTTGAAGAGCGGAAACGTACACATTGCTAATCTGTGCAAGGT

GGCCGCTCCGGCACCTAGCAAGTCGAGGCCCGAACCGGTGGTTGTTTGTCTACGCGGCAAATCAGGTCAA

GGGAAAAGCTTCCTCGCAAACGTTCTCGCGCAGGCAGTTTCCACACATTTCACCGGTAGAACCGACTCAG

TTTGGTACTGCCCGCCCGACCCCGACCACTTCGACGGTTACAATCAACAGGCCGTCGTTGTGATGGACGA

CTTGGGTCAAAACCCCGACGGCAAGGACTTCAAGTATTTTGCCCAAATGGTCTCCACTACGGGGTTCATA

CCACCTATGGCCTCGCTCGAGGACAAGGGCAAACCCTTTAACAGCAAGGTCATAATAGCCACGACCAACC

TGTACTCGGGGTTCACCCCAAGAACCATGGTGTGTCCCGATGCGCTCAACCGGAGGTTCCACTTTGACAT

TGACGTGAGTGCTAAAGACGGGTACAAGGTCAACAACAAACTGGACATAGTCAAGGCACTTGAAGACACC

CACACCAACCCAGTGGCAATGTTCCAGTACGACTGCGCTCTTCTCAACGGGATGGCTGTTGAAATGAAGA

GGATGCAACAGGACATGTTCAAACCACAGCCGCCTCTCCAGAACGTCTACCAACTTGTTCAAGAGGTGAT

TGAGCGGGTGGAACTACACGAGAAAGTGTCGAGCCACCCAATATTCAAGCAA

>HQ632772.1_O_MAY_2007

CCCTGGTACAAACTCATCAAACTCCTGAGCCGCTTGTCATGTATGGCCGCTGTAGCAGCACGGTCCAAGG

ACCCAGTCCTTGTGGCCATCATGCTGGCTGACACCGGCCTTGAGATTCTGGACAGCACGTTTGTCGTGAA

GAAGATCTCCGACTCACTCTCCAGTCTCTTTCACGTGCCGGCCCCTGTCTTCAGTTTCGGAGCTCCGATT

CTGTTGGCCGGGTTGGTCAAGGTCGCCTCGAGTTTCTTCCGGTCAACACCCGAAGACCTTGAGAGAGCAG

AAAAACAGCTCAAAGCACGTGACATCAATGACATTTTCGCCATTCTCAAGAACGGCGAGTGGCTGGTCAA

GCTGATCCTAGCTATCCGCGACTGGATTAAAGCATGGATTGCCTCAGAGGAGAAGTTTGTCACCATGACA

GACTTGGTGCCTGGCATTCTTGAAAAGCAGCGGGACCTTAACGACCCGAGCAAGTACAAGGAAGCCAAGG

AGTGGCTCGACAACGCGCGCCAAGCGTGTCTGAAGAGCGGGAACGTCCACATTGCCAACCTCTGCAAAGT

GGTTGCCCCGGCACCGAGCAAGTCGAGACCCGAGCCCGTGGTCGTTTGCCTCCGAGGCAAATCCGGCCAG

GGCAAGAGTTTCCTTGCGAACGTGCTTGCGCAAGCAATTTCCACCCACTTCACTGGCAGAACCGATTCAG

TTTGGTACTGCCCACCAGACCCTGACCACTTCGACGGTTACAACCAGCAAACTGTCGTAGTGATGGATGA

TTTGGGCCAGAACCCTGATGGCAAGGACTTTAAGTACTTTGCCCAAATGGTGTCAACAACAGGGTTCATC

CCGCCCATGGCCTCGCTCGAAGACAAAGGGAAACCCTTCAACAGCAAAGTCATCATCGCCACCACCAACT

TGTACTCGGGTTTCACCCCGAGGACTATGGTGTGTCCAGACGCGCTGAACCGAAGGTTCCACTTTGACAT

TGATGTGAGTGTCAAGGATGGGTACAAAATTAACAACAAATTGGACATAATCAAAGCTCTTGAGGACACC

CACACCAATCCAGTGGCGATGTTCCAATACGACTGCGCCCTTCTCAACGGCATGGCAGTCGAAATGAAGA

GAATGCAGCAAGACATGTTCAAGCCCCAACCGCCTCTTCAGAATGTGTACCAACTCGTTCAAGAGGTGAT

TGACCGGGTCGAGCTCCACGAAAAAGTGTCGAGCCACCTGATTTTCAAGCAA

>HQ632773.1_A_MAY_2007

CCCTGGTACAAGCTTATCAAGCTCCTAAGCCGCCTGTCATGCATGGCCGCTGTAGCAGCACGGTCCAAGG

ACCCAGTCCTTGTGGCCATCATGCTGGCTGACACCGGCCTTGAGATTCTGGACAGCACGTTTGTCGTGAA

GAAGATTTCCGACTCGCTCTCCAGTCTCTTTCACGTGCCGGCCCCCGTCTTTAGTTTCGGAGCCCCGATC

CTGTTGGCTGGGTTGGTCAAGGTCGCCTCGAGTTTCTTCCGCTCCACGCCCGAAGACCTCGAGAGAGCAG

AAAAACAGCTCAAAGCACGTGACATCAATGACGTATTCGCCATTCTCAAGAACGGCGAGTGGCTGGTCAA

GCTGATCCTAGCTATCCGCGACTGGATTAAAGCATGGATCGCCTCAGAAGAAAAGTTTGTCACCATGACA

GACTTGGTGCCTGGCATCCTTGAAAAGCAGCGGGACCTCAACGACCCGAGCAAGTACAAGGCAGCTAAGG

AGTGGCTCGACAACGCGCGTCAAGCGTGCTTGAAGAGCGGGAACGTCCACATTGCCAACCTCTGCAAAGT

GGTCGCCCCAGCACCAAGCAAGTCAAGACCCGAACCCGTGGTCGTTTGCCTCCGTGGTAAATCCGGCCAG

GGCAAGAGTTTTCTTGCGAACGTGCTCGCACAAGCTATTTCAACCCACTTCACTGGCAGAACCGATTCAG

TTTGGTACTGCCCGCCTGACCCCGACCACTTTGACGGTTACAACCAACAAACCGTTGTTGTGATGGACGA

TTTGGGCCAGAATCCCGACGGCAAGGACTTCAAGTACTTCGCCCAGATGGTCTCAACCACGGGGTTCATC

CCGCCCATGGCCTCACTTGAGGACAAGGGGAAACCCTTCAACAGCAAAGTCATCATTACCACCACTAACC

TCTACTCGGGCTTCACCCCGAGAACCATGGTGTGCCCCGACGCGCTGAACCGGAGGTTCCACTTTGACAT

TGACGTGAGTGCCAAGGATGGGTACAAAATTAACAACAAATTGGACATAATCAAAGCACTTGAAGACACC

CACACCAACCCGGTGGCAATGTTCCAGTACGACTGCGCCCTCCTCAACGGCATGGCAGTCGAGATGAAGA

GATTGCAACAGGATGTGTTTAGTCCCCAACCACCCATACAGAATGTTTACCAACTTGTTCAAGAGGTGAT

TGATCGGGTGGAGCTCCACGAGAAAGTGTCGAGCCACCCGATTTTCAAACAG

>HQ632774.1_Asia1_MAY_1999

CCCTGGTACAAGCTTATCAAGCTCCTGAGCCGCTTGTCATGTATGGCCGCTGTAGCAGCACGGTCCAAGG

ACCCAGTCCTTGTGGCCATCATGCTGGCTGACACCGGCCTCGAGATTCTGGACAGCACGTTTGTCGTGAA

GAAGATCTCCGACTCGCTCTCCAGTCTCTTTCACGTGCCGGCCCCCGTCTTCAGTTTCGGAGCCCCGATT

CTGTTGGCCGGGTTGGTCAAGGTCGCCTCAAGCTTCTTCCGGTGCACACCCGAAGACCTTGAGAGAGCAG

AAAAACAGCTCAAAGCACGTGACATCAATGACATATTCGCCATTCTCAAGAACGGCGAGTGGCTGGTCAA

GTTGATCCTAGCTATCCGCGACTGGGTCAAAGCATGGATCGCCTCAGAGGAGAAGTTCGTCACCATGACG

GACCTGGTGCCTGGCATCCTTGAAAAGCAGCGGGATCTTAACGACCCGAGCAAGTACAAGGAAGCTAAGG

AGTGGCTCGACAACGCGCGCCAAGCGTGTTTGAAGAGTGGGAACGTCCACATTGCCAACCTGTGTAAAGT

GGTCGCCCCGGCACCGAGCAAGTCGAGACCCGAACCCGTGGTCGTTTGTCTCCGTGGCAAATCCGGCCAG

GGTAAGAGTTTCCTTGCGAACGTGATCGCACAAGCAATCTCTACCCACTTCACTGGCAGAACCGATTCAG

TTTGGTATTGTCCGCCAGACCCTGACCACTTCGACGGTTACAACCAACAGACCGTTGTAGTGATGGATGA

TTTGGGCCAGAATCCCGACGGCAAGGACTTCAAGTACTTTGCCCAAATGGTTTCAACTACGGGATTTATC

CCGCCCATGGCCTCGCTTGAAGACAAGGGAAAACCTTTCAACAGCAAAGTCATCATTGCCACCACCAACT

TGTACTCGGGTTTCACCCCGAGGACCATGGTGTGCCCAGACGCGCTGAATCGTAGGTTTCACTTTGACAT

CGATGTGAGTGCCAGGGACGGGTACAAAGTAAACAACAAATTGGACGTAATCAAAGCTCTTGAGGACACC

CACACTAATCCGGTGGCGATGTTTCAGTACGACTGCGCCCTTCTCAACGGCATGGCAGTCGAAATGAAGA

GAATGCAACAAGACGTGTTCAAGCCCCAACCGCCTCTGTTGAACGTTTACCAGCTTGTTCAGGAGGTGAT

TGACCGGGTTGAGCTCCACGAGAAAGTGTCGAGCCACCCGATTTTCAAGCAA

>HQ832576.1_A_IND_1990

CCCTGGTACAAGCTCATCAAACTCCTGAGCCGCTTGTCATGCATGGCCGCTGTAGCAGCACGGTCAAAGG

ACCCAGTCCTTGTGGCCATAATGCTGGCTGACACCGGTCTTGAGATTCTGGACAGCACTTTCGTCGTGAA

GAAGATCTCCGACTCGCTCTCCAGACTCTTTCACGTGCCGGCCCCCGTCTTCAGTTTCGGAGCCCCGATT

CTGTTGGCCGGGTTGGTCAAATTCGCCTCGAGTTTCTTCCGGTCCACACCCGAAGACCTTGAGAGAGCAG

AGAAACAGCTCAAAGCACGTGACATCAATGACATATTCGCCATCCTCAAGAACGGCGAGTGGCTGGTCAA

GCTGATTCTTGCCATCCGCGACTGGATTAAGGCATGGATCGCCTCAGAAGAAAAGTTTGTCACCATGACA

GACTTGGTGCCTGGCATCCTTGAAAAGCAACGGGACCTCAACGACCCGAGCAAGTACAAGGAAGCCAAGG

AGTGGCTCGACAACGCGCGCCAAGCGTGTCTGAAGAGCGGGAACGTCCACATTGCCAACCTGTGCAAAGT

GGTCGCCCCGGCACCTGGCAAGTCGAGACCCGAACCTGTGGTCGTCTGCCTCCGTGGCAAATCCGGCCAG

GGGAAGAGTTTCCTTGCGAATGTGCTCGCACAAGCAATCTCCACCCACTTCACTGGTAGAACTGATTCGG

TTTGGTACTGCCCACCTGACCCTGACCACTTCGACGGTTACAACCAGCAGACCGTTGTAGTGATGGATGA

TTTGGGCCAAAACCCCGACGGCAAGGACTTCAAGTACTTCGCCCAGATGGTGTCGACCACGGGGTTCATC

CCGCCCATGGCTTCGCTCGAAGACAAAGGCAAGCCTTTCAACAGCAAGGTCATCATCGCCACCACCAACC

TCTACGCGGGTTTCACCCCGAGAACCATGGTGTGCCCTGACGCGCTGAACAGAAGGTTTCACTTTGACAT

TGACGTGAGTGCCAAGGACGGGTACAAAATCAACAACAAATTGGACATAATCAAAGCTCTTGAAGACACC

CACACCAACCCTGTGGCAATGTTCCAGTACGATTGTGCCCTTCTCAACGGCATGGCTGTTGAAATGAAGA

GAATGCAACAAGATGTGTTCAATCCCCAACCGCCTCTGCAGAACGTGTACCAACTTGTTCAGGAGGTGAT

TGACCGGGTCGAGCTCCACGAGAAAGTGTCGAGCCACCCGATCTTCAAGCAG

>HQ832577.1_A_IND_1999

CCCTGGTACAAGCTCATCAAACTCCTGAGCCGCCTGTCATGTATGGCCGCTGTAGCAGCACGGTCAAAGG

ACCCAGTCCTTGTGGCTATCATGCTGGCTGACACCGGTCTCGAGATTCTGGACAGCACCTTTGTCGTGAA

GAAGATCTCCGACTCGCTCTCCAGTCTCTTTCACGTGCCGGCCCCCGTCTTCAGTTTCGGAGCCCCGATT

CTGTTGGCCGGGTTGGTCAAAGTCGCCTCGAGTTTCTTCCAGTCAACACCCGAGGAACTTGAGAGAGCAG

AAAAACAGCTCAAAGCACGTGACATCAATGACGTGTTCGCCGTTCTCAAGAACGGCGAGTGGCTGGTCAA

GCTGATTCTTGCCATCCGCGACTGGATCAAGGCATGGATCGCCTCAGAAGAGAAGTTTGTCACCATGACA

GACTTGGTGCCTGGCATCCTTGAAAAGCAGCGGGACCTCAACGACCCGAGCAAGTACAAGGGAGCCAAGG

AGTGGCTCGACAACGCGCGCCAGGCGTGTTTGAAGAGCGGGAACGTCCACATTGCCAACCTCTGCAAAGT

GGTCGCCCCGGCACCCAGCAAGTCGAGACCCGAGCCCGTGGTCGTTTGCCTCCGTGGCAAATCCGGCCAG

GGTAAGAGTTTCCTTGCTAACGTGCTTGCACAAGCAATTTCAACCCACTTTACTGGTAGAACTGATTCAG

TTTGGTACTGCCCACCTGACCCGGACCACTTCGACGGTTACAACCAACAGACCGTTGTAGTGATGGATGA

TTTGGGCCAGAACCCCGATGGCAAGGACTTCAAGTACTTCGCCCAGATGGTGTCAACCACGGGGTTCATC

CCGCCCATGGCTTCACTCGAGGACAAAGGCAAGCCTTTCAACAGCAAGGTCATCATTGCCACCACCAACC

TGTACTCGGGCTTCACCCCGAGGACCATGGTGTGCCCTGATGCACTGAACCGCAGGTTTCACTTTGACAT

CGATGTGAGCGCCAAGGATGGGTACAAAATTAACAATAAATTGGACATAATCAAAGCACTTGAAGACACC

CACACCAATCCAGTGGCGATGTTTCAATATGACTGTGCCCTTCTCAACGGCATGGCCGTTGAAATGAAGA

GAATGCAACAAGACATGTTTAAGCCTCAGCCACCCCTCCAGAACGTGTACCAACTTATTCAGGAGGTGAT

TGACCGGGTCGAGCTCCACGAGAAAGTGTCGAGCCACCCAATCTTCAAGCAG

>HQ832578.1_A_IND_2003

CCCTGGTACAAGCTCATCAAGCTTCTGAGCCGCTTGTCATGCATGGCCGCTGTAGCAGCACGGTCAAAGG

ACCCAGTCCTTGTGGCCATCATGCTAGCTGACACCGGTCTCGAGATTCTGGACAGCACATTTGTCGTGAA

GAAGATCTCCGACTCGCTCTCCAGTCTCTTTCACGTGCCGGCCCCCGTCTTCAGTTTCGGAGCTCCGATT

CTGTTGGCCGGGTTGGTCAAAGTCGCCTCGAGTTTCTTCCGGTCTACACCCGAAGACCTTGAGAGAGCAG

AAAAACAGCTCAAAGCATGGGACAAGAATGACATATTCGCCATTCTCAAGAACGGCGAGTGGCTGGTCAA

GCTGATTCTTGCCATCCGCGACTTGATCAAGGCATGGATCGCCTCAGAAGAAAAGTTTGTCACCATGACA

GACTTGGTGCCTGGCATCCTTGAAAAGCAGCGGGACCTAAACGACCCGAGCAAGTACAAGGAGGCCAAGG

AGTGGCTCGACAACGCGCGCCAAGCGTGTCTGAAGAGTGGGAACATCCACATTGCCAACCTGTGCAAAGT

GGTTGCCCCGGCACCCAGCAAGTCGAGACCCGAACCCGTGGTCGTTTGCCTCCGTGGCAAGTCCGGCCAG

GGCAAGAGTTTCCTTGCGAACGTGCTCGCACAAGCAATCTCCACCCACTTCACTGGCAGAACTGACTCGG

TTTGGTACTGCCCGCCTGACCCTGACCACTTTGACGGCTACAACCAACAGACCGTTGTGGTAATGGATGA

CCTGGGCCAGAACCCTGACGGGAAGGACTTTAAGTACTTCGCCCAAATGGTGTCGACCACAGGGTTCATC

CCGCCCATGGCCTCGCTTGAGGACAAAGGCAAACCTTTCAACAGCAAGGTCATCATTGCCACCACTAACC

TGTACTCGGGGTTCACCCCGAGGACTATGGTGTGCCCCGACGCGCTGAACCGGAGGTTTCACTTTGACAT

CGACGTGAGTGCCAGGGACGAGTACAAGATTAACAACAAACTGGACATAATCAAAGCTCTTGAAGACACC

CACACCAATCCGGTGGCAATGTTTCAATACGACTGTGCCCTTCTCAACGGCATGGCCGTTGAAATGAAGA

GAATGCAACAAGATGTGTTCAAGCCTCAACCACCCCTCCAGAACGTGTACCAGCTAGTTCAGGAGGTGAT

TGATCGGGTTGAGCTCCACGAGAAAGTGTCGAGCCACCCGATCTTCAAGCAG

>HQ832579.1_A_IND_2003

CCCTGGTACAAGCTCATCAAGCTCCTGAGCCGCTTGTCATGCATGGCCGCTGTAGCAGCACGGTCAAAGG

ACCCAGTCCTTGTGGCCATCATGCTAGCTGACACCGGTCTCGAGATTCTGGACAGCACATTTGTCGTGAA

GAAGATCTCCGACTCGCTCTCCAGTCTCTTTCACGTGCCGGCCCCCGTCTTCAGTTTCGGAGCTCCGATT

CTGTTGGCCGGGTTGGTCAAAGTCGCCTCGAGTTTCTTCCGGTCCACACCCGAAGACCTTGAGAGAGCAG

AAAAACAGCTCAAAGCACGTGACATCAATGACATATTCGCCATTCTCAAGAACGGTGAGTGGCTGGTCAA

GCTGATTCTTGCCATCCGCGACTGGATCAAGGCATGGATCGCCTCAGAAGAAAAGTTTGTCACCATGACA

GACTTGGTGCCTGGCATCCTTGAAAAGCAGCGGGACCTAAACGACCCGAGCAAGTACAAGGAGGCCAAGG

AGTGGCTCGACAACGCGCGCCAAGCGTGTCTGAAGAGTGGGAACATCCACATTGCCAACCTGTGCAAAGT

GGTTGTCCCGGCACCCAGCAAGTCGAGACCCGAACCCGTGGTCGTTTGCCTCCGTGGCAAGTCCGGCCAG

GGCAAGAGTTTCCTTGCGAACGTGCTCGCACAAGCAATCTCCACCCACTTCACTGGCAGAACTGACTCGG

TTTGGTACTGCCCGCCTGACCCTGACCACTTTGACGGCTACAACCAACAGACCGTTGTGGTTATGGATGA

CCTGGGCCAGAACCCTGACGGGAAGGACTTCAAGTACTTCGCCCAAATGGTGTCAACTACAGGGTTCATC

CCGCCCATGGCCTCGCTTGAGGACAAAGGCAAACCTTTCAACAGCAAGGTCATCATTGCCACCACCAACC

TGTACTCGGGGTTCACCCCGAGAACTATGGTGTGCCCCGACGCGCTGAACCGGAGGTTTCATTTTGACAT

CGACGTGAGTGCCAGGGACGAGTACAAGATTAACAACAAATTGGACATAATCAAAGCTCTTGAAGACACC

TACACCAATCCGGTGGCAATGTTTCAATACGACTGTGCCCTTCTCAACGGTATGGCCGTTGAAATGAAGA

GAATGCAACAAGATGTGTTCAAGCCTCAACCACCCCTCCAGAACGTGTACCAGCTAGTTCAGGAGGTGAT

TGATCGGGTTGAGCTCCACGAGAAAGTGTCGAGCCACCCGATCTTCAAGCAG

>HQ832580.1_A_IND_2003

CCCTGGTACAAACTCATCAAGCTCCTGAGCCGCTTGTCATGCATGGCCGCTGTAGCAGCACGGTCAAAGG

ACCCAGTCCTTGTGGCCATCATGCTAGCTGACACCGGTCTCGAGATTCTGGACAGCACATTTGTCGTGAA

GAAGATCTCCGACTCGCTCTCCAGTCTCTTTCACGTGCCGGCCCCCGTCTTCAGTTTCGGAGCTCCGATT

CTGTTGGCCGGGTTGGTCAAAGTCGCCTCGAGTTTCTTCCGGTCCACACCCGAAGATCTTGAGAGAGCAG

AAAAACAGCTCAAAGCACGTGACATCAATGACATATTCGCCATTCTCAAGAACGGCGAGTGGCTGGTCAA

GCTGATTCTTGCCATCCGCGACTGGATCAAGGCATGGATCGCCTCAGAAGAAAAGTTTGTCACCATGACA

GACTTGGTGCCTGGCATCCTTGAAAAGCAGCGGGACCTAAACGACCCGAGCAAGTACAAGGAAGCCAAGG

AGTGGCTCGACAACGCGCGCCAAGCGTGTCTGAAAAGTGGGAACATCCACATTGCCAACCTGTGCAAAGT

AGTTGCTCCGGCACCCAGCAAGTCGAGACCCGAACCCGTGGTCGTTTGCCTCCGTGGCAAGTCCGGCCAG

GGCAAGAGTTTCCTTGCGAACGTGCTTGCACAAGCAATCTCCACCCACTTCACTGGCAGAACTGACTCAG

TTTGGTACTGCCCGCCTGACCCTGACCACTTTGACGGCTACAACCAACAGACCGTTGTGGTTATGGACGA

CCTGGGCCAAAACCCTGACGGGAAGGACTTCAAGTACTTCGCCCAAATGGTGTCAACTACAGGGTTCATC

CCGCCCATGGCCTCGCTTGAGGACAAAGGCAAACCTTTCAACAGTAAGGTCATCATTGCCACCACCAACC

TGTACTCGGGGTTCACCCCGAGAACTATGGTGTGCCCCGACGCGCTGAACCGGAGGTTTCACTTTGACAT

CGACGTGAGTGCCAGGGACGGGTACAAGATTAACAACAAGTTGGACATAATCAAAGCTCTTGAGGACACC

CACACCAATCCGGTGGCAATGTTTCAATACGACTGTGCCCTTCTCAACGGTATGGCCGTTGAAATGAAGA

GAATGCAACAAGATGTGTTCAAGCCTCAACCACCCCTCCAGAACGTGTACCAGCTAGTTCAGGAGGTGAT

TGATCGGGTTGAGCTCCACGAGAAAGTGTCGAGCCACCCGATCTTCAAGCAG

>HQ832581.1_A_IND_2004

CCCTGGTACAAACTCATCAAGCTCCTGAGCCGCTTGTCATGCATGGCCGCTGTAGCAGCACGGTCAAAGG

ACCCAGTCCTTGTGGCCATCATGCTAGCTGACACCGGTCTCGAGATTCTGGACAGCACATTTGTCGTGAA

GAAGATCTCCGACTCGCTCTCCAGTCTCTTTCACGTGCCGGCCCCCGTCTTCAGTTTCGGAGCTCCGATT

CTGTTGGCCGGGTTGGTCAAAGTCGCCTCGAGTTTCTTCCGGTCCACACCCGAAGATCTTGAGAGAGCAG

AAAAACAGCTCAAAGCACGTGACATCAATGACATATTCGCCATTCTCAAGAACGGCGAGTGGCTGGTCAA

GCTGATTCTTGCCATCCGCGACTGGATCAAGGCATGGATCGCCTCAGAAGAAAAGTTTGTCACCATGACA

GACTTGGTGCCTGGCATCCTTGAAAAGCAGCGGGACCTAAACGACCCGAGCAAGTACAAGGAAGCCAAGG

AGTGGCTCGACAACGCGCGCCAAGCGTGTCTGAAGAGTGGGAACATCCACATTGCCAACCTGTGCAAAGT

AGTTGCTCCGGCACCCAGCAAGTCGAGACCCGAACCCGTGGTCGTTTGCCTCCGTGGCAAGTCCGGCCAG

GGCAAGAGTTTCCTTGCGAACGTGCTTGCACAAGCAATCTCCACCCACTTCACTGGCAGAACTGACTCAG

TTTGGTACTGCCCGCCTGACCCTGACCACTTTGACGGCTACAACCAACAGACCGTTGTGGTTATGGACGA

CCTGGGCCAGAACCCTGACGGGAAGGACTTCAAGTACTTTGCCCAAATGGTGTCAACTACAGGGTTCATC

CCGCCCATGGCCTCGCTTGAGGACAAAGGCAAACCTTTCAACAGCAAGGTCATCATTGCCACCACCAACC

TGTACTCGGGGTTCACCCCGAGAACTATGGTGTGTCCCGACGCGCTGAACCGGAGGTTTCACTTTGACAT

CGGCGTGAGTGCCAGGGACGAGTACAAGATTAACAACAAATTGGACATAATCAAAGCTCTTGAAGACACC

CACACCAATCCGGTGGCAATGTTTCAATACGACTGCGCCCTTCTCAACGGTATGGCCGTTGAAATGAAGA

GAATGCAACAAGATGTGTTCAAGCCTCAACCACCCCTCCAGAACGTGTACCAGCTAGTTCAGGAGGTGAT

TGATCGGGTTGAGCTCCACGAGAAAGTGTCGAGCCACCCGATCTTCAAGCAG

>HQ832582.1_A_IND_2004

CCCTGGTACAAGCTCATCAAGCTCCTGAGCCGCTTGTCATGCATGGCCGCTGTAGCAGCACGGTCAAAGG

ACCCAGTCCTTGTGGCCATCATGCTAGCTGACACCGGTCTCGAGATTCTGGACAGCACATTTGTCGTGAA

GAAGATCTCCGACTCGCTCTCCAGTCTCTTTCACGTGCCGGCCCCCGTCTTCAGTTTCGGAGCTCCGATT

CTGTTGGCCGGGTTGGTCAAAGTCGCCTCGAGTTTCTTCCGGTCCACACCCGAAGACCTTGAGAGAGCAG

AAAAACAGCTCAAAGCACGTGACATCAATGACGTATTCGCCATTCTCAAGAACGGCGAGTGGCTGGTCAA

GCTGATTCTTGCCATCCGCGACTGGATCAAGGCATGGATCGCCTCAGAGGAAAAGTTTGTCACCATGACA

GACTTGGTGCCTGGCATCCTTGAAAAGCAGCGGGACCTGAACGACCCGAGCAAGTACAAGGAGGCCAAGG

AGTGGCTCGACAACGCGCGCCAAGCGTGTCTGAAGAATGGGAACACCCACATTGCCAGCCTGTGCAAAGT

GGTTGCCCCGGCACCCAGCAAGTCGAGACCCGAACCCGTGGTCGTTTGCCTCCGTGGCAAGTCCGGCCAG

GGCAAGAGTTTCCTTGCGAACGTGCTCGCACAAGCAATTTCCACCCACTTCACTGGCAGAACTGATTCGG

TTTGGTACTGCCCGCCTGACCCTGACCACTTTGACGGCTACAACCAACAGACCGTTGTGGTTATGGATGA

CCTGGGCCAGAACCCTGACGGGAAGGACTTCAAGTACTTCGCCCAAATGGTGTCAACTACAGGGTTCATC

CCGCCCATGGCCTCGCTTGAGGACAAAGGCAAACCTTTCAACAGCAAGGTCATCATCGCCACCACCAACT

TGTACTCGGGGTTCACCCCGAGAACTATGGTGTGCCCTGACGCGCTGAACCGGAGGTTTCACTTTGACAT

CGACGTGAGTGCCAGGGACGAGTACAAAATTAATAACAAATTGGACATAATCAAAGCTCTTGAAGACACC

CACACCAATCCGGTGGCAATGTTTCAGTACGACTGTGCCCTTCTCAACGGTATGGCCGTTGAAATGAAGA

GAATGCAACAAGATGTGTTTAAGCCTCAACCACCCCTCCAGAACGTGTACCAGCTAGTTCAGGAAGTGAT

TGATCGGGTTGAGCTCCACGAGAAAGTGTCGAGCCACCCGATCTTCAAGCAG

>HQ832583.1_A_IND_2005

CCCTGGTACAAGCTCATCAAGCTCCTGAGCCGCTTGTCATGCATGGCCGCTGTAGCAGCACGGTCAAAGG

ACCCAGTCCTTGTGGCCATCATGCTAGCTGACACCGGTCTCGAGATTCTGGACAGCACATTTGTCGTGAA

GAAGATCTCCGACTCGCTCTCCAGTCTCTTTCACGTGCCGGCCCCCGTCTTCAGTTTCGGAGCTCCGATT

CTGTTGGCCGGGTTGGTCAAAGTCGCCTCGAGTTTCTTCCGGTCCACACCCGAAGACCTTGAGAAAGCAG

AAAAACAGCTCAAAGCACGTGACATCAATGACATATTCGCCATTCTCAAGAACGGCGAGTGGCTGGTCAA

GCTGATTCTTGCCATCCGCGACTGGATCAAGGCATGGATCGCCTCAGAAGAAAAGTTTGTCACCATGACA

GACTTGGTGCCTGGCATCCTTGAAAAGCAGCGGGACCTAAACGACCCGAGCAAGTACAAGGAGGCCAAGG

AGTGGCTCGACAACGCGCGCCAAGCGTGTCTGAAGAGTGGGAACATCCACATTGCCAACCTGTGCAAAGT

GGTTGCCCCGGCACCCAGCAAGTCGAGACCCGAACCCGTGGTCGTTTGCCTCCGTGGCAAGTCCGGCCAG

GGCAAGAGTTTCCTTGCGAACGTGCTCGCACAAGCAATCTCCACCCACTTCACTGGCAGAATTGACTCGG

TTTGGTACTGCCCGCCTGACCCTGACCACTTTGACGGCTACAACCAACAGACCGTTGTGGTTATGGACGA

CCTGGGCCAGAACCCTGACGGGAAGGACTTCAAGTACTTCGCCCAAATGGTGTCAACTACAGGGTTCATC

CCGCCCATGGCCTCGCTTGAGGACAAAGGCAAACCTTTCAACAGCAAGGTCATCATTGCCACCACCAACC

TGTACTCGGGGTTCACCCCGAGAACTATGGTGTGCCCCGACGCGCTGAACCGGAGGTTTCACTTTGACAT

CGACGTGAGTGCCAGGGACGAGTACAAGATTAACAACAAATTGGACATAATCAAAGCTCTTGAAGACACC

CACACCAATCCGGTGGCAATGTTTCAATACGACTGTGCCCTTCTCAACGGTATGGCCGTTGAAATGAAGA

GAATGCAACAAGATGTGTTCAAGCCTCAACCACCCCTCCAGAACGTGTACCAGCTAGTTCAGGAGGTGAT

TGATCGGGTTGAGCTCCACGAGAAAGTGTCGAGCCACCCGATCTTCAAGCAG

>HQ832584.1_A_IND_2005

CCCTGGTACAAGCTCATCAAACTACTGAGCCGCCTGTCATGCATGGCCGCTGTAGCAGCACGGTCAAAGG

ACCCAGTCCTTGTGGCCATCATGCTAGCTGACACCGGTCTCGAGATTCTGGACAGCACCTTTGTCGTGAA

GAAAATCTCCGACTCGCTCTCCAGTCTCTTTCACGTGCCGGCCCCCGTCTTCAGTTTTGGAGCCCCAATC

CTGTTGGCCGGGTTGGTCAAGGTCGCCTCGAGTTTCTTCCGGTCCACACCCGAAGACCTCGAGAGAGCAG

AAAAGCAGCTCAAAGCACGTGACATCAATGACATTTTCGCCATTCTCAAGAACGGCGAGTGGCTGGTCAA

GCTGATCCTTGCCATCCGCGACTGGATCAAGGCATGGATCGCCTCAGAAGAAAAGTTTGTCACCATGACA

GACTTGGTGCCCGGCATCCTTGAAAAACAGCGGGAACTCAACGGCCCGAGCAAGTACAAGGAGGCCAAAG

AGTGGCTTGACAACGCGCGCCAGGCGTGTTTAAAGAGCGGGAACGTCTACATTGCCAACCTTTGCAAGGT

GGTCGCCCCGGCACCCAGCAAGTCGAGACCTGAGCCAGTGGTCGTTTGCCTCCGTGGCAAGTCCGGCCAG

GGCAAAAGTTTCCTTGCGAACGTGCTCGCGCAAGAAATCTCTACCCACTTCACTGGCAGAACTGATTCGG

TTTGGTACTGCCCGCCTGACCCCGACCACTTCGACGGTTACAACCAGCAGACTGTTGTTGTGATGGATGA

TTTGGGCCAGAACCCTGACGGCAAGGACTTCAAGTATTTTGCCCAGATGGTTTCAACCACAGGGTTCATC

CCGCCCATGGCCTCACTCGAGGACAAAGGTAAACCTTTCAACAGTAAGGTCATCATAGCCACCACCAACC

TGTACTCGGGGTTCACCCCAAGAACTATGGTGTGCCCTGATGCACTGAACCGCAGGTTTCACTTTGACAT

CGACGTGAGTGCCAAGGATGGGTACAAAATTAACAACAAATTGGACATAATCAAAGCTCTTGAGGACACC

CACACCAATCCCGTGGCAATGTTCCAGTATGACTGTGCCCTTCTCAACGGCATGGCCGTTGAAATGAAGA

GAATGCAACAAGACATGTTCAAGCCTCAGCCGCCCCTCCAGAACGTATACCAGCTCGTTCAGGAGGTGAT

TGAGCGGGTCGAGCTCCACGAGAAAGTGTCGAGCCACCCGATCTTCAAGCAG

>HQ832585.1_A_IND_2005

CCCTGGTACAAGCTCATCAAACTCCTGAGCCGCTTGTCATGCATGGCTGCTGTAGCAGCACGGTCGAAGG

ACCCAGTCCTTGTGGCCATCATGCTAGCTGACACCGGTCTTGAGATTCTGGACAGCACTTTTGTCGTGAA

GAAGATCTCCGACTCACTCTCCAGTCTCTTTCACGTGCCGGCCCCCGTCTTCAGTTTCGGAGCCCCGGTC

CTGTTGGCTGGGTTGGTCAAAGTCGCCTCGAGTTTCTTCCGGTCGACGCCCGAAGACCTTGAGAGAGCCG

AAAAACAGCTCAAAGCACGTGACATTAATGACATTTTCGCCATTCTCAAGAACGGCGAGTGGCTGGTCAA

ACTGATTCTTGCCATCCGCGACTGGATTAAGGCATGGATCGCCTCAGAAGAAAAGTTTGTCACCATGACA

GACTTGGTGCCTGGCATCCTTGAAAAACAACGGGACCTCAACGACCCAAACAAGTATAAGGAGGCCAAGG

AATGGCTTGACAACGCGCGCCAAGCGTGTTTGAAGAGTGGGAACCACCACATTGCCAACCTCTGCAAAGT

GGTCGCCCCGGCACCCAGCAAGTCGAGACCCGAGCCCGTGGTCGTTTGCCTCCGTGGCAAATCTGGCCAG

GGCAAGAGTTTCCTTGCGAACGTGCTCGCGCAGGCAATTTCTACCCACTTTACTGGCAGAACTGATTCAG

TTTGGTACTGCCCACCTGACCCTGACCACTTTGACGGTTACAACCAACAGACCGTTGTAGTGATGGATGA

TTTGGGCCAGAACCCTGACGGCAAGGACTTCAAGTACTTTGCCCAAATGGTGTCAACCACGGGGTTCATC

CCGCCCATGGCCTCACTGGAAGACAAAGGCAAACCTTTCAACAGCAAGGTCATCATTGCCACCACCAACC

TGTACTCGGGTTTCACCCCGAGGACTATGGTGTGCCCTGATGCACTGAACCGTAGGTTTCACTTTGACAT

TGGCGTGAGTGCTAAGGATGGGTACACAATCAACAACAAATTGGACATAATCAAAGCTCTTGAAGACACC

CACACTAACCCAGTGGCAATGTTTCAATACGACTGTGCCCTTCTCAACGGCATGGCCGTTGAAATGAAGA

GAATGCAACAAGACATGTTCAAGCCTCAACCACCCCTCCAGAACGTGTATCAACTCGTCCAGGAGGTGAT

TGAACGGGTCGAGCTCCACGAGAAAGTGTCGAGCCACCCGATCTTCAAGCAG

>HQ832586.1_A_IND_2006

CCCTGGTACAAGCTCATCAAGCTCCTGAGCCGCCTGTCATGCATGGCCGCTGTCGCAGCACGGTCAAAGG

ACCCAGTCCTTGTGGCCATCATGCTAGCAGACACCGGTCTCGAGATTCTGGCCAGCACCTTTGTCGTGAA

GAAGATCTCCGACTCGCTCTCCAGTCTCTTTCACGTGCCGGCCCCCGTCTTCAGTTTCGGAGCCCCGATC

CTGTTGGCTGGGTTCGTCAAAGTCGCCTCGAGTTTCTTCCGGTCCACGCCCGAAGACCTTGAGAGAGCGG

AGAAACAGCTCAAAGCACGTGACATTAATGACATCTTCGCCATTCTCAAGAACGGCGAGTGGCTGGTCAA

GCTGATCCTTGCTATCCGCGACTGGATCAAAGCATGGATCGCCTCAGAAGAGAAGTTTGTCACCATGACA

GACTTGGTGCCTGGCATCCTTGAAAAACAGCGGGACCTCAACGACCCAAGCAAGTACAAGGAGGCCAAGG

AGTGGCTCGACAACGCGCGCCAAGCGTGTTTGAAGAGCGGGAACGTCCACATTGCTAACCTCTGCAAAGT

GGTCGCCCCGGCACCCAGCAGGTCGAGGCCCGAACCTGTGGTCGTTTGCCTCCGTGGCAAATCCGGCCAA

GGAAAGAGTTTCCTTGCGAACGTGCTCGCACAAGCAATCTCCACCCACTTTACTGGCAGAACCGATTCAG

TTTGGTACTGCCCGCCTGACCCCGACCACTTCGACGGTTACAACCAACAGACCGTTGTTGTGATGGACGA

TTTGGGCCAGAACCCTGACGGCAAGGACTTTAAGTACTTCGCCCAGATGGTTTCCACCACAGGGTTCATC

CCGCCCATGGCCTCACTCGAAGACAAAGGTAAACCTTTCAACAGCAAGGTCATCATAGCCACTACCAACC

TGTACTCGGGTTTCACCCCGAGAACCATGGTGTGCCCTGATGCACTGAACCGCAGGTTTCACTTTGACAT

TGACGTGAGCGCCAAGGACGGGTACAAAATTAATAACAAATTGGACATAATCAAAGCTCTTGAAGACACC

CACACCAATCCCGTGGCAATGTTTCAATACGACTGTGCCCTTCTCAACGGCATGGCTGTTGAAATGAAGA

GAATGCAACAAGACATGTTCAAGCCTCAACCGCCCCTCCAAAACGTGTATCAGCTCGTTCAGGAGGTGAT

TGAACGGGTCGAGCTCCACGAGAAAGTGTCGAGCCACCCGATCTTCAAGCAG

>HQ832587.1_A_IND_2005

CCCTGGTACAAGCTCATCAAACTCCTAAGCCGCCTGTCGTGCATGGCCGCTGTAGCAGCACGGTCTAAGG

ACCCAGTCCTTGTGGCCATCATGCTGGCAGACACCGGCCTTGAGATTCTGGACAGCACGTTTGTCGTGAA

GAAAATCTCCGACTCGCTCTCCAGTCTCTTCCACGTGCCGGCCCCCGTCTTCAGTTTCGGAGCCCCGATT

CTCTTGGCCGGGTTGGTCAAAGTCGCCTCGAGCTTCTTCCGGTCCACACCCGAAGACCTCGAGAGAGCAG

AGAAACAGCTCAAAGCACGCGACATCAACGACATATTCGCCATTCTCAAGAACGGCGAGTGGCTGGTCAA

ACTGATTCTTGCCATCCGCGACTGGATCAAGGCATGGATCGCCTCAGAAGAAAAGTTTGTCACCATGACA

GACCTAGTGCCTGGCATCCTTGAAAAGCAGCGGGACCTCAACGACCCGAGCAAGTACAAGGAGGCCAAGG

AATGGCTCGACAACGCGCGTCAAGCGTGTTTGAAGAGCGGGAACGTCCACATTGCCAACCTGTGCAAAGT

GGTCGCCCCGGCACCGAGCAAGTCGAGACCTGAACCCGTGGTTGTTTGCCTCCGTGGCAGATCCGGCCAG

GGGAAGAGTTTCCTTGCAAACGTGCTTGCACAAGCAATCTCCACCCACTTCACCGGCAGAACCGACTCAG

TTTGGTACTGTCCACCTGACCCCGACCACTTCGACGGTTACAACCAACAAACCGTTGTCGTGATGGATGA

TTTGGGCCAAAACCCCGACGGCAAGGACTTCAAGTACTTCGCCCAGATGGTGTCTACCACGGGGTTTATC

CCGCCCATGGCCTCGCTCGAAGACAAAGGCAAGCCCTTCAACAGCAAAGTCATCATCGCTACCACCAACC

TGTACTCGGGCTTCACCCCGAGAACCATGGTGTGTCCTGATGCGCTGAACCGGAGGTTTCACTTTGACAT

TGATGTGAGTGCCAAGGATGGGTACAAAATTAACAACAAATTGGACATAACCAAAGCTCTTGAAGACACC

CACACCAACCCTGTGGCAATGTTTCAGTACGACTGTGCCCTTCTCAACGGCATGGCCGTTGGGGTGAAGA

GAATGCAACAAGATGTGTTCAAGCCCCAACCGCCTCTGGAAAACGTGTACCAACTCGTCCAGGAGGTGAT

TGACCGGGTCGAGCTCCACGAGAAAGTGTCGAGCCACCCGATCTTCAAGCAG

>HQ832588.1_A_IND_2005

CCCTGGTATAAGCTCATCAAGCTCCTGAGCCGCCTGTCATGTATGGCCGCTGTAGCAGCACGGTCAAAGG

ACCCAGTCCTTGTGGCCATCATGCTGGCTGACACCGGCCTTGAGATTCTGGACAGCACTTTTGTCGTGAA

GAAGATCTCCGACTCGCTCTCCAGTCTCTTTCACGTGCCGGCCCCCGTCTTCAGTTTCGGAGCTCCGATT

CTGTTGGCCGGGTTGGTCAAAGTCGCCTCGAGTTTCTTCCGGTCCACACCCGAAGACCTTGAGAGAGCGG

AGAAACAGCTCAAAGCACGTGACATCAATGACATATTCGCCATTCTCAAGAACGGCGAGTGGCTGGTCAA

ACTGATCCTTGCCATCCGCGACTGGATAAAGGCATGGATCGCCTCAGAAGAAAAGTTTGTCACCATGACA

GACCTGGTGCCTGGCATCCTTGAAAAACAGCGGGATCTCAACGATCCTGGCAAATACAAGGAGGCCAAGG

AGTGGCTCGACAACGCGCGCCAAGCGTGTCTGAAGAGTGGGAACGTCCACATTGCAAACCTTTGCAAAGT

GGTTGCCCCAGCACCCAGCAGGTCGAGGCCCGAGCCCGTGGTCGTTTGCCTCCGTGGCAAATCGGGCCAG

GGCAAGAGCTTCCTTGCGAACGTGCTTGCACAAGCAATTTCAACCCACTTCACTGGCAGAACCGACTCGG

TTTGGTACTGCCCGCCTGACCCTGACCACTTCGACGGTTACAACCAACAAACCGTTGTTGTGATGGATGA

TTTGGGCCAGAACCCTGACGGCAAGGACTTCAAGTACTTTGCCCAGATGGTTTCCACCACAGGGTTCATC

CCGCCTATGGCCTCACTTGAAGACAAAGGCAAACCTTTCAACAGCAAGGTCATCATAGCCACCACCAACC

TGTACTCGGGGTTCACCCCGAGGACCATGGTGTGCCCAGATGCACTGAACCGTAGGTTTCACTTTGACAT

TGACGTGAGCGCTAAGGACGGGTACAAAATTAACAACAAATTGGACATAATCAAAGCTCTTGAAGACACC

CACACCAATCCCGTGGCAATGTTTCAATATGACTGTGCCCTTCTCAACGGCATGGCCGTTGAAATGAAGA

GAATGCAACAAGACATATTCAAGCCTCAGCCACCCCTCCAGAACGTGTACCAGCTCGTTCAGGAGGTGAT

TGAAAGGGTCGAGCTCCACGAGAAAGTGTCGAGCCACCCGATCTTCAAGCAA

>HQ832589.1_A_IND_2006

CCCTGGTACAAGCTCATCAAACTCCTAAGCCGCCTGTCATGCATGGCCGCTGTAGCAGCACGGTCAAAGG

ACCCAGTCCTTGTGGCCATCATGCTAGCTGACACCGGTCTCGAGATTCTGGACAGCACCTTTGTCGTGAA

GAAAATCTCCGACTCGCTCTCCAGTCTCTTTCACGTGCCGGCCCCCGTCTTCAGTTTTGGAGCCCCAATT

CTGTTGGCCGGGTTGGTCAAGGTCGCCTCGAGTTTCTTCCGGTCCACACCCGAAGACCTTGAGAGAGCAG

AAAAACAGCTCAAAGCACGTGACATCAATGACATATTCGCCATTCTCAAGAACGGCGAGTGGCTGGTCAA

GCTGATCCTTGCCATCCGCGACTGGATTAAGGCATGGATCGCCTCAGAAGAAAAGTTTGTCACCATGACA

GACTTGGTGCCTGGCATCCTTGAAAAGCAGCGGGACCTCAACGACCCGAGCAAGTACAAGGAGGCCAAGG

AGTGGCTCGACAACGCGCGCCAAGCGTGTTTGAAGAGCGGGAACGTCCACATTGCCAACCTCTGCAAAGT

GGTCGCCCCGGCACCCAGCAAGTCGAGGCCCGAACCTGTGGTCGTTTGCCTCCGTGGCAAGTCCGGCCAG

GGCAAGAGTTTCCTTGCGAACGTGCTCGCGCAAGCAATCTCCACCCACTTCACTGGCAGAACCGACTCGG

TTTGGTACTGCCCGCCTGACCCTGACCACTTCGACGGTTACAACCAACAGACCGTTGTTGTGATGGATGA

TTTGGGCCAGAACCCTGACGGCAAGGACTTCAAGTACTTTGCCCAGATGGTTTCTACCACAGGGTTCATC

CCGCCTATGGCCTCACTCGGAGACAAAGGCAAACCTTTCAACAGCAAGGTCATCATAGCCACCACCAACC

TGTACTCGGGGTTCACCCCGAGGACCATGGTGTGCCCAGATGCACTGAACCGTAGGTTTCACTTTGACAT

TGACGTGAGTGCTAAGGACGGGTACAAAATTAACAACAAATTGGACATAATCAAAGCTCTTGAAGACACC

CACACCAATCCCGTGGCAATGTTTCAATATGACTGTGCCCTTCTCAACGGCATGGCCGTTGAAATGAAGA

GAATGCAACAAGACGTGTTCAAGCCTCAGCCACCCCTCCAGAACGTGTACCAGCTCGTTCAGGAGGTGAT

TGAAAGGGTCGAGCTCCACGAGAAAGTGTCGAGCCACCCGATCTTCAAGCAG

>HQ832590.1_A_IND_2007

CCCTGGTACAAGCTCATCAAACTCCTGAGTCGCCTGTCATGCATGGCCGCTGTAGCAGCACGGTCAAAGG

ACCCAGTCCTTGTGGCCATCATGCTAGCTGACACCGGTCTCGAGATTCTGGACAGCACTTTTGTCGTGAA

GAAGATCTCCGACTCACTCTCCAGTCTCTTTCACGTGCCGGCCCCCGTCTTCAGTTTCGGAGCTCCGATC

CTGTTGGCCGGGTTGGTCAAAGTCGCCTCGAGTTTCTTCCGGTCCACACCCGAAGACCTTGAGAGAGCAG

AGAAACAGCTCAAAGCACGTGACATCAATGACATCTTCGCCATTCTCAAGAACGGCGAGTGGCTGGTCAA

GCTGATCCTTGCTATCCGCGACTGGATCAAAGCATGGATTGCCTCAGAAGAGAAGTTTGTCACCATGACA

GACTTGGTGCCTGGCATCCTTGAAAAGCAGCGGGACCTTAACGACCCAAGTAAGTACAAGGAAGCCAAGG

AGTGGCTCGACAACGCGCGCCAAGCGTGCTTAAAGAGCGGGAACGTCCACATTGCCAACCTCTGCAAAGT

GGTCGCCCCGGCACCCAGCAAGTCGAGACCCGAACCTGTGGTCGTTTGCCTCCGTGGCAAGTCCGGCCAG

GGTAAGAGTTTCCTTGCGAACGTGCTCGCACAAGCAATCTCCACCCACTTCACTGGCAGAACCGATTCAG

TTTGGTACTGCCCGCCTGACCCTGACCACTTCGACGGTTACAACCAGCAGACCGTTGTTGTGATGGACGA

TTTGGGCCAGAACCCCGATGGCAAGGACTTCAAGTACTTCGCCCAGATGGTTTCGACCACGGGGTTCATC

CCGCCCATGGCTTCACTCGAGGACAAAGGCAAACCTTTCAACAGCAAGGTCATCATCGCCACCACCAACC

TGTACTCGGGCTTCACCCCGAGAACTATGGTGTGTCCTGATGCACTGAACCGAAGGTTTCACTTTGACAT

TGATGTGAGTGCCAAGGACGGGTATAAAATTAACAACAAACTGGACATTATCAAAGCTCTTGAGGACACC

CACACCAACTCAGTGGCAATGTTTCAGTACGATTGTGCCCTTCTCAACGGCATGGCCGTTGAAATGAAGA

GAATGCAACAAGATGTGTTCAAGCCTCAACCGCCCCTCCAGAACGTTTACCAGCTTGTTCAGGAGGTGAT

TGACCGGGTCGAGCTCCACGAGAAGGTGTCGAGCCACCCGATCTTCAAGCAG

>HQ832591.1_A_IND_2008

CCCTGGTACAAGCTCATCAAACTCCTGAGTCGCCTGTCATGCATGGCCGCTGTAGCAGCACGGTCAAAGG

ACCCAGTACTTGTGGCCATCATGCTAGCTGACACCGGTCTCGAGATTCTGGACAGCACCTTTGTCGTGAA

GAAGATCTCCGACTCGCTCTCCAGTCTCTTTCACGTGCCGGCCCCCGTCTTCAGTTTCGGAGCTCCGATC

CTGTTGGCCGGGTTGGTCAAAGTCGCCTCGAGCTTCTTCCGGTCTACGCCCGAAGACCTTGAGAGAGCAG

AGAAACAGCTCAAAGCACGTGACATCAATGACATCTTCGCCATTCTCAAGAACGGCGAGTGGCTGGTTAA

GCTGATCCTTGCTATCCGCGACTGGATCAAAGCATGGATTGCCTCAGAAGAGAAGTTTGTCACCATGACA

GACTTGGTGCCTGGCATCCTTGAAAAGCAGCGGGACCTTAACGACCCAAGTAAGTACAAGGAAGCCAAGG

AGTGGCTCGACAACGCGCGCCAAGCGTGCTTGAAGAGCGGGAACGTCCACATTGCCAACCTCTGCAAAGT

GGTCGCCCCGGCACCCAGCAAGTCGAGACCCGAACCTGTGGTCGTTTGCCTCCGTGGCAAGTCCGGCCAG

GGTAAGAGTTTCCTTGCGAACGTGCTCGCACAAGCAATCTCTACCCACTTCACTGGCAGAACCGATTCAG

TTTGGTACTGCCCGCCTGACCCTGACCACTTCGACGGCTACAACCAGCAGACCGTTGTTGTGATGGACGA

TTTGGGCCAGAACCCCGATGGCAAGGACTTCAAGTACTTCGCCCAGATGGTTTCGACCACGGGATTCATC

CCGCCCATGGCTTCACTTGAGGACAAAGGCAAACCCTTCAACAGCAAGGTCATCATCGCCACCACCAACC

TGTACTCGGGCTTCACCCCGAGAACTATGGTGTGTCCTGATGCACTGAACCGGAGGTTTCATTTTGACAT

AGATGTGAGTGCCAAGGACGGGTATAAAATTAACAACAAATTGGACATTATCAAAGCTCTTGAGGACACC

CACACCAACCCAGTGGCAATGTTTCAGTACGATTGTGCCCTTCTCAACGGCATGGCCGTTGAAATGAAGA

GAATGCAACAAGATGTGTTCAAGCCTCAACCGCCCCTCCAGAACGTTTACCAGCTTGTTCAGGAGGTGAT

TGACCGGGTCGAGCTCCACGAGAAGGTGTCGAGCCACCCGATCTTCAAGCAG

>HQ832592.1_A_IND_2009

CCCTGGTACAAGCTCATCAAGCTCCTGAGTCGCCTGTCATGCATGGCCGCTGTAGCAGCACGGTCAAAGG

ACCCAGTCCTTGTGGCCATCATGCTAGCTGACACCGGTCTCGAGATTCTGGACAGCACTTTTGTCGTGAA

GAAGATCTCCGACTCGCTCTCCAGTCTCTTTCACGTGCCGGCCCCCGTCTTCAGTTTTGGAGCCCCAATC

CTGTTGGCCGGGTTGGTCAAGGTCGCCTCGAGTTTCTTCCGGTCCACACCCGAAGACCTTGAGAGAGCAG

AAAAACAGCTCAAAGCACGTGACATCAATGACATATTCGCCATTCTCAAAAACGGCGAGTGGCTGGTCAA

ACTGATTCTTGCCATCCGCGACTGGATTAAGGCATGGATCGCCTCAGAAGAAAAGTTTGTCACCATGACA

GACTTGGTGCCTGGCATCCTTGAAAAGCAGCGGGACCTCAACGACCCAAGCAAGTACAAGGAAGCCAAGG

AGTGGCTCGACAACGCGCGCCAAGCGTGCTTGAAGAGCGGGAACGTCCACATTGCCAACCTTTGCAAAGT

GGTCGCCCCGGCACCCAGCAAGTCGAGGCCCGAACCTGTGGTCGTTTGCCTCCGCGGCAAGTCCGGCCAG

GGTAAGAGTTTCCTTGCGAACGTGCTCGCACAAGCAATCTCCACCCACTTCACTGGCAGGACCGATTCAG

TTTGGTACTGCCCGCCTGATCCCGACCACTTCGACGGTTACAACCAGCAGACCGTTGTTGTGATGGATGA

TTTGGGCCAGAACCCTGATGGCAAGGACTTCAAGTACTTTGCCCAGATGGTTTCCACCACAGGGTTCATC

CCACCCATGGCCTCACTCGAAGACAAAGGTAAGCCTTTTAACAGCAAGGTCATCATAGCCACCACCAACC

TGTACTCGGGGTTCACCCCGAGAACCATGGTGTGCCCTGATGCACTGAACCGCAGGTTTCACTTTGACAT

TGACGTGAGCGCCAAGGACGGGTACAAAATTAACAACAAATTGGACATAATCAAAGCTCTTGAGGACACC

CACACCAATCCCGTGGCAATGTTTCAATATGACTGTGCCCTTCTCAACGGCATGGCCGTTGAAATGAAGA

GAATGCAACAAGACGTGTTCAAGCCACAGCCACCCCTCCAGAACTTGTACCAGCTCGTTCAAGAGGTGAT

TGAACGGGTCGAGCTCCACGAGAAAGTGTCGAGCCACCCGATCTTCAAGCAG

>JF749841.1_A_TUR_2006

CCCTGGTACAAGCTCATTAAGCTCCTGAGCCGCCTGTCATGCATGGCCGCTGTAGCAGCACGGTCAAAGG

ACCCAGTCCTTGTGGCCATCATGCTAGCTGACACCGGTCTCGAGATTCTGGACAGCACCTTTATCGTGAA

GAAGATCTCCGACTCGCTCTCCAGTCTCTTTCACGTGCCGGCCCCCGTCTTCAGTTTCGGAGCTCCGATT

CTGCTGGCCGGGTTGGTCAAAGTCGCCTCGAGTTTCTTCCAGTCCACACCCGAAGACCTTGAGAGAGCAG

AGAAACAGCTCAAAGCACGTGACATCAATGACATTTTCGCCATTCTCAAGAACGGCGAGTGGCTGGTCAA

ACTGATCCTTGCCATCCGCGACTGGATAAAGGCTTGGATCGCCTCAGAAGAGAAGTTTGTCACCATGACA

GACTTGGTGCCCGGCATCCTTGAAAAGCAACGGGATCTCAACGACCCGAGCAAGTACGAGGAGGCCAAGG

AGTGGCTCGACAACGCGCGCCAGGCGTGTCTGAAGAGCGGGAACGTCCACATTGCCAACTTGTGCAAAGT

GGTCGCCCCGGCACCCAGCAAGTCGAGACCCGAACCTGTGGTCGTTTGCCTCCGTGGTAAATCCGGTCAG

GGGAAGAGTTTCCTTGCGAACGTGCTTGCACAAGCAATCTCCACCCACTTCACTGGCAGAACCGATTCAG

TTTGGTACTGCCCGCCTGACCCTGACCACTTCGACGGTTACAACCAGCAGACCGTTGTTGTGATGGACGA

TTTGGGCCAGAATCCCGACGGCAAGGACTTCAAGTACTTCGCCCAGATGGTTTCGACTACGGGGTTCATC

CCGCCCATGGCGTCACTTGAGGACAAAGGCAAACCCTTCAACAGCAAGGTCATCATCGCCACCACCAACC

TGTACTCGGGATTTACCCCGAGAACTATGGTGTGCCCTGATGCCCTGAACCGGAGGTTTCACTTTGACAT

CGACGTGAGCGCCAAGGACGGGTACAAAACTAACAACAAATTGGACATAATCAAAGCTCTTGAAGACACC

CACACCAACCCTGTGGCAATGTTTCAATATGACTGTGCCCTTCTCAACGGCATGGCCGTTGAAATGAAGA

GAATGCAACAAGATGTGTTCAAGCCTCAACCACCCCTCCAGAACGTATACCAACTCGTTCAGGAGGTGAT

CGAACGGGTCGAGCTCCACGAGAAAGTGTCGAGCCACCCGATCTTCAAGCAG

>JF749843.1_A_EGY_2006

CCCTGGTACAAGCTCATCAAACTCCTGAGCCGTTTGTCGTGTATGGCCGCTGTAGCAGCAAGGTCAAAGG

ACCCAGTCCTTGTGGCCATTATGCTGGCTGACACCGGTCTTGAGATTCTGGACAGCACGTTTGTCGTGAA

GAAGATTTCCGACTCGCTCTCCAGTCTCTTTCACGTGCCGGCCCCCGTCTTCAGTTTCGGAGCCCCGATC

CTGCTGGCCGGGTTGGTCAAGGTCGCCTCGAGTTTCTTCCGGTCCACACCCGAGGACCTCGAGAGAGCAG

AAAAACAGCTCAAAGCACGTGACATCAACGACATATTCGCCATTCTCAAGAACGGCGAGTGGCTGGTCAA

ACTGATCCTTGCCATCCGCGACTGGATCAAAGCATGGATCGCCTCAGAAGAAAAGTTCGTCACCATGACG

GACCTGGTGCCTGGCATCCTTGAAAAGCAGCGGGATCTTAACGACCCCGGCAAGTACAAGGAGGCCAAGG

AGTGGCTCGATAGCGCGCGCCAGGCGTGCTTGAAGAGCGGGAATGTTCACATTGCCAACCTGTGCAAAGT

GACCACCCCAGCACCCAGCAAGTCGAGGCCCGAACCAGTGGTCGTTTGCCTTCGCGGCAAATCCGGCCAA

GGGAAGAGCTTCCTTGCGAACGTACTTGCACAAGCAATTTCATCACACTTCACCGGTAGGACAGACTCGG

TCTGGTACTGCCCGCCTGACCCTGACCACTTCGACGGTTACAACCAACAGACCGTCGTAGTAATGGATGA

TTTGGGACAGAACCCCGATGGCAAGGACTTCAAGTACTTCGCCCAGATGGTATCAACCACGGGGTTTATC

CCGCCCATGGCCTCGCTTGAAGACAAAGGCAAGCCTTTCAACAGCAAAGTCATCATTGCCACCACCAACT

TGTACTCGGGTTTCACCCCGAGAACCATGGTGTGCCCTGACGCGCTGAACCGGAGGTTTCACTTTGACAT

TGACGTGAGTGCTAAGGACGGGTACAAAGTTAACAACAGATTGGACATAATCAAAGCTCTTGAAGACACC

CACACCAATCCCGTGGCAATGTTTCAGTACGACTGTGCCCTTCTCAACGGCATGGCCGTTGAGATGAAGA

GGATGCAGCAAGATGTGTTCAAGCCTCAACCGCCCCTCCAGAACGTTTACCAGCTCGTTCAGGAGGTGAT

TGAACGGGTAGAGCTCCACGACAAAGTGTCGAGCCACCCCATTTTCAAACAG

>JF749848.1_A_TUR_2003

CCCTGGTACAAACTCATCAAGCTCCTGAGCCGCTTGTCATGCATGGCCGCTGTAGCAGCACGGTCAAAGG

ACCCAGTCCTTGTGGCTATCATGTTGGCTGACACCGGTCTTGAGATTCTGGACAGCACGTTTGTTGTGAA

GAAGATCTCTGACTCGCTCTCCAGTCTCTTTCACGTGCCGGCCCCTGTCTTCAGTTTCGGAGCCCCGATC

CTGTTGGCTGGACTGGTCAAAGTTGCCTCGAGTTTCTTCCGGTCCACACCCGAAGACCTTGAGAGAGCTG

AGAAACAGCTTAAAGCACGTGACATCAATGACATTTTCGCCATTCTCAAGAACGGCGAGTGGCTGGTCAA

ACTGATTCTTGCCATCCGTGACTGGATCAAGGCATGGATCGCCTCAGAAGAAAAGTTTGTTACCATGACA

GATTTGGTGCCTGGCATCCTTGAGAAGCAACGGGATCTCAACGACCCCAGCAAATACAAGGAGGCCAAGG

AGTGGCTCGACAACGCGCGCCAAGCGTGTTTGAAGAACGGGAACGTCCACATTGCCAACCTGTGCAAAGT

GGTTGCCCCAGCACCCAGCAAGTCGAGACCAGAGCCCGTGGTTGTCTGCCTCCGTGGCAAATCCGGCCAG

GGGAAGAGTTTCCTTGCGAACGTGCTCGCACAAGCAATCTCCACTCACTTCACTGGCAGAACAGACTCTG

TTTGGTACTGCCCGCCTGACCCTGACCACTTCGACGGTTACAACCAACAAACCGTTGTCGTGATGGATGA

TTTGGGCCAGAACCCCGACGGCAAGGACTTTAAGTACTTCGCCCAGATGGTCTCCACCACGGGTTTCATC

CCGCCCATGGCGTCGCTCGAGGACAAAGGCAAGCCTTTCAACAGTAAGGTCATCATCGCCACCACTAACC

TGTACTCGGGTTTTACCCCGAGGACCATGGTGTGCCCTGACGCGCTGAACCGGAGGTTTCACTTTGACAT

TAGCGTGAGTGCCAAGGACGGGTATAAAATTAACAACAAATTGGACATAATTAAAGCTCTTGAAGACACC

CACACTAACCCAGTGGCAATGTTCCAATACGACTGTGCCCTTCTCAACGGCATGGCCGTTGAAATGGAGA

GAATGCAACAAGACATGTTCAAGCCACAACCACCCCTCCAGAACGTGTACCAGCTCGTTCAGGAGGTGAT

TGAGAGGGTCGAGTTCCACGAGAAAGTGTCGAGCCACCCGATCTTCAAGCAG

>JF749849.1_Asia1_PAK_2002

CCTTGGTACAAGCTCGTCAAGCTCCTGAGCCGCCTGTCATGCATGGCCGCTGTAGCAGCACGGTCAAAGG

ACCCAGTCCTTGTGGCCATCATGCTGGCTGACACCGGCCTTGAGATTCTGGACAGCACCTTTGTCGTGAA

GAAGATCTCCGACTCGCTCTCCAGTCTCTTTCACGTGCCGGCCCCCGTCTTCAGTTTCGGAGCCCCGATC

CTGTTGGCTGGGTTGGTCAAAGTCGCCTCGAGTTTCTTCCATTCCACACCCGAAGACCTTGAGAGAGCGG

AGAAACAGCTCAAAGCACGTGACATCAATGACATATTCGCCATTCTCAAGAACGGCGAGTGGCTGGTCAA

ACTGATTCTTGCCATCCGCGACTGGATCAAGGCATGGATCGCCTCAGAAGAAAAGTTTGTCACCGTGACG

GACCTGGTGCCTGGCATCCTTGAAAAGCAGCGGGATCTCAACGACCCAAGCAAGTACAAGGAGGCCAAGG

AGTGGCTCGACAACGCGCGCCAAGCGTGCCTGAAGAGCGGGAACACACACATTGCGAACCTTTGCAAAGT

GATTGCCCCAGCACCCAGCAGGTCGAGACCCGAACCCGTGGTCGTTTGCCTCCGCGGCAAATCGGGCCAG

GGCAAGAGTTTCCTTGCGAACGTGCTTGCACAAGCAATTTCAACCCACTTCACTGGCAGAACCGATTCAG

TTTGGTACTGCCCACCTGACCCTGACCACTTCGACGGTTACAACCAGCAGACCGTCGTAGTGATGGATGA

TTTGGGCCAGAACCCCGACGGGAAGGACTTCAAGTACTTCGCCCAAATGGTTTCAACCACGGGGTTCATC

CCGCCCATGGCTTCACTTGAGGACAAAGGCAAACCTTTCAACAGCAAGGTCATCATCGCCACCACCAACC

TGTACTCGGGCTTCACCCCGAGAACTATGGTGTGCCCTGACGCACTGAACCGAAGGTTCCACTTTGACAT

CGATGTGAGCGCCAAGGACGGGTACAAAATTAACAACAAATTGGACATCATCAAAGCTCTTGAAGATACC

CACACTAACCCAGTGGCAATGTTTCAATACGACTGTGCCCTTCTCAACGGCATGGCCGTTGAAATGAAGA

GAATGCAACAAGATGTGTTCAAGCCTCAACCGCCTCTCCAGAACGTTTACCAGCTTGTCCAGGAGGTGAT

TGACCGGGTCGAGCTCCACGAGAAGGTGTCGAGCCACCCGATTTTCAAGCAA

>JF749851.1_O_IRN_2001

CCCTGGTACAAGCTCATCAAGCTCCTGAGCCGCCTGTCATGCATGGCCGCTGTAGCAGCACGGTCAAAGG

ACCCAGTCCTTGTGGCCATCATGCTGGCTGACACCGGCCTTGAGATTCTGGACAGCACCTTTGTCGTGAA

GAAGATCTCCGACTCGCTCTCCAGTCTCTTTCACGTGCCGGCCCCTGTCTTCAGTTTTGGAGCCCCGATC

TTGTTGGCTGGGTTGGTCAAAGTCGCCTCGAGTTTCTTCCGGTCCACACCCGAAGACCTTGAGAGAGCGG

AGAAACAACTCAAAGCACGTGACATCAATGACATATTCGCCATTCTCAAGAACGGCGAGTGGCTGGTTAA

GCTGATTCTTGCCATCCGCGACTGGATCAAGGCATGGATCGCCTCAGAAGAAAAGTTTGTCACCATGACA

GACCTGGTGCCTGGCATCCTTGAAAAGCAGCGGGATCTCAACGACCCAAGCAAGTACAAGGAGGCCAAGG

AGTGGCTCGACAACGCGCGCCAAGCGTGCCTGAAGAGCGGGAACATCCACATTGCGAACCTTTGCAAAGT

GGTTGCCCCAGCACCCAGCAGGTCGAGACCCGAACCCGTGGTCGTTTGCCTCCGCGGCAAATCAGGCCAG

GGCAAGAGTTTCCTTGCGAACGTGCTTGCACAAGCAATTTCAACCCACTTCACTGGCAGAACCGATTCAG

TTTGGTACTGCCCACCTGACCCTGACCACTTCGACGGTTACAACCAGCAGACCGTCGTAGTGATGGATGA

TTTGGGCCAGAACCCCGACGGGAGGGACTTCAAGTACTTCGCCCAAATGGTTTCAACCACGGGGTTCATC

CCGCCCATGGCTTCACTTGAGGACAAAGGCAAACCTTTCAACAGCAAGGTCATCATCGCCACCACCAACC

TGTACTCGGGCTTCACCCCGAGAACTATGGTGTGCCCTGATGCACTGAACCGAAGGTTCCACTTTGACAT

CGACGTGAGCGCCAAGGACGGGTACAAAATTAACAACAAATTGGACATCATCAAAGCTCTTGAAGATACC

CACACTAACCCAGTGGCAATGTTCCAATACGACTGTGCCCTTCTCAACGGCATGGCCGTTGAAATGAAGA

GAATGCAACAAGATGTGTTCAAGCCTCAACCGCCCCTCCAGAACGTCTACCAGCTTGTCCAGGAGGTGAT

TGACCGGGTCGAGCTCCACGAGAAGGTGTCGAGCCACCCGATTTTCAAGCAA

>JF749852.1_O_MAY_2004

CCCTGGTACAAGCTCATCAAGCTCCTGAGCCGCCTGTCATGCATGGCCGCTGTAGCAGCACGGTCAAAGG

ACCCAGTCCTTGTGGCCATCATGCTAGCTGACACCGGTCTCGAGATTCTAGACAGCACCTTTGTCGTGAA

GAAAATCTCCGACTCGCTCTCTAGTCTCTTCCACGTGCCGGCCCCCGTCTTCAGTTTCGGAGCCCCGATT

CTGTTGGCCGGGTTGGTCAAGGTCGCCTCGAGTTTCTTCCGGTCCACGCCCGAAGACCTCGAGAGAGCAG

AAAAACAGCTCAAAGCACGTGACATCAATGACGTATTCGCCATTCTCAAGAACGGCGAGTGGCTGGTCAA

GCTGATCCTTGCCATCCGCGACTGGATTAAGGCATGGATCGCCTCAGAAGAAAAGTTTGTCACCACGACG

GACTTGGTGCCTGGCATCCTTGAAAAACAGCGGGACCTCAACGACCCAAGCAAGTACAAGGAGGCCAAGG

AGTGGCTCGACAACGCACGCCAAGCGTGTCTGAAGAGCGGGAACGTCCACATTGCCAACCTCTGCAAAGT

GGTCGCCCCGGCGCCCAGCAAGTCGAGGCCCGAACCTGTGGTCGTTTGCCTCCGCGGCAAATCCGGCCAG

GGTAAGAGTTTCCTTGCGAACGTGCTCGCACAAGCAATCTCCACCCACTTCACTGGCAGAACTGATTCAG

TTTGGTACTGCCCGCCTGACCCTGACCACTTCGACGGCTACAACCAACAGGCCGTTGTCGTGATGGACGA

CTTGGGCCAGAACCCTGATGGCAAGGACTTCAAGTACTTTGCCCAGATGGTGTCAACCACAGGGTTCATC

CCGCCAATGGCGTCACTCGAAGACAAAGGTAAACCTTTCAACAGCAAGGTCATCATTGCAACCACCAACT

TGTACTCGGGGTTCACTCCGAGGACCATGGTGTGCCCTGATGCACTGAACCGTAGGTTTCACTTTGACAT

TGACGTGAGTGCCAAAGACGGGTACAAAATTAACAACAAATTGGACATCATCAAAGCTCTTGAAGACACC

CACACCAACCCAGTGGCAATGTTCCAGTACGATTGTGCCCTTCTCAACGGCATGGCCGTTGAAATGAAGA

GAATGCAACAAGATGTGTTCAAGCCTCAACCACCCCTCCAGAACGTGTACCAGCTAGTTCAGGAGGTGAT

TGAACGGGTCGAGCTCCACGAGAAAGTGTCGAGCCACCCGATTTTTAAGCAA

>JF749860.1_SAT1_KEN_2002

CCCTGGTACAAGGTCATCAAGCTCCTTAGCCGCCTGTCGTGCATGGCCGCTGTGGCAGCTCGCTCCAAGG

ACCCTGTCCTTGTGGCGATTATGCTAGCTGACACCGGCCTCGAGATCCTGGACAGCACCTTCGTCGTGAA

GAAAATCTCCGACGCGCTCTCCAGCGTTTTCCACGTCCCGGCCCCTGTCTTCAGTTTCGGAGCCCCGATT

CTGTTGGCAGGCTTGGTCAAGGTCGCCTCCACGTTCTTCCGGTCAACACCCGAGGACTTGGAGAGAGCAG

AAAAACAGCTCAAGGCACGTGACATCAACGACATCTTCGCCATTCTCAAGAACGGCGAGTGGCTGGTCAA

ACTCATCCTGGCAATCCGCGACTGGATCAAAGCCTGGATTTCCTCAGAGGAGAAGTACATCTCCATGACG

GACCTTGTGCCCCGCATTCTTGAATGCCAGCGCAATCTGAACGATCCGTCCAAGTACCAGGAAAGCAAGG

AGTGGCTTGAAAACGCTCGAGAGGCCTGCCTCAAGAACGGCAACATCCACATTGCTAACCTGTGTAAAGT

AAACGCACCAGCACCGAGCAAGTCGAGACCCGAACCAGTGGTCGTCTGCCTCCGCGGCAAGTCCGGCCAG

GGCAAGAGTTTCCTCGCGAACGTGCTTGCACAAGCAATCTCAACCCACTTCACTGGACGGGTGGACTCAG

TCTGGTACTGTCCGCCTGACCCTGACCACTTCGACGGCTACAACCAACAGGCCGTTGTCGTGATGGACGA

TTTGGGCCAGAACCCCGATGGCAAGGACTTCAAGTACTTTGCCCAGATGGTTTCCACCACGGGGTTCATC

CCACCCATGGCCTCGCTCGAAGACAAGGGAAAACCGTTCAACAGCAAGGTGATCATTGCGACGAGCAACC

TTTACTCTGGATTCACACCCAGGACAATGGTCTGCCCCGACGCTCTGAACCGACGGTTCCACTTTGACAT

CGATGTGAGTGCCAAGGATGGGTACAAAGTTAACAACAGATTGGACATCATCAAAGCCTTGGAGGACACG

CACACAAACGCGCCCGCCATGTTCAACTACGACTGTGCCCTTCTCAACGGCTCTGCTGTTGAAATGAAGA

GACTGCAACAAGATGTGTTCAAGCCTCTACCACCTCTCAACAGCCTGTACCAACTGGTTGATGAAGTGAT

AGAGAGGGTGAAGCTCCACGAGAAAGTGTCGAGCCACCCGATTTTCAAACAA

>JF749861.1_SAT2_KEN_2002

CCTTGGTACAAGGTGATCAAGCTCCTCAGCCGCCTGTCGTGCATGGCCGCTGTAGCAGCTCGTTCCAAGG

ACCCGGTCCTTGTGGCGATTATGCTAGCTGACACCGGTCTCGAGATCCTGGACAGCACTTTCGTCGTGAA

GAAAATCTCCGACGCGCTCTCCAGTGTTTTCCACGTCCCGGCCCCTGTCTTCAGTTTCGGAGCCCCGATC

CTGTTGGCAGGTTTGGTCAAGGTCGCCTCCACGTTCTTCCGGTCAACACCCGAAGACTTGGAGAGAGCAG

AAAAACAGCTCAAGGCACGTGACATCAACGACATCTTCGCCATCCTCAAGAACGGCGAATGGCTGGTCAA

ACTCATCCTGGCAATCCGCGACTGGATCAAAGCCTGGATTTCCTCAGAGGAGAAATACATCTCCATGACG

GATCTTGTGCCGCGCATTCTCGAGTGCCAGCGCAACCTGAACGACCCGTCAAAATACCAGGAAAGCAAAG

AGTGGCTTGAAAACGCTCGAGAGGCCTGCCTCAAGAACGGCAACATCCACATCGCTAACCTGTGTAAAGT

GAATGCACCAGCACCAAGCAAGTCGAGACCCGAACCAGTGGTCGTTTGCCTCCGCGGCAAATCCGGCCAG

GGCAAGAGTTTCCTCGCGAACGTGCTCGCACAGGCAATCTCAACCCACTTCACTGGACGGGTGGATTCAG

TCTGGTACTGTCCGCCTGACCCTGACCACTTCGACGGATACAACCAACAAGCCGTTGTTGTGATGGATGA

CTTGGGCCAGAACCCTGACGGCAAGGACTTCAAGTACTTCGCCCAGATGGTTTCCACCACAGGGTTCATC

CCGCCCATGGCTTCGCTCGAGGACAAGGGTAAACCGTTTAACAGCAAGGTGATCATTGCGACGAGCAACC

TTTATTCTGGGTTCACACCCAGGACGATGGTCTGCCCCGACGCTCTGAACCGACGGTTTCACTTTGACAT

CGACGTGAGTGCCAAGGACGGGTACAAAGTCAACAACAGATTGGACATCATCAAAGCCTTGGAGGACACG

CACACAAATGCGCCCGCCATGTTTAACTACGATTGTGCCCTTCTCAATGGCTCCGCCGTTGAAATGAAGA

GACTGCAACAAGATGTGTTCAAGCCTCTACCACCTCTCAACAGCCTCTACCAATTGGTTGATGAAGTGAT

AGAGAGGGTGGAGCTCCACGAGAAGGTGTCGAGCCACCCGATTTTCAAGCAG

>JF749862.1_SAT2_UGA_2002

CCCTGGTATAAAGTCATCAAACTCCTCAGCCGATTGTCATGCATGGCCGCTGTAGCAGCACGCTCAAAGG

ATCCAGTCCTCGTAGCAATAATGCTGGCTGACACCGGTCTTGAAATACTGGACAGCACATTCGTAGTCAA

GAAGATTTCTGACTCCCTCTCCAGTGTTTTCCACGTGCCGGCCCCTGCCTTCAGCTTCGGAGCCCCGATT

CTGTTGGCAGGCTTGGTCAAGGTCGCCTCCACGTTCTTCCGATCAACACCCGAAGACTTGGAGAGAGCTG

AGAAACAGCTCAAAGCACGTGACATCAATGACATCTTCGCCATTCTCAAGAACGGCGAGTGGCTGGTCAA

ACTCATCCTGGCTATCCGCGACTGGATAAAAGCCTGGATCTCCTCAGAAGAGAAATACATAAACATGACT

GACCTGGTCCCGCGCATTCTCGAGTGCCAGCGGAACTTGAACGACCCTTCCAAGTACCAGGAGTCAAAGC

AGTGGCTCGAGAACGCCCGAGAGACGTGTCTCAAGAATGGCAACATTCACATCGCCAACCTCTGCAAAGT

TAATGCACCAGCACCGAGCAAGTCGAGACCCGAGCCGGTGGTCGTCTGCCTACGCGGTAAGTCTGGCCAA

GGCAAGAGTTTCCTTGCTAACGTGCTTGCACAAGCAATTTCAACCCACTTCACTGGGCGTACTGATTCAG

TTTGGTATTGTCCACCTGACCCTGACCACTTTGACGGGTACAACCAACAAACTGTCGTGGTCATGGATGA

TTTGGGCCAGAACCCTGACGGCAAGGACTTCAAGTACTTCGCCCAAATGGTGTCAACCACGGGGTTCATC

CCGCCCATGGCTTCACTTGAAGACAAAGGGAAACCTTTCAACAGCAAAGTCATCATTGCGACCTCCAACC

TGTACTCCGGATTCACTCCGCGCACAATGGTCTGCCCTGATGCACTGAACCGTAGGTTCCACTTTGACAT

CGACGTGAGCGCCAAGGACGGGTACAAAGTAAACAACAAATTGGACATCATCAAAGCACTTGAGGACACC

CACACCAACCCTGTGGCGATGTTCCAGTATGATTGTGCCCTTCTGAACGGCATGGCTGTCGAGATGAAGA

GACTGCAACAAGACATGTTCAAGCCTCAACCACCACTTCAGAGCCTGTACCAACTGGTCGATGAGGTGAT

TGAGAGAGTCAACCTCCATGAGAAAGTTGCCTCACATCCAATCTTCAAACAA

>JF749864.1_SAT2_ZIM_2003

CCCTGGTACAAGGTTATCAAACTCCTCAGCCGCCTGTCGTGCATGGCCGCTGTCGCAGCTCGCTCTAAGG

ATCCCGTCCTTGTGGCGATCATGCTAGCTGACACCGGTCTCGAGATCCTGGACAGCACCTTCGTCGTGAA

GAAAATCTCCGACGCGCTCTCCAGCGTTTTCCACGTCCCGGCCCCTGTCTTCAGCTTCGGAGCCCCGATT

CTGCTGGCAGGTTTGGTCAAGGTCGCCTCCACGTTCTTCCGGTCCACACCCGAAGACTTGGAGAGAGCAG

AGAAACAGCTCAAGGCACGTGACATTAACGACATCTTCGCCATCCTCAAGAACGGCGAATGGCTGGTCAA

ACTCATCCTGGCTATCCGCGACTGGATCAAAGCCTGGATTTCCTCAGAGGAGAAGTACATCTCCATGACG

GACCTTGTGCCGCGCATTCTCGAGTGCCAGCGCAACCTGAACGATCCGTCCAAGTACCAGGAAAGCAAGG

AGTGGCTGGAGAACGCTCGCGAGGCCTGCCTCAAGAGCGGGAACCACCACATCGCCAACTTGTGTAAAGT

GAATGCACCAGCACCCAGCAAGTCGAGACCCGAACCCGTGGTCGTTTGCCTTCGTGGCAAATCCGGCCAG

GGTAAGAGTTTCCTCGCCAACGTGCTCGCACAAGCAATCTCAACCCACTTCACTGGTAGAACAGACTCCG

TCTGGTACTGCCCACCTGACCCCGACCACTTCGATGGCTACAACCAACAAGCTGTCGTTGTCATGGATGA

TTTGGGCCAGAACCCTGACGGCAAAGACTTCAAGTACTTCGCCCAGATGGTCTCCACCACAGGGTTCATC

CCTCCCATGGCCTCACTCGAAGACAAGGGAAAACCGTTCAACAGCAAGGTGATCATTGCGACGAGCAACC

TTTATTCTGGGTTCACACCTAGGACGATGGTTTGTCCCGACGCTCTGAACCGACGGTTCCACTTTGACAT

TGACGTGAGTGCCAAGGATGGGTACAAAGTTAACAACAGATTGGACATTATCAAAGCACTGGAGGACACG

CACACAAACGCGCCCGCCATGTTCAACTATGACTGTGCCCTTCTCAATGGCTCCGCCGTTGAAATGAAGA

GACTGCAACAAGATGTGTTCAAGCCTCTACCACCTCTCAACAGCCTGTACCAACTGGTTGATGAAGTGAT

AGAGAGGGTGAAGCTCCACGAGAAAGTGTCGAGTCACCCGATTTTCAAGCAG

>JN006719.1_Asia1_PAK_2008

CCCTGGTACAAACTCATCAAGCTCTTGAGCCGCTTGTCGTGCATGGCCGCTGTAGCAGCACGGTCAAAGG

ACCCAGTCCTTGTGGCCATCATGCTAGCTGACACCGGTCTCGAGATTCTGGACAGCACCTTCGTCGTGAA

GAAGATCTCCGACTCGCTCTCCAGTCTCTTTCACGTGCCGGCCCCCGTCTTCAGTTTCGGAGCCCCGATT

CTGCTGGCCGGGTTGGTCAAAGTCGCCTCGAGTTTCTTCCGGTCCACACCCGAAGACCTTGAGAGAGCAG

AGAAACAGCTCAAAGCACGTGACATCAACGACATTTTCGCCATTCTCAAGAACGGCGAGTGGCTGGTCAA

GCTGATTCTTGCCATCCGCGACTGGATCAAGGCATGGATCGCCTCAGAAGAAAAGTTTGTCACCATGACG

GACTTGGTGCCTGGCATCCTTGAAAAGCAACGGGACCTCAACGACCCTAGCAAGTACAGTGAGGCCAAGG

AGTGGCTCGACAACGCGCGCCAAGCGTGCTTGAAGAGCGGGAACGTCCACATTGCCAACCTGTGTAAAGT

GGTCGCCCCGGCACCCAGCAAGTCGAGACCTGAGCCCGTGGTCGTTTGCCTCCGTGGCAAATCTGGCCAG

GGTAAGAGTTTCCTTGCCAACGTGCTCGCACAAGCAATTTCAACCCATTTCACTGGCAGAACCGATTCAG

TTTGGTACTGTCCGCCTGACCCTGACCACTTCGACGGTTACAACCAGCAGACCGTTGTAGTGATGGATGA

TTTGGGCCAGAACCCCGACGGGAAGGACTTCAAGTACTTCGCCCAGATGGTTTCAACCACGGGGTTCATC

CCGCCCATGGCTTCACTCGAAGACAAAGGCAAACCTTTCAACAGCAAGGTTATCATTACCACCACCAACC

TGTACTCGGGTTTCACCCCGAGAACTATGGTGTGCCCTGATGCACTGAACCGAAGGTTCCACTTTGACAT

TGACGTGAGCGCCAAGGACGGGTACAAAATCAACAACAAATTGGACATTATCAAAGCTCTTGAAGACACC

CACACCAACCCGGTGGCAATGTTTCAATATGATTGTGCCCTTCTCAACGGCATGGCTGTTGAGATGAAGA

GAATGCAACAAGATGTGTTCAAACCTCAACCGCCCCTCCAGAACGTCTACCAACTCGTTCAGGAGGTGAT

TGACCGGGTCGAGCTCCACGAGAAGGTGTCGAGCCACCCAATTTTCAAGCAG

>JN006720.1_Asia1_PAK_2009

CCCTGGTACAAGCTTATCAAGCTCCTGAGCCGCCTGTCATGCATGGCCGCTGTAGCAGCACGGTCAAAGG

ACCCAGTCCTTGTGGCCATCATGCTAGCTGACACCGGTCTCGAAATTCTGGACAGCACCTTCGTCGTGAA

AAAGATCTCCGACTCGCTCTCCAGTCTCTTTCACGTGCCGGCCCCCGTCTTCAGTTTCGGAGCCCCGATT

CTGTTGGCCGGGTTGGTCAAAGTCGCCTCGAGTTTCTTCCGGTCCACACCCGAAGACCTTGAGAGAGCAG

AGAAACAGCTCAAAGCGCGTGACATCAATGACATTTTTGCCATTCTCAAGAACGGCGAGTGGCTGGTCAA

ACTGATCCTTGCCATCCGCGACTGGATAAAGGCTTGGATCGCCTCAGAAGAGAAGTTTGTCACCATGACA

GACTTGGTGCCTGGCATCCTTGAAAAGCAGCGGGACCTCAACGACCCGAGCAAGTACAAGGAGGCCAAGG

AGTGGCTCGACAATGCACGTCAGGCGTGCCTGAAGAGCGGGAACGTCCACATTGCCAACTTGTGCAAAGT

GGTCGCCCCGGCACCCAGCAAGTCGAGACCCGAGCCTGTGGTCGTTTGCCTCCGTGGTAAATCTGGCCAG

GGGAAGAGTTTCCTCGCGAACGTGCTTGCACAAGCAATCTCCACCCATTTCACTGGCAGAACCGATTCAG

TTTGGTACTGCCCACCTGACCCCGACCACTTCGACGGTTACAACCAACAGACCGTTGTCGTGATGGACGA

TTTGGGCCAGAATCCCGATGGCAAGGACTTCAAGTACTTCGCCCAGATGGTTTCGACCACGGGGTTCATC

CCGCCCATGGCGTCGCTTGAGGACAAAGGCAAACCCTTCAACAGCAAGGTTATCATCGCCACCACCAACC

TGTACTCGGGGTTCACCCCGAGAACCATGGTGTGCCCTGATGCCCTGAACCGGAGGTTTCACTTTGACAT

CGATGTGAGCGCCAAGGACGGGTATAAAATCAACAACAAATTGGACATAATCAAAGCTCTTGAAGACACC

CACACCAACCCTGTTGCAATGTTTCAATATGACTGTGCCCTTCTCAACGGCATGGCCGTTGAAATGAAGA

GAATGCAACAAGATGTGTTCAAGCCTCAGCCACCCCTCCAGAACGTATACCAACTCGTTCAGGAGGTGAT

TGAACGGGTCGAGCTCCACGAGAAAGTGTCGAGCCACCCGATCTTCAAGCAG

>JN006722.1_A_PAK_2008

CCCTGGTACAAGCTTATCAAGCTCCTGAGCCGCCTGTCATGCATGGCCGCTGTAGCAGCACGGTCAAAGG

ACCCAGTCCTTGTGGCCATCATGCTAGCTGACACCGGTCTCGAGATTCTGGACAGCACCTTCGTCGTGAA

GAAGATCTCCGACTCGCTCTCCAGTCTCTTTCACGTGCCGGCCCCCGTCTTCAGTTTCGGAGCCCCGATT

CTGTTGGCCGGGTTGGTCAAAGTCGCCTCGAGTTTCTTCCGGTCCACACCCGAAGACCTTGAGAGAGCAG

AGAAACAGCTCAAAGCGCGTGACATCAATGACATTTTTGCCATTCTCAAGAACGGCGAGTGGCTGGTCAA

ACTGATCCTTGCCATCCGCGACTGGATAAAGGCTTGGATCGCCTCAGAAGAGAAGTTTGTCACCATGACA

GACTTGGTGCCTGGCATCCTTGAAAAGCAGCGGGACCTCAACGACCCGAGCAAGTACAAGGAGGCCAAGG

AGTGGCTCGACAATGCGCGTCAGGCGTGCCTGAAGAGCGGGAACGTCCACATTGCCAACTTGTGCAAAGT

GGTCGCCCCGGCACCCAGCAAGTCGAGACCCGAGCCTGTGGTCGTTTGCCTCCGTGGTAAATCTGGCCAG

GGGAAGAGTTTCCTCGCGAACGTGCTTGCACAAGCAATCTCCACCCATTTCACTGGCAGAACCGATTCAG

TTTGGTACTGCCCACCTGACCCCGACCACTTCGACGGTTACAACCAACAGACCGTTGTCGTGATGGACGA

TTTGGGCCAGAATCCCGATGGCAAGGACTTCAAGTACTTCGCCCAGATGGTTTCGACCACGGGGTTCATC

CCGCCCATGGCGTCACTTGAGGACAAAGGCAAACCCTTCAACAGCAAGGTTATCATCGCCACCACCAACC

TGTACTCGGGGTTCACCCCGAGAACCATGGTGTGCCCTGATGCCCTGAACCGGAGGTTTCACTTTGACAT

CGATGTGAGCGCCAAGGACGGGTATAAAATTAACAACAAACTGGACATAATCAAAGCTCTTGAAGACACC

CACACCAACCCTGTTGCAATGTTTCAATATGACTGTGCCCTTCTCAACGGCATGGCCGTTGAAATGAAGA

GAATGCAACAAGATGTGTTCAAGCCTCAGCCACCCCTCCAGAACGTATACCAACTCGTTCAGGAGGTGAT

TGAACGGGTCGAGCTCCACGAGAAAGTGTCGAGCCACCCGATCTTCAAGCAG

>JN099688.1_A_IRQ_2009

CCCTGGTACAAGCTCATCAAGCTCCTGAGCCGCCTGTCATGCATGGCCGCTGTAGCAGCACGGTCAAAGG

ACCCGGTCCTTGTGGCCATCATGCTAGCTGACACCGGTCTCGAGATTCTGGACAGCACCTTTGTCGTGAA

GAAGATCTCCGATTCGCTCTCCAGTCTTTTTCACGTGCCGGCCCCTGTCTTCAGTTTCGGAGCCCCGATT

CTGTTGGCCGGGTTGGTCAAAGTCGCCTCGAGTTTCTTCCGGTCCACACCCGAAGACCTTGAGAGAGCAG

AGAAACAGCTCAAAGCACGTGACATCAATGACATTTTCGCCATTCTCAAGAACGGCGAGTGGCTGGTAAA

ACTGATCCTTGCCATCCGCGACTGGATAAAGGCTTGGATCGCCTCAGAAGAGAAGTTTGTCACCATGACA

GATTTGGTGCCTGGCATCCTTGAAAAGCAACGGGATCTCAACGACCCAAGTAAGTACAAGGAGGCCAAGG

AGTGGCTCGACAACGCGCGCCAAGCGTGTCTGAAGAGCGGGAATGTCCACATTGCTAACCTTTGCAAGGT

GGTCGCCCCGGCACCCAGCAAGTCGAGACCCGAACCCGTGGTCGTTTGCCTCCGTGGCAAATCCGGCCAG

GGTAAGAGTTTCCTTGCGAACGTGCTCGCACAAGCAATCTCCACCCACTTCACTGGCAGAACTGACTCAG

TTTGGTACTGTCCGCCTGACCCTGACCACTTCGACGGTTACAACCAACAGACCGTTGTCGTGATGGATGA

TTTGGGCCAGAACCCTGACGGCAAGGACTTCAAGTACTTCGCCCAGATGGTATCAACCACAGGGTTCATC

CCGCCAATGGCGTCACTCGAAGACAAAGGTAAACCTTTCAACAGCAAGGTCATCATCGCAACCACCAATC

TGTACTCGGGGTTCACTCCGAGGACCATGGTGTGTCCTGATGCACTGAACCGTAGGTTTCACTTTGACAT

TGACGTAAGCGCCAAAGACGGGTACAAAATTAACAACAAATTGGACATCATCAAAGCTCTTGAAGACACC

CACACCAACCCAGTGGCAATGTTTCAGTACGATTGTGCCCTTCTCAATGGCATGGCCGTTGAAATGAAGA

GAATGCAACAAGATGTGTTCAAGCCTCAACCACCCCTCCAGAACGTGTACCAGTTAGTTCAGGAGGTGAT

TGAACGGGTCGATCTCCACGAGAAAGTGTCGAGCCACCTGATTTTTAAACAA

>JN099694.1_A_IRQ_2009

CCCTGGTACAAGCTCATCAAGCTCCTGAGTCGCCTGTCATGCATGGCCGCTGTAGCAGCACGGTCAAAGG

ACCCGGTCCTTGTGGCCATCATGCTAGCTGACACCGGTCTCGAGATTCTGGACAGCACCTTTGTCGTGAA

GAAGATCTCCGACTCGCTCTCCAGTCTTTTTCACGTGCCGGCCCCCGTCTTCAGTTTCGGAGCCCCGATT

CTGTTGGCCGGGTTGGTCAAAGTCGCCTCGAGTTTCTTCCGGTCCACACCCGAAGACCTTGAGAGAGCAG

AGAAACAGCTCAAAGCACGTGACATCAATGACATTTTCGCCATTCTCAAGAACGGCGAGTGGCTGGTCAA

ACTGATCCTTGCCATCCGCGACTGGATAAAGGCTTGGATCGCCTCAGAAGAGAAGTTTGTCACCATGACA

GATTTGGTGCCTGGCATCCTTGAAAAGCAACGGGATCTCAACGACCCAAGTAAGTACAAGGAGGCCAAGG

AGTGGCTCGACAACGCGCGCCAAGCGTGTCTGAAGAGCGGGAATGTCCACATTGCCAACCTTTGCAAGGT

GGTCGCCCCGGCACCCAGCAAGTCGAGACCCGAACCCGTGGTCGTTTGCCTCCGTGGCAAATCCGGCCAG

GGTAAGAGTTTCCTTGCGAACGTGCTCGCACAAGCAATCTCCACCCACTTCACTGGCAGAACTGACTCAG

TTTGGTACTGTCCGCCTGACCCTGACCACTTCGACGGTTACAACCAACAGACCGTTGTCGTGATGGATGA

TTTGGGCCAGAACCCTGACGGCAAGGACTTCAAGTACTTCGCCCAGATGGTATCAACCACAGGGTTCATC

CCGCCAATGGCGTCACTCGAAGACAAAGGTAAACCTTTCAACAGCAAGGTCATCATCGCAACCACCAATT

TGTACTCGGGGTTCACTCCGAGGACCATGGTGTGCCCTGACGCACTGAACCGTAGGTTTCACTTTGACAT

TGACGTGAGTGCCAAAGACGGGTACAAAATTAACAACAAATTGGACATCATCAAAGCTCTTGAAGACACC

CACACCAACCCAGTGGCAATGTTTCAGTACGATTGTGCCCTTCTCAATGGCATGGCCGTTGAAATGAAGA

GAATGCAACAATGTGTGTTCAAGCCTCAACCACCCCTCCAGAACGTGTACCAGTTAGTTCAGGAGGTGAT

TGAACGGGTCGATCTCCACGAGAAAGTGTCGAGCCACCCGATTTTTAAACAA

>JN099695.1_A_IRQ_2009

CCCTGGTACAAGCTCATCAAGCTCCTGAGCCGCCTGTCATGCATGGCCGCTGTAGCAGCACGGTCAAAGG

ACCCGGTCCTTGTGGCCATCATGCTAGCTGACACCGGTCTTGAGATTCTGGACAGCACCTTTGTCGTGAA

GAAGATCTCCGACTCGCTCTCCAGTCTTTTTCACGTGCCGGCCCCCGTCTTCAGTTTCGGAGCCCCGATT

CTGTTGGCCGGGTTGGTCAAAGTCGCCTCGAGTTTCTTCCGGTCCACACCCGAAGACCTTGAGAGAGCAG

AGAAACAGCTCAAAGCACGTGACATCAATGACATTTTCGCCATTCTCAAGAACGGCGAGTGGCTGGTCAA

ACTGATCCTTGCCATCCGCGACTGGATAAAGGCTTGGATCGCCTCAGAAGAGAAGTTTGTCACCATGACA

GATTTGGTGCCTGGCATCCTTGAAAAGCAACGGGATCTCAACGACCCAAGTAAGTACAAGGAGGCCAAGG

AGTGGCTCGACAACGCGCGCCAAGCGTGTCTGAAGAGCGGGAATGTCCACATTGCCAACCTTTGCAAGGT

GGTCGCCCCGGCACCCAGCAAGTCGAGACCCGAACCCGTGGTCGTTTGCCTCCGTGGCAAATCCGGCCAG

GGTAAGAGTTTCCTTGCGAACGTGCTCGCACAAGCAATCTCCACCCACTTCACTGGCAGAACTGACTCAG

TTTGGTACTGTCCGCCTGACCCTGACCACTTCGACGGTTACAACCAACAGACCGTTGTCGTGATGGATGA

TTTGGGCCAGAACCCTGACGGCAAGGACTTCAAGTACTTCGCCCAGATGGTATCAACCACAGGGTTCATC

CCGCCAATGGCGTCACTCGAAGACAAAGGCAAACCTTTCAACAGCAAGGTCATCATCGCAACCACCAATC

TGTACTCGGGGTTCACTCCGAGGACCATGGTGTGCCCTGATGCACTGAACCGTAGGTTTCACTTTGACAT

TGACGTGAGTGCCAAAGACGGGTACAAAATCAACAACAAATTGGACATCATCAAAGCTCTTGAAGACACC

CACACCAACCCAGTGGCAATGTTTCAGTACGATTGTGCCCTTCTCAATGGCATGGCCGTTGAAATGAAGA

GAATGCAACAAGATGTGTTCAAGCCTCAACCACCCCTCCAGAACGTGTACCAGTTGGTTCAGGAGGTGAT

TGAACGGGTCGATCTCCACGAGAAAGTGTCGAGCCACCTGATTTTTAAGCAA

>JN099697.1_A_IRQ_2009

CCCTGGTACAAGCTCATCAAGCTCCTGAGCCGCCTGTCATGCATGGCCGCTGTAGCAGCACGGTCAAAGG

ACCCGGTCCTTGTGGCCATCATGCTAGCTGACACCGGTCTCGAGATTCTGGACAGCACCTTTGTCGTGAA

GAAGATCTCCGACTCGCTCTCCAGTCTTTTTCACGTGCCGGCCCCCGTCTTCAGTTTCGGAGCCCCGATT

CTGTTGGCCGGGTTGGTCAAAGTCGCCTCGAGTTTCTTCCGGTCCACACCCGAAGACCTTGAGAGAGCAG

AGAAACAGCTCAAAGCACGTGACATCAATGACATTTTCGCCATTCTCAAGAACGGCGAGTGGCTGGTCAA

ACTGATCCTTGCCATCCGCGACTGGATAAAGGCTTGGATCGCCTCAGAAGAGAAGTTTGTCACCATGACA

GATTTGGTGCCTGGCATCCTTGAAAAGCAACGGGATCTCAACGACCCAAGTAAGTACAAGGAGGCCAAGG

AGTGGCTCGACAACGCGCGCCAAGCGTGTCTGAAGAGCGGGAATGTCCACATTGCCAACCTTTGCAAGGT

GGTCGCCCCGGCACCCAGCAAGTCGAGACCCGAACCCGTGGTCGTTTGCCTCCGTGGCAAATCCGGCCAG

GGTAAGAGTTTCCTTGCGAACGTGCTCGCACAAGCAATCTCCACCCACTTCACTGGCAGAACTGACTCAG

TTTGGTACTGTCCGCCTGACCCTGACCACTTCGACGGTTACAACCAACAGACCGTTGTCGTGATGGATGA

TTTGGGCCAGAACCCTGACGGCAAGGACTTCAAGTACTTCGCCCAGATGGTATCAACCACAGGGTTCATC

CCGCCCATGGCGTCACTCGAAGACAAAGGTAAACCTTTCAACAGCAAGGTCATCATCGCAACCACCAATC

TGTACTCGGGGTTCACTCCGAGGACCATGGTGTGCCCTGATGCACTGAACCGTAGGTTTCACTTTGACAT

TGACGTGAGTGCCAAAGACGGGTACAAAATTAACAACAAATTGGACATCATCAAAGCTCTTGAAGACACC

CACACCAACCCAGTGGCAATGTTTCAGTACGACTGTGCCCTTCTCAATGGCATGGCCGTTGAAATGAAGA

GAATGCAACAAGATGTGTTCAAGCCTCAACCACCCCTCCAGAACGTGTACCAGTTAGTTCAGGAGGTGAT

TGAACGGGTCGATCTCCACGAGAAAGTGTCGAGCCACCCGATTTTTAAGCAA

>JN099698.1_A_IRQ_2009

CCCTGGTACAAGCTCATCAAGCTCCTGAGCCGCCTGTCATGCATGGCCGCTGTAGCAGCACGGTCAAAGG

ACCCGGTCCTTGTGGCCATCATGCTAGCTGACACCGGTCTTGAGATTCTGGACAGCACCTTTGTCGTGAA

GAAGATCTCCGACTCGCTCTCCAGTCTTTTTCACGTGCCGGCCCCCGTCTTCAGTTTCGGAGCCCCGATT

CTGTTGGCCGGGTTGGTCAAAGTCGCCTCGAGTTTCTTCCGGTCCACACCCGAAGACCTTGAGAGAGCAG

AGAAACAGCTCAAAGCACGTGACATCAATGACATTTTCGCCATTCTCAAGAACGGCGAGTGGCTGGTCAA

ACTGATCCTTGCCATCCGCGACTGGATAAAGGCTTGGATCGCCTCAGAAGAGAAGTTTGTCACCATGACA

GATTTGGTGCCTGGCATCCTTGAAAAGCAACGGGATCTCAACGACCCAAGTAAGTACAAGGAGGCCAAGG

AGTGGCTCGACAACGCGCGCCAAGCGTGTCTGAAGAGCGGGAATGTCCACATTGCCAACCTTTGCAAGGT

GGTCGCCCCGGCACCCAGCAAGTCGAGACCCGAACCCGTGGTCGTTTGCCTCCGTGGCAAATCCGGCCAG

GGTAAGAGTTTCCTTGCGAACGTGCTCGCACAAGCAATCTCCACCCACTTCACTGGCAGAACTGACTCAG

TTTGGTACTGTCCGCCTGACCCTGACCACTTCGACGGTTACAACCAACAGACCGTTGTCGTGATGGATGA

TTTGGGCCAGAACCCTGACGGCAAGGACTTCAAGTACTTCGCCCAGATGGTATCAACCACAGGGTTCATC

CCGCCAATGGCGTCACTCGAAGACAAAGGCAAACCTTTCAACAGCAAGGTCATCATCGCAACCACCAATC

TGTACTCGGGGTTCACTCCGAGGACCATGGTGTGCCCTGATGCACTGAACCGTAGGTTTCACTTTGACAT

TGACGTGAGTGCCAAAGACGGGTACAAAATCAACAACAAATTGGACATCATCAAAGCTCTTGAAGACACC

CACACCAACCCAGTGGCAATGTTTCAGTACGATTGTGCCCTTCTCAATGGCATGGCCGTTGAAATGAAGA

GAATGCAACAAGATGTGTTCAAGCCTCAACCACCCCTCCAGAACGTGTACCAGTTGGTTCAGGAGGTGAT

TGAACGGGTCGATCTCCACGAGAAAGTGTCGAGCCACCTGATTTTTAAGCAA

>JN099699.1_A_IRQ_2009

CCCTGGTACAAGCTCATCAAGCTCCTGAGCCGCCTGTCATGCATGGCCGCTGTAGCAGCACGGTCAAAGG

ACCCGGTCCTTGTGGCCATCATGCTAGCTGACACCGGTCTCGAGATTCTGGACAGCACCTTTGTCGTGAA

GAAGATCTCCGACTCGCTCTCCAGTCTTTTTCACGTGCCGGCCCCCGTCTTCAGTTTCGGAGCCCCGATT

CTGTTGGCCGGGTTGGTCAAAGTCGCCTCGAGTTTCTTCCGGTCCACACCCGAAGACCTTGAGAGAGCAG

AGAAACAGCTCAAAGCACGTGACATCAATGACATTTTCGCCATTCTCAAGAACGGCGAGTGGCTGGTCAA

ACTGATCCTTGCCATCCGCGACTGGATAAAGGCTTGGATCGCCTCAGAAGAGAAGTTTGTCACCATGACA

GATTTGGTGCCTGGCATCCTTGAAAAGCAACGGGATCTCAACGACCCAAGTAAGTACAAGGAGGCCAAGG

AGTGGCTCGACAACGCGCGCCAAGCGTGTCTGAAGAGCGGGAATGTCCACATTGCCAACCTTTGCAAGGT

GGTCGCCCCGGCACCCAGCAAGTCGAGACCCGAACCCGTGGTCGTTTGCCTCCGTGGCAAATCCGGCCAG

GGTAAGAGTTTCCTTGCGAACGTGCTCGCACAAGCAATCTCCACCCACTTCACTGGCAGAACTGACTCAG

TTTGGTACTGTCCGCCTGACCCTGACCACTTCGACGGTTACAACCAACAGACCGTTGTCGTGATGGATGA

TTTGGGCCAGAACCCTGACGGCAAGGACTTCAAGTACTTCGCCCAGATGGTATCAACCACAGGGTTCATC

CCGCCAATGGCGTCACTCGAAGACAAAGGTAAACCTTTCAACAGCAAGGTCATCATCGCAACCACCAATC

TGTACTCGGGGTTCACTCCGAGGACCATGGTGTGCCCTGATGCACTGAACCGTAGGTTTCACTTTGACAT

TGACGTGAGTGCCAAAGACGGGTACAAAATTAACAACAAATTGGACATCATCAAAGCTCTTGAAGACACC

CACACCAACCCAGTGGCAATGTTTCAGTACGACTGTGCCCTTCTCAATGGCATGGCCGTTGAAATGAAGA

GAATGCAACAAGATGTGTTCAAGCCTCAACCACCCCTCCAGAACGTGTACCAGTTAGTTCAGGAGGTGAT

TGAACGGGTCGATCTCCACGAGAAAGTGTCGAGCCACCCGATTTTTAAGCAA

>JN998085.1_O_CHA_2010

CCCTGGTACAAGCTCATCAAGCTCCTGAGCCGCTTGTCATGCATGGCCGCTGTAGCAGCACGGTCCAAGG

ACCCAGTCCTTGTGGCTATCATGCTGGCTGACACCGGTCTTGAGATTCTGGACAGCACATTTGTCGTGAA

GAAAATCTCCGACTCCCTCTCCAGTCTCTTTCACGTGCCGGCCCCCGTCTTCAGTTTCGGAGCTCCGATT

CTGCTAGCCGGGTTGGTCAAGGTCGCCTCGAGCTTCTTCCGGTCCACACCCGAGGATCTCGAGAGAGCAG

AGAAACAGCTCAAAGCACGTGACATCAATGACATCTTCGCCATTCTCAAGAACGGCGAGTGGCTGGTCAA

GTTGATCCTAGCCATCCGCGACTGGATTAAAGCATGGATCGCCTCAGAAGAGAAGTTTGTCACCGTGACA

GACTTGGTGCCTGGCATCCTTGAAAAGCAGCGGGACCTCAACGACCCGGCCAAGTACAAGGAAGCCAAGG

AATGGCTCGACAACGCGCGCCAAACGTGTTTGAAGAGCGGGAACGTCCACATTGCCAACCTGTGCAAAGT

GGTCGCCCCAGCGCCGAGCAAGTCGAGACCTGAACCCGTGGTCGTGTGCCTCCGCGGCAAATCCGGTCAG

GGTAAGAGTTTCCTTGCGAACGTGCTGGCACAAGCCATCTCTACCCACTTTACCGGCAGGACTGACTCAG

TTTGGTACTGTCCGCCAGACCCTGACCACTTCGACGGTTACAACCAGCAGACCGTTGTTGTGATGGATGA

TTTGGGCCAGAATCCCGACGGCAAGGACTTCAAGTACTTCGCCCAGATGGTCTCGACCACGGGGTTCATC

CCGCCCATGGCTTCACTTGAGGACAAAGGCAAGCCTTTCAACAGCAAAGTCATCATTGCCACCACCAACC

TGTACTCGGGCTTCACCCCGAGAACCATGGTGTGCCCCGATGCGCTGAACCGAAGGTTTCACTTTGACAT

TGACGTGAGTGCCAAGGACGGGTACAAAATTAACAACAAATTGGACATAATCAAAGCTCTCGAGGACACC

CACACCAACCCTGTGGCAATGTTCCAATACGACTGTGCCCTTCTCAACGGCATGGCCGTTGAAATGAAGA

GAATGCAACAAGACATGTTCAAACCCCAGCCGCCTCTGCAGAACATATACCAACTTGTGCAAGAGGTGAT

TGACCGGGTCGAGCTCCACGAGAAAGTGTCGAGCCACCCGATTTTCAAGCAG

>JN998086.1_O_CHA_2010

CCCTGGTACAAGCTCATCAAGCTCCTGAGCCGCTTGTCATGCATGGCCGCTGTAGCAGCACGGTCCAAGG

ACCCAGTCCTTGTGGCCATCATGCTGGCTGACACCGGTCTTGAGATTCTGGACAGCACATTTGTCGTGAA

GAAAATCTCCGACTCCCTCTCCAGTCTCTTTCACGTGCCGGCCCCCGTCTTCAGTTTCGGGGCTCCGATT

CTGCTAGCCGGGTTGGTCAAGGTCGCCTCGAGCTTCTTCCGGTCCACACCCGAGGATCTCGAGAGAGCAG

AGAAACAGCTCAAAGCACGTGACATCAATGACATCTTCGCCATTCTCAAGAACGGCGAGTGGCTGGTCAA

GTTGATCCTAGCCATCCGCGACTGGATTAAAGCATGGATCGCCTCAGAAGAGAAGTTTGTCACCATGACA

GACTTGGTGCCTGGCATCCTTGAAAAGCAGCGGGACCTCAACGACCCGGCCAAGTACAAGGAAGCCAAGG

AATGGCTCGACAACGCGCGCCAAACGTGTTTGAAGAGCGGGAACGTCCACATTGCCAACCTGTGCAAAGT

GGTCGCCCCAGCGCCGAGCAAGTCGAGACCTGAACCCGTGGTCGTTTGCCTCCGCGGCAAATCCGGCCAG

GGTAAGAGTTTCCTTGCGAACGTGCTGGCACAAGCCATCTCTACCCACTTTACCGGCAGGACTGACTCAG

TTTGGTACTGTCCGCCAGACCCTGACCACTTCGACGGTTACAACCAGCAGACCGTTGTTGTGATGGATGA

TTTGGGCCAGAATCCCGACGGCAAGGACTTCAAGTACTTCGCCCAGATGGTCTCGACCACGGGGTTCATC

CCGCCCATGGCTTCACTTGAGGACAAAGGCAAGCCTTTCAACAGCAAAGTCATCATTGCCACCACCAACT

TGTACTCGGGCTTCACCCCGAGAACCATGGTGTGCCCCGATGCGCTGAACCGAAGGTTTCACTTTGACAT

TGACGTGAGTGCCAAGGACGGGTACAAAATTAACAACAAATTGGACATAATCAAAGCTCTCGAGGACACC

CACACCAACCCTGTGGCAATGTTCCAATACGACTGTGCCCTTCTCAACGGCATGGCCGTTGAAATGAAGA

GAATGCAACAAGACATGTTCAAACCCCAACCGCCTTTGCAGAACATATACCAACTTGTGCAAGAGGTGAT

TGACCGGGTCGAGCTCCACGGGAAAGTGTCGAGCCACCCGATTTTCAAGCAG

>JQ900581.1_O_CHA_2010

CCCTGGTACAAGCTCATCAAGCTCCTGAGCCGCTTGTCATGCATGGCCGCTGTAGCAGCACGGTCCAAGG

ATCCAGTCCTTGTGGCCATCATGCTGGCTGACACCGGTCTTGAGATTCTGGACAGCACATTTGTCGTGAA

GAAAATCTCCGACTCCCTCTCCAGTCTCTTTCACGTGCCGGCCCCCGTCTTCAGTTTCGGAGCTCCGATT

CTGCTAGCCGGGTTGGTCAAGGTCGCCTCGAGCTTCTTCCGGTCCACACCCGAGGATCTCGAGAGAGCAG

AGAAACAGCTCAAAGCACGTGACATCAATGACATCTTCGCCATTCTCAAGAACGGCGAGTGGCTGGTCAA

GTTGATCCTAGCCATCCGCGACTGGATTAAAGCATGGATCGCCTCAGAAGAGAAGTTTGTCACCATGACA

GACTTGGTGCCTGGCATCCTTGAAAAGCAGCGGGACCTCAACGACCCGGCCAAGTACAAGGAAGCCAAGG

AATGGCTCGACAACGCGCGCCAAACGTGTTTGAAGAACGGGAACGTCCACATTGCCAACCTGTGCAAAGT

GGTCGCCCCAGCGCCGAGCAAGTCGAGACCTGAACCCGTGGTCGTTTGCCTCCGCGGCAAATCCGGCCAG

GGCAAGAGTTTCCTTGCGAACGTTCTGGCACAAGCCATCTCTACCCACTTTACCGGCAGGACTGACTCAG

TTTGGTACTGTCCGCCAGACCCTGACCACTTCGACGGTTACAACCAGCAGACCGTTGTTGTGATGGATGA

TTTGGGCCAGAATCCCGACGGCAAGGACTTCAAGTACTTCGCCCAGATGGTCTCGACCACGGGGTTCATC

CCGCCCATGGCTTCACTTGAGGACAAAGGCAAGCCTTTCAACAGCAAAGTCATCATTGCCACCACCAACC

TGTACTCGGGCTTCACCCCGAGAACCATGGTGTGCCCCGATGCGCTGAACCGAAGGTTTCACTTTGACAT

TGACGTGAGTGCCAAGGACGGGTACAAAATTAACAACAAATTGGACATAATCAAAGCTCTCGAGGACACC

CACACCAACCCTGTGGCAATGTTCCAATACGACTGTGCCCTTCTCAACGGCATGGCCGTTGAAATGAAGA

GAATGCAACAAGACATGTTCAAACCCCAACCGCCTTTGCAGAACATATACCAACTTGTGCAAGAGGTGAT

TGACCGGGTCGAGCTCCACGAGAAAGTGTCGAGCCACCCGATTTTCAAGCAG

>JQ973889.1_O_CHA_2010

CCCTGGTACAAGCTCATCAAGCTCCTGAGCCGCTTGTCATGCATGGCCGCTGTAGCAGCAAGGTCCAAGG

ACCCAGTCCTTGTGGCCATCATGCTGGCTGACACCGGTCTTGAGATTCTGGACAGCACATTTGTCGTGAA

GAAAATCTCCGACTCCCTCTCCAGTCTCTTTCACGTGCCGGCCCCCGTCTTCAGTTTCGGAGCTCCGATT

CTGCTAGCCGGGTTGGTCAAGGTCGCCTCGAGCTTCTTCCGGTCCACACCCGAGGATCTCGAGAGAGCAG

AGAAACAGCTCAAAGCACGTGACATCAATGACATCTTCGCCATTCTCAAGAACGGCGAGTGGCTGGTCAA

GTTGATCCTAGCCATCCGCGACTGGATTAAAGCATGGATCGCCTCAGAAGAGAAGTTTGTCACCATGACA

GACTTGGTGCCTGGCATCCTTGAAAAGCAGCGGGACCTCAACGACCCGGCCAAGTACAAGGAAGCCAAGG

AATGGCTCGACAACGCGCGCCAAACGTGTTTGAAGAGCGGGAACGTCCACATTGCCAACCTGTGCAAAGT

GGTCGCCCCAGCGCCGAGCAAGTCAAGACCTGAACCCGTGGTCGTTTGCCTCCGCGGCAAATCCGGCCAG

GGTAAGAGTTTCCTTGCTAACGTGCTGGCACAAGCCATCTCTACCCACTTTACCGGCAGGACTGACTCAG

TTTGGTACTGTCCGCCAGACCCTGACCACTTCGACGGTTACAACCAGCAGACCGTTGTTGTGATGGATGA

TTTGGGCCAGAATCCCGACGGCAAGGACTTCAAGTACTTCGCCCAGATGGTCTCGACCACGGGGTTCATC

CCGCCCATGGCTTCACTTGAGGACAAAGGCAAGCCTTTCAACAGCAAAGTCATCATTGCCACCACCAACC

TGTACTCGGGCTTCACCCCGAGAACCATGGTGTGCCCCGATGCGCTGAACCGAAGGTTTCACTTTGACAT

TGACGTGAGTGCCAAGGACGGGTACAAAATTAACAACAAATTGGACATAATCAAAGCTCTCGAGGACACC

CACACCAACCCTGTGGCAATGTTCCAATACGACTGTGCCCTTCTCAACGGCATGGCCGTTGAAATGAAGA

GAATGCAACAAGACATGTTCAAACCCCAACCGCCTTTGCAGAACATATACCAACTTGTGCAAGAGGTGAT

TGACCGGGTCGAGCTCCACGAGAAAGTGTCGAGCCACCCGATTTTCAAGCAG

>JX014255.1_SAT2_EGY_2012

CCCTGGTACAAGGTAATCAAACTCCTCAGCCGTCTGTCGTGCATGGCCGCTGTAGCAGCACGGTCCAAAG

ACCCAGTCCTTGTGGCTATCATGCTAGCTGACACCGGTCTTGAAATCCTGGACAGCACTTTCGTCGTGAA

GAAAATCGCCGACATGCTCTCCAGTGTTTTCCACGTGCCGGCCCCTGTTTTCAGCTTCGGAGCCCCGGTG

CTGTTAGCAGGTTTGGTCAAGGTCGCCTCGAGTTTCTTCCAGTCGACACCCGAAGAACTTGAGAGAGCTG

AGAAGCAGCTCAAGGCGCGTGACATCAACGACATTCTCGCTATCCTCAAGAATGGCGAGTGGCTGGTCAA

ACTCATCCTGGCTATCCGCGACTGGATTAAGGCCTGGATCTCCTCAGAAGAGAAGTTTGTCACCATGACA

GACTTGGTGCCTGGTATCTTGGAAAAGCAGCGAGATCTTTACGATCCTTCCAAGTACCATGAAGCAAAGA

AATGGCTAGAGAACGCCCGCCAGACGTGTCTCAAGAATGGCAACACCAACATTGCCAACCTGTGCAAGGT

CACAGCACCCGCACCGAGCAAGTCGAGACCTGAACCTGTGGTCGTCTGCCTCCGCGGCAAGTCCGGCCAA

GGTAAGAGTTTCCTCGCGAACGTGCTTGCACAAGCCATTTCCACACACTACACTGGCAGAACAGACTCAG

TCTGGTACTGCCCGCCTGACCCCGACCACTTTGACGGTTACAACCAACAGACCGTCGTGGTCATGGATGA

CCTCGGACAAAACCCTGACGGCAAGGACTTCAAGTACTTCGCCCAAATGGTCTCCACAACCGGGTTCATC

CCGCCCATGGCATCGCTCGAAGATAAGGGAAAACCATTCAACAGTAAGGTCATCATCGCGACCTCAAACT

TGTACTCCGGGTTCACCCCGCGTACAATGGTCTGCCCTGATGCACTGAACCGTAGGTTCCACTTTGACAT

CGACGTGAGCGCCAAGGACGGGTACAAAATTGGCAGCAAATTGGACATTATTAAAGCACTTGAAGACACC

CACACAAACCCGGTGGCAATGTTTCAGTACGACTGTGCCCTTCTGAACGGCATGGCTGTTGAGATGAAGA

GACTACAACAAGATATGTTTAAGCCCCAACCACCGATTCTTAACGTGTACCAGCTCGTGGATGAGGTGAT

TGAGAGAGTTAATTTGCATGAAAAGATTTCTTCACAACCCATCTTTAAGCAA

>JX014256.1_SAT2_PAT_2012

CCCTGGTACAAGGTAATCAAACTCCTCAGCCGTCTGTCGTGCATGGCCGCTGTAGCAGCACGGTCCAAAG

ACCCAGTCCTTGTGGCTATCATGCTAGCTGACACCGGTCTTGAAATCCTGGACAGCACTTTCGTCGTGAA

GAAAATCGCCGACATGCTCTCCAGTGTTTTCCACGTGCCGGCCCCTGTTTTCAGCTTCGGAGCCCCGGTG

CTGTTAGCAGGTTTGGTCAAGGTCGCCTCGAGTTTCTTCCAGTCGACACCCGAAGAACTTGAGAGAGCTG

AGAAGCAGCTCAAGGCGCGTGACATCAACGACATTCTCGCTATCCTCAAGAATGGCGAGTGGCTGGTCAA

ACTCATCCTGGCTATCCGCGACTGGATTAAGGCCTGGATCTCCTCAGAAGAGAAGTTTGTCACCATGACA

GACTTGGTGCCTGGTATCTTGGAAAAGCAGCGAGATCTTCACGATCCTTCCAAGTACCATGAAGCAAAGA

AATGGCTAGAGAACGCCCGCCAGACGTGTCTCAAGAACGGCAACACTAACATTGCCAACCTGTGCAAGGT

CACAGCACCCGCACCGAGCAAGTCGAGACCTGAACCTGTGGTCGTCTGCCTCCGCGGCAAGTCCGGCCAA

GGTAAGAGTTTCCTCGCGAACGTGCTTGCACAAGCCATTTCCACACACTACACTGGCAGAACAGACTCAG

TCTGGTACTGCCCGCCTGACCCCGACCACTTTGACGGTTACAACCAACAGACCGTCGTGGTCATGGATGA

CCTCGGACAAAACCCTGACGGCAAGGACTTCAAGTACTTCGCCCAAATGGTCTCCACAACCGGGTTCATC

CCGCCCATGGCATCGCTCGAAGATAAGGGAAAACCATTCAACAGTAAGGTCATCATCGCGACCTCAAACT

TGTACTCCGGGTTCACCCCGCGTACAATGGTCTGCCCTGATGCACTGAACCGTAGGTTCCACTTTGACAT

CGACGTGAGCGCCAAGGACGGGTACAAAATTGGCAACAAATTGGACATTATTAAAGCACTTGAAGACACC

CACACAAACCCGGTGGCGATGTTTCAGTACGATTGTGCCCTTCTGAACGGCATGGCTGTTGAGATGAAGA

GACTACAACAAGATATGTTTAAGCCCCAACCACCGATTCTTAACGTGTACCAGCTCGTGGATGAGGTGAT

TGAGAGAGTTAATTTGCATGAGAAGATTTCTTCACAACCCATCTTCAAGCAA

>JX040485.1_O_BUL_2010

CCCTGGTACAAGCTCATCAAGCTCCTGAGCCGCCTGTCATGCATGGCCGCTGTAGCAGCACGGTCAAAGG

ACCCAGTCCTTGTGGCCATCATGCTAGCTGACACCGGTCTCGAGATTCTGGACAGCACCTTCGTCGTGAA

GAAGATCTCCGACTCGCTCTCCAGTCTCTTTCACGTGCCGGCCCCCGTCTTCAGTTTCGGAGCCCCGATT

CTGTTGGCCGGGTTGGTCAAAGTCGCCTCGAGTTTCTTCCGGTCCACACCCGAAGACCTTGAGAGAGCAG

AGAAACAGCTCAAAGCACGTGACATCAATGACATCTTCGCCGTTCTCAAGAACGGCGAGTGGCTGGTCAA

ACTGATTCTTGCCATCCGCGACTGGATCAAGGCATGGATCGCCTCAGAAGAGAAGTTTGTCACCACGACA

GACTTGGTGCCTGGCATCCTTGAGAAGCAACGGGACCTCAACGACCCTAGCAAGTACAAGGAGGCCAAGG

AGTGGCTCGACAACGCGCGCCAAGCGTGTTTGAAGAGCGGGAATGTCCACATTGCCAACCTGTGCAAAGT

GGTCGCCCCAGCACCCAGCAAGTCGAGACCCGAACCCGTGGTCGTTTGCCTCCGTGGCAAGTCCGGCCAG

GGCAAGAGTTTCCTTGCTAACGTGCTCGCGCAGGCGATTTCATCTCACTTTACCGGCAGAACCGATTCAG

TTTGGTACTGTCCGCCTGACCCAGACCACTTCGACGGTTACAACCAGCAGACCGTTGTAGTGATGGATGA

TTTGGGCCAGAACCCCGACGGGAAGGACTTCAAGTACTTCGCCCAGATGGTTTCAACCACGGGGTTCATC

CCGCCCATGGCTTCACTCGAAGACAAAGGCAAACCTTTCAACAGCAAGGTCATCATTGCCACCACCAACC

TGTACTCGGGCTTCACCCCGAGGACTATGGTGTGCCCTGATGCACTGAACCGAAGGTTCCACTTTGACAT

TGACGTGAGCGCCAAGGACGGGTACAAAATTAACAACAAACTGGACATTATCAAAGCTCTTGAAGACACC

CACACCAACCCGGTGGCAATGTTTCAATATGATTGTGCTCTTCTCAATGGCATGGCCGTTGAAATGAAGA

GAATGCAACAAGATGTGTTCAAGCCTCAACCACCCCTCCAGAACGTGTACCAGCTCGTTCAGGAGGTGAT

TGAACGGGTCGAGCTCCATGAGAAAGTATCGAGCCACCCGATCTTCAAGCAG

>JX040486.1_O_BUL_2011

CCCTGGTACAAGCTCATCAAGCTCCTGAGCCGCCTGTCATGCATGGCCGCTGTAGCAGCACGGTCAAAGG

ACCCAGTCCTTGTGGCCATCATGCTAGCTGACACCGGTCTCGAGATTCTGGACAGCACCTTCGTCGTGAA

GAAGATCTCCGACTCGCTCTCCAGTCTCTTTCACGTGCCGGCCCCCGTCTTCAGTTTCGGAGCCCCGATT

CTGTTGGCCGGGTTGGTCAAAGTCGCCTCGAGTTTCTTCCGGTCCACACCCGAAGACCTTGAGAGAGCAG

AGAAACAGCTCAAAGCACGTGACATCAATGACATCTTCGCCGTTCTCAAGAACGGCGAGTGGCTGGTCAA

ACTGATTCTTGCCATCCGCGACTGGATCAAGGCATGGATCGCCTCAGAAGAGAAGTTTGTCACCACGACA

GACTTGGTGCCTGGCATCCTTGAGAAGCAACGGGACCTCAACGACCCTAGCAAGTACAAGGAGGCCAAGG

AGTGGCTCGACAACGCGCGCCAAGCGTGTTTGAAGAGCGGGAATGTCCACATTGCCAACCTGTGCAAAGT

GGTCGCCCCAGCACCCAGCAAGTCGAGACCCGAACCCGTGGTCGTTTGCCTCCGTGGCAAGTCCGGCCAG

GGCAAGAGTTTCCTTGCTAACGTGCTCGCGCAGGCGATTTCATCTCACTTTACCGGCAGAACCGATTCAG

TTTGGTACTGTCCGCCTGACCCTGACCACTTCGACGGTTACAACCAGCAGACCGTTGTAGTGATGGATGA

TTTGGGCCAGAACCCCGACGGGAAGGACTTCAAGTACTTCGCCCAGATGGTTTCAACCACGGGGTTCATC

CCGCCCATGGCTTCACTCGAAGACAAAGGCAAACCTTTCAACAGCAAGGTCATCATTGCCACCACCAACC

TGTACTCGGGCTTCACCCCGAGGACTATGGTGTGCCCTGATGCACTAAACCGAAGGTTCCACTTTGACAT

TGACGTGAGCGCCAAGGACGGGTACAAAGTTAACAACAAACTGGACATTATCAAAGCTCTTGAAGACACC

CACACCAACCCGGTGGCAATGTTTCAATATGATTGTGCTCTTCTCAATGGCATGGCCGTTGAAATGAAGA

GAATGCAACAAGATGTGTTCAAGCCTCAACCACCCCTCCAGAACGTGTACCAGCTCGTTCAGGAGGTGAT

TGAACGGGTCGAGCTCCATGAGAAAGTATCGAGCCACCCGATCTTCAAGCAG

>JX040487.1_O_BUL_2011

CCCTGGTACAAGCTCATCAAGCTCCTGAGCCGCCTGTCATGCATGGCCGCTGTAGCAGCACGGTCAAAGG

ACCCAGTCCTTGTGGCCATCATGCTAGCTGACACCGGTCTCGAGATTCTGGACAGCACCTTCGTCGTGAA

GAAGATCTCCGACTCGCTCTCCAGTCTCTTTCACGTGCCGGCCCCCGTCTTCAGTTTCGGAGCCCCGATT

CTGTTGGCCGGGTTGGTCAAAGTCGCCTCGAGTTTCTTCCGGTCCACACCCGAAGACCTTGAGAGAGCAG

AGAAACAGCTCAAAGCACGTGACATCAACGACATCTTCGCCGTTCTCAAGAACGGCGAGTGGCTGGTCAA

ACTGATTCTTGCCATCCGCGACTGGATCAAGGCATGGATCGCCTCAGAAGAGAAGTTTGTCACCACGACA

GACTTGGTGCCTGGCATCCTTGAGAAGCAACGGGACCTCAACGACCCTAGCAAGTACAAGGAGGCCAAGG

AGTGGCTCGACAACGCGCGCCAAGCGTGTTTGAAGAGCGGGAATGTCCACATTGCCAACCTGTGCAAAGT

GGTCGCCCCAGCACCCAGCAAGTCGAGACCCGAACCCGTGGTCGTTTGCCTCCGTGGCAAGTCCGGCCAG

GGCAAGAGTTTCCTTGCTAACGTGCTCGCGCAGGCGATTTCATCTCACTTTACCGGCAGAACCGATTCAG

TTTGGTACTGTCCGCCTGACCCTGACCACTTCGACGGTTACAACCAGCAGACCGTTGTAGTGATGGATGA

TTTGGGCCAGAACCCCGACGGGAAGGACTTCAAGTACTTCGCCCAGATGGTTTCAACCACGGGGTTCATC

CCGCCCATGGCTTCACTCGAAGACAAAGGCAAACCTTTCAACAGCAAGGTCATCATTGCCACCACCAACC

TGTACTCGGGCTTCACCCCGAGGACTATGGTGTGCCCTGATGCACTAAACCGAAGGTTCCACTTTGACAT

TGACGTGAGCGCCAAGGACGGGTACAAAATTAACAACAAACTGGACATTATCAAAGCTCTTGAAGACACC

CACACCAACCCGGTGGCAATGTTTCAATATGATTGTGCTCTTCTCAATGGCATGGCCGTTGAAATGAAGA

GAATGCAACAAGATGTGTTCAAGCCTCAACCACCCCTCCAGAACGTGTACCAGCTCGTTCAGGAGGTGAT

TGAACGGGTCGAGCTCCATGAGAAAGTATCGAGCCACCCGATCTTCAAGCAG

>JX040488.1_O_BUL_2011

CCCTGGTACAAGCTCATCAAGCTCCTGAGCCGCCTGTCATGCATGGCCGCTGTAGCAGCACGGTCAAAGG

ACCCAGTCCTTGTGGCCATCATGCTAGCTGACACCGGTCTCGAGATTCTGGACAGCACCTTCGTCGTGAA

GAAGATCTCCGACTCGCTCTCCAGTCTCTTTCACGTGCCGGCCCCCGTCTTCAGTTTCGGAGCCCCGATT

CTGTTGGCCGGGTTGGTCAAAGTCGCCTCGAGTTTCTTCCGGTCCACACCCGAAGACCTTGAGAGAGCAG

AGAAACAGCTCAAAGCACGTGACATCAACGACATCTTCGCCGTTCTCAAGAACGGCGAGTGGCTGGTCAA

ACTGATTCTTGCCATCCGCGACTGGATCAAGGCATGGATCGCCTCAGAAGAGAAGTTTGTCACCACGACA

GACTTGGTGCCTGGCATCCTTGAGAAGCAACGGGACCTCAACGACCCTAGCAAGTACAAGGAGGCCAAGG

AGTGGCTCGACAACGCGCGCCAAGCGTGTTTGAAGAGCGGGAATGTCCACATTGCCAACCTGTGCAAAGT

GGTCGCCCCAGCACCCAGCAAGTCGAGACCCGAACCCGTGGTCGTTTGCCTCCGTGGCAAGTCCGGCCAG

GGCAAGAGTTTCCTTGCTAACGTGCTCGCGCAGGCGATTTCATCTCACTTTACCGGCAGAACCGATTCAG

TTTGGTACTGTCCGCCTGACCCTGACCACTTCGACGGTTACAACCAGCAGACCGTTGTAGTGATGGATGA

TTTGGGCCAGAACCCCGACGGGAAGGACTTCAAGTACTTCGCCCAGATGGTTTCAACCACGGGGTTCATC

CCGCCCATGGCTTCACTCGAAGACAAAGGCAAACCTTTCAACAGCAAGGTCATCATTGCCACCACCAACC

TGTACTCGGGCTTCACCCCGAGGACTATGGTGTGCCCTGATGCACTAAACCGAAGGTTCCACTTTGACAT

TGACGTGAGCGCCAAGGACGGGTACAAAATTAACAACAAACTGGACATTATCAAAGCTCTTGAAGACACC

CACACCAACCCGGTGGCAATGTTTCAATATGATTGTGCTCTTCTCAATGGCATGGCCGTTGAAATGAAGA

GAATGCAACAAGATGTGTTCAAGCCTCAACCACCCCTCCAGAACGTGTACCAGCTCGTTCAGGAGGTGAT

TGAACGGGTCGAGCTCCATGAGAAAGTATCGAGCCACCCGATCTTCAAGCAG

>JX040489.1_O_BUL_2011

CCCTGGTACAAGCTCATCAAGCTCCTGAGCCGCCTGTCATGCATGGCCGCTGTAGCAGCACGGTCAAAGG

ACCCAGTCCTTGTGGCCATCATGCTAGCTGACACCGGTCTCGAGATTCTGGACAGCACCTTCGTCGTGAA

GAAGATCTCCGACTCGCTCTCCAGTCTCTTTCACGTGCCGGCCCCCGTCTTCAGTTTCGGAGCCCCGATT

CTGTTGGCCGGGTTGGTCAAAGTCGCCTCGAGTTTCTTCCGGTCCACACCCGAAGACCTTGAGAGAGCAG

AGAAACAGCTCAAAGCACGTGACATCAACGACATCTTCGCCGTTCTCAAGAACGGCGAGTGGCTGGTCAA

ACTGATTCTTGCCATCCGCGACTGGATCAAGGCATGGATCGCCTCAGAAGAGAAGTTTGTCACCACGACA

GACTTGGTGCCTGGCATCCTTGAGAAGCAACGGGACCTCAACGACCCTAGCAAGTACAAGGAGGCCAAGG

AGTGGCTCGACAACGCGCGCCAAGCGTGTTTGAAGAGCGGGAATGTCCACATTGCCAACCTGTGCAAAGT

GGTCGCCCCAGCACCCAGCAAGTCGAGACCCGAACCCGTGGTCGTTTGCCTCCGTGGCAAGTCCGGCCAG

GGCAAGAGTTTCCTTGCTAACGTGCTCGCGCAGGCGATTTCATCTCACTTTACCGGCAGAACCGATTCAG

TTTGGTACTGTCCGCCTGACCCTGACCACTTCGACGGTTACAACCAGCAGACCGTTGTAGTGATGGATGA

TTTGGGCCAGAACCCCGACGGGAAGGACTTCAAGTACTTCGCCCAGATGGTTTCAACCACGGGGTTCATC

CCGCCCATGGCTTCACTCGAAGACAAAGGCAAACCTTTCAACAGCAAGGTCATCATTGCCACCACCAACC

TGTACTCGGGCTTCACCCCGAGGACTATGGTGTGCCCTGATGCACTAAACCGAAGGTTCCACTTTGACAT

TGACGTGAGCGCCAAGGACGGGTACAAAATTAACAACAAACTGGACATTATCAAAGCTCTTGAAGACACC

CACACCAACCCGGTGGCAATGTTTCAATATGATTGTGCTCTTCTCAATGGCATGGCCGTTGAAATGAAGA

GAATGCAACAAGATGTGTTCAAGCCTCAACCACCCCTCCAGAACGTGTACCAGCTCGTTCAGGAGGTGAT

TGAACGGGTCGAGCTCCATGAGAAAGTATCGAGCCACCCGATCTTCAAGCAG

>JX040490.1_O_BUL_2011

CCCTGGTACAAGCTCATCAAGCTCCTGAGCCGCCTGTCATGCATGGCCGCTGTAGCAGCACGGTCAAAGG

ACCCAGTCCTTGTGGCCATCATGCTAGCTGACACCGGTCTCGAGATTCTGGACAGCACCTTCGTCGTGAA

GAAGATCTCCGACTCGCTCTCCAGTCTCTTTCACGTGCCGGCCCCCGTCTTCAGTTTCGGAGCCCCGATT

CTGTTGGCCGGGTTGGTCAAAGTCGCCTCGAGTTTCTTCCGGTCCACACCCGAAGACCTTGAGAGAGCAG

AGAAACAGCTCAAAGCACGTGACATCAACGACATCTTCGCCGTTCTCAAGAACGGCGAGTGGCTGGTCAA

ACTGATTCTTGCCATCCGCGACTGGATCAAGGCATGGATCGCCTCAGAAGAGAAGTTTGTCACCACGACA

GACTTGGTGCCTGGCATCCTTGAGAAGCAACGGGACCTCAACGACCCTAGCAAGTACAAGGAGGCCAAGG

AGTGGCTCGACAACGCGCGCCAAGCGTGTTTGAAGAGCGGGAATGTCCACATTGCCAACCTGTGCAAAGT

GGTCGCCCCAGCACCCAGCAAGTCGAGACCCGAACCCGTGGTCGTTTGCCTCCGTGGCAAGTCCGGCCAG

GGCAAGAGTTTCCTTGCTAACGTGCTCGCGCAGGCGATTTCATCTCACTTTACCGGCAGAACCGATTCAG

TTTGGTACTGTCCGCCTGACCCTGACCACTTCGACGGTTACAACCAGCAGACCGTTGTAGTGATGGATGA

TTTGGGCCAGAACCCCGACGGGAAGGACTTCAAGTACTTCGCCCAGATGGTTTCAACCACGGGGTTCATC

CCGCCCATGGCTTCACTCGAAGACAAAGGCAAACCTTTCAACAGCAAGGTCATCATTGCCACCACCAACC

TGTACTCGGGCTTCACCCCGAGGACTATGGTGTGCCCTGATGCACTAAACCGAAGGTTCCACTTTGACAT

TGACGTGAGCGCCAAGGACGGGTACAAAATTAACAACAAACTGGACATTATCAAAGCTCTTGAAGACACC

CACACCAACCCGGTGGCAATGTTTCAATATGATTGTGCTCTTCTCAATGGCATGGCCGTTGAAATGAAGA

GAATGCAACAAGATGTGTTCAAGCCTCAACCACCCCTCCAGAACGTGTACCAGCTCGTTCAGGAGGTGAT

TGAACGGGTCGAGCTCCATGAGAAAGTATCGAGCCACCCGATCTTCAAGCAG

>JX040491.1_O_TUR_2010

CCCTGGTACAAGCTCATCAAGCTCCTGAGCCGCCTGTCATGCATGGCCGCTGTAGCAGCACGGTCAAAGG

ACCCAGTCCTTGTGGCCATCATGCTAGCTGACACCGGTCTCGAGATTCTGGACAGCACCTTCGTCGTGAA

GAAGATCTCCGACTCGCTCTCCAGTCTCTTTCACGTGCCGGCCCCCGTCTTCAGTTTCGGAGCCCCGATT

CTGTTGGCCGGGTTGGTCAAAGTCGCCTCGAGTTTCTTCCGGTCCACACCCGAAGACCTTGAGAGAGCAG

AGAAACAGCTCAAAGCACGTGACATCAATGACATCTTCGCCGTTCTCAAGAACGGCGAGTGGCTGGTCAA

ACTGATTCTTGCCATCCGCGACTGGATCAAGGCATGGATCGCCTCAGAAGAAAAGTTTGTCACCATGACA

GACTTGGTGCCTGGCATCCTTGAGAAGCAACGGGACCTCAACGACCCTAGCAAATACAAGGAGGCCAAGG

AGTGGCTCGACAACGCGCGCCAAGCGTGTTTGAAGAGCGGGAATGTCCACATTGCCAACCTGTGCAAAGT

GGTCGCCCCAGCACCCAGCAAGTCGAGACCCGAACCCGTGGTCGTTTGCCTCCGTGGCAAGTCCGGCCAG

GGCAAGAGTTTCCTTGCTAACGTGCTCGCGCAAGCGATTTCATCTCACTTTACCGGCAGAACCGATTCAG

TTTGGTACTGTCCGCCTGACCCTGACCACTTCGACGGTTACAACCAGCAGACCGTTGTAGTGATGGATGA

TTTGGGCCAGAACCCCGACGGGAAGGACTTCAAGTACTTCGCCCAGATGGTTTCAACCACGGGGTTCATC

CCGCCCATGGCTTCACTCGAAGACAAAGGCAAACCTTTCAACAGCAAGGTCATCATTGCCACCACCAACC

TGTACTCGGGCTTCACCCCGAGGACTATGGTGTGCCCTGATGCACTGAACCGAAGGTTCCACTTTGACAT

TGACGTGAGCGCCAAGGACGGGTACAAAATTAACAACAAACTGGACATTATCAAAGCTCTTGAAGACACC

CACACCAACCCGGTGGCAATGTTTCAATATGATTGTGCTCTTCTCAATGGCATGGCCGTTGAAATGAAGA

GAATGCAACAAGATGTGTTCAAGCCTCAACCACCCCTCCAAAACGTGTACCAGCTCGTTCAGGAGGTGAT

TGAACGGGTCGAGCTCCACGAGAAAGTATCGAGCCACCCGATCTTCAAGCAG

>JX040492.1_O_TUR_2010

CCCTGGTACAAGCTCATCAAGCTCCTGAGCCGCCTGTCATGCATGGCCGCTGTAGCAGCACGGTCAAAGG

ACCCAGTCCTTGTGGCCATCATGCTAGCTGACACCGGTCTCGAGATTCTGGACAGCACCTTCGTCGTGAA

GAAGATCTCCGACTCGCTCTCCAGTCTCTTTCACGTGCCGGCCCCCGCCTTCAGTTTCGGAGCCCCGATT

CTGTTGGCCGGGTTGGTCAAAGTCGCCTCGAGTTTCTTCCGGTCCACACCCGAAGACCTTGAGAGAGCAG

AAAAACAGCTCAAAGCACGTGACATCAACGACATCTTCGCCATTCTCAAGAACGGCGAGTGGCTGGTCAA

GCTGATTCTTGCCATCCGCGACTGGATCAAGGCATGGATCGCCTCAGAAGAAAAGTTTGTCACCATGACA

GACTTGGTGCCTGGCATCCTTGAGAAGCAACGGGAGCTCAACGACCCTAGCAAGTACAAGGGGGCCAAGG

AGTGGCTCGACAACGCGCGCCAAGCGTGTTTGAAGAGCGGGAACGTCCACATTGCCAACCTGTGCAAAGT

GGTCGCCCCAGCACCCAGCAAGTCGAGACCCGAACCCGTGGTCGTTTGCCTCCGTGGCAAGTCCGGCCAG

GGCAAGAGTTTCCTTGCTAACGTGCTCGCGCAAGCGATTTCATCTCACTTTACCGGCAGAACCGATTCAG

TTTGGTACTGTCCGCCTGACCCTGACCACTTCGACGGTTACAACCAGCAGACCGTTGTAGTGATGGATGA

TTTGGGCCAGAACCCCGACGGGAAGGACTTCAAGTACTTCGCCCAGATGGTTTCAACCACGGGGTTCATC

CCGCCCATGGCTTCACTCGAAGACAAAGGCAAACCTTTCAACAGCAAGGTCATCATTGCCACCACCAACC

TGTACTCGGGCTTCACCCCGAGGACTATGGTGTGCCCTGATGCACTGAACCGAAGGTTCCACTTTGACAT

TGACGTGAGCGCCAAGGACGGGTACAAAATTAACAACAAACTGGACATTATCAAAGCTCTTGAAGACACC

CACACCAACCCGGTGGCAATGTTTCAATATGATTGTGCTCTTCTCAATGGCATGGCCGTTGAAATGAAGA

GAATGCAACAAGATGTGTTCAAGCCTCAACTACCCCTCCAGAACGTGTACCAGCTCGTTCAGGAGGTGAT

TGAACGGGTCGAGCTCCACGAGAAAGTGTCGAGCCACCCGATCTTCAAACAG

>JX040493.1_O_TUR_2010

CCCTGGTACAAGCTCATCAAGCTCCTGAGCCGCCTGTCATGCATGGCCGCTGTAGCAGCACGGTCAAAGG

ACCCAGTCCTTGTGGCCATCATGCTAGCTGACACCGGTCTCGAGATTCTGGACAGCACCTTCGTCGTGAA

GAAGATCTCCGACTCGCTCTCCAGTCTCTTTCACGTGCCGGCCCCCGTCTTCAGTTTCGGAGCCCCGATT

CTGTTGGCCGGGTTGGTCAAAGTCGCCTCGAGTTTCTTCCGGTCCACACCCGAAGACCTTGAGAGAGCAG

AGAAACAGCTCAAAGCACGTGACATCAATGACATCTTCGCCGTTCTCAAGAACGGCGAGTGGCTGGTCAA

ACTGATTCTTGCCATCCGCGACTGGATCAAGGCATGGATCGCCTCAGAAGAAAAGTTTGTCACCATGACA

GACTTGGTGCCTGGCATCCTTGAGAAGCAACGGGACCTCAACGACCCTAGCAAGTACAAGGAGGCCAAGG

AGTGGCTCGACAACGCGCGCCAAGCGTGTTTGAAGAGCGGGAATGTCCACATTGCCAACCTGTGCAAAGT

GGTCGCCCCAGCACCCAGCAAGTCGAGACCCGAACCCGTGGTCGTTTGCCTCCGTGGCAAGTCCGGCCAG

GGCAAGAGTTTCCTTGCTAACGTGCTCGCGCAAGCGATTTCATCTCACTTTACCGGCAGAACCGATTCAG

TTTGGTACTGTCCGCCTGACCCTGACCACTTCGACGGTTACAACCAGCAGACCGTTGTAGTGATGGATGA

TTTGGGCCAGAACCCCGACGGGAAGGACTTCAAGTACTTCGCCCAGATGGTTTCAACCACGGGGTTCATC

CCGCCCATGGCTTCACTCGAAGACAAAGGCAAACCTTTCAACAGCAAGGTCATCATTGCCACCACCAACC

TGTACTCGGGCTTCACCCCGAGGACTATGGTGTGCCCTGATGCACTGAACCGAAGGTTCCACTTTGACAT

TGACGTGAGCGCCAAGGACGGGTACAAAATTAACAACAAACTGGACATTATCAAAGCTCTTGAAGACACC

CACACCAACCCGGTGGCAATGTTTCAATATGATTGTGCTCTTCTCAATGGCATGGCCGTTGAAATGAAGA

GAATGCAACAAGATGTGTTCAAGCCTCAACCACCCCTCCAGAACGTGTACCAGCTCGTTCAGGAGGTGAT

TGAACGGGTCGAGCTCCACGAGAAAGTATCGAGCCACCCGATCTTCAAGCAG

>JX040494.1_O_TUR_2010

CCCTGGTACAAGCTCATCAAGCTCCTGAGCCGCCTGTCATGCATGGCCGCTGTAGCAGCACGGTCAAAGG

ACCCAGTCCTTGTGGCCATCATGCTAGCTGACACCGGTCTCGAGATTCTGGACAGCACCTTCGTCGTGAA

GAAGATCTCCGACTCGCTCTCCAGTCTCTTTCACGTGCCGGCCCCCGTCTTCAGTTTCGGAGCCCCGATT

CTGTTGGCCGGGTTGGTCAAAGTCGCCTCGAGTTTCTTCCGGTCCACACCCGAAGACCTTGAGAGAGCAG

AGAAACAGCTCAAAGCACGTGACATCAATGACATCTTCGCCGTTCTCAAGAACGGCGAGTGGCTGGTCAA

ACTGATTCTTGCCATCCGCGACTGGATCAAGGCATGGATCGCCTCAGAAGAAAAGTTTGTCACCATGACA

GACTTGGTGCCTGGCATCCTTGAGAAGCAACGGGACCTCAACGACCCTAGCAAGTACAAGGAGGCCAAGG

AGTGGCTCGACAACGCGCGCCAAGCGTGTTTGAAGAGCGGGAATGTCCACATTGCCAACCTGTGCAAAGT

GGTCGCCCCAGCACCCAGCAAGTCGAGACCCGAACCCGTGGTCGTTTGCCTCCGTGGCAAGTCCGGCCAG

GGCAAGAGTTTCCTTGCTAACGTGCTCGCGCAAGCGATTTCATCTCACTTTACCGGCAGAACCGATTCAG

TTTGGTACTGTCCGCCTGACCCTGACCACTTCGACGGTTACAACCAGCAGACCGTTGTAGTGATGGATGA

TTTGGGCCAGAACCCCGACGGGAAGGACTTCAAGTACTTCGCCCAGATGGTTTCAACCACGGGGTTCATC

CCGCCCATGGCTTCACTCGAAGACAAAGGCAAACCTTTCAACAGCAAGGTCATCATTGCCACCACCAACC

TGTACTCGGGCTTCACCCCGAGGACTATGGTGTGCCCTGATGCACTGAACCGAAGGTTCCACTTTGACAT

TGACGTGAGCGCCAAGGACGGGTACAAAATTAACAACAAACTGGACATTATCAAAGCTCTTGAAGACACC

CACACCAACCCGGTGGCAATGTTTCAATATGATTGTGCTCTTCTCAATGGTATGGCCGTTGAAATGAAGA

GAATGCAACAAGATGTGTTCAAGCCTCAACCACCCCTCCAGAACGTGTACCAGCTCGTTCAGGAGGTGAT

TGAACGGGTCGAGCTCCACGAGAAAGTATCGAGCCACCCGATCTTCAAGCAG

>JX040495.1_O_TUR_2010

CCCTGGTACAAGCTCATCAAGCTCCTGAGCCGCCTGTCATGCATGGCCGCTGTAGCAGCACGGTCAAAGG

ACCCAGTCCTTGTGGCCATCATGCTAGCTGACACCGGTCTCGAGATTCTGGACAGCACCTTCGTCGTGAA

GAAGATCTCCGACTCGCTCTCCAGTCTCTTTCACGTGCCGGCCCCCGTCTTCAGTTTCGGAACCCCGATT

CTGTTGGCCGGGTTGGTCAAAGTCGCCTCGAGTTTCTTCCGGTCCACACCCGAAGACCTTGAGAGAGCAG

AGAAACAGCTCAAAGCACGTGACATCAATGACATCTTCGCCGTTCTCAAGAACGGCGAGTGGCTGGTCAA

ACTGATTCTTGCCATCCGCGACTGGATCAAGGCATGGATCGCCTCAGAAGAAAAGTTTGTCACTATGACA

GACTTGGTGCCTGGCATCCTTGAGAAGCAACGGGACCTAAACGACCCTAGCAAGTACAAGGAGGCCAAGG

AGTGGCTCGACAACGCGCGCCAAGCGTGTTTGAAGAGCGGGAATGTCCACATTGCCAACCTGTGCAAAGT

GGTCGCCCCAGCACCCAGCAAGTCGAGACCCGAACCCGTGGTCGTTTGCCTCCGTGGCAAGTCCGGCCAG

GGCAAGAGTTTCCTTGCTAACGTGCTCGCGCAAGCGATTTCATCTCACTTTACCGGCAGAACCGATTCAG

TTTGGTACTGTCCGCCTGACCCTGACCACTTCGACGGTTACAACCAGCAGACCGTTGTAGTGATGGATGA

TTTGGGCCAGAACCCCGACGGGAAGGACTTCAAGTACTTCGCCCAGATGGTTTCAACCACGGGGTTCATC

CCGCCCATGGCTTCACTCGAAGACAAAGGCAAACCTTTCAACAGCAAGGTCATCATTGCCACCACCAACC

TGTACTCGGGCTTCACCCCGAGGACTATGGTGTGCCCTGATGCACTGAACCGAAGGTTCCACTTTGACAT

TGACGTGAGCGCCAAGGACGGGTACAAAATTAACAACAAACTGGACATTATCAAAGCTCTTGAAGACACC

CACACCAACCCGGTGGCAATGTTTCAATATGATTGTGCTCTTCTCAATGGCATGGCCGTTGAAATGAAGA

GAATGCAACAAGACGTGTTCAAGCCTCAACCACCCCTCCAGAACGTGTACCAGCTCGTTCAGGAGGTGAT

TGAACGGGTCGAGCTCCACGAGAAAGTATCGAGCCACCCGATCTTCAAGCAG

>JX040496.1_O_TUR_2010

CCCTGGTACAAGCTCATCAAGCTCCTGAGCCGCCTGTCATGCATGGCCGCTGTAGCAGCACGGTCAAAGG

ACCCAGTCCTTGTGGCCATCATGCTAGCTGACACCGGTCTCGAGATTCTGGACAGCACCTTCGTCGTGAA

GAAGATCTCCGACTCGCTCTCCAGTCTCTTTCACGTGCCGGCCCCCGTCTTCAGTTTCGGAGCCCCGATT

CTGTTGGCCGGGTTGGTCAAAGTCGCCTCGAGTTTCTTCCGGTCCACACCCGAAGACCTTGAGAGAGCAG

AGAAACAGCTCAAAGCACGTGATATCAATGACATCTTCGCCGTTCTCAAGAACGGCGAGTGGCTGGTCAA

ACTGATTCTTGCCATCCGCGACTGGATCAAGGCATGGATCGCCTCAGAAGAAAAGTTTGTCACCATGACA

GACTTGGTGCCTGGCATCCTTGAGAAGCAACGGGACCTCAACGACCCTAGCAAGTACAAGGAGGCCAAGG

AGTGGCTCGACAACGCGCGCCAAGCGTGTTTGAAGAGCGGGAATGTCCACATTGCCAACCTGTGCAAAGT

GGTCGCCCCAGCACCCAGCAAGTCGAGACCCGAACCCGTGGTCGTTTGCCTCCGTGGCAAGTCCGGCCAG

GGCAAGAGTTTCCTTGCTAACGTGCTCGCGCAAGCGATTTCATCTCACTTTACCGGCAGAACCGATTCAG

TTTGGTACTGTCCGCCTGACCCTGACCACTTCGACGGTTACAACCAGCAGACCGTTGTAGTGATGGATGA

TTTGGGCCAGAACCCCGACGGGAAGGACTTCAAGTACTTCGCCCAGATGGTTTCAACCACGGGGTTCATC

CCGCCCATGGCTTCACTCGAAGACAAAGGCAAACCTTTCAACAGCAAGGTCATCATTGCCACCACCAACC

TGTACTCGGGCTTCACCCCGAGGACTATGGTGTGCCCTGATGCACTGAACCGAAGGTTCCACTTTGACAT

TGACGTGAGCGCCAAGGACGGGTACAAAATTAACAACAAACTGGACATTATCAAAGCTCTTGAAGACACC

CACACCAACCCGGTGGCAATGTTTCAATATGATTGTGCTCTTCTCAATGGCATGGCCGTTGAAATGAAGA

GAATGCAACAAGATGTGTTCAAGCCTCAACCACCCCTCCAGAACGTGTACCAGCTCGTTCAGGAGGTGAT

TGAACGGGTCGAGCTCCACGAGAAAGTATCGAGCCACCCGATCTTCAAGCAG

>JX040497.1_O_TUR_2010

CCCTGGTACAAGCTCATCAAGCTCCTGAGCCGCCTGTCATGCATGGCCGCTGTAGCAGCACGGTCAAAGG

ACCCAGTCCTTGTGGCCATCATGCTAGCTGACACCGGTCTCGAGATTCTGGACAGCACCTTCGTCGTGAA

GAAGATCTCCGACTCGCTCTCCAGTCTCTTTCACGTGCCGGCCCCCGTCTTCAGTTTCGGAGCCCCGATT

CTGTTGGCCGGGTTGGTCAAAGTCGCCTCGAGTTTCTTCCGGTCCACACCCGAAGACCTTGAGAGAGCAG

AGAAACAGCTCAAAGCACGTGACATCAATGACATCTTCGCCGTTCTCAAGAACGGCGAGTGGCTGGTCAA

ACTGATTCTTGCCATCCGCGACTGGATCAAGGCATGGATCGCCTCAGAAGAAAAGTTTGTCACCATGACA

GACTTGGTGCCTGGCATCCTTGAGAAGCAACGGGACCTCAACGACCCTAGCAAGTACAAGGAGGCCAAGG

AGTGGCTCGACAACGTGCGCCAAGCGTGTTTGAAGAGCGGGAATGTCCACATTGCCAACCTGTGCAAAGT

GGTCGCCCCAGCACCCAGCAAGTCGAGACCCGAACCCGTGGTCGTTTGCCTCCGTGGCAAGTCCGGCCAG

GGCAAGAGTTTCCTTGCTAACGTGCTCGCGCAAGCGATTTCATCTCACTTTACCGGCAGAACCGATTCAG

TTTGGTACTGTCCGCCTGACCCTGACCACTTCGACGGTTACAACCAGCAGACCGTTGTAGTGATGGATGA

TTTGGGCCAGAACCCCGACGGGAAGGACTTCAAGTACTTCGCCCAGATGGTTTCAACCACGGGGTTCATC

CCGCCCATGGCTTCACTCGAAGACAAAGGCAAACCTTTCAACAGCAAGGTCATCATTGCCACCACCAACC

TGTACTCGGGCTTCACCCCGAGGACTATGGTGTGCCCTGATGCACTGAACCGAAGGTTCCACTTTGACAT

TGACGTGAGCGCCAAGGACGGGTACAAAATTAACAACAAACTGGACATTATCAAAGCTCTTGAAGACACC

CACACCAACCCGGTGGCAATGTTTCAATATGATTGTGCTCTTCTCAATGGCATGGCCGTTGAAATGAAGA

GAATGCAACAAGATGTGTTCAAGCCTCAACCACCCCTCCAGAACGTGTACCAGCTCGTTCAGGAGGTGAT

TGAACGGGTCGAGCTCCACGAGAAAGTATCGAGCCACCCGATCTTCAAGCAG

>JX040498.1_O_TUR_2010

CCCTGGTACAAGCTCATCAAGCTCCTGAGCCGCCTGTCATGCATGGCCGCTGTAGCAGCACGGTCAAAGG

ACCCAGTCCTTGTGGCCATCATGCTAGCTGACACCGGTCTCGAGATTCTGGACAGCACCTTCGTCGTGAA

GAAGATCTCCGACTCGCTCTCCAGTCTCTTTCACGTGCCGGCCCCCGTCTTCAGTTTCGGAGCCCCGATT

CTGTTGGCCGGGTTGGTCAAAGTCGCCTCGAGTTTCTTCCGGTCCACACCCGAAGACCTTGAGAGAGCAG

AGAAACAGCTCAAAGCACGTGACATCAATGACATCTTCGCCGTTCTCAAGAACGGCGAGTGGCTGGTCAA

ACTGATTCTTGCCATCCGCGACTGGATCAAGGCATGGATCGCCTCAGAAGAAAAGTTTGTCACCATGACA

GACTTGGTGCCTGGCATCCTTGAGAAGCAACGGGACCTCAACGACCCTAGCAAGTACAAGGAGGCCAAGG

AGTGGCTCGACAACGCGCGCCAAGCGTGTTTGAAGAGCGGGAATGTCCACATTGCCAACCTGTGCAAAGT

GGTCGCCCCAGCACCCAGCAAGTCGAGACCCGAACCCGTGGTCGTTTGCCTCCGTGGCAAGTCCGGCCAG

GGCAAGAGTTTCCTTGCTAACGTGCTCGCGCAAGCGATTTCATCTCACTTTACCGGCAGAACCGATTCAG

TTTGGTACTGTCCGCCTGACCCTGACCACTTCGACGGTTACAACCAGCAGACCGTTGTAGTGATGGATGA

TTTGGGCCAGAACCCCGACGGGAAGGACTTCAAGTACTTCGCCCAGATGGTTTCAACCACGGGGTTCATC

CCGCCCATGGCTTCACTCGAAGACAAAGGCAAACCTTTCAACAGCAAGGTCATCATTGCCACCACCAACC

TGTACTCGGGCTTCACCCCGAGGACTATGGTGTGCCCTGATGCACTGAACCGAAGGTTCCACTTTGACAT

TGACGTGAGCGCCAAGGACGGGTACAAAATTAACAACAAACTGGACATTATCAAAGCTCTTGAAGACACC

CACACCAACCCGGTGGCAATGTTTCAATATGATTGTGCTCTTCTCAATGGCATGGCCGTTGAAATGAAGA

GAATGCAACAAGATGTGTTCAAGCCTCAACCACCCCTCCAGAACGTGTACCAGCTCGTTCAGGAGGTGAT

TGAACGGGTCGAGCTCCACAAGAAAGTGTCGAGCCACCCGATCTTCAAGCAG

>JX040499.1_O_TUR_2011

CCCTGGTACAAGCTCATCAAGCTCCTGAGCCGCCTGTCATGCATGGCCGCTGTAGCAGCACGGTCAAAGG

ACCCAGTCCTTGTGGCCATCATGCTAGCTGACACCGGTCTCGAGATTCTGGACAGCACCTTCGTCGTGAA

GAAGATCTCCGACTCGCTCTCCAGTCTCTTTCACGTGCCGGCCCCCGTCTTCAGTTTCGGAGCCCCGATT

CTGTTGGCCGGGTTGGTCAAAGTCGCCTCGAGTTTCTTCCGGTCCACACCCGAAGACCTTGAGAGAGCAG

AGAAACAGCTCAAAGCACGTGACATCAATGACATCTTCGCCGTTCTCAAGAACGGCGAGTGGCTGGTCAA

ACTGATTCTTGCCATCCGCGACTGGATCAAGGCATGGATCGCCTCAGAAGAAAAGTTTGTCACCATGACA

GACCTGGTGCCTGGCATCCTTGAAAAGCAACGGGACCTCAACGACCCTAGCAAGTACAAGGAGGCCAAGG

AGTGGCTCGACAACGCGCGCCAAGCGTGTTTGAAGAGCGGGAACGTCCACATTGCCAACCTGTGCAAAGT

GGTCGCCCCAGCACCCAGCAAGTCGAGACCCGAACCCGTGGTCGTTTGCCTCCGTGGCAAGTCCGGCCAG

GGCAAGAGTTTCCTTGCTAACGTGCTCGCGCAAGCGATTTCATCTCACTTTACCGGCAGAACCGATTCAG

TTTGGTACTGTCCGCCTGACCCTGACCACTTTGACGGTTACAACCAGCAGACCGTTGTAGTGATGGATGA

TTTGGGCCAGAACCCCGACGGGAAGGACTTCAAGTACTTCGCCCAGATGGTTTCAACCACGGGGTTCATC

CCGCCCATGGCATCACTCGAAGACAAAGGCAAACCTTTCAACAGCAAGGTCATCATTGCCACCACCAACT

TGTACTCGGGCTTCACCCCGAGGACTATGGTGTGCCCTGATGCGCTGAACCGAAGATTCCACTTTGACAT

TGACTTGAGCGCCAAGGACGGGTACAAAATTAACAACAAGCTGGACATTATCAAAGCTCTTGAAGACACC

CACACCAACCCGGTGGCAATGTTTCAATATGATTGTGCTCTTCTCAATGGCATGGCCGTTGAAATGAAGA

GAATGCAACAAGATGTGTTCAAGCCTCAACCACCCCTCCAGAACGTGTACCAGCTCGTTCAGGAGGTGAT

TGAACGGGTCGAGCTCCACGAGAAAGTATCGAGCCACCCGATCTTCAAGCAG

>JX040500.1_O_TUR_2011

CCCTGGTACAAGCTCATCAAGCTCCTGAGCCGCCTGTCATGCATGGCCGCTGTAGCAGCACGGTCAAAGG

ACCCAGTCCTTGTGGCCATCATGTTAGCTGACACCGGTCTCGAGATTCTGGACAGCACCTTCGTCGTGAA

GAAGATCTCCGACTCGCTCTCCAGTCTCTTTCACGTGCCGGCCCCCGTCTTCAGTTTCGGAGCCCCGATT

CTGTTGGCCGGGTTGGTCAAAGTCGCCTCGAGTTTCTTCCGGTCCACACCCGAAGACCTTGAGAGAGCAG

AGAAACAGCTCAAAGCACGTGACATCAATGACATCTTCGCCGTTCTCAAGAACGGCGAGTGGCTGGTCAA

ACTGATTCTTGCCATCCGCGACTGGATCAAGGCATGGATCGCCTCAGAAGAAAAGTTTGTCACCATGACA

GACTTGGTGCCTGGCATCCTTGAAAAGCAACGGGACCTCAACGACCCTAGCAAGTACAAGGAGGCCAAGG

AGTGGCTCGACAACGCGCGCCAAGCGTGTTTGAAGAGCGGGAATGTCCACATTGCCAACCTGTGCAAAGT

GGTCGCCCCAGCACCCAGCAAGTCGAGACCCGAACCCGTGGTCGTTTGCCTCCGTGGCAAGTCCGGCCAG

GGCAAGAGTTTCCTTGCTAACGTGCTCGCGCAAGCGATTTCGTCTCACTTTACCGGCAGAACCGATTCAG

TTTGGTACTGTCCGCCTGACCCTGACCACTTCGACGGTTACAACCAGCAGACCGTTGTAGTGATGGATGA

TTTGGGCCAGAACCCCGACGGGAAGGACTTCAAGTACTTCGCCCAGATGGTTTCAACCACGGGGTTCATC

CCGCCCATGGCTTCACTCGAAGACAAAGGCAAACCTTTCAACAGCAAGGTCATCATTGCCACCACCAACC

TGTACTCGGGCTTCACCCCGAGGACTATGGTGTGCCCTGATGCACTGAACCGAAGGTTCCACTTTGACAT

TGACTTGAGCGCCAAGGACGGGTACAAAATTAACAACAAACTGGACATTATCAAAGCTCTTGAAGACACC

CACACCAACCCGGTGGCAATGTTTCAATATGATTGTGCTCTTCTCAATGGCATGGCCGTTGAAATGAAGA

GAATGCAACAAGATGTGTTCAAGCCTCAACCACCCCTCCAGAACGTGTACCAGCTCGTCCAGGAGGTGAT

TGAACGGGTCGAGCTCCACGAGAAAGTATCGAGCCACCCGATCTTCAAGCAG

>JX040501.1_O_ISR_2011

CCCTGGTACAAGCTCATCAAGCTCCTGAGCCGCCTGTCATGCATGGCCGCTGTAGCAGCACGGTCAAAGG

ACCCAGTCCTTGTGGCCATCATGCTAGCTGACACCGGTCTCGAGATTCTAGACAGCACCTTCGTCGTGAA

GAAGATCTCCGACTCGCTCTCCAGTCTCTTTCACGTGCCGGCCCCCGTCTTCAGTTTCGGAGCCCCGGTT

CTGTTGGCCGGGTTGGTCAAAGTCGCCTCGAGTTTCTTTCGGTCCACACCCGAAGACCTTGAGAGAGCAG

AAAAACAGCTCAAAGCACGTGACATCAATGACATCTTCGCCATTCTCAAGAACGGCGAGTGGCTGGTCAA

ACTGATTCTTGCCATCCGCGACTGGATCAAGGCATGGGTCGCCTCAGAAGAAAAGTTTGTCACCATGACA

GACTTGGTGCCTGGCATCCTCGAGAAGCAACGGGACCTCAACGACCCTAGCAAGTACAAGGAGGCCAAGG

AGTGGCTCGACAACGCGCGCCAAGCGTGTTTGAAGAGCGGAAACGTCCACATTGCCAACCTGTGCAAAGT

GGTCGCCCCAGCACCCAGCAAGTCGAGACCCGAACCCGTGGTCGTTTGCCTCCGTGGCAAGTCCGGCCAG

GGCAAGAGTTTCCTTGCTAACGTGCTCGCGCAAGCGATTTCATCTCACTTTACCGGCAGAACCGATTCAG

TTTGGTACTGTCCGCCTGACCCTGACCACTTCGACGGTTACAACCAGCAGACCGTTGTAGTGATGGATGA

TTTGGGCCAGAACCCCGACGGGAAGGACTTCAAGTACTTCGCCCAGATGGTTTCGACCACGGGGTTCATC

CCGCCCATGGCTTCACTCGAAGACAAAGGCAAACCTTTCAACAGCAAGGTCATCATTGCCACCACCAACC

TGTACTCGGGCTTCACCCCGAGGACTATGGTGTGCCCTGATGCACTGAACCGAAGGTTCCACTTTGACAT

TGACGTGAGCGCCAAGGACGGGTACAAAATTAACAACAAACTGGACATTATCAAAGCTCTTGAAGACACC

CACACCAACCCGGTGGCAATGTTTCAATATGATTGTGCTCTTCTCAATGGCATGGCCGTTGAAATGAAGA

GAATGCAACAAGATGTGTTCAAGCCTCAACCACCCCTCCAGAACGTGTACCAGCTCGTTCAGGAGGTGAT

TGAACGGGTCGAGCTCCACGAGAAAGTGTCGAGTCACCCGATCTTCAAGCAG

>JX066664.1_O_BUL_2011

CCCTGGTACAAGCTCATCAAGCTCCTGAGCCGCCTGTCATGCATGGCCGCTGTGGCAGCACGGTCAAAGG

ACCCAGTCCTTGTGGCCATCATGCTAGCTGACACCGGTCTCGAGATTCTGGACAGCACCTTCGTCGTGAA

GAAGATCTCCGACTCGCTCTCCAGTCTCTTTCACGTGCCGGCCCCCGTCTTCAGTTTCGGAGCCCCGATT

CTGTTGGCCGGGTTGGTCAAAGTCGCCTCGAGTTTCTTCCGGTCCACACCCGAAGACCTTGAGAGAGCAG

AGAAACAGCTCAAAGCACGTGACATCAATGACATCTTCGCCGTTCTCAAGAACGGCGAGTGGCTGGTCAA

ACTGATTCTTGCCATCCGCGACTGGATCAAGGCATGGATCGCCTCAGAAGAGAAGTTTGTCACCACGACA

GACTTGGTGCCTGGCATCCTTGAGAAGCAACGGGACCTCAACGACCCTAGCAAGTACAAGGAGGCCAAGG

AGTGGCTCGACAACGCGCGCCAAGCGTGTCTGAAGAGCGGGAATGTCCACATTGCCAACCTGTGCAAAGT

GGTCGCCCCAGCACCCAGCAAGTCGAGACCCGAACCCGTGGTCGTTTGCCTCCGTGGCAAGTCCGGCCAG

GGCAAGAGTTTCCTTGCTAACGTGCTCGCGCAGGCGATTTCATCTCACTTTACCGGCAGAACCGATTCAG

TTTGGTACTGTCCGCCTGACCCTGACCACTTCGACGGTTACAACCAGCAGACCGTTGTAGTGATGGATGA

TTTGGGCCAGAACCCCGACGGGAAGGACTTCAAGTACTTCGCCCAGATGGTTTCAACCACGGGGTTCATC

CCGCCCATGGCTTCACTCGAAGACAAAGGCAAACCTTTCAACAGCAAGGTCATCATTGCCACCACCAACC

TGTACTCGGGCTTCACCCCGAGGACTATGGTGTGCCCTGATGCACTGAACCGAAGGTTCCACTTTGACAT

TGACGTGAGCGCCAAGGACGGGTACAAAATTAACAACAAACTGGACATTATCAAAGCTCTTGAAGACACC

CACACCAACCCGGTGGCAATGTTTCAATATGATTGTGCTCTTCTCAATGGCATGGCCGTTGAAATGAAGA

GAATGCAACAAGATGTGTTCAAGCCTCAACCACCCCTCCAGAACGTGTACCAGCTCGTTCAGGAGGTGAT

TGAACGGGTCGAGCTCCATGAGAAAGTATCGAGCCACCCGATCTTCAAGCAG

>JX066665.1_O_BUL_2011

CCCTGGTACAAGCTCATCAAGCTCCTGAGCCGCCTGTCATGCATGGCCGCTGTAGCAGCACGGTCAAAGG

ACCCAGTCCTTGTGGCCATCATGCTAGCTGACACCGGTCTCGAGATTCTGGACAGCACCTTCGTCGTGAA

GAAGATCTCCGACTCGCTCTCCAGTCTCTTTCACGTGCCGGCCCCCGTCTTCAGTTTCGGAGCCCCGATT

CTGTTGGCCGGGTTGGTCAAAGTCGCCTCGAGTTTCTTCCGGTCCACACCCGAAGACCTTGAGAGAGCAG

AGAAACAGCTCAAAGCACGTGACATCAATGACATCTTCGCCGTTCTCAAGAACGGCGAGTGGCTGGTCAA

ACTGATTCTTGCCATCCGCGACTGGATCAAGGCATGGATCGCCTCAGAAGAGAAGTTTGTCACCACGACA

GACTTGGTGCCTGGCATCCTTGAGAAGCAACGGGACCTTAACGACCCTAGCAAGTACAAGGAGGCCAAGG

AGTGGCTCGACAACGCGCGTCAAGCGTGTTTGAAGAGCGGGAATGTCCACATTGCCAACCTGTGCAAAGT

GGTCGCCCCAGCACCCAGCAAGTCGAGACCCGAACCCGTGGTCGTTTGCCTCCGTGGCAAGTCCGGCCAG

GGCAAGAGTTTCCTTGCTAACGTGCTCGCGCAGGCGATTTCATCTCACTTTACCGGCAGAACCGATTCAG

TTTGGTACTGTCCGCCTGACCCTGACCACTTCGACGGTTACAACCAGCAGACCGTTGTAGTGATGGATGA

TTTGGGCCAGAACCCCGACGGGAAGGACTTCAAGTACTTCGCCCAGATGGTTTCAACCACGGGGTTCATC

CCGCCCATGGCTTCACTCGAAGACAAAGGCAAACCTTTCAACAGCAAGGTCATCATTGCCACCACCAACC

TGTACTCGGGCTTCACCCCGAGGACTATGGTGTGCCCTGATGCACTGAACCGAAGGTTCCACTTTGACAT

TGACGTGAGCGCCAGGGACGGGTACAAAATTAACAACAAACTGGACATTATCAAAGCTCTTGAAGACACC

CACACCAACCCGGTGGCAATGTTTCAATATGATTGTGCTCTTCTCAATGGCATGGCCGTTGAAATGAAGA

GAATGCAACAAGATGTGTTCAAGCCTCAACCACCCCTCCAGAACGTGTACCAGCTCGTTCAGGAGGTGAT

TGAACGGGTCGAGCTCCATGAGAAAGTATCGAGCCACCCGATCTTCAAGCAG

>JX570638.1_O_UKG_2007

CCCTGGTACAAGCTTATCAAGCTCCTAAGCCGCCTGTCGTGCATGGCCGCTGTGGCAGCACGGTCCAAGG

ACCCAGTCCTTGTGGCCATCATGCTGGCCGACACCGGTCTCGAGATTCTGGACAGCACCTTCGTCGTGAA

GAAGATCTCCGACTCGCTCTCCAGTCTCTTTCACGTGCCGGCCCCCGTCTTCAGTTTCGGAGCACCGGTC

CTGTTGGCCGGGTTGGTCAAAGTCGCCTCGAGTTTCTTCCGGTCTACACCCGAAGACCTTGAGAGAGCAG

AGAAACAGCTCAAAGCACGTGACATCAACGACATCTTCGCCATTCTCAAGAACGGCGAGTGGCTGGTCAA

ACTGATCCTTGCCATCCGCGACTGGATTAAGGCTTGGATCGCCTCAGAAGAGAAGTTTGTCACCATGACA

GACTTGGTGCCTGGCATCCTTGAAAAGCAGCGGGATCTGAACGACCCGAGCAAGTACAAGGAAGCCAAGG

AGTGGCTCGACAACGCGCGCCAAGCGTGTTTGAAGAGCGGGAACGTCCACATTGCCAACCTGTGTAAAGT

GGTCGCTCCAGCACCCAGCAAGTCGAGGCCCGAACCCGTGGTTGTTTGTCTCCGCGGCAAATCTGGCCAG

GGCAAGAGCTTCCTTGCAAACGTGCTTGCACAGGCAATTTCCGCCCACTTCACCGGCAGAACCGACTCAG

TGTGGTACTGCCCACCTGACCCTGACCACTTCGACGGTTACAACCAGCAAACCGTCGTTGTGATGGATGA

TTTGGGCCAGAACCCTGACGGCAAGGACTTCAAATACTTTGCCCAAATGGTCTCGACCACAGGGTTCATC

CCGCCCATGGCATCACTCGAGGACAAAGGTAAACCTTTCAACAGCAAAGTCATCATCGCGACCACCAACT

TGTACTCGGGCTTCACCCCGAGGACTATGGTGTGTCCCGACGCACTGAACCGGAGGTTTCACTTTGACAT

CGATGTGAGTGCTAAGGATGGGTACAAAATTAACAACAAATTGGACATTATCAAAGCACTTGAAGACACC

CACACCAACCCAGTGGCAATGTTTCAATACGACTGTGCCCTTCTCAACGGCATGGCCGTTGAAATGAAGA

GAATGCAACAAGACATGTTCAAGCCTCAACCACCCCTCCAGAATGTGTACCAGCTTGTTCAGGAGGTGAT

TGATCGGGTCGAGCTCCACGAGAAAGTGTCGAGCCACCCGATCTTCAAGCAG

>JX570639.1_O_UKG_2007

CCCTGGTACAAGCTTATCAAGCTCCTAAGCCGCCTGTCGTGCATGGCCGCTGTGGCAGCACGGTCCAAGG

ACCCAGTCCTTGTGGCCATCATGCTGGCCGACACCGGTCTCGAGATTCTGGACAGCACCTTCGTCGTGAA

GAAGATCTCCGACTCGCTCTCCAGTCTCTTTCACGTGCCGGCCCCCGTCTTCAGTTTCGGAGCACCGGTC

CTGTTGGCCGGGTTGGTCAAAGTCGCCTCGAGTTTCTTCCGGTCTACACCCGAAGACCTTGAGAGAGCAG

AGAAACAGCTCAAAGCACGTGACATCAACGACATCTTCGCCATTCTCAAGAACGGCGAGTGGCTGGTCAA

ACTGATCCTTGCCATCCGCGACTGGATTAAGGCTTGGATCGCCTCAGAAGAGAAGTTTGTCACCATGACA

GACTTGGTGCCTGGCATCCTTGAAAAGCAGCGGGATCTGAACGACCCGAGCAAGTACAAGGAAGCCAAGG

AGTGGCTCGACAACGCGCGCCAAGCGTGTTTGAAGAGCGGGAACGTCCACATTGCCAACCTGTGTAAAGT

GGTCGCTCCAGCACCCAGCAAGTCGAGGCCCGAACCCGTGGTTGTTTGTCTCCGCGGCAAATCTGGCCAG

GGCAAGAGCTTCCTTGCAAACGTGCTTGCACAGGCAATTTCCGCCCACTTCACCGGCAGAACCGACTCAG

TGTGGTACTGCCCACCTGACCCTGACCACTTCGACGGTTACAACCAGCAAACCGTCGTTGTGATGGATGA

TTTGGGCCAGAACCCTGACGGCAAGGACTTCAAATACTTTGCCCAAATGGTCTCGACCACAGGGTTCATC

CCGCCCATGGCATCACTCGAGGACAAAGGTAAACCTTTCAACAGCAAAGTCATCATCGCGACCACCAACT

TGTACTCGGGCTTCACCCCGAGGACTATGGTGTGTCCCGACGCACTGAACCGGAGGTTTCACTTTGACAT

CGATGTGAGTGCTAAGGATGGGTACAAAATTAACAACAAATTGGACATTATCAAAGCACTTGAAGACACC

CACACCAACCCAGTGGCAATGTTTCAATACGACTGTGCCCTTCTCAACGGCATGGCCGTTGAAATGAAGA

GAATGCAACAAGACATGTTCAAGCCTCAACCACCCCTCCAGAATGTGTACCAGCTTGTTCAGGAGGTGAT

TGATCGGGTCGAGCTCCACGAGAAAGTGTCGAGCCACCCGATCTTCAAGCAG
[truncated: 601,752 more chars]
