# Supplementary material for: Early origin and global colonisation of foot-and-mouth disease virus
Source: Sci Rep. 2020 Sep 17;10:15268. doi: 10.1038/s41598-020-72246-6 (PMC7498456; doi:10.1038/s41598-020-72246-6)
Supplement: Supplementary file 7 — Supplementary Data S5. [file 41598_2020_72246_MOESM7_ESM.doc]

>AB079061.1_O_JPN_2000

ATCTCAATTCCTTCCCAAAAGGCTGTACTGTACTTTCTCATTGAGAAGGGCCAGCACGAAGCAGCAATTG

AATTCTTTGAGGGGATGGTGCATGACTCCATCAAGGAGGAGCTCCGGCCTCTCATCCAACAGACCTCATT

TGTGAAGCGCGCTTTTAAGCGCCTGAAGGAAAACTTTGAGATAGTTGCCCTGTGTTTGACTCTTTTGGCA

AACATAGTGATCATGATCCGCGAGACTCGCAAGAGACAGCAGATGGTGGATGATGCAGTGAACGAGTACA

TTGAGAGGGCAAACATCACCACGGATGACAAGACTCTTGACGAGGCGGAAAAGAACCCTCTGGAGACCAG

CGGTGCCACCACTGTTGGTTTCAGAGAGAAAACTCTCCCGGGACACAAGGCGGGTGACGACGTGAACTCC

GAGCCCGCCAAACCCGTGGAAGAACAACCACAAGCTGAAGGACCCTACACCGGTCCACTCGAGCGTCAAA

AACCCCTGAAAGTGAGGGCCAAGCTCCCACAGCAGGAGGGGCCCTACGCTGGCCCGATGGAGAGACAGAA

ACCGCTGAAAGTGAGAGTGAAAGCCCCGGTCGTTAAGGAAGGACCTTACGAAGGACCGGTGAAGAAACCT

GTCGCTTTGAAAGTGAAAGCAAAGAACTTGATTGTCACTGAGAGTGGTGCTCCCCCGACTGACTTGCAAA

AGATGGTCATGGGTAACACCAAGCCTGTTGAGCTCATCCTCGACGGGAAGACGGTGGCCATCTGCTGCGC

CACCGGAGTGTTTGGTACTGCCTACCTTGTTCCTCGTCATCTTTTCGCAGAGAAGTATGACAAGATCATG

TTGGACGGCAGAGCCATGACAGACAGTGACTACAGAGTGTTTGAGTTTGAGATTAAAGTGAAAGGACAGG

ACATGCTCTCAGACGCCGCTCTCATGGTGCTTCACCGTGGGAATCGCGTGCGGGACATCACGAAGCACTT

CCGTGATGTGGCAAGAATGAAGAAAGGCACCCCCGTCGTCGGCGTGATCAACAACGCTGATGTTGGGAGA

CTGATCTTCTCTGGTGAGGCCCTTACCTACAAGGACATTGTAGTGTGCATGGACGGAGACACCATGCCCG

GTCTCTTCGCCTACAAAGCTGCCACCAAGGCGGGTTACTGTGGAGGAGCCGTTCTTGCAAAGGACGGAGC

CGAGACTTTCATCGTCGGCACTCACTCCGCAGGCGGCAATGGAGTTGGATACTGCTCATGCGTTTCCAGG

TCCATGCTGCTCAAAATGAAGGCACACATCGACCCCGAACCACACCACGAG

>AF026168.2_O_TAW_1997

ATTTCAATCCCTTCCCAGAAGTCCGTGTTGTACTTCCTCATTGAGAAGGGTCAGCACGAAGCAGCGATCG

AGTTCTTCGAGGGGATGGTCCACGATTCCATCAAAGAGGAACTCCGACCCCTCATTCAGCAGACCTCGTT

CGTAAAACGCGCCTTCAAGCGCCTGAAAGAGAACTTTGAAGTTGTAGCCCTGTGTTTGACCCTCTTGGCA

AACATAGTGATTATGCTCCGCCAAGCGCGCAAGAGGTACCAATCGGTGGATGACCCACTGGAC-------

-----------------------GGCGACGTAGCTCTTGGCGACGCGGAAAAGAACCCTCTGGAGACGAG

TGCCGCTAGCGCTGTCGGTTTCAGAGAGAGATCCCCCACCGAGCAAGGGACGCGCGAAGACGCGAACGCT

GAGCCCGTCGTGTTCGGTAGGGAACAACCGCGAGCTGAAGGACCCTACGCTGGGCCACTCGAGCGTCAGA

AACCTCTTAAAGTGAAAGCCGAGCTGCCACAACAGGAGGGACCATACGCCGGCCCAATGGAGAGACAGAA

ACCGCTAAAGGTGAAAGCAAAAGCCCCCGTCGTGAAGGAAGGACCTTACGAGGGACCGGTGAAGAAACCT

GTCGCTTTAAAAGTGAAAGCAAAGAACTTGATAGTCACTGAGAGTGGTGCGCCACCGACCGACTTGCAAA

AGATGGTCATGGGCAACACTAAGCCAGTCGAGCTCATCCTCGACGGCAAGACGGTAGCCATTTGCTGTGC

TACCGGAGTGTTCGGCACTGCCTACCTCGTGCCTCGTCATCTCTTCGCGGAAAAGTACGACAAGATCATG

TTGGACGGCAGAGCCTTGACAGACAGTGACTACAGAGTGTTTGAGTTTGAGATTAAAGTAAAAGGACAGG

ACATGCTCTCAGACGCCGCTCTCATGGTGTTGCACCGTGGGAATCGCGTGCGTGACATCACGAAACACTT

TCGTGACGTAGCGAGAATGAAGAAGGGAACCCCCGTCGTCGGTGTGATCAACAATGCTGACGTCGGGAGA

CTCATATTCTCTGGTGTAGCCCTCACTTACAAGGACATCGTCGTGTGTATGGATGGAGACACCATGCCTG

GGCTCTTTGCCTACAGGGCATCCACCAAGGCAGGCTACTGCGGAGGAGCCGTCCTGGCAAAGGACGGGGC

CGAAACGTTCATCGTTGGCACCCACTCCGCAGGTGGAAACGGCATAGGATACTGTTCGTGTGTTTCCCGA

TCAATGCTCCTGAAGATGAAGGCACACATCGACCCTGAACCACACCACGAG

>AF154271.1_O_TAW_1997

ATTTCAATCCCTTCCCAGAAGTCCGTGTTGTACTTCCTCATTGAGAAGGGTCAGCACGAAGCAGCGATCG

AGTTCTTCGAGGGGATGGTCCACGATTCCATCAAAGAGGAACTCCGACCCCTCATTCAGCAGACCTCGTT

CGTAAAACGCGCCTTCAAGCGCCTGAAAGAGAACTTTGAAGTTGTAGCCCTGTGTTTGACCCTCTTGGCA

AACATAGTGATTATGCTCCGCCAAGCGCGCAAGAGGTACCAATCGGTGGATGACCCACTGGAC-------

-----------------------GGCGACGTAACTCTTGGCGACGCGGAAAAGAACCCTCTGGAGACGAG

TGGCGCTAGCGCTGTCGGTTTCAGAGAGAGATCCCCCACCGAGCAAGGGACGCGCGAAGACGCGAACGCT

GAGCCCGTCGTGTTCGGTAGGGAACAACCGCGAGCTGAAGGACCCTACGCTGGGCCACTCGAGCGTCAGA

AACCTCTTAAAGTGAAAGCCGAGCTGCCACAACATGAGGGACCATACGCCGGCCCAATGGAGAGACAGAA

ACCGCTAAAGGTGAAAGCAAAAGCCCCCGTCGTGAAGGAAGGACCTTACGAGGGACCGGTGAAGAAACCT

GTCGCTTTGAAAGTGAAAGCAAAGAACTTGATAGTCACTGAGAGTGGTGCGCCACCGACCGACTTGCAAA

AGATGGTCATGGGCAACACTAAGCCAGTCGAGCTCATCCTCGACGGCAAGACGGTAGCCATTTGCTGTGC

TACCGGAGTGTTCGGCACTGCCTACCTCGTGCCTCGTCATCTCTTCGCGGAAAAGTACGACAAGATCATG

TTGGACGGCAGAGCCTTGACAGACAGTGACTACAGAGTGTTTGAGTTTGAGATTAAAGTAAAAGGACAGG

ACATGCTCTCAGACGCCGCTCTCATGGTGTTGCACCGTGGGAATCGCGTGCGTGACATCACGAAACACTT

TCGTGACGTAGCGAGAATGAAGAAGGGAACCCCCGTCGTCGGTGTGATCAACAATGCTGACGTCGGGAGA

CTCATATTCTCTGGTGAAGCCCTTACTTACAAGGACATCGTCGTGTGTATGGATGGAGACACCATGCCTG

GGCTCTTTGCCTACAGGGCATCCACCAAGGCAGGCTACTGTGGAGGAGCCGTCCTGGCAAAGGACGGGGC

CGAAACGTTCATCGTTGGCACCCACTCCGCAGGTGGAAACGGCATAGGATACTGTTCGTGTGTTTCCCGA

TCAATGCTCCTGAAGATGAAGGCACACATCGACCCTGAACCACACCACGAG

>AF308157.1_O_TAW_1997

ATTTCAATCCCTTCCCAGAAGTCCGTGTTGTACTTCCTCATTGAGAAGGGCCAGCACGAAGCAGCGATCG

AGTTCTTCGAGGGGATGGTCCACGATTCCATCAAAGAGGAACTCCGACCCCTCATTCAGCAGACCTCGTT

CGTAAAACGCGCCTTCAAGCGCCTGAAAGAGAACTTTGAAGTTGTAGCTCTGTGTTTGACCCTCTTGGCA

AACATAGTGATTATGCTCCGCCAAGCGCGCAAGAGGTACCAATCGGTGGATGACCCACTGGAC-------

-----------------------GGCGACGTAACTCTTGGCGACGCGGAAAAGAACCCTCTGGAGACGAG

TGGCGCTAGCGCTGTCGGTTTCCGAGAGAGATCCCCCACCGAGCAAGGGACGCGCGAAGACGCGAACGCT

GAGCCCGTCGTGTTCGGTAGGGAACAACCGCGAGCTGAAGGACCCTACGCTGGGCCACTCGAGCGTCAGA

AACCTCTTAAAGTGAAAGCCGAGCTGCCACAACAGGAGGGACCATACGCCGGCCCAATGGAGAGACAGAA

ACCGCTAAAGGTGAAAGCAAAAGCCCCCGTCGTGAAGGAAGGACCTTACGAGGGACCGGTGAAGAAACCT

GTCGCTTTGAAAGTGAAAGCAAAGAACTTGATAGTCACTGAGAGTGGTGCGCCACCGACCGACTTGCAAA

AGATGGTCATGGGCAACACTAAGCCAGTCGAGCTCATCCTCGACGGCAAGACGGTAGCCATTTGCTGTGC

TACCGGAGTGTTCGGCACTGCCTACCTCGTGCCTCGTCATCTCTTCGCGGAAAAGTACGACAAGATCATG

TTGGACGGCAGAGCCTTGACAGACAGTGACTACAGAGTGTTTGAGTTTGAGATTAAAGTAAAAGGACAGG

ACATGCTCTCAGACGCCGCTCTCATGGTGTTGCACCGTGGGAATCGCGTGCGTGACATCACGAAACACTT

TCGTGACGTAGCGAGAATGAAGAAGGGAACCCCCGTCGTCGGTGTGATCAACAATGCTGACGTCGGGAGA

CTCATATTCTCTGGTGAAGCCCTTACTTACAAGGACATCGTCGTGTGTATGGATGGAGACACCATGCCTG

GGCTCTTTGCCTACAGGGCATCCACCAAGGCAGGCTACTGTGGAGGAGCCGTCCTGGCAAAGGACGGGGC

CGAAACGTTCATCGTTGGCACCCACTCCGCAGGTGGAAACGGCATAGGATACTGTTCGTGTGTTTCCCGA

TCAATGCTCCTGAAGATGAAGGCACACATCGACCCTGAACCACACCACGAG

>AF377945.1_O_SKR_2000

ATCTCAATTCCTTCCCAAAAGGCTGTGCTGTACTTTCTCATTGAGAAGGGCCAGCACGAAGCAGCAATTG

AATTCTTTGAGGGGATGGTGCGTGACTCCGTCAAGGAGGAGCTCCGGCCTCTCATCCAGCAGACCTCATT

TGTGAGGCGCGTTTTTAAGCGCCTGAAGGAAAACTTTGAGATAGTTGCCCTGTGTTTGACCCTTATGGCA

AACATAGTGATCATGATCCGCGAGACTCGTAAGAGACAGCAGATGGTGGATGATGCAGTGAACGAGTACA

TTGAGAAGGCAAACATCACCACGGATGACAAGACTCTTGATGAGGCGGAAAAGAACCCTCTGGAGACCAG

CGGTGCCACCACTGTTGGTTTCAGAGAGAAAACTCTCCCGGGACACAAAGCGAGTAATGACGTGAACTCC

GAGCCCGCCAAACCCGTGGAAGAACAACCACAAGCTGAAGGACCCTACACCGGTCCACTCGAGCGTCAGA

AACCTCTGAAAGTGAGAGCCAAGCTCCCACAGCAGGAGGGGCCCTACGCTGGTCCGATGGAGAGACAGAA

ACCGCTGAAAGTGAAAGTGAAAACCTCGGTCGTGAAGGAAGGACCTTACGAAGGACCGGTGAAGAAACCT

GTCGCTTTGAAAGTGAAAGCTAAGAATTTGATTGTCACTGAGAGTGGTGCTCCCCCGACTGACTTGCAAA

AGATGGTCATGGGTAACACCAAGCCTGTTGAGCTCATCCTCGACGGGAAGACGGTGGCCATTTGCTGCGC

CACCGGAGTGTTTGGTACTGCCTACCTTGTCCCTCGTCATCTTTTCGCAGAGAAGTATGACAAGATCATG

TTGGACGGCAGAGCCATGACAGACAGTGACTACAGAGTGTTTGAGTTTGAGATTAAAGTGAAAGGACAGG

ACATGCTCTCAGACGCCGCGCTCATGGTGCTTCACCGTGGGAATCGCGTGCGGGACATCACGAAGCACTT

CCGTGATGTGGCAAGAATGAAGAAAGGCACCCCCGTCGTCGGCGTGATCAACAACGCTGATGTTGGGAGA

CTAATCTTCTCTGGTGAGGCCCTTACCTACAAGGACATTGTAGTGTGCATGGACGGAGACACCATGCCCG

GTCTCTTCGCCTACAAAGCCGCCACCAAGGCGGGTTACTGTGGAGGAGCCGTTCTTGCCAAAGACGGAGC

CGAGACTTTCATCGTCGGCACTCACTCCGCAGGCGGCAACGGAGTTGGATACTGCTCATGCCTTTCCAGG

TCCATGCTTCTTAAAATGAAGGCACACATTGACCCCGAACCACACCACGAG

>AF506822.2_O_CHA_1999

ATCTCAATTCCTTCCCAAAAGGCTGTGCTGTACTTTCTCATTGAGAAGGGTCAGCACGAAGCAGCAATTG

AATTCTTTGAGGGGATGGTGCATGACTCCATCAAGGAGGAGCTCCGGCCTCTCATCCAACAGACCTCATT

TGTGAAGCGCGCTTTTAAGCGCCTGAAGGAAAACTTTGAGATAGTTGCCCTGTGTTTGACTCTTTTGGCA

AACATAGTGATCATGATCCGCGAGACTCGCAAGAGACAGCAGATGGTGGATGATGCAGTGAACGAGTACA

TTGAGAAGGCAAACATCACCACGGATGACAAGACTCTTGACGAGGCGGAAAAGAACCCTCTGGAGACCAG

CGGTGCCGCCACTGTTGGTTTCAGAGAGAAAACTCTCCCGGGACACAAGGCGAGTGATGACGTGAACTCC

GAGCCCGCCAAACCCGTGGAAGAACAACCACAAGCTGAAGGACCCTACACCGGTCCACTCGAGCGTCAAA

AACCTCTGAAAGTGAGAGCCAAGCTCCCACAGCAGGAGGGGCCCTACGCTGGTCCGATGGAGAGACAGAA

ACCGCTGAAAGTGAAAGTGAAAGCCCCGGTCGTTAAGGAAGGACCTTACGAAGGACCGGTGAAGAAACCT

GTCGCTTTGAAAGTGAAAGCAAAGAACTTGATTGTCACTGAGAGTGGTGCTCCCCCGACTGACTTGCAAA

AGATGGTCATGGGTAACACCAAGCCTGTTGAGCTCATCCTCGACGGGAAGACGGTGGCCATCTGCTGCGC

CACCGGAGTGTTTGGTACTGCCTACCTTGTTCCTCGTCATCTTTTCGCAGAGAAGTATGACAAGATCATG

TTGGACGGCAGAGCCATGACAGACAGTGACTACAGAGTGTTTGAGTTTGAGATTAAAGTGAAAGGACAGG

ACATGCTCTCAGACGCCGCGCTCATGGTGCTTCACCGTGGGAATCGCGTGCGGGACATCACGAAGCACTT

CCGTGATGTGGCAAGAATGAAGAAAGGCACCCCCGTCGTCGGCGTGATCAACAACGCTGATGTTGGGAGA

CTGATCTTCTCTGGTGAGGCCCTTACCTACAAGGACATTGTAGTGTGCATGGACGGAGACACCATGCCCG

GTCTCTTCGCCTACAAAGCCGCCACCAAGGCGGGTTACTGTGGAGGAGCCGTTCTTGCAAAGGACGGAGC

CGAGACTTTCATCGTCGGCACTCACTCCGCAGGCGGCAACGGAGTTGGATACTGCTCATGCGTTTCCAGG

TCTATGCTGCTTAAAATGAAGGCACACATCGATCCCGAACCACACCACGAG

>AH012984.2_O_SKR_2000

ATCTCAATTCCTTCCCAAAAGGCTGTGCTGTACTTTCTCATTGAGAAAGGCCAGCACGAAGCAGCAATTG

AATTCTTTGAGGGGATGGTGTGTGACTCCATCAAGGAGGAGCTCCGGCCTCTCATCCAACAGACCTCATT

TGTGAAGCGCGCTTTTAAGCGCCTGAAGGAAAACTTTGAGATAGTTGCCCTGTGTTTGACTCTAATGGCA

AACATAGTGATCATGATCCGCGAGACTCGCAAGAGACAGCAGATGGTGGATGATGCAGTGAACGAGTACA

CTGAGAAGGTAAACATCACCACGGATGACAAGACTCTTGACGAGGCGGAAAAGAACCCTCTGGAAACCAG

CGGTGCCACCACTGTTGGTTTCAGAGAGAAAACTCTCCCGGGGCACAAGACGGGTGATGACGTGAGCTCC

GAGCCCACCAAACCCGTGGAAGGACAACCACAAGCTGAAGGACCCTACACCGGCCCACTCGAGCGTCAAA

AACCTCTGAAAGTGAGAGCCAAGCTCCCGCAGCAGGAGGGGCCTTATGCTGGTCCCATGGAGAGACAGAA

ACCACTGAAAGTGAAAGCGAAAGCCCCGGTCGTTAAGGAAGGACCTTACGAAGGACCGGTGAAGAAACCT

GTCGCTTTGAAAGTGAAAGCAAAGAACTTGATTGTCACTGAGAGTGGTGCTCCCCCGACTGACTTGCAAA

AGATGGTCATGGGTAACACCAAGCCTGTTGAGCTCGTCCTCGATGGGAAGACGGTGGCCATCTGCTGCGC

CACCGGAGTGTTTGGTACTGCCTACCTTGTTCCTCGTCATCTTTTCGCAGAGAAGTATGACAAGATCATG

TTGGACGGCAGAGCCATGACAGACAGTGACTACAGAGTGTTTGAGTTTGAGATTAAAGTGAAAGGACAGG

ACATGCTCTCAGACGCCGCGCTCATGGTGCTTCACCGTGGGAATCGAGTGCGGGACATCACGAAGCACTT

CCGTGATGTGGCAAGAATGAAGAAAGGCACCCCCGTCGTCGGCGTGATCAACAACGCTGATGTTGGGAGG

CTGATCTTCTCTGGTGAGGCCCTTACCTACAAGGACATTGTAGTGCGCATGGACGGAGACACCATGCCCG

GTCTCTTCGCCTACAAAGCCGCCACCAAGGCGGGCTACTGTGGAGGATCCGTTCTTGCAAAGGACGGAGC

CGATACTTTCATCGTCGGCACTCACTCCGCAGGCGGCAATGGAGTTGGATACTGCTCATGCGTTTCCAGG

TCTATGCTGCTTAAAATGAAGGCACACATCGACCCCGAACCACACCACGAG

>AH012985.2_O_SKR_2000

ATCTCAATACCTTCCCAAAAGGCTGTGCTGTACTTTCTCATTGAGAAGGGCCAGCACGATGCAGCAATTG

AATTCTTTGAGGGGATGGTGCATGACTCCATCAAGGAGGAGCTCCGGCCTCTCATCCAACAGACCTCATT

TGTGAAGCGCGCTTTTAAGCGCCTGAAGGAAAACTTTGAGGTAGTTGCCCTGTGTTTGACCCTTTTGGCA

AACATAGTGATCATGATCCGCGAGACTCGCAAGAGACAGCAGATGGTGGATGACGCAGTGAACGAGTACA

TTGAGAAGGCAAACATCACCACGGATGACAAGACTCTTGACGAGGCGGAAAAGAACCCTCTGGAGACCAG

CGGTGCCACCACTGTTGGTTTTAGAGAGAAAACTCTCCCGGGACACAAGGCGAGTGACGACGTGAACTCC

GAGCCCGCCAAACCCGCGGAAGAACAACCACAAGCTGAAGGACCCTACACCGGTCCACTCGAGCGTCAAA

AACCTCTGAAAGTGAGAGCCAAGCTCCCACAGCAGGAGGGGCCCTACGCTGGTCCGATGGAGAGACAGAA

ACCGCTGAAAGTGAAAGTGAAAGCCCCGGTTGTTAAGGAAGGACCTTACGAAGGACCGGTGAAGAAACCT

GTCGCTTTGAAAGTGAAAGCAAAGAACTTGATTGTCACTGAGAGTGGTGCTCCCCCGACTGACTTGCAAA

AGATGGTCATGGGTAACACCAAGCCTGTTGAGCTCATCCTCGACGGGAAGACGGTGGCCATCTGTTGCGC

CACCGGAGTGTTTGGTACTGCTTACCTTGTCCCTCGTCATCTTTTCGCAGAGAAGTATGACAAGATCATG

TTGGACGGCAGAGCCATGACAGACAGTGACTACAGAGTGTTTGAGTTTGAGATTAAAGTGAAAGGACAGG

ACATGCTCTCAGACGCCGCGCTCATGGTGCTTCACCGTGGGAATCGCGTGCGGGACATCACGAAGCACTT

CCGTGATGTGGCAAGAATGAAGAAAGGCACCCCCGTCGTCGGCGTGGTCAACAACGCTGATGTTGGGAGA

CTGATCTTCTCTGGTGAGGCCCTTACCTACAAGGACATTGTAGTGTGCATGGACGGAGACACCATGCCCG

GTCTCTTCGCCTACAAAGCCGCCACCAAGGCGGGTTACTGTGGAGGAGCCGTTCTTGCAAAGGACGGAGC

CGAGACTTTCATCGTCGGCACTCACTCCGCAGGCGGCAATGGGGTTGGATACTGCTCATGCGTTTCCAGG

TCTATGCTGCTTAAAATGAAGGCACACATCGATCCCGAACCACACCACGAG

>AJ539136.1_O_TAW_1999

ATCTCAATTCCTTCCCAAAAGGCTGTGCTGTACTTTCTCATTGAGAAGGGCCAGCACGAAGCAGCAATTG

AATTCTTTGAGGGGATGGTGCATGACTCCATCAAGGAGGAGCTCCGGCCTCTCATCCAGCAGACCTCATT

TGTGAAGCGCGCTTTTAAGCGCCTGAAGGAAAACTTTGAGATAGTTGCCCTGTGTTTGACTCTTTTGGCA

AACATAGTGATCATGATCCGCGAGACTCGCAAGAGACAGCAGATGGTGGATGATGCAGTGAACGAGTACA

TTGAGAAGGCAAGCATCACCACGGATGACAAGACTCTTGACGAGGCGGAAAAGAACCCTCTGGAGACCAG

CGGTGCCACCACTGTTGGTTTCAGAGAGAAAACTCTCCCGGGACACAAGGCGAGTGATGACGTGAACTCC

GAGCCCGCCAAACCCGTGGAAGAACAACCACAAGCTGAAGGACCCTACACCGGTCCACTCGAGCGTCAAA

AACCTCTGAAAGTGAGAGCCAAGCTCCCACAGCAGGAGGGGCCCTACGCTGGTCCGATGGAGAGACAGAA

ACCGCTGAAAGTGAAAGTGAAAGCCCCGGTCGTTAAGGAAGGACCTTACGAAGGACCGGTGAAGAAACCT

GTCGCTTTGAAAGTGAAAGCAAAGAACTTGATCGTCACTGAGAGTGGTGCTCCCCCGACTGACTTGCAAA

AGATGGTCATGGGTAACACCAAGCCTGTTGAGCTCATCCTCGACGGGAAGACGGTGGCCATCTGCTGCGC

CACTGGAGTGTTTGGTACTGCCTACCTTGTTCCTCGTCATCTTTTCGCAGAGAAGTACGACAAGATCATG

TTGGACGGTAGAGCCATGACAGACAGTGACTACAGAGTGTTTGAGTTTGAGATTAAAGTGAAAGGACAGG

ACATGCTCTCAGACGCCGCGCTCATGGTGCTTCACCGTGGGAATCGCGTGCGGGACATCACGAAGCACTT

CCGTGATGTGGCAAGAATGAAGAAAGGCACCCCCGTCGTCGGCGTGATCAACAACGCTGATGTTGGGAGA

CTGATCTTCTCTGGTGAGGCCCTTACCTACAAGGACATTGTAGTGTGCATGGACGGAGACACCATGCCCG

GTCTCTTCGCCTACAAAGCCGCCACCAAGGCGGGTTACTGTGGAGGAGCCGTTCTTGCAAAGGACGGAGC

CGAGACTTTCATCGTCGGCACTCACTCCGCAGGCGGCAATGGAGTTGGATACTGCTCATGCGTTTCCAGG

TCTATGCTGCTTAAAATGAAGGCACACATCGATCCCGAACCACACCACGAG

>AJ539137.1_O_TAW_1999

ATCTCAATTCCTTCCCAAAAGGCTGTGCTGTACTTTCTCATTGAGAAGGGCCAGCACGAAGCAGCAATTG

AATTCTTTGAGGGGATGGTGCATGACTCCATCAAGGAGGAGCTCCGGCCTCTCATCCAGCAGACCTCATT

TGTGAAGCGCGCTTTTAAGCGCCTGAAGGAAAACTTTGAGATAGTTGCCCTGTGTTTGACTCTTTTGGCA

AACATAGTGATCATGATCCGCGAGACTCGCAAGAGACAGCAGATGGTGGATGATGCAGTGAACGAGTACA

TTGAGAAGGCAAGCATCACCACGGATGACAAGACTCTTGACGAGGCGGAAAAGAACCCTCTGGAGACCAG

CGGTGCCACCACTGTTGGTTTCAGAGAGAAAACTCTCCCGGGACACAAGGCGAGTGATGACGTGAACTCC

GAGCCCGCCAAACCCGTGGAAGAACAACCACAAGCTGAAGGACCCTACACCGGTCCACTCGAGCGTCAAA

AACCTCTGAAAGTGAGAGCCAAGCTCCCACAGCAGGAGGGGCCCTACGCTGGTCCGATGGAGAGACAGAA

ACCGCTGAAAGTGAAAGTGAAAGCCCCGGTCGTTAAGGAAGGACCTTACGAAGGACCGGTGAAGAAACCT

GTCGCTTTGAAAGTGAAAGCAAAGAACTTGATCGTCACTGAGAGTGGTGCTCCCCCGACTGACTTGCAAA

AGATGGTCATGGGTAACACCAAGCCTGTTGAGCTCATCCTCGACGGGAAGACGGTGGCCATCTGCTGCGC

CACTGGAGTGTTTGGTACTGCCTACCTTGTTCCTCGTCATCTTTTCGCAGAGAAGTACGACAAGATCATG

TTGGACGGTAGAGCCATGACAGACAGTGACTACAGAGTGTTTGAGTTTGAGATTAAAGTGAAAGGACAGG

ACATGCTCTCAGACGCCGCGCTCATGGTGCTTCACCGTGGGAATCGCGTGCGGGACATCACGAAGCACTT

CCGTGATGTGGCAAGAATGAAGAAAGGCACCCCCGTCGTCGGCGTGATCAACAACGCTGATGTTGGGAGA

CTGATCTTCTCTGGTGAGGCCCTTACCTACAAGGACATTGTAGTGTGCATGGACGGAGACACCATGCCCG

GTCTCTTCGCCTACAAAGCCGCCACCAAGGCGGGTTACTGTGGAGGAGCCGTTCTTGCAAAGGACGGAGC

CGAGACTTTCATCGTCGGCACTCACTCCGCAGGCGGCAATGGAGTTGGATACTGTTCATGCGTTTCCAGG

TCTATGCTGCTTAAAATGAAGGCACACATCGATCCCGAACCACACCACGAG

>AJ539138.1_O_CHA_1999

ATCTCAATTCCTTCCCAAAAGGCTGTGCTGTACTTTCTCATTGAGAAGGGTCAGCACGAAGCAGCAATTG

AATTCTTTGAGGGGATGGTGCATGACTCCATCAAGGAGGAGCTCCGGCCTCTCATCCAACAGACCTCATT

TGTGAAGCGCGCTTTTAAGCGCCTGAAGGAAAACTTTGAGATAGTTGCCCTGTGTTTGACTCTTTTGGCA

AACATAGTGATCATGATCCGCGAGACTCGCAAGAGACAGCAGATGGTGGATGATGCAGTGAACGAGTACA

TTGAGAAGGCAAACATCACCACGGATGACAAGACTCTTGACGAGGCGGAAAAGAACCCTCTGGAGACCAG

CGGTGCCGCCACTGTTGGTTTCAGAGAGAAAACTCTCCCGGGACACAAGGCGAGTGATGACGTGAACTCC

GAGCCCGCCAAACCCGTGGAAGAACAACCACAAGCTGAAGGACCCTACACCGGTCCACTCGAGCGTCAAA

AACCTCTGAAAGTGAGAGCCAAGCTCCCACAGCAGGAGGGGCCCTACGCTGGTCCGATGGAGAGACAGAA

ACCGCTGAAAGTGAAAGTGAAAGCCCCGGTCGTTAAGGAAGGACCTTACGAAGGACCGGTGAAGAAACCT

GTCGCTTTGAAAGTGAAAGCAAAGAACTTGATTGTCACTGAGAGTGGTGC-CCCCCGACTGACTTGCAAA

AGATGGTCATGGGTAACACCAAGCCTGTTGAGCTCATCCTCGACGGGAAGACGGTGGCCATCTGCTGCGC

CACCGGAGTGTTTGGTACTGCCTACCTTGTTCCTCGTCATCTTTTCGCAGAGAAGTATGACAAGATCATG

TTGGACGGCAGAGCCATGACAGACAGTGACTACAGAGTGTTTGAGTTTGAGATTAAAGTGAAAGGACAGG

ACATGCTCTCAGACGCCGCGCTCATGGTGCTTCACCGTGGGAATCGCGTGCGGGACATCACGAAGCACTT

CCGTGATGTGGCAAGAATGAAGAAAGGCACCCCCGTCGTCGGCGTGATCAACAACGCTGATGTTGGGAGA

CTGATCTTCTCTGGTGAGGCCCTTACCTACAAGGACATTGTAGTGTGCATGGACGGAGACACCATGCCCG

GTCTCTTCGCCTACAAAGCCGCCACCAAGGCGGGTTACTGTGGAGGAGCCGTTCTTGCAAAGGACGGAGC

CGAGACTTTCATCGTCGGCACTCACTCCGCAGGCGGCAACGGAGTTGGATACTGCTCATGCGTTTCCAGG

TCTATGCTGCTTAAAATGAAGGCACACATCGATCCCGAACCACACCACGAG

>AJ539139.1_O_SKR_2000

ATCTCAATACCTTCCCAAAAGGCTGTGCTATACTTTCTCATTGAGAAGGGCCAGCACGATGCAGCAATTG

AATTCTTTGAGGGGATGGTGCATGACTCCATCAAGGAGGAGCTCCGGCCTCTCATCCAACAGACCTCATT

TGTGAAGCGCGCTTTTAAGCGCCTGAAGGAAAACTTTGAGGTAGTTGCCCTGTGTTTGACCCTTTTGGCA

AACATAGTGATCATGATCCGCGAGACTCGCAAGAGACAGCAGATGGTGGATGACGCAGTGAACGAGTACA

TTGAGAAGGCAAACATCACCACGGATGACAAGACTCTTGACGAGGCGGAAAAGAACCCTCTGGAGACCAG

CGGTGCCACCACTGTTGGTTTTAGAGAGAAAACTCTCCCGGGACACAAGGCGAGTGATGACGTGAACTCC

GAGCCCGCCAAACCCGCGGAAGAACAACCACAAGCTGAAGGACCCTACACCGGTCCACTCGAGCGTCAAA

AACCTCTGAAAGTGAGAGCCAAGCTCCCACAGCAGGAGGGGCCCTACGCTGGTCCGATGGAGAGACAGAA

ACCGCTGAAAGTGAAAGTGAAAGCCCCGGTTGTTAAGGAAGGACCTTACGAAGGACCGGTGAAGAAACCT

GTCGCTTTGAAAGTGAAAGCAAAGAACTTGATTGTCACTGAGAGTGGTGCTCCCCCGACTGACTTGCAAA

AGATGGTCATGGGTAACACCAAGCCTGTTGAGCTCATCCTCGACGGGAAGACGGTGGCCATCTGTTGCGC

CACCGGAGTGTTTGGTACTGCTTACCTTGTCCCTCGTCATCTTTTCGCAGAGAAGTATGACAAGATCATG

TTGGACGGCAGAGCCATGACAGACAGTGACTACAGAGTGTTTGAGTTTGAGATTAAAGTGAAAGGACAGG

ACATGCTCTCAGACGCCGCGCTCATGGTGCTTCACCGTGGGAATCGCGTGCGGGACATCACGAAGCACTT

CCGTGATGTGGCAAGAATGAAGAAAGGCACCCCCGTCGTCGGCGTGGTCAACAACGCTGATGTTGGGAGA

CTGATCTTCTCTGGTGAGGCCCTTACCTACAAGGACATTGTAGTGTGCATGGACGGAGACACCATGCCCG

GTCTCTTCGCCTACAAAGCCGCCACCAAGGCGGGTTACTGTGGGGGAGCCGTTCTTGCAAAGGACGGAGC

CGAGACTTTCATCGTCGGCACTCACTCCGCAGGCGGCAATGGGGTTGGATACTGCTCATGCGTTTCCAGG

TCTATGCTGCTTAAAATGAAGGCACACATCGATCCCGAACCACACCACGAG

>AJ539140.1_O_SAR_2000

ATCTCAATTCCTTCCCAAAAGGCTGTGCTGTACTTTCTCATTGAGAAGGGCCAGCACGAAGCAGCAATTG

AGTTCTTTGAGGGGATGGTGCATGACTCCATCAAGGAGGAGCTCCGGCCTCTCATCCAACAGACCTCATT

TGTGAAGCGCGCTTTTAAGCGCCTGAAGGAAAACTTTGAGATAGTTGCCCTGTGTTTGACTCTTTTGGCA

AACATAGTGATCATGATCCGCGAGACTCGCAAGAGACAGCAGATGGTGGATGATGCAGTGAACGAGTACA

TTGAGAAGGCAAACATCACCACGGATGACAAGACTCTTGACGAGGCGGAAAAGAACCCTCTGGAGACCAG

CGGTGCCACCACTGTTGGTTTCAGAGAGAAAACTCTCCCGGGACACAAGGCGGGTGATGACGTGAACTCC

GAGCCCACCAAACCCGTGGAAGAACAACCACAAGCTGAAGGACCCTACACCGGTCCACTCGAGCGTCAAA

AACCCCTGAAAGTGAGGACCAAGCTCCCACAGCAGGAGGGGCCCTACGCTGGTCCGATGGAGAGACAGAA

ACCGCTGAAAGTGAAAGTGAAAGCCCCGGTCGTTAAGGAAGGACCTTACGAAGGACCGGTGAAGAAACCT

GTCGCTTTGAAAGTGAAAGCAAAGAACTTGATTGTCACTGAGAGTGGTGCTCCCCCGACTGACTTGCAAA

AGATGGTCATGGGTAACACCAAGCCTGTTGAGCTCATCCTCGACGGGAAGACGGTGGCCATCTGCTGCGC

CACCGGAGTGTTTGGTACTGCCTACCTAGTTCCTCGTCATCTTTTCGCAGAGAAGTATGACAAGATCATG

TTGGACGGCAGAGCCATGACAGACAGTGACTACAGAGTGTTTGAGTTTGAGACTAAAGTGAAAGGACAGG

ACATGCTCTCAGACGCCGCTCTCATGGTGCTTCACCGTGGGAATCGCGTGCGGGACATCACGAAGCACTT

CCGTGATGTGGCAAGAATGAAGAAAGGCACCCCCGTCGTCGGCGTGATCAACAACGCTGATGTTGGGAGA

CTGATCTTCTCTGGTGAGGCCCTTACCTACAAGGACATTGTAGTGTGCATGGACGGAGACACCATGCCCG

GTCTCTTCGCCTACAAAGCTGCCACCAAGGCGGGTTACTGTGGAGGAGCCGTTCTTGCAAAGGACGGAGC

CGAGACTTTCATCGTCGGCACTCACTCCGCAGGCGGCAATGGAGTTGGATACTGCTCATGCGTTTCCAGG

TCCATGCTGCTTAAAATGAAGGCACACATCGATCCCGAACCACACCACGAG

>AJ539141.1_O_UKG_2001

ATCTCAATTCCTTCCCAAAAGGCTGTGCTGTACTTTCTCATTGAGAAGGGCCAGCACGAAGCAGCAATTG

AGTTCTTTGAGGGGATGGTGCATGACTCCATCAAGGAGGAGCTCCGGCCTCTCATCCAACAGACCTCATT

TGTGAAGCGCGCTTTTAAGCGCCTGAAGGAAAACTTTGAGATAGTTGCCCTGTGTTTGACTCTTTTGGCA

AACATAGTGATCATGATCCGCGAGACTCGCAAGAGACAGCAGATGGTGGATGATGCAGTGAACGAGTACA

TTGAGAAGGCAAACATCACCACGGATGACAAGACTCTTGACGAGGCGGAAAAGAACCCTCTGGAGACCAG

CGGTGCCACCACTGTTGGCTTCAGAGAGAAAACTCTCCCGGGACACAAGGCGGGTGATGACGTGAACTCC

GAGCCCGCCAAACCCGTGGAAGAACAACCACAAGCTGAAGGACCCTACACCGGTCCACTCGAGCGTCAAA

AACCCCTGAAAGTGAGGGCCAAGCTCCCACAGCAGGAGGGGCCCTACGCTGGTCCGATGGAGAGACAGAA

ACCGCTGAAAGTGAAAGTGAAAGCCCCGGTCGTTAAGGAAGGACCTTACGAAGGACCGGTGAAGAAACCT

GTCGCTTTGAAAGTGAAAGCAAAGAACTTGATTGTCACTGAGAGTGGTGCTCCCCCGACTGACTTGCAAA

AGATGGTCATGGGTAACACCAAGCCTGTTGAGCTCATCCTCGACGGGAAGACGGTGGCCATCTGCTGCGC

CACCGGAGTGTTTGGTACTGCCTACCTAGTTCCTCGTCATCTTTTCGCAGAGAAGTATGACAAGATCATG

TTGGACGGCAGAGCCATGACAGACAGTGACTACAGAGTGTTTGAGTTTGAGATTAAAGTGAAAGGACAGG

ACATGCTCTCAGACGCCGCTCTCATGGTGCTTCACCGTGGGAATCGCGTGCGGGACATCACGAAGCACTT

CCGTGATGTGGCAAGAATGAAGAAAGGCACCCCCGTCGTCGGCGTGATCAACAACGCTGATGTTGGGAGA

CTGATCTTCTCTGGTGAGGCCCTTACCTACAAGGACATTGTAGTGTGCATGGACGGAGACACCATGCCCG

GTCTCTTCGCCTACAAAGCTGCCACCAAGGCGGGTTACTGTGGAGGAGCCGTTCTTGCAAAGGACGGAGC

CGAGACTTTCATCGTCGGCACTCACTCCGCAGGCGGCAATGGAGTTGGATACTGCTCATGCGTTTCCAGG

TCCATGCTGCTTAAAATGAAGGCACACATCGATCCCGAACCACACCACGAG

>AJ633821.1_O_FRA_2001

ATCTCAACTCCTTCCCAAAAGGCTGTGCTGTACTTTCTCATTGAGAAGGGCCAGCACGAAGCAGCAATTG

AGTTCTTTGAGGGGATGGTGCATGACTCCATCAAGGAGGAGCTCCGGCCTCTCATCCAACAGACCTCATT

TGTGAAGCGCGCTTTTAAGCGCCTGAAGGAAAACTTTGAGATAGTTGCCCTGTGTTTGACTCTTTTGGCA

AACATAGTGATCATGATCCGCGAGACTCGCAAGAGACAGCAGATGGTGGATGATGCAGTGAACGAGTACA

TTGAGAAGGCAAACATCACCACGGATGACAAGACTCTTGACGAGGCGGAAAAGAACCCTCTGGAGACCAG

CGGTGCCACCACTGTTGGTTTCAGAGAGAAAACTCTCCCGGGACACAAGGCGGGTGATGACGTGAACTCC

GAGCCCGCCAAACCCGTGGAAGAACAACCACAAGCTGAAGGACCCTACACCGGTCCACTCGAGCGTCAAA

AACCCCTGAAAGTGAGGGCCAAGCTCCCACAGCAGGAGGGGCCCTACGCTGGTCCGATGGAGAGACAGAA

ACCGCTGAAAGTGAAAGTGAAAGCCCCGGTCGTTAAGGAAGGACCTTACGAAGGACCGGTGAAGAAACCT

GTCGCTTTGAAAGTGAAAGCAAAGAACTTGATTGTCACTGAGAGTGGTGCTCCCCCGACTGACTTGCAAA

AGATGGTCATGGGTAACACCAAGCCTGTTGAGCTCATCCTCGACGGGAAGACGGTGGCCATCTGCTGCGC

CACCGGAGTGTTTGGTACTGCCTACCTAGTTCCTCGTCATCTTTTCGCAGAGAAGTATGACAAGATCATG

TTGGACGGCAGAGCCATGACAGACAGTGACTACAGAGTGTTTGAGTTTGAGATTAAAGTGAAAGGACAGG

ACATGCTCTCAGACGCCGCTCTCATGGTGCTTCACCGTGGGAATCGCGTGCGGGACATCACGAAGCACTT

CCGTGATGTGGCAAGAATGAAGAAAGGCACCCCCGTCGTCGGCGTGATCAACAACGCTGATGTTGGGAGA

CTGATCTTCTCTGGTGAGGCCCTTACCTACAAGGACATTGTAGTGTGCATGGACGGAGACACCATGCCCG

GTCTCTTCGCCTACAAAGCTGCCACCAAGGCGGGTTACTGTGGAGGAGCCGTTCTTGCAAAGGACGGAGC

CGAGACTTTCATCGTCGGCACTCACTCCGCAGGCGGCAATGGAGTTGGATACTGCTCATGCGTTTCCAGG

TCCATGCTGCTTAAAATGAAGGCACACATCGATCCCGAACCACACCACGAG

>AY317098.1_O_CHA_2002

ATTTCAATCCCTTCCCAGAAGTCTGTGCTGTACTTCCTCATTGAGAAGGGCCAGCACGAAGCAGCAATCG

AGTTCTTCGAGGGTATGGTCCACGACTCCATCAAGGAGGAGCTCCGGCCCCTCATCCAACAGACCTCGTT

TGTAAAACGCGCCTTCAAGCGCCTGAAGGAGAACTTTGAGATCGTTGCTCTGTGTTTAACCCTCTTGGCA

AACATAGTAATTATGCTCCGCCAAGCGCGCAAGAGACGCCAGTCGGTGGATGACTCACCGGAC-------

-----------------------AGCGACATAACTCTTGGCGGCGCGGAAAAGAACCCTCTGGAGACGAG

TGGCGCTAGCGCTGTCGGTTTCAGAGAGAGGCCACCCACCGAGCAAGAGACGTGCGAAGACGCGAACGCT

GAGCCCGTCGTGTTCGGGAGGGAACAACCGCGAGCTGAAGGACCCTACGCTGGGCCACTTGAGCGTCAGA

AACCTCTTAAAGTGAAAGCCAGGCTGCCACAACAGGAGGGACCCTACGCCGGCCCAATGGAGAGACAGAA

ACCGCTAAAGGTGAAAGCAAAAGTCCCCGTCGTGAAGGAAGGACCTTACGAGGGGCCGGTGAAGAAACCT

GTCGCTTTGAAAGTGAAAGCAAAGAACTTGATAGTCACTGAGAGTGGTGCGCCGCCGACCGACTTGCAAA

AGATGGTCATGGGCAACACTAAGCCAGTCGAGCTTATCCTCGACGGTAAGACGGTGGCCATCTGTTGTGC

CACCGGAGTGTTCGGCACTGCCTACCTCGTGCCTCGTCACCTCTTCGCGGAGAAGTATGACAAGATCATG

TTGGACGGTAGAGCCTTAACAGACAGCGACTACAGAGTGTTCGAGTTTGAGATTAAAGTAAAAGGACAGG

ACATGCTCTCAGACGCCGCTCTCATGGTGTTGCACCGTGGGAACCGCGTGCGCGATATCACGAAGCACTT

CCGTGATGTGGCGAGAATGAAGAAGGGTACCCCCGTCGTCGGTGTGATCAATAATGCCGACGTAGGGAGA

CTCATATTCTCTGGTGAAGCCCTTACTTATAAGGACATTGTCGTGTGTATGGACGGAGACACCATGCCTG

GGCTCTTCGCCTACAGAGCATCCACCAAGGCAGGCTACTGTGGAGGAGCCGTCCTAGCAAAGGACGGTGC

CGAGACATTCATCGTTGGCACTCACTCCGCAGGTGGTAACGGTATAGGATACTGTTCATGCGTCTCCCGA

TCGATGCTCATGAAGATGAAGGCACACATCGACCCTGAACCACACCACGAG

>AY593751.1_A_NET_1942

ATCTCGATTCCTTCCCAAAAGTCCGTGTTGTACTTCCTCATTGAGAAAGGCCAACACGAGGCAGCAATTG

AATTCTTTGAGGGCATGGTACACGACTCCGTGAAGGAGGAGCTCCGGCCTCTCATCCAGCAAACCTCATT

TGTGAAACGCGCTTTCAAGCGCCTGAAGGAGAACTTTGAGATTGTTGCCCTATGTTTGACCCTTCTGGCT

AACATAGTGATCATGATCCGCGAAACCCGCAAGAGGCAGAAGATGGTGGATGATGCGGTGAACGATTACA

TCGAGAGGGCAAACATCACCACGGACGACAAGACTCTTGACGAAGCGGAGAAGAACCCTCTGGAAACCAG

CGGTGCCAGTACCGTTGGATTCAGAGAGAGAAGCCTCACAGGCCAAAAGGCGCGCGATGACGTGAACTCC

GAGCCCGCCCAGCCTGCTGAGGATCAACCACAAGCTGAAGGACCCTACGCCGGGCCACTCGAGCGTCAGA

AACCTCTGAAAGTGAGAGCCAAGCTCCCACAGCAGGAGGGACCCTACGCTGGCCCGATGGAGAGACAGAA

ACCGCTGAGAGTAAAAGCAAAAGCCCCGGTCGTTAAGGAAGGACCTTACGAGGGACCGGTGAAGAAGCCT

GTCGCTTTGAAAGTGAAAGCTAGGAACTTGATTGTCACTGAGAGTGGTGCCCCACCGACCGACTTGCAGA

AGCTGGTCATGGGCAACACAAAGCCTGTTGAGCTTATCCTCGACGGGAAGACAGTAGCCATCTGCTGTGC

TACTGGAGTGTTTGGCACTGCTTACCTCGTGCCTCGTCATCTTTTCGCAGAGAAGTATGACAAGATCATG

TTGGAAGGCAGAGCCATGACAGACAGTGATTACAGAGTGTTTGAGTTTGAGATTAAAGTAAAAGGACAGG

ACATGCTCTCAGACGCGGCACTCATGGTGCTTCACCGTGGGAACCGCGTGAGAGACATCACGAAACACTT

TCGTGATACAGCAAGAATGAAGAAAGGCACCCCCGTCGTCGGTGTTGTCAACAACGCCGATGTTGGGAGA

CTGATTTTCTCTGGTGAGGCCCTTACCTACAAGGACATTGTAGTGTGCATGGATGGAGACACCATGCCCG

GCCTCTTTGCCTACAAAGCCGCCACCAAGGCTGGCTACTGTGGAGGAGCCGTTCTTGCCAAGGACGGGGC

TGACACATTTATCGTCGGCACTCACTCTGCAGGTGGCAATGGAGTTGGATACTGCTCATGCGTTTCCAGG

TCCATGCTTCAAAAGATGAAGGCTCACGTCGACCCTGAACCACACCACGAG

>AY593753.1_A_Brazil_1970

ATCTCAATTCCTTCCCAAAAATCCGTGTTGTACTTCCTCATTGAAAAAGGGCAGCACGAGGCAGCAATTG

AATTCTTTGAGGGCATGGTCCACGACTCCATCAAGGAGGAGCTCCGGCCCCTTATCCAGCGAACTTCATT

TGTGAAACGCGCTTTCAAGCGCTTGAAGGAAAACTTTGAGATTGTTGCCCTATGTTTGACCCTCCTGGCC

AACATAGTGATCATGATCCGCGAGACTCGCAAGAGACAGAAGATGGTGGATGATGCGGTGAGTGAGTACA

TTGAGAAAGCAAACATCACCACCGACGACAAGACTCTTGACGAGGCGGAAAAGAACCCTCTGGAAACCAG

CGGTGCCAGCACCGTCGGCTTCAGAGAGAGAACTCTTCCAGGCCAGAAGGCGCGTGGTGAAGAGAGCTCT

GAGCCCGCCCAGCCTATTGAAGAGCAACCGCGAGCTGAAGGACCCTACGCCGGGCCGCTTGAGCGTCAGA

AACCTCTGAAAGTGAGAGCCAAGCTCCCACAGCAAGAGGGGCCCTACGCTGGCCCGATGGAGAGACAGAA

ACCGCTTAAGGTGAAAGCAAAAGCCCCGGTCATTAAGGAAGGACCTTACGAGGGACCGGTGAAGAAGCCT

GTCGCTTTGAAAGTGAAAGCTAAGAACTTGATTGTCACTGAGAGTGGTGCCCCACCGACTGACTTGCAAA

AGATGGTCATGGGCAACACTAAGCCTGTTGAGCTCATTCTCGACGGGAAGACAGTAGCCATCTGCTGTGC

TACTGGAGTGTTTGGCACCGCCTACCTCGTGCCTCGTCATCTTTTCGCTGAGAAGTATGACAAGATTATG

GTGGACGGCAGGGCCATGACAGACAGTGACTACAGAGTGTTTGAGTTTGAGATTAAAGTAAAAGGACAGG

ACATGCTCTCAGACGCTGCGCTCATGGTGCTCCACCGTGGGAACCGCGTGAGAGACATCACGAAACACTT

TCGTGACACAGCAAGAATGAAGAAAGGCACCCCCGTCGTTGGCGTGATCAACAACGCTGATGTCGGGAGA

CTGATTTTCTCTGGTGAAGCCCTTACCTACAAGGACATTGTAGTGTGCATGGATGGTGACACCATGCCTG

GGCTCTTTGCCTACAAAGCCGCAACCAAGGCTGGGTATTGCGGAGGAGCTGTCCTTGCTAAGGACGGGGC

CGACACGTTCATCGTCGGCACTCACTCTGCTGGAGGCAATGGTGTTGGATACTGCTCTTGCGTTTCCAGA

TCCATGCTCCTCAGGATGAAGGCCCACATTGACCCTGAACCGCACCACGAG

>AY593754.1_A_SPA_1959

ATCTCAATTCCTTCCCAAAAATCTGTGTTGTACTTCCTCATTGAGAAAGGGCAGCACGAGGCAGCAATTG

AATTCTTTGAGGGCATGGTGCATGACTCCGTCAAGGAGGAGCTCCGGCCGCTCATCCAACAAACCTCATT

TGTGAAACGCGCGTTTAAGCGCCTGAAGGAAAACTTTGAGATTGTTGCTCTGTGCCTAACACTTTTGGCC

AACATCGTGATCATGATCCGCGAGACCCGTAAGAGACAGAAGATGGTGGACGATGCGGTGAACGAGTACA

TCGAGAAAGCAAACATCACCACCGATGACAAGACTCTTGACGAGGCGGAAAAGAACCCTCTGGAGACTAG

CGGTGCCAGTACCGTTGGTTTCAGAGAGAGAACTCTTCCAGATCAGAAGGCGCGTAATGACGTGAACTCC

GAGCCCGCCCAACCTGCTGAAGAACAACCACAAGCTGAAGGACCCTACGCCGGGCCACTTGAGCGTCAGA

GACCCCTGAAGGTGAGAACCAAGCTCCCACAACAGGAAGGACCCTACGCTGGCCCGATGGAGAGACAGAA

ACCGCTGAAAGTGAAAGCAAAAGCCCCGGTCGTCAAGGAAGGACCTTACGAAGGACCGGTGAAGAAACCT

GTCGCTTTGAAAGTGAAAGCTAAGAATTTGATTGTCACTGAGAGTGGTGCCCCCCCGACCGACCTGCAAA

AGATGGTCATGGGCAACACAAAGCCCGTTGAGCTCATCCTCGACGGGAAGACAGTAGCCATCTGCTGTGC

TACTGGAGTGTTTGGTACTGCCTACCTCGTGCCTCGTCATCTTTTCGCTGAGAAGTATGACAAGATCATG

TTGGACGGCAGAGCCATGACAGACAGTGACTACAGGGTGTTTGAGTTCGAGATCAGAGTAAAAGGACAGG

ACATGCTCTCAGACGCCGCACTCATGGTGCTCCACCGTGGGAACCGCGTGAGAGACATCACGAAGCATTT

CCGTGACACAGCAAGAATGAAGAAAGGCACCCCCATTGTCGGCGTGATTAACAATGCCGATGTCGGGAGA

CTGATTTTCTCTGGCGAAGCTCTTACCTACAAAGACATTGTAGTGTGCATGGACGGAGACACCATGCCCG

GGCTTTTTGCCTACAGAGCCGCCACTAAGGCTGGCTACTGCGGGGGAGCCGTTCTCGCTAAGGACGGGGC

TGACACTTTTATCGTTGGCACTCACTCTGCAGGAGGTAACGGAGTTGGATACTGCTCATGCGTTTCCAGG

TCCATGCTTCTCAAGATGAAGGCACACATTGACCCTGAGCCGCACCACGAG

>AY593755.1_A_TAI_1960

ATCTCGATTCCTTCCCAAAAATCTGTGCTTTACTTCCTCATTGAGAAAGGCCAACACGAAGCAGCAATTG

AATTCTTTGAGGGGATGGTTTGCGACTCTGTCAAGGAGGAGCTCCGGCCCCTCATCCAACAGACCTCATT

TGTGAAACGCGCTTTCAAGCGCCTGAAGGAAAACTTTGAGATTGTTGCCCTGTGTTTGACGCTTCTGGCA

AACATAGTGATCATGATCCGCGAGACTCGCAAGAGACAAAAGATGGTGGATGATGCAGTGAATGACTACA

TTGAGAGAGCGAACATCACCACGGATGACAAGACTCTTGACGAGGCGGAAAAGAACCCTCAGGGGACGAG

CGGTGCCAGCACCGTTGGTTTCAGAGAGAGAACCCTCCCGGGGCAAAAGGCGTGTGATGACGTGAACACT

GAGCCTGCCAAGCCCGTGGGGGAACAACCACAAGCTGAAGGACCCTACGCCGGACCACTCGAGCGTCAGA

AACCTCTTAAAGTGAAAGCCAAGCTGCCACAACAAGAGGGGCCCTACGCTGGTCCGATGGAGAGACAAAA

ACCACTGAAAGTGAAAGCAAAAGCCCCGGTCGTGAAGGAAGGACCTTACGAGGGACCGGTGAAGAAACCT

GTCGCTTTGAAAGTGAAAGCAAAGAACTTGATTGTCACTGAGAGTGGTGCCCCACCGACCGACTTGCAAA

AGATGGTCATGGGCAACACAAAGCCTGTTGAGCTCATTCTCGACGGCAAGACAGTAGCCATCTGCTGTGC

TACTGGAGTGTTTGGCACTGCTTACCTCGTGCCTCGTCACCTTTTCGCGGAAAAGTATGACAAAATCATG

TTGGACGGCAGAGCCATGACAGACAGTGACTACAGAGTGTTTGAGTTTGAGATTAAAGTGAAAGGACAGG

ACATGCTCTCAGACGCCGCCCTCATGGTGCTGCACCGCGGGAATCGCGTGCGTGACATCACGAAACACTT

CCGTGATGTGGCAAAGATGAAGAAAGGAACCCCCGTTGTTGGCGTTATCAACAATGCCGACGTCGGGAGA

CTCATATTCTCTGGTGAGGCCCTCACCTACAAAGACATTGTAGTGTGCATGGATGGAGACACCATGCCTG

GCCTCTTTGCCTACAAGGCCGCCACCAAGGCGGGATACTGTGGAGGAGCCGTTCTGGCAAAGGATGGCGC

TGAGACTTTCATTGTCGGCACTCACTCTGCAGGTGGCAACGGTGTGGGATACTGCTCTTGCGTTTCCCGA

TCGATGCTCCTTAAGATGAAGGCACACGTTGACCCCGAACCACACCACGAG

>AY593756.1_A_Brazil_1959

ATCTCAATTCCTTCCCAAAAATCCGTGTTGTACTTCCTCATTGAGAAAGGACAGCACGAGGCAGCAATTG

AATTCTTTGAGGGAATGGTGCACGACTCCATCAAGGAGGAGCTCCGGCCTCTCATCCAACGAACTTCATT

TGTACAACGCGCTTTCAAGCGCCTGAAGGAAAACTTCGAGATTGTTGCCCTATGTTTGACCCTCTTGGCC

AACATAGTGATCATGATCCGCGAAACTCGCAAGAGGCAGAAGATGGTGGATGATGCGGTGAATGAGTACA

TTGAGAAAGCAAACATCACCACTGATGACAAAACTCTTGACGAGGCGGAAAAGAACCCTCTGGAAACCAG

CGGTGCCAGCACTGTTGGTTTCAGAGAGAGAACACTTCCAGGCCAGAAGGTGCGCGATGACGTGAACTCT

GAGCCCGCCCAACCTGTTGAAGAACAACCGCAAGCTGAAGGACCCTACGCCGGACCACTTGAGCGTCAGA

TACCTCTGAAAGTGAGAGCCAAGCTCCCGCAACAAGAGGGACCTTACGCTGGCCCGATGGAGAGACAGAA

ACCGCTGAAAGTGAAAGTAAGAGCCCCGGTCGTCAAGGAAGGACCTTACGAGGGACCGGTGAAGAAGCCT

GTCGCTTTGAAAGTGAAAGCCAAGAATTTGATTGTCACTGAGAGTGGTGCCCCACCAACCGACTTGCAAA

AGATGGTTATGGGCAACACTAAGCCTGTTGAGCTCATTCTCGACGGGAAGACAGTAGCCATCTGCTGTGC

TACTGGAGTGTTTGGCACTGCTTACCTCGTGCCTCGTCATCTTTTCGCAGAGAAGTATGACAAGATCATG

CTGGACGGCAGAGCCATGACAGACAGTGACTACAGAGTGTTTGAGTTTGAGATTAAAGTAAAAGGACAGG

ACATGCTCTCAGACGCCGCGCTCATGGTGCTCCACCGTGGGAACCGCGTGAGAGACATCACGAAGCACTT

TCGTGACACAGCAAGAATGAAGAAGGGTACCCCCGTCGTTGGCGTAATCAACAACGCTGATGTCGGGAGA

CTGATTTTCTCTGGTGAGGCCCTCACCTACAAGGACATTGTGGTGTGCATGGATGGAGACACCATGCCCG

GTCTCTTTGCCTATAAAGCCGCAACCAAGGCTGGCTATTGCGGAGGAGCTGTTCTTGCTAAGGACGGAGC

TGACACGTTCATCGTCGGCACTCACTCTGCTGGAGGCAATGGAGTTGGTTACTGCTCATGCGTTTCCAGG

TCCATGCTTCTCAAGATGAAGGCACACATTGACCCTGAGCCACACCACGAG

>AY593757.1_A_Brazil_1967

ATCTCAATTCCTTCCCAGAAATCCGTGTTGTACTTCCTCATTGAAAAAGGGCAGCACGAGGCAGCAATTG

AATTCTTTGAGGGCATGGTCCACGACTCCATCAAGGAGGAGCTCCGGCCCCTCATCCAACGAACTTCATT

TGTGAAACGCGCTTTCAAGCGCTTGAAGGAAAACTTTGAGATTGTTGCCCTATGTTTGACCCTCCTGGCC

AACATAGTGATCATGATCCGCGAGACTCGCAAGAGACAGAAGATGGTGGATGATGCGGTGAACGAGTACA

TTGAGAAAGCAAACATCACCACCGATGACAAGACTCTTGACGAGGCGGAAAAGAACCCTCTGGAAACCAG

CGGTGCCAGCACCGTCGGCTTCAGAGAGAGAACTCTTCCAGGCCAGAAGGCGCGTGGCGAAGAGAACTCT

GAGCCCGCCCAGCCTATTGAAGAGCAACCACGAGCTGAAGGACCCTACGCCGGGCCGCTTGAGCGTCAGA

AACCTCTGAAAGTGAGAGCCAAGCTCCCACAGCAAGAGGGGCCTTACGCTGGCCCGATGGAGAGACAGAA

ACCACTTAAGGTGAAAGCAAAAGCCCCGGTCATTAAGGAAGGACCTTACGAGGGACCGGTGAAGAAGCCT

GTCGCTTTGAAAGTGAAAGCTAAGAACCTGATTGTCACTGAGAGTGGTGCCCCACCGACCGACTTGCAAA

AGATGGTCATGGGCAACACTAAGCCTGTTGAGCTCATTCTCGACGGGAAGACAGTAGCCATCTGCTGTGC

TACTGGAGTGTTTGGCACCGCCTACCTCGTGCCACGTCATCTTTTCGCTGAGAAGTATGACAAGATTATG

GTGGATGGCAGGGCCATGACAGACAGTGACTACAGAGTGTTTGAGTTTGAGATTAAAGTAAAAGGACAGG

ACATGCTCTCAGACGCTGCGCTCATGGTGCTCCACCGTGGGAACCGCGTGAGAGACATCACGAAACACTT

TCGTGACACAGCAAGAATGAAGAAAGGCACCCCCGTCGTTGGCGTGATCAACAACGCTGATGTCGGGAGA

CTGATTTTCTCTGGTGAAGCCCTTACCTACAAGGACATTGTAGTGTGCATGGATGGTGACACCATGCCTG

GGCTCTTTGCCTACAAAGCCTCAACCAAGGCTGGGTATTGCGGAGGAGCTGTCCTTGCTAAGGACGGGGC

CGACACGTTCATCGTCGGCACTCACTCTGCTGGAGGCAATGGTGTTGGATACTGCTCTTGCGTTTCCAGA

TCCATGCTTCTCAGGATGAAGGCACACATTGACCCTGAACCACACCACGAG

>AY593758.1_A_VEN_1967

ATCTCAATTCCTTCCCAAAAATCCGTGTTGTACTTCCTCATTGAAAAAGGGCAGCACGAGGCAGCAATTG

AATTCTTTGAGGGCATGGTCCACGACTCCATCAAGGAGGAGCTCCGGCCCCTTATCCAACGAACTTCATT

TGTGAAACGCGCTTTCAAGCGCTTGAAGGAAAACTTTGAGATTGTTGCCCTATGTTTGACCCTCCTGGCC

AACATAGTGATCATGATCCGCGAGACTCGCAAGAGACAGAAGATGGTGGATGATGCGGTGAGTGAGTACA

TTGAGAAAGCAAACATCACCACCGACGACAAGACTCTTGACGAGGCGGAAAAGAACCCTCTGGAAACCAG

CGGTGCCAGCACCGTCGGCTTCAGAGAGAGAACTCTTCCAGGCCAGAAGGCGCGTGGTGAAGAGAGCTCT

GAGCCCGCCCAGCCTATTGAAGAGCAACCGCGAGCTGAAGGACCCTACGCCGGGCCGCTTGAGCGTCAGA

AACCTCTGAAAGTGAGAGCCAAGCTCCCACAGCAAGAGGGGCCCTACGCTGGCCCGATGGAGAGACAGAA

ACCGCTTAAGGTGAAAGCAAAAGCCCCGGTCATTAAGGAAGGACCTTACGAGGGACCGGTGAAGAAGCCT

GTCGCTTTGAAAGTGAAAGCTAAGAACTTGATTGTCACTGAGAGTGGTGCCCCACCGACTGACTTGCAAA

AGATGGTCATGGGCAACACTAAGCCTGTTGAGCTCATTCTCGACGGGAAGACAGTAGCCATCTGCTGTGC

TACTGGAGTGTTTGGCACCGCCTACCTCGTGCCTCGTCATCTTTTCGCTGAGAAGTATGACAAGATTATG

GTGGACGGCAGGGCCATGACAGACAGTGACTACAGAGTGTTTGAGTTTGAGATTAAAGTAAAAGGACAGG

ACATGCTCTCAGACGCTGCGCTCATGGTGCTCCACCGTGGGAACCGCGTGAGAGACATCACGAAACACTT

TCGTGACACAGCAAGAATGAAGAAAGGCACCCCCGTCGTTGGCGTGATCAACAACGCTGATGTCGGGAGA

CTGATTTTCTCTGGTGAAGCCCTTACCTACAAGGACATTGTAGTGTGCATGGATGGTGACACCATGCCTG

GGCTCTTTGCCTACAAAGCCGCAACCAAGGCTGGGTATTGCGGAGGAGCTGTCCTTGCTAAGGACGGGGC

CGACACGTTCATCGTCGGCACTCACTCTGCTGGAGGCAATGGTGTTGGATACTGCTCTTGCGTTTCCAGA

TCCATGCTCCTCAGGATGAAGGCCCACATTGACCCTGAACCGCACCACGAG

>AY593759.1_A_GER_1971

ATCTCAATTCCTTCCCAAAAATCTGTGTTGTACTTCCTCATTGAGAAAGGCCAACACGAGGCAGCAATTG

AATTCTTTGAGGGTATGGTGCATGATTCCATTAAGGAAGAGCTCCGGCCCCTCATCCAACAAACCTCATT

TGTGAAACGCGCTTTCAAGCGCCTGAAGGAAAACTTTGAGATTGTTGCACTGTGCCTGACCCTTCTGGCC

AACATAGTGATCATGGTCCGCGAAACTCGCAAGAGGCAGAAGATGGTGGATGATGCTGTGAATGACTACA

TTGAGAGGGCAAACATCACCACAGATGACAAAACACTTGACGAGGCGGAGAAGAACCCTCTGGAGACCAG

CGGTGCCAGCACCGTTGGTTTCAGAGAGAAAGCTCTGGCAGGACACAAAGCGCGCGACGACGTGAAATCT

GAGCCCGCCCAGCCTACTGAAGAGCAACCACAAGCTGAAGGACCCTACGCCGGGCCACTTGAGCGTCAGA

AACCTCTGAAAGTGAGAGCCAAACTCCCACAACAGGAGGGGCCCTACGCTGGCCCGATGGAGAGACAGAA

GCCGCTGAAAGTAAAAGCAAGAGCCCCGGTCGTCAGGGAAGGACCTTACGAGGGACCGGTGAAGAAGCCT

GTCGCTTTGAAAGTGAAAGCCAAGAACTTGATTGTCACTGAGAGTGGTGCCCCACCGACCGACTTGCAGA

AGTTGGTCATGGGCAACACAAAGCCTGTTGAGCTCATCCTCGACGGGAAGACAGTAGCCATTTGCTGTGC

TACTGGAGTGTTCGGTACTGCTTACCTTGTGCCTCGTCATCTTTTCGCAGAGAAGTATGACAAGATCATG

TTGGACGGTAGAGCCATGACAGACAGTGACTACAGAGTGTTTGAGTTTGAGATTAAAGTAAAAGGACAGG

ACATGCTCTCAGACGCGGCACTCATGGTGCTTCACCGTGGGAACCGCGTGAGAGACATCACGAAACACTT

TCGTGATACAGCAAGAATGAAGAAAGGTACCCCCGTCGTCGGTGTTATTAACAACGCCGATGTCGGGAGA

CTGATTTTCTCTGGTGAGGCCCTTACCTACAAGGACATTGTAGTGTGTATGGACGGAGACACCATGCCCG

GCCTCTTCGCCTACAAAGCCGCCACCAAGGCTGGCTACTGTGGAGGAGCCGTCCTTGCCAAGGACGGGGC

TGACACATTCATCGTCGGCACCCACTCTGCAGGTGGCAACGGAGTTGGATACTGTTCATGTGTTTCCAGG

TCCATGCTTCAAAAGATGAAGGCTCACGTTGACCCTGAACCGCACCACGAG

>AY593760.1_A_USSR_1964

ATCTCAATTCCTTCCCAAAAGTCTGTGTTGTACTTCCTCATTGAGAAAGGCCAACACGAAGCAGCAATTG

AATTCTTCGAGGGGATGGTGCACGATTCCATCAAGGAGGAACTCCGGCCCCTCATCCAACAGACCTCATT

TGTGAAACGCGCTTTCAAGCGCCTGAAGGAAAATTTTGAGATTGTTGCCCTGTGTTTGACTCTTTTGGCA

AACATAGTGATCATGATTCGTGAAACCCGCAAGAGGCAGCAGATGGTGGATGATGCAGTGAATGAGTACA

TTGAGAGAGCAAACATCACCACGGATGATAAAACTCTTGATGAAGCCGAGAAAAACCCTCTGGAAACCAG

CGGTGTCAGCACTGTTGGGTTCAGAGAGAGAACCCTCCCGGGGCAG---GCGAGTGATGACGTGAACTCC

GAGCCCGTCAAACCCGTGGAGGAGCAACCACAAGCTGAAGGACCATACGCCGGACCCCTTGAGCGTCAAA

AACCTCTGAAAGTGAGAGCCAGGCTACCACAACAGGAGGGACCTTACGCTGGTCCGCTGGAGAGACAGAA

ACCTCTGAAAGTGAAAGCAAGAGCCCCGGTCGTCAAGGAAGGACCTTATGAAGGACCGGTGAAGAAACCT

GTTGCTTTGAAAGTGAAAGCTAAGAATTTGATTGTCACTGAGAGTGGTGCCCCCCCGACCGACTTGCAAA

AGATGGTCATGGGCAACACAAAGCCTGTTGAGCTCATTCTCGACGGGAAGACAGTAGCCATTTGCTGTGC

TACTGGAGTATTTGGCACTGCTTACCTCGTACCTCGTCATCTTTTCGCAGAGAAATATGACAAGATCATG

TTGGACGGCAGAGCTATGACAGACAGTGACTACAGAGTGTTTGAGTTTGAGATTAAAGTGAAGGGACAGG

ACATGCTCTCGGACGCCGCTCTCATGGTACTTCATCGTGGGAATCGCGTGAGAGACATCACGAAGCACTT

TCGTGACACGGCAAGAATGAAGAAAGGCACCCCCGTTGTCGGCGTGATTAACAATGCCGATGTCGGGAGA

CTGATTTTCTCCGGTGAGGCCCTCACCTACAAGGACATTGTGGTGTGCATGGATGGAGACACCATGCCCG

GGCTTTTTGCCTACAGAGCCGCCACCAAGGCTGGCTACTGTGGAGGGGCCGTTCTCGCCAAGGACGGAGC

TGACACGTTCATCGTTGGCACTCACTCCGCAGGTGGCAATGGAGTTGGATACTGCTCGTGCGTTTCCAGA

TCCATGCTCTTGAAAATGAAGGCACACATTGACCCTGAGCCACACCACGAG

>AY593761.1_A_KEN_1964

ATCTCAATTCCTTCCCAAAAATCCGTGTTGTACTTTCTCATTGAGAAGGGCCAGCACGAGGCAGCAATCG

AGTTCTTTGAGGGCATGGTTCACGACTCCATCAAAGAAGAACTCCGACCCCTCATTCAACACACCTCATT

TGCGAAACGCGCTTTCAAGCGCCTGAAGGAAAATTTTGAGATCGTTGCCCTGTGTTTGGCTCTCTTGGCC

AACATAGTGATCATGATCCGCGAGACTCACAAGAGGCAGAAGATGGTGGATGACGCGGTGAACGACTACA

TTGAAAAGGCAAACATCACCACAGATGACAAAACCCTTGATGAAGCTGCAAAGAACCCTCTGGAAATCAG

CGGTGCCAGCACCGTCGGTTTCAGAGAGAGGACTCCCGCAGGGCGGGGTGCGTGTGACGACGTGAACTCC

GAGCCCGCACAGCCCAGCGGAGACCAACCGCAAGCTGAAGGACCCTACACCGGGCCACTGGAGCGCCAGA

GACCCCTGAAGGTGAGAGCCAAGCTACCACAACAGGAGGGACCCTACGCCGGCCCGATGGAGAGACAGAA

ACCACTGAAGGTGAAAGTGAAGACGCCCGTCGTCAAGGAAGGTCCTTACGAGGGGCCAGTGAAGAAGCCT

GTCGCTTTGAAAGTGAAAGCCAAGAACCTGATTGTCACTGAGAGTGGTGCCCCACCGACCGACTTGCAAA

AGATGGTCATGGGGAACACAAAGCCTGTTGAGCTCATCCTCGACGGGAAGACAGTAGCCATCTGCTGCGC

CACTGGAGTGTTTGGCACTGCCTACCTCGTGCCTCGTCACCTTTTCGCAGAAAAGTACGACAAGATCATG

TTGGACGGCAGGGCCATGACAGACAGTGACTACAGAGTGTTTGAGTTTGAGATAAAAGTAAAAGGACAGG

ACATGCTCTCGGACGCTGCGCTCATGGTGCTTCACCGTGGGAACCGCGTGCGAGACATCACGAAACACTT

TCGTGACACAGCAAGAATGAAGAAAGGCACCCCCGTTGTCGGTGTTATTAACAACGCCGACGTCGGGAGA

CTCATTTTCTCTGGTGACGCCCTCACTTACAAGGACATTGTTGTGTGCATGGACGGGGACACCATGCCCG

GTCTCTTTGCATACAGAGCAGCCACCAAGGCTGGTTACTGCGGAGGAGCCGTTCTTGCCAAGGACGGAGC

CGACACATTTATCGTGGGCACTCACTCCGCAGGAGGCAATGGAGTTGGGTATTGCTCCTGCGTATCCAGG

TCCATGCTTCTCAAGATGAAGGCACACATCGACCCTGAACCACACCACGAG

>AY593764.1_A_IRQ_1970

ATTTCAATTCCTTCCCAAAAGTCTGTGTTGTACTTTCTCATTGAGAAAGGCCAGCACGAAGCAGCAATTG

AGTTCTTCGAGGGAATGGTACACGACTCCATCAAGGAGGAGCTCCGTCCCCTCATCCAACAAACTTCATT

TGTGAAACGCGCTTTTAAGCGTCTGAAGGAAAACTTTGAGATTGTTGCCCTGTGCTTGACTCTTTTGGCA

AACATAGTGATCATGATCCGCGAGACTCGCAAGAGACAACAGATGGTGGATGATGCAGTGAATGAGTACA

TTGAGAAAGCAAACATCACCACAGACGACAAAACTCTTGACGAGGCGGAAAAGAACCCTTTGGAGACTAG

CGGTGCTAGCACTGTTGGGTTCAGAGAAAGAACTCTCCCAGGACACAAGGCGAGTGGTGACGTGAACTCC

GAGCCTGCCAGACCTGTGGAGGAACAACCACAAGCTGAGGGACCCTACGCCGGGCCACTCGAGCGTCAGA

AGCCTCTGAAAGTGAAAGCCAAGCTGCCACAGCAGGAAGGACCTTACGCTGGCCCGATGGAGAGACAGAA

ACCACTGAAAGTGAAAGTAAAAGCTCCGGTCGTTAAGGAAGGACCTTACGAGGGACCGGTGAAGAAGCCT

GTCGCTTTGAAAGTGAAAGCTAAGAACTTGATTGTCACTGAGAGTGGAGCCCCACCGACCGACTTGCAAA

AGATGGTCATGGGCAACACCAAGCCTGTTGAGCTCATCCTCGACGGGAAGACGGTGGCCATTTGTTGTGC

TACCGGAGTGTTTGGCACTGCGTACCTCGTGCCTCGTCATCTTTTTGCAGAAAAATATGACAAGATCATG

CTGGACGGCAGAGCCATGACAGACAGTGACTACAGAGTGTTTGAGTTTGAGATTAAAGTAAAAGGACAGG

ACATGCTCTCAGACGCTGCGCTCATGGTACTCCACCGTGGGAATCGCGTGAGAGACATCACGAAACACTT

TCGTGACACAGCAAGAATGAAGAAAGGCACCCCTGTTGTCGGAGTGATCAACAATGCCGACGTCGGGAGA

CTGATCTTCTCTGGTGAGGCCCTTACCTACAAGGACATTGTAGTGTGCATGGATGGAGACACCATGCCTG

GCCTGTTTGCCTACAAAGCCGCCACCAAGGCTGGCTACTGTGGGGGAGCCGTTCTTGCTAAGGACGGAGC

TGACACATTCATCGTTGGCACTCACTCCGCAGGCGGCAATGGAGTTGGATACTGCTCATGCGTTTCCAGG

TCCATGTTGCTGAAAATGAAGGCGCACATCGACCCCGAACCACACCACGAG

>AY593765.1_A_TUR_1965

ATTTCAATTCCTTCCCAAAAGTCTGTGTTGTACTTTCTCATTGAGAAAGGCCAGCACGAAGCAGCAATTG

AGTTCTTCGAGGGAATGGTACACGACTCCATCAAGGAGGAGCTCCGTCCCCTCATCCAACAGACTTCATT

TGTGAAACGCGCTTTTAAGCGTCTGAAGGAAAACTTTGAGATTGTTGCCCTATGTTTGACTCTCTTGGCA

AACATAGTGATCATGATCCGCGAGACTCGCAAGAGACAACAGATGGTGGATGATGCAGTGAATGAGTACA

TTGAGAAAGCAAACATCACCACAGATGACAAAACTCTTGACGAGGCGGAAAAGAACCCTTTGGAGACTAG

CGGTGCCAGCACTGTTGGGTTCAGAGAAAGAACTCTCCCAGGACACAAGGCGAGTGATGACGTGAACTCC

GAGCCCGCCAGACCTGTGGAGGAACAACCACAAGCTGAAGGACCCTACGCCGGGCCACTCGAGCGCCAGA

AGCCTCTGAAAGTGAAAGCCAAGCTGCCACAGCAGGAAGGACCTTACGCTGGCCCGATGGAGAGACAGAA

ACCACTGAAAGTGAAAGTAAAAGCTCCGGTCGTTAAGGAAGGACCATACGAGGGACCGGTGAAGAAGCCT

GTCGCTTTGAAAGTGAAAGCTAAGAACTTGATTGTCACTGAGAGTGGAGCCCCACCGACCGACTTGCAAA

AGATGGTCATGGGCAACACCAAGCCTGTTGAGCTCATCCTCGACGGGAAGACGGTGGCCATCTGTTGTGC

TACCGGAGTGTTTGGCACTGCGTACCTCGTGCCTCGTCATCTTTTTGCAGAAAAATATGACAAGATCATG

CTGGACGGTAGAGCCATGACAGACAGTGACTACAGAGTGTTTGAGTTTGAGATTAAAGTAAAAGGACAGG

ACATGCTCTCAGACGCTGCGCTCATGGTACTCCACCGTGGGAATCGCGTGAGAGACATCACGAAACACTT

TCGTGACACAGCAAGAATGAAGAAAGGCACCCCTGTTGTCGGAGTGATCAACAATGCCGACGTCGGGAGA

CTGATTTTCTCTGGTGAGGCCCTTACCTACAAGGACATTGTAGTATGCATGGACGGAGACACCATGCCTG

GCCTGTTTGCCTACAAAGCCGCCACCAAGGCTGGCTACTGTGGGGGAGCCGTTCTTGTTAAGGACGGAGC

TGACACATTCATCGTTGGCACTCACTCCGCAGGCGGCAATGGAGTTGGATACTGCTCATGCGTTTCCAGG

TCCATGTTGCTGAAAATGAAGGCGCACATCGACCCCGAACCACACCACGAG

>AY593766.1_A_KEN_1965

ATCTCAATTCCTTCCCAAAAATCTGTGTTGTACTTCCTCATTGAGAAAGGCCAACACGAAGCAGCAATTG

AATTCTTTGAGGGAATGGTGCACGACTCCATCAAGGAGGAGCTCCGGCCCCTCATCCAACAAACCTCATT

TGTGAAACGCGCTTTCAAGCGCTTGAAGGAAAACTTTGAGATCGTTGCCCTGTGTTTGACCCTCTTGGCA

AACATAGTGATCATGATCCGCGAGACTCGCAAGAGACAGAAGATGGTGGATGATGCAGTGAACGAGTACA

TTGAGAAGGCAAACATCACCACAGATGACAAGACTCTTGATGAGGCGGAAAAGAACCCGCTGGAGACCAG

CGGTGCCAGCACCGTTGGCTTCAGAGAGAGGACTCTCCCGGGACACAAGGCGAGTGATGACGTGAACTCC

GAGCCCGCCCAGCCCCTGGAGAAGCAACCACAAGCTGAAGGACCCTACGCCGGGCCACTCGAGCGTCAGA

AACCTTTGAAAGTGAGAGCCAAGCTACCACAGCAGGAGGGACCTTACGCTGGCCCGATGGAGAGACAAAA

ACCACTGAAGGTGAAAGCAAAAGCCCCGGTCGTCAAGGAAGGACCTTACGAGGGACCGGTGAAGAAGCCT

GTCGCTTTGAAAGTGAAAGCCAAGAATTTGATTGTCACTGAGAGTGGCGCCCCACCGACTGACTTGCAAA

AGATGGTCATGGGCAACACCAAACCTGTCGAACTCATCCTCGACGGGAAGACGGTAGCCATCTGCTGCGC

TACCGGAGTGTTTGGCACTGCTTACCTCGTGCCTCGTCACCTCTTCGCAGAGAAGTATGACAAGATCATG

TTGGACGGTAGAGCCATGACAGACAGTGACTACAGAGTGTTTGAGTTTGAGATTAAAGTAAAAGGACAGG

ACATGCTCTCAGACGCCGCGCTCATGGTGCTTCACCGCGGGAACCGTGTGAGAGACATCACGAAACACTT

TCGTGACACAGCAAGAATGAAGAAAGGCACCCCCGTCGTCGGAGTGATCAACAACGCCGATGTCGGGAGA

CTGATTTTCTCTGGTGAGGCCCTCACCTACAAGGACATTGTAGTGTGCATGGATGGAGACACCATGCCTG

GCCTCTTTGCCTACAGAGCCGCCACCAAGGCTGGCTACTGCGGGGGAGCCGTTCTCGCTAAGGACGGAGC

CGACACATTCATCGTTGGCACTCACTCCGCAGGCGGCAATGGAGTCGGATACTGCTCATGTGTGTCCAGG

TCCATGCTGCTGAAAATGAAGGCACACATCGACCCTGAGCCGCACCACGAG

>AY593767.1_A_ARG_1965

ATCTCAATTCCTTCCCAAAAATCTGTGTTGTACTTTCTCATTGAGAAGGGCCAACATGAGGCAGCAATTG

AATTCTTTGAGGGCATGGTCCACGACTCCATCAAAGAGGAACTCCGACCCCTCATCCAACAAACTTCATT

TGTGAAACGCGCTTTCAAGCGCCTGAAGGAAAATTTTGAGATTGTTGCTCTGTGTTTAACACTTTTGGCG

AACATTGTGATCATGATCCGTGAGACTCGCAAGAGGCAGAAAATGGTGGATGATGCAGTGAATGAGTACA

TTGAGAAAGCAAACATCACCACAGATGACAAGACTCTTGACGAGGCGGAGAAGAGCCCTCTAGAGACCAG

CGGCGCCAGCACCGTTGGCTTTAGAGAGAGAACTCTCCCAGGTCAAAAGGCACGCGATGACGTGAACTCC

GAGCCTGCCCAACCTGTTGAGGAGCAACCACAAGCTGAAGGACCCTACGCCGGACCACTCGAGCGTCAGA

AACCTCTGAAAGTGAGAGCCAAGCTCCCACAGCAGGAGGGGCCTTACGCTGGTCCGATGGAGAGACAGAA

ACCGCTAAAAGTGAAAGCAAAAGCCCCGGTCGTGAAGGAAGGACCTTACGAGGGACCGGTGAAGAAGCCT

GTCGCTTTGAAAGTAAAAGCTAAGAACCTGATTGTCACTGAGAGTGGTGCCCCACCGACCGACTTGCAAA

AGATGGTCATGGGCAACACAAAGCCTGTTGAGCTCATCCTCGACGGGAAGACAGTAGCCATCTGCTGCGC

TACTGGAGTGTTTGGCACTGCTTACCTCGTGCCTCGTCACCTCTTCGCAGAGAAGTACGACAAGATCATG

TTGGACGGCAGAGCCATGACAGACAGTGACTACAGAGTGTTTGAGTTTGAGATCAAAGTAAAAGGACAGG

ACATGCTCTCAGACGCCGCGCTCATGGTGCTCCACCGTGGGAACCGCGTGAGGGACATCACGAAGCACTT

TCGTGACACAGCAAGAATGAAGAAAGGCACCCCCGTTGTCGGTGTGATTAACAACGCCGATGTCGGGAGA

CTGATTTTCTCTGGTGAGGCCCTTACTTACAAGGACATTGTGGTTTGCATGGACGGAGACACCATGCCTG

GCCTCTTTGCCTACAGAGCCGCCACCAAGGCTGGTTACTGCGGAGGAGCCGTTCTTGCCAAAGACGGAGC

TGACACTTTCATCGTCGGCACTCACTCTGCGGGAGGCAACGGAGTTGGATACTGCTCATGCGTTTCCAGG

TCCATGCTTCTTAAAATGAAGGCACACATTGACCCCGAACCACACCACGAG

>AY593768.1_A_Brazil_1955

ATCTCAATTCCTTCCCAAAAATCCGTGTTGTACTTCCTCATTGAGAAAGGACAGCACGAGGCAGCAATTG

AATTCTTTGAGGGCATGGTGCACGACTCCATCAAGGAGGAGCTCCGGCCGCTCATCCAACAAACCTCATT

TGTGAAACGCGCTTTTAAGCGCCTGAAGGAAAACTTTGAGATTGTTGCCCTATGTCTGACCCTCCTGGCC

AACATAGTGATCATGATCCGCGAAACTCGCAAGAGACAGAAGATGGTGGACGATGCAGTGAGTGAGTACA

TTGAGAGAGCAAACATCACCACCGACGACAAGACTCTTGATGAGGCGGAAAAGAACCCTCTGGAAACCAG

CGGTGCCAGCACCGTCGGCTTCAGAGAGAGACCTCTCCCAGGCCAAAAGGCGCGTAATGACGAGAACTCC

GAGCCCGCCCAGCCTGCTGAAGAGCAACCACAAGCTGAAGGACCCTACGCCGGGCCGCTAGAACGACAGA

AACCTCTGAAAGTGAGAGCCAAGCTCCCACAACAAGAGGGACCTTACGCTGGCCCGATGGAGAGACAGAA

ACCACTGAAAGTGAAAGCAAAAGCCCCGGTCGTTAAGGAAGGACCTTACGAGGGACCGGTGAAGAAGCCT

GTTGCTTTGAAAGTGAAAGCTAAGAACTTGATCGTCACTGAGAGTGGTGCCCCACCGACCGACTTGCAAA

AGTTGGTCATGGGCAACACCAAGCCCGTTGAGCTCATCCTTGACGGGAAGACGGTAGCCATTTGCTGTGC

TACTGGAGTTTTCGGCACTGCTTACCTCGTGCCTCGTCATCTTTTCGCAGAAAAGTACGACAAGATCATG

TTGGACGGCAGAGCCATGACAGATAGTGACTACAGAGTGTTTGAGTTTGAGATTAAAGTAAAAGGACAGG

ACATGCTCTCAGACGCTGCGCTCATGGTGCTCCACCGTGGGAATCGCGTGAGAGACATCACGAAACACTT

TCGTGACACAGCAAGAATGAAGAAAGGCACCCCCGTCGTTGGTGTGATCAACAACGCCGATGTCGGGAGA

CTGATTTTCTCTGGTGAAGCCCTTACCTACAAGGACATTGTAGTGTGCATGGATGGAGACACCATGCCTG

GGCTCTTTGCCTACAAAGCCGCAACCAAGGCTGGTTATTGCGGAGGAGCCGTCCTCGCTAAGGACGGGGC

TGACACGTTCATCGTTGGCACCCACTCCGCTGGAGGCAATGGCGTTGGATACTGCTCTTGCGTTTCCAGG

TCCATGCTTCTCAAGATGAAGGCACACGTTGACCCCGAACCACACCACGAG

>AY593769.1_A_ARG_1959

ATCTCAATTCCTTCCCAAAAATCCGTGTTGTACTTCCTCATTGAGAAAGGTCAGCACGAAGCAGCAATTG

AATTCTTTGAGGGAATGGTGCATGACTCCATCAAGGAGGAGCTCCGGCCCCTCATTCAACGGACCTCATT

TGTGAAGCGCGCTTTCAAGCGCCTGAAGGAGAACTTTGAGATTGTTGCCCTGTGTTTGACCCTTTTGGCC

AACATAGTGATCATGATCCGCGAGACTCACAAGAGACAGAAAATGGTGGATGATGCAGTGAATGACTACA

TTGAGAAAGCAAACATCACCACAGATGACAAGACTCTTGACGAGGCGGAAAAGAACCCTCTGGAGACCAG

CGGAGCCAGTACCGTTGGCTTCAGAGAGAGAACTCTCACAGGGCACAAGATGTGCGATGACGTGAACTCC

GAGCCCATCCAACCT---GAAGAGCAACCACAAGCTGAAGGACCCTACGCCGGGCCACTCGAGCGTCAGA

AACCCCTGAAAGTGAGAGCCAAGCTCCCGCAGCAGGAGGGACCTTACGCTGGCCCGATGGAGAGACAGAA

ACCGCTGAAGGTAAAAGTGAAAGCCCCGGTCGTTAAGGAAGGACCTTACGAGGGACCGGTGAGGAAGCCT

GTCGCTTTGAAAGTGAAAGCTAAAAACTTGATAGTCACTGAGAGTGGTGCCCCACCTACTGACTTGCAAA

AGATGGTCATGGGCAACACAAAGCCTGTTGAGCTCATCCTCGACGGGAAGACAGTAGCCATCTGCTGTGC

TACTGGAGTGTTTGGTACTGCCTACCTCGTGCCTCGTCACCTCTTCGCAGAGAAGTATGACAAGATCATG

CTGGATGGTAGAGCCATGACAGACAGTGACTACAGAGTGTTTGAGTTTGAGATTAAAGTAAAAGGACAGG

ACATGCTCTCAGACGCCGCGCTCATGGTGCTCCACCGTGGGAACCGCGTGAGAGACATCACGAAACACTT

TCGTGATACAGCAAGAATGAAGAAAGGAACCCCCGTCGTTGGCGTGATCAACAACGCCGATGTTGGGAGA

CTGATTTTCTCTGGTGAGGCCCTTACCTACAAAGACATTGTGGTGTGCATGGATGGAGACACCATGCCTG

GCCTTTTCGCCTACAAAGCCGCCACCAAGGCTGGCTACTGCGGAGGAGCCGTTCTTGCCAAGGACGGGGC

CGACACTTTCATCGTTGGCACTCACTCCGCAGGAGGTAATGGAGTTGGATACTGCTCGTGCGTTTCCAGG

TCCATGCTCCTCAAGATGAAGGCACACATCGACCCCGAACCACACCACGAG

>AY593770.1_A_ARG_1966

ATCTCAATTCCTTCCCAAAAATCCGTGTTGTACTTCCTCATTGAGAAAGGTCAGCACGAGGCAGCAATTG

AATTCTTTGAGGGGATGGTACACGACTCCATCAAGGAGGAACTCCGACCCCTTATTCAACAAACTTCATT

TGTGAGACGCGCTTTCAAGCGCCTGAAGGAAAACTTTGAGGTCGTTGCCCTATGTCTGACCCTCCTGGCC

AACATAGTGATCATGATCCGCGAGACTCGCAGGAGACAGAAAATGGTGGATGATGCAGTGAACGAGTACA

TTGAGAAAACAAACATCACCACGGACGACAAGACTCTTGACGAGGCGGAAAAGAACCCTCTGGAGACCAG

TGGTGCCAGCACCGTTGGTTTCAGAGAGAGAACTCTTCCAGGTCAGAAGGCGCGCGATGACGTGAACTCC

GAGCCCGCCCAACCTGTTGAAGAACAACCACAAGCTGAAGGACCCTACGCCGGGCCACTTGAGCGCCAGA

AACCTCTGAAAGTGAGAACCAAGCTCCCGCAACAAGAGGGACCCTACGCTGGCCCGATGGAGAGACAGAA

ACCGCTGAAAGTAAAAGCAAAGGCCCCGGTCGTTAAGGAAGGACCTTACGAAGGACCGGTGAAGAAACCT

GTCGCTTTGAAAGTGAAAGCTAGGAACTTGATTGTCACTGAGAGTGGTGCCCCACCGACCGACTTGCAAA

AGATGGTCATGGGCAACACGAAGCCCGTTGAGCTCATCCTCGACGGGAAGACGGTAGCCATCTGTTGTGC

TACTGGAGTGTTCGGCACTGCCTACCTCGTGCCTCGTCACCTTTTCGCAGAGAAGTATGACAAGATCATG

TTGGACGGCAGAGCCTTGACAGACAGTGACTACAGAGTGTTTGAGTTTGAGATTAAAGTAAAAGGACAGG

ACATGCTCTCAGACGCCGCGCTCATGGTGCTCCACCGTGGGAACCGCATGAGAGACATCACGAAGCACTT

TCGTGACACAGCAAGAATGAAAAAGGGTACCCCCGTCGTTGGCGTGATCAACAACGCTGACGTCGGGAGA

CTGATTTTCTCTGGTGAGGCCCTCACCTACAAGGACATTGTAGTGTGCATGGACGGAGACACCATGCCCG

GCCTCTTTGCCTACAAAGCCGCCACCAAGGCCGGCTACTGTGGAGGAGCCGTTCTTGCCAAAGACGGAGC

TGACACGTTCATCGTCGGCACTCACTCCGCTGGAGGCAATGGAGTTGGTTACTGCTCATGCGTTTCCAGG

TCCATGCTACTCAAGATGAAGGCACACATCGACCCTGAGCCACACCACGAG

>AY593771.1_A_COL_1967

ATTTCAATTCCTTCCCAAAAATCCGTTTTGTACTTTCTCATTGAGAAAGGGCAACACGAGGCAGCAATTG

AATTCTTTGAGGGCATGGTGCATGACTCCATTAAGGAGGAGCTCCGGCCGCTCATCCAACGAACCTCATT

TGTGAAACGCGCCTTCAAGCGCCTGAAGGAAAACTTTGAGATTGTTGCCCTTTGCCTAACACTTTTGGCC

AACATTGTGATCATGATCCGCGAGACTCGCAAGAGACAGAAGATGGTGGATGATGCAGTGAATGAGTACA

TTGAGAAAGCAAACATCACCACCGATGACAAGACACTTGACGAGGCGGAAAAGAACCCTCTGGAGACCAG

CGGTGCCAGCACCGTTGGTTTCAGAGAGAGAAGTCTTCCAGGACAGAAGGCGCGCGATGACGTGAACTCT

GAGCCCGCCCAACCTGTTGAAGAACAACCACAAGCTGAAGGACCCTACGCCGGGCCACTCGAGCGTCAGA

GGCCCCTGAAAGTGAGAGCCAAGCTCCCGCAACAGGAGGGACCCTACGCTGGCCCGATGGAGAGACAGAA

ACCGCTGAAAGTGAAAGCAAAAGCCCCGGTCGTTAAGGAAGGACCCTACGAAGGACCGGTGAAGAAGCCT

GTCGCCTTGAAAGTGAAAGCTAAGAACTTGATTGTCACTGAGAGTGGTGCCCCTCCGACCGACTTGCAAA

AGATGGTCATGGGCAACACAAAGCCTGTTGAGCTCATCCTCGATGGGAAGACAGTAGCCATCTGCTGCGC

TACTGGAGTGTTTGGCACTGCTTACCTCGTGCCCCGTCATCTTTTCGCTGAGAAGTATGACAAGATCATG

TTGGACGGCAGAGCCATGACAGACAGTGACTACAGAGTGTTTGAGTTTGAGATTAAAGTAAAAGGACAGG

ACATGCTCTCAGACGCCGCACTCATGGTGCTCCACCGTGGGAACCGCGTGAGAGACATCACGAAGCATTT

TCGTGACACAGCAAGAATGAAGAAAGGCACCCCTGTTGTCGGCGTAATCAACAACGCCGATGTCGGGAGA

CTGATTTTCTCTGGTGAAGCCCTCACCTACAAAGACATTGTGGTGTGTATGGACGGAGACACCATGCCTG

GGCTGTTTGCCTACAGAGCTGCCACCAAGGCTGGCTATTGCGGAGGAGCCGTCCTTGCTAAGGACGGAGC

TGACACCTTCATCGTTGGCACTCACTCTGCAGGAGGCAATGGAGTTGGATACTGCTCATGCGTTTCCAGG

TCCATGCTTCTCAAAATGAAGGCACACATTGACCCTGAGCCACACCACGAG

>AY593772.1_A_TUR_1972

ATTTCAATTCCTTCCCAAAAGTCTGTGTTGTACTTCCTCATTGAGAAAGGCCAACACGAAGCAGCAATTG

AGTTCTTTGAGGGAATGGTACACGACTCCATCAAGGAGGAGCTCCGTCCCCTCATCCAGCGGACTTCATT

TGTGAAACGCGCTTTTAAGCGTCTGAAGGAAAACTTTGAGATTGTTGCCTTGTGTTTGACTCTTTTGGCA

AACATAGTGATCATGATCCGCGAGACTCGCAAGAGACAACAGATGGTGGACGATGCAGTGAATGAGTATA

TTGAGAAAGCAAACATCACCACAGATGACAAAACTCTTGACGAGGCGGAAAAGAACCCTTTGGAGACTAG

CGGTGCCAGCACCGTTGGATTCAGAGAGAGAACTCTCCCAAGACACAAGGCGAGTGATGACGTGAACTCC

GAGCCCGCCGGACCTGTGGAGGAACAACCACAAGCTGAAGGACCCTACGCCGGGCCACTCGAGCGTCAGA

AACCTCTGAAAGTGAAAGCCAAGCTGCCACAGCAGGAAGGACCTTACGCTGGCCCGATGGAGAGACAGAA

ACCACTGAAAGTGAAAGTGAAAGCCCCGGTTGTCAAGGAAGGACCATACGAGGGACCGGTGAAGAAGCCT

GTCGCTTTGAAAGTGAAAGCTAAGAATTTGATTGTCACTGAGAGTGGAGCCCCACCGACCGACTTGCAAA

AGATGGTCATGGGCAACACCAGGCCTGTTGAGCTCATTCTCGACGGGAAGACGGTGGCCATTTGTTGTGC

TACCGGAGTGTTTGGCACTGCCTATCTCGTGCCTCGTCATCTTTTTGCTGAAAAATATGACAAGATCATG

CTGGACGGCAGAGCCATGACAGACAGTGACTACAGAGTGTTTGAGTTTGAGATTAAAGTAAAAGGACAGG

ACATGCTCTCAGACGCTGCGCTCATGGTACTCCACCGTGGGAATCGCGTGAGAGACATCACGAAACACTT

TCGTGACACAGCAAGAATGAAGAAAGGCACCCCCGTTGTCGGAGTGATCAACAACGCCGACGTTGGGAGA

CTGATTTTCTCTGGTGAGGCCCTTACCTACAAGGACATTGTGGTGTGCATGGACGGAGACACCATGCCTG

GCCTGTTTGCCTACAAAGCCGCCACCAAGGCTGGCTACTGTGGGGGAGCCGTTCTTGCTAAGGACGGAGC

TGACACATTCATCGTTGGCACTCACTCCGCAGGTGGCAATGGAGTTGGATACTGCTCATGCGTTTCCAGG

TCCATGTTGCTGAAAATGAAGGCACACCTCGATCCCGAACCACACCACGAG

>AY593773.1_A_PER_1969

ATCTCAATTCCTTCTCAAAAATCTGTGCTGTACTTCCTCATTGAGAAAGGACAACACGAAGCAGCAATTG

AATTCTTTGAGGGCATGGTCCACGACTCCATCAAGGAGGAACTCCGACCCCTCATCCAACGAACTTCATT

TGTGAAACGCGCTTTCAAGCGCCTGAAGGAAAATTTTGAGATTGTTGCTCTGTGCCTAACACTTTTGGCA

AACATTGTGATCATGGTCCGCGAGACTCGCAAGAGGCAGAAAATGGTGGATGATGCAGTGAATGAGTACA

TTGAGAAAGCGAACATCACCACAGATGACAAAACTCTTGACGAGGCGGAGAAGAACCCTCTGGAGGCCAG

CGGCGCTAGCACCGTTGGCTTTAGAGAGAGAACCCCCCCAGGTCAAAAGGCATGTGATGACGTGAACCCC

GAGCCTGCCCAACCTGTTGAGGATCAACCACAAGCTGAAGGACCCTACGCCGGACCACTCGAGCGTCAGA

AACCTCTGAAAGTGAGAGCCAAGCTCCCACAGCAGGAGGGGCCTTACGCTGGTCCGATGGAGAGACAGAA

ACCACTGAAAGTGAAAGCAAAAGCCCCGGTCGTGAAGGAAGGACCCTACGAGGGACCGGTGAAGAAGCCT

GTCGCTTTGAAAGTGAAAGCTAAGAACTTGATTGTCACTGAGAGTGGTGCCCCACCGACCGACTTGCAAA

AGATGGTCATGGGCAACACAAAGCCTGTTGAGCTCATCCTCGACGGGAAGACAGTAGCCATCTGCTGCGC

TACTGGAGTGTTCGGCACTGCTTACCTCGTGCCTCGTCACCTCTTCGCAGAGAAGTATGACAAGATCATG

TTGGACGGCAGAGCCATGACAGACAGTGACTACAGAGTGTTTGAGTTTGAGATTAAAGTAAAAGGACAGG

ACATGCTCTCCGACGCTGCGCTCATGGTGCTCCACCGTGGGAATCGCGTGAGGGACATCACGAAGCACTT

TCGTGATACAGCAAGAATGAAGAAAGGCACCCCCGTTGTCGGTGTGATCAACAACGCTGATGTCGGGAGA

CTGATTTTCTCTGGTGAGGCCCTTACCTACAAGGACATTGTAGTTTGCATGGATGGGGACACCATGCCTG

GCCTCTTTGCCTACAAAGCTGCCACTAAGGCTGGCTACTGTGGAGGAGCCGTTCTTGCTAAAGATGGAGC

TAACACTTTCATCGTTGGCACTCACTCTGCAGGAGGCAATGGAGTTGGATACTGCTCATGCGTTTCCAGG

TCCATGCTTCTTAAAATGAAGGCACACGTCGACCCTGAACCACACCACGAG

>AY593774.1_A_SPA_1969

ATCTCAATTCCTTCCCAAAAATCTGTGTTGTACTTCCTCATTGAGAAAGGCCAACATGAGGCAGCAATTG

AATTCTTTGAGGGTATGGTGCACGATTCCATCAAGGAAGAGCTCCGGCCCCTCATCCAACAAACCTCATT

TGTGAAACGCGCTTTCAAGCGCCTGAAGGAAAATTTTGAGATTGTTGCTCTGTGCCTGACCCTTTTGGCT

AACATAGTGATCATGATCCGCGAAACTCGCAAGAGACAGAAGATGGTGGATGATGCCGTGAACAACTACA

TCGAGAAGGCAAACATCACCACAGATGACAAAACACTTGACGAGGCGGAAAAGAATCCTCTGGAGACCAG

TGGTGCCAGTACCGTTGGTTTCAGAGAGAAAACTCTGTCAGGCCACAAAGCGCGCGATGACGTGAACTCT

GAGCCCGCCCAGCCTGCTGAAGAGCAACCACACGCTGAAGGACCCTACGCCGGGCCACTCGAGCGTCAGA

AACCTCTGAAAGTGAGAGCCAAGCTCCCACAGCAGGAGGGACCCTACGCTGGCCCGATGGAGAGACAGAA

ACCGCTGAAAGTAAAAGCAAAAGCCCCGGTCGTTAAGGAAGGACCTTACGAGGGACCGGTGAAGAAGCCT

GTCGCTTTGAAAGTGAAAGCTAAGAACTTGATTGTCACTGAGAGTGGTGCCCCACCGACCGACTTGCAGA

AGATGGTCATGGGCAACACAAAGCCTGTTGAGCTTATCCTCGACGGGAAGACAGTAGCCATCTGCTGCGC

TACTGGAGTGTTTGGTACTGCTTACCTCGTGCCTCGTCATCTTTTCGCAGAGAAGTATGACAAGATCATG

TTGGACGGCAGAGCCATGACAGACAGTGATTACAGAGTGTTTGAGTTTGAGATTAAAGTAAAAGGACAGG

ACATGCTCTCAGACGCGGCACTCATGGTGCTTCACCGTGGGAACCGCGTGAGGGACATCACGAAACACTT

TCGTGATACAGCAAGAATGAAGAAAGGCACCCCCGTCGTCGGTGTTATCAACAACGCCGATGTTGGGAGA

CTGATTTTCTCTGGTGAGGCCCTTACCTACAAGGACATTGTAGTGTGCATGGATGGAGACACCATGCCCG

GCCTCTTTGCCTACAAAGCCGCCACCAAGGCTGGCTACTGTGGAGGAGCCGTTCTTGCCAAGGACGGGGC

TGACACATTCATCGTCGGCACTCACTCTGCAGGTGGTAATGGGGTTGGATATTGCTCATGCGTTTCCAGG

TCCATGCTTCAAAAGATGAAGGCTCACGTCGACCCTGAACCACACCACGAG

>AY593775.1_A_VEN_1970

ATCTCAATTCCTTCTCAAAAATCTGTGCTGTACTTCCTCATTGAGAAAGGACAACACGAAGCAGCAATTG

AATTCTTTGAGGGCATGGTCCACGACTCCATCAAGGAGGAACTCCGACCCCTCATCCAACGAACTTCATT

TGTGAAACGCGCTTTCAAGCGCCTGAAGGAAAATTTTGAGATTGTTGCTCTGTGCCTAACACTTTTGGCA

AACATTGTGATCATGGTCCGCGAGACTCGCAAGAGGCAGAAAATGGTGGATGATGCAGTGAATGAGTACA

TTGAGAAAGCGAACATCACCACAGATGACAAAACTCTTGACGAGGCGGAGAAGAACCCTCTGGAGGCCAG

CGGCGCTAGCACCGTTGGCTTTAGAGAGAGAACCCCCCCAGGTCAAAAGGCATGTGATGACGTGAACCCC

GAGCCTGCCCAACCTGTTGAGGATCAACCACAAGCTGAAGGACCCTACGCCGGACCGCTTGAGCGTCAGA

AACCTCTGAAAGTGAGAGCCAAGCTCCCACAGCAGGAGGGGCCTTACGCTGGTCCGATGGAGAGACAGAA

ACCACTGAAAGTGAAAGCAAAAGCCCCGGTCGTTAAGGAAGGGCCTTACGAGGGACCGGTGAAGAAGCCT

GTCGCTTTGAAAGTGAAAGCTAAGAACTTGATTGTCACTGAGAGTGGTGCCCCACCGACCGACTTGCAAA

AGATGGTCATGGGCAACACAAAGCCTGTTGAGCTCATCCTCGACGGGAAGACAGTAGCCATCTGCTGCGC

TACTGGAGTGTTCGGCACTGCTTACCTCGTGCCTCGTCACCTCTTCGCAGAGAAGTATGACAAGATCATG

TTGGACGGCAGAGCCATGACAGACAGTGACTACAGAGTGTTTGAGTTTGAGATTAAAGTAAAAGGACAGG

ACATGCTCTCCGACGCTGCGCTCATGGTGCTCCACCGTGGGAACCGCGTGAGGGACATCACGAAGCACTT

TCGTGATACAGCAAGAATGAAGAAAGGCACCCCCGTTGTCGGTGTGATCAACAACGCTGATGTCGGGAGA

CTGATTTTCTCTGGTGAGGCCCTTACCTACAAGGACATTGTAGTTTGCATGGACGGGGACACCATGCCTG

GCCTCTTTGCCTACAAAGCTGCCACTAAGGCTGGCTACTGCGGAGGGGCCGTTCTTGCTAAAGATGGAGC

TAACACTTTCATCGTTGGCACTCACTCTGCAGGAGGCAATGGAGTTGGATACTGCTCATGCGTTTCCAGG

TCCATGCTTCTTAAAATGAAGGCACACGTCGACCCTGAACCACACCACGAG

>AY593776.1_A_GER_1968

ATCTCAATTCCTTCCCAAAAATCTGTGTTGTACTTCCTCATTGAGAAAGGGCAGCACGAGGCAGCAATTG

AATTCTTTGAGGGCATGGTGCATGACTCCGTCAAGGAGGAGCTCCGGCCGCTTATCCAACAAACCTCATT

TGTGAAACGCGCTTTCAAGCGCCTGAAGGAAAACTTTGAGATTGTTGCTCTGTGCCTAACACTTTTGGCC

AACATTGTGATCATGATCCGCGAAACTCGCAAGAGACAGAAGATGGTGGACGATGCGGTGAACGAGTACA

TCGAGAAAGCAAACATCACCACCGATGACAAGACACTTGACGAGGCGGAAAAGAACCCTCTGGAGACTAG

CGGTGCCAGCACCGTTGGTTTCAGAGAGAGAACTCTTCCAGGTCAAAAGGCGCGTGATGACGTGAACTCC

GAGCCCGCCCAACCTGCTGAAGAACAACCACAAGCTGAAGGACCCTACGCCGGGCCACTTGAGCGTCAGA

GACCCCTGAAGGTGAGAGCCAAGCTCCCACAACAGGAAGGACCCTACGCTGGCCCGATGGAGAGACAGAA

ACCGCTGAAAGTGAAAGCAAAAGCCCCGGTCGTCAAGGAAGGACCTTATGAAGGACCGGTGAAGAGACCT

GTCGCTTTGAAAGTGAAAGCTAAGAATTTGATTGTCACTGAGAGTGGTGCCCCCCCGACCGACTTGCAAA

AGATGGTCATGGGCAACACAAAGCCTGTTGAGCTCATCCTCGACGGGAAGACAGTAGCCATCTGCTGTGC

TACTGGAGTGTTTGGCACTGCCTACCTCGTACCTCGTCATCTTTTCGCTGAGAAGTATGACAAGATCATG

TTGGACGGCAGAGCCATGACAGACAGTGACTACAGAGTGTTTGAGTTCGAGATCAAAGTAAAAGGACAGG

ACATGCTCTCAGACGCCGCACTCATGGTGCTCCACCGTGGGAACCGCGTGAGAGACATCACGAAGCACTT

TCGTGACACAGCAAGGATGAAGAAAGGCACCCCCGTTGTCGGCGTGATCAACAATGCCGATGTCGGGAGA

CTGATTTTCTCTGGCGAAGCCCTTACCTACAAAGACATTGTAGTGTGCATGGACGGAGACACCATGCCCG

GGCTTTTTGCCTACAGAGCCGCCACTAAGGCTGGCTACTGCGGGGGAGCCGTTCTCGCTAAGGACGGGGC

TGACACTTTCATCGTTGGCACTCACTCTGCAGGAGGTAATGGAGTTGGATACTGCTCATGCGTTTCCAGG

TCCATGCTTCTCAAGATGAAGGCACACATTGACCCTGAGCCGCACCACGAG

>AY593777.1_A_GER_1972

ATCTCAATTCCTTCCCAAAAATCTGTGTTGTACTTCCTCATTGAGAAAGGCCAACATGAGGCAGCAATTG

AATTCTTTGAGGGTATGGTGCACGATTCCATCAAGGAAGAGCTCCGGCCCCTCATCCAACAAACCTCATT

TGTGAAACGCGCTTTCAAGCGCCTGAAGGAAAATTTTGAGATTGTTGCTCTGTGCCTGACCCTTTTGGCT

AACATAGTGATCATGATCCGCGAAACTCGCAAGAGACAGAAGATGGTGGATGATGCCGTGAACGACTACA

TCGAGAAGGCAAACATCACCACAGATGACAAAACACTTGACGAGGCGGAAAAGAATCCTCTGGAGACCAG

TGGTGCCAGTACCGTTGGTTTCAGAGAGAAAACTCTGTCAGGCCACAAAGCGCGCGATGACGTGAACTCT

GAGCCCGCCCAGCCTGCTGAAGAGCAACCACACGCTGAAGGACCCTACGCCGGGCCACTCGAGCGTCAGA

AACCTCTGAAAGTGAGAGCCAAGCTCCCACAGCAGGAGGGACCCTACGCTGGCCCGATGGAGAGACAGAA

ACCGCTGAAAGTAAAAGCAAAAGCCCCGGTCGTTAAGGAAGGACCTTACGAGGGACCGGTGAAGAAGCCT

GTCGCTTTGAAAGTGAAAGCTAAGAACTTGATTGTCACTGAGAGTGGTGCCCCACCGACCGACTTGCAGA

AGATGGTCATGGGCAACACAAAGCCTGTTGAGCTTATCCTCGACGGGAAGACAGTAGCCATCTGCTGTGC

TACTGGAGTGTTTGGTACTGCTTACCTCGTGCCTCGTCATCTTTTCGCAGAGAAGTATGACAAGATCATG

TTGGACGGCAGAGCCATGACAGACAGTGATTACAGAGTGTTTGAGTTTGAGATTAAAGTAAAAGGACAGG

ACATGCTCTCAGACGCGGCACTCATGGTGCTTCACCGTGGGAACCGCGTGAGGGACATCACGAAACACTT

TCGTGATACAGCAAGAATGAAGAAAGGCACCCCCGTCGTCGGTGTTATCAACAACGCCGATGTTGGGAGA

CTGATTTTCTCTGGTGAGGCCCTTACCTACAAGGACATTGTAGTGTGCATGGATGGAGACACCATGCCCG

GCCTCTTTGCCTACAAAGCCGCCACCAAGGCTGGCTACTGTGGAGGAGCCGTTCTTGCCAAGGACGGGGC

TGACACATTCATCGTCGGCACTCACTCTGCAGGTGGTAATGGGGTTGGATATTGCTCATGCGTTTCCAGG

TCCATGCTTCAAAAGATGAAGGCTCACGTCGACCCTGAACCACACCACGAG

>AY593778.1_A_SPA_1969

ATCTCAATTCCTTCCCAAAAATCTGTGTTGTACTTCCTCATTGAGAAAGGGCAGCACGAGGCAGCAATTG

AATTCTTTGAGGGCATGGTGCATGACTCCGTCAAGGAGGAGCTCCGGCCGCTCATCCAACAAACCTCATT

TGTGAAACGCGCGTTTAAGCGCCTGAAGGAAAACTTTGAGATTGTTGCTCTGTGCCTAACACTTTTGGCC

AACATCGTGATCATGATCCGCGAGACCCGTAAGAGACAGAAGATGGTGGACGATGCGGTGAACGAGTACA

TCGAGAAAGCAAACATCACCACCGATGACAAGACTCTTGACGAGGCGGAAAAGAACCCTCTGGAGACTAG

CGGTGCCAGTACCGTTGGTTTCAGAGAGAGAACTCTTCCAGATCAGAAGGCGCGTAATGACGTGAACTCC

GAGCCCGCCCAACCTGCTGAAGAACAACCACAAGCTGAAGGACCCTACGCCGGGCCACTTGAGCGTCAGA

GACCCCTGAAGGTGAGAACCAAGCTCCCACAACAGGAAGGACCCTACGCTGGCCCGATGGAGAGACAGAA

ACCGCTGAAAGTGAAAGCAAAAGCCCCGGTCGTCAAGGAAGGACCTTACGAAGGACCGGTGAAGAAACCT

GTCGCTTTGAAAGTGAAAGCTAAGAATTTGATTGTCACTGAGAGTGGTGCCCCCCCGACCGACCTGCAAA

AGATGGTCATGGGCAACACAAAGCCCGTTGAGCTCATCCTCGACGGGAAGACAGTAGCCATCTGCTGTGC

TACTGGAGTGTTTGGTACTGCCTACCTCGTGCCTCGTCATCTTTTCGCTGAGAAGTATGACAAGATCATG

TTGGACGGCAGAGCCATGACAGACAGTGACTACAGGGTGTTTGAGTTCGAGATCAGAGTAAAAGGACAGG

ACATGCTCTCAGACGCCGCACTCATGGTGCTCCACCGTGGGAACCGCGTGAGAGACATCACGAAGCATTT

CCGTGACACAGCAAGAATGAAGAAAGGCACCCCCATTGTCGGCGTGATTAACAATGCCGATGTCGGGAGG

CTGATTTTCTCTGGCGAAGCTCTTACCTACAAAGACATTGTAGTGTGCATGGACGGAGACACCATGCCCG

GGCTTTTTGCCTACAGAGCCGCCACTAAGGCTGGCTACTGCGGGGGAGCCGTTCTCGCTAAGGACGGGGC

TGACACTTTTATCGTTGGCACTCACTCTGCAGGAGGTAACGGAGTTGGATACTGCTCATGCGTTTCCAGG

TCCATGCTTCTCAAGATGAAGGCACACATTGACCCTGAGCCGCACCACGAG

>AY593779.1_A_GER_1972

ATCTCAATTCCTTCCCAAAAATCTGTGTTGTACTTCCTCATTGAGAAAGGCCAACATGAGGCAGCAATTG

AATTCTTTGAGGGTATGGTGCACGATTCCATCAAGGAAGAGCTCCGGCCCCTCATCCAACAAACCTCATT

TGTGAAACGCGCTTTCAAGCGCCTGAAGGAAAATTTTGAGATTGTTGCTCTGTGCCTGACCCTTTTGGCT

AACATAGTGATCATGATCCGCGAAACTCGCAAGAGACAGAAGATGGTGGATGATGCCGTGAACGACTACA

TCGAGAAGGCAAACATCACCACAGATGACAAAACACTTGACGAGGCGGAAAAGAATCCTCTGGAGACCAG

TGGTGCCAGTACCGTTGGTTTCAGAGAGAAAACTCTGTCAGGCCACAAAGCGCGCGATGACGTGAACTCT

GAGCCCGCCCAGCCTGCTGAAGAGCAACCACACGCTGAAGGACCCTACGCCGGGCCACTCGAGCGTCAGA

AACCTCTGAAAGTGAGAGCCAAGCTCCCACAGCAGGAGGGACCCTACGCTGGCCCGATGGAGAGACAGAA

ACCGCTGAAAGTAAAAGCAAAAGCCCCGGTCGTTAAGGAAGGACCTTACGAGGGACCGGTGAAGAAGCCT

GTCGCTTTGAAAGTGAAAGCTAAGAACTTGATTGTCACTGAGAGTGGTGCCCCACCGACCGACTTGCAGA

AGATGGTCATGGGCAACACAAAGCCTGTTGAGCTTATCCTCGACGGGAAGACAGTAGCCATCTGCTGTGC

TACTGGAGTGTTTGGTACTGCTTACCTCGTGCCTCGTCATCTTTTCGCAGAGAAGTATGACAAGATCATG

TTGGACGGCAGAGCCATGACAGACAGTGATTACAGAGTGTTTGAGTTTGAGATTAAAGTAAAAGGACAGG

ACATGCTCTCAGACGCGGCACTCATGGTGCTTCACCGTGGGAACCGCGTGAGGGACATCACGAAACACTT

TCGTGATACAGCAAGAATGAAGAAAGGCACCCCCGTCGTCGGTGTTATCAACAACGCCGATGTTGGGAGA

CTGATTTTCTCTGGTGAGGCCCTTACCTACAAGGACATTGTAGTGTGCATGGATGGAGACACCATGCCCG

GCCTCTTTGCCTACAAAGCCGCCACCAAGGCTGGCTACTGTGGAGGAGCCGTTCTTGCCAAGGACGGGGC

TGACACATTCATCGTCGGCACTCACTCTGCAGGTGGTAATGGGGTTGGATATTGCTCATGCGTTTCCAGG

TCCATGCTTCAAAAGATGAAGGCTCACGTCGACCCTGAACCACACCACGAG

>AY593780.1_A_FRA_1960

ATCTCAATTCCTTCCCAAAAATCTGTGTTGTACTTCCTCATTGAGAAAGGGCAGCACGAGGCAGCAATTG

AATTCTTTGAGGGCATGGTGCATGACTCCGTCAAGGAGGAGCTCCGGCCGCTCATCCAACAAACCTCATT

TGTGAAACGCGCTTTCAAGCGCCTGAAGGAAAACTTTGAGATTGTTGCTCTGTGCCTAACACTTTTGGCC

AACATTGTGATCATGATCCGCGAGACCCGCAAGAGACAGAAGATGGTGGACGATGCGGTGAACGAGTACA

TCGAGAAAGCAAACATCACCACCGATGACAAGACACTTGACGAGGCGGAAAAGAACCCTCTGGAGACTAG

CGGTGCCAGCACCGTTGGTTTCAGAGAGAGAACTCTTCCAGGTCAGAAGGCGCGTAATGACGTGAATTCC

GAGCCCGCCCAACCTGCTGAAGAACAACCACAAGCTGAAGGACCCTACGCCGGGCCACTTGAGCGTCAGA

GACCCCTGAAGGTGAGAGCCAAGCTCCCACAACAGGAAGGACCCTACGCTGGCCCGATGGAGAGACAGAA

ACCGCTGAAAGTGAAAGCAAAAGCCCCGGTCGTCAAGGAAGGACCTTACGAAGGACCGGTGAAGAAACCT

GTCGCTTTGAAAGTGAAAGCTAAGAATTTGATTGTCACTGAGAGTGGTGCCCCCCCGACCGACTTGCAAA

AGATGGTCATGGGCAACACAAAGCCTGTTGAGCTCATTCTCGACGGGAAGACAGTAGCCATCTGCTGTGC

TACTGGAGTGTTTGGCACTGCCTACCTCGTGCCTCGTCATCTTTTCGCTGAGAAGTATGACAAGATCATG

TTGGACGGCAGAGCCATGACAGACAGTGACTACAGAGTGTTTGAGTTCGAGATCAAAGTAAAAGGACAGG

ACATGCTCTCAGACGCCGCACTCATGGTGCTCCACCGTGGGAACCGCGTGAGAGACATCACGAAGCATTT

CCGTGACACAGCAAGAATGAAGAAAGGCACCCCCATTGTCGGCGTGATCAACAATGCCGATGTCGGGAGA

CTGATTTTCTCTGGCGAAGCTCTTACCTACAAAGACATTGTAGTGTGCATGGACGGAGATACCATGCCCG

GGCTTTTTGCCTACAGAGCCGCCACTAAGGCTGGCTACTGCGGGGGAGCCGTTCTCGCTAAGGACGGGGC

TGACACTTTCATCGTTGGCACTCACTCTGCAGGAGGTAATGGAGTTGGATACTGCTCATGCGTTTCCAGG

TCCATGCTTCTCAAGATGAAGGCACACATTGACCCTGAGCCGCACCACGAG

>AY593781.1_A_GER_1951

ATCTCAATTCCTTCCCAAAAATCTGTGTTGTACTTCCTCATTAAGAAAGGGCAGCACGAGGCAGCAATTG

AATTCTTTGAGGGCATGGTGCATGACTCCGTCAAGGAGGAGCTCCGGCCGCTCATCCAACAAACCTCATT

TGTGAAACGCGCTTTCAAGCGCCTGAAGGAAAACTTTGAGATTGTTGCTCTGTGCCTAACACTTTTGGCC

AACATTGTGATCATGATCCGCGAAACCCGCAAGAGACAGAAGATGGTGGACGATGCGGTGAACGAGTACA

TCGAGAAAGCAAACATCACCACCGATGACAAGACACTTGACGAGGCGGAAAAGAACCCTCTGGAGACTAG

CGGTGCCAGCACCGTTGGTTTCAGAGAGAGAACTCTTCCAGGTCAGAAGGCGCGTGATGACGTGAACTCC

GAGCCCGCCCAACCTGCTGAAGAACAACCACAAGCTGAAGGACCCTACGCCGGGCCACTTGAGCGTCAGA

GACCCCTGAAGGTGAGAGCCAAGCTCCCACAACAGGAAGGACCCTACGCTGGCCCGATGGAGAGACAGAA

ACCGCTGAAAGTGAAAGCAAAAGCCCCGGTCGTCAAGGAAGGACCTTACGAAGGACCGGTGAAGAAACCT

GTCGCTTTGAAAGTGAGAGCTAAGAATTTGATTGTCACTGAGAGTGGTGCCCCCCCGACCGACTTGCAAA

AGATGGTCATGGGCAACACAAAGCCTGTTGAGCTCATCCTCGACGGGAAGACAGTAGCCATCTGCTGTGC

TACTGGAGTGTTTGGCACTGCCTACCTCGTGCCTCGTCATCTTTTCGCTGAGAAGTATGACAAGATCATG

TTGGACGGCAGAGCCATGACAGACAGTGACTACAGAGTGTTTGAGTTCGAGATCAAAGTAAAAGGACAGG

ACATGCTCTCAGACGCCGCACTCATGGTGCTCCACCGTGGGAACCGCGTGAGAGACATCACGAAGCACTT

TCGTGACACAGCAAGAATGAAGAAAGGCACCCCCGTTGTCGGCGTGATCAACAATGCCGATGTCGGGAGA

CTGATTTTCTCTGGCGAAGCCCTTACCTACAAAGACATTGTAGTGTGCATGGACGGAGACACCATGCCCG

GGCTTTTTGCCTACAGAGCCGCCACTAAGGCAGGCTACTGCGGGGGAGCCGTTCTCGCTAAGGACGGGGC

TGACACTTTCATCGTTGGCACTCACTCTGCAGGAGGTAATGGAGTTGGATACTGCTCATGCGTTTCCAGG

TCCATGCTTCTCAAGATGAAGGCACACATTGACCCTGAGCCGCACCACGAG

>AY593782.1_A_ARG_2000

ATCTCAATTCCTTCCCAAAAGTCTGTGTTGTACTTCCTCATTGAGAAAGGACAACATGAGGCAGCAATTG

AATTCTTTGAGGGGATGGTACACGACTCCATCAAGGAGGAGCTCCGACCTCTCATCCAACAAACCTCATT

TGTGAAACGCGCCTTCAAGCGCTTGAAGGAAAATTTTGAGATGGTTGCTTTGTGCCTGACTCTTCTGGCC

AACATAGTGATCATGATCCGCGAGACTCGCAAGAGACAAAAGATGGTGGATGACGCAGTGGGTGAGTACA

TTGAGAAGACAAACATCACCACCGACGACAAGACTCTTGACGAGGCGGAAAAGAACCCTCTGGAGACTAG

TGGTGCCAGCACCGTTGGTTTCAGAGAGAGAACTCTCCCAGGGCAGAAAGCGAGTGATGACGTGAACACT

GAACCCGCCCAGCCTGCCGGAGAGGAACCAAAAGCTGAAGGACCCTACAGCGGGCCACTTGAGCGTCAGA

AACCTCTGAAAGTGAGAGCCAAATTCCCACAGCAAGAGGGGCCCTACGCTGGTCCGTTGGAGAGACAGAA

ACCACTGAAAGTGAAAGCAAAAGCCCCGGTCGTTAAGGAAGGACCTTACGAAGGGCCGGTGAAGAAACCT

GTCGCTTTGAAAGTGAAAGCCAAGAACTTGATAGTTACTGAGAGTGGTGCCCCCCCGACCGACTTGCAAA

AGATGGTCATGGGCAACACAAAGCCTGTTGAGCTCATCCTTGACGGGAAGACAGTGGCCATCTGCTGTGC

TACTGGAGTGTTTGGTACTGCTTACCTCGTGCCTCGTCATCTTTTCGCAGAGAAGTATGACAAGATCATG

TTGGGCGGTAGAGCCATGACAGACAGTGACTACAGAGTGTTTGAGTTTGAGATTAAAGTAAAAGGACAGG

ACATGCTCTCAGACGCCGCACTCATGGTGCTTCACCGTGGGAACCGCGTGAGAGACATCACGAAACACTT

TCGTGATACTGCAAGAATGAAGAAAGGAACCCCCGTTGTTGGCGTGATCAACAACGCCGACGTCGGGAGA

CTGATTTTCTCTGGTGAGGCCCTTACCTACAAGGACATTGTAGTGTGCATGGACGGAGACACCATGCCCG

GGCTTTTTGCCTACAGAGCTGCAACCAAGGCAGGTTACTGCGGAGGAGCCGTTCTGGCCAAGGACGGCGC

TGACACGTTCATCGTCGGCACTCACTCCGCTGGAGGCAATGGAGTTGGATACTGCTCGTGCGTTTCCAGG

TCCATGCTTCTCAAGATGAAGGCACACATCGACCCTGAACCACACCACGAG

>AY593784.1_A_ARG_2001

ATCTCAATTCCTTCCCAAAAATCTGTGTTGTACTTTCTCATTGAGAAAGGGCAGCACGAAGCAGCAATTG

ATTTCTTTGAGGGCATGGTGCATGACTCCATCAAGGAGGAACTCCGGCCCCTCATCCAACAAACCTCATT

TGTGAAACGCGCTTTCAAACGCTTGAAGGAGAACTTTGAGATTGTTGCCCTATGTTTGACCCTGCTGGCC

AACATAGTGATCATGATCCGCGAGACTCGCAAGAGACAGAAGATGGTGGACGATGCAGTGAACGAGTACA

TCGAGAAGGCAAACATCACCACCGACGACAAGACTCTTGACGAGGCGGAAAAGAACCCTCTGGAGACTAG

CGGTGCCAGTACCGTTGGTTTCAGAGAGAAAACTCTCCCAGGTCACAACGCGCGTGATGACGTGAACTCC

GAGCCCGCCCAACGTGACACAGAGCAACCACAAGCTGAAGGACCCTACGCCGGGCCACTCGAGCGTCAGA

AACCTCTGAAAGTGAGAGCCAAGCTACCACAGCAGGAGGGACCTTACGCTGGCCCGATGGAGAGACAGAA

ACCACTGAAAGTGAAAGCAAAAGCCCCGGTCGTTAAGGAAGGGCCGTACGAAGGACCGGTCAAGAAACCT

GTCGCTTTGAAAGTGAAAGCTAAGAACTTGATTGTCACTGAGAGTGGTGCCCCACCGACCGACCTGCAGA

AGATGGTCATGGGCAACACAAAGCCTGTTGAGCTTATCCTCGACGGGAAGACAGTAGCCATCTGTTGCGC

TACTGGAGTGTTCGGTACTGCCTACCTCGTGCCTCGTCATCTTTTCGCTGAGAAGTACGACAAGATCATG

CTGGACGGCAGAGCCCTGACAGACAGTGACTACAGAGTGTTTGAGTTTGAGATTAAAGTAAAAGGACAGG

ACATGCTCTCAGACGCTGCGCTCATGGTGCTTCACCGTGGGAACCGCGTGAGAGACATCACGAAACACTT

TCGTGACACAGCTAGACTGAAGAAAGGCACCCCCGTTGTTGGCGTTATTAACAATGCTGACGTCGGGAGA

CTGATCTTCTCTGGTGAGGCCCTTACCTACAAGGACATTGTAGTGTGCATGGACGGAGATACCATGCCAG

GGCTTTTTGCCTACAAAGCCGCAACTAAGGCTGGCTATTGCGGAGGGGCCGTTCTCGCAAAGGACGGAGC

CGACACTTTCATCGTTGGCACCCACTCTGCTGGAGGCAATGGAGTTGGTTACTGCTCATGCGTTTCCAGG

TCCATGCTCCAAAAGATGAAGGCACACGTCGACCCGGAGCCACACCACGAA

>AY593785.1_A_ARG_2001

ATCTCAATTCCTTCCCAAAAATCTGTGTTGTACTTTCTCATTGAGAAAGGGCAGCACGAAGCAGCAATTG

ATTTCTTTGAGGGCATGGTGCATGACTCCATCAAGGAGGAACTCCGGCCCCTCATCCAACAAACCTCATT

TGTGAAACGCGCTTTCAAACGCTTGAAGGAGAACTTTGAGATTGTTGCCCTATGTTTGACCCTGCTGGCC

AACATAGTGATCATGATCCGCGAGACTCGCAAGAGACAGAAGATGGTGGACGATGCAGTGAACGAGTACA

TCGAGAAGGCAAACATCACCACCGACGACAAGACTCTTGACGAGGCGGAAAAGAACCCTCTGGAGACTAG

CGGTGCCAGTACCGTTGGTTTCAGAGAGAAAACTCTCCCAGGTCACAACGCGCGTGATGACGTGAACTCC

GAGCCCGCCCAACGTGACACAGAGCAACCACAAGCTGAAGGACCCTACGCCGGGCCACTCGAGCGTCAGA

AACCTCTGAAAGTGAGAGCCAAGCTACCACAGCAGGAGGGACCTTACGCTGGCCCGATGGAGAGACAGAA

ACCACTGAAAGTGAAAGCAAAAGCCCCGGTCGTTAAGGAAGGGCCGTACGAAGGACCGGTCAAGAAACCT

GTCGCTTTGAAAGTGAAAGCTAAGAACTTGATTGTCACTGAGAGTGGTGCCCCACCGACCGACCTGCAGA

AGATGGTCATGGGCAACACAAAGCCTGTTGAGCTTATCCTCGACGGGAAGACAGTAGCCATCTGTTGCGC

TACTGGAGTGTTCGGTACTGCCTACCTCGTGCCTCGTCATCTTTTCGCTGAGAAGTACGACAAGATCATG

CTGGACGGCAGAGCCCTGACAGACAGTGACTACAGAGTGTTTGAGTTTGAGATTAAAGTAAAAGGACAGG

ACATGCTCTCAGACGCTGCGCTCATGGTGCTTCACCGTGGGAACCGCGTGAGAGACATCACGAAACACTT

TCGTGACACAGCTAGACTGAAGAAAGGCACCCCCGTTGTTGGCGTTATTAACAATGCTGACGTCGGGAGA

CTGATCTTCTCTGGTGAGGCCCTTACCTACAAGGACATTGTAGTGTGCATGGACGGAGATACCATGCCAG

GGCTTTTTGCCTACAAAGCCGCAACTAAGGCTGGCTATTGCGGAGGGGCCGTTCTCGCAAAGGACGGAGC

CGACACTTTCATCGTTGGCACCCACTCTGCTGGAGGCAATGGAGTTGGTTACTGCTCATGCGTTTCCAGG

TCCATGCTCCAAAAGATGAAGGCACACGTCGACCCGGAGCCACACCACGAA

>AY593786.1_A_ARG_2001

ATCTCAATTCCTTCCCAAAAATCTGTGTTGTACTTTCTCATTGAGAAAGGGCAGCACGAAGCAGCAATTG

ATTTCTTTGAGGGCATGGTGCATGACTCCATCAAGGAGGAACTCCGGCCCCTCATCCAACAAACCTCATT

TGTGAAACGCGCTTTTAAACGCTTGAAGGAGAACTTTGAGATTGTTGCCCTATGTTTGACCCTGCTGGCC

AACATAGTGATCATGATCCGCGAGACTCGCAAGAGACAGAAGATGGTGGATGATGCAGTGAACGAGTACA

TCGAGAAGGCAAACATCACCACCGACGACAAGACTCTTGACGAGGCGGAAAAGAACCCTCTGGAGACTAG

CGGTGCCAGTACCGTTGGTTTCAGAGAGAAAACTCTCCCAGGTCACAACGCGCGTGATGACGTGAAATCC

GAGCCCGCCCAACGTGACACAGAGCAACCACAAGCTGAGGGACCCTACGCCGGGCCACTCGAGCGTCAGA

AACCTCTGAAAGTGAGAGCCAAGCTACCACAGCAGGAGGGACCTTACGCTGGCCCGATGGAGAGACAGAA

ACCACTGAAAGTGAAAGCAAAAGCCCCGGTCGTCAAGGAAGGACCGTACGAAGGACCGGTCAAGAAACCT

GTCGCTTTGAAAGTGAAAGCTAAGAACTTGATTGTCACTGAGAGTGGTGCCCCACCGACCGACCTGCAGA

AGATGGTCATGGGCAACACAAAGCCTGTTGAGCTTATCCTCGACGGGAAGACAGTAGCCATCTGCTGCGC

TACTGGAGTGTTCGGCACTGCCTACCTCGTGCCTCGTCATCTTTTCGCTGAGAAGTACGACAAGATCATG

CTGGACGGCAGAGCCCTGACAGACAGTGACTACAGAGTGTTTGAGTTTGAGATTAAAGTAAAAGGACAGG

ACATGCTCTCAGACGCTGCGCTCATGGTGCTTCACCGTGGGAACCGCGTGAGAGACATCACGAAACACTT

TCGTGACACAGCTAGAATGAAGAAAGGCACCCCCGTTGTTGGCGTTATCAACAATGCTGACGTCGGGAGA

CTGATCTTCTCTGGTGAGGCCCTTACTTACAAGGACATTGTAGTGTGCATGGACGGAGACACCATGCCAG

GGCTTTTTGCCTACAAAGCCGCAACTAAGGCTGGCTATTGCGGAGGGGCCGTTCTCGCAAAGGACGGAGC

CGACACTTTCATCGTTGGCACCCACTCTGCTGGAGGCAATGGAGTTGGTTACTGCTCATGCGTTTCCAGG

TCCATGCTCCAAAAGATGAAGGCACACGTCGACCCGGAGCCACACCACGAA

>AY593787.1_A_Brazil_1977

ATCTCAATTCCTTCCCAAAAATCCGTGCTGTACTTCCTCATTGAGAAAGGGCAACACGAGGCTGCAATTG

AATTCTTTGAGGGAATGGTGCATGACTCCATCAAGGAGGAACTCCGGCCCCTTATACAACAAACTTCATT

TGTGAAACGCGCTTTTAAGCGCCTGAAGGAAAATTTTGAGATTGTTGCCCTATGTTTGACCCTTCTGGCC

AACATAGTGATCATGATCCGCGAGACACGCAAGAGACAGCAGATGGTGGACGAAGCAGTCAATGAATACA

TTGAGAAAGCAAACATCACCACCGACGACAAGACTCTTGATGAGGCGGAAAAGAACCCTCTAGAGACCAG

CGGTGCCAGCACGGTTGGTTTCAGAGAGAGAACTCTTCCAGGTCAAAAGGCGCGCGATGACGTGAACTCC

GAGCCCGCCAAACCTGCTGAGGAGCAACCACAAGCTGAAGGACCCTACGCCGGGCCACTCGAGCGTCAGA

AACCTCTGAAAGTGAGAGCTAAGCTCCCACAGCAGGAGGGACCCTACGCTGGCCCGATGGAGAGACAGAA

ACCGCTTAAAGTGAAAGCAAAAGCCCCCGTCGTCAAGGAAGGACCTTACGAGGGGCCGGTGAAGAAGCCT

GTCGCTTTGAAAGTGAAAGCCAAGAATTTGATTGTCACTGAGAGTGGTGCCCCACCGACTGATCTTCAAA

AGATGGTCATGGGTAACACCAAGCCCGTTGAGCTCATCCTTGACGGGAAGACAGTAGCCATCTGCTGTGC

TACTGGAGTGTTTGGCACTGCTTACCTCGTGCCTCGTCACCTTTTTGCAGAAAAGTACGACAAGATCATG

TTGGACGGCAGAGCCATGACAGACAGTGACTACAGAGTGTTTGAGTTTGAGATTAAAGTAAAAGGACAGG

ACATGCTCTCAGACGCTGCGCTCATGGTGCTTCACCGCGGGAACCGCGTGAGAGACATCACGAAACACTT

TCGTGATACAGCAAGAATGAAGAAAGGCACCCCCGTCGTTGGTGTGATCAACAACGCTGATGTCGGGAGA

CTGATTTTCTCTGGTGAGGCTCTTACCTACAAGGATATTGTAGTGTGCATGGATGGAGACACCATGCCTG

GCCTCTTTGCCTACAAAGCTGCAACCAAAGCAGGGTACTGCGGAGGAGCCGTTCTGGCCAAGGACGGGGC

TGACACGTTCATCGTCGGCACTCACTCCGCTGGAGGCAATGGAGTTGGATACTGCTCGTGCGTTTCCAGG

TCCATGCTTCTCAAAATGAAGGCACACATTGACCCCGAACCACACCACGAG

>AY593788.1_A_Brazil_1979

ATCTCAATTCCTTCCCAAAAATCCGTGCTGTACTTCCTCATTGAGAAAGGGCAGCACGAGGCTGCTATTG

AATTCTTTGAGGGAATGGTGCATGACTCCATCAAGGAGGAACTCCGGCCCCTTATACAACAAACTTCATT

TGTGAAACGCGCTTTTAAGCGCCTGAAGGAAAATTTTGAGATTGTTGCCCTATGTTTGACCCTTCTGGCC

AACATAGTGATCATGATCCGCGAGACACGCAAGAGACAGCAGATGGTGGACGAAGCAGTCAATGAATACA

TTGAGAAAGCAAACATCACCACCGACGACAAGACTCTTGATGAAGCGGAAAAGAACCCTCTAGAGACCAG

TGGTGCCAGCACGGTTGGTTTCAGAGAGAGAACTCTTCCAGGTCAAAAGGCGCGCGACGACGTGAACTCT

GAGCCCGCCAAACCTGTTGAGGGGCAACCACAAGCTGAAGGACCCTACGCCGGGCCACTCGAGCGTCAGA

AACCTCTGAAAGTGAGAGCTAAGCTCCCACAGCAGGAGGGGCCCTACGCTGGCCCGATGGAGAGACAGAA

ACCGCTTAAAGTGAAAACAAAAGCCCCCGTCGTCAAGGAAGGACCTTATGAGGGGCCGGTGAAGAAGCCT

GTCGCTTTGAAAGTGAAGGCCAAGAATCTGATTGTCACTGAGAGTGGTGCTCCACCGACTGATCTTCAAA

AGATGGTCATGGGTAACACTAAGCCCGTTGAGCTCATCCTTGACGGGAAGACAGTAGCTATCTGCTGTGC

TACTGGAGTGTTTGGCACTGCTTACCTCGTGCCTCGTCACCTTTTTGCAGAAAAGTATGACAAGATCATG

TTGGACGGCAGAGCCATGACAGACAGTGACTACAGAGTGTTTGAGTTTGAGATTAAAGTAAAAGGACAGG

ACATGCTCTCAGACGCTGCGCTCATGGTGCTTCACCGCGGGAACCGCGTGAGAGACATCACGAAACATTT

TCGTGATACAGCAAGAATGAAGAAAGGCACCCCCGTCGTTGGTGTGATCAACAACGCTGATGTCGGGAGA

CTGATCTTCTCTGGTGAGGCTCTTACCTACAAGGATATTGTAGTGTGCATGGATGGAGACACCATGCCTG

GCCTCTTTGCCTACAAAGCTGCAACCAAAGCAGGGTACTGCGGAGGAGCCGTTCTGGCCAAGGACGGGGC

TGACACGTTCATCGTCGGCACTCACTCCGCTGGAGGCAATGGAGTTGGATACTGCTCGTGCGTTTCCAGG

TCCATGCTTCTCAAAATGAAGGCACACATTGACCCCGAACCACACCACGAG

>AY593789.1_A_ARG_1961

ATCTCAATTCCTTCCCAAAAATCCGTGTTGTACTTCCTCATTGAGAAAGGTCAGCACGAAGCAGCAATTG

AATTCTTTGAGGGAATGGTGCATGACTCCATCAAGGAGGAGCTCCGGCCCCTCATTCAACAGACCTCATT

TGTGAAGCGCGCTTTCAAGCGCCTGAAGGAGAACTTTGAGATTGTTGCCCTGTGTTTGACCCTTTTGGCC

AACATAGTGATCATGATCCGCGAGACTCACAAGAGACAGAAAATGGTGGATGATGCAGTGAATGACTACA

TTGAGAAAGCAAACATCACCACAGATGACAAGACTCTTGACGAGGCGGAAAAGAACCCTCTGGAGACCAG

CGGAGCCAGTACCGTTGGCTTCAGAGAGAGAACTCTCACAGGGCACAAGATGTGCGATGACGTGAACTCC

GAGCCCATCCAACCT---GAAGAGCAACCACAAGCTGAAGGACCCTACGCCGGGCCACTCGAGCGTCAGA

AACCCCTGAAAGTGAGAGCCAAGCTCCCGCAGCAGGAGGGACCTTACGCTGGCCCGATGGAGAGACAGAA

ACCGCTGAAGGTAAAAGTGAAAGCCCCGGTCGTTAAGGAAGGACCTTACGAGGGACCGGTGAGGAAGCCT

GTCGCTTTGAAAGTGAAAGCTAAAAACTTGATAGTCACTGAGAGTGGTGCCCCACCTACTGACTTGCAAA

AGATGGTCATGGGCAACACAAAGCCTGTTGAGCTCATCCTCGACGGGAAGACAGTAGCCATCTGCTGTGC

TACTGGAGTGTTTGGTACTGCCTACCTCGTGCCTCGTCACCTCTTCGCAGAGAAGTATGACAAGATCATG

CTGGATGGTAGAGCCATGACAGACAGTGACTACAGAGTGTTTGAGTTTGAGATTAAAGTAAAAGGACAGG

ACATGCTCTCAGACGCCGCGCTCATGGTGCTCCACCGTGGGAACCGCGTGAGAGACATCACGAAACACTT

TCGTGATACAGCAAGAATGAAGAAAGGAACCCCCGTCGTTGGCGTGATCAACAACGCCGATGTTGGGAGA

CTGATTTTCTCTGGTGAGGCCCTTACCTACAAAGACATTGTGGTGTGCATGGATGGAGACACCATGCCTG

GCCTTTTCGCCTACAAAGCCGCCACCAAGGCTGGCTACTGCGGAGGAGCCGTTCTTGCCAAGGACGGGGC

CGACACTTTCATCGTTGGCACTCACTCCGCAGGAGGTAATGGAGTTGGATACTGCTCGTGCGTTTCCAGG

TCCATGCTCCTCAAGATGAAGGCACACATCGACCCCGAACCACACCACGAG

>AY593790.1_A_ARG_2001

ATCTCAATTCCTTCCCAAAAATCTGTGTTGTACTTTCTCATTGAGAAAGGGCAGCACGAAGCAGCAATTG

ATTTCTTTGAGGGCATGGTGCATGACTCCATCAAGGAGGAACTCCGGCCCCTCATCCAACAAACCTCATT

TGTGAAACGCGCTTTTAAACGCTTGAAGGAGAACTTTGAGATTGTTGCCCTATGTTTGACCCTGCTGGCC

AACATAGTGATCATGATCCGCGAGACTCGCAAGAGACAGAAGATGGTGGACGATGCAGTGAACGAGTACA

TCGAGAAGGCAAACATCACCACCGACGACAAGACTCTTGACGAGGCGGAAAAGAACCCTCTGGAGACTAG

CGGTGCCAGTACCGTTGGTTTCAGAGAGAAAACTCTCCCAGGTCACAACGCGCGTGATGACGTGAACTCC

GAGCCCGCCCAACGTGACACAGAGCAACCACAAGCTGAGGGACCCTACGCCGGGCCACTCGAGCGTCAGA

AACCTCTGAAAGTGAGAGCCAAGCTACCACAGCAGGAGGGACCTTACGCTGGCCCGATGGAGAGACAGAA

ACCACTGAAAGTGAAAGCAAAAGCCCCGGTCGTCAAGGAAGGACCGTACGAAGGACCGGTCAAGAAACCT

GCCGCTTTGAAAGTGAAAGCCAAGAACTTGATTGTCACTGAGAGTGGTGCCCCACCGACCGACCTGCAGA

AGATGGTCATGGGCAACACAAAGCCTGTTGAGCTTATCCTCGACGGGAAGACAGTAGCCATCTGCTGCGC

TACTGGAGTGTTCGGCACTGCCTACCTCGTGCCTCGTCATCTTTTCGCTGAGAAGTACGACAAGATCATG

CTGGACGGCAGAGCCCTGACAGACAGTGACTACAGAGTGTTTGAGTTTGAGATTAAAGTAAAAGGACAGG

ACATGCTCTCAGACGCTGCGCTCATGGTGCTTCACCGTGGGAACCGCGTGAGAGACATCACGAAACACTT

TCGTGACACAGCTAGAATGAAGAAAGGCACCCCCGTTGTTGGCGTTATCAACAATGCTGACGTCGGGAGA

CTGATCTTCTCTGGTGAGGCCCTTACTTACAAGGACATTGTAGTGTGCATGGACGGAGACACCATGCCAG

GGCTTTTTGCCTACAAAGCCGCAACTAAGGCTGGCTATTGCGGAGGGGCCGTTCTCGCAAAGGACGGAGC

CGACACTTTCATCGTTGGCACCCACTCTGCTGGAGGCAATGGAGTTGGTTACTGCTCATGCGTTTCCAGG

TCCATGCTCCAAAAGATGAAGGCACACGTCGACCCGGAGCCACACCACGAA

>AY593791.1_A_IRN_1998

ATCTCAATTCCTTCCCAAAAATCTGTGTTGTACTTCCTCATTGAGAAAGGCCAACACGAAGCAGCAATTG

AATTCTTTGAGGGGATGGTGCATGACTCCATCAAGGAGGAACTCCGGCCCCTCATCCAACAGACCTCATT

TGTAAAACGCGCTTTCAAACGCCTGAAGGAGAACTTTGAGATCGTTGCCCTATGTTTGACTCTTCTAGCA

AACATAGTGATCATGATCCGCGAGACTCGCAAGAGACAGCAGATGGTGGATGATGCAGTGAACGAGTACA

TCGAGAAGGCAAACATCACCACAGACGACAAGACTCTTGACGAGGCGGAAAAGAACCCTCTGGAAACTAG

CGGTGCCAGCACCGTCGGTTTCAGAGAGAGAGCCCTCCCGGGACACAAGGCGAGTGGTGACGTGAACTCG

GAGCCCGCCAAACCCGCGGAGGAACAACCACAAGCTGAAGGACCCTACGCCGGGCCACTTGAGCGTCAGA

AGCCTCTGAAAGTGAGAGCCAAGCTACCACAGCAAGAGGGGCCTTACGCCGGCCCGATGGAGAGACAGAA

ACCACTGAAAGTGAAAGCAAAAGCCCCGGTCGTTAAGGAAGGACCTTACGAGGGACCGGTGAAGAAGCCT

GTCGCTTTGAAAGTGAAAGCGAAGAACTTGATTGTCACTGAGAGTGGTGCCCCACCGACCGACTTGCAAA

AGATGGTTATGGGTAACACTAAGCCTGTTGAGCTCATTCTCGACGGGAAGACAGTAGCCATCTGCTGTGC

TACTGGAGTGTTTGGTACTGCCTACCTCGTACCTCGTCACCTTTTCGCGGAGAAGTACGACAAGATCATG

CTGGACGGCAGAGCCATGACAGATAGTGACTACAGAGTGTTTGAGTTTGAGATTAAAGTAAAAGGGCAGG

ACATGCTCTCAGACGCCGCGCTCATGGTGCTCCACCGTGGGAATCGCGTGCGTGACATTACGAAACACTT

TCGTGATGTAGCAAAAATGAAGAAAGGCACCCCCGTCGTCGGTGTGATTAACAACGCTGATGTCGGGAGA

CTGATTTTCTCTGGTGAGGCCCTTACCTACAAAGACATTGTAGTGTGCATGGATGGCGACACCATGCCTG

GCCTCTTTGCCTACAGAGCCGCCACCAAGGCTGGTTACTGCGGAGGGGCTGTTCTTGCCAAAGACGGTGC

TGAAACATTCATCGTTGGCACACACTCCGCAGGTGGCAATGGAGTTGGATACTGCTCATGCGTTTCCAGG

TCCATGCTCTTGAAGATGAAGGCACACATCGACCCTGAACCACACCATGAG

>AY593792.1_A_ITL_1962

ATCTCAATTCCTTCCCAAAAATCTGTGTTGTACTTCCTCATTGAGAAAGGGCAGCACGAGGCAGCAATTG

AATTCTTTGAGGGCATGGTGCATGACTCCGTCAAGGAGGAGCTCCGGCCGCTCATCCAACAGACCTCATT

TGTGAAACGCGCTTTCAAGCGCCTGAAGGAAAACTTTGAGATTGTTGCTCTGTGCCTAACACTTTTGGCC

AACATTGTGATCATGATCCGCGAAACTCGCAAGAGACAGAAGATGGTGGACGATGCGGTGAACGAGTACA

TCGAGAAAGCAAACATCACCACCGATGACAAGACACTTGACGAGGCGGAAAAGAACCCTCTGGAGACTAG

CGGTGCCAGCACCGTTGGTTTCAGAGAGAGAACTCTTCCAGGCCAGAAGGCGCGTGATGACGTGAACTCC

GAGCCCGCCCAACCTGCTGAAGAACAACCACAAGCTGAAGGACCCTACGCCGGGCCACTTGAGCGTCAGA

GACCCCTGAAGGTGAGAGCCAAGCTCCCACAACAGGAAGGACCCTACGCTGGCCCGATGGAGAGACAGAA

ACCGCTGAAAGTGAAAGCAAAAGCCCCGGTCGTCAAGGAAGGACCTTACGAAGGACCGGTGAAGAAACCT

GTCGCTTTGAAAGTGAAAGCTAAGAATTTGATTGTCACTGAGAGTGGTGCCCCCCCGACCGACTTGCAAA

AGATGGTCATGGGCAACACAAAGCCTGTTGAGCTCATCCTCGACGGGAAGACAGTAGCCATCTGCTGTGC

TACTGGAGTGTTTGGCACTGCCTACCTCGTGCCTCGTCATCTTTTCGCTGAGAAGTATGACAAGATCATG

TTGGACGGCAGAGCCATGACAGACAGTGACTACAGAGTGTTTGAGTTCGAGATCAAAGTAAAAGGACAGG

ACATGCTCTCAGACGCCGCACTCATGGTGCTCCACCGTGGGAACCGTGTGAGAGACATCACGAAGCACTT

TCGTGACACAGCAAGAATGAAGAAAGGCACCCCCGTTGTCGGCGTGATCAACAATGCCGATGTCGGGAGA

CTGATTTTCTCTGGCGAAGCCCTTACCTACAAAGACATTGTAGTGTGCATGGACGGAGACACCATGCCCG

GGCTTTTTGCCTACAGAGCCGCCACTAAGGCTGGCTACTGCGGGGGAGCCGTTCTCGCTAAGGACGGGGC

TGACACTTTCATCGTTGGCACTCACTCTGCAGGAGGTAATGGAGTTGGATACTGCTCATGCGTTTCCAGG

TCCATGCTTCTCAAGATGAAGGCACACATTGACCCTGAGCCGCACCACGAG

>AY593793.1_A_PHI_1975

ATTTCAATTCCTTCCCAAAAGTCCGTGTTGTACTTCCTCATTGAGAAAGGGCAACACGAAGCAGCAATTG

AATTCTTTGAGGGGATGGTACACGACTCCATCAAGGAGGAGCTCCGACCTCTCCTCCAACACACCTCATT

TGTGAAACGCGCTTTTAAGCGCCTGAAGGAAAATTTCGAGATTGTTGCCCTGTGTCTGACCCTCTTGGCC

AGTATAGTGGTTATGATCCGCGCGACTCACAAGAGACAGAAGATGGTGGATGATGCAGTGAATGAGTACA

TTGAGAAAGCAAACATCACCACTGACGACAAGACTCTTGATGAGGCGGAAAAGAACCCTCTGGAAACTAG

CGGTGCCAGCACCGTTGGCTTCAGAGAGAGAACTCTCCCAGGCCAGAAAGCGCGCGATGACGTGAACTCT

GAGCCCGCCCAACCTGTTGGAGAACAACCACAAGCTGAAGGACCCTACGCCGGGCCACTTGAACGTCAGA

AACCTCTGAAAGTGAGAGCCAAGCTCCCGCAACAAGAGGGACCTTACGCTGGCCCGATGGAGAGACAGAA

ACCGCTGAAAGTGAAAGCAAAAGCCCCGGTCGTTAAGGAAGGACCCTACGAGGGACCGGTGAAGAAGCCT

GTCGCTTTGAAAGTGAAAGCTAAGAATTTGATTGTCACTGAGAGTGGTGCCCCACCGACCGACTTACAGA

AGATGGTGATGGGTAACACCAAGCCTGTCGAGCTCATCCTTGACGGGAAGACAGTAGCCATCTGCTGTGC

TACTGGGGTGTTTGGCACTGCTTACCTTGTTCCCCGTCATCTTTTTGCGGAAAAGTATGACAAGATCATG

TTGGACGGCAGAGCCATGACAGACAGTGACTACAGAGTGTTTGAGTTTGAGATTAAAGTAAAAGGACAGG

ACATGCTCTCGGACGCCGCGCTCATGGTGCTTCACCGTGGGAACCGCGTGAGAGACATCACGAAACACTT

TCGTGATGTAGCAAGAATGAAGAAAGGAACCCCTGTCGTTGGCGTGATCAACAACGCTGATGTCGGGAGA

CTAATTTTCTCTGGTGAGGCCCTCACCTACAAGGACATTGTAGTGTGCATGGATGGAGACACCATGCCTG

GCCTCTTTGCCTACAGAGCTGCAACCAAGGCAGGTTATTGTGGAGGAGCCGTTCTTGCCAAGGACGGAGC

CGAGACGTTCATCGTCGGCACGCACTCCGCAGGAGGCAATGGAGTTGGATACTGCTCGTGCGTTTCCAGG

TCCATGCTTCTGAAGATGAAGGCACACATCGACCCTGAACCACACCACGAG

>AY593794.1_A_COL_1985

ATCTCAATTCCTTCCCAAAAATCCGTGTTGTACTTCCTCATTGAGAAAGGTCAACATGAGGCAGCAATTG

AATTCTTTGAGGGTATGGTGCATGACTCCATCAAGGAGGAGCTCCGACCGCTCATCCAACAGACCTCATT

TGTGAAACGCGCTTTTAAGCGCTTGAAGGAAAACTTTGAGATCGTGGCCTTGTGTCTGACACTCCTGGCC

AACATTGTCATCATGATCCGCGAAACTCGCAAGAGACAGAAGATGGTGGACGATGCCGTGAACGATTACA

TCGAGAGGGCAAACATCACCACAGATGACAAGACACTTGACGAGGCGGAAAAGAACCCTCTGGAGACCAA

CGGTGCCAGCGCTGTTGGCTTCAGAGAGAAAACCCTCCCAGGCCACGCGGCACGCAATGACGTGAACTCT

GAGCCTGCCCAGCCTGCTGAGGAGCAACCACAAGCTGAAGGACCCTACGCCGGGCCACTCGAGCGTCAGA

AACCCCTGAAGGTGAGAGCCAAGCTCCCACAACAGGAGGGACCCTACGCTGGCCCGATGGAGAGACAGAA

ACCGCTTAAAGTGAAAGCAAGAGCCCCGGTCGTTAAGGAAGGACCCTACGAGGGACCGGTGAAGAGACCT

GTCGCTTTGAAAGTGAAAGAAAAAAGTTTGATTGTCACTGAGAGTGGTGCCCCGCCGACCGACTTACAAA

AGATGGTCATGGGCAACACAAAGCCCGTTGAGCTCATCCTTGACGGGAAGACGGTAGCCATCTGCTGTGC

TACTGGAGTGTTTGGTACTGCTTACCTCGTGCCTCGTCATCTTTTCGCAGAAAAGTATGACAAGATCATG

TTGGACGGCAGAGCCATGACAGACAGTGACTACAGAGTGTTTGAGTTTGAGATTAAAGTAAAAGGACAGG

ACATGCTCTCAGACGCGGCACTCATGGTGCTCCACCGTGGGAACCGCGTGAGAGACATCACGAAGCACTT

TCGTGATACAGCAAGACTGAAGAAAGGCACCCCCGTCGTCGGCGTTATCAACAATGCTGATGTCGGGAGA

CTGATTTTCTCTGGTGAGGCCCTTACCTACAAGGACATTGTAGTGTGCATGGATGGAGACACCATGCCCG

GGCTTTTTGCCTACAAAGCTGCCACCAAGGCTGGCTACTGTGGAGGAGCTGTTCTCGCAAAGGACGGAGC

TGACACGTTCATCGTCGGCACTCACTCTGCAGGTGGCAATGGGGTTGGATACTGCTCATGCGTTTCCAGG

TCCATGCTACAGAGGATGAAAGCACACGTCGACCCTGAACCACACCACGAG

>AY593795.1_Asia1_PAK_1954

ATCTCAATTCCTTCCCAAAAGTCTGTGTTGTACTTTCTCATTGAGAAGGGCCAACACGAAGCAGCAATTG

AATTCTTTGAGGGGATGGTTCACGACTCCATTAAGGAGGAGCTCCGACCCCTTATCCAACAGACCTCATT

TGTGAAACGCGCTTTTAAGCGCCTGAAGGAGAACTTTGAGATTGTTGCCCTGTGTTTGACCCTCTTGGCA

AACATAGTGATCATGATCCGCGAAACTCGCAAGAGACAGAAGATGGTGGATGATGCAGTGAATGAGTACA

TTGAGAAAGCAAACATCACCACGGATGACAAGACTCTTGACGAGGCGGAAAAGAACCCCCTGGAAACCAG

TGGTGCCAGCACCGTTGGCTTCAGAGAGAGAACTCTTCCAGGACAACTAGCGAGTGATGACGTGAACTCC

GAGCCCGCTAAGACCATGGAGGAGCAACCACAAGCTGAAGGACCCTACGCCGGGCCACTCGAGCGTCAGA

AACCTCTGAAAGTGAAAGCCAAGCTGCCACAACAAGAGGGACCCTACGCTGGCCCGATGGAGAGACAGAA

ACCACTGAAAGTGAAAGCAAAAGCCCCGGTCGTGAAGGAAGGACCTTACGAGGGACCGGTGAAGAAGCCT

GTCGCTTTGAAAGTGAAAGCCAAGAACTTGATTGTTACTGAGAGTGGTGCCCCACCGACTGACTTGCAAA

AGATGGTCATGGGCAACACAAAGCCTGTTGAGCTCATACTCGACGGGAAGACAGTAGCCATCTGTTGTGC

TACTGGAGTGTTTGGCACTGCCTACCTTGTGCCTCGTCACCTTTTCGCAGAAAAGTACGACAAGATTATG

CTGGACGGCAGAGCCATGACAGACAGTGACTACAGAGTGTTTGAGTTTGAGATTAAAGTAAAAGGACAGG

ACATGCTCTCAGATGCCGCGCTCATGGTGTTACACCGTGGGAACCGCGTGAGAGACATCACGAAGCACTT

CCGTGATGTGGCAAGAATGAAGAAAGGCACCCCCGTTGTCGGCGTAATCAACAACGCCGATGTCGGGAGG

CTGATATTCTCTGGTGAGGCTCTTACCTACAAAGACATTGTAGTGTGCATGGATGGAGACACCATGCCTG

GCCTCTTTGCCTACAAAGCCGCCACCAAGGCTGGCTACTGCGGAGGAGCTGTCCTTGCCAAGGACGGAGC

TGAGACGTTCATCGTCGGCACTCACTCTGCTGGCGGCAACGGAGTTGGATACTGCTCGTGCGTTTCTAGG

TCCATGCTGCTTAAAATGAAGGCACACATCGATCCTGAACCTCACCACGAG

>AY593796.1_Asia1_ISR_1963

ATCTCAATTCCTTCCCAAAAATCCGTGTTGTACTTTCTCATCGAGAAGGGGCAACACGAAGCAGCAATTG

AATTCTTTGAGGGGATGGTGCACGACTCCATCAAGGAGGAGCTCCGCCCTCTCGTCCAACAAACCTCATT

TGTGAAGCGCGCGTTTAAGCGCCTGAAGGAGAACTTTGAGATTGTTGCTCTGTGTTTGACCCTCTTGGCA

AACATAGTGATAATGATCCGCGAGACTCGCAAGAGACAACAGATGGTGGATGATGCAGTGAATGAGTACA

TTGAGAAAGCAAACATCACCACAGATGACAAGACTCTTGATGAGGCGGAAAAGAACCCTCTGGAGACTAG

TGGTGCCAGCACTGTTGGTTTCAGAGAGAGAACTCTCCCGGGACACAAGGTGAGCAATGACGTGAACTCC

GAGCCCGCCAAGCCCGCGGAGGAGCAACCACAAGCTGAAGGACCCTACGCCGGGCCACTCGAGCGTCAGA

AACCTCTGAAAGTGAAAGCCAAGCTGCCACAGCAGGAGGGACCCTACGCTGGCCCGCTGGAGAGACAGAA

ACCGCTGAAAGTGAAAGCAAAAACCCCGGTCGTGAAGGAAGGACCTTACGAGGGACCGGTGAAGAAACCT

GTCGCTTTGAAAGTGAAAGCTAAGAACCTGATTGTCACTGAGAGTGGTGCCCCACCAACCGACTTGCAAA

AGATGGTTATGGGCAACACAAAGCCTGTTGAGCTCATCCTCGACGGGAAGACAGTAGCCATCTGTTGCGC

TACTGGAGTGTTTGGTACTGCCTACCTCGTGCCTCGTCATCTTTTCGCTGAGAAGTATGACAAGATCATG

TTGGACGGCAGAGCCATGACAGACAGCGACTACAGAGTGTTTGAGTTTGAGATTAAAGTAAAAGGACAGG

ACATGCTCTCAGACGCCGCGCTCATGGTGCTGCACCGTGGGAACCGCGTGAGAGACATCACGAAACATTT

TCGTGATACAGCAAGAATGAAGAAAGGCACCCCCGTCGTCGGCGTGGTCAACAACGCCGATGTTGGGAGA

CTTATTTTCTCTGGTGAGGCCCTTACTTACAAGGACATCGTGGTGTGCATGGATGGAGACACCATGCCTG

GCCTTTTTGCCTACAGAGCCGCCACCAAGGCTGGCTACTGTGGAGGAGCCGTTCTTGCTAAGGATGGCGC

CGACACATTCATCGTTGGCACACACTCCGCAGGCGGCAATGGAGTTGGATACTGCTCCTGCGTTTCCAGG

TCCATGCTTCTGAAGATGAAGGCACACATTGACCCCGAACCGCACCATGAG

>AY593797.1_Asia1_ISR_1963

ATCTCGGTTCCTTCCCAAAAGTCCGTATTGTACTTTCTCATTGAGAAGGGGCAGCACGAAGCAGCAATTG

AATTCTTTGAGGGGATGGTGCACGACTCCATCAAGGAGGAGCTCCGCCCTCTCCTCCAACAGACCTCGTT

TGTGAAGCGCGCGTTTAAGCGCCTGAAGGAGAATTTTGAGGTTGTTGCCCTGTGTTTGACCCTCCTGGCA

AACATAGTGATCATGATCCGCGAGACTCGCAAGAGACAACAG----------------------------

--------------------------AACAAGACTCTCGACGAGGCGGAAAAGAACCCTCTGGAGACTAG

TGGTGCCAGCACTGTTGGCTTCAGAGAGAGAGCTCCCCCGGGACACAAGGTGAGCGATGACGTGAACTCC

GAGCCCACCAAACCCGCGGAGGAGCAACCACAAGCTGAAGGACCCTACGCCGGGCCACTCGAGCGTCAGA

AACCTCTGAAAGTGAAAGCCAAGTTGCCACAGCAGGAGGGACCCTACGCTGGCCCGATGGAGAGACAGAA

ACCACTGAAAGTGAAAGCAAAAGCCCCGGTCGTGAAGGAAGGACCTTACGAGGGACCGGTGAAGAAACCT

GTCGCTCTGAAAGTGAAAGCTAAGAACCTGATTGTCACTGAGAGTGGTGCCCCACCAACCGACTTGCAAA

AGATGGTTATGGGCAACACAAAGCCTGTTGAGCTCATCCTCGACGGGAAGACAGTGGCCATCTGCTGCGC

TACTGGAGTGTTTGGTACCGCCTACCTCGTGCCTCGTCATCTTTTCGCTGAGAAGTACGACAAGATCATG

TTGGACGGCAGAGCCATGACAGATAGTGACTACAGAGTGTTCGAGTTTGAGATTAAAGTAAAAGGACAGG

ACATGCTCTCGGACGCCGCGCTTATGGTGCTCCACCGTGGGAACCGCGTGAGAGACATCACGAAACACTT

TCGTGACACAGCAAGAATGAAGAAAGGCACCCCCGTCGTCGGTGTGATCAACAACGCCGATGTTGGGAGA

CTGATTTTCTCTGGTGAGGCCCTTACCTACAAGGACATTGTGGTGTGTATGGATGGAGACACCATGCCTG

GCCTCTTTGCCTACAGAGCCGCCACCAAGGCTGGCTACTGTGGAGGAGCCGTTCTCGCCAAGGACGGCGC

CGACACATTCATCGTTGGCACACACTCCGCAGGTGGCAATGGAGTTGGATACTGCTCCTGCGTTTCCAGG

TCCATGCTTCTGAAGATGAAGGCACACATTGACCCCGAACCACACCATGAG

>AY593799.1_Asia1_LEB_1983

ATCTCAATTCCTTCCCAAAAGTCCGTTTTGTACTTCCTCATTGAGAAAGGCCAACACGAAGCAGCAATTG

AATTCTTTGAGGGGATGGTGCACGACTCCATTAAGGAGGAGCTCCGGCCCCTCATCCAACAGACGTCATT

TGTGAAACGCGCTTTTAAGCGCCTGAAGGAAAACTTTGAGATTGTTGCCCTGTGTTTGACTCTCCTGGCA

AACATAGTGATCATGATCCGCGAGACTCGCAAGAGACAGCAAATGGTAGATGATGCAGTGAATGAGTACA

TTGAAAAGGCAAACATCACCACGGATGACAAGACCCTTGACGAGGCGGAAAAGAACCCTCTGGAAACCAG

TGGTGCCAGCACCGTTGGCTTCAGAGAGAGAACCCTCCCGGGACACAAGGCGAGTGAAGACGTGAACTCC

GAGCCCGCTAAGACCGTGGAGGAACAACCACAGGCTGAAGGACCCTACGCCGGGCCACTCGAGCGTCAGA

AACCCCTGAAAGTGAGAGCTAAGCTGCCACAACATGAGGGACCTTACGCTGGCCCGATGGAGAGACAGAA

ACCACTGAAAGTGAAAGCAAAAGCCCCGGTCGTTAAGGAAGGACCTTACGAGGGACCGGTGAAGAAGCCT

GTCGCTTTGAAAGTGAAAGCTAAGAACTTGATTGTCACTGAGAGTGGTGCCCCACCGACCGACTTGCAAA

AGATGGTCATGAGCAACACTAAGCCTGTTGAGCTCATCCTTGACGGTAAGACGGTGGCCATCTGCTGCGC

CACCGGAGTGTTTGGTACTGCCTACCTCGTGCCTCGTCACCTTTTCGCAGAAAAGTACGACAGGATCATG

TTGGACGGCAGGGCCATGACAGACAGTGACTACAGAGTGTTTGAGTTTGAGATTAAAGTAAAAGGACAGG

ACATGCTCTCAGACGCTGCGCTCATGGTGCTCCACCGTGGCAACCGTGTGAGAGACATCACGAAACACTT

TCGTGATACAGCAAGAATGAAGAAAGGTACCCCCGTTGTCGGCGTGATCAACAACGCCGACGTTGGGAGA

CTGATTTTCTCCGGTGAGGCCCTCACCTACAAGGACATTGTAGTGTGCATGGATGGAGACACCATGCCGG

GCCTATTTGCCTACAGAGCCGCTACCAAGGCTGGCTACTGTGGAGGAGCCGTTCTTGCCAAGGACGGAGC

TGACACATTTATCGTCGGCACTCACTCCGCAGGAGGCAATGGAGTCGGGTACTGCTCATGCGTATCTAGG

TCCATGCTCTTGAAGATGAAGGCACACATTGACCCCGAACCACACCACGAG

>AY593800.1_Asia1_LEB_1983

ATCTCAATTCCTTCCCAAAAGTCCGTTTTGTACTTCCTCATTGAGAAAGGCCAACACGAAGCAGCAATTG

AATTCTTTGAGGGGATGGTGCACGACTCCATTAAGGAGGAGCTCCGGCCCCTCATCCAACAGACGTCATT

TGTGAAACGCGCTTTTAAGCGCCTGAAGGAAAACTTTGAGATTGTTGCCCTGTGTTTGACTCTCCTGGCA

AACATAGTGATCATGATCCGCGAGACTCGCAAGAGACAGCAAATGGTAGATGATGCAGTGAATGAGTACA

TTGAAAAGGCAAACATCACCACGGATGACAAGACCCTTGACGAGGCGGAAAAGAACCCTCTGGAAACCAG

TGGTGCCAGCACCGTTGGCTTCAGAGAGAGAACCCTCCCGGGACACAAGGCGAGTGAAGACGTGAACTCC

GAGCCCGCTAAGACCGTGGAGGAACAACCACAGGCTGAAGGACCCTACGCCGGGCCACTCGAGCGTCAGA

AACCCCTGAAAGTGAGAGCTAAGCTGCCACAACATGAGGGACCTTACGCTGGCCCGATGGAGAGACAGAA

ACCACTGAAAGTGAAAGCAAAAGCCCCGGTCGTTAAGGAAGGACCTTACGAGGGACCGGTGAAGAAGCCT

GTCGCTTTGAAAGTGAAAGCTAAGAACTTGATTGTCACTGAGAGTGGTGCCCCACCGACCGACTTGCAAA

AGATGGTCATGAGCAACACTAAGCCTGTTGAGCTCATCCTTGACGGTAAGACGGTGGCCATCTGCTGCGC

CACCGGAGTGTTTGGTACTGCCTACCTCGTGCCTCGTCACCTTTTCGCAGAAAAGTACGACAGGATCATG

TTGGACGGCAGGGCCATGACAGACAGTGACTACAGAGTGTTTGAGTTTGAGATTAAAGTAAAAGGACAGG

ACATGCTCTCAGACGCTGCGCTCATGGTGCTCCACCGTGGCAACCGTGTGAGAGACATCACGAAACACTT

TCGTGATACAGCAAGAATGAAGAAAGGTACCCCCGTTGTCGGCGTGATCAACAACGCCGACGTTGGGAGA

CTGATTTTCTCCGGTGAGGCCCTCACCTACAAGGACATTGTAGTGTGCATGGATGGAGACACCATGCCGG

GCCTATTTGCCTACAGAGCCGCTACCAAGGCTGGCTACTGTGGAGGAGCCGTTCTTGCCAAGGACGGAGC

TGACACATTTATCGTCGGCACTCACTCCGCAGGAGGCAATGGAGTCGGGTACTGCTCATGCGTATCTAGG

TCCATGCTCTTGAAGATGAAGGCACACATTGACCCCGAACCACACCACGAG

>AY593802.1_A_URU_2001

ATCTCAATTCCTTCCCAAAAATCTGTGTTGTACTTTCTCATTGAGAAAGGGCAGCACGAAGCAGCAATTG

ATTTCTTTGAGGGCATGGTGCATGACTCCATCAAGGAGGAACTCCGGCCCCTCATCCAACAAACCTCATT

TGTGAAACGCGCTTTCAAACGCTTGAAGGAGAACTTTGAGATTGTTGCCCTATGTTTGACCCTGCTGGCC

AACATAGTGATCATGATCCGCGAGACTCGCAAGAGACAGAAGATGGTGGACGATGCAGTGAACGAGTACA

TCGAGAAGGCAAACATCACCACCGACGACAAGACTCTTGACGAGGCGGAAAAGAACCCTCTGGAGACTAG

CGGTGCCAGTACCGTTGGTTTCAGAGAGAAAACTCTCCCAGGTCACAACGCGCGTGATGACGTGAACTCC

GAGCCCGCCCAACGTGACACAGAGCAACCACAAACTGAAGGACCCTACGCCGGGCCACTCGAGCGTCAGA

AACCTCTGAAAGTGAGAGCCAAGCTACCACAGCAGGAGGGACCTTACGCTGGCCCGATGGAGAGACAGAA

ACCACTGAAAGTGAAAGCAAAAGCCCCGGTCGTTAAGGAAGGGCCGTACGAAGGACCGGTCAAGAAACCT

GTCGCTTTGAAAGTGAAAGCTAAGAACTTGATTGTCACTGAGAGTGGTGCCCCACCGACCGACCTGCAGA

AGATGGTCATGGGCAACACAAAGCCTGTTGAGCTTATCCTCGACGGGAAGACAGTAGCCATCTGCTGCGC

TACTGGAGTGTTCGGTACTGCCTACCTCGTGCCTCGTCATCTTTTCGCTGAGAAGTACGACAAGATCATG

CTGGACGGCAGAGCCCTGACAGACAGTGACTACAGAGTGTTTGAGTTTGAGATTAAAGTAAAAGGACAGG

ACATGCTCTCAGACGCTGCGCTCATGGTGCTTCACCGTGGGAACCGCGTGAGAGACATCACGAAACACTT

TCGTGACACAGCTAGACTGAAGAAAGGCACCCCCGTTGTTGGCGTTATTAACAATGCTGACGTCGGGAGA

CTGATCTTCTCTGGTGAGGCCCTTACCTACAAGGACATTGTAGTGTGCATGGACGGAGATACCATGCCAG

GGCTTTTTGCCTACAAAGCCGCAACTAAGGCTGGCTATTGCGGAGGGGCCGTTCTCGCAAAGGACGGAGC

CGACACTTTCATCGTTGGCACCCACTCTGCTGGAGGCAATGGAGTTGGTTACTGCTCATGCGTTTCCAGG

TCCATGCTCCAAAAGATGAAGGCACACGTCGACCCGGAGCCACACCACGAA

>AY593803.1_A_Brazil_1979

ATCTCAATTCCTTCCCAAAAGTCCGTGCTGTACTTCCTCATTGAGAAAGGCCAGCATGAAGCAGCAATTG

AATTCTTTGAGGGCATGGTGCACGACTCCATCAAGGAGGAGCTCCGCCCCCTCATCCAACAGACCTCATT

TGTGAAACGCGCTTTTAAGCGCCTGAAGGAAAACTTTGAGATTGTTGCCCTATGTTTGACCCTGTTGGCC

AACATAGTGATTATGATCCGCGAAACTCGCAAGAGGCAGAAGATGGTGGATGATGCAGTGAATGAGTACA

TTGAGAAAGCAAACATCACCACCGATGACAAGACTCTTGATGAAGCGGAAAAGAACCCTCTGGAGACCAG

TGGTGCCAGCACCGTTGGTTTCAGAGAGAGAACTCTTCCAGGCCACAAGGCGAGTGATGACGTGAACTCC

GAGCCCGCCCAACCTGTTGGAGAGCAACCACACGCTGAAGGACCCTACGCCGGGCCACTCGAGCGTCAGA

AACCTCTGAAAGTGAGAGCTAAGCTCCCACAGCAGGAGGGACCCTACGCTGGCCCGATGGAGAGACAGAA

ACCACTGAAAGTGAAAGCAAAAGCCCCGGTCGTTAAGGAAGGACCTTACGAGGGACCGGTGAAGAAACCT

GTCGCTTTGAAAGTGAAAGCCAAGAACTTGATCGTCACTGAGAGTGGTGCCCCACCGACCGACTTGCAAA

AGATGGTCATGGGCAACACCAAGCCTGTTGAGCTCATCCTCGACGGGAAGACGGTGGCCATCTGCTGTGC

TACCGGAGTGTTTGGCACCGCCTACCTCGTGCCTCGTCATCTTTTCGCTGAGAAGTATGACAAGATCATG

TTGGACGGCAGAGCCATGACAGACAGTGACTACAGAGTGTTTGAGTTTGAGATTAAAGTAAAAGGACAGG

ACATGCTCTCGGACGCTGCGCTCATGGTGCTTCACCGTGGGAACCGCGTGAGAGACATCACGAAACACTT

TCGTGACACAGCAAGAATGAAGAAAGGCACCCCCGTCGTTGGCGTGATCAACAACGCCGATGTCGGGAGA

CTGATTTTCTCTGGTGAGGCCCTGACCTACAAGGACATTGTAGTGTGCATGGATGGGGACACCATGCCTG

GCCTCTTTGCCTACAGAGCCGCAACCAAAGCTGGTTACTGCGGAGGAGCCGTCCTTGCTAAGGACGGGGC

TGACACGTTCATCGTCGGCACTCACTCTGCTGGAGGCAATGGAGTTGGTTACTGCTCTTGCGTTTCCAGG

TCCATGCTTCTCAAGATGAAGGCACACATTGACCCTGAACCACACCACGAG

>AY593804.1_C_SWZ_1965

ATCTCAATTCCTTCCCAAAAATCTGTGTTGTACTTCCTCATTGAGAAAGGCCAACACGAGGCAGCAATTG

AATTCTTTGAGGGCATGGTACACGACTCCATCAAGGAGGAACTCCGGCCCCTCATCCAACAAACTTCATT

TGTGAAACGCGCTTTTAAGCGCCTGAAAGAGAACTTTGAGATTGTTGCACTGTGCCTAACACTTTTGGCC

AACATTGTCATCATGATCCGCGAGACTCACAAGAGACAGAAGATGGTGGATGATGCGGTGAATGAGTACA

TCGAGAAAGCAAACATCACCACCGATGACAAGACACTTGACGAGGCGGAAAAGAACCCTCTGGAAACCAG

TGGTGCCAGCACCGTTGGCTTCAGAGAGAGAACTCTTCCAGGTCAGAAGGCGCGCGATGACGTGAACTCT

GAGCCCGCCCAGCCGACTGAAGAGCAACCGCAAGCTGAAGGACCCTACGCCGGGCCACTCGAGCGTCAGA

GACCTCTGAAAGTGAGAGCCAAGCTCCCGCAGCAAGAGGGACCTTACGCCGGCCCGATGGAGAGACAGAA

ACCGCTGAAAGTGAAAGCAAGAGCCCCGGTCGTTAAGGAAGGACCTTATGAAGGACCGGTGAAGAAGCCT

GTCGCTTTGAAAGTGAAAGCTAAGAATTTGATTGTCACTGAGAGTGGTGCCCCTCCGACTGACTTGCAAA

AGATGGTCATGGGCAACACGAAGCCTGTTGAGCTCATCCTCGACGGGAAGACAGTAGCCATCTGCTGTGC

TACTGGAGTGTTCGGCACTGCCTACCTCGTGCCTCGTCATCTTTTCGCTGAGAAGTATGACAAGATCATG

TTGGACGGCAGGGCTTTGACAGACAGTGACTACAGAGTGTTTGAGTTTGAGATTAAAGTAAAAGGACAGG

ACATGCTCTCAGACGCTGCGCTCATGGTGCTCCACCGTGGGAACCGCGTGAGAGACATCACGAAACACTT

TCGTGATGTAGCAAGAATGAAGAAAGGTACCCCCGTCGTTGGCGTGATTAACAACGCTGATGTTGGGAGA

CTGATTTTCTCTGGTGAGGCCCTTACCTACAAAGACATTGTAGTGTGCATGGATGGAGACACCATGCCCG

GTCTCTTTGCCTACAAAGCCGCTACCAAGGCTGGCTACTGTGGAGGAGCCGTTCTTGCTAAGGACGGAGC

TGACACCTTCATCGTTGGCACTCACTCTGCTGGAGGCAATGGAGTTGGGTACTGCTCATGCGTATCCAGG

TCCATGCTTCTCAAAATGAAGGCACACATCGACCCTGAACCGCACCACGAG

>AY593805.1_C_GER_1960

ATCTCAATTCCTTCCCAAAAATCTGTGTTGTACTTCCTCATTGAGAAAGGCCAACACGAGGCAGCAATTG

AATTCTTTGAGGGCATGGTACACGACTCCATCAAGGAGGAACTCCGGCCCCTCATCCAACAAACTTCATT

TGTGAAACGCGCTTTTAAGCGCCTGAAAGAGAACTTTGAGATTGTTGCACTGTGCCTAACACTTTTGGCC

AACATTGTCATCATGATCCGCGAGACTCACAAGAGACAGAAGATGGTGGATGATGCGGTGAATGAGTACA

TCGAGAAAGCAAACATCACCACCGATGACAAGACACTTGACGAGGCGGAAAAGAACCCTCTGGAAACCAG

TGGTGCCAGCACCGTTGGCTTCAGAGAGAGAACTCTTCCAGGTCAGAAGGCGCGCGATGACGTGAACTCT

GAGCCCGCCCAGCCGACTGAAGAGCAACCGCAAGCTGAAGGACCCTACGCCGGGCCACTCGAGCGTCAGA

GACCTCTGAAAGTGAGAGCCAAGCTCCCGCAGCAAGAGGGACCTTACGCCGGCCCGATGGAGAGACAGAA

ACCGCTGAAAGTGAAAGCAAGAGCCCCGGTCGTTAAGGAAGGACCTTATGAAGGACCGGTGAAGAAGCCT

GTCGCTTTGAAAGTGAAAGCTAAGAATTTGATTGTCACTGAGAGTGGTGCCCCTCCGACTGACTTGCAAA

AGATGGTCATGGGCAACACGAAGCCTGTTGAGCTCATCCTCGACGGGAAGACAGTAGCCATCTGCTGTGC

TACTGGAGTGTTCGGCACTGCCTACCTCGTGCCTCGTCATCTTTTCGCTGAGAAGTATGACAAGATCATG

TTGGACGGCAGGGCTTTGACAGACAGTGACTACAGAGTGTTTGAGTTTGAGATTAAAGTAAAAGGACAGG

ACATGCTCTCAGACGCTGCGCTCATGGTGCTCCACCGTGGGAACCGCGTGAGAGACATCACGAAACACTT

TCGTGATGTAGCAAGAATGAAGAAAGGTACCCCCGTCGTTGGCGTGATTAACAACGCTGATGTTGGGAGA

CTGATTTTCTCTGGTGAGGCCCTTACCTACAAAGACATTGTAGTGTGCATGGATGGAGACACCATGCCCG

GTCTCTTTGCCTACAAAGCCGCTACCAAGGCTGGCTACTGTGGAGGAGCCGTTCTTGCTAAGGACGGAGC

TGACACCTTCATCGTTGGCACTCACTCTGCTGGAGGCAATGGAGTTGGGTACTGCTCATGCGTATCCAGG

TCCATGCTTCTCAAAATGAAGGCACACATCGACCCTGAACCGCACCACGAG

>AY593806.1_C_Brazil_1971

ATCTCAATTCCTTCCCAAAAATCCGTGTTGTACTTCCTCATTGAGAAAGGACAGCATGAGGCAGCAATTG

AATTTTTTGAGGGAATGGTGCACGACTCCATCAAGGAGGAGCTCCGGCCCCTCATCCAACAAACCTCATT

TGTGAAACGCGCTTTCAAGCGCCTGAAGGAAAACTTTGAGATCGTGGCCTTGTGTTTGACTCTCTTGGCC

AACATTGTGATTATGATCCGCGAAACTCGCAAGAGGCAACAGATGGTGGATGATGCAGTGAATGAGTACA

TCGAGAAAGCAAACATCACCACCGATGACAAGACCCTTGACGAGGCGGAAAAGAACCCCCTGGAGACTAG

CGGTGCCAGCACCGTTGGTTTCAGGGAGAGAACTCTCCCAGGCCACAAGGCGCGCGATGACGTGAATTCT

GAGCCCGCCCAGCCCGTTGAGGAGCAACCACAAGCTGAAGGACCCTACGCCGGGCCTCTTGAGCGTCAGA

AACCTCTGAAAGTGAGAGCCAAGCTCCCGCAACAGGAGGGACCTTACGCTGGCCCGATGGAGAGACAGAA

ACCACTGAAAGTGAAAGCAAAAGCCCCGGTCGTTAAGGAAGGACCTTACGAGGGGCCGGTGAAGAAGCCT

GTCGCTTTGAAAGTGAAAGCCAAGAACTTGATTGTCACTGAGAGCGGTGCCCCACCGACCGACTTGCAAA

AGATGGTCATGGGCAACACCAAGCCTGTTGAGCTGATTCTCGATGGGAAGACGGTGGCCATCTGCTGCGC

CACCGGAGTGTTTGGCACTGCTTACCTCGTGCCGCGTCATCTTTTCGCAGAGAAGTATGACAAGATCATG

TTGGACGGCAGAGCCATGACAGACAGTGACTACAGAGTGTTTGAGTTTGAGATTAAAGTAAAAGGACAGG

ACATGCTCTCGGACGCCGCACTCATGGTGCTACACCGTGGGAATCGCGTGAGAGACATCACGAAGCACTT

TCGTGACACAGCAAGAATGAAGAAAGGCACCCCCGTCGTAGGCGTGATTAACAACGCCGACGTTGGGAGA

CTGATTTTCTCTGGTGAGGCCCTTACCTACAAAGACATTGTGGTGTGCATGGATGGAGACACCATGCCTG

GCCTCTTTGCCTACAGAGCCGCCACCAAGGCTGGTTACTGTGGAGGAGCCGTCCTTGCCAAGGATGGAGC

TGACACATTCATCGTCGGCACTCACTCTGCTGGAGGCAATGGAGTTGGTTACTGCTCATGCGTTTCCAGA

TCCATGCTTCTCAAGATGAAGGCACACATTGACCCTGAGCCACACCACGAG

>AY593807.1_C_Brazil_1955

ATCTCAATTCCTTCCCAAAAATCCGTGCTGTACTTCCTCATTGAGAAAGGGCAACACGAGGCAGCAATTG

AATTCTTTGAGGGAATGGTGCACGACTCCATCAAGGAGGAGCTCCGGCCCCTCATCCAACACACCTCATT

TGCGAAACGCGCTTTTAAGCGCTTGAAGGAAAACTTTGAGATCGTTGCCCTATGTTTGACCCTCTTGGCC

AACATAGTGATCATGGTCCGCGAAACTCGCAAGAGGCAGAAGATGGTGGATGATGCAGTGAATGAGTACA

TTGAGAAAGCAAACATCACCACTGATGACAAGACTCTTGACGAAGCGGAAAAGAACCCTCTGGAAACTAG

CGGTGCCAGCACCGTTGGCTTCAGAGAGAGAACTCTTCCAGGCCAGAAAGCGCGCGATGACGTGAACTCT

GAGCCCGCCCAACCTGTTGAAGAGCAACCACAAGCTGAAGGACCCTACGCCGGGCCACTTGAGCGTCAGA

AACCTCTAAAAGTGAGAGCCAAGCTCCCGCAACAAGAGGGACCTTACGCTGGCCCGATGGAGAGACAGAA

ACCGCTGAAAGTGAAAGCAAAAGCCCCGGTCGTTAAGGAAGGACCTTACGAGGGACCGGTGAAGAAGCCT

GTCGCTTTGAAAGTGAAAGCTAAGAATTTGATTGTCACTGAGAGTGGTGCCCCACCGACCGACTTGCAAA

AGATGGTTATGGGCAACACCAAGCCTGTTGAGCTCATCCTTGACGGGAAGACAGTAGCCATCTGCTGTGC

TACTGGGGTGTTTGGCACTGCTTACCTCGTTCCTCGTCATCTTTTCGCAGAAAAGTATGACAAGATCATG

TTGGACGGCAGAGCCATGACAGACAGTGACTACAGAGTGTTTGAGTTTGAGATCAAAGTAAAAGGACAGG

ACATGCTCTCGGACGCCGCGCTCATGGTGCTCCACCGTGGGAACCGCGTGAGAGACATCACGAAACACTT

CCGTGATGTGGCAAGAATGAAGAAAGGAACCCCCGTCGTTGGCGTGATCAACAACGCTGATGTCGGGAGA

CTGATTTTCTCTGGTGAGGCCCTTACCTACAAAGACATTGTAGTGTGCATGGATGGAGACACCATGCCCG

GCCTTTTTGCCTACAAAGCTGCAACCAAGGCAGGTTACTGCGGAGGAGCCGTTCTTGCTAAGGACGGAGC

CGAGACGTTCATCGTCGGCACACACTCCGCAGGAGGCAATGGAGTTGGATACTGTTCATGCGTTTCCAGA

TCCATGCTTCTGAAGATGAAGGCACACATTGACCCTGAACCACACCACGAG

>AY593808.1_C_ARG_1966

ATCTCAATTCCTTCCCAAAAATCTGTGTTGTACTTCCTCATTGAGAAAGGTCAACACGAGGCAGCAATTG

AATTCTTTGAGGGCATGGTGCATGACTCCATCAAGGAGGAGCTCCGGCCCCTCATCCAACGAACTTCATT

TGTGAAACGCGCTTTTAAGCGCCTGAAGGAGAACTTTGAGATTGTTGCACTGTGCCTAACACTTTTGGCC

AACATTGTCGTCATGATCCGCGAGACTCGCAAGAGGCAGAAGATGGTGGATGATGCGGTGAATGAGTACA

TCGAGAAAGCAAACATCACCACCGACGACAAGACACTTGACGAGGCGGAAAAGAACCCTCTGGAAACCAG

TGGCGCCAGCACCGTTGGCTTCAGGGAGAGAGCTCTTCCAGGTCAGAAGGCGCGCGATGACGTGAACTCC

GAGCCCGCCCAACCAACCGAAGAGCAACCACAAGCTGAAGGACCCTACGCCGGGCCACTCGAGCGTCAGA

GACCTCTGAAAGTGAGAGCCAAGCTCCCGCAACACGAGGGACCTTACGCCGGCCCGATGGAGAGACAGAA

ACCGCTGAAAGTGAAAGCAAGAGCCCCGGTCGTTAAGGAAGGACCTTACGAGGGACCGGTGAAGAAGCCT

GTCGCTTTGAAAGTGAAAGCTAAGAACTTGATTGTCACTGAGAGTGGCGCCCCACCGACCGACTTGCAAA

AGATGGTCATGGGCAACACAAAGCCTGTTGAGCTCATCCTCGACGGGAAGACAGTAGCCATCTGCTGTGC

TACTGGAGTGTTCGGCACCGCCTACCTTGTACCTCGCCATCTTTTCGCTGAGAAGTATGACAAGATTATG

CTGGACGGCAGGGCCATGACAGACAGTGACTACAGAGTGTTTGAGTTTGAGATTAAAGTAAAAGGACAGG

ACATGCTCTCAGACGCTGCGCTCATGGTGCTCCACCGTGGGAACCGCGTGAGAGATATCACGAAACACTT

TCGTGATGTAGCAAGAATGAAGAAAGGTACCCCCGTCGTTGGCGTGATCAACAACGCTGATGTTGGGAGA

CTGATTTTCTCTGGTGAGGCCCTTACCTACAAAGACATTGTAGTGTGCATGGATGGAGACACCATGCCCG

GCCTCTTTGCCTACAAAGCCGCCACCAAGGCTGGTTACTGTGGGGGAGCCGTTCTTGCTAAGGACGGAGC

TGACACCTTCATCGTTGGCACTCACTCTGCTGGAGGCAACGGAGTTGGGTACTGCTCATGTGTATCCAGG

TCCATGCTCCTCAAAATGAAGGCACACATCGACCCTGAACCACACCACGAG

>AY593809.1_C_ARG_1969

ATCTCAATTCCTTCCCAAAAATCCGTGCTGTACTTCCTCATTGAGAAAGGGCAACACGAGGCAGCAATTG

AATTCTTTGAGGGAATGGTGCACGACTCCATCAAGGAGGAGCTCCGGCCCCTCCTCCAACACACCTCATT

TGTGAAACGCGCTTTCAAGCGCTTGAAGGAAAACTTTGAGATCGTTGCCCTATGTTTGACCCTCTTGGCC

AACATAGTGATCATGGTCCGCGAAACTCGCAAGAGGCAGAAGATGGTGGATGATGCAGTGAATGAGTACA

TTGAGAAAGCAAACATCACCACTGATGACAAGACTCTTGACGAAGCGGAAAAGAACCCTCTGGAAACTAG

CGGTGCCAGCACCGTTGGCTTCAGAGAGAGAACTCTTCCAGGCCAGAAAGCGCGCGGTGACATGAACTCT

GAGCCCGCCCAACCTGTTGAAGAGCAACCACAAGCTGAAGGACCCTACGCCGGGCCACTTGAGCGTCAGA

AACCTCTAAAAGTGAGAGCCAAGCTCCCGCAACAAGAGGGACCTTACGCTGGCCCGATGGAGAGACAGAA

ACCGCTGAAGGTGAAAGCAAAAGCCCCGGTCGTTAAGGAAGGACCTTACGAGGGACCGATGAAGAAGCCT

GTCGCTTTGAAAGTGAAAGCTAAGAACTTGATTGTCACTGAGAGTGGTGCCCCACCGACCGACTTGCAAA

AGATGGTTATGGGCAACACCAAGCCTGTTGAGCTCATCCTTGACGGGAAGACAGTAGCCATCTGCTGTGC

TACTGGGGTGTTTGGCACTGCTTACCTCGTTCCTCGTCACCTTTTCGCAGAAAAGTATGACAAGATCATG

TTGGACGGCAGAGCCATGACAGACAGTGACTACAGAGTGTTTGAGTTTGAGATCAAAGTAAAAGGACAGG

ACATGCTCTCGGACGCCGCGCTCATGGTGCTCCACCGTGGGAACCGCGTGAGAGACATCACGAAACACTT

TCGTGATGTGGCAAGAATGAAGAAAGGAACCCCCGTCGTTGGCGTGATCAACAACGCTGATGTCGGGAGA

CTGATTTTCTCTGGTGAGGCCCTTACCTACAAAGACATTGTAGTGTGCATGGATGGAGATACCATGCCCG

GCCTTTTTGCCTACAAAGCTGCAACCAAGGCAGGTTACTGCGGAGGAGCCGTTCTTGCTAAGGACGGAGC

CGAGACGTTCATCGTCGGCACACACTCCGCAGGAGGCAATGGAGTTGGATACTGTTCATGCGTTTCCAGA

TCCATGCTTCTGAAGATGAAGGCACACATTGACCCTGAACCACACCACGAG

>AY593810.1_C_UKG_1970

ATCTCAATTCCTTCCCAAAAATCCGTGTTGTACTTCCTCATTGAGAAAGGCCAGCACGAGGCAGCAATTG

AATTCTTTGACGGCATGGTGCACGACTCCATTAAGGAGGAGCTCCGGCCTCTCATCCAACAAACTTCATT

TGTGAAGCGCGCTTTCAAGCGCTTGAAGGAAAACTTTGAGATTGTTGCCCTATGCTTGACCCTTCTGGCC

AACATAGTGATCATGATCCGCGAAACTCACAAGAGGCAGAAGATGGTGGATGATGCGGTGAACGAGTACA

TTGAGAAGGCAAACATCACCACGGATGACAAGACTCTTGACGAGGCGGAGAAGAACCCTCTGGAGACCAG

TGGCGCCAGCACTGTTGGTTTCAGAGAGAGAACTCTAACGGGTCAAAGGGCGTGCAATGACGTGAACTCC

GAGCCTGCCTGGCCCGCTGAAGAGCAACCACAAGCTGAAGGACCCTATACTGGGCCACTCGAGCGTCAGA

GACCTCTGAAAGTGAGAGCTAAGCTCCCACAGCAGGAAGGACCCTACGCTGGCCCATTGGAGAGACAGAA

ACCGCTGAAAGTGAAAGCAAAAGCCCCGGTCGTCAAGGAAGGACCTTACGAGGGACCGGTGAAGAAGCCT

GTCGCTTTGAAAGTGAAAGCTAAGAACCTGATAGTCACTGAGAGTGGTGCCCCACCGACCGACTTACAAA

AGATGGTCATGGGTAACACAAAGCCTGTTGAGCTCATCCTTGACGGAAAGACAGTAGCCATCTGTTGTGC

TACTGGGGTGTTTGGCACTGCTTACCTCGTGCCTCGTCATCTTTTCGCAGAGAAGTATGACAAGATCATG

CTGGATGGCAGAGCCATGACAGACAGTGACTACAGAGTGTTTGAATTTGAGATTAAAGTAAAAGGACAGG

ACATGCTCTCAGACGCTGCGCTCATGGTGCTCCACCGTGGGAACCGCGTGAGAGACATCACGAAACACTT

TCGTGATGCAGCAAGAATGAAGAAAGGCACCCCCGTTGTCGGTGTGGTCAACAACGCCGACGTTGGGAGA

CTGATTTTCTCTGGTGAGGCCCTTACCTACAAGGATATTGTAGTGTGCATGGACGGAGACACCATGCCTG

GCCTCTTTGCCTACAAAGCCGCCACCAAGGCTGGTTACTGCGGGGGAGCCGTTCTCGCCAAGGACGGGGC

CGACACTTTCATCGTCGGCACTCACTCCGCAGGAGGCAATGGAGTTGGATACTGTTCATGCGTTTCCAGG

TCCATGCTTCTCAGAATGAAGGCACACGTTGACCCTGAACCACACCACGAG

>AY593812.1_O_PHI_1958

ATCTCGATTCCTTCCCAAAAGTCTGTGTTGTACTTCCTCATTGAGAAAGGCCAGCACGAGGCAGCAATTG

AATTCTTTGAGGGGATGGTACATGACTCCATCAAGGAAGAACTCCGGCCCCTCATCCAACAGACCTCATT

TGTGAAGCGCGCTTTTAAGCGCCTGAAGGAGAATTTTGAGATTGTTGCCCTGTGTTTGACTCTTTTGGCA

AACATAGTGATCATGATCCGCGAGACTCGCAAGAGACAGAAGATGGTGGATGACGCAGTGAATGATTACA

TTGAGAAAGCAAACATCACTACAGATGACAAGACTCTTGACGAGGCGGAAAAGAACCCTCTGGAGACTAG

CGGTGCCAGCACCGTGGGTTTCAGAGAGAGAACCCTCCCGGGACACAAGGCGTGCGATGACGTGAACTCC

GAGCCCGCCAAGCCCAGGGAGGAACAACCACAAGCTGAAGGACCCTACACCGGGCCACTCGAACGTCAGA

AACCCCTGAAGGTGAGAGCCAAGCTCCCACAACAGGAGGGACCCTACGTTGGCCCGATGGAGAGACAAAA

ACCCCTCAAAGTCAAAGCAAAAGCCCCGGTCGTGAAGGAAGGACCCTACGAAGGACCGGTGAAGAAGCCT

GTCGCTTTGAAAGTGAAAGCTAAGAACTTGATTGTCACTGAGAGTGGGGCCCCACCGACTGACTTGCAAA

AGTTGGTCATGGGCAACACCAAGCCCGTTGAGCTCATACTCGACGGGAAGACGGTGGCCATCTGTTGCGC

TACCGGAGTGTTTGGCACCGCCTACCTCGTGCCCCGTCACCTTTTCGCAGAAAAGTACGACAAGATCATG

TTGGACGGCAGAGCCATGACAGACAGTGACTACAGAGTGTTTGAGTTTGAGATTAAAGTAAAAGGACAGG

ACATGCTTTCAGACGCCGCGCTCATGGTGCTTCACCGTGGGAACCGCGTGCGAGACATCACGAAGCACTT

CCGTGATGTGGCCAGAATGAAGAAAGGCACCCCCGTTGTTGGCGTGATTAATAATGCTGACGTCGGGAGA

CTGATTTTCTCTGGTGAGGCCCTCACCTACAAGGACATTGTAGTGTGCATGGACGGAGACACCATGCCCG

GCCTCTTTGCCTACAAGGCCGCCACCAAGGCAGGCTACTGTGGAGGAGCCGTTCTCGCAAAGGACGGGGC

CGAGACATTCATTGTCGGCACTCACTCTGCAGGTGGCAACGGAGTTGGATACTGCTCGTGCGTTTCCAGA

TCCATGTTGCAAAAAATGAAGGCACACATTGACCCCGAACCACACCACGAG

>AY593813.1_O_ISA_1962

ATTTCAATTCCTTCACAAAAATCCGTGCTATACTTTCTCATTGAGAAGGGTCAGCACGAGGCAGCAATTG

AATTCTTTGAGGGGATGGTCCACGACTCCATCAAGGAAGAGCTTCGGCCCCTCCTCCAACACACCTCATT

TGTTAAGCGCGCCTTCAAGCGCCTGAAGGAGAACTTTGAGATTGTTGCACTGTGTTTAACTCTTTTGGCT

AACATTGTGATCATGATCCGCGAAACCCGCAAGAGGCAACAAATGGTGGACGATGCAGTCAACGACTACA

TCGAGAGAGCTGGCATCACGACTGATGACAAGACTCTTGCTGACGCGGAGAAGAACCCTCTGGAAACCAG

CGGTGCCAGCACCGTTGGCTTCAGAGAGAGAACCCTGCCGGGACAAACGGTGAGCAATGACGTGAGCTCC

GAGCCCACCGCGCCCGTGGAGGAACAACCACAAGCTGAAGGACCCTATGCCGGGCCCGTGGAGCGTCAGA

AACCCCTGAGAGTGAGAGCCAAGCTACCACAACAGGAGGGACCTTACGCTGGCCCGATGGAGAGACAGAA

ACCGTTAAAAGTAAAAGCAAAAGCCCCGGTCGTTAAGGAAGGACCTTACGAGGGACCGGTGAAGAAGCCT

GTCGCTTTGAAAGTGAAAGCTAAAAATTTGATTGTCACTGAGAGCGGTGCCCCACCGACTGACTTGCAAA

AGATGGTTATGGGGAACACCAAGCCCGTTGAGCTCGTACTCGACGGGAAGACGGTAGCTATCTGCTGTGC

TACTGGAGTGTTTGGTACTGCTTACCTCGTGCCCCGTCATCTTTTCGCTGAGAAGTACGACAAGATCATG

CTAGACGGCAGAGCCCTGACTGACAGTGACTACAGAGTGTTTGAGTTTGAGATTAAAGTAAAAGGACAGG

ACATGCTTTCAGACGCTGCGCTCATGGTGCTGCACCGTGGGAACCGCGTGAGAGACATCACGAAACACTT

TCGTGATACAGCCAGAATGAAGAAGGGCACCCCCGTCGTTGGCGTCATCAACAACGCCGATGTCGGGCGA

TTGATTTTCTCTGGTGAGGCCTTGACCTACAAAGACATTGTAGTGTGCATGGACGGAGACACCATGCCTG

GCCTTTTTGCCTACAAAGCTGCCACCAAGGCAGGATACTGCGGAGGAGCTGTCCTTGCTAAGGACGGGGC

CGACACGTTCATCGTTGGCACTCACTCCGCGGGTGGCAATGGAGTCGGATACTGCTCGTGCGTGTCCAGG

TCCATGCTGCAAAAAATGAAGGCTCACATCGACCCCGAACCACACCACGAG

>AY593814.1_O_ARG_1965

ATCTCAATTCCTTCCCAAAAATCTGTGTTGTACTTTCTCATTGAGAAGGGCCAACATGAGGCAGCAATTG

AATTCTTTGAGGGCATGGTCCACGACTCCATCAAAGAGGAACTCCGACCCCTCATCCAACAAACTTCATT

TGTGAAACGCGCTTTCAAGCGCCTGAAGGAAAATTTTGAGATTGTTGCTCTGTGTTTAACACTTTTGGCA

AACATTGTGATCATGATCCGTGAGACTCGCAAGAGGCAGAAAATGGTGGATGATGCAGTGAATGAGTACA

TTGAGAAAGCAAACATCACCACAGATGACAAGACTCTTGACGAGGCGGAGAAGAGCCCTCTAGAGACCAG

CGGCGCCAGCACCGTTGGCTTTAGAGAGAGAACTCTCCCAGGTCAAAAGGCGCGCGATGACGTGAACTCC

GAGCCTGCCCAACCTGTTGAGGAGCAACCACAAGCTGAAGGACCCTACGCCGGACCACTCGAGCGTCAGA

AACCTCTGAAAGTGAGAGCCAAGCTCCCACAGCAGGAGGGGCCTTACGCTGGTCCGATGGAGAGACAGAA

ACCGCTAAAAGTGAAAGCAAAAGCCCCGGTCGTGAAGGAAGGGCCTTACGAGGGACCGGTGAAGAAGCCT

GTCGCTTTGAAAGTAAAAGCTAAGAACCTGATTGTCACTGAGAGTGGTGCCCCACCGACCGACTTGCAAA

AGATGGTCATGGGCAACACAAAGCCTGTTGAGCTCATCCTCGACGGGAAGACAGTAGCCATCTGCTGCGC

TACTGGAGTGTTTGGCACTGCTTACCTCGTGCCTCGTCATCTCTTCGCAGAGAAGTATGACAAGATCATG

TTGGACGGCAGAGCCATGACAGACAGCGACTATAGAGTGTTTGAGTTTGAGATCAAAGTAAAAGGACAGG

ACATGCTCTCAGACGCCGCGCTCATGGTGCTCCACCGTGGGAACCGCGTGAGGGACATCACGAAGCACTT

TCGTGACACAGCAAGAATGAAGAAAGGCACCCCCGTTGTCGGTGTGATTAACAACGCCGATGTCGGGAGA

CTGATTTTCTCTGGTGAGGCCCTTACTTACAAGGACATTGTGGTTTGCATGGACGGAGACACCATGCCTG

GCCTCTTTGCCTACAGAGCCGCCACCAAGGCTGGTTACTGCGGAGGAGCCGTTCTTGCCAAAGACGGAGC

TGACACTTTCATCGTCGGCACTCACTCTGCGGGAGGCAACGGAGTTGGATACTGCTCATGCGTTTCCAGG

TCCATGCTTCTTAAAATGAAGGCACACATTGACCCCGAACCACACCACGAG

>AY593815.1_O_UKG_1967

ATCTCAATTCCTTCCCAAAAATCTGTGTTGTACTTTCTCATTGAGAAGGGCCAACATGAGGCAGCAATTG

AATTCTTTGAGGGCATGGTCCACGACTCTATTAAAGAGGAACTCCAACCCCTCATCCAACAAACTTCATT

TGTGAAACGCGCTTTCAAGCGCCTGAAGGAAAATTTTGAGATTGTTGCTCTGTGTTTAACACTTTTGGCA

AACATTGTGATCACGGTCCGTGAGACTCGCAAGAGGCAGAAAATGGTGGATGATGCAGTGAATGAGTACA

TTGAGAAAGCAAACATCACCACAGATGACAAGACTCTTGACGAGGCGGAGAAGAGCCCTCTAGAGACCAG

CGGCGCCAGCACCGTTGGCTTTAGAGAGAGAACTCTCCCAGGTCAAAAGGCATGCGATGACGTGAACTCC

GAGCCTGCCCAACCTGTTGAGGAGCAACCACAAGCTGAAGGACCCTACGCCGGACCACTCGAGCGTCAGA

AACCTCTGAAAGTGAGAGCCAAGCTCCCACAGCAGGAGGGGCCTTACGCTGGTCCAATGGAGAGACAGAA

ACCGCTAAAAGTGAAAGCAAAAGCCCCGGTCGTGAAGGAAGGACCTTACGAGGGACCGGTGAAGAAGCCT

GTCGCTTTGAAAGTGAAAGCTAAGAACCTGATTGTCACTGAGAGTGGTGCCCCACCGACCGACTTGCAAA

AGATGGTCATGGGCAACACAAAGCCTGTTGAGCTCATCCTCGACGGGAAGACAGTAGCCATTTGCTGCGC

TACTGGAGTGTTTGGCACTGCTTACCTCGTGCCTCGTCACCTCTTCGCAGAGAAGTATGACAAGATCATG

TTGGACGGCAGAGCCATGACAGACAGTGACTACAGAGTGTTTGAGTTTGAGATCAAAGTAAAAGGACAGG

ACATGCTCTCAGACGCCGCGCTCATGGTGCTCCACCGTGGGAACCGCGTGAGGGACATCACGAAGCACTT

TCGTGACACAGCAAGAATGAAGAAAGGCACCCCCGTTGTCGGTGTGATTAACAACGCCGATGTCGGGAGA

CTGATTTTCTCCGGTGAGGCCCTTACTTACAAGGACATTGTGGTTTGTATGGACGGAGACACCATGCCCG

GCCTCTTTGCCTACAGAGCCGCCACCAAGGCTGGCTACTGCGGAGGAGCCGTTCTCGCCAAAGACGGAGC

TGACACTTTCATCGTCGGCACTCACTCTGCAGGAGGCAACGGAGTTGGATACTGCTCATGCGTTTCCAGG

TCCATGCTTCTTAAAATGAAGGCACACATTGACCCCGAACCACATCACGAG

>AY593816.1_O_UKG_1967

ATCTCAATTCCTTCCCAAAAATCTGTGTTGTACTTTCTCATTGAGAAGGGCCAACATGAGGCAGCAATTG

AATTCTTTGAGGGCATGGTCCACGACTCTATTAAAGAGGAACTCCAACCCCTCATCCAACAAACTTCATT

TGTGAAACGCGCTTTCAAGCGCCTGAAGGAAAATTTTGAGATTGTTGCTCTGTGTTTAACACTTTTGGCA

AACATTGTGATCACGGTCCGTGAGACTCGCAAGAGGCAGAAAATGGTGGATGATGCAGTGAATGAGTACA

TTGAGAAAGCAAACATCACCACAGATGACAAGACTCTTGACGAGGCGGAGAAGAGCCCTCTAGAGACCAG

CGGCGCCAGCACCGTTGGCTTTAGAGAGAGAACTCTCCCAGGTCAAAAGGCATGCGATGACGTGAACTCC

GAGCCTGCCCAACCTGTTGAGGAGCAACCACAAGCTGAAGGACCCTACGCCGGACCACTCGAGCGTCAGA

AACCTCTGAAAGTGAGAGCCAAGCTCCCACAGCAGGAGGGGCCTTACGCTGGTCCAATGGAGAGACAGAA

ACCGCTAAAAGTGAAAGCAAAAGCCCCGGTCGTGAAGGAAGGACCTTACGAGGGACCGGTGAAGAAGCCT

GTCGCTTTGAAAGTGAAAGCTAAGAACCTGATTGTCACTGAGAGTGGTGCCCCACCGACCGACTTGCAAA

AGATGGTCATGGGCAACACAAAGCCTGTTGAGCTCATCCTCGACGGGAAGACAGTAGCCATTTGCTGCGC

TACTGGAGTGTTTGGCACTGCTTACCTCGTGCCTCGTCACCTCTTCGCAGAGAAGTATGACAAGATCATG

TTGGACGGCAGAGCCATGACAGACAGTGACTACAGAGTGTTTGAGTTTGAGATCAAAGTAAAAGGACAGG

ACATGCTCTCAGACGCCGCGCTCATGGTGCTCCACCGTGGGAACCGCGTGAGGGACATCACGAAGCACTT

TCGTGACACAGCAAGAATGAAGAAAGGCACCCCCGTTGTCGGTGTGATTAACAACGCCGATGTCGGGAGA

CTGATTTTCTCCGGTGAGGCCCTTACTTACAAGGACATTGTGGTTTGTATGGACGGAGACACCATGCCCG

GCCTCTTTGCCTACAGAGCCGCCACCAAGGCTGGCTACTGCGGAGGAGCCGTTCTCGCCAAAGACGGAGC

TGACACTTTCATCGTCGGCACTCACTCTGCAGGAGGCAACGGAGTTGGATACTGCTCATGCGTTTCCAGG

TCCATGCTTCTTAAAATGAAGGCACACATTGACCCCGAACCACATCACGAG

>AY593817.1_O_Belgium_1973

ATCTCAATTCCTTCCCAAAAATCCGTGTTGTACTTTCTCATTGAGAAGGGCCAACATGAGGCAGCAATTG

AATTCTTTGAGGGCATGGTCCACGACTCCATCAAAGAGGAACTCCGACCCCTCATCCAACAAACTTCATT

TGTGAAACGCGCTTTCAAGCGCCTGAAGGAAAATTTTGAGATTGTTGCTCTGTGTTTAACACTTTTGGCA

AACATTGTGATCATGATCCGTGAGACTCGCAAGAGGCAGAAAATGGTGGATGATGCAGTGAATGAGTACA

TTGAGAAAGCAAACATCACCACAGATGACAAGACTCTTGACGAGGCGGAGAAGAGCCCTCTAGAGACCAG

CGGCGCCAGCACCGTTGGCTTTAGAGAGAGAACTCTCCCAGGTCAAAAGGCATGCGATGACGTGAACTCC

GAGCCTGCCCAACCTGTTGAGGAGCAACCACAAGCTGAAGGACCCTACGCCGGACCACTCGAGCGTCAGA

AACCTCTGAAAGTGAGAGCCAAGCTCCCACAGCAGGAGGGGCCTTACGCTGGTCCGATGGAGAGACAGAA

ACCGCTAAAAGTGAAAGCAAAAGCCCCGGTCGTGAAGGAAGGACCTTACGAGGGACCGGTGAAGAAGCCT

GTCGCTTTGAAAGTGAAAGCTAAGAACCTGATTGTCACTGAGAGTGGTGCCCCACCGACCGACTTGCAAA

AGATGGTCATGGGCAACACAAAGCCTGTTGAGCTCATCCTCGACGGGAAGACAGTAGCCATCTGCTGCGC

TACTGGAGTGTTTGGCACTGCTTACCTCGTGCCTCGTCACCTCTTCGCAGAGAAGTATGACAAGATCATG

TTGGACGGCAGAGCCATGACAGACAGTGACTATAGAGTGTTTGAGTTTGAGATCAAAGTAAAAGGACAGG

ACATGCTCTCAGACGCCGCGCTCATGGTGCTCCACCGTGGGAACCGCGTGAGGGACATCACGAAGCACTT

TCGTGACACAGCAAGAATGAAGAAAGGCACCCCCGTTGTCGGTGTGATTAATAACGCCGATGTCGGGAGA

CTGATTTTCTCTGGTGAGGCCCTTACTTACAAGGACATTGTGGTTTGCATGGACGGAGACACCATGCCTG

GCCTCTTTGCCTACAGAGCCGCCACCAAGGCTGGTTACTGCGGAGGAGCCGTTCTTGCCAAAGACGGAGC

TGACACTTTCATCGTCGGCACTCACTCTGCAGGAGGCAACGGAGTTGGATACTGTTCATGCGTTTCCAGG

TCCATGCTTCTTAAAATGAAGGCACACATTGACCCCGAACCACACCACGAG

>AY593818.1_O_ARG_1958

ATCTCAATTCCTTCCCAAAAATCTGTGTTGTACTTTCTCATTGAGAAGGGCCAACATGAGGCAGCAATTG

AATTCTTTGAGGGCATGGTCCACGACTCCATCAAAGAGGAACTCCGACCCCTCATCCAACAAACTTCATT

TGTGAAACGCGCTTTCAAGCGCCTGAAGGAAAATTTTGAGATTGTTGCTCTGTGTTTAACACTTTTGGCA

AACATTGTGATCATGATCCGTGAGACTCGCAAGAGGCAGAAAATGGTGGATGATGCAGTGAATGAGTACA

TTGAGAAAGCAAACATCACCACAGATGACAAGACTCTTGATGAGGCGGAGAAGAGCCCTCTAGAGACCAG

CGGCGCCAGCACCGTTGGCTTTAGAGAGAGAACTCTCCCAGGTCAAAAGGCATGCGATGACGTGAACTCC

GAGCCTGCCCAACCTGTTGAGGAGCAACCACAAGCTGAAGGACCCTACGCCGGACCACTCGAGCGTCAGA

AACCTCTGAAAGTGAGAGCCAAGCTCCCACAGCAGGAGGGGCCTTACGCTGGCCCGATGGAGAGACAGAA

ACCGCTAAAAGTGAAAGCAAAAGCCCCGGTCGTGAAGGAAGGACCTTACGAGGGACCGGTGAAGAAGCCT

GTCGCTTTGAAGGTGAAAGCTAAGAACCTGATTGTCACTGAGAGTGGTGCCCCACCGACCGACTTGCAAA

AGATGGTCATGGGCAACACAAAGCCTGTTGAGCTCATCCTCGACGGGAAGACAGTAGCCATCTGCTGCGC

TACTGGAGTGTTTGGCACTGCTTACCTCGTGCCTCGTCACCTCTTCGCAGAGAAGTATGACAAGATCATG

TTGGACGGCAGAGCCATGACAGACAGTGACTACAGAGTGTTTGAGTTTGAGATCAAAGTAAAAGGACAGG

ACATGCTCTCAGACGCCGCGCTCATGGTGCTCCACCGTGGGAACCGCGTGAGGGACATCACGAAGCACTT

TCGTGACACAGCAAGAATGAAGAAAGGCACCCCCGTTGTCGGTGTGATCAACAACGCCGATGTCGGGAGA

CTGATTTTCTCTGGTGAGGCCCTTACTTACAAGGACATTGTGGTTTGCATGGACGGAGACACCATGCCTG

GCCTCTTTGCCTACAGAGCCGCCACCAAGGCTGGCTACTGCGGAGGAGCCGTTCTTGCCAAAGACGGAGC

TGACACTTTCATCGTCGGCACTCACTCTGCAGGAGGCAACGGAGTTGGATACTGCTCATGCGTTTCCAGG

TCCATGCTTCTTAAAATGAAGGCACACATTGACCCCGAACCACACCACGAG

>AY593819.1_O_ARG_1994

ATCTCAATTCCTTCCCAAAAATCTGTGTTGTACTTTCTCATTGAGAAGGGCCAACATGAGGCAGCAATTG

AATTCTTTGAGGGCATGGTCCACGACTCCATCAAAGAGGAACTCCGACCCCTCATCCAACAAACTTCATT

TGTGAAACGCGCTTTCAAGCGCCTGAAGGAAAATTTTGAGATTGTTGCTCTGTGTTTAACACTTTTGGCA

AACATTGTGATCATGATCCGTGAGACTCGCAAGAGGCAGAAAATGGTGGATGATGCAGTGAATGAGTACA

TTGAGAAAGCAAACATCACCACAGATGACAAGACTCTTGACGAGGCGGAGAAGAGCCCTCTAGAGACCAG

CGGCGCCAGCACCGTTGGCTTTAGAGAGAGAACTCTTCCAGGTCAAAAGGCATGCGATGACGTGAACTCC

GAGCCTGCCCAACCTGTTGAGGAGCAACCACAAGCTGAAGGACCCTACGCCGGACCACTCGAGCGTCAGA

AACCTCTGAAAGTGAGAGCCAAGCTCCCACAGCAGGAGGGGCCTTACGCTGGTCCGATGGAGAGACAGAA

ACCGCTAAAAGTGAAAGCAAAAGCCCCGGTCGTGAAGGAAGGACCTTACGAGGGACCGGTGAAGAAGCCT

GTCGCTTTGAAAGTGAAAGCTAAGAACCTGATTGTCACTGAGAGTGGTGCCCCACCGACCGACTTGCAAA

AGATGGTCATGGGCAACACAAAGCCTGTTGAGCTCATCCTCGACGGGAAGACAGTAGCCATCTGCTGCGC

TACTGGAGTGTTTGGCACTGCTTACCTCGTGCCTCGTCACCTCTTCGCAGAGAAGTATGACAAGATCATG

TTGGACGGCAGAGCCATGACAGACAGTGACTACAGAGTGTTTGAGTTTGAGATCAAAGTAAAAGGACAGG

ACATGCTCTCAGACGCCGCGCTCATGGTGCTCCACCGTGGGAACCGCGTGAGGGACATCACGAAGCACTT

TCGTGACACAGCAAGAATGAAGAAAGGCACCCCCGTTGTCGGTGTGATCAACAACGCCGATGTCGGGAGA

CTGATTTTCTCTGGTGAGGCCCTTACTTACAAGGACATTGTGGTTTGCATGGACGGAGACACCATGCCTG

GCCTCTTTGCCTACAGAGCCGCCACCAAGGCTGGCTACTGCGGAGGAGCCGTTCTTGCCAAAGACGGAGC

TGACACTTTCATCGTCGGCACTCACTCTGCAGGAGGCAACGGAGTTGGATACTGCTCATGCGTTTCCAGG

TCCATGCTTCTTAAAATGAAGGCACACATTGACCCCGAACCACACCACGAG

>AY593820.1_O_ARG_1964

ATCTCAATTCCTTCCCAAAAATCTGTGTTGTACTTTCTCATTGAGAAGGGCCAACATGAGGCAGCAATTG

AATTCTTTGAGGGCATGGTCCACGACTCCATCAAAGAGGAACTCCGACCCCTCATCCAACAAACTTCATT

TGTGAAACGCGCTTTCAAGCGCCTGAAGGAAAATTTTGAGATTGTTGCTCTGTGTTTAACACTTTTGGCA

AACATTGTGATCATGATCCGTGAGACTCGCAAGAGGCAGAAAATGGTGGATGATGCAGTGAATGAGTACA

TTGAGAAAGCAAACATCACCACAGATGACAAGACTCTTGACGAGGCGGAGAAGAGCCCTCTAGAGACCAG

CGGCGCCAGCACCGTTGGCTTTAGAGAGAGAACTCTCCCAGGTCAAAAGGCATGCGATGACGTGAACTCC

GAGCCTGCCCAACCTGTTGAGGAGCAACCACAAGCTGAAGGACCCTACGCCGGACCACTCGAGCGTCAGA

AACCTCTGAAAGTGAGAGCCAAGCTCCCACAGCAGGAGGGGCCTTACGCTGGTCCGATGGAGAGACAGAA

ACCGCTAAAAGTGAAAGCAAAAGCCCCGGTCGTGAAGGAAGGACCTTACGAGGGACCGGTGAAGAAGCCT

GTCGCTTTGAAAGTGAAAGCTAAGAACCTGATTGTCACTGAGAGTGGTGCCCCACCGACCGACTTGCAAA

AGATGGTCATGGGCAACACAAAGCCTGTTGAGCTCATCCTCGACGGGAAGACAGTAGCCATCTGCTGCGC

TACTGGAGTGTTTGGCACTGCTTACCTCGTGCCTCGTCACCTCTTCGCAGAGAAGTATGACAAGATCATG

TTGGACGGCAGAGCCATGACAGACAGTGACTACAGAGTGTTTGAGTTTGAGATCAAAGTAAAAGGACAGG

ACATGCTCTCAGACGCCGCGCTCATGGTGCTCCACCGTGGGAACCGCGTGAGGGACATCACGAAGCACTT

TCGTGACACAGCAAGAATGAAGAAAGGCACCCCCGTTGTCGGTGTGATCAACAACGCCGATGTCGGGAGA

CTGATTTTCTCTGGTGAGGCCCTTACTTACAAGGACATCGTGGTTTGCATGGACGGAGACACCATGCCTG

GCCTCTTTGCCTACAGAGCCGCCACCAAGGCTGGCTACTGCGGAGGAGCCGTTCTTGCCAAAGACGGAGC

TGACACTTTCATCGTCGGCACTCACTCTGCAGGAGGCAACGGAGTTGGATACTGCTCATGCGTTTCCAGG

TCCATGCTTCTTAAAATGAAGGCACACATTGACCCCGAACCACACCACGAG

>AY593821.1_O_ARG_1967

ATCTCAATTCCTTCCCAAAAATCCGTGTTGTACTTCCTCATTGAGAAAGGGCAGCACGAGGCAGCAATTG

AATTCTTTGAGGGAATGGTGCACGACTCTATCAAGGAAGAGCTCCGCCCTCTCATCCAACAGACCTCATT

TGTGAAACGCGCTTTCAAGCGCTTGAAGGAAAATTTTGAGATCGTTGCCCTGTGTTTGACCCTCCTGGCC

AACATAGTGATCATGGTCCGCGAGACTCGCAAGAGGCAGAAGATGGTGGATGATGCAGTGAACGAGTACA

TCGAGAAGGCAAACATCACCACTGATGACAAGACTTTTGACGAGGCGGAAAAGAACCCTCTGGAGACTAG

CGGTGCCAGCACCGTTGGCTTTAGAGAGAGAACTCTTCCAGGTCAAAAGGCGTGCGATGACGTGAACTCC

GAGCCCGCCCAACCTGTTGAAGGACAACCACAAGCTGAAGGACCCTACGCCGGGCCACTCGAGCGTCAGA

AACCTCTGAAAGTGAGAGCCAAGCTCCCGCAGCAAGAGGGACCCTACGCTGGCCCGATGGAGAGACAGAA

ACCGCTGAAAGTGAAAGCAAAAGCCCCGGTCGTCAAGGAAGGACCTTACGAGGGACCGGTGAAGAAGCCT

GTCGCTTTGAAAGTGAAAGCTAAGAACTTGATTGTCACTGAGAGTGGTGCCCCACCGACCGACTTGCAAA

AGATGGTCATGGGCAACACCAAGCCTGTTGAGCTCATCCTCGACGGGAAGACGGTAGCCGTCTGCTGCGC

TACCGGAGTGTTCGGCACAGCTTACCTCGTGCCTCGTCATCTCTTCGCAGAGAAGTATGACAAGATCATG

CTGGATGGCAGAGCCATGACAGACAGTGACTACAGAGTGTTTGAGTTTGAGATCAAAGTAAAAGGACAGG

ACATGCTCTCAGACGCCGCGCTCATGGTGCTCCACCGTGGGAACCGCGTGAGAGACATCACGAAGCACTT

TCGTGATACAGCAAGAATGAAGAAAGGCACCCCCGTCGTCGGCGTGATCAACAACGCCGATGTTGGGAGA

CTGATTTTCTCTGGTGAGGCTCTGACCTACAAGGACATTGTAGTGTGCATGGATGGAGACACCATGCCTG

GCCTCTTTGCCTACAAAGCCGCGACAAAGGCTGGCTACTGTGGAGGAGCCGTTCTCGCGAAGGACGGAGC

TGACACATTCATCGTCGGCACTCACTCCGCTGGAGGAAATGGAGTTGGTTACTGCTCATGCGTTTCCAGG

TCCATGCTTCTCAAGATGAAGGCACACATTGACCCTGAACCACACCACGAG

>AY593823.1_O_TUR_1969

ATATCAATTCCTTCCCAAAAGTCTGTGTTGTACTTCCTCATTGAGAAAGGCCAACACGAAGCAGCAATTG

AATTCTTTGAGGGAATGGTGCATGACTCCATCAAGGAAGAGCTCCGGCCCCTCATCCAACAGACCTCATT

TGTGAAACGCGCTTTTAAGCGCCTGAAGGAAAACTTTGAGACTGTTGCCCTGTGTTTGACTCTTTTGGCA

AACATAGTGATCATGATCCGCGAGACTCGCAAGAGACAACAGATGGTGGACGATGCAGTGAATGACTACA

TTGAGAAGGCAAACATCACCACAGATGACAAGACTCTTGACGAGGCGGAAAAGAACCCTCTAGAGACCAG

CGGTGCCAGCACTATTGGTTTCAGAGAGAGAACTCTCCCGGGGCACAAGGCGAGCGATGACGTGAGCACC

GAGCCCGCCAAACCCGTGGAGGACCGACCACAAGCTGAAGGGCCCTACGCCGGACCACTTGAGCGTCAGA

AACCTCTGAGAGTGAAAACCAAGTTGCCACAACAGGAGGGACCCTACGCTGGCCCGATGGATAGACAGAA

ACCGTTGAAAGTGAGAGCAAGAGCCCCGGTCGTGAAGGAGGGACCCTACGAGGGACCGGTGAAGAAGCCT

GTCGCTTTGAAAGTGAAAGCCAAGAACTTGATTGTCACTGAGAGTGGTGCCCCACCGACCGACTTGCAGA

AGATGGTCATGGGCAACACTAAGCCTGTTGAGCTCATCCTCGACGGGAAGACGGTAGCCATCTGCTGTGC

TACCGGAGTGTTTGGCACTGCCTACCTCGTACCTCGTCACCTCTTCGCGGAGAAGTACGACAAGATAATG

TTGGACGGTAGAGCCATGACAGACAGTGACTACAGAGTGTTTGAGTTTGAGATTAAAGTAAAAGGACAGG

ACATGCTCTCAGACGCTGCACTCATGGTGCTTCACCGTGGGAACCGCGTGAGAGACATCACGAAACATTT

TCGTGACACAGCAAGAATGAAGAAAGGCACCCCCGTTGTCGGTGTGATCAACAACGCCGACGTTGGGAGA

CTGATTTTCTCTGGAGAGGCCCTTACCTACAAAGACATTGTAGTGTGCATGGATGGAGACACCATGCCGG

GCCTGTTTGCCTACAGAGCCGCCACCAAGGCTGGTTACTGCGGGGGAGCCGTTCTCGCCAAGGACGGAGC

CGACACATTCATCGTTGGCACTCACTCCGCAGGTGGTAACGGAGTTGGATACTGCTCGTGCGTGTCCAGG

TCCATGCTCCTGAAAATGAAGGCACACATTGACCCTGAACCACACCACGAG

>AY593824.1_O_SKR_2000

ATCTCAATACCTTCCCAAAAGGCTGTGCTATACTTTCTCATTGAGAAGGGCCAGCACGATGCAGCAATTG

AATTCTTTGAGGGGATGGTGCATGACTCCATCAAGGAGGAGCTCCGGCCTCTCATCCAACAGACCTCATT

TGTGAAGCGCGCTTTTAAGCGCCTGAAGGAAAACTTTGAGGTAGTTGCCCTGTGTTTGACCCTTTTGGCA

AACATAGTGATCATGATCCGCGAGACTCGCAAGAGACAGCAGATGGTGGATGACGCAGTGAACGAGTACA

TTGAGAAGGCAAACATCACCACGGATGACAAGACTCTTGACGAGGCGGAAAAGAACCCTCTGGAGACCAG

CGGTGCCACCACTGTTGGTTTTAGAGAGAAAACTCTCCCGGGACACAAGGCGAGTGATGACGTGAACTCC

GAGCCCGCCAAACCCGCGGAAGAACAACCACAAGCTGAAGGACCCTACACCGGTCCACTCGAGCGTCAAA

AACCTCTGAAAGTGAGAGCCAAGCTCCCACAGCAGGAGGGGCCCTACGCTGGTCCGATGGAGAGACAGAA

ACCGCTGAAAGTGAAAGTGAAAGCCCCGGTTGTTAAGGAAGGACCTTACGAAGGACCGGTGAAGAAACCT

GTCGCTTTGAAAGTGAAAGCAAAGAACTTGATTGTCACTGAGAGTGGTGCTCCCCCGACTGACTTGCAAA

AGATGGTCATGGGTAACACCAAGCCTGTTGAGCTCATCCTCGACGGGAAGACGGTGGCCATCTGTTGCGC

CACCGGAGTGTTTGGTACTGCTTACCTTGTCCCTCGTCATCTTTTCGCAGAGAAGTATGACAAGATCATG

TTGGACGGCAGAGCCATGACAGACAGTGACTACAGAGTGTTTGAGTTTGAGATTAAAGTGAAAGGACAGG

ACATGCTCTCAGACGCCGCGCTCATGGTGCTTCACCGTGGGAATCGCGTGCGGGACATCACGAAGCACTT

CCGTGATGTGGCAAGAATGAAGAAAGGCACCCCCGTCGTCGGCGTGGTCAACAACGCTGATGTTGGGAGA

CTGATCTTCTCTGGTGAGGCCCTTACCTACAAGGACATTGTAGTGTGCATGGACGGAGACACCATGCCCG

GTCTCTTCGCCTACAAAGCCGCCACCAAGGCGGGTTACTGTGGGGGAGCCGTTCTTGCAAAGGACGGAGC

CGAGACTTTCATCGTCGGCACTCACTCCGCAGGCGGCAATGGGGTTGGATACTGCTCATGCGTTTCCAGG

TCTATGCTGCTTAAAATGAAGGCACACATCGATCCCGAACCACACCACGAG

>AY593825.1_O_ARG_1939

ATCTCAATCCCTTCTCAAAAGTCCGTGTTGTACTTCCTCATTGAGAAGGGCCAACACGAGGCAGCAATTG

AATTCTTTGAGGGGATGGTCCATGACTCCATCAAGGAGGAACTCCGACCCCTCATCCAGCACACCTCATT

TGTGAAACGCGCGTTCAAGCGCCTGAAGGAAAACTTTGAGATTGTTGCTCTATGTCTGACTCTTTTGGCA

AACATAGTGATCATGATCCGCGAGACTCGCAAGAGACAGAAGATGGTGGATGATGCAGTGAATGAGTACA

TTGAGAAAGCAAACATCACCACAGACGACAGGACTCTCGACGAGGCGGAGAAGAACCCTCTGGAGACTAG

TGGTGCCACCACCGTTGGTTTTAGGGAGAAGACCCTCCCGGGGCACAAAGCGAGTGACGACGTGTTTTCC

GAGCCCGCCAAACCTGTGGAGGAACAACCACAAGCTGAAGGACCCTACGCCGGGCCACTCGAGCGTCAGA

AACCTCTGAAAGTGAGAGCCAAGCTGCCACAACAGGAGGGACCTTACGCTGGCCCGATGGAGAGACAGAA

ACCACTGAAAGTAAAAGCAAGAGCCCCGGTCGTGAAGGAAGGACCTTACGAGGGACCGGTGAAGAAGCCT

GTCGCTTTGAAAGTGAGAGCTAAGAACCTGATTGTCACTGAGAGTGGCGCCCCACCGACCGACTTGCAAA

AGATGGTCATGGGCAACACGAAGCCTGTTGAGCTCATCCTTGACGGGAAGACAGTGGCCATTTGCTGTGC

TACTGGGGTGTTTGGTACTGCTTACCTCGTGCCTCGCCATCTTTTTGCAGAGAAGTATGACAAGATCATG

CTGGACGGCAGAGCCATGACAGACAGTGACTACAGAGTGTTCGAGTTTGAGATTAAAGTAAAAGGACAGG

ACATGCTCTCGGACGCCGCGCTCATGGTGCTTCACCGTGGGAACCGCGTGAGAGACATCACGAAACACTT

TCGTGATACAGCAAGAATGAAGAAAGGCACCCCCGTTGTCGGCGTGATTAATAACGCCGATGTCGGGAGA

CTGATTTTCTCTGGTGAGGCCCTTACCTACAAGGACATTGTAGTGTGCATGGATGGAGACACCATGCCCG

GCCTCTTTGCCTACAAAGCCGCCACCAAGGCTGGGTACTGTGGAGGAGCCGTTCTCGCTAAGGACGGGGC

TGACACGTTCATCGTCGGCACTCACTCTGCAGGAGGCAACGGAGTTGGATACTGTTCATGCGTTTCCAGG

TCCATGCTTCTCAAAATGAAGGCACACATTGACCCTGAACCACACCACGAG

>AY593826.1_O_ITL_1947

ATCTCAATTCCTTCCCAAAAATCTGTGTTGTACTTCCTCATTGAGAAAGGCCAACACGAGGCAGCAATTG

AATTCTTTGAGGGCATGGTGCACGATTCCATCAAGGAAGAACTCCGGCCCCTCATCCAACAAACCTCATT

TGTGAAACGCGCTTTCAAGCGCCTGAAGGAAAACTTTGAGATTGTTGCTCTGTGTCTGACCCTTCTGGCT

AACATAGTGATCATGATCCGCGAAACTCGCAAGAGACAGAAGATGGTGGATGATGCCGTGAATGACTACA

TTGAAAAGGCAAACATCACCACAGATGACAAAACACTTGACGAGGCGGAAAAGAACCCTCTGGAGACCAG

CGGTGCCAGCACCGTTGGTTTCAGAGAGAAAACTCTGCCAGGCCACAAAGCGCGCGATGACGTGAACTCT

GAGCCCGCCCAGCCTGCTGAAGAGCAACCACAAGCTGAAGGACCCTACGTCGGGCCACTTGAGCGTCAGA

AACCTCTGAAAGTGAGAGCCAAGCTCCCACAGCAGGAGGGACCCTACGCTGGCCCGATGGAGAGACAGAA

ACCGCTGAAAGTAAAAGCAAAAGCCCCGGTCGTTAAGGAAGGACCTTACGAGGGACCGGTGAAGAAGCCT

GTCGCTTTGAAAGTGAAAGCTAAGAACTTGATTGTCACTGAGAGTGGTGCCCCACCGACCGACTTGCAGA

AGATGGTCATGGGCAACACAAAGCCTGTTGAGCTCATCCTCGACGGGAAGACAGTAGCCATCTGCTGTGC

TACTGGAGTCTTTGGCACTGCTTACCTCGTGCCTCGTCATCTTTTCGCAGAGAAGTATGACAAGATCATG

TTGGACGGCAGAGCCATGACAGACAGTGACTACAGAGTGTTTGAGTTTGAGATTAAAGTAAAAGGACAGG

ACATGCTCTCAGACGCGGCACTCATGGTGCTCCACCGTGGGAACCGCGTGAGAGACATCACGAAACACTT

TCGTGATACAGCAAGATTGAAGAAAGGCACCCCCGTCGTCGGTGTTATCAACAACGCCGATGTCGGGAGA

CTGATTTTCTCTGGTGAGGCCCTTACCTACAAGGACATTGTAGTGTGCATGGACGGAGACACCATGCCCG

GTCTCTTTGCCTACAAAGCCGCCACCAAGGCTGGCTACTGTGGAGGAGCCGTTCTTGCCAAGGACGGGGC

TGACACATTCATCGTCGGCACTCACTCTGCAGGTGGCAATGGAGTTGGGTACTGCTCATGCGTTTCCAGG

TCCATGCTTCAAAAGATGAAGGCTCACGTCGACCCTGAACCACACCACGAG

>AY593827.1_O_VEN_1971

ATCTCAATTCCTTCCCAAAAATCCGTGTTGTACTTCCTCATTGAGAAAGGCCAACACGAGGCAGCAATTG

AATTCTTTGAGGGTATGGTGCACGAATCCATCAAGGAAGAGCTCCGGCCCCTCATCCAGCAAACCTCATT

TGTGAAGCGCGCTTTCAAGCGCCTAAAGGAAAATTTTGAGATTGTTGCTCTTTGTCTGACCCTTTTGGCT

AACATAGTGATCATGATCCGCGAAACTCGCAAGAGACAAAAGATGGTGGATGATGCCGTGAACGACTACA

TCGAGAAGGCAAACATCACCACAGATGACAAAACACTTGACGAGGCGGAAAAGAACCCTCTGGAGACCAG

TGGTGCCAGCACCGTCGGTTTCAGAGAGAAGACTCTTCCAGGTCACAAAGCGCGTGATGACGTGAACTCT

GAGCCCGCCCAGCCTGCTGAAGAGCAACCACAAGCTGAAGGACCCTACGCCGGGCCACTCGAGCGTCAGA

AACCTCTGAAAGTGAGAGCCAGGCTCCCACAGCAGGAGGGACCCTACGCTGGCCCGATGGAGAGACAGAA

ACCGCTGAAAGTAAAAGTAAAAGCCCCGGTCGTTAAGGAAGGACCTTACGAGGGACCGGTGAAGAAGCCT

GTCGCTTTGAAAGTGAAAGCTAAGAACTTGATTGTCACTGAGAGTGGTGCCCCACCGACCGACTTGCAGA

AGATGGTCATGGGCAACACAAAGCCTGTTGAGCTCATCCTCGACGGGAAGACAGTAGCCATCTGCTGTGC

TACTGGAGTGTTTGGCACTGCTTACCTCGTGCCTCGTCATCTTTTCGCAGAGAAGTATGACAAGATCATG

TTGGACGGCAGAGCCATGACAGACAGTGACTACAGAGTGTTTGAGTTTGAGATTAAAGTAAAAGGACAGG

ACATGCTCTCAGACGCGGCACTCATGGTGCTTCACCGTGGGAACCGCGTGAGAGACATCACGAAACACTT

TCGTGATACAGCGAGGATGAAGAAAGGCACCCCCGTCGTCGGTGTTATCAACAACGCCGATGTTGGGAGA

CTGATTTTCTCTGGTGAGGCCCTTACCTACAAGGACATTGTAGTGTGCATGGATGGAGATACCATGCCCG

GCCTCTTTGCCTACAAAGCCGCCACCAAGGCTGGCTACTGTGGAGGAGCCGTTCTTGCCAAGGACGGGGC

TGACACATTCATCGTCGGCACTCACTCTGCAGGTGGTAATGGAGTTGGGTACTGCTCATGCGTTTCCAGG

TCCATGCTTCAAAAGATGAAGGCTCACGTCGACCCTGAACCACACCACGAG

>AY593828.1_O_IND_1962

ATCTCAATTCCTTCCCAAAAGTCCGTGTTGTACTTTCTCATTGAGAAGGGGCAGCACGAAGCAGCAATTG

AATTCTTCGAGGGGATGGTACACGACTCCATCAAGGAGGAGCTCCGACCTCTCATCCAACGGACCTCATT

TGTGAAGCGCGCGTTCAAGCGCCTGAAGGAGAACTTTGAGATTGTTGCCCTGTGTTTGACTCTTCTGGCA

AACATAGTGATCATGATCCGCGAGACTCGCAAGAGACAACAGATGGTGGATGATGCAGTGAATGAATACA

TTGAGAAAGCAAACATCACCACAGATGACAAGACTCTTGACGAGGCGGAAAAGAACCCTCTGGAGACTAG

TGGTGCCAGCACTGTTGGCTTCAGAGAGAGAACTCTCCCAGGACACAAGGCGTGTGATGACGTGAACTCC

GAGCCCGCCCAACCCGCGGAAGAGCAACCACAAGCTGAAGGACCCTACGCCGGGCCACTTGAGCGTCAGA

AACCTCTGAAAGTGAGAGCCAAGCTCCCACAGCGGGAGGGACCCTACGCTGGCCCGATGGAGAGACAGAA

ACCACTGAAAGTGAAAGCAAAAGCCCCGGTCGTGAAGGAAGGACCTTACGAGGGACCGGTGAAGAAACCT

GTCGCTTTGAAAGTGAAAGCTAAGAACCTGATTGTCACTGAGAGTGGTGCCCCACCGACCGACTTGCAAA

AGATGGTTATGGGCAACACAAAGCCTGTTGAGCTCATCCTCGACGGGAAGACAGTAGCCATCTGCTGCGC

TACTGGAGTGTTTAGCACCGCCTACCTCGTGCCTCGTCATCTCTTCGCTGAGAAGTATGACAAGATCATG

TTGGACGGCAGAGCCATGACAGACAGTGACTACAGAGTGTTTGAGTTTGAGATTAAAGTAAAAGGACAGG

ACATGCTCTCAGACGCCGCGCTCATGGTGCTGCACCGTGGGAACCGCGTGAGAGACATCACGAAGCACTT

TCGTGACACAGCAAGAATGAAGAAAGGCACCCCCGTCGTTGGTGTAATCAACAACGCCGATGTCGGGAGA

CTAATTTTCTCTGGTGAGGCCCTTACGTACAAGGACATTGTAGTGTGCATGGATGGAGACACCATGCCTG

GCCTCTTTGCCTACAGAGCCGCCACCAAGGCTGGCTACTGCGGGGGAGCCGTTCTCGCCAAGGACGGTGC

CGACACATTCATCGTTGGCACACACTCCGCAGGGGGCAATGGAGTTGGATACTGCTCTTGCGTTTCTAGG

TCCATGCTCCTGAAGATGAAGGCACACATCGACCCTGAACCACATCATGAG

>AY593830.1_O_POL_1959

ATCTCAATTCCTTCCCAAAAATCTGTGTTGTACTTTCTCATTGAGAAGGGCCAACATGAGGCAGCAATTG

AATTCTTTGAGGGCATGGTCCACGACTCCATTAAAGAGGAACTCCGACCCCTCATCCAACGAACTTCATT

TGTGAAACGCGCTTTCAAGCGCCTGAAGGAAAATTTTGAGATTGTTGCTCTGTGTTTAACACTTTTGGCA

AACATTGTGATCATGATCCGTGAGACTCGCAAGAGGCAGAAAATGGTGGACGATGCAGTGAATGAGTACA

TTGAGAAAGCAAACATCACCACAGATGACAAGACTCTTGACGAGGCGGAGAAGAGCCCTCTAGAGACCAG

CGGCGCCAGCACCGTTGGCTTTAGAGAGAGAACTCTCCCAGGTCAAAAGGCATGCGATGACGTGAACTCC

GAGCCTGCCCAACCTGTTGAGGAGCAACCACAAGCTGAAGGACCCTACGCCGGACCACTCGAGCGTCAGA

AACCTCTGAAAGTGAGAGCCAAGCTCCCACAGCAGGAGGGGCCTTACGCTGGTCCGATGGAGAGACAGAA

ACCGCTAAAAGTGAAAGCAAAAGCCCCGGTCGTGAAGGAAGGACCTTACGAGGGACCGGTGAAGAAGCCT

GTCGCTTTGAAAGTGAAAGCTAAGAACCTGATTGTCACTGAGAGTGGTGCCCCACCGACCGACTTGCAAA

AGATGGTCATGGGCAACACAAAGCCTGTTGAGCTCATCCTCGACGGGAAGACAGTAGCCATCTGCTGCGC

TACTGGAGTGTTTGGCACTGCTTACCTCGTGCCTCGTCACCTCTTCGCAGAGAAGTATGACAAGATCATG

TTGGACGGCAGAGCCATGACAGACAGTGACTACAGAGTGTTTGAGTTTGAGATCAGAGTAAAAGGACAGG

ACATGCTCTCAGACGCCGCGCTCATGGTGCTCCACCGTGGGAACCGCGTGAGGGACATCACGAAGCACTT

TCGTGACACAGCAAGAATGAAGAAAGGCACCCCCGTTGTCGGTGTGATCAACAACGCCGATGTCGGGAGA

CTGATTTTCTCTGGTGAGGCCCTTACTTACAAGGACATTGTGGTTTGCATGGACGGAGACACCATGCCTG

GCCTCTTTGCCTACAGAGCCGCCACCAAGGCTGGCTACTGCGGAGGAGCCGTTCTTGCCAAAGACGGAGC

TGACACTTTCGTCGTCGGCACTCACTCTGCAGGAGGCAACGGAGTTGGATACTGCTCATGCGTTTCCAGG

TCCATGCTTCTTAAAATGAAGGCACACATTGACCCCGAACCACACCACGAG

>AY593831.1_O_UKG_2002

ATCTCAACTCCTTCCCAAAAGGCTGTGCTGTACTTTCTCATTGAGAAGGGCCAGCACGAAGCAGCAATTG

AGTTCTTTGAGGGGATGGTGCATGACTCCATCAAGGAGGAGCTCCGGCCTCTCATCCAACAGACCTCATT

TGTGAAGCGCGCTTTTAAGCGCCTGAAGGAAAACTTTGAGATAGTTGCCCTGTGTTTGACTCTTTTGGCA

AACATAGTGATCATGATCCGCGAGACTCGCAAGAGACAGCAGATGGTGGATGATGCAGTGAACGAGTACA

TTGAGAAGGCAAACATCACCACGGATGACAAGACTCTTGACGAGGCGGAAAAGAACCCTCTGGAGACCAG

CGGTGCCACCACTGTTGGTTTCAGAGAGAAAACTCTCCCGGGACACAAGGCGGGTGATGACGTGAACTCC

GAGCCCGCCAAACCCGTGGAAGAACAACCACAAGCTGAAGGACCCTACACCGGTCCACTCGAGCGTCAAA

AACCCCTGAAAGTGAGGGCCAAGCTCCCACAGCAGGAGGGGCCCTACGCTGGTCCGATGGAGAGACAGAA

ACCGCTGAAAGTGAAAGTGAAAGCCCCGGTCGTTAAGGAAGGACCTTACGAAGGACCGGTGAAGAAACCT

GTCGCTTTGAAAGTGAAAGCAAAGAACTTGATTGTCACTGAGAGTGGTGCTCCCCCGACTGACTTGCAAA

AGATGGTCATGGGTAACACCAAGCCTGTTGAGCTCATCCTCGACGGGAAGACGGTGGCCATCTGCTGCGC

CACCGGAGTGTTTGGTACTGCCTACCTAGTTCCTCGTCATCTTTTCGCAGAGAAGTATGACAAGATCATG

TTGGACGGCAGAGCCATGACAGACAGTGACTACAGAGTGTTTGAGTTTGAGATTAAAGTGAAAGGACAGG

ACATGCTCTCAGACGCCGCTCTCATGGTGCTTCACCGCGGGAATCGCGTGCGGGACATCACGAAGCACTT

CCGTGATGTGGCAAGAATGAAGAAAGGCACCCCCGTCGTCGGCGTGATCAACAACGCTGATGTTGGGAGA

CTGATCTTCTCTGGTGAGGCCCTTACCTACAAGGACATTGTAGTGTGCATGGACGGAGACACCATGCCCG

GTCTCTTCGCCTACAAAGCTGCCACCAAGGCGGGTTACTGTGGAGGAGCCGTTCTTGCAAAGGACGGAGC

CGAGACTTTCATCGTCGGCACTCACTCCGCAGGCGGCAATGGAGTTGGATACTGCTCATGCGTTTCCAGG

TCCATGCTGCTTAAAATGAAGGCACACATCGATCCCGAACCACACCACGAG

>AY593833.1_O_TAW_1999

ATTTCAATCCCTTCCCAGAAGTCCGTGTTGTACTTCCTCATTGAGAAGGGCCAGCACGAAGCAGCGATCG

AGTTCTTCGAGGGGATGGTCCACGATTCCATCAAAGAGGAACTCCGACCCCTCATTCAGCAGACCTCGTT

CGTAAAACGCGCCTTCAAGCGCCTGAAAGAGAACTTTGAAGTTGTAGCCCTGTGTTTGACCCTCTTGGCA

AACATAGTGATTATGCTCCGCCAAGCGCGCAAGAGGTACCAATCGGTGGATGACCCACTGGAC-------

-----------------------GGCGACGTAACTCTTGGCGACGCGGAAAAGAACCCTCAGGAGACGAG

TGGCGCTAGCGCTGTCGGTTTCAGAGAGAGATCCCCCACCGAGCAAGGGACGCGCGAAGACGCGAACGCT

GAGCCCGTCGTGTTCGGTAGGGAACAACCGCGAGCTGAAGGACCCTACGCTGGGCCACTCGAGCGTCAGA

AACCTCTTAAAGTGAAAGCCGAGCTGCCACAACAGGAGGGACCATACGCCGGCCCAATGGAGAGACAGAA

ACCGCTAAAGGTGAAAGCAAAAGCCCCCGTCGTGAAGGAAGGACCTTACGAGGGACCGGTGAAGAAACCT

GTCGCTTTGAAAGTGAAAGCAAAGAACTTGATAGTCACTGAGAGTGGTGCGCCACCGACCGACTTGCAAA

AGATGGTCATGGGCAACACTAAGCCAGTCGAGCTCATCCTCGACGGCAAGACGGTAGCCATTTGCTGTGC

TACCGGAGTGTTCGGCACTGCCTACCTCGTGCCTCGTCATCTCTTCGCGGAAAAGTACGACAAGATCATG

TTGGACGGCAGAGCCTTGACAGACAGTGACTACAGAGTGTTTGAGTTTGAGATTAAAGTAAAAGGACAGG

ACATGCTCTCAGACGCCGCTCTCATGGTGTTGCACCGTGGGAATCGCGTGCGTGACATCACGAAACACTT

TCGTGACGTAGCGAGAATGAAGAAGGGAACCCCCGTCGTCGGTGTGATCAACAATGCTGACGTCGGGAGA

CTCATATTCTCTGGTGAAGCCCTTACTTACAAGGACATCGTCGTGTGTATGGATGGAGACACCATGCCTG

GGCTCTTTGCCTACAGGGCATCCACCAAGGCAGGCTACTGTGGAGGAGCCGTCCTGGCAAAGGACGGGGC

CGAAACGTTCATCGTTGGCACCCACTCCGCAGGTGGAAACGGCATAGGATACTGTTCGTGTGTTTCCCGA

TCAATGCTCCTGAAGATGAAGGCACACATCGACCCTGAACCACACCACGAG

>AY593834.1_O_IRN_1966

ATCTCAATTCCTTCTCAAAAATCTGTACTGTACTTTCTCATTGAGAGGGGGCAGCACGAAGCAGCAATTG

AATTCTTTGAGGGCATGGTGCACGACTCCATTAAAGAAGAGCTCCGGCCCCTTATCCAACAGACCTCATT

TGTGAAACGCGCTTTCAAGCGCTTGAAGGAAAACTTTGAGATTGTCGCCCTATGTTTGACTCTTTTGGCA

AACATAGTGATCATGATCCGCGAGACTCGCAAGAGACAGCAGATGGTGGATGATGCAGTAAATGAGTACA

TTGAGAAAGCAAACATCACCACAGATGACAAGACTCTTGGCGAGGCGGAAAAGAACCCTCAAGAGACCAG

CGGTGCCAGCACTGTTGGCTTTAGAGAGAGAACTCCTCCCGGGCAGAAGGCGTGCGATGACGTGAACTCC

GAGCCCGCACAACCCGTGGAGGAGCAACCACAAGCTGAAGGACCCTACGCCGGGCCACTTGAGCGTCAGA

AACCTCTGAAAGTGAGAGTCAAGCTACCACAACAGGAGGGGCCTTACGCCGGTCCGATGGAGAGACAGAA

ACCACTGAAAGTGAAAGCAAAAGCCCCGGTCGTTAAGGAAGGGCCTTACGAGGGACCGGTGAAGAAGCCT

GTCGCTTTGAAAGTGAAAGCCAAGAACTTGATTGTCACTGAGAGTGGTGCCCCACCGACTGACTTGCAAA

AGATGGTCATGGGTAACACGAAGCCTGTTGAGCTCATTCTCGACGGGAAGACAGTAGCCATCTGTTGTGC

TACTGGAGTGTTTGGCACTGCCTACCTCGTGCCTCGTCACCTTTTCGCAGAAAAGTACGACAAGATCATG

TTGGACGGCAGAGCCCTGACAGACAGTGACTACAGAGTGTTTGAGTTTGAGATTAAAGTAAAAGGACAGG

ACATGCTCTCAGACGCCGCGCTCATGGTGCTCCACCGTGGGAATCGCGTGAGAGACATCACGAAACACTT

TCGTGACACAGCAAGAATGAAGAAAGGTACCCCCGTCGTCGGCGTCATCAACAACGCCGATGTCGGGAGA

TTGATCTTCTCTGGTGAGGCCCTTACCTACAAAGACATTGTAGTGTGCATGGACGGAGACACCATGCCTG

GCCTCTTTGCCTACAAAGCCGCCACCAAGGCCGGCTACTGTGGAGGAGCCGTTCTCGCGAAGGACGGGGC

CGAGACATTCATCGTCGGCACTCACTCTGCGGGTGGCAATGGAGTTGGGTACTGCTCATGCGTTTCCAGG

TCCATGCTGCTGAAGATGAAGGCACACGTTGACCCCGAACCACACCACGAG

>AY593835.1_O_TAW_1997

ATTTCAATCCCTTCCCAGAAGTCCGTGTTGTACTTCCTCATTGAGAAGGGCCAGCACGAAGCAGCGATCG

AGTTCTTCGAGGGGATGGTCCACGATTCCATCAAAGAGGAACTCCGACCCCTCATTCAGCAGACCTCGTT

CGTAAAACGCGCCTTCAAGCGCCTGAAAGAGAACTTTGAAGTTGTAGCTCTGTGTTTGACCCTCTTGGCA

AACATAGTGATTATGCTCCGCCAAGCGCGCAAGAGGTACCAATCGGTGGATGACCCACTGGAC-------

-----------------------GGCGACGTAACTCTTGGCGACGCGGAAAAGAACCCTCTGGAGACGAG

TGGCGCTAGCGCTGTCGGTTTCCGAGAGAGATCCCCCACCGAGCAAGGGACGCGCGAAGACGCGAACGCT

GAGCCCGTCGTGTTCGGTAGGGAACAACCGCGAGCTGAAGGACCCTACGCTGGGCCACTCGAGCGTCAGA

AACCTCTTAAAGTGAAAGCCGAGCTGCCACAACAGGAGGGACCATACGCCGGCCCAATGGAGAGACAGAA

ACCGCTAAAGGTGAAAGCAAAAGCCCCCGTCGTGAAGGAAGGACCTTACGAGGGACCGGTGAAGAAACCT

GTCGCTTTGAAAGTGAAAGCAAAGAACTTGATAGTCACTGAGAGTGGTGCGCCACCGACCGACTTGCAAA

AGATGGTCATGGGCAACACTAAGCCAGTCGAGCTCATCCTCGACGGCAAGACGGTAGCCATTTGCTGTGC

TACCGGAGTGTTCGGCACTGCCTACCTCGTGCCTCGTCATCTCTTCGCGGAAAAGTACGACAAGATCATG

TTGGACGGCAGAGCCTTGACAGACAGTGACTACAGAGTGTTTGAGTTTGAGATTAAAGTAAAAGGACAGG

ACATGCTCTCAGACGCCGCTCTCATGGTGTTGCACCGTGGGAATCGCGTGCGTGACATCACGAAACACTT

TCGTGACGTAGCGAGAATGAAGAAGGGAACCCCCGTCGTCGGTGTGATCAACAATGCTGACGTCGGGAGA

CTCATATTCTCTGGTGAAGCCCTTACTTACAAGGACATCGTCGTGTGTATGGATGGAGACACCATGCCTG

GGCTCTTTGCCTACAGGGCATCCACCAAGGCAGGCTACTGTGGAGGAGCCGTCCTGGCAAAGGACGGGGC

CGAAACGTTCATCGTTGGCACCCACTCCGCAGGTGGAAACGGCATAGGATACTGTTCGTGTGTTTCCCGA

TCAATGCTCCTGAAGATGAAGGCACACATCGACCCTGAACCACACCACGAG

>AY593836.1_O_UKG_2001

ATCTCAACTCCTTCCCAAAAGGCTGTGCTGTACTTTCTCATTGAGAAGGGCCAGCACGAAGCAGCAATTG

AGTTCTTTGAGGGGATGGTGCATGACTCCATCAAGGAGGAGCTCCGGCCTCTCATCCAACAGACCTCATT

TGTGAAGCGCGCTTTTAAGCGCCTGAAGGAAAACTTTGAGATAGTTGCCCTGTGTTTGACTCTTTTGGCA

AACATAGTGATCATGATCCGCGAGACTCGCAAGAGACAGCAGATGGTGGATGATGCAGTGAACGAGTACA

TTGAGAAGGCAAACATCACCACGGATGACAAGACTCTTGACGAGGCGGAAAAGAACCCTCTGGAGACCAG

CGGTGCCACCACTGTTGGTTTCAGAGAGAAAACTCTCCCGGGACACAAGGCGGGTGATGACGTGAACTCC

GAGCCCGCCAAACCCGTGGAAGAACAACCACAAGCTGAAGGACCCTACACCGGTCCACTCGAGCGTCAAA

AACCCCTGAAAGTGAGGGCCAAGCTCCCACAGCAGGAGGGGCCCTACGCTGGTCCGATGGAGAGACAGAA

ACCGCTGAAAGTGAAAGTGAAAGCCCCGGTCGTTAAGGAAGGACCTTACGAAGGACCGGTGAAGAAACCT

GTCGCTTTGAAAGTGAAAGCAAAGAACTTGATTGTCACTGAGAGTGGTGCTCCCCCGACTGACTTGCAAA

AGATGGTCATGGGTAACACCAAGCCTGTTGAGCTCATCCTCGACGGGAAGACGGTGGCCATCTGCTGCGC

CACCGGAGTGTTTGGTACTGCCTACCTAGTTCCTCGTCATCTTTTCGCAGAGAAGTATGACAAGATCATG

TTGGACGGCAGAGCCATGACAGACAGTGACTACAGAGTGTTTGAGTTTGAGATTAAAGTGAAAGGACAGG

ACATGCTCTCAGACGCCGCTCTCATGGTGCTTCACCGCGGGAATCGCGTGCGGGACATCACGAAGCACTT

CCGTGATGTGGCAAGAATGAAGAAAGGCACCCCCGTCGTCGGCGTGATCAACAACGCTGATGTTGGGAGA

CTGATCTTCTCTGGTGAGGCCCTTACCTACAAGGACATTGTAGTGTGCATGGACGGAGACACCATGCCCG

GTCTCTTCGCCTACAAAGCTGCCACCAAGGCGGGTTACTGTGGAGGAGCCGTTCTTGCAAAGGACGGAGC

CGAGACTTTCATCGTCGGCACTCACTCCGCAGGCGGCAATGGAGTTGGATACTGCTCATGCGTTTCCAGG

TCCATGCTGCTTAAAATGAAGGCACACATCGATCCCGAACCACACCACGAG

>AY593837.1_O_URU_1963

ATCTCAATTCCTTCCCAAAAATCTGTGTTGTACTTTCTCATTGAGAAGGGCCAACATGAGGCAGCAATTG

AATTCTTTGAGGGCATGGTCCACGACTCCATCAAAGAGGAACTCCGACCCCTCATCCAACAAACTTCATT

TGTGAAACGCGCTTTCAAGCGCCTGAAGGAAAATTTTGAGATTGTTGCTCTGTGTTTAACACTTTTGGCA

AACATTGTGATCATGATCCGTGAGACTCACAAGAGGCAGAAAATGGTGGATGATGCAGTGAATGAGTACA

TTGAGAAAGCAAACATCACCACAGATGACAAGACTCTTGACGAGGCGGAGAAGAGCCCTCTAGAGACCAG

CGGCGCCAGCACCGTTGGCTTTAGAGAGAGAACTCTCCCAGGTCAAAAGGCATGCGATGACGTGAACTCC

GAGCCTGCCCAACCTGTTGAGGAGCAACCACAAGCTGAAGGACCCTACGCCGGACCACTCGAGCGTCAGA

AACCTCTGAAAGTGAGAGCCAAGCTCCCACAGCAGGAGGGGCCTTACGCTGGTCCGATGGAGAGACAGAA

ACCGCTAAAAGTGAAAGCAAAAGCCCCGGTCGTGAAGGAAGGACCTTACGAGGGACCGGTGAAGAAGCCT

GTCGCTTTGAAAGTGAAAGCTAAGAACCTGATTGTCACTGAGAGTGGTGCCCCACCGACCGACTTGCAAA

AGATGGTCATGGGCAACACAAAGCCTGTTGAGCTCATCCTCGACGGGAAGACAGTAGCCATCTGCTGCGC

TACTGGAGTGTTTGGCACTGCTTACCTCGTGCCTCGTCACCTCTTCGCAGAGAAGTATGACAAGATCATG

TTGGACGGCAGAGCCATGACAGACAGTGACTACAGAGTGTTTGAGTTTGAGATCAAAGTAAAAGGACAGG

ACATGCTCTCAGACGCCGCGCTCATGGTGCTCCACCGTGGGAACCGCGTGAGGGACATCACGAAGCACTT

TCGTGACACAGCAAGAATGAAGAAAGGCACCCCCGTTGTCGGTGTGATCAACAACGCCGATGTCGGGAGA

CTGATTTTCTCTGGTGAGGCCCTTACTTACAAGGACATTGTGGTTTGCATGGACGGAGACACCATGCCTG

GCCTCTTTGCCTACAGAGCCGCCACCAAGGCTGGCTACTGCGGAGGAGCCGTTCTTGCCAAAGACGGAGC

TGACACTTTCATCGTCGGCACTCACTCTGCAGGAGGCAACGGAGTTGGATACTGCTCATGCGTTTCCAGG

TCCATGCTTCTTAAAATGAAGGCACACATTGACCCCGAACCACACCACGAG

>AY593838.1_SAT1_BOT_1970

ATTTCCATTCCTTCCCAAAAGTCTGTGCTTTACTTCCTCATCGAGAAAGGACAGCACGAAGCGGCAATTG

AATTCTACGAAGGGATGGTACATGACAGCATTAAAGAGGAACTTAAGCCCTTGTTGGAGCAAACCAGCTT

CGCCAAGCGTGCTTTCAAGCGCCTCAAGGAGAACTTCGAGGTCATCACTCTCGTTGTTGTGCTGTTGGCA

AACATCATCATCATGATCCGCGAGACTCGCAAGCGTCAGAAGATGGTCGACGACGCACTCGATGAGTACA

CTGAGAAGGCGAACATCACCACTGACAACAAAACGCTTGAAGAGGCGGAAAAGAACCCTCAGGAGGTTGT

CGACAAACCCACTGTCGGCTTCCGTGAGAGAAAACTCCCCGGGCACAAAACTGACGATGAAGTGAACTCT

GAGCCAGCCAAACCC---GCGGAGAAACCACAAGCTGAAGGACCCTATGCTGGCCCCCTTGAACGACAAC

AGCCGCTGAAACTTAAGGCTAAGCTCCCTCAGGCTGAGGGACCTTACGTCGGGCCACTAGAGAGACAACA

ACCACTGAAATTGAGAGCAAGACTGCCTGTGGCCAAGGAAGGGCCATATGAAGGACCAGTGAAGAAGCCT

GTCGCTTTGAAACTGAAAGCAAAAGCCCCGATTGTCACTGAAAGCGGATGCCCACCGACTGACTTGCAAA

AGATGGTCATGGCAAACGTCAAGCCTGTTGAGCTTATCCTCGACGGGAAGACAGTTGCGCTCTGCTGCGC

GACTGGAGTGTTCGGGACGGCTTACCTCGTGCCTCGTCATCTTTTCGCAGAGAAGTACGACAAGATCATG

CTGGACGGCCGAGCCTTGACAGACAGTGACTTCAGAGTGTTTGAGTTTGAGGTAAAAGTAAAAGGACAGG

ACATGCTCTCAGATGCCGCACTGATGGTTCTCCACTCTGGAAACCGAGTGCGCGACCTCACGAGACACTT

CCGTGACACCATGAAACTGTCGAAAGGCAGTCCCGTCGTTGGCGTGGTCAACAACGCCGACGTCGGAAGA

CTCATCTTCTCAGGAGATGCACTGACCTACAAAGACCTGGTCGTTTGTATGGACGGTGACACCATGCCTG

GACTCTTCGCATACCGCGCAGGGACTAAGGTTGGATACTGCGGAGCTGCCGTTCTCGCGAAGGACGGCGC

CAAGACAGTGATCGTCGGCACGCACTCCGCCGGAGGCAACGGAGTAGGCTACTGCTCCTGTGTCTCAAGA

TCCATGCTCCTGCAAATGAAAGCCCACATCGACCCTCCCCCTCACACCGAG

>AY593839.1_SAT1_UKG_1970

ATTTCCATCCCTTCCCAAAAATCTGTGCTCTACTTCCTCATCGAGAAAGGACAGCATGAAGCAGCAATTG

AATTCTACGAGGGGATGGTGCACGACAGCATCAAGGAAGAGCTTAAACCGCTTCTGGAGCAAACCAGCTT

CGCGAAACGTGCTTTCAAGCGCCTGAAAGATAACTTCGAGATCGTCGCTCTCGTAGTTGTGCTGTTGGCA

AACATCATCATCATGATCCGTGAGACTCGCAAGAGACAGAAGATGGTGGATGACGCCCTCGATGAGTACA

TTGAGAAGGCAAACATCACCACTGATGACAAGACTCTTGACGAGGCGGAAAAGAACCCACAAGAGGTCGT

CGACAAACCCACTGTCGGCTTCCGTGAGAGAAAACTCCCTGGGCACAAGACTGACGATGAAGTGAACTCT

GAGCCAGTCAAGCCC---GTGGAGAAACCACACGCTGAAGGACCCTACGCGGGCCCGCTCGAGCGACAGC

AGCCGCTGAAGCTCAAGGCCAAGCTGCCCAAGGCAGAAGGGCCGTACGCGAGACCGCTAGAGAAGCAACA

ACCACTGAAACTGAAAACAAGATTGCCTGTGGCCAAGGAGGGGCCATATGAAGGACCAGTGAAGAAGCCT

GTCGCTTTGAAAGTGAAAGCAAAAGCCCCGATTGTCACTGAAAGCGGATGTCCACCGACCGACCTGCAGA

GGATGGTCATGGCAAACGTGAAACCCGTTGAGCTCATCCTCGATGGGAAGACCGTTGCGCTCTGCTGCGC

TACGGGAGTGTTCGGGACGGCTTATCTCGTGCCTCGTCATCTTTTCGCAGAGAAGTACGACAAGATCATG

CTGGACGGCCGTGCCCTGACAGACAGTGACTTCAGAGTGTTTGAGTTTGAGGTGAAAGTAAAAGGACAGG

ACATGCTCTCAGATGCCGCGCTCATGGTTCTCCACTCCGGAAACCGTGTGCGCGATCTCACGGGACACTT

CCGTGACACAATGAAACTGTCGAAAGGCAGTCCCGTCGTTGGTGTGGTCAACAACGCCGACGTCGGAAGA

CTCATCTTCTCAGGAGACGCACTGACCTACAAAGACCTGGTCGTTTGTATGGACGGTGACACCATGCCCG

GACTCTTCGCATACCGCGCGGGGACCAAGGTTGGTTATTGTGGAGCAGCCGTTCTTGCAAAAGACGGCGC

CAAGACTGTGATCGTCGGCACCCACTCAGCCGGTGGCAACGGAGTAGGCTACTGCTCCTGTGTCTCACGA

TCCATGCTCCTGCAGATGAAAGCCCACATCGACCCGGCCCCCCACACCGAG

>AY593840.1_SAT1_NMB_1949

ATTTCCATTCCTTCCCAAAAGTCTGTGCTCTACTTCCTCATCGAGAAAGGACAGCACGAAGCAGCAATTG

AGTTCTATGAAGGGATGGTACACGACAGCATCAAAGAGGAGCTCAAGCCCTTGTTGGAGCGAACCAGCTT

CGCCAAGCGCGCTTTCAAACGCCTCAAGGAGAACTTCGAGATCGTCGCTCTCGTTGTTGTGCTGTTGGCA

AACATCATCATCATGATCCGCGAAACTCGCAAGCGTCAGAAGATGGTCGACGACGCTCTCGATGAACACA

TTGAGAAAGCGAACATCACCACTGACGACAAGACACTTGATGAGGCGGAAAAGAACCCTCAGGAGGTTAT

CGACAAACCCACTGTCGGCTTCCGTGGGAGAAAGCTCCCCGGACACAAGACTGACGATGAAGTGAACTCC

GAGCCAGTCAAACCC---GCGGACAAACCACAAGCTGAAGGACCCTACGCTGGCCCCCTCGAGCGACAGC

AGCCGCTGAAACTCAGGGCTAAGCTCCCTCAGGCCGAGGGACCTTATGCTGGGCCACTAGAGAGACAACA

ACCACTGAAACTGAAAGCTAAACTGCCTGTGGCCAAGGAAGGGCCATATGAAGGACCAGTGAAGAAACCT

GTCGCTTTGAAAGTGAAAGCAAAAGCCCCGATTGTCACTGAAAGCGGATGCCCGCCGACCGACTTGCAAA

AGATGGTCATGGCAAACGTGAAGCCCGTTGAGCTCATCCTCGACGGGAAGACAGTTGCGCTCTGCTGCGC

GACTGGAGTGTTCGGGACGGCTTACCTCGTGCCTCGTCATCTTTTCGCAGAGAAGTATGACAAGATCATG

CTGGACGGCCGTGCCCTGACAGACAGTGACTTCAGAGTGTTTGAGTTTGAGGTAAAAGTAAAAGGACAGG

ACATGCTCTCAGATGCCGCGCTGATGGTTCTCCACTCCGGAAACCGTGTGCGCGATCTCACGGGACACTT

CCGTGACACCATGAAGCTGTCGAAAGGCAGCCCCGTCGTTGGCGTGGTCAACAACGCCGATGTCGGAAGA

CTCATCTTCTCAGGAGATGCACTGACCTACAAAGACCTGGTCGTTTGTATGGACGGTGACACCATGCCTG

GACTCTTCGCATACCGCGCAGGGACCAAGGTTGGATACTGCGGAGCTGCTGTTCTCGCAAAGGACGGCGC

CAAGACAGTGATCGTCGGTACCCACTCTGCCGGAGGCAACGGAGTAGGCTACTGCTCCTGCGTCTCACGG

TCCATGCTCCTGCAGATGAAGGCCCACATCGACCCTCCCCCTCACACTGAG

>AY593841.1_SAT1_ZIM_1958

ATTTCCATCCCTTCCCAAAAGTCTGTGCTCTACTTCCTCATTGAGAAAGGACAGCACGAAGCAGCAATTG

AATTCTATGAAGGGATGGTGCACGACAGCATCAAGGAAGAGCTTAAGCCCTTGTTGGAGCAAACCAGCTT

CGCCAAGCGTGCTTTCAAGCGCCTGAAAGAGAACTTCGAGATTGTCGCTCTCGTTGTTGTGCTGTTGGCA

AACATCATCATCATGATCCGCGAAACTCGCAAGCGACAGAAGATGGTTGACGACGCCCTCGATGAGTACA

TTGAGAAGGCAAATATCACCACTGATGACAAAACGCTTGATGAGGCGGAAAAGAACCCTCAAGAGGTTGT

CGACAAACCCACTGTCGGCTTCCGTGAGAGAAAACTCCCCGGGCATAAGACTGACGATGAAGTGAACTCT

GAGCCAGTCAAACCC---GCGGAGAAACCGCAAGCTGAAGGACCCTACGCTGGCCCCCTCGAGCGACAGC

AACCGCTGAAACTCAAGGCTAAGCTCCCTCAGGCCGAGGGACCTTACGCTGGGCCGCTAGAGAAGCAACA

ACCACTGAAACTGAAAGCAAGACTGCCTGTGGCCAAGGAAGGGCCATATGAAGGACCAGTGAAGAAACCT

GTCGCTTTGAAAGTGAAAGCAAAAGCCCCGATTGTCACTGAAAGCGGATGCCCACCGACTGACTTGCAAA

AGATGGTCATGGCAAACGTGAAGCCTGTTGAGCTCATCCTCGACGGGAAGACAGTTGCGCTGTGCTGCGC

GACTGGAGTGTTCGGAACGGCTTACCTCGTGCCTCGTCATCTTTTCGCAGAGAAGTATGACAAGATCATG

TTGGACGGCCGCGCCCTGACAGACAGTGACTTCAGAGTGTTTGAGTTTGAGGTGAAAGTAAAAGGACAGG

ACATGCTCTCAGATGCCGCGCTGATGGTTCTCCACTCCGGAAACCGAGTGCGCGATCTCACGGGACACTT

CCGTGACACCATGAAACTGTCGAAAGGCAGCCCCATCGTTGGCGTGGTCAACAACGCTGACGTCGGAAGA

CTCATCTTCTCAGGAGATGCACTGACCTACAAAGACCTGGTCGTTTGTATGGACGGTGACACCATGCCTG

GACTCTTCGCCTACCGCGCAGGGACCAAGGTTGGATACTGCGGAGCTGCCGTTCTCGCGAAGGACGGCGC

CAAGACAGTGATCGTCGGCACCCACTCTGCCGGAGGCAATGGAGTAGGCTACTGCTCCTGTGTCTCACGA

TCCATGCTCCTACAGATGAAGGCCCACATCGACCCTCCCCCTCACACTGAG

>AY593842.1_SAT1_SAR_1961

ATTTCCATTCCTTCCCAAAAGTCTGTGCTCTACTTCCTCATCGAGAAAGGACAGCACGAAGCAGCAATTG

AATTCTATGAAGGGATGGTGCACGACAGCATCAAGGAAGAGCTTAAGCCCTTGTTGGAGCAAACCAGCTT

CGCCAAGCGTGCTTTCAAGCGCCTCAAAGAAAACTTCGAGATCGTCGCTCTCGTTGTTGTGCTGTTGGCA

AACATCATCATCATGATCCGCGAGACTCGCAAGCGCCAGAAGATGGTTGACGACGTCCTTGATGAGTACA

TTGAGAAGGCGAATATCACCACTGACGACAAAACGCTTGACGAGGCGGAAAAGAACCCTCAGGAGGTTGT

CGACAAACCCACTGTTGGCTTCCGTGAGAGAAAGCTCCCCGGGCACAGGACTGACGATGAAGTGAACTCC

GAGCCAGTCAAACCC---GCGGAGAAACCACAAGCTGAAGGACCCTACGCTGGCCCTCTTGAGCGGCAGC

AACCGCTGAAGCTCAAGGCTAAGCTCCCTCAGGCTGAGGGACCTTACGCTGGGCCACTAGAGAGACAACA

ACCACTGAAACTGAAAGCAAAACTGCCTGTGGCCAAGGAAGGGCCATATGAAGGACCAGTGAAGAAACCT

GTCGCTTTGAAAGTGAAAGCAAAAGCCCCGATTGTCACTGAAAGCGGATGCCCACCGACTGACTTGCAAA

AGATGGTCATGGCAAACGTGAAGCCCGTTGAGCTCATCCTCGACGGGAAGACAGTTGCGCTCTGCTGCGC

GACTGGAGTGTTCGGGACGGCTTACCTCGTGCCTCGTCATCTTTTCGCAGAGAAGTATGACAAGATCATG

CTGGACGGCCGCGCCCTGACAGACAGTGACTTCAGAGTGTTTGAGTTTGAGGTAAAAGTAAAAGGACAGG

ACATGCTCTCAGATGCCGCACTGATGGTTCTCCACTCCGGAAACCGAGTGCGTGATCTCACGGGACATTT

CCGTGACACCATGAAACTGTCGAAAGGCAGTCCCGTCGTTGGTGTGGTCAACAACGCCGACGTCGGAAGA

CTCATCTTCTCAGGAGATGCACTGACCTACAAAGACCTGGTCGTTTGTATGGACGGCGACACCATGCCTG

GACTCTTCGCGTACCGCGCAGGGACCAAAGTTGGATATTGCGGAGCTGCTGTCCTCGCAAAGGATGGCGC

CAAAACAGTGATCGTCGGCACCCACTCTGCCGGAGGTAACGGAGTAGGCTACTGCTCCTGCGTCTCACGA

TCCATGCTCCTGCAAATGAAGGCCCACATCGACCCTCCCCCTCACACTGAG

>AY593843.1_SAT1_NMB_1940

ATCTCTATTCCTTCCCAAAAGTCTGTGCTCTACTTCCTCATCGAGAAAGGACAGCACGAAGCAGCAATTG

AATTCTATGAAGGGATGGTACACGACAGCATCAAGGAAGAGCTTAAGCCCTTGTTGGAGCGAACCAGCTT

CGCCAAGCGTGCTTTCAAGCGCCTTAAGGAAAACTTCGAGATTGTCGCTCTCGTGGTTGTGCTGTTGGCA

AACATCATCATTATGATCCGCGAAACTCGCAAGCGTCAGAAGATGGTCGACGACGCCCTCGATGAGTACA

TTGAGAAGGCGAACATCACCACCGACGACAAAACGCTTGACGAGGCGGAAAAGAACCCTCAAGAGGTAGT

CGACAAACCCACTGTCGGCTTCCGAGAGAGAAAACTCCCTGGGCACAAGACTGACGATGAAGTGAACTCT

GAGCCAGCCAAACCC---GCGGAGAAACCACAAGCTGAAGGACCCTATACTGGCCCGCTCGAGCGACAGC

GGCCGCTGAAGCTCAAGGCTAAGCTCCCTCAGGCTGAGGGGCCTTATGCCGGGCCGCTAGAGAGACAACA

ACCACTGAAACTGAAAGAAAAACTGCCTGTGGCCAAGGAAGGGCCATATGAAGGACCAGTGAAGAAGCCT

GTCGCTTTGAAAGTGAAAGCAAAAGCCCCGATTGTCACTGAAAGCGGATGCCCACCGACCGACTTGCAAA

AGATGGTCATGGCAAACGTGAAGCCTGTTGAGCTCATCCTCGACGGGAAGACAGTTGCGCTCTGCTGCGC

GACTGGAGTGTTCGGGACGGCTTACCTCGTGCCTCGTCATCTTTTCGCTGAGAAGTACGACAAGATCATG

TTGGACGGCCGCGCCCTGACAGACAGTGACTTCAGAGTGTTTGAGTTTGAGGTAAAAGTAAAAGGACAGG

ACATGCTCTCAGATGCCGCGCTGATGGTTCTCCACTCTGGAAACCGTGTGCGCGATCTCACGGGACACTT

CCGTGACACCATGAAACTGTCGAAAGGCAGTCCCGTCGTCGGCGTGGTCAACAACGCCGACGTCGGAAGA

CTCATCTTCTCAGGAGACGCACTGACCTACAAAGACCTGGTCGTTTGTATGGACGGTGACACCATGCCTG

GACTCTTCGCGTACCGCGCAGGGACCAAGGTTGGATACTGCGGAGCTGCCGTTCTCGCGAAGGATGGCGC

CAAAACAGTGATCGTCGGCACCCACTCTGCCGGAGGCAATGGAGTAGGCTACTGCTCCTGCGTCTCACGA

TCCATGCTCCTGCAAATGAAGGCCCACATCGACCCTCCCCCTCACACCGAG

>AY593844.1_SAT1_ISR_1962

ATCTCACTTCCTTCTCAAAAGTCAGTGCTTTACTTCCTCATTGAGAAAGGACAGCACGAAGCTGCAATTG

AATTCTTTGAAGGAATGGTCAGTGACTCCATCAAAGAGGAGCTGCGCCCCCTTCTTCAACAAACTTCATT

TGTGAAACGCGCTTTCAAGCGCCTGAAGGAAAACTTTGAGATCGTTGCACTGGTGTTGGCTCTCCTAGCC

AATATCATCATCATGATCCGCGAAACACGCAAGAGACAAGAAATGGTGGACGCTGCCGTGAACGATTACA

TCGAGCGCGCCGGCATCACCACCGATGACCAAACTCTAGACGAGGCGGAAAAGAACCCTCTGGAAACAAC

TGGTACCAGCACCGTTGGCTTCAGGGAGAGAACTCTCACCGGGCAGAAAGTAGACAATGACGTGAACTCC

GAGCCTGCCGAACCCGTGGAGGAGCAACCACAAGCTGAAGGACCCTACGCCGGACCGATGGAGCGCCAGA

AACCTCTGAAAGTGAAAGCCAAGCTGCCACAACAGGAGGGGCCTTACGCCGGTCCGATGGAGAGACAACA

ACCGCTGAAAGTTAAAGCAAAACCCCCCGTCGTGAAGGAAGGACCTTACGAGGGGCCAGTGAAGAAACCT

GTCGCTTTGAAAGTGAAAGCAAAGAACATGATTGTCACGGAGAGTGGAGCGCCACCCACCGACTTGCAAA

AGATGGTGATGGCCAACACCAAGCCGGTCGAGCTCATACTCGATGGGAAGACAGTGGCAATCTGCTGTGC

CACTGGAGTGTTTGGCACTGCCTATCTCGTGCCTCGTCATCTTTTCGCTGAGAAGTACGACAAGATCATG

GTTGACGGCAGAGCCATGACAGACCGTGATTTCAGAGTGTTTGAGTTTGAGATTAAAGTAAAAGGACAGG

ACATGCTCTCAGACGCTGCTCTCATGGTGCTGCACCGTGGGAACCGCGTGAGAGACATCACGAGACACTT

TCGTGATCAAGCAAGAATGAGGAAAGGAACCCCCGTCGTTGGCGTGATCAACAACGCTGACGTTGGGAGA

CTCATCTTCTCTGGGGAGGCACTCACCTACAAAGACATCGTAGTGTGTATGGATGGCGATACCATGCCAG

GCCTCTTTGCCTACAAGGCCGCCACCAAAGCTGGCTATTGCGGAGGAGCCGTTCTTGCAAAGGACGGGGC

CGAGACTTTTATCGTTGGCACTCACTCCGCTGGAGGTAATGGAGTTGGTTACTGCTCTTGTGTTTCCAAG

TCCATGCTCCTACAAATGAAGGCACACGTTGACCCTGAGCCACACCATGAA

>AY593845.1_SAT1_BOT_1968

ATTTCCATTCCTTCCCAAAAGTCCGTGCTCTACTTCCTCATCGAGAAGGGACAGCACGAAGCAGCAATTG

AATTTTACGAAGGGATGGTTCACGACAGCATCAAGGAAGAGCTTAAGCCCTTGTTGGAGCAAACCAGCTT

CGCCAAGCGTGCTTTCAAGCGCCTTAAGGAAAACTTCGAGATCGTTGCTCTCGTTGTTGTGCTGTTGGCA

AACATCATCATCATGATCCGCGAGACTCGAAAGCGCCAGAAGATGGTGGACGACGCCCTCGATGAATACA

TTGAGAAGGCCAACATCACCACCGACGACAAAACGCTTGAAGAGGCGGAAAAGAACCCTCAAGAGGTTGT

CGACAAACCCACTGTCGGCTTCCGTGAGAGAAAGCTCCCCGGGCACAAGACTGACGATGAAGTGAACTCT

GAGCCAGTTAAACCC---ACGGAGAAACCACAAGCTGAAGGACCCTACGCTGGCCCCCTCGAGCGACAAC

AACCGCTGAAACTCAAGGCTAAGCTCCCTCGGGCAGAGGGACCTTACGCGGGGCCACTAGAGAAGCAACA

ACCGCTGAAACTGAAAGCAAAACTGCCTGTGGCCAAGGAAGGGCCATACGAAGGACCAGTGAAGAAACCT

GTCGCTTTGAAAGTGAAAGCAAAAGCCCCGATTGTCACTGAAAGCGGATGCCCACCGACTGACTTGCAAA

AGATGGTCATGGCAAACGTGAAGCCTGTTGAGCTTATCCTCGACGGGAAGACAGTTGCGCTCTGTTGCGC

GACTGGAGTGTTCGGGACGGCTTACCTCGTGCCTCGTCATCTTTTCGCAGAGAAGTATGACAAGATCATG

CTGGACGGCCGTGCCCTGACAGATAGTGACTTCAGAGTGTTCGAGTTTGAGGTAAAAGTAAAAGGACAGG

ACATGCTCTCAGATGCCGCGCTGATGGTTCTCCACTCCGGAAACCGTGTGCGCGATCTCACGGGACACTT

CCGTGACACCATGAAGCTGTCGAAAGGCAGCCCCGTCGTTGGTGTGGTCAACAACGCCGACGTCGGAAGA

CTCATCTTCTCAGGAGATGCACTGACCTACAAAGACCTGGTCGTTTGTATGGACGGTGACACCATGCCCG

GACTCTTCGCGTACCGCGCAGGGACCAAGGTTGGATACTGCGGAGCTGCCGTTCTTGCAAAAGACGGCGC

CAAGACAGTGATCGTTGGCACCCACTCTGCCGGAGGCAATGGAGTAGGCTACTGCTCCTGCGTCTCAAGA

TCAATGCTCCTGCAGATGAAAGCACACATCGACCCTCCCCCTCACACTGAG

>AY593846.1_SAT1_ZIM_1966

ATTTCCATTCCTTCCCAAAAGCATGTGCTTTACTTCCTCATTGAGAAAGGACAACACGAAGCAGCAATTG

AGTTTTACGAAGGGATGGTGCACGACAGCATCAAGGAGGAGCTTAAGCCGCTTCTGGAGCAAACCAGCTT

CGCGAAGCGCGCTTTCAAGCGCCTAAAGGAGAACTTCGAGATCGTTGCTCTCGTTGTTGTGCTGTTGGCA

AACATCATCATCATGATCCGCGAGACTCGTAAGAGACAGAAGATGGTGGACGACGCTCTTGACGAGTACA

TCGAAAGGGCAAACATCACCACCGACGACAAGACTCTTGACGAGGCGGAAAAGAACCCTCAAGAGGTGGT

CGACAAACCCACTGTCGGCTTCCGTGAGAGAAAACTCCCTGGACACAAGACTGACAATGATGTGAACTCT

GAGCCAGTTAAAACC---GAGGAGCAACCACAAGCTGAAGGACCCTACGCGGGACCGCTCGAGCGCCAGC

AGCCGCTGAAGCTCAAGGCTAAGCTGCCCAAAGCAGAAGGGCCTTACGCGGGACCGTTGGAGAAGCAACA

ACCACTGAAACTGAAAGCAAAGTTGCCTGTGGCCAAGGAAGGGCCATATGAAGGACCAGTGAAGAAACCT

GTCGCTTTGAAAGTGAAAGCAAGAGCCCCGATTGTCACTGAAAGCGGATGCCCACCGACTGACTTGCAAA

AGATGGTCATGGCAAACGTGAAGCCCGTTGAGCTCATCCTCGATGGGAAGACCGTTGCGCTCTGCTGCGC

TACGGGAGTGTTCGGGACAGCCTACCTCGTGCCTCGCCATCTTTTCACAGAGAAGTATGACAAGATCATG

TTGGACGGCCGCGCCCTGACAGACAGTGACTTCAGAGTGTTTGAGTTTGAGGTAAAAGTAAAAGGACAGG

ACATGCTCTCAGATGCCGCGCTCATGGTTCTCCACTCTGGAAACCGCGTGCGCGATCTCACGGGACACTT

CCGTGACACCATGAAACTGTCGAAAGGCAGTCCCGTCGTTGGCGTGGTCAACAACGCCGATGTCGGAAGA

CTCATCTTCTCAGGAGACGCACTGACCTACAAAGACCTGGTCGTTTGCATGGACGGTGACACCATGCCTG

GACTCTTCGCATACCGCGCGGGGACCAAGGTTGGATACTGCGGAGCTGCCGTTCTCGCAAAGGATGGCGC

CAAAACCGTGATCGTCGGCACCCACTCTGCCGGAGGCAATGGAGTAGGCTACTGCTCCTGTGTCTCACGA

TCCATGCTCCTGCAAATGAAGGCTCACATCGACCCTGCTCCACACACCGAG

>AY593847.1_SAT2_ZIM_1948

ATTTCCATTCCTTCCCAAAAGTCTGTGCTCTACTTTCTCATCGAGAAGGGACAGCACGAAGCAGCAATTG

AATTCTACGAGGGAATGGTACACGACAGCATCAAGGAAGAGCTTAAGCCCTTGTTGGAGCAAACCAGTTT

CGCCAAGCGCGCTTTCAAGCGTCTGAAGGAGAACTTCGAGATCGTTGCTCTCATCGTTGTGCTGTTGGCA

AACATCATCATCATGATCCGCGAAACTCGCAAGAGACAGAAGATGGTCGACGACGCCCTCGATGAGTACA

TTGAGAAGGCCAACATCACCACTGATGACAAAACTCTTGACGAGGCGGAAAAGAACCCTCAGGAGGTTGT

CGACAAACCCACTGTCGGCTTCCGTGAGAGAAAGCTCCCCGGGAAAAAAACTGACGACGAAGTGAACTCT

GAGCCAGTTAAACCC---GTGGAGAGACCACAAGCTGAAGGACCCTACGCGGGACCGCTTGAGCGGCAAC

AGCCGCTGAAGCTCAAGGCCAAACTGCCCAAGGCAGAAGGGCCTTACGCTGGACCGCTAGAGAAACAACA

GCCACTGAAACTGAGAGCAAGGCTGCCTGTGGCCAAGGAAGGGCCATATGAAGGACCAGTGAAGAAACCT

GTCGCTTTGAAAGTGAAAGCAAGAGCCCCGATTGTCACTGAGACCGGTTGCCCACCGACCGACTTGCAAA

AGATGGTCATGGCAAACGTGAAGCCCGTTGAACTCATTCTAGACGGGAAGACCGTTGCACTTTGCTGTGC

TACGGGAGTGTTTGGGACAGCTTACCTCGTGCCTCGTCACCTTTTCGCAGAAAAGTATGACAAGATCATG

TTGGACGGCCGCGCCCTGACAGACAGTGACTTCAGGGTGTTTGAGTTCGAGGTAAAAGTAAAAGGACAGG

ACATGCTCTCAGATGCTGCGCTCATGGTTCTCCACTCCGGAAACCGCGTGCGCGACCTCACGGGCCACTT

CCGTGACACCATGAAGCTGTCGAAAGGCAGTCCCGTTGTCGGCATCGTCAACAACGCTGATGTCGGAAGA

CTCGTCTTCTCTGGAGATGCACTGACCTACAAAGACCTGGTCGTTTGTATGGACGGTGACACCATGCCTG

GTCTCTTCGCGTACCGCGCTGGGACCAAGGTTGGATACTGCGGAGCTGCCGTTCTCGCAAAGGACGGCGC

CAAGACAGTGATCGTCGGCACACACTCTGCCGGAGGCAATGGAGTAGGCTACTGCTCCTGTGTCTCACGA

TCCATGCTCCTGCAGATGAAAGCCCACATCGACCCTCCTCCTCACACTGAG

>AY593848.1_SAT2_u_1967

ATCTCCATCCCCTCTCAAAAGTCTGTGCTCTACTTCCTCATCGAGAAAGGACAGCACGAAGCAGCAATTG

AATTTTACGAGGGAATGGTGCACGACAGTATCAAGGAAGAGCTTAAACCGCTTCTGGAGCAAACCAGCTT

CGCGAAGCGCGCTTTTAAGCGCCTGAAGGAAAACTTCGAGATCGTCGCTCTCGTCGTTGTGCTGTTGGCA

AACATCATCATCATGATCCGCGAGACTCGCAAGAGACAGAAGATGGTTGACGACGCCCTCGATGAATACA

TTGAGAAGGCAAACATCACCACTGATGACAAAACTCTTGACGAGGCGGAAAAGAACCCTCAAGAGGTTGT

CGACAAACCCACTGTCGGCTTCCGTGAGAGAAAGCTCCCCGGGCACAAAACTGACGATGAAGTGAACTCT

GAGCCAGTCAAGCCC---GTGGACAAACCACAAGCTGAAGGACCCTACGCAGGACCGCTCGAGCGACAGC

AGCCGCTGAAGCTCAAGGTCAAACTGCCCAAAGCAGAGGGGCCCTACGCGGGACCGCTAGAGAAGCAACA

ACCACTGAGATTGAAAGCTAAACTGCCTGTGGCCAAGGAAGGGCCATATGAAGGACCAGTGAAGAAACCT

GTCGCTTTGAAAGTGAAAGCAAAAGCCCCAATTGTCACTGAAAGCGGATGCCCACCGACCGACTTGCAAA

GAATGGTCATGGCAAACGTGAAGCCCGTTGAGCTCATCCTCGATGGGAAGACCGTTGCGCTCTGCTGCGC

TACGGGAGTGTTCGGGACGGCTTACCTCGTGCCTCGTCATCTTTTCGCAGAGAAGTACGACAAGATCATG

TTGGACGGCCGCGCCCTGACAGACAGTGACTTTAGAGTGTTTGAGTTTGAGGTAAAAGTAAAAGGTCAGG

ACATGCTCTCAGATGCCGCGCTCATGGTTCTCCACTCTGGAAACCGCGTGCGCGATCTCACGGGACACTT

CCGTGACACCATGAAACTGTCGAAAGGCAGTCCCGTCGTTGGTGTGGTCAACAACGCCGATGTCGGGAGA

CTCATCTTCTCCGGGGACGCACTGACCTACAAAGACCTGGTCGTTTGTATGGACGGTGACACCATGCCTG

GACTCTTCGCCTACCGCGCAGGGACTAAGGTTGGTTACTGCGGAGCTGCCGTTCTCGCGAAGGACGGCGC

CAAGACTGTGATCGTTGGCACTCACTCTGCCGGAGGCAACGGAGTAGGCTACTGCTCCTGCGTCTCACGA

TCCATGCTCCTGCAGATGAAGGCCCACATTGACCCGGCCCCTCACAACGAG

>AY593849.1_SAT2_KEN_1960

ATCTCGCTTCCTTCCCAAAAGTCTGTGCTCTACTTCCTCATCGAGAAAGGACAGCACGATGCTGCAATTG

AATTCTTTGAGGGGATGGTGAGTGACTCCATCAAGGAGGAACTCCGTCCTCTCCTCCAACGCACCTCATT

TGTTAAGCGCGCTTTCAAGCGCTTGAAGGAAAACTTTGAGATCGTTGCACTGTGTCTGGCTCTACTCGCC

AACATCGTGATCATGATCCGCGAAACCAACAAGAGGCAACAGATGGTTGATGAAGCCGTGAACGACTACA

TCGAACGCGCCGGCATCACCACCGACGACAAGACTCTTGATGAGGCGGAAAAGAACCCTCTGGAAACTAC

TGGTGCCAGCACCGTTGTGTTCAGAGAGAGAACACTCCCCGGGTACAAAGTGAGTGATGACGTGAACTCC

GAGCCCACTAAACCT---GAGGAGAAACCAAAGGCTGAAGGACCCTACGCCGGGCCGATGGAGCGTCAGA

AACCTCTTAAGGTGAAAGCCAAGCCGCCGCAACAAGAGGGGCCTTACGCCGGTCCGATGGAGAGACAACA

ACCACTGAAGGTTAAAGCCAAAGCCCCCGTCGTGAAGGAAGGACCATACGAGGGGCCAGTGAAGAAGCCT

GTCGCTTTGAAAGTGAAAGCAAAGAACATGATAGTCACGGAGAGTGGAGCGCCACCTACCGACTTGCAAA

AGATGGTAATGGCCAACACAAAGCCTGTTGAGCTCATCCTCGATGGGAAGACGGTGGCCATCTGCTGTGC

CACTGGAGTGTTTGGCACTGCCTATCTCGTGCCTCGTCATCTTTTCGCTGAGAAGTATGACAAGATCATG

ATTGATGGCAGGGCCATGACAGACCGTGATTTCAGAGTGTTTGAGTTTGAGATTAAAGTAAAAGGACAGG

ACATGCTCTCAGACGCTGCGCTCATGGTGTTGCACCGTGGGAACCGCGTGAGAGACATCACGAGACATTT

TCGTGATCAAGCAAGAATGAGGAAAGGAACCCCCGTGGTCGGCGTCATCAACAACGCCGACGTTGGGAGA

CTCATCTTCTCTGGAGAGGCACTCACCTACAAAGACATTGTAGTGTGCATGGACGGCGATACCATGCCTG

GCCTCTTTGCCTACAAAGCCGCCACCAAGGCCGGCTATTGCGGAGGGGCCGTCCTGGCCAAGGACGGAGC

CGAGACATTCATCGTCGGCACCCACTCCGCCGGTGGCAACGGAGTTGGATACTGCTCTTGCGTTTCCAAG

TCGATGCTGCTGCAAATGAAGGCACACGTTGACCCTGAACCACACCACGAG

>AY593850.1_SAT3_SAR_1959

ATTTCCATCCCCTCTCAAAAGTCTGTGCTTTACTTCCTCATCGAGAAGGGACAGCACGAAGCAGCAATTG

AATTCTACGAGGGAATGGTGCACGACAGCATCAAGGAAGAGCTTAAGCCGCTTCTGGAGCGAACCAGCTT

CGCGAAGCGCGCTTTCAAGCGCCTGAAGGAAAACTTCGAGATCGTCGCTCTCGTTGTTGTGCTGTTGGCA

AACATCATTATCATGATCCGCGAGACTCGCAAGAGACAGAAGATGGTTGACGACGCCCTCGATGAATACA

TTGAGAAGGCGAACATCACCACTGACGACAAGACTCTTGACGAAGCGGAAAAGAACCCTCAGGAGGTTGT

CGACAAACCCACTGTCGGCTTCCGTGAGAGAAAGCTCCCCGGGCACAAGACTGACGATGAAGTGAACTCT

GAGCCAGTCAAACCC---GTAGAGAAACCACAAGCTGAAGGACCCTACGCGGGACCGCTTGAGCGACAAC

AGCCGTTGAAGCTCAAGGCTAGACTGCCCAGAGCAGAAGGGCCTTACGCGGGACCGCTAGAGAAGCAACA

ACCACTGAAACTTAAAGCCAAGTTGCCTGTGGCCAAGGAAGGGCCATATGAAGGACCAGTGAAGAAACCT

GTCGCTTTGAAAGTGAAAGCAAAAGCCCCGATTGTCACTGAAAGCGGATGCCCACCGACTGACCTGCAAA

AGATGGTCATGGCGAACGTGAAGCCCGTTGAGCTCATCCTCGACGGTAAGACCGTTGCGCTCTGCTGCGC

CACGGGAGTGTTCGGGACGGCCTACCTCGTGCCTCGTCATCTTTTCGCAGAGAAGTATGACAAGATCATG

CTGGACGGTCGCGCCCTGACAGACAGTGACTTCAGAGTGTTTGAGTTTGAGGTAAAAGTAAAAGGACAGG

ACATGCTTTCGGATGCTGCGCTCATGGTTCTCCACTCTGGAAACCGTGTGCGCGACCTCACGGGACACTT

CCGTGACACCATGAAACTGTCGAAAGGCAGTCCCGTCGTTGGCGTGGTCAACAACGCCGACGTCGGAAGA

CTCATCTTCTCAGGAGACGCACTGACCTACAAAGACTTGGTCGTTTGTATGGACGGTGATACCATGCCTG

GACTCTTCGCCTACCGCGCAGGGACCAAGGTTGGTTACTGTGGAGCTGCCGTTCTCGCGAAGGATGGCGC

CAAGACTGTGATCGTCGGCACCCACTCTGCCGGAGGCAACGGAGTAGGCTACTGCTCCTGCGTCTCACGA

TCCATGCTCCTGCAGATGAAGGCCCACATCGACCCTCCCCCTCACACTGAG

>AY593851.1_SAT3_BOT_1961

ATTTCCATTCCTTCCCAAAAGTCTGTGCTCTACTTCCTCATCGAGAAAGGACAGCACGAAGCAGCAATTG

AATTCTATGAAGGGATGGTGCACGACAGCATCAAGGAAGAGCTTAAGCCCTTGTTGGAGCAAACCAGCTT

CGCCAAGCGTGCTTTCAAGCGCCTCAAAGAGAACTTTGAGATCGTCGCTCTTGTTGTTGTGCTGTTGGCA

AACATTGTCATCATGATCCGCGAGACTCGCAAGCGTCAGAAGATGGTCGACGACGCCCTCGATGAGTACA

TTGAGAAGGCGAACATCACCACAGACGACAAAACGCTTGACGAGGCGGAAAAGAACCCTCAGGAGGTTGT

CGACAAACCCACTGTCGGCTTCCGCAAGAGAGAACTCCCCGGGCAGAAGACTGGCAATGAAGTGAACTCT

GAGCCAACCAAACCC---GTGGAGAAACCACAAGCTGAAGGACCCTACGCTGGCCCCCTCGAGCGACAAC

AGCCGCTGAAGCTCAAGGCCAAGCTCCCTCGGGCCGAGGGACCTTACGCCGGGCCGCTAGAGAAACAACA

ACCATTGAAACTGAAAACAAGACTGCCTGTGGCCAAAGAGGGGCCATATGAAGGACCAGTGAAGAAGCCT

GTCGCTTTGAAAGTGAAAACAAAAGCCCCGATTGTCACTGAAAGCGGATGCCCACCGACTGACTTGCAAA

AGATGGTCATGGCAAACGTGAAGCCTGTTGAGCTTATTCTCGACGGGAAGACAGTTGCGCTGTGCTGCGC

GACTGGAGTGTTCGGGACGGCTTACCTCGTGCCTCGTCATCTTTTCGCAGAGAAGTATGACAAGATCATG

TTGGACGGCCGCGCCCTGACAGACAGTGACTTCAGAGTGTTTGAGTTTGAGGTAAAAGTAAAAGGACAGG

ACATGCTTTCGGACGCCGCGCTGATGGTTCTCCACTCTGGAAACCGTGTGCGCGATCTCACGGGACACTT

TCGCGACACCATGAAACTGTCGAAAGGCAGCCCCATCGTTGGCGTGGTTAATAACGCCGACGTTGGAAGA

CTCATCTTCTCAGGAGATGCACTGACCTACAAAGACCTGGTCGTTTGTATGGACGGTGACACCATGCCTG

GACTCTTCGCGTACCGCGCAGGGACCAAGGTTGGATACTGCGGAGCTGCCGTTCTCGCGAAGGATGGCGC

CAAGACAGTGATCGTCGGCACCCATTCTGCCGGAGGCAATGGAGTAGGCTACTGCTCCTGCGTCTCACGA

TCCATGCTCCTGCAGATGAAGGCCCACATCGACCCTCCCCCTCACACTGAG

>AY593852.1_SAT3_KEN_1960

ATTTCCATTCCTTCCCAAAAGTCTGTGCTCTACTTCCTCATCGAGAAAGGACAGCACGAAGCAGCAATTG

AATTCTATGAAGGGATGGTGCACGACAGCATCAAGGAAGAGCTTAAGCCCTTGTTGGAGCAAACCAGCTT

CGCCAAGCGTGCTTTCAAGCGCCTCAAAGAGAACTTTGAGATCGTCGCTCTTGTTGTTGTGCTGTTGGCA

AACATTGTCATCATGATCCGCGAGACTCGCAAGCGTCAGAAGATGGTCGACGACGCCCTCGATGAGTACA

TTGAGAAGGCGAACATCACCACTGACGACAAAACGCTTGACGAGGCGGAAAAGAACCCTCAGGAGGTTGT

CGACAAACCCACTGTCGGCTTCCGCGAGAGAGAACTCCCCGGGCAGAAGACTGGCAATGAAGTGAACTCT

GAGCCAACCAAACCC---GTGGAGAAACCACAAGCTGAAGGACCCTACGCTGGCCCCCTCGAGCGACAAC

AGCCGCTGAAGCTCAAGGCCAAGCTCCCTCGGGCCGAGGGACCTTACGCCGGGCCGCTAGAGAAACAACA

ACCATTGAAACTGAAAACAAGACTGCCTGTGGCCAAAGAGGGGCCATATGAAGGACCAGTGAAGAAGCCT

GTCGCTTTGAAAGTGAAAACAAAAGCCCCGATTGTCACTGAAAGCGGATGCCCACCGACTGACTTGCAAA

AGATGGTCATGGCAAACGTGAAGCCTGTTGAGCTTATTCTCGACGGGAAGACAGTTGCGCTGTGCTGCGC

GACTGGAGTGTTCGGGACGGCTTACCTCGTGCCTCGTCATCTTTTCGCAGAGAAGTATGACAAGATCATG

TTGGACGGCCGCGCCCTGACAGACAGTGACTTCAGAGTGTTTGAGTTTGAGGTAAAAGTAAAAGGACAGG

ACATGCTTTCGGACGCCGCGCTGATGGTTCTCCACTCTGGAAACCGTGTGCGCGATCTCACGGGACACTT

TCGCGACACCATGAAACTGTCGAAAGGCAGCCCCATCGTTGGCGTGGTTAATAACGCCGACGTTGGAAGA

CTCATCTTCTCAGGAGATGCACTGACCTACAAAGACCTGGTCGTTTGTATGGACGGTGACACCATGCCTG

GACTCTTCGCGTACCGCGCAGGGACCAAGGTTGGATACTGCGGAGCTGCCGTTCTCGCGAAGGATGGCGC

CAAGACAGTGATCGTCGGCACCCATTCTGCCGGAGGCAATGGAGTAGGCTACTGCTCCTGCGTCTCACGA

TCCATGCTCCTGCAGATGAAGGCCCACATCGACCCTCCCCCTCACACTGAG

>AY593853.1_SAT3_BOT_1965

ATTTCCATTCCTTCCCAAAAGTCCGTGCTCTACTTCCTCATCGAGAAAGGACAGCACGAAGCAGCAATTG

AATTCTACGAAGGGATGGTACACGACAGCATCAAGGAAGAGCTTAAGCCCTTGTTGGAGCGAACCAGCTT

CGCCAAGCGTGCTTTCAAGCGCCTCAAGGAAAACTTCGAGATCGTCGCTCTCGTAGTTGTGCTGTTAGCA

AACATCATCATTATGATCCGCGAAACTCGCAAGCGCCAGAAGATGGTCGACGACGCCCTCGATGAGTACA

TTGAGAAGGCTAACATCACCACTGATGATAAAACGCTTGAAGAGGCGGAAAAGAACCCTCAAGAGGCTGT

CGACAAACCCACTGTCGGCTTCCGTGAGAGAAAACTCCCTGAGCACAAGACTGACGATGAAGTGAACTCT

GAGCCAGTCAAGCCT---GCGGAGAAACCACAAGCTGAAGGACCCTACGCCGGCCCTCTTGAGCGACAGC

AGCCGCTGAAGCTCAAAGCTAAGCTCCCTCAGGCTGAGGGACCTTACGCTGGGCCGTTGGAGAGACAGCA

ACCACTGAAACTGAAAGCAAAACTGCCTGTGGCCAAGGAAGGGCCATACGAAGGACCAGTGAAGAAACCT

GTTGCTTTGAAAGTGAAAGCAAGAGCCCCGATTGTCACTGAAAGCGGATGCCCACCGACCGACTTGCAAA

AGATGGTCATGGCAAACGTGAAGCCCGTCGAGCTCATCCTTGACGGGAAGACAGTTGCGCTCTGCTGCGC

GACTGGAGTGTTCGGGACGGCTTACCTCGTGCCTCGTCATCTTTTCGCAGAGAAGTATGACAAGATCATG

TTAGATGGCCGCGCCCTGACAGACAGTGACTTCAGAGTGTTTGAGTTCGAGGTAAAAGTAAAAGGACAGG

ACATGCTCTCAGATGCCGCGCTGATGGTTCTCCACTCCGGGAACCGTGTGCGCGATCTCACGGGACACTT

CCGTGACACCATGAAACTGTCGAAAGGCAGCCCCGTCGTTGGCGTGGTCAACAACGCCGATGTCGGAAGA

CTCATCTTCTCAGGAGACGCACTGACCTACAAAGACCTGGTCGTTTGTATGGACGGTGACACCATGCCTG

GACTCTTCGCCTACCGCGCAGGGACCAAAGTTGGATATTGCGGAGCCGCCGTTCTTGCAAAGGATGGCGC

CAAGACAGTGATCGTCGGCACCCACTCTGCCGGAGGCAACGGAGTAGGCTACTGCTCCTGTGTCTCACGA

TCCATGCTCCTGCAGATGAAGGCCCACATCGACCCTCCCCCTCACACTGAG

>AY686687.1_O_CHA_2001

ATCTCAATCCCTTCACAGAAGTCCGTGTTGTACTTCCTCATTGAGAAAGGACAGCACGAAGCAGCGATCG

AGTTCTTCGAGGGGATGGTCCACGACTCCATCAAGGAGGAGCTCCGGCCCCTCATTCAGCAGACCTCGTT

CGTAAAACGCGCCTTCAAGCGCCTGAAAGAGAACTTCGAGATCGTAGCCCTATGTTTGACCCTCTTGGCA

AACATAGTGATCATGCTCCGCCAATCACGCAAGAGGCGCCAGTCAGTGGAAGACCCACTGGAC-------

-----------------------GGCGGCACAACTCTTGGCGACGCGGAAGAGAACCCTCTGGAGACGAG

TGGCGCTAGCGCTGTCGGTTTCAGAGAGAGATCCCCCGCCGAGCAAGGGACGCGCGAAGACGCGAACGCT

GAGCCCGTCGTGCTCGGCAGGGAGCAACCGCGAGCTGGAGGACCCTACGCTGGGCCACTCGAGCGTCAGA

AACCGCTTAAAGTGAAAGCCAGGCTGCCACAGCAGGAGGGACCATACGCAGGCCCAATGGAGAGACAGAA

ACCGCTAAAGGTGAAAGCAAAAGCCCCCGTCGTGAAGGAAGGACCTTACGAGGGACCGGTGAAGAAACCT

GTCGCTTTGAAAGTGAAAGCAAAGAACTTGATAGTCACTGAGAGTGGTGCGCCGCCGACTGACTTACAAA

AGATGGTCATGGGTAACACTAAGCCAGTCGAGCTCATTCTCGACGGCAAGACGGTAGCCATCTGCTGTGC

TACCGGAGTGTTCGGCACTGCTTACCTCGTGCCTCGTCATCTCTTCGCGGAAAAGTATGACAAGATCATG

TTGGACGGCAGAGCCTTAACAGACAGCGACTACAGAGTGTTTGAGTTTGAGATTAAAGTAAAAGGACAGG

ACATGCTCTCAGACGCCGCTCTCATGGTGCTGCACCGTGGGAACCGCGTGCGCGACATCACGAAACACTT

TCGTGACGTGGCGAGGATGAAGAAAGGAACCCCCGTCGTCGGCGTGATCAACAATGCTGACGTCGGGCGG

CTCATATTCTCTGGTGAAGCCCTTACCTACAAGGACATCGTCGTGTGTATGGATGGAGACACCATGCCTG

GGCTCTTTGCCTACAGGGCATCCACCAAAGCAGGCTACTGTGGAGGAGCCGTCCTGGCGAAGGATGGAGC

CGAGACGTTCATTGTCGGCACCCACTCCGCAGGTGGAAACGGCATAGGATATTGTTCGTGCGTTTCCCGA

TCGATGCTCTTGAGGATGAAGGCACACATCGACCCCGAACCACACCACGAG

>AY687333.1_Asia1_IND_2001

ATCTCAATTCCTTCCCAAAAGTCAGTGTTGTACTTCCTCATCGAGAAAGGCCAACACGAAGCAGCAATTG

AATTCTTTGAGGGGATGGTGCACGACTCCATCAAGGAGGAGCTCCGACCTCTCATTCAACAGACATCATT

TGTGAAACGCGCTTTTAAGCGCCTGAAGGACAACTTTGAGATCGTTGCCCTGTGCTTGACTCTTTTGGCA

AACATTGTGATCATGATCCGCGAGACTCGCAAGAGACAACAGATGGTGGATGATGCAGTGAATGAGTACA

TTGAGAAAGCAAACATCACCACAGATGACAAGACTCTTGACGAGGCGGAAAAGAACCCTCTGGAGACCAG

CGGTGCCAGCACTGTAGGTTTCAGAGAGAGAACCCTCCCGGGACACAAAGCGAGTGATGACGTGAACTCC

GAGCCCGCCAGACCCGTGGAGGAACAACCACAAGCTGAAGGACCCTACGCCGGACCACTCGAGCGTCAGA

AACCTCTGAAAGTGAGAGCCAAGCTCCCACAACATGAGGGACCTTACGCTGGTCCGATGGAGAGGCAGAA

ACCATTGAAAGTGAAAGCAAAAGCCCCGGTCGTTAGAGAAGGACCTTACGAGGGACCGGTGAAGAAGCCT

GTCGCTTTGAAAGTGAAAGCTAAGAACTTGATTGTCACTGAGAGCGGTGCCCCCCCGACCGACTTGCAAA

AGATGGTCATGGGCAACACCAAGCCTGTTGAGCTCATCCTCGACGGGAAGACGGTAGCCATCTGCTGCGC

TACCGGAGTGTTTGGTACTGCCTACCTTGTGCCTCGTCATCTTTTCGCAGAGAAGTACGACAAGATCATG

CTGGACGGCAGAACCATGACAGACAGTGACTACAGAGTGTTTGAGTTTGAGATTAAAGTAAAAGGACAGG

ACATGCTCTCAGACGCCGCGCTCATGGTGCTTCACCGTGGGAACCGCGTGCGGGACATCACAAAACACTT

TCGTGATGTAGCAAGAATGAAGAAAGGCACCCCCGTGGTTGGCGTGATTAACAACGCTGACGTTGGGAGA

CTGATTTTCTCTGGTGAGGCCCTTACCTACAAAGATATTGTAGTGTGTATGGATGGAGACACCATGCCTG

GCCTCTTTGCCTACAAGGCTGCCACCAAGGCAGGCTACTGTGGAGGAGCCGTTCTTGCAAAAGACGGAGC

CGATACTTTCATCGTCGGCACTCACTCCGCAGGTGGTAATGGAGTTGGATACTGCTCGTGCGTTTCCAGG

TCCATGCTACTTAAGATGAAGGCACACATCGACCCTGAACCACACCATGAG

>DQ404158.1_O_UKG_2001

ATCTCAATTCCTTCCCAAAAGGCTGTGCTGTACTTTCTCATTGAGAAGGGCCAGCACGAAGCAGCAATTG

AGTTTTTTGAGGGGATGGTACATGACTCCATCAAGGAGGAGCTCCGGCCTCTCATCCAACAGACCTCATT

TGTGAAGCGCGCTTTTAAGCGCCTGAAGGAAAACTTTGAGATAGTTGCCCTGTGTTTGACTCTTTTGGCA

AACATAGTGATCATGATCCGCGAGACTCGCAAGAGACAGCAGATGGTGGATGATGCAGTGAACGAGTACA

TTGAGAAGGCAAACATCACCACGGATGACAAGACTCTTGACGAGGCGGAAAAGAACCCTCTGGAGACCAG

CGGTGCCACCACTGTTGGTTTCAGAGAGAAAGCTCTCCCGGGACACAAGGCGGGTGACGACGTGAACTCC

GAGCCCGCCAAACCCGTGGAAGAGCAACCACAAGCTGAAGGACCCTACACCGGTCCACTTGAGCGTCAAA

AACCCCTGAAAGTGAGGGCCAAGCTCCCACAGCAGGAGGGGCCCTACGCTGGTCCGATGGAGAGACAGAA

ACCGCTGAAAGTGAAAGTGAAAGCCCCGGTCGTTAAGGAAGGACCTTACGAAGGACCGGTGAAGAAACCC

GTCGCTTTGAAAGTGAAAGCAAAGAACTTGATTGTCACTGAGAGTGGTGCTCCCCCGACTGACTTGCAAA

AGATGGTCATGGGTAACACCAAGCCTGTTGAGCTCATCCTCGACGGGAAGACGGTGGCCATTTGCTGCGC

CACCGGAGTGTTTGGTACTGCCTACCTAGTTCCTCGTCATCTTTTCGCAGAGAAGTATGACAAGATCATG

TTGGACGGCAGAGCCATGACAGACAGTGACTACAGAGTGTTTGAGTTTGAGATTAAAGTGAAAGGACAGG

ACATGCTCTCAGACGCCGCTCTCATGGTGCTTCACCGTGGGAATCGCGTGCGGGACATCACGAAGCACTT

CCGTGATGTGGCAAGAATGAAGAAAGGCACCCCCGTCGTCGGCGTGATTAACAACGCTGATGTTGGGAGA

CTGATCTTCTCTGGTGAGGCCCTTACCTACAAGGACATTGTAGTGTGCATGGACGGAGACACCATGCCCG

GTCTCTTCGCCTACAAAGCTGCCACCAAGGCGGGTTACTGTGGAGGAGCCGTTCTTGCAAAGGACGGAGC

CGAGACTTTCATCGTCGGCACTCACTCCGCAGGCGGCAATGGAGTTGGATACTGCTCATGCGTTTCCAGG

TCCATGCTGCTTAAAATGAAGGCACACATCGATCCCGAACCACACCACGAG

>DQ404159.1_O_UKG_2001

ATCTCAATTCCTTCCCAAAAGGCTGTGCTGTACTTTCTCATTGAGAAGGGCCAGCACGAAGCAGCAATTG

AGTTTTTTGAGGGGATGGTACATGACTCCATCAAGGAGGAGCTCCGGCCTCTCATCCAACAGACCTCATT

TGTGAAGCGCGCTTTTAAGCGCCTGAAGGAAAACTTTGAGATAGTTGCCCTGTGTTTGACTCTTTTGGCA

AACATAGTGATCATGATCCGCGAGACTCGCAAGAGACAGCAGATGGTGGATGATGCAGTGAACGAGTACA

TTGAGAAGGCAAACATCACCACGGATGACAAGACTCTTGACGAGGCGGAAAAGAACCCTCTGGAGACCAG

CGGTGCCACCACTGTTGGTTTCAGAGAGAAAGCTCTCCCGGGACACAAGGCGGGTGACGACGTGAACTCC

GAGCCCGCCAAACCCGTGGAAGAGCAACCACAAGCTGAAGGACCCTACACCGGTCCACTTGAGCGTCAAA

AACCCCTGAAAGTGAGGGCCAAGCTCCCACAGCAGGAGGGGCCCTACGCTGGTCCGATGGAGAGACAGAA

ACCGCTGAAAGTGAAAGTGAAAGCCCCGGTCGTTAAGGAAGGACCTTACGAAGGACCGGTGAAGAAACCC

GTCGCTTTGAAAGTGAAAGCAAAGAACTTGATTGTCACTGAGAGTGGTGCTCCCCCGACTGACTTGCAAA

AGATGGTCATGGGTAACACCAAGCCTGTTGAGCTCATCCTCGACGGGAAGACGGTGGCCATTTGCTGCGC

CACCGGAGTGTTTGGTACTGCCTACCTAGTTCCTCGTCATCTTTTCGCAGAGAAGTATGACAAGATCATG

TTGGACGGCAGAGCCATGACAGACAGTGACTACAGAGTGTTTGAGTTTGAGATTAAAGTGAAAGGACAGG

ACATGCTCTCAGACGCCGCTCTCATGGTGCTTCACCGTGGGAATCGCGTGCGGGACATCACGAAGCACTT

CCGTGATGTGGCAAGAATGAAGAAAGGCACCCCCGTCGTCGGCGTGATTAACAACGCTGATGTTGGGAGA

CTGATCTTCTCTGGTGAGGCCCTTACCTACAAGGACATTGTAGTGTGCATGGACGGAGACACCATGCCCG

GTCTCTTCGCCTACAAAGCTGCCACCAAGGCGGGTTACTGTGGAGGAGCCGTTCTTGCAAAGGACGGAGC

CGAGACTTTCATCGTCGGCACTCACTCCGCAGGCGGCAATGGAGTTGGATACTGCTCATGCGTTTCCAGG

TCCATGCTGCTTAAAATGAAGGCACACATCGATCCCGAACCACACCACGAG

>DQ404160.1_O_UKG_2001

ATCTCAATTCCTTCCCAAAAGGCTGTGCTGTACTTTCTCATTGAGAAGGGCCAGCACGAAGCAGCAATTG

AGTTTTTTGAGGGGATGGTACATGACTCCATCAAGGAGGAGCTCCGGCCTCTCATCCAACAGACCTCATT

TGTGAAGCGCGCTTTTAAGCGCCTGAAGGAAAACTTTGAGATAGTTGCCCTGTGTTTGACTCTTTTGGCA

AACATAGTGATCATGATCCGCGAGACTCGCAAGAGACAGCAGATGGTGGATGATGCAGTGAACGAGTACA

TTGAGAAGGCAAACATCACCACGGATGACAAGACTCTTGACGAGGCGGAAAAGAACCCTCTGGAGACCAG

CGGTGCCACCACTGTTGGTTTCAGAGAGAAAGCTCTCCCGGGACACAAGGCGGGTGACGACGTGAACTCC

GAGCCCGCCAAACCCGTGGAAGAGCAACCACAAGCTGAAGGACCCTACACCGGTCCACTTGAGCGTCAAA

AACCCCTGAAAGTGAGGGCCAAGCTCCCACAGCAGGAGGGGCCCTACGCTGGTCCGATGGAGAGACAGAA

ACCGCTGAAAGTGAAAGTGAAAGCCCCGGTCGTTAAGGAAGGACCTTACGAAGGACCGGTGAAGAAACCC

GTCGCTTTGAAAGTGAAAGCAAAGAACTTGATTGTCACTGAGAGTGGTGCTCCCCCGACTGACTTGCAAA

AGATGGTCATGGGTAACACCAAGCCTGTTGAGCTCATCCTCGACGGGAAGACGGTGGCCATTTGCTGCGC

CACCGGAGTGTTTGGTACTGCCTACCTAGTTCCTCGTCATCTTTTCGCAGAGAAGTATGACAAGATCATG

TTGGACGGCAGAGCCATGACAGACAGTGACTACAGAGTGTTTGAGTTTGAGATTAAAGTGAAAGGACAGG

ACATGCTCTCAGACGCCGCTCTCATGGTGCTTCACCGTGGGAATCGCGTGCGGGACATCACGAAGCACTT

CCGTGATGTGGCAAGAATGAAGAAAGGCACCCCCGTCGTCGGCGTGATTAACAACGCTGATGTTGGGAGA

CTGATCTTCTCTGGTGAGGCCCTTACCTACAAGGACATTGTAGTGTGCATGGACGGAGACACCATGCCCG

GTCTTTTCGCCTACAAAGCTGCCACCAAGGCGGGTTACTGTGGAGGAGCCGTTCTTGCAAAGGACGGAGC

CGAGACTTTCATCGTCGGCACTCACTCCGCAGGCGGCAATGGAGTTGGATACTGCTCATGCGTTTCCAGG

TCCATGCTGCTTAAAATGAAGGCACACATCGATCCCGAACCACACCACGAG

>DQ404161.1_O_UKG_2001

ATCTCAATTCCTTCCCAAAAGGCTGTGCTGTACTTTCTCATTGAGAAGGGCCAGCACGAAGCAGCAATTG

AGTTTTTTGAGGGGATGGTACATGACTCCATCAAGGAGGAGCTCCGGCCTCTCATCCAACAGACCTCATT

TGTGAAGCGCGCTTTTAAGCGCCTGAAGGAAAACTTTGAGATAGTTGCCCTGTGTTTGACTCTTTTGGCA

AACATAGTGATCATGATCCGCGAGACTCGCAAGAGACAGCAGATGGTGGATGATGCAGTGAACGAGTACA

TTGAGAAGGCAAACATCACCACGGATGACAAGACTCTTGACGAGGCGGAAAAGAACCCTCTGGAGACCAG

CGGTGCCACCACTGTTGGTTTCAGAGAGAAAGCTCTCCCGGGACACAAGGCGGGTGACGACGTGAACTCC

GAGCCCGCCAAACCCGTGGAAGAGCAACCACAAGCTGAAGGACCCTACACCGGTCCACTTGAGCGTCAAA

AACCCCTGAAAGTGAGGGCCAAGCTCCCACAGCAGGAGGGGCCCTACGCTGGTCCGATGGAGAGACAGAA

ACCGCTGAAAGTGAAAGTGAAAGCCCCGGTCGTTAAGGAAGGACCTTACGAAGGACCGGTGAAGAAACCC

GTCGCTTTGAAAGTGAAAGCAAAGAACTTGATTGTCACTGAGAGTGGTGCTCCCCCGACTGACTTGCAAA

AGATGGTCATGGGTAACACCAAGCCTGTTGAGCTCATCCTCGACGGGAAGACGGTGGCCATCTGCTGCGC

CACCGGAGTGTTTGGTACTGCCTACCTAGTTCCTCGTCATCTTTTCGCAGAGAAGTATGACAAGATCATG

TTGGACGGCAGAGCCATGACAGACAGTGACTACAGAGTGTTTGAGTTTGAGATTAAAGTGAAAGGACAGG

ACATGCTCTCAGACGCCGCTCTCATGGTGCTTCACCGTGGGAATCGCGTGCGGGACATCACGAAGCACTT

CCGTGATGTGGCAAGAATGAAGAAAGGCACCCCCGTCGTCGGCGTGATCAACAACGCTGATGTTGGGAGA

CTGATCTTCTCTGGTGAGGCCCTTACCTACAAGGACATTGTAGTGTGCATGGACGGAGACACCATGCCCG

GCCTCTTCGCCTACAAAGCTGCCACCAAGGCGGGTTACTGTGGAGGAGCCGTTCTTGCAAAGGACGGAGC

CGAGACTTTCATCGTCGGCACTCACTCCGCAGGCGGCAATGGAGTTGGATACTGCTCATGCGTTTCCAGG

TCCATGCTGCTTAAAATGAAGGCACACATCGATCCCGAACCACACCACGAG

>DQ404162.1_O_UKG_2001

ATCTCAATTCCTTCCCAAAAGGCTGTGCTGTACTTTCTCATTGAGAAGGGCCAGCACGAAGCAGCAATTG

AGTTCTTTGAGGGGATGGTGCATGACTCCATCAAGGAGGAGCTCCGGCCTCTCATCCAACAGACCTCATT

TGTGAAGCGCGCTTTTAAGCGCCTGAAGGAAAACTTTGAGATAGTTGCCCTGTGTTTGACTCTTTTGGCA

AACATAGTGATAATGATCCGCGAGACTCGCAAGAGACAGCAGATGGTGGATGATGCAGTGAACGAGTACA

TTGAGAAGGCAAACATCACCACGGATGACAAGACTCTTGACGAGGCGGAAAAGAACCCTCTGGAGACCAG

CGGTGCCACCACTGTTGGTTTCAGAGAGAAAACTCTCCCAGGACACAAGGCGGGTGACGACGTGAACTCC

GAGCCCGCCAAACCCGTGGAAGAACAACCACAAGCTGAAGGACCCTACACCGGACCACTCGAGCGTCAAA

AACCCCTGAAAGTGAGGGCCAAGCTCCCACAGCAGGAGGGGCCCTACGCTGGTCCGATGGAGAGACAGAA

ACCGCTGAAAGTGAAAGTGAAAGCCCCGGTCGTCAAGGAAGGACCTTACGGAGGACCGGTGAAGAAACCT

GTCGCTTTGAAAGTGAAAGCAAAGAACTTGATTGTCACTGAGAGTGGTGCTCCCCCGACTGACTTGCAAA

AGATGGTCATGGGTAACACCAAGCCTGTTGAGCTCATCCTCGACGGGAAGACGGTGGCCATCTGCTGCGC

CACCGGAGTGTTTGGTACTGCCTACCTAGTTCCTCGTCATCTTTTCGCAGAGAAGTATGACAAGATCATG

TTGGACGGCAGAGCCATGACAGACACTGACTACAGAGTGTTTGAGTTTGAGATTAAAGTGAAAGGACAGG

ACATGCTCTCAGACGCCGCTCTCATGGTGCTTCACCGTGGGAATCGCGTGCGGGACATCACGAAGCACTT

CCGTGATGTGGCAAGAATGAAGAAAGGCACCCCCGTCGTCGGCGTGATCAACAACGCTGATGTTGGGAGA

CTGATCTTCTCTGGTGAGGCCCTTACCTACAAGGACATTGTAGTGTGCATGGACGGAGACACCATGCCCG

GTCTCTTCGCCTACAAAGCTGCCACCAAGGCGGGTTACTGTGGAGGAGCCGTTCTTGCAAAGGACGGAGC

CGAGACTTTCATCGTCGGCACTCACTCCGCAGGCGGCAATGGAGTTGGATACTGCTCATGCGTTTCCAGG

TCCATGCTGCTTAAAATGAAGGCACACATCGATCCCGAACCACACCACGAG

>DQ404163.1_O_UKG_2001

ATCTCAATTCCTTCCCAAAAGGCTGTGCTGTACTTTCTCATTGAGAAGGGCCAGCACGAAGCAGCAATTG

AGTTCTTTGAGGGGATGGTGCATGACTCCATCAAGGAGGAGCTCCGGCCTTTCATCCAACAGACCTCGTT

TGTGAAGCGCGCTTTTAAGCGCCTGAAGGAAAACTTTGAGATAGTTGCCCTGTGTTTGACTCTTTTGGCA

AACATAGTGATTATGATCCGCGAGACTCGCAAGAGACAGCAGATGGTGGATGATGCAGTGAACGAGTACA

TTGAGAAGGCAAACATCACCACGGACGACAAGACTCTTGACGAGGCGGAAAAGAACCCTCTGGAGACCAG

CGGTGCCACCACTGTTGGTTTCAGAGAGAAAACTCTCCCGGGACACAAGGCGGGTGATGACGTGAACTCC

GAGCCCGCCAAACCCGTGGAAGAACAACCACAAGCTGAAGGACCCTACACCGGTCCACTCGAGCGTCAAA

AACCCCTGAAAGTGAGGGTCAAGCTCCCACAGCAGGAGGGGCCCTACGCTGGTCCGATGGAGAGACAGAA

ACCGCTGAAAGTGAAAGTGAAAGCCCCGGTCGTTAAGGAAGGACCTTACGAAGGACCGGTGAAGAAACCT

GTCGCTTTGAAAGTGAAAGCAAAGAACTTGATTGTCACTGAGAGTGGTGCTCCCCCGACTGACTTGCAAA

AGATGGTCATGGGTAACACCAAGCCTGTTGAGCTCGTCCTCGACGGGAAGACGGTGGCCATCTGCTGCGC

CACCGGAGTGTTTGGTACTGCCTACCTAGTTCCTCGTCATCTTTTCGCAGAGAAGTATGACAAGATCATG

TTGGACGGCAGAGCCATGACAGACAGTGACTACAGAGTGTTTGAGTTTGAGATTAAAGTGAAGGGACAGG

ACATGCTCTCAGACGCCGCTCTCATGGTGCTTCACCGTGGGAATCGCGTGCGGGACATCACGAAGCACTT

CCGTGATGTGGCAAGAATGAAGAAAGGCACCCCCGTCGTCGGCGTGATCAACAACGCTGATGTTGGGAGA

CTGATCTTCTCTGGTGAGGCCCTTACCTACAAGGACATTGTAGTGTGCATGGACGGAGACACCATGCCCG

GTCTCTTCGCCTACAAAGCTGCCACCAAGGCGGGTTACTGTGGAGGAGCCGTTCTTGCAAAGGACGGAGC

CGAGACTTTCATCGTCGGCACTCACTCCGCAGGCGGCAATGGAGTTGGATACTGCTCATGCGTTTCCAGG

TCCATGCTGCTTAAAATGAAGGCACACATCGATCCCGAACCACACCACGAG

>DQ404164.1_O_UKG_2001

ATCTCAATTCCTTCCCAAAAGGCTGTGCTGTACTTTCTCATTGAGAAGGGCCAGCACGAAGCAGCAATTG

AGTTCTTTGAGGGGATGGTGCATGACTCCATCAAGGAGGAGCTCCGGCCTCTCATCCAACAGACCTCATT

TGTGAAGCGCGCTTTTAAACGCCTGAAGGAAAACTTTGAGATAGTTGCCCTGTGTTTGACTCTTTTGGCA

AACATAGTGATCATGATCCGCGAGACTCGCAAGAGACAGCAGATGGTGGATGATGCAGTGAACGAGTACA

TTGAGAAGGCAAACATCACCACGGATGACAAGACTCTTGACGAGGCGGAAAAGAACCCTCTGGAGACCAG

CGGTGCCACCACTGTTGGTTTCAGAGAGAAAACTCTCCCGGGACACAAGGCGGGTGATGACGCGAACTCC

GAGCCCGCCAAACCCGTGGAAGAACAACCACAAGCTGAAGGACCCTACACCGGTCCACTCGAGCGTCAAA

AACCCCTGAAAGTGAGGGCCAAGCTCCCACAGCAGGAGGGGCCCTACGCTGGTCCGATGGAGAGACAGAA

ACCGCTGAAAGTGAAAGTGAAAGCCCCGGTCGTTAAGGAAGGACCTTACGAAGGACCGGTGAAGAAACCT

GTCGCTTTGAAAGTGAAAGCAAAGAACTTGATTGTCACTGAGAGTGGTGCTCCCCCGACTGACTTGCAAA

AGATGGTCATGGGTAACACCAAGCCTGTTGAGCTCATCCTCGACGGGAAGACGGTGGCCATCTGCTGCGC

CACCGGAGTGTTTGGTACTGCCTACCTAGTTCCTCGTCATCTTTTCGCAGAGAAGTATGACAAGATCATG

TTGGACGGCAGAGCCATGACAGACAGTGACTACAGAGTGTTTGAGTTTGAGATTAAAGTGAAAGGACAGG

ACATGCTCTCAGACGCCGCTCTCATGGTGCTTCACCGTGGGAATCGCGTGCGGGACATCACGAAGCACTT

CCGTGATGTGGCAAGAATGAAGAAAGGCACCCCCGTCGTCGGCGTGATCAACAACGCCGATGTTGGGAGA

CTGATCTTCTCTGGTGAGGCCCTTACCTACAAGGACATTGTAGTGTGCATGGACGGAGACACCATGCCCG

GTCTCTTCGCCTACAAAGCTGCCACCAAGGCGGGTTACTGTGGAGGAGCCGTTCTTGCAAAGGACGGAGC

CGAGACTTTCATCGTCGGCACTCACTCCGCAGGCGGCAATGGAGTTGGATACTGCTCATGCGTTTCCAGG

TCCATGCTGCTTAAAATGAAGGCACACATCGATCCCGAACCACACCACGAG

>DQ404165.1_O_UKG_2001

ATCTCAATTCCTTCCCAAAAGGCTGTGCTGTACTTTCTCATTGAGAAGGGCCAGCACGAAGCAGCAATTG

AGTTCTTTGAGGGGATGGTGCATGACTCCATCAAGGAGGAGCTCCGGCCTCTCATCCAACAGACCTCATT

TGTGAAGCGCGCTTTTAAGCGCCTGAAGGAAAACTTTGAGATAGTTGCCCTGTGTTTGACTCTTTTGGCA

AACATAGTGATCATGATCCGCGAGACTCGCAAGAGACAGCAGATGATGGATGATGCAGTGAACGAGTACA

TTGAGAAGGCAAACATCACCACGGATGACAAGACTCTTGACGAGGCGGAAAAGAACCCTCTGGAGACCAG

CGGTGCCACCACTGTTGGTTTCAGAGAGAAAACTCTCCCGGGACACAAGGCGGGTGATGACGTGAACTCC

GAGCCCGCCAAACCCGTGGAAGAACAACCACAAGCTGAAGGACCCTACACCGGTCCACTCGAGCGTCAAA

AACCCCTGAAAGTGAGGGCCAAGCTCCCACAGCAGGAGGGGCCCTACGCTGGTCCGATGGAGAGACAGAA

ACCGCTGAAAGTGAAAGTGAAAGCCCCGGTCGTTAAGGAAGGACCTTACGAAGGACCGGTGAAGAAACCT

GTCGCTTTGAAAGTGAAAGCAAAGAACTTGATTGTCACTGAGAGTGGTGCTCCCCCGACTGACTTGCAAA

AGATGGTCATGGGTAACACCAAGCCTGTTGAGCTCATCCTCGACGGGAAGACGGTGGCCATCTGCTGCGC

CACCGGAGTGTTTGGTACTGCCTACCTAGTTCCTCGTCATCTTTTCGCAGAGAAGTATGACAAGATCATG

TTGGACGGCAGAGCCATGACAGACAGTGACTACAGAGTGTTTGAGTTTGAGATTAAAGTGAAAGGACAGG

ACATGCTCTCAGACGCCGCTCTCATGGTGCTTCACCGTGGGAATCGCGTGCGGGACATCACGAAGCACTT

CCGTGATGTGGCAAGAATGAAGAAAGGCACCCCCGTCGTCGGCGTGATCAACAACGCTGATGTTGGGAGA

CTGATCTTCTCTGGTGAGGCCCTTACCTACAAGGACATTGTAGTGTGCATGGACGGAGACACCATGCCCG

GTCTCTTCGCCTATAAAGCTGCCACCAAGGCGGGTTACTGTGGAGGAGCCGTTCTTGCAAAGGACGGAGC

CGAGACTTTCATCGTCGGCACTCACTCCGCAGGCGGCAATGGAGTTGGATACTGCTCATGCGTTTCCAGG

TCCATGCTGCTTAAAATGAAGGCACACATCGATCCCGAACCACACCACGAG

>DQ404166.1_O_UKG_2001

ATCTCAATTCCTTCCCAAAAGGCTGTGCTGTACTTTCTCATTGAGAAGGGCCAGCACGAAGCAGCAATTG

AGTTCTTTGAGGGGATGGTGCATGACTCCATCAAGGAGGAGCTCCGGCCTCTCATCCAACAGACCTCATT

TGTGAAGCGCGCTTTTAAGCGCCTGAAGGAAAACTTTGAGATAGTTGCCCTGTGTTTGACTCTTTTGGCA

AACATAGTGATCATGATCCGCGAGACTCGCAAGAGACAGCAGATGATGGATGATGCAGTGAACGAGTACA

TTGAGAAGGCAAACATCACCACGGATGACAAGACTCTTGACGAGGCGGAAAAGAACCCTCTGGAGACCAG

CGGTGCCACCACTGTTGGTTTCAGAGAGAAAACTCTCCCGGGACACAAGGCGGGTGATGACGTGAACTCC

GAGCCCGCCAAACCCGTGGAAGAACAACCACAAGCTGAAGGACCCTACACCGGTCCACTCGAGCGTCAAA

AACCCCTGAAAGTGAGGGCCAAGCTCCCACAGCAGGAGGGGCCCTACGCTGGTCCGATGGAGAGACAGAA

ACCGCTGAAAGTGAAAGTGAAAGCCCCGGTCGTTAAGGAAGGACCTTACGAAGGACCGGTGAAGAAACCT

GTCGCTTTGAAAGTGAAAGCAAAGAACTTGATTGTCACTGAGAGTGGTGCTCCCCCGACTGACTTGCAAA

AGATGGTCATGGGTAACACCAAGCCTGTTGAGCTCATCCTCGACGGGAAGACGGTGGCCATCTGCTGCGC

CACCGGAGTGTTTGGTACTGCCTACCTAGTTCCTCGTCATCTTTTCGCAGAGAAGTATGACAAGATCATG

TTGGACGGCAGAGCCATGACAGACAGTGACTACAGAGTGTTTGAGTTTGAGATTAAAGTGAAAGGACAGG

ACATGCTCTCAGACGCCGCTCTCATGGTGCTTCACCGTGGGAATCGCGTGCGGGACATCACGAAGCACTT

CCGTGATGTGGCAAGAATGAAGAAAGGCACCCCCGTCGTCGGCGTGATCAACAACGCTGATGTTGGGAGA

CTGATCTTCTCTGGTGAGGCCCTTACCTACAAGGACATTGTAGTGTGCATGGACGGAGACACCATGCCCG

GTCTCTTCGCCTACAAAGCTGCCACCAAGGCGGGTTACTGTGGAGGAGCCGTTCTTGCAAAGGACGGAGC

CGAGACTTTCATCGTCGGCACTCACTCCGCAGGCGGCAATGGAGTTGGATACTGCTCATGCGTTTCCAGG

TCCATGCTGCTTAAAATGAAGGCACACATCGATCCCGAACCACACCACGAG

>DQ404167.1_O_UKG_2001

ATCTCAATTCCTTCCCAAAAGGCTGTGCTGTACTTTCTCATTGAGAAGGGCCAGCACGAAGCAGCAATTG

AGTTCTTTGAGGGGATGGTGCATGACTCCATCAAGGAGGAGCTCCGGCCTCTCATCCAACAGACCTCATT

TGTGAAGCGCGCTTTTAAGCGCCTGAAGGAAAACTTTGAGATAGTTGCCCTGTGTTTGACTCTTTTGGCA

AACATAGTGATCATGATCCGCGAGACTCGCAAGAGACAGCAGATGATGGATGATGCAGTGAACGAGTACA

TTGAGAAGGCAAACATCACCACGGATGACAAGACTCTTGACGAGGCGGAAAAGAACCCTCTGGAGACCAG

CGGTGCCACCACTGTTGGTTTCAGAGAGAAAACTCTCCCGGGACACAAGGCGGGTGATGACGTGAACTCC

GAGCCCGCCAAACCCGTGGAAGAACAACCACAAGCTGAAGGACCCTACACCGGTCCACTCGAGCGTCAAA

AACCCCTGAAAGTGAGGGCCAAGCTCCCACAGCAGGAGGGGCCCTACGCTGGTCCGATGGAGAGACAGAA

ACCGCTGAAAGTGAAAGTGAAAGCCCCGGTCGTTAAGGAAGGACCTTACGAAGGACCGGTGAAGAAACCT

GTCGCTTTGAAAGTGAAAGCAAAGAACTTGATTGTCACTGAGAGTGGTGCTCCCCCGACTGACTTGCAAA

AGATGGTCATGGGTAACACCAAGCCTGTTGAGCTCATCCTCGACGGGAAGACGGTGGCCATCTGCTGCGC

CACCGGAGTGTTTGGTACTGCCTACCTAGTTCCTCGTCATCTTTTCGCAGAGAAGTATGACAAGATCATG

T-GGACGGCAGAGCCATGACAGACAGTGACTACAGAGTGTTTGAGTTTGAGATTAAAGTGAAAGGACAGG

ACATGCTCTCAGACGCCGCTCTCATGGTGCTTCACCGTGGGAATCGCGTGCGGGACATCACGAAGCACTT

CCGTGATGTGGCAAGAATGAAGAAAGGCACCCCCGTCGTCGGCGTGATCAACAACGCTGATGTTGGGAGA

CTGATCTTCTCTGGTGAGGCCCTTACCTACAAGGACATTGTAGTGTGCATGGACGGAGACACCATGCCCG

GTCTCTTCGCCTACAAAGCTGCCACCAAGGCGGGTTACTGTGGAGGAGCCGTTCTTGCAAAGGACGGAGC

CGAGACTTTCATCGTCGGCACTCACTCCGCAGGCGGCAATGGAGTTGGATACTGCTCATGCGTTTCCAGG

TCCATGCTGCTTAAAATGAAGGCACACATCGATCCCGAACCACACCACGAG

>DQ404168.1_O_UKG_2001

ATCTCAATTCCTTCCCAAAAGGCTGTGCTGTACTTTCTCATTGAGAAGGGCCAGCACGAAGCAGCAATTG

AGTTCTTTGAGGGGATGGTGCATGACTCCATCAAGGAGGAGCTCCGGCCTCTCATCCAACAGACCTCATT

TGTGAAGCGCGCTTTTAAGCGCCTGAAGGAAAACTTTGAGATAGTTGCCCTGTGTTTGACTCTTTTGGCA

AACATAGTGATCATGATCCGCGAGACTCGCAAGAGACAGCAGATGGTGGATGATGCAGTGAACGAGTACA

TTGAGAAGGCAAACATCACCACGGATGACAAGACTCTTGACGAGGCGGAAAAGAACCCTCTGGAGACCAG

CGGTGCCACCACTGTTGGTTTCAGAGAGAAAACTCTCCCGGGACACAAGGCGGGTGACGACGTGAACTCC

GAGCCCGCCAAACCCGTGGAAGAACAACCACAAGCTGAAGGACCCTACACCGGTCCACTCGAGCGTCAAA

AACCCCTGAAAGTGAGGGCCAAGCTCCCACAGCAGGAGGGGCCCTACGCTGGTCCGATGGAGAGACAGAA

ACCGCTGAAAGTGAAAGTGAAAGCCCCGGTCGTTAAGGAAGGACCTTACGAAGGACCGGTGAAGAAACCT

GTCGCTTTGAAAGTGAAAGCAAAGAACTTGATTGTCACTGAGAGTGGTGCTCCCCCGACTGACTTGCAAA

AGATGGTCATGGGTAACACCAAGCCTGTTGAGCTCATCCTCGACGGGAAGACGGTGGCCATCTGCTGCGC

CACCGGAGTGTTTGGTACTGCCTACCTAGTTCCTCGTCATCTTTTCGCAGAGAAGTATGACAAGATCATG

TTGGACGGCAGAGCCATGACAGACAGTGACTACAGAGTGTTTGAGTTTGAGATTAAAGTGAAAGGACAGG

ACATGCTCTCAGACGCCGCTCTCATGGTGCTTCACCGTGGGAATCGCGTGCGGGACATCACGAAGCACTT

CCGTGATGTGGCAAGAATGAAGAAAGGCACCCCCGTCGTCGGCGTGATCAACAACGCTGATGTTGGGAGA

CTGATCTTCTCTGGTGAGGCCCTTACCTACAAGGACATTGTAGTGTGCATGGACGGAGACACCATGCCCG

GTCTCTTCGCCTACAAAGCTGCCACCAAGGCGGGTTACTGTGGAGGAGCCGTTCTTGCAAAGGACGGAGC

CGAGACTTTCATCGTCGGCACTCACTCCGCAGGCGGCAATGGAGTTGGATACTGCTCATGCGTTTCCAGG

TCCATGCTGCTTAAAATGAAGGCACACATCGATCCCGAACCACACCACGAG

>DQ404169.1_O_UKG_2001

ATCTCAATTCCTTCCCAAAAGGCTGTGCTGTACTTTCTCATTGAGAAAGGCCAGCACGAAGCAGCAATTG

AGTTCTTTGAGGGGATGGTGCATGACTCCATCAAGGAGGAGCTCCGGCCTCTCATCCAACAGACCTCATT

TGTGAAGCGCGCTTTTAAGCGCCTGAAGGAAAACTTTGAGATAGTTGCCCTGTGTTTGACTCTTTTGGCA

AACATAGTGATCATGATCCGCGAGACTCGCAAGAGACAGCAGATGGTGGATGATGCAGTGAACGAGTACA

TTGAGAAGGCAAACATCACCACGGATGACAAGACTCTTGACGAGGCGGAAAAGAACCCTCTGGAGACCAG

CGGTGCCACCACTGTTGGTTTCAGAGAGAAAACTCTCCCGGGACACAAGGCGGGTGATGACGTGAACTCC

GAGCCCGCCAAACCCGTGGAAGAACAACCACAAGCTGAAGGACCCTACACCGGTCCACTCGAGCGTCAAA

AACCCCTGAAAGTGAGGGCCAAGCTCCCACAGCAGGAGGGGCCCTACGCTGGTCCGATGGAGAGACAGAA

ACCGCTGAAAGTGAAAGTGAAAGCCCCGGTCGTTAAGGAAGGACCTTACGAAGGACCGGTGAAGAAACCT

GTCGCTTTGAAAGTGAAAGCAAAGAACTTGATTGTCACTGAGAGTGGTGCTCCCCCGACTGACTTGCAAA

AGATGGTCATGGGTAACACCAAGCCTGTTGAGCTCATCCTCGACGGGAAGACGGTGGCCATCTGCTGCGC

CACCGGAGTGTTTGGCACTGCCTACCTAGTTCCTCGTCATCTTTTCGCAGAGAAGTATGACAAGATCATG

TTGGACGGCAGAGCCATGACAGACAGTGACTACAGAGTGTTTGAGTTTGAGATTAAAGTGAAAGGACAGG

ACATGCTCTCAGACGCCGCTCTCATGGTGCTTCACCGTGGGAATCGCGTGCGGGACATCACGAAGCACTT

CCGTGATGTGGCAAGAATGAAGAAAGGCACCCCCGTCGTCGGCGTGATCAACAACGCTGATGTTGGGAGA

CTGATCTTCTCTGGTGAGGCCCTTACCTACAAGGACATTGTAGTGTGCATGGACGGAGACACCATGCCCG

GTCTCTTCGCCTACAAAGCTGCCACCAAGGCGGGTTACTGTGGAGGAGCCGTTCTTGCAAAGGACGGAGC

CGAGACTTTCATCGTCGGCACTCACTCCGCAGGCGGCAATGGAGTTGGATACTGCTCATGCGTTTCCAGG

TCCATGCTGCTTAAAATGAAGGCACACATTGATCCCGAACCACACCACGAG

>DQ404170.1_O_UKG_2001

ATCTCAATTCCTTCCCAAAAGGCTGTGCTGTACTTTCTCATTGAGAAGGGCCAGCACGAAGCAGCAATTG

AGTTCTTTGAGGGGATGGTGCATGACTCCATCAAGGAGGAGCTCCGGCCTCTCATCCAACAGACCTCATT

TGTGAAGCGCGCTTTTAAGCGCCTGAAGGAAAACTTTGAGATAGTTGCCCTGTGTTTGACTCTTTTGGCA

AACATAGTGATCATGATCCGCGAGACTCGCAAGAGACAGCAGATGGTGGATGATGCAGTGAACGAGTACA

TTGAGAAGGCAAACATCACCACGGATGACAAGACTCTTGACGAGGCGGAAAAGAACCCTCTGGAGACCAG

CGGTGCCACCACTGTTGGTTTCAGAGAGAAAACTCTCCCGAGACACAAGGCGGGTGATGACGTGAACTCC

GAGCCCGCCAAACCCGTGGAAGAACAACCACAAGCTGAAGGACCCTACACCGGTCCACTCGAGCGTCAAA

AACCCCTGAAAGTGAGGGCCAAGCTCCCACAGCAGGAGGGGCCCTACGCTGGTCCGATGGAGAGACAGAA

ACCGCTGAAAGTGAAAGTGAAAGCCCCGGTCGTTAAGGAAGGACCTTACGAAGGACCGGTGAAGAAACCT

GTCGCTTTGAAAGTGAAAGCAAAGAACTTGATTGTCACTGAGAGTGGTGCTCCCCCGACTGACTTGCAAA

AGATGGTCATGGGTAACACCAAGCCCGTTGAGCTCATCCTCGACGGGAAGACGGTGGCCATCTGCTGCGC

CACCGGAGTGTTTGGTACTGCCTACCTAGTTCCTCGTCATCTTTTCGCAGAGAAGTATGACAAGATCATG

TTGGACGGCAGAGCCATGACAGACAGTGACTACAGAGTGTTTGAGTTTGAGATTAAAGTGAAAGGACAGG

ACATGCTCTCAGACGCCGCTCTCATGGTGCTTCACCGTGGGAATCGCGTGCGGGACATCACGAAGCACTT

CCGTGATGTGGCAAGAATGAAGAAAGGCACCCCCGTCGTCGGCGTGATCAACAACGCTGATGTTGGGAGA

CTGATCTTCTCTGGTGAGGCCCTTACCTACAAGGACATTGTAGTGTGCATGGACGGAGACACCATGCCCG

GTCTCTTCGCCTACAAAGCTGCCACCAAGGCGGGTTACTGTGGAGGAGCCGTTCTTGCAAAGGACGGAGC

CGAGACTTTCATCGTCGGCACTCACTCCGCAGGCGGCAATGGAGTTGGATACTGCTCATGCGTTTCCAGG

TCCATGCTGCTTAAAATGAAGGCACACATCGATCCCGAACCACACCACGAG

>DQ404171.1_O_UKG_2001

ATCTCAATTCCTTCCCAAAAGGCTGTGCTGTACTTTCTCATTGAGAAGGGCCAGCACGAAGCAGCAATTG

AGTTCTTTGAGGGGATGGTGCATGACTCCATCAAGGAGGAGCTCCGGCCTCTCATCCAACAGACCTCATT

TGTGAAGCGCGCTTTTAAGCGCCTGAAGGAAAACTTTGAGATAGTTGCCCTGTGTTTGACTCTTTTGGCA

AACATAGTGATCATGATCCGCGAGACTCGCAAGAGACAGCAGATGGTGGATGATGCAGTGAACGAGTACA

TTGAGAAGGCAAACATCACCACGGATGACAAGACTCTTGACGAGGCGGAAAAGAACCCTCTGGAGACCAG

CGGTGCCACCACTGTTGGTTTCAGAGAGAAAACTCTCCCGGGACACAAGGCGGGTGATGACGTGAACTCC

GAGCCCGCCAAACCCGTGGAAGAACAACCACAAGCTGAAGGACCCTACACCGGTCCACTCGAGCGTCAAA

AACCCCTGAAAGTGAGGGCCAAGCTCCCACAGCAGGAGGGGCCCTACGCTGGTCCGATGGAGAGACAGAA

ACCGCTGAAAGTGAAAGTGAAAGCCCCGGTCGTTAAGGAAGGACCTTACGAAGGACCGGTGAAGAAACCT

GTCGCTTTGAAAGTGAAAGCAAAGAACTTGATTGTCACTGAGAGTGGTGCTCCCCCGACTGACTTGCAAA

AGATGGTCATGGGTAACACCAAGCCTGTTGAGCTCATCCTCGACGGGAAGACGGTGGCCATCTGCTGCGC

CACCGGAGTGTTTGGTACTGCCTACCTAGTTCCTCGTCATCTTTTCGCAGAGAAGTATGACAAGATCATG

TTGGACGGCAGAGCCATGACAGACAGTGACTACAGAGTGTTTGAGTTTGAGATTAAAGTGAAAGGACAGG

ACATGCTCTCAGACGCCGCTCTCATGGTGCTTCACCGTGGGAATCGCGTGCGGGACATCACGAAGCACTT

CCGTGATGTGGCAAGAATGAAGAAAGGCACCCCCGTCGTCGGCGTGATCAACAACGCTGATGTTGGGAGA

CTGATCTTCTCTGGTGAGGCCCTTACCTACAAGGACATTGTAGTGTGCATGGACGGAGACACCATGCCCG

GTCTCTTCGCCTACAAAGCTGCCACCAAGGCGGGTTACTGTGGAGGAGCCGTTCTTGCAAAGGACGGAGC

CGAGACTTTCATCGTCGGCACTCACTCCGCAGGCGGCAATGGAGTTGGATACTGCTCATGCGTTTCCAGG

TCCATGCTGCTTAAAATGAAGGCACACATCGATCCCGAACCACACCACGAG

>DQ404172.1_O_UKG_2001

ATCTCAATTCCTTCCCAAAAGGCTGTGCTGTACTTTCTCATTGAGAAGGGCCAGCACGAAGCAGCAATTG

AGTTCTTTGAGGGGATGGTGCATGACTCCATCAAGGAGGAGCTCCGGCCTCTCATCCAACAGACCTCATT

TGTGAAGCGCGCTTTTAAGCGCCTGAAGGAAAACTTTGAGATAGTTGCCCTGTGTTTGACTCTTTTGGCA

AACATAGTGATCATGATCCGCGAGACTCGCAAGAGACAGCAGATGGTGGATGATGCAGTGAACGAGTACA

TTGAGAAGGCAAACATCACCACGGATGACAAGACTCTTGACGAGGCGGAAAAGAACCCTCTGGAGACCAG

CGGTGCCACCACTGTTGGTTTCAGAGAGAAAACTCTCCCGGGACACAAGGCGGGTGATGACGTGAACTCC

GAGCCCGCCAAACCCGTGGAAGAACAACCACAAGCTGAAGGACCCTACACCGGTCCACTCGAGCGTCAAA

AACCCCTGAAAGTGAGGGCCAAGCTCCCACAGCAGGAGGGGCCCTACGCTGGTCCGATGGAGAGACAGAA

ACCGCTGAAAGTGAAAGTGAAAGCCCCGGTCGTTAAGGAAGGACCTTACGAAGGACCGGTGAAGAAACCT

GTCGCTTTGAAAGTGAAAGCAAAGAACTTGATTGTCACTGAGAGTGGTGCTCCCCCGACTGACTTGCAAA

AGATGGTCATGGGTAACACCAAGCCTGTTGAGCTCATCCTCGACGGGAAGACGGTGGCCATCTGCTGCGC

CACCGGAGTGTTTGGTACTGCCTACCTAGTTCCTCGTCATCTTTTCGCAGAGAAGTATGACAAGATCATG

TTGGACGGCAGAGCCATGACAGACAGTGACTACAGAGTGTTTGAGTTTGAGATTAAAGTGAAAGGACAGG

ACATGCTCTCAGACGCCGCTCTCATGGTGCTTCACCGTGGGAATCGCGTGCGGGACATCACGAAGCACTT

CCGTGATGTGGCAAGAATGAAGAAAGGCACCCCCGTCGTCGGCGTGATCAACAACGCTGATGTTGGGAGA

CTGATCTTCTCTGGTGAGGCCCTTACCTACAAGGACATTGTAGTGTGCATGGACGGAGACACCATGCCCG

GTCTCTTCGCCTACAAAGCTGCCACCAAGGCGGGTTACTGTGGAGGAGCCGTTCTTGCAAAGGACGGAGC

CGAGACTTTCATCGTCGGCACTCACTCCGCAGGCGGCAATGGAGTTGGATACTGCTCATGCGTTTCCAGG

TCCATGCTGCTTAAAATGAAGGCACACATCGATCCCGAACCACACCACGAG

>DQ404173.1_O_UKG_2001

ATCTCAATTCCTTCCCAAAAGGCTGTGCTGTACTTTCTCATTGAGAAGGGCCAGCACGAAGCAGCAATTG

AGTTCTTTGAGGGGATGGTGCATGACTCCATCAAGGAGGAGCTCCGGCCTCTCATCCAACAGACCTCATT

TGTGAAGCGCGCTTTTAAGCGCCTGAAGGAAAACTTTGAGATAGTTGCCCTGTGTTTGACTCTTTTGGCA

AACATAGTGATCATGATCCGCGAGACTCGCAAGAGACAGCAGATGGTGGATGATGCAGTGAACGAGTACA

TTGAGAAGGCAAACATCACCACGGATGACAAGACTCTTGACGAGGCGGAAAAGAACCCTCTGGAGACCAG

CGGTGCCACCACTGTTGGTTTCAGAGAGAAAACTCTCCCGGGACACAAGGCGGGTGATGACGTGAACTCC

GAGCCCGCCAAACCCGTGGAAGAACAACCACAAGCTGAAGGACCCTACACCGGTCCACTCGAGCGTCAAA

AACCCCTGAAAGTGAGGGCCAAGCTCCCACAGCAGGAGGGGCCCTACGCTGGTCCGATGGAGAGACAGAA

ACCGCTGAAAGTGAAAGTGAAAGCCCCGGTCGTTAAGGAAGGACCTTACGAAGGACCGGTGAAGAAACCT

GTCGCTTTGAAAGTGAAAGCAAAGAACTTGATTGTCACTGAGAGTGGTGCTCCCCCGACTGACTTGCAAA

AGATGGTCATGGGTAACACCAAGCCTGTTGAGCTCATCCTCGACGGGAAGACGGTGGCCATCTGCTGCGC

CACCGGAGTGTTTGGTACTGCCTACCTAGTTCCTCGTCATCTTTTCGCAGAGAAGTATGACAAGATCATG

TTGGACGGCAGAGCCATGACAGACAGTGACTACAGAGTGTTTGAGTTTGAGATTAAAGTGAAAGGACAGG

ACATGCTCTCAGACGCCGCTCTCATGGTGCTTCACCGTGGGAATCGCGTGCGGGACATCACGAAGCACTT

CCGTGATGTGGCAAGAATGAAGAAAGGCACCCCCGTCGTCGGCGTGATCAACAACGCTGATGTTGGGAGA

CTGATCTTCTCTGGTGAGGCCCTTACCTACAAGGACATTGTAGTGTGCATGGACGGAGACACCATGCCCG

GTCTCTTCGCCTACAAAGCTGCCACCAAGGCGGGTTACTGTGGAGGAGCCGTTCTTGCAAAGGACGGAGC

CGAGACTTTCATCGTCGGCACTCACTCCGCAGGCGGCAATGGAGTTGGATACTGCTCATGCGTTTCCAGG

TCCATGCTGCTTAAAATGAAGGCACACATCGATCCCGAACCACACCACGAG

>DQ404174.1_O_UKG_2001

ATCTCAATTCCTTCCCAAAAGGCTGTGCTGTACTTTCTCATTGAGAAGGGCCAGCACGAAGCAGCAATTG

AGTTCTTTGAGGGGATGGTGCATGACTCCATCAAGGAGGAGCTCCGGCCTCTCATCCAACAGACCTCATT

TGTGAAGCGCGCTTTTAAGCGCCTGAAGGAAAACTTTGAGATAGTTGCCCTGTGTTTGACTCTTTTGGCA

AACATAGTGATCATGATCCGCGAGACTCGCAAGAGACAGCAGATGGTGGATGATGCAGTGAACGAGTACA

TTGAGAAGGCAAACATCACCACGGATGACAAGACTCTTGACGAGGCGGAAAAGAACCCTCTGGAGACCAG

CGGTGCCACCACTGTTGGTTTCAGAGAGAAAACTCTCCCGGGACACAAGGCGGGTGATGACGTGAACTCC

GAGCCCGCCAAACCCGTGGAAGAACAACCACAAGCTGAAGGACCCTACACCGGTCCACTCGAGCGTCAAA

AACCCCTGAAAGTGAGGGCCAAGCTCCCACAGCAGGAGGGGCCCTACGCTGGTCCGATGGAGAGACAGAA

ACCGCTGAAAGTGAAAGTGAAAGCCCCGGTCGTTAAGGAAGGACCTTACGAAGGACCGGTGAAGAAACCT

GTCGCTTTGAAAGTGAAAGCAAAGAACTTGATTGTCACTGAGAGTGGTGCTCCCCCGACTGACTTGCAAA

AGATGGTCATGGGTAACACCAAGCCTGTTGAGCTCATCCTCGACGGGAAGACGGTGGCCATCTGCTGCGC

CACCGGAGTGTTTGGTACTGCCTACCTAGTTCCTCGTCATCTTTTCGCAGAGAAGTATGACAAGATCATG

TTGGACGGCAGAGCCATGACAGACAGTGACTACAGAGTGTTTGAGTTTGAGATTAAAGTGAAAGGACAGG

ACATGCTCTCAGACGCCGCTCTCATGGTGCTTCACCGTGGGAATCGCGTGCGGGACATCACGAAGCACTT

CCGTGATGTGGCAAGAATGAAGAAAGGCACCCCCGTCGTCGGCGTGATCAACAACGCTGATGTTGGGAGA

CTGATCTTCTCTGGTGAGGCCCTTACCTACAAGGACATTGTAGTGTGCATGGACGGAGACACCATGCCCG

GTCTCTTCGCCTACAAAGCTGCCACCAAGGCGGGTTACTGTGGAGGAGCCGTTCTTGCAAAGGACGGAGC

CGAGACTTTCATCGTCGGCACTCACTCCGCAGGCGGCAATGGAGTTGGATACTGCTCATGCGTTTCCAGG

TCCATGCTGCTTAAAATGAAGGCACACATCGATCCCGAACCACACCACGAG

>DQ404175.1_O_UKG_2001

ATCTCAATTCCTTCCCAAAAGGCTGTGCTGTACTTTCTCATTGAGAAGGGCCAGCACGAAGCAGCAATTG

AGTTCTTTGAGGGGATGGTGCATGACTCCATCAAGGAGGAGCTCCGGCCTCTCATCCAACAGACCTCATT

TGTGAAGCGCGCTTTTAAGCGCCTGAAGGAAAACTTTGAGATAGTTGCCCTGTGTTTGACTCTTTTGGCA

AACATAGTGATCATGATCCGCGAGACTCGCAAGAGACAGCAGATGGTGGATGATGCAGTGAACGAGTACA

TTGAGAAGGCAAACATCACCACGGATGACAAGACTCTTGACGAGGCGGAAAAGAACCCTCTGGAGACCAG

CGGTGCCACCACTGTTGGTTTCAGAGAGAAAACTCTCCCGGGACACAAGGCGGGTGATGACGTGAACTCC

GAGCCCGCCAAACCCGTGGAAGAACAACCACAAGCTGAAGGACCCTACACCGGTCCACTCGAGCGTCAAA

AACCCCTGAAAGTGAGGGCCAAGCTCCCACAGCAGGAGGGGCCCTACGCTGGTCCGATGGAGAGACAGAA

ACCGCTGAAAGTGAAAGTGAAAGCCCCGGTCGTTAAGGAAGGACCTTACGAAGGACCGGTGAAGAAACCT

GTCGCTTTGAAAGTGAAAGCAAAGAACTTGATTGTCACTGAGAGTGGTGCTCCCCCGACTGACTTGCAAA

AGATGGTCATGGGTAACACCAAGCCTGTTGAGCTCATCCTCGACGGGAAGACGGTGGCCATCTGCTGCGC

CACCGGAGTGTTTGGTACTGCCTACCTAGTTCCTCGTCATCTTTTCGCAGAGAAGTATGACAAGATCATG

TTGGACGGCAGAGCCATGACAGACAGTGACTACAGAGTGTTTGAGTTTGAGATTAAAGTGAAAGGACAGG

ACATGCTCTCAGACGCCGCTCTCATGGTGCTTCACCGTGGGAATCGCGTGCGGGACATCACGAAGCACTT

CCGTGATGTGGCAAGAATGAAGAAAGGCACCCCCGTCGTCGGCGTGATCAACAACGCTGATGTTGGGAGA

CTGATCTTCTCTGGTGAGGCCCTTACCTACAAGGACATTGTAGTGTGCATGGACGGAGACACCATGCCCG

GTCTCTTCGCCTACAAAGCTGCCACCAAGGCGGGTTACTGTGGAGGAGCCGTTCTTGCAAAGGACGGAGC

CGAGACTTTCATCGTCGGCACTCACTCCGCAGGCGGCAATGGAGTTGGATACTGCTCATGCGTTTCCAGG

TCCATGCTGCTTAAAATGAAGGCACACATCGATCCCGAACCACACCACGAG

>DQ404176.1_O_UKG_2001

ATCTCAATTCCTTCCCAAAAGGCTGTGCTGTACTTTCTCATTGAGAAGGGCCAGCACGAAGCAGCAATTG

AGTTCTTTGAGGGGATGGTGCATGACTCCATCAAGGAGGAGCTCCGGCCTCTCATCCAACAGACCTCATT

TGTGAAGCGCGCTTTTAAGCGCCTGAAGGAAAACTTTGAGATAGTTGCCCTGTGTTTGACTCTTTTGGCA

AACATAGTGATCATGATCCGCGAGACTCGCAAGAGACAGCAGATGGTGGATGATGCAGTGAACGAGTACA

TTGAGAAGGCAAACATCACCACGGATGACAAGACTCTTGACGAGGCGGAAAAGAACCCTCTGGAGACCAG

CGGTGCCACCACTGTTGGTTTCAGAGAGAAAACTCTCCCGGGACACAAGGCGGGTGATGACGTGAACTCC

GAGCCCGCCAAACCCGTGGAAGAACAACCACAAGCTGAAGGACCCTACACCGGTCCACTCGAGCGTCAAA

AACCCCTGAAAGTGAGGGCCAAGCTCCCACAGCAGGAGGGGCCCTACGCTGGTCCGATGGAGAGACAGAA

ACCGCTGAAAGTGAAAGTGAAAGCCCCGGTCGTTAAGGAAGGACCTTACGAAGGACCGGTGAAGAAACCT

GTCGCTTTGAAAGTGAAAGCAAAGAACTTGATTGTCACTGAGAGTGGTGCTCCCCCGACTGACTTGCAAA

AGATGGTCATGGGTAACACCAAGCCTGTTGAGCTCATCCTCGACGGGAAGACGGTGGCCATCTGCTGCGC

CACCGGAGTGTTTGGTACTGCCTACCTAGTTCCTCGTCATCTTTTCGCAGAGAAGTATGACAAGATCATG

TTGGACGGCAGAGCCATGACAGACAGTGACTACAGAGTGTTTGAGTTTGAGATTAAAGTGAAAGGACAGG

ACATGCTCTCAGACGCCGCTCTCATGGTGCTTCACCGTGGGAATCGCGTGCGGGACATCACGAAGCACTT

CCGTGATGTGGCAAGAATGAAGAAAGGCACCCCCGTCGTCGGCGTGATCAACAACGCTGATGTTGGGAGA

CTGATCTTCTCTGGTGAGGCCCTTACCTACAAGGACATTGTAGTGTGCATGGACGGAGACACCATGCCCG

GTCTCTTCGCCTACAAAGCTGCCACCAAGGCGGGTTACTGTGGAGGAGCCGTTCTTGCAAAGGACGGAGC

CGAGACTTTCATCGTCGGCACTCACTCCGCAGGCGGCAATGGAGTTGGATACTGCTCATGCGTTTCCAGG

TCCATGCTGCTTAAAATGAAGGCACACATCGATCCCGAACCACACCACGAG

>DQ404177.1_O_UKG_2001

ATCTCAATTCCTTCCCAAAAGGCTGTGCTGTACTTTCTCATTGAGAAGGGCCAGCACGAAGCAGCAATTG

AGTTCTTTGAGGGGATGGTGCATGACTCCATCAAGGAGGAGCTCCGGCCTCTCATCCAACAGACCTCATT

TGTGAAGCGCGCTTTTAAGCGCCTGAAGGAAAACTTTGAGATAGTTGCCCTGTGTTTGACTCTTTTGGCA

AACATAGTGATCATGATCCGCGAGACTCGCAAGAGACAGCAGATGGTGGATGATGCAGTGAACGAGTACA

TTGAGAAGGCAAACATCACCACGGATGACAAGACTCTTGACGAGGCGGAAAAGAACCCTCTGGAGACCAG

CGGTGCCACCACTGTTGGTTTCAGAGAGAAAACTCTCCCGGGACACAAGGCGGGTGATGACGTGAACTCC

GAGCCCGCCAAACCCGTGGAAGAACAACCACAAGCTGAAGGACCCTACACCGGTCCACTCGAGCGTCAAA

AACCCCTGAAAGTGAGGGCCAAGCTCCCACAGCAGGAGGGGCCCTACGCTGGTCCGATGGAGAGACAGAA

ACCGCTGAAAGTGAAAGTGAAAGCCCCGGTCGTTAAGGAAGGACCTTACGAAGGACCGGTGAAGAAACCT

GTCGCTTTGAAAGTGAAAGCAAAGAACTTGATTGTCACTGAGAGTGGTGCTCCCCCGACTGACTTGCAAA

AGATGGTCATGGGTAACACCAAGCCTGTTGAGCTCATCCTCGACGGGAAGACGGTGGCCATCTGCTGCGC

CACCGGAGTGTTTGGTACTGCCTACCTAGTTCCTCGTCATCTTTTCGCAGAGAAGTATGACAAGATCATG

TTGGACGGCAGAGCCATGACAGACAGTGACTACAGAGTGTTTGAGTTTGAGATTAAAGTGAAAGGACAGG

ACATGCTCTCAGACGCCGCTCTCATGGTGCTTCACCGTGGGAATCGCGTGCGGGACATCACGAAGCACTT

CCGTGATGTGGCAAGAATGAAGAAAGGCACCCCCGTCGTCGGCGTGATCAACAACGCTGATGTTGGGAGA

CTGATCTTCTCTGGTGAGGCCCTTACCTACAAGGACATTGTAGTGTGCATGGACGGAGACACCATGCCCG

GTCTCTTCGCCTACAAAGCTGCCACCAAGGCGGGTTACTGTGGAGGAGCCGTTCTTGCAAAGGACGGAGC

CGAGACTTTCATCGTCGGCACCCACTCCGCAGGCGGCAATGGAGTTGGATACTGCTCATGCGTTTCCAGG

TCCATGCTGCTTAAAATGAAGGCACACATCGATCCCGAACCACACCACGAG

>DQ404178.1_O_UKG_2001

ATCTCAATTCCTTCCCAAAAGGCTGTGCTGTACTTTCTCATTGAGAAGGGCCAGCACGAAGCAGCAATTG

AGTTCTTTGAGGGGATGGTGCATGACTCCATCAAGGAGGAGCTCCGGCCTCTCATCCAACAGACCTCATT

TGTGAAGCGCGCTTTTAAGCGCCTGAAGGAAAACTTTGAGATAGTTGCCCTGTGTTTGACTCTTTTGGCA

AACATAGTGATCATGATCCGCGAGACTCGCAAGAGACAGCAGATGGTGGATGATGCAGTGAACGAGTACA

TTGAGAAGGCAAACATCACCACGGATGACAAGACTCTTGACGAGGCGGAAAAGAACCCTCTGGAGACCAG

CGGTGCCACCACTGTTGGTTTCAGAGAGAAAACTCTCCCGGGACACAAGGCGGGTGATGACGTGAACTCC

GAGCCCGCCAAACCCGTGGAAGAACAACCACAAGCTGAAGGACCCTACACCGGTCCACTCGAGCGTCAAA

AACCCCTGAAAGTGAGGGCCAAGCTCCCACAGCAGGAGGGGCCCTACGCTGGTCCGATGGAGAGACAGAA

ACCGCTGAAAGTGAAAGTGAAAGCCCCGGTCGTTAAGGAAGGACCTTACGAAGGACCGGTGAAGAAACCT

GTCGCTTTGAAAGTGAAAGCAAAGAACTTGATTGTCACTGAGAGTGGTGCTCCCCCGACTGACTTGCAAA

AGATGGTCATGGGTAACACCAAGCCTGTTGAGCTCATCCTCGACGGGAAGACGGTGGCCATCTGCTGCGC

CACCGGAGTGTTTGGTACTGCCTACCTAGTTCCTCGTCATCTTTTCGCAGAGAAGTATGACAAGATCATG

TTGGACGGCAGAGCCATGACAGACAGTGACTACAGAGTGTTTGAGTTTGAGATTAAAGTGAAAGGACAGG

ACATGCTCTCAGACGCCGCTCTCATGGTGCTTCACCGTGGGAATCGCGTGCGGGACATCACGAAGCACTT

CCGTGATGTGGCAAGAATGAAGAAAGGCACCCCCGTCGTCGGCGTGATCAACAACGCTGATGTTGGGAGA

CTGATCTTCTCTGGTGAGGCCCTTACCTACAAGGACATTGTAGTGTGCATGGACGGAGACACCATGCCCG

GTCTCTTCGCCTACAAAGCTGCCACCAAGGCGGGTTACTGTGGAGGAGCCGTTCTTGCAAAGGACGGAGC

CGAGACTTTCATCGTCGGCACTCACTCCGCAGGCGGCAATGGAGTTGGATACTGCTCATGCGTTTCCAGG

TCCATGCTGCTTAAAATGAAGGCACACATCGATCCCGAACCACACCACGAG

>DQ404179.1_O_UKG_2001

ATCTCAATTCCTTCCCAAAAGGCTGTGCTGTACTTTCTCATTGAGAAGGGCCAGCACGAAGCAGCAATTG

AGTTCTTTGAGGGGATGGTGCATGACTCCATCAAGGAGGAGCTCCGGCCTCTCATCCAACAGACCTCATT

TGTGAAGCGCGCTTTTAAGCGCCTGAAGGAAAACTTTGAGATAGTTGCCCTGTGTTTGACTCTTTTGGCA

AACATAGTGATCATGATCCGCGAGACTCGCAAGAGACAGCAGATGGTGGATGATGCAGTGAACGAGTACA

TTGAGAAGGCAAACATCACCACGGATGACAAGACTCTTGACGAGGCGGAAAAGAACCCTCTGGAGACCAG

CGGTGCCACCACTGTTGGTTTCAGAGAGAAAACTCTCCCGGGACACAAGGCGGGTGATGACGTGAACTCC

GAGCCCGCCAAACCCGTGGAAGAACAACCACAAGCTGAAGGACCCTACACCGGTCCACTCGAGCGTCAAA

AACCCCTGAAAGTGAGGGCCAAGCTCCCACAGCAGGAGGGGCCCTACGCTGGTCCGATGGAGAGACAGAA

ACCGCTGAAAGTGAAAGTGAAAGCCCCGGTCGTTAAGGAAGGACCTTACGAAGGACCGGTGAAGAAACCT

GTCGCTTTGAAAGTGAAAGCAAAGAACTTGATTGTCACTGAGAGTGGTGCTCCCCCGACTGACTTGCAAA

AGATGGTCATGGGTAACACCAAGCCTGTTGAGCTCATCCTCGACGGGAAGACGGTGGCCATCTGCTGCGC

CACCGGAGTGTTTGGTACTGCCTACCTAGTTCCTCGTCATCTTTTCGCAGAGAAGTATGACAAGATCATG

TTGGACGGCAGAGCCATGACAGACAGTGACTACAGAGTGTTTGAGTTTGAGATTAAAGTGAAAGGACAGG

ACATGCTCTCAGACGCCGCTCTCATGGTGCTTCACCGTGGGAATCGCGTGCGGGACATCACGAAGCACTT

CCGTGATGTGGCAAGAATGAAGAAAGGCACCCCCGTCGTCGGCGTGATCAACAACGCTGATGTTGGGAGA

CTGATCTTCTCTGGTGAGGCCCTTACCTACAAGGACATTGTAGTGTGCATGGACGGAGACACCATGCCCG

GTCTCTTCGCCTACAAAGCTGCCACCAAGGCGGGTTACTGTGGAGGAGCCGTTCTTGCAAAGGACGGAGC

CGAGACTTTCATCGTCGGCACTCACTCCGCAGGCGGCAATGGAGTTGGATACTGCTCATGCGTTTCCAGG

TCCATGCTGCTTAAAATGAAGGCACACATCGATCCCGAACCACACCACGAG

>DQ404180.1_O_UKG_2001

ATCTCAATTCCTTCCCAAAAGGCTGTGCTGTACTTTCTCATTGAGAAGGGCCAGCACGAAGCAGCAATTG

AGTTCTTTGAGGGGATGGTGCATGACTCCATCAAGGAGGAGCTCCGGCCTCTCATCCAACAGACCTCATT

TGTGAAGCGCGCTTTTAAGCGCCTGAAGGAAAACTTTGAGATAGTTGCCCTGTGTTTGACTCTTTTGGCA

AACATAGTGATCATGATCCGCGAGACTCGCAAGAGACAGCAGATGGTGGATGATGCAGTGAACGAGTACA

TTGAGAAGGCAAACATCACCACGGATGACAAGACTCTTGACGAGGCGGAAAAGAACCCTCTGGAGACCAG

CGGTGCCACCACTGTTGG-TTCAGAGAGAAAACTCTCCCGGGACACAAGGCGGGTGATGACGTGAACTCC

GAGCCCGCCAAACCCGTGGAAGAACAACCACAAGCTGAAGGACCCTACACCGGTCCACTCGAGCGTCAAA

AACCCCTGAAAGTGAGGGCCAAGCTCCCACAGCAGGAGGGGCCCTACGCTGGTCCGATGGAGAGACAGAA

ACCGCTGAAAGTGAAAGTGAAAGCCCCGGTCGTTAAGGAAGGACCTTACGAAGGACCGGTGAAGAAACCT

GTCGCTTTGAAAGTGAAAGCAAAGAACTTGATTGTCACTGAGAGTGGTGCTCCCCCGACTGACTTGCAAA

AGATGGTCATGGGTAACACCAAGCCTGTTGAGCTCATCCTCGACGGGAAGACGGTGGCCATCTGCTGCGC

CACCGGAGTGTTTGGTACTGCCTACCTAGTTCCTCGTCATCTTTTCGCAGAGAAGTATGACAAGATCATG

TTGGACGGCAGAGCCATGACAGACAGTGACTACAGAGTGTTTGAGTTTGAGATTAAAGTGAAAGGACAGG

ACATGCTCTCAGACGCCGCTCTCATGGTGCTTCACCGTGGGAATCGCGTGCGGGACATCACGAAGCACTT

CCGTGATGTGGCAAGAATGAAGAAAGGCACCCCCGTCGTCGGCGTGATCAACAACGCTGATGTTGGGAGA

CTGATCTTCTCTGGTGAGGCCCTTACCTACAAGGACATTGTAGTGTGCATGGACGGAGACACCATGCCCG

GTCTCTTCGCCTACAAAGCTGCCACCAAGGCGGGTTACTGTGGAGGAGCCGTTCTTGCAAAGGACGGAGC

CGAGACTTTCATCGTCGGCACTCACTCCGCAGGCGGCAATGGAGTTGGATACTGCTCATGCGTTTCCAGG

TCCATGCTGCTTAAAATGAAGGCACACATCGATCCCGAACCACACCACGAG

>DQ989303.1_Asia1_IND_1993

ATCTCAATCCCTTCCCAAAAGTCCGTGTTGTACTTTCTCATCGAGAAGGGGCAGCACGAAGCAGCAATTG

AATTCTTTGAGGGGATGGTGCACGACTCCATCAAGGAAGAGCTCCGACCTCTCATCCAACAGACCTCATT

TGTGAAGCGCGCGTTTAAGCGCCTGAAGGAGAACTTTGAGATTGTTGCCCTATGTTTGACTCTTCTGGCA

AACATAGTGATCATGATCCGCGAGACTCGCAAGAGACAACAGATGGTGGATGATGCAGTGAATGAGTACA

TTGAGAAAGCAAACATCACCACAGATGACAAAACTCTTGACGAAGCGGAAAAGAACCCTCTGGAGACTAG

TGGTGCCAGCACTGTTGGCTTCAGAGAGAGAACTCTCCCGGGACACAAGGTGAGTGATGACGTGAACTCC

GAGCCCACCAAACCCGCGGAAGAGCAACCACAAGCTGAAGGACCCTACGCCGGGCCGCTCGAGCGTCAGA

AACCTCTGAAAGTGAAAGCTAAGCTGCCACAGCAGGAGGGACCCTACGCTGGCCCGATGGAGAGACAGAA

ACCACTGAAAGTGAAAGTGAAAGCCCCGGTCGCGAAGGAAGGACCTTACGAGGGACCGGTGAAGAAGCCT

GTCGCTTTGAAAGTGAAAGCTAAGAACCTGATTGTCACTGAGAGTGGTGCCCCACCAACCGACTTGCAAA

AGATGGTTATGGGCAACACAAAGCCTGTTGAGCTCATCCTCGACGGGAAGACAGTAGCCATCTGCTGTGC

TACTGGAGTGTTTGGCACCGCCTACCTCGTGCCTCGTCATCTTTTCGCTGAGAAGTATGACAAGATCATG

TTGGACGGCAGAGCCATGACAGACAGTGATTACAGAGTGTTTGAGTTTGAGATTAAAGTAAAAGGACAGG

ACATGCTCTCAGACGCCGCGCTCATGGTGCTGCACCGTGGAAACCGCGTGAGAGACATCACGAAACATTT

TCGTGACACAGCAAGAATGAAAAAAGGCACCCCCGTCGTTGGTGTGATTAACAACGCCGACGTCGGGAGA

CTGATTTTCTCTGGTGAGGCCCTTACCTACAAGGACATTGTAGTGTGCATGGATGGAGACACCATGCCTG

GCCTCTTTGCCTACAGAGCCGCCACCAAGGCTGGCTACTGTGGAGGAGCTGTTCTCGCCAAGGACGGCGC

CGACACATTCATCGTTGGCACACACTCCGCAGGTGGCAATGGAGTTGGATACTGCTCTTGTGTTTCCAGG

TCCATGCTCCAGAGGATGAAGGCACACATTGACCCTGAACCACACCACGAG

>DQ989304.1_Asia1_IND_2000

ATCTCAATTCCTTCCCAAAAGTCCGTGTTGTACTTTCTCATCGAGAAGGGGCAGCACGAAGCAGCAATTG

AATTCTTTGAGGGGATGGTGCACGACTCCATCAAGGAAGAGCTCCGACCTCTCATCCAACAGACCTCATT

TGTGAAGCGCGCGTTTAAGCGCCTGAAGGAGAACTTTGAGATTGTTGCCCTATGTTTGACTCTTCTGGCA

AACATAGTGATCATGATCCGCGAGACTCGCAAGAGACAACAGATGGTGGATGATGCAGTGAATGAGTACA

TTGAGAAAGCAAACATCACCACAGATGACAAAACTCTTGACGAAGCGGAAAAGAACCCTCTGGAGACTAG

TGGTGCCAGCACTGTTGGCTTCAGAGAGAGAACTCTCCCGGGACACAAGGTGAGTGATGACGTGAACTCC

GAGCCCACCAAACCCGCGGAAGAGCAACCACAAGCTGAAGGACCCTACGCCGGGCCGCTCGAGCGTCAGA

AACCTCTGAAAGTGAAAGCTAAGCTGCCACAGCAGGAGGGACCCTACGCTGGCCCGATGGAGAGACAGAA

ACCACTGAAAGTGAAAGTGAAAGCCCCGGTCGCGAAGGAAGGACCTTACGAGGGACCGGTGAAGAAGCCT

GTCGCTTTGAAAGTGAAAGCTAAGAACCTGATTGTCACTGAGAGTGGTGCCCCACCAACCGACTTGCAAA

AGATGGTTATGGGCAACACAAAGCCTGTTGAGCTCATCCTCGACGGGAAGACAGTAGCCATCTGCTGTGC

TACTGGAGTGTTTGGCACCGCCTACCTCGTGCCTCGTCATCTTTTCGCTGAGAAGTATGACAAGATCATG

TTGGACGGCAGAGCCATGACAGACAGTGATTACAGAGTGTTTGAGTTTGAGATTAAAGTAAAAGGACAGG

ACATGCTCTCAGACGCCGCGCTCATGGTGCTGCACCGTGGGAACCGCGTGAGAGACATCACGAAACATTT

TCGTGACACAGCAAGAATGAAGAAAGGCACCCCCGTCGTTGGTGTGATTAACAACGCCGACGTCGGGAGA

CTGATTTTCTCTGGTGAGGCCCTTACCTACAAGGACATTGTAGTGTGCATGGATGGAGACACCATGCCTG

GCCTCTTTGCCTACAGAGCCGCCACCAAGGCTGGCTACTGTGGAGGAGCTGTTCTCGCCAAGGACGGCGC

CGACACATTCATCGTTGGCACACACTCCGCAGGTGGCAATGGAGTTGGATACTGCTCTTGTATTTCCAGG

TCCATGCTCCAGAGGATGAAGGCACACATTGACCCTGAACCACACCACGAG

>DQ989305.1_Asia1_IND_1990

ATCTCAATTCCTTCCCAAAAGTCAGTGCTGTACTTCCTCATTGAGAAAGGCCAACACGAAGCAGCAATTG

AATTCTTTGAGGGGATGGTGCATGACTCCATCAAGGAGGAGCTCCGACCTCTCATCCAACAGACATCATT

TGTGCAACGCGCTTTCAAGCGTCTGAAGGAAAACTTTGAGATTGTTGCCCTGTGTTTGACTCTCTTGGCA

AACATAGTGATCATGATCCGCGAGACTCGCAAGAGACAACAGATGGTGGATGATGCAGTGAATGAGTACA

TTGAGAAAGCAAACATCACCACAGATGACAAGACTCTTGACGAGGCGGAAAAGAACCCTCTGGAGACCAG

CGGTGCCAGCACTGTTGGTTTCAGAGAGAGAACTCTCCCGAGACACAAGGCGAGTGATGACGTGAACTCC

GAGCCCGCCCAAACCCTGGAGGAGCAACCACAAGCTGAGGGACCCTACGCCGGGCCACTTGAGCGTCAGA

AACCTCTGAAAGTGAAAGCCAAGCTTCCGCAGCAGGAGGGACCTTACGCTGGCCCGATGGAGAGACAGAA

ACCACTGAAAGTGAAGGCAAAGGCCCCGGTCGTTAAGGAAGGACCTTACGAAGGACCGGTGAAGAAGCCT

GTCGCCTTGAAAGTGAAAGCCAAGAACTTGATTGTCACTGAGAGTGGCGCCCCCCCGACCGACTTGCAAA

AGATGGTCATGGGCAACACCAAGCCCGTTGAGCTCATACTCGACGGGAAGACAGTAGCCATCTGCTGTGC

TACTGGAGTGTTTGGCACTGCTTACCTCGTACCTCGTCATCTTTTCGCAGAGAAGTATGACAAGATCATG

CTGGACGGCAGAGCCATGACGGACAGTGACTACAGAGTGTTTGAGTTTGAGATTAAAGTAAAAGGACAGG

ACATGCTCTCAGACGCCGCCCTCATGGTGCTTCACCGTGGGAACCGCGTGAGAGACATCACGAAACACTT

TCGTGATACAGCAAGAATGAAGAAAGGCACCCCCGTCGTTGGCGTGATCAACAACGCCGACGTTGGGAGA

CTGATTTTCTCTGGTGAGGCCCTCACCTACAAGGACATTGTAGTGTGTATGGATGGTGACACCATGCCTG

GCCTCTTTGCCTACAGGGCCGCTACCAAGGCTGGCTACTGTGGAGGAGCCGTTCTCGCGAAGGACGGAGC

TGACACGTTCATCGTCGGCACTCACTCTGCAGGTGGCAATGGAGTTGGATACTGCTCGTGCGTTTCCAGG

TCCATGCTCTTGAAAATGAAGGCACACATTGACCCTGAACCACACCACGAG

>DQ989306.1_Asia1_IND_1986

ATCTCAATTCCTTCCCAAAAGTCCGTGTTGTACTTTCTCATCGAGAAGGGACAGCACGAAGCAGCAATTG

AATTCTTTGAGGGGATGGTGCACGACTCCATCAAGGAAGAGCTCCGACCTCTCATCCAACAGACCTCATT

TGTGAAGCGCGCGTTTAAGCGCCTGAAGGAGAACTTTGAGATTGTTGCCCTATGTTTGACTCTTCTGGCA

AACATAGTGATCATGATCCGCGAGACTCGCAAGAGACAACAGATGGTGGATGATGCAGTGAATGAGTACA

TTGAGAAAGCAAACATCACCACAGATGACAAAACTCTTGACGAAGCGGAAAAGAACCCTCTGGAGACTAG

TGGTGCCAGCACTGTTGGCTTCAGAGAGAGAACTCTCCCGGGACACAAGGTGAGTGATGACGTGAACTCC

GAGCCCACCAAACCCGCGGAAGAGCAACCACAAGCTGAAGGACCCTACGCCGGGCCGCTCGAGCGTCAGA

AACCTCTGAAAGTGAAAGCTAAGCTGCCACAGCAGGAGGGACCCTACGCTGGCCCGATGGAGAGACAGAA

ACCACTGAAAGTGAAAGTGAAAGCTCCGGTCGCGAAGGAAGGACCTTACGAGGGACCGGTGAAGAAGCCT

GTTGCTTTGAAAGTGAAAGCTAAGAACCTGATTGTCACTGAGAGTGGTGCCCCACCAACCGACTTGCAAA

AGATGGTTATGGGCAACACAAAGCCTGTTGAGCTCATCCTCGACGGGAAGACAGTAGCCATCTGCTGTGC

TACTGGAGTGTTTGGCACCGCCTACCTCGTGCCTCGTCATCTTTTCGCTGAGAAGTATGACAAGATCATG

TTGGACGGCAGAGCCATGACAGACAGTGATTACAGAGTGTTTGAGTTTGAGATTAAAGTAAAAGGACAGG

ACATGCTCTCAGACGCCGCGCTCATGGTGCTGCACCGTGGGAACCGCGTGAGAGACATCACGAAACATTT

TCGTGACACAGCAAGAATGAAGAAAGGCACCCCCGTCGTTGGTGTGATTAACAACGCCGACGTCGGGAGA

CTGATTTTCTCTGGTGAGGCCCTTACCTACAAGGACATTGTAGTGTGCATGGATGGAGACACCATGCCTG

GCCTCTTTGCCTACAGAGCCGCCACCAAGGCTGGCTACTGCGGAGGAGCTGTTCTCGCCAAGGACGGCGC

CGACACATTCATCGTTGGCACACACTCCGCAGGTGGCAATGGAGTTGGATACTGCTCTTGTGTTTCCAGG

TCCATGCTCCAGAGGATGAAGGCACACATTGACCCTGAACCACACCACGAG

>DQ989307.1_Asia1_IND_1992

ATCTCAATTCCTTCCCAAAAGTCAGTGCTGTACTTCCTCATTGAGAAAGGCCAACACGAAGCAGCAATTG

AATTCTTTGAGGGGATGGTGCATGACTCCATCAAGGAGGAGCTCCGACCTCTCATCCAACAGACATCATT

TGTGCAACGCGCTTTCAAGCGTCTGAAGGAAAACTTTGAGATTGTTGCCCTGTGTTTGACTCTCTTGGCA

AACATAGTGATCATGATCCGCGAGACTCGCAAGAGACAACAGATGGTGGATGATGCAGTGAATGAGTACA

TTGAGAAAGCAAACATCACCACAGATGACAAGACTCTTGACGAGGCGGAAAAGAACCCTCTGGAGACCAG

CGGTGCCAGCACTGTTGGTTTCAGAGAGAGAACTCTCCCGAGACACAAGGCGAGTGATGACGTGAACTCC

GAGCCCGCCAAAACCCTGGAGGAGCAACCACAAGCTGAGGGACCCTACGCCGGGCCACTTGAGCGTCAGA

AACCTTTGAAAGTGAAAGCCAAGCTTCCGCAGCAGGAGGGACCTTACGCTGGTCCGATGGTGAGACAGAA

ACCACTGAAAGTGAAAGCAAAAGCCCCGGTCGTTAAGGAAGGACCTTACGAGGGACCGGTAAAGAAGCCT

GTCGCCTTGAAAGTGAAAGCCAAGAACCTGATTGTCACTGAAAGTGGCGCCCCCCCGACCGACTTGCAAA

AAATGGTCATGGGCAACACCAAGCCCGTTGAGCTCATACTCGACGGGAAGACAGTAGCCATCTGCTGTGC

TACTGGAGTGTTTGGCACTGCTTACCTCGTACCTCGTCATCTTTTCGCAGACAAGTATGACAAGATCATG

CTGGACGGCAGAGCCATGACGGACAGTGACTACAGAGTGTTTGAGTTTGAGATTAAAGTAAAAGGACAGG

ACATGCTCTCAGACGCCGCCCTCATGGTGCTTCACCGTGGGAACCGCGTGAGAGACATCACGAAACACTT

TCGTGATACAGCAAGAATGAAGAAAGGCACCCCCGTCGTTGGCGTGATCAACAACGCCGACGTTGGGAGA

CTGATTTTCTCTGGTGAGGCCCTCACCTACAAGGACATTGTAGTGTGTATGGATGGTGACACCATGCCTG

GCCTCTTTGCCTACAGGGCCGCTACCAAGGCTGGCTACTGTGGAGGAGCCGTTCTCGCGAAGGACGGAGC

TGACACGTTCATCGTCGGCACTCACTCTGCAGGTGGCAATGGAGTTGGATACTGCTCGTGCGTTTCCAGG

TCCATGCTCTTGAAAATGAAGGCACACATTGACCCTGAACCACACCACGAG

>DQ989308.1_Asia1_IND_1994

ATCTCAATTCCTTCCCAAAAGTCCGTGCTGTACTTTCTCATCGAGAAAGGTCAACACGAAGCAGCAATTG

AATTCTTTGAGGGGATGGTGCACGACTCCATCAAAGAGGAGCTCCGCCCTCTCATCCAACAGACCTCATT

TGTGAAACGCGCTTTCAAGCGTCTGAAGGAAAATTTTGAGATTGTTGCCTTGTGTTTGACTCTTCTGGCA

AACATAGTGATCATGATCCGCGAGACTCGCAAGAGACAGCAGATGGTGGATGATGCAGTGAATGAATACA

TCGAGAAGGCATACATCACCACAGATGACAAAACTCTAGACGAGGCGGAAAAGAACCCTCTGGAGACCAG

CGGTGCCAGCACTGTCGGTTTCAGAGAGAGAACTCTCCCGGGACAAAAGGTGAGTGGTGACGAGAACTCC

GAGCCCGCCAAACCCGTGGAAGAGCAACCACAAGCTGAAGGACCCTACGCCGGGCCACTCGAGCGTCAGA

AACCTCTGAAAGTAAGAGCCAAGCTACCACAGCAGGAGGGACCCTACGTTGGCCCGATGGAGAGACAGAA

ACCACTGAAAGTGAAAGCAAAAGCCCCGGTCGTGAAGGAAGGACCTTACGAGGGACCGGTGAAGAAGCCT

GTCGCTTTGAAAGTGAAAGCTAAGAACCTGATCGTGACTGAGAGTGGCGCCCCCCCGACCGACTTGCAAA

AGATGGTCATGGGCAACACCAAGCCTGTTGAGCTCATCCTCGATGGGAAGACGGTAGCCATCTGCTGTGC

TACCGGAGTGTTTGGCACTGCTTACCTCGTGCCTCGTCATCTTTTCGCAGAGAAGTATGACAAGATCATG

CTGGACGGCAGAGCCATGACAGACAGTGACTATAGAGTGTTTGAGTTTGAGATTAAAGTGAAAGGACAGG

ACATGCTCTCAGACGCCGCGCTCATGGTGCTCCACCGTGGGAACCGCGTGAGAGACATCACGAAACACTT

TCGTGATACAGCAAGAATGAAGAAAGGCACCCCCGTTGTTGGTGTGGTCAACAACGCCGACGTTGGGAGA

CTGATTTTCTCTGGTGAGGCCCTTACCTACAAGGATATTGTAGTGTGTATGGATGGTGACACCATGCCTG

GCCTCTTTGCCTACAGAGCCGCCACTAAGGCTGGCTACTGCGGAGGAGCCGTTCTCGCGAAGGACGGAGC

TGACACATTCATCGTCGGCACTCACTCTGCGGGTGGCAACGGAGTTGGATACTGCTCATGCGTGTCCAGG

TCCATGCTCCTGAAAATGAAGGCACACATTGACCCCGAACCACACCACGAG

>DQ989309.1_Asia1_IND_1996

ATCTCAATTCCTTCCCAAAAGTCCGTGCTGTACTTTCTCATCGAGAAAGGCCAACACGAAGCAGCAATTG

AATTCTTTGAGGGGATGGTGCACGACTCCATCAAAGAGGAGCTCCGCCCTCTCATCCAACAGACCTCATT

TGTGAAACGCGCTTTCAAGCGTCTGAAGGAAAATTTTGAGATTGTTGCCCTATGTTTGACTCTTCTGGCA

AACATAGTGATCATGATCCGTGAGACTCGCAAGAGGCAGCAGATGGTAGATGACGCAGTGAATGAATACA

TCGAGAAGGCAAACATCACCACAGATGACAAAACTCTTGACGAGGCGGAAAAGAACCCTCTGGAGACCAG

CGGTGCCAGCACTGTCGGTTTCAGAGAGAGAACTCTCCCGGGACAAAAGGTGAGTGATGACGTGAACTCC

GAGCCCGCCAAACCCGTGGAAGAGCAACCACAAGCTGAAGGACCCTACGCCGGGCCACTCGAGCGTCAGA

AACCTCTGAAAGTGAGAGCCAAGCTACCACAGCAGGAGGGACCCTACGCTGGCCCGATGGAGAGACAGAA

ACCACTGAAAGTGAAAGCAAAAGCCCCGGTCGTTAAGGAAGGACCTTACGAGGGACCGGTGAAGAAGCCT

GTCGCTCTGAAAGTGAAAGCTAAGAACCTGATCGTCACTGAGAGTGGCGCCCCACCGACCGACTTGCAAA

AGATGGTCATGGGCAACACCAAGCCTGTCGAGCTCATCCTCGATGGGAAGACGGTAGCCATCTGCTGTGC

TACCGGAGTGTTTGGCACTGCTTACCTCGTGCCTCGTCATCTTTTCGCAGAGAAGTATGACAAGATCATG

CTGGACGGCAGAGCCATGACAGACAGTGACTACAGAGTGTTTGAGTTTGAGATTAAAGTAAAAGGACAGG

ACATGCTCTCAGACGCCGCGCTCATGGTGCTCCACCGTGGGAACCGCGTGAGAGACATCACGAAACACTT

TCGTGACACAGCAAGAATGAAGAAAGGCACCCCCGTTGTCGGTGTGATCAACAACGCCGACGTTGGGAGA

CTGATTTTCTCTGGTGAGGCCCTCACCTACAAGGACATTGTAGTGTGTATGGATGGTGACACCATGCCTG

GTCTCTTTGCCTACAGAGCCGCCACCAAGGCTGGCTACTGCGGAGGAGCCGTTCTCGCGAAGGACGGAGC

TGACACATTCATCGTCGGCACTCACTCTGCGGGTGGCAACGGAGTTGGATACTGCTCGTGCGTGTCCAGG

TCCATGCTCCTCAAAATGAAGGCACACATTGACCCCGAACCACACCACGAG

>DQ989310.1_Asia1_IND_1999

ATCTCAATTCCTTCCCAAAAGTCTGTGTTGTACTTCCTCATTGAGAAAGGCCAGCACGAAGCAGCAATTG

AATTCTTTGAGGGGATGGTGCATGACTCCATTAAGGAGGAGCTCCGCCCTCTCATCCAACAGACCTCATT

TGTGAAACGCGCTTTTAAGCGTCTGAAGGAAAATTTTGAGATTGTTGCCCTGTGTTTGACTCTTCTGGCA

AACATAGTGATCATGATCCGCGAGACTCGCAGGAGACAGCAGATGGTGGATGATGCAGTGAATGAATACA

TTGAGAAAGCAAACATCACCACGGATGACAAGACTCTTGACGAGGCAGAAAAGAACCCTCTGGAGACCAG

TGGTGCTAGCACTGTGGGTTTTAGGGAGAGAACCCTCCCGGGACACAAGGCGAGTGATGACGTGAACTCC

GAGCCCGCCCAGACCGTGGAGGAACAACCACAAGCTGAAGGACCCTACGCCGGACCACTCGAGCGCCAGA

AACCTCTGAAAGTGAGAGCCAAGCTCCCACAACAAGAGGGACCTTACGCTGGTCCGATGGAGAGACAGAA

ACCGCTGAAAGTGAAAGCAAAAGCCCCGGTCGTCAAGGAAGGACCTTACGAGGGACCGGTGAAAAAGCCT

GTCGCTTTGAAAGTTAAAGCTAAGAACCTGATTGTCACTGAGAGTGGTGCCCCACCGACCGACTTGCAAA

AGATGGTCATGGGCAACACCAAGCCTGTTGAGCTCATCCTCGACGGGAAGACGGTTGCCATCTGCTGTGC

TACCGGAGTGTTTGGCACTGCCTACCTCGTGCCTCGTCATCTTTTCGCGGAGAAGTATGACAAGATCATG

CTGGACGGCAGAGCCATGACAGACAGTGACTACAGAGTGTTTGAGTTTGAGATTAAAGTAAAAGGACAGG

ACATGCTCTCAGACGCCGCGCTCATGGTGCTACACCGTGGGAACCGCGTGCGGGACATTACGAAACACTT

TCGTGATGTGGCAAGAATGAAGAAAGGCACCCCCGTGGTTGGCGTGATCAACAACGCTGACGTCGGGAGA

CTGATTTTCTCTGGCGAGGCCCTTACCTACAAGGACATTGTAGTGTGCATGGATGGAGACACTATGCCTG

GCCTCTTTGCCTACAAAGCCGCCACCAAGGCGGGCTATTGTGGAGGAGCTGTTCTTGCAAAGGACGGAGC

CGAGACTTTCATCGTCGGCACTCACTCTGCAGGCGGCAATGGAGTTGGATACTGCTCATGCGTTTCCAGG

TCTATGCTGCTTAAAATGAAGGCACACATCGATCCCGAACCACACCACGAG

>DQ989311.1_Asia1_IND_2002

ATCTCAATTCCTTCCCAAAAGTCCGTGCTGTACTTCCTCATTGAGAAGGGCCAGCACGAAGCAGCAATTG

AATTCTTTGAGGGAATGGTGCACGACTCCATCAAGGAGGAGCTCCGACCCCTCATCCAACAGACCTCATT

TGTGAAACGCGCATTCAAACGCCTGAAGGAAAACTTTGAGATCGTTGCCCTGTGTTTGACTCTCTTGGCA

AACATAGTGATCATGATCCGCGAGACTCGCAAGAGACAGCAAATGGTGGATGATGCAGTGAATGAGTACA

TTGAGAAAGCAAACATCACCACAGATGACAAGACTCTTGATGAGGCGGAAAAGAACCCTCTAGAGACCAG

TGGTGCCAGCACCGTTGGTTTCAGAGAGAGAACTCTCCCGGGACACAAGGTGGGTGATGACGTGAACACC

GAGCCCACCAAACCCGCGGAAGAGCAACCACACGCTGAAGGACCCTACGCCGGGCCACTCGAGCGTCAGA

AACCTCTGAAGGTGAGAGCCAAACTACCACAACAGGAGGGACCCTACGCTGGCCCGATGGAGAGACAGAA

ACCACTGAAAGTGAAAGCAAAAGCCCCGGTCGTGAAGGAAGGACCTTACGAGGGACCGGTGAAGAAACCT

GTCGCTTTGAAAGTGAAAGCTAAGGACTTGATTATCACTGAGAGTGGTGCCCCACCGACCGACTTGCAAA

AGATGGTCATGGGCAACACGAAGCCAGTCGAGCTCATCCTCGACGGGAAGACAGTAGCCATCTGCTGTGC

TACTGGAGTGTTTGGCACTGCCTACCTCGTGCCTCGTCACCTTTTCGCAGAAAAGTACGACAAGATCATG

TTGGACGGCAGAGCCATGACAGACAGTGACTACAGAGTGTTTGAGTTTGAGATTAAAGTAAAAGGACAGG

ACATGCTCTCAGACGCCGCGCTCATGGTGCTTCACCGTGGGAACCGCGTGCGGGACATCACGAAACACTT

CCGTGATGTGGCAAGAATGAAGAAAGGCACCCCCGTCGTTGGCGTGATTAACAACGCTGACGTTGGGAGA

CTGGTTTTCTCTGGTGAGGCCCTTACCTACAAAGACATTGTGGTGTGCATGGATGGAGACACCATGCCTG

GCCTCTTTGCCTACAAAGCCGCCACCAAGGCCGGTTACTGTGGAGGAGCCGTTCTTGCCAAGGACGGTGC

TGAAACATTCATTGTGGGCACGCACTCCGCAGGTGGCAATGGAGTTGGTTACTGCTCTTGCGTTTCCAGG

TCCATGCTCTTGAAAATGAAGGCACACATCGACCCTGAGCCACACCACGAG

>DQ989312.1_Asia1_IND_1990

ATCTCAGTTCCTTCCCAAAAGTCCGTGTTGTATTTTCTCATTGAGAAAGGGCAGCACGAAGCAGCAATTG

AATTCTTTGAGGGGATGGTGCATGACTCCATCAAGGAGGAACTCCGACCCCTTATCCAACAGACCTCATT

TGTGAAACGCGCGTTTAAACGCCTGAAGGAAAACTTTGAGATTGTTGCCCTGTGTTTGACCCTCTTGGCA

AACATAGTGATCATGATCCGCGAGACTCGCAAGAGACAGCAAATGGTGGATGATGCAGTGAATGAGTACA

TCGAGAAAGCAAACATCACCACAGATGACAAGACTCTTGACGAAGCGGAAAAGAACCATCTGGAGACTAG

CGGTGCCAGCACTGTTGGCTTTAGAGAGAGAACTCTCCCAGGACACGGGGCGAACGGCGACGCGAGCCCT

GAGCCCACCAAACCTGTGGGAGAACAACCACAAGCTGAGGGACCCTACGCCGGGCCACTCGAGCGTCAGA

AACCTCTGAAGGTGAGAGCCAAACTGCCACAGCAGGAGGGACCCTACGCTGGCCCGATGGAGAGACAGAA

GCCACTAAAAGTAAAAGCAAAAGCCCCGGCCGTTAAGGAAGGACCTTACGAGGGACCGGTGAAGAAGCCT

GTCGCTTTGAAAGTGAAAGCTAAGAACTTGATTGTCACTGAAAGTGGTGCCCCACCGACGGACTTGCAAA

AGATGGTCATGGGCAACACGAAGCCTGTTGAGCTCATCCTCGACGGGAAGACAGTAGCCATCTGCTGCGC

TACTGGAGTGTTTGGCACTGCTTACCTCGTGCCTCGTCATCTTTTCGCAGAAAAGTACGACAAGATCATG

TTGGACGGCAGAGCTATGACAGACAGTGACTACAGAGTGTTTGAGTTTGAGATTAAAGTAAAAGGACAGG

ACATGCTCTCAGACGCCGCACTCATGGTGCTCCACCGCGGGAATCGCGTGCGGGACATCACGAAGCACTT

CCGTGATGTGGCAAGAATGAAAAAAGGCACCCCCGTCGTTGGTGTGATTAACAACGCTGACGTCGGGAGA

CTGATTTTCTCTGGTGAGGCCCTTACCTACAAAGACATTGTAGTGTGCATGGATGGTGACACCATGCCTG

GTCTCTTTGCCTACAAAGCCGCCACCAAGGCCGGCTACTGTGGAGGAGCCGTTCTTGCTAAGGACGGAGC

TGAGACGTTCATCGTTGGCACACACTCCGCAGGTGGCAATGGAGTTGGATACTGCTCTTGCGTTTCCAGG

TCCATGCTCTTGAAGATGAAGGCACACATCGACCCTGAACCACACCACGAG

>DQ989313.1_Asia1_IND_1986

ATCTCAATTCCTTCCCAAAAGTCTGTGTTGTACTTCCTCATTGAGAAAGGGCAGCACGAAGCAGCAATTG

AATTTTTCGAGGGAATGGTGCACGACTCAATCAAGGAGGAGCTCCGCCCCCTCATTCAACAAACTTCATT

TGTGAAACGCGCTTTTAAGCGCCTGAAGGAAAATTTTGAGATTGTTGCCCTGTGTTTGACTCTTCTGGCA

AACATAGTGATCATGATCCGCGAGACTCGCAAGAGACAACAGATGGTGGATAATGCAGTGAATGAGTACA

TTGAGAAAGCGAACATCACCACGGATGACAAAACTCTTGACGAGGCGGAAAAGAGCCCTCAAGAGACCAA

CAGTGCTGCCACTGTCGGTTTCAGAGAGAGAACCCTCCCGGGACACAAGGCGAGTGATGACGTGAACTCC

GAGCCCGCTGAGCCCGTGGAG---AGACCACAAGCTGAAGGACCCTACGCCGGACCCCTTGAGCGTCAGA

AACCTCTGAAAGTGAGAGCCAAGCTCCCACGACAGGAGGGACCTTACGCTGGTCCGATGGAGAGACAGCA

ACCACTGAAAGTGAGAGTCAAAGCTCCGGTCGTGAAGGAAGGGCCCTACGAGGGACCGGTGAAGAAGCCT

GTCGCTTTGAAAGTGAAAGCTAAGAACTTGATTGTCACTGAGAGTGGTGCCCCCCCGACCGACTTGCAAA

AGATGGTCATGGGCAACACCAAGCCTGTCGAGCTTGTTCTCGATGGGAAGACGGTGGCCATCTGCTGTGC

CACCGGAGTGTTTGGCACTGCCTACCTCGTACCTCGTCACCTTTTCGCAGAGAAGTATGATAAGATTATG

CTGGACGGCAGAGCCATGACAGACAGTGACTACAGAGTGTTTGAGTTTGAGATTAAAGTAAAAGGACAGG

ACATGCTCTCAGACGCCGCGCTCATGGTGCTCCACCGTGGGAACCGCGTGAGAGACATCACGAAACACTT

TCGTGATACAGCAAGAATGAAGAAAGGTACCCCCGTCGTTGGCGTGATCAACAACGCCGATGTCGGGAGA

TTGATTTTCTCTGGTGAGGCCCTCACCTACAAGGACATTGTAGTGTGTATGGATGGTGACACCATGCCTG

GCCTCTTTGCCTACAAAGCTGCCACCAAGGCTGGCTATTGTGGAGGAGCCGTTCTTGCCAAGGACGGGGC

TGACACGTTCATTGTCGGCACTCACTCCGCAGGTGGCAATGGAGTTGGATATTGCTCGTGCGTTTCCAGG

TCCATGCTCCTAAAAATGAAGGCACACATTGACCCTGAACCACACCACGAG

>DQ989314.1_Asia1_IND_2001

ATCTCGATTCCTTCCCAAAAATCTGTGTTGTACTTTCTCATTGAGAAAGGCCAGCACGAAGCAGCAATTG

ACTTCTTTGAGGGGATGGTGCATGACTCCATCAAGGAGGAGCTCCGACCCCTCATCCAACAGACATCATT

TGTGAAACGCGCTTTTAAGCGCCTAAAGGAAAACTTTGAGATTGTCGCCCTGTGTTTGACTCTTTTGGCA

AACATAGTGATCATGATCCGCGAGACTCGCAAGAGACAGCAGATGGTGGATGATGCAGTGAATGAGTACA

TTGAGAAAGCCAACATCACCACAGATGACAAGACTCTTGACGAGGCGGAAAAGAACCCTCTGGAGACCAG

TGGTGCCAGCACCGTTGGTTTCAGAGAGAGAACTCTTCCAGGGCACAAGGTGAGTGATGACGTGAACTCC

GAGCCCACCAAACCTGTGGAAGAGCAACCACAAGCTGAAGGACCCTACGCCGGGCCACTTGAGCGTCAGA

AACCTCTGAAAGTGCGCGCCAAACTGCCACAGCAGGAGGGACCCTACGCTGGCCCCATGGAGAGACAGAA

ACCACTGAAAGTGAAAGCAAAAGCACCGGTCGTTAAGGAAGGACCTTACGAGGGACCGGTGAAGAAGCCT

GTTGCTTTGAAAGTGAAAGCAAAGAACTTGATTGTCACTGAGAGTGGTGCCCCACCGACCGACTTGCAAA

AGATGGTCATGGGCAACACAAAGCCTGTCGAGCTCATTCTTGACGGGAAGACAGTAGCCATCTGCTGTGC

TACTGGAGTGTTTGGTACTGCATACCTCGTGCCTCGTCATCTTTTCGCAGAGAAGTATGACAAGATCATG

CTGGACGGCAGAGCCATGACAGACAGTGACTACAGAGTGTTTGAGTTTGAGATTAAAGTAAAAGGACAGG

ACATGCTCTCAGACGCCGCGCTCATGGTGCTCCACCGTGGGAATCGCGTGCGGGACATCACGAAGCACTT

CCGTGATGTTGCAAGGATGAAGAAAGGCACCCCCGTTGTTGGCGTGATCAACAACGCTGATGTTGGGAGA

CTGATTTTCTCTGGTGAGGCCCTTACCTACAAGGACATTGTAGTGTGCATGGACGGAGACACCATGCCTG

GCCTCTTTGCCTACAAAGCCGCCACCAAGGCTGGTTACTGTGGAGGAGCTGTTCTCGCAAAGGACGGAGC

CGAGACTTTCATCGTCGGCACTCACTCTGCAGGGGGCAATGGAGTTGGATACTGCTCATGCGTCTCCAGG

TCCATGCTTCTCAAGATGAAGGCACACATCGACCCGGAACCACACCACGAG

>DQ989315.1_Asia1_IND_1993

ATCTCAATTCCTTCCCAAAAGTCAGTGTTGTACTTCCTCATTGAGAAAGGCCAACACGAAGCAGCAATTG

AATTCTTTGAGGGGATGGTGCACGACTCCATCAAGGAGGAGCTCCGACCTCTCATCCAACAAACATCATT

TGTGCAACGCGCTTTCAAGCGTCTGAAGGAAAACTTTGAGATTGTTGCCCTATGTTTGACTCTTTTGGCA

AACATTGTGATCATGATCCGCGAGACTCGCAAGAGACAACAGATGGTGGATGATGCAGTGAATGAGTACA

TTGAGAAGGCAAACATCACCACGGATGACAAGACTCTTGACGAGGCGGAAAAGAACCCTCTGGAGACCAG

CGGTGCCAGCACTGTTGGTTTCAGAGAGAGAACTCTCCCGGGGCACAAGGCGAGTGATGACGTGAACTCC

GAGCCCGCCCAGCCCGTGGAGGAGCAACCACAAGCTGAAGGACCCTACGCCGGACCACTCGAGCGCCAGA

AACCTCTGAAAGTGAGAGCCAAGCTCCCACAACAGGAGGGACCTTACGCTGGTCCGATGGAGAGACAGAA

GCCACTGAAAGTGAAAGCAAAAGCCCCGGTCGTTAAGGAAGGACCTTACGAGGGACCGGTGAAGAAGCCT

GTCGCTTTGAAAGTGAAAGCTAAGAACCTGATTGTCACTGAGAGTGGAGCCCCCCCGACCGACTTGCAAA

AGATGGTCATGGGCAACACCAAGCCTGTTGAGCTTAACCTCGACGGGAAGACGGTAGCCATCTGTTGTGC

TACCGGTGTGTTTGGCACTGCCTACCTCGTGCCTCGTCATCTTTTCGCGGAGAAGTACGACAAGATCATG

CTGGACGGCAGAGCCATGACAGACAGTGACTACAGAGTGTTTGAGTTTGAGATTAAAGTGAAAGGACAGG

ACATGCTCTCGGACGCCGCGCTCATGGTGCTCCACCGTGGGAACCGCGTGAGAGACATCACGAAACACTT

TCGTGACACAGCAAGAATGAAGAAGGGCACCCCCGTCGTTGGTGTGATCAACAACGCCGACGTTGGGAGA

CTGATCTTCTCTGGTGAGGCCCTCACCTACAAAGACATTGTAGTGTGCATGGACGGCGACACCATGCCTG

GCCTCTTTGCCTACAGAGCCGCCACCAAGGCTGGCTACTGCGGAGGAGCCGTTCTTGCTAAGGACGGCGC

CGACACATTCATCGTCGGCACTCACTCCGCAGGTGGCAATGGAGTTGGATACTGCTCATGCGTTTCCAGA

TCAATGCTACTGAGAATGAAGGCACACATCGACCCTGAACCACACCACGAG

>DQ989317.1_Asia1_IND_2000

ATCTCGATTCCTTCCCAAAAATCTGTGTTGTACTTTCTCATTGAGAAAGGCCAGCACGAAGCAGCAATTG

ACTTCTTTGAGGGGATGGTGCATGACTCCATCAAGGAGGAGCTCCGACCCCTCATCCAACAGACATCATT

TGTGAAACGCGCTTTCAAGCGCCTGAAGGAAAACTTTGAGATTGTTGCCCTGTGTTTGACTCTTTTGGCA

AACATAGTGATCATGATCCGCGAGACTCGCAAGAGACAGCAGATGGTGGATGATGCAGTGAATGAGTACA

TTGAGAAAGCCAACATCACCACAGATGACAAGACTCTTGACGAGGCGGAAAAGAACCCTCTGGAGACCAG

TGGTGCCAGCACCGTTGGTTTCAGAGAGAGAACTCTCCCAGGACACAAGGTGAGTGATGACGTGAACTCC

GAGCCCACCAAACCTGTGGAAGAGCAACCACAAGTTGAAGGACCCTACGCCGGGCCACTTGAGCGTCAGA

AACCTCTGAAAGTGCGCGCCAAACTGCCACAGCAAGAGGGACCCTACGCTGGCCCGATGGAGAGACAGAA

ACCACTGAAAGTGAAAGCAAAAGCCCCGGTCGTTAAGGAAGGACCTTACGAGGGACCGGTGAGGAAGCCT

GTCGCTTTGAAAGTGAAAGCAAAGAACTTGATTGTCACTGAGAGTGGTGCCCCACCGACCGACTTGCAAA

AGATGGTCATGGGCAACACAAAGCCTGTCGAGCTTATTCTTGACGGGAAGACAGTAGCCATCTGCTGTGC

TACTGGAGTGTTTGGTACTGCCTACCTCGTGCCTCGTCATCTTTTCGCAGAGAAGTATGACAAGATCATG

CTGGACGGCAGAGCCATGACAGACAGTGACTACAGAGTGTTTGAGTTTGAGATTAAAGTAAAAGGACAGG

ACATGCTCTCAGACGCCGCGCTCATGGTGCTCCACCGTGGGAATCGCGTGCGGGACATCACGAAGCACTT

CCGTGATGTTGCAAGGATGAAGAAAGGCACCCCCGTCGTTGGCGTGATCAACAACGCTGATGTTGGGAGA

CTGATATTCTCTGGTGAGGCCCTTACCTACAAGGACATTGTAGTGTGCATGGATGGAGACACCATGCCTG

GCCTCTTTGCCTACAAAGCCGCCACCAAGGCTGGTTACTGTGGAGGAGCTGTTCTCGCAAAGGACGGAGC

CGAGACTTTCATCGTCGGCACTCACTCTGCAGGAGGCAATGGAGTTGGATACTGCTCATGCGTCTCCAGG

TCCATGCTTCTCAAGATGAAGGCACACATCGACCCAGAACCACACCACGAG

>DQ989318.1_Asia1_IND_2002

ATCTCGATTCCTTCCCAAAAATCTGTGTTGTACTTCCTCATTGAGAAAGGCCAGCACGAAGCAGCAATTG

ACTTCTTTGAGGGGATGGTGCATGACTCCATCAAGGAGGAGCTCCGACCCCTCATCCAACAGACATCATT

TGTGAAACGCGCTTTTAAGCGCCTAAAGGAAAACTTTGAGATTGTTGCCCTGTGTTTGACTCTTTTGGCA

AACATAGTGATTATGATCCACGAGACCCGCAAGAGACAGCAGATGGTGGATGATGCAGTGAATGAGTACA

TTGAGAAAGCCAACATCACCACAGATGACAAGACTCTTGACGAGGCGGAAAAGAACCCTCTGGAGACCAG

TGGTGCCAGCACCATTGGTTTCAGAGAAAGAACTCTTCCAGGACACAAGGTGAGTGATGACGTGAACTCC

GAGCCCACCAAACCTGTGGAAGAGCAACCACAAGCTGAAGGACCCTACGCCGGGCCACTTGAGCGTCAGA

AACCTCTGAAAGTGCGCGCCAAACTGCCACAACAGGAGGGACCCTACGCTGGCCCGATGGAGAGACAGAA

ACCACTGAAAGTGAAAGCAAAAGCCCCGGTCGTTAAGGAAGGACCTTACGAGGGACCCGTGAAGAAGCCT

GTAGCTTTGAAAGTGAAAGCAAAGAACTTGATTGTCACTGAGAGTGGTGCCCCACCGACCGACTTGCAAA

AGATGGTCATGGGCAACACAAAGCCTGTCGAACTCATTCCTGACGGGAAGACAGTAGCCATCTGCTGTGC

TACTGGAGTGTTTGGTACTGCCTATCTCGTGCCTCGTCACCTTTTCGCAGAGAAGTACGACAAGATCATG

CTGGACGGCAGAGCCATGACAGACAGTGACTACAGAGTGTTTGAGTTTGAGATTAAAGTAAAAGGACAGG

ACATGCTCTCAGACGCCGCGCTCATGGTGCTCCACCGTGGGAATCGCGTGCGGGACATCACGAAGCACTT

CCGTGATGTTGCAAGGATGAAGAAAGGCACCCCCGTTGTTGGCGTGATCAACAACGCTGATGTTGGGAGA

CTGATTTTCTCTGGTGAGGCCCTTACCTACAAGGACATTGTAGTGTGCATGGATGGAGACACCATGCCTG

GCCTCTTTGCCTACAAAGCCGCCACCAAGGCTGGTTACTGTGGAGGAGCTGTTCTCGCAAAGGACGGAGC

CGAGACTTTCATCGTCGGCACTCACTCTGCAGGGGGCAATGGAGTTGGATACTGCTCATGCGTCTCCAGG

TCCATGCTTCTCAAGATGAAGGCACACATCGACCCAGAACCACACCACGAG

>DQ989319.1_Asia1_IND_2001

ATCTCAATTCCTTCCCAAAAGTCTGTGTTGTACTTTCTCATTGAGAAAGGCCAACACGAAGCAGCAATTG

AATTCTTTGAGGGGATGGTGCATGACTCCATCAAGGAGGAGCTCCGACCTCTCATCCAACAGACATCATT

TGTGAAACGCGCCTTCAAGCGTCTGAAGGAAAATTTTGAGATTGTTGCCCTGTGTTTGACTCTTCTGGCA

AACATAGTGATCATGATCCGCGAAACTCGCAAGAGACAACAGATGGTGGATGATGCAGTGAATGAGTACA

TTGAGAAAGCCAACATCACAACAGATGACAAGACTCTTGACGAGGCGGAAAAGAACCCTCTGGAGACCAG

CGGTGCCAGCACTGTTGGTTTCAGAGAGAGAACTCTCCCAGGGCACAAGGCGTGTGATGACGTGAACTCC

GAGCCCGCCAACCCTGTGGAGGAACAACCACAAGCTGAAGGACCCTACGCCGGGCCACTTGAGCGTCAGA

AGCCCCTGAAAGTGAGAGCCAAGCTCCCACAGCAAGAGGGACCCTACGCTGGCCCGATGGAGAGACAGAA

ACCACTGAAAGTGAAAGCAAAAGCCCCGGTCGTTAAGGAAGGACCTTACGAGGGACCGGTGAAGAAGCCT

GTTGCTTTGAAAGTGAAAGCTAAGAACTTGATTGTCACTGAGAGTGGTGCACCCCCGACCGACTTGCAAA

AGATGGTCATGGGCAACACTAAGCCTGTTGAGCTCATCCTCGACGGGAAGACGGTAGCCATCTGCTGTGC

TACCGGAGTGTTTGGCACCGCCTACCTCGTGCCTCGTCATCTTTTCGCAGAGAAGTACGACAAGATCATG

CTGGACGGCAGAGCCATGACAGACAGTGACTACAGAGTGTTTGAGTTTGAGATTAAAGTAAAAGGGCAGG

ACATGCTCTCAGACGCCGCGCTCATGGTGCTTCACCGTGGGAACCGCGTAAGAGACATCACGAAACACTT

CCGTGATACAGCAAAAATGAAGAAAGGCACCCCCGTCGTTGGCGTGATCAACAACGCCGACGTCGGGAGA

CTAATTTTCTCTGGTGAGGCCCTTACCTACAAAGATATTGTAGTGTGCATGGATGGCGACACCATGCCTG

GCCTCTTTGCCTACAGAGCCGCCACCAAGGCTGGCTACTGCGGAGGAGCCGTTCTTGCAAAAGACGGAGC

CGACACATTCATCGTTGGCACTCACTCTGCAGGTGGCAATGGAGTTGGTTACTGCTCATGCGTTTCCAGG

TCCATGCTTTTGAAAATGAAGGCACACATTGACCCTGAACCACACCATGAG

>DQ989320.1_Asia1_IND_2002

ATCTCAATTCCTTCCCAAAAATCTGTGTTGTACTTTCTCATTGAGAAAGGCCAGCACGAAGCAGCAATTG

ACTTCTTTGAAGGGATGGTGCATGACTCCATCAAGGAGGAGCTCCGACCCCTCATCCAACAGACATCATT

TGTGAAACGCGCTTTTAAGCGCCTAAAGGAAAACTTTGAGATTGTTGCCCTGTGTTTGACTCTTTTGGCA

AACATAGTGATCATGATCCGCGAGACTCGCAAGAGACAGCAGATGGTGGACGATGCAGTGAATGAGTACA

TTGAGAAGGCCAACATCACCACAGATGACAAGACTCTTGACGAGGCGGAAAAGAACCCTCTGGAGACCAG

TGGTGCCAGCACCGTTGGTTTCAGAGAGAGAACTCTTCCAGGACACAAGGTGAGTGATGACGTGAACTCC

GAGCCCACCAAACCTGTGGAAGAGCAACCACAAGCTGAAGGACCCTACGCCGGGCCACTTGAGCGTCAGA

AACCTCTGAAAGTGCGCGCCAAACCGCCACAGCAGGAGGGACCCTACGCTGGCCCGATGGAGAGACAGAA

ACCACTGAAAGTGAAAGCAAAAGCCCCGGTCGTTAAGGAAGGACCTTACGAGGGACCGGTGAAGAAGCCT

GTTGCTTTGAAAGTGAAAGCAAAGAACTTGATTGTCACTGAGAGTGGTGCCCCACCGACCGACTTGCAAA

AGATGGTCATGGGCAACACAAAGCCTGTCGAGCTCAATCTTGACGGGAAGACAGTAGCCATCTGCTGTGC

TACTGGAGTGTTTGGTACTGCCTACCTCGTGCCTCGTCATCTTTTCGCAGAGAAGTATGACAAGATCATG

CTGGACGGCAGAGCCATGGCAGACAGTGACTACAGAGTGTTTGAGTTTGAGATTAAAGTAAAAGGACAGG

ACATGCTCTCAGACGCCGCGCTCATGGTGCTCCACCGTGGGAATCGCGTGCGGGACATCACGAAGCACTT

CCGTGATGTTGCAAGGATGAAGAAAGGCACCCCCGTTGTTGGCGTGATCAACAACGCTGATGTTGGGAGA

CTGATTTTCTCTGGTGAGGCCCTTACCTACAAGGACATTGTAGTGTGCATGGATGGAGACACCATGCCTG

GCCTCTTTGCCTACAAAGCCGCCACCAAGGCTGGTTACTGTGGAGGAGCTGTTCTCGCAAAGGACGGAGC

CGAGACTTTCATCGTCGGCACTCACTCTGCAGGGGCCAATGGAGTTGGATACTGCTCATGCGTCTCCAGG

TCCATGCTTCTCAAGATGAAGGCACACATCGACCCAGAACCACACCACGAG

>DQ989321.1_Asia1_IND_2001

ATCTCGATTCCTTCCCAAAAATCTGTGTTGTACTTTCTCATTGAGAAAGGCCAGCACGAAGCAGCAATTG

ACTTCTTTGAGGGGATGGTACATGACTCCATCAAGGAGGAGCTCCGACCCCTCATCCAACAGACATCATT

TGTGAAACGCGCTTTTAAGCGCCTAAAGGAAAACTTTGAGATTGTTGCCCTGTGTTTGACTCTTTTGGCA

AACATAGTGATCATGATCCGCGAGACTCGCAAGAGACAGCAGATGGTGGATGATGCAGTGAATGAGTATA

TTGAGAAAGCCAACATCACCACAGATGACAAGACTCTTGACGAGGCGGAAAAGAACCCTCTGGAGACCAG

TGGTGCCAGCACCGTTGGTTTCAGAGAGAGAACTCTTCCAGGACACAAGGTGAGTGATGACGTGAACTCC

GAGCCCACCAAACCTGTGGAAGAGCAACCACAAGCTGAAGGACCCTACGCCGGGCCACTTGAGCGTCAGA

AACCTCTGAAAGTGCGCGCCAAACTGCCACAGCAGGAGGGACCTTACGCTGGCCCGATGGAGAGACAGAA

ACCACTGAAAGTGAAAGCAAAAGCCCCGGTCGTTAAGGAAGGACCTTACGAGGGACCGGTGAAGAAGCCT

GTTGCTTTGAAAGTGAAAGCAAAGAACTTGATTGTCACTGAGAGTGGCGCCCCACCGACCGACTTGCAAA

AGATGGTCATGGGCAACACAAAGCCTGTCGAGCTCATTCTTGACGGGAAGACAGTAGCCATCTGCTGTGC

TACTGGAGTGTTTGGTACTGCCTACCTCGTGCCTCGTCATCTTTCCGCAGAGAAGTATGACAAGATCATG

CTGGACGGCAGAGCCATGACAGACAGTGACTACAGAGTGTTTGAGTTTGAGATTAAAGTAAAAGGACAGG

ACATGCTCTCAGACGCCGCGCTCATGGTGCTCCACCGTGGGAATCGCGTGCGGGACATCACGAAGCACTT

CCGTGATGTTGCAAGGATGAAGAAAGGCACCCCCGTTGTTGGCGTGATCAACAACGCTGATGTTGGGAGA

CTGATTTTCTCTGGTGAGGCCCTTACCTACAAGGACATTGTAGTGTGCATGGATGGAGACACCATGCCTG

GCCTCTTTGCCTACAAAGCCGCCACCAAGGCTGGTTACTGTGGAGGAGCTGTTCTCGCAAAGGACGGAGC

CGAGACTTTCATCGTCGGCACTCACTCTGCAGGGGGCAATGGAGTTGGATACTGCTCATGCGTCTCCAGG

TCCATGCTTCTCAAGATGAAGGCACACATCGACCCAGAACCACACCACGAG

>DQ989322.1_Asia1_IND_2002

ATCTCGATTCCTTCCCAAAAATCTGTGCTGTACTTTCTCATTGAGAAAGGCCAGCACGAAGCAGCAATTG

ACTTCTTTGAGGGGATGGTCCATGACTCCATCAAGGAGGAGCTCCGACCCCTCATCCAACAGACATCATT

TGTGAAACGCGCTTTTAAGCGCCTAAAGGAAAACTTTGAGATTGTTGCCCTGTGTTTGACTCTTTTGGCA

AACATAGTGATCATGATCCGCGAGACTCGTAAGAGACAGCAGATGGTGGATGATGCAGTGAATGAGTACA

TTGAGAAAGCCAACATCACCACAGATGACAAGACTCTTGACGAGGCGGAAAAGAACCCTCTGGAGACCAG

TGGTGCCAGCACCGTTGGTTTCAGAGAGAGAACTCTTCCAGGACACAAGGTGAGTGATGACGTGAACTCC

GAGCCCACCAAACCTGTGGAAGAGCAACCACAAGCTGAAGGACCCTACGCCGGGCCACTTGAGCGTCAGA

AACCTCTGAAAGTGCGCGCCAAACTGCCACAGCAGGAGGGACCCTACGCTGGCCCGATGGAGAGACAGAA

ACCACTGAAAGTGAAAGCAAAAGCCCCGGTCGTTAAGGAAGGACCTTACGAGGGACCGGTGAAGAAGCCT

GTTACTTTGAAAGTGAAAGCAAAGAACTTGATTGTCACTGAGAGTGGTGCCCCACCGACCGACTTGCAAA

AGATGGTCATGGGCAACACAAAGCCTGTCGAGCTCATCCTTGACGGGAAGACAGTTGCCATCTGCTGTGC

TACTGGAGTGTTTGGTACTGCCTACCTCGTGCCTCGTCATCTTTTCGCAGAGAATTATGACAAGATCATG

CTGGACGGCAGAGCCATGACAGACAGTGACTACAGAGTGTTTGAGTTTGAGATTAAAGTAAAAGGACAGG

ATATGCTCTCAGACGCCGCGCTCATGGTGCTCCACCGTGGGAATCGCGTGCGGGACATCACGAAGCACTT

TCGTGATGTTGCAAGGATGAAGAAAGGCACCCCCGTTGTTGGCGTGATCAACAACGCTGATGTTGGGAGA

CTGATTTTCTCTGGTGAGGCCCTTACCTACAAGGACATTGTAGTGTGCATGGATGGAGACACCATGCCTG

GCCTCTTTGCCTACAAAGCCGCCACTAAGGCTGGTTACTGTGGAGGAGCTGTTCTCGCAAAGGACGGAGC

CGAGACTTTCATCGTCGGCACTCACTCTGCAGGGGGCAATGGAGTTGGATACTGCTCATGCGTCTCCAGA

TCCATGCTTCTCAAGATGAAGGCACACATCGACCCAGAACCACACCACGAG

>DQ989323.1_Asia1_IND_2002

ATCTCGATTCCTTCCCAAAAATCTGTGTTGTACTTTCTCATTGAGAAAGGCCAGCACGAAGCAGCAATTG

ACTTCTTTGAGGGGATGGTGCATGACTCCATCAAGGAGGAGCTCCGACCCCTCATCCAACAGACATCATT

TGTGAAACGCGCTTTTAAGCGCCTAAAGGAAAACTTTGAGATTGTTGCCCTGTGTTTGACTCTTTTGGCA

AACATAGTGATCATGATCCGCGAGACTCGCAAGAGACAGCAGATGGTGGACGATGCAGTGAATGAGTACA

TTGAGAAAGCCAACATCACCACAGATGACAAGACTCTTGACGAGGCGGAAAAGAACCCTCTGGAGACCAG

TGGTGCCAGCACCGTTGGTTTCAGAGAGAGAACTCTTCCAGGACACAAGGTGAGTGATGACGTGAACTCC

GAGCCCACCAAACCTGTGGAAGAGCAACCACAAGCTGAAGGACCCTACACCGGGCCACTTGAGCGTCAGA

AACCTCTGAAAGTGCGCGCCAAACTGCCACAGCAGGAGGGACCCTACGCTGGCCCGATGGAGAGACAGAA

ACCACTGAAGGTGAAAGCAAAAGCCCCGGTCGTTAAGGAAGGACCTTACGAGGGACCGGTGAAGAAGCCT

GTTGCTTTGAAAGTGAAAGCTAAGAACTTGATTGTCACTGAGAGTGGTGCCCCACCGACCGACTTGCAAA

AGATGGTCATGGGCAACACAAAGCCTGTCGAGCTCATTCTTGACGGGAAGACAGTAGCCATCTGCTGTGC

TACTGGGGTGTTTGGTACTGCCTACCTCGTGCCTCGTCATCTTTTCGCAGAGAAGTATGACAAGATCATG

TTGGACGGCAGAGCCATGACAGACAGTGACTACAGAGTGTTTGAGTTTGAGATTAAAGTAAAAGGACAGG

ACATGCTCTCAGACGCCGCGCTCATGGTGCTCCACCGTGGGAATCGCGTGCGGGACATCACGAAGCACTT

CCGTGATGTTGCAAGGATGAAGAAAGGCACCCCCGTTGTTGGCGTGATCAACAACGCTGATGTTGGGAGA

CTGATTTTCTCTGGTGAGGCCCTCACCTACAAGGACATTGTAGTGTGCATGGATGGAGACACCATGCCTG

GCCTCTTTGCCTACAAAGCCGCCACCAAGGCTGGTTACTGTGGAGGAGCTGTTCTCGCAAAGGACGGAGC

CGAGACTTTCATCGTCGGCACTCACTCTGCAGGGGGCAATGGAGTTGGATACTGCTCATGCGTCTCCAGG

TCCATGCTTCTCAAGATGAAGGCACACATCGACCCAGAACCACACCACGAG

>EF117837.1_A_PAK_2006

ATCTCAATTCCTTCCCAAAAATCTGTGTTGTACTTCCTCATTGAGAAAGCCCAACACGAGGCAGCAATTG

AATTCTTTGAGGGGATGGTTCACGACTCTATTAAGGAGGAACTCCGACCCCTAATCCAACAGACCTCATT

TGTGAAACGCGCTTTCAAGCGCCTGAAGGAAAACTTTGAGATTGTTGCCCTTTGTCTGACTCTACTGGCA

AACATAGTGATCATGATCCGCGAGACTCGCAAGAGACAGCAGATGGTGGATGATGCAGTGAATGAGTACA

TTGAAAAGGCAAACATCACCACAGATGACAAGACTCTTGACGAGGCGGAAAAGAACCCTCAGGAGACAAA

CAGTGCCAGCACTGTTGGCTTCAGAGAGAGAACTCTCCCGGGACACACGGTGAGCAATGACGTGGAATCC

GAGCCCACCGTACCCGTGGAGGGACAACCACAAGCTGAAGGACCCTACGCCGGGCCACTCGAGCGTCAGA

AACCTCTGAAAGTGAGAGCCAAGCTTCCACAGCAGGAGGGGCCCTACGCTGGTCCGATGGAGAGACAAAA

GCCCCTGAAAGTGAAAGCAAAAGCCCCGGTCGTAAAGGAAGGACCTTACGAGGGGCTGGTGAAGAAACCT

GTCGCTTTGAAAGTGAAAGCAAAAAATCTGATTGTCACTGAGAGTGGTGCCCCCCCGACCGACTTGCAAA

AGATGGTCATGGGCAACACCAAGCCTGTTGAGCTCATCCTCGACGGGAAGACGGTAGCCATCTGTTGTGC

TACCGGAGTGTTTGGCACTGCTTACCTCGTACCGCGTCATCTTTTCGCGGAAAAGTATGACAAGATCATG

CTGGACGGCAGGGCCATGACAGACAGTGACTACAGAGTGTTTGAGTTTGAGATTAAAGTAAAAGGACAGG

ACATGCTCTCAGATGCCGCGCTCATGGTGCTCCACCGTGGGAATCGTGTGAGAGATATCACGAAGCACTT

TCGTGATACAGCAAGAATGAAGAAGGGCACCCCCGTTGTCGGTGTTATCAACAACGCCGATGTCGGGAGA

CTGATTTTCTCTGGTGAGGCCCTCACCTACAAGGACATCGTAGTGTGCATGGATGGAGACACCATGCCTG

GCCTCTTTGCCTACAGAGCCGCCACCAAGGCTGGCTATTGTGGAGGAGCTGTTCTTGCAAAGGACGGAGC

CGAGACTTTCATCGTCGGCACTCACTCCGCAGGCGGTAGTGGAGTTGGATACTGTTCATGTGTTTCCAGG

TCCATGCTGCTAAAGATGAAGGCACACATTGACCCTGAGCCACACCACGAG

>EF149009.1_Asia1_CHA_2005

ATCTCAATTCCTTCCCAAAAGTCTGTGCTGTACTTCCTCATCGAGAAAGGCCAGCATGAGGCAGCAATTG

AATTCTTTGAGGGAATGGTGCACGACTCCATTAAGGAGGAACTCCGGCCCCTCATCCAACAGACCTCATT

TGTGAAACGCGCTTTCAAGCGCCTGAAGGAAAACTTTGAGATCGTTGCCCTGTGTTTGACTCTTTTGGCA

AACATAGTGATCATGATCCGCGAGACGCGCAAGAGACAGCAGATGGTGAATGATGCGGTGAACGAGTACA

TCGACAAAGCCAACATCACCACAGATGACAAGACTCTTGACGAGGCGGAAAAGAACCCTCTGGAGACCAG

TGGTGCTAGCACCGTTGGTTTCAGAGAGAGAACCCTCCCGGGGCGCAAGACGAGTGATGACGTGAACTCC

GAGCCCGTCAAATCCGTGGAGGAACAACCACAAGCTGAAGGACCCTACGCCGGGGCATTCGAGCGTCAGA

AAACTCTGAAAGTGAGAGCCAAGCTGCCACAGCAAGAGGGACCTTACGCTGGCCCGATGGAGAGACAGAA

ACCACTGAAAGTGAAAGCAAAAGCCCCGGTCGTGAAGGAAGGACCTTACGAGGGACCGGTGAAGAAGCCT

GTCGCTTTGAAAGTGAAAGCTAAGAACTTGATTGTCACTGAGAGTGGTGCCCCACCGACTGACTTGCAAA

AGATGGTCATGGGCAACACCAAGCCTGTTGAGCTCATCCTCGACGGCAAGACGGTAGCCATCTGCTGCGC

TACCGGAGTCTTTGGTACTGCCTACCTCGTGCCTCGTCACCTTTTCGCAGAGAAGTACGACAAGATCATG

CTGGACGGCAGAGCCATGACAGACAGTGACTACAGAGTGTTTGAGTTTGAGATTAAAGTAAAAGGACAGA

ACATGCTCTCAGACGCCGCACTCATGGTGCTCCACCGTGGGAATCGCGTGCGTGACATCACGAAGCACTT

CCGTGATGTAGCCAAGATGAAGAAAGGAACCCCCGTCGTTGGTGTGATTAACAACGCCGACGTTGGGAGA

CTGATCTTCTCTGGTGAGGCCCTAACCTACAAAGACATTGTAGTGTGCATGGACGGAGACACCATGCCTG

GGCTTTTTGCTTACAAGGCCGTCACCAGGGCGGGCTACTGTGGAGGAGCCGTTCTCGCGAAGGACGGAGC

CGAGACTTTCATCGTCGGCACTCACTCCGCAGGCGGCAACGGAGTTGGATACTGCTCGTGCGTTTCCAGG

TCCATGCTGCTCAAGATGAAGGCTCACATCGACCCCGAACCACACCACGAG

>EF149010.1_Asia1_CHA_2005

ATCTCTATCCCTTCCCAAAAGTCTGTACTGTACTTTCTCATTGAGAAGGGCCAGCACGAAGCAGCAATTG

AGTTCTTTGAGGGGATGGTGCACGACTCCATCAAGGAGGAGCTCCGGCCTCTCATTCAACAGACCTCGTT

TGTGAAGCGCGCTTTCAAGCGCCTGAAGGAAAACTTTGAGATAGTTGCCCTGTGTTTGACCCTTTTGGCA

AACATAGTGATCATGATCCGCGAGACTCGCAAGAGACAGCAAATGGTGGATGATGCAGTGAATGAGTACA

TTGAGAAGGCAAACATCACCACGGATGACAAGACTCTTGACGAGGCGGAAAGGAACCCTTTGGAAACCAG

CGGTGCCACCACTGTTGGTTTCAGAGAGAGAACTCTCCCGGGGCACAAGGCGAGTGATGACGTGAACTCC

GAGCCTGCCAAACTCGTGGAAGAACAACCACAAGCTGAAGGACCCTACGCCGGACCACTCGAGCGTCAGA

AACCTTTGAAAGTGAGAGCCAAGCTCCCGCAGCAGGAGGGGCCCTACGCTGGTCCGATGGAGAGACAGAA

ACCGCTGAAAGTGAAAGCAAAAGCCCCGGTCGTTAAGGAAGGACCTTACGAAGGACCGGTGAAGAAGCCT

GTCGCTTTGAAAGTGAAAGCAAAGAACTTGATTGTCACTGAGAGTGGTGCTCCCCCGACTGACTTGCAAA

AGATGGTCATGGGTAACACCAAGCCTGTTGAGCTCATCCTCGACGGGAAGACGGTGGCCATCTGCTGCGC

TACCGGAGTGTGTGGTACTGCCTACCTTGTTCCTCGTCATCTCTTCGCAGAGAAGTATGACAAGATCATG

CTGGACGGCAGAGCCATGACAGACAGTGACTACAGAGTGTTTGAGTTTGAGATTAAAGTAAAAGGACAGG

ACATGCTCTCAGACGCCGCGCTCATGGTGCTCCACCGTGGGAATCGCGTGCGGGACATCACGAAGCACTT

CCGTGATGTGGCAAGAATGAAGAAAGGCACCCCCGTCGTCGGCGTGATCAACAACGCTGATGTTGGGAGA

CTGATTTTCTCTGGTGAGGCCCTTACCTACAAGGACATTGTAGTGTGCATGGACGGAGACACCATGCCTG

GCCTCTTCGCCTACAAAGCTGCCACCAAGGCAGGCTACTGTGGAGGAGCCGTTCTTGCTAAGGACGGAGC

CGAGACTTTCATCGTCGGCACTCACTCCGCAGGTGGCAATGGAGTTGGATACTGCTCATGCGTTTCCAGG

TCTATGCTGCAAAAAATGAAGGCACACATCGACCCCGAACCACACCACGAG

>EF494486.1_A_TUR_2005

ATCTCAATTCCTTCCCAAAAATCTGTGTTGTACTTCCTCATTGAGAAAGGCCAACACGAGGCAGCAATTG

AATTCTTTGAGGGGATGGTTCACGACTCGATCAAGGAGGAACTCCGACCCCTAATCCAACAGACCTCATT

TGTGAAACGCGCTTTTAAGCGCCTGAAGGAAAACTTTGAGATTGTTGCCCTGTGTCTGACTCTACTGGCA

AACATAGTGATCATGATCCGCGAGACTCGCAAGAGACAGCAGATGGTGGATGATGCAGTGAATGAGTACA

TTGAAAAGGCAAACATCACCACAGATGACAAGACTCTCGACGAGGCGGAAAAGAACCCTCAGGAGACAAA

CAGTGCCAGCACTGTTGGCTTCAGAGAGAGAACTCTCCCGGGACACACGGTGGGCAATGACGTGAACTCC

GAGCCCACCGTACCCGTGGAGGGACAACCACAAGCTGAAGGACCCTACGCCGGGCCACTCGAGCGTCAGA

AACCTCTGAAAGTGAGAGCCAAGCTCCCACAGCAGGAGGGGCCCTACGCTGGTCCGATGGAGAGACAAAA

GCCCCTGAAAGTGAAAGCAAAAGCCCCGGTCGTAAAGGAAGGACCTTACGAGGGGCTTGTGAAGAAACCT

GTCGCTTTGAAAGTGAAAGCCAAAAATTTGATTGTCACTGAGAGTGGTGCCCCCCCGACCGACTTGCAAA

AGATGGTCATGGGCAACACCAAGCCTGTTGAGCTCATCCTCGACGGGAAGACGGTAGCCATTTGTTGCGC

TACCGGAGTGTTTGGCACTGCTTACCTTGTACCACGTCATCTTTTCGCGGAGAAGTATGACAAGATCATG

CTGGACGGCAGAGCCATGACAGACAGTGACTACAGAGTGTTTGAGTTTGAGATTAAAGTAAAAGGACAGG

ACATGCTTTCAGATGCCGCGCTCATGGTGCTCCACCGTGGGAATCGCGTGAGAGATATCACGAAACACTT

TCGTGACACAGCAAGAATGAAGAAGGGCACCCCCGTTGTCGGTGTTATCAACAACGCCGATGTCGGGAGA

CTGATTTTCTCTGGTGAGGCCCTTACCTACAAGGACATTGTAGTGTGTATGGATGGAGACACCATGCCTG

GCCTCTTTGCCTACAGAGCCGCCACCAAGGCTGGCTATTGTGGAGGAGCTGTTCTTGCAAAGGACGGAGC

CGAGACTTTCATCGTCGGCACTCACTCCGCAGGCGGTAATGGAGTTGGATACTGTTCATGCGTTTCCAGG

TCCATGCTGCTAAAGATGAAGGCACACATTGACCCTGAGCCACACCACGAG

>EF494487.1_A_PAK_2006

ATCTCAATTCCTTCCCAAAAATCTGTGTTGTACTTCCTCATTGAGAAAGGCCAACACGAGGCAGCAATTG

AATTCTTTGAGGGGATGGTTCACGACTCTATTAAGGAGGAACTCCGACCCCTAATCCAACAGACCTCATT

TGTGAAACGCGCTTTCAAGCGCCTGAAGGAAAACTTTGAGATTGTTGCCCTTTGTCTGACTCTACTGGCA

AACATAGTGATCATGATCCGCGAGACTCGCAAGAGACAGCAGATGGTGGATGATGCAGTGAATGAGTACA

TTGAAAAGGCAAACATCACCACAGATGACAAGACTCTTGACGAGGCGGAAAAGAACCCTCAGGAGACAAA

CAGTGCCAGCACTGTTGGCTTCAGAGAGAGAACTCTCCCGGGACACACGGTGAGCAATGACGTGGAATCC

GAGCCCACCGTACCCGTGGAGGGACAACCACAAGCTGAAGGACCCTACGCCGGGCCACTCGAGCGTCAGA

AACCTCTGAAAGTGAGAGCCAAGCTTCCACAGCAGGAGGGGCCCTACGCTGGTCCGATGGAGAGACAAAA

GCCCCTGAAAGTGAAAGCAAAAGCCCCGGTCGTAAAGGAAGGACCTTACGAGGGGCTGGTGAAGAAACCT

GTCGCTTTGAAAGTGAAAGCAAAAAATCTGATTGTCACTGAGAGTGGTGCCCCCCCGACCGACTTGCAAA

AGATGGTCATGGGCAACACCAAGCCTGTTGAGCTCATCCTCGACGGGAAGACGGTAGCCATCTGTTGTGC

TACCGGAGTGTTTGGCACTGCTTACCTCGTACCGCGTCATCTTTTCGCGGAAAAGTATGACAAGATCATG

CTGGACGGCAGGGCCATGACAGACAGTGACTACAGAGTGTTTGAGTTTGAGATTAAAGTAAAAGGACAGG

ACATGCTCTCAGATGCCGCGCTCATGGTGCTCCACCGTGGGAATCGTGTGAGAGATATCACGAAGCACTT

TCGTGATACAGCAAGAATGAAGAAGGGCACCCCCGTTGTCGGTGTTATCAACAACGCCGATGTCGGGAGA

CTGATTTTCTCTGGTGAGGCCCTCACCTACAAGGACATCGTAGTGTGCATGGATGGAGACACCATGCCTG

GCCTCTTTGCCTACAGAGCCGCCACCAAGGCTGGCTATTGTGGAGGAGCTGTTCTTGCAAAGGACGGAGC

CGAGACTTTCATCGTCGGCACTCACTCCGCAGGCGGTAATGGAGTTGGATACTGTTCATGTGTTTCCAGG

TCCATGCTGCTAAAGATGAAGGCACACATTGACCCTGAGCCACACCACGAG

>EF494488.1_A_PAK_2006

ATCTCAATTCCTTCCCAAAAATCTGTGTTGTACTTCCTCATTGAGAAAGGCCAACACGAGGCAGCAATTG

AATTCTTTGAGGGGATGGTTCACGACTCTATCAAGGAGGAACTCCGACCCCTAATCCAACAGACCTCATT

TGTGAAACGCGCTTTCAAGCGCCTGAAGGAAAACTTTGAGATCGTTGCCCTGTGTCTGACTCTACTGGCA

AACATAGTGATCATGATCCGCGAGACTCGCAAGAGACAGCAGATGGTGGATGATGCAGTGAATGAGTACA

TTGAAAAGGCAAACATCACCACAGATGACAAGACTCTTGACGAGGCGGAAAAGAACCCTCAGGAGACAAA

CAGTGCCAGCACTGTTGGCTTCAGAGAGAGAACTCTCCCGGGACACACGGTGAGCAATGACGTGAACTCC

GAGCCCACCGTACCCGTGGAGGGACAACCACAAGCTGAAGGACCCTACGCCGGGCCACTCGAGCGTCAGA

AACCTCTGAAAGTGAGAGCCAAGCTCCCACAGCAGGAGGGGCCCTACGCTGGTCCGATGGAGAGACAAAA

GCCCCTGAAAGTGAAAGCAAAAGCCCCGGTCGTAAAGGAAGGACCTTACGAGGGGCCGGTGAAGAAACCT

GTCGCTTTGAAAGTGAAAGCCAAAAATTTGATTGTCACTGAGAGTGGTGCCCCCCCGACCGACTTGCAAA

AGATGGTCATGGGCAACACCAAGCCTGTTGAGCTCATCCTCGACGGGAAGACGGTAGCCATCTGTTGTGC

TACCGGAGTGTTTGGCACTGCTTACCTTGTACCACGTCATCTTTTCGCGGAGAAGTATGACAAGATCATG

CTGGACGGCAGAGCCATGACAGACAGTGACTATAGAGTGTTTGAGTTTGAGATTAAAGTAAAAGGACAGG

ACATGCTCTCAGACGCCGCGCTCATGGTGCTCCACCGTGGGAACCGCGTGAGAGATATCACGAAACACTT

TCGTGATACAGCAAGAATGAAGAAGGGTACCCCCGTTGTCGGTGTTATCAACAACGCCGATGTCGGGAGA

CTGATTTTCTCTGGTGAGGCCCTTACCTACAAGGACATTGTAGTATGCATGGATGGAGACACCATGCCTG

GCCTCTTTGCCTACAGAGCCGCCACCAAGGCTGGCTATTGCGGAGGAGCTGTTCTCGCAAAGGACGGAGC

CGAGACTTTCATCGTCGGCACTCACTCCGCAGGCGGCAATGGAGTTGGATACTGTTCATGCGTTTCCAGG

TCCATGCTGTTAAAGATGAAGGCACACATTGACCCTGAGCCACACCACGAG

>EF552688.1_O_UKG_2001

ATCTCAATTCCTTCCCAAAAGGCTGTGCTGTACTTTCTCATTGAGAAGGGCCAGCACGAAGCAGCAATTG

AGTTCTTTGAGGGGATGGTCCATGACTCCATCAAGGAGGAGCTCCGGCCTCTCATCCAACAGACCTCATT

TGTGAAGCGCGCTTTTAAGCGCCTGAAGGAAAACTTTGAGATAGTTGCCCTGTGTTTGACTCTTTTGGCA

AACATAGTGATCATGATCCGCGAGACTCGCAAGAGACAGCAGATGGTGGATGATGCAGTGAACGAGTACA

TTGAGAAGGCAAACATCACCACGGATGACAAGACTCTTGACGAGGCGGAAAAGAACCCTCTGGAGACCAG

CGGTGCCACCACTGTTGGTTTCAGAGAGAAAACTCTCCCGGGACACAAGGCGGGTGATGACGTGAACTCC

GAGCCCGCCAAACCCGTGGAAGAACAACCACAAGCTGAAGGACCCTACACCGGTCCACTCGAGCGTCAAA

AACCCCTGAAAGTGAGGGCCAAGCTCCCACAGCAGGAGGGGCCCTACGCTGGTCCGATGGAGAGACAGAA

ACCGCTGAAAGTGAAAGTGAAAGCCCCGGTCGTTAAGGAAGGACCTTACGAAGGACCGGTGAAGAAACCT

GTCGCTTTGAAAGTGAAAGCAAAGAACTTGATTGTCACTGAGAGTGGTGCTCCCCCGACTGACTTGCAAA

AGATGGTCATGGGTAACACCAAGCCTGTTGAGCTCATCCTCGACGGGAAGACGGTGGCCATCTGCTGCGC

CACCGGAGTGTTTGGTACTGCCTACCTAGTTCCTCGTCATCTTTTCGCAGAGAAGTATGACAAGATCATG

TTGGACGGCAGAGCCATGACAGACAGTGACTACAGAGTGTTTGAGTTTGAGATTAAAGTGAAAGGACAGG

ACATGCTCTCAGACGCCGCTCTCATGGTGCTTCACCGTGGGAATCGCGTGCGGGACATCACGAAGCACTT

CCGTGATGTGGCAAGAATGAAGAAAGGCACCCCCGTCGTCGGCGTGATCAACAACGCTGATGTTGGGAGA

CTGATCTTCTCTGGTGAGGCCCTTACCTACAAGGACACTGTAGTGTGCATGGACGGAGACACCATGCCCG

GTCTCTTCGCCTACAAAGCTGCCACCAAGGCGGGTTACTGTGGAGGAGCCGTTCTTGCAAAGGACGGAGC

CGAGACTTTCATCGTCGGCACTCACTCCGCAGGCGGCAATGGAGTTGGATACTGCTCATGCGTTTCCAGG

TCCATGCTGCTTAAAATGAAGGCACACATCGATCCCGAACCACACCACGAG

>EF552689.1_O_UKG_2001

ATCTCAATTCCTTCCCAAAAGGCTGTGCTGTACTTTCTCATTGAGAAGGGCCAGCACGAAGCAGCAATTG

AGTTCTTTGAGGGGATGGTGCATGACTCCATCAAGGAGGAGCTCCGGCCTCTCATCCAACAGACCTCATT

TGTGAAGCGCGCTTTTAAGCGCCTGAAGGAAAACTTTGAGATAGTTGCCCTGTGTTTGACTCTTTTGGCA

AACATAGTGATCATGATCCGCGAGACTCGCAAGAGACAGCAGATGGTGGATGATGCAGTGAACGAGTACA

TTGAGAAGGCAAACATCACCACGGATGACAAGACTCTTGACGAGGCGGAAAAGAACCCTCTGGAGACCAG

CGGTGCCACCACTGTTGGTTTCAGAGAGAAAACTCTCCCGGGACACAAGGCGGGTGATGACGTGAACTCC

GAGCCCGCCAAACCCGTGGAAGAACAACCACAAGCTGAAGGACCCTACACCGGTCCACTCGAGCGTCAAA

AACCCCTGAAAGTGAGGGCCAAGCTCCCACAGCAGGAGGGGCCCTACGCTGGTCCGATGGAGAGACAGAA

ACCGCTGAAAGTGAAAGTGAAAGCCCCGGTCGTTAAGGAAGGACCTTACGAAGGACCGGTGAAGAAACCT

GTCGCTTTGAAAGTGAAAGCAAAGAACTTGATTGTCACTGAGAGTGGTGCTCCCCCGACTGACTTGCAAA

AGATGGTCATGGGTAACACCAAGCCTGTTGAGCTCATCCTCGACGGGAAGACGGTGGCCATCTGCTGCGC

CACCGGAGTGTTTGGTACTGCCTACCTAGTTCCTCGTCATCTTTTCGCAGAGAAGTATGACAAGATCATG

TTGGACGGCAGAGCCATGACAGACAGTGACTACAGAGTGTTTGAGTTTGAGATTAAAGTGAAAGGACAGG

ACATGCTCTCAGACGCCGCTCTCATGGTGCTTCACCGTGGGAATCGCGTGCGGGACATCACGAAACACTT

CCGTGATGTGGCAAGAATGAAGAAAGGCACCCCCGTCGTCGGCGTGATCAACAACGCTGATGTTGGGAGA

CTGATCTTCTCTGGTGAGGCCCTTACCTACAAGGACATTGTAGTGTGCATGGACGGAGACACCATGCCCG

GTCTCTTCGCCTACAAAGCTGCCACCAAGGCGGGTTACTGTGGAGGAGCCGTTCTTGCAAAGGACGGAGC

CGAGACTTTCATCGTCGGCACTCACTCCGCAGGCGGCAATGGAGTTGGATACTGCTCATGCGTTTCCAGG

TCCATGCTGCTTAAAATGAAGGCACACATCGATCCCGAACCACACCACGAG

>EF552690.1_O_UKG_2001

ATCTCAATTCCTTCCCAAAAGGCTGTGCTGTACTTTCTCATTGAGAAAGGCCAGCACGAAGCAGCAATTG

AGTTCTTTGAGGGGATGGTGCATGACTCCATCAAGGAGGAGCTCCGGCCTCTCATCCAACAGACCTCATT

TGTGAAGCGCGCTTTTAAGCGCCTGAAGGAAAACTTTGAGATAGTTGCCCTGTGTTTGACTCTTTTGGCA

AACATAGTGATCATGATCCGCGAGACTCGCAAGAGACAGCAGATGGTGGATGATGCAGTGAACGAGTACA

TTGAGAAGGCAAACATCACCACGGATGACAAGACTCTTGACGAGGCGGAAAAGAACCCTCTGGAGACCAG

CGGTGCCACCACTGTTGGTTTCAGAGAGAAAACTCTCCCGGGACACAAGGCGGGTGATGACGTGAACTCC

GAGCCCGCCAAACCCGTGGAAGAACAACCACAAGCTGAAGGACCCTACACCGGTCCACTCGAGCGTCAAA

AACCCCTGAAAGTGAGGGCCAAGCTCCCACAGCAGGAGGGGCCCTACGCTGGTCCGATGGAGAGACAGAA

ACCGCTGAAAGTGAAAGTGAAAGCCCCGGTCGTTAAGGAAGGACCTTACGAAGGACCGGTGAAGAAACCT

GTCGCTTTGAAAGTGAAAGCAAAGAACTTGATTGTCACTGAGAGTGGTGCTCCCCCGACTGACTTGCAAA

AGATGGTCATGGGTAACACCAAGCCTGTTGAGCTCATCCTCGACGGGAAGACGGTGGCCATCTGCTGCGC

CACCGGAGTGTTTGGCACTGCCTACCTAGTTCCTCGTCATCTTTTCGCAGAGAAGTATGACAAGATCATG

TTGGACGGCAGAGCCATGACAGACAGTGACTACAGAGTGTTTGAGTTTGAGATTAAAGTGAAAGGACAGG

ACATGCTCTCAGACGCCGCTCTCATGGTGCTTCACCGTGGGAATCGCGTGCGGGACATCACGAAGCACTT

CCGTGATGTGGCAAGAATGAAGAAAGGCACCCCCGTCGTCGGCGTGATCAACAACGCTGATGTTGGGAGA

CTGATCTTCTCTGGTGAGGCCCTTACCTACAAGGACATTGTAGTGTGCATGGACGGAGACACCATGCCCG

GTCTCTTCGCCTACAAAGCTGCCACCAAGGCGGGTTACTGTGGAGGAGCCGTTCTTGCAAAGGACGGAGC

CGAGACTTTCATCGTCGGCACTCACTCCGCAGGCGGCAATGGAGTTGGATACTGCTCATGCGTTTCCAGG

TCCATGCTGCTTAAAATGAAGGCACACATTGATCCCGAACCACACCACGAG

>EF552691.1_O_UKG_2001

ATCTCAATTCCTTCCCAAAAGGCTGTGCTGTACTTTCTCATTGAGAAAGGCCAGCACGAAGCAGCAATTG

AGTTCTTTGAGGGGATGGTGCATGACTCCATCAAGGAGGAGCTCCGGCCTCTCATCCAACAGACCTCATT

TGTGAAGCGCGCTTTTAAGCGCCTGAAGGAAAACTTTGAGATAGTTGCCCTGTGTTTGACTCTTTTGGCA

AACATAGTGATCATGATCCGCGAGACTCGCAAGAGACAGCAGATGGTGGATGATGCAGTGAACGAGTACA

TTGAGAAGGCAAACATCACCACGGATGACAAGACTCTTGACGAGGCGGAAAAGAACCCTCTGGAGACCAG

CGGTGCCACCACTGTTGGTTTCAGAGAGAAAACTCTCCCGGGACACAAGGCGGGTGATGACGTGAACTCC

GAGCCCGCCAAACCCGTGGAAGAACAACCACAAGCTGAAGGACCCTACACCGGTCCACTCGAGCGTCAAA

AACCCCTGAAAGTGAGGGCCAAGCTCCCACAGCAGGAGGGGCCCTACGCTGGTCCGATGGAGAGACAGAA

ACCGCTGAAAGTGAAAGTGAAAGCCCCGGTCGTTAAGGAAGGACCTTACGAAGGACCGGTGAAGAAACCT

GTCGCTTTGAAAGTGAAAGCAAAGAACTTGATTGTCACTGAGAGTGGTGCTCCCCCGACTGACTTGCAAA

AGATGGTCATGGGTAACACCAAGCCTGTTGAGCTCATCCTCGACGGGAAGACGGTGGCCATCTGCTGCGC

CACCGGAGTGTTTGGTACTGCCTACCTAGTTCCTCGTCATCTTTTCGCAGAGAAGTATGACAAGATCATG

TTGGACGGCAGAGCCATGACAGACAGTGACTACAGAGTGTTTGAGTTTGAGATTAAAGTGAAAGGACAGG

ACATGCTCTCAGACGCCGCTCTCATGGTGCTTCACCGTGGGAATCGCGTGCGGGACATCACGAAGCACTT

CCGTGATGTGGCAAGAATGAAGAAAGGCACCCCCGTCGTCGGCGTGATCAACAACGCTGATGTTGGGAGA

CTGATCTTCTCTGGTGAGGCCCTTACCTACAAGGACATTGTAGTGTGCATGGACGGAGACACCATGCCCG

GTCTCTTCGCCTACAAAGCTGCCACCAAGGCGGGTTACTGTGGAGGAGCCGTTCTTGCAAAGGACGGAGC

CGAGACTTTCATCGTCGGCACTCACTCCGCAGGCGGCAATGGAGTTGGATACTGCTCATGCGTTTCCAGG

TCCATGCTGCTTAAAATGAAGGCACACATCGATCCCGAACCACACCACGAG

>EF552692.1_O_UKG_2001

ATCTCAATTCCTTCCCAAAAGGCTGTGCTGTACTTTCTCATTGAGAAGGGCCAGCACGAAGCAGCAATTG

AGTTCTTTGAGGGGATGGTGCATGACTCCATCAAGGAGGAGCTCCGGCCTCTCATCCAACAGACCTCATT

TGTGAAGCGCGCTTTTAAGCGCCTGAAGGAAAACTTTGAGATAGTTGCCCTGTGTTTGACTCTTTTGGCA

AACATAGTGATCATGATCCGCGAGACTCGCAAGAGACAGCAGATGGTGGATGACGCAGTGAACGAGTACA

TTGAGAAGGCAAACATCACCACGGATGACAAGACTCTTGACGAGGCGGAAAAGAACCCTCTGGAGACCAG

CGGTGCCACCACTGTTGGTTTCAGAGAGAAAACTCTCCCGGGACACAAGGCGGGTGATGACGTGAACTCC

GAGCCCGCCAAACCCGTGGAAGAACAACCACAAGCTGAAGGACCCTACACCGGTCCACTCGAGCGTCAAA

AACCCCTGAAAGTGAGGGCCAAGCTCCCACAGCAGGAGGGGCCCTACGCTGGTCCGATGGAGAGACAGAA

ACCGCTGAAAGTGAAAGTGAAAGCCCCGGTCGTTAAGGAAGGACCTTACGAAGGACCGGTGAAGAAACCT

GTCGCTTTGAAAGTGAAAGCAAAGAACTTGATTGTCACTGAGAGTGGTGCTCCCCCGACTGACTTGCAAA

AGATGGTCATGGGTAACACCAAGCCTGTTGAGCTCATCCTCGACGGGAAGACGGTGGCCATCTGCTGCGC

CACCGGAGTGTTTGGTACTGCCTACCTAGTTCCTCGTCATCTTTTCGCAGAGAAGTATGACAAGATCATG

TTGGACGGCAGAGCCATGACAGACAGTGACTACAGAGTGTTTGAGTTTGAGATTAAAGTGAAAGGACAGG

ACATGCTCTCAGACGCCGCTCTCATGGTGCTTCACCGTGGGAATCGCGTGCGGGACATCACGAAGCACTT

CCGTGATGTGGCAAGAATGAAGAAAGGCACCCCCGTCGTCGGCGTGATCAACAACGCTGATGTTGGGAGA

CTGATCTTCTCTGGTGAGGCCCTTACCTACAAGGACATTGTAGTGTGCATGGACGGAGACACCATGCCCG

GTCTCTTCGCCTACAAAGCTGCCACCAAGGCGGGTTACTGTGGAGGAGCCGTTCTTGCAAAGGACGGAGC

CGAGACTTTCATCGTCGGCACTCACTCCGCAGGCGGCAATGGAGTTGGATACTGCTCATGCGTTTCCAGG

TCCATGCTGCTTAAAATGAAGGCACACATCGATCCCGAACCACACCACGAG

>EF552693.1_O_UKG_2001

ATCTCAATTCCTTCCCAAAAGGCTGTGCTGTACTTTCTCATTGAGAAGGGCCAGCACGAAGCAGCAATTG

AGTTCTTTGAGGGGATGGTGCATGACTCCATCAAGGAGGAGCTCCGGCCTCTCATCCAACAGACCTCATT

TGTGAAGCGCGCTTTTAAGCGCCTGAAGGAAAACTTTGAGATAGTTGCCCTGTGTTTGACTCTTTTGGCA

AACATAGTGATCATGATCCGCGAGACTCGCAAGAGACAGCAGATGGTGGATGATGCAGTGAACGAGTACA

TTGAGAAGGCAAACATCACCACGGATGACAAGACTCTTGACGAGGCGGAAAAGAACCCTCTGGAGACCAG

CGGTGCCACCACTGTTGGTTTCAGAGAGAAAACTCTCCCGGGACACAAGGCGGGTGATGACGTGAACTCC

GAGCCCGCCAAACCCGTGGAAGAACAACCACAAGCTGAAGGACCCTACACCGGTCCACTCGAGCGTCAAA

AACCCCTGAAAGTGAGGGCCAAGCTCCCACAGCAGGAGGGGCCCTACGCTGGTCCGATGGAGAGACAGAA

ACCGCTGAAAGTGAAAGTGAAAGCCCCGGTCGTTAAGGAAGGACCTTACGAAGGACCGGTGAAGAAACCT

GTCGCTTTGAAAGTGAAAGCAAAGAACTTGATTGTCACTGAGAGTGGTGCTCCCCCGACTGACTTGCAAA

AGATGGTCATGGGTAACACCAAGCCTGTTGAGCTCATCCTCGACGGGAAGACGGTGGCCATCTGCTGCGC

CACCGGAGTGTTTGGTACTGCCTACCTAGTTCCTCGTCATCTTTTCGCAGAGAAGTATGACAAGATCATG

TTGGACGGCAGAGCCATGACAGACAGTGACTACAGAGTGTTTGAGTTTGAGATTAAAGTGAAAGGACAGG

ACATGCTCTCAGACGCCGCTCTCATGGTGCTTCACCGTGGGAATCGCGTGCGGGACATCACGAAGCACTT

CCGTGATGTGGCAAGAATGAAGAAAGGCACCCCCGTCGTCGGCGTGATCAACAACGCTGATGTTGGGAGA

CTGATCTTCTCTGGTGAGGCCCTTACCTACAAGGACATTGTAGTGTGCATGGACGGAGACACCATGCCCG

GTCTCTTCGCCTACAAAGCTGCCACCAAGGCGGGTTACTGTGGAGGAGCCGTTCTTGCAAAGGACGGAGC

CGAGACTTTCATCGTCGGCACTCACTCCGCAGGCGGCAATGGAGTTGGATACTGCTCATGCGTTTCCAGG

TCCATGCTGCTTAAAATGAAGGCACACATCGATCCCGAACCACACCACGAG

>EF552695.1_O_UKG_2001

ATCTCAATTCCTTCCCAAAAGGCTGTGCTGTACTTTCTCATTGAGAAAGGCCAGCACGAAGCAGCAATTG

AGTTCTTTGAGGGGATGGTGCATGACTCCATCAAGGAGGAGCTCCGGCCTCTCATCCAACAGACCTCATT

TGTGAAGCGCGCTTTTAAGCGCCTGAAGGAAAACTTTGAGATAGTTGCCCTGTGTTTGACTCTTTTGGCA

AACATAGTGATCATGATCCGCGAGACTCGCAAGAGACAGCAGATGGTGGATGATGCAGTGAACGAGTACA

TTGAGAAGGCAAACATCACCACGGATGACAAGACTCTTGACGAGGCGGAAAAGAACCCTCTGGAGACCAG

CGGTGCCACCACTGTTGGTTTCAGAGAGAAAACTCTCCCGGGACACAAGGCGGGTGATGACGTGAACTCC

GAGCCCGCCAAACCCGTGGAAGAACAACCACAAGCTGAAGGACCCTACACCGGTCCACTCGAGCGTCAAA

AACCCCTGAAAGTGAGGGCCAAGCTCCCACAGCAGGAGGGGCCCTACGCTGGTCCGATGGAGAGACAGAA

ACCGCTGAAAGTGAAAGTGAAAGCCCCGGTCGTTAAGGAAGGACCTTACGAAGGACCGGTGAAGAAACCT

GTCGCTTTGAAAGTGAAAGCAAAGAACTTGATTGTCACTGAGAGTGGTGCTCCCCCGACTGACTTGCAAA

AGATGGTCATGGGTAACACCAAGCCTGTTGAGCTCATCCTCGACGGGAAGACGGTGGCCATCTGCTGCGC

CACCGGAGTGTTTGGTACTGCCTACCTAGTTCCTCGTCATCTTTTCGCAGAGAAGTATGACAAGATCATG

TTGGACGGCAGAGCCATGACAGACAGTGACTACAGAGTGTTTGAGTTTGAGATTAAAGTGAAAGGACAGG

ACATGCTCTCAGACGCCGCTCTCATGGTGCTTCACCGTGGGAATCGCGTGCGGGACATCACGAAGCACTT

CCGTGATGTGGCAAGAATGAAGAAAGGCACCCCCGTCGTCGGCGTGATCAACAACGCTGATGTTGGGAGA

CTGATCTTCTCTGGTGAGGCCCTTACCTACAAGGACATTGTAGTGTGCATGGACGGAGACACCATGCCCG

GTCTCTTCGCCTACAAAGCTGCCACCAAGGCGGGTTACTGTGGAGGAGCCGTTCTTGCAAAGGACGGAGC

CGAGACTTTCATCGTCGGCACTCACTCCGCAGGCGGCAATGGAGTTGGATACTGCTCATGCGTTTCCAGG

TCCATGCTGCTTAAAATGAAGGCACACATCGATCCCGAACCACACCACGAG

>EF552696.1_O_UKG_2001

ATCTCAATTCCTTCCCAAAAGGCTGTGCTGTACTTTCTCATTGAGAAGGGCCAGCACGAAGCAGCAATTG

AGTTCTTTGAGGGGATGGTCCATGACTCCATCAAGGAGGAGCTCCGGCCTCTCATCCAACAGACCTCATT

TGTGAAGCGCGCTTTTAAGCGCCTGAAGGAAAACTTTGAGATAGTTGCCCTGTGTTTGACTCTTTTGGCA

AACATAGTGATCATGATCCGCGAGACTCGCAAGAGACAGCAGATGGTGGATGATGCAGTGAACGAGTACA

TTGAGAAGGCAAACATCACCACGGATGACAAGACTCTTGACGAGGCGGAAAAGAACCCTCTGGAGACCAG

CGGTGCCACCACTGTTGGTTTCAGAGAGAAAACTCTCCCGGGACACAAGGCGGGTGATGACGTGAACTCC

GAGCCCGCCAAACCCGTGGAAGAACAACCACAAGCTGAAGGACCCTACACCGGTCCACTCGAGCGTCAAA

AACCCCTGAAAGTGAGGGCCAAGCTCCCACAGCAGGAGGGGCCCTACGCTGGTCCGATGGAGAGACAGAA

ACCGCTGAAAGTGAAAGTGAAAGCCCCGGTCGTTAAGGAAGGACCTTACGAAGGACCGGTGAAGAAACCT

GTCGCTTTGAAAGTGAAAGCAAAGAACTTGATTGTCACTGAGAGTGGTGCTCCCCCGACTGACTTGCAAA

AGATGGTCATGGGTAACACCAAGCCTGTTGAGCTCATCCTCGACGGGAAGACGGTGGCCATCTGCTGCGC

CACCGGAGTGTTTGGTACTGCCTACCTAGTTCCTCGTCATCTTTTCGCAGAGAAGTATGACAAGATCATG

TTGGACGGCAGAGCCATGACAGACAGTGACTACAGAGTGTTTGAGTTTGAGATTAAAGTGAAAGGACAGG

ACATGCTCTCAGACGCCGCTCTCATGGTGCTTCACCGTGGGAATCGCGTGCGGGACATCACGAAGCACTT

CCGTGATGTGGCAAGAATGAAGAAAGGCACCCCCGTCGTCGGCGTGATCAACAACGCTGATGTTGGGAGA

CTGATCTTCTCTGGTGAGGCCCTTACCTACAAGGACATTGTAGTGTGCATGGACGGAGACACCATGCCCG

GTCTCTTCGCCTACAAAGCTGCCACCAAGGCGGGTTACTGTGGAGGAGCCGTTCTTGCAAAGGACGGAGC

CGAGACTTTCATCGTCGGCACTCACTCCGCAGGCGGCAATGGAGTTGGATACTGCTCATGCGTTTCCAGG

TCCATGCTGCTTAAAATGAAGGCACACATCGATCCCGAACCACACCACGAG

>EF552697.1_O_UKG_2001

ATCTCAATTCCTTCCCAAAAGGCTGTGCTGTACTTTCTCATTGAGAAAGGCCAGCACGAAGCAGCAATTG

AGTTCTTTGAGGGGATGGTGCATGACTCCATCAAGGAGGAGCTCCGGCCTCTCATCCAACAGACCTCATT

TGTGAAGCGCGCTTTTAAGCGCCTGAAGGAAAACTTTGAGATAGTTGCCCTGTGTTTGACTCTTTTGGCA

AACATAGTGATCATGACCCGCGAGACTCGCAAGAGACAGCAGATGGTGGATGATGCAGTGAACGAGTACA

TTGAGAAGGCAAACATCACCACGGATGACAAGACTCTTGACGAGGCGGAAAAGAACCCTCTGGAGACCAG

CGGTGCCACCACTGTTGGTTTCAGAGAGAAAACTCTCCCGGGACACAAGGCGGGTGATGACGTGAACTCC

GAGCCCGCCAAACCCGTGGAAGAACAACCACAAGCTGAAGGACCCTACACCGGTCCACTCGAGCGTCAAA

AACCCCTGAAAGTGAGGGCCAAGCTCCCACAGCAGGAGGGGCCCTACGCTGGTCCGATGGAGAGACAGAA

ACCGCTGAAAGTGAAAGTGAAAGCCCCGGTCGTTAAGGAAGGACCTTACGAAGGACCGGTGAAGAAACCT

GTCGCTTTGAAAGTGAAAGCAAAGAACTTGATTGTCACTGAGAGTGGTGCTCCCCCGACTGACTTGCAAA

AGATGGTCATGGGTAACACCAAGCCTGTTGAGCTCATCCTCGACGGGAAGACGGTGGCCATCTGCTGCGC

CACCGGAGTGTTTGGTACTGCCTACCTAGTTCCTCGTCATCTTTTCGCAGAGAAGTATGACAAGATCATG

TTGGACGGCAGAGCCATGACAGACAGTGACTACAGAGTGTTTGAGTTTGAGATTAAAGTGAAAGGACAGG

ACATGCTCTCAGACGCCGCTCTCATGGTGCTTCACCGTGGGAATCGCATGCGGGACATCACGAAGCACTT

CCGTGATGTGGCAAGAATGAAGAAAGGCACCCCCGTCGTCGGCGTGATCAACAACGCTGATGTTGGGAGA

CTGATCTTCTCTGGTGAGGCCCTTACCTACAAGGACATTGTAGTGTGCATGGACGGAGACACCATGCCCG

GTCTCTTCGCCTACAAAGCTGCCACCAAGGCGGGTTACTGTGGAGGAGCCGTTCTTGCAAAGGACGGAGC

CGAGACTTTCATCGTCGGCACTCACTCCGCAGGCGGCAATGGAGTTGGATACTGCTCATGCGTTTCCAGG

TCCATGCTGCTTAAAATGAAGGCACACATCGATCCCGAACCACACCACGAG

>EF611987.1_O_UGA_2006

ATTTCAATTCCTTCTCAAAAGTCCGTGTTGTACTTCCTCATCGAGAAAGGTCAACACGAAGCAGCAATTG

AATTCTTTGAGGGAATGGTGCACGACTCCATCAAGGAGGAGCTCCGACCTCTCGTTCAACAGACCTCGTT

TGTGAAACGCGCTTTCAAGCGCCTGAAGGAAAACTTTGAAATTGTTGCCCTGTGTTTGACTCTTCTGGCA

AACATAGTGATCATGATCCGTGAAACTCGCAAGAGACAACAGATGGTGGATGACGCAGTGAATGAGTATA

TAGAGAAAGCAAACATCACCACAGATGACAAGACTCTTGACGAGGCGGAAAAGAACCCTCTGGAGACTAG

CGGTGCCAGCACCGTCGGTTTCAGAGAGAGAACCCTCCCGGGTCAAAAGGCGAGTGATGACGTGAACTCC

GAGCCCGCCAAACCCGTGGATGAACAACCACAAGCTGAAGGACCCTACGCCGGGCCACTCGAGCGTCAGA

AACCTCTGAAAGT-AGAGCCAAACTACCACAACAGGAGGGACCTTACGCTGGCCCGATGGAGAGACAGAA

ACCACTAAAAGTGAAAGCAAAAGCCCCGGTCGTTAAGGAAGGACCTTACGAGGGACCGGTGAAGAAACCT

GTCGCTTTGAAAGTGAAAGCTAAGAATCTGATTGTCACTGAGAGTGGCGCCCCACCGACCGACTTGCAAA

AGATGGTCATGGGCAATACCAAGCCTGTTGAGCTCATCCTCGACGGGAAGACGGTAGCCATCTGCTGTGC

TACCGGAGTGTTTGGCACTGCCTACCTCGTGCCTCGTCATCTCTTTGCGGAGAAGTATGACAAGATCATG

TTGGACGGCAGAGCTATGACAGACAGTGACTACAGAGTGTTTGAGTTTGAGATTAAAGTAAAAGGACAGG

ACATGCTCTCAGATGCCGCGCTCATGGTGCTCCACCGCGGGAACCGCGTGAGAGACATCACAAAGCACTT

CCGTGACACGGCAAGAATGAAGAAAGGCACCCCCGTTGTCGGTGTGATCAACAATGCTGACGTCGGGAGA

TTAATTTTCTCTGGTGAGGCCCTCACCTACAAGGACATTGTAGTGTGCATGGATGGAGACACCATGCCAG

GCCTTTTTGCCTACAGAGCCGCCACCAAGGCTGGATACTGCGGAGGGGCCGTTCTCGCTAAGGACGGGGC

TGACACCTTTATCGTCGGCACTCACTCTGCAGGGGGTAATGGAGTTGGATACTGCTCGTGCGTGTCCAGG

TCCATGCTCCTGAAAATGAAGGCACACATTGACCCTGAACCACACCACGAA

>EF614457.1_O_SKR_2002

ATCTCAATTCCTTCCCAAAAGGCTGTGCTGTACTTTCTCATTGAGAAAGGCCAGCACGAAGCAGCAATTG

ACTTCTTTGAGGGGATGGTGTGTGACTCCATCAAGGAGGAGCTCCGGCCTCTCATCCAACAGACCTCATT

TGTGAAGCGCGCTTTTAAGCGCCTGAAGGAAAACTTTGAGATAGTTGCCCTGTGTTTGACTCTAATGGCA

AACATAGTGATCATGATCCGCGAGACTCGCAAGAGACAGCAGATGGTGGATGATGCAGTGAACGAGTACA

TTGAGAAGGTAAACATCACCACGGATGACAAGACTCTTGACGAGGCGGAAAAGAACCCTCTGGAAACCAG

CGGTGCCACCACTGTTGGTTTCAGAGAGAAAACTCTCCCGGGGCACAAGACGGGTGATGACGTGAGCTCC

GAGCCCACCAAACCCGTGGAAGGACAACCACAAGCTGAAGGACCCTACACCGGTCCACTCGAGCGTCAAA

AACCTCTGAAAGTGAGAGCCAAGCTCCCGCAGCAGGAGGGGCCCTATGCTGGCCCCATGGAGAGACAGAA

ACCACTGAAAGTGAAAGCGAAAGCCCCGGTCGTTAAGGAAGGACCTTACGAAGGACCGGTAAAGAAACCT

GTCGCTTTGAAGGTGAAAGCAAAGAACTTGATTGTCACTGAGAGTGGTGCTCCCCCTACTGACTTGCAAA

AGATGGTCATGGGTAACACCAAGCCTGTTGAGCTCGTCCTCGACGGGAAGACGGTGGCCATCTGCTGCGC

CACCGGAGTGTTTGGTACTGCCTACCTTGTTCCTCGTCATCTTTTCGCAGAGAAATATGACAAGATCATG

CTGGACGGCAGGGCCATGACAGACAGTGACTACAGAGTGTTTGAGTTTGAGATTAAAGTGAAAGGACAGG

ACATGCTCTCAGACGCCGCGCTCATGGTGCTTCACCGTGGGAATCGAGTGCGGGACATCACGAAGCACTT

CCGTGATGTGGCAAGAATGAAGAAAGGCACCCCCGTCGTCGGCGTGATCAACAACGCTGATGTTGGGAGG

CTGATCTTCTCTGGTGAGGCCCTTACCTACAAGGACATTGTAGTGTGCATGGACGGAGACACCATGCCCG

GTCTCTTCGCCTACAAAGCCGCCACCAAGGCGGGCTACTGTGGAGGAGCCGTTCTTGCAAAGGACGGAGC

CGAGACTTTCATCGTCGGCACTCACTCCGCAGGCGGCAATGGAGTTGGATACTGCTCATGCGTTTCCAGG

TCTATGCTGCTTAAAATGAAGGCACACATCGATCCCGAACCACACCACGAG

>EU214601.1_O_UKG_2001

ATCTCAATTCCTTCCCAAAAGGCTGTGCTGTACTTTCTCATTGAGAAAGGCCAGCACGAAGCAGCAATTG

AGTTCTTTGAGGGGATGGTGCATGACTCCATCAAGGAGGAGCTCCGGCCTCTCATCCAACAGACCTCATT

TGTGAAGCGCGCTTTTAAGCGCCTGAAGGAAAACTTTGAGGTAGTTGCCCTGTGTTTGACTCTTTTGGCA

AACATAGTGATCATGATCCGCGAGACTCGCAAGAGACAGCAGATGGTGGATGATGCAGTGAACGAGTACA

CTGAGAAGGCAAACATCACCACGGATGACAAGACTCTTGACGAGGCGGAAAAGAACCCTCTGGAGACCAG

CGGTGCCACCACTGTTGGTTTCAGAGAGAAAACTCTCCCGGGACACAAGGCGGGTGATGACGTGAACTCC

GAGCCCGCCAAACCCGTGGAAGAACAACCACAAGCTGAAGGACCCTACACCGGTCCACTCGAGCGTCAAA

AACCCCTGAAAGTGAGGGCCAAGCTCCCACAGCAGGAGGGGCCCTACGCTGGTCCGATGGAGAGACAGAA

ACCGCCGAAAGTGAAAGTGAAAGCCCCGGTCGTTAAGGAAGGACCTTACGAAGGACCGGTGAAGAAACCT

GTCGCTTTGAAAGTGAAAGCAAAGAACTTGATTGTCACTGAGAGTGGTGCTCCCCCGACTGACTTGCAAA

AGATGGTCATGGGTAACACCAAGCCTGTTGAGCTCATCCTCGACGGGAAGACGGTGGCCATCTGCTGCGC

CACCGGAGTGTTTGGTACTGCCTACCTAGTTCCTCGTCATCTTTTCGCAGAGAAGTATGACAAGATCATG

TTGGACGGCAGAGCCATGACAGACAGTGACTACAGAGTGTTTGAGTTTGAGATTAAAGTGAAAGGACAGG

ACATGCTCTCAGACGCCGCTCTCATGGTGCTTCACCGTGGGAATCGCGTGCGGGACATCACGAAGCACTT

CCGTGATGTGGCAAGAATGAAGAAAGGCACCCCCGTCGTCGGCGTGATCAACAACGCTGATGTTGGGAGA

CTGATCTTCTCTGGTGAGGCCCTTACCTACAAGGACATTGTAGTGTGCATGGACGGAGACACCATGCCCG

GTCTCTTCGCCTACAAAGCTGCCACCAAGGCGGGTTACTGTGGAGGAGCCGTTCTTGCAAAGGACGGAGC

CGAGACTTTCATCGTCGGCACTCACTCCGCAGGCGGCAATGGAGTTGGATACTGCTCATGCGTTTCCAGG

TCCATGCTGCTTAAAATGAAGGCACACATCGATCCCGAACCACACCACGAG

>EU400597.1_O_CHA_2001

ATTTCAATCCCTTCCCAGAAGTCTGTGCTGTACTTCCTCATTGAGAAGGGCCAGCACGAAGCAGCAATCG

AGTTCTTCGAGGGTATGGTCCACGACTCCATCAAGGAGGAGCTCCGGCCCCTCATCCAACAGACCTCGTT

TGTGAAACGCGCCTTCAAGCGCCTGAAGGAGAACTTTGAGATCGTTGCTCTGTGTTTAACCCTCTTGGCA

AACATAGTAATTATGCTCCGCCAAGCGCGCAAGAGACGCCAGTCGGTGGATGACTCACTGGAC-------

-----------------------AGCGACATAACTCTTGGCGACGCGGAAAAGAACCCTCTGGAGACGAG

TGGCGCTAGCGCTGTCGGTTTCAGAGAGAGGCCACCCACCGAACAAGAGACGTGCGAAGACGCGAACGCT

GAGCCCGTCGTGTTCGGGAGGGAACAACCGCGAGCTGAAGGACCCTACGCTGGGCCACTTGAGCGTCAGA

AACCTCTTAAAGTGAAAGCCAGGCTGCCACAACAGGAGGGACCCTACGCCGGCCCAATGGAGAGACAGAA

ACCGCTAAAGGTGAAAGCAAAAGCCCCCGTCGTAAAGGAAGGACCTTACGAGGGACCGGTGAAGAAACCT

GTCGCTTTGAAAGTGAAAGCAAAGAACTTGATAGTCACTGAGAGTGGTGCGCCGCCGACCGACTTGCAAA

AGATGGTCATGGGCAACACTAAGCCAGTCGAGCTTATCCTCGACGGCAAGACGGTGGCCATCTGTTGTGC

CACCGGAGTGTTCGGCACTGCCTACCTCGTGCCTCGTCACCTCTTCGCGGAGAAGTATGACAAGATCATG

TTGGACGGTAGAGCCTTGACAGACAGCGACTACAGAGTGTTCGAGTTTGAGATTAAAGTAAAAGGACAGG

ACATGCTCTCAGACGCCGCTCTCATGGTGTTGCACCGTGGGAACCGCGTGCGCGATATCACGAAGCACTT

CCGTGATGTGGCGAGAATGAAGAAGGGAACCCCCGTCGTCGGTGCGATCAATAATGCCGACGTAGGGAGA

CTCATATTCTCTGGTGAAGCCCTTACTTATAAGGACATTGTCGTGTGTATGGACGGAGACACTATGCCTG

GGCTTTTCGCCTACAGAGCATCCACCAAGGCAGGCTACTGTGGAGGAGCCGTCCTAGCAAAGGACGGTGC

TGAGACATTCATCGTTGGCACCCACTCCGCAGGTGGTAACGGTATAGGATACTGTTCATGCGTCTCCCGA

TCGATGCTCATGAAGATGAAGGCACACATCGACCCTGAACCACACCACGAG

>EU448368.1_O_UKG_1967

ATCTCAATTCCTTCCCAAAAATCTGTGTTGTACTTTCTCATTGAGAAGGGCCAACATGAGGCAGCAATTG

AATTCTTTGAGGGCATGGTCCACGACTCTATTAAAGAGGAACTCCAACCCCTCATCCAACAAACTTCATT

TGTGAAACGCGCTTTCAAGCGCCTGAAGGAAAATTTTGAGATTGTTGCTCTGTGTTTAACACTTTTGGCA

AACATTGTGATCATGGTCCGCGAGACTCGCAAGAGGCAGAAAATGGTGGATGATGCAGTGAATGAGTACA

TTGAGAAAGCAAACATCACCACAGATGACAAGACTCTTGACGAGGCGGAGAAGAGCCCTCTAGAGACCAG

CGGCGCCAGCACCGTTGGCTTTAGAGAGAGAACTCTCCCAGGTCAAAAGGCATGCGATGACGTGAACTCC

GAGCCTGCCCAACCTGTTGAGGAGCAACCACAAGCTGAAGGACCCTACGCCGGACCACTCGAGCGTCAGA

AACCTCTGAAAGTGAGAGCCAAGCTCCCACAGCAGGAGGGGCCTTACGCTGGTCCAATGGAGAGACAGAA

ACCGCTAAAAGTGAAAGCAAAAGCCCCGGTCGTGAAGGAAGGACCTTACGAGGGACCGGTGAAGAAGCCT

GTCGCTTTGAAAGTGAAAGCTAAGAACCTGATTGTCACTGAGAGTGGTGCCCCACCGACCGACTTGCAAA

AGATGGTCATGGGCAACACAAAGCCTGTTGAGCTCATCCTCGACGGGAAGACAGTAGCCATTTGCTGCGC

TACTGGAGTGTTTGGCACTGCTTACCTCGTGCCTCGTCACCTCTTCGCAGAGAAGTATGACAAGATCATG

TTGGACGGCAGAGCCATGACAGACAGTGACTACAGAGTGTTTGAGTTTGAGATCAAAGTAAAAGGACAGG

ACATGCTCTCAGACGCCGCGCTCATGGTGCTCCACCGTGGGAACCGCGTGAGGGACATCACGAAGCACTT

TCGTGACACAGCAAGAATGAAGAAAGGCACCCCCGTTGTCGGTGTGATTAACAACGCCGATGTCGGGAGA

CTGATTTTCTCCGGTGAGGCCCTTACTTACAAGGACATTGTGGTTTGTATGGACGGAGACACCATGCCCG

GCCTCTTTGCCTACAGAGCCGCCACCAAGGCTGGCTACTGCGGAGGAGCCGTTCTCGCCAAAGACGGAGC

TGACACTTTCATCGTCGGCACTCACTCTGCAGGAGGCAACGGAGTTGGATACTGCTCATGCGTTTCCAGG

TCCATGCTTCTTAAAATGAAGGCACACATTGACCCCGAACCACATCACGAG

>EU448369.1_O_UKG_1967

ATCTCAATTCCTTCCCAAAAATCTGTGTTGTACTTTCTCATTGAGAAGGGCCAACATGAGGCAGCAATTG

AATTCTTTGAGGGCATGGTCCACGACTCTATTAAAGAGGAACTCCAACCCCTCCTCCAACAAACTTCATT

TGTGAAACGCGCTTTCAAGCGCCTGAAGGAAAATTTTGAGATTGTTGCTCTGTGTTTAACACTTTTGGCA

AACATTGTGATCATGGTCCGTGAGACTCGCAAGAGGCAGAAAATGGTGGATGATGCAGTGAATGAGTACA

TTGAGAAAGCAAACATCACCACAGATGACAAGACTCTTGACGAGGCGGAGAAGAGCCCTCTAGAGACCAG

CGGCGCCAGCACCGTTGGCTTTAGAGAGAGAACTCTCCCAGGTCAAAAGGCATGCGATGACGTGAACTCC

GAGCCTGCCCAACCTGTTGAGGAGCAACCACAAGCTGAAGGACCCTACGCCGGACCACTCGAGCGTCAGA

AACCTCTGAAAGTGAGAGCCAAGCTCCCACAGCAGGAGGGGCCTTACGCTGGTCCAATGGAGAGACAGAA

ACCGCTAAAAGTGAAAGCAAAAGCCCCGGTCGTGAAGGAAGGACCTTACGAGGGACCGGTGAAGAAGCCT

GTCGCTTTGAAAGTGAAAGCTAAGAACCTGATTGTCACTGAGAGTGGTGCCCCACCGACCGACTTGCAAA

AGATGGTCATGGGCAACACAAAGCCTGTTGAGCTCATCCTCGACGGGAAGACAGTAGCCATTTGCTGCGC

TACTGGAGTGTTTGGCACTGCTTACCTCGTGCCTCGTCACCTCTTCGCAGAGAAGTATGACAAGATCATG

TTGGACGGCAGAGCCATGACAGACAGTGACTACAGAGTGTTTGAGTTTGAGATCAAAGTAAAAGGACAGG

ACATGCTCTCAGACGCCGCGCTCATGGTGCTCCACCGTGGGAACCGCGTGAGGGACATCACGAAGCACTT

TCGTGACACAGCAAGAATGAAGAAAGGCACCCCCGTTGTCGGTGTGATTAACAACGCCGATGTCGGGAGA

CTGATTTTCTCCGGTGAGGCCCTTACTTACAAGGACATTGTGGTTTGTATGGACGGAGACACCATGCCCG

GCCTCTTTGCCTACAGAGCCGCCACCAAGGCTGGCTACTGCGGAGGAGCCGTTCTCGCCAAAGACGGAGC

TGACACTTTCATCGTCGGCACTCACTCTGCAGGAGGCAACGGAGTTGGATACTGCTCATGCGTTTCCAGG

TCCATGCTTCTTAAAATGAAGGCACACATTGACCCCGAACCACATCACGAG

>EU448370.1_O_UKG_1967

ATCTCAATTCCTTCCCAAAAATCTGTGTTGTACTTTCTCATTGAGAAGGGCCAACATGAGGCAGCAATTG

AATTCTTTGAGGGCATGGTCCACGACTCTATTAAAGAGGAACTCCAACCCCTCCTCCAACAAACTTCATT

TGTGAAACGCGCTTTCAAGCGCCTGAAGGAAAATTTTGAGATTGTTGCTCTGTGTTTAACACTTTTGGCA

AACATTGTGATCATGGTCCGTGAGACTCGCAAGAGGCAGAAAATGGTGGATGATGCAGTGAATGAGTACA

TTGAGAAAGCAAACATCACCACAGATGACAAGACTCTTGACGAGGCGGAGAAGAGCCCTCTAGAGACCAG

CGGCGCCAGCACCGTTGGCTTTAGAGAGAGAACTCTCCCAGGTCAAAAGGCATGCGATGACGTGAACTCC

GAGCCTGCCCAACCTGTTGAGGAGCAACCACAAGCTGAAGGACCCTACGCCGGACCACTCGAGCGTCAGA

AACCTCTGAAAGTGAGAGCCAAGCTCCCACAGCAGGAGGGGCCTTACGCTGGTCCAATGGAGAGACAGAA

ACCGCTAAAAGTGAAAGCAAAAGCCCCGGTCGTGAAGGAAGGACCTTACGAGGGACCGGTGAAGAAGCCT

GTCGCTTTGAAAGTGAAAGCTAAGAACCTGATTGTCACTGAGAGTGGTGCCCCACCGACCGACTTGCAAA

AGATGGTCATGGGCAACACAAAGCCTGTTGAGCTCATCCTCGACGGGAAGACAGTAGCCATTTGCTGCGC

TACTGGAGTGTTTGGCACTGCTTACCTCGTGCCTCGTCACCTCTTCGCAGAGAAGTATGACAAGATCATG

TTGGACGGCAGAGCCATGACAGACAGTGACTACAGAGTGTTTGAGTTTGAGATCAAAGTAAAAGGACAGG

ACATGCTCTCAGACGCCGCGCTCATGGTGCTCCACCGTGGGAACCGCGTGAGGGACATCACGAAGCACTT

TCGTGACACAGCAAGAATGAAGAAAGGCACCCCCGTTGTCGGTGTGATTAACAACGCCGATGTCGGGAGA

CTGATTTTCTCCGGTGAGGCCCTTACTTACAAGGACATTGTGGTTTGTATGGACGGAGACACCATGCCCG

GCCTCTTTGCCTACAGAGCCGCCACCAAGGCTGGCTACTGCGGAGGAGCCGTTCTCGCCAAAGACGGAGC

TGACACTTTCATCGTCGGCACTCACTCTGCAGGAGGCAACGGAGTTGGATACTGCTCATGCGTTTCCAGG

TCCATGCTTCTTAAAATGAAGGCACACATTGACCCCGAACCACATCACGAG

>EU448371.1_O_UKG_2007

ATCTCAATTCCTTCCCAAAAATCTGTGTTGTACTTTCTCATTGAGAAGGGCCAACATGAGGCAGCAATTG

AATTCTTTGAGGGCATGGTCCACGACTCTATTAAAGAGGAACTCCAACCCCTCCTCCAACAAACTTCATT

TGTGAAACGCGCTTTCAAGCGCCTGAAGGAAAATTTTGAGATTGTTGCTCTGTGTTTAACACTTTTGGCA

AACATTGTGATCATGGTCCGTGAGACTCGCAAGAGGCAGAAAATGGTGGATGATGCAGTGAATGAGTACA

TTGAGAAAGCAAACATCACCACAGATGACAAGACTCTTGACGAGGCGGAGAAGAGCCCTCTAGAGACCAG

CGGCGCCAGCACCGTTGGCTTTAGAGAGAGAACTCTCCCAGGTCAAAAGGCATGCGATGACGTGAACTCC

GAGCCTGCCCAACCTGTTGAGGAGCAACCACAAGCTGAAGGACCCTACGCCGGACCACTCGAGCGTCAGA

AACCTCTGAAAGTGAGAGCCAAGCTCCCACAGCAGGAGGGGCCTTACGCTGGTCCAATGGAGAGACAGAA

ACCGCTAAAAGTGAAAGCAAAAGCCCCGGTCGTGAAGGAAGGACCTTACGAGGGACCGGTGAAGAAGCCT

GTCGCTTTGAAAGTGAAAGCTAAGAACCTGATTGTCACTGAGAGTGGTGCCCCACCGACCGACTTGCAAA

AGATGGTCATGGGCAACACAAAGCCTGTTGAGCTCATCCTCGACGGGAAGACAGTAGCCATTTGCTGCGC

TACTGGAGTGTTTGGCACTGCTTACCTCGTGCCTCGTCACCTCTTCGCAGAGAAGTATGACAAGATCATG

TTGGACGGCAGAGCCATGACAGACAGTGACTACAGAGTGTTTGAGTTTGAGATCAAAGTAAAAGGACAGG

ACATGCTCTCAGACGCCGCGCTCATGGTGCTCCACCGTGGGAACCGCGTGAGGGACATCACGAAGCACTT

TCGTGACACAGCAAGAATGAAGAAAGGCACCCCCGTTGTCGGTGTGATTAACAACGCCGATGTCGGGAGA

CTGATTTTCTCCGGTGAGGCCCTTACTTACAAGGACATTGTGGTTTGTATGGACGGAGACACCATGCCCG

GCCTCTTTGCCTACAGAGCCGCCACCAAGGCTGGCTACTGCGGAGGAGCCGTTCTCGCCAAAGACGGAGC

TGACACTTTCATCGTCGGCACTCACTCTGCAGGAGGCAACGGAGTTGGATACTGCTCATGCGTTTCCAGG

TCCATGCTTCTTAAAATGAAGGCACACATTGACCCCGAACCACATCACGAG

>EU448372.1_O_UKG_2007

ATCTCAATTCCTTCCCAAAAATCTGTGTTGTACTTTCTCATTGAGAAGGGCCAACATGAGGCAGCAATTG

AATTCTTTGAGGGCATGGTCCACGACTCTATTAAAGAGGAACTCCAACCCCTCCTCCAACAAACTTCATT

TGTGAAACGCGCTTTCAAGCGCCTGAAGGAAAATTTTGAGATTGTTGCTCTGTGTTTAACACTTTTGGCA

AACATTGTGATCATGGTCCGTGAGACTCGCAAGAGGCAGAAAATGGTGGATGATGCAGTGAATGAGTACA

TTGAGAAAGCAAACATCACCACAGATGACAAGACTCTTGACGAGGCGGAGAAGAGCCCTCTAGAGACCAG

CGGCGCCAGCACCGTTGGCTTTAGAGAGAGAACTCTCCCAGGTCAAAAGGCATGCGATGACGTGAACTCC

GAGCCTGCCCAACCTGTTGAGGAGCAACCACAAGCTGAAGGACCCTACGCCGGACCACTCGAGCGTCAGA

AACCTCTGAAAGTGAGAGCCAAGCTCCCACAGCAGGAGGGGCCTTACGCTGGTCCAATGGAGAGACAGAA

ACCGCTAAAAGTGAAAGCAAAAGCCCCGGTCGTGAAGGAAGGACCTTACGAGGGACCGGTGAAGAAGCCT

GTCGCTTTGAAAGTGAAAGCTAAGAACCTGATTGTCACTGAGAGTGGTGCCCCACCGACCGACTTGCAAA

AGATGGTCATGGGCAACACAAAGCCTGTTGAGCTCATCCTCGACGGGAAGACAGTAGCCATTTGCTGCGC

TACTGGAGTGTTTGGCACTGCTTACCTCGTGCCTCGTCACCTCTTCGCAGAGAAGTATGACAAGATCATG

TTGGACGGCAGAGCCATGACAGACAGTGACTACAGAGTGTTTGAGTTTGAGATCAAAGTAAAAGGACAGG

ACATGCTCTCAGACGCCGCGCTCATGGTGCTCCACCGTGGGAACCGCGTGAGGGACATCACGAAGCACTT

TCGTGACACAGCAAGAATGAAGAAAGGCACCCCCGTTGTCGGTGTGATTAACAACGCCGATGTCGGGAGA

CTGATTTTCTCCGGTGAGGCCCTTACTTACAAGGACATTGTGGTTTGTATGGACGGAGACACCATGCCCG

GCCTCTTTGCCTACAGAGCCGCCACCAAGGCTGGCTACTGCGGAGGAGCCGTTCTCGCCAAAGACGGAGC

TGACACTTTCATCGTCGGCACTCACTCTGCAGGAGGCAACGGAGTTGGATACTGCTCATGCGTTTCCAGG

TCCATGCTTCTTAAAATGAAGGCACACATTGACCCCGAACCACATCACGAG

>EU448373.1_O_UKG_2007

ATCTCAATTCCTTCCCAAAAATCTGTGTTGTACTTTCTCATTGAGAAGGGCCAACATGAGGCAGCAATTG

AATTCTTTGAGGGCATGGTCCACGACTCTATTAAAGAGGAACTCCAACCCCTCCTCCAACAAACTTCATT

TGTGAAACGCGCTTTCAAGCGCCTGAAGGAAAATTTTGAGATTGTTGCTCTGTGTTTAACACTTTTGGCA

AACATTGTGATCATGGTCCGTGAGACTCGCAAGAGGCAGAAAATGGTGGATGATGCAGTGAATGAGTACA

TTGAGAAAGCAAACATCACCACAGATGACAAGACTCTTGACGAGGCGGAGAAGAGCCCTCTAGAGACCAG

CGGCGCCAGCACCGTTGGCTTTAGAGAGAGAACTCTCCCAGGTCAAAAGGCATGCGATGACGTGAACTCC

GAGCCTGCCCAACCTGTTGAGGAGCAACCACAAGCTGAAGGACCCTACGCCGGACCACTCGAGCGTCAGA

AACCTCTGAAAGTGAGAGCCAAGCTCCCACAGCAGGAGGGGCCTTACGCTGGTCCAATGGAGAGACAGAA

ACCGCTAAAAGTGAAAGCAAAAGCCCCGGTCGTGAAGGAAGGACCTTACGAGGGACCGGTGAAGAAGCCT

GTCGCTTTGAAAGTGAAAGCTAAGAACCTGATTGTCACTGAGAGTGGTGCCCCACCGACCGACTTGCAAA

AGATGGTCATGGGCAACACAAAGCCTGTTGAGCTCATCCTCGACGGGAAGACAGTAGCCATTTGCTGCGC

TACTGGAGTGTTTGGCACTGCTTACCTCGTGCCTCGTCACCTCTTCGCAGAGAAGTATGACAAGATCATG

TTGGACGGCAGAGCCATGACAGACAGTGACTACAGAGTGTTTGAGTTTGAGATCAAAGTAAAAGGACAGG

ACATGCTCTCAGACGCCGCGCTCATGGTGCTCCACCGTGGGAACCGCGTGAGGGACATCACGAAGCACTT

TCGTGACACAGCAAGAATGAAGAAAGGCACCCCCGTTGTCGGTGTGATTAACAACGCCGATGTCGGGAGA

CTGATTTTCTCCGGTGAGGCCCTTACTTACAAGGACATTGTGGTTTGTATGGACGGAGACACCATGCCCG

GCCTCTTTGCCTACAGAGCCGCCACCAAGGCTGGCTACTGCGGAGGAGCCGTTCTCGCCAAAGACGGAGC

TGACACTTTCATCGTCGGCACTCACTCTGCAGGAGGCAACGGAGTTGGATACTGCTCATGCGTTTCCAGG

TCCATGCTTCTTAAAATGAAGGCACACATTGACCCCGAACCACATCACGAG

>EU448374.1_O_UKG_2007

ATCTCAATTCCTTCCCAAAAATCTGTGTTGTACTTTCTCATTGAGAAGGGCCAACATGAGGCAGCAATTG

AATTCTTTGAGGGCATGGTCCACGACTCTATTAAAGAGGAACTCCAACCCCTCCTCCAACAAACTTCATT

TGTGAAACGCGCTTTCAAGCGCCTGAAGGAAAATTTTGAGATTGTTGCTCTGTGTTTAACACTTTTGGCA

AACATTGTGATCATGGTCCGTGAGACTCGCAAGAGGCAGAAAATGGTGGATGATGCAGTGAATGAGTACA

TTGAGAAAGCAAACATCACCACAGATGACAAGACTCTTGACGAGGCGGAGAAGAGCCCTCTAGAGACCAG

CGGCGCCAGCACCGTTGGCTTTAGAGAGAGAACTCTCCCAGGTCAAAAGGCATGCGATGACGTGAACTCC

GAGCCTGCCCAACCTGTTGAGGAGCAACCACAAGCTGAAGGACCCTACGCCGGACCACTCGAGCGTCAGA

AACCTCTGAAAGTGAGAGCCAAGCTCCCACAGCAGGAGGGGCCTTACGCTGGTCCAATGGAGAGACAGAA

ACCGCTAAAAGTGAAAGCAAAAGCCCCGGTCGTGAAGGAAGGACCTTACGAGGGACCGGTGAAGAAGCCT

GTCGCTTTGAAAGTGAAAGCTAAGAACCTGATTGTCACTGAGAGTGGTGCCCCACCGACCGACTTGCAAA

AGATGGTCATGGGCAACACAAAGCCTGTTGAGCTCATCCTCGACGGGAAGACAGTAGCCATTTGCTGCGC

TACTGGAGTGTTTGGCACTGCTTACCTCGTGCCTCGTCACCTCTTCGCAGAGAAGTATGACAAGATCATG

TTGGACGGCAGAGCCATGACAGACAGTGACTACAGAGTGTTTGAGTTTGAGATCAAAGTAAAAGGACAGG

ACATGCTCTCAGACGCCGCGCTCATGGTGCTCCACCGTGGGAACCGCGTGAGGGACATCACGAAGCACTT

TCGTGACACAGCAAGAATGAAGAAAGGCACCCCCGTTGTCGGTGTGATTAACAACGCCGATGTCGGGAGA

CTGATTTTCTCCGGTGAGGCCCTTACTTACAAGGACATTGTGGTTTGTATGGACGGAGACACCATGCCCG

GCCTCTTTGCCTACAGAGCCGCCACCAAGGCTGGCTACTGCGGAGGAGCCGTTCTCGCCAAAGACGGAGC

TGACACTTTCATCGTCGGCACTCACTCTGCAGGAGGCAACGGAGTTGGATACTGCTCATGCGTTTCCAGG

TCCATGCTTCTTAAAATGAAGGCACACATTGACCCCGAACCACATCACGAG

>EU448375.1_O_UKG_2007

ATCTCAATTCCTTCCCAAAAATCTGTGTTGTACTTTCTCATTGAGAAGGGCCAACATGAGGCAGCAATTG

AATTCTTTGAGGGCATGGTCCACGACTCTATTAAGGAGGAACTCCAACCCCTCCTCCAACAAACTTCATT

TGTGAAACGCGCTTTCAAGCGCCTGAAGGAAAATTTTGAGATTGTTGCTCTGTGTTTAACACTTTTGGCA

AACATTGTGATCATGGTCCGTGAGACTCGCAAGAGGCAGAAAATGGTGGATGATGCAGTGAATGAGTACA

TTGAGAAAGCAAACATCACCACAGATGACAAGACTCTTGACGAGGCGGAGAAGAGCCCTCTAGAGACCAG

CGGCGCCAGCACCGTTGGCTTTAGAGAGAGAACTCTCCCAGGTCAAAAGGCATGCGATGACGTGAACTCC

GAGCCTGCCCAACCTGTTGAGGAGCAACCACAAGCTGAAGGACCCTACGCCGGACCACTCGAGCGTCAGA

AACCTCTGAAAGTGAGAGCCAAGCTCCCACAGCAGGAGGGGCCTTACGCTGGTCCAATGGAGAGACAGAA

ACCGCTAAAAGTGAAAGCAAAAGCCCCGGTCGTGAAGGAAGGACCTTACGAGGGACCGGTGAAGAAGCCT

GTCGCTTTGAAAGTGAAAGCTAAGAACCTGATTGTCACTGAGAGTGGTGCCCCACCGACCGACTTGCAAA

AGATGGTCATGGGCAACACAAAGCCTGTTGAGCTCATCCTCGACGGGAAGACAGTAGCCATTTGCTGCGC

TACTGGAGTGTTTGGCACTGCTTACCTCGTGCCTCGCCACCTCTTCGCAGAGAAGTATGACAAGATCATG

TTGGACGGCAGAGCCATGACAGACAGTGACTACAGAGTGTTTGAGTTTGAGATCAAAGTAAAAGGACAGG

ACATGCTCTCAGACGCCGCGCTCATGGTGCTCCACCGTGGGAACCGCGTGAGGGACATCACGAAGCACTT

TCGTGACACAGCAAGAATGAAGAAAGGCACCCCCGTTGTCGGTGTGATTAACAACGCCGATGTCGGGAGA

CTGATTTTCTCCGGTGAGGCCCTTACTTACAAGGACATTGTGGTTTGTATGGACGGAGACACCATGCCCG

GCCTCTTTGCCTACAGAGCCGCCACCAAGGCTGGCTACTGCGGAGGAGCCGTTCTCGCCAAAGACGGAGC

TGACACTTTCATCGTCGGCACTCACTCTGCAGGAGGCAACGGAGTTGGATACTGCTCATGCGTTTCCAGG

TCCATGCTTCTTAAAATGAAGGCACACATTGACCCCGAACCACACCACGAG

>EU448376.1_O_UKG_2007

ATCTCAATTCCTTCCCAAAAATCTGTGTTGTACTTTCTCATTGAGAAGGGCCAACATGAGGCAGCAATTG

AATTCTTTGAGGGCATGGTCCACGACTCTATTAAGGAGGAACTCCAACCCCTCCTCCAACAAACTTCATT

TGTGAAACGCGCTTTCAAGCGCCTGAAGGAAAATTTTGAGATTGTTGCTCTGTGTTTAACACTTTTGGCA

AACATTGTGATCATGGTCCGTGAGACTCGCAAGAGGCAGAAAATGGTGGATGATGCAGTGAATGAGTACA

TTGAGAAAGCAAACATCACCACAGATGACAAGACTCTTGACGAGGCGGAGAAGAGCCCTCTAGAGACCAG

CGGCGCCAGCACCGTTGGCTTTAGAGAGAGAACTCTCCCAGGTCAAAAGGCATGCGATGACGTGAACTCC

GAGCCTGCCCAACCTGTTGAGGAGCAACCACAAGCTGAAGGACCCTACGCCGGACCACTCGAGCGTCAGA

AACCTCTGAAAGTGAGAGCCAAGCTCCCACAGCAGGAGGGGCCTTACGCTGGTCCAATGGAGAGACAGAA

ACCGCTAAAAGTGAAAGCAAAAGCCCCGGTCGTGAAGGAAGGACCTTACGAGGGACCGGTGAAGAAGCCT

GTCGCTTTGAAAGTGAAAGCTAAGAACCTGATTGTCACTGAGAGTGGTGCCCCACCGACCGACTTGCAAA

AGATGGTCATGGGCAACACAAAGCCTGTTGAGCTCATCCTCGACGGGAAGACAGTAGCCATTTGCTGCGC

TACTGGAGTGTTTGGCACTGCTTACCTCGTGCCTCGTCACCTCTTCGCAGAGAAGTATGACAAGATCATG

TTGGACGGCAGAGCCATGACAGACAGTGACTACAGAGTGTTTGAGTTTGAGATCAAAGTAAAAGGACAGG

ACATGCTCTCAGACGCCGCGCTCATGGTGCTCCACCGTGGGAACCGCGTGAGGGACATCACGAAGCACTT

TCGTGACACAGCAAGAATGAAGAAAGGCACCCCCGTTGTCGGTGTGATTAACAACGCCGATGTCGGGAGA

CTGATTTTCTCCGGTGAGGCCCTTACTTACAAGGACATTGTGGTTTGTATGGACGGAGACACCATGCCCG

GCCTCTTTGCCTACAGAGCCGCCACCAAGGCTGGCTACTGCGGAGGAGCCGTTCTCGCCAAAGACGGAGC

TGACACTTTCATCGTCGGCACTCACTCTGCAGGAGGCAACGGAGTTGGATACTGCTCATGCGTTTCCAGG

TCCATGCTTCTTAAAATGAAGGCACACATTGACCCCGAACCACACCACGAG

>EU448377.1_O_UKG_2007

ATCTCAATTCCTTCCCAAAAATCTGTGTTGTACTTTCTCATTGAGAAGGGCCAACATGAGGCAGCAATTG

AATTCTTTGAGGGCATGGTCCACGACTCTATTAAGGAGGAACTCCAACCCCTCCTCCAACAAACTTCATT

TGTGAAACGCGCTTTCAAGCGCCTGAAGGAAAATTTTGAGATTGTTGCTCTGTGTTTAACACTTTTGGCA

AACATTGTGATCATGGTCCGTGAGACTCGCAAGAGGCAGAAAATGGTGGATGATGCAGTGAATGAGTACA

TTGAGAAAGCAAACATCACCACAGATGACAAGACTCTTGACGAGGCGGAGAAGAGCCCTCTAGAGACCAG

CGGCGCCAGCACCGTTGGCTTTAGAGAGAGAACTCTCCCAGGTCAAAAGGCATGCGATGACGTGAACTCC

GAGCCTGCCCAACCTGTTGAGGAGCAACCACAAGCTGAAGGACCCTACGCCGGACCACTCGAGCGTCAGA

AACCTCTGAAAGTGAGAGCCAAGCTCCCACAGCAGGAGGGGCCTTACGCTGGTCCAATGGAGAGACAGAA

ACCGCTAAAAGTGAAAGCAAAAGCCCCGGTCGTGAAGGAAGGACCTTACGAGGGACCGGTGAAGAAGCCT

GTCGCTTTGAAAGTGAAAGCTAAGAACCTGATTGTCACTGAGAGTGGTGCCCCACCGACCGACTTGCAAA

AGATGGTCATGGGCAACACAAAGCCTGTTGAGCTCATCCTCGACGGGAAGACAGTAGCCATTTGCTGCGC

TACTGGAGTGTTTGGCACTGCTTACCTCGTGCCTCGTCACCTCTTCGCAGAGAAGTATGACAAGATCATG

TTGGACGGCAGAGCCATGACAGACAGTGACTACAGAGTGTTTGAGTTTGAGATCAAAGTAAAAGGACAGG

ACATGCTCTCAGACGCCGCGCTCATGGTGCTCCACCGTGGGAACCGCGTGAGGGACATCACGAAGCACTT

TCGTGACACAGCAAGAATGAAGAAAGGCACCCCCGTTGTCGGTGTGATTAACAACGCCGATGTCGGGAGA

CTGATTTTCTCCGGTGAGGCCCTTACTTACAAGGACATTGTGGTTTGTATGGACGGAGACACCATGCCCG

GCCTCTTTGCCTACAGAGCCGCCACCAAGGCTGGCTACTGCGGAGGAGCCGTTCTCGCCAAAGACGGAGC

TGACACTTTCATCGTCGGCACTCACTCTGCAGGAGGCAACGGAGTTGGATACTGCTCATGCGTTTCCAGG

TCCATGCTTCTTAAAATGAAGGCACACATTGACCCCGAACCACACCACGAG

>EU448378.1_O_UKG_2007

ATCTCAATTCCTTCCCAAAAATCTGTGTTGTACTTTCTCATTGAGAAGGGCCAACATGAGGCAGCAATTG

AATTCTTTGAGGGCATGGTCCACGACTCTATTAAGGAGGAACTCCAACCCCTCCTCCAACAAACTTCATT

TGTGAAACGCGCTTTCAAGCGCCTGAAGGAAAATTTTGAGATTGTTGCTCTGTGTTTAACACTTTTGGCA

AACATTGTGATCATGGTCCGTGAGACTCGCAAGAGGCAGAAAATGGTGGATGATGCAGTGAATGAGTACA

TTGAGAAAGCAAACATCACCACAGATGACAAGACTCTTGACGAGGCGGAGAAGAGCCCTCTAGAGACCAG

CGGCGCCAGCACCGTTGGCTTTAGAGAGAGAACTCTCCCAGGTCAAAAGGCATGCGATGACGTGAACTCC

GAGCCTGCCCAACCTGTTGAGGAGCAACCACAAGCTGAAGGACCCTACGCCGGACCACTCGAGCGTCAGA

AACCTCTGAAAGTGAGAGCCAAGCTCCCACAGCAGGAGGGGCCTTACGCTGGTCCAATGGAGAGACAGAA

ACCGCTAAAAGTGAAAGCAAAAGCCCCGGTCGTGAAGGAAGGACCTTACGAGGGACCGGTGAAGAAGCCT

GTCGCTTTGAAAGTGAAAGCTAAGAACCTGATTGTCACTGAGAGTGGTGCCCCACCGACCGACTTGCAAA

AGATGGTCATGGGCAACACAAAGCCTGTTGAGCTCATCCTCGACGGGAAGACAGTAGCCATTTGCTGCGC

TACTGGAGTGTTTGGCACTGCTTACCTCGTGCCTCGTCACCTCTTCGCAGAGAAGTATGACAAGATCATG

TTGGACGGCAGAGCCATGACAGACAGTGACTACAGAGTGTTTGAGTTTGAGATCAAAGTAAAAGGACAGG

ACATGCTCTCAGACGCCGCGCTCATGGTGCTCCACCGTGGGAACCGCGTGAGGGACATCACGAAGCACTT

TCGTGACACAGCAAGAATGAAGAAAGGCACCCCCGTTGTCGGTGTGATTAACAACGCCGATGTCGGGAGA

CTGATTTTCTCCGGTGAGGCCCTTACTTACAAGGACATTGTGGTTTGTATGGACGGAGACACCATGCCCG

GCCTCTTTGCCTACAGAGCCGCCACCAAGGCTGGCTACTGCGGAGGAGCCGTTCTCGCCAAAGACGGAGC

TGACACTTTCATCGTCGGCACTCACTCTGCAGGAGGCAACGGAGTTGGATACTGCTCATGCGTTTCCAGG

TCCATGCTTCTTAAAATGAAGGCACACATTGACCCCGAACCACATCACGAG

>FJ175661.1_O_ISR_2007

GTCTCAATTCCTTCCCAAAAATCCGTGTTGTACTTCCTCATTGAGAAAGGCCAGCACGAGGCTGCAATCG

AATTCTTTGAGGGGATGGTGCATGACTCCATCAAGGAGGAGCTCCGACCCCTCATCCAACAGACATCATT

TGTGAAACGCGCTTTCAAGCGCCTGAAGGAGAACTTTGAGATTGTTGCCCTGTGTTTGACTCTTCTGGCA

AACATAGTGATCATGATTCGCGAAACTCGCAAGAGACAGCAGATGGTGGATGATGCAGTGAGCGAGTACA

TCGAGAAAGCTAACATCACCACGGATGACAAGACTCTTGACGAGGCGGAAAAGAACCCTCTGGAGACCAG

TGGTACCATCGCCGTTGGTTTCAGAGAGAGAGCTCTTCCGGGACACAAGGTGAGTGATGACGTGAACTCC

GAGCCCGCCAAACCTGTGGAAGAGCAACCACAAGCTGAAGGACCCTACGCCGGGCCACTTGAGCGTCAGA

AACCTCTGAAAGTGAGAGCCAAGCTCCCGCAGCAGGAGGGACCTTACGCTGGTCTGATGGAGAGACAGAA

ACCACTGAAAGTAAAAGCAAAAGCCCCGGTCGTTAAGGAAGGACCTTACGAGGGGCCGGTGAAGAAGCCT

GTCGCTTTGAAAGTGAAAGCTAGGAACTTGATTGTCACTGAGAGTGGTGCCCCCCCGACCGACTTGCAAA

AGATGGTCATGGGCAACACTAAGCCTGTTGAGCTCATCCTCGACGGGAAGACGGTGGCCATTTGCTGTGC

TACCGGAGTGTTTGGCACCGCCTACCTCGTGCCTCGTCATCTCTTCGCTGAGAAGTACGACAAGATCATG

TTGGACGGTAGGGCCATGACAGACAGTGACTACAGAGTGTTTGAGTTTGAGGTTAAAGTAAAAGGACAGG

ACATGCTCTCAGACGCCGCGCTCATGGTGCTCCACCGTGGGAACCGCGTGAGAGACATCACGAAACACTT

TCGTGATACAGCAAGAATGAAGAAAGGCACCCCCGTTGTCGGCGTGATTAACAACGCTGATGTCGGGAGA

CTGATTTTCTCTGGTGAGGCCCTCACCTACAAGGACATAGTAGTGTGCATGGATGGAGACACCATGCCTG

GCCTCTTTGCCTACAAAGCCGCCACCAAGGCTGGTTACTGTGGAGGAGCTGTTCTCGCAAAGGACGGAGC

CGAGACTTTCATCCTCGGCACTCACTCTGCAGGGGGCAATGGAGTTGGATACTGCTCATGCGTTTCCAGG

TCCATGCTTCTGAAAATGAAGGCACACATCGACCCTGAACCACATCACGAG

>FJ175662.1_O_ISR_2007

ATCTCAATTCCTTCCCAAAAATCCGTGTTGTACTTCCTCATTGAGAAAGGCCAGCACGAGGCTGCAATCG

AATTCTTTGAGGGGATGGTGCATGACTCCATCAAGGAGGAGCTCCGACCCCTCATCCAACAGACATCATT

TGTGAAACGCGCTTTCAAGCGCCTGAAGGAGAACTTTGAGATTGTTGCCCTGTGTTTGACTCTTCTGGCA

AACATAGTGATCATGATTCGCGAAACTCGCAAGAGACAGCAGATGGTGGATGATGCAGTGAGCGAGTACA

TCGAGAAAGCTAACATCACCACGGATGACAAGACTCTTGACGAGGCGGAAAAGAACCCTCTGGAGACCAG

TGGTACCAGTGCCGTTGGTTTCAGAGAGAGAGCTCTTCCGGGACACAAGGTGAGTGATGACGTGAACTCC

GAGCCCGCCAAACCTGTGGAAGAGCAACCACAAGCTGAAGGACCCTACGCCGGGCCACTTGAGCGTCAGA

AACCTCTGAAAGTGAGAGCCAAGCTCCCGCAGCAGGAGGGACCTTACGCTGGTCTGATGGAGAGACAGAA

ACCACTGAAAGTAAAAGCAAAAGCCCCGGTCGTTAAGGAAGGACCTTACGAGGGGCCGGTGAAGAAGCCT

GTCGCTTTGAAAGTGAAAGCTAGGAACTTGATTGTCACTGAGAGTGGTGCCCCCCCGACCGACTTGCAAA

AGATGGTCATGGGCAACACTAAGCCTGTTGAGCTCATCCTCGACGGGAAGACGGTGGCCATTTGCTGTGC

TACCGGAGTGTTTGGCACCGCCTACCTCGTGCCTCGTCATCTCTTCGCTGAGAAGTACGACAAGATCATG

TTGGACGGTAGGGCCATGACAGACAGTGACTACAGAGTGTTTGAGTTTGAGATTAAAGTAAAAGGACAGG

ACATGCTCTCAGACGCCGCGCTCATGGTGCTCCACCGTGGGAACCGCGTGAGAGACATCACGAAACACTT

TCGTGATACAGCAAGAATGAAGAAAGGCACCCCCGTTGTCGGCGTGATTAACAACGCTGATGTCGGGAGA

CTGATTTTCTCTGGTGAGGCCCTCACCTACAAGGACATAGTAGTGTGCATGGATGGAGACACCATGCCTG

GCCTCTTTGCCTACAAAGCCGCCACCAAGGCTGGTTACTGTGGAGGAGCTGTTCTCGCAAAGGACGGAGC

CGAGACTTTCATCCTCGGCACTCACTCTGCAGGGGGCAATGGAGTTGGATACTGCTCATGCGTTTCCAGG

TCCATGCTTCTGAAAATGAAGGTACACATCGACCCTGAACCACATCACGAG

>FJ175663.1_O_ISR_2007

ATCTCAATTCCTTCCCAAAAATCCGTGTTGTACTTCCTCATTGAGAAAGGCCAGCACGAGGCTGCAATCG

AATTCTTTGAGGGGATGGTGCATGACTCCATCAAGGAGGAGCTCCGACCCCTCATCCAACAGACATCATT

TGTGAAACGCGCTTTCAAGCGCCTGAAGGAGAACTTTGAGATTGTTGCCCTGTGTTTGACTCTTCTGGCA

AACATAGTGATCATGATTCGCGAAACTCGCAAGAGACAGCAGATGGTGGATGATGCAGTGAACGAGTACA

TCGAGAAAGCCAACATCACCACGGATGACAAGACTCTTGACGAGGCGGAAAAGAACCCTCTGGAGACCAG

TGGTACCAGCGCCGTTGGTTTCAGAGAGAGAGCTCTTCCGGGACACAAGGTGAGCGATGACGTGAACTCC

GAGCCCGCCAAACCTGTGGAAGAGCAACCACAAGCTGAAGGACCCTACGCCGGGCCACTTGAGCGTCAGA

AACCTCTGAAAGTGAGAGCCAAGCTCCCACAGCAGGAGGGACCTTACGCTGGTCTGATGGAGAGACAGAA

ACCACTGAAAGTGAAAGCAAAAGCCCCGGTCGTTAAAGAAGGACCTTACGAGGGGCCGGTGAAGAAGCCT

GTCGCCTTGAAAGTGAAAGCTAGGAACTTGATTGTCACTGAGAGTGGTGCCCCCCCGACCGACTTGCAAA

AGATGGTCATGGGCAACACTAAGCCTGTTGAGCTCGTCCTCGACGGGAAGACGGTGGCCATTTGCTGCGC

TACCGGAGTGTTTGGCACCGCCTACCTCGTGCCTCGTCATCTCTTCGCTGAGAAGTACGACAAGATCATG

TTGGACGGTAGGGCCATGACAGACAGTGACTACAGAGTGTTTGAGTTTGAGATCAAAGTAAAAGGACAGG

ACATGCTCTCAGATGCCGCGCTCATGGTGCTCCACCGTGGGAACCGCGTGAGAGACATCACGAAACACTT

TCGTGACACAGCAAGAATGAAGAAAGGCACCCCCGTTGTCGGCGTGATTAACAACGCTGATGTCGGGAGA

CTGATTTTCTCTGGTGAGGCCCTCACCTACAAGGACATAGTAGTGTGCATGGATGGAGACACCATGCCTG

GCCTCTTTGCCTACAAAGCCGCCACCAAGGCTGGTTACTGTGGAGGAGCTGTCCTCGCAAAGGACGGAGC

CGAGACTTTCATCCTCGGCACTCACTCTGCAGGGGGCAATGGAGTTGGATACTGCTCATGCGTTTCCAGG

TCCATGCTTCTGAAAATGAAGGCACACATCGACCCTGAGCCATATCACGAG

>FJ175664.1_O_ISR_2007

ATCTCAATTCCTTCCCAAAAATCCGTGTTGTACTTCCTCATTGAGAAAGGCCAGCACGAGGCTGCAATCG

AATTCTTTGAGGGGATGGTGCATGACTCCATCAAGGAGGAGCTCCGACCCCTCATCCAACAGACATCATT

TGTGAAACGCGCTTTCAAGCGCCTGAAGGAGAACTTTGAGATTGTTGCCCTGTGTTTGACTCTTCTGGCA

AACATAGTGATCATGATTCGCGAAACTCGCAAGAGACAGCAGATGGTGGATGATGCAGTGAACGAGTACA

TCGAGAAAGCCAACATCACCACGGATGACAAGACTCTTGACGAGGCGGAAAAGAACCCTCTGGAGACCAG

TGGTACCAGCGCCGTTGGTTTCAGAGAGAGAGCTCTTCCGGGACACAAGGTGAGCGATGACGTGAACTCC

GAGCCCGCCAAACCTGTGGAAGAGCAACCACAAGCTGAAGGACCCTACGCCGGGCCACTTGAGCGTCAGA

AACCTCTGAAAGTGAGAGCCAAGCTCCCACAGCAGGAGGGACCTTACGCTGGTCTGATGGAGAGACAGAA

ACCACTGAAAGTGAAAGCAAAAGCCCCGGTCGTTAAAGAAGGACCTTACGAGGGGCCGGTGAAGAAGCCT

GTCGCTTTGAAAGTGAAAGCTAGGAACTTGATTGTCACTGAGAGTGGTGCCCCCCCGACCGACTTGCAAA

AGATGGTCATGGGCAACACTAAGCCTGTTGAGCTCGTCCTCGACGGGAAGACGGTGGCCATTTGCTGTGC

TACCGGAGTGTTTGGCACCGCCTACCTCGTGCCTCGTCATCTCTTCGCTGAGAAGTACGACAAGATCATG

TTGGACGGTAGGGCCATGACAGACAGTGACTACAGAGTGTTTGAGTTTGAGATCAAAGTAAAAGGACAGG

ACATGCTCTCAGATGCCGCGCTCATGGTGCTCCACCGTGGGAACCGCGTGAGAGACATCACGAAACACTT

TCGTGACACAGCAAGAATGAAGAAAGGCACCCCCGTTGTCGGCGTGATTAACAACGCTGATGTCGGGAGA

CTGATTTTCTCTGGTGAGGCCCTCACCTACAAGGACATAGTAGTGTGCATGGATGGAGACACCATGCCTG

GCCTCTTTGCCTACAAAGCCGCCACCAAGGCTGGTTACTGTGGAGGAGCTGTCCTCGCAAAGGACGGAGC

CGAGACTTTCATCCTCGGCACTCACTCTGCAGGGGGCAATGGAGTTGGATACTGCTCATGCGTTTCCAGG

TCCATGCTTCTGAAAATGAAGGCACACATCGACCCTGAGCCATATCACGAG

>FJ175665.1_O_ISR_2007

ATCTCAATTCCTTCCCAAAAATCCGTGTTGTACTTCCTCATTGAGAAAGGCCAGCACGAGGCTGCAATCG

AATTCTTTGAGGGGATGGTGCATGACTCCATCAAGGAGGAGCTCCGACCCCTCATCCAACAGACATCATT

TGTGAAACGCGCTTTCAAGCGCCTGAAGGAGAACTTTGAGATTGTTGCCCTGTGTTTGACTCTTCTGGCA

AACATAGTGATCATGATTCGCGAAACTCGCAAGAGACAGCAGATGGTGGATGATGCAGTGAACGAGTGCA

TCGAGAAAGCTAACATCACCACGGATGACAAGACTCTTGACGAGGCGGAAAAGAACCCTCTGGAGACCAG

TGGTACCAGCGCCGTTGGTTTCAGAGAGAGAGCTCTTCCGGGACACAAGGTGAGTGATGACGTGAACTCC

GAGCCCGCCAAACCTGTAGAAGAGCAACCACAAGCTGAAGGACCCTACGCCGGGCCACTTGAGCGTCAGA

AACCTCTGAAAGTGAGAGCCAAGCTCCCGCAGCAGGAGGGACCTTACGCTGGTCTGATGGAGAGACAGAA

ACCACTGAAAGTGAAAGCAAAAGCCCCGGTCGTTAAGGAAGGACCTTACGAGGGGCCGGTGAAGAAGCCT

GTCGCTTTGAAAGTGAAAGCTAGGAACTTGATTGTCACTGAGAGTGGTGCCCCCCCGACCGACTTGCAAA

AGATGGTCATGGGCAACACTAAGCCTGTTGAGCTCGTCCTCGACGGGAAGACGGTGGCCATTTGCTGTGC

TACCGGAGTGTTTGGCACCGCCTACCTCGTGCCTCGTCATCTCTTCGCTGAGAAGTACGACAAGATCATG

TTGGACGGTAGGGCCATGACAGACAGTGACTACAGAGTGTTTGAGTTTGAGATTAAAGTAAAAGGGCAGG

ACATGCTCTCAGACGCCGCGCTCATGGTGCTCCACCGTGGGAACCGCGTGAGAGACATCACGAAACACTT

TCGTGACACAGCAAGAATGAAGAAAGGCACCCCCGTCGTCGGCGTGATTAACAACGCTGATGTCGGGAGA

CTGATTTTCTCTGGTGAGGCCCTCACCTACAAGGACATAGTAGTGTGCATGGATGGAGACACCATGCCTG

GCCTCTTTGCCTACAAAGCCGCCACCAAGGCTGGCTACTGTGGAGGAGCTGTCCTCGCAAAGGACGGAGC

CGAGACTTTCATCCTCGGCACTCACTCTGCAGGGGGCAATGGAGTTGGATACTGCTCATGCGTTTCCAGG

TCCATGCTTCTGAAAATGAAGGCACACATCGACCCTGAACCATATCACGAG

>FJ175666.1_O_ISR_2007

ATCTCAATTCCTTCCCAAAAATCCGTGTTGTACTTCCTCATTGAGAAAGGCCAGCACGAGGCTGCAATCG

AATTCTTTGAGGGGATGGTGCATGACTCCATCAAGGAGGAGCTCCGACCCCTCATCCAACAGACATCATT

TGTGAAGCGCGCTTTCAAGCGCCTGAAGGAGAACTTTGAGATTGTTGCCCTGTGTTTGACTCTTCTGGCA

AACATAGTGATCATGATTCGCGAAACTCGCAAGAGACAGCAGATGGTGGATGATGCAGTGAACGAGTACA

TCGAGAAAGCCAACATCACCACGGATGACAAGACTCTTGACGAGGCGGAAAAGAACCCTCTGGAGACCAG

TGGTACCAGCGCCGTTGGTTTCAGAGAGAGAGCTCTTCCGGGACACAAGGTGAGCGATGACGTGAACTCC

GAGCCCGCCAAACCTGTGGAAGAGCAACCACAAGCTGAAGGACCCTACGCCGGGCCACTTGAGCGTCAGA

AACCTCTGAAAGTGAGAGCCAAGCTCCCACAGCAGGAGGGACCTTACGCTGGTCTGATGGAGAGACAGAA

ACCACTGAAAGTGAAAGCAAAAGCCCCGGTCGTTAAAGAAGGACCTTACGAGGGGCCGGTGAAGAAGCCT

GTCGCTTTGAAAGTGAAAGCTAGGAACTTGATTGTCACTGAGAGTGGTGCCCCCCCGACCGACTTGCAAA

AGATGGTCATGGGCAACACTAAGCCTGTTGAGCTCGTCCTCGACGGGAAGACGGTGGCCATTTGCTGTGC

TACCGGAGTGTTTGGCACCGCCTACCTCGTGCCTCGTCATCTCTTCGCTGAGAAGTACGACAAGATCATG

TTGGACGGTAGGGCCATGACAGACAGTGACTACAGAGTGTTTGAGTTTGAGATCAAAGTAAAAGGACAGG

ACATGCTCTCAGACGCCGCGCTCATGGTGCTCCACCGTGGGAACCGCGTGAGAGACATCACGAAACACTT

TCGTGACACAGCAAGAATGAAGAAAGGCACCCCCGTTGTCGGCGTGATTAACAACGCTGATGTCGGGAGA

CTGATTTTTTCTGGTGAGGCCCTCACCTACAAGGACATAGTAGTGTGCATGGATGGAGACACCATGCCTG

GCCTCTTTGCCTACAAAGCCGCCACCAAGGCTGGTTACTGTGGAGGAGCTGTTCTCGCAAAGGACGGAGC

CGAGACTTTCATCCTCGGCACTCACTCTGCAGGGGGCAATGGAGTTGGATACTGCTCATGCGTTTCCAGG

TCCATGCTTCTGAAAATGAAGGCACACATCGACCCTGAACCACATCACGAG

>FJ461344.1_O_UGA_2002

ATCTCAATTCCTTCCCAAAAGTCCGTGTTGTACTTCCTCATTGAGAAAGGTCAACACGAAGCAGCAATTG

AATTCTTTGAGGGAATGGTGCATGACTCCATCAAGGAGGAGCTCCGACCCCTCATCCAACAGACCTCATT

TGTGAAACGCGCTTTCAAGCGCCTGAAGGAAAACTTTGAAGTTGTCGCTCTGTGTTTGACTCTTTTGGCA

AACATAGTGATCATGATCCGCGAGACTCGCAAGAGACAACAGATGGTGGATGATGCAGTGAATGAGTACA

TAGAGAAAGCAAACATCACCACAGATGACAAAACTCTTGACGAGGCGGAAAAGAACCCTCTGGAGACTAG

CGGTGCCAGTACCGTCGGTTTCAGAGAGAGAACCCTCCCGGGTCAAAGGGCGAGTGATGACGTGAACT-C

GAGCCCGCCAAACCCGTGGAGGAACAACCACAAGCTGAAGGACCCTACGCCGGGCCACTCGAGCGTCAGA

GACCTCTGAAAGTGAAAGCTAAGCTACCACAACAGGAGGGACCTTACGCTGGCCCGATGGAGAGACAGAA

ACCACTGAAAGTGAAAGCAAAAGCCCCGGTCGTCAAGGAAGGACCTTACGAGGGACCGGTGAAGAAACCT

GTCGCTTTGAAAGTGAAAGCTAAGAACCTGATTGTCACTGAGAGTGGCGCCCCACCGACCGACTTGCAAA

AGATGGTCATGGGCAACACCAAGCCTGTTGAGCTCATCCTCGACGGGAAGACGGTAGCCATCTGCTGTGC

TACCGGAGTGTTTGGCACTGCCTACCTTGTGCCTCGTCATCTCTTTGCGGAGAAGTATGACAAGATCATG

TTGGACGGCAGAGCCATGACAGACAGTGATTACAGAGTGTTTGAGTTTGAGATCAAAGTAAAAGGACAGG

ACATGCTCTCAGATGCCGCGCTCATGGTGCTCCACCGCGGGAATCGCGTGAGAGACATCACAAAACACTT

CCGTGACACGGCAAGAATGAAGAAAGGCACCCCCGTTGTCGGTGTGATCAACAACGCTGACGTCGGGAGA

TTGATTTTCTCTGGTGAGGCCCTCACCTACAAGGACATTGTAGTGTGCATGGATGGAGACACCATGCCTG

GCCTTTTTGCCTACAGAGCCGCCACCAAGGCTGGATACTGTGGAGGAGCCGTTCTCGCCAAGGACGGGGC

TGACACCTTCATCGTTGGCACTCACTCTGCAGGGGGTAACGGAGTTGGATACTGCTCGTGCGTGTCCAGG

TCCATGCTCCTGAAAATGAAGGCACACATTGACCCTGAACCACACCATGAA

>FJ461345.1_O_UGA_2002

ATCTCAATTCCTTCCCAAAAGTCTGTGTTGTACTTCCTCATTGAGAAAGGCCAACACGAAGCAGCAATTG

AATTCTTTGAGGGAATGGTGCATGACTCCATCAAGGAGGAGCTCCGACCCCTCATCCAACAGACCTCATT

TGTGAAACGCGCTTTCAAGCGCCTGAAGGAAAACTTTGAAGTTGTTGCCCTGTGCTTGACTCTTTTGGCA

AACATAGTGATCATGATCCGCGAAACTCGCAAGAGACAACAGATGGTGGATGATGCAGTGAATGAGTACA

TAGAGAAAGCAAACATCACCACAGATGACAAAACTCTTGACGAGGCGGAGAAGAACCCTCTGGAGACTAG

CGGTGCCAGCACCGTCGGTTTCAGAGAGAGAACCCTCCCGGGTCAAAAGGCGAGCGGTGACGTGAACTCT

GAGCCCGCCAAACCCGTGGAGGAACAACCACAAGCTGAAGGACCCTACGCCGGGCCACTCGAGCGTCAGA

AACCTCTGAAAGTGAGAGCCAAGCTACCACAACAGGAGGGACCTTACGCTGGCCCGATGGAGAGACAGAA

ACCACTGAAAGTGAAAGCAAAAGCCCCGGTCGTTAAGGAAGGACCTTACGAGGGACCGGTGAAGAAACCT

GTCGCTTTGAAAGTGAAAGCTAAGAACTTGATTGTCACTGAGAGTGGCGCCCCACCGACCGACTTACAAA

AGATGGTCATGGGCAACACCAAGCCTGTTGAGCTCATCCTCGACGGGAAGACGGTAGCCGTCTGCTGTGC

TACCGGAGTGTTTGGCACTGCCTACCTTGTGCCTCGTCATCTCTTTGCGGAGAAGTATGACAAGATCATG

CTGGACGGCAGAGCCATGACAGACAGTGACTACAGAGTGTTTGAGTTTGAGATTAAAGTAAAAGGACAGG

ACATGCTTTCAGACGCCGCGCTCATGGTGCTCCACCGCGGGAACCGCGTGAGAGATATCACAAAACACTT

CCGTGACACGGCAAGAATGAAGAAAGGCACCCCCGTTGTCGGTGTGATCAACAACGCTGACGTCGGGAGA

TTGATTTTCTCTGGTGAGGCCCTCACCTACAAGGACATTGTAGTGTGCATGGATGGAGACACCATGCCTG

GCCTCTTTGCCTACAGAGCCGCCACCAAGGCTGGATACTGTGGAGGAGCCGTTCTCGCTAAGGACGGGGC

TGACACCTTCATCGTCGGCACTCACTCTGCAGGGGGTAACGGAGTTGGATACTGCTCGTGCGTGTCCAGG

TCCATGCTCCTGAAAATGAAGGCACACATTGACCCTGAACCACACCATGAA

>FJ461346.1_SAT2_UGA_2002

ATCTCACTTCCTTCCCAAAAGTCTGTGCTTTACTTTCTCATCGAGAAAGGACAGCATGAGGCAGCAATTG

AATTCTT-GAGGGGATGGTGAGTGACTCCCTCAAGGAGGAACTCCGCCCTCTCCTTCAACAAACCTCATT

TGTGAAGCGCGCTTTTAAGCGCCTGAAGGAAAATTTTGAGATC-TGGCACTGGTGTTGGCTCTCCTGGCC

AACATCATCATCATGTTCCGCGAGACTCGCAAGAGACAGGAAATGGTGGATGCCGCTGTGAACGATTACA

TCGAACGCGCCGGTATCACCACTGACGACCAGACTCTTGACGAGGCGGAGAAGAACCCTCTGGAGACCAG

TGGTACCAGTACCGTTGGATTCAGAGAGAGAACTCTCCCTGGGCACAAAGCACGTGATGACGTGAACTCC

GAGCCTGCTCAACCCAAGGAAGAGAAACCACAAGCTGAAGGACCCTACGCTGGGCCGATGGAGCGTCAGA

AACCTCTGAAAGTGAAAGCCAAGCTTCCACAGCAGGAGGGACCTTACGCCGGTCCAATGGAGAGACAACA

ACCGCTGAAGGTTAGAGCTAAACCCCCCGTCGTGAAGGAAGGACCTTACGAGGGGCCAGTGAAGAAGCCT

GTCGCTTTGAAAGTGAAAGCAAAGAACATGATTGTCACGGAGAGTGGAGCGCCACCCACCGACTTGCAAA

AGATGGTGATGGCCAACACTAAGCCTGTTGAGCTCATACTCGACGGGAAGACAGTGGCAATCTGCTGTGC

CACTGGAGTGTTTGGCACTGCCTATCTCGTGCCTCGTCATCTTTTCGCTGAAAAGTACGACAAGATCATG

ATTGACGGCAGAGCCATGACAGACCGTGACTTCAGAGTGTTTGAGTTTGAGATTAAAGTAAAAGGACAGG

ACATGCTCTCAGACGCCGCTCTCATGGTGCTGCACCGTGGGAACCGCGTGAGAGACATCACGAAACACTT

TCGTGATCAAGCAAGAATGAGGAAAGGAACCCCCGTCGTCGGTGTCATCAACAACGCCGATGTTGGGAGA

CTCATCTTCTCTGGAGAGGCACTCACCTACAAAGACATTGTAGTGTGTATGGATGGTGATACCATGCCAG

GCCTCTTTGCCTACAAAGCCGCCACCAAGGCAGGCTACTGTGGTGGAGCTGTTCTCGCGAAGGACGGAGC

CGAGACTTTCATCGTCGGCACTCACTCCGCAGGTGGCAACGGAGTTGGATACTGCTCATGCGTTTCCAAA

TCCATGTTGCTCCAAATGAAGGCACACGTTGACCCTGAGCCACACCATGAA

>FJ542365.1_O_UKG_2001

ATCTCAATTCCTTCCCAAAAGGCTGTGCTGTACTTTCTCATTGAGAAGGGCCAGCACGAAGCAGCAATTG

AGTTCTTTGAGGGGATGGTGCATGACTCCATCAAGGAGGAGCTCCGGCCTCTCATCCAACAGACCTCATT

TGTGAAGCGCGCTTTTAAGCGCCTGAAGGAAAACTTTGAGATAGTTGCCCTGTGTTTGACTCTTTTGGCA

AACATAGTGATCATGATCCGCGAGACTCGCAAGAGACAGCAGATGGTGGATGATGCAGTGAACGAGTACA

TTGAGAAGGCAAACATCACCACGGATGACAAGACTCTTGACGAGGCGGAAAAGAACCCTCTGGAGACCAG

CGGTGCCACCACTGTTGGTTTCAGAGAGAAAACTCTCCCGGGACACAAGGCGGGTGATGACGTGAACTCC

GAGCCCGCCAAACCCGTGGAAGAACAACCACAAGCTGAAGGACCCTACACCGGTCCACTCGAGCGTCAAA

AACCCCTGAAAGTGAGGGCCAAGCTCCCACAGCAGGAGGGGCCCTACGCTGGTCCGATGGAGAGACAGAA

ACCGCTGAAAGTGAAAGTGAAAGCCCCGGTCGTTAAGGAAGGACCTTACGAAGGACCGGTGAAGAAACCT

GTCGCTTTGAAAGTGAAAGCAAAGAACTTGATTGTCACTGAGAGTGGTGCTCCCCCGACTGACTTGCAAA

AGATGGTCATGGGTAACACCAAGCCTGTTGAGCTCATCCTCGACGGGAAGACGGTGGCCATCTGCTGCGC

CACCGGAGTGTTTGGTACTGCCTACCTAGTTCCTCGTCATCTTTTCGCAGAGAAGTATGACAAGATCATG

TTGGACGGCAGAGCCATGACAGACAGTGACTACAGAGTGTTTGAGTTTGAGATTAAAGTGAAAGGACAGG

ACATGCTCTCAGACGCCGCTCTCATGGTGCTTCACCGTGGGAATCGCGTGCGGGACATCACGAAGCACTT

CCGTGATGTGGCAAGAATGAAGAAAGGCACCCCCGTCGTCGGCGTGATCAACAACGCTGATGTTGGGAGA

CTGATCTTCTCTGGTGAGGCCCTTACCTACAAGGACATTGTAGTGTGCATGGACGGAGACACCATGCCCG

GTCTCTTCGCCTACAAAGCTGCCACCAAGGCGGGTTACTGTGGAGGAGCCGTTCTTGCAAAGGACGGAGC

CGAGACTTTCATCGTCGGCACTCACTCCGCAGGCGGCAATGGAGTTGGATACTGCTCATGCGTTTCCAGG

TCCATGCTGCTTAAAATGAAGGCACACATCGATCCCGAACCACACCACGAG

>FJ542368.1_O_UKG_2001

ATCTCAATTCCTTCCCAAAAGGCTGTGCTGTACTTTCTCATTGAGAAGGGCCAGCACGAAGCAGCAATTG

AGTTCTTTGAGGGGATGGTGCATGACTCCATCAAGGAGGAGCTCCGGCCTCTCATCCAACAGACCTCATT

TGTGAAGCGCGCTTTTAAGCGCCTGAAGGAAAACTTTGAGATAGTTGCCCTGTGTTTGACTCTTTTGGCA

AACATAGTGATCATGATCCGCGAGACTCGCAAGAGACAGCAGATGGTGGATGATGCAGTGAACGAGTACA

TTGAGAAGGCAAACATCACCACGGATGACAAGACTCTTGACGAGGCGGAAAAGAACCCTCTGGAGACCAG

CGGTGCCACCACTGTTGGTTTCAGAGAGAAAACTCTCCCGGGACACAAGGCGGGTGATGACGTGAACTCC

GAGCCCGCCAAACCCGTGGAAGAACAACCACAAGCTGAAGGACCCTACACCGGTCCACTCGAGCGTCAAA

AACCCCTGAAAGTGAGGGCCAAGCTCCCACAGCAGGAGGGGCCCTACGCTGGTCCGATGGAGAGACAGAA

ACCGCTGAAAGTGAAAGTGAAAGCCCCGGTCGTTAAGGAAGGACCTTACGAAGGACCGGTGAAGAAACCT

GTCGCTTTGAAAGTGAAAGCAAAGAACTTGATTGTCACTGAGAGTGGTGCTCCCCCGACTGACTTGCAAA

AGATGGTCATGGGTAACACCAAGCCTGTTGAGCTCATCCTCGACGGGAAGACGGTGGCCATCTGCTGCGC

CACCGGAGTGTTTGGTACTGCCTACCTAGTTCCTCGTCATCTTTTCGCAGAGAAGTATGACAAGATCATG

TTGGACGGCAGAGCCATGACAGACAGTGACTACAGAGTGTTTGAGTTTGAGATTAAAGTGAAAGGACAGG

ACATGCTCTCAGACGCCGCTCTCATGGTGCTTCACCGTGGGAATCGCGTGCGGGACATCACGAAGCACTT

CCGTGATGTGGCAAGAATGAAGAAAGGCACCCCCGTCGTCGGCGTGATCAACAACGCTGATGTTGGGAGA

CTGATCTTCTCTGGTGAGGCCCTTACCTACAAGGACATTGTAGTGTGCATAGACGGAGACACCATGCCCG

GTCTCTTCGCCTACAAAGCTGCCACCAAGGCGGGTTACTGTGGAGGAGCCGTTCTTGCAAAGGACGGAGC

CGAGACTTTCATCGTCGGCACTCACTCCGCAGGCGGCAATGGAGTTGGATACTGCTCATGCGTTTCCAGG

TCCATGCTGCTTAAAATGAAGGCACACATCGATCCCGAACCACACCACGAG

>FJ542369.1_O_UKG_2001

ATCTCAATTCCTTCCCAAAAGGCTGTGCTGTACTTTCTCATTGAGAAGGGCCAGCACGAAGCAGCAATTG

AGTTCTTTGAGGGGATGGTGCATGACTCCATCAAGGAGGAGCTCCGGCCTCTCATCCAACAGACCTCATT

TGTGAAGCGCGCTTTTAAGCGCCTGAAGGAAAACTTTGAGATAGTTGCCCTGTGTTTGACTCTTTTGGCA

AACATAGTGATCATGATCCGCGAGACTCGCAAGAGACAGCAGATGGTGGATGATGCAGTGAACGAGTACA

TTGAGAAGGCAAACATCACCACGGATGACAAGACTCTTGACGAGGCGGAAAAGAACCCTCTGGAGACCAG

CGGTGCCACCACTGTTGGTTTCAGAGAGAAAACTCTCCCGGGACACAAGGCGGGTGATGACGTGAACTCC

GAGCCCGCCAAACCCGTGGAAGAACAACCACAAGCTGAAGGACCCTACACCGGTCCACTCGAGCGTCAAA

AACCCCTGAAAGTGAGGGCCAAGCTCCCACAGCAGGAGGGGCCCTACGCTGGTCCGATGGAGAGACAGAA

ACCGCTGAAAGTGAAAGTGAAAGCCCCGGTCGTTAAGGAAGGACCTTACGAAGGACCGGTGAAGAAACCT

GTCGCTTTGAAAGTGAAAGCAAAGAACTTGATTGTCACTGAGAGTGGTGCTCCCCCGACTGACTTGCAAA

AGATGGTCATGGGTAACACCAAGCCTGTTGAGCTCATCCTCGACGGGAAGACGGTGGCCATCTGCTGCGC

CACCGGAGTGTTTGGTACTGCCTACCTAGTTCCTCGTCATCTTTTCGCAGAGAAGTATGACAAGATCATG

TTGGACGGCAGAGCCATGACAGACAGTGACTACAGAGTGTTTGAGTTTGAGATTAAAGTGAAAGGACAGG

ACATGCTCTCAGACGCCGCTCTCATGGTGCTTCACCGTGGGAATCGCGTGCGGGACATCACGAAGCACTT

CCGTGATGTGGCAAGAATGAAGAAAGGCACCCCCGTCGTCGGCGTGATCAACAACGCTGATGTTGGGAGA

CTGATCTTCTCTGGTGAGGCCCTTACCTACAAGGACATTGTAGTGTGCATGGACGGAGACACCATGCCCG

GTCTCTTCGCCTACAAAGCTGCCACCAAGGCGGGTTACTGTGGAGGAGCCGTTCTTGCGAAGGACGGAGC

CGAGACTTTCATCGTCGGCACTCACTCCGCAGGCGGCAATGGAGTTGGATACTGCTCATGCGTTTCCAGG

TCCATGCTGCTTAAAATGAAGGCACACATCGATCCCGAACCACACCACGAG

>FJ542370.1_O_UKG_2001

ATCTCAACTCCTTCCCAAAAGGCTGTGCTGTACTTTCTCATTGAGAAGGGCCAGCACGAAGCAGCAATTG

AGTTCTTTGAGGGGATGGTGCATGACTCCATCAAGGAGGAGCTCCGGCCTCTCATCCAACAGACCTCATT

TGTGAAGCGCGCTTTTAAGCGCCTGAAGGAAAACTTTGAGATAGTTGCCCTGTGTTTGACTCTTTTGGCA

AACATAGTGATCATGATCCGCGAGACTCGCAAGAGACAGCAGATGGTGGATGATGCAGTGAACGAGTACA

TTGAGAAGGCAAACATCACCACGGATGACAAGACTCTTGACGAGGCGGAAAAGAACCCTCTGGAGACCAG

CGGTGCCACCACTGTTGGTTTCAGAGAGAAAACTCTCCCGGGACACAAGGCGGGTGATGACGTGAACTCC

GAGCCCGCCAAACCCGTGGAAGAACAACCACAAGCTGAAGGACCCTACACCGGTCCACTCGAGCGTCAAA

AACCCCTGAAAGTGAGGGCCAAGCTCCCACAGCAGGAGGGGCCCTACGCTGGTCCGATGGAGAGACAGAA

ACCGCTGAAAGTGAAAGTGAAAGCCCCGGTCGTTAAGGAAGGACCTTACGAAGGACCGGTGAAGAAACCT

GTCGCTTTGAAAGTGAAAGCAAAGAACTTGATTGTCACTGAGAGTGGTGCTCCCCCGACTGACTTGCAAA

AGATGGTCATGGGTAACACCAAGCCTGTTGAGCTCATCCTCGACGGGAAGACGGTGGCCATCTGCTGCGC

CACCGGAGTGTTTGGTACTGCCTACCTAGTTCCTCGCCATCTTTTCGCAGAGAAGTATGACAAGATCATG

TTGGACGGCAGAGCCATGACAGACAGTGACTACAGAGTGTTTGAGTTTGAGATTAAAGTGAAAGGACAGG

ACATGCTCTCAGACGCCGCTCTCATGGTGCTTCACCGTGGGAATCGCGTGCGGGACATCACGAAGCACTT

CCGTGATGTGGCAAGAATGAAGAAAGGCACCCCCGTCGTCGGCGTGATCAACAACGCTGATGTTGGGAGA

CTGATCTTCTCTGGTGAGGCCCTTACCTACAAGGACATTGTAGTGTGCATGGACGGAGACACCATGCCCG

GTCTCTTCGCCTACAAAGCTGCCACCAAGGCGGGTTACTGTGGAGGAGCCGTTCTTGCAAAGGACGGAGC

CGAGACTTTCATCGTCGGCACTCACTCCGCAGGCGGCAATGGAGTTGGATACTGCTCATGCGTTTCCAGG

TCCATGCTGCTTAAAATGAAGGCACACATCGATCCCGAACCACACCACGAG

>FJ542371.1_O_UKG_2001

ATCTCAATTCCTTCCCAAAAGGCTGTGCTGTACTTTCTCATTGAGAAGGGCCAGCACGAAGCAGCAATTG

AGTTCTTTGAGGGGATGGTGCATGACTCCATCAAGGAGGAGCTCCGGCCTCTCATCCAACAGACCTCATT

TGTGAAGCGCGCTTTTAAGCGCCTGAAGGAAAACTTTGAGATAGTTGCCCTGTGTTTGACTCTCTTGGCA

AACATAGTGATCATGATCCGCGAGACTCGCAAGAGACAGCAGATGGTGGATGATGCAGTGAACGAGTACA

TTGAGAAGGCAAACATCACCACGGATGACAAGACTCTTGACGAGGCGGAAAAGAACCCTCTGGAGACCAG

CGGTGCCACCACTGTTGGTTTCAGAGAGAAAACTCTCCCGGGACACAAGGCGGGTGATGACGTGAACTCC

GAGCCCGCCAAACCCGTGGAAGAACAACCACAAGCTGAAGGACCCTACACCGGTCCACTCGAGCGTCAAA

AACCCCTGAAAGTGAGGGCTAAGCTCCCACAGCAGGAGGGGCCCTACGCTGGTCCGATGGAGAGACAGAA

ACCGCTGAAAGTGAAAGTGAAAGCCCCGGTCGTTAAGGAAGGACCTTACGAAGGACCGGTGAAGAAACCT

GTCGCTTTGAAAGTGAAAGCAAAGAACTTGATTGTCACTGAGAGTGGTGCTCCCCCGACTGACTTGCAAA

AGATGGTCATGGGTAACACCAAGCCTGTTGAGCTCATCCTCGACGGGAAGACGGTGGCCATCTGCTGCGC

CACCGGAGTGTTTGGTACTGCCTACCTAGTTCCTCGTCATCTTTTCGCAGAGAAGTATGACAAGATCATG

TTGGACGGCAGAGCCATGACAGACAGTGACTACAGAGTGTTTGAGTTTGAGATTAAAGTGAAAGGACAGG

ACATGCTCTCAGACGCCGCTCTCATGGTGCTTCACCGTGGGAATCGCGTGCGGGACATCACGAAGCACTT

CCGTGATGTGGCAAGAATGAAGAAAGGCACCCCCGTCGTCGGCGTGATCAACAACGCTGATGTTGGGAGA

CTGATCTTCTCTGGTGAGGCCCTTACCTACAAGGACATTGTAGTGTGCATGGACGGAGACACCATGCCCG

GTCTCTTCGCCTACAAAGCTGCCACCAAGGCGGGTTACTGTGGAGGAGCCGTTCTTGCAAAGGACGGAGC

CGAGACTTTCATCGTCGGCACTCACTCCGCAGGCGGCAATGGAGTTGGATACTGCTCATGCGTTTCCAGG

TCCATGCTGCTTAAAATGAAGGCACACATCGATCCCGAACCACACCACGAG

>FJ542372.1_O_UKG_2001

ATCTCAATTCCTTCCCAAAAGGCTGTGCTGTACTTTCTCATTGAGAAGGGCCAGCACGAAGCAGCAATTG

AGTTCTTTGAGGGGATGGTGCATGACTCCATCAAGGAGGAGCTCCGGCCTCTCATCCAACAGACCTCATT

TGTGAAGCGCGCTTTTAAGCGCCTGAAGGAAAACTTTGAGATAGTTGCCCTGTGTTTGACTCTTTTGGCA

AACATAGTGATCATGATCCGCGAGACTCGCAAGAGACAGCAGATGGTGGATGATGCAGTGAACGAGTACA

TTGAGAAGGCAAACATCACCACGGATGACAAGACTCTTGACGAGGCGGAAAAGAACCCTCTGGAGACCAG

CGGTGCCACCACTGTTGGTTTCAGAGAGAAAACTCTCCCGGGACACAAGGCGGGTGATGACGTGAACTCC

GAGCCCGCCAAACCCGTGGAAGAACAACCACAAGCTGAAGGACCCTACACCGGTCCACTCGAGCGTCAAA

AACCCCTGAAAGTGAGGGCCAAGCTCCCACAGCAGGAGGGGCCCTACGCTGGTCCGATGGAGAGACAGAA

ACCGCTGAAAGTGAAAGTGAAAGCCCCGGTCGTTAAGGAAGGACCTTACGAAGGACCGGTGAAGAAACCT

GTCGCTTTGAAAGTGAAAGCAAAGAACTTGATTGTCACTGAGAGTGGTGCTCCCCCGACTGACTTGCAAA

AGATGGTCATGGGTAACACCAAGCCTGTTGAGCTCATCCTCGACGGGAAGACGGTGGCCATCTGCTGCGC

CACCGGAGTGTTTGGTACTGCCTACCTAGTTCCTCGTCATCTTTTCGCAGAGAAGTATGACAAGATCATG

TTGGACGGCAGAGCCATGACAGACAGTGACTACAGAGTGTTTGAGTTTGAGATTAAAGTGAAAGGACAGG

ACATGCTCTCAGACGCCGCTCTCATGGTGCTTCACCGTGGGAATCGCGTGCGGGACATCACGAAGCACTT

CCGTGATGTGGCAAGAATGAAGAAAGGCACCCCCGTCGTCGGCGTGATCAACAACGCTGATGTTGGGAGA

CTGATCTTCTCTGGTGAGGCCCTTACCTACAAGGACATTGTAGTGTGCATGGACGGAGACACCATGCCCG

GTCTCTTCGCCTACAAAGCTGCCACCAAGGCGGGTTACTGTGGAGGAGCCGTTCTTGCAAAGGACGGAGC

CGAGACTTTCATCGTCGGCACTCACTCCGCAGGCGGCAATGGAGTTGGATACTGCTCATGCGTTTCCAGG

TCCATGCTGCTTAAAATGAAGGCACACATCGATCCCGAACCACACCACGAG

>FJ623456.1_A_KAZ_1999

ATTTCAATTCCTTCCCAAAAGTCTGTGTTGTACTTTCTCATTGAGAAAGGCCAGCACGAAGCAGCAATTG

AGTTCTTCGAGGGAATGGTACACGACTCCATCAAGGAGGAGCTTCGTCCCCTCATCCAACAGACTTCATT

TGTCAAACGCGCTTTTAAGCGTCTGAAGGAAAACTTTGAGATTGTTGCCCTATGTTTGACTCTTTTGGCA

AACATAGTGATCATGATCCGCGAGACTCGCAAGAGACAACAGATGGTGGATGATGCAGTGAATGGGTACA

TTGAGAAAGCGAACATCACCACAGATGACAAAACTCTTGACGAGGCGGAAAAGAACCCTTTGGAGACTAG

CGGTGCCAGCACTGTTGGGTTCAGAGAAAGAACTCTCCCAGGACACAGGGCGAGTGATGACGTGAACTCC

GAGCCCGCCAGACCTGTGGAGGAACAACCACAAGCTGAAGGACCCTACACCGGGCCACTCGAGCGTCAGA

AGCCTCTGAAAGTGAAAGCCAAGCTGCCACAGCAGGAAGGACCTTACGCTGGCCCGATGGAGAGACAGAA

ACCACTGAAAGTGAAAGTAAAAGCTCCGGTCGTTAAGGAAGGACCCTACGAGGGACCGGTGAAGAAGCCT

GTCGCTTTGAAAGTGAAAGCTAAGAACTTGATTGTCACTGAGAGTGGAGCCCCACCGACCGACTTGCAAA

AGATGGTCATGGGCAACACCAAGCCTGTTGAGCTCATCCTCGACGGGAAGACGGTGGCCATCTGTTGTGC

TACCGGAGTGTTTGGCACTGCGTACCTCGTGCCTCGTCATCTTTTTACAGAAAAATATGACAAGATCATG

CTGGACGGCAGAGCCATGACAGACAGTGACTACAGAGTGTTTGAGTTTGAGATTAAAGTAAAAGGACAGG

ACATGCTCTCAGACGCTGCGCTCATGGTACTCCACCGTGGGAATCGCGTGAGAGACATCACGAAACACTT

TCGTGACACAGCAAGAATGAAGAAAGGCACCCCTGTTGTCGGAGTGATCAATAATGCCGACGTCGGGAGA

CTGATTTTCTCTGGTGAGGCCCTTACCTACAAGGACATTGTAGTGTGCATGGATGGAGACACCATGCCTG

GCCTGTTTGCCTACAAAGCCGCCACCAAGGCTGGCTACTGTGGGGGAGCCGTTCTTGCTAAGGACGGAGC

TGACACATTCATCGTTGGCACTCACTCCGCAGGCGGCAATGGAGTTGGATACTGCTCATGCGTTTCCAGG

TCCATGTTGCTGAAAATGAAGGCGCACATCGACCCCGAACCACACCACGAG

>FJ824812.1_C_SPA_2009

ATCTCAATACCTTCCCAAAAATCTGTGTTGTACTTCCTCATTGAGAAAGGCCAACACGAGGCAGCAATTG

AATTCTTTGAGGGCATGGTACACGACTCCATTAAGGAGGAACTCCGGCCCCTCATCCAACAAACTTCATT

TGTGAAACGTGCTTTCAAGCGCCTGAAGGAGAACTTTGAAATTGTTGCACTGTGCCTAACACTTTTGGCC

AACATTGTCATCATGATCCGCGAGACTCACAAGAGACAGAAGATGGTGGATGATGCGGTGAATGAGTACA

TCGAGAAAGCAAACATCACCACCGATGGCCAGACACTTGACGAGGCGGAAAAGAACCCTCTGGAAACCAG

TGGTGCCAGCACCGTTGGCTTCAGAGAGAGAACTCTTCCAGGTCAGAAGGCGCGCGATGACGTGAACTCT

GAGCCCGCCCAGCCGACTGAAGAGCAACCGCAAGCTGAAGGACCCTACGCCGGGCCACTCGAGCGTCAGA

GACCTCTGAAAGTGAGAGCCAAGCTCCCGCGACAAGAGGGACCTTACGCCGGTCCGATGGAGAGACAGAA

GCCGCTGAAAGTGAAAGCAAGAGCCCCGGTCGTCAAGGAAGGACCTTATGAAGGACCGGTGAAGAAGCCT

GTCGCTTTGAAAGTGAAAGCTAAGAATTTGATTGTCACTGAGAGTGGTGCCCCCCCGACTGACCTGCAAA

AGATGGTCATGGGCAACACGAAGCCTGTTGAGCTCATCCTCGACGGGAAGACAGTAGCCATCTGCTGTGC

TACTGGAGTGTTCGGCACTGCCTACCTCGTGCCTCGTCATCTTTTCGCTGAGAAGTACGACAAGATCATG

TTGGACGGCAGGGCTTTGACAGACAGTGACTACAGAGTGTTTGAGTTTGAGATTAAAGTAAAAGGACAGG

ACATGCTCTCAGACGCTGCGCTCATGGTGCTCCACCGCGGGAACCGCGTGAGAGACATCACGAAACACTT

TCGTGATGTAGCAAGAATGAAGAAAGGTACCCCCGTCGTTGGCGTGATTAACAACGCTGATGTTGGGAGA

CTGATTTTCTCTGGTGAGGCCCTTACCTACAAAGACATTGTAGTGTGCATGGACGGAGACACCATGCCCG

GCCTCTTTGCCTACAAAGCCGCTACCAAGGCTGGCTACTGTGGAGGAGCCGTTCTTGCTAAGGACGGAGC

TGACACCTTCATCGTTGGCACTCACTCTGCTGGAGGCAATGGAGTTGGGTACTGCTCATGCGTGTCCAGG

TCCATGCTTCTCAAAATGAAGGCACACATCGACCCTGAACCGCACCACGAG

>FJ906802.1_Asia1_CHA_2006

ATCTCAATTCCTTCCCAAAAGTCTGTGCTGTACTTCCTCATCGAGAAAGGCCAGCATGAGGCAGCAATTG

AATTCTTTGAGGGAATGGTGCACGACTCCATTAAGGAGGAACTCCGGCCCCTCATCCAACAGACCTCATT

TGTGAAACGCGCTTTCAAGCGCCTGAAGGAAAACTTTGAGATTGTTGCCCTGTGTTTGACTCTTTTGGCA

AACATAGTGATCATGATCCGCGAGACGCGCAAGAGACAGCAGATGGTGAATGATGCGGTGAACGAGTACA

ACGACAAAGCCAACACCACCACAGACGACAAGACTCTTGACGAGGCGGAAAAGAACCCTCTGGAGACCAG

TGGTGCTAGCACCGTTGGTTTCAGAGAGAGAACCCTCCCGGGGCGCAAGACGAGTGATGACGTGAATTCC

GAGCCCGTCAAACCCGTGGAGGAACAACCACAAGCTGAAGGACCCTACGCCGGGCCACTCGAGCGTCAGA

AACCTCTGAAAGTGAGAGCCAAGCTCCCACAGCAAGAGGGACCTTACGCTGGCCCGATGGAGAGACAGAA

ACCACTGAAAGTGAAAGCAAAAGCCCCGGTCGTTAAGGAAGGGCCTTACGAAGGACCGGTGAAGAAACCT

GTCGCTTTGAAAGTGAAAGCAAAGAATTTGATTGTCACTGAGAGTGGTGCCCCACCGACTGACTTGCAAA

AGATGGTCATGGGCAACACCAAGCCTGTTGAGCTCATCCTCGACGGCAAGACGGTAGCCATCTGCTGCGC

TACCGGAGTCTTTGGTACTGCCTACCTCGTGCCTCGTCACCTTTTCGCAGAGAAGTACGACAAGATCATG

CTGGACGGCAGAGCCATGACAGACAGTGACTACAGAGTGTTTGAGTTTGAGATTAAAGTAAAAGGACAGG

ACATGCTCTCAGACGCCGCACTCATGGTGCTACACCGTGGGAATCGCGTGCGTGACATCACGAAGCACTT

CCGTGATGTAGCCAAGATGAAGAAAGGAACCCCCGTCGTTGGTGTGATCAACAACGCCGACGTTGGGAGA

CTGATTTTCTCCGGTGAGGCCCTAACCTACAAAGACATTGTAGTGTGCATGGATGGAGACACCATGCCTG

GCCTCTTTGCCTACAAGGCCGTCACCAGGGCAGGCTACTGTGGAGGAGCCGTTCTCGCGAAGGACGGAGC

CGAGACTTTCATCGTCGGCACTCACTCTGCAGGCGGCAACGGAGTTGGATACTGCTCGTGCGTTTCCAGG

TCCATGCTGCTCAAGATGAAGGCTCACATTGATCCCGAACCACACCACGAG

>GQ406247.1_A_VIT_2009

ATCTCAATTCCTTCCCAAAAGTCTGTGCTGTACTTTCTCATTGAGAAAGGCCAACACGAGGCAGCAATTG

AATTCTTTGAGGGGATGGTGCATGACTCCATCAAGGAGGAGCTCCGGCCCCTCATCCAGCAAACCTCATT

TGTGAAACGCGCTTTTAAGCGCCTGAAGGAAAACTTTGAGGTCGTTGCCCTATGTTTGACTCTCTTGGCA

AACATAGTGATCATGATCCGCGAGACTCGTAAGAGACAGCAAATGGTGGATGATGCAGTGAATGAGTACA

TTGAGAAGGCAAACATCACCACAGATGACAAAACTCTTGACGAGGCGGAAAAGAACCCGCTGGAGACTAG

CGGTGCCAGTACTGTTGGCTTCAGAGAGAGATCTCTCCCCGGACACAAAGTGAGCGATGACGTGAACTCC

GAGCCCACCAAACCCCCGGGAGGACAACCACAAGCTGAAGGACCCTACGCCGGACCACTCGAGCGTCAGA

AACCTCTGAAAGTGAAAGCCAAGCTGCCACAACAGGAGGGACCCTACGCTGGTCCGATGGAGAGACAGAA

ACCACTGAAAGTGAAAGCAAAAGCCCCGGTCGTGAAGGAAGGACCTTACGAAGGACCGGTGAAGAAGCCT

GTCGCTTTGAAAGTGAAAGCTAAGAACCTGATTGTCACTGAAAGTGGTGCCCCCCCGACCGACTTGCAAA

AGATGGTTATGGGCAACACCAAACCCGTTGAGCTCATCCTCGATGGGAAGACAGTAGCCATCTGCTGCGC

TACTGGAGTGTTCGGCACTGCCTACCTCGTGCCTCGTCATCTTTTCGCTGAGAAGTATGACAAGATCATG

TTGGATGGTAGAGCCATGACAGACAGCGACTACAGAGTGTTTGAGTTTGAGATCAAAGTAAAAGGGCAGG

ACATGCTCTCAGACGCTGCGCTCATGGTGTTACACCGTGGGAATCGCGTGAGAGACATCACGAAACACTT

TCGTGACACAGCAAGAATGAAGAAAGGCACCCCCGTCGTCGGCGTAATAAACAACGCTGATGTTGGTAGA

CTGATTTTCTCTGGTGAGGCCCTTACCTACAAAGACATTGTAGTGTGCATGGATGGAGACACCATGCCGG

GCTTGTTTGCCTACAGAGCCGCCACCAAGGCTGGTTACTGTGGAGGAGCTGTTCTTGCTAAAGACGGAGC

TGACACATTCATCGTCGGCACTCACTCCGCAGGTGGCAATGGGGTTGGGTACTGCTCGTGCGTATCCAGA

TCCATGCTTTTGAAGATGAAGGCACACATCGACCCTGAGCCACACCATGAA

>GQ406248.1_A_VIT_2009

ATCTCAATTCCTTCCCAAAAGTCTGTGCTGTACTTTCTCATTGAGAAAGGCCAACACGAGGCAGCAATTG

AATTCTTTGAGGGGATGGTGCATGACTCCATCAAGGAGGAGCTCCGGCCCCTCATCCAGCAAACCTCATT

TGTGAAACGCGCTTTTAAGCGCCTGAAGGAAAACTTTGAGGTCGTTGCCCTATGTTTGACTCTCTTGGCA

AACATAGTGATCATGATCCGCGAGACTCGTAAGAGACAGCAAATGGTGGATGATGCAGTGAATGAGTACA

TTGAGAAGGCAAACATCACCACAGATGACAAAACTCTTGACGAGGCGGAAAAGAACCCTCTGGAGACTAG

CGGTGCCAGTACTGTTGGCTTCAGAGAGAGATCTCTCCCCGGACACAAAGTGAGCGATGACGTGAACTCC

GAGCCCACCAAACCCGCGGGAGGACAACCACAAGCTGAAGGACCCTACGCCGGACCACTCGAGCGTCAGA

AACCTCTGAAAGTGAAAGCCAAGCTGCCACAACAGGAGGGACCCTACGCTGGTCCGATGGAGAGACAGAA

ACCACTGAAAGTGAAAGCAAAAGCCCCGGTCGTGAAGGAAGGACCTTACGAAGGACCGGTGAAGAAGCCT

GTCGCTTTGAAAGTGAAAGCTAAGAACCTGATTGTCACTGAAAGTGGTGCCCCCCCGACCGACTTGCAAA

AGATGGTTATGGGCAACACCAAACCCGTTGAGCTCATCCTCGATGGGAAGACAGTAGCCATCTGCTGCGC

TACTGGAGTGTTCGGCACTGCCTACCTCGTGCCTCGTCATCTTTTCGCTGAGAAGTATGACAAGATCATG

TTGGATGGTAGAGCCATGACAGACAATGACTACAGAGTGTTTGAGTTTGAGATTAAAGTAAAAGGACAGG

ACATGCTCTCAGACGCTGCGCTCATGGTGTTACACCGTGGGAATCGCGTGAGAGACATCACGAAACACTT

TCGTGACACAGCAAGAATGAAGAAAGGCACCCCCGTCGTCGGCGTAATAAACAACGCTGATGTTGGTAGA

CTGATTTTCTCTGGTGAGGCCCTTACCTACAAAGACATTGTAGTGTGCATGGATGGAGACACCATGCCGG

GCTTGTTTGCCTACAGAGCCGCCACCAAGGCTGGTTACTGTGGAGGAGCTGTTCTTGCTAAAGACGGAGC

TGACACATTCATCGTCGGCACTCACTCCGCAGGTGGCAATGGGGTTGGGTACTGCTCGTGCGTATCCAGA

TCCATGCTTTTGAAGATGAAGGCACACATCGACCCTGAGCCACACCATGAA

>GQ406249.1_A_VIT_2009

ATCTCAATTCCTTCCCAAAAGTCTGTGCTGTATTTTCTCATTGAGAAAGGCCAACACGAGGCAGCAATTG

AATTCTTTGAGGGGATGGTGCATGACTCCATCAAGGAGGAGCTCCGGCCCCTCATCCAACAAACCTCATT

TGTGAAACGCGCTTTCAAGCGCCTGAAGGAAAACTTTGAGGTCGTTGCCCTATGTTTGACTCTCTTGGCA

AACATAGTGATCATGATCCGCGAGACCCGTAAGAGACAGCAAATGGTGGATGATGCAGTGAATGAGTACA

TTGAGAAGGCAAACATCACCACAGATGACAAAACTCTTGACGAGGCGGAAAAGAACCCTCTGGAGACTAG

CGGTGCCAGTACTGTTGGCTTCAGAGAGAGATCTCTCCCCGGACACAAAGTGAGCGATGACGTGAACTCC

GAGCCCACCAAACCCGCGGGAGGACAACCACAAGCTGAAGGACCCTACGCCGGACCACTCGAGCGTCAGA

AACCTCTGAAAGTGAAAGCCAAGCTGCCACAACAGGAGGGACCCTACGCTGGTCCGATGGAGAGACAGAA

ACCACTGAAAGTGAAAGCAAAAGCCCCGGTCGTGAAGGAAGGACCTTACGAAGGACCGGTGAAGAAGCCT

GTCGCTTTGAAAGTGAAAGCTAAGAACCTGATTGTCACTGAAAGTGGTGCCCCCCCGACCGACTTGCAAA

AGATGGTTATGGGCAACACCAAACCCGTTGAGCTCATCCTCGATGGGAAGACAGTAGCCATCTGCTGCGC

TACTGGAGTGTTCGGCACTGCCTACCTCGTGCCTCGTCATCTTTTCGCCGAGAAGTATGACAAGATCATG

TTGGATGGTAGAGCCATGACAGACAGTGACTACAGAGTGTTTGAGTTTGAGATTAAAGTAAAAGGACAGG

ACATGCTCTCAGACGCTGCGCTCATGGTGTTACACCGTGGGAATCGCGTGAGAGACATCACGAAACACTT

TCGTGACACAGCAAGAATGAAGAAAGGCACCCCCGTCGTCGGCGTAATAAACAACGCTGATGTTGGTAGA

CTGATTTTCTCTGGTGAGGCCCTTACCTACAAAGACATTGTAGTGTGCATGGATGGAGACACCATGCCGG

GCTTGTTTGCCTACAGAGCCGCCACCAAGGCTGGTTACTGTGGAGGAGCTGTTCTTGCTAAAGACGGAGC

TGACACATTCATCGTCGGCACTCACTCTGCAGGTGGCAATGGGGTTGGGTACTGCTCGTGCGTATCCAGA

TCCATGCTTTTGAAGATGAAGGCACACATCGACCCTGAGCCACACCATGAA

>GQ406250.1_A_VIT_2009

ATCTCAATTCCTTCCCAAAAATCTGTGCTGTACTTTCTCATTGAGAAAGGCCAACACGAGGCAGCAATTG

AATTCTTTGAGGGGATGGTGCATGACTCCATCAAGGAGGAGCTCCGGCCCCTCATCCAACAAACCTCATT

TGTGAAACGCGCTTTCAAGCGCCTGAAGGAAAACTTTGAGGTCGTTGCCCTATGTTTGACTCTCTTGGCA

AACATAGTGATCATGATCCGCGAGACTCGTAAGAGACAGCAAATGGTGGATGATGCAGTGAATGAGTACA

TTGAGAAGGCAAACATCACCACAGATGACAAAACTCTTGACGAAGCGGAAAAGAACCCTCTGGAGACTAG

CGGTGCCAGTACTGTTGGCTTCAGAGAGAGATCTCTCCCCGGACACAAAGTGAGCGATGACGTGAACTCC

GAACCCACCAAACCCGCGGGAGGACAACCACAAGCTGAAGGACCCTACGCCGGACCACTCGAGCGTCAGA

AACCTCTGAAAGTGAAAGCCAAGCTGCCACAACAGGAGGGACCCTACGCTGGTCCGATGGAGAGACAGAA

ACCACTGAAAGTGAAAGCAAAAGCCCCGGTCGTGAAGGAAGGACCTTACGAAGGACCGGTGAAGAAGCCT

GTCGCTTTGAAAGTGAAAGCTAAGAACCTGATTGTCACTGAAAGTGGTGCCCCCCCGACCGACTTGCAAA

AGATGGTTATGGGCAACACCAAACCCGTTGAGCTCATCCTCGATGGGAAGACAGTAGCCATCTGCTGCGC

TACTGGAGTGTTCGGCACTGCCTACCTCGTGCCTCGTCATCTTTTCGCTGAGAAGTATGACAAGATCATG

TTGGATGGTAGAGCCATGACAGACAGTGACTACAGAGTGTTTGAGTTTGAGATTAAAGTAAAAGGACAGG

ACATGCTCTCAGACGCTGCGCTCATGGTGTTACACCGTGGGAATCGCGTGAGAGACATTACGAAACACTT

TCGTGACACAGCAAGAATGAAGAAAGGCACCCCCGTCGTCGGCGTAATAAACAACGCTGATGTTGGTAGA

CTGATTTTCTCTGGTGAGGCCCTTACCTACAAAGACATTGTAGTGTGCATGGATGGAGACACCATGCCGG

GCTTGTTTGCCTACAGAGCCGCCACCAAGGCTGGTTACTGTGGAGGAGCTGTTCTTGCTAAAGACGGAGC

TGACACATTCATCGTCGGCACTCACTCCGCAGGTGGTAATGGGGTTGGGTACTGCTCGTGCGTATCCAGA

TCCATGCTTTTGAAGATGAAGGCACACATCGACCCTGAGCCACACCATGAA

>GQ406251.1_A_VIT_2009

ATCTCAATTCCTTCCCAAAAGTCTGTGCTGTACTTTCTCATTGAGAAAGGCCAACACGAGGCAGCAATTG

AATTCTTTGAGGGGATGGTGCATGACTCCATCAAGGAGGAGCTCCGGCCCCTCATCCAACAAACCTCATT

TGTGAAACGCGCTTTTAAGCGCCTGAAGGAAAACTTTGAGGTCGTTGCCCTATGTTTGACTCTCTTGGCA

AACATAGTGATCATGATCCGCGAGACTCGTAAGAGACAGCAAATGGTGGATGATGCAGTGAATGAGTACA

TTGAGAAGGCAAACATCACCACAGATGACAAAACTCTTGACGAGGCGGAAAAGAACCCTCTGGAGACTAG

CGGTGCCAGTACTGTTGGCTTCAGAGAGAGATCTCTCCCCGGACACAAAGTGAGCGATGACGTGAACTCC

GAGCCCACCAAACCCGCGGGAGGACAACCACAAGCTGAAGGACCCTACGCCGGACCACTCGAGCGTCAGA

AACCTCTGAAAGTGAAAGCCAAGCTGCCACAACAGGAGGGACCCTACGCTGGTCCGATGGAGAGACAGAA

ACCACTGAAAGTGAAAGCAAAAGCCCCGGTCGTGAAGGAAGGACCTTACGAAGGACCGGTGAAGAAGCCT

GTCGCTTTGAAAGTGAAAGCTAAGAACCTGATTGTCACTGAAAGTGGTGCCCCCCCGACCGACTTGCAAA

AGATGGTTATGGGCAACACCAAACCCGTTGAACTCATCCTCGATGGGAAGACAGTAGCCATCTGCTGCGC

TACTGGAGTGTTCGGCACCGCCTACCTCGTGCCTCGTCATCTTTTCGCTGAGAAGTATGACAAGATCATG

TTGGATGGTAGAGCCATGACAGACAGTGACTACAGAGTGTTTGAGTTTGAGATTAAAGTAAAAGGACAGG

ACATGCTCTCAGACGCTGCGCTCATGGTGTTACACCGTGGGAATCGCGTGAGAGACATCACGAAACACTT

TCGTGACACAGCAAGAATGAAGAAAGGCACCCCCGTCGTCGGCGTAATAAACAACGCTGATGTTGGTAGA

CTGATTTTCTCCGGTGAGGCCCTTACCTACAAAGACATTGTAGTGTGCATGGATGGAGACACCATGCCGG

GCTTGTTTGCCTACAGAGCCGCCACCAAGGCTGGTTACTGTGGAGGAGCTGTTCTTGCTAAAGACGGAGC

TGACACATTCATCGTCGGCACTCACTCCGCAGGTGGCAATGGGGTTGGGTACTGCTCGTGCGTATCCAGA

TCCATGCTTTTGAAGATGAAGGCACACATCGACCCTGAGCCACACCATGAA

>GQ406252.1_A_VIT_2009

ATCTCAATTCCTTCCCAAAAGTCTGTGCTGTACTTTCTCATTGAGAAAGGTCAACACGAGGCAGCAATTG

AATTCTTTGAGGGGATGGTGCATGACTCCATCAAGGAGGAGCTCCGGCCCCTCATCCAACAAACCTCATT

TGTGAAACGCGCTTTTAAGCGCCTGAAGGAAAACTTTGAGGTCGTTGCCCTATGTTTGACTCTCTTGGCA

AACATAGTGATCATGATCCGCGAGACTCGTAAGAGACAGCAAATGGTGGATGATGCAGTGAATGAGTACA

TTGAGAAGGCAAACATCACCACAGATGACAAAACTCTTGACGAGGCGGAAAAGAACCCTCTAGAGACTAG

CGGTGCCAGTACTGTTGGCTTCAGAGAGAGATCTCTCCCCGGACACAAAGCGAGCGATGACGTGAACTCC

GAGCCCACCAAACCCGCGGGAGGACAACCACAAGCTGAAGGACCCTACGCCGGACCACTCGAACGTCAGA

AACCTCTGAAAGTGAAAGCCAAGCTGCCACAACAGGAGGAACCCTACGCTGGTCCGATGGAGAGACAGAA

ACCACTGAAAGTGAAAGCAAAAGCCCCGGTCGTGAAGGAAGGGCCTTACGAAGGACCGGTGAAGAAGCCT

GTCGCTTTGAAAGTGAAAGCTAAGAACCTGATTGTCACTGAAAGTGGTGCCCCCCCGACCGACTTGCAAA

AGATGGTTATGGGCAACACCAAACCCGTTGAGCTCATCCTCGATGGGAAGACAGTAGCCATCTGCTGCGC

TACTGGAGTGTTCGGCACTGCCTACCTCGTGCCTCGTCATCTTTTCGCTGAGAAGTATGACAAGATCATG

TTGGATGGTAGAGCCATGACAGACAATGACTACAGAGTGTTTGAGTTTGAGATTAAAGTAAAAGGACAGG

ACATGCTCTCAGACGCTGCGCTCATGGTGTTACACCGTGGGAATCGCGTGAGAGACATCACGAAACACTT

TCGTGACACAGCAAGAATGAAGAAAGGCACCCCCGTCGTCGGCGTGATAAACAACGCTGATGTTGGTAGA

CTGATTTTCTCTGGTGAAGCCCTTACCTACAAAGACATTGTAGTGTGCATGGACGGAGACACCATGCCGG

GCTTGTTTGCCTACAGAGCCGCCACCAAGGCTGGTTACTGTGGAGGAGCTGTTCTTGCTAAAGACGGAGC

TGACACATTCATCGTCGGCACTCACTCCGCAGGTGGCAATGGGGTTGGGTACTGCTCGTGCGTATCCAGA

TCCATGCTTTTGAAGATGAAGGCACACATCGACCCTGAGCCACACCATGAA

>GQ452295.1_Asia1_VIT_2007

ATCTCAATTCCTTCCCAAAAGTCTGTGTTGTACTTCCTCATCGAGAAAGGCCAGCATGAGGCAGCAATTG

AATTCTTTGAGGGAATGGTGCACGACTCCATTAAGGAGGAACTCCGGCCCCTCATCCAACAGACCTCATT

TGTGAAACGCGCTTTCAAGCGCCTGAAGGAAAACTTTGAGATTGTTGCCCTGTGTTTGACTCTTTTGGCA

AACATAGTGATCATGATCCGCGAGACGCGCAAGAGACAGCAGATGGTGGATGATGCGGTGAACGAGTACA

TCGACAAAGCCAACATCACCACAGATGACAAAACTCTTGACGAGGCGGAAAAGAACCCTCTGGAGACCAG

TGGTGCTAGCACCGTTGGTTTCAGAGAGAGAACCCTCCCGGGGCGCAAGACGAGTGATGACGTGAATTCC

GAGCCCGTCAAACCCGTGGAGGAACAACCACAAGCTGAAGGACCCTACGCCGGGCCACTCGAGCGTCAGA

AACCTCTGAAAGTGAGAGCCAAGCTCCCACAGCAAGAGGGACCTTACGCTGGCCCGATGGAGAGACAGAA

ACCACTGAAAGTGAAAGCAAAAGCCCCGGTCGTTAAGGAAGGGCCTTACGAAGGACCGGTGAAGAAACCT

GTCGCTTTGAAAGTGAAAGCAAAGAATTTGATTGTCACTGAGAGTGGTGCCCCACCGACTGACTTGCAAA

AGATGGTCATGGGCAACACCAAGCCTGTTGAGCTCATCCTCGACGGCAAGACGGTAGCCATCTGCTGCGC

TACCGGAGTCTTTGGTACTGCCTACCTCGTGCCTCGTCACCTTTTCGCGGAGAAGTACGACAAGATCATG

CTGGACGGCAGAGCCATGACAGACAGTGACTACAGAGTGTTTGAGTTTGAGATTAAAGTAAAAGGACAGG

ACATGCTCTCGGACGCCGCACTCATGGTGCTCCACCGTGGGAATCGCGTGCGTGACATCACGAAGCACTT

CCGTGATGTAGCCAAGATGAAGAAAGGAACCCCCGTCGTTGGTGTGATCAACAACGCCGACGTTGGGAGA

CTGATTTTCTCCGGTGAGGCTCTAACCTACAAAGACATTGTAGTGTGCATGGATGGAGACACCATGCCTG

GCCTCTTTGCCTACAAGGCCGTCACCAGGGCAGGCTACTGTGGAGGAGCCGTTCTCGCGAAGGACGGAGC

CGAGACTTTCATCGTCGGCACTCACTCTGCAGGCGGCAACGGAGTTGGATACTGCTCGTGCGTTTCCAGG

TCCATGCTGCTCAAGATAAAGGCTCACATTGATCCCGAACCACACCACGAG

>GU125645.1_Asia1_VIT_2007

ATCTCAATTCCTTCCCAAAAGTCTGTGTTGTACTTCCTCATCGAGAAAGGCCAGCATGAGGCAGCAATTG

AATTCTTTGAGGGAATGGTGCACGACTCCATTAAGGAGGAACTCCGGCCCCTCATCCAACAGACCTCATT

TGTGAAACGCGCTTTCAAGCGCCTGAAGGAAAACTTTGAGATTGTTGCCCTGTGTTTGACTCTTTTGGCA

AACATAGTGATCATGATCCGCGAGACGCGCAAGAGACAGCAGATGGTGGATGATGCGGTGAACGAGTACA

TCGACAAAGCCAACATCACCACAGATGACAAAACTCTTGACGAGGCGGAAAAGAACCCTCTGGAGACCAG

TGGTGCTAGCACCGTTGGTTTCAGAGAGAGAACCCTCCCGGGGCGCAAGACGAGTGATGACGTGAATTCC

GAGCCCGTCAAACCCGTGGAGGAACAACCACAAGCTGAAGGACCCTACGCCGGGCCACTCGAGCGTCAGA

AACCTCTGAAAGTGAGAGCCAAGCTCCCACAGCAAGAGGGACCTTACGCTGGCCCGATGGAGAGACAGAA

ACCACTGAAAGTGAAAGCAAAAGCCCCGGTCGTTAAGGAAGGGCCTTACGAAGGACCGGTGAAGAAACCT

GTCGCTTTGAAAGTGAAAGCAAAGAATTTGATTGTCACTGAGAGTGGTGCCCCACCGACTGACTTGCAAA

AGATGGTCATGGGCAACACCAAGCCTGTTGAGCTCATCCTCGACGGCAAGACGGTAGCCATTTGCTGCGC

TACCGGAGTCTTTGGTACTGCCTACCTCGTGCCTCGTCACCTTTTCGCGGAGAAGTACGACAAGATCATG

CTGGACGGCAGAGCCATGACAGACAGTGACTACAGAGTGTTTGAGTTTGAGATTAAAGTAAAAGGACAGG

ACATGCTCTCGGACGCCGCACTCATGGTGCTCCACCGTGGGAATCGCGTGCGTGACATCACGAAGCACTT

CCGTGATGTAGCCAGGATGAAGAAAGGAACCCCCGTCGTTGGTGTGATCAACAACGCCGACGTTGGGAGA

CTGATTTTCTCCGGTGAGGCTCTAACCTACAAAGACATTGTAGTGTGCATGGATGGAGACACCATGCCTG

GCCTCTTTGCCTACAAGGCCGTCACCAGGGCAGGCTACTGTGGAGGAGCCGTTCTCGCGAAGGACGGAGC

CGAGACTTTCATCGTCGGCACTCACTCTGCAGGCGGCAACGGAGTTGGATACTGCTCGTGCGTTTCCAGG

TCCATGCTGCTCAAGATAAAGGCTCACATTGATCCCGAACCACACCACGAG

>GU125646.1_Asia1_VIT_2005

ATCTCAATTCCTTCCCAAAAGTCTGTGTTGTACTTCCTCATCGAGAAAGGCCAGCATGAGGCAGCAATTG

AATTCTTTGAGGGAATGGTGCACGACTCCATTAAGGAGGAACTCCGGCCCCTCATCCAACAGACCTCATT

TGTGAAACGCGCTTTCAAGCGCCTGAAGGAAAACTTTGAGATTGTTGCCCTGTGTTTGACTCTTTTGGCA

AACATAGTGATCATGATCCGCGAGACGCGCAAGAGACAGCAGATGGTGGATGATGCGGTGAACGAGTACA

TTGAGAAGGCAAACATCACCACAGATGACAAAACTCTTGATGAAGCGGAAAAGAACCCCCTGGAAACCAG

TGGTGCCAGCACCGTTGGCTTCAGGGAGAGGCCTCTCAACGGGCACAAGGCGAGCGATGACGTGAACTCC

GAGCCCGCCAAACCCGTGGAAGAGCAACCACATGCTGAAGGACCCTACGCCGGACCACTCGAGCGTCAGA

AACCTCTGAAAGTGAGAGCCAAGCTGCCACAGCAGGAGGGACCCTACGCCGGTCCGATGGAGAGACAGAA

ACCACTGAAAGTAAAAGCAAAAGCCCCGGTCGTGAAGGAAGGACCCTACGAAGGACCGGTGAAGAAGCCT

GTCGCTTTGAAAGTGAAAGCTAAGAACTTGATTGTCACTGAGAGTGGTGCCCCCCCGACCGACTTGCAAA

AGATGGTCATGGGCAACACCAAGCCTGTTGAGCTCATCCTCGACGGGAAGACGGTAGCCATCTGCTGCGC

TACTGGAGTCTTTGGTACCGCCTACCTCGTGCCTCGCCATCTTTTCGCTGAAAAGTATGACAAGATCATG

CTGGACGGCAGAGCCATGACAGACAGTGACTACAGAGTGTTTGAGTTTGAGATTAAAGTAAAAGGACAGG

ACATGCTCTCAGATGCCGCGCTCATGGTGCTGCACCGTGGGAACCGCGTGAGAGACATCACGAAACACTT

TCGTGATGTGGCAAGAATGAAGAAGGGTACCCCCGTCGTTGGTGTGATTAACAACGCCGACGTCGGGAGA

CTGATCTTTTCTGGTGAGGCCCTCACCTACAAGGATATTGTGGTGTGCATGGACGGGGACACCATGCCGG

GCCTGTTTGCCTACAAAGCCGCCACCAAGGCCGGTTACTGCGGAGGAGCCGTTCTCGCTAAGGACGGAGC

CGACACGTTCATCGTTGGCACTCACTCCGCAGGTGGCAATGGAGTTGGGTACTGCTCATGCGTGTCTAGG

TCCATGCTCCTGAAGATGAAGGCACACATTGACCCTGAACCACACCACGAA

>GU125647.1_O_VIT_2006

ATCTCAATTCCTTCCCAAAAGTCTGTGTTGTACTTCCTCATTGAGAAAGGCCAGCACGAAGCAGCAATTG

AATTCTTTGAGGGGATGGTACACGACTCCATCAAGGACGAGCTCCGACCCCTCATCCAACAGACCTCATT

TGTGAAACGCGCTTTCAAGCGCTTGAGGGAAAACTTTGAGATTGTTGCACTGTGTTTGACTCTTCTGGCA

AACATAGTAATCATGATCCGCGAGACCCGCAAGAGACAGCAAATGGTGGACGATGCAGTGAATGAGTACA

TCGAGAAAGCAAACATCACCACGGATGACAAGACTCTTGACGAGGCGGAAAAGAACCCTCTGGAGACTAG

CGGTGTCAGCACCGTTGGTTTCAGAGAGAAAACTCTCCCAGGACACAGGACGTGTGATGACGTGAACTCC

GAGCCCGTTAAACCTGTGGAAGAGCAACCACAAGCTGAAGGACCCTACGCCGGACCGCTTGAGCGCCAGA

AACCTCTGAAAGTGAGAGCCAAGCTACCACAACAGGAGGGGCCTTACGCTGGTCCGATGGAGAGACAGAA

ACCACTGAAATTGAAAGTGAGCGCCCCGGTCGTGAAGGAAGGACCTTACGAGGGACCGGTGAAGAAGCCT

GTTGCCCTGAAAGTGAAAGCAAAGAACTTGATTGTCACTGAGAGTGGTGCTCCCCCGACCGATCTGCAGA

AGATGGTCATGAGCAACACTAAGCCTGTTGAGCTCATCCTCGACGGGAAGACAGTAGCCATCTGCTGTGC

TACTGGAGTGTTTGGTACTGCCTACCTCGTGCCTCGTCATCTTTTCGCTGAGAAGTACGACAAGATCATG

TTGGACGGTAGAGCCATGACAGACAGTGACTACAGAGTGTTTGAGTTTGAGATTAATGTAAAAGGACAGG

ACATGCTCTCAGACGCCGCTCTCATGGTGTTGCACCGTGGGAACCGCGTAAGGGACATCACGAAACACTT

TCGTGACACAGCAAGAATGAAGAAAGGTACCCCTGTCGTTGGCGTGATCAACAACGCTGACGTTGGGAGA

CTGATTTTCTCTGGTGAGGCCCTTACCTACAAGGACATTGTAGTGTGCATGGACGGAGACGCCATGCCGG

GCCTGTTCGCCTACAAAGCCGCCACCAAGGCTGGCTACTGCGGGGGAGCCGTTCTCGCCAAGGACGGAGC

CGATACGTTCATCGTTGGCACTCACTCCGCAGGTGGCAATGGGGTTGGGTACTGCTCATGTGTATCCAGG

TCCATGCTCCTCAAAATGAAGGCACACATTGACCCCGAACCACACCACGAG

>GU125648.1_O_VIT_2006

ATCTCAATTCCTTCCCAAAAGTCTGTGTTGTACTTCCTCATTGAGAAAGGCCAGCACGAAGCAGCAATTG

AATTCTTTGAGGGGATGGTACACGACTCCATCAAGGACGAGCTCCGACCCCTCATCCAACAGACCTCATT

TGTGAAACGCGCTTTCAAGCGCTTGAGGGAAAACTTTGAGATTGTTGCACTGTGTTTGACTCTTCTGGCA

AACATAGTAATCATGATCCGCGAGACCCGCAAGAGACAGCAAATGGTGGACGATGCAGTGAATGAGTACA

TCGAGAAAGCAAACATCACCACGGATGACAAGACTCTTGACGAGGCGGAAAAGAACCCTCTGGAGACTAG

CGGTGTCAGCACCGTCGGTTTCAGAGAGAAAACTCTCCCAGGACACAGGACGTGTGATGACGTGAACTCC

GAGCCCGTTAAACCTGTGGAAGAGCAACCACAAGCTGAAGGACCCTACGCCGGACCGCTTGAGCGCCAGA

AACCTCTGAAAGTGAGAGCCAAGCTACCACAACAGGAGGGGCCTTACGCTGGTCCGATGGAGAGACAGAA

ACCACTGAAATTGAAAGTGAGCGCCCCGGTCGTGAAGGAAGGACCTTACGAGGGACCGGTGAAGAAGCCT

GTTGCCCTGAAAGTGAAAGCAAAGAACTTGATTGTCACTGAGAGTGGTGCTCCCCCGACCGATCTGCAGA

AGATGGTCATGAGCAACACTAAGCCTGTTGAGCTCATCCTCGACGGGAAGACAGTAGCCATCTGCTGTGC

TACTGGAGTGTTTGGTACTGCCTACCTCGTGCCTCGTCATCTTTTCGCTGAGAAGTACGACAAGATCATG

TTGGACGGTAGAGCCATGACAGACAGTGACTACAGAGTGTTTGAGTTTGAGATTAATGTAAAAGGACAGG

ACATGCTCTCAGACGCCGCTCTCATGGTGTTGCACCGTGGGAACCGCGTGAGGGACATCACGAAACACTT

TCGTGACACAGCAAGAATGAAGAAAGGTACCCCTGTCGTTGGCGTGATCAACAACGCTGACGTTGGGAGA

CTGATTTTCTCTGGTGAGGCCCTTACCTACAAGGACATTGTAGTGTGCATGGACGGAGACGCCATGCCGG

GCCTGTTCGCCTACAAAGCCGCCACCAAGGCTGGCTACTGCGGGGGAGCCGTTCTCGCCAAGGACGGAGC

CGATACGTTCATCGTTGGCACTCACTCCGCAGGTGGCAATGGGGTTGGGTACTGCTCATGTGTATCCAGG

TCCATGCTCCTCAAAATGAAGGCACACATTGACCCCGAACCACACCACGAG

>GU125649.1_O_VIT_2006

ATCTCAATTCCTTCCCAAAAGTCTGTGTTGTACTTCCTCATTGAGAAAGGCCAGCACGAAGCAGCAATTG

AATTCTTTGAGGGGATGGTACACGACTCCATCAAGGACGAGCTCCGACCCCTCATCCAACAGACCTCATT

TGTGAAACGCGCTTTCAAGCGCTTGAGGGAAAACTTTGAGATTGTTGCACTGTGTTTGACTCTTCTGGCA

AACATAGTAATCATGATCCGCGAGACCCGCAAGAGACAGCAAATGGTGGACGATGCAGTGAATGAGTACA

TCGAGAAAACAAACATCACCACGGATGACAAGACTCTTGACGAGGCGGAAAAGAACCCTCTGGAGACTAG

CGGTGTCAGCACCGTTGGTTTCAGAGAGAAAACTCTCCCAGGACACAGGACGTGTGATGACGTGAACTCC

GAGCCCGTTAAACCTGTGGAAGAGCAACCACAAGCTGAAGGACCCTACGCCGGACCGCTTGAGCGCCAGA

AACCTCTGAAAGTGAGAGCCAAGCTACCACAACAGGAGGGGCCTTACGCTGGTCCGATGGAGAGACAGAA

ACCACTGAAATTGAAAGTGAGCGCCCCGGTCGTGAAGGAAGGACCTTACGAGGGACCGGTGAAGAAGCCT

GTTGCCCTGAAAGTGAAAGCAAAGAACTTGATTGTCACTGAGAGTGGTGCTCCCCCGACCGATCTGCAGA

AGATGGTCATGAGCAACACTAAGCCTGTTGAGCTCATCCTCGACGGGAAGACAGTAGCCATCTGCTGTGC

TACTGGAGTGTTTGGTACTGCCTACCTCGTGCCTCGTCATCTTTTCGCTGAGAAGTACGACAAGATCATG

TTGGACGGTAGAGCCATGACAGACAGTGACTACAGAGTGTTTGAGTTTGAGATTAATGTAAAAGGACAGG

ACATGCTCTCAGACGCCGCTCTCATGGTGTTGCACCGTGGGAACCGCGTGAGGGACATCACGAAACACTT

TCGTGACACAGCAAGAATGAAGAAAGGTACCCCTGTCGTTGGCGTGATCAACAACGCTGACGTTGGGAGA

CTGATTTTCTCTGGTGAGGCCCTTACCTACAAGGACATTGTAGTGTGCATGGACGGAGACGCCATGCCGG

GCCTGTTCGCCTACAAAGCCGCCACCAAGGCTGGCTACTGCGGGGGAGCCGTTCTCACCAAGGACGGAGC

CGATACGTTCATCGTTGGCACTCACTCCGCAGGTGGCAATGGGGTTGGGTACTGCTCATGTGTATCCAGG

TCCATGCTCCTCAAAATGAAGGCACACATTGACCCCGAACCACACCACGAG

>GU125650.1_O_VIT_2006

ATCTCAATTCCTTCCCAAAAGTCTGTGTTGTACTTCCTCATTGAGAAAGGCCAGCACGAAGCAGCAATTG

AATTCTTTGAGGGGATGGTGCATGACTCCGTCAAGGAGGAGCTCCGACCCCTCATCCAACAGACCTCATT

TGTGAAACGCGCTTTCAAGCGCCTGAAGGAAAACTTTGAGATTGTTGCCCTGTGTTTGACTCTTTTGGCA

AACATAGTGATTATGATCCGCGAGACTCGCAAGAGACAACAAATGGTGGATGATGCAGTGAATGAGTACA

TTGAGAAAGCAAACATCACCACAGACGACAAGACTCTTGACGAGGCGGAAAAGAACCCTCTGGAGACCAG

CGGTGCCAGCACCGTTGGTTTCAGAGAGAGAACTCTCCCAGGACACAAGACGAGTGATGACGTGAACTCC

GAGCCCGCCAGACCTGTGGAAGAGCAACCACAAGCTGAAGGACCCTACGCCGGACCGCTTGAGCGCCAGA

AACCCCTGCAAGTGAGAGCCAAACTACCACTGCGGGAGGGGCCTTATGCCGGTCCGATGGAGAGACAGAA

ACCACTGAAAGTGAAAGTGAACGCCCCGGTCGTGAAGGAAGGACCTTACGAGGGACCGGTGAGAAAGCCT

GTCGCTTTGAAAGTGAAAGCTAAGAACTTGATTGTCACTGAGAGTGGTGCTCCCCCGACCGATCTGCAAA

AGATGGTCATGGGTAACACTAAGCCCGTTGAACTCATCCTCGACGGGAAGACAGTAGCCATCTGCTGTGC

TACTGGAGTGTTTGGTACTGCCTACCTCGTGCCTCGTCATCTTTTCGCTGAGAAGTACGACAAGATCATG

TTGGACGGCAGAGCCATGACAGACAGTGACTACAGAGTGTTTGAGTTTGAGATTAAAGTAAAAGGACAGG

ACATGCTCTCAGACGCCGCGCTCATGGTGCTGCACCGTGGGAACCGTGTGAGGGACATCACGAAGCACTT

TCGTGACACAGCAAGAATGAAGAAAGGCACCCCCGTCGTTGGCGTGATCAACAATGCTGACGTTGGGAGA

CTGATTTTCTCTGGAGAGGCCCTCACCTACAAGGACATTGTAGTGTGCATGGATGGAGACACCATGCCTG

GCCTGTTCGCCTACAGAGCCGCTACCAAAGCTGGCTATTGCGGGGGAGCCGTTCTCGCTAAGGACGGAGC

CGACACGTTCATCGTTGGCACTCACTCCGCAGGTGGCAATGGGGTTGGGTACTGCTCATGCGTATCCAGG

TCCATGCTCCTCAAAATGAAGGCACACATTGACCCCGAACCACACCACGAG

>GU384682.1_O_PAK_2008

ATCTCAATTCCTTCCCAAAAATCCGTGCTGTACTTCCTCATTGAGAAAGGCCAGCACGAGGCTGCAATTG

AATTCTTTGAGGGGATGGTGCATGACTCCATCAAGGAGGAGCTCCGACCTCTCATCCAACAGACATCATT

TGTGAAACGCGCTTTTAAGCGCTTGAAGGACAATTTTGAGGTTGTTGCCCTGTGTTTGACTCTTTTGGCA

AACATAGTGATCATGATCCGCGAGACTCACAAGAGACAGCAGATGGTGGATGATGCAGTGAATGAGTACA

TTGAGAAAGCCAACATCACCACAGATGACAAGACTCTTGATGAGGCGGAAAAGAACCCACTAGAAACCAG

TGGTGCCAGCACCATTGGTTTCAGAGAGAGAACTCTCCCAGGACACAAGGTGAGTGATGACGTGAACTCC

GAGCCCACCAAACCCGTGGAAGAGCAACCACAAACTGAAGGACCCTACGCCGGGCCACTTGAGCGTCAGA

AACCTCTGAAAGTGAGAGCCAAGCTCCCGCAGCAGGAGGGACCTTACGCTGGTCCGATGGAGAGACAAAA

ACCACTGAAAGTGAAAGCAAGAGCCCCGGTCGTTAAGGAAGGACCTTACGAGGGACCGGTGAAGAAGCCT

GTTGCTTTGAAAGTGAAAGCTAAGAACTTGATTGTCACTGAGAGTGGTGCCCCCCCGACCGACTTGCAAA

AGATGGTCATGGGCAACACTAAGCCTGTTGAGCTCATCCTCGACGGGAAGACGGTGGCCATCTGCTGTGC

TACCGGAGTGTTTGGCACTGCCTACCTCGTGCCTCGTCATCTTTTCGCTGAGAAGTATGACAAGATCATG

TTGGACGGTAGAGCCATGACAGACAGTGACTACAGAGTGTTTGAGTTTGAGATTAAAGTAAAAGGACAGG

ACATGCTCTCAGACGCCGCGCTCATGGTGCTCCACCGTGGGAACCGCGTGAGAGACATCACGAAACACTT

TCGTGATACAGCAAGAATGAAGAAAGGCACCCCCGTTGTCGGCGTGATCAACAATGCTGACGTCGGGAGA

CTGATTTTCTCTGGCGAGGCCCTCACCTACAAAGACATAGTAGTGTGCATGGATGGAGACACCATGCCTG

GCCTCTTTGCCTACAAAGCCGCCACCAAGGCTGGTTACTGTGGAGGAGCTGTTCTCGCAAAGGACGGAGC

CGAGACTTTTATCGTCGGCACTCATTCCGCAGGGGGCAATGGAGTTGGATACTGCTCATGCGTTTCCAGG

TCCATGCTTCTGAAAATGAAGGCGCACATCGACCCCGAACCGCACCACGAG

>GU384683.1_O_PAK_2008

ATCTCAATTCCTTCCCAAAAATCCGTGCTGTACTTCCTCATTGAGAAAGGCCAGCACGAGGCTGCAATTG

AATTCTTTGAGGGGATGGTGCATGACTCCATCAAGGAGGAGCTCCGACCTCTCATCCAACAGACATCATT

TGTGAAACGCGCTTTTAAGCGCTTGAAGGACAATTTTGAGGTTGTTGCCCTGTGTTTGACTCTTTTGGCA

AACATAGTGATCATGATCCGCGAGACTCACAAGAGACAGCAGATGGTGGATGATGCAGTGAATGAGTACA

TTGAGAAAGCCAACATCACCACAGATGACAAGACTCTTGATGAGGCGGAAAAGAACCCACTAGAAACCAG

TGGTGCCAGCACCATTGGTTTCAGAGAGAGAACTCTCCCAGGACACAAGGTGAGTGATGACGTGAACTCC

GAGCCCACCAAACCCGTGGAAGAGCAACCACAAGCTGAAGGACCCTACGCCGGGCCACTTGAGCGTCAGA

AACCTCTGAAAGTGAGAGCCAAGCTCCCGCAGCAGGAGGGACCTTACGCTGGTCCGATGGAGAGACAAAA

ACCACTGAAAGTGAAAGCAAGAGCCCCGGTCGTTAAGGAAGGACCTTACGAGGGACCGGTGAAGAAGCCT

GTTGCTTTGAAAGTGAAAGCTAAGAACTTGATTGTCACTGAGAGTGGTGCCCCCCCGACCGACTTGCAAA

AGATGGTCATGGGCAACACTAAGCCTGTTGAGCTCATCCTCGACGGGAAGACGGTGGCCATCTGCTGTGC

TACCGGAGTGTTTGGCACTGCCTACCTCGTGCCTCGTCATCTTTTCGCTGAGAAGTATGACAAGATCATG

TTGGACGGTAGAGCCATGACAGACAGTGACTACAGAGTGTTTGAGTTTGAGATTAAAGTAAAAGGACAGG

ACATGCTCTCAGACGCCGCGCTCATGGTGCTCCACCGTGGGAACCGCGTGAGAGACATCACGAAACACTT

TCGTGATACAGCAAGAATGAAGAAAGGCACCCCCGTTGTCGGCGTGATCAACAATGCTGACGTCGGGAGA

CTGATTTTCTCTGGCGAGGCCCTCACCTACAAAGACATAGTAGTGTGCATGGATGGAGACACCATGCCTG

GCCTCTTTGCCTACAAAGCCGCCACCAAGGCTGGTTACTGTGGAGGAGCTGTTCTCGCAAAGGACGGAGC

CGAGACTTTTATCGTCGGCACTCATTCCGCAGGGGGCAATGGAGTTGGATACTGCTCATGCGTTTCCAGG

TCCATGCTTCTGAAAATGAAGGCGCACATCGACCCCGAACCGCACCACGAG

>GU582115.1_O_VIT_2009

ATCTCAATCCCTTCCCAAAAGTCTGTGTTGTACTTTCTCATTGAGAAAGGCCAGCACGAAGCAGCAATTG

AATTCTTTGAGGGAATGGTGCACGACTCCATCAAGGAGGAGCTCCGGCCTCTCATCCAACAGACCTCATT

TGTGAAACGCGCCTTTAAGCGCCTGAAGGAAAATTTTGAGATTGTTGCCCTGTGTTTAACTCTTCTGGCA

AACATAGTGATCATGATCCGCGAGACTCGCAAGAGACAACAAATGGTGGACGATGCAGTGAATGAATACA

TTGAGAAGGCAAACATCACCACAGATGACAAGACTCTTGACGAGGCGGAAAAGAACCCTCTGGAGACTAG

CGGTGCCAGCACCGTCGGTTTCAGAGAGAGAACTCTCCCAGGACACAAGACGCGCGATGACGTGAACTCC

GAGCCCGCCAAACCTGTGGAAGAGCAACCACAAGCTGAAGGACCCTACGCCGGACCGCTTGAGCGCCAGA

AACCTCTGAAAGTGAGAGCCAAGCTACCACAGCAGGAGGGGCCTTATGCCGGTCCGATGGAGAGACAGAA

ACCACTGAAAGTGAAAGTGAACGCCCCGGTCGTGAAGGAAGGACCTTACGAGGGACCGGTGAAAAAGCCT

GTCGCTTTGAAAGTGAAAGCTAAGAACCTGATTGTCACTGAGAGTGGTGCTCCCCCGACCGATCTGCAAA

AGATGGTCATGGGCAACACTAAGCCTGTTGAGCTCATTCTCGATGGGAAGACAGTAGCCATTTGCTGTGC

TACTGGAGTGTTTGGTACTGCCTACCTCGTGCCTCGTCACCTCTTCGCCGAGAAGTACGACAAGATCATG

TTGGACGGCAGAGCCATGACAGACAGTGACTACAGAGTGTTTGAGTTTGAGATTAAAGTAAAAGGACAGG

ACATGCTCTCAGACGCCGCGCTCATGGTGCTGCACCGTGGGAACCGCGTGAGGGACATCACGAAACACTT

TCGTGATACAGCAAGAATGAAGAAAGGCACCCCCGTCGTTGGCGTGATCAACAATGCTGACGTTGGGAGA

CTGATCTTCTCTGGTGAGGCCCTTACCTACAAGGACATTGTGGTGTGCATGGACGGAGACACCATGCCGG

GCCTGTTTGCCTACAAAGCCGCTACCAAGGCTGGTTACTGCGGGGGAGCCGTTCTTGCTAAGGACGGAGC

CGACACGTTCATCGTTGGCACTCACTCCGCAGGTGGCAATGGAGTTGGGTACTGCTCATGCGTATCCAGG

TCCATGCTCCTCAAAATGAAGGCACACATTGACCCCGAACCGCACCATGAG

>GU582116.1_O_VIT_2009

ATCTCAATCCCTTCCCAAAAGTCTGTGTTGTACTTTCTCATTGAGAAAGGCCAGCACGAAGCAGCAGTTG

AATTCTTTGAGGGAATGGTGCACGACTCCATCAAGGAGGAGCTCCGGCCTCTCATCCAACAGACCTCATT

TGTGAAACGCGCCTTTAAGCGCCTGAAGGAAAATTTTGAGATTGTTGCCCTGTGTTTAACTCTTCTGGCA

AACATAGTGATCATGATCCGCGAGACTCGCAAGAGACAACAAATGGTGGACGACGCAGTGAATGAATACA

TTGAGAAGGCAAACATCACCACAGATGACAAGACTCTTGACGAGGCGGAAAAGAACCCTCTGGAGACTAG

CGGTGCCAGCACCGTCGGTTTCAGAGAGAGAACTCTCCCAGGACACAAGACGCGCGGTGACGTGAACTCC

GAGCCCGCCAAAACTGTGGAAGAGCAACCACAAGCTGAAGGACCCTACGCCGGACCGCTTGAGCGCCAGA

AACCTCTGAAAGTGAGAGCCAAGCTACCACAGCAGGAGGGGCCTTATGCCGGTCCGATGGAGAGACAGAA

ACCACTGAAAGTGAGAGTGAACGCCCCGGTCGTGAAGGAAGGACCTTACGAGGGACCGGTGAAAAAGCCT

GTCGCTTTGAAAGTGAAAGCTAAGAACCTGATTGTCACTGAGAGTGGTGCTCCCCCGACCGATCTGCAAA

AGATGGTCATGGGCAACACTAAGCCTGTTGAGCTCATTCTCGATGGGAAGACAGTAGCCATTTGCTGTGC

TACTGGAGTGTTTGGTACTGCCTACCTCGTGCCTCGTCACCTCTTCGCCGAGAAGCACGACAAGATCATG

TTGGACGGCAGAGCCATGACAGACAGTGACTACAGAGTGTTTGAGTTTGAGATTAAAGTAAAAGGACAGG

ACATGCTCTCAGACGCCGCGCTCATGGTGCTGCACCGTGGGAACCGCGTGAGGGACATCACGAAACACTT

TCGTGATACAGCAAGAATGAAGAAAGGCACCCCCGTCGTTGGCGTGATCAACAATGCTGACGTTGGGAGA

CTGATCTTCTCTGGTGAGGCCCTTACCTACAAGGACATTGTGGTGTGCATGGACGGAGACACCATGCCGG

GCCTGTTTGCCTACAAAGCCGCTACCAAGGCTGGTTACTGCGGGGGAGCCGTTCTTGCTAAGGACGGAGC

CGACACGTTCATCGTTGGCACTCACTCCGCAGGTGGCAATGGAGTTGGGTACTGCTCATGCGTATCCAGG

TCCATGCTCCTCAAAATGAAGGCACACATTGACCCCGAACCGCACCATGAG

>GU931682.1_Asia1_CHA_2005

ATCTCAATTCCTTCCCAAAAGTCTGTGCTGTACTTCCTCATCGAGAAAGGCCAGCATGAGGCAGCAATTG

AATTCTTTGAGGGAATGGTGCACGACTCCATTAAGGAGGAACTCCGGCCCCTCATCCAACAGACCTCATT

TGTGAAACGCGCTTTCAAGCGCCTGAAGGAAAACTTTGAGATTGTTGCCCTGTGTTTGACTCTTTTGGCA

AACATAGTGATCATGATCCGCGAGACGCGCAAGAGACAGCAGATGGTGAATGATGCGGTGAACGAGTACA

TCGACAAAGCCAACATCACCACAGATGACAAGACTCTTGACGAGGCGGAAAAGAACCCTCTGGAGACCAG

TGGTGCTAGCACCGTTGGTTTCAGAGAGAGAACCCTCCCGGGGCGCAAGACGAGTGATGACGTGTACTCC

GAGCCCGTCAAACCCGTGGAGGAACAACCACAAGCTGAAGGACCCTACGCCGGGCCACTCGAGCGTCAGA

AACCTCTGGAAGTGAGAGCCAAGCTCCCACAGCAAGAGGGACCTTACGCTGGCCCGATGGAGAGACAGAA

ACCACTGAAAGTGAAAGCAAAAGCCCCGGTCGTTAAGGAAGGGCCTTACGAAGGACCGGTGAAGAAACCT

GTCGCTTTGAAAGTGAAAGCAAAGAATTTGATTGTCACTGAGAGTGGTGCCCCACCGACTGACTTGCAAA

AGATGGTCATGGGCAACACCAAGCCTGTTGAGCTCATCCTCGACGGCAAGACGGTAGCCATCTGCTGCGC

TACCGGAGTCTTTGGTACTGCCTACCTCGTGCCTCGTCACCTTTTCGCAGAGAAGTACGACAAGATCATG

CTGGACGGCAGAGCCATGACAGACAGTGACTACAGAGTGTTTGAGTTTGAGATTAAAGTAAAAGGACAGG

ACATGCTCTCAGACGCCGCACTCATGGTGCTCCACCGTGGGAATCGCGTGCGTGACATCACGAAGCACTT

CCGTGATGTAGCCAAGATGAAGAAAGGAACCCCCGTCGTTGGTGTGATTAACAACGCCGACGTTGGGAGA

CTGATTTTCTCTGGTGAGGCCCTAACCTACAAAGACATTGTAGTGTGCATGGACGGAGACACCATGCCTG

GCCTTTTTGCCTACAAGGCCGTCACCAGGGCGGGCTACTGTGGAGGAGCCGTTCTCGCGAAGGACGGAGC

CGAGACTTTCATCGTCGGCACTCACTCCGCAGGCGGCAACGGAGTTGGATACTGCTCGTGCGTTTCCAGG

TCTATGCTGCTCAAGATGAAGGCTCACATTGATCCCGAACCACACCACGAG

>HM008917.1_O_CHA_2005

ATCTCAATTCCTTCCCAAAAGGCTGTGCTGTACTTTCTCATTGAGAAGGGCCAGCACGAAGCAGCAATTG

AATTCTTTGAGGGGATGGTGTGTGACTCCATTAAGGAGGAGCTCCGGCCCCTAATCCAACAGACCTCATT

TGTGAAGCGCGCTTTTAAGCGCCTGAAGGAAAACTTTGAGATTGTCGCTCTGTGTTTGACCCTTTTGGCG

AACATAGTGATCATGATCCGCGGGACTCGCAAGAGACAGCAGATAGTGGACGATGTAGTGGACGAGTACA

CTGAGAAGGCAAACATCGCCACGGATGACAAGACTCTTGACGAGGCGGAAAAGAACCCTCTGGAGGCCAG

TGGTGCCACCACTGTTGGTTTCAGAGAGAAAACTCTCCCGGGACACAAGGCGGGTGATGACGTGAGCTCT

GAGCCCGCCGAACCCGTGGAAGGGCAACCACAGGCTGAAGGACCCTACACCGGTCCACTCGAGCGTCAAA

AACCTCTGAAAGTGAGAGCCAGGCTCCCACAGCAGGAGGGGCCCTACGCTGGCCCGATGGAGAGACAGAA

ACCGCTGAAAGTGAAAGTGAAAGCCCCGGTCGTTAAGGAAGGACCTTACGAAGGACCGGTGAAGAAACCT

GTCGCTCTGAAAGTGAAAGCAAAGAACTTGATTGTCACTGAGAGTGGTGCTCCCCCGACTGACTTGCAAA

AGATGGTCATGGGCAACACCAAGCCTGTTGAGCTCATCCTCGACGGGAAGACGGTGGCCATCTGCTGCGC

CACCGGAGTGTTTGGTACCGCCTACCTTGTTCCTCGCCATCTTTTCGCAGAGAAGTACGACAAGATCATG

TTGGACGGCAGAGCCATGACAGACAGTGACTACAGAGTGTTTGAGTTTGAGATTAAAGTGAAAGGGCAGG

ACATGCTCTCGGACGCCGCGCTCATGGTGCTCCACCGTGGGAATCGCGTGCGGGACATCACGAAGCACTT

CCGTGATGTGGCAAGAATGAAGAAAGGCACCCCCGTCGTCGGCGTGGTCAACAACGCTGATGTTGGGAGA

CTGATCTTCTCTGGTGAGGCCCTTACCTACAAGGACATTGTAGTGTGCATGGACGGAGACACCATGCCCG

GTCTCTTCGCCTACAAAGCCGCCACCAAGGCGGGTTACTGTGGAGGAGCCGTTCTTGCAAAGGACGGAGC

CGAGACTTTCATCGTCGGCACTCACTCCGCAGGCGGCAACGGAGTTGGCTACTGCTCGTGCGTTTCCAGG

TCTATGCTGCTAAAAATGAAGGCACACATCGATCCCGAACCACACCACGAG

>HM055510.1_O_VIT_2009

ATCTCAATTCCTTCCCAAAAGTCAGTGCTGTATTTCCTCATTGAGAAAGGCCAACACGAAGCAGCAATTG

AATTCTTTGAGGGGATGGTCCATGACTCCATCAAGGAAGAGCTCCGACCCCTCATCCAACAGACATCATT

TGTCAAGCGCGCCTTCAAGCGCCTGAAGGAAAACTTTGAGATTGTTGCCCTATGTTTGACTCTCATGGCA

AACATAGTGATCATGATCCGCGAGACTCGCAAGAGACAGCAGATGGTGGATGATGCAGTGAATGAGTACA

TCGAGAAAGCAAACATCACCACAGATGACAAGACTCTTGACGAGGCGGAAAAGAACCCTCTAGAGACTAG

CGGTGCCAGCACTGTTGGTTTCAGAGAGAGAACTCTCCCGGGACACAAGGTGGGTGATGACGTGAACTCC

GAGCCCGCCCACCCCGGGGATGAGCAACCACAAGCTGAAGGACCCTACGCCGGACCACTCGAGCGCCAGA

GACCTCTGAAAGTGAGAGCCAAGCTGCCACAGCAGGAGGGACCTTACGCCGGTCCGATGGAGAGACAGAA

ACCACTGAAAGTGAAAGCGAAAGCCCCGGTCGTGAAGGAAGGACCTTACGAGGGGCCGGTGAAGAAGCCT

GTCGCTTTGAAAGTGAAAGCTAAGAACTTGATCGTCACTGAGAGTGGTGCCCCCCCGACCGACTTGCAAA

AGATGGTCATGGGTAACACCAAGCCCGTTGAGCTCATACTCGACGGGAAGACAGTAGCCATCTGCTGTGC

TACTGGAGTATTTGGCACTGCCTACCTCGTGCCTCGTCATCTTTTCGCTGAGAAGTACGACAAGATCATG

TTGGACGGTAGAGCCATGATAGACAGTGACTACAGAGTGTTTGAGTTTGAGATTAAAGTAAAAGGACAGG

ACATGCTCTCAGACGCTGCGCTCATGGTGCTGCACCGTGGGAACCGCGTGAGAGACATCACGAAACACTT

TCGTGACACAGCAAGAATGAAGAAAGGCACCCCCGTCGTTGGTGTGATCAACAACGCTGACGTCGGGAGA

CTGATTTTCTCAGGTGAGGCCCTCACCTACAAGGACATTGTAGTGTGCATGGATGGAGACACCATGCCGG

GCCTATTTGCCTACAAAGCCGCCACCAAGGCTGGCTACTGCGGGGGAGCCGTCCTTGCTAAGGATGGAGC

CGACACATTCATCGTTGGCACTCACTCTGCAGGTGGCAATGGAGTCGGGTACTGCTCATGCGTATCCAGA

TCCATGCTCCAGAAAATGAAGGCACACATCGACCCTGAACCACACCACGAG

>HM067704.1_SAT2_UGA_2007

ATTTCCATCCCGTCCCAAAAATCTGTGCTCTACTTCCTTATCGAGAAGGGGCAGCACGAAGCGGCAATTG

AATTCTACGAAGGGATGGTACACGACTCCATCAAGGAAGAGCTTAAGCCACTCCTCGAGCAAACCAGCTT

CGCGAAGCGTGCTTTTAAGCGCCTTAAGGAGAACTTTGAGATTGTTGCGCTGGTGGTGGTGCTGTTGGCC

AACATTGTCATCATGATCCGCGAAACTCACAAGAGACAAAAAATGGTGGACGATGCGCTGGACGAGTACA

TCGAGAAGGCAAACATCACCACTGACGACCAGACTCTTGACGAGGCGGAAAAGAGCCCACGGGAGACCAT

TGATAAACCCACTTCAGGTTTCCGTGAGAGAAAGCTCCCGGGCCAAACCTCTGGTGATGAAGTGAACTCC

GAGCCAGGGAAACCC---GCGGACAAACCACAAGCTGAAGGACCCTACGCTGGACCGCTGGAGCGCCAGC

AGCCACTGAAGCTGAGAACTAAGCTTCCTGCGGCTGAAGGCCCGTATGCCGGACCACTAGAGAAACAGCA

ACCACTCAAGCTTAAGACTAAACTGCCTGTGGCCAAAGAAGGGCCATACGAAGGACCAGTGAAGAAGCCT

GTCGCCTTGAAAGTGAAAGCAAAACCAGCAATAGTCACTGAGAGCGGTGCGCCACCGACCGACTTGCAAA

AGATGGTCATGGCCAACGTCAAGCCTGTCGAGCTTATCCTCGACGGGAAGATCGTCGCGCTCTGTTGTGC

GACGGGAGTGTTTGGGACCGCCTATCTTGTGCCTCGTCATCTTTTCGCCGAGAAGTATGACAAGATCATG

TTGGATGGGCGTGCCCTGCAAAACAGTGACTTTAGAGTGTTTGAGTTTGAGGTGAAAGTAAAGGGACAGG

ACATGCTCTCAGACGCCGCCCTCATGGTTCTAAACCGTGGACAGCGCGTTAGAGACATAACAGCCCATTT

CCGCGACACTGTTCGTGTTGCGAAAGGTAACCCCGTTGTCGGTGTGGTCAACAACGCCGACGTCGGGAGA

CTCATCTTCTCAGGAGATGCTCTTACGTACAAGGACGTCGTCGTCTGCATGGACGGCGACACCATGCCTG

GCCTCTTCGCATACCGCGCTGGGACCAAGGTGGGTTACTGCGGAGCAGCCGTGCTGACCAAGAGTGGTAG

CCAGACAGTTATCATTGGCACCCACTCTGCGGGAGGCAACGGCGTGGGCTACTGCTCGTGTGTCTCAAAA

TCGATGCTTGACCAGATGAAAGCCCACATCGACCCAGCGCCCCACACCGAG

>HM067705.1_SAT2_UGA_2007

ATCTCCATCCCATCCCAAAAGTCGGTGCTTTACTTCCTCATTGAGAAAGGACAGCACGAGGCAGCAATTG

AATTCTATGAAGGAATGGTTCATGACTCCATCAAGGAAGAACTTAAGCCGCTTCTCGAGCAAACCAGCTT

CGCGAGGCGAGCCTTCAAGCGCCTGAAGGAGAACTTTGAGCTTGTTGCGCTAGTGGTGGTACTGCTGGCC

AACATTGTCATCATGATCCGCGAAACCCACAAGAGGCAGAAGATGGTCGATGATGCGCTGAACGAGTATA

TCGAGAAGGCCAACATCACCACTGACGACCAAACTCTTGACGAGGCGGAAAAGAACCCTCGCGAAACCAT

CGACAAACCCACTGCTGGTTTTCGTGAGAGAAAACTCCCAGGTCAACCCTCTGACAAGGATGTGAACACT

GAGCCGGAGAAACCT---GAGGAGAAACCGCAAGCTGAAGGACCCTATGCCGGACCGCTGGAGCGCCAAC

AGCCACTGAAGCTCAAAGCTAAGCTTCCCGCGACTGAAGGCCCGTACGCCGGACCGCTAGAGAAACAGCA

ACCACTCAAGCTTAAGACTAAACTGCCTGTGGCCAAGGAAGGGCCATATGAAGGACCAGTCAAGAAGCCT

GTCGCCTTGAAAGTGAAAGCAAAACCAGCGATAGTCACTGAGAGCGGTGCGCCACCGACCGATTTGCAGA

AGATGGTCATGGCCAACGTTAAGCCCGTCGAACTCATCCTCGATGGGAAGATTGTCGCGCTCTGCTGCGC

GACGGGAGTGTTCGGGACCGCCTACCTCGTGCCTCGTCATCTTTTCGCTGAGAAGTATGACAAGATCATG

TTGGACGGGCGTGCCCTAACAAACAGTGACTTCAGGGTGTTTGAGTTTGAGGTGAAAGTAAAGGGACAGG

ACATGCTCTCAGACGCTGCCCTCATGGTTCTGAACCGTGGACAACGCGTTAGAGACATCACAGCCCACTT

CCGCGACACAGTTCGTGTCGCGAAAGGTAACCCCGTTGTCGGCGTGGTCAACAACGCCGACGTCGGGAGG

CTCATCTTCTCAGGAGAAGCCCTCACCTACAAGGACATCGTCGTCTGTATGGACGGTGATACCATGCCTG

GTCTGTTCGCGTACCGCGCCGGGACCAAGGTAGGATACTGTGGGGCCGCCGTGCTAACCAAAAGTGGGAG

CCAAACAGTCATCATTGGCACTCACTCTGCAGGTGGCAACGGCGTGGGCTACTGTTCGTGTGTCTCAAAA

TCGATGCTCGATCAGATGAAAGCCCACATCGACCCGGCACCTCACACTGAG

>HM067706.1_SAT1_UGA_2007

ATTTCCATTCCATCCCAAAAGTCAGTGCTTTACTTCCTCATTGAGAAAGGACAGCACGAAGCAGCAATTG

AGTTTTATGAGGGAATGGTCCATGACTCCATCAAGGAAGAGCTTAGACCGCTTCTCGAGCAGACCAGCTT

CGCGAAGCGAGCTTTCAAGCGCCTGAAGGAAAACTTTGAAATTGTTGCGCTGGTAGTAGTGCTGCTAGCC

AACATTGTAATCATGATCCGCGAGACTCACAAGAGACAGAAGATGGTGGATGATGCGCTGGACGAGTACA

TCGAAAAGGCCAACATCACCACTGATGATCAGACTCTTGACGAAGCGGAAAAGAACCCACGCGAAACCAT

AGACAAACCCACTGCTGGTTTCCGCGAGAGGAAACTCCCAGGCCAACCCTCTGACAAGGACGTGAACACT

GAGCCAGAGCAACCT---GTGGAGAAACCACAAGCTGAAGGACCCTACGCTGGACCACTAGAGCGCCAAC

AACCGCTGAAGCTCAAAACCAAGCTCCCCGCGGCTGAAGGCCCCTACGCCGGACCGCTAGAGAGACAACA

ACCACTCAAGCTTAAGACTAAACTACCTGTGGCCAAGGAAGGGCCATATGAAGGACCAGTCAAGAAACCT

GTCGCCTTGAAAGTGAAAGCAAAACCAGCGATAGTCACTGAGAGCGGTGCGCCGCCGACCGACTTGCAAA

AGATGGTCATGGCCAACGTTAAGCCTGTTGAGCTCATCCTCGACGGGAAGATCGTCGCCCTCTGTTGCGC

GACGGGAGTGTTCGGGACCGCCTACCTCGTGCCTCGTCATCTTTTCGCTGAGAAGTATGACAAGATCATG

TTGGACGGGCGTGCCCTGACAAACAGTGACTTCAGAGTGTTTGAGTTTGAGGTGAAAGTAAAGGGACAGG

ACATGCTCTCAGACGCCGCCCTCATGGTTCTGAACCGTGGACAGCGCGTTAGAGACATCACAGCCCATTT

CCGCGACACCGTTCGTGTTGCGAAAGGAAACCCCGTTGTCGGCGTTGTTAACAATGCTGACGTTGGGAGA

CTCATCTTCTCAGGAGATGCCCTCACGTACAAAGACATCGTCGTCTGTATGGACGGCGATACCATGCCTG

GCCTCTTCGCGTACCGCGCTGGGACTAAGGTAGGTTACTGTGGGGCAGCCGTGCTGACCAAGAGTGGCAG

CCAAACAGTCATCATTGGCACTCACTCTGCGGGAGGCAACGGCGTGGGCTACTGCTCGTGCGTCTCAAAA

TCGATGCTCGATCAGATGAAAGCCCACATCGACCCAGCACCTCACACTGAG

>HM191257.1_O_UGA_2006

ATTTCAATTCCTTCTCAAAAGTCCGTGTTGTACTTCCTCATCGAGAAAGGCCAACACGAAGCAGCAATTG

AATTCTTTGAGGGAATGGTGCACGACTCCATCAAGGACGAGCTCCGACCTCTCGTTCAACAGACCTCATT

TGTGAAACGCGCTTTCAAGCGCCTGAAGGAAAACTTTGAAATTGTTGCCCTGTGTTTGACTCTTCTGGCA

AACATAGTGATCATGATCCGTGAAACTCGCAAGAGACAACAGATGGTGGATGACGCAGTGAATGAGTATA

TAGAGAAAGCAAACATCACCACAGATGACAAGACTCTTGACGAGGCGGAAAAGAACCCTCTGGAGACTAG

CGGTGCCAGCACCGTCGGTTTCAGAGAGAGAACCCTCCCGGGCCAAAAGGCGAGTGATGACGTGAACTCC

GAGCCCGCCAAACCCGTGGATGAACAACCACAAGCTGAAGGACCCTACGCCGGGCCACTCGAGCGTCAGA

AACCTCTGAAAGTGAGAGCCAAACTACCACAACAGGAGGGACCTTACGCTGGCCCGATGGAGAGACAGAA

ACCACTAAAAGTGAAAGCAAAAGCCCCGGTCGTTAAGGAAGGACCTTACGAGGGACCGGTGAAGAAACCT

GTCGCTTTGAAAGTGAAAGCTAAGAATCTGATTGTCACTGAGAGTGGCGCCCCACCGACCGACTTGCAAA

AGATGGTCATGGGCAATACCAAGCCTGTTGAACTCATCCTCGACGGGAAGACGGTAGCCATCTGCTGTGC

TACCGGAGTGTTTGGCACTGCCTACCTCGTGCCTCGTCATCTCTTTGCGGAGAAGTATGACAAGATCATG

TTGGACGGCAGAGCTATGACAGACAGTGACTACAGAGTGTTTGAGTTTGAGATTAAAGTAAAAGGACAGG

ACATGCTCTCAGATGCCGCGCTCATGGTGCTCCACCGCGGGAACCGCGTGAGAGACATCACAAAACACTT

CCGTGACACGGCAAGAATGAAGAAGGGCACCCCCGTTGTCGGTGTGATCAACAATGCTGACGTCGGGAGA

TTAATTTTCTCTGGTGAGGCCCTCACCTACAAGGACATTGTAGTGTGCATGGATGGAGACACCATGCCAG

GCCTCTTTGCCTACAGAGCCGCCACCAAGGCTGGATACTGCGGAGGGGCCGTTCTCGCCAAGGACGGGGC

TGACACCTTCATCGTCGGCACTCACTCTGCAGGGGGTAATGGAGTTGGATACTGCTCGTGCGTGTCCAGG

TCCATGCTCCTGAAAATGAAGGCACACATTGACCCTGAACCACACCACGAA

>HM229661.1_O_HKN_2010

ATCTCAATTCCTTCCCAAAAGTCAGTGCTGTATTTCCTCATTGAGAAAGGCCAACACGAAGCAGCAATTG

AATTCTTTGAGGGGATGGTCCATGACTCCATCAAGGAAGAGCTCCGACCCCTCATCCAACAGACATCATT

TGTCAAGCGCGCCTTCAAGCGCCTGAAGGAAAACTTTGAGATTGTTGCCCTATGTTTGACTCTCATGGCA

AACATAGTGATCATGATCCGCGAGACTCGCAAGAGACAGCAGATGGTGGATGATGCGGTGAACGAGCACA

TCGAGAAAGCAAACATCACCACAGATGACAAGACTCTTGACGAGGCGGAAAAGAACCCTCTAGAGACTAG

CGGTGCCAGCACTGTTGGTTTCAGAGAGAGAACTCTCCCGGGACACAAGGTGGGTGATGACGTGAACTCC

GAGCCCGCCCACCCCGGGGATGAGCAACCACAGGCTGAAGGACCCTACGCCGGACCACTCGAGCGCCAGA

GACCTCTGAAAGTGAGAGCCAAGCTGCCACAGCAGGAGGGACCTTACGCCGGTCCGATGGAGAGACAGAA

ACCACTGAAAGTGAGAGCGAAAGCCCCGGTCGTGAAGGAAGGACCTTACGAGGGACCGGTGAAGAAGCCT

GTCGCTTTGAAAGTGAAAGCTAAGAACTTGATCGTCACTGAGAGTGGTGCCCCCCCGACCGACTTGCAAA

AGATGGTCATGGGTAACACCAAGCCCGTTGAGCTCATACTCGACGGGAAGACAATAGCCATCTGTTGTGC

TACTGGAGTGTTTGGTACTGCCTACCTCGTGCCTCGTCATCTTTTCGCTGAGAAGTACGACAAGATCATG

TTGGACGGTAGAGCCATGATAGACAGTGACTACAGAGTGTTTGAGTTTGAGATCAAAGTAAAAGGACAGG

ACATGCTCTCAGACGCTGCGCTCATGGTGCTGCACCGTGGGAACCGCGTGAGAGACATCACGAAACACTT

TCGTGACACAGCAAGAATGAAGAAAGGCACCCCCGTCGTTGGCGTGATCAACAACGCTGACGTCGGGAGA

CTGATTTTCTCAGGTGAGGCCCTCACCTACAAGGACATTGTAGTGTGCATGGATGGAGACACCATGCCGG

GCCTATTTGCCTACAAAGCCGCCACCAAGGCTGGCTACTGCGGGGGAGCCGTCCTTGCTAAGGATGGAGC

TGACACATTCATCGTTGGCACTCACTCTGCAGGTGGCAATGGAGTTGGGTACTGCTCATGCGTATCCAGA

TCCATGCTCCAGAAAATGAAGGCACACATCGACCCTGAACCACACCACGAG

>HM854021.1_A_IND_2000

ATCTCAATTCCTTCCCAAAAGTCCGTGTTGTACTTTCTCATTGAGAAGGGGCAACACGGAGCAGCAATTG

AATTCTTTGAGGGGATGGTCAGTGACTCCGTCAAGGAGGAGCTCCGACCCCTCATCCAACAGACCTCATT

TGTGAAACGTGCGTTCAAACGCTTGAAGGCAAACTTTGAGATTGTTGCCCTATGTTTGACCCTTTTGGCA

AACATAGTGATCATGATCCGCGAGACTCGTAAGAGGCAACAAATGGTGGATGATGCAGTGAATGAGTACA

TTGAGAAAGCAAACATCACCACAGACGACAAGACTCTTGATGAGGCGGAAAAGAACCCTCAGGAGACTAG

CAGTGCCAGCACTGTTGGCTTCAGAGAGAGGACTCTTCCAGGACAGAAGGTGGGTGATGACGTGAAATCC

GAGCCCACCGAACCTGCGAGAGAGCAACCACAAGCTGAAGGACCCTACGCCGGGCCACTCGAGCGTCAGA

AACCTCTGAAGGTGAGAGCCAAGCTACCACAACAGGAGGGACCTTACGTTGGC---------AGACAGAA

ACCACTTAAAGTGAAAGCAAAAGCCCCGGTCGTTAAGGAAGGACCTTACGAGGGACCGGTGAAGAAACCT

GTCGCTTTGAAAGTGAAAGCTAAGAACTTGATTGTAACTGAGAGTGGAGCCCCACCGACTGACCTGCAAA

AGATGGTCATGGGCAACACGAAGCCTGTTGAGCTCATCCTCGACGGGAAGACAGTAGCCATCTGCTGTGC

TACTGGAGTGTTCGGCACTGCCTACCTCGTGCCTCGTCATCTTTTCGCAGAGAAGTATGACAAGATCATG

TTGGACGGTAGAGCCATGACAGACAGTGACTACAGAGTGTTTGAGTTTGAGATAAAAGTGAAAGGACAAG

ACATGCTCTCAGACGCCGCGCTCATGGTGCTTCACCGTGGGAATCGCGTGCGGGACATCACGAAGCACTT

CCGTGATGTGGCAAAAATGAAGAAAGGCACACCCGTCGTTGGCGTGATCAACAACGCCGATGTCGGGAGA

CTGATTTTCTCTGGTGAGGCCCTCACCTACAAGGACATTGTAGTGTGCATGGACGGAGACACCATGCCTG

GCCTCTTTGCCTACAAAGCCGCCACCAAGGCTGGCTACTGTGGAGGAGCCGTTCTTGCCAAGGACGGTGC

CGAGACATTCATCGTTGGCACTCACTCCGCAGGTGGCAATGGAGTTGGATACTGCTCCTGTGTTTCCAGG

TCCATGCTCATGAAAATGAAGGCACACATCGACCCTGAACCACACCACGAG

>HM854022.1_A_IND_1977

ATCTCAATTCCTTCCCAAAAGTCCGTGTTGTACTTTCTCATCGAGAAGGGGCAGCACGAAGCAGCAATTG

AATTCTTTGAGGGGATGGTGCACGACTCCATCAAGGAAGAACTCCGACCCCTCCTCCAACAGACCTCATT

TGTGAAGCGCGCGTTTAAGCGCCTGAAGGAAAACTTTGAGATTGTTGCCCTGTGTTTGACTCTCCTGGCA

AACATAGTGATTATGATCCGCGAGACTCGCAAGAGACAGCAGATGGTGGATGATGCAGTGAATGAGTACA

TTGAGAAAGCAAACATCACCACAGACGACAAGACTCTTGACGAAGCGGAAAAGAACCCTCTGGAGACTAG

CGGTGCCAGCACTGTTGGCTTTAGAGAGAGAACTCTCCCGGGAAACAAGGTGAGTGATGACGTGAACTCC

GAGCCCACCAAACCCGCGGAAGAACAACCACAAGCTGAAGGACCCTACGCCGGGCCACTCGAGCGTCAGA

AACCTCTGAAAGTGAGAGCCAAGCTCCCACAGCAGGAGGGACCCTACGCTGGCCCGCTGGAGAGACAGAA

ACCACTGAAAGTGAAAGCAAAAGCCCCGGTCGTGAAGGAAGGACCTTACGAGGGACCGGTGAAGAAGCCT

GTCGCTTTGAAAGTGAAAGCTAAGACCTTGATTGTCACTGAGAGTGGTGCCCCACCGACCGACTTGCAAA

AGATGGTCATGGGCAACACAAAGCCTGTTGAGCTCATCCTCGACGGGAAGACAGTAGCCATCTGCTGCGC

TACTGGAGTGTTCGGTACTGCCTACCTCGTGCCTCGCCATCTTTTCGCTGAGAAGTATGACAAGATCATG

TTGGACGGCAGAGTCCTGACAGACAGTGATTATAGAGTGTTTGAGTTTGAGATCAAAGTAAAAGGACAGG

ACATGCTCTCAGACGCCGCGCTCATGGTGCTGCACCGGGGGAACCGCGTGAGAGACATCACGAAACACTT

TCGTGACACAGCAAGAATGAAGAAAGGCACCCCCGTCGTTGGTGTGATCAACAACGCTGATGTCGGGAGA

CTGATTTTCTCTGGTGAGGCCCTTACCTACAAGGACATTGTAGTGTGCATGGATGGAGACACCATGCCTG

GCCTCTTTGCCTACAAAGCCGCCACCAAGGCCGGCTACTGTGGAGGAGCCGTTCTCGCCAAGGACGGAGC

CGACACATTCATCGTTGGCACACACTCCGCAGGTGGCAATGGAGTTGGATACTGTTCTTGCGTTCCCAGG

TCCATGCTTTTGAAGATGAAGGCACACATTGACCCTGAACCACACCACGAG

>HM854023.1_A_IND_1999

ATCTCAATTCCTTCCCAAAAGTCAGTGTTATACTTCCTCATTGAGAAAGGCCAACACGAAGCAGCAATTG

AATTCTTTGAGGGGATGGTGCACGACTCCATCAAGGAGGAGCTCCGACCCCTCATCCAACAGACATCATT

TGTGAAACGCGCTTTCAAGCGCCTGAAGGAAAATTTTGAGATTGTTGCCCTATGTTTGACTCTCCTGGCA

AACATAGTGATCATGATCCGCGAGACTCGCAAGAGACAACGGATGGTGGATGATGCAGTGAATGAGTACA

TTGAGAAAGCAAACATCACCACAGATGACAAGACTCTTGACGAGGCGGAAAAGAACCCTCTAGAGACCAG

TGGTGCCAGCACTGTTGGTTTCAGAGAGAAAGTCCTCCCGGGACACAGGGCGGGTGACGACGTGAATTCC

GAGCCCGCCCAGACCGTGGAGGAACAACCACAAGCTGAAGGACCCTACGGCGGACCACTCGAGCGTCAGA

AACCTCTGAAAGTGAGAGCCAAGCTCCCACAGCAGGAGGGACCTTACGCTGGCCCGATGGAGAGACAGAA

ACCACTGAAAGTGAAAGCAAAAGCCCCGGTCGTTAAGGAAGGACCTTACGAGGGACCGGTGAAGAAGCCT

GTCGCTTTGAAAGTGAAAGCTAAGAACTTGATTGTTACTGAGAGTGGTGCCCCCCCGACCGACTTGCAAA

AGATGGTCATGGGTAACACCAAGCCTGTTGAGCTCATTCTCGACGGGAAGACAGTAGCCATCTGCTGTGC

TACTGGAGTGTTTGGCACAGCCTACCTCGTGCCTCGTCATCTCTTCGCGGAGAAGTATGACAAGATCATG

TTGGACGGCAGAGCCATGACAGACAGTGACTACAGAGTGTTTGAGTTTGAGATTAAAGTAAAAGGACAGG

ACATGCTCTCAGACGCTGCGCTCATGGTGCTCCACCGTGGGAACCGCGTGAGAGACATCACGAAACACTT

TCGTGATGCAGCAAGAATGAAGAAAGGCACCCCCGTCGTTGGTGTGATCAACAACGCCGACGTTGGGAGA

CTGATTTTCTCTGGTGAGGCCCTCACCTACAAAGACATTGTGGTGTGCATGGATGGCGACACCATGCCTG

GCCTCTTTGCCTACAGAGCCGCCACCAAGGCTGGCTACTGCGGAGGAGCCGTTCTCGCGAAGGATGGCGC

TGACACATTCATCGTTGGCACCCACTCTGCAGGTGGCAATGGAGTCGGATACTGCTCATGCGTTTCCAGG

TCCATGCTTCTGAAAATGAAGGCACACATTGACCCTGAACCACACCACGAG

>HQ009509.1_O_CHA_1999

ATCTCAGTTCCCTCCCAAAAGGCTGTGCTGTACTTTCTCATTGAGAAGGGCCAGCACGAAGCAGCAATTG

AATTCTTTGAGGGGATGGTGTGTGACTCCGTCAAGGAGGAGCTCCGGCCTCTCGTCCAACAGACCTCATT

TGTGAAGCGCGCTTTTAAGCGCCTGAAGGACAACTTTGAGATAGTCGCTCTGTGTTTGACCCTTCTGGCG

AACATGGTGATCATGATCCGCGAGACTCGCAAGAGACAGCAGACAGTGGACGATGTAGTGGACGAGTATA

CTGAGAAGGCGAACATCGCCACGGATGACAAGACTCTTGACGGGGCGGAAAAGAGCCCTCTGGAGACCAG

TGGTGCCGCCACTGTCGGTTTCAGAGAGAAAACTCTCCCGGGGCACAAGGCGGGTGATGACGTGAACTCT

GAGCCCGCCGAACCCGTGGAAGGACAACCACAGGCTGAGGGACCTTATACCGGTCCACTCGAGCGCCAGA

AACCTCTGAAAGTGAGAGCCAGGCTCCCACAGCAGGAAGGGCCCTACGCTGGTCCGATGGAGAGACAGAA

ACCGCTGAAAGTGAAAGTGAAAGCCCCGGTCGTCAAGGAAGGACCTTACGAAGGGCCGGTGAAGAAACCT

GTCGCTCTAAAAGTAAAAGCAAAGAACTTGATTGTCACTGAGAGTGGTGCTCCCCCGACTGACTTGCAAA

AGATGGTCATGGGTAACACCAAACCTGTTGAACTCATCCTCGACGGGAAGACGGTAGCCATCTGCTGCGC

CACCGGAGTGTTTGGTACCGCCTACCTTGTTCCTCGTCATCTCTTCGCAGAGAAGTATGACAAGATCATG

TTGGACGGCAGAGCTATGACAGACAGTGACTATAGAGTGTTTGAATTCGAGATTAAAGTGAAAGGACAGG

ATATGCTCTCGGACGCCGCGCTCATGGTGCTCCACCGTGGGAATCGCGTGCGGGACATCACGAAGCACTT

CCGTGATGTGGCAAGAATGAAGAAAGGCACCCCCGTCGTTGGCGTGATCAACAACGCTGATGTTGGGAGA

CTGATCTTCTCTGGTGAGGCCCTTACCTACAAGGACATTGTAGTGTGCATGGATGGAGACACCATGCCCG

GTCTCTTCGCCTACAAAGCCGCCACCAAGGCGGGTTACTGTGGAGGAGCCGTTCTTGCGAAGGACGGAGC

CGAGACTTTCATCGTCGGCACTCACTCCGCGGGCGGCAATGGAGTTGGCTACTGCTCATGCGTTTCCAAG

TCCATGCTGCTCAAAATGAAGGCACACATCGATCCCGAACCACACCACGAG

>HQ113232.1_O_PAK_2009

ATCTCAATTCCTTCCCAAAAATCTGTGTTGTACTTCCTCATTGAGAAAGGACAACACGAGGCAGCAATTG

AATTCTTTGAGGGGATGGTTCATGACTCTATTAAGGAGGAGCTCCGTCCCCTAATCCAACAGACCTCATT

TGTGAAACGCGCTTTTAAGCGCCTGAAGGAAAACTTTGAGATCGTCGCCCTGTGTCTGACCCTGCTGGCA

AACATAGTGATTATGATCCGCGAGACTCGCAAGAGGCAGCAGATGGTGGATGATGCAGTGAATGAGTACA

TTGAAAAGGCAAACATCACCACAGATGACAAGACCCTTGACGAGGCGGAAAAGAACCCTCAGGAGACAAA

CAGTGCCAGCACTGTTGGCTTCAGAGAGAGAACTCTCCCGGGACATACGATGAGCAATGACGTGAACTCC

GAGCCCACCGTACCCGTGGAGGGACAACCACAAGCTGAAGGACCCTACGCCGGGCCACTCGAGCGTCAGA

AACCTCTGAAAGTGAGAGCCAAGCTCCCGCGGCAGGAGGGGCCTTACGCTGGTCCGATGGAGAGACAAAA

ACCACTAAAAGTGAAAGCAAAAGTCCCGGTCGTTAAGGAAGGACCTTACGAGGGACCGGTGAAGAAGCCT

GTCGCTTTGAAAGTGAAAGCTAAGAACTTGATTGTCACTGAGAGTGGTGCTCCCCCGACCGACTTGCAAA

AGATGGTCATGGGCAACACTAAGCCTGTTGAGCTCATCCTCGACGGGAAGACGGTGGCCGTCTGCTGTGC

TACCGGAGTGTTTGGCACTGCCTACCTCGTGCCTCGTCATCTTTTCGCTGAGAAGTATGACAAGATCATG

TTGGACGGTAGAGCCATGACAGACAGTGACTATAGAGTGTTTGAGTTTGAGATCAAAGTAAAAGGACAGG

ACATGCTCTCAGACGCCGCGCTCATGGTGCTCCACCGTGGGAACCGCGTGAGAGACATCACGAAACACTT

TCGTGATACAGCAAGAATGAAGAAAGGCACCCCCGTTGTCGGCGTGATCAACAATGCTGACGTCGGGAGA

CTGATTTTCTCTGGCGAGGCCCTTACCTACAAAGACATAGTAGTGTGCATGGATGGAGACACCATGCCTG

GCCTCTTTGCCTATAAAGCCGCCACCAAGGCTGGTTACTGTGGAGGAGCTGTTCTCGCAAAGGACGGAGC

CGAGACTTTCATCGTCGGCACTCACTCCGCAGGGGGCAATGGAGTCGGATACTGCTCATGCGTTTCCAGG

TCCATGCTTCTGAAAATGAAGGCACACATCGACCCCGAACCGCACCACGAG

>HQ113233.1_Asia1_AFG_2009

ATCTCAATTCCTTCCCAAAAATCTGTGTTGTACTTCCTCATTGAGAAAGGCCAGCACGAGGCTGCAATTG

AATTCTTTGAGGGGATGGTGCACGACTCCATCAAGGAGGAGCTTCGACCCCTCATCCAACAGACATCATT

TGTGAAACGCGCTTTCAAGCGCCTGAAGGAGAACTTTGAGATTGTTGCCCTGTGTTTGACTCTTCTGGCG

AACATAGTGATCATGGTCCGCGAGACTCGCAAGAGACAGCAGATGGTGGATGATGCAGTGAATGAGTACA

TCGAGAAAGCCAATATCACCACGGATGACAAGACTCTTGACGAGGCGGAAAAGAACCCTCTGGAGACCAG

TGGTGCCAGCACCGTTGGTTTCAGAGAGAGAGCTCTTCTGGGACACAAGGTGAGTGATGACGTGAACTCC

GAGCCCGCCGAACCTGTGGAAGAGCAACCACAAGCTGAAGGACCCTACGCCGGGCCACTCGAGCGTCAGA

AACCTCTGAAAGTGAGAGCCAAGCTCCCGCAGCAGGAGGGACCTTACGCTGGTCCGATGGAGAGACAGAA

ACCACTGAAAGTGAAAGCAAAAGCCCCGGTCGTCAAGGAAGGACCTTACGAGGGACCGGTGAAGAAGCCT

GTCGCTTTGAAAGTGAAAGCTAAGAACTTGATTGTCACTGAGAGTGGTGCCCCCCCGACCGACTTGCAAA

AGATGGTCATGGGCAACACTAAGCCTGTTGAGCTCATCCTCGACGGGAAGACGGTGGCCATTTGCTGTGC

TACCGGAGTGTTTGGCACCGCCTACCTCGTGCCTCGTCATCTTTTCGCTGAGAAGTATGACAAGATCATG

TTGGACGGTAGGGCCATGACAGACAGTGACTACAGAGTGTTTGAGTTTGAGATTAAAGTAAAAGGACAGG

ACATGCTCTCAGACGCCGCGCTCATGGTGCTCCACCGTGGGAACCGCGTGAGAGACATCACGAAACACTT

TCGTGATGTAGCAAGAATGAAGAAAGGAACCCCCGTTGTCGGCGTGATTAACAACGCTGATGTCGGGAGA

CTGATTTTCTCTGGCGAGGCCCTCACCTACAAGGACGTAGTAGTGTGCATGGATGGAGACACCATGCCTG

GCCTCTTTGCCTACAAAGCCGCCACCAAGGCTGGTTACTGTGGAGGAGCTGTTCTCGCAAAGGACGGAGC

CGAGACTTTCATTGTCGGCACTCACTCTGCAGGGGGCAATGGAGTTGGATACTGCTCATGCGTTTCCAGG

TCCATGCTTCTGAAAATGAAGGCACACGTCGACCCTGAACCACACCACGAG

>HQ268509.2_A_VIT_2004

ATCTCAATTCCTTCCCAAAAGTCTGTGTTGTACTTTCTCATTGAGAAAGGCCAACACGAGGCGGCAATTG

AATTCTTTGAGGGGATGGTGCATGACTCCATCAAGGAGGAGCTCCGGCCCCTCATCCAACAAACCTCATT

TGTGAAACGCGCTTTTAAGCGCCTGAAGGAAAACTTTGAGATTGTTGCCCTATGTTTGACTCTCTTGGCA

AACATAGTGATCATGATCCGCGAGACTCGTAAGAGACAGCAAATGGTGGATGATGCAGTGAATGAGTACA

TTGAGAAGGCAAACATCACCACAGATGACAAGACTCTTGACGAGGCGGAAAAGAACCCTCTGGAGACCAG

CGGCGCCAGTGCTGTTGGCTTCAGGGAGAGATCTCTCCCCGGACACAAGGTGAGCGATGCCGTGAAATCC

GAGCCCACCAAACCCGCGGAAGAACAACCACAAGCTGAAGGACCCTACGCCGGACCACTCGAGCGTCAGA

AACCTCTGAAAGTGAAAGTCAAACTGCCACAGCAGGAGGGACCCTACGCTGGTCCGATGGAGAGACAGAA

ACCACTGAAAGTGAAGGCGAAAGCCCCGGTCGTGAAGGAAGGACCTTATGAAGGACCGGTGAAGAAGCCT

GTCGCTTTGAAAGTGAAAGCTAAGAACTTGATTGTCACTGAGAGTGGTGCTCCCCCAACCGACTTGCAAA

AGATGGTTATGGGCAACACTAAACCTGTTGAGCTCATCCTCGACGGGAAGACGGTAGCCATCTGCTGCGC

TACTGGAGTGTTTGGCACTGCCTACCTCGTGCCTCGTCACCTTTTCGCTGAGAAGTATGACAAGATCATG

TTGGACGGCAGGGCTATGACAGACAGTGACTACAGAGTGTTTGAGTTTGAGATTAAAGTAAAAGGACAGG

ACATGCTCTCAGACGCTGCGCTCATGGTGCTGCACCGTGGGAACCGCGTGAGAGACATCACGAAACACTT

TCGTGATACAGCAAGAATGAAGAAAGGCACCCCCGTCGTCGGTGTAATCAACAACGCTGATGTTGGGAGA

CTGATTTTCTCTGGTGAGGCCCTTACCTACAAAGACATTGTAGTATGCATGGACGGAGACACCATGCCAG

GCTTGTTTGCCTACAGAGCCGCCACCAAGGCTGGCTATTGCGGAGGAGCTGTTCTTGCCAAAGACGGAGC

TGACACATTCATCGTCGGCACTCACTCCGCAGGTGGCAATGGAGTTGGGTACTGCTCGTGCGTATCCAGA

TCCATGCTCCTGAAGATGAAGGCACACATCGACCCTGAACCACACCATGAA

>HQ268524.1_O_BHU_2004

ATCTCAATTCCTTCCCAAAAATCCGTGCTGTACTTCCTCATTGAGAAAGGCCAGCATGAGGCTGCAATTG

AATTCTTTGAGGGGATGGTGCATGACTCCATCAAGGAGGAGCTCCGACCCCTCATCCAACAGACGTCATT

TGTGAAACGCGCTTTTAAGCGCCTGAAGGAAAACTTTGAGATTGTTGCCCTGTGTTTGACTCTTTTGGCA

AACATAGTGATCATGATCCGCGAGACTCGCAAGAGACAGCAGATGGTGGATGATGCAGTGAATGAGTACA

TTGAGAAAGCTAACATTACCACGGATGACAAGACTCTTGACGAGGCGGAAAAGAACCCTCTAGAGACCAG

TGGTGCCAGCACCGTTGGTTTCAGAGAGAAAACTCTTCCAGGACACAAGGTGAGCGATGACGTGAACTCC

GAGCCCACCAAACCTGTGGAAGAGCAACCACAAGCCGAAGGACCCTACGCCGGGCCACTTGAGCGTCAGA

AACCTCTGAAAGTGAGAGCCAAGCTCCCGCAGCAGGAGGGACCTTACGCTGGTCCGATGGAGAGACAGAA

ACCACTGAAAGTGAAAGCAAAAGCCCCGGTCGTTAAGGAAGGACCTTACGAGGGACCGGTGAAGAAGCCT

GTCGCTTTGAAAGTGAAAGCTAAGAACTTGATTGTCACTGAGAGTGGTGCCCCCCCGACCGACTTGCAAA

AGATGGTCATGGGCAACACTAAGCCTGTTGAGCTCATCCTCGACGGGAAGACGGTGGCCATTTGCTGTGC

TACCGGAGTGTTTGGCACTGCCTACCTCGTGCCTCGTCATCTTTTCGCTGAGAAGTATGACAAGATCATG

CTGGACGGTAGAGCCATGACAGACAGTGACTACAGAGTGTTTGAGTTTGAGATCAAAGTAAAAGGACAGG

ACATGCTCTCAGACGCCGCGCTCATGGTGCTCCACCGTGGGAACCGCGTGAGAGACATCACGAAACACTT

TCGTGATACAGCAAGAATGAAGAAAGGCACCCCCGTTGTCGGCGTGATTAACAACGCTGATGTCGGGAGA

CTGATTTTCTCTGGTGAGGCCCTCACCTACAAGGACATAGTAGTGTGCATGGATGGAGACACCATGCCTG

GCCTCTTTGCCTACAAGGCCGCCACCAAGGCTGGTTACTGTGGAGGGGCTGTTCTCGCAAAGGACGGAGC

CGAGACTTTCATCGTCGGCACTCACTCTGCAGGGGGCAATGGAGTTGGATACTGCTCATGCGTTTCCAGG

TCCATGCTTCTGAAAATGAAGGCACACATCGACCCTGAACCACACCACGAG

>HQ412603.1_O_CHA_2000

ATTTCAATCCCTTCACAGAAATCCGTGTTGTACTTCCTCATTGAGAAAGGTCAGCACGAAGCAGCGATCG

AGTTCTTCGAGGGGATGGTCCACGACTCCATCAAGGAGGAGCTCCGACCCCTCATTCAACAGACCTCGTT

CGTGAAACGCGCCTTTAAACGCTTGAAAGAAAACTTCGAGATCGTAGCTCTGTGTTTAACTCTCTTGGCA

AACATAGTAATTATGCTCCGCCAAACGCGCAGGAGGCACCAGTCAGTAGAAGACCCGCTGGAC-------

-----------------------GACAATGTGACTCCTGACGGCGCGGAAGAGAGCCCTCTGGAGACGAG

TGGTGTTAGCACCTTCGGTTTCAGAGAGAGATCCCATACCGAGCAGAGGGCGAGTGAGGACGCGAACGCT

GAACCCGTTACACTCGGGAGGGAGCAACCGCGGGCTGAAGGGCCCTACGCTGGGCCACTTGAGCGTCAGA

AACCTCTCAAAGTGAAAGCCAAGCTGCCACAACAGGAGGGGCCCTACGCCGGCCCAATGGAGAGACAGAA

ACCGCTGAAAGTGAAAGCTAAAGTCCCCGTCGTGAAGGAAGGACCTTACGAGGGACCGGTGAAGAAACCT

GTCGCTTTGAAAGTGAAAGCAAAGAACTTGATAGTCACTGAGAGTGGTGCACCACCGACCGACTTGCAAA

AGATGGTCATGGGCAACACTAAGCCAGTCGAGCTCATTCTCGACGGCAAGACGGTGGCCATTTGCTGTGC

TACCGGAGTGTTCGGCACTGCCTACCTCGTACCTCGTCATCTCTTTGCGGAGAAGTACGACAAGATCATG

TTGGACGGTAGAGCCTTGACAGACAGTGACTACAGAGTGTTTGAGTTTGAGATTAAAGTAAAAGGACAGG

ACATGCTCTCAGACGCCGCTCTCATGGTGCTTCACCGTGGGAACCGCGTGCGCGACATCACGAAGCACTT

TCGTGACGTGGCGAGAATGAAGAAGGGAACCCCCGTCGTCGGTGTGATCAACAATGCTGACGTTGGGAGA

CTCATATTCTCTGGTGAAGCCCTTACTTACAAGGACATCGTCGTGTGTATGGACGGAGACACCATGCCTG

GGCTCTTTGCCTACAGGGCGTCCACCAAGGCGGGTTACTGTGGAGGAGCCGTTCTAGCAAAGGACGGGGC

CGAAACGTTCATCGTCGGCACCCACTCCGCGGGTGGAAACGGCATAGGGTACTGTTCGTGCGTTTCCCGA

TCGATGCTCCTAAAAATGAAGGCGCACATCGACCCCGAACCACATCACGAG

>HQ631363.1_Asia1_CHA_2006

ATCTCAATTCCTTCCCAAAAGTCTGTGCTGTACTTCCTCATCGAGAAAGGCCAGCATGAGGCAGCAATTG

AATTCTTTGAGGGAATGGTGCACGACTCCATTAAGGAGGAACTCCGGCCCCTCATCCAACAGACCTCATT

TGTGAAACGCGCTTTCAAGCGCCTGAAGGAAAACTTTGAGATTGTTGCCCTGTGTTTGACTCTTTTGGCA

AACATAGTGATCATGATCCGCGAGACGCGCAAGAGACAGCAGATGGTGAATGATGCGGTGAACGAGTACA

TCGACAAAGCCAACATCACCACAGATGACAAGACTCTTGACGAGGCGGAAAAGAACCCTCTGGAGACCAG

TGGTGCTAGCACCGTTGGTTTCAGAGAGAGAACCCTCCCGGGGCGCAAGACGAGTGATGACGTGAATTCC

GAGCCCGTCAAACCCGTGGAGGAACAACCACAAGCTGAAGGACCCTACGCCGGGCCACTCGAGCGTCAGA

AACCTCTGAAAGTGAGAGCCAAGCTCCCACAGCAAGAGGGACCTTACGCTGGCCCGATGGAGAGACAGAA

ACCACTGAAAGTGAAAGCAAAAGCCCCGGTCGTTAAGGAAGGGCCTTACGAAGGACCGGTGAAGAAACCT

GTCGCTTTGAAAGTGAAAGCAAAGAATTTGATTGTCACTGAGAGTGGTGCCCCACCGACTGACTTGCAAA

AGATGGTCATGGGCAACACCAAGCCTGTTGAGCTCATCCTCGACGGCAAGACGGTAGCCATCTGCTGCGC

TACCGGAGTCTTTGGTACTGCCTACCTCGTGCCTCGTCACCTTTTCGCAGAGAAGTACGACAAGATCATG

CTGGACGGCAGAGCCATGACAGACAGTGACTACAGAGTGTTTGAGTTTGAGATTAAAGTAAAAGGACAGG

ACATGCTCTCAGACGCCGCACTCATGGTGCTCCACCGTGGGAATCGCGTGCGTGACATCACGAAGCACTT

CCGTGATGTAGCCAAGATGAAGAAAGGAACCCCCGTCGTTGGTGTGATCAACAACGCCGACGTTGGGAGA

CTGATTTTCTCCGGTGAGGCCCTAACCTACAAAGACATTGTAGTGTGCATGGATGGAGACACCATGCCTG

GCCTCTTTGCCTACAAGGCCGTCACCAGGGCAGGCTACTGTGGAGGAGCCGTTCTCGCGAAGGACGGAGC

CGAGACTTTCATCGTCGGCACTCACTCTGCAGGCGGCAACGGAGTTGGATACTGCTCGTGCGTTTCCAGG

TCCATGCTGCTCAAGATGAAGGCTCACATTGATCCCGAACCACACCACGAG

>HQ632768.1_O_MAY_2000

ATCTCAATTCCTTCCCAAAAGGCTGTGCTGTACTTTCTCATTGAGAAGGGCCAGCACGAAGCAGCAATTG

AGTTCTTTGAGGGGATGGTGCATGACTCCATCAAGGAGGAACTCCGGCCTCTCATCCAGCAGACCTCATT

TGTGAAGCGCGCTTTTAAGCGCCTGAAGGAAAACTTTGAGATAGTTGCCCTGTGTTTGACTCTTTTGGCA

AACATAGTGATCATGATCCGCGAGACTCGCAAGAGACAGCAGATGGTGGATGATGCAGTGAACGAGTACA

TTGAGAAGGCAAACATCACCACGGATGACAAGACTCTTGACGAGGCGGAAAAGAACCCTCTGGAGACCAG

CGGTGCCACCACTGTTGGTTTCAGAGAGAAAACTCTCCCGGGACACAAGGCGAGTGATGACGTGAACTCC

GAGCCCGCCAAACCCGTGGAAGAACAACCACAAGCTGAAGGACCCTACACCGGCCCACTCGAGCGTCAAA

AACCTCTGAAAGTGAGAGCCAAGCTCCCACAGCAGGAGGGGCCCTACGCTGGTCCGATGGAGAGACAGAA

ACCGCTGAAAGTGAAAGTGAAAGCCCCGGTCGTTAAGGAAGGACCTTACGAAGGACCGGTGAAGAAACCT

GTCGCTTTGAAAGTGAAAGCAAAGAACTTGATCGTCACTGAGAGTGGTGCTCCCCCGACTGACTTGCAAA

AGATGGTCATGGGTAACACCAAGCCTGTTGAGCTCATCCTCGACGGGAAGACGGTGGCCATCTGCTGCGC

CACTGGAGTGTTTGGTACTGCCTACCTTGTTCCTCGTCATCTTTTCGCAGAGAAGTATGACAAGATCATG

TTGGACGGCAGAGCCATGACAGACAGTGACTACAGAGTGTTTGAGTTTGAGATTAAAGTGAAAGGACAGG

ACATGCTCTCAGACGCCGCGCTCATGGTGCTTCACCGTGGGAATCGCGTGCGGGACATCACGAAGCACTT

CCGTGATGTGGCAAGAATGAAGAAAGGCACCCCCGTTGTCGGCGTGATCAACAACGCTGATGTTGGGAGA

CTGATCTTCTCTGGTGAGGCCCTTACCTACAAGGATATTGTAGTGTGCATGGACGGAGACACCATGCCCG

GTCTCTTCGCCTACAAAGCCGCCACCAAGGCGGGTTACTGTGGAGGAGCCGTTCTTGCAAAGGACGGAGC

CGAGACTTTCGTCGTCGGCACTCACTCCGCAGGCGGCAATGGAGTTGGATACTGCTCATGCGTTTCCAGG

TCTATGCTGCTTAAAATGAAGGCACACATCGATCCCGAACCACACCACGAG

>HQ632769.1_O_MAY_2001

ATCTCAATTCCTTCCCAAAAGTCCGTACTGTACTTCCTCATTGAGAAAGGCCAGCACGAAGCAGCAATTG

AATTCTTTGAGGGGATGGTGCACGACTCCATCAAGGAGGAGCTCCGGCCTCTCATCCAACAGACCTCATT

TGTGAAGCGCGCCTTCAAGCGCCTGAAGGAAAACTTTGAGATTGTTGTGCTGTGTTTGACTCTCCTGGCA

AACATAGTGATCATGATCCGCGAGACTCACAAGAGACAACAAATGGTGGATGATGCAGTGAATGAGTACA

TTGAGAAAGCAAACATCACCACAGATGACAAGACTCTTGATGAGGCGGAAAAGAACCCTCTGGAAACCAG

CGGCGCTAGCACCGTTGGTTTCAGAGAGAGACCTCTCCCGGGACAC------------------------

---------GACCCCGTGGAAGAGCAACCACAAGCTGAAGGACCCTACGCCGGACCACTCGAGCGTCAGA

AACCTCTGAAAGTGAAAGCCAAGCTGCCACAGCAGGAGGGACCCTACGCTGGTCCGATGGAGAGACAGAA

ACCACTGAAAGTGAAAGCAAAAGCCCCGGTCGTGAAGGAAGGACCTTACGAGGGACCGGTGAAGAAGCCT

GTCGCTTTGAAAGTGAAAGCTAAGAACTTGATTGTCACTGAGAGTGGTGCTCCCCCGACCGACTTGCAAA

AGATGGTCATGGGCAACACCAAGCCTGTTGAGCTCATTCTCGACGGGAAGACAGTAGCCATCTGCTGCGC

TACTGGAGTGTTTGGCACTGCTTACCTCGTGCCTCGTCATCTTTTCGCTGAGAAGTATGACAAGATCATG

TTGGACGGCAGGGCCATGACAGACAGTGACTACAGAGTGTTTGAGTTTGAGATTAAAGTAAAAGGACAGG

ACATGCTCTCAGACGCCGCGCTCATGGTGCTGCACCGTGGGAACCGCGTGAGAGACATCACGAAGCACTT

TCGTGACACAGCAAGAATGAAGAAAGGCACCCCCGTCGTTGGCGTGATCAACAACGCTGACGTTGGGAGA

TTGATTTTCTCTGGTGAGGCCCTCACCTACAAAGACATTGTAGTGTGTATGGATGGAGACACCATGCCAG

GCCTGTTTGCCTACAGAGCCGCCACCAAGGCAGGCTACTGCGGAGGAGCTGTTCTTGCTAAGGACGGTGC

CGACACATTCATCGTTGGCACGCACTCTGCAGGTGGTAATGGAGTTGGATATTGCTCATGCGTGTCCAGA

TCCATGCTCCTCAAGATGAAGGCACACGTTGACCCCGAACCACACCACGAG

>HQ632770.1_O_MAY_2004

ATCTCAATTCCTTCCCAAAAATCCGTGCTGTACTTCCTCATTGAGAAAGGCCAGCACGAGGCTGCAATTG

AATTCTTTGAGGGGATGGTGCATGACTCCATCAAAGAGGAGCTCCGGCCCCTCATCCAGCAGACATCATT

TGTGAAACGCGCTTTTAAGCGCCTGAAGGAAAACTTTGAGATTGTTGCCCTGTGTTTGACTCTTTTGGCA

AACATAGTGATCATGATCCGCGAGACTCACAAGAGACAGCAGATGGTGGATGATGCAGTGAATGAGTACA

TTGAGAAAGCCAACATCACCACGGATGACAAGACTCTTGACGAGGCGGAAAAGAACCCTCTAGAGACCAG

TGGTGCCAGCACCGTTGGTTTCAGAGAGAGAACTCTTCCAGGACACAAGGTGAGCGATGACGTGAACTCC

GAGCCCACCAAACCTGTGGAAGAGCAACCACAAGCCGAAGGACCCTACGCCGGGCCACTTGAGCGTCAGA

AACCTCTGAAAGTGAGAGCCAAGCTCCCGCAGCAGGAGGGGCCTTACGCTGGTCCGATGGAGAGACAGAA

ACCACTGAAAGTGAAAGCAAAAGCCCCGGTCGTTAAGGAAGGACCTTACGAGGGACCGGTGAAGAAGCCT

GTCGCTTTGAAGGTGAAAGCTAAGAACTTGATTGTCACTGAGAGTGGTGCCCCCCCGACCGACTTGCAAA

AGATGGTCATGGGCAACACTAAGCCTGTTGAGCTCATCCTCGACGGGAAGACGGTGGCCATCTGCTGTGC

TACCGGAGTGTTTGGTACTGCCTACCTCGTGCCTCGTCATCTTTTCGCTGAGAAGTATGACAAGATCATG

TTGGACGGTAGAGCCATGACAGACAGTGACTACAGAGTGTTTGAGTTTGAGATTAAAGTAAAAGGACAGG

ACATGCTCTCAGACGCCGCGCTCATGGTGCTCCACCGTGGGAACCGCGTGAGAGACATCACGAAACACTT

TCGTGATACAGCAAGAATGAAGAAAGGCACCCCCGTTGTCGGCGTGATTAACAACGCTGATGTCGGGAGA

CTGATTTTCTCTGGTGAGGCCCTCACCTACAAGGACATAGTGGTGTGCATGGATGGAGACACCATGCCTG

GCCTTTTTGCCTACAAAGCCGCCACCAAGGCTGGTTACTGTGGAGGAGCTGTTCTCGCAAAGGACGGAGC

CGAGACTTTCATCGTCGGCACTCACTCTGCAGGGGGCAATGGAGTTGGATACTGCTCATGCGTTTCTAGG

TCCATGCTTCTGAAAATGAAGGCACACATCGACCCTGAACCACACCACGAG

>HQ632771.1_O_MAY_2005

ATTTCCATCCCTTCCCAGAAGTCTGTGTTGTACTTCCTCATCGAGAAGGGCCAACACGAAGCAGCGATCG

AATTCTTCGAGGGGATGGTCCACGACTCCATTAAGGAGGAACTCCGACCCCTCATTCAACAGACTTCGTT

CGTGAAACGCGCCTTCAAGCGCTTGAAAGAGAACTTTGAGATTGTGGCTCTGTGCTTAACCCTTTTGGCA

AACATAGTGATCATGCTCCGCCAAGCGCGCAAGAGGCGTGAGTCGGTGGATGAACCACTGGAC-------

-----------------------GGTGACATAACTCTTGGTGAAGCGGAAGAGAGCCCTCTGGAGACGAG

TGGCGCTAGCGCCGTCGGTTTCAGAGAGAGATCTCCCGCCGAGCAAGGGACGTGCGAGGACGCGAACGCT

GAGCCCGTCGTGCTCGGGAGGGAACAACCACGAGCTGAAGGACCCTACGCTGGGCCGCTTGAGCGTCAGA

AACCTCTTAAAGTGAAAGCCAAGCTACCACAACAGGAGGGACCCTACGCCGGCCCAATGGAGAGACAGAA

ACCGTTGAAGGTGAAAGTAAAAGCTCCCGTCGTGAAGGAAGGGCCTTACGAGGGACCGGTGAAGAAACCT

GTCGCTTTGAAAGTGAAAGCAAAGAACTTGATAGTCACTGAGAGTGGTGCACCACCGACCGACCTGCAAA

AGATGGTCATGGGCAACACTAAGCCGGTCGAGCTCATTCTCGACGGCAAGACGGTGGCCATCTGTTGTGC

CACCGGAGTGTTTGGCACTGCCTACCTCGTGCCTCGTCATCTCTTTGCGGAGAAGTATGACAAGATCATG

CTGGACGGCAGAGCCATGACAGACAGTGACTACAGAGTGTTTGAGTTTGAGATTAAAGTAAAAGGACAGG

ACATGTTATCGGACGCCGCCCTCATGGTACTACACCGTGGGAACCGCGTGCGCGACATCACGAAACATTT

CCGCGACGTAGCGAGAATGAAGAAGGGAACCCCCGTTGTCGGTGTAATCAACAATGCTGACGTCGGGAGA

CTCATATTCTCTGGCGAAGCCCTCACTTACAAGGACATCGTTGTGTGCATGGACGGAGACACCATGCCCG

GGCTTTTCGCCTACAGGGCATCCACCAAGGCAGGTTACTGTGGAGGAGCCGTCCTGGCAAAAGACGGTGC

CGAGACGTTTATCGTCGGCACTCACTCCGCAGGTGGCAACGGTGTAGGATACTGTTCATGTGTTTCCCGG

TCAATGCTTCTGAAGATGAAGGCACACATCGACCCTGAACCACACCACGAA

>HQ632772.1_O_MAY_2007

ATCTCAATCCCTTCCCAAAAGTCTGTGTTGTACTTCCTCATTGAGAAAGGCCAGCACGAAGCAGCAATTG

AATTTTTTGAGGGAATGGTGCACGACTCCATCAAGGAGGAGCTCCGGCCTCTCATCCAACAGACCTCATT

TGTGAAACGCGCCTTCAAGCGCCTGAAGGAAAATTTTGAGATTGTTGCCCTGTGTTTGACTCTTCTGGCA

AACATAGTGATCATGATCCGCGAGACTCGCAAGAGACAACAAATGGTGGACGATGCAGTGAATGAATACA

TTGAGAAGGCAAACATCACCACAGACGACAAGACTCTTGACGAGGCGGAAAAGAACCCTCTGGAGACTAG

CGGTGCCAGCACCGTCGGTTTCAGAGAGAGAACTCTCCCAGGACACAAGACGAGCGATGACGTGAACTCC

GAGCCCGCCAAATCTGTGGAAGAGCAACCACAAGCTGAAGGGCCCTACGCCGGACCGCTTGAGCGCCAGA

AACCTCTGAAAGTGAGAGCCAGGCTACCACAGCAGGAGGGGCCTTATGCCGGCCCGATGGAGAGACAGAA

ACCACTGAAAGTGAAAGTGAACGCCCCGGTCGTGAAGGAAGGACCTTACGAGGGACCGGTGAAAAAGCCT

GTCGCTTTGAAAGTGAAAGCCAAGAACTTGATTGTCACTGAGAGTGGTGCTCCCCCGACCGATCTGCAAA

AGATGGTCATGGGCAACACCAAGCCTGTTGAGCTTATTCTCGATGGGAAGACAGTAGCCATCTGCTGTGC

TACTGGAGTGTTTGGTACTGCCTACCTCGTGCCTCGTCACCTTTTCGCCGAGAAGTACGACAAGATCATG

TTGGACGGCAGAGCCATGACAGACAGTGACTACAGAGTGTTTGAGTTTGAGATTAAAGTAAAAGGACAGG

ACATGCTCTCAGACGCCGCGCTCATGGTGCTGCACCGTGGGAACCGCGTGAGGGACATTACGAAACACTT

TCGTGATACAGCAAGAATGAAGAAAGGCACCCCCGTCGTTGGCGTGATCAACAACGCTGACGTTGGGAGA

CTGATTTTCTCTGGTGAGGCCCTTACCTACAAGGACATTGTGGTGTGCATGGACGGAGACACCATGCCGG

GCCTGTTTGCCTACAAAGCCGCTACCAAGGCTGGTTACTGCGGGGGAGCCGTTCTCGCTAAGGACGGAGC

CGACACGTTCATCGTTGGCACTCACTCCGCAGGTGGCAATGGAGTTGGGTACTGCTCATGCGTATCCAGG

TCCATGCTCCTCAAAATGAAGGCACACATTGACCCCGAACCGCACCACGAG

>HQ632773.1_A_MAY_2007

ATCTCAATTCCTTCCCAAAAGTCTGTGCTGTACTTTCTCATTGAGAAAGGCCAACACGAGGCAGCAATTG

AATTCTTTGAGGGGATGGTGCATGACTCCATCAAGGAGGAGCTCCGGCCCCTCATCCAACAAACCTCATT

TGTGAAACGCGCTTTCAAGCGCCTGAAGGAAAACTTTGAGGTCGTTGCCCTATGTTTGACTCTCCTGGCA

AACATAGTGATCATGATCCGCGAGACTCGTAAGAGACAGCAAATGGTGGATGATGCAGTGAATGAGTACA

TTGAGAAGGCAAACATCACCACAGATGACAAAACTCTTGACGAGGCGGAAAAGAGCCCTCTGGAGACCAG

CGGCGCTAGTGCTGTTGGCTTCAGAGAGAGATCTTTCCCCGGACACAAGGTGAGCGATGACGTGAACTCT

GAGCCCACCAAACCCGCGGGAGAACAACCACAAGCTGAAGGACCCTACGCCGGGCCACTCGAGCGTCAGA

AACCTCTGAAAGTGAAAGCCAAGCTGCCACAACAGGAGGGACCCTACGCTGGCCCGATGGAGAGACAGAA

ACCACTGAAAGTGAAAGCGAAAGCCCCGGTCGTGAAGGAAGGACCTTACGAAGGACCGGTGAAGAAGCCT

GTCGCTTTGAAAGTGAAAGCTAAGAACCTGATTGTCACTGAGAGTGGTGCTCCCCCAACCGACTTGCAAA

AGATGGTTATGGGCAACACTAAACCCGTTGAGCTCATCCTCGATGGGAAGACAGTAGCCATCTGCTGCGC

TACTGGAGTGTTCGGCACTGCCTACCTCGTGCCTCGTCATCTTTTCGCTGAGAAGTATGACAAGATCATG

TTGGATGGTAGAGCTATGACAGACAGTGACTACAGAGTGTTTGAGTTTGAGATTAAAGTAAGAGGACAGG

ACATGCTCTCAGATGCTGCGCTCATGGTGTTACACCGTGGGAACCGCGTGAGAGACATCACGAAACACTT

TCGTGACACAGCAAGAATGAAGAAAGGCACCCCCGTCGTCGGCGTGATCAACAACGCTGATGTTGGTAGA

CTGATTTTCTCTGGTGAGGCCCTTACCTACAAAGACATTGTAGTGTGCATGGACGGAGACACCATGCCGG

GCTTGTTTGCCTACAGAGCCGCTACTAAGGCTGGTTACTGCGGAGGAGCTGTTCTTGCCAAGGACGGAGC

TGACACTTTCATCGTCGGCACTCACTCTGCAGGTGGCAACGGAGTTGGGTACTGCTCGTGCGTATCCAGA

TCCATGCTTCTGAAGATGAAGGCACACATTGACCCTGAACCACACCATGAA

>HQ632774.1_Asia1_MAY_1999

ATTTCAATTCCTTCCCAAAAATCTGTGTTGTACTTCCTCATTGAGAAAGGTCAGCACGAAGCAGCAATTG

AATTCTTTGAGGGGATGGTGCATGACTCCATCAAGGAGGAACTCCGGCCTCTCATCCAACAGACCTCATT

TGTGAAACGCGCTTTTAAGCGCCTGAAGGAAAACTTTGAGATTGTTGCCCTCTGTTTGACTCTTTTGGCA

AACATAGTGATCATGATCCGCGAGACTCGCAAGAGGCAACAAATGGTGGACGATGCAGTGAACGAGTACA

TCGAGAAGGCAAACATCACCACAGATGACAAGACCCTTGACGAGGCGGAAAAGAACCCTCTGGAGACCAG

TGGTGCCAGCACCGTTGGTTTCAGAGAGAGACCTCTCCCGGGACACAGGACGAGTGACGACGTGAACTCC

GAGCCCGCCCAACCCGTGGGAGAGCAACCACAAGCTGAAGGACCCTACGCCGGACCGCTTGAGCGTCAGA

AACCTCTAAAAGTGAAAGCTAAGCTGCCACAGCAGGAGGGACCCTACGCCGGTCCGATGGAGAGACAGAA

ACCATTGAAAGTGAAAGTGAAAGCCCCGGTCGTGAAGGAAGGACCTTACGAGGGACCGGTGAAGAAGCCT

GTCGCTTTGAAAGTGAAAGCCAAGAACTTGATTGTCACTGAGAGTGGTGCCCCTCCGACCGACTTGCAAA

AGATGGTCATGGGCAACACTAAGCCTGTTGAGCTCATCCTCGACGGGAAGACAGTAGCCATCTGTTGCGC

TACTGGAGTGTTTGGTACTGCCTACCTTGTGCCTCGCCACCTTTTTGCTGAGAAGTATGACAAGATCATG

TTGGACGGCAGAGCCATGACAGACAGTGACTACAGAGTGTTTGAGTTTGAGATTAAAGTAAAAGGACAGG

ACATGCTCTCAGATGCCGCGCTCATGGTGCTCCACCGTGGGAACCGCGTGAGAGACATCACGAAACACTT

TCGTGACACAGCAAGAATGAAGAAAGGTACCCCCGTCGTTGGTGTGATCAACAACGCTGACGTTGGGAGA

CTGATTTTCTCTGGTGAGGCCCTTACCTACAAGGACATTGTGGTGTGCATGGACGGAGACACCATGCCAG

GCCTGTTTGCCTATAGAGCCGCCACCAAGGCTGGCTATTGCGGGGGAGCCGTTCTTGCTAAGGACGGAGC

TGACACATTCATCGTTGGCACTCACTCCGCGGGTGGCAATGGGGTTGGGTACTGCTCGTGCGTATCCAGG

TCCATGCTTCTCAAAATGAAGGCACACATCGACCCCGAACCACATCACGAG

>HQ832576.1_A_IND_1990

ATCTCAATTCCTTCCCAAAAGTCTGTGCTGTACTTCCTCATTGAGAAAGGCCAGCACGAAGCAGCAATTG

AATTCTTTGAGGGAATGGTACACGACTCCATCAAGGAGGAGCTCCGCCCCCTCATCCAACAGACCTCATT

TGTGAAACGCGCTTTTAAGCGCCTGAAGGAAAATTTTGAGATTGTTGCCCTGTGTTTGACTCTTCTGGCA

AACATAGTGATCATGATCCGCGAGACTCGCAAGAGACAGCGGATGGTGGACGATGCAGTGAATGAGTACA

TCGAGAAGGCAAACATCACCACAGATGACAAGACTCTTGACGAGGCGGAAAAGAACCCTCTGGAGACCAG

CGGCGCCAGCACTGTCGGTTTCAGAGAGAGAACTCTCCCGGGACAAAAGGTGAGCGATGACGTGAACTCC

GAGCCCGCCAAACCCGTGGAAAAGCAACCACAAGCTGAAGGACCCTACGCCGGACCACTTGAGCGTCAGA

AACCTCTGAAAGTGAGAGCCAAACTCCCACAGCAAGAGGGACCTTACGCTGGCCCGATGGAGAGACAGAA

ACCACTGAAAGTGAAAGCAAAAGCCCCGGTCGTTAAGGAAGGACCTTACGAGGGACCGGTGAAGAAACCT

GTCGCTTTGAAAGTGAAAGCTAAGAATTTGATTGTCACTGAGAGTGGTGCCCCACCGACTGACTTGCAAA

AGATGGTCATGGGCAACACTAAGCCTGTCGAGCTCATCCTCGATGGGAAGACGGTAGCCATCTGCTGTGC

TACCGGAGTGTTTGGAACTGCCTACCTCGTGCCCCGTCACCTTTTCGCGGAAAAGTATGACAAGATCATG

TTGGACGGTAGAGCCATGACAGACAGTGACTACAGAGTGTTTGAGTTTGAGATTAAAGTAAAAGGACAGG

ACATGCTCTCAGACGCCGCGCTCATGGTCCTTCACCGTGGGAACCGCGTGAGAGACATCACGAAGCACTT

TCGTGACACAGCAAGAATGAAGAAGGGCACCCCCGTTGTTGGCGTGATCAACAACGCCGACGTCGGGAGA

TTGATCTTCTCTGGTGAGGCCCTCACCTACAAGGACATTGTAGTGTGTATGGATGGTGATACCATGCCTG

GCCTCTTTGCCTACAGAGCCGCTACCAAGGCTGGCTACTGCGGAGGAGCCGTTCTCGCGAAGGACGGAGC

TGACACGTTCATCGTCGGCACTCACTCTGCGGGCGGCAACGGAGTTGGATACTGCTCATGCGTGTCCAGG

TCCATGCTCCTGAAAATGAAGGCACACATTGACCCTGAACCACACCACGAG

>HQ832577.1_A_IND_1999

ATCTCAATTCCTTCCCAAAAGTCAGTGTTATACTTCCTCATTGAGAAAGGCCAACACGAAGCAGCAATTG

AATTCTTTGAGGGGATGGTGCACGACTCCATCAAGGAGGAGCTCCGACCTCTCATCCAACAGACATCATT

TGTGAAACGCGCTTTCAAGCGCCTGAAGGAAAATTTTGAGATTGTTGCCCTATGTTTGACTCTCCTGGCA

AACATAGTGATCATGATCCGCGAGACTCGCAAGAGACAACGGATGGTGGACGATGCAGTGAATGAGTACA

TTGAGAAAGCAAACATCACCACAGATGACAAGACTCTTGACGAGGCGGAAAAGAACCCTCTGGAGACCAG

CGGTGCCAGCACTGTTGGTTTCAGAGAGAAAGTCCTCCCGGGACACAAGGCGAGTGATGACGTGAACTCC

GAGCCCGCCCAGACCGTGGAGGAACAACCACAAGCTGAAGGACCCTACGGCGGACCACTCGAGCGTCAGA

AACCTCTGAAAGTGAGAGCCAAGCTCCCACAACAGGAGGGACCTTACGCTGGTCCGATGGAGAGACAGAA

ACCACTGAAAGTGAAAGCAAAAGCCTCGGTCGTTAAGGAAGGACCTTACGAGGGACTGGTGAAGAAGCCT

GTCGCTTTGAAAGTGAAAGCTAAGAACTTGATTGTAACTGAGAGTGGTGCCCCCCCGACCGACTTGCAAA

AGATGGTCATGGGTAACACCAAACCTGTTGAACTCATTCTCGACGGAAAGACAGTAGCCATTTGCTGTGC

TACTGGAGTGTTTGGAACTGCCTACCTCGTGCCCCGTCATCTCTTCGCGGAGAAGTATGACAAGATCATG

TTGGACGGCAGAGCCATGACAGACAGTGACTACAGAGTGTTTGAGTTTGAGATTAAAGTAAAAGGACAGG

ACATGCTCTCAGACGCTGCGCTCATGGTGCTCCACCGTGGGAACCGCGTGAGAGACATCACGAAACACTT

TCGTGATGCAGCAAGAATGAAGAAAGGCACCCCCGTCGTTGGTGTGATCAACAACGCCGACGTTGGGAGA

CTGATTTTCTCTGGTGAGGCCCTCACCTACAAAGACATTGTAGTGTGCATGGACGGCGACACCATGCCTG

GCCTCTTTGCCTACAGAGCCGCCACCAAGGCTGGCTACTGCGGAGGAGCCGTTCTCGCGAAGGATGGCGC

TGACACATTCATCGTTGGCACCCACTCTGCAGGTGGCAATGGAGTTGGATACTGCTCATGCGTTTCCAGG

TCCATGCTTCTGAAAATGAAGGCACACATTGACCCTGAACCACACCACGAG

>HQ832578.1_A_IND_2003

ATCTCAATTCCTTCCCAAAAATCCGTGTTGTACTCCCTCATTGAGAAAGGACAGCACGAGGCTGCAATTG

AATTCTCTGAGGGGATGGTTCACGACTCCATCAAAGAGGAGCTCCGCCCCCTCATCCAACACACCTCATT

TGTGAAGCGCGCTTTCAAGCGCCTGAAGGACATCTTTGAGATTGTTGCCCTGTGTTTGACCCTTTTGGCA

AACATAGTGATCATGATCCGCGAGACTCGCAAGAGACAGCAGATGGTGGATGATGCAGTGAGTGAGTACA

TTGAAAAGGCAAACATCACCACAGATGACAAGACTCTTGACGAGGCGGAAAAGAACCCTCTGGAGACCAG

CGGTGCCAGCACTGTTGGTTTCAGAGAGAGGACTCTCCCGGGGCAGAAAGTGAGCGATGACGTGAACTCC

GAGCCCGCCAAACCCGTGGAAGAGCAACCACAAGCTGAAGGACCCTACGCCGGGCCGCTTGAGCGTCAGA

AACCTCTGAAAGTGAGAGCCAAGCTACCACAGCAGGAGGGACCTTACGCTGGCCCGATGGAGAGACAGAA

GCCACTGAAAGTGAAAGCAAAAGCCCCGGTCGTGAAGGAAGGACCTTACGAGGGACCGGTGAAGAAGCCT

GTCGCTTTGAAAGTGAAAGCCAAGAACTTGATTGTCACTGAGAGTGGCGCCCCCCCGACCGACTTGCAAA

AGATGGTCATGGGCAACACCAAGCCTGTCGAGCTTATCCTCGATGGGAAGACGGTGGCCATCTGCTGTGC

TACCGGAGTGTTTGGCACTGCCTACCTCGTGCCTCGTCATCTTTTCGCAGAGAAGTACGACAAGATCATG

TTGGACGGCAGAGCCATGGCAGACAGTGATTACAGAGTGTTTGAGTTTGAGATTAAAGTAAAAGGACAGG

ACATGCTCTCAGACGCCGCGCTCATGGTGCTCCACCGTGGGAACCGCGTGAGAGACATCACGAAGCACTT

TCGTGACACAGCAAGAATGAAGAAAGGCACCCCCGTTGTCGGTGTGGTTAACAACGCCGACGTTGGGAGA

CTGATTTTCTCTGGTGAGGCCCTCACCTACAAAGACATTGTAGTGTGTATGGACGGTGACACCATGCCTG

GCCTCTTTGCCTACAGAGCCGCCACCAAGGCTGGTTACTGTGGAGGAGCTGTTCTTGCAAAAGACGGAGC

TGACACTTTCATCGTCGGCACCCACTCCGCGGGAGGAAATGGAGTTGGATATTGCTCGTGCGTGTCCAGG

TCCATGCTCCTGAAAATGAAGGCACACATTGACCCTGAACCACACCATGAG

>HQ832579.1_A_IND_2003

ATCTCAATTCCTTCCCAAAAATCCGTGTTGTACTTCCTCATTGAGAAAGGCCAGCACGAGGCTGCAATTG

AATTCTTTGAGGGGATGGTTCACGACTCCATCAAAGAGGAGCTCCGCCCCCTCATCCAACACACCTCATT

TGTGAAGCGCGCTTTCAAGCGCCTGAAGGACAACTTTGAGATTGTTGCCCTGTGTTTGACTCTTTTGGCA

AACATAGTGATCATGATCCGCGAGACTCGCAAGAGACAGCAGATGGTGGATGATGCAGTGAATGAGTACA

TTGAAAAGGCAAACATCACCACAGATGACAAGACTCTTGACGAGGCGGAAAAGAACCCTCTGGAGACCAG

CGGTGCCAGCACTGTTGGTTTCAGAGAGAGGACTCTCCCGGGGCAGAAAGTGAGCGACGACGTGAACTCC

GAGCCCGCCAAACCCGTGGAAGAACAACCACAAGCTGAAGGACCCTACGCCGGACCGCTTGAGCGTCAGA

AACCTCTGAAAGTGAGAGCCAAGCTACCACAGCAGGAGGGACCCTACGCTGGCCCGATGGAGAGACAGAA

ACCACTGAAAGTGAAAGCAAAAGCCCCGGTCGTGAAGGAAGGACCTTACGAAGGACCGGTGAAGAAGCCT

GTCGCTTTGAAAGTGAAAGCCAAGAACTTGATTGTCACTGAGAGTGGCGCCCCCCCGACCGACTTGCAAA

AGATGGTCATGGGCAACACCAAGCCTGTCGAGCTCATCCTCGATGGGAAGACGGTGGCCATCTGCTGTGC

TACCGGAGTGTTTGGCACTGCCTACCTCGTGCCTCGTCATCTTTTCGCAGAGAAGTACGACAAGATCATG

TTGGACGGCAGAGCCATGACAGACAGTGACTACAGAGTGTTTGAGTTTGAGATTAAAGTAAAAGGACAGG

ACATGCTCTCAGACGCCGCGCTCATGGTGCTCCACCGTGGGAACCGCGTGAGAGACATCACGAAGCACTT

TCGTGACACAGCAAGAATGAAGAAAGGCACCCCCGTTGTCGGTGTGATCAACAACGCCGACGTTGGGAGA

CTGATTTTCTCTGGTGAGGCCCTCACCTACAAAGACATTGTAGTGTGTATGGATGGTGACACCATGCCTG

GCCTCTTTGCCTACAGAGCCGCCACCAAGGCTGGTTACTGTGGAGGAGCTGTTCTTGCAAAAGACGGAGC

TGACACTTTCGTCGTCGGCACTCACTCCGCGGGAGGAAATGGAGTTGGATATTGCTCGTGCGTTTCCAGG

TCCATGCTCCTGAAAATGAAGGCACACATTGACCCTGAACCACACCATGAG

>HQ832580.1_A_IND_2003

ATCTCAATTCCTTCCCAAAAATCCGTGTTGTACTTCCTCATTGAGAAAGGCCAGCACGAGGCCGCAATTG

AATTCTTTGAGGGGATGGTTCACGACTCCATCAAAGAGGAGCTCCGCCCCCTCATCCAACACACCTCATT

TGTGAAGCGCGCTTTCAAGCGCCTGAAGGACAACTTTGAGATTGTTGCCCTGTGTTTGACTCTTTTGGCA

AACATAGTGATCATGATCCGCGAGACTCGCAAGAGACAGCAGATGGTGGATGATGCAGTGAACGAGTACA

TTGAAAAGGCAAACATCACCACAGATGACAAGACTCTTGATGAGGCGGAAAAGAACCCTCTGGAGACCAG

CGGTGCCAGCACTGTTGGTTTCAGAGAGAGGACTCTCCCGGGGCAGAAAGTGAGCGATGACGTGAACTCC

GAGCCCGCCAAACCCGTGGAAGAGCAACCACAAGCTGAAGGACCCTACGCCGGGCCGCTTGAGCGTCAGA

AACCTCTGAAAGTGAGAGCCAAGCTACCACAGCAGGAGGGACCTTACGCTGGCCCGATGGAGAGACAGAA

ACCACTGAAAGTGAGAGCAAAAGCCCCGGTCGTGAAGGAAGGACCTTACGAAGGGCCGGTGAAGAAGCCT

GTCGCTTTGAAAGTGAAAACCAAGAACTTGATTGTCACTGAGAGTGGCGCCCCCCCGACCGACTTGCAAA

AGATGGTCATGGGCAACACCAAGCCTGTCGAGCTCATCCTCGATGGAAAGACGGTGGCCATCTGCTGTGC

CACCGGAGTGTTTGGCACTGCCTACCTCGTGCCTCGTCATCTTTTCGCAGAGAAGTACGACAAGATCATG

TTGGACGGTAGAGCCATGGCAGACAGTGACTACAGAGTGTTTGAGTTTGAGATCAAAGTAAAAGGACAGG

ACATGCTCTCAGATGCCGCGCTCATGGTGCTCCACCGTGGGAACCGCGTGAGAGACATCACGAAGCACTT

TCGTGACACAGCAAGAATGAAGAAAGGCACCCCCGTTGTCGGTGTGATCAACAACGCCGACGTTGGGAGA

CTGATTTTCTCTGGTGAGGCCCTCACCTACAAAGACATTGTAGTGTGTATGGATGGTGACACCATGCCTG

GCCTCTTTGCCTACAGAGCCGCCACCAAGGCTGGTTACTGTGGAGGAGCTGTTCTTGCAAAAGACGGAGC

TGACACTTTCATCGTCGGCACTCACTCCGCGGGAGGAAACGGAGTTGGATATTGCTCGTGCGTGTCCAGG

TCCATGCTCCTGAAAATGAAGGCACACATTGACCCTGAACCACACCACGAG

>HQ832581.1_A_IND_2004

ATCTCAATTCCTTCCCAAAAATCCGTGTTGTATTTCCTCATTGAGAAAGGCCAGCACGAGGCTGCAATTG

AATTCTTTGAGGGGATGGTTCACGACTCCATCAAAGAGGAGCTCCGCCCCCTCATCCAACACACCTCATT

TGTGAAGCGCGCTTTCAAGCGCCTGAAGGACAACTTTGAGATTGTTGCCCTGTGTTTGACTCTTTTGGCA

AACATAGTGATCATGATCCGCGAGACTCGCAAGAGACAGCAGATGGTGGATGATGCAGTGAGTGAGTACA

TTGAAAAGGCAAACATCACCACAGATGACAAGACTCTTGACGAGGCGGAAAAGAACCCCTTGGAGACCAG

TGGTGCCAGCACTGTTGGTTTCAGAGAGAGGACTCTCCCGGGGCAGAAAGTGAGCGATGACGTGAACTCC

GAGCCCGCCAAACCCGTGGAAGAGCAACCACAAGCTGAAGGACCCTACGCCGGGCCGCTTGAGCGTCAGA

AACCTCTGAAAGTGAGAGTCAACCTACCACATCAGGAGGGACCCTACGCTGGTCCGATGGAGAGACAGAA

ACCACTGAAAGTGAAAGCAAAAGCCCCGGTCGTGAAGGAAGGACCTTACGAAGGACCGGTGAAGAAGCCT

GCCGCTTTGAGAGTGAAAGCCAAGAACTTGATTGTCACTGAGAGTGGCGCCCCACCGACCGACTTGCAAA

AGATGGTCATGGGCAACCCCAAGCCTGTCGAGCTTATCCTCGATGGGAAGACGGTAGCCATCTGCTGTGC

TGCCGGAGTGTTTGGTACTGCCTCCCTCGTGCCTCGTCATCTCTTTGCAGAGAGGTACGACAAGATCATG

TTGGACGGCAGAGCTATGACAGACAGTGACTACAGAGTGTTTGAGTTCGAGATTAAAGTAAAAGGACAGG

ACATGCTCTCAGACGCCGCGCTCATGGTGCTCCACCGTGGGAACCGCGTGAGAGACATCACGAAGCACTT

TCGTGACACAGCAAGAATGAAAAAAGGCACCCCCGTTGTCGGTGTGATCAACAACGCCGACGTTGGGAGA

CTGATTTTCTCTGGTGAAGCCCTCACCTACAAAGACATTGTAGTGTGTATGGATGGTGACACAATGCCTG

GCCTCTTTGCCTACAGAGCCGCCACTAAGGCTGGTTACTGTGGAGGAGCTGTTCTTGCAAAAGACGGAGC

TGACACTTTTATCGTCGGCACTCACTCCGCGGGAGGAAATGGAGTTGGATATTGCTCGTGCGTGTCCAGG

TCCATGCTCATGAAAATGAAGGCACACATTGACCCTGAACCACACCACGAG

>HQ832582.1_A_IND_2004

ATCTCAATTCCTTCCCAAAAATCCGTGTTGTACTTCCTCATTGAGAAAGGCCAGCACGAGGCTGCAATTG

AATTCTTTGAGGGGATGGTTCACGACTCCATCAAAGAGGAGCTCCGCCCCCTCATCCAACACACCTCATT

TGTGAAGCGCGCTTTCAAGCGCCTGAAGGACAACTTTGAGATTGTTGCCCTATGTTTGACTCTTTTGGCA

AACATAGTGATCATGATCCGCGAGACTCGCAAGAGACAGCAGATGGTGGATGATGCAGTGAATGAGTACA

TTGAAAAGGCAAACATCACCACAGATGACAAGACCCTTGACGAGGCGGAAAAGAACCCCCTGGAGACCAG

CGGTGCCAGCGCTGTTGGTTTCAGAGAGAGGACTCTCCCGGGACAGAAAGTGAGCGATGACGTGAACTCC

GAGCCCGCCAAACCCGTGGAAGAGCAACCACAAGCTGAAGGACCCTACGCCGGGCCGCTTGAGCGTCAGA

AACCTCTGAAAGTGAGAGCCAAGCTACCACAGCAGGAGGGACCCTACGCTGGCCCGATGGAGAGACAGAA

ACCACTGAAAGTGAGAGCAAAAGCCCCGGTCGTGAAGGAAGGACCTTACGAAGGGCCGGTGAAGAAGCCT

GCCGCTTTGAAAGTGAAAGCCAAGAACTTGATTGTCACTGAGAGTGGCGCCCCCCCGACCGACTTACAAA

AGTTGGTCATGGGCAACACCAAGCCTGTCGAGCTCATCCTCGATGGGAAGACGGTGGCCATCTGCTGCGC

TACCGGAGTGTTTGGAACTGCCTACCTCGTGCCTCGTCATCTTTTCACAGAGAAGTACGACAAGATCATG

TTGGACGGCAGAGCCATGATAGACAGTGACTACAGAGTGTTTGAGTTTGAGATTAAAGTAAAAGGACAGG

ACATGCTCTCAGACGCCGCGCTTATGGTGCTCCACCGTGGGAACCGCGTGAGAGACATCACGAAGCACTT

TCGTGACACAGCAAGAATGAAGAAAGGTACCCCCGTTGTCGGTGTGATTAACAACGCCGACGTTGGGAGA

CTGATTTTCTCTGGTGAGGCCCTCACCTACAAAGACATTGTAGTGTGTATGGATGGTGACACCATGCCTG

GCCTCTTCGCCTACAGAGCCGCCACCAAGGCTGGTTACTGTGGAGGAGCCGTTCTTGCAAAAGACGGAGC

TGACACTTTCATCGTCGGCACTCACTCCGCGGGAGGAAATGGTGTTGGATATTGCTCGTGCGTGTCCAGG

TCCATGCTCCTGAAAATGAAGGCACACATTGACCCTGAACCACACCATGAG

>HQ832583.1_A_IND_2005

ATCTCAATTCCTTCCCAAAAATCCGTGTTGTACTTCCTCATTGAGAAAGGCCAGCACGAGGCTGCAATTG

AATTCTTTGAGGGGATGGTTCACGACTCCATCAAAGAGGAGCTCCGCCCCCTCATCCAACACACCTCATT

TGTGAAGCGCGCTTTCAAGCGCCTGAAGGACAACTTTGAGATTGTTGCCCTGTGTTTGACTCTTTTGGCA

AACATAGTGATCATGATCCGCGAGACTCGCAAGAGACAGCAGATGGTGGATGATGCAGTGAATGAGTACA

TTGAAAAGGCAAACATCACCACAGATGACAAGACTCTTGACGAGGCGGAAAAGAACCCTCTGGAGACCAG

CGGTGCTAGCACTGTTGGTTTCAGAGAGAGGACTCTCCCGGGGCAGAAAGTGAGCGATGACGTGAACTCT

GAGCCCGCCAAACCCGTGGAAGAGCAACCACAAGCTGAAGGACCCTACGCCGGGCCGCTTGAGCGTCAGA

AACCTCTGAAAGTGAGAGCCAAGCTACCACAGCAGGAGGGACCCTACGCTGGCCCGATGGAGAGACAGAA

ACCACTGAAAGTGAAAGTAAAAGCCCCGGTCGTGAAGGAAGGACCTTACGAAGGACCGGTGAAGAAGCCT

GTCGCTTTGAAAGTTAAAGCCAAGACCTTGATTGTCACTGAGAGTGGCGCCCCACCGACCGACTTGCAAA

AGATGGTCATGGGCAACACCAAGCCTGTCGAGCTCATCCTCGATGGGAAGACGGTGGCCATCTGCTGTGC

TACCGGAGTGTTTGGCACTGCCTACCTCGTGCCTCGTCATCTTTTCGCAGAGAAGTACGACAAGATCATG

TTGGACGGCAGAGCCATGACAGACAGTGACTACAGAGTGTTTGAGTTTGAGATTAAAGTAAAAGGACAGG

ACATGCTCTCAGACGCCGCGCTCATGGTGCTCCACCGTGGGAACCGCGTGAGAGACATCACGAAGCACTT

TCGTGACACAGCAAGAATGAAGAAAGGCACCCCCGTTGTCGGTGTGATCAACAACGCCGACGTTGGGAGA

CTGATTTTCTCTGGTGAGGCCCTCACCTACAAAGACATTGTAGTGTGTATGGATGGTGACACCATGCCTG

GCCTCTTTGCCTACAGAGCCGCCACTAAGGCTGGTTACTGTGGAGGAGCTGTTCTTGCAAAAGACGGAGC

TGACACTTTCATCGTCGGCACTCACTCCGCGGGAGGAAATGGAGTTGGATATTGCTCGTGCGTGTCCAGG

TCCATGCTCCTGAAAATGAAGGCACACATTGACCCTGAACCACACCATGAG

>HQ832584.1_A_IND_2005

ATCTCAATTCCTTCCCAAAAGTCCGTGTTGTACTTTCTCATTGAGAAGGGGCAGCACGAAGCAGCAATTG

AATTCTACGAGGGGATGGTCAGTGACTCCATCAAGGAGGAGCTCCGGCCCCTCGTCCAACAGACCTCATT

TGTGAAACGTGCGTTTAAACGCCTGAAGGAAAACTTTGAGATTGTTGCCCTATGTCTGACCCTTTTGGCA

AACATAGTGATCATGATCCGCAAGACTCGCAAGAGACAACAAATGGTGGATGATGCAGTGAATGAGTACA

TTGAGAAAGCAAACATCACCACAGATGACAAGACTCTTGACGAGGCGGAAAAGAACCCTCAGGAGACTAG

CAGTGCCAGCACTGTTGGCTTCAGAGAGAGGACTCTCCCAGGGCAGAAGGTGAGCGATGACGTGAACTCT

GAGCCCACCGAACCCGAGAGAGAGCAACCACGAGCAGAAGGACCCTACGCCGGGCCACTCGACCGTCAGA

AACCTCTGAAAGTGAGAGCCAAACTACCACAACAGGAGGGACCCTACGCTGGCCCGATGGAGAGACAGAA

ACCACTAAAAGTGAAAGCAAAAGCCCCGGTCATGAAGGAAGGACCTTACGAGGGACCGGTGAAAAAGCTT

GTCGCTTTGAAAGTGAAAGCCAAGAACTTGATTGTCACTGAGAGTGGAGCCCCACCGACCGACTTGCAAA

AGATGGTCATGGGCAACACGAAGCCTGTTGAGCTCATCCTCGACGGGAAGACAGTAGCCATCTGCTGTGC

TACTGGAGTGTTCGGAACTGCCTACCTCGTGCCTCGTCATCTTTTCGCAGAGAAGTATGACAAGATCATG

TTGGACGGTAGGGCCATGACAGACAGTGACTACAGAGTGTTTGAGTTTGAGATAAAAGTAAAAGGACAGG

ACATGCTCTCAGACGCCGCGCTCATGGTGCTCCACCGTGGGAATCGCGTGCGGGACATCACGAAGCACTT

CCGTGATGTGGCAAAAATGAAGAAAGGCACCCCCGTCGTCGGCGTGGTCAACAACGCTGATGTCGGGAGA

CTGATCTTCTCTGGTGAGGCCCTTACCTACAAAGACATTGTAGTGTGCATGGACGGAGACACCATGCCTG

GCCTCTTTGCCTACAAAGCCGCCACCAAGGCTGGCTACTGTGGAGGAGCCGTTCTTGCCAAGGACGGTGC

CGAGACCTTCATCGTCGGCACTCACTCCGCAGGTGGCAATGGGGTTGGATACTGCTCCTGCGTTTCCAGG

TCCATGCTCATGAAAATGAAGGCACACATCGACCCTGAACCACACCACGAG

>HQ832585.1_A_IND_2005

ATCTCAATTCCTTCCCAAAAGTCAGTGTTGTATTTTCTCATTGAGAAGGGACAGCACGAAGCAGCAATTG

AATTCTTTGAGGGGATGGTGCACGACTCCATCAAGGAGGAACTCCGGCCCCTCATCCAACAGACCTCATT

TGTGAAACGCGCTTTCAAGCGCCTGAAGGAAAATTTTGAGATTGTTGCCCTGTGTTTGACTCTTTTGGCA

AACATAGTGATCATGATCCGCGAGACTCGCAAGAGACAACAAATGGTGGACGACGCAGTGAATGAGTACA

TTGAGAAAGCCAACATCACCACGGACGACAAGACTCTTGACGAGGCGGAAAAGAACCCTCTAGAGACCAA

CGGTACCAGTACTGTCGGTTTCAGAGAGAGAACTCTCCCAGGACACAAGGCGAGTGATGACGTGAACTCC

GAGCCCGCCAACCCTGTGGAGGAGCAACCACAAGCTGAAGGACCCTACGCCGGGCCACTCGAGCGTCAGA

AACCTCTGAAAGTGAGAGCCAAGCTCCCACAGCAAGAGGGACCCTACGCTGGCCCGATGGAGAGACAGAA

ACCACTTAAAGTGAAAGCAAAAGCCCCGGTCGTTAAGGAAGGACCTTACGAGGGACCGGTGAAGAAACCT

GTCGCTTTGAAAGTGAAAGCTAAGAACTTGATTGTCACTGAGAGTGGTGCGCCCCCGACCGACTTGCAGA

AGATGGTCATGGGCAACACCAAGCCTGTTGAGCTCATCCTCGACGGGAAGACGGTGGCCATCTGCTGCGC

CACCGGAGTGTTTGGTACTGCTTACCTCGTGCCTCGTCACCTTTTCGCAGAGAAGTATGACAAGATCATG

TTGGACGGTAGAGCCATGACAGACAGTGACTACAGAGTGTTTGAGTTTGAGATTAGAGTAAAAGGACAGG

ACATGCTCTCAGACGCCGCGCTCATGGTGCTTCACCGTGGGAATCGCGTGCGGGACATCACGAAGCACTT

CCGTGATGTAGCAAGAATGAAGAAAGGCACCCCCGTCGTTGGTGTGATCAACAACGCCGATGTCGGGAGA

CTGATTTTCTCTGGTGAGGCCCTTACCTACAAGGACATTGTAGTGTGCATGGATGGAGACACCATGCCTG

GCCTCTTTGCCTACAAAGCCGCCACCAAGGCTGGCTATTGTGGAGGAGCCGTTCTTGCAAAGGACGGAGC

CGAGACCTTCATCGTCGGTACTCACTCCGCAGGTGGCAATGGAGTTGGATACTGCTCATGCGTTTCCAGG

TCCATGCTTCTGAAAATGAAGGCACACATTGACCCTGAACCACACCACGAG

>HQ832586.1_A_IND_2006

ATCTCAATTCCTTCTCAAAAATCTGTGTTGTACTTTCTCATTGAGAAAGGCCAGCACGAAGCAGCAATTG

ACTTCTTCGAGGGGATGGTGCACGACTCCATCAAGGAGGAGCTCCGACCCCTCATCAAACATACATCATT

TGTGAGACGCGCTTTCAAGCGCCTGAAGGAAAACTTTGAGATTGTTGCCCTGTGTTTGACTCTTCTGGCA

AACATAGTGATCATGATCCGCGAGACTCGCAAGAGACAGCGAATGGTGGATGATGCAGTGAATGAGTACA

TTGAGAAAGCTAACATCACCACAGATGACAAGACTCTTGACGAGGCGGAAAAGAACCCTCTGGAGACCAG

TGGTGCCAGCACCGTTGGTTTCAGAGAGAGAACTCTCCCAAGACACAAGGTGAGTGATGACGTGAACACC

GAGCCCACCAAACCTGTGGAGGAGCAACCACAAGCTGAAGGACCCTACGCCGGGCCACTTGAGCGTCAGA

AACCTCTGAAAGTGCGCACCAAACTGCCACAACAAGAGGGACCCTACGCTGGCCCGATGGAGAGACAGAA

ACCACTGAAAGTGAAAGCAAAAGCCCCGGTCGTCAAGGAAGGACCTTATGAGGGACCGGTGAAGAAGCCT

GTCGCTTTGAAAGTGAAAGCAAAGAACTTGATTGTCACTGAGAGTGGTGCCCCACCGACCGACCTGCAAA

AGATGGTCATGGGCAACACAAAGCCTGTCGAGCTCATTCTCGACGGGAAGACAGTAGCCATCTGCTGTGC

TACTGGAGTGTTTGGCACCGCCTACCTCGTGCCTCGTCATCTTTTCGCTGAGAAGTACGACAAGATCATG

TTGGACGGCAGAGCCATGACAGACAGTGACTACAGAGTGTTTGAGTTTGAGATTAAAGTAAAAGGACAGG

ACATGCTCTCAGACGCCGCGCTCATGGTACTCCACCGTGGGAATCGCGTGCGGGACATCACGAAACACTT

CCGTGATGTAGCAAGAATGAAGAAAGGCACCCCCGTCGTCGGTGTGATCAACAACGCTGATGTCGGGAGA

TTGATCTTCTCTGGTGAGGCCCTTACCTACAAGGACATCGTAGTGTGCATGGACGGAGACACCATGCCTG

GCCTCTTTGCCTACAAAGCCGCCACCAAGGCTGGTTACTGTGGAGGTGCTGTTCTCGCCAAGGACGGAGC

TGACACATTCATCGTCGGCACTCACTCCGCAGGAGGCAATGGAGTTGGATACTGCTCATGCGTTTCCAGG

TCCATGCTTCTCAAGATGAAGGCACACATCGACCCTGAACCACACCACGAG

>HQ832587.1_A_IND_2005

ATCTCAATTCCTTCCCAAAAGTCCGTGTTGTACTTTCTCATTGAGAAGGGCCAGCATGAAGCAGCAATTG

ACTTCTTCGAGGGGATGGTACACGACTCCATCAAGGAGGAGCTCCGCCCTCTCATCCAGCAGACCTCATT

TGTGAAACGCGCTTTTAAGCGCCTGAAGGAAAACTTTGAGATTGTTGCCCTGTGTTTGACTCTCTTGGCA

AACATAGTGATCATGATCCGCGAGACTCGCAAGAGACA---GATGGTGGATGATGCAGTGAATGAGTACA

TTGAGAAAGCAAACATCACCACAGACGACAAAACTCTTGACGAGGCGGAAAAGAACCCTCTGGAGACCAG

CGGTGCCAGCACTGTTGGTTTCAGAGAGAGAACTCTCACGGGGCAAAAAGTGAGTGATGACGTGAACTCC

GAGCCCACCAAACCCGTGGAGGAGCAACCACAAGCTGAAGGACCCTACGCCGGGCCACTCGAGCGTCAGA

AACCTCTGAAAGTGAGAGCCAAGCTACCACAGCAGGAGGGCCCCTACGCTGGCCCGTTGGAGAGACAGAA

ACCACTGAAAGTGAAAGCAAAAGCCCCGGTCGTGAAGGAAGGACCTTACGAGGGACCGGTGAAGAAGCCT

GTCGCTTTGAAAGTGAAAGCCAAGAATTTGATTGTCACTGAGAGTGGCGCCCCCCCGACCGACTTGCAAA

AGATGGTCATGGGCAACACCAAGCCTGTCGAGCTCATCCTCGATGGGAAGACGGTAGCCATCTGCTGTGC

TACCGGAGTGTTTGGCACTGCCTACCTCGTGCCTCGTCATCTTTTCGCAGAGAAGTATGACAAGATCATG

TTGGACGGCAGAGCTATGACAGACAGTGACTACAGAGTGTTTGAGTTTGAGATTAAAGTAAAAGGACAGG

ACATGCTCTCAGACGCCGCGCTCATGGTGCTCCACCGTGGGAACCGCGTGAGAGACATCACGAAACACTT

TCGTGACACAGCAAGAATGAAGAAAGGCACCCCCGTTGTCGGCGTGATCAACAACGCTGACGTTGGGAGA

CTGATTTTCTCTGGTGAGGCCCTTACCTACAAGGACATTGTGGTGTGCATGGATGGCGACACCATGCCTG

GCCTCTTTGCCTACAGAGCCGCCACCAAGGCCGGCTACTGTGGAGGAGCTGTTCTCGCGAAGGACGGAGC

TGACACGTTCATTGTCGGCACCCACTCTGCGGGCGGCAACGGAGTTGGATACTGCTCATGCGTGTCCAGG

TCCATGCTCCTGAAAATGAAGGCACACATTGACCCCGAACCACACCATGAG

>HQ832588.1_A_IND_2005

ATCTCAATTCCTTCCCAAAAGTCCGTGCTGTACTTTCTCATTGAGAAGGGGCAACACGAAGCAGCAATTG

ACTTCTTTGAGGGGATGGTTAGTGACTCCATTAAGGAGGAGCTCCGACCCCTCGTCCAACAGACCTCATT

TGTGAAACGTGCGTTCAGACGCCTGAAGGAAAACTTTGAGATTGTTGCCCTATGTTTGACTCTTCTGGCA

AACATAGTGATCATGATCCGCGAGACTCGCAAGAGACAACAAATGGTGGATGATGCAGTGAATGAGTACA

TTGAGAAAGCAAACATCACCACAGATGACAAGACTCTTGACGAGGCGGAAAAGAACCCTCAGGAGACTAG

CAGTGCCAGCACTGTAGGCTTCAGAGAGAGAACTCTTCCAGGACAGAGAGTGAGCGATGACGTGAACTCC

GAGCCCACCGAACCTGTGAGAGAGCAACCACAAGCTGAAGGACCCTACGCCGGGCCACTCGAGCGTCAGA

AACCTCTGAAGGTGAGAGCCAAGCTACCACAACAGGAGGGACCTTACGCTGGCCCGATGGAGAGACAGAA

ACCACTGAAAGTGAAAACAAAAGCCCCGGTCGTGAAGGAAGGACCTTACGAGGGACCGGTGAAGAAGCCT

GTCGCTTTGAAAGTGAAAGCTAAGAACTTGATTGTCACTGAGAGTGGAGCCCCACCGACTGACTTGCAAA

AGATGGTCATGGGCAACACGAAGCCTGTTGAGCTTATTCTCGACGGGAAGACAGTAGCCATTTGCTGTGC

TACTGGAGTGTTTGGCACTGCCTACCTCGTGCCTCGTCATCTTTTCGCAGAGAAGTATGACAAGATCATG

CTGGACGGCAGGGCCATGACAGACAGTGACTACAGAGTGTTTGAGTTTGAGATAAAAGTAAAGGGACAGG

ACATGCTCTCAGACGCCGCGCTCATGGTGCTCCACCGTGGGAATCGCGTGCGGGACATCACGAAGCATTT

CCGTGATGTAGCAAAATTGAAGAAAGGAACCCCCGTCGTTGGTGTGATTAACAACGCCGACGTCGGGAGA

CTGATTTTCTCTGGTGAGGCCCTTACCTACAAGGACATTGTAGTGTGCATGGACGGAGACACCATGCCTG

GCCTCTTTGCCTACAAAGCCGCCACCAAGGCTGGTTACTGTGGAGGAGCCGTTCTTGCCAAGGACGGTGC

CGAGACATTCATTGTTGGCACTCACTCCGCAGGTGGCAATGGAGTTGGGTACTGCTCTTGCGTTTCCAGA

TCCATGTTAATGAAAATGAAGGCACACATCGACCCTGAACCACACCACGAG

>HQ832589.1_A_IND_2006

ATCTCAATTCCTTCCCAAAAGTCCGTGCTGTACTTTCTCATTGAGAAGGGGCAACACGAGGCAGCAATTG

ACTTCTTTGAGGGGATGGTCAGTGACTCCATCAAGGAGGAGCTCCGGCCCCTCATCCAACAGACCTCATT

TGTGAAACGTGCGTTCAGGCGCCTGAAGGAAAACTTTGAGATTGTTGCCCTATGTTTGACTCTTCTGGCA

AACATAGTGATCATGATCCGCGAGACTCGCAAGAGACAACAAATGGTGGATGATGCAGTGAATGAGTACA

TTGAGAAAGCAAACATCACCACAGATGACAAGACTCTTGACGAGGCGGAAAAGAACCCTCAGGAGACTAG

CAGTGTCAGCACTGTAGGCTTCAGAGAGAGAACTCTTCCAGGACAGAGAGTGAGCGATGACGTGAACTCC

GAGCCCACCGAACCTGTGAGAGAGCAACCACAGGCTGAAGGACCCTACGCCGGGCCACTCGAGCGTCAGA

AACCTCTGAAGGTGAGAGCCAAGCTACCACAACAGGAGGGACCCTACGCTGGCCCGATGGAGAGACAGAA

ACCACTGAAAGTGAAAACAAAAGCCCCGGTCGTGAAGGAAGGACCTTACGAGGGACCGGTGAAGAAGCCT

GTCGCTTTGAAAGTGAAAGCTAAGAACTTGATTGTCACTGAGAGTGGAGCCCCACCGACTGACTTGCAAA

AGATGGTCATGGGCAACACGAAGCCTGTTGAGCTTATTCTCGACGGGAAGACAGTAGCCATCTGCTGTGC

TACTGGAGTGTTTGGCACTGCCTACCTCGTGCCTCGTCATCTTTTCGCAGAGAAGTATGACAAGATCATG

CTGGACGGCAGAGCCATGACAGACAGTGACTACAGAGTGTTTGAGTTTGAGATAAAAGTAAAGGGACAGG

ACATGCTCTCAGACGCCGCGCTCATGGTGCTCCACCGTGGGAATCGCGTGCGGGACATCACGAAGCATTT

CCGTGATGTAGCAAAAATGAAGAAGGGAACCCCCGTCGTTGGTGTGATCAACAACGCCGATGTCGGGAGA

CTGATTTTCTCTGGTGAGGCCCTTACCTACAAGGACATTGTGGTGTGCATGGACGGAGACACCATGCCTG

GCCTCTTTGCCTACAAAGCCGCCACCAAGGCTGGTTACTGTGGAGGAGCCGTTCTTGCCAAGGACGGTGC

CGAGACATTCATTGTTGGCACTCACTCCGCAGGCGGCAATGGAGTTGGGTACTGCTCTTGCGTTTCCAGA

TCCATGTTAATGAAAATGAAGGCACACATCGACCCTGAACCACACCACGAG

>HQ832590.1_A_IND_2007

ATCTCAATTCCTTCCCAAAAGTCTGTGTTGTACTTTCTCATTGAGAAGGGCCAACACGAAGCAGCAATTG

AATTCTTTGAGGGGATGGTGCACGACTCCATTAAGGAGGAGCTCCGGCCCCTCATCCAACAGACATCATT

TGTGAAACGCGCTTTTAAGCGTCTGAAGGAAAACTTTGAGATTGTTGCCCTGTGTTTGACTCTTCTGGCA

AACATAGTGATCATGATCCGCGAGACTCACAAGAGGCAGCAGATGGTGGATGATGCAGTGAATGAGTACA

TCGAGAAAGCAAACATCACCACAGATGACAAGACTCTTGACGAGGCGGAAAAGAACCCTCTGGAAACTAG

TGGCGCCAGCACCGTTGGTTTCAGAGAGAGAACTCTCCCAGGACACAAGGTGAGTGATGACATGAACTCC

GAGCCCACCAAACCTGTGGAAGAGCAACCACAAGCTGAAGGACCCTACGCCGGGCCACTCGAGCGCCAGA

AACCTCTGAAGGTGAGAGCCAAGCTCCCGCAACAAGAGGGACCCTACGCTGGCCCGATGGAGAGACAGAA

ACCATTGAAAGTGAAAGCAAAAGCCCCGGTCGTTAAGGAAGGACCTTACGAAGGACCGGTGAAGAAACCT

GTCGCTTTGAAAGTGAAAGCAAGGAACTTGATTGTCACTGAGAGTGGTGCTCCCCCGACTGACTTGCAAA

AGATGGTCATGGGTAACACCAAGCCTGTCGAGCTCATCCTCGATGGGAAGACGGTAGCCATCTGCTGTGC

TACCGGAGTGTTTGGCACTGCCTACCTCGTGCCTCGTCATCTTTTTGCAGAGAAGTATGACAAGATCATG

TTGGACGGCAGAACCATGACAGACAGTGACTACAGAGTGTTCGAGTTTGAGATTAAAGTAAAAGGACAGG

ACATGCTCTCAGACGCCGCACTCATGGTGCTTCACCGTGGGAATCGCGTGCGGGACATCACGAAGCACTT

CCGTGATGTGGCAAGAATGAAGAAAGGCACCCCCGTCGTCGGCGTGATCAACAACGCTGATGTTGGGAGA

CTGATTTTCTCTGGTGAGGCCCTTACCTACAAGGATATTGTAGTGTGCATGGATGGAGACACCATGCCCG

GTCTCTTTGCCTACAAAGCCGCCACCAAGGCGGGATACTGTGGAGGAGCCGTTCTCGCAAAGGACGGAGC

CGAGACTTTCATCGTCGGCACTCACTCCGCAGGCGGCAACGGAGTTGGATATTGCTCATGCGTGTCCAGG

TCCATGCTGTTTAAAATGAAGGCACACATCGATCCCGAACCGCACCACGAG

>HQ832591.1_A_IND_2008

ATCTCAATTCCTTCCCAAAAGTCTGTGTTGTACTTTCTCATTGAGAAGGGCCAACACGAAGCAGCAATTG

AATTCTTTGAGGGGATGGTGCACGACTCCATCAAGGAGGAGCTCCGGCCCCTCATCCAACAGACATCATT

TGTGAAACGCGCTTTTAAGCGTCTAAAGGAAAACTTTGAGATTGTTGCCCTGTGTTTGACTCTTCTGGCA

AACATAGTGATCATGATCCGCGAGACTCACAAGAGGCAGCAGATGGTGGATGATGCAGTGAATGAGTACA

TCGAGAAAGCAAACATCACCACAGATGACAAGACTCTTGATGAGGCGGAAAAGAACCCTCTGGAAACTAG

TGGTGCCAGCACCGTTGGTTTCAGAGAGAGAACTCTCCCAGGACACAAGGTGAGTGATGACGTGAACTCC

GAGCCCACCAAACCTGTGGAAGAGCAACCACAAGCTGAAGGCCCCTACGCCGGGCCACTCGAGCGTCAGA

AACCTCTGAAGGTGAGAGTCAAGCTCCCGCAACAAGAGGGACCCTACGCTGGCCCGATGGAGAGACAGAA

ACCACTGAAAGTGAAAGCAAAAGCCCCGGTCGTTAAGGAAGGACCTTACGAAGGACCGGTGAAGAAACCT

GTCGCTTTGAAAGTGAAAGCAAAGAACTTGATTGTCACTGAGAGTGGTGCTCCCCCGACTGACTTGCAAA

AGATGGTCATGGGTAACACCAAGCCTGTCGAGCTCATCCTCGATGGGAAGACGGTGGCCATCTGCTGTGC

CACCGGAGTGTTTGGCACTGCATACCTCGTGCCTCGTCATCTTTTTGCAGAGAAGTATGACAAGATCATG

TTGGACGGCAGAGCCATGACAGACAATGACTACAGAGTGTTTGAGTTTGAGATTAAAGTAAAAGGACAGG

ACATGCTCTCAGACGCCGCGCTCATGGTGCTTCACCGTGGGAATCGCGTGCGGGACATCACGAAGCACTT

CCGTGATGTGGCAAGAATGAAGAAAGGCACCCCCGTCGTCGGCGTGATCAACAACGCTGATGTTGGGAGA

CTGATTTTCTCTGGTGAGGCCCTTACCTACAAGGACATTGTAGTGTGCATGGATGGAGACACCATGCCCG

GTCTCTTTGCCTACAAAGCCGCCACCAAGGCGGGATACTGTGGAGGAGCCATTCTCGCAAAGGACGGAGC

CGAGACTTTCATCGTCGGCACTCACTCCGCAGGCGGCAACGGAGTTGGATATTGCTCGTGCGTGTCCAGG

TCCATGCTGTTTAAAATGAAGGCACACATCGATCCCGAACCGCACCACGAG

>HQ832592.1_A_IND_2009

ATCTCAATTCCTTCCCAAAAGTCTGTGTTGTACTTTCTCATTGAGAAGGGGCAGCACGAAGCAGCAATTG

AATTCTTTGAGGGGATGGTTAGTGACTCTGTCAAGGAGGAACTCCGACCCCTTATCCAACAGACCTCATT

TGTGAAACGTGCGTTTAAACGCTTGAAGGAAAATTTTGAGATCGTTGCCCTATGTTTAACCCTCTTGGCA

AACATAGTGATCATGATCCGCGAGACTCGCAAGAGACAGCAAATGGTGGATGATGCGGTGAATGAGTACA

TTGAGAAAGCAAACATCACCACAGATGACAAGACTCTTGACGAAGCGGAAAAGAACCCTCAGGAGACTAA

CAGTGCCAGCACTGTTGGTTTCAGAGAGAGAACTCTCCCAGGGCAGAAGGTGAGTGATGACGTGAACTCC

GAGCCCACCGAACCTGCGGGAGATCAACCACAAGCTGAAGGACCCTACACCGGACCACTCGAGCGTCAGA

AACCTCTGAAAGTGAGAGCCAAGCTACCACAACAGGAGGGACCCTACGCTGGTCCGTTGGAGAGACAGAA

ACCACTAAAGGTGAAAGCAAAAGCCCCGGTAGTGAAGGAAGGACCTTACGAAGGACCGGTGAAGAAGCCT

GTCGCTTTGAAAGTGAAAGCCAAGAACTTGATTGTCACTGAGAGTGGAGCCCCACCGACTGACTTGCAAA

AGATGGTCATGGGCAACACAAAGCCTGTTGAGCTCATCCTCGACGGGAAGACAGTAGCCATCTGCTGTGC

TACTGGAGTGTTCGGCACTGCCTACCTCGTGCCTCGTCATCTCTTCGCAGAGAAATACGACAAGATCATG

TTGGACGGCAGAGCCATGACAGACAGTGACTACAGGGTGTTTGAGTTTGAGATAAAAGTAAAAGGACAGG

ACATGCTCTCAGACGCCGCGCTCATGGTGCTCCACCGTGGGAATCGCGTGCGAGACATCACGAAGCACTT

CCGTGATGTGGCAAAAATGAAGAAAGGCACCCCCGTCGTTGGCGTGATCAACAACGCCGATGTCGGGAGA

CTGATTTTCTCTGGTGAGGCCCTTACTTACAAGGACATTGTTGTGTGCATGGACGGAGACACCATGCCTG

GCCTCTTTGCTTACAAAGCCGCCACCAAGGCGGGCTACTGTGGAGGAGCCGTTCTTGCTAAGGACGGTGC

CGAGACATTCATCGTTGGCACTCACTCTGCAGGAGGCAATGGAGTTGGGTACTGCTCTTGCGTTTCCAGG

TCCATGCTCATGAAAATGAAGGCACACATCGACCCTGAACCACACCACGAG

>JF749841.1_A_TUR_2006

ATCTCAATTCCTTCCCAAAAATCTGTGTTGTACTTCCTCATTGAGAAAGGTCAACACGAGGCAGCAATTG

AATTCTTTGAGGGGATGGTTCACGACTCTATCAAGGAGGAACTCCGACCCCTAATCCAACAGACCTCATT

TGTGAAACGCGCTTTTAAGCGCCTAAAGGAAAACTTTGAGATTGTTGCCCTGTGTCTGACTCTACTGGCA

AACATAGTGATCATGATCCGCGAGACTCGCAAGAGACAGCAGATGGTGGATGATGCAGTGAATGAGTACA

TTGAAAAGGCAAACATCACCACAGATGACAAGACTCTTGACGAGGCGGAAAAGAACCCTCAGGAGACAAG

CAGTGCCAGCACTGTTGGCTTCAGAGAGAGAACTCTCCCGGGACACACGGCGAGCAATGACGTGAACTCC

GAGCCCACCGTACCCGTGGAGGGACAACCACAAGCTGAAGGACCCTACGCCGGGCCACTCGAGCGTCAGA

AACCTCTGAAAGTGAGAGCCAAGCTCCCACAGCAGGAGGGGCCCTACACTGGTCCGATGGAGAGACAAAA

GCCCCTGAAAGTGAAAGCAAAAGCCCCGGTCGTAAAGGAAGGACCTTACGAGGGGCTTGTGAAGAAACCT

GTCGCTTTGAAAGTGAAAGCCAAAAATTTGATTGTCACTGAGAGTGGTGCCCCCCCGACCGACTTGCAAA

AGATGGTCATGGGCAACACCAAGCCTGTTGAGCTCATCCTCGACGGGAAGACGGTAGCCATCTGTTGCGC

TACCGGAGTGTTTGGCACTGCTTACCTTGTGCCACGTCATCTTTTCGCGGAGAAGTATGACAAGATCATG

CTGGACGGCAGAGCCATGACAGACAGTGACTACAGAGTGTTTGAGTTTGAGATTAAAGTAAAAGGGCAGG

ACATGCTCTCAGATGCCGCGCTCATGGTGCTCCACCGTGGGAATCGCGTGAGAGACATCACGAAACACTT

TCGTGATACAGCAAGAATGAAGAAGGGCACCCCCGTTGTCGGTGTTATCAACAACGCTGATGTCGGGAGA

CTGATTTTCTCTGGTGAGGCCCTTACCTACAAGGACATTGTAGTGTGCATGGATGGAGACACCATGCCTG

GCCTCTTTGCCTACAGAGCCGCCACCAAGGCTGGCTATTGTGGAGGAGCTGTTCTTGCAAAGGACGGAGC

CGAGACCTTCATCGTCGGCACTCACTCCGCAGGTGGTAATGGAGTTGGATACTGTTCATGCGTTTCCAGG

TCCATGCTGCTAAAGATGAAGGCACACATCGACCCTGAGCCACACCACGAG

>JF749843.1_A_EGY_2006

ATTTCAATTCCTTCCCAAAAGTCCGTGTTGTACTTCCTCATAGAGAAAGGCCAACATGAAGCAGCAATTG

ACTTCTTTGAGGGGATGGTGCACGACTCCATCAAGGAGGAGCTCCGACCCCTCATCCAACAGACCTCATT

TGTGAAACGCGCTTTCAAACGCTTGAAGGAAAACTTTGAAATTGTTGCCCTGTGTTTGACTCTTTTGGCA

AACATAGTGATCATGATCCGCGAGACTCGCAAGAGGCAAAAGATGGTGGATGACGCAGTGAGTGAGTACA

TTGAGAAGGCAAACATCACCACAGATGACAAGACTCTTGACGAGGCGGAGAAGAACCCTCTGGAGACCAG

TGGTGCCAGCACCGTTGGTTTCAGAGAGAAGACCCTTCGGGGGCAAACGGTGTGCAATGACGTGGACTCC

GAGCCCACCAAACCCGCGGAGGAGCAACCACAAGCTGAAGGACCCTACAACGGACCGCTCGAGCGCCAAA

AACCTCTGAAAGTGAGAGCCAGACTACCACAGCAGGAGGGGCCTTACGCTGGTCCGATGGAGAGACAGAA

ACCACTGAAAGTGAAAGCAAGAGCCCCGGTCGTTAAGGAAGGACCTTACGAGGGACCGGTGAAGAAGCCT

GTCGCTTTGAAAGTGAAAGCCAAGAATTTGATTGTCACTGAGAGTGGAGCCCCACCGACTGACTTGCAAA

AGATGGTCATGGGGAACACTAAACCCGTTGAGCTCATCCTTGACGGAAAGACGGTAGCCATTTGCTGCGC

TACCGGAGTGTTTGGCACTGCCTACCTTGTGCCTCGCCATCTTTTCGCAGAGAAGTATGACAAGATCATG

TTGGACGGCAGAGCCATGACAGACAGTGACTACAGAGTGTTTGAGTTTGAGATTAAAGTAAAAGGACAGG

ACATGCTCTCAGACGCCGCGCTCATGGTGCTCCACCGTGGGAACCGCGTGAGAGACATCACGAAACACTT

TCGTGACACAGCAAGAATGAAGAAAGGAACCCCCGTCGTTGGAGTCATCAACAACGCCGACGTCGGGAGA

CTCATTTTCTCTGGTGAAGCCCTCACCTACAAAGACATTGTAGTGTGTATGGACGGTGACACAATGCCCG

GTCTTTTTGCCTACAGAGCCGCCACCAAGGCCGGTTACTGTGGAGGGGCTGTTCTTGCTAAGGACGGAGC

CGACACGTTCATCGTCGGCACCCACTCCGCAGGAGGCAACGGAGTTGGATACTGCTCGTGTGTGTCCAGA

TCCATGCTTCTGAAAATGAAGGCACACGTTGACCCCGAGCCCCACCACGAG

>JF749848.1_A_TUR_2003

ATCTCAATTCCTTCCCAAAAATCCGTGTTGTACTTTCTCATTGAGAAAGGTCAACACGAAGCAGCAATTG

AATTCTTTGAGGGGATGGTGCATGACTCCATCAAGGAGGAACTCAGGCCCCTCATCCAACAGACCTCATT

TGTGAAACGCGCTTTCAAGCGCCTGAAGGAGAACTTTGAGATTGTCGCCCTATGTTTGACTCTTCTAGCA

AACATAGTGATCATGATCCGCGAGACTCGCAAGAGACAACAGATGGTGGATGATGCAGTGAATGAGTACA

TTGAGAAGGCAAACATCACCACAGATGACAAGACTCTTGACGAGGCGGAGAAGAACCCTCTGGAGACCAG

CGGCGCCAGCACCGTCGGTTTCAGAGAGAGAACCCTCCCGGGACGCATGGCAGGCGATGACGTGAACTCG

GAGCCCGTCAAGCTCGCGGAGGAACAACCACAAGCTGAAGGACCCTACACCGGGCCACTTGAGCGTCAGA

AACCCCTGAAAGTGAGAGCCAAGCTACCACAGCAAGAGGGGCCTTACGCCGGCCCGATGGAGAGACAGAA

ACCACTGAAAGTGAAAGCAAAAGCCCCCGTCGTTAAGGAAGGACCTTACGAGGGACCGGTGAAGAAGCCT

GTCGCTTTGAAAGTGAAAGCTAAGAACTTGATTGTCACTGAGAGTGGTGCCCCACCGACCGACTTACAAA

AGATGGTTATGGGCAACACTAAGCCTGTTGAGCTTATTCTCGACGGGAAGACAGTAGCCATCTGTTGTGC

TACTGGAGTGTTTGGTACTGCCTACCTAGTGCCTCGTCACCTTTTCGCAGAAAAGTACGACAAGATCATG

CTGGACGGCAGAGCCATGACAGACAGTGACTACAGAGTGTTTGAGTTTGAGATTAAAGTAAAAGGACAGG

ACATGCTCTCAGACGCCGCGCTCATGGTGCTTCACCGTGGGAATCGCGTGCGAGACATCACGAAGCACTT

TCGTGACGTAGCAAGAATGAAGAAAGGTACCCCCGTCGTCGGTGTGATTAACAACGCTGACGTCGGGAGA

CTGATTTTCTCTGGTGAGGCCCTTACCTACAAAGACATTGTAGTGTGCATGGATGGCGATACCATGCCTG

GCCTTTTTGCCTACAGAGCCGCCACCAAGGCTGGTTACTGCGGAGGAGCTGTTCTTGCCAAAGATGGTGC

CGAAACGTTCATCGTCGGCACACACTCCGCAGGCGGCAATGGAGTTGGATACTGCTCGTGCGTTTCCAGG

TCCATGCTTTTAAAGATGAAGGCACACATCGACCCCGAACCACACCACGAG

>JF749849.1_Asia1_PAK_2002

ATCTCAATTCCTTCCCAAAAGTCCGTGCTGTACTTTCTCATTGAGAAGGGCCAGCACGAAGCAGCAATTG

AATTCTTCGAGGGGATGGTGCATGACTCCATCAAGGAAGAGCTCCGGCCCCTCATCCAACAGACTTCATT

TGTGAAGCGCGCTTTTAAGCGCCTGAAGGAGAACTTTGAGATAGTTGCCCTGTGTTTGACCCTTCTGGCA

AACATAGTGATCATGATCCGCGAGACTCGCAAGAGACAGCAGATGGTGGATGATGCAGTGAACGAGTACA

TTGAGAAGGCAAACATCACCACGGACGACAAGACTCTTGACGAGGCGGAAAAGAACCCTCTGGAGACCAG

CGGTGCCACCATTGTTGGTTTCAGAGAGAAAACTTTCTCGGGATACAAGTTGAGCGATGACGTGAACTCC

GAGCCCGCCAAACCCGTGGAAGAACAACCACAAGCCGAAGGACCCTACGCCGGACCACTCGAGCGTCAAA

AACCTCTGAAAGTGAGAGCCAAGCTCCCA---CAGGAGGGGCCCTACGCTGGTCCGATGGAGAGACAGAA

ACCGCTGAAAGTGAAAGTGAAAGCCCCGGTCGTTAAGGAAGGACCTTACGAAGGACCGGTGAAGAAACCT

GTCGCTTTGAAAGTGAAAGCAAAGAACCTGATTGTCACTGAGAGTGGTGCTCCCCCGACTGACTTACAAA

AGATGGTCATGGGCAACACCAAGCCTGTTGAGCTCATCCTCGACGGGAAGACGGTGGCCATCTGCTGCGC

CACCGGAGTGTTTGGTACTGCCTACCTTGTCCCTCGTCATCTTTTCGCAGAGAAGTATGACAAGATCATG

GTGGACGGCAGAGCCATGACAGACAGTGACTACAGAGTGTTTGAGTTTGAGATTAAAGTAAAAGGACAGG

ACATGCTCTCGGACGCCGCGCTCATGGTGCTCCACCGTGGGAATCGCGTGCGGGACATCACGAAACACTT

CCGTGATGTGGCAAGAATGAAGAAAGGCACCCCCGTCGTCGGCGTGATTAACAACGCTGATGTTGGGAGA

CTGATCTTCTCTGGTGAGGCCCTTACCTACAAAGACATCGTAGTGTGCATGGACGGAGACACCATGCCCG

GCCTCTTCGCCTACAAAGCCGCCACCAAGGCGGGTTACTGTGGAGGAGCCGTTCTTGCAAAGGACGGAGC

CGAGACTTTCATCGTCGGCACCCACTCCGCAGGCGGCAATGGAGTTGGATACTGCTCATGCGTTTCCAGG

TCTATGCTGCTTAAAATGAAGGCACACATTGATCCCGAACCACACCACGAG

>JF749851.1_O_IRN_2001

ATCTCAATTCCTTCCCAAAAGTCCGTGCTGTACTTTCTCATTGAGAAGGGCCAGCACGAAGCAGCAATTG

AATTCTTCGAGGGGATGGTGCATGACTCCATCAAGGAAGAGCTCCGGCCCCTCATCCAACAGACCTCATT

TGTGAAGCGCGCTTTTAAGCGCCTGAAGGAGAACTTTGAGATAGTTGCCCTGTGTTTGACCCTTCTGGCA

AACATAGTAATCATGATCCGCGAGACTCGCAAGAGACAGCAGATGGTGGATGATGCAGTGAACGAGTACA

TTGAGAAGGCGAACATCACCACGGACGACAAGACTCTTGACGAGGCGGAAAAGAACCCTTTGGAGACCAG

CGGTGCAACCGCTGTTGGTTTCAGAGAGAAAACTCTCCCGGGACACAAGGCGAGCGACGGCGTGAACTCC

GAGCCCGCCAAACCCGTGGAAGAACAACCACAAGCCGAAGGACCCTACGCCGGACCACTCGAGCGTCAAA

AACCTCTGAAAGTGAGAGCCAAGCTCCCACAACAGGAGGGGCCCTACGCTGGTCCGATGGAGAGACAGAA

ACCGCTGAAAGTGAAAATGAAAGCCCCGGTCGTTAAGGAGGGACCTTACGAAGGACCGGCGAAGAAACCT

GTCGCTTTGAAAGTGAAAGCAAAGAACCTGATTGTCACTGAGAGTGGTGCTCCCCCGACTGACTTACAAA

AGATGGTCATGGGCAACACCAAGCCTGTTGAGCTCATCCTCGACGGGAAGACGGTGGCTATCTGCTGCGC

CACCGGAGTGTTTGGTACTGCCTACCTTGTTCCTCGTCATCTTTTCGCAGAGAAGTACGACAAAATCATG

GTGGACGGCAGAGCCATGACAGACAGTGACTACAGAGTGTTTGAGTTTGAGATTAAAGTAAAAGGACAGG

ACATGCTCTCAGACGCCGCGCTCATGGTGCTCCACCGTGGGAATCGCGTGCGGGACATCACGAAACACTT

CCGTGATGTGGCAAGAATGAAGAAAGGCACCCCCGTCGTCGGCGTGATTAACAACGCTGATGTTGGGAGA

CTGATCTTCTCTGGTGAGGCCCTTACCTACAAAGACATTGTAGTGTGCATGGACGGAGACACCATGCCCG

GCCTCTTCGCCTACAAAGCCGCCACCAAGGCGGGTTACTGTGGAGGAGCCGTTCTTGCAAAGGACGGAGC

CGAGACTTTCATCGTCGGCACCCACTCCGCAGGCGGCAATGGAGTTGGATACTGTTCATGCGTTTCCAGG

TCTATGCTGCTTAAAATGAAGGCACACATTGATCCTGAACCACACCACGAG

>JF749852.1_O_MAY_2004

ATCTCAATTCCTTCCCAAAAATCCGTGCTGTACTTCCTCATTGAGAAAGGCCAGCACGAGGCTGCAATTG

AATTCTTTGAGGGGATGGTGCATGACTCCATCAAAGAGGAGCTCCGGCCCCTCATCCAGCAGACATCATT

TGTGAAACGCGCTTTTAAGCGCCTGAAGGAAAACTTTGAGATTGTTGCCCTGTGTTTGACTCTTTTGGCA

AACATAGTGGTCATGATCCGCGAGACTCACAAGAGACAGCAGATGGTGGATGATGCAGTGAATGAGTACA

TTGAGAAAGCCAACATCACCACGGATGACAAGACTCTTGACGAGGCGGAAAAGAACCCTCTAGAGACCAG

TGGTGCCAGCACCGTTGGTTTCAGAGAGAGAACTCTTCCAGGACACAAGGTGAGCGATGACGTGAACTCC

GAGCCCACCAAACCTGTGGAAGAGCAACCACAAGCCGAAGGACCCTACGCCGGGCCACTTGAGCGTCAGA

AACCTCTGAAAGTGAGAGCCAAGCTCCCGCAGCAGGAGGGGCCTTACGCTGGTCCGATGGAGAGACAGAA

ACCACTGAAAGTGAAAGCAAAAGCCCCGGTCGTTAAGGAAGGACCTTACGAGGGACCGGTGAAGAAGCCT

GTCGCTTTGAAGGTGAAAGCTAAGAACTTGATTGTCACTGAGAGTGGTGCCCCCCCGACCGACTTGCAAA

AGATGGTCATGGGCAACACTAAGCCTGTTGAGCTCATCCTCGACGGGAAGACGGTGGCCATCTGCTGTGC

TACCGGAGTGTTTGGTACTGCCTACCTCGTGCCTCGTCATCTTTTCGCTGAGAAGTATGACAAGATCATG

TTGGACGGTAGAGCCATGACAGACAGTGACTACAGAGTGTTTGAGTTTGAGATTAAAGTAAAAGGACAGG

ACATGCTCTCAGACGCCGCGCTCATGGTGCTCCACCGTGGGAACCGCGTGAGAGACATCACGAAACACTT

TCGTGATACAGCAAGAATGAAGAAAGGCACCCCCGTTGTCGGCGTGATTAACAACGCTGATGTCGGGAGA

CTGATTTTCTCTGGTGAGGCCCTCACCTACAAGGACATAGTGGTGTGCATGGATGGAGACACCATGCCTG

GCCTTTTTGCCTACAAAGCCGCCACCAAGGCTGGTTACTGTGGAGGAGCTGTTCTCGCAAAGGACGGAGC

CGAGACTTTCATCGTCGGCACTCACTCTGCAGGGGGCAATGGAGTTGGATACTGCTCATGCGTTTCTAGG

TCCATGCTTCTGAAAATGAAGGCACACATCGACCCTGAACCACACCACGAG

>JF749860.1_SAT1_KEN_2002

ATTTCCATTCCTTCCCAAAAGTCTGTGCTTTACTTCCTCATTGAGAAAGGACAGCACGAGGCTGCAATTG

AGTTCTATGAAGGGATGGTGCACGACAGCATCAAGGAGGAGCTTAAGCCGCTTCTGGAGCAAACCAGCTT

CGCGAAGCGCGCCTTCAAGCGCCTGAAAGAGAACTTCGAGATCGTTGCTCTCGTCGTTGTGCTGTTGGCA

AACATTGTCATCATGCTCCGCGAAACTCGCAAGAGACAACAGATGGTGGACGACGCGCTCAATGAATACA

TTGAGAAGGCGAACATCACTACTGATGACAAAACTCTTGAAGAGGCGGAAAACAACCCTCGTGAGGTCGT

CGACAAACCCACTGTCGGCTTCCGCGAGAGAAAGCTCCCAGGTCAAAAGTCTGACGAGGAAGCGAACGCT

GAACCGGCCAAGCCT---GAAGAGCAACCACAAGCTGAAGGACCCTACGCGGGACCGCTTGAGCGACAGC

AGCCGCTGAAGCTCAAGGCCAAACTGCCCAAAGCAGAAGGGCCTTACGCGGGACCGCTAGAGAAGCAACA

ACCACTGAAACTGAAAGCTAAGCTGCCTGTGGCCAAGGAAGGGCCATATGAAGGACCAGTGAAGAAACCT

GTCGCCTTGAAAGTGAAAGCAAAAGCCCCGATTGTCACTGAAAGCGGATGCCCTCCGACTGACTTGCAAA

AGATGGTCATGGCAAACGTGAAGCCCGTCGAGCTCATCCTTGATGGGAAGACCGTTGCACTGTGCTGTGC

GACGGGAGTGTTTGGGACAGCCTACCTCGTGCCTCGTCATCTTTTCGCAGAGAAGTACGACAAGATAATG

TTGGATGGCCGTGCCCTGACAGACAGTGACTTCAGAGTGTTCGAGTTTGAGGTGAAAGTAAAGGGACAGG

ACATGCTCTCAGATGCCGCGCTGATGGTTCTCCACACTGGAAATCGTGTGCGCGATCTCACGGGACACTT

CCGTGACACCATGAAACTGTCGAAAGGCAGCCCCATCGTTGGCGTAGTCAACAATGCCGACGTTGGAAGA

CTCATCTTCTCAGGTGACGCGCTGACCTACAAAGACCTGGTCGTTTGTATGGACGGTGACACCATGCCTG

GACTCTTCGCGTACCGCGCTGGGACCAAGGTAGGCTACTGTGGGGCCGCTGTTCTCGCAAAGGACGGCGC

CAAGACTGTGATCGTCGGCACTCACTCAGCCGGAGGCAACGGAGTAGGCTACTGCTCCTGTGTCTCGCGA

TCCATGCTCCTGCAGATGAAGGCCCACATTGACCCTCCCCCTCACACTGAG

>JF749861.1_SAT2_KEN_2002

ATCTCCATTCCTTCCCAAAAGTCTGTGCTCTACTTCCTCATTGAGAAAGGACAGCACGATGCGGCAATTG

AGTTCTATGAAGGAATGGTACACGACAGCATCAAAGAGGAACTTAAGCCGCTTCTGGAGCAAACCAGCTT

CGCGAAGCGCGCTTTCAAGCGTCTCAAGGAGAACTTCGAGATCGTCGCTCTCGTCGTTGTGCTGTTGGCA

AACATAGTCATCATGGTCCGCGAAACTCGGAAGAGACAGCAGATGGTGGACGACGCGCTCAATGAGTACA

TTGAGAAGGCGAACATCACCACTGATGACCAGACTCTTGAAGAGGCGGAGAACAACCCTCGTGAGGTTGT

CGACAAACCCACTGTCGGCTTCCGCGAGAGAAAACTCCCAGGGCAAAAGACTGACGAGGAAGTGAACACT

GAGCCAGCCAAACCT---GCGGAACAACCACAAGCTGAAGGACCCTACGCGGGACCGCTTGAGCGTCAGC

AACCGCTGAAACTCAAGGCCAAGCTGCCCAAGGCAGAGGGGCCTTACGCGGGACCGCTAGAGAGACAGCA

ACCACTGAAGCTGAAAGCCAAGCTGCCTGTGGCCAAGGAAGGGCCATATGAAGGACCAGTGAAAAGGCCC

GTCGCCCTGAAAGTGAAAGCAAAAGCCCCGATTGTCACTGAAAGCGGGTGCCCTCCGACCGACTTGCAAA

AGATGGTCATGGCAAACGTGAAGCCCGTCGAGCTCATCCTCGACGGGAAGACCGTTGCACTGTGCTGTGC

GACGGGAGTGTTCGGGACGGCTTACCTCGTGCCTCGTCACCTTTTCGCTGAAAAGTATGACAGGATCATG

TTGGACGGTCGAGCCCTGACAGACAGTGACTTCAGAGTGTTTGAGTTTGAGGTGAAAGTGAAGGGACAGG

ACATGCTCTCAGATGCCGCGCTAATGGTTCTCCACACCGGAAACCGCGTGCGCGATCTCACGGGACACTT

CCGTGACACCATGAAACTGTCGAAAGGCAGCCCCGTCGTTGGTGTGGTCAACAACGCCGACGTCGGAAGA

CTCATCTTCTCAGGTGATGCACTGACCTACAAAGACCTAGTCGTTTGTATGGACGGTGATACCATGCCTG

GTCTCTTCGCATACCGCGCTGGAACCAAGGTAGGTTACTGTGGAGCCGCTGTTCTTGCAAAGGACGGCGC

CAAGACTGTGATCGTCGGCACCCACTCTGCCGGAGGCAACGGAGTAGGCTACTGCTCCTGTGTCTCGCGA

TCCATGCTCCTACAGATGAAGGCCCACATCGACCCTCCCCCTCACACTGAG

>JF749862.1_SAT2_UGA_2002

ATTTCCATTCCATCCCAAAAGTCAGTGCTTTACTTCCTCATTGAGAAAGGACAGCACGAAGCAGCAATTG

AATTCTACGAAGGAATGGTTCATGACTCCATCAAGGAAGAGCTCAAGCCGCTTCTCGAGCAAACCAGCTT

CGCGAAACGTGCTTTCAAGCGCCTGAAGGAGAACTTTGAGATTGTTGCGCTGGTAGTGGTGCTGCTGGCC

AACATCGTCATCATGATCCGCGAAACTCGCAAGAGGCAGAAGATGGTTGATGATGCGCTGGACGAGTACA

TCGAGAAGGCTAACATCACCACTGATGATCAAACTCTTGACGAGGCGGAAAAGAACCCGCGCGAGACCAT

CGACAGACCCACTGCTGGTTTCCGCGAGAGGAAACTCCCAAACCAAACCTCCGACGATGAAGTGAAGACT

GAGCCGGAGAAGGTT---GGTGAGAAACCACAAGCTGAAGGACCCTACGCCGGACCGCTGGAGCGCCAAC

AACCGCTGAAGCTCAAAACTAAGCTTCCCGCGGCTGAAGGCCCGTACGCCGGACCGCTAGAGAAACAGCA

ACCACTCAAGCTTAAGACTAAACTGCCTGTGGCCAAGGAAGGGCCATATGAAGGACCAGTCAAGAAGCCT

GTCGCCTTGAAAGTGAAAGCAAAACCAGCGATAGTCACTGAGAGCGGTGCGCCACCGACCGACTTGCAAA

AGATGGTCATGGCCAACGTCAAGCCCGTCGAGCTCATTCTCGATGGGAAGATCGTCGCGCTTTGCTGTGC

GACGGGAGTGTTTGGGACCGCCTACCTCGTGCCTCGTCATCTTTTCGCTGAGAAGTATGACAAGATCATG

TTGGATGGGCGTGCCCTGACAAACAGTGACTTCAGAGTGTTTGAGTTTGAGGTGAAAGTAAAGGGACAGG

ACATGCTCTCAGACGCTGCTCTCATGGTTCTGAACCGTGGACAACGCGTTAGAGACATCACAGCCCACTT

CCGCGACACCGTCCGTGTTGCGAAAGGCAACCCCGTTGTCGGCGTGGTCAACAATGCCGACGTCGGGAGA

CTCATCTTCTCAGGAGATGCCCTCACCTACAAGGACATCGTCGTCTGTATGGACGGTGATACCATGCCTG

GTCTTTTCGCGTACCGCGCTGGGACCAAGGTAGGATACTGTGGGGCTGCTGTGCTAACCAAGAGTGGAAG

CCAAACAGTCATCATTGGCACTCACTCTGCGGGTGGCAACGGCGTGGGCTACTGCTCGTGCGTCTCAAAA

TCGATGCTTGATCAGATGAAAGCCCACATCGACCCGGCACCTCACACCGAG

>JF749864.1_SAT2_ZIM_2003

ATCTCCATTCCTTCCCAAAAATCTGTGCTCTACTTTCTCATCGAGAAAGGACAGCACGAGGCTGCAATTG

AATTCTACGAAGGGATGGTGCACGACAGCATCAAGGAAGAGATTAGGCCCTTGTTGGAGCAAACCAGCTT

CGCCAAGCGTGCTTTCAAGCGCCTTAAGGAGAACTTCGAGATCGTTGCTCTCGTTGTTGTGCTGTTGGCA

AACATTATCATCATGATCCGCGAAACTCGCAAGCGCCAGAAGATGGTTGACGATGCCCTCGATGAGTACA

TTGAGAAGGCGAACATCACCACTGATGACAAGACACTTGAAGAGGCGGAAAAGAACCCTCAAGAGGTTGT

CGACAAACCCACTGGCGGCTTCCGTGAGAGAAAACTCCCTGGGCAGAAAACTGACGATGAAGTGAACTCT

GAGCCAGCTAAACCC---GCGGAGAAACCACAAGCTGAAGGACCCTACGCTGGCCCCCTCGAGCGACAGC

ATCCGCTGAAGCTCAAGGCTAAGCTCCCTCAGGCAGAGGGACCTTATGTCGGGTCGCTAGAGAGACGACA

ACCACTGAAACTGAAAGCAAAACTGCCTGTGGCCAAGGAAGGGCCATATGAAGGACCAGTGAAGAAGCCT

GTCGCTTTGAAAGTGAAAGCAAAAGCCCCGATTGTCACTGAAAGCGGATGCCCACCGACCGACTTGCAAA

AGATGGTCATGGCAAACGTAAAGCCCGTTGAGCTCATCCTCGATGGGAAGACAGTTGCGCTCTGCTGCGC

GACTGGAGTGTTCGGGACGGCTTACCTCGTGCCTCGTCATCTTTTCGCAGAGAAGTACGACAAGATCATG

CTGGACGGCCGCGCCTTGACAAACAGTGACTTCAGAGTGTTTGAGTTTGAGGTAAAAGTAAAAGGACAGG

ACATGCTCTCAGACGCCGCGCTGATGGTTCTCCACTCTGGAAACCGTGTGCGCGATCTCACGGGACACTT

CCGTGACACCATGAAACTGTCGAAAGGCAGTCCCGTCGTTGGCGTGGTCAACAACGCTGACGTTGGAAGG

CTCATCTTTTCAGGAGATGCACTGACCTACAAAGACCTGGTCGTTTGTATGGACGGTGACACCATGCCTG

GACTCTTCGCATACCGCGCAGGGACCAAGGTTGGATACTGTGGAGCTGCTGTTCTCGCAAAGGACGGCGC

TAAGACAGTGATCGTCGGCACCCACTCTGCCGGAGGCAACGGAGTAGGCTACTGCTCCTGCGTCTCACGA

TCCATGCTCCTGCAGATGAAGGCCCACATTGACCCTCCTCCTCACACCGAG

>JN006719.1_Asia1_PAK_2008

ATCTCAATTCCTTCCCAAAAGTCTGTGTTGTACTTTCTCATTGAGAAGGGCCAGCACGAAGCAGCAATTG

AATTCTTTGAGGGGATGGTGCACGACTCCATCAAGGAGGAGCTCCGGCCTCTCATCCAACAGACTTCATT

TGTGAAACGCGCTTTCAAGCGCCTGAAGGAAAATTTTGAGATAGTTGCCCTGTGTTTGACTCTTCTGGCA

AACATAGTGATCATGATCCGCGAGACTCGCAAGAGGCAGAAAATGGTGGATGATGCAGTGAATGAGTATA

TTGAGAAAGCAAACATCACCACAGACGACAGGACCCTTGACGAGGCGGAAAAGAACCCTCTGGAGACTAG

CGGTGCCAGCACTGTTGGTTTCAGAGAGAGAACTCTCCCAGGACACAAAGCGAGCAATGACGTGAACTCC

GAGCCCGCCAAACCTGTGGAAGAGCAACCACAAGCTGAAGGACCCTACGCCGGGCCACTTGAGCGTCAGA

AACCTCTGAAAGTGAGAGCCAAGCTACCACAGCAAGAGGGACCCTACGCTGGCCCGATGGAGAGACAAAA

GCCACTGAAAGTGAAAGCAAAAGCCCCGGTCGTTAAGGAAGGACCTTACGAGGGACCGGTGAAGAAGCCT

GTCGCTTTGAAAGTGAAAGCAAAGAACTTGATTGTCACTGAGAGTGGTGCCCCACCGACTGACTTGCAAA

AGATGGTTATGGGTAACACTAAGCCCGTTGAGCTCATACTCGACGGGAAGACAGTAGCCATCTGCTGTGC

TACTGGGGTGTTTGGCACCGCCTACCTCGTGCCTCGTCATCTTTTCGCAGAGAAGTATGACAAGATCATG

TTGGACGGCAGAGCCATGACAGACAGTGACTACAGAGTGTTTGAGTTTGAGATTAAAGTAAAAGGACAGG

ACATGCTCTCAGACGCCGCGCTCATGGTGCTCCACCGTGGGAATCGCGTGCGGGACATCACGAAGCACTT

CCGTGATGTGGCAAGAATGAAGAAAGGAACCCCCGTCGTTGGTGTTATCAACAACGCCGATGTTGGGAGA

CTGATTTTCTCTGGTGAGGCCCTCACCTACAAGGACATTGTAGTGTGCATGGATGGAGACACCATGCCCG

GCCTCTTTGCCTATAAAGCCGCCACCAAGGCCGGCTACTGTGGAGGAGCCGTTCTCGCGAAGGACGGGGC

CGAGACTTTCATCGTCGGCACACACTCCGCAGGCGGCAATGGAGTTGGATACTGCTCCTGCGTGTCCAGG

TCCATGCTGTTCAAGATGAAGGCACACATCGATCCTGAACCACACCACGAA

>JN006720.1_Asia1_PAK_2009

ATCTCAATTCCTTCCCAAAAATCCGTGTTGTACTTCCTCATTGAGAAAGGCCAGCACGAGGCTGCAATTG

AATTCTTTGAGGGGATGGTACATGACTCCATCAAGGAGGAGCTCCGACCCCTCATCCAACAGACATCATT

TGTGAAACGCGCCTTTAAGCGCCTGAAGGAGAACTTTGAGATTGTTGCCCTGTGTTTGACCCTTCTGGCA

AACATAGTCATCATGATCCGTGAGACTCGCAAGAGACAGCAGATGGTGGATGATGCAGTGAATGAGTACA

TCGAGAAGGCAAACATCACCACAGATGATAAGACTCTTGACGAGGCGGAAAAGAACCCTCAGGAGACAAA

CAGTGCCAGCGCTGTTGGCTTCAAGGAGAGAACTCCCCCGGGGCACACGGTGAGCGATGACGTGAACCCC

GAGCCCATCGTGCCCGTGGAGGGACAACCACAAGCTGAAGGACCCTACGCCGGACCACTCGAGCGCCAGA

AACCTCTGAAAGTGAGAGCCAAGCTCCCGCAGCAGGAGGGACCTTACGCTGGTCCGATGGAGAGACAGAA

ACCACTGAAAGTGAAAGCAAAACCCCCGGTCGTTAAGGAAGGACCTTACGAGGGACCGGTGAAGAAGCCT

GTCGCTTTGAAAGTGAAAGCTAAGAACTTGATTGTCACTGAGAGTGGTGCCCCCCCGACTGACTTGCAAA

AGATGGTCATGGGCAACACTAAGCCTGTTGAGCTCATCCTTGACGGGAAGACGGTGGCCATCTGCTGTGC

TACCGGAGTGTTTGGCACCGCCTACCTCGTGCCTCGTCATCTTTTCGCTGAGAGGTATGACAAGATCATG

TTGGACGGTAGGGCCATGACAGACAGTGACTACAGAGTGTTTGAGTTTGAGATTAAAGTAAAAGGACAGG

ACATGCTCTCAGACGCCGCGCTCATGGTGCTCCACCGTGGGAACCGCGTGAGAGACATCACGAAACACTT

TCGTGACACAGCAAGAATGAAGAAAGGTACCCCCGTTGTCGGCGTGATCAACAACGCTGATGTCGGGAGA

CTGATTTTCTCTGGTGAGGCCCTCACCTACAAGGACATAGTAGTGTGCATGGATGGAGACACCATGCCTG

GCCTCTTTGCCTACAAAGCCGCCACCAAGGCTGGTTACTGTGGAGGAGCTGTTCTCGCAAAGGACGGAGC

CGAGACTTTCATCGTCGGCACTCACTCTGCAGGGGGCAATGGAGTTGGATACTGTTCATGCGTTTCCAGA

TCCATGCTTCTGAGAATGAAGGCACACGTTGACCCTGAACCACACCACGAG

>JN006722.1_A_PAK_2008

ATCTCAATTCCTTCCCAAAAATCCGTGTTGTACTTCCTCATTGAGAAAGGCCAGCACGAGGCTGCAATTG

AATTCTTTGAGGGGATGGTACATGACTCCATCAAGGAGGAGCTCCGACCCCTCATCCAACAGACATCATT

TGTGAAACGCGCCTTTAAGCGCCTGAAGGAGAACTTTGAGATTGTTGCCCTGTGTTTGACCCTTCTGGCA

AACATAGTGATCATGATCCGTGAGACTCGCAAGAGACAGCAGATGGTGGATGATGCAGTGAATGAGTACA

TCGAGAAGGCAAATATCACCACAGATGACAAGACTCTTGACGAGGCGGAAAAGAACCCTCAGGAGACAAA

CAATGCCAGCACTGTTGGCTTCAGAGAGAGAACTCTCCCGGGGCACACGGTGAGCGATGACGTGAACCCC

GAGCCCATCGTGCCCGTGGAGGGACAACCACAAGCTGAAGGACCCTACGCCGGACCACTCGAGCGCCAGA

AACCTCTGAAAGTGAGAGCCAAGCTCCCGCAGCAGGAGGGACCTTACGCTGGTCCGATGGAGAGACAGAA

ACCACTGAAAGTGAAAGCAAAACCCCCGGTCGTTAAGGAAGGACCTTACGAGGGACCGGTGAAGAAGCCT

GTCGCTTTGAAAGTGAAAGCTAAGAACTTGATTGTCACTGAGAGTGGTGCCCCCCCGACTGACTTGCAAA

AGATGGTCATGGGCAACACTAAGCCTGTTGAGCTCATCCTTGACGGGAAGACGGTGGCCATCTGCTGTGC

TACCGGAGTGTTTGGCACCGCCTACCTCGTGCCTCGTCATCTTTTCGCTGAGAGGTATGACAAGATCATG

TTGGACGGTAGGGCCATGACAGACAGTGACTACAGAGTGTTTGAGTTTGAGATTAAAGTAAAAGGACAGG

ACATGCTCTCAGACGCCGCGCTCATGGTGCTCCACCGTGGGAACCGCGTGAGAGACATCACGAAACACTT

TCGTGACACAGCAAGAATGAAGAAAGGCACCCCCGTTGTCGGCGTGATCAACAACGCTGATGTCGGGAGA

CTGATTTTCTCTGGTGAGGCCCTCACCTACAAGGACATAGTAGTGTGCATGGATGGAGACACCATGCCTG

GCCTCTTTGCCTACAAAGCCGCCACCAAGGCTGGTTACTGTGGAGGAGCTGTTCTCGCAAAGGACGGAGC

CGAGACTTTCATCGTCGGCACTCACTCTGCAGGGGGCAATGGAGTTGGATACTGCTCATGCGTTTCCAGA

TCCATGCTTCTGAAAATGAAGGCACACGTTGACCCTGAACCACACCACGAG

>JN099688.1_A_IRQ_2009

ATCTCAATTCCTTCCCAAAAATCCGTGTTGTACTTCCTCATCGAGAAAGGCCAGCACGAGGCTGCAATTG

AGTTCTATGAGGGGATGGTGCACGACTCCATCAAGGAGGAGCTCCGACCTCTCATCCAACAGACATCATT

TGTGAAACGCGCTTTTAAGCGCTTGAAGGACAATTTTGAAGTTGTTGCCCTGTGTTTGACTCTTTTGGCA

AACATAGTGATCATGATCCGCGAGACTCACAAGAGACAGCAGATGGTGGATGATGCAGTGAATGAGTACA

TTGAGAAAGCCAACATCACCACAGATGACAAGACTCTTGACGAAGCGGAAAAGAACCCTCTGGAAACCAG

TGGTGCCAGCACCGTTGGTTTCAGAGAGAGAACTCTCCCAGGACGCAAGGTGAGTGATGACGTGAACTCC

GAGCCCACCAAGCCCGTGGAAGAGCAACCACAAGCTGAAGGACCCTACGCCGGACCACTTGAGCGTCAGA

AACCTCTGAAAGTGAGAGCCAAGCTCCCGCAGCAGGAGGGGCCTTACGCTGGTCCGATGGAGAGACAAAA

ACCACTGAAAGTGAAAGCAAAAGCCCCGGTCGTTAAGGAAGGACCTTACGAGGGACCGGTGAAGAAGCCT

GTCTCTTTGAAAGTGAAAGCTAAGAACTTGATTGTCACTGAGAGTGGCGCTCCCCCGACCGACTTGCAGA

AGATGGTCATGGGCAACACTAAACCTGTTGAGCTCATCCTCGACGGGAAGACGGTGGCCATCTGCTGTGC

TACCGGAGTGTTTGGCACTGCCTACCTCGTGCCTCGTCATCTTTTCGCTGAGAAGTATGACAAGATCATG

TTGGACGGTAGAGCCATGACAGACAGTGACTATAGAGTGTTTGAGTTTGAGATTAAAGTAAAAGGACAGG

ACATGCTCTCAGACGCCGCGCTCATGGTGCTCCACCGTGGGAACCGCGTGAGAGACATCACGAAACATTT

TCGTGATACAGCAAGAATGAAGAAAGGCACCCCCGTTGTCGGCGTGATCAACAATGCTGACGTCGGGAGA

CTGATTTTCTCTGGTGAGGCCCTCACCTACAAAGACATAGTAGTGTGCATGGATGGAGACACCATGCCTG

GCCTCTTTGCCTACAAGGCCGCCACCAAGGCTGGTTACTGTGGAGGAGCTGTTCTCGCAAAGGACGGAGC

CGAGACTTTTATCGTCGGCACTCACTCCGCAGGGGGCAATGGAGTTGGATACTGCTCATGCGTTTCCAGG

TCCATGCTTCTGAAAATGAAGGCACATATCGACCCCGAACCGCACCACGAG

>JN099694.1_A_IRQ_2009

ATCTCAATTCCTTCCCAAAAATCCGTGTTGTACTTCCTCATCGAGAAAGGCCAGCACGAGGCTGCAATTG

AGTTCTATGAGGGGATGGTGCACGACTCCATCAAGGAGGAGCTCCGACCTCTCATCCAACAGACATCATT

TGTGAAACGCGCTTTTAAGCGCTTGAAGGACAATTTTGAAGTTGTTGCCCTGTGTTTGACTCTTTTGGCA

AATATAGTGATCATGATCCGCGAGACTCACAAGAGACAGCAGATGGTGGATGATGCAGTGAATGAGTACA

TTGAGAAAGCCAACATCACCACAGATGACAAGACTCTTGACGAAGCGGAAAAGAACCCTCTGGAAACCAG

TGGTGCCAGCACCGTTGGTTTCAGAGAGAGAACTTTCCCAGGACGCAAGGTGAGTGATGACGTGAACTCC

GAGCCCACCAAGCCCGTGGAAGAGCAACCACAAGCTGAAGGACCCTACGCCGGACCACTTGAGCGTCAGA

AACCTCTGAAAGTGAGAGCCAAGCTCCCGCAGCAGGAGGGGCCTTACGCTGGTCCGATGGAGAGACAAAA

ACCACTGAAAGTGAAAGCAAAAGCCCCGGTCGTTAAGGAAGGACCTTACGAGGGACCGGTGAAGAAGCCT

GTCGCTTTGAAAGTGAAGGCTAAAAACTTGATTGTCACTGAGAGTGGTGCCCCCCCGACCGACTTGCAGA

AGATGGTCATGGGCAACACTAAGCCTGTTGAGCTCATCCTCGACGGGAAGACGGTGGCCATCTGCTGTGC

TACCGGAGTGTTTGGCACTGCCTACCTCGTGCCTCGTCATCTTTTCGCTGAGAAGTATGACAAGATCATG

TTGGACGGTAGAGCCATGACAGACAGTGACTATAGAGTGTTTGAGTTTGAGATTAAAGTAAAAGGACAGG

ACATGCTCTCAGACGCCGCGCTCATGGTGCTCCACCGTGGGAACCGCGTGAGAGACATCACGAAACATTT

TCGTGATACAGCAAGAATGAAGAAAGGCACCCCCGTTGTCGGCGTGATCAACAATGCTGACGTCGGGAGA

CTGATTTTCTCTGGTGAGGCCCTCACCTACAAAGACATAGTAGTGTGCATGGATGGAGACACCATGCCTG

GCCTCTTTGCCTACAAAGCCGCCACCAAGGCTGGTTACTGTGGAGGAGCTGTTCTCGCAAAGGACGGAGC

CGAGACTTTTATCGTCGGCACTCACTCCGCAGGGGGCAATGGAGTTGGATACTGCTCATGCGTTTCCAGG

TCCATGCTTTTGAAAATGAAGGCACACATCGACCCCGAACCGCACCACGAG

>JN099695.1_A_IRQ_2009

ATCTCAATTCCTTCCCAAAAATCCGTGTTGTACTTCCTCATCGAGAAAGGCCAGCACGAGGCTGCAATTG

AGTTCTATGAGGGGATGGTGCACGACTCCATCAAGGAGGAGCTCCGACCTCTCATCCAACAGACATCATT

TGTGAAACGCGCTTTTAAGCGCTTGAAGGACAATTTTGAAGTTGTTGCCCTGTGTTTGACTCTTTTGGCA

AACATAGTGATCATGATCCGCGAGACTCACAAGAGACAGCAGATGGTGGATGATGCAGTGAATGAGTACA

TTGAGAAAGCCAACATCACCACAGATGACAAGACCCTTGACGAAGCGGAAAAGAACCCTCTGGAAACCAG

TGGTGCCAGCACCGTTGGTTTCAGAGAGAGAACTCTCCCAGGACGCAAGGTGAGTGATGACGTGAACTCC

GAGCCCACCAAGCCCGTGGAAGAGCAACCACAAGCTGAAGGACCCTACGCCGGACCACTTGAGCGTCAGA

AACCTCTGAAAGTGAGAGCCAAGCTCCCGCAGCAGGAGGGGCCTTACGCTGGTCCGATGGAGAGACAAAA

ACCACTGAAAGTGAAAGCAAAAGCCCCGGTCGTTAAGGAAGGACCTTACGAGGGACCGGTGAAGAAGCCT

GTCGCTTTGAAAGTGAAAGCTAAGAACTTGATTGTCACTGAGAGTGGTGCTCCCCCGACCGACTTGCAGA

AGATGGTCATGGGCAACACTAAACCTGTTGAGCTCATCCTCGACGGGAAGACGGTGGCCATCTGCTGTGC

CACCGGAGTGTTTGGCACTGCCTACCTCGTGCCTCGTCATCTTTTCGCTGAGAAGTATGACAAGATCATG

TTGGACGGTAGAGCCATGACAGACAGTGACTATAGAGTGTTTGAGTTTGAGATTAAAGTGAAAGGACAGG

ACATGCTCTCAGACGCCGCGCTCATGGTGCTCCACCGTGGGAACCGCGTGAGAGACATCACGAAACATTT

TCGTGATACAGCAAGAATGAAGAAAGGCACCCCCGTTGTCGGCGTGATTAACAATGCTGACGTCGGGAGA

CTGATTTTCTCTGGTGAGGCCCTCACCTACAAAGACATAGTAGTGTGCATGGATGGAGACACCATGCCTG

GCCTCTTTGCCTACAAAGCCGCCACCAAGGCTGGTTACTGTGGAGGAGCTGTTCTCGCAAAGGACGGAGC

CGAGACTTTTATCGTCGGCACTCACTCCGCAGGGGGCAATGGAGTTGGATACTGCTCATGCGTTTCCAGG

TCCATGCTTCTGAAAATGAAGGCACACATCGACCCCGAACCGCACCACGAG

>JN099697.1_A_IRQ_2009

ATCTCAATTCCTTCCCAAAAATCCGTGTTGTACTTCCTCATCGAGAAAGGCCAGCACGAGGCTGCGATTG

AGTTCTATGAGGGGATGGTGCACGACTCCATCAAGGAGGAGCTCCGACCTCTCATCCAACAGACATCATT

TGTGAAACGCGCTTTTAAGCGCTTGAAGGACAATTTTGAAGTTGTTGCCCTGTGTTTGACTCTTTTGGCA

AACATAGTGATCATGATCCGCGAGACTCACAAGAGACAGCAGATGGTGGATGATGCAGTGAATGAGTACA

TTGAGAAAGCCAACATCACCACAGATGACAAGACTCTTGACGAAGCGGAAAAGAACCCTCTGGAAACCAG

TGGTGCCAGCACCGTTGGTTTCAGAGAGAGAACTCTCCCAGGACGCAAGGTGAGTGATGACGTGAACTTC

GAGCCCACCAAGCCCGTGGAAGAGCAACCACAAGCTGAAGGACCCTACGCCGGACCACTTGAGCGTCAGA

AACCTCTGAAAGTGAGAGCCAAGCTCCCGCAGCAGGAGGGGCCTTACGCTGGTCCGATGGAGAGACAAAA

ACCACTGAAAGTGAAAGCAAAAGCCCCGGTCGTTAAGGAAGGACCTTACGAGGGACCGGTGAAGAAGCCT

GTCGCTTTGAAAGTGAAAGCTAAGAACTTGATTGTCACTGAGAGTGGTGCTCCCCCGACCGACTTGCAGA

AGATGGTCATGGGCAACACCAAGCCTGTTGAGCTCATCCTCGACGGGAAGACGGTGGCCATCTGCTGTGC

CACCGGAGTGTTTGGCACTGCCTACCTCGTGCCTCGTCATCTTTTCGCTGAGAAGTATGACAAGATCATG

TTGGACGGTAGAGCCATGACAGACAGTGACTATAGAGTGTTTGAGTTTGAGATTAAAGTAAAAGGACAGG

ACATGCTCTCAGACGCCGCGCTCATGGTGCTCCACCGTGGGAACCGCGTGAGAGACATCACGAAACATTT

TCGTGATACAGCAAGAATGAAGAAAGGCACCCCCGTTGTCGGCGTGATCAACAACGCTGACGTCGGGAGA

CTGATTTTCTCTGGTGAGGCCCTCACCTACAAAGACATAGTAGTGTGCATGGATGGAGACACCATGCCTG

GCCTCTTTGCCTACAAAGCCGCCACCAAGGCTGGTTACTGTGGAGGAGCTGTTCTCGCAAAGGACGGAGC

CGAGACTTTTATCGTCGGCACTCACTCCGCAGGGGGCAATGGAGTTGGATACTGCTCATGCGTTTCCAGG

TCCATGCTTCTGAAAATGAAGGCACACATCGACCCCGAACCGCACCACGAG

>JN099698.1_A_IRQ_2009

ATCTCAATTCCTTCCCAAAAATCCGTGTTGTACTTCCTCATCGAGAAAGGCCAGCACGAGGCTGCAATTG

AGTTCTATGAGGGGATGGTGCACGACTCCATCAAGGAGGAGCTCCGACCTCTCATCCAACAGACATCATT

TGTGAAACGCGCTTTTAAGCGCTTGAAGGACAATTTTGAAGTTGTTGCCCTGTGTTTGACTCTTTTGGCA

AACATAGTGATCATGATCCGCGAGACTCACAAGAGACAGCAGATGGTGGATGATGCAGTGAATGAGTACA

TTGAGAAAGCCAACATCACCACAGATGACAAGACCCTTGACGAAGCGGAAAAGAACCCTCTGGAAACCAG

TGGTGCCAGCACCGTTGGTTTCAGAGAGAGAACTCTCCCAGGACGCAAGGTGAGTGATGACGTGAACTCC

GAGCCCACCAAGCCCGTGGAAGAGCAACCACAAGCTGAAGGACCCTACGCCGGACCACTTGAGCGTCAGA

AACCTCTGAAAGTGAGAGCCAAGCTCCCGCAGCAGGAGGGGCCTTACGCTGGTCCGATGGAGAGACAAAA

ACCACTGAAAGTGAAAGCAAAAGCCCCGGTCGTTAAGGAAGGACCTTACGAGGGACCGGTGAAGAAGCCT

GTCGCTTTGAAAGTGAAAGCTAAGAACTTGATTGTCACTGAGAGTGGTGCTCCCCCGACCGACTTGCAGA

AGATGGTCATGGGCAACACTAAACCTGTTGAGCTCATCCTCGACGGGAAGACGGTGGCCATCTGCTGTGC

CACCGGAGTGTTTGGCACTGCCTACCTCGTGCCTCGTCATCTTTTCGCTGAGAAGTATGACAAGATCATG

TTGGACGGTAGAGCCATGACAGACAGTGACTATAGAGTGTTTGAGTTTGAGATTAAAGTGAAAGGACAGG

ACATGCTCTCAGACGCCGCGCTCATGGTGCTCCACCGTGGGAACCGCGTGAGAGACATCACGAAACATTT

TCGTGATACAGCAAGAATGAAGAAAGGCACCCCCGTTGTCGGCGTGATTAACAATGCTGACGTCGGGAGA

CTGATTTTCTCTGGTGAGGCCCTCACCTACAAAGACATAGTAGTGTGCATGGATGGAGACACCATGCCTG

GCCTCTTTGCCTACAAAGCCGCCACCAAGGCTGGTTACTGTGGAGGAGCTGTTCTCGCAAAGGACGGAGC

CGAGACTTTTATCGTCGGCACTCACTCCGCAGGGGGCAATGGAGTTGGATACTGCTCATGCGTTTCCAGG

TCCATGCTTCTGAAAATGAAGGCACACATCGACCCCGAACCGCACCACGAG

>JN099699.1_A_IRQ_2009

ATCTCAATTCCTTCCCAAAAATCCGTGTTGTACTTCCTCATCGAGAAAGGCCAGCACGAGGCTGCGATTG

AGTTCTATGAGGGGATGGTGCACGACTCCATCAAGGAGGAGCTCCGACCTCTCATCCAACAGACATCATT

TGTGAAACGCGCTTTTAAGCGCTTGAAGGACAATTTTGAAGTTGTTGCCCTGTGTTTGACTCTTTTGGCA

AACATAGTGATCATGATCCGCGAGACTCACAAGAGACAGCAGATGGTGGATGATGCAGTGAATGAGTACA

TTGAGAAAGCCAACATCACCACAGATGACAAGACTCTTGACGAAGCGGAAAAGAACCCTCTGGAAACCAG

TGGTGCCAGCACCGTTGGTTTCAGAGAGAGAACTCTCCCAGGACGCAAGGTGAGTGATGACGTGAACTTC

GAGCCCACCAAGCCCGTGGAAGAGCAACCACAAGCTGAAGGACCCTACGCCGGACCACTTGAGCGTCAGA

AACCTCTGAAAGTGAGAGCCAAGCTCCCGCAGCAGGAGGGGCCTTACGCTGGTCCGATGGAGAGACAAAA

ACCACTGAAAGTGAAAGCAAAAGCCCCGGTCGTTAAGGAAGGACCTTACGAGGGACCGGTGAAGAAGCCT

GTCGCTTTGAAAGTGAAAGCTAAGAACTTGATTGTCACTGAGAGTGGTGCTCCCCCGACCGACTTGCAGA

AGATGGTCATGGGCAACACTAAGCCTGTTGAGCTCATCCTCGACGGGAAGACGGTGGCCATCTGCTGTGC

TACCGGAGTGTTTGGCACTGCCTACCTCGTGCCTCGTCATCTTTTCGCTGAGAAGTATGACAAGATCATG

TTGGACGGTAGAGCCATGACAGACAGTGACTATAGAGTGTTTGAGTTTGAGATTAAAGTAAAAGGACAGG

ACATGCTCTCAGACGCCGCGCTCATGGTGCTCCACCGTGGGAACCGCGTGAGAGACATCACGAAACATTT

TCGTGATACAGCAAGAATGAAGAAAGGCACCCCCGTTGTCGGCGTGATCAACAATGCTGACGTCGGGAGA

CTGATTTTCTCTGGTGAGGCCCTCACCTACAAAGACATAGTAGTGTGCATGGATGGAGACACCATGCCTG

GCCTCTTTGCCTACAAAGCCGCCACCAAGGCTGGTTACTGTGGAGGAGCTGTTCTCGCAAAGGACGGAGC

CGAGACTTTTATCGTCGGCACTCACTCCGCAGGGGGCAATGGAGTTGGATACTGCTCATGCGTTTCCAGG

TCCATGCTTCTGAAAATGAAGGCACACATCGACCCCGAACCGCACCACGAG

>JN998085.1_O_CHA_2010

ATCTCAATTCCTTCCCAAAAGTCAGTGCTGTATTTCCTCATTGAGAAAGGCCAACACGAAGCAGCAATTG

AATTCTTTGAGGGGATGGTCCATGACTCCATCAAGGAAGAGCTCCGACCCCTCATCCAACAGACATCATT

TGTCAAGCGCGCCTTCAAGCGCCTGAAGGAAAACTTTGAGATTGTTGCCCTATGTTTGACTCTCATGGCA

AACATAGTGATCATGATCCGCGAGACTCGCAAGAGACAGCAGATGGTGGATGATGCAGTGAATGAGTACA

TCGAGAAAGCAAACGTCACCACAGATGACAAGACTCTTGACGAGGCGGAAAAGAACCCTCTAGAGACTAG

CGGTGCCAGCACTGTTGGTTTCAGAGAGAGAACTCTCCCGGGACACAAGGTGGGTGATGACGTGAACTCC

GAGCCCGCCCACCCCGGGGATGAGCAACCACAAGCTGAAGGACCCTACGCCGGACCACTCGAGCGCCAGA

GACCTCTGAAAGTGAGAGCCAAGCTGCCACAGCAGGAGGGACCTTACGCCGGTCCGATGGAGAGACAGAA

ACCACTGAAAGTGAAAGCGAAAGCCCCGGTCGTGAAGGAAGGACCTTACGAGGGACCGGTGAAGAAGCCT

GTCGCTTTGAAAGTGAAAGCTAAGAACTTGATCGTCACTGAGAGTGGTGCCCCCCCGACCGACTTGCAAA

AGATGGTCATGGGTAACACCAAGCCCGTTGAGCTCATACTCGACGGGAAGACAGTAGCCATCTGCTGTGC

TACTGGAGTATTTGGCACTGCCTACCTCGTGCCTCGTCATCTTTTCGCTGAGAAGTACGACAAGATCATG

TTGGACGGTAGAACCATGACAGACAGTGACTACAGAGTGTTTGAGTTTGAGATTAAAGTAAAAGGACAGG

ACATGCTCTCAGACGCTGCGCTCATGGTGCTGCACCGTGGGAACCGCGTGAGAGACATCACGAAACACTT

TCGTGACACAGCAAGAATGAAGAAAGGCACCCCCGTCGTTGGTGTGATCAACAACGCTGACGTCGGGAGA

CTGATTTTCTCAGGTGAGGCCCTCACCTACAAGGACATTGTAGTGTGCATGGATGGAGACACCATGCCGG

GCCTATTTGCCTACAAAGCCGCCACCAAAGCTGGCTACTGCGGGGGAGCCGTCCTTGCTAAGGATGGAGC

CGACACATTCATCGTTGGCACTCACTCTGCAGGTGGCAATGGAGTTGGGTACTGCTCATGCGTATCCAGA

TCCATGCTCCAAAAAATGAAGGCACACATCGACCCTGAACCACACCACGAG

>JN998086.1_O_CHA_2010

ATCTCAATCCCTTCCCAAAAGTCAGTGCTGTATTTCCTCATTGAGAAAGGCCAACACGAAGCAGCAATTG

AGTTCTTTGAGGGGATGGTCCATGACTCCATCAAGGAAGAGCTCCGACCCCTCATCCAACAGACATCATT

TGTCAAGCGCACCTTCAAGCGCCTGAAGGAAAACTTTGAGATTGTTGCCCTATGTTTGACTCTCATGGCA

AACATAGTGATCATGGTCCGCGAAACTCGCAAGAGACAGCAGATGGTGGATGATGCAGTGAATGAGTACA

TCGAGAAAACAAACATCACCACAGATGACAAGACTCTTGACGAGGCGGAAAAGAACCCTCTAGAGACTAG

CGGTGCCAGCACTGTTGGTTTCAGAGAGAGAACTCTCCCGGGACACAAGGCGGGTGATGACGTGAACTCC

GAGCCCGCCCACCCCGGGGATGAGCAACCACAAGCTGAAGGACCCTACGCCGGACCACTCGAGCGCCAGA

GACCTCTGAAAGTGAGAGCCAAGCTGCCACAGCAGGAGGGACCTTACGCCGGTCCGATGGAGAGACAGAA

ACCACTGAAAGTGAAAGCGAAAGCCCCGGTCGTGAAGGAAGGACCTTACGAGGGACCGGTGAAGAAGCCT

GTCGCTTTGAAAGTGAAAGCTAAGAACTTGATCGTCACTGAGAGTGGTGCCCCCCCGACCGACTTGCAAA

AGATGGTCATGGGTAACACCAAGCCCGTTGAGCTCATACTCGACGGGAAGACAGTAGCCATCTGCTGTGC

TACTGGAGTATTTGGCACTGCCTACCTCGTGCCTCGTCATCTTTTCGCTGAGAAGTACGACAAGATCATG

TTGGACGGTAGAGCCATGATAGACAGTGACTACAGAGTGTTTGAGTTTGAGATTAAAGTAAAAGGACAGG

ACATGCTCTCAGACGCTGCGCTCATGGTGCTGCACCGTGGGAACCGCGTGAGAGACATCACGAAACACTT

TCGTGACACAGCAAGAATGAAGAAAGGCACCCCCGTCGTTGGTGTGATCAACAACGCTGACGTCGGGAGA

CTGATTTTCTCAGGTGAGGCCCTCACCTACAAGGACATTGTAGTGTGTATGGATGGGGACACCATGCCGG

GCCTATTTGCCTACAAAGCCGCCACCAAAGCTGGCTACTGCGGGGGAGCCGTCCTTGCTAAGGATGGAGC

CGACACATTCATCGTTGGCACTCACTCTGCAGGTGGCAATGGAGTTGGGTACTGTTCATGCGTATCCAGA

TCCATGCTCCAGAAAATGAAGGCACACATCGACCCTGAACCACACCACGAG

>JQ900581.1_O_CHA_2010

ATCTCAATTCCTTCCCAAAAGTCAGTGCTGTATTTCCTCATTGAGAAAGGCCAACACGAAGCAGCAATTG

AATTCTTTGAGGGGATGGTCCATGACTCCATCAAGGAAGAGCTCCGACCCCTCATCCAACAGACATCATT

TGTCAAGCGCGCCTTCAAGCGCCTGAAGGAAAACTTTGAGATTGTTGCCCTATGTTTGACTCTCATGGCA

AACATAGTGATCATGATCCGCGAGACTCGCAAGAGACAGCAGATGGTGGATGATGCGGTGAACGAGCACA

TCGAGAAAGCAAACATCACCACAGATGACAAGACTCTTGACGAGGCGGAAAAGAACCCTCTAGAGACTAG

CGGTGCCAGCACTGTTGGTTTCAGAGAGAGAACTCTCCCGGGACACAAGGTGGGTGATGACGTGAACTCC

GAGCCCGCCCACCCGGGGGATGAGCAACCACAGGCTGAAGGACCCTACGCCGGACCACTCGAGCGCCAGA

GACCTCTGAAAGTGAGAGCCAAGCTGCCACAGCAGGAGGGACCTTACGCCGGTCCGATGGAGAGACAGAA

ACCACTGAAAGTGAGAGCGAAAGCCCCGGTCGTGAAGGAAGGACCTTACGAGGGACCGGTGAAGAAGCCT

GTCGCTTTGAAAGTGAAAGCTAAGAACTTGATCGTCACTGAGAGTGGTGCCCCCCCGACCGACTTGCAAA

AGATGGTCATGGGTAACACCAAGCCCGTTGAGCTCATACTCGACGGGAAGACAATAGCCATCTGTTGTGC

TACTGGAGTGTTTGGTACTGCCTACCTCGTGCCTCGTCATCTTTTCGCTGAGAAGTACGACAAGATCATG

TTGGACGGTAGAGCCATGATAGACAGTGACTACAGAGTGTTTGAGTTTGAGATTAAAGTAAAAGGACAGG

ACATGCTCTCAGACGCTGCGCTCATGGTGCTGCACCGTGGGAACCGCGTGAGAGACATCACGAAACACTT

TCGTGACACAGCAAGAATGAAGAAAGGCACCCCCGTCGTTGGCGTGATCAACAACGCTGACGTCGGGAGA

CTGATTTTCTCAGGTGAGGCCCTCACCTACAAGGACATTGTAGTGTGCATGGATGGAGACACCATGCCGG

GCCTATTTGCCTACAAAGCCGCCACCAAGGCTGGCTACTGCGGGGGAGCCGTCCTCGCTAAGGATGGGGC

TGACACATTCATCGTTGGCACTCACTCTGCAGGTGGCAATGGAGTTGGGTACTGCTCATGCGTATCCAGA

TCCATGCTCCAGAAAATGAAGGCACACATCGACCCTGAACCACACCACGAG

>JQ973889.1_O_CHA_2010

ATCTCAATTCCTTCCCAAAAGTCAGTGCTGTATTTCCTCATTGAGAAAGGCCAACACGAAGCAGCAATTG

AATTCTTTGAGGGGATGGTCCATGACTCCATCAAGGAAGAGCTCCGACCCCTCATCCAACAGACATCATT

TGTCAAGCGCGCCTTCAAGCGCCTGAAGGAAAACTTTGAGATTGTTGCCCTATGTTTGACTCTCATGGCA

AACATAGTGATCATGATCCGCGAGACTCGCAAGAGACAGCAGATGGTGGATGATGCAGTGAATGAGTACA

TCGAGAAAGCAAACATCACCACAGATGACAAGACTCTTGACGAGGCGGAAAAGAACCCTCTAGAGACTAG

CGGTGCCAGCACTGTTGGTTTCAGAGAGAGAACTCTCCCGGGACACAAGGTGGGCGATGACGTGAACTCC

GAGCCCGCCCACCCCGGGGATGAGCAACCACAAGCTGAAGGACCCTACGCCGGACCACTCGAGCGCCAGA

GACCTCTGAAAGTGAGAGCCAAGCTGCCACAGCAGGAGGGACCTTATGCCGGTCCGATGGAGAGACAGAA

ACCACTGAAAGTGAAAGCGAAAGCCCCGGTCGTGAGGGAAGGACCTTACGAGGGACCGGTGAAGAAGCCT

GTCGCTTTGAAAGTGAAAGCTAAGAACTTGATCGTCACTGAGAGTGGTGCCCCCCCGACCGACTTGCAAA

AGATGGTCATGGGTAATACCAAGCCCGTTGAGCTCATACTCGACGGGAAGACAGTGGCCATCTGCTGTGC

TACTGGAGTATTTGGCACTGCCTACCTCGTGCCTCGTCATCTTTTCGCTGAGAAGTACGACAAGATCATG

TTGGACGGTAGAGCCATGATGGACAGTGACTACAGAGTGTTTGAGTTTGAGATTAAAGTAAAAGGACAGG

ACATGCTCTCAGACGCTGCGCTCATGGTGTTGCACCGTGGGAACCGCGTGAGAGACATTACGAAACACTT

TCGTGACACAGCAAGAATGAAGAAAGGCACCCCCGTCGTTGGTGTGATCAACAACGCTGACGTCGGGAGA

CTGATTTTCTCAGGTGAGGCCCTCACCTACAAGGACATTGTAGTGTGCATGGATGGAGACACCATGCCGG

GCCTATTTGCCTACAAAGCCGCCACCAAGGCTGGCTACTGCGGGGGAGCCGTCCTTGCCAAGGATGGAGC

CGACACATTCATCGTTGGCACTCACTCTGCAGGTGGCAATGGAGTTGGGTACTGCTCATGCGTATCCAGA

TCCATGCTCCAGAAAATGAAGGCACACATCGACCCTGAACCACACCACGAG

>JX014255.1_SAT2_EGY_2012

ATTTCACTTCCTTCCCAAAAGTCTGTGCTTTACTTTCTCATTGAGAAGGGTCAGCACGAAGCTGCAATTG

AGTTCTTTGAGGGGATGGTTAGTGACTCCATCAAAGAGGAACTGCGGCCCCTTATCCAACACACCTCATT

TGTGAAACGCGCTTTCAAGCGCCTGAAGGAAAACTTTGAAGTTGTTGTTTTGGTGCTGGCCCTCTTGGCC

AACATCATCATCATGATCCGCGAGACTCGTAAGAGGCAGGAAATGGTGGACGCTGCTGTGAACGATTACA

TTGAGCGCGCCGGCGTTACCACCGATGACCAGACTCTGGACGAGGCGGAAAAGAACCCCCTGGAAACAAC

CAGTGCCAGCACCGTTGGATTCAGGGAGAGAATCCCACCG---CAGAAAGCAGACGATGACGTGAACTCT

GAGCCTGCCAAACTCGTGGAGGGAAAACCACAAGCTGAAGGACCCTACGCCGGGCCGATGGAGCGTCAGA

AACCTCTGAAAGTGAAAACCAAGCTGCCACTGCAAGAGGGACCGTACGCCGGCCCGATGGAGAGACAACA

ACCGCTGAAAGTTAAAGTGAGACCCCCCGTCGTGAAGGAAGGACCTTACGAGGGGCCAGTGAAGAAGCCT

GTCGCTTTGAAAGTGAAAGCCAAGAACATGATCATCACGGAGAGTGGAGCGCCACCCACCGACTTGCAAA

AGATGGTGATGGCCAACACCAGACCAGTCGAGCTCATACTCGATGGTAAGACAGTGGCGATCTGCTGTGC

CACTGGAGTGTTTGGGACTGCCTATCTCGTGCCTCGTCATCTTTTCGCTGAGAAGTATGACAAGATCATG

ATTGACGGCAGGGCCATGACAGACCGTGATTTCAGAGTGTTTGAGTTTGAGATTAAAGTAAAAGGACAGG

ACATGCTCTCGGACGCCGCCCTCATGGTGCTGCACCGTGGGAACCGCGTGAGAGACATCACGAAGCACTT

TCGTGATCAAGCAAGAATGAGAAAAGGAACCCCCGTGGTTGGCGTGATCAACAACGCCGACGTTGGGAGA

CTCATCTTCTCTGGAGAGGCACTCACCTACAAAGACATTGTAGTGTGTATGGATGGCGACACCATGCCAG

GCCTCTTTGCCTATAAAGCCGCCACCAAAGCTGGCTACTGTGGAGGAGCCGTTCTTGCGAAAGACGGAGC

CGAGACTTTCATCGTCGGCACTCACTCCGCTGGAGGAAACGGAGTTGGTTACTGCTCTTGCGTTTCCAAG

TCCATGCTCCTACAAATGAAGGCACACATTGACCCTGAACCACACCACGAA

>JX014256.1_SAT2_PAT_2012

ATTTCACTTCCTTCCCAAAAGTCTGTGCTTTACTTTCTCATTGAGAAGGGTCAGCACGAAGCTGCAATTG

AGTTCTTTGAGGGGATGGTTAGTGACTCCATTAAAGAGGAACTGCGGCCCCTTATCCAACACACCTCATT

TGTGAAACGCGCTTTCAAGCGCCTGAAGGAAAACTTTGAAGTTGTTGTTCTGGTGCTGGCCCTCTTGGCC

AACATCATCATCATGATCCGCGAGACTCGTAAGAGGCAGGAAATGGTGGACGCTGCTGTGAACGATTACA

TCGAGCGCGCCGGCGTTACCACCGATGACCAGACTCTGGACGAGGCGGAAAAGAACCCCCTGGAAACAAC

CAGTGCCAGCACCGTTGGATTCAGGGAGAGAATCCCACCG---CAGAAAGCAGACGATGACGTGAACTCT

GAGCCTGCCAAACTCGTGGAGGGAAAACCACAAGCTGAAGGACCCTACGCCGGGCCGATGGAGCGTCAGA

AACCTCTGAAAGTGAAAACCAAGCTGCCACTGCAAGAGGGACCGTACGCCGGCCCGATGGAGAGACAACA

ACCGCTGAAAGTTAAAGTGAGACCCCCCGTCGTGAAGGAAGGACCTTACGAGGGGCCAGTGAAGAAGCCT

GTCGCTTTGAAAGTGAAAGCCAAGAACATGATCATCACGGAGAGTGGAGCGCCACCCACCGACTTGCAAA

AGATGGTGATGGCCAACACCAAACCAGTCGAGCTCATACTCGATGGTAAGACAGTGGCGATCTGCTGTGC

CACTGGAGTGTTTGGGACTGCCTATCTCGTGCCTCGTCATCTTTTCGCTGAGAAGTATGACAAGATCATG

ATTGACGGCAGGGCCATGACAGACCGTGATTTCAGAGTGTTTGAGTTTGAGATTAAAGTAAAAGGACAGG

ACATGCTCTCGGACGCCGCCCTCATGGTGCTGCACCGTGGGAACCGCGTGAGAGACATCACGAAGCACTT

TCGTGATCAAGCAAGAATGAGAAAAGGAACCCCCGTGGTTGGCGTGATCAACAACGCCGACGTTGGGAGA

CTCATCTTCTCTGGAGAGGCACTCACCTACAAAGACATTGTAGTGTGTATGGATGGCGACACCATGCCAG

GCCTCTTTGCCTATAAAGCCGCCACCAAAGCTGGCTACTGTGGAGGAGCCGTTCTTGCGAAAGACGGAGC

CGAGACTTTCATCGTCGGCACTCACTCCGCTGGAGGAAACGGAGTTGGTTACTGCTCTTGCGTTTCCAAG

TCCATGCTCCTACAAATGAAGGCACACATTGACCCTGAACCACACCACGAA

>JX040485.1_O_BUL_2010

ATCTCAATTCCCTCCCAAAAATCTGTGTTGTACTTCCTCATTGAGAAAGGCCAACACGAGGCAGCAATTG

AATTCTTTGAGGGGATGGTCCACGACTCCATCAAGGAGGAACTCCGACCCCTGATCCAACAGACCTCATT

TGTGAAACGCGCTTTCAAGCGCCTGAAGGAAAACTTTGAGATCGTTGCCCTGTGTTTGACTCTACTGGCA

AACATAGTGATCATGATCCGCGAGACTCGCAAGAGACAGCAGATGGTGGATGACGCAGTGAATGGGTACA

TTGAAAAGGCAAACATCACCACAGATGACAAGACTCTTGACGAGGCGGAAAAGAACCCTCTGGAAACCAG

TGGTGCCAGCACTGTTGGTTTCAGAGAGAGAGCTCTCCCAGGACACAAGGTGAGTGACGACGTGAACTCC

GAGCCCGCCGAGCCTGTGGAAGAGCAACCACAAGCTGAAGGACCCTACGCCGGGCCACTCGAGCGTCAGA

AACCTCTGAAAGTGAGAGCCAAGCTACCACAGCAAGAGGGACCCTACGCTGGCCCGATGGAGAGACAAAA

GCCACTGAAAGTGAAAGCAAAAGCCCCGGTCGTTAAGGAGGGGCCTTACGAGGGACCAGTGAAGAAGCCT

GTCGCTTTGAAAGTGAAAGCAAAGAACCTGATTGTCACTGAGAGTGGTGCCCCACCGACTGACTTGCAAA

AGTTGGTCATGGGTAACACCAAGCCCGTTGAGCTCATACTTGACGGGAAGACAGTAGCCATCTGCTGTGC

TACTGGAGTGTTTGGCACCGCCTACCTCGTGCCTCGTCATCTTTTCGCAGAGAAGTATGACAAGATCATG

TTGGACGGCAGAGCCATGTCAGACAGTGACTACAGAGTGTTTGAGTTTGAGATTAAAGTAAAAGGACAGG

ACATGCTCTCAGACGCCGCGCTCATGGTGCTCCACCGTGGGAATCGCGTGCGGGACATCACGAAACACTT

CCGTGATGTGGCAAGAATGAAGAAAGGAACCCCCGTCGTTGGTGTTATCAATAACGCCGATGTCGGGAGA

CTGATTTTCTCTGGTGAGGCCCTCACCTACAAGGACATTGTGGTGTGCATGGATGGAGACACCATGCCTG

GCCTCTTTGCCTACAAGGCCGCCACCAAGGCCGGCTACTGTGGAGGAGCCGTTCTCGCAAAGGACGGAGC

CGAGACTTTCATCGTCGGCACGCACTCTGCAGGCGGCAACGGAGTTGGATACTGCTCCTGCGTGTCCAGG

TCCATGCTGTTCAAGATGAAGGCACACATCGATCCTGAACCACACCATGAA

>JX040486.1_O_BUL_2011

ATCTCAATTCCCTCCCAAAAATCTGTGTTGTACTTCCTCATTGAGAAAGGCCAACACGAGGCAGCAATTG

AATTCTTTGAGGGGATGGTCCACGACTCCATCAAGGAGGAACTCCGACCCCTGATCCAACAGACCTCATT

TGTGAAACGCGCTTTCAAGCGCCTGAAGGAAAACTTTGAGATCGTTGCCCTGTGTTTGACTCTACTGGCA

AACATAGTGATCATGATCCGCGAGACTCGCAAGAGACATCAGATGGTGGATGACGCAGTGAATGAGTACA

TTGAAAAGGCAAACATCACCACAGATGACAAGACTCTTGACGAGGCGGAAAAGAACCCTCTGGAAACCAG

TGGTGCCAGCACTGTTGGTTTCAGAGAGAGAGCTCTCCCAGGACACAAGGTGAGTGACGACGTGAACTCC

GAGCCCGCCGAGCCTGTGGAAGAGCAACCACAAGCTGAAGGACCCTACGCCGGGCCACTCGAGCGTCAGA

AACCTCTGAAAGTGAGAGCCAAGCTACCACAGCAAGAGGGACCCTACGCTGGCCCGATGGAGAGACAAAA

GCCACTGAAAGTGAAAGCAAAAGCCCCGGTCGTTAAGGAGGGGCCTTACGAGGGACCAGTGAAGAAGCCT

GTCGCTTTGAAAGTGAAAGCAAAGAACCTGATTGTCACTGAGAGTGGTGCCCCACCGACTGACTTGCAAA

AGTTGGTCATGGGTAACACCAAGCCCGTTGAGCTCATACTTGACGGGAAGACAGTAGCCATCTGCTGTGC

TACTGGAGTGTTTGGCACCGCCTACCTCGTGCCTCGTCATCTTTTCGCAGAGAAGTATGACAAGATCATG

TTGGACGGCAGAGCCATGTCAGACAGTGACTACAGAGTGTTTGAGTTTGAGATTAAAGTAAAAGGACAGG

ACATGCTCTCAGACGCCGCGCTCATGGTGCTCCACCGTGGGAATCGCGTGCGGGACATCACGAAACACTT

CCGTGATGTGGCAAGAATGAAGAAAGGAACCCCCGTCGTTGGTGTTATCAATAACGCCGATGTCGGGAGA

CTGATTTTCTCTGGTGAGGCCCTCACCTACAAGGACATTGTGGTGTGCATGGATGGAGACACCATGCCTG

GCCTCTTTGCCTACAAGGCCGCCACCAAGGCCGGCTACTGTGGAGGAGCCGTTCTCGCAAAGGACGGAGC

CGAGACTTTCATCGTCGGCACGCACTCTGCAGGCGGCAACGGAGTTGGATACTGCTCCTGCGTGTCCAGG

TCCATGCTGTTCAGGATGAAGGCACACATCGATCCTGAACCACACCATGAA

>JX040487.1_O_BUL_2011

ATCTCAATTCCCTCCCAAAAATCTGTGTTGTACTTCCTCATTGAGAAAGGCCAACACGAGGCAGCAATTG

AATTCTTTGAGGGGATGGTCCACGACTCCATCAAGGAGGAACTCCGACCCCTGATCCAACAGACCTCATT

TGTGAAACGCGCTTTCAAGCGCCTGAAGGAAAACTTTGAGATCGTTGCCCTGTGTTTGACTCTACTGGCA

AACATAGTGATCATGATCCGCGAGACTCGCAAGAGACATCAGATGGTGGATGACGCAGTGAATGGGTACA

TTGAAAAGGCAAACATCACCACAGATGACAAGACTCTTGACGAGGCGGAAAAGAACCCTCTGGAAACCAG

TGGTGCCAGCACTGTTGGTTTCAGAGAGAGAGCTCTCCCAGGACGCAAGGTGAGTGACGACGTGAACTCC

GAGCCCGCCGAGCCTGTGGAAGAGCAACCACAAGCTGAAGGACCCTACGCCGGGCCACTCGAGCGTCAGA

AACCTCTGAAAGTGAGAGCCAAGCTACCACAGCAAGAGGGGCCCTACGCTGGCCCGATGGAGAGACAAAA

GCCACTGAAAGTGAAAGCAAAAGCCCCGGTCGTTAAGGAGGGGCCTTACGAGGGACCAGTGAAGAAGCCT

GTCGCTTTGAAAGTGAAAGCAAAGAACCTGATTGTCACTGAGAGTGGTGCCCCACCGACTGACTTGCAAA

AGTTGGTCATGGGTAACACCAAGCCCGTTGAGCTCATACTTGACGGGAAGACAGTAGCCATCTGCTGTGC

TACTGGAGTGTTTGGCACCGCCTACCTCGTGCCTCGTCATCTTTTCGCAGAGAAGTATGACAAGATCATG

TTGGACGGCAGAGCCATGTCAGACAGTGACTACAGAGTGTTTGAGTTTGAGATTAAAGTAAAAGGACAGG

ACATGCTCTCAGACGCCGCGCTCATGGTGCTCCACCGTGGGAATCGCGTGCGGGACATCACGAAACACTT

CCGTGATGTGGCAAGGATGAAGAAAGGAACCCCCGTCGTTGGTGTTATCAATAACGCCGATGTCGGGAGA

CTGATTTTCTCTGGTGAGGCCCTCACCTACAAGGACATTGTGGTGTGCATGGATGGAGACACCATGCCTG

GCCTCTTTGCCTACAAGGCCGCCACCAAGGCCGGCTACTGTGGAGGAGCCGTTCTCGCAAAGGACGGAGC

CGAGACTTTCATCGTCGGCACGCACTCTGCAGGCGGCAACGGAGTTGGATACTGCTCCTGCGTGTCCAGG

TCCATGCTGTTCAGGATGAAGGCACACATCGATCCTGAACCACACCATGAA

>JX040488.1_O_BUL_2011

ATCTCAATTCCCTCCCAAAAATCTGTGTTGTACTTCCTCATTGAGAAAGGCCAACACGAGGCAGCAATTG

AATTCTTTGAGGGGATGGTCCACGACTCCATCAAGGAGGAACTCCGACCCCTGATCCAACAGACCTCATT

TGTGAAACGCGCTTTCAAGCGCCTGAAGGAAAACTTTGAGATCGTTGCCCTGTGTTTGACTCTACTGGCA

AACATAGTGATCATGATCCGCGAGACTCGCAAGAGACATCAGATGGTGGATGACGCAGTGAATGGGTACA

TTGAAAAGGCAAACATCACCACAGATGACAAGACTCTTGACGAGGCGGAAAAGAACCCTCTGGAAACCAG

TGGTGCCAGCACTGTTGGTTTCAGAGAGAGAGCTCTCCCAGGACACAAGGTGAGTGACGACGTGAACTCC

GAGCCCGCCGAGCCTGTGGAAGAGCAACCACAAGCTGAAGGACCCTACGCCGGGCCACTCGAGCGTCAAA

AACCTCTGAAAGTGAGAGCCAAGCTACCACAGCAAGAGGGACCCTACGCTGGCCCGATGGAGAGACAAAA

GCCACTGAAAGTGAAAGCAAAAGCCCCGGTCGTTAAGGAGGGGCCTTACGAGGGACCAGTGAAGAAGCCT

GTCGCTTTGAAAGTGAAAGCAAAGAACCTGATTGTCACTGAGAGTGGTGCCCCACCGACTGACTTGCAAA

AGTTGGTCATGGGTAACACCAAGCCCGTTGAGCTCATACTTGACGGGAAGACAGTAGCCATCTGCTGTGC

TACTGGAGTGTTTGGCACCGCCTACCTCGTGCCTCGTCATCTTTTCGCAGAGAAGTATGACAAGATCATG

TTGGACGGCAGAGCCATGTCAGACAGTGACTACAGAGTGTTTGAGTTTGAGATTAAAGTAAAAGGACAGG

ACATGCTCTCAGACGCCGCGCTCATGGTGCTCCACCGTGGGAATCGCGTGCGGGACATCACGAAACACTT

CCGTGATGTGGCAAGAATGAAGAAAGGAACCCCCGTCGTTGGTGTTATCAATAACGCCGATGTCGGGAGA

CTGATTTTCTCTGGTGAGGCCCTCACCTACAAGGACATTGTGGTGTGCATGGATGGAGACACCATGCCTG

GCCTCTTTGCCTACAAGGCCGCCACCAAGGCCGGCTACTGTGGAGGAGCCGTTCTCGCAAAGGACGGAGC

CGAGACTTTCATCGTCGGCACGCACTCTGCAGGCGGCAACGGAGTTGGATACTGCTCCTGCGTGTCCAGG

TCCATGCTGTTCAGGATGAAGGCACACATCGATCCTGAACCACACCATGAA

>JX040489.1_O_BUL_2011

ATCTCAATTCCCTCCCAAAAATCTGTGTTGTACTTCCTCATTGAGAAAGGCCAACACGAGGCAGCAATTG

AATTCTTTGAGGGGATGGTCCACGACTCCATCAAGGAGGAACTCCGACCCCTGATCCAACAGACCTCATT

TGTGAAACGCGCTTTCAAGCGCCTGAAGGAAAACTTTGAGATCGTTGCCCTGTGTTTGACTCTACTGGCA

AACATAGTGATCATGATCCGCGAGACTCGCAAGAGACATCAGATGGTGGATGACGCAGTGAATGGGTACA

TTGAAAAGGCAAACATCACCACAGATGACAAGACTCTTGACGAGGCGGAAAAGAACCCTCTGGAAACCAG

TGGTGCCAGCACTGTTGGTTTCAGAGAGAGAGCTCTCCCAGGACACAAGGTGAGTGACGACGTGAACTCC

GAGCCCGCCGAGCCTGTGGAAGAGCAACCACAAGCTGAAGGACCCTACGCCGGGCCACTCGAGCGTCAAA

AACCTCTGAAAGTGAGAGCCAAGCTACCACAGCAAGAGGGACCCTACGCTGGCCCGATGGAGAGACAAAA

GCCACTGAAAGTGAAAGCAAAAGCCCCGGTCGTTAAGGAGGGGCCTTACGAGGGACCAGTGAAGAAGCCT

GTCGCTTTGAAAGTGAAAGCAAAGAACCTGATTGTCACTGAGAGTGGTGCCCCACCGACTGACTTGCAAA

AGTTGGTCATGGGTAACACCAAGCCCGTTGAGCTCATACTTGACGGGAAGACAGTAGCCATCTGCTGTGC

TACTGGAGTGTTTGGCACCGCCTACCTCGTGCCTCGTCATCTTTTCGCAGAGAAGTATGACAAGATCATG

TTGGACGGCAGAGCCATGTCAGACAGTGACTACAGAGTGTTTGAGTTTGAGATTAAAGTAAAAGGACAGG

ACATGCTCTCAGACGCCGCGCTCATGGTGCTCCACCGTGGGAATCGCGTGCGGGACATCACGAAACACTT

CCGTGATGTGGCAAGAATGAAGAAAGGAACCCCCGTCGTTGGTGTTATCAATAACGCCGATGTCGGGAGA

CTGATTTTCTCTGGTGAGGCCCTCACCTACAAGGACATTGTGGTGTGCATGGATGGAGACACCATGCCTG

GCCTCTTTGCCTACAAGGCCGCCACCAAGGCCGGCTACTGTGGAGGAGCCGTTCTCGCAAAGGACGGAGC

CGAGACTTTCATCGTCGGCACGCACTCTGCAGGCGGCAACGGAGTTGGATACTGCTCCTGCGTGTCCAGG

TCCATGCTGTTCAGGATGAAGGCACACATCGATCCTGAACCACACCATGAA

>JX040490.1_O_BUL_2011

ATCTCAATTCCCTCCCAAAAATCTGTGTTGTACTTCCTCATTGAGAAAGGCCAACACGAGGCAGCAATTG

AATTCTTTGAGGGGATGGTCCACGACTCCATCAAGGAGGAACTCCGACCCCTGATCCAACAGACCTCATT
[truncated: 656,354 more chars]
