# Supplementary material for: Early origin and global colonisation of foot-and-mouth disease virus
Source: Sci Rep. 2020 Sep 17;10:15268. doi: 10.1038/s41598-020-72246-6 (PMC7498456; doi:10.1038/s41598-020-72246-6)
Supplement: Supplementary file 8 — Supplementary Data S6. [file 41598_2020_72246_MOESM8_ESM.doc]

>AB079061.1_O_JPN_2000

GGATTGATAGTTGACACCAGAGATGTTGAGGAGCGCGTACATGTCATGCGCAAAACCAAGCTCGCACCCA

CCGTGGCACACGGTGTGTTTAACCCCGAATTTGGGCCTGCCGCCTTGTCCAACAAGGACCCGCGCCTGAA

TGAGGGGGTTGTCCTTGATGAAGTCATCTTCTCCAAACACAAAGGAAACACAAAGATGTCTGAGGAGGAC

AAAGCGCTGTTCCGCCGCTGTGCTGCTGACTACGCGTCGCGTCTGCATAGCGTGCTGGGTACGGCAAATG

CCCCACTGAGCGCTTACGAGGCAATCAAGGGCGTCGACGGACTTGACGCCATGGAACCAGACACCGCGCC

TGGTCTCCCCTGGGCTCTCCAGGGGAAACGCCGTGGTGCGCTCATTGACTTCGAGAACGGCACTGTCGGA

CCCGAGGTTGAAGCTGCCTTGAAGCTCATGGAGAAAAGAGAGTACAAGTTTGCATGCCAGACCTTCCTGA

AGGACGAGATTCGCCCGATGGAGAAGGTACGTGCCGGCAAGACTCGCATTGTCGACGTCCTGCCCGTTGA

ACACATTCTTTACACCAGGATGATGATTGGCAGATTTTGTGCTCAAATGCACTCAAACAACGGACCGCAA

ATTGGCTCGGCGGTTGGTTGTAATCCTGATGTTGATTGGCAAAGATTTGGCACGCATTTTGCTCAGTATA

GAAACGTGTGGGATGTGGACTATTCGGCCTTTGATGCCAACCACTGCAGTGACGCAATGAACATTATGTT

TGAGGAGGTGTTTAACACGGATTTCGGTTTCCACCCAAACGCTGAGTGGATCCTGAAAACTCTCGTGAAC

ACTGAACACGCCTATGAGAACAAACGCATCACTGTTGAAGGCGGGATGCCGTCTGGTTGTTCCGCAACAA

GCATCATCAACACAATTTTGAACAACATCTACGTGCTCTACGCCTTGCGTAGACACTATGAGGGAGTTGA

GCTGGACTCTTACACCATGATCTCCTACGGAGACGACATCGTGGTTGCAAGTGATTACGATCTGGACTTT

GAGGCCCTCAAGCCTCACTTCAAATCCCTTGGTCAAACCATTACTCCAGCTGACAAAAGCGACAAAGGTT

TTGTTCTTGGTCACTCCATTACCGATGTCACTTTCCTCAAAAGACACTTCCACATGGACTATGGAACTGG

GTTTTACAAACCTGTGATGGCTTCGAAGACCCTCGAGGCTATCCTCTCCTTCGCACGCCGTGGG

>AF026168.2_O_TAW_1997

GGGTTGATCGTCGACACCAGAGATGTGGAGGAGCGTGTCCACGTGATGCGCAAAACCAAGCTCGCGCCCA

CCGTGGCGCACGGTGTGTTCAACCCTGAGTTCGGGCCTGCCGCTCTGTCCAACAAGGACCCGCGCCTGAA

CGAAGGGGTTGTCCTTGACGATGTCATTTTCTCCAAACACAAAGGAGATACAAGGATGTCTGAAGAGGAC

AAAGCGCTGTTTCGGCGCTGTGCTGCTGACTACGCGTCGCGTCTACACAGTGTGTTGGGGACAGCAAACG

CCCCACTGAGTGTGTATGAAGCCATCAAAGGCGTCGACGGACTTGACGCCATGGAGCCGGACACGCGCCC

CGGTCTCCCCTGGGCTCTCCAAGGGAAACGCCGCGGCGTCCTGATCGACTTCGAAAACGGCACCGTCGGG

CCTGAGGTTGAGGCAGCACTCAAGCTCATGGAAAGCCGCGAGTACAAATTCGTCTGCCAAACCTTCCTGA

AGGACGAAATTCGGCCGCTAGAAAAGGTACGCGCTGGCAAGACACGCATTGTCGACGTGTTGCCTGTTGA

ACACATTCTCTACACCAGAATGATGATTGGCAGATTCTGTGCTCAGATGCATTCAAACAACGGACCGCAA

ATTGGATCAGCGGTCGGTTGTAACCCTGACGTTGATTGGCAAAGATTTGGCACACATTTCGCCCAGTACA

AGAACGTGTGGGATGTGGACTACTCAGCCTTTGATGCAAACCACTGCAGCGATGCGATGAACATCATGTT

CGAGGAAGTGTTCCGCACGGAGTTCGGATTCCACCCGAACGCCGAGTGGATTCTGAAGACTCTAGTGAAC

ACGGAGCACGCTTACGAGAACAAGCGCATTGTTGTTGAAGGTGGAATGCCGTCCGGTTGTTCCGCAACAA

GCATCATCAACACAATTTTGAACAACATCTACGTGCTTTACGCCCTGCGTAGGCACTATGAGGGAGTCGA

GCTGGACACTTACACCATGATCTCTTATGGAGACGACATCGTGGTGGCAAGTGACTACGACCTGGACTTT

GAGGCTCTCAAGCCCCACTTCAAGTCCCTTGGTCAGACTATCACTCCGGCCGACAAAAGCGACAAAGGTT

TTGTTCTTGGTCACTCCATAACCGACGTCACTTTCCTCAAAAGACACTTCCACATGGACTACGGAACTGG

GTTTTACAAACCTGTGATGGCCTCGAAGACCCTCGAGGCCATCCTCTCCTTTGCACGCCGTGGG

>AF154271.1_O_TAW_1997

GGGTTGATCGTCGACACCAGAGATGTGGAGGAGCGTGTCCACGTGATGCGCAAAACTAAGCTCGCGCCCA

CCGTGGCGCACGGTGTGTTCAACCCTGAGTTCGGGCCTGCCGCTCTGTCCAACAAGGACCCGCGCCTGAA

CGAAGGGGTTGTCCTTGACGATGTCATTTTCTCCAAACACAAAGGAGATACAAGGATGTCTGAAGAGGAC

AAAGCGCTGTTTCGGCGCTGTGCTGCTGACTACGCGTCGCGTCTACACAGTGTGTTGGGGACAGCAAACG

CCCCACTGAGTGTGTATGAAGCCATCAAAGGCGTCGACGGACTTGACGCCATGGAGCCGGACACGGCGCC

CGGTCTCCCCTGGGCTCTCCAAGGGAAACGCCGCGGCGCCCTGATCGACTTCGAAAACGGCACCGTCGGG

CCTGAGGTTGAGGCAGCACTCAAGCTCATGGAAAGCCGCGAGTACAAATTCGTCTGCCAAACCTTCCTGA

AGGACGAAATTCGGCCGCTAGAAAAGGTACGCGCTGGCAAGACACGCATTGTCGACGTGTTGCCTGTTGA

ACACATTCTCTACACCAGAATGATGATTGGCAGATTCTGTGCTCAGATGCATTCAAACAACGGACCGCAA

ATTGGATCAGCGGTCGGTTGTAACCCTGACGTTGATTGGCAAAGATTTGGCACACATTTCGCCCAGTACA

AGAACGTGTGGGATGTGGACTACTCAGCCTTTGATGCAAACCACTGCAGCGATGCGATGAACATCATGTT

CGAGGAAGTGTTCCGCACGGAGTTCGGATTCCACCCGAACGCCGAGTGGATTCTGAAGACTCTAGTGAAC

ACGGAGCACGCTTACGGGAACAAGCGCATTGTTGTTGAAGGTGGAATGCCGTCCGGTTGTTCCGCAACAA

GCATCATCAACACAATTTTGAACAACATCTACGTGCTTTACGCCCTGCGTAGGCACTATGAGGGAGTCGA

GCTGGACACTTACACCATGATCTCTTATGGAGACGACATCGTGGTGGCAAGTGACTACGACCTGGACTTT

GAGGCTCTCAAGCCCCACTTCAAGTCCCTTGGTCAGACTATCACTCCGGCCGACAAAAGCGACAAAGGTT

TTGTTCTTGGTCACTCCATAACCGACGTCACTTTCCTCAAAAGACACTTCCACATGGACTACGGAACTGG

GTTTTACAAACCTGTGATGGCCTCGAAGACCCTCGAGGCCATCCTCTCCTTTGCACGCCGTGGG

>AF308157.1_O_TAW_1997

GGGTTGATCGTCGACACCAGAGATGTGGAGGAGCGTGTCCACGTGATGCGCAAAACCAAGCTCGCGCCCA

CCGTGGCGCACGGTGTGTTCAACCCTGAGTTCGGGCCTGCCGCTCTGTCCAACAAGGACCCGCGCCTGAA

CGAAGGGGTTGTCCTTGACGATGTCATTTTCTCCAAACACAAAGGAGATACAAGGATGTCTGAAGAGGAC

AAAGCGCTGTTTCGGCGCTGTGCTGCTGACTACGCGTCGCGTCTACACAGTGTGTTGGGGACAGCAAACG

CCCCACTGAGTGTGTATGAAGCCATCAAAGGCGTCGACGGACTTGACGCCATGGAGCCGGACACGGCGCC

CGGTCTCCCCTGGGCTCTCCAAGGGAAACGCCGCGGCGCCCTGATCGACTTCGAAAACGGCACCGTCGGG

CCTGAGGTTGAGGCAGCACTCAAGCTCATGGAAAGCCGCGAGTACAAATTCGTCTGCCAAACCTTCCTGA

AGGACGAAATTCGGCCGCTAGAAAAGGTACGCGCTGGCAAGACACGCATTGTCGACGTGTTGCCTGTTGA

ACACATTCTCTACACCAGAATGATGATTGGCAGATTCTGTGCTCAGATGCATTCAAACAACGGACCGCAA

ATTGGATCAGCGGTCGGTTGTAACCCTGACGTTGATTGGCAAAGATTTGGCACACATTTCGCCCAGTACA

AAAACGTGTGGGATGTGGACTACTCAGCCTTTGATGCAAACCACTGCAGCGATGCGATGAACATCATGTT

CGAGGAAGTGTTCCGCACGGAGTTCGGATTCCACCCGAACGCCGAGTGGATTCTGAAGACTCTAGTGAAC

ACGGAGCACGCTTACGAGAACAAGCGCATTGTTGTTGAAGGTGGAATGCCGTCCGGTTGTTCCGCAACAA

GCATCATCAACACAATTTTGAACAACATCTACGTGCTTTACGCCCTGCGTAGGCACTATGAGGGAGTCGA

GCTGGACACTTACACCATGATCTCTTATGGAGACGACATCGTGGTGGCAAGTGACTACGACCTGGACTTT

GAGGCTCTCAAGCCCCACTTCAAGTCCCTTGGTCAGACTATCACTCCGGCCGACAAAAGCGACAAAGGTT

TTGTTCTTGGTCACTCCATAACCGACGTCACTTTCCTCAAAAGACACTTCCACATGGACTACGGAACTGG

GTTTTACAAACCTGTGATGGCCTCGAAGACCCTCGAGGCCATCCTCTCCTTTGCACGCCGTGGG

>AF377945.1_O_SKR_2000

GGATTGATAGTTGACACCAGAGATGTTGAGGAGCGCGTACATGTCATGCGCAAAACCAAGCTCGCACCCA

CCGTGGCACACGGTGTGTTTAACCCCGAATTTGGGCCTGCCGCCTTGTCCAACAAGGACCCGCGCCTGAA

TGAGGGGGTTGTCCTCGATGAAGCCATCTTCTCCAAACACAAGGGAAACACAAAGATGTCTGAGGAGGAC

AAAGCGCTGTTCCGCCGCTGTGCTGCTGACTACGCGTCGCGACTGCATAGCGTGCTGGGTACGGCAAACG

CCCCACTGAGCATTTACGAAGCAATCAAGGGCGTCGACGGACTTGACGCCATGGAACCAGACACCGCGCC

TGGTTTACCCTGGGCTCTCCAGGGGAAACGCCGTGGTGCCCTCATTGACTTTGAGAACGGCACAATCGGA

CCCGAAGTTGAAGCTGCCTTGAAGCTCATGGAGAAAAGAGAGTACAAGTTTGTATGTCAGACCTTCCTGA

AGGACGAGATTCGCCCGATGGAGAAAGTAAGTGCCGGCAAGACTCGCATTGTCGACGTCCTGCCTGTTGA

ACACATTCTTTACACCAGGATGATGATTGGCAGATTTTGTGCTCAAATGCACTCCAACAACGGACCGCAA

ATTGGCTCGGCGGTTGGGTGTAATCCTGATGTTGATTGGCAAAGATTTGGCACGCATTTTGCTCAGTACA

GAAACGTGTGGGATGTAGACTATTCGGCCTTTGATGCCAACCACTGCAGTGACGCAATGAACATCATGTT

TGAAGAAGTGTTCAACACGGATTTCGGTTTCCACCCAAACGCTGAGTGGATCCTGAAAACTCTCGTGAAC

ACTGAACACGCCTATGAGAACAAAGGCATCAGTGTTGAAGGCGGGATGCCGTCTGGTTGTTCCGCAACAA

GCATCTTCAACACAATTTCGAACAACATCTACGTGCTCTACGCCTTGCGTAGGCACTATGAGGGAGTTGA

GCTGGACTCTTACACCATGATCTCCTACGGAGACGACATCGTGGTTGCAAGTGATTACGATCTGGACTTT

GAGGCCTTCAAGCCTCACTTCAAATCCCTTGGTCAAACCATTACTCCAGCTGACAAAAGCGACAAAGGTT

TTGTTCTTGGTCACTCCATTACCGATGTCACTTTCCTCAAAAGATCTTTCCACATGGACTATGGAACTGG

GTTTTACAAACCTGTGATGGCTTCGAAGACCCTCGAGGCTATCCTCTCCTTTGCACGCCGTGGG

>AF506822.2_O_CHA_1999

GGATTGATAGTTGACACCAGAGATGTTGAGGAGCGCGTACATGTCATGCGCAAAACCAAGCTCGCACCCA

CCGTGGCACACGGTGTGTTTAACCCCGAATTTGGGCCTGCCGCCTTGTCCAACAAGGGCCCGCGCCTGAA

TGAGGGGGTTGTCCTCGATGAAGCCATCTTCTCCAAACACAAAGGAAACACAAAGATGTCTGAGGAGGAC

AAAGCGCTGTTCCGCCGCTGTGCTGCTGACTACGCGTCGCGTCTACATAGCGTGCTGGGTACGGCAAATG

CCCCACTGAGCACTTACGTGGCAATCAAGGGCGTCGACGGACTTGACGCCATGGAACCAGACACCGCGCC

TGGTCTCCCCTGGGCTCTCCAGGGGAAACGCCGTGGTGCGCTCATTGATTTCGAGAACGGCACTGTCGGA

CCCGAGGTTGAAGCTGCCTTGAAGCTCATGGAGAAAAGAGAGTACAAGTTTGTATGCCAGACCTTCCTGA

AGGACGAGATTCGCCCGATGGAGAAGGTACGTGCCGGCAAGACTCGCATTGTCGACGTCCTGCCTGTTGA

ACACATTCTTTACACCAGGATGATGATTGGCAGATTTTGTGCTCAAATGCACTCAAACAACGGACCGCAA

ATTGGCTCGGCGGTTGGTTGTAATCCTGATGTTGATTGGCAAAGATTTGGCACGCATTTTGCTCAGTACA

GAAACGTGTGGGATGTGGACTATTCGGCCTTTGATGCCAACCACTGCAGTGACGCAATGAACATCATGTT

TGAGGAGGTGTTCAACACGGATTTCGGGTTCCACCCAAACGCTGAGTGGATCCTGAAAACTCTCGTGAAC

ACTGAACACGCCTATGAGAACAAACGCATCACTGTTGAAGGCGGGATGCCGTCTGGTTGTTCCGCAACAA

GCATCATCAACACAATTTTGAACAACATCTACGTGCTCTACGCCTTGCGTAGACACTATGAGGGAGTTGA

GCTGGACTCTTACACCATGATCTCCTACGGAGACGACATCGTGGTTGCAAGTGATTACGATCTGGACTTT

GAGGCCCTCAAGCCTCACTTCAAATCCCTTGGTCAAACCATTACTCCAGCTGACAAAAGCGACAAAGGTT

TTGTTCTTGGTCACTCCATTACCGATGTCACTTTCCTCAAAAGACACTTCCACATGGACTATGGAACTGG

GTTTTACAAACCTGTGATGGCTTCGAAGACCCTCGAGGCTATCCTCTCCTTTGCACGCCGTGGG

>AH012984.2_O_SKR_2000

GGATTGATAGTTGACACCAGAGATGTTGAGGAGCGCGTACATGTCATGCGCAAAACCAAGCTCGCACCCA

CCGTGGCACACGGTGTGTTTAACCCCGAATTTGGGCCTGCCGCCTTGTCCAACAAGGGCCCGCGCCTGAA

TGAGGGGGTTGTCCTCGATGAAGCCATCTTCTCCAAACACAAAGGAAACACAAAGATGTCTGAGGAGGAC

AAAGCGCTGTTCCGCCGCTGTGCTGCTGATTACGCGTCGCGTCTGCACAGTGTGCTGGGTACGGCAAATG

CCCCACTGAGCATTTACGAGGCAATCAAGGGCGTCGACGGACTTGACGCCATGGAACCAGACACCGCGCC

TGGTCTCCCTTGGGCTCTCCAGGGGAAACGCCGGGGTGCGCTCATTGACTTCGAAAACGGCACTGTCGGA

CCCGAGGTTGAAGCTGCCTTGAAGCTCATGGAGAAAAGAGAGTACAAGTTTGTGTGCCAGACCTTCTTGA

AGGACGAGATTCGCCCGATGGAGAAGGTACGTGCCGGTAAGACTCGCATTGTCGACGTCCTGCCTGTTGA

ACACATTCTTTACACCAGGATGATGATTGGCAGATTTTGTGCTCAAATGCACTCAAACAACGGACCGCAA

ATTGGCTCGGCGGTTGGTTGTAATCCTGATGTTGATTGGCAAAGATTTGGCACGCACTTTGCTCAGTACA

GAAACGTGTGGGATGTAGACTATTCGGCCTTCGACGCCAACCACTGCAGTGACGCAATGAACATCATGCT

TGAGGAGGTGTTCAACACGGATTTCGGTTTCCACCCAAACGCTGAGTGGATCCTGAAAACCCTCGTGAAC

ACTGAACACGCCTATGAGAACAAACGCATCACTGTTGAAGGCGGGATGCCGTCTGGTTGTTCCGCGACAA

GCATCATCAACACAATTTTGAACAACATCTACGTGCTCTACGCCTTGCGTAGACACTACGAGGGAGTCGA

GCTGGACTCTTACACCATGATCTCCTACGGAGACGACATCGTGGTTGCAAGTGATCACGATCTGGACTTT

GAGGCCCTCAAGCCTCACTTCAAATCCCTTGGTCAAACCATCACTCCAGCTGACAAAAGCGACAAAGGTT

TTGTTCTTGGTCACTCCATTACCGATGTCACTTTCCTCAAAAGACACTTCCACATGGACTATGGAACTGG

GTTTTACAAACCTGTGATGGCTTCGAAGACCCTCGAAGCTATCCTCTCCTTTGCACGTCGTGGG

>AH012985.2_O_SKR_2000

GGATTGATAGTTGACACCAGAGATGTTGAGGAGCGCGTACATGTCATGCGCAAAACCAAGCTCGCACCCA

CCGTGGCACACGGTGTGTTTAACCCCGAATTTGGGCCTGCCGCCTTGTCCAACAAGGACCCGCGCCTGAA

TGAGGGGGTTGCCCTCGATGAAGCCATCTTCTCCAAACACAAGGGAAACACAAAGATGTCTGAGGAGGAC

AAAGCGCTGTTCCGCCGCTGTGCTGCTGACTACGCGTCGCGTCTGCATAGCGTGCTGGGTACGGCAAACG

CCCCACTGAGCATTTACGAGGCAATCAAGGGCGTCGACGGACTTGACGCCATGGAACCAGACACCGCGCC

TGGTTTACCCTGGGCTCTCCAGGGGAAACGCCGTGGTGCGCTCATTGACTTTGAGAACGGCACAATCGGA

CCCGAGGTTGAAGCTGCCTTGAAGCTCATGGAGAAAAGAGAGTACAAGTTTGTATGTCAGACCTTCCTGA

AGGACGAGATTCGCCCGATGGAGAAGGTACGTGCCGGCAAGACTCGCATTGTCGACGTCCTGCCTGTTGA

ACACACTCTTTACACCAGGATGATGATTGGCAGATTTTGTGCTCAAATGCACTCAAACAACGGACCGCAA

ATTGGCTCGGCGGTTGGGTGTAATCCTGATGTTGATTGGCAAAGATTTGGCACGCATTTTGCTCAGTACA

GAAACGTGTGGGATGTAGACTATTCGGCCTTTGATGCCAACCACTGCAGTGACGCAATGAACATCATGTT

TGAGGAGGTGTTCAACACGGATTTCGGTTTCCACCCAAACGCTGAGTGGATCCTGAAAACTCTCGTGAAC

ACTGAACACGCCTATGAGAACAAACGCATCACTGTTGAAGGCGGGATGCCGTCTGGTTGTTCCGCAACAA

GCATCATCAACACAATTTTGAACAACATCTACGTGCTCTACGCCTTGCGTAGGCACTATGAGGGAGTTGA

GCTGGACTCTTACACCATGATCTCCTACGGAGACGACATCGTGGTTGCAAGTGATTACGATCTGGACTTT

GAGGCCCTCAAGCCTCACTTCAAATCCCTTGGTCAAACCATTACTCCAGCTGACAAAAGCGACAAAGGTT

TTGTTCTTGGTCACTCCATTACCGATGTCACTTTCCTCAAAAGATCTTTCCACATGGACTATGGAACTGG

GTTTTACAAACCTGTGATGGCTTCGAAGACCCTCGAGGCTATCCTCTCCTTTGCACGCCGTGGG

>AJ539136.1_O_TAW_1999

GGATTGATAGTTGACACCAGAGATGTTGAGGAGCGCGTACATGTCATGCGCAAAACCAAGCTCGCACCCA

CCGTGGCACACGGTGTGTTTAACCCCGAATTTGGGCCTGCCGCCTTGTCCAACAAGGACCCGCGCCTGAA

TGAGGGGGTTGTCCTCGATGAAGCCATCTTCTCCAAACACAAAGGAAACACAAAGATGTCTGAGGAGGAC

AAAGCGCTGTTCCGCCGCTGTGCTGCTGACTACGCGTCGCGTCTGCATAGCGTGCTGGGTACGGCAAATG

CCCCACTGAGCACTTACGAGGCAATCAAGGGCGTCGACGGGCTTGACGCCATGGAACCGGACACCGCGCC

TGGTCTCCCCTGGGCTCTCCAGGGGAAACGCCGTGGTGCGCTCATTGACTTCGAGAACGGCACTGTTGGA

CCCGAGGTTGAAGCTGCCTTGAAGCTCATGGAGAAAAGAGAGTACAAGTTTGTATGCCAGACCTTCCTGA

AGGACGAGATTCGCCCGATGGAGAAGGTACGTGCCGGCAAGACTCGCATTGTCGACGTCCTGCCTGTTGA

ACACATTCTTTACACCAGGATGATGATTGGCAGATTTTGTGCTCAAATGCACTTAAACAACGGACCGCAA

ATTGGCTCGGCGGTTGGTTGTAATCCTGATGTTGATTGGCAAAGATTTGGCACGCATTTTGCTCAGTACA

GAAACGTGTGGGATGTGGACTATTCGGCCTTTGATGCCAACCACTGCAGTGACGCAATGAACATCATGTT

TGAGGAGGTGTTCAACACGGATTTCGGTTTCCACCCAAACGCTGAGTGGATCCTGAAAACTCTCGTGAAC

ACTGAACACGCCTATGAGAACAAACGCATCACTGTTGAAGGCGGGATGCCGTCTGGTTGTTCCGCAACAA

GCATCATCAACACAATTTTGAACAACATCTACGTGCTCTACGCCTTGCGTAGACACTATGAGGGAGTTGA

GCTGGACTCTTACACCATGATCTCCTACGGGGACGACATCGTGGTTGCAAGTGATTACGATCTGGACTTT

GAGGCCCTCAGGCCTCACTTCAAATCCCTTGGTCAAACCATTACTCCAGCTGACAAAAGCGACAAAGGTT

TTGTTCTTGGTCACTCCATTACCGATGTCACTTTCCTCAAAAGACACTTCCACATGGACTATGGAACTGG

GTTTTACAAACCTGTGATGGCTTCGAAGACCCTCGAGGCTATCCTCTCCTTTGCACGCCGTGGG

>AJ539137.1_O_TAW_1999

GGATTGATAGTTGACACCAGAGATGTTGAGGAGCGCGTACATGTCATGCGCAAAACCAAGCTCGCACCCA

CCGTGGCACACGGTGTGTTTAACCCCGAATTTGGGCCTGCCGCCTTGTCCAACAAGGACCCGCGCCTGAA

TGAGGGGGTTGTCCTCGATGAAGCCATCTTCTCCAAACACAAAGGAAACACAAAGATGTCTGAGGAGGAC

AAAGCGCTGTTCCGCCGCTGTGCTGCTGACTACGCGTCGCGTCTGCATAGCGTGCTGGGTACGGCAAATG

CCCCACTGAGCACTTACGAGGCAATCAAGGGCGTCGACGGGCTTGACGCCATGGAACCGGACACCGCGCC

TGGTCTCCCCTGGGCTCTCCAGGGGAAACGCCGTGGTGCGCTCATTGACTTCGAGAACGGCACTGTTGGA

CCCGAGGTTGAAGCTGCCTTGAAGCTCATGGAGAAAAGAGAGTACAAGTTTGTATGCCAGACCTTCCTGA

AGGACGAGATTCGCCCGATGGAGAAGGTACGTGCCGGCAAGACTCGCATTGTCGACGTCCTGCCTGTTGA

ACACATTCTTTACACCAGGATGATGATTGGCAGATTTTGTGCTCAAATGCACTTAAACAACGGACCGCAA

ATTGGCTCGGCGGTTGGTTGTAATCCTGATGTTGATTGGCAAAGATTTGGCACGCATTTTGCTCAGTACA

GAAACGTGTGGGATGTGGACTATTCGGCCTTTGATGCCAACCACTGCAGTGACGCAATGAACATCATGTT

TGAGGAGGTGTTCAACACGGATTTCGGTTTCCACCCAAACGCTGAGTGGATCCTGAAAACTCTCGTGAAC

ACTGAACACGCCTATGAGAACAAACGCATCACTGTTGAAGGCGGGATGCCGTCTGGTTGTTCCGCAACAA

GCATCATCAACACAATTTTGAACAACATCTACGTGCTCTACGCCTTGCGTAGACACTATGAGGGAGTTGA

GCTGGACTCTTACACCATGATCTCCTACGGGGACGACATCGTGGTTGCAAGTGATTACGATCTGGACTTT

GAGGCCCTCAGGCCTCACTTCAAATCCCTTGGTCAAACCATTACTCCAGCTGACAAAAGCGACAAAGGTT

TTGTTCTTGGTCACTCCATTACCGATGTCACTTTCCTCAAAAGACACTTCCACATGGACTATGGAACTGG

GTTTTACAAACCTGTGATGGCTTCGAAGACCCTCGAGGCTATCCTCTCCTTTGCACGCCGTGGG

>AJ539138.1_O_CHA_1999

GGATTGATAGTTGACACCAGAGATGTTGAGGAGCGCGTACATGTCATGCGCAAAACCAAGCTCGCACCCA

CCGTGGCACACGGTGTGTTTAACCCCGAATTTGGGCCTGCCGCCTTGTCCAACAAGGACCCGCGCCTGAA

TGAGGGGGTTGTCCTCGATGAAGCCATCTTCTCCAAACACAAAGGAAACACAAAGATGTCTGAGGAGGAC

AAAGCGCTGTTCCGCCGCTGTGCTGCTGACTACGCGTCGCGTCTGCATAGCGTGCTGGGTACGGCAAATG

CCCCACTGAGCACTTACGAGGCAATCAAGGGCGTCGACGGACTTGACGCCATGGAACCAGACACCGCGCC

TGGTCTCCCCTGGGCTCTCCAGGGGAAACGCCGTGGTGCGCTCATTGATTTCGAGAACGGCACTGTCGGA

CCCGAGGTTGAAGCTGCCTTGAAGCTCATGGAGAAAAGAGAGTACAAGTTTGTATGCCAGACCTTCCTGA

AGGACGAGATTCGCCCGATGGAGAAGGTACGTGCCGGCAAGACTCGCATTGTCGACGTCCTGCCTGTTGA

ACACATTCTTTACACCAGGATGATGATTGGCAGATTTTGTGCTCAAATGCACTCAAACAACGGACCGCAA

ATTGGCTCGGCGGTTGGTTGTAATCCTGATGTTGATTGGCAAAGATTTGGCACGCATTTTGCTCAGTACA

GAAACGTGTGGGATGTGGACTATTCGGCCTTTGATGCCAACCACTGCAGTGACGCAATGAACATCATGTT

TGAGGAGGTGTTCAACACGGATTTCGGTTTCCACCCAAACGCTGAGTGGATCCTGAAAACTCTCGTGAAC

ACTGAACACGCCTATGAGAACAAACGCATCACTGTTGAAGGCGGGATGCCGTCTGGTTGTTCCGCAACAA

GCATCATCAACACAATTTTGAACAACATCTACGTGCTCTACGCCTTGCGTAGACACTATGAGGGAGTTGA

GCTGGACTCTTACACCATGATCTCCTACGGAGACGACATCGTGGTTGCAAGTGATTACGATCTGGACTTT

GAGGCCCTCAAGCCTCACTTCAAATCCCTTGGTCAAACCATTACTCCAGCTGACAAAAGCGACAAAGGTT

TTGTTCTTGGTCACTCCATTACCGATGTCACTTTCCTCAAAAGACACTTCCACATGGACTATGGAACTGG

GTTTTACAAACCTGTGATGGCTTCGAAGACCCTCGAGGCTATCCTCTCCTTTGCACGCCGTGGG

>AJ539139.1_O_SKR_2000

GGATTGATAGTTGACACCAGAGATGTTGAGGAGCGCGTACATGTCATGCGCAAAACCAAGCTCGCACCCA

CCGTGGCACACGGTGTGTTTAACCCCGAATTTGGGCCTGCCGCCTTGTCCAACAAGGACCCGCGCCTGAA

TGAGGGGGTTGTCCTCGATGAAGCCATCTTCTCCAAACACAAGGGAAACACAAAGATGTCTGAGGAGGAC

AAAGCGCTGTTCCGCCGCTGTGCTGCTGACTACGCGTCGCGTCTGCATAGCGTGCTGGGTACGGCAAACG

CCCCACTGAGCATTTACGAGGCAATCAAGGGCGTCGACGGACTTGACGCCATGGAACCAGACACCGCGCC

TGGTTTACCCTGGGCTCTCCAGGGGAAACGCCGTGGTGCGCTCATTGACTTTGAGAACGGCACAATCGGA

CCCGAGGTTGAAGCTGCCTTGAAGCTCATGGAGAAAAGAGAGTACAAGTTTGTATGTCAGACCTTCCTGA

AGGACGAGATTCGCCCGATGGAGAAGGTACGTGCCGGCAAGACTCGCATTGTCGACGTCCTGCCTGTTGA

ACACATTCTTTACACCAGGATGATGATTGGCAGATTTTGTGCTCAAATGCACTCAAACAACGGACCGCAA

ATTGGCTCGGCGGTTGGGTGTAATCCTGATGTTGATTGGCAAAGATTTGGCACGCATTTTGCTCAGTACA

GAAACGTGTGGGATGTAGACTATTCGGCCTTTGATGCCAACCACTGCAGTGACGCAATGAACATCATGTT

TGAGGAGGTGTTCAACACGGATTTCGGTTTCCACCCAAACGCTGAGTGGATCCTGAAAACTCTCGTGAAC

ACTGAACACGCCTATGAGAACAAACGCATCACTGTTGAAGGCGGGATGCCGTCTGGTTGTTCCGCAACAA

GCATCATCAACACAATTTTGAACAACATCTACGTGCTCTACGCCTTGCGTAGGCACTATGAGGGAGTTGA

GCTGGACTCTTACACCATGATCTCCTACGGAGACGACATCGTGGTTGCAAGTGATTACGATCTGGACTTT

GAGGCCCTCAAGCCTCACTTCAAATCCCTTGGTCAAACCATTACTCCAGCTGACAAAAGCGACAAAGGTT

TTGTTCTTGGTCACTCCATTACCGATGTCACTTTCCTCAAAAGATCTTTCCACATGGACTATGGAACTGG

GTTTTACAAACCTGTGATGGCTTCGAAGACCCTCGAGGCTATCCTCTCCTTTGCACGCCGTGGG

>AJ539140.1_O_SAR_2000

GGATTGATAGTTGACACCAGAGATGTTGAGGAGCGCGTACATGTCATGCGCAAAACCAAGCTCGCACCCA

CCGTGGCACACGGTGTGTTTAACCCCGAATTTGGGCCTGCCGCCTTGTCCAACAAGGACCCGCGCCTGAA

TGAGGGGGTTGTCCTCGATGAAGTCATCTTCTCCAAACACAAAGGAAACACAAAGATGTCTGAGGAGGAC

AAAGCGCTGTTCCGCCGCTGTGCTGCTGACTACGCGTCGCGTCTGCATAGCGTGCTGGGTACGGCAAATG

CCCCACTGAGCACTTACGAGGCAATCAAGGGCGTCGACGGACTTGACGCCATGGAACCAGACACCGCGCC

TGGTCTCCCCTGGGCTCTCCAGGGGAAACGCCGTGGTGCGCTCATCGACTTCGAGAACGGCACAGTCGGA

CCCGAGGTTGAAGCTGCCTTGAAGCTCATGGAGAAAAGAGAGTACAAGTTTACATGCCAGACCTTCCTGA

AGGACGAGATTCGCCCGATGGAGAAGGTACGTGCCGGCAAGACTCGCATTGTCGACGTCCTGCCCGTTGA

ACACATTCTTTACACTAGGATGATGATTGGCAGATTTTGTGCTCAAATGCACTCAAACAACGGACCGCAA

ATTGGCTCGGCGGTTGGTTGTAATCCTGATGTTGATTGGCAAAGATTTGGCACGCATTTTGCTCAGTATA

GAAACGTGTGGGATGTGGACTATTCGGCCTTTGATGCCAACCACTGCAGTGACGCAATGAACATCATGTT

TGAGGAGGTGTTTAACACGGACTTCGGTTTCCACCCAAACGCTGAGTGGATCCTGAAAACTCTCGTGAAC

ACTGAACACGCCTATGAGAACAAACGCATCACTGTTGAAGGCGGGATGCCGTCTGGTTGTTCCGCAACAA

GCATCATCAACACAATTTTGAACAACATCTACGTGCTCTACGCCTTGCGTAGACACTATGAGGGAGTTGA

GCTGGACTCTTACACCATGATCTCCTACGGAGACGACATCGTGGTTGCAAGTGATTACGATCTGGACTTT

GAGGCCCTCAAGCCTCACTTCAAATCCCTTGGTCAAACCATTACCCCAGCTGACAAAAGCGACAAAGGTT

TTGTTCTTGGTCACTCCATTACCGATGTCACTTTCCTCAAAAGACACTTCCACATGGACTATGGAACTGG

GTTTTACAAACCTGTGATGGCTTCGAAGACCCTCGAGGCTATCCTCTCCTTTGCACGCCGTGGG

>AJ539141.1_O_UKG_2001

GGATTGATAGTTGACACCAGAGATGTTGAGGAGCGCGTACATGTCATGCGCAAAACCAAGCTCGCACCCA

CCGTGGCACACGGTGTGTTTAACCCCGAATTTGGGCCTGCCGCCTTGTCCAACAAGGACCCGCGCCTGAA

TGAGGGGGTTGTCCTCGATGAAGTCATCTTCTCCAAACACAAAGGAAACACAAAGATGTCTGAGGAGGAC

AAAGCGCTGTTCCGCCGCTGTGCTGCTGACTACGCGTCGCGTCTGCATAGCGTGCTGGGTACGGCAAATG

CCCCACTGAGCACTTACGAGGCAATCAAGGGCGTCGACGGACTTGACGCCATGGAACCAGACACCGCGCC

TGGTCTCCCCTGGGCTCTCCAGGGGAAACGCCGTGGTGCGCTCATCGACTTCGAGAACGGCACTGTCGGA

CCCGAGGTTGAAGCTGCCTTGAAGCTCATGGAGAAAAGAGAGTACAAGTTTACATGCCAGACCTTCCTGA

AGGACGAGATTCGCCCGATGGAGAAGGTACGTGCCGGCAAGACTCGCATTGTCGACGTCCTGCCCGTTGA

ACACATTCTTTACACTAGGATGATGATTGGCAGATTTTGTGCTCAAATGCACTCAAACAACGGACCGCAA

ATTGGCTCGGCGGTTGGTTGTAATCCTGATGTTGATTGGCAAAGATTTGGCACGCATTTTGCTCAGTATA

GAAACGTGTGGGATGTGGACTATTCGGCCTTTGATGCCAACCACTGCAGTGACGCAATGAACATCATGTT

TGAGGAGGTGTTTAACACGGACTTCGGTTTCCACCCAAACGCTGAGTGGATCCTGAAAACTCTCGTGAAC

ACTGAACACGCCTATGAGAACAAACGCATCACTGTTGAAGGCGGGATGCCGTCTGGTTGTTCCGCAACAA

GCATCATCAACACAATTTTGAACAACATCTACGTGCTCTACGCCTTGCGTAGACACTATGAGGGGGTTGA

GCTGGACTCTTACACCATGATCTCCTACGGAGACGACATCGTGGTTGCAAGTGATTACGATCTGGACTTT

GAGGCCCTCAAGCCTCACTTCAAATCCCTTGGTCAAACCATTACCCCAGCTGACAAAAGCGACAAAGGTT

TTGTTCTTGGTCACTCCATTACCGATGTCACTTTCCTCAAAAGACACTTCCACATGGACTATGGAACTGG

GTTTTACAAACCTGTGATGGCTTCGAAGACCCTCGAGGCTATCCTCTCCTTTGCACGCCGTGGG

>AJ633821.1_O_FRA_2001

GGATTGATAGTTGACACCAGAGATGTTGAGGAGCGCGTACATGTCATGCGCAAAACCAAGCTCGCACCCA

CCGTGGCACACGGTGTGTTTAACCCCGAATTTGGGCCTGCCGCCTTGTCCAACAAGGACCCGCGCCTGAA

TGAGGGGGTTGTCCTCGATGAAGTCATCTTCTCCAAACACAAAGGAAACACAAAGATGTCTGAGGAGGAC

AAAGCGCTGTTCCGCCGCTGTGCTGCTGACTACGCGTCGCGTCTGCATAGCGTGCTGGGTACGGCAAATA

CCCCACTGAGCACTTACGAGGCAATCAAGGGCGTCGACGGACTTGACGCCATGGAACCAGACACCGCGCC

TGGTCTCCCCTGGGCTCTCCAGGGGAAACGCCGTGGTGCGCTCATCGACTTCGAGAACGGCACTGTCGGA

CCCGAGGTTGAAGCTGCCTTGAAGCTCATGGAGAAAAGAGAGTACAAGTTTACATGCCAGACCTTCCTGA

AGGACGAGATTCGCCCGATGGAGAAGGTACGTGCCGGCAAGACTCGCATTGTCGACGTCCTGCCCGTTGA

ACACATTCTTTACACTAGGATGATGATTGGCAGATTTTGTGCTCAAATGCACTCAAACAACGGACCGCAA

ATTGGCTCGGCGGTTGGTTGTAATCCTGATGTTGATTGGCAAAGATTTGGCACGCATTTTGCTCAGTATA

GAAACGTGTGGGATGTGGACTATTCGGCCTTTGATGCCAACCACTGCAGTGACGCAATGAACATCATGTT

TGAGGAGGTGTTTAACACGGACTTCGGTTTCCACCCAAACGCTGAGTGGATCCTGAAAACTCTCGTGAAC

ACTGAACACGCCTATGAGAACAAACGCATCACTGTTGAAGGCGGGATGCCGTCTGGTTGTTCCGCAACAA

GCATCATCAACACAATTTTGAACAACATCTACGTGCTCTACGCCTTGCGTAGACACTATGAGGGGGTTGA

GCTGGACTCTTACACCATGATCTCCTACGGAGACGACATCGTGGTTGCAAGTGATTACGATCTGGACTTT

GAGGCCCTCAAGCCTCACTTCAAATCCCTTGGTCAAACCATTACCCCAGCTGACAAAAGCGACAAAGGTT

TTGTTCTTGGTCACTCCATTACCGATGTCACTTTCCTCAAAAGACACTTCCACATGGACTATGGAACTGG

GTTTTACAAACCTGTGATGGCTTCGAAGACCCTCGAGGCTATCCTCTCCTTTGCACGCCGTGGG

>AY317098.1_O_CHA_2002

GGGTTGATCGTTGACACCAGAGATGTGGAGGAACGCGTCCACGTGATGCGCAAAACCAAGCTCGCGCCCA

CCGTAGCACACGGTGTGTTCAATCCTGAGTTCGGGCCTGCTGCTCTGTCCAACAAGGACCCGCGTCTGAA

TGAAGGGGTCGTCCTCGACGACGTCATTTTCTCAAAACACAAGGGAGACACGAGGATGTCTGAGGAAGAC

AAAGCGCTGTTCCGGCGCTGTGCTGCCGACTACGCGTCGCGTCTACACAGCGTGCTAGGGACGGCAAACG

CCCCACTGAGTGTATACGAAGCCATCAAAGGCGTCGATGGACTTGACGCCATGGAGCCAGACACCGCACC

CGGTCTCCCCGGGGCTCTCCAAGGAAAACGCCGAGGTGCCCTGATCGACTTCGAAAACGGTACTGTCGGG

CCCGAGGTTGAAGCAGCACTCAAGCTCATGGAAAGCCGTGAGTATAAATTCGTCTGCCAAACCTTTCTGA

AAGACGAAATTCGGCCGCTAGAGAAGGTGCGCGCCGGTAAGACACACATTGTCGACGTTTTGCCTGTTGA

ACACATTCTCTATACCAGAATGATGATTGGTAGATTCTGTGCTCAGATGCACTCAAACAACGGACCGCAA

ATTGGCTCAGCGGTCGGTTGCAACCCTGATGTTGATTGGCAAAGATTTGGCACACATTTCGCCCAGTACA

AGAACGTGTGGGATGTGGACTACTCAGCCTTCGATGCAAACCACTGCAGCGATGCGATGAACATCATCTT

CGAAGAGGTGTTCCGCACGGAGTTTGGGTTCCACCCGAACGCCGAGTGGATTCTGAAGACTCTGGTGAAC

ACGGAGCACGCTTACGAGAACAAGCGCATCACTGTGGAGGGTGGAATGCCGTCCGGTTGTTCCGCAACAA

GCATCATCAACACAATTTTGAACAACATCTACGTGCTCTACGCTCTGCGTAGGCACTATGAAGGAGTTGA

GCTGGACACCTACACAATGATCTCCTATGGAGACGACATCGTGGTGGCTAGTGACTACGACCTGGACTTC

GAGGCTCTCAAGCCCCACTTCAAGTCCCTCGGTCAGACCATCACTCCAGCCGACAAAAGCGACAAAGGTT

TTGTTCTTGGTCACTCCATAACCGATGTCACTTTCCTCAAAAGACACTTCCACATGGACTACGGAACTGG

GTTTTACAAACCTGTGATGGCCTCGAAGACCCTCGAGGCTATCCTCTCCTTTGCACGCCGTGGG

>AY593751.1_A_NET_1942

GGGTTGATTGTTGATACCAGAGATGTGGAAGAGCGCGTCCACGTGATGCGCAAAACAAAGCTTGCACCCA

CCGTTGCACACGGCGTGTTCAACCCTGAGTTTGGGCCTGCCGCCTTGTCAAACAAGGACCCGCGCCTGAA

CGAGGGAGTTGTTCTCGATGAAGTCATTTTCTCCAAACACAAAGGAGACGTAAAGATGACCGAAGAGGAC

AAAGCGCTGTTCCGCCGCTGCGCCGCTGACTACGCGTCACGCCTGCACAGCGTGCTGGGTACGGCAAATG

CCCCATTGAGCATCTACGAGGCAATCAAGGGCGTTGATGGACTCGACGCCATGGAGCCGGACACTGCACC

TGGCCTCCCCTGGGCCCTCCAGGGAAAACGCCGCGGTGCGCTCATCGACTTCGAGAACGGCACGGTCGGA

CCCGAAGTTGAGGCTGCCTTGAAGCTCATGGAGAAAAGAGAATACAAGTTTGCTTGTCAGACCTTCCTGA

AGGACGAGATTCGCCCGATGGAGAAAGTACGCGCCGGCAAGACTCGCATCGTCGATGTTTTGCCTGTTGA

ACACATTCTTTACACCAGGATGATGATTGGCAGGTTCTGTGCACAAATGCACTCGAACAACGGACCACAA

ATTGGCTCTGCGGTCGGTTGCAACCCTGACGTTGATTGGCAAAGATTTGGCACACATTTCGCCCAATACA

GAAACGTGTGGGATGTGGATTACTCGGCCTTTGATGCAAACCACTGCAGTGACGCTATGAACATCATGTT

TGAGGAGGTGTTCCGCACAGACTTTGGCTTCCACCCAAACGCTGAATGGATCCTGAAGACTCTCGTGAAC

ACGGAACACGCCTATGAGAACAAGCGCATCACTGTTGAAGGCGGGATGCCATCTGGTTGTTCCGCAACAA

GCATCATCAACACAATTTTGAACAACATCTACGTGCTCTACGCCTTGCGTAGACACTATGAGGGAGTTGA

GCTGGACACTTACACCATGATCTCCTACGGAGACGACATCGTGGTGGCAAGTGATTACGATCTGGACTTT

GAGGCTCTCAAGCCCCACTTCAAATCTCTTGGCCAAACCATCACTCCAGCTGACAAAAGCGACAAAGGTT

TTGTTCTTGGTCACTCCATTACCGATGTCACTTTCCTCAAAAGACACTTCCACATGGATTATGGAACTGG

GTTTTACAAACCTGTGATGGCCTCAAAGACCCTTGAGGCTATCCTCTCCTTTGCACGCCGTGGG

>AY593753.1_A_Brazil_1970

GGGTTGATTGTTGACACCAGAGATGTGGAAGAGCGCGTCCACGTGATGCGCAAAACCAAGCTTGCACCCA

CCGTCGCACACGGTGTGTTTAACCCTGAGTTCGGGCCCGCCGCCTTGTCCAACAAGGACCCGCGCCTGAA

CGATGGTGTTGTCCTCGACGAAACCATCTTCTCCAAACACAAAGGAGATACAAAGATGTCTGAAGAGGAT

AAAGCGCTGTTCCGCCGCTGCGCCGCTGACTACGCGTCACGCTTGCACAGTGTGTTGGGCACAGCAAATG

CCCCACTGAGCATCTTCGAGGCAATCAAAGGTGTTGATGGGCTCGACGCAATGGAGCCAGACACTGCACC

CGGCCTCCCTTGGGCACTCCAGGGGAAGCGCCGTGGAGCGCTTATCGACTTTGAGAACGGCACTGTCGGA

CCCGAAGTTGAGGCTGCCTTGAAGCTCATGGAGAAAAGAGAGTACAAGTTTGTTTGCCAAACCTTCCTGA

AGGACGAGATTCGCCCGATGGAGAAAGTACGTGCCGGTAAGACTCGCATTGTCGACGTCCTGCCTGTTGA

ACACATTCTCTACACTAGGATGATGATTGGCAGATTCTGTGCACAAATGCACTCAAACAACGGACCCCAA

ATTGGCTCGGCGGTCGGTTGTAACCCTGATGTTGATTGGCAAAGATTTGGCACACACTTCGCCCAATACA

GAAACGTGTGGGATGTGGACTATTCGGCCTTTGATGCTAACCATTGCAGCGATGCCATGAACATCATGTT

TGAGGAGGTGTTCCGCACGGAGTTCGGCTTCCACCCAAACGCGGAGTGGATTCTGAAGACTCTCGTGAAC

ACGGAACACGCCTATGAGAACAAGCGCATCACTGTTGAGGGCGGGATGCCATCTGGCTGTTCCGCAACAA

GCATCATCAACACAATTCTGAACAACATCTACGTGCTCTACGCTTTGCGCAGACACTATGAGGGAGTTGA

GCTGGACACTTACACCATGATCTCCTACGGAGACGACATCGTGGTAGCAAGTGATTACGATTTGGACTTT

GAGGCTCTCAAGCCCCACTTTAAATCCCTTGGTCAAACCATCACTCCAGCTGACAAAAGCGACAAAGGTT

TTGTTCTTGGTCACTCCATTACTGATGTCACTTTCCTCAAAAGACACTTCCACATGGATTACGGAACTGG

GTTTTACAAACCTGTGATGGCCTCAAAGACCCTTGAGGCTATCCTCTCCTTTGCACGCCGTGGG

>AY593754.1_A_SPA_1959

GGGTTGATTGTAGACACCAGAGATGTGGAAGAGCGCGTCCACGTGATGCGCAAAACCAAGCTTGCACCCA

CCGTTGCACACGGTGTGTTCAACCCCGAGTTTGGGCCAGCTGCCTTGTCCAACAAGGACCCGCGTCTGAA

CGAGGGTGTTGTCCTTGATGAAGTCATTTTCTCCAAACACAAGGGAGACACAAAGATGTCTGAGGAGGAC

AAAGCGCTGTTCCGCCGATGTGCTGCTGACTACGCGTCACGCCTGCACAGCGTGCTGGGTACGGCAAATG

CCCCATTGAGCATCTACGAGGCAGTCAAGGGCGTCGACGGACTCGACGCCATGGAGCCAGACACCGCACC

TGGCCTCCCCTGGGCTCTCCAGGGAAAACGCCGTGGTGCGCTCATCGACTTTGAGAACGGCACGGTCGGG

CCCGAAGTCGAGGCTGCCTTGAAGCTCATGGAGAAAAGAGAGTACAAGTTTGTTTGTCAGACCTTCCTGA

AGGACGAGATTCGCCCGATGGAGAAAGTACGTGCCGGCAAGACTCGCATTGTCGACGTCCTGCCCGTTGA

ACACATTCTTTACACCAGGATGATGATTGGCAGATTTTGTGCACAAATGCACTCAAACAACGGACCGCAA

ATTGGCTCGGCGGTCGGTTGTAACCCTGATGTTGATTGGCAAAGATTTGGCACACACTTCGCCCAATACA

GAAACGTGTGGGATGTGGACTATTCGGCCTTTGATGCTAACCACTGCAGTGACGCCATGAACATCATGTT

TGAGGAGGTGTTCCGCACGGACTTCGGGTTCCACCCAAATGCTGAGTGGATCTTGAAGACTCTCGTGCAC

ACGGAACATGCCTATGAGAACAAACGCATCACTGTTGAAGGCGGGATGCCATCTGGTTGTTCCGCAACGA

GCATCATCAACACAATTTTGAACAACATCTACGTGCTCTACGCCTTGCGTAGACACTATGAGGGAGTTGA

GCTGGACACTTACACCATGATCTCCTACGGAGACGACATTGTGGTGGCAAGTGATTATGATCTGGACTTT

GAGGCTCTCAAGCCTCACTTCAAATCTCTTGGTCAAACCATTACTCCAGCTGACAAAAGCGACAAAGGTT

TTGTTCTTGGTCACTCCATCACCGATGTCACTTTCCTCAAAAGACACTTCCACATGGATTATGGAACCGG

GTTTTACAAACCTGTGATGGCCTCAAAGACCCTTGAGGCTATCCTCTCCTTTGCACGCCGTGGG

>AY593755.1_A_TAI_1960

GGGTTGATTGTGGATACCAGAGATGTGGAAGAGCGCGTCCACGTGATGCGCAAAACCAAGCTTGCACCCA

CCGTAGCACACGGTGTGTTCAACCCTGAATTCGGGCCTGCTGCCTTGTCAAACAAGGACCCGCGCCTGAA

CGAGGGAGTTGTCCTAGATGAAGTTATCTTCTCCAAACACAAAGGAGACACAAAGATGACTGATGAGGAC

AAGGCGCTGTTCCGCCGCTGCGCTGCTGACTACGCGTCACGCCTCCACAGCGTGCTGGGGACGGCAAACG

CCCCACTGAGCATTTACGAGGCCATCAAGGGCGTCGACGGACTCGACGCCATGGAGCCGGACACCGCGCC

CGGCCTCCCCTGGGCTCTCCAGGGGAAACGTCGTGGTGCGCTGATTGACTTTGAAAACGGCACAGTTGGC

CCTGAGATTGAGGCTGCCCTTGAGCTCATGGAGAAGCGTGAATACAAGTTTGTTTGTCAGACCTTCCTGA

AGGACGAGATTCGCCCGATGGAGAAAGTGCGTGCCGGCAAGACTCGCATAGTCGACGTTTTGCCTGTTGA

ACACATTCTTTACACCAGGATGATGATAGGCAGATTCTGTGCACAAATGCACTCAAACAACGGACCGCAA

ATTGGCTCAGCGGTCGGTTGCAATCCTGACGTTGACTGGCAAAGATTTGGCACCCATTTTGCCCAGTACA

GAAACGTGTGGGATGTGGACTATTCGGCATTTGATGCTAACCACTGCAGCGATGCAATGAACATCATGTT

TGAGGAGGTCTTTCGCACCGAGTTCGGTTTCCACCCAAATGCTGAGTGGATCCTGAAGACTCTGGTGAAC

ACGGAACACGCCTATGAGAACAAACGCATCACTGTTGAGGGCGGGATGCCGTCAGGCTGTTCCGCAACTA

GCATCATCAACACAATCTTGAACAACATTTACGTGCTCTACGCCTTGCGTAGACACTATGAGGGAGTTGA

GCTGGACACCTACACCATGATCTCCTATGGAGACGATATTGTGGTGGCAAGTGATTACGATTTGGACTTC

GAGGCCCTCAAGCCTCATTTTAAATCTCTTGGTCAAACCATTACCCCAGCTGACAAAAGCGACAAAGGTT

TTGTTCTTGGTCACTCCATCACCGATGTCACTTTCCTCAAAAGACACTTCCACATGGACTATGGAACTGG

GTTTTACAAACCTGTGATGGCTTCGAAGACCCTCGAAGCTATCCTCTCCTTTGCACGCCGTGGG

>AY593756.1_A_Brazil_1959

GGGTTGATCATTGACACCAGAGATGTGGAAGAGCGCGTCCATGTGATGCGCAAAACCAAGCTTGCACCCA

CCGTCGCGCACGGTGTGTTTAACCCTGAGTTTGGGCCCGCCGCCTTGTCTAACAAGGACCCGCGCCTGAA

CGAAGGTGTTGTCCTCGACGAAGTCATCTTCTCCAAACACAAAGGGGACACAAAGATGTCTGAGGAGGAC

AAAGCGCTGTTCCGCCGCTGCGCCGCCGACTACGCGTCGCGCCTGCACAGCGTGCTGGGCACAGCAAATG

CCCCACTGAGCATTTACGAGGCAATCAAGGGCGTTGACGGACTCGACGCAATGGAACCGGACACCGCGCC

CGGTCTTCCCTGGGCGCTCCAGGGGAAGCGCCGCGGTGCGCTCATCGACTTTGAGAACGGCACTGTCGGA

CCCGAAGTTGAGGCTGCCTTGAAGCTCATGGAGAAAAGAGAATACAAATTTGTCTGTCAGACCTTCCTGA

AGGACGAGATTCGCCCGATGGAGAAAGTACGTGCCGGCAAGACTCGCATTGTCGACGTCCTGCCCGTTGA

ACACATTCTTTACACCAGGATGATGATTGGCAGATTTTGTGCACAAATGCACTCAAACAACGGACCGCAG

ATTGGCTCGGCGGTCGGTTGCAACCCTGATGTTGATTGGCAAAGATTTGGCACACACTTCGCCCAATACA

GAAACGTGTGGGACGTGGACTATTCGGCCTTCGATGCTAACCACTGCAGTGACGCCATGAACATCATGTT

TGAGGAGGTGTTTCGCACGGACTTTGGGTTCCACCCGAATGCTGAGTGGATCCTGAAAACTCTCGTGAAC

ACGGAACACGCCTATGAGAACAAACGCATCACTGTTGAGGGCGGGATGCCATCTGGTTGTTCTGCGACAA

GCATCATCAACACAATCCTGAACAACATCTACGTGCTCTACGCCCTGCGTAGACACTATGAGGGAGTTGA

GCTGGACACTTACACCATGATCTCCTACGGAGACGACATTGTGGTGGCAAGTGATTACGATTTGGACTTT

GAGGCTCTCAAGCCCCACTTTAAATCTCTTGGTCAAACCATCACTCCAGCTGACAAAAGCGACAAAGGTT

TTGTTCTTGGTCACTCCATCACCGATGTCACTTTCCTCAAAAGACACTTCCACATGGATTATGGAACTGG

GTTTTACAAACCTGTGATGGCCTCAAAGACCCTTGAGGCTATCCTCTCCTTTGCACGCCGTGGG

>AY593757.1_A_Brazil_1967

GGGTTGATTGTTGACACCAGAGATGTGGAAGAGCGCGTCCACGTGATGCGCAAAACCAAGCTTGCACCCA

CCGTCGCACACGGTGTGTTTAACCCTGAGTTCGGGCCCGCCGCCTTGTCCAACAAGGACCCGCGCCTGAA

CGATGGTGTTGTCCTCGATGAAACCATCTTCTCCAAACACAAAGGAGACACAAAGATGTCTGAGGAGGAC

AAAGCGCTGTTCCGCCGCTGTGCTGCTGACTATGCGTCACGCTTGCACAGTGTGTTGGGCACAGCAAATG

CCCCACTGAGCATCTTCGAGGCAATCAAAGGCGTTGATGGACTCGACGCAATGGAGCCAGACACTGCACC

CGGCCTCCCCTGGGCACTCCAGGGGAAGCGCCGTGGAGCGCTTATCGACTTTGAGAACGGCACTGTCGGA

CCCGAAGTTGAGGCTGCCTTGAAGCTCATGGAGAAAAGAGAGTACAAGTTTGTTTGCCAAACCTTCCTGA

AGGACGAGATTCGCCCAATGGAGAAAGTACGCGCCGGTAAGACTCGCATTGTCGACGTCCTACCTGTTGA

ACACATTCTTTACACTAGGATGATGATTGGCAGATTCTGTGCACAAATGCACTCAAACAACGGACCCCAA

ATTGGCTCGGCGGTCGGTTGTAACCCTGATGTTGATTGGCAAAGATTTGGTACACACTTCGCCCAATACA

GAAACGTGTGGGATGTGGACTACTCGGCCTTTGATGCTAACCACTGCAGCGACGCCATGAACATCATGTT

TGAGGAGGTGTTCCGCACGGAGTTCGGCTTCCACCCAAACGCGGAGTGGATTCTGAAGACTCTCGTGAAC

ACGGAACACGCCTATGAGAACAAGCGCATCACTGTTGAGGGCGGGATGCCATCTGGCTGTTCCGCAACAA

GCATCATCAACACAATTCTGAACAACATCTACGTGCTCTACGCTTTGCGCAGACACTATGAGGGAGTTGA

GCTGGACACTTACACCATGATCTCCTACGGAGACGACATCGTGGTAGCAAGTGATTACGATTTGGACTTT

GAGGCTCTCAAGCCCCACTTTAAATCCCTTGGCCAAACCATCACTCCAGCTGACAAAAGCGACAAAGGTT

TTGTTCTTGGTCACTCCATTACTGATGTCACTTTCCTCAAAAGACACTTCCACATGGATTACGGAACTGG

GTTTTACAAACCTGTGATGGCCTCAAAGACCCTTGAGGCTATCCTCTCCTTTGCACGCCGTGGG

>AY593758.1_A_VEN_1967

GGGTTGATTGTTGACACCAGAGATGTGGAAGAGCGCGTCCACGTGATGCGCAAAACCAAGCTTGCACCCA

CCGTCGCACACGGTGTGTTTAACCCTGAGTTCGGGCCCGCCGCCTTGTCCAACAAGGACCCGCGCCTGAA

CGATGGTGTTGTCCTCGACGAAACCATCTTCTCCAAACACAAAGGAGATACAAAGATGTCTGAAGAGGAT

AAAGCGCTGTTCCGCCGCTGCGCCGCTGACTACGCGTCACGCTTGCACAGTGTGTTGGGCACAGCAAATG

CCCCACTGAGCATCTTCGAGGCAATCAAAGGTGTTGATGGGCTCGACGCAATGGAGCCAGACACTGCACC

CGGCCTCCCTTGGGCACTCCAGGGGAAGCGCCGTGGAGCGCTTATCGACTTTGAGAACGGCACTGTCGGA

CCCGAAGTTGAGGCTGCCTTGAAGCTCATGGAGAAAAGAGAGTACAAGTTTGTTTGCCAAACCTTCCTGA

AGGACGAGATTCGCCCGATGGAGAAAGTACGTGCCGGTAAGACTCGCATTGTCGACGTCCTGCCTGTTGA

ACACATTCTCTACACTAGGATGATGATTGGCAGATTCTGTGCACAAATGCACTCAAACAACGGACCCCAA

ATTGGCTCGGCGGTCGGTTGTAACCCTGATGTTGATTGGCAAAGATTTGGCACACACTTCGCCCAATACA

GAAACGTGTGGGATGTGGACTATTCGGCCTTTGATGCTAACCATTGCAGCGATGCCATGAACATCATGTT

TGAGGAGGTGTTCCGCACGGAGTTCGGCTTCCACCCAAACGCGGAGTGGATTCTGAAGACTCTCGTGAAC

ACGGAACACGCCTATGAGAACAAGCGCATCACTGTTGAGGGCGGGATGCCATCTGGCTGTTCCGCAACAA

GCATCATCAACACAATTCTGAACAACATCTACGTGCTCTACGCTTTGCGCAGACACTATGAGGGAGTTGA

GCTGGACACTTACACCATGATCTCCTACGGAGACGACATCGTGGTAGCAAGTGATTACGATTTGGACTTT

GAGGCTCTCAAGCCCCACTTTAAATCCCTTGGTCAAACCATCACTCCAGCTGACAAAAGCGACAAAGGTT

TTGTTCTTGGTCACTCCATTACTGATGTCACTTTCCTCAAAAGACACTTCCACATGGATTACGGAACTGG

GTTTTACAAACCTGTGATGGCCTCAAAGACCCTTGAGGCTATCCTCTCCTTTGCACGCCGTGGG

>AY593759.1_A_GER_1971

GGGTTGATTGTTGACACCAGAGATGTGGAAGAGCGCGTCCACGTGATGCGCAAAACAAAACTTGCGCCCA

CCGTTGCACACGGTGTGTTCAGCCCTGAGTTTGGGCCTGCCGCCTTGTCAAACAAGGACCCGCGCCTGAA

CGAGGGAGTCGTTCTCGATGAAGTCATTTTCTCCAAACACAAGGGAGACACAAAGATGACCGAGGAGGAC

AAAGCGCTGTTCCGACGCTGTGCCGCTGACTATGCGTCACACCTGCACAGTGTACTGGGTACGGCAAATG

CCCCACTGAGTATCTATGAGGCAATCAAGGGCGTTGATGGACTCGACGCCATGGAGCCGGACACTGCACC

TGGCCTCCCCTGGGCCCTCCAGGGAAAACGCCGCGGTGCGCTTATTGACTTCGAGAACGGCACGGTCGGA

CCCGAAGTTGAGGCTGCCTTGAAGCTCATGGAGAAAAGGGAATTCAAGTTTGTTTGTCAAACCTTCCTGA

AGGACGAGATCCGCCCGATGGGGAAAGTACGCGCCGGCAAGACTCGCATTGTCGATGTTTTGCCTGTTGA

ACATATTCTTTATACCAGGATGATGATTGGTAGATTTTGTGCGCAAATGCACTCAAACAACGGACCACAC

ATTGGCTCTGCGGTCGGTTGTAACCCTGATGTTGATTGGCAAAGATTTGGCACACATTTTGCCCAATACA

GAAACGTGTGGGATGTGGATTATTCGGCCTTTGATGCGAACCACTGCAGTGACGCTATGAACATCATGTT

TGAGGAGGTGTTCCGCACAGACTTTGGCTTCCACCCAAATGCAGAGTGGATCCTGAAAACCCTCGTGAAC

ACGGAACACGCCTATGAGAACAAGCGCATCACTGTTGAAGGCGGGATGCCATCTGGTTGCTCCGCGACAA

GCATCATCAACACAATTTTGAACAACATTTACGTGCTCTACGCCTTGCGTAGACACTATGAGGGAGTTGA

GCTGGACACCTACACCATGATCTCCTATGGAGACGACATCGTGGTGGCAAGTGACTATGATCTGGACTTT

GAGGCTCTCAAGCCCCACTTCAAATCTCTTGGCCAAACTATCACTCCAGCTGACAAAAGCGACAGAGGTT

TTGTTCTTGGACATTCTATTACCGATGTCACCTTCCTCAAAAGACATTTCCACATGGATTATGGAACTGG

GTTTTACAAACCTGTGATGGCCTCAAAGACCCTTGAGGCTATCCTCTCCTTTGCACGCCGTGGG

>AY593760.1_A_USSR_1964

GGGTTGATTGTAGACACCAGAGACGTGGAAGAGCGCGTCCACGTGATGCGCAAAACCAAGCTTGCACCCA

CCGTTGCACACGGTGTGTTCAACCCTGAGTTTGGGCCAGCTGCCTTGTCCAACAAGGACCCGCGTCTGAA

CGAGGGTGTTGTTCTCGATGAAGTCATCTTCTCCAAACACAAGGGAGACACAAAGATGTCTGAGGAGGAC

AAAGCGCTGTTCCGCCGCTGTGCTGCTGATTACGCGTCGCACCTGCACAGCGTACTGGGTACGGCAAATG

CCCCGTTGAGCATCTACGAGGCAATTAAAGGTGTCGACGGACTCGACGCCATGGAGCCAGACACCGCACC

TGGCCTCCCCTGGGCTCTCCAGGGAAAGCGCCGTGGTGCGCTCATCGACTTTGAGAACGGTACGGTCGGA

CCCGAAGTCGAGGCTGCCCTTAAGCTCATGGAGAAAAGAGAGTACAAGTTTGTTTGTCAGACCTTCCTGA

AGGACGAGATTCGTCCGATGGAGAAAGTACGTGCCGGCAAGACTCGCATTGTCGACGTCCTGCCTGTTGA

ACACATTCTTTACACCAGGATGATGATTGGCAGATTTTGTGCACAAATGCACTCAAACAACGGACCGCAA

ATTGGTTCGGCGGTCGGTTGTAACCCTGATGTTGATTGGCAAAGATTTGGCACACACTTCGCCCAATACA

GAAACGTGTGGGATGTGGACTATTCGGCCTTTGATGCTAACCACTGCAGTGACGCCATGAACATCATGTT

TGAGGAGGTGTTCCGCACGGACTTCGGGTTCCACCCAAATGCCGAGTGGATCTTGAAGACTCTCGTGAAT

ACGGAACACGCCTATGAGAACAAACGCATCGCTGTTGAAGGCGGGATGCCATCTGGTTGTTCCGCAACGA

GCATCATCAACACAATTTTGAACAACATCTATGTGCTCTACGCCTTGCGCAGACACTATGAGGGAGTTGA

GCTGGACACTTACACCATGATCTCCTACGGTGACGACATCGTGGTGGCGAGTGATTACGATCTGGACTTT

GAGGCTCTCAAGCCTCACTTCAAATCTCTTGGTCAAACTATTACCCCAGCTGACAAAAGCGACAAAGGTT

TTGTTCTTGGTCACTCCATTACCGATGTCACTTTCCTCAAAAGACACTTCCACATGGACTATGGAACTGG

GTTTTACAAACCTGTGATGGCTTCGAAGACCCTCGAAGCTATCCTCTCCTTTGCACGCCGTGGG

>AY593761.1_A_KEN_1964

GGGTTGATTGTTGACACTAGAGATGTGGAAGAACGCGTCCACGTGATGCGGAAGACAAAGCTTGCACCCA

CCGTCGCACACGGTGTGTTCAACCCTGAATTCGGGCCTGCTGCCTTGTCTAACAAGGACCCGCGCCTGAA

CGAAGGTGTTGTCCTCGATGAAGTCATCTTCTCCAAGCACAAGGGAGACACGAAGATGTCTGAGGAGGAC

AAAGCGCTGTTCCGTCGCTGCGCTGCTGACTACGCGTCACGCCTGCACAGCGTACTGGGCACGGCAAATG

CCCCATTGAGCATCTACGAGGCAATCAAGGGCGTTGACGGACTCGACGCCATGGAACCAGACACTGCGCC

TGGCCTCCCCTGGGCCCTCCAGGGGAAGCGCCGTGGCGCACTCATTGACTTCGAGAACGGCACGGTCGGA

CCCGAGGTCGAGGCTGCACTGAAGCTCATGGAGAACAGAGAATACAAATTTGCTTGTCAAACCTTCCTGA

AGGACGAGATTCGCCCGATGGAGAAAGTGCGTGCCGGCAAGACCCGCATTGTCGACGTTTTGCCTGTTGA

ACATATTCTTTACACCAGGATGATGATTGGCAGATTTTGTGCACAAATGCACTCAAACAATGGACCGCAG

ATTGGTTCAGCGGTCGGATGCAACCCTGATGTTGATTGGCAAAGATTTGGCACCCACTTCGCCCAGTACA

GAAACGTGTGGGATGTGGATTATTCGGCCTTTGATGCTAACCACTGCAGTGACGCCATGAACATCATGTT

CGAGGAGGTCTTCCGCACGGACTTCGGTTTCCACCCAAACGCTGAGTGGATTCTGAAGACTCTTGTGAAC

ACGGAGCACGCCTACGAGAACAAGCGCATCACTGTTGAGGGCGGAATGCCGTCTGGTTGTTCCGCGACTA

GCATCATCAACACAATTTTGAACAACATCTATGTGCTCTACGCCCTGCGTAGACACTATGAGGGAGTTGA

GCTGGACACTTACACCATGATCTCCTACGGAGACGACATCGTGGTAGCAAGTGACTACGATTTGGACTTC

GAGGCCCTCAAGCCTCACTTTAAATCCCTTGGTCAAACCATCACTCCAGCTGACAAAAGCGACAAAGGTT

TTGTACTTGGTCACTCCATCACCGATGTCACTTTCCTCAAAAGACACTTCCACATGGATTATGGAACTGG

GTTTTACAAACCTGTGATGGCCTCGAAGACCCTCGAGGCTATCCTCTCCTTTGCACGCCGTGGG

>AY593764.1_A_IRQ_1970

GGGTTGATCGTTGACACTAGAGATGTTGAAGAGCGTGTGCATGTCATGCGCAAAACCAAGCTTGCACCCA

CCGTGGCTCACGGTGTGTTTAATCCTGAATTTGGTCCCGCCGCCTTGTCCAACAAGGACCCGCGGCTGAA

TGAAGGTGTTGTCCTCGATGAAGTCATTTTCTCCAAACACAAAGGAGACACGAAAATGACCGAGGAGGAC

AAAGCGCTGTTCCGCCGCTGTGCTGCCGACTACGCGTCGCGCTTGCACAACGTGTTGGGTACGGCAAATG

CCCCACTGAGCATCTATGAGGCAATAAAAGGCGTCGACGGCCTTGACGCCATGGAACCAGACACTGCGCC

TGGCCTTCCCTGGGCCCTCCAGGGTAAGCGCCGCGGCGCGTTGATTGACTTCGAGAACGGCACGGTCGGG

CCCGAAGTCGCGGCTGCCTTAGAGCTCATGGAGAAAAGACAATACAAATTTGCTTGTCAGACCTTCCTGA

AGGACGAAATTCGCCCGATGGAAAAAGTACGTGCCGGCAAGACTCGCATCGTCGACGTTTTGCCTGTTGA

ACATATTCTTTACACCAGGATGATGATTGGCAGATTCTGTGCTCAGATGCACTCAAACAACGGACCGCAA

ATTGGCTCAGCGGTTGGCTGCAATCCAGATGTTGATTGGCAGAGATTTGGCACCCATTTTGCTCAGTACA

AAAACGTGTGGGATGTGGACTATTCGGCCTTTGATGCTAACCATTGCAGTGACGCAATGAACATCATGTT

TGAGGAAGTGTTCCGCACGGAGTTTGGTTTCCACCCAAATGCTGAGTGGATCCTGAAAACTCTCGTGAAC

ACGGAACACGCCTACGAGAACAAGCGCATCACTGTTGAGGGCGGGATGCCGTCTGGTTGTTCCGCAACAA

GCATCATCAACACAATTTTGAACAACATCTACGTGCTCTACGCCTTGCGTAGACACTATGAGGGAGTTGA

GCTGGACACCTACACCATGATCTCCTACGGAGACGACATAGTGGTGGCAAGTGATTACGATTTGGATTTT

GAGGCTCTTAAGCCGCACTTTAAATCTCTTGGTCAAACCATCACCCCAGCTGACAAAAGCGACAAAGGTT

TTGTTCTTGGTCACTCCATTACTGATGTCACTTTCCTCAAAAGACACTTCCGCATGGACTATGGAACTGG

GTTTTACAAACCTGTGATGGCTTCGAAGACCCTCGAGGCCATCCTCTCCTTTGCACGCCGTGGG

>AY593765.1_A_TUR_1965

GGGTTGATCGTTGACACTAGAGATGTTGAAGAGCGTGTGCATGTCATGCGCAAAACCAAGCTTGCACCCA

CCGTGGCTCACGGTGTGTTTAATCCTGAATTTGGTCCCGCCGCCTTGTCCAACAAGGACCCGCGGCTGAA

TGAAGGTGTTGTCCTCGATGAAGCCATTTTCTCCAAACACAAAGGAGACACGAAAATGACCGAGGAGGAC

AAAGCGCTGTTCCGCCGCTGTGCTGCCGACTACGCGTCGCGCTTGCACAACGTGTTGGGTACGGCAAATG

CCCCACTGAGCATCTACGAGGCAATAAAAGGCGTCGACGGCCTTGACGCCATGGAACCAGACACTGCGCC

TGGCCTTCCCTGGGCCCTCCAGGGTAAGCGCCGCGGCGCGTTGATTGACTTCGAGAACGGCACGGTCGGG

CCCGAAGTCGCGGCTGCCTTAGAGCTCATGGAGAAAAGACAATACAAATTTGCTTGCCAGACCTTCCTGA

AAGACGAAATTCGCCCGATGGAGAAAGTACGTGCCGGCAAGACTCGCATCGTCGATGTTTTGCCTGTTGA

ACATATTCTTTACACCAGGATGATGATTGGCAGATTCTGTGCTCAGATGCACTCAAACAACGGACCGCAA

ATTGGCTCAGCGGTTGGTTGTAATCCAGATGTTGATTGGCAGAGATTTGGTACCCATTTTGCTCAGTACA

AAAACGTGTGGGATGTGGACTATTCGGCCTTTGATGCTAACCATTGCAGTGACGCAATGAACATCATGTT

TGAGGAAGTGTTCCGCACGGAATTTGGTTTCCACCCAAATGCTGAGTGGATCCTGAAAACTCTCGTGAAC

ACGGAACACGCCTATGAGAACAAGCGCATCACTGTCGAGGGCGGGATGCCGTCTGGTTGTTCCGCAACAA

GCATCATCAACACAATTTTGAACAACATCTACGTGCTCTACGCCTTGCGTAGACACTATGAGGGAGTTGA

GCTGGACACCTACACCATGATCTCCTACGGAGATGACATAGTGGTGGCAAGTGATTACGATTTGGATTTT

GAGGCTCTTAAGCCGCACTTTAAATCTCTTGGTCAAACCATCACCCCAGCTGACAAAAGCGACAAAGGTT

TTGTTCTTGGTCACTCCATTACTGATGTCACTTTCCTCAAAAGACACTTCCGCATGGACTATGGAACTGG

GTTTTACAAACCTGTGATGGCTTCGAAGACCCTCGAGGCCATCCTCTCCTTTGCACGCCGTGGG

>AY593766.1_A_KEN_1965

GGGTTGATCGTTGACACCAGAGATGTGGAAGAACGCGTGCATGTCATGCGCAAAACCAAGCTTGCACCCA

CCGTAGCACACGGTGTGTTCAACCCTGAGTTCGGCCCCGCTGCCCTGTCCAACAAGGACCCGCGACTGAA

CGAAGGTGTTGTCCTCGATGAAGTCATCTTCTCCAAACACAAAGGAGACACAAAGATGACCGAGGAGGAC

AAAGCGCTGTTCCGTCGCTGTGCTGCTGACTACGCGTCGCGCTTGCACAGTGTACTGGGTACGGCAAATG

CCCCACTGAGCATTTACGAGGCAATCAAGGGCGTCGACGGCCTCGACGCCATGGAACCAGACACCGCGCC

TGGTCTCCCCTGGGCCCTCCAGGGGAAACGCCGTGGTGCGCTGATTGACTTCGAGAACGGCACGGTTGGA

CCCGAGGTCGCGGCTGCCCTGAAGCTCATGGAGAAAAGAGAGTACAAATTTGCTTGTCAGACCTTCCTGA

AGGACGAGATTCGCCCGATGGAGAAAGTACGTGCCGGCAAGACTCGCATTGTCGACGTTTTGCCTGTTGA

ACATATTCTTTACACTAGGATGATGATTGGTAGATTCTGTGCTCAAATGCACTCAAACAACGGACCGCAA

ATTGGCTCAGCGGTCGGTTGTAACCCAGATGTTGATTGGCAAAGATTTGGCACACACTTTGCCCAGTACA

GAAATGTGTGGGATGTGGACTATTCGGCCTTTGATGCCAACCATTGCAGCGATGCGATGAACATCATGTT

TGAGGAGGTGTTTCGCACGGATTTTGGTTTCCACCCGAACGCCGAGTGGATCCTGAAGACTCTCGTGAAT

ACGGAGCACGCCTATGAAAACAAACGCATCACTGTTGAGGGCGGGATGCCGTCTGGCTGTTCCGCAACAA

GCATCATCAACACAATTTTGAACAACATCTACGTGCTCTACGCCCTGCGTAGACACTATGAGGGAGTTGA

GCTGGACACTTACACCATGATCTCCTACGGAGATGACATCGTGGTGGCAAGTGATTACGATCTGGACTTT

GAGGCCCTTAGGCCACACTTCAAATCTCTTGGTCAAACCATTACCCCAGCTGACAAAAGCGACAAAGGTT

TTGTTCTTGGCCATTCCATCACCGACGTCACTTTCCTCAAAAGACACTTCCACATGGATTATGGAACTGG

GTTTTACAAACCTGTGATGGCCTCGAAGACCCTCGAGGCTATCCTCTCCTTTGCACGCCGTGGG

>AY593767.1_A_ARG_1965

GGGTTGATTGTGGACATCAGAGATGTGGAAGAGCGCGTTCACGTGATGCGCAAAACCAAGCTTGCACCCA

CCGTTGCACACGGTGTGTTCAACCCCGAGTTTGGGCCCGCTGCCTTGTCCAACAAGGACCCGCGTCTGAA

CGGGGGTGTTGTCCTCGATGAAGTCATCTTCTCCAAACACAAGGGAGACACAAAGATGTCTGAGGAGGAC

AAAGCGCTGTTCCGCCGCTGCGCTGCTGACTACGCGTCACGCCTGCACAGCGTGTTGGGCACAGCAAATG

CCCCACTGAGCATCTACGAGGCAATCAAGGGTGTCGACGGACTCGACGCCATGGAACCAGACACTGCGCC

CGGCCTCCCCTGGGCCCTCCAGGGTAAACGCCGCGGCGCGCTCATCGACTTCGAGAACGGCACGGTCGGA

CCCGAAGTCGAGGCTGCCCTGAAGCTCATGGAGAAGAGAGAATACAAATTTGTTTGTCAGACCTTCCTGA

AGGACGAGATCCGCCCGTTGGAGAAAGTACGTGCCGGCAAGACTCGCATTGTCGACGTCCTGCCCGTTGA

GCATATTCTTTACACCAGGATGATGATTGGCAGATTTTGTGCACAGATGCACTCAAACAACGGACCGCAA

ATTGGCTCAGCGGTCGGTTGCAACCCTGATGTTGATTGGCAGAGATTTGGCACACACTTCGCCCAGTACA

GAAACGTGTGGGATGTGGACTATTCGGCCTTTGATGCTAATCACTGCAGTGATGCCATGAACATCATGTT

TGAGGAGGTGTTTCGCACGGAGTTCGGCTTCCACCCGAATGCCGAGTGGATCCTGAAGACTCTTGTGAAC

ACGGAACACGCCTATGAGAACAAACGCATCACTGTTGAAGGCGGAATGCCGTCTGGTTGTTCCGCGACAA

GCATCATCAACACAATTTTGAACAACATCTACGTGCTTTACGCTCTGCGTAGACACTATGAGGGAGTTGA

GCTGGACACGTACACCATGATCTCCTACGGAGACGACATCGTGGTGGCAAGTGATTATGATTTGGACTTC

GAGGCTCTCAAGCCCCACTTTAAATCCCTTGGTCAAACCATCACTCCAGCTGACAAAAGCGACAAAGGTT

TTGTTCTTGGTCACTCCATTACCGATGTCACTTTCCTCAAAAGACACTTCCACATGGACTATGGAACTGG

GTTTTACAAACCTGTGATGGCCTCAAAGACCCTTGAGGCTATCCTCTCCTTTGCACGCCGTGGG

>AY593768.1_A_Brazil_1955

GGGTTGATTGTTGACACCAGAGATGTGGAAGAGCGCGTTCACGTGATGCGCAAAACCAAGCTTGCACCCA

CCGTTGCGCACGGTGTGTTCAACCCTGAGTTCGGGCCTGCCGCCTTGTCCAACAAGGACCCGCGCCTGAA

CGACGGTGTTGTCCTCGACGAAGTCATCTTCTCCAAACACAAGGGAGACACAAAGATGTCTGAGGAAGAC

AAAGCGCTGTTCCGCCGCTGTGCTGCTGACTACGCGTCACGCCTGCACAGCGTGTTGGGTACGGCAAATG

CCCCACTGAGCATCTACGAGGCAATTAAAGGCGTTGATGGACTCGACGCAATGGAACCAGACACCGCACC

CGGCCTCCCCTGGGCACTCCAGGGGAAGCGCCGTGGCGCGCTCATCGACTTCGAGAACGGCACTGTTGGA

CCCGAAGTTGAGGCTGCCTTGAAGCTCATGGAGAAAAGAGAATACAAGTTTGCTTGCCAAACCTTCCTGA

AGGACGAGATTCGCCCGATGGAGAAAGTACGTGCCGGTAAGACTCGCATTGTCGACGTCCTACCTGTTGA

ACACATCCTCTACACCAGGATGATGATTGGCAGATTTTGTGCACAAATGCACTCAAACAACGGACCCCAA

ATTGGCTCGGCGGTCGGTTGTAACCCTGATGTTGATTGGCAAAGATTTGGCACACACTTCGCCCAATACA

GAAACGTGTGGGATGTGGACTATTCGGCCTTCGATGCTAACCACTGCAGTGACGCCATGAACATCATGTT

TGAGGAAGTGTTTCGCACAGAATTCGGGTTCCACCCAAACGCTGAGTGGATCCTGAAGACTCTCGTGAAC

ACGGAACACGCCTATGAGAACAAACGCATCACTGTTGAAGGCGGGATGCCATCTGGTTGTTCCGCAACAA

GCATCATCAACACAATTTTGAACAACATCTACGTGCTCTACGCTTTGCGTAGACACTATGAGGGAGTTGA

GCTGGACACTTACACCATGATCTCTTACGGAGACGATATCGTGGTGGCAAGTGATTACGATTTGGACTTT

GAGGCTCTCAAGCCCCACTTCAAATCCCTTGGTCAAACCATCACTCCAGCTGACAAAAGCGACAAAGGTT

TTGTTCTTGGTCACTCCATTACTGATGTCACTTTCCTCAAAAGACACTTCCACATGGATTATGGAACTGG

GTTTTACAAACCTGTGATGGCCTCAAAGACCCTTGAGGCTATCCTCTCCTTTGCACGCCGTGGG

>AY593769.1_A_ARG_1959

GGGTTGATTGTGGACACCAGAGATGTGGAAGAGCGCGTCCACGTGATGCGCAAAACCAAGCTCGCACCCA

CCGTTGCACACGGTGTGTTCAACCCCGAGTTCGGGCCTGCCGCCTTGTCCAACAAGGACCCGCGCCTGAA

CGAGGGTGTTGTTCTCGATGAGGTCATCTTCTCCAAACACAAGGGAGACACAAAGATGTCTGAAGAGGAC

AAAGCGCTGTTCCGCCGCTGCGCCGCTGACTACGCGTCACGCCTGCACAGCGTGCTGGGCACAGCAAATG

CCCCACTGAGCATTTATGAGGCAATTAAGGGTGTCGACGGACTCGACGCCATGGAACCAGACACTGCACC

CGGCCTCCCCTGGGCCCTCCAGGGGAAACGTCGTGGTGCGCTCATCGACTTTGAGAACGGCACTGTCGGG

CCCGAAGTCGAGGCTGCCCTAAAGCTCATGGAGAAAAGAGAATACAAGTTTGCTTGTCAGACCTTCCTGA

AGGACGAAATCCGCCCGATGGAGAAAGTACGTGCCGGCAAGACTCGCATTGTCGATGTTTTGCCTGTTGA

ACACATTCTTTACACCAGGATGATGATTGGCAGATTCTGTGCACAAATGCACTCAAACAACGGACCGCAG

ATTGGCTCAGCGGTCGGCTGTAACCCTGATGTTGATTGGCAGAGATTTGGCACACACTTCGCCCAATACA

GAAACGTGTGGGATGTGGACTATTCGGCCTTCGATGCTAACCACTGCAGTGACGCAATGAACATCATGTT

CGAGGAGGTGTTTCGCACAGACTTTGGTTTCCACCCAAATGCTGAGTGGATTCTGAAGACTCTCGTGAAC

ACGGAGCACGCGTACGAGAACAAACGCATCACTGTTGAGGGCGGGATGCCGTCTGGCTGTTCCGCAACAA

GCATCATCAACACAATTCTGAACAACATCTACGTGCTCTACGCTCTGCGTAGACACTATGAGGGAGTTGA

GCTGGACACTTACACCATGATCTCATACGGAGACGACATCGTGGTGGCAAGTGATTACGATTTGGACTTC

GAGGCTCTCAAGCCTCACTTTAAATCCCTTGGTCAAACCATCACTCCAGCTGACAAAAGCGACAAAGGTT

TTGTTCTTGGTCACTCCATTACCGATGTCACTTTCCTCAAAAGACACTTCCACATGGATTATGGAACTGG

GTTTTACAAACCTGTGATGGCCTCAAAGACCCTTGAGGCTATCCTCTCCTTTGCACGCCGTGGG

>AY593770.1_A_ARG_1966

GGGCTGATCGTTGACACCAGAGATGTGGAAGAGCGCGTCCACGTGATGCGCAAAACCAAGCTTGCACCCA

CCGTCGCGCACGGTGTTTTCAACCCTGAGTTTGGGCCCGCCGCCTTGTCCAACAAGGACCCGCGCCTGAA

TGAAGGTGTTGTCCTCGATGAGGTCATCTTCTCCAAGCACAAGGGAGACACAAAGATGTCTGAGGAGGAC

AAAGCGCTGTTCCGCCGCTGTGCTGCTGACTACGCGTCACGTTTGCATAGCGTGCTGGGTACAGCAAACG

CCCCATTGAGCATCTACGAGGCAATCAAGGGCGTTGACGGACTCGACGCCATGGAACCAGACACCGCGCC

TGGCCTTCCTTGGGCCCTCCAGGGGAAACGCCGTGGTGCGCTCATCGACTTCGAGAACGGCACAGTCGGA

CCCGAAGTTGATGCTGCCTTGAAGCTTATGGAGAAAAGAGAATACAAGTTTGCTTGCCAAACCTTCCTGA

AGGACGAGATTCGCCCGATGGAGAAGGTGCGCGCCGGCAAGACTCGCATTGTCGACGTTTTGCCTGTTGA

ACATATTCTTTACACCAGGATGATGATTGGTAGGTTCTGTGCACAAATGCACTCAAACAACGGACCGCAA

ATTGGCTCGGCGGTCGGTTGTAACCCTGATGTTGATTGGCAAAGATTTGGCACACACTTCGCCCAATACA

GAAACGTGTGGGATGTGGACTATTCGGCCTTTGATGCCAACCACTGCAGTGACGCAATGAACATCATGTT

TGAGGAGGTGTTCCGCACGGACTTTGGTTTCCACCCGAACGCCGAGTGGATTCTGAAGACTCTCGTGAAC

ACGGAGCACGCCTATGAGAACAAACGCATCACTGTTGAGGGCGGGATGCCATCCGGTTGTTCCGCGACAA

GCATCATCAACACAATTTTGAACAACATTTACGTGCTTTACGCCCTGCGTAGGCACTATGAAGGAGTTGA

GCTGGACACTTACACCATGATCTCTTACGGAGACGACATCGTGGTGGCAAGTGATTACGATTTGGACTTT

GAGGCTCTCAAGCCTCACTTCAAATCCCTTGGTCAGACCATCACTCCAGCTGACAAAAGTGACAAAGGTT

TCGGTCTTGGTCACTCCATCACCGATGTCACTTTCCTCAAAAGACACTTCCACATGGATTACGGAACTGG

GTTTTACAAACCTGTGATGGCCTCAAAGACCCTTGAGGCTATCCTCTCCTTTGCACGCCGTGGG

>AY593771.1_A_COL_1967

GGGTTGATTGTAGACACCAGAGATGTGGAAGAGCGCGTCCACGTGATGCGCAAAACCAAGCTTGCACCCA

CCGTTGCACACGGTGTGTTCAACCCTGAGTTTGGGCCAGCTGCCTTGTCCAACAAGGACCCGCGTCTGAA

CGAGGGTGTAGTCCTCGATGAAGTCATTTTCTCCAAACACAAAGGAGACACAAAGATGTCTGAGGAGGAC

AAAGCGCTGTTCCGCCGATGTGCTGCTGACTACGCGTCACGCCTGCACAGCGTGCTGGGTACGGCAAATG

CCCCATTGAGCATTTACGAGGCAATCAAGGGCGTCGACGGACTTGACGCCATGGAACCAGACACCGCGCC

TGGTCTTCCCTGGGCTCTCCAGGGAAAACGCCGAGGTGCACTCATTGACTTCGAGAACGGCACTGTCGGA

CCTGAAGTCGCAGAAGCCTTGAAGCTCATGGAGAAAAGAGAGTACAAGTTTGCTTGCCAGACCTTCCTGA

AGGACGAGATTCGCCCGATGGAGAAAGTACGTGCCGGCAAGACTCGCATTGTCGACGTCCTGCCCGTTGA

ACACATTCTTTACACCAGAATGATGATTGGCAGATTCTGCGCACAAATGCACTCAAACAACGGACCGCAA

ATTGGCTCGGCGGTCGGTTGCAACCCTGATGTTGATTGGCAAAGATTTGGCACACACTTCGCCCAGTACA

GAAATGTGTGGGATGTGGACTACTCGGCCTTTGATGCTAACCATTGTAGTGATGCCATGAACATCATGTT

TGAGGAGGTGTTCCGCACGGAATTCGGGTTCCACCCAAACGCTGAGTGGATCTTGAAGACTCTCGTGAAC

ACGGAGCACGCCTATGAGAACAAACGCATCACTGTTGAAGGCGGGATGCCATCCGGCTGTTCCGCAACGA

GCATCATCAACACAATTTTGAACAACATCTACGTGCTCTACGCCTTGCGTAGACACTATGAGGGGGTTGA

GCTGGATACTTACACCATGATCTCCTATGGAGACGACATTGTGGTGGCAAGTGATTACGATTTGGACTTT

GAGGCTCTCAAGCCTCACTTTAAATCCCTTGGTCAAACCATCACTCCAGCTGACAAAAGCGACAAAGGTT

TTGTTCTTGGTCACTCCATCACCGATGTCACTTTCCTCAAAAGACACTTCCACATGGACTATGGAACTGG

GTTTTACAAACCTGTGATGGCCTCAAAGACCCTTGAGGCTATCCTCTCCTTTGCACGCCGTGGG

>AY593772.1_A_TUR_1972

GGGTTGATCGTTGACACCAGAGATGTTGAGGAGCGCGTGCATGTCATGCGCAAAACCAAGCTTGCACCCA

CCGTGGCCCACGGTGTGTTCAACCCTGAATTTGGTCCCGCCGCCTTGTCCAGCAAGGACCCGCGACTGAA

TGAAGGTGTTGTCCTCGATGAGGTCATTTTCTCCAAACACAAGGGAGACACAAAAATGACCGAGGAGGAC

AAAGCGCTGTTCCGCCGCTGTGCTGCCGACTACGCGTCACGCTTGCACAACGTGTTGGGTACGGCAAATG

CCCCACTGAGCATCTATGAGGCGATAAAAGGCGTTGACGGCCTCGACGCCATGGAACCAGACACCGCGCC

TGGCCTTCCCTGGGCCCTCCAGGGTAAACGCCGCGGTGCGTTGATTGACTTTGAGAACGGCACGGTCGGG

CCCGAAGTCGCGGCTGCCTTAGAGCTCATGGAGAAAAGACAATACAAATTTACTTGTCAGACCTTCCTGA

AAGACGAAGTTCGTCCGATGGAGAAAGTACGTGCCGGCAAGACTCGCATCGTCGATGTTTTGCCTGTTGA

ACACATTCTTTACACCAGGATGATGATTGGCAGATTTTGTGCCCAGATGCACTCAAACAACGGACCGCAA

ATTGGCTCGGCGGTTGGTTGCAATCCAGATGTTGATTGGCAGAGATTTGGTACCCATTTTGCTCAGTACA

AAAACGTGTGGGATGTGGACTATTCGGCCTTTGATGCTAACCATTGTAGTGACGCAATGAACATCATGTT

TGAGGAAGTGTTCCGCACGGAATTTGGTTTCCACCCAAATGCTGAGTGGATCCTGAAAACTCTTGTGAAC

ACGGAACACGCCTACGAGAACAAGCGCATCACTATCGAGGGCGGGATGCCGTCTGGTTGTTCCGCAACAA

GCATCATCAACACAATCTTGAACAACATCTACGTGCTCTACGCCTTGCGTAGGCACTATGAGGGAGTTGA

GCTGGACACCTACACCATGATCTCCTACGGAGATGACATAGTGGTGGCAAGTGATCACGATTTGGATTTT

GAGGCTCTCAAGCCGCACTTTAAATCTCTTGGTCAAACCATCACCCCAGCTGACAAAAGCGACAAAGGTT

TTGTTCTTGGTCACTCCATCACCGATGTCACTTTCCTCAAAAGACACTTCCACATGGACTATGGAACTGG

GTTTTACAAACCTGTGATGGCTTCGAAGACCCTCGAGGCCATCCTCTCCTTTGCACGCCGTGGG

>AY593773.1_A_PER_1969

GGGTTGATTGTGGACACCAGAGACGTGGAGGAGCGCGTCCACGTGATGCGCAAAACCAAGCTTGCACCCA

CCGTTGCACACGGTGTGTTCAACCCCGAGTTTGGGCCCGCTGCCTTGTCCAACAAGGACTCGCGTCTGAA

CGAGGGTGTTGTCCTCGATGAAGTCATCTTCTCCAAACACAAGGGAGACACAAAGATGACTGAGGAGGAT

AAAGCGCTGTTCCGCCGCTGCGCTGCTGACTACGCGTCACGCCTGCACAGCGTTCTGGGCACAGCAAATG

CCCCATTGAGCATCTACGAGGCAATTAAGGGTGTTGACGGACTCGACGCTATGGAACCAGACACTGCGCC

TGGCCTCCCCTGGGCCCTCCAGGGTAAACGCCGCGGTGCACTCATCGACTTTGAGAACGGCACGGTCGGA

CCCGAAGTCGAGGCTGCCCTGAAGCTCATGGAGAAGAGAGAATACAAATTTGCTTGTCAGACCTTCCTGA

AGGACGAGATTCGCCCGATGGAGAAAGTACGTGCCGGCAAGACTCGCATTGTCGACGTCCTGCCCGTTGA

ACACATTCTTTACACCAGGATGATGATTGGCAGATTCTGTGCACAAATGCACTCAAACAACGGACCGCAA

ATTGGCTCAGCGGTTGGTTGTAACCCTGATGTTGATTGGCAGAGATTTGGCACACACTTCGCCCAATACA

GAAACGTGTGGGATGTGGACTATTCGGCCTTTGATGCTAACCACTGCAGTGACGCCATGAACATCATGTT

TGAGGAAGTGTTTCGCACGGAGTTCGGTTTCCACCCGAATGCCGAGTGGATTCTGAAGACTCTTGTGAAC

ACGGAACACGCCTATGAGAACAAACGCATCACTGTTGAAGGCGGAATGCCGTCTGGCTGTTCTGCAACAA

GCATCATCAACACAATCTTGAACAACATCTATGTGCTCTACGCCCTGCGTAGACACTATGAGGGAGTTGA

GCTGGACACATACACCATGATCTCCTACGGAGACGACATCGTGGTGGCAAGTGATTATGATTTGGACTTC

GAGGCTCTCAAGCCTCACTTTAAATCCCTTGGTCAAACCATTACTCCAGCTGACAAAAGCGACAAAGGTT

TTGTTCTTGGTCACTCCATTACCGACGTCACTTTCCTCAAAAGACATTTCCACATGGACTATGGAACTGG

GTTTTACAAACCTGTGATGGCCTCAAAGACCCTTGAGGCTATCCTCTCCTTTGCACGCCGTGGG

>AY593774.1_A_SPA_1969

GGGTTGATTGTTGATACCAGAGATGTGGAAGAGCGCGTCCACGTGATGCGCAAAACAAAGCTTGCACCCA

CCGTTGCACACGGTGTGTTCAACCCTGAGTTTGGGCCTGCCGCCTTGTCAAACAAGGACCCGCGCCTGAA

CGAGGGAGTTGTTCTCGATGAAGTCATTTTCTCCAAACACAAAGGAGACGCAAAGATGACCGAAGAGGAC

AAAGCGCTGTTCCGCCGCTGCGCCGCTGACTACGCGTCACGCCTGCACAGCGTGCTGGGTACGGCAAATG

CCCCATTGAGCATCTACGAGGCAATCAAGGGCGTTGACGGACTCGACGCCATGGAGCCGGACACTGCACC

TGGCCTCCCCTGGGCCCTCCAAGGAAAACGCCGCGGTGCGCTCATCGACTTCGAGAACGGCACGGTCGGA

CCCGAAGTTGAGGCTGCCTTGAAGCTCATGGAGAAAAGAGAATACAAGTTTGTTTGTCAGACCTTCCTGA

AGGACGAGATTCGCCCGATGGAGAAAGTACGCGCCGGCAAGACTCGCATCGTCGATGTTTTGCCTGTTGA

ACACATTCTTTACACCAGGATGATGATTGGCAGGTTCTGTGCACAAATGCACTCAAACAACGGACCACAA

ATTGGCTCTGCGGTCGGTTGCAACCCTGACGTTGATTGGCAAAGATTTGGCACACATTTCGCTCAATACA

GAAACGTGTGGGATGTGGATTACTCGGCCTTTGATGCAAACCACTGCAGTGACGCCATGAACATCATGTT

TGAGGAGGTGTTCCGCACAGACTTTGGCTTCCACCCAAATGCTGAGTGGATCCTGAAGACTCTCGTGAAC

ACGGAACACGCCTATGAGAACAAGCGCATCACTGTTGAAGGCGGGATGCCATCTGGTTGTTCCGCAACAA

GCATCATCAACACAATTTTGAACAACATCTACGTGCTCTACGCCTTGCGTAGACACTATGAGGGAGTTGA

GCTGGACACTTACACCATGATCTCCTACGGAGACGACATCGTGGTGGCAAGTGATTACGATCTGGACTTT

GAGGCTCTCAAGCCCCACTTCAAATCTCTTGGCCAAACCATCACTCCAGCTGACAAAAGCGACAAAGGTT

TTGTTCTTGGTCACTCCATTACCGATGTCACTTTCCTCAAAAGACACTTCCACATGGATTATGGAACTGG

GTTTTACAAACCTGTGATGGCCTCAAAGACCCTTGAGGCTATCCTCTCCTTTGCACGCCGTGGG

>AY593775.1_A_VEN_1970

GGGTTGATTGTGGACACCAGAGACGTGGAGGAGCGCGTCCACGTGATGCGCAAAACCAAGCTTGCACCCA

CCGTTGCACACGGTGTGTTCAACCCCGAGTTTGGGCCCGCTGCCTTGTCCAACAAGGACTCGCGTCTGAA

CGAGGGTGTTGTCCTTGATGAAGTCATCTTCTCCAAACACAAGGGAGACACAAAGATGACTGAGGAGGAT

AAAGCGCTGTTCCGCCGCTGCGCTGCTGACTACGCGTCACGCCTGCACAGCGTTCTGGGCACAGCAAATG

CCCCATTGAGCATCTACGAGGCAATTAAGGGTGTTGACGGACTCGACGCTATGGAACCAGACACTGCGCC

TGGCCTCCCCTGGGCCCTCCAGGGTAAACGCCGCGGTGCACTCATCGACTTTGAGAACGGCACGGTCGGA

CCCGAAGTCGAGGCTGCCCTGAAGCTCATGGAGAAGAGAGAATACAAATTTGCTTGTCAGACCTTCCTGA

AGGACGAGATTCGCCCGATGGAGAAAGTACGTGCCGGCAAGACGCGCATTGTCGACGTCCTGCCCGTTGA

ACACATTCTTTACACCAGGATGATGATTGGCAGATTCTGTGCACAAATGCACTCAAACAACGGACCGCAA

ATTGGCTCAGCGGTTGGTTGTAACCCTGATGTTGATTGGCAGAGGTTTGGCACACACTTCGCCCAATACA

GAAACGTGTGGGATGTGGACTATTCGGCCTTTGATGCTAACCACTGCAGTGACGCCATGAACATCATGTT

TGAGGAAGTGTTTCGCACGGAGTTCGGTTTCCACCCGAATGCCGAGTGGATTCTGAAGACTCTTGTGAAC

ACGGAACACGCCTATGAGAACAAACGCATCACTGTTGAAGGCGGAATGCCGTCTGGCTGTTCTGCAACAA

GCATCATCAACACAATCTTGAACAACATCTATGTGCTCTACGCCCTGCGTAGACACTATGAGGGAGTTGA

GCTGGACACATACACCATGATCTCCTACGGAGACGACATCGTGGTGGCAAGTGATTATGATTTGGACTTC

GAGGCTCTCAAGCCTCACTTTAAATCCCTTGGTCAAACCATTACTCCAGCTGACAAAAGCGACAAAGGTT

TTGTTCTTGGTCACTCCATTACCGACGTCACTTTCCTTAAAAGACATTTCCACATGGACTATGGAACTGG

GTTTTACAAACCTGTGATGGCCTCAAAGACCCTTGAGGCTATCCTCTCCTTTGCACGCCGTGGG

>AY593776.1_A_GER_1968

GGGTTGATTGTAGACACCAGAGATGTGGAAGAGCGCGTCCACGTGATGCGCAAAACCAAGCTTGCACCCA

CCGTTGCACACGGTGTGTTCAACCCCGAGTTTGGGCCAGCTGCCTTGTCCAACAAGGACCCGCGTCTGAA

CGAGGGTGTTGTCCTCGATGAAGTCATTTTCTCCAAACACAAGGGAGACACAAAGATGTCTGAGGAGGAC

AAAGCGCTGTTCCGCCGATGTGCAGCTGATTACGCGTCGCGCCTGCACAGCGTGCTGGGTACGGCAAATG

CCCCATTGAGCATCTACGAGGCAATCAAAGGCGTCGACGGACTCGACGCCATGGAGCCAGACACCGCACC

TGGCCTCCCCTGGGCTCTCCAGGGAAAACGCCGTGGTGCGCTCATCGACTTTGAGAACGGCACGGTCGGG

CCCGAAGTCGAGGATGCCTTGAAGCTCATGGAGAAAAGAGAGTACAAGTTTGTTTGTCAGACCTTCCTGA

AGGACGAGATTCGCCCGATGGAGAAAGTACGTGCCGGCAAGACTCGCATTGTCGACGTCCTGCCCGTTGA

ACACATTCTTTACACCAGGATGATGATTGGCAGATTTTGTGCACAAATGCACTCAAACAACGGACCGCAA

ATTGGCTCGGCGGTCGGTTGCAACCCTGATGTTGATTGGCAAAGATTTGGCACACATTTCGCCCAGTACA

GAAACGTGTGGGATGTGGATTATTCGGCCTTTGATGCTAACCACTGTAGTGACGCCATGAACATCATGTT

TGAGGAGGTGTTCCGCACAGACTTTGGCTTCCACCCAAATGCAGAGTGGATCCTGAAGACTCTCGTGAAC

ACGGAACACGCCTATGAGAACAAGCGCATCACTGTTGAAGGCGGGATGCCATCTGGTTGTTCCGCAACAA

GCATCATCAACACAATTTTGAACAACATCTACGTGCTCTACGCCTTGCGTAGACACTATGAGGGAGTTGA

GCTGGACACCTACACCATGATCTCCTACGGAGACGACATCGTGGTGGCAAGTGACTACGATCTGGACTTT

GAGGCTCTCAAGCCCCACTTCAAATCTCTTGGCCAAACTATCACTCCAGCTGACAAAAGCGACAAAGGTT

TTGTTCTTGGACACTCCATTACTGATGTCACCTTCCTCAAAAGACACTTCCACATGGATTATGGGACTGG

GTTTTACAAACCTGTGATGGCCTCAAAGACCCTTGAGGCCATCCTCTCCTTTGCACGCCGTGGG

>AY593777.1_A_GER_1972

GGGTTGATTGTTGATACCAGAGATGTGGAAGAGCGCGTCCACGTGATGCGCAAAACAAAGCTTGCACCCA

CCGTTGCACACGGTGTGTTCAACCCTGAGTTTGGGCCTGCCGCCTTGTCAAACAAGGACCCGCGCCTGAA

CGAGGGAGTTGTTCTCGATGAAGTCATTTTCTCCAAACACAAAGGAGACGCAAAGATGACCGAAGAGGAC

AAAGCGCTGTTCCGCCGCTGCGCCGCTGACTACGCGTCACGCCTGCACAGCGTGCTGGGTACGGCAAATG

CCCCATTGAGCATCTACGAGGCAATCAAGGGCGTTGACGGACTCGACGCCATGGAGCCGGACACTGCACC

TGGCCTCCCCTGGGCCCTCCAAGGAAAACGCCGCGGTGCGCTCATCGACTTCGAGAACGGCACGGTCGGA

CCCGAAGTTGAGGCTGCCTTGAAGCTCATGGAGAAAAGAGAATACAAGTTTGTTTGTCAGACCTTCCTGA

AGGACGAGATTCGCCCGATGGAGAAAGTACGCGCCGGCAAGACTCGCATCGTCGATGTTTTGCCTGTTGA

ACACATTCTTTACACCAGGATGATGATTGGCAGGTTCTGTGCACAAATGCACTCAAACAACGGACCACAA

ATTGGCTCTGCGGTCGGTTGCAACCCTGACGTTGATTGGCAAAGATTTGGCACACATTTCGCTCAATACA

GAAACGTGTGGGATGTGGATTACTCGGCCTTTGATGCAAACCACTGCAGTGACGCCATGAACATCATGTT

TGAGGAGGTGTTCCGCACAGACTTTGGCTTCCACCCAAATGCTGAGTGGATCCTGAAGACTCTCGTGAAC

ACGGAACACGCCTATGAGAACAAGCGCATCACTGTTGAAGGCGGGATGCCATCTGGTTGTTCCGCAACAA

GCATCATCAACACAATTTTGAACAACATCTACGTGCTCTACGCCTTGCGTAGACACTATGAGGGAGTTGA

GCTGGACACTTACACCATGATCTCCTACGGAGACGACATCGTGGTGGCAAGTGATTACGATCTGGACTTT

GAGGCTCTCAAGCCCCACTTCAAATCTCTTGGCCAAACCATCACTCCAGCTGACAAAAGCGACAAAGGTT

TTGTTCTTGGTCACTCCATTACCGATGTCACTTTCCTCAAAAGACACTTCCACATGGATTATGGAACTGG

GTTTTACAAACCTGTGATGGCCTCAAAGACCCTTGAGGCTATCCTCTCCTTTGCACGCCGTGGG

>AY593778.1_A_SPA_1969

GGGTTGATTGTAGACACCAGAGATGTGGAAGAGCGCGTCCACGTGATGCGCAAAACCAAGCTTGCACCCA

CCGTTGCACACGGTGTGTTCAACCCCGAGTTTGGGCCAGCTGCCTTGTCCAACAAGGACCCGCGTCTGAA

CGAGGGTGTTGTCCTTGATGAAGTCATTTTCTCCAAACACAAGGGAGACACAAAGATGTCTGAGGAGGAC

AAAGCGCTGTTCCGCCGATGTGCTGCTGACTACGCGTCACGCCTGCACAGCGTGCTGGGTACGGCAAATG

CCCCATTGAGCATCTACGAGGCAGTCAAGGGCGTCGACGGACTCGACGCCATGGAGCCAGACACCGCACC

TGGCCTCCCCTGGGCTCTCCAGGGAAAACGCCGTGGTGCGCTCATCGACTTTGAGAACGGCACGGTCGGG

CCCGAAGTCGAGGCTGCCTTGAAGCTCATGGAGAAAAGAGAGTACAAGTTTGTTTGTCAGACCTTCCTGA

AGGACGAGATTCGCCCGATGGAGAAAGTACGTGCCGGCAAGACTCGCATTGTCGACGTCCTGCCCGTTGA

ACACATTCTTTACACCAGGATGATGATTGGCAGATTTTGTGCACAAATGCACTCAAACAACGGACCGCAA

ATTGGCTCGGCGGTCGGTTGTAACCCTGATGTTGATTGGCAAAGATTTGGCACACACTTCGCCCAATACA

GAAACGTGTGGGATGTGGACTATTCGGCCTTTGATGCTAACCACTGCAGTGACGCCATGAACATCATGTT

TGAGGAGGTGTTCCGCACGGACTTCGGGTTCCACCCAAATGCTGAGTGGATCTTGAAGACTCTCGTGCAC

ACGGAACATGCCTATGAGAACAAACGCATCACTGTTGAAGGCGGGATGCCATCTGGTTGTTCCGCAACGA

GCATCATCAACACAATTTTGAACAACATCTACGTGCTCTACGCCTTGCGTAGACACTATGAGGGAGTTGA

GCTGGACACTTACACCATGATCTCCTACGGAGACGACATTGTGGTGGCAAGTGATTATGATCTGGACTTT

GAGGCTCTCAAGCCTCACTTCAAATCTCTTGGTCAAACCATTACTCCAGCTGACAAAAGCGACAAAGGTT

TTGTTCTTGGTCACTCCATCACCGATGTCACTTTCCTCAAAAGACACTTCCACATGGATTATGGAACCGG

GTTTTACAAACCTGTGATGGCCTCAAAGACCCTTGAGGCTATCCTCTCCTTTGCACGCCGTGGG

>AY593779.1_A_GER_1972

GGGTTGATTGTTGATACCAGAGATGTGGAAGAGCGCGTCCACGTGATGCGCAAAACAAAGCTTGCACCCA

CCGTTGCACACGGTGTGTTCAACCCTGAGTTTGGGCCTGCCGCCTTGTCAAACAAGGACCCGCGCCTGAA

CGAGGGAGTTGTTCTCGATGAAGTCATTTTCTCCAAACACAAAGGAGACGCAAAGATGACCGAAGAGGAC

AAAGCGCTGTTCCGCCGCTGCGCCGCTGACTACGCGTCACGCCTGCACAGCGTGCTGGGTACGGCAAATG

CCCCATTGAGCATCTACGAGGCAATCAAGGGCGTTGACGGACTCGACGCCATGGAGCCGGACACTGCACC

TGGCCTCCCCTGGGCCCTCCAAGGAAAACGCCGCGGTGCGCTCATCGACTTCGAGAACGGCACGGTCGGA

CCCGAAGTTGAGGCTGCCTTGAAGCTCATGGAGAAAAGAGAATACAAGTTTGTTTGTCAGACCTTCCTGA

AGGACGAGATTCGCCCGATGGAGAAAGTACGCGCCGGCAAGACTCGCATCGTCGATGTTTTGCCTGTTGA

ACACATTCTTTACACCAGGATGATGATTGGCAGGTTCTGTGCACAAATGCACTCAAACAACGGACCACAA

ATTGGCTCTGCGGTCGGTTGCAACCCTGACGTTGATTGGCAAAGATTTGGCACACATTTCGCTCAATACA

GAAACGTGTGGGATGTGGATTACTCGGCCTTTGATGCAAACCACTGCAGTGACGCCATGAACATCATGTT

TGAGGAGGTGTTCCGCACAGACTTTGGCTTCCACCCAAATGCTGAGTGGATCCTGAAGACTCTCGTGAAC

ACGGAACACGCCTATGAGAACAAGCGCATCACTGTTGAAGGCGGGATGCCATCTGGTTGTTCCGCAACAA

GCATCATCAACACAATTTTGAACAACATCTACGTGCTCTACGCCTTGCGTAGACACTATGAGGGAGTTGA

GCTGGACACTTACACCATGATCTCCTACGGAGACGACATCGTGGTGGCAAGTGATTACGATCTGGACTTT

GAGGCTCTCAAGCCCCACTTCAAATCTCTTGGCCAAACCATCACTCCAGCTGACAAAAGCGACAAAGGTT

TTGTTCTTGGTCACTCCATTACCGATGTCACTTTCCTCAAAAGACACTTCCACATGGATTATGGAACTGG

GTTTTACAAACCTGTGATGGCCTCAAAGACCCTTGAGGCTATCCTCTCCTTTGCACGCCGTGGG

>AY593780.1_A_FRA_1960

GGGTTGATTGTAGACACCAGAGATGTGGAAGAGCGCGTCCACGTAATGCGCAAGACCAAGCTTGCACCCA

CCGTTGCACACGGTGTGTTTAACCCCGAGTTTGGGCCAGCTGCCTTGTCCAACAAGGACCCGCGTCTGAA

CGAGGGTGTTGTCCTTGATGAAGTCATTTTCTCCAAACACAAGGGAGACACAAAGATGTCTGAGGAGGAC

AAAGCGCTGTTCCGTCGATGTGCTGCTGACTACGCGTCACGCCTGCACAGCGTGCTGGGTACGGCAAACG

CCCCATTGAGCATCTACGAGGCAATCAAAGGCGTCGACGGACTCGACGCCATGGAGCCAGACACCGCACC

TGGCCTCCCCTGGGCTCTCCAGGGAAAACGCCGTGGTGCGCTCATCGACTTTGAGAACGGCACGGTCGGG

CCCGAAGTCGAGGCTGCCTTGAAGCTCATGGAGAGAAGAGAGTACAAGTTTGTTTGTCAGACCTTCCTGA

AGGACGAGATTCGCCCGATGGAGAAAATACGTGCCGGCAAGACTCGCATTGTCGACGTCCTGCCCGTTGA

ACACATTCTTTACACCAGGATGATGATTGGCAGATTTTGTGCACAAATGCACTCAAACAACGGACCGCAA

ATTGGCTCGGCGGTAGGTTGTAACCCTGATGTTGATTGGCAAAGATTTGGCACACACTTCGCCCAATACA

GAAATGTGTGGGATGTGGACTATTCGGCCTTTGATGCTAACCACTGCAGTGACGCCATGAACATCATGTT

TGAGGAGGTGTTCCACACGGACTTCGGGTTCCACCCAAATGCTGAGTGGATCTTGAAGACTCTCGTGAAC

ACGGAACATGCCTATGAGAACAAACGCATCACTGTTGAAGGCGGGATGCCATCTGGTTGTTCCGCAACGA

GCATCATCAACACAATTTTGAACAACATCTACGTGCTCTACGCCTTGCGTAGACACTATGAGGGAGTTGA

GCTGGACACTTACACCATGATCTCCTACGGAGACGACATTGTGGTGGCAAGTGATTACGATCTGGACTTT

GAGGCTCTCAAGCCCCACTTCAAATCTCTTGGTCAAACCATTACTCCAGCTGACAAAAGCGACAAAGGTT

TTGTTCTTGGTCACTCCATCACCGATGTCACTTTCCTCAAAAGACACTTCCACATGGATTATGGAACTGG

GTTTTACAAACCTGTGATGGCCTCAAAGACCCTTGAGGCTATCCTCTCCTTTGCACGCCGTGGG

>AY593781.1_A_GER_1951

GGGTTGATTGTAGACACCAGAGATGCGGAAGAGCGCGTCCACGTGATGCGCAAAACCAAGCTTGCACCCA

CCGTTGCACACGGTGTGTTCAACCCCGAGTTTGGGCCAGCTGCCTTGTCCAACAAGGACCCGCGTCTGAA

CGAGGGTGTTGTCCTCGATGAAGTCATTTTCTCCAAACACAAGGGAGACACAAAGATGTCTGAGGAGGAC

AAAGCGCTGTTCCGCCGATGTGCTGCTGACTACGCGTCACGCCTGCACAGCGTGCTGGGTACGGCAAATG

CCCCATTGAGCATCTACGAGGCAATCAAAGGCGTCGACGGACTCGACGCCATGGAGCCAGACACCGCACC

TGGCCTCCCCTGGGCTCTCCAGGGAAAACGCCGTGGTGCGCTCATCGACTTTGAGAACGGCACGGTCGGG

CCCGAAGTCGAGGCTGCCTTGAAGCTCATGGAGAAAAGAGAGTACAAGTTTGTTTGTCAGACCTTCCTGA

AGGACGAGATTCGCCCGATGGAGAAAGTACGTGCCGGCAAGACTCGCATTGTCGACGTCCTGCCCGTTGA

ACACATTCTTTACACCAGGATGATGATTGGCAGATTTTGTGCACAAATGCACTCAAACAACGGACCGCAA

ATTGGCTCGGCGGTCGGTTGTAACCCTGATGTTGATTGGCAAAGATTTGGCACACACTTCGCCCAATACA

GAAACGTGTGGGATGTGGACTATTCGGCCTTTGATGCTAACCACTGCAGTGACGCCATGAACATCATGTT

TGAGGAGGTGTTCCGCACGGACTTCGGGTTCCACCCAAATGCTGAGTGGATCTTGAAGACTCTCGTGAAC

ACGGAACATGCCTATGAGAACAAACGCCACACTGTTGAAGGCGGGATGCCATCTGGTTGTTCCGCAACGA

GCATCATCAACACAATTTTGAACAACATCTACGTGCTCTACGCCTTGCGTAGACACTATGAGGGAGTTGA

GCTGGACACTTACACCATGATCTCCTACGGAGACGACATTGTGGTGGCAAGTGATTACGATCTGGACTTT

GAGGCTCTCAAGCCTCACTTCAAATCTCTTGGTCAAACCATTACTCCAGCTGACAAAAGCGACAAAGGTT

TTGTTCTTGGTCACTCCATCACCGATGTCACTTTCCTCAAAAGACACTTCCACATGGATTATGGAACTGG

GTTTTACAAACCTGTGATGGCCTCAAAGACCCTTGAGGCTATCCTCTCCTTTGCACGCCGTGGG

>AY593782.1_A_ARG_2000

GGTTTGATAGTTGACACCAGAGATGTGGAAGAGCGCGTCCACGTGATGCGCAAAACCAAGCTTGCACCCA

CCGTTGCACACGGTGTGTTCAACCCTGAATTCGGGCCCGCTGCCTTGTCCAACAAGGACCCGCGCCTGAA

CGAGGGTGTTGTCCTCGATGAAGTCATCTTTTCCAAACACAAGGGAGACACAAGGATGTCTGAGGAGGAT

AAAGCGCTGTTCCGCCGCTGCGCGGCTGACTACGCGTCACGCCTGCACAGTGTGCTGGGTACAGCAAATG

CCCCATTGAGTATTTACGAGGCGATCAAGGGCGTCGACGGGCTCGACGCCATGGAACCAGACACCGCACC

CGGTCTCCCTTGGGCCCTCCAGGGGAAGCGCCGCGGCGCACTCATCGACTTTGAGAACGGCACGGTCGGA

CCTGAAGTCGAGGCTGCCTTGAAACTTATGGAGAAAAGAGAATACAAGTTTGCTTGCCAGACCTTCCTGA

AGGACGAAATTCGCCCGATGGAGAAAGTGCGCGCCGGCAAGACTCGCATCGTCGATGTCTTGCCTGTTGA

ACATATTCTTTACACCAGGATGATGATTGGCAGATTTTGTGCACAAATGCACATGAACAACGGTCCGCAG

ATTGGCTCGGCGGTCGGTTGTAACCCTGATGTTGATTGGCAAAGATTCGGCACACACTTCGCCCAATACA

AAAACGTGTGGGATGTGGACTATTCGGCCTTTGATGCTAACCATTGCAGTGACGCCATGAACATCATGTT

TGAGGAGGTGTTCCGCACGGATTTCGGGTTCCACCCAAACGCTGAGTGGATTCTGAAGACCCTTGTGAAC

ACGGAACACGCTTATGAAAACAAACGCATCACTGTTGAGGGCGGGATGCCGTCTGGCTGTTCCGCGACAA

GCATCATCAACACAATTATGAACAACATCTACGTGCTCTACGCCCTGCGCAGACACTATGAGGGAGTTGA

GCTGGACACCTACACTATGATCTCCTACGGAGACGACATAGTTGTGGCAAGTGATTATGATTTGGACTTT

GAGGCTCTCAAGCCCCACTTTAAATCTCTTGGTCAAACCATCACCCCAGCTGACAAGAGCGACAAAGGTT

TTGTTGTTGGTCACTCCATTACTGATGTCACTTTCCTCAAAAGACACTTCCACATGGATTATGGAACTGG

GTTTTACAAACCTGTGATGGCCTCAAAGACCCTTGAGGCTATCCTCTCCTTTGCACGCCGTGGG

>AY593784.1_A_ARG_2001

GGGTTGATTGTTGACACCAGAGATGTGGAAGAACGCGTCCACGTGATGCGCAAAACCAAGCTTGCACCCA

CCGTGGCTCACGGTGTGTTCAACCCTGAGTTCGGGCCCGCTGCCTTGTCCAGCAAGGATCCGCGGCTGAA

CGAGGGTGTTGTCCTCGATGAAGTCATTTTCTCCAAACACAAAGGAGACACAAAAATGTCTGAGGAGGAC

AAAGCGCTGTTCCGCCATTGTGCCGCCGACTACGCGTCGCGCTTACACAATGTGTTGGGCACGGCAAACG

CCCCATTGAGCGTTTACGAAGCAATCAAAGGCATCGACGGTCTCGATGCGATGGAACCAGACACCGCGCC

CGGCCTGCCATGGGCACTCCAGGGAAAACGCCGCGGTGCGCTCATCGACTTCGAGAACGGCACTGTCGGG

CCCGAGGTTGAAGCTGCTTTGAAGCTCATGGAGAACAGAGAATACAAATTTGCTTGCCAGACCTTCCTGA

AGGACGAGATTCGCCCGATGGAGAAAGTACGTGCCGGCAAGACTCGCATCGTCGATGTCCTGCCTGTTGA

ACACATTCTTTACACCAGGATGATGATTGGCAGATTTTGTGCACAAATGCACTCAAACAACGGACCGCAG

ATTGGCTCAGCGGTCGGTTGCAACCCTGATGTTGATTGGCAAAGATTTGGCACCCATTTCGCCCAGTACA

GAAACGTGTGGGACGTGGATTATTCGGCCTTCGATGCTAACCACTGCAGTGATGCCATGAACATCATGTT

TGAGGAGGTGTTCCGCACGGAATTTGGATTCCACCCCAACGCTGAGTGGATTCTAAAGACTCTCGTGAAC

ACAGAACACGCCTATGAGAACAAACGCATTGTGGTTGAAGGCGGGATGCCCTCCGGCTGTTCCGCGACAA

GCATCATCAACACGATTTTGAACAACATCTACGTGCTCTACGCCTTGCGTAGACACTATGAGGGAGTTGA

GCTGGACACCTACACCATGATCTCCTACGGAGACGACATCGTGGTGGCAAGTGATTATGATTTGGACTTT

GAGGCTCTCAGGCCCCACTTTAAATCTCTTGGTCAAACCATTACTCCAGCTGACAAAAGCGACAAAGGTT

TTGTTCTTGGTCACTCCATCACCGATGTCACTTTCCTCAAAAGACACTTCCACATGGACTACGGAACTGG

GTTTTACAAACCTGTGATGGCCTCAAAGACCCTTGAAGCCATCCTCTCCTTCGCACGCCGTGGG

>AY593785.1_A_ARG_2001

GGGTTGATTGTTGACACCAGAGATGTGGAAGAACGCGTCCACGTGATGCGCAAAACCAAGCTTGCACCCA

CCGTGGCTCACGGTGTGTTCAACCCTGAGTTCGGGCCCGCTGCCTTGTCCAGCAAGGATCCGCGGCTGAA

CGAGGGTGTTGTCCTCGATGAAGTCATTTTCTCCAAACACAAAGGAGACACAAAAATGTCTGAGGAGGAC

AAAGCGCTGTTCCGCCATTGTGCCGCCGACTACGCGTCGCGCTTACACAATGTGTTGGGCACGGCAAACG

CCCCATTGAGCGTTTACGAAGCAATCAAAGGCATCGACGGTCTCGATGCGATGGAACCAGACACCGCGCC

CGGCCTGCCATGGGCACTCCAGGGAAAACGCCGCGGTGCGCTCATCGACTTCGAGAACGGCACTGTCGGG

CCCGAGGTTGAAGCTGCTTTGAAGCTCATGGAGAACAGAGAATACAAATTTGCTTGCCAGACCTTCCTGA

AGGACGAGATTCGCCCGATGGAGAAAGTACGTGCCGGCAAGACTCGCATCGTCGATGTCCTGCCTGTTGA

ACACATTCTTTACACCAGGATGATGATTGGCAGATTTTGTGCACAAATGCACTCAAACAACGGACCGCAG

ATTGGCTCAGCGGTCGGTTGCAACCCTGATGTTGATTGGCAAAGATTTGGCACCCATTTCGCCCAGTACA

GAAACGTGTGGGACGTGGATTATTCGGCCTTCGATGCTAACCACTGCAGTGATGCCATGAACATCATGTT

TGAGGAGGTGTTCCGCACGGAATTTGGATTCCACCCCAACGCTGAGTGGATTCTAAAGACTCTCGTGAAC

ACAGAACACGCCTATGAGAACAAACGCATTGTGGTTGAAGGCGGGATGCCCTCCGGCTGTTCCGCGACAA

GCATCATCAACACGATTTTGAACAACATCTACGTGCTCTACGCCTTGCGTAGACACTATGAGGGAGTTGA

GCTGGACACCTACACCATGATCTCCTACGGAGACGACATCGTGGTGGCAAGTGATTATGATTTGGACTTT

GAGGCTCTCAGGCCCCACTTTAAATCTCTTGGTCAAACCATTACTCCAGCTGACAAAAGCGACAAAGGTT

TTGTTCTTGGTCACTCCATCACCGATGTCACTTTCCTCAAAAGACACTTCCACATGGACTACGGAACTGG

GTTTTACAAACCTGTGATGGCCTCAAAGACCCTTGAAGCCATCCTCTCCTTCGCACGCCGTGGG

>AY593786.1_A_ARG_2001

GGGTTGATTGTTGACACCAGAGATGTGGAAGAACGCGTCCACGTGATGCGCAAAACCAAGCTTGCACCCA

CCGTGGCTCACGGTGTGTTCAACCCTGAGTTCGGGCCCGCTGCCTTGTCCAGCAAGGATCCGCGGCTGAA

CGAAGGTGTTGTCCTCGATGAAGTCATTTTCTCCAAACACAAAGGAGACACGAAAATGTCTGAGGAGGAC

AAAGCGCTGTTCCGCCGTTGTGCTGCCGACTACGCGTCGCGCTTACACAATGTGCTGGGTACGGCAAACG

CCCCATTGAGCGTTTATGAAGCAATCAAAGGCATCGACGGTCTCGATGCGATGGAACCAGACACCGCGCC

CGGCCTGCCATGGGCACTCCAGGGAAAACGCCGCGGTGCGCTCATCGACTTCGAGAACGGCACTGTCGGG

CCCGAGGTTGAAGCTGCTTTGAAGCTCATGGAGAACAGAGAATACAAATTTGCTTGCCAGACCTTCCTGA

AGGACGAGATTCGCCCGATGGAGAAAGTACGTGCCGGCAAGACTCGCATCGTCGATGTCCTGCCTGTTGA

ACACATTCTTTACACCAGGATGATGATAGGCAGATTTTGTGCACAAATGCACTCAAACAACGGACCGCAG

ATTGGCTCAGCGGTCGGTTGCAACCCCGATGTTGATTGGCAAAGATTTGGCACCCATTTCGCCCAATACA

GAAACGTGTGGGACGTGGATTATTCGGCCTTCGATGCTAACCACTGCAGTGATGCCATGAACATCATGTT

TGAGGAGGTGTTCCGCACGGAATTTGGATTCCACCCCAACGCTGAGTGGATACTAAAGACTCTCGTGAAC

ACAGAACACGCCTATGAGAACAAACGCATTGTGGTTGAAGGCGGGATGCCCTCCGGCTGTTCCGCGACAA

GCATCATCAACACGATTTTGAACAACATCTACGTGCTCTACGCCTTGCGTAGACACTATGAGGGAGTTGA

GCTGGATACCTACACCATGATCTCCTACGGAGACGACATCGTGGTGGCAAGTGATTACGATTTGGACTTT

GAGGCTCTCAGGCCCCACTTTAAATCTCTTGGTCAAACCATTACTCCAGCTGACAAAAGCGACAAAGGTT

TTGTTCTTGGTCACTCCATCACCGATGTCACTTTCCTCAAAAGACACTTCCACATGGACTATGGAACTGG

GTTTTACAAACCTGTGATGGCCTCAAAGACCCTTGAAGCCATCCTCTCCTTTGCACGCCGTGGG

>AY593787.1_A_Brazil_1977

GGGTTGATTGTTGACACCAGAGATGTGGAAGAGCGCGTCCACGTGATGCGCAAGACCAAGCTTGCACCCA

CCGTCGCACACGGTGTGTTCAACCCTGAATTTGGGCCCGCCGCCTTGTCTAACAAGGACCCGCGCCTGAA

TGAAGGTGTTGTCCTCGATGAAGTCATCTTCTCCAAACACAAAGGAGACACAAAGATGTCTGACGAGGAC

AAAGCGCTGTTTCGCCGCTGCGCTGCCGACTACGCGTCGCGCCTGCACAGCGTGCTGGGTACAGCAAATG

CCCCATTGAGCATTTACGAGGCAATCAAAGGCGTCGACGGACTCGACGCCATGGAGCCAGACACTGCACC

TGGCCTTCCCTGGGCACTCCAGGGGAAACGCCGCGGTGCACTCATCGACTTCGAGAACGGCACTGTCGGA

CCCGAAGTTGAGGCTGCCCTGAAGCTCATGGAGAAAAGAGAGTACAAGTTTGCTTGTCAGACCTTCCTGA

AGGACGAGATTCGCCCGATGGAGAAAGTACGTGCCGGTAAGACTCGCATTGTCGACGTCCTGCCTGTTGA

ACACATTCTTTACACCAGGATGATGATTGGCAGATTTTGTGCCCAAATGCACTCAAACAACGGACCGCAG

ATTGGCTCAGCGGTCGGATGCAACCCTGATGTTGATTGGCAAAGATTTGGTACACACTTCGCCCAATACA

GAAACGTGTGGGACGTGGACTATTCGGCCTTTGATGCTAACCACTGCAGTGACGCTATGAACATCATGTT

TGAGGAGGTGTTCCGCACGGAGTTCGGGTTCCACCCCAACGCCGAGTGGATCTTGAAGACTCTCGTGAAC

ACGGAGCACGCCTATGAGAACAAACGCATCACTGTTGAGGGCGGGATGCCGTCTGGTTGTTCCGCGACAA

GCATCATCAACACAATTCTGAACAACATCTACGTGCTCTACGCCCTGCGTAGGCACTATGAGGGAGTTGA

GCTGGACACTTACACCATGATCTCCTACGGAGACGACATCGTGGTGGCGAGTGACTATGATTTGGACTTT

GAGGCCCTCAAGCCCCACTTTAAATCTCTTGGCCAAACTATCACTCCAGCTGACAAAAGCGACAAAGGTT

TTGTTCTTGGTCACTCCATTACCGATGTCACTTTCCTCAAAAGACACTTCCACATGGATTATGGAACTGG

GTTTTACAAACCTGTGATGGCCTCAAAGACCCTTGAGGCTATCCTCTCCTTTGCACGCCGTGGG

>AY593788.1_A_Brazil_1979

GGGTTGATTGTTGACACCAGAGATGTGGAAGAGCGCGTCCACGTGATGCGCAAGACCAAGCTTGCACCCA

CCGTCGCACACGGTGTGTTCAACCCTGAATTTGGGCCCGCCGCCTTGTCTAACAAGGACCCGCGCCTGAA

TGAAGGTGTTGTCCTCGATGAAGTCATCTTCTCCAAACACAAAGGGGACACAAAGATGTCTGAGGAGGAC

AAAGCGCTGTTCCGCCGCTGCGCTGCCGATTACGCATCGCGCCTACACAGCGTGCTGGGCACAGCAAACA

CTCCATTGAGCATTTACGAGGCAATCAAAGGTGTCGACGGCCTCGACGCCATGGAGCCAGACACTGCACC

TGGCCTTCCCTGGGCACTCCAGGGGAAACGCCGCGGTGCACTCATCGACTTCGAGAACGGCACTGTCGGA

CCCGAAGTTGAGGCTGCCCTGAAGCTCATGGAAAAAAGAGAATACAAGTTTGCTTGTCAGACCTTCCTGA

AGGACGAGATTCGCCCGATGGAGAAAGTACGTGCCGGCAAGACTCGCATTGTCGACGTCCTGCCTGTTGA

ACACATTCTTTACACCAGGATGATGATTGGCAGATTTTGTGCCCAAATGCACTCAAACAACGGACCGCAG

ATTGGCTCAGCGGTCGGATGCAACCCTGATGTTGACTGGCAAAGATTTGGTACACACTTCGCCCAATACA

GAAACGTGTGGGACGTGGACTACTCGGCCTTTGATGCCAACCACTGCAGTGACGCTATGAACATCATGTT

TGAGGAGGTGTTCCGCACGGAGTTCGGGTTCCACCCCAACGCCGAGTGGATCTTGAAGACTCTCGTGAAC

ACGGAGCACGCCTATGAGAACAAACGCATCACTGTTGAGGGCGGGATGCCGTCTGGTTGTTCCGCGACAA

GCATCATCAACACAATTTTGAACAACATCTACGTGCTCTACGCTCTGCGTAGGCACTATGAGGGAGTCGA

GCTGGACACTTACACCATGATCTCCTACGGAGACGACATCGTGGTGGCGAGTGACTATGATTTGGACTTT

GAGGCCCTCAAGCCCCACTTCAAATCTCTTGGCCAAACCATCACTCCAGCTGACAAAAGCGACAAAGGTT

TTGTTCTTGGTCACTCCATTACCGATGTCACTTTCCTCAAAAGACACTTCCACATGGATTATGGAACTGG

GTTTTACAAACCTGTGATGGCCTCAAAGACCCTTGAGGCTATCCTCTCCTTTGCACGCCGTGGG

>AY593789.1_A_ARG_1961

GGGTTGATTGTGGACACCAGAGATGTGGAAGAGCGCGTCCACGTGATGCGCAAAACCAAGCTCGCACCCA

CCGTTGCACACGGTGTGTTCAACCCCGAGTTTGGGCCTGCCGCCTTGTCCAACAAGGACCCGCGCCTGAA

CGAGGGTGTTGTTCTCGATGAGGTCATCTTCTCCAAACACAAGGGAGACACAAAGATGTCTGAAGAGGAC

AAAGCGCTGTTCCGCCGCTGCGCCGCTGACTACGCGTCACGCCTGCACAGCGTGCTGGGCACAGCAAATG

CCCCACTGAGCATTTATGAGGCAATTAAGGGTGTCGACGGACTCGACGCCATGGAACCAGACACTGCACC

CGGCCTCCCCTGGGCCCTCCAGGGGAAACGTCGTGGTGCGCTCATCGACTTTGAGAACGGCACTGTCGGG

CCCGAAGTCGAGGCTGCCCTAAAGCTCATGGAGAAAAGAGAATACAAGTTTGCTTGTCAGACCTTCCTGA

AGGACGAAATCCGCCCGATGGAGAAAGTACGTGCCGGCAAGACTCGCATTGTCGATGTTTTGCCTGTTGA

ACACATTCTTTACACCAGGATGATGATTGGCAGATTCTGTGCACAAATGCACTCAAACAACGGACCGCAG

ATTGGCTCAGCGGTCGGCTGTAACCCTGATGTTGATTGGCAGAGATTTGGCACACACTTCGCCCAATACA

GAAACGTGTGGGATGTGGACTATTCGGCCTTCGATGCTAACCACTGCAGTGACGCAATGAACATCATGTT

CGAGGAGGTGTTTCGCACAGACTTTGGTTTCCACCCAAATGCTGAGTGGATTCTGAAGACTCTCGTGAAC

ACGGAGCACGCGTACGAGAACAAACGCATCACTGTTGAGGGCGGGATGCCGTCTGGCTGTTCCGCAACAA

GCATCATCAACACAATTCTGAACAACATCTACGTGCTCTACGCTCTGCGTAGACACTATGAGGGAGTTGA

GCTGGACACTTACACCATGATCTCATACGGAGACGACATCGTGGTGGCAAGTGATTACGATTTGGACTTC

GAGGCTCTCAAGCCTCACTTTAAATCCCTTGGTCAAACCATCACTCCAGCTGACAAAAGCGACAAAGGTT

TTGTTCTTGGTCACTCCATTACCGATGTCACTTTCCTCAAAAGACACTTCCACATGGATTATGGAACTGG

GTTTTACAAACCTGTGATGGCCTCAAAGACCCTTGAGGCTATCCTCTCCTTTGCACGCCGTGGG

>AY593790.1_A_ARG_2001

GGGTTGATTGTTGACACCAGAGATGTGGAAGAACGCGTCCACGTGATGCGCAAAACCAAGCTTGCACCCA

CCGTGGCTCACGGTGTGTTCAACCCTGAGTTCGGGCCCGCTGCCTTGTCCAGCAAGGATCCGCGGCTGAA

CGAAGGTGTTGTCCTCGATGAAGTCATTTTCTCCAAACACAAAGGAGACACGAAAATGTCTGAGGAGGAC

AAAGCGCTGTTCCGCCGTTGTGCTGCCGACTACGCGTCGCGCTTACACAATGTGCTGGGTACGGCAAACG

CCCCATTGAGCGTTTACGAAGCAATCAAAGGCATCGACGGTCTCGATGCGATGGAACCAGACACCGCGCC

CGGCCTGCCATGGGCACTCCAGGGAAAACGCCGCGGTGCGCTCATCGACTTCGAGAACGGCACTGTCGGG

CCCGAGGTTGAAGCTGCTTTGAAGCTCATGGAGAACAGAGAATACAAATTTGCTTGCCAGACCTTCCTGA

AGGACGAGATTCGCCCGATGGAGAAAGTACGTGCCGGCAAGACTCGCATCGTCGATGTCCTGCCTGTTGA

ACACATTCTTTACACCAGGATGATGATAGGCAGATTTTGTGCACAAATGCACTCAAACAACGGACCGCAG

ATTGGCTCAGCGGTCGGTTGCAACCCTGATGTTGATTGGCAAAGATTTGGCACCCATTTCGCCCAATACA

GAAACGTGTGGGACGTGGATTATTCGGCCTTCGATGCTAACCACTGCAGTGATGCCATGAACATCATGTT

TGAGGAGGTGTTCCGCACGGAATTTGGATTCCACCCCAACGCTGAGTGGATTCTAAAGACTCTCGTGAAC

ACAGAACACGCCTATGAGAACAAACGCATTGTGGTTGAAGGCGGGATGCCCTCCGGCTGTTCCGCGACAA

GCATCATCAACACGATTTTGAACAACATCTACGTGCTCTACGCCTTGCGTAGACACTATGAGGGAGTTGA

GCTGGATACCTACACCATGATCTCCTACGGAGACGACATCGTGGTGGCAAGTGATTATGATTTGGACTTT

GAGGCTCTCAGGCCCCACTTTAAATCTCTTGGTCAAACCATTACTCCAGCTGACAAAAGCGACAAAGGTT

TTGTTCTTGGTCACTCCATCACCGATGTCACTTTCCTCAAAAGACACTTCCACATGGACTATGGAACTGG

GTTTTACAAACCTGTGATGGCCTCAAAGACCCTTGAAGCCATCCTCTCCTTTGCACGCCGTGGG

>AY593791.1_A_IRN_1998

GGTTTGATTGTTGACACCAGAGATGTGGAGGAGCGCGTGCATGTCATGCGCAAAACCAAGCTTGCACCCA

CCGTGGCACACGGTGTGTTCAACCCCGAATTTGGGCCTGCTGCCTTGTCCAACAAGGACCCGCGCTTGAG

TGAGGGAGTTGTCCTCGATGAAGTCATCTTCTCTAAACACAAGGGAGACACCAAGATGTCTGAGGAGGAC

AAAGCGCTGTTCCGCCGCTGTGCTGCTGACTACGCGTCACGCTTACACAGTGTGCTGGGCACGGCAAATG

CCCCACTGAGCATCTACGAGGCAATCAAAGGCGTTGACGGCCTCGACGCCATGGAACCAGACACCGCGCC

TGGCCTCCCCTGGGCTCTCCAGGGGAAACGCCGTGGCGCGCTCATCGACTTCGAGAATGGCACAGTCGGA

CCCGAGGTTGAAGCTGCCTTGAAGCTCATGGAGAAAAAGGAATACAAGTTTGCATGCCAGACCTTCTTGA

AAGACGAGATCCGTCCGATGGAGAAAGTACGTGCCGGCAAGACACGCATTGTCGACGTCTTGCCAGTTGA

ACACATTCTTTACACCAGGATGATGATTGGCAGATTCTGTGCTCAGATGCACTCAAACAACGGACCGCAG

ATTGGCTCGGCAGTCGGTTGTAACCCTGATGTTGACTGGCAAAGATTTGGCACACACTTCGCTCAGTACA

GAAACGTGTGGGATGTGGACTATTCGGCCTTTGATGCTAATCACTGCAGCGATGCGATGAACATCATGTT

CGAGGAAGTGTTCTCCACGGAATTTGGTTTCCACCCAAACGCTGAGTGGATCCTGAAAACACTCGTGAAC

ACGGAGCACGCCTATGAAAACAAACGCATCACTGTCGAGGGCGGGATGCCGTCTGGTTGTTCCGCGACAA

GCATCATCAACACAATTCTGAACAACATCTACGTTCTCTACGCTTTGCGTAGACACTATGAGGGAGTTGA

GCTGGACACTTACACCATGATCTCCTACGGAGACGACATCGTGGTTGCAAGCGATTACGATCTGGACTTT

GAGGCCCTCAAGCCTCACTTCAAATCTCTTGGTCAAACCATCACTCCAGCTGACAAAAGCGACAAAGGTT

TTGTTCTTGGTCACTCTATTACTGATGTCACTTTCCTCAAAAGACACTTCCACATGGATTATGGAACTGG

GTTTTACAAACCTGTGATGGCCTCGAAGACCCTCGAGGCTATCCTCTCCTTTGCACGCCGTGGG

>AY593792.1_A_ITL_1962

GGGTTGATTGTAGACACCAGAGATGTGGAAGAGCGCGTCCACGTGATGCGCAAAACCAAGCTTGCACCCA

CCGTTGCACACGGTGTGTTCAACCCCGAGTTTGGGCCAGCTGCCTTGTCCAACAAGGACCCGCGTCTGAA

CGAGGGTGTTGTCCTCGATGAAGTCATTTTCTCCAAACACAAGGGAGACACAAAGATGTCTGAGGAGGAC

AAAGCGCTGTTCCGCCGATGTGCTGCTGATTACGCGTCACGCCTGCACAGCGTGCTGGGTACGGCAAATG

CCCCACTGAGCATCTACGAGGCAATCAAAGGCGTCGACGGACTCGACGCCATGGAGCCAGACACCGCACC

TGGCCTCCCCTGGGCTCTCCAGGGAAAACGCCGTGGTGCGCTCATCGACTTTGAGAACGGCACGGTCGGG

CCCGAAGTCGAGGCTGCCTTGAAGCTCATGGAGAAAAGAGAGTACAAGTTTGTTTGTCAGACCTTCCTGA

AGGACGAGATTCGCCCGATGGAGAAAGTACGTGCCGGCAAGACTCGCATTGTCGACGTCCTGCCCGTTGA

ACACATTCTTTACACCAGGATGATGATTGGCAGATTTTGTGCACAAATGCACTCAAACAATGGACCGCAA

ATTGGCTCGGCGGTCGGTTGTAACCCTGATGTTGATTGGCAAAGATTTGGCACACACTTCGCCCAATACA

GAAACGTGTGGGATGTGGACTATTCGGCCTTTGATGCTAACCACTGCAGTGACGCCATGAACATCATGTT

TGAGGAGGTGTTCCGCACGGACTTCGGGTTCCACCCAAATGCTGAGTGGATCTTGAAGACTCTCGTGAAC

ACGGAACATGCCTATGAGAACAAACGCATCACTGTTGAAGGCGGGATGCCATCTGGTTGTTCCGCAACGA

GCATCATCAACACAATTTTGAACAACATCTACGTGCTCTACGCCTTGCGTAGACACTATGAGGGAGTTGA

GCTGGACACTTACACCATGATCTCCTACGGAGACGACATTGTGGTGGCAAGTGATTACGATCTGGACTTT

GAGGCTCTCAAGCCTCACTTCAGATCTCTTGGTCAAACCATTACTCCAGCTGACAAAAGCGACAAAGGTT

TTGTTCTTGGTCACTCCATCACCGATGTCACTTTCCTCAAAAGACACTTTCACATGGATTATGGAACTGG

GTTTTACAAACCTGTGATGGCCTCAAAGACCCTTGAGGCTATCCTCTCCTTTGCACGCCGTGGG

>AY593793.1_A_PHI_1975

GGGTTGATTGTTGACACCAGAGATGTGGAGGAGCGTGTTCACGTGATGCGCAAAACCAAGCTTGCACCCA

CCGTCGCGCACGGTGTTTTTAACCCCGAATTCGGGCCTGCTGCCTTGTCCAACAAGGACCCACGCCTGAA

TGAAGGTGTTGTCCTCGATGAAGTCATCTTCTCCAAACACAAGGGAGACACAAAGATGTCTGAGGAGGAC

AAAGCGCTGTTCCGCCGCTGTGCCGCTGACTACGCGTCACGTCTACACTGTGTGCTGGGTACAGCAAATG

CCCCACTGAGCATTTACGAGGCAATCAAGGGCGTCGACGGACTCGACGCAATGGAACCGGACACTGCACC

TGGCCTCCCTTGGGCACTCCAGGGGAAACGCCGCGGTGCCCTCATTGACTTTGAGAACGGCACTGTCGGA

CCCGAAGTTGAAGCTGCCTTGAAGCTCATGGAGAAAAGAGAGTACAAATTTGCTTGTCAGACCTTCCTGA

AGGACGAGATCCGCCCGATGGAGAAAGTACGTGCCGGCAAGACTCGCATTGTCGATGTCCTGCCCGTTGA

ACACATTCTTTACACCAGGATGATGATTGGCAGATTCTGTGCACAAATGCACTCAAACAACGGACCGCAA

ATTGGCTCGGCGGTCGGTTGTAACCCTGATGTTGATTGGCAGAGATTTGGCACACACTTCGCCCAATACA

GAAACGTGTGGGACGTGGACTATTCGGCCTTTGATGCTAACCACTGCAGTGACGCCATGAACATCATGTT

TGAGGAGGTGTTCCGCACGGAGTTCGGCTTCCACCCAAACGCTGAGTGGATCTTGAAGACTCTCGTGAAC

ACGGAGCACGCCTATGAGAACAAACGCATCACTGTCGAGGGCGGAATGCCGTCCGGTTGCTCCGCCACAA

GCATCATCAACACAATTTTGAACAACATCTACGTGCTCTACGCCCTGCGTAGACACTATGAGGGAGTTGA

GCTGGACACCTACACCATGATCTCCTATGGAGACGACATCGTGGTGGCAAGTGATTACGATTTGGACTTT

GAGGCTCTCAAGCCCCACTTTAAATCTCTTGGTCAAACCATCACTCCAGCTGACAAAAGCGACAAAGGTT

TTGTTCTTGGTCACTCCATTACCGATGTCACTTTCCTCAAAAGACACTTCCACATGGACTATGGAACTGG

GTTTTACAAACCTGTGATGGCCTCAAAGACCCTTGAGGCTATCCTCTCCTTTGCACGCCGTGGG

>AY593794.1_A_COL_1985

GGGTTGATCGTTGACACCAGAGATGTGGAAGAGCGCGTCCATGTTATGCGCAAAACAAAGCTTGCACCCA

CCGTCGCGCACGGTGTGTTCAACCCTGAGTTTGGGCCCGCTGCCTTGTCAAACAAAGACCCGCGCCTGAA

TGAGGGAGTTGTCCTCGACGAAGTCATCTTTTCCAAGCACAAGGGAGACACAAAGATGACCGAGGAAGAC

AAAGCGCTGTTCCGCCGCTGCGCCGCTGACTACGCGTCACGTCTGCATAGCGTTTTGGGCACAGCAAATG

CCCCACTGAGCATCTATGAGGCAATCAAAGGCGTTGACGGACTCGACGCCATGGAGCCTGACACAGCGCC

AGGCCTCCCTTGGGCCCTCCAGGGGAAGCGCCGCGGTGCGCTCATCGACTTTGAGAACGGCACGGTCGGA

CCCGAGGTTGAAGCTGCCTTGAAGCTCATGGAGAAAAGAGAATACAAGTTTGCTTGTCAGACCTTCCTGA

AAGACGAAATTCGTCCGATGGAGAAGGTGCGCGCTGGCAAAACTCGCATTGTCGACGTCCTGCCTGTCGA

GCACATTCTCTACACCAGGATGATGATTGGCAGGTTCTGCGCGCAAATGCACTCCAACAACGGACCGCAA

ATTGGCTCTGCGGTCGGTTGTAACCCTGATGTTGATTGGCAAAGATTCGGCACACATTTCGCCCAATACA

GGAACGTGTGGGATGTGGACTATTCGGCCTTTGATGCAAACCACTGCAGTGACGCCATGAACATCATGTT

TGAGGAGGTGTTTCGCACGGACTTTGGTTTCCACCCAAACGCTGAGTGGATTCTGAAGACTCTCGTGAAC

ACGGAACACGCCTATGAAAACAAGCGCATCACTGTTGAGGGCGGGATGCCATCTGGTTGTTCCGCAACAA

GCATTATCAACACAATTCTGAACAACATCTACGTGCTCTACGCCCTACGTAGACACTATGAAGGAGTTGA

GCTGGACACTTACACCATGATCTCCTACGGAGACGACATCGTGGTGGCAAGTGATTATGATCTGGACTTT

GAGGCCCTTAAGCCTCACTTCAAATCTCTTGGCCAGACCATCACTCCAGCTGACAAAAGCGACAAAGGTT

TTGTTCTTGGTCACTCCATTACCGATGTCACTTTCCTAAAAAGACACTTCCACATGGATTATGGAACTGG

GTTTTATAAACCTGTGATGGCCTCAAAGACCCTTGAGGCCATCCTCTCCTTTGCACGCCGTGGG

>AY593795.1_Asia1_PAK_1954

GGGCTGATCGTTGACACCAGAGATGTTGAAGAGCGCGTGCATGTCATGCGCAAAACCAAGCTTGCACCCA

CCGTTGCACACGGTGTGTTCAACCCTGAATTCGGGCCTGCTGCTTTATCCAACAAAGACCCGCGCCTGAA

CGAAGGAGTTGTCCTCGATGAAGTCATCTTCTCCAAACACAAGGGAGACACCAAGATGTCTGAGGAGGAC

AAAGCGCTGTTCCGCCGCTGCGCCGCTGACTACGCGTCACGCCTGCACAGTGTGCTGGGTGCGGCAAATG

CCCCACTGAGCATCTACGAGGCTATCAAGGGCGTTGACGGGCTCGACGCCATGGAACCAGACACCGCGCC

TGGCCTTCCCTGGGCCCTCCAGGGGAAACGCCGTGGTGCGCTGATCGACTTCGAGAACGGCACGGTCGGA

CCCGAGGTTGAGGCTGCCTTAAAGCTCATGGAGAAAAGAGAGTACAAGTTTGCTTGCCAGACCTTCCTGA

AGGACGAGATTCGCCCGATGGAGAAAGTACGTGCCGGCAAGACTCGCATTGTCGACGTCTTGCCTGTTGA

ACACGTTCTTTACACCAGGATGATGATTGGCAGATTTTGTGCACAAATGCACTCAAACAACGGACCGCAA

ATTGGATCGGCGGTCGGTTGCAACCCTGATGTTGATTGGCAGAGATTTGGCACCCATTTTTCCCAGTACA

GAAACGTGTGGGATGTGGACTATTCGGCCTTTGATGCTAACCACTGCAGTGATGCAATGAACATCATGTT

TGAGGAGGTGTTCCGCACGGACTTCGGTTTCCACCCAAATGCTGAGTGGATTCTGAAGACTCTCGTGAAC

ACGGAGCATGCCTATGAAAACAAACGCATCACTGTTGAAGGCGGAATGCCGTCCGGCTGTTCCGCGACCA

GCATCATCAACACAATTTTGAACAACATCTACGTGCTCTACGCCCTGCGTAGGCACTATGAGGGAGTTGA

GCTGGACACTTACACCATGATCTCCTACGGAGACGACATCGTGGTAGCAAGTGATTACGATTTGGACTTT

GAGGCTCTCAAGCCCCATTTTAAATCTCTTGGTCAAACCATCACTCCAGCTGACAAAAGCGACAAAGGTT

TTGTTCTTGGTCACTCCATTACCGATGTCACGTTCCTCAAAAGACACTTCCACATGGATTACGGTACTGG

GTTTTACAAACCTGTGATGGCCTCGAAGACCCTCGAGGCTATCCTCTCCTTTGCACGCCGTGGG

>AY593796.1_Asia1_ISR_1963

GGGTTGATCATTGACACCAGAGATGTGGAAGAGCGCGTGCACGTCATGCGCAAAACCAAACTTGCACCCA

CCGTGGCACACGGTGTGTTCAACCCTGATTTTGGGCCCGCCGCCTTGTCTAACAAGGACCCGCGCCTGAA

CGAAGGAGTTGTCCTCGATGAGGTCATCTTCTCCAAACACAAGGGAGACACAAAGATGTCCGAGGAAGAC

AAAGCGCTGTTCCGCCGCTGCGCTGCCGACTACGCGTCGCGCTTGCACAGTGTGCTGGGTACAGCAAATG

CCCCACTGAGCATCTATGAGGCCATCAAAGGCGTCGACGGACTCGACGCCATGGAACCAGACACTGCACC

TGGCCTTCCCTGGGCTCTCCAGGGGAAACGCCGCGGCGCGCTGATCGACTTCGAGAACGGCACGGTTGGA

CCCGAAGTCGAGGCTGCATTGAAGCTCATGGAGAAAAGAGAGTACAAGTTTGCTTGTCAGACCTTCCTGA

AGGATGAAATTCGTCCGATGGAGAAAGTACGTGCCGGTAAGACTCGCATTGTCGACGTCTTGCCCGTTGA

ACACATTCTTTACACCAGGATGATGATTGGCAGGTTCTGTGCTCAAATGCACTCAAACAACGGACCACAA

ATTGGATCAGCGGTCGGTTGCAATCCTGATGTTGATTGGCAAAGATTTGGCACACATTTCGCCCAGTACA

GAAACGTGTGGGATGTGGACTATTCGGCCTTTGATGCGAATCACTGTGGCGATGCGATGAACATCATGTT

CGAGGAGGTGTTCCGCACAGACTTCGGCTTCCACCCAAACGCGGAGTGGATTCTGAAGACTCTCGTGAAC

ACGGAACACGCCTATGAGGACAAACGCATCACTGTTGAAGGCGGGATGCCGTCTGGCTGCTCCGCAACTA

GCATCATCAACACAATTCTGAACAACATCTACGTGCTCTACGCGCTGCGTAGACACTATGAGGGAGTTGA

GCTGGACACTTACACCATGATCTCCTATGGGGACGACATCGTGGTTGCAAGTGATTACGACCTGGACTTT

GAGGCTCTCAAGCCCCACTTCAAATCTCTTGGCCAAACCATCACTCCAGCTGACAAAAGCGACAAAGGTT

TTGTTCTTGGTCACTCCATCACCGATGTCACTTTCCTCAAAAGGCACTTCCACATGGATTACGGAACTGG

GTTTTACAAACCTGTGATGGCTTCGAAGACCCTCGAGGCTATCCTCTCCTTTGCACGCCGTGGG

>AY593797.1_Asia1_ISR_1963

GGGTTGATTGTTGACACCAGAGATGTGGAAGAGCGCGTGCACGTCATGCGTAAAACCAAGCTTGCACCCA

CCGTAGCACACGGTGTGTTCAACCCTGAGTTTGGGCCTGCCGCCTTGTCCAACAAGGACCTGCGCCTGAA

CGAAGGAGTTGTCCTCGATGAGGTCATCTTCTCCAAACACAAGGGAGACGCAAAGATGTCCGAGGAAGAC

AAAGCGCTGTTCCGCCGCTGCGCTGCCGACTACGCGTCGCGCCTGCACAGTGTGCTGGGTATAGCAAATG

CCCCACTGAGCATCTATGAGGCCATCAAAGGCGTCGACGGACTCGACGCCATGGAACCAGACACCGCGCC

CGGCCTTCCCTGGGCTCTCCAGGGGAAACGCCGCGGTGCGCTGATCGACTTCGAGAACGGCACGGTTGGA

CCCGAAGTTGAGGCTGCCTTGAAGCTCATGGAGAGAAGAGAATACAAGTTTGCTTGTCAGACCTTCCTGA

AGGACGAAATTCGCCCGATGGAGAAAGTACGTGCCGGCAAGACTCGCATTGTCGACGTTTTGCCCGTTGA

ACACATTCTTTACACCAGGATGATGATTGGCAGATTCTGTGCTCAAATGCACTTAAACAACGGACCGCAA

ATTGGATCAGCGGTCGGTTGCAATCCTGATGTTGATTGGCAAAGATTTGGCACACATTTCGCCCAATACA

GAAACGTGTGGGATGTGGACTATTCGGCCTTTGATGCTAATCACTGTAGCGATGCGATGAACATCATGTT

CGAGGAGGTGTTCCACACAGACTTTGGCTTCCACCCAAACGCTGAGTGGATTCTGAAGACTCTCGTGAAC

ACGGAACACGCCTATGAGAACAAACGCATCACTGTTGAAGGTGGGATGCCGTCTGGCTGTTCCGCAACTA

GCATCATCAACACAATTCTGAACAACATCTACGTGCTCTACGCGCTGCGTAGACACTATGAGGGAGTTGA

GCTGGACACTTACACCATGATCTCCTACGGGGACGACATCGTGGTTGCAAGTGATAACGATCTGGATTTT

GAGGCTCTCAAGCCCCACTTCAAATCTCTTGGCCAAACCATCACTCCAGCTGACAAAAGCGACAAAGGTT

TTGTTCTTGGTCACTCCATCACCGATGTCACTTTCCTCAAAAGACACTTCCACATGGATTATGGAACTGG

GTTTTACAAACCTGTGATGGCTTCGAAGACCCTCGAGGCTATCCTCTCCTTTGCACGCCGTGGG

>AY593799.1_Asia1_LEB_1983

GGGTTGATCGTTGACACCAGAGATGTGGAAGAGCGCGTGCACGTCATGCGCAAAACCAAGCTTGCACCCA

CCGTGGCACACGGTGTGTTCAACCCTGCATTCGGCCCCGCTGCCTTGTCCAACAAGGACCCGCGGCTGAG

CGAAGGGGTTGTCCTTGATGAAGTCATCTTCTCCAAACACAAAGGAGACACCAAGATGTCTAAGGAGGAC

AAAGCGCTGTTCCGTCGTTGCGCCGCCGACTACGCGTCGCGCCTGCACGGCGTACTGGGTACGGCAAATG

CCCCATTGAGCATCTACGAGGCAATCAAGGGCGTTGACGGACTCGACGCCATGGAACCAGACACTGCGCC

TGGTCTCCCTTGGGCCCTCCAGGGGAAGCGCCGTGGCGCACTGATCGACTTTGAGAACGGCACGGTCGGA

CCCGAGGTTGAGGCTGCCTTGAAGCTTATGGAGAAAAGAGAATACAAGTTTGTTTGTCAGACCTTCCTGA

AAGACGAGATTCGTCCGATGGAGAAAGTACGTGCCGGCAAAACCCGCATTGTCGACGTTTTGCCTGTTGA

ACACATTCTTTACACCAGGATGATGATTGGCAGATTCTGTGCTCAAATGCACTCAAACAACGGACCGCAA

ATTGGCTCGGCGGTCGGTTGCAACCCTGATGTTGATTGGCAAAGATTCGGCACACACTTTTCCCAGTACA

GAAACGTGTGGGATGTGGACTATTCGGCCTTTGATGCTAACCACTGCAGTGACGCAATGAACATCATGTT

TGAGGAGGTGTTTCGCACGGAGTTTGGTTTCCACCCAAATGCTGAGTGGATCCTGAAGACTCTTGTGAAC

ACGGAACACGCCTATGAAAACAAAAGCATCACTGTTGAGGGCGGGATGCCATCTGGTTGTTCCGCGACAA

GCATTATCAACACAATTTTGAACAACATTTACGTGCTCTACGCGTTGCGTAGACACTATGAGGGAGTTGA

GCTGGACACTTACACCATGATCTCCTACGGAGACGACATCGTGGTTGCAAGTGATTACGATCTGGACTTC

GAGGCCCTCAAGCCTCACTTCAAATCTCTTGGTCAAACCATCACCCCAGCTGACAAAAGCGACAAAGGTT

TTGTTCTTGGTCACTCCATCACCGACGTCACTTTCCTCAAAAGACATTTCCACATGGACTATGGAACTGG

GTTTTACAAACCTGTGATGGCCTCGAAGACCCTCGAGGCCATCCTCTCCTTTGCACGCCGTGGG

>AY593800.1_Asia1_LEB_1983

GGGTTGATCGTTGACACCAGAGATGTGGAAGAGCGCGTGCACGTCATGCGCAAAACCAAGCTTGCACCCA

CCGTGGCACACGGTGTGTTCAACCCTGCATTCGGCCCCGCTGCCTTGTCCAACAAGGACCCGCGGCTGAG

CGAAGGGGTTGTCCTTGATGAAGTCATCTTCTCCAAACACAAAGGAGACACCAAGATGTCTAAGGAGGAC

AAAGCGCTGTTCCGTCGTTGCGCCGCCGACTACGCGTCGCGCCTGCACGGCGTACTGGGTACGGCAAATG

CCCCATTGAGCATCTACGAGGCAATCAAGGGCGTTGACGGACTCGACGCCATGGAACCAGACACTGCGCC

TGGTCTCCCTTGGGCCCTCCAGGGGAAGCGCCGTGGCGCACTGATCGACTTTGAGAACGGCACGGTCGGA

CCCGAGGTTGAGGCTGCCTTGAAGCTTATGGAGAAAAGAGAATACAAGTTTGTTTGTCAGACCTTCCTGA

AAGACGAGATTCGTCCGATGGAGAAAGTACGTGCCGGCAAAACCCGCATTGTCGACGTTTTGCCTGTTGA

ACACATTCTTTACACCAGGATGATGATTGGCAGATTCTGTGCTCAAATGCACTCAAACAACGGACCGCAA

ATTGGCTCGGCGGTCGGTTGCAACCCTGATGTTGATTGGCAAAGATTCGGCACACACTTTTCCCAGTACA

GAAACGTGTGGGATGTGGACTATTCGGCCTTTGATGCTAACCACTGCAGTGACGCAATGAACATCATGTT

TGAGGAGGTGTTTCGCACGGAGTTTGGTTTCCACCCAAATGCTGAGTGGATCCTGAAGACTCTTGTGAAC

ACGGAACACGCCTATGAAAACAAAAGCATCACTGTTGAGGGCGGGATGCCATCTGGTTGTTCCGCGACAA

GCATTATCAACACAATTTTGAACAACATTTACGTGCTCTACGCGTTGCGTAGACACTATGAGGGAGTTGA

GCTGGACACTTACACCATGATCTCCTACGGAGACGACATCGTGGTTGCAAGTGATTACGATCTGGACTTC

GAGGCCCTCAAGCCTCACTTCAAATCTCTTGGTCAAACCATCACCCCAGCTGACAAAAGCGACAAAGGTT

TTGTTCTTGGTCACTCCATCACCGACGTCACTTTCCTCAAAAGACATTTCCACATGGACTATGGAACTGG

GTTTTACAAACCTGTGATGGCCTCGAAGACCCTCGAGGCCATCCTCTCCTTTGCACGCCGTGGG

>AY593802.1_A_URU_2001

GGGTTGATTGTTGACACCAGAGATGTGGAAGAACGCGTCCACGTGATGCGCAAAACCAAGCTTGCACCCA

CCGTGGCTCACGGTGTGTTCAACCCTGAGTTCGGGCCCGCTGCCTTGTCCAGCAAGGATCCGCGGCTGAA

CGAGGGTGTTGTCCTCGATGAAGTCATTTTCTCCAAACACAAAGGAGACACAAAAATGTCTGAGGAGGAC

AAAGCGCTGTTCCGCCATTGTGCTGCCGACTACGCGTCGCGCTTACACAATGTGTTGGGCACGGCAAACG

CCCCATTGAGCGTTTACGAAGCAATCAAAGGCATCGACGGTCTCGATGCGATGGAACCAGACACCGCGCC

CGGCCTGCCATGGGCACTCCAGGGAAAACGCCGCGGTGCGCTCATCGACTTCGAGAACGGCACTGTCGGG

CCCGAGGTTGAAGCTGCTTTGAAGCTCATGGAGAACAGAGAATACAAATTTGCTTGTCAGACCTTCCTGA

AGGACGAGATTCGCCCGATGGAGAAAGTACGTGCCGGCAAGACTCGCATCGTCGATGTCCTGCCTGTTGA

ACACATTCTTTACACCAGGATGATGATTGGCAGATTTTGTGCACAAATGCACTCAAACAACGGACCGCAG

ATTGGCTCAGCGGTCGGTTGCAACCCTGATGTTGATTGGCAAAGATTTGGCACCCATTTCGCCCAGTACA

GAAACGTGTGGGACGTGGATTATTCGGCCTTCGATGCTAACCACTGCAGTGATGCCATGAACATCATGTT

TGAGGAGGTGTTCCGCACGGAATTTGGATTCCACCCCAACGCTGAGTGGATTCTAAAGACTCTCGTGAAC

ACAGAACACGCCTATGAGAACAAACGCATTGTGGTTGAAGGCGGGATGCCCTCCGGCTGTTCCGCGACAA

GCATCATCAACACGATTTTGAACAACATCTACGTGCTCTACGCCTTGCGTAGACACTATGAGGGAGTTGA

GCTGGACACCTACACCATGATCTCCTACGGAGACGACATCGTGGTGGCAAGTGATTATGATTTGGACTTT

GAGGCTCTCAGGCCCCACTTTAAATCTCTTGGTCAAACCATTACTCCAGCTGACAAAAGCGACAAAGGTT

TTGTTCTTGGTCACTCCATCACCGATGTCACTTTCCTCAAAAGACACTTCCACATGGACTACGGAACTGG

GTTTTACAAACCTGTGATGGCCTCAAAGACCCTTGAAGCCATCCTCTCCTTCGCACGCCGTGGG

>AY593803.1_A_Brazil_1979

GGGTTGATTGTTGACACCAGAGATGTGGAAGAGCGCGTCCACGTGATGCGCAAAACTAAGCTTGCACCCA

CCGTTGCACACGGTGTGTTCAACCCCGAATTTGGGCCCGCCGCCTTGTCCAACAAGGACCCGCGCCTGAA

TGAGGGTGTTGTCCTCGATGAAGTCATCTTCTCCAAACACAAGGGAGACACAAAGATGTCTGAGGAAGAC

AAAGCGCTGTTCCGCCGCTGCGCTGCTGACTACGCGTCACGCCTGCACAGCGTGCTGGGGACGGCAAATG

CCCCACTGAGCATTTACGAGGCAATCAAAGGCGTCGACGGACTCGACGCCATGGAACCAGACACCGCGCC

TGGTCTTCCCTGGGCCCTCCAGGGAAAGCGCCGTGGCGCGCTCATCGACTTCGAGAACGGCACAGTTGGA

CCCGAAGTTGAAGCTGCCTTGAAGCTCATGGAGAAAAGAGAATACAAGTTTGCTTGCCAGACCTTCCTGA

AGGACGAAATTCGCCCGATGGAGAAAGTACGTGCCGGCAAGACTCGCATTGTCGATGTTTTGCCCGTTGA

ACACATTCTTTACACCAGGATGATGATTGGCAGATTTTGTGCACAAATGCACTCAAACAACGGACCGCAG

ATTGGCTCGGCGGTCGGCTGTAATCCAGATGTTGATTGGCAGAGATTTGGAACACACTTCGCCCAGTACA

GAAACGTGTGGGACGTGGACTATTCGGCCTTTGATGCTAACCACTGCAGTGACGCCATGAACATCATGTT

TGAGGAGGTGTTTCGCACAGAGTTTGGTTTCCACCCGAACGCGGAGTGGATTCTGAAGACTCTTGTGAAC

ACGGAACACGCCTATGAGAACAAACGCATCACTGTCGAGGGCGGGATGCCATCTGGCTGTTCTGCGACAA

GCATCATCAACACAATTTTGAACAACATCTACGTGCTCTACGCTCTGCGTAGACACTATGAGGGAGTTGA

GCTGGACACTTACACCATGATCTCCTACGGAGACGACATCGTGGTGGCAAGTGACTACGATTTGGACTTT

GAGGCTCTCAAGCCCCACTTTAAATCTCTTGGTCAAACCATCACTCCAGCTGACAAAAGCGACAAAGGTT

TTGTTCTTGGTCACTCCATCACCGATGTCACTTTCCTCAAAAGACACTTCCACATGGATTATGGGACTGG

GTTTTACAAACCTGTGATGGCCTCAAAGACCCTTGAGGCTATCCTCTCCTTTGCACGCCGTGGG

>AY593804.1_C_SWZ_1965

GGGTTGATCGTTGATACCAGAGATGTGGAAGAGCGCGTCCATGTAATGCGCAAAACCAAGCTTGCACCCA

CCGTCGCACACGGTGTGTTCAATCCTGAGTTCGGGCCTGCCGCCTTGTCTAATAAGGACCCACGTCTGAA

CGAAGGTGTTGTCCTCGATGAAGTCATCTTCTCCAAGCACAAAGGAGACACAAAGATGTCTGAGGAGGAC

AAAGCGCTGTTCCGCCGCTGCGCTGCTGACTACGCGTCACGCCTGCACAGTGTGCTGGGTACGGCAAATG

CCCCACTGAGCATTTACGAGGCAATCAAGGGCGTTGACGGACTCGACGCCATGGAGCCAGACACCGCACC

TGGCCTTCCCTGGGCCCTCCAGGGGAAACGCCGCGGTGCACTCATCGATTTCGAGAACGGCACGGTCGGA

CCCGAGGTTGAGGCTGCCTTGAAGCTCATGGAGAAAAGAGAATACAAGTTTGCTTGCCAGACCTTCCTGA

AGGACGAAATTCGCCCGATGGAGAAAGTACGTGCCGGCAAGACTCGCATTGTCGACGTTTTGCCTGTTGA

ACACATTCTTTACACCAGGATGATGATTGGCAGATTTTGTGCACAAATGCACTCAAACAACGGACCGCAG

ATTGGCTCAGCGGTCGGTTGCAACCCTGATGTTGATTGGCAGAGATTCGGCACACACTTCGCCCAATACA

GAAACGTGTGGGACGTGGACTATTCGGCCTTTGATGCAAACCACTGCAGCGATGCTATGAACATCATGTT

TGAGGAGGTGTTCCGCACGGAGTTCGGCTTCCACCCGAATGCTGAGTGGATCCTGAAGACTCTCGTGAAC

ACGGAGCACGCCTATGAGAACAAGCGCATCACTGTTGAAGGCGGGATGCCGTCTGGTTGTTCCGCAACAA

GCATCATCAACACAATTTTGAATAACATCTACGTGCTCTACGCCTTGCGTAGACACTATGAGGGGGTTGA

GCTGGACACTTACACCATGATCTCCTATGGAGACGACATCGTGGTGGCAAGTGATTATGATCTGGACTTT

GAGGCCCTCAAGCCTCACTTCAAATCTCTTGGCCAAACCATTACTCCAGCTGACAAAAGCGACAAAGGTT

TTGTTCTTGGTCACTCCATTACTGACGTCACTTTCCTCAAAAGACACTTCCACATGGATTATGGCACTGG

GTTTTACAAACCTGTGATGGCCTCGAAGACCCTCGAGGCTATCCTCTCCTTTGCACGCCGTGGG

>AY593805.1_C_GER_1960

GGGTTGATCGTTGATACCAGAGATGTGGAAGAGCGCGTCCATGTAATGCGCAAAACCAAGCTTGCACCCA

CCGTCGCACACGGTGTGTTCAATCCTGAGTTCGGGCCTGCCGCCTTGTCTAATAAGGACCCACGTCTGAA

CGAAGGTGTTGTCCTCGATGAAGTCATCTTCTCCAAGCACAAAGGAGACACAAAGATGTCTGAGGAGGAC

AAAGCGCTGTTCCGCCGCTGCGCTGCTGACTACGCGTCACGCCTGCACAGTGTGCTGGGTACGGCAAATG

CCCCACTGAGCATTTACGAGGCAATCAAGGGCGTTGACGGACTCGACGCCATGGAGCCAGACACCGCACC

TGGCCTTCCCTGGGCCCTCCAGGGGAAACGCCGCGGTGCACTCATCGATTTCGAGAACGGCACGGTCGGA

CCCGAGGTTGAGGCTGCCTTGAAGCTCATGGAGAAAAGAGAATACAAGTTTGCTTGCCAGACCTTCCTGA

AGGACGAAATTCGCCCGATGGAGAAAGTACGTGCCGGCAAGACTCGCATTGTCGACGTTTTGCCTGTTGA

ACACATTCTTTACACCAGGATGATGATTGGCAGATTTTGTGCACAAATGCACTCAAACAACGGACCGCAG

ATTGGCTCAGCGGTCGGTTGCAACCCTGATGTTGATTGGCAGAGATTCGGCACACACTTCGCCCAATACA

GAAACGTGTGGGACGTGGACTATTCGGCCTTTGATGCAAACCACTGCAGCGATGCTATGAACATCATGTT

TGAGGAGGTGTTCCGCACGGAGTTCGGCTTCCACCCGAATGCTGAGTGGATCCTGAAGACTCTCGTGAAC

ACGGAGCACGCCTATGAGAACAAGCGCATCACTGTTGAAGGCGGGATGCCGTCTGGTTGTTCCGCAACAA

GCATCATCAACACAATTTTGAATAACATCTACGTGCTCTACGCCTTGCGTAGACACTATGAGGGGGTTGA

GCTGGACACTTACACCATGATCTCCTATGGAGACGACATCGTGGTGGCAAGTGATTATGATCTGGACTTT

GAGGCCCTCAAGCCTCACTTCAAATCTCTTGGCCAAACCATTACTCCAGCTGACAAAAGCGACAAAGGTT

TTGTTCTTGGTCACTCCATTACTGACGTCACTTTCCTCAAAAGACACTTCCACATGGATTATGGCACTGG

GTTTTACAAACCTGTGATGGCCTCGAAGACCCTCGAGGCTATCCTCTCCTTTGCACGCCGTGGG

>AY593806.1_C_Brazil_1971

GGGTTGATCGTGGACACCAGAGATGTGGAAGAGCGCGTTCACGTAATGCGCAAAACCAAGCTTGCACCCA

CCGTCGCGCACGGTGTGTTCAACCCTGACTTCGGGCCTGCTGCCTTGTCCAACAGGGACCCTCGCCTGAA

TGAAGGAGTTGTCCTCGATGAAGTCATCTTCTCCAAACACAAGGGAGACACAAAGATGTCCGAGGAGGAC

AAAGCGCTGTTCCGCCGCTGCGCTGCTGACTACGCGTCACGCTTGCACAGCGTACTGGGCACAGCAAATG

CCCCATTGAGCATTTACGAGGCAATCAAGGGCGTTGACGGACTCGACGCCATGGAGCCAGACACCGCACC

CGGCCTTCCCTGGGCCCTCCAGGGGAAACGCCGCGGCGCACTCATCGACTTTGAGAACGGCACGGTCGGG

CCTGAAGTCGAGGCTGCCTTGAAGCTCATGGAGAAAAGAGAATACAAGTTTGCTTGTCAGACCTTCCTGA

AGGACGAGATTCGCCCGATGGAGAAAGTACGTGCCGGCAAGACTCGCATTGTCGACGTTTTGCCTGTTGA

ACACATTCTTTACACCAGGATGATGATTGGCAGATTTTGTGCACAAATGCACTCAAACAACGGACCGCAA

ATTGGCTCAGCGGTCGGTTGCAACCCTGATGTTGATTGGCAAAGATTTGGCACACACTTCGCTCAGTACA

GAAACGTGTGGGATGTGGACTATTCGGCCTTTGATGCCAACCACTGCAGTGACGCCATGAACATCATGTT

CGAGGAGGTGTTCCGCACGGAGTTCGGGTTTCACCCCAACGCGGAGTGGATTCTGAAGACTCTCGTGAAC

ACGGAGCACGCCTATGAGAACAAACGCATCACTGTTGAGGGCGGGATGCCATCTGGTTGTTCCGCGACAA

GCATTATCAACACAATTTTGAACAACATCTATGTGCTCTACGCCTTGCGTAGACACTATGAGGGAGTTGA

GCTGGACACTTACACCATGATCTCCTATGGAGACGACATCGTGGTGGCTAGTGATTATGATCTGGACTTT

GAGGCTCTCAAGCCGCATTTCAAATCTATTGGTCAAACCATTACTCCAGCTGACAAAAGCGACAAAGGTT

TTGTTCTTGGTCACTCCATCACCGATGTCACTTTCCTCAAAAGACACTTCCACATGGATTATGGAACTGG

GTTTTACAAACCTGTGATGGCCTCAAAGACCCTTGAGGCTATCCTCTCCTTTGCACGCCGTGGG

>AY593807.1_C_Brazil_1955

GGGTTGATTGTTGACACCAGAGACGTGGAGGAGCGCGTTCACGTGATGCGCAAAACCAAGCTTGCACCCA

CCGTCGCGCACGGTGTGTTTAACCCTGAATTCGGGCCTGCTGCCTTGTCCAACAGGGACCCGCGCCTGAA

TGAAGGTGTTGTTCTCGATGAAGTCATCTTCTCCAAACACAAGGGAGACACAAAGATGTCTGAGGAGGAC

AAAGCGCTGTTCCGCCGCTGTGCTGCTGACTACGCGTCACGTCTGCATAGTGTGCTGGGCACAGCAAATG

CCCCATTGAGCATTTACGAGGCAATCAAGGGCGTCGACGGACTCGACGCAATGGAGCCAGACACTGCACC

TGGCCTCCCTTGGGCACTCCAGGGGAAGCGCCGCGGTGCGCTCATCGACTTTGAGAACGGCACTGTCGGA

CCCGAAGTTGAGGCTGCCTTGAAGCTCATGGAGAAAAGAGAATACAAATTTGCTTGTCAGACCTTCCTGA

AGGACGAGATTCGCCCGATGGAGAAAGTACGTGCCGGCAAGACTCGCATTGTCGACGTCCTGCCTGTTGA

ACACATTCTTTACACTAGGATGATGATTGGCAGATTTTGTGCACAAATGCACTCAAACAACGGACCGCAA

ATTGGCTCGGCGGTCGGTTGCAACCCTGATGTTGATTGGCAGAGATTTGGCACACACTTCGCCCAATACA

GAAACGTGTGGGACGTGGACTATTCGGCCTTTGATGCTAACCACTGCAGTGACGCCATGAACATCATGTT

TGAGGAGGTGTTTCGCACGGAGTTTGGATTCCACCCGAATGCCGAGTGGATTCTGAAGACTCTCGTGAAC

ACGGAACACGCCTATGAGAACAAACGCATCACTGTTGAGGGTGGAATGCCATCTGGTTGTTCCGCGACAA

GCATCATCAACACAATTTTGAACAACATCTATGTGCTCTACGCCCTGCGCAGACACTATGAGGGAGTTGA

GCTGGACACTTACACCATGATCTCCTACGGAGACGACATCGTGGTGGCAAGTGATTACGATTTGGACTTT

GAGGCTCTCAAGCCCCACTTTAAATCTCTTGGCCAAACCATCACTCCAGCTGACAAAAGCGACAAAGGTT

TTGTTCTTGGTCACTCCATCACCGATGTCACTTTCCTCAAAAGACACTTCCACATGGATTATGGAACTGG

GTTTTACAAACCTGTGATGGCCTCAAAGACCCTTGAGGCTATCCTCTCCTTTGCACGCCGTGGG

>AY593808.1_C_ARG_1966

GGGTTGATCGTTGATACCAGAGATGTGGAAGAGCGCGTTCATGTAATGCGCAAAACCAAGCTTGCACCCA

CCGTCGCACACGGTGTGTTCAACCCTGAGTTCGGGCCTGCCGCCTTGTCCAACAAGGACCCGCGTCTGAA

CGAAGGTGTTGTCCTCGATGAAGTCATCTTCTCCAAGCACAAAGGAGACACAAAGATGTCTGCGGAGGAC

AAAGCGCTGTTCCGCCGCTGCGCTGCTGACTACGCGTCACACCTGCACAGTGTGCTGGGTACGGCAAATG

CCCCACTGAGCATTTACGAGGCAATCAAGGGCGTTGACGGACTCGACGCCATGGAGCCGGACACCGCACC

TGGCCTTCCCTGGGCCCTCCAAGGGAAACGCCGTGGTGCACTCATTGACTTCGAGAACGGCACGGTCGGA

CCCGAAGTTGAGGCTGCCTTGAAGCTCATGGAGAAAAGAGAATACAAGTTTGTTTGCCAGACCTTCCTGA

AAGACGAAATTCGCCCGATGGAGAAAGTACGTGCCGGCAAGACTCGCATTGTCGACGTTTTGCCTGTTGA

ACACATTCTTTACACCAGGATGATGATTGGCAGATTCTGTGCACAAATGCACTCGAACAACGGACCGCAG

ATTGGCTCGGCGGTCGGTTGCAACCCTGATGTTGATTGGCAGAGATTTGGCACACACTTCGCCCAGTACA

GAAATGTGTGGGATGTGGACTATTCGGCCTTTGATGCTAACCATTGTAGTGACGCCATGAACATCATGTT

CGAGGAGGTGTTCCGCACGGAATTCGGTTTCCACCCGAATGCGGAGTGGATTCTGAAGACTCTCGTGAAT

ACGGAGCACGCCTATGAGAACAAGCGCATCACTGTTGAAGGCGGGATGCCGTCTGGTTGTTCCGCAACAA

GCATCATCAACACAATCTTGAACAACATCTATGTGCTCTACGCCTTGCGTAGACACTATGAGGGAGTTGA

GCTGGACACTTACACCATGATCTCCTACGGAGACGACATTGTGGTGGCAAGTGATTACGATCTGGACTTT

GAGGCTCTTAAGCCCCACTTTAAATCTCTTGGTCAAACCATTACTCCAGCTGACAAAAGCGACAAAGGTT

TTGTTCTTGGTCACTCCATCACCGATGTCACTTTCCTCAAAAGACACTTCCACATGGATTATGGCACTGG

GTTTTACAAACCTGTGATGGCCTCGAAGACCCTCGAGGCTATCCTCTCCTTTGCACGCCGTGGG

>AY593809.1_C_ARG_1969

GGGTTGATTGTTGACACCAAAGACGTGGAGGAGCGCGTTCACGTGATGCGCAAAACCAAGCTTGCACCCA

CCGTCGCGCACGGTGTGTTTAACCCTGAATTCGGGCCTGCTGCCTTGTCCAACAAGGACCCGCGCCTGAA

TGAAGGTGTTGTTCTCGATGAAGTCATCTTCTCCAAACACAAGGGAGACACAAAGATGTCTGAGGAGGAC

AAAGCGCTGTTCCGCCGCTGTGCTGCTGACTACGCGTCACGTTTACATAGTGTGCTGGGTACAGCAAATG

CCCCATTGAGCATTTACGAGGCAATCAAGGGCGTCGACGGACTCGACGCAATGGAGCCAGACACTGCACC

TGGCCTCCCTTGGGCACTCCGGGGGAAGCGCCGCGGTGCGCTCATCGACTTTGAGAACGGCACTGTCGGA

CCCGAAGTTGAGGCTGCCTTGAAGCTCATGGAGAAAAGAGAATACAAATTTGTTTGTCAGACCTTCCTGA

AGGACGAGATTCGCCCGATGGAGAAAGTACGTGCCGGCAAGACTCGCATTGTCGACGTCCTGCCTGTTGA

ACACATTCTTTACACTAGGATGATGATTGGCAGATTTTGTGCGCAAATGCACTCAAACAACGGACCGCAA

ATTGGCTCGGCGGTCGGTTGCAACCCTGATGTTGATTGGCAGAGATTTGGCACACACTTCGCCCAATACA

GAAACGTGTGGGATGTGGACTATTCGGCCTTTGATGCTAACCACTGCAGTGACGCCATGAACATCATGTT

TGAGGAGGTGTTTCGCACGGAGTTTGGATTCCACCCGAATGCCGAGTGGATTCTGAAGACTCTCGTGAAC

ACGGAACACGCCTATGAGAACAAACGCATCACTGTTGAGGGTGGAATGCCATCTGGTTGTTCCGCGACAA

GCATCATCAACACAATTTTGAACAACATCTATGTGCTCTACGCCCTGCGCAGACACTATGAGGGAGTTGA

GCTGGACACTTACACCATGATCTCCTATGGAGACGACATCGTGGTGGCAAGTGATTATGATTTGGACTTT

GAGGCTCTCAAGCCCCACTTTAAATCTCTCGGCCAAACCATCACTCCAGCTGACAAAAGCGACAAAGGTT

TTGCTCTCGGTCACTCCATCACCGATGTCACTTTCCTCAAAAGACACTTCCACATGGATCATGGAACTGG

GTTTTACAAACCTGTGATGGCCTCAAAGACCCTTGAGGCTATCCTCTCCTTTGCACGCCGTGGG

>AY593810.1_C_UKG_1970

GGGCTGATCGTTGATACCAGGGACGTGGAAGAGCGCGTTCACGTGATGCGCAAAACCAAGCTTGCACCCA

CCGTTGCACACGGTGTGTTCAACCCTGAGTTTGGGCCCGCTGCCTTGTCCAATAAGGACCCGCGCCTGGA

CGAGGGTGTTGTCCTCGACGAAGTTATCTTCGCCAAACACAAAGGAGACACAAAGATGTCTGCGGAGGAC

AAAGCGCTGTTCCGCCGCTGCGCTGCTGACTACGCGTCACGCTTGCACAGCGTACTGGGTACAGCAAACG

CCCCATTGAGCGTTTACGAGGCAATCAAAGGCGTCGACGGGCTCGATGCCATGGAGTCGGACACCGCGCC

TGGCCTCCCCTGGGCCCTCCAGGGGAAGCGCCGCGGCGCGCTCATTGACTTCGAAAACGGCACGGTTGGA

CCCGAAGTCGAGGCTGCCTTGAAGCTCATGGAGAAAAGAGAGTACAAGTTTGCTTGTCAGACCTTCCTGA

AGGACGAAATTCGCCCGATGGAGAAAGTACGTGCCGGCAAGACTCGCATTGTCGATGTTTTGCCTGTTGA

ACACATTCTTTACACCAGGATGATGATTGGCAGATTTTGTGCACAGATGCACTCGAACAACGGACCGCAA

ATTGGCTCAGCGGTCGGTTGCAACCCTGATGTTGATTGGCAAAGATTTGGCACACACTTCGCCCAGTACA

AAAATGTGTGGGACGTGGACTATTCAGCCTTTGATGCTAACCACTGCAGTGATGCTATGAACATCATGTT

TGAGGAGGTGTTTCGCACGGACTTTGGTTTCCACCCAAATGCTGAGTGGATCCTTAAGACTCTCGTGAAC

ACGGAGCACGCCTATGAGAACAAACGCATTACTGTTGAGGGCGGAATGCCGTCTGGCTGTTCCGCAACAA

GCATTATCAACACAATTTTGAACAACATCTACGTGCTCTACGCCTTGCGTAGACACTATGAGGGAGTTGA

GCTGGACACTTACACCATGATCTCCTACGGAGACGACATCGTGGTGGCAAGTGACTACGATCTGGACTTT

GAGGCTCTTAAGCCCCACTTTAAATCTCTTGGTCAAACTATCACTCCAGCTGACAAAAGCGACAAAGGTT

TTGTTCTTGGTCACTCCATTACCGATGTCACCTTCCTCAAAAGACACTTCCACATGGATTATGGAACTGG

GTTTTATAAACCTGTGATGGCCTCAAAGACCCTTGAGGCTATCCTCTCCTTTGCACGCCGTGGG

>AY593812.1_O_PHI_1958

GGGTTGATTGTTGACACCAGAGATGTTGAAGAGCGTGTGCACGTCATGCGCAAAACCAAGCTTGCACCCA

CCGTGGCACACGGTGTGTTTAACCCCGAGTTTGGGCCTGCCGCCTTGTCTAACAAGGACCCGCGCCTGAA

TGAGGGGGTTGTCCTCGATGAAGTCATCTTCTCCAAACACAAGGGAGACACACGGATGTCTGAGGAGGAC

AAAGCGCTGTTCCGCCGCTGTGCTGCCGACTACGCGTCGCGCCTACACAGCGTGCTGGGGACGGCAAATG

CCCCATTGAGCATTTATGAGGCCATCAAAGGCGTCGACGGGCTCGACGCCATGGAATCAGACACCGCGCC

TGGCCTCCCCTGGGCCCTCCAGGGGAAACGCCGTGGTGCGTTGATTGACTTCGAGAACGGCACAGTTGGA

CCCGAGGTCGAGGCCGCCTTAAAGCTCATGGAGAAGAGAGAATACAAATTTGCCTGCCAGACCTTCCTGA

AAGACGAAATTCGTCCGATGGAGAAAGTACGCGCCGGCAAGACCCGCATTGTCGACGTTTTGCCTGTTGA

ACACATTCTTTACACCAGGATGATGATTGGCAGATTTTGTGCTCAAATGCACTCAAACAACGGACCGCAG

ATTGGCTCAGCGGTCGGCTGTAACCCTGATGTTGATTGGCAAAGATTTGGCACACACTTCGCTCAGTACA

GAAACGTGTGGGATGTGGACTACTCGGCCTTTGATGCTAACCACTGCAGTGATGCGATGAACATTATGTT

TGAGGAGGTGTTTAACACGGACTTCGGTTTCCACCCGAATGCCGAGTGGATTCTGAAAACTCTCGTGAAC

ACAGAACACGCCTATGAAAACAAACGCATCACCGTTGAGGGCGGAATGCCATCTGGTTGTTCCGCGACAA

GCATCATCAACACTATTTTGAACAACATTTACGTGCTCTACGCCTTGCGTAGACACTATGAGGGTGTTGA

GCTGGACACTTACACCATGATCTCCTACGGAGACGACATCGTGGTGGCAAGTGATTACGATTTGGACTTT

GAGGCTCTTAAGCCTCACTTTAAATCTCTTGGTCAAACCATCACTCCAGCTGACAAAAGCGACAAAGGCT

TTGTTCTTGGTCACTCCATTACCGATGTTACTTTCCTCAAAAGACACTTCCACATGGACTATGGAACTGG

GTTTTACAAACCTGTGATGGCCTCAAAGACCCTTGAGGCTATCCTCTCCTTTGCACGCCGTGGG

>AY593813.1_O_ISA_1962

GGGTTGATTGTTGACACCAGAGATGTGGAAGAGCGTGTGCACGTCATGCGCAAAACCAAGCTTGCACCCA

CCGTGGCGCACGGTGTGTTCAGCCCTGACTTCGGCCCTGCTGCCTTGTCCAACAAGGACCCGCGGTTGAA

TGAAGGGGTTGTCCTCGACGAGGTCATCTTCTCCAAACACAAAGGGGATGTGAAAATGTCAGAGGAAGAC

AAGAAGCTGTTCCGTCGCTGTGCTGCTGACTACGCGTCACGCCTGCACAATGAACTGGGTACAGCCAACA

GCCCACTGAGCATCTACGAGGCCATCAAGGGCGTCGACGGACTTGACGCCATGGAACCAGACACTGCCCC

CGGCCTTCCCTGGGCTCTCCAAGGGAAACGCCGTGGTGCTCTGATAGACTTTGAGAACGGCACTGTCGGC

CCTGAAGTGTCGGCAGCTCTGGAGCTCATGGAAAAGAGAGAATACAAATTTGCTTGTCAAACCTTCCTGA

AGGACGAAATTCGCCCGATGGAGAAAGTACGTGCCGGCAAGACTCGCATCGTCGATGTTTTGCCTGTTGA

ACACATTCTTTATACTAGGATGATGATTGGTAGATTTTGTGCTCAAATGCACTCAAACAACGGACCGCGC

ATTGGCTCGGCGGTCGGTTGCAATCCTGATGTTGATTGGCAAAGATTTGGCACACACTTCGCACAGTACA

AAAACGTGTGGGATGTGGATTATTCGGCCTTTGATGCTAACCACTGCAGTGACGCGATGAACATCATGTT

TGAGGAAGTGTTCAGCACGGACTTCGGTTTCCACCCAAATGCTGAGTGGATTCTGAAGACTCTCGTGAAC

ACGGAACACGCTTATGAAAACAAGCGCATCACTGTGGAAGGTGGAATGCCATCTGGTTGTTCCGCCACGA

GCATCATCAACACAATTCTCAACAACATTTATGTCCTCTACGCTCTGCGTAGACACTATGAGGGAGTTGA

GCTGGACACCTACACGATGATTTCCTACGGAGATGACATCGTTGTAGCAAGTGATTATGATTTGGACTTC

GAGGCCCTCAAGCCTCACTTTAAATCCCTTGGTCAAACAATTACTCCAGCTGACAAAAGCGACAAAGGTT

TTGTTCTTGGTCACTCCATCACCGATGTCACTTTTCTCAAAAGACACTTCCACATGGATCATGGAACTGG

GTTTTACAAACCTGTGATGGCCTCAAAGACTCTTGAGGCTATCCTCTCCTTTGCACGCCGTGGG

>AY593814.1_O_ARG_1965

GGGTTGATTGTGGACATCAGAGATGTGGAAGAGCGCGTTCACGTGATGCGCAAAACCAAGCTTACACCCA

CCGTTGCACACGGTGTGTTCAACCCCGAGTTTGGGCCCGCTGCCTTGTCCAACAAGGACCCGCGTCTGAA

CGAGGGTGTTGTCCTCGATGAAGTCATCTTCTCCAAACACAAGGGAGACACAAAGATGTCTGAGGAGGAC

AAAGCGCTGTTCCGCCGCTGCGCTGCTGACTACGCGTCACGCCTGCACAGCGTGTTGGGCACAGCAAATG

CCCCACTGAGCATCTACGAGGCAATCAAGGGTGTCGACGGACTCGACGCCATGGAACCAGACACTGCGCC

CGGCCTCCCCTGGGCCCTCCAGGGTAAACGCCGCGGCGCGCTCATCGACTTCGAGAACGGCACGGTCGGA

CCCGAAGTCGAGGCTGCCCTGAAGCTCATGGAGAAGAGAGAATACAAATTTGCTTGTCAGACCTTCCTGA

AGGACGAGATTCGCCCGTTGGAGAAAGTACGTGCCGGCAAGACTCGCATTGTCGACGTCCTGCCCGTTGA

GCATATTCTTTACACCAGGATGATGATTGGCAGATTTTGTGCACAGATGCACTCAAACAACGGACCGCAA

ATTGGCTCAGCGGTCGGTTGCAACCCTGATGTTGATTGGCAGAGATTTGGCACACACTTCGCCCAGTACA

GAAACGTGTGGGATGTGGACTATTCGGCCTTTGATGCTAATCACTGCAGTGATGCCATGAACATCATGTT

TGAGGAGGTGTTTCGCACGGAGTTCGGCTTCCACCCGAATGCCGAGTGGATCCTGAAGACCCTTGTGAAC

ACGGAACACGCCTATGAGAACAAACGCATCACTGTTGAAGGCGGAATGCCGTCTGGTTGTTCCGCAACAA

GCATCATCAACACAATTTTGAACAACATCTACGTGCTCTACGCTCTGCGTAGACACTACGAGGGAGTTGA

GCTGGACACGTACACCATGATCTCCTACGGAGACGACATCGTGGTGGCAAGTGATCATGATTTAGACTTC

GAGGCTCTCAAGCCCCACTTTAAATCCCTTGGCCAAACCATCACTCCAGCTGACAAAAGCGACAAAGGTT

TTGTTCTTGGTCACTCCATTACCGATGTCACTTTCCTCAAAAGACACTTCCACATGGACTATGGAACTGG

GTTTTACAAACCTGTGATGGCCTCAAAGACCCTTGAGGCTATCCTCTCCTTTGCACGCCGTGGG

>AY593815.1_O_UKG_1967

GGGTTGATTGTGGACACCAGAGATGTGGAAGAGCGCGTTCACGTGATGCGCAAAACCAAGCTTGCACCCA

CCGTTGCACACGGTGTGTTCAACCCCGAGTTTGGGCCCGCTGCCTTGTCCAACAAGGACCCGCGTCTGAA

CGAGGGTGTTGTCCTCGATGAAGTCATCTTCTCCAAACACAAGGGAGACACAAAGATGTCTGAGGAGGAC

AAAGCGCTGTTCCGCCGCTGTGCTGCTGACTACGCGTCACGCCTGCACAGCGTGTTGGGCACAGCAAATG

CCCCATTGAGCATCTACGAGGCAATCAAGGGTGTCGACGGACTCGACGCCATGGAACCAGACACAGCGCC

CGGCCTCCCCTGGGCCCTCCAGGGTAAACGCCGCGGCGCGCTCATCGACTTCGAAAACGGCACGGTCGGA

CCCGAAGTCGAGGCTGCCCTGAAGCTCATGGAGAAGAGAGAATACAAATTTGCTTGTCAGACCTTCCTGA

AGGACGAGATTCGCCCGATGGAGAAAGTACGTGCCGGCAAGACTCGCATTGTCGACGTCTTGCCCGTTGA

GCATATTCTTTACACCAGGATGATGATTGGCAGATTTTGTGCACAGATGCACTCAAACAACGGACCGCAA

ATTGGCTCAGCGGTCGGTTGCAACCCTGATGTTGATTGGCAGAGATTTGGCACACACTTCGCCCAGTACA

GAAACGTGTGGGATGTGGACTATTCGGCCTTTGATGCTAATCACTGCAGTGATGCCATGAACATCATGTT

TGAGGAGGTGTTTCGCACGGAGTTCGGCTTCCACCCGAATGCCGAGTGGATCCTGAAGACTCTTGTGAAC

ACGGAACACGCCTATGAGAACAAACGCATCACTGTTGAAGGCGGAATGCCGTCTGGTTGTTCCGCAACAA

GCATCATCAACACAATTTTGAACAACATCTACGTGCTCTACGCCCTGCGTAGACACTATGAGGGAGTTGA

GCTGGACACATACACCATGATCTCCTACGGAGACGACATCGTGGTGGCAAGTGATTATGACTTGGACTTC

GAGGCTCTCAAGCCCCACTTTAAATCCCTTGGTCAAACCATCACTCCAGCTGACAAAAGCGACAAAGGTT

TTGTTCTTGGTCACTCCATTACCGATGTCACTTTCCTCAAAAGACACTTCCACATGGACTATGGAACTGG

GTTTTACAAACCTGTGATGGCCTCAAAGACCCTTGAGGCTATCCTCTCCTTTGCACGCCGTGGG

>AY593816.1_O_UKG_1967

GGGTTGATTGTGGACACCAGAGATGTGGAAGAGCGCGTTCACGTGATGCGCAAAACCAAGCTTGCACCCA

CCGTTGCACACGGTGTGTTCAACCCCGAGTTTGGGCCCGCTGCCTTGTCCAACAAGGACCCGCGTCTGAA

CGAGGGTGTTGTCCTCGATGAAGTCATCTTCTCCAAACACAAGGGAGACACAAAGATGTCTGAGGAGGAC

AAAGCGCTGTTCCGCCGCTGTGCTGCTGACTACGCGTCACGCCTGCACAGCGTGTTGGGCACAGCAAATG

CCCCATTGAGCATCTACGAGGCAATCAAGGGTGTCGACGGACTCGACGCCATGGAACCAGATACAGCGCC

CGGCCTCCCCTGGGCCCTCCAGGGTAAACGCCGCGGCGCGCTCATCGACTTCGAAAACGGCACGGTCGGA

CCCGAAGTCGAGGCTGCCCTGAAGCTCATGGAGAAGAGAGAATACAAATTTGCTTGTCAGACCTTCCTGA

AGGACGAGATTCGCCCGATGGAGAAAGTACGTGCCGGCAAGACTCGCATTGTCGACGTCTTGCCCGTTGA

GCATATTCTTTACACCAGGATGATGATTGGCAGATTTTGTGCACAGATGCACTCAAACAACGGACCGCAA

ATTGGCTCAGCGGTCGGTTGCAACCCTGATGTTGATTGGCAGAGATTTGGCACACACTTCGCCCAGTACA

GAAACGTGTGGGATGTGGACTATTCGGCCTTTGATGCTAATCACTGCAGTGATGCCATGAACATCATGTT

TGAGGAGGTGTTTCGCACGGAGTTCGGCTTCCACCCGAATGCCGAGTGGATCCTGAAGACTCTTGTGAAC

ACGGAACACGCCTATGAGAACAAACGCATCACTGTTGAAGGCGGAATGCCGTCTGGTTGTTCCGCAACAA

GCATCATCAACACAATTTTGAACAACATCTACGTGCTCTACGCCCTGCGTAGACACTATGAGGGAGTTGA

GCTGGACACATACACCATGATCTCCTACGGAGACGACATCGTGGTGGCAAGTGATTATGACTTGGACTTC

GAGGCTCTCAAGCCCCACTTTAAATCCCTTGGTCAAACCATCACTCCAGCTGACAAAAGCGACAAAGGTT

TTGTTCTTGGTCACTCCATTACCGATGTCACTTTCCTCAAAAGACACTTCCACATGGACTATGGAACTGG

GTTTTACAAACCTGTGATGGCCTCAAAGACCCTTGAGGCTATCCTCTCCTTTGCACGCCGTGGG

>AY593817.1_O_Belgium_1973

GGGTTGATTGTGGACACCAGAGATGTGGAAGAGCGCGTTCACGTGATGCGCAAAACCAAGCTTGCACCCA

CCGTTGCACACGGTGTGTTCAACCCCGAGTTTGGGCCCGCTGCCTTGTCCAACAAGGACCCGCGTCTGAA

CGAGGGTGTTGTCCTCGATGAAGTCATCTTCTCCAAACACAAGGGAGACACAAAGATGTCTGAGGAGGAC

AAAGCGCTGTTCCGCCGCTGCGCTGCTGACTACGCGTCACGCCTGCACAGCGTGTTGGGCACAGCAAATG

CCCCACTGAGCATCTACGAGGCAATCAAGGGTGTCGACGGACTCGACGCCATGGAACCAGACACTGCGCC

CGGCCTCCCCTGGGCCCTCCAGGGTAAACGCCGCGGCGCGCTCATCGACTTCGAGAACGGCACGGTCGGA

CCCGAAGTTGAGGCTGCCCTGAAGCTCATGGAGAAGAGAGAATACAAATTTGTTTGTCAGACCTTCCTGA

AGGACGAGATTCGCCCGTTGGAGAAAGTACGTGCCGGCAAGACTCGCATTGTCGACGTCCTGCCCGTTGA

GCACATTCTTTACACCAGGATGATGATTGGCAGATTTTGTGCACAGATGCACTCAAACAACGGACCGCAA

ATTGGCTCAGCGGTCGGTTGCAACCCTGATGTTGATTGGCAGAGATTTGGCACACACTTCGCCCAATACA

GAAACGTGTGGGATGTGGACTATTCGGCCTTTGATGCTAATCACTGTAGTGATGCCATGAACATCATGTT

TGAGGAGGTGTTTCGCACGGAGTTCGGCTTCCACCCGAATGCCGAGTGGGTACTGAAGACTCTTGTGAAC

ACGGAACACGCCTATGAGAACAAACGCATCACTGTTGGAGGCGGAATGCCGTCTGGTTGCTCCGCAACAA

GCATCATCAACACAATTTTGAACAACATCTACGTGCTCTACGCCCTGCGTAGACACTATGAGGGAGTTGA

GCTGGACACATACACCATGATCTCCTACGGAGACGACATCGTGGTGGCAAGTGATTATGATTTGGACTTC

GAGGCTCTCAAGCCCCACTTTAAATCCCTTGGCCAAACCATCACTCCAGCTGACAAAAGCGACAAAGGTT

TTGTTCTTGGTCACTCCATTACCGATGTCACTTTCCTCAAAAGACACTTCCACATGGACTATGGAACTGG

GTTTTACAAACCTGTGATGGCCTCAAAGACCCTTGAGGCTATCCTCTCCTTTGCACGCCGTGGG

>AY593818.1_O_ARG_1958

GGGTTGATTGTGGACACCAGAGATGTGGAAGAGCGCGTTCACGTGATGCGCAAAACCAAGCTTGCACCCA

CCGTTGCACACGGTGTGTTCAACCCCGAGTTTGGGCCCGCTGCCTTGTCCAACAAGGACCCGCGTCTGAA

CGAGGGTGTTGTCCTCGATGAAGTCATCTTCTCCAAACACAAGGGAGACACAAAGATGTCTGAGGAGGAC

AAAGCGCTGTTCCGCCGCTGCGCTGCTGACTACGCGTCACGCCTGCACAGCGTGTTGGGCACAGCAAATG

CCCCACTGAGCATCTACGAGGCAATCAAGGGTGTCGACGGACTCGACGCCATGGAACCAGACACTGCGCC

CGGCCTCCCCTGGGCCCTCCAGGGTAAACGCCGCGGCGCGCTCATCGACTTCGAGAACGGCACGGTCGGA

CCCGAAGTCGAGGCTGCCCTGAAGCTCATGGAGAAGAGAGAATACAAATTTGCTTGTCAGACCTTCCTGA

AGGACGAGATTCGCCCGATGGAGAAAGTACGTGCCGGCAAGACTCGCATTGTCGACGTCCTGCCCGTTGA

GCATATTCTTTACACCAGGATGATGATTGGCAGATTTTGTGCACAGATGCACTCAAACAACGGACCGCAG

ATTGGCTCAGCGGTCGGTTGCAACCCTGATGTTGATTGGCAGAGATTTGGCACACACTTCGCCCAGTACA

GAAACGTGTGGGATGTGGACTATTCGGCCTTTGATGCTAATCACTGCAGTGATGCCATGAACATCATGTT

TGAGGAGGTGTTTCGCACGGAGTTCGGCTTCCACCCGAATGCTGAGTGGATCCTGAAGACTCTTGCGAAC

ACGGAACACGCCTATGAGAACAAACGCATCACTGTTGAAGGCGGAATGCCGTCTGGTTGTTCCGCAACAA

GCATCATCAACACAATTTTGAACAACATCTACGTGCTCTACGCCCTGCGTAGACACTATGAGGGAGTTGA

GCTGGACACATACACCATGATCTCCTACGGAGACGACATCGTGGTGGCAAGTGATTATGATTTGGACTTC

GAGGCTCTCAAGCCCCACTTTAAATCCCTTGGTCAAACCATCACTCCAGCTGACAAAAGCGACAAAGGTT

TTGTTCTTGGTCACTCCATTACCGATGTCACTTTCCTCAAAAGACACTTCCACATGGACTATGGAACTGG

GTTTTACAAACCTGTGATGGCCTCAAAGACCCTTGAGGCTATCCTCTCCTTTGCACGCCGTGGG

>AY593819.1_O_ARG_1994

GGGTTGATTGTGGACACCAGAGATGTGGAAGAGCGCGTTCACGTGATGCGCAAAACCAAGCTTGCACCCA

CCGTTGCACACGGTGTGTTCAACCCCGAGTTTGGGCCCGCTGCCTTGTCCAACAAGGACCCGCGTCTGAA

CGAGGGTGTTGTCCTCGATGAAGTCATCTTCTCCAAACACAAGGGAGACACAAAGATGTCTGAGGAGGAC

AAAGCGCTGTTCCGCCGCTGCGCTGCTGACTACGCGTCACGCCTGCACAGCGTGTTGGGCACAGCAAATG

CCCCACTGAGCATCTACGAGGCAATCAAGGGTGTCGACGGACTCGACGCCATGGAACCAGACACTGCGCC

CGGCCTCCCCTGGGCCCTCCAGGGTAAACGCCGCGGCGCGCTCATCGACTTCGAGAACGGCACGGTCGGA

CCCGAAGTCGAGGCTGCCCTGAAGCTCATGGAGAAGAGAGAATACAAATTTGCTTGTCAGACCTTCCTGA

AGGACGAGATTCGCCCGATGGAGAAAGTACGTGCCGGCAAGACTCGCATTGTCGACGTCCTGCCCGTTGA

GCATATTCTTTACACCAGGATGATGATTGGCAGATTTTGTGCACAGATGCACTCAAACAACGGACCGCAA

ATTGGCTCAGCGGTCGGTTGCAACCCTGATGTTGATTGGCAGAGATTTGGCACACACTTCGCCCAGTACA

GAAACGTGTGGGATGTGGACTATTCGGCCTTTGATGCTAATCACTGCAGTGATGCCATGAACATCATGTT

TGAGGAGGTGTTTCGCACGGAGTTCGGCTTCCACCCGAATGCCGAGTGGATCCTGAAGACTCTTGTGAAC

ACGGAACACGCCTATGAGAACAAACGCATCACTGTTGAAGGCGGAATGCCGTCTGGTTGTTCCGCAACAA

GCATCATCAACACAATTTTGAACAACATCTACGTGCTCTACGCCCTGCGTAGACACTATGAGGGAGTTGA

GCTGGACACATACACCATGATCTCCTACGGAGACGACATCGTGGTGGCAAGTGATTATGATTTGGACTTC

GAGGCTCTCAAGCCCCACTTTAAATCCCTTGGTCAAACCATCACTCCAGCTGACAAAAGCGACAAAGGTT

TTGTTCTTGGTCACTCCATTACCGATGTCACTTTCCTCAAAAGACACTTCCACATGGACTATGGAACTGG

GTTTTACAAACCTGTGATGGCCTCAAAGACCCTTGAGGCTATCCTCTCCTTTGCACGCCGTGGG

>AY593820.1_O_ARG_1964

GGGTTGATTGTGGACACCAGAGATGTGGAAGAGCGCGTTCACGTGATGCGCAAAACCAAGCTTGCACCCA

CCGTTGCACACGGTGTGTTCAACCCCGAGTTTGGGCCCGCTGCCTTGTCCAACAAGGACCCGCGTCTGAA

CGAGGGTGTTGTCCTCGATGAAGTCATCTTCTCCAAACACAAGGGAGACACAAAGATGTCTGAGGAGGAC

AAAGCGCTGTTCCGCCGCTGCGCTGCTGACTACGCGTCACGCCTGCACAGCGTGTTGGGCACAGCAAATG

CCCCACTGAGCATCTACGAGGCAATCAAGGGTGTCGACGGACTCGACGCCATGGAACCAGACACTGCGCC

CGGCCTCCCCTGGGCCCTCCAGGGTAAACGCCGCGGCGCGCTCATCGACTTCGAGAACGGCACGGTCGGA

CCCGAAGTCGAGGCTGCCCTGAAGCTCATGGAGAAGAGAGAATACAAATTTGCTTGTCAGACCTTCCTGA

AGGACGAGATTCGCCCGATGGAGAAAGTACGTGCCGGCAAGACTCGCATTGTCGACGTCCTGCCCGTTGA

GCATATTCTTTACACCAGGATGATGATTGGCAGATTTTGTGCACAGATGCACTCAAACAACGGACCGCAA

ATTGGCTCAGCGGTCGGTTGCAACCCTGATGTTGATTGGCAGAGATTTGGCACACACTTCGCCCAGTACA

GAAACGTGTGGGATGTGGACTATTCGGCCTTTGATGCTAATCACTGCAGTGATGCCATGAACATTATGTT

TGAGGAGGTGTTTCGCACGGAGTTCGGCTTCCACCCGAATGCCGAGTGGATCCTGAAGACTCTTGTGAAC

ACGGAACACGCCTATGAGAACAAACGCATCACTGTTGAAGGCGGAATGCCGTCTGGTTGTTCCGCAACAA

GCATCATCAACACAATTTTGAACAACATCTACGTGCTCTACGCCCTGCGTAGACACTATGAGGGAGTTGA

GCTGGACACATACACCATGATCTCCTACGGAGACGACATCGTGGTGGCAAGTGATTATGATTTGGACTTC

GAGGCTCTCAAGCCCCACTTTAAATCCCTTGGTCAAACCATCACTCCAGCTGACAAAAGCGACAAAGGTT

TTGTTCTTGGTCACTCCATTACCGATGTCACTTTCCTCAAAAGACACTTCCACATGGACTATGGAACTGG

GTTTTACAAACCTGTGATGGCCTCAAAGACCCTTGAGGCTATCCTCTCCTTTGCACGCCGTGGG

>AY593821.1_O_ARG_1967

GGGTTGATTGTTGACACCAGAGATGTGGAAGAGCGCGTCCACGTGATGCGCAAGACCAAGCTTGCACCCA

CCGTCGCGCACGGTGTGTTCAACCCAGAGTTCGGGCCCGCCGCCTTGTCCAACAAGGACCCGCGCCTGAA

TGAAGGTGTTGTCCTCGATGAAGTCATCTTCTCCAAACACAAAGGAGACACAAAGATGTCTGAGGAGGAC

AAAGCGCTGTTCCGCCGCTGTGCTGCTGACTACGCGTCACGTTTGCACAGTGTTCTGGGTACGGCAAATG

CCCCATTGAGCATTTACGAGGCAATCAAAGGCGTTGACGGACTCGACGCAATGGAGCCAGACACTGCACC

CGGTCTCCCCTGGGCACTCCAGGGGAAGCGCCGCGGCGCGCTCATCGACTTCGAGAACGGCACTGTCGGA

CCTGAAGTTGAGGCTGCCTTGAAGCTCATGGAGAAGAGAGAATACAGATTTGCTTGTCAGACTTTCCTGA

AGGACGAAATTCGCCCGATGGAGAAAGTACGCGCCGGCAAGACTCGCATCGTCGACGTCTTGCCCGTTGA

GCACATTCTTTACACCAGGATGATGATTGGTAGGTTTTGTGCACAAATGCACTCAAACAACGGACCGCAA

ATTGGCTCGGCGGTCGGTTGCAACCCGGATGTTGATTGGCAAAGATTTGGCACACACTTCGCTCAGTACA

GAAACGTGTGGGATGTGGACTATTCGGCCTTTGATGCTAACCATTGCAGCGATGCCATGAACATCATGTT

TGAGGAGGTGTTCCGCACAGACTTTGGTTTCCACCCGAACGCCGAGTGGATCCTGAAGACCCTTGTGAAC

ACGGAGCACGCCTATGAGAACAAACGCATCACTGTTGAGGGCGGGATGCCATCTGGTTGTTCCGCAACAA

GCATCATCAACACAATTTTGAACAACATCTACGTGCTCTACGCCCTGCGTAGACACTATGAGGGAGTTGA

GCTGGACACTTACACCATGATCTCCTACGGAGACGACATCGTGGTGGCAAGTGATTACGATTTGGACTTC

GAGGCCCTCAAGCCTCACTTTAAATCTCTTGGTCAAACCATCACTCCAGCTGACAAAAGCGACAAAGGTT

TTGTTCTTGGTCACTCCATCACCGATGTCACTTTCCTCAAAAGACACTTCCACATGGATTATGGAACTGG

GTTTTACAAACCTGTGATGGCCTCAAAGACCCTTGAGGCTATCCTCTCCTTTGCACGCCGTGGG

>AY593823.1_O_TUR_1969

GGGTTGATTGTTGATACCAGAGATGTGGAAGAGCGCGTGCATGTCATGCGTAAAACCAAGCTTGCACCCA

CCGTGGCACACGGTGTGTTTAACCCTGAATTTGGTCCCGCTGCCTTGTCCAACAAGGACCCGCGGCTGAA

CGAAGGGGTTGTCCTCGATGAAGTCATCTTCTCCAAACACAAGGGAGATACGAAAATGTCTGAGGAGGAC

AAAGCGCTGTTCCGCCGCTGCGCTGCCGACTACGCGTCGCACTTGCACAGCGTGCTGGGGACGGCAAATG

CCCCATTGAGCATCTATGAGGCCATCAAGGGCGTCGACGGGCTCGATGCCATGGAGCCGGACACCGCGCC

CGGCCTCCCCTGGGCCCTCCAGGGGAAACGCCGTGGTGCGTTGATTGACTTCGAGAACGGCACGGTCGGA

CCCGAAGTCGAGGCTGCCCTAAAGCTCATGGAGAAAAGAGAGTACAAATTTGCTTGCCAGACCTTCCTGA

AAGACGAGATTCGTCCGATGGAAAAAGTACGTGCTGGCAAGACTCGCATTGTCGACGTTTTGCCCGTGGA

ACACATTCTTTACACCAGGATGATGATTGGCAGATTCTGTGCTCAAATGCACACAAACAATGGACCGCAG

ATTGGCTCAGCGGTCGGTTGCAATCCTGATGTTGATTGGCAAAGATTTGGCACACATTTTGCTCAGTACA

GAAACGTGTGGGATGTGGACTATTCGGCCTTTGATGCTAACCACTGCAGTGACGCAATGAACATCATGTT

TGAGGAGGTGTTTCGCACAGACTTCGGTTTCCACCCAAATGCTGAGTGGATTCTGAAGACTCTTGTGAAC

ACGGAGCACGCCTATGAGAACAAACGTATCACTGTTGAGGGCGGGATGCCGTCTGGCTGTTCCGCGACAA

GCATCATCAACACAATTTTGAACAACATTTATGTGCTCTACGCTCTTCGTAGACACTATGAGGGAGTTGA

GCTGGACACCTACACCATGATCTCCTACGGAGATGACATCGTGGTTGCAAGTGACTACGATCTGGATTTT

GAGGCTCTCAAACCCCACTTCAAATCTCTTGGTCAAACCATCACTCCAGCTGACAAAAGCGACAAAGGTT

TTGTTCTTGGTCACTCCATTACCGATGTCACTTTCCTCAAAAGACACTTCCACATGGACTATGGAACTGG

GTTTTACAAACCTGTGATGGCCTCAAAGACCCTCGAGGCCATTCTCTCCTTTGCACGCCGTGGG

>AY593824.1_O_SKR_2000

GGATTGATAGTTGACACCAGAGATGTTGAGGAGCGCGTACATGTCATGCGCAAAACCAAGCTCGCACCCA

CCGTGGCACACGGTGTGTTTAACCCCGAATTTGGGCCTGCCGCCTTGTCCAACAAGGACCCGCGCCTGAA

TGAGGGGGTTGTCCTCGATGAAGCCATCTTCTCCAAACACAAGGGAAACACAAAGATGTCTGAGGAGGAC

AAAGCGCTGTTCCGCCGCTGTGCTGCTGACTACGCGTCGCGTCTGCATAGCGTGCTGGGTACGGCAAACG

CCCCACTGAGCATTTACGAGGCAATCAAGGGCGTCGACGGACTTGACGCCATGGAACCAGACACCGCGCC

TGGTTTACCCTGGGCTCTCCAGGGGAAACGCCGTGGTGCGCTCATTGACTTTGAGAACGGCACAATCGGA

CCCGAGGTTGAAGCTGCCTTGAAGCTCATGGAGAAAAGAGAGTACAAGTTTGTATGTCAGACCTTCCTGA

AGGACGAGATTCGCCCGATGGAGAAGGTACGTGCCGGCAAGACTCGCATTGTCGACGTCCTGCCTGTTGA

ACACATTCTTTACACCAGGATGATGATTGGCAGATTTTGTGCTCAAATGCACTCAAACAACGGACCGCAA

ATTGGCTCGGCGGTTGGGTGTAATCCTGATGTTGATTGGCAAAGATTTGGCACGCATTTTGCTCAGTACA

GAAACGTGTGGGATGTAGACTATTCGGCCTTTGATGCCAACCACTGCAGTGACGCAATGAACATCATGTT

TGAGGAGGTGTTCAACACGGATTTCGGTTTCCACCCAAACGCTGAGTGGATCCTGAAAACTCTCGTGAAC

ACTGAACACGCCTATGAGAACAAACGCATCACTGTTGAAGGCGGGATGCCGTCTGGTTGTTCCGCAACAA

GCATCATCAACACAATTTTGAACAACATCTACGTGCTCTACGCCTTGCGTAGGCACTATGAGGGAGTTGA

GCTGGACTCTTACACCATGATCTCCTACGGAGACGACATCGTGGTTGCAAGTGATTACGATCTGGACTTT

GAGGCCCTCAAGCCTCACTTCAAATCCCTTGGTCAAACCATTACTCCAGCTGACAAAAGCGACAAAGGTT

TTGTTCTTGGTCACTCCATTACCGATGTCACTTTCCTCAAAAGATCTTTCCACATGGACTATGGAACTGG

GTTTTACAAACCTGTGATGGCTTCGAAGACCCTCGAGGCTATCCTCTCCTTTGCACGCCGTGGG

>AY593825.1_O_ARG_1939

GGGTTGATTGTCGACACCAGAGATGTGGAAGAGCGCGTTCACGTGATGCGCAAAACCAAGCTTGCACCCA

CCGTTGCACACGGTGTGTTCAACCCTGAGTTTGGGCCTGCCGCCTTGTCTAACAAGGACCCGCGCCTAAA

CGAGGGTGTTGTCCTTGATGAAGTCATCTTCTCCAAACACAAAGGAGACACACAGATGTCCGAGGAAGAC

AAAGCGCTGTTCCGCCGCTGCGCCGCTGACTACGCGTCACGCTTGCACAGCGTACTGGGTACAGCAAACG

CCCCATTGAGCATCTACGAGGCAATCAAGGGCATTGACGGACTCGACGCCATGGAGCCAGACACTGCGCC

TGGCCTCCCCTGGGCTCTCCAGGGGAAGCGTCGTGGCGCGCTCATCGACTTTGAGAACGGCACGGTCGGG

CCCGAAGTCGAGACCGCCTTGGAGCTCATGGAGAAAAGAGAATACAAATTTGCTTGCCAGACCTTCTTGA

AGGACGAAATTCGCCCGATGGAGAAAGTACGTGCCGGCAAGACTCGCATTGTCGACGTTCTGCCCGTTGA

ACACATACTTTACACCAGGATGATGATTGGCAGGTTCTGTGCACAAATGCACTCAAACAACGGACCACAA

ATTGGCTCAGCGGTCGGTTGCAACCCTGATGTTGACTGGCAAAGATTTGGCACACACTTCGCCCAATACA

GAAACGTGTGGGATGTGGACTATTCGGCCTTCGATGCTAACCACTGCAGTGACGCCATGAACATCATGTT

TGAGGAAGTGTTCCGCACGGACTTTGGTTTCCACCCAAACGCTGAGTGGATCCTGAAGACTCTCGTGAAC

ACGGAACACGCCTATGAGAACAAACGCATCACTGTTGAGGGCGGGATGCCATCTGGTTGCTCCGCAACAA

GCATCATTAACACAATCTTGAACAACATCTATGTGCTCTACGCTTTGCGTAGGCACTATGAGGGAGTTGA

GCTGGACACTTACACCATGATCTCCTACGGAGACGACATCGTGGTTGCAAGTGATTACGACTTGGACTTT

GAGGCTCTCAAGCCCCATTTTAAATCTCTTGGCCAAACCATTACTCCAGCTGACAAAAGCGACAAAGGTT

TTGTTCTTGGTCACTCGATCACCGATGTCACTTTCCTCAAAAGACACTTCCACATGGACTATGGAACTGG

GTTTTACAAACCTGTGATGGCCTCAAAGACTCTCGAGGCCATCCTCTCCTTTGCACGCCGTGGG

>AY593826.1_O_ITL_1947

GGGTTGATTGTCGATACCAGAGATGTGGAAGAGCGCGTCCACGTGATGCGCAAAACAAAGCTTGCGCCCA

CCGTTGCACACGGTGTGTTCAATCCTGAGTTTGGGCCTGCCGCCTTGTCAAACAAGGACCCGCGTCTGAA

CGAGGGAGTTGTTCTCGATGAAGTCATTTTCTCCAAACACAAAGGAGACGTAAAGATGACCGAGGAGGAC

AAAGCGCTGTTCCGCCGCTGCGCCGCTGACTACGCGTCACGCCTGCACAGCGTGTTGGGTACGGCAAATG

CCCCATTGAGCGTCTACGAGGCAATCAAGGGCGTTGACGGACTCGACGCCATGGAGCCGGACACTGCACC

CGGTCTCCCCTGGGCCCTCCAGGGAAAACGCCGCGGTGCGCTCATCGACTTCGAGAACGGCACGGTCGGA

CCCGAAGTTGAGGCCGCCTTGAAGCTCATGGAGAAAAGAGAATACAAGTTTGTTTGCCAGACCTTCCTGA

AGGACGAGATTCGCCCGATGGAGAAAGTACGTGCCGGCAAGACTCGCATTGTCGACGTTTTGCCCGTTGA

ACATATTCTTTACACCAGGATGATGATTGGCAGATTCTGTGCACAAATGCACTCAAACAACGGACCGCAG

ATTGGCTCAGCGGTCGGTTGTAACCCTGATGTTGATTGGCAAAGATTTGGCACACACTTCGCCCAATACA

GAAATGTGTGGGACGTGGACTATTCGGCCTTCGATGCTAACCACTGTAGTGACGCGATGAACATCATGTT

TGAGGAAGTGTTCCGCACGGACTTTGGTTTCCACCCAAATGCTGAGTGGATCCTGAAGACCCTCGTGAAC

ACGGAACACGCCTATGAAAACAAACGCATCACTGTAGAGGGCGGGATGCCATCTGGTTGTTCCGCAACAA

GCATCATCAACACAATTCTGAACAACATCTACGTGCTCTACGCCTTGCGTAGACACTATGAGGGAGTTGA

GCTGGACACTTACACCATGATCTCCTACGGAGACGACATCGTGGTGGCAAGTGATTACGATCTGGACTTT

GAGGCTCTCAAGCCCCACTTCAAATCTCTTGGCCAAACCATCACTCCAGCTGACAAAAGCGACAAAGGTT

TTGTTCTTGGTCACTCCATTACCGATGTCACTTTCCTCAAAAGACACTTCCACATGGATTATGGAACTGG

GTTTTACAAACCTGTGATGGCCTCAAAGACCCTTGAGGCTATCCTCTCCTTTGCACGCCGTGGG

>AY593827.1_O_VEN_1971

GGGTTGATTGTTGATACCAGAGATGTGGAAGAGCGCGTCCACGTGATGCGCAAAACAAAGCTTGCGCCCA

CCGTTGCACACGGTGTGTTCAACCCTGAGTTTGGGCCTGCCGCCTTGTCAAACAAGGACCCGCGTCTGAA

CGAGGGGGTTGTTCTCGATGAAGTCATTTTCTCCAAACACAAAGGAGACACAAAGATGACCGAGGAGGAC

AAAGCGCTGTTCCGCCGCTGCGCCGCTGACTACGCGTCACGCCTGCACAGCGTGTTGGGTACGGCAAATG

CCCCATTGAGCATCTACGAGGCAATCAAGGGCGTTGACGGACTCGACGCCATGGAGCCGGACACTGCACC

CGGTCTCCCCTGGGCCCTCCAGGGAAAACGCCGCGGTGCGCTCATCGACTTCGAGAACGGCACGGTCGGA

CCCGAGGTTGAGGCCGCCTTGAAACTCATGGAGAAAAGAGAATATAAGTTTGTTTGCCAGACCTTCCTGA

AGGACGAGATTCGCCCGATGGAGAAAGTACGTGCCGGCAAGACTCGCATTGTCGACGTTTTGCCCGTTGA

ACATATTCTTTACACCAGGATGATGATTGGCAGATTCTGTGCACAAATGCACTCAAACAACGGACCGCAG

ATTGGCTCAGCGGTCGGTTGTAACCCTGATGTTGATTGGCAAAGATTTGGCACACACTTCGCCCAATACA

GAAATGTGTGGGACGTGGACTATTCGGCCTTCGATGCTAACCACTGCAGTGACGCGATGAACATCATGTT

TGAGGAAGTGTTCCGCACGGACTTTGGTTTCCACCCAAATGCTGAGTGGATCCTGAAGACTCTCGTGAAC

ACGGAACACGCCTATGAGAACAAACGCATCACTGTAGAGGGCGGGATGCCATCTGGTTGTTCCGCAACGA

GCATCATCAACACAATTTTGAACAACATATATGTGCTCTACGCCTTGCGTAGACACTATGAGGGAGTTGA

GCTGGACACTTACACCATGATCTCATACGGAGACGATATCGTGGTGGCAAGTGACTACGATTTGGACTTT

GAGGCTCTCAAGCCCCACTTCAAATCTCTTGGTCAAACCATTACTCCAGCTGACAAAAGCGACAAAGGCT

TTGTTCTCGGTCACTCTATCACCGATGTCACCTTCCTCAAAAGACACTTCCACATGGATTATGGAACTGG

GTTTTACAAACCTGTGATGGCCTCAAAGACTCTTGAGGCCATCCTCTCCTTTGCACGCCGTGGG

>AY593828.1_O_IND_1962

GGGTTGATTGTTGACACCAGAGATGTGGAAGAGCGCGTGCACGTCATGCGTAAAACCAAGCTTTCACCCA

CCGTAGCACACGGTGTGTTCAACCCTGAATTTGGGCCCGCCGCCTTGTCCAACAAGGACCCGCGCCTGAA

CGAAGGGGTTGTCCTCGACGAGGTCATCTTTTCCAAACACAAGGGAGACACAAAGATGTCCGAGGAAGAC

AAAGCGCTGTTCCGCCGCTGCGCTGCCGACTACGCGTCGCGCTTGCACAGTGTGTTGGGTACGGCAAATG

CCCCACTGAGCATCTACGAGGCCATCAAAGGCGTCGACGGACTCGACGCCATGGAACCAGACACCGCGCC

TGGCCTTCCCTGGGCTCTCCAGGGGAAGCGCCGCGGCGCGCTGATCGACTTCGAGAACGGCACGGTCGGA

CCCGAGGTTGAGGCTGCCTTGAAGCTCATGGAGAACAGAGAATACAAGTTTGCTTGCCAGACCTTCCTGA

AGGACGAAATTCGCCCGATGGAGAAAGTACGTGCCGGCAAGACTCGCATCGTCGACGTTTTGCCCGTTGA

ACACATTCTTTACACCAGGATGATGATTGGCAGATTCTGTGCTCAAATGCACTCAAACAACGGACCGCAA

ATTGGATCAGCGGTCGGTTGCAACCCTGATGTTGATTGGCAAAGATTTGGCACACATTTTGCTCAGTATA

GAAACGTGTGGGATGTGGACTACTCGGCCTTTGATGCTAATCACTGCAGCGATGCGATGAACATCATGTT

CGAGGAGGTGTTCCGCACAGACTTTGGTTTCCACCCAAACGCTGAGTGGATTCTGAAGACTCTTGTGAAC

ACGGAGCACGCCTATGAGAACAAACGCATCACTGTTGAAGGCGGGATGCCATCCGGCTGTTCCGCAACTA

GCATCATCAACACAATTCTGAACAACATCTACGTGCTCTACGCGCTGCGTAGGCACTACGAGGGAGTTGA

GCTGGACACTTACACCATGATCTCCTACGGGGACGACATCGTGGTTGCAAGTGATTACGATCTGGACTTT

GAGGCTCTCAAGCCCCACTTCAAATCTCTTGGCCAAACCATCACTCCAGCTGACAAAAGCGACAAAGGTT

TTGTTCTTGGTCACTCCATTACCGATGTCACTTTCCTCAAAAGACACTTCCACATGGATTATGGAACTGG

GTTTTACAAACCTGTGATGGCTTCGAAGACCCTCGAAGCCATCCTCTCCTTTGCACGCCGTGGG

>AY593830.1_O_POL_1959

GGGTTGATTGTGGACACCAGAGATGTGGAAGAGCGCGTTCACGTGATGCGCAAAACCAAGCTTGCACCCA

CCGTTGCACACGGTGTGTTCAACCCCGAGTTTGGGCCCGCTGCCTTGTCCAACAAGGACCCGCGTCTGAA

CGAGGGTGTTGTCCTCGATGAAGTCATCTTCTCCAAACACAAGGGAGACACAAAGATGTCTGAGGAGGAC

AAAGCGCTGTTCCGCCGCTGCGCTGCTGACTACGCGTCACGCCTGCACAGCGTGTTGGGCACAGCAAATG

CCCCACTGAGCATCTACGAGGCAATCAAGGGTGTCGACGGACTCGACGCCATGGAACCAGACACTGCGCC

CGGCCTCCCCTGGGCCCTCCAGGGTAAACGCCGCGGCGCGCTCATCGACTTCGAGAACGGCACGGTCGGA

CCCGAAGTCGAGGCTGCCCTGAAGCTCATGGAGAAGAGAGAATACAAATTTGTTTGTCAGACTTTCCTGA

AGGACGAGATTCGCCCGATAGAGAAAGTACGTGCCGGCAAGACTCGCATTGTCGACGTCCTGCCCGTTGA

GCATATTCTTTACACCAGGATGATGATTGGCAGATTTTGCGCACAAATGCACTCAAACAACGGACCGCAA

ATTGGCTCAGCGGTCGGTTGCAACCCTGATGTTGATTGGCAGAGATTTGGCACACACTTCGCCCAGTACA

GAAACGTGTGGGACGTGGACTATTCGGCCTTTGATGCTAATCACTGCAGTGATGCCATGAACATCATGTT

TGAGGAGGTGTTTCGCACGGAGTTCGGCTTCCACCCGAATGCCGAGTGGATCCTGAAGACTCTTGTGAAC

ACGGAACACGCCTATGAGAACAAACGCATCACTGTTGAAGGCGGAATGCCGTCTGGTTGCTCCGCAACAA

GCATCATCAACACAATTTTGAACAACATCTACGTGCTCTACGCCCTGCGTAGACACTATGAGGGAGTTGA

GTTGGACACATACACCATGATCTCCTACGGAGACGACATCGTGGTGGCAAGTGATTATGATTTGGACTTC

GAGGCTCTCAAGCCCCACTTTAAATCCCTTGGTCAAACTATCACTCCAGCTGACAAAAGCGACAAAGGTT

TTGTTCTTGGTCACTCCATTACCGATGTCACTTTCCTTAAAAGACACTTCCACATGGACTATGGAACTGG

GTTTTACAAACCTGTGATGGCCTCAAAGACCCTTGAGGCTATCCTCTCCTTTGCACGCCGTGGG

>AY593831.1_O_UKG_2002

GGATTGATAGTTGACACCAGAGATGTTGAGGAGCGCGTACATGTCATGCGCAAAACCAAGCTCGCACCCA

CCGTGGCACACGGTGTGTTTAACCCCGAATTTGGGCCTGCCGCCTTGTCCAACAAGGACCCGCGCCTGAA

TGAGGGGGTTGTCCTCGATGAAGTCATCTTCTCCAAACACAAAGGAAACACAAAGATGTCTGAGGAGGAC

AAAGCGCTGTTCCGCCGCTGTGCTGCTGACTACGCGTCGCGTCTGCATAGCGTACTGGGTACGGCAAATG

CCCCACTGAGCACTTACGAGGCAATCAAGGGCGTCGACGGACTTGACGCCATGGAACCAGACACCGCGCC

TGGTCTCCCCTGGGCTCTCCAGGGGAAACGCCGTGGTGCGCTCATCGACTTCGAGAACGGCACTGTCGGA

CCCGAGGTTGAAGCTGCCTTGAAGCTCATGGAGAAAAGAGAGTACAAGTTTACATGCCAGACCTTCCTGA

AGGACGAGATTCGCCCGATGGAGAAGGTACGTGCCGGCAAGACTCGCATTGTCGACGTCCTGCCCGTTGA

ACACATTCTTTACACTAGGATGATGATTGGCAGATTTTGTGCTCAAATGCACTCAAACAACGGACCGCAA

ATTGGCTCGGCGGTTGGTTGTAATCCTGATGTTGATTGGCAAAGATTTGGCACGCATTTTGCTCAGTATA

GAAACGTGTGGGATGTGGACTATTCGGCCTTTGATGCCAACCACTGCAGTGACGCAATGAACATCATGTT

TGAGGAGGTGTTTAACACGGACTTCGGTTTCCACCCAAACGCTGAGTGGATCCTGAAAACTCTCGTGAAC

ACTGAACACGCCTATGAGAACAAACGCATCACTGTTGAAGGCGGGATGCCGTCTGGTTGTTCCGCAACAA

GCATCATCAACACAATTTTGAACAACATCTACGTGCTCTACGCCTTGCGTAGACACTATGAGGGGGTTGA

GCTGGACTCTTACACCATGATCTCCTACGGAGACGACATCGTGGTTGCAAGTGATTACGATCTGGACTTT

GAGGCCCTCAAGCCTCACTTCAAATCCCTTGGTCAAACCATTACCCCAGCTGACAAAAGCGACAAAGGTT

TTGTTCTTGGTCACTCCATTACCGATGTCACTTTCCTCAAAAGACACTTCCACATGGACTATGGAACTGG

GTTTTACAAACCTGTGATGGCTTCGAAGACCCTCGAGGCTATCCTCTCCTTTGCACGCCGTGGG

>AY593833.1_O_TAW_1999

GGGTTGATCGTCGACACCAGAGATGTGGAGGAGCGTGTCCACGTGATGCGCAAAACCAAGCTCGCGCCCA

CCGTGGCGCACGGTGTGTTCAACCCAGAGTTCGGGCCTGCCGCTCTGTCCAACAAGGACCCGCGCCTGAA

CGAAGGGGTTGTCCTTGACGATGTCATTTTCTCCAAACACAAAGGAGATACAAGGATGTCTGAAGAGGAC

AAAGCGCTGTTTCGGCGCTGTGCTGCTGACTACGCGTCGCGTCTACACAGTGTGTTGGGGACAGCGAACG

CCCCACTGAGTGTGTATGAAGCCATCAAAGGCGTCGACGGACTTGACGCCATGGAGCCGGACACGGCGCC

CGGTCTCCCCTGGGCTCTCCAAGGGAAACGCCGCGGCGCCCTGATCGACTTCGAAAACGGCACCGTCGGG

CCTGAGGTTGAGGCAGCACTCAAGCTCATGGAAAGCCGCGAGTACAAATTCGTCTGCCAAACCTTCCTGA

AGGACGAAATTCGGCCGCTAGAAAAGGTACGCGCTGGCAAGACACGCATTGTCGACGTGTTGCCTGTTGA

ACACATTCTCTACACCAGAATGATGATTGGCAGATTCTGTGCTCAGATGCATTCAAACAACGGACCGCAA

ATTGGATCAGCGGTCGGTTGTAACCCTGACGTTGATTGGCAAAGATTTGGCACACATTTCGCCCAGTACA

AGAACGTGTGGGATGTGGACTACTCAGCCTTTGATGCAAACCACTGCAGCGATGCGATGAACATCATGTT

CGAGGAAGTGTTCCGCACGGAGTTCGGATTCCACCCGAACGCCGAGTGGATTCTGAAGACTCTAGTGAAC

ACGGAGCACGCTTACGAGAACAAGCGCATTGTTGTTGAAGGTGGAATGCCGTCCGGTTGTTCCGCAACAA

GCATCATCAACACAATTTTGAACAACATCTACGTGCTTTACGCCCTGCGTAGGCACTATGAGGGAGTCGA

GCTGGACACTTACACCATGATCTCTTATGGAGACGACATCGTGGTGGCAAGTGACTACGACCTGGACTTT

GAGGCTCTCAAGCCCCACTTCAAGTCCCTTGGTCAGACTATCACTCCGGCCGACAAAAGCAACAAAGGTT

TTGTTCTTGGTCACTCCATAACCGACGTCACTTTCCTCAAAAGACACTTCCACATGGACTACGGAACTGG

GTTTTACAAACCTGTGATGGCCTCGAAGACCCTCGAGGCCATCCTCTCCTTTGCACGCCGTGGG

>AY593834.1_O_IRN_1966

GGGTTGATTGTTGATACCAGAGATGTGGAAGAGCGCGTTCACGTGATGCGCAAAACCAAGCTTGCACCCA

CTGTGGCACACGGTGTGTTTAACCCTGAGTTCGGGCCTGCCGCCTTGTCCAACAAGGACCCGCGCCTGGA

CGAAGGAGTTGTCCTCGATGAGGTCATCTTCTCCAAGCACAAGGGAGACACAAAGATGTCGGAGGAGGAC

AAAGCGCTGTTCCGCCGCTGTGCTGCCGACTACGCGTCGCGCCTGCACGGTGTGCTGGGTACAGCAAATG

CCCCGTTGAGCGTTTACGAGGCAATCAAGGGCGTTGACGGACTTGACGCCATGGAACCAGACACCGCACC

TGGCCTTCCTTGGGCTCTCCAGGGGAAACGCCGTGGTGCGCTGATCGACTTTGAGAACGGCACTGTCGGA

CCCGAAGTCGAGGCTGCCTTGAAGCTCATGGAGAAAAGAGAGTACAAGTTTGCTTGTCAGACCTTCCTGA

AGGACGAGATTCGCCCGATGGAAAAAGTACGTGCCGGCAAGACTCGCATTGTCGACGTTTTGCCTGTTGA

ACACATTCTTTACACAAGGATGATGATTGGCAGATTTTGTGCACAAATGCACTCAAACAACGGGCCGCAA

ATTGGATCGGCGGTCGGTTGCAACCCTGATGTTGATTGGCAGAGATTTGGCACCCATTTTGCCCAATACA

GAAACGTGTGGGATGTGGACTATTCGGCCTTTGATGCTAACCACTGCAGTGATGCAATGAACATCATGTT

TGAGGAGGTGTTTCGCACGGAGTTTGGCTTCCACCCAAACGCTGAGTGGATCCTGAAGACTCTTGTGAAC

ACGGAGCACGCCTATGAGAACAAACGCATCACTGTTGAAGGCGGAATGCCGTCTGGCTGCTCTGCAACTA

GCATTATTAACACAATTCTGAACAACATTTATGTGCTCTACGCCTTGCGTAGACACTATGAGGGTGTTGA

GCTGGACACCTACACCATGATCTCCTACGGAGACGATATCGTGGTGGCAAGTGATTACGATCTGGACTTT

GAGGCTCTTAAGCCTCACTTCAAATCTCTTGGTCAGACCATTACTCCAGCTGACAAAAGCGACAAAGGTT

TTGTTCTTGGTCACTCCATCACCGATGTCACTTTCCTCAAAAGACACTTCCACATGGATTATGGAACTGG

GTTTTACAAACCTGTGATGGCCTCAAAGACCCTTGAGGCTATCCTCTCCTTTGCACGCCGTGGG

>AY593835.1_O_TAW_1997

GGGTTGATCGTCGACACCAGAGATGTGGAGGAGCGTGTCCACGTGATGCGCAAAACCAAGCTCGCGCCCA

CCGTGGCGCACGGTGTGTTCAACCCTGAGTTCGGGCCTGCCGCTCTGTCCAACAAGGACCCGCGCCTGAA

CGAAGGGGTTGTCCTTGACGATGTCATTTTCTCCAAACACAAAGGAGATACAAGGATGTCTGAAGAGGAC

AAAGCGCTGTTTCGGCGCTGTGCTGCTGACTACGCGTCGCGTCTACACAGTGTGTTGGGGACAGCAAACG

CCCCACTGAGTGTGTATGAAGCCATCAAAGGCGTCGACGGACTTGACGCCATGGAGCCGGACACGGCGCC

CGGTCTCCCCTGGGCTCTCCAAGGGAAACGCCGCGGCGCCCTGATCGACTTCGAAAACGGCACCGTCGGG

CCTGAGGTTGAGGCAGCACTCAAGCTCATGGAAAGCCGCGAGTACAAATTCGTCTGCCAAACCTTCCTGA

AGGACGAAATTCGGCCGCTAGAAAAGGTACGCGCTGGCAAGACACGCATTGTCGACGTGTTGCCTGTTGA

ACACATTCTCTACACCAGAATGATGATTGGCAGATTCTGTGCTCAGATGCATTCAAACAACGGACCGCAA

ATTGGATCAGCGGTCGGTTGTAACCCTGACGTTGATTGGCAAAGATTTGGCACACATTTCGCCCAGTACA

AAAACGTGTGGGATGTGGACTACTCAGCCTTTGATGCAAACCACTGCAGCGATGCGATGAACATCATGTT

CGAGGAAGTGTTCCGCACGGAGTTCGGATTCCACCCGAACGCCGAGTGGATTCTGAAGACTCTAGTGAAC

ACGGAGCACGCTTACGAGAACAAGCGCATTGTTGTTGAAGGTGGAATGCCGTCCGGTTGTTCCGCAACAA

GCATCATCAACACAATTTTGAACAACATCTACGTGCTTTACGCCCTGCGTAGGCACTATGAGGGAGTCGA

GCTGGACACTTACACCATGATCTCTTATGGAGACGACATCGTGGTGGCAAGTGACTACGACCTGGACTTT

GAGGCTCTCAAGCCCCACTTCAAGTCCCTTGGTCAGACTATCACTCCGGCCGACAAAAGCGACAAAGGTT

TTGTTCTTGGTCACTCCATAACCGACGTCACTTTCCTCAAAAGACACTTCCACATGGACTACGGAACTGG

GTTTTACAAACCTGTGATGGCCTCGAAGACCCTCGAGGCCATCCTCTCCTTTGCACGCCGTGGG

>AY593836.1_O_UKG_2001

GGATTGATAGTTGACACCAGAGATGTTGAGGAGCGCGTACATGTCATGCGCAAAACCAAGCTCGCACCCA

CCGTGGCACACGGTGTGTTTAACCCCGAATTTGGGCCTGCCGCCTTGTCCAACAAGGACCCGCGCCTGAA

TGAGGGGGTTGTCCTCGATGAAGTCATCTTCTCCAAACACAAAGGAAACACAAAGATGTCTGAGGAGGAC

AAAGCGCTGTTCCGCCGCTGTGCTGCTGACTACGCGTCGCGTCTGCATAGCGTACTGGGTACGGCAAATG

CCCCACTGAGCACTTACGAGGCAATCAAGGGCGTCGACGGACTTGACGCCATGGAACCAGACACCGCGCC

TGGTCTCCCCTGGGCTCTCCAGGGGAAACGCCGTGGTGCGCTCATCGACTTCGAGAACGGCACTGTCGGA

CCCGAGGTTGAAGCTGCCTTGAAGCTCATGGAGAAAAGAGAGTACAAGTTTACATGCCAGACCTTCCTGA

AGGACGAGATTCGCCCGATGGAGAAGGTACGTGCCGGCAAGACTCGCATTGTCGACGTCCTGCCCGTTGA

ACACATTCTTTACACTAGGATGATGATTGGCAGATTTTGTGCTCAAATGCACTCAAACAACGGACCGCAA

ATTGGCTCGGCGGTTGGTTGTAATCCTGATGTTGATTGGCAAAGATTTGGCACGCATTTTGCTCAGTATA

GAAACGTGTGGGATGTGGACTATTCGGCCTTTGATGCCAACCACTGCAGTGACGCAATGAACATCATGTT

TGAGGAGGTGTTTAACACGGACTTCGGTTTCCACCCAAACGCTGAGTGGATCCTGAAAACTCTCGTGAAC

ACTGAACACGCCTATGAGAACAAACGCATCACTGTTGAAGGCGGGATGCCGTCTGGTTGTTCCGCAACAA

GCATCATCAACACAATTTTGAACAACATCTACGTGCTCTACGCCTTGCGTAGACACTATGAGGGGGTTGA

GCTGGACTCTTACACCATGATCTCCTACGGAGACGACATCGTGGTTGCAAGTGATTACGATCTGGACTTT

GAGGCCCTCAAGCCTCACTTCAAATCCCTTGGTCAAACCATTACCCCAGCTGACAAAAGCGACAAAGGTT

TTGTTCTTGGTCACTCCATTACCGATGTCACTTTCCTCAAAAGACACTTCCACATGGACTATGGAACTGG

GTTTTACAAACCTGTGATGGCTTCGAAGACCCTCGAGGCTATCCTCTCCTTTGCACGCCGTGGG

>AY593837.1_O_URU_1963

GGGTTGATTGTGGACACCAGAGATGTGGAAGAGCGCGTTCACGTGATGCGCAAAACCAAGCTTGCACCCA

CCGTTGCACACGGTGTGTTCAACCCCGAGTTTGGGCCCGCTGCCTTGTCCAACAAGGACCCGCGTCTGAA

CGAGGGTGTTGTCCTCGATGAAGTCATCTTCTCCAAACACAAGGGAGACACAAAGATGTCTGAGGAGGAC

AAAGCGCTGTTCCGCCGCTGCGCTGCTGACTACGCGTCACGCCTGCACAGCGTGTTGGGCACAGCAAATG

CCCCACTGAGCATCTACGAGGCAATCAAGGGTGTCGACGGACTCGACGCCATGGAGCCAGACACTGCGCC

CGGCCTCCCCTGGGCCCTCCAGGGTAAACGCCGCGGCGCGCTCATCGACTTCGAGAACGGCACGGTCGGA

CCCGAAGTCGAGGCTGCCCTGAAGCTCATGGAGAAGAGAGAATACAAATTTGCTTGTCAGACCTTCCTGA

AGGACGAGATTCGCCCGATGGAGAAAGTACGTGCCGGCAAGACTCGCATTGTCGACGTCCTGCCCGTTGA

GCATATTCTTTACACCAGGATGATGATTGGCAGATTTTGTGCACAGATGCACTCAAACAACGGACCGCAA

ATTGGCTCAGCGGTCGGTTGCAACCCTGATGTTGATTGGCAGAGATTTGGCACACACTTCGCCCAGTACA

GAAACGTGTGGGATGTGGACTATTCGGCCTTTGATGCTAATCACTGCAGTGATGCCATGAACATCATGTT

TGAGGAGGTGTTTCGCACGGAGTTCGGCTTCCACCCGAATGCCGAGTGGATCCTGAAGACTCTTGTGAAC

ACGGAACACGCCTATGAGAACAAACGCATCACTGTTGAAGGCGGAATGCCGTCTGGTTGTTCCGCAACAA

GCATCATCAACACAATTTTGAACAACATCTACGTGCTCTACGCTCTGCGTAGACACTATGAGGGAGTTGA

GCTGGACACATACACCATGATCTCCTACGGAGACGACATCGTGGTGGCAAGTGATTATGATTTGGACTTC

GAGGCTCTCAAGCCCCACTTTAAATCCCTTGGTCAAACCATCACTCCAGCTGACAAAAGCGACAAAGGTT

TTGTTCTTGGTCACTCCATTACCGATGTCACTTTCCTCAAAAGACACTTCCACATGGACTATGGAACTGG

GTTTTACAAACCTGTGATGGCCTCAAAGACCCTTGAGGCTATCCTCTCCTTTGCACGCCGTGGG

>AY593838.1_SAT1_BOT_1970

GGGTTGGTAGTGGACACCAGAGAAGTTGAGGAGCGCGTGCATGTCATGCGCAAGACTAAACTTGCACCCA

CCGTGGCTCACGGTGTGTTTCAGCCTGAATTTGGACCCGCCGCCCTGTCGAACAACGACAAACGCCTGAA

CGAAGGCGTGGTTCTGGACGAGGTCATCTTCTCCAAACACAAGGGCGATGCCACAATGTCTGAGGCTGAC

AAAAGACTGTTCCGTCTGTGCGCTGCTGATTATGCCTCGCATCTTCACAATGTGCTGGGGACAGCCAACT

CTCCACTGAGCGTGTTTGAAGCCATCAAGGGCGTCGACGGACTCGACGCAATGGAGCCTGACACAGCACC

CGGTCTCCCCTGGGCACTCCAAGGGAAACGACGTGGAGCTCTCATTGATTTCGAGAACGGCACTGTCGGA

CCCGAGATTGAAGCTGCTCTGAAGCTCATGGAGAAGAAGGAGTACAAGTTCACCTGCCAAACCTTCCTGA

AGGACGAGATTCGCCCTCTGGAGAAAATTAAGGCCGGCAAGACTCGCATTGTCGACGTCTTGCCAGTTGA

GCACATCATCTACACCAGAATGATGATTGGCAGATTCTGTGCACAAATGCACTCCAACAACGGACCGCAA

ATTGGCTCGGCGGTCGGTTGCAACCCTGATGTTGATTGGCAACGATTTGGCACTCACTTTGCCCAGTACA

AAAACGTTTGGGACATTGATTATTCGGCCTTTGATGCTAACCATTGCAGTGACGCCATGAACATCATGTT

CGAGGAGGTCTTCCGTGAGGAATTTGGATTTCATCCGAACGCTGTTTGGATTCTCAAAACTCTCATCAAC

ACGGAACATGCCTACGAAAACAAGCGCATCACTGTTGAAGGTGGAATGCCCTCGGGTTGCTCCGCCACCA

GCATCATCAACACCATTCTCAACAACATCTACGTGCTCTACGCCCTGCGTAGACACTATGAGGGAGTCGA

GCTGTCGCACTACACCATGATTTCCTACGGGGATGACATTGTAGTTGCAAGTGATTACGATTTGGACTTT

GAAGCTCTCAAGCCTCACTTTAAATCTCTTGGTCAAACAATCACTCCAGCCGACAAAAGCGACAAAGGTT

TTGTTCTTGGTCAGTCCATCACCGATGTCACTTTCCTCAAGAGGCATTTTCATTTGGATTATGAAACTGG

GTTTTACAAACCTGTGATGGCTTCGAAGACCCTCGAAGCTATCCTCTCCTTTGCACGCCGTGGG

>AY593839.1_SAT1_UKG_1970

GGGCTGGTAGTGGACACCAGAGAAGTTGAGGAGCGCGTGCACGTCATGCGCAAAACCAAACTTGCACCCA

CCGTGGCTCACGGTGTGTTTCAGCCTGAATTTGGACCTGCCGCCCTGTCAAACAACGACAAACGCCTGAA

TGAAGGTGTGGTCTTGGACGAAGTCATCTTCTCCAAGCACAAGGGCGATGCCAAAATGTCTGAGGCTGAT

AAGAGACTGTTTCGTCTGTGCGCTGCTGATTATGCCTCGCATCTTCACAACGTACTTGGGACAGCAAACT

CTCCACTGAGCGTGTTTGAAGCCATCAAAGGCGTCGACGGACTCGACGCCATGGAGCCTGACACAGCACC

CGGTCTTCCCTGGGCCCTCCAGGGGAAACGCCGCGGAGCTCTCATCGATTTCGAGAACGGCACTGTCGGA

CCCGAGATTGAACAGGCACTGAAGCTCATGGAGAAGAAGGAGTACAAGTTCACCTGTCAAACCTTCCTGA

AGGACGAGATCCGCCCTCTGGAGAAAGTTAAGGCCGGCAAGACTCGCATCGTCGATGTCCTGCCTGTGGA

ACACATCATCTATACCAGAATGATGATTGGCAGATTTTGTGCACAAATGCACTCCAACAACGGACCGCAA

ATTGGCTCGGCGGTCGGTTGCAACCCTGATGTTGATTGGCAAAGATTCGGCTGTCATTTCGCCCAGTACA

AAAATGTTTGGGACATTGATTATTCGGCCTTTGATGCTAACCATTGCAGTGACGCCATGAACATCATGTT

CGAGGAGGTCTTCCGTGAGGAATTTGGATTTCATCCGAACGCTGTTTGGATTCTCAAAACTCTCATCAAC

ACGGAACACGCCTACGAGAACAAGCGCATCACTGTTGAAGGTGGAATGCCCTCGGGTTGCTCCGCCACCA

GCATCATCAACACCATTCTCAACAACATCTATGTGCTCTACGCTCTGCGCAGGCACTATGAGGGAGTCGA

GCTGTCGCACTACACCATGATTTCCTACGGGGATGATATTGTAGTTGCAAGTGATTACGATTTGGACTTT

GAAGCTCTCAAGCCTCACTTTAAATCCCTTGGTCAGACAATCACTCCAGCCGACAAAAGTGACAAAGGTT

TTGTTCTTGGTCAGTCCATTACCGATGTCACTTTCCTCAAGAGGCACTTTCATCTGGATTATGAAACTGG

GTTTTACAAACCTGTAATGGCTTCGAAGACCCTCGAAGCTATCCTCTCCTTTGCACGCCGTGGG

>AY593840.1_SAT1_NMB_1949

GGGTTGGTAGTGGACACCAGAGAAGTTGAGGAGCGCGTGCATGTCATGCGCAAAACCAAGCTTACACCCA

CCGTGGCTCACGGTGTGTTTCAGCCTGAATTTGGACCTGCCGCCCTGTCGAACAACGACAAACGCCTGAA

CGAAGGCGTGGTGCTGGACGAAGTCATCTTCTCCAAACACAAGGGCGATGCCAAAATGTCTGAGGCTGAC

AAGAGACTGTTCCGCCTGTGCGCTGCTGATTACGCCTCGCATCTTCACAACGTACTTGGGACAGCCAACT

CTCCACTGAGCGTGTTTGAAGCCATCAAGGGCGTCGACGGACTCGACGCAATGGAGCCTGACACAGCACC

CGGTCTCCCCTGGGCACTCCAGGGAAAGCGCCGCGGAGCTCTCATCGATTTCGAGAACGGCACTGTCGGA

CCCGAGATTGATGCTGCTCTGAAGCTCATGGAGAAGAAGGAGTACAAGTTCACCTGTCAAACCTTCCTGA

AGGACGAGATTCGCCCTCTGGAGAAAGTCAAGGCCGGCAAGACTCGCATCGTCGACGTCCTGCCCGTTGA

ACACATCATCTACACCAGAATGATGATTGGCAGATTCTGTGCACAAATGCACTCCAACAACGGACCGCAA

ATTGGCTCGGCGGTCGGTTGCAACCCTGATGTTGATTGGCAACGATTTGGCACTCACTTTGCCCAGTACA

AAAATGTTTGGGACATTGATTATTCGGCCTTTGATGCTAACCACTGCAGTGACGCCATGAACATCATGTT

CGAGGAGGTCTTCCGTGAGGAATTTGGATTTCATCCGAACGCCGTTTGGATTCTCAAAACTCTCATCAAC

ACGGAACATGCCTACGAGAATAAGCGCATCACTGTTGAAGGTGGAATGCCCTCGGGTTGCTCCGCCACCA

GCATTATCAACACCATTCTCAACAACATATACGTGCTCTACGCCCTGCGTAGGCACTATGAGGGAGTCGA

GCTGTCGCACTACACCATGATTTCCTACGGGGATGACATTGTAGTTGCAAGTGATTACGATTTGGACTTT

GAAGCTCTCAAGCCTCACTTTAAATCTCTTGGTCAAACAATCACTCCAGCCGACAAAAGTGACAAAGGTT

TTGTTCTTGGTCAGTCCATTACCGATGTTACTTTCCTCAAGAGGCACTTTCATCTGGATTATGAAACTGG

GTTTTACAAACCTGTGATGGCTTCGAAGACCCTCGAAGCTATCCTCTCCTTTGCACGCCGTGGG

>AY593841.1_SAT1_ZIM_1958

GGGTTGGTAGTGGACACCAGAGAAGTTGAGGAGCGCGTGCACGTCATGCGCAAAACCAAGCTCGCACCCA

CCGTGGCTCACGGTGTGTTTCAGCCCGAATTTGGACCTGCCGCCCTGTCGAACAACGACAAACGCCTGAA

CGAAGGCGTGGTGCTTGACGAAGTCATCTTCTCTAAGCACAAGGGCGATGCCAAAATGTCTGAGGCTGAC

AAGAGACTGTTCCGTCTGTGCGCTGCCGACTATGCCTCGCATCTGCACAACGTACTTGGGACAGCCAACT

CTCCACTGAGCGTGTTTGAAGCCATCAAGGGCGTCGACGGACTCGACGCAATGGAGCCTGACACAGCACC

CGGCCTCCCCTGGGCACTCCAGGGAAAGCGCCGAGGAGCTCTCATCGATTTCGAGAACGGCACTGTCGGA

CCCGAGATTGAAGCTGCTCTGAAGCTCATGGAGAAGAAGGAGTACACGTTCACCTGTCAAACCTTTCTAA

AGGACGAGATCCGCCCTTTGGAGAAAGTCAAGGCCGGCAAGACTCGCATTGTCGATGTCTTGCCTGTTGA

GCACATCATCTACACCAGAATGATGATTGGCAGGTTCTGTGCACAAATGCACTCCAGCAACGGACCGCAA

ATTGGCTCGGCGGTCGGTTGCAACCCTGATGTTGATTGGCAACGATTTGGCACTCACTTTGCCCAGTACA

AAAATGTTTGGGACATTGATTATTCGGCCTTTGATGCTAACCATTGCAGTGACGCCATGAACATCATGTT

CGAGGAGGTCTTCCGTGAGGAATTTGGATTTCATCCGAACGCTGTTTGGATTCTCAAAACTCTCATCAAC

ACGGAACACGCCTACGAGAACAAGCGCATCACTGTTGAAGGTGGAATGCCCTCGGGTTGCTCCGCCACCA

GCATCATCAACACCATTCTCAACAACATCTACGTGCTCTACGCCCTACGTAGACACTATGAGGGAGTCGA

GCTGTCGCACTACACCATGATTTCCTACGGGGATGATATTGTAGTTGCAAGTGATTATGATTTGGACTTT

GAAGCTCTCAAGCCTCACTTTAAATCTCTTGGTCAAACAATCACTCCAGCCGACAAAAGCGACAAAGGTT

TTGTTCTTGGTCAGTCCATTACCGATGTTACTTTCCTCAAGAGGCATTTTCATCTGGATTATGAAACTGG

GTTTTACAAACCTGTGATGGCTTCGAAGACCCTCGAAGCTATCCTCTCCTTTGCACGCCGTGGG

>AY593842.1_SAT1_SAR_1961

GGGTTGGTAGTGGACACCAGAGAAGTTGAGGAGCGCGTGCACGTCATGCGCAAAACCAAGCTTGCACCCA

CCGTGGCTCACGGTGTGTTTCAGCCTGAATTTGGACCTGCCGCCCTGTCGAACAACGACAAACGCCTGAA

CGAAGGCGTGGTGCTGGACGAAGTCATCTTCTCCAAGCACAAGGGCGATGTCAAAATGTCTGAGGCTGAC

AAGAGACTGTTCCGTCTGTGCGCTGCTGATTATGCCTCGCATCTTCACAACGTACTTGGGACAGCCAACT

CTCCACTGAGCGTGTTTGAAGCCATCAAGGGCGTCGACGGACTCGACGCAATGGAGCCTGACACAGCACC

CGGCCTCCCCTGGGCACTCCAGGGAAAGCGCCGCGGAGCTCTCATCGATTTCGAGAACGGCACTGTCGGA

CCCGAGATTGAAGCCGCTCTGAAGCTCATGGAGAAGAAGGAGTACAAGTTCACCTGCCAAACCTTCCTGA

AGGACGAGATTCGCCCTCTGGAGAAAGTCAAGGCCGGCAAGACTCGCATCGTCGACGTCCTGCCTGTTGA

GCACATCATCTACACCAGAATGATGATTGGCAGATTCTGTGCACAAATGCACTCCAACAACGGACCGCAA

ATTGGCTCGGCGGTCGGTTGCAACCCTGATGTTGATTGGCAACGATTTGGTACTCACTTTGCCCAGTACA

AAAATGTTTGGGACATTGACTATTCGGCCTTTGATGCTAACCATTGCAGTGACGCCATGAACATCATGTT

CGAGGAGGTTTTCCGTGAGGAATTTGGATTTCATCCGAACGCTATTTGGATTCTCAAAACTCTCATCAAC

ACGGAACACGCCTACGAGAACAAGCGCATCACTGTTGAAGGTGGAATGCCCTCGGGTTGCTCCGCCACCA

GCATCATCAACACCATTCTCAACAACATCTACGTGCTCTACGCCCTGCGTAGACACTATGAGGGAGTCGA

GCTGTCGCACTACACCATGATTTCCTACGGGGATGACATTGTAGTTGCAAGTGATTACGATTTGGACTTT

GAAGCTCTCAAGCCTCACTTTAAATCTCTTGGTCAAACAATCACTCCAGCCGACAAAAGCGACAAAGGTT

TTGTTCTTGGTCAGTCCATTACCGATGTTACTTTCCTCAAGAGGCATTTTCATCTGGATTATGAAACTGG

GTTTTACAAACCTGTGATGGCTTCGAAGACCCTCGAAGCTATCCTCTCCTTTGCACGCCGTGGG

>AY593843.1_SAT1_NMB_1940

GGGTTGGTATTGGACACCAGAGAAGTTGAGGAGCGCGTGCATGTCATGCGCAAAACCAAGCTTGCACCCA

CCGTGGCTCACGGTGTGTTTCAGCCCGATTTTGGGCCTGCCGCCCTGTCGAACAACGACAAACGCCTGAA

CGAAGGCGTGGTGCTGGACGAAGTCATCTTCTCCAAGCACAAGGGTGACGCCAAAATGTCTGAGGCTGAT

AAGAGACTGTTCCGTCTGTGCGCTGCTGATTATGCCTCGCATCTTCACAACGTACTTGGGACAGCCAACT

CTCCACTGAGCGTGTTTGAAGCCATCAAAGGCGTCGACGGACTCGACGCAATGGAGCCTGACACAGCACC

CGGTCTCCCCTGGGCACTCCAGGGAAAGCGCCGCGGAGCTCTCATCGATTTCGAGAACGGCACTGTCGGA

CCCGAGATTGAAGCTGCTCTGAAGCTCATGGAGAAAAAGGAATACAGGTTCACCTGTCAAACCTTCCTGA

AGGACGAGATTCGCCCTCTGGAGAAAGTCAAGGCCGGCAAGACTCGCATCGTCGACGTCCTGCCTGTTGA

GCACATCATCTACACCAGAATGATGATTGGCAGATTCTGTGCACAAATGCACTCCAACAACGGACCGCAA

ACTGGTTCGGCGGTCGGTTGCAACCCTGATATTGATTGGCAACGATTTGGCACTCACTTTGCCCAGTACA

AAAATGTTTGGGACATTGATTATTCGGCCTTTGATGCTAACCATTGCAGTGACGCCATGAACATCATGTT

CGAAGAGGTTTTCCGTGAGGAATTTGGATTTCATCCGAACGCTGTTTGGATTCTCAAAACTCTCATCAAC

ACGGAACACGCCTACGAGAACAAGCGCATCACTGTTGAAGGTGGAATGCCCTCGGGTTGCTCCGCCACCA

GCATCATCAACACCATTCTCAACAACATCTACGTGCTCTACGCCCTGCGTAGGCACTATGAGGGAGTCGA

GCTATCGCACTACACCATGATTTCCTACGGGGATGATATTGTAGTTGCAAGTGATTACGATTTGGACTTT

GAAGCTCTCAAGCCTCACTTTAAATCTCTTGGTCAAACAATCACTCCAGCCGACAAAAGTGACAAAGGTT

TTGTTCTTGGTCAGTCCATTACCGATGTTACTTTCCTCAAGAGGCATTTTCATCTGGATTATGGAACTGG

GTTTTACAAACCTGTGATGGCTTCGAAGACCCTCGAAGCTATCCTCTCCTTTGCACGCCGTGGG

>AY593844.1_SAT1_ISR_1962

GGGTTAATTGTGGACACCAGAGATGTGGAAGAGCGCGTGCACGTCATGCGCAAAACCAAGCTTGCACCCA

CCGTCGCACACGGTGTGTTCAACCCTGACTTTGGACCAGCCGCGTTGTCCAACAACGACAAGCGCCTGAA

CGAAGGTGTCGTCCTCGACGATGTCATCTTTAGTAAACACAAAGGAGACACCAAGATGTCTGAGGAAGAC

AAGAAGCTGTTCAGGCTTTGTGCTGCTGACTACGCGTCGCACCTGCACTCCGTCCTGGGTACAGCGAATG

CTCCATTGACCATCTACGAGGCAATCAAAGGCGTTGACGGACTCGACGCCATGGAGCCTGACACCGCTCC

GGGTTTACCTTGGGCCACCCAGGGCAAACGCCGCGGAGCTCTCATTGATTTCGAAAACGGCACTGTCGGA

CCCGAGATCGAGGAGGCACTGAAGCTCATGGAGAAAAGGGAGTACAAGTTCACATGCCAAACCTTCCTGA

AAGATGAGATTCGTCCGATGGACAAAGTTCGCGCCGGCAAGACTCGCATCGTCGACGTCCTGCCTGTTGA

GCACATCCTCTACACAAGAATGATGATTGGCAAGTTTTGTGCGCAAATGCACTCCAATAACGGGCCGCAA

ATCGGCTCGGCGGTCGGTTGCAACCCAGATGTCGATTGGCAAAGATTCGGAACCCATTTCGCCCAATACA

AAAATGTGTGGGACGTGGATTATTCGGCCTTTGATGCTAACCATTGCAGTGACGCCATGAACATCATGTT

CGAGGAGGTTTTCCGTCCGGAATTCGGTTTCCACCCCAATGCGGAGTGGATCCTGAAAACCCTGGTCAAC

ACGGAACACGCCTACGAGAACAAGCGTATCACTGTTGAAGGCGGTATGCCCTCGGGTTGCTCCGCCACCA

GCATCATTAATACAATTCTCAACAATATCTACGTGCTCTACGCTCTGCGTAGACACTATGAGGGAGTCGA

GCTGTCTAGCTACTCGATGATCTCCTACGGGGATGACATCGTGGTGGCAAGTGATTATGACTTGGACTTT

GAAGCTCTCAAGCCTCACTTCAAGTCCGTCGGCCAGACCATCACCCCAGCCGACAAAAGTGACAAGGGTT

TTGTTCTTGGTCAGTCCATTACCGATGTTACTTTCCTCAAGAGGCACTTCCACTTGGACTTTGGAACTGG

GTTTTACAAACCTGTGATGGCGTCGAAGACCCTCGAAGCTATCCTCTCCTTTGCACGCCGTGGG

>AY593845.1_SAT1_BOT_1968

GGGTTGGTAGTGGACACCAGAGAAGTTGAGGAGCGCGTACACGTTATGCGCAAAACCAAGCTTGCACCCA

CCGTGGCTCACGGTGTGTTTCAGCCTGAATTTGGACCTGCCGCCCTGTCAAACAACGACAAACGCCTGAA

CGAAGGCGTGGTGCTGGACGAAGTCATCTTCTCCAAACACAAGGGCGATGCCAAAATGTCTGAGGCTGAC

AAGAGACTGTTCCGTCTGTGCGCTGCTGACTATGCCTCGCATCTTCACAACGTGCTTGGGACAGCCAACT

CTCCACTGAGCGTGTTTGAAGCCATCAAAGGCGTCGACGGACTCGACGCAATGGAGCCTGACACGGCACC

CGGCCTCCCCTGGGCACTCCAGGGGAAGCGCCGTGGAGCTCTCATCGATTTCGAGAACGGCACTGTCGGA

CCCGAGATTGAAGCGGCTCTGAAGTTCATGGAGAAGAAGGAGTACAAGTTTACCTGTCAAACCTTCCTGA

AGGACGAGATCCGCCCTCTGGAGAAAGTCAAGGCCGGTAAGACTCGCATTGTCGACGTCCTGCCCGTTGA

ACACATCATCTACACCAGAATGATGATTGGCAGATTCTGTGCACAAATGCACTCCAACAACGGACCGCAA

ATTGGCTCGGCGGTCGGTTGCAACCCTGATGTTGATTGGCAACGATTTGGCACCCACTTTGCCCAGTACA

AAAATGTTTGGGACATTGACTATTCGGCCTTTGATGCTAACCATTGCAGTGACGCCATGAACATCATGTT

TGAGGAGGTCTTCCGTGAGGAATTTGGATTTCATCCGAACGCTGTTTGGATTCTCAAAACTCTCATCAAC

ACGGAACACGCCTACGAAAACAAGCGCATCAATGTTGAAGGTGGAATGCCCTCGGGTTGCTCCGCCACCA

GCATCATCAACACCATTCTCAACAACATCTACGTGCTCTACGCCCTGCGTAGGCACTATGAGGGAGTCGA

GCTGTCGCACTACACCATGATTTCCTACGGGGATGATATTGTAGTTGCAAGTGATTACGATTTGGACTTT

GAAGCTCTCAAGCCTCACTTTAAATCTCTTGGTCAAACAATCACTCCAGCCGACAAAAGCGACAAAGGTT

TTGTTCTTGGTCAGTCCATCACCGATGTTACCTTCCTCAAGAGGCATTTTCATCTGGATTATGAAACTGG

GTTTTACAAACCTGTGATGGCTTCGAAGACCCTCGAAGCTATCCTCTCCTTTGCACGCCGTGGG

>AY593846.1_SAT1_ZIM_1966

GGGCTGGTCGTTGACACCAGAGAAGTTGAGGAGCGCGTGCACGTCATGCGCAAAACCAAGCTTGCACCCA

CCGTGGCTCACGGTGTGTTTCAGCCTGAATTTGGACCTGCCGCCCTGTCGAACAACGACAAACGCCTGAA

CGAAGGCGTGGTGCTGGACGAAGTCATCTTCTCCAAGCACAAGGGCGATGCCAAAATGTCTGAGGCTGAC

AAGAAGTTGTTCCGCCTGTGCGCTGCTGACTATGCCTCGCATCTTCACAACGTACTCGGGACAGCAAACT

CTCCACTGAGCGTGTTTGAAGCCATCAAGGGCGTCGACGGACTCGATGCCATGGAGCCTGACACAGCACC

CGGTCTTCCTTGGGCCCTCCAGGGGAAGCGCCGCGGAGCTCTCATCGATTTCGAGAACGGCACTGTCGGA

CCCGAAATTGAACAGGCACTGAAGCTCATGGAGAAGAAGGAGTACAAGTTCACCTGTCAAACCTTCCTGA

AGGACGAGATTCGCCCTCTGGAGAAAGTCAAGGCCGGCAAGACTCGCATCGTCGATGTCCTGCCTGTGGA

ACACATCATCTACACTAGAATGATGATTGGCAGATTTTGTGCACAAATGCACTCCAACAACGGACCGCAA

ATTGGCTCGGCGGTCGGTTGCAACCCTGATGTTGATTGGCAAAGATTCGGCTGTCACTTCGCCCAGTACA

AAAATGTTTGGGACATTGACTATTCGGCCTTTGATGCTAACCATTGCAGTGACGCCATGAACATCATGTT

CGAGGAGGTCTTCCGTGAGGAATTTGGATTTCATCCGAACGCTGTTTGGATTCTCAAAACTCTCATCAAC

ACGGAGCACGCCTACGAGAACAAGCGCATTACTGTTGAAGGTGGAATGCCCTCGGGTTGCTCCGCCACCA

GCATCATCAACACCATTCTCAACAACATCTACGTGCTCTACGCTCTGCGTAGACACTATGAGGGAGTCGA

GCTGTCGCACTACACCATGATTTCCTACGGGGATGACATTGTAGTTGCAAGTGATTACGATTTGGACTTT

GAAGCTCTCAAGCCTCACTTTAAATCTCTTGGTCAGACAATCACTCCAGCCGACAAAAGTGACAAAGGTT

TTGTTCTTGGTCAGTCCATTACTGATGTTACTTTCCTCAAGAGGCATTTTCATCTGGATTATGAAACTGG

GTTTTACAAACCTGTGATGGCTTCGAAGACCCTCGAAGCTATCCTCTCCTTTGCACGCCGTGGG

>AY593847.1_SAT2_ZIM_1948

GGGTTGGTGGTGGACACCAGAGAAGTTGAGGAGCGCGTGCACGTCATGCGCAAAACTAAGCTTGCACCCA

CCGTGGCTCACGGTGTGTTTCAGCCTGAATTTGGACCTGCCGCCCTGTCGAACAACGACAAGCGCCTGAA

CGAAGGCGTGGTCTTAGACGAAGTCATCTTCTCCAAACACAAGGGTGATGCCAAAATGTCTGAGGCTGAT

AAGAAACTGTTCCGCTTGTGCGCTGCTGATTATGCCTCGCATCTTCACAACGTGCTTGGGACAGCCAATT

CTCCATTGAGTGTGTTTGAAGCCATCAAAGGTGTCGACGGACTCGATGCCATGGAGCCTGACACCGCTCC

CGGTCTCCCCTGGGCCCTCCAGGGGAAGCGCCGCGGGGCTCTCATCGATTTCGAGACCGGCACTGTCGGA

CCCGAGATTGAGGAGGCTCTGAAGCTCATGGAGAAGAAGGAGTACAAGTTCACCTGTCAAACCTTCCTCA

AGGACGAGATTCGCCCTTTGGAAAAAGTTAAGGCCGGCAAGACTCGCATCGTCGACGTCCTGCCTGTTGA

GCACATCATCTACACCAGAATGATGATTGGCAGATTCTGTGCACAAATGCACTCCAACAACGGACCGCAA

ATTGGCTCGGCGGTCGGTTGCAACCCTGATGTTGATTGGCAACGATTTGGCACTCATTTTGCCCAGTACA

AAAATGTTTGGGACATTGATTATTCGGCCTTTGATGCTAACCATTGCAGTGACGCCATGAACATCATGTT

CGAGGAGGTCTTCCGTGAGGAATTTGGATTTCATCCGAACGCTGTTTGGATTCTCAAAACTCTCACCAAC

ACGGAACACGCCTATGGGAACAAGCGCATCACTGTTGAAGGTGGAATGCCCTCGGGTTGCTCCGCCACCA

GCATCATTAACACCATTCTCAACAACATCTATGTGCTCTACGCTCTGCGTAGACACTATGAAGGAGTCGA

GCTGTCGCACTACACCATGATTGCCTACGGGGATGACATTGTAGTTGCAAGTGACTACGATTTGGACTTT

GAAGCTCTCAAGCCTCACTTTAAATCTCTTGGTCAAACAATCACTCCAGCCGACAAAAGTGACAAAGGTT

TTGTTCTTGGTCAGTCCATTACTGACGTCACTTTCCTAAAGAGGCATTTCTATCTGGATTATGAAACTGG

GTTTTATAAACCTGTGATGGCTTCGAAGACCCTCGAAGCTATCCTCTCCTTTGCACGCCGTGGG

>AY593848.1_SAT2_u_1967

GGGTTGGTGGTGGACACCAGAGAAGTTGAGGAGCGCGTGCATGTCATGCGCAAAACCAAGCTTGCACCCA

CCGTGGCTCACGGTGTGTTTCAGCCTGAATTTGGACCTGCCGCCCTGTCGAACAACGACAAGCGCCTGAA

CGAAGGCGTGGTGCTAGACGAAGTCATCTTCTCCAAGCACAAGGGCGATGTCAAAATGTCTGAGGCTGAC

AAGAGACTGTTCCGTCTGTGCGCTGCTGACTATGCCTCGCATCTTCACAACGTACTCGGGACAGCCAACT

CTCCATTGAGCGTGTTTGAAGCCATCAAGGGCGTCGACGGACTCGACGCCATGGAGCCTGACACAGCACC

CGGTCTTCCCTGGGCCCTCCAGGGGAAACGCCGCGGAGCTCTCATTGATTTCGAGAACGGCACTGTCGGA

CCCGAGATTGAACAGGCACTGAAGCTTATGGAGAAGAAGGAGTACAAGTTCACCTGTCAAACCTTCCTGA

AGGACGAGATTCGCCCTCTGGAGAAAGTTAAGGCCGGCAAGACTCGCATCGTCGACGTCCTGCCTGTGGA

ACACATCATCTACACTAGAATGATGATTGGCAGATTCTGTGCACAAATGCACTCCAACAATGGACCGCAA

ATTGGCTCGGCGGTCGGTTGCAACCCTGATGTTGATTGGCAAAGGTTTGGCTGTCATTTCGCCCAGTACA

AAAATGTTTGGGACATTGATTATTCGGCCTTTGATGCTAACCACTGCAGTGACGCCATGAACATCATGTT

CGAGGAGGTCTTCCGTGAGGAATTTGGATTTCATCCGAACGCTGTTTGGATTCTCAAAACTCTCATCAAC

ACGGAACACGCCTACGAGAACAAGCGCATCACTGTTGAAGGTGGAATGCCCTCGGGTTGTTCTGCCACCA

GCATCATCAACACCATTCTCAACAACATCTACGTGCTCTACGCCCTGCGTAGACACTATGAGGGAGTCGA

GCTGTCGCACTACACCATGATTTCCTACGGGGATGACATTGTAGTTGCAAGTGATTACGATTTGGACTTT

GAAGCTCTCAAGCCTCACTTTAAATCTCTTGGTCAAACAATCACTCCAGCCGACAAAAGTGACAAAGGTT

TTGTTCTTGGTCAGTCCATCACCGATGTTACTTTCCTCAAGAGGCACTTTCATCTGGATTATGAAACTGG

GTTTTACAAACCTGTGATGGCTTCGAAGACCCTCGAAGCTATCCTCTCCTTTGCACGCCGTGGG

>AY593849.1_SAT2_KEN_1960

GGGTTAATTGTGGACACCAGAGATGTTGAAGAGCGTGTACACGTCATGCGCAAAACCAAACTTGCACCCA

CCGTTGCACACGGTGTGTTCAACCCCGAGTACGGGCCTGCTGCACTGTCCAACAATGACAAGCGCCTGAA

CGAGGGAGTTGTCCTCGACGAGGTGATCTTCTCCAAACACAAGGGAGACACCAAGATGTCTGAGGAAGAC

AAGAAGTTGTTCAGACTTTGTGCTGCTGACTACGCGTCACGTCTGCACTCGGTACTTGGTACAGCAAATG

CCCCACTGAGCATCTATGAGGCCATCAAGGGCGTCGACGGGCTCGACGCCATGGAGCCTGACACCGCTCC

GGGTTTACCTTGGGCCACCCAGGGGAAGCGCCGTGGAGCACTGATCGATTTCGAGAACGGCACTGTCGGA

CCCGAGATTGAGGCAGCACTGAAGCTCATGGAGAAAAGGGAGTACAAGTTCACTTGCCAAACCTTTCTGA

AGGACGAAGTTCGCCCCTTGGAGAAAGTTCGTGCCGGTAAGACTCGCATCGTCGATGTCCTACCCGTTGA

ACACATCCTCTACACTAGAATGATGATTGGCAAATTTTGTGCACAAATGCACTCCAACAACGGACCGCAG

ATTGGCTCAGCGGTCGGTTGCAACCCAGATGTTGATTGGCAGAGATTCGGAACCCATTTTGCCCAGTACA

AAAACGTGTGGGACGTGGACTATTCGGCCTTTGATGCTAACCACTGCAGTGACGCCATGAACATCATGTT

CGAGGAGGTATTCCGTCCGGAATTTGGCTTCCACCCCAATGCGGAGTGGATCCTGAAAACTCTGGTCAAC

ACGGAGCACGCCTACGAGAACAAGCGCATCACTGTTGAAGGCGGGATGCCTTCGGGCTGTTCCGCCACTA

GCATCATCAATACAATTCTCAACAACATCTACGTGCTTTACGCGCTGAGTAGGCACTACGAGGGAGTCAC

GCTGGACAGCTACTCGATGATCTCCTACGGAGATGACATCGTTGTGGCTAGTGATTACGATCTGGACTTT

GAAGCACTCAAGCCTCACTTCAAATCTGTCGGTCAGACCATCACCCCAGCTGACAAAAGTGACAAAGGTT

TTGTTCTTGGTCAGTCCATCACTGACGTCACTTTCCTCAAGAGGCACTTCCACATGGATTACGGAACTGG

GTTTTACAAACCTGTGATGGCTTCGAAGACCCTCGAAGCCATCCTCTCCTTTGCACGCCGTGGG

>AY593850.1_SAT3_SAR_1959

GGGTTGGTAGTGGACACCAGAGAAGTTGAGGAACGCGTGCACGTCATGCGAAAAACCAAGCTTGCACCCA

CCGTGGCTCACGGTGTGTTTCAGCCCGAATTTGGACCTGCCGCCCTGTCGAACAACGACAAGCGCCTGAA

CGAAGGCGTGGTTTTGGACGAAGTCATCTTCTCCAAGCACAAGGGCGATGCCAAAATGTCTGAGGCTGAC

AAGAAACTGTTCCGCCTGTGCGCTGCTGATTATGCCTCGCATCTTCACAACGTACTTGGGACAGCAAACT

CTCCACTGAGCGTGTTTGAAGCCATCAAGGGCGTCGACGGACTCGACGCCATGGAGCCTGACACAGCACC

CGGTCTTCCCTGGGCCCTCCAGGGGAGACGCCGCGGAGCTCTCATCGATTTCGAGAACGGCACTGTCGGA

CCCGAGATTGAACAGGCACTGAAGCTCATGGAGAAGAAGGAGTACAAGTTCACCTGCCAAACCTTCCTGA

AGGACGAGATTCGCCCTCTGGAGAAAGTCAAGGCCGGCAAGACTCGCATCGTCGACGTCCTGCCTGTTGA

GCACATCATCTACACCAGAATGATGATTGGCAGATTCTGTGCACAAATGCACTCCAACAACGGACCGCAA

ATTGGCTCGGCGGTCGGTTGCAACCCTGATGTTGATTGGCAAAGATTCGGCTGTCATTTTGCCCAGTACA

AAAATGTTTGGGACATTGACTATTCGGCCTTTGATGCTAACCATTGCAGTGACGCCATGAACATCATGTT

CGAGGAGGTCTTCCGTGAGGAATTCGGATTTCATCCGAACGCTGTTTGGGTTCTCAAAACTCTCATCAAC

ACGGAACACGCCTACGAGAACAAGCGCATCACTGTTGAAGGTGGAATGCCCTCGGGTTGCTCCGCCACCA

GCATCATCAACACCATTCTCAACAACATCTACGTGCTCTACGCCCTGCGTAGACACTATGAGGGAGTCGA

GCTGTCGCACTACACCATGATTTCCTACGGGGATGACATTGTAGTTGCAAGTGATTACGATTTGGACTTT

GAAGCTCTCAAACCCCACTTTAAATCTCTTGGTCAAACAATCACTCCAGCCGACAAAAGTGACAAAGGTT

TTGTTCTTGGTCAGTCCATTACCGATGTTACTTTCCTCAAGAGGCACTTTCATCTGGATTATGAAACCGG

GTTTTACAAACCTGTGATGGCTTCGAAGACCCTCGAAGCTATCCTCTCCTTTGCACGCCGTGGG

>AY593851.1_SAT3_BOT_1961

GGGTTGGTAGTGGACACCAGAGAAGTTGAGGAGCGCGTGCACGTCATGCGCAAAACCAAGCTTGCACCCA

CCGTGGCTCACGGTGTGTTTCAGCCTGAATTTGGACCTGCCGCCCTGTCAAACAACGACAAACGCCTGAA

CGAAGGCGTGGTTTTGGACGAAGTCATCTTCTCCAAACACAAGGGCGATGCCAAAATGTCTGAGGCTGAC

AAGAGACTGTTCCGCCTGTGCGCTGCTGACTATGCCTCGCATCTTCACAACGTACTTGGGACAGCCAACT

CTCCACTGAGCGTGTTTGAAGCCATTAAGGGCGTCGACGGACTCGACGCAATGGAGCCTGACACAGCGCC

CGGCCTCCCCTGGGCACTCCAGGGAAAGCGCCGCGGAGCTCTCATCGATTTCGAGAACGGCACTGTCGGA

CCCGAGATTGAAGCTGCTCTGAAGCTCATGGAGAAGAAGGAGTACAAGTTTACCTGTCAAACCTTCCTGA

AGGACGAGATTCGCCCTTTGGAAAAAGTCAAAGCCGGCAAGACTCGCATTGTCGATGTCCTGCCTGTTGA

ACACATCATCTACACCAGAATGATGATTGGCAGATTCTGTGCACAAATGCACTCCAACAACGGACCGCAA

ATTGGCTCGGCGGTCGGTTGCAACCCTGATGTTGATTGGCAACGATTTGGCACTCATTTTGCCCAGTACA

AAAATGTTTGGGACATTGACTATTCGGCCTTTGATGCTAACCATTGCAGTGACGCCATGAACATCATGTT

TGAGGAGGTCTTCCGTGAAGAATTTGGATTTCATCCGAACGCTGTTTGGATTCTTAAAACTCTCATCAAC

ACGGAACACGCCTACGAGAACAAGCGCATCACTGTTGAAGGTGGAATGCCCTCGGGTTGCTCCGCCACCA

GCATCATCAACACCATTCTCAACAATATCTACGTGCTCTACGCCCTGCGTAGACACTATGAGGGAGTCGA

GCTGTCGCACTACACCATGATTTCCTACGGGGATGACATTGTAGTTGCAAGTGATTACGATTTGGACTTT

GAAGCTCTCAAGCCTCACTTTAAATCTCTTGGTCAAACAATCACTCCAGCCGACAAAAGTGACAAAGGTT

TTGTTCTTGGTCAGTCCATCACCGATGTTTCTTTCCTCAAGAGGCACTTCCATCTGGATTATGAAACTGG

GTTTTATAAACCTGTGATGGCTTCGAAGACCCTCGAAGCTATCCTCTCCTTTGCACGCCGTGGG

>AY593852.1_SAT3_KEN_1960

GGGTTGGTAGTGGACACCAGAGAAGTTGAGGAGCGCGTGCACGTCATGCGCAAAACCAAGCTTGCACCCA

CCGTGGCTCACGGTGTGTTTCAGCCTGAATTTGGACCTGCCGCCCTGTCAAACAACGACAAACGCCTGAA

CGAAGGCGTGGTTTTGGACGAAGTCATCTTCTCCAAACACAAGGGCGATGCCAAAATGTCTGAGGCTGAC

AAGAGACTGTTCCGCCTGTGCGCTGCTGACTATGCCTCGCATCTTCACAACGTACTTGGGACAGCCAACT

CTCCACTGAGCGTGTTTGAAGCCATTAAGGGCGTCGACGGACTCGACGCAATGGAGCCTGACACAGCGCC

CGGCCTCCCCTGGGCACTCCAGGGAAAGCGCCGCGGAGCTCTCATCGATTTCGAGAACGGCACTGTCGGA

CCCGAGATTGAAGCTGCTCTGAAGCTCATGGAGAAGAAGGAGTACAAGTTTACCTGTCAAACCTTCCTGA

AGGACGAGATTCGCCCTTTGGAAAAAGTCAAAGCCGGCAAGACTCGCATTGTCGATGTCCTGCCTGTTGA

ACACATCATCTACACCAGAATGATGATTGGCAGATTCTGTGCACAAATGCACTCCAACAACGGACCGCAA

ATTGGCTCGGCGGTCGGTTGCAACCCTGATGTTGATTGGCAACGATTTGGCACTCATTTTGCCCAGTACA

AAAATGTTTGGGACATTGACTATTCGGCCTTTGATGCTAACCATTGCAGTGACGCCATGAACATCATGTT

TGAGGAGGTCTTCCGTGAAGAATTTGGATTTCATCCGAACGCTGTTTGGATTCTTAAAACTCTCATCAAC

ACGGAACACGCCTACGAGAACAAGCGCATCACTGTTGAAGGTGGAATGCCCTCGGGTTGCTCCGCCACCA

GCATCATCAACACCATTCTCAACAATATCTACGTGCTCTACGCCCTGCGTAGACACTATGAGGGAGTCGA

GCTGTCGCACTACACCATGATTTCCTACGGGGATGACATTGTAGTTGCAAGTGATTACGATTTGGACTTT

GAAGCTCTCAAGCCTCACTTTAAATCTCTTGGTCAAACAATCACTCCAGCCGACAAAAGTGACAAAGGTT

TTGTTCTTGGTCAGTCCATCACCGATGTTTCTTTCCTCAAGAGGCATTTCCATCTGGATTATGAAACTGG

GTTTTATAAACCTGTGATGGCTTCGAAGACCCTCGAAGCTATCCTCTCCTTTGCACGCCGTGGG

>AY593853.1_SAT3_BOT_1965

GGGTTGGTAGTGGACACTAGAGAAGTTGAGGAGCGCGTGCATGTCATGCGCAAAACCAAGCTTGCACCCA

CCGTGGCTCACGGTGTGTTTCAGCCTGAATTTGGACCTGCCGCCCTGTCGAACAACGACAAGCGCCTGAA

CGAAGGCGTGGTTCTGGACGAAGTCATCTTCTCCAAACACAAGGGTGATGCCAAAATGACTGAGGCTGAC

AAGAGGCTGTTCCGCCTGTGCGCTGCTGACTATGCCTCGCATCTTCACAACGTACTTGGGACAGCCAACT

CTCCACTAAGCGTGTTTGAAGCCATCAAGGGCGTCGACGGACTCGACGCAATGGAGCCTGACACAGCACC

CGGTCTTCCCTGGGCACTCCAGGGAAAACGCCGTGGAGCTCTCATCGATTTCGAGAACGGCACTGTCGGA

CCTGAGATCGAAGCTGCTCTGAAGCTTATGGAGAAGAAGGAGTACAAGTTCACCTGTCAAACCTTCCTGA

AGGACGAGATTCGCCCTCTGGAGAAAGTCAAGGCCGGCAAGACTCGCATCGTCGATGTCCTGCCTGTTGA

GCACATCATCTACACCAGAATGATGATTGGCAGATTCTGTGCACAAATGCACTCCAACAACGGACCGCAA

ATTGGCTCGGCGGTCGGTTGCAACCCTGATGTTGATTGGCAACGATTTGGCACTCACTTTGCCCAGTACA

AAAATGTTTGGGACATTGATTATTCGGCCTTTGATGCTAACCATTGCAGTGACGCCATGAACATCATGTT

CGAGGAGGTTTTCCGTGAGGAATTTGGATTTCATCCGAACGCTGTTTGGATTCTCAAAACTCTCATCAAC

ACGGAACACGCCTACGAGAACAAGCGCATCACTGTTGAAGGTGGAATGCCTTCGGGTTGCTCCGCCACCA

GCATCATCAACACCATTCTCAACAACATCTACGTGCTCTACGCCCTGCGTAGACACTATGAGGGAGTCGA

GCTGTCGCACTACACCATGATTTCCTACGGGGATGACATTGTAGTTGCAAGTGATTACGATTTGGACTTT

GAAGCTCTCAAGCCTCACTTTAAATCTCTTGGTCAAACAATCACTCCAGCCGACAAAAGTGACAAAGGTT

TTGTTCTTGGTCAATCCATCACCGATGTTACTTTCCTCAAGAGGCATTTTCATCTGGATTATGAAACTGG

GTTTTACAAACCTGTGATGGCTTCGAAGACCCTCGAAGCTATCCTCTCCTTTGCACGCCGTGGG

>AY686687.1_O_CHA_2001

GGGCTGATTGTCGACACCAGAGATGTGGAGGAGCGCGTCCACGTGATGCGCAAAACCAAGCTCGCGCCTA

CCGTGGCACACGGTGTGTTCAACCCTGAGTTCGGGCCGGCGGCTTTGTCCAACAAGGACCCGCGCTTGGA

CGAAGGAGTTGTCCTTGACGACGTCATCTTCTCCAAGCACAAAGGAGACACAGGGATGTCTGAAGAGGAC

AAAGCGCTGTTTCGGCGCTGTGCTGCTGATTACGCGTCGCGTCTACACAGTGTGTTAGGGACGGCAAACG

CCCCATTGAGTGTGTATGAGGCCATCAAAGGCGTCGACGGGCTCGATGCCGTGGAGCCGGACACCGCACC

CGGCCTCCCCTGGGCTCTCCAAGGGAAACGCCGTGGTGCCCTGATCGACTTCGAAAACGGTACTGTCGGG

CCCGAGGTTGAGGCAGCACTCAAGCTCATGGAGAAACGTGAGTACAAATTCGTCTGTCAAACCTTCCT--

----------------------------------------------------------------------

----------------------------------------------------------------------

----------------------------------------------------------------------

----------------------------------------------------------------------

----------------------------------------------------------------------

----------------------------------------------------------------------

----------------------------------------------------------------------

----------------------------------------------------------------------

----------------------------------------------------------------------

----------------------------------------------------------------------

-------AAACCTGTGATGGCTTCGAAGACCCTCGAGGCTATCCTCTCCTTTGCACGCCGTGGG

>AY687333.1_Asia1_IND_2001

GGGTTGATTGTTGACACCAGGGATGTGGAAGAGCGCGTGCACGTCATGCGCAAAACCAAGCTTGCACCCA

CCGTGGCACACGGTGTGTTCAACCCCGAGTTTGGGCCCGCCGCCTTGTCCAACAAGGACCCGCGCCTGAG

CGAGGGAGTTGTCCTCGATGAAGTCATCTTCTCCAAACATAAGGGAGACACCAAGATGTCCGAGGAGGAC

AAAGCGCTGTTCCGCCGCTGCGCTGCTGACTACGCGTCACGCTTGCACGGTGTGTTGGGTACGGCAAATG

CCCCACTGAGCATCTACGAGGCAATCAAGGGCGTCGACGGACTCGACGCCATGGAACCAGACACCGCACC

TGGTCTTCCCTGGGCCCTCCAGGGGAAACGCCGAGGCGCGCTGATCGATTTCGAGAACGGCACTGTCGGA

CCCGAAGTCGCTGCTGCTCTAGAGCTCATGGAGAAAAGAGAATACAAGTTTGCATGCCAGACCTTCCTGA

AGGACGAGATTCGCCCGATGGAGAAAGTACGTGCCGGCAAGACTCGCATTGTCGACGTCCTGCCTGTTGA

ACATATTCTTTACACCAGGATGATGATTGGCAGATTTTGTGCTCAAATGCACTCAAACAACGGACCGCAA

ATTGGCTCGGCGGTCGGTTGTAACCCTGATGTTGACTGGCAGAGATTTGGCACAGACTTCGCCCAGTACA

AGAACGTGTGGGATGTGGATTATTCGGCCTTCGATGCTAACCACTGCAGTGACGCGATGAACATCATGTT

CGAGGAGGTGTTCCGCACGGACTTTGGTTTCCACCCAAACGCTGAGTGGATCCTGAAGACTCTCGTGAAC

ACGGAGCACGCCTATGAGAACAAACGCATTACTGTTGAGGGCGGGATGCCGTCTGGTTGTTCCGCGACAA

GCATCATCAACACAATTTTGAACAACATTTACGTGCTCTACGCCCTGCGTAGACACTATGAGGGAGTTGA

GCTGGACACTTACACCATGATCTCCTATGGAGACGACATCGTGGTTGCAAGTGATTACGATTTGGACTTT

GAGGCCCTCAAGCCCCACTTTAAATCTCTTGGTCAAACCATAACTCCAGCTGACAAAAGCGGCAAAGGTT

TTGTTCTTGGTCACTCCATCACCGATGTCACTTTCCTCAAAAGACACTTCTACATGGACTATGGAACTGG

GTTTTACAAACCTGTGATGGCCTCGAAGACCCTCGAGGCTATCCTCTCCTTTGCACGCCGTGGG

>DQ404158.1_O_UKG_2001

GGATTGATAGTTGACACCAGAGATGTTGAGGAGCGCGTACATGTCATGCGCAAAACCAAGCTCGCACCCA

CCGTGGCACACGGTGTGTTTAACCCCGAATTTGGGCCTGCCGCCTTGTCCAACAAGGACCCGCGCCTGAA

CGAGGGGGTTGTCCTCGATGAAGTCATCTTCTCCAAACACAAAGGAAACACAAAGATGTCTGAGGAGGAC

AAAGCGCTCTTCCGCCGCTGTGCTGCTGACTACGCGTCGCGTCTGCATAGCGTGCTGGGTACGGCAAATG

CCCCACTGAGCACTTACGAGGCAATCAAGGGCGTCGACGGACTTGACGCCATGGAACCAGACACCGCGCC

TGGTCTCCCCTGGGCTCTCCAGGGGAAACGCCGTGGTGCGCTCATCGACTTCGAGAACGGCACTGTCGGA

CCCGAGGTTGAAGCTGCCTTGAAGCTCATGGAGAAAAGAGAGTACAAGTTTACATGCCAGACCTTCCTGA

AGGACGAGATTCGCCCGATGGAGAAGGTACGTGCCGGCAAGACTCGCATTGTCGACGTCCTGCCCGTTGA

ACACATTCTTTACACTAGGATGATGATTGGCAGATTTTGTGCTCAAATGCACTCAAACAACGGACCGCAA

ATTGGCTCGGCGGTTGGTTGTAATCCTGATGTTGATTGGCAAAGATTTGGCACGCATTTTGCTCAGTATA

GAAACGTGTGGGATGTGGACTATTCGGCCTTTGATGCCAACCACTGCAGTGACGCAATGAACATCATGTT

TGAGGAGGTGTTTAACACGGACTTCGGTTTCCACCCAAACGCTGAGTGGATCCTGAAAACCCTCGTGAAC

ACTGAACACGCCTATGAGAACAAACGCATCCCTGTTGAAGGCGGGATGCCGTCTGGTTGTTCCGCAACAA

GCATCATCAACACAATTTTGAACAACATCTACGTGCTCTACGCCTTGCGTAGACACTATGAGGGGGTTGA

GCTGGACTCTTACACCATGATCTCCTACGGGGACGACATCGTGGTTGCAAGTGATTACGATCTGGACTTT

GAGGCCCTCAAGCCTCACTTCAAATCCCTTGGTCAAACCATTACCCCAGCTGACAAAAGCGACAAAGGTT

TTGTTCTTGGTCACTCCATTACCGATGTCACTTTCCTCAAAAGACACTTCCACATGGACTATGGAACTGG

GTTTTACAAACCTGTGATGGCTTCGAAGACCCTCGAGGCTATCCTCTCCTTTGCACGCCGTGGG

>DQ404159.1_O_UKG_2001

GGATTGATAGTTGACACCAGAGATGTTGAGGAGCGCGTACATGTCATGCGCAAAACCAAGCTCGCACCCA

CCGTGGCACACGGTGTGTTTAACCCCGAATTTGGGCCTGCCGCCTTGTCCAACAAGGACCCGCGCCTGAA

CGAGGGGGTTGTCCTCGATGAAGTCATCTTCTCCAAACACAAAGGAAACACAAAGATGTCTGAGGAGGAC

AAAGCGCTGTTCCGCCGCTGTGCTGCTGACTACGCGTCGCGTCTGCATAGCGTGCTGGGTACGGCAAATG

CCCCACTGAGCACTTACGAGGCAATCAAGGGCGTCGACGGACTTGACGCCATGGAACCAGACACCGCGCC

TGGTCTCCCCTGGGCTCTCCAGGGGAAACGCCGTGGTGCGCTCATCGACTTCGAGAACGGCACTGTCGGA

CCCGAGGTTGAAGCTGCCTTGAAGCTCATGGAGAAAAGAGAGTACAAGTTTACATGCCAGACCTTCCTGA

AGGACGAGATTCGCCCGATGGAGAAGGTACGTGCCGGCAAGACTCGCATTGTCGACGTCCTGCCCGTTGA

ACACATTCTTTACACTAGGATGATGATTGGCAGATTTTGTGCTCAAATGCACTCAAACAACGGACCGCAA

ATTGGCTCGGCGGTTGGTTGTAATCCTGATGTTGATTGGCAAAGATTTGGCACGCATTTTGCTCAGTATA

GAAACGTGTGGGATGTGGACTATTCGGCCTTTGATGCCAACCACTGCAGTGACGCAATGAACATCATGTT

TGAGGAGGTGTTTAACACGGACTTCGGTTTCCACCCAAACGCTGAGTGGATCCTGAAAACTCTCGTGAAC

ACTGAACACGCCTATGAGAACAAACGCATCACTGTTGAAGGCGGGATGCCGTCTGGTTGTTCCGCAACAA

GCATCATCAACACAATTTTGAACAACATCTACGTGCTCTACGCCTTGCGTAGACACTATGAGGGGGTTGA

GCTGGACTCTTACACCATGATCTCCTACGGAGACGACATCGTGGTTGCAAGTGATTACGATCTGGACTTT

GAGGCCCTCAAGCCTCACTTCAAATCCCTTGGTCAAACCATTACCCCAGCTGACAAAAGCGACAAAGGTT

TTGTTCTTGGTCACTCCATTACCGATGTCACTTTCCTCAAAAGACACTTCCACATGGACTATGGAACTGG

GTTTTACAAACCTGTGATGGCTTCGAAGACCCTCGAGGCTATCCTCTCCTTTGCACGCCGTGGG

>DQ404160.1_O_UKG_2001

GGATTGATAGTTGACACCAGAGATGTTGAGGAGCGCGTACATGTCATGCGCAAAACCAAGCTCGCACCCA

CCGTGGCACACGGTGTGTTTAACCCCGAATTTGGGCCTGCCGCCTTGTCCAACAAGGACCCGCGCCTGAA

CGAGGGGGTTGTCCTCGATGAAGTCATCTTCTCCAAACACAAAGGAAACACAAAGATGTCTGAGGAGGAC

AAAGCGCTGTTCCGCCGCTGTGCTGCTGACTACGCGTCGCGTCTGCATAGCGTGCTGGGTACGGCAAATG

CCCCACTGAGCACTTACGAGGCAATCAAGGGCGTCGACGGACTTGACGCCATGGAACCAGACACCGCGCC

TGGTCTCCCCTGGGCTCTCCAGGGGAAACGCCGTGGTGCGCTCATCGACTTCGAGAACGGCACTGTCGGA

CCCGAGGTTGAAGCTGCCTTGAAGCTCATGGAGAAGAGAGAGTACAAGTTTACATGCCAGACCTTCCTGA

AGGACGAGATTCGCCCGATGGAGAAGGTACGTGCCGGCAAGACTCGCATTGTCGACGTCCTGCCCGTTGA

ACACATTCTTTACACTAGGATGATGATTGGCAGATTTTGTGCTCAAATGCACTCAAACAACGGACCGCAA

ATTGGCTCGGCGGTTGGTTGTAATCCTGATGTTGATTGGCAAAGATTTGGCACGCATTTTGCTCAGTATA

GAAACGTGTGGGATGTGGACTATTCGGCCTTTGATGCCAACCACTGCAGTGACGCAATGAACATCATGTT

TGAGGAGGTGTTTAACACGGACTTCGGTTTCCACCCAAACGCTGAGTGGATCCTGAAAACTCTCGTGAAC

ACTGAACACGCCTATGAGAACAAACGCATCACTGTTGAAGGCGGGATGCCGTCTGGTTGTTCCGCAACAA

GCATCATCAACACAATTTTGAACAACATCTACGTGCTCTACGCCTTGCGTAGACACTATGAGGGGGTTGA

GCTGGACTCTTACACCATGATCTCCTACGGAGACGACATCGTGGTTGCAAGTGATTACGATCTGGACTTT

GAGGCCCTCAAGCCTCACTTCAAATCCCTTGGTCAAACCATTACCCCAGCTGACAAAAGCGACAAAGGTT

TTGTTCTTGGTCACTCCATTACCGATGTCACTTTCCTCAAAAGACACTTCCACATGGACTATGGAACTGG

GTTTTACAAACCTGTGATGGCTTCGAAGACCCTCGAGGCTATCCTCTCCTTTGCACGCCGTGGG

>DQ404161.1_O_UKG_2001

GGATTGATAGTTGACACCAGAGATGTTGAGGAGCGCGTACATGTCATGCGCAAAACCAAGCTCGCACCCA

CCGTGGCACACGGTGTGTTTAACCCCGAATTTGGGCCTGCCGCCTTGTCCAACAAGGACCCGCGCCTGAA

TGAGGGGGTTGTCCTCGATGAAGTCATCTTCTCCAAACACAAAGGAAACACAAAGATGTCTGAGGAGGAC

AAAGCGCTGTTCCGCCGCTGTGCTGCTGACTACGCGTCGCGTCTGCATAGCGTGCTGGGTACGGCAAATG

CCCCACTGAGCACTTACGAGGCAATCAAGGGCGTCGACGGACTTGACGCCATGGAACCAGACACCGCGCC

TGGTCTCCCCTGGGCTCTCCAGGGGAAACGCCGTGGTGCGCTCATCGACTTCGAGAACGGCACTGTCGGA

CCCGAGGTTGAAGCTGCCTTGAAGCTCATGGAGAAAAGAGAGTACAAGTTTACATGCCAGACCTTCCTGA

AGGACGAGATTCGCCCGATGGAGAAGGTACGTGCCGGCAAGACTCGCATTGTCGACGTCCTGCCCGTTGA

ACACATTCTTTACACTAGGATGATGATTGGCAGATTTTGTGCTCAAATGCACTCAAACAACGGACCGCAA

ATTGGCTCGGCGGTTGGTTGTAATCCTGATGTTGATTGGCAAAGATTTGGCACGCATTTTGCTCAGTATA

GAAACGTGTGGGATGTGGACTATTCGGCCTTTGATGCCAACCACTGCAGTGACGCAATGAACATCATGTT

TGAGGAGGTGTTTAACACGGACTTCGGTTTCCACCCAAACGCTGAGTGGATCCTGAAAACTCTCGTGAAC

ACTGAACACGCCTATGAGAACAAACGCATCACTGTTGAAGGCGGGATGCCGTCTGGTTGTTCCGCAACAA

GCATCATCAACACAATTTTGAATAACATCTACGTGCTCTACGCCTTGCGTAGACACTATGAGGGGGTTGA

GCTGGACTCTTACACCATGATCTCCTACGGAGACGACATCGTGGTTGCAAGTGATTACGATCTGGACTTT

GAGGCCCTCAAGCCTCACTTCAAATCCCTTGGTCAAACCATTACCCCAGCTGACAAAAGCGACAAAGGTT

TTGTTCTTGGTCACTCCATTACCGATGTCACTTTCCTCAAAAGACACTTCCACATGGACTATGGAACTGG

GTTTTACAAACCTGTGATGGCTTCGAAGACCCTCGAGGCTATCCTCTCCTTTGCACGCCGTGGG

>DQ404162.1_O_UKG_2001

GGATTGATAGTTGACACCAGAGATGTTGAGGAGCGCGTACATGTCATGCGCAAAACCAAGCTCGCACCCA

CCGTGGCACACGGTGTGTTTAACCCCGAATTTGGGCCTGCCGCCTTGTCCAACAAGGACCCGCGCCTGAA

TGAGGGGGTTGTCCTCGATGAAGTCATCTTCTCCAAACACAAAGGAAACACAAAGATGTCTGAGGAGGAC

AAAGCGCTGTTCCGCCGCTGTGCTGCTGACTACGCGTCGCGTCTGCACAGCGTGCTGGGTACGGCAAATG

CCCCACTGAGCACTTACGAGGCAATCAAGGGCGTCGACGGACTTGACGCCATGGAACCAGACACCGCGCC

TGGTCTCCCCTGGGCTCTCCAGGGGAAACGCCGTGGTGCGCTCATCGACTTCGAGAACGGCACTGTCGGA

CCCGAGGTTGAAGCTGCCTTGAAGCTCATGGAGAAAAGAGAGTACAAGTTTACATGCCAGACCTTCCTGA

AGGACGAGATTCGCCCGATGGAGAAGGTACGTGCCGGCAAGACTCGCATTGTCGACGTCCTGCCCGTTGA

ACACATTCTTTACACTAGGATGATGATTGGCAGATTTTGTGCTCAAATGCACTCAAACAACGGACCGCAA

ATTGGCTCGGCGGTTGGTTGTAATCCTGATGTTGATTGGCAAAGATTTGGCACGCATTTTGCTCAGTATA

GAAACGTGTGGGATGTGGACTATTCGGCCTTTGATGCCAACCACTGCAGTGACGCAATGAACATCATGTT

TGAGGAGGTGTTTAACACGGACTTCGGTTTCCACCCAAACGCTGAGTGGATCCTGAAAACTCTCGTGAAC

ACTGAACACGCCTATGAGAACAAACGCATCACTGTTGAAGGCGGGATGCCGTCTGGTTGTTCCGCAACAA

GCATCATCAACACAATTTTGAACAACATCTACGTGCTCTACGCCTTGCGTAGACACTATGAGGGGGTTGA

GCTGGACTCTTACACCATGATCTCCTACGGAGACGACATCGTGGTTGCAAGTGATTACGATCTGGACTTT

GAGGCCCTCAAGCCTCACTTCAAATCCCTTGGTCAAACCATTACCCCAGCTGACAAAAGCGACAAAGGTT

TTGTTCTTGGTCACTCCATTACCGATGTCACTTTCCTCAAAAGACACTTCCACATGGACTATGGAACTGG

GTTTTACAAACCTGTGATGGCTTCGAAGACCCTCGAGGCTATCCTCTCCTTTGCACGCCGTGGG

>DQ404163.1_O_UKG_2001

GGATTGATAGTTGACACCAGAGATGTTGAGGAGCGCGTACATGTCATGCGCAAAACCAAGCTCGCACCCA

CCGTGGCACACGGTGTGTTTAACCCCGAATTTGGGCCTGCCGCCTTGTCCAACAAGGACCCGCGCCTGAA

TGAGGGGGTTGTCCTCGATGAAGTCATCTTCTCCAAACACAAAGGAAACACAAAGATGTCTGAGGAGGAC

AAAGCGCTGTTCCGCCGCTGTGCTGCTGACTACGCGTCGCGTCTGCATAGCGTGCTGGGTACGGCAAATG

CCCCACTGAGCACTTACGAGGCAATCAAGGGCGTCGACGGACTTGACGCCATGGAACCAGACACCGCGCC

TGGTCTCCCCTGGGCTCTCCAGGGGAAACGCCGTGGTGCGCTCATCGACTTCGAGAACGGCACTGTCGGA

CCCGAGGTTGAAGCTGCCTTGAAGCTCATGGAGAAAAGAGAGTACAAGTTTACATGCCAGACCTTCCTGA

AGGACGAGATTCGCCCGATGGAGAAGGTACGTGCCGGCAAGACCCGCATTGTCGACGTCCTGCCCGTTGA

ACACATTCTTTACACTAGGATGATGATTGGCAGATTTTGTGCTCAAATGCACTCAAACAACGGACCGCAA

ATTGGCTCGGCGGTTGGTTGTAATCCTGATGTTGATTGGCAAAGATTTGGCACGCATTTTGCTCAGTATA

GAAACGTGTGGGATGTGGACTATTCGGCCTTTGATGCCAACCACTGCAGTGACGCAATGAACATCATGTT

TGAGGAGGTGTTTAACACGGACTTCGGTTTCCACCCAAACGCTGAGTGGATCCTGAAAACTCTCGTGAAC

ACTGAACACGCCTATGAGAACAAACGCATCACTGTTGAAGGCGGGATGCCGTCTGGTTGTTCCGCAACAA

GCATCATCAACACAATTTTGAACAACATCTACGTGCTCTACGCCTTGCGTAGACACTATGAGGGGGTTGA

GCTGGACTCTTACACCATGATCTCCTACGGAGACGACATCGTGGTTGCAAGTGATTATGATCTGGACTTT

GAGGCCCTCAAGCCTCACTTCAAATCCCTTGGTCAAACCATTACCCCAGCTGACAAAAGCGACAAAGGTT

TTGTTCTTGGTCACTCCATTACCGACGTCACTTTCCTCAAAAGACACTTCCACATGGACTATGGAACTGG

GTTTTACAAACCTGTGATGGCTTCGAAGACCCTCGAGGCTATCCTCTCCTTTGCACGCCGTGGG

>DQ404164.1_O_UKG_2001

GGATTGATAGTTGACACCAGAGATGTTGAGGAGCGCGTACATGTCATGCGCAAAACCAAGCTCGCACCCA

CCGTGGCACACGGTGTGTTTAACCCCGAATTTGGGCCTGCCGCCCTGTCCAACAAGGACCCGCGCCTGAA

TGAGGGGGTTGTCCTCGATGAAGTCATCTTCTCCAAACACAAAGGAAACACAAAGATGTCTGAGGAGGAC

AAAGCGCTGTTCCGCCGCTGTGCTGCTGACTACGCGTCGCGTCTGCATAGCGTGCTGGGTACGGCAAATG

CCCCACTGAGCACTTACGAGGCAATCAAGGGCGTCGACGGACTTGACGCCATGGAACCAGACACCGCGCC

TGGTCTCCCCTGGGCTCTCCAGGGGAAACGCCGTGGTGCGCTCATCGACTTCGAGAACGGCACTGTCGGA

CCCGAGGTTGAAGCTGCCTTGAAGCTCATGGAGAAAAGAGAGTACAAGTTTACATGCCAGACCTTCCTGA

AGGACGAGATTCGCCCGATGGAGAAGGTACGTGCCGGCAAGACTCGCATTGTCGACGTCCTGCCCGTTGA

ACACATTCTTTACACTAGGATGATGATTGGCAGATTTTGTGCTCAAATGCACTCAAACAACGGACCGCAA

ATTGGCTCGGCGGTTGGTTGTAATCCTGATGTTGATTGGCAAAGATTTGGCACGCATTTTGCTCAGTATA

GAAACGTGTGGGATGTGGACTATTCGGCCTTTGATGCCAACCACTGCAGTGACGCAATGAACATCATGTT

TGAGGAGGTGTTTAACACGGACTTCGGTTTCCACCCAAACGCTGAGTGGATCCTGAAAACTCTCGTGAAC

ACTGAACACGCCTATGAGAACAAACGCATCACTGTTGAAGGCGGGATGCCGTCTGGTTGTTCCGCAACAA

GCATCATCAACACAATTTTGAACAACATCTACGTGCTCTACGCCTTGCGTAGACACTATGAGGGGGTTGA

GCTGGACTCCTACACCATGATCTCCTACGGAGACGACATCGTGGTTGCAAGTGATTACGATCTGGACTTT

GAGGCCCTCAAGCCTCACTTCAAATCCCTTGGTCAAACCATTACCCCAGCTGACAAAAGCGACAAAGGTT

TTGTTCTTGGGCACTCCATTACCGATGTCACTTTCCTCAAAAGACACTTCCACATGGACTATGGAACTGG

GTTTTACAAACCTGTGATGGCTTCGAAGACCCTCGAGGCTATCCTCTCCTTTGCACGCCGTGGG

>DQ404165.1_O_UKG_2001

GGATTGATAGTTGACACCAGAGATGTTGAGGAGCGCGTACATGTCATGCGCAAAACCAAGCTCGCACCCA

CCGTGGCACACGGCGTGTTTAACCCCGAATTTGGGCCTGCCGCCTTGTCCAACAAGGACCCGCGCCTGAA

TGAGGGGGTTGTCCTCGATGAAGTCATCTTCTCCAAACACAAAGGAAACGCAAAGATGTCTGAGGAGGAC

AAAGCGCTGTTCCGCCGCTGTGCTGCTGACTACGCGTCGCGTCTGCATAGCGTGCTGGGTACGGCAAATG

CCCCACTGAGCACTTACGAGGCAATCAAGGGCGTCGACGGACTTGACGCCATGGAACCAGACACCGCGCC

TGGTCTCCCCTGGGCTCTCCAGGGGAAACGCCGTGGTGCGCTCATCGACTTCGAGAACGGCACTGTCGGA

CCCGAGGTTGAAGCTGCCTTGAAGCTCATGGAGAAAAGAGAGTACAAGTTTACATGCCAGACCTTCCTGA

AGGACGAGATTCGCCCGATGGAGAAGGTACGTGCCGGCAAGACTCGCATTGTCGACGTCCTGCCCGTTGA

ACACATTCTTTACACTAGGATGATGATTGGTAGATTTTGTGCTCAAATGCACTCAAACAACGGACCGCAA

ATTGGCTCGGCAGTTGGTTGTAATCCTGATGTTGATTGGCAAAGATTTGGCACGCATTTTGCTCAGTATA

GAAACGTGTGGGATGTGGACTATTCGGCCTTTGATGCCAACCACTGCAGTGACGCAATGAACATCATGTT

TGAGGAGGTGTTTAACACGGACTTCGGTTTCCACCCAAACGCTGAGTGGATCCTGAAAACTCTCGTGAAC

ACTGAACACGCCTACGAGAACAAACGCATCACTGTTGAAGGCGGGATGCCGTCTGGTTGTTCCGCAACAA

GCATCATCAACACAATTTTGAACAACATCTACGTGCTCTACGCCTTGCGTAGACACTATGAGGGGGTTGA

GCTGGACTCTTACACCATGATCTCCTACGGAGACGACATCGTGGTTGCAAGTGATTACGATCTGGACTTT

GAGGCCCTCAAGCCTCACTTCAAATCCCTTGGTCAAACCATTACCCCAGCTGACAAAAGCGACAAAGGTT

TTGTTCTTGGTCACTCCATTACCGATGTCACTTTCCTCAAAAGACACTTCCACATGGACTATGGAACTGG

GTTTTACAAACCTGTGATGGCTTCGAAGACCCTCGAGGCTATCCTCTCCTTTGCACGCCGTGGG

>DQ404166.1_O_UKG_2001

GGATTGATAGTTGACACCAGAGATGTTGAGGAGCGCGTACATGTCATGCGCAAAACCAAGCTCGCACCCA

CCGTGGCACACGGTGTGTTTAACCCCGAATTTGGGCCTGCCGCCTTGTCCAACAAGGACCCGCGCCTGAA

TGAGGGGGTTGTCCTCGATGAAGTCATCTTCTCCAAACACAAAGGAAACGCAAAGATGTCTGAGGAGGAC

AAAGCGCTGTTCCGCCGCTGTGCTGCTGACTACGCGTCGCGTCTGCATAGCGTGCTGGGTACGGCAAATG

CCCCACTGAGCACTTACGAGGCAATCAAGGGCGTCGACGGACTTGACGCCATGGAACCAGACACCGCGCC

TGGTCTCCCCTGGGCTCTCCAGGGGAAACGCCGTGGTGCGCTCATCGACTTCGAGAACGGCACTGTCGGA

CCCGAGGTTGAAGCTGCCTTGAAGCTCATGGAGAAAAGAGAGTACAAGTTTACATGCCAGACCTTCCTGA

AGGACGAGATTCGCCCGATGGAGAAGGTACGTGCCGGCAAGACTCGCATTGTCGACGTCCTGCCCGTTGA

ACACATTCTTTACACTAGGATGATGATTGGTAGATTTTGTGCTCAAATGCACTCAAACAACGGACCGCAA

ATTGGCTCGGCAGTTGGTTGTAATCCTGATGTTGATTGGCAAAGATTTGGCACGCATTTTGCTCAGTATA

GAAACGTGTGGGATGTGGACTATTCGGCCTTTGATGCCAACCACTGCAGTGACGCAATGAACATCATGTT

TGAGGAGGTGTTTAACACGGACTTCGGTTTCCACCCAAACGCTGAGTGGATCCTGAAAACTCTCGTGAAC

ACTGAACACGCCTACGAGAACAAACGCATCACTGTTGAAGGCGGGATGCCGTCTGGTTGTTCCGCAACAA

GCATCATCAACACAATTTTGAACAACATCTACGTGCTCTACGCCTTGCGTAGACACTATGAGGGGGTTGA

GCTGGACTCTTACACCATGATCTCCTACGGAGACGACATCGTGGTTGCAAGTGATTACGATCTGGACTTT

GAGGCCCTCAAGCCTCACTTCAAATCCCTTGGTCAAACCATTACCCCAGCTGACAAAAGCGACAAAGGTT

TTGTTCTTGGTCACTCCATTACCGATGTCACTTTCCTCAAAAGACACTTCCACATGGACTATGGAACTGG

GTTTTACAAACCTGTGATGGCTTCGAAGACCCTCGAGGCTATCCTCTCCTTTGCACGCCGTGGG

>DQ404167.1_O_UKG_2001

GGATTGATAGTTGACACCAGAGATGTTGAGGAGCGCGTACATGTCATGCGCAAAACCAAGCTCGCACCCA

CCGTGGCACACGGTGTGTTTAACCCCGAATTTGGGCCTGCCGCCTTGTCCAACAAGGACCCGCGCCTGAA

TGAGGGGGTTGTCCTCGATGAAGTCATCTTCTCCAAACACAAAGGAAACACAAAGATGTCTGAGGAGGAC

AAAGCGCTGTTCCGCCGCTGTGCTGCTGACTACGCGTCGCGTCTGCATAGCGTGCTGGGTACGGCAAATG

CCCCACTGAGCACTTACGAGGCAATCAAGGGCGTCGACGGACTTGACGCCATGGAACCAGACACCGCGCC

TGGTCTCCCCTGGGCTCTCCAGGGGAAACGCCGTGGTGCGCTCATCGACTTCGAGAACGGCACTGTCGGA

CCCGAGGTTGAAGCTGCCTTGAAGCTCATGGAGAAAAGAGAGTACAAGTTTACATGCCAGACCTTCCTGA

AGGACGAGATTCGCCCGATGGAGAAGGTACGTGCCGGCAAGACTCGCATTGTCGACGTCCTGCCCGTTGA

ACACATTCTTTACACTAGGATGATGATTGGTAGATTTTGTGCTCAAATGCACTCAAACAACGGACCGCAA

ATTGGCTCGGCAGTTGGTTGTAATCCTGATGTTGATTGGCAAAGATTTGGCACGCATTTTGCTCAGTATA

GAAACGTGTGGGATGTGGACTATTCGGCCTTTGATGCCAACCACTGCAGTGACGCAATGAACATCATGTT

TGAGGAGGTGTTTAACACGGACTTCGGTTTCCACCCAAACGCTGAGTGGATCCTGAAAACTCTCGTGAAC

ACTGAACACGCCTATGAGAACAAACGCATCACTGTTGAAGGCGGGATGCCGTCTGGTTGTTCCGCAACAA

GCATCATCAACACAATTTTGAACAACATCTACGTGCTCTACGCCTTGCGTAGACACTATGAGGGGGTTGA

GCTGGACTCTTACACCATGATCTCCTACGGAGACGACATCGTGGTTGCAAGTGATTACGATCTGGACTTT

GAGGCCCTCAAGCCTCACTTCAAATCCCTTGGTCAAACCATTACCCCAGCTGACAAAAGCGACAAAGGTT

TTGTTCTTGGTCACTCCATTACCGATGTCACTTTCCTCAAAAGACACTTCCACATGGACTATGGAACTGG

GTTTTACAAACCTGTGATGGCTTCGAAGACCCTCGAGGCTATCCTCTCCTTTGCACGCCGTGGG

>DQ404168.1_O_UKG_2001

GGATTGATAGTTGACACCAGAGATGTTGAGGAGCGCGTACATGTCATGCGCAAAACCAAGCTCGCACCCA

CCGTGGCACACGGTGTGTTTAACCCCGAATTTGGGCCTGCCGCCTTGTCCAACAAGGACCCGCGCCTGAA

TGAGGGGGTTGTCCTCGATGAAGTCATCTTCTCCAAACACAAAGGAAACACAAAGATGTCTGAGGAGGAC

AAAGCGCTGTTCCGCCGCTGTGCTGCTGACTACGCGTCGCGTCTGCACAGCGTGCTGGGTACGGCAAATG

CCCCACTGAGCACTTACGAGGCAATCAAGGGCGTCGACGGACTTGACGCCATGGAACCAGACACCGCGCC

TGGTCTCCCCTGGGCTCTCCAGGGGAAACGCCGTGGTGCGCTCATCGACTTCGAGAACGGCACTGTCGGA

CCCGAGGTTGAAGCTGCCTTGAAGCTCATGGAGAAAAGAGAGTACAAGTTTACATGCCAGACCTTCCTGA

AGGACGAGATTCGCCCGATGGAGAAGGTACGTGCCGGCAAGACTCGCATTGTCGACGTCCTGCCCGTTGA

ACACATTCTTTACACTAGGATGATGATTGGCAGATTTTGTGCTCAAATGCACTCAAACAACGGACCGCAA

ATTGGCTCGGCGGTTGGTTGTAATCCTGATGTTGATTGGCAAAGATTTGGCACGCATTTTGCTCAGTATA

GAAACGTGTGGGATGTGGACTATTCGGCCTTTGATGCCAACCACTGCAGTGACGCAATGAACATCATGTT

TGAGGAGGTGTTTAACACGGACTTCGGTTTCCACCCAAACGCTGAGTGGATCCTGAAAACTCTCGTGAAC

ACTGAACACGCCTATGAGAACAAACGCATCACTGTTGAAGGCGGGATGCCGTCTGGTTGTTCCGCAACAA

GCATCATCAACACAATTTTGAACAACATCTACGTGCTCTACGCCTTGCGTAGACACTATGAGGGGGTTGA

GCTGGACTCTTACACCATGATCTCCTACGGAGACGACATCGTGGTTGCAAGTGATTACGATCTGGACTTT

GAGGCCCTCAAGCCTCACTTCAAATCCCTTGGTCAAACCATTACCCCAGCTGACAAAAGCGACAAAGGTT

TTGTTCTTGGTCACTCCATTACCGATGTCACTTTCCTCAAAAGACACTTCCACATGGACTATGGAACTGG

GTTTTACAAACCTGTGATGGCTTCGAAGACCCTCGAGGCTATCCTCTCCTTTGCACGCCGTGGG

>DQ404169.1_O_UKG_2001

GGATTGATAGTTGACACCAGAGATGTTGAGGAGCGCGTACATGTCATGCGCAAAACCAAGCTCGCACCCA

CCGTGGCACACGGTGTGTTTAACCCCGAATTTGGGCCTGCCGCCTTGTCCAACAAGGACCCGCGCCTGAA

TGAGGGGGTTGTCCTCGATGAAGTCATCTTCTCCAAACACAAAGGAAACACAAAGATGTCTGAGGAGGAC

AAAGCGCTGTTCCGCCGCTGTGCTGCTGACTACGCGTCGCGTCTGCATAGCGTGCTGGGTACGGCAAATG

CCCCACTGAGCACTTACGAGGCAATCAAGGGCGTCGACGGACTTGACGCCATGGAACCAGACACCGCGCC

TGGTCTCCCCTGGGCTCTCCAGGGGAAACGCCGTGGTGCGCTCATCGACTTCGAGAACGGCACTGTCGGA

CCCGAGGTTGAAGCTGCCTTGAAGCTCATGGAGAAAAGAGAGTACAAGTTTACATGCCAGACCTTCCTGA

AGGACGAGATTCGCCCGATGGAGAAGGTACGTGCCGGCAAGACTCGCATTGTCGACGTCCTGCCCGTTGA

ACACATTCTTTACACTAGGATGATGATTGGCAGATTTTGTGCTCAAATGCACTCAAACAACGGACCGCAA

ATTGGCTCGGCGGTTGGTTGTAATCCTGATGTTGATTGGCAAAGATTTGGCACGCATTTTGCTCAGTATA

GAAACGTGTGGGATGTGGACTATTCGGCCTTTGATGCCAACCACTGCAGTGACGCAATGAACATCATGTT

TGAGGAGGTGTTTAACACGGACTTCGGTTTCCACCCAAACGCTGAGTGGATCCTGAAAACTCTCGTGAAC

ACTGAACACGCCTATGAGAACAAACGCATCACTGTTGAAGGCGGGATGCCGTCTGGTTGTTCCGCAACAA

GCATCATCAACACAATTTTGAACAACATCTACGTGCTCTACGCCTTGCGTAGACACTATGAGGGGGTTGA

GCTGGACTCTTACACCATGATCTCCTACGGAGACGACATCGTGGTTGCAAGTGATTACGATCTGGACTTT

GAGGCCCTCAAGCCTCACTTCAAATCCCTTGGTCAAACCATTACCCCAGCTGACAAAAGCGACAAAGGTT

TTGTTCTTGGTCACTCCATTACCGATGTCACTTTCCTCAAAAGACACTTCCACATGGACTATGGAACTGG

GTTTTACAAACCTGTGATGGCTTCGAAGACCCTCGAGGCTATCCTCTCCTTTGCACGCCGTGGG

>DQ404170.1_O_UKG_2001

GGATTGATAGTTGACACCAGAGATGTTGAGGAGCGCGTACATGTCATGCGCAAAACCAAGCTCGCACCCA

CCGTGGCACACGGTGTGTTTAACCCCGAATTTGGGCCTGCCGCCTTGTCCAACAAGGACCCGCGCCTGAA

TGAGGGGGTTGTCCTCGATGAAGTCATCTTCTCCAAACACAAAGGAAACACAAAGATGTCTGAGGAGGAC

AAAGCGCTGTTCCGCCGCTGTGCTGCTGACTACGCGTCGCGTCTGCATAGCGTGCTGGGTACGGCAAATG

CCCCACTGAGCACTTACGAGGCAATCAAGGGCGTCGACGGACTTGACGCCATGGAACCAGACACCGCGCC

TGGTCTCCCCTGGGCTCTCCAGGGGAAACGCCGTGGTGCGCTCATCGACTTCGAGAACGGCACTGTCGGA

CCCGAGGTTGAAGCTGCCTTGAAGCTCATGGAGAAAAGAGAGTACAAGTTTACATGCCAGACCTTCCTGA

AGGACGAGATTCGCCCGATGGAGAAGGTACGTGCCGGCAAGACTCGCATTGTCGACGTCCTGCCCGTTGA

ACACATTCTTTACACTAGGATGATGATTGGCAGATTTTGTGCTCAAATGCACTCAAACAACGGACCGCAA

ATTGGCTCGGCGGTTGGTTGTAATCCTGATGTTGATTGGCAAAGATTTGGCACGCATTTTGCTCAGTATA

GAAACGTGTGGGATGTGGACTATTCGGCCTTTGATGCCAACCACTGCAGTGACGCAATGAACATCATGTT

TGAGGAGGTGTTTAACACGGACTTCGGTTTCCACCCAAACGCTGAGTGGATCCTGAAAACTCTCGTGAAC

ACTGAACACGCCTATGAGAACAAACGCATCACTGTTGAAGGCGGGATGCCGTCTGGTTGTTCCGCAACAA

GCATCATCAACACAATTTTGAACAACATCTACGTGCTCTACGCCTTGCGTAGACACTATGAGGGGGTTGA

GCTGGACTCTTACACCATGATCTCCTACGGAGACGACATCGTGGTTGCAAGTGATTACGATCTGGACTTT

GAGGCCCTCAAGCCTCACTTCAAATCCCTTGGTCAAACCATTACCCCAGCTGACAAAAGCGACAAAGGTT

TTGTTCTTGGTCACTCCATTACCGATGTCACTTTCCTCAAAAGACACTTCCACATGGACTATGGAACTGG

GTTTTACAAACCTGTGATGGCTTCGAAGACCCTCGAGGCTATCCTCTCCTTTGCACGCCGTGGG

>DQ404171.1_O_UKG_2001

GGGTTGATAGTTGACACCAGAGATGTTGAGGAGCGCGTACATGTCATGCGCAAAACCAAGCTCGCACCCA

CCGTGGCACACGGTGTGTTTAACCCCGAATTTGGGCCTGCCGCCTTGTCCAACAAGGACCCGCGCCTGAA

TGAGGGGGTTGTCCTCGATGAAGTCATCTTCTCCAAACACAAAGGAAACACAAAGATGTCTGAGGAGGAC

AAAGCGCTGTTCCGCCGCTGTGCTGCTGACTACGCGTCGCGTCTGCATAGCGTGCTGGGTACGGCAAATG

CCCCACTGAGCACTTACGAGGCAATCAAGGGCGTCGACGGACTTGACGCCATGGAACCAGACACCGCGCC

TGGTCTCCCCTGGGCTCTCCAGGGGAAACGCCGTGGTGCGCTCATCGACTTCGAGAACGGCACTGTCGGA

CCCGAGGTTGAAGCTGCCTTGAAGCTCATGGAGAAAAGAGAGTACAAGTTTACATGCCAGACCTTCCTGA

AGGACGAGATTCGCCCGATGGAGAAGGTACGTGCCGGCAAGACTCGCATTGTCGACGTCCTGCCCGTTGA

ACACATTCTTTACACTAGGATGATGATTGGCAGATTTTGTGCTCAAATGCACTCAAACAACGGACCGCAA

ATTGGCTCAGCGGTTGGTTGTAATCCTGATGTTGATTGGCAAAGATTTGGCACGCATTTTGCTCAGTATA

GAAACGTGTGGGATGTGGACTATTCGGCCTTTGATGCCAACCACTGCAGTGACGCAATGAACATCATGTT

TGAGGAGGTGTTTAACACGGACTTCGGTTTCCACCCAAACGCTGAGTGGATCCTGAAAACTCTCGTGAAC

ACTGAACACGCCTATGAGAACAAACGCATCACTGTTGAAGGCGGGATGCCGTCTGGTTGTTCCGCAACAA

GCATCATCAACACAATTTTGAACAACATCTACGTGCTCTACGCCTTGCGTAGACACTATGAGGGGGTTGA

GCTGGACTCTTACACCATGATCTCCTACGGAGACGACATCGTGGTTGCAAGTGATTACGATCTGGACTTT

GAGGCCCTCAAGCCTCACTTCAAATCCCTTGGTCAAACCATTACCCCAGCTGACAAAAGCGACAAAGGTT

TTGTTCTTGGTCACTCCATTACCGATGTCACTTTCCTCAAAAGACACTTCCACATGGACTATGGAACTGG

GTTTTACAAACCTGTGATGGCTTCGAAGACCCTCGAGGCTATCCTCTCCTTTGCACGCCGTGGG

>DQ404172.1_O_UKG_2001

GGATTGATAGTTGACACCAGAGATGTTGAGGAGCGCGTACATGTCATGCGCAAAACCAAGCTCGCACCCA

CCGTGGCACACGGTGTGTTTAACCCCGAATTTGGGCCTGCCGCCTTGTCCAACAAGGACCCGCGCCTGAA

TGAGGGGGTTGTCCTCGATGAAGTCATCTTCTCCAAACACAAAGGAAACACAAAGATGTCTGAGGAGGAC

AAAGCGCTGTTCCGCCGCTGTGCTGCTGACTACGCGTCGCGTCTGCATAGCGTGCTGGGTACGGCAAATG

CCCCACTGAGCACTTACGAGGCAATCAAGGGCGTCGACGGACTTGACGCCATGGAACCAGACACCGCGCC

TGGTCTCCCCTGGGCTCTCCAGGGGAAACGCCGTGGTGCGCTCATCGACTTCGAGAACGGCACTGTCGGA

CCCGAGGTTGAAGCTGCCTTGAAGCTCATGGAGAAAAGAGAGTACAAGTTTACATGCCAGACCTTCCTGA

AGGACGAGATTCGCCCGATGGAGAAGGTACGTGCCGGCAAGACTCGCATTGTCGACGTCCTGCCCGTTGA

ACACATTCTTTACACTAGGATGATGATTGGCAGATTTTGTGCTCAAATGCACTCAAACAACGGACCGCAA

ATTGGCTCGGCGGTTGGTTGTAATCCTGATGTTGATTGGCAAAGATTTGGCACGCATTTTGCTCAGTATA

GAAACGTGTGGGATGTGGACTATTCGGCCTTTGATGCCAACCACTGCAGTGACGCAATGAACATCATGTT

TGAGGAGGTGTTTAACACGGACTTCGGTTTCCACCCAAACGCTGAGTGGATCCTGAAAACTCTCGTGAAC

ACTGAACACGCCTATGAGAACAAACGCATCACTGTTGAAGGCGGGATGCCGTCTGGTTGTTCCGCAACAA

GCATCATCAACACAATTTTGAACAACATCTACGTGCTCTACGCCTTGCGTAGACACTATGAGGGGGTTGA

GCTGGACTCTTACACCATGATCTCCTACGGAGACGACATCGTGGTTGCAAGTGATTACGATCTGGACTTT

GAGGCCCTCAAGCCTCACTTCAAATCCCTTGGTCAAACCATTACCCCAGCTGACAAAAGCGACAAAGGTT

TTGTTCTTGGTCACTCCATTACCGATGTCACTTTCCTCAAAAGACACTTCCACATGGACTATGGAACTGG

GTTTTACAAACCTGTGATGGCTTCGAAGACCCTCGAGGCTATCCTCTCCTTTGCACGCCGTGGG

>DQ404173.1_O_UKG_2001

GGATTGATAGTTGACACCAGAGATGTTGAGGAGCGCGTACATGTCATGCGCAAAACCAAGCTCGCACCCA

CCGTGGCACACGGTGTGTTTAACCCCGAATTTGGGCCTGCCGCCTTGTCCAACAAGGACCCGCGCCTGAA

TGAGGGGGTTGTCCTCGATGAAGTCATCTTCTCCAAACACAAAGGAAACACAAAGATGTCTGAGGAGGAC

AAAGCGCTGTTCCGCCGCTGTGCTGCTGACTACGCGTCGCGTCTGCATAGCGTGCTGGGTACGGCAAATG

CCCCACTGAGCACTTACGAGGCAATCAAGGGCGTCGACGGACTTGACGCCATGGAACCAGACACCGCGCC

TGGTCTCCCCTGGGCTCTCCAGGGGAAACGCCGTGGTGCGCTCATCGACTTCGAGAACGGCACTGTCGGA

CCCGAGGTTGAAGCTGCCTTGAAGCTCATGGAGAAAAGAGAGTACAAGTTTACATGCCAGACCTTCCTGA

AGGACGAGATTCGCCCGATGGAGAAGGTACGTGCCGGCAAGACTCGCATTGTCGACGTCCTGCCCGTTGA

ACACATTCTTTACACTAGGATGATGATTGGCAGATTTTGTGCTCAAATGCACTCAAACAACGGACCGCAA

ATTGGCTCGGCGGTTGGTTGTAATCCTGATGTTGATTGGCAAAGATTTGGCACGCATTTTGCTCAGTATA

GAAACGTGTGGGATGTGGACTATTCGGCCTTTGATGCCAACCACTGCAGTGACGCAATGAACATCATGTT

TGAGGAGGTGTTTAACACGGACTTCGGTTTCCACCCAAACGCTGAGTGGATCCTGAAAACTCTCGTGAAC

ACTGAACACGCCTATGAGAACAAACGCATCACTGTTGAAGGCGGGATGCCGTCTGGTTGTTCCGCAACAA

GCATCATCAACACAATTTTGAACAACATCTACGTGCTCTACGCCTTGCGTAGACACTATGAGGGGGTTGA

GCTGGACTCTTACACCATGATCTCCTACGGAGACGACATCGTGGTTGCAAGTGATTACGATCTGGACTTT

GAGGCCCTCAAGCCTCACTTCAAATCCCTTGGTCAAACCATTACCCCAGCTGACAAAAGCGACAAAGGTT

TTGTTCTTGGTCACTCCATTACCGATGTCACTTTCCTCAAAAGACACTTCCACATGGACTATGGAACTGG

GTTTTACAAACCTGTGATGGCTTCGAAGACCCTCGAGGCTATCCTCTCCTTTGCACGCCGTGGG

>DQ404174.1_O_UKG_2001

GGATTGATAGTTGACACCAGAGATGTTGAGGAGCGCGTACATGTCATGCGCAAAACCAAGCTCGCACCCA

CCGTGGCACACGGTGTGTTTAACCCCGAATTTGGGCCTGCCGCCTTGTCCAACAAGGACCCGCGCCTGAA

TGAGGGGGTTGTCCTCGATGAAGTCATCTTCTCCAAACACAAAGGAAACACAAAGATGTCTGAGGAGGAC

AAAGCGCTGTTCCGCCGCTGTGCTGCTGACTACGCGTCGCGTCTGCATAGCGTGCTGGGTACGGCAAATG

CCCCACTGAGCACTTACGAGGCAATCAAGGGCGTCGACGGACTTGACGCCATGGAACCAGACACCGCGCC

TGGTCTCCCCTGGGCTCTCCAGGGGAAACGCCGTGGTGCGCTCATCGACTTCGAGAACGGCACTGTCGGA

CCCGAGGTTGAAGCTGCCTTGAAGCTCATGGAGAAAAGAGAGTACAAGTTTACATGCCAGACCTTCCTGA

AGGACGAGATTCGCCCGATGGAGAAGGTACGTGCCGGCAAGACTCGCATTGTCGACGTCCTGCCCGTTGA

ACACATTCTTTACACTAGGATGATGATTGGCAGATTTTGTGCTCAAATGCACTCAAACAACGGACCGCAA

ATTGGCTCGGCGGTTGGTTGTAATCCTGATGTTGATTGGCAAAGATTTGGCACGCATTTTGCTCAGTATA

GAAACGTGTGGGATGTGGACTATTCGGCCTTTGATGCCAACCACTGCAGTGACGCAATGAACATCATGTT

TGAGGAGGTGTTTAACACGGACTTCGGTTTCCACCCAAACGCTGAGTGGATCCTGAAAACTCTCGTGAAC

ACTGAACACGCCTATGAGAACAAACGCATCACTGTTGAAGGCGGGATGCCGTCTGGTTGTTCCGCAACAA

GCATCATCAACACAATTTTGAACAACATCTACGTGCTCTACGCCTTGCGTAGACACTATGAGGGGGTTGA

GCTGGACTCTTACACCATGATCTCCTACGGAGACGACATCGTGGTTGCAAGTGATTACGATCTGGACTTT

GAGGCCCTCAAGCCTCACTTCAAATCCCTTGGTCAAACCATTACCCCAGCTGACAAAAGCGACAAAGGTT

TTGTTCTTGGTCACTCCATTACCGATGTCACTTTCCTCAAAAGACACTTCCACATGGACTATGGAACTGG

GTTTTACAAACCTGTGATGGCTTCGAAGACCCTCGAGGCTATCCTCTCCTTTGCACGCCGTGGG

>DQ404175.1_O_UKG_2001

GGATTGATAGTTGACACCAGAGATGTTGAGGAGCGCGTACATGTCATGCGCAAAACCAAGCTCGCACCCA

CCGTGGCACACGGTGTGTTTAACCCCGAATTTGGGCCTGCCGCCTTGTCCAACAAGGACCCGCGCCTGAA

TGAGGGGGTTGTCCTCGATGAAGTCATCTTCTCCAAACACAAAGGAAACACAAAGATGTCTGAGGAGGAC

AAAGCGCTGTTCCGCCGCTGTGCTGCTGACTACGCGTCGCGTCTGCATAGCGTGCTGGGTACGGCAAATG

CCCCACTGAGCACTTACGAGGCAATCAAGGGCGTCGACGGACTTGACGCCATGGAACCAGACACCGCGCC

TGGTCTCCCCTGGGCTCTCCAGGGGAAACGCCGTGGTGCGCTCATCGACTTCGAGAACGGCACTGTCGGA

CCCGAGGTTGAAGCTGCCTTGAAGCTCATGGAGAAAAGAGAGTACAAGTTTACATGCCAGACCTTCCTGA

AGGACGAGATTCGCCCGATGGAGAAGGTACGTGCCGGCAAGACTCGCATTGTCGACGTCCTGCCCGTTGA

ACACATTCTTTACACTAGGATGATGATTGGCAGATTTTGTGCTCAAATGCACTCAAACAACGGACCGCAA

ATTGGCTCGGCGGTTGGTTGTAATCCTGATGTTGATTGGCAAAGATTTGGCACGCATTTTGCTCAGTATA

GAAACGTGTGGGATGTGGACTATTCGGCCTTTGATGCCAACCACTGCAGTGACGCAATGAACATCATGTT

TGAGGAGGTGTTTAACACGGACTTCGGTTTCCACCCAAACGCTGAGTGGATCCTGAAAACTCTCGTGAAC

ACTGAACACGCCTATGAGAACAAACGCATCACTGTTGAAGGCGGGATGCCGTCTGGTTGTTCCGCAACAA

GCATCATCAACACAATTTTGAACAACATCTACGTGCTCTACGCCTTGCGTAGACACTATGAGGGGGTTGA

GCTGGACTCTTACACCATGATCTCCTACGGAGACGACATCGTGGTTGCAAGTGATTACGATCTGGACTTT

GAGGCCCTCAAGCCTCACTTCAAATCCCTTGGTCAAACCATTACCCCAGCTGACAAAAGCGACAAAGGTT

TTGTTCTTGGTCACTCCATTACCGATGTCACTTTCCTCAAAAGACACTTCCACATGGACTATGGAACTGG

GTTTTACAAACCTGTGATGGCTTCGAAGACCCTCGAGGCTATCCTCTCCTTTGCACGCCGTGGG

>DQ404176.1_O_UKG_2001

GGATTGATAGTTGACACCAGAGATGTTGAGGAGCGCGTACATGTCATGCGCAAAACCAAGCTCGCACCCA

CCGTGGCACACGGTGTGTTTAACCCCGAATTTGGGCCTGCCGCCTTGTCCAACAAGGACCCGCGCCTGAA

TGAGGGGGTTGTCCTCGATGAAGTCATCTTCTCCAAACACAAAGGAAACACAAAGATGTCTGAGGAGGAC

AAAGCGCTGTTCCGCCGCTGTGCTGCTGACTACGCGTCGCGTCTGCATAGCGTGCTGGGTACGGCAAATG

CCCCACTGAGCACTTACGAGGCAATCAAGGGCGTCGACGGACTTGACGCCATGGAACCAGACACCGCGCC

TGGTCTCCCCTGGGCTCTCCAGGGGAAACGCCGTGGTGCGCTCATCGACTTCGAGAACGGCACTGTCGGA

CCCGAGGTTGAAGCTGCCTTGAAGCTCATGGAGAAAAGAGAGTACAAGTTTACATGCCAGACCTTCCTGA

AGGACGAGATTCGCCCGATGGAGAAGGTACGTGCCGGCAAGACTCGCATTGTCGACGTCCTGCCCGTTGA

ACACATTCTTTACACTAGGATGATGATTGGCAGATTTTGTGCTCAAATGCACTCAAACAACGGACCGCAA

ATTGGCTCGGCGGTTGGTTGTAATCCTGATGTTGATTGGCAAAGATTTGGCACGCATTTTGCTCAGTATA

GAAACGTGTGGGATGTGGACTATTCGGCCTTTGATGCCAACCACTGCAGTGACGCAATGAACATCATGTT

TGAGGAGGTGTTTAACACGGACTTCGGTTTCCACCCAAACGCTGAGTGGATCCTGAAAACTCTCGTGAAC

ACTGAACACGCCTATGAGAACAAACGCATCACTGTTGAAGGCGGGATGCCGTCTGGTTGTTCCGCAACAA

GCATCATCAACACAATTTTGAACAACATCTACGTGCTCTACGCCTTGCGTAGACACTATGAGGGGGTTGA

GCTGGACTCTTACACCATGATCTCCTACGGAGACGACATCGTGGTTGCAAGTGATTACGATCTGGACTTT

GAGGCCCTCAAGCCTCACTTCAAATCCCTTGGTCAAACCATTACCCCAGCTGACAAAAGCGACAAAGGTT

TTGTTCTTGGTCACTCCATTACCGATGTCACTTTCCTCAAAAGACACTTCCACATGGACTATGGAACTGG

GTTTTACAAACCTGTGATGGCTTCGAAGACCCTCGAGGCTATCCTCTCCTTTGCACGCCGTGGG

>DQ404177.1_O_UKG_2001

GGATTGATAGTTGACACCAGAGATGTTGAGGAGCGCGTACATGTCATGCGCAAAACCAAGCTCGCACCCA

CCGTGGCACACGGTGTGTTTAACCCCGAATTTGGGCCTGCCGCCTTGTCCGACAAGGACCCGCGCCTGAA

TGAGGGGGTTGTCCTCGATGAAGTCATCTTCTCCAAACACAAAGGAAACACAAAGATGTCTGAGGAGGAC

AAAGCGCTGTTCCGCCGCTGTGCTGCTGACTACGCGTCGCGTCTGCATAGCGTGCTGGGTACGGCAAATG

CCCCACTGAGCACTTACGAGGCAATCAAGGGCGTCGACGGACTTGACGCCATGGAACCAGACACCGCGCC

TGGTCTCCCCTGGGCTCTCCAGGGGAAACGCCGTGGTGCGCTCATCGACTTCGAGAACGGCACTGTCGGA

CCCGAGGTTGAGGCTGCCTTGAAGCTCATGGAGAAAAGAGAGTACAAGTTTACATGCCAGACCTTCCTGA

AGGACGAGATTCGCCCGATGGAGAAGGTACGTGCCGGCAAGACTCGCATTGTCGACGTCCTGCCCGTTGA

ACACATTCTTTACACTAGGATGATGATTGGCAGATTTTGTGCTCAAATGCACTCAAACAACGGACCGCAA

ATTGGCTCGGCGGTTGGTTGTAATCCTGATGTTGATTGGCAAAGATTTGGCACGCATTTTGCTCAGTATA

GAAACGTGTGGGATGTGGACTATTCGGCCTTTGATGCCAACCACTGCAGTGACGCAATGAACATCATGTT

TGAGGAGGTGTTTAACACGGACTTCGGTTTCCACCCAAACGCTGAGTGGATCCTGAAAACTCTCGTGAAC

ACTGAACACGCCTATGAGAACAAACGCATCACTGTTGAAGGCGGGATGCCGTCTGGTTGTTCCGCAACAA

GCATCATCAACACAATTTTGAACAACATCTACGTGCTCTACGCCTTGCGTAGACACTATGAGGGGGTTGA

GCTGGACTCTTACACCATGATCTCCTACGGAGACGACATCGTGGTTGCAAGTGATTACGATCTGGACTTT

GAGGCCCTCAAGCCTCACTTCAAATCCCTTGGTCAAACCATTACCCCAGCTGACAAAAGCGACAAAGGTT

TTGTTCTTGGTCACTCCATTACCGATGTCACTTTCCTCAAAAGACACTTCCACATGGACTATGGAACTGG

GTTTTACAAACCTGTGATGGCTTCGAAGACCCTCGAGGCTATCCTCTCCTTTGCACGCCGTGGG

>DQ404178.1_O_UKG_2001

GGATTGATAGTTGACACCAGAGATGTTGAGGAGCGCGTACATGTCATGCGCAAAACCAAGCTCGCACCCA

CCGTGGCACACGGTGTGTTTAACCCCGAATTTGGGCCTGCCGCCTTGTCCAACAAGGACCCGCGCCTGAA

TGAGGGGGTTGTCCTCGATGAAGTCATCTTCTCCAAACACAAAGGAAACACAAAGATGTCTGAGGAGGAC

AAAGCGCTGTTCCGCCGCTGTGCTGCTGACTACGCGTCGCGTCTGCATAGCGTGCTGGGTACGGCAAATG

CCCCACTGAGCACTTACGAGGCAATCAAGGGCGTCGACGGACTTGACGCCATGGAACCAGACACCGCGCC

TGGTCTCCCCTGGGCTCTCCAGGGGAAACGCCGTGGTGCGCTCATCGACTTCGAGAACGGTACTGTCGGA

CCCGAGGTTGAGGCTGCCTTGAAGCTCATGGAGAAAAGAGAGTACAAGTTTACATGCCAGACCTTCCTGA

AGGACGAGATTCGCCCGATGGAGAAGGTACGTGCCGGCAAGACTCGCATTGTCGACGTCCTGCCCGTTGA

ACACATTCTTTACACTAGGATGATGATTGGCAGATTTTGTGCTCAAATGCACTCAAACAACGGACCGCAA

ATTGGCTCGGCGGTTGGTTGTAATCCTGATGTTGATTGGCAAAGATTTGGCACGCATTTTGCTCAGTATA

GAAACGTGTGGGATGTGGACTATTCGGCCTTTGATGCCAACCACTGCAGTGACGCAATGAACATCATGTT

TGAGGAGGTGTTTAACACGGACTTCGGTTTCCACCCAAACGCTGAGTGGATCCTGAAAACTCTCGTGAAC

ACTGAACACGCCTATGAGAACAAACGCATCACTGTTGAAGGCGGGATGCCGTCTGGTTGTTCCGCAACAA

GCATCATCAACACAATTTTGAACAACATCTACGTGCTCTACGCCTTGCGTAGACACTATGAGGGGGTTGA

GCTGGACTCTTACACCATGATCTCCTACGGAGACGACATCGTGGTTGCAAGTGATTACGATCTGGACTTT

GAGGCCCTCAAGCCTCACTTCAAATCCCTTGGTCAAACCATTACCCCAGCTGACAAAAGCGACAAAGGTT

TTGTTCTTGGTCACTCCATTACCGATGTCACTTTCCTCAAAAGACACTTCCACATGGACTATGGAACTGG

GTTTTACAAACCTGTGATGGCTTCGAAGACCCTCGAGGCTATCCTCTCCTTTGCACGCCGTGGG

>DQ404179.1_O_UKG_2001

GGATTGATAGTTGACACCAGAGATGTTGAGGAGCGCGTACATGTCATGCGCAAAACCAAGCTCGCACCCA

CCGTGGCACACGGTGTGTTTAACCCCGAATTTGGGCCTGCCGCCTTGTCCAACAAGGACCCGCGCCTGAA

TGAGGGGGTTGTCCTCGATGAAGTCATCTTCTCCAAACACAAAGGAAACACAAAGATGTCTGAGGAGGAC

AAAGCGCTGTTCCGCCGCTGTGCTGCTGACTACGCGTCGCGTCTGCATAGCGTGCTGGGTACGGCAAATG

CCCCACTGAGCACTTACGAGGCAATCAAGGGCGTCGACGGACTTGACGCCATGGAACCAGACACCGCGCC

TGGTCTCCCCTGGGCTCTCCAGGGGAAACGCCGTGGTGCGCTCATCGACTTCGAGAACGGCACTGTCGGA

CCCGAGGTTGAGGCTGCCTTGAAGCTCATGGAGAAAAGAGAGTACAAGTTTACATGCCAGACCTTCCTGA

AGGACGAGATTCGCCCGATGGAGAAGGTACGTGCCGGCAAGACTCGCATTGTCGACGTCCTGCCCGTTGA

ACACATTCTTTACACTAGGATGATGATTGGCAGATTTTGTGCTCAAATGCACTCAAACAACGGACCGCAA

ATTGGCTCGGCGGTTGGTTGTAATCCTGATGTTGATTGGCAAAGATTTGGCACGCATTTTGCTCAGTATA

GAAACGTGTGGGATGTGGACTATTCGGCCTTTGATGCCAACCACTGCAGTGACGCAATGAACATCATGTT

TGAGGAGGTGTTTAACACGGACTTCGGTTTCCACCCAAACGCTGAGTGGATCCTGAAAACTCTCGTGAAC

ACTGAACACGCCTATGAGAACAAACGCATCACTGTTGAAGGCGGGATGCCGTCTGGTTGTTCCGCAACAA

GCATCATCAACACAATTTTGAACAACATCTACGTGCTCTACGCCTTGCGTAGACACTATGAGGGGGTTGA

GCTGGACTCTTACACCATGATCTCCTACGGAGACGACATCGTGGTTGCAAGTGATTACGATCTGGACTTT

GAGGCCCTCAAGCCTCACTTCAAATCCCTTGGTCAAACCATTACCCCAGCTGACAAAAGCGACAAAGGTT

TTGTTCTTGGTCACTCCATTACCGATGTCACTTTCCTCAAAAGACACTTCCACATGGACTATGGAACTGG

GTTTTACAAACCTGTGATGGCTTCGAAGACCCTCGAGGCTATCCTCTCCTTTGCACGCCGTGGG

>DQ404180.1_O_UKG_2001

GGATTGATAGTTGACACCAGAGATGTTGAGGAGCGCGTACATGTCATGCGCAAAACCAAGCTCGCACCCA

CCGTGGCACACGGTGTGTTTAACCCCGAATTTGGGCCTGCCGCCTTGTCCAACAAGGACCCGCGCCTGAA

TGAGGGGGTTGTCCTCGATGAAGTCATCTTCTCCAAACACAAAGGAAACACAAAGATGTCTGAGGAGGAC

AAAGCGCTGTTCCGCCGCTGTGCTGCTGACTACGCGTCGCGTCTGCATAGCGTGCTGGGTACGGCAAATG

CCCCACTGAGCACTTACGAGGCAATCAAGGGCGTCGACGGACTTGACGCCATGGAACCAGACACCGCGCC

TGGTCTCCCCTGGGCTCTCCAGGGGAAACGCCGTGGTGCGCTCATCGACTTCGAGAACGGCACTGTCGGA

CCCGAGGTTGAAGCTGCCTTGAAGCTCATGGAGAAAAGAGAGTACAAGTTTACATGCCAGACCTTCCTGA

AGGACGAGATTCGCCCGATGGAGAAGGTACGTGCCGGCAAGACTCGCATTGTCGACGTCCTGCCCGTTGA

ACACATTCTTTACACTAGGATGATGATTGGCAGATTTTGTGCTCAAATGCACTCAAACAACGGACCGCAA

ATTGGCTCGGCGGTTGGTTGTAATCCTGATGTTGATTGGCAAAGATTTGGCACGCATTTTGCTCAGTATA

GAAACGTGTGGGATGTGGACTATTCGGCCTTTGATGCCAACCACTGCAGTGACGCAATGAACATCATGTT

TGAGGAGGTGTTTAACACGGACTTCGGTTTCCACCCAAACGCTGAGTGGATCCTGAAAACTCTCGTGAAC

ACTGAACACGCCTATGAGAACAAACGCATCACTGTTGAAGGCGGGATGCCGTCTGGTTGTTCCGCAACAA

GCATCATCAACACAATTTTGAACAACATCTACGTGCTCTACGCCTTGCGTAGACACTATGAGGGGGTTGA

GCTGGACTCTTACACCATGATCTCCTACGGAGACGACATCGTGGTTGCAAGTGATTACGATCTGGACTTT

GAGGCCCTCAAGCCTCACTTCAAATCCCTTGGTCAAACCATTACCCCAGCTGACAAAAGCGACAAAGGTT

TTGTTCTTGGTCACTCCATTACCGATGTCACTTTCCTCAAAAGACACTTCCACATGGACTATGGAACTGG

GTTTTACAAACCTGTGATGGCTTCGAAGACCCTCGAGGCTATCCTCTCCTTTGCACGCCGTGGG

>DQ989303.1_Asia1_IND_1993

GGGTTGATCGTTGACACCAGAGATGTGGAAGAGCGTGTGCACGTCATGCGCAAAACCAAGCTTGCACCCA

CCGTGGCACACGGTGTGTTCAACCCTGAGTTTGGGCCCGCCGCCCTGTCCAACAAGGACCCGCGCCTGAA

CGAAGGAGTTGTCCTCGACGAGGTCATCTTCTCCAAACACAAGGGAGACACAAAGATGTCCGCGGAGGAC

AAAGCGTTGTACCGGGCGTGCGCTGCCGACTACGCGTCGCGCCTGCACAGTGTGTTGGGCACGGCAAATG

CCCCACTGAGTATCTATGAGGCCATTAAAGGCGTCGACGGACTCGACGCCATGGAGCCAGACACCGCGCC

TGGCCTTCCCTGGGCTCTCCAGGGGAAGCGCCGCGGCGCACTGATCGACTTCGAGAACGGCACGGTCGGA

CCCGAAGTTGAGGCTGCCTTGAAGCTCATGGAGAAAAGAGAATACAAGTTTGCTTGCCAGACCTTCCTGA

AGGACGAGATTCGCCCGATGGAGAAAGTACGTGCCGGCAAGACTCGCATCGTCGACGTTTTGCCTGTTGA

ACACATTCTTTACACCAGGATGATGATTGGCAGATTCTGTGCTCAAATGCACTCAAACAACGGACCGCAA

ATTGGATCAGCGGTCGGCTGCAATCCTGATGTTGATTGGCAAAGATTTGGCACACATTTTGCCCAATACA

GAAACGTGTGGGATGTGGACTATTCGGCCTTTGATGCTAACCACTGCAGCGACGCGATGAACATCATGTT

TGAGGAGGTGTTCCGCACAGAGTTCGGCTTCCACCCAAACGCTGAGTGGATTCTGAAGACTCTTGTGAAC

ACGGAGCACGCCTACGAGAACAAACGCATCACTGTTGAAGGCGGGATGCCGTCTGGCTGTTCCGCAACTA

GCATCATCAACACAATTCTGAACAACATCTACGTGCTCTACGCGCTGCGTAGACACTATGAGGGAGTTGA

GCTGGACACTTACACCATGATTTCCTACGGGGATGACATCGTGGTCGCAAGTGATTACGATCTGGACTTT

GAGGCTCTTAAGCCCCACTTCAAATCTCTTGGCCAAACCATCACTCCAGCTGACAAAAGCGACAAAGGTT

TTGTTCTTGGTCACTCCATTGCCGATGTCACTTTCCTCAAAAGACACTTCCACATGGATTATGGAACTGG

GTTTTACAAACCTGTGATGGCTTCGAAGACCCTCGAGGCCATCCTCTCCTTTGCACGCCGTGGG

>DQ989304.1_Asia1_IND_2000

GGGTTGATCGTTGACACCAGAGATGTGGAAGAGCGTGTGCACGTCATGCGCAAAACCAAGCTTGCACCCA

CCGTGGCACACGGTGTGTTCAACCCTGAGTTTGGGCCCGCCGCCCTGTCCAACAAGGACCCGCGCCTGAA

CGAAGGAGTTGTCCTCGACGAGGTCATCTTCTCCAAACACAAGGGAGACACAAAGATGTCCGCGGAGGAC

AAAGCGTTGTACCGCCGCTGCGCTGCCGACTACGCGTCGCGCCTGCACAGTGTGTTGGGCACGGCAAATG

CCCCACTGAGTATCTATGAGGCCATTAAAGGCGTCGACGGACTCGACGCCATGGAGCCAGACACCGCGCC

TGGCCTTCCCTGGGCTCTCCAGGGGAAGCGCCGCGGCGCACTGATCGACTTCGAGAACGGCACGGTCGGA

CCCGAAGTTGAGGCTGCCTTGAAGCTCATGGAGAAAAGAGAATACAAGTTTGCTTGCCAGACCTTCCTGA

AGGACGAGATTCGCCCGATGGAGAAAGTACGTGCCGGCAAGACTCGCATCGTCGACGTTTTGCCTGTTGA

ACACATTCTTTACACCAGGATGATGATTGGCAGATTCTGTGCTCAAATGCACTCAAACAACGGACCGCAA

ATTGGATCAGCGGTCGGCTGCAATCCTGATGTTGATTGGCAAAGATTTGGCACACATTTTGCCCAATACA

GAAACGTGTGGGATGTGGACTATTCGGCCTTTGATGCTAACCACTGCAGCGACGCGATGAACATCATGTT

TGAGGAGGTGTTCCGCACAGAGTTCGGCTTCCACCCAAACGCTGAGTGGATTCTGAAGACTCTTGTGAAC

ACGGAGCACGCCTACGAGAACAAGCGCATCACTGTTGAAGGCGGGATGCCGTCTGGCTGTTCCGCAACTA

GCATCATCAACACAATTCTGAACAACATCTACGTGCTCTACGCGCTGCGTAGACACTATGAGGGAGTTGA

GATGGACACTTACACCATGATTTCCTACGGGGATGACATCGTGGTCGCAAGTGATTACGATCTGGACTTT

GAGGCTCTTAAGCCCCACTTCAAATCTCTTGGCCAAACCATCACTCCAGCTGACAAAAGCGACAAAGGTT

TTGTTCTTGGTCACTCCATTGCCGATGTCACTTTCCTCAAAAGACACTTCCACATGGATTATGGAACTGG

GTTTTACAAACCTGTGATGGCTTCGAAGACCCTCGAGGCCATCCTCTCCTTTGCACGCCGTGGG

>DQ989305.1_Asia1_IND_1990

GGGTTGATTGTTGATACCAGAGATGTGGAGGAGCGTGTGCACGTCATGCGCAAAACCAAGCTTGCACCCA

CCGTAGCACACGGTGTGTTCAACCCCGAATTTGGGCCAGCCGCCCTGTCCAACAAGGACCCGCGCCTGAA

TGAGGGAGTTGTCCTCGATGAGGTCATCTTCTCCAAGCACAAAGGAGACACAAAGATGTCTGAGGAGGAC

AAAGCGCTGTTCCGCCGCTGTGCTGCCGACTACGCGTCGCGCCTGCACAGCGTGCTGGGTACAGCCAATG

CCCCATTGAGCATCTACGAGGCCATCAAAGGGGTCGAAGGACTCGACGCCATGGAACCAGACACTGCACC

CGGTCTCCCCTGGGCCCTCCAGGGGAAACGGCGTGGTGCGCTGATCGACTTTGAGAACGGCACTGTCGGA

CCCGAAGTCGCAGCTGCTCTAGAGCTCATGGAGAAAAGGGAATACAAATTTGCTTGTCAGACCTTCCTGA

AGGACGAGATACGCCCGATGGAGAAAGTACGTGCCGGCAAGACTCGCATTGTCGATGTTTTGCCTGTTGA

ACACATTCTTTACACCAGGATGATGATTGGCAGATTTTGTGCTCAAATGCACTCAAACAACGGACCGCAA

ATTGGCTCTGCGGTCGGTTGTAACCCTGATGTTGATTGGCCAAGATTTGGCACACATTTTGCTCAGTACA

GAAACGTGTGGGATGTGGACTATTCGGCCTTTGATGCTAACCACTGCAGTGACGCGATGAACATCATGTT

CGAGGAAGTGTTCCGCACGGACTTTGGTTTCCACCCAAACGCTGTGTGGATTCTGAAGACTCTCGTGAAC

ACGGAGCATGCCTACGAGAACAAACGCATCATTGTTGAGGGCGGGATGCCGTCTGGTTGTTCCGCGACAA

GCATCATCAACACAATTTTGAACAACATCTACGTGCTCTACGCCCTGCGTAGACACTATGAGGGAGTTGA

GCTGGACACTTACACCATGATCTCCTACGGAGACGACATCGTGGTTGCAAGTGATTACGATTTGGACTTT

GAGGCCCTGAAGCCTCACTTTAAATCTCTTGGTCAAACCATTACTCCAGCTGACAAAAGCGACAAAGGTT

TTGTTCTTGGTCACTCCATCACCGATGTCACTTTCCTCAAAAGACACTTCCACATGGATTATGGAACTGG

GTTTTATAAACCTGTGATGGCCTCGAAGACCCTCGAGGCTATCCTCTCCTTTGCACGCCGTGGG

>DQ989306.1_Asia1_IND_1986

GGGTTGATCGTTGACACCAGAGATGTGGAAGAGCGTGTGCACGTCATGCGCAAAACCAAGCTTGCACCCA

CCGTGGCACACGGTGTGTTCAACCCTGAGTTTGGGCCCGCCGCCCTGTCCAACAAGGACCCGCGCCTGAA

CGAAGGAGTTGTCCTCGACGAGGTCATCTTCTCCAAACACAAGGGAGACACAAAGATGTCCGCGGAGGAC

AAAGCGTTGTACCGGCGCTGCGCTGCCGACTACGCGTCGCGCCTGCACAGTGTGTTGGGCACGGCAAATG

CCCCACTGAGTATCTATGAGGCCATTAAAGGCGTCGACGGACTCGACGCCATGGAGCCAGACACCGCGCC

TGGCCTTCCCTGGGCTCTCCAGGGGAAGCGCCGCGGCGCGCTGATCGACTTCGAGAACGGCACGGTCGGA

CCCGAAGTTGAGGCTGCCTTGAAGCTCATGGAGAAAAGAGAATACAAGTTTGCTTGCCAGACCTTCCTGA

AGGACGAGATTCGCCCGATGGAGAAAGTACGTGCCGGCAAGACTCGCATCGTCGACGTTTTGCCTGTTGA

ACACATTCTTTACACCAGGATGATGATTGGCAGATTCTGTGCTCAAATGCACTCAAACAACGGACCGCAA

ATTGGATCAGCGGTCGGCTGCAATCCTGATGTTGATTGGCAAAGATTTGGCACACATTTTGCCCAATACA

GAAACGTGTGGGATGTGGACTATTCGGCCTTTGATGCTAACCACTGCAGCGACGCGATGAACATCATGTT

TGAGGAGGTGTTCCGCACAGAGTTCGGCTTCCACCCAAACGCTGAGTGGATTCTGAAGACTCTTGTGAAC

ACGGAGCACGCCTACGAGAACAAACGCATCACTGTTGAAGGCGGGATGCCGTCTGGCTGTTCCGCAACTA

GCATCATCAACACAATTCTGAACAACATCTACGTGCTCTACGCGCTGCGTAGACACTATGAGGGAGTTGA

GCTGGACACTTACACCATGATTTCCTACGGGGATGACATCGTGGTCGCAAGTGATTACGATCTGGACTTT

GAGGCTCTTAAGCCCCACTTCAAATCTCTTGGCCAAACCATCACTCCAGCTGACAAAAGCGACAAAGGTT

TTGTTCTTGGTCACTCCATTGCCGATGTCACTTTCCTCAAAAGACACTTCCACATGGATTATGGAACTGG

GTTTTACAAACCTGTGATGGCTTCGAAGACCCTCGAGGCCATCCTCTCCTTTGCACGCCGTGGG

>DQ989307.1_Asia1_IND_1992

GGGTTGATTGTTGATACCAGAGATGTGGAGGAGCGTGTGCACGTCATGCGCAAAACCAAGCTCGCACCCA

CCGTGGCACACGGTGTGTTCAACCCTGAATTTGGCCCTGCTGCCTTGTCCAACAAGGACCCACGGCTGAA

CGAAGGAGTTGTCCTTGATGAAGTCATCTTCTCCAAGCACAAGGGAGATACTAAGATGTCTGAGGAGGAC

AAAGCGCTGTTCCGGCGCTGTGCTGCTGACTACGCGTCACGCCTGCATAGCGTGCTGGGCACGGCAAATG

CCCCATTGAGCATTTACGAGGCAATCAAGGGCGTCGACGGGCTCGACGCCATGGAACCAGACACCGCGCC

TGGTCTCCCCTGGGCCCTCCAGGGGAAACGCCGTGGCGCGCTCATCGACTTCGAGAACGGCACAGTCGGA

CCCGAGGTTGAAGCTGCCCTGAAGCTCATGGAGAAAAGAGAATACAGATTTGCCTGTCAGACCTTCCTGA

AGGACGAGATTCGCCCGATGGAGAAAGTACGTGCCGGCAAGACTCGCATTGTCGATGTTTTGCCTGTAGA

ACACATTCTTTACACCAGAATGATGATTGGCAGATTTTGTGCTCAAATGCACTCGAACAACGGACCGCAA

ACTGGTTCAGCGGTCGGTTGTAATCCTGATGTTGATTGGCAAAGATTTGGCACGCATTTTGCCCAGTACA

GAAATGTGTGGGATGTGGACTATTCGGCCTTTGATGCTAACCACTGCAGTGACGCGATGAACATCATGTT

TGAGGAGGTGTTTCGCACGGAGTTCGGTTTCCACCCAAACGCTGAGTGGATTCTGAAAACTCTCGTGAAC

ACGGAACACGCCTATGAAAACAAACGCATCACTGTTGAGGCCGGGATGCCATCTGGTTGTTCCGCTACAA

GCATTATCAACACAATCTTGAACAACATCTACGTGCTTTACGCCCTGCGTAGGCACTATGAGGGAGTTGA

GCTGGACACTTACACCATGATCTCCTACGGAGACGACATTGTGGTCGCAAGTGACTATGATCTGGACTTT

GAGGCTCTTAAGCCCCACTTCAAATCTCTTGGCCAAACTATCACTCCAGCTGACAAAAGCGACAAAGGTT

TTGTTCTTGGTCACTCCATCACCGATGTCACTTTCCTCAAAAGAAGCTTCCACATGGATTATGGAACTGG

GTTTTACAAACCTGTGATGGCCTCGAAGACCCTCGAGGCTATCCTCTCCTTTGCACGCCGTGGG

>DQ989308.1_Asia1_IND_1994

GGGTTGATCGTTGATACCAGAGATGTGGAGGAACGCGTGCACGTCATGCGCAAAACCAAGCTTGCACCCA

CCGTAGCACACGGTGTGTTTAACCCTGAATTTGGCCCCGCTGCCTTGTCAATCAAGGACTCGCGGCTGAA

TGAAGGAGTTGTCCTTGATGAAGTCGTCTTCTCCAAACACAAAGGAGACACCAAGATGTCTGAGGAGGAC

AAAGCGCTGTTCCGCCGCTGTGCTGCCGACTACGCGTCGCGCTTGCATAGTGTGTTGGGTACGGCAAATG

CCCCATTGAGCATCTACGAGCCAATCAAAGGTGTCGACGGGCTCGATGCCATGGAACCAGACACCGCGCC

TGGTCTCCCCTGGGCCCTCCAGGGGAAGCGCCGCGGCGCGCTCATCGACTTTGAGAACGGCACGGTCGGA

CCTGAAATTGAAGCTGCCTTGAAGCTCATGGAGAAAAGAGAATACAAGTTTGCTTGTCAGACCTTCCTGA

AGGACGAAATTCGTCCGATGGAGAAAGTACGTGCCGGCAAGACTCGCATTGTCGATGTTTTGCCTGTTGA

GCATATTCTTTACACCAGAATGATGATTGGCAGATTCTGTGCACAAATGCACTCAAACAACGGACCGCAA

ATTGGCTCAGCGGTCGGCTGCAATCCTGATGTTGACTGGCAAAGATTTGGCACACATTTTGCCCAGTACA

GAAACGTGTGGGATGTGGACTATTCGGCCTTTGATGCTAACCACTGCAGTGACGCAATGAACATCATGTT

CGAGGAGGTGTTTCGCACAGAGTTTGGCTTCCACCCAAACGCTGAGTGGATTCTGAAGACTCTTGTGAAC

ACGGAGCACGCCTATGAGAACAAACGCATCACTGTTGAGGGCGGGATGCCGTCTGGCTGTTCCGCGACAA

GCATCATTAACACAATTCTGAACAACATCTATGTGCTCTACGCCCTGCGTAGACACTATGAGGGAGTTGA

GCTGGACACTTACACCATGATCTCCTACGGAGACGACATCGTGGTTGCAAGTGATTACGATCTGGACTTT

GAGGCCCTTAAGCCTCACTTTAAATCCCTTGGTCAAACCATCACTCCAGCTGACAAAAGCGACAAAGGTT

TTGTTCTTGGTCACTCCATTACCGATGTCACTTTCCTCAAAAGACACTTCCACATGGATTATGGAACTGG

GTTTTACAAACCTGTGATGGCCTCGAAGACCCTCGAGGCTATCCTCTCCTTTGCACGCCGTGGG

>DQ989309.1_Asia1_IND_1996

GGGTTGATTGTTGATACCAGAGATGTGGAAGAGCGCGTGCACGTCATGCGCAAAACCAAGCTTGCACCCA

CCGTAGCACACGGTGTGTTTAACCCTGAATTTGGCCCCGCTGCCTTGTCAAACAAGGACTCGCGGCTGAA

TGAAGGAGTTGTCCTTGATGAAGTCATCTTTTCCAAACACAAAGGAGACACCAAGATGTCTGAGGAGGAC

AAAGCGCTGTTCCGCCGCTGCGCTGCTGACTACGCGTCACGCCTGCACAGTGTGTTGGGTACGGCAAATG

CTCCATTGAGCGTCTACGAGGCAATCAAGGGCGTCGACGGACTCGACGCCATGGAACCAGACACCGCGCC

TGGTCTCCCCTGGGCCCTCCAGGGAAAACGCCGTGGCGCGCTCATTGACTTCGAGAACGGCACGGTCGGA

CCCGAGGTCGAAGCTGCCCTGAAGCTCATGGAGAAAAGAGAATACAAATTTGTTTGCCAGACCTTCCTGA

AGGACGAAATTCGTCCGATGGAGAAAGTACGTGCCGGCAAGACTCGCATTGTCGACGTCTTGCCTGTTGA

ACACATTCTTTACACCAGGATGATGATTGGCAGATTTTGTGCTCAAATGCACTCAAACAACGGACCGCAA

ATTGGCTCGGCGGTCGGTTGTAACCCTGATGTTGATTGGCAAAGATTTGGCACACACTTCGCCCAATACA

GAAACGTGTGGGATGTGGACTATTCGGCCTTCGATGCTAACCACTGCAGTGACGCAATGAACATCATGTT

TGAGGAGGTGTTCCGCACAGAGTTCGGCTTCCACCCAAACGCTGAGTGGATACTGAAGACTCTCGTGAAC

ACGGAGCACGCCTATGAGAACAAACGCATCACTGTGGAAGGCGGGATGCCGTCCGGCTGTTCCGCAACAA

GCATCATCAACACAATCTTGAACAACATCTATGTGCTCTACGCCCTGCGTAGACACTATGAGGGAGTTGA

GCTGGACACTTACACCATGATCTCCTATGGAGACGACATCGTGGTCGCAAGTGATTATGATCTGGACTTC

GAGGCTCTCAAGTCTCACTTCAAATCTCTGGGCCAAACCATCACTCCAGCTGATAAAAGCGACAAAGGTT

TTGTTCTTGGTCACTCCATCACCGATGTCACTTTTCTTAAAAGACACTTCCACATGGACTATGGAACTGG

GTTTTACAAACCTGTGATGGCCTCGAAGACCCTCGAGGCTATCCTCTCCTTTGCACGCCGTGGG

>DQ989310.1_Asia1_IND_1999

GGATTGATAGTTGACACCAGAGATGTTGAGGAGCGCGTACATGTCATGCGCAAAACCAAGCTCGCACCCA

CCGTGGCACACGGTGTGTTTAACCCCGAATTTGGGCCTGCCGCCTTGTCCAACAAGGACCCGCGCCTGAA

TGAGGGAGTTGTCCTCGATGAAGTCATCTTCTCCAAACACAAAGGAAACACAAAGATGTCTGAGGAGGAC

AAAGCGCTGTTCCGCCGCTGTGCTGCTGACTACGCGTCGCGTCTGCATAGCGTGCTAGGCACGGCAAATG

CCCCACTGAGCATTTACGAGGCAATCAAGGCCGTCGACGGACTTGACGCCATGGAACCAGACACCGCGCC

TGGTCTCCCCTGGGCCCTCCAGGGGAAACGCCGTGGTGCGCTCATTGACTTCGAGAACGGCACTGTCGGA

CCCGAGGTAGAAGCTGCCTTGAAGCTCATGGAGAAAAGAGAGTACAAGTTTGCATGCCAGACCTTCCTGA

AGGACGAGATTCTCCCGATGGAGAAAGTACGTGCCGGCAAGACTCGCATTGTCGACGTCCTGCCGGTTGA

ACACATTCTTTACACCAGGATGATGATTGGCAGATTTTGTGCTCAAATGCACTCAAACAACGGACCGCAA

ATTGGCTCGGCGGTTGGTTGTAATCCTGATGTTGATTGGCAAAGATTTGGCACACATTTTGCTCAGTACA

GAAACGTGTGGGATGTGGACTATTCGGCCTTTGATGCCAACCACTGCAGTGACGCAATGAACATCATGTT

TGAGGAGGTGTTCAACACGGATTTCGGTTTCCACCCAAACGCTGAGTGGATCCTGAAAACTCTCGTGAAC

ACTGAACACGCCTATGAGAATAAACGCATCACTGTTGAGGGCGGGATGCCGTCTGGTTGTTCCGCAACAA

GCATCATCAACACAATTTTGAACAACATCTACGTGCTCTACGCCTTGCGTAGACACTATGAGGGAGTTGA

GCTGGACACTTACACCATGATCTCCTACGGAGACGACATCGTGGTTGCAAGTGATTACGATCTGGACTTT

GAGGCCCTCAAGCCTCACTTCAAATCCCTTGGTCAAACCATTACTCCAGCTGACAAAAGCGACAAAGGTT

TTGTTCTTGGTCACTCCATTACCGATGTCACTTTCCTCAAAAGACACTTCCACATGGACTATGGAACTGG

GTTTTACAAACCTGTGATGGCTTCGAAGACCCTCGAGGCTATCCTCTCCTTTGCACGCCGTGGG

>DQ989311.1_Asia1_IND_2002

GGTTTGATTGTTGACACCAGAGATGTGGAAGAGCGCGTGCACGTCATGCGCAAAACCAAGCTTGCACCCA

CCGTGGCACACGGTGTGTTCAACCCTGAATTTGGGCCTGCCGCCTTGTCCAACAAGGACCCGCGCCTGAA

CGAGGGAGTTGTCCTTGATGAAGTCATCTTCTCCAAACACAAAGGAGACACCAAGATGTCCGAGGAGGAC

AAAGCGCTGTTCCGCCGCTGTGCTGCTGACTACGCGTCACGTCTGCATAGTGTGCTGGGTACGGCAAATG

CCCCATTGAGTATCTACGAGGCAATCAAAGGCGTCGACGGACTCGATGCCATGGAACCAGACACCGCGCC

TGGTCTCCCCTGGGCCCTCCAGGGGAAGCGCCGTGGTGCGCTCATCGACTTCGAGAACGGCACTGTCGGA

CCCGAGGTTGAAGCTGCCTTGAAGCTCATGGAGAAAAGAGAATACAAGTTTGCATGCCAGACCTTCCTGA

AGGACGAGATTCGCCCGATGGAGAAAGTACGTGCCGGCAAGACTCGCATTGTCGACGTTTTGCCTGTTGA

ACACATTCTTTACACCAGGATGATGATTGGCAGATTCTGTGCTCAGATGCACTCAAACAACGGACCGCAA

ATTGGCTCAGCGGTCGGTTGCAATCCAGATGTTGATTGGCAGAGATTCGGTACACATTTTGCTCAGTACA

GAAACGTGTGGGATGTGGACTATTCGGCCTTCGATGCTAACCACTGCAGTGACGCGATGAACATCATGTT

CGAAGAGGTGTTCCGCACGGATTACGGTTTCCACCCAAATGCTGAGTGGATTCTGAAGACTCTCGTGAAC

ACGGAGCACGCCTATGAGAACAAACGCATCACTGTTGAGGGCGGAATGCCATCTGGTTGTTCCGCAACTA

GCATCATCAACACAATTCTGAATAACATTTACGTGCTCTACGCCCTGCGTAGACACTATGAGGGAGTTGA

GCTGGACACTTACACCATGATCTCCTACGGAGACGACATCGTGGTTGCAAGTGATTACGATTTGGACTTT

GAGGCCCTGAAGCCTCACTTTAAATCTCTTGGTCAAACCATCACTCCAGCTGACAAAAGCGACAAAGGTT

TTGTTCTTGGTCACTCCATCACCGATGTCACTTTCCTCAAAAGACACTTCCACATGGATCATGGAACTGG

GTTTTACAAACCTGTGATGGCCTCGAAGACCCTCGAGGCTGTCCTCTCCTTTGCACGCCGTGGG

>DQ989312.1_Asia1_IND_1990

GGGTTGATTGTTGACACCAGAGATGTGGAAGAACGCGTCCACGTCATGCGCAAAACTAAGCTGGCACCCA

CCGTGGCACACGGTGTGTTTAACCCTGAATTTGGGCCTGCCGCCTTGTCAAACAAGGACCCGCGCCTGAA

CGAGGGAGTTGTCCTCGATGAAGTCATCTTCTCCAAACACAAGGGAGACACCAAGATGTCCGAGGAGGAC

AAAGCGCTGTTCCGCCGCTGCGCTGCTGACTACGCGTCACGCCTGCACTGTGTGTTGGGTACGGCAAATG

CCCCACTGAGCATCTACGAGGCAATCAAAGGTGTCGACGGGCTCGACGCCATGGAACCAGACACCGCGCC

TGGTCTCCCCTGGGCCCTCCAGGGGAAACGCCGTGGCGCGCTCATCGACTTCGAGAACGGCACTGTCGGG

CCCGAGGTTGAAGCTGCCTTGAAGCTCATGGAGAAAAGAGAATACAAGTTTACATGCCAGACCTTCCTGA

AGGACGAGATTCGCCCGATGGAGAAAGTACGTGCCGGCAAGACTCGCATCGTCGACGTCTTGCCCGTTGA

ACACATTCTTTACACCAGGATGATGATTGGCAGATTCTGTGCTCAGATGCACTCAAACAACGGACCGCAG

ATTGGCTCAGCGGTCGGTTGCAACCCTGATGTTGATTGGCAAAGATTTGGCACACATTTTGCTCAGTACA

GAAACGTGTGGGATGTGGACTATTCGGCCTTTGATGCCAACCACTGCAGTGACGCGATGAACATCATGTT

TGAGGAAGTGTTCCGCACGGACTTCGGCTTCCACCCAAACGCTGAGTGGATCCTGAAGACTCTCGTGAAC

ACGGAGCATGCCTATGAGAACAAACGCGTCACTGTTGAGGGCGGAATGCCGTCTGGCTGTTCCGCGACAA

GCATCATCAACACAATTTTGAACAACATCTACGTGCTCTACGCCCTGCGTAGACACTATGAGGGGGTTGA

GCTGGATACTTACACCATGATCTCCTACGGAGACGATATCGTGGTTGCAAGTGATTACGATTTGGACTTT

GAGGCCCTGAAGCCTCACTTCAAATCTCTTGGTCAAACCATCACTCCAGCCGACAAAAGCGACAAAGGTT

TTGTTCTTGGTCACTCCATCACCGATGTCACTTTCCTCAAAAGACACTTCCACATGGATTATGGAACTGG

GTTTTACAAACCTGTGATGGCCTCGAAGACCCTCGAGGCTATCCTCTCCTTTGCACGCCGTGGG

>DQ989313.1_Asia1_IND_1986

GGGTTGATTGTTGACACCAGAGATGTGGAAGAGCGCGTGCATGTCATGCGTAAAACCAAGCTTGCACCCA

CCGTGGCACACGGTGTGTTCAACCCTGAATTTGGCCCCGCTGCCTTGTCCAACAAGGACCCACGGCTGAA

TGAAGGAGTTGTTCTTGATGAGGTCATCTTCTCCAAACACAAAGGAGACACCAAGATGTCCAAGGAGGAC

AAAGCGCTGTTCCGCCGCTGTGCTGCTGACTACGCGTCGCGCTTGCATAGTGTGCTGGGTACAGCAAATG

CCCCACTGAGCATCTATGAGGCAATCAAGGGTGTCGACGGACTCGACGCCATGGAACCAGACACCGCGCC

TGGTCTTCCCTGGGCCCTCCAGGGGAAACGCCGCGGCGCGCTCATCGACTTCGAGAACGGCACGGTTGGG

CCTGAAGTTGAGGCTGCCTTGAAGCTCATGGAGAAAAGAGAATACAAATTTGCTTGTCAAACCTTCCTGA

AGGACGAGATTCGCCCGATGGAGAAAGTACGTGCCGGCAAGACTCGCATTGTCGACGTTTTGCCTGTAGA

ACACATTCTTTACACCAGAATGATGATTGGCAGATTTTGCGCTCAAATGCACTCAAACAACGGGCCGCAA

ATTGGCTCAGCGGTCGGTTGCAACCCTGATGTTGATTGGCAAAGATTTGGCACCCACTTCGCTCAGTACA

GAAATGTGTGGGATGTGGACTATTCGGCCTTTGATGCCAACCACTGCAGTGATGCGATGAACATCACCTT

TGAGGAGGTGTTTCGCACGGAATTCGGTTTCCACCCAAACGCTGAGTGGATTCTGAAAACTCTTGTGAAC

ACGGAACACGCCTATGAAAACAAACGCATTACTGTTGAGGGCGGGATGCCGTCTGGCTGCTCCGCGACAA

GCATCATCAACACAATTTTGAACAACATCTATGTGCTCTACGCCTTGCGTAGACACTATGAGGGTGTTGA

GCTGGACACTTACACCATGATCTCCTACGGAGACGACATCGTGGTTGCAAGTGATTACGATTTGGACTTT

GAGGCTCTCAAGCCCCACTTCAAATCTCTTGGTCAAACCATCACTCCAGCTGACAAAAGTGACAAAGGTT

TTGTTTTTGGTCACTCCATCACCGATGTCACTTTCCTCAAAAGACACTTCCACATGGATTATGGAACTGG

GTTTTACAAACCTGTGATGGCCTCGAAGACCCTCGAGGCTATCCTCTCCTTTGCACGCCGTGGG

>DQ989314.1_Asia1_IND_2001

GGGTTGATTGTTGACACCAGAGATGTGGAAGAACGCGTCCACGTGATGCGTAAAACCAAGCTTGCGCCCA

CCGTCGCACACGGTGTGTTCAACCCTGAGTTTGGCCCCGCTGCCTTGTCTAACAAGGACCCGCGGCTTAA

CGAGGGCGTTGTCCTCGATGAAGTCATCTTCTCCAAGCACAAAGGGGACACAAAGATGACAGAGGAAGAC

AAGAAGCTGTTCCGGCGCTGTGCTGCTGACTACGCGTCACGCCTGCACTCCGTGCTGGGTACGGCAAATG

CCCCATTGAGCATCTACGAGGCAATCAAAGTTGTTGACGGACTCGACGCCATGGAACCAGACACTGCGCC

TGGTCTCCCCTGGGCCCTCCAGGGGAAGCGCCGTGGTGCCCTGATCGACTTCGAGAACGGCACGGTTGGA

CCCGAAGTTGAGGCTGCCTTAGAGCTCATGGAGAAAAGAGAGTACAAGTTTGCTTGCCAGACCTTCCTGA

AGGACGAAATTCGCCCGATGGAGAAAGTACGCGCCGGCAAGACTCGCATTGTCGATGTTTTGCCCGTTGA

ACACATTCTTTACACCAGGATGATGATTGGCAGATTTTGTGCTCAAATGCACTCAAACAACGGACCGCAA

ATTGGCTCGGCGGTCGGTTGCAACCCTGATGTTGATTGGCAAAGATTTGGCACCCATTTTGCTCAGTACA

GAAACGTGTGGGATGTGGATTATTCGGCCTTTGATGCTAACCACTGCAGTGATGCAATGAATATCATGTT

TGAGGAGGTGTTCCGCACGGAGTTTGGTTTCCACCCCAATGCAGAGTGGATTCTGAAGACTCTCGTGAAC

ACGGAGCATGCCTATGAGAACAAACGCATTGTTGTTGAGGGTGGGATGCCGTCTGGTTGTTCCGCAACAA

GCATCATCAACACAATTTTGAACAACATCTACGTGCTCTACGCCCTGCGTAGACACTACGAGGGAGTTGA

GCTGGACACTTACACCATGATCTCCTACGGAGACGACATCGTGGTGGCAAGTGATTACGATCTGGACTTC

GAGGCTCTCAAGCCTCACTTCAAATCTCTGGGCCAAACCATCACTCCAGCTGACAAAAGTGACAAAGGTT

TTGTTCTTGGTCACTCCATCACCGACGTCACTTTCCTCAAAAGACACTTCCACATGGATTATGGAACTGG

GTTTTACAAACCTGTGATGGCTTCGAAGACCCTCGAAGCTATCCTCTCCTTTGCACGCCGTGGG

>DQ989315.1_Asia1_IND_1993

GGGTTGATCGTTGACACTAGAGATGTGGAAGAGCGCGTTCACGTCATGCGCAAAACCAAGCTTGCACCCA

CCGTTGCACACGGTGTGTTCAACCCTGAATTTGGCCCCGCTGCCTTGTCCAACAAGGACTCGCGGCTGAA

TGAAGGGGTTGTCCTCGATGAAGTCATCTTCTCCAAGCACAAGGGAGACACCAAAATGTCAGAAGAGGAC

AAAGCGCTGTTCCGTCGCTGCGCTGCCGACTACGCGTCGCGCTTGCACAGTGCCCTGGGTACGGCAAATG

CCCCATTGAGCATTTATGAGGCAATCAAGGGCGTCGACGGACTCGATGCCATGGAACCAGACACCGCGCC

TGGTCTTCCCTGGGCCCTCCAGGGGAAACGCCGTGGTGCGCTCATCGACTTCGAGAACGGCACTGTCGGA

CCCGAGGTTGAAGCTGCCCTAAAGCTCATGGAGAAAAGGGAATACAAGTTTGCATGTCAGACCTTCCTGA

AGGACGAGATTCGCCCGATGGAGAAAGTACGCGCCGGCAAGACTCGCATTGTCGATGTCCTGCCTGTTGA

ACACATTCTTTACACCAGGATGATGATTGGCAGATTCTGTGCTCAAATGCACTCAAACAACGGACCGCAA

ATTGGTTCAGCGGTCGGTTGCAACCCTGATGTTGATTGGCAAAGATTTGGCACACACTTCGCTCAGTACA

AAAACGTGTGGGATGTGGACTATTCGGCCTTTGACGCTAACCACTGCAGTGACGCGATGAACATCATGTT

TGAGGAGGTATTCCGCACGGACTTCGGTTTCCACCCAAATGCTGAGTGGATTCTGAAGACTCTTGTGAAC

ACGGAGCACGCCTATGAGAATAAACGCATCACTGTTGAGGGCGGGATGCCGTCTGGTTGTTCCGCGACAA

GCATCATCAACACAATTTTGAACAACATCTACGTGCTCTACGCCTTGCGTAGACACTATGAGGGAGTTGA

GCTGGACACTTACACCATGATCTCCTACGGAGACGACATCGTGGTCGCAAGTGATTACGATCTGGACTTC

GAGGCCCTCAAGCCTCACTTCAAATCCCTTGGTCAAACCATTACTCCAGCTGACAAAAGCGACAAAGGTT

TTGTTCTTGGTCACTCCATTACCGATGTCACTTTCCTCAAAAGACACTTCCACATGGACTATGGAACTGG

GTTTTACAAACCTGTGATGGCCTCGAAGACCCTCGAGGCTATCCTCTCCTTTGCACGCCGTGGG

>DQ989317.1_Asia1_IND_2000

GGGTTGATTGTTGACACCAGAGATGTGGAAGAACGCGTCCACGTGATGCGCAAAACCAAGCTTGCGCCCA

CCGTCGCACACGGTGTGTTCAACCCTGAGTTTGGCCCCGCTGCCTTGTCTAACAAGGACCCGCGACTGAA

CGAAGGCGTTGTCCTCGATGAAGTCATCTTCTCCAAGCACAAAGGGGACACAAAGATGACAGAGGAAGAC

AAGAAGCTGTTCCGGCGCTGTGCTGCTGACTACGCGTCACGCCTGCACTCCGTGCTGGGTACGGCAAATG

CCCCATTGAGCATCTACGAGGCAATCAAAGGTGTTGACGGACTCGACGCCATGGAACCAGACACTGCGCC

TGGTCTCCCCTGGGCCCTCCAGGGGAAGCGCCGTGGTGCCCTGATCGACTTCGAGAACGGCACGGTTGGA

CCCGAAGTTGAGGCTGCCTTAGAACTCATGGAGAAAAGAGAGTACAAGTTTGTTTGCCAGACCTTCCTGA

AGGACGAAATTCGCCCGATGGAGAAAGTACGTGCCGGCAAGACCCGCATTGTCGATGTTTTGCCCGTTGA

ACACATTCTTTACACCAGGATGATGATTGGCAGATTTTGTGCTCAAATGCACTCAAACAACGGACCGCAA

ATTGGCTCGGCGGTCGGTTGCAACCCTGATGTTGATTGGCAAAGATTTGGCACCCATTTTGCTCAGTACA

GAAATGTGTGGGATGTGGATTATTCGGCCTTTGATGCTAACCACTGCAGTGATGCAATGAACATCATGTT

TGAGGAGGTGTTCCGCACGGAGTTTGGTTTCCACCCCAACGCAGAGTGGATTCTGAAGACTCTCGTGAAC

ACGGAGCACGCCTATGAGAACAAACGCATTACTGTTGAGGGCGGGATGCCGTCTGGTTGTTCCGCAACAA

GCATTATCAACACAATTTTGAACAACATCTACGTGCTCTACGCCCTGCGTAGACACTATGAGGGAGTTGA

GCTGGACACTTACACCATGATCTCCTACGGAGACGACATCGTGGTGGCAAGTGATTACGATCTGGACTTC

GAGGCTCTCAAGCCTCACTTCAAATCTCTGGGCCAAACCATCACTCCAGCTGACAAAAGCGACAAAGGTT

TTGTTCTTGGTCACTCCATCACCGATGTCACTTTCCTCAAAAGACACTTCCACATGGATTATGGAACTGG

GTTTTACAAACCTGTGATGGCTTCGAAGACCCTCGAAGCTATCCTCTCCTTTGCACGCCGTGGG

>DQ989318.1_Asia1_IND_2002

GGGTTGATTGTTGACACCAGAGATGTAGAAGAACGCGTCCACGTGATGCGTAAAACCAAGCTTGCGCCCA

CCGTCGCACACGGTGTGTTCAACCCTGAGTTTGGTCCCGCTGCCTTGTCTAACAAGGACCCGCGGCTAAA

CGAAGGCGTTGTCCTCGATGAAGTCATCTTCTCCAAGCACAAAGGGGACACAAAGATGACAGAGGAAGAC

AAGAAGCTGTTCCGGCGCTGTGCTGCTGACTACGCGTCACGCCTGCACTCCGTGCTGGGTACGGCAAATG

CCCCGTTGAGCATCTACGAGGCAATCAAAGGTGTTGACGGACTCGACGCCATGGAACCAGACACTGCGCC

TGGTCTCCCCTGGGCCCTCCAGGGGAAGCGCCGTGGTGCCCTGATCGACTTCGAGAACGGCACGGTTGGA

CCCGAAGTTGAGGCTGCCTTAGAGCTCATGGAGAAAAGAGAGTACAAGTTTGCTTGCCAGACCTTCCTGA

AGGACGAAATTCGCCCGATGGAGAAAGTACGTGCCGGCAAGACTCGCATTGTCGATGTTTTGCCCGTTGA

ACACATTCTTTATACCAGGATGATGATTGGCAGATTTTGTGCTCAAATGCACTCAAACAACGGACCGCAA

ATTGGCTCGGCGGTCGGTTGCAACCCTGATGTTGATTGGCAAAGATTTGGCACCCATTTTGCTCAGTACA

GAAATGTGTGGGATGTGGATTATTCGGCCTTTGATGCTAACCACTGCAGTGATGCAATGAATATCATGTT

TGAGGAGGTGTTCCGCACGGAGTTTGGTTTCCACCCCAATGCAGAGTGGATTCTGAAGACTCTCGTGAAC

ACGGAACATGCCTATGAGAACAAACGCATTGTTGTTGAGGGTGGGATGCCGTCTGGTTGTTCCGCAACAA

GCATCATCAACACAATTTTGAACAACATCTACGTGCTCTACGCCCTGCGTAGACACTATGAGGGAGTTGA

GCTGGACACTTACACCATGATCTCCTACGGAGACGACATTGTGGTGGCAAGTGATTACGATCTGGACTTC

GAGGCTCTCAAGCCTCACTTCAAATCTCTGGGCCAAACCATCACTCCAGCTGACAAAAGTGACAAAGGTT

TTGTTCTTGGTCACTCCATCACCGACGTCACTTTCCTCAAAAGACACTTCCACATGGATTATGGAACTGG

GTTTTACAAACCTGTGATGGCTTCGAAGACCCTCGAAGCTATCCTCTCCTTTGCACGCCGTGGG

>DQ989319.1_Asia1_IND_2001

GGGTTGATCGTTGACACCAGAGATGTGGAAGAGCGCGTGCACGTCATGCGCAAAACCAAGCTTGCACCCA

CCGTTGCGCACGGTGTGTTCAACCCCGAATTTGGCCCCGCTGCCTTGTCCAACAAGGATCCGCGGCTGAA

TGAGGGAGTTGTCCTCGATGAAGTCATCTTCTCCAAACACAAGGGAGACACCAAGATGTCCGAGGAGGAC

AAAGCGCTGTTCCGCCGTTGTGCCGCTGACTACGCGTCACGCCTGCACAGTGTGCTGGGTACAGCAAATG

CCCCACTGAGCATCTACGAGGCAATCAAGGGTGTCGACGGGCTCGATGCCATGGAACCAGACACCGCGCC

CGGGCTCCCCTGGGCCCTCCAGGGGAAGCGCCGTGGTGCGCTCATCGACTTCGAGAACGGCACGGTCGGA

CCCGAGGTTGAAGCTGCCTTGAAGCTCATGGAGAAAAGAGAGTACAAGTTTGCCTGCCAGACCTTCCTCA

AGGATGAAATCCGTCCGATCGAGAAGGTACGCAGCGGCAAGACCCGCATTGTCGACGTCTTGCCCGTTGA

GCACATACTTTACACCAGGATGATGATTGGCAGATTCTGCGCACAAATGCACTCAAACAACGGACCGCAA

ATTGGCTCGGCGGTCGGTTGTAATCCTGATGTTGATTGGCAAAGATTTGGCACACATTTTGCTCAGTACC

GGAACGTGTGGGATGTGGACTATTCGGCCTTTGATGCTAACCACTGCAGTGACGCAATGAACATCATGTT

TGAGGAGGTGTTTCGTACAGAGTTTGGTTTCCACCCAAACGCTGAGTGGATTCTGAAGACTCTTGTGAAC

ACGGAGCACGCCTATGAGAACAAACGCATCACTGTTGAGGGCGGGATGCCGTCTGGTTGTTCCGCGACAA

GCATCATCAACACAATTTTGAACAACATCTATGTGCTCTACGCTCTTCGTAGACACTATGAGGGAGTTGA

GCTGGACACTTACACCATGATCTCCTACGGAGACGACATCGTGGTCGCAAGTGATTATGATCTAGACTTT

GAGGCTCTTAAGCCGCACTTCAAATCTCTTGGCCAAACCATCACTCCAGCTGACAAAAGCGACAAAGGTT

TTGTTCTTGGTCACTCCATCACCGATGTCACTTTCCTCAAAAGACACTTCCACATGGACTATGGAACTGG

GTTTTACAAACCTGTGATGGCCTCGAAGACCCTCGAGGCTATCCTCTCCTTTGCACGCCGTGGG

>DQ989320.1_Asia1_IND_2002

GGGTTGATTGTTGACACCAGAGATGTGGAAGAACGTGTCCACGTGATGCGTAAAACCAAGCTTGCGCCCA

CCGTCGCACACGGTGTGTTCAACCCTGAGTTTGGCCCCGCTGCCTTGTCTAACAAGGACCCTCGGCTAAA

CGAAGGCGTTGTCCTCGATGAAGTCATCTTCTCCAAGCACAAGGGAGACACAAAGATGACAGAGGAAGAC

AAGAAGCTGTTCCGGCGCTGTGCTGCTGACTACGCGTCACGCCTGCACTCCGTGCTGGGTACGGCAAATG

CCCCATTGAGCATCTACGAGGCAATCAAAGGTGTTGACGGACTCGACGCCATGGAACCAGACACTGCGCC

TGGTCTCCCCTGGGCCCTCCAGGGGAAGCGCCGTGGTGCCCTGATCGACTTCGAGAACGGCACGGTTGGA

CCCGAAGTTGAGGCTGCCTTAGAGCTCATGGAGAAAAGAGAGTACAAGTTTGCTTGCCAGACCTTCCTGA

AGGACGAAATTCGCCCGATGGAGAAAGTGCGTGCCGGCAAGACTCGCATTGTCGATGTTCTGCCCGTTGA

ACACATTCTTTATACCAGGATGATGATTGGCAGATTTTGTGCTCAAATGCACTCAAACAACGGGCCGCAA

ATTGGCTCGGCGGTCGGTTGCAACCCTGACGTTGATTGGCAAAGATTTGGCACCCATTTTGCTCAGTACA

GTAATGTGTGGGATGTGGATTATTCGGCCTTTGATGCTAACCACTGCAGTGATGCAATGAATATCATGTT

TGAGGAGGTGTTCCGCACGGAGTTTGGTTTCCACCCCAATGCGGAGTGGATTCTGAAGACTCTCGTGAAC

ACGGAGCATGCCTATGAGAACAAACGCATTGTTGTTGAGGCCGGGATGCCGTCTGGCTGTTCCGCAACAA

GCATCATCAACACAATTTTGAACAACATCTACGTGCTCTACGCCCTGCGTAGACACTATGAGGGGGTTGA

GCTGGACACTTACACCATGATCTCCTACGGAGACGACATCGTGGTGGCAAGTGATTACGATCTGGACTTC

GAGGCTCTCAAGCCTCACTTCAAATCTCTGGGCCAAACCATCACTCCAGCTGACAAAAGTGACAAAGGTT

TTGTTCTTGGTCACTCCATCACCGACGTCACTTTCCTCAAAAGACACTTCCACATGGATTATGGAACTGG

GTTTTACAAACCTGTGATGGCTTCGAAGACCCTCGAAGCTATCCTCTCCTTTGCACGCCGTGGG

>DQ989321.1_Asia1_IND_2001

GGGTTGATTGTTGACACCAGAGATGTGGAAGAACGCGTCCACGTGATGCGTAAAACCAAGCTTGCGCCCA

CCGTCGCACACGGTGTGTTCAACCCTGAGTTTGGCCCCGCTGCCTTGTCTAACAAGGACCCGCGGCTAAA

CGAAGGCGTTGTCCTCGATGAAGTTATCTTCTCCAAGCACAAAGGGGACACAAAGATGACAGAGGAAGAC

AAGAAGCTGTTCCGGCGCTGTGCTGCTGACTACGCGTCACGCCTGCACTCCGTGCTGGGTACGGCAAATG

CCCCATTGAGCATCTACGAGGCAATCAAAGGTGTTGACGGACTCGACGCCATGGAACCAGACACTGCGCC

TGGTCTCCCCTGGGCCCTCCAGGGGAAGCGCCGTGGTGCCCTGATCGACTTTGAGAACGGCACGGTTGGA

CCCGATGTTGAGGCTGCCTTAGAGCTCATGGAGAAAAGAGAGTACAAGTTTGCTTGCCAGACCTTCCTGA

AGGACGAAATTCGCCCGATGGAGAAAGTACGTGCCGGCAAGACTCGCATTGTCGATGTTTTGCCCGTTGA

ACACATTCTTTATACCAGGATGATGATTGGCAGATTTTGTGCTCAAATGCACTCAAACAACGGACCGCAA

ATTGGCTCGGCGGTCGGTTGCAACCCTGATGTTGATTGGCAAAGATTTGGCACCCATTTTGCTCAGTACA

GAAATGTGTGGGATGTGGATTATTCGGCCTTTGATGCTAACCACTGCAGTGATGCAATGAATATCATGTT

TGAAGAGGTGTTCCGCACGGAGTTTGGTTTCCACCCCAATGCAGAGTGGATTCTGAAGACTCTCGTGAAC

ACGGAGCATGCCTATGAGAACAAACGCATTGTTGTTGAGGGCGGGATGCCGTCTGGTTGTTCCGCAACAA

GCATCATCAACACAATTTTAAACAACATCTACGTGCTCTACGCCCTGCGTAGACACTATGAGGGAGTTGA

GCTGGACACTTACACCATGATCTCCTACGGAGACGACATCGTGGTGGCAAGTGATTACGATCTGGACTTC

GAGGCTCTCAAGCCTCACTTCAAATCTCTGGGTCAAACCATCACTCCAGCTGACAAAAGTGACAAAGGTT

TTGTTCTTGGTCACTCCATCACCGACGTCACTTTCCTCAAAAGACACTTCCACATGGATTATGGAACTGG

GTTTTACAAACCTGTGATGGCTTCGAAGACCCTCGAAGCTATCCTCTCCTTTGCACGCCGTGGG

>DQ989322.1_Asia1_IND_2002

GGGTTGATTGTTGACACCAGAGATGTGGAAGAACGCGTCCACGTGATGCGTAAAACCAAGCTTGCGCCCA

CCGTCGCACACGGTGTGTTCAACCCTGAGTTTGGCCCCGCTGCCTTGTCTAACAAGGACCCGCGGCTAAA

CGAAGGCGTTGTCCTCGATGAAGTCATCTTCTCCAAGCATAAAGGGGACACAAAGATGACAGAGGAAGAC

AAGAAGCTGTTCCGGCGCTGTGCTGCTGACTACGCGTCACGCCTGCACTCCGTGCTGGGTACGGCAAATG

CCCCATTGAGCATCTACGAGGCAATCAAAGGTGTTGACGGACTCGACGCCATGGAACCAGACACTGCGCC

TGGTCTCCCCTGGGCCCTCCAGGGGAAGCGCCGTGGTGCCCTGATCGACTTCGAGAACGGCACGGTTGGA

CCCGAAGTTGAGGCTGCCTTAGAGCTCATGGAGAAAAGAGAGTACAAGTTTGCTTGCCAGACCTTCCTGA

AGGACGAAATTCGCCCGATGGAGAAAGTACGTGCCGGCAAGACTCGCATTGTCGATGTTTTGCCCGTTGA

ACACATTCTTTATACCAGGATGATGATTGGCAGATTTTGTGCTCAAATGCACTCAAACAACGGACCGCAA

ATAGGCTCGGCGGTCGGTTGCAACCCTGATGTTGATTGGCAAAGATTTGGCACCCATTTTGCTCAGTACA

GAACCGTGTGGGATGTGGATTATTCGGCCTTTGATGCTAACCACTGCAGTGATGCAATGAATATCATGTT

TGAGGAGGTGTTCCGCACGGAGTTTGGTTTCCACCCCAATGCAGAGTGGATTCTGAAGACTCTCGTGAAC

ACGGAGCACGCCTATGAGAACAAACGCATTGCTGTTGAGGGCGGGATGCCGTCTGGTTGTTCCGCAACAA

GCATCATCAACACAATTTTGAACAACATCTACGTGCTCTACGCCCTGCGTAGACACTATGAGGGAGTTGA

GCTGGACACTTACACCATGATCTCCTACGGAGACGACATCGTGGTGGCAAGTGATTACGATCTGGACTTC

GAGGCTCTCAAGCCTCACTTCAAATCTCTGGGCCAAACCATCACTCCAGCTGACAAAAGTGACAAAGGTT

TTGTTCTTGGTCACTCCATCACCGACGTCACTTTCCTCAAAAGACACTTCCACTTGGATTATGGAACTGG

GTTTTATAAACCTGTGATGGCTTCGAAGACCCTCGAAGCTATCCTCTCCTTTGCACGCCGTGGG

>DQ989323.1_Asia1_IND_2002

GGGTTGATTGTTGATACCAGAGATGTGGAAGAACGCGTCCACGTGATGCGTAAAACCAAGCTTGCGCCCA

CCGTCGCACACGGTGTGTTCAACCCTGCGTTTGGCCCCGCTGCCTTGTCTAACAGGGACCCGCGGCTAAA

CGAAGGCGTTGTCCTCGATGAAGTCATCTTCTCCAAGCACAAAGGGGACACAAAGATGACAGAGGAAGAC

AAGAAGCTGTTCCGGCGCTGTGCTGCTGACTACGCGTCACGCCTACACTCCGTGCTGGGTACGGCAAATG

CCCCACTGAGCATCTACGAGGCAATCAAAGGTGTTGACGGACTCGACGCCATGGAACCAGACACTGCGCC

TGGTCTCCCCTGGGCCCTCCAGGGGAAGCGCCGTGGTGCCCTGATCGACTTCGAGAGCGGCACGGTTGGA

CCCGAAATTGAGGCTGCCTTGGAGCTCATGGAGAAAAGAGAGTACAAGTTTGCTTGCCAGACCTTCCTGA

AGGACGAAATTCGCCCGATGGAGAAAGTGCGTGCCGGCAAGACTCGCATTGTCGATGTTTTGCCCGTTGA

ACACATTCTTTATACCAGGATGATGATTGGCAGATTTTGTGCTCAAATGCACTCAAACAACGGACCGCAA

ATTGGCTCGGCGGTCGGTTGCAACCCTGATGTTGATTGGCAAAGATTTGGCACCCATTTTGCTCAGTACA

GAAATGTGTGGGATGTGGATTACTCGGCCTTTGATGCTAACCACTGCAGTGATGCAATGAATATCATGTT

TGAGGAGGTGTTCCGCACGGAATTTGGTTTCCACCCCAATGCAGAGTGGATTCTGAAGACTCTCGTGAAC

ACGGAGCATGCCTATGAGAACAAACGCATCGTTGTTGAGGGCGGGATGCCGTCTGGCTGTTCCGCAACAA

GCATCATCAACACAATTTTGAACAACATCTACGTGCTCTACGCCCTGCGTAGACACTATGAGGGAGTTGA

GCTGGACACTTACACCATGATCTCCTACGGAGACGACATCGTGGTGGCAAGTGATTACGATCTGGACTTC

GAGGCTCTCAAGCCTCACTTCAAATCTCTGGGCCAAACCATCACTCCAGCTGACAAAAGTGACAAAGGTT

TTGTTCTTGGTGACTCCATCACCGACGTCACTTTCCTCAAAAGACACTTCCACATGGATTATGGAACTGG

GTTTTACAAACCTGTGATGGCTTCGAAGACCCTCGAAGCTATCCTCTCCTTTGCACGCCGTGGG

>EF117837.1_A_PAK_2006

GGGTTGGTCATTGACACCAGGGATGTTGAGGAGCGTGTGCATGTCATGCGCAAAACCAAGCTTGCACCCA

CCGTGGCACACGGTGTGTTTAACCCTGAGTTTGGCCCCGCTGCCTTGTCCAACAAGGACCCGCGGTTAAA

TGAAGGGGTTGTCCTCGATGAGGTCATCTTCTCCAAACACAAAGGAGATACCAAAATGTCTGAGGAGGAC

AAAGCGCTGTTCCGCCGCTGTGCTGCTGACTACGCGTCACGCCTGCACAGTGTGTTGGGTACGGCAAATG

CCCCACTGAGCATCTACGAGGCAATCAAGGGCGTCGACGGACTCGATGCCATGGAACCAGACACCGCGCC

TGGCCTCCCCTGGGCCCTCCAGGGAAAACGCCGCGGCGCGCTCATCGACTTCGAGAACGGCACAGTCGGA

CCCGAAGTTGAATCTGCCTTGAAGCTCATGGAGAAAAGGGAATACAAGTTTGCTTGCCAGACCTTCCTGA

AGGACGAAATCCGCCCGATGGAGAAAGTACGCGCCGGCAAGACTCGCATCGTCGACGTTTTGCCTGTTGA

ACACATTCTTTACACCAGGATGATGATTGGCAGATTTTGTGCTCAAATGCACTCAAACAACGGACCGCAA

ATTGGCTCAGCGGTCGGTTGTAATCCAGATGTTGATTGGCAAAGATTTGGAACACACTTCGCCCAGTACA

AAAACGTGTGGGATGTGGACTATTCGGCCTTTGATGCCAACCACTGCAGTGACGCAATGAACATCATGTT

TGAGGAGGTGTTTCGCACAGAATTCGGTTTCCACCCGAACGCGGAGTGGATTCTGAAGACTCTTGTGAAC

ACGGAGCACGCCTATGAGAACAAGCGCATCACTGTTGAGGGCGGGATGCCATCTGGTTGTTCCGCGACAA

GCATCATCAACACAATTCTGAACAACATCTACGTGCTCTACGCCCTGCGTAGACACTATGAGGGAGTTGA

GCTGGACACTTACACCATGATCTCCTACGGAGACGACATCGTGGTCGCAAGTGATTATGATTTGGACTTT

GAGGCCCTTAAACCTCACTTTAAATCTCTCGGTCAAACCATTACTCCAGCTGACAAAAGCGACAAAGGTT

TTGTTCTTGGTCATTCCATCACCGATGTCACTTTCCTCAAAAGACACTTCCACATGGATTATGGAACTGG

GTTTTACAAACCTGTGATGGCCTCAAAGACCCTTGAGGCTATCCTCTCCTTTGCACGCCGTGGG

>EF149009.1_Asia1_CHA_2005

GGGTTGATCGTTGACACCAGAGATGTGGAGGAGCGCGTGCACGTCATGCGCAAAACCAAGCTTGCACCCA

CCGTAGCACACGGTGTGTTCAACCCTGAATTTGGGCCTGCTGCCTTGTCCAACAAGGACCCGCGCTTGAA

CGAAGGAGTTGTCCTCGATGAAGTCATCTTCTCCAAGCACAAGGGAGACACAAAGATGTCTGAGGAGGAC

AAAGCGCTGTTCCGACGTTGCGCTGCCGACTACGCGTCGCGCTTGCACAGCGTGCTGGGTACAGCAAATG

CCCCATTGAGCATTTACGAGGCAATCAAGGGCGTCGACGGACTCGATGCCATGGAACCAGACACCGCACC

TGGCCTTCCCTGGGCCCTCCAGGGGAAACGCCGTGGTGCGCTGATTGACTTCGAGAACGGCACAGTCGGA

CCCGAGGTCGAGGCTGCCCTAAAGCTTATGGAGAAAAGAGAATACAAGTTTGCTTGTCAGACCTTCCTGA

AGGACGAGATTCGCCCGATGGAGAAAGTACGTGCCGGCAAGACTCGCATTGTCGATGTCTTGCCTGTTGA

ACACATTCTTTACACCAGGATGATGATTGGCAGATTTTGTGCTCAAATGCACTCAAACAACGGACCGCAA

ATTGGCTCGGCGGTCGGTTGCAACCCTGATGTTGATTGGCAACGATTTGGCACACATTTTGCCCAGTACA

GAAACGTGTGGGATGTGGACTATTCGGCCTTTGATGCTAACCACTGCAGCGATGCAATGAACATCATGTT

TGAGGAGGTGTTTCGCACAGAGTTTGGCTTCCACCCGAACGCCGAGTGGATCCTGAAGACTCTTGTGAAT

ACGGAACATGCTTATGAGAACAAACGCATTGTTGTTGAGGGCGGGATGCCGTCTGGCTGTTCCGCGACAA

GCATCATAAACACAATTTTGAACAACATTTACGTGCTCTACGCTTTGCGCAGACACTATGAGGGAGTTGA

GCTGGACACTTACACCATGATCTCCTACGGAGATGACATCGTGGTGGCAAGTGATTACGATCTGGACTTT

GAGGCCCTCAAGCCTCACTTCAAATCTCTGGGCCAAACCATCACTCCAGCTGACAAAAGCGACAAAGGTT

TTGTTCTTGGTCACTCCATTACTGATGTCACTTTCCTCAAAAGACACTTCCACATGGATTATGGAACTGG

GTTTTACAAGCCTGTAATGGCCTCGAAGACTCTCGAGGCTATCCTCTCCTTTGCACGCCGTGGG

>EF149010.1_Asia1_CHA_2005

GGGTTGATAGTTGACACCAGAGATGTTGAGGAGCGCGTGCATGTCATGCGCAAAACCAAGCTTGCACCCA

CTGTGGCACACGGTGTGTTTAACCCTGAATTCGGGCCTGCCGCCTTGTCCAACAAAGACCCGCGCCTGAA

TGAAGGAGTTGTCCTCGATGAAGCCATCTTCTCCAAACACAAGGGAGACACAAAGATGTCTGAGGAGGAC

AAAGCGCTGTTCCGCCGCTGCGCTGCCGACTACGCGTCGCGTCTGCACAGCGTGCTGGGTACGGCAAATG

CCCCACTGAGCATCTACGAGGCAATTAAGGGCGTCGACGGACTTGACGCCATGGAACCAGACACCGCGCC

TGGTCTTCCCTGGGCTCTCCAGGGGAAACGCCGTGGTGCGCTCATTGACTTCGAGAACGGCACTGTCGGA

CCCGAGGTTGAAGCTGCCTTAGAGCTCATGGAGAAAAGAGAGTACAAGTTTGCATGCCAAACCTTCCTGA

AGGACGAGATTCGCCCGATGGAGAAGGTACGTGCCGGCAAGACTCGCATTGTCGACGTCTTGCCTGTTGA

ACACATTCTTTACACCAGGATGATGATTGGCAGATTCTGTGCTCAAATGCACTCAAACAACGGACCGCAA

ATTGGCTCGGCGGTTGGTTGCAATCCTGATGTTGATTGGCAAAGATTTGGCACACATTTTGCTCAGTACA

GAAACGTGTGGGATGTGGACTATTCGGCCTTTGATGCCAACCACTGCAGTGACGCAATGAACATCATGTT

TGAGGAGGTGTTCAGCACGGACTTCGGTTTCCACCCAAACGCTGAGTGGATCCTGAAGACTCTCGTGAAC

ACTGAACACGCCTATGAGAACAAACGCATCACTGTTGAGGGCGGGATGCCATCTGGTTGTTCCGCGACAA

GCATCATCAACACAATTTTGAACAACATCTACGTACTCTACGCCTTGCGTAGACACTATGAGGGAGTTGA

GCTGGACACTTACACCATGATCTCCTACGGAGACGACATCGTGGTTGCAAGTGATTACGATCTGGACTTT

GAGGCCCTCAAGCCTCACTTCAAATCCCTTGGTCAAACCATCACTCCAGCTGACAAAAGCGACAAAGGTT

TTGTTCTTGGTCACTCCATCACCGATGTCACTTTCCTCAAAAGACACTTTCACATGGATTATGGAACTGG

GTTTTACAAACCCGTGATGGCTTCGAAGACCCTCGAGGCTATCCTCTCCTTTGCACGCCGTGGG

>EF494486.1_A_TUR_2005

GGGTTGATCATTGACACCAGGGATGTTGAGGAGCGTGTGCATGTCATGCGCAAAACCAAGCTTGCACCCA

CCGTGGCACACGGTGTGTTTAACCCTGAGTTTGGCCCCGCTGCCTTGTCCAACAAGGACCCGCGGTTGAA

CGAAGGGGTTGTCCTCGATGAGGTCATCTTCTCCAAACACAAGGGAGACACCAAAATGTCTGAGGAGGAC

AAAGCGCTGTTCCGCCGCTGTGCTGCTGACTACGCGTCACACCTGCACAGTGTGTTGGGTACGGCAAATG

CCCCACTGAGCATCTACGAGGCAATCAAGGGCGTCGACGGGCTCGATGCCATGGAACCAGATACCGCGCC

TGGCCTCCCCTGGGCCCTCCAGGGAAAACGCCGCGGCGCGCTCATCGACTTCGAGAACGGCACAGTCGGA

CCCGAAGTTGAATCTGCCTTGAAGCTCATGGAGAAAAGGGAATACGAATTTGCTTGCCAGACCTTCCTGA

AGGACGAAATTCGCCCGATGGAGAAAGTACGCGCCGGCAAGACTCGCATCGTCGACGTTTTGCCTGTTGA

ACACATTCTTTACACCAGGATGATGATTGGCAGATTCTGTGCTCAAATGCACTCAAACAACGGACCGCAA

ATTGGCTCAGCGGTCGGTTGTAATCCAGATGTTGATTGGCAAAGATTTGGAACACACTTCGCCCAGTATA

AAAACGTGTGGGATGTGGACTATTCGGCCTTTGATGCCAACCACTGCAGTGACGCAATGAACATCATGTT

TGAGGAGGTGTTTCGCACAGAATTCGGCTTCCACCCGAACGCGGAGTGGATTCTGAAGACTCTTGTGAAC

ACGGAGCACGCCTATGAGAACAAGCGCATCACTGTTGAGGGCGGGATGCCATCTGGCTGTTCCGCGACAA

GCATCATCAACACAATCCTGAACAACATCTACGTGCTCTACGCCCTGCGTAGACACTATGAGGGAGTTGA

GCTGGACACTTACACCATGATCTCCTACGGAGACGACATCGTGGTCGCAAGTGATTATGATTTGGACTTC

GAGGCCCTCAAGCCTCACTTTAAATCTCTCGGTCAAACCATTACTCCAGCTGACAAAAGCGACAAAGGTT

TTGTTCTTGGTCATTCCATCACCGATGTCACTTTCCTCAAAAGACACTTCCACATGGATTATGGAACTGG

GTTTTACAAACCTGTGATGGCCTCAAAGACCCTTGAGGCTATCCTCTCCTTTGCACGCCGTGGG

>EF494487.1_A_PAK_2006

GGGTTGGTCATTGACACCAGGGATGTTGAGGAGCGTGTGCATGTCATGCGCAAAACCAAGCTTGCACCCA

CCGTGGCACACGGTGTGTTTAACCCTGAGTTTGGCCCCGCTGCCTTGTCCAACAAGGACCCGCGGTTAAA

TGAAGGGGTTGTCCTCGATGAGGTCATCTTCTCCAAACACAAAGGAGATACCAAAATGTCTGAGGAGGAC

AAAGCGCTGTTCCGCCGCTGTGCTGCTGACTACGCGTCACGCCTGCACAGTGTGTTGGGTACGGCAAATG

CCCCACTGAGCATCTACGAGGCAATCAAGGGCGTCGACGGACTCGATGCCATGGAACCAGACACCGCGCC

TGGCCTCCCCTGGGCCCTCCAGGGAAAACGCCGCGGCGCGCTCATCGACTTCGAGAACGGCACAGTCGGA

CCCGAAGTTGAATCTGCCTTGAAGCTCATGGAGAAAAGGGAATACAAGTTTGCTTGCCAGACCTTCCTGA

AGGACGAAATCCGCCCGATGGAGAAAGTACGCGCCGGCAAGACTCGCATCGTCGACGTTTTGCCTGTTGA

ACACATTCTTTACACCAGGATGATGATTGGCAGATTTTGTGCTCAAATGCACTCAAACAACGGACCGCAA

ATTGGCTCAGCGGTCGGTTGTAATCCAGATGTTGATTGGCAAAGATTTGGAACACACTTCGCCCAGTACA

AAAACGTGTGGGATGTGGACTATTCGGCCTTTGATGCCAACCACTGCAGTGACGCAATGAACATCATGTT

TGAGGAGGTGTTTCGCACAGAATTCGGTTTCCACCCGAACGCGGAGTGGATTCTGAAGACTCTTGTGAAC

ACGGAGCACGCCTATGAGAACAAGCGCATCACTGTTGAGGGCGGGATGCCATCTGGTTGTTCCGCGACAA

GCATCATCAACACAATTCTGAACAACATCTACGTGCTCTACGCCCTGCGTAGACACTATGAGGGAGTTGA

GCTGGACACTTACACCATGATCTCCTACGGAGACGACATCGTGGTCGCAAGTGATTATGATTTGGACTTT

GAGGCCCTTAAACCTCACTTTAAATCTCTCGGTCAAACCATTACTCCAGCTGACAAAAGCGACAAAGGTT

TTGTTCTTGGTCATTCCATCACCGATGTCACTTTCCTCAAAAGACACTTCCACATGGATTATGGAACTGG

GTTTTACAAACCTGTGATGGCCTCAAAGACCCTTGAGGCTATCCTCTCCTTTGCACGCCGTGGG

>EF494488.1_A_PAK_2006

GGGTTGATCATTGACACCAGGGATGTTGAGGAGCGTGTGCATGTCATGCGCAAAACCAAGCTTGCACCCA

CCGTGGCACACGGTGTGTTTAACCCTGAGTTTGGCCCCGCTGCCTTGTCCAACAAGGACCCGCGGTTGAA

CGAAGGGGTTGTCCTCGACGAGGTCATCTTCTCCAAACACAAGGGAGATACCAAAATGTCTGAGGAAGAC

AAAGCGCTGTTCCGCCGCTGTGCTGCTGACTACGCGTCACGCCTACACAGTGTATTGGGTACGGCAAATG

CCCCACTGAGCATCTACGAGGCAATCAAGGGCGTCGACGGTCTCGATGCCATGGAACCAGATACCGCGCC

TGGCCTCCCCTGGGCCCTCCAGGGAAAACGCCGCGGCGCGCTCATCGACTTCGAGAACGGCACAGTCGGA

CCCGAAGTTGAATCTGCCTTGAAGCTCATGGAGAAAAGGGAATACAATTTTGTTTGCCAGACCTTCCTGA

AGGACGAAATTCGCCCGATGGAGAAAGTACGCGCCGGCAAGACTCGCATTGTCGACGTTCTGCCTGTTGA

ACACATTCTTTACACCAGGATGATGATTGGCAGATTTTGTGCTCAAATGCACTCAAACAACGGACCGCAA

ATTGGCTCAGCGGTCGGTTGTAATCCAGATGTTGATTGGCAAAGATTTGGAACACACTTCGCCCAGTACA

AAAACGTGTGGGATGTGGACTATTCGGCCTTCGATGCCAACCACTGCAGTGACGCAATGAACATCATGTT

TGAGGAGGTGTTTCGCACAGAATTCGGCTTCCACCCGAACGCGGAGTGGATTCTGAAGACTCTCGTGAAC

ACGGAACACGCCTATGAGAACAAGCGCATCACTGTTGAGGGCGGGATGCCATCTGGCTGTTCCGCGACAA

GCATCATCAACACAATTCTGAATAACATCTACGTGCTCTACGCCCTGCGTAGACACTATGAGGGAGTTGA

GCTGGACACTTACACCATGATCTCCTACGGAGATGACATCGTGGTCGCAAGTGATTATGATTTGGACTTC

GAGGCCCTCAAGCCTCACTTTAAATCCCTTGGTCAAACTATTACTCCAGCTGACAAAAGCGACAAAGGTT

TTGTTCTTGGTCATTCCATCACCGATGTCACTTTCCTCAAAAGACACTTCCACATGGATTATGGAACTGG

GTTTTACAAACCTGTGATGGCCTCAAAGACCCTTGAGGCTATCCTCTCCTTTGCACGCCGTGGG

>EF552688.1_O_UKG_2001

GGATTGATAGTTGACACCAGAGATGTTGAGGAGCGCGTACATGTCATGCGCAAAACCAAGCTCGCACCCA

CCGTGGCACACGGTGTGTTTAACCCCGAATTTGGGCCTGCCGCCTTGTCCAACAAGGACCCGCGCCTGAA

TGAGGGGGTTGTCCTCGATGAAGTCATCTTCTCCAAACACAAAGAAAACACAAAGATGTCTGAGGAGGAC

AAAGCGCTGTTCCGCCGCTGTGCTGCTGACTACGCGTCGCGTCTGCATAGCGTGCTGGGTACGGCAAATG

CCCCACTGAGCACTTACGAGGCAATCAAGGGCGTCGACGGACTTGACGCCATGGAACCAGACACCGCGCC

TGGTCTCCCCTGGGCTCTCCAGGGGAAACGCCGTGGTGCGCTCATCGACTTCGAGAACGGCACTGTCGGA

CCCGAGGTTGAAGCTGCCTTGAAGCTCATGGAGAAAAGAGAGTACAAGTTTACATGCCAGACCTTCCTGA

AGGACGAGATTCGCCCGATGGAGAAGGTACGTGCCGGCAAGACTCGCATTGTCGACGTCCTGCCCGTTGA

ACACATTCTTTACACTAGGATGATGATTGGCAGATTTTGTGCTCAAATGCACTCAAACAACGGACCGCAA

ATTGGCTCGGCGGTTGGTTGTAATCCTGATGTTGATTGGCAAAGATTTGGCACGCATTTTGCTCAGTATA

GAAACGTGTGGGATGTGGACTATTCGGCCTTTGATGCCAACCACTGCAGTGACGCAATGAACATCATGTT

TGAGGAGGTGTTTAACACGGACTTCGGTTTCCACCCAAACGCTGAGTGGATCCTGAAAACTCTCGTGAAC

ACTGAACACGCCTATGAGAACAAACGCATCACTGTTGAAGGCGGGATGCCGTCTGGTTGTTCCGCAACAA

GCATCATCAACACAATTTTGAACAACATCTACGTGCTCTACGCCTTGCGTAGACACTATGAGGGGGTTGA

GCTGGACTCTTACACCATGATCTCCTACGGAGACGACATCGTGGTTGCAAGTGATTACGATCTGGACTTT

GAGGCCCTCAAGCCTCACTTCAAATCCCTTGGTCAAACCATTACCCCAGCTGACAAAAGCGACAAAGGTT

TTGTTCTTGGTCACTCCATTACCGATGTCACTTTCCTCAAAAGACACTTCCACATGGACTATGGAACTGG

GTTTTACAAACCTGTGATGGCTTCGAAGACCCTCGAGGCTATCCTCTCCTTTGCACGCCGTGGG

>EF552689.1_O_UKG_2001

GGATTGATAGTTGACACCAGAGATGTTGAGGAGCGCGTACATGTCATGCGCAAAACCAAGCTCGCACCCA

CCGTGGCACACGGTGTGTTTAACCCCGAATTTGGGCCTGCCGCCTTGTCCAACAAGGACCCGCGCCTGAA

TGAGGGGGTTGTCCTCGATGAAGTCATCTTCTCCAAACACAAAGGAAACACAAAGATGTCCGAGGAGGAC

AAAGCGCTGTTCCGCCGCTGTGCTGCTGACTACGCGTCGCGTCTGCATAGCGTGCTGGGTACGGCAAATG

CCCCACTGAGCACTTACGAGGCAATCAAGGGCGTCGACGGACTTGACGCCATGGAACCAGACACCGCGCC

TGGTCTCCCCTGGGCTCTCCAGGGGAAACGCCGTGGTGCGCTCATCGACTTCGAGAACGGCACTGTCGGA

CCCGAGGTTGAAGCTGCCTTGAAGCTCATGGAGAAAAGAGAGTACAAGTTTACATGCCAGACCTTCCTGA

AGGACGAGATTCGCCCGATGGAGAAGGTACGTGCCGGCAAGACTCGCATTGTCGACGTCCTGCCCGTTGA

ACACATTCTTTACACTAGGATGATGATTGGCAGATTTTGTGCTCAAATGCACTCAAACAACGGACCGCAA

ATTGGCTCGGCGGTTGGTTGTAATCCTGATGTTGATTGGCAAAGATTTGGCACGCATTTTGCTCAGTATA

GAAACGTGTGGGATGTGGACTATTCGGCCTTTGATGCCAACCACTGCAGTGACGCAATGAACATCATGTT

TGAGGAGGTGTTTAACACGGACTTCGGTTTCCACCCAAACGCTGAGTGGATCCTGAAAACTCTCGTGAAC

ACTGAACACGCCTATGAGAACAAACGCATCACTGTTGAAGGCGGGATGCCGTCTGGTTGTTCCGCAACAA

GCATCATCAACACAATTTTGAACAACATCTACGTGCTCTACGCCTTGCGTAGACACTATGAGGGGGTTGA

GCTGGACTCTTACACCATGATCTCCTACGGAGACGACATCGTGGTTGCAAGTGATTACGATCTGGACTTT

GAGGCCCTCAAGCCTCACTTCAAATCCCTTGGTCAAACCATTACCCCAGCTGACAAAAGCGACAAAGGTT

TTGTTCTTGGTCACTCCATTACCGATGTCACTTTCCTCAAAAGACACTTCCACATGGACTATGGAACTGG

GTTTTACAAACCTGTGATGGCTTCGAAGACCCTCGAGGCTATCCTCTCCTTTGCACGCCGTGGG

>EF552690.1_O_UKG_2001

GGATTGATAGTTGACACCAGAGATGTTGAGGAGCGCGTACATGTCATGCGCAAAACCAAGCTCGCACCCA

CCGTGGCACACGGTGTGTTTAACCCCGAATTTGGGCCTGCCGCCTTGTCCAACAAGGACCCGCGCCTGAA

TGAGGGGGTTGTCCTCGATGAAGTCATCTTCTCCAAACACAAAGGAAACACAAAGATGTCTGAGGAGGAC

AAAGCGCTGTTCCGCCGCTGTGCTGCTGACTACGCGTCGCGTCTGCATAGCGTGCTGGGTACGGCAAATG

CCCCACTGAGCACTTACGAGGCAATCAAGGGCGTCGACGGACTTGACGCCATGGAACCAGACACCGCGCC

TGGTCTCCCCTGGGCTCTCCAGGGGAAACGCCGTGGTGCGCTCATCGACTTCGAGAACGGCACTGTCGGA

CCCGAGGTTGAAGCTGCCTTGAAGCTCATGGAGAAAAGAGAGTACAAGTTTACATGCCAGACCTTCCTGA

AGGACGAGATTCGCCCGATGGAGAAGGTACGTGCCGGCAAGACTCGCATTGTCGACGTCCTGCCCGTTGA

ACACATTCTTTACACTAGGATGATGATTGGCAGATTTTGTGCTCAAATGCACTCAAACAACGGACCGCAA

ATTGGCTCGGCGGTTGGTTGTAATCCTGATGTTGATTGGCAAAGATTTGGCACGCATTTTGCTCAGTATA

GAAACGTGTGGGATGTGGACTATTCGGCCTTTGATGCCAACCACTGCAGTGACGCAATGAACATCATGTT

TGAGGAGGTGTTTAACACGGACTTCGGTTTCCACCCAAACGCTGAGTGGATCCTGAAAACTCTCGTGAAC

ACTGAACACGCCTATGAGAACAAACGCATCACTGTTGAAGGCGGGATGCCGTCTGGTTGTTCCGCAACAA

GCATCATCAACACAATTTTGAACAACATCTACGTGCTCTACGCCTTGCGTAGACACTATGAGGGGGTTGA

GCTGGACTCTTACACCATGATCTCCTACGGAGACGACATCGTGGTTGCAAGTGATTACGATCTGGACTTT

GAGGCCCTCAAGCCTCACTTCAAATCCCTTGGTCAAACCATTACCCCAGCTGACAAAAGCGACAAAGGTT

TTGTTCTTGGTCACTCCATTACCGATGTCACTTTCCTCAAAAGACACTTCCACATGGACTATGGAACTGG

GTTTTACAAACCTGTGATGGCTTCGAAGACCCTCGAGGCTATCCTCTCCTTTGCACGCCGTGGG

>EF552691.1_O_UKG_2001

GGATTGATAGTTGACACCAGAGATGTTGAGGAGCGCGTACATGTCATGCGCAAAACCAAGCTCGCACCCA

CCGTGGCACACGGTGTGTTTAACCCCGAATTTGGGCCTGCCGCCTTGTCCAACAAGGACCCGCGCCTGAA

TGAGGGGGTTGTCCTCGATGAAGTCATCTTCTCCAAACACAAAGGAAACACAAAGATGTCTGAGGAGGAC

AAAGCGCTGTTCCGCCGCTGTGCTGCTGACTACGCGTCGCGTCTGCATAGCGTGCTGGGTACGGCAAATG

CCCCACTGAGCACTTACGAGGCAATCAAGGGCGTCGACGGACTTGACGCCATGGAACCAGACACCGCGCC

TGGTCTCCCCTGGGCTCTCCAGGGGAAACGCCGTGGTGCGCTCATCGACTTCGAGAACGGCACTGTCGGA

CCCGAGGTTGAAGCTGCCTTGAAGCTCATGGAGAAAAGAGAGTACAAGTTTACATGCCAGACCTTCCTGA

AGGACGAGATTCGCCCGATGGAGAAGGTACGTGCCGGCAAGACTCGCATTGTCGACGTCCTGCCCGTTGA

ACACATTCTTTACACTAGGATGATGATTGGCAGATTTTGTGCTCAAATGCACTCAAACAACGGACCGCAA

ATTGGCTCGGCGGTTGGTTGTAATCCTGATGTTGATTGGCAAAGATTTGGCACGCATTTTGCTCAGTATA

GAAACGTGTGGGATGTGGACTATTCGGCCTTTGATGCCAACCACTGCAGTGACGCAATGAACATCATGTT

TGAGGAGGTGTTTAACACGGACTTCGGTTTCCACCCAAACGCTGAGTGGATCCTGAAAACTCTCGTGAAC

ACTGAACACGCCTATGAGAACAAACGCATCACTGTTGAAGGCGGGATGCCGTCTGGTTGTTCCGCAACAA

GCATCATCAACACAATTTTGAACAACATCTACGTGCTCTACGCCTTGCGTAGACACTATGAGGGGGTTGA

GCTGGACTCTTACACCATGATCTCCTACGGAGACGACATCGTGGTTGCAAGTGATTACGATCTGGACTTT

GAGGCCCTCAAGCCTCACTTCAAATCCCTTGGTCAAACCATTACCCCAGCTGACAAAAGCGACAAAGGTT

TTGTTCTTGGTCACTCCATTACCGATGTCACTTTCCTCAAAAGACACTTCCACATGGACTATGGAACTGG

GTTTTACAAACCTGTGATGGCTTCGAAGACCCTCGAGGCTATCCTCTCCTTTGCACGCCGTGGG

>EF552692.1_O_UKG_2001

GGATTGATAGTTGACACCAGAGATGTTGAGGAGCGCGTACATGTCATGCGCAAAACCAAGCTCGCACCCA

CCGTGGCACACGGTGTGTTTAACCCCGAATTTGGGCCTGCCGCCTTGTCCAACAAGGACCCGCGCCTGAA

TGAGGGGGTTGTCCTCGATGAAGTCATCTTCTCCAAACACAAAGGAAACACAAAGATGTCTGAGGAGGAC

AAAGCGCTGTTCCGCCGCTGTGCTGCTGACTACGCGTCGCGTCTGCATAGCGTGCTGGGTACGGCAAATG

CCCCACTGAGCACTTACGAGGCAATCAAGGGCGTCGACGGACTTGACGCCATGGAACCAGACACCGCGCC

TGGTCTCCCCTGGGCTCTCCAGGGGAAACGCCGTGGTGCGCTCATCGACTTCGAGAACGGCACTGTCGGA

CCCGAGGTTGAAGCTGCCTTGAAGCTCATGGAGAAAAGAGAGTACAAGTTTACATGCCAGACCTTCCTGA

AGGACGAGATTCGCCCGATGGAGAAGGTACGTGCCGGCAAGACTCGCATTGTCGACGTCCTGCCCGTTGA

ACACATTCTTTACACTAGGATGATGATTGGCAGATTTTGTGCTCAAATGCACTCAAACAACGGACCGCAA

ATTGGCTCGGCGGTTGGTTGTAATCCTGATGTTGATTGGCAAAGATTTGGCACGCATTTTGCTCAGTATA

GAAACGTGTGGGATGTGGACTATTCGGCCTTTGATGCCAACCACTGCAGTGACGCAATGAACATCATGTT

TGAGGAGGTGTTTAACACGGACTTCGGTTTCCACCCAAACGCTGAGTGGATCCTGAAAACTCTCGTGAAC

ACTGAACACGCCTATGAGAACAAACGCATCACTGTTGAAGGCGGGATGCCGTCTGGTTGTTCCGCAACAA

GCATCATCAACACAATTTTGAACAACATCTACGTGCTCTACGCCTTGCGTAGACACTATGAGGGGGTTGA

GCTGGACTCTTACACCATGATCTCCTACGGAGACGACATCGTGGTTGCAAGTGATTACGATCTGGACTTT

GAGGCCCTCAAGCCTCACTTCAAATCCCTTGGTCAAACCATTACCCCAGCTGACAAAAGCGACAAAGGTT

TTGTTCTTGGTCACTCCATTACCGATGTCACTTTCCTCAAAAGACACTTCCACATGGACTATGGAACTGG

GTTTTACAAACCTGTGATGGCTTCGAAGACCCTCGAGGCTATCCTCTCCTTTGCACGCCGTGGG

>EF552693.1_O_UKG_2001

GGATTGATAGTTGACACCAGAGATGTTGAGGAGCGCGTACATGTCATGCGCAAAACCAAGCTCGCACCCA

CCGTGGCACACGGTGTGTTTAACCCCGAATTTGGGCCTGCCGCCTTGTCCAACAAGGACCCGCGCCTGAA

TGAGGGGGTTGTCCTCGATGAAGTCATCTTCTCCAAACACAAAGGAAACACAAAGATGTCTGAGGAGGAC

AAAGCGCTGTTCCGCCGCTGTGCTGCTGACTACGCGTCGCGTCTGCATAGCGTGCTGGGTACGGCAAATG

CCCCACTGAGCACTTACGAGGCAATCAAGGGCGTCGACGGACTTGACGCCATGGAACCAGACACCGCGCC

TGGTCTCCCCTGGGCTCTCCAGGGGAAACGCCGTGGTGCGCTCATCGACTTCGAGAACGGCACTGTCGGA

CCCGAGGTTGAAGCTGCCTTGAAGCTCATGGAGAAAAGAGAGTACAAGTTTACATGCCAGACCTTCCTGA

AGGACGAGATTCGCCCGATGGAGAAGGTACGTGCCGGCAAGACTCGCATTGTCGACGTCCTGCCCGTTGA

ACACATTCTTTACACTAGGATGATGATTGGCAGATTTTGTGCTCAAATGCACTCAAACAACGGACCGCAA

ATTGGCTCGGCGGTTGGTTGTAATCCTGATGTTGATTGGCAAAGATTTGGCACGCATTTTGCTCAGTATA

GAAACGTGTGGGATGTGGACTATTCGGCCTTTGATGCCAACCACTGCAGTGACGCAATGAACATCATGTT

TGAGGAGGTGTTTAACACGGACTTCGGTTTCCACCCAAACGCTGAGTGGATCCTGAAAACTCTCGTGAAC

ACTGAACACGCCTATGAGAACAAACGCATCACTGTTGAAGGCGGGATGCCGTCTGGTTGTTCCGCAACAA

GCATCATCAACACAATTTTGAACAACATCTACGTGCTCTACGCCTTGCGTAGACACTATGAGGGGGTTGA

GCTGGACTCTTACACCATGATCTCCTACGGAGACGACATCGTGGTTGCAAGTGATTACGATCTGGACTTT

GAGGCCCTCAAGCCTCACTTCAAATCCCTTGGTCAAACCATTACCCCAGCTGACAAAAGCGACAAAGGTT

TTGTTCTTGGTCACTCCATTACCGATGTCACTTTCCTCAAAAGACACTTCCACATGGACTATGGAACTGG

GTTTTACAAACCTGTGATGGCTTCGAAGACCCTCGAGGCTATCCTCTCCTTTGCACGCCGTGGG

>EF552695.1_O_UKG_2001

GGATTGATAGTTGACACCAGAGATGTTGAGGAGCGCGTACATGTCATGCGCAAAACCAAGCTCGCACCCA

CCGTGGCACACGGTGTGTTTAACCCCGAATTTGGGCCTGCCGCCTTGTCCAACAAGGACCCGCGCCTGAA

TGAGGGGGTTGTCCTCGATGAAGTCATCTTCTCCAAACACAAAGGAAACACAAAGATGTCTGAGGAGGAC

AAAGCGCTGTTCCGCCGCTGTGCTGCTGACTACGCGTCGCGTCTGCATAGCGTGCTGGGTACGGCAAATG

CCCCACTGAGCACTTACGAGGCAATCAAGGGCGTCGACGGACTTGACGCCATGGAACCAGACACCGCGCC

TGGTCTCCCCTGGGCTCTCCAGGGGAAACGCCGTGGTGCGCTCATCGACTTCGAGAACGGCACTGTCGGA

CCCGAGGTTGAAGCTGCCTTGAAGCTCATGGAGAAAAGAGAGTACAAGTTTACATGCCAGACCTTCCTGA

AGGACGAGATTCGCCCGATGGAGAAGGTACGTGCCGGCAAGACTCGCATTGTCGACGTCCTGCCCGTTGA

ACACATTCTTTACACTAGGATGATGATTGGCAGATTTTGTGCTCAAATGCACTCAAACAACGGACCGCAA

ATTGGCTCGGCGGTTGGTTGTAATCCTGATGTTGATTGGCAAAGATTTGGCACGCATTTTGCTCAGTATA

GAAACGTGTGGGATGTGGACTATTCGGCCTTTGATGCCAACCACTGCAGTGACGCAATGAACATCATGTT

TGAGGAGGTGTTTAACACGGACTTCGGTTTCCACCCAAACGCTGAGTGGATCCTGAAAACTCTCGTGAAC

ACTGAACACGCCTATGAGAACAAACGCATCACTGTTGAAGGCGGGATGCCGTCTGGTTGTTCCGCAACAA

GCATCATCAACACAATTTTGAACAACATCTACGTGCTCTACGCCTTGCGTAGACACTATGAGGGGGTTGA

GCTGGACTCTTACACCATGATCTCCTACGGAGACGACATCGTGGTTGCAAGTGATTACGATCTGGACTTT

GAGGCCCTCAAGCCTCACTTCAAATCCCTTGGTCAAACCATTACCCCAGCTGACAAAAGCGACAAAGGTT

TTGTTCTTGGTCACTCCATTACCGATGTCACTTTCCTCAAAAGACACTTCCACATGGACTATGGAACTGG

GTTTTACAAACCTGTGATGGCTTCGAAGACCCTCGAGGCTATCCTCTCCTTTGCACGCCGTGGG

>EF552696.1_O_UKG_2001

GGATTGATAGTTGACACCAGAGATGTTGAGGAGCGCGTACATGTCATGCGCAAAACCAAGCTCGCACCCA

CCGTGGCACACGGTGTGTTTAACCCCGAATTTGGGCCTGCCGCCTTGTCCAACAAGGACCCGCGCCTGAA

TGAGGGGGTTGTCCTCGATGAAGTCATCTTCTCCAAACACAAAGAAAACACAAAGATGTCTGAGGAGGAC

AAAGCGCTGTTCCGCCGCTGTGCTGCTGACTACGCGTCGCGTCTGCATAGCGTGCTGGGTACGGCAAATG

CCCCACTGAGCACTTACGAGGCAATCAAGGGCGTCGACGGACTTGACGCCATGGAACCAGACACCGCGCC

TGGTCTCCCCTGGGCTCTCCAGGGGAAACGCCGTGGTGCGCTCATCGACTTCGAGAACGGCACTGTCGGA

CCCGAGGTTGAAGCTGCCTTGAAGCTCATGGAGAAAAGAGAGTACAAGTTTACATGTCAGACCTTCCTGA

AGGACGAGATTCGCCCGATGGAGAAGGTACGTGCCGGCAAGACTCGCATTGTCGACGTCCTGCCCGTTGA

ACACATTCTTTACACTAGGATGATGATTGGCAGATTTTGTGCTCAAATGCACTCAAACAACGGACCGCAA

ATTGGCTCGGCGGTTGGTTGTAATCCTGATGTTGATTGGCAAAGATTTGGCACGCATTTTGCTCAGTATA

GAAACGTGTGGGATGTGGACTATTCGGCCTTTGATGCCAACCACTGCAGTGACGCAATGAACATCATGTT

TGAGGAGGTGTTTAACACGGACTTCGGTTTCCACCCAAACGCTGAGTGGATCCTGAAAACTCTCGTGAAC

ACTGAACACGCCTATGAGAACAAACGCATCACTGTTGAAGGCGGGATGCCGTCTGGTTGTTCCGCAACAA

GCATCATCAACACAATTTTGAACAACATCTACGTGCTCTACGCCTTGCGTAGACACTATGAGGGGGTTGA

GCTGGACTCTTACACCATGATCTCCTACGGAGACGACATCGTGGTTGCAAGTGATTACGATCTAGACTTT

GAGGCCCTCAAGCCTCACTTCAAATCCCTTGGTCAAACCATTACCCCAGCTGACAAAAGCGACAAAGGTT

TTGTTCTTGGTCACTCCATTACCGATGTCACTTTCCTCAAAAGACACTTCCACATGGACTATGGAACTGG

GTTTTACAAACCTGTGATGGCTTCGAAGACCCTCGAGGCTATCCTCTCCTTTGCACGCCGTGGG

>EF552697.1_O_UKG_2001

GGATTGATAGTTGACACCAGAGATGTTGAGGAGCGCGTACATGTCATGCGCAAAACCAAGCTCGCACCCA

CCGTGGCACACGGTGTGTTTAACCCCGAATTTGGGCCTGCCGCCTTGTCCAACAAGGACCCGCGCCTGAA

TGAGGGGGTTGTCCTCGATGAAGTCATCTTCTCCAAACACAAAGGAAACACAAAGATGTCTGAGGAGGAC

AAAGCGCTGTTCCGCCGCTGTGCTGCTGACTACGCGTCGCGTCTGCATAGCGTGCTGGGTACGGCAAATG

CCCCACTGAGCACTTACGAGGCAATCAAGGGCGTCGACGGACTTGACGCCATGGAACCAGACACCGCGCC

TGGTCTCCCCTGGGCTCTCCAGGGGAAACGCCGTGGTGCGCTCATCGACTTCGAGAACGGCACTGTCGGA

CCCGAGGTTGAAGCTGCCTTGAAGCTCATGGAGAAAAGAGAGTACAAGTTTACATGCCAGACCTTCCTGA

AGGACGAGATTCGCCCGATGGAGAAGGTACGTGCCGGCAAGACTCGCATTGTCGACGTCCTGCCCGTTGA

ACACATTCTTTACACTAGGATGATGATTGGCAGATTTTGTGCTCAAATGCACTCAAACAACGGACCGCAA

ATTGGCTCGGCGGTTGGTTGTAATCCTGATGTTGATTGGCAAAGATTTGGCACGCATTTTGCTCAGTATA

GAAACGTGTGGGATGTGGACTATTCGGCCTTTGATGCCAACCACTGCAGTGACGCAATGAACATCATGTT

TGAGGAGGTGTTTAACACGGACTTCGGTTTCCACCCAAACGCTGAGTGGATCCTGAAAACTCTCGTGAAC

ACTGAACACGCCTATGAGAACAAACGCATCACTGTTGAAGGCGGGATGCCGTCTGGTTGTTCCGCAACAA

GCATCATCAACACAATTTTGAACAACATCTACGTGCTCTACGCCTTGCGTAGACACTATGAGGGGGTTGA

GCTGGACTCTTACACCATGATCTCCTACGGAGACGACATCGTGGTTGCAAGTGATTACGATCTGGACTTT

GAGGCCCTCAAGCCTCACTTCAAATCCCTTGGTCAAACCATTACCCCAGCTGACAAAAGCGACAAAGGTT

TTGTTCTTGGTCACTCCATTACCGATGTCACTTTCCTCAAAAGACACTTCCACATGGACTATGGAACTGG

GTTTTACAAACCTGTGATGGCTTCGAAGACCCTCGAGGCTATCCTCTCCTTTGCACGCCGTGGG

>EF611987.1_O_UGA_2006

GGGCTGATTATTGACACCAGAGATGTGGAAGAGCGCGTACACGTTATGCGCAAAACAAAGCTTGCACCCA

CCGTGGCACACGGTGTGTTCAACCCTGAGTTTGGCCCCGCCGCCTTGTCCAACAAGGACCCGCGGCTGAA

CGAAGGAGTTGTCCTCGATGAAGTCATCTTCTCCAAGCACAAGGGAGACACGAAGATGTCTGAGGAAGAC

AAAGCGCTGTTCCGTCGTTGTGCTGCTGACTACGCGTCGCGGCTACACAGCGAACTGGGTACGGCGAATG

CCCCGTTGAGCATCTACGAAGCAATCAAAGGCGTTGACGGCCTCGACGCCATGGAACCGGACACTGCTCC

TGGTCTCCCCTGGGCCCTCCAGGGGAAACGCCGTGGAGCTCTGATTGACTTCGAGAACGGCACAGTCGGC

CCTGAGGTCACTGCCGCTCTAGAGCTCATGGAGAAAAGAGAGTACAAATTTGCTTGTCAGACCTTCCTGA

AGGACGAGATTCGCCCGATGGAAAAAGTGCGTGCCGGCAAGACCCGCATTGTCGACGTCCTGCCTGTTGA

ACACATACTTTACACCAGAATGATGATTGGCAGATTTTGTGCCCAAATGCACGCAAACAACGGACCGCGT

ATTGGCTCAGCGGTTGGTTGCAATCCAGATGTTGATTGGCAGAGATTTGGCACACACTTCGCCCAGTACA

GGAATGTGTGGGATGTGGACTATTCGGCCTTCGATGCTAACCACTGCAGTGACGCGATGAACATCATGTT

TGAGGAGGTGTTTCGCACAGAATTTGGCTTCCACCCGAACGCCGAGTGGATCCTGAAAACTCTTGTGAAC

ACGGAACACGCCTATGAGAACAAGCGCATTACTGTTGAAGGCGGGATGCCGTCTGGCTGTTCCGCAACGA

GCATCATCAACACAATTTTGAACAACATCTACGTGCTCTACGCCCTGCGTAGACACTATGAGGGAGTTGA

GTTGGACACCTACACCATGATCTCCTACGGAGACGACATCGTGGTGGCAAGTGATTACGATCTGGACTTC

GAGGCTCTCAAGCCTCATTTCAAATCTCTTGGTCAAACCATCACCCCAGCTGACAAAAGCGACAAAGGTT

TTGTTCTTGGTCACTCCATTACCGATGTCACTTTCCTTAAAAGACACTTCCACATGGACTATGGAACTGG

GTTTTACAAACCTGTGATGGCCTCAAAGACCCTTGAGGCTATCCTCTCCTTTGCACGCCGTGGG

>EF614457.1_O_SKR_2002

GGATTGATAGTTGACACCAGAGATGTTGAGGAGCGCGTACACGTCATGCGCAAAACCAAGCTCGCACCCA

CCGTAGCATACGGTGTGTTTAACCCCGAATTTGGGCCTGCCGCCTTGTCCAACAAGGACCCGCGCCTGAG

TGAGGGGGTTGTCCTCGATGAAGTCATCTTCTCCAAACACAAAGGAAACACAAAGATGTCTGAGGAGGAC

AGAGCGCTGTTCCGCCGCTGTGCTGCTGACTACGCGTCGCGTCTGCACAGTGTGCTGGGTACGGCAAATG

CCCCACTGAGCATTTACGAGGCAATCAAGGGCGTCGACGGACTTGACGCCATGGAACCAGACACCGCGCC

TGGTCTCCCCTGGGCTCTCCAGGGGAAACGCCGTGGTGCGCTCATTGACTTCGAGAACGGCACTGTCGGA

CCCGAGGTTGAAGCTGCCTTGAAGCTCATGGAGAAAAGAGAGTACAAGTTTGTATGCCAGACCTTCTTGA

AGGACGAGATTCGCCCGATGGAGAAGGTACGTGCCGGTAAGACTCGCATTGTCGACGTCCTGCCTGTTGA

ACACATTCTTTACACCAGGATGATGATTGGCAGATTTTGTGCTCAAATGCACTCAAACAACGGACCGCAA

ATTGGCTCGGCGGTTGGTTGTAATCCTGATGTTGATTGGCAAAGATTTGGCACGCACTTTGCTCAGTACA

GAAACGTGTGGGATGTGGACTATTCGGCCTTCGACGCCAACCACTGCAGTGACGCAATGAACATCATGTT

CGAGGAAGTGTTCAACACGGATTTCGGTTTCCACCCAAACGCTGAGTGGATCCTGAAAACTCTCGTGAAC

ACTGAACACGCCTACGAGAACAAACGCATCACTGTTGAAGGCGGGATGCCGTCTGGTTGTTCCGCGACAA

GCATCATCAACACAATTTTGAACAACATCTACGTGCTCTACGCCTTGCGTAGACACTACGAGGGAGTTGA

GCTGGACTCTTACACCATGATCTCCTACGGAGACGACATCGTGGTTGCAAGTGATCACGATCTGGACTTT

GAGGCCCTCAAGCCTCACTTCAAATCCCTTGGTCAAACCATCACTCCAGCTGACAAAAGCGACAAAGGTT

TTGTTCTTGGTCACTCCATTACCGATGTCACTTTCCTCAAAAGACACTTCCACATGGACTATGGAACTGG

GTTTTACAAACCTGTGATGGCTTCGAAGACCCTCGAGGCTATCCTCTCCTTTGCACGTCGTGGG

>EU214601.1_O_UKG_2001

GGATTGATAGTTGACACCAGAGATGTTGAGGAGCGCGTACATGTCATGCGCAAAACCAAGCTCGCACCCA

CCGTGGCACACGGTGTGTTTAACCCCGAATTTGGGCCTGCCGCCTTGTCCAACAAGGACCCGCGCCTGAA

TGAGGGGGTTGTCCTCGATGAAGTCATCTTCTCCAAACACAAAGGAAACACAAAGATGTCTGAGGAGGAC

AAAGCGCTGTTCCGCCGCTGTGCTGCTGACTACGCGTCGCGTCTGCATAGCGTGCTGGGTACGGCAAATG

CCCCACTGAGCACTTACGAGGCAATCAAGGGCGTCGACGGACTTGACGCCATGGAACCAGACACCGCGCC

TGGTCTCCCCTGGGCTCTCCAGGGGAAACGCCGTGGTGCGCTCATCGACTTCGAGAACGGCACTGTCGGA

CCCGAGGTTGAAGCTGCCTTGAAGCTCATGGAGAAAAGAGAGTACAAGTTTACATGCCAGACCTTCCTGA

AGGACGAGATTCGCCCGATGGAGAAGGTACGTGCCGGCAAGACTCGCATTGTCGACGTCCTGCCCGTTGA

ACACATTCTTTACACTAGGATGATGATTGGCAGATTTTGTGCTCAAATGCACTCAAACAACGGACCGCAA

ATTGGCTCGGCGGTTGGTTGTAATCCTGATGTTGATTGGCAAAGATTTGGCACGCATTTTGCTCAGTATA

GAAACGTGTGGGATGTGGACTATTCGGCCTTTGATGCCAACCACTGCAGTGACGCAATGAACATCATGTT

TGAGGAGGTGTTTAACACGGACTTCGGTTTCCACCCAAACGCTGAGTGGATCCTGAAAACTCTCGTGAAC

ACTGAACACGCCTATGAGAACAAACGCATCACTGTTGAAGGCGGGATGCCGTCTGGTTGTTCCGCAACAA

GCATCATCAACACAATTTTGAACAACATCTACGTGCTCTACGCCTTGCGTAGACACTATGAGGGGGTTGA

GCTGGACTCTTACACCATGATCTCCTACGGAGACGACATCGTGGTTGCAAGTGATTACGATCTGGACTTT

GAGGCCCTCAAGCCTCACTTCAAATCCCTTGGTCAAACCATTACCCCAGCTGACAAAAGCGACAAAGGTT

TTGTTCTTGGTCACTCCATTACCGATGTCACTTTCCTCAAAAGACACTTCCACATGGACTATGGAACTGG

GTTTTACAAACCTGTGATGGCTTCGAAGACCCTCGAGGCTATCCTCTCCTTTGCACGCCGTGGG

>EU400597.1_O_CHA_2001

GGGTTGATCGTTGACACCAGAGATGTGGAGGAACGCGTCCACGTGATGCGCAAAACCAAGCTCGCGCCCA

CCGTAGCACACGGTGTGTTCAATCCTGAGTTCGGGCCTGCTGCTCTGTCCAACAAGGACCCGCGTCTGAA

CGAAGGGGTCGTCCTCGACGACGTCATTTTCTCAAAACACAAGGGAGACACGAGGATGTCTGAGGAAGAC

AAAGCGCTGTTCCGGCGTTGTGCTGCCGACTACGCGTCGCGTCTACACAGCGTGCTAGGGACGGCAAACG

CCCCACTGAGTGTATACGAAGCCATCAAAGGCGTCGATGGACTTGACGCCATGGAGCCAGACACCGCACC

CGGTCTCCCCTGGGCTCTCCAAGGAAAACGCCGAGGTGCCCTGATCGACTTCGAAAACGGTACTGTCGGG

CCCGAGGTTGAAGCAGCACTCAAGCTCATGGAAAGCCGTGAGTACAAATTCGTCTGTCAAACCTTTCTGA

AAGACGAAATTCGGCCGCTAGAGAAGGTGCGCGCCGGTAAGACACGCATTGTCGACGTTTTGCCTGTTGA

ACACATTCTCTATACCAGAATGATGATTGGCAGATTCTGTGCTCAGATGCACTCAAACAACGGACCGCAA

ATTGGCTCAGCGGTCGGTTGCAACCCTGATGTTGATTGGCAAAGATTTGGCACACATTTCGCCCAGTACA

AGAACGTGTGGGATGTGGACTACTCAGCCTTCGACGCAAACCACTGCAGCGATGCGATGAACATCATGTT

CGAAGAGGTGTTCCGCACGGAGTTTGGGTTCCACCCGAACGCCGAGTGGATTCTGAAGACTCTGGTGAAC

ACGGAGCACGCTTACGAGAACAAGCGCATCACTGTGGAGGGTGGAATGCCATCCGGTTGTTCCGCAACAA

GCATCATCAACACAATTTTGAACAACATCTACGTGCTCTACGCTCTGCGTAGGCACTATGAGGGAGTTGA

GCTGGACACCTACACCATGATCTCCTATGGAGACGACATCGTGGTGGCTAGTGACTACGACCTGGACTTC

GAGGCTCTCAAGCCCCACTTCAAGTCCCTCGGTCAGACCATCACTCCAGCCGACAAAAGCGACAAAGGTT

TTGTTCTTGGTCACTCCATAACCGATGTCACTTTCCTCAAAAGACACTTCCACATGGACTACGGAACTGG

GTTTTACAAACCTGTGATGGCCTCGAAGACCCTCGAGGCTATCCTCTCCTTTGCACGCCGTGGG

>EU448368.1_O_UKG_1967

GGGTTGATTGTGGACACCAGAGATGTGGAAGAGCGCGTTCACGTGATGCGCAAAACCAAGCTTGCACCCA

CCGTTGCACACGGTGTGTTCAACCCCGAGTTTGGGCCCGCTGCCTTGTCCAACAAGGACCCGCGTCTGAA

CGAGGGTGTTGTCCTCGATGAAGTCATCTTCTCCAAACACAAGGGAGACACAAAGATGTCTGAGGAGGAC

AAAGCGCTGTTCCGCCGCTGTGCTGCTGACTACGCGTCACGCCTGCACAGCGTGTTGGGCACAGCAAATG

CCCCATTGAGCATCTACGAGGCAATCAAGGGTGTCGACGGACTCGACGCCATGGAACCAGACACAGCGCC

CGGCCTCCCCTGGGCCCTCCAGGGTAAACGCCGCGGCGCGCTCATCGACTTCGAAAACGGCACGGTCGGA

CCCGAAGTCGAGGCTGCCCTGAAGCTCATGGAGAAGAGAGAATACAAATTTGCTTGTCAGACCTTCCTGA

AGGACGAGATTCGCCCGATGGAGAAAGTACGTGCCGGCAAGACTCGCATTGTCGACGTCTTGCCCGTTGA

GCATATTCTTTACACCAGGATGATGATTGGCAGATTTTGTGCACAGATGCACTCAAACAACGGACCGCAA

ATTGGCTCAGCGGTCGGTTGCAACCCTGATGTTGATTGGCAGAGATTTGGCACACACTTCGCCCAGTACA

GAAACGTGTGGGATGTGGACTATTCGGCCTTTGATGCTAATCACTGCAGTGATGCCATGAACATCATGTT

TGAGGAGGTGTTTCGCACGGAGTTCGGCTTCCACCCGAATGCCGAGTGGATCCTGAAGACTCTTGTGAAC

ACGGAACACGCCTATGAGAACAAACGCATCACTGTTGAAGGCGGAATGCCGTCTGGTTGTTCCGCAACAA

GCATCATCAACACAATTTTGAACAACATCTACGTGCTCTACGCCCTGCGTAGACACTATGAGGGAGTTGA

GCTGGACACATACACCATGATCTCCTACGGAGACGACATCGTGGTGGCAAGTGATTATGACTTGGACTTC

GAGGCTCTCAAGCCCCACTTTAAATCCCTTGGTCAAACCATCACTCCAGCTGACAAAAGCGACAAAGGTT

TTGTTCTTGGTCACTCCATTACCGATGTCACTTTCCTCAAAAGACACTTCCACATGGACTATGGAACTGG

GTTTTACAAACCTGTGATGGCCTCAAAGACCCTTGAGGCTATCCTCTCCTTTGCACGCCGTGGG

>EU448369.1_O_UKG_1967

GGGTTGATTGTGGACACCAGAGATGTGGAAGAGCGCGTTCACGTGATGCGCAAAACCAAGCTTGCACCCA

CCGTTGCACACGGTGTGTTCAACCCCGAGTTTGGGCCCGCTGCCTTGTCCAACAAGGACCCGCGTCTGAA

CGAGGGTGTTGTCCTCGATGAAGTCATCTTCTCCAAACACAAGGGAGACACAAAGATGTCTGAGGAGGAC

AAAGCGCTGTTCCGCCGCTGTGCTGCTGACTACGCGTCACGCCTGCACAGCGTGTTGGGCACAGCAAATG

CCCCATTGAGCATCTACGAGGCAATCAAGGGTGTCGACGGACTCGACGCCATGGAACCAGATACAGCGCC

CGGCCTCCCCTGGGCCCTCCAGGGTAAACGCCGCGGCGCGCTCATCGACTTCGAAAACGGCACGGTCGGA

CCCGAAGTCGAGGCTGCCCTGAAGCTCATGGAGAAGAGAGAATACAAATTTGCTTGTCAGACCTTCCTGA

AGGACGAGATTCGCCCGATGGAGAAAGTACGTGCCGGCAAGACTCGCATTGTCGACGTCTTGCCCGTTGA

GCATATTCTTTACACCAGGATGATGATTGGCAGATTTTGTGCACAGATGCACTCAAACAACGGACCGCAA

ATTGGCTCAGCGGTCGGTTGCAACCCTGATGTTGATTGGCAGAGATTTGGCACACACTTCGCCCAGTACA

GAAACGTGTGGGATGTGGACTATTCGGCCTTTGATGCTAATCACTGCAGTGATGCCATGAACATCATGTT

TGAGGAGGTGTTTCGCACGGAGTTCGGCTTCCACCCGAATGCCGAGTGGATCCTGAAGACTCTTGTGAAC

ACGGAACACGCCTATGAGAACAAACGCATCACTGTTGAAGGCGGAATGCCGTCTGGTTGTTCCGCAACAA

GCATCATCAACACAATTTTGAACAACATCTACGTGCTCTACGCCCTGCGTAGACACTATGAGGGAGTTGA

GCTGGACACATACACCATGATCTCCTACGGAGACGACATCGTGGTGGCAAGTGATTATGACTTGGACTTC

GAGGCTCTCAAGCCCCACTTTAAATCCCTTGGTCAAACCATCACTCCAGCTGACAAAAGCGACAAAGGTT

TTGTTCTTGGTCACTCCATTACCGATGTCACTTTCCTCAAAAGACACTTCCACATGGACTATGGAACTGG

GTTTTACAAACCTGTGATGGCCTCAAAGACCCTTGAGGCTATCCTCTCCTTTGCACGCCGTGGG

>EU448370.1_O_UKG_1967

GGGTTGATTGTGGACACCAGAGATGTGGAAGAGCGCGTTCACGTGATGCGCAAAACCAAGCTTGCACCCA

CCGTTGCACACGGTGTGTTCAACCCCGAGTTTGGGCCCGCTGCCTTGTCCAACAAGGACCCGCGTCTGAA

CGAGGGTGTTGTCCTCGATGAAGTCATCTTCTCCAAACACAAGGGAGACACAAAGATGTCTGAGGAGGAC

AAAGCGCTGTTCCGCCGCTGTGCTGCTGACTACGCGTCACGCCTGCACAGCGTGTTGGGCACAGCAAATG

CCCCATTGAGCATCTACGAGGCAATCAAGGGTGTCGACGGACTCGACGCCATGGAACCAGATACAGCGCC

CGGCCTCCCCTGGGCCCTCCAGGGTAAACGCCGCGGCGCGCTCATCGACTTCGAAAACGGCACGGTCGGA

CCCGAAGTCGAGGCTGCCCTGAAGCTCATGGAGAAGAGAGAATACAAATTTGCTTGTCAGACCTTCCTGA

AGGACGAGATTCGCCCGATGGAGAAAGTACGTGCCGGCAAGACTCGCATTGTCGACGTCTTGCCCGTTGA

GCATATTCTTTACACCAGGATGATGATTGGCAGATTTTGTGCACAGATGCACTCAAACAACGGACCGCAA

ATTGGCTCAGCGGTCGGTTGCAACCCTGATGTTGATTGGCAGAGATTTGGCACACACTTCGCCCAGTACA

GAAACGTGTGGGATGTGGACTATTCGGCCTTTGATGCTAATCACTGCAGTGATGCCATGAACATCATGTT

TGAGGAGGTGTTTCGCACGGAGTTCGGCTTCCACCCGAATGCCGAGTGGATCCTGAAGACTCTTGTGAAC

ACGGAACACGCCTATGAGAACAAACGCATCACTGTTGAAGGCGGAATGCCGTCTGGTTGTTCCGCAACAA

GCATCATCAACACAATTTTGAACAACATCTACGTGCTCTACGCCCTGCGTAGACACTATGAGGGAGTTGA

GCTGGACACATACACCATGATCTCCTACGGAGACGACATCGTGGTGGCAAGTGATTATGACTTGGACTTC

GAGGCTCTCAAGCCCCACTTTAAATCCCTTGGTCAAACCATCACTCCAGCTGACAAAAGCGACAAAGGTT

TTGTTCTTGGTCACTCCATTACCGATGTCACTTTCCTCAAAAGACACTTCCACATGGACTATGGAACTGG

GTTTTACAAACCTGTGATGGCCTCAAAGACCCTTGAGGCTATCCTCTCCTTTGCACGCCGTGGG

>EU448371.1_O_UKG_2007

GGGTTGATTGTGGACACCAGAGATGTGGAAGAGCGCGTTCACGTGATGCGCAAAACCAAGCTTGCACCCA

CCGTTGCACACGGTGTGTTCAACCCCGAGTTTGGGCCCGCTGCCTTGTCCAACAAGGACCCGCGTCTGAA

CGAGGGTGTTGTCCTCGATGAAGTCATCTTCTCCAAACACAAGGGAGACACAAAGATGTCTGAGGAGGAC

AAAGCGCTGTTCCGCCGCTGTGCTGCTGACTACGCGTCACGCCTGCACAGCGTGTTGGGCACAGCAAATG

CCCCATTGAGCATCTACGAGGCAATCAAGGGTGTCGACGGACTCGACGCCATGGAACCAGATACAGCGCC

CGGCCTCCCCTGGGCCCTCCAGGGTAAACGCCGCGGCGCGCTCATCGACTTCGAAAACGGCACGGTCGGA

CCCGAAGTCGAGGCTGCCCTGAAGCTCATGGAGAAGAGAGAATACAAATTTGCTTGTCAGACCTTCCTGA

AGGACGAGATTCGCCCGATGGAGAAAGTACGTGCCGGCAAGACTCGCATTGTCGACGTCTTGCCCGTTGA

GCATATTCTTTACACCAGGATGATGATTGGCAGATTTTGTGCACAGATGCACTCAAACAACGGACCGCAA

ATTGGCTCAGCGGTCGGTTGCAACCCTGATGTTGATTGGCAGAGATTTGGCACACACTTCGCCCAGTACA

GAAACGTGTGGGATGTGGACTATTCGGCCTTTGATGCTAATCACTGCAGTGATGCCATGAACATCATGTT

TGAGGAGGTGTTTCGCACGGAGTTCGGCTTCCACCCGAATGCCGAGTGGATCCTGAAGACTCTTGTGAAC

ACGGAACACGCCTATGAGAACAAACGCATCACTGTTGAAGGCGGAATGCCGTCTGGTTGTTCCGCAACAA

GCATCATCAACACAATTTTGAACAACATCTACGTGCTCTACGCCCTGCGTAGACACTATGAGGGAGTTGA

GCTGGACACATACACCATGATCTCCTACGGAGACGACATCGTGGTGGCAAGTGATTATGACTTGGACTTC

GAGGCTCTCAAGCCCCACTTTAAATCCCTTGGTCAAACCATCACTCCAGCTGACAAAAGCGACAAAGGTT

TTGTTCTTGGTCACTCCATTACCGATGTCACTTTCCTCAAAAGACACTTCCACATGGACTATGGAACTGG

GTTTTACAAACCTGTGATGGCCTCAAAGACCCTTGAGGCTATCCTCTCCTTTGCACGCCGTGGG

>EU448372.1_O_UKG_2007

GGGTTGATTGTGGACACCAGAGATGTGGAAGAGCGCGTTCACGTGATGCGCAAAACCAAGCTTGCACCCA

CCGTTGCACACGGTGTGTTCAACCCCGAGTTTGGGCCCGCTGCCTTGTCCAACAAGGACCCGCGTCTGAA

CGAGGGTGTTGTCCTCGATGAAGTCATCTTCTCCAAACACAAGGGAGACACAAAGATGTCTGAGGAGGAC

AAAGCGCTGTTCCGCCGCTGTGCTGCTGACTACGCGTCACGCCTGCACAGCGTGTTGGGCACAGCAAATG

CCCCATTGAGCATCTACGAGGCAATCAAGGGTGTCGACGGACTCGACGCCATGGAACCAGATACAGCGCC

CGGCCTCCCCTGGGCCCTCCAGGGTAAACGCCGCGGCGCGCTCATCGACTTCGAAAACGGCACGGTCGGA

CCCGAAGTCGAGGCTGCCCTGAAGCTCATGGAGAAGAGAGAATACAAATTTGCTTGTCAGACCTTCCTGA

AGGACGAGATTCGCCCGATGGAGAAAGTACGTGCCGGCAAGACTCGCATTGTCGACGTCTTGCCCGTTGA

GCATATTCTTTACACCAGGATGATGATTGGCAGATTTTGTGCACAGATGCACTCAAACAACGGACCGCAA

ATTGGCTCAGCGGTCGGTTGCAACCCTGATGTTGATTGGCAGAGATTTGGCACACACTTCGCCCAGTACA

GAAACGTGTGGGATGTGGACTATTCGGCCTTTGATGCTAATCACTGCAGTGATGCCATGAACATCATGTT

TGAGGAGGTGTTTCGCACGGAGTTCGGCTTCCACCCGAATGCCGAGTGGATCCTGAAGACTCTTGTGAAC

ACGGAACACGCCTATGAGAACAAACGCATCACTGTTGAAGGCGGAATGCCGTCTGGTTGTTCCGCAACAA

GCATCATCAACACAATTTTGAACAACATCTACGTGCTCTACGCCCTGCGTAGACACTATGAGGGAGTTGA

GCTGGACACATACACCATGATCTCCTACGGAGACGACATCGTGGTGGCAAGTGATTATGACTTGGACTTC

GAGGCTCTCAAGCCCCACTTTAAATCCCTTGGTCAAACCATCACTCCAGCTGACAAAAGCGACAAAGGTT

TTGTTCTTGGTCACTCCATTACCGATGTCACTTTCCTCAAAAGACACTTCCACATGGACTATGGAACTGG

GTTTTACAAACCTGTGATGGCCTCAAAGACCCTTGAGGCTATCCTCTCCTTTGCACGCCGTGGG

>EU448373.1_O_UKG_2007

GGGTTGATTGTGGACACCAGAGATGTGGAAGAGCGCGTTCACGTGATGCGCAAAACCAAGCTTGCACCCA

CCGTTGCACACGGTGTGTTCAACCCCGAGTTTGGGCCCGCTGCCTTGTCCAACAAGGACCCGCGTCTGAA

CGAGGGTGTTGTCCTCGATGAAGTCATCTTCTCCAAACACAAGGGAGACACAAAGATGTCTGAGGAGGAC

AAAGCGCTGTTCCGCCGCTGTGCTGCTGACTACGCGTCACGCCTGCACAGCGTGTTGGGCACAGCAAATG

CCCCATTGAGCATCTACGAGGCAATCAAGGGTGTCGACGGACTCGACGCCATGGAACCAGATACAGCGCC

CGGCCTCCCCTGGGCCCTCCAGGGTAAACGCCGCGGCGCGCTCATCGACTTCGAAAACGGCACGGTCGGA

CCCGAAGTCGAGGCTGCCCTGAAGCTCATGGAGAAGAGAGAATACAAATTTGCTTGTCAGACCTTCCTGA

AGGACGAGATTCGCCCGATGGAGAAAGTACGTGCCGGCAAGACTCGCATTGTCGACGTCTTGCCCGTTGA

GCATATTCTTTACACCAGGATGATGATTGGCAGATTTTGTGCACAGATGCACTCAAACAACGGACCGCAA

ATTGGCTCAGCGGTCGGTTGCAACCCTGATGTTGATTGGCAGAGATTTGGCACACACTTCGCCCAGTACA

GAAACGTGTGGGATGTGGACTATTCGGCCTTTGATGCTAATCACTGCAGTGATGCCATGAACATCATGTT

TGAGGAGGTGTTTCGCACGGAGTTCGGCTTCCACCCGAATGCCGAGTGGATCCTGAAGACTCTTGTGAAC

ACGGAACACGCCTATGAGAACAAACGCATCACTGTTGAAGGCGGAATGCCGTCTGGTTGTTCCGCAACAA

GCATCATCAACACAATTTTGAACAACATCTACGTGCTCTACGCCCTGCGTAGACACTATGAGGGAGTTGA

GCTGGACACATACACCATGATCTCCTACGGAGACGACATCGTGGTGGCAAGTGATTATGACTTGGACTTC

GAGGCTCTCAAGCCCCACTTTAAATCCCTTGGTCAAACCATCACTCCAGCTGACAAAAGCGACAAAGGTT

TTGTTCTTGGTCACTCCATTACCGATGTCACTTTCCTCAAAAGACACTTCCACATGGACTATGGAACTGG

GTTTTACAAACCTGTGATGGCCTCAAAGACCCTTGAGGCTATCCTCTCCTTTGCACGCCGTGGG

>EU448374.1_O_UKG_2007

GGGTTGATTGTGGACACCAGAGATGTGGAAGAGCGCGTTCACGTGATGCGCAAAACCAAGCTTGCACCCA

CCGTTGCACACGGTGTGTTCAACCCCGAGTTTGGGCCCGCTGCCTTGTCCAACAAGGACCCGCGTCTGAA

CGAGGGTGTTGTCCTCGATGAAGTCATCTTCTCCAAACACAAGGGAGACACAAAGATGTCTGAGGAGGAC

AAAGCGCTGTTCCGCCGCTGTGCTGCTGACTACGCGTCACGCCTGCACAGCGTGTTGGGCACAGCAAATG

CCCCATTGAGCATCTACGAGGCAATCAAGGGTGTCGACGGACTCGACGCCATGGAACCAGATACAGCGCC

CGGCCTCCCCTGGGCCCTCCAGGGTAAACGCCGCGGCGCGCTCATCGACTTCGAAAACGGCACGGTCGGA

CCCGAAGTCGAGGCTGCCCTGAAGCTCATGGAGAAGAGAGAATACAAATTTGCTTGTCAGACCTTCCTGA

AGGACGAGATTCGCCCGATGGAGAAAGTACGTGCCGGCAAGACTCGCATTGTCGACGTCTTGCCCGTTGA

GCATATTCTTTACACCAGGATGATGATTGGCAGATTTTGTGCACAGATGCACTCAAACAACGGACCGCAA

ATTGGCTCAGCGGTCGGTTGCAACCCTGATGTTGATTGGCAGAGATTTGGCACACACTTCGCCCAGTACA

GAAACGTGTGGGATGTGGACTATTCGGCCTTTGATGCTAATCACTGCAGTGATGCCATGAACATCATGTT

TGAGGAGGTGTTTCGCACGGAGTTCGGCTTCCACCCGAATGCCGAGTGGATCCTGAAGACTCTTGTGAAC

ACGGAACACGCCTATGAGAACAAACGCATCACTGTTGAAGGCGGAATGCCGTCTGGTTGTTCCGCAACAA

GCATCATCAACACAATTTTGAACAACATCTACGTGCTCTACGCCCTGCGTAGACACTATGAGGGAGTTGA

GCTGGACACATACACCATGATCTCCTACGGAGACGACATCGTGGTGGCAAGTGATTATGACTTGGACTTC

GAGGCTCTCAAGCCCCACTTTAAATCCCTTGGTCAAACCATCACTCCAGCTGACAAAAGCGACAAAGGTT

TTGTTCTTGGTCACTCCATTACCGATGTCACTTTCCTCAAAAGACACTTCCACATGGACTATGGAACTGG

GTTTTACAAACCTGTGATGGCCTCAAAGACCCTTGAGGCTATCCTCTCCTTTGCACGCCGTGGG

>EU448375.1_O_UKG_2007

GGGTTGATTGTGGACACCAGAGATGTGGAAGAGCGCGTTCACGTGATGCGCAAAACCAAGCTTGCACCCA

CCGTTGCACACGGTGTGTTCAACCCCGAGTTTGGGCCCGCTGCCTTGTCCAACAAGGACCCGCGTCTGAA

CGAGGGTGTTGTCCTCGATGAAGTCATCTTCTCCAAACACAAGGGAGACACAAAGATGTCTGAGGAGGAC

AAAGCGCTGTTCCGCCGCTGTGCTGCTGACTACGCGTCACGCCTGCACAGCGTGTTGGGCACAGCAAATG

CCCCATTGAGCATCTACGAGGCAATCAAGGGTGTCGACGGACTCGACGCCATGGAACCAGATACAGCGCC

CGGCCTCCCCTGGGCCCTCCAGGGTAAACGCCGCGGCGCGCTCATCGACTTCGAAAACGGCACGGTCGGA

CCCGAAGTCGAGGCTGCCCTGAAGCTCATGGAGAAGAGAGAATACAAATTTGCTTGTCAGACTTTCCTGA

AGGACGAGATTCGCCCGATGGAGAAAGTACGTGCCGGCAAGACTCGCATTGTCGACGTCTTGCCCGTTGA

GCATATTCTTTACACCAGGATGATGATTGGCAGATTTTGTGCACAGATGCACTCAAACAACGGACCGCAA

ATTGGCTCAGCGGTCGGTTGCAACCCTGATGTTGATTGGCAGAGATTTGGCACACACTTCGCCCAGTACA

GAAACGTGTGGGATGTGGACTATTCGGCCTTTGATGCTAATCACTGCAGTGATGCCATGAACATCATGTT

TGAGGAGGTGTTTCGCACGGAGTTCGGCTTCCACCCGAATGCCGAGTGGATCCTGAAGACTCTTGTGAAC

ACGGAACACGCCTATGAGAACAAACGCATCACTGTTGAAGGCGGAATGCCGTCTGGTTGTTCCGCAACAA

GCATCATCAACACAATTTTGAACAACATCTACGTGCTCTACGCCCTGCGTAGACACTATGAGGGAGTTGA

GCTGGACACATACACCATGATCTCCTACGGAGACGACATCGTGGTGGCAAGTGATTATGACTTGGACTTT

GAGGCTCTCAAGCCCCACTTCAAATCCCTTGGTCAAACCATCACTCCAGCTGACAAAAGCGACAAAGGTT

TTGTTCTTGGTCACTCCATTACCGATGTCACTTTCCTCAAAAGACACTTCCACATGGACTATGGAACTGG

GTTTTACAAACCTGTGATGGCCTCAAAGACCCTTGAGGCTATCCTCTCCTTTGCACGCCGTGGG

>EU448376.1_O_UKG_2007

GGGTTGATTGTGGACACCAGAGATGTGGAAGAGCGCGTTCACGTGATGCGCAAAACCAAGCTTGCACCCA

CCGTTGCACACGGTGTGTTCAACCCCGAGTTTGGGCCCGCTGCCTTGTCCAACAAGGACCCGCGTCTGAA

CGAGGGTGTTGTCCTCGATGAAGTCATCTTCTCCAAACACAAGGGAGACACAAAGATGTCTGAGGAGGAC

AAAGCGCTGTTCCGCCGCTGTGCTGCTGACTACGCGTCACGCCTGCACAGCGTGTTGGGCACAGCAAATG

CCCCATTGAGCATCTACGAGGCAATCAAGGGTGTCGACGGACTCGACGCCATGGAACCAGATACAGCGCC

CGGCCTCCCCTGGGCCCTCCAGGGTAAACGCCGCGGCGCGCTCATCGACTTCGAAAACGGCACGGTCGGA

CCCGAAGTCGAGGCTGCCCTGAAGCTCATGGAGAAGAGAGAATACAAATTTGCTTGTCAGACCTTCCTGA

AGGACGAGATTCGCCCGATGGAGAAAGTACGTGCCGGCAAGACTCGCATTGTCGACGTCTTGCCCGTTGA

GCATATTCTTTACACCAGGATGATGATTGGCAGATTTTGTGCACAGATGCACTCAAACAACGGACCGCAA

ATTGGCTCAGCGGTCGGTTGCAACCCTGATGTTGATTGGCAGAGATTTGGCACACACTTCGCCCAGTACA

GAAACGTGTGGGATGTGGACTATTCGGCCTTTGATGCTAATCACTGCAGTGATGCCATGAACATCATGTT

TGAGGAGGTGTTTCGCACGGAGTTCGGCTTCCACCCGAATGCCGAGTGGATCCTGAAGACTCTTGTGAAC

ACGGAACACGCCTATGAGAACAAACGCATCACTGTTGAAGGCGGAATGCCGTCTGGTTGTTCCGCAACAA

GCATCATCAACACAATTTTGAACAACATCTACGTGCTCTACGCCCTGCGTAGACACTATGAGGGAGTTGA

GCTGGACACATACACCATGATCTCCTACGGAGACGACATCGTGGTGGCAAGTGATTATGACTTGGACTTT

GAGGCTCTCAAGCCCCACTTCAAATCCCTTGGTCAAACCATCACTCCAGCTGACAAAAGCGACAAAGGTT

TTGTTCTTGGTCACTCCATTACCGATGTCACTTTCCTCAAAAGACACTTCCACATGGACTATGGAACTGG

GTTTTACAAACCTGTGATGGCCTCAAAGACCCTTGAGGCTATCCTCTCCTTTGCACGCCGTGGG

>EU448377.1_O_UKG_2007

GGGTTGATTGTGGACACCAGAGATGTGGAAGAGCGCGTTCACGTGATGCGCAAAACCAAGCTTGCACCCA

CCGTTGCACACGGTGTGTTCAACCCCGAGTTTGGGCCCGCTGCCTTGTCCAACAAGGACCCGCGTCTGAA

CGAGGGTGTTGTCCTCGATGAAGTCATCTTCTCCAAACACAAGGGAGACACAAAGATGTCTGAGGAGGAC

AAAGCGCTGTTCCGCCGCTGTGCTGCTGACTACGCGTCACGCCTGCACAGCGTGTTGGGCACAGCAAATG

CCCCATTGAGCATCTACGAGGCAATCAAGGGTGTCGACGGACTCGACGCCATGGAACCAGATACAGCGCC

CGGCCTCCCCTGGGCCCTCCAGGGTAAACGCCGCGGCGCGCTCATCGACTTCGAAAACGGCACGGTCGGA

CCCGAAGTCGAGGCTGCCCTGAAGCTCATGGAGAAGAGAGAATACAAATTTGCTTGTCAGACCTTCCTGA

AGGACGAGATTCGCCCGATGGAGAAAGTACGTGCCGGCAAGACTCGCATTGTCGACGTCTTGCCCGTTGA

GCATATTCTTTACACCAGGATGATGATTGGCAGATTTTGTGCACAGATGCACTCAAACAACGGACCGCAA

ATTGGCTCAGCGGTCGGTTGCAACCCTGATGTTGATTGGCAGAGATTTGGCACACACTTCGCCCAGTACA

GAAACGTGTGGGATGTGGACTATTCGGCCTTTGATGCTAATCACTGCAGTGATGCCATGAACATCATGTT

TGAGGAGGTGTTTCGCACGGAGTTCGGCTTCCACCCGAATGCCGAGTGGATCCTGAAGACTCTTGTGAAC

ACGGAACACGCCTATGAGAACAAACGCATCACTGTTGAAGGCGGAATGCCGTCTGGTTGTTCCGCAACAA

GCATCATCAACACAATTTTGAACAACATCTACGTGCTCTACGCCCTGCGTAGACACTATGAGGGAGTTGA

GCTGGACACATACACCATGATCTCCTACGGAGACGACATCGTGGTGGCAAGTGATTATGACTTGGACTTT

GAGGCTCTCAAGCCCCACTTCAAATCCCTTGGTCAAACCATCACTCCAGCTGACAAAAGCGACAAAGGTT

TTGTTCTTGGTCACTCCATTACCGATGTCACTTTCCTCAAAAGACACTTCCACATGGACTATGGAACTGG

GTTTTACAAACCTGTGATGGCCTCAAAGACCCTTGAGGCTATCCTCTCCTTTGCACGCCGTGGG

>EU448378.1_O_UKG_2007

GGGTTGATTGTGGACACCAGAGATGTGGAAGAGCGCGTTCACGTGATGCGCAAAACCAAGCTTGCACCCA

CCGTTGCACACGGTGTGTTCAACCCCGAGTTTGGGCCCGCTGCCTTGTCCAACAAGGACCCGCGTCTGAA

CGAGGGTGTTGTCCTCGATGAAGTCATCTTCTCCAAACACAAGGGAGACACAAAGATGTCTGAGGAGGAC

AAAGCGCTGTTCCGCCGCTGTGCTGCTGACTACGCGTCACGCCTGCACAGCGTGTTGGGCACAGCAAATG

CCCCATTGAGCATCTACGAGGCAATCAAGGGTGTCGACGGACTCGACGCCATGGAACCAGATACAGCGCC

CGGCCTCCCCTGGGCCCTCCAGGGTAAACGCCGCGGCGCGCTCATCGACTTCGAAAACGGCACGGTCGGA

CCCGAAGTCGAGGCTGCCCTGAAGCTCATGGAGAAGAGAGAATACAAATTTGCTTGTCAGACCTTCCTGA

AGGACGAGATTCGCCCGATGGAGAAAGTACGTGCCGGCAAGACTCGCATTGTCGACGTCTTGCCCGTTGA

GCATATTCTTTACACCAGGATGATGATTGGCAGATTTTGTGCACAGATGCACTCAAACAACGGACCGCAA

ATTGGCTCAGCGGTCGGTTGCAACCCTGATGTTGATTGGCAGAGATTTGGCACACACTTCGCCCAGTACA

GAAACGTGTGGGATGTGGACTATTCGGCCTTTGATGCTAATCACTGCAGTGATGCCATGAACATCATGTT

TGAGGAGGTGTTTCGCACGGAGTTCGGCTTCCACCCGAATGCCGAGTGGATCCTGAAGACTCTTGTGAAC

ACGGAACACGCCTATGAGAACAAACGCATCACTGTTGAAGGCGGAATGCCGTCTGGTTGTTCCGCAACAA

GCATCATCAACACAATTTTGAACAACATCTACGTGCTCTACGCCCTGCGTAGACACTATGAGGGAGTTGA

GCTGGACACATACACCATGATCTCCTACGGAGACGACATCGTGGTGGCAAGTGATTATGACTTGGACTTC

GAGGCTCTCAAGCCCCACTTTAAATCCCTTGGTCAAACCATCACTCCAGCTGACAAAAGCGACAAAGGTT

TTGTTCTTGGTCACTCCATTACCGATGTCACTTTCCTCAAAAGACACTTCCACATGGACTATGGAACTGG

GTTTTACAAACCTGTGATGGCCTCAAAGACCCTTGAGGCTATCCTCTCCTTTGCACGCCGTGGG

>FJ175661.1_O_ISR_2007

GGGTTGATCGTTGACACCAGAGACGTAGAAGAGCGCGTGCACGTCATGCGCAAAACCAAGCTTGCACCCA

CCGTTGCACACGGTGTGTTCAACCCTGAATTCGGCCCCGCCGCTCTGTCCAACAGAGACCCGCGGCTGAA

TGAAGGGGTTGCCCTTGATGAAGTCATCTTCTCCAAGCACAAGGGAGACACCAAGATGTCGGAAGAGGAC

AAAGCGCTGTTCCGTCGCTGCGCTGCCGACTACGCGTCGCGCTTGCACAGTGTTCTGGGTACGGCAAATG

CCCCACTGAGCATTTACGAGGCAATCAAGGGCGTCGACGGACTCGACGCCATGGAACCAGACACCGCGCC

TGGCCTCCCCTGGGCCCTCCAGGGGAAACGCCGTGGCGCGCTCATCGACTTCGAGAACGGCACTGTCGGA

CCCGAGGTTGAAGCTGCCTTGAAGCTCATGGAGAAAAGAGAATACAAGTTTGCATGTCAGACCTTCCTGA

AGGACGAGATTCGCCCGATGGAGAAAGTACGTGCCGGCAAGACTCGCATTGTCGATGTCCTGCCTGTTGA

ACACATTCTTTACACTAGGATGATGATTGGCCGATTCTGTGCTCAAATGCACTCAAACAACGGACCGCAA

ATTGGCTCAGCGGTCGGATGCAATCCTGACGTTGATTGGCAGAGATTTGGCACACATTTTGCTCAGTACA

GAAACGTGTGGGATGTGGATTATTCGGCCTTTGATGCTAACCATTGCAGTGATGCCATGAACATCATGTT

TGAGGAGGTGTTCCGCACGGAATTCGGTTTCCACCCTAACGCTGAGTGGATCCTGAAGACTCTCGTGAAC

ACGGAGCACGCGTACGAAAACAAACGCATCACTATTGAGGGCGGGATGCCGTCTGGTTGTTCCGCAACAA

GCATCATCAACACAATTCTGAACAACATCTACGTGCTCTACGCTTTGCGTAGACACTATGAGGGAGTCGA

GCTGGACACTTACACCATGATCTCCTACGGAGACGACATCGTGGTTGCAAGTGATTACGACCTGGACTTT

GAGGCCCTCAAGCCTCACTTCAAATCTCTTGGTCAAACCATCACCCCAGCCGACAAAAGCGACAAAGGTT

TTGTTCTTGGTCACTCCATTACCGACGTCACTTTCCTCAAAAGACACTTCCACATGGACTATGGAACTGG

GTTTTACAAACCTGTGATGGCCTCGAAGACCCTCGAGGCTATCCTCTCCTTTGCACGCCGTGGG

>FJ175662.1_O_ISR_2007

GGGTTGATCGTTGACACCAGAGACGTAGAAGAGCGCGTGCACGTCATGCGCAAAACCAAGCTTGCACCCA

CCGTTGCACACGGTGTGTTCAACCCTGAATTCGGCCCCGCCGCTCTGTCCAACAGAGACCCGCGGCTGAA

TGAAGGGGTTGCCCTTGATGAAGTCATCTTCTCCAAGCACAAGGGAGACACCAAGATGTCGGAAGAGGAC

AAAGCGCTGTTCCGTCGCTGCGCTGCCGACTACGCGTCGCGCTTGCACAGTGTTCTGGGTACGGCAAATG

CCCCACTGAGCATTTACGAGGCAATCAAGGGCGTCGACGGACTCGACGCCATGGAACCAGACACCGCGCC

TGGCCTCCCCTGGGCCCTCCAGGGGAAACGCCGTGGCGCGCTCATCGACTTCGAGAACGGCACTGTCGGA

CCCGAGGTTGAAGCTGCCTTGGAGCTCATGGAGAAAAGAGAATACAAGTTTGCATGTCAGACCTTCCTGA

AGGACGAGATTCGCCCGATGGAGAAAGTACGTGCCGGCAAGACTCGCATTGTCGATGTCCTGCCTGTTGA

ACACATTCTTTACACTAGGATGATGATTGGCCGATTCTGTGCTCAAATGCACTCAAACAACGGACCGCAA

ATTGGCTCAGCGGTCGGATGCAATCCTGACGTTGATTGGCAGAGATTTGGCACACATTTTGCTCAGTACA

GAAACGTGTGGGATGTGGATTATTCGGCCTTTGATGCTAACCATTGCAGTGATGCCATGAACATCATGTT

TGAGGAGGTGTTCCGCACGGAATTCGGTTTCCACCCTAACGCTGAGTGGATCCTGAAGACTCTCGTGAAC

ACGGAGCACGCGTACGAAAACAAACGCATTACTGTTGAGGGCGGGATGCCGTCTGGTTGTTCCGCAACAA

GCATCATCAACACAATTCTGAACAACATCTACGTGCTCTACGCTTTGCGTAGACACTATGAGGGAGTCGA

GCTGGACACTTACACCATGATCTCCTACGGAGACGACATCGTGGTTGCAAGTGATTACGACCTGGACTTT

GAGGCCCTCAAGCCTCACTTCAAATCTCTTGGTCAAACCATCACCCCAGCCGACAAAAGCGACAAAGGTT

TTGTTCTTGGTCACTCCATTACCGACGTCACTTTCCTCAAAAGACACTTCCACATGGACTATGGAACTGG

GTTTTACAAACCTGTGATGGCCTCGAAGACCCTCGAGGCTATCCTCTCCTTTGCACGCCGTGGG

>FJ175663.1_O_ISR_2007

GGGTTGATCGTTGACACCAGAGACGTAGAAGAGCGCGTGCACGTCATGCGCAAAACCAAGCTTGCACCCA

CCGTTGCACACGGTGTGTTCAACCCTGAATTCGGCCCCGCCGCTCTGTCCAACGGAGACCCGCGGCTGAA

TGAAGGGGTTGCCCTTGATGAAGTCATCTTCTCCAAGCACAAGGGAGACACCAAGATGTCGGAAGAGGAC

AAAGCGCTGTTCCGTCGCTGCGCTGCCGACTACGCGTCGCGCTTGCACAGTGTTCTGGGTACGGCAAATG

CCCCACTGAGCATTTACGAGGCAATCAAGGGCGTTGACGGACTCGACGCCATGGAACCAGACACCGCGCC

TGGCCTCCCCTGGGCCCTCCAGGGGAAACGCCGTGGCGCGCTCATCGACTTCGAGAACGGCACTGTCGGA

ACCGAGGTTGAAGCTGCCTTGAAGCTCATGGAGAAAAGAGAATACAAGTTTGCATGTCAGACCTTCCTGA

AGGACGAGATTCGCCCGATGGAGAAAGTACGTGCCGGCAAGACTCGCATTGTCGATGTCCTGCCTGTTGA

ACACATTCTTTACACTAGGATGATGATTGGCCGATTCTGTGCTCAAATGCACTTAAACAACGGACCGCAA

ATTGGCTCAGCGGTCGGATGCAATCCTGACGTTGACTGGCAGAGATTTGGCACACATTTTGCTCAGTACA

GAAACGTGTGGGATGTGGATTATTCGGCCTTTGATGCTAACCATTGCAGTGATGCCATGAACATCATGTT

TGAGGAGGTGTTCCGCACGGAATTCGGTTTCCACCCTAACGCTGAGTGGGTCCTGAAGACTCTCGTGAAC

ACGGAGCACGCGTACGAAAACAAACGCATCACTGTTGAGGGCGGGATGCCGTCTGGTTGTTCCGCAACAA

GCATCATCAACACAATTCTGAACAACATCTACGTGCTCTACGCCTTGCGTAGACACTATGAGGGAGTCGA

GCTGGACACTTACACCATGATCTCCTACGGAGACGACATCGTGGTTGCAAGTGATTACGACCTGGACTTT

GAGGCCCTCAAGCCTCACTTCAAATCTCTTGGTCAAACCATCACCCCAGCCGACAAAAGCGACAAAGGTT

TTGTTCTTGGTCACTCCATTACCGACGTCACTTTCCTCAAAAGACACTTCCACATGGACTATGGAACTGG

GTTTTACAAACCTGTGATGGCCTCGAAGACCCTCGAGGCTATCCTCTCCTTTGCACGCCGTGGG

>FJ175664.1_O_ISR_2007

GGGTTGATCGTTGACACCAGAGACGTAGAAGAGCGCGTGCACGTCATGCGCAAAACCAAGCTTGCACCCA

CCGTTGCACACGGTGTGTTCAACCCTGAATTCGGCCCCGCCGCTCTGTCCAACGGAGACCCGCGGCTGAA

TGAAGGGGTTGCCCTTGATGAAGTCATCTTCTCCAAGCACAAGGGAGACACCAAGATGTCGGAAGAGGAC

AAAGCGCTGTTCCGTCGCTGCGCTGCCGACTACGCGTCGCGCTTGCACAGTGTTCTGGGTACGGCAAATG

CCCCACTGAGCATTTACGAGGCAATCAAGGGCGTTGACGGACTCGACGCCATGGAACCAGACACCGCGCC

TGGCCTCCCCTGGGCCCTCCAGGGGAAACGCCGTGGCGCGCTCATCGACTTCGAGAACGGCACTGTCGGA

ACCGAGGTTGAAGCTGCCTTGAAGCTCATGGAGAAAAGAGAATACAAGTTTGCATGTCAGACCTTCCTGA

AGGACGAGATTCGCCCGATGGAGAAAGTACGTGCCGGCAAGACTCGCATTGTCGATGTCCTGCCTGTTGA

ACACATTCTTTACACTAGGATGATGATTGGCCGATTCTGTGCTCAAATGCACTTAAACAACGGACCGCAA

ATTGGCTCAGCGGTCGGATGCAATCCTGACGTTGACTGGCAGAGATTTGGCACACATTTTGCTCAGTACA

GAAACGTGTGGGATGTGGATTATTCGGCCTTTGATGCTAACCATTGCAGTGATGCCATGAACATCATGTT

TGAGGAGGTGTTCCGCACGGAATTCGGTTTCCACCCTAACGCTGAGTGGGTCCTGAAGACTCTCGTGAAC

ACGGAGCACGCGTACGAAAACAAACGCATCACTGTTGAGGGCGGGATGCCGTCTGGTTGTTCCGCAACAA

GCATCATCAACACAATTCTGAACAACATCTACGTGCTCTACGCCTTGCGTAGACACTATGAGGGAGTCGA

GCTGGACACTTACACCATGATCTCCTACGGAGACGACATCGTGGTTGCAAGTGATTACGACCTGGACTTT

GAGGCCCTCAAGCCTCACTTCAAATCTCTTGGTCAAACCATCACCCCAGCCGACAAAAGCGACAAAGGTT

TTGTTCTTGGTCACTCCATTACCGACGTCACTTTCCTCAAAAGACACTTCCACATGGACTATGGAACTGG

GTTTTACAAACCTGTGATGGCCTCGAAGACCCTCGAGGCTATCCTCTCCTTTGCACGCCGTGGG

>FJ175665.1_O_ISR_2007

GGGTTGATCGTTGACACCAGAGACGTAGAAGAGCGCGTGCACGTCATGCGCAAAACCAAGCTTGCACCCA

CCGTTGCACACGGTGTGTTCAACCCTGAATTCGGCCCCGCCGCTCTGTCCAACAGAGACCCGCGGCTGAA

TGAAGGGGTTGCCCTTGATGAAGTCATCTTCTCCAAGCACAAGGGAGACACCAAGATGTCGGAAGAGGAC

AAAGCGCTGTTCCGTCGCTGCGCTGCCGACTACGCGTCGCGCTTGCACAGTGTTCTGGGTACGGCAAATG

CCCCACTGAGCATTTACGAGGCAATCAAGGGCGTTGACGGACTCGACGCCATGGAACCAGACACCGCGCC

TGGCCTCCCCTGGGCCCTCCAGGGGAAACGCCGTGGCGCGCTCATCGACTTCGAGAACGGCACTGTCGGA

CCCGAGGTTGAAGCTGCCTTGAAGCTCATGGAGAAAAGAGAATACAAGTTTGCATGTCAGACCTTCCTGA

AGGACGAGATTCGCCCGATGGAGAAAGTACGTGCCGGCAAGACTCGCATTGTCGATGTCCTGCCTGTTGA

ACACATTCTTTACACTAGGATGATGATTGGCCGATTCTGTGCTCAAATGCACTCAAACAACGGACCGCAA

ATTGGCTCAGCGGTCGGATGCAATCCTGACGTTGATTGGCAGAGATTTGGCACACATTTTGCTCAGTACA

GAAACGTGTGGGATGTGGATTATTCGGCCTTTGATGCTAACCATTGCAGTGACGCCATGAACATCATGTT

TGAGGAGGTGTTCCGCACGGAATTCGGTTTCCACCCTAACGCTGAGTGGATCCTGAAGACTCTCGTGAAC

ACGGAGCACGCGTACGAAAACAAACGCATCACTGTTGAGGGCGGGATGCCGTCTGGTTGTTCCGCAACAA

GCATCATCAACACAATTTTGAACAACATCTACGTGCTCTACGCTTTGCGTAGACACTATGAGGGAGTCGA

GCTGGACACTTACACCATGATCTCCTACGGAGACGACATCGTGGTTGCAAGTGATTACGACCTGGACTTT

GAGGCCCTCAAGCCTCACTTCAAATCTCTTGGCCAAACCATCACCCCAGCCGACAAAAGCGACAAAGGTT

TTGTTCTTGGTCACTCCATTACCGACGTCACTTTCCTCAAAAGACACTTCCACATGGATTATGGAACTGG

GTTTTACAAACCTGTGATGGCCTCGAAGACCCTCGAGGCTATCCTCTCCTTTGCACGCCGTGGG

>FJ175666.1_O_ISR_2007

GGGTTGATCGTTGACACCAGAGACGTAGAAGAGCGCGTGCACGTCATGCGCAAAACCAAGCTTGCACCCA

CCGTTGCACACGGTGTGTTCAACCCTGAATTCGGCCCCGCCGCTCTGTCCAACAGAGACCCGCGGCTGAA

TGAAGGGGTTGCCCTTGATGAAGTCATCTTCTCCAAGCACAAGGGAGACACCAAGATGTCGGAAGAGGAC

AAAGCGCTGTTCCGTCGCTGCGCTGCCGACTACGCGTCGCGCTTGCACAGTGTTCTGGGTACGGCAAATG

CCCCACTGAGCATTTACGAGGCAATCAAGGGCGTCGACGGACTCGACGCCATGGAACCAGACACCGCGCC

TGGCCTCCCCTGGGCCCTCCAGGGGAAACGCCGTGGCGCGCTCATCGACTTCGAGAACGGCACTGTCGGA

CCCGAGGTTGAAGCTGCCTTGAAGCTCATGGAGAAAAGAGAATACAAGTTTGCATGTCAGACCTTCCTGA

AGGACGAGATTCGCCCGATGGAGAAAGTACGTGCCGGCAAGACTCGCATTGTCGATGTCCTGCCTGTTGA

ACACATTCTTTACACTAGGATGATGATTGGCCGATTCTGTGCTCAAATGCACTCAAACAACGGACCGCAA

ATTGGCTCAGCGGTCGGATGCAATCCTGACGTTGATTGGCAGAGATTTGGCACACATTTTGCTCAGTACA

GAAACGTGTGGGATGTGGATTATTCGGCCTTTGATGCTAACCATTGCAGTGATGCCATGAACATCATGTT

TGAGGAGGTGTTCCGCACGGAATTCGGTTTCCACCCTAACGCTGAGTGGATCCTGAAGACTCTCGTGAAC

ACGGAGCACGCGTACGAAAACAAACGCATCACTATTGAGGGCGGGATGCCGTCTGGTTGTTCCGCAACAA

GCATCATCAACACAATTCTGAACAACATCTACGTGCTCTACGCTTTGCGTAGACACTATGAGGGAGTCGA

GCTGGACACTTACACCATGATCTCCTACGGAGACGACATCGTGGTTGCAAGTGATTACGACCTGGACTTT

GAGGCCCTCAAGCCTCACTTCAAATCTCTTGGTCAAACCATCACCCCAGCCGACAAAAGCGACAAAGGTT

TTGTTCTTGGTCACTCCATTACCGACGTCACTTTCCTCAAAAGACACTTCCACATGGACTATGGAACTGG

GTTTTACAAACCTGTGATGGCCTCGAAGACCCTCGAGGCTATCCTCTCCTTTGCACGCCGTGGG

>FJ461344.1_O_UGA_2002

GGGTTGATTGTTGACACCAGAGATGTGGAAGAGCGCGTACACGTTATGCGCAAAACAAAGCTTGCACCCA

CAGTGGCGCACGGTGTGTTCAACCCTGAGTTTGGCCCCGCTGCCTTGTCCAACAAGGACCCGCGGCTGAG

CGAAGGTGTTGTCCTCGATGAAGTCATCTTCTCCAAGCACAAGGGAGACACGAAGATGTCCGAGGAGGAC

AAAGCGCTGTTCCGTCGCTGTGCTGCTGACTACGCGTCGCGCCTACACAGCGTACTGGGTACAGCGAATG

CCCCACTGAGCATCTACGAAGCAATCAAAGGCGTTGACGGTCTCGACGCCATGGAACCAGACACTGCTCC

TGGCCTCCCCTGGGCCCTCCAGGGGAAACGTCGTGGAGTTCTGATTGACTTCGAGAACGGCACAGTCGGC

CCTGAGGTCGCTGCCGCTCTAGATCTCATGGAGAAGAGAGAGTACAAATTTGCTTGTCAGACCTTCCTGA

AGGACGAGATTCGCCCGATGGAAAAAGTGCGTGCCGGCAAGACTCGCATTGTCGACGTCCTGCCTGTTGA

ACACATACTTTACACTAGAATGATGATTGGTAGATTTTGTGCCCAAATGCACACAAACAACGGACCGCAA

ATCGGCTCAGCGGTCGGTTGCAACCCAGATGTTGATTGGCAGAGATTTGGCACACACTTTGCCCAGTACA

GGAACGTGTGGGACGTGGACTATTCGGCCTTTGATGCTAACCACTGCAGTGACGCGATGAACATCATGTT

TGAGGAGGTGTTTCGCACAGAGTTTGGCTTTCACCCGAACGCCGAGTGGATCCTGAAAACTCTTGTGAAC

ACGGAACACGCCTATGAGAACAAGCGCATTACTGTTGAAGGCGGGATGCCGTCTGGCTGTTCCGCAACAA

GCATCATCAACACGATTTTAAACAACATCTACGTGCTCTACGCTCTGCGCAGACACTATGAGGGAGTTGA

GCTGGACACCTACACCATGATCTCCTATGGAGACGACATCGTGGTGGCAAGTGATTACGATCTGGACTTT

GAGGCTCTCAAGCCTCATTTCAAATCTCTTGGTCAAACCATCACCCCAGCTGACAAAAGCGACAAAGGTT

TTGTTCTTGGTCACTCCATCACCGATGTCACTTTCCTCAAAAGACACTTCCACATGGACTATGGAACTGG

GTTTTACAAACCTGTGATGGCCTCGAAGACCCTCGAGGCTATCCTCTCCTTTGCACGCCGTGGG

>FJ461345.1_O_UGA_2002

GGGTTGATTGTTGACACCAGAGATGTGGAAGAGCGCGTACACGTTATGCGCAAAACAAAGCTTGCACCCA

CCGTGGCACACGGTGTGTTCAACCCTGAGTTTGGCCCCGCTGCCTTGTCCAACAAGGACCCGCGGCTGAA

TGAAGGTGTTGTCCTCGATGAAGTCATCTTCTCCAAGCACAAGGGAGACACGAAGATGTCCGAGGAAGAC

AAAGCGCTGTTCCGTCGCTGTGCTGCTGACTACGCGTCGCGCTTACACAGCGAACTGGGTACAGCAAATG

CCCCACTGAGTATTTATGAAGCAATCAAAGGCGTTGACGGTCTCGACGCCATGGAACCAGACACTGCTCC

TGGTCTCCCCTGGGCCCTCCAGGGGAAACGCCGTGGAGCTCTGATTGACTTCGAGAACGGCACAGTTGGC

CCTGAGGTCGCCGCCGCTCTAAAGCTCATGGAGAAGAGAGAGTACAAATTTGCTTGTCAGACCTTCCTGA

AGGACGAGATTCGCCCGATGGAAAAAGTGCGTGCCGGCAAGACTCGCATTGTCGACGTCCTGCCTGTTGA

ACACATACTTTACACCAGAATGATGATTGGCAGATTTTGTGCCCAAATGCACGCAAATAACGGACCGCGA

ATTGGCTCAGCGGTCGGTTGCAACCCAGATGTTGATTGGCAAAGATTTGGCACACACTTCGCCCAGTACA

GGAATGTGTGGGATGTGGACTATTCGGCCTTTGATGCTAACCACTGCAGTGATGCGATGAACATCATGTT

TGAGGAGGTGTTTCGCACAGAATTTGGCTTCCACCCGAACGCCGAATGGATTCTGAAAACTCTTGTGAAC

ACGGAACACGCCTATGAGAACAAGCGCATTACTGTTGAAGGCGGGATGCCGTCTGGCTGTTCTGCAACAA

GCATCATCAACACAATTTTGAACAACATCTACGTGCTCTACGCTCTGCGCAGACACTATGAGGGAGTTGA

GCTGGACACCTACACCATGATCTCCTACGGAGACGACATCGTGGTGGCAAGTGATTATGATTTGGACTTC

GAGGCTCTCAAGCCTCATTTTAAATCTCTTGGTCAAACCATCACCCCAGCTGACAAAAGCGACAAAGGTT

TTGTTCTTGGTCACTCCATTACCGATGTCACTTTCCTCAAAAGACACTTCCACATGGACTATGGAACTGG

GTTTTACAAACCTGTGATGGCCTCGAAGACCCTCGAGGCTATCCTCTCCTTTGCACGCCGTGGG

>FJ461346.1_SAT2_UGA_2002

GGGTTGATCGTGGACACCAGAGATGTGGAAGAGCGTGTGCACGTCATGCGCAAAACCAAGCTTGCACCC-

C-GTCGCACACGGTGTGTTCAACCCTGATTTCGGGCCAGCTGCTTTGTCCAACAACGACAAACGCCTGAA

TGAAGGGGTCATCCTCGACGACGTCATCTTCTCCAAACACAAGGGAGACACGAAGATGTCTGAGGAGGA-

AAGAAGCTGTTCAGAATGTGTGCTGCTGACTACGCGTCACAC-TGCACTCAGTTCTTGGCACAGCAAATG

CCCCA-TGAGCATCTA-GAGGCCATCAAGGGCGTCGACGGACTCGACGCCATGGAGCCTGACACCGCTCC

TGGCTTGCCCTGGGCCACCCAGGGGAAACGCCGTGGAGCCCTCATTGA-TTCGAGAACGGCACTGTCGGA

CCCGAGATTGAAGAGGCACTGAAGCTCATGGAGAAAAGGGAGTACAAGTTCACATGCCAAACCTTCCTGA

AGGACGA-ATTCGCCCGATGGAAAAAGTTCGCGCCGGCAAGACTCGCATCGTCGATGTCCTGCCTGTTGA

GCACATCCTCTACACTAGAATGAT-ATTGGCAAGTTCTGTGCACAAATGCACTCCAACAACGGACCGCAA

ATTGGCTCAGCGGTCGGTTGCAACCCAGATGTTGATTGGCAAAGATTCGGAACCCATTTCGCCCAGTACA

AAAACGTGTGGGACGTGGATTATTCGGCCTTTGATGCTAACCATTGCAGTGACGCCATGAACATCATGTT

CGAGGAGGTTTTC-GCCCAGAATTCGGGTTCCACCCCAACGCGGAGTGGAT-CTGAAAACTCTGGTCAAC

ACGGAACACGCCTACGAGAACAAGCGCATCACTGTCGAAGGTGGAATGCCCTCGGGTTGCTCCGCCACTA

GCATCATCAACACAATTCTCAACAACATCTACGTGCTCTACGCTTTGCGTAGACACTATGAGGGAGTCGA

GCTGTCGAGCTACTCGATGATTTTCTACGGGGATGACATCGTCGTGGCAAGTGATTACGACTTGGATTTT

GAAGCTCTCAAGCCTCACTTCAAGTCCGTTGGTCAGACCATCACTCCAGCCGACAAAAGTGACAAAGGTT

TTGTTCTTGGTCAGTCCATTACCGATGTTACTTTCCTCAAGAGGCACTTCCACTTGGACTTTGGAACTGG

GTTTTACAAACCTGTGATGGCTTCGAAGACCCTCGAAGCTATCCTCTCCTTTGCACGCCGTGGG

>FJ542365.1_O_UKG_2001

GGATTGATAGTTGACACCAGAGATGTTGAGGAGCGCGTACATGTCATGCGCAAAACCAAGCTCGCACCCA

CCGTGGCACACGGTGTGTTTAACCCCGAATTTGGGCCTGCCGCCTTGTCCAACAAGGACCCGCGCCTGAA

TGAGGGGGTTGTCCTCGATGAAGTCATCTTCTCCAAACACAAAGGAAACACAAAGATGTCTGAGGAGGAC

AAAGCGCTGTTCCGCCGCTGTGCTGCTGACTACGCGTCGCGTCTGCATAGCGTGCTGGGTACGGCAAATG

CCCCACTGAGCACTTACGAGGCAATCAAGGGCGTCGACGGACTTGACGCCATGGAACCAGACACCGCGCC

TGGTCTCCCCTGGGCTCTCCAGGGGAAACGCCGTGGTGCGCTCATCGACTTCGAGAACGGCACTGTCGGA

CCCGAGGTTGAAGCTGCCTTGAAGCTCATGGAGAAAAGAGAGTACAAGTTTACATGCCAGACCTTCCTGA

AGGACGAGATTCGCCCGATGGAGAAGGTACGTGCCGGCAAGACTCGCATTGTCGACGTCCTGCCCGTTGA

ACACATTCTTTACACTAGGATGATGATTGGCAGATTTTGTGCTCAAATGCACTCAAACAACGGACCGCAA

ATTGGCTCGGCGGTTGGTTGTAATCCTGATGTTGATTGGCAAAGATTTGGCACGCATTTTGCTCAGTATA

GAAACGTGTGGGATGTGGACTATTCGGCCTTTGATGCCAACCACTGCAGTGACGCAATGAACATCATGTT

TGAGGAGGTGTTTAACACGGACTTCGGTTTCCACCCAAACGCTGAGTGGATCCTGAAAACTCTCGTGAAC

ACTGAACACGCCTATGAGAACAAACGCATCACTGTTGAAGGCGGGATGCCGTCTGGTTGTTCCGCAACAA

GCATCATCAACACAATTTTGAACAACATCTACGTGCTCTACGCCTTGCGTAGACACTATGAGGGGGTTGA

GCTGGACTCTTACACCATGATCTCCTACGGAGACGACATCGTGGTTGCAAGTGATTACGATCTGGACTTT

GAGGCCCTCAAGCCTCACTTCAAATCCCTTGGTCAAACCATTACCCCAGCTGACAAAAGCGACAAAGGTT

TTGTTCTTGGTCACTCCATTACCGATGTCACTTTCCTCAAAAGACACTTCCACATGGACTATGGAACTGG

GTTTTACAAACCTGTGATGGCTTCGAAGACCCTCGAGGCTATCCTCTCCTTTGCACGCCGTGGG

>FJ542368.1_O_UKG_2001

GGATTGATAGTTGACACCAGAGATGTTGAGGAGCGCGTACATGTCATGCGCAAAACCAAGCTCGCACCCA

CCGTGGCACACGGTGTGTTTAACCCCGAATTTGGGCCTGCCGCCTTGTCCAACAAGGACCCGCGCCTGAA

TGAGGGGGTTGTCCTCGATGAAGTCATCTTCTCCAAACACAAAGGAAACACAAAGATGTCTGAGGAGGAC

AAAGCGCTGTTCCGCCGCTGTGCTGCTGACTACGCGTCGCGTCTGCATAGCGTGCTGGGTACGGCAAATG

CCCCACTGAGCACTTACGAGGCAATCAAGGGCGTCGACGGACTTGACGCCATGGAACCAGACACCGCGCC

TGGTCTCCCCTGGGCTCTCCAGGGGAAACGCCGTGGTGCGCTCATCGACTTCGAGAACGGCACTGTCGGA

CCCGAGGTTGAAGCTGCCTTGAAGCTCATGGAGAAAAGAGAGTACAAGTTTACATGCCAGACCTTCCTGA

AGGACGAGATTCGCTCGATGGAGAAGGTACGTGCCGGCAAGACTCGCATTGTCGACGTCCTGCCCGTTGA

ACACATTCTTTACACTAGGATGATGATTGGCAGATTTTGTGCTCAAATGCACTCAAACAACGGACCGCAA

ATTGGCTCGGCGGTTGGTTGTAATCCTGATGTTGATTGGCAAAGATTTGGCACGCATTTTGCTCAGTATA

GAAACGTGTGGGATGTGGACTATTCGGCCTTTGATGCCAACCACTGCAGTGACGCAATGAACATCATGTT

TGAGGAGGTGTTTAACACGGACTTCGGTTTCCACCCAAACGCTGAGTGGATCCTGAAAACTCTCGTGAAC

ACTGAACACGCCTATGAGAACAAACGCATCACTGTTGAAGGCGGGATGCCGTCTGGTTGTTCCGCAACAA

GCATCATCAACACAATTTTGAACAACATCTACGTGCTCTACGCCTTGCGTAGACACTATGAGGGGGTTGA

GCTGGACTCTTACACCATGATCTCCTACGGAGACGACATCGTGGTTGCAAGTGATTACGATCTGGACTTT

GAGGCCCTCAAGCCTCACTTCAAATCCCTTGGTCAAACCATTACCCCAGCTGACAAAAGCGACAAAGGTT

TTGTTCTTGGTCACTCCATTACCGATGTCACTTTCCTCAAAAGACACTTCCACATGGACTATGGAACTGG

GTTTTACAAACCTGTGATGGCTTCGAAGACCCTCGAGGCTATCCTCTCCTTTGCACGCCGTGGG

>FJ542369.1_O_UKG_2001

GGATTGATAGTTGACACCAGAGATGTTGAGGAGCGCGTACATGTCATGCGCAAAACCAAGCTCGCACCCA

CCGTGGCACACGGTGTGTTTAACCCCGAATTTGGGCCTGCCGCCTTGTCCAACAAGGACCCGCGCCTGAA

TGAGGGGGTTGTCCTCGATGAAGTCATCTTCTCCAAACACAAAGGAAACACAAAGATGTCTGAGGAGGAC

AAAGCGCTGTTCCGCCGCTGTGCTGCTGACTACGCGTCGCGTCTGCATAGCGTGCTGGGTACGGCAAATG

CCCCACTGAGCACTTACGAGGCAATCAAGGGCGTCGACGGACTTGACGCCATGGAACCAGACACCGCGCC

TGGTCTCCCCTGGGCTCTCCAGGGGAAACGCCGTGGTGCGCTCATCGACTTCGAGAACGGCACTGTCGGA

CCCGAGGTTGAAGCTGCCTTGAAGCTCATGGAGAAAAGAGAGTACAAGTTTACATGCCAGACCTTCCTGA

AGGACGAGATTCGCCCGATGGAGAAGGTACGTGCCGGCAAGACTCGCATTGTCGACGTCCTGCCCGTTGA

ACACATTCTTTACACTAGGATGATGATTGGCAGATTTTGTGCTCAAATGCACTCAAACAACGGACCGCAA

ATTGGCTCGGCGGTTGGTTGTAATCCTGATGTTGATTGGCAAAGATTTGGCACGCATTTTGCTCAGTATA

GAAACGTGTGGGATGTGGACTATTCGGCCTTTGATGCCAACCACTGCAGTGACGCAATGAACATCATGTT

TGAGGAGGTGTTTAACACGGACTTCGGTTTCCACCCAAACGCTGAGTGGATCCTGAAAACTCTCGTGAAC

ACTGAACACGCCTATGAGAACAAACGCATCACTGTTGAAGGCGGGATGCCGTCTGGTTGTTCCGCAACAA

GCATCATCAACACAATTTTGAACAACATCTACGTGCTCTACGCCTTGCGTAGACACTATGAGGGGGTTGA

GCTGGACTCTTACACCATGATCTCCTACGGAGACGACATCGTGGTTGCAAGTGATTACGATCTGGACTTT

GAGGCCCTCAAGCCTCACTTCAAATCCCTTGGTCAAACCATTACCCCAGCTGACAAAAGCGACAAAGGTT

TTGTTCTTGGTCACTCCATTACCGATGTCACTTTCCTCAAAAGACACTTCCACATGGACTATGGAACTGG

GTTTTACAAACCTGTGATGGCTTCGAAGACCCTCGAGGCTATCCTCTCCTTTGCACGCCGTGGG

>FJ542370.1_O_UKG_2001

GGATTGATAGTTGACACCAGAGATGTTGAGGAGCGCGTACATGTCATGCGCAAAACCAAGCTCGCACCCA

CCGTGGCACACGGTGTGTTTAACCCCGAATTTGGGCCTGCCGCCTTGTCCAACAAGGACCCGCGCCTGAA

TGAGGGGGTTGTCCTCGATGAAGTCATCTTCTCCAAACACAAAGGAAACACAAAGATGTCTGAGGAGGAC

AAAGCGCTGTTCCGCCGCTGTGCTGCTGACTACGCGTCGCGTCTGCATAGCGTGCTGGGTACGGCAAATG

CCCCACTGAGCACTTACGAGGCAATCAAGGGCGTCGACGGACTTGACGCCATGGAACCAGACACCGCGCC

TGGTCTCCCCTGGGCTCTCCAGGGGAAACGCCGTGGTGCGCTCATCGACTTCGAGAACGGCACTGTCGGA

CCCGAGGTTGAAGCTGCCTTGAAGCTCATGGAGAAAAGAGAGTACAAGTTTACATGCCAGACCTTCCTGA

AGGACGAGATTCGCCCGATGGAGAAGGTACGTGCCGGCAAGACTCGCATTGTCGACGTCCTGCCCGTTGA

ACACATTCTTTACACTAGGATGATGATTGGCAGATTTTGTGCTCAAATGCACTCAAACAACGGACCGCAA

ATTGGCTCGGCGGTTGGTTGTAATCCTGATGTTGATTGGCAAAGATTTGGCACGCATTTTGCTCAGTATA

GAAACGTGTGGGATGTGGACTATTCGGCCTTTGATGCCAACCACTGCAGTGACGCAATGAACATCATGTT

TGAGGAGGTGTTTAACACGGACTTCGGTTTCCACCCAAACGCTGAGTGGATCCTGAAAACTCTCGTGAAC

ACTGAACACGCCTATGAGAACAAACGCATCACTGTTGAAGGCGGGATGCCGTCTGGTTGTTCCGCAACAA

GCATCATCAACACAATTTTGAACAACATCTACGTGCTCTACGCCTTGCGTAGACACTATGAGGGGGTTGA

GCTGGACTCTTACACCATGATCTCCTACGGAGACGACATCGTGGTTGCAAGTGATTACGATCTGGACTTT

GAGGCCCTCAAGCCTCACTTCAAATCCCTTGGTCAAACCATTACCCCAGCTGACAAAAGCGACAAAGGTT

TTGTTCTTGGTCACTCCATTACCGATGTCACTTTCCTCAAAAGACACTTCCACATGGACTATGGAACTGG

GTTTTACAAACCTGTGATGGCTTCGAAGACCCTCGAGGCTATCCTCTCCTTTGCACGCCGTGGG

>FJ542371.1_O_UKG_2001

GGATTGATAGTTGACACCAGAGATGTTGAGGAGCGCGTACATGTCATGCGCAAAACCAAGCTCGCACCCA

CCGTGGCACACGGTGTGTTTAACCCCGAATTTGGGCCTGCCGCCTTGTCCAACAAGGACCCGCGCCTGAA

TGAGGGGGTTGTCCTCGATGAAGTCATCTTCTCCAAACACAAAGGAAACACAAAGATGTCTGAGGAGGAC

AAAGCGCTGTTCCGCCGCTGTGCTGCTGACTACGCGTCGCGTCTGCATAGCGTGCTGGGTACGGCAAATG

CCCCACTGAGCACTTACGAGGCAATCAAGGGCGTCGACGGACTTGACGCCATGGAACCAGACACCGCGCC

TGGTCTCCCCTGGGCTCTTCAGGGGAAACGCCGTGGTGCGCTCATCGACTTCGAGAACGGCACTGTCGGA

CCCGAGGTTGAAGCTGCCTTGAAGCTCATGGAGAAAAGAGAGTACAAGTTTACATGCCAGACCTTCCTGA

AGGACGAGATTCGCCCGATGGAGAAGGTACGTGTCGGCAAGACTCGCATTGTCGACGTCCTGCCCGTTGA

ACACATTCTTTACACTAGGATGATGATTGGCAGATTTTGTGCTCAAATGCACTCAAACAACGGACCGCAA

ATTGGCTCGGCGGTTGGTTGTAATCCTGATGTTGATTGGCAAAGATTTGGCACGCATTTTGCTCAGTATA

GAAACGTGTGGGATGTGGACTATTCGGCCTTTGATGCCAACCACTGCAGTGACGCAATGAACATCATGTT

TGAGGAGGTGTTTAACACGGACTTCGGTTTCCACCCAAACGCTGAGTGGATCCTGAAAACTCTCGTGAAT

ACTGAACACGCCTATGAGAACAAACGCATCACTGTTGAAGGCGGGATGCCGTCTGGTTGTTCCGCAACAA

GCATCATCAACACAATTTTGAACAACATCTACGTGCTCTACGCCTTGCGTAGACACTATGAGGGGGTTGA

GCTGGACTCTTACACCATGATCTCCTACGGAGACGACATCGTGGTTGCAAGTGATTACGATCTGGACTTT

GAGGCCCTCAAGCCTCACTTCAAATCCCTTGGTCAAACCATTACCCCAGCTGACAAAAGCGACAAAGGTT

TTGTTCTTGGTCACTCCATTACCGATGTCACTTTCCTCAAAAGACACTTCCACATGGACTATGGAACTGG

GTTTTACAAACCTGTGATGGCTTCGAAGACCCTCGAGGCTATCCTCTCCTTTGCACGCCGTGGG

>FJ542372.1_O_UKG_2001

GGATTGATAGTTGACACCAGAGATGTTGAGGAGCGCGTACATGTCATGCGCAAAACCAAGCTCGCACCCA

CCGTGGCACACGGTGTGTTTAACCCCGAATTTGGGCCTGCCGCCTTGTCCAACAAGGACCCGCGCCTGAA

TGAGGGGGTTGTCCTCGATGAAGTCATCTTCTCCAAACACAAAGGAAACACAAAGATGTCTGAGGAGGAC

AAAGCGCTGTTCCGCCGCTGTGCTGCTGACTACGCGTCGCGTCTGCATAGCGTGCTGGGTACGGCAAATG

CCCCACTGAGCACTTACGAGGCAATCAAGGGCGTCGACGGACTTGACGCCATGGAACCAGACACCGCGCC

TGGTCTCCCCTGGGCTCTCCAGGGGAAACGCCGTGGTGCGCTCATCGACTTCGAGAACGGCACTGTCGGA

CCCGAGGTTGAAGCTGCCTTGAAGCTCATGGAGAAAAGAGAGTACAAGTTTACATGCCAGACCTTCCTGA

AGGACGAGATTCGCCCGATGGAGAAGGTACGTGCCGGCAAGACTCGCATTGTCGACGTCCTGCCCGTTGA

ACACATTCTTTACACTAGGATGATGATTGGCAGATTTTGTGCTCAAATGCACTCAAACAACGGACCGCAA

ATTGGCTCGGCGGTTGGTTGTAATCCTGATGTTGATTGGCAAAGATTTGGTACGCATTTTGCTCAGTATA

GAAACGTGTGGGATGTGGACTATTCGGCCTTTGATGCCAACCACTGCAGTGACGCAATGAACATCATGTT

TGAGGAGGTGTTTAACACGGACTTCGGTTTCCACCCAAACGCTGAGTGGATCCTGAAAACTCTCGTGAAC

ACTGAACACGCCTATGAGAACAAACGCATCACTGTTGAAGGCGGGATGCCGTCTGGTTGTTCCGCAACAA

GCATCATCAACACAATTTTGAACAACATCTACGTGCTCTACGCCTTGCGTAGACACTATGAGGGGGTTGA

GCTGGACTCTTACACCATGATCTCCTACGGAGACGACATCGTGGTTGCAAGTGATTACGATCTGGACTTT

GAGGCCCTCAAGCCTCACTTCAAATCCCTTGGTCAAACCATTACCCCAGCTGACAAAAGCGACAAAGGTT

TTGTTCTTGGTCACTCCATTACCGATGTCACTTTCCTCAAAAGACACTTCCACATGGACTATGGAACTGG

GTTTTACAAACCTGTGATGGCTTCGAAGACCCTCGAGGCTATCCTCTCCTTTGCACGCCGTGGG

>FJ623456.1_A_KAZ_1999

GGGTTGATCGTTGACACTAGAGATGTTGAAGAGCGTGTGCATGTCATGCGCAAAACCAAGCTTGCACCCA

CCGTGGCTCACGGTGTGTTTAATCCTGAATTTGGTCCCGCCGCTTTGTCCAACAAGGACCCGCGGCTGAA

TGAAGGTGTTGTCCTCGATGAAGTCATTTTCTCCAAGCACAAAGGAGACACGAAAATGACCGAGGAGGAC

AAAGCGCTGTTCCGCCGCTGTGCTGCCGACTACGCGTCGCGCTTGCACAACGTGTTGGGTACGGCAAATG

CCCCACTGAGCATCTACGAGGCAATAAAAGGCGTTGACGGCCTTGACGCCATGGAACCAGACACTGCGCC

TGGCCTTCCCTGGGCCCTCCAGGGTAAGCGCCGCGGCGCGTTGATTGACTTCGAGAACGGCACGGTCGGG

CCCGAAGTCGCGTCTGCCTTAGAGCTCATGGAGAAAAGACAATACAAATTTACCTGTCAGACCTTCCTGA

AGGACGAAGTTCGCCCGATGGAGAAAGTACGTGCCGGCAAGACTCGCATCGTCGACGTTTTGCCTGTTGA

ACATATTCTTTACACCAGGATGATGATTGGCAGATTCTGTGCTCAGATGCACTCAAACAATGGACCGCAA

ATTGGCTCAGCGGTTGGTTGTAATCCAGATGTTGATTGGCAGAGATTTGGCACCCATTTTGCTCAGTACA

AAAACGTGTGGGATGTGGACTATTCGGCCTTTGATGCTAACCATTGCAGTGACGCAATGAACATCATGTT

TGAGGAAGTGTTCCGCACGGAATTTGGTTTCCACCCAAATGCTGAGTGGATCCTGAAAACTCTCGTGAAC

ACGGAACACGCCTATGAGAACAAGCGCATCACTGTTGAGGGCGGGATGCCGTCTGGTTGTTCCGCAACAA

GCATCATCAACACAATTTTGAACAACATCTACGTGCTCTACGCCTTGCGTAGACACTATGAGGGAGTTGA

GCTGGACACCTACACCATGATCTCCTACGGAGACGACATAGTGGTGGCAAGTGATTACGACTTGGATTTT

GAGGCTCTTAAGCCGCACTTTAAATCTCTTGGTCAAACCATCACCCCAGCTGACAAAAGCGACAAAGGTT

TTGTTCTTGGTCAATCCATTACTGATGTCACTTTCCTCAAAAGACACTTCCGCATGGACTATGGAACTGG

GTTTTACAAACCTGTGATGGCTTCGAAGACCCTCGAGGCCATCCTCTCCTTTGCACGCCGTGGG

>FJ824812.1_C_SPA_2009

GGGTTGATCGTTGATACCAGAGATGTGGAAGAGCGCGTCCATGTAATGCGCAAAACCAAGCTTGCACCCA

CCGTCGCGCACGGTGTGTTCAATCCTGAGTTCGGGCCTGCCGCCTTGTCTAACAAGGACCCACGTCTGAA

CGAAGGTGTTGTCCTCGATGAAGTCATTTTCTCCAAGCATAAAGGAGACACAAAGATGTCTGCGGAGGAC

AAAGCGCTGTTCCGCCGCTGCGCTGCTGACTACGCGTCACGCCTGCACAGTGTGCTGGGTACGGCAAATG

CCCCACTGAGCATTTACGAGGCAATCAAGGGCGTTGACGGACTCGACGCCATGGAGCCAGACACCGCACC

TGGCCTTCCCTGGGCCCTCCAGGGGAAACGCCGCGGTGCACTTATCGATTTCGAGAACGGCACGGTCGGA

CCCGAGGTTGAGGCTGCCTTGAAGCTCATGGAGAAAAGAGAATACAAGTTTGCTTGCCAGACCTTCCTGA

AGGACGAAATTCGCCCGATGGAGAAAGTACGTGCCGGCAAGACTCGCATTGTCGACGTTTTGCCTGTTGA

ACACATTCTTTACACCAGGATGATGATTGGCAGATTTTGTGCACAAATGCACTCAAACAACGGGCCGCAG

ATTGGCTCAGCGGTCGGTTGCAACCCTGATGTTGATTGGCAGAGATTCGGCACACACTTCGCCCAATACA

GAAACGTGTGGGACGTGGACTATTCGGCCTTTGATGCAAACCACTGCAGCGATGCCATGAACATCATGTT

TGAAGAGGTGTTCCGCACGGAGTTCGGCTTCCACCCGAATGCTGAGTGGATCCTGAAGACTCTCGTGAAC

ACGGAACACGCCTATGAGAACAAGCGCATCACTGTTGAAGGCGGGATGCCATCTGGCTGTTCCGCAACAA

GCATCATCAACACAATTTTGAATAACATCTACGTGCTCTACGCCTTGCGTAGACACTATGAGGGGGTTGA

GCTGGACACCTACACCATGATCTCCTATGGAGACGACATCGTGGTGGCAAGCGATTATGATCTGGACTTT

GAGGCCCTCAAGCCTCACTTCAAATCTCTTGGCCAAACCATTACTCCAGCTGACAAAAGCGACAAAGGTT

TTGTTCTTGGTCACTCCATTACTGACGTCACTTTCCTCAAAAGACACTTCCACATGGATTATGGCACTGG

GTTTTACAAACCTGTGATGGCCTCGAAGACCCTCGAGGCTATCCTCTCCTTTGCACGCCGTGGG

>FJ906802.1_Asia1_CHA_2006

GGATTGGTTGTTGACACCAGAGATGTGGAGGAGCGCGTGCACGTCATGCGCAAAACCAAGCTTGCACCCA

CCGTAGCACACGGTGTGTTCAACCCTGAATTTGGGCCTGCTGCCTTGTCCAACAAGGACCCGCGCTTGAA

CGGAGGAGTTGTCCTCGATGAAGTCATCTTCTCCAAGCACAAGGGAGACACAAAGATGTCTGAGGAGGAC

AAAGCGCTGTTCCGACGTTGCGCTGCCGACTACGCGTCGCGCTTGCACAGCGTGCTGGGTACAGCAAATG

CCCCATTGAGCATTTACGAGGCAATCAAGGGCGTCGACGGACTCGATGCCATGGAACCAGACACCGCACC

TGGCCTTCCCTGGGCCCTCCAGGGGAAACGCCGTGGTGCGCTGATTGACTTCGAGAACGGCACAGTCGGA

CCCGAGGTCGAGGCTGCCCTAAAGCTCATGGAGAAAAGAGAATACAAGTTCGCTTGTCAGGCCTTCCTGA

AGGACGAGATTCGCCCGATGGAGAAAGTACGTGCCGGCAAGACTCGCATTGTCGATGTCTTGCCTGTTGA

ACACATTCTATACACCAGGATGATGATTGGCAGATTTTGTGCTCAAATGCACTCAAACAACGGACCGCAA

ATTGGCTCGGCGGTCGGTTGCAACCCTGATGTTGATTGGCAACGATTTGGCACACATTTTGCCCAGTACA

GAAACGTGTGGGATGTGGACTATTCGGCCTTTGATGCTAACCACTGCAGCGATGCAATGAACATCATGCT

TGAGGAGGTGTTTCGCACAGAGTTTGGCTTCCACCCGAACGCCGAGTGGATCCTGAAGACTCTTGTGAAT

ACGGAACATGCTTATGAGAACAAACGCATTGTTGTCGAGGGCGGGATGCCGTCTGGCTGTTCCGCGACAA

GCATCATAAACACAATTTTGAACAACATTTACGTGCTCTACGCTTTGCGCAGACACTATGAGGGAGTTGA

GCTGGACACTTACACCATGATCTCCTACGGAGATGACATCGTGGTGGCAAGTGATTACGATCTGGACTTT

GAGGCCCTCAAGCCTCACTTCAAATCTCTGGGCCAAACCATCACTCCAGCTGACAAAAGCGACAAAGGTT

TTGTTCTTGGTCACTCCATTACTGATGTCACTTTCCTCAAAAGACACTTCCACATGGATTATGGAACTGG

GTTTTACAAACCTGTAATGGCCTCGAAGACCCTCGAGGCTATCCTCTCCTTTGCACGCCGTGGG

>GQ406247.1_A_VIT_2009

GGGTTGATTGTTGACACCAGAGATGTGGAAGAGCGCGTGCACGTCATGCGTAAAACCAAGCTTGCGCCCA

CCGTGGCTCACGGTGTGTTCAACCCCGAGTACGGCCCCGCTGCCTTGTCCAACAAGGATCCGCGGCTGAA

TGAGGGAGTTGTCCTCGACGAGGTCATCTTCTCCAAACACAAGGGAGACACAAAGATGTCACCGGAGGAT

AAAGCGCTGTTCCGCCGCTGTGCTGCCGACTACGCGTCGCGTTTGCACGGTGTGCTGGGTACGGCAAATG

CCCCATTGAGCGTCTATGAGGCCATCAAAGGTGTCGACGGACTCGACGCCATGGAACCTGACACAGCACC

TGGCCTTCCCTGGGCCCTCCAGGGGAAGCGTCGTGGCACGCTGATTGACTTTGAGAACGGCACGGTCGGG

CCCGAAGTCCAGGCTGCTCTAGAGCTCATGGAGAAAAGAGAATACAAGTTTGCTTGCCAGACCTTCCTGA

AGGACGAGATTCGCCCGATGGAAAAAGTACGTGCCGGCAAGACTCGCATCGTCGATGTCTTGCCTGTTGA

ACACATTCTTTACACTAGGATGATGATTGGCAGATTTTGTGCACAAATGCACTCAAACAACGGCCCGCAA

ATTGGATCAGCGGTCGGTTGTAATCCTGATGTTGATTGGCAAAGATTTGGAACACACTTCGCCCAATACA

GGAACGTGTGGGATGTGGACTATTCGGCCTTTGATGCTAACCACTGCGGTGATGCAATGAACATCATGTT

TGAGGAGGTGTTTCGCACAGACTTTGGTTTCCACCCGAACGCTGAGTGGATTCTGAAGACTCTTGTGAAC

ACGGAACACGCCTATGAAAACAAACGCATTACTGTTGAAGGCGGGATGCCATCAGGTTGTTCCGCGACTA

GCATCATCAACACAATTTTGAACAACATTTACGTGCTCTACGCCCTGCGTAGACACTATGAGGGAGTTGA

GCTGGACACTTACACCATGATCTCCTACGGGGACGACATCGTGGTCGCAAGTGATTACGATCTGGACTTT

GAGGCTCTCAAGCCCCACTTCAAATCTCTTGGTCAAACCATCACCCCAGCCGACAAAAGCGACAAAGGTT

TTGTTCTTGGTCACTCCATCACCGATGTCACTTTCCTCAAAAGACACTTCCACATGGACTACGGAACTGG

GTTTTACAAACCTGTGATGGCTTCGAAGACCCTCGAAGCTATCCTCTCCTTTGCACGCCGTGGG

>GQ406248.1_A_VIT_2009

GGGTTGATTGTTGACACCAGAGATGTGGAAGAGCGCGTGCACGTCATGCGTAAAACCAAGCTTGCGCCCA

CCGTGGCTCACGGTGTGTTCAACCCCGAGTACGGCCCCGCTGCCTTGTCCAACAAGGATCCGCGGCTGAA

TGAGGGAGTTGTCCTCGACGAGGTCATCTTCTCCAAACACAAGGGAGACACAAAGATGTCACCGGAGGAT

AAAGCGCTGTTCCGCCGCTGTGCTGCCGACTACGCGTCGCGTTTGCACGGTGTGCTGGGTACGGCAAATG

CCCCATTGAGCGTCTATGAGGCCATCAAAGGTGTCGACGGACTCGACGCCATGGAACCTGACACAGCACC

TGGCCTTCCCTGGGCCCTCCAGGGGAAGCGTCGTGGCACGCTGATTGACTTTGAGAACGGCACGGTCGGG

CCCGAAGTCCAGGCTGCTCTAGAGCTCATGGAGAAAAGAGAATACAAGTTTGCTTGCCAGACCTTCCTGA

AGGACGAGATTCGCCCGATGGAAAAAGTACGTGCCGGCAAGACTCGCATCGTCGATGTCTTGCCTGTTGA

ACACATTCTTTACACTAGGATGATGATTGGCAGATTTTGTGCACAAATGCACTCAAACAACGGCCCGCAA

ATTGGATCAGCGGTCGGTTGTAATCCTGATGTTGATTGGCAAAGATTTGGAACACACTTCGCCCAATACA

GGAACGTGTGGGATGTGGACTATTCGGCCTTTGATGCTAACCACTGCAGTGATGCAATGAACATCATGTT

TGAGGAGGTGTTTCGCACAGACTTTGGTTTCCACCCGAACGCTGAGTGGATTCTGAAGACTCTTGTGAAC

ACGGAACACGCCTATGAAAACAAACGCATTACTGTTGAAGGCGGGATGCCATCAGGTTGTTCCGCGACTA

GCATCATCAACACAATTTTGAACAACATTTACGTGCTCTACGCCCTGCGTAGACACTATGAGGGAGTTGA

GCTGGACACTTACACCATGATCTCCTACGGGGACGACATCGTGGTCGCAAGTGATTACGATCTGGACTTT

GAGGCTCTCAAGCCCCACTTCAAATCTCTTGGTCAAACCATCACCCCAGCTGACAAAAGCGACAAAGGTT

TTGTTCTTGGTCACTCCATCACCGATGTCACTTTCCTCAAAAGACACTTCCACATGGACTACGGAACTGG

GTTTTACAAACCTGTGATGGCTTCGAAGACCCTCGAAGCTATCCTCTCCTTTGCACGCCGTGGG

>GQ406249.1_A_VIT_2009

GGGTTGATTGTTGACACCAGAGATGTGGAAGAGCGCGTGCACGTCATGCGTAAAACCAAGCTTGCGCCCA

CCGTGGCTCACGGTGTGTTCAACCCCGAGTACGGCCCCGCTGCCTTGTCCAACAAGGATCCGCGGCTGAA

TGAGGGAGTTGTCCTCGACGAGGTCATCTTCTCCAAACACAAGGGAGACACAAAGATGTCACCGGAGGAT

AAAGCGCTGTTCCGCCGCTGTGCTGCCGACTACGCGTCGCGTTTGCACGGTGTGCTGGGTACGGCAAATG

CCCCATTGAGCGTCTATGAGGCCATCAAAGGTGTCGACGGACTCGACGCCATGGAACCTGACACAGCACC

TGGCCTTCCCTGGGCCCTCCAGGGGAAGCGTCGTGGCACGCTGATTGACTTTGAGAACGGCACGGTCGGG

CCCGAAGTCCAGGCTGCTCTAGAGCTCATGGAGAAACGAGAATACAGGTTTGCTTGCCAGACCTTCCTGA

AGGACGAGATTCGCCCGATGGAAAAAGTACGTGCCGGCAAGACTCGCATCGTCGATGTCTTGCCTGTTGA

ACACATTCTTTACACTAGGATGATGATTGGCAGATTTTGTGCACAAATGCACTCAAACAGCGGCCCGCAA

ATTGGATCAGCGGTCGGTTGTAACCCTGATGTTGATTGGCAAAGATTTGGAACACACTTCGCCCAATACA

GGAACGTGTGGGATGTGGACTATTCGGCCTTTGATGCTAACCACTGCAGTGATGCAATGAACATCATGTT

TGAGGAGGTGTTTCGCACAGACTTTGGTTTCCACCCGAACGCTGAGTGGATTCTGAAGACTCTTGTGAAC

ACGGAACACGCCTATGAAAACAAACGCATTACTGTTGAAGGCGGGATGCCATCAGGTTGTTCCGCGACTA

GCATCATTAACACAATTTTGAACAACATTTACGTGCTCTACGCCCTGCGTAGACACTATGAGGGAGTTGA

GCTGGACACTTACACCATGATCTCCTACGGGGACGACATCGTGGTTGCAAGTGATTACGATCTGGACTTT

GAGGCTCTCAAGCCCCACTTCAAATCTCTTGGTCAAACCATCACCCCAGCTGACAAAAGCGACAAAGGTT

TTGTTCTTGGTCACTCCATCACCGATGTCACTTTCCTCAAAAGACACTTCCACATGGACTACGGAACTGG

GTTTTACAAACCTGTGATGGCTTCGAAGACCCTCGAAGCTATCCTCTCCTTTGCACGCCGTGGG

>GQ406250.1_A_VIT_2009

GGGTTGATTGTTGACACCAGAGATGTGGAAGAGCGCGTGCACGTCATGCGTAAAACCAAGCTTGCGCCCA

CCGTGGCTCACGGTGTGTTCAACCCCGAGTACGGCCCCGCTGCCTTGTCCAACAAGGATCCGCGGCTGAA

TGAGGGAGTTGTCCTCGACGAGGTCATCTTCTCCAAACACAAGGGAGACACAAAGATGTCACCGGAGGAC

AAAGCGCTGTTCCGCCGCTGTGCTGCCGACTACGCGTCGCGTTTGCACGGTGTGCTGGGTACGGCAAATG

CCCCATTGAGCGTCTATGAGGCCATCAAAGGTGTCGACGGGCTCGACGCCATGGAACCTGACACAGCACC

TGGCCTTCCCTGGGCCCTCCAGGGGAAGCGTCGTGGCACGCTGATTGACTTTGAGAACGGCACGGTCGGG

CCCGAAGTCCAGGCTGCTCTAGAGCTCATGGAGAAAAGAGAATACAAGTTTGCTTGCCAGACCTTCCTGA

AGGACGAGATTCGCCCGATGGAAAAAGTACGTGCCGGCAAGACTCGCATCGTCGATGTCTTGCCTGTTGA

ACACATTCTTTACACTAGGATGATGATTGGCAGATTTTGTGCACAAATGCACTTAAACAACGGCCCGCAA

ATTGGATCAGCGGTCGGTTGTAACCCTGATGTTGATTGGCAAAGGTTTGGAACACACTTCGCCCAGTACA

GGAACGTGTGGGATGTGGACTATTCGGCCTTTGATGCCAACCACTGCAGTGATGCAATGAACATCATGTT

TGAGGAGGTGTTTCGCACAGACTTTGGTTTCCACCCGAACGCTGAGTGGATTCTGAAGACTCTTGTGAAC

ACGGAACACGCCTATGAAAACAAACGCATTGCTGTTGAAGGCGGGATGCCATCAGGTTGTTCCGCGACTA

GCATCATCAACACAATTTTGAACAACATTTACGTGCTCTACGCCCTGCGTAGACACTATGAGGGAGTTGA

GCTGGACACTTACACCATGATCTCCTACGGGGACGACATCGTGGTCGCAAGTGATTACGATCTGGACTTT

GAGGCCCTCAAGCCCCACTTCAAATCTCTTGGTCAAACCATCACCCCAGCTGACAAAAGCGACAAAGGTT

TTGTTCTTGGTCACTCCATCACCGATGTCACTTTCCTCAAAAGACACTTCCACATGGACTACGGAACTGG

GTTTTACAAACCTGTGATGGCTTCGAAGACCCTCGAAGCTATCCTCTCCTTTGCACGCCGTGGG

>GQ406251.1_A_VIT_2009

GGGTTGATTGTTGACACCAGAGATGTGGAAGAGCGCGTGCACGTCATGCGTAAAACCAAGCTTGCGCCCA

CCGTGGCTCACGGTGTGTTCAACCCCGAGTACGGCCCCGCTGCCTTGTCCAACAAGGATCCGCGGCTGAA

TGAGGGAGTTGTCCTCGACGAGGTCATCTTCTCCAAACACAAGGGAGACACAAAGATGTCACCGGAGGAT

AAAGCGCTGTTCCGCCGCTGTGCTGCCGACTACGCGTCGCGTTTGCACGGTGTGCTGGGTACGGCAAATG

CCCCATTGAGCGTCTACGAGGCCATCAAAGGTGTCGACGGACTCGACGCCATGGAACCTGACACAGCACC

TGGCCTCCCCTGGGCCCTCCAGGGGAAGCGTCGTGGCACGCTGATTGACTTTGAGAACGGCACGGTCGGG

CCCGAAGTCCAGGCTGCTCTAGAGCTCATGGACAAAAGAGAATACAAGTTTGCTTGCCAGACCTTCCTGA

AGGACGAGATTCGCCCGATGGAAAAAGTACGTGCCGGCAAGACTCGCATCGTCGATGTCTTGCCTGTTGA

ACACATTCTTTACACTAGGATGATGATTGGCAGATTTTGTGCACAAATGCACTCAAACAACGGCCCGCAA

ATTGGATCAGCGGTCGGTTGTAATCCTGATGTTGATTGGCAAAGATTTGGAACACACTTCGCCCAATACA

GGAACGTGTGGGATGTGGACTATTCGGCCTTTGATGCTAACCACTGCAGTGATGCAATGAACATCATGTT

TGAGGAGGTGTTTCGCACAGACTTTGGTTTCCACCCGAACGCTGAGTGGATTCTGAAGACTCTTGTGAAC

ACGGAACACGCCTATGAAAACAAACGCATTACTGTTGAAGGCGGGATGCCATCAGGTTGTTCCGCGACTA

GCATCATCAACACAATTTTGAACAACATTTACGTGCTCTACGCCCTGCGTAGACACTATGAGGGAGTTGA

GCTGGACACTTACACCATGATCTCCTACGGGGACGACATCGTGGTCGCAAGTGATTACGATCTGGACTTT

GAGGCTCTCAAGCCCCACTTCAAATCTCTTGGTCAAACCATCACCCCAGCCGACAAAAGCGACAAAGGTT

TTGTTCTTGGTCACTCCATCACCGATGTCACTTTCCTCAAAAGACACTTCCACATGGACTACGGAACTGG

GTTTTACAAACCTGTGATGGCTTCGAAGACCCTCGAAGCTATCCTCTCCTTTGCACGCCGTGGG

>GQ406252.1_A_VIT_2009

GGGTTGATTGTTGACACCAGAGATGTGGAAGAGCGCGTGCACGTCATGCGTAAAACCAAGCTTGCGCCCA

CCGTGGCTCACGGTGTGTTCAACCCCGAGTACGGCCCCGCTGCCTTGTCCAACAAGGATCCGCGGCTGAA

TGAGGGAGTTGTCCTCGACGAGGTCATCTTCTCCAAACACAAGGGAGACACAAAGATGTCACCGGAGGAT

AAAGCGCTGTTCCGCCGCTGTGCTGCCGACTACGCGTCGCGTTTGCACGGTGTGCTGGGTACGGCAAATG

CCCCATTGAGCGTCTATGAGGCCATCAAAGGTGTCGACGGACTCGACGCCATGGAACCTGACACGGCACC

TGGCCTTCCCTGGGCCCTCCAGGGGAAGCGTCGTGGCACGCTGATTGACTTTGAGAACGGCACGGTCGGG

CCCGAAGTCCAGGCTGCTCTAGAGCTCATGGAGAAAAGAGAATACAAGTTTGCTTGCCAGACCTTCCTGA

AGGACGAGATTCGCCCGATGGAAAAAGTACGTGCCGGCAAGACTCGCATCGTCGATGTCTTACCTGTTGA

ACACATTCTTTACACTAGGATGATGATTGGCAGATTTTGTGCACAAATGCACTCAAACAACGGCCCGCAA

ATTGGATCAGCGGTCGGTTGTAATCCTGATGTTGATTGGCAAAGATTTGGAACACACTTCGCCCAATACA

GGAACGTGTGGGATGTGGACTATTCGGCCTTTGATGCTAACCACTGCAGTGATGCAATGAACATCATGTT

TGAGGAGGTGTTTCGCACAGACTTTGGTTTCCACCCGAACGCTGAGTGGATTCTGAAGACTCTTGTGAAC

ACGGAACACGCCTATGAAAACAAACGCATTACTGTTGAAGGCGGGATGCCATCAGGTTGTTCCGCGACTA

GCATCATCAACACAATTTTGAACAACATTTACGTGCTCTACGCCCTGCGTAGACACTATGAGGGAGTTGA

GCTGGACACTTACACCATGATCTCCTACGGGGACGACATCGTGGTCGCAAGTGATTACGATCTGGACTTT

GAGGCTCTCAAGCCCCACTTCAAATCTCTTGGTCAAACCATCACCCCAGCTGACAAAAGCGACAAAGGTT

TTGTTCTTGGTCACTCCATCACCGATGTCACTTTCCTCAAAAGACACTTCCACATGGACTACGGAACTGG

GTTTTACAAACCTGTGATGGCTTCGAAGACCCTCGAAGCTATCCTCTCCTTTGCACGCCGTGGG

>GQ452295.1_Asia1_VIT_2007

GGATTGGTTGTTGACACCAGAGATGTGGAGGAGCGCGTGCACGTCATGCGCAAAACCAAGCTCGCACCCA

CCGTAGCACACGGTGTGTTCAACCCTGAATTTGGGCCTGCTGCCTTGTCCAACAAGGACCCGCGCTTGAA

CGAAGGAGTTGTCCTCGACGAAGTCATCTTCTCCAAGCACAAGGGAGACACAAAGATGTCTGAGGAGGAC

AAAGCGCTGTTCCGACGTTGCGCTGCCGACTACGCGTCGCGCTTGCACAGCGTGCTGGGTACAGCAAATG

CCCCGTTGAGCATTTACGAGGCAATCAAGGGCGTCGACGGACTCGATGCCATGGAACCAGACACCGCACC

TGGCCTTCCCTGGGCCCTCCAGGGGAAACGCCGTGGTGCGCTGATTGACTTCGAGAACGGCACAGTCGGA

CCCGAGGTCGAGGCTGCCCTAAAGCTCATGGAGAAAAGAGAATACAAGTTTGCTTGTCAGACCTTCCTGA

AGGACGAGATTCGCCCGATGGAGAAAGTACGAGCCGGCAAGACTCGCATTGTCGATGTCTTGCCTGTTGA

ACACATTCTTTACACTAGGATGATGATTGGCAGATTTTGTGCTCAAATGCACTCAAACAACGGACCGCGA

ATTGGCTCGGCGGTCGGTTGCAACCCTGATGTTGATTGGCAACGATTTGGCACACATTTCGCCCAGTACA

GAAACGTGTGGGATGTGGACTACTCGGCCTTTGATGCTAACCACTGCAGCGATGCAATGAACATCATGTT

TGAGGAGGTGTTTCGCACAGAGTTTGGCTTCCACCCGAACGCCGAGTGGATCCTGAAGACTCTTGTGAAT

ACGGAACATGCTTATGAGAACAAACGCATTGTTGTCGAGGGCGGGATGCCGTCTGGCTGTTCCGCGACAA

GCATCATAAACACAATTTTGAACAACATTTACGTGCTCTACGCTTTGCGCAGACACTATGAGGGAGTTGA

GCTGGACACTTACACCATGATCTCCTACGGAGATGACATCGTGGTGGCAAGTGACTACGATCTGGACTTT

GAGGCCCTCAAGCCTCACTTCAAATCTCTGGGCCAAACCATCACTCCAGCTGACAAAAGCGACAAAGGTT

TTGTTCTTGGTCACTCCATTACTGATGTCACTTTCCTCAAAAGACACTTCCACATGGATTATGGAACTGG

GTTTTACAAACCTGTGATGGCCTCGAAGACCCTCGAGGCTATCCTCTCCTTTGCACGCCGTGGG

>GU125645.1_Asia1_VIT_2007

GGATTGGTTGTTGACACCAGAGATGTGGAGGAGCGCGTGCACGTCATGCGCAAAACCAAGCTCGCACCCA

CCGTAGCACACGGTGTGTTCAACCCTGAATTTGGGCCTGCTGCTTTGTCCAACAAGGACCCGCGCTTGAA

CGAAGGAGTTGTCCTCGACGAAGTCATCTTCTCCAAGCACAAGGGAGACACAAAGATGTCTGAGGAGGAC

AAAGCGCTGTTCCGACGTTGCGCTGCCGACTACGCGTCGCGCTTGCACAGCGTGCTGGGTACAGCAAATG

CCCCGTTGAGCATTTACGAGGCAATCAAGGGCGTCGACGGACTCGATGCCATGGAACCAGACACCGCACC

TGGCCTTCCCTGGGCCCTCCAGGGGAAACGCCGTGGTGCGCTGATTGACTTCGAGAACGGCACAGTCGGA

CCCGAGGTCGAGGCTGCCCTAAAGCTCATGGAGAAAAGAGAATACAAGTTTGCTTGTCAGACCTTCCTGA

AGGACGAGATTCGCCCGATGGAGAAAGTACGAGCCGGCAAGACTCGCATTGTCGATGTCTTGCCTGTTGA

ACACATTCTTTACACTAGGATGATGATTGGCAGATTTTGTGCTCAAATGCACTCAAACAACGGACCGCGA

ATTGGCTCGGCGGTCGGTTGCAACCCTGATGTTGATTGGCAACGATTTGGCACACATTTCGCCCAGTACA

GAAACGTGTGGGATGTGGACTACTCGGCCTTTGATGCTAACCACTGCAGCGATGCAATGAACATCATGTT

TGAGGAGGTGTTTCGCACAGAGTTTGGCTTCCACCCGAACGCCGAGTGGATCCTGAAGACTCTTGTGAAT

ACGGAACATGCTTATGAGAACAAACGCATTGTTGTCGAGGGCGGGATGCCGTCTGGCTGTTCCGCGACAA

GCATCATAAACACAATTTTGAACAACATTTACGTGCTCTACGCTTTGCGCAGACACTATGAAGGAGTGGA

ACTGGACACTTACACCATGATCTCCTACGGAGACGACGTTGTGGTCGCAAGTGACTATGATTTGGACTTT

GAGGCTCTCAAGCCCCACTTTAAATCTCTTGGTCAAACCATCACTCCGGCTGACAAAAGCGACAAAGGTT

TTGTTCTTGGTCACTCCATCACCGATGTCACTTTCCTCAAAAGACACTTCCACATGGATTATGGAACTGG

GTTTTACAAACCTGTGATGGCCTCGAAGACCCTCGAGGCTATCCTCTCCTTTGCACGCCGTGGG

>GU125646.1_Asia1_VIT_2005

GGGCTGATTGTTGACACCAGAGATGTGGAAGAGCGCGTCCATGTTATGCGCAAAACCAAGCTTGCACCCA

CCGTGGCACACGGTGTGTTTAACCCTGAGTACGGCCCCGCCGCCTTGTCTAACAAAGACCCGCGGCTGAA

CGAAGGGGTTGTCCTCGACGAGGTTATCTTCTCCAAACACAAGGGAGACGCAAAGATGTCACCGGAGGAC

AAAGAGCTGTTCCGCCGCTGTGCCGCCGACTACGCGTCGCGTCTTCACGGTGTGCTGGGTACGGCGAACG

CCCCATTGAGCATCTACGAGGCCATTAAAGGTGTCGACGGACTCGACGCCATGGAACCAGACACAGCGCC

TGGTCTCCCCTGGGCCCTCCAGGGGAAACGCCGCGGCGCGCTGATTGACTTCGAGAACGGCACTGTCGGG

CCCGAAGTTCAGACTGCCCTAGATCTCATGGAGAAGAGAGAATACAAGTTTGCTTGTCAGACCTTCCTGA

AAGACGAGATTCGTTCGATGGAGAAAGTCCGTGCCGGCAAGACTCGCATCGTCGACGTCCTGCCTGTTGA

ACATATTCTTTACACCATGATGATGATTGGCAGATTCTGTGCTCAAATGCACTCAAACAACGGACCGCAA

ATTGGATCGGCGGTTGGTTGTAACCCTGATGTTGACTGGCAAAGATTTGGAACACACTTCGCTCAATACA

GGAACGTGTGGGACGTGGACTATCGCGCCTTTGATGCTAA------------------------------

----------------------------------------------------------------------

----------------------------------------------------------------------

----------------------------------------------------------------------

----------------------------------------------------------------------

----------------------------------------------------------------------

----------------------------------------------------------------------

------------------------------------------CCTCTCCTTTGCACGCCGTGGG

>GU125647.1_O_VIT_2006

GGGCTGATTGTTGACACCAGAGATGTGGAAGAACGCGTGCACGTCATGCGTAAAACCAAGCTTGCACCCA

CCGTGGCGCACGGTGTGTTCAACCCCGAATACGGTCCCGCTGCCTTGTCCAACAAGGACCCGCGACTGAA

TGAAGGGATTGTCCTCGACGAGGTCATCTTCTCCAAGCATAAAGGAGACACACACATGTCACCAGAGGAC

AAAGCGCTGTTCCGCCGCTGCGCTGCCGACTACGCGTCGCGCTTGCACAGTGTTTTGGGTACGGCAAATG

CCCCGTTGAGCATCTACGAGGCCATCAAAGGCGTCGACGGACTCGACGCCATGGAGCCGGACACAGCGCC

CGGCCTTCCCTGGGCTCTTCAGGGGAAGCGCCGCGGTGCGCTCATTGACTTCGAGAACGGCACGGTCGGA

CCCGAAGTCCAGGCTGCACTAGAGCTCATGGAGAAAAGAGAATACAAGTTTGCTTGCCAGACCTTCCTGA

AGGACGAAATTCGCCCGATGGAGAAAGTGCGTGCCGGCAAGACTCGCATTGTCGATGTCCTGCCTGTTGA

ACACATTCTTTACACCAGGATGATGATTGGCAGATTTTGTGCACAGATGCACTCAAACAACGGCCCGCAA

ATTGGATCCGCGGTCGGTTGTAACCCTGATGTTGATTGGCAAAGATTTGGCACACACTTCGCCCAATACA

GAAACGTGTGGGATGTGGACTATTCGGCCTTTGATGCTAACCACTGCAGCGATGCAATGAACATCATGTT

TGAGGAGGTGTTTCGCACAGACTTTGGCTTCCACCCGAACGCTGAGTGGATACTGAAGACCCTTGTGAAC

ACGGAACACGCCTATGAGAACAAACGCATTACTGTTGAGGGCGGAATGCCTTCTGGTTGTTCTGCGACTA

GCATCATCAACACAATTCTGAACAACATCTACGTGCTCTACGCGCTGCGTAGACACTATGAGGGTGTTGA

GCTGGACACTTACACCATGATCTCTTACGGAGACGATATCGTGGTTGCGAGTGATTACGATCTGGACTTT

GAGGCTCTCAAGCCTCACTTCAAATCTCTTGGTCAAACCATTACTCCAGCTGACAAAAGTGACAAAGGTT

TTGTTCTTGGTCATTCCATCACCGACGTTACTTTCCTCAAAAGACACTTCCACATGGATTATGGAACTGG

GTTTTACAAACCTGTGATGGCCTCGAAGACCCTCGAGGCTATCCTCTCCTTTGCACGCCGTGGG

>GU125648.1_O_VIT_2006

GGGCTGATTGTTGACACCAGAGATGTGGAAGAACGCGTGCACGTCATGCGTAAAACCAAGCTTGCACCCA

CCGTGGCGCACGGTGTGTTCAACCCCGAATACGGTCCCGCTGCCTTGTCCAACAAGGACCCGCGACTGAA

TGAAGGGATTGTCCTCGACGAGGTCATCTTCTCCAAGCATAAAGGAGACACACACATGTCACCAGAGGAC

AAAGCGCTGTTCCGCCGCTGCGCTGCCGACTACGCGTCGCGCTTGCACAGTGTTTTGGGTACGGCAAATG

CCCCGTTGAGCATCTACGAGGCCATCAAAGGCGTCGACGGACTCGACGCCATGGAGCCGGACACAGCGCC

CGGCCTTCCCTGGGCTCTTCAGGGGAAGCGCCGCGGTGCGCTCATTGACTTCGAGAACGGCACGGTCGGA

CCCGAAGTCCAGGCTGCACTAGAGCTCATGGAGAAAAGAGAATACAAGTTTGCTTGCCAGACCTTCCTAA

AGGACGAAATTCGCCCGATGGAGAAAGTACGTGCTGGCAAGACTCGCATTGTCGATGTCCTGCCTGTTGA

ACACATTCTTTACACCAGGATGATGATTGGCAGATTTTGTGCACAGATGCACTCAAACAACGGCCCGCAA

ATTGGATCCGCGGTCGGTTGTAACCCTGATGTTGATTGGCAAAGATTTGGCACACACTTCGCCCAATACA

GAAACGTGTGGGATGTGGACTATTCGGCCTTTGATGCTAACCACTGCAGCGATGCAATGAACATCATGTT

TGAGGAGGTGTTTCGCACAGACTTTGGCTTCCACCCGAACGCTGAGTGGATACTGAAGACCCTTGTGAAC

ACGGAACACGCCTATGAGAACAAACGCATTACTGTTGAGGGCGGAATGCCTTCTGGTTGTTCTGCGACTA

GCATCATCAACACAATTCTGAACAACATCTACGTGCTCTACGCGCTGCGTAGACACTATGAGGGTGTTGA

GCTGGACACTTACACCATGATCTCTTACGGAGACGATATCGTGGTTGCGAGTGATTACGATCTGGACTTT

GAGGCTCTCAAGCCTCACTTCAAATCTCTTGGTCAAACCATTACTCCAGCTGACAAAAGTGACAAAGGTT

TTGTTCTTGGTCATTCCATCACCGACGTTACTTTCCTCAAAAGACACTTCCACATGGATTATGGAACTGG

GTTTTACAAACCTGTGATGGCCTCGAAGACCCTCGAGGCTATCCTCTCCTTTGCACGCCGTGGG

>GU125649.1_O_VIT_2006

GGGCTGATTGTTGACACCAGAGATGTGGAAGAACGCGTGCACGTCATGCGTAAAACCAAGCTTGCACCCA

CCGTGGCGCACGGTGTGTTCAACCCCGAGTACGGTCCCGCTGCCTTGTCCAACAAGGACCCGCGACTGAA

TGAAGGGATTGTCCTCGACGAGGTCATCTTCTCCAAGCATAAAGGAGACACACACATGTCACCAGAGGAC

AAAGCGCTGTTCCGCCGCTGCGCTGCCGACTACGCGTCGCGCTTGCACAGTGTTTTGGGTACGGCAAATG

CCCCGTTGAGCATCTACGAGGCCATCAAAGGCGTCGACGGACTCGACGCCATGGAGCCGGACACAGCGCC

CGGCCTTCCCTGGGCTCTTCAGGGGAAGCGCCGCGGTGCGCTCATTGACTTCGAGAACGGCACGGTCGGA

CCCGAAGTCCAGGCTGCACTAGAGCTCATGGAGAAAAGAGAATACAAGTTTGCTTGCCAGACCTTCCTGA

AGGACGAAATTCGCCCGATGGAGAAAGTACGTGCCGGCAAGACTCGCATTGTCGATGTCCTGCCTGTTGA

ACACATTCTTTACACCAGGATGATGATTGGCAGATTTTGTGCACAGATGCACTCAAACAACGGCCCGCAA

ATTGGATCCGCGGTCGGTTGTAACCCTGATGTTGATTGGCAAAGATTTGGCACACACTTCGCCCAATACA

GAAACGTGTGGGATGTGGACTATTCGGCCTTTGATGCTAACCACTGCAGCGATGCAATGAACATCATGTT

TGAGGAGGTGTTTCGCACAGACTTTGGCTTCCACCCGAACGCTGAGTGGATACTGAAGACCCTTGTGAAC

ACGGAACACGCCTATGAGAACAAACGCATTACTGTTGAGGGCGGAATGCCTTCTGGTTGTTCTGCGACTA

GCATCATCAACACAATTCTGAACAACATCTACGTGCTCTACGCGCTGCGTAGACACTATGAGGGTGTTGA

GCTGGACACTTACACCATGATCTCTTACGGAGACGATATCGTGGTTGCGAGTGATTACGATCTGGACTTT

GAGGCTCTCAAGCCTCACTTCAAATCTCTTGGTCAAACCATTACTCCAGCTGACAAAAGTGACAAAGGTT

TTGTTCTTGGTCATTCCATCACCGATGTTACTTTCCTCAAAAGACACTTCCACATGGATTATGGAACTGG

GTTTTACAAACCTGTGATGGCCTCGAAGACCCTCGAGGCTATCCTCTCCTTTGCACGCCGTGGG

>GU125650.1_O_VIT_2006

GGGTTGATTGTTGACACCAGAGATGTGGAAGAGCGCGTGCACGTCATGCGCAAAACCAAGCTTGCACCCA

CCGTGGCGCACGGTGTGTTCAATCCCCAATACGGTCCCGCTGCCTTGTCCAACAAGGACCCGCGACTGAA

TGAAGGGGTTGTCCTCGACGAGGTCATCTTCTCCAAGCACAAAGGAGACACCAAGATGTCACCAGAGGAT

AAAGCGCTGTTCCGCCGCTGTGCCGCCGACTACGCGTCGCGCTTACACAGTGTGTTGGGGACGGCAAATG

CCCCATTGAGCATCTACGAGGCCATCAAGGGTGTCGACGGACTCGACGCCATGGAGCCGGACACAGCGCC

TGGTCTCCCCTGGGCCCTCCAGGGGAAGCGCCGCGGTGCGTTGATTGACTTCGAGAACGGCACGGTCGGA

CCCGAAGTTCAGGCTGCCTTAGAGCTCATGGAGAAAAGAGAATACAAGTTTGCTTGCCAGACCTTCCTGA

AGGACGAAATTCGCCCGATGGAGAAAGTACGTGCCGGCAAGACTCGCATTGTCGACGTCCTGCCTGTTGA

ACACATTCTTTACACTAGGATGATGATTGGCAGATTTTGTGCACAAATGCACTCAAACAACGGCCCGCAA

ATTGGATCCGCGGTCGGTTGTAACCCTGATGTTGATTGGCAAAGATTTGGCACACACTTCGCCCAGTACA

GAAACGTGTGGGACGTGGACTATTCGGCCTTTGATGCTAACCACTGTAGTGATGCAATGAACATCATGTT

TGAGGAGGTGTTTCGCACAGACTTTGGCTTCCACCCGAATGCTGAGTGGATTCTGAAGACCCTCGTGAAC

ACGGAGCACGCCTATGAGAACAAACGCATTACTGTTGAAGGCGGAATGCCCTCCGGTTGTTCCGCGACTA

GCATCATCAACACAATTTTGAACAACATCTACATGCTCTACGCGCTGCGTAGACACTATGAGGGTGTTGA

GCTGGACACTTACACCATGATCTCTTACGGAGACGATATCGTGGTTGCGAGTGATTACGATCTGGACTTT

GAGGCTCTCAAGCCTCACTTCAAATCTCTTGGTCAAACCATTACTCCAGCTGACAAAAGTGACAAAGGTT

TTGTTCTTGGTCATTCCATCACCGATGTTACTTTCCTCAAAAGACACTTCCACATGGATTATGGAACTGG

GTTTTACAAACCTGTGATGGCCTCGAAGACCCTCGAGGCTATCCTCTCCTTTGCACGCCGTGGG

>GU384682.1_O_PAK_2008

GGGTTGATCGTTGACACCAGAGATGTGGAAGAGCGCGTACACGTCATGCGCAAAACCAAGCTTGCACCCA

CCGTTGCACACGGTGTGTTCAACCCTGAATTCGGCCCCGCTGCTTTGTCTAACAAAGACCCGCGGCTGAA

TGAAGGGGTTGTCCTTGACGAAGTCATCTTCTCCAAGCACAAGGGAGACGCCAAGATGTCGGAAGAGGAC

AAAGCGCTGTTCCGTCGCTGCGCTGCCGACTACGCGTCGCGCTTACACAGTGTTCTGGGTACGGCAAATG

CCCCATTGAGCATCTACGAGGCAATCAAGGGCATCGACGGACTCGACGCCATGGAACCAGACACCGCGCC

TGGCCTCCCCTGGGCCCTCCAGGGGAAGCGCCGTGGTGCGCTCATCGACTTCGAGAACGGCACTGTCGGA

CCCGAGGTTGAGGCTGCCTTGAAGCTCATGGAGAAAAGAGAATACAAGTTTGCATGTCAGACCTTCCTGA

AGGACGAAATTCGCCCGATGGAGAAAGTACGTGCCGGCAAGACTCGCATCGTCGATGTCCTGCCTGTTGA

ACACATTCTTTACACCAGAATGATGATTGGTAGATTCTGTGCTCAAATGCACTCAAACAACGGACCGCAA

ATTGGCTCAGCGGTCGGATGCAACCCTGACGTTGATTGGCAGAGATTTGGCACACATTTTGCTCAGTACA

GAAACGTGTGGGATGTGGATTATTCGGCCTTTGATGCTAACCATTGCAGTGATGCCATGAACATCATGTT

TGAGGAGGTGTTCCGCACGGAGTTCGGTTTCCACCCTAACGCTGAGTGGATCCTGAAGACTCTTGTGAAC

ACGGAACACGCGTACGAAAACAAACGCGTCACTGTTGAGGGCGGGATGCCGTCTGGTTGTTCCGCAACAA

GCATCATCAACACAATTTTGAACAACATTTACGTGCTCTACGCCTTGCGCAGACACTATGAGGGAGTTGA

GCTGGACACTTACACCATGATCTCCTATGGAGACGACATCGTGGTTGCAAGTGATTACGATCTGGACTTT

GAGGCCCTCAAGCCTCACTTCAAATCTCTTGGTCAAACCATTACCCCAGCTGACAAAAGCGACAAAGGTT

TTGTTCTTGGTCACTCCATTACCGACGTCACTTTCCTCAAAAGACACTTCCACATGGACTATGGAACTGG

GTTTTACAAACCTGTGATGGCCTCGAAGACCCTCGAGGCTATCCTCTCCTTTGCACGCCGTGGG

>GU384683.1_O_PAK_2008

GGGTTGATCGTTGACACCAGAGATGTGGAAGAGCGCGTACACGTCATGCGCAAAACCAAGCTTGCACCCA

CCGTTGCACACGGTGTGTTCAACCCTGAATTCGGCCCCGCTGCTTTGTCTAACAAAGACCCGCGGCTGAA

TGAAGGGGTTGTCCTTGACGAAGTCATCTTCTCCAAGCACAAGGGAGACGCCAAGATGTCGGAAGAGGAC

AAAGCGCTGTTCCGTCGCTGCGCTGCCGACTACGCGTCGCGCTTACACAGTGTTCTGGGTACGGCAAATG

CCCCATTGAGCATCTACGAGGCAATCAAGGGCATCGACGGACTCGACGCCATGGAACCAGACACCGCGCC

TGGCCTCCCCTGGGCCCTCCAGGGGAAGCGCCGTGGTGCGCTCATCGACTTCGAGAACGGCACTGTCGGA

CCCGAGGTTGAGGCTGCCTTGAAGCTCATGGAGAAAAGAGAATACAAGTTTGCATGTCAGACCTTCCTGA

AGGACGAAATTCGCCCGATGGAGAAAGTACGTGCCGGCAAGACTCGCATCGTCGATGTCCTGCCTGTTGA

ACACATTCTTTACACCAGAATGATGATTGGTAGATTCTGTGCTCAAATGCACTCAAACAACGGACCGCAA

ATTGGCTCAGCGGTCGGATGCAACCCTGACGTTGATTGGCAGAGATTTGGCACACATTTTGCTCAGTACA

GAAACGTGTGGGATGTGGATTATTCGGCCTTTGATGCTAACCATTGCAGTGATGCCATGAACATCATGTT

TGAGGAGGTGTTCCGCACGGAGTTCGGTTTCCACCCTAACGCTGAGTGGATCCTGAAGACTCTTGTGAAC

ACGGAACACGCGTACGAAAACAAACGCGTCACTGTTGAGGGCGGGATGCCGTCTGGTTGTTCCGCAACAA

GCATCATCAACACAATTTTGAACAACATTTACGTGCTCTACGCCTTGCGCAGACACTATGAGGGAGTTGA

GCTGGACACTTACACCATGATCTCCTATGGAGACGACATCGTGGTTGCAAGTGATTACGATCTGGACTTT

GAGGCCCTCAAGCCTCACTTCAAATCTCTTGGTCAAACCATTACCCCAGCTGACAAAAGCGACAAAGGTT

TTGTTCTTGGTCACTCCATTACCGACGTCACTTTCCTCAAAAGACACTTCCACATGGACTATGGAACTGG

GTTTTACAAACCTGTGATGGCCTCGAAGACCCTCGAGGCTATCCTCTCCTTTGCACGCCGTGGG

>GU582115.1_O_VIT_2009

GGGTTGATTGTTGACACCAGAGATGTGGAAGAGCGCGTGCACGTCATGCGCAAAACCAAGCTTGCACCCA

CCGTGGCGCACGGTGTGTTCAACCCCGAATACGGTCCAGCTGCCTTGTCCAACAAGGATCCGCGACTGAA

TGAAGGGGTTGTCCTCGACGAAGTCATTTTCTCCAAACACAAAGGAGACACAAAGATGACACCAGAGGAC

AAAGCGCTGTTCCGCCGCTGCGCTGCTGACTACGCGTCACGTTTGCACAGTGTGCTGGGTACGGCAAATG

CCCCATTGAGCGTCTATGAGGCCATCAAAGGTGTCGACGGACTCGACGCCATGGAGCCGGACACAGCGCC

TGGTCTCCCCTGGGCCCTCCAGGGGAAGCGCCGCGGCGCGCTGATTGACTTCGAGAACGGCACGGTCGGA

CCCGAAGTTCAGGCTGCCTTAGAGCTCATGGAGAAAAGAGAATACAAGTTTGCTTGTCAGACCTTCCTGA

AGGACGAGATTCGCCCGATGGAGAAAGTGCGTGCCGGCAAGACCCGCATTGTCGATGTCCTGCCTGTTGA

ACATATTCTTTACACCAGGATGATGATTGGCAGATTTTGTGCACAAATGCACTCAAACAACGGCCCGCAA

ATTGGATCTGCGGTTGGCTGTAACCCTGATGTTGATTGGCAAAGATTTGGCACACATTTCGCCCAATACA

GAAACGTGTGGGATGTGGACTATTCGGCTTTTGATGCTAACCACTGCAGTGACGCAATGAACATTATGTT

TGAGGAGGTGTTCCGCACAGACTTCGGCTTCCACCCGAACGCTGAGTGGATACTGAAGACCCTTGTGAAC

ACGGAGCATGCCTATGAGAACAAACGCATCACTGTTGAAGGCGGAATGCCCTCTGGTTGTTCCGCGACTA

GCATCATCAATACAATTTTGAACAACATCTACGTGCTCTACGCGTTGCGTAGACACTATGAGGGTGTTGA

GCTGGACACTTACACCATGATCTCTTACGGAGACGACATCGTGGTGGCGAGTGATTATGATCTGGACTTT

GAGGCTCTCAAGCCTCACTTCAAATCTCTTGGCCAAACCATTACTCCAGCTGACAAAAGTGACAAAGGTT

TTGTTCTTGGTCATTCCATTACTGATGTTACTTTCCTCAAAAGACACTTCCACATGGACTATGGAATTGG

GTTTTACAAACCTGTGATGGCCTCGAAGACCCTCGAGGCCATCCTCTCCTTTGCACGCCGTGGG

>GU582116.1_O_VIT_2009

GGGTTGATTGTTGACACCAGAGATGTGGAAGAGCGCGTGCACGTCATGCGCAAAACCAAGCTTGCACCCA

CCGTGGCGCACGGTGTGTTCAACCCCGAATACGGTCCAGCTGCCTTGTCCAACAAGGATCCGCGACTGAA

TGAAGGGGTTGTCCTCGACGAAGTCATTTTCTCCAAACACAAAGGAGACACAAAGATGACACCAGAGGAC

AAAGCGCTGTTCCGCCGCTGCGCTGCTGACTACGCGTCACGTTTGCACAGTGTGCTGGGTACGGCAAATG

CCCCATTGAGCATCTATGAGGCCATCAAAGGTGTCGACGGACTCGACGCCATGGAGCCGGACACAGCGCC

TGGTCTCCCCTGGGCCCTCCAGGGGAAGCGCCGCGGCGCGCTGATTGACTTCGAGAACGGCACGGTCGGA

CCCGAAGTTCAGGCTGCCTTAGAGCTCATGGAGAAAAGAGAATACAAGTTTGCTTGTCAGACCTTCCTGA

AGGACGAGATTCGCCCGATGGAGAAAGTGCGTGCCGGCAAGACCCGCATTGTCGATGTCCTGCCTGTTGA

ACATATTCTTTACACCAGGATGATGATTGGCAGATTTTGTGCACAAATGCACTCAAACAACGGCCCGCAA

ATTGGATCTGCGGTTGGCTGTAACCCTGATGTTGACTGGCAAAGATTTGGCACACATTTCGCCCAATACA

GAAACGTGTGGGATGTGGACTATTCGGCTTTTGATGCTAACCACTGCAGTGACGCAATGAACATTATGTT

TGAGGAGGTGTTCCGCACAGACTTTGGCTTCCACCCGAACGCTGAGTGGATACTGAAGACCCTTGTGAAC

ACGGAGCATGCCTATGAGAACAAACGCATCACTGTTGAAGGCGGAATGCCCTCTGGTTGTTCCGCGACTA

GCATCATCAATACAATTTTGAACAACATCTACGTGCTCTACGCGTTGCGTAGACACTATGAGGGTGTTGA

GCTGGACACTTACACCATGATCTCTTACGGAGACGACATCGTGGTGGCGAGTGATTATGATCTGGACTTT

GAGGCTCTCAAGCCTCACTTCAAATCTCTTGGCCAAACCATTACTCCAGCTGACAAAAGTGACAAAGGTT

TTGTTCTTGGTCATTCCATTACTGATGTTACTTTCCTCAAAAGACACTTCCACATGGACTATGGAACTGG

GTTTTACAAACCTGTGATGGCCTCGAAGACCCTCGAGGCCATCCTCTCCTTTGCACGCCGTGGG

>GU931682.1_Asia1_CHA_2005

GGATTGGTTGTTGACACCAGAGATGTGGAGGAGCGCGTGCACGTCATGCGCAAAACCAAGCTTGCACCCA

CCGTAGCACACGGTGTGTTCAACCCTGAATTTGGGCCTGCTGCCTTGTCCAACAAGGACCCGCGCTTGAA

CGAAGGAGTTGTCCTCGATGAGGTCATCTTCTCCAAGCACAAGGGAGACACAAAGATGTCTGAGGAGGAC

AAAGCGCTGTTCCGACGTTGCGCTGCCGACTACGCGTCGCGCTTGCACAGCGTGCTGGGTACAGCAAATG

CCCCATTGAGCATTTACGAGGCAATCAAGGGCGTCGACGGACTCGATGCCATGGAACCAGACACCGCACC

TGGCCTTCCCTGGGCCCTCCAGGGGAAACGCCGTGGTGCGCTGATTGACTTCGAGAACGGCACAGTCGGA

CCCGAGGTCGAGGCTGCCCTAAAGCTCATGGAGAAAAGAGAATACAAGTTTGCTTGTCAGACCTTCCTGA

AGGACGAGATTCGCCCGATGGAGAAAGTACGTGCCGGCAAGACTCGCATTGTCGATGTCTTGCCTGTTGA

ACACATTCTTTACACCAGGATGATGATTGGCAGATTTTGTGCTCAAATGCACTCAAACAACGGACCGCAA

ATTGGCTCGGCGGTCGGTTGCAACCCTGATATTGATTGGCAAAGATTTGGCACACATTTTGCCCAGTACA

GAAACGTGTGGGATGTGGACTATTCGGCCTTTGATGCTAACCACTGCAGCGATGCAATGAACATCATGTT

CGAGGAGGTGTTCCGCACAGAGTTTGGCTTCCACCCGAACGCCGAGTGGATCCTGAAGACTCTTGTGAAT

ACGGAACATGCTTATGAGAACAAACGCATTGTTGTTGAGGGCGGGATGCCGTCTGGCTGTTCCGCGACAA

GCATCATAAACACAATTTTGAACAACATTTACGTGCTCTACGCTTTGCGCAGACACTATGAGGGAGTTGA

GCTGGACACTTACACCATGATCTCCTACGGAGATGACATCGTGGTGGCAAGTGATTACGATCTGGACTTT

GAGGCTCTCAAGCCTCACTTCAAATCTCTGGGCCAAACCATCACTCCAGCTGACAAAAGCGACAAAGGTT

TTGTTCTTGGTCACTCCATTACTGATGTCACTTTCCTCAAAAGACACTTCCACATGGATTATGGAACTGG

GTTTTACAAACCTGTAATGGCCTCGAAGACCCTCGAGGCTATCCTCTCCTTTGCACGCCGTGGG

>HM008917.1_O_CHA_2005

GGATTGATAGTTGACACCAGAGATGTTGAGGAGCGCGTACATGTCATGCGCAAAACCAAGCTCGCACCCA

CCGTGGCATACGGTGTATTCAACCCCGAATTTGGGCCTGCCGCCTTGTCCAACCAGGACCCGCGCCTGAA

TGAAGGGGTTGTCCTCGATGAAGTTATCTTCTCTAAACACAAGGAAAACACAAAGATGTCTGAGGAGGAC

AAAGCGCTGTTCCGCCGCTGTGCTGCTGACTACGCGTCCCGCCTGCACAGCGTGCTGGGTACGGCAAACG

CCCCACTGAGCATTTACGAGGCAATTAAGGGTGTCGACGGACTTGACGCCATGGAACCAGACACCGCGCC

TGGCCTCCCCTGGGCCCTCCAGGGGAAACGCCGTGGTGCGCTCATTGACTTCGAGAACGGCACTGTCGGA

CCCGAGGTTGAGGCTGCTTTGAAGCTCATGGAGAAAAGAGAGTACAAGTTTGTATGCCAGACCTTTCTGA

AGGACGAGATCCGTCCGATGGAAAAGGTACGTGCCGGTAAGACTCGCATTGTCGACGTCCTGCCTGTTGA

ACACATTCTTTACACCAGGATGATGATTGGTAGATTTTGCGCTCAAATGCACTCAAACAACGGACCGCAA

ATTGGTTCGGCGGTTGGTTGTAATCCTGATGTTGATTGGCAAAGATTTGGCACGCACTTTGCTCAGTACA

AAAACGTGTGGGATGTGGACTATTCGGCCTTTGACGCCAACCACTGTAGTGATGCAATGAACATCATGTT

TGAGGAGGTGTTCAACACGGATTTCGGTTTCCACCCAAACGCTGAGTGGATCTTGAAAACTCTCGTGGAC

ACTGAACACGCCTATGAGAACAAACGCATCACTGTTGAAGGCGGGATGCCGTCTGGTTGTTCCGCGACAA

GCATCATCAACACAATTTTGAACAACATCTACGTGCTCTACGCCTTGCGCAGACACTATGAGGGAGTTGA

GCTGGACTCTTACACCATGATCTCCTACGGAGACGACATCGTGGTTGCAAGTGATCACGATCTGGACTTT

GAGGCCCTCAAGCCTCACTTCAAATCCCTTGGTCAAACCATCACTCCAGCTGACAAAAGTGACAAAGGTT

TTGTTCTTGGTCACTCCATTACCGATGTCACTTTCCTCAAAAGACACTTCCACATGGACTATGGAACTGG

GTTTTACAAACCTGTGATGGCTTCGAAGACCCTCGAGGCTATCCTCTCCTTTGCACGTCGTGGG

>HM055510.1_O_VIT_2009

GGGTTGATCGTTGACACCAGAGATGTGGAAGAGCGCGTGCACGTCATGCGCAAAACCAAGCTTGCACCCA

CCGTGGCACACGGTGTGTTCAACCCTGAGTACGGCCCCGCTGCCTTGTCCAACAAGGACCCGCGGCTGAA

TGAAGGAGTTGTCCTCGATGAGGTCATCTTCTCCAAACACAAGGGGGACACAAAGATGTCACCGGAAGAC

AAAGCGCTGTTCCGCCGCTGCGCTGCCGACTACGCGTCGCGTCTTCACAGTGTGCTGGGTACAGCAAATG

CCCCATTGAGCATCTACGAGGCCATTAAAGGCGTTGACGGACTCGACGCCATGGAACCAGACACAGCGCC

TGGCCTTCCCTGGGCACTCCAGGGGAAACGCCGCGGCGCGCTGATTGACTTCGAGAACGGCACTGTCGGA

CCCGAAGTCCAGGCTGCCTTGGAGCTCATGGAGAAAAGAGAATACAAGTTTGCCTGTCAGACCTTCCTGA

AGGACGAAATTCGCCCGATGGAAAAAGTACGTGCCGGCAAGACGCGCATCGTCGATGTTTTGCCTGTTGA

ACACATTCTTTACACCAGGATGATGATTGGCAGATTTTGTGCTCAAATGCACTCAAACAACGGACCGCAA

ATTGGATCAGCGGTCGGTTGTAATCCTGATGTTGATTGGCAAAGATTTGGAACACACTTCGCCCAATACA

GAAACGTGTGGGATGTGGACTATTCGGCCTTTGATGCTAATCACTGTAGTGATGCAATGAACATCATGTT

TGAGGAGGTGTTTCGCACAGACTTTGGTTTCCACCCGAATGCTGAGTGGATTCTGAAGACCCTCGTGAAC

ACGGAACACGCCTATGAGAACAAACGCATTACAGTTGAAGGTGGAATGCCGTCCGGCTGTTCCGCGACCA

GCATCATCAACACAATTCTGAACAACATCTACGTGCTCTACGCGCTGCGTAGACACTATGAGGGAGTTGA

GCTGGACACTTACACCATGATCTCCTACGGAGACGACATCGTGGTTGCTAGTGATTATGACTTGGACTTT

GAGGCTCTCAAGCCCCACTTTAAATCTCTTGGTCAAACCATTACTCCAGCTGACAAAAGCGACAAAGGTT

TTGTTCTTGGTCACTCCATTACCGATGTCACTTTCCTCAAAAGACACTTCCACATGGATTATGGAACTGG

GTTTTACAAACCTGTGATGGCTTCGAAGACCCTCGAGGCTATCCTCTCCTTTGCACGCCGTGGG

>HM067704.1_SAT2_UGA_2007

GGACTGATCGTCGACACAAGAGAAGTTGAGGAGCGCGTACACGTTATGCGCAAAACAAAGCTCGCGCCCA

CCGCTGCACACGGTGTGTTTCAGCCTGAATACGGACCTGCTGCGCTGTCGAACAACGACAAGCGCCTCAA

CGAGGGCGTCGTCCTGGACGACGTCATCTTCTCCAAGCACAAAGGAGACACAAAGATGTCCGAGGCTGAC

AAGAAGCTGTTCCGCATGTGCGCTGCCGACTACGCGTCGCACTTGCACAATGTGCTCGGGACAGCAAACT

CTCCACTGAGTGTGTTTGAAGCCATCAAGGGCGTCGACGGACTCGACGCAATGGAGCCTGACACCGCACC

CGGCCTGCCCTGGGCAATCCAAGGCAAGCGCCGCGGAGCTCTCATCGATTTCGAGAACGGCACTGTCGGA

CCCGAGATTGAACAGGCTCTCAAGCTCATGGAGAACAAGGAGTACAAGTTTGTGTGCCAAACCTTCCTGA

AGGACGAGATTCGCCCTATGGAGAAAGTACGTGCCGGCAAGACTCGCATCGTCGATGTCCTGCCTGTTGA

ACACATCCTCTACACTAGAATGATGATTGGCAGATTCTGTGCAAACATGCACTCCAACAACGGACCGCAA

ATTGGCTCGGCGGTCGGTTGCAACCCAGATGTTGATTGGCAGAGATTCGGAACCCACTTTGCCCAGTACA

AGAACGTGTGGGACGTGGATTATTCGGCCTTTGACGCTAACCATTGCAGTGATGCCATGAACATCATGTT

CGAGGAGGTCTTCCGGCCAGAATTTGGTTTCCACCCCAACGCGGAGTGGATTCTGAAGACCCTGGTTAAC

ACGGAACACGCCTACGAGAACAAGCGCATTACTGTTGAAGGTGGAATGCCCTCAGGCTGTTCCGCCACCA

GCATCATCAATACGATTCTCAACAACATCTACGTGCTCTACGCTCTGCGCAGGCACTATGAGGGAGTCGA

GCTGTCGCACTACACCATGATCTCCTACGGAGATGATATTGTGGTCGCAAGTGATTACGACTTGGACTTT

GAAGCTCTCAAGCCTCACTTCAAGTCCGTCGGTCAAACCATCACTCCAGCCGACAAAAGTGACAAAGGTT

TTGTTCTTGGTCAGTCCATCACCGATGTTACTTTCCTCAAGAGGCACTTCCATCTGGACTACGGAACTGG

GTTTTACAAACCTGTGATGGCCTCGAAGACCCTCGAAGCCATCCTCTCCTTTGCACGCCGTGGG

>HM067705.1_SAT2_UGA_2007

GGGCTGATTGTCGACACAAGAGAAGTTGAGGAGCGCGTGCACGTCATGCGCAAAACAAAGCTCGCGCCCA

CCGTTGCACACGGTGTGTTTCAGCCTGAGTACGGACCTGCTGCGCTGTCAAACAACGACAAGCGCCTGGA

CGAGGGCGTCGTTTTGGACGACGTCATCTTCTCCAAGCACAAGGGAGACGCAAAGATGTCTGAGGCTGAC

AAGAAGCTGTTCCGTCTGTGTGCTGCTGACTACGCGTCACATCTGCACAATGTGCTCGGAACTGCGAACT

CTCCACTGACAGTGTTTGAAGCCATCAAGGGCGTCGACGGACTCGACGCCATGGAGCCTGACACCGCACC

CGGGCTGCCCTGGGCAATCCAAGGGAAGCGCCGCGGGGCCCTCATCGATTTCGAGAACGGCACCGTCGGA

CCCGAGATCGAACAGGCTTTAAAGCTCATGGAGAGTAAGGAATACAAGTTTGTATGCCAAACCTTCCTGA

AGGACGAGATTCGCCCGATGGAGAAAGTACGTGCCGGCAAGACTCGCATCGTCGATGTCCTGCCCGTTGA

ACACATCCTGTACACCAGAATGATGATTGGCAGATTTTGTGCAAACATGCATTCCAACAACGGACCGCAA

ATTGGCTCGGCGGTCGGTTGCAACCCAGATGTTGATTGGCAAAGATTTGGAACCCACTTCGCCCAGTACA

AGAACGTGTGGGACGTGGACTACTCGGCCTTTGATGCCAACCATTGCAGTGACGCCATGAACATCATGTT

TGAAGAGGTCTTCCGACCGGAATTCGGGTTCCACCCCAACGCGGAGTGGATCTTGAAAACTCTGGTCAAC

ACGGAACACGCCTACGAAAACAAGCGCATCACTGTTGAAGGTGGAATGCCCTCGGGTTGCTCCGCCACCA

GCATCATTAACACAATTCTCAACAACATCTACGTGCTCTACGCGCTGCGTAGACACTATGAGGGAGTCGA

GCTGTCGCACTACACCATGATTTCCTACGGGGATGACATTGTAGTTGCAAGTGACTACGACTTGGATTTT

GAAGCTCTTAAGCCTCACTTCAAATCCGTTGGTCAAACCATCACTCCAGCCGACAAAAGTGACAAAGGTT

TTGTTCTTGGTCAGTCCATCACCGATGTTACTTTCCTCAAGAGGCATTTCCATCTGGACTACGGAACTGG

GTTTTACAAACCTGTGATGGCCTCGAAGACCCTCGAAGCTATCCTCTCCTTTGCACGCCGTGGG

>HM067706.1_SAT1_UGA_2007

GGACTGATTGTCGACACAAGGGAAGTTGAGGAGCGCGTGCACGTCATGCGTAAGACAAAGCTCGCACCCA

CCGTTGCACACGGTGTGTTTCAGCCTGAGTACGGACCTGCTGCACTGTCGAACAACGACAAGCGGCTGAA

TGAGGGCGTCGTCCTGGACGACGTCATCTTCTCCAAACACAAAGGAGATGCAAAGATGTCTGAGGCTGAC

AAGAAGTTGTTCCGTCTGTGTGCCGCCGACTACGCGTCGCACCTGCACAACGTGCTCGGAACTGCAAACT

CTCCACTGACTGTGTTTGAAGCCATCAAGGGTGTCGACGGACTCGACGCCATGGAGCCAGACACTGCACC

CGGACTGCCTTGGGCGACCCAGGGTAAGCGCCGAGGAGCTCTCATCGATTTCGAGAACGGCACTGTCGGA

CCCGAGATTGAACAGGCCTTGAAGCTCATGGAGAACAAGGAATACAAGTTTGTGTGCCAAACCTTCCTGA

AAGACGAGATTCGTCCGATGGAGAAAGTACGTGCCGGCAAGACTCGAATTGTCGATGTCCTGCCTGTTGA

ACACATCCTCTACACCAGAATGATGATTGGCAGATTTTGTGCAAACATGCATTCCAACAACGGACCGCAA

ATTGGCTCGGCGGTCGGTTGCAACCCAGATGTTGATTGGCAAAGATTTGGAACCCACTTCGCCCAGTACA

AAAACGTGTGGGACGTGGACTATTCGGCCTTTGATGCTAACCATTGCAGTGACGCCATGAACATCATGTT

CGAAGAGGTTTTCCGCCCGGAGTTCGGATTCCACCCCAACGCAGAGTGGATTCTGAAGACTCTTGTCAAC

ACGGAACACGCCTATGAGAACAAGCGCATCACTGTTGAAGGCGGGATGCCCTCGGGTTGTTCCGCCACCA

GCATCATCAACACAATTCTCAACAACATCTACGTGCTCTACGCTCTGCGTAGACACTATGAGGGAGTCGA

GCTGTCGCACTACACCATGATTTCCTACGGAGATGATATTGTAGTTGCAAGTGATTACGACTTGGATTTT

GAAGCTCTCAAGCCTCACTTCAAATCCGTTGGTCAGACCATCACTCCAGCCGACAAGAGTGACAAAGGTT

TTGTTCTTGGTCAGTCCATCACCGATGTTACTTTCCTCAAGAGGCATTTCCATCTGGACTACGGAACTGG

GTTTTACAAACCTGTGATGGCCTCGAAGACCCTCGAGGCCATCCTCTCCTTTGCACGCCGTGGG

>HM191257.1_O_UGA_2006

GGGCTGATTGTTGACACCAGAGACGTGGAAGAGCGCGTGCACGTTATGCGCAAAACAAAGCTTGCACCCA

CCGTGGCACACGGTGTGTTCAACCCTGAGTTTGGCCCCGCCGCCTTGTCCAACAAGGACCCGCGGCTGAA

CGAAGGAGTTGTCCTCGATGAAGTCATCTTCTCCAAGCACAAGGGAGACACGAAGATGTCTGAGGAAGAC

AAAGCGCTGTTCCGCCGTTGTGCTGCTGACTACGCGTCGCGGCTACACAGCGAACTGGGTACGGCGAATG

CCCCATTGAGCATCTACGAAGCAATCAAAGGCGTTGACGGCCTCGACGCCATGGAACCAGACACTGCTCC

TGGTCTCCCCTGGGCCCTCCAGGGGAAACGCCGTGGAGCTCTGATTGACTTCGAGAACGGCACAGTCGGC

CCTGAGGTCACTGCCGCTCTAGAGCTCATGGAGAAAAGAGAGTACAAATTTGCTTGTCAGACCTTCCTGA

AGGACGAGATTCGCCCGATGGAAAAAGTGCGTGCCGGCAAGACCCGCATTGTCGACGTCCTGCCTGTTGA

ACATATACTTTACACCAGAATGATGATTGGCAGATTTTGTGCCCAAATGCACGCAAACAACGGACCGCGT

ATTGGCTCAGCGGTTGGTTGCAATCCAGATGTTGATTGGCAAAGATTTGGCACACACTTCGCCCAGTACA

GGAATGTGTGGGATGTGGACTATTCGGCCTTCGATGCTAACCACTGCAGTGACGCGATGAACATCATGTT

TGAGGAGGTGTTTCGCACAGAATTTGGCTTCCACCCGAACGCCGAGTGGATCCTGAAAACTCTTGTGAAC

ACGGAACACGCCTATGAGAACAAGCGCATTACTGTTGAAGGCGGGATGCCGTCTGGCTGTTCCGCAACGA

GCATCATCAACACAATTTTGAACAACATCTACGTGCTCTACGCCCTGCGTAGACACTATGAGGGAGTTGA

GTTGGACACCTACACCATGATCTCCTACGGAGACGACATCGTGGTGGCAAGTGATTACGATCTGGACTTC

GAGGCTCTCAAGCCTCATTTTAAATCTCTTGGTCAAACCATCACCCCAGCTGACAAAAGCGACAAAGGTT

TTGTTCTTGGTCACTCCATTACCGATGTCACTTTCCTTAAAAGACACTTCCACATGGACTATGGAACTGG

GTTTTACAAACCTGTGATGGCCTCAAAGACCCTTGAGGCTATCCTCTCCTTCGCACGCCGTGGG

>HM229661.1_O_HKN_2010

GGGTTGATCGTTGACACCAGAGACGTGGAAGAGCGCGTGCACGTCATGCGCAAAACCAAGCTTGCACCTA

CCGTGGCACACGGTGTGTTCAACCCTGAGTACGGCCCCGCTGCCTTGTCCAACAAGGATCCGCGGCTGAA

TGAAGGAGTTGTCCTCGATGAGGTCATCTTCTCCAAACACAAGGGGGACACAAAGATGTCACCGGAAGAC

AAAGCGCTGTTCCGCCGCTGCGCTGCCGACTACGCGTCGCGTCTTCACAGTGTGCTGGGTACAGCAAATG

CCCCATTGAGCATCTACGAGGCCATTAAAGGCGTTGACGGACTCGACGCCATGGAACCAGACACAGCGCC

TGGCCTTCCCTGGGCACTCCAGGGGAAACGCCGCGGCGCGCTGATTGACTTCGAGAACGGCACCGTCGGA

CCCGAAGTCCAGGCTGCCTTGGAGCTCATGGAGAAAAGAGAATACAAGTTTGCCTGTCAGACCTTCCTGA

AGGACGAAATTCGCCCGATGGAAAAAGTACGTGCCGGCAAGACGCGCATCGTCGATGTTTTGCCTGTTGA

ACACATTCTTTACACCAGGATGATGATTGGCAGATTTTGTGCTCAAATGCACTCAAACAACGGACCGCAA

ATTGGATCAGCGGTCGGTTGTAATCCTGATGTTGATTGGCAAAGATTTGGCACACACTTCGCCCAATACA

GAAACGTGTGGGATGTGGACTATTCGGCCTTTGATGCTAATCACTGTAGTGATGCAATGAACATCATGTT

TGAGGAGGTGTTTCGCACAGACTTCGGTTTCCACCCGAATGCTGAGTGGATTCTGAAGACCCTCGTGAAC

ACGGAACACGCCTATGAGAACAAACGCATTACAGTTGAAGGTGGAATGCCGTCCGGCTGTTCCGCAACCA

GCATCATCAACACAATTCTGAACAACATCTACGTGCTCTACGCGCTGCGTAGACACTATGAGGGAGTTGA

GCTGGACACTTACACCATGATCTCCTACGGAGACGACATCGTGGTTGCTAGTGATTATGACTTGGACTTT

GAGGCTCTCAAGCCCCACTTTAAATCTCTTGGTCAAACCATTACTCCAGCTGACAAAAGCGACAAAGGTT

TTGTTCTTGGTCACTCCATTACCGATGTCACTTTCCTCAAAAGACACTTCCACATGGATTATGGAACTGG

GTTTTACAAACCTGTGATGGCTTCGAAGACCCTCGAGGCTATCCTCTCCTTTGCACGCCGTGGG

>HM854021.1_A_IND_2000

GGTTTGATTGTTGACACCAGAGATGTGGAAGAGCGCGTGCACGTCATGCGCAAAACCAAGCTTGCACCCA

CCGTCGCGCACGGTGTGTTCAACCCTGAATTCGGGCCTGCCGCCTTGTCCAGCAAGGATCCGCGCCTGAA

CGACGGGGTCGTCCTCGACGAAGTCATCTTCTCCAAACACAAGGGAGACACTAAGATGTCTGAGGAGGAC

AAAGCGCTGTTCCGCCGCTGTGCTGCTGACTACGCGTCACGCCTGCATAGTGTGTTGGGTACAGCAAATG

CCCCACTGAGCATTTACGAGGCAATCAAAGGCGTTGACGGGCTCGACGCCATGGAGCCAGACACTGCACC

TGGCCTTCCCTGGGCCCTCCAGGGAAAGCGCCGTGGCGCACTCATCGACTTCGAGAACGGCACGGTCGGA

CCCGAGGTTGAAGCTGCCTTGAAGCTCATGGAGAAAAGGGAATACAAATTTGCTTGTCAGACCTTCCTGA

AGGACGAAATTCGCCCAATGGAGAAAGTCCGTGCCGGCAAGACTCGCATTGTCGACGTCCTGCCCGTTGA

ACACATTCTTTACACCAGGATGATGATTGGCAGATTTTGTGCTCAAATGCACTCGAACAACGGACCGCAA

ATTGGCTCTGCGGTCGGCTGTAATCCTGATGTTGATTGGCAAAGATTCGGAACCCATTTTGCTCAGTACA

GAAATGTGTGGGATGTGGACTATTCGGCCTTTGATGCTAACCACTGCAGTGACGCGATGAACATCATGTT

TGAGGAAGTGTTTTGCACGGAGTTCGGTTTCCACCCAAACGCTGAGTGGATTCTGAAGACTCTAGTGAAC

ACGGAGCACGCCTATGAGAACAAACGCATCACTGTTGAGGGCGGGATGCCGTCTGGCTGTTCCGCGACAA

GCATTATCAACACAATTTTGAACAACATTTACGTGCTCTACGCGCTGCGTAGACACTATGAGGGAGTTGA

GCTGGACACTTACACCATGATCTCCTACGGAGACGACATCGTGGTTGCAAGTGATCACGATTTGGACTTT

GAGGCCCTCAAGCCTCACTTTAAATCTCTTGGTCAAACCATCACTCCAGCTGACAAAAGCGACAAAGGTT

TTGTTCTTGGTCACTCCATCACTGATGTCACTTTCCTCAAAAGACACTTCCACATGGATTATGGAACTGG

GTTTTACAAACCTGTGATGGCCTCAAAGACCCTTGAGGCTATCCTCTCCTTTGCACGCCGTGGG

>HM854022.1_A_IND_1977

GGGTTGATTGTTGATACCAGAGATGTGGAAGAACGCGTCCACGTCATGCGTAAAACCAAGCTTGCACCCA

CCGTGGCACACGGTGTGTTCAACCCTGAATTTGGGCCCGCCGCCTTGTCCAACAAGGACTCGCGCCTGAA

TGAAGGAGTTGTCCTCGACGAGGTCATCTTCTCCAAACACAAGGGAGATACAAAGATGTCTGAAGAGGAC

AAAGCGCTGTACCGCCGCTGCGCCGCCGACTACGCGTCACGCCTGCACAGCGTGCTGGGTACGGCAAATG

CCCCACTGAGCATCTACGAGGCAATCAAAGGCGTCGACGGACTCGACGCCATGGAACCAGACACTGCACC

TGGCCTTCCCTGGGCCCTCCAGGGAAAACGCCGTGGCGCACTCATCGACTTCGAGAACGGCACGGTCGGA

CCCGAGGTTGAAGCTGCCCTGAAGCTCATGGAGAAAAGAGAATACAAGTTTGCTTGTCAGACCTTCCTGA

AGGACGAGATTCGCCCGATGGAGAAAGTACGTGCCGGCAAGACTCGCATCGTCGATGTTTTGCCTGTTGA

ACACATTCTTTACACCAGGATGATGATTGGCAGATTTTGTGCTCAAATGCACTCAAACAACGGACCGCAA

ATTGGCTCAGCGGTAGGTTGCAACCCTGATGTTGATTGGCAAAGATTTGGCACACACTTCGCTCAGTACA

GAAACGTGTGGGATGTGGACTATTCGGCCTTTGATGCTAACCACTGCAGTGACGCAATGAACATCATGTT

TGAGGAGGTGTTTCGCACGGAATTTGGTTTCCACCCAAACGCTGAGTGGATCCTGAAGACTCTCGTGAAC

ACGGAACACGCCTATGAGAACAAACGCATCACTGTTGAAGGCGGGATGCCGTCCGGTTGTTCTGCGACAA

GCATCATCAACACAATTTTGAACAACATCTATGTGCTCTACGCCCTGCGTAGACACTATGAGGGAGTTGA

GCTGGACACTTACACCATGATCTCCTACGGAGACGACATCGTGGTTGCGAGTGATTACGATCTGGACTTT

GAGGCCCTCAAGCCTCACTTCAAATCTCTTGGTCAAACCATCACCCCAGCTGACAAAAGCGACAAAGGTT

TTGTCCTTGGTCACTCCATCACCGATGTCACTTTCCTCAAAAGACACTTCCACATGGACTATGGAACTGG

GTTTTACAAACCTGTGATGGCCTCAAAGACCCTTGAGGCTATCCTCTCCTTTGCACGCCGTGGG

>HM854023.1_A_IND_1999

GGGTTGATTGTTGACACCAGAGATGTCGAAGAGCGCGTGCACGTCATGCGCAAAACCAAGCTTGCACCCA

CCGTTGCACACGGTGTGTTCAACCCTGAATTCGGCCCCGCTGCCTTGTCCAACAAGGACCCGCGGCTAAA

TGAAGGGGTTGTCCTCGATGAGGTCATCTTCTCCAAACACAAGGGAGACACCAAGATGTCAGAGGAGGAC

AAAGCTCTGTTCCGAGCCTGCGCTGCCGACTACGCGTCGCGCTTGCACAGTGTTTTGGGTACAGCAAATG

CCCCATTGAGCATTTACGAGGCAATCAAAGGCGTCGACGGACTCGATGCCATGGAACCAGACACCGCGCC

TGGTCTTCCCTGGGCCCTCCAGGGAAAACGCCGTGGTGCGCTCATCGACTTCGAGAACGGCACTGTCGGA

CCCGAGGTTGAAGCTGCCCTAAAGCTGATGGAGAAAAGAGAGTACAAATTTGCTTGCCAGACCTTCCTGA

AGGACGAGATCCGCCCGATGGAGAAAGTGCGTGCCGGCAAGACTCGCATCGTCGATGTCCTGCCTGTTGA

ACACATTCTTTACACCAGGATGATGATTGGTAGATTCTGTGCTCAAATGCACTCAAACAACGGACCGCAG

ATTGGCTCAGCGGTCGGTTGTAATCCTGATGTTGATTGGCAAAGATTTGGCACACATTTCGCTCAGTACA

AAAACGTGTGGGATGTGGATTATTCGGCCTTTGATGCTAACCACTGCAGTGACGCGATGAACATCATGTT

TGAGGAGGTGTTCCGCACGGAATTCGGTTTCCACCCAAACGCTGAGTGGATCCTGAAAACTCTCGTGAAC

ACGGAACACGCCTATGAGAACAAACGCATCAATGTTGAGGGCGGGATGCCATCTGGCTGTTCCGCGACAA

GCATTATCAACACAATTCTGAACAACATCTACGTGCTCTACGCCCTGCGTAGACACTATGAGGGAGTTGA

GCTGGACACCTACACCATGATCTCCTACGGAGACGACATCGTGGTTGCAAGTGATTACGACCTGGACTTT

GAGGCTCTCAGACCTCACTTCAAATCTCTTGGTCAAACCATCACTCCAGCTGACAAAAGCGACAAAGGTT

TTGTTCTTGGTCACTCCATCACCGATGTCACTTTCCTCAAAAGGCACTTCCACATGGATTATGGAACTGG

GTTTTACAAACCTGTGATGGCCTCGAAGACCCTCGAGGCTATCCTCTCCTTTGCACGCCGTGGG

>HQ009509.1_O_CHA_1999

GGATTGATAATTGACACCAGAGATGTTGAGGAGCGGGTACACGTCATGCGCAAAACCAAGCTCGCACCCA

CCGTCGCATACGGTGTATTCAACCCCGAATTTGGGCCTGCCGCCTTGTCCAACCAGGACCCGCGCCTGAA

TGAAGGGGTTGTCCTTGACGAGGTCATTTTCTCCAAACACAAAGAAGACACAAAGATGTCTGAGGAGGAC

AAAGCACTGTTCCGCCGCTGTGCTGCTGACTACGCGTCGCGTCTGCACAGCGTGCTGGGTACGGCAAACG

CCCCACTGAGCATTTACGAGGCAATTAAGGGCGTCGACGGACTTGACGCCATGGAACCAGACACCGCGCC

TGGTCTCCCCTGGGCTCTCCAGGGGAAACGCCGTGGTGCGCTTATTGACTTCGAGAACGGCACTGTCGGA

CCCGAGGTTGAGGCTGCCTTGAAGCTCATGGAGAAAAGAGAGTACAAGTTCGTATGCCAGACCTTCCTGA

AGGACGAGATCCGCCCGATGGAAAAGGTACGTGCCGGTAAGACTCGCATAGTCGACGTCCTGCCTGTTGA

ACACATTCTGTACACCAGGATGATGATTGGTAGATTTTGTGCCCAAATGCACTCAAACAACGGACCGCGA

ATCGGCTCGGCGGTTGGTTGTAATCCTGATGTTGACTGGCAAAGATTTGGCACACACTTTGCCCAGTACA

AAAATGTGTGGGATGTGGACTATTCGGCCTTTGACGCCAACCACTGCAGTGATGCAATGAACATCATGTT

CGAGGAGGTGTTCAACACGGATTTCGGTTTCCACCCAAACGCTGAGTGGATCTTGAAAACTCTCGTGAAC

ACTGAACACGCCTATGAGAACAAACGCATCACTGTTGAAGGTGGAATGCCGTCTGGTTGTTCCGCGACAA

GTATCATCAACACAATTTTGAACAACATCTACGTGCTCTACGCCTTGCGCAGACACTATGAGGGAGTTGA

GCTGGACTCTTACACCATGATCTCCTACGGGGACGACATCGTGGTTGCAAGTGATTACGATTTGGACTTT

GAGGCCCTCAAGCCTCACTTCAAATCCCTTGGCCAAACCATCACTCCAGCTGACAAAAGCGACAAAGGTT

TTGTTCTTGGTCACTCCATTACCGATGTCACTTTCCTCAAAAGACACTTCCACATGGACTATGGAACCGG

GTTTTACAAACCTGTGATGGCTTCGAAGACCCTCGAGGCTATCCTCTCCTTTGCACGTCGTGGG

>HQ113232.1_O_PAK_2009

GGGTTGATCGTTGACACCAGAGATGTGGAAGAGCGCGTGCACGTCATGCGCAAGACCAAGCTTGCACCCA

CCGTCGCACACGGTGTGTTCAACCCTGAATTCGGCCCCGCTGCTTTGTCCAACAAAGACCCGCGGCTGAA

TGAAGGGGTTGTCCTCGACGAAGTCATCTTCTCCAAGCACAAGGGAGACACCAAGATGTCGGAAGAGGAC

AAAGCGCTGTACCGTCGCTGCGCCGCCGACTACGCGTCGCGCTTGCACAGTGTTCTGGGTACGGCAAATG

CCCCATTGAGCATCTACGAGGCAATCAAGGGCGTCGACGGACTCGACGCCATGGAACCAGACACCGCGCC

TGGCCTCCCCTGGGCCCTCCAGGGAAAACGCCGTGGTGCGCTCATCGACTTCGAGAACGGCACTGTCGGA

CCCGAGGTTGAGGCTGCCTTGAAGCTCATGGAGAAAAGAGAATACAAGTTTGCATGTCAGACCTTCCTGA

AGGACGAAATTCGCCCGATGGCGAAAGTGCGTGCCGGCAAGACTCGCATTGTCGACGTCCTGCCTGTTGA

ACATATTCTTTACACCAGAATGATGATTGGTAGGTTCTGTGCTCAAATGCACTCAAACAACGGACCGCAA

ATTGGCTCAGCGGTCGGATGCAACCCTGACGTTGATTGGCAGAGATTTGGCACACATTTTGCTCAGTACA

GAAACGTGTGGGATGTGGACTATTCGGCCTTTGATGCCAACCACTGCAGCGATGCCATGAACATCATGTT

TGAGGAGGTGTTCCGCACGGAGTTCGGTTTCCACCCTAACGCTGAGTGGATCCTGAAGACTCTTGTGAAT

ACGGAGCACGCGTACGAGAACAAACGCATCACTGTTGAGGGCGGGATGCCGTCTGGCTGTTCCGCAACAA

GCATCATCAACACAATTTTGAACAACATTTACGTGCTCTACGCCTTGCGTAGACACTATGAGGGAGTTGA

GCTGGACACTTACACCATGATCTCCTATGGAGACGACATCGTGGTTGCAAGTGATTACGATCTGGACTTT

GAGGCCCTCAAGCCTCACTTCAAATCTCTTGGTCAAACCATTACCCCAGCTGACAAAAGCGACAAAGGTT

TTGTTCTTGGTCACTCCATTACCGATGTCACTTTCCTCAAAAGACACTTCCACATGGACTATGGAACTGG

GTTCTACAAACCTGTGATGGCCTCGAAGACCCTCGAGGCTATCCTCTCCTTTGCACGCCGTGGG

>HQ113233.1_Asia1_AFG_2009

GGGTTGATCGTTGACACCAGAGATGTGGAAGAGCGCGTGCACGTCATGCGCAAAACCAAGCTTGCACCCA

CCGTTGCACACGGTGTGTTCAACCCTGCATTCGGCCCCGCCGCTCTGTCCAACAGAGACCCGCGGCTGAA

CGAAGGGGTTGTCCTTGATGAAGTCATCTTCTCCAAGCACAAGGGAGACACCAAGATGTCGGAAGAGGAC

AAAGCGCTGTTCCGTCGCTGCGCTGCCGACTACGCGTCGCGCTTGCACGGTGTTCTGGGTACGGCAAATG

CCCCACTGAGCATTTATGAGGCAATCAAGGGCGTCGAAGGACTCGACGCCATGGAACCAGACACCGCGCC

TGGCCTCCCCTGGGCCCTCCAGGGGAAACGCCGTGGTGTGCTCATCGACTTCGAGAACGGCACTGTCGGA

CCCGAGGTTGAAGCTGCCTTGAAGCTCATGGAGAAAAGAGAATACAAGTTTGCATGTCAGACCTTCCTGA

AGGACGAGATTCGCCCGATGGAGAAAGTACGTGCCGGCAAGACTCGCATTGTCGATGTCCTGCCTGTTGA

ACACATTCTTTACACTAGGATGATGATTGGCAGATTCTGTGCTCAAATGCACTCAAACAACGGACCGCAA

ATTGGCTCAGCGGTCGGTTGCAACCCTGACGTTGATTGGCAGAGATTTGGCACACATTTTGCTCAGTACA

GAAACGTGTGGGATGTGGATTATTCGGCCTTTGATGCTAACCATTGCAGTGACGCCATGAACATCATGTT

TGAGGAGGTGTTCCGCACGGAATTCGGTTTCCACCCTAACGCTGAGTGGATTCTGAAGACTCTCGTGAAC

ACGGAGCACGCGTACGAAAACAAACGCATCACTGTTGAGGGCGGGATGCCGTCTGGTTGTTCCGCAACAA

GCATCATCAACACAATTCTGAACAACATCTACGTGCTCTACGCCTTGCGTAGACACTATGAGGGAGTTGA

GCTGGACACTTACACCATGATCTCCTACGGGGACGACATCGTGGTTGCAAGTGATTACGATCTGGACTTC

GAGGCCCTTAAGCCTCACTTTAAATCTCTTGGTCAAACCATCACCCCAGCTGACAAAAGCGACAAAGGTT

TTGTTCTTGGTCACTCCATCACCGATGTCACTTTCCTCAAAAGACACTTCCACATGGACTATGGAACTGG

GTTTTACAAACCTGTGATGGCCTCGAAGACCCTCGAGGCTATCCTCTCCTTTGCACGCCGTGGG

>HQ268509.2_A_VIT_2004

GGGTTGATTGTCGACACCAGAGACGTGGAAGAGCGCGTGCACGTCATGCGCAAAACCAAGCTTGCGCCCA

CCGTGGCTCACGGTGTGTTCAACCCTGACTACGGCCCCGCTGCCTTGTCCAACAAGGACCCGCGGCTGAA

TGAGGGAGTTGTCCTCGACGAGGTCATCTTCTCCAAACACAAGGGAGACACAAAGATGTCACCGGAGGAC

AAAGCGCTGTTCCGCCGCTGTGCTGCCGACTACGCGTCGCGTTTGCACGGTGTGCTGGGTACGGCAAATG

CCCCATTGAGTATCTACGAGGCCATCAAAGGCGTCGACGGACTCGACGCTATGGAACCAGACACAGCGCC

TGGCCTCCCCTGGGCCCTCCAGGGGAAGCGTCGCGGCGCGCTGATTGACTTCGAGAACGGCACGGTCGGG

TCCGAAGTCCAGGCTGCTCTAGAGCTCATGGAGAAAAGAGAATACAAGTTTGCTTGCCAGACCTTCCTGA

AGGACGAGATTCGCCCGATGGAAAAAGTACGTGCCGGCAAGACTCGCATTGTCGATGTCTTGCCTGTTGA

ACACATTCTTTACACTAGGATGATGATTGGCAGATTCTGTGCACAAATGCACTCAAACAACGGTCCGCAA

ATTGGATCAGCGGTCGGTTGTAATCCTGATGTTGATTGGCAAAGATTTGGAACACACTTCGCCCAATACA

GAAACGTGTGGGACGTGGACTATT-GGCCTTTGATGCTAACCACTGCAGTGATGCAATGAACATCATGTT

TGAGGAGGTGTTTCGCACAGACTTTGGCTTCCACCCGAACGCTGAGTGGATTCTGAAGACTCTTGTGAAC

ACGGAACACGCCTATGAAAACAAACGCATTACTGTTGAGGGCGGGATGCCGTCTGGTTGTTCTGCAACTA

GCATCATCAACACAATTTTGAACAACATCTACGTGCTCTACGCCTTGCGTAGGCACTATGAGGGAGTTGA

GCTGGACACTTATACCATGATCTCCTACGGGGACGACATCGTGGTCGCAAGTGATTACGATCTGGACTTT

GAGGCTCTTAAGCCTCACTTCAAATCTCTTGGTCAAACCATCACCCCAGCTGACAAAAGCGACAAAGGTT

TTGTTCTTGGTCACTCCATCACCGATGTCACTTTCCTCAAAAGATACTTCCACATGGATTATGGAACTGG

GTTTTACAAACCTGTGATGGCTTCGAAGACCCTCGAAGCTATCCTCTCCTTTGCACGCCGTGGG

>HQ268524.1_O_BHU_2004

GGGTTGATCGTTGACACCAGAGATGTGGAAGAGCGCGTGCACGTCATGCGCAAAACCAAGCTTGCACCCA

CCGTTGCACACGGTGTGTTCAACCCTGAATTCGGCCCCGCCGCTTTGTCCAACAAAGACCCGCGGCTGAA

TGAAGGAGTTGTCCTTGATGAAGTCATCTTCTCCAAGCATAAGGGAGACACCAAGATGTCGGAAGAGGAC

AAAGCGCTGTTCCGTCGCTGCGCTGCCGACTACGCGTCGCGCTTACACAGTGTTCTGGGTACGGCAAATG

CCCCATTGAGCATTTACGAGGCAATCAAGGGCGTCGACGGACTCGATGCCATGGAACCAGACACCGCGCC

TGGCCTCCCCTGGGCCCTCCAGGGGAAACGCCGTGGTGCGCTCGTCGACTTCGAGAACGGCACTGTCGGA

CCCGAGGTTGAAGCTGCCTTGAAGCTCATGGAGAAAAGAGAATACAAGTTTGCATGTCAGACCTTCCTGA

AGGACGAAATTCGCCCGATGGAGAAAGTACGTGCCGGCAAGACTCGCATCGTCGATGTCCTGCCTGTTGA

ACACATTCTTTACACCAGAATGATGATTGGCAGATTCTGTGCTCAAATGCACTCAAACAACGGACCGCAA

ATTGGCTCAGCGGTCGGATGCAACCCTGACGTTGATTGGCAGAGATTTGGCACACATTTTGCTCAGTACA

GAAACGTGTGGGATGTGGATTATTCGGCCTTTGATGCTAACCATTGCAGTGACGCCATGAACATCATGTT

TGAGGAGGTGTTCCGCACGGAATTCGGTTTCCACCCTAACGCTGAGTGGATCCTGAAGACTCTCGTGAAT

ACGGAGCACGCATACGAAAACAAACGCATCACTGTCGAGGGCGGGATGCCGTCTGGCTGTTCCGCAACAA

GCATCATCAACACAATTCTGAACAACATTTACGTGCTCTACGCCCTGCGTAGACACTATGAGGGAGTTGA

GCTGGACACTTACACCATGATCTCCTACGGAGACGACATCGTGGTTGCAAGTGATTACGATCTGGACTTT

GAGGCCCTCAAGCCTCACTTCAAATCTCTTGGTCAAACCATCACCCCAGCCGACAAAAGCGACAAAGGTT

TTGTTCTTGGTCACTCCATTACCGACGTCACTTTCCTCAAAAGACACTTCCACATGGACTATGGAACTGG

GTTTTACAAACCTGTGATGGCCTCGAAGACCCTCGAGGCTATCCTCTCCTTTGCACGCCGTGGG

>HQ412603.1_O_CHA_2000

GGGTTGATCGTTGACACCAGAGATGTCGAGGAGCGCGTCCACGTGATGCGTAAAACCAAGCTCGCGCCCA

CCGTGGCACACGGTGTGTTCAACCCTGAATTTGGGCCTGCTGCTCTGTCCAACAAGGACCCGCGCCTGAA

CGAAGGGGTTGTCCTTGACGACACCATTTTCTCCAAACACAAAGGAGATACAAAGATGTCTAAGGAGGAC

AAAGCGCTGTTCCGGCGCTGCGCTGCTGACTACGCGTCGCGTTTGCACAGCGTGTTGGGGACAGCGAACG

CCCCACTGAGTGTGTACGAGGCCATCAAAGGTGTCGAGGGGCTCGACGCCATGGAGCCGGACACCGCACC

TGGTCTCCCCTGGGCCCTCCAGGGAAAACGCCGCGGTGCTCTGATCGACTTTGAAAACGGTACGGTCGGG

CCCGAGGTTGAGGCAGCACTCAAGCTTATGGAAAACCGTGAGTACAAATTCGTCTGCCAGACCTTCCTGA

AGGACGAAATTCGACCGTTAGAAAAGGTGCGCGCTGGCAAGACACGCATTGTCGACGTCTTGCCCGTTGA

ACACATTCTCTACACCAGAATGATGATTGGCAGGTTCTGTGCCCAGATGCACTCGAACAACGGACCACAA

ATTGGATCAGCGGTCGGTTGTAACCCTGATGTTGATTGGCAAAGATTTGGCACACATTTCGCCCAGTACA

AAAACGTGTGGGATGTGGACTACTCAGCCTTTGATGCAAACCACTGCAGTGATGCGATGAACATCATGTT

CGAAGAGGTGTTCCGTACGGAGTTCGGATTCCACCCGAACGCCGAGTGGATTCTAAAGACTCTTGTGAAC

ACGGAGCACGCTTACGAGAACAAGCGTATTGCTGTCGAAGGTGGAATGCCGTCTGGTTGTTCCGCGACAA

GCATCATCAACACAATTCTGAACAACATTTACGTGCTTTATGCTCTGCGCAGGCACTACGAGGGAGTCGA

GCTGGACACTTACACCATGATCTCTTATGGAGACGACATCGTGGTGGCAAGTGACTATGACCTGGACTTT

GAGGCTCTCAAGCCCCACTTCAAGTCCCTTGGCCAGACTATCACTCCGGCCGACAAAAGCGACAAAGGTT

TTGCTCTTGGTCACTCCATAACCGATGTCACTTTCCTCAAAAGACATTTCCACATGGACTACGGAACTGG

GTTTTACAAACCTGTGATGGCCTCGAAGACCCTCGAGGCTATCCTCTCCTTTGCACGCCGTGGG

>HQ631363.1_Asia1_CHA_2006

GGATTGGTTGTTGACACCAGAGATGTGGAGGAGCGCGTGCACGTCATGCGCAAAACCAAGCTTGCACCCA

CCGTAGCACACGGTGTGTTCAACCCTGAATTTGGGCCTGCTGCCTTGTCCAACAAGGACCCGCGCTTGAA

CGAAGGAGTTGTCCTCGATGAAGTCATCTTCTCCAAGCACAAGGGAGACACAAAGATGTCTGAGGAGGAC

AAAGCGCTGTTCCGACGTTGCGCTGCCGACTACGCGTCGCGCTTGCACAGCGTGCTGGGTACAGCAAATG

CCCCATTGAGCATTTACGAGGCAATCAAGGGCGTCGACGGACTCGATGCCATGGAACCAGACACCGCACC

TGGCCTTCCCTGGGCCCTCCAGGGGAAACGCCGTGGTGCGCTGATTGACTTCGAGAACGGCACAGTCGGA

CCCGAGGTCGAGGCTGCCCTAAAGCTCATGGAGAAAAGAGAATACAAGTTTGCTTGTCAGACCTTCCTGA

AGGACGAGATTCGCCCGATGGAGAAAGTACGTGCCGGCAAGACTCGCATTGTCGATGTCTTGCCTGTTGA

ACACATTCTTTACACCAGGATGATGATTGGCAGATTTTGCGCTCAAATGCACTCAAACAACGGACCGCAA

ATTGGCTCGGCGGTCGGTTGCAACCCTGATGTTGATTGGCAACGATTTGGCACACATTTTGCCCAGTACA

GAAACGTGTGGGATGTGGACTATTCGGCCTTTGATGCTAACCACTGCAGCGATGCAATGAACATCATGTT

TGAGGAGGTGTTTCGCACAGAGTTTGGCTTCCACCCGAACGCCGAGTGGATCCTGAAGACTCTTGTGAAT

ACGGAACATGCTTATGAGAACAAACGCATTGTTGTCGAGGGCGGGATGCCGTCTGGCTGTTCCGCGACAA

GCATCATAAACACAATTTTGAACAACATTTACGTGCTCTACGCTTTGCGCAGACACTATGAGGGAGTTGA

GCTGGACACTTACACCATGATCTCCTACGGAGATGACATCGTGGTGGCAAGTGACTACGATCTGGACTTT

GAGGCCCTCAAGCCTCACTTCAAATCTCTGGGCCAAACCATCACTCCAGCTGACAAAAGCGACAAAGGTT

TTGTTCTTGGTCACTCCATTACTGATGTCACTTTCCTCAAAAGACACTTCCACATGGATTATGGAACTGG

GTTTTACAAACCTGTAATGGCCTCGAAGACCCTCGAGGCTATCCTCTCCTTTGCACGCCGTGGG

>HQ632768.1_O_MAY_2000

GGATTGATAGTTGACACCAGAGATGTTGAGGAGCGCGTACACGTCATGCGCAAAACCAAGCTCGCACCCA

CCGTGGCACACGGTGTGTTTAACCCCGAATTTGGGCCTGCCGCCTTGTCCAACAAGGACCCGCGCCTGAA

TGAGGGGGTTGTCCTCGATGAAGCCATCTTCTCCAAACACAAAGGAAACACAAAGATGTCTGAGGAGGAC

AAAGCGCTGTTCCGCCGCTGTGCTGCTGACTACGCGTCGCGTCTGCATAGCGTGCTGGGTACTGCAAATG

CCCCACTGAGCACTTACGAGGCAATCAAGGGCGTCGACGGACTTGACGCCATGGAACCGGACACCGCGCC

TGGTCTCCCCTGGGCTCTCCAGGGGAAACGCCGTGGTGCGCTCATTGACTTCGAGAACGGCACTGTTGGA

CCCGAGGTTGAAGCTGCCTTGAAGCTCATGGAGAAAAGAGAGTACAAGTTTGTATGCCAGACCTTCCTGA

AGGACGAGATTCGCCCGATGGAGAAAGTACGTGCCGGCAAGACTCGCATTGTCGACGTCCTGCCTGTTGA

ACACATTCTTTACACCAGGATGATGATTGGCAGATTTTGTGCTCAAATGCACTCAAACAACGGACCGCAA

ATTGGCTCGGCGGTTGGTTGTAATCCTGATGTTGATTGGCAAAGATTTGGCACGCATTTTGCTCAGTACA

GAAACGTGTGGGATGTGGACTATTCGGCCTTTGATGCCAACCACTGCAGTGACGCAATGAACATCATGTT

TGAGGAGGTGTTCAACACGGATTTCGGTTTCCACCCAAACGCTGAGTGGATCCTGAAAACTCTCGTGAAC

ACTGAACACGCCTATGAGAACAAACGCATCACTGTTGAAGGCGGGATGCCGTCTGGTTGTTCCGCAACAA

GCATCATCAACACAATTTTGAACAACATCTACGTGCTCTACGCCTTGCGTAGACACTACGAGGGAGTTGA

GCTGGACTCTTACACCATGATCTCCTACGGAGACGACATCGTGGTTGCAAGTGATTACGATCTGGACTTT

GAGGCCCTCAAGCCTCACTTCAAATCCCTTGGTCAAACCATTACTCCGGCTGACAAAAGCGACAAAGGTT

TTGTTCTTGGTCACTCCATTACCGATGTCACTTTCCTCAAAAGACACTTCCACATGGACTATGGAACTGG

GTTTTACAAACCTGTGATGGCTTCGAAGACCCTCGAGGCTATCCTCTCCTTTGCACGCCGTGGG

>HQ632769.1_O_MAY_2001

GGGTTGATTGTTGACACCAGAGATGTGGAAGAGCGCGTACACGTTATGCGCAAAACCAAGCTTGCACCCA

CCGTGGCACACGGTGTGTTCAATCCTGAATACGGCCCCGCTGCCTTGTCCAACAAGGACCCGCGGCTGAA

TGAGGGAGTCGTCCTCGACGAGGTCATTTTCTCCAAACACAAAGGAGACACAAAGATGTCCCCGGAGGAC

AAAGAGCTGTTTCGCCGCTGTGCTGCCGACTACGCGTCGCGCCTACACAGTGTGCTGGGTACAGCAAATG

CCCCACTGAGCATCTACGAGGCCATCAAAGGTGTCGACGGACTCGACGCCATGGAACCAGACACAGCGCC

TGGCCTCCCCTGGGCCCTCCAGGGGAAGCGCCGTGGTGCGTTGATTGACTTCGAGAACGGCACAGTCGGA

CCCGAAGTTCAGGCTGCCTTAGAGCTCATGGAGAAGAGAGAATACAAATTTGCTTGCCAGACCTTCCTGA

AGGACGAGATTCGCCCGATGGAGAAAGTACGTGCCGGCAAGACTCGCATTGTCGATGTCCTGCCTGTTGA

ACACATTCTTTACACCAGGATGATGATTGGCAGATTTTGTGCACAGATGCACTCAAACAACGGCCCGCAA

ATTGGATCAGCGGTCGGTTGTAATCCTGATGTTGATTGGCAGAGATTTGGCACACACTTCGCCCAATACA

GAAACGTGTGGGATGTGGACTATTCGGCCTTTGATGCTAACCACTGCAGTGACGCGATGAACATCATGTT

TGAGGAGGTGTTTCGCACAGACTTTGGCTTCCACCCAAACGCTGAGTGGATTCTGAAGACCCTTGTGAAC

ACGGAACACGCCTATGAAAACAAACGCATCACTGTTGAAGGCGGGATGCCGTCTGGTTGTTCCGCAACTA

GCATCATCAACACAATTTTGAACAACATCTACGTGCTCTACGCGCTGCGTAGACACTATGAGGGAGTTGA

GCTGGACACTTACACCATGATCTCCTATGGAGACGACATCGTGGTTGCAAGTGATTACGATCTGGACTTT

GAGGCTCTCAAGCCCCACTTCAAATCTCTTGGTCAAACCATCACTCCAGCTGACAAAAGCGACAAAGGTT

TTGTTCTTGGTCACTCCATCACCGATGTCACTTTCCTCAAAAGACACTTCCACATGGATTATGGAACTGG

GTTTTACAAACCTGTGATGGCTTCGAAGACCCTCGAGGCTATCCTCTCCTTTGCACGCCGTGGG

>HQ632770.1_O_MAY_2004

GGGTTGATCGTTGACACCAGAGATGTGGAAGAGCGCGTGCACGTCATGCGCAAAACCAAGCTTGCACCCA

CCGTTGCACACGGTGTGTTCAACCCTGAATTCGGCCCCGCCGCTTTGTCCAACAAAGACCCGCGGCTGAA

TGAAGGGGTTGTCCTTGATGAAGTCATCTTCTCCAAGCATAAGGGAGACACCAAGATGTCGGAAGAGGAC

AAAGCGCTGTTCCGTCGCTGCGCTGCCGACTACGCGTCGCGCTTGCACAGTGTTCTGGGTACGGCAAATG

CCCCATTGAGCATTTACGAGGCAATCAAGGGCGTCGACGGACTCGATGCCATGGAACCAGACACCGCGCC

TGGCCTCCCCTGGTCCCTCCAGGGGAAACGCCGTGGTGCGCTCATCGACTTCGAGAACGGCACTGTCGGA

CCCGAGGTTGAAGCTGCCTTGAAGCTCATGGAGAAAAGAGAATACAAGTTTGCATGTCAGACCTTCCTGA

AGGACGAAATTCGCCCGATGGAGAAAGTACGTGCCGGCAAGACTCGCATCGTCGATGTCCTGCCTGTTGA

ACACATTCTTTACACCAGAATGATGATTGGCAGATTCTGTGCTCAAATGCACTCAAACAACGGGCCGCGA

ATTGGCTCAGCGGTCGGATGCAACCCTGACGTTGATTGGCAGAGATTTGGCACACATTTTGCTCAGTACA

GAAACGTGTGGGATGTGGATTATTCGGCCTTTGATGCTAACCATTGCAGTGACGCCATGAACATCATGTT

TGAGGAGGTGTTCCGCACGGAATTCGGTTTCCACCCTAACGCTGAGTGGATCCTGAAGACTCTCGTGAAT

ACGGAGCACGCGTACGAAAACAAACGCATCACTGTCGAGGGCGGGATGCCGTCAGGCTGTTCCGCAACAA

GCATCATCAACACAATTCTGAACAACATTTACATGCTCTACGCCTTGCGTAGACACTATGAGGGAGTTGA

GCTGGACACTTACACCATGATCTCCTACGGAGACGACATCGTGGTTGCAAGTGATTACGATCTGGACTTT

GAGGCCCTCAAGCCTCACTTCAAATCTCTTGGTCAAACCATCACCCCAGCCGACAAAAGCGACAAAGGTT

TTGTTCTTGGTCACTCCATTACCGACGTCACTTTCCTCAAAAGACACTTCCACATGGACTATGGAACTGG

GTTTTACAAACCTGTGATGGCCTCGAAGACCCTCGAGGCTATCCTCTCCTTTGCACGCCGTGGG

>HQ632771.1_O_MAY_2005

GGGTTGATCGTTGACACCAGAGACGTGGAGGAGCGTGTCCACGTGATGCGCAAAACCAAGCTTGCACCCA

CCGTGGCACACGGTGTGTTCAACCCTGAGTTCGGGCCTGCTGCTCTGTCCAACAGGGACCCGCGCCTAAA

CGAAGGGGTTGTCCTCGACGACGTCATTTTCTCCAAACACAAGGGAGACACGAGGATGTCTGAGGAGGAT

AAAGTGCTGTTCCGGCGCTGTGCTGCCGACTACGCGTCGCGCCTGCACAGCGTGTTGGGGACAGCAAACG

CCCCACTAAGCGTCTACGAGGCCATCAAAGGGGTCGACGGGCTTGACGCCATGGAACCGGACACCGCACC

CGGCCTCCCCTGGGCTCTCCAGGGGAAACGCCGCGGTGCCCTAATCGACTTCGAGAACGGTACCGTCGGA

CCGGAAGTTGAGGCAGCACTCAAGCTCATGGAAAAACGTGAGTATAGATTCGTCTGCCAAACCTTCTTGA

AGGACGAAATTCGGCCGCTGGAAAAGGTGCGCGCTGGTAAGACACGCATCGTCGATGTCTTGCCTGTTGA

ACACATTCTCTACACTAGAATGATGATTGGCAGATTTTGTGCCCAGATACACTCAAACAACGGACCGCAA

ATTGGATCGGCGGTCGGTTGTAACCCTGACGTTGATTGGCAAAGATTCGGCACACATTTCGCCCAATACA

AGAACGTCTGGGATGTGGACTACTCGGCCTTTGATGCAAACCACTGCAGCGATGCGATGAACATTATGTT

CGAAGAGGTGTTCCGCACGGAGTTTGGGTTTCACCCGAATGCTGAGTGGATCCTGAAAACTTTGGTGAAC

ACTGAGCACGCTTACGAGAACAAGCGCATCGTTGTGGAAGGCGGAATGCCGTCCGGCTGTTCCGCAACGA

GCATCATCAACACAATTTTAAACAACATCTACGTGCTTTACGCTCTGCGCAGGCACTACGAGGGAGTCGA

GCTGGACACTTACACCATGATCTCTTATGGAGACGACATCGTGGTGGCAAGTGACTACGACCTGGACTTT

GAGGCTCTCAAGCCCCACTTCAAGTCCCTCGGTCAGACAATCACCCCAGCCGACAAAAGTGACAAGGGTT

TTGTTCTTGGTCACTCCATAACTGATGTCACTTTCCTCAAAAGACACTTCCACATGGACTACGGAACTGG

GTTTTACAAACCTGTGATGGCCTCGAAGACCCTCGAGGCTATCCTCTCCTTTGCACGCCGTGGG

>HQ632772.1_O_MAY_2007

GGGTTGATTGTTGACACCAGAGACGTGGAAGAGCGCGTGCACGTCATGCGCAAAACCAAGCTCGCACCCA

CCGTGGCGCACGGTGTGTTCAACCCCGAATACGGTCCCGCTGCCTTGTCCAACAAGGATCCGCGACTGGA

CGAAGGGGTTGTCCTCGACGAAGTCATTTTCTCCAAGCACAAAGGAGACACAAAGATGACACCAGAGGAC

AAAGCGCTGTTCCGCCGTTGCGCTGCCGACTACGCGTCACGTTTGCACAGTGTGCTGGGTACGGCAAATG

CCCCATTGAGCATCTATGAGGCCATCAAAGGTGTCGACGGACTCGACGCCATGGAGCCGGACACAGCGCC

TGGTCTCCCCTGGGCCCTCCAGGGGAAGCGCCGCGGCGCGCTGATTGACTTCGAGAACGGCACGGTCGGA

CCCGAAGTTCAGGCTGCCTTAGAGCTCATGGAGAAAAGAGAATACAAGTTTGTTTGTCAGACCTTCCTGA

AGGACGAGATTCGCCCGATGGAGAAAGTGCGTGCCGGCAAGACCCGCATTGTCGATGTCCTGCCTGTTGA

ACATATTCTTTACACCAGGATGATGATTGGCAGGTTTTGTGCACAAATGCACTCAAACAACGGCCCGCAA

ATTGGATCTGCGGTTGGCTGTAACCCTGATGTTGATTGGCAAAGATTTGGCACACATTTCGCCCAATACA

GAAACGTGTGGGATGTGGACTATTCGGCCTTTGATGCTAACCACTGCAGTGACGCAATGAACATCATGTT

TGAGGAGGTGTTTCGCACAGACTTTGGCTTCCACCCGAACGCTGAGTGGATACTGAAGACCCTTGTGAAC

ACGGAACACGCCTATGAGAACAAACGCATCACTGTTGAAGGCGGAATGCCCTCTGGTTGTTCCGCGACTA

GCATCATTAACACAATTTTGAACAACATCTACGTGCTCTACGCGTTGCGTAGACACTATGAGGGTGTTGA

GCTGGACACTTACAACATGATCTCTTACGGAGACGACATCGTGGTGGCGAGTGATTATGATCTGGACTTT

GAGGCTCTCAAGCCTCACTTCAAATCTCTTGGTCAAACCATTACTCCAGCTGACAAAAGTGACAAAGGTT

TTGTTCTTGGTCATTCCATTACTGATGTTACTTTCCTCAAAAGACACTTCCACATGGACTATGGAACTGG

GTTTTACAAACCTGTGATGGCCTCGAAGACCCTCGAGGCCATCCTCTCCTTTGCACGCCGTGGG

>HQ632773.1_A_MAY_2007

GGGTTGATTGTTGACACCAGAGACGTGGAAGAGCGCGTGCACGTCATGCGTAAAACCAAGCTTGCGCCCA

CCGTGGCTCACGGTGTGTTCAACCCCGAGTACGGCCCCGCTGCCTTGTCCAACAAGGACCCGCGGCTGAA

TGAGGGAGTTGTCCTCGACGAGGTCATCTTCTCCAAACACAAGGGAGACACAAAGATGTCACCGGAGGAC

AAAGCGCTGTTCCGCCGCTGTGCTGCCGACTACGCGTCGCGTTTGCACGGTGTGCTGGGTACGGCAAATG

CCCCATTGAGCATCTACGAGGCCATCAAAGGTGTCGACGGACTCGACGCCATGGAACCTGACACAGCGCC

CGGCCTCCCCTGGGCCCTCCAGGGGAAGCGTCGTGGTGCGCTGATTGACTTTGAGAACGGCACGGTCGGG

CCCGAAGTCCAGGCTGCTCTAGAGCTCATGGAGAAAAGAGAATACAAGTTTGCTTGCCAGACCTTCCTGA

AGGACGAGATTCGCCCGTTGGAAAAAGTACGTGCCGGCAAGACTCGCATTGTCGATGTCTTGCCTGTTGA

ACACATTCTTTACACTAGGATGATGATTGGCAGATTCTGTGCACAAATGCACTCAAACAACGGCCCTCAA

ATTGGATCAGCGGTCGGTTGTAATCCTGATGTTGATTGGCAAAGATTTGGAACACACTTCGCCCAATACA

GAAACGTGTGGGATGTGGACTATTCGGCCTTTGATGCTAACCACTGCAGTGACGCAATGAACATCATGTT

TGAGGAGGTGTTTCGCACAGACTTTGGCTTCCACCCGAACGCTGAGTGGATTCTGAAGACTCTTGTGAAC

ACGGAACACGCCTATGAAAACAAACGCATTACTGTTGAAGGCGGGATGCCGTCCGGCTGTTCCGCAACTA

GCATCATCAACACAATTTTGAACAACATCTACGTGCTCTACGCCCTGCGCAGACACTATGAGGGAGTTGA

GCTGGACACTTACACCATGATCTCCTACGGGGACGACATCGTGGTCGCAAGTGATTACGATCTGGACTTT

GAGGCTCTCAAGCCCCACTTCAAATCTCTTGGTCAAACCATCACCCCAGCCGACAAAAGCGACAAAGGTT

TTGTTCTTGGTCACTCCATCACCGATGTCACTTTCCTCAAAAGACACTTCCACATGGATTATGGAACTGG

GTTTTACAAACCTGTGATGGCTTCGAAGACCCTCGAAGCTATCCTCTCCTTTGCACGCCGTGGG

>HQ632774.1_Asia1_MAY_1999

GGGTTGATCGTTGATACCAGAGATGTGGAAGAGCGCGTGCACGTCATGCGCAAAACTAAGCTTGCACCCA

CCGTGGCCCACGGTGTGTTCAACCCTGAATACGGCCCCGCTGCCTTGTCCAACAAGGACCCGCGGCTGCA

TGAAGGAGTCAGCCTCGACGAGGTCATTTTCTCCAAACACAAAGGAGACACGAAGATGTCACTCGAGGAT

AAAGCGCTGTTTCGCCGCTGTGCTGCCGACTACGCGTCGCACCTACACAGCGTGCTGGGCACAGCAAATG

CCCCACTGAGCACCTATGAGGCCATCAAGGGTGTCGATGGACTCGACGCCATGGAGCCAGACACAGCGCC

TGGTCTCCCCTGGGCCCTTCAGGGGAAACGCCGTGGTGCGCTGATCGACTTCGAGAACGGCACAGTCGGA

CCTGAAGTTCAGGCTGCCTTGGAGCTCATGGAGAAAAGAGAATACAAATTTGTTTGCCAGACCTTCCTGA

AGGACGAAATTCGTCCGATGGAGAAAGTGCGTGCCGGCAAGACTCGCATTGTCGATGTCCTGCCTGTTGA

ACATATTCTTTACACCAGGATGATGATTGGCAGATTTTGTGCGCAGATGCACTTAAACAACGGCCCGCAA

ATTGGATCAGCGGTCGGTTGTAACCCTGATGTTGATTGGCAAAGATTTGGCACACATTTTGCCCAATACA

GAAACGTGTGGGATGTGGACTATTCGGCCTTTGATGCTAACCACTGCAGTGACGCGATGAACATCATGTT

TGAGGAGGTGTTTCGCACAGACTTTGGCTTCCACCCAAACGCTGAGTGGATTCTGAAGACTCTTGTGAAC

ACGGAACACGCCTATGAGAACAAACGCATCACCGTTGAAGGCGGGATGCCGTCTGGCTGTTCCGCAACTA

GCATCATCAACACAATTCTGAACAACATTTACGTGCTCTACGCGCTGCGTAGACACTATGAGGGAGTTGA

GCTGGACACTTACACCATGATCTCCTACGGAGACGACATCGTGGTTGCAAGTGATTACGATCTGGACTTT

GAGGCTCTTAAGCCCCACTTCAAATCTCTTGGTCAAACCATCACTCCGGCTGACAAAAGCGACAAAGGTT

TTGTTTTTGGTCACTCCATTACCGATGTCACTTTCCTCAAAAGACACTTCCACATGGATTACGGAACCGG

GTTTTACAAACCTGTGATGGCTTCGAAGACCCTCGAGGCTATCCTCTCCTTTGCACGCCGTGGG

>HQ832576.1_A_IND_1990

GGGTTGATTGTTGACACCAGAGATGTGGAAGAGCGCGTGCATGTCATGCGCAAAACCAAGCTTGCACCCA

CCGTGGCACACGGTGTGTTCAACCCTGAATTTGGCCCCGCCGCCTTGTCCAACAAGGACCCGCGGCTGAA

TGAAGGAGTTGTCCTTGATGAGGTCATCTTCTCCAAGCACAAGGGAGACACCAAGATGTCTGAGGAGGAC

AAAGCGCTGTTCCGCCGCTGCGCTGCTGACTACGCGTCACGCTTGCACAGTGTGCTGGGTACGGCAAATG

CCCCATTGAGCATCTACGAGGCAATCAAGGGCGTTGACGGACTCGACGCCATGGAACCAGACACCGCGCC

TGGCCTCCCCTGGGCCCTCCAGGGGAAACGCCGTGGTGCGCTCATCGACTTCGAGAACGGCACGGTCGGA

CCCGAGGTTGAAGCTGCCTTGAAGCTCATGGAGAAAAGAGAATACAAATTTGCTTGTCAGACTTTCCTGA

AGGACGAAATTCGCCCGATGGAGAAAGTACGTGCCGGCAAGACTCGCATTGTCGATGTGTTGCCTGTTGA

ACATATTCTTTACACCAGGATGATGATTGGTAGATTTTGTGCTCAAATGCACTCAAACAACGGACCGCAA

ATTGGCTCGGCGGTCGGTTGCAACCCTGATGTTGATTGGCAAAGATTTGGCACACATTTCGCTCAGTAC-

--AACGTGTGGGATGTGGATTATTCGGCCTTTGATGCTAACCACTGCAGTGACGCGATGAACATCATGTT

TGAGGAGGTGTTCCGCACGGAATTCGGTTTCCACCCAAACGCTGAGTGGATCCTGAAAACTCTCGTGAAC

ACGGAACACGCCTATGAGAACAAACGCATCACTGTTGAGGGCGGGATGCCATCTGGCTGTTCCGCGACAA

GCATCATCAACACAATTCTGAACAACATCTACGTGCTCTACGCTCTGCGTAGACACTATGAGGGAGTTGA

GCTGGACACCTACACCATGATCTCCTACGGAGACGACATCGTGGTTGCAAGTGATTACGATCTGGACTTT

GAGGCCCTCAGACCTCACTTCAAATCTCTTGGTCAAACCATCACTCCAGCTGACAAAAGCGACAAAGGTT

TTGTTCTTGGTGACTCCATCACCGATGTCACTTTCCTCAAAAGGCATTTCCACATGGATTATGGAACTGG

GTTTTACAAACCTGTGATGGCCTCGAAGACCCTCGAGGCTATCCTCTCCTTTGCACGCCGTGGG

>HQ832577.1_A_IND_1999

GGGTTGATTGTTGACACCAGAGATGTGGAAGAGCGCGTGCACATCATGCGCAAAACAAAGCTTGCACCCA

CCGTTGCACACGGCGTGTTCAACCCTGAATTTGGCCCCGCTGCCTTGTCCAACAAGGACCCGCGGCTAAA

TGAAGGGGTTGTCCTCGATGAGGTCATCTTCTCCAAACACAAGGGAGACACCAGGATGTCAGAGGAGGAC

AAAGCGCTGTTCCGCTGCTGCGCTGCCGACTACGCGTCGCGCTTGCACAGTGTTTTGGGTACAGCAAATG

CCCCATTGAGCATCTACGAGGCAATCAAAGGCGTCGACGGACTCGATGCCATGGAACCAGACACCGCGCC

TGGTCTTCCCTGGGCCCTCCAGGGGAAACGCCGTGGTGCGCTCATCGACTTCGAGAACGGCACTGTCGGA

CCCGAGGTTGAAGCTGCCCTAAAGCTCATGGAGAAAAGAGAGTACAAATTTGCTTGCCAGACCTTCCTGA

AGGACGAGATCCGCCCGATGGAGAAAGTGCGTGCCGGCAAGACTCGCATCGTCGATGTCCTGCCTGTTGA

ACACATTCTTTACACCAGGATGATGATTGGCAGATTCTGTGCTCAAATGCACTCAAACAACGGACCGCAG

ATTGGCTCAGCGGTCGGTTGCAATCCTGATGTTGATTGGCAAAGATTTGGCACACATTTCGCTCAGTACA

AGAACGTGTG------------------------------------------------------------

----------------------------------------------------------------------

----------------------------------------------------------------------

----------------------------------------------------------------------

----------------------------------------------------------------------

----------------------------------------------------------------------

----------------------------------------------------------------------

-------AAACCTGTGATGGCCTCGAAGACCCTCGAGGCTATCCTCTCCTTTGCACGCCGTGGG

>HQ832578.1_A_IND_2003

GGGTTGATTGTTGACACCAGAGATGTGGAGGAGCGCGTGCATGTCATGCGCAAAACCAAGCTTGCACCCA

CCGTAGCACACGGTGTGTTCAACCCTGAATTTGGCCCCGCTGCCTTGTCAAACAAGGACCCGCGGCTGAA

CGAAGGAGTTGTCCTCGATGAGGTCATCTTCTCCAAACACAAGGGAGACACCAAGATGTCTGAGGAGGAC

AAAGCGCTGTTCCGCCGCTGCGCTGCTGACTACGCGTCACGCTTGCACAGTGTGTTGGGTACGGCAAATG

CCCCACTGAGCATATACGAGGCAATCAAAGGCGTCGACGGACTCGACGCCATGGAACCAGACACCGCACC

CGGTCTCCCTTGGGCCCTCCAAGGGAAACGCCGTGGTGCGCTCATCGACTTCGAGAACGGCACTGTCGGA

CCCGAGGTTGAAGCTGCCCTGAAGCTCATGGAGAAAAGAGAATACGAGTTTGCTTGTCAGACCTTCCTGA

AGGACGAAATTCGCCCGATGGAGAAAGTACGTGCCGGCAAGACTCGCATTGTCGACGTTTTGCCCGTTGA

ACACATTCTCTACACTAGGATGATGATTGGCAGATTTTGTGCACAAATGCACTTAAACAACGGACCGCAA

ATTGGCTCGGCGGTCGGCTGTAATCCTGATGTTGATTGGCAAAGATTTGGCACACATTTCGCTCAGTACA

GAAACGTGTGGGATGTGGATTATTCGGCCTTTGATGCTAACCACTGCAGTGACGCGATGAACATCATGTT

TGAGGAGGTGTTTCGCACAGAGTTCGGCTTCCACCCAAATGCTGAGTGGATTCTGAAGACTCTTGTGAAC

ACGGAACACGCCTATGAGAACAAACGCATCACTGTGGAGGCCGGGATGCCATCTGGCTGTTCCGCAACGA

GCATCATCAACACAATCCTGAACAACATTTACGTGCTCTACGCCCTGCGTAGACACTATGAGGGAGTTGA

GCTGGACACTTACACCATGATCTCCTACGGAGACGACATCGTGGTTGCAAGTGATTACGACCTGGACTTT

GAGGCTCTCAAGCCTCACTTCAAATCTTTGGGCCAGACCATCACTCCAGCTGACAAAAGCGACAAAGGTT

TTGTTCTTGGTCACTCCATCACCGATGTCACTTTCCTCAAAAGACACTTCCACATGGATTATGGAACTGG

GTTTTACAAACCTGTGATGGCCTCGAAGACCCTCGAGGCTATCCTCTCCTTTGCACGCCGTGGG

>HQ832579.1_A_IND_2003

GGGTTGATCGTTGACACCAGAGATGTGGAGGAGCGCGTGCATGTCATGCGCAAAACCAAGCTTGCACCCA

CCGTAGCACACGGTGTGTTCAACCCTGAATTTGGCCCCGCTGCCTTGTCAAACAAGGACCCGCGGCTGAA

CGAAGGAGTTGTCCTCGATGAGGTCATCTTCTCCAAACACAAGGGAGACACCAAGATGTCTGAGGAGGAC

AAAGCGCTGTTCCGCCGCTGCGCTGCTGACTACGCGTCACGCTTGCACAGTGTGTTGGGTACGGCAAATG

CCCCATTGAGCATATACGAGGCAATCAAGGGCGTCGACGGACTCGACGCCATGGAACCAGACACCGCACC

CGGTCTCCCTTGGGCCCTCCAAGGGAAACGCCGTGGTGCGCTCATCGACTTCGAGAACGGCACTGTCGGA

CCCGAGGTTGAAGCCGCCCTGAAGCTCATGGAGAAAAGAGAATACAAGTTTGCTTGTCAGACCTTCCTGA

AGGACGAAATTCGCCCGATGGAGAAAGTACGTGCCGGCAAGACTCGCATTGTCGACGTTTTGCCCGTTGA

ACACATTCTCTACACTAGGATGATGATTGGCAGATTTTGTGCACAAATGCACTCAAACAACGGACCGCAA

ATTGGCTCGGCGGTCGGCTGTAATCCTGATGTTGATTGGCAAAGATTTGGCACACATTTCGCTCAGTACA

GAAACGTGTGGGATGTGGATTATTCGGCCTTTGATGCTAACCACTGCAGTGACGCGATGAACATCATGTT

TGAGGAGGTGTTTCGCACAGAGTTCGGCTTCCACCCAAATGCTGAGTGGATTCTGAAGACTCTCGTGAAC

ACGGAGCACGCCTATGAGAACAAACGCATCACTGTGGAGGGCGGGATGCCATCTGGCTGTTCCGCAACGA

GCATCATCAACACAATCCTGAACAACATTTACGTGCTCTACGCCCTGCGTAGACACTATGAGGGAGTTGA

GCTGGACACTTACACCATGATCTCCTACGGAGACGACATCGTGGTTGCAAGTGATTACGATCTGGACTTT

GAGGCTCTCAAGCCTCACTTCAAATCTTTGGGCCAAACCATCACTCCAGCTGACAAAAGCGACAAAGGTT

TTGTTCTTGGTCACTCCATCACCGATGTCACTTTCCTCAAAAGACACTTCCACATGGATTATGGAACTGG

GTTTTACAAACCTGTGATGGCTTCGAAGACCCTCGAGGCTATCCTCTCCTTTGCACGCCGTGGG

>HQ832580.1_A_IND_2003

GGGTTGATTGTTGACACCAGAGATGTGGAGGAGCGCGTGCATGTCATGCGCAAAACCAAGCTTGCACCCA

CCGTAGCACACGGTGTGTTCAACCCTGAATTTGGCCCCGCTGCCTTGTCAAACAAGGACCCGCGGCTGAA

CGAAGGAGTTGTCCTCGATGAGGTCATCTTCTCCAAACACAAGGGAGACACCAAGATGTCTGAGGAGGAC

AAAGCGCTGTTCCGCCGCTGCGCTGCTGACTACGCGTCACGCTTGCATAGTGTGTTGGGTACGGCAAATG

CCCCATTGAGCATATACGAGGCAATCAAGGGCGTTGACGGACTCGACGCCATGGAACCAGACACCGCACC

CGGTCTCCCTTGGGCCCTCCAAGGGAAACGCCGTGGTGCGCTCATCGACTTCGAGAACGGCACTGTCGGA

CCCGAGGTTGAAGCTGTCCTGAAGCTCATGGAGAAAAGAGAATACAAGTTTGCTTGTCAGACCTTCCTGA

AGGACGAAATTCGCCCGATGGAGAAAGTACGTGCCGGCAAGACTCGCATTGTCGACGTTTTGCCCGTTGA

ACACATTCTCTACACTAGGATGATGATTGGCAGATTTTGTGCACAAATGCACTTAAACAACGGACCACGA

ATTGGCTCGGCGGTCGGCTGTAATCCTGATGTTGACTGGCAAAGATTTGGCACACATTTCGCTCAATACA

GAAACGTGTGGGATGTGGATTATTCGGCCTTTGACGCTAACCACTGCAGTGACGCGATGAACATCATGTT

TGAGGAAGTGTTTCGCACAGAGTTCGGCTTCCACCCAAATGCTGAGTGGATTCTGAAGACTCTCGTGAAC

ACGGAACACGCCTATGAGAACAAACGCATCACTGTGGAGGGCGGGATGCCATCTGGCTGTTCCGCAACGA

GCATCATCAACACAATCCTGAACAACATTTACGTGCTCTACGCCCTGCGTAGACACTATGAGGGAGTTGA

GCTGGACACTTACACCATGATCTCCTACGGAGACGACATCGTGGTTGCAAGTGATTACGATCTGGACTTT

GAGGCTCTCAAGCCTCACTTCAAATCTTTGGGCCAAACCATCACTCCAGCTGACAAAAGCGACAAAGGTT

TTGTTCTTGGTCACTCCATCACCGATGTCACTTTCCTCAAAAGACACTTCCACATGGATTATGGAACTGG

GTTTTACAAACCTGTGATGGCCTCAAAGACCCTCGAGGCTATCCTCTCCTTTGCACGCCGTGGG

>HQ832581.1_A_IND_2004

GGGTTGATCGTTGACACCAGAGATGTGGAGGAGCGCGTGCATGTCATGCGAAAAACCAAGCTTGTACCCA

CCGTAGCACACGGTGTGTTCAACCCTGAATTTGGCCCCGCTGCCTTGTCAAACAAGGACCCGCGGCTGAA

CGAAGGAGTTGTCCTCGATGAGGTCATCTTCTCCAAACACAAGGGAGACACCAAGATGTCTGAGGAGGAC

AAAGCGCTGTTCCGCCGCTGCGCTGCTGACTACGCGTCACGCTTGCACAGTGTGTTGGGTACGGCAAATG

CCCCATTGAGTATATACGAGGCAATCAAGGGCGTCGACGGACTCGACGCCATGGAACCAGACACCGCACC

CGGTCTCCCTTGGGCCCTCCAAGGGAAACGCCGTGGTGCGCTCATCGACTTCGAGAACGGCACTGTCGGG

CCCGAGGTTGAAGCTGCCCTGAAGCTCATGGAGAAAAGAGAATATAAGTTTGCTTGTCAGACCTTCTTGA

AGGACGAAATTCGCCCGATGGAGAAAGTACGTGCCGGCAAGACTCGCATTGTCGACGTTTTGCCCGTTGA

ACACATTCTCTACACTAGGATGATGATTGGCAGATTTTGTGCACAAATGCACTCAAACAACGGACCGCAA

ATTGGCTCGGCGGTCGGCTGTAATCCTGATGTTGATTGGCAAAGATTTGGCACACATTTCGCTCAGTACA

AAAACGTGTGGGATGTGGATTATTCGGCCTTTGATGCTAACCACTGCAGTGACGCGATGAACATCATGTT

TGAGGAGGTGTTTCGCACAGAGTTCGGCTTCCACCCAAATGCTGAGTGGATTCTGAAGACTCTCGTGAAC

ACGGAGCACGCCTATGAGAACAAACGCATCACTGTGGAGGGCGGGATGCCATCTGGCTGTTCCGCAACGA

GCATCATCAACACAATCCTGAACAACATTTACGTGCTCTACGCCCTGCGTAGACACTATGAGGGAGTTGA

GCTGGACACTTACACCATGATCTCCTACGGAGACGACATCGTGGTTGCAAGTGATTACGATCTGGACTTT

GAGGCTCTCAAGCCTCACTTCAAATCTTTGGGCCAAACCATTACTCCAGCTGACAAAAGCGACAAAGGTT

TTGTTCTTGGTCACTCCATCACCGATGTCACTTTCCTCAAAAGACACTTCCACATGGATTATGGAACTGG

GTTTTACAAACCTGTGATGGCTTCGAAGACCCTCGAGGCTATCCTCTCCTTTGCACGCCGTGGG

>HQ832582.1_A_IND_2004

GGTTTGATTGTTGACACCAGAGATGTGGAGGAGCGCGTGCACGTCATGCGCAAAACCAAGCTTGCACCCA

CCGTAGCACACGGTGTGTTCAACCCTGAATTTGGCCCCGCTGCCTTGTCAAACAAAGACCCGCGGCTGAA

CGAAGGGGTTGTCCTCGATGAGGTCATCTTCTCCAAACACAAGGGAGACACCAAGATGTCTGTGGAGGAC

AAAGCGCTGTTCCGCCGCTGCGCTGCTGACTACGCGTCGCGCTTGCACAGTGTGTTGGGTACGGCAAATG

CCCCATTGAGCATATACGAGGCAATCAAGGGCGTTGACGGACTCGACGCCATGGAACCAGACACCGCACC

CGGTCTCCCTTGGGCCCTCCAAGGGAAACGCCGTGGTGCGCTCATCGACTTCGAGAACGGCACTGTCGGA

CCCGAGGTTGAAGCTGCCCTGAAGCTCATGGAGAAAAGAGAATACAAGTTTGCCTGTCAGACCTTCCTGA

AGGACGAAATTCGCCCGATGGAAAAAGTACGTGCCGGCAAGACTCGCATTGTCGACGTTTTGCCCGTTGA

ACACATTCTCTACACTAGGATGATGATTGGCAGATTTTGTGCACAAATGCACTCAAATAACGGACCGCAA

ATTGGCTCGGCGGTCGGCTGTAATCCTGATGTTGATTGGCAAAGATTTGGCACACATTTCGCTCAGTACA

GAAACGTGTGGGATGTGGATTATTCGGCCTTTGATGCTAACCACTGCAGTGACGCGATGAACATCATGTT

TGAGGAGGTGTTTCGCACAGAGTTCGGCTTCCACCCAAATGCTGAGTGGATCCTGAAGACTCTCGTGAAC

ACGGAGCACGCCTATGAGAACAAACGCATCACTGTGGAGGGCGGGATGCCATCTGGCTGTTCCGCAACGA

GCATCATCAACACAATCCTGAACAACATTTACGTGCTCTACGCCCTGCGTAGACACTATGAGGGAGTTGA

GCTGGACACTTACACCATGATCTCCTACGGAGACGACGTCGTGGTTGCAAGTGATTACGATTTGGACTTT

GAGGCTCTCAAGCCTCACTTCAAATCTTTTGGCCAAACCATCACTCCAGCTGACAAAAGCGACAAAGGTT

TTGTTCTTGGTCACTCCATCACCGATGTCACTTTCCTCAAAAGACACTTCCACATGGATTATGGAACTGG

GTTTTACAAACCTGTGATGGCCTCGAAGACCCTCGAGGCTATCCTCTCCTTTGCACGCCGTGGG

>HQ832583.1_A_IND_2005

GGGTTGATTGTTGACACCAGAGATGTGGAGGAGCGCGTGCATGTCATGCGCAAAACTAAGCTTGCACCCA

CCGTAGCACACGGTGTGTTCAACCCTGAATTTGGCCCCGCTGCCTTGTCAAACAAGGACCCGCGGCTGAA

CGAAGGAGTTGTCCTCGATGAGGTCATCTTCTCCAAACACAAGGGAGACACCAAGATGTCTGAGGAGGAC

AAAGCGCTGTTCCGGCGCTGCGCTGCTGACTACGCGTCACGCTTGCACAGTGTATTGGGTACGGCAAATG

CCCCATTGAGCATATACGAGGCAATCAAGGGCGTCGACGGACTCGACGCCATGGAACCAGACACCGCACC

CGGTCTCCCTTGGGCCCTCCAAGGGAAACGCCGTGGTGCGCTCATCGACTTCGAGAACGGCACTGTCGGA

CCCGAGGTTGAAGCTGCCCTCAAGCTCATGGAGAAAAGAGAATACAAGTTTGCTTGTCAGACCTTCCTGA

AGGACGAAATTCGCCCGATGGAGAAAGTACGTGCCGGCAAGACTCGCATTGTCGACGTTTTGCCCGTTGA

ACACATTCTCTACACTAGGATGATGATTGGCAGATTTTGTGCACAAATGCACTCAAACAACGGACCGCAA

ATTGGCTCGGCGGTCGGCTGTAACCCTGATGTTGATTGGCAAAGATTTGGCACACATTTCGCTCAGTACA

GAACCGTGTGGGATGTGGATTATTCGGCCTTTGATGCTAACCACTGCAGTGACGCGATGAACATCATGTT

TGAGGAGGTGTTTCGCACAGAGTTCGGCTTCCACCCAAATGCTGAGTGGATTCTGAAGACTCTCGTGAAC

ACGGAGCACGCCTATGAGAGCAAACGCATCACTGTAGAGGGCGGGATGCCATCTGGCTGTTCCGCAACGA

GCATCATCAACACAATCCTGAACAACATTTACGTGCTCTACGCCCTGCGTAGACACTATGAGGGAGTAGA

GCTGGACACTTACACCATGATCTCCTACGGAGACGACATCGTGGTTGCAAGTGATTACGATCTGGACTTT

GAGGCTCTTAAGCCTCACTTCAAATCTTTGGGCCAAACCATCACTCCCGCTGACAAAAGCGACAAAGGTT

TTGTTCTTGGTCACTCCATCACCGATGTCACTTTCCTCAAAAGACACTTCCACATGGATTATGGAACTGG

GTTTTACAAACCTGTGATGGCCTCGAAGACCCTCGAGGCTATCCTCTCCTTTGCACGCCGTGGG

>HQ832584.1_A_IND_2005

GGCTTGATTGTTGACACCAGAGATGTGGAAGAGCGCGTGCACGTCATGCGCAAAACCAAACTTGCACCCA

CCGTCGCGCACGGTGTGTTCAACCCTGAATTCGGGCCTGCCGCCTTGTCTAACAAGGATCCGCGCCTGAA

CGAGGGGGTCGTCCTCGATGAGGTCATCTTCTCCAAACACAAGGGAGACACCAAGATGTCTGAGGAGGAC

AAAGTGCTGTTCCGCCGCTGTGCTGCTGACTACGCGTCACGCCTGCATAGTGTGCTGGGTACGGCAAATG

CCCCACTGAGCATTTACGAGGCAATCAAAGGCGTTGACGGACTCGACGCCATGGAGCCAGACACCGCACC

TGGCCTTCCCTGGGCCCTCCAGGGAAAGCGCCGCGGCGCACTCATCGACTTCGAGAACGGCACGGTCGGA

CCCGAGGTTGAAGCTGCCTTGAAGCTCATGGAGAAAAGGGAATACAAATTTGCTTGTCAGACCTTCCTGA

AGGACGAAATTCGCCCAATGGAGAAAGTGCGTGCCGGTAAGACTCGCATCGTCGACGTCCTGCCCGTTGA

ACACATTCTTTACACCAGAATGATGATTGGCAGATTTTGTGCCCAAATGCACTCGAACAACGGACCGCAA

ATTGGCTCAGCGGTCGGTTGTAATCCTGATGTTGATTGGCAAAGATTTGGAACCCATTTTGCTCAGTACA

GAAATGTGTGGGATGTGGACTATTCGGCCTTTGATGCTAACCACTGCAGTGATGCGATGAACATCATGTT

TGAGGAGGTGTTTCGCACGGAGTTCGGCTTCCATCCAAACGCTGAGTGGATCCTGAAGACTCTAGTGAAC

ACGGAACACGCCTATGAGAACAAACGCATCACCGTTGAAGGCGGGATGCCGTCTGGCTGTTCCGCGACAA

GCATCATCAACACAATCTTGAACAACATTTACGTGCTCTACGCGCTGCGTAGACACTATGAGGGAGTTGA

GCTGGACACTTACACCATGATCTCCTACGGAGACGACATCGTGGTTGCAAGTGATTACGATTTGGACTTC

GAGGCCCTCAAGCCTCACTTTAAATCTCTTGGTCAAACCATCACTCCAGCTGACAAAAGCGACACAGGTT

TTGTTCTCGGTCACTCCATTACCGATGTCACTTTCCTCAAAAGACACTTCCACATGGATTATGGAACTGG

GTTTTACAAACCTGTGATGGCCTCAAAGACCCTTGAGGCTATCCTCTCCTTTGCACGCCGTGGG

>HQ832585.1_A_IND_2005

GGTTTGATTGTTGACACCAGAGATGTGGAAGAGCGCGTGCACGTCATGCGCAAAACCAAGCTTGCACCCA

CCGTCGCGCACGGTGTGTTCAACCCCGAGTTTGGCCCCGCTGCCTTGTCCAACAAGGACCCGCGGTTGAA

CGAGGGAGTTGTCCTCGATGAGGTCATCTTCTCCAAACACAAGGGAGACACAAAGATGTCTGAGGAGGAC

AAAGCGCTGTTCCGCCGCTGCGCTGCCGACTATGCGTCGCGCCTGCACAGTGTGTTGGGTACAGCAAATG

CCCCACTGAGCATTTACGAGGCGATCAAAGGCGTCGACGGACTCGATGCTATGGAACCAGACACCGCGCC

TGGCCTCCCCTGGGCTCTCCAGGGGAAACGCCGTGGTGCGCTCATCGACTTCGAGAACGGCACTGTCGGA

CCCGAGGTTGAGGCTGCCTTGAAGCTCATGGAGAAGAGAGAATACAAGTTTGCATGCCAGACCTTCCTGA

AGGACGAAATTCGCCCGATGGAGAAAGTACGTGCCGGCAAGACTCGCATTGTCGACGTCCTGCCCGTTGA

ACACATTCTTTACACTAGGATGATGATTGGCAGATTTTGTGCTCAAATGCACTCAAACAATGGACCGCAG

ATTGGCTCAGCGGTCGGCTGTAACCCTGATGTTGATTGGCAAAGATGCGGCACACATTGCGCTCAGTACA

GAAACGTGTGGGATGTGGATTATTCGGCCTTTGATGCTAACCACTGCAGTGACGCAATGAACATCATGTT

CGAGGAGGTGTTCCGCACGGACTTCGGGTTCCACCCAAACGCTGAGTGGATCCTGAAAACTCTCGTGAAC

ACGGAGCACGCCTATGAGAACAAGCGCATCACTGTTGAGGGCGGGATGCCGTCTGGTTGTTCCGCGACAA

GCATCATCAACACAATTTTGAACAACATTTACGTGCTCTACGCCTTGCGTAGACACTATGAGGGAGTTGA

GCTGGACACTTACACCATGATCTCCTACGGAGACGACATCGTGGTTGCGAGTGACTACGACTTGGACTTT

GAGGCTCTCAAGCCCCACTTCAAATCTCTTGGTCAAACCATCACTCCAGCTGACAAAAGCGACAAAGGTT

TTGTTCTTGGTCACTCCATTACCGATGTCACTTTCCTCAAAAGACACTTCCACATGGATTATGGAACTGG

GTTTTACAAACCTGTGATGGCCTCGAAGACCCTCGAGGCTATCCTCTCCTTTGCACGCCGTGGG

>HQ832586.1_A_IND_2006

GGGTTGATTGTTGACACCAGAGATGCGGAAGAGCGTGTGCACGTCACGCGCAAAACCAAGCTTGCACCCA

CCGTTGCACACGGTGTGTTCAACCCCGAATTCGGCCCCGCTGCCTTGTCCAACAAGGACCCGCGGTTGAA

TGAGGGAGTTGTCCTCGATGAAGTCATCTTCTCCAAACACAAGGGAGACACCAAGATGTCTGAGGAGGAC

AAAGCGCTGTTCCGCCGCTGCGCTGCTGACTACGCGTCACGCCTGCACAGTGTGTTGGGTACGGCAAATG

CCCCACTGAGCATTTACGAGGCGATCAAAGGCGTCGACGGACTCGATGCTATGGAACCAGACACCGCGCC

TGGCCTCCCCTGGGCTCTCCAGGGGAAACGCCGTGGTGCGCTCATCGACTTCGAGAACGGCACTGTCGGA

CCCGAGGTTGAGGCTGCCTTGAAGCTCATGGAGAAGAGAGAATACAAGTTTGCATGCCAGACCTTCCTGA

AGGACGAAATTCGCCCGATGGAGAAAGTACGTGCCGGCAAGACTCGCATTGTCGACGTCCTGCCCGTTGA

ACACATTCTTTACACTAGGATGATGATTGGCAGATTTTGTGCACAAATGCACTCAAACAACGGACCGCAA

ATTGGCTCAGCGGTCGGTTGTAACCCTGATGTTGATTGGCAAAGATTTGGCACACATTTTGCTCAGTACC

GAAACGTGTGGGATGTGGACTATTCGGCCTTTGATGCTAACCACTGCAGCGATGCAATGAACATCATGTT

CGAGGAGGTGTTCCACAAAGACTTCGGTTTCCACCCAAACGCTGAGTGGATTCTGAAGACTCTTGTGAAC

ACAGAGCACGCCTACGAGAACAAACGCATCATTGTTGAGGGCGGTATGCCGTCTGGTTGTTCCGCGACAA

GCATCATCAACACAATTTTGAACAACATCTACGTGCTCTACGCTCTTCGTAGACACTATGAAGGAGTTGA

GCTGGACACTTACACCATGATCGCCTATGGAGACGACATCGTGGTCGCAAGTGATTATGATCTGGACTTT

GAGGCTCTCAAGCCACACTTCAAATCTCTTGGCCAAACCATTACTCCAGCTGACAAAAGCGACAAAGGTT

TTGTTCTTGGTCACTCCATTACTGATGTCACTTTCCTCAAAAGACACTTCCACATGGACTATGGAACTGG

GTTTTACAAACCTGTGATGGCCTCGAAGACCCTCGAGGCTATCCTCTCCTTTGCACGCCGTGGG

>HQ832587.1_A_IND_2005

GGGTTGATTGTTGACACCAGAGATGTGGAGGAACGCGTGCACGTCATGCGTAAAACCAAGCTTGCACCCA

CCGTAGCACACGGCGTGTTCAACCCTGAATTTGGCCCCGCCGCCTTGTCAAACAAGGACCCACGGCTGAA

CGAAGGAGTTGTCCTCGATGAAGTCATCTTTTCCAAACACAAAGGAGACACCAAGATGTCTGAGGAGGAC

AAAGCGCTGTTCCGCCGCTGCGCTGCTGACTACGCATCACGCCTGCACAGCGTGTTGGGGACGGCAAATG

CCCGATTGAGCATCTACGAGGCAATCAAAGGCGTTGACGGACTCGACGCCATGGAACCAGACACCGCACC

TGGTCTCCCCTGGGCCCTCCAGGGAAAACGCCGTGGTGCGCTCATTGACTTTGAGAACGGCACGGTTGGA

CCCGAGGTTGCAGCTGCCTTAGAGCTCATGGAGAAAAGAGAATACACATTCACATGCCAGACCTTCCTGA

AGGACGAGATTCGCCCGATGGAGAAAGTACGCGCCGGCAAGACTCGCATCGTCGATGTCCTGCCTGTTGA

ACACATTCTTTACACCAGGATGATGATTGGCAGATTTTGTGCTCAAATGCACTCAAACAACGGACCGCAA

ATTGGCTCGGCGGTCGGTTGTAACCCTGATGTTGATTGGCAGAGATTCGGCACACACTTCGCCCAATACA

GAAACGTGTGGGACGTGGACTATTCGGCCTTTGATGCTAACCACTGCAGTGACGCAATGAACATCATGTT

TGAGGAGGTGTTTCGCACAGAGTTCGGTTTCCACCCAAACGCTGAGTGGATACTGAAGACTCTCGTGGAC

ACGGAGCACGCCTATGAGAACAAACGCATCACTGTGGCAGGCGGAATGCCGTCTGGTTGTTCCGCAACAA

GCATTATCAACACAATTTTGAACAACATCTACGTGCTCTACGCCCTGCGTAGACACTATGAGGGAGTTGA

GCTGGACACTTACACCATGATCTCCTATGGAGACGACATCGTGGTCGCAAGTGACTATGATCTGGACTTC

GAGGCCCTCAAGCCTCACTTCAAATCCCTGGGTCAAACCATCACTCCAGCTGACAAAAGTGACAAAGGTT

TTGTTCTTGGTCATTCCATCACCGATGTTACTTTCCTCAAAAGACACTTCCACATGGACTATGGAACTGG

GTTTTACAAACCTGTGATGGCCTCGAAGACCCTCGAGGCTATCCTCTCCTTTGCACGCCGTGGG

>HQ832588.1_A_IND_2005

GGTTTGATCGTTGACACCAGAGATGTTGAGGAACGCGTGCACGTTATGCGCAAAACCAAGCTTGCGCCCA

CCGTGGCACACGGTGTGTTTAACCCCGAATTTGGGCCTGCCGCCTTGTCTAACAAGGACCCACGCCTGAA

TGAGGGAGTTGTCCTCGATGAAGTCATCTTCTCCAAACACAAAGGAAACACAGAGATGTCTGAGGAGGAC

AAAGCGCTGTTCCGCCGTTGTGCTGCTGACTACGCGTCGCGTTTGCATAGCGTGCTGGGTACGGCAAATG

CCCCACTGAGCATTTACGAGGCAATCAAGGCCGTCGATGGACTTGACGCCATGGAACCAGACACCGCGCC

TGGTCTCCCCTGGGCCCTCCAGGGGAAACGCCGTGGCGTGCTCATCGACTTCGAGAACGGCACCGTCGGA

CCCGAGGTTGAAGCTGCCTTGAAGCTCATGGAGACAAGAGAATACAAGTTTACATGCCAGACCTTCCTGA

AGGACGAGATTCGCCCGATGGAGAAAGTACGTGCCGGCAAGACTCGCATTGTCGATGTCCTGCCTGTTGA

ACACATTCTTTACACCAGGATGATGATTGGCAGATTTTGTGCTCAGATGCACTCAAACAACGGACCGCAA

ATTGGTTCGGCGGTCGGTTGTAACCCTGATGTTGATTGGCAAAGATTTGGCACGCATTTTGCTCAGTACA

GAAACGTGTGGGATGTGGACTATTCGGCCTTCGATGCTAACCACTGCAGTGACGCAATGAACATCATGTT

TGAGGAGGTGTTTAACACGGACTTTGGTTTCCACCCAAATGCTGAGTGGATCCTGAAAACTCTCGTGAAC

ACTGAACACGCCTATGAGAACAAACGCATCACTGTTGAGGCCGGGATGCCGTCTGGTTGTTCCGCAACAA

GCATCATCAACACAATTTTGAACAACATCTACGTGCTCTACGCCTTGCGTAGACACTATGAGGGAGTTGA

GCTGGACACTTACACCATGATCTCCTACGGAGACGACATCGTGGTTGCGAGTGATTACGATTTGGACTTT

GAGGCCCTCAAGCCTCACTTTAAATCTTTGGGTCAAACCATTACACCAGCTGACAAAAGTGACAAAGGTT

TTGTTCTTGGTCACTCCATCACCGACGTCACTTTCCTCAAAAGACACTTCCACATGGACTATGGAACTGG

GTTTTACAAACCTGTGATGGCTTCGAAGACCCTCGAGGCTATCCTCTCCTTTGCACGCCGTGGG

>HQ832589.1_A_IND_2006

GGTTTGATTGTTGACACCAGAGATGTGGAAGAGCGCGTGCACGTCATGCGCAAAACCAAGCTTGCACCCA

CCGTCGCGCACGGTGTGTTCAACCCTGAATTCGGGCCTGCCGCCTTGTCCAACAAGGACCCGCGCCTGAA

CGATGGGGTCGTCCTCGACGAAGTCATCTTCTCCAAACACAAGGGAAACACCAAGATGTCTGAGGAGGAC

AAAGCGCTGTTCCGCCGCTGTGCTGCTGACTACGCGTCACGCCTACATAACGTGCTGGGTACAGCAAATG

CCCCACTGAGCATTTACGAGGCAATCAAAGGCGTTGACGGACTCGACGCCATGGAACCAGACACTGCACC

TGGTCTTCCCTGGGCCCTCCAGGGAAAGCGCCGTGGCGCACTCATCGACTTCGAGAACGGAACGGTCGGA

CCCGAGGTTGAAGCTGCCTTGAAGCTCATGGAGAAAAGAGAATACAAATTTGCTTGTCAGACTTTCCTGA

AGGACGAAATTCGCCCGATGGAGAAAGTGCGTGCCGGCAAGACTCGCATCGTCGACGTCCTGCCCGTTGA

ACACATTCTTTACACCAGAATGATGATTGGCAGATTTTGTGCTCAGATGCACTCAAACAATGGACCGCAA

ATTGGCTCAGCGGTCGGCTGTAACCCTGACGTTGATTGGCAGAGATTCGGAACTCATTTTGCTCAGTACA

GAAATGTGTGGGATGTGGACTATTCGGCCTTTGATGCTAACCACTGCAGTGACGCGATGAACATCATGTT

TGAGGAGGTGTTTCGCACGGAGTACGGTTTCCACCCAAACGCTGAGTGGATTCTGAAGACTCTAGTGAAC

ACGGAGCACGCCTATGAGAACAAACGCATCACTGTTGAGGGCGGGATGCCGTCTGGCTGTTCCGCGACAA

GCATCATCAACACAATTTTAAACAACATTTATGTGCTCTACGCGCTGCGTAGACACTATGAGGGAGTTGA

GCTGGACACTTACACCATGATCTCCTACGGAGACGACATCGTGGTTGCAAGTGATTACGATTTGGACTTT

GAGGCCCTCAAGCCTCACTTCAAGTCTCTTGGTCAAACCATCACTCCAGCTGACAAAAGCGACAAAGGTT

TTGTTCTTGGTCACTCCATCACCGATGTCACTTTCCTCAAAAGACACTTCCACATGGATTATGGAACTGG

GTTTTACAAACCTGTGATGGCCTCAAAGACCCTTGAGGCTATCCTCTCCTTTGCACGCCGTGGG

>HQ832590.1_A_IND_2007

GGATTGATAGTTGATACCAGAGATGTTGAGGAGCGCGTGCACGTCATGCGCAAAACCAAGCTCGCACCCA

CCGTGGCACATGGTGTGTTCAACCCTGAATTTGGGCCTGCCGCTTTGTCCAACAAGGACCCGCGCCTGAA

TGAAGGTGTCGTCCTCGATGAAGTCATCTTCTCCAAGCACAAGGGAGACACCAACATGTCAGAGGAAGAC

AAAGCGCTGTTCCGCCGCTGCGCTGCCGACTACGCGTCGCGCTTGCACAGTGTTTTGGGCACAGCAAATG

CCCCATTGAGCATTTACGAGGCAATCAAGGGCGTCGACGGACTCGACGCCATGGAACCAGACACCGCACC

CGGTCTCCCTTGGGCCCTCCAAGGGAAACGCCGTGGTGCGCTCATCGACTTCGAGAACGGCACTGTCGGA

CCCGAGGTTGAAGCTGCCTTGAAGCTCATGGAGAAAAGAGAATACAAGTTTGCTTGTCAGACCTTCCTGA

AGGACGAAATTCGCCCGATGGAGAAAGTACGTGCCGGCAAGACTCGCATTGTCGACGTTTTGCCCGTTGA

ACACATTCTTTACACCAGGATGATGATTGGCAGATTTTGTGCTCAAATGCACTCAAACAACGGACCGCAA

ATTGGCTCGGCGGTCGGCTGTAATCCTGATGTTGATTGGCAAAGATTTGGCACACATTTCGCTCAGTACA

GAAACGTGTGGGATGTGGACTATTCGGCCTTTGATGCTAACCACTGCAGTGACGCGATGAACATCATGTT

TGAGGAGGTGTTTCGCACGGAGTTCGGTTTCCACCCAAATGCTGAGTGGATTCTGAAGACTCTCGTGAAC

ACGGAGCACGCCTATGAGAACAAACGCATCACTGTTGAGGGCGGGATGCCATCTGGCTGTTCCGCAACAA

GCATCATCAACACAATTCTGAACAACATTTACGTGCTCTACGCCCTGCGTAGACACTATGAGGGAGTTGA

GCTGGACACTTACACCATGATCTCCTACGGAGACGACATCGTGGTTGCAAGTGATTACGATCTGGACTTT

GAGGCTCTCAAGCCTCACTTCAAATCTTTGGGCCAAACCATCACTCCAGCTGACAAAAGCGACAAAGGTT

TTGTTCTTGGTCACTCCATCACCGATGTCACTTTCCTCAAAAGACACTTCCACATGGATTATGGAACTGG

GTTTTACAAACCTGTGATGGCCTCGAAGACCCTCGAGGCTATCCTCTCCTTTGCACGCCGTGGG

>HQ832591.1_A_IND_2008

GGATTGATAGTTGATACCAGAGATGTTGAGGAGCGCGTGCACGTCATGCGCAAAACCAAGCTCGCACCCA

CCGTGGCACATGGTGTGTTCAACCCTGAATTTGGGCCTGCCGCCTTGTCCAACAAGGACCCGCGCCTGAA

TGAAGGTGTCGTCCTCGACGAAGTCATCTTCTCCAAGCACAAGGGGGACACCAACATGTCAGAGGAAGAC

AAAGCGCTGTTCCGCCGCTGCGCTGCCGACTACGCGTCGCGCTTGCACAGTGTTTTGGGTACAGCAAATG

CCCCATTGAGCATCTACGAGGCAATCAAAGGCGTCGATGGACTCGACGCCATGGAACCAGACACCGCGCC

CGGCCTACCCTGGGCTCTCCAGGGGAAGCGCCGTGGTGCGCTCATTGACTTCGAGAACGGCACTGTCGGA

CCCGAGGTTGAAGCTGCCTTGAAGCTCATGGAGAAAAGAGAGTACAAGTTTGTATGCCAGACCTTCCTGA

AGGACGAGATTCGCCCGATGGAGAAAGTACGTGCCGGCAAGACTCGCATTGTCGACGTCTTGCCTGTTGA

ACACATTCTTTACACCAGGATGATGATTGGCAGATTCTGTGCTCAAATGCACTCAAACAACGGACCGCAG

ATTGGCTCAGCGGTTGGTTGCAATCCTGATGTTGATTGGCAAAGATTTGGCACACATTTTGCTCAGTACG

GAAATGTGTGGGATGTGGACTATTCGGCCTTTGATGCCAACCACTGCAGTGATGCGATGAACATCATGTT

TGAGGAGGTGTTTAACACGGACTTCGGTTTCCACCCAAATGCTGAGTGGATCCTGAAAACTCTCGTGAAT

ACTGAACACGCCTATGAGAACAAACGCATCACTGTTGAGGGCGGGATGCCATCTGGGTGTTCCGCAACAA

GCATCATCAACACAATTCTGAACAACATCTACGTGCTCTACGCCTTGCGCAGGCACTATGAGGGAGTTGA

GCTGGACACTTACACCATGATCTCCTACGGAGACGACATCGTGGTTGCGAGTGATTACGATCTGGACTTT

GAGGCTCTCAAGCCTCACTTCAAATCTTTGGGCCAAACCATCACTCCAGCTGACAAAAGCGACAAAGGTT

TTGTTCTTGGTCGCTCCATTACCGACGTCACCTTCCTCAAAAGACACTTCCACATGGATTATGGAACTGG

GTTTTATAAACCTGTGATGGCCTCAAAGACCCTCGAGGCCATCCTCTCCTTTGCACGCCGTGGG

>HQ832592.1_A_IND_2009

GGTTTGATTGTTGACACCAGAGATGTGGAAGAGCGCGTGCACGTCATGCGCAAAACCAAGCTTGCACCCA

CCGTTGCACACGGTGTGTTCAACCCTGAATTCGGGCCTGCCGCCTTGTCCAACAAGGATCCACGCCTGAA

TGACGGGGTCGTCCTCGATGAAGTCATCTTCTCCAAACACAAGGGAGACACCAAGATGTCTGAGGAGGAC

AAAGCGCTGTTCCGCCGCTGTGCTGCTGACTACGCGTCGCACCTGCATAGTGTGTTGGGCACAGCAAATG

CCCCATTGAGCATTTACGAGGCAATCAAAGGCGTTGACGGACTCGACGCCATGGAGCCAGACACTGCACC

CGGTCTTCCCTGGGCCCTCCAGGGGAAACGCCGCGGCGCGCTCATCGACTTCGAGAACGGCACGGTCGGG

CCCGAGGTTGAAGCTGCCTTGAAGCTCATGGAGAAAAGAGAATACAAATTTGTTTGCCAAACCTTCCTGA

AGGACGAGATTCGCCCGATGGAGAAAGTGCGTGCCGGCAAGACTCGCATTGTCGACGTCCTGCCCGTTGA

ACACATTCTTTACACCAGAATGATGATTGGCAGATTTTGTGCTCAAATGCACTCGAACAACGGACCGCAA

ATTGGCTCGGCGGTCGGTTGCAATCCTGATGTTGATTGGCAAAGATTCGGAATCCATTTCTCTCAGTACA

GAAACGTGTGGGATGTGGACTATTCGGCCTTTGATGCTAACCACTGCAGTGATGCAATGAACATCATGTT

TGAGGAAGTGTTCCGCACGGAGTTCGGCTTCCACCCAAATGCTGAGTGGATCCTGAAGACTCTAGTGAAC

ACGGAGCACGCCTATGAAAACAAACGTGTCACTGTTGAGGGCGGGATGCCATCTGGCTGTTCCGCAACAA

GCATCATCAACACAATTTTGAACAACATCTACGTGCTCTACGCGCTGCGTAGACACTATGAGGGAGTTGA

GCTGGACACTTACACCATGATCTCCTACGGAGACGACATCGTGGTTGCAAGTGATTACGATTTGGACTTC

GAGGCCCTCAAGCCTCACTTTAAATCTCTTGGTCAAACCATCACTCCAGCTGACAAAAGCGACAAAGGTT

TTGTTCTTGGTCACTCCATTACTGATGTCACTTTCCTCAAAAGACACTTCCACATGGATTATGGAACTGG

GTTTTACAAACCTGTGATGGCCTCAAAGACCCTTGAGGCTATCCTCTCCTTTGCACGCCGTGGG

>JF749841.1_A_TUR_2006

GGGTTGATCATTGACACCAGGGATGTTGAGGAGCGTGTGCATGTCATGCGCAAAACCAAGCTTGCACCCA

CCGTGGCACACGGTGTGTTTAACCCTGAGTTTGGCCCCGCTGCCTTGTCCAACAAAGACCCGCGGTTGAA

CGAAGGGGTTGTCCTCGATGAGGTCATCTTCTCCAAACACAAGGGAGATACCAAAATGTCTGAGGAGGAC

AAAGCGCTGTTCCGCCGCTGTGCTGCTGACTACGCGTCACACCTGCACAGTGTGTTGGGTACGGCGAATG

CCCCACTGAGCATCTACGAGGCAATCAAGGGCGTCGACGGGCTCGATGCCATGGAACCAGACACCGCGCC

TGGCCTCCCCTGGGCCCTCCAGGGAAAACGCCGCGGCGCGCTCATCGACTTCGAGAACGGCACAGTCGGA

CCCGAAGTTGAATCTGCCTTGAAGCTCATGGAGAAAAGGGAGTACAAATTTGCTTGCCAGACCTTCCTGA

AGGACGAAATTCGCCCGATGGAGAAAGTACGCGCCGGCAAGACTCGCATTGTCGACGTTTTGCCTGTTGA

ACACATTCTTTACACCAGGATGATGATTGGCAGATTTTGTGCTCAAATGCACTCAAACAACGGACCGCAA

ATTGGCTCAGCGGTCGGTTGCAACCCGGATGTTGATTGGCAAAGATTTGGAACACACTTCGCCCAGTACA

AAAACGTGTGGGATGTGGACTATTCGGCCTTTGATGCCAACCACTGCAGTGACGCAATGAACATCATGTT

TGAGGAGGTGTTTCGCACAGAATTCGGCTTCCACCCGAATGCGGAGTGGATTCTGAAGACTCTTGTGAAC

ACGGAGCACGCCTATGAGAACAAGCGCATCACTGTTGAGGGCGGGATGCCATCTGGCTGTTCCGCGACAA

GCATCATCAATACAATCCTGAATAACATCTACGTGCTCTACGCCCTGCGTAGACACTATGAGGGAATTGA

GCTAGACACTTACACCATGATCTCCTACGGAGACGACATCGTGGTCGCAAGTGATTATGATTTGGACTTC

GAGGCCCTCAAGCCTCACTTTAAATCTCTCGGTCAAACCATTACTCCAGCTGACAAAAGCGACAAAGGTT

TTGTTCTTGGTCATTCCATCACCGATGTCACTTTCCTCAAAAGACACTTCCACATGGATTATGGAACTGG

GTTTTACAAACCTGTGATGGCCTCGAAGACCCTTGAGGCTATCCTCTCCTTTGCACGCCGTGGG

>JF749843.1_A_EGY_2006

GGGTTGATTGTTGACACCAGAGAAGTGGAGGAGCGCGTGCACGTCATGCGCAAAACCAAGCTTGCACCCA

CCGTGGCACACGGTGTGTTTAACCCTGAATTTGGCCCTGCTGCTTTGTCCAACAAGGACCCGCGACTGAA

CGAGGGCGTCGTCCTCGATGAAGTCATTTTCTCCAAACACAAAGGGGACACAAAGATGAGCGAGGAGGAC

AAGGCGCTGTTCCGTCGCTGCGCGGCCGACTACGCGTCGCGACTGCATAGTGTGCTGGGCACAGCGAACG

CCCCACTGAGCATCTACGAGGCAATCAAAGGAGTTGACGGCCTCGACGCCATGGAGCCGGACACTGCGCC

CGGCCTCCCCTGGGCCCTCCAGGGGAAACGCCGCGGTGCTCTGATTGACTTCGAGAATGGCACGGTCGGA

CCCGAGGTCACGGCCGCACTGGAGCTCATGGAGAAAAGAGAGTACAAGTTTGCATGCCAAACCTTCCTGA

AGGACGAGATTCGCCCTATGGAGAAAGTACGCGCCGGCAAGACTCGCATTGTCGATGTTTTGCCTGTTGA

ACACATCCTCTACACTAGAATGATGATTGGCAGATTTTGTGCTCAAATGCACTCAAACAATGGACCGCAG

ATAGGCTCAGCGGTCGGATGCAACCCAGATGTTGATTGGCAGAGATTCGGCACACATTTCGCCCAATACA

GGAACGTGTGGGACGTTGACTATTCGGCCTTCGATGCTAACCACTGCAGCGATGCCATGAACATCATGTT

TGAGGAGGTGTTCCGCACAGACTTTGGCTTCCACCCGAACGCCGAGTGGATACTGAAGACTCTTGTGAAC

ACGGAGCATGCTTACGAAAACAAACGCATCACTGTTGAGGGCGGGATGCCATCCGGTTGTTCCGCAACAA

GCATCATCAACACAATTTTGAACAACATCTACGTGCTCTACGCCCTGCGTAGACACTATGAGGGAGTTGA

GCTGGACACATACACCATGATCTCCTACGGAGACGACATCGTGGTGGCAAGTGATTATGACTTGGACTTT

GAAGCTCTCAAACCACACTTTAAATCCCTTGGTCAAACCATCACCCCAGCTGACAAAAGCGACAAAGGTT

TTGTTCTTGGTCACTCCATTACCGATGTCACTTTCCTAAAAAGACACTTCCACATGGACTATGGAACTGG

GTTTTACAAACCTGTGATGGCCTCGAAGACCCTCGAGGCTATCCTCTCCTTTGCACGCCGTGGG

>JF749848.1_A_TUR_2003

GGTTTGATTGTTGACACCAGAGATGTGGAAGAGCGCGTGCACGTCATGCGCAAAACCAAGCTTGCACCCA

CCGTAGCACACGGTGTGTTCAACCCCGAATTTGGGCCTGCTGCCTTGTCCAACAAGGACCCACGCCTGAA

CGAAGGGGTTGTCCTCGATGAAGTCATTTTCTCCAAGCACAAGGGAGACACCAAAATGTCTGAGGAGGAC

AAAGCGCTGTTCCGCCGCTGTGCTGCTGACTACGCGTCACGCTTACACAGTGTGTTGGGTACGGCAAATG

CCCCACTGAGCATTTACGAGGCAATCAAAGGCGTTGACGGTCTCGACGCCATGGAACCAGACACCGCACC

TGGCCTCCCCTGGGCTCTCCAGGGGAAACGCCGTGGCGCGCTCATCGACTTTGAGAACGGCACAGTCGGA

CCCGAGGTTGAAGCTGCCTTGAAGCTCATGGAGAAGAAGGAATACAAGTTTGCATGCCAGACCTTCCTGA

AAGACGAGATCCGTCCGATGGAGAAAGTACGTGCCGGCAAGACTCGCATTGTCGACGTCTTACCAGTTGA

ACACATTCTTTACACCAGGATGATGATTGGCAGGTTCTGTGCTCAAATGCACTCAAACAACGGACCGCAG

ATTGGCTCGGCGGTTGGTTGTAACCCTGATGTTGATTGGCAAAGATTTGGCACACACTTCGCTCAGTACA

GAAACGTGTGGGATGTGGACTATTCGGCCTTTGACGCTAATCACTGCAGTGATGCGATGAACATTATGTT

TGAGGAAGTGTTCTCCACGGAATTTGGTTTCCACCCGAACGCTGAGTGGATCCTGAAAACACTCGTGGAC

ACGGAGCACGCCTATGAAAACAAACGCATCACTGTTGAGGGCGGGATGCCGTCTGGCTGTTCCGCTACAA

GCATCATCAACACAATTTTGAACAACATCTACGTGCTCTACGCCCTGCGTAGACACTATGATGGAGTTGA

GCTGGACACTTACACCATGATCTCCTACGGAGATGACATCGTGGTTGCAAGTGATTATGATCTGGACTTC

GAGGCCCTCAAGCCTCACTTCAAATCTCTTGGTCAAACCATTACTCCAGCTGACAAAAGCGACAAAGGTT

TTGTTCTTGGTCACTCCATTACCGACGTCACTTTCCTTAAAAGACACTTCCACATGGATTATGGAACTGG

GTTTTACAAACCTGTGATGGCCTCGAAGACCCTCGAGGCCATCCTCTCCTTTGCACGCCGTGGG

>JF749849.1_Asia1_PAK_2002

GGATTGATAGTTGACACCAGAGATGTTGAGGAGCGCGTGCATGTCATGCGCAAAACCAAGCTCGCACCCA

CTGTGGCACACGGTGTGTTTGACCCCGAATTTGGGCCCGCCGCCTTGTCTAACAAGGACCCGCGCCTGAG

TGAGGGGGTTGTCCTCGATGAAGTCATCTTCTCCAAACACAAAGGAGACACAAAGATGTCTGAGGAGGAC

AAAGCGCTGTTCCGCCGCTGTGCTGCTGACTACGCGTCACGTCTGCATAGTGTGTTGGGTACGGCAAATG

CCCCACTGAGCATTTACGAGGCAATCAAGGGCGTCGACGGGCTCGACGCCATGGAACCAGACACCGCGCC

TGGACTCCCCTGGGCTCTCCAGGGGAAGCGCCGTGGTGCGCTCATTGACTTCGAGAACGGTACTGTCGGA

CCCGAGGTTGAAGCTGCCTTGAAACTCATGGAGAAAAGAGAGTACAAGTTTGCATGCCAGACCTTCCTGA

AGGACGAGATTCGCCCGATGGAGAAGGTGCGTGCCGGCAAGACTCGCATTGTCGATGTCCTGCCTGTTGA

ACACATTCTTTACACCAGGATGATGATTGGCAGATTTTGTGCTCAAATGCACTCAAACAACGGACCGCAA

ATTGGCTCGGCGGTTGGTTGCAATCCTGATGTTGATTGGCAAAGATTTGGCACACACTTTGCTCAGTACA

GAAACGTGTGGGATGTGGACTATTCGGCCTTTGATGCCAACCACTGCAGTGACGCAATGAACATCATGTT

TGAGGAGGTGTTCAGCACGGATTTCGGTTTCCACCCAAACGCTGAGTGGATCCTGAAAACTCTCGTGAAC

ACTGAACACGCCTATGAGAACAAACGCATCACTGTTGAGGGCGGGATGCCGTCTGGTTGTTCCGCAACAA

GCATCATCAACACAATTCTGAACAACATCTACGTGCTCTACGCCTTGCGTAGACACTACGAGGGAGTTGA

GCTGGACACCTACACCATGATCTCCTACGGAGACGACATCGTGGTTGCAAGCGATTACGATCTGGACTTT

GAGGCCCTCAAGCCTCACTTCAAATCCCTTGGTCAAACCATTACTCCAGCTGACAAAAGCGACAAAGGTT

TTGTTCTTGGTCACTCCATTACCGATGTCACTTTCCTCAAAAGACACTTCCACATGGACTATGGAACTGG

GTTTTACAAACCTGTGATGGCCTCGAAGACCCTCGAGGCCATCCTCTCCTTTGCACGCCGTGGG

>JF749851.1_O_IRN_2001

GGATTGATAGTTGACACCAGAGATGTTGAGGAGCGCGTGCATGTCATGCGCAAAACCAAGCTCGCACCCA

CCGTGGCACACGGTGTGTTTAACCCCGAATTTGGGCCCGCCGCCTTGTCTAACAAGGACCCGCGCCTGAG

TGAGGGGGTTGTCCTCGATGAAGTCATCTTCTCCAAACACAAAGGAGACACAAAGATGTCTGAGGAGGAC

AAAGCGCTGTTCCGCCGCTGTGCTGCTGACTACGCGTCACGTCTGCATAGTGTGTTGGGTACGGCAAATG

CCCCACTGAGCATTTACGAGGCAATCAAGGGCGTCGACGGGCTCGACGCCATGGAGCCAGACACCGCGCC

TGGCCTCCCCTGGGCTCTCCAGGGGAAGCGCCGTGGTGCGCTCATTGACTTCGAGAACGGCACTGTCGGA

CCCGAGGTTGAAGCTGCCTTGAAGCTCATGGAGAAAAGAGAGTACAAGTTTGCATGCCAGACCTTCCTGA

AGGACGAGATTCGCCCAATGGAGAAGGTACGTGCCGGCAAGACTCGCATTGTCGACGTCCTGCCTGTTGA

ACACATTCTTTACACCAGGATGATGATTGGCAGATTTTGTGCTCAAATGCACTCAAACAACGGACCGCAA

ATTGGCTCGGCGGTTGGTTGTAATCCTGATGTTGATTGGCAAAGATTTGGCACACATTTTGCTCAGTACA

GAAACGTGTGGGATGTGGACTATTCGGCCTTTGATGCCAACCACTGCAGTGACGCAATGAACATCATGTT

TGAGGAGGTGTTCAGCACGGATTTCGGTTTCCACCCAAATGCTGAGTGGATCCTGAAAACTCTCGTGAAC

ACTGAACACGCCTATGAGAACAAACGCATCACTGTTGAGGGCGGAATGCCGTCTGGTTGTTCCGCAACAA

GCATCATCAACACAATTTTGAACAACATCTACGTGCTCTACGCCTTGCGTAGACACTACGAGGGAGTTGA

GCTGGACACCTACACCATGATCTCCTACGGAGACGACATCGTGGTTGCAAGTGATTACGACCTGGACTTT

GAGGCCCTCAAGCCTCACTTCAAATCCCTTGGCCAAACCATTACTCCAGCTGACAAAAGCGACAAAGGTT

TTGTTCTTGGTCACTCCATTACCGATGTCACTTTCCTCAAAAGACACTTCCACATGGACTATGGAACTGG

GTTTTACAAACCTGTGATGGCCTCGAAGACCCTCGAGGCTATCCTCTCCTTTGCACGCCGTGGG

>JF749852.1_O_MAY_2004

GGGTTGATCGTTGACACCAGAGATGTGGAAGAGCGCGTGCACGTCATGCGCAAAACCAAGCTTGCACCCA

CCGTTGCACACGGTGTGTTCAACCCTGAATTCGGCCCCGCCGCTTTGTCCAACAAAGACCCGCGGCTGAA

TGAAGGGGTTGTCCTTGATGAAGTCATCTTCTCCAAGCATAAGGGAGACACCAAGATGTCGGAAGAGGAC

AAAGCGCTGTTCCGTCGCTGCGCTGCCGACTACGCGTCGCGCTTGCACAGTGTTCTGGGTACGGCAAATG

CCCCATTGAGCATTTACGAGGCAATCAAGGGCGTCGACGGACTCGATGCCATGGAACCAGACACCGCGCC

TGGCCTCCCCTGGTCCCTCCAGGGGAAACGCCGTGGTGCGCTCATCGACTTCGAGAACGGCACTGTCGGA

CCCGAGGTTGAAGCTGCCTTGAAGCTCATGGAGAAAAGAGAATACAAGTTTGCATGTCAGACCTTCCTGA

AGGACGAAATTCGCCCGATGGAGAAAGTACGTGCCGGCAAGACTCGCATCGTCGATGTCCTGCCTGTTGA

ACACATTCTTTACACCAGAATGATGATTGGCAGATTCTGTGCTCAAATGCACTCAAACAACGGGCCGCGA

ATTGGCTCAGCGGTCGGATGCAACCCTGACGTTGATTGGCAGAGATTTGGCACACATTTTGCTCAGTACA

GAAACGTGTGGGATGTGGATTATTCGGCCTTTGATGCTAACCATTGCAGTGACGCCATGAACATCATGTT

TGAGGAGGTGTTCCGCACGGAATTCGGTTTCCACCCTAACGCTGAGTGGATCCTGAAGACTCTCGTGAAT

ACGGAGCACGCGTACGAAAACAAACGCATCACTGTTGAGGGCGGGATGCCGTCAGGCTGTTCCGCAACAA

GCATCATCAACACAATTCTGAACAACATTTACATGCTCTACGCCTTGCGTAGACACTATGAGGGAGTTGA

GCTGGACACTTACACCATGATCTCCTACGGAGACGACATCGTGGTTGCAAGTGATTACGATCTGGACTTT

GAGGCCCTCAAGCCTCACTTCAAATCTCTTGGTCAAACCATCACCCCAGCCGACAAAAGCGACAAAGGTT

TTGTTCTTGGTCACTCCATTACCGACGTCACTTTCCTCAAAAGACACTTCCACATGGACTATGGAACTGG

GTTTTACAAACCTGTGATGGCCTCGAAGACCCTCGAGGCTATCCTCTCCTTTGCACGCCGTGGG

>JF749860.1_SAT1_KEN_2002

GGGTTGGTAGTGGACACCAGAGAGGTTGAGGAGCGCGTGCACGTCATGCGCAAAACCAAGCTTGCACCCA

CCGTGGCTCACGGTGTGTTTCAGCCTGAATTTGGACCTGCCGCCCTGTCTAACAACGACAAGCGCCTGAA

CGAAGGCGTAGTCTTAGATGAAGTCATCTTCTCTAAGCACAAGGGCGATGCCAAAATGTCTGAGGCTGAC

AAGAGATTGTTCCGCCTGTGCGCTGCTGATTATGCCTCGCATCTTCACAACGTGCTTGGGACAGCAAACT

CTCCATTGAGTGTGTTTGAAGCCATCAAGGGCGTCGACGGGCTCGACGCCATGGAGCCTGACACGGCACC

CGGCCTTCCCTGGGCTCTCCAGGGGAAACGCCGTGGCGCTCTCATCGATTTCGAGAACGGCACTGTCGGA

CCCGAGATTGAACAGGCCCTGAAGCTCATGGAGAAGAAGGAGTACAAGTTCACCTGTCAAACCTTCCTGA

AGGACGAGATCCGTCCCATGGAGAAAGTTAAGGCCGGCAAGACTCGCATTGTCGATGTCCTGCCTGTTGA

ACACATCATCTATACCAGAATGATGATTGGCAGATTTTGTGCACAGATGCACTCCAACAACGGACCGCAA

ATTGGCTCGGCGGTCGGTTGCAACCCTGATGTTGATTGGCAACGATTTGGCACACACTTTGCCCAGTACA

AAAATGTTTGGGACATCGATTATTCGGCCTTTGATGCTAACCATTGCAGTGACGCCATGAACATCATGTT

CGAAGAGGTCTTCCGTGAGGAATTTGGATTTCATCCGAACGCTGTTTGGATACTCAAAACACTTATCAAC

ACGGAACACGCCTACGAGAACAAGCGCATCACTGTTGAAGGTGGAATGCCCTCGGGTTGCTCCGCCACCA

GCATCATCAACACCATTCTCAACAACATCTACGTGCTCTACGCCCTGCGTAGACATTATGAGGGAGTCGA

GCTGTCGCACTACACCATGATTTCCTACGGGGATGACATTGTAGTTGCAAGTGATTACGATTTGGACTTT

GAAGCTCTCAAGCCTCACTTCAGATCCCTTGGTCAAACAATCACTCCAGCCGACAAAAGTGACAAAGGTT

TTGTTCTTGGTCAGTCCATTACCGATGTTACTTTCCTCAAGAGGCATTTCCATCTGGATTATGAAACTGG

GTTTTACAAACCTGTGATGGCTTCGAAGACCCTCGAAGCTATCCTCTCCTTTGCACGCCGTGGG

>JF749861.1_SAT2_KEN_2002

GGGTTGGTAGTGGACACCAGAGAGGTTGAGGAACGCGTGCACGTCATGCGCAAAACCAAGCTTGCACCCA

CCGTGGCTTACGGTGTGTTTCAGCCTGAATTTGGACCTGCCGCCCTGTCGAACAACGACAAGCGCCTGAA

TGAAGGTGTGGTCTTAGACGAAGTCATCTTCTCTAAGCACAAGGGCGATGCCAAAATGTCTGAGGCTGAC

AAGAGACTGTTCCGCCTGTGCGCTGCTGATTATGCCTCGCATCTTCACAACGTGCTTGGGACAGCAAACT

CTCCATTGAGTGTGTTTGAAGCCATCAAGGGCGTCGACGGACTCGACGCCATGGAGCCTGACACGGCACC

CGGACTCCCCTGGGCCCTCCAGGGGAAACGCCGCGGAGCCCTCATCGATTTCGAGAACGGCACTGTCGGA

CCCGAGATTGAGCAGGCACTGGAACTCATGGAGAAGAAGGAGTACAAGTTCACCTGTCAAACCTTCCTGA

AGGACGAGATTCGTCCCATGGAGAAAGTTAAGGCCGGCAAGACCCGCATCGTCGATGTCCTGCCTGTCGA

GCACATCATTTACACCAGAATGATGATTGGTAGATTCTGTGCACAGATGCACTCCAACAACGGACCGCAA

ATTGGCTCGGCGGTCGGTTGCAACCCTGATGTCGATTGGCAACGATTTGGCACACACTTTGCCCAGTACA

AAAATGTTTGGGACATTGATTATTCGGCCTTTGATGCCAACCATTGCAGTGACGCCATGAACATCATGTT

CGAGGAGGTCTTCCGTGAGGAATTTGGATTTCACCCGAACGCTGTTTGGATACTCAAAACACTCATCAAC

ACGGAACACGCCTACGAGAACAAGCGCATCACTGTTGAAGGTGGAATGCCCTCGGGTTGCTCCGCCACCA

GCATCATCAACACCATTCTCAACAACATCTACGTGCTCTACGCTCTGCGTAGACACTATGAGGGTGTCGA

GCTGTCGCACTACACCATGATTTCCTACGGGGATGACATTGTAGTTGCAAGTGATTACGATTTGGACTTT

GAAGCTCTTAAGCCTCACTTTAAATCTCTTGGTCAGACAATCACTCCAGCCGACAAAAGTGACAAAGGTT

TTGTTCTTGGTCAGTCCATCACCGATGTTACTTTCCTCAAGAGGCATTTTCATCTGGATTATGAAACTGG

GTTTTACAAACCTGTGATGGCTTCGAAGACCCTTGAAGCTATCCTCTCCTTTGCACGCCGTGGG

>JF749862.1_SAT2_UGA_2002

GGGCTGATTGTCGACACAAGAGAAGTTGAGGAGCGCGTGCACGTCATGCGCAAAACAAAGCTCGCACCCA

CCGTTGCACACGGTGTGTTTCAGCCTGAGTACGGACCTGCTGCGCTGTCAAACAACGACAAGCGCCTGAA

CGAGGGCGTTGTCCTGGACGACGTCATCTTCTCCAAACACAAGGGAGACACAAAGATGTCTGAGGCTGAT

AAGAAGCTGTTCCGTCTGTGTGCTGCTGATTACGCGTCGCACTTGCACAACGTACTCGGAACCGCAAACT

CTCCATTGACTGTGTTTGAAGCCATCAAGGGCGTCGACGGGCTCGACGCCATGGAGCCTGACACCGCACC

CGGACTGCCCTGGGCAACCCAAGGGAAGCGCCGCGGAGCCCTCATCGACTTCGAGAACGGCACTGTCGGA

CCCGAGATCGAACAGGCCCTAAAGCTCATGGAGAGTAAGGAATACAAGTTTGTGTGCCAAACCTTCCTGA

AGGATGAGATTCGTCCAATGGAGAAAGTACGTGCCGGTAAGACTCGCATCGTCGATGTCCTGCCTGTTGA

ACACATCCTGTACACCAGGATGATGATTGGCAGATTCTGTGCAAACATGCATTCCAACAACGGACCGCAA

ATTGGCTCGGCGGTCGGTTGCAACCCAGATGTTGATTGGCAAAGATTTGGAACCCACTTCGCCCAGTACA

AAAACGTATGGGACGTGGATTATTCGGCCTTTGATGCTAACCATTGCAGTGACGCCATGAACATCATGTT

TGAAGAGGTTTTCCGACCGGAATTCGGGTTCCACCCCAACGCGGAGTGGATCTTGAAAACTCTGGTCAAC

ACGGAACACGCCTACGAAAACAAGCGTATCACTGTTGAAGGTGGAATGCCCTCGGGTTGCTCCGCCACCA

GCATCATTAACACAATTCTCAACAACATCTACGTGCTCTACGCGCTGCGTAGACACTATGAGGGAGTCGA

GCTGTCGCACTACACCATGATTTCCTACGGGGATGACATTGTAGTTGCAAGTGACTACGACTTGGATTTT

GAAGCTCTCAAGCCTCACTTCAAATCCGTTGGTCAAACCATCACTCCAGCCGACAAAAGTGACAAAGGTT

TTGTTCTTGGTCAGTCCATCACCGATGTTACTTTCCTCAAGAGGCATTTCCATCTGGACTACGGAACTGG

GTTTTACAAACCTGTGATGGCCTCGAAGACCCTCGAAGCCATCCTCTCCTTTGCACGCCGTGGG

>JF749864.1_SAT2_ZIM_2003

GGGTTGGTAGTGGACATCAGAGAAGTAGAGGAGCGCGTGCATGTCATGCGCAAAACCAAGCTTGCACCCA

CCGTGGCTCACGGTGTGTTTCAGCCTGAATTTGGACCTGCCGCCCTGTCGAACAACGACAAGCGCCTGAA

TGAAGGTGTGGTGTTGGATGAAGTCATCTTCTCCAAGCACAAGGGCGATGCCAAAATGTCTGAGGCTGAT

AAGAGATTGTTCCGCCTGTGCGCTGCTGATTATGCCTCACATCTTCACAACGTACTTGGGACAGCCAACT

CTCCACTGAGCGTGTTTGAAGCCATCAAGGGCGTCGACGGACTCGACGCCATGGAGCCTGACACAGCACC

TGGCCTCCCCTGGGCACTCCAGGGTAAGCGCCGCGGAGCTCTCATCGATTTCGAGAACGGCACTGTCGGA

CCCGAGATTGAAGCTGTTCTGAAGCTCATGGAGAACAAGGAGTACAAGTTCACCTGTCAAACCTTCCTGA

AGGACGAGATTCGCCCTCTGGAGAAAGTCAAGGCCGGTAAGACTCGCATTGTCGACGTCCTGCCTGTTGA

GCACATCCTCTACACAAGAATGATGATTGGCAGATTCTGTGCACAAATGCACTCCAACAACGGACCGCAA

ATTGGCTCGGCGGTCGGTTGTAACCCTGATGTTGATTGGCAACGATTTGGCACTCACTTTGCCCAGTACA

AAAATGTTTGGGACATTGATTATTCGGCCTTTGATGCTAACCATTGCAGTGACGCCATGAACATCATGTT

CGAGGAGGTCTTCCGTGAGGAATTTGGATTCCATCCGAACGCTGTTTGGATTCTCAAAACTCTCGTCAAC

ACGGAACACGCCTATGAGAACAAGCGCATCACTGTTGAAGGTGGAATGCCCTCGGGTTGCTCCGCCACCA

GCATCATCAACACCATTCTCAACAACATCTACGTGCTCTACGCTCTGCGCAGGCACTATGAGGGAGTCGA

GCTGTCGCACTACACCATGATTTCCTACGGGGATGATATTGTAGTTGCAAGTGATTACGATTTGGACTTT

GAAGCTCTCAAGCCCCACTTTAAATCTCTTGGTCAAACAATCACTCCAGCCGACAAAAGTGACAAAGGTT

TTGTTCTTGGTCAGTCCATTACCGATGTTACTTTCCTCAAGAGGCATTTCCATCTGGATTATGAAACTGG

GTTTTACAAACCTGTGATGGCTTCGAAGACCCTCGAAGCTATCCTCTCCTTTGCACGCCGTGGG

>JN006719.1_Asia1_PAK_2008

GGACTGATCGTTGACACCAGAGATGTTGAGGAGCGCGTGCACGTCATGCGCAAAACCAAGCTCGCACCCA

CCGTTGCACACGGTGTGTTCAATCCTGAGTTTGGCCCCGCTGCCTTGTCTAACAAGGACCCGCGGCTGAA

CGAGGGAGTTGTCCTCGATGAAGTCATCTTCTCCAAACACAAGGGAGACACCAAAATGTCTGAGGAGGAC

AAAGCGCTTTTCCGCCGCTGTGCTGCTGACTACGCGTCACGCCTACACAGTGTGTTGGGAACAGCAAATG

CCCCACTGAGCATTTACGAGGCAATCAAAGGCGTTGACGGACTCGACGCCATGGAACCAGACACCGCGCC

TGGCCTCCCCTGGGCCCTCCAGGGGAAGCGTCGCGGCGCGCTCATCGACTTCGAGAACGGCACCGTCGGA

CCCGAGGTTGAAGCAGCCTTGAAGCTCATGGAGAAAAGAGAATACAAATTTGCTTGCCAGACCTTCCTGA

AGGACGAGATTCGCCCGATGGAGAAAGTACGTGCCGGCAAGACGCGCATTGTCGATGTCTTGCCTGTTGA

ACACATTCTTTACACCAGGATGATGATTGGCAGATTTTGTGCGCAAATGCACTCAAACAACGGACCGCAA

ATTGGCTCGGCGGTCGGTTGCAATCCTGATGTTGATTGGCAGAGATTCGGCACACATTTTGCTCAGTACA

GAAACGTGTGGGACGTGGACTATTCGGCCTTTGATGCTAACCACTGCAGTGACGCAATGAACATCATGTT

TGAGGAGGTGTTCCGCACGGAGTTCGGCTTCCACCCAAATGCTGAGTGGATTCTGAAAACTCTTGTGAAC

ACGGAACACGCCTATGAGAACAAGCGCATCACTGTTGAGGGCGGGATGCCTTCTGGGTGTTCTGCGACAA

GCATCATCAACACAATTTTGAACAACATCTACGTGCTCTACGCACTGCGTAGACACTATGAGGGAGTTGA

GCTGGACACTTACACCATGATCTCCTACGGAGACGACATCGTGGTTGCAAGTGATTATGATCTGGACTTT

GAGGCTCTCAAGCCTCACTTCAAATCTCTTGGCCAAACTATCACTCCAGCTGACAAAAGCGACAAAGGTT

TTGTTCTTGGTCACTCCATTACCGATGTCACTTTCCTCAAAAGACACTTCCACATGGACTATGAAACTGG

GTTTTACAAACCTGTGATGGCTTCGAAGACCCTCGAGGCTATCCTCTCCTTTGCACGCCGTGGG

>JN006720.1_Asia1_PAK_2009

GGGTTGATCGTTGACACCAGAGATGTGGAAGAGCGCGTGCACGTCATGCGAAAAACCAAGCTTGCGCCCA

CCGTTGCACACGGTGTGTTCAACCCTGAATTCGGCCCCGCCGCTCTGTCTAACAAGGACCCGCGGCTGAA

TGAAGGGGTTGTCCTTGATGAAGTCATCTTCTCCAAGCACAAGGGAGACACCAAGATGTCGGAAGAGGAC

AAAGCGCTGTTTCGTCGCTGCGCTGCCGACTACGCGTCGCGCTTGCACAGTGTTCTGGGTACGGCAAATG

CTCCACTGAGCATTTACGAGGCAATCAAGGGCGTCGACGGACTCGACGCCATGGAACCAGACACCGCGCC

TGGCCTCCCCTGGGCCCTCCAAGGGAAACGCCGTGGTGCGCTCATCGACTTCGAGAACGGCACTGTCGGA

CCCGATGTTGAAGCTGCCTTGAAGCTCATGGAGAAAAGAGAATACAAGTTTGCATGTCAGACTTTCCTGA

AGGACGAGATTCGCCCGATGGAGAAAGTACGTGCCGGCAAGACTCGCATTGTCGATGTCCTGCCTGTTGA

ACACATTCTTTACACTAGGATGATGATTGGCAGATTCTGTGCTCAAATGCACTCAAACAACGGACCGCAA

ATTGGCTCAGCGGTCGGATGCAACCCTGACGTTGATTGGCAGAGATTTGGCACACATTTTGCTCAGTACA

GAAACGTGTGGGATGTGGATTATTCGGCCTTTGATGCTAACCATTGCAGTGACGCCATGAACATCATGTT

TGAGGAGGTGTTCCGCACAGAATTCGGTTTTCACCCTAATGCTGAGTGGATTCTGAAGACTCTCGTGAAC

ACGGAGCACGCGTACGAAAACAAACGCATCACTGTTGAGGGCGGGATGCCATCTGGTTGTTCCGCGACAA

GCATCATCAACACAATTCTGAACAACATCTACGTGCTCTACGCCCTGCGTAGACACTACGAGGGAGTTGA

GCTGGACACTTACACCATGATCTCCTACGGAGACGACATCGTGGTTGCAAGTGATTACGATCTGGACTTT

GAGGCCCTCAAGCCTCACTTCAAATCTCTTGGTCAAACCATTACTCCAGCTGACAAAAGTGATAAAGGTT

TTGTTCTTGGTCACTCCATTACCGATGTCACTTTCCTCAAAAGACACTTCCACATGGACTATGGAACTGG

GTTTTACAAACCTGTGATGGCCTCGAAGACCCTCGAGGCTATCCTCTCCTTTGCACGCCGTGGG

>JN006722.1_A_PAK_2008

GGGTTGATCGTTGACACCAGAGATGTGGAAGAGCGCGTGCACGTCATGCGCAAAACCAAGCTTGCGCCCA

CCGTTGCACACGGTGTGTTCAACCCTGAATTCGGCCCCGCCGCTCTGTCTAACAAAGACCCGCGGCTGAA

TGAAGGGGTTGTCCTTGATGAAGTCATCTTCTCCAAGCACAAGGGAGACACCAAGATGTCGGAAGAGGAC

AAAGCGCTGTTTCGTCGCTGCGCTGCCGACTACGCGTCGCGCTTGCACAGTGTTCTGGGTACGGCAAATG

CCCCACTGAGCATTTACGAGGCAATCAAGGGCGTCGACGGACTCGACGCCATGGAACCAGACACCGCGCC

TGGCCTCCCCTGGGCCCTCCAAGGGAAACGCCGTGGTGCGCTCATCGACTTCGAGAACGGCACTGTCGGA

CCCGATGTTGAAGCTGCCTTGAAGCTCATGGAGAAAAGAGAATACAAGTTTGCATGTCAGACTTTCCTGA

AGGACGAGATTCGCCCGATGGAGAAAGTACGTGCCGGCAAGACTCGCATTGTCGATGTCCTGCCTGTTGA

ACACATTCTTTACACTAGGATGATGATTGGCAGATTCTGTGCTCAAATGCACTCAAACAACGGACCGCAA

ATTGGCTCAGCGGTCGGATGCAACCCTGACGTTGATTGGCAGAGATTTGGCACACATTTTGCTCAGTACA

GAAACGTGTGGGATGTGGATTATTCGGCCTTTGATGCTAACCATTGCAGTGACGCCATGAACATCATGTT

TGAGGAGGTGTTCCGCACGGAATTCGGTTTCCACCCTAATGCTGAGTGGATCCTGAAGACTCTCGTGAAC

ACGGAGCACGCGTACGAAAACAAACGCATCACTGTTGAGGGCGGGATGCCATCTGGTTGTTCCGCGACAA

GCATCATCAACACAATTCTGAACAACATCTACGTGCTCTACGCCCTGCGTAGACACTACGAGGGAGTTGA

GCTGGACACTTACACCATGATCTCCTACGGAGACGACATCGTGGTTGCAAGTGATTACGATCTGGACTTT

GAGGCCCTCAAGCCTCACTTCAAATCTCTTGGTCAAACCATTACTCCAGCTGACAAAAGTGATAAAGGTT

TTGTTCTTGGTCACTCCATTACCGATGTCACTTTCCTCAAAAGACACTTCCACATGGACTATGGAACTGG

GTTTTACAAACCTGTGATGGCCTCGAAGACCCTCGAGGCTATCCTCTCCTTTGCACGCCGTGGG

>JN099688.1_A_IRQ_2009

GGGTTGATCGTTGACACCAGAGATGTGGAAGAGCGTGTGCACGTCATGCGCAAAACTAAGCTTGCACCCA

CCGTTGCACACGGTGTGTTCAACCCTGAATTCGGCCCCGCTGCTTTGTCCAACAAAGACCCGCGGCTGAA

TGAAGGGGTTGTCCTTGATGAAGTCATCTTCTCCAAACACAAGGGAGACACCAAGATGTCGGAAGAGGAC

AAAGCGCTGTTCCGACGCTGCGCTGCCGACTACGCGTCGCGCTTGCACAGCGTTCTGGGTACAGCAAATG

CCCCATTGAGCATCTACGAGGCTATCAAGGGCGTCGACGGACTCGACGCCATGGAACCAGACACCGCGCC

TGGCCTCCCCTGGGCCCTCCAGGGGAAACGCCGTGGTGCGCTCATCGACTTCGAGAACGGCACTGCCGGA

CCCGAGGTTGAGGCTGCCTTGAAGCTCATGGAGAAAAGAGAATACAAGTTTGCATGCCAGACCTTCCTGA

AGGACGAAATTCGCCCGATGGAGAAAGTACGTGCCGGCAAGACTCGCATTGTCGACGTCCTGCCTGTTGA

ACACATTCTTTACACTAGAATGATGATTGGCAGATTCTGTGCTCAAATGCACTCAAACAACGGACCGCAA

ATTGGCTCAGCGGTCGGATGTAACCCTGACGTTGATTGGCAGAGATTTGGCACACATTTTGCTCAGTACA

GAAACGTGTGGGATGTGGATTATTCGGCCTTTGATGCTAACCACTGCAGTGATGCCATGAACATCATGTT

TGAGGAGGTGTTCCGCACGGAGTTCGGTTTCCACCCTAACGCTGAGTGGATCCTGAAGACTCTTGTGAAC

ACGGAGCACGCCTACGAGAACAAACGCATCACTGTTGAGGGCGGGATGCCGTCTGGTTGTTCCGCAACAA

GCATCATCAACACAATTTTGAACAACATTTACGTGCTCTACGCCTTGCGTAGACACTATGAGGGAGTTGA

GCTGGACACTTACACCATGATCTCCTATGGAGACGACATCGTGGTTGCAAGTGATTACGATCTGGACTTT

GAGGCCCTCAAGCCTCACTTCAAATCTCTTGGTCAAACCATCACCCCAGCTGACAAAAGCGACAAAGGTT

TTGTTCTTGGTCACTCCATCACCGATGTCACGTTCCTCAAAAGACACTTCCACATGGACTATGGAACTGG

GTTTTACAAACCTGTGATGGCCTCGAAGACCCTCGAGGCTATCCTCTCCTTTGCACGCCGTGGG

>JN099694.1_A_IRQ_2009

GGGTTGATCGTTGACACCAGAGATGTGGAAGAGCGCGTGCACGTCATGCGCAAAACTAAGCTTGCACCCA

CCGTTGCACACGGTGTGTTCAACCCTGAATTCGGCCCCGCTGCTTTGTCCAACAAAGACCCGCGGCTGAA

TGAAGGGGTTGTCCTTGATGAAGTCATCTTCTCCAAACACAAGGGAGACACCAAGATGTCGGAAGAGGAC

AAAGCGCTGTTCCGACGCTGCGCTGCCGACTACGCGTCGCGCTTGCACAGCGTTCTGGGTGCAGCAAATG

CCCCATTGAGCATCTACGAGGCTATCAAGGGCGTCGACGGACTCGACGCCATGGAACCAGACACCGCGCC

TGGCCTCCCCTGGGCCCTCCAGGGGAAACGCCGTGGTGCGCTCATCGACTTCGAGAACGGCACTGTCGGA

CCCGAGGTTGAGGCTGCCTTGAAGCTCATGGAGAAAAGAGAATACAAGTTTGCATGCCAGACCTTCCTGA

AGGACGAAATTCGCCCGATGGAGAAAGTACGTGCCGGCAAGACTCGCATTGTCGATGTCCTGCCTGTTGA

ACACATTCTTTACACTAGAATGATGATTGGCAGATTCTGTGCTCAAATGCACTCAAACAACGGACCGCAA

ATTGGCTCAGCGGTCGGATGTAACCCTGACGTTGATTGGCAGAGATTTGGCACACATTTTGCTCAGTACA

GAAACGTGTGGGATGTGGATTATTCGGCCTTTGATGCTAACCACTGCAGTGATGCCATGAACATCATGTT

TGAAGAGGTGTTCCGCACGGAGTTCGGTTTCCACCCTAACGCTGAGTGGATCCTGAAGACTCTTGTGAAC

ACGGAGCACGCCTACGAGAACAAACGCATCACTGTTGAGGGCGGGATGCCGTCTGGTTGTTCCGCAACAA

GCATCATCAACACAATTTTGAACAACATTTACGTGCTCTACGCCTTGCGTAGACACTATGAGGGAGTTGA

GCTGGACACTTACACCATGATCTCCTATGGAGACGACATCGTGGTTGCAAGTGATTACGATCTGGACTTT

GAGGCCCTCAAGCCTCACTTCAAATCTCTTGGTCAAACCATCACCCCAGCTGACAAAAGCGACAAAGGTT

TTGTTCTTGGTCACTCCATCACCGATGTCACGTTCCTCAAAAGACACTTCCACATGGACTATGGAACTGG

GTTTTACAAACCTGTGATGGCCTCGAAGACCCTCGAGGCTATCCTCTCCTTTGCACGCCGTGGG

>JN099695.1_A_IRQ_2009

GGGTTGATCGTTGACACCAGAGATGTGGAAGAGCGCGTGCACGTCATGCGCAAAACTAAGCTTGCACCCA

CCGTTGCACACGGTGTGTTCAACCCTGAATTCGGCCCCGCTGCTTTGTCCAACAAAGACCCCCGGCTGAA

TGAAGGGGTTGTCCTTGATGAAGTCATCTTCTCCAAACACAAGGGAGACACCAAGATGTCGGAAGAGGAC

AAAGCGCTGTTCCGACGCTGCGCTGCCGACTACGCGTCGCGCTTGCACAGCGTTCTGGGTACAGCAAATG

CCCCATTGAGCATCTACGAGGCTATCAAGGGCGTCGACGGACTCGACGCCATGGAACCAGACACCGCGCC

TGGCCTCCCCTGGGCCCTCCAGGGGAAACGCCGTGGTGCGCTCATCGACTTCGAGAACGGCACTGTCGGA

CCCGAGGTTGAGGCTGCCTTGAAGCTCATGGAGAAAAGAGAATACAAGTTTGCATGCCAGACCTTCCTGA

AGGACGAAATTCGCCCGATGGAGAAAGTACGTGCCGGCAAGACTCGCATTGTCGATGTCCTGCCTGTTGA

ACACATTCTTTACACTAGAATGATGATTGGCAGATTCTGTGCTCAAATGCACTCAAACAACGGACCGCAA

ATTGGTTCAGCGGTCGGATGTAACCCTGACGTTGATTGGCAGAGATTTGGCACACATTTTGCTCAGTACA

GAAACGTGTGGGATGTGGATTATTCGGCCTTTGATGCTAACCACTGCAGTGATGCCATGAACATCATGTT

TGAGGAGGTGTTCCGCACGGAGTTCGGTTTCCACCCTAACGCTGAGTGGATCCTGAAGACTCTTGTGAAC

ACGGAGCACGCCTACGAGAACAAACGCATCACTGTTGAGGGCGGGATGCCGTCTGGTTGTTCCGCAACAA

GCATCATCAACACAATTTTGAACAACATTTACGTGCTCTACGCCTTGCGTAGACACTATGAGGGAGTTGA

GCTGGACATTTACACCATGATCTCCTATGGAGACGACATCGTGGTTGCAAGTGATTACGATCTGGACTTT

GAGGCCCTCAAGCCTCACTTCAAATCTCTTGGTCAAACCATCACCCCAGCTGACAAAAGCGACAAAGGTT

TTGTTCTTGGTCACTCCATCACCGATGTCACGTTCCTCAAAAGACACTTCCACATGGACTATGGAACTGG

GTTTTACAAACCTGTGATGGCCTCGAAGACCCTCGAGGCTATCCTCTCCTTTGCACGCCGTGGG

>JN099697.1_A_IRQ_2009

GGGTTGATCGTTGACACCAGAGATGTGGAAGAGCGCGTGCACGTCATGCGCAAAACTAAGCTTGCACCCA

CCGTTGCACACGGTGTGTTCAACCCTGAATTCGGCCCCGCTGCTTTGTCCAACAAAGACCCGCGGCTGAA

TGAAGGGGTTGTCCTTGATGAAGTCATCTTCTCCAAACACAAGGGAGACACCAAGATGTCGGAAGAGGAC

AAAGCGCTGTTCCGACGCTGCGCTGCCGACTACGCGTCGCGCTTGCACAGCGTTCTGGGTACAGCAAATG

CCCCATTGAGCATCTACGAGGCTGTCAAGGGCGTCGACGGACTCGACGCCATGGAACCAGACACCGCGCC

TGGCCTCCCCTGGGCCCTCCAGGGGAAACGCCGTGGTGCGCTCATCGACTTCGAGAACGGCACTGTCGGA

CCCGAGGTTGAGGCTGCCTTGAAGCTCATGGAGAAAAGAGAATACAAGTTTGCATGCCAGACCTTCCTGA

AGGACGAAATTCGCCCGATGGAGAAAGTACGTGCCGGCAAGACTCGCATTGTCGATGTCCTGCCTGTTGA

ACACATTCTTTACACTAGAATGATGATTGGCAGATTCTGTGCTCAAATGCACTCAAACAACGGACCGCAA

ATTGGCTCAGCGGTCGGATGTAACCCTGACGTTGATTGGCAGAGATTTGGCACACATTTTGCTCAGTACA

GAAACGTGTGGGATGTGGATTATTCGGCCTTTGATGCTAACCACTGCAGTGATGCCATGAACATCATGTT

TGAGGAGGTGTTCCGCACGGAGTTCGGTTTCCACCCTAACGCCGAGTGGATCCTGAAGACTCTTGTGAAC

ACGGAGCACGCCTACGAGAACAAACGCATCACTGTTGAGGGCGGGATGCCGTCTGGTTGTTCCGCAACAA

GCATCATCAACACAATTTTGAACAACATTTACGTGCTCTACGCCTTGCGTAGACACTATGAGGGAGTTGA

GCTGGACACTTACACCATGATCTCCTATGGAGACGACATCGTGGTTGCAAGTGATTACGATCTGGACTTT

GAGGCCCTCAAGCCTCACTTCAAATCTCTTGGTCAAACCATCACCCCAGCTGACAAAAGCGACAAAGGTT

TTGTTCTTGGTCACTCCATCACCGATGTCACGTTCCTCAAAAGACACTTCCACATGGACTATGGAACTGG

GTTTTACAAACCTGTGATGGCCTCGAAGACCCTCGAGGCTATCCTCTCCTTTGCACGCCGTGGG

>JN099698.1_A_IRQ_2009

GGGTTGATCGTTGACACCAGAGATGTGGAAGAGCGCGTGCACGTCATGCGCAAAACTAAGCTTGCACCCA

CCGTTGCACACGGTGTGTTCAACCCTGAATTCGGCCCCGCTGCTTTGTCCAACAAAGACCCCCGGCTGAA

TGAAGGGGTTGTCCTTGATGAAGTCATCTTCTCCAAACACAAGGGAGACACCAAGATGTCGGAAGAGGAC

AAAGCGCTGTTCCGACGCTGCGCTGCCGACTACGCGTCGCGCTTGCACAGCGTTCTGGGTACAGCAAATG

CCCCATTGAGCATCTACGAGGCTATCAAGGGCGTCGACGGACTCGACGCCATGGAACCAGACACCGCGCC

TGGCCTCCCCTGGGCCCTCCAGGGGAAACGCCGTGGTGCGCTCATCGACTTCGAGAACGGCACTGTCGGA

CCCGAGGTTGAGGCTGCCTTGAAGCTCATGGAGAAAAGAGAATACAAGTTTGCATGCCAGACCTTCCTGA

AGGACGAAATTCGCCCGATGGAGAAAGTACGTGCCGGCAAGACTCGCATTGTCGATGTCCTGCCTGTTGA

ACACATTCTTTACACTAGAATGATGATTGGCAGATTCTGTGCTCAAATGCACTCAAACAACGGACCGCAA

ATTGGTTCAGCGGTCGGATGTAACCCTGACGTTGATTGGCAGAGATTTGGCACACATTTTGCTCAGTACA

GAAACGTGTGGGATGTGGATTATTCGGCCTTTGATGCTAACCACTGCAGTGATGCCATGAACATCATGTT

TGAGGAGGTGTTCCGCACGGAGTTCGGTTTCCACCCTAACGCTGAGTGGATCCTGAAGACTCTTGTGAAC

ACGGAGCACGCCTACGAGAACAAACGCATCACTGTTGAGGGCGGGATGCCGTCTGGTTGTTCCGCAACAA

GCATCATCAACACAATTTTGAACAACATTTACGTGCTCTACGCCTTGCGTAGACACTATGAGGGAGTTGA

GCTGGACATTTACACCATGATCTCCTATGGAGACGACATCGTGGTTGCAAGTGATTACGATCTGGACTTT

GAGGCCCTCAAGCCTCACTTCAAATCTCTTGGTCAAACCATCACCCCAGCTGACAAAAGCGACAAAGGTT

TTGTTCTTGGTCACTCCATCACCGATGTCACGTTCCTCAAAAGACACTTCCACATGGACTATGGAACTGG

GTTTTACAAACCTGTGATGGCCTCGAAGACCCTCGAGGCTATCCTCTCCTTTGCACGCCGTGGG

>JN099699.1_A_IRQ_2009

GGGTTGATCGTTGACACCAGAGATGTGGAAGAGCGCGTGCACGTCATGCGCAAAACTAAGCTTGCACCCA

CCGTTGCACACGGTGTGTTCAACCCTGAATTCGGCCCCGCTGCTTTGTCCAACAAAGACCCGCGGCTGAA

TGAAGGGGTTGTCCTTGATGAAGTCATCTTCTCCAAACACAAGGGAGACACCAAGATGTCGGAAGAGGAC

AAAGCGCTGTTCCGACGCTGCGCTGCCGACTACGCGTCGCGCTTGCACAGCGTTCTGGGTACAGCAAATG

CCCCATTGAGCATCTACGAGGCTGTCAAGGGCGTCGACGGACTCGACGCCATGGAACCAGACACCGCGCC

TGGCCTCCCCTGGGCCCTCCAGGGGAAACGCCGTGGTGCGCTCATCGACTTCGAGAACGGCACTGTCGGA

CCCGAGGTTGAGGCTGCCTTGAAGCTCATGGAGAAAAGAGAATACAAGTTTGCATGCCAGACCTTCCTGA

AGGACGAAATTCGCCCGATGGAGAAAGTACGTGCCGGCAAGACTCGCATTGTCGATGTCCTGCCTGTTGA

ACACATTCTTTACACTAGAATGATGATTGGCAGATTCTGTGCTCAAATGCACTCAAACAACGGACCGCAA

ATTGGCTCAGCGGTCGGATGTAACCCTGACGTTGATTGGCAGAGATTTGGCACACATTTTGCTCAGTACA

GAAACGTGTGGGATGTGGATTATTCGGCCTTTGATGCTAACCACTGCAGTGATGCCATGAATATCATGTT

TGAGGAGGTGTTCCGCACGGAGTTCGGTTTCCACCCTAACGCCGAGTGGATCCTGAAGACTCTTGTGAAC

ACGGAGCACGCCTACGAGAACAAACGCATCACTGTTGAGGGCGGGATGCCGTCTGGTTGTTCCGCAACAA

GCATCATCAACACAATTTTGAACAACATTTACGTGCTCTACGCCTTGCGTAGACACTATGAGGGAGTTGA

GCTGGACACTTACACCATGATCTCCTATGGAGACGACATCGTGGTTGCAAGTGATTACGATCTGGACTTT

GAGGCCCTCAAGCCTCACTTCAAATCTCTTGGTCAAACCATCACCCCAGCTGACAAAAGCGACAAAGGTT

TTGTTCTTGGTCACTCCATCACCGATGTCACGTTCCTCAAAAGACACTTCCACATGGACTATGGAACTGG

GTTTTACAAACCTGTGATGGCCTCGAAGACCCTCGAGGCTATCCTCTCCTTTGCACGCCGTGGG

>JN998085.1_O_CHA_2010

GGGTTGATCGTTGACACCAGAGATGTGGAAGAGCGCGTGCACGTCATGCGCAAAACCAAGCTTGCACCTA

CCGTGGCACACGGTGTGTTCAACCCTGAGTACGGCCCCGCTGCCTTGTCCAACAAGGACCCGCGGCTGAA

TGAGGGAGTTGTCCTCGATGAGGTCATCTTCTCCAAACACAAGGGGGACACAAAGATGTCACCGGAAGAC

AAAGCGCTGTTCCGCCGCTGCGCTGCCGACTACGCGTCGCGTCTTCACAGCGTGCTGGGTACAGCAAATG

CCCCATTGAGCATCTACGAGGCCATTAAAGGCGTTGACGGACTCGACGCCATGGAACCAGACACAGCGCC

TGGCCTTCCCTGGGCACTCCAGGGGAAACGCCGCGGCGCGCTGATTGACTTCGAGAACGGCACTGTCGGA

CCCGAAGTCCAGGCTGCCTTGGAGCTCATGGAGAAAAGAGAATACAAGTTTGCCTGTCAGACCTTCCTGA

AGGACGAAATTCGCCCGATGGAAAAAGTACGTGCCGGCAAGACGCGCATCGTCGATGTTTTGCCTGTTGA

ACACATTCTTTACACCAGGATGATGATTGGCAGATTTTGTGCTCAAATGCACTCAAACAACGGACCACAA

ATTGGATCAGCGGTCGGTTGTAATCCTGATGTTGATTGGCAAAGATTTGGCACACACTTCGCCCAATACA

GAAACGTGTGGGATGTGGACTATTCGGCCTTTGATGCTAACCACTGTAGTGATGCAATGAACATCATGTT

TGAGGAGGTGTTTCGCACAGACTTTGGTTTCCACCCGAATGCTGAGTGGATTCTGAAGACCCTCGTGAAC

ACGGAACACGCCTATGAGAACAAACGCATTACAGTTGAAGGTGGAATGCCGTCCGGCTGTTCCGCAACCA

GCATCATCAACACAATTCTGAACAACATCTACGTGCTCTACGCGCTGCGTAGACACTATGAGGGAGTTGA

GCTGGACACTTACACCATGATCTCCTACGGAGACGACATCGTGGTTGCTAGTGATTATGACTTGGACTTT

GAGGCTCTCAAGCCCCACTTTAAATCTCTTGGTCAAACCATTACTCCAGCTGACAAAAGCGACAAAGGTT

TTGTTCTTGGTCACTCCATTACCGATGTCACTTTCCTCAAAAGACACTTCCACATGGATTATGGAACTGG

GTTTTACAAACCTGTGATGGCTTCGAAGACCCTCGAGGCTATCCTCTCCTTTGCACGCCGTGGG

>JN998086.1_O_CHA_2010

GGGTTGATCGTTGACACCAGAGATGTGGAAGAGCGCGTGCACGTCATGCGCAAAACCAAGCTTGCACCTA

CCGTGGCACACGGTGTGTTCAACCCCGAGTACGGCCCCGCTGCCTTGTCCAACAAGGACCCGCGGCTGAA

TGAAGGAGTTGTCCTCGATGAGGTCATTTTTTCCAAACACAAGGGGGACACAAAGATGTCACCGGAAGAC

AAAGCGCTGTTCCGCCGCTGCGCTGCCGATTACGCGTCGCGTCTTCACAGTGTGCTGGGTACAGCAAATG

CCCCATTGAGCATCTACGAGGCCATTAAAGGCGTTGACGGACTCGACGCCATGGAACCAGACACAGCGCC

TGGCCTTCCCTGGGCACTCCAGGGGAAACGCCGCGGCGCGCTGATTGACTTCGAGAACGGCACTGTCGGA

CCCGAAGTCCAGGCTGCCTTGGAGCTCATGGAGAAAAGAGAATACAAGTTTGCCTGTCAGACCTTCCTGA

AGGACGAAATTCGCCCGATGGAAAAAGTACGTGCCGGCAAGACGCGCATCGTCGATGTTTTGCCTGTTGA

ACACATTCTTTACACCAGGATGATGATTGGCAGATTTTGTGCTCAAATGCACTCAAACAACGGACCGCAA

ATTGGATCAGCGGTCGGTTGTAATCCTGATGTTGATTGGCAAAGATTTGGCACACACTTCGCCCAATACA

GAAACGTGTGGGATGTGGACTATTCGGCCTTTGATGCTAATCACTGTAGTGATGCAATGAACATCATGTT

TGAGGAGGTGTTTCGCACAGACTTTGGTTTCCACCCGAATGCTGAGTGGATTCTGAAGACCCTCGTGAAC

ACGGAACACGCCTATGAGAACAAACGCATTACAGTTGAAGGTGGAATGCCGTCCGGCTGTTCCGCAACCA

GCATCATCAACACAATTCTGAACAACATCTACGTGCTCTACGCACTGCGTAGACACTATGAGGGAGTTGA

GCTGGACACTTACACCATGATCTCCTACGGAGACGACATCGTGGTTGCTAGTGATTATGACTTGGACTTT

GAGGCTCTCAAGCCCCACTTTAAATCTCTTGGTCAAACCATTACTCCAGCTGACAAAAGCGACAAAGGTT

TTGTTCTTGGTCACTCCATTACCGATGTCACTTTCCTCAAAAGACACTTCCACATGGATTATGGAACTGG

GTTTTACAAACCTGTGATGGCTTCGAAGACCCTCGAGGCTATCCTCTCCCTTGCACGCCGTGGG

>JQ900581.1_O_CHA_2010

GGGTTGATCGTTGACACCAGAGATGTGGAAGAGCGCGTGCACGTCATGCGCAAAACCAAGCTTGCACCTA

CCGTGGCACACGGTGTGTTCAACCCTGAGTACGGCCCCGCTGCCTTGTCCAACAAGGACCCGCGGCTGAA

TGAAGGAGTTGTCCTCGATGAGGTCATCTTCTCCAAACACAAAGGGGACACGAAGATGTCGCCGGAAGAC

AAAGCGCTGTTCCGCCGCTGCGCTGCCGACTACGCGTCGCGTCTTCACAGTGTGCTGGGTACAGCAAATG

CCCCATTGAGCATCTACGAGGCCATTAAAGGCGTTGACGGACTCGATGCCATGGAACCAGACACAGCGCC

TGGCCTTCCCTGGGCACTCCAGGGGAAACGCCGTGGCGCGCTGATTGACTTCGAGAACGGCACCGTCGGA

CCCGAAGTCCAGGCTGCCTTGGAGCTCATGGAGAAAAGAGAATACAAGTTTGTCTGTCAGACCTTCCTGA

AGGACGAAATTCGCCCGATGGAAAAAGTACGTGCCGGCAAGACGCGCATCGTCGATGTTTTGCCTGTTGA

ACACATTCTTTACACCAGGATGATGATTGGCAGATTTTGTGCTCAAATGCACTCAAACAACGGACCGCAA

ATTGGATCAGCGGTCGGTTGTAACCCTGATGTTGATTGGCAAAGATTTGGCACACACTTCGCCCAATACA

GAAACGTGTGGGATGTGGACTATTCGGCCTTTGATGCTAATCACTGTGGTGATGCAATGAACATCATGTT

TGAGGAGGTGTTTCGCACAGACTTCGGTTTCCACCCGAATGCTGAGTGGATTCTGAAGACCCTCGTGAAC

ACGGAACACGCCTATGAGAACAAACGCATTACAGTTGAAGGTGGAATGCCGTCCGGCTGTTCCGCAACCA

GCATCATCAACACAATTCTGAACAACATCTACGTGCTCTACGCGTTGCGTAGACACTATGAGGGAGTTGA

GCTGGACACTTACACCATGATCTCCTACGGAGACGACATCGTGGTTGCTAGTGATTATGACTTGGACTTT

GAGGCTCTCAAGCCCCACTTTAAATCTCTTGGTCAAACCATTACCCCAGCTGACAAAAGCGACAAAGGTT

TTGTTCTTGGTCACTCCATTACCGATGTCACTTTCCTCAAAAGACACTTCCACATGGATTATGGAACTGG

GTTTTACAAACCTGTGATGGCTTCGAAGACCCTCGAGGCTATCCTCTCCTTTGCACGCCGTGGG

>JQ973889.1_O_CHA_2010

GGGTTGATCGTTGACACCAGAGATGTGGAAGAGCGCGTGCACGTCATGCGCAAAACCAAGCTTGCACCTA

CCGTGGCACACGGTGTGTTCAACCCTGAGTACGGCCCCGCTGCCTTGTCCAACAAGGACCCGCGGCTGAA

TGAAGGAGTTGTCCTCGATGAGGTCATCTTCTCCAAACACAAGGGGGATACAAAGATGTCACCGGAAGAC

AAAGCGCTGTTCCGCCGCTGCGCTGCCGACTACGCGTCGCGTCTTCACAGTGTGCTGGGTACAGCAAATG

CCCCATTGAGCATCTACGAGGCCATTAAAGGCGTTGACGGACTCGACGCCATGGAACCAGACACAGCACC

TGGCCTTCCCTGGGCACTCCAGGGGAAACGCCGCGGCGCGCTGATTGACTTCGAGAACGGCACTGTCGGA

CCCGAAGTCCAGGCTGCCTTGAAGCTCATGGAGAAAAGAGAATACAAGTTTGCCTGTCAGACCTTCCTGA

AGGACGAAATTCGCCCGATGGAAAAAGTACGTGCCGGCAAGACGCGCATCGTCGATGTTTTGCCTGTTGA

ACACATTCTTTACACCAGGATGATGATTGGCAGATTTTGTGCTCAAATGCACTCAAACAACGGACCGCAA

ATTGGATCAGCGGTCGGTTGTAATCCTGATGTTGATTGGCAAAGATTTGGCACACACTTCGCCCAATACA

GAAACGTGTGGGATGTGGACTATTCGGCCTTTGATGCTAATCACTGTAGTGATGCAATGAACATCATGTT

TGAGGAGGTGTTTCGCACAGACTTTGGTTTCCACCCGAATGCTGAGTGGATTCTGAAGACCCTCGTGAAC

ACGGAACACGCCTATGAGAACAAACGCATTACAGTTGAAGGTGGAATGCCGTCCGGCTGTTCCGCAACCA

GCATCATCAACACAATTCTGAACAACATCTACGTGCTCTACGCGCTGCGTAGACACTATGAGGGAGTTGA

GCTGGACACTTATACCATGATCTCCTACGGAGACGATATCGTGGTTGCTAGTGATTATGACTTGGACTTT

GAGGCTCTCAAGCCCCACTTTAAATCTCTTGGTCAAACCATTACTCCAGCTGACAAAAGCGACAAAGGTT

TTGTTCTTGGTCACTCCATTACCGATGTCACTTTCCTCAAAAGACACTTCCACATGGATTATGGAACTGG

GTTTTACAAACCTGTGATGGCTTCGAAGACCCTCGAGGCTATCCTCTCCTTTGCACGCCGTGGG

>JX014255.1_SAT2_EGY_2012

GGGTTGATCGTGGACACCAGAGATGTGGAAGAGCGTGTGCACGTCATGCGCAAAACCAAGCTTGCACCCA

CCGTTGCACACGGTGTATTCAACCCCGACTTCGGACCTGCCGCACTGTCCAACAACGACAAGCGCCTGGA

CGAAGGGGTTATCCTCGACGACGTCATCTTTTCTAAGCACAAGGGAGACAAGAAGATGTCAGAGGAAGAC

AAGAAGCTGTTCAGAACATGTGCTGCTGACTACGCGTCACACCTGCACTCAGTTCTCGGCACAGCAAATG

CCCCATTGAGCATCTACGAGGCCATCAAGGGCATCGATGGACTTGACGCCATGGAGCCTGACACCGCTCC

AGGTCTGCCTTGGGCAACCCAGGGGAAACGCCGCGGAGCCCTCATTGACTTCGAGAACGGCACCGTCGGA

CCCGAGGTCGAAGAGGCGTTGAAGCTCATGGAGAAAAGAGAATACAAATTCACATGCCAGACATTCCTGA

AGGACGAAATTCGCCCGATGGAAAAAGTTCGCGCCGGTAAGACCCGCATCGTCGACGTCCTGCCTGTTGA

GCACATCCTTTACACCAGAATGATGATTGGCAAATTCTGTGCACAAATGCACTCCAACAACGGACCGCGA

ATTGGTTCGGCGGTCGGTTGCAACCCAGATGTTGATTGGCAGAGATTTGGAACCCATTTTGCCCAGTACC

GGAACGTGTGGGACGTGGACTATTCGGCCTTTGATGCTAACCACTGCAGTGATGCCATGAACATCATGTT

CGAGGAGGTTTTCCGTCCAGAATTCGGTTTCCACCCCAACGCGGAGTGGATCCTGAAGACTCTGGTTAAT

ACGGAACACGCCTATGAGAACAAGCGCATTACAGTGGAAGGCGGTATGCCCTCGGGTTGTTCCGCCACCA

GCATAATCAACACAATTCTTAACAACATTTATGTGCTCTACGCCTTGCGTAGACACTATGAGGGAGTCGA

GCTGTCTACCTACTCGATGATTTCCTACGGGGATGACATCGTGGTAGCAAGTGACTTTGACCTGGACTTT

GAATCTCTTAAGCCTCACTTCAAGTCCGTTGGCCAGACCATCACTCCAGCCGACAAAAGTGACAAAGGTT

TTGTCCTCGGTCAGTCCATCACTGACGTCACTTTCCTCAAGAGGCACTTCCACATGGACTATGGAACTGG

ATTTTATAAACCTGTGATGGCTTCGAAGACCCTCGAAGCTATCCTCTCCTTTGCACGCCGTGGG

>JX014256.1_SAT2_PAT_2012

GGGTTGATCGTGGACACCAGAGATGTGGAAGAGCGTGTGCACGTCATGCGCAAAACCAAGCTTGCACCCA

CCGTTGCACACGGTGTATTCAACCCCGACTTCGGACCTGCCGCACTGTCCAACAACGACAAGCGCCTGGA

CGAAGGGGTTGTCCTCGACGACGTCATCTTTTCTAAGCACAAGGGGGACAAGAAGATGTCAGAGGAAGAT

AAGAAGCTGTTCAGAACATGTGCTGCTGACTACGCGTCACACCTGCACTCAGTTCTCGGCACAGCAAATG

CCCCATTGAGCATCTACGAGGCCATCAAGGGCATCGATGGACTTGACGCCATGGAGCCTGACACCGCTCC

AGGTCTGCCTTGGGCAACCCAGGGGAAACGCCGCGGAGCCCTCATTGACTTCGAGAACGGCACCGTCGGA

CCCGAGGTCGAAGAGGCGTTGAAGCTCATGGAGAAAAGAGAATACAAATTCACATGCCAGACATTCCTGA

AGGACGAAATTCGCCCGATGGAAAAAGTTCGCGCCGGTAAGACTCGCATCGTCGACGTCCTGCCTGTTGA

GCACATCCTTTACACCAGAATGATGATTGGCAAGTTCTGTGCACAAATGCACTCCAACAACGGACCGCGA

ATTGGTTCGGCGGTCGGTTGCAACCCAGATGTTGATTGGCAGAGATTTGGAACCCATTTTGCCCAGTACC

GGAACGTGTGGGACGTGGACTATTCGGCCTTTGATGCTAACCACTGCAGTGATGCCATGAACATCATGTT

CGAGGAGGTTTTCCGTCCAGAATTCGGTTTCCACCCCAACGCGGAGTGGATCCTGAAAACTCTGGTTAAC

ACGGAACACGCCTATGAGAACAAGCGCATTACAGTGGAAGGCGGTATGCCCTCGGGTTGTTCCGCCACCA

GCATAATCAACACAATTCTTAACAACATTTACGTGCTCTACGCCTTGCGTAGACACTATGAGGGAGTCGA

GCTGTCTACCTACTCGATGATTTCCTACGGGGATGACATCGTGGTAGCAAGTGACTTTGACCTGGACTTT

GAAGCTCTTAAGCCTCACTTCAAGTCCGTTGGCCAGACCATCACTCCAGCCGACAAAAGTGACAAAGGTT

TTGTCCTCGGTCAGTCCATCACTGACGTCACTTTCCTCAAGAGGCACTTCCACATGGACTATGGAACTGG

ATTTTACAAACCTGTGATGGCTTCGAAGACCCTCGAAGCTATCCTCTCCTTTGCACGCCGTGGG

>JX040485.1_O_BUL_2010

GGATTGATCGTTGACACCAGAGATGTTGAGGAACGCGTGCACGTCATGCGCAAAACTAAGCTTGCACCCA

CCGTTGCACACGGTGTGTTCAATCCTGAGTTTGGCCCCGCTGCCTTGTCCAACAAGGACCCGCGGCTGAA

CGAGGGAGTTGTCCTCGATGAAGTCATCTTATCCAAACACAAGGGAGACACCAAAATGTCTGAGGAGGAC

AAAGCGCTATTCCGCCGCTGTGCTGCTGACTACGCGTCACGCCTACACAGTGTGTTGGGAACAGCAAATG

CCCCACTGAGCATCTATGAGGCAATCAAGGGCGTTGACGGACTCGACGCCATGGAACCAGACACCGCACC

CGGTCTCCCCTGGGCCCTCCAGGGGAAGCGCCGCGGAGCGCTCATCGACTTCGAGAACGGCACTGTCGGA

CCCGAGGTTGAAGCAGCCTTGAAGCTCATGGAGAAAAGAGAATACAAGTTTGTTTGCCAGACCTTCCTGA

AGGACGAGATTCGCCCGATGGAGAAAGTACGTGCCGGTAAGACTCGCATTGTCGACGTCTTGCCTGTTGA

ACACATTCTTTACACCAGGATGATGATTGGCAGATTTTGTGCGCAAATGCACTCAAACAACGGACCGCAA

ATTGGCTCAGCGGTCGGTTGCAACCCTGATGTTGATTGGCAGAGATTTGGCACACACTTCGCCCAGTACA

GAAACGTGTGGGACGTGGACTATTCGGCCTTTGATGCTAACCACTGCAGTGACGCAATGAACATCATGTT

TGAGGAGGTGTTCCGCACGGAGTTCGGCTTCCACCCAAACGCTGAGTGGATTCTGAAAACTCTTGTGAAC

ACGGAGCATGCCTATGAGAACAAACGCATCACTGTTGAGGGCGGGATGCCTTCCGGGTGTTCTGCGACAA

GTATCATCAACACAATCTTGAACAACATCTACGTGCTCTACGCGCTGCGTAGACACTACGAGGGAGTTGA

ACTGGACACTTATACCATGATCTCCTACGGAGACGACATCGTGGTTGCAAGTGATTACGATCTGGACTTC

GAGGCTCTCAAGCCTCACTTCAAATCTCTTGGCCAGACCATCACTCCAGCTGACAAAAGCGACAAAGGTT

TTGTTCTTGGTCACTCCATTACCGATGTCACTTTCCTCAAAAGACACTTCCACATGGACTATGGAACTGG

GTTTTACAAACCTGTGATGGCCTCGAAGACCCTCGAGGCTGTCCTCTCCTTTGCACGCCGTGGG

>JX040486.1_O_BUL_2011

GGATTGATCGTTGACACCAGAGATGTTGAGGAGCGCGTGCACGTCATGCGCAAAACTAAGCTTGCACCCA

CCGTTGCACACGGTGTGTTCAATCCTGAGTTTGGCCCCGCTGCCTTGTCCAACAAGGACCCGCGGCTGAA

CGAGGGAGTTGTCCTCGATGAAGTCATCTTCTCCAAACACAAGGGAGACACCAAAATGTCTGAGGAGGAC

AAAGCGCTGTTCCGCCGCTGTGCTGCTGACTACGCGTCACGCCTACACAGTGTGTTGGGAACAGCAAATG

CCCCACTGAGCATCTATGAGGCAATCAAGGGCGTTGACGGACTCGACGCCATGGAACCAGACACCGCACC

CGGTCTCCCCTGGGCCCTCCAGGGGAAGCGCCGCGGAGCGCTCATCGACTTCGAGAACGGCACTGTCGGA

CCCGAGGTTGAAGCAGCCTTGAAGCTCATGGAGAAAAGAGAATACAAGTTTGTTTGCCAGACCTTCCTGA

AGGACGAGATTCGCCCGATGGAGAAAGTACGTGCCGGTAAGACTCGCATTGTCGACGTCTTGCCTGTTGA

ACACATTCTTTACACCAGGATGATGATTGGCAGATTTTGTGCGCAAATGCACTCAAACAACGGACCGCAA

ATTGGCTCAGCGGTCGGTTGCAACCCTGATGTTGATTGGCAGAGATTTGGCACACACTTCGCCCAGTACA

GAAACGTGTGGGACGTGGACTATTCGGCCTTTGATGCTAACCACTGCAGTGACGCAATGAACATCATGTT

TGAGGAGGTGTTCCGCACGGAGTTCGGCTTCCACCCGAACGCTGAGTGGATTCTGAAAACTCTTGTGAAC

ACGGAGCATGCCTATGAGAACAAACGCATCACTGTTGAGGGCGGGATGCCTTCCGGGTGTTCTGCGACAA

GTATCATCAACACAATCTTGAACAACATCTACGTGCTCTACGCGCTGCGTAGACACTACGAGGGAGTTGA

ACTGGACACTTATACCATGATCTCCTACGGAGACGACATCGTGGTTGCAAGTGATTACGATCTGGACTTC

GAGGCTCTCAAGCCTCACTTCAAATCTCTTGGCCAGACCATCACTCCAGCTGACAAAAGCGACAAAGGTT

TTGTTCTTGGCCACTCCATTACCGATGTCACTTTCCTCAAAAGACACTTCCACATGGACTATGGAACTGG

GTTTTACAAACCTGTGATGGCCTCGAAGACCCTCGAGGCTGTCCTCTCCTTTGCACGCCGTGGG

>JX040487.1_O_BUL_2011

GGATTGATCGTTGACACCAGAGATGTTGAGGAACGCGTGCACGTCATGCGCAAAACTAAGCTTGCACCCA

CCGTTGCACACGGTGTGTTCAATCCTGAGTTTGGCCCCGCTGCCTTGTCCAACAAGGACCCGCGGCTGGA

CGAGGGAGTTGTCCTCGATGAAGTCATCTTCTCCAAACACAAGGGAGACACCAAAATGTCTGAGGAGGAC

AAAGCGCTGTTCCGCCGCTGTGCTGCTGACTACGCGTCACGCCTACACAGTGTGTTGGGAACAGCAAATG

CCCCACTGAGCATCTATGAGGCAATCAAGGGCGTTGACGGACTCGACGCCATGGAACCAGACACCGCACC

CGGTCTCCCCTGGGCCCTCCAGGGGAAGCGCCGCGGAGCGCTCATCGACTTCGAGAACGGCACTGTCGGA

CCCGAGGTTGAAGCAGCCTTGAAGCTCATGGAGAAAAGAGAATACAAGTTTGTTTGCCAGACCTTCCTGA

AGGACGAGATTCGCCCGATGGAGAAAGTACGTGCCGGTAAGACTCGCATTGTCGACGTCTTGCCTGTTGA

ACACATTCTTTACACCAGGATGATGATTGGCAGATTTTGTGCGCAAATGCACTCAAACAACGGACCGCAA

ATTGGCTCAGCGGTCGGTTGCAACCCTGATGTTGATTGGCAGAGATTTGGCACACACTTCGCCCAGTACA

GAAACGTGTGGGACGTGGACTATTCGGCCTTTGATGCTAACCACTGCAGTGACGCAATGAACATCATGTT

TGAGGAGGTGTTCCGCACGGAGTTCGGCTTCCACCCGAACGCTGAGTGGATTCTGAAAACTCTTGTGAAC

ACAGAGCACGCCTATGAGAACAAACGCATCACTGTTGAGGGCGGGATGCCTTCCGGGTGTTCTGCGACAA

GTATCATCAACACAATCTTGAACAACATCTACGTGCTCTACGCGCTGCGTAGACACTACGAGGGAGTTGA

ACTGGACACTTATACCATGATCTCCTACGGAGACGACATCGTGGTTGCAAGTGATTACGATCTGGACTTC

GAGGCTCTCAAGCCTCACTTCAAATCTCTTGGCCAGACCATCACTCCAGCTGACAAAAGCGACAAAGGTT

TTGTTCTTGGTCACTCCATTACCGATGTCACTTTCCTCAAAAGACACTTCCACATGGACTATGGAACTGG

GTTTTACAAACCTGTGATGGCCTCGAAGACCCTCGAGGCTGTCCTCTCCTTTGCACGCCGTGGG

>JX040488.1_O_BUL_2011

GGATTGATCGTTGACACCAGAGATGTTGAGGAACGCGTGCACGTCATGCGCAAAACTAAGCTTGCACCCA

CCGTTGCACACGGTGTGTTCAATCCTGAGTTTGGCCCCGCTGCCTTGTCCAACAAGGACCCGCGGCTGGA

CGAGGGAGTTGTCCTCGATGAAGTCATCTTCTCCAAACACAAGGGAGACACCAAAATGTCTGAGGAGGAC

AAAGCGCTGTTCCGCCGCTGTGCTGCTGACTACGCGTCACGCCTACACAGTGTGTTGGGAACAGCAAATG

CCCCACTGAGCATCTATGAGGCAATCAAGGGCGTTGACGGACTCGACGCCATGGAACCAGACACCGCACC

CGGTCTCCCCTGGGCCCTCCAGGGGAAGCGCCGCGGAGCGCTCATCGACTTCGAGAACGGCACTGTCGGA

CCCGAGGTTGAAGCAGCCTTGAAGCTCATGGAGAAAAGAGAATACAAGTTTGTTTGCCAGACCTTCCTGA

AGGACGAGATTCGCCCGATGGAGAAAGTACGTGCCGGTAAGACTCGCATTGTCGACGTCTTGCCTGTTGA

ACACATTCTTTACACCAGGATGATGATTGGCAGATTTTGTGCGCAAATGCACTCAAACAACGGACCGCAA

ATTGGCTCAGCGGTCGGTTGCAACCCTGATGTTGATTGGCAGAGATTTGGCACACACTTCGCCCAGTACA

GAAACGTGTGGGACGTGGACTATTCGGCCTTTGATGCTAACCACTGCAGTGACGCAATGAACATCATGTT

TGAGGAGGTGTTCCGCACGGAGTTCGGCTTCCACCCGAACGCTGAGTGGATTCTGAAAACTCTTGTGAAC

ACGGAGCATGCCTATGAGAACAAACGCATCACTGTTGAGGGCGGGATGCCTTCCGGGTGTTCTGCGACAA

GTATCATCAACACAATCTTGAACAACATCTACGTGCTCTACGCGCTGCGTAGACACTACGAGGGAGTTGA

ACTGGACACTTATACCATGATCTCCTACGGAGACGACATCGTGGTTGCAAGTGATTACGATCTGGACTTC

GAGGCTCTCAAGCCTCACTTCAAATCTCTTGGCCAGACCATCACTCCAGCTGACAAAAGCGACAAAGGTT

TTGTTCTTGGTCACTCCATTACCGATGTCACTTTCCTCAAAAGACACTTCCACATGGACTATGGAACTGG

GTTTTACAAACCTGTGATGGCCTCGAAGACCCTCGAGGCTGTCCTCTCCTTTGCACGCCGTGGG

>JX040489.1_O_BUL_2011

GGATTGATCGTTGACACCAGAGATGTTGAGGAACGCGTGCACGTCATGCGCAAAACTAAGCTTGCACCCA

CCGTTGCACACGGTGTGTTCAATCCTGAGTTTGGCCCCGCTGCCTTGTCCAACAAGGACCCGCGGCTGGA

CGAGGGAGTTGTCCTCGATGAAGTCATCTTCTCCAAACACAAGGGAGACACCAAAATGTCTGAGGAGGAC

AAAGCGCTGTTCCGCCGCTGTGCTGCTGACTACGCGTCACGCCTACACAGTGTGTTGGGAACAGCAAATG

CCCCACTGAGCATCTATGAGGCAATCAAGGGCGTTGACGGACTCGACGCCATGGAACCAGACACCGCACC

CGGTCTCCCCTGGGCCCTCCAGGGGAAGCGCCGCGGAGCGCTCATCGACTTCGAGAACGGCACTGTCGGA

CCCGAGGTTGAAGCAGCCTTGAAGCTCATGGAGAAAAGAGAATACAAGTTTGTTTGCCAGACCTTCCTGA

AGGACGAGATTCGCCCGATGGAGAAAGTACGTGCCGGTAAGACTCGCATTGTCGACGTCTTGCCTGTTGA

ACACATTCTTTACACCAGGATGATGATTGGCAGATTTTGTGCGCAAATGCACTCAAACAACGGACCGCAA

ATTGGCTCAGCGGTCGGTTGCAACCCTGATGTTGATTGGCAGAGATTTGGCACACACTTCGCCCAGTACA

GAAACGTGTGGGACGTGGACTATTCGGCCTTTGATGCTAACCACTGCAGTGACGCAATGAACATCATGTT

TGAGGAGGTGTTCCGCACGGAGTTCGGCTTCCACCCGAACGCTGAGTGGATTCTGAAAACTCTTGTGAAC

ACGGAGCATGCCTATGAGAACAAACGCATCACTGTTGAGGGCGGGATGCCTTCCGGGTGTTCTGCGACAA

GTATCATCAACACAATCTTGAACAACATCTACGTGCTCTACGCGCTGCGTAGACACTACGAGGGAGTTGA

ACTGGACACTTATACCATGATCTCCTACGGAGACGACATCGTGGTTGCAAGTGATTACGATCTGGACTTC

GAGGCTCTCAAGCCTCACTTCAAATCTCTTGGCCAGACCATCACTCCAGCTGACAAAAGCGACAAAGGTT

TTGTTCTTGGTCACTCCATTACCGATGTCACTTTCCTCAAAAGACACTTCCACATGGACTATGGAACTGG

GTTTTACAAACCTGTGATGGCCTCGAAGACCCTCGAGGCTGTCCTCTCCTTTGCACGCCGTGGG

>JX040490.1_O_BUL_2011

GGATTGATCGTTGACACCAGAGATGTTGAGGAACGCGTGCACGTCATGCGCAAAACTAAGCTTGCACCCA

CCGTTGCACACGGTGTGTTCAATCCTGAGTTTGGCCCCGCTGCCTTGTCCAACAAGGACCCGCGGCTGGA

CGAGGGAGTTGTCCTCGATGAAGTCATCTTCTCCAAACACAAGGGAGACACCAAAATGTCTGAGGAGGAC

AAAGCGCTGTTCCGCCGCTGTGCTGCTGACTACGCGTCACGCCTACACAGTGTGTTGGGAACAGCAAATG

CCCCACTGAGCATCTATGAGGCAATCAAGGGCGTTGACGGACTCGACGCCATGGAACCAGACACCGCACC

CGGTCTCCCCTGGGCCCTCCAGGGGAAGCGCCGCGGAGCGCTCATCGACTTCGAGAACGGCACTGTCGGA

CCCGAGGTTGAAGCAGCCTTGAAGCTCATGGAGAAAAGAGAATACAAGTTTGTTTGCCAGACCTTCCTGA

AGGACGAGATTCGCCCGATGGAGAAAGTACGTGCCGGTAAGACTCGCATTGTCGACGTCTTGCCTGTTGA

ACACATTCTTTACACCAGGATGATGATTGGCAGATTTTGTGCGCAAATGCACTCAAACAACGGACCGCAA

ATTGGCTCAGCGGTCGGTTGCAACCCTGATGTTGATTGGCAGAGATTTGGCACACACTTCGCCCAGTACA

GAAACGTGTGGGACGTGGACTATTCGGCCTTTGATGCTAACCACTGCAGTGACGCAATGAACATCATGTT

TGAGGAGGTGTTCCGCACGGAGTTCGGCTTCCACCCGAACGCTGAGTGGATTCTGAAAACTCTTGTGAAC

ACAGAGCACGCCTATGAGAACAAACGCATCACTGTTGAGGGCGGGATGCCTTCCGGGTGTTCTGCGACAA

GTATCATCAACACAATCTTGAACAACATCTACGTGCTCTACGCGCTGCGTAGACACTACGAGGGAGTTGA

ACTGGACACTTATACCATGATCTCCTACGGAGACGACATCGTGGTTGCAAGTGATTACGATCTGGACTTC

GAGGCTCTCAAGCCTCACTTCAAATCTCTTGGCCAGACCATCACTCCAGCTGACAAAAGCGACAAAGGTT

TTGTTCTTGGTCACTCCATTACCGATGTCACTTTCCTCAAAAGACACTTCCACATGGACTATGGAACTGG

GTTTTACAAACCTGTGATGGCCTCGAAGACCCTCGAGGCTGTCCTCTCCTTTGCACGCCGTGGG

>JX040491.1_O_TUR_2010

GGATTGATCGTTGACACCAGAGATGTTGAGGAACGCGTGCACGTCATGCGCAAAACTAAGCTTGCACCCA

CCGTTGCACACGGTGTGTTCAATCCTGAGTTTGGCCCCGCTGCCTTGTCCAACAAGGACCCGCGGCTGAA

CGAGGGAGTTGCCCTCGATGAAGTCATCTTCTCCAAACACAAGGGAGACACCAAAATGTCTGAGGAGGAC

AAAGCGCTATTCCGCCGCTGTGCTGCTGACTACGCGTCACGCCTACACAGTGTGTTGGGAACAGCAAATG

CCCCACTGAGCATCTATGAGGCAATCAAGGGCGTTGACGGACTCGACGCCATGGAACCAGACACCGCACC

CGGTCTCCCCTGGGCCCTCCAGGGGAAGCGCCGCGGAGCGCTCATCGACTTCGAGAACGGCACTGTCGGA

CCCGAGGTTGAAGCAGCCTTGAAGCTCATGGAGAAAAGAGAATACAAGTTTGTTTGCCAGACCTTCCTGA

AGGACGAGATTCGCCCAATGGAGAAAGTACGTGCCGGTAAGACCCGTATTGTCGACGTCTTGCCTGTTGA

ACACATTCTTTACACCAGGATGATGATTGGCAGATTTTGTGCGCAAATGCACTCAAACAACGGACCGCAA

ATTGGCTCAGCGGTCGGTTGCAACCCTGATGTTGATTGGCAGAGATTTGGCACACACTTCGCCCAGTACA

GAAACGTGTGGGACGTGGACTATTCGGCCTTTGATGCTAACCACTGCAGTGACGCAATGAACATCATGTT

TGAGGAGGTGTTCCGCACGGAGTTCGGCTTCCACCCAAACGCTGAGTGGATTCTGAAAACTCTTGTGAAC

ACGGAGCATGCCTATGAGAACAAACGCATCACTGTTGAGGGCGGGATGCCTTCCGGGTGTTCTGCGACAA

GTATCATCAACACAATCTTGAACAACATCTACGTGCTCTACGCGCTGCGTAGACACTACGAGGGAGTTGA

GCTGGACACTTATACCATGATCTCCTACGGAGACGACATCGTGGTTGCAAGTGATTACGATCTGGACTTC

GAGGCTCTCAAGCCTCACTTCAAATCTCTTGGCCAAACCATCACTCCAGCTGACAAAAGCGACAAAGGTT

TTGTTCTTGGTCACTCCATTACCGATGTCACTTTCCTCAAAAGACACTTCCACATGGACTATGGAACTGG

GTTTTACAAACCTGTGATGGCCTCGAAGACCCTCGAGGCTGTCCTCTCCTTTGCACGCCGTGGG

>JX040492.1_O_TUR_2010

GGATTGATCGTTGACACCAGAGATGTTGAGGAACGCGTGCACGTCATGCGCAAAACCAAGCTTGCACCCA

CCGTTGCACACGGTGTGTTCAATCCTGAGTTTGGCCCCGCTGCATTGTCCAACAAGGACCCGCGGCTGAA

CGAGGGAGTTGTCCTTGATGAAGTCATCTTCTCCAAACACAAGGGAGACGCCAAAATGTCTGAGGAGGAC

AAAGCGCTATTCCGCCGCTGTGCTGCTGACTACGCGTCACGCCTACACAGTGTGTTGGGAACAGCAAATG

CCCCACTGAGCATCTATGAGGCAATCAAGGGCGTTGACGGACTCGACGCCATGGAACCAGACACCGCACC

CGGTCTCCCCTGGGCCCTCCAGGGGAAGCGCCGCGGAGCGCTCATCGACTTCGAGAACGGCACTGTCGGA

CCCGAGGTTGAAGCAGCCTTGAAGCTCATGGAGAAAAGAGAATACAAGTTTGCTTGCCAGACCTTCCTGA

AGGACGAGATTCGCCCGATGGAGAAAGTACGTGCCGGTAAGACTCGCATTGTCGACGTCTTGCCTGTTGA

ACACATTCTTTACACCAGGATGATGATTGGCAGATTTTGTGCGCAAATGCACTTAAACAACGGACCGCAA

ATTGGCTCAGCGGTCGGTTGCAACCCTGATGTTGATTGGCAGAGATTTGGCACACACTTCGCCCAGTACA

GAAACGTGTGGGACGTGGACTATTCGGCTTTTGATGCTAACCACTGCAGTGACGCAATGAACATCATGTT

TGAGGAGGTGTTCCGCACGGAGTTCGGCTTCCACCCAAACGCTGAGTGGATTCTGAAAACTCTTGTGAAC

ACGGAGCATGCCTATGAGAACAAACGCATCACTGTTGATGGCGGGATGCCTTCCGGGTGTTCTGCGACAA

GTATCATCAACACAATCTTGAACAACATCTACGTGCTCTACGCGCTGCGTAGACACTATGAGGGAGTTGA

GCTGGACACTTACACCATGATCTCCTACGGAGACGACATCGTGGTTGCAAGTGATTACGATCTGGACTTC

GAGGCTCTTAAGCCTCACTTCAAATCTCTTGGCCAAACCATCACTCCAGCTGACAAAAGCGACAAAGGTT

TTGTTCTTGGTCACTCCATTACCGATGTCACTTTCCTCAAAAGACACTTCCACATGGATTATGGAACTGG

GTTTTATAAACCTGTGATGGCCTCGAAGACCCTCGAGGCTATCCTCTCCTTTGCACGCCGTGGG

>JX040493.1_O_TUR_2010

GGATTGATCGTTGACACCAGAGATGTTGAGGAGCGCGTGCACGTCATGCGCAAAACTAAGCTTGCACCCA

CCGTTGCACACGGTGTGTTCAATCCTGAGTTTGGCCCCGCTGCCTTGTCCAACAAGGACCCGCGGCTGAA

CGAGGGAGTTGTCCTCGATGAAGTCATCTTCTCCAAACACAAGGGAGACACCAAAATGTCTGAGGAGGAC

AAAGCGCTATTCCGCCGCTGTGCTGCTGACTACGCGTCACGCCTACACAGTGTGTTGGGAACAGCAAATG

CCCCACTGAGCATCTATGAGGCAATCAAGGGCGTTGACGGACTCGACGCCATGGAACCAGACACCGCACC

CGGTCTCCCCTGGGCCCTCCAGGGGAAGCGCCGCGGAGCGCTCATCGACTTCGAGAACGGCACTGTCGGA

CCCGAGGTTGAAGCAGCCTTGAAGCTCATGGAGAAAAGAGAATACAAGTTTGTTTGCCAGACCTTCCTGA

AGGACGAGATTCGCCCGATGGAGAAAGTACGTGCCGGTAAGACTCGCATTGTCGACGTCTTGCCTGTTGA

ACACATTCTTTACACCAGGATGATGATTGGCAGATTTTGTGCGCAAATGCACTCAAACAACGGACCGCAA

ATTGGCTCAGCGGTCGGTTGCAACCCTGATGTTGATTGGCAGAGATTTGGCACACACTTCGCCCAGTACA

GAAACGTGTGGGACGTGGACTATTCGGCCTTTGATGCTAACCACTGCAGTGACGCAATGAACATCATGTT

TGAGGAGGTGTTCCGCACGGAGTTCGGCTTCCACCCAAACGCTGAGTGGATTCTGAAAACTCTTGTGAAC

ACGGAGCATGCCTATGAGAACAAACGCATCACTGTTGAGGGCGGGATGCCTTCCGGGTGTTCTGCGACAA

GTATCATCAACACAATCTTGAACAACATCTACGTGCTCTACGCGCTGCGTAGACACTACGAGGGAGTTGA

GCTGGACACTTATACCATGATCTCCTACGGAGACGACATCGTGGTTGCAAGTGATTACGATCTGGACTTC

GAGGCTCTCAAGCCTCACTTCAAATCTCTTGGCCAAACCATCACTCCAGCTGACAAAAGCGACAAAGGTT

TTGTTCTTGGTCACTCCATTACCGATGTCACTTTCCTCAAAAGACACTTCCACATGGACTATGGAACTGG

GTTTTACAAACCTGTGATGGCCTCGAAGACCCTCGAGGCTGTCCTCTCCTTTGCACGCCGTGGG

>JX040494.1_O_TUR_2010

GGATTGATCGTTGACACCAGAGATGTTGAGGAACGCGTGCACGTCATGCGCAAAACTAAGCTTGCACCCA

CCGTTGCACACGGTGTGTTCAATCCTGAGTTTGGCCCCGCTGCCTTGTCCAACAAGGACCCGCGGCTGAA

CGAGGGAGTTGTCCTCGACGAAGTCATCTTCTCCAAACACAAGGGAGACACCAAAATGTCTGAGGAGGAC

AAAGCGCTATTCCGCCGCTGTGCTGCTGACTACGCGTCACGCCTACACAGTGTGTTGGGAACAGCAAATG

CCCCACTGAGCATCTATGAGGCAATCAAGGGCGTTGACGGACTCGACGCCATGGAACCAGACACCGCACC

CGGTCTCCCCTGGGCCCTCCAGGGGAAGCGCCGCGGAGCGCTCATCGACTTCGAGAACGGCACTGTCGGA

CCCGAGGTTGAAGCAGCCTTGAAGCTCATGGAGAAAAGAGAATACAAGTTTGTTTGCCAGACCTTCCTGA

AGGACGAGATTCGCCCGATGGAGAAAGTACGTGCCGGTAAGACTCGCATTGTCGACGTCTTGCCTGTTGA

ACACATTCTTTACACCAGGATGATGATTGGCAGATTTTGTGCGCAAATGCACTCAAACAACGGACCGCGA

ATTGGCTCAGCGGTCGGTTGCAACCCTGATGTTGATTGGCAGAGATTTGGCACACACTTCGCCCAGTACA

GAAACGTGTGGGACGTGGACTATTCGGCCTTTGATGCTAACCACTGCAGTGACGCAATGAACATCATGTT

TGAGGAGGTGTTCCGCACGGAGTTCGGCTTCCACCCAAACGCTGAGTGGATTCTGAAAACTCTTGTGAAC

ACGGAGCATGCCTATGAGAACAAACGCATCACTGTTGAGGGCGGGATGCCTTCCGGGTGTTCTGCGACAA

GTATCATCAACACAATCTTGAACAACATCTACGTGCTCTACGCGCTGCGTAGACACTACGAGGGAGTTGA

GCTGGACACTTATACCATGATCTCCTACGGAGACGACATCGTGGTTGCAAGTGATTACGATCTGGACTTC

GAGGCTCTCAAGCCTCACTTCAAATCTCTTGGCCAAACCATCACTCCAGCTGACAAAAGCGACAAAGGTT

TTGTTCTTGGTCACTCCATTACCGATGTCACTTTCCTCAAAAGACACTTCCACATGGACTATGGAACTGG

GTTTTACAAACCTGTGATGGCCTCGAAGACCCTCGAGGCTGTCCTCTCCTTTGCACGCCGTGGG

>JX040495.1_O_TUR_2010

GGATTGATCGTTGACACCAGAGATGTTGAGGAACGCGTGCACGTCATGCGCAAAACCAAGCTTGCACCCA

CCGTTGCACACGGTGTGTTCAATCCTGAGTTTGGCCCCGCTGCCTTGTCCAACAAGGACCCGCGGCTGAA

CGAGGGAGTTGTCCTCGATGAAGTCATCTTCTCCAAACACAAGGGAGACACCAAAATGTCTGAGGAGGAC

AAAGCGCTATTCCGCCGCTGTGCTGCTGACTACGCGTCACGCCTACACAGTGTGTTGGGGACAGCAAATG

CCCCACTGAGCATCTATGAGGCAATCAAGGGCGTTGACGGACTCGACGCCATGGAACCAGACACCGCACC

CGGTCTCCCCTGGGCCCTCCAGGGGAAGCGCCGCGGAGCGCTCATCGACTTCGAGAACGGCACTGTCGGA

CCCGAGGTTGAAGCAGCCTTGAAGCTCATGGAGAAAAGAGAATACAAGTTTGTTTGCCAGACCTTCCTGA

AGGACGAGATTCGCCCGATGGAGAAAGTACGTGCCGGTAAGACTCGCATTGTCGACGTCTTGCCTGTTGA

ACACATTCTTTACACCAGGATGATGATTGGCAGATTTTGTGCGCAAATGCACTCAAACAACGGACCGCAA

ATTGGCTCAGCGGTCGGTTGCAACCCTGATGTTGATTGGCAGAGATTTGGCACACACTTCGCCCAGTACA

GAAACGTGTGGGACGTGGACTATTCGGCCTTTGATGCTAACCACTGCAGTGACGCAATGAACATTATGTT

TGAGGAGGTGTTCCGCACGGAGTTCGGCTTCCACCCAAACGCTGAGTGGATTCTGAAAACTCTTGTGAAC

ACGGAGCATGCCTATGAGAACAAACGCATCACTGTTGAGGGCGGGATGCCTTCCGGGTGTTCTGCGACAA

GTATCATCAACACAATCTTGAACAACATCTACGTGCTCTACGCGCTGCGTAGACACTACGAGGGAGTTGA

GCTGGACACTTATACCATGATCTCCTACGGAGACGACATCGTGGTTGCAAGTGATTACGATCTGGACTTC

GAGGCTCTCAAGCCTCACTTCAAATCTCTTGGCCAAACCATCACTCCAGCTGACAAAAGCGACAAAGGTT

TTGTTCTTGGTCACTCCATTACCGATGTCACTTTCCTCAAAAGACACTTCCACATGGACTATGGAACTGG

GTTTTACAAACCTGTGATGGCCTCGAAGACCCTCGAGGCTGTCCTCTCCTTTGCACGCCGTGGG

>JX040496.1_O_TUR_2010

GGATTGATCGTTGACACCAGAGATGTTGAGGAACGCGTGCACGTCATGCGCAAAACTAAGCTTGCACCCA

CCGTTGCACACGGTGTGTTCAATCCTGGGTTTGGCCCCGCTGCCTTGTCCAACAAGGACCCGCGGCTGAA

CGAGGGAGTTGTCCTCGATGAAGTCATCTTCTCCAAACACAAGGGAGACACCAAAATGTCTGAGGAGGAC

AAAGCGCTATTCCGCCGCTGCGCTGCTGACTACGCGTCACGCCTACACAGTGTGTTGGGAACAGCAAATG

CCCCACTGAGCATCTATGAGGCAATCAAGGGCGTTGACGGACTCGACGCCATGGAACCAGACACCGCACC

CGGTCTCCCCTGGGCCCTCCAGGGGAAGCGCCGCGGAGCGCTCATCGACTTCGAGAACGGCACTGTCGGA

CCCGAGGTTGAAGCAGCCTTGAAGCTCATGGAGAAAAGAGAATACAAGTTTGTTTGCCAGACCTTCCTGA

AGGACGAGATTCGCCCGATGGAGAAAGTACGTGCCGGTAAGACTCGCATTGTCGACGTCTTGCCTGTTGA

ACACATTCTTTACACCAGGATGATGATTGGCAGATTTTGTGCGCAAATGCACTCAAACAACGGACCGCAA

ATTGGCTCAGCGGTCGGTTGCAACCCTGATGTTGATTGGCAGAGATTTGGCACACACTTCGCCCAGTACA

GAAACGTGTGGGACGTGGACTATTCGGCCTTTGATGCTAACCACTGCAGTGACGCAATGAACATCATGTT

TGAGGAGGTGTTCCGCACGGAGTTCGGCTTCCACCCAAACGCTGAGTGGATTCTGAAAACTCTTGTGAAC

ACGGAGCATGCCTATGAGAACAAACGCATCACTGTTGAGGGCGGGATGCCTTCCGGGTGTTCTGCGACAA

GTATCATCAACACAATCTTGAACAACATCTACGTGCTCTACGCGCTGCGTAGACACTACGAGGGAGTTGA

GCTGGACACTTATACCATGATCTCCTACGGAGACGACATCGTGGTTGCAAGTGATTACGATCTGGACTTC

GAGGCTCTCAAGCCTCACTTCAAATCTCTTGGCCAGACCATCACTCCAGCTGACAAAAGCGACAAAGGTT

TTGTTCTTGGTCACTCCATTACCGATGTCACTTTCCTCAAAAGACACTTCCACATGGACTATGGAACTGG

GTTTTACAAACCTGTGATGGCCTCGAAGACCCTCGAGGCTGTCCTCTCCTTTGCACGCCGTGGG

>JX040497.1_O_TUR_2010

GGATTGATCGTTGACACCAGAGATGTTGAGGAACGCGTGCACGTCATGCGCAAAACTAAGCTTGCACCCA

CCGTTGCACACGGTGTGTTCAATCCTGAGTTTGGCCCCGCTGCCTTGTCCAACAAGGACCCGCGGCTGAA

CGAGGGAGTTGTCCTCGATGAAGTCATCTTCTCCAAACACAAGGGAGACACCAAAATGTCTGAGGAGGAC

AAAGCGCTATTCCGCCGCTGTGCTGCTGACTACGCGTCACGCCTACACAGCGTGTTGGGAACAGCAAATG

CCCCACTGAGCATCTATGAGGCAATCAAGGGCGTTGACGGACTCGACGCCATGGAACCAGACACCGCACC

CGGTCTCCCCTGGGCCCTCCAGGGGAAGCGCCGCGGAGCGCTCATCGACTTCGAGAACGGCACTGTCGGA

CCCGAGGTTGAAGCAGCCTTGAAGCTCATGGAGAAAAGAGAATACAAGTTTGTTTGCCAGACCTTCCTGA

AGGACGAGATTCGCCCGATGGAGAAAGTACGTGCCGGTAAGACTCGCATTGTCGACGTCTTGCCTGTTGA

ACACATTCTTTACACCAGGATGATGATTGGCAGATTTTGTGCGCAAATGCACTCAAACAACGGACCGCAA

ATTGGCTCAGCGGTCGGTTGTAACCCTGATGTTGATTGGCAGAGATTTGGCACACACTTCGCCCAGTACA

GAAACGTGTGGGACGTGGACTATTCGGCCTTTGATGCTAACCACTGCAGTGACGCAATGAACATCATGTT

TGAGGAGGTGTTCCGCACGGAGTTCGGCTTCCACCCAAACGCTGAGTGGATTCTGAAAACTCTTGTGAAC

ACGGAGCATGCCTATGAGAACAAACGCACCACTGTTGAGGGCGGGATGCCTTCCGGGTGTTCTGCGACAA

GTATCATCAACACAATCTTGAACAACATCTACGTGCTCTACGCGCTGCGTAGACACTACGAGGGAGTTGA

GCTGGACACTTATACCATGATCTCCTACGGAGACGACATCGTGGTTGCAAGTGATTACGATCTGGACTTC

GAGGCTCTCAAGCCTCACTTCAAATCTCTTGGCCAAACCATCACTCCAGCTGACAAAAGCGACAAAGGCT

TTGTTCTTGGTCACTCCATTACCGATGTCACTTTCCTCAAAAGACACTTCCACATGGACTATGGAACTGG

GTTTTACAAACCTGTGATGGCCTCGAAGACCCTCGAGGCTGTCCTCTCCTTTGCACGCCGTGGG

>JX040498.1_O_TUR_2010

GGATTGATCGTTGACACCAGAGATGTTGAGGAACGCGTGCACGTCATGCGCAAAACCAAGCTTGCGCCCA

CCGTTGCACACGGTGTGTTCAATCCTGAGTTTGGCCCCGCTGCCTTGTCCAACAAGGACCCGCGGCTGAA

CGAGGGAGTTGTCCTCGATGAAGTCATCTTCTCCAAACACAAGGGAGACACCAAAATGTCTGAGGAGGAC

AAAGCGCTATTCCGCCGCTGTGCTGCTGACTACGCGTCACGCCTACACAGTGTGTTGGGAACAGCAAATG

CCCCACTGAGCATCTATGAGGCAATCAAGGGCGTTGACGGACTCGACGCCATGGAACCAGACACCGCACC

CGGTCTCCCCTGGGCCCTCCAGGGGAAGCGCCGCGGAGCGCTCATCGACTTCGAGAACGGCACTGTCGGA

CCCGAGGTTGAAGCAGCCTTGAAGCTCATGGAGAAAAGAGAATACAAGTTTGTTTGCCAGACCTTCCTGA

AGGACGAGATTCGCCCGATGGAGAAAGTACGTGCCGGTAAGACTCGCATTGTCGACGTCTTGCCTGTTGA

ACACATTCTTTACACCAGGATGATGATTGGCAGATTTTGTGCGCAAATGCACTCAAACAACGGACCGCAA

ATTGGCTCAGCGGTCGGTTGCAACCCTGATGTTGATTGGCAGAGATTTGGCACGCACTTCGCCCAGTACA

GAAACGTGTGGGACGTGGACTATTCGGCCTTTGATGCTAACCACTGCAGTGACGCAATGAACATCATGTT

TGAGGAGGTGTTCCGCACGGAGTTCGGCTTCCACCCAAACGCTGAGTGGATTCTGAAAACTCTTGTGAAC

ACGGAGCATGCCTATGAGAACAAACGCATCACTGTTGAGGGCGGGATGCCTTCCGGGTGTTCTGCGACAA

GTATCATCAATACAATCTTGAACAACATCTACGTGCTCTACGCGCTGCGTAGACACTACGAGGGAGTTGA

GCTGGACACTTATACCATGATCTCCTACGGAGACGACATCGTGGTTGCAAGTGATTACGATCTGGACTTC

GAGGCTCTCAAGCCTCACTTCAAATCTCTTGGCCAAACCATCACTCCAGCTGACAAAAGCGACAAAGGTT

TTGTTCTTGGTCACTCCATTACCGATGTCACTTTCCTCAAAAGACACTTCCACATGGACTATGGAACTGG

GTTTTATAAACCTGTGATGGCCTCGAAGACCCTCGAGGCTGTCCTCTCCTTTGCACGCCGTGGG

>JX040499.1_O_TUR_2011

GGATTGATCGTTGACACCAGAGATGTTGAGGAACGCGTGCACGTCATGCGCAAAACTAAGCTTGCACCCA

CCGTTGCACACGGTGTGTTCAATCCTGAGTTTGGCCCCGCTGCCTTGTCCAACAAGGACCCGCGGCTGAA

CGAGGGAGTTGTCCTCGATGAAGTCATCTTCTCCAAACACAAGGGAGACACCAAAATGTCTGAGGAGGAC

AAAGCGCTATTCCGCCGCTGTGCTGCTGACTACGCGTCACGCCTACACAGTGTGTTGGGAACAGCAAATG

CCCCACTGAGTATCTATGAGGCAATCAAGGGCGTTGACGGACTCGACGCCATGGAACCAGACACCGCACC

CGGTCTCCCCTGGGCCCTCCAGGGGAAGCGCCGCGGAGCGCTCATCGACTTCGAGAACGGCACTGTCGGA

CCCGAGGTCGAAGCAGCCTTGAAGCTCATGGAGAAAAGAGAATACAAGTTTGTTTGCCAGACCTTCCTGA

AGGACGAGATTCGCCCGATGGAGAAAGTACGTGCCGGTAAGACTCGCATTGTCGACGTCTTGCCTGTTGA

ACACATTCTTTACACCAGGATGATGATTGGCAGATTTTGTGCGCAAATGCACTCAAACAACGGACCGCAA

ATTGGTTCAGCGGTCGGTTGTAACCCTGATGTTGATTGGCAGAGATTTGGCACACACTTCGCCCAGTACA

GAAACGTGTGGGACGTGGACTATTCGGCCTTTGATGCTAACCACTGCAGTGACGCAATGAACATCATGTT

TGAGGAGGTGTTCCGCACGGAGTTCGGCTTCCACCCAAACGCTGAGTGGATTCTGAAAACTCTTGTGAAC

ACGGAGCATGCCTATGAGAACAAACGCATCACTGTTGAGGGCGGGATGCCTTCCGGGTGTTCTGCGACAA

GTATCATCAACACAATCTTGAACAACATCTACGTGCTCTACGCGCTGCGTAGACACTACGAGGGAGTTGA

GCTGGACACTTATACCATGATCTCCTACGGAGACGACATCGTGGTTGCAAGTGATTACGATTTGGACTTC

GAGGCTCTCAAGCCTCACTTCAAATCTCTTGGCCAAACCATCACTCCAGCTGATAAAAGCGACAAAGGTT

TTGTTCTTGGTCACGCCATTACCGACGTCACTTTCCTCAAAAGACACTTCCACTTGGACTATGGAACTGG

GTTTTACAAACCTGTGATGGCCTCGAAGACCCTCGAGGCTGTCCTCTCCTTTGCACGCCGTGGG

>JX040500.1_O_TUR_2011

GGATTGATCGTTGACACCAGAGATGTTGAGGAACGCGTGCACGTCATGCGCAAAACTAAGCTTGCACCCA

CCGTTGCACACGGTGTGTTCAATCCTGAGTTTGGCCCCGCTGCCTTGTCCAACAAGGACCCGCGGCTGAA

CGAGGGAGTTGTCCTCGATGAAGTCATCTTCTCCAAACACAAGGGAGACACCAAAATGTCTGAGGAGGAC

AAAGCGCTATTCCGCCGCTGTGCTGCTGACTACGCGTCACGCCTACACAGTGTGTTGGGAACAACAAATG

CCCCACTGAGCATCTATGAGGCAATCAAGGGCGTTGACGGACTCGACGCCATGGAACCAGACACCGCTCC

CGGTCTCCCCTGGGCCCTCCAGGGGAAGCGCCGCGGAGCGCTCATCGACTTCGAGAACGGCACTGTCGGA

CCCGAGGTTGAAGCAGCCTTGAAGCTCATGGAGAAAAGAGAATACAAGTTTGTTTGCCAGACCTTCTTGA

AGGACGAGATTCGCCCGATGGAGAAAGTACGTGCCGGTAAGACTCGCATTGTCGACGTCTTGCCTGTTGA

ACACATTCTTTACACCAGGATGATGATTGGCAGATTTTGTGCGCAAATGCACTCAAACAACGGACCGCAA

ATTGGTTCAGCGGTCGGTTGTAACCCTGATGTTGATTGGCAGAGATTTGGCACACACTTCGCCCAGTACA

GAAACGTGTGGGACGTGGACTATTCGGCCTTTGATGCTAACCACTGCAGTGACGCAATGAACATCATGTT

TGAGGAGGTGTTCCACACGGAGTTCGGCTTCCACCCAAACGCTGAGTGGATTCTGAAAACTCTTGTGAAC

ACGGAGCATGCCTATGAGAACAAACGCATCACTGTTGAGGGCGGGATGCCTTCCGGGTGTTCTGCGACAA

GTATCATCAACACAATCTTGAACAACATCTACGTGCTCTACGCGCTGCGTAGACACTACGAGGGAGTTGA

GCTGGACACTTATACCATGATCTCCTACGGAGACGACATCGTGGTTGCAAGTGATTACGATTTGGACTTC

GAGGCCCTCAAGCCTCACTTCAAATCTCTTGGCCAAACCATCACTCCAGCTGATAAAAGCGACAAAGGTT

TTGTTCTTGGTCACTCCATTACCGATGTCACTTTCCTCAAAAGACACTTCCACTTGGACTATGGAACTGG

GTTTTACAAACCTATGATGGCCTCGAAGACCCTCGAGGCTGTCCTCTCCTTTGCACGCCGTGGG

>JX040501.1_O_ISR_2011

GGATTGATCGTTGACACCAGAGATGTTGAGGAACGCGTGCACGTCATGCGCAAAACCAAGCTTGCACCCA

CCGTTGCACACGGTGTGTTCAATCCTGAGTTTGGCCCCGCTGCCTTGTCCAACAAGGACCCGCGGCTGAA

CGAGGGAGTTGTCCTCGATGAAGTCATCTTCTCCAAACACAAGGGAGACACCAAAATGTCTGAGGAGGAC

AAAGCGCTATTCCGCCGCTGTGCTGCTGACTACGCGTCACGCCTACACAGTGTGTTGGGAACAGCAAATG

CCCCACTGAGCATCTATGAGGCAATCAAGGGCGTTGACGGACTCGACGCCATGGAACCAGACACCGCACC

CGGTCTCCCCTGGGCCCTCCAGGGGAAGCGCCGCGGAGCGCTCATCGACTTCGAGAACGGCACTGTCGGA

CCCGAGGTTGAAGCAGCCTTGGAGCTCATGGGGAAAAGAGAATACAAGTTTGCTTGCCAGACCTTCCTGA

AGGACGAGATTCGCCCGATGGAGAAAGTACGTGCCGGTAAGACTCGCATTGTCGACGTCTTGCCTGTTGA

ACACATTCTTTACACCAGGATGATGATTGGCAGATTTTGTGCGCAAATGCACTCAAACAACGGACCGCAA

ATTGGCTCAGCGGTCGGTTGCAACCCTGATGTTGATTGGCAGAGATTTGGCACACACTTCGCCCAGTACA

GAAACGTGTGGGACGTGGACTACTCGGCCTTTGATGCCAACCACTGCAGTGACGCAATGAACATCATGTT

TGAGGAGGTGTTCCGCACGGAGTTCGGCTTCCACCCAAACGCTGAGTGGATTCTGAAAACTCTTGTGAAC

ACGGAGCATGCCTATGAGAACAAACGCATCACTGTTGAGGGCGGGATGCCTTCCGGGTGTTCTGCGACAA

GTATCATCAACACAATCTTGAACAACATCTACGTGCTCTACGCGCTGCGTAGACACTATGAGGGAGTCGA

GCTGGACACTTACACCATGATCTCCTACGGAGACGACATCGTGGTTGCAAGTGATTACGATCTGGACTTC

GAGGCTCTCAAGCCCCACTTCAAATCTCTTGGCCAAACCATCACTCCAGCTGACAAAAGCGACAAAGGTT

TTGTTCTTGGTCACTCCATTACCGATGTCACTTTCCTCAAAAGACACTTCCACATGGACTATGGAACTGG

GTTTTATAAACCTGTGATGGCCTCGAAGACCCTCGAGGCTATCCTCTCCTTTGCACGCCGTGGG

>JX066664.1_O_BUL_2011

GGATTGATCGTTGACACCAGAGATGTTGAGGAACGCGTGCACGTCATGCGCAAAACTAAGCTTGCACCCA

CCGTTGCACACGGTGTGTTCAATCCTGAGTTTGGCCCCGCTGCCTTGTCCAACAAGGACCCGCGGCTGAA

CGAGGGAGTTGTCCTCGATGAAGTCATCTTCTCCAAACACAAGGGAGACACCAAAATGTCTGAGGAGGAC

AAAGCGCTATTCCGCCGCTGTGCTGCTGACTACGCGTCACGCCTACACAGTGTGTTGGGAACAGCAAATG

CCCCACTGAGCATCTATGAGGCAATCAAGGGCGTTGACGGACTCGACGCCATGGAACCAGACACCGCACC

CGGTCTCCCCTGGGCCCTCCAGGGGAAGCGCCGCGGAGCGCTCATCGACTTCGAGAACGGCACTGTCGGA

CCCGAGGTTGAAGCAGCCTTGAAGCTCATGGAGAAAAGAGAATACAAGTTTGTTTGCCAGACCTTCCTGA

AGGACGAGATTCGCCCGATGGAGAAAGTACGTGCCGGTAAGACTCGCATTGTCGACGTCTTGCCTGTTGA

ACACATTCTTTACACCAGGATGATGATTGGCAGATTTTGTGCGCAAATGCACTCAAACAACGGACCGCAA

ATTGGCTCAGCGGTCGGTTGCAACCCTGATGTTGATTGGCAGAGATTTGGCACACACTTCGCCCAGTACA

GAAACGTGTGGGACGTGGACTATTCGGCCTTTGATGCTAACCACTGCAGTGACGCAATGAACATCATGTT

TGAGGAGGTGTTCCGCACGGAGTTCGGCTTCCACCCAAACGCTGAGTGGATTCTGAAAACTCTTGTGAAC

ACGGAGCATGCCTATGAGAATAAACGCATCACTGTTGAGGGCGGGATGCCTTCCGGGTGTTCTGCGACAA

GTATCATCAACACAATCTTGAACAACATCTACGTGCTCTACGCGCTGCGTAGACACTACGAGGGAGTTGA

ACTGGACACTTATACCATGATCTCCTACGGAGACGACATCGTGGTTGCAAGTGATTACGATCTGGACTTC

GAGGCTCTCAAGCCTCACTTCAAATCTCTTGGCCAGACCATCACTCCAGCTGACAAAAGCGACAAAGGTT

TTGTTCTTGGTCACTCCATTACCGATGTCACTTTCCTCAAAAGACACTTCCACATGGACTATGGAACTGG

GTTTTACAAACCTGTGATGGCCTCGAAGACCCTCGAGGCTGTCCTCTCCTTTGCACGCCGTGGG

>JX066665.1_O_BUL_2011

GGATTGATCGTTGACACCAGAGATGTTGAGGAACGCGTGCACGTCATGCGCAAAACTAAGCTTGCACCCA

CCGCTGCACACGGTGTGTTCAATCCTGAGTTTGGCCCCGCTGCCTTGTCCAACAAGGACCCGCGGCTGAA

CGAGGGAGTTGTCCTCGATGAAGTCATCTTCTCCAAACACAAGGGAGACACCAAAATGTCTGAGGAGGAC
[truncated: 610,998 more chars]
