# Supplementary material for: Early origin and global colonisation of foot-and-mouth disease virus
Source: Sci Rep. 2020 Sep 17;10:15268. doi: 10.1038/s41598-020-72246-6 (PMC7498456; doi:10.1038/s41598-020-72246-6)
Supplement: Supplementary file 9 — Supplementary Tree S1. [file 41598_2020_72246_MOESM9_ESM.doc]

#NEXUS

begin trees;

tree tree_1 = [&R] ((((((((((((((((((((((((((((((('MG983740.1_O_VIT_2015':0.004577,'MG983693.1_O_LAO_2015':1.0E-6)[&label=0.294643]:1.0E-6,'KY657269.1_O_VIT_2015':0.006883)[&label=0.975694]:0.011676,(('MG983686.1_O_BAR_2015':0.0045,'MG983690.1_O_BHU_2016':0.007074)[&label=0.990079]:0.014211,'MG983714.1_O_NEP_2013':0.006883)[&label=0.606647]:1.0E-6)[&label=0.487897]:0.002274,'MG983715.1_O_NEP_2014':0.002269)[&label=0.408234]:1.0E-6,(((('KJ825805.1_O_IND_2013':0.002285,'KJ825806.1_O_IND_2013':0.002297)[&label=0.535714]:0.002285,'KJ825804.1_O_IND_2013':0.00227)[&label=0.308532]:1.0E-6,('MG983731.1_O_SRL_2013':0.006891,'KJ825807.1_O_IND_2014':0.004561)[&label=0.073413]:1.0E-6)[&label=0.200893]:1.0E-6,'KJ825809.1_O_IND_2013':1.0E-6)[&label=0.179365]:1.0E-6)[&label=0.345486]:1.0E-6,'MG983732.1_O_SRL_2014':1.0E-6)[&label=0.429106]:1.0E-6,(('MG983685.1_O_BAR_2015':0.009281,'MG983733.1_O_SRL_2014':0.00232)[&label=0.607143]:0.002233,('MG983736.1_O_UAE_2014':1.0E-6,'MG983716.1_O_NEP_2014':1.0E-6)[&label=0.832341]:0.002268)[&label=0.364418]:1.0E-6)[&label=0.735819]:1.0E-6,'MG983734.1_O_SRL_2014':0.004559)[&label=0.797564]:0.002222,'MG983711.1_O_NEP_2013':0.009334)[&label=0.829939]:0.002341,('KJ825801.1_O_IND_2013':1.0E-6,'KJ825808.1_O_IND_2013':1.0E-6)[&label=0.99504]:0.009244)[&label=0.859505]:1.0E-6,'KJ825803.1_O_IND_2013':0.006886)[&label=0.890061]:0.006897,(('MG983709.1_O_NEP_2012':1.0E-6,'MG983708.1_O_NEP_2012':0.002276)[&label=0.87004]:0.002302,('MG983713.1_O_NEP_2013':1.0E-6,'MG983712.1_O_NEP_2013':1.0E-6)[&label=0.96131]:0.004614)[&label=0.634921]:0.002319)[&label=0.869238]:1.0E-6,('KJ206908.1_O_BHU_2013':0.01678,'MG983717.1_O_NEP_2015':0.009352)[&label=0.730159]:0.004655)[&label=0.87826]:0.004203,(((((((('KJ206909.1_O_LIB_2013':1.0E-6,'MG983694.1_O_LIB_2013':1.0E-6)[&label=0.858135]:0.002287,'MG983697.1_O_LIB_2013':0.006946)[&label=0.488591]:1.0E-6,('MG983695.1_O_LIB_2013':0.002288,'MG983696.1_O_LIB_2013':0.002289)[&label=0.194444]:1.0E-6)[&label=0.760665]:0.002291,'KU291242.1_O_MOR_2015':0.00463)[&label=0.738492]:1.0E-6,'MG983683.1_O_ALG_2014':0.002294)[&label=0.764385]:1.0E-6,'MG983735.1_O_TUN_2014':0.002295)[&label=0.842404]:0.002326,(((('MG983721.1_O_SAU_2013':1.0E-6,'KJ206910.1_O_SAU_2013':1.0E-6)[&label=0.325397]:1.0E-6,'MG983722.1_O_SAU_2013':1.0E-6)[&label=0.992063]:0.006939,(('MG983723.1_O_SAU_2013':1.0E-6,'MG983724.1_O_SAU_2013':1.0E-6)[&label=0.97619]:0.006923,'MG983726.1_O_SAU_2014':1.0E-6)[&label=0.829861]:0.002288)[&label=0.792857]:1.0E-6,'MG983725.1_O_SAU_2014':0.006957)[&label=0.844081]:0.002296)[&label=0.932398]:0.002295,'KJ825802.1_O_IND_2013':1.0E-6)[&label=0.950066]:0.002778)[&label=0.882508]:0.00894,((((((((((((('MG983698.1_O_MUR_2016':1.0E-6,'MG983702.1_O_MUR_2016':0.002265)[&label=0.041667]:1.0E-6,'MG983701.1_O_MUR_2016':1.0E-6)[&label=0.164187]:1.0E-6,('MG983699.1_O_MUR_2016':1.0E-6,'MG983700.1_O_MUR_2016':1.0E-6)[&label=0.178571]:1.0E-6)[&label=0.980655]:0.009225,('MG983727.1_O_SAU_2015':0.00458,'MG983728.1_O_SAU_2016':1.0E-6)[&label=0.834325]:0.004576)[&label=0.684028]:1.0E-6,(('MG983739.1_O_UAE_2016':1.0E-6,'MG983738.1_O_UAE_2016':1.0E-6)[&label=0.882937]:0.002279,'MG983691.1_O_BHU_2016':1.0E-6)[&label=0.862599]:0.002279)[&label=0.603064]:1.0E-6,('MG983718.1_O_NEP_2015':0.004572,'MG983729.1_O_SAU_2016':0.004575)[&label=0.116071]:1.0E-6)[&label=0.696338]:1.0E-6,'MG983719.1_O_NEP_2015':0.011598)[&label=0.749173]:0.002213,((((('LC438823.1_O_MYA_2016':0.00229,'MH891503.1_O_VIT_2017':0.011651)[&label=0.535714]:0.002271,'LC438822.1_O_MYA_2016':0.009169)[&label=0.568452]:1.0E-6,'MG983741.1_O_VIT_2016':0.004552)[&label=0.602844]:1.0E-6,((('MG983720.1_O_RUS_2016':1.0E-6,'MF461724.1_O_CHA_2017':1.0E-6)[&label=0.786706]:1.0E-6,'LC320038.1_O_MOG_2015':0.002264)[&label=0.904266]:0.002274,'MG983730.1_O_SKR_2017':0.00698)[&label=0.863095]:0.002274)[&label=0.989087]:0.014037,'KX712091.1_O_BAN_2015':1.0E-6)[&label=0.941592]:0.002345)[&label=0.93287]:0.002343,'MG983703.1_O_MYA_2016':0.004618)[&label=0.950938]:0.012229,(('MG983689.1_O_BHU_2012':0.004582,'MG983707.1_O_NEP_2012':0.009194)[&label=0.241071]:1.0E-6,'MG983710.1_O_NEP_2012':0.011661)[&label=0.789187]:0.006824)[&label=0.940516]:0.016897,(((('MG983687.1_O_BHU_2009':0.002272,'MG983684.1_O_BAN_2009':0.006877)[&label=0.065476]:1.0E-6,('MG983706.1_O_NEP_2010':0.009293,'MG983692.1_O_IRN_2009':0.004631)[&label=0.486111]:0.002263)[&label=0.568783]:1.0E-6,('MG983688.1_O_BHU_2009':1.0E-6,'MG983705.1_O_NEP_2010':0.006872)[&label=0.595238]:0.002273)[&label=0.907143]:0.009302,'MG983704.1_O_NEP_2008':0.002334)[&label=0.867394]:0.002338)[&label=0.823506]:0.002843,'KF985189.1_O_BAN_2013':0.016893)[&label=0.814755]:0.003394,((('HQ832592.1_A_IND_2009':1.0E-6,'HQ832590.1_A_IND_2007':0.002279)[&label=0.974206]:0.002417,'HQ832591.1_A_IND_2008':0.016502)[&label=0.934524]:0.009054,('KJ754939.1_A_BAN_2013':0.015282,'KU127247.1_A_SAU_2015':0.026124)[&label=0.623016]:0.014808)[&label=0.774306]:0.01437)[&label=0.768771]:0.00478)[&label=0.958883]:0.038813,(((((((('DQ989323.1_Asia1_IND_2002':0.002259,'DQ989320.1_Asia1_IND_2002':0.006906)[&label=0.094246]:1.0E-6,('DQ989317.1_Asia1_IND_2000':0.004554,'DQ989319.1_Asia1_IND_2001':0.01151)[&label=0.669643]:0.002301)[&label=0.442791]:1.0E-6,'DQ989322.1_Asia1_IND_2002':0.002259)[&label=0.674355]:1.0E-6,'DQ989318.1_Asia1_IND_2002':0.016421)[&label=0.844444]:0.002256,'DQ989314.1_Asia1_IND_2001':1.0E-6)[&label=0.915509]:0.002252,'DQ989321.1_Asia1_IND_2001':1.0E-6)[&label=0.99263]:0.047708,'DQ989315.1_Asia1_IND_1993':0.052601)[&label=0.914683]:0.008121,((('DQ989309.1_Asia1_IND_1996':1.0E-6,'MF372126.1_Asia1_IND_1994':1.0E-6)[&label=0.677579]:0.002239,'DQ989308.1_Asia1_IND_1994':1.0E-6)[&label=0.997024]:0.01446,'HQ832587.1_A_IND_2005':0.070244)[&label=0.74041]:0.020973)[&label=0.72338]:0.011773)[&label=0.895968]:0.011709,((((((((((('FJ175664.1_O_ISR_2007':1.0E-6,'FJ175663.1_O_ISR_2007':0.002238)[&label=0.322421]:1.0E-6,'FJ175666.1_O_ISR_2007':0.002238)[&label=0.767857]:0.002235,'FJ175665.1_O_ISR_2007':0.01383)[&label=0.554563]:1.0E-6,('FJ175662.1_O_ISR_2007':0.002233,'FJ175661.1_O_ISR_2007':0.002231)[&label=0.199405]:1.0E-6)[&label=0.895635]:0.003996,'KC440883.1_O_EGY_2011':0.04841)[&label=0.955192]:0.008849,'KM268895.1_O_TUR_2013':0.034379)[&label=0.875992]:0.006559,('JN006722.1_A_PAK_2008':1.0E-6,'JN006720.1_Asia1_PAK_2009':0.008974)[&label=0.97123]:0.017067)[&label=0.842593]:0.008783,((('KM268896.1_A_TUR_2013':0.037806,'HQ113232.1_O_PAK_2009':0.020631)[&label=0.460317]:0.00265,('GU384683.1_O_PAK_2008':1.0E-6,'GU384682.1_O_PAK_2008':1.0E-6)[&label=1.0]:0.007482)[&label=0.902778]:0.018701,('JF749852.1_O_MAY_2004':1.0E-6,'HQ632770.1_O_MAY_2004':0.002216)[&label=0.880952]:0.002402)[&label=0.680159]:0.002093)[&label=0.862831]:1.0E-6,'HQ268524.1_O_BHU_2004':0.009093)[&label=0.915737]:0.021688,((('HQ832586.1_A_IND_2006':0.056625,'HQ832589.1_A_IND_2006':0.029614)[&label=0.323413]:0.007865,('HM854021.1_A_IND_2000':0.008877,'HQ832584.1_A_IND_2005':0.045485)[&label=0.498016]:0.008885)[&label=0.718915]:0.02104,'HQ832585.1_A_IND_2005':0.064626)[&label=0.725694]:0.019664)[&label=0.912793]:0.020466,(('DQ989310.1_Asia1_IND_1999':1.0E-6,'MF372125.1_Asia1_IND_2016':1.0E-6)[&label=1.0]:0.019952,('DQ989311.1_Asia1_IND_2002':0.05042,'MF782478.1_Asia1_BAN_2013':0.091432)[&label=0.515873]:0.008629)[&label=0.920304]:0.019923)[&label=0.897262]:0.041436)[&label=0.84867]:1.0E-6,((((('HQ832580.1_A_IND_2003':1.0E-6,'HQ832581.1_A_IND_2004':0.013881)[&label=0.62996]:0.002254,'HQ832582.1_A_IND_2004':0.018594)[&label=0.604167]:1.0E-6,('HQ832579.1_A_IND_2003':0.00225,'HQ832583.1_A_IND_2005':0.00453)[&label=0.077381]:1.0E-6)[&label=0.839286]:0.001981,'HQ832578.1_A_IND_2003':0.009557)[&label=0.976984]:0.062495,('HM854023.1_A_IND_1999':0.014214,'HQ832577.1_A_IND_1999':0.01)[&label=0.940476]:0.021653)[&label=0.81746]:0.022145)[&label=0.859127]:0.014764,('AY593797.1_Asia1_ISR_1963':0.013528,'AY593796.1_Asia1_ISR_1963':0.049955)[&label=0.848214]:0.028691)[&label=0.848913]:0.005488,(('AY593791.1_A_IRN_1998':0.033611,'JF749848.1_A_TUR_2003':0.028084)[&label=0.993056]:0.044551,'HM854022.1_A_IND_1977':0.017714)[&label=0.954365]:0.042203)[&label=0.836045]:1.0E-6,((((((((((((('EF611987.1_O_UGA_2006':1.0E-6,'HM191257.1_O_UGA_2006':0.004526)[&label=1.0]:0.009354,'KU821591.1_O_ZAM_2010':0.02998)[&label=0.99256]:0.03017,('MH053318.1_O_UGA_2002':0.007034,'FJ461344.1_O_UGA_2002':0.014306)[&label=0.722222]:0.004607)[&label=0.829365]:6.53E-4,'FJ461345.1_O_UGA_2002':0.016507)[&label=0.977183]:0.038256,'MH053307.1_A_ZAM_1990':0.052069)[&label=0.964947]:0.019001,'MH053316.1_O_UGA_1996':0.030434)[&label=0.973214]:0.035099,(('MH053311.1_O_ETH_2004':0.024539,'MH053314.1_O_ETH_2007':0.050702)[&label=0.96627]:0.027994,'MH053313.1_O_ETH_2006':0.067842)[&label=0.967758]:0.057072)[&label=0.868353]:0.008245,'MH053315.1_O_SUD_1976':0.095557)[&label=0.852092]:0.011125,(('KM268897.1_C_KEN_2004':1.0E-6,'MH053309.1_C_KEN_1967':1.0E-6)[&label=1.0]:0.055619,'MH053308.1_C_ETH_1971':0.02377)[&label=0.996032]:0.081795)[&label=0.897676]:0.016874,((('AY593764.1_A_IRQ_1970':0.00456,'FJ623456.1_A_KAZ_1999':0.004604)[&label=0.94246]:0.005869,('AY593772.1_A_TUR_1972':0.034882,'AY593765.1_A_TUR_1965':0.004263)[&label=0.355159]:0.001465)[&label=0.999339]:0.060988,(('AY593828.1_O_IND_1962':0.031094,'AY593812.1_O_PHI_1958':0.023745)[&label=0.933532]:0.023177,'AY593823.1_O_TUR_1969':0.046501)[&label=0.846726]:0.015779)[&label=0.68998]:0.014593)[&label=0.871977]:0.01915,'KY072818.1_O_CHA_1959':0.1737)[&label=0.837347]:0.001329,(('KY825718.1_Asia1_ISR_1989':1.0E-6,'AY593799.1_Asia1_LEB_1983':1.0E-6)[&label=0.702381]:1.0E-6,'AY593800.1_Asia1_LEB_1983':0.002234)[&label=0.998512]:0.084788)[&label=0.766905]:0.011164,(((((('KC462884.1_Asia1_CHA_2006':1.0E-6,'KC412634.1_Asia1_CHA_2006':1.0E-6)[&label=0.757937]:1.0E-6,'KU360085.1_Asia1_CHA_2015':0.002253)[&label=0.945437]:0.004551,('EF149009.1_Asia1_CHA_2005':0.016155,'GU931682.1_Asia1_CHA_2005':1.0E-6)[&label=0.600198]:0.002241)[&label=0.809276]:0.002279,(('KY446901.1_Asia1_PAK_2006':1.0E-6,'FJ906802.1_Asia1_CHA_2006':1.0E-6)[&label=0.302579]:1.0E-6,'HQ631363.1_Asia1_CHA_2006':1.0E-6)[&label=0.869544]:1.0E-6)[&label=0.947704]:0.004531,('GU125645.1_Asia1_VIT_2007':1.0E-6,'GQ452295.1_Asia1_VIT_2007':1.0E-6)[&label=0.945437]:1.0E-6)[&label=0.994489]:0.053531,'AY593834.1_O_IRN_1966':0.050051)[&label=0.985813]:0.043302)[&label=0.616292]:0.013405)[&label=0.723716]:0.01973,(((((((('AF308157.1_O_TAW_1997':1.0E-6,'AY593835.1_O_TAW_1997':1.0E-6)[&label=0.735119]:1.0E-6,'AF154271.1_O_TAW_1997':0.002297)[&label=0.90129]:1.0E-6,'AF026168.2_O_TAW_1997':0.0094)[&label=0.915675]:0.004624,'AY593833.1_O_TAW_1999':1.0E-6)[&label=0.94122]:0.015335,(('KU204893.1_O_CHA_2013':0.046184,'KU204894.1_O_CHA_2013':0.008457)[&label=0.999008]:0.061193,('AY317098.1_O_CHA_2002':0.006935,'EU400597.1_O_CHA_2001':1.0E-6)[&label=0.916667]:0.020544)[&label=1.0]:0.05744)[&label=0.903398]:0.01156,'AY686687.1_O_CHA_2001':0.044364)[&label=0.910384]:0.004321,'HQ412603.1_O_CHA_2000':0.07747)[&label=0.958631]:0.032238,'HQ632771.1_O_MAY_2005':0.080924)[&label=0.981331]:0.14418)[&label=0.683122]:1.0E-6,(((((((((((((((((((((((((((('DQ404174.1_O_UKG_2001':1.0E-6,'DQ404173.1_O_UKG_2001':1.0E-6)[&label=0.012897]:1.0E-6,'FJ542372.1_O_UKG_2001':1.0E-6)[&label=0.017857]:1.0E-6,'FJ542371.1_O_UKG_2001':1.0E-6)[&label=0.021825]:1.0E-6,(('DQ404178.1_O_UKG_2001':1.0E-6,'AJ539141.1_O_UKG_2001':1.0E-6)[&label=0.017857]:1.0E-6,'DQ404180.1_O_UKG_2001':1.0E-6)[&label=0.020833]:1.0E-6)[&label=0.040509]:1.0E-6,'FJ542369.1_O_UKG_2001':1.0E-6)[&label=0.057398]:1.0E-6,'DQ404179.1_O_UKG_2001':1.0E-6)[&label=0.074529]:1.0E-6,'DQ404175.1_O_UKG_2001':1.0E-6)[&label=0.0819]:1.0E-6,(('AJ633821.1_O_FRA_2001':0.002258,'FJ542368.1_O_UKG_2001':1.0E-6)[&label=9.92E-4]:1.0E-6,'DQ404172.1_O_UKG_2001':1.0E-6)[&label=0.00744]:1.0E-6)[&label=0.116898]:1.0E-6,(('AY593831.1_O_UKG_2002':1.0E-6,'DQ404177.1_O_UKG_2001':1.0E-6)[&label=0.014881]:1.0E-6,'DQ404168.1_O_UKG_2001':1.0E-6)[&label=0.02381]:1.0E-6)[&label=0.211177]:1.0E-6,(((((('KM257061.1_O_UKG_2001':1.0E-6,'FJ542365.1_O_UKG_2001':1.0E-6)[&label=0.008929]:1.0E-6,'FJ542370.1_O_UKG_2001':1.0E-6)[&label=0.022817]:1.0E-6,('DQ404176.1_O_UKG_2001':1.0E-6,'EF552696.1_O_UKG_2001':1.0E-6)[&label=0.011905]:1.0E-6)[&label=0.03373]:1.0E-6,'EF552688.1_O_UKG_2001':1.0E-6)[&label=0.041468]:1.0E-6,('AY593836.1_O_UKG_2001':0.002255,'DQ404164.1_O_UKG_2001':0.004563)[&label=0.139881]:1.0E-6)[&label=0.051304]:1.0E-6,'DQ404171.1_O_UKG_2001':1.0E-6)[&label=0.059028]:1.0E-6)[&label=0.670552]:1.0E-6,'KM257062.1_O_UKG_2001':1.0E-6)[&label=0.718333]:1.0E-6,'KM257063.1_O_UKG_2001':1.0E-6)[&label=0.764042]:1.0E-6,((((('DQ404158.1_O_UKG_2001':1.0E-6,'KM257065.1_O_UKG_2001':1.0E-6)[&label=0.296627]:1.0E-6,'DQ404159.1_O_UKG_2001':1.0E-6)[&label=0.869544]:1.0E-6,'DQ404160.1_O_UKG_2001':1.0E-6)[&label=0.976521]:0.004623,'DQ404161.1_O_UKG_2001':0.002307)[&label=0.901538]:0.002231,'DQ404163.1_O_UKG_2001':0.006887)[&label=0.787103]:1.0E-6)[&label=0.892764]:0.002258,((((((('DQ404170.1_O_UKG_2001':1.0E-6,'DQ404166.1_O_UKG_2001':1.0E-6)[&label=0.092262]:1.0E-6,'EF552693.1_O_UKG_2001':1.0E-6)[&label=0.176091]:1.0E-6,'DQ404165.1_O_UKG_2001':1.0E-6)[&label=0.267196]:1.0E-6,(('EF552692.1_O_UKG_2001':1.0E-6,'DQ404167.1_O_UKG_2001':1.0E-6)[&label=0.063492]:1.0E-6,'KM257064.1_O_UKG_2001':1.0E-6)[&label=0.12252]:1.0E-6)[&label=0.950728]:0.002261,'EF552691.1_O_UKG_2001':0.002258)[&label=0.829507]:1.0E-6,(((('EF552690.1_O_UKG_2001':1.0E-6,'DQ404169.1_O_UKG_2001':1.0E-6)[&label=0.118056]:1.0E-6,'EF552695.1_O_UKG_2001':1.0E-6)[&label=0.229167]:1.0E-6,'EF552689.1_O_UKG_2001':1.0E-6)[&label=0.49041]:1.0E-6,'EF552697.1_O_UKG_2001':1.0E-6)[&label=0.725446]:1.0E-6)[&label=0.67915]:1.0E-6,('DQ404162.1_O_UKG_2001':0.00226,'EU214601.1_O_UKG_2001':0.002257)[&label=0.160714]:1.0E-6)[&label=0.686295]:1.0E-6)[&label=0.985731]:0.007045,'AJ539140.1_O_SAR_2000':0.009348)[&label=0.98175]:0.002129,'AB079061.1_O_JPN_2000':0.006861)[&label=0.972607]:1.0E-6,(((((('MF947141.1_O_VIT_2012':0.009226,'MF947126.1_O_VIT_2011':0.004551)[&label=0.360119]:1.0E-6,'MF947123.1_O_VIT_2011':1.0E-6)[&label=0.190476]:1.0E-6,('MF947128.1_O_VIT_2010':1.0E-6,'KY234501.1_O_CHA_2011':1.0E-6)[&label=0.392857]:1.0E-6)[&label=0.714534]:1.0E-6,(('MF947127.1_O_VIT_2012':1.0E-6,'MF947137.1_O_VIT_2012':1.0E-6)[&label=0.999008]:0.014188,('MF947124.1_O_VIT_2012':0.002366,'MF947129.1_O_VIT_2014':0.018842)[&label=0.808532]:0.006846)[&label=0.665013]:0.002319)[&label=0.81126]:0.004521,(((('MF143577.1_O_VIT_2013':1.0E-6,'MF143578.1_O_VIT_2013':1.0E-6)[&label=0.790675]:0.002267,'MF143576.1_O_VIT_2013':1.0E-6)[&label=0.871032]:1.0E-6,'MF143574.1_O_VIT_2012':0.011585)[&label=0.719577]:0.002343,'MF143575.1_O_VIT_2012':0.002231)[&label=0.840774]:0.007162)[&label=0.837302]:0.001946,(((('KY234502.1_O_CHA_2015':0.011285,'MF947132.1_O_VIT_2015':0.022335)[&label=0.992063]:0.017442,('MF947142.1_O_VIT_2013':1.0E-6,'MF947143.1_O_VIT_2013':1.0E-6)[&label=0.766865]:0.002262)[&label=0.536045]:1.0E-6,'MF947131.1_O_VIT_2013':1.0E-6)[&label=0.71255]:0.002785,('MF143572.1_O_VIT_2012':0.00271,'MF143573.1_O_VIT_2012':0.008891)[&label=0.577381]:0.006943)[&label=0.59127]:0.004508)[&label=0.999504]:0.048882)[&label=0.781448]:0.002272,('AJ539136.1_O_TAW_1999':1.0E-6,'AJ539137.1_O_TAW_1999':1.0E-6)[&label=0.902778]:0.00226)[&label=0.765212]:1.0E-6,(('AJ539138.1_O_CHA_1999':1.0E-6,'AF506822.2_O_CHA_1999':1.0E-6)[&label=0.887897]:0.00226,'HQ632768.1_O_MAY_2000':0.002258)[&label=0.545635]:1.0E-6)[&label=0.771944]:1.0E-6,(((((((((('KF694739.1_O_SKR_2002':1.0E-6,'KF694745.1_O_SKR_2002':1.0E-6)[&label=0.036706]:1.0E-6,'KF694737.1_O_SKR_2002':1.0E-6)[&label=0.080357]:1.0E-6,'KF694740.1_O_SKR_2002':1.0E-6)[&label=0.125992]:1.0E-6,'KF694734.1_O_SKR_2002':1.0E-6)[&label=0.19246]:1.0E-6,('KF694744.1_O_SKR_2002':1.0E-6,'KF694743.1_O_SKR_2002':1.0E-6)[&label=0.043651]:1.0E-6)[&label=0.345073]:1.0E-6,((('EF614457.1_O_SKR_2002':1.0E-6,'KF694731.1_O_SKR_2002':1.0E-6)[&label=0.084325]:1.0E-6,'KF694735.1_O_SKR_2002':1.0E-6)[&label=0.08631]:1.0E-6,('KF694741.1_O_SKR_2002':1.0E-6,'KF694736.1_O_SKR_2002':1.0E-6)[&label=0.056548]:1.0E-6)[&label=0.152282]:1.0E-6)[&label=0.97105]:1.0E-6,'KF694742.1_O_SKR_2002':0.002265)[&label=0.973876]:1.0E-6,'AH012984.2_O_SKR_2000':0.006859)[&label=0.980006]:0.004653,('KF694738.1_O_SKR_2002':1.0E-6,'KF694732.1_O_SKR_2002':1.0E-6)[&label=0.998016]:0.006938)[&label=0.997884]:0.006598,('HQ009509.1_O_CHA_1999':0.033419,'HM008917.1_O_CHA_2005':0.013102)[&label=0.999008]:0.027558)[&label=0.984127]:0.009726)[&label=0.940081]:1.0E-6,((('AY593824.1_O_SKR_2000':1.0E-6,'AJ539139.1_O_SKR_2000':1.0E-6)[&label=0.386905]:1.0E-6,'AH012985.2_O_SKR_2000':0.009204)[&label=0.478671]:1.0E-6,'MG372730.1_O_SKR_2000':1.0E-6)[&label=0.967593]:0.009174)[&label=0.974063]:0.002097,'AF377945.1_O_SKR_2000':0.004784)[&label=0.982882]:0.011349,'HQ832588.1_A_IND_2005':0.028893)[&label=0.982794]:0.006313,((('HQ113233.1_Asia1_AFG_2009':0.010572,'EF149010.1_Asia1_CHA_2005':0.020891)[&label=0.289683]:0.005534,'JF749849.1_Asia1_PAK_2002':0.01223)[&label=0.551091]:0.005409,'JF749851.1_O_IRN_2001':0.011227)[&label=0.748347]:0.012006)[&label=0.979282]:0.016302,'JN006719.1_Asia1_PAK_2008':1.0E-6)[&label=0.975093]:6.15E-4,'KM268898.1_Asia1_TUR_2013':0.047897)[&label=0.976153]:0.012703,((((((((((('JX040490.1_O_BUL_2011':1.0E-6,'JX040487.1_O_BUL_2011':1.0E-6)[&label=0.81746]:0.002295,('JX040489.1_O_BUL_2011':1.0E-6,'JX040488.1_O_BUL_2011':0.002294)[&label=0.424603]:1.0E-6)[&label=0.821429]:0.002296,'JX066665.1_O_BUL_2011':1.0E-6)[&label=0.627728]:1.0E-6,('JX040486.1_O_BUL_2011':1.0E-6,'JX066664.1_O_BUL_2011':1.0E-6)[&label=0.220238]:1.0E-6)[&label=0.716931]:1.0E-6,'JX040485.1_O_BUL_2010':0.002295)[&label=0.802721]:0.002297,('JX040491.1_O_TUR_2010':0.002311,'JX040497.1_O_TUR_2010':1.0E-6)[&label=0.685516]:0.002297)[&label=0.664462]:1.0E-6,((('JX040495.1_O_TUR_2010':0.002295,'JX040493.1_O_TUR_2010':0.004627)[&label=0.133929]:1.0E-6,'JX040499.1_O_TUR_2011':0.002323)[&label=0.135913]:1.0E-6,(('JX040498.1_O_TUR_2010':1.0E-6,'JX040494.1_O_TUR_2010':1.0E-6)[&label=0.178571]:1.0E-6,'JX040496.1_O_TUR_2010':1.0E-6)[&label=0.537202]:1.0E-6)[&label=0.365476]:1.0E-6)[&label=0.86746]:1.0E-6,'JX040492.1_O_TUR_2010':0.002298)[&label=0.928385]:1.0E-6,'JX040500.1_O_TUR_2011':0.0023)[&label=0.956116]:0.00358,'JX040501.1_O_ISR_2011':0.018057)[&label=0.958719]:0.008018,(('MH784403.1_O_PAK_2016':0.002299,'MH784404.1_O_PAK_2017':1.0E-6)[&label=0.922619]:0.011728,'MH784405.1_O_PAK_2017':0.002436)[&label=0.991071]:0.027743)[&label=0.916667]:0.002477)[&label=0.975245]:0.024018,(((((((('JN099699.1_A_IRQ_2009':1.0E-6,'JN099698.1_A_IRQ_2009':0.002234)[&label=0.365079]:1.0E-6,'KC440882.1_A_EGY_2012':0.013808)[&label=0.457341]:0.002235,'JN099697.1_A_IRQ_2009':1.0E-6)[&label=0.518849]:1.0E-6,(('JN099695.1_A_IRQ_2009':1.0E-6,'JN099694.1_A_IRQ_2009':1.0E-6)[&label=0.955357]:0.002201,'JN099688.1_A_IRQ_2009':0.004582)[&label=0.857143]:0.004571)[&label=0.998512]:0.042524,'EF494488.1_A_PAK_2006':0.004362)[&label=0.913549]:0.007225,'EF494486.1_A_TUR_2005':0.006908)[&label=0.816468]:1.0E-6,'JF749841.1_A_TUR_2006':0.013991)[&label=0.835317]:0.004535,('EF117837.1_A_PAK_2006':1.0E-6,'EF494487.1_A_PAK_2006':1.0E-6)[&label=0.990079]:1.0E-6)[&label=0.99991]:0.105302)[&label=0.925852]:0.019323)[&label=0.496381]:1.0E-6,((((((((((((((((((((('MG725872.1_A_NIG_2013':0.028535,'MG725874.1_A_NIG_2015':0.037008)[&label=0.987103]:0.054697,'MG725873.1_A_NIG_2015':1.0E-6)[&label=0.656746]:0.002129,('MG725876.1_A_NIG_2015':1.0E-6,'MG725875.1_A_NIG_2015':0.002257)[&label=0.959325]:0.00474)[&label=0.646577]:0.007165,(('MG913340.1_A_ALG_2017':1.0E-6,'MG923579.1_A_ALG_2017':1.0E-6)[&label=0.895833]:1.0E-6,'MG923580.1_A_ALG_2017':0.004546)[&label=0.982143]:0.014467)[&label=0.9786]:0.05567,('KP940474.1_A_EGY_2014':1.0E-6,'KC440881.1_A_EGY_2011':1.0E-6)[&label=1.0]:0.098744)[&label=0.907959]:0.018761,(('JX014255.1_SAT2_EGY_2012':1.0E-6,'KC440884.1_SAT2_EGY_2012':1.0E-6)[&label=0.6875]:1.0E-6,'JX014256.1_SAT2_PAT_2012':0.002254)[&label=0.998512]:0.058867)[&label=0.990741]:0.046026,'MH053326.1_SAT1_UGA_1970':0.114078)[&label=0.982448]:0.041312,(('MH053325.1_SAT1_UGA_1978':0.101808,'MH053323.1_SAT1_TCH_1972':0.059381)[&label=0.608135]:0.020891,'AY593844.1_SAT1_ISR_1962':0.06718)[&label=0.464286]:0.008415)[&label=0.921379]:0.011242,((((('HM067706.1_SAT1_UGA_2007':0.079159,'MH053327.1_SAT1_UGA_1970':0.021045)[&label=0.391865]:0.015665,'MH053337.1_SAT2_UGA_1970':0.02082)[&label=0.615079]:0.022524,(('KJ820999.1_SAT3_UGA_2013':0.051027,'MH053341.1_SAT3_UGA_1970':0.068736)[&label=0.455357]:0.01859,'HM067704.1_SAT2_UGA_2007':0.06002)[&label=0.368552]:8.14E-4)[&label=0.749008]:0.021631,(('HM067705.1_SAT2_UGA_2007':0.030555,'JF749862.1_SAT2_UGA_2002':0.022155)[&label=0.982143]:0.048306,'MH053336.1_SAT2_UGA_1970':0.057568)[&label=0.739583]:0.009707)[&label=0.827753]:0.02465,(('FJ461346.1_SAT2_UGA_2002':0.038472,'AY593849.1_SAT2_KEN_1960':0.10487)[&label=0.502976]:0.012414,'MH053324.1_SAT1_UGA_1971':0.09222)[&label=0.686508]:0.016946)[&label=0.702832]:0.003416)[&label=0.976084]:0.137547,(('AY593756.1_A_Brazil_1959':0.058068,'AY593770.1_A_ARG_1966':0.038121)[&label=0.924603]:0.032463,('AY593775.1_A_VEN_1970':1.0E-6,'AY593773.1_A_PER_1969':1.0E-6)[&label=1.0]:0.092708)[&label=0.611111]:0.005596)[&label=0.868118]:0.004048,(('AY593787.1_A_Brazil_1977':0.004253,'AY593788.1_A_Brazil_1979':0.021283)[&label=0.85119]:0.006469,'AY593803.1_A_Brazil_1979':0.029327)[&label=0.989087]:0.04994)[&label=0.821259]:0.00838,(((((((((((((('AY593785.1_A_ARG_2001':1.0E-6,'AY593784.1_A_ARG_2001':1.0E-6)[&label=0.305556]:1.0E-6,'KX002203.1_A_ARG_2001':1.0E-6)[&label=0.852679]:0.002303,'KX002186.1_A_ARG_2001':0.002304)[&label=0.587632]:1.0E-6,'KX002177.1_A_ARG_2001':1.0E-6)[&label=0.453125]:1.0E-6,(('KX002194.1_A_ARG_2001':1.0E-6,'KX002199.1_A_ARG_2001':0.004629)[&label=0.649802]:0.002303,'KX002201.1_A_ARG_2001':0.002307)[&label=0.356647]:1.0E-6)[&label=0.297761]:1.0E-6,'KX002195.1_A_ARG_2001':1.0E-6)[&label=0.284474]:1.0E-6,(('KX002202.1_A_ARG_2001':0.009362,'AY593802.1_A_URU_2001':0.002307)[&label=0.051587]:1.0E-6,'KX002185.1_A_ARG_2001':1.0E-6)[&label=0.032242]:1.0E-6)[&label=0.32233]:1.0E-6,(('KX002188.1_A_ARG_2001':0.002318,'KX002190.1_A_ARG_2001':0.002335)[&label=0.514881]:0.002318,'KX002197.1_A_ARG_2001':0.002303)[&label=0.28373]:1.0E-6)[&label=0.363308]:1.0E-6,'KX002179.1_A_ARG_2001':1.0E-6)[&label=0.423413]:1.0E-6,('KX002181.1_A_ARG_2001':0.00702,'KX002200.1_A_ARG_2001':0.002303)[&label=0.063492]:1.0E-6)[&label=0.463527]:1.0E-6,((((((((('KX002182.1_A_ARG_2001':1.0E-6,'KX002180.1_A_ARG_2001':1.0E-6)[&label=0.517857]:1.0E-6,'KX002198.1_A_ARG_2001':0.002301)[&label=0.463294]:1.0E-6,'KX002184.1_A_ARG_2001':0.002298)[&label=0.593585]:1.0E-6,'KX002176.1_A_ARG_2001':0.002302)[&label=0.755704]:0.002301,'KX002178.1_A_ARG_2001':0.00463)[&label=0.633532]:1.0E-6,((('KX002205.1_A_ARG_2001':1.0E-6,'AY593790.1_A_ARG_2001':1.0E-6)[&label=0.205357]:1.0E-6,'AY593786.1_A_ARG_2001':1.0E-6)[&label=0.525794]:1.0E-6,'KX002204.1_A_ARG_2001':1.0E-6)[&label=0.825728]:1.0E-6)[&label=0.691248]:1.0E-6,('KX002192.1_A_ARG_2001':0.004662,'KX002189.1_A_ARG_2001':1.0E-6)[&label=0.659722]:0.002303)[&label=0.844877]:1.0E-6,'KX002183.1_A_ARG_2001':0.009325)[&label=0.897983]:1.0E-6,'KX002187.1_A_ARG_2001':0.00709)[&label=0.912469]:0.004628)[&label=0.953917]:0.007069,'KX002196.1_A_ARG_2001':0.002285)[&label=0.954334]:0.002335,('KX002193.1_A_ARG_2001':0.004679,'KX002191.1_A_ARG_2001':0.007015)[&label=0.588294]:1.0E-6)[&label=0.990371]:0.066094,'AY593806.1_C_Brazil_1971':0.06058)[&label=0.982511]:0.032332)[&label=0.699726]:0.010618,'AY593810.1_C_UKG_1970':0.047161)[&label=0.698316]:0.007826,('AY593809.1_C_ARG_1969':0.009433,'AY593807.1_C_Brazil_1955':0.004661)[&label=0.998016]:0.069817)[&label=0.688023]:0.006559,((((('AY593759.1_A_GER_1971':0.0294,'AY593776.1_A_GER_1968':0.031856)[&label=0.519841]:0.008281,'AY593751.1_A_NET_1942':0.030291)[&label=0.639385]:0.004387,('AY593826.1_O_ITL_1947':0.019261,'AY593827.1_O_VEN_1971':0.004694)[&label=0.97123]:0.022194)[&label=0.96255]:0.033339,(('AY593779.1_A_GER_1972':1.0E-6,'AY593774.1_A_SPA_1969':1.0E-6)[&label=0.324405]:1.0E-6,'AY593777.1_A_GER_1972':1.0E-6)[&label=1.0]:0.043867)[&label=0.726757]:0.02403,('NC_039210.1_O_UKG_1965':0.087536,'AY593825.1_O_ARG_1939':0.043848)[&label=0.305556]:0.009107)[&label=0.625992]:0.012361)[&label=0.630693]:0.002857,((((((((((((('MH559800.1_A_USA_2016':1.0E-6,'AY593768.1_A_Brazil_1955':1.0E-6)[&label=0.012897]:1.0E-6,'MH559799.1_A_USA_2016':1.0E-6)[&label=0.037698]:1.0E-6,'MH559793.1_A_USA_2016':1.0E-6)[&label=0.054894]:1.0E-6,'MH559783.1_A_USA_2016':1.0E-6)[&label=0.085565]:1.0E-6,('MH559796.1_A_USA_2016':1.0E-6,'MH559805.1_A_USA_2016':1.0E-6)[&label=0.052579]:1.0E-6)[&label=0.197917]:1.0E-6,(((('MH559786.1_A_USA_2016':1.0E-6,'MH559785.1_A_USA_2016':1.0E-6)[&label=0.031746]:1.0E-6,'MH559780.1_A_USA_2016':1.0E-6)[&label=0.043155]:1.0E-6,'MH559804.1_A_USA_2016':1.0E-6)[&label=0.063492]:1.0E-6,'MH559791.1_A_USA_2016':1.0E-6)[&label=0.089038]:1.0E-6)[&label=0.82224]:1.0E-6,'MH559788.1_A_USA_2016':1.0E-6)[&label=0.918485]:1.0E-6,('MH559801.1_A_USA_2016':1.0E-6,'MH559781.1_A_USA_2016':1.0E-6)[&label=0.806548]:0.002308)[&label=0.974277]:0.002311,'MH559798.1_A_USA_2016':1.0E-6)[&label=1.0]:0.069437,(((('AY593805.1_C_GER_1960':1.0E-6,'AY593804.1_C_SWZ_1965':1.0E-6)[&label=0.993056]:1.0E-6,'FJ824812.1_C_SPA_2009':0.011642)[&label=0.99256]:0.02727,'AY593808.1_C_ARG_1966':0.035259)[&label=0.990741]:0.048958,(('AY593753.1_A_Brazil_1970':1.0E-6,'AY593757.1_A_Brazil_1967':0.014146)[&label=0.489087]:0.002289,'AY593758.1_A_VEN_1967':1.0E-6)[&label=0.99256]:0.049106)[&label=0.73297]:0.01624)[&label=0.981241]:0.006796,'AY593793.1_A_PHI_1975':0.157145)[&label=0.960576]:0.012069,'AY593782.1_A_ARG_2000':0.193073)[&label=0.925885]:0.006609,((((((('AY593754.1_A_SPA_1959':1.0E-6,'AY593778.1_A_SPA_1969':1.0E-6)[&label=0.99504]:0.011875,'AY593780.1_A_FRA_1960':0.004671)[&label=0.793155]:0.004635,'AY593781.1_A_GER_1951':1.0E-6)[&label=0.785714]:0.002538,('AY593767.1_A_ARG_1965':0.056189,'AY593771.1_A_COL_1967':0.074343)[&label=0.457341]:0.015423)[&label=0.846429]:0.002326,'AY593760.1_A_USSR_1964':0.029622)[&label=0.898644]:0.002013,'AY593792.1_A_ITL_1962':0.00493)[&label=0.971939]:0.028961,(('AY593769.1_A_ARG_1959':1.0E-6,'AY593789.1_A_ARG_1961':1.0E-6)[&label=1.0]:0.075389,'AY593794.1_A_COL_1985':0.041794)[&label=0.666667]:0.005515)[&label=0.824901]:0.016011)[&label=0.778231]:0.011059)[&label=0.704588]:0.01219,(((((((((((((((((((((((((((((((((('JX570643.1_O_UKG_2007':1.0E-6,'AY593815.1_O_UKG_1967':1.0E-6)[&label=0.00496]:1.0E-6,'EU448368.1_O_UKG_1967':1.0E-6)[&label=0.00744]:1.0E-6,'JX869182.1_O_UKG_1968':0.004606)[&label=0.006614]:1.0E-6,('JX869183.1_O_UKG_1968':1.0E-6,'KJ560299.1_O_UKG_2007':1.0E-6)[&label=0.0]:1.0E-6)[&label=0.013889]:1.0E-6,((('JX570651.1_O_UKG_2007':1.0E-6,'JX869188.1_O_UKG_1968':1.0E-6)[&label=0.003968]:1.0E-6,'EU448369.1_O_UKG_1967':1.0E-6)[&label=0.006944]:1.0E-6,('JX570642.1_O_UKG_2007':1.0E-6,'EU448378.1_O_UKG_2007':1.0E-6)[&label=0.0]:1.0E-6)[&label=0.011409]:1.0E-6)[&label=0.020437]:1.0E-6,(((((('JX570652.1_O_UKG_2007':1.0E-6,'JX570649.1_O_UKG_2007':1.0E-6)[&label=0.0]:1.0E-6,'KJ560304.1_O_UKG_2007':1.0E-6)[&label=0.0]:1.0E-6,'KJ560277.1_O_UKG_2007':1.0E-6)[&label=0.0]:1.0E-6,'KJ560285.1_O_UKG_2007':1.0E-6)[&label=0.0]:1.0E-6,(('JX570648.1_O_UKG_2007':1.0E-6,'JX869187.1_O_UKG_1968':1.0E-6)[&label=0.001984]:1.0E-6,'JX570655.1_O_UKG_2007':1.0E-6)[&label=0.005952]:1.0E-6)[&label=0.012188]:1.0E-6,'KJ560303.1_O_UKG_2007':1.0E-6)[&label=0.012153]:1.0E-6)[&label=0.047776]:1.0E-6,'JX570640.1_O_UKG_2007':1.0E-6)[&label=0.054712]:1.0E-6,('EU448371.1_O_UKG_2007':1.0E-6,'JX570638.1_O_UKG_2007':1.0E-6)[&label=0.003968]:1.0E-6)[&label=0.066153]:1.0E-6,('AY593816.1_O_UKG_1967':0.002291,'JX869185.1_O_UKG_1968':0.002294)[&label=0.08631]:1.0E-6)[&label=0.079654]:1.0E-6,((('JX869179.1_O_UKG_1967':1.0E-6,'KJ560281.1_O_UKG_2007':1.0E-6)[&label=0.00496]:1.0E-6,'KJ560296.1_O_UKG_2007':1.0E-6)[&label=0.006944]:1.0E-6,('KJ560302.1_O_UKG_2007':1.0E-6,'JX570646.1_O_UKG_2007':1.0E-6)[&label=0.0]:1.0E-6)[&label=0.006448]:1.0E-6)[&label=0.155617]:1.0E-6,'EU448374.1_O_UKG_2007':1.0E-6)[&label=0.187202]:1.0E-6,'KJ560307.1_O_UKG_2007':1.0E-6)[&label=0.226863]:1.0E-6,('JX570650.1_O_UKG_2007':1.0E-6,'JX570654.1_O_UKG_2007':1.0E-6)[&label=0.009921]:1.0E-6)[&label=0.305886]:1.0E-6,'KJ560283.1_O_UKG_2007':1.0E-6)[&label=0.349119]:1.0E-6,'EU448375.1_O_UKG_2007':1.0E-6)[&label=0.391156]:1.0E-6,'KJ560298.1_O_UKG_2007':1.0E-6)[&label=0.433008]:1.0E-6,('JX869184.1_O_UKG_1968':1.0E-6,'EU448376.1_O_UKG_2007':1.0E-6)[&label=0.0]:1.0E-6)[&label=0.510182]:1.0E-6,('JX869186.1_O_UKG_1968':1.0E-6,'JX869181.1_O_UKG_1967':0.002292)[&label=0.001984]:1.0E-6)[&label=0.572892]:1.0E-6,'JX570644.1_O_UKG_2007':1.0E-6)[&label=0.606175]:1.0E-6,'EU448373.1_O_UKG_2007':1.0E-6)[&label=0.638416]:1.0E-6,'JX570639.1_O_UKG_2007':1.0E-6)[&label=0.668305]:1.0E-6,'EU448372.1_O_UKG_2007':1.0E-6)[&label=0.697443]:1.0E-6,(('KJ560308.1_O_UKG_2007':1.0E-6,'KJ560294.1_O_UKG_2007':1.0E-6)[&label=0.0]:1.0E-6,'JX570645.1_O_UKG_2007':1.0E-6)[&label=0.0]:1.0E-6)[&label=0.778875]:1.0E-6,'KJ560300.1_O_UKG_2007':1.0E-6)[&label=0.80353]:1.0E-6,'JX869180.1_O_UKG_1967':1.0E-6)[&label=0.82732]:1.0E-6,(('EU448377.1_O_UKG_2007':1.0E-6,'JX570647.1_O_UKG_2007':1.0E-6)[&label=0.0]:1.0E-6,'JX570653.1_O_UKG_2007':1.0E-6)[&label=0.003472]:1.0E-6)[&label=0.89446]:1.0E-6,('EU448370.1_O_UKG_1967':1.0E-6,'KJ560276.1_O_UKG_2007':1.0E-6)[&label=0.005952]:1.0E-6)[&label=0.933899]:1.0E-6,'JX570641.1_O_UKG_2007':1.0E-6)[&label=0.953084]:1.0E-6,'KJ560287.1_O_UKG_2007':1.0E-6)[&label=0.970504]:1.0E-6,'KJ560297.1_O_UKG_2007':0.002292)[&label=0.985833]:0.004607,((((('AY593830.1_O_POL_1959':0.011764,'AY593814.1_O_ARG_1965':0.006925)[&label=0.271825]:1.0E-6,('JX869177.1_O_UKG_1967':0.002222,'JX869178.1_O_UKG_1967':0.016583)[&label=0.631944]:0.002383)[&label=0.383267]:1.0E-6,'AY593819.1_O_ARG_1994':1.0E-6)[&label=0.293403]:1.0E-6,'AY593837.1_O_URU_1963':1.0E-6)[&label=0.274802]:1.0E-6,('AY593818.1_O_ARG_1958':1.0E-6,'AY593820.1_O_ARG_1964':1.0E-6)[&label=0.22123]:1.0E-6)[&label=0.567885]:1.0E-6)[&label=0.99591]:0.006948,'AY593817.1_O_Belgium_1973':1.0E-6)[&label=0.998407]:0.036255,'KP940473.1_O_EGY_2014':0.069473)[&label=0.994744]:0.032075,'AY593821.1_O_ARG_1967':0.080499)[&label=0.981574]:0.006141)[&label=0.921553]:0.007037,'MH053310.1_C_UGA_1970':0.131113)[&label=0.926128]:0.006834,'AY593813.1_O_ISA_1962':0.184221)[&label=0.929205]:0.03315,((((('MH053317.1_O_UGA_1998':0.06541,'MH053312.1_O_ETH_2005':0.06524)[&label=0.988095]:0.053753,'JF749843.1_A_EGY_2006':0.098765)[&label=0.857639]:0.028413,('MH053305.1_A_EGY_1972':0.056798,'AY593761.1_A_KEN_1964':0.04818)[&label=0.935516]:0.045463)[&label=0.613591]:0.016385,'MH053306.1_A_TCH_1973':0.067319)[&label=0.800992]:0.005864,'AY593766.1_A_KEN_1965':0.141697)[&label=0.929233]:0.053897)[&label=0.904326]:0.003784,('AY593795.1_Asia1_PAK_1954':0.079611,'AY593755.1_A_TAI_1960':0.131396)[&label=0.28869]:0.014739)[&label=0.898671]:0.01391)[&label=0.717069]:0.003715,(((((('DQ989307.1_Asia1_IND_1992':0.006862,'DQ989305.1_Asia1_IND_1990':0.009527)[&label=0.999008]:0.041678,'DQ989313.1_Asia1_IND_1986':0.057781)[&label=0.768849]:0.012674,('KT003716.1_O_PAK_2005':1.0E-6,'KY446903.1_O_PAK_2005':1.0E-6)[&label=1.0]:0.068041)[&label=0.767113]:0.011262,'HQ832576.1_A_IND_1990':0.066901)[&label=0.85754]:0.010255,'KY446902.1_A_PAK_2005':0.061162)[&label=0.909226]:0.036865,(('DQ989312.1_Asia1_IND_1990':0.029642,'KU726614.1_O_GRE_1994':0.021049)[&label=0.818452]:0.017309,'AY687333.1_Asia1_IND_2001':0.066291)[&label=0.613095]:0.008199)[&label=0.672729]:0.008833)[&label=0.741187]:0.017149,(((((((((((((((((((((((((((((((((((((('LC149661.1_O_JPN_2010':1.0E-6,'LC149715.1_O_JPN_2010':1.0E-6)[&label=0.12004]:1.0E-6,'LC149639.1_O_JPN_2010':1.0E-6)[&label=0.381944]:1.0E-6,'LC149685.1_O_JPN_2010':1.0E-6)[&label=0.853505]:0.002308,(('LC149633.1_O_JPN_2010':1.0E-6,'LC149647.1_O_JPN_2010':1.0E-6)[&label=0.819444]:1.0E-6,'LC149655.1_O_JPN_2010':0.002307)[&label=0.828373]:0.002308)[&label=0.465112]:1.0E-6,(('LC149638.1_O_JPN_2010':1.0E-6,'LC149625.1_O_JPN_2010':1.0E-6)[&label=9.92E-4]:1.0E-6,'LC149682.1_O_JPN_2010':1.0E-6)[&label=0.001488]:1.0E-6)[&label=0.312169]:1.0E-6,(('LC149659.1_O_JPN_2010':1.0E-6,'LC149656.1_O_JPN_2010':0.002337)[&label=0.673611]:0.002309,'LC149704.1_O_JPN_2010':0.002309)[&label=0.344246]:1.0E-6)[&label=0.249008]:1.0E-6,('LC149654.1_O_JPN_2010':1.0E-6,'LC149714.1_O_JPN_2010':1.0E-6)[&label=0.006944]:1.0E-6)[&label=0.213435]:1.0E-6,'LC149617.1_O_JPN_2010':1.0E-6)[&label=0.199206]:1.0E-6,'LC149720.1_O_JPN_2010':1.0E-6)[&label=0.186756]:1.0E-6,'LC149620.1_O_JPN_2010':1.0E-6)[&label=0.17577]:1.0E-6,'LC149718.1_O_JPN_2010':1.0E-6)[&label=0.166116]:1.0E-6,('LC149698.1_O_JPN_2010':1.0E-6,'LC149681.1_O_JPN_2010':1.0E-6)[&label=0.006944]:1.0E-6)[&label=0.150099]:1.0E-6,'LC149674.1_O_JPN_2010':1.0E-6)[&label=0.143093]:1.0E-6,'KF112889.1_O_HKN_2010':1.0E-6)[&label=0.136724]:1.0E-6,(((('LC149667.1_O_JPN_2010':1.0E-6,'LC149684.1_O_JPN_2010':1.0E-6)[&label=0.0]:1.0E-6,'LC149668.1_O_JPN_2010':1.0E-6)[&label=0.004464]:1.0E-6,'LC149687.1_O_JPN_2010':1.0E-6)[&label=0.002976]:1.0E-6,('LC149653.1_O_JPN_2010':1.0E-6,'LC149693.1_O_JPN_2010':1.0E-6)[&label=0.0]:1.0E-6)[&label=0.002976]:1.0E-6)[&label=0.10817]:1.0E-6,'LC149650.1_O_JPN_2010':1.0E-6)[&label=0.10444]:1.0E-6,'LC149648.1_O_JPN_2010':1.0E-6)[&label=0.101058]:1.0E-6,((((((((('KF112886.1_O_SKR_2010':0.009426,'KR401158.1_O_SKR_2010':1.0E-6)[&label=0.849206]:0.007005,'HM055510.1_O_VIT_2009':0.00231)[&label=0.521825]:1.0E-6,('JN998086.1_O_CHA_2010':0.004644,'LC149707.1_O_JPN_2010':1.0E-6)[&label=0.125992]:1.0E-6)[&label=0.582341]:0.00231,'LC149691.1_O_JPN_2010':0.002306)[&label=0.470635]:1.0E-6,('LC149676.1_O_JPN_2010':1.0E-6,'LC149660.1_O_JPN_2010':1.0E-6)[&label=0.0]:1.0E-6)[&label=0.336168]:1.0E-6,'LC149680.1_O_JPN_2010':1.0E-6)[&label=0.294147]:1.0E-6,'LC149629.1_O_JPN_2010':1.0E-6)[&label=0.261795]:1.0E-6,'LC149632.1_O_JPN_2010':1.0E-6)[&label=0.235615]:1.0E-6,'LC149688.1_O_JPN_2010':0.002308)[&label=0.216901]:1.0E-6)[&label=0.101592]:1.0E-6,'LC149706.1_O_JPN_2010':1.0E-6)[&label=0.099737]:1.0E-6,(((((((('LC149636.1_O_JPN_2010':1.0E-6,'LC149622.1_O_JPN_2010':1.0E-6)[&label=0.001984]:1.0E-6,'LC149678.1_O_JPN_2010':0.002308)[&label=0.001488]:1.0E-6,'LC149644.1_O_JPN_2010':1.0E-6)[&label=0.007275]:1.0E-6,'LC149670.1_O_JPN_2010':1.0E-6)[&label=0.010417]:1.0E-6,'LC149630.1_O_JPN_2010':1.0E-6)[&label=0.01131]:1.0E-6,'HM229661.1_O_HKN_2010':1.0E-6)[&label=0.013724]:1.0E-6,'LC149695.1_O_JPN_2010':1.0E-6)[&label=0.012613]:1.0E-6,'LC149626.1_O_JPN_2010':1.0E-6)[&label=0.015749]:1.0E-6)[&label=0.09043]:1.0E-6,'LC149645.1_O_JPN_2010':1.0E-6)[&label=0.090315]:1.0E-6,('LC149671.1_O_JPN_2010':1.0E-6,'LC149683.1_O_JPN_2010':1.0E-6)[&label=0.014881]:1.0E-6)[&label=0.091108]:1.0E-6,('LC149658.1_O_JPN_2010':1.0E-6,'LC149697.1_O_JPN_2010':1.0E-6)[&label=0.0]:1.0E-6)[&label=0.092157]:1.0E-6,(((((((((((((((((((('KX534089.1_O_SKR_2016':1.0E-6,'KY086465.1_O_SKR_2016':0.00231)[&label=0.994048]:0.011625,('KX162590.1_O_SKR_2014':0.02213,'KY086466.1_O_SKR_2016':0.004753)[&label=0.643849]:1.0E-6)[&label=0.995701]:0.019828,'MH845413.2_O_VIT_2014':0.012522)[&label=0.996528]:0.004038,'KY322674.1_O_SKR_2014':0.017412)[&label=0.997619]:0.015391,'KF112879.1_O_TAI_2009':0.012265)[&label=0.885747]:0.002072,'LC149717.1_O_JPN_2010':1.0E-6)[&label=0.793651]:0.002305,'LC149702.1_O_JPN_2010':1.0E-6)[&label=0.704117]:1.0E-6,'LC036265.1_O_JPN_2010':1.0E-6)[&label=0.625882]:1.0E-6,((('LC149651.1_O_JPN_2010':1.0E-6,'JN998085.1_O_CHA_2010':0.007007)[&label=0.0]:1.0E-6,'LC149618.1_O_JPN_2010':1.0E-6)[&label=0.0]:1.0E-6,'LC149679.1_O_JPN_2010':0.00231)[&label=0.00463]:1.0E-6)[&label=0.458333]:1.0E-6,'LC149677.1_O_JPN_2010':1.0E-6)[&label=0.425595]:1.0E-6,'LC149642.1_O_JPN_2010':1.0E-6)[&label=0.397222]:1.0E-6,'LC149666.1_O_JPN_2010':1.0E-6)[&label=0.372396]:1.0E-6,('LC149709.1_O_JPN_2010':0.002306,'JQ900581.1_O_CHA_2010':0.002338)[&label=0.02381]:1.0E-6)[&label=0.332892]:1.0E-6,('LC149652.1_O_JPN_2010':1.0E-6,'LC149696.1_O_JPN_2010':1.0E-6)[&label=0.0]:1.0E-6)[&label=0.299603]:1.0E-6,('LC149649.1_O_JPN_2010':1.0E-6,'KF112885.1_O_JPN_2010':1.0E-6)[&label=9.92E-4]:1.0E-6)[&label=0.272457]:1.0E-6,'LC149672.1_O_JPN_2010':1.0E-6)[&label=0.260611]:1.0E-6,('LC149699.1_O_JPN_2010':1.0E-6,'LC149701.1_O_JPN_2010':1.0E-6)[&label=0.003968]:1.0E-6)[&label=0.239762]:1.0E-6,('LC149711.1_O_JPN_2010':1.0E-6,'LC149708.1_O_JPN_2010':1.0E-6)[&label=0.0]:1.0E-6)[&label=0.222075]:1.0E-6,((((('LC149703.1_O_JPN_2010':0.00231,'LC149675.1_O_JPN_2010':0.002306)[&label=0.005952]:1.0E-6,'LC149712.1_O_JPN_2010':1.0E-6)[&label=0.002976]:1.0E-6,('LC149710.1_O_JPN_2010':0.002307,'JQ973889.1_O_CHA_2010':0.006994)[&label=0.014881]:1.0E-6)[&label=0.024058]:1.0E-6,('LC149643.1_O_JPN_2010':1.0E-6,'LC149694.1_O_JPN_2010':1.0E-6)[&label=0.005952]:1.0E-6)[&label=0.017857]:1.0E-6,((('LC149624.1_O_JPN_2010':1.0E-6,'LC149621.1_O_JPN_2010':1.0E-6)[&label=0.006944]:1.0E-6,'LC149673.1_O_JPN_2010':1.0E-6)[&label=0.003472]:1.0E-6,'LC149623.1_O_JPN_2010':1.0E-6)[&label=0.003638]:1.0E-6)[&label=0.01627]:1.0E-6)[&label=0.160897]:1.0E-6,'LC149619.1_O_JPN_2010':1.0E-6)[&label=0.156771]:1.0E-6)[&label=0.6349]:1.0E-6,((((('LC149665.1_O_JPN_2010':1.0E-6,'LC149631.1_O_JPN_2010':1.0E-6)[&label=0.0]:1.0E-6,'LC149716.1_O_JPN_2010':1.0E-6)[&label=0.004464]:1.0E-6,('LC149641.1_O_JPN_2010':1.0E-6,'LC149664.1_O_JPN_2010':1.0E-6)[&label=9.92E-4]:1.0E-6)[&label=0.00372]:1.0E-6,'LC149627.1_O_JPN_2010':1.0E-6)[&label=0.009921]:1.0E-6,'LC149657.1_O_JPN_2010':0.002309)[&label=0.012235]:1.0E-6)[&label=0.725227]:1.0E-6,(((((((('KF112887.1_O_SKR_2010':1.0E-6,'KC503937.1_O_SKR_2010':1.0E-6)[&label=0.21131]:1.0E-6,'KR401159.1_O_SKR_2010':1.0E-6)[&label=0.540179]:1.0E-6,'KF501486.1_O_SKR_2010':1.0E-6)[&label=0.948082]:0.002305,('KF112883.1_O_RUS_2010':0.002301,'KF112888.1_O_DRK_2011':0.009343)[&label=0.181548]:1.0E-6)[&label=0.670833]:1.0E-6,(('KF501488.1_O_SKR_2010':0.002311,'KF501487.1_O_SKR_2010':1.0E-6)[&label=0.631944]:0.002309,'KR401160.1_O_SKR_2011':1.0E-6)[&label=0.881944]:0.006985)[&label=0.842014]:0.004635,'LC149692.1_O_JPN_2010':0.002307)[&label=0.751102]:1.0E-6,'LC149700.1_O_JPN_2010':1.0E-6)[&label=0.675992]:1.0E-6,'LC149690.1_O_JPN_2010':1.0E-6)[&label=0.614538]:1.0E-6)[&label=0.843485]:1.0E-6,('LC149640.1_O_JPN_2010':1.0E-6,'LC149669.1_O_JPN_2010':1.0E-6)[&label=0.0]:1.0E-6)[&label=0.863087]:1.0E-6,('LC149719.1_O_JPN_2010':1.0E-6,'LC149713.1_O_JPN_2010':1.0E-6)[&label=0.0]:1.0E-6)[&label=0.881978]:1.0E-6,('LC149662.1_O_JPN_2010':1.0E-6,'LC149634.1_O_JPN_2010':1.0E-6)[&label=0.005952]:1.0E-6)[&label=0.900298]:1.0E-6,'LC149646.1_O_JPN_2010':1.0E-6)[&label=0.909222]:1.0E-6,((('LC149705.1_O_JPN_2010':1.0E-6,'LC149628.1_O_JPN_2010':1.0E-6)[&label=0.330357]:1.0E-6,'LC149637.1_O_JPN_2010':1.0E-6)[&label=0.926587]:0.002311,'LC149635.1_O_JPN_2010':0.002311)[&label=0.625661]:1.0E-6)[&label=0.943038]:1.0E-6,(('LC149689.1_O_JPN_2010':1.0E-6,'LC149663.1_O_JPN_2010':1.0E-6)[&label=0.713294]:1.0E-6,'LC149686.1_O_JPN_2010':0.002306)[&label=0.740575]:0.002306)[&label=0.964957]:0.002326,('KR401152.1_O_MYA_2009':0.007033,'KF112880.1_O_MYA_2009':0.004622)[&label=0.039683]:1.0E-6)[&label=0.976363]:0.002236,('KR401153.1_O_MYA_2009':1.0E-6,'KR401155.1_O_MYA_2007':1.0E-6)[&label=0.989087]:0.004784)[&label=0.987658]:0.009234,('KR401156.1_O_MYA_2009':1.0E-6,'KR401157.1_O_MYA_2009':0.002329)[&label=0.99504]:2.22E-4)[&label=0.996973]:0.016746,'KY322672.1_O_MAY_2014':0.049949)[&label=0.998494]:0.007138,'KR401154.1_O_MYA_1998':0.005255)[&label=0.999303]:0.042479,(((((((('KF112882.1_O_MOG_2010':1.0E-6,'KF112884.1_O_RUS_2010':1.0E-6)[&label=0.330357]:1.0E-6,'KF112881.1_O_MOG_2010':1.0E-6)[&label=0.983631]:0.006879,('GU582116.1_O_VIT_2009':0.004562,'GU582115.1_O_VIT_2009':1.0E-6)[&label=0.882937]:1.0E-6)[&label=0.890625]:0.005949,(('KY322671.1_O_MAY_2014':0.002368,'MF947130.1_O_VIT_2014':0.004534)[&label=0.986111]:0.012713,'KY322670.1_O_LAO_2013':0.003775)[&label=0.970238]:0.026368)[&label=0.916241]:0.004755,'KY322673.1_O_MAY_2014':0.051873)[&label=0.979291]:0.013274,'HQ632772.1_O_MAY_2007':1.0E-6)[&label=0.997354]:0.041037,(('GU125648.1_O_VIT_2006':1.0E-6,'GU125649.1_O_VIT_2006':1.0E-6)[&label=0.327381]:1.0E-6,'GU125647.1_O_VIT_2006':1.0E-6)[&label=0.999504]:0.035876)[&label=0.897404]:0.01024,'GU125650.1_O_VIT_2006':0.03416)[&label=0.927274]:0.014352)[&label=0.978109]:0.013766)[&label=0.710606]:0.00487,((((((((((('GQ406248.1_A_VIT_2009':1.0E-6,'GQ406247.1_A_VIT_2009':1.0E-6)[&label=0.985119]:0.004556,'GQ406251.1_A_VIT_2009':0.004641)[&label=0.758433]:0.002261,(('GQ406252.1_A_VIT_2009':0.00684,'GQ406250.1_A_VIT_2009':0.002255)[&label=0.159722]:1.0E-6,'GQ406249.1_A_VIT_2009':1.0E-6)[&label=0.399802]:1.0E-6)[&label=0.88373]:1.0E-6,'KC588943.1_A_SKR_2010':0.013831)[&label=0.987434]:0.017479,'HQ632773.1_A_MAY_2007':0.013654)[&label=0.998866]:0.030239,'HQ268509.2_A_VIT_2004':0.012619)[&label=0.972966]:0.010412,((('KT968663.1_A_CHA_2013':0.028909,'KY322676.1_A_MAY_2013':0.024714)[&label=0.521825]:0.006923,'KJ608371.1_A_VIT_2013':0.016369)[&label=0.75248]:0.00934,'KY322678.1_A_MAY_2013':0.027506)[&label=0.967262]:0.026472)[&label=0.905589]:0.01131,(('KY322679.1_A_TAI_2014':0.00693,'KY322680.1_A_VIT_2013':0.002298)[&label=0.811508]:0.002222,('KY322675.1_A_LAO_2014':1.0E-6,'KY322677.1_A_MAY_2013':1.0E-6)[&label=0.946429]:0.002325)[&label=0.997354]:0.042983)[&label=0.937686]:0.006232,'KJ933864.1_A_MAY_1997':0.017334)[&label=0.962885]:0.031474,('HQ632774.1_Asia1_MAY_1999':0.066253,'HQ632769.1_O_MAY_2001':0.064031)[&label=0.358135]:0.009675)[&label=0.889881]:0.008078,'GU125646.1_Asia1_VIT_2005':0.076592)[&label=0.855208]:0.002075)[&label=0.959189]:0.038813,'DQ989303.1_Asia1_IND_1993':0.080193)[&label=0.971404]:0.010947,'DQ989306.1_Asia1_IND_1986':0.029186)[&label=0.984552]:0.008291,'DQ989304.1_Asia1_IND_2000':1.0E-6)[&label=1.0]:1.101755,(((((((((((((((((((((('KU821592.1_SAT2_ZAM_2009':0.035565,'MH053351.1_SAT3_ZIM_1984':0.033902)[&label=0.141865]:0.004604,'AY593843.1_SAT1_NMB_1940':0.016741)[&label=0.28621]:0.005811,('MH053328.1_SAT2_BOT_1968':0.028235,'MH053331.1_SAT2_BOT_1972':0.031471)[&label=0.345238]:0.013518)[&label=0.447421]:0.006827,('JF749864.1_SAT2_ZIM_2003':0.049044,'MH053319.1_SAT1_BOT_1974':0.048744)[&label=0.03373]:1.0E-6)[&label=0.485615]:0.001902,'MH053329.1_SAT2_BOT_1969':0.021085)[&label=0.633362]:0.007264,'AY593845.1_SAT1_BOT_1968':0.029004)[&label=0.740203]:0.005574,'KU821590.1_SAT1_NMB_2010':0.032545)[&label=0.832892]:0.005331,'AY593842.1_SAT1_SAR_1961':0.054585)[&label=0.890278]:0.005441,'MH053322.1_SAT1_NMB_1989':0.049578)[&label=0.906205]:0.007405,'MH053332.1_SAT2_BOT_1974':0.039453)[&label=0.892857]:0.010769,'AY593840.1_SAT1_NMB_1949':0.040775)[&label=0.864164]:0.001847,((('MH053338.1_SAT3_BOT_1966':0.012146,'AY593853.1_SAT3_BOT_1965':0.00177)[&label=0.979167]:0.034835,'AY593841.1_SAT1_ZIM_1958':0.011477)[&label=0.883929]:0.021255,'MH053339.1_SAT3_BOT_1970':0.031407)[&label=0.766865]:0.011022)[&label=0.879143]:0.011573,(('AY593852.1_SAT3_KEN_1960':1.0E-6,'AY593851.1_SAT3_BOT_1961':1.0E-6)[&label=1.0]:0.064064,'AY593838.1_SAT1_BOT_1970':0.049017)[&label=0.801587]:0.014285)[&label=0.956796]:0.004523,'MH053330.1_SAT2_BOT_1969':0.035169)[&label=0.981529]:0.028834,(((((((('MH053348.1_SAT3_ZIM_1977':0.002266,'MH053346.1_SAT3_ZIM_1976':1.0E-6)[&label=1.0]:0.052713,'KX375417.1_SAT3_ZIM_1981':0.017374)[&label=0.916171]:0.026097,'AY593846.1_SAT1_ZIM_1966':0.053441)[&label=0.875]:0.024178,'MH053342.1_SAT3_ZAM_1996':0.052075)[&label=0.681796]:0.005431,'MH053335.1_SAT2_ZIM_1965':0.065692)[&label=0.592659]:0.002854,(('MH053334.1_SAT2_ZAM_1964':0.064448,'AY593847.1_SAT2_ZIM_1948':0.049676)[&label=0.624008]:0.034697,('AY593850.1_SAT3_SAR_1959':0.036126,'MH053321.1_SAT1_MOZ_1981':0.039026)[&label=0.904762]:0.023815)[&label=0.406415]:0.00439)[&label=0.569224]:0.011502,'MH053352.1_SAT3_ZIM_1990':0.059627)[&label=0.544147]:0.014892,((((('MH053347.1_SAT3_ZIM_1976':0.002243,'MH053345.1_SAT3_ZIM_1975':1.0E-6)[&label=0.962302]:0.025959,('MH053349.1_SAT3_ZIM_1983':1.0E-6,'MH053350.1_SAT3_ZIM_1983':0.002384)[&label=0.941468]:0.028574)[&label=0.971892]:0.042538,('MH053340.1_SAT3_MAL_1976':0.101756,'KM268901.1_SAT3_ZIM_1991':0.034478)[&label=0.134921]:0.007247)[&label=0.751786]:0.011615,'MH053343.1_SAT3_ZIM_1934':0.043781)[&label=0.699405]:0.004911,'KR108950.1_SAT3_SAR_2009':0.050204)[&label=0.623724]:0.00496)[&label=0.529486]:0.012652)[&label=0.857912]:0.004172,(('JF749861.1_SAT2_KEN_2002':0.032362,'KM268899.1_SAT1_TAN_2012':0.041733)[&label=0.510913]:0.016154,'KM268900.1_SAT2_TAN_2012':0.058093)[&label=0.619048]:0.0685)[&label=0.873523]:0.01188,(('MH053320.1_SAT1_KEN_1983':0.073542,'JF749860.1_SAT1_KEN_2002':0.074155)[&label=0.71627]:0.030587,'MH053333.1_SAT2_ETH_1989':0.107839)[&label=0.747024]:0.028814)[&label=0.910326]:0.009982,'MH053344.1_SAT3_ZIM_1974':0.08286)[&label=0.929437]:0.006761,('AY593839.1_SAT1_UKG_1970':0.051176,'KR108949.1_SAT2_SAR_2009':0.032086)[&label=0.243056]:0.008126)[&label=0.966331]:0.01564,'AY593848.1_SAT2_u_1967':0.057635)[&label=0.982302]:0.019334,'KR108948.1_SAT1_SAR_2009':1.0E-6)[&label=0.99858]:0.751141,(('MF678826.1_SAT1_NIG_2015':1.0E-6,'MF678825.1_SAT1_NIG_2015':1.0E-6)[&label=0.946429]:1.0E-6,('MF678824.1_SAT1_NIG_2015':0.002288,'MF678823.1_SAT1_NIG_2015':1.0E-6)[&label=0.907738]:0.00693)[&label=1.0]:0.81313)[&label=1.0]:0.434226);

end;

begin figtree;

set appearance.backgroundColorAttribute="Default";

set appearance.backgroundColour=#ffffff;

set appearance.branchColorAttribute="User selection";

set appearance.branchColorGradient=false;

set appearance.branchLineWidth=1.0;

set appearance.branchMinLineWidth=0.0;

set appearance.branchWidthAttribute="Fixed";

set appearance.foregroundColour=#000000;

set appearance.hilightingGradient=false;

set appearance.selectionColour=#2d3680;

set branchLabels.colorAttribute="User selection";

set branchLabels.displayAttribute="Branch times";

set branchLabels.fontName="Calibri";

set branchLabels.fontSize=12;

set branchLabels.fontStyle=0;

set branchLabels.isShown=false;

set branchLabels.significantDigits=4;

set layout.expansion=0;

set layout.layoutType="RECTILINEAR";

set layout.zoom=0;

set legend.attribute="label";

set legend.fontSize=10.0;

set legend.isShown=false;

set legend.significantDigits=4;

set nodeBars.barWidth=4.0;

set nodeBars.displayAttribute=null;

set nodeBars.isShown=false;

set nodeLabels.colorAttribute="User selection";

set nodeLabels.displayAttribute="label";

set nodeLabels.fontName="Arial";

set nodeLabels.fontSize=12;

set nodeLabels.fontStyle=0;

set nodeLabels.isShown=true;

set nodeLabels.significantDigits=4;

set nodeShapeExternal.colourAttribute="User selection";

set nodeShapeExternal.isShown=false;

set nodeShapeExternal.minSize=10.0;

set nodeShapeExternal.scaleType=Width;

set nodeShapeExternal.shapeType=Circle;

set nodeShapeExternal.size=4.0;

set nodeShapeExternal.sizeAttribute="Fixed";

set nodeShapeInternal.colourAttribute="User selection";

set nodeShapeInternal.isShown=false;

set nodeShapeInternal.minSize=10.0;

set nodeShapeInternal.scaleType=Width;

set nodeShapeInternal.shapeType=Circle;

set nodeShapeInternal.size=4.0;

set nodeShapeInternal.sizeAttribute="Fixed";

set polarLayout.alignTipLabels=false;

set polarLayout.angularRange=0;

set polarLayout.rootAngle=0;

set polarLayout.rootLength=100;

set polarLayout.showRoot=true;

set radialLayout.spread=0.0;

set rectilinearLayout.alignTipLabels=true;

set rectilinearLayout.curvature=0;

set rectilinearLayout.rootLength=100;

set scale.offsetAge=0.0;

set scale.rootAge=1.0;

set scale.scaleFactor=1.0;

set scale.scaleRoot=false;

set scaleAxis.automaticScale=true;

set scaleAxis.fontSize=8.0;

set scaleAxis.isShown=false;

set scaleAxis.lineWidth=1.0;

set scaleAxis.majorTicks=1.0;

set scaleAxis.minorTicks=0.5;

set scaleAxis.origin=0.0;

set scaleAxis.reverseAxis=false;

set scaleAxis.showGrid=true;

set scaleBar.automaticScale=true;

set scaleBar.fontSize=12.0;

set scaleBar.isShown=true;

set scaleBar.lineWidth=1.0;

set scaleBar.scaleRange=0.0;

set tipLabels.colorAttribute="User selection";

set tipLabels.displayAttribute="Names";

set tipLabels.fontName="Arial";

set tipLabels.fontSize=12;

set tipLabels.fontStyle=0;

set tipLabels.isShown=true;

set tipLabels.significantDigits=4;

set trees.order=true;

set trees.orderType="increasing";

set trees.rooting=false;

set trees.rootingType="User Selection";

set trees.transform=false;

set trees.transformType="cladogram";

end;
