# Supplementary material for: Early origin and global colonisation of foot-and-mouth disease virus
Source: Sci Rep. 2020 Sep 17;10:15268. doi: 10.1038/s41598-020-72246-6 (PMC7498456; doi:10.1038/s41598-020-72246-6)
Supplement: Supplementary file 10 — Supplementary Tree S2. [file 41598_2020_72246_MOESM10_ESM.doc]

#NEXUS

begin trees;

tree tree_1 = [&R] ((((((((((((((((((((((((((((((((('KM257062.1_O_UKG_2001':1.0E-6,'FJ542365.1_O_UKG_2001':1.0E-6)[&label=0.026786]:1.0E-6,'AJ633821.1_O_FRA_2001':1.0E-6)[&label=0.041667]:1.0E-6,'FJ542369.1_O_UKG_2001':1.0E-6)[&label=0.086971]:1.0E-6,'FJ542370.1_O_UKG_2001':1.0E-6)[&label=0.105407]:1.0E-6,'DQ404178.1_O_UKG_2001':1.0E-6)[&label=0.175992]:1.0E-6,'DQ404176.1_O_UKG_2001':1.0E-6)[&label=0.231316]:1.0E-6,'DQ404179.1_O_UKG_2001':1.0E-6)[&label=0.326531]:1.0E-6,(((('AJ539141.1_O_UKG_2001':5.24E-4,'DQ404180.1_O_UKG_2001':1.0E-6)[&label=0.774802]:1.0E-6,'DQ404175.1_O_UKG_2001':5.25E-4)[&label=0.446925]:1.0E-6,'DQ404177.1_O_UKG_2001':1.0E-6)[&label=0.304894]:1.0E-6,'FJ542372.1_O_UKG_2001':1.0E-6)[&label=0.239087]:1.0E-6)[&label=0.467758]:1.0E-6,(((((('DQ404172.1_O_UKG_2001':1.0E-6,'DQ404173.1_O_UKG_2001':1.0E-6)[&label=0.907738]:1.0E-6,'DQ404163.1_O_UKG_2001':0.004772)[&label=0.762897]:5.25E-4,'FJ542371.1_O_UKG_2001':0.001052)[&label=0.544312]:1.0E-6,(('AY593836.1_O_UKG_2001':1.0E-6,'AY593831.1_O_UKG_2002':1.0E-6)[&label=0.949405]:0.001052,'DQ404171.1_O_UKG_2001':5.25E-4)[&label=0.510913]:1.0E-6)[&label=0.33912]:1.0E-6,('DQ404164.1_O_UKG_2001':0.002108,'DQ404174.1_O_UKG_2001':5.25E-4)[&label=0.154762]:1.0E-6)[&label=0.298611]:1.0E-6,'KM257061.1_O_UKG_2001':1.0E-6)[&label=0.268078]:1.0E-6)[&label=0.688763]:1.0E-6,(('FJ542368.1_O_UKG_2001':1.0E-6,'KM257063.1_O_UKG_2001':1.0E-6)[&label=0.962302]:5.25E-4,('EF552696.1_O_UKG_2001':5.25E-4,'EF552688.1_O_UKG_2001':1.0E-6)[&label=0.982143]:0.002108)[&label=0.414352]:1.0E-6)[&label=0.805327]:1.0E-6,((((((((('DQ404167.1_O_UKG_2001':1.0E-6,'DQ404166.1_O_UKG_2001':1.0E-6)[&label=0.296627]:1.0E-6,'DQ404165.1_O_UKG_2001':1.0E-6)[&label=0.985615]:0.001582,('EF552692.1_O_UKG_2001':5.25E-4,'KM257064.1_O_UKG_2001':0.001058)[&label=0.899802]:0.001057)[&label=0.733383]:1.0E-6,'DQ404170.1_O_UKG_2001':1.0E-6)[&label=0.744246]:1.0E-6,'EF552693.1_O_UKG_2001':5.27E-4)[&label=0.813161]:5.27E-4,('EF552697.1_O_UKG_2001':0.001054,'EF552689.1_O_UKG_2001':0.001054)[&label=0.110119]:1.0E-6)[&label=0.671999]:1.0E-6,(('EU214601.1_O_UKG_2001':1.0E-6,'EF552690.1_O_UKG_2001':1.0E-6)[&label=0.265873]:1.0E-6,'DQ404169.1_O_UKG_2001':1.0E-6)[&label=0.608135]:1.0E-6)[&label=0.747655]:1.0E-6,('EF552695.1_O_UKG_2001':1.0E-6,'EF552691.1_O_UKG_2001':1.0E-6)[&label=0.969246]:0.001053)[&label=0.842796]:5.26E-4,(((('KM257065.1_O_UKG_2001':1.0E-6,'DQ404159.1_O_UKG_2001':5.26E-4)[&label=0.912698]:0.001053,('DQ404158.1_O_UKG_2001':0.001583,'DQ404160.1_O_UKG_2001':0.001054)[&label=0.500992]:1.0E-6)[&label=0.998016]:0.002654,'DQ404161.1_O_UKG_2001':0.001603)[&label=0.934772]:5.15E-4,('DQ404168.1_O_UKG_2001':1.0E-6,'DQ404162.1_O_UKG_2001':0.002115)[&label=0.656746]:5.26E-4)[&label=0.894511]:5.29E-4)[&label=0.922817]:0.001053)[&label=0.989087]:5.27E-4,'AJ539140.1_O_SAR_2000':5.25E-4)[&label=0.999897]:0.003624,'AB079061.1_O_JPN_2000':0.007512)[&label=0.997591]:0.001161,(((('MG372730.1_O_SKR_2000':0.002691,'AY593824.1_O_SKR_2000':5.27E-4)[&label=0.378968]:1.0E-6,'AJ539139.1_O_SKR_2000':1.0E-6)[&label=0.845238]:5.12E-4,'AH012985.2_O_SKR_2000':0.003732)[&label=0.998347]:0.006979,((('AJ539136.1_O_TAW_1999':5.26E-4,'AJ539137.1_O_TAW_1999':5.24E-4)[&label=0.97619]:0.001075,'HQ632768.1_O_MAY_2000':0.005308)[&label=0.845238]:9.94E-4,'AF377945.1_O_SKR_2000':0.028047)[&label=0.910714]:0.001124)[&label=0.580074]:5.07E-4)[&label=0.917241]:5.26E-4,('AJ539138.1_O_CHA_1999':1.0E-6,'AF506822.2_O_CHA_1999':0.001579)[&label=0.915675]:0.001053)[&label=0.905704]:4.21E-4,((((((((((('KF694742.1_O_SKR_2002':1.0E-6,'KF694740.1_O_SKR_2002':1.0E-6)[&label=0.098214]:1.0E-6,'KF694743.1_O_SKR_2002':1.0E-6)[&label=0.159226]:1.0E-6,'KF694745.1_O_SKR_2002':1.0E-6)[&label=0.258598]:1.0E-6,('KF694734.1_O_SKR_2002':1.0E-6,'KF694735.1_O_SKR_2002':1.0E-6)[&label=0.066468]:1.0E-6)[&label=0.56369]:1.0E-6,'KF694731.1_O_SKR_2002':1.0E-6)[&label=0.683201]:1.0E-6,('KF694739.1_O_SKR_2002':5.28E-4,'KF694737.1_O_SKR_2002':0.001058)[&label=0.146825]:1.0E-6)[&label=0.671503]:1.0E-6,'KF694736.1_O_SKR_2002':1.0E-6)[&label=0.815476]:1.0E-6,'KF694741.1_O_SKR_2002':5.28E-4)[&label=0.879464]:1.0E-6,('KF694744.1_O_SKR_2002':1.0E-6,'EF614457.1_O_SKR_2002':0.001057)[&label=0.983135]:0.002118)[&label=0.994957]:0.001718,(('KF694738.1_O_SKR_2002':5.25E-4,'KF694732.1_O_SKR_2002':5.3E-4)[&label=0.998016]:0.003079,'AH012984.2_O_SKR_2000':0.002244)[&label=0.878472]:0.001585)[&label=1.0]:0.009834,('HQ009509.1_O_CHA_1999':0.02683,'HM008917.1_O_CHA_2005':0.012131)[&label=1.0]:0.017946)[&label=0.997666]:0.006585)[&label=0.958101]:0.003781,(((((((('MF947127.1_O_VIT_2012':0.001667,'MF947137.1_O_VIT_2012':4.42E-4)[&label=1.0]:0.02498,'MF947123.1_O_VIT_2011':5.09E-4)[&label=0.700397]:5.28E-4,'KY234501.1_O_CHA_2011':0.003179)[&label=0.585979]:4.25E-4,(('MF947129.1_O_VIT_2014':0.022369,'MF947124.1_O_VIT_2012':0.005818)[&label=0.986111]:0.007161,'MF947126.1_O_VIT_2011':0.002606)[&label=0.811508]:0.00122)[&label=0.887566]:0.002757,'MF947128.1_O_VIT_2010':0.001582)[&label=0.889739]:1.0E-6,'MF947141.1_O_VIT_2012':0.010182)[&label=0.914807]:0.00248,((((('MF143578.1_O_VIT_2013':0.001593,'MF143577.1_O_VIT_2013':0.001584)[&label=0.996032]:0.004651,'MF143575.1_O_VIT_2012':0.002294)[&label=0.775794]:0.001752,'MF143576.1_O_VIT_2013':0.007736)[&label=0.983135]:0.007283,'MF143574.1_O_VIT_2012':0.001934)[&label=0.849454]:0.003503,('MF143573.1_O_VIT_2012':0.003048,'MF143572.1_O_VIT_2012':0.003341)[&label=0.954365]:0.001996)[&label=0.999339]:0.011612)[&label=0.857474]:0.004224,(((('MF947132.1_O_VIT_2015':0.011793,'KY234502.1_O_CHA_2015':0.016488)[&label=1.0]:0.012498,'MF947131.1_O_VIT_2013':0.002368)[&label=0.962302]:0.002446,'MF947143.1_O_VIT_2013':0.006439)[&label=0.951389]:8.34E-4,'MF947142.1_O_VIT_2013':0.004468)[&label=1.0]:0.011669)[&label=0.999653]:0.043292)[&label=0.955134]:0.00413,'JF749851.1_O_IRN_2001':0.02284)[&label=0.959907]:0.007771,((((((((((((((((('JX040487.1_O_BUL_2011':1.0E-6,'JX040490.1_O_BUL_2011':5.27E-4)[&label=0.718254]:5.27E-4,('JX040488.1_O_BUL_2011':1.0E-6,'JX040489.1_O_BUL_2011':1.0E-6)[&label=0.710317]:1.0E-6)[&label=0.925926]:1.0E-6,'JX040486.1_O_BUL_2011':0.001056)[&label=0.99752]:0.002645,(('JX066664.1_O_BUL_2011':5.27E-4,'JX066665.1_O_BUL_2011':5.27E-4)[&label=0.271825]:1.0E-6,'JX040485.1_O_BUL_2010':1.0E-6)[&label=0.806052]:1.0E-6)[&label=0.996032]:0.002115,'JX040496.1_O_TUR_2010':0.001586)[&label=0.985987]:0.001058,'JX040493.1_O_TUR_2010':0.001584)[&label=0.892416]:1.0E-6,(('JX040500.1_O_TUR_2011':0.002121,'JX040499.1_O_TUR_2011':5.23E-4)[&label=0.96131]:0.001589,'JX040494.1_O_TUR_2010':5.27E-4)[&label=0.568948]:1.0E-6)[&label=0.838542]:1.0E-6,'JX040491.1_O_TUR_2010':1.0E-6)[&label=0.918269]:1.0E-6,'JX040497.1_O_TUR_2010':0.001055)[&label=0.982426]:0.001055,'JX040495.1_O_TUR_2010':0.001583)[&label=0.960582]:1.0E-6,'JX040498.1_O_TUR_2010':0.001583)[&label=0.996776]:0.002091,'JX040492.1_O_TUR_2010':0.003746)[&label=0.993114]:0.001205,'JX040501.1_O_ISR_2011':0.005792)[&label=0.999008]:0.011068,(('MH784404.1_O_PAK_2017':0.001857,'MH784403.1_O_PAK_2016':0.002385)[&label=0.999008]:0.012436,'MH784405.1_O_PAK_2017':0.015174)[&label=1.0]:0.028283)[&label=1.0]:0.018663,(((((('FJ175666.1_O_ISR_2007':1.0E-6,'FJ175664.1_O_ISR_2007':1.0E-6)[&label=0.996032]:1.0E-6,'FJ175663.1_O_ISR_2007':0.003164)[&label=1.0]:0.004783,'FJ175665.1_O_ISR_2007':0.004242)[&label=0.973214]:1.0E-6,('FJ175662.1_O_ISR_2007':0.001587,'FJ175661.1_O_ISR_2007':0.001053)[&label=0.834325]:0.002083)[&label=0.945635]:0.002498,'KC440883.1_O_EGY_2011':0.035247)[&label=0.988095]:0.004415,'KM268895.1_O_TUR_2013':0.022403)[&label=0.985686]:0.003723)[&label=0.996682]:0.006859,(('GU384683.1_O_PAK_2008':1.0E-6,'GU384682.1_O_PAK_2008':1.0E-6)[&label=1.0]:0.006432,'HQ113232.1_O_PAK_2009':0.016534)[&label=1.0]:0.010576)[&label=0.990978]:0.004443,(('JF749852.1_O_MAY_2004':5.2E-4,'HQ632770.1_O_MAY_2004':0.001063)[&label=1.0]:0.005246,'HQ268524.1_O_BHU_2004':0.006567)[&label=0.830357]:0.002934)[&label=1.0]:0.020361)[&label=0.998773]:0.02278,((((((((((((((((((((((('MG983732.1_O_SRL_2014':6.34E-4,'MG983734.1_O_SRL_2014':0.003091)[&label=0.477183]:5.16E-4,'MG983733.1_O_SRL_2014':0.002028)[&label=0.985615]:0.00339,(('MG983740.1_O_VIT_2015':0.003186,'KY657269.1_O_VIT_2015':0.003711)[&label=0.982143]:9.64E-4,'MG983693.1_O_LAO_2015':0.004327)[&label=1.0]:0.008749)[&label=0.688889]:4.44E-4,((('KJ825806.1_O_IND_2013':0.005837,'KJ825809.1_O_IND_2013':0.003172)[&label=0.242063]:1.0E-6,('KJ825804.1_O_IND_2013':5.26E-4,'KJ825807.1_O_IND_2014':0.004776)[&label=0.077381]:1.0E-6)[&label=0.490079]:1.0E-6,'KJ825805.1_O_IND_2013':0.002112)[&label=0.646577]:5.27E-4)[&label=0.594048]:1.0E-6,(('MG983714.1_O_NEP_2013':0.007522,'MG983736.1_O_UAE_2014':0.003241)[&label=0.515873]:5.04E-4,'MG983715.1_O_NEP_2014':0.002652)[&label=0.539187]:5.3E-4)[&label=0.75931]:1.0E-6,'MG983716.1_O_NEP_2014':0.005302)[&label=0.827523]:5.27E-4,'MG983685.1_O_BAR_2015':0.011285)[&label=0.860582]:1.0E-6,(('MG983686.1_O_BAR_2015':0.00383,'MG983690.1_O_BHU_2016':0.005868)[&label=0.998016]:0.005443,'MG983731.1_O_SRL_2013':0.004224)[&label=0.895833]:0.001647)[&label=0.96164]:5.11E-4,'MG983711.1_O_NEP_2013':0.007006)[&label=0.975042]:0.001075,(('KJ825801.1_O_IND_2013':0.001053,'KJ825808.1_O_IND_2013':5.31E-4)[&label=0.955357]:0.002648,'KJ825803.1_O_IND_2013':0.003181)[&label=0.758433]:1.0E-6)[&label=1.0]:0.008686,'KJ206908.1_O_BHU_2013':0.007093)[&label=0.981453]:0.001602,('MG983708.1_O_NEP_2012':0.001062,'MG983709.1_O_NEP_2012':1.0E-6)[&label=1.0]:0.007995)[&label=0.938532]:5.73E-4,((((('KJ206909.1_O_LIB_2013':5.29E-4,'MG983694.1_O_LIB_2013':5.3E-4)[&label=0.62996]:0.001059,'MG983696.1_O_LIB_2013':0.004794)[&label=0.742063]:1.0E-6,('MG983695.1_O_LIB_2013':5.29E-4,'MG983697.1_O_LIB_2013':0.002122)[&label=0.123016]:1.0E-6)[&label=0.617063]:1.0E-6,(('MG983683.1_O_ALG_2014':0.001058,'MG983735.1_O_TUN_2014':1.0E-6)[&label=1.0]:0.001004,'KU291242.1_O_MOR_2015':0.008688)[&label=1.0]:0.004873)[&label=1.0]:0.00432,(((((('KJ206910.1_O_SAU_2013':1.0E-6,'MG983722.1_O_SAU_2013':5.29E-4)[&label=0.640873]:1.0E-6,'MG983721.1_O_SAU_2013':5.29E-4)[&label=0.995536]:0.002149,('MG983723.1_O_SAU_2013':5.32E-4,'MG983724.1_O_SAU_2013':0.001058)[&label=1.0]:0.004297)[&label=0.903274]:0.001041,'MG983725.1_O_SAU_2014':0.00425)[&label=0.82619]:1.0E-6,'MG983726.1_O_SAU_2014':0.007471)[&label=0.903439]:5.24E-4,'KJ825802.1_O_IND_2013':0.002126)[&label=0.956916]:0.001023)[&label=0.998942]:0.007074)[&label=0.94592]:5.64E-4,('MG983712.1_O_NEP_2013':0.001034,'MG983713.1_O_NEP_2013':0.00109)[&label=1.0]:0.013056)[&label=0.980136]:0.001608,'MG983717.1_O_NEP_2015':0.017491)[&label=0.998084]:0.017438,'MG983692.1_O_IRN_2009':0.009438)[&label=0.986596]:8.02E-4,'MG983688.1_O_BHU_2009':0.00535)[&label=0.973106]:1.0E-6,'MG983687.1_O_BHU_2009':0.00212)[&label=0.961352]:1.0E-6,'MG983706.1_O_NEP_2010':0.00534)[&label=0.971582]:5.28E-4,('MG983705.1_O_NEP_2010':0.001593,'MG983684.1_O_BAN_2009':0.003728)[&label=0.262897]:1.0E-6)[&label=0.992282]:0.00816,'MG983704.1_O_NEP_2008':0.008886)[&label=0.99076]:0.00572,'KF985189.1_O_BAN_2013':0.026659)[&label=0.985234]:0.006112,((((((((((('MG983720.1_O_RUS_2016':5.25E-4,'LC320038.1_O_MOG_2015':0.001588)[&label=0.668651]:5.29E-4,'MF461724.1_O_CHA_2017':0.002113)[&label=0.707341]:1.0E-6,'MG983730.1_O_SKR_2017':0.003708)[&label=0.999339]:0.003173,('LC438822.1_O_MYA_2016':0.003762,'MG983741.1_O_VIT_2016':0.004833)[&label=0.759921]:4.76E-4)[&label=0.899802]:1.0E-6,('MH891503.1_O_VIT_2017':0.007531,'LC438823.1_O_MYA_2016':0.006431)[&label=0.895833]:0.002106)[&label=0.98356]:0.002116,'KX712091.1_O_BAN_2015':0.001057)[&label=0.97247]:0.001607,'MG983719.1_O_NEP_2015':0.006415)[&label=0.920525]:5.09E-4,(((((('MG983699.1_O_MUR_2016':5.24E-4,'MG983701.1_O_MUR_2016':5.24E-4)[&label=0.079365]:1.0E-6,'MG983698.1_O_MUR_2016':1.0E-6)[&label=0.31002]:1.0E-6,'MG983700.1_O_MUR_2016':5.24E-4)[&label=0.784722]:1.0E-6,'MG983702.1_O_MUR_2016':5.24E-4)[&label=1.0]:0.005308,'MG983729.1_O_SAU_2016':0.005859)[&label=0.914087]:5.23E-4,('MG983703.1_O_MYA_2016':0.005835,'MG983718.1_O_NEP_2015':0.003172)[&label=0.265873]:1.0E-6)[&label=0.695295]:1.0E-6)[&label=0.875058]:5.5E-4,('MG983727.1_O_SAU_2015':0.002099,'MG983728.1_O_SAU_2016':0.003216)[&label=0.997024]:0.004281)[&label=0.904501]:9.09E-4,(('MG983738.1_O_UAE_2016':0.001052,'MG983739.1_O_UAE_2016':1.0E-6)[&label=1.0]:0.003744,'MG983691.1_O_BHU_2016':0.004272)[&label=1.0]:0.004983)[&label=0.999955]:0.016597,(('MG983707.1_O_NEP_2012':0.004854,'MG983710.1_O_NEP_2012':0.008128)[&label=0.938492]:6.28E-4,'MG983689.1_O_BHU_2012':0.003554)[&label=0.996032]:0.006387)[&label=0.997103]:0.010126)[&label=0.997278]:0.037106)[&label=0.994743]:0.018819,'KU726614.1_O_GRE_1994':0.080324)[&label=0.993088]:0.004982,('KT003716.1_O_PAK_2005':1.0E-6,'KY446903.1_O_PAK_2005':1.0E-6)[&label=1.0]:0.048553)[&label=0.992914]:0.008708,('KP940473.1_O_EGY_2014':0.043796,'AY593823.1_O_TUR_1969':0.028567)[&label=1.0]:0.029884)[&label=0.990134]:0.004103,(((((((((((((((((((((((((((((((((((((((('LC149701.1_O_JPN_2010':1.0E-6,'LC149695.1_O_JPN_2010':1.0E-6)[&label=0.027778]:1.0E-6,'LC149675.1_O_JPN_2010':1.0E-6)[&label=0.032738]:1.0E-6,'LC149623.1_O_JPN_2010':1.0E-6)[&label=0.031085]:1.0E-6,'LC149694.1_O_JPN_2010':1.0E-6)[&label=0.05878]:1.0E-6,'LC149635.1_O_JPN_2010':1.0E-6)[&label=0.078571]:1.0E-6,'LC149633.1_O_JPN_2010':1.0E-6)[&label=0.07705]:1.0E-6,'LC149628.1_O_JPN_2010':1.0E-6)[&label=0.085034]:1.0E-6,((((('LC149672.1_O_JPN_2010':1.0E-6,'LC149637.1_O_JPN_2010':1.0E-6)[&label=0.262897]:1.0E-6,'LC149636.1_O_JPN_2010':1.0E-6)[&label=0.745536]:1.0E-6,('LC149704.1_O_JPN_2010':5.26E-4,'LC149638.1_O_JPN_2010':5.26E-4)[&label=0.166667]:1.0E-6)[&label=0.766617]:5.25E-4,'LC149654.1_O_JPN_2010':5.27E-4)[&label=0.67123]:1.0E-6,('LC149650.1_O_JPN_2010':1.0E-6,'LC149688.1_O_JPN_2010':0.001582)[&label=0.477183]:1.0E-6)[&label=0.48852]:1.0E-6)[&label=0.232209]:1.0E-6,(('LC149664.1_O_JPN_2010':1.0E-6,'LC149671.1_O_JPN_2010':1.0E-6)[&label=0.016865]:1.0E-6,'LC149712.1_O_JPN_2010':1.0E-6)[&label=0.010913]:1.0E-6)[&label=0.198743]:1.0E-6,((((((('LC149669.1_O_JPN_2010':1.0E-6,'LC149644.1_O_JPN_2010':1.0E-6)[&label=0.815476]:1.0E-6,'LC149713.1_O_JPN_2010':0.001584)[&label=0.634921]:5.21E-4,'LC149639.1_O_JPN_2010':5.27E-4)[&label=0.494709]:1.0E-6,'LC149714.1_O_JPN_2010':0.001055)[&label=0.416171]:1.0E-6,'LC149632.1_O_JPN_2010':5.27E-4)[&label=0.452183]:1.0E-6,'LC149653.1_O_JPN_2010':1.0E-6)[&label=0.528108]:1.0E-6,'LC149645.1_O_JPN_2010':1.0E-6)[&label=0.61352]:1.0E-6)[&label=0.212912]:1.0E-6,(('LC149640.1_O_JPN_2010':5.26E-4,'LC149692.1_O_JPN_2010':0.001583)[&label=0.063492]:1.0E-6,('LC149620.1_O_JPN_2010':5.27E-4,'LC149709.1_O_JPN_2010':5.27E-4)[&label=0.012897]:1.0E-6)[&label=0.049272]:1.0E-6)[&label=0.191303]:1.0E-6,(((('LC149686.1_O_JPN_2010':1.0E-6,'LC149663.1_O_JPN_2010':5.27E-4)[&label=0.640873]:1.0E-6,'LC149689.1_O_JPN_2010':0.001054)[&label=0.880456]:0.001584,'LC149622.1_O_JPN_2010':5.27E-4)[&label=0.60086]:1.0E-6,('LC149717.1_O_JPN_2010':0.001054,'LC149715.1_O_JPN_2010':0.001054)[&label=0.036706]:1.0E-6)[&label=0.374603]:1.0E-6)[&label=0.174796]:1.0E-6,('LC149655.1_O_JPN_2010':5.27E-4,'LC149682.1_O_JPN_2010':0.001054)[&label=0.019841]:1.0E-6)[&label=0.172593]:1.0E-6,'LC149652.1_O_JPN_2010':1.0E-6)[&label=0.171347]:1.0E-6,(((((((((((((('LC149698.1_O_JPN_2010':1.0E-6,'LC149679.1_O_JPN_2010':1.0E-6)[&label=0.122024]:1.0E-6,'LC149711.1_O_JPN_2010':1.0E-6)[&label=0.217262]:1.0E-6,('LC149674.1_O_JPN_2010':1.0E-6,'LC149641.1_O_JPN_2010':1.0E-6)[&label=0.097222]:1.0E-6)[&label=0.55754]:1.0E-6,('LC149684.1_O_JPN_2010':5.27E-4,'LC149697.1_O_JPN_2010':5.27E-4)[&label=0.160714]:1.0E-6)[&label=0.586144]:1.0E-6,'LC149687.1_O_JPN_2010':1.0E-6)[&label=0.751984]:1.0E-6,'LC149651.1_O_JPN_2010':5.27E-4)[&label=0.809028]:4.18E-4,'LC149658.1_O_JPN_2010':5.27E-4)[&label=0.749118]:1.2E-5,'LC149678.1_O_JPN_2010':1.0E-6)[&label=0.701786]:1.0E-6,'LC149662.1_O_JPN_2010':1.0E-6)[&label=0.697691]:9.5E-5,'LC149683.1_O_JPN_2010':5.27E-4)[&label=0.642444]:1.0E-6,('LC149700.1_O_JPN_2010':1.0E-6,'LC149656.1_O_JPN_2010':1.0E-6)[&label=0.006944]:1.0E-6)[&label=0.551729]:1.0E-6,('LC149648.1_O_JPN_2010':5.26E-4,'LC149706.1_O_JPN_2010':5.26E-4)[&label=0.009921]:1.0E-6)[&label=0.486855]:1.0E-6,(('LC149702.1_O_JPN_2010':5.26E-4,'LC149720.1_O_JPN_2010':0.001055)[&label=0.02877]:1.0E-6,'LC149643.1_O_JPN_2010':1.0E-6)[&label=0.015873]:1.0E-6)[&label=0.414265]:1.0E-6,((('LC149618.1_O_JPN_2010':1.0E-6,'LC149624.1_O_JPN_2010':1.0E-6)[&label=0.859127]:0.001054,'LC149666.1_O_JPN_2010':1.0E-6)[&label=0.740079]:5.26E-4,'LC149681.1_O_JPN_2010':5.27E-4)[&label=0.499339]:1.0E-6)[&label=0.348818]:1.0E-6)[&label=0.395534]:1.0E-6,'LC149659.1_O_JPN_2010':1.0E-6)[&label=0.418728]:1.0E-6,'LC149625.1_O_JPN_2010':1.0E-6)[&label=0.441392]:1.0E-6,'LC149661.1_O_JPN_2010':1.0E-6)[&label=0.463519]:1.0E-6,('LC149680.1_O_JPN_2010':1.0E-6,'LC149705.1_O_JPN_2010':1.0E-6)[&label=0.010913]:1.0E-6)[&label=0.507324]:1.0E-6,(((('LC149718.1_O_JPN_2010':1.0E-6,'LC149634.1_O_JPN_2010':1.0E-6)[&label=0.997024]:0.002114,('LC149673.1_O_JPN_2010':1.0E-6,'LC149660.1_O_JPN_2010':0.001054)[&label=0.550595]:1.0E-6)[&label=0.865741]:0.001054,'LC149647.1_O_JPN_2010':1.0E-6)[&label=0.766121]:5.27E-4,(('LC149691.1_O_JPN_2010':5.27E-4,'LC149676.1_O_JPN_2010':1.0E-6)[&label=0.198413]:1.0E-6,'LC149657.1_O_JPN_2010':5.26E-4)[&label=0.593254]:5.26E-4)[&label=0.452239]:1.0E-6)[&label=0.651433]:1.0E-6,'LC149693.1_O_JPN_2010':1.0E-6)[&label=0.668561]:1.0E-6,(((((((('LC149677.1_O_JPN_2010':1.0E-6,'LC149710.1_O_JPN_2010':1.0E-6)[&label=0.098214]:1.0E-6,'LC149667.1_O_JPN_2010':1.0E-6)[&label=0.442956]:1.0E-6,('LC149685.1_O_JPN_2010':5.27E-4,'LC149646.1_O_JPN_2010':5.27E-4)[&label=0.095238]:1.0E-6)[&label=0.491319]:1.0E-6,('LC149642.1_O_JPN_2010':0.001055,'LC149708.1_O_JPN_2010':5.26E-4)[&label=0.222222]:1.0E-6)[&label=0.656581]:5.22E-4,'LC149649.1_O_JPN_2010':0.002113)[&label=0.595947]:3.0E-6,'LC149619.1_O_JPN_2010':1.0E-6)[&label=0.582589]:1.0E-6,'LC149665.1_O_JPN_2010':1.0E-6)[&label=0.62985]:1.0E-6,'LC149707.1_O_JPN_2010':5.27E-4)[&label=0.570437]:1.0E-6)[&label=0.827482]:1.0E-6,((((((((('LC149716.1_O_JPN_2010':1.0E-6,'LC036265.1_O_JPN_2010':0.001054)[&label=0.802579]:0.001054,'LC149629.1_O_JPN_2010':0.001054)[&label=0.472718]:1.0E-6,('KF112885.1_O_JPN_2010':0.001057,'LC149617.1_O_JPN_2010':5.26E-4)[&label=0.416667]:5.28E-4)[&label=0.346974]:1.0E-6,'LC149621.1_O_JPN_2010':1.0E-6)[&label=0.429762]:1.0E-6,'LC149631.1_O_JPN_2010':5.28E-4)[&label=0.533069]:5.23E-4,'LC149630.1_O_JPN_2010':1.0E-6)[&label=0.484977]:1.0E-6,'LC149626.1_O_JPN_2010':1.0E-6)[&label=0.4969]:1.0E-6,'LC149627.1_O_JPN_2010':1.0E-6)[&label=0.551257]:1.0E-6,((('LC149703.1_O_JPN_2010':1.0E-6,'LC149690.1_O_JPN_2010':1.0E-6)[&label=0.19246]:1.0E-6,'LC149696.1_O_JPN_2010':1.0E-6)[&label=0.764881]:1.0E-6,('LC149668.1_O_JPN_2010':0.001054,'LC149699.1_O_JPN_2010':5.27E-4)[&label=0.181548]:1.0E-6)[&label=0.671627]:5.26E-4)[&label=0.405187]:1.0E-6)[&label=0.983771]:1.0E-6,'LC149670.1_O_JPN_2010':5.27E-4)[&label=0.993418]:5.27E-4,'LC149719.1_O_JPN_2010':1.0E-6)[&label=0.998375]:0.002648,(((('KF501488.1_O_SKR_2010':0.002639,'KF501487.1_O_SKR_2010':5.26E-4)[&label=0.662698]:5.29E-4,'KR401160.1_O_SKR_2011':0.001054)[&label=1.0]:0.005924,'KF112888.1_O_DRK_2011':0.007015)[&label=0.911706]:4.58E-4,'KF112883.1_O_RUS_2010':0.005332)[&label=0.870536]:0.00106)[&label=0.970148]:1.0E-6,((('KC503937.1_O_SKR_2010':5.26E-4,'KF501486.1_O_SKR_2010':0.001582)[&label=0.349206]:1.0E-6,'KF112887.1_O_SKR_2010':1.0E-6)[&label=0.813492]:1.0E-6,'KR401159.1_O_SKR_2010':0.001054)[&label=0.997024]:0.003711)[&label=0.991585]:5.28E-4,'JN998085.1_O_CHA_2010':0.002643)[&label=0.994928]:5.54E-4,(((((('KX534089.1_O_SKR_2016':1.0E-6,'KY086465.1_O_SKR_2016':5.29E-4)[&label=1.0]:0.00708,'KY086466.1_O_SKR_2016':0.010354)[&label=0.998016]:0.003247,'KX162590.1_O_SKR_2014':4.64E-4)[&label=1.0]:0.013646,'KY322674.1_O_SKR_2014':0.015501)[&label=0.870784]:0.001845,'MH845413.2_O_VIT_2014':0.014586)[&label=1.0]:0.020136,'KF112889.1_O_HKN_2010':6.46E-4)[&label=0.958499]:0.0036)[&label=0.987762]:0.001574,(('KR401158.1_O_SKR_2010':5.28E-4,'KF112886.1_O_SKR_2010':1.0E-6)[&label=0.998016]:0.002657,'JQ973889.1_O_CHA_2010':0.003203)[&label=0.99504]:0.003183)[&label=0.987198]:5.46E-4,('JN998086.1_O_CHA_2010':0.003717,'HM055510.1_O_VIT_2009':0.001062)[&label=0.772817]:5.27E-4)[&label=0.98965]:4.75E-4,('JQ900581.1_O_CHA_2010':0.002664,'HM229661.1_O_HKN_2010':0.004265)[&label=0.985119]:0.004318)[&label=0.999862]:0.004324,(('KF112880.1_O_MYA_2009':1.0E-6,'KR401156.1_O_MYA_2009':0.001586)[&label=0.969246]:0.003744,('KR401153.1_O_MYA_2009':0.008582,'KR401152.1_O_MYA_2009':0.005354)[&label=0.65873]:5.31E-4)[&label=0.927579]:0.001604)[&label=0.999142]:0.001326,'KF112879.1_O_TAI_2009':0.00946)[&label=0.999919]:0.008431,'KR401157.1_O_MYA_2009':0.018641)[&label=1.0]:0.023138,'KY322672.1_O_MAY_2014':0.048968)[&label=0.996681]:0.01145,'KR401154.1_O_MYA_1998':0.013595)[&label=0.993946]:0.008228,((((((((('KF112881.1_O_MOG_2010':0.002114,'KF112884.1_O_RUS_2010':0.001584)[&label=0.882937]:5.15E-4,'KF112882.1_O_MOG_2010':0.001586)[&label=0.976687]:0.001588,'GU582115.1_O_VIT_2009':0.002107)[&label=0.878307]:1.0E-6,'GU582116.1_O_VIT_2009':0.002638)[&label=0.916915]:0.00289,(('KY322671.1_O_MAY_2014':5.04E-4,'MF947130.1_O_VIT_2014':0.003717)[&label=0.996032]:0.005878,'KY322670.1_O_LAO_2013':0.007082)[&label=0.998512]:0.022497)[&label=0.99915]:0.012494,'KY322673.1_O_MAY_2014':0.03503)[&label=0.989087]:0.002971,'HQ632772.1_O_MAY_2007':0.003328)[&label=0.998677]:0.012945,'KR401155.1_O_MYA_2007':0.002475)[&label=0.99881]:0.024631,((('GU125647.1_O_VIT_2006':1.0E-6,'GU125648.1_O_VIT_2006':1.0E-6)[&label=0.680556]:1.0E-6,'GU125649.1_O_VIT_2006':5.22E-4)[&label=1.0]:0.011734,'GU125650.1_O_VIT_2006':0.005472)[&label=1.0]:0.024541)[&label=0.991213]:0.010583)[&label=1.0]:0.042078,'HQ632769.1_O_MAY_2001':0.069541)[&label=0.999857]:0.025416,('AY593812.1_O_PHI_1958':0.047124,'AY593828.1_O_IND_1962':0.020264)[&label=0.992063]:0.014302)[&label=0.998259]:0.01188)[&label=0.986775]:0.013445,'AY593834.1_O_IRN_1966':0.065021)[&label=0.985797]:0.010473,(((('MH053317.1_O_UGA_1998':0.042281,'MH053312.1_O_ETH_2005':0.05569)[&label=1.0]:0.051398,'MH053315.1_O_SUD_1976':0.059479)[&label=0.773313]:0.010834,(('MH053314.1_O_ETH_2007':0.028073,'MH053311.1_O_ETH_2004':0.035359)[&label=1.0]:0.034773,'MH053313.1_O_ETH_2006':0.088453)[&label=0.804067]:0.014638)[&label=0.618056]:0.001997,'MH053316.1_O_UGA_1996':0.095894)[&label=0.624339]:0.006843)[&label=0.984194]:0.018271,((((('EF611987.1_O_UGA_2006':0.006984,'HM191257.1_O_UGA_2006':0.003411)[&label=0.835317]:0.004446,'KU821591.1_O_ZAM_2010':0.023376)[&label=1.0]:0.026408,'MH053318.1_O_UGA_2002':0.0158)[&label=0.842923]:0.004571,'FJ461344.1_O_UGA_2002':0.018925)[&label=0.895337]:0.004335,'FJ461345.1_O_UGA_2002':0.012089)[&label=1.0]:0.074238)[&label=0.99067]:0.016583,((((((((((((((((((((((((((('KJ560299.1_O_UKG_2007':1.0E-6,'KJ560307.1_O_UKG_2007':1.0E-6)[&label=0.022817]:1.0E-6,'EU448375.1_O_UKG_2007':1.0E-6)[&label=0.06002]:1.0E-6,('KJ560302.1_O_UKG_2007':1.0E-6,'EU448377.1_O_UKG_2007':1.0E-6)[&label=0.022817]:1.0E-6)[&label=0.180804]:1.0E-6,'KJ560285.1_O_UKG_2007':1.0E-6)[&label=0.258532]:1.0E-6,((('KJ560300.1_O_UKG_2007':1.0E-6,'KJ560294.1_O_UKG_2007':1.0E-6)[&label=0.020833]:1.0E-6,'KJ560304.1_O_UKG_2007':1.0E-6)[&label=0.030754]:1.0E-6,'KJ560303.1_O_UKG_2007':1.0E-6)[&label=0.064815]:1.0E-6)[&label=0.655423]:1.0E-6,('EU448376.1_O_UKG_2007':5.25E-4,'KJ560298.1_O_UKG_2007':0.002641)[&label=0.309524]:1.0E-6)[&label=0.640602]:1.0E-6,'KJ560281.1_O_UKG_2007':1.0E-6)[&label=0.688244]:1.0E-6,('EU448378.1_O_UKG_2007':5.26E-4,'KJ560297.1_O_UKG_2007':5.26E-4)[&label=0.058532]:1.0E-6)[&label=0.762755]:1.0E-6,'KJ560308.1_O_UKG_2007':5.26E-4)[&label=0.814749]:1.0E-6,'KJ560296.1_O_UKG_2007':5.25E-4)[&label=0.863591]:5.26E-4,'KJ560283.1_O_UKG_2007':0.001054)[&label=0.841912]:1.0E-6,(('EU448374.1_O_UKG_2007':1.0E-6,'KJ560287.1_O_UKG_2007':1.0E-6)[&label=0.9375]:5.26E-4,'EU448373.1_O_UKG_2007':5.26E-4)[&label=0.512401]:1.0E-6)[&label=0.807044]:1.0E-6,(('EU448371.1_O_UKG_2007':1.0E-6,'KJ560276.1_O_UKG_2007':1.0E-6)[&label=0.112103]:1.0E-6,'EU448372.1_O_UKG_2007':1.0E-6)[&label=0.520833]:1.0E-6)[&label=0.887638]:1.0E-6,'KJ560277.1_O_UKG_2007':5.26E-4)[&label=0.917576]:1.0E-6,'JX570643.1_O_UKG_2007':1.0E-6)[&label=0.928889]:0.001052,((((((((((('JX570655.1_O_UKG_2007':1.0E-6,'JX570650.1_O_UKG_2007':1.0E-6)[&label=0.292659]:1.0E-6,'JX570653.1_O_UKG_2007':1.0E-6)[&label=0.782738]:1.0E-6,('JX570654.1_O_UKG_2007':1.0E-6,'JX570645.1_O_UKG_2007':1.0E-6)[&label=0.928571]:5.25E-4)[&label=0.858631]:5.25E-4,'JX570649.1_O_UKG_2007':5.25E-4)[&label=0.758333]:1.0E-6,(('JX570646.1_O_UKG_2007':1.0E-6,'JX570640.1_O_UKG_2007':1.0E-6)[&label=0.069444]:1.0E-6,'JX570641.1_O_UKG_2007':1.0E-6)[&label=0.147817]:1.0E-6)[&label=0.494172]:1.0E-6,('JX570638.1_O_UKG_2007':1.0E-6,'JX570639.1_O_UKG_2007':1.0E-6)[&label=0.039683]:1.0E-6)[&label=0.536706]:1.0E-6,'JX570644.1_O_UKG_2007':1.0E-6)[&label=0.629509]:1.0E-6,('JX570642.1_O_UKG_2007':1.0E-6,'JX570647.1_O_UKG_2007':1.0E-6)[&label=0.027778]:1.0E-6)[&label=0.811889]:1.0E-6,('JX570652.1_O_UKG_2007':5.25E-4,'JX570651.1_O_UKG_2007':1.0E-6)[&label=0.723214]:5.25E-4)[&label=0.90959]:5.24E-4,'JX570648.1_O_UKG_2007':1.0E-6)[&label=0.927579]:1.0E-6,('EU448370.1_O_UKG_1967':1.0E-6,'EU448369.1_O_UKG_1967':1.0E-6)[&label=0.843254]:1.0E-6)[&label=0.951058]:5.26E-4)[&label=0.908099]:5.26E-4,'JX869180.1_O_UKG_1967':5.25E-4)[&label=0.903153]:1.0E-6,((((('JX869185.1_O_UKG_1968':0.001596,'JX869181.1_O_UKG_1967':0.00212)[&label=0.654762]:5.16E-4,'JX869184.1_O_UKG_1968':0.002107)[&label=0.43254]:1.0E-6,(('JX869179.1_O_UKG_1967':1.0E-6,'JX869182.1_O_UKG_1968':0.002106)[&label=0.645833]:5.25E-4,'JX869183.1_O_UKG_1968':0.002108)[&label=0.391369]:1.0E-6)[&label=0.315873]:1.0E-6,(('AY593816.1_O_UKG_1967':5.25E-4,'EU448368.1_O_UKG_1967':1.0E-6)[&label=0.651786]:5.25E-4,'JX869186.1_O_UKG_1968':0.001052)[&label=0.395337]:1.0E-6)[&label=0.306052]:1.0E-6,'AY593815.1_O_UKG_1967':1.0E-6)[&label=0.318011]:1.0E-6)[&label=0.968867]:1.0E-6,('JX869188.1_O_UKG_1968':1.0E-6,'JX869187.1_O_UKG_1968':1.0E-6)[&label=0.912698]:5.25E-4)[&label=0.995701]:0.004238,('AY593814.1_O_ARG_1965':0.004295,'AY593817.1_O_Belgium_1973':0.006452)[&label=0.531746]:4.82E-4)[&label=0.983034]:5.3E-4,((('JX869177.1_O_UKG_1967':0.003232,'JX869178.1_O_UKG_1967':0.012504)[&label=0.956349]:0.003234,'AY593830.1_O_POL_1959':0.003741)[&label=0.770833]:5.11E-4,'AY593820.1_O_ARG_1964':0.001056)[&label=0.734127]:5.28E-4)[&label=0.968081]:1.0E-6,'AY593818.1_O_ARG_1958':0.003174)[&label=0.979058]:5.28E-4,'AY593837.1_O_URU_1963':0.001056)[&label=0.987012]:5.26E-4,'AY593819.1_O_ARG_1994':1.0E-6)[&label=0.996453]:0.065525,'AY593821.1_O_ARG_1967':0.09526)[&label=0.98885]:0.010669,(('NC_039210.1_O_UKG_1965':0.050617,'AY593825.1_O_ARG_1939':0.036749)[&label=0.977183]:0.017975,('AY593826.1_O_ITL_1947':0.025942,'AY593827.1_O_VEN_1971':0.022372)[&label=1.0]:0.037386)[&label=0.843915]:0.013711)[&label=0.988752]:0.036285,'AY593813.1_O_ISA_1962':0.114713)[&label=0.981385]:0.02196)[&label=0.974919]:0.04229,'KY072818.1_O_CHA_1959':0.058026)[&label=0.976647]:0.034383,(((((((('AF308157.1_O_TAW_1997':0.002144,'AY593835.1_O_TAW_1997':1.0E-6)[&label=0.853175]:0.001603,'AY593833.1_O_TAW_1999':0.002679)[&label=0.887897]:1.0E-6,'AF026168.2_O_TAW_1997':0.004847)[&label=0.922619]:0.001005,'AF154271.1_O_TAW_1997':0.002219)[&label=1.0]:0.01182,'HQ412603.1_O_CHA_2000':0.034817)[&label=0.995833]:0.009934,'AY686687.1_O_CHA_2001':0.030284)[&label=0.993221]:0.012852,'HQ632771.1_O_MAY_2005':0.07559)[&label=0.988662]:0.009625,(('AY317098.1_O_CHA_2002':0.005122,'EU400597.1_O_CHA_2001':0.010378)[&label=1.0]:0.017467,('KU204893.1_O_CHA_2013':0.021184,'KU204894.1_O_CHA_2013':0.018558)[&label=1.0]:0.052763)[&label=0.997354]:0.023188)[&label=0.994048]:0.056473)[&label=1.0]:0.394457,((((((((((((('DQ989310.1_Asia1_IND_1999':0.011944,'DQ989311.1_Asia1_IND_2002':0.025285)[&label=0.631944]:0.004151,('MF372125.1_Asia1_IND_2016':0.057203,'MF782478.1_Asia1_BAN_2013':0.030773)[&label=0.99504]:0.020734)[&label=0.999008]:0.020539,(('DQ989309.1_Asia1_IND_1996':0.003434,'MF372126.1_Asia1_IND_1994':0.005226)[&label=0.997024]:0.007411,'DQ989308.1_Asia1_IND_1994':0.018737)[&label=0.922123]:0.003627)[&label=0.962467]:0.005251,'AY687333.1_Asia1_IND_2001':0.037019)[&label=0.980584]:0.007478,(('HQ113233.1_Asia1_AFG_2009':0.015661,'JF749849.1_Asia1_PAK_2002':0.015122)[&label=0.474206]:0.008118,'EF149010.1_Asia1_CHA_2005':0.011033)[&label=0.999504]:0.021486)[&label=0.996528]:0.015445,((((((('DQ989320.1_Asia1_IND_2002':0.01588,'DQ989323.1_Asia1_IND_2002':0.007387)[&label=0.957341]:0.004424,'DQ989322.1_Asia1_IND_2002':0.028578)[&label=0.766369]:5.72E-4,'DQ989318.1_Asia1_IND_2002':0.006719)[&label=0.632606]:1.0E-6,('DQ989319.1_Asia1_IND_2001':0.012472,'DQ989321.1_Asia1_IND_2001':0.009844)[&label=0.258929]:9.79E-4)[&label=0.887103]:8.3E-4,'DQ989314.1_Asia1_IND_2001':0.003807)[&label=0.973876]:0.003292,'DQ989317.1_Asia1_IND_2000':0.007843)[&label=1.0]:0.040521,'DQ989315.1_Asia1_IND_1993':0.032897)[&label=0.992312]:0.010977)[&label=0.997807]:0.019034,(('DQ989305.1_Asia1_IND_1990':0.004609,'DQ989307.1_Asia1_IND_1992':0.00297)[&label=0.805556]:0.005787,'DQ989312.1_Asia1_IND_1990':0.044952)[&label=0.999504]:0.01713)[&label=0.998106]:0.026679,(('KY825718.1_Asia1_ISR_1989':1.0E-6,'AY593800.1_Asia1_LEB_1983':1.0E-6)[&label=0.329365]:1.0E-6,'AY593799.1_Asia1_LEB_1983':1.0E-6)[&label=1.0]:0.049837)[&label=0.997381]:0.028093,((('JN006719.1_Asia1_PAK_2008':0.003614,'JN006720.1_Asia1_PAK_2009':0.014664)[&label=0.99504]:0.011136,'KM268898.1_Asia1_TUR_2013':0.030171)[&label=0.990079]:0.065424,'DQ989313.1_Asia1_IND_1986':0.045492)[&label=0.923942]:0.033275)[&label=0.962746]:0.023101,(((((('GQ452295.1_Asia1_VIT_2007':1.0E-6,'GU125645.1_Asia1_VIT_2007':0.001605)[&label=1.0]:0.004867,('FJ906802.1_Asia1_CHA_2006':0.002678,'HQ631363.1_Asia1_CHA_2006':1.0E-6)[&label=0.755952]:5.2E-4)[&label=0.948743]:0.001081,'EF149009.1_Asia1_CHA_2005':0.002142)[&label=0.818204]:1.0E-6,((('KC412634.1_Asia1_CHA_2006':5.32E-4,'KC462884.1_Asia1_CHA_2006':5.32E-4)[&label=0.977183]:1.0E-6,'KU360085.1_Asia1_CHA_2015':0.002132)[&label=1.0]:0.007017,'GU931682.1_Asia1_CHA_2005':1.0E-6)[&label=0.999008]:0.003213)[&label=0.957217]:0.001346,'KY446901.1_Asia1_PAK_2006':0.00187)[&label=1.0]:0.055939,((('DQ989304.1_Asia1_IND_2000':5.23E-4,'DQ989303.1_Asia1_IND_1993':0.001088)[&label=0.990079]:0.002125,'DQ989306.1_Asia1_IND_1986':0.017035)[&label=0.99504]:0.046765,('AY593796.1_Asia1_ISR_1963':0.024166,'AY593797.1_Asia1_ISR_1963':0.022111)[&label=0.999008]:0.01619)[&label=0.912946]:0.013578)[&label=0.989938]:0.029485)[&label=0.974432]:0.015367,'AY593795.1_Asia1_PAK_1954':0.0852)[&label=0.984127]:0.029732,('HQ632774.1_Asia1_MAY_1999':0.054627,'GU125646.1_Asia1_VIT_2005':0.046569)[&label=0.997024]:0.036371)[&label=1.0]:0.402423,((((((('KM268897.1_C_KEN_2004':0.003157,'MH053309.1_C_KEN_1967':5.57E-4)[&label=1.0]:0.021799,'MH053308.1_C_ETH_1971':0.035195)[&label=0.958829]:0.018963,'MH053310.1_C_UGA_1970':0.049572)[&label=1.0]:0.061638,'AY593810.1_C_UKG_1970':0.053151)[&label=0.945188]:0.034514,'AY593806.1_C_Brazil_1971':0.065634)[&label=0.820833]:0.01019,('AY593809.1_C_ARG_1969':0.015203,'AY593807.1_C_Brazil_1955':0.002961)[&label=0.999008]:0.04054)[&label=0.758362]:0.021587,((('AY593805.1_C_GER_1960':5.38E-4,'AY593804.1_C_SWZ_1965':0.001065)[&label=0.991071]:0.00195,'FJ824812.1_C_SPA_2009':0.011941)[&label=0.99504]:0.028668,'AY593808.1_C_ARG_1966':0.032242)[&label=0.915675]:0.008187)[&label=1.0]:0.408022)[&label=0.875908]:0.079535)[&label=0.889573]:0.049039,(((((((((((((((((((((('KX002203.1_A_ARG_2001':1.0E-6,'AY593784.1_A_ARG_2001':1.0E-6)[&label=0.732143]:1.0E-6,'AY593785.1_A_ARG_2001':5.3E-4)[&label=0.853671]:5.3E-4,'AY593802.1_A_URU_2001':0.001594)[&label=0.703704]:1.0E-6,'KX002179.1_A_ARG_2001':0.002666)[&label=0.815724]:5.29E-4,'KX002195.1_A_ARG_2001':1.0E-6)[&label=0.756151]:1.0E-6,'KX002181.1_A_ARG_2001':0.003735)[&label=0.661376]:1.0E-6,(((('KX002200.1_A_ARG_2001':0.001596,'KX002201.1_A_ARG_2001':0.001061)[&label=0.159722]:1.0E-6,'KX002188.1_A_ARG_2001':0.004815)[&label=0.707837]:5.3E-4,'KX002177.1_A_ARG_2001':0.002127)[&label=0.564484]:1.0E-6,('KX002190.1_A_ARG_2001':0.00213,'KX002186.1_A_ARG_2001':0.001595)[&label=0.166667]:1.0E-6)[&label=0.770437]:5.3E-4)[&label=0.856812]:1.0E-6,'KX002185.1_A_ARG_2001':5.3E-4)[&label=0.93605]:0.001061,(('KX002199.1_A_ARG_2001':0.002658,'KX002194.1_A_ARG_2001':5.3E-4)[&label=0.727183]:5.35E-4,'KX002202.1_A_ARG_2001':0.00643)[&label=0.888393]:0.001059)[&label=0.922247]:5.32E-4,'KX002197.1_A_ARG_2001':0.004268)[&label=0.890815]:1.0E-6,((((((('KX002189.1_A_ARG_2001':0.00203,'KX002192.1_A_ARG_2001':0.002781)[&label=0.522817]:5.05E-4,'KX002187.1_A_ARG_2001':0.004723)[&label=0.970734]:0.001196,'KX002183.1_A_ARG_2001':0.004818)[&label=0.878968]:5.19E-4,('KX002205.1_A_ARG_2001':1.0E-6,'AY593786.1_A_ARG_2001':1.0E-6)[&label=0.988095]:0.001592)[&label=0.659524]:1.0E-6,'KX002198.1_A_ARG_2001':1.0E-6)[&label=0.562335]:1.0E-6,((('KX002176.1_A_ARG_2001':1.0E-6,'KX002180.1_A_ARG_2001':1.0E-6)[&label=0.638889]:1.0E-6,('KX002182.1_A_ARG_2001':5.3E-4,'KX002184.1_A_ARG_2001':0.00106)[&label=0.27381]:1.0E-6)[&label=0.752315]:5.3E-4,('AY593790.1_A_ARG_2001':1.0E-6,'KX002204.1_A_ARG_2001':1.0E-6)[&label=0.939484]:0.001062)[&label=0.575595]:1.0E-6)[&label=0.961971]:5.29E-4,'KX002178.1_A_ARG_2001':0.002134)[&label=0.985958]:0.00213)[&label=0.995936]:0.004499,('KX002191.1_A_ARG_2001':0.011304,'KX002193.1_A_ARG_2001':0.003955)[&label=0.877976]:0.002107)[&label=0.993356]:0.004626,'KX002196.1_A_ARG_2001':0.003345)[&label=1.0]:0.064841,'AY593782.1_A_ARG_2000':0.090682)[&label=1.0]:0.043686,(('AY593775.1_A_VEN_1970':1.0E-6,'AY593773.1_A_PER_1969':1.0E-6)[&label=1.0]:0.03993,'AY593793.1_A_PHI_1975':0.052155)[&label=0.980655]:0.015527)[&label=0.998042]:0.020711,(((((((((((('MH559796.1_A_Brazil_2016':1.0E-6,'MH559801.1_A_Brazil_2016':1.0E-6)[&label=0.760913]:1.0E-6,'MH559781.1_A_Brazil_2016':5.3E-4)[&label=0.772817]:5.3E-4,'AY593768.1_A_Brazil_1955':0.001597)[&label=0.617063]:1.0E-6,'MH559798.1_A_Brazil_2016':5.3E-4)[&label=0.512649]:1.0E-6,'MH559786.1_A_Brazil_2016':1.0E-6)[&label=0.475198]:1.0E-6,'MH559788.1_A_Brazil_2016':1.0E-6)[&label=0.543651]:1.0E-6,((((('MH559800.1_A_Brazil_2016':1.0E-6,'MH559805.1_A_Brazil_2016':1.0E-6)[&label=0.144841]:1.0E-6,'MH559793.1_A_Brazil_2016':1.0E-6)[&label=0.309524]:1.0E-6,('MH559791.1_A_Brazil_2016':1.0E-6,'MH559804.1_A_Brazil_2016':1.0E-6)[&label=0.115079]:1.0E-6)[&label=0.90005]:1.0E-6,'MH559780.1_A_Brazil_2016':5.3E-4)[&label=0.875198]:1.0E-6,'MH559785.1_A_Brazil_2016':0.001064)[&label=0.865741]:5.3E-4)[&label=0.937653]:4.9E-4,('MH559799.1_A_Brazil_2016':0.001069,'MH559783.1_A_Brazil_2016':0.001054)[&label=0.85119]:0.001114)[&label=1.0]:0.018434,'AY593794.1_A_COL_1985':0.102413)[&label=0.992808]:0.012156,'AY593756.1_A_Brazil_1959':0.04135)[&label=0.989146]:0.010633,'AY593770.1_A_ARG_1966':0.079451)[&label=0.96875]:0.005735,(('AY593788.1_A_Brazil_1979':0.012331,'AY593787.1_A_Brazil_1977':0.002346)[&label=1.0]:0.021976,'AY593803.1_A_Brazil_1979':0.022988)[&label=1.0]:0.058964)[&label=0.998347]:0.016911)[&label=0.997999]:0.027404,(('AY593758.1_A_VEN_1967':0.001046,'AY593753.1_A_Brazil_1970':5.55E-4)[&label=1.0]:0.020032,'AY593757.1_A_Brazil_1967':0.013885)[&label=1.0]:0.061879)[&label=0.994914]:0.014911,(((((((('AY593778.1_A_SPA_1969':5.35E-4,'AY593754.1_A_SPA_1959':1.0E-6)[&label=1.0]:0.012067,'AY593780.1_A_FRA_1960':0.003361)[&label=0.962302]:0.004172,'AY593781.1_A_GER_1951':0.002232)[&label=0.958995]:0.003109,'AY593792.1_A_ITL_1962':0.008752)[&label=0.981399]:0.006896,'AY593760.1_A_USSR_1964':0.029238)[&label=0.941468]:0.005752,'AY593767.1_A_ARG_1965':0.048943)[&label=0.99537]:0.018185,'AY593771.1_A_COL_1967':0.056575)[&label=1.0]:0.042691,(((('AY593777.1_A_GER_1972':1.0E-6,'AY593779.1_A_GER_1972':1.0E-6)[&label=0.994048]:0.002099,'AY593774.1_A_SPA_1969':5.65E-4)[&label=0.991071]:0.008797,('AY593759.1_A_GER_1971':0.022366,'AY593776.1_A_GER_1968':0.035909)[&label=0.785714]:0.014963)[&label=0.894841]:0.008361,'AY593751.1_A_NET_1942':0.023578)[&label=1.0]:0.041332)[&label=0.865385]:0.013466)[&label=0.988495]:0.00788,('AY593789.1_A_ARG_1961':1.0E-6,'AY593769.1_A_ARG_1959':5.33E-4)[&label=1.0]:0.088795)[&label=0.997124]:0.046882,((((((((('MG923579.1_A_ALG_2017':0.001612,'MG913340.1_A_ALG_2017':5.36E-4)[&label=0.636905]:5.38E-4,'MG923580.1_A_ALG_2017':1.0E-6)[&label=0.999504]:0.01077,(('MG725875.1_A_NIG_2015':0.001076,'MG725876.1_A_NIG_2015':1.0E-6)[&label=1.0]:0.002828,'MG725873.1_A_NIG_2015':0.001495)[&label=0.994544]:0.004857)[&label=1.0]:0.035214,('MG725872.1_A_NIG_2013':0.028626,'MG725874.1_A_NIG_2015':0.035563)[&label=0.678571]:0.008404)[&label=1.0]:0.041284,('KC440881.1_A_EGY_2011':1.0E-6,'KP940474.1_A_EGY_2014':1.0E-6)[&label=1.0]:0.080449)[&label=1.0]:0.04827,'MH053306.1_A_TCH_1973':0.051942)[&label=0.944048]:0.012824,(('JF749843.1_A_EGY_2006':0.118135,'MH053305.1_A_EGY_1972':0.074141)[&label=0.281746]:0.005281,'AY593761.1_A_KEN_1964':0.050227)[&label=0.579365]:0.010604)[&label=0.981303]:0.01598,'MH053307.1_A_ZAM_1990':0.108423)[&label=0.995961]:0.024046,'AY593766.1_A_KEN_1965':0.120906)[&label=0.987765]:0.019095)[&label=0.976671]:0.038505,((((((((((((('GQ406252.1_A_VIT_2009':0.002712,'GQ406251.1_A_VIT_2009':0.007061)[&label=0.756944]:5.04E-4,'GQ406249.1_A_VIT_2009':0.001068)[&label=0.731151]:1.0E-6,(('GQ406247.1_A_VIT_2009':0.001066,'GQ406248.1_A_VIT_2009':1.0E-6)[&label=0.948413]:0.0016,'GQ406250.1_A_VIT_2009':0.001605)[&label=0.729167]:5.35E-4)[&label=0.966071]:0.001569,'KC588943.1_A_SKR_2010':0.009335)[&label=1.0]:0.020229,'HQ632773.1_A_MAY_2007':0.012871)[&label=0.994189]:0.008323,(('KY322677.1_A_MAY_2013':0.002694,'KY322675.1_A_LAO_2014':0.004307)[&label=0.956349]:0.003156,('KY322679.1_A_TAI_2014':0.002118,'KY322680.1_A_VIT_2013':0.001625)[&label=0.72123]:0.001231)[&label=1.0]:0.044443)[&label=0.852273]:0.001051,'HQ268509.2_A_VIT_2004':0.01762)[&label=0.890708]:0.003092,((('KY322678.1_A_MAY_2013':0.029745,'KT968663.1_A_CHA_2013':0.019238)[&label=0.874008]:0.004895,'KY322676.1_A_MAY_2013':0.02132)[&label=0.860119]:0.002964,'KJ608371.1_A_VIT_2013':0.01874)[&label=1.0]:0.031591)[&label=0.995722]:0.016113,'KJ933864.1_A_MAY_1997':0.033673)[&label=1.0]:0.075755,'HM854022.1_A_IND_1977':0.050436)[&label=0.960207]:0.009474,((((((((('JN099695.1_A_IRQ_2009':1.0E-6,'JN099694.1_A_IRQ_2009':1.0E-6)[&label=0.999008]:0.00322,'JN099688.1_A_IRQ_2009':0.002684)[&label=0.845734]:0.001068,('JN099697.1_A_IRQ_2009':1.0E-6,'JN099699.1_A_IRQ_2009':1.0E-6)[&label=0.974206]:1.0E-6)[&label=0.733135]:1.0E-6,'JN099698.1_A_IRQ_2009':0.002669)[&label=0.893254]:1.68E-4,'KC440882.1_A_EGY_2012':0.011281)[&label=0.990906]:0.014903,(((('EF117837.1_A_PAK_2006':0.002134,'EF494487.1_A_PAK_2006':1.0E-6)[&label=1.0]:0.008866,('EF494486.1_A_TUR_2005':0.005306,'JF749841.1_A_TUR_2006':0.0061)[&label=0.688492]:0.001387)[&label=0.986111]:0.010714,'EF494488.1_A_PAK_2006':0.004792)[&label=0.882937]:0.003354,'JN006722.1_A_PAK_2008':0.025429)[&label=0.807738]:0.00379)[&label=0.951472]:0.008503,'KM268896.1_A_TUR_2013':0.052116)[&label=0.999924]:0.074142,'KY446902.1_A_PAK_2005':0.043566)[&label=0.990859]:0.019991,(('HQ832577.1_A_IND_1999':0.003062,'HM854023.1_A_IND_1999':0.01459)[&label=1.0]:0.079709,'HQ832576.1_A_IND_1990':0.047505)[&label=0.96131]:0.023474)[&label=0.914683]:0.014201)[&label=0.710703]:0.005889,((('AY593765.1_A_TUR_1965':0.005187,'FJ623456.1_A_KAZ_1999':0.00939)[&label=0.915675]:0.002105,'AY593764.1_A_IRQ_1970':0.006159)[&label=0.995536]:0.011869,'AY593772.1_A_TUR_1972':0.02699)[&label=1.0]:0.052626)[&label=0.740774]:0.010267,((((((('HQ832579.1_A_IND_2003':0.004254,'HQ832578.1_A_IND_2003':0.001595)[&label=0.544643]:5.32E-4,('HQ832583.1_A_IND_2005':0.008186,'HQ832580.1_A_IND_2003':0.008184)[&label=0.335317]:3.88E-4)[&label=0.69246]:5.33E-4,'HQ832582.1_A_IND_2004':0.012431)[&label=0.908234]:0.00234,'HQ832581.1_A_IND_2004':0.010655)[&label=0.999802]:0.011361,((('HQ832592.1_A_IND_2009':0.011658,'HQ832590.1_A_IND_2007':0.006315)[&label=0.78869]:0.007954,'HQ832591.1_A_IND_2008':0.0126)[&label=0.978671]:0.009421,('KJ754939.1_A_BAN_2013':0.014421,'KU127247.1_A_SAU_2015':0.036462)[&label=1.0]:0.031081)[&label=0.932292]:0.013871)[&label=1.0]:0.034327,((((('HQ832584.1_A_IND_2005':0.043952,'HM854021.1_A_IND_2000':0.016541)[&label=0.77877]:0.005722,'HQ832586.1_A_IND_2006':0.054753)[&label=0.570437]:0.00293,'HQ832587.1_A_IND_2005':0.04544)[&label=0.740079]:0.005686,'HQ832585.1_A_IND_2005':0.049965)[&label=0.691716]:0.005135,('HQ832588.1_A_IND_2005':0.004892,'HQ832589.1_A_IND_2006':0.014147)[&label=1.0]:0.029444)[&label=0.916501]:0.012793)[&label=1.0]:0.047867,('JF749848.1_A_TUR_2003':0.037935,'AY593791.1_A_IRN_1998':0.019646)[&label=1.0]:0.061006)[&label=0.99269]:0.023698)[&label=0.964732]:0.035287)[&label=0.99827]:0.04193,'AY593755.1_A_TAI_1960':0.05029)[&label=1.0]:0.263497)[&label=1.0]:0.684809,((((((((('MH053329.1_SAT2_BOT_1969':0.055036,'KU821592.1_SAT2_ZAM_2009':0.082603)[&label=0.75496]:0.020227,'MH053328.1_SAT2_BOT_1968':0.068512)[&label=0.99256]:0.025696,('MH053334.1_SAT2_ZAM_1964':0.077031,'AY593847.1_SAT2_ZIM_1948':0.067013)[&label=0.999008]:0.032736)[&label=1.0]:0.057804,(('JF749861.1_SAT2_KEN_2002':0.063103,'KM268900.1_SAT2_TAN_2012':0.058803)[&label=0.90377]:0.017492,'MH053333.1_SAT2_ETH_1989':0.091511)[&label=1.0]:0.047441)[&label=0.810516]:0.017953,((('AY593848.1_SAT2_u_1967':0.05285,'MH053335.1_SAT2_ZIM_1965':0.072197)[&label=0.991071]:0.03039,'KR108949.1_SAT2_SAR_2009':0.073665)[&label=1.0]:0.047053,'JF749864.1_SAT2_ZIM_2003':0.12078)[&label=0.965939]:0.028453)[&label=0.973936]:0.046031,(('MH053331.1_SAT2_BOT_1972':0.02017,'MH053330.1_SAT2_BOT_1969':0.015574)[&label=0.999008]:0.050216,'MH053332.1_SAT2_BOT_1974':0.049574)[&label=1.0]:0.089737)[&label=1.0]:0.102945,((((('MH053336.1_SAT2_UGA_1970':0.043548,'HM067704.1_SAT2_UGA_2007':0.13067)[&label=0.485119]:0.021266,('JF749862.1_SAT2_UGA_2002':0.040325,'HM067705.1_SAT2_UGA_2007':0.047477)[&label=1.0]:0.047854)[&label=0.882937]:0.03465,'MH053337.1_SAT2_UGA_1970':0.102953)[&label=0.971726]:0.052036,'AY593849.1_SAT2_KEN_1960':0.117799)[&label=1.0]:0.122907,((('JX014255.1_SAT2_EGY_2012':0.00268,'JX014256.1_SAT2_PAT_2012':0.002682)[&label=0.613095]:8.86E-4,'KC440884.1_SAT2_EGY_2012':0.001797)[&label=1.0]:0.101763,'FJ461346.1_SAT2_UGA_2002':0.144513)[&label=1.0]:0.126952)[&label=0.995591]:0.091292)[&label=1.0]:0.527798,((((((((((('MH053348.1_SAT3_ZIM_1977':0.002682,'MH053346.1_SAT3_ZIM_1976':0.001621)[&label=1.0]:0.037508,'KX375417.1_SAT3_ZIM_1981':0.041295)[&label=1.0]:0.044773,'MH053344.1_SAT3_ZIM_1974':0.091338)[&label=0.797619]:0.016225,'AY593850.1_SAT3_SAR_1959':0.07086)[&label=0.754216]:0.019152,'KR108950.1_SAT3_SAR_2009':0.094146)[&label=0.654167]:0.010412,((('MH053345.1_SAT3_ZIM_1975':0.004643,'MH053347.1_SAT3_ZIM_1976':0.015175)[&label=1.0]:0.026528,('MH053350.1_SAT3_ZIM_1983':5.38E-4,'MH053349.1_SAT3_ZIM_1983':1.0E-6)[&label=1.0]:0.044017)[&label=0.898479]:0.010188,'KM268901.1_SAT3_ZIM_1991':0.060944)[&label=0.94122]:0.015305)[&label=1.0]:0.087051,'MH053343.1_SAT3_ZIM_1934':0.145368)[&label=0.930195]:0.02229,('MH053352.1_SAT3_ZIM_1990':0.097854,'MH053340.1_SAT3_MAL_1976':0.092174)[&label=1.0]:0.114193)[&label=0.866377]:0.029826,(((('MH053338.1_SAT3_BOT_1966':0.010617,'AY593853.1_SAT3_BOT_1965':0.003123)[&label=1.0]:0.052218,'MH053339.1_SAT3_BOT_1970':0.046039)[&label=0.979167]:0.026001,'MH053351.1_SAT3_ZIM_1984':0.067583)[&label=0.861111]:0.025284,('AY593852.1_SAT3_KEN_1960':1.0E-6,'AY593851.1_SAT3_BOT_1961':0.001597)[&label=1.0]:0.066456)[&label=1.0]:0.094205)[&label=0.96768]:0.044066,'MH053342.1_SAT3_ZAM_1996':0.151882)[&label=0.999901]:0.189201,('MH053341.1_SAT3_UGA_1970':0.14687,'KJ820999.1_SAT3_UGA_2013':0.11567)[&label=1.0]:0.302273)[&label=0.99982]:0.297692)[&label=0.667933]:0.088486,((((((('KR108948.1_SAT1_SAR_2009':0.064028,'MH053321.1_SAT1_MOZ_1981':0.065553)[&label=0.96131]:0.022024,('AY593839.1_SAT1_UKG_1970':0.038601,'AY593846.1_SAT1_ZIM_1966':0.075204)[&label=1.0]:0.039556)[&label=1.0]:0.089774,(('JF749860.1_SAT1_KEN_2002':0.070559,'MH053320.1_SAT1_KEN_1983':0.059074)[&label=0.441468]:0.013017,'KM268899.1_SAT1_TAN_2012':0.05237)[&label=1.0]:0.113757)[&label=0.996528]:0.054136,(('MH053327.1_SAT1_UGA_1970':0.066236,'HM067706.1_SAT1_UGA_2007':0.143585)[&label=0.888889]:0.028095,'MH053326.1_SAT1_UGA_1970':0.125633)[&label=1.0]:0.128257)[&label=0.965608]:0.044024,((((((('AY593843.1_SAT1_NMB_1940':0.028185,'AY593845.1_SAT1_BOT_1968':0.039264)[&label=0.952381]:0.015201,'MH053319.1_SAT1_BOT_1974':0.054499)[&label=0.707837]:0.005792,'MH053322.1_SAT1_NMB_1989':0.084569)[&label=0.874339]:0.011991,('KU821590.1_SAT1_NMB_2010':0.085636,'AY593842.1_SAT1_SAR_1961':0.034731)[&label=0.475198]:0.008124)[&label=0.789087]:0.016349,'AY593841.1_SAT1_ZIM_1958':0.070632)[&label=0.810185]:0.010402,'AY593840.1_SAT1_NMB_1949':0.079749)[&label=0.93424]:0.018401,'AY593838.1_SAT1_BOT_1970':0.086047)[&label=1.0]:0.096171)[&label=0.999118]:0.089254,(('MH053323.1_SAT1_TCH_1972':0.1625,'AY593844.1_SAT1_ISR_1962':0.163789)[&label=0.746032]:0.033263,('MH053325.1_SAT1_UGA_1978':0.085787,'MH053324.1_SAT1_UGA_1971':0.059118)[&label=1.0]:0.070614)[&label=1.0]:0.119043)[&label=0.999098]:0.100949,(('MF678823.1_SAT1_NIG_2015':0.001076,'MF678824.1_SAT1_NIG_2015':1.0E-6)[&label=0.923611]:0.005389,('MF678826.1_SAT1_NIG_2015':1.0E-6,'MF678825.1_SAT1_NIG_2015':5.38E-4)[&label=1.0]:0.003893)[&label=1.0]:0.294007)[&label=1.0]:0.127622)[&label=1.0]:0.537892);

end;

begin figtree;

set appearance.backgroundColorAttribute="Default";

set appearance.backgroundColour=#ffffff;

set appearance.branchColorAttribute="User selection";

set appearance.branchColorGradient=false;

set appearance.branchLineWidth=1.0;

set appearance.branchMinLineWidth=0.0;

set appearance.branchWidthAttribute="Fixed";

set appearance.foregroundColour=#000000;

set appearance.hilightingGradient=false;

set appearance.selectionColour=#2d3680;

set branchLabels.colorAttribute="User selection";

set branchLabels.displayAttribute="Branch times";

set branchLabels.fontName="Calibri";

set branchLabels.fontSize=12;

set branchLabels.fontStyle=0;

set branchLabels.isShown=false;

set branchLabels.significantDigits=4;

set layout.expansion=301;

set layout.layoutType="RECTILINEAR";

set layout.zoom=0;

set legend.attribute="label";

set legend.fontSize=10.0;

set legend.isShown=false;

set legend.significantDigits=4;

set nodeBars.barWidth=4.0;

set nodeBars.displayAttribute=null;

set nodeBars.isShown=false;

set nodeLabels.colorAttribute="User selection";

set nodeLabels.displayAttribute="label";

set nodeLabels.fontName="Arial";

set nodeLabels.fontSize=12;

set nodeLabels.fontStyle=0;

set nodeLabels.isShown=true;

set nodeLabels.significantDigits=4;

set nodeShapeExternal.colourAttribute="User selection";

set nodeShapeExternal.isShown=false;

set nodeShapeExternal.minSize=10.0;

set nodeShapeExternal.scaleType=Width;

set nodeShapeExternal.shapeType=Circle;

set nodeShapeExternal.size=4.0;

set nodeShapeExternal.sizeAttribute="Fixed";

set nodeShapeInternal.colourAttribute="User selection";

set nodeShapeInternal.isShown=false;

set nodeShapeInternal.minSize=10.0;

set nodeShapeInternal.scaleType=Width;

set nodeShapeInternal.shapeType=Circle;

set nodeShapeInternal.size=4.0;

set nodeShapeInternal.sizeAttribute="Fixed";

set polarLayout.alignTipLabels=false;

set polarLayout.angularRange=0;

set polarLayout.rootAngle=0;

set polarLayout.rootLength=100;

set polarLayout.showRoot=true;

set radialLayout.spread=0.0;

set rectilinearLayout.alignTipLabels=true;

set rectilinearLayout.curvature=0;

set rectilinearLayout.rootLength=100;

set scale.offsetAge=0.0;

set scale.rootAge=1.0;

set scale.scaleFactor=1.0;

set scale.scaleRoot=false;

set scaleAxis.automaticScale=true;

set scaleAxis.fontSize=8.0;

set scaleAxis.isShown=false;

set scaleAxis.lineWidth=1.0;

set scaleAxis.majorTicks=1.0;

set scaleAxis.minorTicks=0.5;

set scaleAxis.origin=0.0;

set scaleAxis.reverseAxis=false;

set scaleAxis.showGrid=true;

set scaleBar.automaticScale=true;

set scaleBar.fontSize=12.0;

set scaleBar.isShown=true;

set scaleBar.lineWidth=1.0;

set scaleBar.scaleRange=0.0;

set tipLabels.colorAttribute="User selection";

set tipLabels.displayAttribute="Names";

set tipLabels.fontName="Arial";

set tipLabels.fontSize=12;

set tipLabels.fontStyle=0;

set tipLabels.isShown=true;

set tipLabels.significantDigits=4;

set trees.order=true;

set trees.orderType="increasing";

set trees.rooting=false;

set trees.rootingType="User Selection";

set trees.transform=false;

set trees.transformType="cladogram";

end;
