# Supplementary material for: Early origin and global colonisation of foot-and-mouth disease virus
Source: Sci Rep. 2020 Sep 17;10:15268. doi: 10.1038/s41598-020-72246-6 (PMC7498456; doi:10.1038/s41598-020-72246-6)
Supplement: Supplementary file 11 — Supplementary Tree S3. [file 41598_2020_72246_MOESM11_ESM.doc]

#NEXUS

begin trees;

tree tree_1 = [&R] (((((((((((((((((((((((((((((((((((((((((((((((((((((((((((((((((((((((('LC149686.1_O_JPN_2010':1.0E-6,'LC149701.1_O_JPN_2010':1.0E-6)[&label=0.006]:1.0E-6,'LC149699.1_O_JPN_2010':1.0E-6)[&label=0.0045]:1.0E-6,(('LC149620.1_O_JPN_2010':1.0E-6,'LC149621.1_O_JPN_2010':1.0E-6)[&label=0.0]:1.0E-6,'LC149618.1_O_JPN_2010':1.0E-6)[&label=0.0065]:1.0E-6)[&label=0.0152]:1.0E-6,((('LC149633.1_O_JPN_2010':1.0E-6,'LC149708.1_O_JPN_2010':1.0E-6)[&label=0.01]:1.0E-6,'LC149697.1_O_JPN_2010':1.0E-6)[&label=0.007]:1.0E-6,'LC149665.1_O_JPN_2010':1.0E-6)[&label=0.006]:1.0E-6)[&label=0.018222]:1.0E-6,('LC149683.1_O_JPN_2010':1.0E-6,'LC149642.1_O_JPN_2010':1.0E-6)[&label=0.006]:1.0E-6)[&label=0.018182]:1.0E-6,((((('LC149705.1_O_JPN_2010':1.0E-6,'LC149637.1_O_JPN_2010':1.0E-6)[&label=0.336]:1.0E-6,'LC149628.1_O_JPN_2010':1.0E-6)[&label=0.9895]:0.002351,('LC149634.1_O_JPN_2010':1.0E-6,'LC149718.1_O_JPN_2010':1.0E-6)[&label=0.987]:0.002355)[&label=0.5495]:1.0E-6,('LC149623.1_O_JPN_2010':0.001173,'LC149702.1_O_JPN_2010':0.001174)[&label=0.037]:1.0E-6)[&label=0.3815]:1.0E-6,((('LC149663.1_O_JPN_2010':0.001173,'LC149664.1_O_JPN_2010':1.0E-6)[&label=0.343]:1.0E-6,'LC149671.1_O_JPN_2010':0.001174)[&label=0.1885]:1.0E-6,('LC149640.1_O_JPN_2010':1.0E-6,'LC149645.1_O_JPN_2010':1.0E-6)[&label=0.001]:1.0E-6)[&label=0.1005]:1.0E-6)[&label=0.221273]:1.0E-6)[&label=0.110391]:1.0E-6,((((((('LC149688.1_O_JPN_2010':1.0E-6,'LC149695.1_O_JPN_2010':1.0E-6)[&label=0.196]:1.0E-6,'LC149668.1_O_JPN_2010':1.0E-6)[&label=0.4395]:1.0E-6,'LC149682.1_O_JPN_2010':1.0E-6)[&label=0.814333]:1.0E-6,'LC149662.1_O_JPN_2010':0.001172)[&label=0.75875]:1.0E-6,(('LC149691.1_O_JPN_2010':1.0E-6,'LC149676.1_O_JPN_2010':1.0E-6)[&label=0.461]:1.0E-6,'LC149657.1_O_JPN_2010':0.001178)[&label=0.7015]:0.001173)[&label=0.827]:0.001173,'LC149619.1_O_JPN_2010':0.001174)[&label=0.730375]:1.0E-6,(('LC036265.1_O_JPN_2010':0.001174,'LC149716.1_O_JPN_2010':1.0E-6)[&label=0.535]:0.001174,'LC149720.1_O_JPN_2010':0.001174)[&label=0.2865]:1.0E-6)[&label=0.549455]:1.0E-6)[&label=0.252771]:1.0E-6,'LC149622.1_O_JPN_2010':1.0E-6)[&label=0.246722]:1.0E-6,'LC149660.1_O_JPN_2010':1.0E-6)[&label=0.240892]:1.0E-6,('LC149677.1_O_JPN_2010':1.0E-6,'LC149684.1_O_JPN_2010':1.0E-6)[&label=0.0]:1.0E-6)[&label=0.230821]:1.0E-6,(('LC149631.1_O_JPN_2010':1.0E-6,'LC149666.1_O_JPN_2010':1.0E-6)[&label=0.004]:1.0E-6,'LC149678.1_O_JPN_2010':1.0E-6)[&label=0.0085]:1.0E-6)[&label=0.220738]:1.0E-6,'LC149629.1_O_JPN_2010':1.0E-6)[&label=0.219488]:1.0E-6,'LC149636.1_O_JPN_2010':1.0E-6)[&label=0.217591]:1.0E-6,'LC149656.1_O_JPN_2010':1.0E-6)[&label=0.2178]:1.0E-6,(((('LC149644.1_O_JPN_2010':1.0E-6,'LC149638.1_O_JPN_2010':1.0E-6)[&label=0.0]:1.0E-6,'LC149625.1_O_JPN_2010':1.0E-6)[&label=0.006]:1.0E-6,'LC149710.1_O_JPN_2010':1.0E-6)[&label=0.004]:1.0E-6,'LC149661.1_O_JPN_2010':1.0E-6)[&label=0.003]:1.0E-6)[&label=0.2224]:1.0E-6,'LC149693.1_O_JPN_2010':1.0E-6)[&label=0.227569]:1.0E-6,'LC149698.1_O_JPN_2010':1.0E-6)[&label=0.233923]:1.0E-6,((('LC149658.1_O_JPN_2010':1.0E-6,'LC149647.1_O_JPN_2010':1.0E-6)[&label=0.013]:1.0E-6,'LC149679.1_O_JPN_2010':1.0E-6)[&label=0.01]:1.0E-6,'LC149709.1_O_JPN_2010':1.0E-6)[&label=0.006667]:1.0E-6)[&label=0.279339]:1.0E-6,'LC149624.1_O_JPN_2010':1.0E-6)[&label=0.297088]:1.0E-6,('LC149670.1_O_JPN_2010':1.0E-6,'LC149641.1_O_JPN_2010':1.0E-6)[&label=0.0]:1.0E-6)[&label=0.334746]:1.0E-6,'LC149685.1_O_JPN_2010':1.0E-6)[&label=0.353917]:1.0E-6,'LC149707.1_O_JPN_2010':1.0E-6)[&label=0.374885]:1.0E-6,(((((('LC149703.1_O_JPN_2010':0.003554,'LC149717.1_O_JPN_2010':0.001174)[&label=0.032]:1.0E-6,'LC149704.1_O_JPN_2010':0.001179)[&label=0.0495]:1.0E-6,'LC149626.1_O_JPN_2010':1.0E-6)[&label=0.033]:1.0E-6,'LC149673.1_O_JPN_2010':1.0E-6)[&label=0.02825]:1.0E-6,(('LC149672.1_O_JPN_2010':1.0E-6,'LC149719.1_O_JPN_2010':1.0E-6)[&label=0.006]:1.0E-6,'LC149632.1_O_JPN_2010':1.0E-6)[&label=0.0095]:1.0E-6)[&label=0.020857]:1.0E-6,((('LC149681.1_O_JPN_2010':0.001174,'LC149687.1_O_JPN_2010':0.001174)[&label=0.022]:1.0E-6,'LC149652.1_O_JPN_2010':1.0E-6)[&label=0.016]:1.0E-6,'LC149627.1_O_JPN_2010':1.0E-6)[&label=0.010667]:1.0E-6)[&label=0.035455]:1.0E-6)[&label=0.60989]:1.0E-6,'LC149692.1_O_JPN_2010':1.0E-6)[&label=0.627635]:1.0E-6,'LC149649.1_O_JPN_2010':1.0E-6)[&label=0.645053]:1.0E-6,(('LC149713.1_O_JPN_2010':1.0E-6,'LC149700.1_O_JPN_2010':1.0E-6)[&label=0.007]:1.0E-6,'LC149667.1_O_JPN_2010':1.0E-6)[&label=0.0035]:1.0E-6)[&label=0.695731]:1.0E-6,'LC149655.1_O_JPN_2010':1.0E-6)[&label=0.711861]:1.0E-6,(((('LC149659.1_O_JPN_2010':1.0E-6,'LC149639.1_O_JPN_2010':1.0E-6)[&label=0.008]:1.0E-6,'LC149706.1_O_JPN_2010':1.0E-6)[&label=0.0095]:1.0E-6,('LC149648.1_O_JPN_2010':1.0E-6,'LC149654.1_O_JPN_2010':1.0E-6)[&label=0.002]:1.0E-6)[&label=0.01225]:1.0E-6,'LC149694.1_O_JPN_2010':1.0E-6)[&label=0.0102]:1.0E-6)[&label=0.798694]:1.0E-6,('LC149680.1_O_JPN_2010':1.0E-6,'LC149669.1_O_JPN_2010':1.0E-6)[&label=0.0]:1.0E-6)[&label=0.825747]:1.0E-6,'LC149650.1_O_JPN_2010':1.0E-6)[&label=0.839045]:1.0E-6,('LC149689.1_O_JPN_2010':0.001173,'LC149696.1_O_JPN_2010':0.003539)[&label=0.092]:1.0E-6)[&label=0.861356]:1.0E-6,(((('LC149714.1_O_JPN_2010':1.0E-6,'LC149674.1_O_JPN_2010':1.0E-6)[&label=0.721]:0.001174,'LC149675.1_O_JPN_2010':0.001178)[&label=0.38]:1.0E-6,'LC149712.1_O_JPN_2010':1.0E-6)[&label=0.258]:1.0E-6,'LC149653.1_O_JPN_2010':1.0E-6)[&label=0.19375]:1.0E-6)[&label=0.916632]:1.0E-6,'LC149630.1_O_JPN_2010':1.0E-6)[&label=0.927438]:1.0E-6,'LC149690.1_O_JPN_2010':1.0E-6)[&label=0.938495]:1.0E-6,'LC149646.1_O_JPN_2010':1.0E-6)[&label=0.949122]:1.0E-6,('LC149651.1_O_JPN_2010':0.001174,'LC149711.1_O_JPN_2010':0.001174)[&label=0.017]:1.0E-6)[&label=0.96555]:0.001171,'LC149643.1_O_JPN_2010':1.0E-6)[&label=0.97101]:1.0E-6,'LC149635.1_O_JPN_2010':1.0E-6)[&label=0.978324]:1.0E-6,'LC149715.1_O_JPN_2010':1.0E-6)[&label=0.986893]:1.0E-6,('KF112885.1_O_JPN_2010':1.0E-6,'LC149617.1_O_JPN_2010':1.0E-6)[&label=0.771]:1.0E-6)[&label=0.998667]:0.003554,'JN998085.1_O_CHA_2010':0.003546)[&label=0.992858]:1.0E-6,((((('KF501487.1_O_SKR_2010':1.0E-6,'KF501488.1_O_SKR_2010':0.002363)[&label=0.678]:0.001178,'KR401160.1_O_SKR_2011':1.0E-6)[&label=0.938]:0.002363,'KF112883.1_O_RUS_2010':0.007167)[&label=0.713]:1.0E-6,'KF112888.1_O_DRK_2011':0.010812)[&label=0.82275]:0.001168,((('KC503937.1_O_SKR_2010':1.0E-6,'KF501486.1_O_SKR_2010':0.001175)[&label=0.08]:1.0E-6,'KF112887.1_O_SKR_2010':1.0E-6)[&label=0.5255]:1.0E-6,'KR401159.1_O_SKR_2010':1.0E-6)[&label=0.993667]:0.004753)[&label=0.7885]:0.001189)[&label=0.9818]:0.001187,'KF112889.1_O_HKN_2010':0.003548)[&label=0.982879]:0.002356,(('KR401158.1_O_SKR_2010':0.001176,'KF112886.1_O_SKR_2010':1.0E-6)[&label=0.901]:0.002353,'JQ973889.1_O_CHA_2010':0.002359)[&label=0.81]:0.001182)[&label=0.964739]:1.0E-6,((((((('KY086465.1_O_SKR_2016':1.0E-6,'KX534089.1_O_SKR_2016':1.0E-6)[&label=0.999]:0.008408,'KY086466.1_O_SKR_2016':0.010837)[&label=0.825]:0.001142,'KX162590.1_O_SKR_2014':1.0E-6)[&label=1.0]:0.013534,'MH845413.2_O_VIT_2014':0.012348)[&label=0.93075]:0.002996,'KY322674.1_O_SKR_2014':0.020518)[&label=1.0]:0.020262,'HM229661.1_O_HKN_2010':0.002357)[&label=0.855667]:1.0E-6,'JQ900581.1_O_CHA_2010':0.003545)[&label=0.926571]:0.00118)[&label=0.975118]:1.0E-6,('HM055510.1_O_VIT_2009':0.003548,'JN998086.1_O_CHA_2010':0.002387)[&label=0.664]:0.001165)[&label=0.988496]:0.002357,((('KF112880.1_O_MYA_2009':1.0E-6,'KR401156.1_O_MYA_2009':1.0E-6)[&label=1.0]:0.005938,('KR401155.1_O_MYA_2007':1.0E-6,'KR401153.1_O_MYA_2009':1.0E-6)[&label=1.0]:0.005924)[&label=0.828667]:0.001211,'KR401152.1_O_MYA_2009':0.008373)[&label=0.89525]:1.0E-6)[&label=0.997209]:0.002742,'KF112879.1_O_TAI_2009':0.006794)[&label=0.999911]:0.01309,'KR401157.1_O_MYA_2009':0.020003)[&label=0.999949]:0.016838,'KY322672.1_O_MAY_2014':0.062065)[&label=1.0]:0.035524,'KR401154.1_O_MYA_1998':0.035082)[&label=0.998449]:0.0051,(((((((((('GQ406249.1_A_VIT_2009':0.002351,'GQ406252.1_A_VIT_2009':0.003537)[&label=0.203]:1.0E-6,'GQ406250.1_A_VIT_2009':0.00354)[&label=0.6565]:0.001173,'GQ406251.1_A_VIT_2009':1.0E-6)[&label=0.694667]:1.0E-6,('GQ406247.1_A_VIT_2009':1.0E-6,'GQ406248.1_A_VIT_2009':1.0E-6)[&label=0.983]:0.004726)[&label=0.8716]:0.001107,'KC588943.1_A_SKR_2010':0.01847)[&label=0.999833]:0.012751,'HQ632773.1_A_MAY_2007':0.025861)[&label=0.971]:0.007406,(((('KY322675.1_A_LAO_2014':0.005911,'KY322677.1_A_MAY_2013':0.004804)[&label=0.385]:0.001307,('KY322680.1_A_VIT_2013':0.005939,'KY322679.1_A_TAI_2014':0.007287)[&label=0.481]:0.00114)[&label=1.0]:0.061158,('KJ608371.1_A_VIT_2013':0.023075,'KY322678.1_A_MAY_2013':0.035924)[&label=1.0]:0.032867)[&label=0.8512]:0.001681,'HQ268509.2_A_VIT_2004':0.013668)[&label=0.839333]:0.006838)[&label=0.996286]:0.018546,'KJ933864.1_A_MAY_1997':0.029871)[&label=0.9976]:0.021814,'KY322676.1_A_MAY_2013':0.058934)[&label=0.97775]:0.026487,(((((((((('KF112882.1_O_MOG_2010':0.003521,'KF112884.1_O_RUS_2010':0.002341)[&label=0.491]:1.0E-6,'KF112881.1_O_MOG_2010':0.005902)[&label=0.9785]:0.00471,'GU582116.1_O_VIT_2009':0.003523)[&label=0.863333]:1.0E-6,'GU582115.1_O_VIT_2009':0.003501)[&label=0.98775]:0.013063,('HQ632772.1_O_MAY_2007':0.007832,'KY322673.1_O_MAY_2014':0.057803)[&label=0.828]:0.006458)[&label=0.861667]:0.015764,(('MF947130.1_O_VIT_2014':0.00584,'KY322671.1_O_MAY_2014':0.001252)[&label=0.974]:0.00795,'KY322670.1_O_LAO_2013':0.006603)[&label=0.998]:0.018641)[&label=0.986667]:0.013505,'KT968663.1_A_CHA_2013':0.072727)[&label=0.931]:0.014122,(('GU125648.1_O_VIT_2006':1.0E-6,'GU125647.1_O_VIT_2006':1.0E-6)[&label=0.331]:1.0E-6,'GU125649.1_O_VIT_2006':1.0E-6)[&label=1.0]:0.032509)[&label=0.877923]:1.0E-6,'GU125650.1_O_VIT_2006':0.043084)[&label=0.909]:0.015372,('HQ632774.1_Asia1_MAY_1999':0.070989,'HQ632769.1_O_MAY_2001':0.063142)[&label=0.312]:7.93E-4)[&label=0.865687]:0.009132)[&label=0.971758]:0.024089)[&label=0.995076]:0.011783,'GU125646.1_Asia1_VIT_2005':0.05375)[&label=0.995098]:0.017661,((((('KC412634.1_Asia1_CHA_2006':1.0E-6,'KC462884.1_Asia1_CHA_2006':1.0E-6)[&label=0.714]:1.0E-6,'KU360085.1_Asia1_CHA_2015':0.00118)[&label=0.977]:0.003474,'EF149009.1_Asia1_CHA_2005':0.038518)[&label=0.774667]:0.001273,'GU931682.1_Asia1_CHA_2005':1.0E-6)[&label=0.775]:0.002549,(((('GU125645.1_Asia1_VIT_2007':1.0E-6,'GQ452295.1_Asia1_VIT_2007':1.0E-6)[&label=0.989]:0.004765,'HQ631363.1_Asia1_CHA_2006':1.0E-6)[&label=0.828]:1.0E-6,'FJ906802.1_Asia1_CHA_2006':0.008397)[&label=0.885667]:0.002292,'KY446901.1_Asia1_PAK_2006':0.004851)[&label=0.8125]:0.002466)[&label=0.997889]:0.027521)[&label=0.951044]:0.00366,(('HQ832587.1_A_IND_2005':0.044085,'DQ989312.1_Asia1_IND_1990':0.041448)[&label=0.966]:0.027557,'HQ832576.1_A_IND_1990':0.044167)[&label=0.83]:0.014634)[&label=0.941839]:0.001758,(('KT003716.1_O_PAK_2005':0.003226,'KY446903.1_O_PAK_2005':0.001455)[&label=0.991]:0.015723,'KY446902.1_A_PAK_2005':0.008016)[&label=0.9985]:0.04306)[&label=0.929852]:0.005433,((((('DQ989309.1_Asia1_IND_1996':1.0E-6,'MF372126.1_Asia1_IND_1994':1.0E-6)[&label=1.0]:0.046822,'KU726614.1_O_GRE_1994':0.052703)[&label=0.8945]:0.01714,(('AY593800.1_Asia1_LEB_1983':1.0E-6,'AY593799.1_Asia1_LEB_1983':1.0E-6)[&label=0.687]:1.0E-6,'KY825718.1_Asia1_ISR_1989':0.001174)[&label=1.0]:0.0499)[&label=0.7748]:0.00179,'HM854022.1_A_IND_1977':0.068807)[&label=0.835]:0.011725,'DQ989313.1_Asia1_IND_1986':0.048485)[&label=0.752286]:0.008964)[&label=0.908701]:0.005983,(((((((((((((('AF308157.1_O_TAW_1997':1.0E-6,'AY593835.1_O_TAW_1997':1.0E-6)[&label=0.979]:0.00242,'AY593833.1_O_TAW_1999':0.002427)[&label=0.871]:0.001217,'AF154271.1_O_TAW_1997':0.001209)[&label=0.962]:6.02E-4,'AF026168.2_O_TAW_1997':0.022414)[&label=0.99975]:0.035599,'HQ632771.1_O_MAY_2005':0.148036)[&label=0.9094]:0.007749,('HQ412603.1_O_CHA_2000':0.048246,'AY686687.1_O_CHA_2001':0.05377)[&label=0.806]:0.014088)[&label=0.948714]:0.020891,((('KU204893.1_O_CHA_2013':0.031855,'KU204894.1_O_CHA_2013':0.013047)[&label=1.0]:0.087424,'EU400597.1_O_CHA_2001':0.01045)[&label=0.7745]:0.002966,'AY317098.1_O_CHA_2002':0.005452)[&label=0.973333]:0.043639)[&label=1.0]:0.164525,(((((('MG725875.1_A_NIG_2015':1.0E-6,'MG725876.1_A_NIG_2015':0.003526)[&label=0.615]:0.003227,'MG725873.1_A_NIG_2015':0.005058)[&label=0.9655]:0.00909,(('MG913340.1_A_ALG_2017':1.0E-6,'MG923580.1_A_ALG_2017':0.001172)[&label=0.652]:0.001172,'MG923579.1_A_ALG_2017':1.0E-6)[&label=0.999]:0.022836)[&label=1.0]:0.055341,('MG725872.1_A_NIG_2013':0.020997,'MG725874.1_A_NIG_2015':0.05582)[&label=0.999]:0.044383)[&label=1.0]:0.063843,'MH053315.1_O_SUD_1976':0.054089)[&label=0.97525]:0.022262,'MH053305.1_A_EGY_1972':0.077473)[&label=0.914333]:1.0E-6)[&label=0.590333]:0.011907,(((('MH053314.1_O_ETH_2007':0.035539,'MH053311.1_O_ETH_2004':0.051268)[&label=0.998]:0.05028,('JF749843.1_A_EGY_2006':0.082028,'MH053313.1_O_ETH_2006':0.091213)[&label=0.473]:0.008799)[&label=0.832667]:0.025663,('MH053312.1_O_ETH_2005':0.074657,'MH053317.1_O_UGA_1998':0.041605)[&label=0.996]:0.040331)[&label=0.634]:0.009956,('AY593766.1_A_KEN_1965':0.052293,'MH053316.1_O_UGA_1996':0.088768)[&label=0.373]:0.007915)[&label=0.534]:0.002225)[&label=0.617103]:0.005076,(((('EF611987.1_O_UGA_2006':0.003714,'HM191257.1_O_UGA_2006':0.001069)[&label=1.0]:0.01364,'KU821591.1_O_ZAM_2010':0.024884)[&label=0.974]:0.011914,(('FJ461345.1_O_UGA_2002':0.019066,'FJ461344.1_O_UGA_2002':0.018816)[&label=0.529]:0.004317,'MH053318.1_O_UGA_2002':0.012488)[&label=0.827]:0.00395)[&label=1.0]:0.03369,'MH053307.1_A_ZAM_1990':0.045959)[&label=0.994]:0.031439)[&label=0.605194]:0.002629,((('MH053309.1_C_KEN_1967':1.0E-6,'KM268897.1_C_KEN_2004':0.003496)[&label=0.973]:0.035232,'MH053310.1_C_UGA_1970':0.079722)[&label=0.998]:0.037847,'MH053308.1_C_ETH_1971':0.046607)[&label=0.865]:0.026246)[&label=0.6079]:0.006381,(('AY593791.1_A_IRN_1998':0.022032,'JF749848.1_A_TUR_2003':0.045399)[&label=1.0]:0.042737,'AY593812.1_O_PHI_1958':0.074515)[&label=0.6025]:0.002187)[&label=0.589116]:0.003157,((((((('DQ989303.1_Asia1_IND_1993':0.003515,'DQ989304.1_Asia1_IND_2000':0.001187)[&label=0.59]:8.62E-4,'DQ989308.1_Asia1_IND_1994':0.038645)[&label=0.799]:0.003898,'DQ989306.1_Asia1_IND_1986':1.0E-6)[&label=0.998]:0.03733,'AY593796.1_Asia1_ISR_1963':0.033896)[&label=0.84375]:0.00381,'AY593797.1_Asia1_ISR_1963':0.029166)[&label=0.9752]:0.023066,'AY593828.1_O_IND_1962':0.026577)[&label=0.926167]:0.011903,(((('AY593765.1_A_TUR_1965':0.008165,'AY593764.1_A_IRQ_1970':0.004773)[&label=0.765]:0.002385,'FJ623456.1_A_KAZ_1999':0.010793)[&label=0.8645]:0.004586,'AY593772.1_A_TUR_1972':0.036974)[&label=1.0]:0.071259,'AY593760.1_A_USSR_1964':0.07759)[&label=0.7655]:0.005646)[&label=0.596091]:0.008669)[&label=0.591164]:0.00267,('AY593823.1_O_TUR_1969':0.036109,'KP940473.1_O_EGY_2014':0.02993)[&label=0.979]:0.027827)[&label=0.594123]:0.006002)[&label=0.849502]:0.004193,(((((((('HQ832581.1_A_IND_2004':0.00472,'HQ832580.1_A_IND_2003':0.007304)[&label=0.989]:0.008569,'HQ832583.1_A_IND_2005':0.002449)[&label=0.82]:0.001108,'HQ832579.1_A_IND_2003':0.00476)[&label=0.814]:1.0E-6,'HQ832578.1_A_IND_2003':0.018465)[&label=0.917]:0.002962,'HQ832582.1_A_IND_2004':0.016576)[&label=1.0]:0.047706,('DQ989305.1_Asia1_IND_1990':0.02561,'DQ989307.1_Asia1_IND_1992':0.02189)[&label=0.909]:0.025167)[&label=0.745143]:0.006933,'DQ989310.1_Asia1_IND_1999':0.075024)[&label=0.661625]:0.002253,(((('HM854023.1_A_IND_1999':0.003554,'HQ832577.1_A_IND_1999':0.012292)[&label=0.999]:0.024672,'HM854021.1_A_IND_2000':0.040224)[&label=0.665]:0.005513,'DQ989315.1_Asia1_IND_1993':0.034515)[&label=0.823]:0.01353,'HQ832585.1_A_IND_2005':0.068971)[&label=0.6995]:0.007732)[&label=0.439846]:0.007814)[&label=0.839037]:0.005436,(((((((((((((((((((((((((((((((((((('EU448373.1_O_UKG_2007':1.0E-6,'KJ560300.1_O_UKG_2007':1.0E-6)[&label=0.012]:1.0E-6,('EU448377.1_O_UKG_2007':1.0E-6,'KJ560296.1_O_UKG_2007':1.0E-6)[&label=0.025]:1.0E-6)[&label=0.064667]:1.0E-6,'KJ560277.1_O_UKG_2007':1.0E-6)[&label=0.0785]:1.0E-6,'KJ560299.1_O_UKG_2007':1.0E-6)[&label=0.1136]:1.0E-6,'KJ560294.1_O_UKG_2007':1.0E-6)[&label=0.1845]:1.0E-6,'EU448374.1_O_UKG_2007':1.0E-6)[&label=0.263143]:1.0E-6,(('KJ560298.1_O_UKG_2007':1.0E-6,'EU448371.1_O_UKG_2007':1.0E-6)[&label=0.015]:1.0E-6,'EU448372.1_O_UKG_2007':1.0E-6)[&label=0.0235]:1.0E-6)[&label=0.5007]:1.0E-6,'EU448376.1_O_UKG_2007':1.0E-6)[&label=0.582091]:1.0E-6,'KJ560283.1_O_UKG_2007':1.0E-6)[&label=0.659083]:1.0E-6,((((('KJ560307.1_O_UKG_2007':1.0E-6,'KJ560303.1_O_UKG_2007':1.0E-6)[&label=0.303]:1.0E-6,'KJ560308.1_O_UKG_2007':1.0E-6)[&label=0.933]:0.001177,(('KJ560304.1_O_UKG_2007':1.0E-6,'KJ560302.1_O_UKG_2007':1.0E-6)[&label=0.284]:1.0E-6,'EU448375.1_O_UKG_2007':1.0E-6)[&label=0.835]:1.0E-6)[&label=0.8552]:0.001177,'KJ560276.1_O_UKG_2007':0.001177)[&label=0.730667]:1.0E-6,(('KJ560281.1_O_UKG_2007':1.0E-6,'KJ560285.1_O_UKG_2007':1.0E-6)[&label=0.957]:0.001177,'EU448378.1_O_UKG_2007':0.001176)[&label=0.5385]:1.0E-6)[&label=0.579556]:1.0E-6)[&label=0.815318]:1.0E-6,'KJ560287.1_O_UKG_2007':1.0E-6)[&label=0.851739]:1.0E-6,'KJ560297.1_O_UKG_2007':0.001177)[&label=0.882667]:0.001177,'JX869183.1_O_UKG_1968':0.001177)[&label=0.85476]:1.0E-6,(('JX869187.1_O_UKG_1968':1.0E-6,'JX570640.1_O_UKG_2007':1.0E-6)[&label=0.011]:1.0E-6,'JX869188.1_O_UKG_1968':1.0E-6)[&label=0.019]:1.0E-6)[&label=0.764571]:1.0E-6,(((((((((((((('JX869184.1_O_UKG_1968':1.0E-6,'JX570652.1_O_UKG_2007':1.0E-6)[&label=0.013]:1.0E-6,'EU448368.1_O_UKG_1967':1.0E-6)[&label=0.0295]:1.0E-6,('JX570642.1_O_UKG_2007':1.0E-6,'JX570643.1_O_UKG_2007':1.0E-6)[&label=0.002]:1.0E-6)[&label=0.02975]:1.0E-6,'JX570646.1_O_UKG_2007':1.0E-6)[&label=0.0332]:1.0E-6,('JX869180.1_O_UKG_1967':1.0E-6,'EU448370.1_O_UKG_1967':1.0E-6)[&label=0.023]:1.0E-6)[&label=0.053714]:1.0E-6,'JX869182.1_O_UKG_1968':1.0E-6)[&label=0.07325]:1.0E-6,'JX570638.1_O_UKG_2007':1.0E-6)[&label=0.087778]:1.0E-6,'AY593816.1_O_UKG_1967':1.0E-6)[&label=0.1039]:1.0E-6,(('JX570648.1_O_UKG_2007':1.0E-6,'EU448369.1_O_UKG_1967':1.0E-6)[&label=0.015]:1.0E-6,'AY593815.1_O_UKG_1967':1.0E-6)[&label=0.033]:1.0E-6)[&label=0.176]:1.0E-6,'JX570641.1_O_UKG_2007':1.0E-6)[&label=0.215071]:1.0E-6,'JX570644.1_O_UKG_2007':1.0E-6)[&label=0.258067]:1.0E-6,('JX570651.1_O_UKG_2007':1.0E-6,'JX570639.1_O_UKG_2007':1.0E-6)[&label=0.006]:1.0E-6)[&label=0.298529]:1.0E-6,'JX869181.1_O_UKG_1967':1.0E-6)[&label=0.333167]:1.0E-6,'JX869186.1_O_UKG_1968':1.0E-6)[&label=0.364632]:1.0E-6)[&label=0.850542]:1.0E-6,((((((('JX570649.1_O_UKG_2007':1.0E-6,'JX570645.1_O_UKG_2007':1.0E-6)[&label=0.127]:1.0E-6,'JX570650.1_O_UKG_2007':1.0E-6)[&label=0.26]:1.0E-6,'JX570654.1_O_UKG_2007':1.0E-6)[&label=0.588667]:1.0E-6,'JX570655.1_O_UKG_2007':1.0E-6)[&label=0.887]:1.0E-6,'JX570653.1_O_UKG_2007':0.001174)[&label=0.923]:0.001176,'JX570647.1_O_UKG_2007':1.0E-6)[&label=0.934833]:0.001176,'JX869179.1_O_UKG_1967':0.001178)[&label=0.812]:1.0E-6)[&label=0.978214]:1.0E-6,'JX869185.1_O_UKG_1968':0.005962)[&label=0.991772]:0.002364,('AY593767.1_A_ARG_1965':0.016175,'AY593814.1_O_ARG_1965':0.004857)[&label=0.607]:0.00232)[&label=0.976695]:1.0E-6,'AY593817.1_O_Belgium_1973':0.007178)[&label=0.983267]:0.001183,'AY593830.1_O_POL_1959':0.003578)[&label=0.980705]:0.001183,'AY593819.1_O_ARG_1994':1.0E-6)[&label=0.967387]:1.0E-6,(('AY593818.1_O_ARG_1958':1.0E-6,'AY593837.1_O_URU_1963':1.0E-6)[&label=0.211]:1.0E-6,'AY593820.1_O_ARG_1964':1.0E-6)[&label=0.5585]:1.0E-6)[&label=0.99]:1.0E-6,'JX869177.1_O_UKG_1967':0.00596)[&label=0.999288]:0.02051,'JX869178.1_O_UKG_1967':0.031041)[&label=0.999522]:0.023265,'AY593793.1_A_PHI_1975':0.074281)[&label=0.992529]:0.00994,('AY593775.1_A_VEN_1970':1.0E-6,'AY593773.1_A_PER_1969':1.0E-6)[&label=1.0]:0.067221)[&label=0.989429]:0.018371,((('AY593827.1_O_VEN_1971':0.00833,'AY593826.1_O_ITL_1947':0.012447)[&label=0.969]:0.014703,'AY593751.1_A_NET_1942':0.040693)[&label=0.89]:0.00835,'AY593759.1_A_GER_1971':0.050553)[&label=0.934333]:0.011577)[&label=0.956351]:0.007069,((('AY593774.1_A_SPA_1969':0.001165,'AY593779.1_A_GER_1972':1.0E-6)[&label=0.815]:1.0E-6,'AY593777.1_A_GER_1972':0.002336)[&label=1.0]:0.027654,'AY593810.1_C_UKG_1970':0.03037)[&label=0.988667]:0.017436)[&label=0.93609]:0.005293,(((('MH053306.1_A_TCH_1973':0.081423,'AY593761.1_A_KEN_1964':0.050058)[&label=0.992]:0.03354,'AY593825.1_O_ARG_1939':0.075477)[&label=0.736]:0.009987,'NC_039210.1_O_UKG_1965':0.068338)[&label=0.517]:1.0E-6,('AY593789.1_A_ARG_1961':1.0E-6,'AY593769.1_A_ARG_1959':0.002361)[&label=1.0]:0.065605)[&label=0.447]:0.007472)[&label=0.942679]:0.008313,'AY593794.1_A_COL_1985':0.0701)[&label=0.946459]:0.011135,('AY593795.1_Asia1_PAK_1954':0.070258,'AY593834.1_O_IRN_1966':0.064697)[&label=0.125]:0.006516)[&label=0.930874]:0.002117,((((((((((((((((((('AY593785.1_A_ARG_2001':1.0E-6,'KX002203.1_A_ARG_2001':1.0E-6)[&label=0.312]:1.0E-6,'AY593784.1_A_ARG_2001':1.0E-6)[&label=0.9035]:1.0E-6,'AY593802.1_A_URU_2001':0.003551)[&label=0.829667]:0.001179,'KX002181.1_A_ARG_2001':0.005956)[&label=0.64975]:1.0E-6,('KX002188.1_A_ARG_2001':0.001179,'KX002177.1_A_ARG_2001':0.003555)[&label=0.128]:1.0E-6)[&label=0.514333]:1.0E-6,((('KX002201.1_A_ARG_2001':0.007217,'KX002200.1_A_ARG_2001':0.001195)[&label=0.382]:0.001175,'KX002186.1_A_ARG_2001':0.005947)[&label=0.395]:1.0E-6,'KX002185.1_A_ARG_2001':0.002365)[&label=0.325667]:1.0E-6)[&label=0.8532]:1.0E-6,'KX002190.1_A_ARG_2001':0.007182)[&label=0.869545]:0.001182,'KX002179.1_A_ARG_2001':0.008419)[&label=0.88175]:0.001181,'KX002195.1_A_ARG_2001':1.0E-6)[&label=0.876308]:1.0E-6,'KX002197.1_A_ARG_2001':0.005952)[&label=0.856286]:1.0E-6,('KX002194.1_A_ARG_2001':0.001162,'KX002199.1_A_ARG_2001':0.004778)[&label=0.688]:0.001199)[&label=0.88325]:0.00118,(((((((('KX002192.1_A_ARG_2001':0.00354,'KX002189.1_A_ARG_2001':0.001599)[&label=0.981]:0.004365,'KX002187.1_A_ARG_2001':0.002365)[&label=0.854]:0.001198,'KX002184.1_A_ARG_2001':0.002368)[&label=0.812333]:0.001172,'KX002183.1_A_ARG_2001':0.004758)[&label=0.66325]:1.0E-6,'KX002182.1_A_ARG_2001':0.00118)[&label=0.549]:1.0E-6,(('KX002205.1_A_ARG_2001':1.0E-6,'AY593786.1_A_ARG_2001':1.0E-6)[&label=0.781]:0.001179,'KX002178.1_A_ARG_2001':0.002367)[&label=0.4305]:1.0E-6)[&label=0.412375]:1.0E-6,(('KX002180.1_A_ARG_2001':1.0E-6,'KX002198.1_A_ARG_2001':1.0E-6)[&label=0.112]:1.0E-6,'KX002176.1_A_ARG_2001':1.0E-6)[&label=0.3605]:1.0E-6)[&label=0.562182]:1.0E-6,('KX002204.1_A_ARG_2001':1.0E-6,'AY593790.1_A_ARG_2001':1.0E-6)[&label=0.941]:0.002364)[&label=0.638308]:0.001179)[&label=0.9488]:1.0E-6,'KX002202.1_A_ARG_2001':0.012072)[&label=0.968323]:0.004763,(('KX002191.1_A_ARG_2001':0.01588,'KX002193.1_A_ARG_2001':0.004835)[&label=0.68]:0.00115,'KX002196.1_A_ARG_2001':0.018437)[&label=0.491]:1.0E-6)[&label=1.0]:0.069853,'AY593782.1_A_ARG_2000':0.063069)[&label=0.9846]:0.017553,'AY593806.1_C_Brazil_1971':0.07978)[&label=0.961583]:0.005328,(('AY593788.1_A_Brazil_1979':0.018988,'AY593787.1_A_Brazil_1977':5.45E-4)[&label=0.999]:0.024357,'AY593803.1_A_Brazil_1979':0.03885)[&label=0.9465]:0.022561)[&label=0.895077]:0.005356,(((((((((((('MH559780.1_A_Brazil_2016':1.0E-6,'MH559788.1_A_Brazil_2016':1.0E-6)[&label=0.021]:1.0E-6,'MH559796.1_A_Brazil_2016':1.0E-6)[&label=0.089]:1.0E-6,'MH559805.1_A_Brazil_2016':1.0E-6)[&label=0.215667]:1.0E-6,(('MH559781.1_A_Brazil_2016':1.0E-6,'MH559793.1_A_Brazil_2016':1.0E-6)[&label=0.045]:1.0E-6,'MH559786.1_A_Brazil_2016':1.0E-6)[&label=0.0965]:1.0E-6)[&label=0.783667]:1.0E-6,'MH559798.1_A_Brazil_2016':0.001184)[&label=0.775857]:1.0E-6,(('MH559791.1_A_Brazil_2016':1.0E-6,'MH559804.1_A_Brazil_2016':1.0E-6)[&label=0.191]:1.0E-6,('MH559785.1_A_Brazil_2016':1.0E-6,'MH559800.1_A_Brazil_2016':1.0E-6)[&label=0.198]:1.0E-6)[&label=0.893333]:0.001184)[&label=0.894909]:0.001184,'MH559801.1_A_Brazil_2016':0.001184)[&label=0.875083]:1.0E-6,('MH559783.1_A_Brazil_2016':1.0E-6,'MH559799.1_A_Brazil_2016':1.0E-6)[&label=0.445]:1.0E-6)[&label=0.9455]:1.0E-6,'AY593768.1_A_Brazil_1955':0.001184)[&label=0.998867]:0.033789,(((('AY593778.1_A_SPA_1969':0.001257,'AY593754.1_A_SPA_1959':0.00118)[&label=1.0]:0.029495,'AY593780.1_A_FRA_1960':0.003475)[&label=0.945]:0.00612,(('AY593792.1_A_ITL_1962':0.009808,'AY593776.1_A_GER_1968':0.007285)[&label=0.917]:0.003511,'AY593781.1_A_GER_1951':0.004812)[&label=0.7615]:1.0E-6)[&label=0.9948]:0.017094,'AY593771.1_A_COL_1967':0.047763)[&label=0.999333]:0.031625)[&label=0.896591]:0.004576,(('AY593758.1_A_VEN_1967':1.0E-6,'AY593753.1_A_Brazil_1970':1.0E-6)[&label=1.0]:0.014515,'AY593757.1_A_Brazil_1967':0.008878)[&label=1.0]:0.040858)[&label=0.94528]:0.013441,(((('AY593807.1_C_Brazil_1955':1.0E-6,'AY593809.1_C_ARG_1969':0.013167)[&label=1.0]:0.031189,'AY593821.1_O_ARG_1967':0.075125)[&label=0.6465]:0.008687,'AY593770.1_A_ARG_1966':0.071631)[&label=0.547333]:0.004301,'AY593756.1_A_Brazil_1959':0.045502)[&label=0.56575]:0.005976)[&label=0.8467]:0.00928)[&label=0.8409]:0.002111,((('AY593805.1_C_GER_1960':1.0E-6,'FJ824812.1_C_SPA_2009':0.019571)[&label=0.831]:0.001265,'AY593804.1_C_SWZ_1965':0.003471)[&label=1.0]:0.020357,'AY593808.1_C_ARG_1966':0.027448)[&label=0.994667]:0.022246)[&label=0.869054]:0.011414)[&label=0.863907]:0.002056,'AY593755.1_A_TAI_1960':0.118223)[&label=0.866957]:0.007351,('KY072818.1_O_CHA_1959':0.097153,'AY593813.1_O_ISA_1962':0.10829)[&label=0.314]:0.016358)[&label=0.866885]:0.008802,'DQ989311.1_Asia1_IND_2002':0.056475)[&label=0.862789]:0.0034,((((('EF494487.1_A_PAK_2006':1.0E-6,'EF117837.1_A_PAK_2006':1.0E-6)[&label=1.0]:0.007403,('JF749841.1_A_TUR_2006':0.006023,'EF494486.1_A_TUR_2005':0.004806)[&label=0.957]:0.002144)[&label=0.998]:0.008214,'EF494488.1_A_PAK_2006':0.005308)[&label=0.9385]:0.004177,('JN006720.1_Asia1_PAK_2009':0.006016,'JN006722.1_A_PAK_2008':0.001139)[&label=1.0]:0.025337)[&label=0.998]:0.022358,'KM268896.1_A_TUR_2013':0.04706)[&label=0.943]:0.020906)[&label=0.829724]:0.004596)[&label=0.585152]:0.007981,((((((((((((((('KY657269.1_O_VIT_2015':0.001158,'MG983740.1_O_VIT_2015':0.003482)[&label=0.912]:0.002276,'MG983693.1_O_LAO_2015':0.001194)[&label=1.0]:0.012029,'MG983716.1_O_NEP_2014':0.007201)[&label=0.862]:0.003334,'MG983714.1_O_NEP_2013':0.008163)[&label=0.7145]:1.0E-6,((('MG983715.1_O_NEP_2014':0.001109,'MG983685.1_O_BAR_2015':0.018035)[&label=0.723]:0.002359,'KJ825806.1_O_IND_2013':0.004636)[&label=0.67]:1.0E-6,'MG983736.1_O_UAE_2014':0.002304)[&label=0.786667]:0.00115)[&label=0.703375]:0.001152,('KJ825805.1_O_IND_2013':0.011781,'KJ825809.1_O_IND_2013':1.0E-6)[&label=0.611]:0.001151)[&label=0.636]:1.0E-6,(((('MG983733.1_O_SRL_2014':0.001152,'MG983734.1_O_SRL_2014':0.004673)[&label=0.788]:0.001152,'MG983732.1_O_SRL_2014':1.0E-6)[&label=0.9805]:0.003483,'KJ825807.1_O_IND_2014':0.003504)[&label=0.963333]:0.002295,'KJ825804.1_O_IND_2013':0.00347)[&label=0.79125]:1.0E-6)[&label=0.933933]:0.003471,'MG983731.1_O_SRL_2013':0.004645)[&label=0.92425]:1.0E-6,'MG983711.1_O_NEP_2013':0.003473)[&label=0.941176]:0.001152,'KJ825803.1_O_IND_2013':0.003475)[&label=0.944444]:1.0E-6,'KJ825808.1_O_IND_2013':0.005827)[&label=0.988211]:0.008211,('MG983709.1_O_NEP_2012':1.0E-6,'MG983708.1_O_NEP_2012':1.0E-6)[&label=1.0]:0.008262)[&label=0.920857]:1.0E-6,((((((('MG983721.1_O_SAU_2013':1.0E-6,'MG983722.1_O_SAU_2013':1.0E-6)[&label=0.707]:1.0E-6,'KJ206910.1_O_SAU_2013':0.001165)[&label=0.999]:0.007448,'MG983726.1_O_SAU_2014':0.012171)[&label=0.834]:8.75E-4,(('MG983723.1_O_SAU_2013':0.001206,'MG983724.1_O_SAU_2013':0.001149)[&label=0.999]:0.0138,'MG983725.1_O_SAU_2014':0.01005)[&label=0.9015]:7.15E-4)[&label=0.8095]:0.001196,('KJ825801.1_O_IND_2013':1.0E-6,'KJ825802.1_O_IND_2013':1.0E-6)[&label=1.0]:0.007154)[&label=0.812625]:0.001067,(((('MG983694.1_O_LIB_2013':0.001163,'KJ206909.1_O_LIB_2013':0.00233)[&label=0.067]:1.0E-6,('MG983695.1_O_LIB_2013':1.0E-6,'MG983697.1_O_LIB_2013':0.004682)[&label=0.523]:0.001162)[&label=0.727]:1.0E-6,'MG983696.1_O_LIB_2013':0.00233)[&label=0.96075]:0.002456,(('MG983683.1_O_ALG_2014':0.007123,'MG983735.1_O_TUN_2014':0.001146)[&label=0.985]:0.002368,'KU291242.1_O_MOR_2015':0.010789)[&label=0.9925]:0.008528)[&label=0.974857]:0.003481)[&label=0.953313]:0.007171,(('MG983713.1_O_NEP_2013':0.001191,'MG983712.1_O_NEP_2013':0.002317)[&label=0.999]:0.013721,'KJ206908.1_O_BHU_2013':0.011181)[&label=0.8375]:6.71E-4)[&label=0.855421]:0.001167)[&label=0.980317]:0.006297,'MG983717.1_O_NEP_2015':0.024532)[&label=0.987786]:0.006302,(((('MG983688.1_O_BHU_2009':0.014331,'MG983706.1_O_NEP_2010':0.005878)[&label=0.242]:1.0E-6,'MG983705.1_O_NEP_2010':0.003495)[&label=0.3105]:1.0E-6,'MG983692.1_O_IRN_2009':0.010622)[&label=0.369333]:1.0E-6,('MG983684.1_O_BAN_2009':0.00943,'MG983687.1_O_BHU_2009':1.0E-6)[&label=0.861]:0.002324)[&label=0.995]:0.014754)[&label=0.998583]:0.044133)[&label=0.522591]:1.0E-6,(((((((((((((((((((((((((('DQ404159.1_O_UKG_2001':1.0E-6,'KM257065.1_O_UKG_2001':1.0E-6)[&label=0.921]:0.001181,'DQ404160.1_O_UKG_2001':0.002367)[&label=0.6485]:1.0E-6,'DQ404158.1_O_UKG_2001':0.002366)[&label=0.910667]:0.001168,'DQ404161.1_O_UKG_2001':0.002386)[&label=0.98]:0.003582,'DQ404168.1_O_UKG_2001':0.001182)[&label=0.8466]:1.0E-6,'DQ404162.1_O_UKG_2001':0.00237)[&label=0.8795]:0.00118,'KM257064.1_O_UKG_2001':0.001179)[&label=0.760143]:1.0E-6,((('DQ404163.1_O_UKG_2001':0.001193,'DQ404172.1_O_UKG_2001':0.002376)[&label=0.658]:0.001177,'DQ404173.1_O_UKG_2001':1.0E-6)[&label=0.768]:0.00118,('FJ542368.1_O_UKG_2001':1.0E-6,'KM257063.1_O_UKG_2001':1.0E-6)[&label=0.717]:0.001178)[&label=0.438]:1.0E-6)[&label=0.514833]:1.0E-6,(('DQ404169.1_O_UKG_2001':1.0E-6,'EF552690.1_O_UKG_2001':1.0E-6)[&label=0.834]:0.001179,('EF552691.1_O_UKG_2001':1.0E-6,'EF552695.1_O_UKG_2001':1.0E-6)[&label=0.964]:0.001179)[&label=0.368333]:1.0E-6)[&label=0.434125]:1.0E-6,(((((('FJ542369.1_O_UKG_2001':1.0E-6,'FJ542365.1_O_UKG_2001':1.0E-6)[&label=0.142]:1.0E-6,'FJ542372.1_O_UKG_2001':1.0E-6)[&label=0.2865]:1.0E-6,'FJ542371.1_O_UKG_2001':1.0E-6)[&label=0.689667]:1.0E-6,'KM257061.1_O_UKG_2001':1.0E-6)[&label=0.93]:0.001179,((('DQ404166.1_O_UKG_2001':1.0E-6,'DQ404165.1_O_UKG_2001':1.0E-6)[&label=0.272]:1.0E-6,'DQ404167.1_O_UKG_2001':1.0E-6)[&label=0.876]:0.001179,'DQ404170.1_O_UKG_2001':1.0E-6)[&label=0.880333]:0.001179)[&label=0.51525]:1.0E-6,('DQ404164.1_O_UKG_2001':0.002362,'EU214601.1_O_UKG_2001':0.001178)[&label=0.08]:1.0E-6)[&label=0.4301]:1.0E-6)[&label=0.693148]:1.0E-6,'DQ404171.1_O_UKG_2001':1.0E-6)[&label=0.729571]:1.0E-6,((((('DQ404174.1_O_UKG_2001':1.0E-6,'EF552697.1_O_UKG_2001':1.0E-6)[&label=0.059]:1.0E-6,'DQ404175.1_O_UKG_2001':1.0E-6)[&label=0.122]:1.0E-6,'EF552693.1_O_UKG_2001':1.0E-6)[&label=0.223333]:1.0E-6,'EF552689.1_O_UKG_2001':1.0E-6)[&label=0.301]:1.0E-6,'EF552692.1_O_UKG_2001':1.0E-6)[&label=0.4432]:1.0E-6)[&label=0.941088]:0.001179,'KM257062.1_O_UKG_2001':1.0E-6)[&label=0.944886]:0.001179,((((('DQ404179.1_O_UKG_2001':1.0E-6,'AJ633821.1_O_FRA_2001':1.0E-6)[&label=0.047]:1.0E-6,('AY593831.1_O_UKG_2002':1.0E-6,'DQ404177.1_O_UKG_2001':1.0E-6)[&label=0.058]:1.0E-6)[&label=0.159667]:1.0E-6,('AJ539141.1_O_UKG_2001':0.001178,'DQ404180.1_O_UKG_2001':1.0E-6)[&label=0.794]:1.0E-6)[&label=0.208]:1.0E-6,(('EF552688.1_O_UKG_2001':1.0E-6,'DQ404176.1_O_UKG_2001':1.0E-6)[&label=0.071]:1.0E-6,'FJ542370.1_O_UKG_2001':1.0E-6)[&label=0.096]:1.0E-6)[&label=0.529625]:1.0E-6,(('DQ404178.1_O_UKG_2001':1.0E-6,'EF552696.1_O_UKG_2001':0.001179)[&label=0.004]:1.0E-6,'AY593836.1_O_UKG_2001':1.0E-6)[&label=0.0295]:1.0E-6)[&label=0.798455]:1.0E-6)[&label=0.97583]:0.001177,'AJ539140.1_O_SAR_2000':0.00118)[&label=0.978708]:0.001227,'AB079061.1_O_JPN_2000':0.010864)[&label=0.97351]:0.001135,'AF377945.1_O_SKR_2000':0.035737)[&label=0.95902]:1.0E-6,((('MG372730.1_O_SKR_2000':1.0E-6,'AJ539139.1_O_SKR_2000':1.0E-6)[&label=0.766]:1.0E-6,'AY593824.1_O_SKR_2000':0.001172)[&label=0.983]:0.001176,'AH012985.2_O_SKR_2000':0.005926)[&label=0.999667]:0.012085)[&label=0.929056]:1.0E-6,('AF506822.2_O_CHA_1999':1.0E-6,'AJ539138.1_O_CHA_1999':1.0E-6)[&label=0.99]:0.002359)[&label=0.937018]:0.001176,((((((((('KF694740.1_O_SKR_2002':1.0E-6,'KF694745.1_O_SKR_2002':0.001176)[&label=0.733]:0.001177,'KF694737.1_O_SKR_2002':0.001177)[&label=0.587]:1.0E-6,'KF694743.1_O_SKR_2002':1.0E-6)[&label=0.417]:1.0E-6,('KF694734.1_O_SKR_2002':1.0E-6,'KF694739.1_O_SKR_2002':1.0E-6)[&label=0.268]:1.0E-6)[&label=0.8608]:1.0E-6,('EF614457.1_O_SKR_2002':0.001175,'KF694744.1_O_SKR_2002':1.0E-6)[&label=0.983]:0.004752)[&label=0.921857]:0.002359,((('KF694736.1_O_SKR_2002':1.0E-6,'KF694742.1_O_SKR_2002':1.0E-6)[&label=0.289]:1.0E-6,'KF694741.1_O_SKR_2002':1.0E-6)[&label=0.7725]:1.0E-6,('KF694735.1_O_SKR_2002':0.001177,'KF694731.1_O_SKR_2002':0.00118)[&label=0.207]:1.0E-6)[&label=0.795]:1.0E-6)[&label=0.982833]:0.001531,(('KF694738.1_O_SKR_2002':1.0E-6,'KF694732.1_O_SKR_2002':1.0E-6)[&label=0.999]:0.001223,'AH012984.2_O_SKR_2000':0.010833)[&label=0.876]:0.003248)[&label=0.992267]:0.006931,('HM008917.1_O_CHA_2005':0.010999,'HQ009509.1_O_CHA_1999':0.044071)[&label=1.0]:0.020151)[&label=0.973]:0.003713,'HQ632768.1_O_MAY_2000':0.010993)[&label=0.957]:0.001033)[&label=0.980227]:1.0E-6,('AJ539137.1_O_TAW_1999':0.002363,'AJ539136.1_O_TAW_1999':0.002365)[&label=0.535]:0.001181)[&label=0.998169]:0.009761,'EF149010.1_Asia1_CHA_2005':0.067849)[&label=0.991167]:0.001211,('JF749849.1_Asia1_PAK_2002':0.017804,'JF749851.1_O_IRN_2001':0.010073)[&label=0.988]:0.016324)[&label=0.9978]:0.015056,((((((('MF143572.1_O_VIT_2012':1.0E-6,'MF143573.1_O_VIT_2012':0.004685)[&label=0.355]:0.001092,'MF143574.1_O_VIT_2012':0.003611)[&label=0.9775]:0.007346,('MF143575.1_O_VIT_2012':0.005993,'MF143576.1_O_VIT_2013':0.006021)[&label=0.824]:0.003529)[&label=0.6065]:0.002344,('MF143577.1_O_VIT_2013':0.003645,'MF143578.1_O_VIT_2013':0.001098)[&label=0.998]:0.010856)[&label=0.767167]:1.0E-6,'MF947141.1_O_VIT_2012':0.020818)[&label=0.770714]:0.001231,((((('MF947127.1_O_VIT_2012':1.0E-6,'MF947137.1_O_VIT_2012':1.0E-6)[&label=1.0]:0.02239,'MF947123.1_O_VIT_2011':0.004951)[&label=0.8525]:8.95E-4,'KY234501.1_O_CHA_2011':0.010875)[&label=0.814]:0.001226,(('MF947124.1_O_VIT_2012':0.013566,'MF947129.1_O_VIT_2014':0.017307)[&label=0.989]:0.012396,'MF947126.1_O_VIT_2011':0.003533)[&label=0.8135]:0.001183)[&label=0.877167]:0.001168,'MF947128.1_O_VIT_2010':0.002363)[&label=0.911714]:0.002352)[&label=0.9198]:0.00185,((('MF947132.1_O_VIT_2015':0.021521,'KY234502.1_O_CHA_2015':0.011997)[&label=0.998]:0.013816,'MF947131.1_O_VIT_2013':0.003139)[&label=0.9725]:0.005091,('MF947142.1_O_VIT_2013':0.00597,'MF947143.1_O_VIT_2013':0.008348)[&label=0.76]:1.0E-6)[&label=0.99875]:0.008842)[&label=1.0]:0.030605)[&label=0.998525]:0.016415,((((((((((((((('JX040490.1_O_BUL_2011':1.0E-6,'JX040488.1_O_BUL_2011':1.0E-6)[&label=0.237]:1.0E-6,'JX040489.1_O_BUL_2011':1.0E-6)[&label=0.598]:1.0E-6,'JX040487.1_O_BUL_2011':1.0E-6)[&label=0.961333]:0.001172,'JX040486.1_O_BUL_2011':0.001173)[&label=0.91675]:0.001178,(('JX066665.1_O_BUL_2011':0.003527,'JX066664.1_O_BUL_2011':0.002351)[&label=0.198]:1.0E-6,'JX040485.1_O_BUL_2010':0.001176)[&label=0.261]:1.0E-6)[&label=0.992571]:0.004721,'JX040491.1_O_TUR_2010':0.002348)[&label=0.89225]:1.0E-6,((('JX040494.1_O_TUR_2010':0.001171,'JX040495.1_O_TUR_2010':0.004738)[&label=0.232]:1.0E-6,'JX040496.1_O_TUR_2010':0.001171)[&label=0.1615]:1.0E-6,'JX040493.1_O_TUR_2010':1.0E-6)[&label=0.207333]:1.0E-6)[&label=0.807]:1.0E-6,'JX040497.1_O_TUR_2010':0.001171)[&label=0.881385]:1.0E-6,('JX040500.1_O_TUR_2011':0.003543,'JX040499.1_O_TUR_2011':0.00964)[&label=0.848]:0.002364)[&label=0.965267]:0.001172,'JX040498.1_O_TUR_2010':0.001176)[&label=0.989]:0.003525,'JX040501.1_O_ISR_2011':0.009565)[&label=0.958294]:1.0E-6,'JX040492.1_O_TUR_2010':0.008399)[&label=0.985111]:0.003935,(('MH784403.1_O_PAK_2016':0.005587,'MH784404.1_O_PAK_2017':0.005206)[&label=1.0]:0.023078,'MH784405.1_O_PAK_2017':0.020181)[&label=0.999]:0.017086)[&label=0.999286]:0.013652,'KM268898.1_Asia1_TUR_2013':0.027973)[&label=0.971273]:0.003278,'JN006719.1_Asia1_PAK_2008':0.040237)[&label=0.995913]:0.022141)[&label=0.927552]:0.008613,'AY687333.1_Asia1_IND_2001':0.081508)[&label=0.921333]:0.003306)[&label=0.667544]:0.021491,(('HQ832590.1_A_IND_2007':0.005904,'HQ832591.1_A_IND_2008':0.016081)[&label=1.0]:0.018127,('KJ754939.1_A_BAN_2013':0.025788,'KU127247.1_A_SAU_2015':0.021944)[&label=0.924]:0.013035)[&label=0.911667]:0.007291)[&label=0.67135]:0.008665,((((((((((((((((((('LC320038.1_O_MOG_2015':0.004711,'MG983730.1_O_SKR_2017':0.010751)[&label=0.194]:1.0E-6,'MG983720.1_O_RUS_2016':0.004704)[&label=0.884]:0.003527,'MF461724.1_O_CHA_2017':0.004746)[&label=0.970333]:0.003558,'LC438823.1_O_MYA_2016':0.009517)[&label=0.85475]:1.0E-6,'MG983741.1_O_VIT_2016':0.011905)[&label=0.8466]:0.001866,'LC438822.1_O_MYA_2016':0.008829)[&label=0.908167]:0.002433,'MH891503.1_O_VIT_2017':0.015268)[&label=0.957143]:0.001574,'KX712091.1_O_BAN_2015':1.0E-6)[&label=0.93325]:0.001168,('MG983727.1_O_SAU_2015':0.004782,'MG983728.1_O_SAU_2016':0.003464)[&label=0.995]:0.00969)[&label=0.8332]:1.0E-6,'MG983703.1_O_MYA_2016':0.007106)[&label=0.864818]:0.001168,((('MG983702.1_O_MUR_2016':1.0E-6,'MG983700.1_O_MUR_2016':1.0E-6)[&label=0.174]:1.0E-6,'MG983701.1_O_MUR_2016':1.0E-6)[&label=0.3895]:1.0E-6,('MG983699.1_O_MUR_2016':1.0E-6,'MG983698.1_O_MUR_2016':1.0E-6)[&label=0.134]:1.0E-6)[&label=1.0]:0.010794)[&label=0.830937]:1.0E-6,('MG983719.1_O_NEP_2015':0.005985,'MG983729.1_O_SAU_2016':0.015753)[&label=0.6]:0.001135)[&label=0.910444]:1.0E-6,'MG983718.1_O_NEP_2015':0.008297)[&label=0.940526]:0.002007,(('MG983739.1_O_UAE_2016':1.0E-6,'MG983738.1_O_UAE_2016':0.001174)[&label=1.0]:0.003483,'MG983691.1_O_BHU_2016':0.00724)[&label=0.9985]:0.008896)[&label=0.999955]:0.020378,((('MG983707.1_O_NEP_2012':0.008594,'MG983689.1_O_BHU_2012':0.008419)[&label=0.432]:8.55E-4,'MF372125.1_Asia1_IND_2016':0.028676)[&label=0.745]:0.001786,'MG983710.1_O_NEP_2012':0.011856)[&label=0.764333]:0.00116)[&label=0.999962]:0.01899,'MG983704.1_O_NEP_2008':0.021318)[&label=0.985222]:7.62E-4,'KF985189.1_O_BAN_2013':0.03155)[&label=0.990964]:0.010341,('DQ989319.1_Asia1_IND_2001':0.023795,'DQ989322.1_Asia1_IND_2002':0.016847)[&label=0.876]:0.006355)[&label=0.975033]:0.007126,(((('HQ832588.1_A_IND_2005':0.057787,'HQ832589.1_A_IND_2006':0.007885)[&label=0.787]:0.017276,'HQ832592.1_A_IND_2009':0.027497)[&label=0.5715]:0.002601,'HQ832584.1_A_IND_2005':0.05746)[&label=0.763333]:0.01455,'HQ832586.1_A_IND_2006':0.038086)[&label=0.659]:0.007843)[&label=0.909143]:0.011611)[&label=0.784056]:0.010753,(((('DQ989314.1_Asia1_IND_2001':0.013256,'DQ989320.1_Asia1_IND_2002':0.009514)[&label=0.865]:0.002396,('DQ989321.1_Asia1_IND_2001':0.005919,'DQ989323.1_Asia1_IND_2002':0.013128)[&label=0.398]:1.0E-6)[&label=0.852]:1.0E-6,'DQ989318.1_Asia1_IND_2002':0.004693)[&label=0.96]:0.006617,'DQ989317.1_Asia1_IND_2000':0.005424)[&label=0.99]:0.016998)[&label=0.810554]:0.010675,'MF782478.1_Asia1_BAN_2013':0.043419)[&label=0.8156]:0.020323,((((((('FJ175663.1_O_ISR_2007':1.0E-6,'FJ175664.1_O_ISR_2007':1.0E-6)[&label=0.892]:1.0E-6,'FJ175666.1_O_ISR_2007':0.00235)[&label=0.98]:0.002353,'FJ175665.1_O_ISR_2007':0.004774)[&label=0.91]:0.001388,('FJ175662.1_O_ISR_2007':0.001177,'FJ175661.1_O_ISR_2007':0.001172)[&label=0.999]:0.008134)[&label=0.9338]:0.004813,(('KC440882.1_A_EGY_2012':0.035503,'KC440883.1_O_EGY_2011':0.034251)[&label=0.671]:0.015129,'KM268895.1_O_TUR_2013':0.024882)[&label=0.5655]:6.35E-4)[&label=0.894625]:0.006733,(('JF749852.1_O_MAY_2004':0.001177,'HQ632770.1_O_MAY_2004':0.002378)[&label=1.0]:0.009185,'HQ268524.1_O_BHU_2004':0.013109)[&label=0.9885]:0.007584)[&label=0.937818]:0.006727,('GU384683.1_O_PAK_2008':1.0E-6,'GU384682.1_O_PAK_2008':1.0E-6)[&label=1.0]:0.018635)[&label=0.955]:0.008561)[&label=0.892535]:0.004445,((((('JN099698.1_A_IRQ_2009':1.0E-6,'JN099695.1_A_IRQ_2009':1.0E-6)[&label=0.996]:0.004703,'JN099688.1_A_IRQ_2009':0.009522)[&label=0.807]:0.001192,('JN099699.1_A_IRQ_2009':1.0E-6,'JN099697.1_A_IRQ_2009':0.001171)[&label=0.852]:0.001172)[&label=0.84025]:1.0E-6,'JN099694.1_A_IRQ_2009':0.007077)[&label=0.9944]:0.021202,'HQ113232.1_O_PAK_2009':0.018862)[&label=0.9695]:0.007145)[&label=0.968241]:0.010065,'HQ113233.1_Asia1_AFG_2009':0.029514)[&label=0.980551]:0.03107,('MG983690.1_O_BHU_2016':0.010175,'MG983686.1_O_BAR_2015':0.005451)[&label=0.998]:0.034771)[&label=1.0]:0.141874,(('MF678825.1_SAT1_NIG_2015':1.0E-6,'MF678826.1_SAT1_NIG_2015':1.0E-6)[&label=1.0]:0.00372,('MF678824.1_SAT1_NIG_2015':0.001173,'MF678823.1_SAT1_NIG_2015':1.0E-6)[&label=1.0]:0.008333)[&label=1.0]:0.195483)[&label=0.995222]:0.036888,(((((((((((((((((((('AY593851.1_SAT3_BOT_1961':1.0E-6,'AY593852.1_SAT3_KEN_1960':1.0E-6)[&label=1.0]:0.031998,('AY593853.1_SAT3_BOT_1965':0.003244,'MH053338.1_SAT3_BOT_1966':0.011361)[&label=1.0]:0.025029)[&label=0.631667]:0.010502,(('AY593840.1_SAT1_NMB_1949':0.026995,'MH053351.1_SAT3_ZIM_1984':0.022973)[&label=0.276]:0.002253,'MH053331.1_SAT2_BOT_1972':0.030576)[&label=0.236]:0.004856)[&label=0.4195]:1.0E-6,((('MH053328.1_SAT2_BOT_1968':0.034967,'MH053339.1_SAT3_BOT_1970':0.023623)[&label=0.339]:0.005614,'AY593845.1_SAT1_BOT_1968':0.044045)[&label=0.372]:0.004594,'MH053329.1_SAT2_BOT_1969':0.009997)[&label=0.365333]:0.004004)[&label=0.5576]:0.002536,((((('KU821592.1_SAT2_ZAM_2009':0.039561,'JF749864.1_SAT2_ZIM_2003':0.025895)[&label=0.412]:0.008571,'KU821590.1_SAT1_NMB_2010':0.027771)[&label=0.4075]:0.00418,('AY593843.1_SAT1_NMB_1940':0.033029,'AY593841.1_SAT1_ZIM_1958':0.034358)[&label=0.1]:0.003734)[&label=0.368]:0.0048,'MH053332.1_SAT2_BOT_1974':0.016939)[&label=0.3858]:0.002763,'MH053322.1_SAT1_NMB_1989':0.028559)[&label=0.366]:0.003796)[&label=0.770882]:0.004355,('AY593842.1_SAT1_SAR_1961':0.026446,'AY593838.1_SAT1_BOT_1970':0.076736)[&label=0.203]:0.008568)[&label=0.868789]:0.003356,'MH053330.1_SAT2_BOT_1969':0.028584)[&label=0.9102]:0.001613,'MH053319.1_SAT1_BOT_1974':0.017147)[&label=0.942048]:0.032243,(('MH053344.1_SAT3_ZIM_1974':0.070065,'KR108950.1_SAT3_SAR_2009':0.027674)[&label=0.52]:0.007889,'MH053343.1_SAT3_ZIM_1934':0.052265)[&label=0.414]:0.002301)[&label=0.848542]:0.007228,('AY593850.1_SAT3_SAR_1959':0.031846,'MH053342.1_SAT3_ZAM_1996':0.024097)[&label=0.178]:0.006208)[&label=0.800192]:0.002244,'KM268901.1_SAT3_ZIM_1991':0.051397)[&label=0.784556]:1.0E-6,'MH053321.1_SAT1_MOZ_1981':0.046017)[&label=0.777]:0.002204,('AY593848.1_SAT2_u_1967':0.042713,'KR108948.1_SAT1_SAR_2009':0.032433)[&label=0.523]:0.011288)[&label=0.758133]:0.005257,((((('MH053334.1_SAT2_ZAM_1964':0.049473,'AY593847.1_SAT2_ZIM_1948':0.048619)[&label=0.885]:0.019927,'MH053340.1_SAT3_MAL_1976':0.027478)[&label=0.7005]:0.007522,'MH053352.1_SAT3_ZIM_1990':0.054683)[&label=0.591667]:0.004588,((('MH053346.1_SAT3_ZIM_1976':0.003157,'MH053348.1_SAT3_ZIM_1977':0.004005)[&label=1.0]:0.042545,'AY593846.1_SAT1_ZIM_1966':0.041928)[&label=0.729]:0.010683,'AY593839.1_SAT1_UKG_1970':0.039891)[&label=0.651667]:0.009122)[&label=0.543857]:0.01134,'KR108949.1_SAT2_SAR_2009':0.024263)[&label=0.507]:1.0E-6)[&label=0.836487]:0.005961,'KX375417.1_SAT3_ZIM_1981':0.035299)[&label=0.857275]:0.007625,(('MH053350.1_SAT3_ZIM_1983':1.0E-6,'MH053349.1_SAT3_ZIM_1983':0.001189)[&label=1.0]:0.025145,'MH053335.1_SAT2_ZIM_1965':0.041003)[&label=0.8315]:0.00807)[&label=0.908047]:0.007465,('MH053347.1_SAT3_ZIM_1976':0.008765,'MH053345.1_SAT3_ZIM_1975':0.002053)[&label=1.0]:0.024221)[&label=0.934756]:0.014853,(((('KM268899.1_SAT1_TAN_2012':0.032088,'JF749861.1_SAT2_KEN_2002':0.02856)[&label=0.658]:0.008179,'MH053320.1_SAT1_KEN_1983':0.060245)[&label=0.515]:0.00766,('MH053333.1_SAT2_ETH_1989':0.066775,'JF749860.1_SAT1_KEN_2002':0.046227)[&label=0.418]:0.012754)[&label=0.75525]:0.003556,'KM268900.1_SAT2_TAN_2012':0.057254)[&label=0.8896]:0.010769)[&label=1.0]:0.261892,((((('MH053327.1_SAT1_UGA_1970':0.014078,'MH053337.1_SAT2_UGA_1970':0.02632)[&label=1.0]:0.045863,'KJ820999.1_SAT3_UGA_2013':0.061155)[&label=0.7945]:0.014257,'MH053341.1_SAT3_UGA_1970':0.041704)[&label=0.764333]:0.010819,(('HM067705.1_SAT2_UGA_2007':0.081348,'JF749862.1_SAT2_UGA_2002':0.051353)[&label=0.719]:0.014979,('MH053326.1_SAT1_UGA_1970':0.066304,'MH053336.1_SAT2_UGA_1970':0.051946)[&label=0.591]:0.018102)[&label=0.556667]:0.010329)[&label=0.863571]:0.009492,('HM067704.1_SAT2_UGA_2007':0.067005,'HM067706.1_SAT1_UGA_2007':0.052947)[&label=0.696]:0.016627)[&label=0.998556]:0.059882)[&label=0.989344]:0.082524,((((((('JX014255.1_SAT2_EGY_2012':0.00472,'JX014256.1_SAT2_PAT_2012':0.004713)[&label=0.629]:0.001255,'KC440884.1_SAT2_EGY_2012':0.001138)[&label=1.0]:0.103938,('KP940474.1_A_EGY_2014':0.002483,'KC440881.1_A_EGY_2011':0.00232)[&label=1.0]:0.132368)[&label=0.98825]:0.051667,'AY593844.1_SAT1_ISR_1962':0.092034)[&label=0.9298]:0.005959,'MH053323.1_SAT1_TCH_1972':0.086071)[&label=0.983333]:0.030264,'AY593849.1_SAT2_KEN_1960':0.128529)[&label=0.948429]:0.009011,(('MH053324.1_SAT1_UGA_1971':0.037425,'FJ461346.1_SAT2_UGA_2002':0.034836)[&label=0.527]:0.016765,'MH053325.1_SAT1_UGA_1978':0.075401)[&label=0.5915]:0.020562)[&label=0.919]:0.023373)[&label=0.995222]:0.041346);

end;

begin figtree;

set appearance.backgroundColorAttribute="Default";

set appearance.backgroundColour=#ffffff;

set appearance.branchColorAttribute="User selection";

set appearance.branchColorGradient=false;

set appearance.branchLineWidth=1.0;

set appearance.branchMinLineWidth=0.0;

set appearance.branchWidthAttribute="Fixed";

set appearance.foregroundColour=#000000;

set appearance.hilightingGradient=false;

set appearance.selectionColour=#2d3680;

set branchLabels.colorAttribute="User selection";

set branchLabels.displayAttribute="Branch times";

set branchLabels.fontName="Calibri";

set branchLabels.fontSize=12;

set branchLabels.fontStyle=0;

set branchLabels.isShown=false;

set branchLabels.significantDigits=4;

set layout.expansion=374;

set layout.layoutType="RECTILINEAR";

set layout.zoom=0;

set legend.attribute="label";

set legend.fontSize=10.0;

set legend.isShown=false;

set legend.significantDigits=4;

set nodeBars.barWidth=4.0;

set nodeBars.displayAttribute=null;

set nodeBars.isShown=false;

set nodeLabels.colorAttribute="User selection";

set nodeLabels.displayAttribute="label";

set nodeLabels.fontName="Arial";

set nodeLabels.fontSize=12;

set nodeLabels.fontStyle=0;

set nodeLabels.isShown=true;

set nodeLabels.significantDigits=4;

set nodeShapeExternal.colourAttribute="User selection";

set nodeShapeExternal.isShown=false;

set nodeShapeExternal.minSize=10.0;

set nodeShapeExternal.scaleType=Width;

set nodeShapeExternal.shapeType=Circle;

set nodeShapeExternal.size=4.0;

set nodeShapeExternal.sizeAttribute="Fixed";

set nodeShapeInternal.colourAttribute="User selection";

set nodeShapeInternal.isShown=false;

set nodeShapeInternal.minSize=10.0;

set nodeShapeInternal.scaleType=Width;

set nodeShapeInternal.shapeType=Circle;

set nodeShapeInternal.size=4.0;

set nodeShapeInternal.sizeAttribute="Fixed";

set polarLayout.alignTipLabels=false;

set polarLayout.angularRange=0;

set polarLayout.rootAngle=0;

set polarLayout.rootLength=100;

set polarLayout.showRoot=true;

set radialLayout.spread=0.0;

set rectilinearLayout.alignTipLabels=true;

set rectilinearLayout.curvature=0;

set rectilinearLayout.rootLength=100;

set scale.offsetAge=0.0;

set scale.rootAge=1.0;

set scale.scaleFactor=1.0;

set scale.scaleRoot=false;

set scaleAxis.automaticScale=true;

set scaleAxis.fontSize=8.0;

set scaleAxis.isShown=false;

set scaleAxis.lineWidth=1.0;

set scaleAxis.majorTicks=1.0;

set scaleAxis.minorTicks=0.5;

set scaleAxis.origin=0.0;

set scaleAxis.reverseAxis=false;

set scaleAxis.showGrid=true;

set scaleBar.automaticScale=true;

set scaleBar.fontSize=12.0;

set scaleBar.isShown=true;

set scaleBar.lineWidth=1.0;

set scaleBar.scaleRange=0.0;

set tipLabels.colorAttribute="User selection";

set tipLabels.displayAttribute="Names";

set tipLabels.fontName="Arial";

set tipLabels.fontSize=12;

set tipLabels.fontStyle=0;

set tipLabels.isShown=true;

set tipLabels.significantDigits=4;

set trees.order=true;

set trees.orderType="increasing";

set trees.rooting=false;

set trees.rootingType="User Selection";

set trees.transform=false;

set trees.transformType="cladogram";

end;
