# Supplementary material for: Early origin and global colonisation of foot-and-mouth disease virus
Source: Sci Rep. 2020 Sep 17;10:15268. doi: 10.1038/s41598-020-72246-6 (PMC7498456; doi:10.1038/s41598-020-72246-6)
Supplement: Supplementary file 12 — Supplementary Tree S4. [file 41598_2020_72246_MOESM12_ESM.doc]

#NEXUS

begin trees;

tree tree_1 = [&R] ((((((((((((((((((((((((((((((((((((((('KJ560285.1_O_UKG_2007':1.0E-6,'EU448369.1_O_UKG_1967':1.0E-6)[&label=0.031]:1.0E-6,'KJ560276.1_O_UKG_2007':1.0E-6)[&label=0.0335]:1.0E-6,'EU448373.1_O_UKG_2007':1.0E-6)[&label=0.042667]:1.0E-6,(('JX570647.1_O_UKG_2007':1.0E-6,'JX570638.1_O_UKG_2007':1.0E-6)[&label=0.012]:1.0E-6,'JX570639.1_O_UKG_2007':1.0E-6)[&label=0.0195]:1.0E-6)[&label=0.076333]:1.0E-6,((('JX570641.1_O_UKG_2007':1.0E-6,'KJ560281.1_O_UKG_2007':1.0E-6)[&label=0.015]:1.0E-6,'JX570649.1_O_UKG_2007':1.0E-6)[&label=0.031]:1.0E-6,'KJ560277.1_O_UKG_2007':1.0E-6)[&label=0.049333]:1.0E-6)[&label=0.1426]:1.0E-6,'JX570648.1_O_UKG_2007':1.0E-6)[&label=0.169]:1.0E-6,('JX570642.1_O_UKG_2007':1.0E-6,'EU448372.1_O_UKG_2007':1.0E-6)[&label=0.011]:1.0E-6)[&label=0.258]:1.0E-6,((('EU448370.1_O_UKG_1967':1.0E-6,'JX570654.1_O_UKG_2007':1.0E-6)[&label=0.017]:1.0E-6,'JX570650.1_O_UKG_2007':1.0E-6)[&label=0.0245]:1.0E-6,'EU448374.1_O_UKG_2007':1.0E-6)[&label=0.039]:1.0E-6)[&label=0.462647]:1.0E-6,'KJ560287.1_O_UKG_2007':1.0E-6)[&label=0.507778]:1.0E-6,'JX570645.1_O_UKG_2007':1.0E-6)[&label=0.542053]:1.0E-6,'EU448371.1_O_UKG_2007':1.0E-6)[&label=0.58345]:1.0E-6,((((((((((('KJ560308.1_O_UKG_2007':1.0E-6,'KJ560303.1_O_UKG_2007':1.0E-6)[&label=0.257]:1.0E-6,'KJ560307.1_O_UKG_2007':1.0E-6)[&label=0.8875]:0.001062,'KJ560304.1_O_UKG_2007':1.0E-6)[&label=0.888333]:0.001061,'EU448375.1_O_UKG_2007':1.0E-6)[&label=0.76275]:1.0E-6,'KJ560302.1_O_UKG_2007':0.002127)[&label=0.7782]:0.001061,((('KJ560296.1_O_UKG_2007':1.0E-6,'EU448377.1_O_UKG_2007':1.0E-6)[&label=0.232]:1.0E-6,'EU448376.1_O_UKG_2007':1.0E-6)[&label=0.5385]:1.0E-6,'KJ560294.1_O_UKG_2007':1.0E-6)[&label=0.835667]:1.0E-6)[&label=0.737556]:0.001061,'KJ560298.1_O_UKG_2007':0.001061)[&label=0.7532]:1.0E-6,('EU448378.1_O_UKG_2007':1.0E-6,'KJ560299.1_O_UKG_2007':1.0E-6)[&label=0.202]:1.0E-6)[&label=0.754667]:1.0E-6,'KJ560300.1_O_UKG_2007':1.0E-6)[&label=0.794308]:0.00106,'KJ560297.1_O_UKG_2007':1.0E-6)[&label=0.782643]:1.0E-6,'KJ560283.1_O_UKG_2007':0.001061)[&label=0.740733]:1.0E-6)[&label=0.731972]:1.0E-6,((('JX570644.1_O_UKG_2007':1.0E-6,'JX570646.1_O_UKG_2007':0.001059)[&label=0.906]:0.002124,'JX570640.1_O_UKG_2007':1.0E-6)[&label=0.7575]:1.0E-6,'JX570643.1_O_UKG_2007':0.002126)[&label=0.526667]:1.0E-6)[&label=0.8152]:1.0E-6,('JX570651.1_O_UKG_2007':1.0E-6,'JX570652.1_O_UKG_2007':0.003195)[&label=0.634]:0.001062)[&label=0.863571]:1.0E-6,('JX570653.1_O_UKG_2007':1.0E-6,'JX570655.1_O_UKG_2007':1.0E-6)[&label=0.927]:0.001061)[&label=0.908977]:0.001064,'EU448368.1_O_UKG_1967':0.001061)[&label=0.903333]:1.0E-6,((((('AY593815.1_O_UKG_1967':1.0E-6,'AY593816.1_O_UKG_1967':1.0E-6)[&label=0.879]:0.001061,('JX869187.1_O_UKG_1968':0.001062,'JX869184.1_O_UKG_1968':0.002126)[&label=0.047]:1.0E-6)[&label=0.330667]:1.0E-6,('JX869181.1_O_UKG_1967':0.002127,'JX869185.1_O_UKG_1968':0.002126)[&label=0.131]:1.0E-6)[&label=0.2498]:1.0E-6,'JX869180.1_O_UKG_1967':1.0E-6)[&label=0.2315]:1.0E-6,'JX869179.1_O_UKG_1967':1.0E-6)[&label=0.296571]:1.0E-6)[&label=0.939208]:1.0E-6,('JX869183.1_O_UKG_1968':0.008595,'JX869186.1_O_UKG_1968':0.002127)[&label=0.221]:1.0E-6)[&label=0.973364]:1.0E-6,'JX869188.1_O_UKG_1968':0.001061)[&label=0.990982]:0.001062,'JX869182.1_O_UKG_1968':1.0E-6)[&label=0.999018]:0.01088,(('AY593767.1_A_ARG_1965':0.002287,'AY593814.1_O_ARG_1965':0.005242)[&label=0.842]:0.003238,'AY593817.1_O_Belgium_1973':0.004152)[&label=0.9495]:0.001199)[&label=0.98475]:0.001065,'AY593830.1_O_POL_1959':0.005359)[&label=0.973967]:1.0E-6,(('AY593819.1_O_ARG_1994':0.001063,'JX869177.1_O_UKG_1967':0.010836)[&label=0.083]:1.0E-6,'AY593820.1_O_ARG_1964':0.001064)[&label=0.1335]:1.0E-6)[&label=0.967516]:1.0E-6,'AY593837.1_O_URU_1963':0.001063)[&label=0.9826]:1.0E-6,'AY593818.1_O_ARG_1958':0.003203)[&label=0.992076]:0.004371,'JX869178.1_O_UKG_1967':0.048463)[&label=0.998433]:0.015565,('AY593773.1_A_PER_1969':0.005055,'AY593775.1_A_VEN_1970':0.004605)[&label=0.999]:0.038137)[&label=0.999101]:0.036999,((((((('AY593778.1_A_SPA_1969':0.001064,'AY593754.1_A_SPA_1959':1.0E-6)[&label=1.0]:0.0179,'AY593780.1_A_FRA_1960':0.003008)[&label=0.9995]:0.006751,'AY593781.1_A_GER_1951':0.003262)[&label=0.888]:0.001031,'AY593776.1_A_GER_1968':0.006454)[&label=0.8505]:1.0E-6,'AY593792.1_A_ITL_1962':0.003207)[&label=1.0]:0.035822,'AY593771.1_A_COL_1967':0.037482)[&label=0.996833]:0.016889,((('AY593805.1_C_GER_1960':1.0E-6,'AY593804.1_C_SWZ_1965':1.0E-6)[&label=1.0]:0.002499,'FJ824812.1_C_SPA_2009':0.019488)[&label=1.0]:0.019506,'AY593808.1_C_ARG_1966':0.03114)[&label=1.0]:0.042813)[&label=0.9643]:0.017683)[&label=0.939975]:0.008556,((((((((((((((((('KX002203.1_A_ARG_2001':1.0E-6,'AY593784.1_A_ARG_2001':1.0E-6)[&label=0.235]:1.0E-6,'AY593785.1_A_ARG_2001':1.0E-6)[&label=0.907]:0.001064,'KX002181.1_A_ARG_2001':0.006452)[&label=0.657667]:1.0E-6,(('KX002179.1_A_ARG_2001':0.003228,'KX002201.1_A_ARG_2001':0.00107)[&label=0.467]:0.001062,'KX002186.1_A_ARG_2001':0.007548)[&label=0.3485]:1.0E-6)[&label=0.434167]:1.0E-6,(('KX002190.1_A_ARG_2001':0.004298,'KX002188.1_A_ARG_2001':0.003217)[&label=0.706]:0.001069,'KX002185.1_A_ARG_2001':0.005356)[&label=0.402]:1.0E-6)[&label=0.670333]:1.0E-6,'KX002200.1_A_ARG_2001':1.0E-6)[&label=0.7817]:1.0E-6,'AY593802.1_A_URU_2001':0.001064)[&label=0.870273]:0.001072,'KX002177.1_A_ARG_2001':0.003224)[&label=0.877583]:0.001059,'KX002202.1_A_ARG_2001':0.005368)[&label=0.832846]:1.0E-6,((('KX002194.1_A_ARG_2001':0.001068,'KX002199.1_A_ARG_2001':0.001067)[&label=0.644]:0.001068,'KX002197.1_A_ARG_2001':0.007563)[&label=0.718]:1.0E-6,'KX002195.1_A_ARG_2001':1.0E-6)[&label=0.66]:0.001066)[&label=0.909588]:0.001077,(((((((('KX002184.1_A_ARG_2001':0.002141,'KX002180.1_A_ARG_2001':0.002141)[&label=0.369]:1.0E-6,'KX002176.1_A_ARG_2001':0.001069)[&label=0.9325]:0.002142,'KX002182.1_A_ARG_2001':1.0E-6)[&label=0.955333]:0.002141,'KX002198.1_A_ARG_2001':1.0E-6)[&label=0.98775]:0.003224,('KX002204.1_A_ARG_2001':1.0E-6,'AY593790.1_A_ARG_2001':1.0E-6)[&label=0.955]:0.002139)[&label=0.7255]:1.0E-6,(('AY593786.1_A_ARG_2001':1.0E-6,'KX002205.1_A_ARG_2001':1.0E-6)[&label=0.954]:0.002139,'KX002183.1_A_ARG_2001':0.001067)[&label=0.5555]:1.0E-6)[&label=0.707556]:1.0E-6,(('KX002189.1_A_ARG_2001':1.0E-6,'KX002192.1_A_ARG_2001':0.003206)[&label=0.958]:0.003209,'KX002187.1_A_ARG_2001':0.008657)[&label=0.732]:1.0E-6)[&label=0.941167]:0.001064,'KX002178.1_A_ARG_2001':0.004327)[&label=0.946692]:0.005388)[&label=0.967129]:0.002138,'KX002193.1_A_ARG_2001':0.008701)[&label=0.969219]:0.001119,('KX002191.1_A_ARG_2001':0.022164,'KX002196.1_A_ARG_2001':0.005392)[&label=0.161]:1.0E-6)[&label=1.0]:0.094508,'AY593803.1_A_Brazil_1979':0.039252)[&label=0.977571]:0.007758,((((((('MH559805.1_A_Brazil_2016':1.0E-6,'MH559796.1_A_Brazil_2016':1.0E-6)[&label=0.211]:1.0E-6,('MH559786.1_A_Brazil_2016':1.0E-6,'MH559781.1_A_Brazil_2016':1.0E-6)[&label=0.058]:1.0E-6)[&label=0.381667]:1.0E-6,'AY593768.1_A_Brazil_1955':1.0E-6)[&label=0.521]:1.0E-6,'MH559799.1_A_Brazil_2016':1.0E-6)[&label=0.7574]:1.0E-6,(('MH559804.1_A_Brazil_2016':1.0E-6,'MH559783.1_A_Brazil_2016':1.0E-6)[&label=0.284]:1.0E-6,'MH559801.1_A_Brazil_2016':1.0E-6)[&label=0.9]:0.00107)[&label=0.84275]:0.00107,(((('MH559785.1_A_Brazil_2016':1.0E-6,'MH559800.1_A_Brazil_2016':1.0E-6)[&label=0.03]:1.0E-6,'MH559798.1_A_Brazil_2016':1.0E-6)[&label=0.1635]:1.0E-6,('MH559780.1_A_Brazil_2016':1.0E-6,'MH559788.1_A_Brazil_2016':1.0E-6)[&label=0.058]:1.0E-6)[&label=0.41475]:1.0E-6,('MH559793.1_A_Brazil_2016':1.0E-6,'MH559791.1_A_Brazil_2016':1.0E-6)[&label=0.105]:1.0E-6)[&label=0.864667]:1.0E-6)[&label=1.0]:0.049003,(('AY593758.1_A_VEN_1967':1.0E-6,'AY593753.1_A_Brazil_1970':0.00106)[&label=1.0]:0.011206,'AY593757.1_A_Brazil_1967':0.008511)[&label=1.0]:0.039975)[&label=0.991111]:0.013139)[&label=0.833907]:0.008836,((((('AY593807.1_C_Brazil_1955':0.002863,'AY593809.1_C_ARG_1969':0.009107)[&label=0.948]:0.017323,'AY593793.1_A_PHI_1975':0.055452)[&label=0.9995]:0.029223,'AY593782.1_A_ARG_2000':0.101295)[&label=0.756667]:0.003234,('AY593788.1_A_Brazil_1979':0.020921,'AY593787.1_A_Brazil_1977':1.0E-6)[&label=1.0]:0.072209)[&label=0.664]:0.008211,((('AY593756.1_A_Brazil_1959':0.046327,'AY593770.1_A_ARG_1966':0.061063)[&label=0.336]:0.005176,'AY593806.1_C_Brazil_1971':0.073611)[&label=0.3175]:0.002646,'AY593821.1_O_ARG_1967':0.05669)[&label=0.478333]:0.007317)[&label=0.600778]:0.002507)[&label=0.852688]:0.014071,((('MH053306.1_A_TCH_1973':0.107484,'AY593761.1_A_KEN_1964':0.068704)[&label=0.999]:0.104321,'NC_039210.1_O_UKG_1965':0.1155)[&label=0.6615]:0.007713,(('AY593789.1_A_ARG_1961':1.0E-6,'AY593769.1_A_ARG_1959':0.00107)[&label=1.0]:0.061559,'AY593810.1_C_UKG_1970':0.079671)[&label=0.6485]:0.008637)[&label=0.4696]:0.004998)[&label=0.7943]:0.003321)[&label=0.925947]:0.004216,(((((('AY593779.1_A_GER_1972':1.0E-6,'AY593777.1_A_GER_1972':1.0E-6)[&label=0.865]:1.0E-6,'AY593774.1_A_SPA_1969':0.002121)[&label=1.0]:0.013796,'AY593827.1_O_VEN_1971':0.020651)[&label=0.888667]:0.006402,('AY593826.1_O_ITL_1947':0.010431,'AY593759.1_A_GER_1971':0.049925)[&label=0.755]:0.002449)[&label=0.9688]:0.008437,'AY593794.1_A_COL_1985':0.095559)[&label=0.952667]:0.00792,'AY593751.1_A_NET_1942':0.044757)[&label=0.984]:0.027281)[&label=0.967805]:0.008853,(('MH053309.1_C_KEN_1967':1.0E-6,'KM268897.1_C_KEN_2004':0.00107)[&label=1.0]:0.049662,('MH053308.1_C_ETH_1971':0.036399,'MH053310.1_C_UGA_1970':0.075252)[&label=0.932]:0.015138)[&label=0.952667]:0.022204)[&label=0.963331]:0.011836,'AY593834.1_O_IRN_1966':0.084935)[&label=0.959494]:0.007454,'AY593760.1_A_USSR_1964':0.102246)[&label=0.954006]:0.003009,((((((((((((((((((((((((('MG983733.1_O_SRL_2014':0.006487,'MG983734.1_O_SRL_2014':0.005426)[&label=0.969]:0.002075,'MG983732.1_O_SRL_2014':0.002124)[&label=0.973]:0.003274,'MG983711.1_O_NEP_2013':0.006465)[&label=0.844667]:0.001014,'MG983685.1_O_BAR_2015':0.018581)[&label=0.71925]:1.0E-6,('MG983731.1_O_SRL_2013':0.004262,'MG983736.1_O_UAE_2014':0.002126)[&label=0.086]:1.0E-6)[&label=0.497333]:1.0E-6,(('KJ825805.1_O_IND_2013':0.002156,'KJ825809.1_O_IND_2013':0.007547)[&label=0.646]:0.001034,'KJ825806.1_O_IND_2013':0.006427)[&label=0.3715]:1.0E-6)[&label=0.428778]:1.0E-6,(((('MG983740.1_O_VIT_2015':0.001476,'KY657269.1_O_VIT_2015':0.00496)[&label=0.927]:0.005421,'MG983693.1_O_LAO_2015':0.005041)[&label=0.9925]:0.009129,'MG983716.1_O_NEP_2014':0.005356)[&label=0.859667]:0.001078,'KJ825804.1_O_IND_2013':0.003199)[&label=0.66375]:1.0E-6)[&label=0.816]:1.0E-6,('MG983714.1_O_NEP_2013':0.005342,'MG983715.1_O_NEP_2014':0.007571)[&label=0.102]:1.0E-6)[&label=0.95275]:1.0E-6,'KJ825807.1_O_IND_2014':0.006461)[&label=0.988118]:0.003654,'KJ825803.1_O_IND_2013':0.006012)[&label=0.972556]:0.001056,'KJ825808.1_O_IND_2013':0.003835)[&label=0.996474]:0.005934,('MG983709.1_O_NEP_2012':1.0E-6,'MG983708.1_O_NEP_2012':1.0E-6)[&label=1.0]:0.008676)[&label=0.97881]:0.002176,'KJ206908.1_O_BHU_2013':0.015272)[&label=0.975455]:0.001095,((((((('MG983724.1_O_SAU_2013':1.0E-6,'MG983723.1_O_SAU_2013':1.0E-6)[&label=1.0]:0.007646,'MG983725.1_O_SAU_2014':0.00546)[&label=0.751]:9.69E-4,'MG983726.1_O_SAU_2014':0.008636)[&label=0.581]:1.0E-6,('KJ825802.1_O_IND_2013':1.0E-6,'KJ825801.1_O_IND_2013':1.0E-6)[&label=1.0]:0.006443)[&label=0.5516]:1.0E-6,(('KJ206910.1_O_SAU_2013':1.0E-6,'MG983721.1_O_SAU_2013':0.001065)[&label=0.582]:1.0E-6,'MG983722.1_O_SAU_2013':0.001065)[&label=0.997]:0.00536)[&label=0.5865]:9.07E-4,(((('MG983695.1_O_LIB_2013':0.001069,'MG983697.1_O_LIB_2013':0.002132)[&label=0.695]:0.001062,'MG983696.1_O_LIB_2013':0.004277)[&label=0.567]:1.0E-6,('KJ206909.1_O_LIB_2013':0.00213,'MG983694.1_O_LIB_2013':0.001065)[&label=0.685]:0.001063)[&label=0.98375]:0.002085,(('KU291242.1_O_MOR_2015':0.013187,'MG983735.1_O_TUN_2014':1.0E-6)[&label=0.876]:0.002175,'MG983683.1_O_ALG_2014':0.003208)[&label=0.999]:0.007648)[&label=0.999143]:0.010082)[&label=0.993125]:0.006901,('MG983713.1_O_NEP_2013':0.002174,'MG983712.1_O_NEP_2013':0.003181)[&label=1.0]:0.01688)[&label=0.9345]:8.02E-4)[&label=0.919268]:0.001723,'MG983717.1_O_NEP_2015':0.022767)[&label=0.934905]:0.004939,(((('MG983692.1_O_IRN_2009':0.011197,'MG983706.1_O_NEP_2010':0.010081)[&label=0.839]:7.31E-4,'MG983705.1_O_NEP_2010':0.011989)[&label=0.843]:0.001022,('MG983687.1_O_BHU_2009':0.004278,'MG983684.1_O_BAN_2009':0.004308)[&label=0.965]:0.003251)[&label=0.9855]:0.003047,'MG983688.1_O_BHU_2009':0.011128)[&label=1.0]:0.023015)[&label=0.995833]:0.037122,((((((((('FJ175663.1_O_ISR_2007':0.002135,'FJ175664.1_O_ISR_2007':1.0E-6)[&label=0.984]:0.00394,'FJ175666.1_O_ISR_2007':0.002523)[&label=0.87]:0.005131,'FJ175665.1_O_ISR_2007':0.007511)[&label=0.915667]:0.002267,('FJ175662.1_O_ISR_2007':0.002128,'FJ175661.1_O_ISR_2007':0.003209)[&label=0.95]:0.002608)[&label=0.9544]:0.008582,('KC440882.1_A_EGY_2012':0.021876,'KC440883.1_O_EGY_2011':0.019582)[&label=1.0]:0.028685)[&label=0.995286]:0.007603,'HQ113233.1_Asia1_AFG_2009':0.019449)[&label=0.99375]:0.009595,(('JF749852.1_O_MAY_2004':0.001056,'HQ632770.1_O_MAY_2004':1.0E-6)[&label=1.0]:0.011431,'HQ268524.1_O_BHU_2004':0.01064)[&label=0.921]:0.004842)[&label=0.927636]:0.004063,((((('JN099695.1_A_IRQ_2009':1.0E-6,'JN099698.1_A_IRQ_2009':1.0E-6)[&label=0.998]:0.004262,'JN099688.1_A_IRQ_2009':0.004271)[&label=0.866]:0.001063,('JN099699.1_A_IRQ_2009':1.0E-6,'JN099697.1_A_IRQ_2009':0.003188)[&label=0.838]:0.002126)[&label=0.938]:7.96E-4,'JN099694.1_A_IRQ_2009':0.005607)[&label=0.9998]:0.014742,('GU384682.1_O_PAK_2008':0.001057,'GU384683.1_O_PAK_2008':1.0E-6)[&label=1.0]:0.011132)[&label=0.999571]:0.018269)[&label=0.884263]:0.002135,((((('EF117837.1_A_PAK_2006':0.002126,'EF494487.1_A_PAK_2006':1.0E-6)[&label=1.0]:0.018381,('JF749841.1_A_TUR_2006':0.013222,'EF494486.1_A_TUR_2005':0.007686)[&label=0.94]:0.002606)[&label=0.971]:0.006542,'EF494488.1_A_PAK_2006':0.006107)[&label=1.0]:0.049637,'HQ113232.1_O_PAK_2009':0.030182)[&label=0.9988]:0.01947,('JN006722.1_A_PAK_2008':0.002155,'JN006720.1_Asia1_PAK_2009':0.009806)[&label=1.0]:0.023304)[&label=0.995714]:0.022491)[&label=0.973519]:0.0243)[&label=0.710895]:0.013267,('MG983690.1_O_BHU_2016':0.007651,'MG983686.1_O_BAR_2015':0.006435)[&label=1.0]:0.047629)[&label=0.695641]:0.003038,'DQ989319.1_Asia1_IND_2001':0.045447)[&label=0.689304]:0.007419,((('HQ832577.1_A_IND_1999':0.014608,'HM854023.1_A_IND_1999':0.011373)[&label=1.0]:0.028645,'DQ989315.1_Asia1_IND_1993':0.04178)[&label=0.6745]:0.0097,('DQ989307.1_Asia1_IND_1992':0.009378,'DQ989305.1_Asia1_IND_1990':0.00375)[&label=1.0]:0.040571)[&label=0.7965]:0.004098)[&label=0.66194]:9.79E-4,'DQ989313.1_Asia1_IND_1986':0.097992)[&label=0.655753]:0.007856,((((((('HQ832582.1_A_IND_2004':0.020177,'HQ832580.1_A_IND_2003':0.014333)[&label=0.496]:0.002097,'HQ832578.1_A_IND_2003':0.01763)[&label=0.572]:1.0E-6,'HQ832579.1_A_IND_2003':0.005398)[&label=0.532333]:8.22E-4,('HQ832583.1_A_IND_2005':0.005447,'HQ832581.1_A_IND_2004':0.03195)[&label=0.582]:0.001284)[&label=1.0]:0.038085,'HQ832587.1_A_IND_2005':0.039718)[&label=0.878]:0.007826,((('MF372126.1_Asia1_IND_1994':1.0E-6,'DQ989309.1_Asia1_IND_1996':1.0E-6)[&label=1.0]:0.010515,'DQ989308.1_Asia1_IND_1994':0.023039)[&label=0.9825]:0.016624,'HQ832576.1_A_IND_1990':0.047591)[&label=0.868333]:0.006378)[&label=0.7064]:0.010308,(('KY446903.1_O_PAK_2005':1.0E-6,'KY446902.1_A_PAK_2005':0.001072)[&label=0.918]:0.005438,'KT003716.1_O_PAK_2005':0.00439)[&label=1.0]:0.05722)[&label=0.587846]:0.008377)[&label=0.670182]:0.0057,((((((('EF611987.1_O_UGA_2006':0.004873,'HM191257.1_O_UGA_2006':0.006086)[&label=1.0]:0.013158,'KU821591.1_O_ZAM_2010':0.030427)[&label=0.9985]:0.015033,(('MH053318.1_O_UGA_2002':0.017682,'FJ461344.1_O_UGA_2002':0.013151)[&label=0.92]:0.007256,'FJ461345.1_O_UGA_2002':0.012736)[&label=0.9625]:0.006125)[&label=1.0]:0.03058,'MH053307.1_A_ZAM_1990':0.046975)[&label=0.882333]:0.004727,('AY593825.1_O_ARG_1939':0.078487,'MH053316.1_O_UGA_1996':0.068202)[&label=0.124]:0.009156)[&label=0.75925]:0.005164,(('KY825718.1_Asia1_ISR_1989':1.0E-6,'AY593799.1_Asia1_LEB_1983':1.0E-6)[&label=0.331]:1.0E-6,'AY593800.1_Asia1_LEB_1983':1.0E-6)[&label=1.0]:0.073717)[&label=0.583636]:0.002728,(((('MH053313.1_O_ETH_2006':0.056697,'JF749843.1_A_EGY_2006':0.07128)[&label=0.994]:0.029111,('MH053314.1_O_ETH_2007':0.039147,'MH053311.1_O_ETH_2004':0.034375)[&label=1.0]:0.035471)[&label=0.864]:0.017972,(('MH053312.1_O_ETH_2005':0.068139,'MH053317.1_O_UGA_1998':0.057263)[&label=1.0]:0.04808,'AY593813.1_O_ISA_1962':0.180791)[&label=0.629]:0.00944)[&label=0.5145]:0.003087,'MH053305.1_A_EGY_1972':0.07771)[&label=0.443857]:1.0E-6)[&label=0.478842]:0.004518)[&label=0.618941]:0.004445,(((('FJ623456.1_A_KAZ_1999':0.00987,'AY593765.1_A_TUR_1965':0.007546)[&label=0.797]:9.95E-4,'AY593764.1_A_IRQ_1970':0.009958)[&label=0.9605]:0.012912,'AY593772.1_A_TUR_1972':0.026956)[&label=1.0]:0.041715,('AY593823.1_O_TUR_1969':0.031799,'KP940473.1_O_EGY_2014':0.05086)[&label=1.0]:0.040968)[&label=0.6888]:0.008148)[&label=0.610816]:0.004715,('MH053315.1_O_SUD_1976':0.057245,'AY593766.1_A_KEN_1965':0.052562)[&label=0.166]:0.008914)[&label=0.609976]:0.003643)[&label=0.626659]:0.001939,((((((((((((((((((((((((('DQ404167.1_O_UKG_2001':1.0E-6,'DQ404166.1_O_UKG_2001':1.0E-6)[&label=0.732]:1.0E-6,'DQ404165.1_O_UKG_2001':0.001063)[&label=0.7865]:0.001063,('EF552688.1_O_UKG_2001':0.001063,'EF552696.1_O_UKG_2001':1.0E-6)[&label=0.67]:0.001067)[&label=0.43475]:1.0E-6,('FJ542368.1_O_UKG_2001':1.0E-6,'KM257063.1_O_UKG_2001':1.0E-6)[&label=0.903]:0.001063)[&label=0.325167]:1.0E-6,('FJ542369.1_O_UKG_2001':0.001063,'KM257062.1_O_UKG_2001':0.001063)[&label=0.02]:1.0E-6)[&label=0.260125]:1.0E-6,((((('EF552690.1_O_UKG_2001':1.0E-6,'DQ404169.1_O_UKG_2001':1.0E-6)[&label=0.954]:0.00213,'EU214601.1_O_UKG_2001':0.003201)[&label=0.647]:1.0E-6,('EF552695.1_O_UKG_2001':1.0E-6,'EF552691.1_O_UKG_2001':1.0E-6)[&label=0.718]:1.0E-6)[&label=0.6355]:1.0E-6,'EF552697.1_O_UKG_2001':0.00213)[&label=0.711]:0.001063,'FJ542371.1_O_UKG_2001':0.002131)[&label=0.606833]:1.0E-6)[&label=0.306]:1.0E-6,((('AJ539141.1_O_UKG_2001':0.001062,'DQ404180.1_O_UKG_2001':1.0E-6)[&label=0.813]:1.0E-6,'AJ539140.1_O_SAR_2000':0.003201)[&label=0.433]:1.0E-6,('DQ404163.1_O_UKG_2001':0.007528,'DQ404177.1_O_UKG_2001':0.001063)[&label=0.061]:1.0E-6)[&label=0.2355]:1.0E-6)[&label=0.30035]:1.0E-6,((((((((((((((('EF552689.1_O_UKG_2001':0.001063,'DQ404170.1_O_UKG_2001':0.002131)[&label=0.045]:1.0E-6,('EF552692.1_O_UKG_2001':0.001063,'DQ404164.1_O_UKG_2001':0.003203)[&label=0.075]:1.0E-6)[&label=0.113667]:1.0E-6,'DQ404179.1_O_UKG_2001':1.0E-6)[&label=0.08775]:1.0E-6,'DQ404172.1_O_UKG_2001':1.0E-6)[&label=0.0772]:1.0E-6,'DQ404176.1_O_UKG_2001':1.0E-6)[&label=0.0745]:1.0E-6,'DQ404175.1_O_UKG_2001':1.0E-6)[&label=0.081429]:1.0E-6,'FJ542365.1_O_UKG_2001':1.0E-6)[&label=0.082375]:1.0E-6,'DQ404178.1_O_UKG_2001':1.0E-6)[&label=0.094444]:1.0E-6,'DQ404173.1_O_UKG_2001':1.0E-6)[&label=0.1337]:1.0E-6,'DQ404171.1_O_UKG_2001':1.0E-6)[&label=0.158455]:1.0E-6,'EF552693.1_O_UKG_2001':1.0E-6)[&label=0.18975]:1.0E-6,'DQ404174.1_O_UKG_2001':1.0E-6)[&label=0.236]:1.0E-6,'KM257061.1_O_UKG_2001':1.0E-6)[&label=0.265929]:1.0E-6,'KM257064.1_O_UKG_2001':1.0E-6)[&label=0.312]:1.0E-6,'FJ542372.1_O_UKG_2001':1.0E-6)[&label=0.353313]:1.0E-6)[&label=0.776541]:1.0E-6,((('AY593831.1_O_UKG_2002':1.0E-6,'AY593836.1_O_UKG_2001':1.0E-6)[&label=0.791]:0.001063,'FJ542370.1_O_UKG_2001':0.001063)[&label=0.581]:1.0E-6,'AJ633821.1_O_FRA_2001':1.0E-6)[&label=0.761]:0.001063)[&label=0.880976]:1.0E-6,((((('KM257065.1_O_UKG_2001':1.0E-6,'DQ404158.1_O_UKG_2001':1.0E-6)[&label=0.305]:1.0E-6,'DQ404159.1_O_UKG_2001':1.0E-6)[&label=0.829]:1.0E-6,'DQ404160.1_O_UKG_2001':0.00106)[&label=0.963333]:0.002145,'DQ404161.1_O_UKG_2001':0.001054)[&label=0.999]:0.006497,('DQ404162.1_O_UKG_2001':0.006484,'DQ404168.1_O_UKG_2001':1.0E-6)[&label=0.101]:1.0E-6)[&label=0.846833]:0.001063)[&label=0.989708]:0.002117,'AB079061.1_O_JPN_2000':0.007593)[&label=0.996612]:0.006521,('AF506822.2_O_CHA_1999':1.0E-6,'AJ539138.1_O_CHA_1999':1.0E-6)[&label=0.998]:0.003205)[&label=0.961647]:1.0E-6,(('AJ539137.1_O_TAW_1999':0.001063,'AJ539136.1_O_TAW_1999':1.0E-6)[&label=0.975]:0.003203,'HQ632768.1_O_MAY_2000':0.00649)[&label=0.9565]:0.003245)[&label=0.934685]:9.93E-4,(((((((((((((('KF694740.1_O_SKR_2002':1.0E-6,'KF694745.1_O_SKR_2002':0.001069)[&label=0.722]:0.001069,'KF694735.1_O_SKR_2002':0.001069)[&label=0.46]:1.0E-6,'KF694741.1_O_SKR_2002':1.0E-6)[&label=0.311]:1.0E-6,'KF694739.1_O_SKR_2002':1.0E-6)[&label=0.24525]:1.0E-6,'KF694736.1_O_SKR_2002':1.0E-6)[&label=0.2236]:1.0E-6,('KF694743.1_O_SKR_2002':1.0E-6,'KF694742.1_O_SKR_2002':1.0E-6)[&label=0.085]:1.0E-6)[&label=0.529571]:1.0E-6,'KF694731.1_O_SKR_2002':1.0E-6)[&label=0.6975]:1.0E-6,'KF694734.1_O_SKR_2002':1.0E-6)[&label=0.838556]:1.0E-6,'KF694737.1_O_SKR_2002':0.001069)[&label=0.9246]:1.0E-6,('KF694744.1_O_SKR_2002':1.0E-6,'EF614457.1_O_SKR_2002':1.0E-6)[&label=0.987]:0.003227)[&label=0.999833]:0.010844,'AH012984.2_O_SKR_2000':0.003377)[&label=0.982692]:0.004249,('KF694738.1_O_SKR_2002':1.0E-6,'KF694732.1_O_SKR_2002':1.0E-6)[&label=1.0]:0.001174)[&label=1.0]:0.020709,('HQ009509.1_O_CHA_1999':0.05047,'HM008917.1_O_CHA_2005':0.018876)[&label=1.0]:0.021412)[&label=0.988]:0.004827,'AF377945.1_O_SKR_2000':0.029573)[&label=0.954667]:0.00114)[&label=0.970795]:1.0E-6,((('AJ539139.1_O_SKR_2000':1.0E-6,'AY593824.1_O_SKR_2000':1.0E-6)[&label=0.959]:1.0E-6,'MG372730.1_O_SKR_2000':1.0E-6)[&label=0.9315]:0.002193,'AH012985.2_O_SKR_2000':0.001013)[&label=1.0]:0.01326)[&label=0.995169]:0.0073,('JF749849.1_Asia1_PAK_2002':0.011273,'JF749851.1_O_IRN_2001':0.01782)[&label=0.994]:0.020962)[&label=0.990215]:0.008783,'EF149010.1_Asia1_CHA_2005':0.034727)[&label=0.984425]:0.006269,((((((('MF143572.1_O_VIT_2012':0.003452,'MF143573.1_O_VIT_2012':0.007477)[&label=0.69]:0.003249,'MF143574.1_O_VIT_2012':0.0024)[&label=0.577]:8.83E-4,('MF143578.1_O_VIT_2013':0.003223,'MF143577.1_O_VIT_2013':1.0E-6)[&label=0.97]:0.010927)[&label=0.6785]:0.003348,('MF143576.1_O_VIT_2013':0.010187,'MF143575.1_O_VIT_2012':0.008624)[&label=0.58]:0.002883)[&label=0.796667]:0.005423,((('MF947128.1_O_VIT_2010':0.00431,'MF947126.1_O_VIT_2011':0.004312)[&label=0.368]:1.0E-6,('KY234501.1_O_CHA_2011':0.004425,'MF947123.1_O_VIT_2011':0.006586)[&label=0.423]:0.001008)[&label=0.491333]:1.0E-6,(('MF947124.1_O_VIT_2012':0.005213,'MF947129.1_O_VIT_2014':0.027563)[&label=0.996]:0.01043,'MF947141.1_O_VIT_2012':0.029933)[&label=0.752]:8.73E-4)[&label=0.701333]:0.001667)[&label=0.857615]:0.001461,('MF947137.1_O_VIT_2012':1.0E-6,'MF947127.1_O_VIT_2012':0.001069)[&label=1.0]:0.031881)[&label=0.976533]:0.008212,((('KY234502.1_O_CHA_2015':0.019467,'MF947132.1_O_VIT_2015':0.008872)[&label=1.0]:0.021907,'MF947131.1_O_VIT_2013':0.004809)[&label=1.0]:0.011224,('MF947143.1_O_VIT_2013':0.002132,'MF947142.1_O_VIT_2013':0.006598)[&label=0.999]:0.005294)[&label=1.0]:0.011562)[&label=1.0]:0.033286)[&label=0.998564]:0.025883,('HQ832590.1_A_IND_2007':0.011669,'HQ832591.1_A_IND_2008':0.010604)[&label=1.0]:0.038767)[&label=0.992534]:0.009067,(((((((((('AF154271.1_O_TAW_1997':0.001135,'AF026168.2_O_TAW_1997':0.006768)[&label=0.628]:0.001095,'AY593833.1_O_TAW_1999':0.001121)[&label=0.7645]:5.28E-4,('AF308157.1_O_TAW_1997':1.0E-6,'AY593835.1_O_TAW_1997':1.0E-6)[&label=0.953]:0.001702)[&label=0.99975]:0.025108,'AY686687.1_O_CHA_2001':0.064331)[&label=0.9032]:0.012606,((('EU400597.1_O_CHA_2001':0.005087,'AY317098.1_O_CHA_2002':0.012061)[&label=0.866]:0.00856,('KU204894.1_O_CHA_2013':0.010465,'KU204893.1_O_CHA_2013':0.036834)[&label=1.0]:0.074188)[&label=1.0]:0.050127,'HQ632771.1_O_MAY_2005':0.094668)[&label=0.86575]:0.010725)[&label=0.9478]:0.018758,'HQ412603.1_O_CHA_2000':0.091643)[&label=1.0]:0.184246,'KY072818.1_O_CHA_1959':0.066617)[&label=0.975917]:0.008557,'AY593755.1_A_TAI_1960':0.070676)[&label=0.997846]:0.045572,(((((('KU360085.1_Asia1_CHA_2015':1.0E-6,'KC462884.1_Asia1_CHA_2006':1.0E-6)[&label=0.321]:1.0E-6,'KC412634.1_Asia1_CHA_2006':1.0E-6)[&label=0.9895]:1.0E-6,'EF149009.1_Asia1_CHA_2005':0.019111)[&label=0.871667]:0.002166,'GU931682.1_Asia1_CHA_2005':0.002173)[&label=0.97075]:0.006539,((('GU125645.1_Asia1_VIT_2007':0.002151,'GQ452295.1_Asia1_VIT_2007':1.0E-6)[&label=0.968]:0.007612,'FJ906802.1_Asia1_CHA_2006':0.00434)[&label=0.7305]:1.0E-6,'HQ631363.1_Asia1_CHA_2006':1.0E-6)[&label=0.859]:0.002185)[&label=0.96875]:0.003119,'KY446901.1_Asia1_PAK_2006':0.004541)[&label=0.999444]:0.053484)[&label=0.746957]:0.009905,(('DQ989310.1_Asia1_IND_1999':0.045298,'AY687333.1_Asia1_IND_2001':0.057832)[&label=0.586]:0.007862,'AY593812.1_O_PHI_1958':0.09091)[&label=0.4535]:0.005675)[&label=0.696077]:0.008334)[&label=0.877731]:0.010711,((((((((((((((('JX040489.1_O_BUL_2011':1.0E-6,'JX040488.1_O_BUL_2011':1.0E-6)[&label=0.905]:0.001073,'JX040486.1_O_BUL_2011':0.001073)[&label=0.666]:1.0E-6,('JX040487.1_O_BUL_2011':0.001081,'JX040490.1_O_BUL_2011':0.001068)[&label=0.893]:0.002158)[&label=0.8065]:0.002159,(('JX066664.1_O_BUL_2011':0.003234,'JX066665.1_O_BUL_2011':0.005412)[&label=0.612]:1.0E-6,'JX040485.1_O_BUL_2010':1.0E-6)[&label=0.6335]:1.0E-6)[&label=0.995]:0.004346,'JX040494.1_O_TUR_2010':0.00217)[&label=0.951875]:0.00106,'JX040495.1_O_TUR_2010':0.007623)[&label=0.859889]:1.0E-6,(('JX040496.1_O_TUR_2010':0.001075,'JX040498.1_O_TUR_2010':0.003239)[&label=0.078]:1.0E-6,('JX040493.1_O_TUR_2010':0.001075,'JX040497.1_O_TUR_2010':0.001075)[&label=0.161]:1.0E-6)[&label=0.195333]:1.0E-6)[&label=0.790769]:1.0E-6,('JX040499.1_O_TUR_2011':0.003248,'JX040491.1_O_TUR_2010':0.001075)[&label=0.085]:1.0E-6)[&label=0.9356]:1.0E-6,'JX040500.1_O_TUR_2011':0.004327)[&label=0.995687]:0.004435,('JX040492.1_O_TUR_2010':0.00998,'JX040501.1_O_ISR_2011':0.01307)[&label=0.745]:0.001128)[&label=0.997556]:0.006019,('KM268895.1_O_TUR_2013':0.025559,'KM268898.1_Asia1_TUR_2013':0.028485)[&label=0.852]:0.013821)[&label=0.9501]:1.0E-6,'KM268896.1_A_TUR_2013':0.032872)[&label=0.97381]:0.005903,(('MH784404.1_O_PAK_2017':0.005562,'MH784403.1_O_PAK_2016':0.008752)[&label=1.0]:0.017002,'MH784405.1_O_PAK_2017':0.022928)[&label=0.9945]:0.009527)[&label=0.999208]:0.021176,'JN006719.1_Asia1_PAK_2008':0.031832)[&label=0.99876]:0.022537,'AY593795.1_Asia1_PAK_1954':0.08608)[&label=0.962962]:0.001327)[&label=0.834051]:0.004404,((((((((((((('LC320038.1_O_MOG_2015':0.007559,'MG983720.1_O_RUS_2016':0.003205)[&label=0.878]:0.002138,'MG983730.1_O_SKR_2017':0.004281)[&label=0.734]:1.0E-6,'MF461724.1_O_CHA_2017':0.004276)[&label=0.903]:0.002122,('LC438823.1_O_MYA_2016':0.009809,'MH891503.1_O_VIT_2017':0.014443)[&label=0.847]:0.005485)[&label=0.8696]:0.001094,('MG983741.1_O_VIT_2016':0.007707,'LC438822.1_O_MYA_2016':0.005507)[&label=0.689]:9.46E-4)[&label=0.952429]:0.003234,'KX712091.1_O_BAN_2015':0.004287)[&label=0.938125]:0.003228,(('MG983727.1_O_SAU_2015':9.38E-4,'MG983728.1_O_SAU_2016':0.008801)[&label=0.989]:0.00771,'MG983703.1_O_MYA_2016':0.007542)[&label=0.7]:1.0E-6)[&label=0.699909]:1.0E-6,(((('MG983738.1_O_UAE_2016':1.0E-6,'MG983739.1_O_UAE_2016':1.0E-6)[&label=1.0]:0.005457,'MG983691.1_O_BHU_2016':0.006482)[&label=0.953]:0.005493,'MG983719.1_O_NEP_2015':0.005438)[&label=0.792333]:0.001015,'MG983718.1_O_NEP_2015':0.004287)[&label=0.6065]:1.0E-6)[&label=0.695125]:1.0E-6,'MG983729.1_O_SAU_2016':0.015423)[&label=0.762235]:3.03E-4,((('MG983702.1_O_MUR_2016':1.0E-6,'MG983701.1_O_MUR_2016':1.0E-6)[&label=0.363]:1.0E-6,'MG983700.1_O_MUR_2016':1.0E-6)[&label=0.974]:0.001032,('MG983698.1_O_MUR_2016':1.0E-6,'MG983699.1_O_MUR_2016':1.0E-6)[&label=0.976]:0.002166)[&label=0.98625]:0.005096)[&label=0.991136]:0.012277,((('MG983689.1_O_BHU_2012':0.007416,'MG983707.1_O_NEP_2012':0.006744)[&label=0.498]:7.32E-4,'MG983710.1_O_NEP_2012':0.011348)[&label=0.859]:0.005066,'MF372125.1_Asia1_IND_2016':0.030568)[&label=0.851333]:0.005594)[&label=0.988885]:0.010229,('MG983704.1_O_NEP_2008':0.030088,'KF985189.1_O_BAN_2013':0.032924)[&label=0.78]:0.004639)[&label=0.998071]:0.018909,((((((('DQ989321.1_Asia1_IND_2001':0.005326,'DQ989322.1_Asia1_IND_2002':0.011928)[&label=0.758]:1.0E-6,'DQ989314.1_Asia1_IND_2001':0.007528)[&label=0.537]:1.0E-6,('DQ989323.1_Asia1_IND_2002':0.006583,'DQ989320.1_Asia1_IND_2002':0.007672)[&label=0.684]:9.06E-4)[&label=0.9145]:1.0E-6,'DQ989318.1_Asia1_IND_2002':0.015222)[&label=0.9944]:0.007491,'DQ989317.1_Asia1_IND_2000':0.004389)[&label=0.9945]:0.008952,'HQ832586.1_A_IND_2006':0.043091)[&label=0.952]:0.005417,(('KJ754939.1_A_BAN_2013':0.026685,'KU127247.1_A_SAU_2015':0.031554)[&label=1.0]:0.02643,'MF782478.1_Asia1_BAN_2013':0.049138)[&label=0.8335]:0.010076)[&label=0.8044]:0.00245)[&label=0.980282]:0.020486)[&label=0.873228]:0.005418,'HQ832585.1_A_IND_2005':0.052125)[&label=0.875404]:0.004587,((((('HQ832589.1_A_IND_2006':0.009369,'HQ832588.1_A_IND_2005':0.009365)[&label=1.0]:0.032619,('HQ832584.1_A_IND_2005':0.039559,'HM854021.1_A_IND_2000':0.031751)[&label=0.477]:0.002187)[&label=0.830667]:0.002806,'HQ832592.1_A_IND_2009':0.050322)[&label=0.999]:0.025437,('DQ989312.1_Asia1_IND_1990':0.036287,'KU726614.1_O_GRE_1994':0.031191)[&label=1.0]:0.029396)[&label=0.912167]:0.005719,'DQ989311.1_Asia1_IND_2002':0.051871)[&label=0.976286]:0.021634)[&label=0.867015]:0.013053,('JF749848.1_A_TUR_2003':0.047344,'AY593791.1_A_IRN_1998':0.029612)[&label=0.981]:0.048673)[&label=0.864582]:0.00919)[&label=0.719117]:0.00846,(((((((((((((((((((((((((((((((((((((((((('LC149654.1_O_JPN_2010':1.0E-6,'LC149699.1_O_JPN_2010':1.0E-6)[&label=0.116]:1.0E-6,'LC149643.1_O_JPN_2010':1.0E-6)[&label=0.747]:1.0E-6,('LC149716.1_O_JPN_2010':1.0E-6,'LC036265.1_O_JPN_2010':1.0E-6)[&label=0.949]:0.002147)[&label=0.70575]:0.001071,'LC149630.1_O_JPN_2010':1.0E-6)[&label=0.7246]:1.0E-6,'LC149694.1_O_JPN_2010':0.001072)[&label=0.614]:1.0E-6,(((('LC149686.1_O_JPN_2010':0.001072,'LC149689.1_O_JPN_2010':0.001071)[&label=0.269]:1.0E-6,'LC149663.1_O_JPN_2010':1.0E-6)[&label=0.701]:0.001072,'LC149691.1_O_JPN_2010':0.001072)[&label=0.482]:1.0E-6,('LC149644.1_O_JPN_2010':0.001073,'LC149657.1_O_JPN_2010':0.002149)[&label=0.044]:1.0E-6)[&label=0.3198]:1.0E-6)[&label=0.368167]:1.0E-6,('LC149711.1_O_JPN_2010':0.001072,'LC149669.1_O_JPN_2010':0.001073)[&label=0.021]:1.0E-6)[&label=0.334857]:1.0E-6,'LC149707.1_O_JPN_2010':1.0E-6)[&label=0.3154]:1.0E-6,'LC149696.1_O_JPN_2010':1.0E-6)[&label=0.296375]:1.0E-6,('LC149677.1_O_JPN_2010':1.0E-6,'LC149656.1_O_JPN_2010':1.0E-6)[&label=0.016]:1.0E-6)[&label=0.264389]:1.0E-6,(('LC149692.1_O_JPN_2010':0.001073,'LC149720.1_O_JPN_2010':0.001072)[&label=0.026]:1.0E-6,'LC149674.1_O_JPN_2010':1.0E-6)[&label=0.014]:1.0E-6)[&label=0.243]:1.0E-6,'LC149635.1_O_JPN_2010':1.0E-6)[&label=0.232682]:1.0E-6,('LC149660.1_O_JPN_2010':1.0E-6,'LC149675.1_O_JPN_2010':1.0E-6)[&label=0.003]:1.0E-6)[&label=0.214833]:1.0E-6,'LC149641.1_O_JPN_2010':1.0E-6)[&label=0.20756]:1.0E-6,'LC149666.1_O_JPN_2010':1.0E-6)[&label=0.201769]:1.0E-6,'LC149659.1_O_JPN_2010':1.0E-6)[&label=0.19637]:1.0E-6,'LC149703.1_O_JPN_2010':1.0E-6)[&label=0.192214]:1.0E-6,(((('LC149719.1_O_JPN_2010':1.0E-6,'LC149693.1_O_JPN_2010':1.0E-6)[&label=0.008]:1.0E-6,'LC149708.1_O_JPN_2010':1.0E-6)[&label=0.01]:1.0E-6,('LC149682.1_O_JPN_2010':1.0E-6,'LC149683.1_O_JPN_2010':1.0E-6)[&label=0.006]:1.0E-6)[&label=0.021]:1.0E-6,'LC149697.1_O_JPN_2010':1.0E-6)[&label=0.0204]:1.0E-6)[&label=0.208559]:1.0E-6,'LC149687.1_O_JPN_2010':1.0E-6)[&label=0.223514]:1.0E-6,'LC149681.1_O_JPN_2010':1.0E-6)[&label=0.241028]:1.0E-6,((((((((('LC149638.1_O_JPN_2010':1.0E-6,'LC149690.1_O_JPN_2010':1.0E-6)[&label=0.002]:1.0E-6,'LC149650.1_O_JPN_2010':1.0E-6)[&label=0.002]:1.0E-6,('LC149679.1_O_JPN_2010':1.0E-6,'LC149640.1_O_JPN_2010':1.0E-6)[&label=0.006]:1.0E-6)[&label=0.01825]:1.0E-6,'LC149684.1_O_JPN_2010':1.0E-6)[&label=0.0258]:1.0E-6,'LC149627.1_O_JPN_2010':1.0E-6)[&label=0.0325]:1.0E-6,'LC149702.1_O_JPN_2010':1.0E-6)[&label=0.036857]:1.0E-6,('LC149652.1_O_JPN_2010':1.0E-6,'LC149704.1_O_JPN_2010':1.0E-6)[&label=0.007]:1.0E-6)[&label=0.042889]:1.0E-6,'LC149648.1_O_JPN_2010':1.0E-6)[&label=0.0489]:1.0E-6,'LC149645.1_O_JPN_2010':1.0E-6)[&label=0.053091]:1.0E-6)[&label=0.578708]:1.0E-6,('LC149709.1_O_JPN_2010':0.001075,'LC149667.1_O_JPN_2010':0.001072)[&label=0.02]:1.0E-6)[&label=0.61784]:1.0E-6,('LC149673.1_O_JPN_2010':1.0E-6,'LC149651.1_O_JPN_2010':1.0E-6)[&label=0.004]:1.0E-6)[&label=0.666077]:1.0E-6,'LC149713.1_O_JPN_2010':1.0E-6)[&label=0.689358]:1.0E-6,((('LC149668.1_O_JPN_2010':1.0E-6,'LC149662.1_O_JPN_2010':1.0E-6)[&label=0.884]:0.001076,'LC149717.1_O_JPN_2010':0.001072)[&label=0.4555]:1.0E-6,('LC149680.1_O_JPN_2010':0.002148,'LC149706.1_O_JPN_2010':0.001073)[&label=0.045]:1.0E-6)[&label=0.248]:1.0E-6)[&label=0.773017]:1.0E-6,(('LC149688.1_O_JPN_2010':1.0E-6,'LC149695.1_O_JPN_2010':0.002151)[&label=0.592]:0.001072,'LC149710.1_O_JPN_2010':0.001072)[&label=0.314]:1.0E-6)[&label=0.822902]:0.001072,(((((('LC149661.1_O_JPN_2010':1.0E-6,'LC149715.1_O_JPN_2010':1.0E-6)[&label=0.419]:1.0E-6,'LC149639.1_O_JPN_2010':1.0E-6)[&label=0.9425]:1.0E-6,'LC149685.1_O_JPN_2010':0.002147)[&label=0.943333]:0.002155,('LC149718.1_O_JPN_2010':1.0E-6,'LC149634.1_O_JPN_2010':1.0E-6)[&label=0.989]:0.002154)[&label=0.6816]:1.0E-6,('LC149671.1_O_JPN_2010':1.0E-6,'LC149714.1_O_JPN_2010':1.0E-6)[&label=0.237]:1.0E-6)[&label=0.621286]:1.0E-6,'LC149676.1_O_JPN_2010':1.0E-6)[&label=0.664125]:1.0E-6)[&label=0.877629]:0.001072,(((((((('LC149623.1_O_JPN_2010':1.0E-6,'LC149632.1_O_JPN_2010':1.0E-6)[&label=0.021]:1.0E-6,'LC149629.1_O_JPN_2010':1.0E-6)[&label=0.0525]:1.0E-6,'LC149631.1_O_JPN_2010':1.0E-6)[&label=0.081667]:1.0E-6,('LC149712.1_O_JPN_2010':1.0E-6,'LC149670.1_O_JPN_2010':1.0E-6)[&label=0.022]:1.0E-6)[&label=0.1266]:1.0E-6,'LC149636.1_O_JPN_2010':1.0E-6)[&label=0.1955]:1.0E-6,(('LC149672.1_O_JPN_2010':1.0E-6,'LC149653.1_O_JPN_2010':1.0E-6)[&label=0.062]:1.0E-6,('LC149649.1_O_JPN_2010':1.0E-6,'LC149678.1_O_JPN_2010':1.0E-6)[&label=0.019]:1.0E-6)[&label=0.127333]:1.0E-6)[&label=0.4804]:1.0E-6,((((('LC149628.1_O_JPN_2010':1.0E-6,'LC149705.1_O_JPN_2010':1.0E-6)[&label=0.78]:1.0E-6,'LC149637.1_O_JPN_2010':1.0E-6)[&label=0.8055]:0.001072,'LC149620.1_O_JPN_2010':0.001072)[&label=0.546667]:1.0E-6,('LC149701.1_O_JPN_2010':0.001073,'LC149665.1_O_JPN_2010':0.001072)[&label=0.061]:1.0E-6)[&label=0.3508]:1.0E-6,((('LC149618.1_O_JPN_2010':1.0E-6,'LC149624.1_O_JPN_2010':1.0E-6)[&label=0.997]:0.004312,'LC149700.1_O_JPN_2010':0.001072)[&label=0.5495]:1.0E-6,('LC149642.1_O_JPN_2010':0.001072,'LC149664.1_O_JPN_2010':0.002161)[&label=0.083]:1.0E-6)[&label=0.33675]:1.0E-6)[&label=0.2798]:1.0E-6)[&label=0.444476]:1.0E-6,((('LC149621.1_O_JPN_2010':1.0E-6,'LC149617.1_O_JPN_2010':1.0E-6)[&label=0.299]:1.0E-6,'KF112885.1_O_JPN_2010':1.0E-6)[&label=0.821]:0.001072,'LC149698.1_O_JPN_2010':0.001073)[&label=0.557667]:1.0E-6)[&label=0.46424]:0.001072)[&label=0.928458]:1.0E-6,'LC149619.1_O_JPN_2010':0.001072)[&label=0.933866]:1.0E-6,((((('LC149647.1_O_JPN_2010':1.0E-6,'LC149626.1_O_JPN_2010':1.0E-6)[&label=0.076]:1.0E-6,'LC149658.1_O_JPN_2010':1.0E-6)[&label=0.182]:1.0E-6,('LC149646.1_O_JPN_2010':1.0E-6,'LC149622.1_O_JPN_2010':1.0E-6)[&label=0.07]:1.0E-6)[&label=0.51025]:1.0E-6,'LC149633.1_O_JPN_2010':1.0E-6)[&label=0.715]:1.0E-6,'LC149655.1_O_JPN_2010':1.0E-6)[&label=0.7135]:1.0E-6)[&label=0.989029]:1.0E-6,'LC149625.1_O_JPN_2010':0.001072)[&label=0.997286]:0.006508,(((((('KF501488.1_O_SKR_2010':0.001078,'KF501487.1_O_SKR_2010':1.0E-6)[&label=0.716]:0.001074,'KR401160.1_O_SKR_2011':1.0E-6)[&label=0.998]:0.005435,'KF112888.1_O_DRK_2011':0.004323)[&label=0.947667]:1.0E-6,'KF112883.1_O_RUS_2010':1.0E-6)[&label=0.967]:0.002166,((('KC503937.1_O_SKR_2010':1.0E-6,'KR401159.1_O_SKR_2010':1.0E-6)[&label=0.646]:1.0E-6,'KF501486.1_O_SKR_2010':0.001072)[&label=0.834]:1.0E-6,'KF112887.1_O_SKR_2010':0.001072)[&label=0.999333]:0.007625)[&label=0.98225]:0.003242,'JN998085.1_O_CHA_2010':0.002152)[&label=0.933333]:1.0E-6)[&label=0.981339]:0.002153,(((('KR401158.1_O_SKR_2010':1.0E-6,'KF112886.1_O_SKR_2010':1.0E-6)[&label=0.997]:0.001044,'JQ973889.1_O_CHA_2010':0.006573)[&label=0.949]:0.003274,(('JQ900581.1_O_CHA_2010':0.003338,'HM229661.1_O_HKN_2010':9.96E-4)[&label=1.0]:0.012166,'JN998086.1_O_CHA_2010':0.012044)[&label=0.5475]:1.0E-6)[&label=0.5524]:1.0E-6,'HM055510.1_O_VIT_2009':0.002152)[&label=0.608667]:0.001075)[&label=0.979598]:1.0E-6,((((('KY086465.1_O_SKR_2016':1.0E-6,'KX534089.1_O_SKR_2016':0.001072)[&label=1.0]:0.006516,'KY086466.1_O_SKR_2016':0.011051)[&label=0.975]:0.003152,'KX162590.1_O_SKR_2014':0.0012)[&label=0.999333]:0.017449,'MH845413.2_O_VIT_2014':0.016191)[&label=0.91575]:0.003471,'KY322674.1_O_SKR_2014':0.017002)[&label=1.0]:0.03307)[&label=0.995008]:0.001045,'KF112889.1_O_HKN_2010':0.003276)[&label=0.999822]:0.006593,((('KR401153.1_O_MYA_2009':1.0E-6,'KR401155.1_O_MYA_2007':1.0E-6)[&label=1.0]:0.008716,'KR401152.1_O_MYA_2009':0.003226)[&label=0.646]:1.0E-6,('KR401156.1_O_MYA_2009':0.001056,'KF112880.1_O_MYA_2009':0.001097)[&label=0.996]:0.006532)[&label=0.88475]:0.002167)[&label=0.997963]:0.001185,'KF112879.1_O_TAI_2009':0.014299)[&label=0.999719]:0.00659,'KR401157.1_O_MYA_2009':0.011067)[&label=0.99986]:0.017877,'KY322672.1_O_MAY_2014':0.03544)[&label=0.999664]:0.01557,'KR401154.1_O_MYA_1998':0.031881)[&label=0.998638]:0.010688,((((((((((('KF112882.1_O_MOG_2010':0.002136,'KF112881.1_O_MOG_2010':0.006463)[&label=0.573]:1.0E-6,'KF112884.1_O_RUS_2010':0.009784)[&label=0.8815]:0.002171,('GU582116.1_O_VIT_2009':0.006473,'GU582115.1_O_VIT_2009':1.0E-6)[&label=0.735]:0.00104)[&label=0.99225]:0.004871,(('MF947130.1_O_VIT_2014':0.00866,'KY322671.1_O_MAY_2014':0.005607)[&label=0.986]:0.005146,'KY322670.1_O_LAO_2013':0.015193)[&label=0.991]:0.015598)[&label=0.890571]:0.008354,'HQ632772.1_O_MAY_2007':0.012104)[&label=0.932]:1.86E-4,'KY322673.1_O_MAY_2014':0.050961)[&label=0.999667]:0.020732,'GU125650.1_O_VIT_2006':0.033457)[&label=0.9737]:0.004507,(('GU125647.1_O_VIT_2006':0.001066,'GU125649.1_O_VIT_2006':0.002135)[&label=0.586]:1.0E-6,'GU125648.1_O_VIT_2006':0.001062)[&label=1.0]:0.045806)[&label=0.992769]:0.009053,'KT968663.1_A_CHA_2013':0.066926)[&label=0.989786]:0.011406,'HQ632774.1_Asia1_MAY_1999':0.060382)[&label=0.981067]:0.011109,'HQ632769.1_O_MAY_2001':0.055879)[&label=0.945812]:0.005694)[&label=0.945839]:0.002491,(((((((((('GQ406248.1_A_VIT_2009':1.0E-6,'GQ406252.1_A_VIT_2009':0.010817)[&label=0.492]:0.001066,'GQ406247.1_A_VIT_2009':0.005374)[&label=0.6295]:0.001067,'GQ406251.1_A_VIT_2009':0.003207)[&label=0.722]:1.0E-6,(('GQ406250.1_A_VIT_2009':0.004553,'KC588943.1_A_SKR_2010':0.025831)[&label=0.748]:8.05E-4,'GQ406249.1_A_VIT_2009':0.004309)[&label=0.766]:0.001057)[&label=0.999833]:0.017032,'HQ632773.1_A_MAY_2007':0.02585)[&label=0.999143]:0.01883,'HQ268509.2_A_VIT_2004':0.02398)[&label=0.96]:0.002443,'KJ933864.1_A_MAY_1997':0.027469)[&label=0.982333]:0.010317,(('KJ608371.1_A_VIT_2013':0.018583,'KY322678.1_A_MAY_2013':0.036165)[&label=0.837]:0.005853,'KY322676.1_A_MAY_2013':0.037297)[&label=0.9625]:0.014468)[&label=0.949833]:0.014523,(('KY322675.1_A_LAO_2014':0.002152,'KY322677.1_A_MAY_2013':0.003227)[&label=0.709]:0.001063,('KY322679.1_A_TAI_2014':0.008661,'KY322680.1_A_VIT_2013':0.001069)[&label=0.349]:1.0E-6)[&label=1.0]:0.02982)[&label=0.999313]:0.023985,'GU125646.1_Asia1_VIT_2005':0.072239)[&label=0.987588]:0.012789)[&label=0.994162]:0.013491)[&label=0.907011]:0.007111,((((('DQ989303.1_Asia1_IND_1993':0.003217,'DQ989304.1_Asia1_IND_2000':0.001068)[&label=0.903]:1.0E-6,'DQ989306.1_Asia1_IND_1986':0.004297)[&label=1.0]:0.023075,('AY593797.1_Asia1_ISR_1963':0.029523,'AY593796.1_Asia1_ISR_1963':0.037676)[&label=0.934]:0.013122)[&label=0.90275]:0.003137,'AY593828.1_O_IND_1962':0.032022)[&label=0.9418]:0.00946,'HM854022.1_A_IND_1977':0.035699)[&label=0.9685]:0.017648)[&label=0.973476]:0.069331,((((((((('MG725876.1_A_NIG_2015':1.0E-6,'MG725875.1_A_NIG_2015':1.0E-6)[&label=1.0]:0.004695,'MG725873.1_A_NIG_2015':0.004121)[&label=0.9395]:0.003725,(('MG913340.1_A_ALG_2017':0.002171,'MG923579.1_A_ALG_2017':0.001084)[&label=0.71]:0.001132,'MG923580.1_A_ALG_2017':0.002133)[&label=1.0]:0.027996)[&label=1.0]:0.079812,('MG725872.1_A_NIG_2013':0.056881,'MG725874.1_A_NIG_2015':0.045674)[&label=1.0]:0.048628)[&label=0.890286]:0.014689,('KP940474.1_A_EGY_2014':0.00108,'KC440881.1_A_EGY_2011':1.0E-6)[&label=1.0]:0.066296)[&label=0.931111]:0.030614,(('JX014256.1_SAT2_PAT_2012':1.0E-6,'KC440884.1_SAT2_EGY_2012':0.002161)[&label=0.709]:2.13E-4,'JX014255.1_SAT2_EGY_2012':0.004125)[&label=1.0]:0.06845)[&label=0.996167]:0.033291,'AY593844.1_SAT1_ISR_1962':0.054437)[&label=0.997154]:0.030745,((('MH053324.1_SAT1_UGA_1971':0.031026,'MH053325.1_SAT1_UGA_1978':0.096172)[&label=0.748]:0.021238,'FJ461346.1_SAT2_UGA_2002':0.04146)[&label=0.8625]:0.0142,'MH053323.1_SAT1_TCH_1972':0.074951)[&label=0.828667]:0.014577)[&label=0.978059]:0.037838,'AY593849.1_SAT2_KEN_1960':0.098462)[&label=0.978278]:0.094814)[&label=1.0]:0.312461,((((((((((((('MH053319.1_SAT1_BOT_1974':0.022417,'MH053331.1_SAT2_BOT_1972':0.017502)[&label=0.628]:0.00307,'MH053330.1_SAT2_BOT_1969':0.017098)[&label=0.772]:0.004104,('AY593853.1_SAT3_BOT_1965':0.006,'MH053338.1_SAT3_BOT_1966':0.018741)[&label=1.0]:0.044421)[&label=0.6195]:0.004729,'MH053322.1_SAT1_NMB_1989':0.036909)[&label=0.6092]:0.004365,((('AY593845.1_SAT1_BOT_1968':0.043757,'MH053329.1_SAT2_BOT_1969':0.01462)[&label=0.663]:0.011629,'AY593840.1_SAT1_NMB_1949':0.040995)[&label=0.4525]:0.003592,'AY593842.1_SAT1_SAR_1961':0.037768)[&label=0.354]:0.003001)[&label=0.526889]:0.004458,(((('AY593852.1_SAT3_KEN_1960':1.0E-6,'AY593851.1_SAT3_BOT_1961':0.002172)[&label=1.0]:0.040104,'AY593841.1_SAT1_ZIM_1958':0.030842)[&label=0.6535]:0.006641,'MH053342.1_SAT3_ZAM_1996':0.031363)[&label=0.528]:0.002596,'MH053332.1_SAT2_BOT_1974':0.024055)[&label=0.51875]:0.005911)[&label=0.517571]:0.001578,(('JF749864.1_SAT2_ZIM_2003':0.046847,'MH053339.1_SAT3_BOT_1970':0.034623)[&label=0.164]:0.010672,'AY593838.1_SAT1_BOT_1970':0.057992)[&label=0.156]:0.002134)[&label=0.728]:0.001611,((('KU821592.1_SAT2_ZAM_2009':0.024481,'KU821590.1_SAT1_NMB_2010':0.013663)[&label=0.998]:0.029447,'MH053351.1_SAT3_ZIM_1984':0.024684)[&label=0.522]:0.002256,('MH053328.1_SAT2_BOT_1968':0.035178,'AY593843.1_SAT1_NMB_1940':0.026779)[&label=0.285]:0.009047)[&label=0.29725]:0.003757)[&label=0.993727]:0.036412,'AY593847.1_SAT2_ZIM_1948':0.08429)[&label=0.970478]:0.013243,((((((((('KM268900.1_SAT2_TAN_2012':0.062515,'JF749860.1_SAT1_KEN_2002':0.062349)[&label=0.392]:0.015002,'JF749861.1_SAT2_KEN_2002':0.049955)[&label=0.6385]:0.011712,'KM268899.1_SAT1_TAN_2012':0.027991)[&label=0.772333]:0.017338,('MH053333.1_SAT2_ETH_1989':0.085637,'MH053320.1_SAT1_KEN_1983':0.055485)[&label=0.538]:0.017908)[&label=0.993]:0.032712,'MH053340.1_SAT3_MAL_1976':0.061285)[&label=0.9675]:0.014959,'MH053334.1_SAT2_ZAM_1964':0.111022)[&label=0.869143]:0.003083,'MH053352.1_SAT3_ZIM_1990':0.05788)[&label=0.82475]:0.003619,(('AY593850.1_SAT3_SAR_1959':0.043336,'AY593848.1_SAT2_u_1967':0.047973)[&label=0.312]:0.004769,('KR108948.1_SAT1_SAR_2009':0.008356,'KR108949.1_SAT2_SAR_2009':0.016529)[&label=0.754]:0.007041)[&label=0.749]:0.011129)[&label=0.707583]:0.003512,'MH053321.1_SAT1_MOZ_1981':0.051366)[&label=0.678692]:0.002113)[&label=0.857514]:0.003012,((((('MH053348.1_SAT3_ZIM_1977':0.007134,'MH053346.1_SAT3_ZIM_1976':0.002856)[&label=1.0]:0.032634,'KX375417.1_SAT3_ZIM_1981':0.033116)[&label=0.8945]:0.009827,'AY593839.1_SAT1_UKG_1970':0.070407)[&label=0.702333]:0.002785,('KM268901.1_SAT3_ZIM_1991':0.065131,'MH053343.1_SAT3_ZIM_1934':0.052729)[&label=0.562]:0.021224)[&label=0.4506]:0.002469,('KR108950.1_SAT3_SAR_2009':0.039628,'AY593846.1_SAT1_ZIM_1966':0.059468)[&label=0.183]:0.010313)[&label=0.516714]:0.006955)[&label=0.908044]:0.009462,((('MH053350.1_SAT3_ZIM_1983':0.003363,'MH053349.1_SAT3_ZIM_1983':1.0E-6)[&label=1.0]:0.029781,'MH053335.1_SAT2_ZIM_1965':0.056511)[&label=0.7275]:0.010204,(('MH053345.1_SAT3_ZIM_1975':0.006767,'MH053347.1_SAT3_ZIM_1976':0.009919)[&label=0.986]:0.036109,'MH053344.1_SAT3_ZIM_1974':0.047727)[&label=0.5795]:0.008152)[&label=0.6106]:0.001352)[&label=0.999725]:0.149363,(((((('HM067704.1_SAT2_UGA_2007':0.133799,'HM067706.1_SAT1_UGA_2007':0.058489)[&label=0.426]:0.018439,('JF749862.1_SAT2_UGA_2002':0.045379,'HM067705.1_SAT2_UGA_2007':0.063149)[&label=0.589]:0.013056)[&label=0.673667]:0.005648,'MH053326.1_SAT1_UGA_1970':0.078538)[&label=0.66675]:0.008362,(('MH053337.1_SAT2_UGA_1970':0.013182,'MH053327.1_SAT1_UGA_1970':0.020431)[&label=0.992]:0.023399,('KJ820999.1_SAT3_UGA_2013':0.044772,'MH053341.1_SAT3_UGA_1970':0.022255)[&label=0.602]:0.009022)[&label=0.907333]:0.02096)[&label=0.922875]:0.010347,'MH053336.1_SAT2_UGA_1970':0.034085)[&label=0.998222]:0.158349,((('MF678825.1_SAT1_NIG_2015':1.0E-6,'MF678826.1_SAT1_NIG_2015':1.0E-6)[&label=0.999]:0.005734,'MF678823.1_SAT1_NIG_2015':0.002825)[&label=0.8375]:0.003219,'MF678824.1_SAT1_NIG_2015':0.002517)[&label=1.0]:0.633283)[&label=0.838308]:0.131161)[&label=1.0]:0.209045);

end;

begin figtree;

set appearance.backgroundColorAttribute="Default";

set appearance.backgroundColour=#ffffff;

set appearance.branchColorAttribute="User selection";

set appearance.branchColorGradient=false;

set appearance.branchLineWidth=1.0;

set appearance.branchMinLineWidth=0.0;

set appearance.branchWidthAttribute="Fixed";

set appearance.foregroundColour=#000000;

set appearance.hilightingGradient=false;

set appearance.selectionColour=#2d3680;

set branchLabels.colorAttribute="User selection";

set branchLabels.displayAttribute="Branch times";

set branchLabels.fontName="Calibri";

set branchLabels.fontSize=12;

set branchLabels.fontStyle=0;

set branchLabels.isShown=false;

set branchLabels.significantDigits=4;

set layout.expansion=0;

set layout.layoutType="RECTILINEAR";

set layout.zoom=0;

set legend.attribute=null;

set legend.fontSize=10.0;

set legend.isShown=false;

set legend.significantDigits=4;

set nodeBars.barWidth=4.0;

set nodeBars.displayAttribute=null;

set nodeBars.isShown=false;

set nodeLabels.colorAttribute="User selection";

set nodeLabels.displayAttribute="label";

set nodeLabels.fontName="Arial";

set nodeLabels.fontSize=12;

set nodeLabels.fontStyle=0;

set nodeLabels.isShown=true;

set nodeLabels.significantDigits=4;

set nodeShapeExternal.colourAttribute=null;

set nodeShapeExternal.isShown=false;

set nodeShapeExternal.minSize=10.0;

set nodeShapeExternal.scaleType=Width;

set nodeShapeExternal.shapeType=Circle;

set nodeShapeExternal.size=4.0;

set nodeShapeExternal.sizeAttribute=null;

set nodeShapeInternal.colourAttribute=null;

set nodeShapeInternal.isShown=false;

set nodeShapeInternal.minSize=10.0;

set nodeShapeInternal.scaleType=Width;

set nodeShapeInternal.shapeType=Circle;

set nodeShapeInternal.size=4.0;

set nodeShapeInternal.sizeAttribute=null;

set polarLayout.alignTipLabels=false;

set polarLayout.angularRange=0;

set polarLayout.rootAngle=0;

set polarLayout.rootLength=100;

set polarLayout.showRoot=true;

set radialLayout.spread=0.0;

set rectilinearLayout.alignTipLabels=true;

set rectilinearLayout.curvature=0;

set rectilinearLayout.rootLength=100;

set scale.offsetAge=0.0;

set scale.rootAge=1.0;

set scale.scaleFactor=1.0;

set scale.scaleRoot=false;

set scaleAxis.automaticScale=true;

set scaleAxis.fontSize=8.0;

set scaleAxis.isShown=false;

set scaleAxis.lineWidth=1.0;

set scaleAxis.majorTicks=1.0;

set scaleAxis.minorTicks=0.5;

set scaleAxis.origin=0.0;

set scaleAxis.reverseAxis=false;

set scaleAxis.showGrid=true;

set scaleBar.automaticScale=true;

set scaleBar.fontSize=12.0;

set scaleBar.isShown=true;

set scaleBar.lineWidth=1.0;

set scaleBar.scaleRange=0.0;

set tipLabels.colorAttribute="User selection";

set tipLabels.displayAttribute="Names";

set tipLabels.fontName="Arial";

set tipLabels.fontSize=12;

set tipLabels.fontStyle=0;

set tipLabels.isShown=true;

set tipLabels.significantDigits=4;

set trees.order=true;

set trees.orderType="increasing";

set trees.rooting=false;

set trees.rootingType="User Selection";

set trees.transform=false;

set trees.transformType="cladogram";

end;
