# Supplementary material for: Early origin and global colonisation of foot-and-mouth disease virus
Source: Sci Rep. 2020 Sep 17;10:15268. doi: 10.1038/s41598-020-72246-6 (PMC7498456; doi:10.1038/s41598-020-72246-6)
Supplement: Supplementary file 13 — Supplementary Tree S5. [file 41598_2020_72246_MOESM13_ESM.doc]

#NEXUS

begin trees;

tree tree_1 = [&R] (((((((((((((((((((((((((((((((((((('AY593831.1_O_UKG_2002':1.0E-6,'AY593836.1_O_UKG_2001':1.0E-6)[&label=0.845]:0.001025,'DQ404171.1_O_UKG_2001':0.002055)[&label=0.447]:1.0E-6,('DQ404161.1_O_UKG_2001':0.001025,'AJ633821.1_O_FRA_2001':0.001025)[&label=0.031]:1.0E-6)[&label=0.24325]:1.0E-6,('DQ404169.1_O_UKG_2001':1.0E-6,'DQ404173.1_O_UKG_2001':1.0E-6)[&label=0.014]:1.0E-6)[&label=0.164667]:1.0E-6,((('DQ404165.1_O_UKG_2001':0.001025,'DQ404166.1_O_UKG_2001':1.0E-6)[&label=0.882]:0.002054,'DQ404167.1_O_UKG_2001':1.0E-6)[&label=0.928]:0.002054,(('DQ404177.1_O_UKG_2001':0.001025,'DQ404179.1_O_UKG_2001':1.0E-6)[&label=0.419]:1.0E-6,'DQ404178.1_O_UKG_2001':0.001025)[&label=0.695]:0.001025)[&label=0.436]:1.0E-6)[&label=0.236083]:1.0E-6,'DQ404180.1_O_UKG_2001':1.0E-6)[&label=0.218538]:1.0E-6,(((((((('EF552691.1_O_UKG_2001':1.0E-6,'DQ404176.1_O_UKG_2001':1.0E-6)[&label=0.011]:1.0E-6,'KM257062.1_O_UKG_2001':1.0E-6)[&label=0.021]:1.0E-6,('EF552690.1_O_UKG_2001':1.0E-6,'EU214601.1_O_UKG_2001':1.0E-6)[&label=0.028]:1.0E-6)[&label=0.05825]:1.0E-6,'EF552695.1_O_UKG_2001':1.0E-6)[&label=0.0674]:1.0E-6,'EF552692.1_O_UKG_2001':1.0E-6)[&label=0.077833]:1.0E-6,'FJ542365.1_O_UKG_2001':1.0E-6)[&label=0.089286]:1.0E-6,'FJ542370.1_O_UKG_2001':1.0E-6)[&label=0.104]:1.0E-6,('DQ404170.1_O_UKG_2001':1.0E-6,'DQ404174.1_O_UKG_2001':1.0E-6)[&label=0.033]:1.0E-6)[&label=0.137]:1.0E-6)[&label=0.231792]:1.0E-6,((('EF552697.1_O_UKG_2001':1.0E-6,'FJ542369.1_O_UKG_2001':1.0E-6)[&label=0.019]:1.0E-6,'AJ539141.1_O_UKG_2001':1.0E-6)[&label=0.017]:1.0E-6,'DQ404175.1_O_UKG_2001':1.0E-6)[&label=0.022333]:1.0E-6)[&label=0.433857]:1.0E-6,'EF552693.1_O_UKG_2001':1.0E-6)[&label=0.485241]:1.0E-6,'DQ404172.1_O_UKG_2001':1.0E-6)[&label=0.531433]:1.0E-6,((('DQ404162.1_O_UKG_2001':1.0E-6,'DQ404168.1_O_UKG_2001':1.0E-6)[&label=0.825]:0.001025,('EF552696.1_O_UKG_2001':0.002054,'EF552688.1_O_UKG_2001':1.0E-6)[&label=0.642]:0.001025)[&label=0.347333]:1.0E-6,'KM257064.1_O_UKG_2001':1.0E-6)[&label=0.26125]:1.0E-6)[&label=0.694657]:1.0E-6,(('FJ542372.1_O_UKG_2001':0.001025,'DQ404163.1_O_UKG_2001':0.00309)[&label=0.032]:1.0E-6,'KM257061.1_O_UKG_2001':0.001025)[&label=0.0405]:1.0E-6)[&label=0.776553]:1.0E-6,((((('DQ404159.1_O_UKG_2001':1.0E-6,'KM257065.1_O_UKG_2001':1.0E-6)[&label=0.72]:1.0E-6,'DQ404160.1_O_UKG_2001':0.001026)[&label=0.729]:1.0E-6,'DQ404158.1_O_UKG_2001':0.004137)[&label=0.713333]:0.001025,'DQ404164.1_O_UKG_2001':0.003092)[&label=0.576]:1.0E-6,('EF552689.1_O_UKG_2001':0.001025,'FJ542371.1_O_UKG_2001':0.003088)[&label=0.065]:1.0E-6)[&label=0.419333]:1.0E-6)[&label=0.948178]:1.0E-6,('FJ542368.1_O_UKG_2001':1.0E-6,'KM257063.1_O_UKG_2001':1.0E-6)[&label=0.96]:0.001025)[&label=0.991191]:0.001026,'AJ539140.1_O_SAR_2000':0.001033)[&label=0.998792]:0.00519,'AB079061.1_O_JPN_2000':0.004184)[&label=0.997898]:0.004179,((((((((('KF694745.1_O_SKR_2002':1.0E-6,'KF694743.1_O_SKR_2002':0.001025)[&label=0.012]:1.0E-6,'KF694740.1_O_SKR_2002':1.0E-6)[&label=0.083]:1.0E-6,(('KF694731.1_O_SKR_2002':1.0E-6,'KF694735.1_O_SKR_2002':1.0E-6)[&label=0.02]:1.0E-6,'KF694734.1_O_SKR_2002':1.0E-6)[&label=0.113]:1.0E-6)[&label=0.4512]:1.0E-6,'KF694737.1_O_SKR_2002':0.001026)[&label=0.478]:1.0E-6,('KF694744.1_O_SKR_2002':1.0E-6,'KF694739.1_O_SKR_2002':1.0E-6)[&label=0.078]:1.0E-6)[&label=0.82625]:1.0E-6,((('KF694742.1_O_SKR_2002':1.0E-6,'KF694741.1_O_SKR_2002':1.0E-6)[&label=0.361]:1.0E-6,'KF694736.1_O_SKR_2002':1.0E-6)[&label=0.978]:0.002055,'EF614457.1_O_SKR_2002':0.002056)[&label=0.716]:1.0E-6)[&label=0.996167]:0.00416,(('KF694738.1_O_SKR_2002':1.0E-6,'KF694732.1_O_SKR_2002':1.0E-6)[&label=0.999]:1.0E-6,'AH012984.2_O_SKR_2000':0.010491)[&label=0.981]:0.004169)[&label=0.9978]:0.004864,('HQ009509.1_O_CHA_1999':0.034686,'HM008917.1_O_CHA_2005':0.01854)[&label=0.999]:0.016644)[&label=0.998706]:0.010022,(((('AJ539137.1_O_TAW_1999':1.0E-6,'AJ539136.1_O_TAW_1999':1.0E-6)[&label=1.0]:0.004172,'HQ632768.1_O_MAY_2000':0.005232)[&label=0.9525]:0.002023,('AF506822.2_O_CHA_1999':0.004134,'AJ539138.1_O_CHA_1999':1.0E-6)[&label=0.66]:0.001025)[&label=0.8145]:0.001003,(((('AY593824.1_O_SKR_2000':1.0E-6,'MG372730.1_O_SKR_2000':1.0E-6)[&label=0.316]:1.0E-6,'AH012985.2_O_SKR_2000':0.002056)[&label=0.5085]:1.0E-6,'AF377945.1_O_SKR_2000':0.014932)[&label=0.666667]:1.0E-6,'AJ539139.1_O_SKR_2000':1.0E-6)[&label=1.0]:0.01495)[&label=0.692333]:0.001046)[&label=0.792111]:9.56E-4)[&label=0.993494]:0.00384,(('DQ989310.1_Asia1_IND_1999':1.0E-6,'MF372125.1_Asia1_IND_2016':1.0E-6)[&label=1.0]:0.007004,'HQ832588.1_A_IND_2005':0.050857)[&label=0.783]:0.003112)[&label=0.9843]:0.003789,('JF749849.1_Asia1_PAK_2002':0.013042,'JF749851.1_O_IRN_2001':0.006656)[&label=1.0]:0.014121)[&label=0.990646]:0.01218,'EF149010.1_Asia1_CHA_2005':0.02363)[&label=0.990602]:0.012701,(((((((('MF947127.1_O_VIT_2012':1.0E-6,'MF947137.1_O_VIT_2012':0.003087)[&label=1.0]:0.017044,('MF947123.1_O_VIT_2011':0.002063,'KY234501.1_O_CHA_2011':0.003114)[&label=0.898]:0.002048)[&label=0.600667]:0.00105,(('MF947129.1_O_VIT_2014':0.012801,'MF947124.1_O_VIT_2012':0.006316)[&label=0.902]:0.005446,'MF947126.1_O_VIT_2011':0.008486)[&label=0.5535]:9.03E-4)[&label=0.6825]:1.0E-6,'MF947141.1_O_VIT_2012':0.012667)[&label=0.710571]:0.001028,(((('MF143578.1_O_VIT_2013':0.002077,'MF143577.1_O_VIT_2013':0.002058)[&label=1.0]:0.005294,'MF143576.1_O_VIT_2013':0.005268)[&label=0.9185]:0.004267,'MF143575.1_O_VIT_2012':0.006336)[&label=0.816333]:0.001559,(('MF143572.1_O_VIT_2012':0.002417,'MF143573.1_O_VIT_2012':0.009124)[&label=0.54]:0.001498,'MF143574.1_O_VIT_2012':0.004426)[&label=0.9365]:0.008887)[&label=0.732667]:0.003628)[&label=0.765357]:7.09E-4,'MF947128.1_O_VIT_2010':0.004485)[&label=0.826]:0.003034,('MF947132.1_O_VIT_2015':0.018417,'KY234502.1_O_CHA_2015':0.024342)[&label=1.0]:0.03791)[&label=0.910118]:0.005287,(('MF947143.1_O_VIT_2013':0.008357,'MF947142.1_O_VIT_2013':0.003097)[&label=0.669]:1.0E-6,'MF947131.1_O_VIT_2013':0.013729)[&label=0.999]:0.012829)[&label=0.99995]:0.028286)[&label=0.998952]:0.021529,(((('DQ989312.1_Asia1_IND_1990':0.028039,'KU726614.1_O_GRE_1994':0.028713)[&label=0.555]:0.003977,'DQ989311.1_Asia1_IND_2002':0.035477)[&label=0.7145]:0.004751,('AY593791.1_A_IRN_1998':0.026801,'JF749848.1_A_TUR_2003':0.035485)[&label=1.0]:0.038507)[&label=0.629]:0.004374,(('AY593813.1_O_ISA_1962':0.155094,'DQ989305.1_Asia1_IND_1990':0.037558)[&label=0.278]:0.01631,'AY687333.1_Asia1_IND_2001':0.047387)[&label=0.397]:0.013723)[&label=0.504143]:0.00232)[&label=0.956562]:0.004388,(((('HQ832584.1_A_IND_2005':0.028658,'HM854021.1_A_IND_2000':0.015333)[&label=0.356]:0.00248,'HQ832592.1_A_IND_2009':0.046119)[&label=0.7145]:0.007194,'HQ832589.1_A_IND_2006':0.020942)[&label=0.997333]:0.038398,'HM854022.1_A_IND_1977':0.042241)[&label=0.7795]:0.011326)[&label=0.933658]:0.00525,(('HM854023.1_A_IND_1999':0.013169,'HQ832577.1_A_IND_1999':0.009699)[&label=0.998]:0.033231,'HQ832576.1_A_IND_1990':0.017464)[&label=0.7625]:0.019928)[&label=0.9106]:0.004517,((((((((((((((((((('JX040488.1_O_BUL_2011':1.0E-6,'JX040489.1_O_BUL_2011':1.0E-6)[&label=0.906]:1.0E-6,('JX040490.1_O_BUL_2011':1.0E-6,'JX040487.1_O_BUL_2011':1.0E-6)[&label=0.944]:0.002071)[&label=0.909333]:0.00103,'JX040486.1_O_BUL_2011':0.002079)[&label=0.946]:0.002079,'JX066664.1_O_BUL_2011':0.001032)[&label=0.7964]:1.0E-6,('JX066665.1_O_BUL_2011':0.002069,'JX040485.1_O_BUL_2010':0.001033)[&label=0.093]:1.0E-6)[&label=0.913]:0.001038,'JX040496.1_O_TUR_2010':0.002074)[&label=0.906875]:0.00103,'JX040491.1_O_TUR_2010':0.004158)[&label=0.823111]:1.0E-6,(((('JX040500.1_O_TUR_2011':0.006355,'JX040499.1_O_TUR_2011':0.004141)[&label=0.984]:0.004233,'JX040497.1_O_TUR_2010':0.003152)[&label=0.8625]:0.001002,'JX040493.1_O_TUR_2010':0.001032)[&label=0.595333]:1.0E-6,'JX040494.1_O_TUR_2010':0.00207)[&label=0.513]:1.0E-6)[&label=0.964]:0.001029,'JX040495.1_O_TUR_2010':0.002079)[&label=0.976067]:0.001037,'JX040498.1_O_TUR_2010':0.003123)[&label=0.989062]:0.00419,'JX040501.1_O_ISR_2011':0.006292)[&label=0.960118]:1.0E-6,'JX040492.1_O_TUR_2010':0.00842)[&label=0.978222]:0.001894,'KM268896.1_A_TUR_2013':0.031371)[&label=0.983263]:0.002871,('KM268895.1_O_TUR_2013':0.018914,'KM268898.1_Asia1_TUR_2013':0.018361)[&label=0.873]:0.008993)[&label=0.974667]:0.004823,(('MH784403.1_O_PAK_2016':0.005227,'MH784404.1_O_PAK_2017':0.002065)[&label=1.0]:0.016391,'MH784405.1_O_PAK_2017':0.021784)[&label=0.9815]:0.017214)[&label=0.986542]:0.011749,'JN006719.1_Asia1_PAK_2008':0.022931)[&label=0.99896]:0.021417,(('KY446902.1_A_PAK_2005':0.00554,'KT003716.1_O_PAK_2005':0.014823)[&label=0.807]:0.004385,'KY446903.1_O_PAK_2005':0.004242)[&label=0.9965]:0.023126)[&label=0.989571]:0.014306,(('HQ832586.1_A_IND_2006':0.029501,'HQ832585.1_A_IND_2005':0.039731)[&label=0.604]:0.019223,'DQ989319.1_Asia1_IND_2001':0.056309)[&label=0.606]:0.006621)[&label=0.911613]:0.006319,(((((((((((('FJ175666.1_O_ISR_2007':1.0E-6,'FJ175661.1_O_ISR_2007':1.0E-6)[&label=0.981]:0.00103,'FJ175662.1_O_ISR_2007':0.002063)[&label=0.9175]:1.0E-6,(('FJ175663.1_O_ISR_2007':1.0E-6,'FJ175664.1_O_ISR_2007':1.0E-6)[&label=1.0]:0.006344,'FJ175665.1_O_ISR_2007':0.004207)[&label=0.752]:9.73E-4)[&label=0.9924]:0.006234,'KC440883.1_O_EGY_2011':0.037643)[&label=0.968]:0.006465,'HQ113233.1_Asia1_AFG_2009':0.014249)[&label=0.908857]:0.001087,('JN006720.1_Asia1_PAK_2009':0.006232,'JN006722.1_A_PAK_2008':1.0E-6)[&label=1.0]:0.016131)[&label=0.906333]:0.0065,(('JF749852.1_O_MAY_2004':0.001029,'HQ632770.1_O_MAY_2004':1.0E-6)[&label=0.972]:0.005347,'HQ268524.1_O_BHU_2004':0.005192)[&label=0.999]:0.008654)[&label=0.882583]:0.00872,('GU384683.1_O_PAK_2008':1.0E-6,'GU384682.1_O_PAK_2008':1.0E-6)[&label=1.0]:0.014392)[&label=0.827571]:0.002302,'HQ113232.1_O_PAK_2009':0.021154)[&label=0.841133]:0.001403,(((('JN099694.1_A_IRQ_2009':0.002057,'JN099688.1_A_IRQ_2009':0.003093)[&label=0.092]:1.0E-6,('JN099697.1_A_IRQ_2009':1.0E-6,'JN099699.1_A_IRQ_2009':0.001027)[&label=0.922]:0.002057)[&label=0.498]:1.0E-6,('JN099698.1_A_IRQ_2009':1.0E-6,'JN099695.1_A_IRQ_2009':1.0E-6)[&label=0.991]:0.003104)[&label=0.9998]:0.010303,'KC440882.1_A_EGY_2012':0.026905)[&label=0.913333]:0.002267)[&label=0.997273]:0.021828,'DQ989315.1_Asia1_IND_1993':0.035809)[&label=0.970348]:0.008666,'MF782478.1_Asia1_BAN_2013':0.04016)[&label=0.974458]:0.015247)[&label=0.570125]:0.00246)[&label=0.711056]:0.005156,('DQ989313.1_Asia1_IND_1986':0.051246,'DQ989307.1_Asia1_IND_1992':0.054177)[&label=0.606]:0.012019)[&label=0.704564]:1.0E-6,(((((((((((((((((((((('MG983733.1_O_SRL_2014':0.002309,'MG983734.1_O_SRL_2014':0.002857)[&label=0.905]:0.003128,'MG983732.1_O_SRL_2014':0.001277)[&label=0.994]:0.003907,'KJ825807.1_O_IND_2014':0.004183)[&label=0.954333]:0.003088,'KJ825804.1_O_IND_2013':0.003089)[&label=0.7485]:1.0E-6,(('MG983731.1_O_SRL_2013':0.006305,'MG983711.1_O_NEP_2013':0.003147)[&label=0.256]:9.77E-4,'MG983716.1_O_NEP_2014':0.007266)[&label=0.186]:1.0E-6)[&label=0.553429]:0.001025,('KJ825803.1_O_IND_2013':0.007275,'MG983714.1_O_NEP_2013':0.005176)[&label=0.161]:1.0E-6)[&label=0.516]:1.0E-6,'KJ825809.1_O_IND_2013':1.0E-6)[&label=0.5434]:1.0E-6,'KJ825805.1_O_IND_2013':0.002055)[&label=0.588636]:0.001026,((('MG983715.1_O_NEP_2014':0.007513,'MG983685.1_O_BAR_2015':0.008546)[&label=0.301]:8.36E-4,'KJ825806.1_O_IND_2013':0.002062)[&label=0.2345]:1.0E-6,('KJ825801.1_O_IND_2013':0.011573,'KJ825808.1_O_IND_2013':1.0E-6)[&label=0.724]:0.00622)[&label=0.344]:0.001026)[&label=0.805875]:1.0E-6,'MG983736.1_O_UAE_2014':0.003089)[&label=0.850882]:0.001022,(('MG983740.1_O_VIT_2015':0.001027,'KY657269.1_O_VIT_2015':0.002057)[&label=0.846]:0.00103,'MG983693.1_O_LAO_2015':0.003101)[&label=1.0]:0.01495)[&label=0.9651]:0.004914,'KJ206908.1_O_BHU_2013':0.017688)[&label=0.944333]:8.54E-4,('MG983709.1_O_NEP_2012':1.0E-6,'MG983708.1_O_NEP_2012':0.001025)[&label=0.999]:0.006608)[&label=0.956391]:0.002816,(((((((('MG983722.1_O_SAU_2013':1.0E-6,'KJ206910.1_O_SAU_2013':0.002057)[&label=0.603]:1.0E-6,'MG983721.1_O_SAU_2013':0.001023)[&label=1.0]:0.007268,'MG983726.1_O_SAU_2014':0.010455)[&label=0.731]:1.0E-6,'MG983725.1_O_SAU_2014':0.00938)[&label=0.83075]:0.001025,'KJ825802.1_O_IND_2013':0.006221)[&label=0.8226]:0.001049,('MG983723.1_O_SAU_2013':0.002057,'MG983724.1_O_SAU_2013':1.0E-6)[&label=1.0]:0.006242)[&label=0.811571]:9.94E-4,(((('MG983694.1_O_LIB_2013':0.001026,'MG983697.1_O_LIB_2013':0.001026)[&label=0.162]:1.0E-6,'KJ206909.1_O_LIB_2013':1.0E-6)[&label=0.3925]:1.0E-6,('MG983696.1_O_LIB_2013':0.003093,'MG983695.1_O_LIB_2013':0.001027)[&label=0.214]:1.0E-6)[&label=0.7335]:1.0E-6,(('MG983735.1_O_TUN_2014':1.0E-6,'MG983683.1_O_ALG_2014':0.002055)[&label=0.613]:1.0E-6,'KU291242.1_O_MOR_2015':0.014935)[&label=0.999]:0.00728)[&label=0.851143]:0.002083)[&label=0.983067]:0.005257,('MG983712.1_O_NEP_2013':0.008332,'MG983713.1_O_NEP_2013':1.0E-6)[&label=0.999]:0.010533)[&label=0.898882]:0.001022)[&label=0.963415]:4.62E-4,'MG983717.1_O_NEP_2015':0.026728)[&label=0.980167]:0.01074,(((('MG983687.1_O_BHU_2009':1.0E-6,'MG983684.1_O_BAN_2009':0.005193)[&label=0.951]:0.00309,'MG983692.1_O_IRN_2009':0.006236)[&label=0.586]:1.0E-6,('MG983688.1_O_BHU_2009':0.009447,'MG983705.1_O_NEP_2010':0.00413)[&label=0.145]:1.0E-6)[&label=0.93275]:7.65E-4,'MG983706.1_O_NEP_2010':0.006525)[&label=1.0]:0.012872)[&label=0.987292]:0.015691,('MG983690.1_O_BHU_2016':0.010917,'MG983686.1_O_BAR_2015':0.008523)[&label=1.0]:0.026416)[&label=0.97212]:0.0069,(((('HQ832581.1_A_IND_2004':0.008469,'HQ832579.1_A_IND_2003':0.001009)[&label=0.894]:0.002094,'HQ832583.1_A_IND_2005':0.01171)[&label=0.556]:1.0E-6,('HQ832580.1_A_IND_2003':0.010647,'HQ832578.1_A_IND_2003':0.007404)[&label=0.848]:0.002016)[&label=0.887]:6.65E-4,'HQ832582.1_A_IND_2004':0.014243)[&label=0.9708]:0.008221)[&label=0.988679]:0.007421,('HQ832591.1_A_IND_2008':0.06391,'HQ832590.1_A_IND_2007':1.0E-6)[&label=0.935]:0.032967)[&label=0.989828]:0.017269,(('DQ989309.1_Asia1_IND_1996':1.0E-6,'MF372126.1_Asia1_IND_1994':1.0E-6)[&label=1.0]:0.027087,'HQ832587.1_A_IND_2005':0.052119)[&label=0.9885]:0.02108)[&label=0.965066]:0.007539,'DQ989308.1_Asia1_IND_1994':0.054618)[&label=0.951323]:0.001482,((('EF494486.1_A_TUR_2005':0.005639,'JF749841.1_A_TUR_2006':0.013591)[&label=0.494]:0.002921,'EF494488.1_A_PAK_2006':0.018293)[&label=0.8065]:0.003691,('EF494487.1_A_PAK_2006':1.0E-6,'EF117837.1_A_PAK_2006':1.0E-6)[&label=1.0]:0.009359)[&label=1.0]:0.057981)[&label=0.896373]:0.006358)[&label=0.728838]:0.004245,(('KY825718.1_Asia1_ISR_1989':1.0E-6,'AY593800.1_Asia1_LEB_1983':1.0E-6)[&label=0.334]:1.0E-6,'AY593799.1_Asia1_LEB_1983':1.0E-6)[&label=1.0]:0.06231)[&label=0.727864]:0.00525,((((((((((((((((((((((((((((((((((('LC149669.1_O_JPN_2010':1.0E-6,'LC149667.1_O_JPN_2010':1.0E-6)[&label=0.186]:1.0E-6,'LC149681.1_O_JPN_2010':1.0E-6)[&label=0.378]:1.0E-6,'LC149666.1_O_JPN_2010':1.0E-6)[&label=0.583]:1.0E-6,'LC149694.1_O_JPN_2010':1.0E-6)[&label=0.81025]:1.0E-6,('LC149707.1_O_JPN_2010':0.001024,'LC149720.1_O_JPN_2010':0.001024)[&label=0.097]:1.0E-6)[&label=0.734667]:1.0E-6,('LC149717.1_O_JPN_2010':0.002051,'LC149711.1_O_JPN_2010':1.0E-6)[&label=0.424]:0.001023)[&label=0.744875]:0.001027,(((('LC149699.1_O_JPN_2010':1.0E-6,'LC036265.1_O_JPN_2010':1.0E-6)[&label=0.154]:1.0E-6,'LC149654.1_O_JPN_2010':1.0E-6)[&label=0.531]:1.0E-6,'LC149716.1_O_JPN_2010':1.0E-6)[&label=0.919667]:0.001025,'LC149630.1_O_JPN_2010':1.0E-6)[&label=0.827]:1.0E-6)[&label=0.497538]:1.0E-6,(((((('LC149703.1_O_JPN_2010':1.0E-6,'LC149697.1_O_JPN_2010':1.0E-6)[&label=0.904]:0.001026,'LC149715.1_O_JPN_2010':1.0E-6)[&label=0.471]:1.0E-6,'LC149661.1_O_JPN_2010':1.0E-6)[&label=0.355333]:1.0E-6,('LC149685.1_O_JPN_2010':1.0E-6,'LC149639.1_O_JPN_2010':1.0E-6)[&label=0.196]:1.0E-6)[&label=0.8254]:0.001025,'LC149623.1_O_JPN_2010':0.001026)[&label=0.691667]:1.0E-6,'LC149658.1_O_JPN_2010':0.001025)[&label=0.597]:1.0E-6)[&label=0.366762]:1.0E-6,(('LC149668.1_O_JPN_2010':1.0E-6,'LC149682.1_O_JPN_2010':1.0E-6)[&label=0.019]:1.0E-6,'LC149619.1_O_JPN_2010':1.0E-6)[&label=0.0135]:1.0E-6)[&label=0.321667]:1.0E-6,(((((((('LC149706.1_O_JPN_2010':1.0E-6,'LC149704.1_O_JPN_2010':0.003091)[&label=0.273]:0.001025,'LC149672.1_O_JPN_2010':0.001024)[&label=0.181]:1.0E-6,'LC149705.1_O_JPN_2010':1.0E-6)[&label=0.142]:1.0E-6,('LC149691.1_O_JPN_2010':1.0E-6,'LC149629.1_O_JPN_2010':1.0E-6)[&label=0.223]:1.0E-6)[&label=0.5098]:0.001025,'LC149687.1_O_JPN_2010':0.001026)[&label=0.427833]:1.0E-6,(((('LC149695.1_O_JPN_2010':1.0E-6,'LC149688.1_O_JPN_2010':1.0E-6)[&label=0.931]:0.001025,'LC149641.1_O_JPN_2010':0.002055)[&label=0.4775]:1.0E-6,'LC149632.1_O_JPN_2010':1.0E-6)[&label=0.322333]:1.0E-6,'LC149631.1_O_JPN_2010':1.0E-6)[&label=0.24225]:1.0E-6)[&label=0.248]:1.0E-6,('LC149648.1_O_JPN_2010':1.0E-6,'LC149625.1_O_JPN_2010':1.0E-6)[&label=0.011]:1.0E-6)[&label=0.210385]:1.0E-6,'LC149678.1_O_JPN_2010':1.0E-6)[&label=0.195429]:1.0E-6)[&label=0.305846]:1.0E-6,('LC149638.1_O_JPN_2010':1.0E-6,'LC149674.1_O_JPN_2010':1.0E-6)[&label=0.01]:1.0E-6)[&label=0.294463]:1.0E-6,((('LC149643.1_O_JPN_2010':0.001025,'LC149710.1_O_JPN_2010':0.002062)[&label=0.339]:1.0E-6,'LC149642.1_O_JPN_2010':0.001025)[&label=0.195]:1.0E-6,'LC149689.1_O_JPN_2010':1.0E-6)[&label=0.130333]:1.0E-6)[&label=0.293822]:1.0E-6,'LC149683.1_O_JPN_2010':1.0E-6)[&label=0.290261]:1.0E-6,(((((((((('LC149653.1_O_JPN_2010':1.0E-6,'LC149646.1_O_JPN_2010':1.0E-6)[&label=0.002]:1.0E-6,'LC149679.1_O_JPN_2010':1.0E-6)[&label=0.016]:1.0E-6,'LC149702.1_O_JPN_2010':1.0E-6)[&label=0.017333]:1.0E-6,'LC149647.1_O_JPN_2010':1.0E-6)[&label=0.0205]:1.0E-6,'LC149664.1_O_JPN_2010':1.0E-6)[&label=0.0254]:1.0E-6,'LC149644.1_O_JPN_2010':1.0E-6)[&label=0.028333]:1.0E-6,((((('LC149671.1_O_JPN_2010':1.0E-6,'LC149659.1_O_JPN_2010':1.0E-6)[&label=0.008]:1.0E-6,'LC149684.1_O_JPN_2010':1.0E-6)[&label=0.0045]:1.0E-6,('LC149655.1_O_JPN_2010':1.0E-6,'LC149651.1_O_JPN_2010':1.0E-6)[&label=0.0]:1.0E-6)[&label=0.01225]:1.0E-6,'LC149700.1_O_JPN_2010':1.0E-6)[&label=0.0142]:1.0E-6,'LC149620.1_O_JPN_2010':1.0E-6)[&label=0.016]:1.0E-6)[&label=0.045615]:1.0E-6,('LC149673.1_O_JPN_2010':1.0E-6,'LC149663.1_O_JPN_2010':1.0E-6)[&label=0.005]:1.0E-6)[&label=0.048333]:1.0E-6,(('LC149637.1_O_JPN_2010':1.0E-6,'LC149713.1_O_JPN_2010':1.0E-6)[&label=0.006]:1.0E-6,'LC149657.1_O_JPN_2010':1.0E-6)[&label=0.0075]:1.0E-6)[&label=0.051722]:1.0E-6,((((((('LC149677.1_O_JPN_2010':1.0E-6,'LC149693.1_O_JPN_2010':0.001026)[&label=0.027]:1.0E-6,'LC149692.1_O_JPN_2010':1.0E-6)[&label=0.2305]:1.0E-6,'LC149675.1_O_JPN_2010':1.0E-6)[&label=0.614333]:1.0E-6,'LC149690.1_O_JPN_2010':0.001026)[&label=0.613]:1.0E-6,'LC149708.1_O_JPN_2010':0.001026)[&label=0.7272]:0.001026,'LC149701.1_O_JPN_2010':0.001026)[&label=0.611]:1.0E-6,'LC149698.1_O_JPN_2010':1.0E-6)[&label=0.526857]:1.0E-6)[&label=0.148308]:1.0E-6)[&label=0.613959]:1.0E-6,('LC149652.1_O_JPN_2010':1.0E-6,'LC149622.1_O_JPN_2010':0.001026)[&label=0.01]:1.0E-6)[&label=0.644573]:1.0E-6,'LC149676.1_O_JPN_2010':1.0E-6)[&label=0.661263]:1.0E-6,((((((((((((('LC149709.1_O_JPN_2010':1.0E-6,'LC149645.1_O_JPN_2010':1.0E-6)[&label=0.005]:1.0E-6,'LC149618.1_O_JPN_2010':1.0E-6)[&label=0.008]:1.0E-6,'LC149665.1_O_JPN_2010':1.0E-6)[&label=0.009667]:1.0E-6,'LC149649.1_O_JPN_2010':1.0E-6)[&label=0.01425]:1.0E-6,'LC149628.1_O_JPN_2010':1.0E-6)[&label=0.0142]:1.0E-6,'LC149635.1_O_JPN_2010':1.0E-6)[&label=0.015667]:1.0E-6,'LC149633.1_O_JPN_2010':1.0E-6)[&label=0.019429]:1.0E-6,('LC149719.1_O_JPN_2010':1.0E-6,'LC149686.1_O_JPN_2010':1.0E-6)[&label=0.018]:1.0E-6)[&label=0.025222]:1.0E-6,'LC149624.1_O_JPN_2010':1.0E-6)[&label=0.0274]:1.0E-6,'LC149640.1_O_JPN_2010':1.0E-6)[&label=0.03]:1.0E-6,(((('LC149696.1_O_JPN_2010':1.0E-6,'LC149656.1_O_JPN_2010':1.0E-6)[&label=0.0]:1.0E-6,'LC149680.1_O_JPN_2010':1.0E-6)[&label=0.012]:1.0E-6,'LC149670.1_O_JPN_2010':1.0E-6)[&label=0.008333]:1.0E-6,'LC149714.1_O_JPN_2010':1.0E-6)[&label=0.01]:1.0E-6)[&label=0.0495]:1.0E-6,'LC149627.1_O_JPN_2010':1.0E-6)[&label=0.052471]:1.0E-6,'LC149662.1_O_JPN_2010':1.0E-6)[&label=0.053167]:1.0E-6)[&label=0.922558]:1.0E-6,(('LC149626.1_O_JPN_2010':0.001026,'LC149660.1_O_JPN_2010':0.002055)[&label=0.047]:1.0E-6,'LC149636.1_O_JPN_2010':0.001026)[&label=0.0585]:1.0E-6)[&label=0.952153]:1.0E-6,('LC149634.1_O_JPN_2010':1.0E-6,'LC149718.1_O_JPN_2010':1.0E-6)[&label=0.772]:0.001025)[&label=0.97249]:1.0E-6,'LC149650.1_O_JPN_2010':0.001025)[&label=0.981851]:0.001025,(('KF112885.1_O_JPN_2010':0.001026,'LC149617.1_O_JPN_2010':1.0E-6)[&label=0.966]:0.003091,'LC149712.1_O_JPN_2010':0.003091)[&label=0.703]:1.0E-6)[&label=0.979606]:1.0E-6,'LC149621.1_O_JPN_2010':1.0E-6)[&label=0.986695]:0.00103,'JN998085.1_O_CHA_2010':0.002054)[&label=0.985613]:0.001025,(((('KF501488.1_O_SKR_2010':1.0E-6,'KF501487.1_O_SKR_2010':1.0E-6)[&label=0.91]:1.0E-6,'KR401160.1_O_SKR_2011':0.002055)[&label=0.9385]:0.002056,('KF112888.1_O_DRK_2011':0.00728,'KF112883.1_O_RUS_2010':0.003089)[&label=0.199]:1.0E-6)[&label=0.996]:0.006455,((('KC503937.1_O_SKR_2010':1.0E-6,'KR401159.1_O_SKR_2010':1.0E-6)[&label=0.236]:1.0E-6,'KF112887.1_O_SKR_2010':1.0E-6)[&label=0.5915]:1.0E-6,'KF501486.1_O_SKR_2010':1.0E-6)[&label=1.0]:0.01071)[&label=0.804375]:8.18E-4)[&label=0.961461]:1.0E-6,'KF112889.1_O_HKN_2010':0.004141)[&label=0.96281]:0.001024,(((((((('KX534089.1_O_SKR_2016':0.001031,'KY086465.1_O_SKR_2016':1.0E-6)[&label=1.0]:0.017131,'KY086466.1_O_SKR_2016':0.007335)[&label=0.79]:0.00106,'KX162590.1_O_SKR_2014':1.0E-6)[&label=1.0]:0.023245,'MH845413.2_O_VIT_2014':0.017034)[&label=0.95425]:0.004326,'KY322674.1_O_SKR_2014':0.020854)[&label=1.0]:0.029231,('JQ900581.1_O_CHA_2010':0.01047,'HM229661.1_O_HKN_2010':0.002061)[&label=0.443]:1.0E-6)[&label=0.912857]:0.002052,'JN998086.1_O_CHA_2010':0.006211)[&label=0.850125]:1.0E-6,(('KF112886.1_O_SKR_2010':1.0E-6,'KR401158.1_O_SKR_2010':1.0E-6)[&label=0.989]:0.002071,'JQ973889.1_O_CHA_2010':0.003087)[&label=0.9095]:0.00205)[&label=0.654455]:1.0E-6)[&label=0.975594]:1.0E-6,'HM055510.1_O_VIT_2009':0.00309)[&label=0.982093]:9.9E-4,((('KF112879.1_O_TAI_2009':0.011552,'KR401152.1_O_MYA_2009':0.005217)[&label=0.698]:0.001007,('KR401156.1_O_MYA_2009':0.003088,'KF112880.1_O_MYA_2009':1.0E-6)[&label=0.953]:0.004149)[&label=0.806]:0.001034,('KR401153.1_O_MYA_2009':1.0E-6,'KR401155.1_O_MYA_2007':1.0E-6)[&label=1.0]:0.013732)[&label=0.8612]:0.002093)[&label=0.998904]:0.011732,'KR401157.1_O_MYA_2009':0.00989)[&label=0.998956]:0.010773,'KY322672.1_O_MAY_2014':0.026446)[&label=0.998715]:0.014952,'KR401154.1_O_MYA_1998':0.034354)[&label=0.99858]:0.008076,'GU125646.1_Asia1_VIT_2005':0.099399)[&label=0.99705]:0.011873,((((((((((('KF112884.1_O_RUS_2010':0.004136,'KF112881.1_O_MOG_2010':0.005183)[&label=0.353]:1.0E-6,'KF112882.1_O_MOG_2010':0.003098)[&label=0.9045]:0.003097,('GU582116.1_O_VIT_2009':0.001028,'GU582115.1_O_VIT_2009':0.003098)[&label=0.499]:1.0E-6)[&label=0.99925]:0.007296,'HQ632772.1_O_MAY_2007':0.013276)[&label=0.887]:3.53E-4,'KY322673.1_O_MAY_2014':0.042852)[&label=0.803333]:0.004105,(('MF947130.1_O_VIT_2014':0.007372,'KY322671.1_O_MAY_2014':0.002072)[&label=0.943]:0.002059,'KY322670.1_O_LAO_2013':0.007403)[&label=0.996]:0.012326)[&label=0.937222]:0.007694,'KT968663.1_A_CHA_2013':0.045677)[&label=0.9736]:0.019838,((('GU125648.1_O_VIT_2006':0.002072,'GU125647.1_O_VIT_2006':0.001031)[&label=0.748]:9.99E-4,'GU125649.1_O_VIT_2006':0.00106)[&label=1.0]:0.030618,'GU125650.1_O_VIT_2006':0.023951)[&label=0.890667]:0.003155)[&label=1.0]:0.032043,('HQ632774.1_Asia1_MAY_1999':0.053953,'HQ632769.1_O_MAY_2001':0.014693)[&label=0.931]:0.013837)[&label=0.928312]:1.0E-6,(((((((((('GQ406249.1_A_VIT_2009':0.005442,'KC588943.1_A_SKR_2010':0.017336)[&label=0.301]:7.76E-4,'GQ406250.1_A_VIT_2009':0.009413)[&label=0.5585]:1.0E-6,'GQ406248.1_A_VIT_2009':1.0E-6)[&label=0.454]:1.0E-6,'GQ406252.1_A_VIT_2009':0.002056)[&label=0.71925]:0.001026,'GQ406247.1_A_VIT_2009':0.001031)[&label=0.8894]:0.001997,'GQ406251.1_A_VIT_2009':0.001099)[&label=1.0]:0.016978,'HQ632773.1_A_MAY_2007':0.008023)[&label=0.972571]:0.0044,(('KJ608371.1_A_VIT_2013':0.027351,'KY322678.1_A_MAY_2013':0.024983)[&label=0.998]:0.023793,'HQ268509.2_A_VIT_2004':0.013519)[&label=0.7915]:0.0092)[&label=0.8778]:0.007044,'KY322676.1_A_MAY_2013':0.048788)[&label=0.909364]:0.009621,((('KY322680.1_A_VIT_2013':0.003097,'KY322679.1_A_TAI_2014':0.007362)[&label=0.6]:0.001057,'KY322677.1_A_MAY_2013':0.001031)[&label=0.644]:1.0E-6,'KY322675.1_A_LAO_2014':0.004161)[&label=1.0]:0.038172)[&label=0.988733]:0.01711)[&label=0.942094]:0.009327,'KJ933864.1_A_MAY_1997':0.049117)[&label=0.943333]:0.002983)[&label=0.999769]:0.018743,((('DQ989303.1_Asia1_IND_1993':0.003404,'DQ989306.1_Asia1_IND_1986':7.86E-4)[&label=0.608]:0.001079,'DQ989304.1_Asia1_IND_2000':0.002306)[&label=0.998]:0.030921,(('AY593797.1_Asia1_ISR_1963':0.016916,'AY593796.1_Asia1_ISR_1963':0.032859)[&label=0.906]:0.009838,'AY593828.1_O_IND_1962':0.025894)[&label=0.809]:0.006105)[&label=0.9342]:0.021864)[&label=0.995179]:0.014936,('AY593823.1_O_TUR_1969':0.019547,'KP940473.1_O_EGY_2014':0.035243)[&label=1.0]:0.047194)[&label=0.986569]:0.008158)[&label=0.775164]:0.002086,((((((((((((((('LC320038.1_O_MOG_2015':0.007251,'MG983730.1_O_SKR_2017':0.003079)[&label=0.55]:1.0E-6,'MG983720.1_O_RUS_2016':0.002049)[&label=0.801]:0.001037,'MF461724.1_O_CHA_2017':0.003076)[&label=0.979]:0.004266,('MG983741.1_O_VIT_2016':0.012951,'LC438822.1_O_MYA_2016':0.007575)[&label=0.262]:6.33E-4)[&label=0.8264]:0.001048,'LC438823.1_O_MYA_2016':0.004213)[&label=0.911]:0.002074,'MH891503.1_O_VIT_2017':0.010615)[&label=0.967429]:0.005106,'KX712091.1_O_BAN_2015':0.001046)[&label=0.876125]:0.001005,'MG983719.1_O_NEP_2015':0.004124)[&label=0.790778]:1.0E-6,((((('MG983701.1_O_MUR_2016':1.0E-6,'MG983702.1_O_MUR_2016':1.0E-6)[&label=0.348]:1.0E-6,'MG983700.1_O_MUR_2016':1.0E-6)[&label=0.8435]:0.001023,('MG983698.1_O_MUR_2016':1.0E-6,'MG983699.1_O_MUR_2016':1.0E-6)[&label=0.697]:1.0E-6)[&label=1.0]:0.007311,('MG983727.1_O_SAU_2015':0.00199,'MG983728.1_O_SAU_2016':0.005282)[&label=1.0]:0.009601)[&label=0.8005]:0.001009,('MG983729.1_O_SAU_2016':0.008315,'MG983703.1_O_MYA_2016':0.013693)[&label=0.298]:1.0E-6)[&label=0.708]:1.0E-6)[&label=0.882167]:1.0E-6,'MG983718.1_O_NEP_2015':0.008337)[&label=0.915]:0.001045,(('MG983739.1_O_UAE_2016':1.0E-6,'MG983738.1_O_UAE_2016':0.001026)[&label=1.0]:0.006388,'MG983691.1_O_BHU_2016':0.008485)[&label=0.9725]:0.005131)[&label=0.999773]:0.017243,(('MG983689.1_O_BHU_2012':0.019247,'MG983707.1_O_NEP_2012':0.004162)[&label=0.611]:1.0E-6,'MG983710.1_O_NEP_2012':0.011562)[&label=0.7565]:0.001997)[&label=0.99896]:0.013603,('KF985189.1_O_BAN_2013':0.027876,'MG983704.1_O_NEP_2008':0.016979)[&label=0.68]:0.005104)[&label=1.0]:0.025918,(((((('DQ989320.1_Asia1_IND_2002':0.012058,'DQ989323.1_Asia1_IND_2002':0.013086)[&label=0.893]:0.00167,('DQ989314.1_Asia1_IND_2001':0.007397,'DQ989318.1_Asia1_IND_2002':0.005266)[&label=0.503]:9.32E-4)[&label=0.594667]:1.0E-6,'DQ989321.1_Asia1_IND_2001':0.006219)[&label=0.90625]:0.001958,'DQ989322.1_Asia1_IND_2002':0.006365)[&label=0.992]:0.0074,'DQ989317.1_Asia1_IND_2000':0.0065)[&label=0.927667]:0.006032,('KU127247.1_A_SAU_2015':0.017462,'KJ754939.1_A_BAN_2013':0.023335)[&label=0.999]:0.027517)[&label=0.955875]:0.006792)[&label=0.99925]:0.049579,((((((((('EF611987.1_O_UGA_2006':0.004638,'HM191257.1_O_UGA_2006':0.00585)[&label=0.988]:0.008945,'KU821591.1_O_ZAM_2010':0.017881)[&label=0.977]:0.012149,(('FJ461344.1_O_UGA_2002':0.026681,'MH053318.1_O_UGA_2002':0.010835)[&label=0.62]:0.005646,'FJ461345.1_O_UGA_2002':0.01787)[&label=0.6135]:0.005848)[&label=0.9944]:0.031553,'MH053307.1_A_ZAM_1990':0.046118)[&label=0.893167]:0.004901,'MH053316.1_O_UGA_1996':0.087465)[&label=0.987286]:0.027226,('JF749843.1_A_EGY_2006':0.092196,'MH053313.1_O_ETH_2006':0.050145)[&label=0.616]:0.02076)[&label=0.820889]:0.007072,(('MH053305.1_A_EGY_1972':0.06014,'MH053315.1_O_SUD_1976':0.066889)[&label=0.892]:0.025306,'AY593766.1_A_KEN_1965':0.047811)[&label=0.551]:0.001267)[&label=0.75475]:0.006065,(((('FJ623456.1_A_KAZ_1999':0.01099,'AY593764.1_A_IRQ_1970':0.005989)[&label=0.285]:0.00351,'AY593765.1_A_TUR_1965':0.004063)[&label=0.767]:0.016037,'AY593772.1_A_TUR_1972':0.029435)[&label=1.0]:0.064096,('MH053312.1_O_ETH_2005':0.050759,'MH053317.1_O_UGA_1998':0.044031)[&label=1.0]:0.046758)[&label=0.7534]:0.010799)[&label=0.9295]:0.009488,('MH053311.1_O_ETH_2004':0.03603,'MH053314.1_O_ETH_2007':0.02551)[&label=0.998]:0.04485)[&label=0.9707]:0.008401)[&label=0.727947]:0.006839)[&label=0.798805]:0.008373,(((((((((('AY593835.1_O_TAW_1997':1.0E-6,'AF308157.1_O_TAW_1997':1.0E-6)[&label=0.949]:0.001047,'AF026168.2_O_TAW_1997':0.004235)[&label=0.492]:1.0E-6,'AF154271.1_O_TAW_1997':0.002098)[&label=0.703]:1.0E-6,'AY593833.1_O_TAW_1999':0.003165)[&label=0.99925]:0.034426,'HQ412603.1_O_CHA_2000':0.064907)[&label=0.8558]:0.004944,'HQ632771.1_O_MAY_2005':0.086447)[&label=0.792167]:0.015919,(('AY317098.1_O_CHA_2002':0.009811,'EU400597.1_O_CHA_2001':0.003065)[&label=0.981]:0.012825,('KU204894.1_O_CHA_2013':0.017791,'KU204893.1_O_CHA_2013':0.024631)[&label=1.0]:0.086517)[&label=0.934]:0.030149)[&label=0.9227]:0.018886,'AY686687.1_O_CHA_2001':0.057685)[&label=1.0]:0.127303,('KY072818.1_O_CHA_1959':0.061879,'AY593755.1_A_TAI_1960':0.071323)[&label=0.82]:0.014868)[&label=0.954308]:0.015144,'AY593812.1_O_PHI_1958':0.0824)[&label=0.892714]:0.006998)[&label=0.798595]:0.002598,(((((('GU125645.1_Asia1_VIT_2007':0.019402,'GQ452295.1_Asia1_VIT_2007':1.0E-6)[&label=0.982]:0.009509,'HQ631363.1_Asia1_CHA_2006':0.00106)[&label=0.849]:0.001005,'FJ906802.1_Asia1_CHA_2006':0.005241)[&label=0.899]:9.98E-4,'EF149009.1_Asia1_CHA_2005':0.006288)[&label=0.8655]:0.00211,((('KC412634.1_Asia1_CHA_2006':1.0E-6,'KC462884.1_Asia1_CHA_2006':1.0E-6)[&label=0.764]:1.0E-6,'KU360085.1_Asia1_CHA_2015':0.001029)[&label=0.999]:0.006263,'GU931682.1_Asia1_CHA_2005':0.00105)[&label=0.981333]:0.003102)[&label=0.897875]:0.001026,'KY446901.1_Asia1_PAK_2006':1.0E-6)[&label=0.998222]:0.054921)[&label=0.824091]:0.004655,('AY593834.1_O_IRN_1966':0.066465,'AY593795.1_Asia1_PAK_1954':0.044603)[&label=0.132]:0.006735)[&label=0.82526]:0.00977,((((((((((((((((((((((((((((((((('KJ560308.1_O_UKG_2007':1.0E-6,'KJ560302.1_O_UKG_2007':1.0E-6)[&label=0.065]:1.0E-6,'KJ560307.1_O_UKG_2007':1.0E-6)[&label=0.1745]:1.0E-6,'EU448375.1_O_UKG_2007':1.0E-6)[&label=0.435333]:1.0E-6,'KJ560303.1_O_UKG_2007':1.0E-6)[&label=0.73625]:1.0E-6,'KJ560304.1_O_UKG_2007':1.0E-6)[&label=0.955]:0.001034,((('KJ560294.1_O_UKG_2007':1.0E-6,'EU448377.1_O_UKG_2007':1.0E-6)[&label=0.161]:1.0E-6,'EU448376.1_O_UKG_2007':1.0E-6)[&label=0.5075]:1.0E-6,'KJ560296.1_O_UKG_2007':1.0E-6)[&label=0.879333]:1.0E-6)[&label=0.968333]:0.002073,'JX570645.1_O_UKG_2007':0.002074)[&label=0.8923]:1.0E-6,((('JX570638.1_O_UKG_2007':1.0E-6,'AY593816.1_O_UKG_1967':1.0E-6)[&label=0.027]:1.0E-6,'KJ560276.1_O_UKG_2007':1.0E-6)[&label=0.033]:1.0E-6,'KJ560285.1_O_UKG_2007':1.0E-6)[&label=0.037333]:1.0E-6)[&label=0.638286]:1.0E-6,'EU448372.1_O_UKG_2007':1.0E-6)[&label=0.595867]:1.0E-6,('JX570644.1_O_UKG_2007':1.0E-6,'JX570643.1_O_UKG_2007':1.0E-6)[&label=0.002]:1.0E-6)[&label=0.526529]:1.0E-6,'KJ560298.1_O_UKG_2007':1.0E-6)[&label=0.498]:1.0E-6,('KJ560281.1_O_UKG_2007':1.0E-6,'EU448373.1_O_UKG_2007':1.0E-6)[&label=0.002]:1.0E-6)[&label=0.44945]:1.0E-6,('EU448369.1_O_UKG_1967':1.0E-6,'EU448374.1_O_UKG_2007':1.0E-6)[&label=0.008]:1.0E-6)[&label=0.410545]:1.0E-6,'KJ560299.1_O_UKG_2007':1.0E-6)[&label=0.395348]:1.0E-6,((((((('JX570648.1_O_UKG_2007':1.0E-6,'EU448378.1_O_UKG_2007':1.0E-6)[&label=0.004]:1.0E-6,'KJ560300.1_O_UKG_2007':1.0E-6)[&label=0.018]:1.0E-6,('JX570654.1_O_UKG_2007':1.0E-6,'KJ560287.1_O_UKG_2007':1.0E-6)[&label=0.002]:1.0E-6)[&label=0.0315]:1.0E-6,'EU448370.1_O_UKG_1967':1.0E-6)[&label=0.04]:1.0E-6,('JX570650.1_O_UKG_2007':1.0E-6,'KJ560277.1_O_UKG_2007':1.0E-6)[&label=0.014]:1.0E-6)[&label=0.052571]:1.0E-6,'EU448371.1_O_UKG_2007':1.0E-6)[&label=0.060125]:1.0E-6,'JX570640.1_O_UKG_2007':1.0E-6)[&label=0.072778]:1.0E-6)[&label=0.641152]:1.0E-6,(('JX570646.1_O_UKG_2007':1.0E-6,'JX570649.1_O_UKG_2007':1.0E-6)[&label=0.008]:1.0E-6,'JX570647.1_O_UKG_2007':1.0E-6)[&label=0.0135]:1.0E-6)[&label=0.750778]:1.0E-6,((('JX570651.1_O_UKG_2007':1.0E-6,'JX570652.1_O_UKG_2007':1.0E-6)[&label=0.985]:0.001034,'KJ560283.1_O_UKG_2007':0.001034)[&label=0.5265]:1.0E-6,'JX570639.1_O_UKG_2007':1.0E-6)[&label=0.366333]:1.0E-6)[&label=0.8453]:1.0E-6,(('JX570642.1_O_UKG_2007':1.0E-6,'JX570641.1_O_UKG_2007':0.001033)[&label=0.489]:1.0E-6,'KJ560297.1_O_UKG_2007':0.001034)[&label=0.2725]:1.0E-6)[&label=0.900465]:1.0E-6,('JX570655.1_O_UKG_2007':1.0E-6,'JX570653.1_O_UKG_2007':1.0E-6)[&label=0.983]:0.001034)[&label=0.942556]:0.001034,'JX869185.1_O_UKG_1968':0.002072)[&label=0.931739]:1.0E-6,'JX869186.1_O_UKG_1968':0.003114)[&label=0.915894]:1.0E-6,'JX869181.1_O_UKG_1967':1.0E-6)[&label=0.904542]:1.0E-6,(((('EU448368.1_O_UKG_1967':1.0E-6,'JX869179.1_O_UKG_1967':1.0E-6)[&label=0.15]:1.0E-6,'JX869182.1_O_UKG_1968':0.001034)[&label=0.0945]:1.0E-6,'JX869187.1_O_UKG_1968':1.0E-6)[&label=0.142]:1.0E-6,(('AY593815.1_O_UKG_1967':1.0E-6,'JX869184.1_O_UKG_1968':0.002074)[&label=0.009]:1.0E-6,'JX869188.1_O_UKG_1968':1.0E-6)[&label=0.0545]:1.0E-6)[&label=0.437667]:1.0E-6)[&label=0.963182]:1.0E-6,('JX869183.1_O_UKG_1968':0.005216,'JX869180.1_O_UKG_1967':1.0E-6)[&label=0.839]:0.002073)[&label=0.996632]:0.005387,(((((('JX869178.1_O_UKG_1967':0.01346,'JX869177.1_O_UKG_1967':0.006269)[&label=0.794]:1.0E-6,'AY593837.1_O_URU_1963':1.0E-6)[&label=0.8035]:0.001038,('AY593767.1_A_ARG_1965':0.005223,'AY593814.1_O_ARG_1965':0.006323)[&label=0.838]:0.003164)[&label=0.6815]:0.001032,'AY593820.1_O_ARG_1964':0.001034)[&label=0.5688]:1.0E-6,'AY593819.1_O_ARG_1994':1.0E-6)[&label=0.573833]:1.0E-6,(('AY593817.1_O_Belgium_1973':0.009713,'AY593830.1_O_POL_1959':0.0086)[&label=0.296]:0.001945,'AY593818.1_O_ARG_1958':0.003117)[&label=0.167]:1.0E-6)[&label=0.707667]:9.08E-4)[&label=0.997851]:0.015947,('AY593773.1_A_PER_1969':1.0E-6,'AY593775.1_A_VEN_1970':0.004152)[&label=1.0]:0.024184)[&label=0.999942]:0.024418,('AY593769.1_A_ARG_1959':0.001031,'AY593789.1_A_ARG_1961':1.0E-6)[&label=1.0]:0.040822)[&label=0.98969]:0.012503,(((((((((('AY593778.1_A_SPA_1969':1.0E-6,'AY593754.1_A_SPA_1959':1.0E-6)[&label=1.0]:0.005382,'AY593780.1_A_FRA_1960':0.011801)[&label=0.828]:8.65E-4,'AY593781.1_A_GER_1951':0.00311)[&label=0.748667]:1.0E-6,'AY593792.1_A_ITL_1962':0.005192)[&label=0.8705]:0.001694,'AY593771.1_A_COL_1967':0.053163)[&label=0.8254]:0.001959,'AY593760.1_A_USSR_1964':0.0381)[&label=0.880167]:0.006358,'AY593776.1_A_GER_1968':0.025399)[&label=0.989]:0.021068,(((((('AY593779.1_A_GER_1972':1.0E-6,'AY593774.1_A_SPA_1969':1.0E-6)[&label=0.336]:1.0E-6,'AY593777.1_A_GER_1972':1.0E-6)[&label=0.9975]:0.002039,'AY593751.1_A_NET_1942':0.008484)[&label=0.982333]:0.008561,'AY593759.1_A_GER_1971':0.058393)[&label=0.96475]:0.00598,'AY593794.1_A_COL_1985':0.091524)[&label=0.96]:0.007371,('AY593827.1_O_VEN_1971':0.022483,'AY593826.1_O_ITL_1947':0.009857)[&label=0.989]:0.01889)[&label=0.982143]:0.015947)[&label=0.6844]:0.00801,'AY593810.1_C_UKG_1970':0.071866)[&label=0.66]:1.0E-6,('AY593825.1_O_ARG_1939':0.035332,'NC_039210.1_O_UKG_1965':0.039524)[&label=0.971]:0.027075)[&label=0.6555]:0.003583)[&label=0.882411]:0.010411,'AY593770.1_A_ARG_1966':0.053751)[&label=0.875363]:0.002638,'AY593803.1_A_Brazil_1979':0.048866)[&label=0.870913]:0.00466,((((((((((((((((((((('KX002200.1_A_ARG_2001':1.0E-6,'KX002201.1_A_ARG_2001':0.003096)[&label=0.622]:0.001028,'KX002186.1_A_ARG_2001':0.004143)[&label=0.3925]:1.0E-6,('KX002185.1_A_ARG_2001':0.002067,'KX002177.1_A_ARG_2001':0.002061)[&label=0.097]:1.0E-6)[&label=0.41625]:1.0E-6,('KX002188.1_A_ARG_2001':0.004141,'KX002190.1_A_ARG_2001':0.002061)[&label=0.141]:1.0E-6)[&label=0.764333]:1.0E-6,'KX002181.1_A_ARG_2001':0.002061)[&label=0.899429]:0.002061,(('KX002203.1_A_ARG_2001':1.0E-6,'AY593785.1_A_ARG_2001':1.0E-6)[&label=0.344]:1.0E-6,'AY593784.1_A_ARG_2001':1.0E-6)[&label=0.939]:0.001028)[&label=0.8589]:1.0E-6,'AY593802.1_A_URU_2001':0.001027)[&label=0.923818]:0.001031,'KX002179.1_A_ARG_2001':0.005201)[&label=0.948667]:0.004154,'KX002195.1_A_ARG_2001':1.0E-6)[&label=0.936615]:1.0E-6,'KX002197.1_A_ARG_2001':0.002058)[&label=0.883857]:1.0E-6,('KX002194.1_A_ARG_2001':0.001037,'KX002199.1_A_ARG_2001':0.005186)[&label=0.828]:0.002062)[&label=0.906125]:1.0E-6,'KX002202.1_A_ARG_2001':0.003094)[&label=0.91]:0.003088,((((((('KX002189.1_A_ARG_2001':1.0E-6,'KX002192.1_A_ARG_2001':0.0031)[&label=0.986]:0.00414,'KX002183.1_A_ARG_2001':0.002058)[&label=0.5885]:1.0E-6,('AY593786.1_A_ARG_2001':1.0E-6,'KX002205.1_A_ARG_2001':1.0E-6)[&label=0.992]:0.00415)[&label=0.409]:1.0E-6,'AY593790.1_A_ARG_2001':1.0E-6)[&label=0.3362]:1.0E-6,'KX002204.1_A_ARG_2001':1.0E-6)[&label=0.36]:1.0E-6,(((('KX002176.1_A_ARG_2001':0.001028,'KX002198.1_A_ARG_2001':0.001028)[&label=0.118]:1.0E-6,'KX002182.1_A_ARG_2001':0.003096)[&label=0.232]:1.0E-6,('KX002180.1_A_ARG_2001':0.003098,'KX002184.1_A_ARG_2001':0.002059)[&label=0.145]:1.0E-6)[&label=0.6945]:0.001027,'KX002187.1_A_ARG_2001':0.006252)[&label=0.6042]:1.0E-6)[&label=0.905833]:0.001021,'KX002178.1_A_ARG_2001':0.002064)[&label=0.921923]:0.001049)[&label=0.974323]:0.004137,'KX002196.1_A_ARG_2001':0.005322)[&label=0.965281]:5.51E-4,('KX002193.1_A_ARG_2001':0.007325,'KX002191.1_A_ARG_2001':0.006334)[&label=0.513]:0.00148)[&label=1.0]:0.089406,((('AY593809.1_C_ARG_1969':0.013832,'AY593807.1_C_Brazil_1955':0.002082)[&label=0.995]:0.014355,'AY593793.1_A_PHI_1975':0.040261)[&label=0.9585]:0.016893,'AY593756.1_A_Brazil_1959':0.039581)[&label=0.709]:0.002546)[&label=0.931158]:1.0E-6,'AY593821.1_O_ARG_1967':0.048933)[&label=0.925]:0.003964,(((((((((((('MH559793.1_A_Brazil_2016':1.0E-6,'MH559804.1_A_Brazil_2016':1.0E-6)[&label=0.039]:1.0E-6,'MH559799.1_A_Brazil_2016':1.0E-6)[&label=0.095]:1.0E-6,'AY593768.1_A_Brazil_1955':1.0E-6)[&label=0.084]:1.0E-6,'MH559800.1_A_Brazil_2016':1.0E-6)[&label=0.12375]:1.0E-6,'MH559796.1_A_Brazil_2016':1.0E-6)[&label=0.2066]:1.0E-6,('MH559798.1_A_Brazil_2016':1.0E-6,'MH559788.1_A_Brazil_2016':1.0E-6)[&label=0.065]:1.0E-6)[&label=0.460714]:1.0E-6,'MH559805.1_A_Brazil_2016':1.0E-6)[&label=0.6115]:1.0E-6,('MH559785.1_A_Brazil_2016':1.0E-6,'MH559780.1_A_Brazil_2016':1.0E-6)[&label=0.05]:1.0E-6)[&label=0.7193]:1.0E-6,(('MH559801.1_A_Brazil_2016':1.0E-6,'MH559783.1_A_Brazil_2016':1.0E-6)[&label=0.875]:0.001028,'MH559791.1_A_Brazil_2016':1.0E-6)[&label=0.442]:1.0E-6)[&label=0.891846]:1.0E-6,'MH559786.1_A_Brazil_2016':1.0E-6)[&label=0.970429]:1.0E-6,'MH559781.1_A_Brazil_2016':0.001027)[&label=0.9996]:0.025951,(('AY593753.1_A_Brazil_1970':1.0E-6,'AY593758.1_A_VEN_1967':1.0E-6)[&label=1.0]:0.012907,'AY593757.1_A_Brazil_1967':0.007522)[&label=1.0]:0.03208)[&label=0.998]:0.023223)[&label=0.92431]:0.007345,('AY593787.1_A_Brazil_1977':0.005607,'AY593788.1_A_Brazil_1979':0.019604)[&label=1.0]:0.036632)[&label=0.936683]:0.008413,'AY593782.1_A_ARG_2000':0.075826)[&label=0.925967]:1.0E-6,'AY593806.1_C_Brazil_1971':0.050563)[&label=0.915661]:0.004951)[&label=0.913381]:0.00886,('AY593761.1_A_KEN_1964':0.029301,'MH053306.1_A_TCH_1973':0.09392)[&label=0.932]:0.033442)[&label=0.909726]:1.0E-6,((('AY593804.1_C_SWZ_1965':1.0E-6,'AY593805.1_C_GER_1960':1.0E-6)[&label=1.0]:0.001774,'FJ824812.1_C_SPA_2009':0.013121)[&label=0.998]:0.029212,'AY593808.1_C_ARG_1966':0.026302)[&label=0.987]:0.028398)[&label=0.914565]:0.007248)[&label=0.971932]:0.019934,((('MH053309.1_C_KEN_1967':1.0E-6,'KM268897.1_C_KEN_2004':0.001036)[&label=1.0]:0.053806,'MH053310.1_C_UGA_1970':0.058243)[&label=0.7475]:0.00569,'MH053308.1_C_ETH_1971':0.035609)[&label=0.968667]:0.02113)[&label=1.0]:0.113322,(((((((((((((((((((('KU821590.1_SAT1_NMB_2010':0.011638,'KU821592.1_SAT2_ZAM_2009':0.017591)[&label=0.57]:0.003014,'MH053351.1_SAT3_ZIM_1984':0.015001)[&label=0.5985]:0.002924,('MH053332.1_SAT2_BOT_1974':0.00877,'MH053322.1_SAT1_NMB_1989':0.016316)[&label=0.348]:7.36E-4)[&label=0.39625]:0.001692,'JF749864.1_SAT2_ZIM_2003':0.032255)[&label=0.3592]:0.001166,'AY593843.1_SAT1_NMB_1940':0.022399)[&label=0.332667]:1.0E-6,'AY593841.1_SAT1_ZIM_1958':0.022841)[&label=0.33]:0.001076,'AY593842.1_SAT1_SAR_1961':0.008425)[&label=0.339125]:0.001086,'AY593840.1_SAT1_NMB_1949':0.017065)[&label=0.328556]:0.00102,((((('MH053338.1_SAT3_BOT_1966':0.008379,'AY593853.1_SAT3_BOT_1965':0.006373)[&label=0.991]:0.011847,'MH053328.1_SAT2_BOT_1968':0.025551)[&label=0.693]:0.001867,(('AY593851.1_SAT3_BOT_1961':0.001019,'AY593852.1_SAT3_KEN_1960':1.0E-6)[&label=1.0]:0.018374,'MH053342.1_SAT3_ZAM_1996':0.007663)[&label=0.6435]:0.001891)[&label=0.5912]:0.002214,'MH053339.1_SAT3_BOT_1970':0.010991)[&label=0.571167]:8.27E-4,'AY593838.1_SAT1_BOT_1970':0.033211)[&label=0.610857]:0.001219)[&label=0.74]:0.001756,(('MH053319.1_SAT1_BOT_1974':0.01546,'MH053329.1_SAT2_BOT_1969':0.006795)[&label=0.606]:0.001658,'AY593845.1_SAT1_BOT_1968':0.02189)[&label=0.6455]:0.00477)[&label=0.87985]:0.002809,('MH053330.1_SAT2_BOT_1969':0.006392,'MH053331.1_SAT2_BOT_1972':0.005284)[&label=0.437]:0.001692)[&label=0.935818]:0.008878,'AY593847.1_SAT2_ZIM_1948':0.045707)[&label=0.920435]:0.005853,((((('MH053320.1_SAT1_KEN_1983':0.025226,'KM268899.1_SAT1_TAN_2012':0.013293)[&label=0.54]:0.004656,'JF749860.1_SAT1_KEN_2002':0.029044)[&label=0.528]:0.002573,('JF749861.1_SAT2_KEN_2002':0.019807,'MH053333.1_SAT2_ETH_1989':0.087078)[&label=0.315]:0.002979)[&label=0.9105]:0.010158,'KM268900.1_SAT2_TAN_2012':0.027913)[&label=0.9836]:0.010134,'MH053352.1_SAT3_ZIM_1990':0.029267)[&label=0.861333]:0.001147)[&label=0.843367]:0.004209,(((((((((((('MH053349.1_SAT3_ZIM_1983':1.0E-6,'MH053350.1_SAT3_ZIM_1983':0.002178)[&label=1.0]:0.015544,'MH053335.1_SAT2_ZIM_1965':0.039307)[&label=0.775]:0.005584,'KM268901.1_SAT3_ZIM_1991':0.032615)[&label=0.576667]:0.001317,'AY593846.1_SAT1_ZIM_1966':0.022396)[&label=0.5685]:0.001646,'AY593848.1_SAT2_u_1967':0.020421)[&label=0.4862]:0.001627,'MH053343.1_SAT3_ZIM_1934':0.026672)[&label=0.442333]:1.0E-6,(('MH053348.1_SAT3_ZIM_1977':1.0E-6,'MH053346.1_SAT3_ZIM_1976':1.0E-6)[&label=1.0]:0.025302,'KX375417.1_SAT3_ZIM_1981':0.024479)[&label=0.66]:0.007451)[&label=0.539]:0.002064,'MH053321.1_SAT1_MOZ_1981':0.022175)[&label=0.5695]:1.0E-6,'AY593839.1_SAT1_UKG_1970':0.026494)[&label=0.595364]:0.001216,('KR108949.1_SAT2_SAR_2009':0.005216,'KR108948.1_SAT1_SAR_2009':0.009559)[&label=0.383]:0.003375)[&label=0.688846]:0.003734,'AY593850.1_SAT3_SAR_1959':0.015436)[&label=0.684071]:0.002253,(('MH053345.1_SAT3_ZIM_1975':0.001936,'MH053347.1_SAT3_ZIM_1976':0.00653)[&label=0.995]:0.017721,'KR108950.1_SAT3_SAR_2009':0.024539)[&label=0.546]:0.001544)[&label=0.689647]:0.001132)[&label=0.953917]:0.00298,'MH053334.1_SAT2_ZAM_1964':0.035749)[&label=0.970163]:0.005411,'MH053344.1_SAT3_ZIM_1974':0.026412)[&label=0.98682]:0.013161,'MH053340.1_SAT3_MAL_1976':0.021963)[&label=1.0]:0.094882,(('MF678823.1_SAT1_NIG_2015':0.001023,'MF678824.1_SAT1_NIG_2015':1.0E-6)[&label=0.999]:0.00251,('MF678826.1_SAT1_NIG_2015':1.0E-6,'MF678825.1_SAT1_NIG_2015':1.0E-6)[&label=0.999]:0.004786)[&label=1.0]:0.083326)[&label=0.972291]:0.017018,(((((('MH053337.1_SAT2_UGA_1970':0.015366,'MH053327.1_SAT1_UGA_1970':0.018752)[&label=0.987]:0.017054,'KJ820999.1_SAT3_UGA_2013':0.039476)[&label=0.724]:0.00358,'MH053341.1_SAT3_UGA_1970':0.020625)[&label=0.904333]:0.016294,('HM067705.1_SAT2_UGA_2007':0.028575,'JF749862.1_SAT2_UGA_2002':0.02079)[&label=0.991]:0.026519)[&label=0.6216]:0.002291,(('MH053336.1_SAT2_UGA_1970':0.013709,'MH053326.1_SAT1_UGA_1970':0.050317)[&label=0.886]:0.016397,'HM067706.1_SAT1_UGA_2007':0.039044)[&label=0.8915]:0.015109)[&label=0.946125]:0.01745,'HM067704.1_SAT2_UGA_2007':0.057292)[&label=0.999889]:0.042376)[&label=0.998923]:0.05902,(((((((('KC440884.1_SAT2_EGY_2012':0.001037,'JX014255.1_SAT2_EGY_2012':0.006296)[&label=0.574]:0.001052,'JX014256.1_SAT2_PAT_2012':0.003142)[&label=1.0]:0.044444,'MG725872.1_A_NIG_2013':0.03628)[&label=0.877]:0.00436,('KC440881.1_A_EGY_2011':1.0E-6,'KP940474.1_A_EGY_2014':1.0E-6)[&label=1.0]:0.090072)[&label=0.9158]:0.0157,'MG725874.1_A_NIG_2015':0.051612)[&label=0.901333]:0.009749,((('MG923579.1_A_ALG_2017':0.002088,'MG913340.1_A_ALG_2017':0.002082)[&label=0.407]:1.0E-6,'MG923580.1_A_ALG_2017':0.001039)[&label=1.0]:0.012061,(('MG725875.1_A_NIG_2015':1.0E-6,'MG725876.1_A_NIG_2015':1.0E-6)[&label=0.993]:0.001772,'MG725873.1_A_NIG_2015':0.003456)[&label=0.847]:0.003284)[&label=0.9996]:0.064213)[&label=0.991167]:0.032161,((('MH053325.1_SAT1_UGA_1978':0.070609,'MH053324.1_SAT1_UGA_1971':0.019878)[&label=0.267]:0.004872,'AY593844.1_SAT1_ISR_1962':0.093959)[&label=0.459]:0.003008,('MH053323.1_SAT1_TCH_1972':0.065043,'FJ461346.1_SAT2_UGA_2002':0.026149)[&label=0.329]:0.005099)[&label=0.686]:0.013815)[&label=0.875588]:0.006172,'AY593849.1_SAT2_KEN_1960':0.126426)[&label=0.893667]:0.035454)[&label=1.0]:0.048932);

end;

begin figtree;

set appearance.backgroundColorAttribute="Default";

set appearance.backgroundColour=#ffffff;

set appearance.branchColorAttribute="User selection";

set appearance.branchColorGradient=false;

set appearance.branchLineWidth=1.0;

set appearance.branchMinLineWidth=0.0;

set appearance.branchWidthAttribute="Fixed";

set appearance.foregroundColour=#000000;

set appearance.hilightingGradient=false;

set appearance.selectionColour=#2d3680;

set branchLabels.colorAttribute="User selection";

set branchLabels.displayAttribute="Branch times";

set branchLabels.fontName="Calibri";

set branchLabels.fontSize=12;

set branchLabels.fontStyle=0;

set branchLabels.isShown=false;

set branchLabels.significantDigits=4;

set layout.expansion=0;

set layout.layoutType="RECTILINEAR";

set layout.zoom=0;

set legend.attribute=null;

set legend.fontSize=10.0;

set legend.isShown=false;

set legend.significantDigits=4;

set nodeBars.barWidth=4.0;

set nodeBars.displayAttribute=null;

set nodeBars.isShown=false;

set nodeLabels.colorAttribute="User selection";

set nodeLabels.displayAttribute="label";

set nodeLabels.fontName="Arial";

set nodeLabels.fontSize=12;

set nodeLabels.fontStyle=0;

set nodeLabels.isShown=true;

set nodeLabels.significantDigits=4;

set nodeShapeExternal.colourAttribute=null;

set nodeShapeExternal.isShown=false;

set nodeShapeExternal.minSize=10.0;

set nodeShapeExternal.scaleType=Width;

set nodeShapeExternal.shapeType=Circle;

set nodeShapeExternal.size=4.0;

set nodeShapeExternal.sizeAttribute=null;

set nodeShapeInternal.colourAttribute=null;

set nodeShapeInternal.isShown=false;

set nodeShapeInternal.minSize=10.0;

set nodeShapeInternal.scaleType=Width;

set nodeShapeInternal.shapeType=Circle;

set nodeShapeInternal.size=4.0;

set nodeShapeInternal.sizeAttribute=null;

set polarLayout.alignTipLabels=false;

set polarLayout.angularRange=0;

set polarLayout.rootAngle=0;

set polarLayout.rootLength=100;

set polarLayout.showRoot=true;

set radialLayout.spread=0.0;

set rectilinearLayout.alignTipLabels=true;

set rectilinearLayout.curvature=0;

set rectilinearLayout.rootLength=100;

set scale.offsetAge=0.0;

set scale.rootAge=1.0;

set scale.scaleFactor=1.0;

set scale.scaleRoot=false;

set scaleAxis.automaticScale=true;

set scaleAxis.fontSize=8.0;

set scaleAxis.isShown=false;

set scaleAxis.lineWidth=1.0;

set scaleAxis.majorTicks=1.0;

set scaleAxis.minorTicks=0.5;

set scaleAxis.origin=0.0;

set scaleAxis.reverseAxis=false;

set scaleAxis.showGrid=true;

set scaleBar.automaticScale=true;

set scaleBar.fontSize=12.0;

set scaleBar.isShown=true;

set scaleBar.lineWidth=1.0;

set scaleBar.scaleRange=0.0;

set tipLabels.colorAttribute="User selection";

set tipLabels.displayAttribute="Names";

set tipLabels.fontName="Arial";

set tipLabels.fontSize=12;

set tipLabels.fontStyle=0;

set tipLabels.isShown=true;

set tipLabels.significantDigits=4;

set trees.order=true;

set trees.orderType="increasing";

set trees.rooting=false;

set trees.rootingType="User Selection";

set trees.transform=false;

set trees.transformType="cladogram";

end;
